# Genomic alterations caused by HPV integration in a cohort of Chinese endocervical adenocarcinomas

**DOI:** 10.1038/s41417-020-00283-4

**Published:** 2021-01-04

**Authors:** Wenhui Li, Wanjun Lei, Xiaopei Chao, Xiaochen Song, Yalan Bi, Huanwen Wu, Ming Wu, Lei Li

**Affiliations:** 1 Department of Obstetrics and Gynecology, Peking Union Medical College Hospital, Beijing 100730, China; 2 Department of Obstetrics and Gynecology, China-Japan Friendship Hospital, Beijing 100029, China; 3 Novogene Co., Ltd, Beijing 100142, China; 4 Department of Pathology, Peking Union Medical College Hospital, Beijing 100730, China; 5 Department of Dermatology, Beijing Tsinghua Changgung Hospital, Beijing 102218, China

**Keywords:** Cancer genetics, Cervical cancer

## Abstract

The association between human papillomavirus (HPV) integration and relevant genomic changes in uterine cervical adenocarcinoma is poorly understood. This study is to depict the genomic mutational landscape in a cohort of 20 patients. HPV+ and HPV− groups were defined as patients with and without HPV integration in the host genome. The genetic changes between these two groups were described and compared by whole-genome sequencing (WGS) and whole-exome sequencing (WES). WGS identified 2916 copy number variations and 743 structural variations. WES identified 6113 somatic mutations, with a mutational burden of 2.4 mutations/Mb. Six genes were predicted as driver genes: PIK3CA, KRAS, TRAPPC12, NDN, GOLGA6L4 and BAIAP3. PIK3CA, NDN, GOLGA6L4, and BAIAP3 were recognized as significantly mutated genes (SMGs). HPV was detected in 95% (19/20) of patients with cervical adenocarcinoma, 7 of whom (36.8%) had HPV integration (HPV+ group). In total, 1036 genes with somatic mutations were confirmed in the HPV+ group, while 289 genes with somatic mutations were confirmed in the group without HPV integration (HPV− group); only 2.1% were shared between the two groups. In the HPV+ group, GOLGA6L4 and BAIAP3 were confirmed as SMGs, while PIK3CA, NDN, KRAS, FUT1, and GOLGA6L64 were identified in the HPV− group. ZDHHC3, PKD1P1, and TGIF2 showed copy number amplifications after HPV integration. In addition, the HPV+ group had significantly more neoantigens. HPV integration rather than HPV infection results in different genomic changes in cervical adenocarcinoma.

## Introdution

Cervical adenocarcinoma accounts for nearly 15–25% of all cervical cancers worldwide [1]. In contrast to cervical squamous carcinoma, the incidence of cervical adenocarcinoma shows an obvious increase, especially in young women [2, 3]. Patients with cervical adenocarcinoma often present with a series of characteristic clinical features, such as high positive lymph node, distant metastasis, and high recurrence rates, corresponding to a poor prognosis [1, 4]. There is a much lower prevalence of human papillomavirus (HPV) infection in adenocarcinomas than in squamous type adenocarcinoma (100%) [5–9], as the cervical glandular epithelium does not support HPV proliferation to the extent that the squamous epithelium does [6]. HPV-negative endocervical adenocarcinomas of the usual type are found and vary in frequency from 4.8 to 40.0% across China [8, 10]. The early diagnosis of HPV-negative cervical adenocarcinomas still continues to pose a challenge [11].

HPV infection is responsible for almost all cases of cervical squamous cancers [2, 12] and most cervical adenocarcinomas [13]. The International Endocervical Adenocarcinoma Criteria and Classification (IECC) distinguish between HPV-associated adenocarcinoma (HPVA) and no or limited HPVA features (NHPVA) [14]. These criteria have been shown to be prognostically valuable in retrospective studies. Compared with HPVAs, NHPVAs are more likely to have a more advanced stage and are associated with more aggressive clinical behavior, with higher recurrence rates and worse overall survival [15]. All NHPVA endocervical adenocarcinomas belong to the most invasive Silva pattern [16]. However, these criteria are based on HPV infection rather than HPV integration. In the study of Baalbergen et al. [17], with the exception of HPV-45, HPV positivity, or type in endocervical adenocarcinoma had no significant influence on survival. A classification based on the destructiveness of stromal invasion found an association between the invasion degree and genomic alterations in endocervical adenocarcinomas [18]. Data from vulvar and non-gynecological squamous carcinomas suggest that a classification based on molecular pathogenesis is clinically informative and reproducible [19, 20]. These findings suggest that HPV integration may play an important role in genotype and phenotype alterations.

It has been reported that at least 83% [21, 22] of cervical cancers with HPV infection have HPV integration, which can occur at any chromosome but more frequently occurs at certain fragile sites [23]. Structural alterations of the host genome are frequently observed at the integration site of HPV DNA in cervical cancer and may act in oncogenesis [24]. However, most of the work on the genomic alterations in cervical cancer caused by HPV integration has been performed in the squamous or mixed types rather than adenocarcinoma.

In this study, we aimed to explore the role of HPV integration in the genomic changes of endocervical adenocarcinomas or adenocarcinomas of the usual type, the most common subtype of adenocarcinomas, by whole-genome (WGS) and whole-exome sequencing (WES). These genomic changes included copy number variations (CNVs), structural variations (SVs), somatic mutations, and neoantigens.

## Methods

### Study subjects

This is a preliminary analysis of the study “A multi-omics study on the human papillomavirus integration and tumorigenesis of uterine cervical adenocarcinoma (HITA)” (registration no. NCT03742869, *clinicaltrials.gov*). Primary tumor tissues and matched adjacent normal tissues from 20 patients confirmed with endocervical adenocarcinoma were obtained from the study center from September 1, 2017 to September 1, 2018. No patient received radiation or chemotherapy prior to sample collection, and they or their deputies provided informed consent to participate in the study. Data on clinical characteristics were collected from medical records. This study was approved by the Institutional Review Board (IRB) of the study center (registration no. JS-1696).

Six pairs of samples were snap-frozen, and fourteen pairs were formalin fixed and paraffin embedded so that they could be macrodissected to reach a tumor cell purity of up to 75% or more if needed. Genomic DNA (gDNA) from the matched tumor tissues and corresponding normal tissues was extracted. The DNA samples underwent further DNA quantification and qualification, genomic library preparation, clustering and sequencing, and bioinformatics analysis with Novogene (Tianjin, China).

### Next-generation sequencing

gDNA was extracted from paired fresh-frozen specimens or formalin-fixed paraffin-embedded sections using a Qiagen DNeasy Blood and Tissue Kit (Qiagen, Hilden, Germany). The quality of the isolated gDNA was verified by using agarose gel electrophoresis and a Qubit® DNA Assay Kit on a Qubit® 2.0 Fluorometer (Invitrogen, USA) to monitor DNA degradation, contamination, and concentration.

A total amount of 0.5 μg DNA per sample was used as input material for the DNA library preparations for WGS. A sequencing library was generated using a TruSeq Nano DNA HT Sample Prep Kit (Illumina, USA) following the manufacturer’s recommendations, and index codes were added to each sample. Briefly, the gDNA sample was fragmented by sonication to a size of 350 bp. Then, DNA fragments were end polished, A-tailed, and ligated with the full-length adapter for Illumina sequencing, followed by further PCR amplification. After PCR products were purified (AMPure XP system), libraries were analysed for size distribution on an Agilent 2100 Bioanalyzer (Agilent, USA) and quantified by real-time PCR (3 nM).

A total amount of 0.6 μg genomic DNA per sample was used as input material for WES. Sequencing libraries were generated using the Agilent SureSelect Human All Exon V6 Kit (Agilent Technologies, CA, USA) following the manufacturer’s recommendations, and index codes were added to each sample. Briefly, fragmentation was carried out with a hydrodynamic shearing system (Covaris, Massachusetts, USA) to generate 180–280 bp fragments. The remaining overhangs were converted into blunt ends via exonuclease/polymerase activities. After adenylation of the 3′ ends of DNA fragments, adapter oligonucleotides were ligated. DNA fragments with ligated adapter molecules on both ends were selectively enriched in a PCR. After PCR analysis, libraries were hybridized with the liquid phase of a biotin-labeled probe. Then, magnetic beads with streptomycin were used to capture the exons. Captured libraries were enriched in a PCR to add index tags to prepare for sequencing. Products were purified using the AMPure XP system (Beckman Coulter, Beverly, USA) and quantified using the Agilent High Sensitivity DNA Assay on the Agilent Bioanalyzer 2100 system.

Clustering of the index-coded samples for WGS and WES was performed on a cBot Cluster Generation System using the HiSeq PE Cluster Kit V2.5 (Illumina) according to the manufacturer’s instructions. After cluster generation, the DNA libraries were sequenced on the Illumina HiSeq platform, and 150 bp paired-end reads were generated.

### Sequencing data analysis and mutation calling

Samtools and bcftools were used to perform variant calling and identify single nucleotide polymorphisms (SNPs) and InDels [25]. Control-FREEC was utilized to perform CNV detection, while Crest was utilized for SV discovery [26, 27].

ANNOVAR was used to annotate the variant call format (VCF) obtained in a previous study [28]. dbSNP, 1000 Genomes and other related databases were applied to characterize the detected variants. Given the significance of exonic variants, gene transcript annotation databases (Consensus CDS, RefSeq, Ensembl and UCSC) were also used to determine amino acid alterations. Somatic SNVs were detected by MuTect [29], somatic InDels were detected by Strelka [30], and somatic SVs were detected by Crest. Control-FREEC was used to detect somatic CNVs.

#### Driver genes and SMGs

Driver genes were identified by comparing the somatic mutations to known driver genes published in open databases. In addition, considering that functional mutations in driver genes often cluster together while synonymous mutations often distribute randomly in the genomic region, a mutational model was built to cooperate with OncodriveClust, OncodriveFM, and MuSiC software to predict potential driver genes in our study [31]. This model was used for the comprehensive analysis, as it takes SMGs, cluster bias, and functional impact into account when considering the driver functions of mutated genes.

SMGs that had obviously higher mutation rates than the background mutation rate were obtained by comprehensively analysing somatic SNVs and InDels. MuSiC software was used to perform convolution tests to identify SMGs [32].

### HPV infection and integration status

ViFi software, which combines phylogenetic methods and reference sequence alignment to detect viral integration, was used to analyse the integration status of HPV present in the cervical adenocarcinoma samples based on WGS data [33]. The algorithm generates a set of papillomavirus reference genomes for cervical cancer sample analysis containing 208 HPVs, including 18 common high- and intermediate-risk HPVs that have been proven to be closely related to cervical cancer (e.g., HPV16, 18, 26, 31, 33, 35, 39, 45, 51, 52, 53, 56, 58, 59, 66, 68, 73, and 82).

### Statistical analysis

The comparison of clinicopathological characteristics and overall survival between patients with and without HPV integration utilized nonparametric tests and the Kaplan–Meier method, respectively. Unless otherwise stated, all analyses were performed with a two-sided significance level of 0.2 due to limited sample sized and mutation numbers. The analysis was conducted with the use of the software Statistical Product and Service Solutions (SPSS) Statistics 20.0 (IBM Corporation, Armonk, NY, USA).

## Results

### Samples and clinical data

Primary endocervical adenocarcinoma tissues and paired normal tissues were obtained from 20 patients who had not received prior radiation or chemotherapy after radical hysterectomy (Supplementary Table 1). The mean patient age was 43.7 years (range, 33–62 years). In total, 14 patients were in stage I, and 6 patients were in stage II (International Federation of Gynecology and Obstetrics [FIGO] 2009). Postoperative pathology was confirmed by two pathologists (YB and HW). Of the 20 patients, five were diagnosed with deep stromal invasion, five were diagnosed with lymphovascular space involvement (LVSI), three were diagnosed with lymph node metastasis, two were diagnosed with parametrial infiltration, and one was diagnosed with a positive vaginal margin. After a median follow-up of 8 months (range, 3–14 months), one patient (5.0%) died of disease recurrence.

### Somatic genomic alterations

#### Somatic single nucleotide variations (SNVs) and insertions and deletions (InDels)

**Fig. 1:**
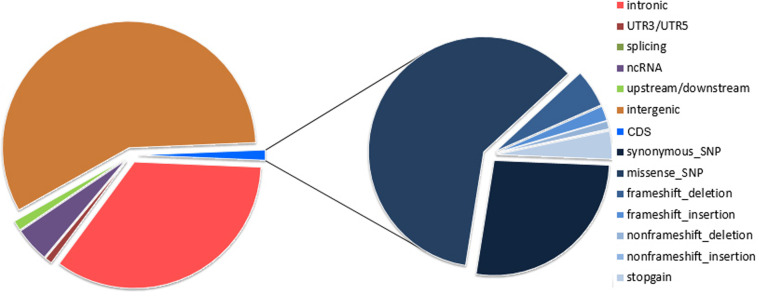
Distribution of somatic mutations identified in cervical adenocarcinoma by WGS and WES. Left, the circle represents the distribution of genomic mutations by WGS; right, the circle represents the types of mutations in the coding sequence by WES. CDS coding sequence, WES whole-exome sequencing, WGS whole-genome sequencing, SNP single-nucleotide polymorphism.

A concatenated quality report of the WGS and WES data refers Supplementary Table 2. WGS covered the whole genomic region with a median depth of 31× (range: 25–39×) in the 40 samples. In the WGS analysis, the 20 paired samples contained a total of 100,491 somatic mutations, most of which were present in the intergenic regions (57.5%), intronic regions (34.46%) and noncoding RNA (ncRNA) regions (4.5%). The coding sequence (CDS) regions contained 1320 (1.3%) somatic mutations, including 1305 SNVs and 15 InDels (Supplementary Tables 3–5 and Fig. 1).

WES covered the target exons with a median depth of 204× (range: 185–276×) for the 20 tumor samples and 106× (range: 96–139×) for the normal samples. The 20 pairs of samples contained a total of 6113 somatic mutations, with an average of 305.7 mutations per cervical tumor. In total, 1955 mutations were located in CDS regions, 69 were located in splice sites, 2296 were located in intronic regions, and 1029 were located in intergenic regions. The WES analysis disclosed 1788 SNVs and 167 InDels in CDS regions, including 1143 missense mutations, 507 synonymous mutations, 139 frameshift InDels, 24 nonframeshift InDels, and 74 stop-gains. Regarding somatic mutations in CDS regions, the most common type identified was a missense mutation, which accounted for 58.5% of all mutations (Supplementary Tables 6–8 and Fig. 1). The aggregate mutation density was calculated based on non-silent mutations in the WES data. One tumor with outlier mutation frequencies (>600 per sample) was defined as “hypermutant”. The aggregate mutation density was 2.19 mutations per Mb across all cervical adenocarcinoma tumors and 0.94 when the hypermutant tumor was excluded.

#### Copy number variations (CNVs) and structural variations (SVs)

**Fig. 2:**
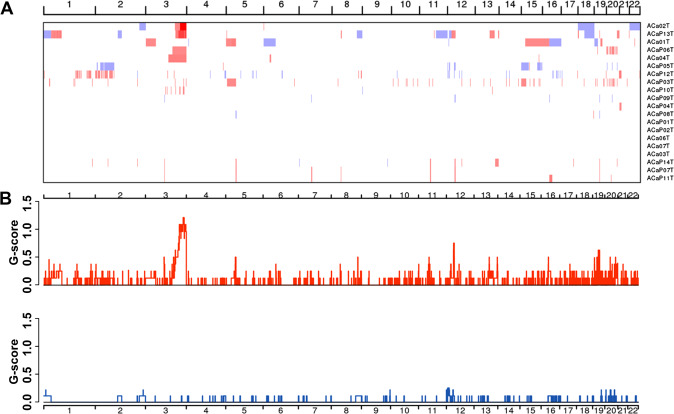
Distribution of copy number variations in cervical adenocarcinomas. **A** Clustered heatmap of samples. In the heatmap, blue represents downregulated expression, red represents upregulated expression, and white represents no change in expression. **B** Distribution of CNV in the chromosomes. The *y*-axis shows the variation scores of GISTIC software; the higher the score is, the higher the significance is. CNV copy number variants.

In total, 2916 CNVs were identified among the in 20 samples, with an average of 145.8 CNVs per tumor, including 2633 copy number amplifications and 283 copy number deletions. GISTIC2.0 analysis (with a threshold of *q* < 0.20) revealed eight focal amplifications and seven focal deletions in addition to four recurrently altered whole arms (Supplementary Tables 9, 10 and Fig. 2). The chromosomal regions or arms with recurrent copy number amplifications were 3q27.1 (30%), 12q11 (30%), 19q11 (35%), 19q13.11 (20%), 3p11.1 (20%), 5p11 (20%), 8q24.21 (15%), 11p11.12 (15%), 13q22.1 (20%), 16p11.1 (20%), 19p12 (25%), and 20q13.2 (30%), while the regions with recurrent copy number deletions were 12p13.31 (10%), 1p36.22 (5%), 2q37.1 (10%), 9q34.3 (10%), 12p12.3 (5%), 19q13.2 (10%), and 20p11.1 (15%). 3q27.1 was confirmed as the most significant region with recurrent copy number amplifications and was verified to be present in six tumors. The shortest common sequence in the 3q27.1 region with CNVs covered six genes, namely, *LINC00888*, *MCF2L2*, *KLHL6*, *KLHL24*, *YEATS2*, and *MAP6D1*.

SVs were identified by Crest software based on the analysis of WGS data, and 734(743) SVs, including 74 inversions, 375 large fragment copy number deletions, 3 large fragment insertions, 251 intrachromosomal translocations (ITXs), and 40 interchromosomal translocations (CTXs), were confirmed in the 20 samples with cervical adenocarcinoma. The breakpoints were mostly located in intergenic regions (49.1%), followed by intronic regions (40.7%), and intronic regions of ncRNAs (4.1%). The SVs with the breakpoints located in the gene regions were analysed with inhouse software, and the fusion genes were detected. There were 52 genetic fusions, forming 34 kinds of fusion genes, including *KRT37-KRT38*, *SDHAP2-SDHAP1*, *ZNF714-ZNF431*, *NBPF25P-LOC654342*, *NBPF20-NUDT4*, *HLA-H-HLA-A*, *FAM99A-FAM99B*, *APOL4-APOL1*, *LINC00893-LINC00894*, and *ZNF160-ZNF415*.

#### Mutational spectrum and mutational signature

**Fig. 3:**
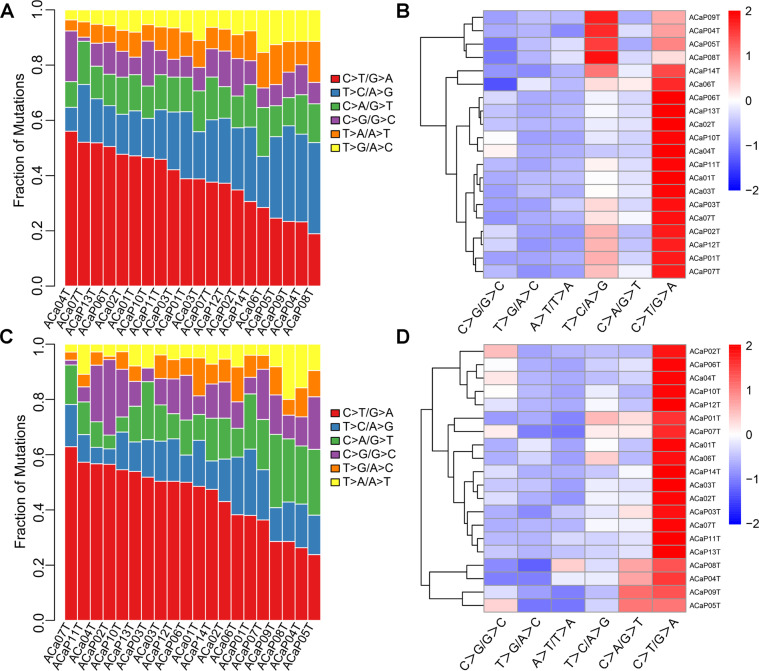
Distribution of single-nucleotide variations in different adenocarcinoma samples. **A** Column diagram of various mutations identified by WGS. **B** Clustered heatmap of various types mutations in 20 cases by WGS. In the heatmap, blue represents downregulated expression, red represents upregulated expression, and white represents no change in expression. **C** Column diagram of various mutations identified by WES. **D** Clustered heatmap of various types mutations in 20 cases by WES. WES whole-exome sequencing, WGS whole-genome sequencing.

According to the type of single nucleotide substitution, the most common kind of point mutation was the replacement of C with T, consistent with the WGS and WES data (Fig. 3). Point mutations were classified into 96 different types according to the base types at each 1 bp upstream and downstream of the point mutation site. By means of nonnegative matrix factorization (NMF), three signatures were confirmed in cervical adenocarcinoma. Cluster analysis was performed on the three signatures with the 30 known signatures summarized and presented in the Catalog of Somatic Mutations in Cancer (COSMIC, https://cancer.sanger.ac.uk/cosmic/signatures_v2.) database to help understand the mutational characteristics identified in our study. These three signatures found in cervical adenocarcinoma corresponded to the known signatures 5, 2, and 6, and they were confirmed in both the WGS and WES analyses (Supplementary Fig. 1).

#### Driver genes and significantly mutated genes (SMGs)

In this cohort, *PIK3CA*, *KRAS*, *TRAPPC12*, *NDN*, *GOLGA6L4*, and *BAIAP3* were predicted as driver genes by WES data. Among the predicted driver genes, *PIK3CA* and *KRAS* have been reported previously (Supplementary Fig. 2) [22], and the other four genes are novel.

**Fig. 4:**
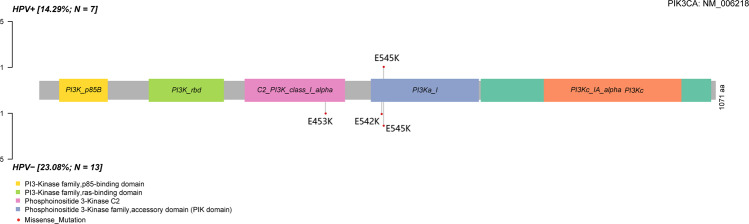
Mutational characteristic of *PIK3CA*. The red circle represents non-silent mutations, and the size represents the number of mutations.

In total, 1298 genes were confirmed to have non-silent somatic mutations, 50 of which had a mutation frequency of more than 10%. Four SMGs with a false discovery rate (FDR) < 0.2 from the WES data were found using the MutSiC algorithm (Supplementary Table 11). We identified *PIK3CA*, *NDN*, *GOLGA6L4*, and *BAIAP3* as SMGs in cervical adenocarcinoma and confirmed that *NDN*, *GOLGA6L4*, and *BAIAP3* were novel SMGs. *PIK3CA* was considered the most prominent mutant gene in cervical adenocarcinoma, consistent with previous studies on cervical cancer (Fig. 4) [34, 35]. *PIK3CA* has mostly activating helical domain *E542K* and *E545K* mutations, with a marked relative decrease in mutations elsewhere in the gene. Five missense mutations were revealed, of which three were located in the *E545K* domain, one in the *E542K* domain, and one in the *E453K* domain, which had not been reported before.

### HPV infection and integration

Based on the ViFi algorithm and WGS data, HPV infection and integration were found in 19 (95%) and 7 (36.8%) of 19 patients (Supplementary Table 1). According to the state of HPV integration, the 20 patients were divided into the HPV+ group and the HPV− group.

**Fig. 5:**
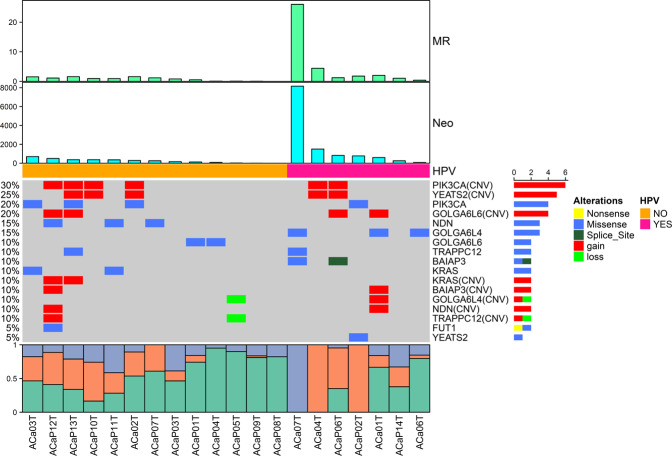
The mutational landscape identified in the HPV+ group and HPV− group of cervical adenocarcinomas. Clustered heatmaps of significantly mutated genes and driver genes in the samples are shown in groups. Clusters presented from left to right include HPV− (orange) and HPV+ (pink). Tracks for mutational rates (MR) and neoantigens (Neo) are shown on top of the figure. In the heatmap, blue represents downregulated expression, red represents upregulated expression, and white represents no change in expression. The column diagram on the bottom is the summary of mutational spectrum in each sample. HPV human papillomavirus.

The aggregate mutation density of the HPV+ group was 5.28 mutations per Mb across all tumors and 1.82 when the hypermutant tumor was excluded, both of which were significantly higher than those of the HPV− group (0.53 mutations per Mb) (*P* = 0.002), based on which the neoantigens were predicted and compared between the two groups. The median number of neoantigens was markedly higher in the HPV+ group (772) than in the HPV− group (259) (*P* = 0.024), which might suggest a more promising prospect for immunotherapy (Fig. 5).

**Fig. 6:**
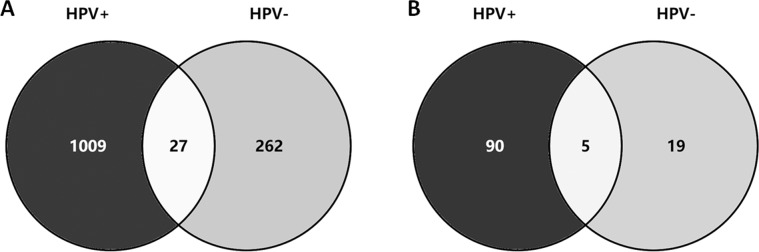
Distribution of mutated genes (A) and driver genes (B) in the HPV+ group and HPV− group. HPV, human papillomavirus.

In the HPV+ group, 1036 genes with 1109 non-silent somatic mutations were identified, including 868 missense mutations, 47 splice site mutations, 52 nonsense mutations, 129 frameshift indels and 13 in-frameshift indels. However, in the HPV-group, only 289 genes with 303 non-silent mutations were identified, including 242 missense mutations, 20 splice site mutations, 20 nonsense mutations, 10 frameshift indels, and 11 in-frameshift indels. Among the mutated genes confirmed, only 27 (2.1%) were shared between the two groups (Fig. 6A).

In the HPV+ group, 95 driver genes with somatic mutations were detected, whereas in the HPV− group, 24 driver genes with somatic mutations were detected. Only 5 of the 114 (4.4%) driver genes with mutations were shared between the two groups (Fig. 6B).

Concerning the grouping analysis of SMGs, *GOLGA6L4*, and *BAIAP3* were confirmed as SMGs in the HPV+ group, while *PIK3CA*, *NDN*, *KRAS*, *FUT1*, and *GOLGA6L6* were confirmed as SMGs in the HPV− group. No common genes were shared between the two groups (Supplementary Table 12 and Fig. 5).

**Fig. 7:**
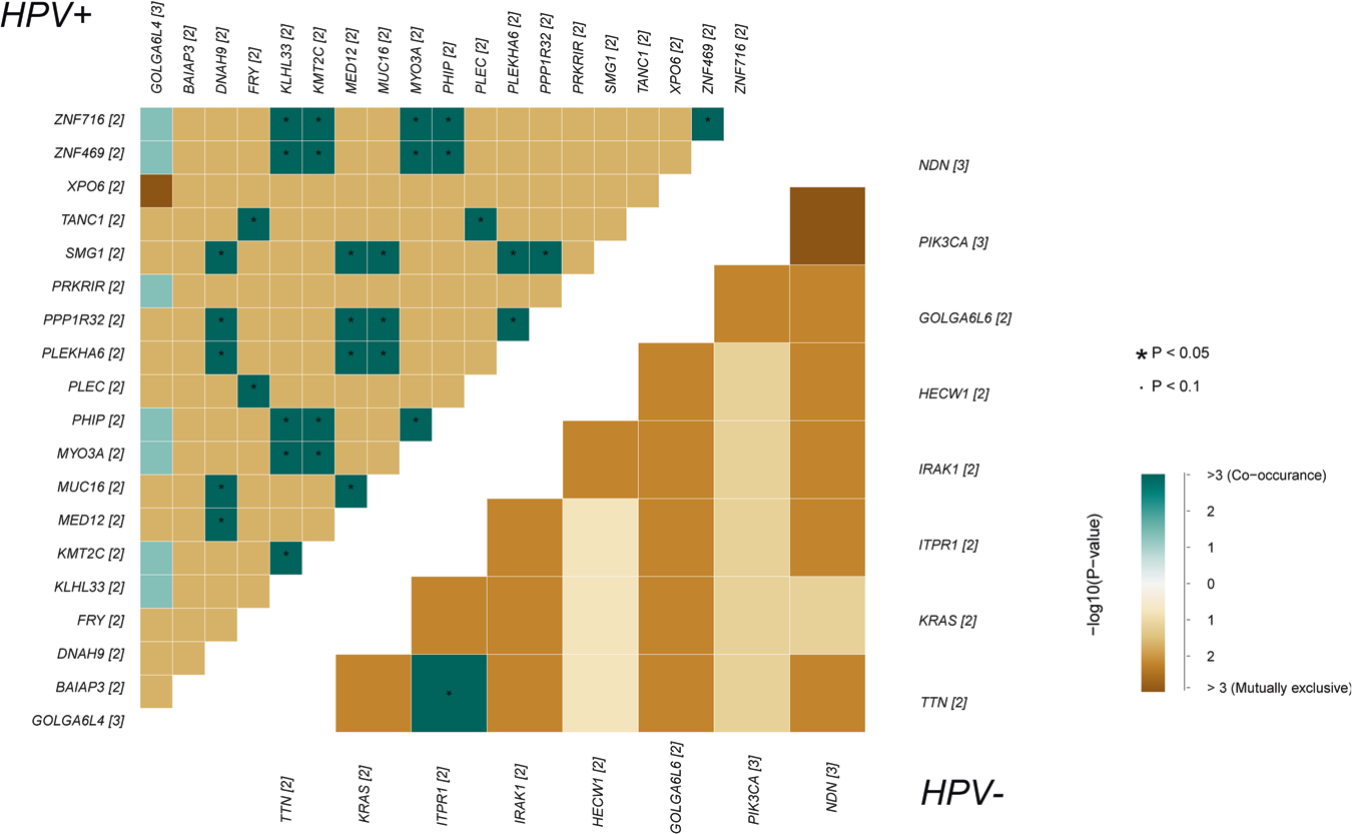
Correlation analysis of mutated genes in HPV+ and HPV− groups.

The mutated genes were examined after correlation analysis in both groups (Fig. 7). In the HPV+ group, *ZNF* family genes showed a significant positive correlation with *KLHL33*, *KMT2C*, *MYO3A*, and *PHIP*, which are enriched in lysine degradation and mRNA surveillance pathways; in the HPV− group, *TTN*’s were obviously related to *ITPR1*.

**Fig. 8:**
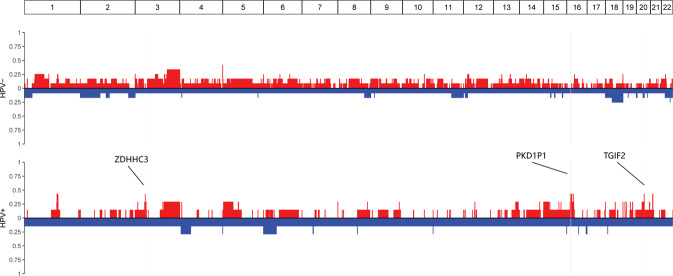
Remarkable copy number amplifications of ZDHHC3, PKD1P1 and TGIF2 after HPV integration.

After further comparing the sequences for the two groups, we determined notably different CNV distribution for three regions. *ZDHHC3*, *PKD1P1*, and *TGIF2* on chromosomes 3, 16, and 20, respectively, were predisposed towards remarkable copy number amplifications after HPV integration (Fig. 8).

Between the HPV+ and HPV− groups, there was no significant difference either in the clinicopathological characteristics listed in Supplementary Table 1 (all *P* values greater than 0.05) or in overall survival (*P* = 0.463) according to Kaplan–Meier analysis.

## Discussion

Cervical adenocarcinomas consist of a diverse group of tumors with several distinct histological tumor types. A growing body of literature indicates that these tumor types have type-specific pathogenesis, clinicopathological characteristics, and prognoses. We performed a genomic analysis in endocervical adenocarcinoma, the most common type of adenocarcinoma arising from the mucinous endocervical epithelium, which is also referred to as the “usual type” [36, 37], accounting for approximately 75% of all adenocarcinomas [5]. In our study, specifically, a tumor cell purity of up to 75% or more was needed in formalin fixed and paraffin embedded samples, since other cell types may cause substantial bias in the analysis of genomic changes, especially in the study of a small sample size. The TCGA data has showed different features of tumor purity (*P* = 9.5 × 10^−3^) between two squamous-carcinoma-enriched groups (keratin-low and keratin-high) and one adenocarcinoma-enriched group, however, the database had a large sample size of 178 core-set samples [22]. Based on stringent criteria, by WGS and WES, we revealed that endocervical adenocarcinoma had specific genomic characteristics. These findings provide both biomarkers for potential targeted therapy and a foundation for the analysis of the role of HPV integration.

First, in our study, 6113 somatic mutations were identified among 20 samples of cervical adenocarcinoma, with an average of 305.65 mutations per tumor, which was higher than the 225.6 mutations per tumor on average from The Cancer Genome Atlas (TCGA) database [22]. Most genetic mutations identified were located in intergenic and intronic regions, and the most common type of mutation was a missense mutation, which accounted for more than 50% of all mutations. However, the analysis of non-silent mutations revealed a tumor mutational burden of 2.4 mutations per Mb in our study, which was much lower than the mutational burden of 4.04 mutations per Mb from the TCGA [22], in which only 31 of 228 (13.6%) belonged to adenocarcinoma. This discrepancy suggests, as reported previously [38], that cervical adenocarcinoma has a lower mutational burden than cervical squamous cell cancer.

Second, we identified an average of 145.8 CNVs in each tumor sample of cervical adenocarcinoma, which is higher than that in unclassified cervical cancers from the TCGA [22] and in endometrioid endometrial carcinomas [39] but similar to the average in head and neck squamous cancers (141 CNVs per tumor) [40]. GISTIC2.0 analysis revealed eight focal amplifications and seven focal deletions in addition to four recurrently altered whole arms. Copy number alterations were observed in 5–30% of all tumor specimens, which was in accordance with pan-cancer research (1.5–14%) [41]. Six of the 20 patients were certified to have copy number amplifications at 3q27.1, among which the shortest repetition covered six genes: *LINC00888*, *MCF2L2*, *KLHL6*, *KLHL24*, *YEATS2*, and *MAP6D1*. *YEATS2* is considered a scaffolding subunit of the Ada-two-A-containing (ATAC) complex that binds to acetylated histone H3 via its YEATS domain [42, 43]. The YEATS2-containing ATAC complex co-localizes with H3K27 acetylation (H3K27ac) on the promoters of actively transcribed genes. The *YEATS2* gene has been confirmed to be highly amplified in human nonsmall cell lung cancer (NSCLC) and is required for cancer cell growth and survival [44]. It was believed that the identification of *YEATS2* amplification could be used as a novel therapeutic target for the targeted therapy of NSCLC [44]. However, whether it works similarly in the development of cervical adenocarcinoma still requires further study.

Third, Crest software was applied to detect a total of 743 SVs in 20 samples of cervical adenocarcinoma, revealing 34 fusion genes (e.g., HLA-H-HLA-A, APOL4-APOL1, and LINC00893-LINC00894), all with a positive rate among all samples of 15%. In the TCGA, ZC3H7A-BCAR4 was the most frequently detected fusion gene in cervical cancer, accompanied by amplifications of *BCAR4* [22]. However, we did not detect this fusion gene in cervical adenocarcinoma.

Fourth, in our study, after the evaluation of WES data in 20 cervical adenocarcinomas, six genes (i.e., *PIK3CA*, *KRAS*, *TRAPPC12*, *NDN*, *GOLGA6L4*, and *BAIAP3*) were predicted as driver genes that could promote tumor formation and development. Except for *PIK3CA* and *KRAS*, the four other genes were identified as novel driver genes in cervical adenocarcinoma, which might need further validation. Cervical squamous cell carcinoma and adenocarcinoma have distinct molecular profiles. The *PIK3CA* mutation rates did not differ significantly between adenocarcinomas and squamous cell carcinomas, and *KRAS* mutations were identified only in adenocarcinomas [45]. Previous reports have uncovered plentiful SMGs in studies on cervical cancers [22, 45]. Based on WES data, we identified four SMGs that may be associated with cervical adenocarcinoma: *PIK3CA*, *NDN*, *GOLGA6L4*, and *BAIAP3*. Typically, the mutations occurred in the E542K and E545K domains of PIK3CA [46]. In our study, *PIK3CA* appeared to be a frequently altered gene in cervical adenocarcinoma, with missense mutations in 4 of 20 (20%) tumors, consistent with previous reports (8–37.5%) [45, 47]. We identified five mutations located in the E542K and E545K domains as well as in the E453 domain. However, *NDN*, *GOLGA6L4*, and *BAIAP3* were revealed as novel SMGs in our study and were also predicted to have their own driver functions. Therefore, these genes may play an important role in cervical adenocarcinoma and need further research. In contrast, other genes frequently mutated in previous studies [22, 38, 45], such as *HLA-B*, *NFE2L2, EP300*, *FBXW7*, *ARID1A*, *SHKBP1*, *ERBB3*, and *CASP8*, were not confirmed in our study, probably because of the heterogeneity of tumor origins in previous studies.

Last, in our cervical adenocarcinoma samples, three signatures were identified in both WES and WGS analyses, corresponding to signatures 2, 5, and 6 from the COSMIC database. According to Henderson’s research, virus infection can activate *APOBEC*, and more than 99% of *APOBEC* mutations are caused by persistent HPV infection [46]. This suggests that APOBEC mutagenesis may also be the predominant source of mutations in cervical adenocarcinoma under the action of HPV, as reported previously in cervical cancer [22].

In cervical cancer as a whole, gene expression levels at HPV integration sites are significantly higher in tumors with HPV integration than in tumors without viral integration at the same site [47]. The integration of HPV was recently observed to be associated with structural aberrations and increased target gene expression [22]. In our study on adenocarcinomas, these findings were further confirmed. According to CNV analysis, *ZDHHC3*, *PKD1P1*, and *TGIF2* showed copy number amplifications after HPV integration. Among which, *ZDHHC3* (zinc finger DHHC-type containing 3) was associated with protein palmitoylation, a common post-translational lipid modification, with a significant role in protein trafficking and the progression of cell division. Research published in 2018 revealed that *ZDHHC3* can promote herpes simplex virus infectivity in vitro and viral pathogenesis in vivo [48]. Thus, we explored whether it has similar functions in the formation or development of cervical adenocarcinoma by HPV integration. After HPV integration, the mutated genes *KLHL33*, *KMT2C*, *MYO3A*, and *PHIP* were confirmed to be positively related to ZNF families, which have been reported to affect inflammatory reactions to some viruses [49]. They were found to be enriched in pathways such as the lysine degradation pathway and mRNA surveillance pathway. Thus, HPV integration promoted the accumulation of certain metabolites in these pathways, resulting in the development of cervical adenocarcinoma.

In summary, in our study, the HPV+ group had several distinct genomic characteristics relative to the HPV− group, as follows: (1) higher aggregate mutation density, (2) more neoantigen expression, (3) more non-silent somatic mutations, (4) more specific driver genes with somatic mutations, and (5) specific SMGs. These characteristics make HPV+ adenocarcinoma a unique subpopulation that could benefit from targeted therapy and/or immunotherapy, since HPV16 vaccination together with PD-1 inhibitors could afford a promising survival outcomes for incurable HPV16-raleted cancers [50]. WES revealed HPV sequences in 13 tumors (86.7%) in women from Hong Kong, in which the HPV genome might have integrated into and hence disrupted the functions of certain exons [38].

On a genome-wide scale, the squamous type shows significantly more gains than the adenocarcinoma type [51], which determines the different responses to treatment and survival outcomes in these two major types. Therefore, genomic alterations may be associated with survival outcomes, which awaits long follow-up in a large cohort. However, based on our findings, cervical adenocarcinoma with and without HPV integration are probably two entities with different treatment protocols and survival outcomes. There is some evidence supporting our hypothesis. As mentioned previously, the subclassification of adenocarcinomas addressed the role of HPV infection and/or genomic alterations in the survival outcomes of patients with cervical adenocarcinoma [15, 18], a miscellaneous complex. In addition, specific HPV16 variant sublineages identified by a genome-wide association study (GWAS) strongly influence the risk of the histological types of cervical precancer and cancer [52]. These findings need both long-term follow-up in a cohort population and combined omics analyses, which have revealed global changes caused by HPV oncogenes in a less biased way, and allow the identification of novel factors and key cellular networks that may promote malignant transformation [53].

## Conclusions

Based on our findings, HPV integration rather than HPV infection results in different genomic changes in cervical adenocarcinoma, which suggested cervical adenocarcinoma with and without HPV integration may be two entities with different treatment protocols and survival outcomes.

## Supplementary information

Legends of Supplementary Tables and Figures

Supplementary Table 1

Supplementary Table 2

Supplementary Table 3

Supplementary Table 4

Supplementary Table 5

Supplementary Table 6

Supplementary Table 7

Supplementary Table 8

Supplementary Table 9

Supplementary Table 10

Supplementary Table 11

Supplementary Table 12

Supplementary Figure 2

## Data Availability

All data of this study has been contained in the text and its supplementary materials.
