# Supplementary material for: Genomic alterations caused by HPV integration in a cohort of Chinese endocervical adenocarcinomas
Source: Cancer Gene Ther. 2021 Jan 4;28(12):1353–64. doi: 10.1038/s41417-020-00283-4 (PMC8636260; doi:10.1038/s41417-020-00283-4)
Supplement: Supplementary file 1 — Legends of Supplementary Tables and Figures [file 41417_2020_283_MOESM1_ESM.docx]

**Legends of Supplementary Tables**

Supplementary Table 1

Clinical characteristics of patients enrolled (n=20). HPV, human papillomavirus. LN, lymph nodes. LVSI, lymph-vascular space invasion. NA, not available. OS, overall survival.

Supplementary Table 2

A concatenated quality report of the WGS and WES data.

Supplementary Table 3

Somatic mutations detected in 20 cases by whole genome sequencing.

CDS, coding sequence. UTR, untranslated region. SNV, single nucleotide variant. ncRNA, noncoding RNA. INDEL, insertion and deletion.

Supplementary Table 4

Detail of somatic SNV detected in each case by whole genome sequencing.

SNV, single nucleotide variant. CHROM, chromosome. POS, position. REF, reference base. ALT, alternative base. Func, function. AAChange, amino acid change. QUAL: Quality value under the Phred standard. wgRna, the annotation of microRNAs and snoRNAs related to variation point. Cytoband, chromosomal region of variants. targetScanS, annotation from targetScanS database. tfbsConsSites, the score to assess the conservation of variants based on transfac database. genomicSuperDups, screening for segmental duplication. avsnp150, ID of variants from dbSNP150. cosmic82, annotation from cosmic database. clinvar_20170905, annotation of variants on relationship with human health by NCBI format. gwasCatalognew, annotation from Genome-wide association studies. 1000g2015aug_Chinese, allele frequency of mutated base in Chinese population from the 1000-genome project. 1000g2015aug_eas, allele frequency of mutated base in East Asian population from the 1000-genome project. 1000g2015aug_all, allele frequency of mutated base in all population from the 1000-genome project. esp6500siv2_all, allele frequency from NHLBI-ESP project in China. ExAC_ALL, allele frequency in all population from Exome Aggregation Consortium database. ExAC_EAS, allele frequency in East Asian population from Exome Aggregation Consortium database. SIFT, SIFT score showing the effect of variants on protein sequence. Polyphen2_HVAR, score assessing the effect of variants on protein sequence by HumanVar database by PolyPhen2. Polyphen2_HDIV, score assessing the effect of variants on protein sequence by HumanDiv database by PolyPhen2. dbNSFP version3.0, index assessing the effect of variants on protein sequence by dbNSFP version3.0. gerp++gt2, annotation of conservation of variants by ANNOVAR. CADD, deleterious score of SNVs and InDels. INFO, variants’ information detected from mutation software. FORMAT: GT, Genotype; PL, Standardized likelihood value of the genotype; DP, sequencing depth; DV, sequencing coverage; SP, likelihood value of chain preference p-value. Normal_samplename, FORMAT information of normal samples. Tumor_samplename, FORMAT information of tumor samples. Ori_REF, value of REF column from VCF file. Ori_ALT, value of ALT column from VCF file. shared_hom, number of samples with homozygous mutation. shared_het, number of samples with heterozygous mutation. OMIM, annotation from Mendelian database. GWAS_Pubmed_pValue, p-value of relativity between the variant and diseases from previous researches. HGMD_ID_Diseasename, ID and related diseases of the variant from HGMD database. GO_BP：annotation of biological progress from Gene Ontology. GO_CC, annotation of cell component from Gene Ontology. GO_MF, annotation of molecular function from Gene Ontology. KEGG_PATHWAY, annotation of signal pathways from KEGG. PID_PATHWAY, annotation of signal pathways from PID. BIOCARTA_PATHWAY, annotation of signal pathways from BIOCARTA. REACTOME_PATHWAY, annotation of signal pathways from REACTOME.

Supplementary Table 5

Detail of somatic INDELs detected in each case by whole genome sequencing.

INDEL, insertion and deletion. CHROM, chromosome. POS, position. REF, reference base. ALT, alternative base. Func, function. AAChange, amino acid change. QUAL: Quality value under the Phred standard. wgRna, the annotation of microRNAs and snoRNAs related to variation point. Cytoband, chromosomal region of variants. targetScanS, annotation from targetScanS database. tfbsConsSites, the score to assess the conservation of variants based on transfac database. genomicSuperDups, screening for segmental duplication. avsnp150, ID of variants from dbSNP150. cosmic82, annotation from cosmic database. clinvar_20170905, annotation of variants on relationship with human health by NCBI format. gwasCatalognew, annotation from Genome-wide association studies. 1000g2015aug_Chinese, allele frequency of mutated base in Chinese population from the 1000-genome project. 1000g2015aug_eas, allele frequency of mutated base in East Asian population from the 1000-genome project. 1000g2015aug_all, allele frequency of mutated base in all population from the 1000-genome project. esp6500siv2_all, allele frequency from NHLBI-ESP project in China. ExAC_ALL, allele frequency in all population from Exome Aggregation Consortium database. ExAC_EAS, allele frequency in East Asian population from Exome Aggregation Consortium database. SIFT, SIFT score showing the effect of variants on protein sequence. Polyphen2_HVAR, score assessing the effect of variants on protein sequence by HumanVar database by PolyPhen2. Polyphen2_HDIV, score assessing the effect of variants on protein sequence by HumanDiv database by PolyPhen2. dbNSFP version3.0, index assessing the effect of variants on protein sequence by dbNSFP version3.0. gerp++gt2, annotation of conservation of variants by ANNOVAR. CADD, deleterious score of SNVs and InDels. INFO, variants’ information detected from mutation software. FORMAT: GT, Genotype; PL, Standardized likelihood value of the genotype; DP, sequencing depth; DV, sequencing coverage; SP, likelihood value of chain preference p-value. Normal_samplename, FORMAT information of normal samples. Tumor_samplename, FORMAT information of tumor samples. Ori_REF, value of REF column from VCF file. Ori_ALT, value of ALT column from VCF file. shared_hom, number of samples with homozygous mutation. shared_het, number of samples with heterozygous mutation. OMIM, annotation from Mendelian database. GWAS_Pubmed_pValue, p-value of relativity between the variant and diseases from previous researches. HGMD_ID_Diseasename, ID and related diseases of the variant from HGMD database. GO_BP：annotation of biological progress from Gene Ontology. GO_CC, annotation of cell component from Gene Ontology. GO_MF, annotation of molecular function from Gene Ontology. KEGG_PATHWAY, annotation of signal pathways from KEGG. PID_PATHWAY, annotation of signal pathways from PID. BIOCARTA_PATHWAY, annotation of signal pathways from BIOCARTA. REACTOME_PATHWAY, annotation of signal pathways from REACTOME.

Supplementary Table 6

Somatic mutations detected in each case by whole exome sequencing.

CDS, coding sequence. UTR, untranslated region. SNV, single nucleotide variant. ncRNA, noncoding RNA. INDEL, insertion and deletion.

Supplementary Table 7

Detail of somatic SNV detected in each case by whole exome sequencing.

SNV, single nucleotide variant. CHROM, chromosome. POS, position. REF, reference base. ALT, alternative base. Func, function. AAChange, amino acid change. QUAL: Quality value under the Phred standard. wgRna, the annotation of microRNAs and snoRNAs related to variation point. Cytoband, chromosomal region of variants. targetScanS, annotation from targetScanS database. tfbsConsSites, the score to assess the conservation of variants based on transfac database. genomicSuperDups, screening for segmental duplication. avsnp150, ID of variants from dbSNP150. cosmic82, annotation from cosmic database. clinvar_20170905, annotation of variants on relationship with human health by NCBI format. gwasCatalognew, annotation from Genome-wide association studies. 1000g2015aug_Chinese, allele frequency of mutated base in Chinese population from the 1000-genome project. 1000g2015aug_eas, allele frequency of mutated base in East Asian population from the 1000-genome project. 1000g2015aug_all, allele frequency of mutated base in all population from the 1000-genome project. esp6500siv2_all, allele frequency from NHLBI-ESP project in China. ExAC_ALL, allele frequency in all population from Exome Aggregation Consortium database. ExAC_EAS, allele frequency in East Asian population from Exome Aggregation Consortium database. SIFT, SIFT score showing the effect of variants on protein sequence. Polyphen2_HVAR, score assessing the effect of variants on protein sequence by HumanVar database by PolyPhen2. Polyphen2_HDIV, score assessing the effect of variants on protein sequence by HumanDiv database by PolyPhen2. dbNSFP version3.0, index assessing the effect of variants on protein sequence by dbNSFP version3.0. gerp++gt2, annotation of conservation of variants by ANNOVAR. CADD, deleterious score of SNVs and InDels. INFO, variants’ information detected from mutation software. FORMAT: GT, Genotype; PL, Standardized likelihood value of the genotype; DP, sequencing depth; DV, sequencing coverage; SP, likelihood value of chain preference p-value. Normal_samplename, FORMAT information of normal samples. Tumor_samplename, FORMAT information of tumor samples. Ori_REF, value of REF column from VCF file. Ori_ALT, value of ALT column from VCF file. shared_hom, number of samples with homozygous mutation. shared_het, number of samples with heterozygous mutation. OMIM, annotation from Mendelian database. GWAS_Pubmed_pValue, p-value of relativity between the variant and diseases from previous researches. HGMD_ID_Diseasename, ID and related diseases of the variant from HGMD database. GO_BP：annotation of biological progress from Gene Ontology. GO_CC, annotation of cell component from Gene Ontology. GO_MF, annotation of molecular function from Gene Ontology. KEGG_PATHWAY, annotation of signal pathways from KEGG. PID_PATHWAY, annotation of signal pathways from PID. BIOCARTA_PATHWAY, annotation of signal pathways from BIOCARTA. REACTOME_PATHWAY, annotation of signal pathways from REACTOME.

Supplementary Table 8

Detail of somatic INDELs detected in each case by whole exome sequencing.

INDEL, insertion and deletion. CHROM, chromosome. POS, position. REF, reference base. ALT, alternative base. Func, function. AAChange, amino acid change. QUAL: Quality value under the Phred standard. wgRna, the annotation of microRNAs and snoRNAs related to variation point. Cytoband, chromosomal region of variants. targetScanS, annotation from targetScanS database. tfbsConsSites, the score to assess the conservation of variants based on transfac database. genomicSuperDups, screening for segmental duplication. avsnp150, ID of variants from dbSNP150. cosmic82, annotation from cosmic database. clinvar_20170905, annotation of variants on relationship with human health by NCBI format. gwasCatalognew, annotation from Genome-wide association studies. 1000g2015aug_Chinese, allele frequency of mutated base in Chinese population from the 1000-genome project. 1000g2015aug_eas, allele frequency of mutated base in East Asian population from the 1000-genome project. 1000g2015aug_all, allele frequency of mutated base in all population from the 1000-genome project. esp6500siv2_all, allele frequency from NHLBI-ESP project in China. ExAC_ALL, allele frequency in all population from Exome Aggregation Consortium database. ExAC_EAS, allele frequency in East Asian population from Exome Aggregation Consortium database. SIFT, SIFT score showing the effect of variants on protein sequence. Polyphen2_HVAR, score assessing the effect of variants on protein sequence by HumanVar database by PolyPhen2. Polyphen2_HDIV, score assessing the effect of variants on protein sequence by HumanDiv database by PolyPhen2. dbNSFP version3.0, index assessing the effect of variants on protein sequence by dbNSFP version3.0. gerp++gt2, annotation of conservation of variants by ANNOVAR. CADD, deleterious score of SNVs and InDels. INFO, variants’ information detected from mutation software. FORMAT: GT, Genotype; PL, Standardized likelihood value of the genotype; DP, sequencing depth; DV, sequencing coverage; SP, likelihood value of chain preference p-value. Normal_samplename, FORMAT information of normal samples. Tumor_samplename, FORMAT information of tumor samples. Ori_REF, value of REF column from VCF file. Ori_ALT, value of ALT column from VCF file. shared_hom, number of samples with homozygous mutation. shared_het, number of samples with heterozygous mutation. OMIM, annotation from Mendelian database. GWAS_Pubmed_pValue, p-value of relativity between the variant and diseases from previous researches. HGMD_ID_Diseasename, ID and related diseases of the variant from HGMD database. GO_BP：annotation of biological progress from Gene Ontology. GO_CC, annotation of cell component from Gene Ontology. GO_MF, annotation of molecular function from Gene Ontology. KEGG_PATHWAY, annotation of signal pathways from KEGG. PID_PATHWAY, annotation of signal pathways from PID. BIOCARTA_PATHWAY, annotation of signal pathways from BIOCARTA. REACTOME_PATHWAY, annotation of signal pathways from REACTOME.

Supplementary Table 9

CNVs identified in 20 cases of cervical adenocarcinoma.

CNV, Copy number variation.

Supplementary Table 10

Detail of CNVs detected in each case by whole genome sequencing.

CNV, Copy number variation. Chrom, chromosome. Start, start site of CNV. End, end site of CNV. Genename, genes related to CNV. Ref, reference genotype. ALT, alternative genotype. Func, function. Gene, CNV related transcript. AAChange, amino acid change. Gencode, transcript ID from Ensembl database. cpgIslandExt, predicted CpG island. Cytoband, chromosomal region of CNV. genomicSuperDups, screening for segmental duplication. dgvMerged, published CNV from the Database of Genomic Variants. GTConfidence, degree of uncertainty about the predicted genotype.

Supplementary Table 11

Significantly mutated genes identified by whole exome sequencing.

SNV, single nucleotide variants.

Supplementary Table 12

Significantly mutated genes identified in the HPV+ group and the HPV- group.

HPV, human papillomavirus.

**Legends of Supplementary Figures**

Supplementary Figure 1

All 30 patterns of mutational signatures validated from COSMIC database.

Supplementary Figure 2

Significantly mutated genes identified in the TCGA database including *NDN*, *GOLGA6L4*, and *BAIAP3*.
