# Supplementary material for: Genomic alterations caused by HPV integration in a cohort of Chinese endocervical adenocarcinomas
Source: Cancer Gene Ther. 2021 Jan 4;28(12):1353–64. doi: 10.1038/s41417-020-00283-4 (PMC8636260; doi:10.1038/s41417-020-00283-4)
Supplement: Supplementary file 2 — Supplementary Table 1 [file 41417_2020_283_MOESM2_ESM.docx]

Supplementary Table 1

Clinical characteristics of patients enrolled (n=20). HPV, human papillomavirus. LN, lymph nodes. LVSI, lymph-vascular space invasion. NA, not available. OS, overall survival.

| **No.** | **Stage** | **Differentiation** | **Stromal invasion** | **LVSI** | **LN involvement** | **Parametrium** | **Vaginal margin** | **Recurrence or death** | **OS (months)** | **HPV infection** | **HPV integration** |
| --- | --- | --- | --- | --- | --- | --- | --- | --- | --- | --- | --- |
| ACaP01 | IB1 | High | ＜1/2 | No | No | Negative | Negative | No | 14 | hpv16, 40 | - |
| ACaP02 | IB1 | NA | ＜1/2 | No | No | Negative | Negative | No | 13 | hpv16, 59 | hpv16 |
| ACaP03 | IB1 | High | ＜1/2 | No | No | Negative | Negative | No | 12 | hpv18, 62, 127 | - |
| ACaP04 | IB1 | High | ＜1/2 | No | No | Negative | Negative | No | 12 | - | - |
| ACaP05 | IIA2 | Low | ＜1/2 | No | Yes | Negative | Negative | No | 8 | hpv18, 21, 62, 81, etc | - |
| ACaP06 | IB1 | High-middle | ＜1/2 | No | No | Negative | Negative | No | 8 | hpv18, 27, 61, 123 | hpv18 |
| ACaP07 | IB1 | High | ＜1/2 | No | No | Negative | Negative | No | 8 | hpv16 | - |
| ACaP08 | IB1 | High | ＜1/2 | No | No | Negative | Negative | No | 7 | hpv16, 60, 106, 127 | - |
| ACaP09 | IB1 | High | ＜1/2 | No | No | Negative | Negative | No | 7 | hpv18, 51, 52, 73, etc | - |
| ACaP10 | IB1 | High-middle | ＜1/2 | No | No | Negative | Negative | No | 6 | hpv18, 56, 139, 141, etc | - |
| ACaP11 | IB1 | Low | ＞1/2 | Yes | No | Negative | Negative | No | 11 | hpv16, 41, 94, 123, etc | - |
| ACaP12 | IIA1 | High-middle | ＞1/2 | Yes | Yes | Positive | Negative | Yes | 3 | hpv18, 52, 72, 200, etc | - |
| ACaP13 | IB1 | NA | ＜1/2 | No | No | Negative | Negative | No | 8 | hpv16, 21, 81, 123, etc | - |
| ACaP14 | IB1 | High-middle | ＜1/2 | No | No | Negative | Negative | No | 7 | hpv16, 32, 38, 42 | hpv16 |
| ACa01 | IB1 | High | ＜1/2 | Yes | No | Negative | Negative | No | 5 | hpv18 | hpv18 |
| ACa02 | IB1 | High | ＞1/2 | No | No | Negative | Negative | No | 14 | hpv16 | - |
| ACa03 | IB1 | Low | ＜1/2 | No | No | Negative | Negative | No | 11 | hpv16, 56 | - |
| ACa04 | IB2 | High-middle | ＜1/2 | No | No | Negative | Negative | No | 6 | hpv18 | hpv18 |
| ACa06 | IIA1 | Middle | ＞1/2 | Yes | Yes | Positive | Negative | No | 6 | hpv57 | viral_hmm* |
| ACa07 | IIA1 | NA | ＞1/2 | No | No | Negative | Positive | No | 6 | hpv13, 162, 180 | viral_hmm |

* Viral_hmm denotes mutated type of HPV
