# Supplementary material for: Genomic alterations caused by HPV integration in a cohort of Chinese endocervical adenocarcinomas
Source: Cancer Gene Ther. 2021 Jan 4;28(12):1353–64. doi: 10.1038/s41417-020-00283-4 (PMC8636260; doi:10.1038/s41417-020-00283-4)
Supplement: Supplementary file 3 — Supplementary Table 2 [file 41417_2020_283_MOESM3_ESM.docx]

Supplementary Table 2

A concatenated quality report of the WGS and WES data.

| Sample name | % GC | % Dups | % Aligned | Average sequencing depth on target | % Coverage of target region | % Coverage of target region was at least 100x | Sequencing strategy |
| --- | --- | --- | --- | --- | --- | --- | --- |
| ACaP10N | 53.53% | 23.26% | 99.86% | 119 | 99.62% | 40.34% | WES |
| ACaP10T | 52.17% | 23.76% | 99.80% | 198 | 99.64% | 64.51% | WES |
| ACaP11N | 51.92% | 23.01% | 99.83% | 119 | 99.63% | 43.43% | WES |
| ACaP11T | 52.08% | 22.87% | 99.86% | 197 | 99.65% | 69.54% | WES |
| ACaP12N | 52.28% | 23.82% | 99.81% | 113 | 99.62% | 39.70% | WES |
| ACaP12T | 51.81% | 26.64% | 99.70% | 206 | 99.65% | 74.18% | WES |
| ACaP13N | 53.74% | 26.83% | 99.80% | 140 | 99.66% | 44.47% | WES |
| ACaP13T | 53.95% | 24.28% | 99.84% | 231 | 99.67% | 67.75% | WES |
| ACaP14N | 53.91% | 24.35% | 99.83% | 102 | 99.64% | 30.23% | WES |
| ACaP14T | 51.73% | 24.71% | 99.83% | 186 | 99.64% | 64.96% | WES |
| ACa01N | 51.57% | 19.56% | 99.91% | 109 | 99.64% | 42.53% | WES |
| ACa01T | 50.93% | 21.71% | 99.94% | 241 | 99.66% | 85.48% | WES |
| ACaP01N | 54.62% | 24.13% | 99.84% | 112 | 99.63% | 37.26% | WES |
| ACaP01T | 53.58% | 24.78% | 99.76% | 224 | 99.65% | 63.80% | WES |
| ACa02N | 51.65% | 19.27% | 99.92% | 103 | 99.64% | 38.53% | WES |
| ACa02T | 50.79% | 23.31% | 99.94% | 281 | 99.66% | 90.33% | WES |
| ACaP02N | 53.28% | 25.83% | 99.71% | 105 | 99.63% | 33.10% | WES |
| ACaP02T | 51.72% | 29.64% | 99.81% | 188 | 99.65% | 68.86% | WES |
| ACa03N | 51.66% | 20.22% | 99.94% | 117 | 99.64% | 46.33% | WES |
| ACa03T | 51.11% | 22.86% | 99.92% | 238 | 99.66% | 86.77% | WES |
| ACaP03N | 52.38% | 22.63% | 99.77% | 101 | 99.61% | 33.89% | WES |
| ACaP03T | 50.80% | 25.95% | 99.83% | 201 | 99.63% | 76.02% | WES |
| ACa04N | 52.21% | 18.30% | 99.93% | 99 | 99.62% | 36.05% | WES |
| ACa04T | 51.35% | 20.82% | 99.95% | 205 | 99.65% | 79.64% | WES |
| ACaP04N | 51.00% | 23.13% | 99.82% | 100 | 99.61% | 34.72% | WES |
| ACaP04T | 49.98% | 28.53% | 99.81% | 203 | 99.65% | 77.82% | WES |
| ACaP05N | 51.70% | 25.96% | 99.85% | 96 | 99.60% | 32.67% | WES |
| ACaP05T | 51.85% | 27.88% | 99.81% | 200 | 99.65% | 71.43% | WES |
| ACa06N | 50.97% | 22.24% | 99.88% | 97 | 99.63% | 34.97% | WES |
| ACa06T | 50.88% | 23.73% | 99.89% | 207 | 99.65% | 80.40% | WES |
| ACaP06N | 54.19% | 25.43% | 99.84% | 125 | 99.63% | 39.81% | WES |
| ACaP06T | 52.38% | 32.95% | 99.89% | 266 | 99.66% | 77.18% | WES |
| ACa07N | 51.14% | 23.85% | 99.90% | 107 | 99.62% | 41.44% | WES |
| ACa07T | 50.40% | 21.15% | 99.91% | 203 | 99.65% | 78.53% | WES |
| ACaP07N | 51.54% | 25.50% | 99.81% | 101 | 99.62% | 34.41% | WES |
| ACaP07T | 51.89% | 26.29% | 99.82% | 190 | 99.65% | 69.83% | WES |
| ACaP08N | 53.25% | 25.25% | 99.81% | 127 | 99.63% | 44.50% | WES |
| ACaP08T | 53.97% | 24.50% | 99.85% | 244 | 99.66% | 64.76% | WES |
| ACaP09N | 53.49% | 23.66% | 99.84% | 97 | 99.63% | 31.85% | WES |
| ACaP09T | 54.34% | 33.96% | 99.87% | 276 | 99.67% | 74.33% | WES |
| ACa01N | 40.52% | 17.35% | 99.84% | 34.47 | 99.16% | 95.81% | WGS |
| ACa01T | 40.52% | 17.17% | 99.85% | 30.37 | 99.15% | 88.21% | WGS |
| ACa02N | 40.54% | 17.42% | 99.83% | 33.54 | 99.16% | 95.09% | WGS |
| ACa02T | 40.49% | 18.70% | 99.85% | 38.73 | 99.16% | 95.23% | WGS |
| ACa03N | 40.68% | 17.06% | 99.85% | 36.51 | 99.17% | 97.01% | WGS |
| ACa03T | 40.72% | 16.63% | 99.86% | 31.21 | 99.16% | 92.54% | WGS |
| ACa04N | 40.72% | 17.26% | 99.80% | 29.56 | 99.15% | 89.69% | WGS |
| ACa04T | 40.46% | 16.70% | 99.81% | 37.56 | 99.16% | 96.59% | WGS |
| ACa06N | 41.19% | 7.67% | 99.84% | 35.42 | 99.16% | 97.33% | WGS |
| ACa06T | 40.94% | 12.35% | 99.83% | 30.05 | 99.15% | 92.82% | WGS |
| ACa07N | 41.12% | 8.28% | 99.84% | 31.21 | 99.17% | 94.74% | WGS |
| ACa07T | 41.16% | 8.22% | 99.87% | 31.74 | 99.17% | 95.24% | WGS |
| ACaP01N | 44.71% | 18.45% | 98.82% | 35.68 | 99.21% | 77.33% | WGS |
| ACaP01T | 44.34% | 13.94% | 99.66% | 35.87 | 99.20% | 78.49% | WGS |
| ACaP02N | 43.14% | 12.81% | 97.99% | 29.42 | 99.18% | 75.21% | WGS |
| ACaP02T | 41.53% | 15.79% | 99.39% | 24.81 | 99.21% | 63.70% | WGS |
| ACaP03N | 42.17% | 12.74% | 97.61% | 29.5 | 99.18% | 82.73% | WGS |
| ACaP03T | 41.98% | 13.02% | 99.70% | 30.32 | 99.16% | 82.46% | WGS |
| ACaP04N | 41.60% | 12.72% | 99.18% | 31.28 | 99.15% | 85.82% | WGS |
| ACaP04T | 40.39% | 16.18% | 98.65% | 27.6 | 99.09% | 74.58% | WGS |
| ACaP05N | 42.78% | 13.05% | 99.76% | 32.04 | 99.11% | 85.24% | WGS |
| ACaP05T | 41.77% | 12.56% | 99.62% | 28.26 | 99.15% | 78.64% | WGS |
| ACaP06N | 43.63% | 12.26% | 99.63% | 32.1 | 99.18% | 74.55% | WGS |
| ACaP06T | 43.42% | 11.84% | 99.88% | 30.09 | 99.18% | 71.71% | WGS |
| ACaP07N | 42.19% | 12.79% | 99.51% | 30.64 | 99.17% | 84.24% | WGS |
| ACaP07T | 42.41% | 12.65% | 99.72% | 27.73 | 99.18% | 77.88% | WGS |
| ACaP08N | 44.51% | 11.99% | 99.76% | 36.39 | 99.17% | 76.53% | WGS |
| ACaP08T | 44.63% | 12.74% | 99.66% | 36.59 | 99.18% | 78.97% | WGS |
| ACaP09N | 43.18% | 12.76% | 99.82% | 29.06 | 99.18% | 75.83% | WGS |
| ACaP09T | 42.90% | 11.91% | 99.76% | 27.86 | 99.16% | 73.78% | WGS |
| ACaP10N | 44.90% | 12.74% | 99.74% | 31.25 | 99.00% | 69.31% | WGS |
| ACaP10T | 44.18% | 15.86% | 99.81% | 30.89 | 99.01% | 68.25% | WGS |
| ACaP11N | 42.68% | 12.99% | 98.36% | 28.46 | 99.17% | 78.38% | WGS |
| ACaP11T | 43.63% | 11.88% | 99.84% | 28.34 | 99.03% | 73.45% | WGS |
| ACaP12N | 42.84% | 11.96% | 99.33% | 27.48 | 99.16% | 69.75% | WGS |
| ACaP12T | 40.93% | 12.77% | 99.69% | 32.03 | 99.16% | 83.28% | WGS |
| ACaP13N | 43.12% | 14.06% | 99.72% | 31.89 | 99.16% | 81.23% | WGS |
| ACaP13T | 42.53% | 15.52% | 99.53% | 26.82 | 99.11% | 65.97% | WGS |
| ACaP14N | 42.20% | 12.63% | 99.77% | 28.35 | 99.19% | 77.36% | WGS |
| ACaP14T | 43.02% | 13.09% | 99.85% | 27.66 | 99.11% | 71.39% | WGS |
