# Supplementary material for: Genomic alterations caused by HPV integration in a cohort of Chinese endocervical adenocarcinomas
Source: Cancer Gene Ther. 2021 Jan 4;28(12):1353–64. doi: 10.1038/s41417-020-00283-4 (PMC8636260; doi:10.1038/s41417-020-00283-4)
Supplement: Supplementary file 4 — Supplementary Table 3 [file 41417_2020_283_MOESM4_ESM.docx]

Supplementary Table 3

Somatic mutations detected in 20 cases by whole genome sequencing. CDS, coding sequence. UTR, untranslated region.

| **Sample** | **Total** | | **SNV** | | **INDEL** | |
| --- | --- | --- | --- | --- | --- | --- |
|  | Number | Ratio | Number | Ratio | Number | Ratio |
| CDS | 1,320 | 1.31% | 1,305 | 1.33% | 15 | 0.69% |
| Synonymous_SNP | 389 | 0.39% | 389 | 0.40% | - | - |
| Missense_SNP | 838 | 0.83% | 838 | 0.85% | - | - |
| Frameshift_deletion | 6 | 0.01% | - | - | 6 | 0.28% |
| Frameshift_insertion | 2 | 0.00% | - | - | 2 | 0.09% |
| Nonframeshift_deletion | 5 | 0.00% | - | - | 5 | 0.23% |
| Nonframeshift_insertion | 1 | 0.00% | - | - | 1 | 0.05% |
| Stopgain | 60 | 0.06% | 59 | 0.06% | 1 | 0.05% |
| Stoploss | 0 | 0.00% | 0 | 0.00% | 0 | 0.00% |
| Unknown | 19 | 0.02% | 19 | 0.02% | 0 | 0.00% |
| Intronic | 34,631 | 34.46% | 33,823 | 34.40% | 808 | 37.34% |
| UTR3 | 716 | 0.71% | 695 | 0.71% | 21 | 0.97% |
| UTR5 | 197 | 0.20% | 193 | 0.20% | 4 | 0.18% |
| Splicing | 39 | 0.04% | 38 | 0.04% | 1 | 0.05% |
| ncRNA_exonic | 292 | 0.29% | 285 | 0.29% | 7 | 0.32% |
| ncRNA_intronic | 4,192 | 4.17% | 4,093 | 4.16% | 99 | 4.57% |
| ncRNA_UTR3 | 0 | 0.00% | 0 | 0.00% | 0 | 0.00% |
| ncRNA_UTR5 | 0 | 0.00% | 0 | 0.00% | 0 | 0.00% |
| ncRNA_splicing | 3 | 0.00% | 3 | 0.00% | 0 | 0.00% |
| Upstream | 699 | 0.70% | 679 | 0.69% | 20 | 0.92% |
| Downstream | 583 | 0.58% | 570 | 0.58% | 13 | 0.60% |
| Intergenic | 57,792 | 57.51% | 56,619 | 57.58% | 1,173 | 54.21% |
| Others | 27 | 0.03% | 24 | 0.02% | 3 | 0.14% |
| Total | 100,491 | 100.00% | 98,327 | 100% | 2,164 | 100.00% |
