# Supplementary material for: Genomic alterations caused by HPV integration in a cohort of Chinese endocervical adenocarcinomas
Source: Cancer Gene Ther. 2021 Jan 4;28(12):1353–64. doi: 10.1038/s41417-020-00283-4 (PMC8636260; doi:10.1038/s41417-020-00283-4)
Supplement: Supplementary file 6 — Supplementary Table 5 [file 41417_2020_283_MOESM6_ESM.pdf]

Supplementary Table 5
Detail of somatic INDELs detected in each case by whole genome sequencing.

Patient	CHF	POS	ID	REF	ALT	QUAL	FILTER	GeneNam	Func
ACa01	2	1.22E+08	.	AC	A	.	PASS	TFCP2L1	intronic
ACa01	2	2.05E+08	.	G	GT	.	PASS	CTLA4	exonic
ACa01	6	58777054	rs6078884	TTGGAG	T	.	PASS	.	intergenic
ACa01	7	93623657	.	GTTT	G	.	PASS	BET1	exonic
ACa01	12	1.14E+08	rs3773783	T	TC	.	PASS	SLC8B1	intronic
ACa01	15	62173088	.	AT	A	.	PASS	VPS13C	exonic
ACa01	17	3030310	.	CAGA	C	.	PASS	OR1G1	exonic
ACa01	19	39760071	.	T	TC	.	PASS	IFNL2	intronic
ACa01	21	45668946	.	C	CAG	.	PASS	DNMT3L	exonic
ACa01	X	53461076	rs7821786	T	TAGAC	.	PASS	HSD17B1(	intronic
ACa02	5	10618545	.	ACACAGC	A	.	PASS	ANKRD33I	exonic
ACa02	13	28973403	rs9190283	G	GT	.	PASS	FLT1	intronic
ACa02	14	69445857	.	T	TTGGGC	.	PASS	ACTN1	UTR5
ACa02	19	17397481	rs7640343	GTGTGTG	G	.	PASS	ANKLE1	exonic
ACa02	22	30734626	rs5844903	TC	T	.	PASS	SF3A1	intronic
ACa03	3	50005225	.	GGGGAT1	G	.	PASS	RBM6	exonic
ACa04	2	33500956	.	CG	C	.	PASS	LTBP1	exonic
ACa04	3	1.96E+08	.	CGT	C	.	PASS	.	intergenic
ACa04	16	23224248	.	GC	G	.	PASS	SCNN1G	intronic
ACa06	16	90233007	.	CCTCTTT.	C	.	PASS	.	ncRNA_int
ACa06	17	6563152	.	C	CTCTCTC	.	PASS	ALOX15P1	ncRNA_int
ACa06	17	72337184	.	CTG	C	.	PASS	KIF19	intronic
ACa07	1	889573	rs7760984	AC	A	.	PASS	NOC2L	intronic
ACa07	1	1078309	rs9376078	TC	T	.	PASS	LINC0134	ncRNA_ex
ACa07	1	1256093	.	AC	A	.	PASS	CPSF3L	intronic
ACa07	1	1356387	rs7790390	T	TG	.	PASS	ANKRD65	splicing
ACa07	1	1391388	.	AC	A	.	PASS	ATAD3C	intronic
ACa07	1	12253249	.	CG	C	.	PASS	TNFRSF1I	intronic
ACa07	1	19467922	.	TG	T	.	PASS	UBR4	exonic
ACa07	1	27106800	.	CA	C	.	PASS	ARID1A	exonic
ACa07	1	39734616	.	A	AC	.	PASS	MACF1	intronic
ACa07	1	39919366	.	C	CT	.	PASS	MACF1	intronic
ACa07	1	41990610	rs7712392	GA	G	.	PASS	HIVEP3	intronic
ACa07	1	62190602	rs7686883	T	TG	.	PASS	TM2D1	intronic
ACa07	1	86361739	.	CT	C	.	PASS	COL24A1	exonic
ACa07	1	1.1E+08	rs7700977	GT	G	.	PASS	SARS	intronic
ACa07	1	1.13E+08	.	AG	A	.	PASS	RHOC	intronic
ACa07	1	1.5E+08	.	TG	T	.	PASS	APH1A	intronic
ACa07	1	1.55E+08	.	GT	G	.	PASS	UBE2Q1	exonic
ACa07	1	1.55E+08	.	TA	T	.	PASS	CLK2	exonic
ACa07	1	1.56E+08	.	GA	G	.	PASS	GON4L	intronic
ACa07	1	1.57E+08	.	CAG	C	.	PASS	BCAN	intronic
ACa07	1	1.77E+08	.	TC	T	.	PASS	PAPPA2	intronic
ACa07	1	1.79E+08	rs7584166	G	GA	.	PASS	AXDND1	exonic
ACa07	1	1.83E+08	rs7537986	GA	G	.	PASS	DHX9	UTR3
ACa07	1	1.99E+08	rs5395449	AT	A	.	PASS	PTPRC	intronic
ACa07	1	2.04E+08	.	AC	A	.	PASS	PLEKHA6	exonic
ACa07	1	2.26E+08	rs7594247	TA	T	.	PASS	LIN9	intronic
ACa07	1	2.27E+08	rs7703770	TA	T	.	PASS	CDC42BP	intronic
ACa07	1	2.32E+08	.	CG	C	.	PASS	TSNAX-DI	ncRNA_ex
ACa07	2	7421609	rs7968975	CTG	C	.	PASS	.	intergenic

ACa07	2	11389836	.	C	CA	.	PASS	ROCK2	exonic
ACa07	2	24406265	rs7808912	AT	A	.	PASS	FAM228A	intronic
ACa07	2	28464145	.	TA	T	.	PASS	BRE	intronic
ACa07	2	41384161	rs1997131	CA	C	.	PASS	.	intergenic
ACa07	2	46841951	.	GA	G	.	PASS	PIGF	intronic
ACa07	2	55464460	rs7703599	A	AT	.	PASS	MTIF2	exonic
ACa07	2	74763923	rs7789371	G	GC	.	PASS	LOXL3	exonic
ACa07	2	86433450	.	G	GT	.	PASS	MRPL35	intronic
ACa07	2	1E+08	.	TA	T	.	PASS	AFF3	intronic
ACa07	2	1.22E+08	.	CG	C	.	PASS	TFCP2L1	intronic
ACa07	2	1.49E+08	rs7647197	TA	T	.	PASS	ACVR2A	exonic
ACa07	2	1.67E+08	.	AG	A	.	PASS	CSRNP3	exonic
ACa07	2	1.67E+08	.	A	AT	.	PASS	LOC10272	ncRNA_int
ACa07	2	1.68E+08	rs7456483	T	TG	.	PASS	XIRP2	exonic
ACa07	2	1.76E+08	.	CT	C	.	PASS	ATF2	exonic
ACa07	2	1.79E+08	.	AT	A	.	PASS	PDE11A	intronic
ACa07	2	1.79E+08	rs7470964	CT	C	.	PASS	MIR548N	ncRNA_int
ACa07	2	1.79E+08	.	AT	A	.	PASS	TTN	exonic
ACa07	2	1.92E+08	.	A	AT	.	PASS	STAT1	exonic
ACa07	2	1.99E+08	.	CA	C	.	PASS	BOLL	intronic
ACa07	2	2.03E+08	.	T	TA	.	PASS	CDK15	intronic
ACa07	2	2.07E+08	.	AC	A	.	PASS	ADAM23	exonic
ACa07	2	2.08E+08	.	AG	A	.	PASS	FASTKD2	exonic
ACa07	2	2.08E+08	.	GA	G	.	PASS	METTL21A	intronic
ACa07	2	2.2E+08	.	TA	T	.	PASS	RESP18	splicing
ACa07	2	2.29E+08	.	TG	T	.	PASS	SPHKAP	exonic
ACa07	2	2.35E+08	rs5440185	A	AC	.	PASS	DNAJB3	exonic
ACa07	2	2.42E+08	.	CT	C	.	PASS	FARP2	intronic
ACa07	3	1363509	.	AT	A	.	PASS	CNTN6	exonic
ACa07	3	14974794	rs9042896	C	CT	.	PASS	FGD5	UTR3
ACa07	3	25820209	rs7608755	TA	T	.	PASS	NGLY1	intronic
ACa07	3	45765061	.	GAA	G	.	PASS	SACM1L	intronic
ACa07	3	47449380	.	TGAG	T	.	PASS	PTPN23	exonic
ACa07	3	50005144	rs7647092	A	AG	.	PASS	RBM6	exonic
ACa07	3	51909483	.	CA	C	.	PASS	IQCF5-AS1	ncRNA_ex
ACa07	3	53220652	.	CG	C	.	PASS	PRKCD	exonic
ACa07	3	53810811	.	CT	C	.	PASS	CACNA1D	intronic
ACa07	3	1.13E+08	.	AAG	A	.	PASS	C3orf17	intronic
ACa07	3	1.29E+08	.	T	TG	.	PASS	ACAD9	intronic
ACa07	3	1.38E+08	rs7755820	GA	G	.	PASS	FAIM	exonic
ACa07	3	1.49E+08	.	T	TC	.	PASS	ANKUB1	intronic
ACa07	3	1.52E+08	.	CTTCT	C	.	PASS	MBNL1	intronic
ACa07	3	1.74E+08	.	A	AT	.	PASS	NLGN1	intronic
ACa07	3	1.84E+08	.	TG	T	.	PASS	CHRD	intronic
ACa07	4	1194388	.	G	GC	.	PASS	LOC10013	ncRNA_int
ACa07	4	1379730	rs7704742	TTAAC	T	.	PASS	UVSSA	exonic
ACa07	4	8358662	rs7480610	GAGA	G	.	PASS	.	intergenic
ACa07	4	37863107	rs7646973	AT	A	.	PASS	PGM2	intronic
ACa07	4	41672908	rs9281864	GA	G	.	PASS	LIMCH1	intronic
ACa07	4	54231548	.	AAGG	A	.	PASS	SCFD2	exonic
ACa07	4	56448411	rs7525252	AAAAAG	A	.	PASS	PDCL2	intronic
ACa07	4	81123328	.	TC	T	.	PASS	PRDM8	exonic
ACa07	4	95192048	.	C	CT	.	PASS	SMARCAD	intronic
ACa07	4	1.11E+08	.	CG	C	.	PASS	EGF	intronic

ACa07	4	1.14E+08	.	C	CA	.	PASS	CAMK2D	intronic
ACa07	4	1.15E+08	rs7641185	C	CT	.	PASS	ARSJ	exonic
ACa07	4	1.26E+08	rs7639462	TCTC	T	.	PASS	FAT4	exonic
ACa07	4	1.78E+08	.	A	AT	.	PASS	NEIL3	exonic
ACa07	4	1.91E+08	.	C	CA	.	PASS	FRG2	intronic
ACa07	5	1576160	.	AG	A	.	PASS	SDHAP3	ncRNA_int
ACa07	5	14487780	rs7466549	AC	A	.	PASS	TRIO	exonic
ACa07	5	14711208	rs7717298	C	CA	.	PASS	ANKH	UTR3
ACa07	5	33683110	.	GA	G	.	PASS	ADAMTS1	intronic
ACa07	5	37239071	.	GA	G	.	PASS	C5orf42	intronic
ACa07	5	38903970	rs7625018	CT	C	.	PASS	OSMR	intronic
ACa07	5	74054640	rs7658478	T	TG	.	PASS	GFM2	intronic
ACa07	5	77425138	.	G	GA	.	PASS	AP3B1	intronic
ACa07	5	1.4E+08	.	ACT	A	.	PASS	SRA1	intronic
ACa07	5	1.41E+08	rs7778584	GT	G	.	PASS	PCDHGA3	exonic
ACa07	5	1.54E+08	rs7693384	G	GC	.	PASS	LARP1	exonic
ACa07	5	1.68E+08	.	AG	A	.	PASS	SLIT3	exonic
ACa07	5	1.68E+08	rs7768326	CG	C	.	PASS	SLIT3	intronic
ACa07	6	28494004	rs3743843	CT	C	.	PASS	GPX5	intronic
ACa07	6	31473742	.	TG	T	.	PASS	MICB	intronic
ACa07	6	31931561	rs7666007	CT	C	.	PASS	SKIV2L	intronic
ACa07	6	32149321	rs7805014	TC	T	.	PASS	AGER	exonic
ACa07	6	32156482	rs7489955	CG	C	.	PASS	PBX2	intronic
ACa07	6	32905244	rs7612855	G	GA	.	PASS	HLA-DMB	intronic
ACa07	6	36867370	rs7600208	AC	A	.	PASS	C6orf89	exonic
ACa07	6	41555084	.	GC	G	.	PASS	FOXP4	exonic
ACa07	6	76028720	.	GA	G	.	PASS	FILIP1	intronic
ACa07	6	99848190	.	GA	G	.	PASS	PNISR	UTR3
ACa07	6	1.01E+08	rs7792033	C	CA	.	PASS	SIM1	intronic
ACa07	6	1.03E+08	rs7668254	CA	C	.	PASS	GRIK2	exonic
ACa07	6	1.07E+08	.	TC	T	.	PASS	BEND3	exonic
ACa07	6	1.11E+08	.	A	AG	.	PASS	SLC16A10	intronic
ACa07	6	1.12E+08	.	G	GT	.	PASS	TRAF3IP2	exonic
ACa07	6	1.12E+08	rs1490463	ATG	A	.	PASS	LAMA4	intronic
ACa07	6	1.22E+08	.	AT	A	.	PASS	TBC1D32	exonic
ACa07	6	1.35E+08	rs7570813	CT	C	.	PASS	HBS1L	exonic
ACa07	6	1.44E+08	rs7468588	C	CA	.	PASS	PHACTR2	exonic
ACa07	6	1.6E+08	.	TAG	T	.	PASS	PNLDC1	intronic
ACa07	6	1.6E+08	.	A	AT	.	PASS	IGF2R	intronic
ACa07	7	35473	.	T	TGCC	.	PASS	.	intergenic
ACa07	7	1703082	rs1443745	T	TG	.	PASS	.	intergenic
ACa07	7	4874484	.	TC	T	.	PASS	RADIL	exonic
ACa07	7	5401258	rs7542333	C	CG	.	PASS	TNRC18	exonic
ACa07	7	15666546	rs3701666	GA	G	.	PASS	MEOX2	splicing
ACa07	7	27498345	.	A	AG	.	PASS	.	intergenic
ACa07	7	30472801	rs7470634	GA	G	.	PASS	NOD1	intronic
ACa07	7	30491509	.	AC	A	.	PASS	NOD1	exonic
ACa07	7	55433871	.	T	TC	.	PASS	LANCL2	exonic
ACa07	7	57528541	rs7819467	TA	T	.	PASS	ZNF716	exonic
ACa07	7	72952223	.	C	CA	.	PASS	BCL7B	intronic
ACa07	7	73480937	.	TG	T	.	PASS	ELN	intronic
ACa07	7	99672965	.	GA	G	.	PASS	ZNF3	intronic
ACa07	7	1E+08	.	TG	T	.	PASS	NYAP1	exonic
ACa07	7	1E+08	rs5592025	CG	C	.	PASS	EPHB4	intronic

ACa07	7	1.02E+08	.	GCCAGC(	G	.		PASS	CUX1	exonic
ACa07	7	1.23E+08	rs7594121	A	AGAG	.		PASS	LMOD2	exonic
ACa07	7	1.34E+08	rs7708715	TA	T	.		PASS	AKR1B10	intronic
ACa07	7	1.51E+08	.	G	GC	.		PASS	ATG9B	exonic
ACa07	7	1.55E+08	rs7618495	CT	C	.		PASS	DPP6	intronic
ACa07	8	15977886	.	A	AAAC	.		PASS	MSR1	intronic
ACa07	8	18656880	rs7790763	GA	G	.		PASS	PSD3	intronic
ACa07	8	22017278	.	G	GC	.		PASS	.	intergenic
ACa07	8	23340737	rs1126292	CG	C	.		PASS	.	intergenic
ACa07	8	32607049	rs3832550	C	CT	.		PASS	NRG1	intronic
ACa07	8	38458195	.	CA	C	.		PASS	RNF5P1	ncRNA_ex
ACa07	8	56866371	.	AT	A	.		PASS	LYN	intronic
ACa07	8	80678509	rs7782900	CT	C	.		PASS	HEY1	UTR5
ACa07	8	95686610	rs7759500	TA	T	.		PASS	ESRP1	exonic
ACa07	8	1.05E+08	.	CT	C	.		PASS	RIMS2	intronic
ACa07	8	1.33E+08	rs1462533	TAC	T	.		PASS	KCNQ3	intronic
ACa07	8	1.44E+08	.	T	TC	.		PASS	BAI1	intronic
ACa07	8	1.45E+08	rs7562540	A	AC	.		PASS	CYC1	exonic
ACa07	9	27359243	rs7648900	GA	G	.		PASS	MOB3B	intronic
ACa07	9	34997368	rs7766165	CA	C	.		PASS	DNAJB5	UTR3
ACa07	9	35707140	.	CG	C	.		PASS	TLN1	exonic
ACa07	9	44078574	rs5303481	AT	A	.		PASS	.	intergenic
ACa07	9	96438893	.	G	GA	.		PASS	PHF2	exonic
ACa07	9	98209616	.	TG	T	.		PASS	PTCH1	exonic
ACa07	9	98278828	.	CT	C	.		PASS	PTCH1	UTR5
ACa07	9	1.14E+08	rs7606317	C	CA	.		PASS	KIAA0368	intronic
ACa07	9	1.25E+08	rs7792498	AT	A	.		PASS	OR1L3	exonic
ACa07	9	1.26E+08	rs1028985	CCTT	C	.		PASS	STRBP	exonic
ACa07	9	1.28E+08	rs7703285	CT	C	.		PASS	RABEPK	intronic
ACa07	9	1.32E+08	rs3675892	AC	A	.		PASS	C9orf50,N	intronic
ACa07	9	1.34E+08	.	TG	T	.		PASS	PPAPDC3	intronic
ACa07	9	1.39E+08	.	TG	T	.		PASS	PMPCA	exonic
ACa07	9	1.4E+08	.	G	GC	.		PASS	LCN8	intronic
ACa07	9	1.4E+08	rs7517698	A	AC	.		PASS	CCDC183	intronic
ACa07	9	1.4E+08	.	AC	A	.		PASS	C9orf142	exonic
ACa07	10	7269835	.	A	AG	.		PASS	SFMBT2	exonic
ACa07	10	26534837	rs7625775	AT	A	.		PASS	GAD2	intronic
ACa07	10	29169287	.	CA	C	.		PASS	C10orf126	exonic
ACa07	10	47381004	.	TA	T	.		PASS	FAM35DP	ncRNA_ex
ACa07	10	64977094	rs7582351	T	TA	.		PASS	JMJD1C	splicing
ACa07	10	70731843	.	G	GT	.		PASS	DDX21	intronic
ACa07	10	72520489	rs7542104	C	CG	.		PASS	ADAMTS1	exonic
ACa07	10	75560463	rs7696631	T	TC	.		PASS	ZSWIM8	exonic
ACa07	10	81072274	.	CT	C	.		PASS	ZMIZ1	intronic
ACa07	10	81927077	rs7488695	GACAA	G	.		PASS	ANXA11	intronic
ACa07	10	88768816	rs7664186	GGAA	G	.		PASS	AGAP11	exonic
ACa07	10	95400667	rs7585686	AT	A	.		PASS	PDE6C	intronic
ACa07	10	1.05E+08	.	CA	C	.		PASS	INA	intronic
ACa07	10	1.24E+08	rs5534658	G	GC	.		PASS	TACC2	intronic
ACa07	10	1.34E+08	.	GT	G	.		PASS	BNIP3	intronic
ACa07	11	320191	.	T	TG	.		PASS	IFITM3	intronic
ACa07	11	375699	.	TG	T	.		PASS	B4GALNT	exonic
ACa07	11	1478186	rs7518721	AC	A	.		PASS	BRSK2	exonic
ACa07	11	4712970	rs9766384	CA	C	.		PASS	OR51E2	intronic

ACa07	11	7110948	.	GC	G	.	PASS	RBMXL2	exonic
ACa07	11	8716961	.	CCCTT	C	.	PASS	ST5	intronic
ACa07	11	20409548	rs7632273	T	TTG	.	PASS	PRMT3	intronic
ACa07	11	26584821	rs7778008	C	CA	.	PASS	MUC15,AN	intronic
ACa07	11	43740330	rs1049395	CA	C	.	PASS	HSD17B12	intronic
ACa07	11	45907401	.	AG	A	.	PASS	MAPK8IP1	exonic
ACa07	11	62378941	.	AG	A	.	PASS	EML3	exonic
ACa07	11	66433146	.	GA	G	.	PASS	RBM4B,RE	intronic
ACa07	11	67049843	rs7588677	GC	G	.	PASS	ADRBK1	intronic
ACa07	11	71238177	rs8987047	GA	G	.	PASS	.	upstream
ACa07	11	77920855	rs7647351	AC	A	.	PASS	USP35	exonic
ACa07	11	83344240	.	AC	A	.	PASS	DLG2	intronic
ACa07	11	92531965	.	AT	A	.	PASS	FAT3	exonic
ACa07	11	93439895	rs5739889	AT	A	.	PASS	CEP295	intronic
ACa07	11	1.34E+08	rs7766671	AAAGT	A	.	PASS	JAM3	exonic
ACa07	12	416952	rs7749641	CT	C	.	PASS	KDM5A	exonic
ACa07	12	4877574	rs7666743	C	CT	.	PASS	GALNT8	intronic
ACa07	12	6765122	.	CT	C	.	PASS	ING4	intronic
ACa07	12	7064305	.	TC	T	.	PASS	PTPN6	intronic
ACa07	12	7967145	.	GA	G	.	PASS	SLC2A14	intronic
ACa07	12	9885640	.	A	AAG	.	PASS	CLECL1	exonic
ACa07	12	12510357	rs7566997	GTTC	G	.	PASS	LOH12CR1	UTR5
ACa07	12	27786283	rs7649880	AT	A	.	PASS	PPFIBP1	intronic
ACa07	12	27803090	.	TCTC	T	.	PASS	PPFIBP1	intronic
ACa07	12	40878124	.	TG	T	.	PASS	MUC19	exonic
ACa07	12	46385784	.	T	TC	.	PASS	.	intergenic
ACa07	12	49433406	rs7636694	CA	C	.	PASS	KMT2D	intronic
ACa07	12	50475320	.	TC	T	.	PASS	ASIC1	intronic
ACa07	12	51402108	.	GTA	G	.	PASS	SLC11A2	intronic
ACa07	12	52200420	.	GC	G	.	PASS	SCN8A	exonic
ACa07	12	53400448	.	TG	T	.	PASS	EIF4B	intronic
ACa07	12	54426885	rs5532896	AT	A	.	PASS	HOXC5	UTR5
ACa07	12	56827355	.	CA	C	.	PASS	TIMELESS	exonic
ACa07	12	57422572	rs7706124	CT	C	.	PASS	MYO1A	exonic
ACa07	12	57522679	rs5359317	T	TG	.	PASS	LRP1	UTR5
ACa07	12	57921731	rs7763171	TG	T	.	PASS	MBD6	exonic
ACa07	12	1.04E+08	rs1999629	GT	G	.	PASS	HCFC2	intronic
ACa07	12	1.05E+08	.	TAAAG	T	.	PASS	NFYB	intronic
ACa07	12	1.24E+08	.	C	CT	.	PASS	DNAH10	exonic
ACa07	13	27255386	rs7643750	GC	G	.	PASS	WASF3	exonic
ACa07	13	36855327	.	A	AG	.	PASS	CCDC169,	intronic
ACa07	13	1.11E+08	rs7166969	TG	T	.	PASS	CARS2	intronic
ACa07	14	55236839	.	G	GA	.	PASS	SAMD4A	exonic
ACa07	14	58934608	.	GA	G	.	PASS	KIAA0586	exonic
ACa07	14	59781977	rs1134746	CT	C	.	PASS	DAAM1	intronic
ACa07	14	69354434	rs5526989	TA	T	.	PASS	ACTN1	intronic
ACa07	14	94120292	.	CT	C	.	PASS	UNC79	exonic
ACa07	14	96798757	.	AT	A	.	PASS	ATG2B	intronic
ACa07	14	1.06E+08	.	AC	A	.	PASS	CRIP1	intronic
ACa07	15	23021238	rs1451472	CT	C	.	PASS	NIPA2	exonic
ACa07	15	42930851	.	CT	C	.	PASS	STARD9	intronic
ACa07	15	43705010	rs7643803	CA	C	.	PASS	RNU6-28P	ncRNA_int
ACa07	15	43748660	.	CT	C	.	PASS	TP53BP1	exonic
ACa07	15	49574201	.	CTG	C	.	PASS	GALK2	exonic

ACa07	15	54003202	rs5635626	GT	G	.	PASS	WDR72	intronic
ACa07	15	72072854	rs7515595	T	TA	.	PASS	THSD4	UTR3
ACa07	15	73558645	rs5561116	C	CT	.	PASS	NEO1	intronic
ACa07	15	73995992	.	GC	G	.	PASS	CD276	intronic
ACa07	15	85405928	.	TG	T	.	PASS	ALPK3	exonic
ACa07	15	86807692	rs7643461	CA	C	.	PASS	AGBL1	exonic
ACa07	15	88472356	.	GA	G	.	PASS	NTRK3	intronic
ACa07	15	90190139	.	AC	A	.	PASS	KIF7	exonic
ACa07	15	91434151	.	GC	G	.	PASS	FES	intronic
ACa07	16	3599228	rs7781350	G	GA	.	PASS	NLRC3	splicing
ACa07	16	4387550	rs7809656	GC	G	.	PASS	GLIS2	UTR3
ACa07	16	4862228	rs7491504	G	GC	.	PASS	GLYR1	exonic
ACa07	16	12536764	.	CT	C	.	PASS	SNX29	intronic
ACa07	16	15122677	.	G	GT	.	PASS	PDXDC1	intronic
ACa07	16	30392609	.	AC	A	.	PASS	SEPT1,ZN	intronic
ACa07	16	30983080	rs7570296	T	TGA	.	PASS	SETD1A	intronic
ACa07	16	50340876	.	GT	G	.	PASS	ADCY7	intronic
ACa07	16	66436882	rs7817135	G	GC	.	PASS	CDH5	exonic
ACa07	16	67325657	rs7679025	CG	C	.	PASS	KCTD19	exonic
ACa07	16	68321838	.	ACT	A	.	PASS	SLC7A6	intronic
ACa07	16	88494628	.	T	TC	.	PASS	ZNF469	exonic
ACa07	17	663583	.	TA	T	.	PASS	GLOD4	intronic
ACa07	17	2597572	.	TG	T	.	PASS	CLUH	splicing
ACa07	17	7297382	.	TG	T	.	PASS	TMEM256	ncRNA_int
ACa07	17	7832616	rs7574964	AC	A	.	PASS	KCNAB3	exonic
ACa07	17	8380302	.	GCTT	G	.	PASS	MYH10	exonic
ACa07	17	29687463	rs5285990	TA	T	.	PASS	NF1	intronic
ACa07	17	37318860	.	ATC	A	.	PASS	ARL5C	intronic
ACa07	17	37687471	.	TG	T	.	PASS	CDK12	exonic
ACa07	17	40458083	.	GA	G	.	PASS	STAT5A	intronic
ACa07	17	41004673	.	TC	T	.	PASS	AOC3	exonic
ACa07	17	42477138	.	CT	C	.	PASS	GPATCH8	exonic
ACa07	17	43322182	.	CCA	C	.	PASS	FMNL1	splicing
ACa07	17	45291237	rs1380173	T	TGGA	.	PASS	MYL4	intronic
ACa07	17	45915469	.	AC	A	.	PASS	SCRN2	UTR3
ACa07	17	46878603	.	CG	C	.	PASS	TTLL6	intronic
ACa07	17	76794615	.	AT	A	.	PASS	USP36	exonic
ACa07	17	77081973	rs7634794	GC	G	.	PASS	ENGASE	intronic
ACa07	17	78942662	.	ATGGAAT	A	.	PASS	.	intergenic
ACa07	17	80012761	rs7487058	GC	G	.	PASS	GPS1	intronic
ACa07	18	2724882	rs1429731	AT	A	.	PASS	SMCHD1	intronic
ACa07	18	12274075	rs7500268	TC	T	.	PASS	CIDEA	intronic
ACa07	18	13643281	.	GT	G	.	PASS	LDLRAD4	intronic
ACa07	18	21140211	.	C	CA	.	PASS	NPC1	exonic
ACa07	18	23845110	rs7661354	CT	C	.	PASS	TAF4B	intronic
ACa07	18	33553014	.	GA	G	.	PASS	C18orf21	intronic
ACa07	18	44101049	rs5710814	G	GC	.	PASS	LOXHD1	intronic
ACa07	18	46476715	rs1019291	CCCT	C	.	PASS	SMAD7	exonic
ACa07	18	56246441	rs7630535	T	TC	.	PASS	ALPK2	exonic
ACa07	19	1128144	.	GA	G	.	PASS	SBNO2	intronic
ACa07	19	1434835	.	GC	G	.	PASS	DAZAP1	exonic
ACa07	19	3831692	rs7644591	CG	C	.	PASS	ZFR2	exonic
ACa07	19	3941222	.	G	GC	.	PASS	NMRK2	intronic
ACa07	19	4697751	.	TC	T	.	PASS	DPP9	intronic

ACa07	19	4909388	.	GC	G	.	PASS	.	intronic
ACa07	19	5218843	rs7590798	GA	G	.	PASS	PTPRS	intronic
ACa07	19	5240270	.	CG	C	.	PASS	PTPRS	exonic
ACa07	19	6387617	rs7708014	GC	G	.	PASS	GTF2F1	intronic
ACa07	19	6719395	.	TG	T	.	PASS	C3	exonic
ACa07	19	8601567	.	TG	T	.	PASS	MYO1F	intronic
ACa07	19	13081255	.	GT	G	.	PASS	DAND5	intronic
ACa07	19	17941276	.	GAGA	G	.	PASS	JAK3	intronic
ACa07	19	18887992	rs7799007	TC	T	.	PASS	CRTC1	exonic
ACa07	19	33638508	rs7677659	C	CA	.	PASS	WDR88	intronic
ACa07	19	34941384	.	CA	C	.	PASS	UBA2	intronic
ACa07	19	35843419	.	CG	C	.	PASS	.	downstrea
ACa07	19	36211898	.	AC	A	.	PASS	KMT2B	exonic
ACa07	19	37118180	rs9414440	GA	G	.	PASS	ZNF382	exonic
ACa07	19	38023216	.	A	AT	.	PASS	ZNF793	intronic
ACa07	19	38573182	.	GC	G	.	PASS	SIPA1L3	exonic
ACa07	19	43808555	.	T	TC	.	PASS	.	intergenic
ACa07	19	43991218	rs7662018	TC	T	.	PASS	PHLDB3	exonic
ACa07	19	45655750	.	TG	T	.	PASS	NKPD1	exonic
ACa07	19	45803093	.	CG	C	.	PASS	MARK4	exonic
ACa07	19	46124694	rs7520653	C	CG	.	PASS	EML2	intronic
ACa07	19	46190720	.	GA	G	.	PASS	SNRPD2	UTR3
ACa07	19	46998234	.	AC	A	.	PASS	PNMAL2	exonic
ACa07	19	48197890	rs7555061	A	AC	.	PASS	GLTSCR1	exonic
ACa07	19	49458970	rs3981228	T	TG	.	PASS	BAX	exonic
ACa07	19	50139128	.	CG	C	.	PASS	RRAS	intronic
ACa07	19	50862108	.	TC	T	.	PASS	NAPSA	intronic
ACa07	19	51955893	rs7666986	CAG	C	.	PASS	SIGLEC8	intronic
ACa07	19	54754576	.	T	TG	.	PASS	LILRB5	UTR3
ACa07	19	54947243	.	TC	T	.	PASS	TTYH1	intronic
ACa07	19	55815035	rs7811378	A	AC	.	PASS	BRSK1	exonic
ACa07	19	59069508	.	AC	A	.	PASS	UBE2M	intronic
ACa07	20	2730208	.	GC	G	.	PASS	EBF4	intronic
ACa07	20	3944693	rs7575646	GA	G	.	PASS	RNF24	intronic
ACa07	20	13698200	rs3605176	CT	C	.	PASS	ESF1	intronic
ACa07	20	17596265	.	GC	G	.	PASS	RRBP1	intronic
ACa07	20	31022441	rs1085307	A	AG	.	PASS	ASXL1	exonic
ACa07	20	33501331	rs7539299	C	CG	.	PASS	ACSS2	intronic
ACa07	20	35155634	.	CA	C	.	PASS	DLGAP4-A	ncRNA_int
ACa07	20	35284816	rs7511829	TG	T	.	PASS	NDRG3	splicing
ACa07	20	45644793	rs7684989	C	CT	.	PASS	EYA2	intronic
ACa07	20	47858503	rs7667143	CA	C	.	PASS	DDX27	exonic
ACa07	20	62070158	rs9359712	AC	A	.	PASS	KCNQ2	intronic
ACa07	20	62193605	.	GC	G	.	PASS	HELZ2	intronic
ACa07	21	21112410	rs3687317	AG	A	.	PASS	.	intergenic
ACa07	21	47571590	rs7538174	GC	G	.	PASS	FTCD	exonic
ACa07	21	47663396	rs7755389	C	CG	.	PASS	MCM3AP	exonic
ACa07	22	20137447	rs2001464	TC	T	.	PASS	.	upstream
ACa07	22	25853204	.	TC	T	.	PASS	CRYBB2P	ncRNA_int
ACa07	22	28195488	.	AG	A	.	PASS	MN1	exonic
ACa07	22	31927025	.	CT	C	.	PASS	SFI1	intronic
ACa07	22	37769103	rs7686708	AG	A	.	PASS	ELFN2	UTR3
ACa07	22	37964382	.	AC	A	.	PASS	CDC42EP	exonic
ACa07	22	42905256	.	GC	G	.	PASS	SERHL	ncRNA_int

ACa07	22	46712113	rs7743082	A	AC	.	PASS	GTSE1	exonic
ACa07	22	50636198	.	AC	A	.	PASS	TRABD	intronic
ACa07	X	302051	.	GT	G	.	PASS	PPP2R3B	exonic
ACa07	X	7243363	rs7631509	CT	C	.	PASS	STS	intronic
ACa07	X	20134812	.	G	GC	.	PASS	MAP7D2	intronic
ACa07	X	41203475	rs7752182	A	AT	.	PASS	DDX3X	intronic
ACa07	X	47500595	rs7515575	AG	A	.	PASS	ELK1	intronic
ACa07	X	48763589	rs7821251	A	AC	.	PASS	SLC35A2	intronic
ACa07	X	49055509	.	T	TG	.	PASS	SYP-AS1	ncRNA_ex
ACa07	X	49961701	.	G	GC	.	PASS	AKAP4	intronic
ACa07	X	75004753	.	TG	T	.	PASS	MAGEE2	exonic
ACa07	X	1.35E+08	rs1064795	CG	C	.	PASS	SLC9A6	UTR5
ACa07	X	1.53E+08	rs7823430	A	AG	.	PASS	PLXNB3	exonic
ACaP0	6	1611718	.	CCCG	C	.	PASS	FOXC1	exonic
ACaP0	11	75279877	.	A	AGGTGCT	.	PASS	SERPINH1	splicing
ACaP0	19	1128220	.	C	CCTGAAG	.	PASS	SBNO2	intronic
ACaP0	1	43916716	.	CCATCTT	C	.	PASS	HYI,SZT2	UTR3
ACaP0	2	1.6E+08	rs7485648	AGCACTG	A	.	PASS	DAPL1	UTR5
ACaP0	3	19554555	.	GTCTCCC	G	.	PASS	KCNH8	exonic
ACaP0	13	47263423	.	CATTTGT	C	.	PASS	LRCH1	intronic
ACaP0	14	70040199	rs9344233	GGAGGC(	G	.	PASS	CCDC177	exonic
ACaP0	15	23690415	rs4036589	T	TTGTGC	.	PASS	.	intergenic
ACaP0	1	54691919	rs1062553	G	GGAA	.	PASS	SSBP3	UTR3
ACaP0	2	1.19E+08	.	A	AGGAGG	.	PASS	INSIG2	intronic
ACaP0	2	1.19E+08	rs3707030	AGGAGG/	A	.	PASS	INSIG2	intronic
ACaP0	6	90121644	.	ATCCTCC	A	.	PASS	RRAGD	exonic
ACaP0	9	1.28E+08	.	T	TTCACGG	.	PASS	WDR38	intronic
ACaP0	17	78450008	.	CGGATG(	C	.	PASS	NPTX1	exonic
ACaP0	18	33877739	.	GCGTCC(	G	.	PASS	FHOD3	UTR5
ACaP0	2	1.74E+08	.	ACTCAAT	A	.	PASS	ZAK	exonic
ACaP0	4	25162105	rs8860593	AAAAAC	A	.	PASS	SEPSECS	UTR5
ACaP0	7	6226691	.	TCCATGT	T	.	PASS	CYTH3	exonic
ACaP0	11	87011	.	AGGTGTC	A	.	PASS	.	intergenic
ACaP0	14	21993501	rs9488701	ACTCCT	A	.	PASS	SALL2	exonic
ACaP0	17	7811126	rs1047246	CCAGTA	C	.	PASS	CHD3	intronic
ACaP0	1	1.48E+08	.	AGAGTT	A	.	PASS	NBPF14,N	exonic
ACaP0	2	1.73E+08	.	CCGGGA(	C	.	PASS	PDK1	intronic
ACaP0	6	1.58E+08	.	GCCAAG(	G	.	PASS	SYNJ2	exonic
ACaP0	12	1.33E+08	.	GA	G	.	PASS	POLE	exonic
ACaP0	17	7578525	.	G	GCAA	.	PASS	TP53	exonic
ACaP0	19	49691954	.	A	ACGC	.	PASS	TRPM4	exonic
ACaP0	19	51172433	.	TGGGGA(	T	.	PASS	SHANK1	exonic
ACaP0	22	36960223	rs3980370	G	GT	.	PASS	CACNG2	UTR3
ACaP0	3	1.85E+08	.	AAGTTGC	A	.	PASS	IGF2BP2	intronic
ACaP0	4	71554619	rs7562167	CGAGGA(	C	.	PASS	UTP3	exonic
ACaP0	10	21805804	rs7459278	GGCCGC(	G	.	PASS	SKIDA1	exonic
ACaP0	16	845968	.	AC	A	.	PASS	CHTF18	exonic
ACaP0	17	46673923	.	CGCCGC(	C	.	PASS	HOXB6	exonic
ACaP0	19	36237318	.	A	AG	.	PASS	PSENEN	splicing
ACaP0	MT	9531	rs2676066	A	AC	.	PASS	.	intergenic
ACaP0	20	13765944	.	C	CGCGGG(	.	PASS	NDUFAF5	intronic
ACaP1	17	18159768	.	GC	G	.	PASS	FLII	intronic
ACaP1	1	43770851	rs7519299	C	CG	.	PASS	TIE1	intronic
ACaP1	8	14095086	.	CCCCAAA	C	.	PASS	SGCZ	exonic

ACaP1:	9	1.4E+08	.	T	TG	.	PASS	ENTPD2	exonic
ACaP1:	11	1.32E+08	.	ATCT	A	.	PASS	NTM	intronic
ACaP1:	14	68162286	.	T	TA	.	PASS	RDH11	intronic
ACaP1:	22	42557265	rs7666083	CGGGCG	C	.	PASS	TCF20	UTR3
ACaP1:	2	23925992	.	C	CTTG	.	PASS	KLHL29	intronic
ACaP1:	5	56719286	.	CAAGGG	C	.	PASS	.	intergenic
ACaP1:	9	1.31E+08	.	G	GC	.	PASS	ODF2	intronic
ACaP1:	11	6662745	rs7788921	CCAGCAG	C	.	PASS	DCHS1	exonic
ACaP1:	12	9907649	.	TA	T	.	PASS	CD69	intronic
ACaP1:	13	99461550	rs7743913	TTTTTG	T	.	PASS	DOCK9	intronic
ACaP1:	14	88892767	rs7758009	CG	C	.	PASS	SPATA7	exonic
ACaP1:	16	67063717	.	G	GT	.	PASS	CBFB	splicing
ACaP1:	17	15410163	.	A	AATTCT	.	PASS	TVP23C-C	intronic
ACaP1:	17	27044730	.	AGAGCGC	A	.	PASS	NARR,RAI	UTR5
ACaP1:	21	46900013	rs7645628	CCCAGGA	C	.	PASS	COL18A1	exonic
ACaP1:	22	17414848	.	AGAGCCC	A	.	PASS	.	intergenic
ACaP1:	X	16888095	.	GCCGCGC	G	.	PASS	RBBP7	intronic
ACaP1:	7	6682259	.	CGAGGTC	C	.	PASS	ZNF316	exonic
ACaP1:	8	1.08E+08	.	TTGATCA	T	.	PASS	ANGPT1	intronic
ACaP1:	11	1.02E+08	rs6015769	TA	T	.	PASS	BIRC3	intronic
ACaP1:	16	69153951	rs7596499	GGGGCCT	G	.	PASS	CHTF8	UTR3
ACaP1:	19	46366371	.	TCCGCCC	T	.	PASS	SYMPK	UTR5
ACaP1:	19	58002863	.	G	GCTGT	.	PASS	ZNF419	exonic
ACaP1:	X	48163160	.	C	CT	.	PASS	SSX9	ncRNA_int
ACaP1:	X	1.07E+08	.	A	AT	.	PASS	COL4A6	intronic
ACaP1	3	51392370	.	AGG	A	.	PASS	DOCK3	exonic
ACaP1	3	1.02E+08	.	CTT	C	.	PASS	NFKBIZ	exonic
ACaP1	15	93632120	.	GTCTGCC	G	.	PASS	RGMA	intronic

Gene	GeneDetai	ExonicFun	AAChange	Genecode:	wgRna	cytoBand	targetScan	tfbsConsSi
NM_01455	.	.	.	ENST0000	.	2q14.2	.	.
NM_00521	.	frameshift	CTLA4:NM	ENST0000	.	2q33.2	.	Score=999
NR_00366	dist=48933	.	.	.	.	6p11.1	.	.
NM_00586	.	nonframes	BET1:NM_	ENST0000	.	7q21.3	.	.
NM_02495	.	.	.	ENST0000	.	12q24.13	.	.
NM_00101	.	frameshift	VPS13C:N	ENST0000	.	15q22.2	.	.
NM_00355	.	nonframes	OR1G1:NM	ENST0000	.	17p13.3	.	.
NM_17213	.	.	.	ENST0000	.	19q13.2	.	.
NM_01336	.	frameshift	DNMT3L:N	ENST0000	.	21q22.3	.	.
NM_00103	.	.	.	ENST0000	.	Xp11.22	.	.
NM_00116	.	nonframes	ANKRD33I	ENST0000	.	5p15.2	.	Score=807
NM_00115	.	.	.	ENST0000	.	13q12.3	.	.
NM_00110	NM_00110	.	.	ENST0000	.	14q24.1	.	.
NM_00127	.	frameshift	ANKLE1:N	ENST0000	.	19p13.11	.	.
NM_00587	.	.	.	ENST0000	.	22q12.2	.	.
NM_00577	.	nonframes	RBM6:NM_	ENST0000	.	3p21.31	.	Score=905
NM_00062	.	frameshift	LTBP1:NM	ENST0000	.	2p22.3	.	Score=979
NM_00578	dist=2844;	.	.	ENST0000	.	3q29	.	.
NM_00103	.	.	.	ENST0000	.	16p12.2	.	.
NR_12616	.	.	.	ENST0000	.	16q24.3	.	.
NR_04598	.	.	.	ENST0000	.	17p13.1	.	.
NM_15320	.	.	.	ENST0000	.	17q25.1	.	.
NM_01565	.	.	.	ENST0000	.	1p36.33	.	.
NR_03886	.	.	.	ENST0000	.	1p36.33	.	.
NM_00125	.	.	.	ENST0000	.	1p36.33	.	.
NM_00114	.	.	.	ENST0000	.	1p36.33	.	.
NM_00103	.	.	.	ENST0000	.	1p36.33	.	.
NM_00106	.	.	.	ENST0000	.	1p36.22	.	.
NM_02076	.	frameshift	UBR4:NM_	ENST0000	.	1p36.13	.	.
NM_00601	.	frameshift	ARID1A:NI	ENST0000	.	1p36.11	.	Score=894
NM_01209	.	.	.	ENST0000	.	1p34.3	.	.
NM_01209	.	.	.	ENST0000	.	1p34.3	.	.
NM_00112	.	.	.	ENST0000	.	1p34.2	.	.
NM_03202	.	.	.	ENST0000	.	1p31.3	.	.
NM_15289	.	frameshift	COL24A1:	ENST0000	.	1p22.3	.	.
NM_00651	.	.	.	ENST0000	.	1p13.3	.	.
NM_00104	.	.	.	ENST0000	.	1p13.2	.	.
NM_00107	.	.	.	ENST0000	.	1q21.2	.	.
NM_01758	.	frameshift	UBE2Q1:N	ENST0000	.	1q21.3	.	Score=833
NM_00129	.	frameshift	CLK2:NM_	ENST0000	.	1q22	.	Score=851
NM_00128	.	.	.	ENST0000	.	1q22	.	.
NM_02194	.	.	.	ENST0000	.	1q23.1	.	.
NM_02031	.	.	.	ENST0000	.	1q25.2	.	.
NM_14469	.	frameshift	AXDND1:N	ENST0000	.	1q25.2	.	.
NM_00135	NM_00135	.	.	ENST0000	.	1q25.3	.	.
NM_00283	.	.	.	ENST0000	.	1q31.3	.	.
NM_01493	.	frameshift	PLEKHA6:	ENST0000	.	1q32.1	.	Score=888
NM_00127	.	.	.	ENST0000	.	1q42.12	.	.
NM_00360	.	.	.	ENST0000	.	1q42.13	.	.
NR_02839	.	.	.	ENST0000	.	1q42.2	.	.
NR_11025	dist=20359	.	.	.	.	2p25.1	.	.

NM_00485	.	frameshift	ROCK2:NM	ENST0000	.	2p25.1	.	Score=935
NM_00104	.	.	.	ENST0000	.	2p23.3	.	.
NM_00126	.	.	.	ENST0000	.	2p23.2	.	.
NM_00111	dist=64458	.	.	ENST0000	.	2p22.1	.	.
NM_00264	.	.	.	ENST0000	.	2p21	.	.
NM_00100	.	frameshift	MTIF2:NM_	ENST0000	.	2p16.1	.	.
NM_03260	.	frameshift	LOXL3:NM	ENST0000	.	2p13.1	.	.
NM_01662	.	.	.	ENST0000	.	2p11.2	.	.
NM_00102	.	.	.	ENST0000	.	2q11.2	.	.
NM_01455	.	.	.	ENST0000	.	2q14.2	.	.
NM_00127	.	frameshift	ACVR2A:N	ENST0000	.	2q22.3	.	.
NM_00117	.	frameshift	CSRNP3:N	ENST0000	.	2q24.3	.	.
NR_11059	.	.	.	ENST0000	.	2q24.3	.	.
NM_00119	.	frameshift	XIRP2:NM_	ENST0000	.	2q24.3	.	.
NM_00125	.	frameshift	ATF2:NM_	ENST0000	.	2q31.1	.	.
NM_00107	.	.	.	ENST0000	.	2q31.2	.	.
NR_03166	.	.	.	ENST0000	.	2q31.2	.	.
NM_00125	.	stopgain	TTN:NM_0	ENST0000	.	2q31.2	.	.
NM_00731	.	frameshift	STAT1:NM	ENST0000	.	2q32.2	.	.
NM_00128	.	.	.	ENST0000	.	2q33.1	.	.
NM_00126	.	.	.	ENST0000	.	2q33.1	.	.
NM_00381	.	frameshift	ADAM23:N	ENST0000	.	2q33.3	.	.
NM_00113	.	frameshift	FASTKD2:	ENST0000	.	2q33.3	.	.
NM_00112	.	.	.	ENST0000	.	2q33.3	.	.
NM_00100	NM_00100	.	.	ENST0000	.	2q35	.	.
NM_00114	.	frameshift	SPHKAP:N	ENST0000	.	2q36.3	.	.
NM_00100	.	frameshift	DNAJB3:N	ENST0000	.	2q37.1	.	.
NM_01480	.	.	.	ENST0000	.	2q37.3	.	.
NM_00128	.	frameshift	CNTN6:NM	ENST0000	.	3p26.3	.	Score=844
NM_15253	NM_15253	.	.	ENST0000	.	3p25.1	.	.
NM_00114	.	.	.	ENST0000	.	3p24.2	.	.
NM_01401	.	.	.	ENST0000	.	3p21.31	.	.
NM_01546	.	nonframes	PTPN23:N	ENST0000	.	3p21.31	.	.
NM_00577	.	frameshift	RBM6:NM_	ENST0000	.	3p21.31	.	.
NR_10998	.	.	.	ENST0000	.	3p21.2	.	.
NM_00625	.	frameshift	PRKCD:NM	ENST0000	.	3p21.1	.	.
NM_00072	.	.	.	ENST0000	.	3p21.1	.	.
NM_01541	.	.	.	ENST0000	.	3q13.2	.	.
NM_01404	.	.	.	ENST0000	.	3q21.3	.	.
NM_00103	.	frameshift	FAIM:NM_	ENST0000	.	3q22.3	.	.
NM_00114	.	.	.	ENST0000	.	3q25.1	.	.
NM_02103	.	.	.	ENST0000	.	3q25.1	.	.
NM_01493	.	.	.	ENST0000	.	3q26.31	.	.
NM_00374	.	.	.	ENST0000	.	3q27.1	.	.
NR_02456	.	.	.	ENST0000	.	4p16.3	.	.
NM_02089	.	frameshift	UVSSA:NM	ENST0000	.	4p16.3	.	.
NM_05304	dist=49825	.	.	ENST0000	.	4p16.1	.	.
NM_01829	.	.	.	ENST0000	.	4p14	.	.
NM_00111	.	.	.	ENST0000	.	4p13	.	.
NM_15254	.	nonframes	SCFD2:NM	ENST0000	.	4q12	.	.
NM_15240	.	.	.	ENST0000	.	4q12	.	.
NM_00109	.	frameshift	PRDM8:NM	ENST0000	.	4q21.21	.	.
NM_00112	.	.	.	ENST0000	.	4q22.3	.	.
NM_00117	.	.	.	ENST0000	.	4q25	.	.

NM_00122	.	.	.	ENST0000	.	4q26	.	.
NM_02459	.	frameshift	ARSJ:NM_	ENST0000	.	4q26	.	.
NM_00129	.	nonframes	FAT4:NM_	ENST0000	.	4q28.1	.	Score=956
NM_01824	.	frameshift	NEIL3:NM_	ENST0000	.	4q34.3	.	.
NM_00100	.	.	.	ENST0000	.	4q35.2	.	.
NR_00326	.	.	.	ENST0000	.	5p15.33	.	.
NM_00711	.	frameshift	TRIO:NM_	ENST0000	.	5p15.2	.	.
NM_05402	NM_05402	.	.	ENST0000	.	5p15.2	.	.
NM_03095	.	.	.	ENST0000	.	5p13.3	.	.
NM_02307	.	.	.	ENST0000	.	5p13.2	.	.
NM_00399	.	.	.	ENST0000	.	5p13.1	.	.
NM_00128	.	.	.	ENST0000	.	5q13.3	.	.
NM_00127	.	.	.	ENST0000	.	5q14.1	.	.
NM_00103	.	.	.	ENST0000	.	5q31.3	.	.
NM_01891	.	frameshift	PCDHGA3	ENST0000	.	5q31.3	.	.
NM_01531	.	frameshift	LARP1:NM	ENST0000	.	5q33.2	.	.
NM_00127	.	frameshift	SLIT3:NM_	ENST0000	.	5q34	.	Score=781
NM_00127	.	.	.	ENST0000	.	5q34	.	.
NM_00150	.	.	.	ENST0000	.	6p22.1	.	.
NM_00128	.	.	.	ENST0000	.	6p21.33	.	.
NM_00692	.	.	.	ENST0000	.	6p21.33	.	.
NM_00120	.	frameshift	AGER:NM_	ENST0000	.	6p21.32	.	.
NM_00258	.	.	.	ENST0000	.	6p21.32	.	.
NM_00211	.	.	.	ENST0000	.	6p21.32	.	.
NM_00128	.	frameshift	C6orf89:NI	ENST0000	.	6p21.2	.	Score=724
NM_00101	.	frameshift	FOXP4:NM	ENST0000	.	6p21.1	.	Score=998
NM_00128	.	.	.	ENST0000	.	6q14.1	.	.
NM_01549	NM_01549	.	.	ENST0000	.	6q16.2	.	.
NM_00506	.	.	.	ENST0000	.	6q16.3	.	.
NM_00116	.	frameshift	GRIK2:NM	ENST0000	.	6q16.3	.	Score=835
NM_00108	.	frameshift	BEND3:NM	ENST0000	.	6q21	.	.
NM_01859	.	.	.	ENST0000	.	6q21	.	.
NM_00116	.	frameshift	TRAF3IP2	ENST0000	.	6q21	.	.
NM_00110	.	.	.	ENST0000	.	6q21	.	.
NM_15273	.	frameshift	TBC1D32:	ENST0000	.	6q22.31	.	Score=837
NM_00114	.	frameshift	HBS1L:NM	ENST0000	.	6q23.3	.	.
NM_00110	.	frameshift	PHACTR2	ENST0000	.	6q24.2	.	.
NM_00127	.	.	.	ENST0000	.	6q25.3	.	.
NM_00087	.	.	.	ENST0000	.	6q25.3	.	.
NONE,NR_	dist=NONE	.	.	ENST0000	.	7p22.3	.	.
NR_00128	dist=46754	.	.	.	.	7p22.3	.	.
NM_01805	.	frameshift	RADIL:NM	ENST0000	.	7p22.1	.	.
NM_00108	.	frameshift	TNRC18:N	ENST0000	.	7p22.1	.	Score=858
NM_00592	NM_00592	.	.	ENST0000	.	7p21.2	.	.
NR_12050	dist=21149	.	.	ENST0000	.	7p15.2	.	.
NM_00609	.	.	.	ENST0000	.	7p14.3	.	.
NM_00609	.	frameshift	NOD1:NM_	ENST0000	.	7p14.3	.	.
NM_01869	.	frameshift	LANCL2:N	ENST0000	.	7p11.2	.	.
NM_00115	.	frameshift	ZNF716:NI	ENST0000	.	7p11.2	.	.
NM_00119	.	.	.	ENST0000	.	7q11.23	.	.
NM_00050	.	.	.	ENST0000	.	7q11.23	.	.
NM_00127	.	.	.	ENST0000	.	7q22.1	.	.
NM_17356	.	frameshift	NYAP1:NM	ENST0000	.	7q22.1	.	.
NM_00444	.	.	.	ENST0000	.	7q22.1	.	.

NM_00120	.	frameshift	CUX1:NM_	ENST0000	.	7q22.1	.	Score=824
NM_20716	.	nonframes	LMOD2:NM	ENST0000	.	7q31.32	.	.
NM_02029	.	.	.	ENST0000	.	7q33	.	.
NM_17368	.	unknown	UNKNOWI	ENST0000	.	7q36.1	.	Score=822
NM_00103	.	.	.	ENST0000	.	7q36.2	.	.
NM_13871	.	.	.	ENST0000	.	8p22	.	.
NM_01531	.	.	.	ENST0000	.	8p22	.	.
NM_13927	dist=2934;	.	.	ENST0000	.	8p21.3	.	Score=946
NM_00112	dist=25494	.	.	ENST0000	.	8p21.2	.	.
NM_00115	.	.	.	ENST0000	.	8p12	.	.
NR_00312	.	.	.	ENST0000	.	8p11.22	.	.
NM_00111	.	.	.	ENST0000	.	8q12.1	.	.
NM_00128	NM_00128	.	.	ENST0000	.	8q21.13	.	.
NM_00103	.	frameshift	ESRP1:NM	ENST0000	.	8q22.1	.	.
NM_00110	.	.	.	ENST0000	.	8q22.3	.	.
NM_00120	.	.	.	ENST0000	.	8q24.22	.	.
NM_00170	.	.	.	ENST0000	.	8q24.3	.	.
NM_00191	.	frameshift	CYC1:NM_	ENST0000	.	8q24.3	.	Score=904
NM_02476	.	.	.	ENST0000	.	9p21.2	.	.
NM_00113	NM_00113	.	.	ENST0000	.	9p13.3	Score=98;I	.
NM_00628	.	frameshift	TLN1:NM_	ENST0000	.	9p13.3	.	.
NM_00120	dist=15610	.	.	ENST0000	.	9p11.2	.	.
NM_00539	.	frameshift	PHF2:NM_	ENST0000	.	9q22.31	.	.
NM_00026	.	frameshift	PTCH1:NM	ENST0000	.	9q22.32	.	.
NM_00108	NM_00108	.	.	ENST0000	.	9q22.32	.	.
NM_00108	.	.	.	ENST0000	.	9q31.3	.	.
NM_00100	.	frameshift	OR1L3:NM	ENST0000	.	9q33.2	.	.
NM_00117	.	nonframes	STRBP:NM	ENST0000	.	9q33.3	.	.
NM_00117	.	.	.	ENST0000	.	9q33.3	.	.
NM_00128	.	.	.	ENST0000	.	9q34.11	.	.
NM_03272	.	.	.	ENST0000	.	9q34.13	.	.
NM_00128	.	frameshift	PMPCA:NI	ENST0000	.	9q34.3	.	.
NM_17846	.	.	.	ENST0000	.	9q34.3	.	.
NM_00103	.	.	.	ENST0000	.	9q34.3	.	.
NM_18324	.	frameshift	C9orf142:N	ENST0000	.	9q34.3	.	.
NM_00101	.	frameshift	SFMBT2:N	ENST0000	.	10p14	.	.
NM_00081	.	.	.	ENST0000	.	10p12.1	.	.
NM_00127	.	frameshift	C10orf126	ENST0000	.	10p12.1	.	.
NR_02763	.	.	.	ENST0000	.	10q11.22	.	.
NM_00128	NM_00128	.	.	ENST0000	.	10q21.3	.	.
NM_00125	.	.	.	ENST0000	.	10q22.1	.	.
NM_08072	.	frameshift	ADAMTS1	ENST0000	.	10q22.1	.	.
NM_00124	.	frameshift	ZSWIM8:N	ENST0000	.	10q22.2	.	Score=991
NM_02033	.	.	.	ENST0000	.	10q22.3	.	.
NM_00115	.	.	.	ENST0000	.	10q22.3	.	.
NM_13344	.	nonframes	AGAP11:N	ENST0000	.	10q23.2	.	.
NM_00620	.	.	.	ENST0000	.	10q23.33	.	.
NM_03272	.	.	.	ENST0000	.	10q24.33	.	.
NM_00129	.	.	.	ENST0000	.	10q26.13	.	.
NM_00405	.	.	.	ENST0000	.	10q26.3	.	.
NM_02103	.	.	.	ENST0000	.	11p15.5	.	.
NM_17853	.	frameshift	B4GALNT4	ENST0000	.	11p15.5	.	.
NM_00125	.	frameshift	BRSK2:NM	ENST0000	.	11p15.5	.	.
NM_03077	.	.	.	ENST0000	.	11p15.4	.	.

NM_01446	.	frameshift	RBMXL2:N	ENST0000	.	11p15.4	.	.
NM_00541	.	.	.	ENST0000	.	11p15.4	.	.
NM_00114	.	.	.	ENST0000	.	11p15.1	.	.
NM_00113	.	.	.	ENST0000	.	11p14.2	.	.
NM_01614	.	.	.	ENST0000	.	11p11.2	.	.
NM_00545	.	frameshift	MAPK8IP1	ENST0000	.	11p11.2	.	.
NM_00130	.	frameshift	EML3:NM_	ENST0000	.	11q12.3	.	.
NM_00119	.	.	.	ENST0000	.	11q13.2	.	.
NM_00161	.	.	.	ENST0000	.	11q13.2	.	.
NM_00101	.	.	.	.	.	11q13.4	.	.
NM_02079	.	frameshift	USP35:NM	ENST0000	.	11q14.1	.	Score=882
NM_00114	.	.	.	ENST0000	.	11q14.1	.	.
NM_00100	.	frameshift	FAT3:NM_	ENST0000	.	11q14.3	.	.
NM_03339	.	.	.	ENST0000	.	11q21	.	.
NM_00120	.	frameshift	JAM3:NM_	ENST0000	.	11q25	.	Score=856
NM_00104	.	frameshift	KDM5A:NM	ENST0000	.	12p13.33	.	.
NM_01741	.	.	.	ENST0000	.	12p13.32	.	.
NM_00112	.	.	.	ENST0000	.	12p13.31	.	.
NM_00283	.	.	.	ENST0000	.	12p13.31	.	.
NM_00128	.	.	.	ENST0000	.	12p13.31	.	.
NM_00125	.	frameshift	CLECL1:N	ENST0000	.	12p13.31	.	.
NM_05816	NM_05816	.	.	ENST0000	.	12p13.2	.	.
NM_00119	.	.	.	ENST0000	.	12p11.23	.	.
NM_00119	.	.	.	ENST0000	.	12p11.22	.	.
NM_17360	.	unknown	UNKNOWI	ENST0000	.	12q12	.	.
NM_00471	dist=1383;	.	.	ENST0000	.	12q12	.	.
NM_00348	.	.	.	ENST0000	.	12q13.12	.	.
NM_00109	.	.	.	ENST0000	.	12q13.12	.	.
NM_00061	.	.	.	ENST0000	.	12q13.12	.	.
NM_00117	.	frameshift	SCN8A:NM	ENST0000	.	12q13.13	.	Score=836
NM_00130	.	.	.	ENST0000	.	12q13.13	.	.
NM_01895	NM_01895	.	.	ENST0000	.	12q13.13	.	.
NM_00392	.	frameshift	TIMELESS	ENST0000	.	12q13.3	.	Score=866
NM_00125	.	frameshift	MYO1A:NM	ENST0000	.	12q13.3	.	.
NM_00233	NM_00233	.	.	ENST0000	.	12q13.3	.	.
NM_05289	.	frameshift	MBD6:NM_	ENST0000	.	12q13.3	.	Score=879
NM_01332	.	.	.	ENST0000	.	12q23.3	.	.
NM_00616	.	.	.	ENST0000	.	12q23.3	.	.
NM_20743	.	frameshift	DNAH10:N	ENST0000	.	12q24.31	.	.
NM_00129	.	frameshift	WASF3:NM	ENST0000	.	13q12.13	.	.
NM_00114	.	.	.	ENST0000	.	13q13.3	.	.
NM_02453	.	.	.	ENST0000	.	13q34	.	.
NM_00116	.	frameshift	SAMD4A:N	ENST0000	.	14q22.2	.	.
NM_00124	.	frameshift	KIAA0586:	ENST0000	.	14q23.1	.	.
NM_00127	.	.	.	ENST0000	.	14q23.1	.	.
NM_00110	.	.	.	ENST0000	.	14q24.1	.	.
NM_02081	.	frameshift	UNC79:NM	ENST0000	.	14q32.12	.	.
NM_01803	.	.	.	ENST0000	.	14q32.2	.	.
NM_00131	.	.	.	ENST0000	.	14q32.33	.	.
NM_00100	.	frameshift	NIPA2:NM	ENST0000	.	15q11.2	.	.
NM_02075	.	.	.	ENST0000	.	15q15.2	.	.
NR_04648	.	.	.	ENST0000	.	15q15.3	.	.
NM_00114	.	frameshift	TP53BP1:I	ENST0000	.	15q15.3	.	.
NM_00100	.	frameshift	GALK2:NM	ENST0000	.	15q21.2	.	Score=807

NM_18275	.	.	.	ENST0000	.	15q21.3	.	.
NM_00128	NM_00128	.	.	ENST0000	.	15q23	.	.
NM_00117	.	.	.	ENST0000	.	15q24.1	.	.
NM_00102	.	.	.	ENST0000	.	15q24.1	.	.
NM_02077	.	frameshift	ALPK3:NM	ENST0000	.	15q25.3	.	Score=872
NM_15233	.	frameshift	AGBL1:NM	ENST0000	.	15q25.3	.	.
NM_00101	.	.	.	ENST0000	.	15q25.3	.	.
NM_19852	.	frameshift	KIF7:NM_1	ENST0000	.	15q26.1	.	Score=920
NM_00114	.	.	.	ENST0000	.	15q26.1	.	.
NM_17884	NM_17884	.	.	ENST0000	.	16p13.3	.	.
NM_03257	NM_03257	.	.	ENST0000	.	16p13.3	.	.
NM_03256	.	frameshift	GLYR1:NM	ENST0000	.	16p13.3	.	Score=782
NM_03216	.	.	.	ENST0000	.	16p13.13	.	.
NM_00128	.	.	.	ENST0000	.	16p13.11	.	.
NM_00121	.	.	.	ENST0000	.	16p11.2	.	.
NM_01471	.	.	.	ENST0000	.	16p11.2	.	.
NM_00111	.	.	.	ENST0000	.	16q12.1	.	.
NM_00179	.	frameshift	CDH5:NM_	ENST0000	.	16q21	.	.
NM_00110	.	frameshift	KCTD19:N	ENST0000	.	16q22.1	.	Score=899
NM_00107	.	.	.	ENST0000	.	16q22.1	.	.
NM_00112	.	frameshift	ZNF469:NI	ENST0000	.	16q24.2	.	Score=800
NM_01608	.	.	.	ENST0000	.	17p13.3	.	.
NM_01522	NM_01522	.	.	ENST0000	.	17p13.3	.	.
NR_03771	.	.	.	ENST0000	.	17p13.1	.	.
NM_00473	.	frameshift	KCNAB3:N	ENST0000	.	17p13.1	.	Score=942
NM_00125	.	nonframes	MYH10:NM	ENST0000	.	17p13.1	.	Score=826
NM_00026	.	.	.	ENST0000	.	17q11.2	.	.
NM_00114	.	.	.	ENST0000	.	17q12	.	.
NM_01508	.	frameshift	CDK12:NM	ENST0000	.	17q12	.	Score=922
NM_00128	.	.	.	ENST0000	.	17q21.2	.	.
NM_00127	.	frameshift	AOC3:NM_	ENST0000	.	17q21.31	.	.
NM_00100	.	frameshift	GPATCH8	ENST0000	.	17q21.31	.	.
NM_00589	.	.	.	ENST0000	.	17q21.31	.	Score=968
NM_00100	.	.	.	ENST0000	.	17q21.32	.	.
NM_00114	NM_00114	.	.	ENST0000	.	17q21.32	.	.
NM_00113	.	.	.	ENST0000	.	17q21.32	.	.
NM_02509	.	frameshift	USP36:NM	ENST0000	.	17q25.3	.	.
NM_00104	.	.	.	ENST0000	.	17q25.3	.	.
NM_00116	dist=2490;	.	.	ENST0000	.	17q25.3	.	.
NM_00412	.	.	.	ENST0000	.	17q25.3	.	.
NM_01529	.	.	.	ENST0000	.	18p11.32	.	.
NM_00127	.	.	.	ENST0000	.	18p11.21	.	.
NM_00100	.	.	.	ENST0000	.	18p11.21	.	.
NM_00027	.	frameshift	NPC1:NM_	ENST0000	.	18q11.2	.	.
NM_00129	.	.	.	ENST0000	.	18q11.2	.	.
NM_00120	.	.	.	ENST0000	.	18q12.2	.	.
NM_00114	.	.	.	ENST0000	.	18q21.1	.	.
NM_00119	.	nonframes	SMAD7:NI	ENST0000	.	18q21.1	.	.
NM_05294	.	frameshift	ALPK2:NM	ENST0000	.	18q21.32	.	.
NM_00110	.	.	.	ENST0000	.	19p13.3	.	.
NM_01895	.	frameshift	DAZAP1:N	ENST0000	.	19p13.3	.	Score=879
NM_01517	.	frameshift	ZFR2:NM_	ENST0000	.	19p13.3	.	.
NM_00128	.	.	.	ENST0000	.	19p13.3	.	.
NM_13915	.	.	.	ENST0000	.	19p13.3	.	.

NM_00129	.	.	.	ENST0000	.	19p13.3	.	.
NM_00285	.	.	.	ENST0000	.	19p13.3	.	.
NM_00285	.	frameshift	PTPRS:NM	ENST0000	.	19p13.3	.	.
NM_00209	.	.	.	ENST0000	.	19p13.3	.	.
NM_00006	.	frameshift	C3:NM_00	ENST0000	.	19p13.3	.	.
NM_01233	.	.	.	ENST0000	.	19p13.2	.	.
NM_15265	.	.	.	ENST0000	.	19p13.2	.	.
NM_00021	.	.	.	ENST0000	.	19p13.11	.	.
NM_00109	.	frameshift	CRTC1:NM	ENST0000	.	19p13.11	.	.
NM_17347	.	.	.	ENST0000	.	19q13.11	.	.
NM_00549	.	.	.	ENST0000	.	19q13.11	.	.
NM_00530	.	.	.	ENST0000	.	19q13.12	.	.
NM_01472	.	frameshift	KMT2B:NM	ENST0000	.	19q13.12	.	.
NM_00125	.	frameshift	ZNF382:NI	ENST0000	.	19q13.12	.	.
NM_00101	.	.	.	ENST0000	.	19q13.12	.	.
NM_01507	.	unknown	UNKNOWI	ENST0000	.	19q13.13	.	Score=755
NM_00130	dist=34840	.	.	ENST0000	.	19q13.31	.	.
NM_19885	.	frameshift	PHLDB3:N	ENST0000	.	19q13.31	.	.
NM_19847	.	frameshift	NKPD1:NM	ENST0000	.	19q13.32	.	.
NM_00119	.	frameshift	MARK4:NM	ENST0000	.	19q13.32	.	Score=755
NM_00119	.	.	.	ENST0000	.	19q13.32	.	.
NM_00459	NM_00459	.	.	ENST0000	.	19q13.32	.	.
NM_02070	.	frameshift	PNMAL2:N	ENST0000	.	19q13.32	.	Score=766
NM_01571	.	frameshift	GLTSCR1:	ENST0000	.	19q13.33	.	.
NM_00129	.	frameshift	BAX:NM_0	ENST0000	.	19q13.33	.	Score=836
NM_00627	.	.	.	ENST0000	.	19q13.33	.	.
NM_00485	.	.	.	ENST0000	.	19q13.33	.	.
NM_01444	.	.	.	ENST0000	.	19q13.41	.	.
NM_00108	NM_00108	.	.	ENST0000	.	19q13.42	.	.
NM_00100	.	.	.	ENST0000	.	19q13.42	.	.
NM_03243	.	frameshift	BRSK1:NM	ENST0000	.	19q13.42	.	.
NM_00396	.	.	.	ENST0000	.	19q13.43	.	.
NM_00111	.	.	.	ENST0000	.	20p13	.	.
NM_00113	.	.	.	ENST0000	.	20p13	.	.
NM_00127	.	.	.	ENST0000	.	20p12.1	.	.
NM_00104	.	.	.	ENST0000	.	20p12.1	.	.
NM_01533	.	frameshift	ASXL1:NM	ENST0000	.	20q11.21	.	Score=888
NM_00107	.	.	.	ENST0000	.	20q11.22	.	.
NR_10993	.	.	.	ENST0000	.	20q11.23	.	.
NM_02247	NM_02247	.	.	ENST0000	.	20q11.23	.	.
NM_00524	.	.	.	ENST0000	.	20q13.12	.	.
NM_01789	.	frameshift	DDX27:NM	ENST0000	.	20q13.13	.	.
NM_00451	.	.	.	ENST0000	.	20q13.33	.	.
NM_00103	.	.	.	ENST0000	.	20q13.33	.	.
NR_10992	dist=98028	.	.	ENST0000	.	21q21.1	.	.
NM_00665	.	frameshift	FTCD:NM_	ENST0000	.	21q22.3	.	.
NM_00390	.	frameshift	MCM3AP:I	ENST0000	.	21q22.3	.	Score=976
NM_00124	.	.	.	ENST0000	.	22q11.21	.	.
NR_03373	.	.	.	ENST0000	.	22q11.23	.	.
NM_00243	.	frameshift	MN1:NM_0	ENST0000	.	22q12.1	.	Score=869
NM_00100	.	.	.	ENST0000	.	22q12.2	.	.
NM_05290	NM_05290	.	.	ENST0000	.	22q13.1	.	.
NM_15224	.	frameshift	CDC42EP1	ENST0000	.	22q13.1	.	Score=888
NR_02778	.	.	.	ENST0000	.	22q13.2	.	.

NM_01642	.	frameshift	GTSE1:NM	ENST0000	.	22q13.31	.	.
NM_02520	.	.	.	ENST0000	.	22q13.33	.	.
NM_01323	.	frameshift	PPP2R3B:	ENST0000	.	Xp22.33	.	.
NM_00035	.	.	.	ENST0000	.	Xp22.31	.	.
NM_00116	.	.	.	ENST0000	.	Xp22.12	.	.
NM_00119	.	.	.	ENST0000	.	Xp11.4	.	.
NM_00111	.	.	.	ENST0000	.	Xp11.23	.	.
NM_00103	.	.	.	ENST0000	.	Xp11.23	.	.
NR_04664	.	.	.	ENST0000	.	Xp11.23	.	.
NM_00388	.	.	.	ENST0000	.	Xp11.22	.	.
NM_13870	.	frameshift	MAGEE2:N	ENST0000	.	Xq13.3	.	.
NM_00104	NM_00104	.	.	ENST0000	.	Xq26.3	.	.
NM_00116	.	frameshift	PLXNB3:N	ENST0000	.	Xq28	.	.
NM_00145	.	nonframes	FOXC1:NM	ENST0000	.	6p25.3	.	.
NM_00120	NM_00120	.	.	ENST0000	.	11q13.5	.	.
NM_00110	.	.	.	ENST0000	.	19p13.3	.	.
NM_00119	NM_00119	.	.	ENST0000	.	1p34.2	.	.
NM_00101	NM_00101	.	.	ENST0000	.	2q24.1	.	.
NM_14463	.	frameshift	KCNH8:NM	ENST0000	.	3p24.3	.	.
NM_00116	.	.	.	ENST0000	.	13q14.13	.	.
NM_00127	.	nonframes	CCDC177:	ENST0000	.	14q24.1	.	.
NR_03884	dist=76944	.	.	ENST0000	.	15q11.2	.	.
NM_00100	NM_00100	.	.	ENST0000	.	1p32.3	.	.
NM_01613	.	.	.	ENST0000	.	2q14.2	.	.
NM_01613	.	.	.	ENST0000	.	2q14.2	.	.
NM_02124	.	nonframes	RRAGD:NI	ENST0000	.	6q15	.	.
NM_00104	.	.	.	ENST0000	.	9q33.3	.	.
NM_00252	.	nonframes	NPTX1:NM	ENST0000	.	17q25.3	.	Score=699
NM_00128	NM_00128	.	.	ENST0000	.	18q12.2	.	.
NM_01665	.	frameshift	ZAK:NM_0	ENST0000	.	2q31.1	.	.
NM_01695	NM_01695	.	.	.	.	4p15.2	.	.
NM_00422	.	nonframes	CYTH3:NM	ENST0000	.	7p22.1	.	.
NONE,NR_	dist=NONE	.	.	ENST0000	.	11p15.5	.	.
NM_00540	.	frameshift	SALL2:NM	ENST0000	.	14q11.2	.	.
NM_00100	.	.	.	ENST0000	.	17p13.1	.	.
NM_00103	.	frameshift	NBPF8:NM	ENST0000	.	1q21.2	.	.
NM_00127	.	.	.	ENST0000	.	2q31.1	.	.
NM_00389	.	frameshift	SYNJ2:NM	ENST0000	.	6q25.3	.	.
NM_00623	.	frameshift	POLE:NM_	ENST0000	.	12q24.33	.	.
NM_00054	.	nonframes	TP53:NM_	ENST0000	.	17p13.1	.	.
NM_00119	.	nonframes	TRPM4:NM	ENST0000	.	19q13.33	.	.
NM_01614	.	frameshift	SHANK1:N	ENST0000	.	19q13.33	.	Score=985
NM_00607	NM_00607	.	.	ENST0000	.	22q12.3	.	.
NM_00100	.	.	.	ENST0000	.	3q27.2	.	.
NM_02036	.	nonframes	UTP3:NM_	ENST0000	.	4q13.3	.	.
NM_20737	.	nonframes	SKIDA1:NI	ENST0000	.	10p12.31	.	.
NM_02209	.	frameshift	CHTF18:N	ENST0000	.	16p13.3	.	.
NM_01895	.	frameshift	HOXB6:NM	ENST0000	.	17q21.32	.	.
NM_00128	NM_00128	.	.	ENST0000	.	19q13.12	.	Score=926
NONE,NO	dist=NONE	.	.	.	.	.	.	.
NM_00103	.	.	.	ENST0000	.	20p12.1	.	.
NM_00125	.	.	.	ENST0000	.	17p11.2	.	.
NM_00125	.	.	.	ENST0000	.	1p34.2	.	.
NM_13916	.	frameshift	SGCZ:NM_	ENST0000	.	8p22	.	.

NM_00124	.	frameshift	ENTPD2:N	ENST0000	.	9q34.3	.	.
NM_00104	.	.	.	ENST0000	.	11q25	.	.
NM_00125	.	.	.	ENST0000	.	14q24.1	.	.
NM_00565	NM_00565	.	.	ENST0000	.	22q13.2	.	.
NM_05292	.	.	.	ENST0000	.	2p24.1	.	.
NM_02291	dist=15878	.	.	ENST0000	.	5q11.2	.	.
NM_00124	.	.	.	ENST0000	.	9q34.11	.	.
NM_00373	.	nonframes	DCHS1:NM	ENST0000	.	11p15.4	.	.
NM_00178	.	.	.	ENST0000	.	12p13.31	.	.
NM_00113	.	.	.	ENST0000	.	13q32.3	.	.
NM_00104	.	frameshift	SPATA7:N	ENST0000	.	14q31.3	.	.
NM_00175	NM_00175	.	.	ENST0000	.	16q22.1	.	.
NM_00120	.	.	.	ENST0000	.	17p12	.	.
NM_00114	NM_00114	.	.	ENST0000	.	17q11.2	.	.
NM_03058	.	nonframes	COL18A1:	ENST0000	.	21q22.3	.	.
NR_00360	dist=10462	.	.	ENST0000	.	22q11.1	.	.
NM_00289	.	.	.	ENST0000	.	Xp22.2	.	Score=911
NM_00127	.	nonframes	ZNF316:NI	ENST0000	.	7p22.1	.	.
NM_00114	.	.	.	ENST0000	.	8q23.1	.	.
NM_00116	.	.	.	ENST0000	.	11q22.2	.	.
NM_00103	NM_00103	.	.	ENST0000	.	16q22.1	.	.
NM_00481	NM_00481	.	.	ENST0000	.	19q13.32	.	.
NM_00109	.	frameshift	ZNF419:NI	ENST0000	.	19q13.43	.	.
NR_07339	.	.	.	ENST0000	.	Xp11.23	.	.
NM_00128	.	.	.	ENST0000	.	Xq22.3	.	.
NM_00494	.	frameshift	DOCK3:NM	ENST0000	.	3p21.2	.	.
NM_00100	.	frameshift	NFKBIZ:NI	ENST0000	.	3q12.3	.	.
NM_02021	.	.	.	ENST0000	.	15q26.1	.	.

genomicSu	Repeat	avsnp150	cosmic82	clinvar_20	gwasCatal	1000g2015	1000g2015	1000g2015
.	.	.	.	.	.	.	.	.
.	.	.	ID=COSM1	.	.	.	.	.
.	Score=118	rs6078884	.	.	.	.	.	.
Score=0.9;	.	.	.	.	.	.	.	.
.	.	rs3773783	.	.	.	.	.	.
.	.	.	.	.	.	.	.	.
.	.	.	.	.	.	.	.	.
Score=0.9	Score=239	.	.	.	.	.	.	.
.	.	.	.	.	.	.	.	.
.	Score=344	rs7821786	.	.	.	.	0.0039	0.001589
.	.	.	.	.	.	.	.	.
.	.	rs9190283	.	.	.	.	.	.
.	.	.	.	.	.	.	.	.
.	Score=561	rs7640343	.	.	.	.	.	.
.	.	rs5844903	.	.	.	.	0.1448	0.153754
.	.	.	.	.	.	.	.	.
.	.	.	.	.	.	.	.	.
.	.	.	.	.	.	.	.	.
.	.	.	ID=COSN1	.	.	.	.	.
Score=0.9	.	.	.	.	.	.	.	.
Score=0.9	Score=446	.	.	.	.	.	.	.
.	Score=366	.	.	.	.	.	.	.
.	.	rs7760984	.	.	.	.	.	.
.	.	rs9376078	ID=COSN2	.	.	.	.	.
.	.	.	.	.	.	.	.	.
.	.	rs7790390	.	.	.	.	.	.
.	.	.	.	.	.	.	.	.
.	.	.	.	.	.	.	.	.
.	.	.	ID=COSM1	.	.	.	.	.
.	.	.	.	.	.	.	.	.
.	.	.	.	.	.	.	.	.
.	.	.	.	.	.	.	.	.
.	.	rs7712392	ID=COSN5	.	.	.	.	.
.	.	rs7686883	.	.	.	.	.	.
.	.	.	.	.	.	.	.	.
.	.	rs7700977	ID=COSN2	.	.	.	.	.
.	.	.	.	.	.	.	.	.
.	.	.	.	.	.	.	.	.
.	.	.	ID=COSM5	.	.	.	.	.
.	.	.	.	.	.	.	.	.
.	.	.	ID=COSM1	.	.	.	.	.
.	.	.	.	.	.	.	.	.
.	.	.	.	.	.	.	.	.
.	.	rs7584166	ID=COSM5	.	.	.	.	.
.	.	rs7537986	ID=COSN1	.	.	.	.	.
.	.	rs5395449	.	.	.	.	.	0.000599
.	.	.	ID=COSM2	.	.	.	.	.
.	.	rs7594247	ID=COSM3	.	.	.	.	.
.	.	rs7703770	.	.	.	.	.	.
.	.	.	.	.	.	.	.	.
.	Score=390	rs7968975	.	.	.	.	.	.

.	.	.	ID=COSM3	.	.	.	.	.
.	.	rs7808912	.	.	.	.	.	.
.	Score=625	.	.	.	.	.	.	.
Score=0.92	.	rs1997131	ID=COSN1	.	.	.	0.005	0.001597
.	.	.	.	.	.	.	.	.
.	.	rs7703599	ID=COSM3	.	.	.	.	.
.	.	rs7789371	ID=COSM2	.	.	.	.	.
.	Score=121	.	.	.	.	.	.	.
.	.	.	ID=COSN2	.	.	.	.	.
.	.	.	.	.	.	.	.	.
.	.	rs7647197	ID=COSM2	.	.	.	.	.
.	.	.	.	.	.	.	.	.
.	.	.	.	.	.	.	.	.
.	.	rs7456483	ID=COSM1	.	.	.	.	.
.	.	.	.	.	.	.	.	.
.	.	.	ID=COSN2	.	.	.	.	.
.	.	rs7470964	.	.	.	.	.	.
.	.	.	.	.	.	.	.	.
.	.	.	.	.	.	.	.	.
.	.	.	ID=COSM3	.	.	.	.	.
.	.	.	.	.	.	.	.	.
.	.	.	ID=COSM4	.	.	.	.	.
.	.	.	.	.	.	.	.	.
.	.	.	.	.	.	.	.	.
.	.	.	.	.	.	.	.	.
.	.	.	ID=COSM5	.	.	.	.	.
Score=0.92	.	rs5440185	ID=COSN2	.	.	.	.	0.00619
.	.	.	.	.	.	.	.	.
.	.	.	ID=COSM4	.	.	.	.	.
.	.	rs9042896	.	.	.	.	.	.
.	.	rs7608755	ID=COSN1	.	.	.	.	.
.	.	.	.	.	.	.	.	.
.	.	.	.	.	.	.	.	.
.	.	rs7647092	ID=COSM5	.	.	.	.	.
.	.	.	.	.	.	.	.	.
.	.	.	ID=COSM1	.	.	.	.	.
.	.	.	.	.	.	.	.	.
.	.	.	.	.	.	.	.	.
.	.	.	.	.	.	.	.	.
.	.	rs7755820	.	.	.	.	.	.
.	.	.	.	.	.	.	.	.
.	.	.	.	.	.	.	.	.
.	.	.	.	.	.	.	.	.
.	.	.	ID=COSN1	.	.	.	.	.
.	.	.	.	.	.	.	.	.
.	.	rs7704742	.	.	.	.	.	.
.	.	rs7480610	.	.	.	.	.	.
.	.	rs7646973	ID=COSN2	.	.	.	.	.
.	.	rs9281864	.	.	.	.	.	.
.	.	.	.	.	.	.	.	.
.	.	rs7525252	.	.	.	.	.	.
.	.	.	.	.	.	.	.	.
.	.	.	.	.	.	.	.	.
.	.	.	ID=COSN2	.	.	.	.	.

.	.	.	.	.	.	.	.	.
.	Score=234	rs7641185	ID=COSM1	.	.	.	.	.
.	.	rs7639462	.	.	.	.	.	.
.	.	.	ID=COSM4	.	.	.	.	.
Score=0.99	.	.	.	.	.	.	.	.
Score=0.95	.	.	.	.	.	.	.	.
.	.	rs7466549	ID=COSM1	.	.	.	.	.
.	.	rs7717298	.	.	.	.	.	.
.	.	.	.	.	.	.	.	.
.	.	.	.	.	.	.	.	.
.	.	rs7625018	ID=COSN2	.	.	.	.	.
.	.	rs7658478	.	.	.	.	.	.
.	.	.	.	.	.	.	.	.
.	.	.	.	.	.	.	.	.
.	.	rs7778584	.	.	.	.	.	.
.	.	rs7693384	ID=COSM2	.	.	.	.	.
.	.	.	.	.	.	.	.	.
.	.	rs7768326	.	.	.	.	.	.
.	.	rs3743843	.	.	.	.	0.003	0.000599
Score=0.92	.	.	.	.	.	.	.	.
.	.	rs7666007	.	.	.	.	.	.
.	.	rs7805014	.	.	.	.	.	.
Score=0.97	.	rs7489955	ID=COSN2	.	.	.	.	.
.	.	rs7612855	.	.	.	.	.	.
.	.	rs7600208	ID=COSM3	.	.	.	.	.
.	.	.	ID=COSM5	.	.	.	.	.
Score=0.92	.	.	ID=COSN1	.	.	.	.	.
.	.	.	.	.	.	.	.	.
.	.	rs7792033	.	.	.	.	.	.
.	.	rs7668254	ID=COSM4	.	.	.	.	.
.	.	.	ID=COSM1	.	.	.	.	.
.	.	.	.	.	.	.	.	.
.	.	.	.	.	.	.	.	.
.	.	rs1490463	.	.	.	.	.	.
.	.	.	ID=COSM3	.	.	.	.	.
Score=0.95	.	rs7570813	.	.	.	.	.	.
.	.	rs7468588	ID=COSM3	.	.	.	.	.
.	.	.	.	.	.	.	.	.
.	.	.	.	.	.	.	.	.
Score=0.99	Score=321	.	.	.	.	.	.	.
.	.	rs1443745	.	.	.	.	.	.
.	.	.	.	.	.	.	.	.
.	.	rs7542333	ID=COSM5	.	.	.	.	.
.	.	rs3701666	ID=COSM1	.	.	.	.	.
Score=0.89	.	.	.	.	.	.	.	.
.	.	rs7470634	ID=COSM1	.	.	.	.	.
.	.	.	.	.	.	.	.	.
.	.	.	.	.	.	.	.	.
Score=0.89	.	rs7819467	.	.	.	.	.	.
.	Score=217	.	.	.	.	.	.	.
.	.	.	.	.	.	.	.	.
.	.	.	.	.	.	.	.	.
.	.	.	.	.	.	.	.	.
.	.	rs5592025	.	.	.	.	0.004	0.000799

.	.	.	.	.	.	.	.	.
.	.	rs7594121	.	.	.	.	.	.
Score=0.92	Score=568	rs7708715	.	.	.	.	.	.
.	.	.	.	.	.	.	.	.
.	.	rs7618495	.	.	.	.	.	.
.	Score=232	.	.	.	.	.	.	.
.	.	rs7790763	.	.	.	.	.	.
.	.	.	.	.	.	.	.	.
.	.	rs1126292	.	.	.	.	0.0198	0.04972
.	.	rs3832550	.	.	.	.	.	.
.	.	.	.	.	.	.	.	.
.	.	.	ID=COSN5	.	.	.	.	.
.	.	rs7782900	ID=COSN2	.	.	.	.	.
.	.	rs7759500	ID=COSM6	.	.	.	.	.
.	.	.	.	.	.	.	.	.
.	Score=198	rs1462533	.	.	.	.	.	.
.	.	.	.	.	.	.	.	.
.	.	rs7562540	ID=COSM1	.	.	.	.	.
.	.	rs7648900	.	.	.	.	.	.
.	.	rs7766165	ID=COSN5	.	.	.	.	.
.	.	.	ID=COSM1	.	.	.	.	.
Score=0.98	.	rs5303481	.	.	.	.	0.002	0.000599
.	.	.	ID=COSM1	.	.	.	.	.
.	.	.	ID=COSM1	.	.	.	.	.
.	.	.	.	.	.	.	.	.
.	Score=261	rs7606317	.	.	.	.	.	.
.	.	rs7792498	ID=COSM1	.	.	.	.	.
.	.	rs1028985	.	.	.	.	.	.
.	.	rs7703285	.	.	.	.	.	.
.	.	rs3675892	.	.	.	.	0.003	0.000599
.	Score=402	.	.	.	.	.	.	.
.	.	.	.	.	.	.	.	.
.	.	.	.	.	.	.	.	.
.	.	rs7517698	.	.	.	.	.	.
.	.	.	ID=COSM4	.	.	.	.	.
.	.	.	.	.	.	.	.	.
.	.	rs7625775	ID=COSN2	.	.	.	.	.
.	.	.	.	.	.	.	.	.
Score=0.99	.	.	ID=COSN1	.	.	.	.	.
.	.	rs7582351	.	.	.	.	.	.
.	.	.	.	.	.	.	.	0.008387
.	.	rs7542104	.	.	.	.	.	.
.	.	rs7696631	ID=COSM1	.	.	.	.	.
.	.	.	ID=COSN1	.	.	.	.	.
.	.	rs7488695	.	.	.	.	.	.
Score=0.97	.	rs7664186	.	.	.	.	.	.
.	.	rs7585686	.	.	.	.	.	.
.	.	.	.	.	.	.	.	.
.	.	rs5534658	.	.	.	.	0.0069	0.002196
.	.	.	.	.	.	.	.	.
Score=0.97	.	.	.	.	.	.	.	.
.	.	.	ID=COSM4	.	.	.	.	.
.	.	rs7518721	ID=COSM5	.	.	.	.	.
.	.	rs9766384	.	.	.	.	.	.

.	.	.	.	.	.	.	.	.
.	.	.	.	.	.	.	.	.
.	.	rs7632273	.	.	.	.	.	.
.	.	rs7778008	.	.	.	.	.	.
.	.	rs1049395	.	.	.	.	.	.
.	.	.	.	.	.	.	.	.
.	.	.	ID=COSM1	.	.	.	.	.
Score=0.92	.	.	ID=COSN2	.	.	.	.	.
.	.	rs7588677	.	.	.	.	.	.
Score=0.93	.	rs8987047	.	.	.	.	.	.
.	.	rs7647351	ID=COSM1	.	.	.	.	.
.	.	.	.	.	.	.	.	.
.	.	.	ID=COSM6	.	.	.	.	.
.	.	rs5739889	ID=COSN2	.	.	.	.	0.000799
.	.	rs7766671	.	.	.	.	.	.
.	.	rs7749641	ID=COSM1	.	.	.	.	.
.	.	rs7666743	.	.	.	.	.	.
.	Score=214	.	.	.	.	.	.	.
.	.	.	.	.	.	.	.	.
.	.	.	.	.	.	.	.	.
.	.	.	.	.	.	.	.	.
.	.	rs7566997	.	.	.	.	.	.
Score=0.93	.	rs7649880	.	.	.	.	.	.
Score=0.93	Score=200	.	.	.	.	.	.	.
.	.	.	.	.	.	.	.	.
.	.	.	.	.	.	.	.	.
.	.	rs7636694	.	.	.	.	.	.
.	.	.	.	.	.	.	.	.
.	Score=216	.	.	.	.	.	.	.
.	.	.	.	.	.	.	.	.
.	.	.	.	.	.	.	.	.
.	.	rs5532896	.	.	.	.	.	0.008986
.	.	.	.	.	.	.	.	.
.	.	rs7706124	ID=COSM1	.	.	.	.	.
.	.	rs5359317	.	.	.	.	.	.
.	.	rs7763171	ID=COSM1	.	.	.	.	.
.	.	rs1999629	.	.	.	.	.	.
.	.	.	.	.	.	.	.	.
.	.	.	.	.	.	.	.	.
.	.	rs7643750	ID=COSM1	.	.	.	.	.
.	Score=481	.	.	.	.	.	.	.
.	.	rs7166969	.	.	.	.	.	0.026957
.	.	.	.	.	.	.	.	.
.	.	.	ID=COSM1	.	.	.	.	.
.	.	rs1134746	.	.	.	.	0.003	0.103235
Score=0.94	.	rs5526989	.	.	.	.	.	.
.	.	.	.	.	.	.	.	.
.	.	.	ID=COSN2	.	.	.	.	.
.	.	.	.	.	.	.	.	.
.	.	rs1451472	ID=COSM1	.	.	.	.	.
.	Score=215	.	.	.	.	.	.	.
.	.	rs7643803	.	.	.	.	.	.
.	.	.	ID=COSM4	.	.	.	.	.
.	.	.	.	.	.	.	.	.

.	.	rs5635626	ID=COSN2	.	.	.	0.001	0.000799
.	.	rs7515595	.	.	.	.	.	.
.	.	rs5561116	ID=COSN1	.	.	.	.	0.000799
.	.	.	.	.	.	.	.	.
.	.	.	.	.	.	.	.	.
.	.	rs7643461	ID=COSM4	.	.	.	.	.
.	.	.	.	.	.	.	.	.
.	.	.	ID=COSM2	.	.	.	.	.
.	.	.	.	.	.	.	.	.
.	.	rs7781350	.	.	.	.	.	.
.	.	rs7809656	.	.	.	.	.	.
.	.	rs7491504	ID=COSM2	.	.	.	.	.
.	Score=119	.	.	.	.	.	.	.
Score=0.97	.	.	.	.	.	.	.	.
.	.	.	.	.	.	.	.	.
.	.	rs7570296	.	.	.	.	.	.
.	.	.	.	.	.	.	.	.
.	.	rs7817135	ID=COSM1	.	.	.	.	.
.	.	rs7679025	ID=COSM1	.	.	.	.	.
.	.	.	.	.	.	.	.	.
.	.	.	.	.	.	.	.	.
.	.	.	.	.	.	.	.	.
.	.	.	.	.	.	.	.	.
.	.	.	.	.	.	.	.	.
.	.	rs7574964	ID=COSM1	.	.	.	.	.
.	.	.	ID=COSM4	.	.	.	.	.
.	.	rs5285990	ID=COSN2	.	.	.	.	0.002796
.	Score=276	.	.	.	.	.	.	.
.	.	.	ID=COSM2	.	.	.	.	.
.	.	.	.	.	.	.	.	.
Score=0.94	.	.	.	.	.	.	.	.
.	.	.	.	.	.	.	.	.
.	.	.	.	.	.	.	.	.
.	.	rs1380173	.	.	.	.	.	0.026558
.	.	.	ID=COSN1	.	.	.	.	.
.	.	.	.	.	.	.	.	.
.	.	.	.	.	.	.	.	.
.	.	rs7634794	.	.	.	.	.	.
.	.	.	.	.	.	.	.	.
.	.	rs7487058	.	.	.	.	.	.
.	.	rs1429731	ID=COSN1	Likely beni	.	.	.	.
.	.	rs7500268	.	.	.	.	.	.
.	.	.	.	.	.	.	.	.
.	.	.	.	.	.	.	.	.
.	.	rs7661354	.	.	.	.	.	.
.	.	.	.	.	.	.	.	.
.	.	rs5710814	.	.	.	.	.	.
.	Score=235	rs1019291	.	.	.	.	.	.
.	.	rs7630535	ID=COSM4	.	.	.	.	.
.	Score=346	.	.	.	.	.	.	.
.	Score=23;I	.	.	.	.	.	.	.
.	.	rs7644591	ID=COSM4	.	.	.	.	.
.	.	.	.	.	.	.	.	.
.	Score=856	.	.	.	.	.	.	.

.	.	.	.	.	.	.	.	.
.	.	rs7590798	.	.	.	.	.	.
.	.	.	.	.	.	.	.	.
.	.	rs7708014	ID=COSN2	.	.	.	.	.
.	.	.	.	.	.	.	.	.
.	.	.	.	.	.	.	.	.
.	Score=620	.	.	.	.	.	.	.
.	.	.	.	.	.	.	.	.
.	.	rs7799007	ID=COSM1	.	.	.	.	.
.	Score=225	rs7677659	.	.	.	.	.	.
.	Score=289	.	.	.	.	.	.	.
.	.	.	.	.	.	.	.	.
.	.	.	ID=COSM3	.	.	.	.	.
.	.	rs9414440	ID=COSM6	.	.	.	.	.
.	.	.	.	.	.	.	.	.
.	.	.	.	.	.	.	.	.
.	.	.	.	.	.	.	.	.
.	.	rs7662018	.	.	.	.	.	.
.	.	.	.	.	.	.	.	.
.	.	.	.	.	.	.	.	.
.	.	rs7520653	.	.	.	.	.	.
.	.	.	.	.	.	.	.	.
.	.	.	.	.	.	.	.	.
.	.	rs7555061	ID=COSM1	.	.	.	.	.
.	.	rs3981228	ID=COSM1	Pathogenic	.	.	.	.
.	.	.	.	.	.	.	.	.
.	.	.	.	.	.	.	.	.
.	.	rs7666986	.	.	.	.	.	.
.	.	.	.	.	.	.	.	.
.	.	.	.	.	.	.	.	.
.	.	rs7811378	ID=COSM1	.	.	.	.	.
.	.	.	.	.	.	.	.	.
.	.	.	.	.	.	.	.	.
.	.	rs7575646	ID=COSN2	.	.	.	.	.
.	.	rs3605176	.	.	.	.	.	.
.	.	.	.	.	.	.	.	.
.	.	rs1085307	ID=COSM4	Pathogenic	.	.	.	.
.	.	rs7539299	.	.	.	.	.	.
.	.	.	.	.	.	.	.	.
.	.	rs7511829	ID=COSM3	.	.	.	.	.
.	.	rs7684989	.	.	.	.	.	.
.	.	rs7667143	ID=COSM2	.	.	.	.	.
.	.	rs9359712	ID=COSN1	.	.	.	.	.
.	.	.	.	.	.	.	.	.
.	Score=308	rs3687317	ID=COSN2	.	.	.	.	.
.	.	rs7538174	.	.	.	.	.	.
.	.	rs7755389	ID=COSM4	.	.	.	.	.
Score=0.9	.	rs2001464	ID=COSN1	.	.	.	0.0794	0.049121
Score=0.9	.	.	.	.	.	.	.	.
.	Score=241	.	.	.	.	.	.	.
.	.	.	.	.	.	.	.	.
.	.	rs7686708	ID=COSN1	.	.	.	.	.
.	.	.	.	.	.	.	.	.
Score=0.9	.	.	.	.	.	.	.	.

.	.	rs7743082	.	.	.	.	.	.
.	.	.	.	.	.	.	.	.
Score=0.9	.	.	ID=COSM	.	.	.	.	.
.	.	rs7631509	.	.	.	.	.	.
.	Score=22;	.	.	.	.	.	.	.
.	.	rs7752182	.	.	.	.	.	.
.	.	rs7515575	ID=COSN	.	.	.	.	.
.	.	rs7821251	.	.	.	.	.	.
.	.	.	.	.	.	.	.	.
.	.	.	.	.	.	.	.	.
.	.	.	ID=COSM	.	.	.	.	.
.	.	rs1064795	.	Likely beni	.	.	.	.
.	.	rs7823430	ID=COSM	.	.	.	.	.
.	.	.	.	.	.	.	.	.
.	.	.	.	.	.	.	.	.
.	.	.	.	.	.	.	.	.
.	.	.	.	.	.	.	.	.
.	.	rs7485648	.	.	.	.	.	.
.	.	.	.	.	.	.	.	.
.	.	.	.	.	.	.	.	.
.	.	rs9344233	.	.	.	.	.	.
Score=0.9	.	rs4036589	ID=COSN1	.	.	.	.	.
.	.	rs1062553	.	.	.	.	0.3085	0.441094
.	Score=207	.	.	.	.	.	.	.
.	.	rs3707030	.	.	.	.	.	.
.	Score=204	.	.	.	.	.	.	.
.	.	.	.	.	.	.	.	.
.	.	.	.	.	.	.	.	.
.	Score=28;	.	.	.	.	.	.	.
.	.	.	.	.	.	.	.	.
.	Score=275	rs8860593	.	Uncertain s	.	.	.	.
.	.	.	.	.	.	.	.	.
Score=0.9	.	.	.	.	.	.	.	.
.	.	rs9488701	ID=COSM	.	.	.	.	.
.	.	rs1047246	.	.	.	.	.	.
Score=0.9	.	.	.	.	.	.	.	.
.	.	.	.	.	.	.	.	.
.	.	.	.	.	.	.	.	.
.	.	.	.	.	.	.	.	.
.	.	.	.	.	.	.	.	.
.	.	.	.	.	.	.	.	.
.	.	.	.	.	.	.	.	.
.	Score=405	rs3980370	.	.	.	.	0.2173	0.234625
.	.	.	.	.	.	.	.	.
.	Score=192	rs7562167	.	.	.	.	.	.
.	Score=366	rs7459278	.	.	.	.	.	.
.	.	.	.	.	.	.	.	.
.	.	.	.	.	.	.	.	.
.	.	.	.	.	.	.	.	.
.	.	rs2676066	.	Pathogenic	.	.	.	.
.	Score=32;	.	.	.	.	.	.	.
.	.	.	.	.	.	.	.	.
.	.	rs7519299	.	.	.	.	.	.
.	.	.	.	.	.	.	.	.

.	.	.	.	.	.	.	.	.
.	.	.	.	.	.	.	.	.
.	.	.	.	.	.	.	.	.
.	Score=210	rs7666083	.	.	.	.	.	.
.	.	.	.	.	.	.	.	.
.	.	.	.	.	.	.	.	.
.	Score=255	.	.	.	.	.	.	.
.	Score=240	rs7788921	.	.	.	.	.	.
.	.	.	.	.	.	.	.	.
.	Score=224	rs7743913	.	.	.	.	.	.
.	.	rs7758009	.	.	.	.	.	.
.	.	.	ID=COSM5	.	.	.	.	.
.	Score=917	.	.	.	.	.	.	.
.	Score=22;I	.	.	.	.	.	.	.
.	.	rs7645628	.	.	.	.	.	.
.	.	.	.	.	.	.	.	.
.	Score=51;I	.	.	.	.	.	.	.
.	Score=450	.	.	.	.	.	.	.
.	.	.	.	.	.	.	.	.
.	Score=207	rs6015769	ID=COSN1	.	.	.	0.7262	0.701877
Score=0.94	.	rs7596499	.	.	.	.	.	.
.	Score=180	.	.	.	.	.	.	.
Score=0.92	.	.	.	.	.	.	.	.
Score=0.95	.	.	.	.	.	.	.	.
.	.	.	.	.	.	.	.	.
.	.	.	.	.	.	.	.	.
.	.	.	.	.	.	.	.	.
.	.	.	.	.	.	.	.	.

esp6500siv	ExAC_ALL	ExAC_EAS	SIFT	Polyphen2_	Polyphen2_	MutationTa	gerp++gt2	CADD
.	.	.	.	.	.	.	.	.
.	.	.	.	.	.	.	.	.
.	.	.	.	.	.	.	.	.
.	.	.	.	.	.	.	.	.
0.0068	0.0017	0.0004	.	.	.	.	.	-0.015565,
.	.	.	.	.	.	.	.	.
.	.	.	.	.	.	.	.	.
.	.	.	.	.	.	.	.	.
.	.	.	.	.	.	.	.	.
.	.	.	.	.	.	.	.	-0.171358,
.	.	.	.	.	.	.	.	.
.	.	.	.	.	.	.	.	.
.	.	.	.	.	.	.	.	.
.	0.1944	0.069	.	.	.	.	.	0.721048,7
.	.	.	.	.	.	.	.	0.640624,7
.	.	.	.	.	.	.	.	.
.	.	.	.	.	.	.	.	.
.	.	.	.	.	.	.	.	.
.	.	.	.	.	.	.	.	.
.	.	.	.	.	.	.	.	.
.	.	.	.	.	.	.	.	.
.	.	.	.	.	.	.	.	.
.	.	.	.	.	.	.	.	.
.	.	.	.	.	.	.	.	.
.	.	.	.	.	.	.	.	.
.	.	.	.	.	.	.	.	-0.055266,
.	.	.	.	.	.	.	.	.
.	.	.	.	.	.	.	.	.
.	.	.	.	.	.	.	.	8.456148,4
.	.	.	.	.	.	.	.	.
.	.	.	.	.	.	.	.	.
.	.	.	.	.	.	.	.	.
0.0021	0.0001	0	.	.	.	.	.	3.824671,1
.	0.0005	0.0003	.	.	.	.	.	.
.	.	.	.	.	.	.	.	.
0.0011	8.25E-06	0.0001	.	.	.	.	.	1.220399,9
.	.	.	.	.	.	.	.	.
.	.	.	.	.	.	.	.	.
.	.	.	.	.	.	.	.	.
.	.	.	.	.	.	.	.	.
.	.	.	.	.	.	.	.	.
.	.	.	.	.	.	.	.	0.318819,5
.	.	.	.	.	.	.	.	.
0.0015	0.0005	0	.	.	.	.	.	3.318747,1
.	2.22E-05	0	.	.	.	.	.	.
0.0124	0.0002	0	.	.	.	.	.	-0.262035,
.	.	.	.	.	.	.	.	.
0.0007	0.0003	0.0007	.	.	.	.	.	0.831281,8
.	7.87E-05	0	.	.	.	.	.	.
.	.	.	.	.	.	.	.	.
.	.	.	.	.	.	.	.	.

.	.	.	.	.	.	.	.	.
.	0.0007	0	.	.	.	.	.	.
.	.	.	.	.	.	.	.	.
.	.	.	.	.	.	.	.	-0.366243,
.	.	.	.	.	.	.	.	-0.344064,
.	2.47E-05	0	.	.	.	.	.	.
0.0042	0.0033	0.0002	.	.	.	.	.	2.599557,1
.	.	.	.	.	.	.	.	.
.	.	.	.	.	.	.	.	.
.	.	.	.	.	.	.	.	.
.	3.31E-05	0	.	.	.	.	.	.
.	.	.	.	.	.	.	.	.
.	.	.	.	.	.	.	.	.
.	2.50E-05	0	.	.	.	.	.	.
.	.	.	.	.	.	.	.	.
.	.	.	.	.	.	.	.	.
.	0.0003	0	.	.	.	.	.	.
.	.	.	.	.	.	.	.	.
.	.	.	.	.	.	.	.	.
.	.	.	.	.	.	.	.	.
.	.	.	.	.	.	.	.	-0.745937,
.	.	.	.	.	.	.	.	.
.	.	.	.	.	.	.	.	.
.	.	.	.	.	.	.	.	.
.	.	.	.	.	.	.	.	.
.	.	.	.	.	.	.	.	.
0.0068	0.0019	0.0002	.	.	.	.	.	-1.033226,
.	.	.	.	.	.	.	.	.
.	.	.	.	.	.	.	.	.
.	.	.	.	.	.	.	.	.
.	3.49E-05	0	.	.	.	.	.	.
.	.	.	.	.	.	.	.	.
.	.	.	.	.	.	.	.	.
.	4.13E-05	0	.	.	.	.	.	2.045653,1
.	.	.	.	.	.	.	.	.
.	.	.	.	.	.	.	.	.
.	.	.	.	.	.	.	.	.
.	.	.	.	.	.	.	.	.
.	.	.	.	.	.	.	.	.
.	3.30E-05	0	.	.	.	.	.	.
.	.	.	.	.	.	.	.	.
.	.	.	.	.	.	.	.	.
.	.	.	.	.	.	.	.	.
.	.	.	.	.	.	.	.	.
.	.	.	.	.	.	.	.	.
8.00E-05	4.14E-05	0	.	.	.	.	.	6.272893,3
.	0.0013	0	.	.	.	.	.	.
.	7.43E-05	0	.	.	.	.	.	0.215872,5
.	.	.	.	.	.	.	.	.
.	.	.	.	.	.	.	.	.
.	6.67E-05	0	.	.	.	.	.	.
.	.	.	.	.	.	.	.	.
.	.	.	.	.	.	.	.	.
.	.	.	.	.	.	.	.	.

.	.	.	.	.	.	.	.	.
0.0018	0.0002	0.0001	.	.	.	.	.	3.925528,1
.	3.31E-05	0	.	.	.	.	.	.
.	.	.	.	.	.	.	.	.
.	.	.	.	.	.	.	.	.
.	.	.	.	.	.	.	.	.
0.0062	0.0003	0.0003	.	.	.	.	.	12.104388
.	.	.	.	.	.	.	.	0.699566,7
.	.	.	.	.	.	.	.	.
.	.	.	.	.	.	.	.	.
.	2.54E-05	0	.	.	.	.	.	.
.	0.0003	0.0002	.	.	.	.	.	.
.	.	.	.	.	.	.	.	.
.	.	.	.	.	.	.	.	.
.	8.29E-06	0	.	.	.	.	.	.
.	0.0018	0.0003	.	.	.	.	.	3.668062,1
.	.	.	.	.	.	.	.	.
.	0.0013	0.0003	.	.	.	.	.	-0.354890,
.	.	.	.	.	.	.	.	-0.012894,
.	.	.	.	.	.	.	.	.
.	3.91E-05	0.0005	.	.	.	.	.	.
.	8.60E-06	0	.	.	.	.	.	.
0.0108	0.0132	0.0002	.	.	.	.	.	0.779711,8
.	8.55E-06	0	.	.	.	.	.	.
.	0.0014	0.0002	.	.	.	.	.	2.174337,1
.	.	.	.	.	.	.	.	.
.	.	.	.	.	.	.	.	.
.	.	.	.	.	.	.	.	.
.	0.0003	0	.	.	.	.	.	.
.	1.67E-05	0	.	.	.	.	.	.
.	.	.	.	.	.	.	.	.
.	.	.	.	.	.	.	.	.
8.00E-05	.	.	.	.	.	.	.	4.835186,2
.	.	.	.	.	.	.	.	0.253508,5
.	.	.	.	.	.	.	.	.
.	0.0027	0	.	.	.	.	.	.
.	2.51E-05	0	.	.	.	.	.	.
.	.	.	.	.	.	.	.	.
.	.	.	.	.	.	.	.	.
.	.	.	.	.	.	.	.	-0.792134,
.	.	.	.	.	.	.	.	0.066003,4
.	.	.	.	.	.	.	.	.
.	0.0011	0	.	.	.	.	.	7.541577,3
.	0.0002	0	.	.	.	.	.	0.414848,6
.	.	.	.	.	.	.	.	.
.	7.45E-05	0	.	.	.	.	.	.
.	.	.	.	.	.	.	.	.
.	.	.	.	.	.	.	.	.
.	8.41E-06	0	.	.	.	.	.	.
.	.	.	.	.	.	.	.	.
.	.	.	.	.	.	.	.	.
.	.	.	.	.	.	.	.	.
.	.	.	.	.	.	.	.	.
.	0.0006	0.0026	.	.	.	.	.	-0.337970,

.	.	.	.	.	.	.	.	.
.	0.0075	0.0013	.	.	.	.	.	1.449740,1
.	0.0042	0.0001	.	.	.	.	.	-0.171168,
.	.	.	.	.	.	.	.	.
.	1.66E-05	0	.	.	.	.	.	.
.	.	.	.	.	.	.	.	.
.	0.0012	0	.	.	.	.	.	0.332589,5
.	.	.	.	.	.	.	.	.
.	.	.	.	.	.	.	.	-1.012315,
0.0011	0.0002	.	.	.	.	.	.	3.590050,1
.	.	.	.	.	.	.	.	.
.	.	.	.	.	.	.	.	.
.	0.0006	0	.	.	.	.	.	.
.	8.28E-06	0	.	.	.	.	.	.
.	.	.	.	.	.	.	.	.
.	.	.	.	.	.	.	.	0.781695,8
.	.	.	.	.	.	.	.	.
.	0.0003	0	.	.	.	.	.	.
.	8.31E-06	0	.	.	.	.	.	.
.	0.0005	0.0004	.	.	.	.	.	.
.	.	.	.	.	.	.	.	.
.	.	.	.	.	.	.	.	.
.	.	.	.	.	.	.	.	.
.	.	.	.	.	.	.	.	.
0.0076	.	.	.	.	.	.	.	0.848277,8
0.0061	0.0384	0.1569	.	.	.	.	.	-0.953411,
.	9.89E-05	0.001	.	.	.	.	.	4.370603,2
.	.	.	.	.	.	.	.	2.454346,1
.	4.37E-05	0	.	.	.	.	.	.
.	.	.	.	.	.	.	.	-0.201835,
.	.	.	.	.	.	.	.	.
.	.	.	.	.	.	.	.	.
.	.	.	.	.	.	.	.	.
.	1.38E-05	0	.	.	.	.	.	.
.	.	.	.	.	.	.	.	.
.	.	.	.	.	.	.	.	.
.	9.35E-05	0	.	.	.	.	.	.
.	.	.	.	.	.	.	.	.
.	.	.	.	.	.	.	.	.
8.80E-05	0.0002	0.0005	.	.	.	.	.	-0.750302,
0.0086	0.0025	0.0001	.	.	.	.	.	0.948278,8
.	8.28E-06	.	.	.	.	.	.	.
0.0043	0.0031	0.0005	.	.	.	.	.	5.924892,3
.	.	.	.	.	.	.	.	.
.	1.66E-05	0.0001	.	.	.	.	.	.
.	8.28E-06	0	.	.	.	.	.	-0.266141,
.	2.62E-05	0	.	.	.	.	.	.
.	.	.	.	.	.	.	.	.
0.005	0.0046	0.0122	.	.	.	.	.	0.334116,5
.	.	.	.	.	.	.	.	.
.	.	.	.	.	.	.	.	.
0.0021	.	.	.	.	.	.	.	1.247081,1
0.0175	0.0006	0.0004	.	.	.	.	.	1.443171,1
.	.	.	.	.	.	.	.	1.184019,9

.	.	.	.	.	.	.	.	.
.	.	.	.	.	.	.	.	.
0.0003	0.0037	0.0036	.	.	.	.	.	1.940664,1
.	7.67E-05	0	.	.	.	.	.	.
.	.	.	.	.	.	.	.	.
.	.	.	.	.	.	.	.	.
.	.	.	.	.	.	.	.	.
.	.	.	.	.	.	.	.	-1.180968,
.	1.66E-05	0	.	.	.	.	.	.
.	.	.	.	.	.	.	.	.
0.0159	0.0016	0	.	.	.	.	.	.
.	.	.	.	.	.	.	.	.
.	.	.	.	.	.	.	.	.
0.0031	0.0006	0.0018	.	.	.	.	.	0.076681,4
8.00E-05	8.24E-06	0	.	.	.	.	.	2.368741,1
.	1.66E-05	0	.	.	.	.	.	.
.	0.0004	0	.	.	.	.	.	.
.	.	.	.	.	.	.	.	.
.	.	.	.	.	.	.	.	.
.	.	.	.	.	.	.	.	.
.	.	.	.	.	.	.	.	.
.	9.44E-06	0	.	.	.	.	.	.
.	2.54E-05	0	.	.	.	.	.	.
.	.	.	.	.	.	.	.	.
.	.	.	.	.	.	.	.	.
.	.	.	.	.	.	.	.	.
0.0036	0.0035	0.0001	.	.	.	.	.	1.973183,1
.	.	.	.	.	.	.	.	.
.	.	.	.	.	.	.	.	-1.068213,
.	.	.	.	.	.	.	.	.
.	.	.	.	.	.	.	.	.
0.001	0.0106	0	.	.	.	.	.	1.306697,1
.	.	.	.	.	.	.	.	.
.	1.65E-05	0	.	.	.	.	.	5.434959,3
.	.	.	.	.	.	.	.	1.657264,1
0.007	0.0023	0.0008	.	.	.	.	.	6.333780,3
0.0346	0.001	0.0068	.	.	.	.	.	-0.644200,
.	.	.	.	.	.	.	.	.
.	.	.	.	.	.	.	.	.
.	0.001	0.0002	.	.	.	.	.	.
.	.	.	.	.	.	.	.	.
0.0355	0.0018	0.0009	.	.	.	.	.	-0.629183,
.	.	.	.	.	.	.	.	.
.	.	.	.	.	.	.	.	.
0.1221	0.0684	0.0073	.	.	.	.	.	0.792157,8
.	.	.	.	.	.	.	.	.
.	.	.	.	.	.	.	.	.
.	.	.	.	.	.	.	.	.
.	.	.	.	.	.	.	.	.
.	.	.	.	.	.	.	.	.
.	.	.	.	.	.	.	.	.
.	0.0002	0	.	.	.	.	.	.
.	.	.	.	.	.	.	.	.
.	.	.	.	.	.	.	.	.

.	0.0001	0	.	.	.	.	.	-0.838457,
.	0	0	.	.	.	.	.	-0.979719,
.	0.0002	0	.	.	.	.	.	0.629140,7
.	.	.	.	.	.	.	.	.
.	.	.	.	.	.	.	.	.
.	0.0001	0.0008	.	.	.	.	.	.
.	.	.	.	.	.	.	.	.
.	.	.	.	.	.	.	.	.
.	.	.	.	.	.	.	.	.
0.0063	0.0002	0.0007	.	.	.	.	.	-0.538121,
.	0.0001	0	.	.	.	.	.	-0.925102,
0.0057	0.0052	0.001	.	.	.	.	.	3.748789,1
.	.	.	.	.	.	.	.	.
.	.	.	.	.	.	.	.	-0.233735,
.	.	.	.	.	.	.	.	-0.195350,
.	0.0114	0.0117	.	.	.	.	.	-0.102010,
.	.	.	.	.	.	.	.	.
.	0.0001	0	.	.	.	.	.	.
.	0.0006	0.0001	.	.	.	.	.	.
.	.	.	.	.	.	.	.	.
.	.	.	.	.	.	.	.	.
.	.	.	.	.	.	.	.	.
.	.	.	.	.	.	.	.	.
0.0045	.	.	.	.	.	.	.	-0.230199,
.	0.0002	0.0022	.	.	.	.	.	.
.	.	.	.	.	.	.	.	.
0.002	0.0003	0	.	.	.	.	.	0.144172,4
.	.	.	.	.	.	.	.	.
.	.	.	.	.	.	.	.	.
.	.	.	.	.	.	.	.	.
.	.	.	.	.	.	.	.	.
.	.	.	.	.	.	.	.	5.822128,3
.	.	.	.	.	.	.	.	.
0.0226	0.0064	0	.	.	.	.	.	0.090218,4
.	.	.	.	.	.	.	.	.
.	.	.	.	.	.	.	.	.
.	.	.	.	.	.	.	.	.
.	0	0	.	.	.	.	.	.
.	.	.	.	.	.	.	.	.
.	0.001	0.0001	.	.	.	.	.	.
.	0.0003	0	.	.	.	.	.	.
.	1.68E-05	0	.	.	.	.	.	.
.	.	.	.	.	.	.	.	.
.	.	.	.	.	.	.	.	.
0.0009	7.63E-05	0	.	.	.	.	.	0.764831,8
.	.	.	.	.	.	.	.	.
0.002	0.0005	0	.	.	.	.	.	3.326528,1
.	.	.	.	.	.	.	.	.
.	7.42E-05	0	.	.	.	.	.	.
.	.	.	.	.	.	.	.	.
.	.	.	.	.	.	.	.	.
.	4.65E-05	0	.	.	.	.	.	.
.	.	.	.	.	.	.	.	.
.	.	.	.	.	.	.	.	.

.	.	.	.	.	.	.	.	.
.	4.39E-05	0	.	.	.	.	.	.
.	.	.	.	.	.	.	.	.
.	0.0002	0.0001	.	.	.	.	.	.
.	.	.	.	.	.	.	.	.
.	.	.	.	.	.	.	.	.
.	.	.	.	.	.	.	.	.
.	.	.	.	.	.	.	.	.
0.006	0.0002	0	.	.	.	.	.	6.184515,3
0.0002	0.0004	0.0004	.	.	.	.	.	0.091208,4
.	.	.	.	.	.	.	.	.
.	.	.	.	.	.	.	.	.
.	.	.	.	.	.	.	.	.
.	.	.	.	.	.	.	.	.
.	.	.	.	.	.	.	.	.
.	.	.	.	.	.	.	.	.
.	.	.	.	.	.	.	.	.
.	3.27E-05	0	.	.	.	.	.	.
.	.	.	.	.	.	.	.	.
.	.	.	.	.	.	.	.	.
0.0106	0.0088	0.0091	.	.	.	.	.	-0.310612,
.	.	.	.	.	.	.	.	.
.	.	.	.	.	.	.	.	.
0.0075	0.0002	0.0003	.	.	.	.	.	2.258493,1
0.0064	0.0016	0.0001	.	.	.	.	.	1.111180,9
.	.	.	.	.	.	.	.	.
.	.	.	.	.	.	.	.	-0.232107,
.	0.0003	0.0002	.	.	.	.	.	.
.	.	.	.	.	.	.	.	.
.	.	.	.	.	.	.	.	.
.	0.0015	0.0005	.	.	.	.	.	.
.	.	.	.	.	.	.	.	.
.	.	.	.	.	.	.	.	.
.	0.0003	0.0005	.	.	.	.	.	.
.	5.83E-05	0	.	.	.	.	.	.
.	.	.	.	.	.	.	.	.
0.0252	0.0016	0.0005	.	.	.	.	.	4.966242,2
0.0048	0.0003	0.0001	.	.	.	.	.	-1.576300,
.	.	.	.	.	.	.	.	.
.	0.0002	0	.	.	.	.	.	.
.	0.0003	0.0001	.	.	.	.	.	.
.	0.0004	0.0006	.	.	.	.	.	4.399397,2
.	.	.	.	.	.	.	.	-0.789274,
.	.	.	.	.	.	.	.	.
.	.	.	.	.	.	.	.	-1.711714,
.	8.36E-06	0	.	.	.	.	.	.
.	0.0002	0.0002	.	.	.	.	.	.
.	0.0166	0.0104	.	.	.	.	.	0.846469,8
.	.	.	.	.	.	.	.	.
.	.	.	.	.	.	.	.	.
.	.	.	.	.	.	.	.	.
.	0.0002	0.0009	.	.	.	.	.	0.118246,4
.	.	.	.	.	.	.	.	.
.	.	.	.	.	.	.	.	.

.	8.48E-06	0	.	.	.	.	.	.
.	.	.	.	.	.	.	.	.
.	.	.	.	.	.	.	.	1.295933,1
.	.	.	.	.	.	.	.	.
.	.	.	.	.	.	.	.	.
0.0009	3.49E-05	0	.	.	.	.	.	-1.137313,
0.0034	0.0059	0.0016	.	.	.	.	.	-0.278162,
.	0.0009	0	.	.	.	.	.	-0.002064,
.	.	.	.	.	.	.	.	.
.	.	.	.	.	.	.	.	.
.	.	.	.	.	.	.	.	.
.	.	.	.	.	.	.	.	.
.	0.0014	0	.	.	.	.	.	6.926590,3
.	.	.	.	.	.	.	.	.
.	.	.	.	.	.	.	.	.
.	.	.	.	.	.	.	.	.
.	.	.	.	.	.	.	.	.
0.0226	9.62E-05	0	.	.	.	.	.	0.044422,4
.	.	.	.	.	.	.	.	.
.	.	.	.	.	.	.	.	.
.	.	.	.	.	.	.	.	.
.	0.0131	0.0007	.	.	.	.	.	-0.967556,
.	.	.	.	.	.	.	.	-0.708287,
.	.	.	.	.	.	.	.	.
.	.	.	.	.	.	.	.	-0.621617,
.	.	.	.	.	.	.	.	.
.	.	.	.	.	.	.	.	.
.	.	.	.	.	.	.	.	.
.	.	.	.	.	.	.	.	.
.	.	.	.	.	.	.	.	.
.	.	.	.	.	.	.	.	2.489362,1
.	.	.	.	.	.	.	.	.
.	.	.	.	.	.	.	.	.
.	.	.	.	.	.	.	.	.
.	.	.	.	.	.	.	.	.
.	.	.	.	.	.	.	.	.
.	.	.	.	.	.	.	.	.
.	.	.	.	.	.	.	.	.
.	.	.	.	.	.	.	.	.
.	.	.	.	.	.	.	.	.
.	.	.	.	.	.	.	.	.
.	.	.	.	.	.	.	.	.
.	.	.	.	.	.	.	.	2.267836,1
.	.	.	.	.	.	.	.	.
0.0006	0.0201	0.0152	.	.	.	.	.	2.539731,1
.	0.0006	0	.	.	.	.	.	.
.	.	.	.	.	.	.	.	.
.	.	.	.	.	.	.	.	.
.	.	.	.	.	.	.	.	.
.	.	.	.	.	.	.	.	.
.	0.0159	0.0034	.	.	.	.	.	1.032582,9
.	.	.	.	.	.	.	.	.
.	8.54E-06	0.0001	.	.	.	.	.	.
.	.	.	.	.	.	.	.	.

.	.	.	.	.	.	.	.	.
.	.	.	.	.	.	.	.	.
.	.	.	.	.	.	.	.	.
0.0085	.	.	.	.	.	.	.	.
.	.	.	.	.	.	.	.	.
.	.	.	.	.	.	.	.	.
.	.	.	.	.	.	.	.	.
.	0.1054	0.2256	.	.	.	.	.	.
.	.	.	.	.	.	.	.	.
.	0.0006	0	.	.	.	.	.	-2.866279,
.	8.26E-06	0	.	.	.	.	.	.
.	.	.	.	.	.	.	.	.
.	.	.	.	.	.	.	.	.
.	.	.	.	.	.	.	.	.
.	1.70E-05	0	.	.	.	.	.	.
.	.	.	.	.	.	.	.	.
.	.	.	.	.	.	.	.	.
.	.	.	.	.	.	.	.	.
.	.	.	.	.	.	.	.	.
.	0.0829	0.0719	.	.	.	.	.	-1.762378,
.	0.0002	0.0051	.	.	.	.	.	.
.	.	.	.	.	.	.	.	.
.	.	.	.	.	.	.	.	.
.	.	.	.	.	.	.	.	.
.	.	.	.	.	.	.	.	.
.	.	.	.	.	.	.	.	.
.	.	.	.	.	.	.	.	.
.	.	.	.	.	.	.	.	.

NovoDb_W	NovoDb_W	INFO	FORMAT	ACa01N	ACa01T	Ori_REF	Ori_ALT	shared_ho
.	.	IC=2;IHP=	DP:DP2:T	24:24:24,2	47:47:23,2	AC	A	0
.	.	IC=8;IHP=	DP:DP2:T	62:62:54,5	114:114:69	G	GT	0
0.001063	.	IC=0;IHP=	DP:DP2:T	74:74:73,7	236:236:20	TTGGAG	T	0
.	.	IC=1;IHP=	DP:DP2:T	51:51:51,5	101:101:69	GTTT	G	0
.	.	IC=9;IHP=	DP:DP2:T	77:77:75,7	154:154:99	T	TC	0
.	.	IC=0;IHP=	DP:DP2:T	96:96:94,9	334:334:28	AT	A	0
.	.	IC=1;IHP=	DP:DP2:T	93:93:92,9	166:166:10	CAGA	C	0
0.000389	.	IC=5;IHP=	DP:DP2:T	145:145:13	576:576:23	T	TC	0
.	.	IC=1;IHP=	DP:DP2:T	106:106:10	234:234:13	C	CAG	0
0.001392	.	IC=6;IHP=	DP:DP2:T	51:51:48,4	116:116:85	T	TAGAC	0
.	.	IC=0;IHP=	DP:DP2:T	107:107:11	245:245:16	ACACAGC	A	0
.	.	IC=9;IHP=	DP:DP2:T	38:38:31,3	72:72:32,3	G	GT	0
0.011341	.	IC=1;IHP=	DP:DP2:T	70:70:64,6	176:176:14	T	TTGGGC	0
0.002806	.	IC=0;IHP=	DP:DP2:T	78:78:74,7	205:205:19	GTGTGTG	G	0
0.090962	0.147788	IC=2;IHP=	DP:DP2:T	12:12:12,1	29:29:0,0:2	TC	T	0
.	.	IC=0;IHP=	DP:DP2:T	97:97:95,9	152:152:13	GGGGATT	G	0
.	.	IC=2;IHP=	DP:DP2:T	79:79:76,7	167:167:11	CG	C	0
.	.	IC=0;IHP=	DP:DP2:T	65:65:61,6	254:254:19	CGT	C	0
.	.	IC=4;IHP=	DP:DP2:T	41:41:41,4	83:83:53,5	GC	G	0
.	.	IC=0;IHP=	DP:DP2:T	83:83:83,9	172:172:15	CCTCTTT	C	0
0.005265	.	IC=1;IHP=	DP:DP2:T	69:69:68,6	144:144:12	C	CTCTCTC	0
.	.	IC=2;IHP=	DP:DP2:T	70:70:69,6	177:177:14	CTG	C	0
.	.	IC=5;IHP=	DP:DP2:T	118:118:11	220:220:16	AC	A	0
.	.	IC=7;IHP=	DP:DP2:T	152:152:14	304:304:24	TC	T	0
.	.	IC=4;IHP=	DP:DP2:T	67:67:68,6	194:194:13	AC	A	0
.	.	IC=8;IHP=	DP:DP2:T	103:103:10	193:193:15	T	TG	0
.	.	IC=5;IHP=	DP:DP2:T	42:42:42,4	77:77:56,5	AC	A	0
.	.	IC=5;IHP=	DP:DP2:T	89:89:82,8	173:173:12	CG	C	0
.	.	IC=6;IHP=	DP:DP2:T	86:86:83,8	209:209:16	TG	T	0
.	.	IC=0;IHP=	DP:DP2:T	165:165:16	317:317:26	CA	C	0
.	.	IC=8;IHP=	DP:DP2:T	105:105:99	191:191:16	A	AC	0
.	.	IC=7;IHP=	DP:DP2:T	50:50:50,5	85:85:64,6	C	CT	0
.	.	IC=7;IHP=	DP:DP2:T	24:24:24,2	53:53:35,3	GA	G	0
.	.	IC=8;IHP=	DP:DP2:T	108:108:10	203:203:15	T	TG	0
.	.	IC=4;IHP=	DP:DP2:T	56:56:56,5	84:84:56,5	CT	C	0
.	.	IC=6;IHP=	DP:DP2:T	90:90:88,8	153:153:11	GT	G	0
.	.	IC=4;IHP=	DP:DP2:T	60:60:60,6	137:137:10	AG	A	0
.	.	IC=5;IHP=	DP:DP2:T	51:51:49,4	68:68:52,5	TG	T	0
.	.	IC=6;IHP=	DP:DP2:T	94:94:91,9	237:237:19	GT	G	0
.	.	IC=4;IHP=	DP:DP2:T	93:93:92,9	198:198:15	TA	T	0
.	.	IC=7;IHP=	DP:DP2:T	63:63:57,5	106:106:79	GA	G	0
.	.	IC=2;IHP=	DP:DP2:T	58:58:59,5	108:108:84	CAG	C	0
.	.	IC=4;IHP=	DP:DP2:T	71:71:68,6	114:114:87	TC	T	0
.	.	IC=9;IHP=	DP:DP2:T	59:59:59,5	97:97:72,7	G	GA	0
.	.	IC=6;IHP=	DP:DP2:T	56:56:52,5	105:105:82	GA	G	0
.	.	IC=7;IHP=	DP:DP2:T	52:52:51,5	92:92:65,6	AT	A	0
.	.	IC=5;IHP=	DP:DP2:T	217:217:21	458:458:36	AC	A	0
.	.	IC=7;IHP=	DP:DP2:T	73:73:70,7	133:133:10	TA	T	0
.	.	IC=7;IHP=	DP:DP2:T	59:59:55,5	86:86:61,6	TA	T	0
.	.	IC=5;IHP=	DP:DP2:T	52:52:48,5	79:79:57,5	CG	C	0
.	0.000883	IC=5;IHP=	DP:DP2:T	31:31:32,3	73:73:49,4	CTG	C	0

.	.	IC=8;IHP=	DP:DP2:T/	50:50:49,4	79:79:62,6	C	CA	0
.	.	IC=6;IHP=	DP:DP2:T/	72:72:68,6	114:114:87	AT	A	0
.	.	IC=3;IHP=	DP:DP2:T/	41:41:41,4	54:54:34,3	TA	T	0
0.001131	0.001416	IC=7;IHP=	DP:DP2:T/	52:52:49,4	85:85:61,6	CA	C	0
.	.	IC=4;IHP=	DP:DP2:T/	25:25:25,2	48:48:30,3	GA	G	0
.	.	IC=9;IHP=	DP:DP2:T/	40:40:38,3	61:61:43,4	A	AT	0
.	.	IC=9;IHP=	DP:DP2:T/	131:131:12	324:324:24	G	GC	0
.	.	IC=7;IHP=	DP:DP2:T/	71:71:67,6	101:101:77	G	GT	0
.	.	IC=7;IHP=	DP:DP2:T/	47:47:47,4	73:73:50,5	TA	T	0
.	.	IC=5;IHP=	DP:DP2:T/	55:55:53,5	108:108:82	CG	C	0
.	.	IC=7;IHP=	DP:DP2:T/	103:103:98	125:125:70	TA	T	0
.	.	IC=4;IHP=	DP:DP2:T/	97:97:95,9	182:182:14	AG	A	0
.	.	IC=7;IHP=	DP:DP2:T/	109:109:10	187:187:14	A	AT	0
0.000194	.	IC=8;IHP=	DP:DP2:T/	70:70:64,6	136:136:10	T	TG	0
.	.	IC=5;IHP=	DP:DP2:T/	66:66:63,6	110:110:80	CT	C	0
.	.	IC=7;IHP=	DP:DP2:T/	58:58:55,5	108:108:85	AT	A	0
.	.	IC=7;IHP=	DP:DP2:T/	86:86:86,8	131:131:10	CT	C	0
.	.	IC=4;IHP=	DP:DP2:T/	73:73:71,7	145:145:11	AT	A	0
.	.	IC=6;IHP=	DP:DP2:T/	51:51:49,4	104:104:73	A	AT	0
.	.	IC=6;IHP=	DP:DP2:T/	72:72:67,6	105:105:79	CA	C	0
.	.	IC=9;IHP=	DP:DP2:T/	59:59:59,5	118:118:88	T	TA	0
.	.	IC=6;IHP=	DP:DP2:T/	62:62:60,6	111:111:81	AC	A	0
.	.	IC=0;IHP=	DP:DP2:T/	106:106:10	152:152:13	AG	A	0
.	.	IC=4;IHP=	DP:DP2:T/	96:96:92,9	137:137:10	GA	G	0
.	.	IC=3;IHP=	DP:DP2:T/	67:67:67,6	140:140:11	TA	T	0
.	.	IC=4;IHP=	DP:DP2:T/	136:136:13	304:304:23	TG	T	0
.	.	IC=8;IHP=	DP:DP2:T/	181:181:17	310:310:25	A	AC	0
.	.	IC=6;IHP=	DP:DP2:T/	119:119:11	176:176:14	CT	C	0
.	.	IC=5;IHP=	DP:DP2:T/	71:71:66,6	120:120:98	AT	A	0
.	.	IC=7;IHP=	DP:DP2:T/	39:39:35,3	96:96:65,6	C	CT	0
.	.	IC=7;IHP=	DP:DP2:T/	49:49:44,4	66:66:51,5	TA	T	0
.	.	IC=6;IHP=	DP:DP2:T/	101:101:97	131:131:10	GAA	G	0
.	.	IC=1;IHP=	DP:DP2:T/	102:102:10	225:225:18	TGAG	T	0
.	.	IC=8;IHP=	DP:DP2:T/	90:90:88,8	174:174:13	A	AG	0
.	.	IC=3;IHP=	DP:DP2:T/	79:79:77,7	122:122:97	CA	C	0
.	.	IC=6;IHP=	DP:DP2:T/	251:251:24	394:394:32	CG	C	0
.	.	IC=6;IHP=	DP:DP2:T/	79:79:75,7	158:158:12	CT	C	0
.	.	IC=0;IHP=	DP:DP2:T/	35:35:35,3	78:78:52,5	AAG	A	0
.	.	IC=7;IHP=	DP:DP2:T/	122:122:11	214:214:17	T	TG	0
.	.	IC=5;IHP=	DP:DP2:T/	68:68:68,6	81:81:64,6	GA	G	0
.	.	IC=5;IHP=	DP:DP2:T/	58:58:57,5	125:125:97	T	TC	0
0.045347	.	IC=0;IHP=	DP:DP2:T/	38:38:37,3	92:92:70,7	CTTCT	C	0
.	.	IC=9;IHP=	DP:DP2:T/	68:68:63,6	98:98:76,7	A	AT	0
.	.	IC=6;IHP=	DP:DP2:T/	183:183:17	449:449:33	TG	T	0
.	.	IC=6;IHP=	DP:DP2:T/	105:105:10	177:177:13	G	GC	0
.	.	IC=0;IHP=	DP:DP2:T/	91:91:91,9	227:227:18	TTAAC	T	0
0.000518	0.00088	IC=2;IHP=	DP:DP2:T/	166:166:17	280:280:21	GAGA	G	0
.	.	IC=6;IHP=	DP:DP2:T/	79:79:79,7	157:157:13	AT	A	0
.	.	IC=5;IHP=	DP:DP2:T/	77:77:69,7	125:125:93	GA	G	0
.	.	IC=1;IHP=	DP:DP2:T/	148:148:14	282:282:23	AAGG	A	0
.	.	IC=2;IHP=	DP:DP2:T/	65:65:69,6	141:141:12	AAAAAG	A	0
.	.	IC=4;IHP=	DP:DP2:T/	207:207:20	405:405:33	TC	T	0
.	.	IC=9;IHP=	DP:DP2:T/	46:46:44,4	67:67:46,4	C	CT	0
.	.	IC=6;IHP=	DP:DP2:T/	55:55:52,5	104:104:73	CG	C	0

.	.	IC=8;IHP=!	DP:DP2:T/	52:52:51,5	85:85:67,6	C	CA	0
.	.	IC=9;IHP=.	DP:DP2:T/	84:84:75,7	113:113:8{	C	CT	0
.	.	IC=1;IHP=4	DP:DP2:T/	67:67:69,6	137:137:11	TCTC	T	0
.	.	IC=8;IHP=!	DP:DP2:T/	54:54:46,4	122:122:8{	A	AT	0
.	.	IC=8;IHP=.	DP:DP2:T/	177:177:17	348:348:2{	C	CA	0
.	.	IC=4;IHP=!	DP:DP2:T/	183:183:1{	351:351:2{	AG	A	0
.	.	IC=6;IHP=!	DP:DP2:T/	52:52:49,4	137:137:9{	AC	A	0
.	.	IC=9;IHP=.	DP:DP2:T/	80:80:77,7	133:133:1(	C	CA	0
.	.	IC=5;IHP=(	DP:DP2:T/	35:35:36,3	66:66:51,5	GA	G	0
.	.	IC=7;IHP=!	DP:DP2:T/	54:54:43,4	91:91:61,6	GA	G	0
.	.	IC=7;IHP=.	DP:DP2:T/	84:84:81,8	131:131:1(	CT	C	0
.	.	IC=8;IHP=.	DP:DP2:T/	91:91:88,8	174:174:1{	T	TG	0
.	.	IC=8;IHP=!	DP:DP2:T/	37:37:32,3	87:87:60,6	G	GA	0
0.000195	.	IC=2;IHP=:	DP:DP2:T/	77:77:73,7	134:134:1(	ACT	A	0
.	.	IC=4;IHP=(	DP:DP2:T/	77:77:76,7	140:140:1(	GT	G	0
.	.	IC=9;IHP=.	DP:DP2:T/	176:176:1(	391:391:2{	G	GC	0
.	.	IC=4;IHP=!	DP:DP2:T/	117:117:11	188:188:1{	AG	A	0
.	.	IC=6;IHP=7	DP:DP2:T/	39:39:38,3	108:108:7(	CG	C	0
0.001761	0.005587	IC=7;IHP=!	DP:DP2:T/	40:40:37,3	91:91:69,6	CT	C	0
.	.	IC=4;IHP=!	DP:DP2:T/	62:62:61,6	86:86:61,6	TG	T	0
.	.	IC=7;IHP=!	DP:DP2:T/	90:90:86,8	133:133:1(	CT	C	0
.	.	IC=5;IHP=(	DP:DP2:T/	162:162:1{	396:396:31	TC	T	0
.	.	IC=7;IHP=!	DP:DP2:T/	122:122:11	204:204:14	CG	C	0
.	.	IC=8;IHP=!	DP:DP2:T/	43:43:40,4	122:122:92	G	GA	0
.	.	IC=7;IHP=!	DP:DP2:T/	70:70:59,5	106:106:6{	AC	A	0
.	.	IC=4;IHP=!	DP:DP2:T/	133:133:1{	274:274:21	GC	G	0
.	.	IC=7;IHP=!	DP:DP2:T/	31:31:31,3	58:58:33,3	GA	G	0
.	.	IC=7;IHP=!	DP:DP2:T/	62:62:57,5	106:106:7{	GA	G	0
.	.	IC=9;IHP=.	DP:DP2:T/	59:59:58,5	108:108:84	C	CA	0
.	.	IC=7;IHP=!	DP:DP2:T/	77:77:74,7	151:151:72	CA	C	0
.	.	IC=6;IHP=7	DP:DP2:T/	131:131:12	243:243:1{	TC	T	0
.	.	IC=7;IHP=(	DP:DP2:T/	56:56:51,5	89:89:62,6	A	AG	0
.	.	IC=9;IHP=.	DP:DP2:T/	109:109:1(	179:179:1{	G	GT	0
0.028386	0.003559	IC=7;IHP=:	DP:DP2:T/	62:62:63,6	107:107:8{	ATG	A	0
.	.	IC=6;IHP=7	DP:DP2:T/	37:37:37,3	59:59:43,4	AT	A	0
.	.	IC=7;IHP=!	DP:DP2:T/	52:52:50,5	55:55:35,3	CT	C	0
.	.	IC=9;IHP=.	DP:DP2:T/	50:50:49,4	82:82:66,6	C	CA	0
.	.	IC=1;IHP=:	DP:DP2:T/	61:61:59,5	87:87:62,6	TAG	T	0
.	.	IC=7;IHP=.	DP:DP2:T/	69:69:69,6	146:146:1(	A	AT	0
0.010551	0.00354	IC=6;IHP=:	DP:DP2:T/	29:29:24,2	59:59:28,3	T	TGCC	0
.	.	IC=9;IHP=.	DP:DP2:T/	42:42:38,3	90:90:63,6	T	TG	0
.	.	IC=3;IHP=!	DP:DP2:T/	88:88:86,8	145:145:1(	TC	T	0
.	.	IC=8;IHP=!	DP:DP2:T/	31:31:30,3	77:77:53,5	C	CG	0
.	.	IC=7;IHP=!	DP:DP2:T/	47:47:43,4	65:65:46,4	GA	G	0
.	.	IC=7;IHP=(	DP:DP2:T/	61:61:59,5	129:129:9(	A	AG	0
.	.	IC=7;IHP=!	DP:DP2:T/	103:103:97	202:202:1(	GA	G	0
.	.	IC=4;IHP=(	DP:DP2:T/	144:144:1{	244:244:1{	AC	A	0
.	.	IC=6;IHP=!	DP:DP2:T/	107:107:9{	222:222:17	T	TC	0
.	.	IC=6;IHP=7	DP:DP2:T/	90:90:87,8	172:172:1{	TA	T	0
.	.	IC=8;IHP=.	DP:DP2:T/	111:111:1(	261:261:2(	C	CA	0
.	.	IC=6;IHP=!	DP:DP2:T/	100:100:1(	182:182:1{	TG	T	0
.	.	IC=4;IHP=!	DP:DP2:T/	91:91:89,8	161:161:11	GA	G	0
.	.	IC=3;IHP=4	DP:DP2:T/	117:117:11	271:271:1{	TG	T	0
0.002138	0.001767	IC=6;IHP=!	DP:DP2:T/	103:103:92	214:214:1{	CG	C	0

.	.	IC=1;IHP=	DP:DP2:T	123:123:12	256:256:22	GCCAGCC	G	0
0.000278	.	IC=7;IHP=	DP:DP2:T	42:42:38,3	65:65:40,4	A	AGAG	0
.	.	IC=7;IHP=	DP:DP2:T	77:77:77,7	156:156:12	TA	T	0
.	.	IC=6;IHP=	DP:DP2:T	80:80:76,7	137:137:11	G	GC	0
.	.	IC=7;IHP=	DP:DP2:T	75:75:67,6	153:153:12	CT	C	0
.	.	IC=5;IHP=	DP:DP2:T	92:92:86,9	203:203:15	A	AAAC	0
0.001942	.	IC=7;IHP=	DP:DP2:T	75:75:71,7	137:137:11	GA	G	0
.	.	IC=7;IHP=	DP:DP2:T	154:154:14	336:336:27	G	GC	0
.	.	IC=1;IHP=	DP:DP2:T	23:23:21,2	48:48:26,2	CG	C	0
.	.	IC=9;IHP=	DP:DP2:T	111:111:10	187:187:13	C	CT	0
.	.	IC=6;IHP=	DP:DP2:T	90:90:89,9	209:209:17	CA	C	0
.	.	IC=4;IHP=	DP:DP2:T	68:68:64,6	130:130:98	AT	A	0
.	.	IC=7;IHP=	DP:DP2:T	35:35:30,3	54:54:37,3	CT	C	0
.	.	IC=7;IHP=	DP:DP2:T	86:86:85,8	162:162:12	TA	T	0
.	.	IC=6;IHP=	DP:DP2:T	37:37:34,3	83:83:60,6	CT	C	0
0.000194	0.003565	IC=6;IHP=	DP:DP2:T	91:91:86,8	164:164:11	TAC	T	0
.	.	IC=7;IHP=	DP:DP2:T	150:150:14	221:221:16	T	TC	0
.	.	IC=8;IHP=	DP:DP2:T	116:116:11	236:236:19	A	AC	0
.	.	IC=6;IHP=	DP:DP2:T	66:66:63,6	122:122:79	GA	G	0
.	.	IC=7;IHP=	DP:DP2:T	94:94:88,8	225:225:16	CA	C	0
.	.	IC=5;IHP=	DP:DP2:T	104:104:97	223:223:17	CG	C	0
.	.	IC=7;IHP=	DP:DP2:T	50:50:43,4	80:80:55,5	AT	A	0
.	.	IC=9;IHP=	DP:DP2:T	49:49:48,4	98:98:76,7	G	GA	0
.	.	IC=6;IHP=	DP:DP2:T	72:72:65,6	155:155:11	TG	T	0
.	.	IC=7;IHP=	DP:DP2:T	132:132:12	304:304:22	CT	C	0
0.000197	.	IC=9;IHP=	DP:DP2:T	61:61:55,5	87:87:59,6	C	CA	0
0.000705	.	IC=7;IHP=	DP:DP2:T	75:75:68,6	193:193:15	AT	A	0
.	.	IC=1;IHP=	DP:DP2:T	67:67:70,7	72:72:61,6	CCTT	C	0
.	.	IC=3;IHP=	DP:DP2:T	227:227:22	425:425:35	CT	C	0
0.005479	0.012433	IC=5;IHP=	DP:DP2:T	71:71:71,7	123:123:98	AC	A	0
.	.	IC=5;IHP=	DP:DP2:T	47:47:47,4	98:98:75,7	TG	T	0
.	.	IC=4;IHP=	DP:DP2:T	101:101:99	170:170:12	TG	T	0
.	.	IC=7;IHP=	DP:DP2:T	99:99:95,9	183:183:13	G	GC	0
0.000389	.	IC=8;IHP=	DP:DP2:T	91:91:86,8	125:125:86	A	AC	0
.	.	IC=5;IHP=	DP:DP2:T	90:90:88,8	197:197:14	AC	A	0
.	.	IC=5;IHP=	DP:DP2:T	40:40:40,4	77:77:58,5	A	AG	0
.	.	IC=7;IHP=	DP:DP2:T	41:41:39,3	90:90:62,6	AT	A	0
.	.	IC=6;IHP=	DP:DP2:T	87:87:83,8	154:154:12	CA	C	0
0.001391	.	IC=6;IHP=	DP:DP2:T	77:77:71,7	120:120:91	TA	T	0
0.001206	.	IC=9;IHP=	DP:DP2:T	49:49:46,4	73:73:52,5	T	TA	0
.	.	IC=9;IHP=	DP:DP2:T	95:95:81,8	176:176:13	G	GT	0
.	.	IC=7;IHP=	DP:DP2:T	270:270:25	556:556:42	C	CG	0
.	.	IC=9;IHP=	DP:DP2:T	64:64:61,6	164:164:11	T	TC	0
.	.	IC=6;IHP=	DP:DP2:T	93:93:93,9	139:139:10	CT	C	0
0.000389	.	IC=0;IHP=	DP:DP2:T	85:85:81,8	178:178:15	GACAA	G	0
.	.	IC=3;IHP=	DP:DP2:T	115:115:11	308:308:24	GGAA	G	0
.	.	IC=6;IHP=	DP:DP2:T	73:73:71,7	123:123:96	AT	A	0
.	.	IC=6;IHP=	DP:DP2:T	82:82:81,8	131:131:10	CA	C	0
0.00529	0.002833	IC=9;IHP=	DP:DP2:T	49:49:43,4	80:80:57,5	G	GC	0
.	.	IC=7;IHP=	DP:DP2:T	75:75:75,7	165:165:12	GT	G	0
.	.	IC=5;IHP=	DP:DP2:T	41:41:39,6	80:80:55,6	T	TG	0
.	.	IC=6;IHP=	DP:DP2:T	159:159:15	362:362:27	TG	T	0
.	.	IC=7;IHP=	DP:DP2:T	75:75:74,7	181:181:14	AC	A	0
.	.	IC=7;IHP=	DP:DP2:T	48:48:45,4	80:80:60,6	CA	C	0

.	.	IC=4;IHP=	DP:DP2:T	95:95:95,9	176:176:13	GC	G	0
.	.	IC=1;IHP=	DP:DP2:T	64:64:64,6	124:124:10	CCCTT	C	0
0.000777	.	IC=7;IHP=	DP:DP2:T	93:93:86,8	204:204:15	T	TTG	0
0.000229	.	IC=9;IHP=	DP:DP2:T	46:46:40,4	51:51:32,3	C	CA	0
.	.	IC=7;IHP=	DP:DP2:T	62:62:60,6	88:88:69,6	CA	C	0
.	.	IC=6;IHP=	DP:DP2:T	57:57:56,5	120:120:84	AG	A	0
.	.	IC=4;IHP=	DP:DP2:T	95:95:93,9	189:189:15	AG	A	0
.	0.00088	IC=7;IHP=	DP:DP2:T	125:125:11	214:214:16	GA	G	0
.	.	IC=5;IHP=	DP:DP2:T	101:101:93	206:206:16	GC	G	0
.	.	IC=7;IHP=	DP:DP2:T	52:52:51,5	77:77:54,5	GA	G	0
.	.	IC=7;IHP=	DP:DP2:T	119:119:10	230:230:17	AC	A	0
.	.	IC=0;IHP=	DP:DP2:T	116:116:11	228:228:18	AC	A	0
.	.	IC=5;IHP=	DP:DP2:T	96:96:90,9	130:130:98	AT	A	0
0.00136	.	IC=5;IHP=	DP:DP2:T	59:59:59,6	114:114:92	AT	A	0
.	.	IC=1;IHP=	DP:DP2:T	70:70:69,7	116:116:95	AAAGT	A	0
.	.	IC=7;IHP=	DP:DP2:T	98:98:93,9	156:156:11	CT	C	0
.	.	IC=9;IHP=	DP:DP2:T	47:47:46,4	97:97:74,7	C	CT	0
.	.	IC=7;IHP=	DP:DP2:T	59:59:54,5	101:101:70	CT	C	0
.	.	IC=5;IHP=	DP:DP2:T	84:84:81,8	187:187:14	TC	T	0
.	.	IC=5;IHP=	DP:DP2:T	56:56:55,5	112:112:85	GA	G	0
.	.	IC=5;IHP=	DP:DP2:T	80:80:78,7	129:129:95	A	AAG	0
.	.	IC=1;IHP=	DP:DP2:T	53:53:54,5	110:110:88	GTTC	G	0
.	.	IC=7;IHP=	DP:DP2:T	54:54:51,5	107:107:77	AT	A	0
.	.	IC=1;IHP=	DP:DP2:T	64:64:66,6	64:64:47,4	TCTC	T	0
.	.	IC=3;IHP=	DP:DP2:T	916:916:90	1551:1551	TG	T	0
.	.	IC=5;IHP=	DP:DP2:T	194:194:18	363:363:27	T	TC	0
.	.	IC=7;IHP=	DP:DP2:T	85:85:81,8	203:203:16	CA	C	0
.	.	IC=4;IHP=	DP:DP2:T	120:120:11	237:237:18	TC	T	0
0.010408	0.006184	IC=6;IHP=	DP:DP2:T	28:28:26,2	51:51:29,3	GTA	G	0
.	.	IC=5;IHP=	DP:DP2:T	180:180:17	314:314:26	GC	G	0
.	.	IC=6;IHP=	DP:DP2:T	124:124:11	245:245:18	TG	T	0
.	.	IC=6;IHP=	DP:DP2:T	83:83:84,8	167:167:13	AT	A	0
.	.	IC=5;IHP=	DP:DP2:T	78:78:76,7	164:164:11	CA	C	0
.	.	IC=7;IHP=	DP:DP2:T	135:135:12	259:259:19	CT	C	0
.	.	IC=9;IHP=	DP:DP2:T	56:56:52,5	105:105:81	T	TG	0
.	.	IC=7;IHP=	DP:DP2:T	113:113:11	269:269:20	TG	T	0
0.006646	0.00499	IC=5;IHP=	DP:DP2:T	107:107:97	175:175:12	GT	G	0
.	.	IC=0;IHP=	DP:DP2:T	41:41:38,3	66:66:50,5	TAAAG	T	0
.	.	IC=7;IHP=	DP:DP2:T	68:68:67,6	130:130:93	C	CT	0
.	.	IC=7;IHP=	DP:DP2:T	119:119:10	243:243:18	GC	G	0
.	.	IC=5;IHP=	DP:DP2:T	55:55:54,5	72:72:50,5	A	AG	0
.	.	IC=7;IHP=	DP:DP2:T	47:47:46,4	77:77:53,5	TG	T	0
.	.	IC=8;IHP=	DP:DP2:T	129:129:12	239:239:18	G	GA	0
.	.	IC=6;IHP=	DP:DP2:T	81:81:77,7	142:142:10	GA	G	0
0.00312	0.001767	IC=6;IHP=	DP:DP2:T	61:61:60,6	114:114:88	CT	C	0
.	.	IC=5;IHP=	DP:DP2:T	43:43:42,4	74:74:55,5	TA	T	0
.	.	IC=5;IHP=	DP:DP2:T	115:115:11	241:241:18	CT	C	0
.	.	IC=7;IHP=	DP:DP2:T	74:74:71,7	97:97:70,7	AT	A	0
.	.	IC=4;IHP=	DP:DP2:T	77:77:77,7	182:182:15	AC	A	0
.	.	IC=7;IHP=	DP:DP2:T	51:51:50,5	118:118:82	CT	C	0
.	.	IC=6;IHP=	DP:DP2:T	33:33:31,3	52:52:36,3	CT	C	0
.	.	IC=7;IHP=	DP:DP2:T	40:40:37,3	110:110:85	CA	C	0
.	.	IC=6;IHP=	DP:DP2:T	67:67:66,6	133:133:10	CT	C	0
.	.	IC=1;IHP=	DP:DP2:T	72:72:73,7	129:129:10	CTG	C	0

.	.	IC=7;IHP=	DP:DP2:T/	74:74:70,7	176:176:13	GT	G	0
.	.	IC=8;IHP=	DP:DP2:T/	75:75:74,7	149:149:11	T	TA	0
.	.	IC=9;IHP=	DP:DP2:T/	56:56:54,5	144:144:10	C	CT	0
.	.	IC=4;IHP=	DP:DP2:T/	144:144:14	278:278:21	GC	G	0
.	.	IC=5;IHP=	DP:DP2:T/	85:85:84,8	178:178:14	TG	T	0
.	.	IC=7;IHP=	DP:DP2:T/	82:82:81,8	156:156:11	CA	C	0
.	.	IC=4;IHP=	DP:DP2:T/	74:74:74,7	126:126:10	GA	G	0
.	.	IC=5;IHP=	DP:DP2:T/	99:99:97,9	198:198:15	AC	A	0
.	.	IC=4;IHP=	DP:DP2:T/	66:66:65,6	176:176:13	GC	G	0
.	.	IC=9;IHP=	DP:DP2:T/	119:119:11	300:300:22	G	GA	0
.	.	IC=6;IHP=	DP:DP2:T/	80:80:74,7	156:156:12	GC	G	0
.	.	IC=9;IHP=	DP:DP2:T/	114:114:10	192:192:11	G	GC	0
.	.	IC=7;IHP=	DP:DP2:T/	69:69:68,6	115:115:91	CT	C	0
.	.	IC=9;IHP=	DP:DP2:T/	78:78:78,7	154:154:12	G	GT	0
.	.	IC=5;IHP=	DP:DP2:T/	93:93:88,8	212:212:15	AC	A	0
0.000224	.	IC=8;IHP=	DP:DP2:T/	56:56:47,4	107:107:75	T	TGA	0
.	.	IC=7;IHP=	DP:DP2:T/	55:55:53,5	105:105:80	GT	G	0
.	.	IC=8;IHP=	DP:DP2:T/	168:168:16	416:416:33	G	GC	0
.	.	IC=6;IHP=	DP:DP2:T/	139:139:13	233:233:19	CG	C	0
.	.	IC=1;IHP=	DP:DP2:T/	83:83:83,8	134:134:10	ACT	A	0
.	.	IC=7;IHP=	DP:DP2:T/	139:139:12	250:250:19	T	TC	0
.	.	IC=6;IHP=	DP:DP2:T/	44:44:40,4	57:57:34,3	TA	T	0
.	.	IC=5;IHP=	DP:DP2:T/	92:92:91,9	181:181:14	TG	T	0
.	.	IC=6;IHP=	DP:DP2:T/	57:57:54,5	109:109:79	TG	T	0
0.000389	0.002427	IC=5;IHP=	DP:DP2:T/	129:129:12	215:215:16	AC	A	0
.	.	IC=2;IHP=	DP:DP2:T/	107:107:10	195:195:15	GCTT	G	0
.	.	IC=7;IHP=	DP:DP2:T/	26:26:27,2	45:45:29,3	TA	T	0
.	.	IC=1;IHP=	DP:DP2:T/	45:45:44,4	95:95:71,7	ATC	A	0
.	.	IC=6;IHP=	DP:DP2:T/	109:109:99	276:276:21	TG	T	0
.	.	IC=6;IHP=	DP:DP2:T/	77:77:76,7	121:121:93	GA	G	0
.	.	IC=4;IHP=	DP:DP2:T/	111:111:10	213:213:15	TC	T	0
.	.	IC=4;IHP=	DP:DP2:T/	99:99:96,9	229:229:18	CT	C	0
.	.	IC=0;IHP=	DP:DP2:T/	100:100:10	166:166:13	CCA	C	0
0.000469	.	IC=8;IHP=	DP:DP2:T/	82:82:67,6	149:149:95	T	TGGA	0
.	0.00088	IC=5;IHP=	DP:DP2:T/	79:79:78,7	158:158:12	AC	A	0
.	.	IC=5;IHP=	DP:DP2:T/	53:53:54,5	99:99:79,7	CG	C	0
.	.	IC=5;IHP=	DP:DP2:T/	61:61:55,5	145:145:10	AT	A	0
.	.	IC=4;IHP=	DP:DP2:T/	71:71:69,7	108:108:83	GC	G	0
.	.	IC=1;IHP=	DP:DP2:T/	160:160:15	296:296:23	ATGGAAT	A	0
.	0.000885	IC=6;IHP=	DP:DP2:T/	176:176:17	456:456:35	GC	G	0
.	.	IC=7;IHP=	DP:DP2:T/	31:31:31,3	47:47:32,3	AT	A	0
.	.	IC=5;IHP=	DP:DP2:T/	48:48:49,4	87:87:61,6	TC	T	0
.	.	IC=4;IHP=	DP:DP2:T/	144:144:14	272:272:21	GT	G	0
.	.	IC=7;IHP=	DP:DP2:T/	71:71:68,6	167:167:12	C	CA	0
.	.	IC=7;IHP=	DP:DP2:T/	33:33:32,3	58:58:37,3	CT	C	0
.	.	IC=4;IHP=	DP:DP2:T/	45:45:44,4	54:54:33,3	GA	G	0
.	.	IC=8;IHP=	DP:DP2:T/	114:114:11	197:197:14	G	GC	0
.	.	IC=2;IHP=	DP:DP2:T/	78:78:75,7	143:143:11	CCCT	C	0
.	.	IC=1;IHP=	DP:DP2:T/	187:187:18	337:337:25	T	TC	0
.	.	IC=6;IHP=	DP:DP2:T/	90:90:89,8	197:197:14	GA	G	0
.	.	IC=6;IHP=	DP:DP2:T/	79:79:78,8	193:193:15	GC	G	0
.	.	IC=5;IHP=	DP:DP2:T/	55:55:52,5	131:131:95	CG	C	0
0.00039	.	IC=1;IHP=	DP:DP2:T/	57:57:33,3	88:88:26,2	G	GC	0
.	.	IC=6;IHP=	DP:DP2:T/	81:81:76,7	175:175:12	TC	T	0

.	.	IC=5;IHP=	DP:DP2:T	74:74:69,6	151:151:11	GC	G	0
0.000194	.	IC=6;IHP=	DP:DP2:T	69:69:62,6	124:124:92	GA	G	0
.	.	IC=5;IHP=	DP:DP2:T	33:33:32,3	64:64:46,4	CG	C	0
.	.	IC=6;IHP=	DP:DP2:T	228:228:21	467:467:35	GC	G	0
.	.	IC=4;IHP=	DP:DP2:T	86:86:83,8	215:215:16	TG	T	0
.	.	IC=5;IHP=	DP:DP2:T	19:19:20,2	24:24:9,9:7	TG	T	0
.	.	IC=6;IHP=	DP:DP2:T	154:154:14	271:271:22	GT	G	0
.	.	IC=1;IHP=	DP:DP2:T	61:61:63,6	112:112:86	GAGA	G	0
.	.	IC=7;IHP=	DP:DP2:T	129:129:11	265:265:18	TC	T	0
.	.	IC=9;IHP=	DP:DP2:T	107:107:10	225:225:17	C	CA	0
.	.	IC=6;IHP=	DP:DP2:T	27:27:24,2	52:52:30,3	CA	C	0
.	.	IC=5;IHP=	DP:DP2:T	44:44:41,4	69:69:51,5	CG	C	0
.	.	IC=6;IHP=	DP:DP2:T	240:240:23	562:562:43	AC	A	0
.	.	IC=4;IHP=	DP:DP2:T	75:75:71,7	131:131:10	GA	G	0
.	.	IC=9;IHP=	DP:DP2:T	77:77:72,7	127:127:94	A	AT	0
.	.	IC=5;IHP=	DP:DP2:T	267:267:25	479:479:38	GC	G	0
.	.	IC=5;IHP=	DP:DP2:T	54:54:54,5	83:83:59,5	T	TC	0
.	.	IC=4;IHP=	DP:DP2:T	122:122:11	196:196:15	TC	T	0
.	.	IC=3;IHP=	DP:DP2:T	129:129:13	247:247:18	TG	T	0
.	.	IC=4;IHP=	DP:DP2:T	67:67:65,6	152:152:12	CG	C	0
.	.	IC=9;IHP=	DP:DP2:T	95:95:93,9	188:188:14	C	CG	0
.	.	IC=6;IHP=	DP:DP2:T	45:45:47,4	74:74:52,5	GA	G	0
.	.	IC=3;IHP=	DP:DP2:T	224:224:22	547:547:44	AC	A	0
.	.	IC=9;IHP=	DP:DP2:T	100:100:84	192:192:12	A	AC	0
.	.	IC=9;IHP=	DP:DP2:T	185:185:17	384:384:29	T	TG	0
.	.	IC=4;IHP=	DP:DP2:T	26:26:34,3	38:38:21,2	CG	C	0
.	.	IC=6;IHP=	DP:DP2:T	83:83:74,7	196:196:15	TC	T	0
.	.	IC=4;IHP=	DP:DP2:T	39:39:34,3	71:71:43,4	CAG	C	0
.	.	IC=6;IHP=	DP:DP2:T	82:82:81,8	191:191:14	T	TG	0
.	.	IC=4;IHP=	DP:DP2:T	226:226:21	418:418:33	TC	T	0
.	.	IC=9;IHP=	DP:DP2:T	246:246:23	516:516:40	A	AC	0
.	.	IC=7;IHP=	DP:DP2:T	104:104:10	207:207:15	AC	A	0
.	.	IC=5;IHP=	DP:DP2:T	156:156:14	370:370:27	GC	G	0
.	.	IC=7;IHP=	DP:DP2:T	39:39:36,3	84:84:62,6	GA	G	0
.	.	IC=7;IHP=	DP:DP2:T	65:65:62,6	144:144:10	CT	C	0
.	.	IC=6;IHP=	DP:DP2:T	78:78:72,7	175:175:14	GC	G	0
.	.	IC=9;IHP=	DP:DP2:T	136:136:12	223:223:17	A	AG	0
.	.	IC=8;IHP=	DP:DP2:T	86:86:78,7	141:141:10	C	CG	0
.	.	IC=7;IHP=	DP:DP2:T	34:34:33,3	51:51:37,3	CA	C	0
.	.	IC=5;IHP=	DP:DP2:T	65:65:61,6	93:93:72,7	TG	T	0
.	.	IC=9;IHP=	DP:DP2:T	95:95:90,9	161:161:12	C	CT	0
.	.	IC=7;IHP=	DP:DP2:T	104:104:10	230:230:17	CA	C	0
.	.	IC=7;IHP=	DP:DP2:T	52:52:46,4	74:74:48,4	AC	A	0
.	.	IC=1;IHP=	DP:DP2:T	97:97:96,9	158:158:13	GC	G	0
0.003326	0.001393	IC=0;IHP=	DP:DP2:T	26:26:25,2	48:48:28,2	AG	A	0
.	.	IC=5;IHP=	DP:DP2:T	95:95:94,9	155:155:12	GC	G	0
.	.	IC=8;IHP=	DP:DP2:T	149:149:14	242:242:16	C	CG	0
0.003911	.	IC=7;IHP=	DP:DP2:T	127:127:11	299:299:22	TC	T	0
.	.	IC=5;IHP=	DP:DP2:T	72:72:72,7	137:137:10	TC	T	0
.	.	IC=4;IHP=	DP:DP2:T	47:47:44,4	102:102:77	AG	A	0
.	.	IC=6;IHP=	DP:DP2:T	46:46:46,4	91:91:63,6	CT	C	0
.	.	IC=7;IHP=	DP:DP2:T	86:86:83,8	133:133:10	AG	A	0
.	.	IC=5;IHP=	DP:DP2:T	75:75:71,7	176:176:12	AC	A	0
.	.	IC=5;IHP=	DP:DP2:T	129:129:11	262:262:19	GC	G	0

.	.	IC=7;IHP=7	DP:DP2:T/	137:137:12	240:240:18	A	AC	0
.	.	IC=6;IHP=.	DP:DP2:T/	51:51:49,5	82:82:46,4	AC	A	0
.	.	IC=6;IHP=8	DP:DP2:T/	48:48:48,4	135:135:10	GT	G	0
.	.	IC=5;IHP=7	DP:DP2:T/	88:88:87,8	136:136:11	CT	C	0
.	.	IC=8;IHP=.	DP:DP2:T/	94:94:91,9	200:200:14	G	GC	0
.	.	IC=9;IHP=8	DP:DP2:T/	72:72:66,6	123:123:99	A	AT	0
.	.	IC=7;IHP=8	DP:DP2:T/	81:81:79,7	113:113:78	AG	A	0
.	.	IC=8;IHP=9	DP:DP2:T/	118:118:11	203:203:15	A	AC	0
.	.	IC=5;IHP=5	DP:DP2:T/	167:167:16	372:372:28	T	TG	0
.	.	IC=7;IHP=6	DP:DP2:T/	74:74:72,7	134:134:10	G	GC	0
0.000194	.	IC=5;IHP=6	DP:DP2:T/	118:118:11	268:268:21	TG	T	0
.	.	IC=5;IHP=8	DP:DP2:T/	53:53:53,5	74:74:58,5	CG	C	0
.	.	IC=9;IHP=.	DP:DP2:T/	81:81:77,7	159:159:10	A	AG	0
.	.	IC=0;IHP=6	DP:DP2:T/	46:46:46,4	81:81:61,6	CCCG	C	0
.	.	IC=2;IHP=3	DP:DP2:T/	124:124:12	212:212:19	A	AGGTGCT	0
.	.	IC=2;IHP=3	DP:DP2:T/	125:125:10	237:237:18	C	CCTGAAG	0
.	.	IC=0;IHP=4	DP:DP2:T/	81:81:90,9	161:161:17	CCATCTT	C	0
.	.	IC=2;IHP=3	DP:DP2:T/	90:90:86,8	149:149:13	AGCACTG	A	0
.	.	IC=0;IHP=4	DP:DP2:T/	73:73:73,7	165:165:15	GTCTCCC	G	0
.	.	IC=0;IHP=3	DP:DP2:T/	95:95:81,8	169:169:14	CATTTGT/	C	0
0.000451	.	IC=2;IHP=4	DP:DP2:T/	145:145:13	152:152:13	GGAGGC(	G	0
0.002163	0.004448	IC=2;IHP=4	DP:DP2:T/	90:90:87,9	122:122:11	T	TTGTGC	0
0.180922	0.286092	IC=1;IHP=5	DP:DP2:T/	16:16:16,1	7:7:1,1:6,6	G	GGAA	0
.	.	IC=1;IHP=3	DP:DP2:T/	77:77:76,9	107:107:91	A	AGGAGG	0
.	.	IC=0;IHP=4	DP:DP2:T/	80:80:84,9	149:149:13	AGGAGG/	A	0
.	.	IC=5;IHP=4	DP:DP2:T/	171:171:16	229:229:20	ATCCTCC	A	0
.	.	IC=1;IHP=5	DP:DP2:T/	100:100:10	133:133:11	T	TTCACGG	0
.	.	IC=0;IHP=3	DP:DP2:T/	219:219:20	189:189:16	CGGATGC	C	0
.	.	IC=0;IHP=5	DP:DP2:T/	76:76:106,	108:108:12	GCGTCCC	G	0
.	.	IC=0;IHP=3	DP:DP2:T/	81:81:89,8	149:149:15	ACTCAAT	A	0
.	0.00088	IC=4;IHP=.	DP:DP2:T/	114:114:89	243:243:18	AAAAAC	A	0
.	.	IC=0;IHP=3	DP:DP2:T/	82:82:82,8	148:148:14	TCCATGT	T	0
.	.	IC=0;IHP=3	DP:DP2:T/	83:83:79,9	240:240:23	AGGTGTC	A	0
.	.	IC=2;IHP=3	DP:DP2:T/	90:90:89,8	159:159:14	ACTCCT	A	0
.	0.00088	IC=2;IHP=3	DP:DP2:T/	130:130:12	158:158:14	CCAGTA	C	0
.	.	IC=1;IHP=3	DP:DP2:T/	116:116:12	241:241:22	AGAGTT	A	0
.	.	IC=0;IHP=5	DP:DP2:T/	123:123:12	248:248:24	CCGGGA(	C	0
.	.	IC=0;IHP=3	DP:DP2:T/	147:147:16	194:194:17	GCCAAGC	G	0
.	.	IC=1;IHP=3	DP:DP2:T/	78:78:78,7	159:159:12	GA	G	0
.	.	IC=2;IHP=4	DP:DP2:T/	98:98:91,9	200:200:16	G	GCAA	0
.	.	IC=2;IHP=3	DP:DP2:T/	101:101:99	232:232:17	A	ACGC	0
.	.	IC=0;IHP=7	DP:DP2:T/	65:65:75,7	121:121:12	TGGGGA(	T	0
0.039913	0.190813	IC=7;IHP=7	DP:DP2:T/	20:20:18,1	35:35:11,1	G	GT	0
.	.	IC=0;IHP=3	DP:DP2:T/	60:60:70,7	72:72:67,6	AAGTTGC	A	0
.	.	IC=4;IHP=4	DP:DP2:T/	115:115:10	186:186:15	CGAGGA(	C	0
0.000235	.	IC=0;IHP=4	DP:DP2:T/	40:40:45,4	51:51:41,4	GGCCGC(	G	0
.	.	IC=2;IHP=4	DP:DP2:T/	111:111:10	223:223:15	AC	A	0
.	.	IC=0;IHP=3	DP:DP2:T/	499:499:54	706:706:63	CGCCGC(	C	0
.	.	IC=7;IHP=6	DP:DP2:T/	92:92:91,9	219:219:17	A	AG	0
.	.	IC=7;IHP=6	DP:DP2:T/	106:106:99	71:71:44,4	A	AC	0
0.00366	0.048893	IC=5;IHP=5	DP:DP2:T/	90:90:64,6	234:234:16	C	CGCGGG(	0
.	.	IC=2;IHP=3	DP:DP2:T/	75:75:74,7	102:102:80	GC	G	0
.	.	IC=7;IHP=8	DP:DP2:T/	225:225:21	298:298:22	C	CG	0
.	.	IC=0;IHP=4	DP:DP2:T/	114:114:13	178:178:19	CCCCAAA	C	0

.	.	IC=4;IHP=:	DP:DP2:T/	77:77:75,7	180:180:1	T	TG	0
0.000194	.	IC=1;IHP=7	DP:DP2:T/	54:54:52,5	56:56:37,3	ATCT	A	0
.	.	IC=2;IHP=:	DP:DP2:T/	76:76:76,7	117:117:92	T	TA	0
0.000194	0.003521	IC=2;IHP=8	DP:DP2:T/	76:76:77,7	151:151:14	CGGGCG	C	0
.	.	IC=2;IHP=:	DP:DP2:T/	74:74:72,7	93:93:79,7	C	CTTG	0
.	.	IC=2;IHP=:	DP:DP2:T/	72:72:73,7	68:68:58,5	CAAGGG	C	0
.	.	IC=3;IHP=:	DP:DP2:T/	90:90:88,8	60:60:47,5	G	GC	0
.	.	IC=6;IHP=!	DP:DP2:T/	101:101:9(	151:151:12	CCAGCAG	C	0
.	.	IC=0;IHP=.	DP:DP2:T/	29:29:29,2	82:82:55,5	TA	T	0
0.002622	0.002727	IC=0;IHP=.	DP:DP2:T/	191:191:19	202:202:19	TTTTTG	T	0
.	.	IC=2;IHP=4	DP:DP2:T/	82:82:78,7	198:198:15	CG	C	0
.	.	IC=2;IHP=:	DP:DP2:T/	67:67:65,6	175:175:12	G	GT	0
.	.	IC=1;IHP=:	DP:DP2:T/	109:109:1(	88:88:76,7	A	AATTCT	0
.	.	IC=0;IHP=:	DP:DP2:T/	77:77:82,8	242:242:23	AGAGCG(	A	0
.	.	IC=0;IHP=!	DP:DP2:T/	84:84:89,8	178:178:16	CCCAGG/	C	0
.	.	IC=1;IHP=:	DP:DP2:T/	81:81:78,7	77:77:63,6	AGAGCC(	A	0
.	.	IC=0;IHP=8	DP:DP2:T/	73:73:88,8	81:81:72,7	GCCGCG(	G	0
.	.	IC=1;IHP=4	DP:DP2:T/	93:93:97,9	163:163:15	CGAGGT(	C	0
.	.	IC=0;IHP=7	DP:DP2:T/	19:19:20,2	50:50:28,2	TTGATCA	T	0
0.023056	.	IC=0;IHP=.	DP:DP2:T/	48:48:46,4	33:33:24,2	TA	T	0
0.000389	0.001393	IC=2;IHP=7	DP:DP2:T/	148:148:14	279:279:25	GGGGCC	G	0
.	0.000882	IC=4;IHP=4	DP:DP2:T/	161:161:14	222:222:17	TCCGCCG	T	0
.	.	IC=2;IHP=:	DP:DP2:T/	126:126:12	145:145:72	G	GCTGT	0
.	.	IC=2;IHP=:	DP:DP2:T/	81:81:76,8	104:104:52	C	CT	0
.	.	IC=8;IHP=7	DP:DP2:T/	118:118:1(	295:295:16	A	AT	0
.	.	IC=0;IHP=:	DP:DP2:T/	53:53:55,5	182:182:13	AGG	A	0
.	.	IC=3;IHP=!	DP:DP2:T/	113:113:11	232:232:16	CTT	C	0
.	.	IC=0;IHP=4	DP:DP2:T/	77:77:79,8	69:69:62,6	GTCTGC(	G	0

shared_het	OMIM	GWAS_Pu	HGMD_ID_	GO_BP	GO_CC	GO_MF	KEGG_PA	PID_PATH
0	.	NA	.	MULTI_OF	.	TRANSCR	.	.
0	{Celiac dis	NA	.	IMMUNE_I	INTRINSIC	.	KEGG_CE	PID_NFAT
0	.	NA	.	.	.	.	.	.
0	.	NA	.	ESTABLIS	ORGANEL	.	KEGG_SN	.
0	.	NA	.	.	.	.	.	.
0	.	NA	.	.	.	.	.	.
0	.	NA	.	.	.	.	KEGG_OL	.
0	.	NA	.	.	.	.	.	.
0	.	NA	.	.	CYTOPLA	ENZYME_	KEGG_CY	.
0	17-beta-hy	NA	.	LIPID_ME	CYTOPLA	STEROID_	KEGG_VA	.
0	.	NA	.	.	.	.	.	.
0	.	NA	.	MULTI_OF	INTRINSIC	TRANSME	KEGG_CY	PID_GLYP
0	Bleeding d	NA	.	FOCAL_AI	ADHEREN	INTEGRIN	KEGG_FO	PID_ILK_F
0	.	NA	.	.	.	.	.	.
0	.	NA	.	RNA_MET	ORGANEL	.	KEGG_SP	.
0	.	NA	.	RNA_MET	NUCLEUS	RNA_BIND	.	.
0	.	NA	.	.	.	.	KEGG_TG	.
0	.	NA	.	.	.	.	.	.
0	Bronchiect	NA	.	ESTABLIS	INTRINSIC	CATION_T	KEGG_TA	.
0	.	NA	.	.	.	.	.	.
0	.	NA	.	.	.	.	.	.
0	.	NA	.	.	.	.	.	.
0	.	NA	.	.	.	.	.	PID_TAP6
0	.	NA	.	.	.	.	.	.
0	.	NA	.	RNA_MET	NUCLEOP	.	.	.
0	.	NA	.	.	.	.	.	.
0	.	NA	.	.	.	.	.	.
0	.	NA	.	.	.	RECEPTO	KEGG_CY	PID_TNFP
0	.	NA	.	.	.	.	.	.
0	Mental reta	NA	.	RNA_MET	NUCLEOP	TRANSCR	.	.
0	.	NA	.	.	CYTOPLA	.	.	.
0	.	NA	.	.	CYTOPLA	.	.	.
0	.	NA	.	NUCLEOB	.	TRANSCR	.	.
0	.	NA	.	.	.	.	.	.
0	.	NA	.	.	.	.	.	PID_ATF2_
0	.	NA	.	TRNA_PR	.	LIGASE_A	KEGG_AM	.
0	.	NA	.	POSITIVE_	.	HYDROLA	.	PID_PRLS
0	.	NA	.	GLYCOPR	INTRINSIC	.	KEGG_NC	PID_NOTC
0	.	NA	.	.	.	.	KEGG_UB	.
0	.	NA	.	.	.	PHOSPHO	.	.
0	.	NA	.	.	.	.	.	.
0	.	NA	.	.	.	.	.	.
0	.	NA	.	PROTEOL	.	METALLO	.	.
0	.	NA	.	.	.	.	.	.
0	.	NA	.	.	CYTOPLA	HYDROLA	.	.
0	Severe cor	NA	.	REGULAT	INTRINSIC	PROTEIN_	KEGG_CE	PID_BCR_
0	.	NA	.	.	.	.	.	.
0	.	NA	.	.	.	.	.	.
0	.	NA	.	BIOPOLYM	CELL_JUN	NUCLEOT	.	PID_CDC4
0	.	NA	.	.	.	.	.	.
0	.	NA	.	.	.	.	.	.

0	.	NA	.	BIOPOLYN	.	PHOSPHC	KEGG_CH	PID_RHO/
0	.	NA	.	.	.	.	.	.
0	.	NA	.	CELL_DE\	ORGANEL	PEPTIDE_	.	.
0	.	NA	.	.	.	.	.	.
0	.	NA	.	PHOSPHC	ORGANEL	TRANSFE	KEGG_GL	.
0	.	NA	.	MACROM(	CYTOPLA:	RIBONUCI	.	.
0	.	NA	.	.	EXTRACE	.	.	.
0	.	NA	.	.	.	.	.	.
0	.	NA	.	MULTICEL	NUCLEUS	.	.	.
0	.	NA	.	MULTI_OF	.	TRANSCR	.	.
0	.	NA	.	REGULAT	INTRINSIC	TRANSME	KEGG_CY	PID_ALK1I
0	.	NA	.	.	.	.	.	.
0	.	NA	.	.	.	.	.	.
0	.	NA	.	.	.	.	.	.
0	.	NA	.	.	.	RNA_POL'	KEGG_MA	PID_SMAD
0	Pigmented	NA	.	SIGNAL_T	.	.	KEGG_PU	.
0	.	NA	.	.	.	.	.	.
0	Cardiomyo	NA	.	MUSCLE_	ORGANEL	NUCLEOT	KEGG_HY	.
0	Candidiasi:	NA	.	RNA_MET	CYTOPLA:	RECEPTO	KEGG_CH	PID_GMC!
0	.	NA	.	POSITIVE_	CYTOPLA:	TRANSLA'	.	.
0	.	NA	.	.	.	.	.	.
0	.	NA	.	SYSTEM_I	INTRINSIC	INTEGRIN	.	.
0	Mitochondı	NA	.	.	.	.	.	.
0	.	NA	.	.	.	.	.	.
0	.	NA	.	.	.	.	.	.
0	.	NA	.	.	.	.	.	.
0	.	NA	.	.	.	.	.	.
0	.	NA	.	SYSTEM_I	CYTOPLA:	RAS_GUA	KEGG_AD	PID_NECT
0	.	NA	.	SYSTEM_I	.	.	.	PID_NOTC
0	.	NA	.	REGULAT	CYTOPLA:	GTPASE_I	.	.
0	?Congenit:	NA	.	GLYCOPR	CYTOPLA:	.	.	.
0	.	NA	.	.	CYTOPLA:	.	.	.
0	.	NA	.	.	.	.	.	.
0	.	NA	.	RNA_MET	NUCLEUS	RNA_BINE	.	.
0	.	NA	.	.	.	.	.	.
0	.	NA	.	BIOPOLYN	CYTOPLA:	ENZYME_.	KEGG_CH	PID_ENDC
0	Sinoatrial r	NA	.	ESTABLIS	MEMBRAN	CATION_T	KEGG_MA	.
0	.	NA	.	.	.	.	.	.
0	ACAD9 de	NA	.	.	.	.	.	.
0	.	NA	.	.	.	.	.	PID_TRKF
0	.	NA	.	.	.	.	.	.
0	.	NA	.	MUSCLE_	CYTOPLA:	RNA_BINE	.	.
0	.	NA	.	REGULAT	INTRINSIC	.	KEGG_CE	.
0	.	NA	.	SYSTEM_I	.	.	KEGG_TG	PID_BMPF
0	.	NA	.	.	.	.	.	.
0	UV-sensitiv	NA	.	.	.	.	.	.
0	.	NA	.	.	.	.	.	.
0	.	NA	.	.	.	.	KEGG_GL	.
0	.	NA	.	.	.	.	.	.
0	.	NA	.	.	.	.	.	.
0	.	NA	.	.	.	.	.	.
0	.	NA	.	.	.	.	.	.
0	Adermatog	NA	.	DNA_MET	.	.	.	.
0	Hypomagn	NA	.	DNA_MET	MEMBRAN	RECEPTO	KEGG_MA	PID_PTP1

0	.	NA	.	.	MACROM(	.	KEGG_ER	PID_IFNGI
0	.	NA	.	.	.	ARYLSULI	.	.
0	.	NA	.	.	.	.	.	.
0	.	NA	.	.	.	.	KEGG_BA	.
0	.	NA	.	.	.	.	.	.
0	.	NA	.	.	.	.	.	.
0	.	NA	.	ENZYME_	.	PHOSPHC	.	PID_RHOA
0	Chondroca	NA	.	SYSTEM_I	MEMBRAN	ACTIVE_T	.	.
0	.	NA	.	.	.	.	.	.
0	Joubert syı	NA	.	.	.	.	.	.
0	Amyloidosi	NA	.	SIGNAL_T	INTRINSIC	RECEPTO	KEGG_CY	.
0	.	NA	.	.	.	.	.	.
0	Hermansky	NA	.	ESTABLIS	CYTOPLA:	.	KEGG_LY:	.
0	Acampome	NA	.	CELL_DEV	RIBONUCI	.	.	PID_ERA_
0	.	NA	.	.	.	.	.	.
0	.	NA	.	.	.	.	.	.
0	.	NA	.	SYSTEM_I	EXTRACE	.	KEGG_AX	.
0	.	NA	.	SYSTEM_I	EXTRACE	.	KEGG_AX	.
0	.	NA	.	.	.	.	KEGG_GL	.
0	.	NA	.	DEFENSE_	.	.	KEGG_NA	.
0	Trichohepa	NA	.	.	.	HYDROLA	KEGG_RN	.
0	.	NA	.	BIOPOLYN	INTRINSIC	RECEPTO	.	PID_AMB2
0	.	NA	.	.	.	.	.	.
0	.	NA	.	.	.	.	KEGG_CE	.
0	.	NA	.	.	.	.	.	.
0	.	NA	.	.	.	.	.	.
0	.	NA	.	.	.	.	.	.
0	.	NA	.	.	.	.	.	.
0	Obesity, se	NA	.	SYSTEM_I	.	TRANSCR	.	.
0	Mental reta	NA	.	REGULAT	INTRINSIC	GLUTAMA	KEGG_NE	.
0	.	NA	.	.	.	.	.	.
0	.	NA	.	ESTABLIS	MEMBRAN	.	.	.
0	{Psoriasis :	NA	.	POSITIVE_	.	.	.	.
0	Cardiomyo	NA	.	.	PROTEIN/	STRUCTU	KEGG_FO	PID_INTE(
0	.	NA	.	.	.	.	.	.
0	.	NA	.	SIGNAL_T	.	GTP_BINE	.	.
0	.	NA	.	.	.	.	.	.
0	.	NA	.	.	.	.	.	.
0	Hepatocell	NA	.	ESTABLIS	ORGANEL	TRANSME	KEGG_LY:	.
0	.	NA	.	.	.	.	.	.
0	.	NA	.	.	.	.	.	.
0	.	NA	.	.	.	.	.	.
0	.	NA	.	.	.	.	.	.
0	.	NA	.	MULTICEL	.	.	.	.
0	.	NA	.	.	.	.	.	.
0	.	NA	.	PROTEIN_	CYTOPLA:	ENZYME_	KEGG_NC	.
0	.	NA	.	PROTEIN_	CYTOPLA:	ENZYME_	KEGG_NC	.
0	.	NA	.	NEGATIVE	CYTOPLA:	.	.	.
0	.	NA	.	.	.	.	.	.
0	.	NA	.	.	.	.	.	.
0	Cutis laxa,	NA	.	SYSTEM_I	.	.	.	.
0	.	NA	.	.	.	.	.	.
0	.	NA	.	.	.	.	.	.
0	.	NA	.	SYSTEM_I	INTRINSIC	.	KEGG_AX	PID_EPHE

0	.	NA	.	RNA_MET	.	RNA_POL	.	.
0	.	NA	.	.	.	.	.	.
0	.	NA	.	STEROID_	.	ALDO_KE	KEGG_FR	.
0	.	NA	.	ORGANEL	.	.	.	.
0	Ventricular	NA	.	.	.	.	.	.
0	Barrett esc	NA	.	ESTABLIS	INTRINSIC	RECEPTO	.	.
0	.	NA	.	.	.	.	KEGG_EN	.
0	.	NA	.	.	.	.	.	.
0	.	NA	.	.	.	.	.	.
0	{?Schizoph	NA	.	NEGATIVE	.	TRANSCR	KEGG_ER	PID_ERBB
0	.	NA	.	.	.	.	.	.
0	.	NA	.	REGULAT	MEMBRAN	PHOSPHO	KEGG_CH	PID_FCER
0	.	NA	.	SYSTEM_I	.	TRANSCR	.	PID_HES_
0	.	NA	.	.	.	.	.	.
0	.	NA	.	.	.	.	.	.
0	Seizures, b	NA	.	ESTABLIS	INTRINSIC	CATION_T	.	.
0	.	NA	.	SYSTEM_I	INTRINSIC	G_PROTE	KEGG_P5	.
0	.	NA	.	.	CYTOPLA	ELECTRO	KEGG_OX	.
0	.	NA	.	.	.	.	.	.
0	.	NA	.	RESPONS	.	.	.	.
0	.	NA	.	MACROMO	CELL_PRO	PROTEIN_	KEGG_FO	PID_RHOA
0	.	NA	.	.	.	.	.	.
0	.	NA	.	.	NUCLEUS	.	.	.
0	Basal cell c	NA	.	SYSTEM_I	INTRINSIC	RECEPTO	KEGG_HE	PID_HEDG
0	Basal cell c	NA	.	SYSTEM_I	INTRINSIC	RECEPTO	KEGG_HE	PID_HEDG
0	.	NA	.	BIOPOLYM	ORGANEL	.	.	.
0	.	NA	.	.	.	.	KEGG_OL	.
0	.	NA	.	.	NUCLEUS	.	.	.
0	.	NA	.	ESTABLIS	CYTOPLA	.	.	.
0	.	NA	.	.	.	.	.	.
0	.	NA	.	.	.	.	.	.
0	.	NA	.	PROTEOL	ORGANEL	METALLO	.	.
0	.	NA	.	.	.	.	.	.
0	.	NA	.	.	.	.	.	.
0	.	NA	.	.	.	.	.	.
0	.	NA	.	.	.	.	.	.
0	.	NA	.	CARBOXY	.	.	KEGG_AL	.
0	.	NA	.	.	.	.	.	.
0	.	NA	.	.	.	.	.	.
0	.	NA	.	RNA_MET	.	NUCLEAR	.	.
0	.	NA	.	.	ORGANEL	HYDROLA	.	.
0	.	NA	.	.	.	.	.	.
0	.	NA	.	.	.	.	.	.
0	.	NA	.	.	.	.	.	PID_AR_P
0	.	NA	.	IMMUNE_I	CYTOPLA	.	.	.
0	.	NA	.	.	.	.	.	.
0	Cone dystr	NA	.	SENSORY	.	.	KEGG_PU	PID_CONE
0	.	NA	.	.	ORGANEL	STRUCTU	.	.
0	.	NA	.	.	.	NUCLEAR	.	.
0	.	NA	.	REGULAT	NUCLEOP	PROTEIN_	.	PID_MTOR
0	{Influenza,	NA	.	IMMUNE_I	MEMBRAN	.	.	.
0	.	NA	.	.	.	.	.	.
0	.	NA	.	SYSTEM_I	.	NUCLEOT	.	PID_LKB1_
0	.	NA	.	.	.	.	KEGG_OL	.

0	.	NA	.	.	.	.	.	.
0	.	NA	.	.	.	.	.	.
0	.	NA	.	.	.	.	.	.
0	Dystonia 2	NA	.	.	.	.	.	.
0	.	NA	.	.	.	.	KEGG_ST	.
0	{Diabetes	NA	.	ESTABLIS	CYTOPLA	KINASE_B	KEGG_MA	PID_REEL
0	.	NA	.	.	.	.	.	.
0	.	NA	.	.	.	RNA_BIND	.	.
0	.	NA	.	SIGNAL_T	CYTOPLA	.	KEGG_CH	PID_HDAC
0	.	NA	.	.	.	.	.	.
0	.	NA	.	.	.	.	.	.
0	.	NA	.	.	MEMBRAN	PHOSPHO	.	.
0	.	NA	.	.	.	.	.	.
0	.	NA	.	.	.	.	.	.
0	Hemorrhag	NA	.	.	.	.	KEGG_CE	PID_AMB2
0	.	NA	.	RNA_MET	ORGANEL	TRANSCR	.	.
0	.	NA	.	.	.	TRANSFE	KEGG_O_	.
0	.	NA	.	RNA_MET	NUCLEUS	.	.	.
0	.	NA	.	CELL_DEV	MEMBRAN	PROTEIN_	KEGG_AD	PID_BCR_
0	.	NA	.	.	.	.	.	.
0	.	NA	.	.	.	.	.	.
0	.	NA	.	.	.	.	.	.
0	.	NA	.	.	.	.	.	.
0	.	NA	.	.	.	.	.	.
0	.	NA	.	.	.	.	.	.
0	.	NA	.	.	.	.	.	.
0	.	NA	.	.	.	.	.	.
0	.	NA	.	.	.	.	.	.
0	.	NA	.	ESTABLIS	INTRINSIC	CATION_T	KEGG_LY	PID_HIF2F
0	Cognitive i	NA	.	SYSTEM_	INTRINSIC	.	.	.
0	.	NA	.	MACROMO	CYTOPLA	TRANSLAT	KEGG_MT	PID_MTOR
0	.	NA	.	RNA_MET	.	TRANSCR	.	.
0	.	NA	.	NEGATIVE	ORGANEL	PROTEIN_	.	PID_ATR_
0	Deafness,	NA	.	SENSORY	ORGANEL	.	.	.
0	.	NA	.	LIPID_MET	INTRINSIC	CATION_E	KEGG_AL	PID_AMB2
0	.	NA	.	.	.	.	.	.
0	.	NA	.	RNA_MET	CYTOPLA	RNA_POL	.	.
0	.	NA	.	RNA_MET	NUCLEOP	TRANSCR	KEGG_AN	PID_P53D
0	.	NA	.	.	.	.	.	.
0	.	NA	.	MACROMO	.	.	KEGG_AD	.
0	.	NA	.	.	.	.	.	.
0	.	NA	.	.	.	.	KEGG_AM	.
0	.	NA	.	POSITIVE_	.	TRANSLAT	.	.
0	.	NA	.	.	.	.	.	.
0	.	NA	.	.	.	.	KEGG_WN	PID_WNT_
0	Bleeding d	NA	.	FOCAL_AI	ADHEREN	INTEGRIN	KEGG_FO	PID_ILK_F
0	.	NA	.	.	.	.	.	.
0	.	NA	.	.	.	.	.	.
0	.	NA	.	CELL_PRO	CYTOPLA	CATION_E	.	.
0	.	NA	.	.	.	.	.	.
0	.	NA	.	.	.	.	.	.
0	.	NA	.	.	.	.	.	.
0	.	NA	.	.	NUCLEOP	.	.	PID_ATM_
0	.	NA	.	.	.	PHOSPHO	KEGG_GA	.

0	Amelogene	NA	.	.	.	.	.	.
0	.	NA	.	.	.	.	.	.
0	.	NA	.	.	INTRINSIC	.	KEGG_CE	.
0	.	NA	.	POSITIVE_	.	.	KEGG_CE	.
0	.	NA	.	SYSTEM_I	NUCLEUS	.	.	.
0	.	NA	.	.	.	.	.	.
0	.	NA	.	ENZYME_	INTRINSIC	GROWTH_	KEGG_NE	PID_SHP2
0	Acrocallosa	NA	.	.	.	.	.	.
0	.	NA	.	BIOPOLYM	.	.	KEGG_AX	PID_IL4_2
0	.	NA	.	REGULAT	CYTOPLA	.	.	.
0	Nephronop	NA	.	NEGATIVE	NUCLEOP	TRANSCR	.	.
0	.	NA	.	.	.	.	.	.
0	.	NA	.	.	.	.	.	.
0	.	NA	.	.	.	.	.	.
0	.	NA	.	.	.	.	.	PID_AURO
0	.	NA	.	.	.	.	KEGG_LYS	.
0	.	NA	.	NUCLEOB	INTRINSIC	PHOSPHO	KEGG_PU	PID_ENDO
0	.	NA	.	HOMOPHI	MEMBRAN	.	KEGG_CE	PID_VEGF
0	.	NA	.	.	.	.	.	.
0	.	NA	.	ESTABLIS	INTRINSIC	ACTIVE_T	.	.
0	Brittle corn	NA	.	.	.	.	.	.
0	.	NA	.	.	.	.	.	.
0	.	NA	.	.	.	.	.	.
0	.	NA	.	.	.	.	.	.
0	.	NA	.	ESTABLIS	.	AUXILIARY	.	.
0	.	NA	.	CYTOKINE	ORGANEL	HYDROLA	KEGG_TIG	.
0	Leukemia,	NA	.	REGULAT	AXON;CYT	RAS_GTP	KEGG_MA	PID_HNF3
0	.	NA	.	.	.	.	.	.
0	.	NA	.	PROTEIN_	NUCLEUS	.	.	.
0	.	NA	.	JAK_STAT	.	TRANSCR	KEGG_ER	PID_ERBE
0	.	NA	.	DEFENSE_	CELL_SUF	COPPER_	KEGG_GL	.
0	.	NA	.	.	.	.	.	.
0	.	NA	.	.	.	.	.	.
0	.	NA	.	.	ORGANEL	.	.	.
0	.	NA	.	.	.	.	.	.
0	.	NA	.	.	.	.	.	.
0	.	NA	.	.	.	.	.	.
0	.	NA	.	.	.	.	KEGG_OT	.
0	.	NA	.	.	.	.	.	.
0	.	NA	.	REGULAT	.	GTPASE_I	.	.
0	Fascioscap	NA	.	.	.	.	.	.
0	.	NA	.	DNA_MET	CYTOPLA	PROTEIN_	.	.
0	.	NA	.	.	.	.	.	.
0	Niemann-P	NA	.	REGULAT	ORGANEL	SUBSTRA	KEGG_LYS	.
0	.	NA	.	.	.	NF_KAPPA	KEGG_BA	PID_MYC_
0	.	NA	.	.	.	.	.	.
0	Deafness,	NA	.	.	.	.	.	.
0	{Colorectal	NA	.	ENZYME_	.	RECEPTO	KEGG_TG	PID_SMAD
0	.	NA	.	.	.	.	.	.
0	.	NA	.	.	.	.	.	.
0	.	NA	.	GAMETE_	.	RNA_BIND	.	.
0	.	NA	.	.	.	.	.	.
0	.	NA	.	.	.	.	.	.
0	.	NA	.	.	.	.	.	.

0	.	NA	.	.	.	.	.	.
0	.	NA	.	.	INTRINSIC	PROTEIN_	.	.
0	.	NA	.	.	INTRINSIC	PROTEIN_	.	.
0	.	NA	.	RNA_MET	NUCLEOP	RNA_POL	KEGG_BA	.
0	C3 deficier	NA	.	SIGNAL_T	.	RECEPTO	KEGG_CC	PID_ERB_
0	.	NA	.	.	.	.	.	.
0	.	NA	.	.	.	.	.	.
0	SCID, autc	NA	.	BIOPOLYM	.	.	KEGG_CH	PID_IL4_2
0	Mucoepide	NA	.	RNA_MET	NUCLEUS	TRANSCR	.	PID_AP1_
0	.	NA	.	.	.	.	.	.
0	.	NA	.	.	.	ENZYME_	KEGG_UB	.
0	.	NA	.	.	.	.	.	.
0	.	NA	.	.	.	.	.	.
0	.	NA	.	.	.	.	.	.
0	.	NA	.	.	.	.	.	.
0	.	NA	.	.	.	.	.	.
0	.	NA	.	.	.	.	.	.
0	.	NA	.	.	.	.	.	.
0	.	NA	.	.	.	.	.	.
0	.	NA	.	SYSTEM_	ORGANEL	PHOSPHO	.	PID_LKB1_
0	.	NA	.	SENSORY	ORGANEL	.	.	.
0	.	NA	.	RNA_MET	ORGANEL	.	KEGG_SP	.
0	.	NA	.	.	.	.	.	.
0	.	NA	.	.	.	.	.	.
0	Colorectal	NA	.	REGULAT	ORGANEL	PROTEIN_	KEGG_P5	PID_P73P
0	.	NA	.	SIGNAL_T	.	HYDROLA	KEGG_MA	PID_EPHE
0	.	NA	.	PROTEOL	.	.	KEGG_LY	.
0	.	NA	.	SIGNAL_T	INTRINSIC	CARBOHY	.	.
0	.	NA	.	SIGNAL_T	.	RECEPTO	.	.
0	.	NA	.	.	INTRINSIC	.	.	.
0	.	NA	.	MITOTIC_	CYTOPLA	PHOSPHO	.	PID_LKB1_
0	.	NA	.	.	.	ACID_AMI	KEGG_UB	PID_ERB_
0	.	NA	.	.	.	.	.	.
0	.	NA	.	.	.	.	.	.
0	.	NA	.	.	.	.	.	.
0	.	NA	.	BIOSYNTH	RIBONUCL	RECEPTO	.	.
0	Bohring-O	NA	.	.	.	.	.	.
0	.	NA	.	.	.	LIGASE_A	KEGG_GL	PID_HDAC
0	.	NA	.	.	.	.	.	.
0	.	NA	.	.	CYTOPLA	.	.	.
0	.	NA	.	SYSTEM_	.	.	.	.
0	.	NA	.	.	.	.	.	.
0	Epileptic e	NA	.	SYSTEM_	INTRINSIC	CATION_T	.	.
0	.	NA	.	.	.	.	.	.
0	.	NA	.	.	.	.	.	.
0	Glutamate	NA	.	COFACTO	CYTOPLA	.	KEGG_HIS	.
0	.	NA	.	DNA_MET	CYTOPLA	DNA_BIND	.	.
0	.	NA	.	.	.	.	.	.
0	.	NA	.	.	.	.	.	.
0	Meningiom	NA	.	.	.	.	.	.
0	.	NA	.	.	.	.	.	.
0	.	NA	.	.	.	.	.	.
0	.	NA	.	REGULAT	.	.	.	.
0	.	NA	.	.	.	.	.	.

0	.	NA	.	.	.	.	KEGG_P53	.
0	.	NA	.	.	.	.	.	.
0	.	NA	.	BIOPOLYM	PROTEIN_	PROTEIN_	.	PID_RB_1
0	Ichthyosis,	NA	.	LIPID_CAT	VESICULA	HYDROLA	KEGG_ST	.
0	.	NA	.	.	.	.	.	.
0	.	NA	.	.	CYTOPLAS	HYDROLA	KEGG_RIC	.
0	.	NA	.	RNA_MET	.	TRANSCR	KEGG_MA	PID_BCR_
0	Congenital	NA	.	ESTABLIS	.	SUBSTRA	.	.
0	.	NA	.	.	.	.	.	.
0	.	NA	.	SIGNAL_T	NON_MEM	KINASE_B	.	.
0	.	NA	.	.	.	.	.	.
0	Mental reta	NA	.	.	ORGANEL	CATION_T	KEGG_CA	.
0	.	NA	.	.	.	.	KEGG_AX	.
0	Axenfeld-R	NA	.	SYSTEM_I	ORGANEL	SEQUENC	.	.
0	Osteogene	NA	.	RESPONS	CYTOPLAS	PROTEAS	.	.
0	.	NA	.	.	.	.	.	.
0	.	NA	.	.	.	.	KEGG_GL	.
0	.	NA	.	.	.	.	.	.
0	.	NA	.	.	.	.	.	.
0	.	NA	.	.	.	.	.	.
0	.	NA	.	.	.	.	.	.
0	.	NA	.	.	.	.	.	.
0	.	NA	.	.	.	.	.	.
0	.	NA	.	.	.	.	.	.
0	.	NA	.	.	.	.	.	.
0	.	NA	.	.	CYTOPLAS	.	.	PID_MTOR
0	.	NA	.	.	.	.	.	.
0	.	NA	.	SYSTEM_I	.	.	.	.
0	.	NA	.	.	.	.	.	.
0	.	NA	.	CELL_DEV	CYTOPLAS	NUCLEOT	KEGG_MA	PID_P38G
0	Pontocereb	NA	.	BIOPOLYM	CYTOPLAS	RNA_BIND	KEGG_AM	.
0	.	NA	.	ESTABLIS	MEMBRAN	LIPID_BIN	.	PID_ARF6
0	.	NA	.	.	.	.	.	.
0	.	NA	.	.	.	.	.	.
0	.	NA	.	RNA_MET	.	HYDROLA	.	PID_HDAC
0	.	NA	.	.	.	.	.	.
0	.	NA	.	GLUCOSE	CYTOPLAS	PHOSPHO	KEGG_T_C	.
0	.	NA	.	.	.	.	KEGG_INC	.
0	.	NA	.	DNA_MET	NUCLEUS	NUCLEOT	KEGG_PU	.
0	Adrenal co	NA	.	REGULAT	NUCLEOP	NUCLEOT	KEGG_MA	PID_HDAC
0	Progressiv	NA	.	.	.	.	.	.
0	.	NA	.	.	CELL_FRA	.	.	.
0	Mental reta	NA	.	.	MEMBRAN	CATION_T	KEGG_MA	.
0	{Diabetes m	NA	.	BIOSYNTH	CYTOPLAS	RNA_BIND	.	.
0	.	NA	.	SYSTEM_I	ORGANEL	.	.	.
0	.	NA	.	.	.	.	.	.
0	.	NA	.	.	.	.	.	.
0	.	NA	.	.	.	.	.	.
0	Acne inver	NA	.	GLYCOPR	INTRINSIC	.	KEGG_NC	PID_NOTC
0	.	NA	.	.	.	.	.	.
0	Mitochondr	NA	.	.	.	.	.	.
0	.	NA	.	SYSTEM_I	.	ACTIN_BIN	.	.
0	.	NA	.	SYSTEM_I	INTRINSIC	TRANSME	.	.
0	.	NA	.	.	.	.	.	.

0	.	NA	.	.	.	.	KEGG_PU	.	
0	.	NA	.	CELL_REC	.	.	.	.	
0	.	NA	.	ISOPRENC	.	OXIDOREI	KEGG_RE	.	
0	.	NA	.	.	.	.	.	.	
0	.	NA	.	.	.	.	.	.	
0	.	NA	.	.	.	.	.	.	
0	.	NA	.	.	.	STRUCTU	.	PID_PLK1_	
0	.	NA	.	.	.	.	.	.	
0	.	NA	.	.	INTRINSIC	RECEPTO	.	.	
0	.	NA	.	.	.	.	.	PID_CDC4	
0	Leber cong	NA	.	.	.	.	.	.	
0	Myeloid leu	NA	.	RNA_MET	.	RNA_POL	.	PID_SMAD	
0	.	NA	.	.	.	.	.	.	
0	.	NA	.	.	.	.	.	.	
0	Knobloch s	NA	.	SYSTEM_I	PROTEINA	.	.	PID_INTEC	
0	.	NA	.	.	.	.	.	.	
0	.	NA	.	MULTICEL	.	.	.	PID_SMAD	
0	.	NA	.	.	.	.	.	.	
0	.	NA	.	SIGNAL_T	.	RECEPTO	.	PID_ANGI	
0	.	NA	.	CELL_DEV	CYTOPLAS	ACID_AMI	KEGG_UB	PID_CD40	
0	.	NA	.	.	.	.	.	.	
0	.	NA	.	.	NUCLEOP	.	KEGG_TIC	.	
0	.	NA	.	.	.	.	.	.	
0	.	NA	.	.	.	.	.	.	
0	Leiomyoma	NA	.	.	.	.	KEGG_FO	PID_INTEC	
0	.	NA	.	.	.	.	.	.	
0	.	NA	.	.	.	.	.	.	
0	.	NA	.	.	.	.	.	PID_BMPF	

BIOCART/	REACTOME_PATHWAY
.	.
BIOCART/	REACTOME_COSTIMULATION_BY_THE_CD28_FAMILY;REACTOME_CTLA4_INHIBIT
.	.
.	.
.	.
.	.
.	REACTOME_SIGNALING_BY_GPCR;REACTOME_OLFACTORY_SIGNALING_PATHW
.	.
.	.
.	REACTOME_METABOLISM_OF_AMINO_ACIDS_AND_DERIVATIVES;REACTOME_BR
.	.
BIOCART/	REACTOME_VEGF_LIGAND_RECEPTOR_INTERACTIONS
BIOCART/	REACTOME_CELL_CELL_COMMUNICATION;REACTOME_RESPONSE_TO_ELEVATE
.	.
.	REACTOME_PROCESSING_OF_CAPPED_INTRON_CONTAINING_PRE_MRNA;REAC
.	.
.	.
.	.
.	.
.	.
.	.
.	.
.	.
.	.
.	.
.	.
.	.
BIOCART/	.
.	REACTOME_IMMUNE_SYSTEM;REACTOME_ADAPTIVE_IMMUNE_SYSTEM;REACTO
.	.
.	.
.	.
.	.
.	.
.	REACTOME_EXTRACELLULAR_MATRIX_ORGANIZATION;REACTOME_COLLAGEN_
.	REACTOME_CYTOSOLIC_TRNA_AMINOACYLATION;REACTOME_TRNA_AMINOACY
.	REACTOME_SIGNALING_BY_RHO_GTPASES;REACTOME_DEVELOPMENTAL_BIOL
.	REACTOME_SIGNALLING_BY_NGF;REACTOME_SIGNALING_BY_ERBB4;REACTOM
.	REACTOME_IMMUNE_SYSTEM;REACTOME_ADAPTIVE_IMMUNE_SYSTEM;REACTO
.	.
.	.
.	REACTOME_CS_DS_DEGRADATION;REACTOME_CHONDROITIN_SULFATE_BIOSY
.	REACTOME_DIABETES_PATHWAYS;REACTOME_REGULATION_OF_INSULIN_LIKE_
.	.
.	REACTOME_PROCESSING_OF_CAPPED_INTRON_CONTAINING_PRE_MRNA;REAC
BIOCART/	REACTOME_DEVELOPMENTAL_BIOLOGY;REACTOME_TCR_SIGNALING;REACTOM
.	.
.	REACTOME_G0_AND_EARLY_G1;REACTOME_CELL_CYCLE;REACTOME_CELL_CY
.	.
.	.
.	.

BIOCARTA	REACTOME_DEVELOPMENTAL_BIOLOGY;REACTOME_SIGNALING_BY_GPCR;REA
.	.
.	.
.	.
.	REACTOME_METABOLISM_OF_PROTEINS;REACTOME_POST_TRANSLATIONAL_M
.	.
.	.
.	.
.	.
.	.
.	REACTOME_DEVELOPMENTAL_BIOLOGY;REACTOME_SIGNALING_BY_NODAL;RE
.	.
.	.
.	.
BIOCARTA	REACTOME_TRIF_MEDIATED_TLR3_SIGNALING;REACTOME_MAP_KINASE_ACTIV
.	REACTOME_SIGNALING_BY_GPCR;REACTOME_GPCR_DOWNSTREAM_SIGNALIN
.	.
.	.
BIOCARTA	REACTOME_SIGNALING_BY_SCF_KIT;REACTOME_GROWTH_HORMONE_RECEPT
.	.
.	.
.	.
.	.
.	.
.	.
BIOCARTA	.
.	.
.	REACTOME_DEVELOPMENTAL_BIOLOGY;REACTOME_AXON_GUIDANCE;REACTO
.	REACTOME_DEVELOPMENTAL_BIOLOGY;REACTOME_AXON_GUIDANCE;REACTO
.	.
.	.
.	REACTOME_SYNTHESIS_OF_PIPS_AT_THE_GOLGI_MEMBRANE;REACTOME_PHO
.	.
.	.
.	.
BIOCARTA	REACTOME_APOPTOTIC_CLEAVAGE_OF_CELLULAR_PROTEINS;REACTOME_SIG
.	.
.	.
.	.
.	.
.	.
.	.
.	.
BIOCARTA	.
.	.
.	.
.	.
.	REACTOME_GLYCOGEN_BREAKDOWN_GLYCOGENOLYSIS;REACTOME_METABO
.	.
.	.
.	.
.	.
.	.
BIOCARTA	REACTOME_SIGNALING_BY_ERBB4;REACTOME_SIGNALING_BY_ERBB2;REACTO

BIOCARTA	REACTOME_TRANSMISSION_ACROSS_CHEMICAL_SYNAPSES;REACTOME_NEUR(
.	REACTOME_PTM_GAMMA_CARBOXYLATION_HYPUSINE_FORMATION_AND_ARYL
.	.
.	.
.	.
.	.
BIOCARTA	REACTOME_SIGNALING_BY_RHO_GTPASES;REACTOME_SIGNALLING_BY_NGF;RI
.	.
.	.
.	.
.	.
.	.
.	REACTOME_MEMBRANE_TRAFFICKING;REACTOME_TRANS_GOLGI_NETWORK_V
BIOCARTA	.
.	.
.	.
.	REACTOME_DEVELOPMENTAL_BIOLOGY;REACTOME_AXON_GUIDANCE;REACTOI
.	REACTOME_DEVELOPMENTAL_BIOLOGY;REACTOME_AXON_GUIDANCE;REACTOI
.	.
.	.
.	.
.	REACTOME_TRIF_MEDIATED_TLR3_SIGNALING;REACTOME_RIP_MEDIATED_NFKI
.	.
.	REACTOME_MHC_CLASS_II_ANTIGEN_PRESENTATION;REACTOME_IMMUNE_SYS
.	.
.	.
.	.
.	.
.	.
.	REACTOME_TRANSMISSION_ACROSS_CHEMICAL_SYNAPSES;REACTOME_NEUR(
.	.
.	REACTOME_AMINO_ACID_TRANSPORT_ACROSS_THE_PLASMA_MEMBRANE;REA
.	.
BIOCARTA	.
.	.
.	.
.	.
.	.
.	REACTOME_MEMBRANE_TRAFFICKING;REACTOME_TRANS_GOLGI_NETWORK_V
.	.
.	.
.	.
.	.
.	.
.	.
.	REACTOME_TRIF_MEDIATED_TLR3_SIGNALING;REACTOME_TAK1_ACTIVATES_NI
.	REACTOME_TRIF_MEDIATED_TLR3_SIGNALING;REACTOME_TAK1_ACTIVATES_NI
.	.
.	.
.	.
.	.
.	REACTOME_GENERIC_TRANSCRIPTION_PATHWAY
.	.
.	.

.	REACTOME_SIGNALING_BY_FGFR_IN_DISEASE;REACTOME_SIGNALING_BY_FGFI
.	.
.	.
.	.
.	.
.	.
.	.
.	.
.	.
BIOCARTA	REACTOME_SIGNALING_BY_ERBB4;REACTOME_DOWNREGULATION_OF_ERBB2_
.	.
BIOCARTA	REACTOME_SIGNALING_BY_SCF_KIT;REACTOME_GROWTH_HORMONE_RECEPT
.	REACTOME_NOTCH1_INTRACELLULAR_DOMAIN_REGULATES_TRANSCRIPTION;F
.	.
.	.
.	REACTOME_DEVELOPMENTAL_BIOLOGY;REACTOME_NEURONAL_SYSTEM;REAC
.	.
.	REACTOME_TCA_CYCLE_AND_RESPIRATORY_ELECTRON_TRANSPORT;REACTO
.	.
.	.
BIOCARTA	REACTOME_DEVELOPMENTAL_BIOLOGY;REACTOME_RESPONSE_TO_ELEVATED
.	.
.	.
BIOCARTA	REACTOME_SIGNALING_BY_GPCR;REACTOME_CLASS_B_2_SECRETIN_FAMILY_F
BIOCARTA	REACTOME_SIGNALING_BY_GPCR;REACTOME_CLASS_B_2_SECRETIN_FAMILY_F
.	.
.	REACTOME_SIGNALING_BY_GPCR;REACTOME_OLFACTORY_SIGNALING_PATHW
.	.
.	.
.	.
.	.
.	REACTOME_MITOCHONDRIAL_PROTEIN_IMPORT;REACTOME_METABOLISM_OF_I
.	.
.	.
.	.
.	.
.	REACTOME_TRANSMISSION_ACROSS_CHEMICAL_SYNAPSES;REACTOME_NEUR(
.	.
.	.
.	REACTOME_FACTORS_INVOLVED_IN_MEGAKARYOCYTE_DEVELOPMENT_AND_P
.	.
.	REACTOME_EXTRACELLULAR_MATRIX_ORGANIZATION;REACTOME_COLLAGEN_
.	.
.	.
.	.
.	.
.	.
.	.
.	.
.	.
.	REACTOME_INTERFERON_ALPHA_BETA_SIGNALING;REACTOME_INTERFERON_S
.	.
.	.
.	REACTOME_SIGNALING_BY_GPCR;REACTOME_OLFACTORY_SIGNALING_PATHW

.	.
.	.
.	.
.	REACTOME_O_LINKED_GLYCOSYLATION_OF_MUCINS;REACTOME_TERMINATION
.	REACTOME_TRIGLYCERIDE_BIOSYNTHESIS;REACTOME_FATTY_ACYL_COA_BIOS
.	.
.	.
.	.
.	REACTOME_SIGNALLING_BY_NGF;REACTOME_DAG_AND_IP3_SIGNALING;REACT
.	.
.	.
.	.
.	.
.	.
.	REACTOME_CELL_SURFACE_INTERACTIONS_AT_THE_VASCULAR_WALL;REACTO
.	.
.	REACTOME_O_LINKED_GLYCOSYLATION_OF_MUCINS;REACTOME_METABOLISM_
.	.
BIOCARTA	REACTOME_SIGNALING_BY_SCF_KIT;REACTOME_GROWTH_HORMONE_RECEPT
.	.
.	.
.	.
.	.
.	.
.	REACTOME_O_LINKED_GLYCOSYLATION_OF_MUCINS;REACTOME_TERMINATION
.	.
.	.
.	.
.	.
.	REACTOME_DEVELOPMENTAL_BIOLOGY;REACTOME_AXON_GUIDANCE;REACTO
BIOCARTA	REACTOME_TRANSLATION;REACTOME_INSULIN_RECEPTOR_SIGNALLING_CASC
.	.
.	.
.	.
.	.
.	.
.	.
.	REACTOME_PPARA_ACTIVATES_GENE_EXPRESSION;REACTOME_DIABETES_PAT
.	.
BIOCARTA	.
.	.
.	REACTOME_MITOCHONDRIAL_TRNA_AMINOACYLATION;REACTOME_TRNA_AMIN
.	.
.	.
.	.
BIOCARTA	REACTOME_CELL_CELL_COMMUNICATION;REACTOME_RESPONSE_TO_ELEVATE
.	.
.	.
.	.
.	.
.	.
.	.
.	REACTOME_HOMOLOGOUS_RECOMBINATION_REPAIR_OF_REPLICATION_INDEP
.	.

.	.
.	.
.	REACTOME_DEVELOPMENTAL_BIOLOGY;REACTOME_AXON_GUIDANCE;REACTO
.	.
.	.
.	.
.	.
.	.
.	REACTOME_SIGNALING_BY_SCF_KIT;REACTOME_DEVELOPMENTAL_BIOLOGY;RE
.	.
.	.
.	.
.	.
.	.
.	.
.	.
.	REACTOME_SIGNALLING_BY_NGF;REACTOME_DAG_AND_IP3_SIGNALING;REACT
.	REACTOME_CELL_CELL_COMMUNICATION;REACTOME_ADHERENS_JUNCTIONS_
.	.
.	REACTOME_CELL_SURFACE_INTERACTIONS_AT_THE_VASCULAR_WALL;REACTC
.	.
.	.
.	.
.	.
.	REACTOME_NEURONAL_SYSTEM;REACTOME_VOLTAGE_GATED_POTASSIUM_CH
.	REACTOME_DEVELOPMENTAL_BIOLOGY;REACTOME_AXON_GUIDANCE;REACTO
.	.
.	.
.	.
BIOCART	REACTOME_SIGNALING_BY_SCF_KIT;REACTOME_GROWTH_HORMONE_RECEPT
.	.
.	.
.	.
.	REACTOME_STRIATED_MUSCLE_CONTRACTION;REACTOME_MUSCLE_CONTRAC
.	.
.	.
.	.
.	.
.	.
.	.
.	.
.	.
.	.
.	.
.	REACTOME_RNA_POL_II_TRANSCRIPTION;REACTOME_RNA_POL_II_TRANSCRIPT
.	.
.	.
BIOCART	REACTOME_SIGNALING_BY_BMP;REACTOME_DOWNREGULATION_OF_TGF_BET
.	.
.	.
.	.
.	.
.	.
.	.

.	.
.	.
.	.
BIOCART	REACTOME_PROCESSING_OF_CAPPED_INTRON_CONTAINING_PRE_MRNA;REAC
BIOCART	REACTOME_IMMUNOREGULATORY_INTERACTIONS_BETWEEN_A_LYMPHOID_AN
.	.
.	.
BIOCART	REACTOME_IL_7_SIGNALING;REACTOME_SIGNALING_BY_ILS;REACTOME_IL_3_5_
.	.
.	.
.	REACTOME_IMMUNE_SYSTEM;REACTOME_ADAPTIVE_IMMUNE_SYSTEM;REACTO
.	.
.	.
.	REACTOME_GENERIC_TRANSCRIPTION_PATHWAY
.	.
.	.
.	.
.	.
.	.
.	.
.	.
.	REACTOME_METABOLISM_OF_NON_CODING_RNA;REACTOME_PROCESSING_OF
.	.
.	.
BIOCART	REACTOME_APOPTOSIS;REACTOME_INTRINSIC_PATHWAY_FOR_APOPTOSIS
.	REACTOME_DEVELOPMENTAL_BIOLOGY;REACTOME_TRANSMISSION_ACROSS_
.	.
.	.
.	REACTOME_IMMUNOREGULATORY_INTERACTIONS_BETWEEN_A_LYMPHOID_AN
.	.
.	.
BIOCART	REACTOME_IMMUNE_SYSTEM;REACTOME_ADAPTIVE_IMMUNE_SYSTEM;REACTO
.	.
.	.
.	.
.	.
.	.
.	REACTOME_BIOLOGICAL_OXIDATIONS;REACTOME_PHASE1_FUNCTIONALIZATIO
.	.
.	.
.	.
.	.
.	REACTOME_DEVELOPMENTAL_BIOLOGY;REACTOME_NEURONAL_SYSTEM;REAC
.	.
.	.
.	REACTOME_METABOLISM_OF_AMINO_ACIDS_AND_DERIVATIVES
.	.
.	.
.	.
.	.
.	.
.	.
.	.
.	.

.	.
.	.
BIOCART/	REACTOME_CELL_CYCLE;REACTOME_CELL_CYCLE_MITOTIC;REACTOME_G1_PH
.	REACTOME_PTM_GAMMA_CARBOXYLATION_HYPUSINE_FORMATION_AND_ARYL
.	.
.	.
BIOCART/	REACTOME_SIGNALLING_BY_NGF;REACTOME_TRIF_MEDIATED_TLR3_SIGNALIN(
.	REACTOME_TRANSMEMBRANE_TRANSPORT_OF_SMALL_MOLECULES;REACTOM
.	.
.	.
.	.
.	REACTOME_TRANSMEMBRANE_TRANSPORT_OF_SMALL_MOLECULES;REACTOM
.	REACTOME_DEVELOPMENTAL_BIOLOGY;REACTOME_AXON_GUIDANCE;REACTOI
.	.
.	REACTOME_EXTRACELLULAR_MATRIX_ORGANIZATION;REACTOME_COLLAGEN_
.	.
.	.
.	.
.	REACTOME_NEURONAL_SYSTEM;REACTOME_VOLTAGE_GATED_POTASSIUM_CH
.	.
.	.
.	.
.	.
.	.
.	.
.	.
.	.
.	.
.	.
.	.
.	.
BIOCART/	.
.	.
.	.
.	.
.	.
.	REACTOME_PYRUVATE_METABOLISM_AND_CITRIC_ACID_TCA_CYCLE;REACTOM
BIOCART/	REACTOME_PHOSPHOLIPID_METABOLISM;REACTOME_SYNTHESIS_OF_PIPS_AT_
.	REACTOME_ACTIVATION_OF_THE_PRE_REPLICATIVE_COMPLEX;REACTOME_CE
BIOCART/	REACTOME_CELL_CYCLE;REACTOME_PRE_NOTCH_TRANSCRIPTION_AND_TRAN
.	.
.	.
.	REACTOME_TRANSMISSION_ACROSS_CHEMICAL_SYNAPSES;REACTOME_NEUR(
.	REACTOME_DIABETES_PATHWAYS
.	.
.	.
.	.
.	.
.	REACTOME_SIGNALLING_BY_NGF;REACTOME_SIGNALING_BY_ERBB4;REACTOM
.	.
.	.
.	.
.	.
.	.

.	.
.	.
.	REACTOME_METABOLISM_OF_STEROID_HORMONES_AND_VITAMINS_A_AND_D;
.	.
.	.
.	.
.	.
.	.
.	.
.	REACTOME_FACTORS_INVOLVED_IN_MEGAKARYOCYTE_DEVELOPMENT_AND_P
.	.
.	.
.	.
.	.
.	.
.	.
.	REACTOME_CELL_CYCLE;REACTOME_CHROMOSOME_MAINTENANCE;REACTOM
.	.
.	REACTOME_CELL_SURFACE_INTERACTIONS_AT_THE_VASCULAR_WALL;REACT
BIOCART	REACTOME_INNATE_IMMUNE_SYSTEM;REACTOME_IMMUNE_SYSTEM;REACTOM
.	.
.	.
.	REACTOME_GENERIC_TRANSCRIPTION_PATHWAY
.	.
BIOCART	REACTOME_EXTRACELLULAR_MATRIX_ORGANIZATION;REACTOME_COLLAGEN_
.	REACTOME_FACTORS_INVOLVED_IN_MEGAKARYOCYTE_DEVELOPMENT_AND_P
.	.
.	REACTOME_DEVELOPMENTAL_BIOLOGY;REACTOME_AXON_GUIDANCE;REACTO

TORY_SIGNALING;REACTOME_IMMUNE_SYSTEM;REACTOME_ADAPTIVE_IMMUNE_SYSTEM

AY;REACTOME_GPCR_DOWNSTREAM_SIGNALING

RANCHED_CHAIN_AMINO_ACID_CATABOLISM

ED_PLATELET_CYTOSOLIC_CA2_;REACTOME_CELL_EXTRACELLULAR_MATRIX_INTERACTIO

CTOME_MRNA_PROCESSING;REACTOME_MRNA_SPLICING

OME_CLASS_I_MHC_MEDIATED_ANTIGEN_PROCESSING_PRESENTATION;REACTOME_ANTIG

FORMATION
LATION
OGY;REACTOME_SIGNALING_BY_GPCR;REACTOME_AXON_GUIDANCE;REACTOME_G_ALPH
E_NUCLEAR_SIGNALING_BY_ERBB4;REACTOME_ACTIVATED_NOTCH1_TRANSMITS_SIGNAL
OME_CLASS_I_MHC_MEDIATED_ANTIGEN_PROCESSING_PRESENTATION;REACTOME_ANTIG

NTHESIS;REACTOME_CHONDROITIN_SULFATE_DERMATAN_SULFATE_METABOLISM;REACT
_GROWTH_FACTOR_IGF_ACTIVITY_BY_INSULIN_LIKE_GROWTH_FACTOR_BINDING_PROTEI

CTOME_MRNA_PROCESSING;REACTOME_MRNA_SPLICING
E_PHOSPHORYLATION_OF_CD3_AND_TCR_ZETA_CHAINS;REACTOME_AXON_GUIDANCE;R

CLE_MITOTIC;REACTOME_MITOTIC_G1_G1_S_PHASES

CTOME_AXON_GUIDANCE;REACTOME_G_ALPHA1213_SIGNALLING_EVENTS;REACTOME_GF

ODIFICATION_SYNTHESIS_OF_GPI_ANCHORED_PROTEINS;REACTOME_POST_TRANSLATIO

ACTOME_SIGNALING_BY_BMP

ATION_IN_TLR_CASCADE;REACTOME_ACTIVATION_OF_THE_AP1_FAMILY_OF_TRANSCRIPTI
G;REACTOME_G_ALPHA_S_SIGNALLING_EVENTS;REACTOME_CGMP_EFFECTS;REACTOME_

OR_SIGNALING;REACTOME_ANTIVIRAL_MECHANISM_BY_IFN_STIMULATED_GENES;REACTO

ME_SEMAPHORIN_INTERACTIONS;REACTOME_SEMA3A_PLEXIN_REPULSION_SIGNALING_B
ME_L1CAM_INTERACTIONS

SPHOLIPID_METABOLISM;REACTOME_PI_METABOLISM;REACTOME_METABOLISM_OF_LIPID

NALLING_BY_NGF;REACTOME_DAG_AND_IP3_SIGNALING;REACTOME_SIGNALING_BY_ERBB

LISM_OF_CARBOHYDRATES;REACTOME_GLUCOSE_METABOLISM

ME_SIGNALING_BY_CONSTITUTIVELY_ACTIVE_EGFR;REACTOME_GRB2_EVENTS_IN_ERBB2_

ONAL_SYSTEM;REACTOME_NEUROTRANSMITTER_RECEPTOR_BINDING_AND_DOWNSTREA
SULFATASE_ACTIVATION;REACTOME_GLYCOSPHINGOLIPID_METABOLISM;REACTOME_PHO

EACTOME_DEVELOPMENTAL_BIOLOGY;REACTOME_GASTRIN_CREB_SIGNALLING_PATHWA

ESICLE_BUDDING;REACTOME_GOLGI_ASSOCIATED_VESICLE_BIOGENESIS

ME_NETRIN1_SIGNALING
ME_NETRIN1_SIGNALING

B_ACTIVATION_VIA_DAI;REACTOME_TAK1_ACTIVATES_NFKB_BY_PHOSPHORYLATION_AND

TEM;REACTOME_ADAPTIVE_IMMUNE_SYSTEM

ONAL_SYSTEM;REACTOME_NEUROTRANSMITTER_RECEPTOR_BINDING_AND_DOWNSTREA

CTOME_TRANSMEMBRANE_TRANSPORT_OF_SMALL_MOLECULES;REACTOME_SLC_MEDIAT

ESICLE_BUDDING;REACTOME_GOLGI_ASSOCIATED_VESICLE_BIOGENESIS

FKB_BY_PHOSPHORYLATION_AND_ACTIVATION_OF_IKKS_COMPLEX;REACTOME_MAP_KINA
FKB_BY_PHOSPHORYLATION_AND_ACTIVATION_OF_IKKS_COMPLEX;REACTOME_MAP_KINA

R1_MUTANTS;REACTOME_SIGNALING_BY_FGFR1_FUSION_MUTANTS;REACTOME_SIGNALIN

_ERBB3_SIGNALING;REACTOME_SIGNALING_BY_ERBB2;REACTOME_GRB2_EVENTS_IN_ERB

OR_SIGNALING;REACTOME_REGULATION_OF_KIT_SIGNALING;REACTOME_ANTIGEN_ACTIV
REACTOME_SIGNALING_BY_NOTCH1;REACTOME_SIGNALING_BY_NOTCH

TOME_AXON_GUIDANCE;REACTOME_L1CAM_INTERACTIONS;REACTOME_INTERACTION_BE

ME_MITOCHONDRIAL_PROTEIN_IMPORT;REACTOME_METABOLISM_OF_PROTEINS;REACTO

_PLATELET_CYTOSOLIC_CA2_;REACTOME_INTEGRIN_CELL_SURFACE_INTERACTIONS;REA

RECEPTORS;REACTOME_GPCR_LIGAND_BINDING
RECEPTORS;REACTOME_GPCR_LIGAND_BINDING

AY;REACTOME_GPCR_DOWNSTREAM_SIGNALING

PROTEINS

ONAL_SYSTEM;REACTOME_NEUROTRANSMITTER_RELEASE_CYCLE;REACTOME_GABA_SYN

LATELET_PRODUCTION;REACTOME_HEMOSTASIS

FORMATION

SIGNALING;REACTOME_IMMUNE_SYSTEM;REACTOME_CYTOKINE_SIGNALING_IN_IMMUNE_S

AY;REACTOME_GPCR_DOWNSTREAM_SIGNALING

[illegible]_OF_O_GLYCAN_BIOSYNTHESIS;REACTOME_METABOLISM_OF_PROTEINS;REACTOME_PO[illegible]
[illegible]YNTHESIS;REACTOME_METABOLISM_OF_LIPIDS_AND_LIPOPROTEINS;REACTOME_FATTY_[illegible]

[illegible]OME_SIGNALING_BY_ERBB2;REACTOME_SIGNALING_BY_EGFR_IN_CANCER;REACTOME_N[illegible]

[illegible]ME_INTEGRIN_CELL_SURFACE_INTERACTIONS;REACTOME_HEMOSTASIS

_OF_PROTEINS;REACTOME_POST_TRANSLATIONAL_PROTEIN_MODIFICATION

OR_SIGNALING;REACTOME_CELL_CELL_COMMUNICATION;REACTOME_REGULATION_OF_K[illegible]

[illegible]_OF_O_GLYCAN_BIOSYNTHESIS;REACTOME_METABOLISM_OF_PROTEINS;REACTOME_PO[illegible]

ME_L1CAM_INTERACTIONS;REACTOME_INTERACTION_BETWEEN_L1_AND_ANKYRINS
ADE;REACTOME_ACTIVATION_OF_THE_MRNA_UPON_BINDING_OF_THE_CAP_BINDING_CO[illegible]

[illegible]THWAYS;REACTOME_PERK_REGULATED_GENE_EXPRESSION;REACTOME_ACTIVATION_OF[illegible]

OACYLATION

[illegible]D_PLATELET_CYTOSOLIC_CA2_;REACTOME_CELL_EXTRACELLULAR_MATRIX_INTERACTIO[illegible]

ENDENT_DOUBLE_STRAND_BREAKS;REACTOME_DOUBLE_STRAND_BREAK_REPAIR;REACT[illegible]

ME_MYOGENESIS;REACTOME_NETRIN1_SIGNALING

EACTOME_AXON_GUIDANCE;REACTOME_CRMPS_IN_SEMA3A_SIGNALING;REACTOME_SEM

OME_SIGNALING_BY_ERBB2;REACTOME_SIGNALING_BY_EGFR_IN_CANCER;REACTOME_N
_INTERACTIONS;REACTOME_CELL_CELL_JUNCTION_ORGANIZATION;REACTOME_CELL_JUN

OME_BASIGIN_INTERACTIONS;REACTOME_AMINO_ACID_TRANSPORT_ACROSS_THE_PLASM

HANNELS;REACTOME_POTASSIUM_CHANNELS
ME_SEMA4D_IN_SEMAPHORIN_SIGNALING;REACTOME_SEMAPHORIN_INTERACTIONS;REAC

OR_SIGNALING;REACTOME_IL_7_SIGNALING;REACTOME_SIGNALING_BY_ERBB4;REACTOM

CTION

TION_PRE_INITIATION_AND_PROMOTER_OPENING;REACTOME_TRANSCRIPTION;REACTOME

A_RECEPTOR_SIGNALING;REACTOME_SMAD2_SMAD3_SMAD4_HETEROTRIMER_REGULATE

:TOME_RNA_POL_II_TRANSCRIPTION;REACTOME_MRNA_CAPPING;REACTOME_RNA_POL_II_
D_A_NON_LYMPHOID_CELL;REACTOME_REGULATION_OF_COMPLEMENT_CASCADE;REACT

_AND_GM_CSF_SIGNALING;REACTOME_IL_RECEPTOR_SHC_SIGNALING;REACTOME_IL_2_S

)ME_CLASS_I_MHC_MEDIATED_ANTIGEN_PROCESSING_PRESENTATION;REACTOME_ANTIG

_CAPPED_INTRON_CONTAINING_PRE_MRNA;REACTOME_MRNA_PROCESSING;REACTOME_

CHEMICAL_SYNAPSES;REACTOME_NEURONAL_SYSTEM;REACTOME_NEUROTRANSMITTER_

D_A_NON_LYMPHOID_CELL;REACTOME_IMMUNE_SYSTEM;REACTOME_ADAPTIVE_IMMUNE_

)ME_CLASS_I_MHC_MEDIATED_ANTIGEN_PROCESSING_PRESENTATION;REACTOME_ANTIG

N_OF_COMPOUNDS;REACTOME_ETHANOL_OXIDATION

TOME_AXON_GUIDANCE;REACTOME_L1CAM_INTERACTIONS;REACTOME_INTERACTION_BE

IASE;REACTOME_G1_S_TRANSITION;REACTOME_MITOTIC_G1_G1_S_PHASES;REACTOME_I
SULFATASE_ACTIVATION;REACTOME_GLYCOSPHINGOLIPID_METABOLISM;REACTOME_PHC

G;REACTOME_NGF_SIGNALLING_VIA_TRKA_FROM_THE_PLASMA_MEMBRANE;REACTOME_N
IE_SLC_MEDIATED_TRANSMEMBRANE_TRANSPORT;REACTOME_TRANSPORT_OF_VITAMINS

IE_SLC_MEDIATED_TRANSMEMBRANE_TRANSPORT;REACTOME_TRANSPORT_OF_INORGAN
ME_OTHER_SEMAPHORIN_INTERACTIONS;REACTOME_SEMAPHORIN_INTERACTIONS

FORMATION

HANNELS;REACTOME_POTASSIUM_CHANNELS

IE_TCA_CYCLE_AND_RESPIRATORY_ELECTRON_TRANSPORT;REACTOME_REGULATION_OI
_THE_PLASMA_MEMBRANE;REACTOME_PI_METABOLISM;REACTOME_METABOLISM_OF_LIP
LL_CYCLE;REACTOME_CELL_CYCLE_MITOTIC;REACTOME_TRANSCRIPTION_COUPLED_NER
ISLATION;REACTOME_PRE_NOTCH_EXPRESSION_AND_PROCESSING;REACTOME_CELL_CY

ONAL_SYSTEM;REACTOME_NEUROTRANSMITTER_RECEPTOR_BINDING_AND_DOWNSTREA

E_NUCLEAR_SIGNALING_BY_ERBB4;REACTOME_ACTIVATED_NOTCH1_TRANSMITS_SIGNAL

ᴚEACTOME_METABOLISM_OF_LIPIDS_AND_LIPOPROTEINS

LATELET_PRODUCTION;REACTOME_HEMOSTASIS

E_DEPOSITION_OF_NEW_CENPA_CONTAINING_NUCLEOSOMES_AT_THE_CENTROMERE

OME_TIE2_SIGNALING;REACTOME_HEMOSTASIS
E_NOD1_2_SIGNALING_PATHWAY;REACTOME_NUCLEOTIDE_BINDING_DOMAIN_LEUCINE_R

FORMATION
LATELET_PRODUCTION;REACTOME_HEMOSTASIS

ME_NETRIN1_SIGNALING

ONS;REACTOME_CELL_JUNCTION_ORGANIZATION;REACTOME_NEPHRIN_INTERACTIONS;RE

GEN_PROCESSING_UBIQUITINATION_PROTEASOME_DEGRADATION

A1213_SIGNALLING_EVENTS;REACTOME_GPCR_DOWNSTREAM_SIGNALING;REACTOME_SE
_TO_THE_NUCLEUS;REACTOME_SIGNALING_BY_NOTCH4;REACTOME_SIGNALING_BY_NOT
GEN_PROCESSING_UBIQUITINATION_PROTEASOME_DEGRADATION

OME_HEPARAN_SULFATE_HEPARIN_HS_GAG_METABOLISM;REACTOME_GLYCOSAMINOGL
NS_IGFBPS

EACTOME_SEMA4D_IN_SEMAPHORIN_SIGNALING;REACTOME_SEMAPHORIN_INTERACTION

PCR_DOWNSTREAM_SIGNALING;REACTOME_SEMA4D_IN_SEMAPHORIN_SIGNALING;REACTO

ONAL_PROTEIN_MODIFICATION;REACTOME_SYNTHESIS_OF_GLYCOSYLPHOSPHATIDYLINOS

ION_FACTORS;REACTOME_MAPK_TARGETS_NUCLEAR_EVENTS_MEDIATED_BY_MAP_KINAS
_NITRIC_OXIDE_STIMULATES_GUANYLATE_CYCLASE;REACTOME_PLATELET_HOMEOSTASIS

OME_SIGNALING_BY_FGFR_IN_DISEASE;REACTOME_SIGNALING_BY_FGFR1_MUTANTS;REA

Y_INHIBITING_INTEGRIN_ADHESION

OS_AND_LIPOPROTEINS

32;REACTOME_SIGNALING_BY_EGFR_IN_CANCER;REACTOME_NGF_SIGNALLING_VIA_TRKA_

_SIGNALING;REACTOME_SIGNALING_BY_EGFR_IN_CANCER;REACTOME_PI3K_EVENTS_IN_

M_TRANSMISSION_IN_THE_POSTSYNAPTIC_CELL;REACTOME_TRAFFICKING_OF_AMPA_RE
SPHOLIPID_METABOLISM;REACTOME_THE_ACTIVATION_OF_ARYLSULFATASES;REACTOM

Y_VIA_PKC_AND_MAPK;REACTOME_NRAGE_SIGNALS_DEATH_THROUGH_JNK;REACTOME_

_ACTIVATION_OF_IKKS_COMPLEX;REACTOME_TRAF6_MEDIATED_NFKB_ACTIVATION;REAC

M_TRANSMISSION_IN_THE_POSTSYNAPTIC_CELL;REACTOME_ACTIVATION_OF_KAINATE_

TED_TRANSMEMBRANE_TRANSPORT;REACTOME_TRANSPORT_OF_INORGANIC_CATIONS_

SE_ACTIVATION_IN_TLR_CASCADE;REACTOME_JNK_C_JUN_KINASES_PHOSPHORYLATIO
SE_ACTIVATION_IN_TLR_CASCADE;REACTOME_JNK_C_JUN_KINASES_PHOSPHORYLATIO

IG_BY_FGFR_MUTANTS

B2_SIGNALING;REACTOME_PI3K_EVENTS_IN_ERBB4_SIGNALING;REACTOME_SHC1_EVENT

/ATES_B_CELL_RECEPTOR_LEADING_TO_GENERATION_OF_SECOND_MESSENGERS;REACT

ETWEEN_L1_AND_ANKYRINS;REACTOME_VOLTAGE_GATED_POTASSIUM_CHANNELS;REACT

ME_RESPIRATORY_ELECTRON_TRANSPORT;REACTOME_RESPIRATORY_ELECTRON_TRAN

CTOME_DIABETES_PATHWAYS;REACTOME_P130CAS_LINKAGE_TO_MAPK_SIGNALING_FOF

NTHESIS_RELEASE_REUPTAKE_AND_DEGRADATION

SYSTEM

ST_TRANSLATIONAL_PROTEIN_MODIFICATION
ACID_TRIACYLGLYCEROL_AND_KETONE_BODY_METABOLISM;REACTOME_SYNTHESIS_OF_

GF_SIGNALLING_VIA_TRKA_FROM_THE_PLASMA_MEMBRANE;REACTOME_SIGNALING_BY_F

IT_SIGNALING;REACTOME_CELL_SURFACE_INTERACTIONS_AT_THE_VASCULAR_WALL;REA

ST_TRANSLATIONAL_PROTEIN_MODIFICATION

MPLEX_AND_EIFS_AND_SUBSEQUENT_BINDING_TO_43S;REACTOME_METABOLISM_OF_PRO

_CHAPERONES_BY_ATF6_ALPHA;REACTOME_ACTIVATION_OF_GENES_BY_ATF4;REACTOM

ONS;REACTOME_CELL_JUNCTION_ORGANIZATION;REACTOME_NEPHRIN_INTERACTIONS;RE

TOME_DNA_REPAIR

A3A_PAK_DEPENDENT_AXON_REPULSION;REACTOME_SEMAPHORIN_INTERACTIONS;REAC

GF_SIGNALLING_VIA_TRKA_FROM_THE_PLASMA_MEMBRANE;REACTOME_SIGNALING_BY_F
CTION_ORGANIZATION

MA_MEMBRANE;REACTOME_TRANSMEMBRANE_TRANSPORT_OF_SMALL_MOLECULES;REAC

CTOME_SEMA4D_INDUCED_CELL_MIGRATION_AND_GROWTH_CONE_COLLAPSE

E_PROLACTIN_RECEPTOR_SIGNALING;REACTOME_NUCLEAR_SIGNALING_BY_ERBB4;REAC

E_RNA_POL_II_PRE_TRANSCRIPTION_EVENTS;REACTOME_HIV_INFECTION;REACTOME_HIV

S_TRANSCRIPTION;REACTOME_TGF_BETA_RECEPTOR_SIGNALING_ACTIVATES_SMADS;RE

_TRANSCRIPTION_PRE_INITIATION_AND_PROMOTER_OPENING;REACTOME_MRNA_PROCE
TOME_SIGNALING_BY_GPCR;REACTOME_PEPTIDE_LIGAND_BINDING_RECEPTORS;REACTO

IGNALING;REACTOME_IMMUNE_SYSTEM;REACTOME_CYTOKINE_SIGNALING_IN_IMMUNE_S

GEN_PROCESSING_UBIQUITINATION_PROTEASOME_DEGRADATION

_MRNA_SPLICING;REACTOME_MRNA_SPLICING_MINOR_PATHWAY;REACTOME_METABOLIS

_RECEPTOR_BINDING_AND_DOWNSTREAM_TRANSMISSION_IN_THE_POSTSYNAPTIC_CELL

_SYSTEM

GEN_PROCESSING_UBIQUITINATION_PROTEASOME_DEGRADATION

ETWEEN_L1_AND_ANKYRINS;REACTOME_VOLTAGE_GATED_POTASSIUM_CHANNELS;REACT

NHIBITION_OF_REPLICATION_INITIATION_OF_DAMAGED_DNA_BY_RB1_E2F1;REACTOME_E2
OSPHOLIPID_METABOLISM;REACTOME_THE_ACTIVATION_OF_ARYLSULFATASES;REACTOME

NUCLEAR_EVENTS_KINASE_AND_TRANSCRIPTION_FACTOR_ACTIVATION;REACTOME_ERK_
S_NUCLEOSIDES_AND_RELATED_MOLECULES

NIC_CATIONS_ANIONS_AND_AMINO_ACIDS_OLIGOPEPTIDES

F_PYRUVATE_DEHYDROGENASE_PDH_COMPLEX;REACTOME_PYRUVATE_METABOLISM
IDS_AND_LIPOPROTEINS
R_TC_NER;REACTOME_M_G1_TRANSITION;REACTOME_G1_S_TRANSITION;REACTOME_NUC
CLE_CHECKPOINTS;REACTOME_P53_DEPENDENT_G1_DNA_DAMAGE_RESPONSE;REACTO

M_TRANSMISSION_IN_THE_POSTSYNAPTIC_CELL;REACTOME_TRAFFICKING_OF_AMPA_RE

_TO_THE_NUCLEUS;REACTOME_SIGNALING_BY_NOTCH4;REACTOME_SIGNALING_BY_NOT

ICH_REPEAT_CONTAINING_RECEPTOR_NLR_SIGNALING_PATHWAYS

:ACTOME_HEMOSTASIS;REACTOME_PLATELET_ACTIVATION_SIGNALING_AND_AGGREGATI(

EMA4D_IN_SEMAPHORIN_SIGNALING;REACTOME_SEMAPHORIN_INTERACTIONS;REACTOME
CH2;REACTOME_SIGNALING_BY_NOTCH1;REACTOME_SIGNALING_BY_NOTCH3;REACTOME

YCAN_METABOLISM;REACTOME_A_TETRASACCHARIDE_LINKER_SEQUENCE_IS_REQUIRED

S;REACTOME_IMMUNE_SYSTEM;REACTOME_ADAPTIVE_IMMUNE_SYSTEM

OME_SEMAPHORIN_INTERACTIONS;REACTOME_SEMA4D_INDUCED_CELL_MIGRATION_AND

SITOL_GPI

SES;REACTOME_CIRCADIAN_CLOCK;REACTOME_TRAF6_MEDIATED_INDUCTION_OF_NFKB_
S;REACTOME_HEMOSTASIS

CTOME_SIGNALING_BY_FGFR1_FUSION_MUTANTS;REACTOME_SIGNALING_BY_FGFR_MUT

_FROM_THE_PLASMA_MEMBRANE;REACTOME_SIGNALING_BY_FGFR_IN_DISEASE;REACTO

ERBB2_SIGNALING;REACTOME_EGFR_DOWNREGULATION;REACTOME_GAB1_SIGNALOSOM

CEPTORS;REACTOME_RAS_ACTIVATION_UOPN_CA2_INFUX_THROUGH_NMDA_RECEPTOR
E_METABOLISM_OF_PROTEINS;REACTOME_SPHINGOLIPID_METABOLISM;REACTOME_POST

CELL_DEATH_SIGNALLING_VIA_NRAGE_NRIF_AND_NADE;REACTOME_P75_NTR_RECEPTOR

CTOME_TRAF6_MEDIATED_INDUCTION_OF_NFKB_AND_MAP_KINASES_UPON_TLR7_8_OR_9_

RECEPTORS_UPON_GLUTAMATE_BINDING;REACTOME_IONOTROPIC_ACTIVITY_OF_KAINATE

ANIONS_AND_AMINO_ACIDS_OLIGOPEPTIDES;REACTOME_AMINO_ACID_AND_OLIGOPEPTID

N_AND_ACTIVATION_MEDIATED_BY_ACTIVATED_HUMAN_TAK1;REACTOME_ACTIVATED_TAK
N_AND_ACTIVATION_MEDIATED_BY_ACTIVATED_HUMAN_TAK1;REACTOME_ACTIVATED_TAK

S_IN_ERBB4_SIGNALING;REACTOME_PI3K_EVENTS_IN_ERBB2_SIGNALING;REACTOME_NU

TOME_SIGNALING_BY_THE_B_CELL_RECEPTOR_BCR;REACTOME_CELL_SURFACE_INTERA

TOME_POTASSIUM_CHANNELS

SPORT_ATP_SYNTHESIS_BY_CHEMIOSMOTIC_COUPLING_AND_HEAT_PRODUCTION_BY_U

_INTEGRINS;REACTOME_GRB2_SOS_PROVIDES_LINKAGE_TO_MAPK_SIGNALING_FOR_INT

.VERY_LONG_CHAIN_FATTY_ACYL_COAS

FGFR_IN_DISEASE;REACTOME_GASTRIN_CREB_SIGNALLING_PATHWAY_VIA_PKC_AND_MAP

ACTOME_PECAM1_INTERACTIONS;REACTOME_PD1_SIGNALING;REACTOME_COSTIMULATIO

OTEINS;REACTOME_3_UTR_MEDIATED_TRANSLATIONAL_REGULATION;REACTOME_DEADEN

E_UNFOLDED_PROTEIN_RESPONSE;REACTOME_ACTIVATION_OF_CHAPERONE_GENES_BY

EACTOME_HEMOSTASIS;REACTOME_PLATELET_ACTIVATION_SIGNALING_AND_AGGREGATIO

:TOME_SEMA3A_PLEXIN_REPULSION_SIGNALING_BY_INHIBITING_INTEGRIN_ADHESION

FGFR_IN_DISEASE;REACTOME_TRANSMISSION_ACROSS_CHEMICAL_SYNAPSES;REACTOME

:TOME_SLC_MEDIATED_TRANSMEMBRANE_TRANSPORT;REACTOME_TRANSPORT_OF_INO

:TOME_SIGNALING_BY_FGFR_IN_DISEASE;REACTOME_SIGNALING_BY_FGFR1_MUTANTS;RE

_LIFE_CYCLE;REACTOME_LATE_PHASE_OF_HIV_LIFE_CYCLE

EACTOME_TRANSCRIPTIONAL_ACTIVITY_OF_SMAD2_SMAD3_SMAD4_HETEROTRIMER;REAC

SSING;REACTOME_MRNA_SPLICING;REACTOME_MRNA_SPLICING_MINOR_PATHWAY;REAC
ME_CLASS_A1_RHODOPSIN_LIKE_RECEPTORS;REACTOME_GPCR_DOWNSTREAM_SIGNALI

SYSTEM

M_OF_RNA

;REACTOME_AXON_GUIDANCE;REACTOME_SEMA4D_IN_SEMAPHORIN_SIGNALING;REACTC

TOME_POTASSIUM_CHANNELS

2F_MEDIATED_REGULATION_OF_DNA_REPLICATION
E_METABOLISM_OF_PROTEINS;REACTOME_SPHINGOLIPID_METABOLISM;REACTOME_POST

_MAPK_TARGETS;REACTOME_MAP_KINASE_ACTIVATION_IN_TLR_CASCADE;REACTOME_MA

CLEOTIDE_EXCISION_REPAIR;REACTOME_REPAIR_SYNTHESIS_FOR_GAP_FILLING_BY_DNA_
ME_AUTODEGRADATION_OF_THE_E3_UBIQUITIN_LIGASE_COP1;REACTOME_FACTORS_INV

ECEPTORS

CH2;REACTOME_SIGNALING_BY_NOTCH1;REACTOME_SIGNALING_BY_NOTCH3;REACTOME

ON

E_SEMA4D_INDUCED_CELL_MIGRATION_AND_GROWTH_CONE_COLLAPSE
_REGULATED_PROTEOLYSIS_OF_P75NTR;REACTOME_NRIF_SIGNALS_CELL_DEATH_FROM

_FOR_GAG_SYNTHESIS;REACTOME_METABOLISM_OF_CARBOHYDRATES

)_GROWTH_CONE_COLLAPSE

_AND_MAP_KINASES_UPON_TLR7_8_OR_9_ACTIVATION;REACTOME_NFKB_AND_MAP_KINAS

ANTS;REACTOME_SIGNALING_BY_PDGF;REACTOME_DOWNSTREAM_SIGNAL_TRANSDUCTI

ME_GASTRIN_CREB_SIGNALLING_PATHWAY_VIA_PKC_AND_MAPK;REACTOME_SIGNALING_

ИE;REACTOME_SHC1_EVENTS_IN_EGFR_SIGNALING;REACTOME_RESPONSE_TO_ELEVATEI

;REACTOME_ACTIVATION_OF_NMDA_RECEPTOR_UPON_GLUTAMATE_BINDING_AND_POST
T_TRANSLATIONAL_PROTEIN_MODIFICATION;REACTOME_METABOLISM_OF_LIPIDS_AND_LI

R_MEDIATED_SIGNALLING;REACTOME_SIGNALING_BY_GPCR;REACTOME_AXON_GUIDANCE

_ACTIVATION;REACTOME_ADVANCED_GLYCOSYLATION_ENDPRODUCT_RECEPTOR_SIGNA

E_RECEPTORS

DE_SLC_TRANSPORTERS

K1_MEDIATES_P38_MAPK_ACTIVATION;REACTOME_SIGNALING_BY_ILS;REACTOME_IL1_SIG
K1_MEDIATES_P38_MAPK_ACTIVATION;REACTOME_SIGNALING_BY_ILS;REACTOME_IL1_SIG

CLEAR_SIGNALING_BY_ERBB4

CTIONS_AT_THE_VASCULAR_WALL;REACTOME_PLATELET_ADHESION_TO_EXPOSED_COLL

NCOUPLING_PROTEINS_

ERGRINS_;REACTOME_INTEGRIN_ALPHAIIB_BETA3_SIGNALING;REACTOME_MUSCLE_CON

PK;REACTOME_SIGNALING_BY_GPCR;REACTOME_OPIOID_SIGNALLING;REACTOME_CA_DEI

ON_BY_THE_CD28_FAMILY;REACTOME_SIGNALING_BY_ILS;REACTOME_IL_3_5_AND_GM_CS

NYLATION_OF_MRNA;REACTOME_METABOLISM_OF_MRNA;REACTOME_DEADENYLATION_D

Y_ATF6_ALPHA;REACTOME_METABOLISM_OF_LIPIDS_AND_LIPOPROTEINS;REACTOME_FAT

ON

E_NEURONAL_SYSTEM;REACTOME_SIGNALING_BY_GPCR;REACTOME_INTEGRATION_OF_E

RGANIC_CATIONS_ANIONS_AND_AMINO_ACIDS_OLIGOPEPTIDES;REACTOME_AMINO_ACID_

EACTOME_SIGNALING_BY_FGFR1_FUSION_MUTANTS;REACTOME_SIGNALING_BY_FGFR_MU

CTOME_GENERIC_TRANSCRIPTION_PATHWAY;REACTOME_SIGNALING_BY_TGF_BETA_REC

TOME_TRANSCRIPTION;REACTOME_FORMATION_OF_RNA_POL_II_ELONGATION_COMPLEX
NG;REACTOME_G_ALPHA_I_SIGNALLING_EVENTS;REACTOME_GPCR_LIGAND_BINDING;REA

ME_SEMAPHORIN_INTERACTIONS;REACTOME_SEMA3A_PLEXIN_REPULSION_SIGNALING_

T_TRANSLATIONAL_PROTEIN_MODIFICATION;REACTOME_METABOLISM_OF_LIPIDS_AND_LI

APK_TARGETS_NUCLEAR_EVENTS_MEDIATED_BY_MAP_KINASES;REACTOME_TRAF6_MEDIA

_POL_IN_TC_NER;REACTOME_SYNTHESIS_OF_DNA;REACTOME_MITOTIC_G1_G1_S_PHASE
VOLVED_IN_MEGAKARYOCYTE_DEVELOPMENT_AND_PLATELET_PRODUCTION;REACTOME_

_REGULATED_PROTEOLYSIS_OF_P75NTR;REACTOME_NRIF_SIGNALS_CELL_DEATH_FROM

_THE_NUCLEUS;REACTOME_CELL_DEATH_SIGNALLING_VIA_NRAGE_NRIF_AND_NADE;REA

SES_ACTIVATION_MEDIATED_BY_TLR4_SIGNALING_REPERTOIRE;REACTOME_MYD88_MAL_

ON;REACTOME_SIGNALING_BY_ILS;REACTOME_REGULATION_OF_IFNG_SIGNALING;REACT

_BY_GPCR;REACTOME_OPIOID_SIGNALLING;REACTOME_CA_DEPENDENT_EVENTS;REACTO

D_PLATELET_CYTOSOLIC_CA2_;REACTOME_HEMOSTASIS;REACTOME_PLATELET_ACTIVATI

SYNAPTIC_EVENTS;REACTOME_CREB_PHOSPHORYLATION_THROUGH_THE_ACTIVATION_(
POPROTEINS

:;REACTOME_G_ALPHA_Q_SIGNALLING_EVENTS;REACTOME_G_ALPHA1213_SIGNALLING_EV

LING;REACTOME_NFKB_AND_MAP_KINASES_ACTIVATION_MEDIATED_BY_TLR4_SIGNALING

NALING;REACTOME_TRAF6_MEDIATED_INDUCTION_OF_NFKB_AND_MAP_KINASES_UPON_T
NALING;REACTOME_TRAF6_MEDIATED_INDUCTION_OF_NFKB_AND_MAP_KINASES_UPON_T

.AGEN;REACTOME_PECAM1_INTERACTIONS;REACTOME_GPVI_MEDIATED_ACTIVATION_CAS

TRACTION;REACTOME_AXON_GUIDANCE;REACTOME_ACTIVATION_OF_CHAPERONE_GENE

PENDENT_EVENTS;REACTOME_PLC_BETA_MEDIATED_EVENTS;REACTOME_SIGNALING_BY_

F_SIGNALING;REACTOME_PLATELET_HOMEOSTASIS;REACTOME_PLATELET_SENSITIZATIO

EPENDENT_MRNA_DECAY;REACTOME_METABOLISM_OF_RNA;REACTOME_PKB_MEDIATED_

TY_ACID_TRIACYLGLYCEROL_AND_KETONE_BODY_METABOLISM

NERGY_METABOLISM;REACTOME_OPIOID_SIGNALLING;REACTOME_CA_DEPENDENT_EVEN

_AND_OLIGOPEPTIDE_SLC_TRANSPORTERS;REACTOME_HEMOSTASIS

JTANTS;REACTOME_SIGNALING_BY_PDGF;REACTOME_DOWNSTREAM_SIGNAL_TRANSDUC

EPTOR_COMPLEX

L_;REACTOME_ELONGATION_ARREST_AND_RECOVERY;REACTOME_RNA_POL_II_PRE_TRAN
ACTOME_INNATE_IMMUNE_SYSTEM;REACTOME_IMMUNE_SYSTEM;REACTOME_COMPLEME

BY_INHIBITING_INTEGRIN_ADHESION;REACTOME_ACTIVATION_OF_NMDA_RECEPTOR_UPON

POPROTEINS

ATED_INDUCTION_OF_NFKB_AND_MAP_KINASES_UPON_TLR7_8_OR_9_ACTIVATION;REACT[illegible]

S;REACTOME_MITOTIC_M_M_G1_PHASES;REACTOME_DNA_REPAIR;REACTOME_CHROMOS[illegible]
SIGNALING_BY_NOTCH;REACTOME_APOPTOSIS;REACTOME_HEMOSTASIS;REACTOME_ACT[illegible]

_THE_NUCLEUS;REACTOME_CELL_DEATH_SIGNALLING_VIA_NRAGE_NRIF_AND_NADE;REA[illegible]

CTOME_P75_NTR_RECEPTOR_MEDIATED_SIGNALLING;REACTOME_SIGNALING_BY_NOTCH

CASCADE_INITIATED_ON_PLASMA_MEMBRANE;REACTOME_INNATE_IMMUNE_SYSTEM;REA

OME_INTERFERON_GAMMA_SIGNALING;REACTOME_INTERFERON_ALPHA_BETA_SIGNALIN

ME_PLC_BETA_MEDIATED_EVENTS;REACTOME_SIGNALING_BY_PDGF;REACTOME_DOWNS

ON_SIGNALING_AND_AGGREGATION

OF_RAS;REACTOME_POST_NMDA_RECEPTOR_ACTIVATION_EVENTS;REACTOME_UNBLOCK

VENTS;REACTOME_GPCR_DOWNSTREAM_SIGNALING;REACTOME_NETRIN1_SIGNALING;RE

_REPERTOIRE;REACTOME_RIG_I_MDA5_MEDIATED_INDUCTION_OF_IFN_ALPHA_BETA_PAT

TLR7_8_OR_9_ACTIVATION;REACTOME_NFKB_AND_MAP_KINASES_ACTIVATION_MEDIATED_
TLR7_8_OR_9_ACTIVATION;REACTOME_NFKB_AND_MAP_KINASES_ACTIVATION_MEDIATED_

SCADE;REACTOME_CD28_CO_STIMULATION;REACTOME_COSTIMULATION_BY_THE_CD28_F

S_BY_XBP1S;REACTOME_UNFOLDED_PROTEIN_RESPONSE;REACTOME_SEMAPHORIN_INT

_PDGF;REACTOME_DOWNSTREAM_SIGNAL_TRANSDUCTION;REACTOME_G_ALPHA_Q_SIG[illegible]

N_BY_LDL;REACTOME_IL_RECEPTOR_SHC_SIGNALING;REACTOME_SIGNAL_REGULATORY_[illegible]

_EVENTS;REACTOME_SIGNALING_BY_INSULIN_RECEPTOR;REACTOME_MTORC1_MEDIATE[illegible]

ITS;REACTOME_ADENYLATE_CYCLASE_ACTIVATING_PATHWAY;REACTOME_ADENYLATE_C

CTION;REACTOME_SIGNALING_BY_ILS;REACTOME_IL_3_5_AND_GM_CSF_SIGNALING;REACT

NSCRIPTION_EVENTS;REACTOME_INFLUENZA_LIFE_CYCLE;REACTOME_INFLUENZA_VIRAL_
ENT_CASCADE;REACTOME_ADAPTIVE_IMMUNE_SYSTEM;REACTOME_INITIAL_TRIGGERING_

N_GLUTAMATE_BINDING_AND_POSTSYNAPTIC_EVENTS;REACTOME_CREB_PHOSPHORYLA

OME_NFKB_AND_MAP_KINASES_ACTIVATION_MEDIATED_BY_TLR4_SIGNALING_REPERTOIF

SOME_MAINTENANCE;REACTOME_GLOBAL_GENOMIC_NER_GG_NER;REACTOME_DNA_REP
TIVATION_OF_BH3_ONLY_PROTEINS;REACTOME_INTRINSIC_PATHWAY_FOR_APOPTOSIS

CTOME_P75_NTR_RECEPTOR_MEDIATED_SIGNALLING;REACTOME_SIGNALING_BY_NOTCH

CTOME_ACTIVATED_TLR4_SIGNALLING;REACTOME_IMMUNE_SYSTEM;REACTOME_TOLL_R

G;REACTOME_REGULATION_OF_IFNA_SIGNALING;REACTOME_INTERFERON_SIGNALING;RE

STREAM_SIGNAL_TRANSDUCTION;REACTOME_G_ALPHA_Q_SIGNALLING_EVENTS;REACTOM

:ING_OF_NMDA_RECEPTOR_GLUTAMATE_BINDING_AND_ACTIVATION;REACTOME_CREB_PI

:ACTOME_DCC_MEDIATED_ATTRACTIVE_SIGNALING

'HWAYS;REACTOME_MYD88_MAL_CASCADE_INITIATED_ON_PLASMA_MEMBRANE;REACTOM

_BY_TLR4_SIGNALING_REPERTOIRE;REACTOME_MYD88_MAL_CASCADE_INITIATED_ON_PL/
_BY_TLR4_SIGNALING_REPERTOIRE;REACTOME_MYD88_MAL_CASCADE_INITIATED_ON_PL/

FAMILY;REACTOME_CTLA4_INHIBITORY_SIGNALING;REACTOME_SIGNALING_BY_ILS;REACTO

ERACTIONS;REACTOME_SEMA3A_PLEXIN_REPULSION_SIGNALING_BY_INHIBITING_INTEGR

NALLING_EVENTS;REACTOME_GPCR_DOWNSTREAM_SIGNALING;REACTOME_DOWNSTREA

_PROTEIN_SIRP_FAMILY_INTERACTIONS;REACTOME_REGULATION_OF_IFNG_SIGNALING;RI

D_SIGNALLING;REACTOME_PI3K_CASCADE

YCLASE_INHIBITORY_PATHWAY;REACTOME_NEUROTRANSMITTER_RECEPTOR_BINDING_A

OME_IL_2_SIGNALING;REACTOME_IMMUNE_SYSTEM;REACTOME_CYTOKINE_SIGNALING_I

_RNA_TRANSCRIPTION_AND_REPLICATION;REACTOME_HIV_INFECTION;REACTOME_HIV_LIF
OF_COMPLEMENT

TION_THROUGH_THE_ACTIVATION_OF_RAS;REACTOME_POST_NMDA_RECEPTOR_ACTIVAT

RE;REACTOME_MYD88_MAL_CASCADE_INITIATED_ON_PLASMA_MEMBRANE;REACTOME_INN

LICATION;REACTOME_TELOMERE_MAINTENANCE;REACTOME_EXTENSION_OF_TELOMERES

ECEPTOR_CASCADES

EACTOME_IL_6_SIGNALING;REACTOME_IMMUNE_SYSTEM;REACTOME_CYTOKINE_SIGNALIN

ME_GPCR_DOWNSTREAM_SIGNALING;REACTOME_G_ALPHA_Z_SIGNALLING_EVENTS;REAC

HOSPHORYLATION_THROUGH_THE_ACTIVATION_OF_CAMKII;REACTOME_INTERFERON_GA

ME_INNATE_IMMUNE_SYSTEM;REACTOME_ACTIVATED_TLR4_SIGNALLING;REACTOME_IMMU

ASMA_MEMBRANE;REACTOME_INNATE_IMMUNE_SYSTEM;REACTOME_ACTIVATED_TLR4_S
ASMA_MEMBRANE;REACTOME_INNATE_IMMUNE_SYSTEM;REACTOME_ACTIVATED_TLR4_S

OME_REGULATION_OF_SIGNALING_BY_CBL;REACTOME_IL_3_5_AND_GM_CSF_SIGNALING;R

IN_ADHESION;REACTOME_SMOOTH_MUSCLE_CONTRACTION;REACTOME_PLATELET_AGGR

M_SIGNALING_OF_ACTIVATED_FGFR;REACTOME_PHOSPHOLIPASE_C_MEDIATED_CASCAD

EACTOME_INTERFERON_GAMMA_SIGNALING;REACTOME_INTERFERON_ALPHA_BETA_SIG

ND_DOWNSTREAM_TRANSMISSION_IN_THE_POSTSYNAPTIC_CELL;REACTOME_PLC_BETA_

N_IMMUNE_SYSTEM

FE_CYCLE;REACTOME_ABORTIVE_ELONGATION_OF_HIV1_TRANSCRIPT_IN_THE_ABSENCE_

TION_EVENTS

NATE_IMMUNE_SYSTEM;REACTOME_ACTIVATED_TLR4_SIGNALLING;REACTOME_IMMUNE_S

S;REACTOME_S_PHASE

NG_IN_IMMUNE_SYSTEM

TOME_METABOLISM_OF_MRNA;REACTOME_METABOLISM_OF_RNA;REACTOME_DOWNSTR

MMA_SIGNALING;REACTOME_INTERFERON_SIGNALING;REACTOME_IMMUNE_SYSTEM;REA

UNE_SYSTEM;REACTOME_TOLL_RECEPTOR_CASCADES

IGNALLING;REACTOME_IMMUNE_SYSTEM;REACTOME_TOLL_RECEPTOR_CASCADES;REAC
IGNALLING;REACTOME_IMMUNE_SYSTEM;REACTOME_TOLL_RECEPTOR_CASCADES;REAC

REACTOME_HEMOSTASIS;REACTOME_IMMUNE_SYSTEM;REACTOME_ADAPTIVE_IMMUNE_S

REGATION_PLUG_FORMATION;REACTOME_HEMOSTASIS;REACTOME_PLATELET_ACTIVATIO

E;REACTOME_SIGNALING_BY_FGFR

NALING;REACTOME_REGULATION_OF_IFNA_SIGNALING;REACTOME_INTERFERON_SIGNALIN

_MEDIATED_EVENTS;REACTOME_PKA_MEDIATED_PHOSPHORYLATION_OF_CREB;REACTO[illegible]

_OF_TAT;REACTOME_FORMATION_OF_THE_HIV1_EARLY_ELONGATION_COMPLEX;REACTO[illegible]

[illegible]YSTEM;REACTOME_TOLL_RECEPTOR_CASCADES

EAM_SIGNALING_OF_ACTIVATED_FGFR;REACTOME_PHOSPHOLIPASE_C_MEDIATED_CASC

CTOME_CYTOKINE_SIGNALING_IN_IMMUNE_SYSTEM

TOME_NOD1_2_SIGNALING_PATHWAY;REACTOME_CYTOKINE_SIGNALING_IN_IMMUNE_SYS
TOME_NOD1_2_SIGNALING_PATHWAY;REACTOME_CYTOKINE_SIGNALING_IN_IMMUNE_SYS

YSTEM;REACTOME_CYTOKINE_SIGNALING_IN_IMMUNE_SYSTEM;REACTOME_PLATELET_A

N_SIGNALING_AND_AGGREGATION

NG;REACTOME_IL_2_SIGNALING;REACTOME_HEMOSTASIS;REACTOME_IMMUNE_SYSTEM;R

ME_TRANSMEMBRANE_TRANSPORT_OF_SMALL_MOLECULES;REACTOME_GLUCAGON_SIGI

ME_VIRAL_MESSENGER_RNA_SYNTHESIS;REACTOME_LATE_PHASE_OF_HIV_LIFE_CYCLE

ADE;REACTOME_EFFECTS_OF_PIP2_HYDROLYSIS;REACTOME_REGULATION_OF_MRNA_ST

TEM;REACTOME_NUCLEOTIDE_BINDING_DOMAIN_LEUCINE_RICH_REPEAT_CONTAINING_R
TEM;REACTOME_NUCLEOTIDE_BINDING_DOMAIN_LEUCINE_RICH_REPEAT_CONTAINING_R

CTIVATION_SIGNALING_AND_AGGREGATION

EACTOME_ADAPTIVE_IMMUNE_SYSTEM;REACTOME_CYTOKINE_SIGNALING_IN_IMMUNE_S

NALING_IN_METABOLIC_REGULATION;REACTOME_SIGNALING_BY_PDGF;REACTOME_DOWN

TABILITY_BY_PROTEINS_THAT_BIND_AU_RICH_ELEMENTS;REACTOME_INTERFERON_GAMM

ECEPTOR_NLR_SIGNALING_PATHWAYS
ECEPTOR_NLR_SIGNALING_PATHWAYS

YSTEM

NSTREAM_SIGNAL_TRANSDUCTION;REACTOME_GPCR_DOWNSTREAM_SIGNALING;REACTC

ΛA_SIGNALING;REACTOME_INTERFERON_SIGNALING;REACTOME_APOPTOSIS;REACTOME_I

)ME_G_ALPHA_I_SIGNALLING_EVENTS;REACTOME_G_ALPHA_S_SIGNALLING_EVENTS;REA(

HEMOSTASIS;REACTOME_IMMUNE_SYSTEM;REACTOME_CYTOKINE_S

CTOME_G_ALPHA_Z_SIGNALLING_EVENTS;REACTOME_DOWNSTREAM_SIG
