# Supplementary material for: Genomic alterations caused by HPV integration in a cohort of Chinese endocervical adenocarcinomas
Source: Cancer Gene Ther. 2021 Jan 4;28(12):1353–64. doi: 10.1038/s41417-020-00283-4 (PMC8636260; doi:10.1038/s41417-020-00283-4)
Supplement: Supplementary file 7 — Supplementary Table 6 [file 41417_2020_283_MOESM7_ESM.docx]

Supplementary Table 6

Somatic mutations detected in each case by whole exome sequencing.

CDS, coding sequence. UTR, untranslated region. SNV, single nucleotide variant. ncRNA, noncoding RNA. INDEL, insertion and deletion.

| **Sample** | **Total** | | **SNV** | | **INDEL** | |
| --- | --- | --- | --- | --- | --- | --- |
|  | Number | Ratio | Number | Ratio | Number | Ratio |
| CDS | 1,955 | 31.98% | 1,788 | 31.61% | 167 | 36.54% |
| Synonymous_SNP | 507 | 8.29% | 507 | 8.96% | - | - |
| Missense_SNP | 1,143 | 18.70% | 1,143 | 20.21% | - | - |
| Frameshift_deletion | 100 | 1.64% | - | - | 100 | 21.88% |
| Frameshift_insertion | 39 | 0.64% | - | - | 39 | 8.53% |
| Nonframeshift_deletion | 21 | 0.34% | - | - | 21 | 4.60% |
| Nonframeshift_insertion | 3 | 0.05% | - | - | 3 | 0.66% |
| Stopgain | 74 | 1.21% | 73 | 1.29% | 1 | 0.22% |
| Stoploss | 0 | 0.00% | 0 | 0.00% | 0 | 0.00% |
| unknown | 68 | 1.11% | 65 | 1.15% | 3 | 0.66% |
| Intronic | 2,296 | 37.56% | 2,086 | 36.88% | 210 | 45.95% |
| UTR3 | 150 | 2.45% | 134 | 2.37% | 16 | 3.50% |
| UTR5 | 99 | 1.62% | 87 | 1.54% | 12 | 2.63% |
| Splicing | 69 | 1.13% | 58 | 1.03% | 11 | 2.41% |
| ncRNA_exonic | 132 | 2.16% | 126 | 2.23% | 6 | 1.31% |
| ncRNA_intronic | 261 | 4.27% | 249 | 4.40% | 12 | 2.63% |
| ncRNA_UTR3 | 0 | 0.00% | 0 | 0.00% | 0 | 0.00% |
| ncRNA_UTR5 | 0 | 0.00% | 0 | 0.00% | 0 | 0.00% |
| ncRNA_splicing | 1 | 0.02% | 1 | 0.02% | 0 | 0.00% |
| Upstream | 79 | 1.29% | 77 | 1.36% | 2 | 0.44% |
| Downstream | 36 | 0.59% | 35 | 0.62% | 1 | 0.22% |
| Intergenic | 1,029 | 16.83% | 1,009 | 17.84% | 20 | 4.38% |
| Others | 6 | 0.10% | 6 | 0.11% | 0 | 0.00% |
| Total | 6,113 | 100.00% | 5,656 | 100.00% | 457 | 100.00% |
