# Supplementary material for: Genomic alterations caused by HPV integration in a cohort of Chinese endocervical adenocarcinomas
Source: Cancer Gene Ther. 2021 Jan 4;28(12):1353–64. doi: 10.1038/s41417-020-00283-4 (PMC8636260; doi:10.1038/s41417-020-00283-4)
Supplement: Supplementary file 9 — Supplementary Table 8 [file 41417_2020_283_MOESM9_ESM.pdf]

Supplementary Table 8

Detail of somatic INDELs detected in each case by whole exome sequencing.

Patient	CHRO	POS	ID	REF	ALT	QUAL	FILTER	GeneNam	Func	Gene
ACa01	2	###	.	AC	A	.	PASS	TFCP2L1	intronic	NM_01455
ACa01	2	###	.	G	GT	.	PASS	CTLA4	exonic	NM_00521
ACa01	6	###	rs6078884	TTGGAG	T	.	PASS	.	intergenic	NR_00366
ACa01	7	###	.	GTTT	G	.	PASS	BET1	exonic	NM_00586
ACa01	12	###	rs3773783	T	TC	.	PASS	SLC8B1	intronic	NM_02495
ACa01	15	###	.	AT	A	.	PASS	VPS13C	exonic	NM_00101
ACa01	17	###	.	CAGA	C	.	PASS	OR1G1	exonic	NM_00355
ACa01	19	###	.	T	TC	.	PASS	IFNL2	intronic	NM_17213
ACa01	21	###	.	C	CAG	.	PASS	DNMT3L	exonic	NM_01336
ACa01	X	###	rs7821786	T	TAGAC	.	PASS	HSD17B1	intronic	NM_00103
ACa02	5	###	.	ACACAGC	A	.	PASS	ANKRD33	exonic	NM_00116
ACa02	13	###	rs9190283	G	GT	.	PASS	FLT1	intronic	NM_00115
ACa02	14	###	.	T	TTGGGC	.	PASS	ACTN1	UTR5	NM_00110
ACa02	19	###	rs7640343	GTGTGTG	G	.	PASS	ANKLE1	exonic	NM_00127
ACa02	22	###	rs5844903	TC	T	.	PASS	SF3A1	intronic	NM_00587
ACa03	3	###	.	GGGGATT	G	.	PASS	RBM6	exonic	NM_00577
ACa04	2	###	.	CG	C	.	PASS	LTBP1	exonic	NM_00062
ACa04	3	###	.	CGT	C	.	PASS	.	intergenic	NM_00578
ACa04	16	###	.	GC	G	.	PASS	SCNN1G	intronic	NM_00103
ACa06	16	###	.	CCTCTTT	C	.	PASS	.	ncRNA_int	NR_12616
ACa06	17	###	.	C	CTCTCTC	.	PASS	ALOX15P1	ncRNA_int	NR_04598
ACa06	17	###	.	CTG	C	.	PASS	KIF19	intronic	NM_15320
ACa07	1	###	rs7760984	AC	A	.	PASS	NOC2L	intronic	NM_01565
ACa07	1	###	rs9376078	TC	T	.	PASS	LINC0134	ncRNA_ex	NR_03886
ACa07	1	###	.	AC	A	.	PASS	CPSF3L	intronic	NM_00125
ACa07	1	###	rs7790390	T	TG	.	PASS	ANKRD65	splicing	NM_00114
ACa07	1	###	.	AC	A	.	PASS	ATAD3C	intronic	NM_00103
ACa07	1	###	.	CG	C	.	PASS	TNFRSF1	intronic	NM_00106
ACa07	1	###	.	TG	T	.	PASS	UBR4	exonic	NM_02076
ACa07	1	###	.	CA	C	.	PASS	ARID1A	exonic	NM_00601
ACa07	1	###	.	A	AC	.	PASS	MACF1	intronic	NM_01209
ACa07	1	###	.	C	CT	.	PASS	MACF1	intronic	NM_01209
ACa07	1	###	rs7712392	GA	G	.	PASS	HIVEP3	intronic	NM_00112
ACa07	1	###	rs7686883	T	TG	.	PASS	TM2D1	intronic	NM_03202
ACa07	1	###	.	CT	C	.	PASS	COL24A1	exonic	NM_15289
ACa07	1	###	rs7700977	GT	G	.	PASS	SARS	intronic	NM_00651
ACa07	1	###	.	AG	A	.	PASS	RHOC	intronic	NM_00104
ACa07	1	###	.	TG	T	.	PASS	APH1A	intronic	NM_00107
ACa07	1	###	.	GT	G	.	PASS	UBE2Q1	exonic	NM_01758
ACa07	1	###	.	TA	T	.	PASS	CLK2	exonic	NM_00129
ACa07	1	###	.	GA	G	.	PASS	GON4L	intronic	NM_00128
ACa07	1	###	.	CAG	C	.	PASS	BCAN	intronic	NM_02194
ACa07	1	###	.	TC	T	.	PASS	PAPPA2	intronic	NM_02031
ACa07	1	###	rs7584166	G	GA	.	PASS	AXDND1	exonic	NM_14469
ACa07	1	###	rs7537986	GA	G	.	PASS	DHX9	UTR3	NM_00135
ACa07	1	###	rs5395449	AT	A	.	PASS	PTPRC	intronic	NM_00283
ACa07	1	###	.	AC	A	.	PASS	PLEKHA6	exonic	NM_01493
ACa07	1	###	rs7594247	TA	T	.	PASS	LIN9	intronic	NM_00127
ACa07	1	###	rs7703770	TA	T	.	PASS	CDC42BP	intronic	NM_00360
ACa07	1	###	.	CG	C	.	PASS	TSNAX-DI	ncRNA_ex	NR_02839
ACa07	2	###	rs7968975	CTG	C	.	PASS	.	intergenic	NR_11025

ACa07	2	###	.	C	CA	.	PASS	ROCK2	exonic	NM_00485
ACa07	2	###	rs7808912	AT	A	.	PASS	FAM228A	intronic	NM_00104
ACa07	2	###	.	TA	T	.	PASS	BRE	intronic	NM_00126
ACa07	2	###	rs1997131	CA	C	.	PASS	.	intergenic	NM_00111
ACa07	2	###	.	GA	G	.	PASS	PIGF	intronic	NM_00264
ACa07	2	###	rs7703599	A	AT	.	PASS	MTIF2	exonic	NM_00100
ACa07	2	###	rs7789371	G	GC	.	PASS	LOXL3	exonic	NM_03260
ACa07	2	###	.	G	GT	.	PASS	MRPL35	intronic	NM_01662
ACa07	2	###	.	TA	T	.	PASS	AFF3	intronic	NM_00102
ACa07	2	###	.	CG	C	.	PASS	TFCP2L1	intronic	NM_01455
ACa07	2	###	rs7647197	TA	T	.	PASS	ACVR2A	exonic	NM_00127
ACa07	2	###	.	AG	A	.	PASS	CSRNP3	exonic	NM_00117
ACa07	2	###	.	A	AT	.	PASS	LOC10272	ncRNA_int	NR_11059
ACa07	2	###	rs7456483	T	TG	.	PASS	XIRP2	exonic	NM_00119
ACa07	2	###	.	CT	C	.	PASS	ATF2	exonic	NM_00125
ACa07	2	###	.	AT	A	.	PASS	PDE11A	intronic	NM_00107
ACa07	2	###	rs7470964	CT	C	.	PASS	MIR548N	ncRNA_int	NR_03166
ACa07	2	###	.	AT	A	.	PASS	TTN	exonic	NM_00125
ACa07	2	###	.	A	AT	.	PASS	STAT1	exonic	NM_00731
ACa07	2	###	.	CA	C	.	PASS	BOLL	intronic	NM_00128
ACa07	2	###	.	T	TA	.	PASS	CDK15	intronic	NM_00126
ACa07	2	###	.	AC	A	.	PASS	ADAM23	exonic	NM_00381
ACa07	2	###	.	AG	A	.	PASS	FASTKD2	exonic	NM_00113
ACa07	2	###	.	GA	G	.	PASS	METTL21A	intronic	NM_00112
ACa07	2	###	.	TA	T	.	PASS	RESP18	splicing	NM_00100
ACa07	2	###	.	TG	T	.	PASS	SPHKAP	exonic	NM_00114
ACa07	2	###	rs5440185	A	AC	.	PASS	DNAJB3	exonic	NM_00100
ACa07	2	###	.	CT	C	.	PASS	FARP2	intronic	NM_01480
ACa07	3	###	.	AT	A	.	PASS	CNTN6	exonic	NM_00128
ACa07	3	###	rs9042896	C	CT	.	PASS	FGD5	UTR3	NM_15253
ACa07	3	###	rs7608755	TA	T	.	PASS	NGLY1	intronic	NM_00114
ACa07	3	###	.	GAA	G	.	PASS	SACM1L	intronic	NM_01401
ACa07	3	###	.	TGAG	T	.	PASS	PTPN23	exonic	NM_01546
ACa07	3	###	rs7647092	A	AG	.	PASS	RBM6	exonic	NM_00577
ACa07	3	###	.	CA	C	.	PASS	IQCF5-AS1	ncRNA_ex	NR_10998
ACa07	3	###	.	CG	C	.	PASS	PRKCD	exonic	NM_00625
ACa07	3	###	.	CT	C	.	PASS	CACNA1D	intronic	NM_00072
ACa07	3	###	.	AAG	A	.	PASS	C3orf17	intronic	NM_01541
ACa07	3	###	.	T	TG	.	PASS	ACAD9	intronic	NM_01404
ACa07	3	###	rs7755820	GA	G	.	PASS	FAIM	exonic	NM_00103
ACa07	3	###	.	T	TC	.	PASS	ANKUB1	intronic	NM_00114
ACa07	3	###	.	CTTCT	C	.	PASS	MBNL1	intronic	NM_02103
ACa07	3	###	.	A	AT	.	PASS	NLGN1	intronic	NM_01493
ACa07	3	###	.	TG	T	.	PASS	CHRD	intronic	NM_00374
ACa07	4	###	.	G	GC	.	PASS	LOC10013	ncRNA_int	NR_02456
ACa07	4	###	rs7704742	TTAAC	T	.	PASS	UVSSA	exonic	NM_02089
ACa07	4	###	rs7480610	GAGA	G	.	PASS	.	intergenic	NM_05304
ACa07	4	###	rs7646973	AT	A	.	PASS	PGM2	intronic	NM_01829
ACa07	4	###	rs9281864	GA	G	.	PASS	LIMCH1	intronic	NM_00111
ACa07	4	###	.	AAGG	A	.	PASS	SCFD2	exonic	NM_15254
ACa07	4	###	rs7525252	AAAAAG	A	.	PASS	PDCL2	intronic	NM_15240
ACa07	4	###	.	TC	T	.	PASS	PRDM8	exonic	NM_00109
ACa07	4	###	.	C	CT	.	PASS	SMARCAD	intronic	NM_00112
ACa07	4	###	.	CG	C	.	PASS	EGF	intronic	NM_00117

ACa07	4	###	.	C	CA	.	PASS	CAMK2D	intronic	NM_00122
ACa07	4	###	rs7641185	C	CT	.	PASS	ARSJ	exonic	NM_02459
ACa07	4	###	rs7639462	TCTC	T	.	PASS	FAT4	exonic	NM_00129
ACa07	4	###	.	A	AT	.	PASS	NEIL3	exonic	NM_01824
ACa07	4	###	.	C	CA	.	PASS	FRG2	intronic	NM_00100
ACa07	5	###	.	AG	A	.	PASS	SDHAP3	ncRNA_int	NR_00326
ACa07	5	###	rs7466549	AC	A	.	PASS	TRIO	exonic	NM_00711
ACa07	5	###	rs7717298	C	CA	.	PASS	ANKH	UTR3	NM_05402
ACa07	5	###	.	GA	G	.	PASS	ADAMTS1	intronic	NM_03095
ACa07	5	###	.	GA	G	.	PASS	C5orf42	intronic	NM_02307
ACa07	5	###	rs7625018	CT	C	.	PASS	OSMR	intronic	NM_00399
ACa07	5	###	rs7658478	T	TG	.	PASS	GFM2	intronic	NM_00128
ACa07	5	###	.	G	GA	.	PASS	AP3B1	intronic	NM_00127
ACa07	5	###	.	ACT	A	.	PASS	SRA1	intronic	NM_00103
ACa07	5	###	rs7778584	GT	G	.	PASS	PCDHGA3	exonic	NM_01891
ACa07	5	###	rs7693384	G	GC	.	PASS	LARP1	exonic	NM_01531
ACa07	5	###	.	AG	A	.	PASS	SLIT3	exonic	NM_00127
ACa07	5	###	rs7768326	CG	C	.	PASS	SLIT3	intronic	NM_00127
ACa07	6	###	rs3743843	CT	C	.	PASS	GPX5	intronic	NM_00150
ACa07	6	###	.	TG	T	.	PASS	MICB	intronic	NM_00128
ACa07	6	###	rs7666007	CT	C	.	PASS	SKIV2L	intronic	NM_00692
ACa07	6	###	rs7805014	TC	T	.	PASS	AGER	exonic	NM_00120
ACa07	6	###	rs7489955	CG	C	.	PASS	PBX2	intronic	NM_00258
ACa07	6	###	rs7612855	G	GA	.	PASS	HLA-DMB	intronic	NM_00211
ACa07	6	###	rs7600208	AC	A	.	PASS	C6orf89	exonic	NM_00128
ACa07	6	###	.	GC	G	.	PASS	FOXP4	exonic	NM_00101
ACa07	6	###	.	GA	G	.	PASS	FILIP1	intronic	NM_00128
ACa07	6	###	.	GA	G	.	PASS	PNISR	UTR3	NM_01549
ACa07	6	###	rs7792033	C	CA	.	PASS	SIM1	intronic	NM_00506
ACa07	6	###	rs7668254	CA	C	.	PASS	GRIK2	exonic	NM_00116
ACa07	6	###	.	TC	T	.	PASS	BEND3	exonic	NM_00108
ACa07	6	###	.	A	AG	.	PASS	SLC16A10	intronic	NM_01859
ACa07	6	###	.	G	GT	.	PASS	TRAF3IP2	exonic	NM_00116
ACa07	6	###	rs1490463	ATG	A	.	PASS	LAMA4	intronic	NM_00110
ACa07	6	###	.	AT	A	.	PASS	TBC1D32	exonic	NM_15273
ACa07	6	###	rs7570813	CT	C	.	PASS	HBS1L	exonic	NM_00114
ACa07	6	###	rs7468588	C	CA	.	PASS	PHACTR2	exonic	NM_00110
ACa07	6	###	.	TAG	T	.	PASS	PNLDC1	intronic	NM_00127
ACa07	6	###	.	A	AT	.	PASS	IGF2R	intronic	NM_00087
ACa07	7	###	.	T	TGCC	.	PASS	.	intergenic	NONE,NR_
ACa07	7	###	rs1443745	T	TG	.	PASS	.	intergenic	NR_00128
ACa07	7	###	.	TC	T	.	PASS	RADIL	exonic	NM_01805
ACa07	7	###	rs7542333	C	CG	.	PASS	TNRC18	exonic	NM_00108
ACa07	7	###	rs3701666	GA	G	.	PASS	MEOX2	splicing	NM_00592
ACa07	7	###	.	A	AG	.	PASS	.	intergenic	NR_12050
ACa07	7	###	rs7470634	GA	G	.	PASS	NOD1	intronic	NM_00609
ACa07	7	###	.	AC	A	.	PASS	NOD1	exonic	NM_00609
ACa07	7	###	.	T	TC	.	PASS	LANCL2	exonic	NM_01869
ACa07	7	###	rs7819467	TA	T	.	PASS	ZNF716	exonic	NM_00115
ACa07	7	###	.	C	CA	.	PASS	BCL7B	intronic	NM_00119
ACa07	7	###	.	TG	T	.	PASS	ELN	intronic	NM_00050
ACa07	7	###	.	GA	G	.	PASS	ZNF3	intronic	NM_00127
ACa07	7	###	.	TG	T	.	PASS	NYAP1	exonic	NM_17356
ACa07	7	###	rs5592025	CG	C	.	PASS	EPHB4	intronic	NM_00444

ACa07	7	###	.	GCCAGC(	G	.	PASS	CUX1	exonic	NM_00120
ACa07	7	###	rs7594121	A	AGAG	.	PASS	LMOD2	exonic	NM_20716
ACa07	7	###	rs7708715	TA	T	.	PASS	AKR1B10	intronic	NM_02029
ACa07	7	###	.	G	GC	.	PASS	ATG9B	exonic	NM_17368
ACa07	7	###	rs7618495	CT	C	.	PASS	DPP6	intronic	NM_00103
ACa07	8	###	.	A	AAAC	.	PASS	MSR1	intronic	NM_13871
ACa07	8	###	rs7790763	GA	G	.	PASS	PSD3	intronic	NM_01531
ACa07	8	###	.	G	GC	.	PASS	.	intergenic	NM_13927
ACa07	8	###	rs1126292	CG	C	.	PASS	.	intergenic	NM_00112
ACa07	8	###	rs3832550	C	CT	.	PASS	NRG1	intronic	NM_00115
ACa07	8	###	.	CA	C	.	PASS	RNF5P1	ncRNA_ex	NR_00312
ACa07	8	###	.	AT	A	.	PASS	LYN	intronic	NM_00111
ACa07	8	###	rs7782900	CT	C	.	PASS	HEY1	UTR5	NM_00128
ACa07	8	###	rs7759500	TA	T	.	PASS	ESRP1	exonic	NM_00103
ACa07	8	###	.	CT	C	.	PASS	RIMS2	intronic	NM_00110
ACa07	8	###	rs1462533	TAC	T	.	PASS	KCNQ3	intronic	NM_00120
ACa07	8	###	.	T	TC	.	PASS	BAI1	intronic	NM_00170
ACa07	8	###	rs7562540	A	AC	.	PASS	CYC1	exonic	NM_00191
ACa07	9	###	rs7648900	GA	G	.	PASS	MOB3B	intronic	NM_02476
ACa07	9	###	rs7766165	CA	C	.	PASS	DNAJB5	UTR3	NM_00113
ACa07	9	###	.	CG	C	.	PASS	TLN1	exonic	NM_00628
ACa07	9	###	rs5303481	AT	A	.	PASS	.	intergenic	NM_00120
ACa07	9	###	.	G	GA	.	PASS	PHF2	exonic	NM_00539
ACa07	9	###	.	TG	T	.	PASS	PTCH1	exonic	NM_00026
ACa07	9	###	.	CT	C	.	PASS	PTCH1	UTR5	NM_00108
ACa07	9	###	rs7606317	C	CA	.	PASS	KIAA0368	intronic	NM_00108
ACa07	9	###	rs7792498	AT	A	.	PASS	OR1L3	exonic	NM_00100
ACa07	9	###	rs1028985	CCTT	C	.	PASS	STRBP	exonic	NM_00117
ACa07	9	###	rs7703285	CT	C	.	PASS	RABEPK	intronic	NM_00117
ACa07	9	###	rs3675892	AC	A	.	PASS	C9orf50,N	intronic	NM_00128
ACa07	9	###	.	TG	T	.	PASS	PPAPDC3	intronic	NM_03272
ACa07	9	###	.	TG	T	.	PASS	PMPCA	exonic	NM_00128
ACa07	9	###	.	G	GC	.	PASS	LCN8	intronic	NM_17846
ACa07	9	###	rs7517698	A	AC	.	PASS	CCDC183	intronic	NM_00103
ACa07	9	###	.	AC	A	.	PASS	C9orf142	exonic	NM_18324
ACa07	10	###	.	A	AG	.	PASS	SFMBT2	exonic	NM_00101
ACa07	10	###	rs7625775	AT	A	.	PASS	GAD2	intronic	NM_00081
ACa07	10	###	.	CA	C	.	PASS	C10orf126	exonic	NM_00127
ACa07	10	###	.	TA	T	.	PASS	FAM35DP	ncRNA_ex	NR_02763
ACa07	10	###	rs7582351	T	TA	.	PASS	JMJD1C	splicing	NM_00128
ACa07	10	###	.	G	GT	.	PASS	DDX21	intronic	NM_00125
ACa07	10	###	rs7542104	C	CG	.	PASS	ADAMTS1	exonic	NM_08072
ACa07	10	###	rs7696631	T	TC	.	PASS	ZSWIM8	exonic	NM_00124
ACa07	10	###	.	CT	C	.	PASS	ZMIZ1	intronic	NM_02033
ACa07	10	###	rs7488695	GACAA	G	.	PASS	ANXA11	intronic	NM_00115
ACa07	10	###	rs7664186	GGAA	G	.	PASS	AGAP11	exonic	NM_13344
ACa07	10	###	rs7585686	AT	A	.	PASS	PDE6C	intronic	NM_00620
ACa07	10	###	.	CA	C	.	PASS	INA	intronic	NM_03272
ACa07	10	###	rs5534658	G	GC	.	PASS	TACC2	intronic	NM_00129
ACa07	10	###	.	GT	G	.	PASS	BNIP3	intronic	NM_00405
ACa07	11	###	.	T	TG	.	PASS	IFITM3	intronic	NM_02103
ACa07	11	###	.	TG	T	.	PASS	B4GALNT4	exonic	NM_17853
ACa07	11	###	rs7518721	AC	A	.	PASS	BRSK2	exonic	NM_00125
ACa07	11	###	rs9766384	CA	C	.	PASS	OR51E2	intronic	NM_03077

ACa07	11	###	.	GC	G	.	PASS	RBMXL2	exonic	NM_01446
ACa07	11	###	.	CCCTT	C	.	PASS	ST5	intronic	NM_00541
ACa07	11	###	rs7632273	T	TTG	.	PASS	PRMT3	intronic	NM_00114
ACa07	11	###	rs7778008	C	CA	.	PASS	MUC15,AN	intronic	NM_00113
ACa07	11	###	rs1049395	CA	C	.	PASS	HSD17B12	intronic	NM_01614
ACa07	11	###	.	AG	A	.	PASS	MAPK8IP1	exonic	NM_00545
ACa07	11	###	.	AG	A	.	PASS	EML3	exonic	NM_00130
ACa07	11	###	.	GA	G	.	PASS	RBM4B,RE	intronic	NM_00119
ACa07	11	###	rs7588677	GC	G	.	PASS	ADRBK1	intronic	NM_00161
ACa07	11	###	rs8987047	GA	G	.	PASS	.	upstream	NM_00101
ACa07	11	###	rs7647351	AC	A	.	PASS	USP35	exonic	NM_02079
ACa07	11	###	.	AC	A	.	PASS	DLG2	intronic	NM_00114
ACa07	11	###	.	AT	A	.	PASS	FAT3	exonic	NM_00100
ACa07	11	###	rs5739889	AT	A	.	PASS	CEP295	intronic	NM_03339
ACa07	11	###	rs7766671	AAAGT	A	.	PASS	JAM3	exonic	NM_00120
ACa07	12	###	rs7749641	CT	C	.	PASS	KDM5A	exonic	NM_00104
ACa07	12	###	rs7666743	C	CT	.	PASS	GALNT8	intronic	NM_01741
ACa07	12	###	.	CT	C	.	PASS	ING4	intronic	NM_00112
ACa07	12	###	.	TC	T	.	PASS	PTPN6	intronic	NM_00283
ACa07	12	###	.	GA	G	.	PASS	SLC2A14	intronic	NM_00128
ACa07	12	###	.	A	AAG	.	PASS	CLECL1	exonic	NM_00125
ACa07	12	###	rs7566997	GTTC	G	.	PASS	LOH12CR1	UTR5	NM_05816
ACa07	12	###	rs7649880	AT	A	.	PASS	PPFIBP1	intronic	NM_00119
ACa07	12	###	.	TCTC	T	.	PASS	PPFIBP1	intronic	NM_00119
ACa07	12	###	.	TG	T	.	PASS	MUC19	exonic	NM_17360
ACa07	12	###	.	T	TC	.	PASS	.	intergenic	NM_00471
ACa07	12	###	rs7636694	CA	C	.	PASS	KMT2D	intronic	NM_00348
ACa07	12	###	.	TC	T	.	PASS	ASIC1	intronic	NM_00109
ACa07	12	###	.	GTA	G	.	PASS	SLC11A2	intronic	NM_00061
ACa07	12	###	.	GC	G	.	PASS	SCN8A	exonic	NM_00117
ACa07	12	###	.	TG	T	.	PASS	EIF4B	intronic	NM_00130
ACa07	12	###	rs5532896	AT	A	.	PASS	HOXC5	UTR5	NM_01895
ACa07	12	###	.	CA	C	.	PASS	TIMELESS	exonic	NM_00392
ACa07	12	###	rs7706124	CT	C	.	PASS	MYO1A	exonic	NM_00125
ACa07	12	###	rs5359317	T	TG	.	PASS	LRP1	UTR5	NM_00233
ACa07	12	###	rs7763171	TG	T	.	PASS	MBD6	exonic	NM_05289
ACa07	12	###	rs1999629	GT	G	.	PASS	HCFC2	intronic	NM_01332
ACa07	12	###	.	TAAAG	T	.	PASS	NFYB	intronic	NM_00616
ACa07	12	###	.	C	CT	.	PASS	DNAH10	exonic	NM_20743
ACa07	13	###	rs7643750	GC	G	.	PASS	WASF3	exonic	NM_00129
ACa07	13	###	.	A	AG	.	PASS	CCDC169,	intronic	NM_00114
ACa07	13	###	rs7166969	TG	T	.	PASS	CARS2	intronic	NM_02453
ACa07	14	###	.	G	GA	.	PASS	SAMD4A	exonic	NM_00116
ACa07	14	###	.	GA	G	.	PASS	KIAA0586	exonic	NM_00124
ACa07	14	###	rs1134746	CT	C	.	PASS	DAAM1	intronic	NM_00127
ACa07	14	###	rs5526989	TA	T	.	PASS	ACTN1	intronic	NM_00110
ACa07	14	###	.	CT	C	.	PASS	UNC79	exonic	NM_02081
ACa07	14	###	.	AT	A	.	PASS	ATG2B	intronic	NM_01803
ACa07	14	###	.	AC	A	.	PASS	CRIP1	intronic	NM_00131
ACa07	15	###	rs1451472	CT	C	.	PASS	NIPA2	exonic	NM_00100
ACa07	15	###	.	CT	C	.	PASS	STARD9	intronic	NM_02075
ACa07	15	###	rs7643803	CA	C	.	PASS	RNU6-28P	ncRNA_int	NR_04648
ACa07	15	###	.	CT	C	.	PASS	TP53BP1	exonic	NM_00114
ACa07	15	###	.	CTG	C	.	PASS	GALK2	exonic	NM_00100

ACa07	15	###	rs5635626	GT	G	.	PASS	WDR72	intronic	NM_18275
ACa07	15	###	rs7515595	T	TA	.	PASS	THSD4	UTR3	NM_00128
ACa07	15	###	rs5561116	C	CT	.	PASS	NEO1	intronic	NM_00117
ACa07	15	###	.	GC	G	.	PASS	CD276	intronic	NM_00102
ACa07	15	###	.	TG	T	.	PASS	ALPK3	exonic	NM_02077
ACa07	15	###	rs7643461	CA	C	.	PASS	AGBL1	exonic	NM_15233
ACa07	15	###	.	GA	G	.	PASS	NTRK3	intronic	NM_00101
ACa07	15	###	.	AC	A	.	PASS	KIF7	exonic	NM_19852
ACa07	15	###	.	GC	G	.	PASS	FES	intronic	NM_00114
ACa07	16	###	rs7781350	G	GA	.	PASS	NLRC3	splicing	NM_17884
ACa07	16	###	rs7809656	GC	G	.	PASS	GLIS2	UTR3	NM_03257
ACa07	16	###	rs7491504	G	GC	.	PASS	GLYR1	exonic	NM_03256
ACa07	16	###	.	CT	C	.	PASS	SNX29	intronic	NM_03216
ACa07	16	###	.	G	GT	.	PASS	PDXDC1	intronic	NM_00128
ACa07	16	###	.	AC	A	.	PASS	SEPT1,ZN	intronic	NM_00121
ACa07	16	###	rs7570296	T	TGA	.	PASS	SETD1A	intronic	NM_01471
ACa07	16	###	.	GT	G	.	PASS	ADCY7	intronic	NM_00111
ACa07	16	###	rs7817135	G	GC	.	PASS	CDH5	exonic	NM_00179
ACa07	16	###	rs7679025	CG	C	.	PASS	KCTD19	exonic	NM_00110
ACa07	16	###	.	ACT	A	.	PASS	SLC7A6	intronic	NM_00107
ACa07	16	###	.	T	TC	.	PASS	ZNF469	exonic	NM_00112
ACa07	17	###	.	TA	T	.	PASS	GLOD4	intronic	NM_01608
ACa07	17	###	.	TG	T	.	PASS	CLUH	splicing	NM_01522
ACa07	17	###	.	TG	T	.	PASS	TMEM256-	ncRNA_int	NR_03771
ACa07	17	###	rs7574964	AC	A	.	PASS	KCNAB3	exonic	NM_00473
ACa07	17	###	.	GCTT	G	.	PASS	MYH10	exonic	NM_00125
ACa07	17	###	rs5285990	TA	T	.	PASS	NF1	intronic	NM_00026
ACa07	17	###	.	ATC	A	.	PASS	ARL5C	intronic	NM_00114
ACa07	17	###	.	TG	T	.	PASS	CDK12	exonic	NM_01508
ACa07	17	###	.	GA	G	.	PASS	STAT5A	intronic	NM_00128
ACa07	17	###	.	TC	T	.	PASS	AOC3	exonic	NM_00127
ACa07	17	###	.	CT	C	.	PASS	GPATCH8	exonic	NM_00100
ACa07	17	###	.	CCA	C	.	PASS	FMNL1	splicing	NM_00589
ACa07	17	###	rs1380173	T	TGGA	.	PASS	MYL4	intronic	NM_00100
ACa07	17	###	.	AC	A	.	PASS	SCRN2	UTR3	NM_00114
ACa07	17	###	.	CG	C	.	PASS	TTLL6	intronic	NM_00113
ACa07	17	###	.	AT	A	.	PASS	USP36	exonic	NM_02509
ACa07	17	###	rs7634794	GC	G	.	PASS	ENGASE	intronic	NM_00104
ACa07	17	###	.	ATGGAAT	A	.	PASS	.	intergenic	NM_00116
ACa07	17	###	rs7487058	GC	G	.	PASS	GPS1	intronic	NM_00412
ACa07	18	###	rs1429731	AT	A	.	PASS	SMCHD1	intronic	NM_01529
ACa07	18	###	rs7500268	TC	T	.	PASS	CIDEA	intronic	NM_00127
ACa07	18	###	.	GT	G	.	PASS	LDLRAD4	intronic	NM_00100
ACa07	18	###	.	C	CA	.	PASS	NPC1	exonic	NM_00027
ACa07	18	###	rs7661354	CT	C	.	PASS	TAF4B	intronic	NM_00129
ACa07	18	###	.	GA	G	.	PASS	C18orf21	intronic	NM_00120
ACa07	18	###	rs5710814	G	GC	.	PASS	LOXHD1	intronic	NM_00114
ACa07	18	###	rs1019291	CCCT	C	.	PASS	SMAD7	exonic	NM_00119
ACa07	18	###	rs7630535	T	TC	.	PASS	ALPK2	exonic	NM_05294
ACa07	19	###	.	GA	G	.	PASS	SBNO2	intronic	NM_00110
ACa07	19	###	.	GC	G	.	PASS	DAZAP1	exonic	NM_01895
ACa07	19	###	rs7644591	CG	C	.	PASS	ZFR2	exonic	NM_01517
ACa07	19	###	.	G	GC	.	PASS	NMRK2	intronic	NM_00128
ACa07	19	###	.	TC	T	.	PASS	DPP9	intronic	NM_13915

ACa07	19	###	.	GC	G	.	PASS	.	intronic	NM_00129
ACa07	19	###	rs7590798	GA	G	.	PASS	PTPRS	intronic	NM_00285
ACa07	19	###	.	CG	C	.	PASS	PTPRS	exonic	NM_00285
ACa07	19	###	rs7708014	GC	G	.	PASS	GTF2F1	intronic	NM_00209
ACa07	19	###	.	TG	T	.	PASS	C3	exonic	NM_00006
ACa07	19	###	.	TG	T	.	PASS	MYO1F	intronic	NM_01233
ACa07	19	###	.	GT	G	.	PASS	DAND5	intronic	NM_15265
ACa07	19	###	.	GAGA	G	.	PASS	JAK3	intronic	NM_00021
ACa07	19	###	rs7799007	TC	T	.	PASS	CRTC1	exonic	NM_00109
ACa07	19	###	rs7677659	C	CA	.	PASS	WDR88	intronic	NM_17347
ACa07	19	###	.	CA	C	.	PASS	UBA2	intronic	NM_00549
ACa07	19	###	.	CG	C	.	PASS	.	downstrea	NM_00530
ACa07	19	###	.	AC	A	.	PASS	KMT2B	exonic	NM_01472
ACa07	19	###	rs9414440	GA	G	.	PASS	ZNF382	exonic	NM_00125
ACa07	19	###	.	A	AT	.	PASS	ZNF793	intronic	NM_00101
ACa07	19	###	.	GC	G	.	PASS	SIPA1L3	exonic	NM_01507
ACa07	19	###	.	T	TC	.	PASS	.	intergenic	NM_00130
ACa07	19	###	rs7662018	TC	T	.	PASS	PHLDB3	exonic	NM_19885
ACa07	19	###	.	TG	T	.	PASS	NKPD1	exonic	NM_19847
ACa07	19	###	.	CG	C	.	PASS	MARK4	exonic	NM_00119
ACa07	19	###	rs7520653	C	CG	.	PASS	EML2	intronic	NM_00119
ACa07	19	###	.	GA	G	.	PASS	SNRPD2	UTR3	NM_00459
ACa07	19	###	.	AC	A	.	PASS	PNMAL2	exonic	NM_02070
ACa07	19	###	rs7555061	A	AC	.	PASS	GLTSCR1	exonic	NM_01571
ACa07	19	###	rs3981228	T	TG	.	PASS	BAX	exonic	NM_00129
ACa07	19	###	.	CG	C	.	PASS	RRAS	intronic	NM_00627
ACa07	19	###	.	TC	T	.	PASS	NAPSA	intronic	NM_00485
ACa07	19	###	rs7666986	CAG	C	.	PASS	SIGLEC8	intronic	NM_01444
ACa07	19	###	.	T	TG	.	PASS	LILRB5	UTR3	NM_00108
ACa07	19	###	.	TC	T	.	PASS	TTYH1	intronic	NM_00100
ACa07	19	###	rs7811378	A	AC	.	PASS	BRSK1	exonic	NM_03243
ACa07	19	###	.	AC	A	.	PASS	UBE2M	intronic	NM_00396
ACa07	20	###	.	GC	G	.	PASS	EBF4	intronic	NM_00111
ACa07	20	###	rs7575646	GA	G	.	PASS	RNF24	intronic	NM_00113
ACa07	20	###	rs3605176	CT	C	.	PASS	ESF1	intronic	NM_00127
ACa07	20	###	.	GC	G	.	PASS	RRBP1	intronic	NM_00104
ACa07	20	###	rs1085307	A	AG	.	PASS	ASXL1	exonic	NM_01533
ACa07	20	###	rs7539299	C	CG	.	PASS	ACSS2	intronic	NM_00107
ACa07	20	###	.	CA	C	.	PASS	DLGAP4-A	ncRNA_int	NR_10993
ACa07	20	###	rs7511829	TG	T	.	PASS	NDRG3	splicing	NM_02247
ACa07	20	###	rs7684989	C	CT	.	PASS	EYA2	intronic	NM_00524
ACa07	20	###	rs7667143	CA	C	.	PASS	DDX27	exonic	NM_01789
ACa07	20	###	rs9359712	AC	A	.	PASS	KCNQ2	intronic	NM_00451
ACa07	20	###	.	GC	G	.	PASS	HELZ2	intronic	NM_00103
ACa07	21	###	rs3687317	AG	A	.	PASS	.	intergenic	NR_10992
ACa07	21	###	rs7538174	GC	G	.	PASS	FTCD	exonic	NM_00665
ACa07	21	###	rs7755389	C	CG	.	PASS	MCM3AP	exonic	NM_00390
ACa07	22	###	rs2001464	TC	T	.	PASS	.	upstream	NM_00124
ACa07	22	###	.	TC	T	.	PASS	CRYBB2P	ncRNA_int	NR_03373
ACa07	22	###	.	AG	A	.	PASS	MN1	exonic	NM_00243
ACa07	22	###	.	CT	C	.	PASS	SFI1	intronic	NM_00100
ACa07	22	###	rs7686708	AG	A	.	PASS	ELFN2	UTR3	NM_05290
ACa07	22	###	.	AC	A	.	PASS	CDC42EP	exonic	NM_15224
ACa07	22	###	.	GC	G	.	PASS	SERHL	ncRNA_int	NR_02778

ACa07		22	###	rs7743082	A	AC	.	PASS	GTSE1	exonic	NM_01642
ACa07		22	###	.	AC	A	.	PASS	TRABD	intronic	NM_02520
ACa07	X		###	.	GT	G	.	PASS	PPP2R3B	exonic	NM_01323
ACa07	X		###	rs7631509	CT	C	.	PASS	STS	intronic	NM_00035
ACa07	X		###	.	G	GC	.	PASS	MAP7D2	intronic	NM_00116
ACa07	X		###	rs7752182	A	AT	.	PASS	DDX3X	intronic	NM_00119
ACa07	X		###	rs7515575	AG	A	.	PASS	ELK1	intronic	NM_00111
ACa07	X		###	rs7821251	A	AC	.	PASS	SLC35A2	intronic	NM_00103
ACa07	X		###	.	T	TG	.	PASS	SYP-AS1	ncRNA_ex	NR_04664
ACa07	X		###	.	G	GC	.	PASS	AKAP4	intronic	NM_00388
ACa07	X		###	.	TG	T	.	PASS	MAGEE2	exonic	NM_13870
ACa07	X		###	rs1064795	CG	C	.	PASS	SLC9A6	UTR5	NM_00104
ACa07	X		###	rs7823430	A	AG	.	PASS	PLXNB3	exonic	NM_00116
ACaP01		6	###	.	CCCG	C	.	PASS	FOXC1	exonic	NM_00145
ACaP01		11	###	.	A	AGGTGCT	.	PASS	SERPINH1	splicing	NM_00120
ACaP01		19	###	.	C	CCTGAAG	.	PASS	SBNO2	intronic	NM_00110
ACaP02		1	###	.	CCATCTT	C	.	PASS	HYI,SZT2	UTR3	NM_00119
ACaP02		2	###	rs7485648	AGCACTG	A	.	PASS	DAPL1	UTR5	NM_00101
ACaP02		3	###	.	GTCTCCC	G	.	PASS	KCNH8	exonic	NM_14463
ACaP02		13	###	.	CATTTGT	C	.	PASS	LRCH1	intronic	NM_00116
ACaP02		14	###	rs9344233	GGAGGC(	G	.	PASS	CCDC177	exonic	NM_00127
ACaP02		15	###	rs4036589	T	TTGTGC	.	PASS	.	intergenic	NR_03884
ACaP03		1	###	rs1062553	G	GGAA	.	PASS	SSBP3	UTR3	NM_00100
ACaP03		2	###	.	A	AGGAGG	.	PASS	INSIG2	intronic	NM_01613
ACaP03		2	###	rs3707030	AGGAGG/	A	.	PASS	INSIG2	intronic	NM_01613
ACaP03		6	###	.	ATCCTCC	A	.	PASS	RRAGD	exonic	NM_02124
ACaP03		9	###	.	T	TTCACGG	.	PASS	WDR38	intronic	NM_00104
ACaP03		17	###	.	CGGATG(	C	.	PASS	NPTX1	exonic	NM_00252
ACaP03		18	###	.	GCGTCC(	G	.	PASS	FHOD3	UTR5	NM_00128
ACaP04		2	###	.	ACTCAAT	A	.	PASS	ZAK	exonic	NM_01665
ACaP04		4	###	rs8860593	AAAAAC	A	.	PASS	SEPSECS	UTR5	NM_01695
ACaP04		7	###	.	TCCATGT	T	.	PASS	CYTH3	exonic	NM_00422
ACaP04		11	###	.	AGGTGTC	A	.	PASS	.	intergenic	NONE,NR_
ACaP04		14	###	rs9488701	ACTCCT	A	.	PASS	SALL2	exonic	NM_00540
ACaP04		17	###	rs1047246	CCAGTA	C	.	PASS	CHD3	intronic	NM_00100
ACaP05		1	###	.	AGAGTT	A	.	PASS	NBPF14,N	exonic	NM_00103
ACaP06		2	###	.	CCGGGA(	C	.	PASS	PDK1	intronic	NM_00127
ACaP06		6	###	.	GCCAAG(	G	.	PASS	SYNJ2	exonic	NM_00389
ACaP06		12	###	.	GA	G	.	PASS	POLE	exonic	NM_00623
ACaP06		17	###	.	G	GCAA	.	PASS	TP53	exonic	NM_00054
ACaP06		19	###	.	A	ACGC	.	PASS	TRPM4	exonic	NM_00119
ACaP06		19	###	.	TGGGGA(	T	.	PASS	SHANK1	exonic	NM_01614
ACaP06		22	###	rs3980370	G	GT	.	PASS	CACNG2	UTR3	NM_00607
ACaP07		3	###	.	AAGTTGC	A	.	PASS	IGF2BP2	intronic	NM_00100
ACaP07		4	###	rs7562167	CGAGGA(	C	.	PASS	UTP3	exonic	NM_02036
ACaP07		10	###	rs7459278	GGCCGC(	G	.	PASS	SKIDA1	exonic	NM_20737
ACaP07		16	###	.	AC	A	.	PASS	CHTF18	exonic	NM_02209
ACaP07		17	###	.	CGCCGC(	C	.	PASS	HOXB6	exonic	NM_01895
ACaP07		19	###	.	A	AG	.	PASS	PSENEN	splicing	NM_00128
ACaP07	MT		###	rs2676066	A	AC	.	PASS	.	intergenic	NONE,NO
ACaP09		20	###	.	C	CGCGGG(	.	PASS	NDUFAF5	intronic	NM_00103
ACaP10		17	###	.	GC	G	.	PASS	FLII	intronic	NM_00125
ACaP11		1	###	rs7519299	C	CG	.	PASS	TIE1	intronic	NM_00125
ACaP11		8	###	.	CCCCAAA	C	.	PASS	SGCZ	exonic	NM_13916

ACaP11	9	###	.	T	TG	.	PASS	ENTPD2	exonic	NM_00124
ACaP11	11	###	.	ATCT	A	.	PASS	NTM	intronic	NM_00104
ACaP11	14	###	.	T	TA	.	PASS	RDH11	intronic	NM_00125
ACaP11	22	###	rs7666083	CGGGCG	C	.	PASS	TCF20	UTR3	NM_00565
ACaP12	2	###	.	C	CTTG	.	PASS	KLHL29	intronic	NM_05292
ACaP12	5	###	.	CAAGGG	C	.	PASS	.	intergenic	NM_02291
ACaP12	9	###	.	G	GC	.	PASS	ODF2	intronic	NM_00124
ACaP12	11	###	rs7788921	CCAGCAC	C	.	PASS	DCHS1	exonic	NM_00373
ACaP12	12	###	.	TA	T	.	PASS	CD69	intronic	NM_00178
ACaP12	13	###	rs7743913	TTTTTG	T	.	PASS	DOCK9	intronic	NM_00113
ACaP12	14	###	rs7758009	CG	C	.	PASS	SPATA7	exonic	NM_00104
ACaP12	16	###	.	G	GT	.	PASS	CBFB	splicing	NM_00175
ACaP12	17	###	.	A	AATTCT	.	PASS	TVP23C-C	intronic	NM_00120
ACaP12	17	###	.	AGAGCG(	A	.	PASS	NARR,RAI	UTR5	NM_00114
ACaP12	21	###	rs7645628	CCCAGG/	C	.	PASS	COL18A1	exonic	NM_03058
ACaP12	22	###	.	AGAGCCC	A	.	PASS	.	intergenic	NR_00360
ACaP12	X	###	.	GCCGCG(	G	.	PASS	RBBP7	intronic	NM_00289
ACaP13	7	###	.	CGAGGT(	C	.	PASS	ZNF316	exonic	NM_00127
ACaP13	8	###	.	TTGATCA	T	.	PASS	ANGPT1	intronic	NM_00114
ACaP13	11	###	rs6015769	TA	T	.	PASS	BIRC3	intronic	NM_00116
ACaP13	16	###	rs7596499	GGGGCC	G	.	PASS	CHTF8	UTR3	NM_00103
ACaP13	19	###	.	TCCGCC(	T	.	PASS	SYMPK	UTR5	NM_00481
ACaP13	19	###	.	G	GCTGT	.	PASS	ZNF419	exonic	NM_00109
ACaP13	X	###	.	C	CT	.	PASS	SSX9	ncRNA_int	NR_07339
ACaP13	X	###	.	A	AT	.	PASS	COL4A6	intronic	NM_00128
ACaP14	3	###	.	AGG	A	.	PASS	DOCK3	exonic	NM_00494
ACaP14	3	###	.	CTT	C	.	PASS	NFKBIZ	exonic	NM_00100
ACaP14	15	###	.	GTCTGCC	G	.	PASS	RGMA	intronic	NM_02021

GeneDetai	ExonicFun	AAChange	Genecode:	wgRna	cytoBand	targetScan	tfbsConsSi	genomicSu
.	.	.	ENST0000	.	2q14.2	.	.	.
.	frameshift	CTLA4:NM	ENST0000	.	2q33.2	.	Score=999	.
dist=48933	.	.	.	.	6p11.1	.	.	.
.	nonframes	BET1:NM_	ENST0000	.	7q21.3	.	.	Score=0.9
.	.	.	ENST0000	.	12q24.13	.	.	.
.	frameshift	VPS13C:N	ENST0000	.	15q22.2	.	.	.
.	nonframes	OR1G1:NM	ENST0000	.	17p13.3	.	.	.
.	.	.	ENST0000	.	19q13.2	.	.	Score=0.9
.	frameshift	DNMT3L:N	ENST0000	.	21q22.3	.	.	.
.	.	.	ENST0000	.	Xp11.22	.	.	.
.	nonframes	ANKRD33I	ENST0000	.	5p15.2	.	Score=807	.
.	.	.	ENST0000	.	13q12.3	.	.	.
NM_00110	.	.	ENST0000	.	14q24.1	.	.	.
.	frameshift	ANKLE1:N	ENST0000	.	19p13.11	.	.	.
.	.	.	ENST0000	.	22q12.2	.	.	.
.	nonframes	RBM6:NM_	ENST0000	.	3p21.31	.	Score=905	.
.	frameshift	LTBP1:NM	ENST0000	.	2p22.3	.	Score=979	.
dist=2844;	.	.	ENST0000	.	3q29	.	.	.
.	.	.	ENST0000	.	16p12.2	.	.	.
.	.	.	ENST0000	.	16q24.3	.	.	Score=0.9
.	.	.	ENST0000	.	17p13.1	.	.	Score=0.9
.	.	.	ENST0000	.	17q25.1	.	.	.
.	.	.	ENST0000	.	1p36.33	.	.	.
.	.	.	ENST0000	.	1p36.33	.	.	.
.	.	.	ENST0000	.	1p36.33	.	.	.
.	.	.	ENST0000	.	1p36.33	.	.	.
.	.	.	ENST0000	.	1p36.33	.	.	.
.	.	.	ENST0000	.	1p36.22	.	.	.
.	frameshift	UBR4:NM_	ENST0000	.	1p36.13	.	.	.
.	frameshift	ARID1A:NI	ENST0000	.	1p36.11	.	Score=894	.
.	.	.	ENST0000	.	1p34.3	.	.	.
.	.	.	ENST0000	.	1p34.3	.	.	.
.	.	.	ENST0000	.	1p34.2	.	.	.
.	.	.	ENST0000	.	1p31.3	.	.	.
.	frameshift	COL24A1:	ENST0000	.	1p22.3	.	.	.
.	.	.	ENST0000	.	1p13.3	.	.	.
.	.	.	ENST0000	.	1p13.2	.	.	.
.	.	.	ENST0000	.	1q21.2	.	.	.
.	frameshift	UBE2Q1:N	ENST0000	.	1q21.3	.	Score=833	.
.	frameshift	CLK2:NM_	ENST0000	.	1q22	.	Score=851	.
.	.	.	ENST0000	.	1q22	.	.	.
.	.	.	ENST0000	.	1q23.1	.	.	.
.	.	.	ENST0000	.	1q25.2	.	.	.
.	frameshift	AXDND1:N	ENST0000	.	1q25.2	.	.	.
NM_00135	.	.	ENST0000	.	1q25.3	.	.	.
.	.	.	ENST0000	.	1q31.3	.	.	.
.	frameshift	PLEKHA6:	ENST0000	.	1q32.1	.	Score=888	.
.	.	.	ENST0000	.	1q42.12	.	.	.
.	.	.	ENST0000	.	1q42.13	.	.	.
.	.	.	ENST0000	.	1q42.2	.	.	.
dist=20359	.	.	.	.	2p25.1	.	.	.

.	frameshift	ROCK2:NI	ENST000C	.	2p25.1	.	Score=935	.
.	.	.	ENST000C	.	2p23.3	.	.	.
.	.	.	ENST000C	.	2p23.2	.	.	.
dist=64458	.	.	ENST000C	.	2p22.1	.	.	Score=0.92
.	.	.	ENST000C	.	2p21	.	.	.
.	frameshift	MTIF2:NM_	ENST000C	.	2p16.1	.	.	.
.	frameshift	LOXL3:NM	ENST000C	.	2p13.1	.	.	.
.	.	.	ENST000C	.	2p11.2	.	.	.
.	.	.	ENST000C	.	2q11.2	.	.	.
.	.	.	ENST000C	.	2q14.2	.	.	.
.	frameshift	ACVR2A:N	ENST000C	.	2q22.3	.	.	.
.	frameshift	CSRNP3:N	ENST000C	.	2q24.3	.	.	.
.	.	.	ENST000C	.	2q24.3	.	.	.
.	frameshift	XIRP2:NM_	ENST000C	.	2q24.3	.	.	.
.	frameshift	ATF2:NM_	ENST000C	.	2q31.1	.	.	.
.	.	.	ENST000C	.	2q31.2	.	.	.
.	.	.	ENST000C	.	2q31.2	.	.	.
.	stopgain	TTN:NM_C	ENST000C	.	2q31.2	.	.	.
.	frameshift	STAT1:NM	ENST000C	.	2q32.2	.	.	.
.	.	.	ENST000C	.	2q33.1	.	.	.
.	.	.	ENST000C	.	2q33.1	.	.	.
.	frameshift	ADAM23:N	ENST000C	.	2q33.3	.	.	.
.	frameshift	FASTKD2:	ENST000C	.	2q33.3	.	.	.
.	.	.	ENST000C	.	2q33.3	.	.	.
NM_00100	.	.	ENST000C	.	2q35	.	.	.
.	frameshift	SPHKAP:N	ENST000C	.	2q36.3	.	.	.
.	frameshift	DNAJB3:N	ENST000C	.	2q37.1	.	.	Score=0.92
.	.	.	ENST000C	.	2q37.3	.	.	.
.	frameshift	CNTN6:NM	ENST000C	.	3p26.3	.	Score=844	.
NM_15253	.	.	ENST000C	.	3p25.1	.	.	.
.	.	.	ENST000C	.	3p24.2	.	.	.
.	.	.	ENST000C	.	3p21.31	.	.	.
.	nonframes	PTPN23:N	ENST000C	.	3p21.31	.	.	.
.	frameshift	RBM6:NM_	ENST000C	.	3p21.31	.	.	.
.	.	.	ENST000C	.	3p21.2	.	.	.
.	frameshift	PRKCD:NI	ENST000C	.	3p21.1	.	.	.
.	.	.	ENST000C	.	3p21.1	.	.	.
.	.	.	ENST000C	.	3q13.2	.	.	.
.	.	.	ENST000C	.	3q21.3	.	.	.
.	frameshift	FAIM:NM_	ENST000C	.	3q22.3	.	.	.
.	.	.	ENST000C	.	3q25.1	.	.	.
.	.	.	ENST000C	.	3q25.1	.	.	.
.	.	.	ENST000C	.	3q26.31	.	.	.
.	.	.	ENST000C	.	3q27.1	.	.	.
.	.	.	ENST000C	.	4p16.3	.	.	.
.	frameshift	UVSSA:NM	ENST000C	.	4p16.3	.	.	.
dist=49825	.	.	ENST000C	.	4p16.1	.	.	.
.	.	.	ENST000C	.	4p14	.	.	.
.	.	.	ENST000C	.	4p13	.	.	.
.	nonframes	SCFD2:NM	ENST000C	.	4q12	.	.	.
.	.	.	ENST000C	.	4q12	.	.	.
.	frameshift	PRDM8:NI	ENST000C	.	4q21.21	.	.	.
.	.	.	ENST000C	.	4q22.3	.	.	.
.	.	.	ENST000C	.	4q25	.	.	.

.	.	.	ENST0000	.	4q26	.	.	.
.	frameshift	ARSJ:NM_	ENST0000	.	4q26	.	.	.
.	nonframes	FAT4:NM_	ENST0000	.	4q28.1	.	Score=956	.
.	frameshift	NEIL3:NM_	ENST0000	.	4q34.3	.	.	.
.	.	.	ENST0000	.	4q35.2	.	.	Score=0.9
.	.	.	ENST0000	.	5p15.33	.	.	Score=0.9
.	frameshift	TRIO:NM_	ENST0000	.	5p15.2	.	.	.
NM_05402	.	.	ENST0000	.	5p15.2	.	.	.
.	.	.	ENST0000	.	5p13.3	.	.	.
.	.	.	ENST0000	.	5p13.2	.	.	.
.	.	.	ENST0000	.	5p13.1	.	.	.
.	.	.	ENST0000	.	5q13.3	.	.	.
.	.	.	ENST0000	.	5q14.1	.	.	.
.	.	.	ENST0000	.	5q31.3	.	.	.
.	frameshift	PCDHGA3	ENST0000	.	5q31.3	.	.	.
.	frameshift	LARP1:NM	ENST0000	.	5q33.2	.	.	.
.	frameshift	SLIT3:NM_	ENST0000	.	5q34	.	Score=781	.
.	.	.	ENST0000	.	5q34	.	.	.
.	.	.	ENST0000	.	6p22.1	.	.	.
.	.	.	ENST0000	.	6p21.33	.	.	Score=0.9
.	.	.	ENST0000	.	6p21.33	.	.	.
.	frameshift	AGER:NM_	ENST0000	.	6p21.32	.	.	.
.	.	.	ENST0000	.	6p21.32	.	.	Score=0.9
.	.	.	ENST0000	.	6p21.32	.	.	.
.	frameshift	C6orf89:N	ENST0000	.	6p21.2	.	Score=724	.
.	frameshift	FOXP4:NM	ENST0000	.	6p21.1	.	Score=998	.
.	.	.	ENST0000	.	6q14.1	.	.	Score=0.9
NM_01549	.	.	ENST0000	.	6q16.2	.	.	.
.	.	.	ENST0000	.	6q16.3	.	.	.
.	frameshift	GRIK2:NM	ENST0000	.	6q16.3	.	Score=835	.
.	frameshift	BEND3:NM	ENST0000	.	6q21	.	.	.
.	.	.	ENST0000	.	6q21	.	.	.
.	frameshift	TRAF3IP2	ENST0000	.	6q21	.	.	.
.	.	.	ENST0000	.	6q21	.	.	.
.	frameshift	TBC1D32:	ENST0000	.	6q22.31	.	Score=837	.
.	frameshift	HBS1L:NM	ENST0000	.	6q23.3	.	.	Score=0.9
.	frameshift	PHACTR2	ENST0000	.	6q24.2	.	.	.
.	.	.	ENST0000	.	6q25.3	.	.	.
.	.	.	ENST0000	.	6q25.3	.	.	.
dist=NONE	.	.	ENST0000	.	7p22.3	.	.	Score=0.9
dist=46754	.	.	.	.	7p22.3	.	.	.
.	frameshift	RADIL:NM	ENST0000	.	7p22.1	.	.	.
.	frameshift	TNRC18:N	ENST0000	.	7p22.1	.	Score=858	.
NM_00592	.	.	ENST0000	.	7p21.2	.	.	.
dist=21149	.	.	ENST0000	.	7p15.2	.	.	Score=0.8
.	.	.	ENST0000	.	7p14.3	.	.	.
.	frameshift	NOD1:NM_	ENST0000	.	7p14.3	.	.	.
.	frameshift	LANCL2:N	ENST0000	.	7p11.2	.	.	.
.	frameshift	ZNF716:N	ENST0000	.	7p11.2	.	.	Score=0.8
.	.	.	ENST0000	.	7q11.23	.	.	.
.	.	.	ENST0000	.	7q11.23	.	.	.
.	.	.	ENST0000	.	7q22.1	.	.	.
.	frameshift	NYAP1:NM	ENST0000	.	7q22.1	.	.	.
.	.	.	ENST0000	.	7q22.1	.	.	.

.	frameshift	CUX1:NM_	ENST0000	.	7q22.1	.	Score=824	.	
.	nonframes	LMOD2:NM	ENST0000	.	7q31.32	.	.	.	
.	.	.	ENST0000	.	7q33	.	.	Score=0.9	
.	unknown	UNKNOWN	ENST0000	.	7q36.1	.	Score=822	.	
.	.	.	ENST0000	.	7q36.2	.	.	.	
.	.	.	ENST0000	.	8p22	.	.	.	
.	.	.	ENST0000	.	8p22	.	.	.	
dist=2934;	.	.	ENST0000	.	8p21.3	.	Score=946	.	
dist=25494	.	.	ENST0000	.	8p21.2	.	.	.	
.	.	.	ENST0000	.	8p12	.	.	.	
.	.	.	ENST0000	.	8p11.22	.	.	.	
.	.	.	ENST0000	.	8q12.1	.	.	.	
NM_00128	.	.	ENST0000	.	8q21.13	.	.	.	
.	frameshift	ESRP1:NM	ENST0000	.	8q22.1	.	.	.	
.	.	.	ENST0000	.	8q22.3	.	.	.	
.	.	.	ENST0000	.	8q24.22	.	.	.	
.	.	.	ENST0000	.	8q24.3	.	.	.	
.	frameshift	CYC1:NM_	ENST0000	.	8q24.3	.	Score=904	.	
.	.	.	ENST0000	.	9p21.2	.	.	.	
NM_00113	.	.	ENST0000	.	9p13.3	Score=98;I	.	.	
.	frameshift	TLN1:NM_	ENST0000	.	9p13.3	.	.	.	
dist=15610	.	.	ENST0000	.	9p11.2	.	.	Score=0.9	
.	frameshift	PHF2:NM_	ENST0000	.	9q22.31	.	.	.	
.	frameshift	PTCH1:NM	ENST0000	.	9q22.32	.	.	.	
NM_00108	.	.	ENST0000	.	9q22.32	.	.	.	
.	.	.	ENST0000	.	9q31.3	.	.	.	
.	frameshift	OR1L3:NM	ENST0000	.	9q33.2	.	.	.	
.	nonframes	STRBP:NM	ENST0000	.	9q33.3	.	.	.	
.	.	.	ENST0000	.	9q33.3	.	.	.	
.	.	.	ENST0000	.	9q34.11	.	.	.	
.	.	.	ENST0000	.	9q34.13	.	.	.	
.	frameshift	PMPCA:NI	ENST0000	.	9q34.3	.	.	.	
.	.	.	ENST0000	.	9q34.3	.	.	.	
.	.	.	ENST0000	.	9q34.3	.	.	.	
.	frameshift	C9orf142:N	ENST0000	.	9q34.3	.	.	.	
.	frameshift	SFMBT2:N	ENST0000	.	10p14	.	.	.	
.	.	.	ENST0000	.	10p12.1	.	.	.	
.	frameshift	C10orf126	ENST0000	.	10p12.1	.	.	.	
.	.	.	ENST0000	.	10q11.22	.	.	Score=0.9	
NM_00128	.	.	ENST0000	.	10q21.3	.	.	.	
.	.	.	ENST0000	.	10q22.1	.	.	.	
.	frameshift	ADAMTS1	ENST0000	.	10q22.1	.	.	.	
.	frameshift	ZSWIM8:N	ENST0000	.	10q22.2	.	Score=991	.	
.	.	.	ENST0000	.	10q22.3	.	.	.	
.	.	.	ENST0000	.	10q22.3	.	.	.	
.	nonframes	AGAP11:N	ENST0000	.	10q23.2	.	.	Score=0.9	
.	.	.	ENST0000	.	10q23.33	.	.	.	
.	.	.	ENST0000	.	10q24.33	.	.	.	
.	.	.	ENST0000	.	10q26.13	.	.	.	
.	.	.	ENST0000	.	10q26.3	.	.	.	
.	.	.	ENST0000	.	11p15.5	.	.	Score=0.9	
.	frameshift	B4GALNT	ENST0000	.	11p15.5	.	.	.	
.	frameshift	BRSK2:NM	ENST0000	.	11p15.5	.	.	.	
.	.	.	ENST0000	.	11p15.4	.	.	.	

.	frameshift	RBMXL2:N	ENST0000	.	11p15.4	.	.	.	
.	.	.	ENST0000	.	11p15.4	.	.	.	
.	.	.	ENST0000	.	11p15.1	.	.	.	
.	.	.	ENST0000	.	11p14.2	.	.	.	
.	.	.	ENST0000	.	11p11.2	.	.	.	
.	frameshift	MAPK8IP1	ENST0000	.	11p11.2	.	.	.	
.	frameshift	EML3:NM_	ENST0000	.	11q12.3	.	.	.	
.	.	.	ENST0000	.	11q13.2	.	.	Score=0.9	
.	.	.	ENST0000	.	11q13.2	.	.	.	
.	.	.	.	.	11q13.4	.	.	Score=0.9	
.	frameshift	USP35:NM	ENST0000	.	11q14.1	.	Score=882	.	
.	.	.	ENST0000	.	11q14.1	.	.	.	
.	frameshift	FAT3:NM_	ENST0000	.	11q14.3	.	.	.	
.	.	.	ENST0000	.	11q21	.	.	.	
.	frameshift	JAM3:NM_	ENST0000	.	11q25	.	Score=856	.	
.	frameshift	KDM5A:NM	ENST0000	.	12p13.33	.	.	.	
.	.	.	ENST0000	.	12p13.32	.	.	.	
.	.	.	ENST0000	.	12p13.31	.	.	.	
.	.	.	ENST0000	.	12p13.31	.	.	.	
.	.	.	ENST0000	.	12p13.31	.	.	.	
.	frameshift	CLECL1:N	ENST0000	.	12p13.31	.	.	.	
NM_05816	.	.	ENST0000	.	12p13.2	.	.	.	
.	.	.	ENST0000	.	12p11.23	.	.	Score=0.9	
.	.	.	ENST0000	.	12p11.22	.	.	Score=0.9	
.	unknown	UNKNOWN	ENST0000	.	12q12	.	.	.	
dist=1383;	.	.	ENST0000	.	12q12	.	.	.	
.	.	.	ENST0000	.	12q13.12	.	.	.	
.	.	.	ENST0000	.	12q13.12	.	.	.	
.	.	.	ENST0000	.	12q13.12	.	.	.	
.	frameshift	SCN8A:NM	ENST0000	.	12q13.13	.	Score=836	.	
.	.	.	ENST0000	.	12q13.13	.	.	.	
NM_01895	.	.	ENST0000	.	12q13.13	.	.	.	
.	frameshift	TIMELESS	ENST0000	.	12q13.3	.	Score=866	.	
.	frameshift	MYO1A:NM	ENST0000	.	12q13.3	.	.	.	
NM_00233	.	.	ENST0000	.	12q13.3	.	.	.	
.	frameshift	MBD6:NM_	ENST0000	.	12q13.3	.	Score=879	.	
.	.	.	ENST0000	.	12q23.3	.	.	.	
.	.	.	ENST0000	.	12q23.3	.	.	.	
.	frameshift	DNAH10:N	ENST0000	.	12q24.31	.	.	.	
.	frameshift	WASF3:NM	ENST0000	.	13q12.13	.	.	.	
.	.	.	ENST0000	.	13q13.3	.	.	.	
.	.	.	ENST0000	.	13q34	.	.	.	
.	frameshift	SAMD4A:N	ENST0000	.	14q22.2	.	.	.	
.	frameshift	KIAA0586:	ENST0000	.	14q23.1	.	.	.	
.	.	.	ENST0000	.	14q23.1	.	.	.	
.	.	.	ENST0000	.	14q24.1	.	.	Score=0.9	
.	frameshift	UNC79:NM	ENST0000	.	14q32.12	.	.	.	
.	.	.	ENST0000	.	14q32.2	.	.	.	
.	.	.	ENST0000	.	14q32.33	.	.	.	
.	frameshift	NIPA2:NM_	ENST0000	.	15q11.2	.	.	.	
.	.	.	ENST0000	.	15q15.2	.	.	.	
.	.	.	ENST0000	.	15q15.3	.	.	.	
.	frameshift	TP53BP1:I	ENST0000	.	15q15.3	.	.	.	
.	frameshift	GALK2:NM	ENST0000	.	15q21.2	.	Score=807	.	

.	.	.	ENST000C	.	15q21.3	.	.	.
NM_00128	.	.	ENST000C	.	15q23	.	.	.
.	.	.	ENST000C	.	15q24.1	.	.	.
.	.	.	ENST000C	.	15q24.1	.	.	.
.	frameshift	ALPK3:NM	ENST000C	.	15q25.3	.	Score=872	.
.	frameshift	AGBL1:NM	ENST000C	.	15q25.3	.	.	.
.	.	.	ENST000C	.	15q25.3	.	.	.
.	frameshift	KIF7:NM_	ENST000C	.	15q26.1	.	Score=920	.
.	.	.	ENST000C	.	15q26.1	.	.	.
NM_17884	.	.	ENST000C	.	16p13.3	.	.	.
NM_03257	.	.	ENST000C	.	16p13.3	.	.	.
.	frameshift	GLYR1:NM	ENST000C	.	16p13.3	.	Score=782	.
.	.	.	ENST000C	.	16p13.13	.	.	.
.	.	.	ENST000C	.	16p13.11	.	.	Score=0.9
.	.	.	ENST000C	.	16p11.2	.	.	.
.	.	.	ENST000C	.	16p11.2	.	.	.
.	.	.	ENST000C	.	16q12.1	.	.	.
.	frameshift	CDH5:NM_	ENST000C	.	16q21	.	.	.
.	frameshift	KCTD19:N	ENST000C	.	16q22.1	.	Score=899	.
.	.	.	ENST000C	.	16q22.1	.	.	.
.	frameshift	ZNF469:N	ENST000C	.	16q24.2	.	Score=800	.
.	.	.	ENST000C	.	17p13.3	.	.	.
NM_01522	.	.	ENST000C	.	17p13.3	.	.	.
.	.	.	ENST000C	.	17p13.1	.	.	.
.	frameshift	KCNAB3:N	ENST000C	.	17p13.1	.	Score=942	.
.	nonframes	MYH10:NM	ENST000C	.	17p13.1	.	Score=826	.
.	.	.	ENST000C	.	17q11.2	.	.	.
.	.	.	ENST000C	.	17q12	.	.	.
.	frameshift	CDK12:NM	ENST000C	.	17q12	.	Score=922	.
.	.	.	ENST000C	.	17q21.2	.	.	.
.	frameshift	AOC3:NM_	ENST000C	.	17q21.31	.	.	Score=0.9
.	frameshift	GPATCH8	ENST000C	.	17q21.31	.	.	.
.	.	.	ENST000C	.	17q21.31	.	Score=968	.
.	.	.	ENST000C	.	17q21.32	.	.	.
NM_00114	.	.	ENST000C	.	17q21.32	.	.	.
.	.	.	ENST000C	.	17q21.32	.	.	.
.	frameshift	USP36:NM	ENST000C	.	17q25.3	.	.	.
.	.	.	ENST000C	.	17q25.3	.	.	.
dist=2490;	.	.	ENST000C	.	17q25.3	.	.	.
.	.	.	ENST000C	.	17q25.3	.	.	.
.	.	.	ENST000C	.	18p11.32	.	.	.
.	.	.	ENST000C	.	18p11.21	.	.	.
.	.	.	ENST000C	.	18p11.21	.	.	.
.	frameshift	NPC1:NM_	ENST000C	.	18q11.2	.	.	.
.	.	.	ENST000C	.	18q11.2	.	.	.
.	.	.	ENST000C	.	18q12.2	.	.	.
.	.	.	ENST000C	.	18q21.1	.	.	.
.	nonframes	SMAD7:N	ENST000C	.	18q21.1	.	.	.
.	frameshift	ALPK2:NM	ENST000C	.	18q21.32	.	.	.
.	.	.	ENST000C	.	19p13.3	.	.	.
.	frameshift	DAZAP1:N	ENST000C	.	19p13.3	.	Score=879	.
.	frameshift	ZFR2:NM_	ENST000C	.	19p13.3	.	.	.
.	.	.	ENST000C	.	19p13.3	.	.	.
.	.	.	ENST000C	.	19p13.3	.	.	.

.	.	.	ENST0000	.	19p13.3	.	.	.
.	.	.	ENST0000	.	19p13.3	.	.	.
.	frameshift	PTPRS:NM	ENST0000	.	19p13.3	.	.	.
.	.	.	ENST0000	.	19p13.3	.	.	.
.	frameshift	C3:NM_00	ENST0000	.	19p13.3	.	.	.
.	.	.	ENST0000	.	19p13.2	.	.	.
.	.	.	ENST0000	.	19p13.2	.	.	.
.	.	.	ENST0000	.	19p13.11	.	.	.
.	frameshift	CRTC1:NM	ENST0000	.	19p13.11	.	.	.
.	.	.	ENST0000	.	19q13.11	.	.	.
.	.	.	ENST0000	.	19q13.11	.	.	.
.	.	.	ENST0000	.	19q13.12	.	.	.
.	frameshift	KMT2B:NM	ENST0000	.	19q13.12	.	.	.
.	frameshift	ZNF382:NI	ENST0000	.	19q13.12	.	.	.
.	.	.	ENST0000	.	19q13.12	.	.	.
.	unknown	UNKNOWI	ENST0000	.	19q13.13	.	Score=755	.
dist=3484C	.	.	ENST0000	.	19q13.31	.	.	.
.	frameshift	PHLDB3:N	ENST0000	.	19q13.31	.	.	.
.	frameshift	NKPD1:NM	ENST0000	.	19q13.32	.	.	.
.	frameshift	MARK4:NM	ENST0000	.	19q13.32	.	Score=755	.
.	.	.	ENST0000	.	19q13.32	.	.	.
NM_00459	.	.	ENST0000	.	19q13.32	.	.	.
.	frameshift	PNMAL2:N	ENST0000	.	19q13.32	.	Score=766	.
.	frameshift	GLTSCR1:	ENST0000	.	19q13.33	.	.	.
.	frameshift	BAX:NM_(	ENST0000	.	19q13.33	.	Score=836	.
.	.	.	ENST0000	.	19q13.33	.	.	.
.	.	.	ENST0000	.	19q13.33	.	.	.
.	.	.	ENST0000	.	19q13.41	.	.	.
NM_00108	.	.	ENST0000	.	19q13.42	.	.	.
.	.	.	ENST0000	.	19q13.42	.	.	.
.	frameshift	BRSK1:NM	ENST0000	.	19q13.42	.	.	.
.	.	.	ENST0000	.	19q13.43	.	.	.
.	.	.	ENST0000	.	20p13	.	.	.
.	.	.	ENST0000	.	20p13	.	.	.
.	.	.	ENST0000	.	20p12.1	.	.	.
.	.	.	ENST0000	.	20p12.1	.	.	.
.	frameshift	ASXL1:NM	ENST0000	.	20q11.21	.	Score=888	.
.	.	.	ENST0000	.	20q11.22	.	.	.
.	.	.	ENST0000	.	20q11.23	.	.	.
NM_02247	.	.	ENST0000	.	20q11.23	.	.	.
.	.	.	ENST0000	.	20q13.12	.	.	.
.	frameshift	DDX27:NM	ENST0000	.	20q13.13	.	.	.
.	.	.	ENST0000	.	20q13.33	.	.	.
.	.	.	ENST0000	.	20q13.33	.	.	.
dist=98028	.	.	ENST0000	.	21q21.1	.	.	.
.	frameshift	FTCD:NM_	ENST0000	.	21q22.3	.	.	.
.	frameshift	MCM3AP:I	ENST0000	.	21q22.3	.	Score=976	.
.	.	.	ENST0000	.	22q11.21	.	.	Score=0.9
.	.	.	ENST0000	.	22q11.23	.	.	Score=0.9
.	frameshift	MN1:NM_(	ENST0000	.	22q12.1	.	Score=869	.
.	.	.	ENST0000	.	22q12.2	.	.	.
NM_05290	.	.	ENST0000	.	22q13.1	.	.	.
.	frameshift	CDC42EP:	ENST0000	.	22q13.1	.	Score=888	.
.	.	.	ENST0000	.	22q13.2	.	.	Score=0.9

.	frameshift	GTSE1:NM	ENST0000	.	22q13.31	.	.	.	
.	.	.	ENST0000	.	22q13.33	.	.	.	
.	frameshift	PPP2R3B:	ENST0000	.	Xp22.33	.	.	Score=0.9	
.	.	.	ENST0000	.	Xp22.31	.	.	.	
.	.	.	ENST0000	.	Xp22.12	.	.	.	
.	.	.	ENST0000	.	Xp11.4	.	.	.	
.	.	.	ENST0000	.	Xp11.23	.	.	.	
.	.	.	ENST0000	.	Xp11.23	.	.	.	
.	.	.	ENST0000	.	Xp11.23	.	.	.	
.	.	.	ENST0000	.	Xp11.22	.	.	.	
.	frameshift	MAGEE2:N	ENST0000	.	Xq13.3	.	.	.	
NM_00104	.	.	ENST0000	.	Xq26.3	.	.	.	
.	frameshift	PLXNB3:N	ENST0000	.	Xq28	.	.	.	
.	nonframes	FOXC1:NM	ENST0000	.	6p25.3	.	.	.	
NM_00120	.	.	ENST0000	.	11q13.5	.	.	.	
.	.	.	ENST0000	.	19p13.3	.	.	.	
NM_00119	.	.	ENST0000	.	1p34.2	.	.	.	
NM_00101	.	.	ENST0000	.	2q24.1	.	.	.	
.	frameshift	KCNH8:NM	ENST0000	.	3p24.3	.	.	.	
.	.	.	ENST0000	.	13q14.13	.	.	.	
.	nonframes	CCDC177:	ENST0000	.	14q24.1	.	.	.	
dist=76944	.	.	ENST0000	.	15q11.2	.	.	Score=0.9	
NM_00100	.	.	ENST0000	.	1p32.3	.	.	.	
.	.	.	ENST0000	.	2q14.2	.	.	.	
.	.	.	ENST0000	.	2q14.2	.	.	.	
.	nonframes	RRAGD:NI	ENST0000	.	6q15	.	.	.	
.	.	.	ENST0000	.	9q33.3	.	.	.	
.	nonframes	NPTX1:NM	ENST0000	.	17q25.3	.	Score=699	.	
NM_00128	.	.	ENST0000	.	18q12.2	.	.	.	
.	frameshift	ZAK:NM_0	ENST0000	.	2q31.1	.	.	.	
NM_01695	.	.	.	.	4p15.2	.	.	.	
.	nonframes	CYTH3:NM	ENST0000	.	7p22.1	.	.	.	
dist=NONE	.	.	ENST0000	.	11p15.5	.	.	Score=0.9	
.	frameshift	SALL2:NM	ENST0000	.	14q11.2	.	.	.	
.	.	.	ENST0000	.	17p13.1	.	.	.	
.	frameshift	NBPF8:NM	ENST0000	.	1q21.2	.	.	Score=0.9	
.	.	.	ENST0000	.	2q31.1	.	.	.	
.	frameshift	SYNJ2:NM	ENST0000	.	6q25.3	.	.	.	
.	frameshift	POLE:NM_	ENST0000	.	12q24.33	.	.	.	
.	nonframes	TP53:NM_	ENST0000	.	17p13.1	.	.	.	
.	nonframes	TRPM4:NM	ENST0000	.	19q13.33	.	.	.	
.	frameshift	SHANK1:N	ENST0000	.	19q13.33	.	Score=985	.	
NM_00607	.	.	ENST0000	.	22q12.3	.	.	.	
.	.	.	ENST0000	.	3q27.2	.	.	.	
.	nonframes	UTP3:NM_	ENST0000	.	4q13.3	.	.	.	
.	nonframes	SKIDA1:NI	ENST0000	.	10p12.31	.	.	.	
.	frameshift	CHTF18:N	ENST0000	.	16p13.3	.	.	.	
.	frameshift	HOXB6:NM	ENST0000	.	17q21.32	.	.	.	
NM_00128	.	.	ENST0000	.	19q13.12	.	Score=926	.	
dist=NONE	.	.	.	.	.	.	.	.	
.	.	.	ENST0000	.	20p12.1	.	.	.	
.	.	.	ENST0000	.	17p11.2	.	.	.	
.	.	.	ENST0000	.	1p34.2	.	.	.	
.	frameshift	SGCZ:NM_	ENST0000	.	8p22	.	.	.	

.	frameshift	ENTPD2:N	ENST000C	.	9q34.3	.	.	.
.	.	.	ENST000C	.	11q25	.	.	.
.	.	.	ENST000C	.	14q24.1	.	.	.
NM_00565	.	.	ENST000C	.	22q13.2	.	.	.
.	.	.	ENST000C	.	2p24.1	.	.	.
dist=15878	.	.	ENST000C	.	5q11.2	.	.	.
.	.	.	ENST000C	.	9q34.11	.	.	.
.	nonframes	DCHS1:NM	ENST000C	.	11p15.4	.	.	.
.	.	.	ENST000C	.	12p13.31	.	.	.
.	.	.	ENST000C	.	13q32.3	.	.	.
.	frameshift	SPATA7:N	ENST000C	.	14q31.3	.	.	.
NM_00175	.	.	ENST000C	.	16q22.1	.	.	.
.	.	.	ENST000C	.	17p12	.	.	.
NM_00114	.	.	ENST000C	.	17q11.2	.	.	.
.	nonframes	COL18A1:	ENST000C	.	21q22.3	.	.	.
dist=10462	.	.	ENST000C	.	22q11.1	.	.	.
.	.	.	ENST000C	.	Xp22.2	.	Score=911	.
.	nonframes	ZNF316:NI	ENST000C	.	7p22.1	.	.	.
.	.	.	ENST000C	.	8q23.1	.	.	.
.	.	.	ENST000C	.	11q22.2	.	.	.
NM_00103	.	.	ENST000C	.	16q22.1	.	.	Score=0.9
NM_00481	.	.	ENST000C	.	19q13.32	.	.	.
.	frameshift	ZNF419:NI	ENST000C	.	19q13.43	.	.	Score=0.9
.	.	.	ENST000C	.	Xp11.23	.	.	Score=0.9
.	.	.	ENST000C	.	Xq22.3	.	.	.
.	frameshift	DOCK3:NM	ENST000C	.	3p21.2	.	.	.
.	frameshift	NFKBIZ:NI	ENST000C	.	3q12.3	.	.	.
.	.	.	ENST000C	.	15q26.1	.	.	.

Repeat	avsnp150	cosmic82	clinvar_201	gwasCatal	1000g2015	1000g2015	1000g2015	esp6500siv
.	.	.	.	.	.	.	.	.
.	.	ID=COSM1	.	.	.	.	.	.
Score=118	rs6078884	.	.	.	.	.	.	.
.	.	.	.	.	.	.	.	.
.	rs3773783	.	.	.	.	.	.	0.0068
.	.	.	.	.	.	.	.	.
.	.	.	.	.	.	.	.	.
Score=239	.	.	.	.	.	.	.	.
.	.	.	.	.	.	.	.	.
Score=344	rs7821786	.	.	.	.	0.0039	0.001589	.
.	.	.	.	.	.	.	.	.
.	rs9190283	.	.	.	.	.	.	.
.	.	.	.	.	.	.	.	.
Score=561	rs7640343	.	.	.	.	.	.	.
.	rs5844903	.	.	.	.	0.1448	0.153754	.
.	.	.	.	.	.	.	.	.
.	.	.	.	.	.	.	.	.
.	.	.	.	.	.	.	.	.
.	.	ID=COSN1	.	.	.	.	.	.
.	.	.	.	.	.	.	.	.
Score=446	.	.	.	.	.	.	.	.
Score=366	.	.	.	.	.	.	.	.
.	rs7760984	.	.	.	.	.	.	.
.	rs9376078	ID=COSN2	.	.	.	.	.	.
.	.	.	.	.	.	.	.	.
.	rs7790390	.	.	.	.	.	.	.
.	.	.	.	.	.	.	.	.
.	.	.	.	.	.	.	.	.
.	.	ID=COSM1	.	.	.	.	.	.
.	.	.	.	.	.	.	.	.
.	.	.	.	.	.	.	.	.
.	.	.	.	.	.	.	.	.
.	rs7712392	ID=COSN5	.	.	.	.	.	0.0021
.	rs7686883	.	.	.	.	.	.	.
.	.	.	.	.	.	.	.	.
.	rs7700977	ID=COSN2	.	.	.	.	.	0.0011
.	.	.	.	.	.	.	.	.
.	.	.	.	.	.	.	.	.
.	.	ID=COSM5	.	.	.	.	.	.
.	.	.	.	.	.	.	.	.
.	.	ID=COSM1	.	.	.	.	.	.
.	.	.	.	.	.	.	.	.
.	.	.	.	.	.	.	.	.
.	rs7584166	ID=COSM5	.	.	.	.	.	0.0015
.	rs7537986	ID=COSN1	.	.	.	.	.	.
.	rs5395449	.	.	.	.	.	0.000599	0.0124
.	.	ID=COSM2	.	.	.	.	.	.
.	rs7594247	ID=COSM3	.	.	.	.	.	0.0007
.	rs7703770	.	.	.	.	.	.	.
.	.	.	.	.	.	.	.	.
Score=390	rs7968975	.	.	.	.	.	.	.

.	.	ID=COSM3	.	.	.	.	.	.
.	rs7808912	.	.	.	.	.	.	.
Score=625	.	.	.	.	.	.	.	.
.	rs1997131	ID=COSN1	.	.	.	0.005	0.001597	.
.	.	.	.	.	.	.	.	.
.	rs7703599	ID=COSM3	.	.	.	.	.	.
.	rs7789371	ID=COSM2	.	.	.	.	.	0.0042
Score=121	.	.	.	.	.	.	.	.
.	.	ID=COSN2	.	.	.	.	.	.
.	.	.	.	.	.	.	.	.
.	rs7647197	ID=COSM2	.	.	.	.	.	.
.	.	.	.	.	.	.	.	.
.	.	.	.	.	.	.	.	.
.	rs7456483	ID=COSM1	.	.	.	.	.	.
.	.	.	.	.	.	.	.	.
.	.	ID=COSN2	.	.	.	.	.	.
.	rs7470964	.	.	.	.	.	.	.
.	.	.	.	.	.	.	.	.
.	.	.	.	.	.	.	.	.
.	.	ID=COSM3	.	.	.	.	.	.
.	.	.	.	.	.	.	.	.
.	.	ID=COSM4	.	.	.	.	.	.
.	.	.	.	.	.	.	.	.
.	.	.	.	.	.	.	.	.
.	.	.	.	.	.	.	.	.
.	.	ID=COSM5	.	.	.	.	.	.
.	rs5440185	ID=COSN2	.	.	.	.	0.00619	0.0068
.	.	.	.	.	.	.	.	.
.	.	ID=COSM4	.	.	.	.	.	.
.	rs9042896	.	.	.	.	.	.	.
.	rs7608755	ID=COSN1	.	.	.	.	.	.
.	.	.	.	.	.	.	.	.
.	.	.	.	.	.	.	.	.
.	rs7647092	ID=COSM5	.	.	.	.	.	.
.	.	.	.	.	.	.	.	.
.	.	ID=COSM1	.	.	.	.	.	.
.	.	.	.	.	.	.	.	.
.	.	.	.	.	.	.	.	.
.	.	.	.	.	.	.	.	.
.	rs7755820	.	.	.	.	.	.	.
.	.	.	.	.	.	.	.	.
.	.	.	.	.	.	.	.	.
.	.	.	.	.	.	.	.	.
.	.	ID=COSN1	.	.	.	.	.	.
.	.	.	.	.	.	.	.	.
.	rs7704742	.	.	.	.	.	.	8.00E-05
.	rs7480610	.	.	.	.	.	.	.
.	rs7646973	ID=COSN2	.	.	.	.	.	.
.	rs9281864	.	.	.	.	.	.	.
.	.	.	.	.	.	.	.	.
.	rs7525252	.	.	.	.	.	.	.
.	.	.	.	.	.	.	.	.
.	.	.	.	.	.	.	.	.
.	.	ID=COSN2	.	.	.	.	.	.

.	.	.	.	.	.	.	.	.	
Score=234	rs7641185	ID=COSM1	.	.	.	.	.		0.0018
.	rs7639462	.	.	.	.	.	.	.	
.	.	ID=COSM4	.	.	.	.	.	.	
.	.	.	.	.	.	.	.	.	
.	.	.	.	.	.	.	.	.	
.	rs7466549	ID=COSM1	.	.	.	.	.		0.0062
.	rs7717298	.	.	.	.	.	.	.	
.	.	.	.	.	.	.	.	.	
.	.	.	.	.	.	.	.	.	
.	rs7625018	ID=COSN2	.	.	.	.	.	.	
.	rs7658478	.	.	.	.	.	.	.	
.	.	.	.	.	.	.	.	.	
.	.	.	.	.	.	.	.	.	
.	rs7778584	.	.	.	.	.	.	.	
.	rs7693384	ID=COSM2	.	.	.	.	.	.	
.	.	.	.	.	.	.	.	.	
.	rs7768326	.	.	.	.	.	.	.	
.	rs3743843	.	.	.	.	0.003	0.000599	.	
.	.	.	.	.	.	.	.	.	
.	rs7666007	.	.	.	.	.	.	.	
.	rs7805014	.	.	.	.	.	.	.	
.	rs7489955	ID=COSN2	.	.	.	.	.		0.0108
.	rs7612855	.	.	.	.	.	.	.	
.	rs7600208	ID=COSM3	.	.	.	.	.	.	
.	.	ID=COSM5	.	.	.	.	.	.	
.	.	ID=COSN1	.	.	.	.	.	.	
.	.	.	.	.	.	.	.	.	
.	rs7792033	.	.	.	.	.	.	.	
.	rs7668254	ID=COSM4	.	.	.	.	.	.	
.	.	ID=COSM1	.	.	.	.	.	.	
.	.	.	.	.	.	.	.	.	
.	.	.	.	.	.	.	.		8.00E-05
.	rs1490463	.	.	.	.	.	.	.	
.	.	ID=COSM3	.	.	.	.	.	.	
.	rs7570813	.	.	.	.	.	.	.	
.	rs7468588	ID=COSM3	.	.	.	.	.	.	
.	.	.	.	.	.	.	.	.	
.	.	.	.	.	.	.	.	.	
Score=321	.	.	.	.	.	.	.	.	
.	rs1443745	.	.	.	.	.	.	.	
.	.	.	.	.	.	.	.	.	
.	rs7542333	ID=COSM5	.	.	.	.	.	.	
.	rs3701666	ID=COSM1	.	.	.	.	.	.	
.	.	.	.	.	.	.	.	.	
.	rs7470634	ID=COSM1	.	.	.	.	.	.	
.	.	.	.	.	.	.	.	.	
.	.	.	.	.	.	.	.	.	
.	rs7819467	.	.	.	.	.	.	.	
Score=217	.	.	.	.	.	.	.	.	
.	.	.	.	.	.	.	.	.	
.	.	.	.	.	.	.	.	.	
.	.	.	.	.	.	.	.	.	
.	rs5592025	.	.	.	.	0.004	0.000799	.	

.	.	.	.	.	.	.	.	.
.	rs7594121	.	.	.	.	.	.	.
Score=568	rs7708715	.	.	.	.	.	.	.
.	.	.	.	.	.	.	.	.
.	rs7618495	.	.	.	.	.	.	.
Score=232	.	.	.	.	.	.	.	.
.	rs7790763	.	.	.	.	.	.	.
.	.	.	.	.	.	.	.	.
.	rs1126292	.	.	.	.	0.0198	0.04972	.
.	rs3832550	.	.	.	.	.	.	0.0011
.	.	.	.	.	.	.	.	.
.	.	ID=COSN5	.	.	.	.	.	.
.	rs7782900	ID=COSN2	.	.	.	.	.	.
.	rs7759500	ID=COSM(	.	.	.	.	.	.
.	.	.	.	.	.	.	.	.
Score=198	rs1462533	.	.	.	.	.	.	.
.	.	.	.	.	.	.	.	.
.	rs7562540	ID=COSM1	.	.	.	.	.	.
.	rs7648900	.	.	.	.	.	.	.
.	rs7766165	ID=COSN5	.	.	.	.	.	.
.	.	ID=COSM1	.	.	.	.	.	.
.	rs5303481	.	.	.	.	0.002	0.000599	.
.	.	ID=COSM1	.	.	.	.	.	.
.	.	ID=COSM1	.	.	.	.	.	.
.	.	.	.	.	.	.	.	0.0076
Score=261	rs7606317	.	.	.	.	.	.	0.0061
.	rs7792498	ID=COSM1	.	.	.	.	.	.
.	rs1028985	.	.	.	.	.	.	.
.	rs7703285	.	.	.	.	.	.	.
.	rs3675892	.	.	.	.	0.003	0.000599	.
Score=402	.	.	.	.	.	.	.	.
.	.	.	.	.	.	.	.	.
.	.	.	.	.	.	.	.	.
.	rs7517698	.	.	.	.	.	.	.
.	.	ID=COSM4	.	.	.	.	.	.
.	.	.	.	.	.	.	.	.
.	rs7625775	ID=COSN2	.	.	.	.	.	.
.	.	.	.	.	.	.	.	.
.	.	ID=COSN1	.	.	.	.	.	.
.	rs7582351	.	.	.	.	.	.	8.80E-05
.	.	.	.	.	.	.	0.008387	0.0086
.	rs7542104	.	.	.	.	.	.	.
.	rs7696631	ID=COSM1	.	.	.	.	.	0.0043
.	.	ID=COSN1	.	.	.	.	.	.
.	rs7488695	.	.	.	.	.	.	.
.	rs7664186	.	.	.	.	.	.	.
.	rs7585686	.	.	.	.	.	.	.
.	.	.	.	.	.	.	.	.
.	rs5534658	.	.	.	.	0.0069	0.002196	0.005
.	.	.	.	.	.	.	.	.
.	.	.	.	.	.	.	.	.
.	.	ID=COSM4	.	.	.	.	.	0.0021
.	rs7518721	ID=COSM5	.	.	.	.	.	0.0175
.	rs9766384	.	.	.	.	.	.	.

.	.	.	.	.	.	.	.	.
.	.	.	.	.	.	.	.	.
.	rs7632273	.	.	.	.	.	.	0.0003
.	rs7778008	.	.	.	.	.	.	.
.	rs1049395	.	.	.	.	.	.	.
.	.	.	.	.	.	.	.	.
.	.	ID=COSM1	.	.	.	.	.	.
.	.	ID=COSN2	.	.	.	.	.	.
.	rs7588677	.	.	.	.	.	.	.
.	rs8987047	.	.	.	.	.	.	.
.	rs7647351	ID=COSM1	.	.	.	.	.	0.0159
.	.	.	.	.	.	.	.	.
.	.	ID=COSM6	.	.	.	.	.	.
.	rs5739889	ID=COSN2	.	.	.	.	0.000799	0.0031
.	rs7766671	.	.	.	.	.	.	8.00E-05
.	rs7749641	ID=COSM1	.	.	.	.	.	.
.	rs7666743	.	.	.	.	.	.	.
Score=214	.	.	.	.	.	.	.	.
.	.	.	.	.	.	.	.	.
.	.	.	.	.	.	.	.	.
.	.	.	.	.	.	.	.	.
.	rs7566997	.	.	.	.	.	.	.
.	rs7649880	.	.	.	.	.	.	.
Score=200	.	.	.	.	.	.	.	.
.	.	.	.	.	.	.	.	.
.	.	.	.	.	.	.	.	.
.	rs7636694	.	.	.	.	.	.	0.0036
.	.	.	.	.	.	.	.	.
Score=216	.	.	.	.	.	.	.	.
.	.	.	.	.	.	.	.	.
.	.	.	.	.	.	.	.	.
.	rs5532896	.	.	.	.	.	0.008986	0.001
.	.	.	.	.	.	.	.	.
.	rs7706124	ID=COSM1	.	.	.	.	.	.
.	rs5359317	.	.	.	.	.	.	.
.	rs7763171	ID=COSM1	.	.	.	.	.	0.007
.	rs1999629	.	.	.	.	.	.	0.0346
.	.	.	.	.	.	.	.	.
.	.	.	.	.	.	.	.	.
.	rs7643750	ID=COSM1	.	.	.	.	.	.
Score=481	.	.	.	.	.	.	.	.
.	rs7166969	.	.	.	.	.	0.026957	0.0355
.	.	.	.	.	.	.	.	.
.	.	ID=COSM1	.	.	.	.	.	.
.	rs1134746	.	.	.	.	0.003	0.103235	0.1221
.	rs5526989	.	.	.	.	.	.	.
.	.	.	.	.	.	.	.	.
.	.	ID=COSN2	.	.	.	.	.	.
.	.	.	.	.	.	.	.	.
.	rs1451472	ID=COSM1	.	.	.	.	.	.
Score=215	.	.	.	.	.	.	.	.
.	rs7643803	.	.	.	.	.	.	.
.	.	ID=COSM4	.	.	.	.	.	.
.	.	.	.	.	.	.	.	.

.	rs5635626	ID=COSN2	.	.	.	0.001	0.000799	.
.	rs7515595	.	.	.	.	.	.	.
.	rs5561116	ID=COSN1	.	.	.	.	0.000799	.
.	.	.	.	.	.	.	.	.
.	.	.	.	.	.	.	.	.
.	rs7643461	ID=COSM4	.	.	.	.	.	.
.	.	.	.	.	.	.	.	.
.	.	ID=COSM2	.	.	.	.	.	.
.	.	.	.	.	.	.	.	.
.	rs7781350	.	.	.	.	.	.	0.0063
.	rs7809656	.	.	.	.	.	.	.
.	rs7491504	ID=COSM2	.	.	.	.	.	0.0057
Score=119	.	.	.	.	.	.	.	.
.	.	.	.	.	.	.	.	.
.	.	.	.	.	.	.	.	.
.	rs7570296	.	.	.	.	.	.	.
.	.	.	.	.	.	.	.	.
.	rs7817135	ID=COSM1	.	.	.	.	.	.
.	rs7679025	ID=COSM1	.	.	.	.	.	.
.	.	.	.	.	.	.	.	.
.	.	.	.	.	.	.	.	.
.	.	.	.	.	.	.	.	.
.	.	.	.	.	.	.	.	.
.	.	.	.	.	.	.	.	0.0045
.	rs7574964	ID=COSM1	.	.	.	.	.	.
.	.	ID=COSM4	.	.	.	.	.	.
.	rs5285990	ID=COSN2	.	.	.	.	0.002796	0.002
Score=276	.	.	.	.	.	.	.	.
.	.	ID=COSM2	.	.	.	.	.	.
.	.	.	.	.	.	.	.	.
.	.	.	.	.	.	.	.	.
.	.	.	.	.	.	.	.	.
.	.	.	.	.	.	.	.	.
.	rs1380173	.	.	.	.	.	0.026558	0.0226
.	.	ID=COSN1	.	.	.	.	.	.
.	.	.	.	.	.	.	.	.
.	.	.	.	.	.	.	.	.
.	rs7634794	.	.	.	.	.	.	.
.	.	.	.	.	.	.	.	.
.	rs7487058	.	.	.	.	.	.	.
.	rs1429731	ID=COSN1	Likely beni	.	.	.	.	.
.	rs7500268	.	.	.	.	.	.	.
.	.	.	.	.	.	.	.	.
.	.	.	.	.	.	.	.	.
.	rs7661354	.	.	.	.	.	.	0.0009
.	.	.	.	.	.	.	.	.
.	rs5710814	.	.	.	.	.	.	0.002
Score=235	rs1019291	.	.	.	.	.	.	.
.	rs7630535	ID=COSM4	.	.	.	.	.	.
Score=346	.	.	.	.	.	.	.	.
Score=23;I	.	.	.	.	.	.	.	.
.	rs7644591	ID=COSM4	.	.	.	.	.	.
.	.	.	.	.	.	.	.	.
Score=856	.	.	.	.	.	.	.	.

.	.	.	.	.	.	.	.	.
.	rs7590798	.	.	.	.	.	.	.
.	.	.	.	.	.	.	.	.
.	rs7708014	ID=COSN2	.	.	.	.	.	.
.	.	.	.	.	.	.	.	.
.	.	.	.	.	.	.	.	.
Score=620	.	.	.	.	.	.	.	.
.	.	.	.	.	.	.	.	.
.	rs7799007	ID=COSM1	.	.	.	.	.	0.006
Score=225	rs7677659	.	.	.	.	.	.	0.0002
Score=289	.	.	.	.	.	.	.	.
.	.	.	.	.	.	.	.	.
.	.	ID=COSM3	.	.	.	.	.	.
.	rs9414440	ID=COSM(	.	.	.	.	.	.
.	.	.	.	.	.	.	.	.
.	.	.	.	.	.	.	.	.
.	.	.	.	.	.	.	.	.
.	rs7662018	.	.	.	.	.	.	.
.	.	.	.	.	.	.	.	.
.	.	.	.	.	.	.	.	.
.	rs7520653	.	.	.	.	.	.	0.0106
.	.	.	.	.	.	.	.	.
.	.	.	.	.	.	.	.	.
.	rs7555061	ID=COSM1	.	.	.	.	.	0.0075
.	rs3981228	ID=COSM1	Pathogenic	.	.	.	.	0.0064
.	.	.	.	.	.	.	.	.
.	.	.	.	.	.	.	.	.
.	rs7666986	.	.	.	.	.	.	.
.	.	.	.	.	.	.	.	.
.	.	.	.	.	.	.	.	.
.	rs7811378	ID=COSM1	.	.	.	.	.	.
.	.	.	.	.	.	.	.	.
.	.	.	.	.	.	.	.	.
.	rs7575646	ID=COSN2	.	.	.	.	.	.
.	rs3605176	.	.	.	.	.	.	.
.	.	.	.	.	.	.	.	.
.	rs1085307	ID=COSM4	Pathogenic	.	.	.	.	0.0252
.	rs7539299	.	.	.	.	.	.	0.0048
.	.	.	.	.	.	.	.	.
.	rs7511829	ID=COSM3	.	.	.	.	.	.
.	rs7684989	.	.	.	.	.	.	.
.	rs7667143	ID=COSM2	.	.	.	.	.	.
.	rs9359712	ID=COSN1	.	.	.	.	.	.
.	.	.	.	.	.	.	.	.
Score=308	rs3687317	ID=COSN2	.	.	.	.	.	.
.	rs7538174	.	.	.	.	.	.	.
.	rs7755389	ID=COSM4	.	.	.	.	.	.
.	rs2001464	ID=COSN1	.	.	.	0.0794	0.049121	.
.	.	.	.	.	.	.	.	.
Score=241	.	.	.	.	.	.	.	.
.	.	.	.	.	.	.	.	.
.	rs7686708	ID=COSN1	.	.	.	.	.	.
.	.	.	.	.	.	.	.	.
.	.	.	.	.	.	.	.	.

.	rs7743082	.	.	.	.	.	.	.
.	.	.	.	.	.	.	.	.
.	.	ID=COSM1	.	.	.	.	.	.
.	rs7631509	.	.	.	.	.	.	.
Score=22;I	.	.	.	.	.	.	.	.
.	rs7752182	.	.	.	.	.	.	0.0009
.	rs7515575	ID=COSN5	.	.	.	.	.	0.0034
.	rs7821251	.	.	.	.	.	.	.
.	.	.	.	.	.	.	.	.
.	.	.	.	.	.	.	.	.
.	.	ID=COSM2	.	.	.	.	.	.
.	rs1064795	.	Likely beni	.	.	.	.	.
.	rs7823430	ID=COSM1	.	.	.	.	.	.
.	.	.	.	.	.	.	.	.
.	.	.	.	.	.	.	.	.
.	.	.	.	.	.	.	.	.
.	.	.	.	.	.	.	.	.
.	rs7485648	.	.	.	.	.	.	0.0226
.	.	.	.	.	.	.	.	.
.	.	.	.	.	.	.	.	.
.	rs9344233	.	.	.	.	.	.	.
.	rs4036589	ID=COSN1	.	.	.	.	.	.
.	rs1062553	.	.	.	.	0.3085	0.441094	.
Score=207	.	.	.	.	.	.	.	.
.	rs3707030	.	.	.	.	.	.	.
Score=204	.	.	.	.	.	.	.	.
.	.	.	.	.	.	.	.	.
.	.	.	.	.	.	.	.	.
Score=28;I	.	.	.	.	.	.	.	.
.	.	.	.	.	.	.	.	.
Score=275	rs8860593	.	Uncertain s	.	.	.	.	.
.	.	.	.	.	.	.	.	.
.	.	.	.	.	.	.	.	.
.	rs9488701	ID=COSM4	.	.	.	.	.	.
.	rs1047246	.	.	.	.	.	.	.
.	.	.	.	.	.	.	.	.
.	.	.	.	.	.	.	.	.
.	.	.	.	.	.	.	.	.
.	.	.	.	.	.	.	.	.
.	.	.	.	.	.	.	.	.
.	.	.	.	.	.	.	.	.
.	.	.	.	.	.	.	.	.
Score=405	rs3980370	.	.	.	.	0.2173	0.234625	.
.	.	.	.	.	.	.	.	.
Score=192	rs7562167	.	.	.	.	.	.	0.0006
Score=366	rs7459278	.	.	.	.	.	.	.
.	.	.	.	.	.	.	.	.
.	.	.	.	.	.	.	.	.
.	.	.	.	.	.	.	.	.
.	rs2676066	.	Pathogenic	.	.	.	.	.
Score=32;I	.	.	.	.	.	.	.	.
.	.	.	.	.	.	.	.	.
.	rs7519299	.	.	.	.	.	.	.
.	.	.	.	.	.	.	.	.

.	.	.	.	.	.	.	.	.
.	.	.	.	.	.	.	.	.
.	.	.	.	.	.	.	.	.
Score=210	rs7666083	.	.	.	.	.	.	0.0085
.	.	.	.	.	.	.	.	.
.	.	.	.	.	.	.	.	.
Score=255	.	.	.	.	.	.	.	.
Score=240	rs7788921	.	.	.	.	.	.	.
.	.	.	.	.	.	.	.	.
Score=224	rs7743913	.	.	.	.	.	.	.
.	rs7758009	.	.	.	.	.	.	.
.	.	ID=COSM!	.	.	.	.	.	.
Score=917	.	.	.	.	.	.	.	.
Score=22;I	.	.	.	.	.	.	.	.
.	rs7645628	.	.	.	.	.	.	.
.	.	.	.	.	.	.	.	.
Score=51;I	.	.	.	.	.	.	.	.
Score=450	.	.	.	.	.	.	.	.
.	.	.	.	.	.	.	.	.
Score=207	rs6015769	ID=COSN1	.	.	.	0.7262	0.701877	.
.	rs7596499	.	.	.	.	.	.	.
Score=180	.	.	.	.	.	.	.	.
.	.	.	.	.	.	.	.	.
.	.	.	.	.	.	.	.	.
.	.	.	.	.	.	.	.	.
.	.	.	.	.	.	.	.	.
.	.	.	.	.	.	.	.	.
.	.	.	.	.	.	.	.	.

ExAC_ALL	ExAC_EAS	SIFT	Polyphen2_	Polyphen2_	MutationTa	gerp++gt2	CADD	NovoDb_W
.	.	.	.	.	.	.	.	.
.	.	.	.	.	.	.	.	.
.	.	.	.	.	.	.	.	0.001063
.	.	.	.	.	.	.	.	.
0.0017	0.0004	.	.	.	.	.	-0.015565,	.
.	.	.	.	.	.	.	.	.
.	.	.	.	.	.	.	.	.
.	.	.	.	.	.	.	.	0.000389
.	.	.	.	.	.	.	.	.
.	.	.	.	.	.	.	-0.171358,	0.001392
.	.	.	.	.	.	.	.	.
.	.	.	.	.	.	.	.	.
.	.	.	.	.	.	.	.	0.011341
0.1944	0.069	.	.	.	.	.	0.721048,7	0.002806
.	.	.	.	.	.	.	0.640624,7	0.090962
.	.	.	.	.	.	.	.	.
.	.	.	.	.	.	.	.	.
.	.	.	.	.	.	.	.	.
.	.	.	.	.	.	.	.	.
.	.	.	.	.	.	.	.	.
.	.	.	.	.	.	.	.	0.005265
.	.	.	.	.	.	.	.	.
.	.	.	.	.	.	.	.	.
.	.	.	.	.	.	.	.	.
.	.	.	.	.	.	.	.	.
.	.	.	.	.	.	.	-0.055266,	.
.	.	.	.	.	.	.	.	.
.	.	.	.	.	.	.	.	.
.	.	.	.	.	.	.	8.456148,4	.
.	.	.	.	.	.	.	.	.
.	.	.	.	.	.	.	.	.
.	.	.	.	.	.	.	.	.
0.0001	0	.	.	.	.	.	3.824671,1	.
0.0005	0.0003	.	.	.	.	.	.	.
.	.	.	.	.	.	.	.	.
8.25E-06	0.0001	.	.	.	.	.	1.220399,9	.
.	.	.	.	.	.	.	.	.
.	.	.	.	.	.	.	.	.
.	.	.	.	.	.	.	.	.
.	.	.	.	.	.	.	.	.
.	.	.	.	.	.	.	.	.
.	.	.	.	.	.	.	0.318819,5	.
.	.	.	.	.	.	.	.	.
0.0005	0	.	.	.	.	.	3.318747,1	.
2.22E-05	0	.	.	.	.	.	.	.
0.0002	0	.	.	.	.	.	-0.262035,	.
.	.	.	.	.	.	.	.	.
0.0003	0.0007	.	.	.	.	.	0.831281,8	.
7.87E-05	0	.	.	.	.	.	.	.
.	.	.	.	.	.	.	.	.
.	.	.	.	.	.	.	.	.

.	.	.	.	.	.	.	.	.
0.0007	0	.	.	.	.	.	.	.
.	.	.	.	.	.	.	.	.
.	.	.	.	.	.	.	-0.366243,	0.001131
.	.	.	.	.	.	.	-0.344064,	.
2.47E-05	0	.	.	.	.	.	.	.
0.0033	0.0002	.	.	.	.	.	2.599557,1	.
.	.	.	.	.	.	.	.	.
.	.	.	.	.	.	.	.	.
.	.	.	.	.	.	.	.	.
3.31E-05	0	.	.	.	.	.	.	.
.	.	.	.	.	.	.	.	.
.	.	.	.	.	.	.	.	.
2.50E-05	0	.	.	.	.	.	.	0.000194
.	.	.	.	.	.	.	.	.
.	.	.	.	.	.	.	.	.
0.0003	0	.	.	.	.	.	.	.
.	.	.	.	.	.	.	.	.
.	.	.	.	.	.	.	.	.
.	.	.	.	.	.	.	.	.
.	.	.	.	.	.	.	-0.745937,	.
.	.	.	.	.	.	.	.	.
.	.	.	.	.	.	.	.	.
.	.	.	.	.	.	.	.	.
.	.	.	.	.	.	.	.	.
.	.	.	.	.	.	.	.	.
0.0019	0.0002	.	.	.	.	.	-1.033226,	.
.	.	.	.	.	.	.	.	.
.	.	.	.	.	.	.	.	.
.	.	.	.	.	.	.	.	.
3.49E-05	0	.	.	.	.	.	.	.
.	.	.	.	.	.	.	.	.
.	.	.	.	.	.	.	.	.
4.13E-05	0	.	.	.	.	.	2.045653,1	.
.	.	.	.	.	.	.	.	.
.	.	.	.	.	.	.	.	.
.	.	.	.	.	.	.	.	.
.	.	.	.	.	.	.	.	.
.	.	.	.	.	.	.	.	.
3.30E-05	0	.	.	.	.	.	.	.
.	.	.	.	.	.	.	.	.
.	.	.	.	.	.	.	.	0.045347
.	.	.	.	.	.	.	.	.
.	.	.	.	.	.	.	.	.
.	.	.	.	.	.	.	.	.
4.14E-05	0	.	.	.	.	.	6.272893,3	.
0.0013	0	.	.	.	.	.	.	0.000518
7.43E-05	0	.	.	.	.	.	0.215872,5	.
.	.	.	.	.	.	.	.	.
.	.	.	.	.	.	.	.	.
6.67E-05	0	.	.	.	.	.	.	.
.	.	.	.	.	.	.	.	.
.	.	.	.	.	.	.	.	.
.	.	.	.	.	.	.	.	.

.	.	.	.	.	.	.	.	.
0.0002	0.0001	.	.	.	.	.	3.925528,1	.
3.31E-05	0	.	.	.	.	.	.	.
.	.	.	.	.	.	.	.	.
.	.	.	.	.	.	.	.	.
.	.	.	.	.	.	.	.	.
0.0003	0.0003	.	.	.	.	.	12.104388	.
.	.	.	.	.	.	.	0.699566,7	.
.	.	.	.	.	.	.	.	.
.	.	.	.	.	.	.	.	.
2.54E-05	0	.	.	.	.	.	.	.
0.0003	0.0002	.	.	.	.	.	.	.
.	.	.	.	.	.	.	.	.
.	.	.	.	.	.	.	.	0.000195
8.29E-06	0	.	.	.	.	.	.	.
0.0018	0.0003	.	.	.	.	.	3.668062,1	.
.	.	.	.	.	.	.	.	.
0.0013	0.0003	.	.	.	.	.	-0.354890,	.
.	.	.	.	.	.	.	-0.012894,	0.001761
.	.	.	.	.	.	.	.	.
3.91E-05	0.0005	.	.	.	.	.	.	.
8.60E-06	0	.	.	.	.	.	.	.
0.0132	0.0002	.	.	.	.	.	0.779711,8	.
8.55E-06	0	.	.	.	.	.	.	.
0.0014	0.0002	.	.	.	.	.	2.174337,1	.
.	.	.	.	.	.	.	.	.
.	.	.	.	.	.	.	.	.
.	.	.	.	.	.	.	.	.
0.0003	0	.	.	.	.	.	.	.
1.67E-05	0	.	.	.	.	.	.	.
.	.	.	.	.	.	.	.	.
.	.	.	.	.	.	.	.	.
.	.	.	.	.	.	.	4.835186,2	.
.	.	.	.	.	.	.	0.253508,5	0.028386
.	.	.	.	.	.	.	.	.
0.0027	0	.	.	.	.	.	.	.
2.51E-05	0	.	.	.	.	.	.	.
.	.	.	.	.	.	.	.	.
.	.	.	.	.	.	.	.	.
.	.	.	.	.	.	.	-0.792134,	0.010551
.	.	.	.	.	.	.	0.066003,4	.
.	.	.	.	.	.	.	.	.
0.0011	0	.	.	.	.	.	7.541577,3	.
0.0002	0	.	.	.	.	.	0.414848,6	.
.	.	.	.	.	.	.	.	.
7.45E-05	0	.	.	.	.	.	.	.
.	.	.	.	.	.	.	.	.
.	.	.	.	.	.	.	.	.
8.41E-06	0	.	.	.	.	.	.	.
.	.	.	.	.	.	.	.	.
.	.	.	.	.	.	.	.	.
.	.	.	.	.	.	.	.	.
.	.	.	.	.	.	.	.	.
0.0006	0.0026	.	.	.	.	.	-0.337970,	0.002138

.	.	.	.	.	.	.	.	.
0.0075	0.0013	.	.	.	.	.	1.449740,1	0.000278
0.0042	0.0001	.	.	.	.	.	-0.171168,	.
.	.	.	.	.	.	.	.	.
1.66E-05	0	.	.	.	.	.	.	.
.	.	.	.	.	.	.	.	.
0.0012	0	.	.	.	.	.	0.332589,5	0.001942
.	.	.	.	.	.	.	.	.
.	.	.	.	.	.	.	-1.012315,	.
0.0002	.	.	.	.	.	.	3.590050,1	.
.	.	.	.	.	.	.	.	.
.	.	.	.	.	.	.	.	.
0.0006	0	.	.	.	.	.	.	.
8.28E-06	0	.	.	.	.	.	.	.
.	.	.	.	.	.	.	.	.
.	.	.	.	.	.	.	0.781695,8	0.000194
.	.	.	.	.	.	.	.	.
0.0003	0	.	.	.	.	.	.	.
8.31E-06	0	.	.	.	.	.	.	.
0.0005	0.0004	.	.	.	.	.	.	.
.	.	.	.	.	.	.	.	.
.	.	.	.	.	.	.	.	.
.	.	.	.	.	.	.	.	.
.	.	.	.	.	.	.	.	.
.	.	.	.	.	.	.	0.848277,8	.
0.0384	0.1569	.	.	.	.	.	-0.953411,	0.000197
9.89E-05	0.001	.	.	.	.	.	4.370603,2	0.000705
.	.	.	.	.	.	.	2.454346,1	.
4.37E-05	0	.	.	.	.	.	.	.
.	.	.	.	.	.	.	-0.201835,	0.005479
.	.	.	.	.	.	.	.	.
.	.	.	.	.	.	.	.	.
.	.	.	.	.	.	.	.	.
1.38E-05	0	.	.	.	.	.	.	0.000389
.	.	.	.	.	.	.	.	.
.	.	.	.	.	.	.	.	.
9.35E-05	0	.	.	.	.	.	.	.
.	.	.	.	.	.	.	.	.
.	.	.	.	.	.	.	.	0.001391
0.0002	0.0005	.	.	.	.	.	-0.750302,	0.001206
0.0025	0.0001	.	.	.	.	.	0.948278,8	.
8.28E-06	.	.	.	.	.	.	.	.
0.0031	0.0005	.	.	.	.	.	5.924892,3	.
.	.	.	.	.	.	.	.	.
1.66E-05	0.0001	.	.	.	.	.	.	0.000389
8.28E-06	0	.	.	.	.	.	-0.266141,	.
2.62E-05	0	.	.	.	.	.	.	.
.	.	.	.	.	.	.	.	.
0.0046	0.0122	.	.	.	.	.	0.334116,5	0.00529
.	.	.	.	.	.	.	.	.
.	.	.	.	.	.	.	.	.
.	.	.	.	.	.	.	1.247081,1	.
0.0006	0.0004	.	.	.	.	.	1.443171,1	.
.	.	.	.	.	.	.	1.184019,9	.

.	.	.	.	.	.	.	.	.
.	.	.	.	.	.	.	.	.
0.0037	0.0036	.	.	.	.	.	1.940664,1	0.000777
7.67E-05	0	.	.	.	.	.	.	0.000229
.	.	.	.	.	.	.	.	.
.	.	.	.	.	.	.	.	.
.	.	.	.	.	.	.	.	.
.	.	.	.	.	.	.	-1.180968,	.
1.66E-05	0	.	.	.	.	.	.	.
.	.	.	.	.	.	.	.	.
0.0016	0	.	.	.	.	.	.	.
.	.	.	.	.	.	.	.	.
.	.	.	.	.	.	.	.	.
0.0006	0.0018	.	.	.	.	.	0.076681,4	0.00136
8.24E-06	0	.	.	.	.	.	2.368741,1	.
1.66E-05	0	.	.	.	.	.	.	.
0.0004	0	.	.	.	.	.	.	.
.	.	.	.	.	.	.	.	.
.	.	.	.	.	.	.	.	.
.	.	.	.	.	.	.	.	.
.	.	.	.	.	.	.	.	.
9.44E-06	0	.	.	.	.	.	.	.
2.54E-05	0	.	.	.	.	.	.	.
.	.	.	.	.	.	.	.	.
.	.	.	.	.	.	.	.	.
.	.	.	.	.	.	.	.	.
0.0035	0.0001	.	.	.	.	.	1.973183,1	.
.	.	.	.	.	.	.	.	.
.	.	.	.	.	.	.	-1.068213,	0.010408
.	.	.	.	.	.	.	.	.
.	.	.	.	.	.	.	.	.
0.0106	0	.	.	.	.	.	1.306697,1	.
.	.	.	.	.	.	.	.	.
1.65E-05	0	.	.	.	.	.	5.434959,3	.
.	.	.	.	.	.	.	1.657264,1	.
0.0023	0.0008	.	.	.	.	.	6.333780,3	.
0.001	0.0068	.	.	.	.	.	-0.644200,	0.006646
.	.	.	.	.	.	.	.	.
.	.	.	.	.	.	.	.	.
0.001	0.0002	.	.	.	.	.	.	.
.	.	.	.	.	.	.	.	.
0.0018	0.0009	.	.	.	.	.	-0.629183,	.
.	.	.	.	.	.	.	.	.
.	.	.	.	.	.	.	.	.
0.0684	0.0073	.	.	.	.	.	0.792157,8	0.00312
.	.	.	.	.	.	.	.	.
.	.	.	.	.	.	.	.	.
.	.	.	.	.	.	.	.	.
.	.	.	.	.	.	.	.	.
.	.	.	.	.	.	.	.	.
.	.	.	.	.	.	.	.	.
0.0002	0	.	.	.	.	.	.	.
.	.	.	.	.	.	.	.	.
.	.	.	.	.	.	.	.	.

0.0001	0	.	.	.	.	.	-0.838457,	.
0	0	.	.	.	.	.	-0.979719,	.
0.0002	0	.	.	.	.	.	0.629140,7	.
.	.	.	.	.	.	.	.	.
.	.	.	.	.	.	.	.	.
0.0001	0.0008	.	.	.	.	.	.	.
.	.	.	.	.	.	.	.	.
.	.	.	.	.	.	.	.	.
.	.	.	.	.	.	.	.	.
0.0002	0.0007	.	.	.	.	.	-0.538121,	.
0.0001	0	.	.	.	.	.	-0.925102,	.
0.0052	0.001	.	.	.	.	.	3.748789,1	.
.	.	.	.	.	.	.	.	.
.	.	.	.	.	.	.	-0.233735,	.
.	.	.	.	.	.	.	-0.195350,	.
0.0114	0.0117	.	.	.	.	.	-0.102010,	0.000224
.	.	.	.	.	.	.	.	.
0.0001	0	.	.	.	.	.	.	.
0.0006	0.0001	.	.	.	.	.	.	.
.	.	.	.	.	.	.	.	.
.	.	.	.	.	.	.	.	.
.	.	.	.	.	.	.	.	.
.	.	.	.	.	.	.	.	.
.	.	.	.	.	.	.	-0.230199,	.
0.0002	0.0022	.	.	.	.	.	.	0.000389
.	.	.	.	.	.	.	.	.
0.0003	0	.	.	.	.	.	0.144172,4	.
.	.	.	.	.	.	.	.	.
.	.	.	.	.	.	.	.	.
.	.	.	.	.	.	.	.	.
.	.	.	.	.	.	.	.	.
.	.	.	.	.	.	.	5.822128,3	.
.	.	.	.	.	.	.	.	.
0.0064	0	.	.	.	.	.	0.090218,4	0.000469
.	.	.	.	.	.	.	.	.
.	.	.	.	.	.	.	.	.
.	.	.	.	.	.	.	.	.
0	0	.	.	.	.	.	.	.
.	.	.	.	.	.	.	.	.
0.001	0.0001	.	.	.	.	.	.	.
0.0003	0	.	.	.	.	.	.	.
1.68E-05	0	.	.	.	.	.	.	.
.	.	.	.	.	.	.	.	.
.	.	.	.	.	.	.	.	.
7.63E-05	0	.	.	.	.	.	0.764831,8	.
.	.	.	.	.	.	.	.	.
0.0005	0	.	.	.	.	.	3.326528,1	.
.	.	.	.	.	.	.	.	.
7.42E-05	0	.	.	.	.	.	.	.
.	.	.	.	.	.	.	.	.
.	.	.	.	.	.	.	.	.
4.65E-05	0	.	.	.	.	.	.	.
.	.	.	.	.	.	.	.	0.00039
.	.	.	.	.	.	.	.	.

.	.	.	.	.	.	.	.	.
4.39E-05	0	.	.	.	.	.	.	0.000194
.	.	.	.	.	.	.	.	.
0.0002	0.0001	.	.	.	.	.	.	.
.	.	.	.	.	.	.	.	.
.	.	.	.	.	.	.	.	.
.	.	.	.	.	.	.	.	.
.	.	.	.	.	.	.	.	.
0.0002	0	.	.	.	.	.	6.184515,3	.
0.0004	0.0004	.	.	.	.	.	0.091208,4	.
.	.	.	.	.	.	.	.	.
.	.	.	.	.	.	.	.	.
.	.	.	.	.	.	.	.	.
.	.	.	.	.	.	.	.	.
.	.	.	.	.	.	.	.	.
.	.	.	.	.	.	.	.	.
.	.	.	.	.	.	.	.	.
3.27E-05	0	.	.	.	.	.	.	.
.	.	.	.	.	.	.	.	.
.	.	.	.	.	.	.	.	.
0.0088	0.0091	.	.	.	.	.	-0.310612,	.
.	.	.	.	.	.	.	.	.
.	.	.	.	.	.	.	.	.
0.0002	0.0003	.	.	.	.	.	2.258493,1	.
0.0016	0.0001	.	.	.	.	.	1.111180,9	.
.	.	.	.	.	.	.	.	.
.	.	.	.	.	.	.	-0.232107,	.
0.0003	0.0002	.	.	.	.	.	.	.
.	.	.	.	.	.	.	.	.
.	.	.	.	.	.	.	.	.
0.0015	0.0005	.	.	.	.	.	.	.
.	.	.	.	.	.	.	.	.
.	.	.	.	.	.	.	.	.
0.0003	0.0005	.	.	.	.	.	.	.
5.83E-05	0	.	.	.	.	.	.	.
.	.	.	.	.	.	.	.	.
0.0016	0.0005	.	.	.	.	.	4.966242,2	.
0.0003	0.0001	.	.	.	.	.	-1.576300,	.
.	.	.	.	.	.	.	.	.
0.0002	0	.	.	.	.	.	.	.
0.0003	0.0001	.	.	.	.	.	.	.
0.0004	0.0006	.	.	.	.	.	4.399397,2	.
.	.	.	.	.	.	.	-0.789274,	.
.	.	.	.	.	.	.	.	.
.	.	.	.	.	.	.	-1.711714,	0.003326
8.36E-06	0	.	.	.	.	.	.	.
0.0002	0.0002	.	.	.	.	.	.	.
0.0166	0.0104	.	.	.	.	.	0.846469,8	0.003911
.	.	.	.	.	.	.	.	.
.	.	.	.	.	.	.	.	.
.	.	.	.	.	.	.	.	.
0.0002	0.0009	.	.	.	.	.	0.118246,4	.
.	.	.	.	.	.	.	.	.
.	.	.	.	.	.	.	.	.

8.48E-06	0	.	.	.	.	.	.	.
.	.	.	.	.	.	.	.	.
.	.	.	.	.	.	.	1.295933,1	.
.	.	.	.	.	.	.	.	.
.	.	.	.	.	.	.	.	.
3.49E-05	0	.	.	.	.	.	-1.137313,	.
0.0059	0.0016	.	.	.	.	.	-0.278162,	.
0.0009	0	.	.	.	.	.	-0.002064,	.
.	.	.	.	.	.	.	.	.
.	.	.	.	.	.	.	.	.
.	.	.	.	.	.	.	.	0.000194
.	.	.	.	.	.	.	.	.
0.0014	0	.	.	.	.	.	6.926590,3	.
.	.	.	.	.	.	.	.	.
.	.	.	.	.	.	.	.	.
.	.	.	.	.	.	.	.	.
.	.	.	.	.	.	.	.	.
9.62E-05	0	.	.	.	.	.	0.044422,4	.
.	.	.	.	.	.	.	.	.
.	.	.	.	.	.	.	.	.
.	.	.	.	.	.	.	.	0.000451
0.0131	0.0007	.	.	.	.	.	-0.967556,	0.002163
.	.	.	.	.	.	.	-0.708287,	0.180922
.	.	.	.	.	.	.	.	.
.	.	.	.	.	.	.	-0.621617,	.
.	.	.	.	.	.	.	.	.
.	.	.	.	.	.	.	.	.
.	.	.	.	.	.	.	.	.
.	.	.	.	.	.	.	.	.
.	.	.	.	.	.	.	.	.
.	.	.	.	.	.	.	2.489362,1	.
.	.	.	.	.	.	.	.	.
.	.	.	.	.	.	.	.	.
.	.	.	.	.	.	.	.	.
.	.	.	.	.	.	.	.	.
.	.	.	.	.	.	.	.	.
.	.	.	.	.	.	.	.	.
.	.	.	.	.	.	.	.	.
.	.	.	.	.	.	.	.	.
.	.	.	.	.	.	.	.	.
.	.	.	.	.	.	.	.	.
.	.	.	.	.	.	.	.	.
.	.	.	.	.	.	.	2.267836,1	0.039913
.	.	.	.	.	.	.	.	.
0.0201	0.0152	.	.	.	.	.	2.539731,1	.
0.0006	0	.	.	.	.	.	.	0.000235
.	.	.	.	.	.	.	.	.
.	.	.	.	.	.	.	.	.
.	.	.	.	.	.	.	.	.
.	.	.	.	.	.	.	.	.
0.0159	0.0034	.	.	.	.	.	1.032582,9	0.00366
.	.	.	.	.	.	.	.	.
8.54E-06	0.0001	.	.	.	.	.	.	.
.	.	.	.	.	.	.	.	.

.	.	.	.	.	.	.	.	.
.	.	.	.	.	.	.	.	0.000194
.	.	.	.	.	.	.	.	.
.	.	.	.	.	.	.	.	0.000194
.	.	.	.	.	.	.	.	.
.	.	.	.	.	.	.	.	.
.	.	.	.	.	.	.	.	.
0.1054	0.2256	.	.	.	.	.	.	.
.	.	.	.	.	.	.	.	.
0.0006	0	.	.	.	.	.	-2.866279,	0.002622
8.26E-06	0	.	.	.	.	.	.	.
.	.	.	.	.	.	.	.	.
.	.	.	.	.	.	.	.	.
.	.	.	.	.	.	.	.	.
1.70E-05	0	.	.	.	.	.	.	.
.	.	.	.	.	.	.	.	.
.	.	.	.	.	.	.	.	.
.	.	.	.	.	.	.	.	.
.	.	.	.	.	.	.	.	.
0.0829	0.0719	.	.	.	.	.	-1.762378,	0.023056
0.0002	0.0051	.	.	.	.	.	.	0.000389
.	.	.	.	.	.	.	.	.
.	.	.	.	.	.	.	.	.
.	.	.	.	.	.	.	.	.
.	.	.	.	.	.	.	.	.
.	.	.	.	.	.	.	.	.
.	.	.	.	.	.	.	.	.
.	.	.	.	.	.	.	.	.

NovoDb_W	INFO	FORMAT	ACa01N	ACa01T	Ori_REF	Ori_ALT	shared_ho	shared_he
.	IC=2;IHP=:	DP:DP2:T/	24:24:24,2	47:47:23,2	AC	A	0	0
.	IC=8;IHP=!	DP:DP2:T/	62:62:54,5	114:114:69	G	GT	0	0
.	IC=0;IHP=4	DP:DP2:T/	74:74:73,7	236:236:20	TTGGAG	T	0	0
.	IC=1;IHP=4	DP:DP2:T/	51:51:51,5	101:101:69	GTTT	G	0	0
.	IC=9;IHP=:	DP:DP2:T/	77:77:75,7	154:154:99	T	TC	0	0
.	IC=0;IHP=4	DP:DP2:T/	96:96:94,9	334:334:28	AT	A	0	0
.	IC=1;IHP=4	DP:DP2:T/	93:93:92,9	166:166:10	CAGA	C	0	0
.	IC=5;IHP=!	DP:DP2:T/	145:145:13	576:576:23	T	TC	0	0
.	IC=1;IHP=:	DP:DP2:T/	106:106:10	234:234:13	C	CAG	0	0
.	IC=6;IHP=:	DP:DP2:T/	51:51:48,4	116:116:85	T	TAGAC	0	0
.	IC=0;IHP=:	DP:DP2:T/	107:107:11	245:245:16	ACACAGC	A	0	0
.	IC=9;IHP=!	DP:DP2:T/	38:38:31,3	72:72:32,3	G	GT	0	0
.	IC=1;IHP=:	DP:DP2:T/	70:70:64,6	176:176:14	T	TTGGGC	0	0
.	IC=0;IHP=4	DP:DP2:T/	78:78:74,7	205:205:19	GTGTGTG	G	0	0
0.147788	IC=2;IHP=4	DP:DP2:T/	12:12:12,1	29:29:0,0:2	TC	T	0	0
.	IC=0;IHP=4	DP:DP2:T/	97:97:95,9	152:152:13	GGGGATT	G	0	0
.	IC=2;IHP=4	DP:DP2:T/	79:79:76,7	167:167:11	CG	C	0	0
.	IC=0;IHP=:	DP:DP2:T/	65:65:61,6	254:254:19	CGT	C	0	0
.	IC=4;IHP=!	DP:DP2:T/	41:41:41,4	83:83:53,5	GC	G	0	0
.	IC=0;IHP=:	DP:DP2:T/	83:83:83,9	172:172:15	CCTCTTT.	C	0	0
.	IC=1;IHP=:	DP:DP2:T/	69:69:68,6	144:144:12	C	CTCTCTC	0	0
.	IC=2;IHP=:	DP:DP2:T/	70:70:69,6	177:177:14	CTG	C	0	0
.	IC=5;IHP=(	DP:DP2:T/	118:118:11	220:220:16	AC	A	0	0
.	IC=7;IHP=8	DP:DP2:T/	152:152:14	304:304:24	TC	T	0	0
.	IC=4;IHP=!	DP:DP2:T/	67:67:68,6	194:194:13	AC	A	0	0
.	IC=8;IHP=8	DP:DP2:T/	103:103:10	193:193:15	T	TG	0	0
.	IC=5;IHP=(	DP:DP2:T/	42:42:42,4	77:77:56,5	AC	A	0	0
.	IC=5;IHP=(	DP:DP2:T/	89:89:82,8	173:173:12	CG	C	0	0
.	IC=6;IHP=7	DP:DP2:T/	86:86:83,8	209:209:16	TG	T	0	0
.	IC=0;IHP=7	DP:DP2:T/	165:165:16	317:317:26	CA	C	0	0
.	IC=8;IHP=7	DP:DP2:T/	105:105:99	191:191:16	A	AC	0	0
.	IC=7;IHP=!	DP:DP2:T/	50:50:50,5	85:85:64,6	C	CT	0	0
.	IC=7;IHP=8	DP:DP2:T/	24:24:24,2	53:53:35,3	GA	G	0	0
.	IC=8;IHP=8	DP:DP2:T/	108:108:10	203:203:15	T	TG	0	0
.	IC=4;IHP=!	DP:DP2:T/	56:56:56,5	84:84:56,5	CT	C	0	0
.	IC=6;IHP=7	DP:DP2:T/	90:90:88,8	153:153:11	GT	G	0	0
.	IC=4;IHP=:	DP:DP2:T/	60:60:60,6	137:137:10	AG	A	0	0
.	IC=5;IHP=(	DP:DP2:T/	51:51:49,4	68:68:52,5	TG	T	0	0
.	IC=6;IHP=7	DP:DP2:T/	94:94:91,9	237:237:19	GT	G	0	0
.	IC=4;IHP=(	DP:DP2:T/	93:93:92,9	198:198:15	TA	T	0	0
.	IC=7;IHP=8	DP:DP2:T/	63:63:57,5	106:106:79	GA	G	0	0
.	IC=2;IHP=4	DP:DP2:T/	58:58:59,5	108:108:84	CAG	C	0	0
.	IC=4;IHP=(	DP:DP2:T/	71:71:68,6	114:114:87	TC	T	0	0
.	IC=9;IHP=!	DP:DP2:T/	59:59:59,5	97:97:72,7	G	GA	0	0
.	IC=6;IHP=7	DP:DP2:T/	56:56:52,5	105:105:82	GA	G	0	0
.	IC=7;IHP=!	DP:DP2:T/	52:52:51,5	92:92:65,6	AT	A	0	0
.	IC=5;IHP=(	DP:DP2:T/	217:217:21	458:458:36	AC	A	0	0
.	IC=7;IHP=:	DP:DP2:T/	73:73:70,7	133:133:10	TA	T	0	0
.	IC=7;IHP=8	DP:DP2:T/	59:59:55,5	86:86:61,6	TA	T	0	0
.	IC=5;IHP=(	DP:DP2:T/	52:52:48,5	79:79:57,5	CG	C	0	0
0.000883	IC=5;IHP=:	DP:DP2:T/	31:31:32,3	73:73:49,4	CTG	C	0	0

.	IC=8;IHP=7	DP:DP2:T/	50:50:49,4	79:79:62,6	C	CA	0	0
.	IC=6;IHP=7	DP:DP2:T/	72:72:68,6	114:114:87	AT	A	0	0
.	IC=3;IHP=5	DP:DP2:T/	41:41:41,4	54:54:34,3	TA	T	0	0
0.001416	IC=7;IHP=9	DP:DP2:T/	52:52:49,4	85:85:61,6	CA	C	0	0
.	IC=4;IHP=6	DP:DP2:T/	25:25:25,2	48:48:30,3	GA	G	0	0
.	IC=9;IHP=3	DP:DP2:T/	40:40:38,3	61:61:43,4	A	AT	0	0
.	IC=9;IHP=8	DP:DP2:T/	131:131:12	324:324:24	G	GC	0	0
.	IC=7;IHP=3	DP:DP2:T/	71:71:67,6	101:101:77	G	GT	0	0
.	IC=7;IHP=9	DP:DP2:T/	47:47:47,4	73:73:50,5	TA	T	0	0
.	IC=5;IHP=7	DP:DP2:T/	55:55:53,5	108:108:82	CG	C	0	0
.	IC=7;IHP=9	DP:DP2:T/	103:103:98	125:125:70	TA	T	0	0
.	IC=4;IHP=5	DP:DP2:T/	97:97:95,9	182:182:14	AG	A	0	0
.	IC=7;IHP=7	DP:DP2:T/	109:109:10	187:187:14	A	AT	0	0
.	IC=8;IHP=7	DP:DP2:T/	70:70:64,6	136:136:10	T	TG	0	0
.	IC=5;IHP=6	DP:DP2:T/	66:66:63,6	110:110:80	CT	C	0	0
.	IC=7;IHP=8	DP:DP2:T/	58:58:55,5	108:108:85	AT	A	0	0
.	IC=7;IHP=3	DP:DP2:T/	86:86:86,8	131:131:10	CT	C	0	0
.	IC=4;IHP=7	DP:DP2:T/	73:73:71,7	145:145:11	AT	A	0	0
.	IC=6;IHP=6	DP:DP2:T/	51:51:49,4	104:104:73	A	AT	0	0
.	IC=6;IHP=8	DP:DP2:T/	72:72:67,6	105:105:79	CA	C	0	0
.	IC=9;IHP=8	DP:DP2:T/	59:59:59,5	118:118:88	T	TA	0	0
.	IC=6;IHP=8	DP:DP2:T/	62:62:60,6	111:111:81	AC	A	0	0
.	IC=0;IHP=3	DP:DP2:T/	106:106:10	152:152:13	AG	A	0	0
.	IC=4;IHP=8	DP:DP2:T/	96:96:92,9	137:137:10	GA	G	0	0
.	IC=3;IHP=6	DP:DP2:T/	67:67:67,6	140:140:11	TA	T	0	0
.	IC=4;IHP=5	DP:DP2:T/	136:136:13	304:304:23	TG	T	0	0
.	IC=8;IHP=7	DP:DP2:T/	181:181:17	310:310:25	A	AC	0	0
.	IC=6;IHP=7	DP:DP2:T/	119:119:11	176:176:14	CT	C	0	0
.	IC=5;IHP=7	DP:DP2:T/	71:71:66,6	120:120:98	AT	A	0	0
.	IC=7;IHP=6	DP:DP2:T/	39:39:35,3	96:96:65,6	C	CT	0	0
.	IC=7;IHP=9	DP:DP2:T/	49:49:44,4	66:66:51,5	TA	T	0	0
.	IC=6;IHP=8	DP:DP2:T/	101:101:97	131:131:10	GAA	G	0	0
.	IC=1;IHP=3	DP:DP2:T/	102:102:10	225:225:18	TGAG	T	0	0
.	IC=8;IHP=8	DP:DP2:T/	90:90:88,8	174:174:13	A	AG	0	0
.	IC=3;IHP=5	DP:DP2:T/	79:79:77,7	122:122:97	CA	C	0	0
.	IC=6;IHP=7	DP:DP2:T/	251:251:24	394:394:32	CG	C	0	0
.	IC=6;IHP=8	DP:DP2:T/	79:79:75,7	158:158:12	CT	C	0	0
.	IC=0;IHP=9	DP:DP2:T/	35:35:35,3	78:78:52,5	AAG	A	0	0
.	IC=7;IHP=7	DP:DP2:T/	122:122:11	214:214:17	T	TG	0	0
.	IC=5;IHP=7	DP:DP2:T/	68:68:68,6	81:81:64,6	GA	G	0	0
.	IC=5;IHP=4	DP:DP2:T/	58:58:57,5	125:125:97	T	TC	0	0
.	IC=0;IHP=3	DP:DP2:T/	38:38:37,3	92:92:70,7	CTTCT	C	0	0
.	IC=9;IHP=9	DP:DP2:T/	68:68:63,6	98:98:76,7	A	AT	0	0
.	IC=6;IHP=7	DP:DP2:T/	183:183:17	449:449:33	TG	T	0	0
.	IC=6;IHP=6	DP:DP2:T/	105:105:10	177:177:13	G	GC	0	0
.	IC=0;IHP=3	DP:DP2:T/	91:91:91,9	227:227:18	TTAAC	T	0	0
0.00088	IC=2;IHP=4	DP:DP2:T/	166:166:17	280:280:21	GAGA	G	0	0
.	IC=6;IHP=7	DP:DP2:T/	79:79:79,7	157:157:13	AT	A	0	0
.	IC=5;IHP=6	DP:DP2:T/	77:77:69,7	125:125:93	GA	G	0	0
.	IC=1;IHP=4	DP:DP2:T/	148:148:14	282:282:23	AAGG	A	0	0
.	IC=2;IHP=9	DP:DP2:T/	65:65:69,6	141:141:12	AAAAAG	A	0	0
.	IC=4;IHP=9	DP:DP2:T/	207:207:20	405:405:33	TC	T	0	0
.	IC=9;IHP=8	DP:DP2:T/	46:46:44,4	67:67:46,4	C	CT	0	0
.	IC=6;IHP=9	DP:DP2:T/	55:55:52,5	104:104:73	CG	C	0	0

.	IC=8;IHP=8	DP:DP2:T/	52:52:51,5	85:85:67,6	C	CA	0	0
.	IC=9;IHP=1	DP:DP2:T/	84:84:75,7	113:113:88	C	CT	0	0
.	IC=1;IHP=4	DP:DP2:T/	67:67:69,6	137:137:11	TCTC	T	0	0
.	IC=8;IHP=8	DP:DP2:T/	54:54:46,4	122:122:89	A	AT	0	0
.	IC=8;IHP=1	DP:DP2:T/	177:177:17	348:348:28	C	CA	0	0
.	IC=4;IHP=5	DP:DP2:T/	183:183:18	351:351:29	AG	A	0	0
.	IC=6;IHP=9	DP:DP2:T/	52:52:49,4	137:137:93	AC	A	0	0
.	IC=9;IHP=1	DP:DP2:T/	80:80:77,7	133:133:10	C	CA	0	0
.	IC=5;IHP=6	DP:DP2:T/	35:35:36,3	66:66:51,5	GA	G	0	0
.	IC=7;IHP=9	DP:DP2:T/	54:54:43,4	91:91:61,6	GA	G	0	0
.	IC=7;IHP=1	DP:DP2:T/	84:84:81,8	131:131:10	CT	C	0	0
.	IC=8;IHP=1	DP:DP2:T/	91:91:88,8	174:174:13	T	TG	0	0
.	IC=8;IHP=8	DP:DP2:T/	37:37:32,3	87:87:60,6	G	GA	0	0
.	IC=2;IHP=1	DP:DP2:T/	77:77:73,7	134:134:10	ACT	A	0	0
.	IC=4;IHP=6	DP:DP2:T/	77:77:76,7	140:140:10	GT	G	0	0
.	IC=9;IHP=1	DP:DP2:T/	176:176:16	391:391:28	G	GC	0	0
.	IC=4;IHP=5	DP:DP2:T/	117:117:11	188:188:13	AG	A	0	0
.	IC=6;IHP=7	DP:DP2:T/	39:39:38,3	108:108:76	CG	C	0	0
0.005587	IC=7;IHP=9	DP:DP2:T/	40:40:37,3	91:91:69,6	CT	C	0	0
.	IC=4;IHP=9	DP:DP2:T/	62:62:61,6	86:86:61,6	TG	T	0	0
.	IC=7;IHP=9	DP:DP2:T/	90:90:86,8	133:133:10	CT	C	0	0
.	IC=5;IHP=6	DP:DP2:T/	162:162:15	396:396:31	TC	T	0	0
.	IC=7;IHP=8	DP:DP2:T/	122:122:11	204:204:14	CG	C	0	0
.	IC=8;IHP=8	DP:DP2:T/	43:43:40,4	122:122:92	G	GA	0	0
.	IC=7;IHP=9	DP:DP2:T/	70:70:59,5	106:106:69	AC	A	0	0
.	IC=4;IHP=5	DP:DP2:T/	133:133:13	274:274:21	GC	G	0	0
.	IC=7;IHP=8	DP:DP2:T/	31:31:31,3	58:58:33,3	GA	G	0	0
.	IC=7;IHP=8	DP:DP2:T/	62:62:57,5	106:106:75	GA	G	0	0
.	IC=9;IHP=1	DP:DP2:T/	59:59:58,5	108:108:84	C	CA	0	0
.	IC=7;IHP=8	DP:DP2:T/	77:77:74,7	151:151:72	CA	C	0	0
.	IC=6;IHP=7	DP:DP2:T/	131:131:12	243:243:19	TC	T	0	0
.	IC=7;IHP=6	DP:DP2:T/	56:56:51,5	89:89:62,6	A	AG	0	0
.	IC=9;IHP=1	DP:DP2:T/	109:109:10	179:179:13	G	GT	0	0
0.003559	IC=7;IHP=1	DP:DP2:T/	62:62:63,6	107:107:88	ATG	A	0	0
.	IC=6;IHP=7	DP:DP2:T/	37:37:37,3	59:59:43,4	AT	A	0	0
.	IC=7;IHP=8	DP:DP2:T/	52:52:50,5	55:55:35,3	CT	C	0	0
.	IC=9;IHP=1	DP:DP2:T/	50:50:49,4	82:82:66,6	C	CA	0	0
.	IC=1;IHP=3	DP:DP2:T/	61:61:59,5	87:87:62,6	TAG	T	0	0
.	IC=7;IHP=1	DP:DP2:T/	69:69:69,6	146:146:10	A	AT	0	0
0.00354	IC=6;IHP=1	DP:DP2:T/	29:29:24,2	59:59:28,3	T	TGCC	0	0
.	IC=9;IHP=1	DP:DP2:T/	42:42:38,3	90:90:63,6	T	TG	0	0
.	IC=3;IHP=5	DP:DP2:T/	88:88:86,8	145:145:10	TC	T	0	0
.	IC=8;IHP=8	DP:DP2:T/	31:31:30,3	77:77:53,5	C	CG	0	0
.	IC=7;IHP=8	DP:DP2:T/	47:47:43,4	65:65:46,4	GA	G	0	0
.	IC=7;IHP=6	DP:DP2:T/	61:61:59,5	129:129:96	A	AG	0	0
.	IC=7;IHP=8	DP:DP2:T/	103:103:97	202:202:16	GA	G	0	0
.	IC=4;IHP=6	DP:DP2:T/	144:144:13	244:244:18	AC	A	0	0
.	IC=6;IHP=5	DP:DP2:T/	107:107:98	222:222:17	T	TC	0	0
.	IC=6;IHP=7	DP:DP2:T/	90:90:87,8	172:172:13	TA	T	0	0
.	IC=8;IHP=1	DP:DP2:T/	111:111:10	261:261:20	C	CA	0	0
.	IC=6;IHP=8	DP:DP2:T/	100:100:10	182:182:13	TG	T	0	0
.	IC=4;IHP=5	DP:DP2:T/	91:91:89,8	161:161:11	GA	G	0	0
.	IC=3;IHP=4	DP:DP2:T/	117:117:11	271:271:19	TG	T	0	0
0.001767	IC=6;IHP=9	DP:DP2:T/	103:103:92	214:214:15	CG	C	0	0

.	IC=1;IHP=!	DP:DP2:T/	123:123:12	256:256:22	GCCAGCC	G	0	0
.	IC=7;IHP=:	DP:DP2:T/	42:42:38,3	65:65:40,4	A	AGAG	0	0
.	IC=7;IHP=!	DP:DP2:T/	77:77:77,7	156:156:12	TA	T	0	0
.	IC=6;IHP=(	DP:DP2:T/	80:80:76,7	137:137:11	G	GC	0	0
.	IC=7;IHP=!	DP:DP2:T/	75:75:67,6	153:153:12	CT	C	0	0
.	IC=5;IHP=!	DP:DP2:T/	92:92:86,9	203:203:15	A	AAAC	0	0
.	IC=7;IHP=:	DP:DP2:T/	75:75:71,7	137:137:11	GA	G	0	0
.	IC=7;IHP=(	DP:DP2:T/	154:154:14	336:336:27	G	GC	0	0
.	IC=1;IHP=7	DP:DP2:T/	23:23:21,2	48:48:26,2	CG	C	0	0
.	IC=9;IHP=:	DP:DP2:T/	111:111:10	187:187:13	C	CT	0	0
.	IC=6;IHP=!	DP:DP2:T/	90:90:89,9	209:209:17	CA	C	0	0
.	IC=4;IHP=!	DP:DP2:T/	68:68:64,6	130:130:98	AT	A	0	0
.	IC=7;IHP=8	DP:DP2:T/	35:35:30,3	54:54:37,3	CT	C	0	0
.	IC=7;IHP=!	DP:DP2:T/	86:86:85,8	162:162:12	TA	T	0	0
.	IC=6;IHP=!	DP:DP2:T/	37:37:34,3	83:83:60,6	CT	C	0	0
0.003565	IC=6;IHP=:	DP:DP2:T/	91:91:86,8	164:164:11	TAC	T	0	0
.	IC=7;IHP=(	DP:DP2:T/	150:150:14	221:221:16	T	TC	0	0
.	IC=8;IHP=8	DP:DP2:T/	116:116:11	236:236:19	A	AC	0	0
.	IC=6;IHP=!	DP:DP2:T/	66:66:63,6	122:122:79	GA	G	0	0
.	IC=7;IHP=8	DP:DP2:T/	94:94:88,8	225:225:16	CA	C	0	0
.	IC=5;IHP=(	DP:DP2:T/	104:104:97	223:223:17	CG	C	0	0
.	IC=7;IHP=!	DP:DP2:T/	50:50:43,4	80:80:55,5	AT	A	0	0
.	IC=9;IHP=:	DP:DP2:T/	49:49:48,4	98:98:76,7	G	GA	0	0
.	IC=6;IHP=7	DP:DP2:T/	72:72:65,6	155:155:11	TG	T	0	0
.	IC=7;IHP=8	DP:DP2:T/	132:132:12	304:304:22	CT	C	0	0
.	IC=9;IHP=:	DP:DP2:T/	61:61:55,5	87:87:59,6	C	CA	0	0
.	IC=7;IHP=!	DP:DP2:T/	75:75:68,6	193:193:15	AT	A	0	0
.	IC=1;IHP=4	DP:DP2:T/	67:67:70,7	72:72:61,6	CCTT	C	0	0
.	IC=3;IHP=4	DP:DP2:T/	227:227:22	425:425:35	CT	C	0	0
0.012433	IC=5;IHP=8	DP:DP2:T/	71:71:71,7	123:123:98	AC	A	0	0
.	IC=5;IHP=7	DP:DP2:T/	47:47:47,4	98:98:75,7	TG	T	0	0
.	IC=4;IHP=(	DP:DP2:T/	101:101:99	170:170:12	TG	T	0	0
.	IC=7;IHP=(	DP:DP2:T/	99:99:95,9	183:183:13	G	GC	0	0
.	IC=8;IHP=8	DP:DP2:T/	91:91:86,8	125:125:86	A	AC	0	0
.	IC=5;IHP=7	DP:DP2:T/	90:90:88,8	197:197:14	AC	A	0	0
.	IC=5;IHP=7	DP:DP2:T/	40:40:40,4	77:77:58,5	A	AG	0	0
.	IC=7;IHP=!	DP:DP2:T/	41:41:39,3	90:90:62,6	AT	A	0	0
.	IC=6;IHP=7	DP:DP2:T/	87:87:83,8	154:154:12	CA	C	0	0
.	IC=6;IHP=7	DP:DP2:T/	77:77:71,7	120:120:91	TA	T	0	0
.	IC=9;IHP=!	DP:DP2:T/	49:49:46,4	73:73:52,5	T	TA	0	0
.	IC=9;IHP=:	DP:DP2:T/	95:95:81,8	176:176:13	G	GT	0	0
.	IC=7;IHP=7	DP:DP2:T/	270:270:25	556:556:42	C	CG	0	0
.	IC=9;IHP=!	DP:DP2:T/	64:64:61,6	164:164:11	T	TC	0	0
.	IC=6;IHP=7	DP:DP2:T/	93:93:93,9	139:139:10	CT	C	0	0
.	IC=0;IHP=4	DP:DP2:T/	85:85:81,8	178:178:15	GACAA	G	0	0
.	IC=3;IHP=4	DP:DP2:T/	115:115:11	308:308:24	GGAA	G	0	0
.	IC=6;IHP=!	DP:DP2:T/	73:73:71,7	123:123:96	AT	A	0	0
.	IC=6;IHP=8	DP:DP2:T/	82:82:81,8	131:131:10	CA	C	0	0
0.002833	IC=9;IHP=8	DP:DP2:T/	49:49:43,4	80:80:57,5	G	GC	0	0
.	IC=7;IHP=:	DP:DP2:T/	75:75:75,7	165:165:12	GT	G	0	0
.	IC=5;IHP=!	DP:DP2:T/	41:41:39,6	80:80:55,6	T	TG	0	0
.	IC=6;IHP=8	DP:DP2:T/	159:159:15	362:362:27	TG	T	0	0
.	IC=7;IHP=:	DP:DP2:T/	75:75:74,7	181:181:14	AC	A	0	0
.	IC=7;IHP=:	DP:DP2:T/	48:48:45,4	80:80:60,6	CA	C	0	0

.	IC=4;IHP=	DP:DP2:T	95:95:95,9	176:176:13	GC	G	0	0
.	IC=1;IHP=	DP:DP2:T	64:64:64,6	124:124:10	CCCTT	C	0	0
.	IC=7;IHP=	DP:DP2:T	93:93:86,8	204:204:15	T	TTG	0	0
.	IC=9;IHP=	DP:DP2:T	46:46:40,4	51:51:32,3	C	CA	0	0
.	IC=7;IHP=	DP:DP2:T	62:62:60,6	88:88:69,6	CA	C	0	0
.	IC=6;IHP=	DP:DP2:T	57:57:56,5	120:120:84	AG	A	0	0
.	IC=4;IHP=	DP:DP2:T	95:95:93,9	189:189:15	AG	A	0	0
0.00088	IC=7;IHP=	DP:DP2:T	125:125:11	214:214:16	GA	G	0	0
.	IC=5;IHP=	DP:DP2:T	101:101:93	206:206:16	GC	G	0	0
.	IC=7;IHP=	DP:DP2:T	52:52:51,5	77:77:54,5	GA	G	0	0
.	IC=7;IHP=	DP:DP2:T	119:119:10	230:230:17	AC	A	0	0
.	IC=0;IHP=	DP:DP2:T	116:116:11	228:228:18	AC	A	0	0
.	IC=5;IHP=	DP:DP2:T	96:96:90,9	130:130:98	AT	A	0	0
.	IC=5;IHP=	DP:DP2:T	59:59:59,6	114:114:92	AT	A	0	0
.	IC=1;IHP=	DP:DP2:T	70:70:69,7	116:116:95	AAAGT	A	0	0
.	IC=7;IHP=	DP:DP2:T	98:98:93,9	156:156:11	CT	C	0	0
.	IC=9;IHP=	DP:DP2:T	47:47:46,4	97:97:74,7	C	CT	0	0
.	IC=7;IHP=	DP:DP2:T	59:59:54,5	101:101:70	CT	C	0	0
.	IC=5;IHP=	DP:DP2:T	84:84:81,8	187:187:14	TC	T	0	0
.	IC=5;IHP=	DP:DP2:T	56:56:55,5	112:112:85	GA	G	0	0
.	IC=5;IHP=	DP:DP2:T	80:80:78,7	129:129:95	A	AAG	0	0
.	IC=1;IHP=	DP:DP2:T	53:53:54,5	110:110:88	GTTC	G	0	0
.	IC=7;IHP=	DP:DP2:T	54:54:51,5	107:107:77	AT	A	0	0
.	IC=1;IHP=	DP:DP2:T	64:64:66,6	64:64:47,4	TCTC	T	0	0
.	IC=3;IHP=	DP:DP2:T	916:916:90	1551:1551	TG	T	0	0
.	IC=5;IHP=	DP:DP2:T	194:194:18	363:363:27	T	TC	0	0
.	IC=7;IHP=	DP:DP2:T	85:85:81,8	203:203:16	CA	C	0	0
.	IC=4;IHP=	DP:DP2:T	120:120:11	237:237:18	TC	T	0	0
0.006184	IC=6;IHP=	DP:DP2:T	28:28:26,2	51:51:29,3	GTA	G	0	0
.	IC=5;IHP=	DP:DP2:T	180:180:17	314:314:26	GC	G	0	0
.	IC=6;IHP=	DP:DP2:T	124:124:11	245:245:18	TG	T	0	0
.	IC=6;IHP=	DP:DP2:T	83:83:84,8	167:167:13	AT	A	0	0
.	IC=5;IHP=	DP:DP2:T	78:78:76,7	164:164:11	CA	C	0	0
.	IC=7;IHP=	DP:DP2:T	135:135:12	259:259:19	CT	C	0	0
.	IC=9;IHP=	DP:DP2:T	56:56:52,5	105:105:81	T	TG	0	0
.	IC=7;IHP=	DP:DP2:T	113:113:11	269:269:20	TG	T	0	0
0.00499	IC=5;IHP=	DP:DP2:T	107:107:97	175:175:12	GT	G	0	0
.	IC=0;IHP=	DP:DP2:T	41:41:38,3	66:66:50,5	TAAAG	T	0	0
.	IC=7;IHP=	DP:DP2:T	68:68:67,6	130:130:93	C	CT	0	0
.	IC=7;IHP=	DP:DP2:T	119:119:10	243:243:18	GC	G	0	0
.	IC=5;IHP=	DP:DP2:T	55:55:54,5	72:72:50,5	A	AG	0	0
.	IC=7;IHP=	DP:DP2:T	47:47:46,4	77:77:53,5	TG	T	0	0
.	IC=8;IHP=	DP:DP2:T	129:129:12	239:239:18	G	GA	0	0
.	IC=6;IHP=	DP:DP2:T	81:81:77,7	142:142:10	GA	G	0	0
0.001767	IC=6;IHP=	DP:DP2:T	61:61:60,6	114:114:88	CT	C	0	0
.	IC=5;IHP=	DP:DP2:T	43:43:42,4	74:74:55,5	TA	T	0	0
.	IC=5;IHP=	DP:DP2:T	115:115:11	241:241:18	CT	C	0	0
.	IC=7;IHP=	DP:DP2:T	74:74:71,7	97:97:70,7	AT	A	0	0
.	IC=4;IHP=	DP:DP2:T	77:77:77,7	182:182:15	AC	A	0	0
.	IC=7;IHP=	DP:DP2:T	51:51:50,5	118:118:82	CT	C	0	0
.	IC=6;IHP=	DP:DP2:T	33:33:31,3	52:52:36,3	CT	C	0	0
.	IC=7;IHP=	DP:DP2:T	40:40:37,3	110:110:85	CA	C	0	0
.	IC=6;IHP=	DP:DP2:T	67:67:66,6	133:133:10	CT	C	0	0
.	IC=1;IHP=	DP:DP2:T	72:72:73,7	129:129:10	CTG	C	0	0

.	IC=7;IHP=8	DP:DP2:T/	74:74:70,7	176:176:13	GT	G	0	0
.	IC=8;IHP=1	DP:DP2:T/	75:75:74,7	149:149:11	T	TA	0	0
.	IC=9;IHP=1	DP:DP2:T/	56:56:54,5	144:144:10	C	CT	0	0
.	IC=4;IHP=9	DP:DP2:T/	144:144:14	278:278:21	GC	G	0	0
.	IC=5;IHP=0	DP:DP2:T/	85:85:84,8	178:178:14	TG	T	0	0
.	IC=7;IHP=8	DP:DP2:T/	82:82:81,8	156:156:11	CA	C	0	0
.	IC=4;IHP=0	DP:DP2:T/	74:74:74,7	126:126:10	GA	G	0	0
.	IC=5;IHP=7	DP:DP2:T/	99:99:97,9	198:198:15	AC	A	0	0
.	IC=4;IHP=0	DP:DP2:T/	66:66:65,6	176:176:13	GC	G	0	0
.	IC=9;IHP=8	DP:DP2:T/	119:119:11	300:300:22	G	GA	0	0
.	IC=6;IHP=7	DP:DP2:T/	80:80:74,7	156:156:12	GC	G	0	0
.	IC=9;IHP=9	DP:DP2:T/	114:114:10	192:192:11	G	GC	0	0
.	IC=7;IHP=9	DP:DP2:T/	69:69:68,6	115:115:91	CT	C	0	0
.	IC=9;IHP=9	DP:DP2:T/	78:78:78,7	154:154:12	G	GT	0	0
.	IC=5;IHP=0	DP:DP2:T/	93:93:88,8	212:212:15	AC	A	0	0
.	IC=8;IHP=2	DP:DP2:T/	56:56:47,4	107:107:75	T	TGA	0	0
.	IC=7;IHP=9	DP:DP2:T/	55:55:53,5	105:105:80	GT	G	0	0
.	IC=8;IHP=7	DP:DP2:T/	168:168:16	416:416:33	G	GC	0	0
.	IC=6;IHP=8	DP:DP2:T/	139:139:13	233:233:19	CG	C	0	0
.	IC=1;IHP=1	DP:DP2:T/	83:83:83,8	134:134:10	ACT	A	0	0
.	IC=7;IHP=8	DP:DP2:T/	139:139:12	250:250:19	T	TC	0	0
.	IC=6;IHP=7	DP:DP2:T/	44:44:40,4	57:57:34,3	TA	T	0	0
.	IC=5;IHP=0	DP:DP2:T/	92:92:91,9	181:181:14	TG	T	0	0
.	IC=6;IHP=8	DP:DP2:T/	57:57:54,5	109:109:79	TG	T	0	0
0.002427	IC=5;IHP=8	DP:DP2:T/	129:129:12	215:215:16	AC	A	0	0
.	IC=2;IHP=4	DP:DP2:T/	107:107:10	195:195:15	GCTT	G	0	0
.	IC=7;IHP=8	DP:DP2:T/	26:26:27,2	45:45:29,3	TA	T	0	0
.	IC=1;IHP=1	DP:DP2:T/	45:45:44,4	95:95:71,7	ATC	A	0	0
.	IC=6;IHP=7	DP:DP2:T/	109:109:99	276:276:21	TG	T	0	0
.	IC=6;IHP=7	DP:DP2:T/	77:77:76,7	121:121:93	GA	G	0	0
.	IC=4;IHP=7	DP:DP2:T/	111:111:10	213:213:15	TC	T	0	0
.	IC=4;IHP=5	DP:DP2:T/	99:99:96,9	229:229:18	CT	C	0	0
.	IC=0;IHP=4	DP:DP2:T/	100:100:10	166:166:13	CCA	C	0	0
.	IC=8;IHP=4	DP:DP2:T/	82:82:67,6	149:149:95	T	TGGA	0	0
0.00088	IC=5;IHP=0	DP:DP2:T/	79:79:78,7	158:158:12	AC	A	0	0
.	IC=5;IHP=9	DP:DP2:T/	53:53:54,5	99:99:79,7	CG	C	0	0
.	IC=5;IHP=0	DP:DP2:T/	61:61:55,5	145:145:10	AT	A	0	0
.	IC=4;IHP=9	DP:DP2:T/	71:71:69,7	108:108:83	GC	G	0	0
.	IC=1;IHP=1	DP:DP2:T/	160:160:15	296:296:23	ATGGAAT	A	0	0
0.000885	IC=6;IHP=9	DP:DP2:T/	176:176:17	456:456:35	GC	G	0	0
.	IC=7;IHP=8	DP:DP2:T/	31:31:31,3	47:47:32,3	AT	A	0	0
.	IC=5;IHP=1	DP:DP2:T/	48:48:49,4	87:87:61,6	TC	T	0	0
.	IC=4;IHP=0	DP:DP2:T/	144:144:14	272:272:21	GT	G	0	0
.	IC=7;IHP=0	DP:DP2:T/	71:71:68,6	167:167:12	C	CA	0	0
.	IC=7;IHP=1	DP:DP2:T/	33:33:32,3	58:58:37,3	CT	C	0	0
.	IC=4;IHP=5	DP:DP2:T/	45:45:44,4	54:54:33,3	GA	G	0	0
.	IC=8;IHP=7	DP:DP2:T/	114:114:11	197:197:14	G	GC	0	0
.	IC=2;IHP=5	DP:DP2:T/	78:78:75,7	143:143:11	CCCT	C	0	0
.	IC=1;IHP=0	DP:DP2:T/	187:187:18	337:337:25	T	TC	0	0
.	IC=6;IHP=1	DP:DP2:T/	90:90:89,8	197:197:14	GA	G	0	0
.	IC=6;IHP=9	DP:DP2:T/	79:79:78,8	193:193:15	GC	G	0	0
.	IC=5;IHP=9	DP:DP2:T/	55:55:52,5	131:131:95	CG	C	0	0
.	IC=1;IHP=1	DP:DP2:T/	57:57:33,3	88:88:26,2	G	GC	0	0
.	IC=6;IHP=7	DP:DP2:T/	81:81:76,7	175:175:12	TC	T	0	0

.	IC=5;IHP=!	DP:DP2:T/	74:74:69,6	151:151:11	GC	G	0	0
.	IC=6;IHP=!	DP:DP2:T/	69:69:62,6	124:124:92	GA	G	0	0
.	IC=5;IHP=7	DP:DP2:T/	33:33:32,3	64:64:46,4	CG	C	0	0
.	IC=6;IHP=7	DP:DP2:T/	228:228:21	467:467:35	GC	G	0	0
.	IC=4;IHP=(	DP:DP2:T/	86:86:83,8	215:215:16	TG	T	0	0
.	IC=5;IHP=(	DP:DP2:T/	19:19:20,2	24:24:9,9:7	TG	T	0	0
.	IC=6;IHP=8	DP:DP2:T/	154:154:14	271:271:22	GT	G	0	0
.	IC=1;IHP=5	DP:DP2:T/	61:61:63,6	112:112:86	GAGA	G	0	0
.	IC=7;IHP=8	DP:DP2:T/	129:129:11	265:265:18	TC	T	0	0
.	IC=9;IHP=1	DP:DP2:T/	107:107:10	225:225:17	C	CA	0	0
.	IC=6;IHP=8	DP:DP2:T/	27:27:24,2	52:52:30,3	CA	C	0	0
.	IC=5;IHP=7	DP:DP2:T/	44:44:41,4	69:69:51,5	CG	C	0	0
.	IC=6;IHP=7	DP:DP2:T/	240:240:23	562:562:43	AC	A	0	0
.	IC=4;IHP=5	DP:DP2:T/	75:75:71,7	131:131:10	GA	G	0	0
.	IC=9;IHP=8	DP:DP2:T/	77:77:72,7	127:127:94	A	AT	0	0
.	IC=5;IHP=(	DP:DP2:T/	267:267:25	479:479:38	GC	G	0	0
.	IC=5;IHP=4	DP:DP2:T/	54:54:54,5	83:83:59,5	T	TC	0	0
.	IC=4;IHP=(	DP:DP2:T/	122:122:11	196:196:15	TC	T	0	0
.	IC=3;IHP=5	DP:DP2:T/	129:129:13	247:247:18	TG	T	0	0
.	IC=4;IHP=5	DP:DP2:T/	67:67:65,6	152:152:12	CG	C	0	0
.	IC=9;IHP=8	DP:DP2:T/	95:95:93,9	188:188:14	C	CG	0	0
.	IC=6;IHP=8	DP:DP2:T/	45:45:47,4	74:74:52,5	GA	G	0	0
.	IC=3;IHP=4	DP:DP2:T/	224:224:22	547:547:44	AC	A	0	0
.	IC=9;IHP=1	DP:DP2:T/	100:100:84	192:192:12	A	AC	0	0
.	IC=9;IHP=1	DP:DP2:T/	185:185:17	384:384:29	T	TG	0	0
.	IC=4;IHP=(	DP:DP2:T/	26:26:34,3	38:38:21,2	CG	C	0	0
.	IC=6;IHP=1	DP:DP2:T/	83:83:74,7	196:196:15	TC	T	0	0
.	IC=4;IHP=2	DP:DP2:T/	39:39:34,3	71:71:43,4	CAG	C	0	0
.	IC=6;IHP=5	DP:DP2:T/	82:82:81,8	191:191:14	T	TG	0	0
.	IC=4;IHP=(	DP:DP2:T/	226:226:21	418:418:33	TC	T	0	0
.	IC=9;IHP=8	DP:DP2:T/	246:246:23	516:516:40	A	AC	0	0
.	IC=7;IHP=!	DP:DP2:T/	104:104:10	207:207:15	AC	A	0	0
.	IC=5;IHP=(	DP:DP2:T/	156:156:14	370:370:27	GC	G	0	0
.	IC=7;IHP=!	DP:DP2:T/	39:39:36,3	84:84:62,6	GA	G	0	0
.	IC=7;IHP=8	DP:DP2:T/	65:65:62,6	144:144:10	CT	C	0	0
.	IC=6;IHP=7	DP:DP2:T/	78:78:72,7	175:175:14	GC	G	0	0
.	IC=9;IHP=1	DP:DP2:T/	136:136:12	223:223:17	A	AG	0	0
.	IC=8;IHP=1	DP:DP2:T/	86:86:78,7	141:141:10	C	CG	0	0
.	IC=7;IHP=!	DP:DP2:T/	34:34:33,3	51:51:37,3	CA	C	0	0
.	IC=5;IHP=(	DP:DP2:T/	65:65:61,6	93:93:72,7	TG	T	0	0
.	IC=9;IHP=8	DP:DP2:T/	95:95:90,9	161:161:12	C	CT	0	0
.	IC=7;IHP=8	DP:DP2:T/	104:104:10	230:230:17	CA	C	0	0
.	IC=7;IHP=!	DP:DP2:T/	52:52:46,4	74:74:48,4	AC	A	0	0
.	IC=1;IHP=5	DP:DP2:T/	97:97:96,9	158:158:13	GC	G	0	0
0.001393	IC=0;IHP=1	DP:DP2:T/	26:26:25,2	48:48:28,2	AG	A	0	0
.	IC=5;IHP=(	DP:DP2:T/	95:95:94,9	155:155:12	GC	G	0	0
.	IC=8;IHP=8	DP:DP2:T/	149:149:14	242:242:16	C	CG	0	0
.	IC=7;IHP=1	DP:DP2:T/	127:127:11	299:299:22	TC	T	0	0
.	IC=5;IHP=(	DP:DP2:T/	72:72:72,7	137:137:10	TC	T	0	0
.	IC=4;IHP=7	DP:DP2:T/	47:47:44,4	102:102:77	AG	A	0	0
.	IC=6;IHP=7	DP:DP2:T/	46:46:46,4	91:91:63,6	CT	C	0	0
.	IC=7;IHP=8	DP:DP2:T/	86:86:83,8	133:133:10	AG	A	0	0
.	IC=5;IHP=(	DP:DP2:T/	75:75:71,7	176:176:12	AC	A	0	0
.	IC=5;IHP=(	DP:DP2:T/	129:129:11	262:262:19	GC	G	0	0

.	IC=7;IHP=	DP:DP2:T	137:137:12	240:240:18	A	AC	0	0
.	IC=6;IHP=	DP:DP2:T	51:51:49,5	82:82:46,4	AC	A	0	0
.	IC=6;IHP=	DP:DP2:T	48:48:48,4	135:135:10	GT	G	0	0
.	IC=5;IHP=	DP:DP2:T	88:88:87,8	136:136:11	CT	C	0	0
.	IC=8;IHP=	DP:DP2:T	94:94:91,9	200:200:14	G	GC	0	0
.	IC=9;IHP=	DP:DP2:T	72:72:66,6	123:123:99	A	AT	0	0
.	IC=7;IHP=	DP:DP2:T	81:81:79,7	113:113:78	AG	A	0	0
.	IC=8;IHP=	DP:DP2:T	118:118:11	203:203:15	A	AC	0	0
.	IC=5;IHP=	DP:DP2:T	167:167:16	372:372:28	T	TG	0	0
.	IC=7;IHP=	DP:DP2:T	74:74:72,7	134:134:10	G	GC	0	0
.	IC=5;IHP=	DP:DP2:T	118:118:11	268:268:21	TG	T	0	0
.	IC=5;IHP=	DP:DP2:T	53:53:53,5	74:74:58,5	CG	C	0	0
.	IC=9;IHP=	DP:DP2:T	81:81:77,7	159:159:10	A	AG	0	0
.	IC=0;IHP=	DP:DP2:T	46:46:46,4	81:81:61,6	CCCG	C	0	0
.	IC=2;IHP=	DP:DP2:T	124:124:12	212:212:19	A	AGGTGCT	0	0
.	IC=2;IHP=	DP:DP2:T	125:125:10	237:237:18	C	CCTGAAG	0	0
.	IC=0;IHP=	DP:DP2:T	81:81:90,9	161:161:17	CCATCTT	C	0	0
.	IC=2;IHP=	DP:DP2:T	90:90:86,8	149:149:13	AGCACTG	A	0	0
.	IC=0;IHP=	DP:DP2:T	73:73:73,7	165:165:15	GTCTCCC	G	0	0
.	IC=0;IHP=	DP:DP2:T	95:95:81,8	169:169:14	CATTTGT	C	0	0
.	IC=2;IHP=	DP:DP2:T	145:145:13	152:152:13	GGAGGC	G	0	0
0.004448	IC=2;IHP=	DP:DP2:T	90:90:87,9	122:122:11	T	TTGTGC	0	0
0.286092	IC=1;IHP=	DP:DP2:T	16:16:16,1	7:7:1,1:6,6	G	GGAA	0	0
.	IC=1;IHP=	DP:DP2:T	77:77:76,9	107:107:91	A	AGGAGG	0	0
.	IC=0;IHP=	DP:DP2:T	80:80:84,9	149:149:13	AGGAGG	A	0	0
.	IC=5;IHP=	DP:DP2:T	171:171:16	229:229:20	ATCCTCC	A	0	0
.	IC=1;IHP=	DP:DP2:T	100:100:10	133:133:11	T	TTCACGG	0	0
.	IC=0;IHP=	DP:DP2:T	219:219:20	189:189:16	CGGATGC	C	0	0
.	IC=0;IHP=	DP:DP2:T	76:76:106,	108:108:12	GCGTCCC	G	0	0
.	IC=0;IHP=	DP:DP2:T	81:81:89,8	149:149:15	ACTCAAT	A	0	0
0.00088	IC=4;IHP=	DP:DP2:T	114:114:89	243:243:18	AAAAAC	A	0	0
.	IC=0;IHP=	DP:DP2:T	82:82:82,8	148:148:14	TCCATGT	T	0	0
.	IC=0;IHP=	DP:DP2:T	83:83:79,9	240:240:23	AGGTGTC	A	0	0
.	IC=2;IHP=	DP:DP2:T	90:90:89,8	159:159:14	ACTCCT	A	0	0
0.00088	IC=2;IHP=	DP:DP2:T	130:130:12	158:158:14	CCAGTA	C	0	0
.	IC=1;IHP=	DP:DP2:T	116:116:12	241:241:22	AGAGTT	A	0	0
.	IC=0;IHP=	DP:DP2:T	123:123:12	248:248:24	CCGGGA	C	0	0
.	IC=0;IHP=	DP:DP2:T	147:147:16	194:194:17	GCCAAGC	G	0	0
.	IC=1;IHP=	DP:DP2:T	78:78:78,7	159:159:12	GA	G	0	0
.	IC=2;IHP=	DP:DP2:T	98:98:91,9	200:200:16	G	GCAA	0	0
.	IC=2;IHP=	DP:DP2:T	101:101:99	232:232:17	A	ACGC	0	0
.	IC=0;IHP=	DP:DP2:T	65:65:75,7	121:121:12	TGGGGA	T	0	0
0.190813	IC=7;IHP=	DP:DP2:T	20:20:18,1	35:35:11,1	G	GT	0	0
.	IC=0;IHP=	DP:DP2:T	60:60:70,7	72:72:67,6	AAGTTGC	A	0	0
.	IC=4;IHP=	DP:DP2:T	115:115:10	186:186:15	CGAGGA	C	0	0
.	IC=0;IHP=	DP:DP2:T	40:40:45,4	51:51:41,4	GGCCGC	G	0	0
.	IC=2;IHP=	DP:DP2:T	111:111:10	223:223:15	AC	A	0	0
.	IC=0;IHP=	DP:DP2:T	499:499:54	706:706:63	CGCCGC	C	0	0
.	IC=7;IHP=	DP:DP2:T	92:92:91,9	219:219:17	A	AG	0	0
.	IC=7;IHP=	DP:DP2:T	106:106:99	71:71:44,4	A	AC	0	0
0.048893	IC=5;IHP=	DP:DP2:T	90:90:64,6	234:234:16	C	CGCGGG	0	0
.	IC=2;IHP=	DP:DP2:T	75:75:74,7	102:102:80	GC	G	0	0
.	IC=7;IHP=	DP:DP2:T	225:225:21	298:298:22	C	CG	0	0
.	IC=0;IHP=	DP:DP2:T	114:114:13	178:178:19	CCCCAAA	C	0	0

.	IC=4;IHP=:	DP:DP2:T/	77:77:75,7	180:180:14	T	TG	0	0
.	IC=1;IHP='	DP:DP2:T/	54:54:52,5	56:56:37,3	ATCT	A	0	0
.	IC=2;IHP=:	DP:DP2:T/	76:76:76,7	117:117:92	T	TA	0	0
0.003521	IC=2;IHP=8	DP:DP2:T/	76:76:77,7	151:151:14	CGGGCG	C	0	0
.	IC=2;IHP=:	DP:DP2:T/	74:74:72,7	93:93:79,7	C	CTTG	0	0
.	IC=2;IHP=:	DP:DP2:T/	72:72:73,7	68:68:58,5	CAAGGG	C	0	0
.	IC=3;IHP=:	DP:DP2:T/	90:90:88,8	60:60:47,5	G	GC	0	0
.	IC=6;IHP=!	DP:DP2:T/	101:101:90	151:151:12	CCAGCAG	C	0	0
.	IC=0;IHP=:	DP:DP2:T/	29:29:29,2	82:82:55,5	TA	T	0	0
0.002727	IC=0;IHP=:	DP:DP2:T/	191:191:19	202:202:19	TTTTTG	T	0	0
.	IC=2;IHP=4	DP:DP2:T/	82:82:78,7	198:198:15	CG	C	0	0
.	IC=2;IHP=:	DP:DP2:T/	67:67:65,6	175:175:12	G	GT	0	0
.	IC=1;IHP=:	DP:DP2:T/	109:109:10	88:88:76,7	A	AATTCT	0	0
.	IC=0;IHP=:	DP:DP2:T/	77:77:82,8	242:242:23	AGAGCGC	A	0	0
.	IC=0;IHP=!	DP:DP2:T/	84:84:89,8	178:178:16	CCCAGGA	C	0	0
.	IC=1;IHP=:	DP:DP2:T/	81:81:78,7	77:77:63,6	AGAGCCC	A	0	0
.	IC=0;IHP=8	DP:DP2:T/	73:73:88,8	81:81:72,7	GCCGCGC	G	0	0
.	IC=1;IHP=4	DP:DP2:T/	93:93:97,9	163:163:15	CGAGGTC	C	0	0
.	IC=0;IHP='	DP:DP2:T/	19:19:20,2	50:50:28,2	TTGATCA	T	0	0
.	IC=0;IHP=:	DP:DP2:T/	48:48:46,4	33:33:24,2	TA	T	0	0
0.001393	IC=2;IHP='	DP:DP2:T/	148:148:14	279:279:25	GGGGCCT	G	0	0
0.000882	IC=4;IHP=4	DP:DP2:T/	161:161:14	222:222:17	TCCGCCC	T	0	0
.	IC=2;IHP=:	DP:DP2:T/	126:126:12	145:145:72	G	GCTGT	0	0
.	IC=2;IHP=:	DP:DP2:T/	81:81:76,8	104:104:52	C	CT	0	0
.	IC=8;IHP='	DP:DP2:T/	118:118:10	295:295:16	A	AT	0	0
.	IC=0;IHP=:	DP:DP2:T/	53:53:55,5	182:182:13	AGG	A	0	0
.	IC=3;IHP=!	DP:DP2:T/	113:113:11	232:232:16	CTT	C	0	0
.	IC=0;IHP=4	DP:DP2:T/	77:77:79,8	69:69:62,6	GTCTGCC	G	0	0

OMIM	GWAS_Pu	HGMD_ID_	GO_BP	GO_CC	GO_MF	KEGG_PA	PID_PATH	BIOCARTA
.	NA	.	MULTI_OF	.	TRANSCR	.	.	.
{Celiac dis	NA	.	IMMUNE_I	INTRINSIC	.	KEGG_CE	PID_NFAT	BIOCARTA
.	NA	.	.	.	.	.	.	.
.	NA	.	ESTABLIS	ORGANEL	.	KEGG_SN	.	.
.	NA	.	.	.	.	.	.	.
.	NA	.	.	.	.	.	.	.
.	NA	.	.	.	.	KEGG_OL	.	.
.	NA	.	.	.	.	.	.	.
.	NA	.	.	CYTOPLA	ENZYME_	KEGG_CY	.	.
17-beta-hy	NA	.	LIPID_ME	CYTOPLA	STEROID_	KEGG_VA	.	.
.	NA	.	.	.	.	.	.	.
.	NA	.	MULTI_OF	INTRINSIC	TRANSME	KEGG_CY	PID_GLYP	BIOCARTA
Bleeding d	NA	.	FOCAL_A	ADHEREN	INTEGRIN	KEGG_FO	PID_ILK_F	BIOCARTA
.	NA	.	.	.	.	.	.	.
.	NA	.	RNA_MET	ORGANEL	.	KEGG_SP	.	.
.	NA	.	RNA_MET	NUCLEUS	RNA_BIND	.	.	.
.	NA	.	.	.	.	KEGG_TG	.	.
.	NA	.	.	.	.	.	.	.
Bronchiect	NA	.	ESTABLIS	INTRINSIC	CATION_T	KEGG_TA	.	.
.	NA	.	.	.	.	.	.	.
.	NA	.	.	.	.	.	.	.
.	NA	.	.	.	.	.	.	.
.	NA	.	.	.	.	.	PID_TAP6	.
.	NA	.	.	.	.	.	.	.
.	NA	.	RNA_MET	NUCLEOP	.	.	.	.
.	NA	.	.	.	.	.	.	.
.	NA	.	.	.	.	.	.	.
.	NA	.	.	.	RECEPTO	KEGG_CY	PID_TNFP	BIOCARTA
.	NA	.	.	.	.	.	.	.
Mental reta	NA	.	RNA_MET	NUCLEOP	TRANSCR	.	.	.
.	NA	.	.	CYTOPLA	.	.	.	.
.	NA	.	.	CYTOPLA	.	.	.	.
.	NA	.	NUCLEOB	.	TRANSCR	.	.	.
.	NA	.	.	.	.	.	.	.
.	NA	.	.	.	.	.	PID_ATF2	.
.	NA	.	TRNA_PR	.	LIGASE_A	KEGG_AM	.	.
.	NA	.	POSITIVE_	.	HYDROLA	.	PID_PRLS	.
.	NA	.	GLYCOPR	INTRINSIC	.	KEGG_NO	PID_NOTC	.
.	NA	.	.	.	.	KEGG_UB	.	.
.	NA	.	.	.	PHOSPHO	.	.	.
.	NA	.	.	.	.	.	.	.
.	NA	.	.	.	.	.	.	.
.	NA	.	PROTEOL	.	METALLO	.	.	.
.	NA	.	.	.	.	.	.	.
.	NA	.	.	CYTOPLA	HYDROLA	.	.	.
Severe cor	NA	.	REGULAT	INTRINSIC	PROTEIN_	KEGG_CE	PID_BCR_	BIOCARTA
.	NA	.	.	.	.	.	.	.
.	NA	.	.	.	.	.	.	.
.	NA	.	BIOPOLYM	CELL_JUN	NUCLEOT	.	PID_CDC4	.
.	NA	.	.	.	.	.	.	.
.	NA	.	.	.	.	.	.	.

.	NA	.	BIOPOLYM	.	PHOSPHO	KEGG_CH	PID_RHOA	BIOCARTA
.	NA	.	.	.	.	.	.	.
.	NA	.	CELL_DEV	ORGANEL	PEPTIDE_	.	.	.
.	NA	.	.	.	.	.	.	.
.	NA	.	PHOSPHO	ORGANEL	TRANSFE	KEGG_GL	.	.
.	NA	.	MACROMO	CYTOPLA	RIBONUCL	.	.	.
.	NA	.	.	EXTRACE	.	.	.	.
.	NA	.	.	.	.	.	.	.
.	NA	.	MULTICEL	NUCLEUS	.	.	.	.
.	NA	.	MULTI_OF	.	TRANSCR	.	.	.
.	NA	.	REGULAT	INTRINSIC	TRANSME	KEGG_CY	PID_ALK1	.
.	NA	.	.	.	.	.	.	.
.	NA	.	.	.	.	.	.	.
.	NA	.	.	.	.	.	.	.
.	NA	.	.	.	RNA_POL	KEGG_MA	PID_SMAD	BIOCARTA
Pigmented	NA	.	SIGNAL_T	.	.	KEGG_PU	.	.
.	NA	.	.	.	.	.	.	.
Cardiomyo	NA	.	MUSCLE_	ORGANEL	NUCLEOT	KEGG_HY	.	.
Candidiasi	NA	.	RNA_MET	CYTOPLA	RECEPTO	KEGG_CH	PID_GMCS	BIOCARTA
.	NA	.	POSITIVE_	CYTOPLA	TRANSLA	.	.	.
.	NA	.	.	.	.	.	.	.
.	NA	.	SYSTEM_I	INTRINSIC	INTEGRIN	.	.	.
Mitochondi	NA	.	.	.	.	.	.	.
.	NA	.	.	.	.	.	.	.
.	NA	.	.	.	.	.	.	.
.	NA	.	.	.	.	.	.	BIOCARTA
.	NA	.	.	.	.	.	.	.
.	NA	.	SYSTEM_I	CYTOPLA	RAS_GUA	KEGG_AD	PID_NECT	.
.	NA	.	SYSTEM_I	.	.	.	PID_NOTC	.
.	NA	.	REGULAT	CYTOPLA	GTPASE_I	.	.	.
?Congenita	NA	.	GLYCOPR	CYTOPLA	.	.	.	.
.	NA	.	.	CYTOPLA	.	.	.	.
.	NA	.	.	.	.	.	.	.
.	NA	.	RNA_MET	NUCLEUS	RNA_BIND	.	.	.
.	NA	.	.	.	.	.	.	.
.	NA	.	BIOPOLYM	CYTOPLA	ENZYME_	KEGG_CH	PID_ENDO	BIOCARTA
Sinoatrial r	NA	.	ESTABLIS	MEMBRAN	CATION_T	KEGG_MA	.	.
.	NA	.	.	.	.	.	.	.
ACAD9 de	NA	.	.	.	.	.	.	.
.	NA	.	.	.	.	.	PID_TRKR	.
.	NA	.	.	.	.	.	.	.
.	NA	.	MUSCLE_	CYTOPLA	RNA_BIND	.	.	.
.	NA	.	REGULAT	INTRINSIC	.	KEGG_CE	.	.
.	NA	.	SYSTEM_I	.	.	KEGG_TG	PID_BMPR	BIOCARTA
.	NA	.	.	.	.	.	.	.
UV-sensitiv	NA	.	.	.	.	.	.	.
.	NA	.	.	.	.	.	.	.
.	NA	.	.	.	.	KEGG_GL	.	.
.	NA	.	.	.	.	.	.	.
.	NA	.	.	.	.	.	.	.
.	NA	.	.	.	.	.	.	.
.	NA	.	.	.	.	.	.	.
Adermatog	NA	.	DNA_MET	.	.	.	.	.
Hypomagn	NA	.	DNA_MET	MEMBRAN	RECEPTO	KEGG_MA	PID_PTP1	BIOCARTA

.	NA	.	.	MACROMO	.	KEGG_ER	PID_IFNGI	BIOCARTA
.	NA	.	.	.	ARYLSULI	.	.	.
.	NA	.	.	.	.	.	.	.
.	NA	.	.	.	.	KEGG_BA	.	.
.	NA	.	.	.	.	.	.	.
.	NA	.	.	.	.	.	.	.
.	NA	.	ENZYME_	.	PHOSPHO	.	PID_RHOA	BIOCARTA
Chondroca	NA	.	SYSTEM_I	MEMBRAN	ACTIVE_T	.	.	.
.	NA	.	.	.	.	.	.	.
Joubert syı	NA	.	.	.	.	.	.	.
Amyloidosi	NA	.	SIGNAL_T	INTRINSIC	RECEPTO	KEGG_CY	.	.
.	NA	.	.	.	.	.	.	.
Hermansky	NA	.	ESTABLIS	CYTOPLAS	.	KEGG_LYS	.	.
Acampome	NA	.	CELL_DEV	RIBONUCI	.	.	PID_ERA_	BIOCARTA
.	NA	.	.	.	.	.	.	.
.	NA	.	.	.	.	.	.	.
.	NA	.	SYSTEM_I	EXTRACE	.	KEGG_AX	.	.
.	NA	.	SYSTEM_I	EXTRACE	.	KEGG_AX	.	.
.	NA	.	.	.	.	KEGG_GL	.	.
.	NA	.	DEFENSE	.	.	KEGG_NA	.	.
Trichohepa	NA	.	.	.	HYDROLA	KEGG_RN	.	.
.	NA	.	BIOPOLYM	INTRINSIC	RECEPTO	.	PID_AMB2	.
.	NA	.	.	.	.	.	.	.
.	NA	.	.	.	.	KEGG_CE	.	.
.	NA	.	.	.	.	.	.	.
.	NA	.	.	.	.	.	.	.
.	NA	.	.	.	.	.	.	.
.	NA	.	.	.	.	.	.	.
Obesity, se	NA	.	SYSTEM_I	.	TRANSCR	.	.	.
Mental reta	NA	.	REGULAT	INTRINSIC	GLUTAMA	KEGG_NE	.	.
.	NA	.	.	.	.	.	.	.
.	NA	.	ESTABLIS	MEMBRAN	.	.	.	.
{Psoriasis :	NA	.	POSITIVE	.	.	.	.	.
Cardiomyo	NA	.	.	PROTEINA	STRUCTU	KEGG_FO	PID_INTEG	BIOCARTA
.	NA	.	.	.	.	.	.	.
.	NA	.	SIGNAL_T	.	GTP_BIND	.	.	.
.	NA	.	.	.	.	.	.	.
.	NA	.	.	.	.	.	.	.
Hepatocell	NA	.	ESTABLIS	ORGANEL	TRANSME	KEGG_LYS	.	.
.	NA	.	.	.	.	.	.	.
.	NA	.	.	.	.	.	.	.
.	NA	.	.	.	.	.	.	.
.	NA	.	.	.	.	.	.	.
.	NA	.	MULTICEL	.	.	.	.	.
.	NA	.	.	.	.	.	.	.
.	NA	.	PROTEIN_	CYTOPLAS	ENZYME_	KEGG_NO	.	.
.	NA	.	PROTEIN_	CYTOPLAS	ENZYME_	KEGG_NO	.	.
.	NA	.	NEGATIVE	CYTOPLAS	.	.	.	.
.	NA	.	.	.	.	.	.	.
.	NA	.	.	.	.	.	.	.
Cutis laxa,	NA	.	SYSTEM_I	.	.	.	.	.
.	NA	.	.	.	.	.	.	.
.	NA	.	.	.	.	.	.	.
.	NA	.	SYSTEM_I	INTRINSIC	.	KEGG_AX	PID_EPHB	.

.	NA	.	RNA_MET	.	RNA_POL	.	.	.
.	NA	.	.	.	.	.	.	.
.	NA	.	STEROID_	.	ALDO_KE	KEGG_FR	.	.
.	NA	.	ORGANEL	.	.	.	.	.
Ventricular	NA	.	.	.	.	.	.	.
Barrett esc	NA	.	ESTABLIS	INTRINSIC	RECEPTO	.	.	.
.	NA	.	.	.	.	KEGG_EN	.	.
.	NA	.	.	.	.	.	.	.
.	NA	.	.	.	.	.	.	.
{?Schizoph	NA	.	NEGATIVE	.	TRANSCR	KEGG_ER	PID_ERBE	BIOCARTA
.	NA	.	.	.	.	.	.	.
.	NA	.	REGULAT	MEMBRAN	PHOSPHO	KEGG_CH	PID_FCER	BIOCARTA
.	NA	.	SYSTEM_I	.	TRANSCR	.	PID_HES_	.
.	NA	.	.	.	.	.	.	.
.	NA	.	.	.	.	.	.	.
Seizures, b	NA	.	ESTABLIS	INTRINSIC	CATION_T	.	.	.
.	NA	.	SYSTEM_I	INTRINSIC	G_PROTE	KEGG_P5	.	.
.	NA	.	.	CYTOPLA	ELECTRO	KEGG_OX	.	.
.	NA	.	.	.	.	.	.	.
.	NA	.	RESPONS	.	.	.	.	.
.	NA	.	MACROM	CELL_PRO	PROTEIN_	KEGG_FO	PID_RHOA	BIOCARTA
.	NA	.	.	.	.	.	.	.
.	NA	.	.	NUCLEUS	.	.	.	.
Basal cell c	NA	.	SYSTEM_I	INTRINSIC	RECEPTO	KEGG_HE	PID_HEDG	BIOCARTA
Basal cell c	NA	.	SYSTEM_I	INTRINSIC	RECEPTO	KEGG_HE	PID_HEDG	BIOCARTA
.	NA	.	BIOPOLYM	ORGANEL	.	.	.	.
.	NA	.	.	.	.	KEGG_OL	.	.
.	NA	.	.	NUCLEUS	.	.	.	.
.	NA	.	ESTABLIS	CYTOPLA	.	.	.	.
.	NA	.	.	.	.	.	.	.
.	NA	.	.	.	.	.	.	.
.	NA	.	PROTEOL	ORGANEL	METALLO	.	.	.
.	NA	.	.	.	.	.	.	.
.	NA	.	.	.	.	.	.	.
.	NA	.	.	.	.	.	.	.
.	NA	.	.	.	.	.	.	.
.	NA	.	CARBOXY	.	.	KEGG_AL	.	.
.	NA	.	.	.	.	.	.	.
.	NA	.	.	.	.	.	.	.
.	NA	.	RNA_MET	.	NUCLEAR	.	.	.
.	NA	.	.	ORGANEL	HYDROLA	.	.	.
.	NA	.	.	.	.	.	.	.
.	NA	.	.	.	.	.	.	.
.	NA	.	.	.	.	.	PID_AR_P	.
.	NA	.	IMMUNE_I	CYTOPLA	.	.	.	.
.	NA	.	.	.	.	.	.	.
Cone dystr	NA	.	SENSORY	.	.	KEGG_PU	PID_CONE	.
.	NA	.	.	ORGANEL	STRUCTU	.	.	.
.	NA	.	.	.	NUCLEAR	.	.	.
.	NA	.	REGULAT	NUCLEOP	PROTEIN_	.	PID_MTOF	.
{Influenza,	NA	.	IMMUNE_I	MEMBRAN	.	.	.	.
.	NA	.	.	.	.	.	.	.
.	NA	.	SYSTEM_I	.	NUCLEOT	.	PID_LKB1	.
.	NA	.	.	.	.	KEGG_OL	.	.

.	NA	.	.	.	.	.	.	.
.	NA	.	.	.	.	.	.	.
.	NA	.	.	.	.	.	.	.
Dystonia 2	NA	.	.	.	.	.	.	.
.	NA	.	.	.	.	KEGG_ST	.	.
{Diabetes r	NA	.	ESTABLIS	CYTOPLA	KINASE_B	KEGG_MA	PID_REEL	.
.	NA	.	.	.	.	.	.	.
.	NA	.	.	.	RNA_BIND	.	.	.
.	NA	.	SIGNAL_T	CYTOPLA	.	KEGG_CH	PID_HDAC	.
.	NA	.	.	.	.	.	.	.
.	NA	.	.	.	.	.	.	.
.	NA	.	.	MEMBRAN	PHOSPHO	.	.	.
.	NA	.	.	.	.	.	.	.
.	NA	.	.	.	.	.	.	.
Hemorrhag	NA	.	.	.	.	KEGG_CE	PID_AMB2	.
.	NA	.	RNA_MET	ORGANEL	TRANSCR	.	.	.
.	NA	.	.	.	TRANSFE	KEGG_O_	.	.
.	NA	.	RNA_MET	NUCLEUS	.	.	.	.
.	NA	.	CELL_DEV	MEMBRAN	PROTEIN_	KEGG_AD	PID_BCR_	BIOCARTA
.	NA	.	.	.	.	.	.	.
.	NA	.	.	.	.	.	.	.
.	NA	.	.	.	.	.	.	.
.	NA	.	.	.	.	.	.	.
.	NA	.	.	.	.	.	.	.
.	NA	.	.	.	.	.	.	.
.	NA	.	.	.	.	.	.	.
.	NA	.	.	.	.	.	.	.
.	NA	.	.	.	.	.	.	.
.	NA	.	ESTABLIS	INTRINSIC	CATION_T	KEGG_LY	PID_HIF2F	.
Cognitive i	NA	.	SYSTEM_	INTRINSIC	.	.	.	.
.	NA	.	MACROMO	CYTOPLA	TRANSLA	KEGG_MT	PID_MTOF	BIOCARTA
.	NA	.	RNA_MET	.	TRANSCR	.	.	.
.	NA	.	NEGATIVE	ORGANEL	PROTEIN_	.	PID_ATR_	.
Deafness,	NA	.	SENSORY	ORGANEL	.	.	.	.
.	NA	.	LIPID_ME	INTRINSIC	CATION_E	KEGG_AL	PID_AMB2	.
.	NA	.	.	.	.	.	.	.
.	NA	.	RNA_MET	CYTOPLA	RNA_POL	.	.	.
.	NA	.	RNA_MET	NUCLEOP	TRANSCR	KEGG_AN	PID_P53D	.
.	NA	.	.	.	.	.	.	.
.	NA	.	MACROM	.	.	KEGG_AD	.	BIOCARTA
.	NA	.	.	.	.	.	.	.
.	NA	.	.	.	.	KEGG_AM	.	.
.	NA	.	POSITIVE_	.	TRANSLA	.	.	.
.	NA	.	.	.	.	.	.	.
.	NA	.	.	.	.	KEGG_WN	PID_WNT_	.
Bleeding d	NA	.	FOCAL_A	ADHEREN	INTEGRIN	KEGG_FO	PID_ILK_F	BIOCARTA
.	NA	.	.	.	.	.	.	.
.	NA	.	.	.	.	.	.	.
.	NA	.	CELL_PRO	CYTOPLA	CATION_E	.	.	.
.	NA	.	.	.	.	.	.	.
.	NA	.	.	.	.	.	.	.
.	NA	.	.	.	.	.	.	.
.	NA	.	.	NUCLEOP	.	.	PID_ATM_	.
.	NA	.	.	.	PHOSPHO	KEGG_GA	.	.

Amelogene	NA	.	.	.	.	.	.	.
.	NA	.	.	.	.	.	.	.
.	NA	.	.	INTRINSIC	.	KEGG_CE	.	.
.	NA	.	POSITIVE_	.	.	KEGG_CE	.	.
.	NA	.	SYSTEM_I	NUCLEUS	.	.	.	.
.	NA	.	.	.	.	.	.	.
.	NA	.	ENZYME_	INTRINSIC	GROWTH_	KEGG_NE	PID_SHP2	.
Acrocallosa	NA	.	.	.	.	.	.	.
.	NA	.	BIOPOLYM	.	.	KEGG_AX	PID_IL4_2	.
.	NA	.	REGULAT	CYTOPLAS	.	.	.	.
Nephronop	NA	.	NEGATIVE	NUCLEOP	TRANSCR	.	.	.
.	NA	.	.	.	.	.	.	.
.	NA	.	.	.	.	.	.	.
.	NA	.	.	.	.	.	.	.
.	NA	.	.	.	.	.	PID_AURC	.
.	NA	.	.	.	.	KEGG_LYS	.	.
.	NA	.	NUCLEOB	INTRINSIC	PHOSPHO	KEGG_PU	PID_ENDC	.
.	NA	.	HOMOPHI	MEMBRAN	.	KEGG_CE	PID_VEGF	.
.	NA	.	.	.	.	.	.	.
.	NA	.	ESTABLIS	INTRINSIC	ACTIVE_T	.	.	.
Brittle corn	NA	.	.	.	.	.	.	.
.	NA	.	.	.	.	.	.	.
.	NA	.	.	.	.	.	.	.
.	NA	.	.	.	.	.	.	.
.	NA	.	ESTABLIS	.	AUXILIARY	.	.	.
.	NA	.	CYTOKINE	ORGANEL	HYDROLA	KEGG_TIG	.	.
Leukemia,	NA	.	REGULAT	AXON;CYT	RAS_GTP	KEGG_MA	PID_HNF3	.
.	NA	.	.	.	.	.	.	.
.	NA	.	PROTEIN_	NUCLEUS	.	.	.	.
.	NA	.	JAK_STAT	.	TRANSCR	KEGG_ER	PID_ERBB	BIOCARTA
.	NA	.	DEFENSE	CELL_SUF	COPPER_	KEGG_GL	.	.
.	NA	.	.	.	.	.	.	.
.	NA	.	.	.	.	.	.	.
.	NA	.	.	ORGANEL	.	.	.	.
.	NA	.	.	.	.	.	.	.
.	NA	.	.	.	.	.	.	.
.	NA	.	.	.	.	.	.	.
.	NA	.	.	.	.	KEGG_OT	.	.
.	NA	.	.	.	.	.	.	.
.	NA	.	REGULAT	.	GTPASE_I	.	.	.
Fascioscap	NA	.	.	.	.	.	.	.
.	NA	.	DNA_MET	CYTOPLAS	PROTEIN_	.	.	.
.	NA	.	.	.	.	.	.	.
Niemann-P	NA	.	REGULAT	ORGANEL	SUBSTRA	KEGG_LYS	.	.
.	NA	.	.	.	NF_KAPPA	KEGG_BA	PID_MYC_	.
.	NA	.	.	.	.	.	.	.
Deafness,	NA	.	.	.	.	.	.	.
{Colorectal	NA	.	ENZYME_	.	RECEPTO	KEGG_TG	PID_SMAD	BIOCARTA
.	NA	.	.	.	.	.	.	.
.	NA	.	.	.	.	.	.	.
.	NA	.	GAMETE_	.	RNA_BIND	.	.	.
.	NA	.	.	.	.	.	.	.
.	NA	.	.	.	.	.	.	.
.	NA	.	.	.	.	.	.	.

.	NA	.	.	.	.	.	.	.
.	NA	.	.	INTRINSIC	PROTEIN_	.	.	.
.	NA	.	.	INTRINSIC	PROTEIN_	.	.	.
.	NA	.	RNA_MET	NUCLEOP	RNA_POL	KEGG_BA	.	BIOCART/
C3 deficier	NA	.	SIGNAL_T	.	RECEPTO	KEGG_CC	PID_ERB_	BIOCART/
.	NA	.	.	.	.	.	.	.
.	NA	.	.	.	.	.	.	.
SCID, auto	NA	.	BIOPOLYN	.	.	KEGG_CH	PID_IL4_2	BIOCART/
Mucoepide	NA	.	RNA_MET	NUCLEUS	TRANSCR	.	PID_AP1_	.
.	NA	.	.	.	.	.	.	.
.	NA	.	.	.	ENZYME_	KEGG_UB	.	.
.	NA	.	.	.	.	.	.	.
.	NA	.	.	.	.	.	.	.
.	NA	.	.	.	.	.	.	.
.	NA	.	.	.	.	.	.	.
.	NA	.	.	.	.	.	.	.
.	NA	.	.	.	.	.	.	.
.	NA	.	.	.	.	.	.	.
.	NA	.	.	.	.	.	.	.
.	NA	.	SYSTEM_I	ORGANEL	PHOSPHC	.	PID_LKB1	.
.	NA	.	SENSORY	ORGANEL	.	.	.	.
.	NA	.	RNA_MET	ORGANEL	.	KEGG_SP	.	.
.	NA	.	.	.	.	.	.	.
.	NA	.	.	.	.	.	.	.
Colorectal	NA	.	REGULAT	ORGANEL	PROTEIN_	KEGG_P5	PID_P73P	BIOCART/
.	NA	.	SIGNAL_T	.	HYDROLA	KEGG_MA	PID_EPHE	.
.	NA	.	PROTEOL	.	.	KEGG_LY	.	.
.	NA	.	SIGNAL_T	INTRINSIC	CARBOHY	.	.	.
.	NA	.	SIGNAL_T	.	RECEPTO	.	.	.
.	NA	.	.	INTRINSIC	.	.	.	.
.	NA	.	MITOTIC_	CYTOPLA	PHOSPHC	.	PID_LKB1	.
.	NA	.	.	.	ACID_AMI	KEGG_UB	PID_ERB_	BIOCART/
.	NA	.	.	.	.	.	.	.
.	NA	.	.	.	.	.	.	.
.	NA	.	.	.	.	.	.	.
.	NA	.	BIOSYNTH	RIBONUCI	RECEPTO	.	.	.
Bohring-O	NA	.	.	.	.	.	.	.
.	NA	.	.	.	LIGASE_A	KEGG_GL	PID_HDAC	.
.	NA	.	.	.	.	.	.	.
.	NA	.	.	CYTOPLA	.	.	.	.
.	NA	.	SYSTEM_I	.	.	.	.	.
.	NA	.	.	.	.	.	.	.
Epileptic er	NA	.	SYSTEM_I	INTRINSIC	CATION_T	.	.	.
.	NA	.	.	.	.	.	.	.
.	NA	.	.	.	.	.	.	.
Glutamate	NA	.	COFACTC	CYTOPLA	.	KEGG_HIS	.	.
.	NA	.	DNA_MET	CYTOPLA	DNA_BINC	.	.	.
.	NA	.	.	.	.	.	.	.
.	NA	.	.	.	.	.	.	.
Meningiom	NA	.	.	.	.	.	.	.
.	NA	.	.	.	.	.	.	.
.	NA	.	.	.	.	.	.	.
.	NA	.	REGULAT	.	.	.	.	.
.	NA	.	.	.	.	.	.	.

.	NA	.	.	.	.	KEGG_P5	.	.	
.	NA	.	.	.	.	.	.	.	
.	NA	.	BIOPOLYN	PROTEIN_	PROTEIN_	.	PID_RB_1	BIOCARTA	
Ichthyosis,	NA	.	LIPID_CAT	VESICULA	HYDROLA	KEGG_ST	.	.	
.	NA	.	.	.	.	.	.	.	
.	NA	.	.	CYTOPLA	HYDROLA	KEGG_RI(	.	.	
.	NA	.	RNA_MET	.	TRANSCR	KEGG_MA	PID_BCR_	BIOCARTA	
Congenital	NA	.	ESTABLIS	.	SUBSTRA	.	.	.	
.	NA	.	.	.	.	.	.	.	
.	NA	.	SIGNAL_T	NON_MEN	KINASE_B	.	.	.	
.	NA	.	.	.	.	.	.	.	
Mental reta	NA	.	.	ORGANEL	CATION_T	KEGG_CA	.	.	
.	NA	.	.	.	.	KEGG_AX	.	.	
Axenfeld-R	NA	.	SYSTEM_I	ORGANEL	SEQUENC	.	.	.	
Osteogene	NA	.	RESPONS	CYTOPLA	PROTEAS	.	.	.	
.	NA	.	.	.	.	.	.	.	
.	NA	.	.	.	.	KEGG_GL	.	.	
.	NA	.	.	.	.	.	.	.	
.	NA	.	.	.	.	.	.	.	
.	NA	.	.	.	.	.	.	.	
.	NA	.	.	.	.	.	.	.	
.	NA	.	.	.	.	.	.	.	
.	NA	.	.	.	.	.	.	.	
.	NA	.	.	.	.	.	.	.	
.	NA	.	.	.	.	.	.	.	
.	NA	.	.	CYTOPLA	.	.	PID_MTOF	.	
.	NA	.	.	.	.	.	.	.	
.	NA	.	SYSTEM_I	.	.	.	.	.	
.	NA	.	.	.	.	.	.	.	
.	NA	.	CELL_DEV	CYTOPLA	NUCLEOT	KEGG_MA	PID_P38G	.	
Pontocereb	NA	.	BIOPOLYN	CYTOPLA	RNA_BIND	KEGG_AM	.	.	
.	NA	.	ESTABLIS	MEMBRAN	LIPID_BIN	.	PID_ARF6	BIOCARTA	
.	NA	.	.	.	.	.	.	.	
.	NA	.	.	.	.	.	.	.	
.	NA	.	RNA_MET	.	HYDROLA	.	PID_HDAC	.	
.	NA	.	.	.	.	.	.	.	
.	NA	.	GLUCOSE	CYTOPLA	PHOSPHC	KEGG_T_(	.	.	
.	NA	.	.	.	.	KEGG_INC	.	BIOCARTA	
.	NA	.	DNA_MET	NUCLEUS	NUCLEOT	KEGG_PU	.	.	
Adrenal co	NA	.	REGULAT	NUCLEOP	NUCLEOT	KEGG_MA	PID_HDAC	BIOCARTA	
Progressiv	NA	.	.	.	.	.	.	.	
.	NA	.	.	CELL_FRA	.	.	.	.	
Mental reta	NA	.	.	MEMBRAN	CATION_T	KEGG_MA	.	.	
{Diabetes r	NA	.	BIOSYNTH	CYTOPLA	RNA_BIND	.	.	.	
.	NA	.	SYSTEM_I	ORGANEL	.	.	.	.	
.	NA	.	.	.	.	.	.	.	
.	NA	.	.	.	.	.	.	.	
.	NA	.	.	.	.	.	.	.	
Acne inver	NA	.	GLYCOPR	INTRINSIC	.	KEGG_NC	PID_NOTC	.	
.	NA	.	.	.	.	.	.	.	
Mitochondı	NA	.	.	.	.	.	.	.	
.	NA	.	SYSTEM_I	.	ACTIN_BII	.	.	.	
.	NA	.	SYSTEM_I	INTRINSIC	TRANSME	.	.	.	
.	NA	.	.	.	.	.	.	.	

.	NA	.	.	.	.	KEGG_PU	.	.
.	NA	.	CELL_REC	.	.	.	.	.
.	NA	.	ISOPRENC	.	OXIDOREI	KEGG_RE	.	.
.	NA	.	.	.	.	.	.	.
.	NA	.	.	.	.	.	.	.
.	NA	.	.	.	.	.	.	.
.	NA	.	.	.	STRUCTU	.	PID_PLK1	.
.	NA	.	.	.	.	.	.	.
.	NA	.	.	INTRINSIC	RECEPTO	.	.	.
.	NA	.	.	.	.	.	PID_CDC4	.
Leber cong	NA	.	.	.	.	.	.	.
Myeloid leu	NA	.	RNA_MET	.	RNA_POL	.	PID_SMAD	.
.	NA	.	.	.	.	.	.	.
.	NA	.	.	.	.	.	.	.
Knobloch s	NA	.	SYSTEM_I	PROTEIN/	.	.	PID_INTEC	.
.	NA	.	.	.	.	.	.	.
.	NA	.	MULTICEL	.	.	.	PID_SMAD	.
.	NA	.	.	.	.	.	.	.
.	NA	.	SIGNAL_T	.	RECEPTO	.	PID_ANGI	.
.	NA	.	CELL_DEV	CYTOPLAS	ACID_AMI	KEGG_UB	PID_CD40	BIOCARTA
.	NA	.	.	.	.	.	.	.
.	NA	.	.	NUCLEOP	.	KEGG_TIC	.	.
.	NA	.	.	.	.	.	.	.
.	NA	.	.	.	.	.	.	.
Leiomyoma	NA	.	.	.	.	KEGG_FO	PID_INTEC	BIOCARTA
.	NA	.	.	.	.	.	.	.
.	NA	.	.	.	.	.	.	.
.	NA	.	.	.	.	.	PID_BMPF	.

REACTOME_PATHWAY
.
REACTOME_COSTIMULATION_BY_THE_CD28_FAMILY;REACTOME_CTLA4_INHIBITORY_SIG
.
.
.
.
REACTOME_SIGNALING_BY_GPCR;REACTOME_OLFACTORY_SIGNALING_PATHWAY;REACT
.
.
REACTOME_METABOLISM_OF_AMINO_ACIDS_AND_DERIVATIVES;REACTOME_BRANCHED_
.
REACTOME_VEGF_LIGAND_RECEPTOR_INTERACTIONS
REACTOME_CELL_CELL_COMMUNICATION;REACTOME_RESPONSE_TO_ELEVATED_PLATEL
.
REACTOME_PROCESSING_OF_CAPPED_INTRON_CONTAINING_PRE_MRNA;REACTOME_MR
.
.
.
.
.
.
.
.
.
.
.
.
.
REACTOME_IMMUNE_SYSTEM;REACTOME_ADAPTIVE_IMMUNE_SYSTEM;REACTOME_CLAS
.
.
.
.
.
REACTOME_EXTRACELLULAR_MATRIX_ORGANIZATION;REACTOME_COLLAGEN_FORMATIO
REACTOME_CYTOSOLIC_TRNA_AMINOACYLATION;REACTOME_TRNA_AMINOACYLATION
REACTOME_SIGNALING_BY_RHO_GTPASES;REACTOME_DEVELOPMENTAL_BIOLOGY;REAC
REACTOME_SIGNALLING_BY_NGF;REACTOME_SIGNALING_BY_ERBB4;REACTOME_NUCLEA
REACTOME_IMMUNE_SYSTEM;REACTOME_ADAPTIVE_IMMUNE_SYSTEM;REACTOME_CLAS
.
.
REACTOME_CS_DS_DEGRADATION;REACTOME_CHONDROITIN_SULFATE_BIOSYNTHESIS;
REACTOME_DIABETES_PATHWAYS;REACTOME_REGULATION_OF_INSULIN_LIKE_GROWTH
.
REACTOME_PROCESSING_OF_CAPPED_INTRON_CONTAINING_PRE_MRNA;REACTOME_MR
REACTOME_DEVELOPMENTAL_BIOLOGY;REACTOME_TCR_SIGNALING;REACTOME_PHOSP
.
REACTOME_G0_AND_EARLY_G1;REACTOME_CELL_CYCLE;REACTOME_CELL_CYCLE_MITO
.
.
.

REACTOME_DEVELOPMENTAL_BIOLOGY;REACTOME_SIGNALING_BY_GPCR;REACTOME_AX
.
.
.
REACTOME_METABOLISM_OF_PROTEINS;REACTOME_POST_TRANSLATIONAL_MODIFICATI
.
.
.
.
.
REACTOME_DEVELOPMENTAL_BIOLOGY;REACTOME_SIGNALING_BY_NODAL;REACTOME_S
.
.
.
REACTOME_TRIF_MEDIATED_TLR3_SIGNALING;REACTOME_MAP_KINASE_ACTIVATION_IN_
REACTOME_SIGNALING_BY_GPCR;REACTOME_GPCR_DOWNSTREAM_SIGNALING;REACTO
.
.
REACTOME_SIGNALING_BY_SCF_KIT;REACTOME_GROWTH_HORMONE_RECEPTOR_SIGNA
.
.
.
.
.
.
.
.
REACTOME_DEVELOPMENTAL_BIOLOGY;REACTOME_AXON_GUIDANCE;REACTOME_SEMAI
REACTOME_DEVELOPMENTAL_BIOLOGY;REACTOME_AXON_GUIDANCE;REACTOME_L1CAM
.
.
REACTOME_SYNTHESIS_OF_PIPS_AT_THE_GOLGI_MEMBRANE;REACTOME_PHOSPHOLIPID
.
.
.
REACTOME_APOPTOTIC_CLEAVAGE_OF_CELLULAR_PROTEINS;REACTOME_SIGNALLING_E
.
.
.
.
.
.
.
.
.
.
.
REACTOME_GLYCOGEN_BREAKDOWN_GLYCOGENOLYSIS;REACTOME_METABOLISM_OF_C
.
.
.
.
.
REACTOME_SIGNALING_BY_ERBB4;REACTOME_SIGNALING_BY_ERBB2;REACTOME_SIGNAI

REACTOME_TRANSMISSION_ACROSS_CHEMICAL_SYNAPSES;REACTOME_NEURONAL_SYS
REACTOME_PTM_GAMMA_CARBOXYLATION_HYPUSINE_FORMATION_AND_ARYLSULFATAS
.
.
.
.
REACTOME_SIGNALING_BY_RHO_GTPASES;REACTOME_SIGNALLING_BY_NGF;REACTOME_
.
.
.
.
.
REACTOME_MEMBRANE_TRAFFICKING;REACTOME_TRANS_GOLGI_NETWORK_VESICLE_BU
.
.
.
REACTOME_DEVELOPMENTAL_BIOLOGY;REACTOME_AXON_GUIDANCE;REACTOME_NETRI
REACTOME_DEVELOPMENTAL_BIOLOGY;REACTOME_AXON_GUIDANCE;REACTOME_NETRI
.
.
.
REACTOME_TRIF_MEDIATED_TLR3_SIGNALING;REACTOME_RIP_MEDIATED_NFKB_ACTIVAT
.
REACTOME_MHC_CLASS_II_ANTIGEN_PRESENTATION;REACTOME_IMMUNE_SYSTEM;REAC
.
.
.
.
.
REACTOME_TRANSMISSION_ACROSS_CHEMICAL_SYNAPSES;REACTOME_NEURONAL_SYS
.
REACTOME_AMINO_ACID_TRANSPORT_ACROSS_THE_PLASMA_MEMBRANE;REACTOME_TI
.
.
.
.
.
.
REACTOME_MEMBRANE_TRAFFICKING;REACTOME_TRANS_GOLGI_NETWORK_VESICLE_BU
.
.
.
.
.
.
REACTOME_TRIF_MEDIATED_TLR3_SIGNALING;REACTOME_TAK1_ACTIVATES_NFKB_BY_PI
REACTOME_TRIF_MEDIATED_TLR3_SIGNALING;REACTOME_TAK1_ACTIVATES_NFKB_BY_PI
.
.
.
.
REACTOME_GENERIC_TRANSCRIPTION_PATHWAY
.
.

REACTOME_SIGNALING_BY_FGFR_IN_DISEASE;REACTOME_SIGNALING_BY_FGFR1_MUTAN
.
.
.
.
.
.
.
.
REACTOME_SIGNALING_BY_ERBB4;REACTOME_DOWNREGULATION_OF_ERBB2_ERBB3_SI
.
REACTOME_SIGNALING_BY_SCF_KIT;REACTOME_GROWTH_HORMONE_RECEPTOR_SIGNA
REACTOME_NOTCH1_INTRACELLULAR_DOMAIN_REGULATES_TRANSCRIPTION;REACTOME
.
.
REACTOME_DEVELOPMENTAL_BIOLOGY;REACTOME_NEURONAL_SYSTEM;REACTOME_AX
.
REACTOME_TCA_CYCLE_AND_RESPIRATORY_ELECTRON_TRANSPORT;REACTOME_MITOC
.
.
REACTOME_DEVELOPMENTAL_BIOLOGY;REACTOME_RESPONSE_TO_ELEVATED_PLATELE
.
.
REACTOME_SIGNALING_BY_GPCR;REACTOME_CLASS_B_2_SECRETIN_FAMILY_RECEPTOR
REACTOME_SIGNALING_BY_GPCR;REACTOME_CLASS_B_2_SECRETIN_FAMILY_RECEPTOR
.
REACTOME_SIGNALING_BY_GPCR;REACTOME_OLFACTORY_SIGNALING_PATHWAY;REACT
.
.
.
.
REACTOME_MITOCHONDRIAL_PROTEIN_IMPORT;REACTOME_METABOLISM_OF_PROTEINS
.
.
.
.
REACTOME_TRANSMISSION_ACROSS_CHEMICAL_SYNAPSES;REACTOME_NEURONAL_SYS
.
.
REACTOME_FACTORS_INVOLVED_IN_MEGAKARYOCYTE_DEVELOPMENT_AND_PLATELET_
.
REACTOME_EXTRACELLULAR_MATRIX_ORGANIZATION;REACTOME_COLLAGEN_FORMATIC
.
.
.
.
.
.
.
.
REACTOME_INTERFERON_ALPHA_BETA_SIGNALING;REACTOME_INTERFERON_SIGNALING
.
.
REACTOME_SIGNALING_BY_GPCR;REACTOME_OLFACTORY_SIGNALING_PATHWAY;REACT

.
.
.
REACTOME_O_LINKED_GLYCOSYLATION_OF_MUCINS;REACTOME_TERMINATION_OF_O_G
REACTOME_TRIGLYCERIDE_BIOSYNTHESIS;REACTOME_FATTY_ACYL_COA_BIOSYNTHESIS
.
.
.
REACTOME_SIGNALLING_BY_NGF;REACTOME_DAG_AND_IP3_SIGNALING;REACTOME_SIG
.
.
.
.
.
REACTOME_CELL_SURFACE_INTERACTIONS_AT_THE_VASCULAR_WALL;REACTOME_INTE
.
REACTOME_O_LINKED_GLYCOSYLATION_OF_MUCINS;REACTOME_METABOLISM_OF_PRO
.
REACTOME_SIGNALING_BY_SCF_KIT;REACTOME_GROWTH_HORMONE_RECEPTOR_SIGN
.
.
.
.
.
REACTOME_O_LINKED_GLYCOSYLATION_OF_MUCINS;REACTOME_TERMINATION_OF_O_G
.
.
.
.
REACTOME_DEVELOPMENTAL_BIOLOGY;REACTOME_AXON_GUIDANCE;REACTOME_L1CA
REACTOME_TRANSLATION;REACTOME_INSULIN_RECEPTOR_SIGNALLING_CASCADE;REAC
.
.
.
.
.
.
REACTOME_PPARA_ACTIVATES_GENE_EXPRESSION;REACTOME_DIABETES_PATHWAYS;R
.
.
.
REACTOME_MITOCHONDRIAL_TRNA_AMINOACYLATION;REACTOME_TRNA_AMINOACYLAT
.
.
.
REACTOME_CELL_CELL_COMMUNICATION;REACTOME_RESPONSE_TO_ELEVATED_PLATE
.
.
.
.
.
.
REACTOME_HOMOLOGOUS_RECOMBINATION_REPAIR_OF_REPLICATION_INDEPENDENT_
.

.
.
REACTOME_DEVELOPMENTAL_BIOLOGY;REACTOME_AXON_GUIDANCE;REACTOME_MYOG
.
.
.
.
.
REACTOME_SIGNALING_BY_SCF_KIT;REACTOME_DEVELOPMENTAL_BIOLOGY;REACTOME_
.
.
.
.
.
.
.
REACTOME_SIGNALLING_BY_NGF;REACTOME_DAG_AND_IP3_SIGNALING;REACTOME_SIGN
REACTOME_CELL_CELL_COMMUNICATION;REACTOME_ADHERENS_JUNCTIONS_INTERACT
.
REACTOME_CELL_SURFACE_INTERACTIONS_AT_THE_VASCULAR_WALL;REACTOME_BASIC
.
.
.
.
REACTOME_NEURONAL_SYSTEM;REACTOME_VOLTAGE_GATED_POTASSIUM_CHANNELS;R
REACTOME_DEVELOPMENTAL_BIOLOGY;REACTOME_AXON_GUIDANCE;REACTOME_SEMA4
.
.
.
REACTOME_SIGNALING_BY_SCF_KIT;REACTOME_GROWTH_HORMONE_RECEPTOR_SIGNA
.
.
.
REACTOME_STRIATED_MUSCLE_CONTRACTION;REACTOME_MUSCLE_CONTRACTION
.
.
.
.
.
.
.
.
.
.
REACTOME_RNA_POL_II_TRANSCRIPTION;REACTOME_RNA_POL_II_TRANSCRIPTION_PRE_
.
.
REACTOME_SIGNALING_BY_BMP;REACTOME_DOWNREGULATION_OF_TGF_BETA_RECEPT
.
.
.
.
.
.

.
.
.
REACTOME_PROCESSING_OF_CAPPED_INTRON_CONTAINING_PRE_MRNA;REACTOME_RN
REACTOME_IMMUNOREGULATORY_INTERACTIONS_BETWEEN_A_LYMPHOID_AND_A_NON_
.
.
REACTOME_IL_7_SIGNALING;REACTOME_SIGNALING_BY_ILS;REACTOME_IL_3_5_AND_GM_
.
.
REACTOME_IMMUNE_SYSTEM;REACTOME_ADAPTIVE_IMMUNE_SYSTEM;REACTOME_CLAS
.
.
REACTOME_GENERIC_TRANSCRIPTION_PATHWAY
.
.
.
.
.
.
.
REACTOME_METABOLISM_OF_NON_CODING_RNA;REACTOME_PROCESSING_OF_CAPPED_
.
.
REACTOME_APOPTOSIS;REACTOME_INTRINSIC_PATHWAY_FOR_APOPTOSIS
REACTOME_DEVELOPMENTAL_BIOLOGY;REACTOME_TRANSMISSION_ACROSS_CHEMICAL_
.
.
REACTOME_IMMUNOREGULATORY_INTERACTIONS_BETWEEN_A_LYMPHOID_AND_A_NON_
.
.
REACTOME_IMMUNE_SYSTEM;REACTOME_ADAPTIVE_IMMUNE_SYSTEM;REACTOME_CLAS
.
.
.
.
.
REACTOME_BIOLOGICAL_OXIDATIONS;REACTOME_PHASE1_FUNCTIONALIZATION_OF_CO
.
.
.
.
REACTOME_DEVELOPMENTAL_BIOLOGY;REACTOME_NEURONAL_SYSTEM;REACTOME_AX
.
.
REACTOME_METABOLISM_OF_AMINO_ACIDS_AND_DERIVATIVES
.
.
.
.
.
.
.
.

.
.
REACTOME_CELL_CYCLE;REACTOME_CELL_CYCLE_MITOTIC;REACTOME_G1_PHASE;REAC
REACTOME_PTM_GAMMA_CARBOXYLATION_HYPUSINE_FORMATION_AND_ARYLSULFATAS
.
.
REACTOME_SIGNALLING_BY_NGF;REACTOME_TRIF_MEDIATED_TLR3_SIGNALING;REACTO
REACTOME_TRANSMEMBRANE_TRANSPORT_OF_SMALL_MOLECULES;REACTOME_SLC_ME
.
.
.
REACTOME_TRANSMEMBRANE_TRANSPORT_OF_SMALL_MOLECULES;REACTOME_SLC_ME
REACTOME_DEVELOPMENTAL_BIOLOGY;REACTOME_AXON_GUIDANCE;REACTOME_OTHE
.
REACTOME_EXTRACELLULAR_MATRIX_ORGANIZATION;REACTOME_COLLAGEN_FORMATIO
.
.
.
REACTOME_NEURONAL_SYSTEM;REACTOME_VOLTAGE_GATED_POTASSIUM_CHANNELS;
.
.
.
.
.
.
.
.
.
.
.
.
.
.
.
.
.
REACTOME_PYRUVATE_METABOLISM_AND_CITRIC_ACID_TCA_CYCLE;REACTOME_TCA_C
REACTOME_PHOSPHOLIPID_METABOLISM;REACTOME_SYNTHESIS_OF_PIPS_AT_THE_PLA
REACTOME_ACTIVATION_OF_THE_PRE_REPLICATIVE_COMPLEX;REACTOME_CELL_CYCLE
REACTOME_CELL_CYCLE;REACTOME_PRE_NOTCH_TRANSCRIPTION_AND_TRANSLATION;
.
.
REACTOME_TRANSMISSION_ACROSS_CHEMICAL_SYNAPSES;REACTOME_NEURONAL_SYS
REACTOME_DIABETES_PATHWAYS
.
.
.
.
REACTOME_SIGNALLING_BY_NGF;REACTOME_SIGNALING_BY_ERBB4;REACTOME_NUCLE
.
.
.
.
.

.
.
REACTOME_METABOLISM_OF_STEROID_HORMONES_AND_VITAMINS_A_AND_D;REACTOMI
.
.
.
.
.
.
REACTOME_FACTORS_INVOLVED_IN_MEGAKARYOCYTE_DEVELOPMENT_AND_PLATELET_
.
.
.
.
.
.
REACTOME_CELL_CYCLE;REACTOME_CHROMOSOME_MAINTENANCE;REACTOME_DEPOSI
.
REACTOME_CELL_SURFACE_INTERACTIONS_AT_THE_VASCULAR_WALL;REACTOME_TIE2_
REACTOME_INNATE_IMMUNE_SYSTEM;REACTOME_IMMUNE_SYSTEM;REACTOME_NOD1_2
.
.
REACTOME_GENERIC_TRANSCRIPTION_PATHWAY
.
REACTOME_EXTRACELLULAR_MATRIX_ORGANIZATION;REACTOME_COLLAGEN_FORMATIC
REACTOME_FACTORS_INVOLVED_IN_MEGAKARYOCYTE_DEVELOPMENT_AND_PLATELET_
.
REACTOME_DEVELOPMENTAL_BIOLOGY;REACTOME_AXON_GUIDANCE;REACTOME_NETRI

NALING;REACTOME_IMMUNE_SYSTEM;REACTOME_ADAPTIVE_IMMUNE_SYSTEM

TOME_GPCR_DOWNSTREAM_SIGNALING

CHAIN_AMINO_ACID_CATABOLISM

LET_CYTOSOLIC_CA2_;REACTOME_CELL_EXTRACELLULAR_MATRIX_INTERACTIONS;REACT

RNA_PROCESSING;REACTOME_MRNA_SPLICING

S_I_MHC_MEDIATED_ANTIGEN_PROCESSING_PRESENTATION;REACTOME_ANTIGEN_PROC

ON

CTOME_SIGNALING_BY_GPCR;REACTOME_AXON_GUIDANCE;REACTOME_G_ALPHA1213_SIG
AR_SIGNALING_BY_ERBB4;REACTOME_ACTIVATED_NOTCH1_TRANSMITS_SIGNAL_TO_THE_
S_I_MHC_MEDIATED_ANTIGEN_PROCESSING_PRESENTATION;REACTOME_ANTIGEN_PROC

REACTOME_CHONDROITIN_SULFATE_DERMATAN_SULFATE_METABOLISM;REACTOME_HEPA
_FACTOR_IGF_ACTIVITY_BY_INSULIN_LIKE_GROWTH_FACTOR_BINDING_PROTEINS_IGFBP

RNA_PROCESSING;REACTOME_MRNA_SPLICING
HORYLATION_OF_CD3_AND_TCR_ZETA_CHAINS;REACTOME_AXON_GUIDANCE;REACTOME_

TIC;REACTOME_MITOTIC_G1_G1_S_PHASES

XON_GUIDANCE;REACTOME_G_ALPHA1213_SIGNALLING_EVENTS;REACTOME_GPCR_DOWN

ON_SYNTHESIS_OF_GPI_ANCHORED_PROTEINS;REACTOME_POST_TRANSLATIONAL_PRO

SIGNALING_BY_BMP

TLR_CASCADE;REACTOME_ACTIVATION_OF_THE_AP1_FAMILY_OF_TRANSCRIPTION_FACT
ME_G_ALPHA_S_SIGNALLING_EVENTS;REACTOME_CGMP_EFFECTS;REACTOME_NITRIC_O

LING;REACTOME_ANTIVIRAL_MECHANISM_BY_IFN_STIMULATED_GENES;REACTOME_SIGN

PHORIN_INTERACTIONS;REACTOME_SEMA3A_PLEXIN_REPULSION_SIGNALING_BY_INHIBITI
M_INTERACTIONS

D_METABOLISM;REACTOME_PI_METABOLISM;REACTOME_METABOLISM_OF_LIPIDS_AND_LI

BY_NGF;REACTOME_DAG_AND_IP3_SIGNALING;REACTOME_SIGNALING_BY_ERBB2;REACTO

CARBOHYDRATES;REACTOME_GLUCOSE_METABOLISM

LING_BY_CONSTITUTIVELY_ACTIVE_EGFR;REACTOME_GRB2_EVENTS_IN_ERBB2_SIGNALIN

;TEM;REACTOME_NEUROTRANSMITTER_RECEPTOR_BINDING_AND_DOWNSTREAM_TRANS
;E_ACTIVATION;REACTOME_GLYCOSPHINGOLIPID_METABOLISM;REACTOME_PHOSPHOLIPI

_DEVELOPMENTAL_BIOLOGY;REACTOME_GASTRIN_CREB_SIGNALLING_PATHWAY_VIA_PK(

JDDING;REACTOME_GOLGI_ASSOCIATED_VESICLE_BIOGENESIS

N1_SIGNALING
N1_SIGNALING

TION_VIA_DAI;REACTOME_TAK1_ACTIVATES_NFKB_BY_PHOSPHORYLATION_AND_ACTIVATI

:TOME_ADAPTIVE_IMMUNE_SYSTEM

;TEM;REACTOME_NEUROTRANSMITTER_RECEPTOR_BINDING_AND_DOWNSTREAM_TRANS

RANSMEMBRANE_TRANSPORT_OF_SMALL_MOLECULES;REACTOME_SLC_MEDIATED_TRAN

JDDING;REACTOME_GOLGI_ASSOCIATED_VESICLE_BIOGENESIS

HOSPHORYLATION_AND_ACTIVATION_OF_IKKS_COMPLEX;REACTOME_MAP_KINASE_ACTIV
HOSPHORYLATION_AND_ACTIVATION_OF_IKKS_COMPLEX;REACTOME_MAP_KINASE_ACTIV

ITS;REACTOME_SIGNALING_BY_FGFR1_FUSION_MUTANTS;REACTOME_SIGNALING_BY_FGI

GNALING;REACTOME_SIGNALING_BY_ERBB2;REACTOME_GRB2_EVENTS_IN_ERBB2_SIGNA

ALING;REACTOME_REGULATION_OF_KIT_SIGNALING;REACTOME_ANTIGEN_ACTIVATES_B_C
E_SIGNALING_BY_NOTCH1;REACTOME_SIGNALING_BY_NOTCH

ON_GUIDANCE;REACTOME_L1CAM_INTERACTIONS;REACTOME_INTERACTION_BETWEEN_L

CHONDRIAL_PROTEIN_IMPORT;REACTOME_METABOLISM_OF_PROTEINS;REACTOME_RESPI

T_CYTOSOLIC_CA2_;REACTOME_INTEGRIN_CELL_SURFACE_INTERACTIONS;REACTOME_D

RS;REACTOME_GPCR_LIGAND_BINDING
RS;REACTOME_GPCR_LIGAND_BINDING

TOME_GPCR_DOWNSTREAM_SIGNALING

STEM;REACTOME_NEUROTRANSMITTER_RELEASE_CYCLE;REACTOME_GABA_SYNTHESIS_F

PRODUCTION;REACTOME_HEMOSTASIS

ON

;REACTOME_IMMUNE_SYSTEM;REACTOME_CYTOKINE_SIGNALING_IN_IMMUNE_SYSTEM

TOME_GPCR_DOWNSTREAM_SIGNALING

LYCAN_BIOSYNTHESIS;REACTOME_METABOLISM_OF_PROTEINS;REACTOME_POST_TRANS
;;REACTOME_METABOLISM_OF_LIPIDS_AND_LIPOPROTEINS;REACTOME_FATTY_ACID_TRIA

NALING_BY_ERBB2;REACTOME_SIGNALING_BY_EGFR_IN_CANCER;REACTOME_NGF_SIGNAI

GRIN_CELL_SURFACE_INTERACTIONS;REACTOME_HEMOSTASIS

TEINS;REACTOME_POST_TRANSLATIONAL_PROTEIN_MODIFICATION

ALING;REACTOME_CELL_CELL_COMMUNICATION;REACTOME_REGULATION_OF_KIT_SIGNAL

LYCAN_BIOSYNTHESIS;REACTOME_METABOLISM_OF_PROTEINS;REACTOME_POST_TRANS

M_INTERACTIONS;REACTOME_INTERACTION_BETWEEN_L1_AND_ANKYRINS
TOME_ACTIVATION_OF_THE_MRNA_UPON_BINDING_OF_THE_CAP_BINDING_COMPLEX_AN

EACTOME_PERK_REGULATED_GENE_EXPRESSION;REACTOME_ACTIVATION_OF_CHAPER(

ON

LET_CYTOSOLIC_CA2_;REACTOME_CELL_EXTRACELLULAR_MATRIX_INTERACTIONS;REACT

DOUBLE_STRAND_BREAKS;REACTOME_DOUBLE_STRAND_BREAK_REPAIR;REACTOME_DNA

ENESIS;REACTOME_NETRIN1_SIGNALING

_AXON_GUIDANCE;REACTOME_CRMPS_IN_SEMA3A_SIGNALING;REACTOME_SEMA3A_PAK_

NALING_BY_ERBB2;REACTOME_SIGNALING_BY_EGFR_IN_CANCER;REACTOME_NGF_SIGNAL
TIONS;REACTOME_CELL_CELL_JUNCTION_ORGANIZATION;REACTOME_CELL_JUNCTION_OR

GIN_INTERACTIONS;REACTOME_AMINO_ACID_TRANSPORT_ACROSS_THE_PLASMA_MEMBR

REACTOME_POTASSIUM_CHANNELS
4D_IN_SEMAPHORIN_SIGNALING;REACTOME_SEMAPHORIN_INTERACTIONS;REACTOME_SE

ALING;REACTOME_IL_7_SIGNALING;REACTOME_SIGNALING_BY_ERBB4;REACTOME_PROLAC

_INITIATION_AND_PROMOTER_OPENING;REACTOME_TRANSCRIPTION;REACTOME_RNA_PO

OR_SIGNALING;REACTOME_SMAD2_SMAD3_SMAD4_HETEROTRIMER_REGULATES_TRANS(

A_POL_II_TRANSCRIPTION;REACTOME_MRNA_CAPPING;REACTOME_RNA_POL_II_TRANSCF
_LYMPHOID_CELL;REACTOME_REGULATION_OF_COMPLEMENT_CASCADE;REACTOME_SIGI

_CSF_SIGNALING;REACTOME_IL_RECEPTOR_SHC_SIGNALING;REACTOME_IL_2_SIGNALING;

S_I_MHC_MEDIATED_ANTIGEN_PROCESSING_PRESENTATION;REACTOME_ANTIGEN_PROC

_INTRON_CONTAINING_PRE_MRNA;REACTOME_MRNA_PROCESSING;REACTOME_MRNA_SF

_SYNAPSES;REACTOME_NEURONAL_SYSTEM;REACTOME_NEUROTRANSMITTER_RECEPTC

_LYMPHOID_CELL;REACTOME_IMMUNE_SYSTEM;REACTOME_ADAPTIVE_IMMUNE_SYSTEM

S_I_MHC_MEDIATED_ANTIGEN_PROCESSING_PRESENTATION;REACTOME_ANTIGEN_PROC

MPOUNDS;REACTOME_ETHANOL_OXIDATION

ON_GUIDANCE;REACTOME_L1CAM_INTERACTIONS;REACTOME_INTERACTION_BETWEEN_L

:TOME_G1_S_TRANSITION;REACTOME_MITOTIC_G1_G1_S_PHASES;REACTOME_INHIBITION
;E_ACTIVATION;REACTOME_GLYCOSPHINGOLIPID_METABOLISM;REACTOME_PHOSPHOLIPI

ME_NGF_SIGNALLING_VIA_TRKA_FROM_THE_PLASMA_MEMBRANE;REACTOME_NUCLEAR_
EDIATED_TRANSMEMBRANE_TRANSPORT;REACTOME_TRANSPORT_OF_VITAMINS_NUCLEC

EDIATED_TRANSMEMBRANE_TRANSPORT;REACTOME_TRANSPORT_OF_INORGANIC_CATIO
R_SEMAPHORIN_INTERACTIONS;REACTOME_SEMAPHORIN_INTERACTIONS

)N

REACTOME_POTASSIUM_CHANNELS

YCLE_AND_RESPIRATORY_ELECTRON_TRANSPORT;REACTOME_REGULATION_OF_PYRUVA
SMA_MEMBRANE;REACTOME_PI_METABOLISM;REACTOME_METABOLISM_OF_LIPIDS_AND_I
;REACTOME_CELL_CYCLE_MITOTIC;REACTOME_TRANSCRIPTION_COUPLED_NER_TC_NER
REACTOME_PRE_NOTCH_EXPRESSION_AND_PROCESSING;REACTOME_CELL_CYCLE_CHE(

TEM;REACTOME_NEUROTRANSMITTER_RECEPTOR_BINDING_AND_DOWNSTREAM_TRANS

AR_SIGNALING_BY_ERBB4;REACTOME_ACTIVATED_NOTCH1_TRANSMITS_SIGNAL_TO_THE_

E_METABOLISM_OF_LIPIDS_AND_LIPOPROTEINS

PRODUCTION;REACTOME_HEMOSTASIS

TION_OF_NEW_CENPA_CONTAINING_NUCLEOSOMES_AT_THE_CENTROMERE

_SIGNALING;REACTOME_HEMOSTASIS
_SIGNALING_PATHWAY;REACTOME_NUCLEOTIDE_BINDING_DOMAIN_LEUCINE_RICH_REPE

ON
PRODUCTION;REACTOME_HEMOSTASIS

N1_SIGNALING

TOME_CELL_JUNCTION_ORGANIZATION;REACTOME_NEPHRIN_INTERACTIONS;REACTOME_

ESSING_UBIQUITINATION_PROTEASOME_DEGRADATION

GNALLING_EVENTS;REACTOME_GPCR_DOWNSTREAM_SIGNALING;REACTOME_SEMA4D_IN_
_NUCLEUS;REACTOME_SIGNALING_BY_NOTCH4;REACTOME_SIGNALING_BY_NOTCH2;REAC
ESSING_UBIQUITINATION_PROTEASOME_DEGRADATION

ARAN_SULFATE_HEPARIN_HS_GAG_METABOLISM;REACTOME_GLYCOSAMINOGLYCAN_MET
S

_SEMA4D_IN_SEMAPHORIN_SIGNALING;REACTOME_SEMAPHORIN_INTERACTIONS;REACTO

NSTREAM_SIGNALING;REACTOME_SEMA4D_IN_SEMAPHORIN_SIGNALING;REACTOME_SEMA

TEIN_MODIFICATION;REACTOME_SYNTHESIS_OF_GLYCOSYLPHOSPHATIDYLINOSITOL_GPI

ORS;REACTOME_MAPK_TARGETS_NUCLEAR_EVENTS_MEDIATED_BY_MAP_KINASES;REAC
XIDE_STIMULATES_GUANYLATE_CYCLASE;REACTOME_PLATELET_HOMEOSTASIS;REACTO

ALING_BY_FGFR_IN_DISEASE;REACTOME_SIGNALING_BY_FGFR1_MUTANTS;REACTOME_SI

NG_INTEGRIN_ADHESION

POPROTEINS

ME_SIGNALING_BY_EGFR_IN_CANCER;REACTOME_NGF_SIGNALLING_VIA_TRKA_FROM_TH

IG;REACTOME_SIGNALING_BY_EGFR_IN_CANCER;REACTOME_PI3K_EVENTS_IN_ERBB2_SIG

MISSION_IN_THE_POSTSYNAPTIC_CELL;REACTOME_TRAFFICKING_OF_AMPA_RECEPTORS
D_METABOLISM;REACTOME_THE_ACTIVATION_OF_ARYLSULFATASES;REACTOME_METABC

C_AND_MAPK;REACTOME_NRAGE_SIGNALS_DEATH_THROUGH_JNK;REACTOME_CELL_DEA

ION_OF_IKKS_COMPLEX;REACTOME_TRAF6_MEDIATED_NFKB_ACTIVATION;REACTOME_TR

MISSION_IN_THE_POSTSYNAPTIC_CELL;REACTOME_ACTIVATION_OF_KAINATE_RECEPTOR

ISMEMBRANE_TRANSPORT;REACTOME_TRANSPORT_OF_INORGANIC_CATIONS_ANIONS_AI

/ATION_IN_TLR_CASCADE;REACTOME_JNK_C_JUN_KINASES_PHOSPHORYLATION_AND_AC
/ATION_IN_TLR_CASCADE;REACTOME_JNK_C_JUN_KINASES_PHOSPHORYLATION_AND_AC

=R_MUTANTS

LING;REACTOME_PI3K_EVENTS_IN_ERBB4_SIGNALING;REACTOME_SHC1_EVENTS_IN_ERBI

CELL_RECEPTOR_LEADING_TO_GENERATION_OF_SECOND_MESSENGERS;REACTOME_SIG

.1_AND_ANKYRINS;REACTOME_VOLTAGE_GATED_POTASSIUM_CHANNELS;REACTOME_POT

IRATORY_ELECTRON_TRANSPORT;REACTOME_RESPIRATORY_ELECTRON_TRANSPORT_AT

IABETES_PATHWAYS;REACTOME_P130CAS_LINKAGE_TO_MAPK_SIGNALING_FOR_INTEGRI

RELEASE_REUPTAKE_AND_DEGRADATION

;LATIONAL_PROTEIN_MODIFICATION
ACYLGLYCEROL_AND_KETONE_BODY_METABOLISM;REACTOME_SYNTHESIS_OF_VERY_LON

LLING_VIA_TRKA_FROM_THE_PLASMA_MEMBRANE;REACTOME_SIGNALING_BY_FGFR_IN_D

LING;REACTOME_CELL_SURFACE_INTERACTIONS_AT_THE_VASCULAR_WALL;REACTOME_F

;LATIONAL_PROTEIN_MODIFICATION

D_EIFS_AND_SUBSEQUENT_BINDING_TO_43S;REACTOME_METABOLISM_OF_PROTEINS;RE

ONES_BY_ATF6_ALPHA;REACTOME_ACTIVATION_OF_GENES_BY_ATF4;REACTOME_UNFOLD

TOME_CELL_JUNCTION_ORGANIZATION;REACTOME_NEPHRIN_INTERACTIONS;REACTOME_

_REPAIR

DEPENDENT_AXON_REPULSION;REACTOME_SEMAPHORIN_INTERACTIONS;REACTOME_SE

LLING_VIA_TRKA_FROM_THE_PLASMA_MEMBRANE;REACTOME_SIGNALING_BY_FGFR_IN_D
RGANIZATION

RANE;REACTOME_TRANSMEMBRANE_TRANSPORT_OF_SMALL_MOLECULES;REACTOME_SL

MA4D_INDUCED_CELL_MIGRATION_AND_GROWTH_CONE_COLLAPSE

CTIN_RECEPTOR_SIGNALING;REACTOME_NUCLEAR_SIGNALING_BY_ERBB4;REACTOME_SIG

L_II_PRE_TRANSCRIPTION_EVENTS;REACTOME_HIV_INFECTION;REACTOME_HIV_LIFE_CYC

CRIPTION;REACTOME_TGF_BETA_RECEPTOR_SIGNALING_ACTIVATES_SMADS;REACTOME_

RIPTION_PRE_INITIATION_AND_PROMOTER_OPENING;REACTOME_MRNA_PROCESSING;REA
NALING_BY_GPCR;REACTOME_PEPTIDE_LIGAND_BINDING_RECEPTORS;REACTOME_CLASS

;REACTOME_IMMUNE_SYSTEM;REACTOME_CYTOKINE_SIGNALING_IN_IMMUNE_SYSTEM

ESSING_UBIQUITINATION_PROTEASOME_DEGRADATION

PLICING;REACTOME_MRNA_SPLICING_MINOR_PATHWAY;REACTOME_METABOLISM_OF_RNA

OR_BINDING_AND_DOWNSTREAM_TRANSMISSION_IN_THE_POSTSYNAPTIC_CELL;REACTOM

ESSING_UBIQUITINATION_PROTEASOME_DEGRADATION

.1_AND_ANKYRINS;REACTOME_VOLTAGE_GATED_POTASSIUM_CHANNELS;REACTOME_POT

_OF_REPLICATION_INITIATION_OF_DAMAGED_DNA_BY_RB1_E2F1;REACTOME_E2F_MEDIAT
D_METABOLISM;REACTOME_THE_ACTIVATION_OF_ARYLSULFATASES;REACTOME_METABO

EVENTS_KINASE_AND_TRANSCRIPTION_FACTOR_ACTIVATION;REACTOME_ERK_MAPK_TAR
)SIDES_AND_RELATED_MOLECULES

)NS_ANIONS_AND_AMINO_ACIDS_OLIGOPEPTIDES

\TE_DEHYDROGENASE_PDH_COMPLEX;REACTOME_PYRUVATE_METABOLISM
LIPOPROTEINS
;REACTOME_M_G1_TRANSITION;REACTOME_G1_S_TRANSITION;REACTOME_NUCLEOTIDE_
CKPOINTS;REACTOME_P53_DEPENDENT_G1_DNA_DAMAGE_RESPONSE;REACTOME_AUTOI

MISSION_IN_THE_POSTSYNAPTIC_CELL;REACTOME_TRAFFICKING_OF_AMPA_RECEPTORS

_NUCLEUS;REACTOME_SIGNALING_BY_NOTCH4;REACTOME_SIGNALING_BY_NOTCH2;REAC

AT_CONTAINING_RECEPTOR_NLR_SIGNALING_PATHWAYS

HEMOSTASIS;REACTOME_PLATELET_ACTIVATION_SIGNALING_AND_AGGREGATION

_SEMAPHORIN_SIGNALING;REACTOME_SEMAPHORIN_INTERACTIONS;REACTOME_SEMA4D_
TOME_SIGNALING_BY_NOTCH1;REACTOME_SIGNALING_BY_NOTCH3;REACTOME_REGULA

TABOLISM;REACTOME_A_TETRASACCHARIDE_LINKER_SEQUENCE_IS_REQUIRED_FOR_GA

ME_IMMUNE_SYSTEM;REACTOME_ADAPTIVE_IMMUNE_SYSTEM

APHORIN_INTERACTIONS;REACTOME_SEMA4D_INDUCED_CELL_MIGRATION_AND_GROWTH

TOME_CIRCADIAN_CLOCK;REACTOME_TRAF6_MEDIATED_INDUCTION_OF_NFKB_AND_MAP
ME_HEMOSTASIS

GNALING_BY_FGFR1_FUSION_MUTANTS;REACTOME_SIGNALING_BY_FGFR_MUTANTS;REA

HE_PLASMA_MEMBRANE;REACTOME_SIGNALING_BY_FGFR_IN_DISEASE;REACTOME_GAST

GNALING;REACTOME_EGFR_DOWNREGULATION;REACTOME_GAB1_SIGNALOSOME;REACTO

;REACTOME_RAS_ACTIVATION_UOPN_CA2_INFUX_THROUGH_NMDA_RECEPTOR;REACTOM
OLISM_OF_PROTEINS;REACTOME_SPHINGOLIPID_METABOLISM;REACTOME_POST_TRANSL

ATH_SIGNALLING_VIA_NRAGE_NRIF_AND_NADE;REACTOME_P75_NTR_RECEPTOR_MEDIATE

AF6_MEDIATED_INDUCTION_OF_NFKB_AND_MAP_KINASES_UPON_TLR7_8_OR_9_ACTIVATI

RS_UPON_GLUTAMATE_BINDING;REACTOME_IONOTROPIC_ACTIVITY_OF_KAINATE_RECEPT

ND_AMINO_ACIDS_OLIGOPEPTIDES;REACTOME_AMINO_ACID_AND_OLIGOPEPTIDE_SLC_TF

TIVATION_MEDIATED_BY_ACTIVATED_HUMAN_TAK1;REACTOME_ACTIVATED_TAK1_MEDIAT
TIVATION_MEDIATED_BY_ACTIVATED_HUMAN_TAK1;REACTOME_ACTIVATED_TAK1_MEDIAT

B4_SIGNALING;REACTOME_PI3K_EVENTS_IN_ERBB2_SIGNALING;REACTOME_NUCLEAR_SIG

NALING_BY_THE_B_CELL_RECEPTOR_BCR;REACTOME_CELL_SURFACE_INTERACTIONS_AT

TASSIUM_CHANNELS

TP_SYNTHESIS_BY_CHEMIOSMOTIC_COUPLING_AND_HEAT_PRODUCTION_BY_UNCOUPLIN

NS;REACTOME_GRB2_SOS_PROVIDES_LINKAGE_TO_MAPK_SIGNALING_FOR_INTERGRINS_

NG_CHAIN_FATTY_ACYL_COAS

ISEASE;REACTOME_GASTRIN_CREB_SIGNALLING_PATHWAY_VIA_PKC_AND_MAPK;REACT(

PECAM1_INTERACTIONS;REACTOME_PD1_SIGNALING;REACTOME_COSTIMULATION_BY_THE

ACTOME_3_UTR_MEDIATED_TRANSLATIONAL_REGULATION;REACTOME_DEADENYLATION_

ED_PROTEIN_RESPONSE;REACTOME_ACTIVATION_OF_CHAPERONE_GENES_BY_ATF6_AL

HEMOSTASIS;REACTOME_PLATELET_ACTIVATION_SIGNALING_AND_AGGREGATION

MA3A_PLEXIN_REPULSION_SIGNALING_BY_INHIBITING_INTEGRIN_ADHESION

ISEASE;REACTOME_TRANSMISSION_ACROSS_CHEMICAL_SYNAPSES;REACTOME_NEURON

C_MEDIATED_TRANSMEMBRANE_TRANSPORT;REACTOME_TRANSPORT_OF_INORGANIC_C

NALING_BY_FGFR_IN_DISEASE;REACTOME_SIGNALING_BY_FGFR1_MUTANTS;REACTOME_

LE;REACTOME_LATE_PHASE_OF_HIV_LIFE_CYCLE

TRANSCRIPTIONAL_ACTIVITY_OF_SMAD2_SMAD3_SMAD4_HETEROTRIMER;REACTOME_GE

ACTOME_MRNA_SPLICING;REACTOME_MRNA_SPLICING_MINOR_PATHWAY;REACTOME_TRA
S_A1_RHODOPSIN_LIKE_RECEPTORS;REACTOME_GPCR_DOWNSTREAM_SIGNALING;REACT

A

ME_AXON_GUIDANCE;REACTOME_SEMA4D_IN_SEMAPHORIN_SIGNALING;REACTOME_SEMA

TASSIUM_CHANNELS

TED_REGULATION_OF_DNA_REPLICATION
OLISM_OF_PROTEINS;REACTOME_SPHINGOLIPID_METABOLISM;REACTOME_POST_TRANSL

RGETS;REACTOME_MAP_KINASE_ACTIVATION_IN_TLR_CASCADE;REACTOME_MAPK_TARG

_EXCISION_REPAIR;REACTOME_REPAIR_SYNTHESIS_FOR_GAP_FILLING_BY_DNA_POL_IN_T
DEGRADATION_OF_THE_E3_UBIQUITIN_LIGASE_COP1;REACTOME_FACTORS_INVOLVED_IN

TOME_SIGNALING_BY_NOTCH1;REACTOME_SIGNALING_BY_NOTCH3;REACTOME_REGULA

_INDUCED_CELL_MIGRATION_AND_GROWTH_CONE_COLLAPSE
TED_PROTEOLYSIS_OF_P75NTR;REACTOME_NRIF_SIGNALS_CELL_DEATH_FROM_THE_NUC

G_SYNTHESIS;REACTOME_METABOLISM_OF_CARBOHYDRATES

H_CONE_COLLAPSE

_KINASES_UPON_TLR7_8_OR_9_ACTIVATION;REACTOME_NFKB_AND_MAP_KINASES_ACTIV

CTOME_SIGNALING_BY_PDGF;REACTOME_DOWNSTREAM_SIGNAL_TRANSDUCTION;REACT

RIN_CREB_SIGNALLING_PATHWAY_VIA_PKC_AND_MAPK;REACTOME_SIGNALING_BY_GPCR

OME_SHC1_EVENTS_IN_EGFR_SIGNALING;REACTOME_RESPONSE_TO_ELEVATED_PLATELE

ME_ACTIVATION_OF_NMDA_RECEPTOR_UPON_GLUTAMATE_BINDING_AND_POSTSYNAPTIC_
ATIONAL_PROTEIN_MODIFICATION;REACTOME_METABOLISM_OF_LIPIDS_AND_LIPOPROTEI

ED_SIGNALLING;REACTOME_SIGNALING_BY_GPCR;REACTOME_AXON_GUIDANCE;REACTOM

ON;REACTOME_ADVANCED_GLYCOSYLATION_ENDPRODUCT_RECEPTOR_SIGNALING;REAC

TORS

RANSPORTERS

TES_P38_MAPK_ACTIVATION;REACTOME_SIGNALING_BY_ILS;REACTOME_IL1_SIGNALING;RE
TES_P38_MAPK_ACTIVATION;REACTOME_SIGNALING_BY_ILS;REACTOME_IL1_SIGNALING;RE

;NALING_BY_ERBB4

T_THE_VASCULAR_WALL;REACTOME_PLATELET_ADHESION_TO_EXPOSED_COLLAGEN;REA

G_PROTEINS_

_;REACTOME_INTEGRIN_ALPHAIIB_BETA3_SIGNALING;REACTOME_MUSCLE_CONTRACTION

OME_SIGNALING_BY_GPCR;REACTOME_OPIOID_SIGNALLING;REACTOME_CA_DEPENDENT_

E_CD28_FAMILY;REACTOME_SIGNALING_BY_ILS;REACTOME_IL_3_5_AND_GM_CSF_SIGNALI

_OF_MRNA;REACTOME_METABOLISM_OF_MRNA;REACTOME_DEADENYLATION_DEPENDEN

PHA;REACTOME_METABOLISM_OF_LIPIDS_AND_LIPOPROTEINS;REACTOME_FATTY_ACID_

IAL_SYSTEM;REACTOME_SIGNALING_BY_GPCR;REACTOME_INTEGRATION_OF_ENERGY_M

CATIONS_ANIONS_AND_AMINO_ACIDS_OLIGOPEPTIDES;REACTOME_AMINO_ACID_AND_OLI

_SIGNALING_BY_FGFR1_FUSION_MUTANTS;REACTOME_SIGNALING_BY_FGFR_MUTANTS;R

NERIC_TRANSCRIPTION_PATHWAY;REACTOME_SIGNALING_BY_TGF_BETA_RECEPTOR_C

ANSCRIPTION;REACTOME_FORMATION_OF_RNA_POL_II_ELONGATION_COMPLEX_;REACTO
TOME_G_ALPHA_I_SIGNALLING_EVENTS;REACTOME_GPCR_LIGAND_BINDING;REACTOME_I

PHORIN_INTERACTIONS;REACTOME_SEMA3A_PLEXIN_REPULSION_SIGNALING_BY_INHIBIT

ATIONAL_PROTEIN_MODIFICATION;REACTOME_METABOLISM_OF_LIPIDS_AND_LIPOPROTEI

ETS_NUCLEAR_EVENTS_MEDIATED_BY_MAP_KINASES;REACTOME_TRAF6_MEDIATED_INDU

C_NER;REACTOME_SYNTHESIS_OF_DNA;REACTOME_MITOTIC_G1_G1_S_PHASES;REACTO
_MEGAKARYOCYTE_DEVELOPMENT_AND_PLATELET_PRODUCTION;REACTOME_SIGNALING

TED_PROTEOLYSIS_OF_P75NTR;REACTOME_NRIF_SIGNALS_CELL_DEATH_FROM_THE_NUC

CLEUS;REACTOME_CELL_DEATH_SIGNALLING_VIA_NRAGE_NRIF_AND_NADE;REACTOME_P

'ATION_MEDIATED_BY_TLR4_SIGNALING_REPERTOIRE;REACTOME_MYD88_MAL_CASCADE_

'OME_SIGNALING_BY_ILS;REACTOME_REGULATION_OF_IFNG_SIGNALING;REACTOME_INTE

;REACTOME_OPIOID_SIGNALLING;REACTOME_CA_DEPENDENT_EVENTS;REACTOME_PLC_I

ET_CYTOSOLIC_CA2_;REACTOME_HEMOSTASIS;REACTOME_PLATELET_ACTIVATION_SIGNA

_EVENTS;REACTOME_CREB_PHOSPHORYLATION_THROUGH_THE_ACTIVATION_OF_RAS;RE
NS

ME_G_ALPHA_Q_SIGNALLING_EVENTS;REACTOME_G_ALPHA1213_SIGNALLING_EVENTS;REA

CTOME_NFKB_AND_MAP_KINASES_ACTIVATION_MEDIATED_BY_TLR4_SIGNALING_REPERTO

EACTOME_TRAF6_MEDIATED_INDUCTION_OF_NFKB_AND_MAP_KINASES_UPON_TLR7_8_OR
EACTOME_TRAF6_MEDIATED_INDUCTION_OF_NFKB_AND_MAP_KINASES_UPON_TLR7_8_OR

ACTOME_PECAM1_INTERACTIONS;REACTOME_GPVI_MEDIATED_ACTIVATION_CASCADE;REA

;REACTOME_AXON_GUIDANCE;REACTOME_ACTIVATION_OF_CHAPERONE_GENES_BY_XBP

_EVENTS;REACTOME_PLC_BETA_MEDIATED_EVENTS;REACTOME_SIGNALING_BY_PDGF;REA

NG;REACTOME_PLATELET_HOMEOSTASIS;REACTOME_PLATELET_SENSITIZATION_BY_LDL

T_MRNA_DECAY;REACTOME_METABOLISM_OF_RNA;REACTOME_PKB_MEDIATED_EVENTS;F

TRIACYLGLYCEROL_AND_KETONE_BODY_METABOLISM

ETABOLISM;REACTOME_OPIOID_SIGNALLING;REACTOME_CA_DEPENDENT_EVENTS;REACT

GOPEPTIDE_SLC_TRANSPORTERS;REACTOME_HEMOSTASIS

EACTOME_SIGNALING_BY_PDGF;REACTOME_DOWNSTREAM_SIGNAL_TRANSDUCTION;REA

OMPLEX

OME_ELONGATION_ARREST_AND_RECOVERY;REACTOME_RNA_POL_II_PRE_TRANSCRIPTIC
NNATE_IMMUNE_SYSTEM;REACTOME_IMMUNE_SYSTEM;REACTOME_COMPLEMENT_CASC/

ING_INTEGRIN_ADHESION;REACTOME_ACTIVATION_OF_NMDA_RECEPTOR_UPON_GLUTAM

NS

JCTION_OF_NFKB_AND_MAP_KINASES_UPON_TLR7_8_OR_9_ACTIVATION;REACTOME_NFKB

ME_MITOTIC_M_M_G1_PHASES;REACTOME_DNA_REPAIR;REACTOME_CHROMOSOME_MAI
G_BY_NOTCH;REACTOME_APOPTOSIS;REACTOME_HEMOSTASIS;REACTOME_ACTIVATION_

CLEUS;REACTOME_CELL_DEATH_SIGNALLING_VIA_NRAGE_NRIF_AND_NADE;REACTOME_P

75_NTR_RECEPTOR_MEDIATED_SIGNALLING;REACTOME_SIGNALING_BY_NOTCH

_INITIATED_ON_PLASMA_MEMBRANE;REACTOME_INNATE_IMMUNE_SYSTEM;REACTOME_A[illegible]

[illegible]RFERON_GAMMA_SIGNALING;REACTOME_INTERFERON_ALPHA_BETA_SIGNALING;REACT[illegible]

[illegible]BETA_MEDIATED_EVENTS;REACTOME_SIGNALING_BY_PDGF;REACTOME_DOWNSTREAM_S[illegible]

[illegible]LING_AND_AGGREGATION

EACTOME_POST_NMDA_RECEPTOR_ACTIVATION_EVENTS;REACTOME_UNBLOCKING_OF_N

ACTOME_GPCR_DOWNSTREAM_SIGNALING;REACTOME_NETRIN1_SIGNALING;REACTOME_

OIRE;REACTOME_RIG_I_MDA5_MEDIATED_INDUCTION_OF_IFN_ALPHA_BETA_PATHWAYS;RI

R_9_ACTIVATION;REACTOME_NFKB_AND_MAP_KINASES_ACTIVATION_MEDIATED_BY_TLR4_
R_9_ACTIVATION;REACTOME_NFKB_AND_MAP_KINASES_ACTIVATION_MEDIATED_BY_TLR4_

ACTOME_CD28_CO_STIMULATION;REACTOME_COSTIMULATION_BY_THE_CD28_FAMILY;RE/

1S;REACTOME_UNFOLDED_PROTEIN_RESPONSE;REACTOME_SEMAPHORIN_INTERACTION

ACTOME_DOWNSTREAM_SIGNAL_TRANSDUCTION;REACTOME_G_ALPHA_Q_SIGNALLING_E

;REACTOME_IL_RECEPTOR_SHC_SIGNALING;REACTOME_SIGNAL_REGULATORY_PROTEIN_

REACTOME_SIGNALING_BY_INSULIN_RECEPTOR;REACTOME_MTORC1_MEDIATED_SIGNALL

'OME_ADENYLATE_CYCLASE_ACTIVATING_PATHWAY;REACTOME_ADENYLATE_CYCLASE_I

CTOME_SIGNALING_BY_ILS;REACTOME_IL_3_5_AND_GM_CSF_SIGNALING;REACTOME_IL_2

ON_EVENTS;REACTOME_INFLUENZA_LIFE_CYCLE;REACTOME_INFLUENZA_VIRAL_RNA_TRAI
ADE;REACTOME_ADAPTIVE_IMMUNE_SYSTEM;REACTOME_INITIAL_TRIGGERING_OF_COMP

MATE_BINDING_AND_POSTSYNAPTIC_EVENTS;REACTOME_CREB_PHOSPHORYLATION_THR

3_AND_MAP_KINASES_ACTIVATION_MEDIATED_BY_TLR4_SIGNALING_REPERTOIRE;REACTO

NTENANCE;REACTOME_GLOBAL_GENOMIC_NER_GG_NER;REACTOME_DNA_REPLICATION;
OF_BH3_ONLY_PROTEINS;REACTOME_INTRINSIC_PATHWAY_FOR_APOPTOSIS

75_NTR_RECEPTOR_MEDIATED_SIGNALLING;REACTOME_SIGNALING_BY_NOTCH

CTIVATED_TLR4_SIGNALLING;REACTOME_IMMUNE_SYSTEM;REACTOME_TOLL_RECEPTOR_

OME_REGULATION_OF_IFNA_SIGNALING;REACTOME_INTERFERON_SIGNALING;REACTOME_

IGNAL_TRANSDUCTION;REACTOME_G_ALPHA_Q_SIGNALLING_EVENTS;REACTOME_GPCR_

IMDA_RECEPTOR_GLUTAMATE_BINDING_AND_ACTIVATION;REACTOME_CREB_PHOSPHORY

DCC_MEDIATED_ATTRACTIVE_SIGNALING

EACTOME_MYD88_MAL_CASCADE_INITIATED_ON_PLASMA_MEMBRANE;REACTOME_INNATE

_SIGNALING_REPERTOIRE;REACTOME_MYD88_MAL_CASCADE_INITIATED_ON_PLASMA_MEI
_SIGNALING_REPERTOIRE;REACTOME_MYD88_MAL_CASCADE_INITIATED_ON_PLASMA_MEI

ACTOME_CTLA4_INHIBITORY_SIGNALING;REACTOME_SIGNALING_BY_ILS;REACTOME_REG[illegible]

S;REACTOME_SEMA3A_PLEXIN_REPULSION_SIGNALING_BY_INHIBITING_INTEGRIN_ADHES

EVENTS;REACTOME_GPCR_DOWNSTREAM_SIGNALING;REACTOME_DOWNSTREAM_SIGNAL

_SIRP_FAMILY_INTERACTIONS;REACTOME_REGULATION_OF_IFNG_SIGNALING;REACTOME_

LING;REACTOME_PI3K_CASCADE

NHIBITORY_PATHWAY;REACTOME_NEUROTRANSMITTER_RECEPTOR_BINDING_AND_DOWN

_SIGNALING;REACTOME_IMMUNE_SYSTEM;REACTOME_CYTOKINE_SIGNALING_IN_IMMUNE

\NSCRIPTION_AND_REPLICATION;REACTOME_HIV_INFECTION;REACTOME_HIV_LIFE_CYCLE;
LEMENT

OUGH_THE_ACTIVATION_OF_RAS;REACTOME_POST_NMDA_RECEPTOR_ACTIVATION_EVEI

OME_MYD88_MAL_CASCADE_INITIATED_ON_PLASMA_MEMBRANE;REACTOME_INNATE_IMM

REACTOME_TELOMERE_MAINTENANCE;REACTOME_EXTENSION_OF_TELOMERES;REACTO

_CASCADES

_IL_6_SIGNALING;REACTOME_IMMUNE_SYSTEM;REACTOME_CYTOKINE_SIGNALING_IN_IMM

_DOWNSTREAM_SIGNALING;REACTOME_G_ALPHA_Z_SIGNALLING_EVENTS;REACTOME_ME

YLATION_THROUGH_THE_ACTIVATION_OF_CAMKII;REACTOME_INTERFERON_GAMMA_SIGN

E_IMMUNE_SYSTEM;REACTOME_ACTIVATED_TLR4_SIGNALLING;REACTOME_IMMUNE_SYST

MBRANE;REACTOME_INNATE_IMMUNE_SYSTEM;REACTOME_ACTIVATED_TLR4_SIGNALLING
MBRANE;REACTOME_INNATE_IMMUNE_SYSTEM;REACTOME_ACTIVATED_TLR4_SIGNALLING

JLATION_OF_SIGNALING_BY_CBL;REACTOME_IL_3_5_AND_GM_CSF_SIGNALING;REACTOMI

ION;REACTOME_SMOOTH_MUSCLE_CONTRACTION;REACTOME_PLATELET_AGGREGATION_

.ING_OF_ACTIVATED_FGFR;REACTOME_PHOSPHOLIPASE_C_MEDIATED_CASCADE;REACTC

_INTERFERON_GAMMA_SIGNALING;REACTOME_INTERFERON_ALPHA_BETA_SIGNALING;RE

NSTREAM_TRANSMISSION_IN_THE_POSTSYNAPTIC_CELL;REACTOME_PLC_BETA_MEDIATEI

_SYSTEM

;REACTOME_ABORTIVE_ELONGATION_OF_HIV1_TRANSCRIPT_IN_THE_ABSENCE_OF_TAT;R

NTS

UNE_SYSTEM;REACTOME_ACTIVATED_TLR4_SIGNALLING;REACTOME_IMMUNE_SYSTEM;RE

ME_S_PHASE

IUNE_SYSTEM

TABOLISM_OF_MRNA;REACTOME_METABOLISM_OF_RNA;REACTOME_DOWNSTREAM_SIGN

JALING;REACTOME_INTERFERON_SIGNALING;REACTOME_IMMUNE_SYSTEM;REACTOME_C[illegible]

EM;REACTOME_TOLL_RECEPTOR_CASCADES

;;REACTOME_IMMUNE_SYSTEM;REACTOME_TOLL_RECEPTOR_CASCADES;REACTOME_NO[illegible]
;;REACTOME_IMMUNE_SYSTEM;REACTOME_TOLL_RECEPTOR_CASCADES;REACTOME_NO[illegible]

E_HEMOSTASIS;REACTOME_IMMUNE_SYSTEM;REACTOME_ADAPTIVE_IMMUNE_SYSTEM;RE

_PLUG_FORMATION;REACTOME_HEMOSTASIS;REACTOME_PLATELET_ACTIVATION_SIGNAL

)ME_SIGNALING_BY_FGFR

ACTOME_REGULATION_OF_IFNA_SIGNALING;REACTOME_INTERFERON_SIGNALING;REACT(

D_EVENTS;REACTOME_PKA_MEDIATED_PHOSPHORYLATION_OF_CREB;REACTOME_TRANS

REACTOME_FORMATION_OF_THE_HIV1_EARLY_ELONGATION_COMPLEX;REACTOME_VIRAL_

EACTOME_TOLL_RECEPTOR_CASCADES

IALING_OF_ACTIVATED_FGFR;REACTOME_PHOSPHOLIPASE_C_MEDIATED_CASCADE;REAC

YTOKINE_SIGNALING_IN_IMMUNE_SYSTEM

D1_2_SIGNALING_PATHWAY;REACTOME_CYTOKINE_SIGNALING_IN_IMMUNE_SYSTEM;REAC
D1_2_SIGNALING_PATHWAY;REACTOME_CYTOKINE_SIGNALING_IN_IMMUNE_SYSTEM;REAC

:ACTOME_CYTOKINE_SIGNALING_IN_IMMUNE_SYSTEM;REACTOME_PLATELET_ACTIVATION

.ING_AND_AGGREGATION

OME_IL_2_SIGNALING;REACTOME_HEMOSTASIS;REACTOME_IMMUNE_SYSTEM;REACTOME_

SMEMBRANE_TRANSPORT_OF_SMALL_MOLECULES;REACTOME_GLUCAGON_SIGNALING_IN

_MESSENGER_RNA_SYNTHESIS;REACTOME_LATE_PHASE_OF_HIV_LIFE_CYCLE

:TOME_EFFECTS_OF_PIP2_HYDROLYSIS;REACTOME_REGULATION_OF_MRNA_STABILITY_B

:TOME_NUCLEOTIDE_BINDING_DOMAIN_LEUCINE_RICH_REPEAT_CONTAINING_RECEPTOR_
:TOME_NUCLEOTIDE_BINDING_DOMAIN_LEUCINE_RICH_REPEAT_CONTAINING_RECEPTOR_

[illegible]_SIGNALING_AND_AGGREGATION

_ADAPTIVE_IMMUNE_SYSTEM;REACTOME_CYTOKINE_SIGNALING_IN_IMMUNE_SYSTEM

_METABOLIC_REGULATION;REACTOME_SIGNALING_BY_PDGF;REACTOME_DOWNSTREAM_

Y_PROTEINS_THAT_BIND_AU_RICH_ELEMENTS;REACTOME_INTERFERON_GAMMA_SIGNAL

_NLR_SIGNALING_PATHWAYS
_NLR_SIGNALING_PATHWAYS

_SIGNAL_TRANSDUCTION;REACTOME_GPCR_DOWNSTREAM_SIGNALING;REACTOME_G_AL

.ING;REACTOME_INTERFERON_SIGNALING;REACTOME_APOPTOSIS;REACTOME_HEMOSTAS

PHA_I_SIGNALLING_EVENTS;REACTOME_G_ALPHA_S_SIGNALLING_EVENTS;REACTOME_G_

SIS;REACTOME_IMMUNE_SYSTEM;REACTOME_CYTOKINE_S

_ALPHA_Z_SIGNALLING_EVENTS;REACTOME_DOWNSTREAM_SIG
