# Supplementary material for: Genomic alterations caused by HPV integration in a cohort of Chinese endocervical adenocarcinomas
Source: Cancer Gene Ther. 2021 Jan 4;28(12):1353–64. doi: 10.1038/s41417-020-00283-4 (PMC8636260; doi:10.1038/s41417-020-00283-4)
Supplement: Supplementary file 10 — Supplementary Table 9 [file 41417_2020_283_MOESM10_ESM.docx]

Supplementary Table 9

CNVs identified in 20 cases of cervical adenocarcinoma.

CNV, Copy number variation.

| **CNV** | **Cytoband** | **Wide Peak Boundaries** | **Q values** | **Residual q values** | **Percent** |
| --- | --- | --- | --- | --- | --- |
| Amplication | 3q27.1 | chr3:164412001-198022430 | 8.62E-05 | 8.62E-05 | 30% |
| Amplication | 12q11 | chr12:34779001-38249999 | 0.017509 | 0.017509 | 30% |
| Amplication | 19q11 | chr19:24383001-28124999 | 0.054242 | 0.054242 | 35% |
| Amplication | 19q13.11 | chr19:33465001-33571999 | 0.054242 | 0.054242 | 20% |
| Amplication | 3p11.1 | chr3:90310001-93518999 | 0.17566 | 0.17566 | 20% |
| Amplication | 5p11 | chr5:45909001-49552999 | 0.17566 | 0.17566 | 20% |
| Amplication | 8q24.21 | chr8:128836001-128932999 | 0.17566 | 0.17566 | 15% |
| Amplication | 11p11.12 | chr11:50474001-55025999 | 0.17566 | 0.17566 | 15% |
| Amplication | 13q22.1 | chr13:73355001-73832999 | 0.17566 | 0.17566 | 20% |
| Amplication | 16p11.1 | chr16:35182001-46437999 | 0.17566 | 0.17566 | 20% |
| Amplication | 19p12 | chr19:21046001-21053999 | 0.17566 | 0.17566 | 25% |
| Amplication | 20q13.2 | chr20:52084001-52420999 | 0.17566 | 0.17566 | 30% |
| Deletion | 12p13.31 | chr12:5604001-13153999 | 0.017498 | 0.017498 | 30% |
| Deletion | 1p36.22 | chr1:1-35135999 | 0.12185 | 0.12185 | 30% |
| Deletion | 2q37.1 | chr2:213403001-243199373 | 0.12185 | 0.12185 | 35% |
| Deletion | 9q34.3 | chr9:137029001-139440999 | 0.12185 | 0.12185 | 20% |
| Deletion | 12p12.3 | chr12:1-38710999 | 0.12185 | 0.12185 | 20% |
| Deletion | 19q13.2 | chr19:42192001-42300999 | 0.12185 | 0.12185 | 20% |
| Deletion | 20p11.1 | chr20:5485001-47988999 | 0.12185 | 0.12185 | 15% |
