# Supplementary material for: Genomic alterations caused by HPV integration in a cohort of Chinese endocervical adenocarcinomas
Source: Cancer Gene Ther. 2021 Jan 4;28(12):1353–64. doi: 10.1038/s41417-020-00283-4 (PMC8636260; doi:10.1038/s41417-020-00283-4)
Supplement: Supplementary file 11 — Supplementary Table 10 [file 41417_2020_283_MOESM11_ESM.pdf]

Supplementary Table 10
Detail of CNVs detected in each case by whole genome sequencing.

Patient	chro	Start	End	Ref	Alt	GeneName	Func	Gene	GeneDetai	ExonicFun	AAChange	Gencode	cpgIslandE	cytoBand
ACa01	1	85860000	85870000	0	0	DDAH1	intronic	NM_00113	.	.	.	ENST0000	.	1p22.3
ACa01	1	2.32E+08	2.32E+08	0	0	TSNAX-DI	exonic	NM_00101	.	.	.	ENST0000	.	1q42.2
ACa01	2	30810000	30830000	0	0	LCLAT1	intronic	NM_0010C	.	.	.	ENST0000	.	2p23.1
ACa01	2	63400000	63410000	0	0	WDPCP	exonic	NM_00104	.	.	.	ENST0000	.	2p15
ACa01	2	1.61E+08	1.61E+08	0	0	ITGB6	exonic	NM_00088	.	.	.	ENST0000	.	2q24.2
ACa01	2	1.98E+08	1.98E+08	0	0	RFTN2	exonic	NM_14462	.	.	.	ENST0000	.	2q33.1
ACa01	3	0	230000	0	0	.	ncRNA_ex	NR_11082	.	.	.	ENST0000	.	3p26.3
ACa01	3	230000	32240000	0	0	C3orf83,LI	exonic	NM_00006	.	.	.	ENST0000	CpG: 22,C	3p25.2-p25
ACa01	3	32240000	32280000	0	0	.	upstream	NM_17886	.	.	.	ENST0000	CpG: 118	3p22.3
ACa01	3	32280000	33270000	0	0	CMTM7,CI	exonic	NM_0004C	.	.	.	ENST0000	CpG: 118,(	3p22.3
ACa01	3	33270000	33310000	0	0	.	intergenic	NM_01555	dist=9293;(	.	.	.	.	3p22.3
ACa01	3	33310000	33360000	0	0	FBXL2	exonic	NM_00117	.	.	.	ENST0000	CpG: 60	3p22.3
ACa01	3	33360000	49040000	0	0	ATRIP,AC'	exonic	NM_00009	.	.	.	ENST0000	CpG: 104,(	3p22.1-p21
ACa01	4	5980000	48550000	0	0	TLR1,NKX	exonic	NM_00008	.	.	.	ENST0000	CpG: 181,(	4p15.32-p1
ACa01	5	0	140000	0	0	.	upstream	NM_0529C	.	.	.	ENST0000	CpG: 87,C	5p15.33
ACa01	5	140000	31460000	0	0	LOC40117	exonic	NM_0010C	.	.	.	ENST0000	CpG: 47,C	5p13.3-p15
ACa01	5	31460000	41190000	0	0	GOLPH3,L	exonic	NM_00006	.	.	.	ENST0000	CpG: 187,(	5p13.3-p13
ACa01	5	41190000	43510000	0	0	SEPP1,C5	exonic	NM_00006	.	.	.	ENST0000	CpG: 116,(	5p13.1-p12
ACa01	5	43510000	43530000	0	0	C5orf34,P/	exonic	NM_00645	.	.	.	ENST0000	CpG: 17	5p12
ACa01	5	43530000	43550000	0	0	PAIP1	exonic	NM_00645	.	.	.	ENST0000	.	5p12
ACa01	5	43550000	45700000	0	0	FGF10-AS	exonic	NM_00446	.	.	.	ENST0000	CpG: 139,(	5p12
ACa01	6	390000	57260000	0	0	PSORS1C	exonic	NM_00006	.	.	.	ENST0000	CpG: 96,C	6p24.1-p24
ACa01	7	27700000	27710000	0	0	HIBADH	exonic	NM_15274	.	.	.	ENST0000	CpG: 66	7p15.2
ACa01	7	43360000	43450000	0	0	HECW1	exonic	NM_00128	.	.	.	ENST0000	.	7p13
ACa01	7	1.07E+08	1.07E+08	0	0	COG5	exonic	NM_00116	.	.	.	ENST0000	.	7q22.3
ACa01	7	1.35E+08	1.35E+08	0	0	CALD1	exonic	NM_00434	.	.	.	ENST0000	.	7q33
ACa01	8	0	150000	0	0	OR4F21	exonic	NM_0010C	.	.	.	ENST0000	CpG: 109	8p23.3
ACa01	8	150000	170000	0	0	RPL23AP5	ncRNA_ex	NR_00357	.	.	.	ENST0000	.	8p23.3
ACa01	9	14630000	14640000	0	0	ZDHHC21	exonic	NM_17856	.	.	.	ENST0000	.	9p22.3
ACa01	9	36820000	36830000	0	0	MIR4475	ncRNA_ex	NR_03968	.	.	.	ENST0000	.	9p13.2
ACa01	11	61340000	61350000	0	0	SYT7	exonic	NM_00125	.	.	.	ENST0000	CpG: 100	11q12.2
ACa01	12	1.18E+08	1.18E+08	0	0	TESC	exonic	NM_00116	.	.	.	ENST0000	CpG: 108	12q24.22
ACa01	13	25540000	25550000	0	0	TPTE2P1	ncRNA_ex	NR_02673	.	.	.	ENST0000	.	13q12.13

ACa01	15	0	20160000	0	0	.	intergenic	NONE,NR_	dist=NONE	.	.	ENST0000	CpG: 55,C	15p13-p11
ACa01	15	20160000	21970000	0	0	POTEB2,C	exonic	NM_00114	.	.	.	ENST0000	CpG: 81,C	15q11.2-q1
ACa01	15	22670000	25310000	0	0	SNORD11	exonic	NM_00100	.	.	.	ENST0000	CpG: 49,C	15q11.2
ACa01	15	25310000	25410000	0	0	SNORD11	ncRNA_ex	NR_00129	.	.	.	ENST0000	.	15q11.2
ACa01	15	25410000	30270000	0	0	SNORD10	exonic	NM_00027	.	.	.	ENST0000	CpG: 32,C	15q13.1-q1
ACa01	15	30270000	30370000	0	0	.	intergenic	NM_00130	dist=8998;(	.	.	ENST0000	CpG: 45	15q13.2-q1
ACa01	15	30370000	30390000	0	0	GOLGA8J	exonic	NM_00128	.	.	.	ENST0000	.	15q13.2
ACa01	15	30390000	30520000	0	0	DKFZP434	ncRNA_ex	NR_02677	.	.	.	ENST0000	CpG: 74,C	15q13.2
ACa01	15	30520000	30650000	0	0	.	intergenic	NR_02677	dist=13257	.	.	ENST0000	.	15q13.2
ACa01	15	30650000	30890000	0	0	ULK4P2,C	exonic	NM_00128	.	.	.	ENST0000	CpG: 73,C	15q13.2
ACa01	15	30890000	32330000	0	0	MTMR10,F	exonic	NM_00074	.	.	.	ENST0000	CpG: 18,C	15q13.2-q1
ACa01	15	32330000	32390000	0	0	CHRNA7	intronic	NM_00074	.	.	.	ENST0000	.	15q13.3
ACa01	15	32390000	32790000	0	0	ULK4P1,U	exonic	NM_00074	.	.	.	ENST0000	CpG: 46,C	15q13.3
ACa01	15	32790000	32810000	0	0	.	intergenic	NM_00127	dist=42165	.	.	.	.	15q13.3
ACa01	15	32810000	36950000	0	0	GOLGA8A	exonic	NM_00102	.	.	.	ENST0000	CpG: 115,(	15q13.3-q1
ACa01	15	36950000	37020000	0	0	C15orf41	exonic	NM_00113	.	.	.	ENST0000	.	15q14
ACa01	15	37020000	38640000	0	0	MEIS2,C1!	exonic	NM_00113	.	.	.	ENST0000	CpG: 36,C	15q14
ACa01	15	38640000	38800000	0	0	RASGRP1	exonic	NM_00112	.	.	.	ENST0000	CpG: 42	15q14
ACa01	15	38800000	64500000	0	0	GANC,LDI	exonic	NM_00007	.	.	.	ENST0000	CpG: 31,C	15q21.1-q1
ACa01	15	64500000	67630000	0	0	MAP2K1,S	exonic	NM_00096	.	.	.	ENST0000	CpG: 89,C	15q22.33-(
ACa01	15	67630000	67840000	0	0	C15orf61,I	exonic	NM_00103	.	.	.	ENST0000	CpG: 103,(	15q23
ACa01	15	67840000	68470000	0	0	PIAS1,MAI	exonic	NM_00120	.	.	.	ENST0000	CpG: 112,(	15q23
ACa01	15	68470000	1.02E+08	0	0	MESDC1,F	exonic	NM_00005	.	.	.	ENST0000	CpG: 17,C	15q26.1-q2
ACa01	15	1.02E+08	1.02E+08	0	0	TARSL2	exonic	NM_15233	.	.	.	ENST0000	CpG: 82	15q26.3
ACa01	15	1.02E+08	1.03E+08	0	0	OR4F15,O	exonic	NM_00100	.	.	.	ENST0000	CpG: 806,(	15q26.3
ACa01	15	1.03E+08	1.03E+08	0	0	.	upstream	NR_03409	.	.	.	.	.	15q26.3
ACa01	16	0	60000	0	0	.	intergenic	NONE,NR_	dist=NONE	.	.	.	.	16p13.3
ACa01	16	60000	260000	0	0	DDX11L10	exonic	NM_00051	.	.	.	ENST0000	CpG: 68,C	16p13.3
ACa01	16	260000	270000	0	0	LUC7L	intronic	NM_01803	.	.	.	ENST0000	.	16p13.3
ACa01	16	270000	2380000	0	0	MRPL28,P	exonic	NM_00029	.	.	.	ENST0000	CpG: 39,C	16p13.3
ACa01	16	2380000	2470000	0	0	ABCA17P,	ncRNA_ex	NR_00357	.	.	.	ENST0000	CpG: 247	16p13.3
ACa01	16	2470000	2500000	0	0	CCNF,AB(	exonic	NM_00176	.	.	.	ENST0000	CpG: 146	16p13.3
ACa01	16	2500000	2540000	0	0	MIR6768,C	exonic	NM_00176	.	.	.	ENST0000	CpG: 108,(	16p13.3
ACa01	16	2540000	14790000	0	0	MIR4718,N	exonic	NM_00024	.	.	.	ENST0000	CpG: 59,C	16p13.3-p1
ACa01	16	14790000	14800000	0	0	.	intergenic	NM_00356	dist=1474;(	.	.	ENST0000	CpG: 70	16p13.12

ACa01	16	14800000	15100000	0	0	ABCC6P2,	exonic	NM_00127	.	.	.	ENST0000	CpG:	69,C	16p13.12-p
ACa01	16	15100000	15120000	0	0	PDXDC1	exonic	NM_00128	.	.	.	ENST0000	.		16p13.11
ACa01	16	15120000	16360000	0	0	MYH11,KI/	exonic	NM_00100	.	.	.	ENST0000	CpG:	74,C	16p13.11
ACa01	16	16360000	18830000	0	0	XYLT1,MIF	exonic	NM_00100	.	.	.	ENST0000	CpG:	140,(	16p12.3-p1
ACa01	16	18830000	18840000	0	0	SMG1	exonic	NM_01509	.	.	.	ENST0000	.		16p12.3
ACa01	16	20420000	20480000	0	0	ACSM5,AC	exonic	NM_00101	.	.	.	ENST0000	.		16p12.3
ACa01	16	20480000	21400000	0	0	ERI2,ACSI	exonic	NM_00101	.	.	.	ENST0000	CpG:	46,C	16p12.2-p1
ACa01	16	21400000	21500000	0	0	NPIPB3,LC	exonic	NM_13046	.	.	.	ENST0000	CpG:	26,C	16p12.2
ACa01	16	21500000	21510000	0	0	LOC10027	ncRNA_int	NR_02715	.	.	.	.	.		16p12.2
ACa01	16	21510000	21740000	0	0	IGSF6,SL(	exonic	NM_00107	.	.	.	ENST0000	CpG:	108,(	16p12.2
ACa01	16	21740000	21930000	0	0	OTOA,RRI	exonic	NM_00116	.	.	.	ENST0000	CpG:	25,C	16p12.2
ACa01	16	21930000	21940000	0	0	.	intergenic	NR_00337	dist=99505	.	.	.	.		16p12.2
ACa01	16	21940000	22340000	0	0	VWA3A,P(	exonic	NM_00116	.	.	.	ENST0000	CpG:	115,(	16p12.2
ACa01	16	22340000	22360000	0	0	CDR2,POL	exonic	NM_00125	.	.	.	ENST0000	.		16p12.2
ACa01	16	22360000	22630000	0	0	LOC65378	exonic	NM_00113	.	.	.	ENST0000	CpG:	26,C	16p12.2
ACa01	16	22630000	22820000	0	0	MIR548AA	ncRNA_ex	NR_03038	.	.	.	ENST0000	.		16p12.2
ACa01	16	22820000	28200000	0	0	AQP8,HS3	exonic	NM_00033	.	.	.	ENST0000	CpG:	97,C	16p12.1-p1
ACa01	16	28200000	28340000	0	0	XPO6,SBK	exonic	NM_00102	.	.	.	ENST0000	CpG:	63,C	16p11.2
ACa01	16	28340000	28790000	0	0	NUPR1,SU	exonic	NM_00008	.	.	.	ENST0000	CpG:	43,C	16p11.2
ACa01	16	28790000	28830000	0	0	.	intergenic	NM_00128	dist=5856;(	.	.	ENST0000	.		16p11.2
ACa01	16	28830000	29350000	0	0	LOC10028	exonic	NM_00101	.	.	.	ENST0000	CpG:	90,C	16p11.2
ACa01	16	29350000	29360000	0	0	SNX29P2	ncRNA_int	NR_00293	.	.	.	ENST0000	.		16p11.2
ACa01	16	29360000	29560000	0	0	SLX1A-SU	exonic	NM_00101	.	.	.	ENST0000	CpG:	26,C	16p11.2
ACa01	16	29560000	29910000	0	0	SPN,KIF22	exonic	NM_00103	.	.	.	ENST0000	CpG:	109,(	16p11.2
ACa01	16	29910000	30250000	0	0	ASPHD1,T	exonic	NM_00003	.	.	.	ENST0000	CpG:	77,C	16p11.2
ACa01	16	30250000	32020000	0	0	CLUHP3,Z	exonic	NM_00029	.	.	.	ENST0000	CpG:	112,(	16p11.2
ACa01	16	32020000	32170000	0	0	.	intergenic	NM_00341	dist=91371	.	.	ENST0000	CpG:	147,(	16p11.2
ACa01	16	32170000	32490000	0	0	HERC2P4,	exonic	NM_00124	.	.	.	ENST0000	CpG:	64,C	16p11.2
ACa01	16	32490000	32680000	0	0	.	intergenic	NR_03386	dist=18869	.	.	ENST0000	CpG:	36,C	16p11.2
ACa01	16	32680000	33970000	0	0	RNU6-76P	exonic	NM_00109	.	.	.	ENST0000	CpG:	128,(	16p11.2
ACa01	18	25750000	25760000	0	0	CDH2	exonic	NM_00179	.	.	.	ENST0000	CpG:	223	18q12.1
ACa01	19	30930000	33080000	0	0	PDCD5,DF	exonic	NM_00113	.	.	.	ENST0000	CpG:	131,(	19q13.11-(
ACa01	19	33080000	33990000	0	0	RGS9BP,N	exonic	NM_00028	.	.	.	ENST0000	CpG:	40,C	19q13.11
ACa01	19	33990000	34930000	0	0	GPI,KCTD	exonic	NM_00017	.	.	.	ENST0000	CpG:	71,C	19q13.11
ACa01	19	34930000	36400000	0	0	AC002511	exonic	NM_0070	.	.	.	ENST0000	CpG:	37,C	19q13.12-(

ACa01	19	36400000	36430000	0	0	LRFN3	UTR5	NM_0245C	NM_0245C	.	.	ENST0000	CpG:	55,C	19q13.12
ACa01	19	36430000	36700000	0	0	ALKBH6,P	exonic	NM_0010C	.	.	.	ENST0000	CpG:	39,C	19q13.12
ACa01	19	36700000	36720000	0	0	ZNF565,ZI	UTR5	NM_00104	NM_00104	.	.	ENST0000	CpG:	75	19q13.12
ACa01	19	36720000	38630000	0	0	ZNF850,L(	exonic	NM_00101	.	.	.	ENST0000	CpG:	57,C	19q13.12-(
ACa01	19	38630000	39530000	0	0	SIPA1L3,F	exonic	NM_00054	.	.	.	ENST0000	CpG:	26,C	19q13.13-(
ACa01	19	39530000	39570000	0	0	.	intergenic	NM_17882	dist=6802;(	.	.	ENST0000	.		19q13.2
ACa01	19	39570000	39910000	0	0	PAK4,SAN	exonic	NM_0010C	.	.	.	ENST0000	CpG:	44,C	19q13.2
ACa01	19	39910000	40510000	0	0	LGALS17A	exonic	NM_0010C	.	.	.	ENST0000	CpG:	57,C	19q13.2
ACa01	19	42770000	43430000	0	0	PRR19,TM	exonic	NM_00102	.	.	.	ENST0000	CpG:	102,(	19q13.2-q1
ACa01	20	1560000	1590000	0	0	SIRPB1	exonic	NM_00113	.	.	.	ENST0000	.		20p13
ACa01	21	22580000	22590000	0	0	NCAM2	intronic	NM_00454	.	.	.	ENST0000	.		21q21.1
ACa01	21	35490000	35520000	0	0	MRPS6	exonic	NM_03247	.	.	.	ENST0000	.		21q22.11
ACa01	22	19260000	19280000	0	0	CLTCL1	exonic	NM_00183	.	.	.	ENST0000	CpG:	88	22q11.21
ACa01	22	26350000	26360000	0	0	MYO18B	exonic	NM_0326C	.	.	.	ENST0000	.		22q12.1
ACa01	22	38770000	38780000	0	0	LOC40092	ncRNA_ex	NR_00282	.	.	.	ENST0000	.		22q13.1
ACa01	22	47020000	47040000	0	0	GRAMD4	exonic	NM_01512	.	.	.	ENST0000	CpG:	33,C	22q13.31
ACa01	X	43510000	43560000	0	0	MAOA	exonic	NM_00024	.	.	.	ENST0000	CpG:	52,C	Xp11.3
ACa01	X	1.09E+08	1.1E+08	0	0	GUCY2F,A	exonic	NM_00102	.	.	.	ENST0000	CpG:	39,C	Xq23-Xq22
ACa02	1	0	10000	0	0	.	intergenic	NONE,NR_	dist=NONE	.	.	.	.		1p36.33
ACa02	1	10000	40000	0	0	WASH7P,I	ncRNA_ex	NR_02454	.	.	.	ENST0000	CpG:	116	1p36.33
ACa02	1	1.63E+08	1.63E+08	0	0	RGS5	ncRNA_ex	NR_11069	.	.	.	ENST0000	.		1q23.3
ACa02	2	2.14E+08	2.43E+08	0	0	COL6A3,U	exonic	NM_00003	.	.	.	ENST0000	CpG:	174,(	2q37.3-q36
ACa02	3	1.4E+08	1.64E+08	0	0	LINC0121:	exonic	NM_00009	.	.	.	ENST0000	CpG:	53,C	3q23-q25.3
ACa02	3	1.64E+08	1.65E+08	0	0	.	ncRNA_ex	NR_12640	.	.	.	ENST0000	.		3q26.1
ACa02	3	1.65E+08	1.73E+08	0	0	SERPINI1,	exonic	NM_00005	.	.	.	ENST0000	CpG:	39,C	3q26.2-q26
ACa02	3	1.73E+08	1.73E+08	0	0	.	downstreai	NM_00125	.	.	.	.	.		3q26.31
ACa02	3	1.73E+08	1.81E+08	0	0	ACTL6A,M	exonic	NM_00101	.	.	.	ENST0000	CpG:	98,C	3q26.33-q2
ACa02	3	1.81E+08	1.88E+08	0	0	SNORA81	exonic	NM_00041	.	.	.	ENST0000	CpG:	32,C	3q27.3-q27
ACa02	3	1.88E+08	1.88E+08	0	0	LPP	intronic	NM_00116	.	.	.	ENST0000	.		3q28
ACa02	3	1.88E+08	1.89E+08	0	0	LPP,TPRG	exonic	NM_00111	.	.	.	ENST0000	CpG:	25	3q28
ACa02	3	1.89E+08	1.9E+08	0	0	TP63	exonic	NM_00111	.	.	.	ENST0000	.		3q28
ACa02	3	1.9E+08	1.98E+08	0	0	PPP1R2,A	exonic	NM_00099	.	.	.	ENST0000	CpG:	138,(	3q29-q28
ACa02	3	1.98E+08	1.98E+08	0	0	.	intergenic	NM_00114	dist=2272;(	.	.	ENST0000	.		3q29
ACa02	3	1.98E+08	1.98E+08	0	0	.	intergenic	NM_00114	dist=32272	.	.	.	.		3q29
ACa02	3	1.98E+08	1.98E+08	0	0	.	intergenic	NM_00114	dist=52272	.	.	.	.		3q29

ACa02	5	9220000	9240000	0	0	SEMA5A	exonic	NM_00396	.	.	.	ENST000C	.	5p15.31
ACa02	5	81280000	81290000	0	0	ATG10	exonic	NM_00113	.	.	.	ENST000C	.	5q14.1
ACa02	5	1.15E+08	1.15E+08	0	0	COMMD1(	exonic	NM_01614	.	.	.	ENST000C	.	5q23.1
ACa02	5	1.4E+08	1.4E+08	0	0	PCDHA12,	exonic	NM_01889	.	.	.	ENST000C	.	5q31.3
ACa02	5	1.45E+08	1.45E+08	0	0	PRELID2	exonic	NM_13849	.	.	.	ENST000C	.	5q32
ACa02	5	1.72E+08	1.72E+08	0	0	NEURL1B	exonic	NM_00114	.	.	.	ENST000C	CpG: 94	5q35.1
ACa02	6	0	110000	0	0	.	intergenic	NONE,NR_	dist=NONE	.	.	.	.	6p25.3
ACa02	6	130000	150000	0	0	LINC0026(	ncRNA_ex	NR_10981	.	.	.	ENST000C	.	6p25.3
ACa02	6	37970000	38050000	0	0	ZFAND3	exonic	NM_02194	.	.	.	ENST000C	.	6p21.2
ACa02	7	1.54E+08	1.54E+08	0	0	DPP6	exonic	NM_00103	.	.	.	ENST000C	.	7q36.2
ACa02	8	39040000	39050000	0	0	ADAM32	exonic	NM_14500	.	.	.	ENST000C	.	8p11.22
ACa02	9	20000	40000	0	0	FAM138C,	exonic	NM_18290	.	.	.	ENST000C	CpG: 69	9p24.3
ACa02	9	86360000	86370000	0	0	GKAP1	exonic	NM_00113	.	.	.	ENST000C	.	9q21.32
ACa02	9	1.04E+08	1.04E+08	0	0	LPPR1	exonic	NM_01775	.	.	.	ENST000C	.	9q31.1
ACa02	9	1.14E+08	1.14E+08	0	0	LPAR1	intronic	NM_00140	.	.	.	ENST000C	.	9q31.3
ACa02	9	1.41E+08	1.41E+08	0	0	FAM157B	exonic	NM_00114	.	.	.	ENST000C	CpG: 27	9q34.3
ACa02	10	20290000	20360000	0	0	PLXDC2	exonic	NM_00128	.	.	.	ENST000C	.	10p12.31
ACa02	10	1.12E+08	1.12E+08	0	0	MXI1	exonic	NM_00596	.	.	.	ENST000C	.	10q25.2
ACa02	11	1.01E+08	1.01E+08	0	0	ARHGAP4	exonic	NM_15243	.	.	.	ENST000C	.	11q22.1
ACa02	12	14690000	14730000	0	0	PLBD1	exonic	NM_02482	.	.	.	ENST000C	CpG: 65	12p13.1
ACa02	12	1.31E+08	1.31E+08	0	0	RIMBP2,R	exonic	NM_00119	.	.	.	ENST000C	CpG: 341,(	12q24.33
ACa02	13	1.13E+08	1.13E+08	0	0	TUBGCP3	exonic	NM_00128	.	.	.	ENST000C	.	13q34
ACa02	14	80120000	80140000	0	0	NRXN3	exonic	NM_00110	.	.	.	ENST000C	.	14q31.1
ACa02	14	89690000	89700000	0	0	FOXN3	intronic	NM_00108	.	.	.	ENST000C	.	14q31.3
ACa02	15	57350000	57490000	0	0	TCF12	exonic	NM_00320	.	.	.	ENST000C	.	15q21.3
ACa02	15	1.02E+08	1.03E+08	0	0	WASH3P,(	exonic	NM_00100	.	.	.	ENST000C	CpG: 116	15q26.3
ACa02	15	1.03E+08	1.03E+08	0	0	.	upstream	NR_03409	.	.	.	.	.	15q26.3
ACa02	16	23880000	23890000	0	0	PRKCB	intronic	NM_00273	.	.	.	ENST000C	.	16p12.2
ACa02	16	29970000	30140000	0	0	ALDOA,FA	exonic	NM_00003	.	.	.	ENST000C	CpG: 17,C	16p11.2
ACa02	16	30140000	30190000	0	0	.	intergenic	NM_00274	dist=5370;(	.	.	.	.	16p11.2
ACa02	16	30190000	30200000	0	0	CORO1A	exonic	NM_00119	.	.	.	ENST000C	CpG: 49	16p11.2
ACa02	16	47170000	47260000	0	0	ITFG1,NE	exonic	NM_00120	.	.	.	ENST000C	CpG: 186	16q12.1
ACa02	16	56550000	56560000	0	0	BBS2	exonic	NM_03188	.	.	.	ENST000C	CpG: 59	16q12.2
ACa02	18	0	40000	0	0	.	ncRNA_ex	NR_11079	.	.	.	ENST000C	CpG: 67	18p11.32
ACa02	18	40000	120000	0	0	ROCK1P1	ncRNA_ex	NR_03377	.	.	.	ENST000C	CpG: 51,C	18p11.32

ACa02	18	120000	2970000	0	0	THOC1,C1	exonic	NM_00101	.	.	.	ENST000C	CpG: 146,(	18p11.31-p
ACa02	18	3060000	6950000	0	0	DLGAP1-A	exonic	NM_0010C	.	.	.	ENST000C	CpG: 241,(	18p11.31
ACa02	18	6950000	34530000	0	0	C18orf61,T	exonic	NM_00022	.	.	.	ENST000C	CpG: 88,C	18p11.23-c
ACa02	18	34530000	34810000	0	0	KIAA1328	exonic	NM_02077	.	.	.	ENST000C	.	18q12.2
ACa02	18	34810000	61660000	0	0	SNORD58	exonic	NM_00014	.	.	.	ENST000C	CpG: 119,(	18q12.2-q2
ACa02	18	61760000	77480000	0	0	LINC0030£	exonic	NM_00102	.	.	.	ENST000C	CpG: 52,C	18q23-q22
ACa02	18	77620000	77930000	0	0	PARD6G,T	exonic	NM_00113	.	.	.	ENST000C	CpG: 114,(	18q23
ACa02	18	77930000	78077248	0	0	PARD6G-A	exonic	NM_03251	.	.	.	ENST000C	CpG: 122	18q23
ACa02	19	0	60000	0	0	.	downstrear	NR_03326	.	.	.	.	.	19p13.3
ACa02	19	60000	80000	0	0	FAM138A,'	ncRNA_ex	NR_02681	.	.	.	ENST000C	CpG: 117	19p13.3
ACa02	19	80000	100000	0	0	.	intergenic	NR_02682	dist=2310;(	.	.	.	.	19p13.3
ACa02	19	100000	120000	0	0	OR4F17	exonic	NM_0010C	.	.	.	ENST000C	.	19p13.3
ACa02	19	120000	150000	0	0	.	intergenic	NM_0010C	dist=8404;(	.	.	.	.	19p13.3
ACa02	19	150000	160000	0	0	.	intergenic	NM_0010C	dist=38404	.	.	ENST000C	.	19p13.3
ACa02	19	160000	180000	0	0	.	intergenic	NM_0010C	dist=48404	.	.	ENST000C	.	19p13.3
ACa02	19	180000	210000	0	0	LINC0100:	ncRNA_ex	NR_02832	.	.	.	ENST000C	.	19p13.3
ACa02	19	10280000	10420000	0	0	DNMT1,MI	exonic	NM_0002C	.	.	.	ENST000C	CpG: 80,C	19p13.2
ACa02	19	31830000	32100000	0	0	TSHZ3,TH	exonic	NM_0012C	.	.	.	ENST000C	CpG: 31,C	19q12
ACa02	19	32100000	32110000	0	0	.	intergenic	NM_00127	dist=15544	.	.	.	.	19q12
ACa02	19	35270000	35280000	0	0	.	intergenic	NM_0010C	dist=5866;(	.	.	.	.	19q13.11
ACa02	20	13460000	13550000	0	0	TASP1	exonic	NM_01771	.	.	.	ENST000C	.	20p12.1
ACa02	20	48180000	48190000	0	0	PTGIS	exonic	NM_00096	.	.	.	ENST000C	CpG: 53	20q13.13
ACa02	22	16950000	16970000	0	0	.	intergenic	NM_0010C	dist=50019	.	.	.	.	22q11.1
ACa02	22	20340000	20610000	0	0	LOC72944	exonic	NM_00124	.	.	.	ENST000C	CpG: 129,(	22q11.21
ACa02	22	20610000	21660000	0	0	THAP7-AS	exonic	NM_00018	.	.	.	ENST000C	CpG: 248,(	22q11.21
ACa02	22	22580000	22590000	0	0	.	intergenic	NM_00393	dist=24276	.	.	.	.	22q11.22
ACa02	22	22590000	24270000	0	0	CES5AP1,	exonic	NM_0010C	.	.	.	ENST000C	CpG: 30,C	22q11.22-c
ACa02	22	24270000	24330000	0	0	GSTT2,GS	exonic	NM_00085	.	.	.	ENST000C	CpG: 88,C	22q11.23
ACa02	22	24550000	24960000	0	0	ADORA2A	exonic	NM_00067	.	.	.	ENST000C	CpG: 24,C	22q11.23
ACa02	22	24960000	24970000	0	0	SNRPD3	exonic	NM_00127	.	.	.	ENST000C	.	22q11.23
ACa02	22	25060000	29490000	0	0	MYO18B,N	exonic	NM_00049	.	.	.	ENST000C	CpG: 30,C	22q12.1-q1
ACa02	22	29490000	30940000	0	0	SDC4P,MT	exonic	NM_00026	.	.	.	ENST000C	CpG: 95,C	22q12.2-q1
ACa02	22	30940000	30970000	0	0	SEC14L6,(	exonic	NM_00119	.	.	.	ENST000C	CpG: 113	22q12.2
ACa02	22	30970000	31860000	0	0	SMTN,SEI	exonic	NM_00035	.	.	.	ENST000C	CpG: 54,C	22q12.2
ACa02	22	31860000	31880000	0	0	EIF4ENIF1	exonic	NM_00116	.	.	.	ENST000C	.	22q12.2

ACa02	22	32000000	36640000	0	0	DEPDC5,A	exonic	NM_00034	.	.	.	ENST000C	CpG: 29,C	22q12.2-q1
ACa02	22	36670000	37340000	0	0	IFT27,MIR	exonic	NM_00039	.	.	.	ENST000C	CpG: 150,(	22q12.3
ACa02	22	37340000	37380000	0	0	LL22NC01	ncRNA_ex	NR_03895	.	.	.	ENST000C	.	22q12.3
ACa02	22	37500000	41300000	0	0	LOC10013	exonic	NM_00002	.	.	.	ENST000C	CpG: 159,(	22q13.1-q1
ACa02	22	42070000	46840000	0	0	NUP50,TC	exonic	NM_0001C	.	.	.	ENST000C	CpG: 176,(	22q13.2-q1
ACa02	22	46840000	49150000	0	0	MIR3201,L	exonic	NM_00108	.	.	.	ENST000C	CpG: 26,C	22q13.31-(
ACa02	22	50010000	51190000	0	0	SCO2,PIM	exonic	NM_00048	.	.	.	ENST000C	CpG: 44,C	22q13.33
ACa02	22	51190000	51200000	0	0	RPL23AP8	ncRNA_ex	NR_02698	.	.	.	.	.	22q13.33
ACa02	X	24620000	24640000	0	0	PCYT1B	exonic	NM_00116	.	.	.	ENST000C	.	Xp22.11
ACa02	X	37880000	37900000	0	0	SYTL5	exonic	NM_00116	.	.	.	ENST000C	.	Xp11.4
ACa02	X	1.12E+08	1.12E+08	0	0	LHFPL1	exonic	NM_17817	.	.	.	ENST000C	.	Xq23
ACa02	X	1.35E+08	1.35E+08	0	0	SLC9A6,FI	exonic	NM_00104	.	.	.	ENST000C	CpG: 32,C	Xq26.3
ACa03	1	33200000	33220000	0	0	KIAA1522	exonic	NM_02088	.	.	.	ENST000C	CpG: 61,C	1p35.1
ACa03	1	1.97E+08	1.97E+08	0	0	CRB1	exonic	NM_00119	.	.	.	ENST000C	.	1q31.3
ACa03	1	2.32E+08	2.32E+08	0	0	DISC2,TSI	exonic	NM_00101	.	.	.	ENST000C	.	1q42.2
ACa03	2	12330000	12340000	0	0	AC096559	ncRNA_int	NR_11019	.	.	.	ENST000C	.	2p24.3
ACa03	2	98450000	98620000	0	0	TMEM131	exonic	NM_01534	.	.	.	ENST000C	CpG: 146	2q11.2
ACa03	2	2.19E+08	2.19E+08	0	0	TNS1	UTR5	NM_02264	NM_02264	.	.	ENST000C	CpG: 22	2q35
ACa03	3	1.31E+08	1.31E+08	0	0	NEK11	exonic	NM_00114	.	.	.	ENST000C	.	3q22.1
ACa03	3	1.69E+08	1.69E+08	0	0	.	intergenic	NM_0012C	dist=38437	.	.	.	.	3q26.2
ACa03	3	1.71E+08	1.71E+08	0	0	TNIK	exonic	NM_00116	.	.	.	ENST000C	.	3q26.31
ACa03	4	24890000	24900000	0	0	CCDC149	exonic	NM_17346	.	.	.	ENST000C	.	4p15.2
ACa03	4	81860000	81870000	0	0	C4orf22	exonic	NM_0012C	.	.	.	ENST000C	.	4q21.21
ACa03	5	58880000	58890000	0	0	PDE4D	exonic	NM_0062C	.	.	.	ENST000C	.	5q11.2
ACa03	5	1.08E+08	1.08E+08	0	0	FBXL17	exonic	NM_00116	.	.	.	ENST000C	.	5q21.3
ACa03	6	1.22E+08	1.22E+08	0	0	TBC1D32	exonic	NM_15273	.	.	.	ENST000C	.	6q22.31
ACa03	7	2180000	2190000	0	0	MAD1L1	exonic	NM_00101	.	.	.	ENST000C	CpG: 30	7p22.3
ACa03	7	37310000	37320000	0	0	ELMO1	exonic	NM_0012C	.	.	.	ENST000C	.	7p14.1
ACa03	7	81670000	82080000	0	0	CACNA2D	exonic	NM_00072	.	.	.	ENST000C	CpG: 143	7q21.11
ACa03	7	1.35E+08	1.35E+08	0	0	CALD1	exonic	NM_00434	.	.	.	ENST000C	.	7q33
ACa03	7	1.4E+08	1.4E+08	0	0	PARP12	exonic	NM_02275	.	.	.	ENST000C	CpG: 124	7q34
ACa03	8	850000	970000	0	0	ERICH1-A:	ncRNA_ex	NR_07339	.	.	.	ENST000C	CpG: 61,C	8p23.3
ACa03	9	80430000	80440000	0	0	GNAQ	exonic	NM_00207	.	.	.	ENST000C	.	9q21.2
ACa03	9	1.27E+08	1.27E+08	0	0	DENND1A	exonic	NM_02094	.	.	.	ENST000C	.	9q33.3
ACa03	10	18290000	18300000	0	0	RP11-110I	exonic	NM_00114	.	.	.	ENST000C	.	10p12.33

ACa03	10	62490000	62500000	0	0	ANK3	exonic	NM_0012C	.	.	.	ENST000C	CpG: 52	10q21.2
ACa03	10	68280000	68290000	0	0	CTNNA3	exonic	NM_00112	.	.	.	ENST000C	.	10q21.3
ACa03	10	1.19E+08	1.19E+08	0	0	PDZD8	exonic	NM_17379	.	.	.	ENST000C	.	10q25.3
ACa03	10	1.29E+08	1.29E+08	0	0	NPS	exonic	NM_00103	.	.	.	ENST000C	.	10q26.2
ACa03	11	74500000	74510000	0	0	RNF169	exonic	NM_00109	.	.	.	ENST000C	.	11q13.4
ACa03	11	79130000	79140000	0	0	MIR5579,T	ncRNA_ex	NR_04984	.	.	.	ENST000C	.	11q14.1
ACa03	11	83540000	83810000	0	0	DLG2	exonic	NM_00114	.	.	.	ENST000C	.	11q14.1
ACa03	11	92430000	92440000	0	0	FAT3	exonic	NM_0010C	.	.	.	ENST000C	.	11q14.3
ACa03	11	1.15E+08	1.15E+08	0	0	CADM1	intronic	NM_00109	.	.	.	ENST000C	.	11q23.3
ACa03	11	1.25E+08	1.25E+08	0	0	SPA17	exonic	NM_01742	.	.	.	ENST000C	.	11q24.2
ACa03	12	29780000	29790000	0	0	TMTC1	exonic	NM_00119	.	.	.	ENST000C	.	12p11.22
ACa03	12	47580000	47590000	0	0	MIR4698,F	ncRNA_ex	NR_03984	.	.	.	ENST000C	.	12q13.11
ACa03	12	97220000	97260000	0	0	.	intergenic	NM_00117	dist=42563	.	.	ENST000C	.	12q23.1
ACa03	13	50840000	50850000	0	0	DLEU1	ncRNA_int	NR_10997	.	.	.	ENST000C	.	13q14.2
ACa03	14	25340000	25350000	0	0	STXBP6	intronic	NM_01417	.	.	.	ENST000C	.	14q12
ACa03	14	33140000	33150000	0	0	AKAP6	exonic	NM_00427	.	.	.	ENST000C	.	14q12
ACa03	14	63460000	63520000	0	0	KCNH5	exonic	NM_13931	.	.	.	ENST000C	CpG: 92	14q23.2
ACa03	14	73240000	73250000	0	0	DPF3	intronic	NM_00128	.	.	.	ENST000C	.	14q24.2
ACa03	14	80290000	80300000	0	0	NRXN3	intronic	NM_0011C	.	.	.	ENST000C	.	14q31.1
ACa03	14	91440000	91470000	0	0	RPS6KA5	exonic	NM_00475	.	.	.	ENST000C	.	14q32.11
ACa03	15	58430000	58440000	0	0	AQP9	exonic	NM_02098	.	.	.	ENST000C	.	15q21.3
ACa03	17	480000	490000	0	0	VPS53	exonic	NM_00112	.	.	.	ENST000C	.	17p13.3
ACa03	17	61340000	61350000	0	0	TANC2	exonic	NM_02518	.	.	.	ENST000C	.	17q23.3
ACa03	18	21770000	21870000	0	0	OSBPL1A	exonic	NM_00124	.	.	.	ENST000C	CpG: 86	18q11.2
ACa03	19	42060000	42070000	0	0	CEACAM2	intronic	NM_00128	.	.	.	ENST000C	.	19q13.2
ACa03	19	51730000	51750000	0	0	CD33	exonic	NM_00108	.	.	.	ENST000C	.	19q13.41
ACa03	21	24450000	24460000	0	0	.	intergenic	NR_03840	dist=96115	.	.	ENST000C	.	21q21.2
ACa03	21	27480000	27520000	0	0	APP	exonic	NM_00048	.	.	.	ENST000C	.	21q21.3
ACa03	21	30830000	30840000	0	0	.	intergenic	NR_04656	dist=8370C	.	.	.	.	21q21.3
ACa03	21	44370000	44380000	0	0	.	intergenic	NM_02107	dist=40227	.	.	ENST000C	.	21q22.3
ACa03	22	44110000	44140000	0	0	EFCAB6	exonic	NM_02278	.	.	.	ENST000C	.	22q13.2
ACa03	X	31670000	31680000	0	0	DMD	exonic	NM_0001C	.	.	.	ENST000C	.	Xp21.1
ACa03	X	34670000	34680000	0	0	TMEM47	exonic	NM_03144	.	.	.	ENST000C	CpG: 113	Xp21.1
ACa04	2	20790000	20800000	0	0	HS1BP3-IT	ncRNA_ex	NR_04683	.	.	.	ENST000C	.	2p24.1
ACa04	2	35690000	35700000	0	0	.	intergenic	NR_12640	dist=74237	.	.	ENST000C	.	2p22.3

ACa04	2	62900000	63100000	0	0	EHBP1	exonic	NM_00114	.	.	.	ENST0000	CpG: 64	2p15
ACa04	2	68790000	68810000	0	0	APLF	exonic	NM_17354	.	.	.	ENST0000	.	2p13.3
ACa04	2	1.63E+08	1.63E+08	0	0	KCNH7	exonic	NM_03327	.	.	.	ENST0000	.	2q24.2
ACa04	2	2.14E+08	2.14E+08	0	0	SPAG16	exonic	NM_02453	.	.	.	ENST0000	.	2q34
ACa04	3	1.11E+08	1.3E+08	0	0	HEG1,CST	exonic	NM_00017	.	.	.	ENST0000	CpG: 71,C	3q13.13-q2
ACa04	3	1.3E+08	1.3E+08	0	0	COL6A4P2	ncRNA_ex	NR_02425	.	.	.	ENST0000	CpG: 111,(	3q22.1
ACa04	3	1.3E+08	1.95E+08	0	0	SPSB4,TM	exonic	NM_00005	.	.	.	ENST0000	CpG: 94,C	3q27.3-q2
ACa04	3	1.95E+08	1.98E+08	0	0	MIR922,ZI	exonic	NM_00099	.	.	.	ENST0000	CpG: 56,C	3q29
ACa04	3	1.98E+08	1.98E+08	0	0	FAM157A	exonic	NM_00114	.	.	.	.	.	3q29
ACa04	3	1.98E+08	1.98E+08	0	0	FAM157A	exonic	NM_00114	.	.	.	ENST0000	CpG: 27	3q29
ACa04	3	1.98E+08	1.98E+08	0	0	.	intergenic	NM_00114	dist=52272	.	.	.	.	3q29
ACa04	4	3380000	3390000	0	0	RGS12	exonic	NM_00292	.	.	.	ENST0000	.	4p16.3
ACa04	4	1.14E+08	1.14E+08	0	0	MIR1243,A	exonic	NM_00114	.	.	.	ENST0000	.	4q25
ACa04	4	1.68E+08	1.68E+08	0	0	SPOCK3	exonic	NM_00104	.	.	.	ENST0000	.	4q32.3
ACa04	4	1.76E+08	1.76E+08	0	0	GLRA3	exonic	NM_00104	.	.	.	ENST0000	.	4q34.1
ACa04	6	35420000	35760000	0	0	TULP1,RP	exonic	NM_00101	.	.	.	ENST0000	CpG: 23,C	6p21.31
ACa04	6	1.58E+08	1.58E+08	0	0	SNX9	exonic	NM_01622	.	.	.	ENST0000	.	6q25.3
ACa04	7	29150000	29170000	0	0	CPVL	exonic	NM_01902	.	.	.	ENST0000	.	7p14.3
ACa04	8	1.34E+08	1.34E+08	0	0	PHF20L1,T	exonic	NM_00114	.	.	.	ENST0000	CpG: 53	8q24.22
ACa04	8	1.44E+08	1.44E+08	0	0	TOP1MT	intronic	NM_00125	.	.	.	ENST0000	.	8q24.3
ACa04	9	80400000	80420000	0	0	GNAQ	exonic	NM_00207	.	.	.	ENST0000	.	9q21.2
ACa04	9	1.03E+08	1.03E+08	0	0	STX17,ER	exonic	NM_01505	.	.	.	ENST0000	.	9q31.1
ACa04	9	1.28E+08	1.28E+08	0	0	MAPKAP1	exonic	NM_00100	.	.	.	ENST0000	.	9q33.3
ACa04	10	32660000	32670000	0	0	LOC10203	exonic	NM_00128	.	.	.	ENST0000	.	10p11.22
ACa04	10	34550000	34580000	0	0	PARD3	exonic	NM_00118	.	.	.	ENST0000	.	10p11.21
ACa04	10	60460000	60470000	0	0	BICC1	exonic	NM_00108	.	.	.	ENST0000	.	10q21.1
ACa04	10	70540000	70590000	0	0	CCAR1,ST	exonic	NM_00113	.	.	.	ENST0000	CpG: 169	10q21.3
ACa04	10	98470000	98480000	0	0	PIK3AP1	intronic	NM_15230	.	.	.	ENST0000	CpG: 62	10q24.1
ACa04	10	1.18E+08	1.18E+08	0	0	CCDC172	exonic	NM_19851	.	.	.	ENST0000	.	10q25.3
ACa04	10	1.28E+08	1.28E+08	0	0	.	intergenic	NM_00100	dist=11999	.	.	ENST0000	.	10q26.2
ACa04	12	0	60000	0	0	.	intergenic	NONE,NR_	dist=NONE	.	.	.	.	12p13.33
ACa04	12	60000	80000	0	0	.	ncRNA_ex	NR_02682	.	.	.	ENST0000	CpG: 112	12p13.33
ACa04	12	80000	100000	0	0	LOC10028	ncRNA_ex	NR_02826	.	.	.	.	.	12p13.33
ACa04	12	80260000	80270000	0	0	PPP1R12A	exonic	NM_00114	.	.	.	ENST0000	.	12q21.2
ACa04	13	20790000	21020000	0	0	GJB6,CRY	exonic	NM_00111	.	.	.	ENST0000	CpG: 87,C	13q12.11

ACa04	13	45820000	45860000	0	0	GTF2F2	exonic	NM_00412	.	.	.	ENST0000	.	13q14.13
ACa04	13	46030000	46040000	0	0	COG3	exonic	NM_03143	.	.	.	ENST0000	CpG: 56	13q14.13
ACa04	13	46780000	46790000	0	0	LRRC63	exonic	NM_00128	.	.	.	ENST0000	.	13q14.13
ACa04	13	1E+08	1E+08	0	0	CLYBL	exonic	NM_20680	.	.	.	ENST0000	.	13q32.3
ACa04	13	1.06E+08	1.06E+08	0	0	DAOA,DA	exonic	NM_00116	.	.	.	ENST0000	.	13q33.2
ACa04	14	36030000	36050000	0	0	RALGAPA	exonic	NM_00128	.	.	.	ENST0000	.	14q13.2
ACa04	15	27570000	27730000	0	0	GABRG3	exonic	NM_00127	.	.	.	ENST0000	.	15q12
ACa04	15	64540000	64600000	0	0	CSNK1G1	exonic	NM_02204	.	.	.	ENST0000	.	15q22.31
ACa04	16	90170000	90290000	0	0	.	ncRNA_ex	NR_12616	.	.	.	ENST0000	CpG: 26,C	16q24.3
ACa04	16	90290000	90354753	0	0	.	intergenic	NR_12616	dist=4598	.	.	.	.	16q24.3
ACa04	17	930000	940000	0	0	ABR	exonic	NM_00125	.	.	.	ENST0000	CpG: 59,C	17p13.3
ACa04	17	60630000	60640000	0	0	TLK2	exonic	NM_00128	.	.	.	ENST0000	.	17q23.2
ACa04	17	67310000	67320000	0	0	ABCA5	exonic	NM_01867	.	.	.	ENST0000	.	17q24.3
ACa04	18	0	40000	0	0	.	ncRNA_ex	NR_11079	.	.	.	ENST0000	CpG: 67	18p11.32
ACa04	18	40000	120000	0	0	ROCK1P1	ncRNA_ex	NR_03377	.	.	.	ENST0000	CpG: 21,C	18p11.32
ACa04	18	2670000	2690000	0	0	SMCHD1	exonic	NM_01529	.	.	.	ENST0000	.	18p11.32
ACa04	18	9250000	9360000	0	0	ANKRD12,	exonic	NM_00108	.	.	.	ENST0000	CpG: 30,C	18p11.22
ACa04	18	18600000	18980000	0	0	GREB1L,F	exonic	NM_00114	.	.	.	ENST0000	CpG: 118,	18q11.1
ACa04	18	43300000	43820000	0	0	C18orf25,F	exonic	NM_00100	.	.	.	ENST0000	CpG: 47,C	18q21.1-q
ACa04	18	45390000	45430000	0	0	SMAD2	exonic	NM_00100	.	.	.	ENST0000	.	18q21.1
ACa04	18	53170000	53310000	0	0	TCF4	exonic	NM_00108	.	.	.	ENST0000	CpG: 30	18q21.2
ACa04	18	53310000	53330000	0	0	.	intergenic	NM_00124	dist=6776;	.	.	.	.	18q21.2
ACa04	18	53330000	54820000	0	0	RP11-456	exonic	NM_00125	.	.	.	ENST0000	CpG: 24,C	18q21.2-q
ACa04	18	55210000	56040000	0	0	RP11-35G	exonic	NM_00014	.	.	.	ENST0000	CpG: 32,C	18q21.31
ACa04	18	56040000	56120000	0	0	NEDD4L,M	exonic	NM_00114	.	.	.	ENST0000	.	18q21.31
ACa04	19	0	60000	0	0	.	downstrea	NR_03326	.	.	.	.	.	19p13.3
ACa04	19	60000	210000	0	0	FAM138A,	exonic	NM_00100	.	.	.	ENST0000	CpG: 117	19p13.3
ACa04	21	9860000	10800000	0	0	TEKT4P2	ncRNA_ex	NR_03832	.	.	.	ENST0000	CpG: 20,C	21p11.2
ACa04	21	15010000	15040000	0	0	POTED	exonic	NM_17498	.	.	.	ENST0000	CpG: 24,C	21q11.2
ACa04	21	22780000	22790000	0	0	NCAM2	exonic	NM_00454	.	.	.	ENST0000	.	21q21.1
ACa04	21	41170000	41310000	0	0	IGSF5,PC	exonic	NM_00108	.	.	.	ENST0000	.	21q22.2
ACa04	21	45010000	45040000	0	0	MIR6070,H	exonic	NM_00703	.	.	.	ENST0000	.	21q22.3
ACa04	22	21920000	21930000	0	0	UBE2L3	exonic	NM_00125	.	.	.	ENST0000	CpG: 85	22q11.21
ACa04	X	32530000	32540000	0	0	DMD	exonic	NM_00010	.	.	.	ENST0000	.	Xp21.1
ACa04	X	67450000	67460000	0	0	OPHN1	exonic	NM_00254	.	.	.	ENST0000	.	Xq12

ACa04	X	1.51E+08	1.51E+08	0	0	GABRA3	exonic	NM_0008C	.	.	.	ENST000C	.	Xq28
ACa06	2	2.14E+08	2.14E+08	0	0	MIR4776-2	ncRNA_ex	NR_03993	.	.	.	ENST000C	.	2q34
ACa06	3	1.24E+08	1.24E+08	0	0	KALRN,MI	ncRNA_ex	NR_04979	.	.	.	ENST000C	.	3q21.2
ACa06	4	25140000	25240000	0	0	SEPSECS	exonic	NM_01695	.	.	.	ENST000C	CpG: 52,C	4p15.2
ACa06	4	1.07E+08	1.07E+08	0	0	.	ncRNA_ex	NR_12592	.	.	.	ENST000C	.	4q24
ACa06	4	1.13E+08	1.13E+08	0	0	AP1AR	exonic	NM_00112	.	.	.	ENST000C	CpG: 106	4q25
ACa06	5	11360000	11400000	0	0	CTNND2	exonic	NM_00128	.	.	.	ENST000C	CpG: 70	5p15.2
ACa06	5	26980000	26990000	0	0	CDH9	exonic	NM_01627	.	.	.	ENST000C	.	5p14.1
ACa06	5	1.7E+08	1.7E+08	0	0	KCNIP1	exonic	NM_00103	.	.	.	ENST000C	.	5q35.1
ACa06	6	84380000	84390000	0	0	SNAP91	exonic	NM_00125	.	.	.	ENST000C	.	6q14.2
ACa06	6	1.43E+08	1.43E+08	0	0	GPR126	exonic	NM_00103	.	.	.	ENST000C	CpG: 91	6q24.1
ACa06	6	1.71E+08	1.71E+08	0	0	.	intergenic	NM_00259	dist=14622	.	.	ENST000C	.	6q27
ACa06	6	1.71E+08	1.71E+08	0	0	.	intergenic	NM_00259	dist=16622	.	.	.	.	6q27
ACa06	7	57140000	57150000	0	0	.	intergenic	NR_03624	dist=11642	.	.	.	.	7p11.2
ACa06	8	1.32E+08	1.32E+08	0	0	ADCY8	exonic	NM_00111	.	.	.	ENST000C	CpG: 208	8q24.22
ACa06	9	89760000	89780000	0	0	C9orf170	exonic	NM_0010C	.	.	.	ENST000C	CpG: 40	9q21.33
ACa06	9	1.26E+08	1.27E+08	0	0	DENND1A	exonic	NM_02094	.	.	.	ENST000C	.	9q33.3
ACa06	10	49480000	49490000	0	0	FRMPD2	exonic	NM_00101	.	.	.	ENST000C	.	10q11.22
ACa06	12	40220000	40430000	0	0	SLC2A13	exonic	NM_05288	.	.	.	ENST000C	.	12q12
ACa06	12	95770000	95780000	0	0	.	intergenic	NR_03745	dist=6624C	.	.	ENST000C	.	12q22
ACa06	12	1.06E+08	1.06E+08	0	0	.	intergenic	NM_00114	dist=14704	.	.	ENST000C	.	12q23.3
ACa06	13	61140000	61150000	0	0	TDRD3	exonic	NM_00114	.	.	.	ENST000C	.	13q21.2
ACa06	13	74380000	74390000	0	0	KLF12	exonic	NM_00724	.	.	.	ENST000C	.	13q22.1
ACa06	15	58780000	58790000	0	0	LIPC	ncRNA_ex	NR_12033	.	.	.	ENST000C	.	15q21.3
ACa06	15	67980000	68000000	0	0	MAP2K5	exonic	NM_0012C	.	.	.	ENST000C	.	15q23
ACa06	15	68870000	68880000	0	0	CORO2B	exonic	NM_00609	.	.	.	ENST000C	CpG: 135	15q23
ACa06	15	77360000	77370000	0	0	TSPAN3	exonic	NM_00116	.	.	.	ENST000C	CpG: 82	15q24.3
ACa06	17	28570000	28580000	0	0	BLMH	exonic	NM_00038	.	.	.	ENST000C	.	17q11.2
ACa06	17	57190000	57210000	0	0	SKA2	exonic	NM_0011C	.	.	.	ENST000C	.	17q22
ACa06	18	5630000	5640000	0	0	.	intergenic	NM_00128	dist=1010;(	.	.	.	CpG: 241	18p11.31
ACa06	19	34010000	34020000	0	0	PEPD	exonic	NM_00028	.	.	.	ENST000C	CpG: 71	19q13.11
ACa06	19	35270000	35280000	0	0	.	intergenic	NM_0010C	dist=5866;(	.	.	.	.	19q13.11
ACa06	19	35870000	35880000	0	0	.	intergenic	NM_0053C	dist=18611	.	.	.	.	19q13.12
ACa06	20	8130000	8140000	0	0	PLCB1	exonic	NM_01519	.	.	.	ENST000C	.	20p12.3
ACa06	20	20390000	20400000	0	0	RALGAPA	exonic	NM_02034	.	.	.	ENST000C	.	20p11.23

ACa06	21	35440000	35520000	0	0	SLC5A3,M	exonic	NM_00693	.	.	.	ENST0000	CpG: 96	21q22.11
ACa06	X	22200000	22210000	0	0	PHEX	exonic	NM_00044	.	.	.	ENST0000	.	Xp22.11
ACa06	X	78150000	78160000	0	0	MIR4328	ncRNA_ex	NR_03625	.	.	.	.	.	Xq21.1
ACa06	X	1.04E+08	1.04E+08	0	0	IL1RAPL2	exonic	NM_01741	.	.	.	ENST0000	.	Xq22.3
ACa06	X	1.14E+08	1.14E+08	0	0	LRCH2	exonic	NM_00124	.	.	.	ENST0000	CpG: 31	Xq23
ACa07	1	15250000	15260000	0	0	KAZN	exonic	NM_00101	.	.	.	ENST0000	CpG: 135	1p36.21
ACa07	1	71340000	71360000	0	0	PTGER3	intronic	NM_19871	.	.	.	ENST0000	.	1p31.1
ACa07	1	2.4E+08	2.41E+08	0	0	FMN2	exonic	NM_02006	.	.	.	ENST0000	CpG: 26	1q43
ACa07	2	87280000	87290000	0	0	LOC28507	ncRNA_ex	NR_02684	.	.	.	.	.	2p11.2
ACa07	3	11420000	11750000	0	0	VGLL4,AT	exonic	NM_00112	.	.	.	ENST0000	CpG: 22,C	3p25.3
ACa07	3	57190000	57210000	0	0	IL17RD	exonic	NM_01756	.	.	.	ENST0000	CpG: 100,	3p14.3
ACa07	3	1.23E+08	1.23E+08	0	0	PTPLB,MY	exonic	NM_19840	.	.	.	ENST0000	CpG: 64,C	3q21.1
ACa07	4	39000000	39050000	0	0	KLHL5,TM	exonic	NM_02494	.	.	.	ENST0000	CpG: 83	4p14
ACa07	4	1.06E+08	1.07E+08	0	0	ARHGEF3	exonic	NM_00124	.	.	.	ENST0000	.	4q24
ACa07	6	38250000	38530000	0	0	BTBD9	exonic	NM_00109	.	.	.	ENST0000	.	6p21.2
ACa07	6	47270000	47530000	0	0	TNFRSF2	exonic	NM_01212	.	.	.	ENST0000	CpG: 106,	6p12.3
ACa07	6	1.11E+08	1.11E+08	0	0	METTL24	exonic	NM_00112	.	.	.	ENST0000	CpG: 45,C	6q21
ACa07	9	340000	350000	0	0	DOCK8	exonic	NM_00119	.	.	.	ENST0000	.	9p24.3
ACa07	9	6620000	7170000	0	0	KDM4C,GI	exonic	NM_00017	.	.	.	ENST0000	CpG: 154,	9p24.1
ACa07	9	7170000	7180000	0	0	KDM4C	exonic	NM_00114	.	.	.	ENST0000	.	9p24.1
ACa07	9	1.36E+08	1.36E+08	0	0	AK8	exonic	NM_15257	.	.	.	ENST0000	.	9q34.13
ACa07	11	1410000	1420000	0	0	BRSK2	exonic	NM_00125	.	.	.	ENST0000	CpG: 170	11p15.5
ACa07	11	68080000	68090000	0	0	LRP5	exonic	NM_00233	.	.	.	ENST0000	CpG: 72	11q13.2
ACa07	11	1.11E+08	1.12E+08	0	0	SIK2	exonic	NM_01519	.	.	.	ENST0000	CpG: 135	11q23.1
ACa07	11	1.3E+08	1.3E+08	0	0	ST14	exonic	NM_02197	.	.	.	ENST0000	CpG: 64	11q24.3
ACa07	12	46140000	46170000	0	0	ARID2	intronic	NM_15264	.	.	.	ENST0000	.	12q12
ACa07	12	48210000	48220000	0	0	HDAC7	exonic	NM_00109	.	.	.	ENST0000	CpG: 94	12q13.11
ACa07	12	1.31E+08	1.31E+08	0	0	RIMBP2	exonic	NM_01534	.	.	.	ENST0000	.	12q24.33
ACa07	13	1.15E+08	1.15E+08	0	0	RASA3	exonic	NM_00736	.	.	.	ENST0000	CpG: 137	13q34
ACa07	14	63510000	63520000	0	0	KCNH5	exonic	NM_13931	.	.	.	ENST0000	CpG: 92	14q23.2
ACa07	14	89640000	89820000	0	0	FOXN3	exonic	NM_00108	.	.	.	ENST0000	.	14q31.3-q3
ACa07	14	91280000	91290000	0	0	TTC7B	exonic	NM_00101	.	.	.	ENST0000	CpG: 42	14q32.11
ACa07	14	1.01E+08	1.01E+08	0	0	.	intergenic	NM_02083	dist=13869	.	.	ENST0000	CpG: 37	14q32.2
ACa07	15	21960000	21970000	0	0	.	intergenic	NR_02705	dist=19261	.	.	ENST0000	CpG: 84	15q11.2
ACa07	15	60820000	60860000	0	0	RORA	exonic	NM_00294	.	.	.	ENST0000	.	15q22.2

ACa07	15	76760000	76960000	0	0	SCAPER	exonic	NM_00114	.	.	.	ENST0000	.	15q24.3
ACa07	15	77170000	77180000	0	0	SCAPER	exonic	NM_02084	.	.	.	ENST0000	.	15q24.3
ACa07	15	1.02E+08	1.02E+08	0	0	PCSK6	exonic	NM_00129	.	.	.	ENST0000	CpG: 176	15q26.3
ACa07	16	12370000	12380000	0	0	SNX29	exonic	NM_03216	.	.	.	ENST0000	.	16p13.13
ACa07	17	3860000	3870000	0	0	ATP2A3	exonic	NM_00517	.	.	.	ENST0000	CpG: 104	17p13.2
ACa07	17	55470000	55480000	0	0	MSI2	exonic	NM_13896	.	.	.	ENST0000	.	17q22
ACa07	18	77940000	78010000	0	0	PARD6G	exonic	NM_03251	.	.	.	ENST0000	CpG: 122	18q23
ACa07	18	78010000	78077248	0	0	.	intergenic	NM_03251	dist=4603;	.	.	.	.	18q23
ACa07	19	340000	350000	0	0	MIER2	exonic	NM_01755	.	.	.	ENST0000	CpG: 27,C	19p13.3
ACa07	19	2010000	2020000	0	0	BTBD2	exonic	NM_01779	.	.	.	ENST0000	.	19p13.3
ACa07	19	2450000	2580000	0	0	LMNB2,GN	exonic	NM_01567	.	.	.	ENST0000	CpG: 180,	19p13.3
ACa07	19	3700000	3710000	0	0	TJP3,PIP5	exonic	NM_00119	.	.	.	ENST0000	CpG: 81	19p13.3
ACa07	19	13100000	13110000	0	0	NFIX	exonic	NM_00250	.	.	.	ENST0000	CpG: 96	19p13.2
ACa07	19	29700000	29710000	0	0	UQCRFS1	exonic	NM_00600	.	.	.	ENST0000	CpG: 91	19q12
ACa07	19	37180000	37190000	0	0	ZNF567	exonic	NM_00130	.	.	.	ENST0000	.	19q13.12
ACa07	19	44310000	44320000	0	0	LYPD5	intronic	NM_18257	.	.	.	ENST0000	.	19q13.31
ACa07	19	46460000	46480000	0	0	NOVA2	exonic	NM_00251	.	.	.	ENST0000	.	19q13.32
ACa07	20	17550000	17560000	0	0	DSTN	exonic	NM_00687	.	.	.	ENST0000	CpG: 101,	20p12.1
ACa07	20	58880000	60080000	0	0	LOC10050	exonic	NM_00125	.	.	.	ENST0000	CpG: 34,C	20q13.33
ACa07	21	9860000	10800000	0	0	TEKT4P2	ncRNA_ex	NR_03832	.	.	.	ENST0000	CpG: 30,C	21p11.2
ACa07	21	19190000	19200000	0	0	C21orf91	exonic	NM_00110	.	.	.	ENST0000	CpG: 108	21q21.1
ACa07	21	33240000	33250000	0	0	HUNK	exonic	NM_01458	.	.	.	ENST0000	CpG: 169	21q22.11
ACa07	21	35440000	35450000	0	0	MRPS6,SL	exonic	NM_03247	.	.	.	ENST0000	CpG: 96	21q22.11
ACa07	21	44070000	44080000	0	0	PDE9A	exonic	NM_00100	.	.	.	ENST0000	CpG: 114	21q22.3
ACa07	21	45130000	45140000	0	0	PDXK	exonic	NM_00368	.	.	.	ENST0000	CpG: 156	21q22.3
ACa07	22	36420000	36430000	0	0	RBFOX2	exonic	NM_00108	.	.	.	ENST0000	CpG: 102	22q12.3
ACa07	22	44250000	44260000	0	0	SULT4A1	exonic	NM_01435	.	.	.	ENST0000	CpG: 68	22q13.31
ACa07	22	47020000	47040000	0	0	GRAMD4	exonic	NM_01512	.	.	.	ENST0000	CpG: 14,C	22q13.31
ACa07	X	2410000	2430000	0	0	DHRSX,ZE	exonic	NM_14517	.	.	.	ENST0000	CpG: 55	Xp22.33
ACaP0	1	12770000	13650000	0	0	PRAMEF1	exonic	NM_00100	.	.	.	ENST0000	.	1p36.21
ACaP0	2	1.12E+08	1.13E+08	0	0	BCL2L11,A	exonic	NM_00120	.	.	.	ENST0000	CpG: 24,C	2q13
ACaP0	4	1.09E+08	1.09E+08	0	0	RPL34-AS	ncRNA_ex	NR_02696	.	.	.	ENST0000	.	4q25
ACaP0	5	80410000	80430000	0	0	RASGRF2	exonic	NM_00690	.	.	.	ENST0000	.	5q14.1
ACaP0	5	90440000	90460000	0	0	GPR98	exonic	NM_03211	.	.	.	ENST0000	.	5q14.3
ACaP0	6	1.49E+08	1.49E+08	0	0	SASH1	exonic	NM_01527	.	.	.	ENST0000	.	6q24.3

ACaP0:	7	1.38E+08	1.38E+08	0	0	SVOPL	exonic	NM_00113	.	.	.	ENST000C	.	7q34
ACaP0:	8	3430000	3450000	0	0	CSMD1	exonic	NM_03322	.	.	.	ENST000C	.	8p23.2
ACaP0:	8	1.32E+08	1.32E+08	0	0	ADCY8	exonic	NM_00111	.	.	.	ENST000C	.	8q24.22
ACaP0:	9	1.15E+08	1.15E+08	0	0	PTBP3,MII	exonic	NM_00116	.	.	.	ENST000C	CpG: 79	9q31.3-q32
ACaP0:	10	68130000	68140000	0	0	CTNNA3	exonic	NM_00112	.	.	.	ENST000C	.	10q21.3
ACaP0:	10	96520000	96750000	0	0	CYP2C19,	exonic	NM_00076	.	.	.	ENST000C	.	10q23.33
ACaP0:	11	4590000	4610000	0	0	OR52I2,C1	exonic	NM_0010C	.	.	.	ENST000C	.	11p15.4
ACaP0:	11	21550000	21560000	0	0	NELL1	exonic	NM_00128	.	.	.	ENST000C	.	11p15.1
ACaP0:	11	33060000	33080000	0	0	TCP11L1	exonic	NM_00114	.	.	.	ENST000C	CpG: 123	11p13
ACaP0:	11	33080000	33170000	0	0	LINC0029	exonic	NM_00103	.	.	.	ENST000C	.	11p13
ACaP0:	12	8040000	8080000	0	0	SLC2A3,SI	exonic	NM_00128	.	.	.	ENST000C	.	12p13.31
ACaP0:	12	70910000	70970000	0	0	PTPRB	exonic	NM_0011C	.	.	.	ENST000C	.	12q15
ACaP0:	12	93880000	93900000	0	0	MRPL42	exonic	NM_01405	.	.	.	ENST000C	.	12q22
ACaP0:	12	1.13E+08	1.13E+08	0	0	PTPN11	exonic	NM_00283	.	.	.	ENST000C	.	12q24.13
ACaP0:	12	1.17E+08	1.17E+08	0	0	FBXW8	exonic	NM_01217	.	.	.	ENST000C	.	12q24.22
ACaP0:	13	21520000	21530000	0	0	LINC0036	ncRNA_ex	NR_10405	.	.	.	ENST000C	CpG: 26	13q12.11
ACaP0:	13	21530000	21610000	0	0	LATS2	exonic	NM_01457	.	.	.	ENST000C	CpG: 58,C	13q12.11
ACaP0:	13	24550000	24560000	0	0	SPATA13	exonic	NM_00128	.	.	.	ENST000C	CpG: 28	13q12.12
ACaP0:	13	24560000	24590000	0	0	SPATA13	intronic	NM_00128	.	.	.	ENST000C	.	13q12.12
ACaP0:	13	24590000	25550000	0	0	ATP12A,SI	exonic	NM_00116	.	.	.	ENST000C	CpG: 78,C	13q12.13-c
ACaP0:	13	25550000	25800000	0	0	AMER2,PA	exonic	NM_03097	.	.	.	ENST000C	CpG: 184,(	13q12.13
ACaP0:	13	36370000	36690000	0	0	MIR548F5	exonic	NM_00119	.	.	.	ENST000C	.	13q13.3
ACaP0:	13	36930000	36950000	0	0	SPG20OS	ncRNA_ex	NR_04518	.	.	.	ENST000C	.	13q13.3
ACaP0:	13	73460000	73490000	0	0	PIBF1	exonic	NM_00634	.	.	.	ENST000C	.	13q22.1
ACaP0:	14	31600000	31610000	0	0	HECTD1	exonic	NM_01538	.	.	.	ENST000C	.	14q12
ACaP0:	14	31610000	31760000	0	0	HECTD1	exonic	NM_01538	.	.	.	ENST000C	CpG: 144	14q12
ACaP0:	14	63240000	63320000	0	0	KCNH5	exonic	NM_13931	.	.	.	ENST000C	.	14q23.2
ACaP0:	14	64550000	64690000	0	0	MIR548AZ	exonic	NM_01518	.	.	.	ENST000C	.	14q23.2
ACaP0:	14	64690000	64810000	0	0	SYNE2,ES	exonic	NM_00104	.	.	.	ENST000C	CpG: 48,C	14q23.2-q2
ACaP0:	14	68200000	68590000	0	0	ZFYVE26,I	exonic	NM_00287	.	.	.	ENST000C	.	14q24.1
ACaP0:	14	70800000	70920000	0	0	COX16,AD	exonic	NM_0012C	.	.	.	ENST000C	CpG: 25	14q24.2
ACaP0:	14	73620000	73640000	0	0	PSEN1	exonic	NM_00002	.	.	.	ENST000C	.	14q24.2
ACaP0:	14	74520000	74540000	0	0	ALDH6A1,	exonic	NM_00127	.	.	.	ENST000C	.	14q24.3
ACaP0:	14	74540000	74750000	0	0	ALDH6A1,	exonic	NM_00102	.	.	.	ENST000C	CpG: 196,(	14q24.3
ACaP0:	14	76180000	76350000	0	0	TTLL5	exonic	NM_01507	.	.	.	ENST000C	.	14q24.3

ACaP0	14	77300000	77320000	0	0	C14orf166	exonic	NM_19428	.	.	.	ENST0000	.	14q24.3
ACaP0	14	92080000	92100000	0	0	CATSPER	exonic	NM_02476	.	.	.	ENST0000	.	14q32.12
ACaP0	15	74930000	74970000	0	0	EDC3	exonic	NM_00114	.	.	.	ENST0000	.	15q24.1
ACaP0	16	70860000	71110000	0	0	HYDIN	exonic	NM_00119	.	.	.	ENST0000	.	16q22.2
ACaP0	17	44320000	44630000	0	0	LRRC37A2	exonic	NM_00100	.	.	.	ENST0000	CpG: 17,C	17q21.31
ACaP0	17	65890000	65950000	0	0	BPTF	exonic	NM_00445	.	.	.	ENST0000	.	17q24.2
ACaP0	18	12460000	12510000	0	0	SPIRE1	exonic	NM_00112	.	.	.	ENST0000	.	18p11.21
ACaP0	18	48500000	48520000	0	0	ELAC1	exonic	NM_01869	.	.	.	ENST0000	.	18q21.2
ACaP0	18	50960000	51010000	0	0	DCC	exonic	NM_00521	.	.	.	ENST0000	.	18q21.2
ACaP0	18	51010000	51030000	0	0	DCC	exonic	NM_00521	.	.	.	ENST0000	.	18q21.2
ACaP0	19	14900000	15000000	0	0	OR7A17,C	exonic	NM_00100	.	.	.	ENST0000	.	19p13.12
ACaP0	19	48370000	48380000	0	0	SULT2A1	exonic	NM_00316	.	.	.	ENST0000	.	19q13.33
ACaP0	19	55270000	55290000	0	0	KIR2DL1	exonic	NM_01421	.	.	.	ENST0000	.	19q13.42
ACaP0	22	24750000	24770000	0	0	SPECC1L-	exonic	NM_00114	.	.	.	ENST0000	.	22q11.23
ACaP0	22	40850000	40860000	0	0	MKL1	exonic	NM_00128	.	.	.	ENST0000	.	22q13.1
ACaP0	X	37910000	37920000	0	0	SYTL5	exonic	NM_00116	.	.	.	ENST0000	.	Xp11.4
ACaP0	X	49170000	49370000	0	0	GAGE12J,	exonic	NM_00104	.	.	.	ENST0000	CpG: 42,C	Xp11.23
ACaP0	1	1.44E+08	1.5E+08	0	0	HIST2H2B	exonic	NM_00056	.	.	.	ENST0000	CpG: 27,C	1q21.2-q2
ACaP0	1	1.5E+08	1.5E+08	0	0	OTUD7B,V	exonic	NM_00127	.	.	.	ENST0000	CpG: 35,C	1q21.2
ACaP0	1	1.55E+08	1.56E+08	0	0	CHRNB2,C	exonic	NM_00015	.	.	.	ENST0000	CpG: 125,	1q22-q21.3
ACaP0	1	1.57E+08	1.58E+08	0	0	ETV3,FCR	exonic	NM_00114	.	.	.	ENST0000	CpG: 57,C	1q23.1
ACaP0	3	16970000	16980000	0	0	PLCL2,MII	ncRNA_ex	NR_03746	.	.	.	ENST0000	.	3p24.3
ACaP0	4	9200000	9370000	0	0	USP17L22	exonic	NM_00124	.	.	.	ENST0000	.	4p16.1
ACaP0	6	1.61E+08	1.61E+08	0	0	PLG,LPA	exonic	NM_00030	.	.	.	ENST0000	CpG: 36	6q26
ACaP0	6	1.61E+08	1.61E+08	0	0	PLG	exonic	NM_00030	.	.	.	ENST0000	.	6q26
ACaP0	8	10920000	10930000	0	0	XKR6	intronic	NM_17368	.	.	.	ENST0000	CpG: 16	8p23.1
ACaP0	10	1.3E+08	1.3E+08	0	0	MKI67	exonic	NM_00114	.	.	.	ENST0000	.	10q26.2
ACaP0	13	20980000	21080000	0	0	MIR4499,C	exonic	NM_01597	.	.	.	ENST0000	CpG: 72,C	13q12.11
ACaP0	13	25270000	25290000	0	0	ATP12A	exonic	NM_00118	.	.	.	ENST0000	.	13q12.12
ACaP0	14	1.06E+08	1.06E+08	0	0	KIAA0125	ncRNA_ex	NR_02680	.	.	.	ENST0000	.	14q32.33
ACaP0	14	1.06E+08	1.07E+08	0	0	ADAM6,LII	ncRNA_ex	NR_00222	.	.	.	ENST0000	CpG: 22,C	14q32.33
ACaP0	14	1.07E+08	1.07E+08	0	0	.	intergenic	NR_02745	dist=33847	.	.	.	.	14q32.33
ACaP0	15	1.02E+08	1.02E+08	0	0	.	intergenic	NM_15233	dist=15355	.	.	ENST0000	CpG: 87,C	15q26.3
ACaP0	16	52580000	52590000	0	0	CASC16,T	exonic	NM_00108	.	.	.	ENST0000	CpG: 98,C	16q12.1
ACaP0	18	10450000	10460000	0	0	APCDD1	exonic	NM_15300	.	.	.	ENST0000	CpG: 91,C	18p11.22

ACaP0:	20	25590000	31840000	0	0	DEFB116,I	exonic	NM_0010C	.	.	.	ENST000C	CpG: 43,C	20p11.21-p
ACaP0:	20	35150000	35890000	0	0	RBL1,TGIF	exonic	NM_00104	.	.	.	ENST000C	CpG: 38,C	20q11.23
ACaP0:	X	49170000	49370000	0	0	GAGE12C	exonic	NM_00104	.	.	.	ENST000C	CpG: 42,C	Xp11.23
ACaP0:	1	1380000	1450000	0	0	ATAD3C,A	exonic	NM_00103	.	.	.	ENST000C	CpG: 27,C	1p36.33
ACaP0:	1	1.44E+08	1.45E+08	0	0	NBPF8,NB	exonic	NM_00103	.	.	.	ENST000C	CpG: 86,C	1q21.1
ACaP0:	1	1.45E+08	1.45E+08	0	0	NBPF8,NB	UTR3	NM_00103	NM_00103	.	.	ENST000C	.	1q21.1
ACaP0:	1	1.45E+08	1.45E+08	0	0	NBPF20,N	exonic	NM_0010C	.	.	.	ENST000C	CpG: 116,(	1q21.1
ACaP0:	1	1.45E+08	1.45E+08	0	0	NBPF9,PD	intronic	NM_00127	.	.	.	ENST000C	CpG: 42	1q21.1
ACaP0:	1	1.45E+08	1.45E+08	0	0	NBPF10,N	exonic	NM_00103	.	.	.	ENST000C	CpG: 41,C	1q21.1
ACaP0:	1	1.45E+08	1.45E+08	0	0	NBPF20,N	ncRNA_ex	NR_10421	.	.	.	ENST000C	CpG: 27,C	1q21.1
ACaP0:	1	1.5E+08	1.5E+08	0	0	HIST2H2A	exonic	NM_0010C	.	.	.	ENST000C	CpG: 288,(	1q21.2
ACaP0:	1	1.5E+08	1.5E+08	0	0	HIST2H2B	exonic	NM_00103	.	.	.	ENST000C	CpG: 86,C	1q21.2
ACaP0:	1	1.55E+08	1.55E+08	0	0	ADAR,CHI	exonic	NM_00102	.	.	.	ENST000C	.	1q21.3
ACaP0:	1	1.56E+08	1.56E+08	0	0	MSTO2P,Y	exonic	NM_00119	.	.	.	ENST000C	CpG: 63	1q22
ACaP0:	1	1.56E+08	1.56E+08	0	0	DAP3,YY1	exonic	NM_00119	.	.	.	ENST000C	CpG: 128	1q22
ACaP0:	1	2.28E+08	2.28E+08	0	0	GJC2,C1o	exonic	NM_00085	.	.	.	ENST000C	CpG: 24,C	1q42.13
ACaP0:	1	2.28E+08	2.28E+08	0	0	OBSCN,C1	exonic	NM_00109	.	.	.	ENST000C	CpG: 23,C	1q42.13
ACaP0:	2	2.2E+08	2.2E+08	0	0	NHEJ1,ZF	exonic	NM_0010C	.	.	.	ENST000C	CpG: 68,C	2q35
ACaP0:	3	1.72E+08	1.72E+08	0	0	TNFSF10,I	exonic	NM_00114	.	.	.	ENST000C	.	3q26.31
ACaP0:	4	9200000	9210000	0	0	.	intergenic	NM_00104	dist=24787	.	.	.	.	4p16.1
ACaP0:	4	9210000	9370000	0	0	USP17L19	exonic	NM_00124	.	.	.	ENST000C	.	4p16.1
ACaP0:	4	13460000	13540000	0	0	LINC0109	exonic	NM_00101	.	.	.	ENST000C	CpG: 132,(	4p15.33
ACaP0:	4	13540000	13590000	0	0	BOD1L1,N	exonic	NM_00118	.	.	.	ENST000C	CpG: 218,(	4p15.33
ACaP0:	4	1.91E+08	1.91E+08	0	0	DUX4,DUX	exonic	NM_00112	.	.	.	ENST000C	CpG: 2005	4q35.2
ACaP0:	5	150000	320000	0	0	LOC10246	exonic	NM_00108	.	.	.	ENST000C	CpG: 117,(	5p15.33
ACaP0:	5	3610000	31930000	0	0	PRDM9,PI	exonic	NM_00103	.	.	.	ENST000C	CpG: 54,C	5p15.1-p15
ACaP0:	5	31930000	32060000	0	0	PDZD2,MI	exonic	NM_17814	.	.	.	ENST000C	.	5p13.3
ACaP0:	5	32060000	45470000	0	0	C5orf28,U	exonic	NM_00006	.	.	.	ENST000C	CpG: 129,(	5p13.2-p12
ACaP0:	6	26010000	26430000	0	0	BTN3A1,H	exonic	NM_00041	.	.	.	ENST000C	CpG: 35,C	6p22.2
ACaP0:	6	26430000	26630000	0	0	ABT1,BTN	exonic	NM_00119	.	.	.	ENST000C	CpG: 72,C	6p22.2
ACaP0:	6	26630000	27120000	0	0	ZNF322,HI	exonic	NM_00124	.	.	.	ENST000C	CpG: 74,C	6p22.1-p22
ACaP0:	6	33150000	33290000	0	0	RXRB,COI	exonic	NM_00107	.	.	.	ENST000C	CpG: 97,C	6p21.32
ACaP0:	6	80810000	80840000	0	0	BCKDHB	exonic	NM_00005	.	.	.	ENST000C	CpG: 53	6q14.1
ACaP0:	6	1.61E+08	1.61E+08	0	0	LPA	exonic	NM_00557	.	.	.	ENST000C	.	6q26
ACaP0:	7	5680000	6190000	0	0	USP42,AN	exonic	NM_00053	.	.	.	ENST000C	CpG: 59,C	7p22.1

ACaP0	7	56180000	56190000	0	0	NUPR1L	exonic	NM_00114	.	.	.	ENST0000	CpG: 63	7p11.2
ACaP0	7	56190000	56450000	0	0	.	intergenic	NM_00114	dist=5910;	.	.	ENST0000	CpG: 40,C	7p11.2
ACaP0	7	1.44E+08	1.44E+08	0	0	ARHGEF3	exonic	NM_00100	.	.	.	ENST0000	CpG: 52,C	7q35
ACaP0	8	6870000	7140000	0	0	DEFA11P,	exonic	NM_00104	.	.	.	ENST0000	CpG: 60,C	8p23.1
ACaP0	8	37410000	37460000	0	0	.	intergenic	NM_00103	dist=61635	.	.	ENST0000	.	8p11.23
ACaP0	8	39130000	39340000	0	0	ADAM32,A	exonic	NM_14500	.	.	.	ENST0000	.	8p11.22
ACaP0	8	62480000	62500000	0	0	ASPH	exonic	NM_00116	.	.	.	ENST0000	.	8q12.3
ACaP0	8	62500000	63160000	0	0	ASPH,MIR	exonic	NM_00116	.	.	.	ENST0000	CpG: 37,C	8q12.3
ACaP0	8	86550000	86840000	0	0	REXO1L2I	ncRNA_ex	NR_00359	.	.	.	ENST0000	CpG: 130,	8q21.2
ACaP0	8	1.25E+08	1.25E+08	0	0	TMEM65	exonic	NM_19429	.	.	.	ENST0000	CpG: 158	8q24.13
ACaP0	8	1.46E+08	1.46E+08	0	0	RECQL4,G	exonic	NM_00100	.	.	.	ENST0000	CpG: 104,	8q24.3
ACaP0	8	1.46E+08	1.46E+08	0	0	ARHGAP3	intronic	NM_02525	.	.	.	ENST0000	CpG: 20,C	8q24.3
ACaP0	9	35650000	35830000	0	0	SPAG8,TL	exonic	NM_00101	.	.	.	ENST0000	CpG: 42,C	9p13.3
ACaP0	9	1.4E+08	1.4E+08	0	0	LCN15,SN	exonic	NM_00100	.	.	.	ENST0000	CpG: 21,C	9q34.3
ACaP0	11	57060000	57960000	0	0	OR9I1,TM	exonic	NM_00006	.	.	.	ENST0000	CpG: 90,C	11q12.1
ACaP0	11	1.19E+08	1.19E+08	0	0	VPS11,TR	exonic	NM_00019	.	.	.	ENST0000	CpG: 88,C	11q23.3
ACaP0	14	23340000	23380000	0	0	LRP10,RE	exonic	NM_00107	.	.	.	ENST0000	CpG: 64,C	14q11.2
ACaP0	14	23380000	23440000	0	0	RP11-298I	exonic	NM_00103	.	.	.	ENST0000	CpG: 50,C	14q11.2
ACaP0	14	24560000	24710000	0	0	PCK2,TINI	exonic	NM_00100	.	.	.	ENST0000	CpG: 102,	14q11.2-q1
ACaP0	15	67700000	67790000	0	0	IQCH,IQCI	exonic	NM_00103	.	.	.	ENST0000	.	15q23
ACaP0	15	75540000	75590000	0	0	GOLGA6C	exonic	NM_00114	.	.	.	ENST0000	.	15q24.2
ACaP0	15	90770000	90790000	0	0	CIB1,GDP	exonic	NM_00101	.	.	.	ENST0000	CpG: 82	15q26.1
ACaP0	15	1.02E+08	1.02E+08	0	0	.	intergenic	NM_15233	dist=5355;	.	.	ENST0000	.	15q26.3
ACaP0	16	14330000	14350000	0	0	MKL2	exonic	NM_01404	.	.	.	ENST0000	.	16p13.12
ACaP0	16	29380000	29550000	0	0	BOLA2B,S	exonic	NM_00101	.	.	.	ENST0000	CpG: 159,	16p11.2
ACaP0	16	30200000	30350000	0	0	LOC38824	exonic	NM_00101	.	.	.	ENST0000	CpG: 94,C	16p11.2
ACaP0	16	30350000	30380000	0	0	CD2BP2,T	exonic	NM_00124	.	.	.	ENST0000	CpG: 52	16p11.2
ACaP0	16	74360000	74460000	0	0	CLEC18B,	exonic	NM_00101	.	.	.	ENST0000	CpG: 110,	16q23.1
ACaP0	17	0	60000	0	0	LOC10050	exonic	NM_00358	.	.	.	ENST0000	CpG: 212,	17p13.3
ACaP0	19	33430000	33670000	0	0	WDR88,CI	exonic	NM_01802	.	.	.	ENST0000	CpG: 124,	19q13.11
ACaP0	19	55240000	55290000	0	0	KIR2DL3,K	exonic	NM_01421	.	.	.	ENST0000	.	19q13.42
ACaP0	20	17630000	17650000	0	0	RRBP1	exonic	NM_00104	.	.	.	ENST0000	.	20p12.1
ACaP0	20	35710000	35870000	0	0	MROH8,RI	exonic	NM_00113	.	.	.	ENST0000	CpG: 70,C	20q11.23
ACaP0	20	35870000	36010000	0	0	RPN2,GHI	exonic	NM_00100	.	.	.	ENST0000	CpG: 65,C	20q11.23
ACaP0	22	21470000	21830000	0	0	POM121L8	exonic	NM_00112	.	.	.	ENST0000	CpG: 48,C	22q11.21

ACaP03	22	25010000	25030000	0	0	GGT1,BCF	exonic	NM_00103	.	.	.	ENST000C	.	22q11.23
ACaP03	22	36350000	36930000	0	0	MYH9,APC	exonic	NM_00108	.	.	.	ENST000C	CpG: 48,C	22q12.3
ACaP03	X	31080000	37550000	0	0	XK,CHDC2	exonic	NM_00010	.	.	.	ENST000C	CpG: 20,C	Xp21.2-Xp
ACaP04	1	55530000	1.1E+08	0	0	PPAP2B,L	exonic	NM_00001	.	.	.	ENST000C	CpG: 90,C	1p31.2-p32
ACaP04	1	1.48E+08	1.5E+08	0	0	RP11-14N	exonic	NM_00103	.	.	.	ENST000C	CpG: 63,C	1q21.2
ACaP04	1	1.84E+08	2.01E+08	0	0	CDC73,RC	exonic	NM_00018	.	.	.	ENST000C	CpG: 55,C	1q31.2-q3
ACaP04	2	29110000	29160000	0	0	SNORD53	exonic	NM_01513	.	.	.	ENST000C	CpG: 79	2p23.2
ACaP04	2	31750000	70150000	0	0	AC007392	exonic	NM_00010	.	.	.	ENST000C	CpG: 180,(	2p13.3-p22
ACaP04	2	96500000	96650000	0	0	.	intergenic	NR_10373	dist=7271;(	.	.	ENST000C	.	2q11.1
ACaP04	2	97770000	98200000	0	0	AC159540	exonic	NM_00116	.	.	.	ENST000C	CpG: 105	2q11.2
ACaP04	2	1.09E+08	1.09E+08	0	0	RANBP2,L	exonic	NM_00119	.	.	.	ENST000C	CpG: 105,(	2q12.3
ACaP04	2	1.09E+08	1.1E+08	0	0	CCDC138,	exonic	NM_00626	.	.	.	ENST000C	CpG: 73	2q12.3
ACaP04	2	1.14E+08	1.16E+08	0	0	AC104653	exonic	NM_00100	.	.	.	ENST000C	CpG: 51,C	2q14.1-q1
ACaP04	2	1.39E+08	1.61E+08	0	0	EPC2,ZEB	exonic	NM_00040	.	.	.	ENST000C	CpG: 92,C	2q22.2-q2
ACaP04	2	1.61E+08	1.61E+08	0	0	LY75-CD3	exonic	NM_00119	.	.	.	ENST000C	CpG: 74	2q24.2
ACaP04	2	1.61E+08	1.61E+08	0	0	PLA2R1	exonic	NM_00100	.	.	.	ENST000C	.	2q24.2
ACaP04	2	1.88E+08	1.9E+08	0	0	MIR1245B	exonic	NM_00009	.	.	.	ENST000C	CpG: 108	2q32.2-q32
ACaP04	2	2.03E+08	2.03E+08	0	0	CDK15	exonic	NM_00126	.	.	.	ENST000C	.	2q33.1
ACaP04	2	2.03E+08	2.03E+08	0	0	CDK15,FZ	exonic	NM_00126	.	.	.	ENST000C	CpG: 338	2q33.1
ACaP04	3	0	7790000	0	0	CHL1,CNT	exonic	NM_00056	.	.	.	ENST000C	CpG: 67,C	3p26.1-p26
ACaP04	3	14970000	37830000	0	0	SLC4A7,Z(	exonic	NM_00006	.	.	.	ENST000C	CpG: 84,C	3p22.3-p24
ACaP04	3	53880000	1E+08	0	0	STX19,ZN	exonic	NM_00009	.	.	.	ENST000C	CpG: 105,(	3p14.1-p12
ACaP04	3	1E+08	1E+08	0	0	TFG,ABI3E	exonic	NM_00100	.	.	.	ENST000C	.	3q12.2
ACaP04	3	1E+08	1.01E+08	0	0	ABI3BP	exonic	NM_01542	.	.	.	ENST000C	.	3q12.2
ACaP04	3	1.3E+08	1.32E+08	0	0	NPHP3-AC	exonic	NM_00100	.	.	.	ENST000C	CpG: 29,C	3q22.1
ACaP04	4	0	50000	0	0	.	intergenic	NONE,NM	dist=NONE	.	.	ENST000C	.	4p16.3
ACaP04	4	50000	290000	0	0	ZNF876P,Z	exonic	NM_00103	.	.	.	ENST000C	CpG: 46,C	4p16.3
ACaP04	4	290000	360000	0	0	ZNF141	exonic	NM_00344	.	.	.	ENST000C	CpG: 156,(	4p16.3
ACaP04	4	360000	440000	0	0	ZNF721,ZI	exonic	NM_00344	.	.	.	ENST000C	CpG: 38	4p16.3
ACaP04	4	5520000	5530000	0	0	C4orf6	exonic	NM_00575	.	.	.	ENST000C	.	4p16.2
ACaP04	4	10440000	17720000	0	0	FGFBP1,B	exonic	NM_00032	.	.	.	ENST000C	CpG: 24,C	4p15.32-p1
ACaP04	4	17790000	17910000	0	0	DCAF16,N	exonic	NM_00116	.	.	.	ENST000C	CpG: 107	4p15.32-p1
ACaP04	4	40930000	40950000	0	0	APBB2	exonic	NM_00116	.	.	.	ENST000C	.	4p14
ACaP04	4	42020000	42060000	0	0	SLC30A9	exonic	NM_00634	.	.	.	ENST000C	.	4p13
ACaP04	4	42060000	42140000	0	0	BEND4,SL	exonic	NM_00115	.	.	.	ENST000C	.	4p13

ACaP0	4	47860000	47980000	0	0	CNGA1,NF	exonic	NM_00008	.	.	.	ENST0000	CpG: 86	4p12
ACaP0	4	47980000	48060000	0	0	NIPAL1,CN	exonic	NM_20733	.	.	.	ENST0000	CpG: 73	4p12
ACaP0	4	48610000	48630000	0	0	FRYL	exonic	NM_01503	.	.	.	ENST0000	.	4p11
ACaP0	4	68680000	68800000	0	0	TMPRSS1	exonic	NM_00111	.	.	.	ENST0000	.	4q13.2
ACaP0	4	68800000	68910000	0	0	TMPRSS1	exonic	NM_00111	.	.	.	ENST0000	.	4q13.2
ACaP0	4	68910000	69320000	0	0	SYT14L,L(	exonic	NM_00103	.	.	.	ENST0000	CpG: 27,C	4q13.2
ACaP0	4	71680000	71770000	0	0	MOB1B,GI	exonic	NM_00109	.	.	.	ENST0000	CpG: 107,(	4q13.3
ACaP0	4	76570000	76740000	0	0	USO1,G3E	exonic	NM_00371	.	.	.	ENST0000	CpG: 126,(	4q21.1
ACaP0	4	76740000	76870000	0	0	NAAA,PPE	exonic	NM_00104	.	.	.	ENST0000	CpG: 133	4q21.1
ACaP0	4	77910000	77940000	0	0	11-Sep	exonic	NM_01824	.	.	.	ENST0000	.	4q21.1
ACaP0	4	77940000	78650000	0	0	CXCL13,C	exonic	NM_00128	.	.	.	ENST0000	CpG: 147,(	4q21.1
ACaP0	4	96190000	1.06E+08	0	0	SLC9B1,C	exonic	NM_00025	.	.	.	ENST0000	CpG: 155,(	4q24-q22.3
ACaP0	4	1.09E+08	1.09E+08	0	0	HADH	exonic	NM_00118	.	.	.	ENST0000	.	4q25
ACaP0	4	1.1E+08	1.11E+08	0	0	CFI,SEC24	exonic	NM_00020	.	.	.	ENST0000	CpG: 117,(	4q25
ACaP0	4	1.11E+08	1.11E+08	0	0	GAR1,RRH	exonic	NM_00658	.	.	.	ENST0000	CpG: 23	4q25
ACaP0	4	1.13E+08	1.21E+08	0	0	PDE5A,NE	exonic	NM_00013	.	.	.	ENST0000	CpG: 28,C	4q26-q25
ACaP0	4	1.21E+08	1.22E+08	0	0	TNIP3,NDI	exonic	NM_00112	.	.	.	ENST0000	CpG: 112,(	4q27
ACaP0	4	1.22E+08	1.24E+08	0	0	CCNA2,IL2	exonic	NM_00058	.	.	.	ENST0000	CpG: 160,(	4q27
ACaP0	4	1.24E+08	1.24E+08	0	0	NUDT6,FG	exonic	NM_00200	.	.	.	ENST0000	.	4q28.1-q27
ACaP0	4	1.24E+08	1.24E+08	0	0	NUDT6	exonic	NM_00708	.	.	.	ENST0000	.	4q28.1
ACaP0	4	1.41E+08	1.41E+08	0	0	SCOC,RP1	exonic	NM_00115	.	.	.	ENST0000	CpG: 55	4q31.1
ACaP0	4	1.44E+08	1.44E+08	0	0	USP38,GA	exonic	NM_00203	.	.	.	ENST0000	CpG: 18,C	4q31.21
ACaP0	4	1.53E+08	1.58E+08	0	0	KIAA0922,	exonic	NM_00050	.	.	.	ENST0000	CpG: 21,C	4q31.3-q32
ACaP0	4	1.58E+08	1.58E+08	0	0	GRIA2	exonic	NM_00082	.	.	.	ENST0000	.	4q32.1
ACaP0	4	1.65E+08	1.65E+08	0	0	1-Mar	exonic	NM_00116	.	.	.	ENST0000	.	4q32.3
ACaP0	4	1.69E+08	1.69E+08	0	0	DDX60L,D	exonic	NM_00101	.	.	.	ENST0000	CpG: 27	4q32.3
ACaP0	4	1.69E+08	1.69E+08	0	0	DDX60L	exonic	NM_00101	.	.	.	ENST0000	.	4q32.3
ACaP0	4	1.71E+08	1.71E+08	0	0	AADAT	exonic	NM_00128	.	.	.	ENST0000	.	4q33
ACaP0	4	1.78E+08	1.78E+08	0	0	VEGFC	exonic	NM_00542	.	.	.	ENST0000	CpG: 113	4q34.3
ACaP0	4	1.91E+08	1.91E+08	0	0	LOC10028	exonic	NM_00100	.	.	.	ENST0000	CpG: 95,C	4q35.2
ACaP0	4	1.91E+08	1.91E+08	0	0	.	intergenic	NM_03317	dist=6558;(	.	.	.	.	4q35.2
ACaP0	5	36100000	68720000	0	0	LOC10192	exonic	NM_00006	.	.	.	ENST0000	CpG: 100,(	5p12-q12.1
ACaP0	5	70750000	1.31E+08	0	0	ST8SIA4,N	exonic	NM_00003	.	.	.	ENST0000	CpG: 33,C	5q21.2-q21
ACaP0	5	1.7E+08	1.71E+08	0	0	FGF18,NP	exonic	NM_00103	.	.	.	ENST0000	CpG: 96,C	5q35.1
ACaP0	6	17600000	17780000	0	0	NUP153,K	exonic	NM_00110	.	.	.	ENST0000	CpG: 86,C	6p22.3

ACaP0	6	42530000	42650000	0	0	UBR2	exonic	NM_00118	.	.	.	ENST0000	CpG: 29	6p21.1
ACaP0	6	44280000	55920000	0	0	SLC25A27	exonic	NM_00025	.	.	.	ENST0000	CpG: 50,C	6p12.1-p12
ACaP0	6	55920000	57060000	0	0	KIAA1586,	exonic	NM_00103	.	.	.	ENST0000	CpG: 73,C	6p11.2-p12
ACaP0	6	64380000	89550000	0	0	CNR1,COL	exonic	NM_00005	.	.	.	ENST0000	CpG: 58,C	6q14.3-q13
ACaP0	6	89550000	89830000	0	0	RNGTT,SF	exonic	NM_00128	.	.	.	ENST0000	CpG: 60,C	6q15
ACaP0	6	89830000	89960000	0	0	PM20D2,G	exonic	NM_00101	.	.	.	ENST0000	CpG: 109	6q15
ACaP0	6	1.08E+08	1.38E+08	0	0	SNX3,SER	exonic	NM_00004	.	.	.	ENST0000	CpG: 152,(	6q23.3-q22
ACaP0	6	1.39E+08	1.5E+08	0	0	SF3B5,GP	exonic	NM_00100	.	.	.	ENST0000	CpG: 44,C	6q24.1-q24
ACaP0	7	1.02E+08	1.03E+08	0	0	FAM185A,	exonic	NM_00111	.	.	.	ENST0000	CpG: 51	7q22.1
ACaP0	7	1.07E+08	1.25E+08	0	0	ST7,CAV1	exonic	NM_00010	.	.	.	ENST0000	CpG: 59,C	7q31.1-q31
ACaP0	8	38850000	39680000	0	0	ADAM32,L	exonic	NM_00102	.	.	.	ENST0000	CpG: 47,C	8p11.22
ACaP0	8	48950000	86390000	0	0	MRPL15,C	exonic	NM_00006	.	.	.	ENST0000	CpG: 34,C	8q21.13-q1
ACaP0	8	88220000	95540000	0	0	FAM92A1,	exonic	NM_00100	.	.	.	ENST0000	CpG: 44,C	8q21.3-q22
ACaP0	9	0	10000	0	0	.	intergenic	NONE,NR_	dist=NONE	.	.	.	.	9p24.3
ACaP0	9	10000	180000	0	0	DDX11L5,I	exonic	NM_00114	.	.	.	ENST0000	CpG: 69,C	9p24.3
ACaP0	9	180000	13100000	0	0	INSL6,SLC	exonic	NM_00017	.	.	.	ENST0000	CpG: 39,C	9p24.1-p24
ACaP0	9	13100000	13260000	0	0	MPDZ	exonic	NM_00126	.	.	.	ENST0000	.	9p23
ACaP0	9	13260000	32510000	0	0	ZDHHC21,	exonic	NM_00007	.	.	.	ENST0000	CpG: 61,C	9p21.1-p21
ACaP0	9	42360000	42720000	0	0	FOXD4L4,	exonic	NM_00101	.	.	.	ENST0000	CpG: 112,(	9p12
ACaP0	9	42750000	44410000	0	0	RP11-381(	exonic	NM_00101	.	.	.	ENST0000	CpG: 27,C	9p11.2-p12
ACaP0	9	67920000	71090000	0	0	FOXD4L3,	exonic	NM_00101	.	.	.	ENST0000	CpG: 29,C	9q13-q21.1
ACaP0	9	94970000	95300000	0	0	ASPN,MIR	exonic	NM_00101	.	.	.	ENST0000	CpG: 64,C	9q22.31
ACaP0	9	1.02E+08	1.12E+08	0	0	OR13C9,N	exonic	NM_00003	.	.	.	ENST0000	CpG: 31,C	9q31.2-q22
ACaP0	10	15550000	22680000	0	0	STAM,EBL	exonic	NM_00072	.	.	.	ENST0000	CpG: 112,(	10p12.2-p1
ACaP0	10	31130000	38270000	0	0	ZEB1-AS1	exonic	NM_00102	.	.	.	ENST0000	CpG: 20,C	10p11.22-p
ACaP0	10	51020000	51050000	0	0	PARG	exonic	NM_00363	.	.	.	ENST0000	.	10q11.23
ACaP0	10	51050000	51250000	0	0	AGAP8,PA	exonic	NM_00107	.	.	.	ENST0000	CpG: 18	10q11.23
ACaP0	10	1.06E+08	1.23E+08	0	0	MIR4483,S	exonic	NM_00014	.	.	.	ENST0000	CpG: 57,C	10q26.11-c
ACaP0	11	20380000	43970000	0	0	CCDC73,L	exonic	NM_00028	.	.	.	ENST0000	CpG: 20,C	11p13-p14
ACaP0	11	1.11E+08	1.17E+08	0	0	NCAM1-AS	exonic	NM_00003	.	.	.	ENST0000	CpG: 110,(	11q23.3-q2
ACaP0	12	8320000	8810000	0	0	LINC0093	exonic	NM_00100	.	.	.	ENST0000	CpG: 75,C	12p13.31
ACaP0	12	8810000	8980000	0	0	MFAP5,RI	exonic	NM_00348	.	.	.	ENST0000	CpG: 171	12p13.31
ACaP0	12	8980000	9750000	0	0	MIR1244-2	exonic	NM_00001	.	.	.	ENST0000	CpG: 41,C	12p13.31
ACaP0	12	14590000	24050000	0	0	SLCO1B1,	exonic	NM_00041	.	.	.	ENST0000	CpG: 121,(	12p12.2-p1
ACaP0	12	44400000	45280000	0	0	TMEM117,	exonic	NM_00114	.	.	.	ENST0000	CpG: 82	12q12

ACaP04	12	58330000	1.07E+08	0	0	PLXNC1,G	exonic	NM_00023	.	.	.	ENST0000	CpG: 64,C	12q21.32-q
ACaP04	13	32690000	38230000	0	0	LINC00423	exonic	NM_00005	.	.	.	ENST0000	CpG: 43,C	13q13.2-q1
ACaP04	13	80090000	80130000	0	0	NDFIP2	exonic	NM_00116	.	.	.	ENST0000	.	13q31.1
ACaP04	13	96320000	96690000	0	0	DNAJC3,U	exonic	NM_00626	.	.	.	ENST0000	CpG: 117	13q32.1
ACaP04	14	25320000	35330000	0	0	AKAP6,SC	exonic	NM_00103	.	.	.	ENST0000	CpG: 50,C	14q12-q13
ACaP04	14	35330000	35530000	0	0	SRP54,IGE	exonic	NM_00107	.	.	.	ENST0000	CpG: 183,(	14q13.2
ACaP04	14	35530000	50300000	0	0	MIR4503,F	exonic	NM_00100	.	.	.	ENST0000	CpG: 123,(	14q21.3-q2
ACaP04	14	76170000	76250000	0	0	TTLL5	exonic	NM_01507	.	.	.	ENST0000	.	14q24.3
ACaP04	14	80120000	91600000	0	0	EFCAB11,	exonic	NM_00015	.	.	.	ENST0000	CpG: 240,(	14q31.3-q3
ACaP04	14	1.01E+08	1.01E+08	0	0	SNORD11	ncRNA_ex	NR_00319	.	.	.	ENST0000	.	14q32.2-q3
ACaP04	15	42480000	42500000	0	0	VPS39,MIF	exonic	NM_01528	.	.	.	ENST0000	.	15q15.1
ACaP04	15	42500000	42550000	0	0	TMEM87A	exonic	NM_00128	.	.	.	ENST0000	CpG: 35	15q15.1
ACaP04	16	33950000	48650000	0	0	LINC00273	exonic	NM_00029	.	.	.	ENST0000	CpG: 117,(	16p11.2-q1
ACaP04	17	18670000	18750000	0	0	FBXW10,T	exonic	NM_00126	.	.	.	ENST0000	CpG: 42	17p11.2
ACaP04	17	18750000	18800000	0	0	PRPSAP2	exonic	NM_00124	.	.	.	ENST0000	CpG: 49	17p11.2
ACaP04	18	14120000	20740000	0	0	ANKRD20A	exonic	NM_00110	.	.	.	ENST0000	CpG: 23,C	18p11.21-q
ACaP04	18	20740000	20930000	0	0	TMEM241,	exonic	NM_00110	.	.	.	ENST0000	.	18q11.2
ACaP04	18	20930000	30530000	0	0	AQP4-AS1	exonic	NM_00022	.	.	.	ENST0000	CpG: 89,C	18q11.2-q1
ACaP04	19	36800000	38380000	0	0	ZNF540,ZF	exonic	NM_00101	.	.	.	ENST0000	CpG: 36,C	19q13.13-q
ACaP04	20	2670000	2690000	0	0	EBF4	exonic	NM_00111	.	.	.	ENST0000	CpG: 178	20p13
ACaP04	20	5980000	6000000	0	0	LOC10192	exonic	NM_00112	.	.	.	ENST0000	CpG: 104	20p12.3
ACaP04	20	58410000	58520000	0	0	PHACTR3	exonic	NM_00119	.	.	.	ENST0000	CpG: 154,(	20q13.33
ACaP04	21	34000000	34150000	0	0	SYNJ1,C21	exonic	NM_00116	.	.	.	ENST0000	CpG: 109,(	21q22.11
ACaP04	22	18650000	18880000	0	0	USP18,GG	exonic	NM_01741	.	.	.	ENST0000	CpG: 41,C	22q11.21
ACaP05	2	0	87140000	0	0	VAMP8,RN	exonic	NM_00010	.	.	.	ENST0000	CpG: 98,C	2p22.2-p23
ACaP05	3	0	240000	0	0	CHL1	UTR5	NM_00125	NM_00125	.	.	ENST0000	CpG: 171	3p26.3
ACaP05	3	64420000	64440000	0	0	.	intergenic	NM_19885	dist=20886	.	.	ENST0000	CpG: 67	3p14.1
ACaP05	4	1.11E+08	1.11E+08	0	0	ENPEP	exonic	NM_00197	.	.	.	ENST0000	.	4q25
ACaP05	4	1.75E+08	1.78E+08	0	0	ADAM29,A	exonic	NM_00086	.	.	.	ENST0000	CpG: 34,C	4q34.1-q34
ACaP05	5	1.56E+08	1.56E+08	0	0	HAVCR1	exonic	NM_00109	.	.	.	ENST0000	.	5q33.3
ACaP05	5	1.56E+08	1.57E+08	0	0	HAVCR2	exonic	NM_03278	.	.	.	ENST0000	.	5q33.3
ACaP05	8	87380000	87430000	0	0	WWP1	exonic	NM_00701	.	.	.	ENST0000	.	8q21.3
ACaP05	12	8050000	8340000	0	0	ZNF705A,I	exonic	NM_00100	.	.	.	ENST0000	CpG: 42,C	12p13.31
ACaP05	12	8340000	8370000	0	0	FAM66C	ncRNA_ex	NR_02678	.	.	.	ENST0000	.	12p13.31
ACaP05	13	96670000	96690000	0	0	UGGT2	exonic	NM_02012	.	.	.	ENST0000	.	13q32.1

ACaP0!	14	26910000	27070000	0	0	NOVA1	exonic	NM_00251	.	.	.	ENST0000	CpG: 24	14q12
ACaP0!	14	94670000	94680000	0	0	PPP4R4	exonic	NM_02095	.	.	.	ENST0000	.	14q32.12
ACaP0!	14	1.04E+08	1.04E+08	0	0	CKB,TRM	exonic	NM_00182	.	.	.	ENST0000	CpG: 44,C	14q32.33-(
ACaP0!	15	30370000	31100000	0	0	DKFZP434	exonic	NM_00103	.	.	.	ENST0000	CpG: 274,(	15q13.2
ACaP0!	15	32450000	32900000	0	0	GOLGA8N	exonic	NM_00074	.	.	.	ENST0000	CpG: 78,C	15q13.3
ACaP0!	15	36870000	36880000	0	0	C15orf41	exonic	NM_00113	.	.	.	ENST0000	CpG: 35	15q14
ACaP0!	15	36880000	36900000	0	0	C15orf41	intronic	NM_00113	.	.	.	ENST0000	.	15q14
ACaP0!	15	36900000	36990000	0	0	C15orf41	exonic	NM_00113	.	.	.	ENST0000	.	15q14
ACaP0!	15	36990000	37010000	0	0	C15orf41	exonic	NM_00113	.	.	.	ENST0000	.	15q14
ACaP0!	15	82720000	83200000	0	0	RPS17,UB	exonic	NM_00102	.	.	.	ENST0000	CpG: 21,C	15q25.2
ACaP0!	15	83200000	83350000	0	0	RPS17,RP	exonic	NM_00102	.	.	.	ENST0000	CpG: 95,C	15q25.2
ACaP0!	15	84830000	85060000	0	0	GOLGA6L	exonic	NM_00124	.	.	.	ENST0000	CpG: 88,C	15q25.2
ACaP0!	15	1.02E+08	1.02E+08	0	0 .		intergenic	NM_15233	dist=15355	.	.	ENST0000	CpG: 87	15q26.3
ACaP0!	18	50760000	50770000	0	0	DCC	intronic	NM_00521	.	.	.	ENST0000	.	18q21.2
ACaP0!	20	29940000	35890000	0	0	GHRH,PSI	exonic	NM_00017	.	.	.	ENST0000	CpG: 47,C	20q11.21-(
ACaP0!	21	9820000	9830000	0	0	MIR3648,M	ncRNA_ex	NR_03742	.	.	.	ENST0000	CpG: 120	21p11.2
ACaP0!	X	8990000	31830000	0	0	ASB9,RPS	exonic	NM_0001C	.	.	.	ENST0000	CpG: 86,C	Xp22.12-X
ACaP0!	X	31830000	33150000	0	0	DMD	exonic	NM_0001C	.	.	.	ENST0000	.	Xp21.1
ACaP0!	X	33150000	44390000	0	0	CXorf38,M	exonic	NM_0001C	.	.	.	ENST0000	CpG: 54,C	Xp11.4-Xp
ACaP0!	X	49290000	52940000	0	0	XAGE1D,N	exonic	NM_00008	.	.	.	ENST0000	CpG: 60,C	Xp11.23-X
ACaP0(	1	12850000	12860000	0	0	PRAMEF1	exonic	NM_02301	.	.	.	ENST0000	.	1p36.21
ACaP0(	1	12860000	12880000	0	0	.	intergenic	NM_02301	dist=3223;(	.	.	.	.	1p36.21
ACaP0(	1	12880000	13720000	0	0	LOC64933	exonic	NM_0010C	.	.	.	ENST0000	.	1p36.21
ACaP0(	1	91730000	91860000	0	0	HFM1	exonic	NM_00101	.	.	.	ENST0000	.	1p22.2
ACaP0(	1	1.2E+08	1.2E+08	0	0	HSD3BP4,	exonic	NM_00019	.	.	.	ENST0000	CpG: 127,(	1p12
ACaP0(	1	1.2E+08	1.21E+08	0	0	ADAM30,N	exonic	NM_0012C	.	.	.	ENST0000	CpG: 21,C	1p12
ACaP0(	1	1.21E+08	1.48E+08	0	0	GJA5,RNF	exonic	NM_0010C	.	.	.	ENST0000	CpG: 19,C	1p12-q12
ACaP0(	1	1.48E+08	1.48E+08	0	0	NBPF8,MI	ncRNA_ex	NR_02746	.	.	.	ENST0000	CpG: 16,C	1q21.2
ACaP0(	1	1.48E+08	1.5E+08	0	0	NBPF15,N	exonic	NM_00056	.	.	.	ENST0000	CpG: 94,C	1q21.2
ACaP0(	1	1.5E+08	1.5E+08	0	0	HIST2H2B	exonic	NM_00103	.	.	.	ENST0000	CpG: 86,C	1q21.2
ACaP0(	1	1.5E+08	1.52E+08	0	0	CTSK,C1o	exonic	NM_00039	.	.	.	ENST0000	CpG: 53,C	1q21.2-q2
ACaP0(	1	1.58E+08	1.58E+08	0	0	FCRL5	exonic	NM_00119	.	.	.	ENST0000	.	1q23.1
ACaP0(	1	2.45E+08	2.49E+08	0	0	OR2L5,AH	exonic	NM_0010C	.	.	.	ENST0000	CpG: 65,C	1q44
ACaP0(	2	87120000	88330000	0	0	RGPD1,AN	exonic	NM_00102	.	.	.	ENST0000	CpG: 31,C	2p11.2
ACaP0(	2	88330000	88360000	0	0	KRCC1	UTR5	NM_01661	NM_01661	.	.	ENST0000	CpG: 60	2p11.2

ACaP0	2	89310000	89320000	0	0	.	intergenic	NR_03963	dist=19803	.	.	ENST000	.	2p11.2
ACaP0	2	89320000	95520000	0	0	FAM95A,G	ncRNA_ex	NR_00336	.	.	.	ENST000	CpG: 121,	2p11.1-q1
ACaP0	2	95520000	95530000	0	0	ANKRD20	ncRNA_ex	NR_00336	.	.	.	ENST000	CpG: 120,	2q11.1
ACaP0	2	1.09E+08	1.09E+08	0	0	RANBP2,C	exonic	NM_00626	.	.	.	ENST000	CpG: 73	2q12.3
ACaP0	2	1.09E+08	1.1E+08	0	0	EDAR,CC	exonic	NM_02233	.	.	.	ENST000	.	2q12.3
ACaP0	2	1.33E+08	1.61E+08	0	0	RIF1,AC01	exonic	NM_00040	.	.	.	ENST000	CpG: 187,	2q21.3-q2
ACaP0	2	1.71E+08	1.71E+08	0	0	UBR3,PH	exonic	NM_00119	.	.	.	ENST000	CpG: 72,C	2q31.1
ACaP0	2	1.71E+08	1.78E+08	0	0	OLA1,AC0	exonic	NM_00007	.	.	.	ENST000	CpG: 47,C	2q31.2-q3
ACaP0	2	2.01E+08	2.02E+08	0	0	CASP10,S	exonic	NM_00108	.	.	.	ENST000	CpG: 53,C	2q33.1
ACaP0	2	2.21E+08	2.33E+08	0	0	LINC0047	exonic	NM_00009	.	.	.	ENST000	CpG: 242,	2q37.1-q3
ACaP0	3	42130000	45150000	0	0	CLEC3B,Z	exonic	NM_00072	.	.	.	ENST000	CpG: 29,C	3p21.33-p
ACaP0	3	45150000	45750000	0	0	CDCP1,S	exonic	NM_01401	.	.	.	ENST000	CpG: 21,C	3p21.31
ACaP0	3	45750000	45860000	0	0	SACM1L,S	exonic	NM_01401	.	.	.	ENST000	CpG: 88	3p21.31
ACaP0	3	57610000	57850000	0	0	SLMAP,DE	exonic	NM_00715	.	.	.	ENST000	CpG: 116,	3p14.3
ACaP0	3	57850000	57990000	0	0	SLMAP	exonic	NM_00715	.	.	.	ENST000	.	3p14.3
ACaP0	3	1.09E+08	1.09E+08	0	0	MORC1,F	exonic	NM_01442	.	.	.	ENST000	CpG: 36	3q13.13
ACaP0	3	1.23E+08	1.24E+08	0	0	CCDC14,	exonic	NM_02275	.	.	.	ENST000	CpG: 68,C	3q21.1
ACaP0	3	1.28E+08	1.29E+08	0	0	KIAA1257,	exonic	NM_00017	.	.	.	ENST000	CpG: 37,C	3q22.1-q2
ACaP0	3	1.29E+08	1.29E+08	0	0	TMCC1	UTR3	NM_00101	NM_00101	.	.	ENST000	CpG: 65	3q22.1
ACaP0	3	1.29E+08	1.93E+08	0	0	PIK3CA,L	exonic	NM_00005	.	.	.	ENST000	CpG: 136,	3q22.2-q2
ACaP0	3	1.93E+08	1.93E+08	0	0	ATP13A5,	exonic	NM_03227	.	.	.	ENST000	.	3q29
ACaP0	3	1.93E+08	1.93E+08	0	0	.	upstream	NM_01556	.	.	.	ENST000	.	3q29
ACaP0	3	1.93E+08	1.96E+08	0	0	FAM43A,L	exonic	NM_00101	.	.	.	ENST000	CpG: 108,	3q29
ACaP0	3	1.96E+08	1.96E+08	0	0	.	intergenic	NR_00326	dist=1285	.	.	.	.	3q29
ACaP0	3	1.96E+08	1.98E+08	0	0	UBXN7,LR	exonic	NM_00099	.	.	.	ENST000	CpG: 57,C	3q29
ACaP0	3	1.98E+08	1.98E+08	0	0	ANKRD18	ncRNA_ex	NR_00329	.	.	.	ENST000	CpG: 25,C	3q29
ACaP0	3	1.98E+08	1.98E+08	0	0	FAM157A	exonic	NM_00114	.	.	.	ENST000	CpG: 27,C	3q29
ACaP0	3	1.98E+08	1.98E+08	0	0	.	intergenic	NM_00114	dist=52272	.	.	.	.	3q29
ACaP0	4	9270000	9380000	0	0	USP17L25	exonic	NM_00124	.	.	.	ENST000	.	4p16.1
ACaP0	4	76520000	76740000	0	0	CDKL2,US	exonic	NM_00371	.	.	.	ENST000	CpG: 76,C	4q21.1
ACaP0	4	76740000	83740000	0	0	CXCL9,NU	exonic	NM_00100	.	.	.	ENST000	CpG: 111,	4q21.22-q
ACaP0	4	83740000	83910000	0	0	THAP9,LI	exonic	NM_00107	.	.	.	ENST000	CpG: 34,C	4q21.22
ACaP0	4	83910000	84210000	0	0	COPS4,C	exonic	NM_00113	.	.	.	ENST000	CpG: 64,C	4q21.23-q
ACaP0	4	1.29E+08	1.29E+08	0	0	LARP1B	exonic	NM_00127	.	.	.	ENST000	.	4q28.2
ACaP0	4	1.43E+08	1.44E+08	0	0	INPP4B,U	exonic	NM_00110	.	.	.	ENST000	CpG: 121,	4q31.21

ACaP0(	5	3500000	68870000	0	0	GAPT,EGF	exonic	NM_00006	.	.	.	ENST0000	CpG: 134,(	5p15.33-q1
ACaP0(	7302	3	65370000	gain	AAA	-1								
ACaP0(	5	68870000	70960000	0	0	GUSBP9,S	exonic	NM_00034	.	.	.	ENST0000	CpG: 61,C	5q13.2
ACaP0(	5	70960000	82770000	0	0	CKMT2-AS	exonic	NM_00004	.	.	.	ENST0000	CpG: 27,C	5q14.2-q13
ACaP0(	5	82770000	82790000	0	0	VCAN	exonic	NM_00112	.	.	.	ENST0000	CpG: 39	5q14.2
ACaP0(	5	1.56E+08	1.57E+08	0	0	TIMD4,FAI	exonic	NM_00103	.	.	.	ENST0000	CpG: 36,C	5q33.3
ACaP0(	5	1.8E+08	1.8E+08	0	0	BTNL8	exonic	NM_00104	.	.	.	ENST0000	.	5q35.3
ACaP0(	5	1.81E+08	1.81E+08	0	0	LOC10013	exonic	NM_0010C	.	.	.	ENST0000	CpG: 243,(	5q35.3
ACaP0(	5	1.81E+08	1.81E+08	0	0	.	intergenic	NM_0010C	dist=10477	.	.	.	.	5q35.3
ACaP0(	6	10430000	25990000	0	0	TDP2,TRII	exonic	NM_00033	.	.	.	ENST0000	CpG: 34,C	6p23-p24.3
ACaP0(	6	70380000	1.56E+08	0	0	GSTM2P1	exonic	NM_00004	.	.	.	ENST00000606998.1,ENST0000(		
ACaP0(	,EN!	CpG: 93,C	6q14.1-q2	Score=0.9	Score=650	esv2656528,esv2678613,esv2732520,esv2732451,esv2665536,nsv510902,esv28643,esv2669643,es								
ACaP0(	8237	3	85360000	gain	AAA	-1								
ACaP0(	7	57020000	63360000	0	0	MIR4283-2	exonic	NM_00115	.	.	.	ENST0000	CpG: 139,(	7q11.1-p11
ACaP0(	7	72430000	72720000	0	0	LOC10010	exonic	NM_00116	.	.	.	ENST0000	CpG: 77,C	7q11.23
ACaP0(	7	74180000	75150000	0	0	NCF1,PMS	exonic	NM_00026	.	.	.	ENST0000	CpG: 51,C	7q11.23
ACaP0(	7	75930000	76690000	0	0	ZP3,UPK3	exonic	NM_0011C	.	.	.	ENST0000	CpG: 22,C	7q11.23
ACaP0(	7	1.02E+08	1.02E+08	0	0	SPDYE2B,	exonic	NM_00103	.	.	.	ENST0000	CpG: 37,C	7q22.1
ACaP0(	7	1.02E+08	1.03E+08	0	0	PSMC2,PN	exonic	NM_00103	.	.	.	ENST0000	CpG: 99,C	7q22.1
ACaP0(	7	1.03E+08	1.03E+08	0	0	SLC26A5	exonic	NM_00116	.	.	.	ENST0000	.	7q22.1
ACaP0(	7	1.4E+08	1.4E+08	0	0	TBXAS1	exonic	NM_00106	.	.	.	ENST0000	.	7q34
ACaP0(	8	0	10000	0	0	.	intergenic	NONE,NM_	dist=NONE	.	.	.	.	8p23.3
ACaP0(	8	10000	190000	0	0	OR4F21,R	exonic	NM_0010C	.	.	.	ENST0000	CpG: 109,(	8p23.3
ACaP0(	8	190000	280000	0	0	ZNF596	exonic	NM_00104	.	.	.	ENST0000	CpG: 40,C	8p23.3
ACaP0(	8	19190000	19460000	0	0	CSGALNA	exonic	NM_00113	.	.	.	ENST0000	CpG: 20,C	8p21.3
ACaP0(	8	25040000	25720000	0	0	DOCK5,EE	exonic	NM_00082	.	.	.	ENST0000	CpG: 97,C	8p21.2
ACaP0(	8	87060000	1.42E+08	0	0	OC90,MIR	exonic	NM_00012	.	.	.	ENST0000	CpG: 55,C	8q22.2-q24
ACaP0(	9	33530000	34180000	0	0	UBE2R2,L	exonic	NM_00117	.	.	.	ENST0000	CpG: 18,C	9p13.3
ACaP0(	9	34180000	34240000	0	0	UBAP1	exonic	NM_00117	.	.	.	ENST0000	.	9p13.3
ACaP0(	9	39070000	70920000	0	0	LINC0118(	exonic	NM_00101	.	.	.	ENST0000	CpG: 103,(	9p11.1-q13
ACaP0(	9	70920000	70990000	0	0	FOXD4L3,	exonic	NM_02196	.	.	.	ENST0000	CpG: 100,(	9q21.11
ACaP0(	9	70990000	77360000	0	0	TRPM3,PF	exonic	NM_00014	.	.	.	ENST0000	CpG: 177,(	9q21.13-q2
ACaP0(	9	1.02E+08	1.14E+08	0	0	GRIN3A,TI	exonic	NM_00003	.	.	.	ENST0000	CpG: 49,C	9q31.1-q31
ACaP0(	9	1.14E+08	1.14E+08	0	0	OR2K2,KI/	exonic	NM_00108	.	.	.	ENST0000	.	9q31.3
ACaP0(	9	1.17E+08	1.29E+08	0	0	DENND1A	exonic	NM_00017	.	.	.	ENST0000	CpG: 89,C	9q33.3-q33

ACaP0(	9	1.33E+08	1.33E+08	0	0	FNBP1	exonic	NM_01503	.	.	.	ENST0000	CpG: 26,C	9q34.11
ACaP0(	10	48030000	49620000	0	0	FRMPD2P	exonic	NM_00101	.	.	.	ENST0000	CpG: 111,(	10q11.22
ACaP0(	10	49620000	49680000	0	0	MAPK8,AF	exonic	NM_00125	.	.	.	ENST0000	CpG: 86,C	10q11.22
ACaP0(	10	96490000	96750000	0	0	CYP2C19,	exonic	NM_00076	.	.	.	ENST0000	.	10q23.33
ACaP0(	10	1.16E+08	1.16E+08	0	0	ABLIM1	exonic	NM_00231	.	.	.	ENST0000	.	10q25.3
ACaP0(	10	1.3E+08	1.3E+08	0	0	MKI67	exonic	NM_00114	.	.	.	ENST0000	.	10q26.2
ACaP0(	11	0	80000	0	0	.	intergenic	NONE,NR_	dist=NONE	.	.	.	.	11p15.5
ACaP0(	11	80000	140000	0	0	LINC0100	ncRNA_ex	NR_02832	.	.	.	ENST0000	CpG: 30	11p15.5
ACaP0(	11	18250000	18590000	0	0	SAA1,SAA	exonic	NM_00033	.	.	.	ENST0000	CpG: 52,C	11p15.1
ACaP0(	11	18590000	18720000	0	0	SPTY2D1,	exonic	NM_00104	.	.	.	ENST0000	CpG: 38,C	11p15.1
ACaP0(	11	62850000	63170000	0	0	SLC22A9,I	exonic	NM_00103	.	.	.	ENST0000	.	11q12.3
ACaP0(	11	63170000	63520000	0	0	RARRES3	exonic	NM_00112	.	.	.	ENST0000	CpG: 83,C	11q12.3-q1
ACaP0(	11	72600000	72800000	0	0	FCHSD2	exonic	NM_01482	.	.	.	ENST0000	.	11q13.4
ACaP0(	11	77730000	77850000	0	0	THRSP,NI	exonic	NM_0010C	.	.	.	ENST0000	CpG: 64	11q14.1
ACaP0(	11	77850000	78170000	0	0	GAB2,USF	exonic	NM_0010C	.	.	.	ENST0000	CpG: 105,(	11q14.1
ACaP0(	12	7550000	8090000	0	0	NANOG,C	exonic	NM_00114	.	.	.	ENST0000	CpG: 52,C	12p13.31
ACaP0(	12	50560000	51100000	0	0	FAM186A,	exonic	NM_00111	.	.	.	ENST0000	CpG: 31,C	12q13.12
ACaP0(	12	51100000	51250000	0	0	DIP2B,ATF	exonic	NM_00517	.	.	.	ENST0000	CpG: 27,C	12q13.12
ACaP0(	12	51250000	51390000	0	0	HIGD1C,M	exonic	NM_00061	.	.	.	ENST0000	.	12q13.12
ACaP0(	12	51390000	51420000	0	0	SLC11A2	exonic	NM_00061	.	.	.	ENST0000	CpG: 73	12q13.12
ACaP0(	12	1.2E+08	1.2E+08	0	0	CIT,LINC0	exonic	NM_00113	.	.	.	ENST0000	CpG: 31,C	12q24.23
ACaP0(	13	19770000	20630000	0	0	LINC0042	exonic	NM_00103	.	.	.	ENST0000	CpG: 102,(	13q12.11
ACaP0(	13	20630000	20640000	0	0	ZMYM2	exonic	NM_00119	.	.	.	ENST0000	.	13q12.11
ACaP0(	13	24320000	24470000	0	0	C1QTNF9I	exonic	NM_0010C	.	.	.	ENST0000	CpG: 53	13q12.12
ACaP0(	13	25000000	25030000	0	0	PARP4	exonic	NM_00643	.	.	.	ENST0000	.	13q12.12
ACaP0(	13	28550000	28640000	0	0	FLT3,URA	exonic	NM_0011C	.	.	.	ENST0000	CpG: 42,C	13q12.2
ACaP0(	13	28640000	28800000	0	0	PAN3,FLT	exonic	NM_00411	.	.	.	ENST0000	CpG: 119,(	13q12.2
ACaP0(	13	28800000	28860000	0	0	PAN3	exonic	NM_17585	.	.	.	ENST0000	.	13q12.2
ACaP0(	13	33740000	33770000	0	0	STARD13	exonic	NM_00124	.	.	.	ENST0000	.	13q13.1
ACaP0(	13	36130000	36340000	0	0	NBEA,MIR	exonic	NM_0012C	.	.	.	ENST0000	.	13q13.3
ACaP0(	13	38350000	38360000	0	0	TRPC4	exonic	NM_00113	.	.	.	ENST0000	.	13q13.3
ACaP0(	13	50020000	50850000	0	0	ARL11,MIF	exonic	NM_0010C	.	.	.	ENST0000	CpG: 101,(	13q14.2
ACaP0(	13	53240000	53320000	0	0	SUGT1,LE	exonic	NM_00101	.	.	.	ENST0000	CpG: 80	13q14.3
ACaP0(	13	53320000	60540000	0	0	LINC0055	exonic	NM_00104	.	.	.	ENST0000	CpG: 88,C	13q14.3-q2
ACaP0(	13	73630000	73640000	0	0	KLF5	exonic	NM_00128	.	.	.	ENST0000	CpG: 149	13q22.1

ACaP0(	13	80910000	1.1E+08	0	0	HS6ST3,S	exonic	NM_00012	.	.	.	ENST0000	CpG:	391,(	13q31.2-q3
ACaP0(	14	19110000	20350000	0	0	LOC10050	exonic	NM_0010C	.	.	.	ENST0000	CpG:	49,C	14q11.2
ACaP0(	14	21770000	21850000	0	0	RPGRIP1,:	exonic	NM_00719	.	.	.	ENST0000	.		14q11.2
ACaP0(	14	31530000	92630000	0	0	RP11-840I	exonic	NM_00002	.	.	.	ENST0000	CpG:	92,C	14q13.3-q3
ACaP0(	15	0	20160000	0	0	.	intergenic	NONE,NR_	dist=NONE	.	.	ENST0000	CpG:	55,C	15p13-p11
ACaP0(	15	20160000	23380000	0	0	MIR4509-3	exonic	NM_0010C	.	.	.	ENST0000	CpG:	49,C	15q11.2-q1
ACaP0(	15	67670000	67770000	0	0	IQCH,IQCI	exonic	NM_00103	.	.	.	ENST0000	.		15q23
ACaP0(	16	14570000	15110000	0	0	LOC10028	exonic	NM_00113	.	.	.	ENST0000	CpG:	73,C	16p13.11-p
ACaP0(	16	16330000	18600000	0	0	MIR3180-3	exonic	NM_0010C	.	.	.	ENST0000	CpG:	140,(	16p13.11-p
ACaP0(	16	20540000	21060000	0	0	ERI2,THUI	exonic	NM_0011C	.	.	.	ENST0000	CpG:	87,C	16p12.3
ACaP0(	16	21060000	21080000	0	0	DNAH3	exonic	NM_01753	.	.	.	ENST0000	.		16p12.3
ACaP0(	16	21080000	21970000	0	0	NPIPB3,LC	exonic	NM_00101	.	.	.	ENST0000	CpG:	102,(	16p12.2-p1
ACaP0(	16	21970000	21980000	0	0	UQCRC2	exonic	NM_00336	.	.	.	ENST0000	.		16p12.2
ACaP0(	16	22460000	22630000	0	0	SMG1P1,L	exonic	NM_00113	.	.	.	ENST0000	CpG:	26,C	16p12.2
ACaP0(	16	29350000	29580000	0	0	SLX1A-SU	exonic	NM_00101	.	.	.	ENST0000	CpG:	159,(	16p11.2
ACaP0(	16	70850000	71170000	0	0	HYDIN	exonic	NM_00119	.	.	.	ENST0000	.		16q22.2
ACaP0(	16	71170000	71190000	0	0	HYDIN	exonic	NM_00119	.	.	.	ENST0000	.		16q22.2
ACaP0(	17	44120000	44380000	0	0	LRRC37A,	exonic	NM_0011C	.	.	.	ENST0000	CpG:	51,C	17q21.31
ACaP0(	17	44380000	44630000	0	0	ARL17B,LI	exonic	NM_0010C	.	.	.	ENST0000	CpG:	68,C	17q21.31
ACaP0(	17	44630000	44850000	0	0	LRRC37A2	exonic	NM_0010C	.	.	.	ENST0000	CpG:	71,C	17q21.31
ACaP0(	17	48800000	58060000	0	0	MRPS23,L	exonic	NM_00025	.	.	.	ENST0000	CpG:	46,C	17q22-q21
ACaP0(	18	0	40000	0	0	.	intergenic	NONE,NR_	dist=NONE	.	.	ENST0000	CpG:	67	18p11.32
ACaP0(	18	40000	130000	0	0	MIR8078,F	ncRNA_ex	NR_03377	.	.	.	ENST0000	CpG:	21,C	18p11.32
ACaP0(	18	130000	190000	0	0	USP14	exonic	NM_00103	.	.	.	ENST0000	CpG:	70	18p11.32
ACaP0(	18	21520000	21540000	0	0	LAMA3	exonic	NM_00022	.	.	.	ENST0000	.		18q11.2
ACaP0(	18	21540000	44540000	0	0	DTNA,KCT	exonic	NM_00037	.	.	.	ENST0000	CpG:	37,C	18q12.1-q1
ACaP0(	18	50970000	51030000	0	0	DCC	exonic	NM_00521	.	.	.	ENST0000	.		18q21.2
ACaP0(	19	0	60000	0	0	.	downstreal	NR_03326	.	.	.	.	.		19p13.3
ACaP0(	19	60000	300000	0	0	WASH5P,I	exonic	NM_0010C	.	.	.	ENST0000	CpG:	117,(	19p13.3
ACaP0(	19	8950000	9880000	0	0	MBD3L1,C	exonic	NM_0010C	.	.	.	ENST0000	CpG:	54,C	19p13.2
ACaP0(	19	14680000	15060000	0	0	EMR2,NDI	exonic	NM_0010C	.	.	.	ENST0000	CpG:	29,C	19p13.12
ACaP0(	19	42940000	43930000	0	0	PSG6,CEA	exonic	NM_00102	.	.	.	ENST0000	CpG:	20,C	19q13.2-q1
ACaP0(	19	53720000	53740000	0	0	ZNF677	UTR3	NM_1826C	NM_1826C	.	.	ENST0000	.		19q13.42
ACaP0(	19	53740000	54300000	0	0	MIR520H,I	exonic	NM_0010C	.	.	.	ENST0000	CpG:	51,C	19q13.42
ACaP0(	20	0	120000	0	0	DEFB125	exonic	NM_15332	.	.	.	ENST0000	.		20p13

ACaP0	20	120000	3220000	0	0	SIRPG,RS	exonic	NM_00049	.	.	.	ENST0000	CpG:	102, 20p13
ACaP0	20	3220000	3230000	0	0	C20orf194	UTR3	NM_00100	NM_00100	.	.	ENST0000	CpG:	47,C 20p13
ACaP0	20	3230000	3630000	0	0	ATRN,C20	exonic	NM_00100	.	.	.	ENST0000	CpG:	93,C 20p13
ACaP0	20	3630000	3640000	0	0	GFRA4,AT	UTR3	NM_02213	NM_02213	.	.	ENST0000	.	20p13
ACaP0	20	3640000	3870000	0	0	CDC25B,C	exonic	NM_00103	.	.	.	ENST0000	CpG:	73,C 20p13
ACaP0	20	3870000	3880000	0	0	PANK2	exonic	NM_15363	.	.	.	ENST0000	CpG:	128 20p13
ACaP0	20	3880000	17620000	0	0	SNAP25-A	exonic	NM_00021	.	.	.	ENST0000	CpG:	71,C 20p13-p12
ACaP0	20	17620000	17650000	0	0	RRBP1	exonic	NM_00104	.	.	.	ENST0000	.	20p12.1
ACaP0	20	17650000	17700000	0	0	BANF2,RF	exonic	NM_00115	.	.	.	ENST0000	CpG:	177 20p12.1
ACaP0	20	17700000	21150000	0	0	SLC24A3,	exonic	NM_00101	.	.	.	ENST0000	CpG:	67,C 20p12.1-p1
ACaP0	20	21150000	21180000	0	0	PLK1S1,R	ncRNA_ex	NR_10995	.	.	.	ENST0000	.	20p11.23
ACaP0	20	21180000	26050000	0	0	FAM182A,	exonic	NM_00009	.	.	.	ENST0000	CpG:	111, 20p11.1-p1
ACaP0	20	26050000	31300000	0	0	DEFB116,	exonic	NM_00100	.	.	.	ENST0000	CpG:	24,C 20q11.21-
ACaP0	20	31300000	31310000	0	0	COMMD7	intronic	NM_00109	.	.	.	ENST0000	.	20q11.21
ACaP0	20	31310000	31320000	0	0	COMMD7	exonic	NM_00109	.	.	.	ENST0000	.	20q11.21
ACaP0	20	31320000	31330000	0	0	COMMD7	intronic	NM_00109	.	.	.	ENST0000	.	20q11.21
ACaP0	20	31330000	34460000	0	0	MMP24-AS	exonic	NM_00017	.	.	.	ENST0000	CpG:	149, 20q11.23-
ACaP0	20	34460000	34480000	0	0	PHF20	intronic	NM_01643	.	.	.	ENST0000	.	20q11.23
ACaP0	20	34480000	34490000	0	0	PHF20	exonic	NM_01643	.	.	.	ENST0000	.	20q11.23
ACaP0	20	34490000	34500000	0	0	PHF20	intronic	NM_01643	.	.	.	ENST0000	.	20q11.23
ACaP0	20	34500000	35540000	0	0	SCAND1,T	exonic	NM_00104	.	.	.	ENST0000	CpG:	209, 20q11.23
ACaP0	20	35540000	35850000	0	0	RBL1,SAM	exonic	NM_00113	.	.	.	ENST0000	CpG:	70,C 20q11.23
ACaP0	20	35850000	50420000	0	0	WFDC11,	exonic	NM_00002	.	.	.	ENST0000	CpG:	100, 20q13.13-
ACaP0	20	50420000	50660000	0	0	RP5-1112	ncRNA_ex	NR_11001	.	.	.	ENST0000	.	20q13.2
ACaP0	20	50660000	50670000	0	0	.	intergenic	NR_11001	dist=18054	.	.	ENST0000	.	20q13.2
ACaP0	20	50670000	50700000	0	0	.	downstrea	NM_19942	.	.	.	ENST0000	.	20q13.2
ACaP0	20	50700000	55030000	0	0	MC3R,FAM	exonic	NM_00078	.	.	.	ENST0000	CpG:	27,C 20q13.31-
ACaP0	20	55030000	55040000	0	0	CASS4	exonic	NM_00116	.	.	.	ENST0000	.	20q13.31
ACaP0	20	55040000	55120000	0	0	GCNT7,RT	exonic	NM_00101	.	.	.	ENST0000	CpG:	37 20q13.31
ACaP0	20	55120000	55200000	0	0	.	intergenic	NM_00101	dist=8426;	.	.	ENST0000	.	20q13.31
ACaP0	20	55200000	55220000	0	0	TFAP2C	exonic	NM_00322	.	.	.	ENST0000	CpG:	475 20q13.31
ACaP0	20	55220000	55740000	0	0	.	intergenic	NM_00322	dist=5662;	.	.	ENST0000	CpG:	59,C 20q13.31
ACaP0	20	55740000	60080000	0	0	CTCFL,LO	exonic	NM_00011	.	.	.	ENST0000	CpG:	86,C 20q13.32-
ACaP0	20	62920000	62960000	0	0	LINC0026	ncRNA_ex	NR_04041	.	.	.	ENST0000	CpG:	78,C 20q13.33
ACaP0	20	62960000	63025520	0	0	.	intergenic	NR_04041	dist=25293	.	.	.	.	20q13.33

ACaP0(	21	0	9750000	0	0	.	intergenic	NONE,NR_	dist=NONE	.	.	ENST0000	CpG: 165,(	21p12-p11
ACaP0(	21	9750000	11100000	0	0	TPTE,MIR	exonic	NM_00118	.	.	.	ENST0000	CpG: 46,C	21p11.2-p1
ACaP0(	21	11100000	14410000	0	0	.	upstream	NR_02691	.	.	.	ENST0000	CpG: 38,C	21q11.2-p1
ACaP0(	21	41420000	41430000	0	0	DSCAM	exonic	NM_00127	.	.	.	ENST0000	.	21q22.2
ACaP0(	22	0	16090000	0	0	.	intergenic	NONE,NR_	dist=NONE	.	.	ENST0000	.	22p11.2-p1
ACaP0(	22	16090000	16970000	0	0	POTEH,BN	exonic	NM_00100	.	.	.	ENST0000	CpG: 37,C	22q11.1
ACaP0(	22	51180000	51240000	0	0	RPL23AP8	exonic	NM_00100	.	.	.	ENST0000	CpG: 63	22q13.33
ACaP0(	22	51240000	51304566	0	0	.	intergenic	NR_02698	dist=1935;(	.	.	.	.	22q13.33
ACaP0(	X	48040000	48250000	0	0	SSX3,SSX	exonic	NM_00103	.	.	.	ENST0000	.	Xp11.23
ACaP0(	X	49150000	49170000	0	0	GAGE10	exonic	NM_00109	.	.	.	ENST0000	.	Xp11.23
ACaP0(	X	49170000	49360000	0	0	GAGE12C	exonic	NM_00109	.	.	.	ENST0000	CpG: 43,C	Xp11.23
ACaP0(	X	53640000	54080000	0	0	PHF8,HUV	exonic	NM_00118	.	.	.	ENST0000	CpG: 90,C	Xp11.22
ACaP0(	X	54080000	70810000	0	0	OGT,PAGI	exonic	NM_00003	.	.	.	ENST0000	CpG: 23,C	Xp11.21-X
ACaP0(	X	1.18E+08	1.35E+08	0	0	SMIM10,M	exonic	NM_00019	.	.	.	ENST0000	CpG: 32,C	Xq25-Xq26
ACaP0(	X	1.35E+08	1.35E+08	0	0	CT45A6,C	exonic	NM_00100	.	.	.	ENST0000	CpG: 31,C	Xq26.3
ACaP0(	X	1.54E+08	1.55E+08	0	0	H2AFB2,S	exonic	NM_00013	.	.	.	ENST0000	CpG: 29,C	Xq28
ACaP0(	X	1.55E+08	1.55E+08	0	0	.	intergenic	NM_00218	dist=19518	.	.	.	.	Xq28
ACaP0'	1	9990000	10080000	0	0	RBP7,NMN	exonic	NM_02278	.	.	.	ENST0000	CpG: 39,C	1p36.22
ACaP0'	1	23900000	23920000	0	0	.	intergenic	NM_00216	dist=13715	.	.	ENST0000	.	1p36.11-p3
ACaP0'	1	1.09E+08	1.09E+08	0	0	NBPF4,NB	exonic	NM_00114	.	.	.	ENST0000	CpG: 51	1p13.3
ACaP0'	1	2E+08	2E+08	0	0	NR5A2	exonic	NM_00127	.	.	.	ENST0000	.	1q32.1
ACaP0'	1	2.24E+08	2.24E+08	0	0	FBXO28,N	exonic	NM_00113	.	.	.	ENST0000	CpG: 121	1q42.11
ACaP0'	3	10070000	10280000	0	0	FANCD2,V	exonic	NM_00055	.	.	.	ENST0000	CpG: 43,C	3p25.3
ACaP0'	5	1.56E+08	1.56E+08	0	0	HAVCR1	exonic	NM_00109	.	.	.	ENST0000	.	5q33.3
ACaP0'	5	1.56E+08	1.57E+08	0	0	HAVCR1,H	exonic	NM_00109	.	.	.	ENST0000	.	5q33.3
ACaP0'	8	1.29E+08	1.29E+08	0	0	MIR1204	ncRNA_ex	NR_03160	.	.	.	ENST0000	CpG: 79	8q24.21
ACaP0'	11	18250000	18500000	0	0	SAA1,SAA	exonic	NM_00033	.	.	.	ENST0000	CpG: 60,C	11p15.1
ACaP0'	11	73510000	73720000	0	0	MRPL48,C	exonic	NM_00126	.	.	.	ENST0000	CpG: 95,C	11q13.4
ACaP0'	12	7850000	7860000	0	0	.	intergenic	NM_02063	dist=1640;(	.	.	.	CpG: 24	12p13.31
ACaP0'	12	7860000	8220000	0	0	SLC2A14,I	exonic	NM_00114	.	.	.	ENST0000	CpG: 52,C	12p13.31
ACaP0'	15	41630000	41670000	0	0	NUSAP1	exonic	NM_00124	.	.	.	ENST0000	.	15q15.1
ACaP0'	21	10790000	10800000	0	0	.	intergenic	NR_03832	dist=82140	.	.	.	.	21p11.2
ACaP0'	X	1.19E+08	1.19E+08	0	0	MIR766,SE	exonic	NM_01512	.	.	.	ENST0000	CpG: 18,C	Xq24
ACaP08	1	1.52E+08	1.52E+08	0	0	FLG	exonic	NM_00201	.	.	.	ENST0000	.	1q21.3
ACaP08	4	9220000	9250000	0	0	USP17L21	exonic	NM_00125	.	.	.	ENST0000	.	4p16.1

ACaP08	4	9250000	9270000	0	0	USP17L19	exonic	NM_00125	.	.	.	ENST0000	.	4p16.1
ACaP08	4	9270000	9350000	0	0	USP17L20	exonic	NM_00124	.	.	.	ENST0000	.	4p16.1
ACaP08	4	9350000	9360000	0	0	USP17L5,	exonic	NM_00124	.	.	.	ENST0000	.	4p16.1
ACaP08	4	1.91E+08	1.91E+08	0	0	DUX4L2,D	exonic	NM_00112	.	.	.	ENST0000	CpG: 2005	4q35.2
ACaP08	4	1.91E+08	1.91E+08	0	0	.	intergenic	NM_03317	dist=6558;	.	.	.	.	4q35.2
ACaP08	5	80930000	80950000	0	0	SSBP2	exonic	NM_00125	.	.	.	ENST0000	.	5q14.1
ACaP08	5	1.79E+08	1.79E+08	0	0	ADAMTS2	exonic	NM_01424	.	.	.	ENST0000	CpG: 19,C	5q35.3
ACaP08	7	1.01E+08	1.01E+08	0	0	MUC12	exonic	NM_00116	.	.	.	ENST0000	.	7q22.1
ACaP08	7	1.01E+08	1.01E+08	0	0	MUC17	exonic	NM_00104	.	.	.	ENST0000	.	7q22.1
ACaP08	12	18520000	18660000	0	0	PIK3C2G	exonic	NM_00128	.	.	.	ENST0000	.	12p12.3
ACaP08	14	1.05E+08	1.05E+08	0	0	AHNAK2	exonic	NM_13842	.	.	.	ENST0000	.	14q32.33
ACaP08	15	1.02E+08	1.02E+08	0	0	.	intergenic	NM_15233	dist=15355	.	.	ENST0000	CpG: 87	15q26.3
ACaP08	20	0	70000	0	0	DEFB125	exonic	NM_15332	.	.	.	ENST0000	.	20p13
ACaP08	22	43550000	43690000	0	0	TTLL12,TS	exonic	NM_00071	.	.	.	ENST0000	CpG: 44,C	22q13.2
ACaP08	22	47360000	50180000	0	0	LOC90834	exonic	NM_00103	.	.	.	ENST0000	CpG: 64,C	22q13.32-
ACaP09	1	1.52E+08	1.52E+08	0	0	FLG	exonic	NM_00201	.	.	.	ENST0000	.	1q21.3
ACaP09	1	2.24E+08	2.24E+08	0	0	CAPN8	intronic	NM_00114	.	.	.	ENST0000	CpG: 213	1q41
ACaP09	2	45160000	45240000	0	0	SIX3-AS1,	exonic	NM_00541	.	.	.	ENST0000	CpG: 115,	2p21
ACaP09	2	1.2E+08	1.2E+08	0	0	EN1	exonic	NM_00142	.	.	.	ENST0000	CpG: 41,C	2q14.2
ACaP09	2	1.91E+08	1.91E+08	0	0	TMEM194	exonic	NM_00114	.	.	.	ENST0000	CpG: 73	2q32.2
ACaP09	2	2.25E+08	2.25E+08	0	0	CUL3	exonic	NM_00125	.	.	.	ENST0000	CpG: 106	2q36.2
ACaP09	3	71800000	71840000	0	0	EIF4E3,GF	exonic	NM_00112	.	.	.	ENST0000	CpG: 193,	3p13
ACaP09	4	42640000	42660000	0	0	ATP8A1	exonic	NM_00110	.	.	.	ENST0000	CpG: 110	4p13
ACaP09	4	1.91E+08	1.91E+08	0	0	DUX4L4,D	exonic	NM_00112	.	.	.	ENST0000	CpG: 2005	4q35.2
ACaP09	4	1.91E+08	1.91E+08	0	0	.	intergenic	NM_03317	dist=6558;	.	.	.	.	4q35.2
ACaP09	5	1.11E+08	1.11E+08	0	0	CAMK4	exonic	NM_00174	.	.	.	ENST0000	CpG: 95	5q22.1
ACaP09	6	0	110000	0	0	.	intergenic	NONE,NR_	dist=NONE	.	.	.	.	6p25.3
ACaP09	7	1.01E+08	1.01E+08	0	0	MUC12	exonic	NM_00116	.	.	.	ENST0000	.	7q22.1
ACaP09	8	6830000	8660000	0	0	DEFA1B,D	exonic	NM_00103	.	.	.	ENST0000	CpG: 60,C	8p23.1
ACaP09	8	86550000	86840000	0	0	REXO1L2	ncRNA_ex	NR_00359	.	.	.	ENST0000	CpG: 130,	8q21.2
ACaP09	8	1.05E+08	1.05E+08	0	0	RIMS2	exonic	NM_00110	.	.	.	ENST0000	CpG: 242	8q22.3
ACaP09	10	11040000	11060000	0	0	CELF2	exonic	NM_00102	.	.	.	ENST0000	CpG: 131	10p14
ACaP09	10	1.35E+08	1.36E+08	0	0	DUX4L3,D	exonic	NM_00108	.	.	.	ENST0000	CpG: 1534	10q26.3
ACaP09	11	1.07E+08	1.07E+08	0	0	GUCY1A2	exonic	NM_00085	.	.	.	ENST0000	CpG: 134	11q22.3
ACaP09	14	1.05E+08	1.05E+08	0	0	AHNAK2	exonic	NM_13842	.	.	.	ENST0000	CpG: 178	14q32.33

ACaP0	15	73650000	73740000	0	0	C15orf60,I	exonic	NM_00104	.	.	.	ENST0000	CpG: 21,C	15q24.1
ACaP0	15	1.02E+08	1.02E+08	0	0	.	intergenic	NM_15233	dist=15355	.	.	ENST0000	CpG: 87	15q26.3
ACaP0	16	51180000	51190000	0	0	SALL1	exonic	NM_00296	.	.	.	ENST0000	CpG: 47,C	16q12.1
ACaP0	16	75010000	75610000	0	0	ZNRF1,TM	exonic	NM_00102	.	.	.	ENST0000	CpG: 98,C	16q23.1
ACaP0	17	19870000	21550000	0	0	CCDC144I	exonic	NM_00100	.	.	.	ENST0000	CpG: 47,C	17p11.2
ACaP0	18	40000	120000	0	0	MIR8078,F	ncRNA_ex	NR_03377	.	.	.	ENST0000	CpG: 59,C	18p11.32
ACaP0	18	7560000	7570000	0	0	PTPRM	exonic	NM_00110	.	.	.	ENST0000	CpG: 232	18p11.23
ACaP0	18	29070000	29080000	0	0	DSG2	exonic	NM_00194	.	.	.	ENST0000	CpG: 80	18q12.1
ACaP0	18	34390000	34420000	0	0	KIAA1328,	exonic	NM_00127	.	.	.	ENST0000	CpG: 81	18q12.2
ACaP0	18	77150000	77670000	0	0	PQLC1,KC	exonic	NM_00114	.	.	.	ENST0000	CpG: 73,C	18q23
ACaP0	18	77670000	78010000	0	0	ADNP2,PC	exonic	NM_00113	.	.	.	ENST0000	CpG: 105,	18q23
ACaP0	19	22830000	22950000	0	0	ZNF492,ZI	exonic	NM_00108	.	.	.	ENST0000	CpG: 22	19p12
ACaP0	19	22950000	23410000	0	0	ZNF730,ZI	exonic	NM_00108	.	.	.	ENST0000	CpG: 40,C	19p12
ACaP0	20	3950000	3960000	0	0	RNF24	exonic	NM_00113	.	.	.	ENST0000	.	20p13
ACaP0	20	26180000	26190000	0	0	MIR663A,L	ncRNA_ex	NR_03038	.	.	.	ENST0000	CpG: 201	20p11.1
ACaP0	20	61800000	61810000	0	0	MIR124-3	ncRNA_ex	NR_02967	.	.	.	ENST0000	CpG: 424	20q13.33
ACaP0	21	42530000	42540000	0	0	BACE2,MI	ncRNA_ex	NR_03616	.	.	.	ENST0000	CpG: 149	21q22.2
ACaP0	22	18650000	18930000	0	0	USP18,GG	exonic	NM_00119	.	.	.	ENST0000	CpG: 97,C	22q11.21
ACaP0	22	20320000	20730000	0	0	PI4KAP1,F	exonic	NM_00124	.	.	.	ENST0000	CpG: 131,	22q11.21
ACaP0	22	20730000	20740000	0	0	.	intergenic	NM_01567	dist=26821	.	.	ENST0000	.	22q11.21
ACaP0	22	21470000	21750000	0	0	BCRP2,FA	exonic	NM_00112	.	.	.	ENST0000	CpG: 29,C	22q11.21
ACaP0	X	1.2E+08	1.2E+08	0	0	CT47A11,	exonic	NM_00108	.	.	.	ENST0000	CpG: 75,C	Xq24
ACaP1	1	12830000	12860000	0	0	PRAMEF1	exonic	NM_00108	.	.	.	ENST0000	.	1p36.21
ACaP1	1	12860000	12880000	0	0	.	intergenic	NM_02301	dist=3223;	.	.	.	.	1p36.21
ACaP1	1	12880000	13370000	0	0	PRAMEF1	exonic	NM_00100	.	.	.	ENST0000	.	1p36.21
ACaP1	1	13370000	13680000	0	0	PRAMEF8	exonic	NM_00101	.	.	.	ENST0000	.	1p36.21
ACaP1	1	45960000	53510000	0	0	CYP4A11,	exonic	NM_00077	.	.	.	ENST0000	CpG: 39,C	1p34.1-p3
ACaP1	1	1.44E+08	1.45E+08	0	0	PPIAL4A,F	exonic	NM_00103	.	.	.	ENST0000	CpG: 86,C	1q21.1
ACaP1	1	1.45E+08	1.45E+08	0	0	NBPF25P,	exonic	NM_00103	.	.	.	ENST0000	.	1q21.1
ACaP1	1	1.46E+08	1.46E+08	0	0	NBPF12,G	exonic	NM_00103	.	.	.	ENST0000	CpG: 16,C	1q21.1
ACaP1	1	1.48E+08	1.49E+08	0	0	NBPF9,PP	exonic	NM_00103	.	.	.	ENST0000	CpG: 26,C	1q21.2
ACaP1	1	1.49E+08	1.5E+08	0	0	.	intergenic	NR_02748	dist=81697	.	.	ENST0000	CpG: 25,C	1q21.2
ACaP1	1	1.5E+08	1.5E+08	0	0	PPIAL4B,F	exonic	NM_00113	.	.	.	ENST0000	.	1q21.2
ACaP1	1	1.5E+08	1.52E+08	0	0	CTSK,SEL	exonic	NM_00039	.	.	.	ENST0000	CpG: 103,	1q21.3-q2
ACaP1	1	2.48E+08	2.49E+08	0	0	OR2T3,OF	exonic	NM_00100	.	.	.	ENST0000	.	1q44

ACaP1(	1	2.49E+08	2.49E+08	0	0	SH3BP5L,(	exonic	NM_00100	.	.	.	ENST0000	CpG: 86,C	1q44
ACaP1(	1	2.49E+08	2.49E+08	0	0	PGBD2	exonic	NM_00101	.	.	.	ENST0000	.	1q44
ACaP1(	2	1.38E+08	1.38E+08	0	0	THSD7B	exonic	NM_00108	.	.	.	ENST0000	.	2q22.1
ACaP1(	2	1.99E+08	1.99E+08	0	0	PLCL1	exonic	NM_00622	.	.	.	ENST0000	.	2q33.1
ACaP1(	2	2.07E+08	2.07E+08	0	0	.	intergenic	NM_02092	dist=10085	.	.	.	.	2q33.3
ACaP1(	3	89480000	1.82E+08	0	0	ABI3BP,RI	exonic	NM_00005	.	.	.	ENST0000	CpG: 105,(	3q26.31-q2
ACaP1(	3	1.82E+08	1.82E+08	0	0	FLJ46066	ncRNA_ex	NR_04010	.	.	.	ENST0000	.	3q26.33
ACaP1(	3	1.82E+08	1.83E+08	0	0	FLJ46066	ncRNA_ex	NR_04010	.	.	.	ENST0000	CpG: 62	3q26.33
ACaP1(	3	1.83E+08	1.98E+08	0	0	DGKG,SN(	exonic	NM_00041	.	.	.	ENST0000	CpG: 108,(	3q29-q26.3
ACaP1(	3	1.98E+08	1.98E+08	0	0	.	intergenic	NM_00114	dist=52272	.	.	.	.	3q29
ACaP1(	4	71220000	71240000	0	0	SMR3A	exonic	NM_01239	.	.	.	ENST0000	.	4q13.3
ACaP1(	4	86490000	86500000	0	0	ARHGAP2	exonic	NM_00102	.	.	.	ENST0000	.	4q21.23
ACaP1(	5	90440000	90460000	0	0	GPR98	exonic	NM_03211	.	.	.	ENST0000	.	5q14.3
ACaP1(	6	28960000	29150000	0	0	LOC10012	exonic	NM_00100	.	.	.	ENST0000	CpG: 23,C	6p22.1
ACaP1(	6	34570000	34630000	0	0	C6orf106	exonic	NM_02275	.	.	.	ENST0000	.	6p21.31
ACaP1(	6	51770000	51880000	0	0	PKHD1	exonic	NM_13869	.	.	.	ENST0000	.	6p12.2-p12
ACaP1(	6	73040000	73120000	0	0	RIMS1	exonic	NM_00116	.	.	.	ENST0000	.	6q13
ACaP1(	6	1.17E+08	1.17E+08	0	0	FAM26E,T	exonic	NM_00113	.	.	.	ENST0000	.	6q22.1
ACaP1(	6	1.61E+08	1.61E+08	0	0	SLC22A3,I	exonic	NM_00030	.	.	.	ENST0000	CpG: 36	6q25.3-q26
ACaP1(	7	36710000	37060000	0	0	ELMO1-AS	exonic	NM_00103	.	.	.	ENST0000	.	7p14.2
ACaP1(	7	64990000	69600000	0	0	KCTD7,TY	exonic	NM_00004	.	.	.	ENST0000	CpG: 50,C	7q11.21-q1
ACaP1(	7	74180000	75150000	0	0	NCF1C,TF	exonic	NM_00026	.	.	.	ENST0000	CpG: 60,C	7q11.23
ACaP1(	8	1.04E+08	1.04E+08	0	0	ODF1	exonic	NM_02441	.	.	.	ENST0000	.	8q22.3
ACaP1(	8	1.22E+08	1.22E+08	0	0	SNTB1	exonic	NM_02102	.	.	.	ENST0000	.	8q24.12
ACaP1(	10	68930000	71060000	0	0	SLC25A16	exonic	NM_00103	.	.	.	ENST0000	CpG: 74,C	10q22.1-q2
ACaP1(	10	96470000	96750000	0	0	CYP2C9,C	exonic	NM_00076	.	.	.	ENST0000	.	10q23.33
ACaP1(	11	6120000	6200000	0	0	OR56B4,C	exonic	NM_00100	.	.	.	ENST0000	.	11p15.4
ACaP1(	11	1.24E+08	1.25E+08	0	0	OR8B12,C	exonic	NM_00100	.	.	.	ENST0000	CpG: 44	11q24.2
ACaP1(	12	32520000	32540000	0	0	BICD1	exonic	NM_00100	.	.	.	ENST0000	.	12p11.21
ACaP1(	13	73420000	74390000	0	0	KLF12,PIB	exonic	NM_00128	.	.	.	ENST0000	CpG: 149	13q22.1
ACaP1(	13	74390000	74410000	0	0	KLF12	intronic	NM_00724	.	.	.	ENST0000	.	13q22.1
ACaP1(	13	74410000	74420000	0	0	KLF12	exonic	NM_00724	.	.	.	ENST0000	.	13q22.1
ACaP1(	14	59730000	59740000	0	0	DAAM1	exonic	NM_00127	.	.	.	ENST0000	.	14q23.1
ACaP1(	14	61790000	62130000	0	0	RP11-47I2	exonic	NM_00625	.	.	.	ENST0000	.	14q23.1-q2
ACaP1(	15	43850000	44020000	0	0	CATSPER	exonic	NM_00101	.	.	.	ENST0000	CpG: 45,C	15q15.3

ACaP1(	16	10850000	10870000	0	0	TVP23A,N	exonic	NM_00107	.	.	.	ENST0000	.	16p13.13
ACaP1(	16	21000000	21220000	0	0	TMEM159,	exonic	NM_00346	.	.	.	ENST0000	CpG: 50	16p12.3-p1
ACaP1(	16	33360000	33510000	0	0	RNU6-76P	ncRNA_ex	NR_04694	.	.	.	ENST0000	CpG: 27	16p11.2
ACaP1(	16	56780000	56840000	0	0	NUP93	exonic	NM_00124	.	.	.	ENST0000	.	16q13
ACaP1(	16	68070000	68250000	0	0	NFATC3,D	exonic	NM_00127	.	.	.	ENST0000	CpG: 104	16q22.1
ACaP1(	16	70880000	71170000	0	0	HYDIN	exonic	NM_00119	.	.	.	ENST0000	.	16q22.2
ACaP1(	16	71170000	71180000	0	0	HYDIN	exonic	NM_00119	.	.	.	ENST0000	.	16q22.2
ACaP1(	16	73170000	74460000	0	0	AC009120	exonic	NM_00101	.	.	.	ENST0000	CpG: 65,C	16q23.1-q2
ACaP1(	18	42440000	42460000	0	0	SETBP1	exonic	NM_00113	.	.	.	ENST0000	.	18q12.3
ACaP1(	20	1350000	1360000	0	0	FKBP1A-S	exonic	NM_0080	.	.	.	ENST0000	.	20p13
ACaP1(	20	23530000	23540000	0	0	.	intergenic	NR_00127	dist=7345;(	.	.	.	.	20p11.21
ACaP1(	20	35290000	35330000	0	0	NDRG3	exonic	NM_02247	.	.	.	ENST0000	.	20q11.23
ACaP1(	20	35330000	35340000	0	0	NDRG3	exonic	NM_03201	.	.	.	ENST0000	.	20q11.23
ACaP1(	20	36360000	36390000	0	0	CTNNBL1	exonic	NM_00128	.	.	.	ENST0000	.	20q11.23
ACaP1(	21	27310000	27390000	0	0	APP	exonic	NM_00048	.	.	.	ENST0000	.	21q21.3
ACaP1(	21	27390000	27860000	0	0	CYYR1,AF	exonic	NM_00048	.	.	.	ENST0000	CpG: 168	21q21.3
ACaP1(	21	34650000	34780000	0	0	IFNGR2,IF	exonic	NM_00062	.	.	.	ENST0000	CpG: 110,(	21q22.11
ACaP1(	21	37710000	37740000	0	0	MORC3	exonic	NM_01535	.	.	.	ENST0000	.	21q22.12
ACaP1(	21	37740000	37750000	0	0	MORC3	exonic	NM_01535	.	.	.	ENST0000	.	21q22.12
ACaP1(	21	37750000	37790000	0	0	CHAF1B	exonic	NM_00544	.	.	.	ENST0000	CpG: 68	21q22.12
ACaP1(	21	39420000	39430000	0	0	DSCR4	exonic	NM_00586	.	.	.	ENST0000	.	21q22.13
ACaP1(	22	24750000	24770000	0	0	SPECC1L-	exonic	NM_00114	.	.	.	ENST0000	.	22q11.23
ACaP1(	22	29560000	29570000	0	0	KREMEN1	exonic	NM_03204	.	.	.	ENST0000	.	22q12.1
ACaP1(	22	41170000	41320000	0	0	ST13,SLC:	exonic	NM_00120	.	.	.	ENST0000	CpG: 88,C	22q13.2
ACaP1(	22	41320000	41460000	0	0	RBX1,XPN	exonic	NM_01424	.	.	.	ENST0000	CpG: 93,C	22q13.2
ACaP1(	X	1730000	8550000	0	0	VCX2,MIR	exonic	NM_00004	.	.	.	ENST0000	CpG: 146,(	Xp22.32-X
ACaP1(	X	8550000	8570000	0	0	KAL1	exonic	NM_00021	.	.	.	ENST0000	.	Xp22.31
ACaP1(	X	49150000	49970000	0	0	USP27X-A	exonic	NM_00008	.	.	.	ENST0000	CpG: 91,C	Xp11.23-X
ACaP1:	3	51490000	51520000	0	0	VPRBP	exonic	NM_00117	.	.	.	ENST0000	.	3p21.2
ACaP1:	3	1.14E+08	1.14E+08	0	0	GRAMD1C	exonic	NM_01757	.	.	.	ENST0000	CpG: 28	3q13.31
ACaP1:	4	9190000	9200000	0	0	.	intergenic	NM_00104	dist=23787	.	.	.	.	4p16.1
ACaP1:	4	39550000	39580000	0	0	SMIM14,M	exonic	NM_17492	.	.	.	ENST0000	.	4p14
ACaP1:	4	1.09E+08	1.09E+08	0	0	RPL34-AS	ncRNA_ex	NR_02696	.	.	.	ENST0000	.	4q25
ACaP1:	4	1.29E+08	1.29E+08	0	0	LARP1B,C	exonic	NM_00103	.	.	.	ENST0000	CpG: 186	4q28.2
ACaP1:	6	20400000	21240000	0	0	E2F3,CDK	exonic	NM_00124	.	.	.	ENST0000	CpG: 156,(	6p22.3

ACaP1	6	31560000	31570000	0	0	NCR3	exonic	NM_00114	.	.	.	ENST000C	.	6p21.33
ACaP1	6	33860000	34790000	0	0	LINC0101	exonic	NM_00084	.	.	.	ENST000C	CpG: 37,C	6p21.31
ACaP1	7	43430000	43450000	0	0	HECW1	exonic	NM_00128	.	.	.	ENST000C	.	7p13
ACaP1	9	45080000	45090000	0	0	.	intergenic	NR_02742	dist=88508	.	.	.	.	9p11.2
ACaP1	9	74560000	74690000	0	0	C9orf57,C	exonic	NM_00112	.	.	.	ENST000C	.	9q21.13
ACaP1	11	1250000	1280000	0	0	MIR6744,N	exonic	NM_00245	.	.	.	ENST000C	CpG: 48,C	11p15.5
ACaP1	11	1280000	1290000	0	0	MUC5B	exonic	NM_00245	.	.	.	ENST000C	.	11p15.5
ACaP1	11	1.19E+08	1.19E+08	0	0	CBL	exonic	NM_00518	.	.	.	ENST000C	.	11q23.3
ACaP1	12	7800000	7990000	0	0	APOBEC1	exonic	NM_00114	.	.	.	ENST000C	CpG: 24	12p13.31
ACaP1	13	28630000	28650000	0	0	FLT3	exonic	NM_00411	.	.	.	ENST000C	.	13q12.2
ACaP1	13	99220000	99380000	0	0	SLC15A1,S	exonic	NM_00103	.	.	.	ENST000C	CpG: 122	13q32.2-q3
ACaP1	13	1.13E+08	1.13E+08	0	0	ATP11A,C	exonic	NM_0152C	.	.	.	ENST000C	CpG: 131	13q34
ACaP1	15	47870000	50340000	0	0	SLC12A1,S	exonic	NM_00013	.	.	.	ENST000C	CpG: 47,C	15q21.1-q2
ACaP1	17	29810000	29830000	0	0	RAB11FIP	intronic	NM_03293	.	.	.	ENST000C	CpG: 91	17q11.2
ACaP1	18	90000	120000	0	0	ROCK1P1	ncRNA_ex	NR_03377	.	.	.	ENST000C	CpG: 21	18p11.32
ACaP1	19	5820000	5850000	0	0	FUT6,NRT	exonic	NM_00014	.	.	.	ENST000C	CpG: 36,C	19p13.3
ACaP1	19	15130000	15140000	0	0	CCDC105	exonic	NM_17348	.	.	.	ENST000C	CpG: 42	19p13.12
ACaP1	20	31940000	32670000	0	0	PXMP4,AC	exonic	NM_00102	.	.	.	ENST000C	CpG: 97,C	20q11.22-c
ACaP1	20	32670000	32680000	0	0	EIF2S2,RA	exonic	NM_0039C	.	.	.	ENST000C	.	20q11.22
ACaP1	20	35650000	35770000	0	0	RBL1,MRC	exonic	NM_00289	.	.	.	ENST000C	CpG: 52	20q11.23
ACaP1	21	40690000	40700000	0	0	BRWD1-A	ncRNA_ex	NR_04665	.	.	.	ENST000C	.	21q22.2
ACaP1	21	45130000	45140000	0	0	PDXK	exonic	NM_00368	.	.	.	ENST000C	CpG: 156	21q22.3
ACaP1	X	47220000	47250000	0	0	SNORA11	exonic	NM_00344	.	.	.	ENST000C	.	Xp11.23
ACaP1	X	1E+08	1E+08	0	0	TRMT2B,A	exonic	NM_00116	.	.	.	ENST000C	CpG: 27,C	Xq22.1
ACaP1	X	1.19E+08	1.19E+08	0	0	NDUFA1,N	exonic	NM_0010C	.	.	.	ENST000C	CpG: 45,C	Xq24
ACaP1	1	860000	3650000	0	0	MEGF6,MI	exonic	NM_00081	.	.	.	ENST000C	CpG: 27,C	1p36.33-p3
ACaP1	1	47480000	53060000	0	0	CYP4A22,S	exonic	NM_0010C	.	.	.	ENST000C	CpG: 480,(	1p32.3-p33
ACaP1	1	55520000	1E+08	0	0	DNAJB4,T	exonic	NM_00001	.	.	.	ENST000C	CpG: 139,(	1p22.2-p32
ACaP1	1	1E+08	1E+08	0	0	AGL	exonic	NM_00002	.	.	.	ENST000C	.	1p21.2
ACaP1	1	1E+08	1.01E+08	0	0	TRMT13,S	exonic	NM_00113	.	.	.	ENST000C	CpG: 36,C	1p21.2
ACaP1	1	1.01E+08	1.09E+08	0	0	RNU6-31P	exonic	NM_00069	.	.	.	ENST000C	CpG: 21,C	1p21.2-p13
ACaP1	1	1.09E+08	1.1E+08	0	0	CLCC1,FN	exonic	NM_00104	.	.	.	ENST000C	CpG: 61,C	1p13.3
ACaP1	1	1.18E+08	1.19E+08	0	0	WDR3,SP/	exonic	NM_00113	.	.	.	ENST000C	CpG: 136,(	1p12
ACaP1	1	1.2E+08	1.21E+08	0	0	HIST2H2B	exonic	NM_0010C	.	.	.	ENST000C	CpG: 108,(	1p11.2-p12
ACaP1	1	1.21E+08	1.21E+08	0	0	EMBP1,SF	ncRNA_ex	NR_00395	.	.	.	ENST000C	CpG: 132,(	1p11.2

ACaP1:	1	1.21E+08	1.5E+08	0	0	LINC0086	exonic	NM_00056	.	.	.	ENST0000	CpG:	59,C	1q12-q11
ACaP1:	1	1.5E+08	1.58E+08	0	0	SPRR1A,K	exonic	NM_00015	.	.	.	ENST0000	CpG:	103,(	1q21.2-q22
ACaP1:	1	1.58E+08	1.6E+08	0	0	APCS,OR1	exonic	NM_00100	.	.	.	ENST0000	CpG:	46,C	1q23.1-q23
ACaP1:	1	1.6E+08	1.6E+08	0	0	.	intergenic	NM_00163	dist=1339;(	.	.	.	.		1q23.2
ACaP1:	1	1.6E+08	1.62E+08	0	0	PEX19,RP	exonic	NM_00030	.	.	.	ENST0000	CpG:	25,C	1q23.3-q23
ACaP1:	1	1.62E+08	1.69E+08	0	0	RCSD1,MI	exonic	NM_00069	.	.	.	ENST0000	CpG:	115,(	1q24.2-q24
ACaP1:	1	1.69E+08	1.74E+08	0	0	METTL11E	exonic	NM_00013	.	.	.	ENST0000	CpG:	60,C	1q24.3-q24
ACaP1:	1	1.74E+08	1.85E+08	0	0	GM140,C1	exonic	NM_00043	.	.	.	ENST0000	CpG:	67,C	1q25.1-q25
ACaP1:	1	1.85E+08	2.01E+08	0	0	PTGS2,PL	exonic	NM_00018	.	.	.	ENST0000	CpG:	80,C	1q31.2-q31
ACaP1:	1	2.01E+08	2.07E+08	0	0	BTG2,KISS	exonic	NM_00006	.	.	.	ENST0000	CpG:	22,C	1q32.2-q32
ACaP1:	1	2.07E+08	2.08E+08	0	0	C4BPA,CF	exonic	NM_00057	.	.	.	ENST0000	CpG:	72	1q32.2
ACaP1:	1	2.08E+08	2.15E+08	0	0	LINC0053	exonic	NM_00022	.	.	.	ENST0000	CpG:	207,(	1q32.2-q41
ACaP1:	1	2.15E+08	2.15E+08	0	0	PTPN14	UTR5	NM_00540	NM_00540	.	.	ENST0000	CpG:	88,C	1q41
ACaP1:	1	2.15E+08	2.17E+08	0	0	CENPF,KC	exonic	NM_00101	.	.	.	ENST0000	CpG:	79,C	1q41
ACaP1:	1	2.17E+08	2.25E+08	0	0	SLC30A10	exonic	NM_00103	.	.	.	ENST0000	CpG:	77,C	1q42.12-q4
ACaP1:	1	2.25E+08	2.26E+08	0	0	DNAH14	exonic	NM_00114	.	.	.	ENST0000	.		1q42.12
ACaP1:	1	2.26E+08	2.49E+08	0	0	MIR6741,N	exonic	NM_00002	.	.	.	ENST0000	CpG:	149,(	1q42.12-q4
ACaP1:	2	0	210000	0	0	FAM110C	exonic	NM_00107	.	.	.	ENST0000	CpG:	107,(	2p25.3
ACaP1:	2	210000	12890000	0	0	LOC10050	exonic	NM_00054	.	.	.	ENST0000	CpG:	76,C	2p25.2-p25
ACaP1:	2	12890000	13530000	0	0	LOC10050	ncRNA_ex	NR_03843	.	.	.	ENST0000	.		2p24.3
ACaP1:	2	13530000	20650000	0	0	MSGN1,LA	exonic	NM_00100	.	.	.	ENST0000	CpG:	163,(	2p24.3-p24
ACaP1:	2	20650000	31630000	0	0	PLB1,TRIN	exonic	NM_00018	.	.	.	ENST0000	CpG:	114,(	2p23.3-p24
ACaP1:	2	31630000	44120000	0	0	SULT6B1,(	exonic	NM_00010	.	.	.	ENST0000	CpG:	44,C	2p22.1-p22
ACaP1:	2	44120000	45830000	0	0	SIX3,SLC3	exonic	NM_00034	.	.	.	ENST0000	CpG:	18,C	2p21
ACaP1:	2	45830000	47660000	0	0	MCFD2,AC	exonic	NM_00025	.	.	.	ENST0000	CpG:	40,C	2p21
ACaP1:	2	47660000	68870000	0	0	LOC33980	exonic	NM_00014	.	.	.	ENST0000	CpG:	124,(	2p14-p13.3
ACaP1:	2	68870000	75750000	0	0	EVA1A,SP	exonic	NM_00018	.	.	.	ENST0000	CpG:	75,C	2p13.2-p12
ACaP1:	2	75750000	75870000	0	0	AC007099	ncRNA_ex	NR_11028	.	.	.	ENST0000	CpG:	23,C	2p12
ACaP1:	2	75870000	85200000	0	0	AC073628	exonic	NM_00100	.	.	.	ENST0000	CpG:	32,C	2p12-p11.2
ACaP1:	2	85200000	85250000	0	0	KCMF1	intronic	NM_02012	.	.	.	ENST0000	.		2p11.2
ACaP1:	2	85250000	86570000	0	0	ATOH8,GC	exonic	NM_00054	.	.	.	ENST0000	CpG:	47,C	2p11.2
ACaP1:	2	86570000	86660000	0	0	.	intergenic	NM_00116	dist=4794;(	.	.	ENST0000	.		2p11.2
ACaP1:	2	86660000	86950000	0	0	CHMP3,KI	exonic	NM_00100	.	.	.	ENST0000	CpG:	135,(	2p11.2
ACaP1:	2	86950000	86970000	0	0	RMND5A	exonic	NM_02278	.	.	.	ENST0000	.		2p11.2
ACaP1:	2	86970000	89200000	0	0	FOXI3,LO(	exonic	NM_00102	.	.	.	ENST0000	CpG:	34,C	2p11.2

ACaP1:	2	89200000	89240000	0	0	.	intergenic	NR_03963	dist=88032	.	.	.	.		2p11.2
ACaP1:	2	89240000	96610000	0	0	LINC00342	exonic	NM_00101	.	.	.	ENST0000	CpG:	65,C	2q11.1-p1
ACaP1:	2	97610000	98290000	0	0	FAHD2B,L	exonic	NM_00112	.	.	.	ENST0000	CpG:	48,C	2q11.2
ACaP1:	2	98290000	1.29E+08	0	0	RALB,SUL	exonic	NM_00012	.	.	.	ENST0000	CpG:	69,C	2q12.2-q1
ACaP1:	2	1.29E+08	1.31E+08	0	0	UGGT1,RA	exonic	NM_00109	.	.	.	ENST0000	CpG:	34,C	2q21.1-q1
ACaP1:	2	1.31E+08	1.31E+08	0	0	MED15P9,	ncRNA_ex	NR_03390	.	.	.	ENST0000	.		2q21.1
ACaP1:	2	1.31E+08	1.32E+08	0	0	TUBA3D,C	exonic	NM_00100	.	.	.	ENST0000	CpG:	59,C	2q21.1
ACaP1:	2	1.32E+08	1.38E+08	0	0	MGAT5,LC	exonic	NM_00100	.	.	.	ENST0000	CpG:	52,C	2q21.2-q2
ACaP1:	2	1.38E+08	1.59E+08	0	0	MIR4773-2	exonic	NM_00040	.	.	.	ENST0000	CpG:	69,C	2q22.3-q2
ACaP1:	2	1.6E+08	1.98E+08	0	0	OSGEPL1-	exonic	NM_00007	.	.	.	ENST0000	CpG:	93,C	2q31.1-q2
ACaP1:	2	1.98E+08	2.11E+08	0	0	BMPR2,IC	exonic	NM_00100	.	.	.	ENST0000	CpG:	94,C	2q33.2-q3
ACaP1:	2	2.11E+08	2.16E+08	0	0	CPS1,CPS	exonic	NM_00046	.	.	.	ENST0000	CpG:	180,(	2q34-q35
ACaP1:	3	0	9010000	0	0	EGOT,CN	exonic	NM_00056	.	.	.	ENST0000	CpG:	43,C	3p26.3-p2
ACaP1:	3	15710000	31700000	0	0	TBC1D5,R	exonic	NM_00046	.	.	.	ENST0000	CpG:	129,(	3p25.1-p2
ACaP1:	3	31700000	36940000	0	0	DYNC1LI1	exonic	NM_00040	.	.	.	ENST0000	CpG:	68,C	3p23-p22.2
ACaP1:	3	36940000	37070000	0	0	EPM2AIP1	exonic	NM_00024	.	.	.	ENST0000	CpG:	93,C	3p22.2
ACaP1:	3	37070000	37080000	0	0	MLH1	exonic	NM_00024	.	.	.	ENST0000	.		3p22.2
ACaP1:	3	43590000	44890000	0	0	TCAIM,ZN	exonic	NM_00102	.	.	.	ENST0000	CpG:	24,C	3p22.1-p2
ACaP1:	3	52460000	52480000	0	0	SEMA3G	exonic	NM_02016	.	.	.	ENST0000	.		3p21.1
ACaP1:	3	54150000	75720000	0	0	GPR27,TM	exonic	NM_00024	.	.	.	ENST0000	CpG:	66,C	3p21.1-p1
ACaP1:	3	75720000	1E+08	0	0	DCBLD2,T	exonic	NM_00009	.	.	.	ENST0000	CpG:	27,C	3p12.1-p1
ACaP1:	3	1E+08	1.09E+08	0	0	SENP7,DZ	exonic	NM_00098	.	.	.	ENST0000	CpG:	38,C	3q12.2-q1
ACaP1:	3	1.09E+08	1.23E+08	0	0	LINC0048	exonic	NM_00018	.	.	.	ENST0000	CpG:	56,C	3q13.33-q
ACaP1:	3	1.3E+08	1.33E+08	0	0	SNORA58	exonic	NM_00100	.	.	.	ENST0000	CpG:	83,C	3q22.1
ACaP1:	3	1.33E+08	1.41E+08	0	0	TOPBP1,F	exonic	NM_00053	.	.	.	ENST0000	CpG:	24,C	3q22.1-q2
ACaP1:	3	1.41E+08	1.58E+08	0	0	PLOD2,PA	exonic	NM_00009	.	.	.	ENST0000	CpG:	60,C	3q25.2-q2
ACaP1:	3	1.58E+08	1.58E+08	0	0	RP11-538	exonic	NM_00113	.	.	.	ENST0000	CpG:	44,C	3q25.32
ACaP1:	3	1.58E+08	1.59E+08	0	0	RARRES1	exonic	NM_00288	.	.	.	ENST0000	CpG:	98	3q25.32
ACaP1:	3	1.59E+08	1.83E+08	0	0	GPR160,M	exonic	NM_00005	.	.	.	ENST0000	CpG:	55,C	3q26.2-q2
ACaP1:	3	1.84E+08	1.89E+08	0	0	RPL39L,A	exonic	NM_00041	.	.	.	ENST0000	CpG:	32,C	3q27.2-q2
ACaP1:	3	1.89E+08	1.91E+08	0	0	MIR944,G	exonic	NM_00111	.	.	.	ENST0000	CpG:	63,C	3q28
ACaP1:	3	1.91E+08	1.92E+08	0	0	FGF12,CC	exonic	NM_00108	.	.	.	ENST0000	CpG:	24	3q28
ACaP1:	3	1.92E+08	1.92E+08	0	0	FGF12	exonic	NM_00411	.	.	.	ENST0000	.		3q28
ACaP1:	3	1.92E+08	1.92E+08	0	0	FGF12	intronic	NM_00411	.	.	.	ENST0000	.		3q28
ACaP1:	3	1.92E+08	1.94E+08	0	0	ATP13A5,I	exonic	NM_00108	.	.	.	ENST0000	CpG:	42,C	3q28-q29

ACaP1:	3	1.94E+08	1.94E+08	0	0	LINC0088	ncRNA_ex	NR_03392	.	.	.	ENST0000	CpG:	129	3q29
ACaP1:	3	1.95E+08	1.96E+08	0	0	MUC20,AC	exonic	NM_00128	.	.	.	ENST0000	CpG:	38,C	3q29
ACaP1:	3	1.96E+08	1.96E+08	0	0	.	downstrea	NM_00101	.	.	.	ENST0000	CpG:	27	3q29
ACaP1:	3	1.96E+08	1.97E+08	0	0	MIR4797,F	exonic	NM_00101	.	.	.	ENST0000	CpG:	137,(	3q29
ACaP1:	3	1.97E+08	1.97E+08	0	0	FYTTD1,K	exonic	NM_00114	.	.	.	ENST0000	CpG:	94,C	3q29
ACaP1:	3	1.97E+08	1.98E+08	0	0	LRCH3,LM	exonic	NM_00099	.	.	.	ENST0000	CpG:	160,(	3q29
ACaP1:	3	1.98E+08	1.98E+08	0	0	.	intergenic	NM_00114	dist=52272	.	.	.	.		3q29
ACaP1:	4	0	50000	0	0	.	intergenic	NONE,NM	dist=NONE	.	.	ENST0000	.		4p16.3
ACaP1:	4	50000	1820000	0	0	PDE6B,FG	exonic	NM_00014	.	.	.	ENST0000	CpG:	123,(	4p16.3
ACaP1:	4	3430000	3510000	0	0	RGS12,HG	exonic	NM_00116	.	.	.	ENST0000	CpG:	24,C	4p16.3
ACaP1:	4	9210000	9470000	0	0	USP17L22	exonic	NM_00104	.	.	.	ENST0000	CpG:	34	4p16.1
ACaP1:	4	10110000	41360000	0	0	LOC10050	exonic	NM_00032	.	.	.	ENST0000	CpG:	94,C	4p15.33-p1
ACaP1:	4	41360000	65140000	0	0	ERVMER3	exonic	NM_00008	.	.	.	ENST0000	CpG:	79,C	4q11-q12
ACaP1:	4	65140000	71210000	0	0	CENPC,TM	exonic	NM_00020	.	.	.	ENST0000	CpG:	51,C	4q13.1-q13
ACaP1:	4	71210000	90830000	0	0	AREG,NA	exonic	NM_00029	.	.	.	ENST0000	CpG:	47,C	4q21.3-q2
ACaP1:	4	90830000	94440000	0	0	CCSER1,C	exonic	NM_00114	.	.	.	ENST0000	CpG:	122,(	4q22.1-q22
ACaP1:	4	94440000	1.59E+08	0	0	PP12613,L	exonic	NM_00013	.	.	.	ENST0000	CpG:	95,C	4q24-q28.1
ACaP1:	4	1.59E+08	1.84E+08	0	0	NPY1R,HP	exonic	NM_00002	.	.	.	ENST0000	CpG:	41,C	4q34.2-q32
ACaP1:	4	1.91E+08	1.91E+08	0	0	DUX2,DU)	exonic	NM_00112	.	.	.	ENST0000	CpG:	2005	4q35.2
ACaP1:	4	1.91E+08	1.91E+08	0	0	.	intergenic	NM_03317	dist=6558;(	.	.	.	.		4q35.2
ACaP1:	5	0	150000	0	0	PLEKHG4I	exonic	NM_05290	.	.	.	ENST0000	CpG:	108,(	5p15.33
ACaP1:	5	150000	35050000	0	0	SNHG18,C	exonic	NM_00090	.	.	.	ENST0000	CpG:	470,(	5p15.31-p1
ACaP1:	5	35050000	35060000	0	0	PRLR	UTR3	NM_00094	NM_00094	.	.	ENST0000	.		5p13.2
ACaP1:	5	35060000	66490000	0	0	ESM1,C5o	exonic	NM_00006	.	.	.	ENST0000	CpG:	48,C	5q12.1-p13
ACaP1:	5	66490000	70540000	0	0	SMN1,MAI	exonic	NM_00034	.	.	.	ENST0000	CpG:	34,C	5q12.3-q13
ACaP1:	5	70540000	80560000	0	0	PDE8B,BT	exonic	NM_00004	.	.	.	ENST0000	CpG:	148,(	5q13.2-q13
ACaP1:	5	80560000	1.31E+08	0	0	COX7C,SS	exonic	NM_00003	.	.	.	ENST0000	CpG:	39,C	5q21.3-q15
ACaP1:	5	1.31E+08	1.46E+08	0	0	UQCRQ,W	exonic	NM_00017	.	.	.	ENST0000	CpG:	102,(	5q32-q31.3
ACaP1:	5	1.46E+08	1.48E+08	0	0	SPINK1,H	exonic	NM_00087	.	.	.	ENST0000	CpG:	67,C	5q32
ACaP1:	5	1.51E+08	1.7E+08	0	0	MAT2B,NU	exonic	NM_00017	.	.	.	ENST0000	CpG:	27,C	5q33.1-q35
ACaP1:	5	1.7E+08	1.71E+08	0	0	MIR3912,N	exonic	NM_00103	.	.	.	ENST0000	CpG:	411,(	5q35.1
ACaP1:	5	1.71E+08	1.8E+08	0	0	ERGIC1,E	exonic	NM_00050	.	.	.	ENST0000	CpG:	128,(	5q35.2-q35
ACaP1:	6	0	8050000	0	0	C6orf195,E	exonic	NM_00012	.	.	.	ENST0000	CpG:	392,(	6p25.3-p25
ACaP1:	6	8050000	10410000	0	0	TFAP2A,B	exonic	NM_00103	.	.	.	ENST0000	CpG:	35,C	6p24.3
ACaP1:	6	10410000	28980000	0	0	HIST1H1T	exonic	NM_00033	.	.	.	ENST0000	CpG:	32,C	6p23-p22.3

ACaP1	6	28980000	29010000	0	0	LOC10012	UTR5	NM_00119	NM_00119	.	.	.	CpG: 23	6p22.1
ACaP1	6	29010000	29370000	0	0	OR2W1,O	exonic	NM_0010	.	.	.	ENST0000	CpG: 42	6p22.1
ACaP1	6	38700000	38980000	0	0	DNAH8,RF	exonic	NM_0012	.	.	.	ENST0000	.	6p21.2
ACaP1	6	44310000	54180000	0	0	OPN5,LOC	exonic	NM_00025	.	.	.	ENST0000	CpG: 165,(	6p12.1-p2
ACaP1	6	54180000	54190000	0	0	TINAG	exonic	NM_01446	.	.	.	ENST0000	.	6p12.1
ACaP1	6	54190000	66020000	0	0	LGSN,HCF	exonic	NM_00094	.	.	.	ENST0000	CpG: 83,C	6q11.2-q1
ACaP1	6	66020000	81060000	0	0	OGFRL1,N	exonic	NM_00005	.	.	.	ENST0000	CpG: 32,C	6q12-q13
ACaP1	6	81060000	82450000	0	0	.	intergenic	NM_00005	dist=4013;(	.	.	ENST0000	.	6q14.1
ACaP1	6	82450000	83080000	0	0	IBTK,TPB(	exonic	NM_00116	.	.	.	ENST0000	CpG: 49,C	6q14.1
ACaP1	6	83080000	83770000	0	0	UBE3D	exonic	NM_19892	.	.	.	ENST0000	.	6q14.1
ACaP1	6	83770000	83860000	0	0	DOPEY1,U	exonic	NM_00119	.	.	.	ENST0000	CpG: 48,C	6q14.1
ACaP1	6	83860000	83920000	0	0	RWDD2A,I	exonic	NM_00119	.	.	.	ENST0000	CpG: 113	6q14.1-q1
ACaP1	6	91300000	1.05E+08	0	0	FBXL4,EP	exonic	NM_00101	.	.	.	ENST0000	CpG: 51,C	6q15-q16.
ACaP1	6	1.05E+08	1.17E+08	0	0	LINC0022	exonic	NM_00049	.	.	.	ENST0000	CpG: 147,(	6q21-q16.
ACaP1	6	1.17E+08	1.39E+08	0	0	ENPP3,PE	exonic	NM_00004	.	.	.	ENST0000	CpG: 88,C	6q23.2-q2
ACaP1	6	1.39E+08	1.43E+08	0	0	NHSL1,MI	exonic	NM_00107	.	.	.	ENST0000	CpG: 94,C	6q24.1-q2
ACaP1	6	1.43E+08	1.48E+08	0	0	HIVEP2,SI	exonic	NM_00101	.	.	.	ENST0000	CpG: 92,C	6q24.2-q2
ACaP1	6	1.48E+08	1.48E+08	0	0	SAMD5	intronic	NM_00103	.	.	.	ENST0000	.	6q24.3
ACaP1	6	1.48E+08	1.71E+08	0	0	SNORA29	exonic	NM_00012	.	.	.	ENST0000	CpG: 105,(	6q27-q25.
ACaP1	6	1.71E+08	1.71E+08	0	0	.	intergenic	NM_00259	dist=16622	.	.	.	.	6q27
ACaP1	7	0	10000	0	0	.	intergenic	NONE,NR_	dist=NONE	.	.	.	.	7p22.3
ACaP1	7	10000	300000	0	0	LOC10050	exonic	NM_02022	.	.	.	ENST0000	CpG: 35,C	7p22.3
ACaP1	7	300000	320000	0	0	FAM20C	UTR3	NM_02022	NM_02022	.	.	ENST0000	CpG: 25,C	7p22.3
ACaP1	7	320000	2060000	0	0	PSMG3,TF	exonic	NM_00101	.	.	.	ENST0000	CpG: 25,C	7p22.3
ACaP1	7	7100000	21860000	0	0	NDUFA4,N	exonic	NM_00047	.	.	.	ENST0000	CpG: 39,C	7p15.3-p2
ACaP1	7	21860000	41540000	0	0	NEUROD6	exonic	NM_00052	.	.	.	ENST0000	CpG: 22,C	7p15.1-p1
ACaP1	7	48230000	50170000	0	0	CDC14C,Z	exonic	NM_00115	.	.	.	ENST0000	CpG: 251,(	7p12.3-p1
ACaP1	7	56420000	65120000	0	0	ERV3-1,ZN	exonic	NM_0010	.	.	.	ENST0000	CpG: 62,C	7q11.1-p1
ACaP1	7	65120000	72340000	0	0	WBSCR17	exonic	NM_00004	.	.	.	ENST0000	CpG: 79,C	7q11.21-q
ACaP1	7	76710000	97500000	0	0	PTPN12,R	exonic	NM_00007	.	.	.	ENST0000	CpG: 59,C	7q21.13-q
ACaP1	7	1.02E+08	1.03E+08	0	0	DPY19L2F	exonic	NM_00103	.	.	.	ENST0000	CpG: 78,C	7q22.1
ACaP1	7	1.03E+08	1.21E+08	0	0	TES,RELN	exonic	NM_0010	.	.	.	ENST0000	CpG: 98,C	7q31.1-q2
ACaP1	7	1.21E+08	1.27E+08	0	0	LOC15487	exonic	NM_00084	.	.	.	ENST0000	CpG: 81,C	7q31.31-q
ACaP1	7	1.4E+08	1.42E+08	0	0	TAS2R38,I	exonic	NM_0010	.	.	.	ENST0000	CpG: 34,C	7q34
ACaP1	7	1.44E+08	1.47E+08	0	0	CNTNAP2	exonic	NM_00104	.	.	.	ENST0000	CpG: 48,C	7q35

ACaP1:	8	6830000	8650000	0	0	ZNF705B,I	exonic	NM_00103	.	.	.	ENST0000	CpG:	56,C	8p23.1
ACaP1:	8	8650000	8990000	0	0	MFHAS1,M	exonic	NM_00422	.	.	.	ENST0000	CpG:	197,(	8p23.1
ACaP1:	8	12550000	18500000	0	0	DLC1,RP1	exonic	NM_00001	.	.	.	ENST0000	CpG:	83,C	8p22-p23.1
ACaP1:	8	24160000	24390000	0	0	ADAM28,A	exonic	NM_00114	.	.	.	ENST0000	.		8p21.2
ACaP1:	8	24390000	38840000	0	0	MIR4288,E	exonic	NM_00002	.	.	.	ENST0000	CpG:	82,C	8p21.1-p12
ACaP1:	8	38840000	39680000	0	0	ADAM9,AD	exonic	NM_00102	.	.	.	ENST0000	CpG:	47,C	8p11.22
ACaP1:	8	49630000	86550000	0	0	SNTG1,PF	exonic	NM_00006	.	.	.	ENST0000	CpG:	80,C	8q21.13-q1
ACaP1:	8	86550000	86840000	0	0	REXO1L2I	ncRNA_ex	NR_00359	.	.	.	ENST0000	CpG:	129,(	8q21.2
ACaP1:	8	86840000	87220000	0	0	REXO1L2I	exonic	NM_03312	.	.	.	ENST0000	CpG:	130,(	8q21.2-q2
ACaP1:	8	87220000	1.11E+08	0	0	ABRA,RGS	exonic	NM_00098	.	.	.	ENST0000	CpG:	98,C	8q22.2-q22
ACaP1:	8	1.11E+08	1.13E+08	0	0	.	intergenic	NM_01437	dist=3041;(	.	.	ENST0000	.		8q23.2-q23
ACaP1:	8	1.13E+08	1.14E+08	0	0	MIR2053,C	exonic	NM_05290	.	.	.	ENST0000	.		8q23.3
ACaP1:	8	1.14E+08	1.34E+08	0	0	ADCY8,MI	exonic	NM_00012	.	.	.	ENST0000	CpG:	21,C	8q24.22-q2
ACaP1:	8	1.42E+08	1.44E+08	0	0	ZFP41,LY6	exonic	NM_00049	.	.	.	ENST0000	CpG:	29,C	8q24.3
ACaP1:	8	1.44E+08	1.44E+08	0	0	TOP1MT,F	ncRNA_ex	NR_02678	.	.	.	ENST0000	CpG:	68	8q24.3
ACaP1:	8	1.44E+08	1.46E+08	0	0	CYC1,PUF	exonic	NM_00044	.	.	.	ENST0000	CpG:	95,C	8q24.3
ACaP1:	8	1.46E+08	1.46E+08	0	0	ARHGAP3	intronic	NM_02525	.	.	.	ENST0000	CpG:	20,C	8q24.3
ACaP1:	9	0	10000	0	0	.	intergenic	NONE,NR_	dist=NONE	.	.	.	.		9p24.3
ACaP1:	9	10000	180000	0	0	WASH1,DI	exonic	NM_00114	.	.	.	ENST0000	CpG:	210,(	9p24.3
ACaP1:	9	2160000	16440000	0	0	RLN2,RAN	exonic	NM_00017	.	.	.	ENST0000	CpG:	84,C	9p24.1-p24
ACaP1:	9	16440000	16690000	0	0	BNC2	exonic	NM_01763	.	.	.	ENST0000	.		9p22.3-p22
ACaP1:	9	16870000	32640000	0	0	IFNA17,AC	exonic	NM_00007	.	.	.	ENST0000	CpG:	54,C	9p21.1-p22
ACaP1:	9	38610000	41530000	0	0	SPATA31A	exonic	NM_00104	.	.	.	ENST0000	CpG:	37,C	9p12-p13.1
ACaP1:	9	41530000	41580000	0	0	.	intergenic	NM_00111	dist=23075	.	.	ENST0000	.		9p12
ACaP1:	9	41580000	71010000	0	0	SPATA31A	exonic	NM_00101	.	.	.	ENST0000	CpG:	29,C	9q21.11-q1
ACaP1:	9	71010000	71110000	0	0	PGM5	exonic	NM_02196	.	.	.	ENST0000	.		9q21.11
ACaP1:	9	71110000	95470000	0	0	SEMA4D,T	exonic	NM_00014	.	.	.	ENST0000	CpG:	70,C	9q21.13-q2
ACaP1:	9	95470000	95780000	0	0	ANKRD19I	exonic	NM_00100	.	.	.	ENST0000	CpG:	219,(	9q22.31
ACaP1:	9	95780000	95850000	0	0	FGD3,SUS	exonic	NM_00108	.	.	.	ENST0000	CpG:	84	9q22.31
ACaP1:	9	95850000	96870000	0	0	C9orf129,E	exonic	NM_00109	.	.	.	ENST0000	CpG:	49,C	9q22.31-q2
ACaP1:	9	96870000	1.01E+08	0	0	LOC44145	exonic	NM_00013	.	.	.	ENST0000	CpG:	53,C	9q22.32-q2
ACaP1:	9	1.02E+08	1.02E+08	0	0	TGFBR1	exonic	NM_00113	.	.	.	ENST0000	.		9q22.33
ACaP1:	9	1.02E+08	1.03E+08	0	0	NAMA,ERI	exonic	NM_00113	.	.	.	ENST0000	CpG:	70,C	9q22.33-q3
ACaP1:	9	1.03E+08	1.15E+08	0	0	SVEP1,TX	exonic	NM_00003	.	.	.	ENST0000	CpG:	115,(	9q31.3-q31
ACaP1:	9	1.15E+08	1.27E+08	0	0	ZNF618,AI	exonic	NM_00003	.	.	.	ENST0000	CpG:	239,(	9q32-q33.3

ACaP1:	9	1.39E+08	1.39E+08	0	0	SOHLH1,K	exonic	NM_00101	.	.	.	ENST0000	CpG: 56,C	9q34.3
ACaP1:	9	1.39E+08	1.39E+08	0	0	.	intergenic	NM_01617	dist=6774;(	.	.	.	CpG: 58,C	9q34.3
ACaP1:	9	1.39E+08	1.4E+08	0	0	PTGDS,TF	exonic	NM_0006C	.	.	.	ENST0000	CpG: 147,(	9q34.3
ACaP1:	9	1.41E+08	1.41E+08	0	0	FAM157B,	exonic	NM_00114	.	.	.	ENST0000	CpG: 27,C	9q34.3
ACaP1:	9	1.41E+08	1.41E+08	0	0	.	intergenic	NM_00114	dist=5828;(	.	.	ENST0000	.	9q34.3
ACaP1:	10	0	90000	0	0	.	intergenic	NONE,NM_	dist=NONE	.	.	.	.	10p15.3
ACaP1:	10	90000	100000	0	0	TUBB8	exonic	NM_17798	.	.	.	ENST0000	CpG: 64	10p15.3
ACaP1:	10	100000	280000	0	0	ZMYND11	exonic	NM_0012C	.	.	.	ENST0000	CpG: 165,(	10p15.3
ACaP1:	10	280000	11660000	0	0	PRR26,ITI	exonic	NM_00041	.	.	.	ENST0000	CpG: 97,C	10p14-p15
ACaP1:	10	11890000	12080000	0	0	PROSER2	exonic	NM_01554	.	.	.	ENST0000	CpG: 80,C	10p14
ACaP1:	10	12080000	12150000	0	0	UPF2,DHT	exonic	NM_0187C	.	.	.	ENST0000	CpG: 96,C	10p14
ACaP1:	10	15560000	27070000	0	0	MLLT10,P	exonic	NM_00072	.	.	.	ENST0000	CpG: 71,C	10p12.33-p
ACaP1:	10	27070000	27140000	0	0	ABI1	exonic	NM_00101	.	.	.	ENST0000	.	10p12.1
ACaP1:	10	27140000	28290000	0	0	ACBD5,PT	exonic	NM_00101	.	.	.	ENST0000	CpG: 72,C	10p12.1
ACaP1:	10	28290000	28340000	0	0	MPP7	UTR3	NM_17349	NM_17349	.	.	ENST0000	.	10p12.1
ACaP1:	10	28340000	28910000	0	0	MIR5586,V	exonic	NM_01662	.	.	.	ENST0000	CpG: 60,C	10p12.1
ACaP1:	10	31160000	43310000	0	0	LINC0083	exonic	NM_0010C	.	.	.	ENST0000	CpG: 238,(	10p11.23-p
ACaP1:	10	43310000	46130000	0	0	LINC0061	exonic	NM_0006C	.	.	.	ENST0000	CpG: 21,C	10q11.22-(
ACaP1:	10	46130000	46170000	0	0	ZFAND4	exonic	NM_00112	.	.	.	ENST0000	CpG: 104	10q11.22
ACaP1:	10	46170000	49380000	0	0	LOC10099	exonic	NM_00101	.	.	.	ENST0000	CpG: 102,(	10q11.22
ACaP1:	10	49380000	49690000	0	0	FRMPD2,A	exonic	NM_00101	.	.	.	ENST0000	CpG: 16,C	10q11.22
ACaP1:	10	50570000	68290000	0	0	AGAP7,CD	exonic	NM_00012	.	.	.	ENST0000	CpG: 87,C	10q21.2-q2
ACaP1:	10	68290000	69360000	0	0	CTNNA3,L	exonic	NM_00112	.	.	.	ENST0000	.	10q21.3
ACaP1:	10	69360000	69910000	0	0	DNAJC12,	exonic	NM_00112	.	.	.	ENST0000	CpG: 39,C	10q21.3
ACaP1:	10	89260000	93810000	0	0	LINC0086	exonic	NM_00004	.	.	.	ENST0000	CpG: 101,(	10q23.31-(
ACaP1:	10	93810000	98090000	0	0	SORBS1,C	exonic	NM_00076	.	.	.	ENST0000	CpG: 26,C	10q23.33-(
ACaP1:	10	1.06E+08	1.21E+08	0	0	PDCD4,CA	exonic	NM_00049	.	.	.	ENST0000	CpG: 172,(	10q25.1-q2
ACaP1:	10	1.21E+08	1.21E+08	0	0	EIF3A	exonic	NM_00375	.	.	.	ENST0000	CpG: 93	10q26.11
ACaP1:	10	1.21E+08	1.34E+08	0	0	TEX36-AS	exonic	NM_00014	.	.	.	ENST0000	CpG: 54,C	10q26.13-(
ACaP1:	10	1.34E+08	1.35E+08	0	0	MIR202HG	exonic	NM_00101	.	.	.	ENST0000	CpG: 16,C	10q26.3
ACaP1:	10	1.35E+08	1.35E+08	0	0	SCART1,S	exonic	NM_00077	.	.	.	ENST0000	CpG: 84,C	10q26.3
ACaP1:	10	1.35E+08	1.36E+08	0	0	FRG2B,DU	exonic	NM_00108	.	.	.	ENST0000	CpG: 73,C	10q26.3
ACaP1:	10	1.36E+08	1.36E+08	0	0	.	intergenic	NM_00112	dist=21542	.	.	.	.	10q26.3
ACaP1:	11	0	110000	0	0	.	intergenic	NONE,NR_	dist=NONE	.	.	.	.	11p15.5
ACaP1:	11	110000	2960000	0	0	MIR483,IR	exonic	NM_00007	.	.	.	ENST0000	CpG: 116,(	11p15.5-p1

ACaP1:	11	20690000	31680000	0	0	MIR8087,N	exonic	NM_00051	.	.	.	ENST0000	CpG:	57,C	11p14.1-p1
ACaP1:	11	31680000	43910000	0	0	CD59,TTC	exonic	NM_00028	.	.	.	ENST0000	CpG:	154,(	11p11.2-p1
ACaP1:	11	48350000	48360000	0	0	.	intergenic	NM_0010C	dist=2518;(	.	.	.	.		11p11.2
ACaP1:	11	48360000	56240000	0	0	OR5T2,LO	exonic	NM_0010C	.	.	.	ENST0000	CpG:	38,C	11q12.1-p1
ACaP1:	11	56240000	56340000	0	0	OR5M11,C	exonic	NM_0010C	.	.	.	ENST0000	.		11q12.1
ACaP1:	11	56340000	56500000	0	0	OR9G9,OF	exonic	NM_0010C	.	.	.	ENST0000	.		11q12.1
ACaP1:	11	56500000	56810000	0	0	OR5AK4P,	exonic	NM_0010C	.	.	.	ENST0000	.		11q12.1
ACaP1:	11	57970000	60300000	0	0	TCN1,OR1	exonic	NM_00013	.	.	.	ENST0000	CpG:	36,C	11q12.1-q1
ACaP1:	11	62840000	63180000	0	0	SLC22A9,S	exonic	NM_00103	.	.	.	ENST0000	.		11q12.3
ACaP1:	11	81600000	89170000	0	0	RAB30-AS	exonic	NM_00037	.	.	.	ENST0000	CpG:	89,C	11q14.2-q1
ACaP1:	11	89170000	89830000	0	0	TRIM77,TF	exonic	NM_0011C	.	.	.	ENST0000	CpG:	69,C	11q14.3
ACaP1:	11	89830000	1.11E+08	0	0	JRKL,LINC	exonic	NM_00001	.	.	.	ENST0000	CpG:	37,C	11q23.1-q2
ACaP1:	12	8290000	11340000	0	0	A2M,KLRD	exonic	NM_00001	.	.	.	ENST0000	CpG:	35,C	12p13.2-p1
ACaP1:	12	14950000	30900000	0	0	MGP,PDE3	exonic	NM_00041	.	.	.	ENST0000	CpG:	95,C	12p12.3-p1
ACaP1:	12	30900000	33540000	0	0	DNM1L,FL	exonic	NM_0010C	.	.	.	ENST0000	CpG:	86,C	12p11.21-p
ACaP1:	12	33540000	38600000	0	0	ALG10,SY	exonic	NM_03283	.	.	.	ENST0000	CpG:	44,C	12p11.1-q1
ACaP1:	12	38600000	39290000	0	0	CPNE8,AL	exonic	NM_00101	.	.	.	ENST0000	CpG:	25	12q12
ACaP1:	12	39290000	48090000	0	0	CPNE8,LIN	exonic	NM_0010C	.	.	.	ENST0000	CpG:	165,(	12q13.11-c
ACaP1:	12	55230000	55970000	0	0	OR6C6,OF	exonic	NM_0010C	.	.	.	ENST0000	.		12q13.2
ACaP1:	12	58230000	80000000	0	0	LRIG3,HEI	exonic	NM_00023	.	.	.	ENST0000	CpG:	83,C	12q14.1-q1
ACaP1:	12	80000000	91540000	0	0	KERA,LIN(	exonic	NM_00089	.	.	.	ENST0000	CpG:	34,C	12q21.32-c
ACaP1:	12	91540000	1.08E+08	0	0	KRT19P2,(	exonic	NM_00027	.	.	.	ENST0000	CpG:	80,C	12q23.3-q2
ACaP1:	12	1.23E+08	1.24E+08	0	0	VPS33A,M	exonic	NM_0010C	.	.	.	ENST0000	CpG:	48,C	12q24.31
ACaP1:	12	1.24E+08	1.24E+08	0	0	TMED2,DI	exonic	NM_00681	.	.	.	ENST0000	CpG:	80,C	12q24.31
ACaP1:	12	1.32E+08	1.34E+08	0	0	NOC4L,MI	exonic	NM_0010C	.	.	.	ENST0000	CpG:	24,C	12q24.33
ACaP1:	12	1.34E+08	1.34E+08	0	0	ANHX	exonic	NM_00119	.	.	.	ENST0000	CpG:	79	12q24.33
ACaP1:	13	0	34410000	0	0	RPL21P28	exonic	NM_00005	.	.	.	ENST0000	CpG:	113,(	13p13-p11
ACaP1:	13	34410000	36030000	0	0	NBEA,LIN(	exonic	NM_00291	.	.	.	ENST0000	.		13q13.2-q1
ACaP1:	13	36030000	41130000	0	0	SPG20OS	exonic	NM_00053	.	.	.	ENST0000	CpG:	30,C	13q14.11-c
ACaP1:	13	42190000	42240000	0	0	VWA8	intronic	NM_01505	.	.	.	ENST0000	.		13q14.11
ACaP1:	13	42240000	43530000	0	0	EPSTI1,FA	exonic	NM_0010C	.	.	.	ENST0000	CpG:	105,(	13q14.11
ACaP1:	13	43530000	52280000	0	0	DNAJC15,	exonic	NM_00032	.	.	.	ENST0000	CpG:	44,C	13q14.3-q1
ACaP1:	13	53010000	70380000	0	0	LECT1,DIA	exonic	NM_00101	.	.	.	ENST0000	CpG:	216,(	13q21.1-q2
ACaP1:	13	70380000	72040000	0	0	LINC0034{	exonic	NM_00128	.	.	.	ENST0000	CpG:	42	13q21.33
ACaP1:	13	72040000	72430000	0	0	DACH1	exonic	NM_00439	.	.	.	ENST0000	CpG:	26	13q21.33

ACaP1	13	72430000	98120000	0	0	KLF12,MIF	exonic	NM_00011	.	.	.	ENST0000	CpG: 49,C	13q31.1-q2
ACaP1	13	98120000	98600000	0	0	RAP2A	UTR3	NM_02103	NM_02103	.	.	ENST0000	.	13q32.1-q3
ACaP1	13	98600000	1.02E+08	0	0	RNF113B,	exonic	NM_00028	.	.	.	ENST0000	CpG: 70,C	13q32.2-q3
ACaP1	13	1.02E+08	1.1E+08	0	0	SLC10A2,(	exonic	NM_00012	.	.	.	ENST0000	CpG: 32,C	13q33.1-q3
ACaP1	13	1.1E+08	1.1E+08	0	0	MYO16	intronic	NM_00119	.	.	.	ENST0000	.	13q33.3
ACaP1	14	0	19110000	0	0	.	intergenic	NONE,NM	dist=NONE	.	.	.	.	14q11.2-p1
ACaP1	14	19110000	20410000	0	0	POTEG,OI	exonic	NM_0010C	.	.	.	ENST0000	CpG: 209,(	14q11.2
ACaP1	14	20410000	20440000	0	0	.	intergenic	NM_0010C	dist=5158;(	.	.	.	.	14q11.2
ACaP1	14	20440000	20510000	0	0	OR4K14,O	exonic	NM_0010C	.	.	.	ENST0000	.	14q11.2
ACaP1	14	20510000	20520000	0	0	.	intergenic	NM_0010C	dist=7083;(	.	.	.	.	14q11.2
ACaP1	14	20520000	20530000	0	0	OR4L1	exonic	NM_0010C	.	.	.	ENST0000	.	14q11.2
ACaP1	14	20530000	20580000	0	0	.	downstrea	NM_0010C	.	.	.	ENST0000	.	14q11.2
ACaP1	14	20580000	20720000	0	0	OR4N5,OF	exonic	NM_0010C	.	.	.	ENST0000	.	14q11.2
ACaP1	14	22300000	22340000	0	0	.	intergenic	NM_0010C	dist=16576	.	.	ENST0000	.	14q11.2
ACaP1	14	22340000	22350000	0	0	.	intergenic	NM_0010C	dist=20576	.	.	.	.	14q11.2
ACaP1	14	22350000	23320000	0	0	MMP14,SL	exonic	NM_00112	.	.	.	ENST0000	CpG: 25,C	14q11.2
ACaP1	14	26930000	33680000	0	0	GPR33,DT	exonic	NM_00103	.	.	.	ENST0000	CpG: 24,C	14q13.1-q1
ACaP1	14	33680000	33690000	0	0	NPAS3	exonic	NM_00116	.	.	.	ENST0000	.	14q13.1
ACaP1	14	33690000	38200000	0	0	PPP2R3C,	exonic	NM_00107	.	.	.	ENST0000	CpG: 88,C	14q13.2-q2
ACaP1	14	38200000	48150000	0	0	LINC0063	exonic	NM_0010C	.	.	.	ENST0000	CpG: 108,(	14q21.1-q2
ACaP1	14	48150000	48220000	0	0	.	intergenic	NM_00111	dist=5843;(	.	.	.	.	14q21.3
ACaP1	14	48220000	50180000	0	0	MGAT2,RF	exonic	NM_0010C	.	.	.	ENST0000	CpG: 167,(	14q21.3
ACaP1	14	50180000	50280000	0	0	NEMF,KLH	exonic	NM_00471	.	.	.	ENST0000	CpG: 121	14q21.3
ACaP1	14	52520000	52800000	0	0	PTGDR,PT	exonic	NM_00095	.	.	.	ENST0000	CpG: 111,(	14q22.1
ACaP1	14	52800000	60390000	0	0	MIR4308,F	exonic	NM_00016	.	.	.	ENST0000	CpG: 145,(	14q23.1-q2
ACaP1	14	60390000	60950000	0	0	PCNXL4,L	exonic	NM_01602	.	.	.	ENST0000	CpG: 85,C	14q23.1
ACaP1	14	60950000	64010000	0	0	HIF1A,SIX	exonic	NM_00101	.	.	.	ENST0000	CpG: 135,(	14q23.2-q2
ACaP1	14	64010000	64060000	0	0	PPP2R5E	UTR5	NM_00128	NM_00128	.	.	ENST0000	CpG: 89	14q23.2
ACaP1	14	64060000	64460000	0	0	WDR89,M	exonic	NM_0010C	.	.	.	ENST0000	CpG: 29,C	14q23.2
ACaP1	14	64460000	64550000	0	0	MIR548AZ	exonic	NM_01518	.	.	.	ENST0000	.	14q23.2
ACaP1	14	66020000	66490000	0	0	FUT8	exonic	NM_00448	.	.	.	ENST0000	.	14q23.3
ACaP1	14	66490000	66960000	0	0	LINC0023	ncRNA_ex	NR_02433	.	.	.	ENST0000	.	14q23.3
ACaP1	14	66960000	67830000	0	0	FAM71D,G	exonic	NM_00102	.	.	.	ENST0000	CpG: 97,C	14q23.3
ACaP1	14	67830000	67900000	0	0	EIF2S1,PL	exonic	NM_00409	.	.	.	ENST0000	CpG: 67	14q23.3
ACaP1	14	74340000	74410000	0	0	PTGR2,FA	exonic	NM_00114	.	.	.	ENST0000	CpG: 59	14q24.3

ACaP1:	14	80120000	81570000	0	0	NRXN3,CE	exonic	NM_00036	.	.	.	ENST0000C	CpG: 25,C	14q31.1
ACaP1:	14	81570000	81670000	0	0	GTF2A1,S	exonic	NM_00036	.	.	.	ENST0000C	.	14q31.1
ACaP1:	14	81670000	81980000	0	0	RP11-299I	exonic	NM_00124	.	.	.	ENST0000C	CpG: 112,(	14q31.1
ACaP1:	14	81980000	85990000	0	0	SEL1L,RP:	exonic	NM_00124	.	.	.	ENST0000C	CpG: 52	14q31.1-q3
ACaP1:	14	85990000	89110000	0	0	KCNK10,F	exonic	NM_00015	.	.	.	ENST0000C	CpG: 29,C	14q31.3
ACaP1:	14	89110000	89260000	0	0	EML5	exonic	NM_18338	.	.	.	ENST0000C	CpG: 129	14q31.3
ACaP1:	14	89260000	89300000	0	0	TTC8	exonic	NM_00128	.	.	.	ENST0000C	CpG: 41	14q31.3
ACaP1:	14	89300000	89600000	0	0	TTC8	exonic	NM_00128	.	.	.	ENST0000C	CpG: 108	14q31.3
ACaP1:	14	1.05E+08	1.05E+08	0	0	MIR3545,II	exonic	NM_00100	.	.	.	ENST0000C	CpG: 42,C	14q32.33
ACaP1:	14	1.05E+08	1.05E+08	0	0	ZBTB42	UTR3	NM_00113	NM_00113	.	.	ENST0000C	.	14q32.33
ACaP1:	14	1.05E+08	1.05E+08	0	0	LINC0063	ncRNA_ex	NR_02439	.	.	.	ENST0000C	CpG: 136	14q32.33
ACaP1:	14	1.05E+08	1.05E+08	0	0	LINC0063	ncRNA_ex	NR_02439	.	.	.	ENST0000C	CpG: 22,C	14q32.33
ACaP1:	14	1.05E+08	1.06E+08	0	0	C14orf79,J	exonic	NM_00111	.	.	.	ENST0000C	CpG: 166,(	14q32.33
ACaP1:	14	1.06E+08	1.06E+08	0	0	MTA1,TEX	exonic	NM_00110	.	.	.	ENST0000C	CpG: 94,C	14q32.33
ACaP1:	14	1.06E+08	1.06E+08	0	0	C14orf80,(	exonic	NM_00113	.	.	.	ENST0000C	CpG: 25,C	14q32.33
ACaP1:	14	1.06E+08	1.06E+08	0	0	C14orf80,T	exonic	NM_00113	.	.	.	ENST0000C	CpG: 23,C	14q32.33
ACaP1:	14	1.06E+08	1.06E+08	0	0	MIR8071-2	ncRNA_ex	NR_04621	.	.	.	ENST0000C	CpG: 46,C	14q32.33
ACaP1:	15	0	20160000	0	0	.	intergenic	NONE,NR_	dist=NONE	.	.	ENST0000C	CpG: 170,(	15p11.1-p1
ACaP1:	15	20160000	23890000	0	0	GOLGA6L	exonic	NM_00100	.	.	.	ENST0000C	CpG: 69,C	15q11.1-q1
ACaP1:	15	23890000	24920000	0	0	PWRN2,P\	exonic	NM_00248	.	.	.	ENST0000C	CpG: 32,C	15q11.2
ACaP1:	15	32900000	35150000	0	0	MIR1233-1	exonic	NM_00102	.	.	.	ENST0000C	CpG: 71,C	15q13.3-q1
ACaP1:	15	35150000	40570000	0	0	DPH6,MIR	exonic	NM_00100	.	.	.	ENST0000C	CpG: 109,(	15q14-q15
ACaP1:	15	45850000	62460000	0	0	UNC13C,L	exonic	NM_00010	.	.	.	ENST0000C	CpG: 180,(	15q22.1-q2
ACaP1:	15	67490000	68470000	0	0	SKOR1,M/	exonic	NM_00103	.	.	.	ENST0000C	CpG: 112,(	15q22.33-c
ACaP1:	15	70370000	71350000	0	0	UACA,TH/	exonic	NM_00100	.	.	.	ENST0000C	CpG: 42,C	15q23
ACaP1:	15	71350000	73860000	0	0	ADPGK-A!	exonic	NM_00052	.	.	.	ENST0000C	CpG: 39,C	15q24.1-q2
ACaP1:	15	76110000	77370000	0	0	ISL2,FBXC	exonic	NM_00012	.	.	.	ENST0000C	CpG: 94,C	15q24.2-q2
ACaP1:	15	82630000	83200000	0	0	RPS17,GC	exonic	NM_00102	.	.	.	ENST0000C	CpG: 21,C	15q25.2
ACaP1:	15	93440000	98520000	0	0	NR2F2,CH	exonic	NM_00104	.	.	.	ENST0000C	CpG: 145,(	15q26.2-q2
ACaP1:	16	60000	190000	0	0	MIR6859-1	exonic	NM_00101	.	.	.	ENST0000C	CpG: 33,C	16p13.3
ACaP1:	16	200000	580000	0	0	TMEM8A,L	exonic	NM_00051	.	.	.	ENST0000C	CpG: 34,C	16p13.3
ACaP1:	16	580000	1590000	0	0	HAGHL,LM	exonic	NM_00100	.	.	.	ENST0000C	CpG: 172,(	16p13.3
ACaP1:	16	1800000	2290000	0	0	ZNF598,H/	exonic	NM_00029	.	.	.	ENST0000C	CpG: 37,C	16p13.3
ACaP1:	16	21380000	21570000	0	0	SLC7A5P2	exonic	NM_13046	.	.	.	ENST0000C	CpG: 26,C	16p12.2
ACaP1:	16	22360000	22630000	0	0	RRN3P3,N	exonic	NM_00113	.	.	.	ENST0000C	CpG: 139,(	16p12.2

ACaP1:	16	30190000	31110000	0	0	ZNF668,L(	exonic	NM_00029	.	.	.	ENST000C	CpG: 62,C	16p11.2
ACaP1:	16	31110000	31190000	0	0	PRSS8,KA	exonic	NM_00112	.	.	.	ENST000C	CpG: 115,(	16p11.2
ACaP1:	16	33810000	55620000	0	0	FTO-IT1,N	exonic	NM_00029	.	.	.	ENST000C	CpG: 28,C	16p11.2-p1
ACaP1:	16	88050000	89270000	0	0	SNAI3-AS:	exonic	NM_0010C	.	.	.	ENST000C	CpG: 85,C	16q24.2-q2
ACaP1:	17	0	130000	0	0	RPH3AL,L	exonic	NM_00119	.	.	.	ENST000C	CpG: 40,C	17p13.3
ACaP1:	17	29480000	29710000	0	0	NF1,EVI2A	exonic	NM_00026	.	.	.	ENST000C	CpG: 29	17q11.2
ACaP1:	17	39200000	39390000	0	0	KRTAP4-6	exonic	NM_00112	.	.	.	ENST000C	.	17q21.2
ACaP1:	17	44120000	45210000	0	0	KANSL1,M	exonic	NM_0010C	.	.	.	ENST000C	CpG: 140,(	17q21.32-(
ACaP1:	17	45210000	45230000	0	0	CDC27	exonic	NM_00111	.	.	.	ENST000C	.	17q21.32
ACaP1:	17	45230000	49260000	0	0	MIR152,SI	exonic	NM_00002	.	.	.	ENST000C	CpG: 56,C	17q21.33-(
ACaP1:	17	49260000	54940000	0	0	ANKFN1,P	exonic	NM_00108	.	.	.	ENST000C	CpG: 97,C	17q22-q21
ACaP1:	17	54940000	54960000	0	0	DGKE	exonic	NM_00364	.	.	.	ENST000C	.	17q22
ACaP1:	17	54960000	58060000	0	0	MKS1,MT\	exonic	NM_00025	.	.	.	ENST000C	CpG: 17,C	17q22-q23
ACaP1:	17	58060000	60370000	0	0	USP32,TB	exonic	NM_00071	.	.	.	ENST000C	CpG: 43,C	17q23.1-q2
ACaP1:	17	60370000	60440000	0	0	.	intergenic	NR_02748	dist=16984	.	.	ENST000C	.	17q23.2
ACaP1:	17	60440000	66990000	0	0	PSMD12,F	exonic	NM_00004	.	.	.	ENST000C	CpG: 198,(	17q24.2-q2
ACaP1:	17	66990000	68130000	0	0	MAP2K6,A	exonic	NM_00127	.	.	.	ENST000C	CpG: 116	17q24.3-q2
ACaP1:	17	78920000	80060000	0	0	ARHGDIA,	exonic	NM_00016	.	.	.	ENST000C	CpG: 23,C	17q25.3
ACaP1:	18	0	40000	0	0	.	intergenic	NONE,NR_	dist=NONE	.	.	ENST000C	CpG: 67	18p11.32
ACaP1:	18	40000	120000	0	0	ROCK1P1	ncRNA_ex	NR_03377	.	.	.	ENST000C	CpG: 51,C	18p11.32
ACaP1:	18	120000	11600000	0	0	C18orf56,L	exonic	NM_0010C	.	.	.	ENST000C	CpG: 79,C	18p11.23-p
ACaP1:	18	18540000	19020000	0	0	GREB1L,R	exonic	NM_00114	.	.	.	ENST000C	CpG: 195,(	18q11.2-q1
ACaP1:	18	21960000	30340000	0	0	DSC1,WBI	exonic	NM_00037	.	.	.	ENST000C	CpG: 66,C	18q12.1-q1
ACaP1:	18	30340000	32420000	0	0	DTNA,ASX	exonic	NM_0011C	.	.	.	ENST000C	CpG: 43,C	18q12.1
ACaP1:	18	48430000	50460000	0	0	RP11-267(	exonic	NM_00116	.	.	.	ENST000C	CpG: 107,(	18q21.2
ACaP1:	18	50460000	51680000	0	0	DCC,MBD:	exonic	NM_00521	.	.	.	ENST000C	.	18q21.2
ACaP1:	18	51680000	54630000	0	0	TCF4,MIR4	exonic	NM_00108	.	.	.	ENST000C	CpG: 59,C	18q21.2-q2
ACaP1:	18	54630000	54680000	0	0	WDR7	intronic	NM_01528	.	.	.	ENST000C	.	18q21.31
ACaP1:	18	56820000	70930000	0	0	RP11-909I	exonic	NM_00063	.	.	.	ENST000C	CpG: 120,(	18q21.33-(
ACaP1:	18	70930000	71740000	0	0	LOC10050	ncRNA_ex	NR_03413	.	.	.	ENST000C	.	18q22.3
ACaP1:	18	71740000	78077248	0	0	TSHZ1,AT	exonic	NM_00102	.	.	.	ENST000C	CpG: 151,(	18q22.3-q2
ACaP1:	19	0	60000	0	0	.	downstreal	NR_03326	.	.	.	.	.	19p13.3
ACaP1:	19	60000	210000	0	0	FAM138A,	exonic	NM_0010C	.	.	.	ENST000C	CpG: 117	19p13.3
ACaP1:	19	210000	360000	0	0	MIER2,PP,	exonic	NM_00371	.	.	.	ENST000C	CpG: 70,C	19p13.3
ACaP1:	19	360000	2360000	0	0	DAZAP1,R	exonic	NM_00015	.	.	.	ENST000C	CpG: 18,C	19p13.3

ACaP1:	19	2360000	6000000	0	0	CREB3L3,	exonic	NM_00014	.	.	.	ENST0000	CpG:	40,C	19p13.3
ACaP1:	19	6000000	6210000	0	0	ACSBG2,F	exonic	NM_00063	.	.	.	ENST0000	CpG:	23,C	19p13.3
ACaP1:	19	18330000	22610000	0	0	JUND,KIA	exonic	NM_00009	.	.	.	ENST0000	CpG:	191,(	19p12-p13
ACaP1:	19	22610000	22830000	0	0	LOC10192	ncRNA_ex	NR_03389	.	.	.	ENST0000	CpG:	82,C	19p12
ACaP1:	19	22830000	23550000	0	0	ZNF730,ZI	exonic	NM_00108	.	.	.	ENST0000	CpG:	32,C	19p12
ACaP1:	20	1540000	1560000	0	0	SIRPB1	exonic	NM_00108	.	.	.	ENST0000	.		20p13
ACaP1:	20	1560000	1580000	0	0	SIRPB1	exonic	NM_00113	.	.	.	ENST0000	.		20p13
ACaP1:	20	5520000	17620000	0	0	SNRPB2,S	exonic	NM_00021	.	.	.	ENST0000	CpG:	43,C	20p12.3-p1
ACaP1:	20	17670000	23410000	0	0	SLC24A3,I	exonic	NM_00036	.	.	.	ENST0000	CpG:	61,C	20p12.1-p1
ACaP1:	20	44160000	44360000	0	0	WFDC8,SI	exonic	NM_00119	.	.	.	ENST0000	.		20q13.12
ACaP1:	20	44360000	44630000	0	0	NEURL2,V	exonic	NM_00030	.	.	.	ENST0000	CpG:	46,C	20q13.12
ACaP1:	20	60870000	62210000	0	0	PPDPF,MI	exonic	NM_00074	.	.	.	ENST0000	CpG:	54,C	20q13.33
ACaP1:	20	62210000	62220000	0	0	GMEB2	UTR3	NM_01238	NM_01238	.	.	ENST0000	.		20q13.33
ACaP1:	20	62220000	62430000	0	0	RTEL1-TN	exonic	NM_00108	.	.	.	ENST0000	CpG:	17,C	20q13.33
ACaP1:	21	0	9750000	0	0	.	intergenic	NONE,NR_	dist=NONE	.	.	ENST0000	CpG:	31,C	21p13-p12
ACaP1:	21	9750000	9830000	0	0	MIR3648,I	ncRNA_ex	NR_03742	.	.	.	ENST0000	CpG:	120	21p11.2
ACaP1:	21	9830000	14420000	0	0	TPTE,BAG	exonic	NM_00118	.	.	.	ENST0000	CpG:	24,C	21p11.1-q1
ACaP1:	21	14420000	15450000	0	0	ANKRD30I	exonic	NM_17498	.	.	.	ENST0000	CpG:	92,C	21q11.2
ACaP1:	21	15450000	27140000	0	0	NRIP1,CH	exonic	NM_00100	.	.	.	ENST0000	CpG:	138,(	21q11.2-q2
ACaP1:	21	27140000	31670000	0	0	GRIK1-AS	exonic	NM_00048	.	.	.	ENST0000	CpG:	46,C	21q21.3-q2
ACaP1:	21	34000000	34150000	0	0	C21orf49,F	exonic	NM_00116	.	.	.	ENST0000	CpG:	100,(	21q22.11
ACaP1:	21	45700000	45760000	0	0	PFKL,C21	exonic	NM_00038	.	.	.	ENST0000	CpG:	55,C	21q22.3
ACaP1:	21	45760000	45770000	0	0	.	upstream	NM_00127	.	.	.	ENST0000	.		21q22.3
ACaP1:	21	45770000	45870000	0	0	TRPM2-AS	exonic	NM_00330	.	.	.	ENST0000	CpG:	20,C	21q22.3
ACaP1:	21	45870000	45910000	0	0	LRRC3,LR	exonic	NM_03089	.	.	.	ENST0000	CpG:	28,C	21q22.3
ACaP1:	21	45910000	45930000	0	0	TSPEAR-A	exonic	NM_00127	.	.	.	ENST0000	CpG:	19	21q22.3
ACaP1:	21	45930000	47590000	0	0	COL18A1-	exonic	NM_00021	.	.	.	ENST0000	CpG:	142,(	21q22.3
ACaP1:	21	47770000	47790000	0	0	PCNT	exonic	NM_00603	.	.	.	ENST0000	.		21q22.3
ACaP1:	21	47790000	47820000	0	0	PCNT	exonic	NM_00603	.	.	.	ENST0000	CpG:	34,C	21q22.3
ACaP1:	22	0	16090000	0	0	.	intergenic	NONE,NR_	dist=NONE	.	.	ENST0000	.		22p12-q11
ACaP1:	22	16090000	17160000	0	0	OR11H1,B	exonic	NM_00100	.	.	.	ENST0000	CpG:	37,C	22q11.1
ACaP1:	22	18650000	18870000	0	0	GGT3P,US	exonic	NM_01741	.	.	.	ENST0000	CpG:	130,(	22q11.21
ACaP1:	22	19500000	20140000	0	0	TBX1,MIR	exonic	NM_00040	.	.	.	ENST0000	CpG:	73,C	22q11.21
ACaP1:	22	20140000	20190000	0	0	LOC28486	ncRNA_ex	NR_03846	.	.	.	ENST0000	CpG:	21	22q11.21
ACaP1:	22	20190000	20740000	0	0	LOC72944	exonic	NM_00124	.	.	.	ENST0000	CpG:	140,(	22q11.21

ACaP1:	22	20740000	20790000	0	0	SCARF2,Z	exonic	NM_00125	.	.	.	ENST0000	CpG:	248,(	22q11.21
ACaP1:	22	21470000	21910000	0	0	POM121L8	exonic	NM_00112	.	.	.	ENST0000	CpG:	170,(	22q11.21
ACaP1:	22	21910000	21970000	0	0	UBE2L3	exonic	NM_00125	.	.	.	ENST0000	CpG:	85	22q11.21
ACaP1:	22	22900000	22990000	0	0	GGTLC2,F	exonic	NM_00128	.	.	.	ENST0000	CpG:	28,C	22q11.22
ACaP1:	22	22990000	23040000	0	0	GGTLC2	exonic	NM_00128	.	.	.	ENST0000	.		22q11.22
ACaP1:	22	45210000	50600000	0	0	C22orf34,\	exonic	NM_00100	.	.	.	ENST0000	CpG:	84,C	22q13.31-(
ACaP1:	22	50600000	50620000	0	0	PANX2,MC	exonic	NM_00116	.	.	.	ENST0000	CpG:	46,C	22q13.33
ACaP1:	22	50620000	50630000	0	0	TRABD	UTR5	NM_0252C	NM_0252C	.	.	ENST0000	CpG:	25,C	22q13.33
ACaP1:	22	50630000	51170000	0	0	MAPK12,P	exonic	NM_00048	.	.	.	ENST0000	CpG:	30,C	22q13.33
ACaP1:	X	20200000	46960000	0	0	LOC39245	exonic	NM_0010C	.	.	.	ENST0000	CpG:	154,(	Xp21.1-Xp
ACaP1:	X	71680000	73050000	0	0	LINC0068	exonic	NM_00101	.	.	.	ENST0000	CpG:	34,C	Xq13.1-Xq
ACaP1:	X	73050000	74370000	0	0	RLIM,SLC:	exonic	NM_0010C	.	.	.	ENST0000	CpG:	29,C	Xq13.3-Xq
ACaP1:	X	74370000	76910000	0	0	ZDHHC15,	exonic	NM_00048	.	.	.	ENST0000	CpG:	60,C	Xq21.1-Xq
ACaP1:	X	80060000	1.48E+08	0	0	IL13RA2,P	exonic	NM_00006	.	.	.	ENST0000	CpG:	102,(	Xq24-Xq27
ACaP1:	X	1.48E+08	1.55E+08	0	0	ZFP92,FAI	exonic	NM_00003	.	.	.	ENST0000	CpG:	69,C	Xq28
ACaP1:	X	1.55E+08	1.55E+08	0	0	.	intergenic	NM_00218	dist=19518	.	.	.	.		Xq28
ACaP1;	1	0	10000	0	0	.	intergenic	NONE,NR_	dist=NONE	.	.	.	.		1p36.33
ACaP1;	1	10000	34680000	0	0	CAMK2N1	exonic	NM_00008	.	.	.	ENST0000	CpG:	161,(	1p35.1-p3(
ACaP1;	1	34680000	34690000	0	0	C1orf94	exonic	NM_00113	.	.	.	ENST0000	.		1p34.3
ACaP1;	1	34690000	47260000	0	0	C1orf122,(	exonic	NM_00031	.	.	.	ENST0000	CpG:	93,C	1p34.3-p34
ACaP1;	1	47260000	47660000	0	0	CYP4A22,(	exonic	NM_00077	.	.	.	ENST0000	CpG:	40	1p33
ACaP1;	1	47660000	86190000	0	0	ASB17,GL	exonic	NM_00001	.	.	.	ENST0000	CpG:	21,C	1p32.1-p31
ACaP1;	1	86190000	86530000	0	0	COL24A1	exonic	NM_15289	.	.	.	ENST0000	.		1p22.3
ACaP1;	1	86530000	86550000	0	0	COL24A1	intronic	NM_15289	.	.	.	ENST0000	.		1p22.3
ACaP1;	1	86550000	87370000	0	0	SEP15,CO	exonic	NM_0010C	.	.	.	ENST0000	CpG:	106,(	1p22.3
ACaP1;	1	87370000	87390000	0	0	SEP15,HS	exonic	NM_00113	.	.	.	ENST0000	CpG:	101	1p22.3
ACaP1;	2	89080000	96270000	0	0	TRIM43,AI	exonic	NM_00101	.	.	.	ENST0000	CpG:	55,C	2q11.1-p1:
ACaP1;	2	96270000	96280000	0	0	.	intergenic	NM_1388C	dist=4531;(	.	.	.	.		2q11.1
ACaP1;	2	1.14E+08	1.28E+08	0	0	CLASP1,IN	exonic	NM_00012	.	.	.	ENST0000	CpG:	54,C	2q14.2-q1:
ACaP1;	3	1.43E+08	1.58E+08	0	0	SUCNR1,F	exonic	NM_00009	.	.	.	ENST0000	CpG:	303,(	3q25.2-q25
ACaP1;	3	1.58E+08	1.58E+08	0	0	RSRC1	intronic	NM_00127	.	.	.	ENST0000	.		3q25.32
ACaP1;	3	1.58E+08	1.98E+08	0	0	FLJ46066,	exonic	NM_00005	.	.	.	ENST0000	CpG:	25,C	3q29-q27.:
ACaP1;	3	1.98E+08	1.98E+08	0	0	.	intergenic	NM_00114	dist=52272	.	.	.	.		3q29
ACaP1;	4	3240000	3320000	0	0	HTT,MSAI	exonic	NM_00104	.	.	.	ENST0000	CpG:	20,C	4p16.3
ACaP1;	4	3320000	3480000	0	0	DOK7,HGI	exonic	NM_00116	.	.	.	ENST0000	CpG:	22,C	4p16.3

ACaP1:	4	1.91E+08	1.91E+08	0	0	DUX4L2,D	exonic	NM_00112	.	.	.	ENST0000	CpG: 2005	4q35.2
ACaP1:	4	1.91E+08	1.91E+08	0	0	.	intergenic	NM_03317	dist=6558;(	.	.	.	.	4q35.2
ACaP1:	5	520000	660000	0	0	SLC9A3,M	exonic	NM_00128	.	.	.	ENST0000	CpG: 87,C	5p15.33
ACaP1:	5	660000	1040000	0	0	TPPP,LOC	exonic	NM_0010C	.	.	.	ENST0000	CpG: 23,C	5p15.33
ACaP1:	5	1040000	1050000	0	0	.	downstreai	NM_00659	.	.	.	.	.	5p15.33
ACaP1:	5	1120000	1150000	0	0	.	intergenic	NM_00659	dist=7828;(	.	.	.	.	5p15.33
ACaP1:	5	1150000	1430000	0	0	CLPTM1L,	exonic	NM_0010C	.	.	.	ENST0000	CpG: 17,C	5p15.33
ACaP1:	5	6730000	6750000	0	0	PAPD7	exonic	NM_00117	.	.	.	ENST0000	CpG: 21	5p15.31
ACaP1:	5	19470000	31330000	0	0	LINC0102:	exonic	NM_00116	.	.	.	ENST0000	CpG: 93,C	5p13.3-p14
ACaP1:	5	1.02E+08	1.02E+08	0	0	SLCO6A1	exonic	NM_00128	.	.	.	ENST0000	.	5q21.1
ACaP1:	5	1.76E+08	1.76E+08	0	0	LOC10050	exonic	NM_00126	.	.	.	ENST0000	.	5q35.2
ACaP1:	6	1.31E+08	1.31E+08	0	0	AKAP7	exonic	NM_01637	.	.	.	ENST0000	CpG: 71	6q23.2
ACaP1:	7	1.16E+08	1.16E+08	0	0	TFEC	exonic	NM_00101	.	.	.	ENST0000	.	7q31.2
ACaP1:	8	53470000	53480000	0	0	FAM150A	exonic	NM_20741	.	.	.	ENST0000	CpG: 119	8q11.23
ACaP1:	8	1.04E+08	1.05E+08	0	0	RIMS2,DC	exonic	NM_0011C	.	.	.	ENST0000	CpG: 242	8q22.3
ACaP1:	8	1.19E+08	1.42E+08	0	0	CASC8,RF	exonic	NM_00012	.	.	.	ENST0000	CpG: 92,C	8q24.12-q2
ACaP1:	8	1.42E+08	1.46E+08	0	0	TIGD5,ZNI	exonic	NM_00044	.	.	.	ENST0000	CpG: 209,(	8q24.3
ACaP1:	8	1.46E+08	1.46E+08	0	0	ZNF252P,Z	exonic	NM_02308	.	.	.	ENST0000	CpG: 94,C	8q24.3
ACaP1:	9	16720000	16740000	0	0	BNC2	exonic	NM_01763	.	.	.	ENST0000	CpG: 46	9p22.2
ACaP1:	9	38520000	38620000	0	0	ANKRD18/	exonic	NM_14719	.	.	.	ENST0000	CpG: 228,(	9p13.1
ACaP1:	9	38620000	39070000	0	0	ANKRD18/	exonic	NM_14719	.	.	.	ENST0000	CpG: 228	9p13.1
ACaP1:	9	39070000	39380000	0	0	SPATA31/	exonic	NM_00104	.	.	.	ENST0000	CpG: 43,C	9p13.1
ACaP1:	9	1.14E+08	1.14E+08	0	0	KIAA0368	exonic	NM_00108	.	.	.	ENST0000	CpG: 183	9q31.3
ACaP1:	10	1.3E+08	1.3E+08	0	0	FOXI2,PTI	exonic	NM_15231	.	.	.	ENST0000	CpG: 238,(	10q26.2
ACaP1:	10	1.35E+08	1.35E+08	0	0	GPR123,L	exonic	NM_00108	.	.	.	ENST0000	CpG: 38,C	10q26.3
ACaP1:	10	1.35E+08	1.36E+08	0	0	DUX4L6,D	exonic	NM_00112	.	.	.	ENST0000	CpG: 1534	10q26.3
ACaP1:	10	1.36E+08	1.36E+08	0	0	.	intergenic	NM_00112	dist=21542	.	.	.	.	10q26.3
ACaP1:	11	82530000	1.35E+08	0	0	THYN1,OF	exonic	NM_00001	.	.	.	ENST0000	CpG: 119,(	11q23.3-q2
ACaP1:	12	0	60000	0	0	.	intergenic	NONE,NR_	dist=NONE	.	.	.	.	12p13.33
ACaP1:	12	60000	100000	0	0	LOC10028	ncRNA_ex	NR_02826	.	.	.	ENST0000	CpG: 112	12p13.33
ACaP1:	12	8930000	18430000	0	0	ART4,TAS	exonic	NM_00001	.	.	.	ENST0000	CpG: 19,C	12p13.1-p1
ACaP1:	12	18430000	18480000	0	0	PIK3C2G	exonic	NM_00128	.	.	.	ENST0000	.	12p12.3
ACaP1:	12	18480000	18490000	0	0	PIK3C2G	intronic	NM_00128	.	.	.	ENST0000	.	12p12.3
ACaP1:	12	18490000	20730000	0	0	AEBP2,RP	exonic	NM_00092	.	.	.	ENST0000	CpG: 97,C	12p12.2-p1
ACaP1:	12	20730000	20760000	0	0	PDE3A	intronic	NM_00092	.	.	.	ENST0000	.	12p12.2

ACaP1:	12	20760000	21540000	0	0	IAPP,SLC(	exonic	NM_00041	.	.	.	ENST000C	.	12p12.1-p1
ACaP1:	12	21540000	21590000	0	0	SLCO1A2	UTR5	NM_13443	NM_13443	.	.	ENST000C	.	12p12.1
ACaP1:	12	21590000	22840000	0	0	KCNJ8,C2	exonic	NM_00103	.	.	.	ENST000C	CpG: 21,C	12p12.1
ACaP1:	12	22840000	23680000	0	0	ETNK1	UTR3	NM_01863	NM_01863	.	.	ENST000C	.	12p12.1
ACaP1:	12	23680000	25360000	0	0	BCAT1,KR	exonic	NM_0010C	.	.	.	ENST000C	CpG: 98,C	12p12.1
ACaP1:	12	25360000	25800000	0	0	IFLTD1,KF	exonic	NM_00114	.	.	.	ENST000C	CpG: 69,C	12p12.1
ACaP1:	12	25800000	33540000	0	0	MRPS35,F	exonic	NM_0010C	.	.	.	ENST000C	CpG: 90,C	12p11.21-p
ACaP1:	13	67790000	80130000	0	0	LINC0033	exonic	NM_00011	.	.	.	ENST000C	CpG: 177,(	13q21.32-c
ACaP1:	13	1.11E+08	1.12E+08	0	0	LINC0034(	exonic	NM_00111	.	.	.	ENST000C	CpG: 206,(	13q34
ACaP1:	13	1.12E+08	1.13E+08	0	0	SOX1,TEX	exonic	NM_00111	.	.	.	ENST000C	CpG: 201,(	13q34
ACaP1:	13	1.13E+08	1.14E+08	0	0	TUBGCP3	exonic	NM_00128	.	.	.	ENST000C	CpG: 38,C	13q34
ACaP1:	13	1.14E+08	1.14E+08	0	0	.	intergenic	NM_03218	dist=38518	.	.	ENST000C	CpG: 79	13q34
ACaP1:	13	1.14E+08	1.14E+08	0	0	MCF2L,M(	exonic	NM_00111	.	.	.	ENST000C	CpG: 26,C	13q34
ACaP1:	13	1.14E+08	1.15E+08	0	0	CUL4A,LIN	exonic	NM_00013	.	.	.	ENST000C	CpG: 137,(	13q34
ACaP1:	13	1.15E+08	1.15E+08	0	0	UPF3A,CH	exonic	NM_00116	.	.	.	ENST000C	CpG: 100	13q34
ACaP1:	14	0	19110000	0	0	.	intergenic	NONE,NM	dist=NONE	.	.	.	.	14p11.2-p1
ACaP1:	14	19110000	20410000	0	0	DUXAP10,	exonic	NM_0010C	.	.	.	ENST000C	CpG: 42,C	14q11.2
ACaP1:	14	20410000	20470000	0	0	OR4K15	exonic	NM_0010C	.	.	.	ENST000C	.	14q11.2
ACaP1:	14	1.04E+08	1.05E+08	0	0	ASPG,MIR	exonic	NM_00108	.	.	.	ENST000C	CpG: 23,C	14q32.33
ACaP1:	14	1.06E+08	1.06E+08	0	0	.	intergenic	NM_02526	dist=53461	.	.	ENST000C	CpG: 15,C	14q32.33
ACaP1:	14	1.06E+08	1.06E+08	0	0	MIR8071-1	ncRNA_ex	NR_10703	.	.	.	ENST000C	.	14q32.33
ACaP1:	14	1.06E+08	1.06E+08	0	0	MIR8071-1	ncRNA_ex	NR_02680	.	.	.	ENST000C	CpG: 19,C	14q32.33
ACaP1:	15	20160000	22570000	0	0	OR4M2,OF	exonic	NM_0010C	.	.	.	ENST000C	CpG: 141,(	15q11.1-q1
ACaP1:	15	25350000	25500000	0	0	PWAR4,SI	ncRNA_ex	NR_00129	.	.	.	ENST000C	.	15q11.2
ACaP1:	16	32060000	33790000	0	0	TP53TG3D	exonic	NM_00109	.	.	.	ENST000C	CpG: 107,(	16p11.2
ACaP1:	16	33790000	34250000	0	0	LINC0027:	ncRNA_ex	NR_03836	.	.	.	ENST000C	CpG: 142,(	16p11.2
ACaP1:	16	34250000	34480000	0	0	UBE2MP1	ncRNA_ex	NR_00283	.	.	.	ENST000C	CpG: 93,C	16p11.2
ACaP1:	17	34440000	34490000	0	0	.	intergenic	NM_00298	dist=6986;(	.	.	ENST000C	.	17q12
ACaP1:	17	34490000	34810000	0	0	TBC1D3G,	exonic	NM_0010C	.	.	.	ENST000C	CpG: 103,(	17q12
ACaP1:	18	0	40000	0	0	.	intergenic	NONE,NR_	dist=NONE	.	.	ENST000C	CpG: 67	18p11.32
ACaP1:	18	40000	510000	0	0	MIR8078,(	exonic	NM_00103	.	.	.	ENST000C	CpG: 21,C	18p11.32
ACaP1:	18	510000	580000	0	0	.	upstream	NM_00406	.	.	.	ENST000C	CpG: 99	18p11.32
ACaP1:	18	580000	3890000	0	0	DLGAP1-A	exonic	NM_0010C	.	.	.	ENST000C	CpG: 330,(	18p11.31-p
ACaP1:	18	29970000	78077248	0	0	CCDC102I	exonic	NM_00014	.	.	.	ENST000C	CpG: 149,(	18q12.3-q2
ACaP1:	19	100000	210000	0	0	LINC0100:	exonic	NM_0010C	.	.	.	ENST000C	.	19p13.3

ACaP1:	19	59100000	59120000	0	0	.	intergenic	NR_02605	dist=4238;(	.	.	ENST000C	.	19q13.43
ACaP1:	19	59120000	59128983	0	0	.	intergenic	NR_02605	dist=24238	.	.	.	.	19q13.43
ACaP1:	20	1350000	1360000	0	0	SDCBP2-A	exonic	NM_0008C	.	.	.	ENST000C	.	20p13
ACaP1:	20	49300000	49310000	0	0	.	intergenic	NM_08082	dist=46574	.	.	ENST000C	.	20q13.13
ACaP1:	20	50090000	59900000	0	0	MTRNR2L	exonic	NM_00011	.	.	.	ENST000C	CpG: 29,C	20q13.2-q1
ACaP1:	20	59900000	59910000	0	0	CDH4	intronic	NM_00179	.	.	.	ENST000C	.	20q13.33
ACaP1:	20	59910000	60070000	0	0	CDH4	intronic	NM_00179	.	.	.	ENST000C	CpG: 16	20q13.33
ACaP1:	20	60070000	61850000	0	0	LOC10192	exonic	NM_00102	.	.	.	ENST000C	CpG: 44,C	20q13.33
ACaP1:	20	61850000	61860000	0	0	.	intergenic	NM_01779	dist=2462;(	.	.	.	.	20q13.33
ACaP1:	20	61860000	62200000	0	0	HELZ2,PT	exonic	NM_00074	.	.	.	ENST000C	CpG: 134,(	20q13.33
ACaP1:	20	62200000	62930000	0	0	DNAJC5,T	exonic	NM_00091	.	.	.	ENST000C	CpG: 43,C	20q13.33
ACaP1:	20	62930000	63025520	0	0	LINC0026(	ncRNA_ex	NR_04041	.	.	.	ENST000C	CpG: 78,C	20q13.33
ACaP1:	21	42840000	43170000	0	0	LINC0011z	exonic	NM_00113	.	.	.	ENST000C	CpG: 152,(	21q22.3
ACaP1:	22	0	16090000	0	0	.	intergenic	NONE,NR_	dist=NONE	.	.	ENST000C	.	22p12-q11
ACaP1:	22	16090000	16920000	0	0	POTEH-AS	exonic	NM_0010C	.	.	.	ENST000C	CpG: 37,C	22q11.1
ACaP1:	22	16920000	16950000	0	0	.	intergenic	NM_0010C	dist=47019	.	.	.	.	22q11.1
ACaP1:	22	16950000	17470000	0	0	GAB4,TPT	exonic	NM_00103	.	.	.	ENST000C	CpG: 24,C	22q11.1
ACaP1:	22	39360000	39390000	0	0	APOBEC3	exonic	NM_00119	.	.	.	ENST000C	.	22q13.1
ACaP1:	22	46920000	46940000	0	0	CELSR1	exonic	NM_01424	.	.	.	ENST000C	CpG: 476	22q13.31
ACaP1:	X	210000	310000	0	0	LINC0068E	exonic	NM_01222	.	.	.	ENST000C	CpG: 75,C	Xp22.33
ACaP1:	X	83310000	84640000	0	0	RPS6KA6,	exonic	NM_00101	.	.	.	ENST000C	CpG: 46,C	Xq21.2-Xq
ACaP1·	1	25540000	25560000	0	0	SYF2	exonic	NM_01548	.	.	.	ENST000C	CpG: 45	1p36.11
ACaP1·	1	43880000	43910000	0	0	SZT2	exonic	NM_01528	.	.	.	ENST000C	.	1p34.2
ACaP1·	1	1.45E+08	1.46E+08	0	0	POLR3GL,	exonic	NM_00101	.	.	.	ENST000C	CpG: 31,C	1q21.1
ACaP1·	1	1.48E+08	1.5E+08	0	0	PPIAL4E,F	exonic	NM_00056	.	.	.	ENST000C	CpG: 34,C	1q21.2
ACaP1·	1	1.5E+08	1.5E+08	0	0	HIST2H4A	exonic	NM_00103	.	.	.	ENST000C	CpG: 133,(	1q21.2
ACaP1·	2	2.2E+08	2.2E+08	0	0	TUBA4A,S	exonic	NM_0010C	.	.	.	ENST000C	CpG: 90,C	2q35
ACaP1·	3	44910000	44930000	0	0	TGM4	exonic	NM_00324	.	.	.	ENST000C	.	3p21.31
ACaP1·	3	44930000	45120000	0	0	TGM4,CLE	exonic	NM_00113	.	.	.	ENST000C	CpG: 54,C	3p21.31
ACaP1·	3	48280000	49710000	0	0	MIR711,C(	exonic	NM_00009	.	.	.	ENST000C	CpG: 35,C	3p21.31
ACaP1·	3	49710000	49940000	0	0	TRAIP,GM	exonic	NM_0010C	.	.	.	ENST000C	CpG: 82,C	3p21.31
ACaP1·	3	1E+08	1E+08	0	0	TFG,ABI3E	exonic	NM_0010C	.	.	.	ENST000C	.	3q12.2
ACaP1·	3	1E+08	1.01E+08	0	0	ABI3BP	exonic	NM_01542	.	.	.	ENST000C	.	3q12.2
ACaP1·	4	9200000	9220000	0	0	USP17L18	exonic	NM_00125	.	.	.	ENST000C	.	4p16.1
ACaP1·	4	9220000	9370000	0	0	USP17L5,l	exonic	NM_00124	.	.	.	ENST000C	.	4p16.1

ACaP1	4	1.91E+08	1.91E+08	0	0	DUX4L4,D	exonic	NM_00112	.	.	.	ENST0000	CpG: 2005	4q35.2
ACaP1	5	68910000	70530000	0	0	GTF2H2B,	exonic	NM_00034	.	.	.	ENST0000	CpG: 61,C	5q13.2
ACaP1	5	1.32E+08	1.32E+08	0	0	SLC22A5,(	exonic	NM_00219	.	.	.	ENST0000	CpG: 72,C	5q31.1
ACaP1	5	1.56E+08	1.57E+08	0	0	HAVCR1,H	exonic	NM_00109	.	.	.	ENST0000	.	5q33.3
ACaP1	6	25980000	26480000	0	0	HIST1H4G	exonic	NM_00041	.	.	.	ENST0000	CpG: 39,C	6p22.2
ACaP1	6	30450000	30980000	0	0	MUC21,HL	exonic	NM_00101	.	.	.	ENST0000	CpG: 161,(	6p21.33
ACaP1	6	30980000	30990000	0	0	MUC22	intronic	NM_00119	.	.	.	ENST0000	.	6p21.33
ACaP1	6	30990000	31000000	0	0	MUC22	exonic	NM_00119	.	.	.	ENST0000	.	6p21.33
ACaP1	6	31000000	31960000	0	0	MIR4646,S	exonic	NM_00006	.	.	.	ENST0000	CpG: 19,C	6p21.33
ACaP1	6	33130000	33290000	0	0	RXRB,TAF	exonic	NM_00107	.	.	.	ENST0000	CpG: 31,C	6p21.32
ACaP1	6	41870000	42040000	0	0	MED20,CC	exonic	NM_00113	.	.	.	ENST0000	CpG: 41,C	6p21.1
ACaP1	6	42040000	42100000	0	0	C6orf132,T	exonic	NM_00116	.	.	.	ENST0000	CpG: 69	6p21.1
ACaP1	6	42100000	42180000	0	0	GUCA1B,C	exonic	NM_00040	.	.	.	ENST0000	CpG: 19,C	6p21.1
ACaP1	6	42180000	42200000	0	0	MRPS10,T	exonic	NM_01814	.	.	.	ENST0000	.	6p21.1
ACaP1	7	48520000	50140000	0	0	C7orf72,V	exonic	NM_00115	.	.	.	ENST0000	CpG: 251,(	7p12.2-p12
ACaP1	7	50140000	50350000	0	0	IKZF1,C7o	exonic	NM_00116	.	.	.	ENST0000	CpG: 46,C	7p12.2
ACaP1	7	1.01E+08	1.01E+08	0	0	MUC12	intronic	NM_00116	.	.	.	ENST0000	.	7q22.1
ACaP1	7	1.44E+08	1.44E+08	0	0	ARHGEF5	exonic	NM_00100	.	.	.	ENST0000	CpG: 44,C	7q35
ACaP1	8	17470000	17490000	0	0	PDGFRL	exonic	NM_00620	.	.	.	ENST0000	.	8p22
ACaP1	8	86550000	86840000	0	0	REXO1L2I	ncRNA_ex	NR_00359	.	.	.	ENST0000	CpG: 130,(	8q21.2
ACaP1	9	35680000	35820000	0	0	FAM221B,	exonic	NM_00101	.	.	.	ENST0000	CpG: 54,C	9p13.3
ACaP1	10	1.35E+08	1.36E+08	0	0	DUX4L3,L(	exonic	NM_00112	.	.	.	ENST0000	CpG: 1534	10q26.3
ACaP1	10	1.36E+08	1.36E+08	0	0	.	intergenic	NM_00112	dist=21542	.	.	.	.	10q26.3
ACaP1	12	6090000	7300000	0	0	TAPBPL,C	exonic	NM_00036	.	.	.	ENST0000	CpG: 74,C	12p13.31
ACaP1	12	49220000	51220000	0	0	LIMA1,RNI	exonic	NM_00048	.	.	.	ENST0000	CpG: 21,C	12q13.12
ACaP1	12	56210000	58230000	0	0	MIR6758,K	exonic	NM_00007	.	.	.	ENST0000	CpG: 86,C	12q14.1-q1
ACaP1	12	1.08E+08	1.08E+08	0	0	BTBD11	exonic	NM_00101	.	.	.	ENST0000	.	12q23.3
ACaP1	13	24880000	25170000	0	0	TPTE2P6,I	exonic	NM_00643	.	.	.	ENST0000	CpG: 62,C	13q12.12
ACaP1	13	31280000	31290000	0	0	ALOX5AP	exonic	NM_00120	.	.	.	.	.	13q12.3
ACaP1	13	97990000	99670000	0	0	RNF113B,I	exonic	NM_00100	.	.	.	ENST0000	CpG: 22,C	13q32.2-q3
ACaP1	13	99670000	99730000	0	0	DOCK9	intronic	NM_00113	.	.	.	ENST0000	.	13q32.3
ACaP1	13	99730000	1.11E+08	0	0	TPP2,UBA	exonic	NM_00012	.	.	.	ENST0000	CpG: 60,C	13q33.1-q3
ACaP1	13	1.11E+08	1.15E+08	0	0	F10,SPAC	exonic	NM_00013	.	.	.	ENST0000	CpG: 81,C	13q34
ACaP1	13	1.15E+08	1.15E+08	0	0	CHAMP1,U	exonic	NM_00116	.	.	.	ENST0000	CpG: 100	13q34
ACaP1	14	74000000	74010000	0	0	ACOT1,HE	exonic	NM_00103	.	.	.	ENST0000	CpG: 75	14q24.3

ACaP1	15	1.02E+08	1.02E+08	0	0	.	intergenic	NM_15233	dist=5355;	.	.	ENST0000	.	15q26.3
ACaP1	16	16330000	19040000	0	0	SMG1,ARI	exonic	NM_00100	.	.	.	ENST0000	CpG: 51,C	16p12.3-p1
ACaP1	16	19040000	19070000	0	0	TMC7	exonic	NM_00116	.	.	.	ENST0000	.	16p12.3
ACaP1	16	21390000	21500000	0	0	LOC10019	exonic	NM_13046	.	.	.	ENST0000	CpG: 26,C	16p12.2
ACaP1	16	21500000	21510000	0	0	LOC10027	ncRNA_int	NR_02715	.	.	.	.	.	16p12.2
ACaP1	16	21510000	31580000	0	0	ZKSCAN2,	exonic	NM_00003	.	.	.	ENST0000	CpG: 23,C	16p11.2-p1
ACaP1	16	85950000	89270000	0	0	GALNS,MY	exonic	NM_00010	.	.	.	ENST0000	CpG: 39,C	16q24.1-q2
ACaP1	17	0	60000	0	0	LOC10050	exonic	NM_00358	.	.	.	ENST0000	CpG: 212,	17p13.3
ACaP1	17	34390000	34810000	0	0	CCL3L1,C	exonic	NM_00100	.	.	.	ENST0000	CpG: 16,C	17q12
ACaP1	17	78270000	78710000	0	0	LOC10029	exonic	NM_00116	.	.	.	ENST0000	CpG: 18,C	17q25.3
ACaP1	19	6410000	6440000	0	0	MIR3940,K	exonic	NM_00368	.	.	.	ENST0000	CpG: 28,C	19p13.3
ACaP1	19	8840000	9100000	0	0	ZNF558,M	exonic	NM_00100	.	.	.	ENST0000	CpG: 51	19p13.2
ACaP1	19	14680000	14870000	0	0	NDUFB7,Z	exonic	NM_00120	.	.	.	ENST0000	CpG: 29,C	19p13.12
ACaP1	19	33440000	33670000	0	0	C19orf40,F	exonic	NM_01802	.	.	.	ENST0000	CpG: 34,C	19q13.11
ACaP1	19	43670000	43780000	0	0	LOC28434	exonic	NM_00113	.	.	.	ENST0000	.	19q13.31
ACaP1	20	2540000	2560000	0	0	TMC2	exonic	NM_08075	.	.	.	ENST0000	.	20p13
ACaP1	20	2560000	2650000	0	0	SNORD57	exonic	NM_00125	.	.	.	ENST0000	CpG: 121,	20p13
ACaP1	20	34550000	35420000	0	0	TGIF2-C2	exonic	NM_00104	.	.	.	ENST0000	CpG: 37,C	20q11.23
ACaP1	X	17730000	19860000	0	0	PPEF1,RA	exonic	NM_00028	.	.	.	ENST0000	CpG: 56,C	Xp22.12-X
ACaP1	X	23390000	30270000	0	0	PRDX4,M	exonic	NM_00101	.	.	.	ENST0000	CpG: 74,C	Xp21.2-Xp
ACaP1	X	47050000	47230000	0	0	CDK16,ZN	exonic	NM_00117	.	.	.	ENST0000	CpG: 65,C	Xp11.23
ACaP1	X	49150000	49770000	0	0	GAGE2A,	exonic	NM_00104	.	.	.	ENST0000	CpG: 42,C	Xp11.23
ACaP1	X	49770000	50050000	0	0	MIR502,M	exonic	NM_00008	.	.	.	ENST0000	CpG: 16	Xp11.22-X
ACaP1	X	1.55E+08	1.55E+08	0	0	IL9R	exonic	NM_00218	.	.	.	ENST0000	CpG: 29	Xq28
ACaP1	X	1.55E+08	1.55E+08	0	0	.	intergenic	NM_00218	dist=19518	.	.	.	.	Xq28

genomicSu	Repeat	dgvMergec	CopyNumb	Size	CNVType	Genotype	GTConfidence
.	Score=254	nsv830470	3	10000	gain	-1	-1
.	Score=298	esv265735	4	20000	gain	AABB	95.0698
.	Score=230	esv271986	3	20000	gain	AAB	55.2336
.	Score=136	nsv7313,ns	3	10000	gain	-1	-1
.	Score=390	.	3	10000	gain	AAB	100
.	Score=192	.	3	20000	gain	AAB	95.5602
Score=0.9!	Score=645	nsv513029	3	230000	gain	AAB	-1
Score=0.98	Score=642	esv266686	3	32010000	gain	AAB	0.616557
.	Score=634	nsv876676	3	40000	gain	AAB	-1
Score=0.9:	Score=268	nsv3752,es	3	990000	gain	AAB	13.5536
.	Score=200	esv239877	3	40000	gain	AAB	-1
.	Score=239	nsv876679	4	50000	gain	-1	-1
Score=0.9!	Score=507	esv26141,e	3	15680000	gain	AAA	-1
Score=0.9!	Score=627	nsv523112	1	42570000	loss	A	-1
Score=0.9!	Score=238	nsv822925	3	140000	gain	AAB	-1
Score=0.9!	Score=668	esv272937	3	31320000	gain	AAB	0.668971
Score=0.98	Score=510	nsv4785,ns	3	9730000	gain	AAB	2.70045
Score=0.96	Score=442	esv265950	3	2320000	gain	AAB	12.7818
Score=0.9!	Score=143	esv273014	3	20000	gain	AAB	-1
.	Score=274	nsv823062	3	20000	gain	-1	-1
Score=0.94	Score=285	nsv880483	4	2150000	gain	AABB	100
Score=0.9!	Score=607	nsv526252	1	56870000	loss	A	1.46254
.	Score=434	nsv7393	3	10000	gain	-1	-1
.	Score=229	esv266471	3	90000	gain	AAB	97.2606
.	Score=240	dgv53n47,e	3	10000	gain	-1	-1
.	Score=223	.	4	10000	gain	AABB	62.2499
Score=0.9!	Score=229	nsv831205	5	150000	gain	AAAAB	-1
Score=0.9!	Score=801	nsv524542	5	20000	gain	AAAAB	14.9411
.	Score=145	nsv892611	3	10000	gain	AAB	8.06487
.	Score=225	nsv893055	3	10000	gain	-1	-1
.	Score=759	nsv468582	3	10000	gain	-1	-1
.	Score=314	nsv519118	3	30000	gain	-1	-1
Score=0.96	Score=105	nsv964,nsv	4	10000	gain	AAAB	100

Score=0.9	Score=263	esv993979	3	20160000	gain	AAB	-1
Score=0.9	Score=270	esv5154,dg	3	1810000	gain	AAB	0.455651
Score=0.9	Score=282	nsv94485,e	3	2640000	gain	AAB	0.624254
.	Score=142	dgv426n67	3	100000	gain	AAB	-1
Score=0.9	Score=342	esv274948	3	4860000	gain	AAB	2.17404
Score=0.9	Score=182	nsv832946	3	100000	gain	AAB	-1
Score=0.9	Score=252	dgv2292n7	3	20000	gain	AAA	4.01767
Score=0.9	Score=733	nsv832946	3	130000	gain	AAA	6.27554
Score=0.9	Score=281	nsv514775	3	130000	gain	AAB	-1
Score=0.9	Score=641	esv997659	3	240000	gain	AAB	5.55805
Score=0.9	Score=270	esv27055,e	3	1440000	gain	AAB	2.87274
.	Score=255	dgv725e1,n	3	60000	gain	AAB	-1
Score=0.9	Score=281	dgv2324n7	3	400000	gain	AAB	3.95639
Score=0.9	Score=584	nsv903959	3	20000	gain	AAB	-1
Score=0.9	Score=276	nsv903983	3	4140000	gain	AAB	1.50755
.	Score=199	nsv94945,e	3	70000	gain	AAB	-1
Score=0.9	Score=275	esv266432	3	1620000	gain	AAB	43.3513
.	Score=158	nsv523391	3	160000	gain	AAB	-1
Score=0.9	Score=631	esv268625	3	25700000	gain	AAB	0.188277
Score=0.9	Score=472	nsv904326	3	3130000	gain	AAB	1.62298
.	Score=273	esv268324	3	210000	gain	AAB	-1
Score=0.9	Score=162	nsv498848	3	630000	gain	AAB	100
Score=0.9	Score=444	esv260862	3	33730000	gain	AAB	0.395269
.	Score=965	dgv2453n7	3	70000	gain	AAB	-1
Score=0.9	Score=222	dgv453n67	3	250000	gain	AAA	1.25927
Score=0.9	Score=544	nsv9315,ns	3	11392	gain	AAA	-1
.	.	.	3	60000	gain	AAB	-1
Score=0.9	Score=923	dgv2459n7	3	200000	gain	AAB	5.92928
.	Score=241	nsv904652	3	10000	gain	AAB	-1
Score=0.9	Score=102	esv136097	3	2110000	gain	AAB	1.37245
.	Score=113	esv272992	3	90000	gain	AAB	-1
.	Score=492	nsv905139	3	30000	gain	AAA	54.5752
.	Score=250	nsv905139	3	40000	gain	AAA	-1
Score=0.9	Score=304	nsv827537	3	12250000	gain	AAB	1.36397
Score=0.9	Score=428	dgv812e1,e	3	10000	gain	AAB	-1

Score=0.9	Score=170	nsv433435	3	300000	gain	AAB		5.60356
Score=0.9	Score=284	nsv9357,n	3	20000	gain	AAB		-1
Score=0.9	Score=100	esv198505	3	1240000	gain	AAB		3.16905
Score=0.9	Score=295	nsv9375,d	3	2470000	gain	AAB		1.69366
.	Score=276	esv216908	3	10000	gain	AAB		-1
Score=0.9	Score=177	dgv346n27	3	60000	gain	AAA		78.4217
Score=0.9	Score=252	nsv514798	3	920000	gain	AAB		3.11094
Score=0.9	Score=689	nsv905577	3	100000	gain	AAB		60.6909
Score=0.9	Score=247	nsv905580	3	10000	gain	AAB		-1
Score=0.9	Score=319	nsv820589	3	230000	gain	AAB		38.0299
Score=0.9	Score=737	nsv435668	3	190000	gain	AAB		3.37336
Score=0.9	Score=254	esv34235,	3	10000	gain	AAB		-1
Score=0.9	Score=128	esv267281	3	400000	gain	AAB		15.2144
.	Score=536	nsv511047	3	20000	gain	AAB		-1
Score=0.9	Score=891	esv995324	3	270000	gain	AAB		3.18502
.	Score=830	nsv905600	3	190000	gain	AAB		-1
Score=0.9	Score=383	esv271415	3	5380000	gain	AAB		50.4084
Score=0.9	Score=298	nsv526174	3	140000	gain	AAB		-1
Score=0.9	Score=110	nsv1776,n	3	450000	gain	AAB		5.2646
Score=0.9	Score=103	dgv13n14,	3	40000	gain	AAB		-1
Score=0.9	Score=294	nsv9421,e	3	520000	gain	AAB		3.62776
Score=0.9	Score=265	nsv905711	3	10000	gain	AAB		-1
Score=0.9	Score=796	nsv820959	3	200000	gain	AAB		7.82512
Score=0.9	Score=128	nsv9429,e	3	350000	gain	AAB		27.12
Score=0.9	Score=129	nsv9432,n	3	340000	gain	AAA		10.7904
Score=0.9	Score=409	esv243613	3	1770000	gain	AAB		2.53953
Score=0.9	Score=186	dgv2679n7	3	150000	gain	AAB		-1
Score=0.9	Score=242	dgv2706n7	3	320000	gain	AAB		13.2731
Score=0.9	Score=112	esv33069,	3	190000	gain	AAB		-1
Score=0.9	Score=280	esv9440,n	3	1290000	gain	AAB		0.972516
.	Score=227	nsv9622	3	10000	gain		-1	-1
Score=0.9	Score=274	esv271844	3	2150000	gain	AAB		43.5935
Score=0.9	Score=274	esv24627,	3	910000	gain	AAB		11.1928
.	Score=143	nsv526512	3	940000	gain	AAB		9.66075
Score=0.9	Score=283	esv5547,e	3	1470000	gain	AAB		1.04992

.	Score=261	esv271850	3	30000	gain	AAB	-1
Score=0.9	Score=154	nsv138090	3	270000	gain	AAB	1.46849
.	Score=262	nsv509739	3	20000	gain	AAB	-1
Score=0.9	Score=551	nsv138463	3	1910000	gain	AAB	3.66486
Score=0.9	Score=319	nsv833823	3	900000	gain	AAB	0.338089
.	Score=576	nsv522530	3	40000	gain	AAB	-1
Score=0.9	Score=115	esv271854	3	340000	gain	AAB	6.76229
Score=0.9	Score=266	esv271855	3	600000	gain	AAB	3.01197
Score=0.9	Score=273	nsv911910	3	660000	gain	AAB	18.2638
Score=0.9	Score=751	nsv438306	3	30000	gain	AAA	7.79637
.	Score=325	.	3	10000	gain	AAB	100
.	Score=775	nsv3499	3	30000	gain	AAB	100
.	Score=290	nsv914272	3	20000	gain	-1	-1
.	Score=232	nsv7350,n	3	10000	gain	AAB	46.4057
Score=0.9	Score=735	esv29271,	3	10000	gain	AAB	44.3973
.	Score=259	nsv915368	3	20000	gain	AAB	15.3225
.	Score=259	nsv517873	3	50000	gain	AAB	7.82891
Score=0.9	Score=271	dgv2463e1	1	1340000	loss	A	65.9659
.	.	.	4	10000	gain	AAAA	-1
Score=0.9	Score=405	dgv1e1,dg	4	30000	gain	AAAA	3.60996
.	Score=198	.	4	10000	gain	AABB	100
Score=0.9	Score=434	esv992038	1	29329373	loss	A	-1
Score=0.9	Score=626	esv114226	3	23940000	gain	AAB	0.225795
.	Score=274	nsv509860	3	630000	gain	AAB	-1
Score=0.9	Score=385	nsv10352,	7	7850000	gain	AAAAABB	4.30696
.	Score=120	nsv877990	8	60000	gain	AAAAAAB	-1
Score=0.9	Score=536	nsv527268	8	8050000	gain	AAAAAAB	12.6957
Score=0.9	Score=504	esv216788	7	7480000	gain	AAAAABB	4.01554
.	Score=223	nsv822370	7	70000	gain	AAAAABB	-1
.	Score=265	esv28218,	8	1260000	gain	AAAAAAB	100
.	Score=134	nsv878092	6	60000	gain	AAAABB	-1
Score=0.9	Score=443	nsv10378,	6	8390000	gain	AAAABB	51.9435
Score=0.9	Score=719	esv26652,	6	30000	gain	AAAABB	-1
Score=0.9	Score=360	dgv1562e1	4	20000	gain	AAAA	4.87173
Score=0.9	Score=571	esv26652,	4	62430	gain	AAAA	-1

.	Score=277	esv272972	3	20000	gain		-1	-1
.	Score=734	nsv328970	3	10000	gain		-1	-1
.	Score=231	dgv6297n7	4	10000	gain	AABB		39.3356
.	Score=269	dgv1857e1	3	20000	gain		-1	-1
.	Score=675	.	3	10000	gain		-1	-1
.	Score=243	nsv883108	4	10000	gain	AABB		46.8243
Score=0.9	Score=223	nsv428134	4	110000	gain		-1	-1
Score=0.9	Score=415	dgv1n41,d	3	20000	gain	AAB		7.17626
.	Score=103	esv273194	3	80000	gain		-1	-1
.	Score=571	esv129459	4	10000	gain		-1	-1
.	Score=167	.	3	10000	gain		-1	-1
Score=0.9	Score=401	esv989554	4	20000	gain	AAAB		23.033
.	Score=234	nsv893513	3	10000	gain	AAB		100
.	Score=308	.	3	10000	gain	AAB		100
.	Score=215	.	3	10000	gain		-1	-1
Score=0.9	Score=466	esv100910	3	40000	gain	AAB		24.3583
.	Score=105	dgv228e1,	3	70000	gain	AAB		31.0088
.	Score=620	nsv7211,n	3	10000	gain		-1	-1
.	Score=135	nsv520995	3	30000	gain	AAB		86.3897
.	Score=543	nsv618	3	40000	gain	AAB		100
.	Score=109	esv274676	3	450000	gain	AAB		2.75218
.	Score=145	esv274816	4	10000	gain	AABB		100
.	Score=248	nsv1370	3	20000	gain		-1	-1
.	Score=256	nsv832851	4	10000	gain	AAAB		100
.	Score=955	nsv94861,	3	140000	gain		-1	-1
Score=0.9	Score=736	nsv9315,n	3	60000	gain	AAB		8.83873
Score=0.9	Score=544	nsv9315,n	3	11392	gain	AAB		-1
.	Score=321	nsv103651	3	10000	gain		-1	-1
.	Score=628	esv265974	3	170000	gain	AAB		2.95149
.	Score=633	nsv905722	3	50000	gain	AAB		-1
Score=0.9	Score=254	nsv821543	3	10000	gain	AAB		28.6278
.	Score=258	nsv525170	3	90000	gain	AAB		7.56032
.	Score=265	nsv833243	3	10000	gain	AAB		100
Score=0.9	Score=277	nsv9599,n	1	40000	loss	A		-1
Score=0.9	Score=277	esv3749,e	1	80000	loss	A		18.082

Score=0.98	Score=471	esv260317	1	2850000	loss	A		4.62697
Score=0.97	Score=345	esv266498	1	3890000	loss	A		8.06019
Score=0.99	Score=523	esv256195	1	27580000	loss	A		-1
.	Score=268	nsv909546	1	280000	loss	A		31.234
Score=0.99	Score=477	nsv909685	1	26850000	loss	A		-1
Score=0.99	Score=417	esv3212,es	1	15720000	loss	A		-1
Score=0.89	Score=588	nsv131578	1	310000	loss	A		39.6818
.	Score=240	esv267818	1	147248	loss	A		-1
.	.	.	3	60000	gain	AAB		-1
Score=0.99	Score=424	nsv910070	3	20000	gain	AAB		45.5978
Score=0.99	Score=765	esv22059,n	5	20000	gain	AAAAB		-1
Score=0.99	Score=153	nsv910070	5	20000	gain	AAAAB		100
Score=0.99	Score=224	nsv821117	5	30000	gain	AAAAB		-1
Score=0.99	Score=224	nsv9651,ns	6	10000	gain	AAAAAB		100
Score=0.99	Score=112	nsv821117	3	20000	gain	AAA		-1
Score=0.99	Score=354	nsv471520	3	30000	gain	AAA		10.7192
.	Score=265	nsv911030	3	140000	gain	AAB		7.35528
Score=0.94	Score=113	nsv137976	3	270000	gain		-1	-1
.	Score=318	.	4	10000	gain	AABB		100
.	Score=346	esv267175	3	10000	gain	AAB		100
.	Score=147	nsv912687	3	90000	gain	AAB		69.3148
.	Score=247	.	3	10000	gain	AAB		8.89668
Score=0.99	Score=860	esv272392	1	20000	loss	A		-1
Score=0.99	Score=838	dgv16n31,n	1	270000	loss	A		-1
Score=0.99	Score=193	nsv438327	1	1050000	loss	A		25.0685
.	Score=421	esv242237	1	10000	loss	A		-1
Score=0.98	Score=327	esv266829	1	1680000	loss	A		5.3014
Score=0.98	Score=107	nsv820435	1	60000	loss	A		-1
Score=0.98	Score=147	nsv834157	1	410000	loss	A		40.1088
.	Score=377	nsv914629	1	10000	loss	A		-1
Score=0.99	Score=294	dgv4931n7	1	4430000	loss	A		-1
Score=0.97	Score=121	nsv191239	1	1450000	loss	A		9.60673
Score=0.96	Score=249	nsv829171	1	30000	loss	A		-1
Score=0.98	Score=194	esv215914	1	890000	loss	A		4.67827
.	Score=361	nsv483006	1	20000	loss	A		-1

Score=0.9	Score=282	nsv829181	1	4640000	loss	A		-1
Score=0.9	Score=135	nsv834185	1	670000	loss	A		5.79616
.	Score=125	nsv915010	1	40000	loss	A		-1
Score=0.9	Score=331	nsv834200	1	3800000	loss	A		-1
Score=0.9	Score=442	esv261809	1	4770000	loss	A		-1
Score=0.9	Score=223	esv173082	1	2310000	loss	A		49.2376
Score=0.9	Score=253	esv33533,n	1	1180000	loss	A		5.55441
Score=0.9	Score=253	essv13762	1	10000	loss	A		-1
.	Score=593	.	3	20000	gain		-1	-1
.	Score=111	nsv518029	3	20000	gain		-1	-1
.	Score=161	.	3	10000	gain		-1	-1
Score=0.9	Score=676	esv130737	3	230000	gain	AAB		7.56032
.	Score=266	nsv509158	3	20000	gain	AAB		100
.	Score=582	nsv528460	3	10000	gain		-1	-1
.	Score=253	nsv4855,ns	3	270000	gain	AAB		28.2067
.	Score=238	.	3	10000	gain		-1	-1
.	Score=214	esv268146	3	170000	gain	AAB		100
Score=0.8	Score=650	nsv834541	3	70000	gain		-1	-1
.	Score=826	nsv877499	3	10000	gain		-1	-1
Score=0.9	Score=117	nsv7356,es	3	10000	gain		-1	-1
.	Score=248	nsv7356	3	10000	gain		-1	-1
.	Score=216	nsv829887	4	10000	gain	AABB		36.5748
.	Score=511	nsv525865	4	10000	gain		-1	-1
.	Score=407	nsv881708	4	10000	gain		-1	-1
.	Score=246	nsv882683	3	10000	gain	AAB		63.058
.	Score=141	esv26352,n	3	30000	gain		-1	-1
.	Score=327	dgv7108n7	4	10000	gain		-1	-1
.	Score=567	.	3	10000	gain	AAB		100
.	Score=800	esv101044	3	410000	gain	AAB		56.1048
.	Score=379	nsv521781	3	30000	gain		-1	-1
.	Score=712	esv273521	4	10000	gain	AABB		31.1743
Score=0.9	Score=836	esv273612	3	120000	gain	AAB		100
.	Score=472	nsv893491	4	10000	gain		-1	-1
.	Score=535	.	3	10000	gain	AAB		7.5058
.	Score=400	.	4	10000	gain	AABB		100

.	Score=181	.	4	10000	gain	AABB		100
.	Score=727	dgv753n71	4	10000	gain	AAAB		100
.	Score=225	nsv825578	4	10000	gain	AABB		100
.	Score=602	nsv7607,es	3	20000	gain	AAB		100
.	Score=846	dgv397e1,(	3	10000	gain		-1	-1
.	Score=223	nsv832216	4	10000	gain	AABB		32.8279
Score=0.9	Score=269	dgv1242n7	3	270000	gain	AAB		100
.	Score=197	esv107357	4	10000	gain	AABB		23.1335
.	Score=285	nsv832278	3	10000	gain		-1	-1
.	Score=143	.	3	20000	gain	AAA		100
.	Score=340	nsv469190	3	10000	gain		-1	-1
.	Score=325	nsv832399	3	10000	gain		-1	-1
.	Score=103	esv29777,(	3	40000	gain	AAB		44.0877
.	Score=529	nsv900076	3	10000	gain		-1	-1
.	Score=161	nsv901506	3	10000	gain	AAB		100
.	Score=224	.	3	10000	gain		-1	-1
.	Score=141	esv21487,(	3	60000	gain	AAB		100
.	Score=412	.	3	10000	gain	AAB		74.1514
.	Score=248	nsv902113	3	10000	gain		-1	-1
.	Score=517	nsv832856	3	30000	gain		-1	-1
.	Score=245	nsv457172	3	10000	gain		-1	-1
.	Score=241	dgv521e19	3	10000	gain		-1	-1
.	Score=612	nsv833509	3	10000	gain		-1	-1
.	Score=218	nsv2229,ns	3	100000	gain	AAB		67.9837
.	Score=149	esv266855	3	10000	gain	AAB		100
.	Score=150	.	3	20000	gain	AAA		100
.	Score=109	nsv913579	3	10000	gain	AAB		100
.	Score=277	.	3	40000	gain		-1	-1
.	Score=240	nsv834073	4	10000	gain	AABB		72.6802
.	Score=357	nsv913820	3	10000	gain		-1	-1
.	Score=790	esv272432	3	30000	gain	AAB		72.0264
.	Score=269	nsv520230	3	10000	gain		-1	-1
Score=0.9	Score=346	esv266443	4	10000	gain		-1	-1
.	Score=323	dgv56n68	4	10000	gain	AABB		52.9243
.	Score=246	nsv519522	3	10000	gain	AAB		51.8194

Score=0.9	Score=276	esv272013	3	200000	gain		-1	-1
.	Score=737	.	3	20000	gain		-1	-1
.	Score=575	dgv4353n7	4	10000	gain		-1	-1
.	Score=258	nsv428406	3	30000	gain		-1	-1
Score=0.9	Score=590	dgv5235n7	3	19000000	gain	AAB		1.02154
Score=0.9	Score=632	nsv822252	3	260000	gain	AAB		-1
Score=0.9	Score=626	esv257722	3	65400000	gain	AAB		0.525643
Score=0.9	Score=289	nsv4189,e	3	2360000	gain	AAB		3.32391
Score=0.9	Score=113	dgv5383n7	3	30000	gain	AAB		-1
Score=0.9	Score=719	nsv471429	3	80000	gain	AAB		19.7034
Score=0.9	Score=571	esv26652,	3	62430	gain	AAB		-1
.	Score=104	esv272688	4	10000	gain	AAAB		100
.	Score=112	nsv879725	4	70000	gain	AABB		50.1201
.	Score=109	dgv1712e1	4	10000	gain		-1	-1
.	Score=451	nsv880584	3	20000	gain	AAB		100
.	Score=974	esv136246	3	340000	gain	AAB		10.1621
.	Score=292	esv273298	3	20000	gain		-1	-1
.	Score=260	.	3	20000	gain		-1	-1
.	Score=245	nsv831463	3	20000	gain	AAB		8.0889
.	Score=247	nsv891675	3	10000	gain	AAB		17.804
.	Score=546	dgv8253n7	3	20000	gain		-1	-1
Score=0.9	Score=243	nsv831668	3	30000	gain		-1	-1
.	Score=261	.	3	10000	gain		-1	-1
.	Score=269	.	3	10000	gain		-1	-1
.	Score=626	esv272084	3	30000	gain		-1	-1
.	Score=291	nsv831886	3	10000	gain		-1	-1
.	Score=616	esv259044	3	50000	gain	AAB		7.56032
.	Score=556	nsv508601	3	10000	gain	AAB		100
.	Score=476	nsv7211,n	3	10000	gain		-1	-1
.	Score=256	.	4	10000	gain	AAAB		100
.	.	.	3	60000	gain	AAB		-1
Score=0.9	Score=670	.	3	20000	gain	AAB		100
Score=0.9	Score=372	.	3	20000	gain	AAB		10.4263
.	Score=228	nsv899336	3	10000	gain		-1	-1
.	Score=191	nsv899873	3	230000	gain	AAA		100

.	Score=550	nsv518593	3	40000	gain	AAB		100
.	Score=259	dgv250e20	4	10000	gain	AABB		100
.	Score=233	dgv250e20	4	10000	gain		-1	-1
.	Score=825	esv274787	3	10000	gain	AAB		100
.	Score=246	nsv519018	3	30000	gain	AAB		100
.	Score=261	nsv901620	3	20000	gain	AAB		42.6508
.	Score=115	esv32599,e	3	160000	gain		-1	-1
.	Score=896	nsv904306	3	60000	gain		-1	-1
Score=0.99	Score=724	nsv9484,es	3	120000	gain	AAB		17.7699
Score=0.99	Score=350	esv24801,n	3	64753	gain	AAB		-1
.	Score=219	essv8,esv2	3	10000	gain	AAB		100
Score=0.97	Score=471	.	4	10000	gain	AABB		95.0698
.	Score=238	nsv457890	3	10000	gain	AAB		100
Score=0.97	Score=277	nsv9599,ns	3	40000	gain	AAB		-1
Score=0.98	Score=277	esv8460,ns	3	80000	gain	AAB		7.72808
.	Score=430	esv267763	4	20000	gain	AAAB		100
.	Score=258	nsv828176	3	110000	gain	AAA		100
.	Score=147	nsv833595	3	380000	gain		-1	-1
Score=0.95	Score=269	nsv909588	3	520000	gain	AAB		7.56778
.	Score=265	nsv909614	4	40000	gain		-1	-1
.	Score=766	nsv131347	4	140000	gain	AABB		25.8795
.	Score=726	esv267866	3	20000	gain	AAB		-1
.	Score=347	nsv132192	3	1490000	gain	AAB		100
Score=0.95	Score=286	esv264390	3	830000	gain	AAB		31.3463
.	Score=624	esv271716	3	80000	gain	AAB		-1
.	.	.	3	60000	gain	AAB		-1
Score=0.99	Score=224	nsv9651,es	3	150000	gain	AAB		7.12562
Score=0.99	Score=274	esv274273	3	940000	gain	AAB		2.63737
Score=0.99	Score=825	dgv1353e1	4	30000	gain	AAAB		48.6559
.	Score=130	esv259744	3	10000	gain		-1	-1
.	Score=175	nsv834099	3	140000	gain	AAB		100
.	Score=769	nsv470903	3	30000	gain		-1	-1
.	Score=243	nsv834145	4	10000	gain	AAAB		100
.	Score=265	nsv521825	3	10000	gain		-1	-1
.	Score=237	esv158407	3	10000	gain		-1	-1

.	Score=276	esv274061	3	20000	gain		-1	-1
.	Score=285	nsv834528	4	10000	gain		-1	-1
.	Score=714	nsv460837	4	10000	gain	AABB		84.4308
.	Score=105	nsv4277,es	3	100000	gain	AAB		58.9542
.	Score=158	nsv522792	3	10000	gain		-1	-1
.	Score=255	.	3	10000	gain	AAB		100
.	Score=239	esv29160,e	3	40000	gain		-1	-1
.	Score=228	nsv462021	4	10000	gain	AABB		26.3282
.	Score=189	nsv883106	3	10000	gain	AAB		100
.	Score=372	nsv886330	4	10000	gain	AAAB		100
.	Score=195	nsv830825	3	20000	gain		-1	-1
Score=0.9	Score=468	nsv5606,ns	3	20000	gain	AAB		30.4257
.	.	.	3	55067	gain	AAB		-1
Score=0.9	Score=244	nsv8095,es	3	10000	gain	AAB		16.6468
.	Score=781	esv100059	3	250000	gain	AAB		11.3057
.	Score=474	nsv466430	3	20000	gain	AAB		7.56032
.	Score=122	nsv893828	3	260000	gain		-1	-1
.	Score=119	nsv467190	3	10000	gain		-1	-1
.	Score=281	esv267885	3	210000	gain	AAB		31.9552
.	Score=101	esv266599	3	10000	gain	AAB		18.022
.	Score=227	nsv899500	4	10000	gain	AABB		100
.	Score=780	.	4	10000	gain	AAAB		100
.	Score=233	esv274764	4	10000	gain	AABB		43.1713
.	Score=261	nsv904254	3	10000	gain		-1	-1
.	Score=667	nsv1576	3	20000	gain	AAB		100
.	Score=242	esv274981	3	10000	gain	AAB		58.1509
.	Score=261	nsv517068	3	10000	gain		-1	-1
.	Score=608	nsv2023	3	10000	gain		-1	-1
.	Score=331	nsv833499	3	20000	gain	AAB		7.71883
.	Score=483	dgv184n21	3	10000	gain	AAB		100
.	Score=241	dgv1073e1	3	10000	gain	AAB		95.5602
.	Score=346	dgv1074e1	3	10000	gain	AAB		27.5256
Score=0.9	Score=121	nsv2471,ns	3	10000	gain	AAB		87.2075
.	Score=118	nsv912653	3	10000	gain		-1	-1
.	Score=188	nsv912812	3	10000	gain	AAB		70.5974

.	Score=775	nsv438323	3	80000	gain	AAB		100
.	Score=544	.	4	10000	gain	AABB		81.2481
.	Score=155	.	3	10000	gain		-1	-1
.	Score=192	.	3	10000	gain		-1	-1
.	Score=139	nsv520351	4	10000	gain		-1	-1
.	Score=258	nsv820102	4	10000	gain	AABB		9.11279
.	Score=179	nsv830148	4	20000	gain	AABB		100
.	Score=131	esv266234	3	190000	gain	AAB		84.4527
Score=0.9	Score=360	nsv874473	5	10000	gain	AAAAB		12.2095
Score=0.9	Score=157	dgv5107n7	3	330000	gain	AAB		100
.	Score=286	nsv3835,n	3	20000	gain	AAB		62.1648
.	Score=100	nsv877395	4	70000	gain	AABB		30.8865
.	Score=138	esv272745	3	50000	gain	AAB		100
.	Score=149	esv271243	3	70000	gain		-1	-1
.	Score=222	esv27759,	3	280000	gain	AAB		3.92557
.	Score=229	nsv5290,e	3	260000	gain	AAB		30.1359
.	Score=647	.	4	40000	gain	AABB		35.9746
.	Score=371	dgv254e55	4	10000	gain	AAAB		100
Score=0.9	Score=244	nsv818679	3	550000	gain	AAB		61.5232
.	Score=225	nsv892190	4	10000	gain	AABB		100
.	Score=554	esv273913	5	10000	gain	AAABB		34.3634
.	Score=737	esv274391	5	10000	gain		-1	-1
Score=0.9	Score=234	nsv825963	5	10000	gain	AAABB		100
.	Score=631	esv274507	3	30000	gain		-1	-1
.	Score=250	dgv446e1,	5	10000	gain	AAABB		65.6953
.	Score=145	dgv508e1,	3	30000	gain		-1	-1
.	Score=214	dgv94n21,	4	10000	gain	AABB		100
.	Score=234	esv274676	5	10000	gain	AAABB		36.9759
.	Score=202	dgv1810n7	4	10000	gain	AABB		100
.	Score=234	dgv137n21	4	10000	gain		-1	-1
.	Score=464	esv274900	3	180000	gain	AAB		63.0321
.	Score=755	nsv832855	4	10000	gain	AAAB		100
.	Score=221	nsv456427	4	10000	gain		-1	-1
Score=0.9	Score=236	dgv2148n7	4	10000	gain	AABB		96.6962
Score=0.9	Score=300	nsv7266,n	3	40000	gain	AAB		100

.	Score=260	nsv510668	3	200000	gain		-1	-1
.	Score=239	esv274989	4	10000	gain		-1	-1
.	Score=235	nsv904605	4	10000	gain		-1	-1
.	Score=232	nsv833141	4	10000	gain		-1	-1
.	Score=215	nsv833343	5	10000	gain	AAABB		100
.	Score=329	esv33941	4	10000	gain		-1	-1
.	Score=240	nsv910068	3	70000	gain	AAB		55.8333
.	Score=723	nsv910069	3	67248	gain	AAB		-1
.	Score=266	nsv833702	3	10000	gain	AAB		100
.	Score=255	nsv470107	3	10000	gain	AAB		26.839
.	Score=268	esv274471	3	130000	gain	AAB		90.9651
.	Score=250	dgv3656n7	3	10000	gain	AAB		100
.	Score=252	nsv833754	4	10000	gain		-1	-1
.	Score=621	nsv2452,dg	4	10000	gain	AABB		65.4883
.	Score=278	nsv470139	4	10000	gain	AABB		37.3501
.	Score=361	nsv509745	3	10000	gain	AAB		30.0415
.	Score=242	nsv2508	4	20000	gain	AABB		77.2089
.	Score=223	nsv510781	4	10000	gain	AABB		100
.	Score=230	esv100777	3	1200000	gain	AAB		20.0564
Score=0.9	Score=274	esv272306	3	940000	gain	AAB		2.10058
.	Score=401	nsv834055	3	10000	gain		-1	-1
.	Score=259	nsv913680	4	10000	gain		-1	-1
.	Score=243	esv28721	4	10000	gain	AABB		56.8538
.	Score=114	nsv3534,es	3	10000	gain	AAB		100
.	Score=464	dgv4761n7	3	10000	gain	AAB		77.5138
.	Score=257	nsv915003	4	10000	gain		-1	-1
.	Score=246	nsv834217	4	10000	gain		-1	-1
.	Score=259	esv272444	4	20000	gain	AABB		23.8127
Score=0.9	Score=277	esv275231	4	20000	gain	AABB		100
Score=0.9	Score=616	esv106316	3	880000	gain	AAB		4.46373
Score=0.9	Score=166	esv270761	3	710000	gain	AAB		44.673
.	Score=142	.	5	20000	gain		-1	-1
.	Score=594	nsv830360	3	20000	gain	AAB		100
.	Score=976	.	4	20000	gain	AABB		41.1754
.	Score=639	esv267703	3	20000	gain	AAB		40.545

.	Score=566	esv273521	4	20000	gain	AABB		100
.	Score=514	dgv7631n7	4	20000	gain	AABB		71.8887
.	Score=398	nsv6397	4	10000	gain	AABB		71.9763
.	Score=149	esv255542	3	350000	gain	AAB		8.47197
.	Score=237	nsv467312	4	10000	gain	AABB		100
Score=0.9	Score=654	esv267244	4	230000	gain	AABB		16.6802
.	Score=394	nsv519327	3	20000	gain	AAA		34.6358
.	Score=150	nsv832086	4	10000	gain		-1	-1
.	Score=561	nsv521130	3	20000	gain	AAA		100
Score=0.9	Score=307	esv265839	3	90000	gain	AAA		-1
Score=0.9	Score=399	dgv454e1,	3	40000	gain	AAA		54.5785
.	Score=531	nsv8986,e	3	60000	gain		-1	-1
Score=0.9	Score=553	nsv7234,d	3	20000	gain	AAB		100
.	Score=457	nsv889	3	10000	gain		-1	-1
.	Score=618	nsv900,ns	3	50000	gain		-1	-1
Score=0.9	Score=247	nsv483010	3	10000	gain	AAA		67.7827
Score=0.9	Score=476	nsv515960	3	80000	gain	AAA		-1
.	Score=562	nsv832566	4	10000	gain		-1	-1
.	Score=361	nsv899931	3	30000	gain	AAB		-1
Score=0.9	Score=274	esv274715	3	960000	gain	AAB		14.0941
Score=0.9	Score=231	nsv899941	3	250000	gain	AAB		-1
.	Score=271	nsv510948	3	320000	gain	AAB		6.98223
.	Score=305	nsv899993	4	20000	gain		-1	-1
.	Score=224	nsv900551	3	30000	gain		-1	-1
.	Score=260	esv33189,	3	10000	gain	AAA		100
.	Score=158	nsv832763	3	150000	gain	AAA		-1
Score=0.9	Score=151	nsv902015	3	80000	gain		-1	-1
.	Score=102	esv269192	3	140000	gain	AAB		100
.	Score=184	nsv1307,n	3	120000	gain	AAB		-1
Score=0.9	Score=277	nsv85157,	3	390000	gain	AAA		100
Score=0.9	Score=378	esv274883	3	120000	gain		-1	-1
.	Score=350	nsv902081	4	20000	gain	AABB		100
.	Score=254	.	3	20000	gain	AAA		100
.	Score=432	esv269970	3	210000	gain	AAA		-1
.	Score=212	nsv518383	3	170000	gain	AAB		52.509

.	Score=291	nsv522389	3	20000	gain	AAA		36.8066
.	Score=561	esv258944	3	20000	gain		-1	-1
.	Score=909	nsv817698	4	40000	gain	AABB		63.7447
Score=0.9	Score=286	dgv897e1,	3	250000	gain	AAB		6.14723
Score=0.9	Score=965	dgv3213n7	3	310000	gain	AAB		18.1366
Score=0.9	Score=303	esv271613	3	60000	gain		-1	-1
.	Score=524	esv28832,	3	50000	gain	AAB		23.7256
.	Score=106	.	4	20000	gain		-1	-1
.	Score=145	esv268292	4	50000	gain		-1	-1
.	Score=999	nsv2311,n	3	20000	gain	AAA		100
.	Score=106	esv271822	4	100000	gain	AAAB		100
.	Score=270	esv100658	3	10000	gain	AAB		77.1146
Score=0.9	Score=303	essv8523,	3	20000	gain	AAA		100
.	Score=147	nsv7350,e	4	20000	gain	AAAB		100
.	Score=255	.	5	10000	gain	AAABB		28.5494
.	Score=720	nsv519012	5	10000	gain	AAABB		100
Score=0.9	Score=233	nsv515203	3	200000	gain	AAB		51.4323
Score=0.9	Score=287	esv100763	3	5770000	gain	AAB		0.195804
.	Score=255	esv271776	3	130000	gain	AAB		-1
Score=0.9	Score=507	esv33703,	3	1290000	gain	AAB		56.8813
Score=0.9	Score=434	esv5765,e	3	690000	gain	AAB		23.8929
.	Score=224	.	5	10000	gain		-1	-1
Score=0.9	Score=114	nsv515016	3	170000	gain	AAB		31.6153
Score=0.9	Score=291	nsv515029	3	120000	gain	AAA		6.82657
.	Score=539	esv130271	3	10000	gain	AAA		-1
.	Score=136	esv173299	4	10000	gain	AABB		100
Score=0.9	Score=245	esv267691	4	30000	gain	AABB		5.7772
.	Score=904	dgv545e1,	3	100000	gain		-1	-1
.	Score=294	esv274714	3	20000	gain	AAA		100
Score=0.9	Score=348	dgv394n67	3	10000	gain	AAB		-1
Score=0.9	Score=717	nsv817656	3	890000	gain	AAB		2.89565
.	.	.	3	59540	gain	AAB		-1
Score=0.9	Score=288	esv27669,	0	30000	loss		-1	-1
.	Score=809	nsv833230	1	10000	loss		-1	-1
.	Score=961	esv33029	1	10000	loss		-1	-1

Score=0.9!	Score=274	esv250329	3	6250000	gain	AAB		6.04995
Score=0.9!	Score=112	esv267773	3	740000	gain	AAB		45.8354
Score=0.9!	Score=233	nsv515201	3	200000	gain	AAA		15.732
Score=0.9	Score=821	dgv41n71,i	1	70000	loss	A		23.8957
Score=0.9!	Score=220	dgv66e199	3	680000	gain	AAB		5.22178
Score=0.9!	Score=257	nsv7181,dg	3	20000	gain	AAB		-1
Score=0.9!	Score=220	dgv16n16,i	3	190000	gain	AAB		3.66037
.	Score=263	dgv344n71	4	30000	gain	AAAB		-1
Score=0.9!	Score=107	nsv2655,dg	4	300000	gain	AAAB		3.24742
Score=0.9!	Score=423	esv271720	4	40000	gain	AAAB		-1
Score=0.9!	Score=241	nsv7182,es	3	30000	gain	AAB		87.2075
Score=0.9!	Score=273	dgv117e1,i	3	40000	gain	AAB		-1
.	Score=533	esv265858	3	20000	gain	AAA		100
Score=0.9:	Score=507	nsv428246	3	70000	gain	AAB		100
Score=0.9	Score=192	nsv428246	3	60000	gain	AAB		-1
Score=0.9!	Score=113	dgv537n71	1	220000	loss	A		28.1882
.	Score=184	esv272428	1	70000	loss	A		-1
.	Score=108	nsv834543	4	130000	gain	AABB		16.5094
Score=0.9	Score=133	nsv520203	4	140000	gain	AABB		14.8115
Score=0.9!	Score=114	dgv922e19	3	10000	gain		-1	-1
Score=0.9!	Score=381	esv272713	3	160000	gain	AAA		1.98731
.	Score=492	.	1	80000	loss	A		54.5738
.	Score=980	nsv4239	1	50000	loss	A		-1
Score=0.9!	Score=219	esv23426,i	1	174276	loss	A		-1
Score=0.9(	Score=252	esv147606	3	170000	gain	AAB		14.8928
Score=0.9!	Score=668	esv272974	3	28320000	gain	AAA		-1
.	Score=642	dgv1789e1	4	130000	gain	AABB		9.48625
Score=0.9!	Score=510	esv265790	3	13410000	gain	AAB		3.32473
Score=0.9	Score=429	nsv525347	4	420000	gain	AABB		6.55705
Score=0.9:	Score=789	esv266401	4	200000	gain	AABB		-1
Score=0.9!	Score=251	dgv6441n7	3	490000	gain	AAB		100
.	Score=634	nsv823665	4	140000	gain	AABB		3.17938
.	Score=127	nsv5371	1	30000	loss		-1	-1
Score=0.9!	Score=238	nsv5567,es	3	50000	gain	AAA		7.90423
Score=0.9!	Score=113	nsv830894	3	510000	gain	AAB		68.1946

.	Score=278	esv158407	1	10000	loss	A		100
Score=0.98	Score=270	esv100554	1	260000	loss	A		-1
Score=0.99	Score=121	esv33253,r	3	200000	gain	AAB		6.44546
Score=0.99	Score=328	esv273655	1	270000	loss	A		80.3384
.	Score=444	nsv831293	4	50000	gain		-1	-1
.	Score=219	nsv527334	1	210000	loss	A		75.7586
Score=0.94	Score=916	.	1	20000	loss	A		100
Score=0.89	Score=273	esv22308,e	1	660000	loss	A		-1
Score=0.99	Score=192	nsv820555	1	290000	loss	A		7.60458
.	Score=318	nsv527751	1	10000	loss		-1	-1
.	Score=150	esv273807	1	90000	loss	A		45.5097
.	Score=239	dgv1296e2	1	20000	loss	A		-1
.	Score=562	esv257175	4	180000	gain	AABB		3.17343
.	Score=819	dgv8413n7	1	40000	loss	A		100
Score=0.95	Score=283	esv163799	3	900000	gain	AAB		21.1497
.	Score=108	esv267007	3	130000	gain	AAB		3.0607
.	Score=233	dgv385e19	3	40000	gain	AAA		100
.	Score=262	.	3	60000	gain	AAA		-1
.	Score=847	nsv7249,ns	4	150000	gain	AABB		4.85282
.	Score=273	.	3	90000	gain	AAB		95.6719
Score=0.98	Score=374	nsv1613,es	3	50000	gain	AAB		8.28201
.	Score=256	nsv442384	3	20000	gain	AAA		100
Score=0.97	Score=260	nsv521896	3	10000	gain	AAB		96.4156
.	Score=267	esv275519	3	20000	gain	AAA		100
Score=0.99	Score=796	nsv9426,ns	3	170000	gain	AAA		86.678
Score=0.99	Score=803	nsv9431,dg	3	150000	gain	AAA		100
Score=0.95	Score=358	dgv847e1,r	3	30000	gain	AAA		-1
Score=0.98	Score=253	nsv7285,ns	3	100000	gain	AAA		26.3026
.	Score=439	esv21587,e	1	60000	loss	A		-1
Score=0.94	Score=961	nsv9705,ns	3	240000	gain	AAB		3.33038
Score=0.98	Score=303	esv266146	3	50000	gain	AAB		100
.	Score=270	nsv180652	3	20000	gain	AAA		6.60573
.	Score=771	esv272238	3	160000	gain	AAA		55.3167
.	Score=817	dgv510n27	3	140000	gain	AAA		-1
Score=0.99	Score=976	nsv471490	1	360000	loss	A		3.31062

Score=0.97	Score=656	esv272406	1	20000	loss	A		-1
Score=0.90	Score=167	nsv817991	3	580000	gain	AAB		60.331
Score=0.90	Score=453	esv266719	1	6470000	loss	A		-1
Score=0.99	Score=554	esv260613	3	54040000	gain	AAA		-1
Score=0.99	Score=268	esv2796,n	3	2120000	gain	AAB		0.791925
Score=0.97	Score=456	nsv819563	3	17260000	gain	AAA		-1
.	Score=264	esv266010	3	50000	gain	AAA		100
Score=0.99	Score=468	nsv527241	3	38400000	gain	AAA		-1
Score=0.90	Score=267	nsv834303	3	150000	gain	AAB		34.6755
Score=0.98	Score=269	dgv1229e1	3	430000	gain	AAB		28.4753
Score=0.98	Score=403	esv267770	3	270000	gain	AAA		36.3816
Score=0.97	Score=144	esv266200	3	160000	gain	AAA		-1
Score=0.98	Score=300	nsv10134,r	3	1720000	gain	AAB		3.83068
Score=0.99	Score=438	esv32911,e	3	21950000	gain	AAA		-1
.	Score=232	nsv875344	3	130000	gain	AAA		100
.	Score=707	nsv875345	3	30000	gain	AAA		-1
Score=0.92	Score=460	nsv459989	4	1680000	gain	AABB		100
.	Score=458	nsv524548	3	40000	gain	AAA		100
.	Score=786	esv100423	3	210000	gain	AAA		-1
Score=0.97	Score=443	nsv876460	3	7790000	gain	AAA		-1
Score=0.99	Score=642	esv996170	3	22860000	gain	AAA		-1
Score=0.99	Score=470	nsv822151	3	46570000	gain	AAA		-1
.	Score=600	nsv460787	3	20000	gain	AAA		100
.	Score=507	dgv183e55	3	50000	gain	AAA		-1
Score=0.99	Score=442	esv204094	3	2350000	gain	AAA		-1
Score=0.99	Score=152	nsv4192,n	3	50000	gain	AAB		-1
Score=0.90	Score=241	nsv516451	3	240000	gain	AAB		6.70249
Score=0.93	Score=164	dgv5386n7	3	70000	gain	AAB		-1
Score=0.92	Score=217	nsv461144	3	80000	gain	AAA		18.1897
.	Score=267	nsv522680	3	10000	gain		-1	-1
Score=0.97	Score=295	nsv527745	3	7280000	gain	AAA		-1
Score=0.94	Score=117	nsv819190	3	120000	gain		-1	-1
.	Score=315	nsv10486	3	20000	gain		-1	-1
Score=0.91	Score=228	dgv5530n7	3	40000	gain	AAB		100
.	Score=110	nsv878960	3	80000	gain	AAB		-1

.	Score=233	esv249861	3	120000	gain	AAA		80.6793
.	Score=239	nsv509895	3	80000	gain	AAA		-1
.	Score=342	nsv829925	3	20000	gain	AAA		73.0136
Score=0.9	Score=222	dgv658n27	3	120000	gain	AAB		18.5294
Score=0.9	Score=278	nsv470043	3	110000	gain	AAB		-1
Score=0.9	Score=268	dgv5581n7	3	410000	gain		-1	-1
.	Score=135	nsv4381,n	3	90000	gain	AAA		80.6793
.	Score=107	esv272803	3	170000	gain	AAA		100
Score=0.9	Score=485	nsv7363,n	3	130000	gain	AAA		-1
.	Score=348	nsv7363	3	30000	gain		-1	-1
Score=0.9	Score=486	esv272785	3	710000	gain	AAB		62.8971
Score=0.9	Score=485	esv267564	3	10200000	gain	AAA		-1
.	Score=521	.	3	20000	gain	AAA		100
Score=0.9	Score=248	esv272340	3	340000	gain	AAA		100
.	Score=228	nsv290561	3	60000	gain	AAA		-1
Score=0.9	Score=439	nsv509924	3	7450000	gain	AAA		-1
.	Score=276	nsv879854	3	1140000	gain		-1	-1
Score=0.9	Score=295	nsv528104	4	1620000	gain	AABB		4.39222
.	Score=166	nsv879867	4	80000	gain	AABB		-1
.	Score=233	nsv879867	3	10000	gain		-1	-1
.	Score=302	.	3	10000	gain		-1	-1
Score=0.9	Score=270	esv272847	3	300000	gain		-1	-1
Score=0.9	Score=288	nsv289607	3	4980000	gain	AAA		-1
.	Score=230	dgv1700e1	3	20000	gain	AAA		100
.	Score=564	.	3	10000	gain		-1	-1
.	Score=211	esv272871	3	210000	gain	AAA		100
.	Score=222	nsv881291	3	10000	gain	AAA		-1
.	Score=498	nsv4601,n	3	20000	gain	AAA		100
.	Score=119	nsv822850	3	10000	gain		-1	-1
Score=0.9	Score=234	dgv5873n7	3	160000	gain	AAA		5.54674
Score=0.9	Score=219	nsv10641,	3	134276	gain	AAA		-1
Score=0.9	Score=510	nsv4804,n	3	32620000	gain	AAA		-1
Score=0.9	Score=700	nsv882271	3	60300000	gain	AAA		-1
Score=0.9	Score=273	nsv830552	3	590000	gain	AAA		100
Score=0.8	Score=135	nsv883455	3	180000	gain	AAA		12.1314

.	Score=568	esv267121	4	120000	gain	AABB		11.3155
Score=0.99	Score=419	dgv1052e2	3	11640000	gain	AAA		-1
Score=0.98	Score=266	dgv1053e2	4	1140000	gain	AABB		100
Score=0.99	Score=650	esv273516	3	25170000	gain	AAA		-1
Score=0.93	Score=262	esv267176	3	280000	gain	AAA		81.7294
.	Score=285	esv23341,e	3	130000	gain	AAA		-1
Score=0.99	Score=523	nsv886546	3	29360000	gain	AAA		-1
Score=0.99	Score=451	nsv520992	3	10900000	gain	AAA		-1
Score=0.98	Score=261	nsv5880,es	3	190000	gain	AAB		31.6192
Score=0.99	Score=533	nsv889060	3	17780000	gain	AAA		-1
Score=0.99	Score=255	esv273689	3	830000	gain	AAB		84.0358
Score=0.99	Score=660	esv3679,ns	3	37440000	gain	AAA		-1
Score=0.97	Score=446	nsv831394	3	7320000	gain	AAA		-1
.	.	.	3	10000	gain	AAB		-1
Score=0.99	Score=275	esv266845	3	170000	gain	AAB		5.10012
Score=0.99	Score=413	dgv1321e1	3	12920000	gain	AAA		-1
.	Score=143	esv242221	3	160000	gain	AAB		58.6855
Score=0.98	Score=685	nsv892759	3	19250000	gain	AAA		-1
Score=0.99	Score=276	esv23416,e	4	360000	gain	AAAB		28.7068
Score=0.99	Score=245	dgv8208n7	3	1660000	gain	AAB		3.64829
Score=0.99	Score=282	esv273850	3	3170000	gain	AAB		1.00653
Score=0.93	Score=281	esv101910	3	330000	gain	AAB		100
Score=0.99	Score=412	nsv893655	3	10020000	gain	AAA		-1
Score=0.99	Score=310	esv3464,ns	3	7130000	gain	AAA		-1
Score=0.99	Score=602	nsv24936,n	3	7140000	gain	AAA		-1
.	Score=929	essv75,nsv	3	30000	gain		-1	-1
Score=0.99	Score=748	nsv514557	3	200000	gain	AAB		100
Score=0.99	Score=449	esv265646	3	17420000	gain	AAA		-1
Score=0.99	Score=510	dgv200n67	3	23590000	gain	AAA		-1
Score=0.98	Score=328	esv275439	3	6350000	gain	AAA		-1
Score=0.96	Score=598	esv274550	3	490000	gain	AAB		20.302
Score=0.96	Score=417	esv251215	3	170000	gain	AAB		-1
Score=0.99	Score=274	nsv527404	3	770000	gain	AAB		3.88904
Score=0.99	Score=402	nsv898865	3	9460000	gain	AAB		0.576937
Score=0.96	Score=287	nsv826356	3	880000	gain		-1	-1

Score=0.9	Score=651	nsv805,esv	3	49160000	gain	AAA		-1
Score=0.9	Score=275	esv268414	3	5540000	gain	AAA		-1
.	Score=152	nsv832657	4	40000	gain	AABB		43.4011
.	Score=264	nsv507726	3	370000	gain	AAB		100
Score=0.9	Score=360	dgv612e1,r	3	10010000	gain	AAA		-1
Score=0.9	Score=984	esv267568	3	200000	gain	AAA		100
Score=0.9	Score=436	esv7179,ns	3	14770000	gain	AAA		-1
.	Score=777	nsv1358,es	3	80000	gain	AAB		16.0097
Score=0.9	Score=479	nsv819254	3	11480000	gain	AAA		-1
.	Score=627	nsv902241	4	70000	gain	AABB		2.96889
.	Score=920	nsv471683	3	20000	gain	AAA		100
.	Score=226	esv100630	3	50000	gain	AAA		-1
Score=0.9	Score=476	esv267423	3	14700000	gain	AAA		-1
Score=0.9	Score=424	nsv9504,ns	3	80000	gain	AAA		15.324
Score=0.9	Score=683	esv104146	3	50000	gain	AAA		-1
Score=0.9	Score=389	dgv70e19,r	3	6620000	gain	AAB		57.6394
.	Score=254	esv100839	3	190000	gain	AAB		-1
Score=0.9	Score=365	esv265117	3	9600000	gain	AAA		-1
Score=0.9	Score=551	nsv511620	3	1580000	gain	AAB		14.8447
.	Score=278	nsv458852	1	20000	loss		-1	-1
.	Score=254	.	3	20000	gain	AAA		100
.	Score=999	dgv4583n7	4	110000	gain	AABB		100
.	Score=360	nsv509793	3	150000	gain	AAB		100
Score=0.9	Score=789	esv242229	3	230000	gain	AAB		1.99044
Score=0.9	Score=623	esv271943	1	87140000	loss	A		-1
Score=0.9	Score=645	nsv514129	3	240000	gain		-1	-1
.	Score=121	esv275269	4	20000	gain	AAAB		100
Score=0.9	Score=405	nsv830036	4	20000	gain	AABB		100
Score=0.9	Score=480	nsv881270	4	2310000	gain	AABB		27.7768
.	Score=645	esv273095	1	30000	loss	A		100
.	Score=804	esv273095	1	70000	loss	A		-1
.	Score=102	.	3	50000	gain		-1	-1
Score=0.9	Score=221	dgv1355n7	1	290000	loss	A		100
Score=0.9	Score=932	nsv433419	1	30000	loss	A		-1
.	Score=732	nsv7246	4	20000	gain	AABB		100

.	Score=762	nsv901522	3	160000	gain	AAB	100
.	Score=658	nsv832862	4	10000	gain	-1	-1
.	Score=637	nsv9170,n	3	30000	gain	AAB	100
Score=0.9	Score=281	nsv903831	1	730000	loss	A	4.77996
Score=0.9	Score=281	nsv903975	1	450000	loss	A	5.51424
.	Score=383	.	3	10000	gain	-1	-1
.	Score=404	.	3	20000	gain	AAB	-1
Score=0.9	Score=199	nsv94945,	3	90000	gain	AAB	11.205
.	Score=271	esv164372	3	20000	gain	AAB	-1
Score=0.9	Score=274	nsv510669	1	480000	loss	A	23.101
Score=0.9	Score=141	dgv764e1,	1	150000	loss	A	-1
Score=0.9	Score=265	dgv766e1,	1	230000	loss	A	29.4137
Score=0.9	Score=275	nsv469708	6	10000	gain	AAAAAA	18.536
.	Score=285	.	4	10000	gain	-1	-1
Score=0.9	Score=235	esv991017	1	5950000	loss	A	-1
Score=0.9	Score=511	esv272301	4	10000	gain	AABB	100
Score=0.9	Score=463	nsv520612	3	22840000	gain	AAA	-1
Score=0.9	Score=453	nsv525602	4	1320000	gain	AABB	18.0486
Score=0.9	Score=560	nsv829366	3	11240000	gain	AAA	-1
Score=0.9	Score=660	nsv527767	3	3650000	gain	AAB	8.00259
Score=0.9	Score=631	dgv150n71	3	10000	gain	AAB	7.67682
Score=0.9	Score=201	esv28644,	3	20000	gain	AAB	-1
Score=0.9	Score=232	dgv169n71	3	840000	gain	AAB	1.55138
Score=0.9	Score=269	nsv871685	3	130000	gain	-1	-1
Score=0.9	Score=376	nsv437971	4	470000	gain	AABB	5.81909
Score=0.9	Score=167	nsv831215	4	130000	gain	AABB	-1
Score=0.9	Score=288	esv9701,e	3	27290000	gain	AAB	0.211117
Score=0.9	Score=120	esv275080	3	140000	gain	AAB	-1
Score=0.9	Score=268	nsv160066	3	1880000	gain	AAB	3.5166
Score=0.9	Score=273	nsv831525	3	40000	gain	AAB	-1
Score=0.9	Score=385	esv266198	3	1790000	gain	AAA	-1
Score=0.9	Score=676	nsv3143,e	7	10000	gain	AAAABBB	51.695
Score=0.9	Score=425	esv272678	3	4600000	gain	AAB	2.75933
Score=0.9	Score=195	esv272030	3	1210000	gain	AAB	6.08566
.	Score=662	dgv61n16,	3	30000	gain	AAB	-1

Score=0.98	Score=134	dgv645n67	3	10000	gain	AAB	-1
Score=0.99	Score=320	nsv874619	3	6200000	gain	AAB	2.37073
Score=0.97	Score=441	essv23304	3	10000	gain	AAB	-1
Score=0.97	Score=139	nsv2872	3	110000	gain	AAA	54.6576
.	Score=144	nsv508854	3	70000	gain	AAA	-1
Score=0.99	Score=438	nsv441785	3	28160000	gain	AAA	-1
Score=0.88	Score=149	nsv875407	3	330000	gain	AAB	31.7779
Score=0.99	Score=499	nsv528929	3	7410000	gain	AAA	-1
Score=0.99	Score=262	esv192612	4	990000	gain	AABB	5.91581
Score=0.99	Score=404	esv272152	3	12590000	gain	AAA	-1
Score=0.98	Score=442	esv272520	3	3020000	gain	AAA	-1
.	Score=237	nsv236202	3	600000	gain	AAB	50.1841
.	Score=117	esv272522	3	110000	gain	AAB	-1
.	Score=119	nsv834701	3	240000	gain	AAA	100
.	Score=325	esv266521	3	140000	gain	AAA	-1
.	Score=904	esv266947	3	60000	gain	-1	-1
Score=0.95	Score=177	esv266347	3	210000	gain	AAB	7.56032
Score=0.99	Score=212	esv272589	3	1040000	gain	AAB	1.80726
.	Score=673	dgv5254n7	5	40000	gain	AAABB	-1
Score=0.99	Score=632	esv274484	5	63700000	gain	AAABB	0.731165
.	Score=168	nsv437908	6	210000	gain	AAAABB	14.1377
.	Score=495	esv172712	3	30000	gain	AAB	-1
Score=0.98	Score=443	esv273412	3	2420000	gain	AAB	1.40044
Score=0.97	Score=758	nsv10385,	3	20000	gain	AAB	-1
Score=0.99	Score=289	esv272662	5	2020000	gain	AAABB	1.57407
Score=0.91	Score=786	dgv5381n7	5	70000	gain	AAABB	-1
Score=0.99	Score=113	nsv508984	3	120000	gain	AAA	21.2368
Score=0.99	Score=571	esv26652,	3	62430	gain	AAA	-1
Score=0.99	Score=829	dgv1570e1	3	110000	gain	AAA	2.09064
.	Score=107	esv272803	3	220000	gain	AAA	80.6793
Score=0.99	Score=486	esv272787	3	7000000	gain	AAA	-1
.	Score=817	esv266806	3	170000	gain	AAA	73.6761
.	Score=321	esv266311	3	300000	gain	AAA	-1
Score=0.95	Score=273	nsv879915	3	150000	gain	AAA	100
.	Score=285	nsv292163	3	980000	gain	-1	-1

Score=0.9	Score=668	nsv880457,esv2729999,esv2421732,nsv527109,esv1281279,nsv327785,esv23623,dgv1789e1,esv2670678,esv25636,dgv1755e1,esv2					
Score=0.9	Score=283	nsv433373	3	2090000	gain	AAB	1.4389
Score=0.9	Score=700	dgv1811e1	3	11810000	gain	AAA	-1
.	Score=215	nsv4903,n	5	20000	gain	-1	-1
Score=0.9	Score=235	esv100952	4	380000	gain	AABB	21.3994
.	Score=283	dgv6n29,n	4	20000	gain	AABB	23.7481
Score=0.9	Score=225	nsv509114	3	160000	gain	AAA	100
Score=0.9	Score=406	esv28275,	3	15260	gain	AAA	-1
Score=0.9	Score=479	esv269341	3	15560000	gain	AAA	-1
0384579.1,ENST00000517282.1,ENST00000251691.4,ENST00000531410.1,ENST00000423099.1,ENST00000341338.6,ENST00000483931.1,ENST00000							
sv6877,nsv7968,esv2732391,nsv886606,nsv510039,nsv435987,nsv7962,esv2482492,nsv514374,esv2732526,nsv470836,esv2732692,esv2732691,nsv8307							
Score=0.9	Score=270	nsv888219	3	6340000	gain	AAB	1.19195
Score=0.9	Score=104	nsv821246	3	290000	gain	AAB	26.448
Score=0.9	Score=105	dgv120n16	3	970000	gain	AAB	7.5422
Score=0.9	Score=277	dgv63n64,	3	760000	gain	AAB	4.43405
Score=0.9	Score=257	nsv436086	3	450000	gain	AAB	1.08415
Score=0.9	Score=261	esv273530	3	620000	gain	AAA	100
.	Score=247	nsv5885	3	10000	gain	AAA	-1
.	Score=243	nsv831156	7	10000	gain	AAAABBB	100
.	.	.	3	10000	gain	AAA	-1
Score=0.9	Score=229	nsv465264	3	180000	gain	AAA	100
Score=0.9	Score=131	nsv6034,e	3	90000	gain	AAA	-1
.	Score=157	nsv6105,e	4	270000	gain	AABB	18.4935
Score=0.9	Score=279	esv25667,	4	680000	gain	AABB	6.53755
Score=0.9	Score=496	esv243682	3	55090000	gain	AAA	-1
Score=0.9	Score=236	esv265724	3	650000	gain	AAB	100
.	Score=144	nsv509300	3	60000	gain	AAB	-1
Score=0.9	Score=303	nsv821355	3	31850000	gain	AAB	0.48834
Score=0.9	Score=826	esv273852	3	70000	gain	AAB	-1
Score=1;N	Score=390	esv266112	3	6370000	gain	AAA	-1
Score=0.9	Score=412	esv273891	3	12190000	gain	AAA	-1
.	Score=238	nsv416941	3	160000	gain	AAA	100
Score=0.9	Score=327	esv267113	3	11770000	gain	AAA	-1

.	Score=543	nsv831732	3	160000	gain	AAA	80.6793
Score=0.9!	Score=239	esv267046	3	1590000	gain	AAB	2.14333
.	Score=400	nsv519701	3	60000	gain	AAB	-1
Score=0.9(	Score=654	nsv516555	4	260000	gain	AABB	24.4739
.	Score=234	nsv528140	6	10000	gain	AAABBB	74.585
Score=0.9:	Score=245	esv266073	4	30000	gain	AABB	10.7153
Score=0.9!	Score=224	dgv910n71	3	80000	gain	AAB	-1
Score=0.9!	Score=224	esv22231,r	3	60000	gain	AAB	58.7147
Score=0.9(	Score=104	esv266303	3	340000	gain	AAB	74.6732
Score=0.9(	Score=313	nsv897028	3	130000	gain	AAB	-1
.	Score=276	nsv897625	3	320000	gain	-1	-1
Score=0.9!	Score=261	nsv349,nsv	3	350000	gain	AAB	100
.	Score=223	esv242241	4	200000	gain	AABB	54.4751
Score=0.9(	Score=947	nsv516638	3	120000	gain	AAB	77.9109
Score=0.9(	Score=120	esv33345,r	3	320000	gain	AAB	-1
Score=0.9!	Score=260	dgv179n27	4	540000	gain	AABB	10.589
Score=0.9(	Score=404	esv158346	3	540000	gain	AAB	8.83875
.	Score=148	esv266193	3	150000	gain	AAB	-1
.	Score=720	esv143399	3	140000	gain	AAA	100
.	Score=552	esv34039	3	30000	gain	AAA	-1
Score=0.9:	Score=249	esv275239	3	690000	gain	AAB	24.4642
Score=0.9:	Score=276	esv274705	3	860000	gain	AAB	6.31397
.	Score=235	esv268973	3	10000	gain	AAB	-1
Score=0.9:	Score=245	esv5325,es	3	150000	gain	AAB	12.669
Score=0.9:	Score=372	esv275113	3	30000	gain	AAB	9.1331
.	Score=577	esv123251	4	90000	gain	AABB	7.37222
Score=0.9(	Score=128	nsv525459	3	160000	gain	AAB	100
.	Score=739	nsv64676,r	3	60000	gain	AAB	-1
.	Score=274	esv274724	5	30000	gain	AAABB	96.6605
.	Score=131	esv265900	3	210000	gain	-1	-1
.	Score=222	esv33246	3	10000	gain	AAA	73.0136
Score=0.9(	Score=267	esv266653	3	830000	gain	AAB	24.0729
.	Score=239	nsv820041	3	80000	gain	AAB	31.4844
Score=0.9!	Score=461	esv266793	3	7220000	gain	-1	-1
.	Score=237	nsv820122	3	10000	gain	AAA	100

Score=0.9‹	Score=434	esv274780	3	28800000	gain	AAA	-1
Score=0.9‹	Score=263	nsv901215	3	1240000	gain	AAB	0.999551
Score=0.9‹	Score=433	esv274845	4	80000	gain	AABB	40.9549
Score=0.9‹	Score=479	esv267118	3	61100000	gain	AAA	-1
Score=0.9‹	Score=263	dgv2076n7	3	20160000	gain	AAB	-1
Score=1;N	Score=270	dgv2195n7	3	3220000	gain	AAB	0.585623
.	Score=273	.	4	100000	gain	AAAB	100
Score=0.9‹	Score=170	nsv905370	3	540000	gain	AAB	3.04855
Score=0.9‹	Score=295	nsv509600	3	2270000	gain	AAB	4.0923
Score=0.9‹	Score=242	esv258249	3	520000	gain	AAA	20.0517
.	Score=234	nsv457444	3	20000	gain	AAA	-1
Score=0.9‹	Score=319	nsv436835	3	890000	gain	AAB	4.93729
.	Score=128	esv998989	3	10000	gain	AAB	-1
Score=0.9‹	Score=735	dgv834e1,‹	4	170000	gain	AAAB	18.6372
Score=0.9‹	Score=796	esv265877	3	230000	gain	AAB	7.40663
Score=0.9‹	Score=286	esv268722	3	320000	gain	AAB	3.49193
Score=0.9‹	Score=611	esv27528,‹	3	20000	gain	AAB	-1
Score=0.9‹	Score=101	nsv908327	3	260000	gain	AAB	14.4441
Score=0.9‹	Score=697	dgv975e1,‹	3	250000	gain	AAB	19.0072
Score=0.9‹	Score=643	nsv908562	3	220000	gain	AAB	-1
Score=0.9‹	Score=510	esv273661	3	9260000	gain	AAA	-1
Score=0.9‹	Score=277	nsv909285	3	40000	gain	AAB	-1
Score=0.9‹	Score=277	nsv909286	3	90000	gain	AAB	6.93135
Score=0.9‹	Score=132	nsv909285	3	60000	gain	AAB	-1
.	Score=258	nsv2228	3	20000	gain	AAA	100
Score=0.9‹	Score=442	esv230713	3	23000000	gain	AAA	-1
.	Score=145	nsv510457	5	60000	gain	AAABB	100
.	.	.	3	60000	gain	AAB	-1
Score=0.9‹	Score=224	nsv910081	3	240000	gain	AAB	9.64635
Score=0.9‹	Score=638	nsv911020	4	930000	gain	AABB	2.56782
Score=0.9‹	Score=106	esv275428	4	380000	gain	AABB	6.48553
Score=0.9‹	Score=278	nsv911819	4	990000	gain	AABB	4.56473
.	Score=110	esv271881	3	20000	gain	AAA	100
Score=0.9‹	Score=294	esv268330	3	560000	gain	AAB	1.43999
.	Score=270	dgv4507n7	3	120000	gain	AAB	-1

Score=0.98	Score=463	nsv833898	3	3100000	gain	AAB	1.62352
.	Score=253	nsv528355	3	10000	gain	AAB	-1
Score=0.99	Score=216	esv24766,n	5	400000	gain	AAABB	10.4212
.	Score=222	dgv4521n7	5	10000	gain	AAABB	-1
.	Score=622	nsv912615	3	230000	gain	AAB	55.9202
.	Score=348	dgv776e19	3	10000	gain	AAB	-1
Score=0.98	Score=286	esv272212	3	13740000	gain	AAB	5.18667
.	Score=270	esv152139	3	30000	gain	AAA	12.1589
.	Score=242	.	4	50000	gain	AABB	-1
Score=0.91	Score=294	nsv912811	4	3450000	gain	AABB	17.3108
.	Score=150	esv267044	3	30000	gain	AAB	-1
Score=0.99	Score=462	nsv510784	3	4870000	gain	AAB	5.34341
Score=0.99	Score=274	esv25843,e	3	5250000	gain	AAB	8.95248
.	Score=266	nsv833956	3	10000	gain	AAB	-1
.	Score=905	esv255213	6	10000	gain	AAAABB	4.15705
.	Score=242	nsv512611	3	10000	gain	AAB	-1
Score=0.99	Score=235	esv223499	3	3130000	gain	AAB	2.14461
Score=0.91	Score=259	nsv3367,ns	3	20000	gain	AAB	-1
.	Score=244	nsv526188	7	10000	gain	AAAAABB	100
.	Score=248	nsv526188	3	10000	gain	AAB	-1
Score=0.98	Score=935	esv272237	3	1040000	gain	AAB	2.29134
Score=0.98	Score=112	nsv178626	5	310000	gain	AAABB	5.74584
Score=0.98	Score=287	nsv9811,ns	3	14570000	gain	AAB	0.81314
.	Score=373	esv272252	3	240000	gain	AAB	-1
.	Score=250	nsv912928	7	10000	gain	-1	-1
.	Score=436	nsv912928	3	30000	gain	AAB	-1
Score=0.96	Score=283	nsv912928	3	4330000	gain	AAB	4.83312
.	Score=330	nsv525425	3	10000	gain	AAB	-1
.	Score=146	esv29810,n	3	80000	gain	AAA	96.2339
.	Score=460	esv272257	3	80000	gain	AAA	-1
.	Score=237	nsv834015	3	20000	gain	AAB	100
.	Score=151	esv265975	3	520000	gain	AAB	-1
Score=0.98	Score=340	esv272262	3	4340000	gain	AAB	8.27479
Score=0.98	Score=598	nsv9836,es	3	40000	gain	AAB	43.0455
Score=0.98	Score=125	nsv9836,es	3	65520	gain	AAB	-1

Score=0.9!	Score=263	esv272301	3	9750000	gain	AAB		-1
Score=0.9!	Score=274	esv272303	3	1350000	gain	AAB		0.572513
Score=0.9{	Score=254	esv7291,es	3	3310000	gain	AAB		-1
.	Score=242	nsv7346,ns	3	10000	gain		-1	-1
Score=0.9!	Score=799	nsv469815	3	16090000	gain	AAB		-1
Score=0.9!	Score=262	dgv772e20	3	880000	gain	AAB		7.60149
Score=0.9{	Score=253	esv25306,e	3	60000	gain	AAB		36.6149
Score=0.9{	Score=400	esv25306	3	64566	gain	AAB		-1
Score=0.9	Score=116	esv275232	4	210000	gain	AABB		7.58168
Score=0.9	Score=233	nsv469734	4	20000	gain	AABB		28.3551
Score=0.9!	Score=233	nsv6897,ns	3	190000	gain	AAB		3.26537
Score=0.9	Score=269	esv267875	3	440000	gain	AAA		55.3104
Score=1;N	Score=430	esv266797	3	16730000	gain	AAA		-1
Score=0.9!	Score=662	esv274045	3	17340000	gain	AAA		-1
Score=0.9!	Score=247	esv153107	3	550000	gain	AAB		32.9414
Score=0.9!	Score=662	nsv436632	3	1360000	gain	AAB		30.7263
Score=0.9!	Score=162	nsv10003	3	10560	gain	AAB		-1
.	Score=458	esv266461	3	90000	gain	AAB		20.2272
.	Score=237	nsv834435	3	20000	gain		-1	-1
Score=0.9!	Score=245	nsv10550,r	3	250000	gain	AAB		33.5651
.	Score=258	nsv873093	4	10000	gain	AABB		100
.	Score=342	esv3940,ns	3	110000	gain	AAB		64.3991
Score=0.9(	Score=100	esv267470	3	210000	gain	AAB		21.9586
.	Score=645	esv273095	3	20000	gain	AAA		54.57
.	Score=804	esv266149	3	80000	gain	AAA		-1
.	Score=217	nsv499795	5	10000	gain	AAABB		73.8733
Score=0.9(	Score=104	nsv519650	3	250000	gain	AAB		6.43682
.	Score=377	esv270609	3	210000	gain	AAB		100
.	Score=402	dgv6e196,(	3	10000	gain	AAB		-1
Score=0.9!	Score=168	esv29743,e	3	360000	gain	AAB		19.2092
.	Score=370	esv267170	4	40000	gain	AAAB		100
.	Score=109	dgv69n68,(	3	10000	gain	AAB		12.5965
.	Score=292	.	3	80000	gain	AAB		35.5408
Score=0.9;	Score=100	esv266957	91	10000	gain	AAAAAAA		100
Score=0.9!	Score=350	dgv922e19	7	30000	gain	AAAAAAB		100

Score=0.9ʼ	Score=348	dgv922e19	3	20000	gain	AAA	-1
Score=0.9ʼ	Score=381	esv101094	3	80000	gain	AAA	4.10631
Score=0.9ʼ	Score=303	dgv1569e1	17	10000	gain	AAAAAAA	100
Score=0.9ʼ	Score=769	nsv471487	3	40000	gain	AAA	4.57632
Score=0.9ʼ	Score=219	nsv10641,ε	3	134276	gain	AAA	-1
.	Score=691	dgv6189n7	3	20000	gain	-1	-1
.	Score=876	nsv328622	3	240000	gain	AAB	23.1518
Score=0.9ʼ	Score=220	nsv8,nsv58	6	10000	gain	AAAAAA	30.3547
Score=0.9(	Score=243	dgv2120e1	18	10000	gain	AAAAAAA	87.1734
.	Score=216	nsv8926,ns	3	140000	gain	AAB	7.56032
Score=0.9:	.	esv266469	12	10000	gain	AAAAAABI	14.3222
Score=0.9ʼ	Score=275	dgv788e1,r	5	10000	gain	AAAAA	3.99866
.	Score=826	dgv4506n7	1	70000	loss	-1	-1
Score=0.9(	Score=378	esv272430	3	140000	gain	AAB	28.8997
Score=0.9ʼ	Score=279	nsv191166	3	2820000	gain	AAB	100
Score=0.9ʼ	Score=100	esv271811	7	10000	gain	AAAABBB	22.2976
Score=0.9ʼ	Score=239	nsv832681	4	20000	gain	AABB	100
.	Score=678	nsv526386	3	80000	gain	AAB	47.9616
.	Score=366	nsv874956	3	20000	gain	-1	-1
.	Score=208	.	4	10000	gain	AABB	36.7053
.	Score=126	nsv875909	4	20000	gain	AABB	100
.	Score=269	nsv524847	3	40000	gain	AAB	100
.	Score=244	esv272749	4	20000	gain	AABB	68.972
Score=0.9ʼ	Score=769	nsv820863	4	40000	gain	AAAB	21.2442
Score=0.9ʼ	Score=219	nsv820863	4	134276	gain	AAAB	-1
.	Score=153	nsv882709	4	10000	gain	-1	-1
Score=0.9ʼ	Score=223	esv581,nsv	3	110000	gain	-1	-1
Score=0.9ʼ	Score=244	nsv824240	112	10000	gain	AAAAAAA	100
Score=0.9ʼ	Score=697	dgv7671n7	3	1830000	gain	AAB	1.11149
Score=0.9ʼ	Score=192	nsv6284,ns	3	290000	gain	AAA	4.65699
.	Score=192	.	4	10000	gain	-1	-1
.	Score=276	.	4	20000	gain	-1	-1
Score=0.9ʼ	Score=607	dgv8n64,dg	3	90000	gain	AAB	5.7394
.	Score=265	.	4	10000	gain	AABB	100
Score=0.9:	Score=181	esv274925	6	10000	gain	AAABBB	2.49043

.	Score=913	esv265694	3	90000	gain	-1	-1
Score=0.9	Score=275	dgv113e55	7	10000	gain	AAAAAAA	3.70457
.	Score=312	esv265888	4	10000	gain	AABB	47.1736
Score=0.9	Score=145	nsv906908	3	600000	gain	AAB	16.2996
Score=0.9	Score=206	esv210377	3	1680000	gain	AAB	11.4161
Score=0.9	Score=277	esv143586	3	80000	gain	AAB	9.36616
.	Score=273	esv258673	5	10000	gain	-1	-1
.	Score=129	nsv833615	5	10000	gain	-1	-1
.	Score=521	nsv909545	4	30000	gain	AAAB	100
Score=0.9	Score=129	nsv512551	3	520000	gain	AAB	18.4463
.	Score=588	esv114874	3	340000	gain	AAB	59.4207
Score=0.9	Score=294	esv265751	1	120000	loss	A	21.8288
Score=0.9	Score=392	nsv9686,es	1	460000	loss	A	-1
.	Score=146	nsv828673	1	10000	loss	-1	-1
Score=0.9	Score=123	dgv1322e1	3	10000	gain	-1	-1
.	Score=441	dgv519n27	4	10000	gain	-1	-1
.	Score=523	nsv7347	4	10000	gain	AABB	33.7258
Score=0.9	Score=452	nsv914231	3	280000	gain	AAB	1.60347
Score=0.9	Score=838	nsv436867	3	410000	gain	AAB	3.8425
Score=0.9	Score=351	nsv914393	3	10000	gain	AAB	-1
Score=0.9	Score=976	nsv914437	3	280000	gain	AAB	1.35125
Score=0.9	Score=130	esv27639,r	3	190000	gain	AAB	100
Score=0.9	Score=276	nsv471539	3	30000	gain	AAB	14.5204
Score=0.9	Score=201	nsv832869	3	20000	gain	AAB	-1
Score=0.9	Score=232	dgv20e1,ns	3	490000	gain	AAB	3.40302
Score=0.9	Score=232	dgv22e1,ns	3	310000	gain	AAB	6.47336
Score=0.9	Score=501	nsv871961	3	7550000	gain	AAA	-1
Score=0.9	Score=220	nsv8003,dg	4	690000	gain	AAAB	22.9415
Score=0.9	Score=107	dgv104e1,(	4	100000	gain	AAAB	6.50785
Score=0.9	Score=287	dgv46e201	3	550000	gain	AAB	6.0595
Score=0.9	Score=268	nsv818422	3	1900000	gain	AAB	0.658195
Score=0.9	Score=143	nsv428235	3	90000	gain	AAB	-1
Score=0.9	Score=229	nsv428235	7	10000	gain	AAAAABB	50.5523
Score=0.9	Score=385	nsv441714	3	2110000	gain	AAA	-1
Score=0.9	Score=204	dgv573n71	3	210000	gain	AAA	4.8288

Score=0.9{	Score=268	dgv575n71	3	560000	gain	AAB	15.5062
Score=0.9{	Score=162	.	3	40621	gain	AAB	-1
.	Score=226	esv168311	4	150000	gain	AABB	21.6267
.	Score=467	nsv834507	5	10000	gain	-1	-1
Score=0.94	Score=203	nsv834516	6	10000	gain	AAAABB	100
Score=0.9!	Score=632	esv267346	3	92680000	gain	AAA	-1
.	Score=493	nsv878047	8	10000	gain	-1	-1
Score=0.9:	Score=238	esv273410	3	340000	gain	AAB	-1
Score=0.9!	Score=504	nsv509877	3	15450000	gain	AAB	0.478959
Score=0.9!	Score=571	dgv1562e1	3	62430	gain	AAB	-1
.	Score=106	nsv517266	4	20000	gain	AABB	29.307
.	Score=252	esv222858	6	10000	gain	AAAABB	83.5582
.	Score=976	.	4	20000	gain	AABB	68.4669
Score=0.9	Score=254	nsv830623	4	190000	gain	AABB	9.58797
.	Score=779	nsv885792	5	60000	gain	AAABB	100
.	Score=172	esv149335	3	110000	gain	AAB	62.0865
.	Score=102	nsv5349	4	80000	gain	AABB	100
.	Score=224	nsv830783	4	70000	gain	AABB	14.5473
Score=0.9(	Score=291	nsv428155	4	330000	gain	AABB	5.83793
Score=0.9(	Score=251	nsv366743	4	350000	gain	AAAB	100
Score=0.9!	Score=275	esv273461	3	4610000	gain	AAB	53.3505
Score=0.9!	Score=105	nsv482102	3	970000	gain	AAB	3.58914
.	Score=253	esv22177,(	3	20000	gain	AAA	36.379
.	Score=413	dgv7872n7	5	20000	gain	-1	-1
Score=0.9!	Score=272	nsv508589	3	2130000	gain	AAA	-1
Score=0.9(	Score=654	esv274193	4	280000	gain	AAAB	100
.	Score=260	nsv522884	4	80000	gain	-1	-1
Score=0.94	Score=193	nsv898435	3	480000	gain	AAB	3.78482
.	Score=255	esv275109	5	20000	gain	AAABB	13.0171
.	Score=260	esv274764	3	970000	gain	AAB	15.9343
.	Score=250	.	3	20000	gain	AAB	-1
.	Score=216	.	5	10000	gain	AAABB	100
.	Score=300	esv274874	6	10000	gain	-1	-1
.	Score=101	nsv517477	3	340000	gain	AAB	19.7
Score=0.9{	Score=278	dgv2371n7	3	170000	gain	AAB	17.4097

.	Score=228	esv271396	3	20000	gain	AAA		100
.	Score=234	nsv1762,ns	4	220000	gain	AABB		9.39762
Score=0.98	Score=224	nsv906545	3	150000	gain	AAB		0.367671
.	Score=637	nsv833244	3	60000	gain	AAB		9.03301
.	Score=233	nsv1846,ns	4	180000	gain	AABB		60.2008
Score=0.99	Score=286	esv260390	3	290000	gain	AAB		5.53685
Score=0.99	Score=235	nsv1859,ns	3	10000	gain	AAB		-1
Score=0.98	Score=293	esv100279	3	1290000	gain	AAB		2.57139
.	Score=221	esv100571	4	20000	gain	AABB		100
Score=0.96	Score=244	nsv522008	3	10000	gain		-1	-1
.	Score=201	nsv458949	3	10000	gain		-1	-1
.	Score=636	esv992784	3	40000	gain	AAA		100
.	Score=245	.	3	10000	gain	AAA		-1
.	Score=566	.	5	30000	gain	AAABB		100
.	Score=463	esv130196	3	80000	gain	AAB		100
Score=0.95	Score=226	nsv913657	4	470000	gain	AABB		100
.	Score=119	nsv913716	3	130000	gain	AAB		100
.	Score=742	nsv3505,es	3	30000	gain	AAA		100
.	Score=242	nsv3505,es	3	10000	gain	AAA		-1
.	Score=106	esv33578	3	40000	gain	AAB		17.2872
.	Score=222	esv272341	3	10000	gain		-1	-1
.	Score=147	esv272404	4	20000	gain	AABB		84.8236
.	Score=302	nsv817986	3	10000	gain		-1	-1
Score=0.96	Score=488	nsv428389	3	150000	gain	AAA		100
.	Score=622	nsv524585	3	140000	gain	AAA		-1
Score=0.99	Score=515	esv265949	3	6820000	gain	AAA		-1
.	Score=366	nsv518704	4	20000	gain	AAAB		100
Score=0.99	Score=274	esv33289,n	3	820000	gain	AAA		9.18906
.	Score=271	nsv508920	3	30000	gain		-1	-1
.	Score=631	nsv516703	3	20000	gain	AAB		8.06487
Score=0.95	Score=229	esv267819	3	10000	gain	AAA		100
.	Score=316	nsv878947	3	30000	gain	AAB		41.6544
.	Score=142	.	3	20000	gain		-1	-1
.	Score=952	esv24873,n	3	110000	gain		-1	-1
Score=0.95	Score=232	nsv883468	3	840000	gain	AAB		92.2585

.	Score=252	nsv884418	3	10000	gain	AAB		8.5861
Score=0.9	Score=189	nsv527079	3	930000	gain	AAB		13.6491
.	Score=558	.	3	20000	gain		-1	-1
Score=0.9	Score=416	nsv8483,d	4	10000	gain	AABB		100
.	Score=219	nsv521672	3	130000	gain	AAB		100
Score=0.9	Score=233	dgv964n71	3	30000	gain	AAA		58.7741
.	Score=117	dgv968n71	3	10000	gain	AAA		-1
.	Score=571	.	4	10000	gain		-1	-1
Score=0.9	Score=777	dgv1361n7	3	190000	gain	AAB		40.6964
.	Score=385	esv34052,	3	20000	gain	AAB		12.7274
.	Score=320	nsv64073,	3	160000	gain	AAB		21.0881
.	Score=878	esv274816	4	20000	gain	AABB		66.0696
Score=0.9	Score=360	esv22168,	1	2470000	loss	A		-1
.	Score=253	esv253668	3	20000	gain	AAB		100
Score=0.9	Score=637	esv24223,	3	30000	gain	AAB		8.98935
Score=0.9	Score=740	nsv910903	3	30000	gain	AAB		66.8917
.	Score=300	nsv828463	3	10000	gain		-1	-1
Score=0.9	Score=921	esv175459	3	730000	gain	AAA		43.4713
.	Score=242	.	3	10000	gain	AAA		-1
.	Score=988	esv26419	3	120000	gain	AAB		10.942
.	Score=241	nsv913744	3	10000	gain	AAB		51.1179
.	Score=464	nsv913873	4	10000	gain	AAAB		100
.	Score=445	.	4	30000	gain	AABB		65.9194
Score=0.9	Score=389	nsv7013,n	3	400000	gain	AAB		100
Score=0.9	Score=294	esv274042	3	520000	gain	AAB		37.2752
Score=0.9	Score=217	esv274480	4	2790000	gain	AABB		6.61232
Score=0.9	Score=501	nsv516709	4	5580000	gain	AAAA		-1
Score=0.9	Score=554	nsv871854	4	44850000	gain	AABB		3.85467
.	Score=459	dgv38e201	4	60000	gain	AABB		-1
.	Score=223	nsv522987	4	330000	gain	AAAA		36.9381
Score=0.9	Score=438	esv274476	4	8470000	gain	AAAA		-1
.	Score=101	esv148805	4	310000	gain	AABB		7.1396
Score=0.9	Score=393	nsv526227	6	760000	gain	AAABBB		100
Score=0.9	Score=156	nsv435845	3	660000	gain	AAB		4.25847
Score=0.9	Score=281	nsv820880	4	160000	gain	AAAB		-1

Score=0.9	Score=288	esv271685		4	28540000	gain	AAAB	1.93683
Score=0.9	Score=507	esv271852		3	8470000	gain	AAB	0.467238
Score=0.9	Score=275	esv242216		5	1260000	gain	AAAAB	8.72821
.	Score=257	nsv522601		3	120000	gain	AAB	-1
Score=0.9	Score=481	nsv508042		3	2020000	gain	AAB	1.63236
Score=0.9	Score=418	nsv442271		4	7370000	gain	AAAA	-1
Score=0.9	Score=505	nsv508044		5	4620000	gain	AAAAA	-1
Score=0.9	Score=497	nsv528675		4	11380000	gain	AAAA	-1
Score=0.9	Score=456	esv265822		6	15520000	gain	AAAAAA	-1
Score=0.9	Score=441	esv26033,		3	6680000	gain	AAB	0.503676
Score=0.9	Score=262	esv267177		5	390000	gain	AAABB	34.1463
Score=0.9	Score=451	esv267247		4	6980000	gain	AAAB	4.69502
Score=0.9	Score=116	esv272260		4	130000	gain	AAAB	-1
Score=0.9	Score=270	esv29119,		6	1930000	gain	AAAABB	50.1259
Score=0.9	Score=294	esv267094		4	8440000	gain	AAAA	-1
.	Score=335	dgv184e1,		7	410000	gain	AAAAABB	6.31945
Score=0.9	Score=498	esv261371		4	23020000	gain	AAAB	1.1735
Score=0.9	Score=302	esv143238		3	210000	gain	AAB	-1
Score=0.9	Score=437	esv271912		3	12680000	gain	AAB	0.847781
Score=0.9	Score=221	nsv518954		3	640000	gain	AAB	-1
Score=0.9	Score=460	dgv685e19		6	7120000	gain	AAAABB	56.4343
Score=0.9	Score=290	esv25159,		3	10980000	gain	AAA	-1
Score=0.9	Score=468	esv271995		4	12490000	gain	AAAA	-1
Score=0.9	Score=284	nsv833936		5	1710000	gain	AAABB	69.0339
Score=0.9	Score=397	esv272001		3	1830000	gain	AAB	3.65124
Score=0.9	Score=426	esv266098		5	21210000	gain	AAAAA	-1
Score=0.9	Score=485	esv33940,		3	6880000	gain	AAA	-1
.	Score=222	nsv438356		5	120000	gain	AAABB	-1
Score=0.9	Score=623	nsv874383		5	9330000	gain	AAABB	37.5719
.	Score=307	dgv4201n7		5	50000	gain	AAABB	-1
Score=0.9	Score=270	dgv4204n7		3	1320000	gain	AAB	7.53434
.	Score=168	esv25769,		3	90000	gain	AAB	-1
Score=0.9	Score=276	nsv829489		6	290000	gain	AAABBB	100
Score=0.9	Score=360	nsv10063,		5	20000	gain	AAABB	-1
Score=0.9	Score=213	dgv59n17,		5	2230000	gain	AAABB	6.63492

Score=0.9	Score=144	dgv61n16,	3	40000	gain	AAB	-1
Score=0.99	Score=320	esv141733	3	7370000	gain	AAB	44.0202
Score=0.98	Score=269	nsv441770	4	680000	gain	AABB	17.9881
Score=1;N	Score=451	dgv86e180	3	30610000	gain	AAA	-1
Score=0.98	Score=293	esv100471	3	1980000	gain	AAB	58.4431
Score=0.98	Score=113	dgv4306n7	3	10000	gain	AAB	-1
Score=0.99	Score=274	nsv514976	3	1350000	gain	AAB	22.0657
Score=0.98	Score=414	nsv819183	4	5690000	gain	AABB	1.22073
Score=0.99	Score=438	nsv470491	4	21530000	gain	AAAA	-1
Score=0.99	Score=554	esv100492	4	37610000	gain	AAAA	-1
Score=0.99	Score=339	esv259284	4	13090000	gain	AABB	1.4904
Score=0.99	Score=430	nsv213622	4	5140000	gain	AABB	10.7903
Score=0.97	Score=443	esv272486	4	9010000	gain	AAAA	-1
Score=0.98	Score=642	esv267443	4	15990000	gain	AAAA	-1
Score=0.95	Score=419	esv134604	3	5240000	gain	AAA	-1
.	Score=179	nsv834663	3	130000	gain	AAA	100
.	Score=130	nsv834662	3	10000	gain	AAA	-1
Score=0.98	Score=442	nsv3789,e	4	1300000	gain	AABB	100
.	Score=436	nsv834697	3	20000	gain	AAA	100
Score=0.99	Score=470	esv266505	3	21570000	gain	AAA	-1
Score=0.99	Score=411	nsv3897,e	4	24620000	gain	AAAA	-1
Score=0.97	Score=523	esv25892,r	5	8450000	gain	AAAAA	-1
Score=0.99	Score=507	nsv515688	4	13810000	gain	AAAA	-1
Score=0.99	Score=442	esv265835	5	3290000	gain	AAAAA	-1
Score=0.99	Score=410	dgv1513e1	3	7830000	gain	AAA	-1
Score=0.99	Score=626	nsv877687	4	16800000	gain	AAAA	-1
.	Score=272	esv114142	4	410000	gain	AAAB	88.8711
.	Score=786	nsv877692	4	90000	gain	AAAB	-1
Score=0.99	Score=536	nsv236646	5	24170000	gain	AAABB	12.1685
Score=0.98	Score=504	esv179126	3	5350000	gain	AAA	-1
Score=0.97	Score=316	esv32747,	4	2190000	gain	AABB	90.2248
.	Score=257	nsv437360	4	750000	gain	AABB	-1
.	Score=280	esv267523	5	30000	gain	-1	-1
.	Score=209	nsv819527	5	160000	gain	AAABB	-1
Score=0.96	Score=443	dgv309n21	5	2140000	gain	AAABB	25.9309

.	Score=372	nsv516867	5	20000	gain	AAABB	-1
Score=0.98	Score=128	esv5061,e	3	440000	gain	AAB	1.33559
.	Score=407	nsv878167	3	50000	gain	AAB	-1
Score=0.99	Score=289	esv267767	3	1850000	gain	AAB	7.00977
.	Score=854	esv266310	3	40000	gain	AAB	-1
Score=0.99	Score=113	esv118129	3	480000	gain	AAB	36.4281
Score=0.99	Score=571	esv26652,	3	62430	gain	AAB	-1
Score=0.99	Score=152	nsv507147	4	50000	gain	AABB	-1
Score=0.97	Score=241	nsv878278	4	1770000	gain	AABB	1.21347
.	Score=388	nsv433362	5	80000	gain	AAABB	18.3198
Score=0.99	Score=283	nsv428437	1	260000	loss	A	-1
Score=0.98	Score=521	nsv527095	3	31250000	gain	AAA	-1
Score=0.99	Score=581	dgv97e19,	4	23780000	gain	AAAA	-1
Score=0.98	Score=422	esv265850	5	6070000	gain	AAAAA	-1
Score=0.99	Score=486	esv4642,d	3	19620000	gain	AAA	-1
Score=0.96	Score=528	dgv885n67	5	3610000	gain	AAABB	3.47917
Score=0.99	Score=659	esv269003	4	64640000	gain	AAAA	-1
Score=0.99	Score=483	esv993671	3	24500000	gain	AAA	-1
Score=0.99	Score=769	esv23426,	6	40000	gain	AAAAAA	14.3123
Score=0.99	Score=219	nsv471487	6	134276	gain	AAAAAA	-1
Score=0.95	Score=238	esv21891,	3	150000	gain	-1	-1
Score=0.99	Score=668	nsv880520	3	34900000	gain	AAB	0.247004
.	Score=327	dgv6032n7	3	10000	gain	AAB	-1
Score=0.99	Score=510	dgv6073n7	4	31430000	gain	AAAA	-1
Score=0.99	Score=272	nsv882003	3	4050000	gain	AAB	2.1441
Score=0.99	Score=700	dgv6179n7	4	10020000	gain	AAAA	-1
Score=0.99	Score=659	esv273077	4	50730000	gain	AAAB	0.767163
Score=0.98	Score=477	esv267448	3	14550000	gain	AAB	0.91928
Score=0.97	Score=294	esv270273	4	2210000	gain	AAAB	7.58891
Score=0.98	Score=476	nsv830540	3	19050000	gain	AAA	-1
Score=0.95	Score=273	nsv328840	5	560000	gain	AAABB	69.7505
Score=0.99	Score=294	nsv521607	3	9190000	gain	AAB	0.938385
Score=0.99	Score=421	nsv883397	3	8050000	gain	AAA	-1
Score=0.97	Score=445	esv27793,	4	2360000	gain	AAAB	100
Score=0.99	Score=607	nsv349842	3	18570000	gain	AAB	0.518409

.	Score=907	nsv830622	3	30000	gain	AAB		-1
Score=0.9	Score=261	esv273176	4	360000	gain	AAAB		22.1237
.	Score=166	nsv823676	4	280000	gain	AAAB		100
Score=0.9	Score=419	esv273203	4	9870000	gain	AAAA		-1
.	Score=260	nsv885888	4	10000	gain		-1	-1
Score=0.9	Score=642	esv6929,e	3	11830000	gain	AAB		36.6399
Score=0.9	Score=650	dgv59n64,	3	15040000	gain	AAA		-1
.	Score=340	esv267769	3	1390000	gain	AAB		-1
.	Score=259	nsv509142	3	630000	gain	AAB		6.04952
.	Score=365	esv128530	3	690000	gain	AAB		-1
.	Score=509	nsv886329	4	90000	gain	AAAA		53.0365
.	Score=100	esv266485	4	60000	gain	AAAA		-1
Score=0.9	Score=412	nsv516048	4	13960000	gain	AAAA		-1
Score=0.9	Score=523	esv273255	3	11530000	gain	AAA		-1
Score=0.9	Score=449	esv273270	4	21770000	gain	AAAA		-1
Score=0.9	Score=432	esv273278	3	3960000	gain	AAB		4.67522
Score=0.9	Score=451	esv124163	4	5320000	gain	AAAB		96.7645
.	Score=397	esv266221	3	40000	gain	AAB		-1
Score=0.9	Score=495	nsv823862	3	23180000	gain	AAB		0.356559
.	.	.	3	55067	gain	AAB		-1
.	.	.	4	10000	gain	AAAB		-1
Score=0.9	Score=168	dgv1176e1	4	290000	gain	AAAB		100
.	Score=429	dgv7078n7	4	20000	gain	AABB		-1
Score=0.9	Score=107	esv273374	4	1740000	gain	AABB		4.58457
Score=0.9	Score=519	esv273408	4	14760000	gain	AAAA		-1
Score=0.9	Score=437	esv8983,e	3	19680000	gain	AAA		-1
Score=0.9	Score=272	nsv524173	5	1940000	gain	AAABB		100
Score=0.9	Score=559	esv273458	4	8700000	gain	AABB		9.93203
Score=0.9	Score=288	nsv511358	3	7220000	gain	AAB		32.2646
Score=0.9	Score=711	esv28195,	4	20790000	gain	AAAA		-1
Score=0.9	Score=261	esv27075,	3	520000	gain	AAB		9.67469
Score=0.9	Score=533	esv34648,	4	17800000	gain	AAAA		-1
Score=0.9	Score=431	esv23955,	5	6330000	gain	AAABB		63.6451
Score=0.9	Score=307	nsv818560	4	1540000	gain	AABB		1.7339
Score=0.9	Score=280	esv273531	4	3190000	gain	AABB		100

Score=0.9!	Score=697	esv273655	3	1820000	gain	AAB	1.34122
.	Score=209	esv275541	3	340000	gain	AAB	-1
Score=0.9!	Score=325	nsv6086,es	4	5950000	gain	AAAA	-1
.	Score=270	nsv831271	6	230000	gain	AAABBB	100
Score=0.9!	Score=442	esv6612,es	3	14450000	gain	AAA	-1
Score=0.9!	Score=255	esv273690	5	840000	gain	AAABB	65.1304
Score=0.9!	Score=660	dgv7810n7	4	36920000	gain	AAAA	-1
Score=0.9!	Score=192	nsv891123	5	290000	gain	AAAAA	4.49722
Score=0.9!	Score=236	nsv6290,es	5	380000	gain	AAAAA	-1
Score=0.9	Score=496	esv266909	4	23770000	gain	AABB	1.34446
.	Score=424	esv24736,i	4	2240000	gain	AABB	-1
Score=0.9	Score=275	nsv511390	6	890000	gain	AAABBB	87.2215
Score=0.9!	Score=370	nsv527309	4	19740000	gain	AAAA	-1
Score=0.9!	Score=240	esv100376	5	2740000	gain	AAABB	9.49948
.	Score=314	dgv7924n7	5	20000	gain	AAABB	-1
Score=0.9!	Score=260	nsv891722	5	1360000	gain	AAABB	18.0115
.	Score=239	dgv7969n7	5	20000	gain	AAABB	-1
.	.	.	3	10000	gain	AAB	-1
Score=0.9!	Score=275	nsv891943	3	170000	gain	AAB	14.9511
Score=0.9!	Score=413	nsv819362	4	14280000	gain	AABB	1.25419
.	Score=112	nsv892647	4	250000	gain	AABB	-1
Score=0.9	Score=685	esv273834	4	15770000	gain	AAAA	-1
Score=0.9!	Score=246	dgv8173n7	3	2920000	gain	AAB	5.71603
Score=0.9!	Score=235	nsv831565	3	50000	gain	AAB	-1
Score=0.9!	Score=303	esv266935	3	29430000	gain	AAB	0.634081
Score=0.9!	Score=695	dgv8242n7	3	100000	gain	AAB	-1
Score=1;N	Score=709	nsv821495	3	24360000	gain	AAA	-1
Score=0.9!	Score=277	esv267385	3	310000	gain	AAA	44.6482
.	Score=374	esv267769	3	70000	gain	AAA	-1
Score=0.9!	Score=281	nsv509311	3	1020000	gain	AAB	3.69067
Score=0.9!	Score=295	nsv525113	3	3880000	gain	AAA	-1
.	Score=325	nsv831666	4	20000	gain	AABB	62.2491
Score=0.9	Score=303	esv273883	3	1320000	gain	-1	-1
Score=0.9!	Score=412	esv273890	4	11920000	gain	AABB	0.803186
Score=0.9!	Score=327	nsv8560,es	3	12210000	gain	AAA	-1

.	Score=238	nsv517335	6	110000	gain	AAABBB	17.7702
.	Score=270	dgv8372n7	4	30000	gain	AABB	-1
Score=0.9	Score=192	nsv894386	4	1510000	gain	AABB	2.62756
Score=0.9	Score=159	esv100910	3	80000	gain	AAB	9.22149
Score=0.9	Score=374	dgv2430e1	3	73431	gain	AAB	-1
Score=0.9	Score=280	nsv894693	3	90000	gain	AAA	-1
Score=0.9	Score=583	dgv209e1,	3	10000	gain	AAA	10.0867
Score=0.9	Score=190	nsv894694	3	180000	gain	AAB	-1
Score=0.9	Score=749	nsv894814	3	11380000	gain	AAB	0.373387
.	Score=120	nsv894879	4	190000	gain	AAAB	72.8113
.	Score=278	nsv831785	4	70000	gain	AAAB	-1
Score=0.9	Score=311	esv228663	4	11510000	gain	AABB	1.95127
.	Score=123	nsv6132,e	4	70000	gain	AABB	-1
Score=0.9	Score=283	esv271932	3	1150000	gain	AAB	100
.	Score=516	nsv831816	3	50000	gain	AAB	-1
Score=0.9	Score=148	esv266995	3	570000	gain	AAB	62.3355
Score=0.9	Score=676	esv265103	4	12150000	gain	AAAA	-1
Score=0.9	Score=446	dgv251e1,	3	2820000	gain	AAB	2.61171
Score=0.9	Score=134	dgv254e1,	3	40000	gain	AAB	-1
Score=0.9	Score=239	dgv86n27,	3	3210000	gain	AAB	2.43501
Score=0.9	Score=225	nsv467190	3	310000	gain	AAB	7.40631
Score=0.9	Score=694	esv270045	4	17720000	gain	AABB	0.790409
.	Score=272	dgv743n71	4	1070000	gain	AABB	-1
Score=0.9	Score=268	esv267645	4	550000	gain	AABB	47.3675
Score=0.9	Score=418	esv167788	4	4550000	gain	AAAA	-1
Score=0.9	Score=654	nsv7497,e	3	4280000	gain	AAA	-1
Score=0.9	Score=449	nsv525892	4	15010000	gain	AABB	1.12191
.	Score=263	.	4	20000	gain	AABB	-1
Score=0.9	Score=499	esv100502	3	13050000	gain	AAA	-1
Score=0.9	Score=180	esv266077	4	1330000	gain	AABB	1.54964
Score=0.9	Score=434	dgv8n64,n	4	190000	gain	AABB	-1
Score=0.9	Score=607	nsv896428	3	90000	gain	AAA	35.8881
Score=0.9	Score=118	nsv896428	3	14747	gain	AAA	-1
Score=0.9	Score=224	esv22231,	4	110000	gain	AABB	-1
Score=0.9	Score=265	nsv896601	4	2850000	gain	AABB	0.519937

Score=0.9	Score=383	esv274431	4	10990000	gain	AAAA		-1
Score=0.9	Score=510	esv195123	3	12230000	gain	AAA		-1
.	Score=843	dgv1091n7	5	10000	gain	AAABB		-1
Score=0.9	Score=472	dgv375e1,	5	7880000	gain	AAABB		85.5912
Score=0.9	Score=269	dgv69n21,	5	100000	gain	AAABB		-1
Score=0.9	Score=273	esv267290	4	160000	gain		-1	-1
.	Score=271	nsv482981	5	310000	gain	AAABB		63.2719
Score=0.9	Score=490	esv24876,	4	2330000	gain	AABB		4.16081
.	Score=276	nsv517447	5	340000	gain	AAABB		89.5407
Score=0.9	Score=383	nsv898062	3	7570000	gain	AAA		-1
Score=0.9	Score=276	nsv898088	5	660000	gain	AAABB		33.552
Score=0.9	Score=681	nsv437721	4	20870000	gain	AAAA		-1
Score=0.9	Score=598	nsv598,ns	3	3050000	gain	AAB		5.70413
Score=0.9	Score=424	nsv826263	3	15950000	gain	AAB		1.83095
Score=0.9	Score=273	esv266627	3	2640000	gain	AAB		4.68876
Score=0.9	Score=703	dgv279e19	3	5060000	gain	AAB		-1
Score=0.9	Score=285	esv22995,	5	690000	gain		-1	-1
Score=0.9	Score=419	esv29183,	4	8800000	gain	AABB		10.8835
Score=0.9	Score=276	nsv82,esv	4	740000	gain	AABB		57.318
Score=0.9	Score=651	nsv510302	4	21770000	gain	AAAA		-1
Score=0.9	Score=578	nsv899421	4	11540000	gain	AABB		12.3746
Score=0.9	Score=563	esv267473	4	16180000	gain	AABB		0.682372
Score=0.9	Score=111	esv24540,	3	1290000	gain	AAB		24.0245
Score=0.9	Score=414	esv265666	3	60000	gain	AAB		-1
Score=0.9	Score=270	esv274697	4	1530000	gain	AABB		1.51713
Score=0.9	Score=262	.	4	51895	gain	AABB		-1
Score=0.9	Score=417	nsv899934	3	34410000	gain	AAA		-1
Score=0.9	Score=263	esv270120	4	1620000	gain	AABB		84.919
Score=0.9	Score=284	nsv525432	4	5100000	gain	AAAA		-1
.	Score=175	esv24878,	5	50000	gain	AAABB		-1
Score=0.9	Score=268	nsv519614	5	1290000	gain	AAABB		77.4909
Score=0.9	Score=461	esv114009	4	8750000	gain	AABB		2.03579
Score=0.9	Score=618	esv261414	5	17370000	gain	AAABB		27.1885
Score=0.9	Score=271	esv274759	5	1660000	gain	AAABB		-1
Score=0.9	Score=506	esv274761	4	390000	gain		-1	-1

Score=0.98	Score=407	esv996694	4	25690000	gain	AABB	2.17513
Score=0.91	Score=434	nsv1139,ns	4	480000	gain	AABB	-1
Score=0.96	Score=365	esv265745	3	3140000	gain	AAA	-1
Score=0.91	Score=379	nsv820338	4	8060000	gain	AABB	8.86675
.	Score=199	nsv832718	4	10000	gain	AABB	-1
Score=0.95	Score=275	esv6572,ns	3	19110000	gain	AAB	-1
Score=0.99	Score=263	nsv428292	3	1300000	gain	AAB	4.99326
Score=0.95	Score=101	dgv602e1,(	3	30000	gain	AAB	-1
Score=0.95	Score=269	esv270175	4	70000	gain	AABB	4.3386
.	Score=269	nsv832738	4	10000	gain	AABB	-1
.	Score=117	dgv599e1,(	6	10000	gain	-1	-1
.	Score=268	nsv818998	4	50000	gain	AABB	-1
Score=0.94	Score=259	esv257172	4	140000	gain	AABB	100
.	Score=759	nsv524527	3	40000	gain	AAB	83.822
.	Score=727	nsv1208,es	3	10000	gain	AAB	-1
Score=0.92	Score=463	esv7166,es	3	970000	gain	AAB	1.95539
Score=0.99	Score=360	esv212580	4	6750000	gain	AAAA	-1
.	Score=183	dgv1869n7	4	10000	gain	-1	-1
Score=0.99	Score=274	esv29451,(	4	4510000	gain	AAAA	-1
Score=0.97	Score=436	nsv819994	4	9950000	gain	AABB	0.815804
.	Score=184	nsv456304	4	70000	gain	AABB	-1
Score=0.97	Score=284	nsv516268	3	1960000	gain	AAA	100
.	Score=117	esv28926,(	3	100000	gain	AAA	-1
Score=0.95	Score=351	nsv1278,es	4	280000	gain	AAAA	100
Score=0.98	Score=441	nsv820177	4	7590000	gain	AAAA	-1
Score=0.98	Score=281	dgv638e1,r	5	560000	gain	AAABB	100
Score=0.95	Score=287	nsv470650	4	3060000	gain	AABB	14.9179
.	Score=411	dgv137n21	4	50000	gain	AABB	-1
Score=0.93	Score=139	nsv1305,ns	4	400000	gain	AAAA	90.8007
.	Score=150	nsv456326	4	90000	gain	AAAA	-1
Score=0.96	Score=275	esv266469	5	470000	gain	AAABB	67.758
.	Score=270	nsv521739	4	470000	gain	AAAB	-1
Score=0.92	Score=309	esv267118	4	870000	gain	AAAB	100
.	Score=716	nsv1319,ns	4	70000	gain	AAAB	-1
.	Score=452	nsv826992	3	70000	gain	-1	-1

Score=0.94	Score=479	esv22714,e	4	1450000	gain	AABB	25.2804
.	Score=265	nsv832841	4	100000	gain	AABB	-1
.	Score=187	esv274893	3	310000	gain	AAA	80.6793
Score=0.97	Score=308	esv266708	5	4010000	gain	AAABB	-1
Score=0.95	Score=323	nsv516702	5	3120000	gain	AAABB	72.0789
.	Score=232	nsv902181	5	150000	gain	AAABB	-1
.	Score=681	nsv832850	4	40000	gain	AABB	-1
.	Score=238	esv996160	4	300000	gain	AABB	66.8635
Score=0.90	Score=242	nsv827073	4	720000	gain	AABB	16.0236
.	Score=262	dgv2017n7	4	10000	gain	AABB	-1
.	Score=239	nsv902416	3	10000	gain	-1	-1
.	Score=313	nsv902389	4	10000	gain	AABB	-1
Score=0.99	Score=138	esv2953,es	4	460000	gain	AABB	1.2812
.	Score=124	dgv2034n7	4	160000	gain	AABB	-1
.	Score=234	nsv832890	6	40000	gain	AAABBB	100
Score=0.97	Score=997	esv267327	3	90000	gain	AAB	-1
Score=0.96	Score=195	esv266874	3	290000	gain	AAB	42.0905
Score=0.97	Score=263	dgv2076n7	3	20160000	gain	AAB	-1
Score=1;N	Score=270	nsv509560	3	3730000	gain	AAB	0.793584
Score=0.96	Score=282	nsv513423	3	1030000	gain	AAB	-1
Score=0.98	Score=246	esv266572	3	2250000	gain	AAB	19.3165
Score=0.99	Score=285	nsv498844	4	5420000	gain	AABB	2.02105
Score=0.98	Score=570	nsv904279	4	16610000	gain	AAAA	-1
Score=0.94	Score=273	esv274980	4	980000	gain	AABB	100
Score=0.90	Score=277	esv267264	5	980000	gain	AAABB	8.8789
Score=0.99	Score=293	nsv469595	3	2510000	gain	AAA	-1
Score=0.93	Score=321	nsv1617,ns	4	1260000	gain	AABB	8.31453
Score=0.99	Score=274	dgv764e1,c	3	570000	gain	AAB	1.50873
Score=0.98	Score=279	esv275009	4	5080000	gain	AABB	4.47228
Score=0.98	Score=923	nsv518291	3	130000	gain	AAB	6.24972
Score=0.97	Score=933	nsv471063	4	380000	gain	AABB	1.30504
Score=0.98	Score=733	esv265795	4	1010000	gain	AABB	2.69159
Score=0.97	Score=102	esv275037	4	490000	gain	AABB	3.64152
Score=0.99	Score=689	nsv436835	3	190000	gain	AAA	15.674
Score=0.99	Score=891	nsv428324	3	270000	gain	AAA	12.2404

Score=0.99	Score=409	nsv1782,dg	3	920000	gain	AAB		40.172
.	Score=735	esv227328	3	80000	gain	AAB		-1
Score=0.99	Score=476	esv250512	3	21810000	gain	AAA		-1
Score=0.97	Score=102	dgv2979n7	3	1220000	gain	AAB		6.6248
.	Score=846	esv25469,e	3	130000	gain	AB		-1
Score=0.99	Score=231	nsv833413	5	230000	gain	AAABB		87.8293
Score=0.97	Score=284	dgv552e19	4	190000	gain	AAAB		12.4503
Score=0.99	Score=133	nsv833461	3	1090000	gain	AAB		2.00638
Score=0.93	Score=419	esv8272,es	4	20000	gain	AAAA		61.4822
Score=0.99	Score=429	esv4896,es	3	4030000	gain	AAB		0.615072
Score=0.97	Score=510	esv5462,es	5	5680000	gain	AAABB		77.6361
Score=0.92	Score=259	nsv828055	5	20000	gain	AAABB		-1
Score=0.99	Score=240	esv271607	3	3100000	gain	AAA		-1
Score=0.99	Score=280	nsv507850	3	2310000	gain	AAB		100
Score=0.98	Score=232	dgv997e1,n	4	70000	gain	AABB		-1
Score=0.98	Score=294	esv273813	4	6550000	gain	AABB		2.26523
.	Score=263	esv271615	5	1140000	gain	AAABB		28.9818
Score=0.96	Score=107	esv137642	5	1140000	gain	AAABB		42.1055
Score=0.97	Score=277	nsv909286	4	40000	gain	AAAB		-1
Score=0.98	Score=277	nsv821099	4	80000	gain	AAAB		100
Score=0.98	Score=523	dgv3383n7	3	11480000	gain	AAA		-1
.	Score=147	esv266805	3	480000	gain		-1	-1
Score=0.99	Score=365	esv145257	4	8380000	gain	AAAA		-1
.	Score=282	nsv520131	5	2080000	gain	AAABB		100
Score=0.94	Score=368	esv268983	4	2030000	gain	AABB		17.6138
.	Score=269	esv271711	4	1220000	gain	AABB		-1
.	Score=347	nsv833660	4	2950000	gain	AABB		7.61169
.	Score=272	esv269189	4	50000	gain	AABB		-1
Score=0.99	Score=477	esv271732	4	14110000	gain	AABB		0.666739
.	Score=254	nsv131514	4	810000	gain	AABB		-1
Score=0.97	Score=588	esv271747	3	6337248	gain	AAA		-1
.	.	.	3	60000	gain	AAB		-1
Score=0.99	Score=224	esv994482	3	150000	gain	AAB		5.01727
Score=0.99	Score=792	esv139957	3	150000	gain	AAB		-1
Score=0.98	Score=129	esv25510,n	3	2000000	gain	AAB		6.48469

Score=0.99	Score=280	esv177461	3	3640000	gain	AAB	14.0224
.	Score=750	dgv3697n7	3	210000	gain	AAB	-1
Score=0.99	Score=685	dgv3791n7	3	4280000	gain	AAB	1.3688
Score=0.95	Score=236	esv271835	3	220000	gain	AAB	-1
Score=0.97	Score=392	esv100718	3	720000	gain	AAA	11.9811
.	Score=471	dgv1302e1	1	20000	loss	A	100
.	Score=751	esv995112	1	20000	loss	A	-1
Score=0.97	Score=286	nsv498967	3	12100000	gain	AAA	-1
Score=0.96	Score=295	nsv912812	3	5740000	gain	AAA	-1
.	Score=276	esv32588,e	3	200000	gain	AAA	100
Score=0.94	Score=114	esv100010	3	270000	gain	AAA	-1
.	Score=771	esv272289	4	1340000	gain	AABB	4.2254
.	Score=249	dgv4643n7	4	10000	gain	AABB	-1
Score=0.97	Score=824	nsv179256	4	210000	gain	AABB	2.03747
Score=0.99	Score=263	esv272301	5	9750000	gain	AAAAA	-1
Score=0.99	Score=618	esv272301	5	80000	gain	AAAAA	100
Score=0.99	Score=274	dgv742e20	4	4590000	gain	AABB	-1
Score=0.99	Score=412	nsv913398	4	1030000	gain	AABB	2.79713
Score=0.99	Score=576	esv143389	4	11690000	gain	AAAA	-1
Score=0.97	Score=423	esv997049	4	4530000	gain	AABB	3.49289
.	Score=360	nsv834086	4	150000	gain	AABB	21.9682
.	Score=697	dgv4771n7	4	60000	gain	AABB	40.3325
.	Score=553	esv272363	4	10000	gain	AABB	-1
.	Score=111	esv263677	4	100000	gain	AABB	33.3609
.	Score=148	nsv913946	4	40000	gain	AABB	-1
.	Score=148	nsv834109	3	20000	gain	AAA	74.5606
Score=0.95	Score=195	esv272372	4	1660000	gain	AABB	1.01238
.	Score=246	nsv519904	4	20000	gain	AAAA	100
Score=0.90	Score=255	esv991505	4	30000	gain	AAAA	-1
Score=0.99	Score=799	nsv428384	4	16090000	gain	AABB	-1
Score=0.99	Score=262	esv100256	4	1070000	gain	AABB	56.4349
Score=0.99	Score=789	dgv74n16,r	3	220000	gain	AAB	1.26346
Score=0.91	Score=232	dgv4850n7	4	640000	gain	AABB	1.35682
Score=0.91	Score=407	esv992649	4	50000	gain	AABB	-1
Score=0.99	Score=838	dgv1387e1	3	550000	gain	AAB	41.2963

Score=0.9	Score=778	nsv914390	4	50000	gain	AABB	11.8148
Score=0.9	Score=976	esv266782	3	440000	gain	AAB	1.11226
Score=0.9	Score=273	nsv7350,ns	3	60000	gain	AAB	-1
Score=0.9	Score=389	esv242248	3	90000	gain	AAB	29.1344
Score=0.9	Score=676	nsv828979	3	50000	gain	AAB	-1
Score=0.9	Score=442	esv272445	3	5390000	gain	AAB	0.624221
.	Score=266	esv272475	7	20000	gain	AAAABBB	81.8992
.	Score=239	nsv915525	4	10000	gain	AABB	-1
Score=0.8	Score=919	dgv781n67	4	540000	gain	AABB	0.533598
Score=0.9	Score=560	esv25002,e	3	26760000	gain	AAB	1.04541
Score=0.9	Score=447	nsv507970	3	1370000	gain	AAB	9.11466
Score=0.9	Score=338	esv267850	3	1320000	gain	AAB	-1
Score=0.9	Score=491	esv274023	4	2540000	gain	AAAB	100
Score=0.9	Score=662	essv6070,r	3	68000000	gain	AAB	0.50492
Score=0.9	Score=662	dgv1405e1	3	7200000	gain	AAB	3.0817
Score=0.9	Score=162	nsv10003	3	10560	gain	AAB	-1
.	.	.	1	10000	loss	A	-1
Score=0.9	Score=616	dgv11e1,es	1	34670000	loss	A	0.494588
.	Score=287	nsv834669	1	10000	loss	A	-1
Score=0.9	Score=454	nsv461350	3	12570000	gain	AAA	-1
Score=0.9	Score=286	esv267087	4	400000	gain	AAAB	16.7432
Score=0.9	Score=554	nsv932,nsv	4	38530000	gain	AAAA	-1
.	Score=287	esv242133	5	340000	gain	AAABB	37.7124
.	Score=221	nsv525678	3	20000	gain	AAB	-1
Score=0.9	Score=288	nsv525045	3	820000	gain	AAB	32.0364
.	Score=116	.	3	20000	gain	AAB	-1
Score=0.9	Score=320	nsv436703	3	7190000	gain	AAB	2.811
Score=0.9	Score=516	nsv834300	3	10000	gain	AAB	-1
Score=0.9	Score=421	esv267702	1	13920000	loss	A	-1
Score=0.9	Score=626	essv20845	4	15190000	gain	AAAA	-1
.	Score=255	esv274065	4	70000	gain	AAAB	-1
Score=0.9	Score=536	esv21640,e	4	40040000	gain	AAAB	0.491575
Score=0.9	Score=571	esv26652,e	4	62430	gain	AAAB	-1
.	Score=507	nsv878455	3	80000	gain	AAA	100
.	Score=388	nsv441855	3	160000	gain	AAA	-1

Score=0.9	Score=769	nsv820863	3	40000	gain	AAA		10.9205
Score=0.9	Score=219	nsv820863	3	134276	gain	AAA		-1
Score=0.9	Score=612	esv121866	3	140000	gain		-1	-1
Score=0.9	Score=172	dgv1741e1	3	380000	gain	AAB		9.74695
.	Score=215	nsv428459	3	10000	gain	AAB		-1
.	Score=365	nsv881117	3	30000	gain	AAB		-1
Score=0.9	Score=172	nsv511283	3	280000	gain	AAB		2.37159
.	Score=219	nsv830196	3	20000	gain	AAA		100
Score=0.9	Score=668	dgv5993n7	3	11860000	gain	AAB		31.4077
.	Score=227	dgv6250n7	3	40000	gain		-1	-1
Score=0.9	Score=249	nsv883139	3	30000	gain	AAB		99.4079
.	Score=248	nsv886664	3	10000	gain		-1	-1
.	Score=247	nsv831103	3	20000	gain	AAB		100
.	Score=228	nsv470213	5	10000	gain		-1	-1
.	Score=275	nsv891250	3	730000	gain	AAB		27.9433
Score=0.9	Score=367	esv992210	1	23360000	loss	A		-1
Score=0.9	Score=391	esv273794	1	4040000	loss	A		0.987943
Score=0.9	Score=279	esv266940	1	154022	loss	A		-1
.	Score=264	nsv892650	1	20000	loss		-1	-1
Score=0.9	Score=721	dgv2313e1	3	100000	gain	AAB		100
Score=0.9	Score=243	esv273844	3	450000	gain	AAB		-1
Score=0.9	Score=213	nsv893080	3	310000	gain	AAB		55.0266
.	Score=396	esv265940	3	20000	gain		-1	-1
.	Score=318	nsv7608,e	3	240000	gain		-1	-1
Score=0.9	Score=138	esv274376	3	380000	gain	AAB		3.11286
Score=0.9	Score=487	dgv909n71	3	50000	gain	AAB		54.4664
Score=0.9	Score=118	esv28562,	3	14747	gain	AAB		-1
Score=0.9	Score=681	nsv428267	1	52476516	loss	A		-1
.	.	.	3	60000	gain	AAA		-1
Score=0.9	Score=670	.	3	40000	gain	AAA		46.6712
Score=0.9	Score=439	esv267435	1	9500000	loss	A		-1
.	Score=963	nsv898846	6	50000	gain	AAAABB		100
.	Score=274	nsv832344	4	10000	gain	AAAB		-1
Score=0.9	Score=287	esv271722	4	2240000	gain	AAAB		63.7764
.	Score=254	.	4	30000	gain	AAAB		-1

.	Score=365	esv275049	5	780000	gain	AAAAB		16.1148
.	Score=275	nsv469162	4	50000	gain	AAAB		-1
Score=0.99	Score=271	nsv528556	4	1250000	gain	AAAB		4.4111
Score=0.94	Score=274	dgv92n21,	3	840000	gain	AAB		-1
Score=0.91	Score=272	nsv898915	3	1680000	gain	AAB		7.59037
Score=0.91	Score=392	esv272970	3	440000	gain	AAB		-1
Score=0.99	Score=424	esv266088	3	7740000	gain	AAB		1.84158
Score=0.98	Score=618	esv274765	3	12340000	gain	AAA		-1
Score=0.97	Score=693	esv220587	3	730000	gain	AAB		12.929
Score=0.97	Score=307	esv274808	4	1210000	gain	AAAB		27.1503
Score=0.91	Score=145	esv274816	3	430000	gain	AAB		7.56677
.	Score=940	esv274821	3	40000	gain	AAB		-1
.	Score=501	esv26193,	4	130000	gain	AAAB		32.6814
Score=0.98	Score=227	esv274824	3	1320000	gain	AAB		2.09557
Score=0.98	Score=669	nsv516205	3	99878	gain	AAB		-1
Score=0.95	Score=275	nsv510626	3	19110000	gain	AAB		-1
Score=0.99	Score=263	dgv1836n7	3	1300000	gain	AAB		1.45547
Score=0.95	Score=165	nsv428292	3	60000	gain	AAB		-1
Score=0.90	Score=242	dgv304e20	3	510000	gain	AAB		4.41611
Score=0.96	Score=142	dgv2045n7	4	20000	gain	AABB		3.49704
Score=0.96	Score=663	nsv902569	3	20000	gain	AAB		-1
Score=0.96	Score=195	nsv827154	3	320000	gain	AAB		1.39934
Score=1;N	Score=270	dgv2099n7	3	2410000	gain	AAB		0.764695
Score=0.93	Score=142	esv999310	3	150000	gain	AAB		100
Score=0.99	Score=280	dgv874e1,	3	1730000	gain	AAB		0.822878
Score=0.98	Score=213	dgv883e1,	3	460000	gain	AAB		-1
Score=0.94	Score=476	esv266094	3	230000	gain		-1	-1
Score=0.99	Score=177	esv29998,	3	50000	gain	AAB		-1
Score=0.99	Score=379	esv29264,	3	320000	gain	AAB		4.95936
Score=0.97	Score=277	nsv909286	4	40000	gain	AAAB		-1
Score=0.98	Score=277	esv4511,dg	4	470000	gain	AAAB		19.0016
.	Score=292	nsv909289	3	70000	gain	AAB		-1
Score=0.97	Score=471	esv267875	3	3310000	gain	AAB		7.16881
Score=0.99	Score=588	nsv526596	1	48107248	loss	A		-1
Score=0.99	Score=224	nsv821117	3	110000	gain	AAA		20.1103

Score=0.98	Score=115	esv28703	3	20000	gain	AAA	29.5738
.	.	.	3	8983	gain	AAA	-1
Score=0.90	Score=244	nsv522008	1	10000	loss	-1	-1
.	Score=240	nsv834001	1	10000	loss	-1	-1
Score=0.98	Score=340	esv170890	3	9810000	gain	AAA	-1
.	Score=519	esv141189	6	10000	gain	AAAABB	100
.	Score=188	nsv912983	3	160000	gain	AAB	-1
Score=0.90	Score=104	dgv4624n7	3	1780000	gain	AAB	2.10077
.	Score=250	esv272289	3	10000	gain	AAB	-1
.	Score=659	nsv517702	4	340000	gain	AABB	2.76928
Score=0.98	Score=106	dgv4653n7	3	730000	gain	AAB	2.01143
Score=0.98	Score=125	esv24036,n	3	95520	gain	AAB	-1
.	Score=292	esv267191	4	330000	gain	AABB	10.1381
Score=0.99	Score=799	nsv469815	3	16090000	gain	AAB	-1
Score=0.99	Score=262	esv21839,n	3	830000	gain	AAB	3.82082
Score=0.97	Score=238	nsv527637	3	30000	gain	AAB	-1
Score=0.99	Score=296	esv272393	3	520000	gain	AAB	46.6362
Score=0.94	Score=265	nsv915082	1	30000	loss	A	-1
.	Score=331	nsv834227	4	20000	gain	-1	-1
Score=1;N	Score=256	esv267399	3	100000	gain	AAB	6.84711
Score=0.94	Score=538	esv274026	3	1330000	gain	AAB	100
.	Score=318	nsv7187	6	20000	gain	AAAAAB	61.4059
.	Score=690	esv274759	5	30000	gain	AAABB	14.3496
Score=0.99	Score=107	essv16633	3	320000	gain	AAB	9.54857
Score=0.99	Score=268	nsv499132	3	2030000	gain	AAB	0.803109
Score=0.99	Score=273	nsv499132	3	40000	gain	AAB	-1
.	Score=108	nsv436272	3	130000	gain	AAB	17.8052
.	Score=239	nsv3792,ns	3	20000	gain	AAA	100
Score=0.92	Score=124	nsv834676	3	190000	gain	AAA	-1
Score=0.91	Score=359	esv995444	3	1430000	gain	AAA	-1
Score=0.97	Score=624	nsv876766	3	230000	gain	AAA	38.2855
.	Score=600	nsv877220	3	20000	gain	AAA	100
.	Score=507	nsv822202	3	70000	gain	AAA	-1
Score=0.98	Score=114	dgv1569e1	5	20000	gain	-1	-1
Score=0.99	Score=381	dgv5467n7	5	150000	gain	AAABB	16.4136

Score=0.99	Score=219	nsv10641,n	1	174276	loss	A	-1
Score=0.99	Score=232	nsv509072	3	1620000	gain	AAB	3.18956
.	Score=242	nsv5002,ns	4	120000	gain	AABB	41.5836
.	Score=804	esv266149	3	80000	gain	AAB	61.5362
Score=0.94	Score=429	nsv5230,es	4	500000	gain	AABB	2.90822
Score=0.97	Score=366	dgv1917e1	3	530000	gain	AAB	1.03727
.	Score=108	nsv884129	3	10000	gain	AAB	-1
.	Score=108	esv267026	6	10000	gain	AAABBB	24.6938
Score=0.99	Score=443	nsv884405	4	960000	gain	AABB	0.986513
.	Score=634	esv270331	4	160000	gain	AABB	2.6962
.	Score=202	esv230123	4	170000	gain	AABB	7.38949
.	Score=268	dgv753n27	4	60000	gain	AABB	-1
Score=0.96	Score=263	nsv830649	3	80000	gain	AAA	100
.	Score=243	esv6714,ns	3	20000	gain	AAA	-1
Score=0.98	Score=272	esv271615	1	1620000	loss	A	100
.	Score=152	nsv510080	1	210000	loss	A	-1
.	Score=377	nsv5871,es	4	10000	gain	AAAB	17.4148
Score=0.99	Score=120	nsv515042	3	140000	gain	AAB	6.63343
.	Score=266	nsv831246	5	20000	gain	AAABB	80.3055
Score=0.99	Score=192	nsv891139	1	290000	loss	A	2.19623
.	Score=562	esv273841	3	140000	gain	AAB	9.51239
Score=0.99	Score=487	nsv482152	1	50000	loss	A	7.22425
Score=0.97	Score=118	nsv896428	1	14747	loss	A	-1
Score=0.96	Score=223	esv274548	3	1210000	gain	AAB	1.16231
Score=0.96	Score=404	esv27744,n	3	2000000	gain	AAB	13.1799
Score=0.97	Score=274	esv141454	4	2020000	gain	AABB	0.742839
.	Score=179	nsv899507	5	30000	gain	-1	-1
Score=0.96	Score=265	nsv520280	3	290000	gain	AAB	27.5981
.	Score=346	nsv832574	5	10000	gain	-1	-1
Score=0.96	Score=434	nsv1140,es	3	1680000	gain	AAB	1.04609
.	Score=764	esv274787	3	60000	gain	AAB	-1
Score=0.92	Score=418	dgv239n27	3	11270000	gain	AAB	2.98909
Score=0.98	Score=693	nsv826821	3	4070000	gain	AAB	31.9803
Score=0.98	Score=669	nsv518491	3	99878	gain	AAB	-1
Score=0.95	Score=248	esv242196	5	10000	gain	AAABB	59.7169

Score=0.9	Score=260	dgv113e55	4	10000	gain	AAAA	33.787
Score=0.9	Score=295	dgv2640n7	3	2710000	gain	AAB	8.52517
.	Score=351	nsv905565	3	30000	gain	AAB	-1
Score=0.9	Score=689	nsv433438	3	110000	gain	AAA	9.33868
Score=0.9	Score=247	nsv433437	3	10000	gain	AAB	-1
Score=0.9	Score=409	dgv490e19	3	10070000	gain	AAB	4.54035
Score=0.9	Score=266	nsv907362	1	3320000	loss	A	-1
.	Score=439	esv23611,r	1	60000	loss	A	-1
Score=0.9	Score=379	nsv523265	3	420000	gain	AAB	3.87842
.	Score=317	esv271639	3	440000	gain	AAB	19.0976
.	Score=247	nsv910922	3	30000	gain	AAA	54.7302
Score=0.9	Score=276	esv24941,r	4	260000	gain	AABB	1.00561
Score=0.9	Score=851	nsv820830	4	190000	gain	AABB	14.618
Score=0.9	Score=961	esv207792	4	230000	gain	AABB	21.0259
Score=0.9	Score=278	dgv421n27	3	110000	gain	AAA	53.2496
.	Score=951	nsv470535	3	20000	gain	AAA	100
.	Score=711	esv118841	3	90000	gain	AAB	7.31916
Score=0.9	Score=935	esv272237	3	870000	gain	AAB	100
Score=0.9	Score=289	nsv6826,ns	3	2130000	gain	AAB	3.76978
Score=0.9	Score=463	esv272941	3	6880000	gain	AAB	15.3501
Score=0.9	Score=569	esv174687	4	180000	gain	AABB	18.9151
Score=0.9	Score=270	esv270725	3	620000	gain	AAB	8.13278
.	Score=274	nsv513763	3	280000	gain	AAB	-1
Score=0.9	Score=964	esv24353,e	3	20000	gain	AAB	9.15435
Score=0.9	Score=162	nsv10003	3	10560	gain	AAB	-1

2675278,nsv4694,esv2729955,esv2729948,esv1361510,esv2730020,nsv4859,esv2729783,nsv818335,nsv499762,dgv6062n71,nsv881076,nsv462210,nsv483

416123.2,ENST00000523087.1,ENST00000409948.1,ENST00000368268.2,ENST00000449449.2,ENST00000518273.1,ENST00000472914.2,ENST0000041
79,esv2666897,esv1003005,esv2732609,esv2732942,esv2732939,esv267507,nsv830801,esv2732571,nsv464075,esv2668901,nsv349422,nsv886509,esv273

31,nsv830209,nsv525360,esv2526226,esv2730260,nsv4708,esv2730131,nsv881153,esv2665095,nsv881022,esv7370,esv2729669,nsv880361,dgv1771e1,nsv

14028.2,ENST00000430428.1,ENST00000392282.1,ENST00000520886.2,ENST00000369220.4,ENST00000422023.1,ENST00000452675.1,ENST00000264
32377,nsv511325,esv2732912,esv1791114,esv275229,esv2658177,esv1113556,esv2656633,esv2732502,nsv349212,nsv886161,esv32969,esv2663486,esv2

v517060,nsv471724,nsv830267,esv2729892,nsv4802,esv23114,dgv108n17,nsv819474,esv2730278,esv2586588,esv28799,esv1988123,esv2729879,esv991

839.7,ENST00000392348.2,ENST00000362224.1,ENST00000528577.1,ENST00000438797.1,ENST00000283977.4,ENST00000490177.1,ENST00000356
72696,nsv886676,esv2660189,nsv823728,esv2732877,nsv522813,dgv6938n71,esv2732351,nsv511898,nsv830841,esv2669946,esv2732291,esv2057606,ns

019,esv2449363,dgv5998n71,nsv4800,esv2393790,nsv4796,esv28441,nsv526042,nsv823069,essv19140,esv2729876,nsv830214,nsv880530,esv2729935,ns

35.4,ENST00000525849.1,ENST00000368961.5,ENST00000265198.4,ENST00000456137.2,ENST00000424296.2,ENST00000506649.1,ENST0000040863
sv830743,esv2663059,esv268396,nsv517329,nsv349993,esv2732560,dgv1972e1,nsv348864,esv269648,esv2600122,esv2671329,nsv520330,esv2069832,es

v820142,nsv4798,nsv4704,esv2671278,esv1264464,nsv442950,esv3407,nsv881586,dgv6024n71,dgv1778e1,nsv10700,nsv513220,nsv462187,esv1398165,[illegible]

[illegible]2.2,ENST00000392587.2,ENST00000302071.2,ENST00000530481.1,ENST00000602399.1,ENST00000391285.1,ENST00000370050.5,ENST00000367598.[illegible]
[illegible]v2662175,dgv1060e201,nsv516738,esv2666990,nsv514369,esv27575,esv2670835,nsv524944,esv32923,esv2732886,esv2662356,nsv510058,nsv5440,nsv8[illegible]

dgv6027n71,dgv1011e199,esv2729858,esv274597,nsv881027,esv2107734,nsv471740,esv29149,nsv511306,nsv881577,dgv705n27,esv2730198,esv2674155

5,ENST00000529453.1,ENST00000417502.1,ENST00000392482.2,ENST00000411390.2,ENST00000461487.1,ENST00000437067.1,ENST00000369070.1,
30833,nsv442016,nsv510937,nsv463999,esv2732689,nsv5427,nsv520490,nsv5372,esv2732725,nsv5375,esv2422431,esv2676114,nsv514378,esv

[illegible],esv1966970,dgv946n67,esv2674555,esv32808,esv1124892,nsv527103,esv2730023,nsv881311,esv274670,esv

ENST00000369123.3,ENST00000589192.1,ENST00000459080.1,ENST00000367748.1,ENST00000520034.1,ENST00000452973.
