# Supplementary material for: Genomic alterations caused by HPV integration in a cohort of Chinese endocervical adenocarcinomas
Source: Cancer Gene Ther. 2021 Jan 4;28(12):1353–64. doi: 10.1038/s41417-020-00283-4 (PMC8636260; doi:10.1038/s41417-020-00283-4)
Supplement: Supplementary file 12 — Supplementary Table 11 [file 41417_2020_283_MOESM12_ESM.docx]

Supplementary Table 11

Significantly mutated genes identified by whole exome sequencing. SNV, single nucleotide variants.

| **Gene** | **Indels** | **SNVs** | **Tot Muts*** | **Sample Affect** | **Sample Percent(%)** | **P-value CT†** | **FDR CT‡** |
| --- | --- | --- | --- | --- | --- | --- | --- |
| PIK3CA | 0 | 5 | 5 | 4 | 20 | 5.56E-10 | 1.07E-05 |
| NDN | 0 | 3 | 3 | 3 | 15 | 4.50E-07 | 0.004 |
| GOLGA6L4 | 0 | 3 | 3 | 3 | 15 | 2.72E-06 | 0.017 |
| BAIAP3 | 0 | 4 | 4 | 2 | 10 | 4.81E-06 | 0.023 |

*Tot Muts denotes the total mutations occurred in certain genes.

† P-value CT denotes p value obtained from hypothesis testing of convolution.

‡ FDR CT denotes corrected p value.
