# Supplementary material for: Genomic alterations caused by HPV integration in a cohort of Chinese endocervical adenocarcinomas
Source: Cancer Gene Ther. 2021 Jan 4;28(12):1353–64. doi: 10.1038/s41417-020-00283-4 (PMC8636260; doi:10.1038/s41417-020-00283-4)
Supplement: Supplementary file 13 — Supplementary Table 12 [file 41417_2020_283_MOESM13_ESM.docx]

Supplementary Table 12

Significantly mutated genes identified in the HPV+ group and the HPV- group. HPV, human papillomavirus.

| Group | Genes | Mutations | Sample Affect | P-value CT* | FDR CT† |
| --- | --- | --- | --- | --- | --- |
| HPV Integration | *GOLGA6L4* | 3 | 3/7 | 1.22E-06 | 0.014 |
| HPV Integration | *BAIAP3* | 4 | 2/7 | 1.42E-06 | 0.014 |
| No HPV Integration | *PIK3CA* | 4 | 3/13 | 7.98E-10 | 1.53E-05 |
| No HPV Integration | *NDN* | 3 | 3/13 | 3.78E-09 | 3.63E-05 |
| No HPV Integration | *KRAS* | 2 | 2/13 | 2.38E-05 | 0.095 |
| No HPV Integration | *FUT1* | 2 | 1/13 | 2.24E-05 | 0.095 |
| No HPV Integration | *GOLGA6L6* | 2 | 2/13 | 2.49E-05 | 0.095 |

* P-value CT means p value obtained from hypothesis testing of convolution.

† FDR CT means corrected p value.
