# Supplementary figures and images for: Genomic alterations caused by HPV integration in a cohort of Chinese endocervical adenocarcinomas
Source: Cancer Gene Ther. 2021 Jan 4;28(12):1353–64. doi: 10.1038/s41417-020-00283-4 (PMC8636260; doi:10.1038/s41417-020-00283-4)

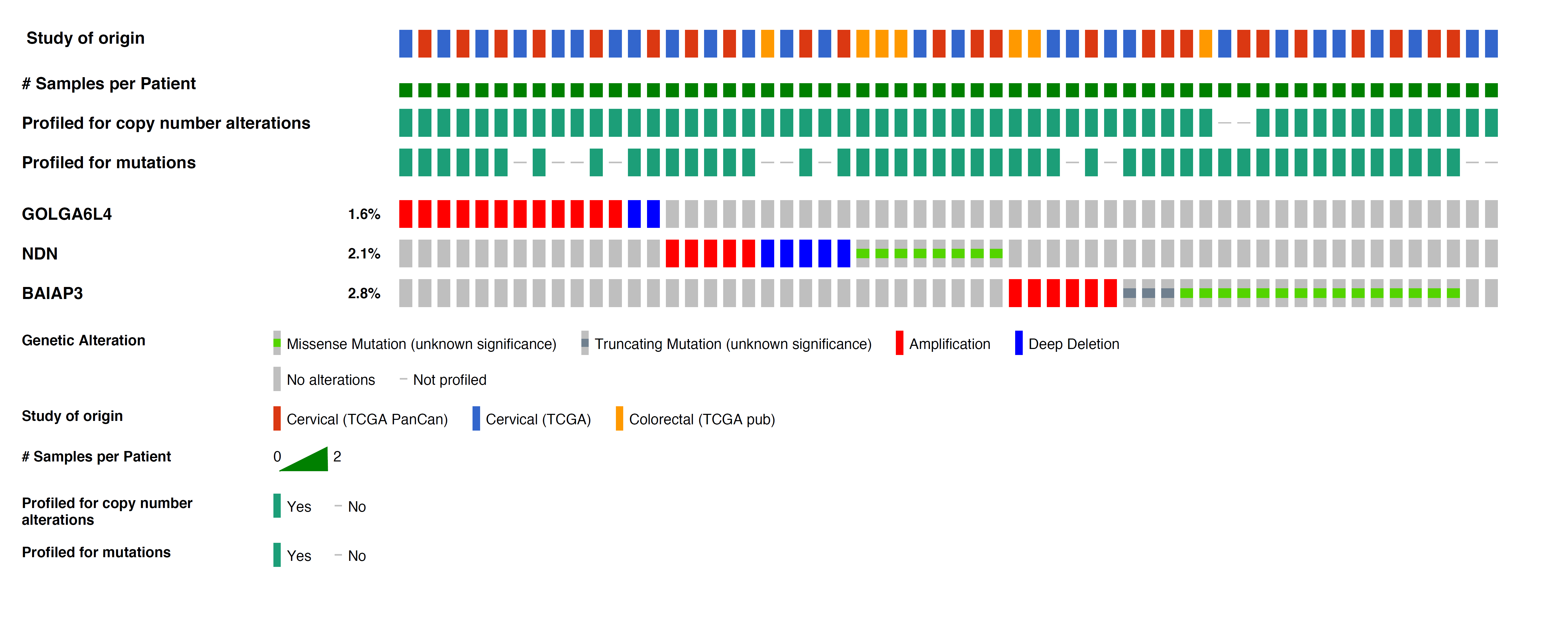

Supplement: Supplementary file 14 — Supplementary Figure 2 [file 41417_2020_283_MOESM14_ESM.tif]
