# Supplementary material for: Genomic alterations caused by HPV integration in a cohort of Chinese endocervical adenocarcinomas
Source: Cancer Gene Ther. 2021 Jan 4;28(12):1353–64. doi: 10.1038/s41417-020-00283-4 (PMC8636260; doi:10.1038/s41417-020-00283-4)
Supplement: Supplementary file 5 — Supplementary Table 4 [file 41417_2020_283_MOESM5_ESM.pdf]

Supplementary Table 4

Detail of somatic SNV detected in each case by whole genome sequencing.

Patient	CHRC	POS	ID	REF	ALT	QU	FILTER	GeneNa	Fun	Gen	Ge	ExonicF
ACa01	1	135040	rs6264213	T	C	.	PASS	LINC01(	ncR	NR_	.	.
ACa01	1	137263	rs8798212	A	G	.	PASS	LINC01(	ncR	NR_	.	.
ACa01	1	934378	.	C	T	.	PASS	HES4	UTF	NM_	NM	.
ACa01	1	8126760	rs2011489	C	T	.	PASS	.	inte	NM_	dis	.
ACa01	1	11589972	rs7706792	G	A	.	PASS	PTCHD.	exo	NM_	.	missens
ACa01	1	11856167	.	C	T	.	PASS	MTHFR	intro	NM_	.	.
ACa01	1	13036141	rs7573447	A	G	.	PASS	PRAME	intro	NM_	.	.
ACa01	1	16891786	.	G	T	.	PASS	NBPF1	intro	NM_	.	.
ACa01	1	16911827	rs643011	C	G	.	PASS	NBPF1	intro	NM_	.	.
ACa01	1	17007664	rs1891369	T	C	.	PASS	.	ups	NR_	.	.
ACa01	1	19071199	rs7967920	G	A	.	PASS	PAX7	intro	NM_	.	.
ACa01	1	28930226	.	A	C	.	PASS	TAF12	intro	NM_	.	.
ACa01	1	38433845	rs3699237	G	A	.	PASS	SF3A3	intro	NM_	.	.
ACa01	1	56673667	.	G	A	.	PASS	.	inte	NR_	dis	.
ACa01	1	60381458	.	A	C	.	PASS	CYP2J2	intro	NM_	.	.
ACa01	1	1.12E+08	.	G	A	.	PASS	CEPT1	intro	NM_	.	.
ACa01	1	1.21E+08	.	A	T	.	PASS	.	inte	NR_	dis	.
ACa01	1	1.43E+08	rs1809861	G	A	.	PASS	.	inte	NON	dis	.
ACa01	1	1.43E+08	rs3742168	A	C	.	PASS	.	inte	NR_	dis	.
ACa01	1	1.43E+08	rs1409433	T	C	.	PASS	.	inte	NR_	dis	.
ACa01	1	1.43E+08	rs7967822	G	A	.	PASS	.	ncR	NR_	.	.
ACa01	1	1.43E+08	rs7763460	C	G	.	PASS	.	ncR	NR_	.	.
ACa01	1	1.43E+08	.	A	T	.	PASS	.	inte	NR_	dis	.
ACa01	1	1.43E+08	rs1694653	C	G	.	PASS	.	inte	NR_	dis	.
ACa01	1	1.45E+08	rs1129555	G	A	.	PASS	NBPF8,	intro	NM_	.	.
ACa01	1	1.45E+08	rs3732644	C	T	.	PASS	NBPF8,	intro	NM_	.	.
ACa01	1	1.45E+08	.	T	C	.	PASS	PDE4DI	exo	NM_	.	missens
ACa01	1	1.45E+08	rs2691756	T	C	.	PASS	NBPF9,	intro	NM_	.	.
ACa01	1	1.45E+08	.	A	G	.	PASS	NBPF2!	ncR	NR_	.	.
ACa01	1	1.45E+08	.	G	T	.	PASS	NBPF2!	ncR	NR_	.	.
ACa01	1	1.46E+08	.	G	A	.	PASS	NBPF2!	ncR	NR_	.	.
ACa01	1	1.47E+08	rs1932337	C	T	.	PASS	.	inte	NR_	dis	.
ACa01	1	1.48E+08	.	C	G	.	PASS	NBPF8	intro	NM_	.	.
ACa01	1	1.48E+08	rs2837929	G	A	.	PASS	NBPF8	intro	NM_	.	.
ACa01	1	1.49E+08	rs2983273	A	C	.	PASS	.	inte	NR_	dis	.
ACa01	1	1.53E+08	rs3740307	C	T	.	PASS	PGLYRI	exo	NM_	.	synonyr
ACa01	1	1.62E+08	rs1674785	C	T	.	PASS	.	UTF	NM_	NM	.
ACa01	1	1.62E+08	rs1485348	T	G	.	PASS	FCGR2I	exo	NM_	.	missens
ACa01	1	1.67E+08	rs7598068	G	A	.	PASS	POU2F:	exo	NM_	.	missens
ACa01	1	2.1E+08	.	G	T	.	PASS	DIEXF	intro	NM_	.	.
ACa01	1	2.26E+08	.	A	C	.	PASS	LBR	intro	NM_	.	.
ACa01	1	2.38E+08	.	A	C	.	PASS	LOC10(	ncR	NR_	.	.
ACa01	1	2.43E+08	rs2006447	A	G	.	PASS	CEP17(	exo	NM_	.	synonyr
ACa01	1	2.49E+08	rs3739868	A	G	.	PASS	.	ups	NM_	.	.
ACa01	1	2.49E+08	rs1782199	G	T	.	PASS	.	ups	NM_	.	.
ACa01	2	83738306	rs3461159	C	A	.	PASS	.	inte	NR_	dis	.
ACa01	2	91744332	rs8689209	G	A	.	PASS	.	inte	NON	dis	.
ACa01	2	91837965	.	C	T	.	PASS	LOC65<	ncR	NR_	.	.
ACa01	2	1.03E+08	.	A	G	.	PASS	IL1R1	exo	NM_	.	missens
ACa01	2	1.14E+08	rs3877497	T	G	.	PASS	FAM13E	ncR	NR_	.	.

ACa01	2	1.25E+08	rs1037863	A	G	.	PASS	CNTNA	intro	NM_	.	.
ACa01	2	1.31E+08	rs1839843	T	C	.	PASS	POTEF	exo	NM_	.	synonyr
ACa01	2	1.31E+08	.	C	A	.	PASS	POTEF	exo	NM_	.	missens
ACa01	2	1.35E+08	.	C	T	.	PASS	MGAT5	intro	NM_	.	.
ACa01	2	1.45E+08	.	A	T	.	PASS	ZEB2	exo	NM_	.	missens
ACa01	2	1.74E+08	.	T	G	.	PASS	CDCA7	intro	NM_	.	.
ACa01	2	1.99E+08	.	C	T	.	PASS	MARS2	exo	NM_	.	missens
ACa01	2	2.09E+08	.	T	G	.	PASS	PLEKHI	exo	NM_	.	missens
ACa01	2	2.09E+08	rs7812258	C	T	.	PASS	PTH2R	intro	NM_	.	.
ACa01	2	2.2E+08	rs1010785	C	T	.	PASS	DNAJB2	intro	NM_	.	.
ACa01	2	2.34E+08	rs7526847	A	C	.	PASS	.	inte	NM_	dis	.
ACa01	3	15070036	.	T	G	.	PASS	NR2C2	intro	NM_	.	.
ACa01	3	15275297	.	T	G	.	PASS	CAPN7	intro	NM_	.	.
ACa01	3	20153132	.	C	G	.	PASS	KAT2B	exo	NM_	.	missens
ACa01	3	38050749	.	G	A	.	PASS	PLCD1	intro	NM_	.	.
ACa01	3	38753882	rs1450320	C	T	.	PASS	SCN10A	exo	NM_	.	missens
ACa01	3	38770232	rs1398610	C	T	.	PASS	SCN10A	exo	NM_	.	missens
ACa01	3	73673407	.	G	A	.	PASS	PDZRN	exo	NM_	.	synonyr
ACa01	3	97868559	rs1126455	G	T	.	PASS	OR5H14	exo	NM_	.	synonyr
ACa01	3	1.02E+08	.	C	G	.	PASS	NFKBIZ	intro	NM_	.	.
ACa01	3	1.02E+08	rs7541975	C	T	.	PASS	NFKBIZ	intro	NM_	.	.
ACa01	3	1.08E+08	rs3757741	G	A	.	PASS	IFT57	exo	NM_	.	missens
ACa01	3	1.92E+08	.	C	T	.	PASS	.	inte	NR_	dis	.
ACa01	3	1.95E+08	rs6228513	T	C	.	PASS	.	inte	NM_	dis	.
ACa01	3	1.95E+08	.	C	T	.	PASS	.	inte	NM_	dis	.
ACa01	4	49846	rs4970287	C	T	.	PASS	.	inte	NON	dis	.
ACa01	4	84398700	.	T	G	.	PASS	FAM175	intro	NM_	.	.
ACa01	4	1.21E+08	rs3764069	C	T	.	PASS	.	inte	NR_	dis	.
ACa01	4	1.33E+08	rs7963572	G	A	.	PASS	.	inte	NR_	dis	.
ACa01	4	1.45E+08	rs9542709	C	T	.	PASS	FREM3	exo	NM_	.	missens
ACa01	4	1.59E+08	rs3721454	C	T	.	PASS	FAM198	intro	NM_	.	.
ACa01	4	1.89E+08	.	C	T	.	PASS	TRIML1	exo	NM_	.	synonyr
ACa01	5	840233	rs649883	T	C	.	PASS	ZDHHC	intro	NM_	.	.
ACa01	5	1593082	rs1467072	G	A	.	PASS	SDHAP	ncR	NR_	.	.
ACa01	5	21196870	.	C	A	.	PASS	.	inte	NM_	dis	.
ACa01	5	21492197	rs1500138	A	G	.	PASS	GUSBP	ncR	NR_	.	.
ACa01	5	37299166	.	T	G	.	PASS	NUP155	intro	NM_	.	.
ACa01	5	65028907	rs7962294	T	C	.	PASS	NLN	intro	NM_	.	.
ACa01	5	69784758	.	C	G	.	PASS	LOC441	ncR	NR_	.	.
ACa01	5	1.02E+08	.	A	G	.	PASS	PPIP5K	intro	NM_	.	.
ACa01	5	1.11E+08	rs7609290	C	T	.	PASS	CAMK4	exo	NM_	.	synonyr
ACa01	5	1.34E+08	.	C	T	.	PASS	PPP2CA	intro	NM_	.	.
ACa01	5	1.41E+08	rs7647428	C	T	.	PASS	PCDHG	exo	NM_	.	missens
ACa01	5	1.76E+08	rs1407436	A	C	.	PASS	.	inte	NR_	dis	.
ACa01	5	1.81E+08	rs7380674	G	C	.	PASS	.	inte	NR_	dis	.
ACa01	6	7517870	rs5568001	G	A	.	PASS	.	inte	NM_	dis	.
ACa01	6	52656696	rs3977792	T	G	.	PASS	GSTA1	exo	NM_	.	missens
ACa01	6	57841232	.	T	C	.	PASS	.	inte	NM_	dis	.
ACa01	6	79724810	.	A	G	.	PASS	PHIP	exo	NM_	.	missens
ACa01	6	80663139	rs9844362	C	T	.	PASS	.	inte	NM_	dis	.
ACa01	6	1.01E+08	.	T	A	.	PASS	.	inte	NM_	dis	.
ACa01	6	1.08E+08	rs9992807	T	C	.	PASS	PDSS2	intro	NM_	.	.
ACa01	6	1.09E+08	.	C	T	.	PASS	ARMC2	exo	NM_	.	missens
ACa01	6	1.34E+08	rs7554849	G	A	.	PASS	SLC2A1	exo	NM_	.	synonyr

ACa01	6	1.45E+08	.	G	A	.	PASS	UTRN	intrc	NM_	.	.
ACa01	6	1.47E+08	rs7747387	C	T	.	PASS	ADGB	exoı	NM_	.	synonyr
ACa01	6	1.5E+08	rs1467177	C	T	.	PASS	RAET1L	intrc	NM_	.	.
ACa01	7	2418057	.	G	T	.	PASS	EIF3B	intrc	NM_	.	.
ACa01	7	17904468	rs7308136	G	C	.	PASS	SNX13	intrc	NM_	.	.
ACa01	7	44149192	.	G	A	.	PASS	AEBP1	intrc	NM_	.	.
ACa01	7	56439839	.	C	T	.	PASS	.	inteı	NM_	dis	.
ACa01	7	56891402	rs7911953	C	T	.	PASS	.	inteı	NR_	dis	.
ACa01	7	56891487	.	G	C	.	PASS	.	inteı	NR_	dis	.
ACa01	7	57008817	.	C	T	.	PASS	.	inteı	NR_	dis	.
ACa01	7	57529608	.	A	C	.	PASS	ZNF716	exoı	NM_	.	missens
ACa01	7	61970351	rs4311554	G	T	.	PASS	.	inteı	NON	dis	.
ACa01	7	63019476	.	G	T	.	PASS	.	inteı	NR_	dis	.
ACa01	7	63096227	.	G	A	.	PASS	.	inteı	NR_	dis	.
ACa01	7	63210059	rs5467260	G	A	.	PASS	.	inteı	NR_	dis	.
ACa01	7	63210067	.	C	G	.	PASS	.	inteı	NR_	dis	.
ACa01	7	63213633	rs8688020	G	A	.	PASS	.	inteı	NR_	dis	.
ACa01	7	74237397	rs8799757	G	T	.	PASS	GTF2IR	intrc	NM_	.	.
ACa01	7	92845041	rs7686126	G	A	.	PASS	HEPAC.	intrc	NM_	.	.
ACa01	7	1.02E+08	rs3727203	G	C	.	PASS	POLR2.	intrc	NM_	.	.
ACa01	7	1.25E+08	rs7809364	G	A	.	PASS	POT1	exoı	NM_	.	missens
ACa01	7	1.3E+08	.	G	A	.	PASS	SSMEM	exoı	NM_	.	synonyr
ACa01	7	1.52E+08	rs7754267	A	G	.	PASS	KMT2C	exoı	NM_	.	synonyr
ACa01	7	1.52E+08	rs1499922	C	T	.	PASS	KMT2C	exoı	NM_	.	missens
ACa01	7	1.52E+08	.	C	T	.	PASS	KMT2C	UTF	NM_	NM	.
ACa01	7	1.59E+08	rs1407758	C	T	.	PASS	.	inteı	NM_	dis	.
ACa01	7	1.59E+08	rs1482637	T	C	.	PASS	.	inteı	NM_	dis	.
ACa01	8	7877648	.	C	A	.	PASS	.	inteı	NR_	dis	.
ACa01	8	17570524	.	A	G	.	PASS	MTUS1	intrc	NM_	.	.
ACa01	8	21859882	.	A	C	.	PASS	XPO7	intrc	NM_	.	.
ACa01	8	25296822	.	T	G	.	PASS	KCTD9	exoı	NM_	.	missens
ACa01	8	39742519	rs1813428	G	A	.	PASS	.	inteı	NM_	dis	.
ACa01	8	43822531	rs2022368	A	G	.	PASS	.	inteı	NM_	dis	.
ACa01	8	73137623	.	T	C	.	PASS	LOC392	ncR	NR_	.	.
ACa01	8	82588523	rs1837720	A	T	.	PASS	IMPA1	intrc	NM_	.	.
ACa01	9	39174437	rs1329860	T	C	.	PASS	CNTNA	intrc	NM_	.	.
ACa01	9	65602674	rs2954515	T	C	.	PASS	.	inteı	NM_	dis	.
ACa01	9	66500520	rs1968548	C	A	.	PASS	PTGER	ncR	NR_	.	.
ACa01	9	66501146	rs2019611	G	A	.	PASS	PTGER	ncR	NR_	.	.
ACa01	9	68415282	rs4928839	A	C	.	PASS	.	inteı	NM_	dis	.
ACa01	9	96051030	.	G	A	.	PASS	WNK2	intrc	NM_	.	.
ACa01	9	1.16E+08	rs2868900	G	A	.	PASS	.	inteı	NM_	dis	.
ACa01	9	1.31E+08	.	C	T	.	PASS	ENG	exoı	NM_	.	synonyr
ACa01	9	1.33E+08	.	T	G	.	PASS	FNBP1	exoı	NM_	.	synonyr
ACa01	9	1.36E+08	.	A	G	.	PASS	ADAMT	intrc	NM_	.	.
ACa01	9	1.37E+08	rs7694907	C	T	.	PASS	DBH	exoı	NM_	.	synonyr
ACa01	10	14909181	.	G	A	.	PASS	HSPA14	exoı	NM_	.	missens
ACa01	10	15177202	rs1051909	T	G	.	PASS	NMT2	intrc	NM_	.	.
ACa01	10	26443696	.	C	T	.	PASS	MYO3A	exoı	NM_	.	missens
ACa01	10	26491918	.	G	A	.	PASS	MYO3A	exoı	NM_	.	missens
ACa01	10	28260161	rs7531795	G	A	.	PASS	ARMC4	exoı	NM_	.	unknow
ACa01	10	29822153	rs5566342	C	T	.	PASS	SVIL	exoı	NM_	.	synonyr
ACa01	10	37442619	rs1759036	G	T	.	PASS	ANKRD	intrc	NM_	.	.
ACa01	10	38356181	.	C	T	.	PASS	.	inteı	NM_	dis	.

ACa01	10	38938279	rs1494650	A	C	.	PASS	.	inte	NR_	dis	.
ACa01	10	38990567	rs5524339	C	T	.	PASS	ACTR3	ncR	NR_	.	.
ACa01	10	47207765	rs7827248	T	A	.	PASS	AGAP9	intr	NM_	.	.
ACa01	10	48389068	rs7821206	C	T	.	PASS	RBP3	exo	NM_	.	missens
ACa01	10	62498154	.	C	A	.	PASS	.	inte	NM_	dis	.
ACa01	10	69991321	.	C	T	.	PASS	ATOH7	exo	NM_	.	synony
ACa01	10	70451076	rs1810812	C	T	.	PASS	TET1	exo	NM_	.	synony
ACa01	10	70646055	rs1852770	G	A	.	PASS	STOX1	exo	NM_	.	missens
ACa01	10	75457579	.	G	T	.	PASS	.	ups	NM_	.	.
ACa01	10	90983620	.	A	C	.	PASS	LIPA	intr	NM_	.	.
ACa01	10	98510422	rs9002476	C	T	.	PASS	.	inte	NM_	dis	.
ACa01	10	1.06E+08	.	A	G	.	PASS	COL17	intr	NM_	.	.
ACa01	10	1.17E+08	rs1028763	G	A	.	PASS	TRUB1	intr	NM_	.	.
ACa01	11	4880803	.	G	T	.	PASS	.	inte	NM_	dis	.
ACa01	11	28332552	rs7615769	A	T	.	PASS	METTL	intr	NM_	.	.
ACa01	11	36514292	.	G	A	.	PASS	TRAF6	intr	NM_	.	.
ACa01	11	49071349	.	T	G	.	PASS	.	inte	NM_	dis	.
ACa01	11	60539049	.	G	A	.	PASS	MS4A1	intr	NM_	.	.
ACa01	11	76062520	.	T	A	.	PASS	PRKRI	exo	NM_	.	missens
ACa01	11	77614843	.	T	G	.	PASS	INTS4	intr	NM_	.	.
ACa01	11	77614844	.	T	G	.	PASS	INTS4	intr	NM_	.	.
ACa01	11	77916959	rs7614089	C	T	.	PASS	USP35	exo	NM_	.	synony
ACa01	11	93778909	rs1438612	C	T	.	PASS	HEPHL	exo	NM_	.	missens
ACa01	11	1.12E+08	.	G	T	.	PASS	SIK2	exo	NM_	.	synony
ACa01	11	1.24E+08	rs7757345	C	T	.	PASS	OR10S	exo	NM_	.	missens
ACa01	11	1.24E+08	rs2512286	T	C	.	PASS	.	inte	NM_	dis	.
ACa01	12	68657	rs5382343	T	C	.	PASS	.	ncR	NR_	.	.
ACa01	12	88453	rs8799828	C	G	.	PASS	LOC10	ncR	NR_	.	.
ACa01	12	330603	.	A	C	.	PASS	SLC6A1	exo	NM_	.	missens
ACa01	12	1100428	rs8681434	T	C	.	PASS	ERC1	UT	NM_	NM	.
ACa01	12	31353627	rs2575282	A	G	.	PASS	.	inte	NM_	dis	.
ACa01	12	41871792	.	G	A	.	PASS	PDZRN	intr	NM_	.	.
ACa01	12	46589903	.	G	C	.	PASS	SLC38A	UT	NM_	NM	.
ACa01	12	51693046	.	C	T	.	PASS	BIN2	exo	NM_	.	synony
ACa01	12	68946858	.	A	C	.	PASS	.	inte	NR_	dis	.
ACa01	12	75700028	.	G	A	.	PASS	CAPS2	intr	NM_	.	.
ACa01	12	1.05E+08	rs1043870	C	T	.	PASS	CHST1	exo	NM_	.	synony
ACa01	12	1.11E+08	.	C	T	.	PASS	CCDC6	intr	NM_	.	.
ACa01	12	1.2E+08	.	C	T	.	PASS	CIT	intr	NM_	.	.
ACa01	13	58299300	.	C	A	.	PASS	PCDH1	exo	NM_	.	missens
ACa01	14	19475264	.	G	A	.	PASS	.	inte	NR_	dis	.
ACa01	14	19475298	rs2014705	C	T	.	PASS	.	inte	NR_	dis	.
ACa01	14	19720608	rs8794601	G	A	.	PASS	.	inte	NR_	dis	.
ACa01	14	19962075	.	C	T	.	PASS	.	inte	NR_	dis	.
ACa01	14	20145911	rs8674109	G	T	.	PASS	.	inte	NM_	dis	.
ACa01	14	20897283	.	C	T	.	PASS	KLHL33	exo	NM_	.	missens
ACa01	14	22958314	.	A	C	.	PASS	.	inte	NM_	dis	.
ACa01	14	22958315	.	A	C	.	PASS	.	inte	NM_	dis	.
ACa01	14	39628570	.	G	A	.	PASS	TRAPP	intr	NM_	.	.
ACa01	14	53173723	.	G	A	.	PASS	.	ups	NM_	.	.
ACa01	14	58917202	.	T	G	.	PASS	KIAA05	intr	NM_	.	.
ACa01	14	64687233	rs7732738	G	A	.	PASS	SYNE2	exo	NM_	.	missens
ACa01	14	1.01E+08	.	G	T	.	PASS	WDR25	intr	NM_	.	.
ACa01	14	1.06E+08	rs2897118	C	T	.	PASS	.	inte	NM_	dis	.

ACa01	14	1.06E+08	rs3681255	T	A	.	PASS	.	ups	NR_	.	.
ACa01	14	1.07E+08	rs6199969	T	C	.	PASS	.	inte	NR_	dis	.
ACa01	15	20433937	.	C	A	.	PASS	.	inte	NON	dis	.
ACa01	15	20446879	.	T	G	.	PASS	.	inte	NON	dis	.
ACa01	15	20832999	rs3749240	G	A	.	PASS	.	inte	NR_	dis	.
ACa01	15	21009303	.	C	T	.	PASS	.	inte	NR_	dis	.
ACa01	15	22015966	.	C	T	.	PASS	CXADR	ncR	NR_	.	.
ACa01	15	22705227	.	C	T	.	PASS	GOLGA	ncR	NR_	.	.
ACa01	15	23377558	rs7964394	T	C	.	PASS	HERC2I	ncR	NR_	.	.
ACa01	15	23402623	.	A	G	.	PASS	.	inte	NR_	dis	.
ACa01	15	23512812	rs7126232	C	T	.	PASS	.	intro	NM_	.	.
ACa01	15	24922007	.	G	A	.	PASS	NPAP1	exo	NM_	.	synonyr
ACa01	15	25958879	.	G	A	.	PASS	ATP10A	exo	NM_	.	synonyr
ACa01	15	28952979	rs7695754	C	T	.	PASS	GOLGA	exo	NM_	.	missens
ACa01	15	29367166	.	G	A	.	PASS	APBA2	exo	NM_	.	missens
ACa01	15	30874998	.	T	C	.	PASS	ULK4P2	ncR	NR_	.	.
ACa01	15	31092738	.	C	A	.	PASS	.	inte	NR_	dis	.
ACa01	15	32738450	rs8658676	G	A	.	PASS	GOLGA	intro	NM_	.	.
ACa01	15	32738540	.	G	T	.	PASS	GOLGA	intro	NM_	.	.
ACa01	15	40650467	rs3683923	G	A	.	PASS	DISP2	UTF	NM_	NM	.
ACa01	15	62315589	rs3741666	G	A	.	PASS	VPS13C	intro	NM_	.	.
ACa01	15	80050960	.	C	T	.	PASS	.	inte	NM_	dis	.
ACa01	15	84859986	rs5743805	G	A	.	PASS	.	ups	NR_	.	.
ACa01	15	84908877	rs1998088	A	G	.	PASS	GOLGA	exo	NM_	.	synonyr
ACa01	15	84909416	rs3775649	A	G	.	PASS	GOLGA	exo	NM_	.	missens
ACa01	15	1.03E+08	rs6202866	A	G	.	PASS	WASH3	ncR	NR_	.	.
ACa01	16	319032	rs7561426	G	A	.	PASS	RGS11	UTF	NM_	NM	.
ACa01	16	21732577	.	C	T	.	PASS	OTOA	intro	NM_	.	.
ACa01	16	30196569	rs7464587	C	T	.	PASS	CORO1	exo	NM_	.	synonyr
ACa01	16	31091068	.	G	A	.	PASS	ZNF646	exo	NM_	.	synonyr
ACa01	16	32437180	rs2018870	C	T	.	PASS	.	inte	NR_	dis	.
ACa01	16	46388675	rs4643347	T	C	.	PASS	.	inte	NON	dis	.
ACa01	16	46392276	rs4535017	T	C	.	PASS	.	inte	NON	dis	.
ACa01	16	46393953	rs2128471	T	C	.	PASS	.	inte	NON	dis	.
ACa01	16	46394039	rs2888787	C	G	.	PASS	.	inte	NON	dis	.
ACa01	16	46395236	rs4355081	T	A	.	PASS	.	inte	NON	dis	.
ACa01	16	46395893	rs4249097	T	C	.	PASS	.	inte	NON	dis	.
ACa01	16	46399631	rs4232920	C	T	.	PASS	.	inte	NON	dis	.
ACa01	16	46401781	rs7970299	T	G	.	PASS	.	inte	NON	dis	.
ACa01	16	46401891	rs1483332	A	T	.	PASS	.	inte	NON	dis	.
ACa01	16	46402061	rs9972801	A	G	.	PASS	.	inte	NON	dis	.
ACa01	16	46402155	rs2878607	A	T	.	PASS	.	inte	NON	dis	.
ACa01	16	46402193	rs4249050	C	G	.	PASS	.	inte	NON	dis	.
ACa01	16	46405336	rs4250282	T	C	.	PASS	.	inte	NON	dis	.
ACa01	16	46405873	rs7198709	T	C	.	PASS	.	inte	NON	dis	.
ACa01	16	46407028	rs4445923	A	T	.	PASS	.	inte	NON	dis	.
ACa01	16	46407951	rs4450416	A	G	.	PASS	.	inte	NON	dis	.
ACa01	16	46408218	.	G	A	.	PASS	.	inte	NON	dis	.
ACa01	16	46408282	rs8056404	C	G	.	PASS	.	inte	NON	dis	.
ACa01	16	46420425	rs8056672	A	C	.	PASS	.	inte	NON	dis	.
ACa01	16	46421747	rs6152895	G	A	.	PASS	.	inte	NON	dis	.
ACa01	16	46421915	rs2884801	T	C	.	PASS	.	inte	NON	dis	.
ACa01	16	53844261	.	A	C	.	PASS	FTO	intro	NM_	.	.
ACa01	16	67063672	.	C	T	.	PASS	CBFB	exo	NM_	.	stopgai

ACa01	16	70237719	.	C	G	.	PASS	.	inte	NM_	dis	.
ACa01	16	88493891	.	C	T	.	PASS	ZNF469	exo	NM_	.	missens
ACa01	17	1578542	rs7801100	G	A	.	PASS	PRPF8	exo	NM_	.	synony
ACa01	17	3343563	.	T	C	.	PASS	SPATA	exo	NM_	.	missens
ACa01	17	7814784	rs3687584	G	A	.	PASS	CHD3	exo	NM_	.	missens
ACa01	17	11778655	.	A	C	.	PASS	DNAH9	intr	NM_	.	.
ACa01	17	15690511	rs1845094	C	T	.	PASS	MEIS3P	ncR	NR_	.	.
ACa01	17	18045131	.	G	A	.	PASS	MYO15	intr	NM_	.	.
ACa01	17	21904565	.	G	T	.	PASS	FLJ360	ncR	NR_	.	.
ACa01	17	21906266	.	C	G	.	PASS	FLJ360	ncR	NR_	.	.
ACa01	17	21906963	rs4503851	G	T	.	PASS	FLJ360	ncR	NR_	.	.
ACa01	17	22252147	rs1462263	C	A	.	PASS	.	inte	NM_	dis	.
ACa01	17	22253154	rs4362423	T	G	.	PASS	.	inte	NM_	dis	.
ACa01	17	22253158	rs6565430	T	G	.	PASS	.	inte	NM_	dis	.
ACa01	17	22253167	rs1434103	G	A	.	PASS	.	inte	NM_	dis	.
ACa01	17	22254209	.	G	C	.	PASS	.	inte	NM_	dis	.
ACa01	17	26348946	rs8916657	T	A	.	PASS	.	inte	NM_	dis	.
ACa01	17	37426807	rs1167082	A	T	.	PASS	FBXL20	intr	NM_	.	.
ACa01	17	40123777	.	T	G	.	PASS	CNP	intr	NM_	.	.
ACa01	17	40762339	rs1459625	G	A	.	PASS	TUBG1	intr	NM_	.	.
ACa01	17	43663933	rs1724395	T	C	.	PASS	.	inte	NR_	dis	.
ACa01	17	56690735	.	G	A	.	PASS	TEX14	intr	NM_	.	.
ACa01	17	66535309	.	T	G	.	PASS	FAM20	intr	NM_	.	.
ACa01	17	72522246	.	A	C	.	PASS	CD300L	intr	NM_	.	.
ACa01	18	5397057	.	C	T	.	PASS	EPB41L	exo	NM_	.	synony
ACa01	18	11633266	rs5374020	T	C	.	PASS	.	inte	NM_	dis	.
ACa01	18	11644538	rs2012257	C	G	.	PASS	.	inte	NM_	dis	.
ACa01	19	36759691	.	G	A	.	PASS	.	inte	NM_	dis	.
ACa01	19	37785301	rs7125437	C	G	.	PASS	.	inte	NR_	dis	.
ACa01	19	37785451	.	C	T	.	PASS	.	inte	NR_	dis	.
ACa01	19	41631873	.	A	G	.	PASS	CYP2F1	intr	NM_	.	.
ACa01	19	42012297	.	G	T	.	PASS	.	inte	NR_	dis	.
ACa01	19	42620764	.	G	A	.	PASS	POU2F	intr	NM_	.	.
ACa01	19	43858238	.	G	A	.	PASS	CD177	intr	NM_	.	.
ACa01	19	49439523	.	G	T	.	PASS	DHDH	intr	NM_	.	.
ACa01	19	56538348	.	G	A	.	PASS	NLRP5	exo	NM_	.	missens
ACa01	19	57522507	rs8677343	T	C	.	PASS	.	inte	NR_	dis	.
ACa01	20	34568263	.	G	A	.	PASS	CNBD2	intr	NM_	.	.
ACa01	20	36770283	.	C	T	.	PASS	TGM2	intr	NM_	.	.
ACa01	21	9752424	rs8795581	C	T	.	PASS	.	inte	NO	dis	.
ACa01	21	9907668	rs8661396	G	A	.	PASS	TEKT4	ncR	NR_	.	.
ACa01	21	10624265	.	G	T	.	PASS	.	inte	NR_	dis	.
ACa01	21	11040737	rs7532385	G	A	.	PASS	BAGE2,	intr	NM_	.	.
ACa01	21	11047253	rs3737231	A	T	.	PASS	BAGE2,	intr	NM_	.	.
ACa01	21	11114884	.	G	A	.	PASS	.	inte	NM_	dis	.
ACa01	22	16272032	rs2003402	C	A	.	PASS	POTEH	intr	NM_	.	.
ACa01	22	16352391	rs2818474	G	A	.	PASS	.	inte	NM_	dis	.
ACa01	22	17976597	rs5464921	C	T	.	PASS	CECR2	exo	NM_	.	unknow
ACa01	22	24621744	rs9350299	A	G	.	PASS	GGT5	intr	NM_	.	.
ACa01	22	24644673	rs4050109	C	G	.	PASS	.	inte	NM_	dis	.
ACa01	22	24644674	.	T	G	.	PASS	.	inte	NM_	dis	.
ACa01	22	25054076	.	A	C	.	PASS	POM12	ncR	NR_	.	.
ACa01	22	26294165	.	C	T	.	PASS	MYO18	intr	NM_	.	.
ACa01	22	29907312	.	A	C	.	PASS	THOC5	intr	NM_	.	.

ACa01	22	30782805	.	G	A	.	PASS	RNF21	intr	NM_	.	.
ACa01	22	42536924	rs1425675	C	T	.	PASS	CYP2D	ncR	NR_	.	.
ACa01	22	46761386	rs1054069	G	A	.	PASS	CELSR	intr	NM_	.	.
ACa01	22	46858756	.	G	A	.	PASS	CELSR	intr	NM_	.	.
ACa01	X	7811224	rs7661994	C	T	.	PASS	VCX	UT	NM_	NM	.
ACa01	X	9377823	.	C	T	.	PASS	.	inte	NM_	dis	.
ACa01	X	25087714	.	T	C	.	PASS	.	inte	NM_	dis	.
ACa01	X	48888030	.	G	A	.	PASS	TFE3	exo	NM_	.	missen
ACa01	X	48890958	.	G	A	.	PASS	TFE3	intr	NM_	.	.
ACa01	X	49084983	.	C	T	.	PASS	CACNA	intr	NM_	.	.
ACa01	X	50653733	rs7822715	C	T	.	PASS	.	ups	NM_	.	.
ACa01	X	53112190	rs7821690	C	T	.	PASS	TSPYL	exo	NM_	.	synony
ACa01	X	61718822	.	A	T	.	PASS	.	inte	NO	dis	.
ACa01	X	66789118	.	T	G	.	PASS	AR	intr	NM_	.	.
ACa01	X	76712110	.	C	T	.	PASS	.	dow	NM_	.	.
ACa01	X	80064380	.	C	T	.	PASS	BRWD	intr	NM_	.	.
ACa01	X	92477284	.	C	T	.	PASS	.	inte	NM_	dis	.
ACa01	X	1E+08	.	G	A	.	PASS	CSTF2	exo	NM_	.	synony
ACa01	X	1.03E+08	rs7818697	G	A	.	PASS	ZCCHC	exo	NM_	.	missen
ACa01	X	1.32E+08	.	C	T	.	PASS	HS6ST	exo	NM_	.	missen
ACa01	X	1.39E+08	.	C	T	.	PASS	MCF2	exo	NM_	.	missen
ACa01	X	1.5E+08	.	C	T	.	PASS	CD99L2	intr	NM_	.	.
ACa01	MT	7830	rs8788971	G	A	.	PASS	.	inte	NO	dis	.
ACa02	1	11317067	.	C	T	.	PASS	MTOR	exo	NM_	.	missen
ACa02	1	11317177	.	C	T	.	PASS	MTOR	exo	NM_	.	missen
ACa02	1	12898846	rs5506852	G	A	.	PASS	.	inte	NM_	dis	.
ACa02	1	12921642	rs2994093	G	A	.	PASS	PRAME	UT	NM_	NM	.
ACa02	1	13413890	.	C	T	.	PASS	.	intr	NM_	.	.
ACa02	1	17024409	rs1390932	C	T	.	PASS	ESPNP	ncR	NR_	.	.
ACa02	1	20992955	.	A	C	.	PASS	KIF17	intr	NM_	.	.
ACa02	1	52867128	rs3686746	G	A	.	PASS	ORC1	exo	NM_	.	synony
ACa02	1	70625174	.	T	C	.	PASS	LRRC4	intr	NM_	.	.
ACa02	1	91384072	.	T	C	.	PASS	ZNF644	intr	NM_	.	.
ACa02	1	1.11E+08	.	C	T	.	PASS	KCNA2	exo	NM_	.	synony
ACa02	1	1.43E+08	rs2010827	G	C	.	PASS	.	inte	NO	dis	.
ACa02	1	1.43E+08	.	C	A	.	PASS	.	inte	NO	dis	.
ACa02	1	1.43E+08	rs2018849	T	G	.	PASS	.	inte	NO	dis	.
ACa02	1	1.43E+08	rs3724010	A	T	.	PASS	.	inte	NR_	dis	.
ACa02	1	1.43E+08	.	T	C	.	PASS	.	inte	NR_	dis	.
ACa02	1	1.43E+08	rs3772379	G	C	.	PASS	.	inte	NR_	dis	.
ACa02	1	1.44E+08	rs8799699	G	A	.	PASS	NBPF2	intr	NM_	.	.
ACa02	1	1.45E+08	.	C	A	.	PASS	NBPF9,	intr	NM_	.	.
ACa02	1	1.45E+08	.	A	C	.	PASS	NBPF8,	intr	NM_	.	.
ACa02	1	1.45E+08	rs4649849	A	G	.	PASS	NBPF9,	intr	NM_	.	.
ACa02	1	1.45E+08	rs8799650	A	C	.	PASS	NBPF2	ncR	NR_	.	.
ACa02	1	1.45E+08	rs7668601	C	G	.	PASS	NBPF2	ncR	NR_	.	.
ACa02	1	1.45E+08	rs7823737	C	T	.	PASS	NBPF2	ncR	NR_	.	.
ACa02	1	1.48E+08	.	C	T	.	PASS	NBPF8	intr	NM_	.	.
ACa02	1	1.48E+08	rs2003436	G	A	.	PASS	NBPF8	intr	NM_	.	.
ACa02	1	1.48E+08	rs6181174	G	A	.	PASS	NBPF8	intr	NM_	.	.
ACa02	1	1.48E+08	rs6181174	G	A	.	PASS	NBPF8	intr	NM_	.	.
ACa02	1	1.73E+08	.	A	C	.	PASS	LOC10	ncR	NR_	.	.
ACa02	1	1.75E+08	rs7494818	C	T	.	PASS	TNR	exo	NM_	.	missen
ACa02	1	1.79E+08	.	C	T	.	PASS	SOAT1	intr	NM_	.	.

ACa02	1	1.79E+08	rs8790889	C	A	.	PASS	AXDND	intrc	NM_	.	.
ACa02	1	2.2E+08	.	G	T	.	PASS	RNU5F-	ncR	NR_	.	.
ACa02	1	2.28E+08	.	G	A	.	PASS	ZNF678	intrc	NM_	.	.
ACa02	1	2.28E+08	.	A	G	.	PASS	.	inte	NM_	dis	.
ACa02	1	2.41E+08	.	C	A	.	PASS	RGS7	intrc	NM_	.	.
ACa02	2	24920686	.	A	G	.	PASS	NCOA1	intrc	NM_	.	.
ACa02	2	87581132	rs1020943	C	A	.	PASS	.	inte	NR_	dis	.
ACa02	2	91836143	.	C	T	.	PASS	LOC654	ncR	NR_	.	.
ACa02	2	91896050	rs2004118	A	T	.	PASS	.	inte	NR_	dis	.
ACa02	2	96561474	rs2021466	A	T	.	PASS	.	inte	NR_	dis	.
ACa02	2	97875309	rs3694799	T	C	.	PASS	ANKRD	intrc	NM_	.	.
ACa02	2	1.08E+08	rs5416674	G	A	.	PASS	RGPD4	exoı	NM_	.	missens
ACa02	2	1.09E+08	rs1053772	A	C	.	PASS	LIMS1	intrc	NM_	.	.
ACa02	2	1.14E+08	.	C	T	.	PASS	WASH2	ncR	NR_	.	.
ACa02	2	1.32E+08	rs5382277	C	T	.	PASS	RNU6-8	ncR	NR_	.	.
ACa02	2	1.61E+08	.	G	C	.	PASS	7-Mar	intrc	NM_	.	.
ACa02	2	1.7E+08	rs1875087	G	A	.	PASS	BBS5	intrc	NM_	.	.
ACa02	2	1.8E+08	.	G	T	.	PASS	TTN	exoı	NM_	.	missens
ACa02	2	2.04E+08	.	A	G	.	PASS	CARF	intrc	NM_	.	.
ACa02	2	2.16E+08	.	T	C	.	PASS	ATIC	UTF	NM_	NM	.
ACa02	2	2.28E+08	rs1509794	C	T	.	PASS	COL4A4	exoı	NM_	.	missens
ACa02	2	2.34E+08	.	C	T	.	PASS	NGEF	exoı	NM_	.	missens
ACa02	2	2.43E+08	rs7970263	T	C	.	PASS	.	inte	NR_	dis	.
ACa02	3	4733029	.	G	A	.	PASS	ITPR1	splic	NM_	NM	.
ACa02	3	31677754	.	G	A	.	PASS	.	UTF	NM_	NM	.
ACa02	3	49723881	rs6777426	G	C	.	PASS	MST1	exoı	NM_	.	missens
ACa02	3	49724014	rs6779963	G	A	.	PASS	MST1	intrc	NM_	.	.
ACa02	3	69225746	rs5472981	G	A	.	PASS	FRMD4	exoı	NM_	.	unknow
ACa02	3	69988398	.	C	T	.	PASS	MITF	intrc	NM_	.	.
ACa02	3	1.25E+08	.	C	G	.	PASS	.	inte	NM_	dis	.
ACa02	3	1.79E+08	rs1219132	G	A	.	PASS	PIK3CA	exoı	NM_	.	missens
ACa02	3	1.79E+08	.	C	G	.	PASS	MFN1	intrc	NM_	.	.
ACa02	3	1.91E+08	.	A	G	.	PASS	GMNC	exoı	NM_	.	synonyr
ACa02	3	1.95E+08	.	G	T	.	PASS	.	ncR	NR_	.	.
ACa02	3	1.97E+08	rs4857535	A	G	.	PASS	LOC220	ncR	NR_	.	.
ACa02	4	1230342	.	C	T	.	PASS	CTBP1	intrc	NM_	.	.
ACa02	4	9212542	.	G	T	.	PASS	USP17L	exoı	NM_	.	missens
ACa02	4	9241015	.	G	T	.	PASS	.	inte	NM_	dis	.
ACa02	4	9245326	rs7961122	C	G	.	PASS	.	ups	NM_	.	.
ACa02	4	39328028	.	C	T	.	PASS	MIR127	ncR	NR_	.	.
ACa02	4	49539207	.	A	G	.	PASS	.	inte	NM_	dis	.
ACa02	4	54794073	.	G	A	.	PASS	.	inte	NR_	dis	.
ACa02	4	81123234	rs3705413	C	T	.	PASS	PRDM8	exoı	NM_	.	synonyr
ACa02	4	1E+08	.	C	T	.	PASS	ADH1C	exoı	NM_	.	unknow
ACa02	4	1.04E+08	.	T	C	.	PASS	SLC9B2	UTF	NM_	NM	.
ACa02	4	1.14E+08	.	G	A	.	PASS	ANK2	intrc	NM_	.	.
ACa02	4	1.45E+08	rs2016625	T	G	.	PASS	GYPB	exoı	NM_	.	missens
ACa02	4	1.77E+08	.	G	A	.	PASS	WDR17	intrc	NM_	.	.
ACa02	4	1.89E+08	rs7619626	C	T	.	PASS	ZFP42	exoı	NM_	.	synonyr
ACa02	5	7301667	rs8684061	C	G	.	PASS	LOC442	ncR	NR_	.	.
ACa02	5	14692852	.	C	T	.	PASS	OTULIN	intrc	NM_	.	.
ACa02	5	17527667	.	G	A	.	PASS	.	inte	NR_	dis	.
ACa02	5	34182503	.	G	A	.	PASS	.	inte	NR_	dis	.
ACa02	5	49410264	rs8659304	A	G	.	PASS	.	inte	NON	dis	.

ACa02	5	49437291	rs1335780	A	C	.	PASS	.	inte	NON	dis	.	
ACa02	5	49440809	rs1483787	G	C	.	PASS	.	inte	NON	dis	.	
ACa02	5	63802402	.	C	A	.	PASS	RGS7BI	UTF	NM_	NM	.	
ACa02	5	79598356	rs1416874	T	G	.	PASS	.	inte	NR_	dis	.	
ACa02	5	1.48E+08	.	G	A	.	PASS	SH3TC2	intro	NM_	.	.	
ACa02	5	1.69E+08	rs7284112	G	A	.	PASS	DOCK2	intro	NM_	.	.	
ACa02	5	1.79E+08	rs1829546	C	A	.	PASS	MAML1	intro	NM_	.	.	
ACa02	6	31948477	rs7743469	A	G	.	PASS	STK19	exo	NM_	.	synony	
ACa02	6	31973462	rs1403232	T	C	.	PASS	CYP21A	ncR	NR_	.	.	
ACa02	6	39278875	rs7596914	C	T	.	PASS	KCNK1	intro	NM_	.	.	
ACa02	6	58778766	rs4928651	T	A	.	PASS	.	inte	NR_	dis	.	
ACa02	6	1.17E+08	.	G	T	.	PASS	DSE	exo	NM_	.	stopgai	
ACa02	7	1518653	.	A	C	.	PASS	INTS1	intro	NM_	.	.	
ACa02	7	37890219	rs7467237	A	G	.	PASS	NME8	intro	NM_	.	.	
ACa02	7	45123761	rs7762925	C	T	.	PASS	NACAD	exo	NM_	.	missens	
ACa02	7	55826532	.	G	T	.	PASS	.	inte	NR_	dis	.	
ACa02	7	56887905	.	A	G	.	PASS	.	inte	NR_	dis	.	
ACa02	7	57082051	rs1422600	C	T	.	PASS	.	inte	NR_	dis	.	
ACa02	7	61969020	rs4460255	G	C	.	PASS	.	inte	NON	dis	.	
ACa02	7	63210111	.	C	A	.	PASS	.	inte	NR_	dis	.	
ACa02	7	63212664	rs3740384	G	A	.	PASS	.	inte	NR_	dis	.	
ACa02	7	63213718	rs8672068	G	A	.	PASS	.	inte	NR_	dis	.	
ACa02	7	63214682	.	C	G	.	PASS	.	inte	NR_	dis	.	
ACa02	7	63219127	.	G	A	.	PASS	.	inte	NR_	dis	.	
ACa02	7	63222486	.	G	T	.	PASS	.	inte	NR_	dis	.	
ACa02	7	72604281	rs5541523	C	T	.	PASS	GTF2IP	ncR	NR_	.	.	
ACa02	7	1.02E+08	rs3757000	T	C	.	PASS	.	inte	NM_	dis	.	
ACa02	7	1.13E+08	.	A	G	.	PASS	GPR85	exo	NM_	.	synony	
ACa02	7	1.51E+08	.	T	G	.	PASS	SLC4A2	intro	NM_	.	.	
ACa02	7	1.51E+08	.	T	G	.	PASS	ABCF2	intro	NM_	.	.	
ACa02	7	1.51E+08	.	T	G	.	PASS	ABCF2	intro	NM_	.	.	
ACa02	7	1.58E+08	rs8681384	G	C	.	PASS	PTPRN2	intro	NM_	.	.	
ACa02	8	1678575	.	C	T	.	PASS	.	inte	NM_	dis	.	
ACa02	8	7461251	rs3989791	C	G	.	PASS	.	inte	NR_	dis	.	
ACa02	8	7468906	.	G	A	.	PASS	.	inte	NR_	dis	.	
ACa02	8	10467390	rs7469289	G	A	.	PASS	RP1L1	exo	NM_	.	synony	
ACa02	8	12272503	rs7595500	C	G	.	PASS	FAM90A	ncR	NR_	.	.	
ACa02	8	12584753	.	G	A	.	PASS	MIR392	ncR	NR_	.	.	
ACa02	8	17125921	.	G	T	.	PASS	VPS37A	intro	NM_	.	.	
ACa02	8	46841804	.	G	C	.	PASS	.	inte	NON	dis	.	
ACa02	8	46849744	rs4440644	C	T	.	PASS	.	inte	NON	dis	.	
ACa02	8	81599998	.	G	T	.	PASS	ZNF704	intro	NM_	.	.	
ACa02	8	1.14E+08	.	G	A	.	PASS	CSMD3	intro	NM_	.	.	
ACa02	8	1.44E+08	.	C	T	.	PASS	LY6H	intro	NM_	.	.	
ACa02	9	19596939	rs5356927	G	A	.	PASS	SLC24A	intro	NM_	.	.	
ACa02	9	37015284	rs5620843	C	T	.	PASS	PAX5	intro	NM_	.	.	
ACa02	9	39085808	rs1412360	T	C	.	PASS	CNTNA	exo	NM_	.	missens	
ACa02	9	41954395	rs3755065	C	A	.	PASS	GLIDR	ncR	NR_	.	.	
ACa02	9	41955952	rs5368523	T	A	.	PASS	.	ups	NR_	.	.	
ACa02	9	66499856	rs7792676	G	T	.	PASS	PTGER	ncR	NR_	.	.	
ACa02	9	66500520	rs1968548	C	A	.	PASS	PTGER	ncR	NR_	.	.	
ACa02	9	68438589	rs6254382	A	G	.	PASS	LOC642	ncR	NR_	.	.	
ACa02	9	92995229	.	G	T	.	PASS	.	inte	NR_	dis	.	
ACa02	9	1.23E+08	rs5781745	G	A	.	PASS	.	inte	NM_	dis	.	

ACa02	9	1.36E+08	.	G	A	.	PASS	RALGD	intrc	NM_	.	.
ACa02	9	1.41E+08	.	G	A	.	PASS	CACNA	exoı	NM_	.	missens
ACa02	10	16962027	rs7660547	C	T	.	PASS	CUBN	exoı	NM_	.	synonyı
ACa02	10	22006660	rs1930137	C	T	.	PASS	MLLT1C	intrc	NM_	.	.
ACa02	10	43326310	.	T	G	.	PASS	BMS1	splic	NM_	NM	.
ACa02	10	47405478	rs7819664	T	A	.	PASS	FAM35[	ncR	NR_	.	.
ACa02	10	51470151	rs6185008	G	C	.	PASS	TIMM23	intrc	NM_	.	.
ACa02	10	75451923	.	T	A	.	PASS	AGAP5	intrc	NM_	.	.
ACa02	10	81606558	.	G	A	.	PASS	.	inteı	NR_	dis	.
ACa02	10	1.02E+08	.	C	T	.	PASS	PKD2L1	intrc	NM_	.	.
ACa02	10	1.2E+08	rs2928119	C	G	.	PASS	RAB11F	intrc	NM_	.	.
ACa02	10	1.3E+08	.	C	G	.	PASS	.	inteı	NR_	dis	.
ACa02	10	1.35E+08	rs1021891	C	T	.	PASS	SCART	ncR	NR_	.	.
ACa02	11	1092858	rs6263724	A	C	.	PASS	MUC2	exoı	NM_	.	synonyı
ACa02	11	1277292	rs1494932	T	C	.	PASS	MUC5B	intrc	NM_	.	.
ACa02	11	8715859	.	T	G	.	PASS	ST5	intrc	NM_	.	.
ACa02	11	9406464	.	G	A	.	PASS	IPO7	intrc	NM_	.	.
ACa02	11	44125218	.	G	A	.	PASS	EXT2	intrc	NM_	.	.
ACa02	11	61310123	.	G	A	.	PASS	SYT7	intrc	NM_	.	.
ACa02	11	64006105	rs9265024	G	C	.	PASS	VEGFB	UTF	NM_	NM	.
ACa02	11	64899489	.	T	C	.	PASS	SYVN1	intrc	NM_	.	.
ACa02	11	66034763	.	C	T	.	PASS	KLC2	UTF	NM_	NM	.
ACa02	11	67140900	.	G	A	.	PASS	LOC10C	ncR	NR_	.	.
ACa02	11	72406877	rs3679844	G	A	.	PASS	ARAP1	exoı	NM_	.	synonyı
ACa02	11	72409035	.	G	A	.	PASS	ARAP1	exoı	NM_	.	synonyı
ACa02	11	75154422	.	C	A	.	PASS	GDPD5	intrc	NM_	.	.
ACa02	11	76901624	rs3831451	A	G	.	PASS	MYO7A	intrc	NM_	.	.
ACa02	11	77698429	.	A	T	.	PASS	INTS4	intrc	NM_	.	.
ACa02	11	1.05E+08	.	C	T	.	PASS	.	inteı	NM_	dis	.
ACa02	11	1.17E+08	rs1441056	C	T	.	PASS	BACE1	intrc	NM_	.	.
ACa02	11	1.24E+08	rs5379167	G	T	.	PASS	OR8B8	exoı	NM_	.	missens
ACa02	11	1.34E+08	.	T	C	.	PASS	.	inteı	NM_	dis	.
ACa02	12	93080	rs1482401	C	G	.	PASS	.	inteı	NR_	dis	.
ACa02	12	3047379	.	T	A	.	PASS	TULP3	exoı	NM_	.	missens
ACa02	12	9707317	rs2005141	T	C	.	PASS	.	inteı	NR_	dis	.
ACa02	12	27066602	.	G	A	.	PASS	ASUN	exoı	NM_	.	synonyı
ACa02	12	57011270	.	T	C	.	PASS	BAZ2A	exoı	NM_	.	synonyı
ACa02	12	57396918	rs3737629	G	A	.	PASS	ZBTB39	exoı	NM_	.	missens
ACa02	12	80604519	.	C	T	.	PASS	OTOGL	intrc	NM_	.	.
ACa02	12	82147793	.	G	A	.	PASS	PPFIA2	exoı	NM_	.	stopgaiı
ACa02	12	1.02E+08	.	C	G	.	PASS	.	inteı	NM_	dis	.
ACa02	12	1.08E+08	.	C	T	.	PASS	BTBD11	exoı	NM_	.	missens
ACa02	12	1.11E+08	.	G	A	.	PASS	PPTC7	intrc	NM_	.	.
ACa02	12	1.11E+08	rs3495666	A	G	.	PASS	PPTC7	intrc	NM_	.	.
ACa02	12	1.18E+08	.	G	C	.	PASS	NOS1	intrc	NM_	.	.
ACa02	12	1.25E+08	.	C	G	.	PASS	NCOR2	exoı	NM_	.	missens
ACa02	13	31035409	.	A	G	.	PASS	HMGB1	UTF	NM_	NM	.
ACa02	13	42352245	.	A	C	.	PASS	VWA8	intrc	NM_	.	.
ACa02	13	44621118	rs3772091	T	C	.	PASS	.	inteı	NR_	dis	.
ACa02	13	78236166	.	G	T	.	PASS	.	inteı	NM_	dis	.
ACa02	13	1.03E+08	.	C	G	.	PASS	TPP2	intrc	NM_	.	.
ACa02	14	19806361	rs2259899	C	A	.	PASS	.	inteı	NR_	dis	.
ACa02	14	20444114	rs7532438	G	A	.	PASS	OR4K15	exoı	NM_	.	missens
ACa02	14	22386739	.	T	A	.	PASS	.	inteı	NM_	dis	.

ACa02	14	23573949	.	G	T	.	PASS	.	inte	NM_	dis	.	
ACa02	14	50559357	.	T	C	.	PASS	C14orf1	exo	NM_	.	missens	
ACa02	14	73572961	.	G	C	.	PASS	RBM25	exo	NM_	.	missens	
ACa02	14	75052544	.	G	A	.	PASS	LTBP2	intro	NM_	.	.	
ACa02	14	1.01E+08	.	A	C	.	PASS	SLC25A	intro	NM_	.	.	
ACa02	14	1.01E+08	rs9310230	C	T	.	PASS	MEG3	ncR	NR_	.	.	
ACa02	14	1.04E+08	.	C	T	.	PASS	TRMT6	intro	NM_	.	.	
ACa02	15	21195945	rs2259724	A	G	.	PASS	LINC01	ncR	NR_	NR	.	
ACa02	15	30850293	rs2648130	G	T	.	PASS	.	inte	NM_	dis	.	
ACa02	15	31089768	rs2015085	C	A	.	PASS	.	inte	NR_	dis	.	
ACa02	15	32607256	rs2007357	A	G	.	PASS	.	inte	NM_	dis	.	
ACa02	15	33927852	.	C	A	.	PASS	RYR3	exo	NM_	.	unknow	
ACa02	15	41193053	.	A	G	.	PASS	VPS18	exo	NM_	.	synonyr	
ACa02	15	48529260	.	G	A	.	PASS	SLC12A	intro	NM_	.	.	
ACa02	15	63481764	.	C	T	.	PASS	RAB8B	UTF	NM_	NM	.	
ACa02	15	72493057	.	C	T	.	PASS	PKM	intro	NM_	.	.	
ACa02	15	73418657	.	C	A	.	PASS	NEO1	intro	NM_	.	.	
ACa02	15	75561115	.	C	T	.	PASS	GOLGA	intro	NM_	.	.	
ACa02	15	76077724	rs1401448	T	C	.	PASS	.	inte	NR_	dis	.	
ACa02	15	76763454	rs8788873	C	A	.	PASS	SCAPEI	intro	NM_	.	.	
ACa02	15	84859111	rs2870850	G	A	.	PASS	.	inte	NM_	dis	.	
ACa02	15	92397621	.	C	T	.	PASS	SLCO3	intro	NM_	.	.	
ACa02	15	1.01E+08	rs7724699	C	T	.	PASS	ADAMT	exo	NM_	.	synonyr	
ACa02	15	1.03E+08	.	G	A	.	PASS	WASH3	ncR	NR_	.	.	
ACa02	16	15032227	rs7760391	C	T	.	PASS	NPIPA1	intro	NM_	.	.	
ACa02	16	15744027	.	C	G	.	PASS	NDE1	intro	NM_	.	.	
ACa02	16	30674106	.	G	A	.	PASS	.	inte	NM_	dis	.	
ACa02	16	30750888	.	C	T	.	PASS	SRCAP	exo	NM_	.	missens	
ACa02	16	32265903	rs2111615	A	G	.	PASS	TP53TC	intro	NM_	.	.	
ACa02	16	32265931	rs1123661	T	C	.	PASS	TP53TC	intro	NM_	.	.	
ACa02	16	34569998	.	C	A	.	PASS	.	inte	NR_	dis	.	
ACa02	16	34877417	rs5547762	C	T	.	PASS	.	inte	NR_	dis	.	
ACa02	16	35141389	rs3718897	C	T	.	PASS	.	inte	NR_	dis	.	
ACa02	16	46388964	rs4967312	G	T	.	PASS	.	inte	NON	dis	.	
ACa02	16	46391668	rs4270195	G	C	.	PASS	.	inte	NON	dis	.	
ACa02	16	46391690	rs4347658	A	T	.	PASS	.	inte	NON	dis	.	
ACa02	16	46393134	rs4246399	T	C	.	PASS	.	inte	NON	dis	.	
ACa02	16	46398753	rs5908263	A	G	.	PASS	.	inte	NON	dis	.	
ACa02	16	46401863	rs4249045	T	C	.	PASS	.	inte	NON	dis	.	
ACa02	16	46401949	rs7188365	G	T	.	PASS	.	inte	NON	dis	.	
ACa02	16	46402193	rs4249050	C	G	.	PASS	.	inte	NON	dis	.	
ACa02	16	46404687	rs4505355	C	T	.	PASS	.	inte	NON	dis	.	
ACa02	16	46404715	rs1244825	T	G	.	PASS	.	inte	NON	dis	.	
ACa02	16	46405534	rs1393031	A	G	.	PASS	.	inte	NON	dis	.	
ACa02	16	46405934	rs5614462	A	C	.	PASS	.	inte	NON	dis	.	
ACa02	16	46407482	rs4967197	T	G	.	PASS	.	inte	NON	dis	.	
ACa02	16	46407951	rs4450416	A	G	.	PASS	.	inte	NON	dis	.	
ACa02	16	46408282	rs8056404	C	G	.	PASS	.	inte	NON	dis	.	
ACa02	16	46408483	rs4246356	C	T	.	PASS	.	inte	NON	dis	.	
ACa02	16	46408518	rs8052624	G	T	.	PASS	.	inte	NON	dis	.	
ACa02	16	46408534	rs4249654	A	T	.	PASS	.	inte	NON	dis	.	
ACa02	16	46413328	rs2885782	G	T	.	PASS	.	inte	NON	dis	.	
ACa02	16	46413340	rs6153388	C	T	.	PASS	.	inte	NON	dis	.	
ACa02	16	46413364	.	G	C	.	PASS	.	inte	NON	dis	.	

ACa02	16	46420361	rs2875315	T	C	.	PASS	.	inte	NON	dis	.
ACa02	16	46423135	.	G	A	.	PASS	.	inte	NON	dis	.
ACa02	16	46433334	rs1479181	C	G	.	PASS	.	inte	NON	dis	.
ACa02	16	46433422	rs8677676	C	T	.	PASS	.	inte	NON	dis	.
ACa02	16	70917747	.	C	G	.	PASS	HYDIN	intr	NM_	.	.
ACa02	16	72094757	rs1891151	G	C	.	PASS	HP	exo	NM_	.	missens
ACa02	16	72097225	rs1291890	T	C	.	PASS	HPR	intr	NM_	.	.
ACa02	16	76389509	.	G	T	.	PASS	CNTNA	intr	NM_	.	.
ACa02	16	89167385	.	C	A	.	PASS	ACSF3	exo	NM_	.	missens
ACa02	16	89167409	rs7697160	C	T	.	PASS	ACSF3	exo	NM_	.	missens
ACa02	16	90229186	rs7945920	C	A	.	PASS	.	ncR	NR_	.	.
ACa02	16	90234858	rs3697515	C	A	.	PASS	.	ncR	NR_	.	.
ACa02	16	90234879	rs5596034	A	G	.	PASS	.	ncR	NR_	.	.
ACa02	17	6663752	.	G	A	.	PASS	XAF1	exo	NM_	.	missens
ACa02	17	7164992	.	A	C	.	PASS	CLDN7	intr	NM_	.	.
ACa02	17	7818722	.	C	T	.	PASS	LOC28	ncR	NR_	.	.
ACa02	17	10263519	.	G	A	.	PASS	MYH13	intr	NM_	.	.
ACa02	17	11584352	rs9806119	C	T	.	PASS	DNAH9	intr	NM_	.	.
ACa02	17	11725588	rs7571703	C	T	.	PASS	DNAH9	intr	NM_	.	.
ACa02	17	16649883	rs1460005	T	A	.	PASS	CCDC1	intr	NM_	.	.
ACa02	17	18365605	rs2168782	A	G	.	PASS	.	inte	NR_	dis	.
ACa02	17	21906266	.	C	G	.	PASS	FLJ360	ncR	NR_	.	.
ACa02	17	22253154	rs4362423	T	G	.	PASS	.	inte	NM_	dis	.
ACa02	17	22253204	rs4283255	A	G	.	PASS	.	inte	NM_	dis	.
ACa02	17	22260691	rs1403689	T	G	.	PASS	.	inte	NM_	dis	.
ACa02	17	22260798	rs1405621	C	T	.	PASS	.	inte	NM_	dis	.
ACa02	17	25982780	.	G	C	.	PASS	.	inte	NM_	dis	.
ACa02	17	34585231	rs7126069	C	T	.	PASS	TBC1D	intr	NM_	.	.
ACa02	17	36349205	rs573849	T	C	.	PASS	.	ups	NM_	.	.
ACa02	17	36350145	rs9693530	T	A	.	PASS	.	inte	NM_	dis	.
ACa02	17	36371573	.	T	A	.	PASS	LOC44	ncR	NR_	.	.
ACa02	17	36498855	.	C	G	.	PASS	GPR17	intr	NM_	.	.
ACa02	17	50235479	.	G	A	.	PASS	CA10	UTF	NM_	NM	.
ACa02	17	60347096	rs7516213	G	T	.	PASS	TBC1D	ncR	NR_	.	.
ACa02	17	63533091	rs7607356	G	A	.	PASS	AXIN2	exo	NM_	.	synony
ACa02	17	75187303	.	C	T	.	PASS	SEC14L	exo	NM_	.	missens
ACa02	17	77166111	.	G	A	.	PASS	RBFOX	intr	NM_	.	.
ACa02	17	79005358	rs1138327	C	T	.	PASS	BAIAP2	ncR	NR_	.	.
ACa02	17	80735912	rs8791354	A	T	.	PASS	TBCD	intr	NM_	.	.
ACa02	18	99943	.	G	C	.	PASS	.	inte	NR_	dis	.
ACa02	18	14143809	rs8658406	G	A	.	PASS	.	inte	NM_	dis	.
ACa02	18	14143811	rs8676712	C	T	.	PASS	.	inte	NM_	dis	.
ACa02	19	1397560	.	C	A	.	PASS	GAMT	intr	NM_	.	.
ACa02	19	2389762	rs3756184	G	A	.	PASS	.	ups	NM_	.	.
ACa02	19	2785626	.	G	A	.	PASS	THOP1	UTF	NM_	NM	.
ACa02	19	2785627	.	G	T	.	PASS	THOP1	UTF	NM_	NM	.
ACa02	19	7621418	rs5460066	G	A	.	PASS	PNPLA	exo	NM_	.	missens
ACa02	19	15063677	.	T	C	.	PASS	SLC1A	intr	NM_	.	.
ACa02	19	33870095	.	C	T	.	PASS	CEBPG	UTF	NM_	NM	.
ACa02	19	36270883	.	G	A	.	PASS	ARHGA	intr	NM_	.	.
ACa02	19	37772012	rs2385185	G	C	.	PASS	.	inte	NR_	dis	.
ACa02	19	37785301	rs7125437	C	G	.	PASS	.	inte	NR_	dis	.
ACa02	19	39026511	.	G	C	.	PASS	RYR1	intr	NM_	.	.
ACa02	19	41531320	rs1390553	T	A	.	PASS	.	inte	NM_	dis	.

ACa02	19	52249443	rs7714460	G	A	.	PASS	FPR1	exo	NM_	.	missens
ACa02	19	53945837	rs1785512	T	C	.	PASS	TPM3P	ncR	NR_	.	.
ACa02	19	55032858	rs9207323	G	A	.	PASS	.	inte	NM_	dis	.
ACa02	20	17605301	.	T	G	.	PASS	RRBP1	exo	NM_	.	missens
ACa02	20	23168959	.	G	A	.	PASS	.	inte	NR_	dis	.
ACa02	20	25003624	rs7787265	G	A	.	PASS	ACSS1	exo	NM_	.	synony
ACa02	20	25003759	rs3775467	G	A	.	PASS	ACSS1	intro	NM_	.	.
ACa02	20	29612803	.	C	T	.	PASS	FRG1B	ncR	NR_	.	.
ACa02	20	42144110	.	C	A	.	PASS	L3MBTL	intro	NM_	.	.
ACa02	20	44511079	.	G	A	.	PASS	ZSWIM	intro	NM_	.	.
ACa02	20	61870764	.	G	A	.	PASS	BIRC7	exo	NM_	.	missens
ACa02	21	9674240	rs3747993	C	G	.	PASS	.	inte	NON	dis	.
ACa02	21	9723335	rs3730978	G	A	.	PASS	.	inte	NON	dis	.
ACa02	21	10139840	rs2927801	G	A	.	PASS	.	inte	NR_	dis	.
ACa02	21	11026580	rs1996169	T	C	.	PASS	BAGE2,	intro	NM_	.	.
ACa02	21	11047309	rs8789866	G	T	.	PASS	BAGE4,	intro	NM_	.	.
ACa02	21	11114478	rs4358221	G	A	.	PASS	.	inte	NM_	dis	.
ACa02	21	11123779	.	T	A	.	PASS	.	inte	NM_	dis	.
ACa02	21	34144206	rs8664333	A	C	.	PASS	.	ups	NM_	.	.
ACa02	21	37443162	rs7967544	T	G	.	PASS	LOC10	ncR	NR_	.	.
ACa02	21	45504131	.	G	A	.	PASS	TRAPP	exo	NM_	.	synony
ACa02	21	47976176	.	G	A	.	PASS	DIP2A	intro	NM_	.	.
ACa02	21	48103323	.	C	T	.	PASS	.	inte	NM_	dis	.
ACa02	22	19748622	.	G	A	.	PASS	TBX1	exo	NM_	.	missens
ACa02	22	20502354	rs3702204	C	T	.	PASS	.	inte	NM_	dis	.
ACa02	22	20502806	rs1119554	G	T	.	PASS	.	inte	NM_	dis	.
ACa02	22	21044704	rs1880252	C	T	.	PASS	POM12	ncR	NR_	.	.
ACa02	22	21663503	.	G	A	.	PASS	.	inte	NR_	dis	.
ACa02	X	7811824	rs1637792	G	C	.	PASS	VCX	exo	NM_	.	missens
ACa02	X	10450722	.	G	A	.	PASS	MID1	intro	NM_	.	.
ACa02	X	27765544	.	G	A	.	PASS	DCAF8L	exo	NM_	.	missens
ACa02	X	30035259	.	C	A	.	PASS	.	inte	NM_	dis	.
ACa02	X	34150216	rs7740495	G	A	.	PASS	FAM47	exo	NM_	.	synony
ACa02	X	47082411	.	G	C	.	PASS	CDK16	intro	NM_	.	.
ACa02	X	47082419	.	G	T	.	PASS	CDK16	UTF	NM_	NM	.
ACa02	X	61685369	.	G	T	.	PASS	.	inte	NON	dis	.
ACa02	X	61696571	rs8686279	A	C	.	PASS	.	inte	NON	dis	.
ACa02	X	61718935	.	C	T	.	PASS	.	inte	NON	dis	.
ACa02	X	61718971	.	G	C	.	PASS	.	inte	NON	dis	.
ACa02	X	70357408	.	C	T	.	PASS	MED12	exo	NM_	.	stopgai
ACa02	X	1.02E+08	.	T	A	.	PASS	GPRAS	exo	NM_	.	synony
ACa02	X	1.03E+08	.	G	A	.	PASS	.	inte	NM_	dis	.
ACa02	X	1.11E+08	.	A	G	.	PASS	DCX	intro	NM_	.	.
ACa02	X	1.29E+08	.	C	A	.	PASS	OCRL	UTF	NM_	NM	.
ACa02	X	1.3E+08	.	C	T	.	PASS	IGSF1	intro	NM_	.	.
ACa02	X	1.35E+08	rs9883463	T	G	.	PASS	MAP7D	intro	NM_	.	.
ACa02	X	1.38E+08	.	G	C	.	PASS	FGF13	intro	NM_	.	.
ACa02	X	1.46E+08	rs7821231	G	A	.	PASS	.	ups	NR_	.	.
ACa02	X	1.54E+08	.	G	A	.	PASS	FLNA	intro	NM_	.	.
ACa02	Y	59015673	rs3769023	C	A	.	PASS	.	inte	NON	dis	.
ACa02	MT	759	.	C	T	.	PASS	.	inte	NON	dis	.
ACa02	MT	14563	rs1057516	C	T	.	PASS	.	inte	NON	dis	.
ACa03	1	674109	.	A	G	.	PASS	.	inte	NR_	dis	.
ACa03	1	1644945	.	G	A	.	PASS	CDK11	intro	NM_	.	.

ACa03	1	3644226	.	C	T	.	PASS	TP73	exo	NM_	.	missens
ACa03	1	12423089	rs7579432	C	T	.	PASS	VPS13D	intro	NM_	.	.
ACa03	1	16890922	rs5565927	T	C	.	PASS	NBPF1	intro	NM_	.	.
ACa03	1	16891696	rs7619917	T	C	.	PASS	NBPF1	intro	NM_	.	.
ACa03	1	16893407	rs663828	T	C	.	PASS	NBPF1	intro	NM_	.	.
ACa03	1	16908275	rs646524	T	G	.	PASS	NBPF1	intro	NM_	.	.
ACa03	1	16931160	rs4596945	G	C	.	PASS	NBPF1	intro	NM_	.	.
ACa03	1	16957414	rs2296161	C	T	.	PASS	.	ups	NR_	.	.
ACa03	1	19549286	rs5616387	C	T	.	PASS	EMC1	exo	NM_	.	missens
ACa03	1	37947479	.	G	A	.	PASS	ZC3H12	intro	NM_	.	.
ACa03	1	45472818	rs9490484	G	T	.	PASS	HECTD	intro	NM_	.	.
ACa03	1	63070550	.	T	G	.	PASS	ANGPT	UTF	NM_	NM	.
ACa03	1	75097583	.	G	T	.	PASS	ERICH3	exo	NM_	.	missens
ACa03	1	76252058	.	C	T	.	PASS	RABGG	intro	NM_	.	.
ACa03	1	1.16E+08	.	G	A	.	PASS	NHLH2	exo	NM_	.	missens
ACa03	1	1.43E+08	rs1409433	T	C	.	PASS	.	inte	NR_	dis	.
ACa03	1	1.43E+08	rs2022010	G	A	.	PASS	.	inte	NR_	dis	.
ACa03	1	1.43E+08	rs7763460	C	G	.	PASS	.	ncR	NR_	.	.
ACa03	1	1.43E+08	rs1457064	G	A	.	PASS	.	inte	NR_	dis	.
ACa03	1	1.44E+08	.	C	G	.	PASS	NBPF20	intro	NM_	.	.
ACa03	1	1.45E+08	rs1148852	G	C	.	PASS	NBPF9,	intro	NM_	.	.
ACa03	1	1.45E+08	rs3746294	A	G	.	PASS	NBPF20	intro	NM_	.	.
ACa03	1	1.45E+08	rs3675661	C	G	.	PASS	NBPF25	ncR	NR_	.	.
ACa03	1	1.48E+08	rs7986155	T	A	.	PASS	NBPF8	intro	NM_	.	.
ACa03	1	1.56E+08	rs7593611	G	A	.	PASS	MEX3A	exo	NM_	.	synonyr
ACa03	1	1.59E+08	.	G	T	.	PASS	OR6P1	exo	NM_	.	missens
ACa03	1	2.01E+08	.	G	C	.	PASS	LAD1	intro	NM_	.	.
ACa03	1	2.03E+08	rs7508890	G	A	.	PASS	PPFIA4	exo	NM_	.	missens
ACa03	1	2.26E+08	rs1454313	A	G	.	PASS	LEFTY1	exo	NM_	.	missens
ACa03	1	2.48E+08	rs3720052	C	T	.	PASS	OR6F1	exo	NM_	.	missens
ACa03	1	2.49E+08	rs8789457	A	G	.	PASS	.	ups	NM_	.	.
ACa03	2	17773489	.	C	A	.	PASS	VSNL1	exo	NM_	.	missens
ACa03	2	77749254	.	T	A	.	PASS	LRRTM	UTF	NM_	NM	.
ACa03	2	80530489	.	G	A	.	PASS	LRRTM	exo	NM_	.	synonyr
ACa03	2	87131091	rs2013675	G	A	.	PASS	.	inte	NR_	dis	.
ACa03	2	88071329	.	G	C	.	PASS	RGPD1	intro	NM_	.	.
ACa03	2	1.38E+08	.	T	C	.	PASS	.	inte	NM_	dis	.
ACa03	2	1.71E+08	rs1473449	G	T	.	PASS	UBR3	intro	NM_	.	.
ACa03	2	1.82E+08	.	C	T	.	PASS	ITGA4	exo	NM_	.	missens
ACa03	2	2.16E+08	.	C	T	.	PASS	ATIC	splic	NM_	NM	.
ACa03	2	2.43E+08	.	C	A	.	PASS	.	inte	NR_	dis	.
ACa03	3	47297292	rs1050893	G	C	.	PASS	KIF9	intro	NM_	.	.
ACa03	3	47297297	.	C	T	.	PASS	KIF9	intro	NM_	.	.
ACa03	3	55108112	rs1178604	G	A	.	PASS	CACNA	intro	NM_	.	.
ACa03	3	74663610	rs3744125	G	A	.	PASS	.	inte	NM_	dis	.
ACa03	3	77459682	rs1451899	C	T	.	PASS	ROBO2	intro	NM_	.	.
ACa03	3	1.27E+08	.	G	A	.	PASS	MGLL	intro	NM_	.	.
ACa03	3	1.31E+08	.	A	C	.	PASS	NUDT10	UTF	NM_	NM	.
ACa03	3	1.37E+08	.	A	C	.	PASS	IL20RB	intro	NM_	.	.
ACa03	3	1.38E+08	.	T	G	.	PASS	ARMC8	exo	NM_	.	synonyr
ACa03	3	1.79E+08	rs1057519	G	A	.	PASS	PIK3CA	exo	NM_	.	missens
ACa03	3	1.79E+08	rs1048860	G	A	.	PASS	PIK3CA	exo	NM_	.	missens
ACa03	3	1.97E+08	.	C	A	.	PASS	FYTTD1	intro	NM_	.	.
ACa03	4	2306307	rs3725657	G	A	.	PASS	ZFYVE2	exo	NM_	.	missens

ACa03	4	6080728	.	A	G	.	PASS	JAKMIP	splic	NM_	NM	.	
ACa03	4	9245302	rs8796734	A	G	.	PASS	.	ups	NM_	.	.	
ACa03	4	49261257	rs5941177	C	T	.	PASS	.	inte	NM_	dis	.	
ACa03	4	73013446	.	C	T	.	PASS	NPFFR	exo	NM_	.	missens	
ACa03	4	1.68E+08	.	A	C	.	PASS	SPOCK	intrc	NM_	.	.	
ACa03	5	34180054	rs4288119	G	A	.	PASS	.	inte	NR_	dis	.	
ACa03	5	34183130	rs8669155	C	T	.	PASS	.	inte	NR_	dis	.	
ACa03	5	1.34E+08	rs7765625	G	A	.	PASS	JADE2	exo	NM_	.	missens	
ACa03	5	1.71E+08	rs5593800	C	T	.	PASS	.	inte	NM_	dis	.	
ACa03	5	1.77E+08	.	G	A	.	PASS	.	inte	NR_	dis	.	
ACa03	6	3138322	.	A	C	.	PASS	BPHL	intrc	NM_	.	.	
ACa03	6	31324628	rs1513411	G	C	.	PASS	HLA-B	exo	NM_	.	missens	
ACa03	6	31852180	.	C	A	.	PASS	EHMT2	exo	NM_	.	missens	
ACa03	6	35391921	rs7668272	C	T	.	PASS	PPARD	exo	NM_	.	missens	
ACa03	6	47251575	.	G	A	.	PASS	TNFRSI	intrc	NM_	.	.	
ACa03	6	52617512	.	T	A	.	PASS	GSTA2	intrc	NM_	.	.	
ACa03	6	75904503	.	T	G	.	PASS	COL12A	intrc	NM_	.	.	
ACa03	7	11860631	.	C	T	.	PASS	THSD7A	intrc	NM_	.	.	
ACa03	7	25208115	.	C	T	.	PASS	C7orf31	intrc	NM_	.	.	
ACa03	7	43590116	.	G	A	.	PASS	HECW1	exo	NM_	.	missens	
ACa03	7	44191820	.	T	G	.	PASS	GCK	intrc	NM_	.	.	
ACa03	7	56439590	.	C	T	.	PASS	.	inte	NM_	dis	.	
ACa03	7	56871105	rs1439657	G	A	.	PASS	.	inte	NR_	dis	.	
ACa03	7	56886784	.	C	T	.	PASS	.	inte	NR_	dis	.	
ACa03	7	56888186	rs8799634	G	T	.	PASS	.	inte	NR_	dis	.	
ACa03	7	56891058	.	A	G	.	PASS	.	inte	NR_	dis	.	
ACa03	7	56891160	rs2002309	C	G	.	PASS	.	inte	NR_	dis	.	
ACa03	7	56891593	.	C	A	.	PASS	.	inte	NR_	dis	.	
ACa03	7	62956286	rs2018986	T	C	.	PASS	.	inte	NR_	dis	.	
ACa03	7	75128472	.	C	T	.	PASS	SPDYE5	intrc	NM_	.	.	
ACa03	7	75128489	.	C	T	.	PASS	SPDYE5	intrc	NM_	.	.	
ACa03	7	76166588	.	G	A	.	PASS	.	inte	NM_	dis	.	
ACa03	7	79842137	.	G	A	.	PASS	GNAI1	exo	NM_	.	missens	
ACa03	7	92731585	.	C	T	.	PASS	SAMD9	exo	NM_	.	missens	
ACa03	7	95216347	rs2676016	C	T	.	PASS	PDK4	exo	NM_	.	missens	
ACa03	7	1E+08	rs1826826	G	A	.	PASS	NYAP1	intrc	NM_	.	.	
ACa03	7	1.17E+08	rs1409779	C	T	.	PASS	ST7	intrc	NM_	.	.	
ACa03	7	1.44E+08	.	A	C	.	PASS	NOBOX	intrc	NM_	.	.	
ACa03	7	1.52E+08	.	G	C	.	PASS	KMT2C	intrc	NM_	.	.	
ACa03	7	1.59E+08	rs7962502	C	T	.	PASS	.	inte	NM_	dis	.	
ACa03	8	6371222	.	G	C	.	PASS	ANGPT	exo	NM_	.	synonyr	
ACa03	8	7468654	rs3763515	G	A	.	PASS	.	inte	NR_	dis	.	
ACa03	8	7885536	rs5673338	T	A	.	PASS	.	inte	NR_	dis	.	
ACa03	8	17942458	.	C	G	.	PASS	LOC101	ncR	NR_	.	.	
ACa03	8	92346512	.	C	T	.	PASS	SLC26A	intrc	NM_	.	.	
ACa03	8	1.01E+08	.	G	A	.	PASS	VPS13E	intrc	NM_	.	.	
ACa03	8	1.33E+08	rs5609552	T	C	.	PASS	KCNQ3	intrc	NM_	.	.	
ACa03	9	20978329	.	G	A	.	PASS	FOCAD	intrc	NM_	.	.	
ACa03	9	34658334	rs1463382	C	T	.	PASS	IL11RA	intrc	NM_	.	.	
ACa03	9	46843601	rs7495042	G	A	.	PASS	.	ups	NR_	.	.	
ACa03	9	66516655	.	C	T	.	PASS	.	ncR	NR_	.	.	
ACa03	9	67274240	rs7714176	G	A	.	PASS	AQP7P	ncR	NR_	.	.	
ACa03	9	68415355	rs4452915	C	T	.	PASS	.	inte	NM_	dis	.	
ACa03	9	68415359	rs7492487	T	C	.	PASS	.	inte	NM_	dis	.	

ACa03	9	70993081	rs391727	G	C	.	PASS	PGM5	intrc	NM_	.	.
ACa03	9	74622718	.	G	A	.	PASS	.	inte	NM_	dis	.
ACa03	9	86900782	.	G	T	.	PASS	SLC28A	intrc	NM_	.	.
ACa03	9	1.36E+08	rs2855049	T	C	.	PASS	CEL	intrc	NM_	.	.
ACa03	9	1.39E+08	rs7771119	G	A	.	PASS	NOTCH	exol	NM_	.	synonyr
ACa03	10	12802960	.	G	A	.	PASS	CAMK1	exol	NM_	.	missens
ACa03	10	18430225	.	G	A	.	PASS	CACNB	intrc	NM_	.	.
ACa03	10	35858114	rs7747854	G	A	.	PASS	CCNY	UTF	NM_	NM	.
ACa03	10	37442393	rs1759035	A	G	.	PASS	ANKRD	intrc	NM_	.	.
ACa03	10	49200211	rs3125109	G	A	.	PASS	.	inte	NR_	dis	.
ACa03	10	72539624	.	G	A	.	PASS	TBATA	intrc	NM_	.	.
ACa03	10	75854181	rs2018088	G	A	.	PASS	VCL	exol	NM_	.	missens
ACa03	10	85841351	.	A	G	.	PASS	.	inte	NM_	dis	.
ACa03	10	88988731	rs3128226	C	G	.	PASS	NUTM2	intrc	NM_	.	.
ACa03	10	98510597	.	G	A	.	PASS	.	inte	NM_	dis	.
ACa03	10	1.2E+08	.	C	G	.	PASS	RAB11F	UTF	NM_	NM	.
ACa03	10	1.24E+08	rs5495128	C	T	.	PASS	.	inte	NM_	dis	.
ACa03	10	1.26E+08	.	C	A	.	PASS	CPXM2	exol	NM_	.	missens
ACa03	11	44950537	.	G	T	.	PASS	TSPAN:	intrc	NM_	.	.
ACa03	11	66512484	.	C	T	.	PASS	C11orf8	intrc	NM_	.	.
ACa03	11	89665513	.	A	T	.	PASS	MIR569	ncR	NR_	.	.
ACa03	12	89716	rs7827727	T	C	.	PASS	LOC10(	ncR	NR_	.	.
ACa03	12	4758160	.	C	A	.	PASS	AKAP3	UTF	NM_	NM	.
ACa03	12	6709098	.	G	C	.	PASS	CHD4	exol	NM_	.	missens
ACa03	12	6778145	.	G	C	.	PASS	ZNF384	intrc	NM_	.	.
ACa03	12	8327717	.	C	A	.	PASS	ZNF705	intrc	NM_	.	.
ACa03	12	25398284	rs1219135	C	T	.	PASS	KRAS	exol	NM_	.	missens
ACa03	12	26848567	rs3731379	C	T	.	PASS	ITPR2	exol	NM_	.	missens
ACa03	12	31242433	rs4031316	T	C	.	PASS	DDX11	intrc	NM_	.	.
ACa03	12	44124214	rs5661873	C	T	.	PASS	PUS7L	exol	NM_	.	missens
ACa03	12	50790087	rs9815586	G	A	.	PASS	FAM186	exol	NM_	.	stopgair
ACa03	12	56231265	rs1409167	G	A	.	PASS	MMP19	intrc	NM_	.	.
ACa03	12	1.23E+08	.	C	A	.	PASS	HIP1R	intrc	NM_	.	.
ACa03	12	1.24E+08	.	G	A	.	PASS	DNAH1(	intrc	NM_	.	.
ACa03	12	1.31E+08	rs1707911	T	C	.	PASS	.	inte	NM_	dis	.
ACa03	12	1.33E+08	rs7662808	G	A	.	PASS	DDX51	exol	NM_	.	missens
ACa03	13	19539354	rs9079318	C	T	.	PASS	.	inte	NR_	dis	.
ACa03	13	28367362	.	A	C	.	PASS	GSX1	intrc	NM_	.	.
ACa03	13	28623894	rs7666774	G	A	.	PASS	FLT3	exol	NM_	.	stopgair
ACa03	13	36410222	.	C	T	.	PASS	DCLK1	exol	NM_	.	missens
ACa03	13	1.13E+08	.	C	T	.	PASS	SPACA7	intrc	NM_	.	.
ACa03	14	19563657	rs2018046	T	G	.	PASS	LOC101	ncR	NR_	.	.
ACa03	14	19624885	rs4982820	G	A	.	PASS	.	inte	NM_	dis	.
ACa03	14	19644287	rs2005137	C	T	.	PASS	.	inte	NM_	dis	.
ACa03	14	19694665	.	T	G	.	PASS	.	inte	NR_	dis	.
ACa03	14	20106847	.	T	C	.	PASS	.	inte	NM_	dis	.
ACa03	14	20148329	rs3763109	C	T	.	PASS	.	inte	NM_	dis	.
ACa03	14	22434146	.	G	T	.	PASS	.	inte	NM_	dis	.
ACa03	14	24523589	rs7592315	G	A	.	PASS	LRRC16	intrc	NM_	.	.
ACa03	14	1.06E+08	.	T	A	.	PASS	.	inte	NM_	dis	.
ACa03	14	1.06E+08	rs7473295	G	A	.	PASS	.	inte	NR_	dis	.
ACa03	15	20216196	rs5686085	C	T	.	PASS	.	inte	NON	dis	.
ACa03	15	20448387	rs5761409	G	C	.	PASS	.	inte	NON	dis	.
ACa03	15	20756984	rs3684417	G	T	.	PASS	.	inte	NM_	dis	.

ACa03	15	21137759	rs1130343	A	C	.	PASS	.	inte	NR_	dis	.
ACa03	15	21370826	rs1995519	C	G	.	PASS	.	inte	NR_	dis	.
ACa03	15	22546388	rs1836337	A	G	.	PASS	.	ups	NR_	.	.
ACa03	15	23478648	rs3700524	G	A	.	PASS	.	intrc	NM_	.	.
ACa03	15	23606350	rs3766397	G	A	.	PASS	GOLGA	ncR	NR_	.	.
ACa03	15	30659755	.	G	A	.	PASS	CHRFA	intrc	NM_	.	.
ACa03	15	41136788	rs5573362	C	T	.	PASS	SPINT1	exo	NM_	.	synonyr
ACa03	15	42646731	.	C	T	.	PASS	.	dow	NM_	.	.
ACa03	15	72954595	rs7443087	A	G	.	PASS	GOLGA	exo	NM_	.	missens
ACa03	15	82636965	.	G	A	.	PASS	GOLGA	intrc	NM_	.	.
ACa03	16	1614158	.	T	C	.	PASS	IFT140	exo	NM_	.	missens
ACa03	16	3119290	.	A	T	.	PASS	IL32	exo	NM_	.	synonyr
ACa03	16	12571730	.	C	T	.	PASS	SNX29	intrc	NM_	.	.
ACa03	16	29115328	.	G	A	.	PASS	RRN3P:	ncR	NR_	.	.
ACa03	16	46387441	rs2887413	C	G	.	PASS	.	inte	NON	dis	.
ACa03	16	46388702	rs4966607	C	T	.	PASS	.	inte	NON	dis	.
ACa03	16	46389631	rs4359628	T	G	.	PASS	.	inte	NON	dis	.
ACa03	16	46391941	rs4249500	G	A	.	PASS	.	inte	NON	dis	.
ACa03	16	46392009	rs4372866	C	T	.	PASS	.	inte	NON	dis	.
ACa03	16	46392046	rs4396558	T	A	.	PASS	.	inte	NON	dis	.
ACa03	16	46392187	rs4249160	C	T	.	PASS	.	inte	NON	dis	.
ACa03	16	46394150	rs4967780	G	C	.	PASS	.	inte	NON	dis	.
ACa03	16	46394809	rs7203681	C	T	.	PASS	.	inte	NON	dis	.
ACa03	16	46400019	rs7826866	G	A	.	PASS	.	inte	NON	dis	.
ACa03	16	46401863	rs4249045	T	C	.	PASS	.	inte	NON	dis	.
ACa03	16	46401949	rs7188365	G	T	.	PASS	.	inte	NON	dis	.
ACa03	16	46402061	rs9972801	A	G	.	PASS	.	inte	NON	dis	.
ACa03	16	46402080	rs4249048	G	C	.	PASS	.	inte	NON	dis	.
ACa03	16	46402947	rs7823469	C	A	.	PASS	.	inte	NON	dis	.
ACa03	16	46405534	rs1393031	A	G	.	PASS	.	inte	NON	dis	.
ACa03	16	46406765	rs4090116	C	T	.	PASS	.	inte	NON	dis	.
ACa03	16	46406884	rs4090115	C	T	.	PASS	.	inte	NON	dis	.
ACa03	16	46407723	rs4459567	C	T	.	PASS	.	inte	NON	dis	.
ACa03	16	46407894	rs6133865	A	T	.	PASS	.	inte	NON	dis	.
ACa03	16	46408354	rs4341908	A	C	.	PASS	.	inte	NON	dis	.
ACa03	16	46408513	rs4451975	A	C	.	PASS	.	inte	NON	dis	.
ACa03	16	46420402	rs7187636	T	C	.	PASS	.	inte	NON	dis	.
ACa03	16	70020483	.	C	A	.	PASS	PDXDC	ncR	NR_	.	.
ACa03	16	70154480	rs2004697	A	G	.	PASS	PDPR	exo	NM_	.	missens
ACa03	16	70473074	.	A	C	.	PASS	.	ups	NM_	.	.
ACa03	16	89858338	.	C	G	.	PASS	FANCA	exo	NM_	.	missens
ACa03	16	89902722	.	C	A	.	PASS	SPIRE2	intrc	NM_	.	.
ACa03	17	1715169	.	T	G	.	PASS	SMYD4	intrc	NM_	.	.
ACa03	17	9862686	.	G	A	.	PASS	GAS7	UTF	NM_	NM	.
ACa03	17	17942355	.	G	T	.	PASS	ATPAF:	UTF	NM_	NM	.
ACa03	17	22253129	rs1440233	G	A	.	PASS	.	inte	NM_	dis	.
ACa03	17	22253154	rs4362423	T	G	.	PASS	.	inte	NM_	dis	.
ACa03	17	34813271	rs1409172	A	G	.	PASS	.	inte	NM_	dis	.
ACa03	17	36281954	rs3737234	C	A	.	PASS	.	inte	NR_	dis	.
ACa03	17	36636008	.	C	T	.	PASS	ARHGA	exo	NM_	.	missens
ACa03	17	36646848	.	A	G	.	PASS	ARHGA	intrc	NM_	.	.
ACa03	17	38126930	rs8679073	A	T	.	PASS	GSDMA	intrc	NM_	.	.
ACa03	17	42646434	.	G	A	.	PASS	.	inte	NM_	dis	.
ACa03	17	44144162	.	G	A	.	PASS	KANSL:	intrc	NM_	.	.

ACa03	17	64881187	rs7651139	C	T	.	PASS	CACNG	exo	NM_	.	missens
ACa03	17	73592837	rs7754213	G	A	.	PASS	MYO15I	ncR	NR_	.	.
ACa03	17	74898771	.	G	A	.	PASS	MGAT5	intr	NM_	.	.
ACa03	17	75303430	rs5408783	C	T	.	PASS	9-Sep	intr	NM_	.	.
ACa03	17	79639149	.	G	A	.	PASS	CCDC1	exo	NM_	.	synony
ACa03	17	79769449	.	C	T	.	PASS	GCGR	intr	NM_	.	.
ACa03	17	80039454	rs3732207	C	T	.	PASS	FASN	intr	NM_	.	.
ACa03	18	12264368	.	C	T	.	PASS	CIDEA	exo	NM_	.	synony
ACa03	19	4544051	rs7575217	C	T	.	PASS	SEMA6I	exo	NM_	.	synony
ACa03	19	6415948	.	G	A	.	PASS	KHSRP	intr	NM_	.	.
ACa03	19	7685588	.	G	T	.	PASS	XAB2	intr	NM_	.	.
ACa03	19	7999266	.	G	T	.	PASS	TIMM44	intr	NM_	.	.
ACa03	19	8173984	.	C	A	.	PASS	FBN3	intr	NM_	.	.
ACa03	19	8880680	rs1492924	G	A	.	PASS	.	inte	NM_	dis	.
ACa03	19	33418071	rs1999730	T	C	.	PASS	CEP89	intr	NM_	.	.
ACa03	19	37780243	rs3762437	G	T	.	PASS	.	inte	NR_	dis	.
ACa03	19	37785301	rs7125437	C	G	.	PASS	.	inte	NR_	dis	.
ACa03	19	41018712	.	C	T	.	PASS	SPTBN	exo	NM_	.	synony
ACa03	19	41893613	.	A	C	.	PASS	EXOSC	intr	NM_	.	.
ACa03	19	43787795	.	C	G	.	PASS	.	inte	NM_	dis	.
ACa03	19	48558299	.	C	A	.	PASS	PLA2G	exo	NM_	.	missens
ACa03	19	49551897	rs6212787	C	A	.	PASS	CGB8	intr	NM_	.	.
ACa03	19	51331278	.	A	C	.	PASS	KLK15	intr	NM_	.	.
ACa03	19	53945648	rs5492748	T	C	.	PASS	TPM3P	ncR	NR_	.	.
ACa03	19	54225561	.	T	C	.	PASS	.	dow	NR_	.	.
ACa03	19	54404000	rs7600928	G	A	.	PASS	PRKCG	exo	NM_	.	synony
ACa03	19	58151254	.	G	T	.	PASS	ZNF211	intr	NM_	.	.
ACa03	19	58385467	rs7704038	T	G	.	PASS	ZNF814	exo	NM_	.	missens
ACa03	19	59083815	.	C	T	.	PASS	MZF1-A	ncR	NR_	.	.
ACa03	20	2380513	.	C	T	.	PASS	TGM6	intr	NM_	.	.
ACa03	20	5909667	.	G	T	.	PASS	.	inte	NM_	dis	.
ACa03	20	26174148	.	C	A	.	PASS	MIR663	ncR	NR_	.	.
ACa03	20	29628491	rs9569348	A	G	.	PASS	FRG1B	ncR	NR_	.	.
ACa03	20	44099904	.	C	A	.	PASS	WFDC2	intr	NM_	.	.
ACa03	21	10535397	rs3772733	C	T	.	PASS	.	inte	NR_	dis	.
ACa03	21	11059912	rs9184726	C	T	.	PASS	BAGE,E	intr	NM_	.	.
ACa03	21	11114515	rs9266067	A	G	.	PASS	.	inte	NM_	dis	.
ACa03	21	11164201	.	G	A	.	PASS	.	inte	NM_	dis	.
ACa03	21	34954231	.	C	T	.	PASS	DONSC	intr	NM_	.	.
ACa03	21	37443162	rs7967544	T	G	.	PASS	LOC10	ncR	NR_	.	.
ACa03	22	16405002	rs2019683	C	T	.	PASS	.	inte	NM_	dis	.
ACa03	22	21349411	rs7543647	C	T	.	PASS	LZTR1	intr	NM_	.	.
ACa03	22	28392112	rs5711604	G	A	.	PASS	TTC28-	ncR	NR_	.	.
ACa03	22	29804684	rs1856371	C	T	.	PASS	.	inte	NM_	dis	.
ACa03	22	35811911	rs3713772	C	T	.	PASS	MCM5	exo	NM_	.	synony
ACa03	22	38621350	.	G	A	.	PASS	TMEM1	intr	NM_	.	.
ACa03	22	39218542	.	G	A	.	PASS	NPTXR	UTF	NM_	NM	.
ACa03	22	48023174	.	C	A	.	PASS	LINC00	ncR	NR_	.	.
ACa03	X	1010738	rs6644312	G	T	.	PASS	.	inte	NM_	dis	.
ACa03	X	9791221	rs2010857	T	C	.	PASS	SHROC	intr	NM_	.	.
ACa03	X	10062212	.	C	T	.	PASS	WWC3	exo	NM_	.	missens
ACa03	X	38008990	rs7457127	C	T	.	PASS	SRPX	exo	NM_	.	missens
ACa03	X	48544044	.	G	A	.	PASS	WAS	intr	NM_	.	.
ACa03	X	49069162	rs7825308	G	A	.	PASS	CACNA	exo	NM_	.	missens

ACa03	X	61687463	.	G	C	.	PASS	.	inte	NON	dis	.	
ACa03	X	61712615	.	T	C	.	PASS	.	inte	NON	dis	.	
ACa03	X	61712692	rs7756689	A	G	.	PASS	.	inte	NON	dis	.	
ACa03	X	72797180	rs2486782	A	T	.	PASS	CHIC1	intro	NM_	.	.	
ACa03	X	1.12E+08	.	G	A	.	PASS	AMOT	exo	NM_	.	missens	
ACa03	X	1.2E+08	.	T	C	.	PASS	C1GAL	exo	NM_	.	missens	
ACa03	X	1.24E+08	.	C	T	.	PASS	.	ups	NM_	.	.	
ACa03	X	1.39E+08	rs1016097	C	A	.	PASS	MCF2	intro	NM_	.	.	
ACa03	X	1.52E+08	.	G	A	.	PASS	NSDHL	intro	NM_	.	.	
ACa03	X	1.55E+08	rs7802961	G	A	.	PASS	.	inte	NM_	dis	.	
ACa03	MT	4451	.	T	C	.	PASS	.	inte	NON	dis	.	
ACa03	MT	5777	rs3868289	G	A	.	PASS	.	inte	NON	dis	.	
ACa03	MT	12474	.	C	A	.	PASS	.	inte	NON	dis	.	
ACa04	1	1396169	rs7466465	C	T	.	PASS	ATAD3(	exo	NM_	.	synonyr	
ACa04	1	1582548	.	C	G	.	PASS	CDK11E	intro	NM_	.	.	
ACa04	1	11710616	rs1454709	C	T	.	PASS	FBXO2	exo	NM_	.	missens	
ACa04	1	11740656	.	C	T	.	PASS	MAD2L	exo	NM_	.	missens	
ACa04	1	12921635	rs1440990	G	A	.	PASS	PRAME	UTF	NM_	NM	.	
ACa04	1	12936256	rs1157686	G	C	.	PASS	.	inte	NM_	dis	.	
ACa04	1	17007400	.	T	A	.	PASS	.	ups	NR_	.	.	
ACa04	1	17183526	rs7511577	C	T	.	PASS	.	inte	NM_	dis	.	
ACa04	1	21048338	.	G	C	.	PASS	SH2D5	exo	NM_	.	missens	
ACa04	1	23743493	rs5551428	C	T	.	PASS	TCEA3	intro	NM_	.	.	
ACa04	1	24657930	rs1422489	G	A	.	PASS	GRHL3	exo	NM_	.	missens	
ACa04	1	25616915	.	G	A	.	PASS	RHD	intro	NM_	.	.	
ACa04	1	32160745	.	G	A	.	PASS	COL16/	intro	NM_	.	.	
ACa04	1	33402492	rs7753177	G	A	.	PASS	RNF19E	exo	NM_	.	unknow	
ACa04	1	38003606	.	C	T	.	PASS	SNIP1	exo	NM_	.	missens	
ACa04	1	39906714	.	G	C	.	PASS	MACF1	exo	NM_	.	missens	
ACa04	1	39906811	.	G	T	.	PASS	MACF1	intro	NM_	.	.	
ACa04	1	39907024	.	G	T	.	PASS	MACF1	intro	NM_	.	.	
ACa04	1	39908309	.	G	C	.	PASS	MACF1	intro	NM_	.	.	
ACa04	1	41583066	.	A	G	.	PASS	SCMH1	intro	NM_	.	.	
ACa04	1	44595584	rs7580073	C	T	.	PASS	KLF17	exo	NM_	.	missens	
ACa04	1	46707594	rs8662033	C	A	.	PASS	.	inte	NM_	dis	.	
ACa04	1	54656279	.	C	T	.	PASS	CYB5RI	intro	NM_	.	.	
ACa04	1	62503734	.	C	G	.	PASS	INADL	intro	NM_	.	.	
ACa04	1	70144225	.	G	A	.	PASS	.	inte	NR_	dis	.	
ACa04	1	74790728	.	G	A	.	PASS	FPGT-T	intro	NM_	.	.	
ACa04	1	93222477	.	G	A	.	PASS	EVI5	intro	NM_	.	.	
ACa04	1	94484293	.	C	A	.	PASS	ABCA4	intro	NM_	.	.	
ACa04	1	99225562	.	G	A	.	PASS	SNX7	intro	NM_	.	.	
ACa04	1	1.11E+08	rs7598590	G	A	.	PASS	CD53	intro	NM_	.	.	
ACa04	1	1.13E+08	.	G	A	.	PASS	LOC64	exo	NM_	.	synonyr	
ACa04	1	1.21E+08	rs2004173	G	A	.	PASS	FCGR1	UTF	NM_	NM	.	
ACa04	1	1.43E+08	rs1043712	C	T	.	PASS	.	inte	NON	dis	.	
ACa04	1	1.43E+08	.	C	T	.	PASS	.	inte	NR_	dis	.	
ACa04	1	1.43E+08	rs2002777	G	A	.	PASS	.	inte	NR_	dis	.	
ACa04	1	1.43E+08	rs7506054	A	G	.	PASS	.	inte	NR_	dis	.	
ACa04	1	1.43E+08	rs1694653	C	G	.	PASS	.	inte	NR_	dis	.	
ACa04	1	1.45E+08	rs4125366	A	T	.	PASS	NBPF2(	intro	NM_	.	.	
ACa04	1	1.45E+08	.	T	G	.	PASS	NBPF25	ncR	NR_	.	.	
ACa04	1	1.46E+08	.	A	G	.	PASS	NBPF25	ncR	NR_	.	.	
ACa04	1	1.48E+08	rs6181199	G	A	.	PASS	NBPF11	intro	NM_	.	.	

ACa04	1	1.48E+08	rs8799948	C	A	.	PASS	NBPF8	intro	NM_	.	.
ACa04	1	1.48E+08	rs482979	C	T	.	PASS	NBPF25	ncR	NR_	.	.
ACa04	1	1.49E+08	rs2021032	T	G	.	PASS	NBPF25	ncR	NR_	.	.
ACa04	1	1.49E+08	rs2009700	C	G	.	PASS	NBPF25	ncR	NR_	.	.
ACa04	1	1.49E+08	rs2003739	G	A	.	PASS	NBPF25	ncR	NR_	.	.
ACa04	1	1.49E+08	rs3705433	G	A	.	PASS	NBPF25	ncR	NR_	.	.
ACa04	1	1.49E+08	rs226753	G	C	.	PASS	NBPF25	ncR	NR_	.	.
ACa04	1	1.49E+08	.	G	A	.	PASS	NBPF25	ncR	NR_	.	.
ACa04	1	1.49E+08	rs2787777	C	T	.	PASS	.	inte	NR_	dis	.
ACa04	1	1.53E+08	rs3750007	T	A	.	PASS	.	dow	NM_	.	.
ACa04	1	1.54E+08	.	C	T	.	PASS	.	inte	NM_	dis	.
ACa04	1	1.54E+08	.	C	G	.	PASS	INTS3	intro	NM_	.	.
ACa04	1	1.54E+08	.	C	A	.	PASS	SLC27A	intro	NM_	.	.
ACa04	1	1.55E+08	.	A	T	.	PASS	SLC50A	exo	NM_	.	synonyr
ACa04	1	1.56E+08	.	G	T	.	PASS	ARHGE	intro	NM_	.	.
ACa04	1	1.57E+08	.	C	T	.	PASS	PEAR1	intro	NM_	.	.
ACa04	1	1.57E+08	.	C	T	.	PASS	PEAR1	exo	NM_	.	synonyr
ACa04	1	1.73E+08	.	C	T	.	PASS	TNFSF4	intro	NM_	.	.
ACa04	1	1.97E+08	.	C	G	.	PASS	ASPM	exo	NM_	.	missens
ACa04	1	2.01E+08	rs1997462	G	A	.	PASS	KIF21B	intro	NM_	.	.
ACa04	1	2.04E+08	rs3692540	G	A	.	PASS	PLEKHA	exo	NM_	.	missens
ACa04	1	2.21E+08	.	G	A	.	PASS	C1orf11	exo	NM_	.	missens
ACa04	1	2.24E+08	.	C	T	.	PASS	CAPN2	exo	NM_	.	missens
ACa04	1	2.24E+08	.	G	A	.	PASS	.	inte	NM_	dis	.
ACa04	1	2.29E+08	rs7793949	G	A	.	PASS	OBSCN	intro	NM_	.	.
ACa04	2	9347094	.	C	T	.	PASS	ASAP2	UTF	NM_	NM	.
ACa04	2	9991574	.	C	G	.	PASS	TAF1B	intro	NM_	.	.
ACa04	2	24290671	.	G	C	.	PASS	SF3B6	exo	NM_	.	synonyr
ACa04	2	27445559	.	G	C	.	PASS	CAD	intro	NM_	.	.
ACa04	2	27763222	.	C	A	.	PASS	.	inte	NM_	dis	.
ACa04	2	44547496	.	C	T	.	PASS	SLC3A1	exo	NM_	.	synonyr
ACa04	2	44547872	.	C	T	.	PASS	SLC3A1	UTF	NM_	NM	.
ACa04	2	46787755	.	A	G	.	PASS	RHOQ	intro	NM_	.	.
ACa04	2	65729306	.	C	G	.	PASS	.	inte	NM_	dis	.
ACa04	2	69009288	.	C	G	.	PASS	ARHGA	intro	NM_	.	.
ACa04	2	71839619	.	C	T	.	PASS	DYSF	intro	NM_	.	.
ACa04	2	85570645	rs3764475	C	T	.	PASS	RETSA	intro	NM_	.	.
ACa04	2	86362060	rs1425097	C	T	.	PASS	PTCD3	exo	NM_	.	synonyr
ACa04	2	86440603	.	C	T	.	PASS	.	dow	NM_	.	.
ACa04	2	86851036	.	G	A	.	PASS	RNF103	intro	NM_	.	.
ACa04	2	87211799	rs8671368	A	G	.	PASS	RGPD2	intro	NM_	.	.
ACa04	2	87211809	rs8674281	C	A	.	PASS	RGPD1	intro	NM_	.	.
ACa04	2	87276887	.	C	T	.	PASS	LOC285	ncR	NR_	.	.
ACa04	2	90473577	rs1210470	G	A	.	PASS	.	inte	NR_	dis	.
ACa04	2	95858692	rs2015850	T	C	.	PASS	.	inte	NM_	dis	.
ACa04	2	1.03E+08	.	C	T	.	PASS	SLC9A2	exo	NM_	.	synonyr
ACa04	2	1.12E+08	.	G	A	.	PASS	ACOXL	intro	NM_	.	.
ACa04	2	1.13E+08	.	G	C	.	PASS	POLR1I	intro	NM_	.	.
ACa04	2	1.19E+08	rs6216365	A	T	.	PASS	INSIG2	intro	NM_	.	.
ACa04	2	1.28E+08	.	C	T	.	PASS	IWS1	UTF	NM_	NM	.
ACa04	2	1.31E+08	.	C	T	.	PASS	POTEF	intro	NM_	.	.
ACa04	2	1.31E+08	rs1839843	T	C	.	PASS	POTEF	exo	NM_	.	synonyr
ACa04	2	1.32E+08	rs2001821	A	G	.	PASS	POTEE	intro	NM_	.	.
ACa04	2	1.32E+08	.	C	T	.	PASS	LOC401	ncR	NR_	.	.

ACa04	2	1.54E+08	.	G	T	.	PASS	RPRM	UTF	NM_	NM	.
ACa04	2	1.6E+08	.	G	C	.	PASS	WDSUE	intrc	NM_	.	.
ACa04	2	1.78E+08	.	T	G	.	PASS	.	inte	NM_	dis	.
ACa04	2	1.8E+08	.	C	T	.	PASS	CCDC1	exo	NM_	.	missens
ACa04	2	1.98E+08	.	T	G	.	PASS	GTF3C	intrc	NM_	.	.
ACa04	2	2.11E+08	.	T	C	.	PASS	CPS1	intrc	NM_	.	.
ACa04	2	2.17E+08	.	C	T	.	PASS	TMEM1	intrc	NM_	.	.
ACa04	2	2.31E+08	.	G	A	.	PASS	SP140	exo	NM_	.	missens
ACa04	2	2.31E+08	.	G	A	.	PASS	SP140	intrc	NM_	.	.
ACa04	2	2.4E+08	.	C	T	.	PASS	HDAC4	intrc	NM_	.	.
ACa04	3	12892058	.	G	A	.	PASS	.	inte	NM_	dis	.
ACa04	3	15117322	.	G	A	.	PASS	ZFYVE	intrc	NM_	.	.
ACa04	3	15286887	.	G	A	.	PASS	CAPN7	intrc	NM_	.	.
ACa04	3	31837795	.	G	T	.	PASS	OSBPL	intrc	NM_	.	.
ACa04	3	32586427	.	C	G	.	PASS	DYNC1	exo	NM_	.	missens
ACa04	3	47038617	.	C	T	.	PASS	NBEAL	intrc	NM_	.	.
ACa04	3	47270077	.	G	A	.	PASS	KIF9-AS	ncR	NR_	.	.
ACa04	3	49060155	.	C	T	.	PASS	NDUFA	exo	NM_	.	synonyr
ACa04	3	50386165	.	C	T	.	PASS	NPRL2	exo	NM_	.	missens
ACa04	3	50413184	.	G	A	.	PASS	CACNA	intrc	NM_	.	.
ACa04	3	52552723	rs7801345	C	T	.	PASS	STAB1	intrc	NM_	.	.
ACa04	3	52836359	rs3677973	G	A	.	PASS	ITIH3	exo	NM_	.	missens
ACa04	3	55508349	rs7454520	G	A	.	PASS	WNT5A	intrc	NM_	.	.
ACa04	3	97439031	.	A	T	.	PASS	EPHA6	intrc	NM_	.	.
ACa04	3	99643257	rs1911937	G	A	.	PASS	MIR548	ncR	NR_	.	.
ACa04	3	1.14E+08	.	C	T	.	PASS	ZNF80	exo	NM_	.	missens
ACa04	3	1.2E+08	.	C	G	.	PASS	GPR15	exo	NM_	.	missens
ACa04	3	1.23E+08	.	C	T	.	PASS	SEMA5	intrc	NM_	.	.
ACa04	3	1.29E+08	.	C	T	.	PASS	H1FX	exo	NM_	.	missens
ACa04	3	1.29E+08	.	C	T	.	PASS	RPL32F	ncR	NR_	.	.
ACa04	3	1.29E+08	.	C	T	.	PASS	MBD4	exo	NM_	.	missens
ACa04	3	1.29E+08	.	C	T	.	PASS	MBD4	intrc	NM_	.	.
ACa04	3	1.3E+08	.	G	A	.	PASS	COL6A	exo	NM_	.	synonyr
ACa04	3	1.31E+08	.	C	T	.	PASS	ATP2C1	exo	NM_	.	synonyr
ACa04	3	1.38E+08	rs1469630	G	A	.	PASS	NME9	intrc	NM_	.	.
ACa04	3	1.39E+08	.	C	A	.	PASS	FOXL2	exo	NM_	.	stopgai
ACa04	3	1.41E+08	.	G	A	.	PASS	GRK7	exo	NM_	.	synonyr
ACa04	3	1.42E+08	.	T	G	.	PASS	ATR	intrc	NM_	.	.
ACa04	3	1.51E+08	rs7613736	G	C	.	PASS	P2RY12	intrc	NM_	.	.
ACa04	3	1.58E+08	.	C	T	.	PASS	MLF1	intrc	NM_	.	.
ACa04	3	1.67E+08	.	G	A	.	PASS	ZBBX	intrc	NM_	.	.
ACa04	3	1.71E+08	.	G	C	.	PASS	SLC2A	exo	NM_	.	synonyr
ACa04	3	1.72E+08	.	C	T	.	PASS	FNDC3	exo	NM_	.	synonyr
ACa04	3	1.84E+08	.	C	G	.	PASS	HTR3E	exo	NM_	.	missens
ACa04	3	1.85E+08	.	G	A	.	PASS	SENP2	intrc	NM_	.	.
ACa04	3	1.87E+08	.	G	A	.	PASS	RFC4	intrc	NM_	.	.
ACa04	3	1.93E+08	.	C	T	.	PASS	HRASL	intrc	NM_	.	.
ACa04	3	1.95E+08	rs8795450	C	T	.	PASS	.	inte	NM_	dis	.
ACa04	3	1.95E+08	.	C	T	.	PASS	.	inte	NM_	dis	.
ACa04	3	1.96E+08	rs4927838	G	A	.	PASS	SDHAP	ncR	NR_	.	.
ACa04	3	1.96E+08	rs1739998	C	T	.	PASS	SDHAP	ncR	NR_	.	.
ACa04	4	67919	rs4022005	C	T	.	PASS	ZNF595	intrc	NM_	.	.
ACa04	4	983923	rs7518089	C	T	.	PASS	SLC26A	exo	NM_	.	synonyr
ACa04	4	23814675	rs7529426	G	A	.	PASS	PPARG	exo	NM_	.	missens

ACa04	4	28824738	.	G	A	.	PASS	.	inte	NR_	dis	.	
ACa04	4	40099192	.	G	A	.	PASS	N4BP2	splic	NM_	NM	.	
ACa04	4	41608010	.	C	G	.	PASS	LIMCH1	exo	NM_	.	missens	
ACa04	4	49237957	rs7541707	G	T	.	PASS	.	inte	NM_	dis	.	
ACa04	4	57220835	.	G	A	.	PASS	AASDH	intrc	NM_	.	.	
ACa04	4	75067120	rs5423520	C	T	.	PASS	MTHFD	intrc	NM_	.	.	
ACa04	4	79507362	.	G	C	.	PASS	ANXA3	intrc	NM_	.	.	
ACa04	4	1.04E+08	rs8789415	C	A	.	PASS	CISD2	UTF	NM_	NM	.	
ACa04	4	1.1E+08	rs8900187	C	T	.	PASS	COL25A	intrc	NM_	.	.	
ACa04	4	1.19E+08	.	G	A	.	PASS	SNHG8	ncR	NR_	.	.	
ACa04	4	1.29E+08	rs1378897	A	T	.	PASS	MFSD8	intrc	NM_	.	.	
ACa04	4	1.34E+08	.	G	C	.	PASS	PCDH1(	exo	NM_	.	missens	
ACa04	4	1.45E+08	rs3676041	A	G	.	PASS	GYPB	intrc	NM_	.	.	
ACa04	4	1.57E+08	.	T	G	.	PASS	GUCY1.	intrc	NM_	.	.	
ACa04	4	1.7E+08	.	T	C	.	PASS	NEK1	intrc	NM_	.	.	
ACa04	4	1.88E+08	.	G	C	.	PASS	.	ups	NR_	.	.	
ACa04	5	311434	.	G	A	.	PASS	PDCD6	exo	NM_	.	missens	
ACa04	5	12795084	.	C	T	.	PASS	LINC01	ncR	NR_	.	.	
ACa04	5	42467975	.	G	A	.	PASS	GHR	intrc	NM_	.	.	
ACa04	5	55439821	.	A	C	.	PASS	ANKRD	intrc	NM_	.	.	
ACa04	5	56718954	.	G	A	.	PASS	.	inte	NM_	dis	.	
ACa04	5	66085342	.	T	A	.	PASS	MAST4	intrc	NM_	.	.	
ACa04	5	67861845	.	C	T	.	PASS	.	inte	NM_	dis	.	
ACa04	5	69716102	.	G	C	.	PASS	GTF2H2	ncR	NR_	.	.	
ACa04	5	70503586	rs2848141	C	T	.	PASS	.	inte	NR_	dis	.	
ACa04	5	74011331	.	C	T	.	PASS	HEXB	splic	NM_	NM	.	
ACa04	5	74012431	.	C	T	.	PASS	HEXB	exo	NM_	.	stopgai	
ACa04	5	1.19E+08	.	C	T	.	PASS	HSD17B	exo	NM_	.	missens	
ACa04	5	1.4E+08	.	C	A	.	PASS	HARS	intrc	NM_	.	.	
ACa04	5	1.4E+08	.	C	A	.	PASS	HARS	intrc	NM_	.	.	
ACa04	5	1.51E+08	rs1024566	A	C	.	PASS	G3BP1	intrc	NM_	.	.	
ACa04	5	1.51E+08	.	C	T	.	PASS	GLRA1	intrc	NM_	.	.	
ACa04	5	1.57E+08	.	G	C	.	PASS	ADAM1'	intrc	NM_	.	.	
ACa04	5	1.58E+08	.	G	A	.	PASS	EBF1	intrc	NM_	.	.	
ACa04	5	1.68E+08	rs5341551	G	A	.	PASS	TENM2	intrc	NM_	.	.	
ACa04	5	1.76E+08	.	C	T	.	PASS	EIF4E1I	intrc	NM_	.	.	
ACa04	5	1.79E+08	.	G	C	.	PASS	CBY3	exo	NM_	.	synonyr	
ACa04	6	335156	rs2013466	C	G	.	PASS	DUSP22	exo	NM_	.	missens	
ACa04	6	3138326	.	C	G	.	PASS	BPHL	intrc	NM_	.	.	
ACa04	6	4892397	.	C	G	.	PASS	CDYL	exo	NM_	.	missens	
ACa04	6	26158679	rs7489137	G	A	.	PASS	HIST1H	exo	NM_	.	synonyr	
ACa04	6	27279024	.	C	T	.	PASS	POM12	exo	NM_	.	missens	
ACa04	6	27279395	.	C	G	.	PASS	POM12	exo	NM_	.	missens	
ACa04	6	28255115	rs9402071	C	T	.	PASS	PGBD1	intrc	NM_	.	.	
ACa04	6	30627926	.	G	C	.	PASS	DHX16	exo	NM_	.	synonyr	
ACa04	6	30629422	.	G	C	.	PASS	DHX16	intrc	NM_	.	.	
ACa04	6	31239998	.	G	A	.	PASS	.	ups	NM_	.	.	
ACa04	6	31544686	.	G	A	.	PASS	TNF	intrc	NM_	.	.	
ACa04	6	31868501	.	G	A	.	PASS	ZBTB12	exo	NM_	.	synonyr	
ACa04	6	31948488	.	C	T	.	PASS	STK19	exo	NM_	.	missens	
ACa04	6	31973995	rs5421901	C	T	.	PASS	CYP21A	ncR	NR_	.	.	
ACa04	6	31974685	.	C	T	.	PASS	CYP21A	exo	NM_	.	unknow	
ACa04	6	31974901	.	C	T	.	PASS	CYP21A	ncR	NR_	.	.	
ACa04	6	36341267	.	C	A	.	PASS	ETV7	exo	NM_	.	synonyr	

ACa04	6	37420800	.	T	G	.	PASS	CMTR1	intrc	NM_	.	.
ACa04	6	44279287	rs3746609	A	C	.	PASS	AARS2	intrc	NM_	.	.
ACa04	6	46826299	rs5482384	C	T	.	PASS	GPR11(	exoı	NM_	.	missens
ACa04	6	50011361	rs1442276	G	A	.	PASS	DEFB11	exoı	NM_	.	missens
ACa04	6	80737801	rs9008212	C	G	.	PASS	TTK	intrc	NM_	.	.
ACa04	6	90562798	.	C	T	.	PASS	CASP8/	intrc	NM_	.	.
ACa04	6	1.1E+08	rs7466151	C	T	.	PASS	AK9	intrc	NM_	.	.
ACa04	6	1.19E+08	.	C	T	.	PASS	CEP85L	exoı	NM_	.	missens
ACa04	6	1.54E+08	rs5411063	G	A	.	PASS	.	inteı	NM_	dis	.
ACa04	6	1.6E+08	.	G	C	.	PASS	LOC10(	ncR	NR_	.	.
ACa04	7	1513164	rs3695415	G	A	.	PASS	INTS1	intrc	NM_	.	.
ACa04	7	4829784	rs9183664	C	T	.	PASS	AP5Z1	intrc	NM_	.	.
ACa04	7	7106766	.	G	T	.	PASS	.	inteı	NM_	dis	.
ACa04	7	29742438	.	C	T	.	PASS	.	inteı	NR_	dis	.
ACa04	7	45016694	rs1485954	C	T	.	PASS	MYO1G	intrc	NM_	.	.
ACa04	7	56881682	rs3677059	G	A	.	PASS	.	inteı	NR_	dis	.
ACa04	7	56886709	.	C	G	.	PASS	.	inteı	NR_	dis	.
ACa04	7	56886816	.	A	T	.	PASS	.	inteı	NR_	dis	.
ACa04	7	56891472	.	T	C	.	PASS	.	inteı	NR_	dis	.
ACa04	7	62956286	rs2018986	T	C	.	PASS	.	inteı	NR_	dis	.
ACa04	7	63040357	rs1860492	C	T	.	PASS	.	inteı	NR_	dis	.
ACa04	7	63216281	.	C	A	.	PASS	.	inteı	NR_	dis	.
ACa04	7	63219061	.	G	C	.	PASS	.	inteı	NR_	dis	.
ACa04	7	66648249	rs2687038	T	C	.	PASS	TYW1	intrc	NM_	.	.
ACa04	7	70881071	.	A	C	.	PASS	WBSCF	intrc	NM_	.	.
ACa04	7	75240989	.	G	T	.	PASS	HIP1	intrc	NM_	.	.
ACa04	7	97558116	.	A	G	.	PASS	.	inteı	NM_	dis	.
ACa04	7	1E+08	rs3730249	G	A	.	PASS	SRRT	intrc	NM_	.	.
ACa04	7	1.28E+08	rs3721622	G	A	.	PASS	RBM28	exoı	NM_	.	missens
ACa04	7	1.28E+08	.	G	C	.	PASS	RBM28	exoı	NM_	.	missens
ACa04	7	1.29E+08	rs1030263	C	T	.	PASS	KCP	intrc	NM_	.	.
ACa04	7	1.42E+08	.	C	T	.	PASS	.	inteı	NM_	dis	.
ACa04	7	1.49E+08	.	G	C	.	PASS	EZH2	intrc	NM_	.	.
ACa04	7	1.52E+08	rs8678229	G	T	.	PASS	KMT2C	intrc	NM_	.	.
ACa04	8	7438248	.	G	A	.	PASS	.	inteı	NR_	dis	.
ACa04	8	7468775	rs6264068	C	T	.	PASS	.	inteı	NR_	dis	.
ACa04	8	11410882	.	G	A	.	PASS	BLK	intrc	NM_	.	.
ACa04	8	12045755	.	A	G	.	PASS	FAM86E	intrc	NM_	.	.
ACa04	8	12293612	.	G	A	.	PASS	FAM86E	intrc	NM_	.	.
ACa04	8	23104273	rs7607907	C	T	.	PASS	CHMP7	exoı	NM_	.	missens
ACa04	8	32406297	.	G	A	.	PASS	NRG1	exoı	NM_	.	missens
ACa04	8	37732570	rs3736343	G	A	.	PASS	RAB11F	exoı	NM_	.	missens
ACa04	8	54628641	.	C	T	.	PASS	ATP6V1	intrc	NM_	.	.
ACa04	8	73937164	.	G	A	.	PASS	TERF1	exoı	NM_	.	synonyr
ACa04	8	80677293	rs9872967	T	A	.	PASS	HEY1	UTF	NM_	NM	.
ACa04	8	89497390	.	G	A	.	PASS	.	inteı	NM_	dis	.
ACa04	8	90426559	.	G	A	.	PASS	.	inteı	NM_	dis	.
ACa04	8	91586038	.	T	A	.	PASS	.	inteı	NR_	dis	.
ACa04	8	1.01E+08	.	G	C	.	PASS	RGS22	intrc	NM_	.	.
ACa04	8	1.14E+08	.	G	A	.	PASS	CSMD3	intrc	NM_	.	.
ACa04	8	1.21E+08	.	G	T	.	PASS	TAF2	exoı	NM_	.	missens
ACa04	8	1.24E+08	.	G	A	.	PASS	.	inteı	NR_	dis	.
ACa04	8	1.26E+08	rs1436548	G	C	.	PASS	KIAA01!	exoı	NM_	.	missens
ACa04	8	1.26E+08	.	G	C	.	PASS	KIAA01!	intrc	NM_	.	.

ACa04	8	1.41E+08	.	G	A	.	PASS	TRAPP(	intrc	NM_	.	.
ACa04	9	14997152	.	C	T	.	PASS	LOC389	ncR	NR_	.	.
ACa04	9	19078284	rs7682516	C	T	.	PASS	HAUS6	exoı	NM_	.	missens
ACa04	9	21077312	.	C	T	.	PASS	IFNB1	exoı	NM_	.	missens
ACa04	9	33923025	.	G	C	.	PASS	UBAP2	exoı	NM_	.	stopgaiı
ACa04	9	39144453	rs3764507	T	C	.	PASS	CNTNA	intrc	NM_	.	.
ACa04	9	45733695	.	C	G	.	PASS	LOC102	inteı	NR_	dis	.
ACa04	9	67786147	.	G	C	.	PASS	FAM27E	ncR	NR_	.	.
ACa04	9	69452504	.	G	A	.	PASS	.	inteı	NM_	dis	.
ACa04	9	72832084	.	C	G	.	PASS	SMC5-A	ncR	NR_	.	.
ACa04	9	79946883	.	C	G	.	PASS	VPS13A	intrc	NM_	.	.
ACa04	9	82336825	.	T	G	.	PASS	TLE4	intrc	NM_	.	.
ACa04	9	98678009	.	G	C	.	PASS	ERCC6I	exoı	NM_	.	missens
ACa04	9	1.15E+08	rs9619494	C	T	.	PASS	HSDL2	splic	NM_	NM	.
ACa04	9	1.25E+08	.	C	T	.	PASS	DAB2IP	exoı	NM_	.	synonyr
ACa04	9	1.29E+08	.	G	A	.	PASS	LMX1B	exoı	NM_	.	synonyr
ACa04	9	1.31E+08	.	G	A	.	PASS	CERCA	intrc	NM_	.	.
ACa04	9	1.32E+08	rs7589091	C	T	.	PASS	IER5L	UTF	NM_	NM	.
ACa04	9	1.33E+08	.	C	A	.	PASS	GPR107	splic	NM_	NM	.
ACa04	9	1.34E+08	.	G	T	.	PASS	RAPGE	intrc	NM_	.	.
ACa04	9	1.4E+08	.	C	T	.	PASS	.	inteı	NM_	dis	.
ACa04	9	1.4E+08	.	C	T	.	PASS	PNPLA7	exoı	NM_	.	unknow
ACa04	10	11971934	.	C	A	.	PASS	UPF2	exoı	NM_	.	stopgaiı
ACa04	10	21805480	rs2018361	T	C	.	PASS	SKIDA1	exoı	NM_	.	synonyr
ACa04	10	45487624	.	T	G	.	PASS	RASSF4	intrc	NM_	.	.
ACa04	10	45487625	.	T	G	.	PASS	RASSF4	intrc	NM_	.	.
ACa04	10	71025668	.	G	T	.	PASS	HKDC1	intrc	NM_	.	.
ACa04	10	71026465	rs3760330	G	A	.	PASS	HKDC1	exoı	NM_	.	synonyr
ACa04	10	71027074	.	G	A	.	PASS	HKDC1	UTF	NM_	NM	.
ACa04	10	73039805	.	G	C	.	PASS	UNC5B	splic	NM_	NM	.
ACa04	10	73053568	rs7593379	G	A	.	PASS	UNC5B	exoı	NM_	.	missens
ACa04	10	73330675	rs7698966	G	A	.	PASS	CDH23	exoı	NM_	.	synonyr
ACa04	10	81292524	.	C	T	.	PASS	.	inteı	NM_	dis	.
ACa04	10	81292525	.	A	G	.	PASS	.	inteı	NM_	dis	.
ACa04	10	94409977	.	C	G	.	PASS	KIF11	intrc	NM_	.	.
ACa04	10	1.05E+08	.	G	A	.	PASS	PDCD1	intrc	NM_	.	.
ACa04	10	1.24E+08	.	G	A	.	PASS	PLEKHA	intrc	NM_	.	.
ACa04	10	1.35E+08	rs7966467	A	G	.	PASS	.	inteı	NM_	dis	.
ACa04	11	1026075	rs7657851	G	A	.	PASS	MUC6	exoı	NM_	.	synonyr
ACa04	11	4351281	rs8667539	C	G	.	PASS	.	inteı	NR_	dis	.
ACa04	11	9990085	.	G	A	.	PASS	SBF2	exoı	NM_	.	missens
ACa04	11	25004735	.	C	T	.	PASS	LUZP2	exoı	NM_	.	stopgaiı
ACa04	11	32456855	.	C	T	.	PASS	WT1	exoı	NM_	.	missens
ACa04	11	46397105	.	A	G	.	PASS	DGKZ	exoı	NM_	.	missens
ACa04	11	47194231	.	G	T	.	PASS	ARFGA	intrc	NM_	.	.
ACa04	11	56229791	.	C	A	.	PASS	.	dow	NM_	.	.
ACa04	11	61249334	rs7463740	C	T	.	PASS	PPP1R3	exoı	NM_	.	missens
ACa04	11	61687361	.	T	G	.	PASS	RAB3IL	intrc	NM_	.	.
ACa04	11	63033390	.	C	T	.	PASS	MIR368	ncR	NR_	.	.
ACa04	11	63681628	rs3704854	C	T	.	PASS	RCOR2	exoı	NM_	.	missens
ACa04	11	63992577	.	C	G	.	PASS	TRPT1	intrc	NM_	.	.
ACa04	11	63995110	.	C	G	.	PASS	NUDT22	exoı	NM_	.	missens
ACa04	11	64004869	.	G	T	.	PASS	VEGFB	intrc	NM_	.	.
ACa04	11	64010930	.	G	A	.	PASS	FKBP2	exoı	NM_	.	missens

ACa04	11	64011526	.	G	A	.	PASS	FKBP2	UTF	NM_	NM	.
ACa04	11	64119499	.	C	T	.	PASS	CCDC8	intrc	NM_	.	.
ACa04	11	65315334	rs7459229	C	T	.	PASS	LTBP3	intrc	NM_	.	.
ACa04	11	73997380	.	C	G	.	PASS	P4HA3	exoı	NM_	.	missens
ACa04	11	1.13E+08	.	G	A	.	PASS	NCAM1	intrc	NM_	.	.
ACa04	11	1.17E+08	rs3521580	T	C	.	PASS	PCSK7	intrc	NM_	.	.
ACa04	11	1.17E+08	rs3580670	C	T	.	PASS	PCSK7	intrc	NM_	.	.
ACa04	11	1.25E+08	rs9203240	C	T	.	PASS	HEPAC	UTF	NM_	NM	.
ACa04	11	1.3E+08	.	C	T	.	PASS	ZBTB44	exoı	NM_	.	missens
ACa04	12	7031400	.	C	G	.	PASS	ENO2	intrc	NM_	.	.
ACa04	12	7031852	.	C	T	.	PASS	ENO2	intrc	NM_	.	.
ACa04	12	8234860	.	C	T	.	PASS	NECAP	UTF	NM_	NM	.
ACa04	12	31242999	rs7123030	G	A	.	PASS	DDX11	exoı	NM_	.	missens
ACa04	12	32135759	.	G	A	.	PASS	KIAA15	exoı	NM_	.	missens
ACa04	12	41585427	.	G	C	.	PASS	PDZRN	intrc	NM_	.	.
ACa04	12	46321767	.	C	G	.	PASS	SCAF11	exoı	NM_	.	missens
ACa04	12	58158669	.	C	T	.	PASS	CYP27E	exoı	NM_	.	missens
ACa04	12	58160118	.	C	G	.	PASS	CYP27E	intrc	NM_	.	.
ACa04	12	63153019	rs8674713	G	A	.	PASS	PPM1H	intrc	NM_	.	.
ACa04	12	68709753	rs9109827	C	T	.	PASS	MDM1	intrc	NM_	.	.
ACa04	12	80848802	rs9642294	C	T	.	PASS	PTPRQ	intrc	NM_	.	.
ACa04	12	88429683	.	T	G	.	PASS	C12orf2	intrc	NM_	.	.
ACa04	12	94132221	.	C	A	.	PASS	CRADD	intrc	NM_	.	.
ACa04	12	1.02E+08	.	G	C	.	PASS	MYBPC	exoı	NM_	.	missens
ACa04	12	1.14E+08	.	C	T	.	PASS	RBM19	intrc	NM_	.	.
ACa04	12	1.16E+08	.	C	T	.	PASS	MED13I	exoı	NM_	.	synonyr
ACa04	12	1.21E+08	.	G	A	.	PASS	GCN1L	intrc	NM_	.	.
ACa04	12	1.21E+08	rs7540482	G	A	.	PASS	PLA2G1	intrc	NM_	.	.
ACa04	12	1.24E+08	.	G	A	.	PASS	SBNO1	intrc	NM_	.	.
ACa04	12	1.33E+08	.	C	G	.	PASS	EP400	intrc	NM_	.	.
ACa04	13	21244946	.	A	C	.	PASS	IFT88	intrc	NM_	.	.
ACa04	13	35716455	.	G	C	.	PASS	NBEA	exoı	NM_	.	missens
ACa04	13	43462522	.	G	C	.	PASS	EPSTI1	exoı	NM_	.	stopgaiı
ACa04	13	99554177	.	C	A	.	PASS	DOCK9	intrc	NM_	.	.
ACa04	13	1.03E+08	rs7720033	G	T	.	PASS	FGF14	exoı	NM_	.	synonyr
ACa04	14	19750514	rs1450473	C	T	.	PASS	.	inteı	NR_	dis	.
ACa04	14	22237385	.	A	C	.	PASS	.	inteı	NM_	dis	.
ACa04	14	24422863	.	G	A	.	PASS	DHRS4	ncR	NR_	.	.
ACa04	14	24423433	.	G	A	.	PASS	DHRS4	ncR	NR_	.	.
ACa04	14	38218126	.	C	T	.	PASS	.	inteı	NM_	dis	.
ACa04	14	50033101	.	G	C	.	PASS	.	inteı	NR_	dis	.
ACa04	14	55326401	rs1501582	C	T	.	PASS	GCH1	exoı	NM_	.	synonyr
ACa04	14	60427856	.	C	A	.	PASS	LRRC9	ncR	NR_	.	.
ACa04	14	65488560	.	C	A	.	PASS	MAX,CH	intrc	NM_	.	.
ACa04	14	65488830	.	C	A	.	PASS	MAX,CH	intrc	NM_	.	.
ACa04	14	70346418	rs7676169	G	A	.	PASS	SMOC1	exoı	NM_	.	missens
ACa04	14	73718454	.	C	T	.	PASS	PAPLN	exoı	NM_	.	synonyr
ACa04	14	77257611	.	G	A	.	PASS	ANGEL	intrc	NM_	.	.
ACa04	14	89591578	.	G	C	.	PASS	.	inteı	NM_	dis	.
ACa04	14	93177988	.	G	A	.	PASS	LGMN	intrc	NM_	.	.
ACa04	14	95111247	rs3756754	G	A	.	PASS	SERPIN	ncR	NR_	.	.
ACa04	14	1.06E+08	.	T	G	.	PASS	MTA1	intrc	NM_	.	.
ACa04	14	1.06E+08	rs5537870	G	A	.	PASS	.	inteı	NR_	dis	.
ACa04	14	1.07E+08	rs1289410	G	A	.	PASS	.	inteı	NR_	dis	.

ACa04	15	21021529	.	C	T	.	PASS	.	inte	NR_	dis	.
ACa04	15	23105942	.	C	A	.	PASS	LOC283	ncR	NR_	.	.
ACa04	15	28408339	rs5409237	C	T	.	PASS	HERC2	exo	NM_	.	synonyr
ACa04	15	28649661	.	T	G	.	PASS	.	inte	NR_	dis	.
ACa04	15	29064347	.	T	G	.	PASS	PDCD6I	ncR	NR_	.	.
ACa04	15	30874998	.	T	C	.	PASS	ULK4P1	ncR	NR_	.	.
ACa04	15	31089768	rs2015085	C	A	.	PASS	.	inte	NR_	dis	.
ACa04	15	41822216	rs7481072	C	T	.	PASS	RPAP1	intrc	NM_	.	.
ACa04	15	41827886	.	C	T	.	PASS	RPAP1	intrc	NM_	.	.
ACa04	15	41828755	.	C	G	.	PASS	RPAP1	exo	NM_	.	missens
ACa04	15	42154421	rs7754448	G	A	.	PASS	SPTBN!	exo	NM_	.	synonyr
ACa04	15	43927718	.	G	T	.	PASS	CATSPI	intrc	NM_	.	.
ACa04	15	44100887	rs1922871	C	T	.	PASS	MFAP1	intrc	NM_	.	.
ACa04	15	44849933	.	C	T	.	PASS	EIF3J	intrc	NM_	.	.
ACa04	15	45120967	rs6202600	G	A	.	PASS	.	inte	NM_	dis	.
ACa04	15	45697652	.	G	A	.	PASS	SPATA!	exo	NM_	.	synonyr
ACa04	15	56390588	.	G	A	.	PASS	RFX7	intrc	NM_	.	.
ACa04	15	60678134	.	C	T	.	PASS	ANXA2	intrc	NM_	.	.
ACa04	15	65041446	rs5478938	C	T	.	PASS	RBPMS	intrc	NM_	.	.
ACa04	15	66774038	.	C	A	.	PASS	MAP2K	intrc	NM_	.	.
ACa04	15	67920461	.	G	C	.	PASS	MAP2K!	intrc	NM_	.	.
ACa04	15	74468572	.	G	A	.	PASS	ISLR	UTF	NM_	NM	.
ACa04	15	75518573	.	C	T	.	PASS	.	inte	NM_	dis	.
ACa04	15	78922067	.	C	T	.	PASS	CHRNB	exo	NM_	.	missens
ACa04	15	79586294	.	C	G	.	PASS	ANKRD	exo	NM_	.	missens
ACa04	15	84924696	rs2401418	T	C	.	PASS	.	inte	NM_	dis	.
ACa04	15	89828324	.	C	T	.	PASS	FANCI	splic	NM_	NM	.
ACa04	15	91551123	.	C	G	.	PASS	VPS33E	exo	NM_	.	missens
ACa04	15	94884003	.	C	G	.	PASS	MCTP2	intrc	NM_	.	.
ACa04	15	1E+08	.	C	T	.	PASS	MEF2A	intrc	NM_	.	.
ACa04	15	1.02E+08	rs7510253	C	T	.	PASS	TARSL2	exo	NM_	.	missens
ACa04	16	138813	rs7504825	G	A	.	PASS	NPRL3	exo	NM_	.	unknow
ACa04	16	1498194	.	C	T	.	PASS	CLCN7	intrc	NM_	.	.
ACa04	16	7703685	.	G	A	.	PASS	RBFOX	intrc	NM_	.	.
ACa04	16	10775977	.	G	A	.	PASS	TEKT5	exo	NM_	.	stopgai
ACa04	16	12061615	.	C	T	.	PASS	TNFRSI	exo	NM_	.	missens
ACa04	16	14932478	.	G	A	.	PASS	NOMO1	intrc	NM_	.	.
ACa04	16	15460754	rs5522924	C	T	.	PASS	NPIPA5	intrc	NM_	.	.
ACa04	16	16465395	rs6203288	T	C	.	PASS	.	inte	NR_	dis	.
ACa04	16	18840681	rs7654396	C	T	.	PASS	SMG1	exo	NM_	.	missens
ACa04	16	22274612	.	G	A	.	PASS	EEF2K	intrc	NM_	.	.
ACa04	16	22314516	.	C	T	.	PASS	POLR3I	intrc	NM_	.	.
ACa04	16	22492403	.	A	G	.	PASS	SMG1P	ncR	NR_	.	.
ACa04	16	28181173	.	C	G	.	PASS	XPO6	exo	NM_	.	missens
ACa04	16	28188845	.	G	A	.	PASS	XPO6	intrc	NM_	.	.
ACa04	16	29634159	.	G	A	.	PASS	.	inte	NR_	dis	.
ACa04	16	30777461	.	T	A	.	PASS	RNF40	intrc	NM_	.	.
ACa04	16	31283023	.	C	T	.	PASS	ITGAM	intrc	NM_	.	.
ACa04	16	33393614	rs7896651	A	C	.	PASS	.	inte	NR_	dis	.
ACa04	16	48311245	rs7515102	C	G	.	PASS	LONP2	splic	NM_	NM	.
ACa04	16	58571009	.	C	T	.	PASS	CNOT1	exo	NM_	.	missens
ACa04	16	67650756	.	C	G	.	PASS	CTCF	exo	NM_	.	missens
ACa04	16	71917174	.	T	A	.	PASS	ZNF821	UTF	NM_	NM	.
ACa04	16	74366694	rs2911030	A	G	.	PASS	LOC283	ncR	NR_	.	.

ACa04	16	89748999	.	G	A	.	PASS	.	inte	NM_	dis	.	
ACa04	16	90239923	.	C	T	.	PASS	.	ncR	NR_	.	.	
ACa04	17	3819296	rs7805194	C	T	.	PASS	P2RX1	intrc	NM_	.	.	
ACa04	17	6905777	.	C	T	.	PASS	ALOX12	ncR	NR_	.	.	
ACa04	17	7452538	.	C	G	.	PASS	TNFSF1	exo	NM_	.	missens	
ACa04	17	7644304	.	C	T	.	PASS	DNAH2	exo	NM_	.	synonyr	
ACa04	17	10261063	.	A	G	.	PASS	MYH13	exo	NM_	.	missens	
ACa04	17	11502002	.	G	C	.	PASS	DNAH9	exo	NM_	.	missens	
ACa04	17	15905433	rs5595610	G	A	.	PASS	TTC19	intrc	NM_	.	.	
ACa04	17	17715857	.	C	G	.	PASS	SREBF	UTF	NM_	NM	.	
ACa04	17	18580745	.	G	C	.	PASS	ZNF286	intrc	NM_	.	.	
ACa04	17	20239326	.	C	T	.	PASS	CCDC1	ncR	NR_	.	.	
ACa04	17	20361478	.	G	A	.	PASS	LGALS	intrc	NM_	.	.	
ACa04	17	21904546	rs7841783	G	A	.	PASS	FLJ360	ncR	NR_	.	.	
ACa04	17	27051414	.	C	T	.	PASS	TLCD1	UTF	NM_	NM	.	
ACa04	17	27186144	.	G	C	.	PASS	ERAL1	intrc	NM_	.	.	
ACa04	17	27188767	.	G	A	.	PASS	.	ups	NR_	.	.	
ACa04	17	30267462	.	C	G	.	PASS	SUZ12	exo	NM_	.	missens	
ACa04	17	30267521	.	C	A	.	PASS	SUZ12	intrc	NM_	.	.	
ACa04	17	30509668	.	G	C	.	PASS	RHOT1	intrc	NM_	.	.	
ACa04	17	34585113	.	G	T	.	PASS	LOC101	intrc	NM_	.	.	
ACa04	17	34585273	rs8795183	C	T	.	PASS	TBC1D	intrc	NM_	.	.	
ACa04	17	34959090	.	G	A	.	PASS	MRM1	intrc	NM_	.	.	
ACa04	17	35696532	.	G	C	.	PASS	ACACA	intrc	NM_	.	.	
ACa04	17	35879278	.	G	A	.	PASS	SYNRG	intrc	NM_	.	.	
ACa04	17	36349424	.	C	A	.	PASS	.	ups	NM_	.	.	
ACa04	17	37564752	.	G	C	.	PASS	MED1	exo	NM_	.	missens	
ACa04	17	39563642	rs1035039	C	T	.	PASS	LOC100	ncR	NR_	.	.	
ACa04	17	40189689	.	G	A	.	PASS	ZNF385	intrc	NM_	.	.	
ACa04	17	42336474	.	C	T	.	PASS	SLC4A1	intrc	NM_	.	.	
ACa04	17	48456415	.	C	T	.	PASS	EME1	intrc	NM_	.	.	
ACa04	17	48695858	.	C	T	.	PASS	CACNA	intrc	NM_	.	.	
ACa04	17	56382745	.	C	G	.	PASS	BZRAP	splic	NM_	NM	.	
ACa04	17	61875411	.	G	C	.	PASS	DDX42	exo	NM_	.	missens	
ACa04	17	61875421	.	G	T	.	PASS	DDX42	exo	NM_	.	missens	
ACa04	17	63685153	.	T	G	.	PASS	CEP112	intrc	NM_	.	.	
ACa04	17	73830621	.	G	C	.	PASS	UNC13	intrc	NM_	.	.	
ACa04	17	78210752	rs1405528	C	T	.	PASS	SLC26A	exo	NM_	.	synonyr	
ACa04	17	79077457	.	C	T	.	PASS	BAIAP2	exo	NM_	.	synonyr	
ACa04	17	81180181	rs3677917	A	G	.	PASS	FLJ436	ncR	NR_	.	.	
ACa04	18	6241463	.	G	C	.	PASS	L3MBTL	intrc	NM_	.	.	
ACa04	18	7231454	.	G	A	.	PASS	LRRC3	exo	NM_	.	synonyr	
ACa04	18	9561991	.	C	G	.	PASS	PPP4R	exo	NM_	.	missens	
ACa04	18	9885754	.	G	C	.	PASS	TXNDC	UTF	NM_	NM	.	
ACa04	18	10682288	.	C	G	.	PASS	PIEZO2	exo	NM_	.	missens	
ACa04	18	13734582	.	G	A	.	PASS	RNMT	exo	NM_	.	missens	
ACa04	18	14186351	.	C	T	.	PASS	ANKRD	ncR	NR_	.	.	
ACa04	18	24173671	.	G	T	.	PASS	KCTD1	intrc	NM_	.	.	
ACa04	18	44773328	.	C	T	.	PASS	SKOR2	exo	NM_	.	missens	
ACa04	18	53252369	.	G	A	.	PASS	TCF4	intrc	NM_	.	.	
ACa04	18	53695975	.	C	T	.	PASS	.	inte	NR_	dis	.	
ACa04	18	60027077	rs7468202	C	T	.	PASS	TNFRSI	intrc	NM_	.	.	
ACa04	18	66564563	.	G	A	.	PASS	CCDC1	exo	NM_	.	synonyr	
ACa04	18	77132914	.	A	G	.	PASS	ATP9B	intrc	NM_	.	.	

ACa04	19	544031	rs9545783	C	T	.	PASS	.	ups	NM_	.	.
ACa04	19	1067640	.	G	A	.	PASS	HMHA1	intrc	NM_	.	.
ACa04	19	1068927	.	G	C	.	PASS	HMHA1	intrc	NM_	.	.
ACa04	19	1625821	.	G	T	.	PASS	TCF3	intrc	NM_	.	.
ACa04	19	1627347	rs3742787	G	A	.	PASS	TCF3	intrc	NM_	.	.
ACa04	19	1923847	rs1418930	A	T	.	PASS	SCAMP	intrc	NM_	.	.
ACa04	19	2805172	.	G	T	.	PASS	THOP1	exoı	NM_	.	stopgaiı
ACa04	19	2806807	rs7627736	G	A	.	PASS	THOP1	intrc	NM_	.	.
ACa04	19	3359586	.	C	T	.	PASS	NFIC	UTF	NM_	NM	.
ACa04	19	4423978	.	G	C	.	PASS	CHAF1/	intrc	NM_	.	.
ACa04	19	6161470	.	G	A	.	PASS	ACSBG	intrc	NM_	.	.
ACa04	19	7707610	.	T	A	.	PASS	STXBP2	intrc	NM_	.	.
ACa04	19	8430554	.	A	G	.	PASS	ANGPT	intrc	NM_	.	.
ACa04	19	8444864	.	G	C	.	PASS	RAB11E	ncR	NR_	.	.
ACa04	19	8604992	.	C	G	.	PASS	MYO1F	intrc	NM_	.	.
ACa04	19	9049466	.	G	T	.	PASS	MUC16	exoı	NM_	.	stopgaiı
ACa04	19	9049499	.	G	A	.	PASS	MUC16	exoı	NM_	.	missens
ACa04	19	9071765	.	G	A	.	PASS	MUC16	exoı	NM_	.	synonyr
ACa04	19	9072600	rs7741875	G	A	.	PASS	MUC16	exoı	NM_	.	missens
ACa04	19	10812558	.	C	T	.	PASS	QTRT1	intrc	NM_	.	.
ACa04	19	12460871	.	G	C	.	PASS	ZNF442	exoı	NM_	.	missens
ACa04	19	12876605	.	G	A	.	PASS	HOOK2	intrc	NM_	.	.
ACa04	19	12902551	.	G	T	.	PASS	JUNB	UTF	NM_	NM	.
ACa04	19	16890254	.	C	G	.	PASS	NWD1	exoı	NM_	.	missens
ACa04	19	17657528	rs7797020	G	A	.	PASS	FAM129	exoı	NM_	.	missens
ACa04	19	17989080	.	G	A	.	PASS	SLC5A5	intrc	NM_	.	.
ACa04	19	19643635	.	A	T	.	PASS	YJEFN3	intrc	NM_	.	.
ACa04	19	20002691	.	C	T	.	PASS	ZNF253	exoı	NM_	.	missens
ACa04	19	22379589	.	G	A	.	PASS	ZNF676	UTF	NM_	NM	.
ACa04	19	30156809	.	C	G	.	PASS	PLEKHF	intrc	NM_	.	.
ACa04	19	33302189	.	G	A	.	PASS	.	inteı	NM_	dis	.
ACa04	19	36108349	.	G	C	.	PASS	HAUS5	intrc	NM_	.	.
ACa04	19	36759849	rs8683521	T	G	.	PASS	.	inteı	NM_	dis	.
ACa04	19	36759850	rs8793125	T	C	.	PASS	.	inteı	NM_	dis	.
ACa04	19	37240872	rs7653982	C	T	.	PASS	ZNF850	exoı	NM_	.	missens
ACa04	19	39869313	rs9896621	C	T	.	PASS	SAMD4	intrc	NM_	.	.
ACa04	19	40370173	.	G	A	.	PASS	FCGBP	intrc	NM_	.	.
ACa04	19	42406617	rs1408143	C	T	.	PASS	ARHGE	intrc	NM_	.	.
ACa04	19	42558106	.	G	C	.	PASS	GRIK5	intrc	NM_	.	.
ACa04	19	42569637	rs1444115	C	T	.	PASS	GRIK5	intrc	NM_	.	.
ACa04	19	43349628	.	G	A	.	PASS	PSG10F	ncR	NR_	.	.
ACa04	19	44156138	.	G	C	.	PASS	PLAUR	intrc	NM_	.	.
ACa04	19	44156387	rs4251912	G	A	.	PASS	PLAUR	exoı	NM_	.	synonyr
ACa04	19	46417852	.	C	G	.	PASS	NANOS	exoı	NM_	.	missens
ACa04	19	47545927	.	G	T	.	PASS	NPAS1	intrc	NM_	.	.
ACa04	19	50160962	.	C	A	.	PASS	SCAF1	intrc	NM_	.	.
ACa04	19	52496934	.	G	A	.	PASS	ZNF615	exoı	NM_	.	synonyr
ACa04	19	53059406	.	T	G	.	PASS	.	dow	NM_	.	.
ACa04	19	55344025	.	G	A	.	PASS	.	ups	NM_	.	.
ACa04	19	55421386	.	G	C	.	PASS	NCR1	exoı	NM_	.	missens
ACa04	19	57641484	.	G	C	.	PASS	USP29	exoı	NM_	.	missens
ACa04	19	57641504	.	G	C	.	PASS	USP29	exoı	NM_	.	missens
ACa04	20	1569372	.	C	T	.	PASS	SIRPB1	intrc	NM_	.	.
ACa04	20	2378367	.	T	G	.	PASS	TGM6	intrc	NM_	.	.

ACa04	20	2575258	.	G	A	.	PASS	TMC2	intrc	NM_	.	.
ACa04	20	3674136	.	G	A	.	PASS	SIGLEC	exoı	NM_	.	missens
ACa04	20	8722139	rs7610831	C	G	.	PASS	PLCB1	exoı	NM_	.	missens
ACa04	20	17705827	.	C	G	.	PASS	BANF2	intrc	NM_	.	.
ACa04	20	17943516	.	G	A	.	PASS	SNORD	ncR	NR_	.	.
ACa04	20	29623957	rs8120738	C	G	.	PASS	FRG1B	ncR	NR_	.	.
ACa04	20	29624070	rs7791580	G	A	.	PASS	FRG1B	ncR	NR_	.	.
ACa04	20	30640006	.	C	T	.	PASS	HCK	UTF	NM_	NM	.
ACa04	20	33328131	.	C	G	.	PASS	NCOA6	intrc	NM_	.	.
ACa04	20	33328684	rs1904867	C	T	.	PASS	NCOA6	exoı	NM_	.	missens
ACa04	20	48562018	.	C	T	.	PASS	RNF114	exoı	NM_	.	stopgaiı
ACa04	20	53226961	.	C	T	.	PASS	DOK5	exoı	NM_	.	unknow
ACa04	20	57767593	.	C	T	.	PASS	ZNF831	exoı	NM_	.	synonyı
ACa04	20	60859560	.	G	T	.	PASS	OSBPL2	intrc	NM_	.	.
ACa04	20	61942626	.	G	A	.	PASS	COL20A	intrc	NM_	.	.
ACa04	20	62843563	rs1874434	G	A	.	PASS	MYT1	intrc	NM_	.	.
ACa04	21	9873531	.	C	T	.	PASS	.	inteı	NR_	dis	.
ACa04	21	9892201	.	A	C	.	PASS	.	inteı	NR_	dis	.
ACa04	21	10139683	.	T	G	.	PASS	.	inteı	NR_	dis	.
ACa04	21	10624133	rs3707796	G	A	.	PASS	.	inteı	NR_	dis	.
ACa04	21	11058377	rs7469658	C	T	.	PASS	BAGE2,	intrc	NM_	.	.
ACa04	21	11119368	rs4638882	C	A	.	PASS	.	inteı	NM_	dis	.
ACa04	21	37510089	.	T	C	.	PASS	CBR3-A	ncR	NR_	.	.
ACa04	21	39325007	.	G	A	.	PASS	.	inteı	NM_	dis	.
ACa04	21	43833080	.	G	A	.	PASS	UBASH	intrc	NM_	.	.
ACa04	21	44485591	.	G	C	.	PASS	CBS	exoı	NM_	.	missens
ACa04	21	46392916	rs7276130	G	A	.	PASS	FAM207	intrc	NM_	.	.
ACa04	21	46931982	.	C	G	.	PASS	COL18A	intrc	NM_	.	.
ACa04	22	16123397	.	C	T	.	PASS	.	inteı	NON	dis	.
ACa04	22	16350457	.	T	A	.	PASS	.	inteı	NM_	dis	.
ACa04	22	16402051	.	G	C	.	PASS	.	inteı	NM_	dis	.
ACa04	22	18769491	rs3769174	G	T	.	PASS	GGT3P	ncR	NR_	.	.
ACa04	22	18905842	rs2870983	C	T	.	PASS	PRODH	exoı	NM_	.	missens
ACa04	22	20946316	.	G	A	.	PASS	.	inteı	NM_	dis	.
ACa04	22	21680779	rs1389774	C	G	.	PASS	.	inteı	NR_	dis	.
ACa04	22	25043098	rs3731564	G	C	.	PASS	POM12	ncR	NR_	.	.
ACa04	22	29804684	rs1856371	C	T	.	PASS	.	inteı	NM_	dis	.
ACa04	22	30772838	.	C	G	.	PASS	KIAA16	ncR	NR_	.	.
ACa04	22	30975994	rs5669283	G	A	.	PASS	PES1	intrc	NM_	.	.
ACa04	22	35729221	.	G	T	.	PASS	TOM1	intrc	NM_	.	.
ACa04	22	37868474	rs7606577	G	A	.	PASS	MFNG	intrc	NM_	.	.
ACa04	22	39387762	.	G	C	.	PASS	APOBE	ncR	NR_	.	.
ACa04	22	39760283	.	G	C	.	PASS	SYNGR	exoı	NM_	.	missens
ACa04	22	41278055	rs1409901	C	T	.	PASS	XPNPEI	exoı	NM_	.	missens
ACa04	22	41574519	.	G	C	.	PASS	EP300	exoı	NM_	.	missens
ACa04	22	42263232	rs3731131	G	A	.	PASS	SREBF2	exoı	NM_	.	synonyı
ACa04	22	45754721	.	C	G	.	PASS	SMC1B	intrc	NM_	.	.
ACa04	22	49834699	.	C	T	.	PASS	.	inteı	NR_	dis	.
ACa04	22	50582294	.	G	A	.	PASS	MOV10I	intrc	NM_	.	.
ACa04	22	50727449	rs7711497	G	A	.	PASS	PLXNB2	exoı	NM_	.	synonyı
ACa04	X	1584248	.	C	T	.	PASS	P2RY8	UTF	NM_	NM	.
ACa04	X	2132123	rs8673094	G	A	.	PASS	.	inteı	NM_	dis	.
ACa04	X	8433469	rs7643909	T	C	.	PASS	VCX3B	UTF	NM_	NM	.
ACa04	X	9373412	rs6640384	G	A	.	PASS	.	inteı	NM_	dis	.

ACa04	X	12903684	.	C	G	.	PASS	TLR7	exoı	NM_	.	missens
ACa04	X	12938436	.	C	A	.	PASS	TLR8	exoı	NM_	.	stopgaiı
ACa04	X	32834767	.	G	T	.	PASS	DMD	intrc	NM_	.	.
ACa04	X	43591975	.	G	A	.	PASS	MAOA	exoı	NM_	.	missens
ACa04	X	48123486	.	G	A	.	PASS	SSX1	intrc	NM_	.	.
ACa04	X	48690443	.	G	A	.	PASS	PCSK1N	exoı	NM_	.	synonyr
ACa04	X	49643021	.	C	G	.	PASS	USP27X	ncR	NR_	.	.
ACa04	X	54099413	.	G	A	.	PASS	FAM12(	UTF	NM_	NM	.
ACa04	X	54184236	.	A	G	.	PASS	FAM12(	intrc	NM_	.	.
ACa04	X	54577415	.	G	A	.	PASS	GNL3L	exoı	NM_	.	synonyr
ACa04	X	56763695	.	C	T	.	PASS	UQCRB	ncR	NR_	.	.
ACa04	X	69898849	.	T	C	.	PASS	TEX11	intrc	NM_	.	.
ACa04	X	70348196	.	C	G	.	PASS	MED12	exoı	NM_	.	stopgaiı
ACa04	X	70367605	.	G	A	.	PASS	NLGN3	exoı	NM_	.	stopgaiı
ACa04	X	70814750	.	C	G	.	PASS	BCYRN	ncR	NR_	.	.
ACa04	X	1.17E+08	.	G	A	.	PASS	.	inteı	NM_	dis	.
ACa04	X	1.29E+08	.	C	T	.	PASS	SASH3	exoı	NM_	.	stopgaiı
ACa04	X	1.29E+08	.	G	C	.	PASS	UTP14A	exoı	NM_	.	missens
ACa04	X	1.29E+08	.	G	A	.	PASS	BCORL	exoı	NM_	.	missens
ACa04	X	1.31E+08	.	G	A	.	PASS	FIRRE	ncR	NR_	.	.
ACa04	X	1.36E+08	.	C	T	.	PASS	ARHGE	exoı	NM_	.	missens
ACa04	X	1.5E+08	.	G	A	.	PASS	HMGB3	exoı	NM_	.	unknow
ACa04	X	1.53E+08	rs7824625	G	A	.	PASS	TREX2	exoı	NM_	.	missens
ACa04	X	1.53E+08	.	A	C	.	PASS	BCAP31	intrc	NM_	.	.
ACa06	1	674136	.	T	C	.	PASS	.	inteı	NR_	dis	.
ACa06	1	674146	rs7810569	G	A	.	PASS	.	inteı	NR_	dis	.
ACa06	1	1454505	rs1149269	C	T	.	PASS	ATAD3A	intrc	NM_	.	.
ACa06	1	2518366	.	G	T	.	PASS	FAM213	exoı	NM_	.	synonyr
ACa06	1	3410162	.	G	A	.	PASS	MEGF6	intrc	NM_	.	.
ACa06	1	9165837	.	A	C	.	PASS	GPR157	intrc	NM_	.	.
ACa06	1	16363042	rs2008876	C	T	.	PASS	.	inteı	NM_	dis	.
ACa06	1	16890922	rs5565927	T	C	.	PASS	NBPF1	intrc	NM_	.	.
ACa06	1	16904073	rs6177234	C	A	.	PASS	NBPF1	intrc	NM_	.	.
ACa06	1	16957316	rs1008195	C	G	.	PASS	CROCC	ncR	NR_	.	.
ACa06	1	17044324	rs5388409	G	A	.	PASS	ESPNP	ncR	NR_	.	.
ACa06	1	17280821	rs6669627	G	C	.	PASS	CROCC	exoı	NM_	.	missens
ACa06	1	18962986	.	A	C	.	PASS	PAX7	intrc	NM_	.	.
ACa06	1	27994920	rs7661411	C	T	.	PASS	IFI6	intrc	NM_	.	.
ACa06	1	42049019	rs7802118	C	T	.	PASS	HIVEP3	exoı	NM_	.	missens
ACa06	1	46707738	rs8664767	C	T	.	PASS	.	inteı	NM_	dis	.
ACa06	1	51525497	.	G	C	.	PASS	.	inteı	NM_	dis	.
ACa06	1	1.43E+08	rs7581372	T	C	.	PASS	.	inteı	NON	dis	.
ACa06	1	1.43E+08	rs7697846	A	C	.	PASS	.	inteı	NON	dis	.
ACa06	1	1.43E+08	rs2016919	T	A	.	PASS	.	inteı	NR_	dis	.
ACa06	1	1.43E+08	.	C	A	.	PASS	.	inteı	NR_	dis	.
ACa06	1	1.43E+08	rs3703375	C	A	.	PASS	.	inteı	NR_	dis	.
ACa06	1	1.43E+08	rs1996515	T	A	.	PASS	.	inteı	NR_	dis	.
ACa06	1	1.43E+08	.	A	T	.	PASS	.	inteı	NR_	dis	.
ACa06	1	1.43E+08	.	C	G	.	PASS	.	inteı	NR_	dis	.
ACa06	1	1.44E+08	.	G	A	.	PASS	LINC01	ncR	NR_	.	.
ACa06	1	1.45E+08	.	T	A	.	PASS	NBPF9,	intrc	NM_	.	.
ACa06	1	1.45E+08	.	G	T	.	PASS	NBPF25	ncR	NR_	.	.
ACa06	1	1.48E+08	rs8799960	G	C	.	PASS	NBPF8	intrc	NM_	.	.
ACa06	1	1.49E+08	rs2003739	G	A	.	PASS	NBPF25	ncR	NR_	.	.

ACa06	1	1.49E+08	rs3705433	G	A	.	PASS	NBPF25	ncR	NR_	.	.
ACa06	1	1.58E+08	.	T	G	.	PASS	FCRL4	intro	NM_	.	.
ACa06	1	2.23E+08	rs1839036	T	A	.	PASS	.	inter	NR_	dis	.
ACa06	1	2.24E+08	.	T	C	.	PASS	.	inter	NM_	dis	.
ACa06	1	2.24E+08	.	A	G	.	PASS	.	inter	NM_	dis	.
ACa06	1	2.26E+08	.	A	C	.	PASS	ENAH	intro	NM_	.	.
ACa06	1	2.28E+08	rs1828179	T	C	.	PASS	.	inter	NM_	dis	.
ACa06	2	87581867	rs4096246	A	C	.	PASS	.	inter	NR_	dis	.
ACa06	2	88071329	.	G	C	.	PASS	RGPD1	intro	NM_	.	.
ACa06	2	91692625	.	G	A	.	PASS	.	inter	NON	dis	.
ACa06	2	91894128	.	G	T	.	PASS	.	inter	NR_	dis	.
ACa06	2	1.13E+08	rs2011280	G	A	.	PASS	ANAPC	exon	NM_	.	missens
ACa06	2	1.14E+08	rs8685337	G	A	.	PASS	.	ups	NR_	.	.
ACa06	2	1.14E+08	rs2747968	A	G	.	PASS	WASH2	ncR	NR_	.	.
ACa06	2	1.19E+08	rs1029282	G	T	.	PASS	INSIG2	intro	NM_	.	.
ACa06	2	1.32E+08	rs2001821	A	G	.	PASS	POTEE	intro	NM_	.	.
ACa06	2	1.33E+08	rs7800646	C	T	.	PASS	.	inter	NM_	dis	.
ACa06	2	1.7E+08	.	T	C	.	PASS	DHRS9	intro	NM_	.	.
ACa06	2	1.73E+08	.	A	T	.	PASS	SLC25A	intro	NM_	.	.
ACa06	2	1.82E+08	rs1875561	C	T	.	PASS	UBE2E3	intro	NM_	.	.
ACa06	2	2.14E+08	rs8671576	C	G	.	PASS	IKZF2	intro	NM_	.	.
ACa06	3	47384995	.	T	G	.	PASS	KLHL18	intro	NM_	.	.
ACa06	3	1.48E+08	.	G	A	.	PASS	AGTR1	exon	NM_	.	synonyr
ACa06	3	1.49E+08	.	T	G	.	PASS	CP	intro	NM_	.	.
ACa06	3	1.49E+08	.	T	G	.	PASS	CP	intro	NM_	.	.
ACa06	3	1.95E+08	rs3760511	G	A	.	PASS	.	inter	NM_	dis	.
ACa06	3	1.96E+08	rs8677455	A	G	.	PASS	LINC00	ncR	NR_	.	.
ACa06	3	1.97E+08	rs1128840	A	G	.	PASS	.	inter	NR_	dis	.
ACa06	4	60459	.	C	T	.	PASS	ZNF595	intro	NM_	.	.
ACa06	4	20568852	rs1443656	G	A	.	PASS	SLIT2	intro	NM_	.	.
ACa06	5	16483589	.	G	A	.	PASS	FAM134	intro	NM_	.	.
ACa06	5	34179578	.	C	T	.	PASS	.	inter	NR_	dis	.
ACa06	5	69206624	rs3759258	G	T	.	PASS	.	inter	NR_	dis	.
ACa06	5	85581332	rs8790446	T	C	.	PASS	NBPF22	ncR	NR_	.	.
ACa06	5	99715839	.	G	A	.	PASS	LOC100	ncR	NR_	.	.
ACa06	5	1.51E+08	.	A	C	.	PASS	SLC36A	intro	NM_	.	.
ACa06	5	1.63E+08	.	A	G	.	PASS	CCNG1	intro	NM_	.	.
ACa06	5	1.78E+08	.	C	A	.	PASS	COL23A	exon	NM_	.	missens
ACa06	5	1.79E+08	.	C	A	.	PASS	ADAMT	intro	NM_	.	.
ACa06	6	26020676	rs3719775	T	C	.	PASS	.	ups	NM_	.	.
ACa06	6	26104023	rs9202904	C	G	.	PASS	.	ups	NM_	.	.
ACa06	6	58776826	rs4292554	A	G	.	PASS	.	inter	NR_	dis	.
ACa06	6	58779162	rs4437505	G	T	.	PASS	.	inter	NR_	dis	.
ACa06	6	1.39E+08	rs7508774	C	T	.	PASS	KIAA124	intro	NM_	.	.
ACa06	6	1.52E+08	.	A	C	.	PASS	SYNE1	intro	NM_	.	.
ACa06	6	1.68E+08	rs1436754	G	A	.	PASS	TCP10L	intro	NM_	.	.
ACa06	6	1.7E+08	.	A	G	.	PASS	WDR27	intro	NM_	.	.
ACa06	7	4153191	.	A	C	.	PASS	SDK1	intro	NM_	.	.
ACa06	7	6005457	rs2016650	T	A	.	PASS	RSPH10	intro	NM_	.	.
ACa06	7	36543628	.	T	G	.	PASS	.	inter	NM_	dis	.
ACa06	7	56881190	.	C	G	.	PASS	.	inter	NR_	dis	.
ACa06	7	56883751	.	C	G	.	PASS	.	inter	NR_	dis	.
ACa06	7	56887007	.	C	G	.	PASS	.	inter	NR_	dis	.
ACa06	7	56887955	.	C	A	.	PASS	.	inter	NR_	dis	.

ACa06	7	56888086	.	G	T	.	PASS	.	inte	NR_	dis	.	
ACa06	7	56888134	.	C	T	.	PASS	.	inte	NR_	dis	.	
ACa06	7	56902845	.	A	C	.	PASS	.	inte	NR_	dis	.	
ACa06	7	61969095	rs4596537	C	A	.	PASS	.	inte	NON	dis	.	
ACa06	7	61970255	rs2167825	A	C	.	PASS	.	inte	NON	dis	.	
ACa06	7	62956351	rs586765	G	T	.	PASS	.	inte	NR_	dis	.	
ACa06	7	63213633	rs8688020	G	A	.	PASS	.	inte	NR_	dis	.	
ACa06	7	76130717	rs2462259	C	T	.	PASS	DTX2	intro	NM_	.	.	
ACa06	7	1.02E+08	rs6248349	G	A	.	PASS	POLR2J	intro	NM_	.	.	
ACa06	7	1.28E+08	rs3741264	C	G	.	PASS	LINC01	ncR	NR_	.	.	
ACa06	7	1.52E+08	.	T	C	.	PASS	KMT2C	intro	NM_	.	.	
ACa06	8	7386635	rs2077351	A	C	.	PASS	.	inte	NM_	dis	.	
ACa06	8	38123857	.	T	G	.	PASS	PPAPD	intro	NM_	.	.	
ACa06	8	43828037	rs7503847	T	G	.	PASS	.	inte	NM_	dis	.	
ACa06	8	43838571	rs7967153	G	A	.	PASS	.	inte	NM_	dis	.	
ACa06	8	43838646	.	A	G	.	PASS	.	inte	NM_	dis	.	
ACa06	8	46844372	rs4363208	C	G	.	PASS	.	inte	NON	dis	.	
ACa06	8	46849129	rs3740584	G	C	.	PASS	.	inte	NON	dis	.	
ACa06	8	1.12E+08	.	C	T	.	PASS	.	ncR	NR_	.	.	
ACa06	8	1.44E+08	.	G	A	.	PASS	ARC	exo	NM_	.		missens
ACa06	8	1.45E+08	rs7821104	T	G	.	PASS	OPLAH	intro	NM_	.	.	
ACa06	9	46843601	rs7495042	G	A	.	PASS	.	ups	NR_	.	.	
ACa06	9	68415282	rs4928839	A	C	.	PASS	.	inte	NM_	dis	.	
ACa06	9	68427877	rs4568707	G	A	.	PASS	LOC642	ncR	NR_	.	.	
ACa06	9	70731624	rs1435617	T	C	.	PASS	.	inte	NM_	dis	.	
ACa06	9	70731789	rs3773733	T	C	.	PASS	.	inte	NM_	dis	.	
ACa06	9	91150674	.	C	G	.	PASS	NXNL2	intro	NM_	.	.	
ACa06	9	1.27E+08	.	T	G	.	PASS	.	inte	NM_	dis	.	
ACa06	9	1.28E+08	.	C	T	.	PASS	MAPKA	intro	NM_	.	.	
ACa06	10	23639834	rs1397322	T	C	.	PASS	.	inte	NM_	dis	.	
ACa06	10	24564647	.	G	T	.	PASS	MIR603	ncR	NR_	.	.	
ACa06	10	33338230	rs9631413	T	C	.	PASS	.	inte	NM_	dis	.	
ACa06	10	35437448	.	T	C	.	PASS	CREM	intro	NM_	.	.	
ACa06	10	46952565	rs694157	A	G	.	PASS	.	inte	NR_	dis	.	
ACa06	10	79540724	rs5275172	G	A	.	PASS	.	inte	NM_	dis	.	
ACa06	10	79603188	rs1888127	G	A	.	PASS	DLG5	intro	NM_	.	.	
ACa06	10	81270971	rs2573327	T	C	.	PASS	.	inte	NM_	dis	.	
ACa06	10	88988731	rs3128226	C	G	.	PASS	NUTM2	intro	NM_	.	.	
ACa06	11	49896522	.	T	C	.	PASS	.	inte	NR_	dis	.	
ACa06	11	49896523	.	G	A	.	PASS	.	inte	NR_	dis	.	
ACa06	11	67386723	rs9035709	C	T	.	PASS	.	inte	NR_	dis	.	
ACa06	11	71373513	rs1874489	T	C	.	PASS	.	inte	NM_	dis	.	
ACa06	11	76062600	rs9666739	G	T	.	PASS	PRKRIF	exo	NM_	.		missens
ACa06	11	76320147	rs5688517	G	A	.	PASS	.	inte	NM_	dis	.	
ACa06	11	1.24E+08	.	G	A	.	PASS	OR8G5	exo	NM_	.		missens
ACa06	11	1.32E+08	.	T	G	.	PASS	NTM	intro	NM_	.	.	
ACa06	12	52883962	rs7606061	T	C	.	PASS	KRT6A	intro	NM_	.	.	
ACa06	12	56481671	.	T	C	.	PASS	ERBB3	exo	NM_	.		missens
ACa06	12	1.04E+08	rs7597010	C	T	.	PASS	TDG	UTF	NM_	NM	.	
ACa06	13	64320876	rs2004528	C	T	.	PASS	.	inte	NR_	dis	.	
ACa06	13	1.1E+08	rs1510074	G	A	.	PASS	MYO16	exo	NM_	.		synonyr
ACa06	14	19890942	rs7446578	T	C	.	PASS	DUXAP	ncR	NR_	.	.	
ACa06	14	19962107	.	C	T	.	PASS	.	inte	NR_	dis	.	
ACa06	14	20073097	rs2006543	C	T	.	PASS	.	inte	NM_	dis	.	

ACa06	14	20073103	rs2020426	T	C	.	PASS	.	inte	NM_	dis	.
ACa06	14	20158519	rs7704695	G	T	.	PASS	.	inte	NM_	dis	.
ACa06	14	62014518	.	G	A	.	PASS	PRKCH	exo	NM_	.	unknow
ACa06	14	77794476	.	A	T	.	PASS	GSTZ1	intro	NM_	.	.
ACa06	14	1.06E+08	.	C	T	.	PASS	.	inte	NR_	dis	.
ACa06	15	20358726	rs6200967	C	T	.	PASS	.	inte	NON	dis	.
ACa06	15	23265062	.	C	T	.	PASS	.	inte	NR_	dis	.
ACa06	15	25304994	.	A	C	.	PASS	.	dow	NR_	.	.
ACa06	15	30433484	rs7751825	G	C	.	PASS	GOLGA	ncR	NR_	.	.
ACa06	15	31048067	rs2449652	T	C	.	PASS	LOC10C	ncR	NR_	.	.
ACa06	15	31048088	rs2449653	T	C	.	PASS	LOC10C	ncR	NR_	.	.
ACa06	15	32685477	.	G	A	.	PASS	GOLGA	intro	NM_	.	.
ACa06	15	32695794	.	G	A	.	PASS	.	ups	NM_	.	.
ACa06	15	45120967	rs6202600	G	A	.	PASS	.	inte	NM_	dis	.
ACa06	15	50521102	.	A	C	.	PASS	SLC27A	intro	NM_	.	.
ACa06	15	64388133	.	T	A	.	PASS	SNX1	UTF	NM_	NM	.
ACa06	15	84909514	.	C	T	.	PASS	GOLGA	exo	NM_	.	missens
ACa06	15	84917347	rs3743150	T	G	.	PASS	.	inte	NM_	dis	.
ACa06	15	85053791	rs9700260	C	G	.	PASS	GOLGA	ncR	NR_	.	.
ACa06	15	85060248	rs364085	T	C	.	PASS	.	ups	NR_	.	.
ACa06	16	16456691	rs2009400	G	A	.	PASS	.	inte	NR_	dis	.
ACa06	16	16456731	rs2016432	C	T	.	PASS	.	inte	NR_	dis	.
ACa06	16	18478257	.	C	T	.	PASS	.	inte	NR_	dis	.
ACa06	16	21844945	rs5667377	T	C	.	PASS	.	inte	NR_	dis	.
ACa06	16	29110458	rs5618411	T	C	.	PASS	RRN3P	ncR	NR_	.	.
ACa06	16	29538827	rs8792106	A	T	.	PASS	.	dow	NR_	.	.
ACa06	16	46392004	rs4319791	C	T	.	PASS	.	inte	NON	dis	.
ACa06	16	46395310	rs4349321	G	A	.	PASS	.	inte	NON	dis	.
ACa06	16	46395945	rs4246344	G	A	.	PASS	.	inte	NON	dis	.
ACa06	16	46399446	.	A	T	.	PASS	.	inte	NON	dis	.
ACa06	16	46399518	rs8799648	A	G	.	PASS	.	inte	NON	dis	.
ACa06	16	46402061	rs9972801	A	G	.	PASS	.	inte	NON	dis	.
ACa06	16	46403685	rs4324300	G	A	.	PASS	.	inte	NON	dis	.
ACa06	16	46405090	rs9328621	T	C	.	PASS	.	inte	NON	dis	.
ACa06	16	46405110	rs4380209	C	T	.	PASS	.	inte	NON	dis	.
ACa06	16	46405469	rs4291933	A	G	.	PASS	.	inte	NON	dis	.
ACa06	16	46405548	rs4011858	G	C	.	PASS	.	inte	NON	dis	.
ACa06	16	46406714	.	G	C	.	PASS	.	inte	NON	dis	.
ACa06	16	46406854	rs7205231	T	A	.	PASS	.	inte	NON	dis	.
ACa06	16	46408278	rs4093117	C	A	.	PASS	.	inte	NON	dis	.
ACa06	16	46408358	rs4249029	G	A	.	PASS	.	inte	NON	dis	.
ACa06	16	46409112	.	C	T	.	PASS	.	inte	NON	dis	.
ACa06	16	90224905	rs6205366	G	A	.	PASS	.	ncR	NR_	.	.
ACa06	17	20254635	rs2543804	G	A	.	PASS	CCDC1	ncR	NR_	.	.
ACa06	17	20319816	rs7126379	T	C	.	PASS	.	ups	NR_	.	.
ACa06	17	20319971	rs7126379	G	A	.	PASS	.	ups	NR_	.	.
ACa06	17	21904546	rs7841783	G	A	.	PASS	FLJ360	ncR	NR_	.	.
ACa06	17	22251757	.	T	A	.	PASS	.	inte	NM_	dis	.
ACa06	17	22258648	rs1437650	T	G	.	PASS	.	inte	NM_	dis	.
ACa06	17	22258694	.	G	A	.	PASS	.	inte	NM_	dis	.
ACa06	17	34795273	rs6207957	C	T	.	PASS	.	intro	NM_	.	.
ACa06	17	39432066	rs7482024	G	T	.	PASS	KRTAP	exo	NM_	.	synonyr
ACa06	17	40762339	rs1459625	G	A	.	PASS	TUBG1	intro	NM_	.	.
ACa06	17	43663697	.	A	T	.	PASS	.	inte	NR_	dis	.

ACa06	18	11644538	rs2012257	C	G	.	PASS	.	inte	NM_	dis	.
ACa06	18	11644705	rs3734996	A	T	.	PASS	.	inte	NM_	dis	.
ACa06	18	61256074	rs7520640	G	A	.	PASS	SERPIN	intro	NM_	.	.
ACa06	19	4902496	.	A	C	.	PASS	ARRDC	intro	NM_	.	.
ACa06	19	35828631	.	T	A	.	PASS	CD22	intro	NM_	.	.
ACa06	19	37780300	rs7641371	G	T	.	PASS	.	inte	NR_	dis	.
ACa06	19	37784701	.	G	A	.	PASS	.	inte	NR_	dis	.
ACa06	19	43422207	.	C	A	.	PASS	.	ups	NM_	.	.
ACa06	19	46118992	.	T	A	.	PASS	EML2	intro	NM_	.	.
ACa06	19	53788121	rs3499068	A	G	.	PASS	.	dow	NR_	.	.
ACa06	19	53788159	rs5615613	C	T	.	PASS	.	dow	NR_	.	.
ACa06	19	54745775	rs1334652	G	A	.	PASS	LILRA6,	intro	NM_	.	.
ACa06	19	56755845	rs3751555	G	A	.	PASS	.	inte	NM_	dis	.
ACa06	20	33345744	.	C	T	.	PASS	NCOA6	exo	NM_	.	synonyr
ACa06	21	9649976	rs7984422	A	G	.	PASS	.	inte	NON	dis	.
ACa06	21	11124384	rs2017188	T	C	.	PASS	.	inte	NM_	dis	.
ACa06	22	16350628	rs3753995	G	A	.	PASS	.	inte	NM_	dis	.
ACa06	22	16400056	.	A	T	.	PASS	.	inte	NM_	dis	.
ACa06	22	16401857	rs2021509	A	G	.	PASS	.	inte	NM_	dis	.
ACa06	22	20339179	.	T	G	.	PASS	DGCR6	inte	NM_	dis	.
ACa06	22	20715169	rs6221896	C	T	.	PASS	.	inte	NM_	dis	.
ACa06	22	21579897	rs2016329	G	T	.	PASS	.	inte	NR_	dis	.
ACa06	22	25045452	rs6223125	C	T	.	PASS	POM12	ncR	NR_	.	.
ACa06	22	29818992	rs367416	T	C	.	PASS	.	inte	NM_	dis	.
ACa06	22	42538399	rs3609392	C	T	.	PASS	CYP2D	ncR	NR_	.	.
ACa06	22	42910604	.	C	A	.	PASS	RRP7A	intro	NM_	.	.
ACa06	22	44579135	.	G	A	.	PASS	PARVG	intro	NM_	.	.
ACa06	X	53254115	.	C	T	.	PASS	KDM5C	UTF	NM_	NM	.
ACa06	X	61687463	.	G	C	.	PASS	.	inte	NON	dis	.
ACa06	X	61712648	.	C	T	.	PASS	.	inte	NON	dis	.
ACa06	X	61712657	rs7520049	T	C	.	PASS	.	inte	NON	dis	.
ACa06	X	61712692	rs7756689	A	G	.	PASS	.	inte	NON	dis	.
ACa06	X	61718950	.	A	C	.	PASS	.	inte	NON	dis	.
ACa06	X	70280889	rs1048883	C	T	.	PASS	SNX12	exo	NM_	.	missens
ACa06	X	71091139	.	T	A	.	PASS	.	inte	NM_	dis	.
ACa06	X	71358615	.	G	A	.	PASS	NHSL2	exo	NM_	.	missens
ACa06	X	1.39E+08	rs1423249	G	A	.	PASS	.	inte	NM_	dis	.
ACa06	X	1.52E+08	.	C	T	.	PASS	.	inte	NM_	dis	.
ACa07	1	135163	rs3744991	C	T	.	PASS	LINC01	ncR	NR_	.	.
ACa07	1	888176	.	C	T	.	PASS	NOC2L	intro	NM_	.	.
ACa07	1	897337	rs1159856	C	T	.	PASS	KLHL17	exo	NM_	.	synonyr
ACa07	1	915454	.	G	A	.	PASS	PERM1	exo	NM_	.	unknow
ACa07	1	949634	.	C	T	.	PASS	ISG15	exo	NM_	.	missens
ACa07	1	1177864	rs5413521	C	T	.	PASS	FAM13	UTF	NM_	NM	.
ACa07	1	1192565	rs7680451	C	T	.	PASS	UBE2J2	intro	NM_	.	.
ACa07	1	1262554	.	G	A	.	PASS	CPTP	intro	NM_	.	.
ACa07	1	1290074	.	C	T	.	PASS	MXRA8	exo	NM_	.	missens
ACa07	1	1323140	rs7800811	C	T	.	PASS	CCNL2	intro	NM_	.	.
ACa07	1	1559099	.	G	A	.	PASS	MIB2	intro	NM_	.	.
ACa07	1	1573264	rs2014492	C	T	.	PASS	CDK11	intro	NM_	.	.
ACa07	1	1575704	rs7463384	G	A	.	PASS	CDK11	exo	NM_	.	unknow
ACa07	1	1580887	.	C	T	.	PASS	CDK11	intro	NM_	.	.
ACa07	1	1581607	.	C	T	.	PASS	CDK11	intro	NM_	.	.
ACa07	1	1961685	.	C	A	.	PASS	GABRD	exo	NM_	.	synonyr

ACa07	1	1987788	.	G	A	.	PASS	PRKCZ	intrc	NM_	.	.
ACa07	1	2341796	.	G	A	.	PASS	PEX10	intrc	NM_	.	.
ACa07	1	3410459	.	A	G	.	PASS	MEGF6	exoı	NM_	.	synonyr
ACa07	1	3777167	.	C	A	.	PASS	DFFB	intrc	NM_	.	.
ACa07	1	5947402	.	G	T	.	PASS	NPHP4	exoı	NM_	.	unknow
ACa07	1	6008389	.	G	A	.	PASS	NPHP4	intrc	NM_	.	.
ACa07	1	6183992	rs9430027	C	T	.	PASS	CHD5	intrc	NM_	.	.
ACa07	1	6185193	.	C	T	.	PASS	CHD5	exoı	NM_	.	missens
ACa07	1	6190317	.	C	T	.	PASS	CHD5	exoı	NM_	.	missens
ACa07	1	6272914	rs9864160	G	A	.	PASS	RNF207	intrc	NM_	.	.
ACa07	1	6647232	rs7562596	C	T	.	PASS	ZBTB48	intrc	NM_	.	.
ACa07	1	7724209	rs1431018	C	T	.	PASS	CAMTA	exoı	NM_	.	synonyr
ACa07	1	7731947	.	T	A	.	PASS	CAMTA	intrc	NM_	.	.
ACa07	1	7811261	rs7748471	C	T	.	PASS	CAMTA	exoı	NM_	.	synonyr
ACa07	1	9085255	rs9446685	C	T	.	PASS	SLC2A7	intrc	NM_	.	.
ACa07	1	9165694	rs1476580	G	A	.	PASS	GPR157	exoı	NM_	.	missens
ACa07	1	9642260	.	C	T	.	PASS	SLC25A	intrc	NM_	.	.
ACa07	1	11896347	.	C	T	.	PASS	CLCN6	intrc	NM_	.	.
ACa07	1	11939929	.	A	G	.	PASS	.	inteı	NM_	dis	.
ACa07	1	12018717	rs7934532	C	T	.	PASS	PLOD1	intrc	NM_	.	.
ACa07	1	12123731	.	C	T	.	PASS	TNFRSI	intrc	NM_	.	.
ACa07	1	12372069	.	G	T	.	PASS	VPS13D	intrc	NM_	.	.
ACa07	1	12520438	.	C	T	.	PASS	VPS13D	exoı	NM_	.	missens
ACa07	1	12942392	rs2021163	G	C	.	PASS	PRAME	intrc	NM_	.	.
ACa07	1	13497520	rs5876978	C	T	.	PASS	PRAME	intrc	NM_	.	.
ACa07	1	15844727	rs4646008	G	A	.	PASS	CASP9	exoı	NM_	.	missens
ACa07	1	16451683	rs3745685	C	T	.	PASS	EPHA2	UTF	NM_	NM	.
ACa07	1	16534709	.	G	A	.	PASS	ARHGE	exoı	NM_	.	missens
ACa07	1	16895623	rs3734377	G	A	.	PASS	NBPF1	exoı	NM_	.	unknow
ACa07	1	16904073	rs6177234	C	A	.	PASS	NBPF1	intrc	NM_	.	.
ACa07	1	16916702	rs4112521	T	C	.	PASS	NBPF1	intrc	NM_	.	.
ACa07	1	16918693	rs1213433	A	T	.	PASS	NBPF1	UTF	NM_	NM	.
ACa07	1	17007589	rs3930814	G	A	.	PASS	.	ups	NR_	.	.
ACa07	1	17318448	rs5780915	G	A	.	PASS	ATP13A	intrc	NM_	.	.
ACa07	1	18618364	rs3774826	C	T	.	PASS	IGSF21	exoı	NM_	.	missens
ACa07	1	19180811	rs3766882	C	T	.	PASS	TAS1R2	exoı	NM_	.	missens
ACa07	1	19447882	rs3471023	G	A	.	PASS	UBR4	exoı	NM_	.	synonyr
ACa07	1	19465611	.	G	A	.	PASS	UBR4	exoı	NM_	.	synonyr
ACa07	1	19582577	.	A	G	.	PASS	MRTO4	intrc	NM_	.	.
ACa07	1	19665997	rs3712996	C	T	.	PASS	CAPZB	UTF	NM_	NM	.
ACa07	1	20027202	.	G	A	.	PASS	TMCO4	intrc	NM_	.	.
ACa07	1	21928363	rs1001195	G	A	.	PASS	RAP1GA	intrc	NM_	.	.
ACa07	1	22201078	.	C	T	.	PASS	HSPG2	intrc	NM_	.	.
ACa07	1	22207859	.	A	G	.	PASS	HSPG2	exoı	NM_	.	unknow
ACa07	1	22303276	rs7584776	C	T	.	PASS	.	ups	NM_	.	.
ACa07	1	22332199	.	C	A	.	PASS	CELA3A	exoı	NM_	.	missens
ACa07	1	23285608	.	G	A	.	PASS	.	intrc	NM_	.	.
ACa07	1	24122428	.	G	T	.	PASS	GALE	UTF	NM_	NM	.
ACa07	1	24133341	rs1391690	C	T	.	PASS	HMGCL	intrc	NM_	.	.
ACa07	1	25228982	rs7518873	G	A	.	PASS	RUNX3	exoı	NM_	.	synonyr
ACa07	1	25725774	.	G	T	.	PASS	RHCE	intrc	NM_	.	.
ACa07	1	26104493	.	G	A	.	PASS	MAN1C	intrc	NM_	.	.
ACa07	1	26232774	.	G	A	.	PASS	STMN1	intrc	NM_	.	.
ACa07	1	26487992	.	C	T	.	PASS	FAM11(	exoı	NM_	.	synonyr

ACa07	1	26510587	rs7737670	C	T	.	PASS	CNKSR	exo	NM_	.	missens
ACa07	1	26612349	rs7767332	C	T	.	PASS	UBXN1	exo	NM_	.	missens
ACa07	1	26881685	.	G	A	.	PASS	RPS6K	exo	NM_	.	missens
ACa07	1	27106354	rs8792552	C	T	.	PASS	ARID1A	exo	NM_	.	stopgai
ACa07	1	27158954	.	C	T	.	PASS	ZDHHC	exo	NM_	.	missens
ACa07	1	27432696	rs1003250	G	A	.	PASS	SLC9A1	intr	NM_	.	.
ACa07	1	27440796	rs7625790	C	T	.	PASS	SLC9A1	intr	NM_	.	.
ACa07	1	27629951	rs9468364	G	A	.	PASS	WDTC1	intr	NM_	.	.
ACa07	1	27652113	.	C	T	.	PASS	LOC64	ncR	NR_	.	.
ACa07	1	28315229	.	G	A	.	PASS	EYA3	intr	NM_	.	.
ACa07	1	29063869	.	G	C	.	PASS	YTHDF	intr	NM_	.	.
ACa07	1	31845811	rs7596455	G	A	.	PASS	FABP3	exo	NM_	.	synony
ACa07	1	32280661	rs9232513	G	A	.	PASS	SPOCD	exo	NM_	.	missens
ACa07	1	32542285	.	G	A	.	PASS	TMEM3	intr	NM_	.	.
ACa07	1	32751255	.	C	A	.	PASS	LCK	exo	NM_	.	missens
ACa07	1	32914631	.	G	A	.	PASS	.	inte	NM_	dis	.
ACa07	1	33318809	.	C	T	.	PASS	S100PB	intr	NM_	.	.
ACa07	1	35865003	rs9651423	A	G	.	PASS	ZMYM4	intr	NM_	.	.
ACa07	1	38082341	rs7723940	G	A	.	PASS	RSPO1	exo	NM_	.	missens
ACa07	1	38155909	.	G	A	.	PASS	C1orf10	intr	NM_	.	.
ACa07	1	38200891	rs1814745	C	T	.	PASS	EPHA1	intr	NM_	.	.
ACa07	1	39923930	.	C	T	.	PASS	MACF1	intr	NM_	.	.
ACa07	1	39930863	.	G	A	.	PASS	MACF1	intr	NM_	.	.
ACa07	1	39951571	.	A	C	.	PASS	MACF1	UTF	NM_	NM	.
ACa07	1	39989003	rs5523408	C	T	.	PASS	PPIEL	ncR	NR_	.	.
ACa07	1	40030304	.	G	T	.	PASS	PABPC	intr	NM_	.	.
ACa07	1	40562796	.	G	A	.	PASS	PPT1	exo	NM_	.	missens
ACa07	1	40705849	.	T	C	.	PASS	RLF	exo	NM_	.	synony
ACa07	1	40706184	.	G	A	.	PASS	RLF	UTF	NM_	NM	.
ACa07	1	41283715	rs9093579	G	T	.	PASS	KCNQ4	intr	NM_	.	.
ACa07	1	41303941	.	T	A	.	PASS	KCNQ4	intr	NM_	.	.
ACa07	1	42654359	rs1179985	C	T	.	PASS	FOXJ3	intr	NM_	.	.
ACa07	1	43166550	rs7456140	G	A	.	PASS	YBX1	exo	NM_	.	missens
ACa07	1	43852750	.	G	A	.	PASS	MED8	intr	NM_	.	.
ACa07	1	44166300	.	C	T	.	PASS	KDM4A	ncR	NR_	.	.
ACa07	1	45267625	.	C	T	.	PASS	PLK3	intr	NM_	.	.
ACa07	1	46088738	.	C	A	.	PASS	CCDC1	exo	NM_	.	missens
ACa07	1	46726082	rs7290104	C	T	.	PASS	RAD54L	intr	NM_	.	.
ACa07	1	46874979	.	A	G	.	PASS	FAAH	intr	NM_	.	.
ACa07	1	47025920	rs5508458	C	T	.	PASS	MKNK1	exo	NM_	.	synony
ACa07	1	47358142	.	C	T	.	PASS	CYP4Z	ncR	NR_	.	.
ACa07	1	50307777	.	G	T	.	PASS	AGBL4	intr	NM_	.	.
ACa07	1	51808840	rs7570389	C	T	.	PASS	TTC39	intr	NM_	.	.
ACa07	1	53720726	rs9512571	C	T	.	PASS	LRP8	intr	NM_	.	.
ACa07	1	53990329	rs7759188	C	T	.	PASS	GLIS1	intr	NM_	.	.
ACa07	1	54661138	.	G	A	.	PASS	CYB5R	exo	NM_	.	missens
ACa07	1	55518417	rs3769455	G	A	.	PASS	PCSK9	exo	NM_	.	missens
ACa07	1	57157158	rs7815069	C	T	.	PASS	PRKAA	exo	NM_	.	missens
ACa07	1	58522063	.	C	T	.	PASS	DAB1	intr	NM_	.	.
ACa07	1	65312146	.	T	A	.	PASS	JAK1	intr	NM_	.	.
ACa07	1	67390207	.	C	T	.	PASS	WDR78	intr	NM_	.	.
ACa07	1	74667202	.	C	A	.	PASS	FPGT,F	intr	NM_	.	.
ACa07	1	77661094	.	A	G	.	PASS	PIGK	intr	NM_	.	.
ACa07	1	79383715	rs1785229	G	A	.	PASS	ELTD1	exo	NM_	.	synony

ACa07	1	79521735	.	G	A	.	PASS	.	inte	NM_	dis	.	
ACa07	1	82431836	rs1394863	C	T	.	PASS	LPHN2	exo	NM_	.	synonyr	
ACa07	1	85279635	rs7636045	C	T	.	PASS	LPAR3	exo	NM_	.	missens	
ACa07	1	86119846	.	C	A	.	PASS	ZNHIT6	intro	NM_	.	.	
ACa07	1	92251861	.	G	T	.	PASS	TGFBR:	intro	NM_	.	.	
ACa07	1	94485309	.	G	C	.	PASS	ABCA4	exo	NM_	.	synonyr	
ACa07	1	94520782	rs6174943	G	A	.	PASS	ABCA4	exo	NM_	.	synonyr	
ACa07	1	1.01E+08	.	T	A	.	PASS	LRRC39	intro	NM_	.	.	
ACa07	1	1.03E+08	.	A	T	.	PASS	COL11A	intro	NM_	.	.	
ACa07	1	1.09E+08	.	A	C	.	PASS	PRPF38	exo	NM_	.	missens	
ACa07	1	1.1E+08	rs9363214	C	T	.	PASS	CELSR2	intro	NM_	.	.	
ACa07	1	1.11E+08	rs7774328	G	A	.	PASS	STRIP1	intro	NM_	.	.	
ACa07	1	1.11E+08	.	C	T	.	PASS	RBM15	exo	NM_	.	missens	
ACa07	1	1.12E+08	.	T	C	.	PASS	CHIA	UTF	NM_	NM	.	
ACa07	1	1.12E+08	.	C	T	.	PASS	C1orf16	exo	NM_	.	unknow	
ACa07	1	1.12E+08	.	T	C	.	PASS	FAM212	UTF	NM_	NM	.	
ACa07	1	1.13E+08	rs7556469	C	T	.	PASS	MOV10	exo	NM_	.	missens	
ACa07	1	1.13E+08	.	G	A	.	PASS	RHOC	exo	NM_	.	synonyr	
ACa07	1	1.14E+08	.	G	T	.	PASS	MAGI3	exo	NM_	.	missens	
ACa07	1	1.15E+08	.	T	G	.	PASS	AMPD1	intro	NM_	.	.	
ACa07	1	1.15E+08	rs3724512	C	T	.	PASS	AMPD1	exo	NM_	.	missens	
ACa07	1	1.18E+08	.	G	A	.	PASS	TTF2	intro	NM_	.	.	
ACa07	1	1.18E+08	.	G	T	.	PASS	SPAG17	intro	NM_	.	.	
ACa07	1	1.2E+08	.	C	T	.	PASS	NBPF7	intro	NM_	.	.	
ACa07	1	1.2E+08	rs1041798	C	T	.	PASS	NOTCH	intro	NM_	.	.	
ACa07	1	1.2E+08	.	A	G	.	PASS	NOTCH	splic	NM_	NM	.	
ACa07	1	1.43E+08	.	T	G	.	PASS	ANKRD	ncR	NR_	.	.	
ACa07	1	1.45E+08	rs9441133	T	G	.	PASS	NBPF9,	exo	NM_	.	unknow	
ACa07	1	1.45E+08	rs4067693	G	C	.	PASS	NBPF20	intro	NM_	.	.	
ACa07	1	1.45E+08	.	A	G	.	PASS	NBPF9,	exo	NM_	.	unknow	
ACa07	1	1.45E+08	rs7819390	C	T	.	PASS	SEC22E	exo	NM_	.	unknow	
ACa07	1	1.45E+08	rs7515424	T	A	.	PASS	NBPF25	ncR	NR_	.	.	
ACa07	1	1.45E+08	.	C	T	.	PASS	NBPF25	ncR	NR_	.	.	
ACa07	1	1.46E+08	.	C	T	.	PASS	POLR30	exo	NM_	.	missens	
ACa07	1	1.46E+08	.	G	A	.	PASS	NBPF25	ncR	NR_	.	.	
ACa07	1	1.49E+08	.	G	A	.	PASS	LOC101	ncR	NR_	.	.	
ACa07	1	1.49E+08	.	C	T	.	PASS	NBPF25	ncR	NR_	.	.	
ACa07	1	1.5E+08	.	C	T	.	PASS	TARS2	intro	NM_	.	.	
ACa07	1	1.51E+08	.	G	A	.	PASS	PI4KB	exo	NM_	.	stopgai	
ACa07	1	1.51E+08	.	T	C	.	PASS	POGZ	exo	NM_	.	missens	
ACa07	1	1.52E+08	.	G	A	.	PASS	FLG2	exo	NM_	.	synonyr	
ACa07	1	1.53E+08	rs9479104	C	T	.	PASS	PGLYRI	intro	NM_	.	.	
ACa07	1	1.54E+08	.	C	T	.	PASS	S100A1	intro	NM_	.	.	
ACa07	1	1.54E+08	.	A	G	.	PASS	INTS3	intro	NM_	.	.	
ACa07	1	1.54E+08	rs7704251	G	A	.	PASS	DENND	exo	NM_	.	missens	
ACa07	1	1.55E+08	.	C	A	.	PASS	UBE2Q:	splic	NM_	NM	.	
ACa07	1	1.55E+08	.	C	T	.	PASS	MTX1	intro	NM_	.	.	
ACa07	1	1.55E+08	.	C	A	.	PASS	ASH1L	exo	NM_	.	missens	
ACa07	1	1.56E+08	.	C	A	.	PASS	YY1AP1	intro	NM_	.	.	
ACa07	1	1.56E+08	rs9321901	C	T	.	PASS	PMF1	UTF	NM_	NM	.	
ACa07	1	1.57E+08	.	T	C	.	PASS	ARHGE	intro	NM_	.	.	
ACa07	1	1.57E+08	rs3718046	C	T	.	PASS	ETV3L	exo	NM_	.	missens	
ACa07	1	1.58E+08	rs2000983	A	G	.	PASS	.	inte	NM_	dis	.	
ACa07	1	1.58E+08	rs7734794	G	A	.	PASS	CD1B	exo	NM_	.	synonyr	

ACa07	1	1.6E+08	rs7622475	C	T	.	PASS	CRP	intrc	NM_	.	.
ACa07	1	1.61E+08	.	G	A	.	PASS	ADAMT	exoı	NM_	.	missens
ACa07	1	1.62E+08	rs2010794	C	T	.	PASS	C1orf22	exoı	NM_	.	synonyr
ACa07	1	1.63E+08	.	C	A	.	PASS	RGS4	exoı	NM_	.	missens
ACa07	1	1.65E+08	.	A	G	.	PASS	PBX1	exoı	NM_	.	missens
ACa07	1	1.67E+08	.	C	T	.	PASS	POU2F1	exoı	NM_	.	synonyr
ACa07	1	1.69E+08	.	G	A	.	PASS	CCDC1	intrc	NM_	.	.
ACa07	1	1.7E+08	.	A	G	.	PASS	SELP	intrc	NM_	.	.
ACa07	1	1.7E+08	.	C	T	.	PASS	SCYL3	intrc	NM_	.	.
ACa07	1	1.74E+08	.	G	A	.	PASS	RABGA	intrc	NM_	.	.
ACa07	1	1.77E+08	.	T	G	.	PASS	PAPPA2	intrc	NM_	.	.
ACa07	1	1.77E+08	rs7763360	C	T	.	PASS	ASTN1	exoı	NM_	.	synonyr
ACa07	1	1.79E+08	.	C	A	.	PASS	ABL2	exoı	NM_	.	stopgaiı
ACa07	1	1.8E+08	rs1014592	C	T	.	PASS	NPHS2	UTF	NM_	NM	.
ACa07	1	1.8E+08	.	C	T	.	PASS	CEP350	intrc	NM_	.	.
ACa07	1	1.82E+08	rs7733795	C	A	.	PASS	CACNA	exoı	NM_	.	missens
ACa07	1	1.82E+08	rs7640679	G	A	.	PASS	ZNF648	exoı	NM_	.	missens
ACa07	1	1.82E+08	.	G	A	.	PASS	TEDDM	exoı	NM_	.	missens
ACa07	1	1.83E+08	rs7702036	C	T	.	PASS	NPL	exoı	NM_	.	missens
ACa07	1	1.86E+08	.	C	A	.	PASS	TPR	exoı	NM_	.	missens
ACa07	1	1.97E+08	.	C	T	.	PASS	CRB1	intrc	NM_	.	.
ACa07	1	2.01E+08	.	A	G	.	PASS	CAMSA	intrc	NM_	.	.
ACa07	1	2.01E+08	rs3712350	G	A	.	PASS	KIF21B	exoı	NM_	.	missens
ACa07	1	2.01E+08	rs3723002	C	T	.	PASS	CACNA	intrc	NM_	.	.
ACa07	1	2.02E+08	rs3694010	G	A	.	PASS	NAV1	exoı	NM_	.	missens
ACa07	1	2.02E+08	.	G	T	.	PASS	TIMM17	intrc	NM_	.	.
ACa07	1	2.04E+08	rs1044866	C	T	.	PASS	ETNK2	intrc	NM_	.	.
ACa07	1	2.05E+08	.	C	T	.	PASS	CNTN2	intrc	NM_	.	.
ACa07	1	2.07E+08	.	T	G	.	PASS	C4BPA	exoı	NM_	.	synonyr
ACa07	1	2.08E+08	.	G	A	.	PASS	CR1L	intrc	NM_	.	.
ACa07	1	2.08E+08	.	T	C	.	PASS	PLXNA2	intrc	NM_	.	.
ACa07	1	2.1E+08	.	C	G	.	PASS	HSD11E	intrc	NM_	.	.
ACa07	1	2.11E+08	.	T	C	.	PASS	RCOR3	intrc	NM_	.	.
ACa07	1	2.13E+08	rs9188348	C	T	.	PASS	TATDN3	intrc	NM_	.	.
ACa07	1	2.13E+08	.	C	T	.	PASS	FLVCR1	exoı	NM_	.	missens
ACa07	1	2.13E+08	.	G	T	.	PASS	RPS6K(	exoı	NM_	.	missens
ACa07	1	2.15E+08	.	A	G	.	PASS	PTPN14	intrc	NM_	.	.
ACa07	1	2.15E+08	.	G	A	.	PASS	PTPN14	intrc	NM_	.	.
ACa07	1	2.16E+08	rs9106912	T	C	.	PASS	USH2A	exoı	NM_	.	missens
ACa07	1	2.16E+08	.	G	T	.	PASS	USH2A	intrc	NM_	.	.
ACa07	1	2.16E+08	rs7564262	C	T	.	PASS	USH2A	exoı	NM_	.	missens
ACa07	1	2.17E+08	rs1909372	C	T	.	PASS	ESRRG	UTF	NM_	NM	.
ACa07	1	2.2E+08	.	C	T	.	PASS	SLC30A	splic	NM_	NM	.
ACa07	1	2.2E+08	.	C	T	.	PASS	IARS2	exoı	NM_	.	synonyr
ACa07	1	2.2E+08	rs7799500	C	T	.	PASS	IARS2	exoı	NM_	.	missens
ACa07	1	2.21E+08	.	C	A	.	PASS	2-Mar	exoı	NM_	.	missens
ACa07	1	2.23E+08	.	T	G	.	PASS	DISP1	exoı	NM_	.	missens
ACa07	1	2.24E+08	.	C	T	.	PASS	NVL	intrc	NM_	.	.
ACa07	1	2.26E+08	.	G	A	.	PASS	TMEM6	intrc	NM_	.	.
ACa07	1	2.26E+08	.	G	A	.	PASS	LEFTY2	exoı	NM_	.	missens
ACa07	1	2.27E+08	rs9303868	C	T	.	PASS	.	inteı	NM_	dis	.
ACa07	1	2.27E+08	.	G	A	.	PASS	ADCK3	intrc	NM_	.	.
ACa07	1	2.29E+08	.	A	T	.	PASS	OBSCN	exoı	NM_	.	missens
ACa07	1	2.31E+08	.	G	A	.	PASS	TRIM67	exoı	NM_	.	missens

ACa07	1	2.33E+08	.	C	T	.	PASS	KIAA18(	intrc	NM_	.	.
ACa07	1	2.34E+08	rs1006991	C	T	.	PASS	KCNK1	exoı	NM_	.	synonyr
ACa07	1	2.38E+08	rs3676265	C	T	.	PASS	RYR2	intrc	NM_	.	.
ACa07	1	2.43E+08	.	C	T	.	PASS	PLD5	intrc	NM_	.	.
ACa07	1	2.44E+08	.	C	T	.	PASS	AKT3	splic	NM_	NM	.
ACa07	1	2.47E+08	rs1000082	G	A	.	PASS	.	inteı	NM_	dis	.
ACa07	1	2.47E+08	rs1825931	C	T	.	PASS	ZNF496	intrc	NM_	.	.
ACa07	1	2.49E+08	rs7580154	C	T	.	PASS	ZNF692	exoı	NM_	.	missens
ACa07	2	1426925	rs3724090	G	A	.	PASS	TPO	intrc	NM_	.	.
ACa07	2	3212531	rs7778730	G	A	.	PASS	TSSC1	intrc	NM_	.	.
ACa07	2	3391996	.	A	G	.	PASS	TRAPP(	exoı	NM_	.	missens
ACa07	2	3428371	rs5690508	G	A	.	PASS	TRAPP(	exoı	NM_	.	missens
ACa07	2	7594616	.	T	C	.	PASS	.	inteı	NR_	dis	.
ACa07	2	9002811	rs7490370	G	A	.	PASS	MBOAT	exoı	NM_	.	missens
ACa07	2	9630641	rs3691590	C	T	.	PASS	ADAM1	exoı	NM_	.	missens
ACa07	2	11311984	rs9653906	G	A	.	PASS	PQLC3	intrc	NM_	.	.
ACa07	2	16736197	.	C	T	.	PASS	FAM49/	intrc	NM_	.	.
ACa07	2	16740691	rs3765736	C	T	.	PASS	FAM49/	intrc	NM_	.	.
ACa07	2	17830745	rs3680831	C	T	.	PASS	VSNL1	exoı	NM_	.	synonyr
ACa07	2	18112529	rs7556987	C	T	.	PASS	KCNS3	exoı	NM_	.	missens
ACa07	2	23916391	.	G	A	.	PASS	KLHL29	exoı	NM_	.	missens
ACa07	2	24432146	rs5650507	G	A	.	PASS	ITSN2	intrc	NM_	.	.
ACa07	2	24980875	rs7736455	G	A	.	PASS	NCOA1	exoı	NM_	.	synonyr
ACa07	2	27305031	rs1479767	G	A	.	PASS	EMILIN:	exoı	NM_	.	missens
ACa07	2	27456909	.	G	A	.	PASS	CAD	exoı	NM_	.	missens
ACa07	2	27462259	.	A	T	.	PASS	CAD	exoı	NM_	.	missens
ACa07	2	27716532	.	G	A	.	PASS	FNDC4	intrc	NM_	.	.
ACa07	2	30142703	.	G	A	.	PASS	ALK	intrc	NM_	.	.
ACa07	2	32750576	rs7664313	G	A	.	PASS	BIRC6	exoı	NM_	.	missens
ACa07	2	32929615	rs1047800	G	A	.	PASS	TTC27	intrc	NM_	.	.
ACa07	2	33127368	.	A	G	.	PASS	LINC00	ncR	NR_	.	.
ACa07	2	37085088	.	C	T	.	PASS	STRN	exoı	NM_	.	missens
ACa07	2	40655592	rs7725290	C	T	.	PASS	SLC8A1	intrc	NM_	.	.
ACa07	2	44204020	.	T	G	.	PASS	LRPPR(	intrc	NM_	.	.
ACa07	2	48741989	.	T	C	.	PASS	PPP1R2	UTF	NM_	NM	.
ACa07	2	49003359	.	C	A	.	PASS	STON1	intrc	NM_	.	.
ACa07	2	54875965	.	C	T	.	PASS	SPTBN:	intrc	NM_	.	.
ACa07	2	54876819	.	C	A	.	PASS	SPTBN:	exoı	NM_	.	missens
ACa07	2	55237295	.	T	C	.	PASS	RTN4	UTF	NM_	NM	.
ACa07	2	56103922	rs7787019	T	C	.	PASS	EFEMP	intrc	NM_	.	.
ACa07	2	56420574	rs7608139	C	T	.	PASS	CCDC8	exoı	NM_	.	synonyr
ACa07	2	60679661	.	T	C	.	PASS	BCL11A	UTF	NM_	NM	.
ACa07	2	63660799	.	C	T	.	PASS	WDPCF	intrc	NM_	.	.
ACa07	2	64114789	.	A	T	.	PASS	UGP2	intrc	NM_	.	.
ACa07	2	64321781	.	A	C	.	PASS	PELI1	UTF	NM_	NM	.
ACa07	2	69040538	.	C	T	.	PASS	ARHGA	exoı	NM_	.	missens
ACa07	2	69420334	.	A	G	.	PASS	ANTXR:	intrc	NM_	.	.
ACa07	2	70905828	.	C	A	.	PASS	ADD2	intrc	NM_	.	.
ACa07	2	71304538	.	C	T	.	PASS	NAGK	intrc	NM_	.	.
ACa07	2	71795020	.	C	A	.	PASS	DYSF	intrc	NM_	.	.
ACa07	2	75745201	rs7670256	C	T	.	PASS	EVA1A	exoı	NM_	.	synonyr
ACa07	2	84811352	.	A	G	.	PASS	DNAH6	exoı	NM_	.	missens
ACa07	2	85262073	rs1883943	G	A	.	PASS	KCMF1	intrc	NM_	.	.
ACa07	2	85578699	.	G	A	.	PASS	RETSA	intrc	NM_	.	.

ACa07	2	85781324	.	G	T	.	PASS	GGCX	exo	NM_	.	synonyr
ACa07	2	85819135	.	T	C	.	PASS	VAMP5	intro	NM_	.	.
ACa07	2	87169353	.	C	T	.	PASS	RGPD2	intro	NM_	.	.
ACa07	2	87357125	.	A	C	.	PASS	.	inte	NR_	dis	.
ACa07	2	87373787	rs1915368	G	A	.	PASS	.	inte	NR_	dis	.
ACa07	2	87580866	rs7778874	G	A	.	PASS	.	inte	NR_	dis	.
ACa07	2	87580910	rs2923588	G	A	.	PASS	.	inte	NR_	dis	.
ACa07	2	89292023	rs3763447	C	T	.	PASS	.	inte	NR_	dis	.
ACa07	2	89399384	.	T	G	.	PASS	.	inte	NR_	dis	.
ACa07	2	90048195	.	T	C	.	PASS	.	inte	NR_	dis	.
ACa07	2	91744332	rs8689209	G	A	.	PASS	.	inte	NON	dis	.
ACa07	2	91746820	rs3759446	C	A	.	PASS	.	inte	NON	dis	.
ACa07	2	91812093	rs1210521	G	A	.	PASS	.	inte	NON	dis	.
ACa07	2	95482920	rs2931782	G	A	.	PASS	ANKRD	ncR	NR_	.	.
ACa07	2	96148540	.	A	G	.	PASS	TRIM43	intro	NM_	.	.
ACa07	2	96798403	rs7784367	C	T	.	PASS	ASTL	exo	NM_	.	synonyr
ACa07	2	96964625	.	C	T	.	PASS	SNRNP	exo	NM_	.	synonyr
ACa07	2	97311987	rs5704884	G	A	.	PASS	FER1L5	intro	NM_	.	.
ACa07	2	97494583	.	T	C	.	PASS	CNNM3	intro	NM_	.	.
ACa07	2	97784149	rs5571075	G	A	.	PASS	ANKRD	exo	NM_	.	synonyr
ACa07	2	98128563	.	T	C	.	PASS	ANKRD	intro	NM_	.	.
ACa07	2	98373844	.	C	A	.	PASS	TMEM1	exo	NM_	.	synonyr
ACa07	2	1E+08	rs7682782	C	T	.	PASS	AFF3	exo	NM_	.	missens
ACa07	2	1.03E+08	.	G	A	.	PASS	IL18R1	exo	NM_	.	missens
ACa07	2	1.06E+08	rs7516119	G	A	.	PASS	TGFBR	exo	NM_	.	synonyr
ACa07	2	1.07E+08	rs2018112	C	T	.	PASS	RGPD3	exo	NM_	.	missens
ACa07	2	1.11E+08	rs7589510	C	T	.	PASS	BUB1	exo	NM_	.	missens
ACa07	2	1.13E+08	.	G	A	.	PASS	.	inte	NM_	dis	.
ACa07	2	1.14E+08	.	A	G	.	PASS	.	dow	NM_	.	.
ACa07	2	1.14E+08	.	A	G	.	PASS	IL36A	exo	NM_	.	missens
ACa07	2	1.14E+08	.	G	A	.	PASS	.	inte	NM_	dis	.
ACa07	2	1.17E+08	.	T	C	.	PASS	DPP10	intro	NM_	.	.
ACa07	2	1.21E+08	rs7642264	C	T	.	PASS	RALB	exo	NM_	.	synonyr
ACa07	2	1.28E+08	.	T	A	.	PASS	PROC	intro	NM_	.	.
ACa07	2	1.28E+08	.	G	A	.	PASS	MYO7B	intro	NM_	.	.
ACa07	2	1.31E+08	rs7817298	C	T	.	PASS	SMPD4	intro	NM_	.	.
ACa07	2	1.31E+08	rs1340756	G	C	.	PASS	.	inte	NM_	dis	.
ACa07	2	1.33E+08	rs1820004	C	T	.	PASS	.	inte	NM_	dis	.
ACa07	2	1.33E+08	.	A	G	.	PASS	GPR39	intro	NM_	.	.
ACa07	2	1.34E+08	.	C	T	.	PASS	MIR785	ncR	NR_	.	.
ACa07	2	1.42E+08	.	C	A	.	PASS	LRP1B	intro	NM_	.	.
ACa07	2	1.52E+08	.	G	A	.	PASS	NEB	exo	NM_	.	missens
ACa07	2	1.53E+08	.	G	T	.	PASS	STAM2	intro	NM_	.	.
ACa07	2	1.53E+08	.	G	A	.	PASS	FMNL2	intro	NM_	.	.
ACa07	2	1.54E+08	rs1012824	C	T	.	PASS	PRPF40	intro	NM_	.	.
ACa07	2	1.54E+08	rs9859742	A	C	.	PASS	ARL6IP	intro	NM_	.	.
ACa07	2	1.6E+08	rs2003571	C	T	.	PASS	TANC1	exo	NM_	.	missens
ACa07	2	1.63E+08	rs3721419	G	A	.	PASS	SLC4A1	exo	NM_	.	missens
ACa07	2	1.63E+08	.	G	T	.	PASS	DPP4	intro	NM_	.	.
ACa07	2	1.63E+08	rs1029714	C	T	.	PASS	DPP4	intro	NM_	.	.
ACa07	2	1.66E+08	.	G	A	.	PASS	SCN3A	intro	NM_	.	.
ACa07	2	1.7E+08	.	T	G	.	PASS	ABCB1	intro	NM_	.	.
ACa07	2	1.7E+08	.	G	T	.	PASS	BBS5	UTF	NM_	NM	.
ACa07	2	1.72E+08	.	A	G	.	PASS	LOC101	ncR	NR_	.	.

ACa07	2	1.72E+08	rs9215367	C	T	.	PASS	GORAS	exo	NM_	.	stopgai
ACa07	2	1.73E+08	.	T	A	.	PASS	DYNC1I	intro	NM_	.	.
ACa07	2	1.74E+08	rs9638533	G	A	.	PASS	RAPGE	intro	NM_	.	.
ACa07	2	1.75E+08	.	T	C	.	PASS	WIPF1	intro	NM_	.	.
ACa07	2	1.76E+08	.	T	C	.	PASS	CHRNA	intro	NM_	.	.
ACa07	2	1.76E+08	.	G	A	.	PASS	.	inte	NM_	dis	.
ACa07	2	1.78E+08	.	A	T	.	PASS	AGPS	intro	NM_	.	.
ACa07	2	1.79E+08	.	G	A	.	PASS	PDE11A	intro	NM_	.	.
ACa07	2	1.8E+08	rs9603080	T	A	.	PASS	TTN	intro	NM_	.	.
ACa07	2	1.84E+08	rs3772721	C	T	.	PASS	DNAJC:	exo	NM_	.	missens
ACa07	2	1.87E+08	rs3766275	T	C	.	PASS	FSIP2	exo	NM_	.	missens
ACa07	2	1.87E+08	.	A	G	.	PASS	FSIP2	exo	NM_	.	missens
ACa07	2	1.88E+08	.	T	C	.	PASS	ITGAV	intro	NM_	.	.
ACa07	2	1.9E+08	rs3768239	G	A	.	PASS	COL5A2	intro	NM_	.	.
ACa07	2	1.9E+08	rs7715235	C	T	.	PASS	WDR75	intro	NM_	.	.
ACa07	2	1.92E+08	.	G	A	.	PASS	STAT1	intro	NM_	.	.
ACa07	2	1.93E+08	.	G	A	.	PASS	SDPR	exo	NM_	.	missens
ACa07	2	1.97E+08	.	T	A	.	PASS	STK17E	exo	NM_	.	missens
ACa07	2	2.01E+08	.	C	T	.	PASS	KCTD18	exo	NM_	.	missens
ACa07	2	2.02E+08	.	T	C	.	PASS	CLK1	exo	NM_	.	missens
ACa07	2	2.03E+08	rs5481928	G	A	.	PASS	KIAA20	exo	NM_	.	synonyr
ACa07	2	2.04E+08	rs1901033	T	A	.	PASS	NBEAL1	intro	NM_	.	.
ACa07	2	2.04E+08	rs7778048	A	G	.	PASS	RAPH1	exo	NM_	.	synonyr
ACa07	2	2.07E+08	rs1477917	G	A	.	PASS	EEF1B2	exo	NM_	.	synonyr
ACa07	2	2.07E+08	rs5379398	G	A	.	PASS	ADAM2	intro	NM_	.	.
ACa07	2	2.09E+08	.	G	C	.	PASS	CRYGD	exo	NM_	.	missens
ACa07	2	2.1E+08	.	C	A	.	PASS	.	inte	NM_	dis	.
ACa07	2	2.11E+08	.	G	A	.	PASS	UNC80	exo	NM_	.	missens
ACa07	2	2.11E+08	.	C	T	.	PASS	UNC80	intro	NM_	.	.
ACa07	2	2.11E+08	.	A	G	.	PASS	UNC80	intro	NM_	.	.
ACa07	2	2.11E+08	.	T	C	.	PASS	CPS1	intro	NM_	.	.
ACa07	2	2.19E+08	.	G	T	.	PASS	TNS1	intro	NM_	.	.
ACa07	2	2.19E+08	.	A	T	.	PASS	.	inte	NM_	dis	.
ACa07	2	2.19E+08	.	C	A	.	PASS	CATIP-A	ncR	NR_	.	.
ACa07	2	2.19E+08	.	G	T	.	PASS	SLC11A	splic	NM_	NM	.
ACa07	2	2.19E+08	rs5756405	G	A	.	PASS	VIL1	intro	NM_	.	.
ACa07	2	2.2E+08	.	G	T	.	PASS	TTLL4	intro	NM_	.	.
ACa07	2	2.2E+08	rs3732211	G	A	.	PASS	CCDC1	intro	NM_	.	.
ACa07	2	2.2E+08	.	G	A	.	PASS	DNAJB2	exo	NM_	.	synonyr
ACa07	2	2.2E+08	rs7469670	C	T	.	PASS	DES	intro	NM_	.	.
ACa07	2	2.2E+08	rs5290584	C	T	.	PASS	SPEG	splic	NM_	NM	.
ACa07	2	2.22E+08	.	T	A	.	PASS	EPHA4	exo	NM_	.	missens
ACa07	2	2.28E+08	.	C	T	.	PASS	IRS1	exo	NM_	.	synonyr
ACa07	2	2.28E+08	.	A	T	.	PASS	LOC654	ncR	NR_	.	.
ACa07	2	2.31E+08	rs499449	C	T	.	PASS	FBXO36	intro	NM_	.	.
ACa07	2	2.32E+08	rs7533248	G	A	.	PASS	NMUR1	exo	NM_	.	missens
ACa07	2	2.33E+08	.	C	A	.	PASS	DIS3L2	splic	NM_	NM	.
ACa07	2	2.33E+08	.	G	T	.	PASS	DIS3L2	intro	NM_	.	.
ACa07	2	2.33E+08	.	G	A	.	PASS	ALPPL2	intro	NM_	.	.
ACa07	2	2.34E+08	rs2020267	G	A	.	PASS	INPP5D	exo	NM_	.	unknow
ACa07	2	2.35E+08	rs1053770	C	T	.	PASS	MROH2	intro	NM_	.	.
ACa07	2	2.35E+08	.	C	T	.	PASS	MROH2	exo	NM_	.	synonyr
ACa07	2	2.37E+08	.	C	T	.	PASS	IQCA1	intro	NM_	.	.
ACa07	2	2.39E+08	rs7483373	C	T	.	PASS	PER2	exo	NM_	.	missens

ACa07	2	2.41E+08	.	C	T	.	PASS	LOC150	ncR	NR_	.	.
ACa07	2	2.42E+08	rs3776284	G	A	.	PASS	KIF1A	exo	NM_	.	synonyr
ACa07	2	2.42E+08	rs1500761	C	T	.	PASS	KIF1A	intrc	NM_	.	.
ACa07	2	2.42E+08	.	G	T	.	PASS	.	ups	NM_	.	.
ACa07	2	2.42E+08	.	C	T	.	PASS	.	inte	NR_	dis	.
ACa07	2	2.42E+08	.	G	A	.	PASS	SNED1	intrc	NM_	.	.
ACa07	3	439920	rs3731564	C	T	.	PASS	CHL1	exo	NM_	.	synonyr
ACa07	3	1363137	rs5694312	C	T	.	PASS	CNTN6	intrc	NM_	.	.
ACa07	3	8574667	.	C	T	.	PASS	LMCD1	intrc	NM_	.	.
ACa07	3	9594406	.	G	A	.	PASS	LHFPL4	UTF	NM_	NM	.
ACa07	3	9895229	rs7502023	C	T	.	PASS	.	inte	NM_	dis	.
ACa07	3	9972567	.	T	C	.	PASS	IL17RC	intrc	NM_	.	.
ACa07	3	9979307	rs7567892	C	T	.	PASS	CRELD	exo	NM_	.	missens
ACa07	3	10102136	.	C	T	.	PASS	FANCD	intrc	NM_	.	.
ACa07	3	10953794	.	C	T	.	PASS	SLC6A1	exo	NM_	.	missens
ACa07	3	11832172	.	G	A	.	PASS	TAMM4	intrc	NM_	.	.
ACa07	3	12510284	.	G	A	.	PASS	.	inte	NM_	dis	.
ACa07	3	12572765	rs9925095	C	T	.	PASS	TSEN2	intrc	NM_	.	.
ACa07	3	12632551	.	G	T	.	PASS	RAF1	intrc	NM_	.	.
ACa07	3	12957408	.	C	A	.	PASS	IQSEC1	intrc	NM_	.	.
ACa07	3	15281998	.	T	A	.	PASS	CAPN7	exo	NM_	.	missens
ACa07	3	17209148	.	T	C	.	PASS	TBC1D5	intrc	NM_	.	.
ACa07	3	19556925	.	G	T	.	PASS	KCNH8	intrc	NM_	.	.
ACa07	3	24003631	.	G	A	.	PASS	NR1D2	exo	NM_	.	synonyr
ACa07	3	25654229	.	C	T	.	PASS	TOP2B	intrc	NM_	.	.
ACa07	3	27047435	.	A	G	.	PASS	.	inte	NM_	dis	.
ACa07	3	27478853	rs8972401	A	G	.	PASS	SLC4A7	intrc	NM_	.	.
ACa07	3	30713346	rs1124655	G	A	.	PASS	TGFBR	exo	NM_	.	missens
ACa07	3	31203246	.	C	T	.	PASS	.	inte	NM_	dis	.
ACa07	3	31621381	rs7802104	C	T	.	PASS	STT3B	exo	NM_	.	unknow
ACa07	3	36526355	.	G	T	.	PASS	STAC	intrc	NM_	.	.
ACa07	3	36779885	rs5407838	G	A	.	PASS	DCLK3	exo	NM_	.	missens
ACa07	3	37045772	rs5599017	G	A	.	PASS	MLH1	intrc	NM_	.	.
ACa07	3	37340490	.	T	C	.	PASS	GOLGA	exo	NM_	.	synonyr
ACa07	3	38153245	rs3732183	G	A	.	PASS	DLEC1	intrc	NM_	.	.
ACa07	3	38540225	.	C	T	.	PASS	EXOG	intrc	NM_	.	.
ACa07	3	38753883	rs7485706	G	A	.	PASS	SCN10	exo	NM_	.	synonyr
ACa07	3	44621509	.	G	A	.	PASS	ZKSCA	intrc	NM_	.	.
ACa07	3	45943055	rs7745693	G	A	.	PASS	CCR9	exo	NM_	.	missens
ACa07	3	46786468	.	G	A	.	PASS	.	ups	NM_	.	.
ACa07	3	47058834	.	G	A	.	PASS	SETD2	intrc	NM_	.	.
ACa07	3	48476363	rs7512571	C	T	.	PASS	CCDC5	exo	NM_	.	missens
ACa07	3	48613210	rs5304720	G	A	.	PASS	COL7A	intrc	NM_	.	.
ACa07	3	48716575	rs3713830	C	T	.	PASS	NCKIPS	exo	NM_	.	missens
ACa07	3	49050939	.	C	T	.	PASS	WDR6	exo	NM_	.	missens
ACa07	3	49095171	rs2001906	C	T	.	PASS	QRICH1	exo	NM_	.	synonyr
ACa07	3	49362425	.	G	A	.	PASS	USP4	exo	NM_	.	missens
ACa07	3	49689519	.	C	T	.	PASS	BSN	exo	NM_	.	stopgai
ACa07	3	49897525	.	G	T	.	PASS	CAMKV	intrc	NM_	.	.
ACa07	3	50416564	rs7596904	C	T	.	PASS	CACNA	exo	NM_	.	unknow
ACa07	3	51909508	rs1821535	G	A	.	PASS	IQCF5-	ncR	NR_	.	.
ACa07	3	51993350	.	A	G	.	PASS	PCBP4	intrc	NM_	.	.
ACa07	3	51995196	rs7780907	G	A	.	PASS	PCBP4	exo	NM_	.	missens
ACa07	3	52242097	.	A	G	.	PASS	ALAS1	splic	NM_	NM	.

ACa07	3	52412482	.	G	A	.	PASS	DNAH1	intrc	NM_	.	.
ACa07	3	52415487	.	A	G	.	PASS	DNAH1	intrc	NM_	.	.
ACa07	3	52425181	.	C	T	.	PASS	DNAH1	intrc	NM_	.	.
ACa07	3	52476458	rs9624625	C	T	.	PASS	SEMA3(	intrc	NM_	.	.
ACa07	3	52675780	.	A	T	.	PASS	PBRM1	intrc	NM_	.	.
ACa07	3	52713682	.	T	G	.	PASS	PBRM1	exoı	NM_	.	missens
ACa07	3	53766890	.	C	T	.	PASS	CACNA	exoı	NM_	.	missens
ACa07	3	53910929	rs2000990	C	T	.	PASS	ACTR8	intrc	NM_	.	.
ACa07	3	54952366	rs9858893	T	C	.	PASS	CACNA	intrc	NM_	.	.
ACa07	3	54952367	rs1042360	T	C	.	PASS	CACNA	intrc	NM_	.	.
ACa07	3	57168155	.	G	A	.	PASS	IL17RD	intrc	NM_	.	.
ACa07	3	57269719	.	C	A	.	PASS	APPL1	intrc	NM_	.	.
ACa07	3	57613858	.	G	T	.	PASS	DENND	UTF	NM_	NM	.
ACa07	3	58383459	.	G	T	.	PASS	PXK	intrc	NM_	.	.
ACa07	3	64547317	rs7458693	C	T	.	PASS	ADAMT	exoı	NM_	.	synonyr
ACa07	3	69168127	.	A	G	.	PASS	LMOD3	exoı	NM_	.	missens
ACa07	3	69987019	.	C	T	.	PASS	MITF	exoı	NM_	.	missens
ACa07	3	75478300	rs7794528	C	T	.	PASS	FAM86[	ncR	NR_	.	.
ACa07	3	97666222	.	G	A	.	PASS	MINA	exoı	NM_	.	missens
ACa07	3	1.04E+08	.	A	G	.	PASS	.	inteı	NR_	dis	.
ACa07	3	1.08E+08	.	G	T	.	PASS	KIAA15:	intrc	NM_	.	.
ACa07	3	1.09E+08	.	C	T	.	PASS	MORC1	intrc	NM_	.	.
ACa07	3	1.13E+08	.	G	T	.	PASS	CFAP4	exoı	NM_	.	synonyr
ACa07	3	1.19E+08	.	A	C	.	PASS	TMEM3	splic	NM_	NM	.
ACa07	3	1.19E+08	.	C	A	.	PASS	TMEM3	intrc	NM_	.	.
ACa07	3	1.21E+08	rs3684939	G	A	.	PASS	STXBP!	exoı	NM_	.	missens
ACa07	3	1.23E+08	rs1001693	G	A	.	PASS	SEMA5I	intrc	NM_	.	.
ACa07	3	1.25E+08	rs1465223	G	A	.	PASS	.	inteı	NM_	dis	.
ACa07	3	1.26E+08	.	C	A	.	PASS	UROC1	intrc	NM_	.	.
ACa07	3	1.27E+08	rs5528272	G	A	.	PASS	PLXNA:	exoı	NM_	.	synonyr
ACa07	3	1.27E+08	rs7511092	G	A	.	PASS	TPRA1	exoı	NM_	.	synonyr
ACa07	3	1.28E+08	.	A	G	.	PASS	.	inteı	NR_	dis	.
ACa07	3	1.29E+08	rs5631647	C	T	.	PASS	LOC65:	ncR	NR_	.	.
ACa07	3	1.29E+08	.	A	G	.	PASS	RPL32F	ncR	NR_	.	.
ACa07	3	1.31E+08	.	G	A	.	PASS	NUDT1(	exoı	NM_	.	missens
ACa07	3	1.32E+08	.	C	A	.	PASS	ACPP	UTF	NM_	NM	.
ACa07	3	1.39E+08	.	G	A	.	PASS	PRR23/	exoı	NM_	.	missens
ACa07	3	1.39E+08	.	C	T	.	PASS	PRR23(	exoı	NM_	.	synonyr
ACa07	3	1.4E+08	rs1408440	C	T	.	PASS	CLSTN:	exoı	NM_	.	synonyr
ACa07	3	1.41E+08	.	G	A	.	PASS	RASA2	exoı	NM_	.	synonyr
ACa07	3	1.42E+08	.	C	T	.	PASS	TFDP2	intrc	NM_	.	.
ACa07	3	1.42E+08	rs7685217	A	T	.	PASS	ATR	intrc	NM_	.	.
ACa07	3	1.43E+08	rs7705095	C	T	.	PASS	PCOLC	exoı	NM_	.	missens
ACa07	3	1.45E+08	.	G	A	.	PASS	.	inteı	NM_	dis	.
ACa07	3	1.5E+08	.	C	T	.	PASS	.	inteı	NR_	dis	.
ACa07	3	1.5E+08	rs7605662	C	T	.	PASS	EIF2A	exoı	NM_	.	synonyr
ACa07	3	1.58E+08	.	C	A	.	PASS	GFM1	intrc	NM_	.	.
ACa07	3	1.6E+08	.	G	A	.	PASS	MIR16-:	ncR	NR_	.	.
ACa07	3	1.67E+08	.	G	A	.	PASS	.	inteı	NM_	dis	.
ACa07	3	1.7E+08	.	G	C	.	PASS	.	inteı	NM_	dis	.
ACa07	3	1.7E+08	rs5388049	C	T	.	PASS	SKIL	exoı	NM_	.	missens
ACa07	3	1.77E+08	rs9368630	G	A	.	PASS	.	inteı	NM_	dis	.
ACa07	3	1.79E+08	.	G	T	.	PASS	KCNMB	UTF	NM_	NM	.
ACa07	3	1.79E+08	rs7652687	C	T	.	PASS	USP13	exoı	NM_	.	synonyr

ACa07	3	1.81E+08	rs5714458	C	T	.	PASS	LOC101	ncR	NR_	.	.
ACa07	3	1.83E+08	.	T	G	.	PASS	MCCC1	exo	NM_	.	missens
ACa07	3	1.83E+08	.	C	T	.	PASS	KLHL6	exo	NM_	.	missens
ACa07	3	1.84E+08	rs9015268	C	T	.	PASS	HTR3D	intro	NM_	.	.
ACa07	3	1.84E+08	.	T	C	.	PASS	VWA5B	intro	NM_	.	.
ACa07	3	1.84E+08	rs3748943	G	A	.	PASS	ECE2	exo	NM_	.	missens
ACa07	3	1.87E+08	.	T	A	.	PASS	SST	intro	NM_	.	.
ACa07	3	1.88E+08	rs7531929	C	T	.	PASS	LPP	exo	NM_	.	synonyr
ACa07	3	1.94E+08	rs7777314	G	A	.	PASS	TMEM4	intro	NM_	.	.
ACa07	3	1.95E+08	.	C	T	.	PASS	.	inte	NM_	dis	.
ACa07	3	1.95E+08	.	C	T	.	PASS	.	inte	NM_	dis	.
ACa07	3	1.95E+08	.	C	T	.	PASS	MUC4	intro	NM_	.	.
ACa07	3	1.96E+08	.	C	T	.	PASS	.	inte	NM_	dis	.
ACa07	3	1.96E+08	rs8866852	G	A	.	PASS	NRROS	intro	NM_	.	.
ACa07	3	1.98E+08	rs7637750	A	G	.	PASS	.	inte	NR_	dis	.
ACa07	4	429436	.	G	T	.	PASS	ABCA1	ncR	NR_	.	.
ACa07	4	952132	rs7620115	G	A	.	PASS	TMEM1	exo	NM_	.	unknow
ACa07	4	1215618	.	C	A	.	PASS	CTBP1	intro	NM_	.	.
ACa07	4	1646117	rs9125004	C	T	.	PASS	FAM53	intro	NM_	.	.
ACa07	4	1739773	.	C	T	.	PASS	TACC3	intro	NM_	.	.
ACa07	4	1834370	.	G	A	.	PASS	LETM1	intro	NM_	.	.
ACa07	4	1953047	rs5508099	G	A	.	PASS	WHSC1	intro	NM_	.	.
ACa07	4	2260660	.	T	C	.	PASS	MXD4	intro	NM_	.	.
ACa07	4	3478009	.	G	A	.	PASS	DOK7	intro	NM_	.	.
ACa07	4	3498489	rs9333127	G	A	.	PASS	.	intro	NM_	.	.
ACa07	4	3769294	.	C	T	.	PASS	ADRA2	exo	NM_	.	stopgai
ACa07	4	6303946	rs5603533	C	T	.	PASS	WFS1	exo	NM_	.	synonyr
ACa07	4	6415457	.	G	A	.	PASS	PPP2R2	intro	NM_	.	.
ACa07	4	7663381	.	G	A	.	PASS	SORCS	intro	NM_	.	.
ACa07	4	7783107	rs3763511	G	A	.	PASS	AFAP1	exo	NM_	.	missens
ACa07	4	8099021	rs2006520	C	T	.	PASS	ABLIM2	intro	NM_	.	.
ACa07	4	8218865	rs7521101	C	T	.	PASS	SH3TC	exo	NM_	.	synonyr
ACa07	4	10447435	rs7804064	G	A	.	PASS	ZNF518	exo	NM_	.	missens
ACa07	4	13615962	.	C	A	.	PASS	BOD1L	exo	NM_	.	missens
ACa07	4	15581724	.	G	A	.	PASS	CC2D2	exo	NM_	.	missens
ACa07	4	20728844	.	A	T	.	PASS	PACRG	intro	NM_	.	.
ACa07	4	24854707	.	T	C	.	PASS	CCDC1	exo	NM_	.	missens
ACa07	4	33744074	rs1420745	A	G	.	PASS	.	inte	NR_	dis	.
ACa07	4	39739166	rs5344165	C	T	.	PASS	UBE2K	intro	NM_	.	.
ACa07	4	39771917	.	C	T	.	PASS	UBE2K	intro	NM_	.	.
ACa07	4	40022163	.	G	T	.	PASS	.	inte	NM_	dis	.
ACa07	4	40245476	rs7759148	G	A	.	PASS	RHOH	exo	NM_	.	missens
ACa07	4	40776896	.	A	T	.	PASS	NSUN7	exo	NM_	.	missens
ACa07	4	47033791	rs2856467	C	T	.	PASS	GABRB	intro	NM_	.	.
ACa07	4	49244763	rs1451863	C	T	.	PASS	.	inte	NM_	dis	.
ACa07	4	49261257	rs5941177	C	T	.	PASS	.	inte	NM_	dis	.
ACa07	4	52861933	.	C	T	.	PASS	LRRC6	exo	NM_	.	missens
ACa07	4	53578826	.	C	T	.	PASS	DANCR	ncR	NR_	.	.
ACa07	4	56738008	.	G	C	.	PASS	EXOC1	intro	NM_	.	.
ACa07	4	57460905	.	C	A	.	PASS	THEGL	intro	NM_	.	.
ACa07	4	68693318	.	G	A	.	PASS	TMPRS	intro	NM_	.	.
ACa07	4	70361565	.	C	T	.	PASS	UGT2B	exo	NM_	.	stopgai
ACa07	4	70803516	.	A	T	.	PASS	CSN1S	splic	NM_	NM	.
ACa07	4	72412020	rs7619680	C	T	.	PASS	SLC4A4	intro	NM_	.	.

ACa07	4	73169633	.	C	T	.	PASS	ADAMT	splic	NM_	NM	.
ACa07	4	74365007	rs1194702	T	A	.	PASS	AFM	intrc	NM_	.	.
ACa07	4	74477637	.	G	T	.	PASS	RASSF(	intrc	NM_	.	.
ACa07	4	76804222	rs3691209	C	T	.	PASS	PPEF2	exoı	NM_	.	missens
ACa07	4	77290615	.	G	T	.	PASS	CCDC1	exoı	NM_	.	synonyr
ACa07	4	77651986	rs7609961	C	T	.	PASS	SHROC	exoı	NM_	.	missens
ACa07	4	79291009	.	G	T	.	PASS	FRAS1	exoı	NM_	.	stopgaiı
ACa07	4	83931968	.	A	G	.	PASS	LIN54	UTF	NM_	NM	.
ACa07	4	85660018	rs8867538	T	C	.	PASS	WDFY3	intrc	NM_	.	.
ACa07	4	89671588	.	G	T	.	PASS	FAM13/	exoı	NM_	.	missens
ACa07	4	1.04E+08	.	C	A	.	PASS	NFKB1	intrc	NM_	.	.
ACa07	4	1.06E+08	.	G	A	.	PASS	TET2	exoı	NM_	.	missens
ACa07	4	1.07E+08	rs3719859	C	T	.	PASS	TBCK	exoı	NM_	.	unknow
ACa07	4	1.11E+08	.	C	T	.	PASS	ENPEP	exoı	NM_	.	synonyr
ACa07	4	1.13E+08	.	A	G	.	PASS	.	inteı	NM_	dis	.
ACa07	4	1.19E+08	.	A	G	.	PASS	NDST3	intrc	NM_	.	.
ACa07	4	1.2E+08	.	A	G	.	PASS	LOC72(	ncR	NR_	.	.
ACa07	4	1.2E+08	.	G	T	.	PASS	METTL	exoı	NM_	.	stopgaiı
ACa07	4	1.22E+08	.	C	A	.	PASS	NDNF	exoı	NM_	.	missens
ACa07	4	1.26E+08	rs7744530	T	C	.	PASS	ANKRD	exoı	NM_	.	missens
ACa07	4	1.29E+08	.	G	T	.	PASS	C4orf29	exoı	NM_	.	missens
ACa07	4	1.29E+08	rs7741072	A	G	.	PASS	C4orf29	exoı	NM_	.	missens
ACa07	4	1.33E+08	.	C	A	.	PASS	.	inteı	NR_	dis	.
ACa07	4	1.39E+08	.	G	A	.	PASS	.	inteı	NR_	dis	.
ACa07	4	1.42E+08	.	G	T	.	PASS	TBC1D(	exoı	NM_	.	synonyr
ACa07	4	1.42E+08	.	G	A	.	PASS	TBC1D(	exoı	NM_	.	synonyr
ACa07	4	1.43E+08	.	A	G	.	PASS	IL15	exoı	NM_	.	synonyr
ACa07	4	1.45E+08	.	C	A	.	PASS	GYPB	intrc	NM_	.	.
ACa07	4	1.54E+08	rs8661228	T	C	.	PASS	TRIM2	intrc	NM_	.	.
ACa07	4	1.66E+08	rs4095550	G	A	.	PASS	KLHL2	intrc	NM_	.	.
ACa07	4	1.73E+08	.	G	A	.	PASS	GALNTI	intrc	NM_	.	.
ACa07	4	1.84E+08	.	A	G	.	PASS	TENM3	exoı	NM_	.	missens
ACa07	4	1.86E+08	rs9420962	C	T	.	PASS	CCDC1	intrc	NM_	.	.
ACa07	4	1.87E+08	.	G	A	.	PASS	SORBS	exoı	NM_	.	synonyr
ACa07	4	1.87E+08	rs7781796	G	A	.	PASS	F11-AS	ncR	NR_	.	.
ACa07	5	482582	.	A	G	.	PASS	SLC9A3	intrc	NM_	.	.
ACa07	5	620233	.	G	A	.	PASS	CEP72	exoı	NM_	.	missens
ACa07	5	833995	rs412221	G	C	.	PASS	ZDHHC	intrc	NM_	.	.
ACa07	5	1089337	rs1006578	C	T	.	PASS	SLC12A	intrc	NM_	.	.
ACa07	5	1879655	.	C	A	.	PASS	IRX4	exoı	NM_	.	missens
ACa07	5	4420496	rs9030458	G	A	.	PASS	.	inteı	NM_	dis	.
ACa07	5	5461158	rs7491497	G	A	.	PASS	ICE1	exoı	NM_	.	missens
ACa07	5	6491435	.	G	T	.	PASS	UBE2QI	exoı	NM_	.	missens
ACa07	5	6748634	.	A	G	.	PASS	PAPD7	exoı	NM_	.	missens
ACa07	5	11159846	rs1497666	G	A	.	PASS	CTNND	exoı	NM_	.	synonyr
ACa07	5	13820656	rs6554817	G	A	.	PASS	DNAH5	intrc	NM_	.	.
ACa07	5	14145003	rs9140889	G	C	.	PASS	TRIO	intrc	NM_	.	.
ACa07	5	14461080	rs7661668	G	A	.	PASS	TRIO	intrc	NM_	.	.
ACa07	5	32522898	rs9251299	G	A	.	PASS	.	inteı	NM_	dis	.
ACa07	5	33642089	rs5697598	G	A	.	PASS	ADAMT	intrc	NM_	.	.
ACa07	5	34182050	rs8799835	G	C	.	PASS	.	inteı	NR_	dis	.
ACa07	5	34182071	rs9765292	G	T	.	PASS	.	inteı	NR_	dis	.
ACa07	5	34182201	rs9765293	C	T	.	PASS	.	inteı	NR_	dis	.
ACa07	5	38557659	.	C	A	.	PASS	MIR365	ncR	NR_	.	.

ACa07	5	40762835	.	T	A	.	PASS	PRKAA:	UTF	NM_	NM	.
ACa07	5	41794029	.	A	G	.	PASS	OXCT1	intrc	NM_	.	.
ACa07	5	43296059	.	G	T	.	PASS	HMGCS	intrc	NM_	.	.
ACa07	5	49410188	.	T	C	.	PASS	.	inte	NON	dis	.
ACa07	5	49410224	rs4327635	T	C	.	PASS	.	inte	NON	dis	.
ACa07	5	49410231	.	A	G	.	PASS	.	inte	NON	dis	.
ACa07	5	56778002	rs2005764	C	T	.	PASS	ACTBL2	exoi	NM_	.	missens
ACa07	5	59189136	.	C	T	.	PASS	PDE4D	exoi	NM_	.	missens
ACa07	5	62073158	rs5582099	C	T	.	PASS	.	inte	NM_	dis	.
ACa07	5	64817356	.	C	A	.	PASS	CENPK	splic	NM_	NM	.
ACa07	5	65324199	.	G	A	.	PASS	ERBB2I	intrc	NM_	.	.
ACa07	5	66430158	rs5672287	G	A	.	PASS	MAST4	intrc	NM_	.	.
ACa07	5	66461072	.	A	G	.	PASS	MAST4	exoi	NM_	.	missens
ACa07	5	66461884	rs2016310	C	T	.	PASS	MAST4	exoi	NM_	.	missens
ACa07	5	67592074	.	C	T	.	PASS	PIK3R1	exoi	NM_	.	synonyr
ACa07	5	68670689	.	A	G	.	PASS	RAD17	intrc	NM_	.	.
ACa07	5	69826392	.	C	G	.	PASS	GUSBP	ncR	NR_	.	.
ACa07	5	71482425	.	C	A	.	PASS	MAP1B	intrc	NM_	.	.
ACa07	5	74948776	.	A	G	.	PASS	ANKDD	intrc	NM_	.	.
ACa07	5	78808788	.	C	T	.	PASS	HOMEF	UTF	NM_	NM	.
ACa07	5	80626348	.	G	A	.	PASS	ACOT1:	exoi	NM_	.	synonyr
ACa07	5	82352890	.	G	A	.	PASS	TMEM1	UTF	NM_	NM	.
ACa07	5	82868186	rs3717547	C	T	.	PASS	VCAN	intrc	NM_	.	.
ACa07	5	86811590	rs7815826	C	T	.	PASS	.	inte	NM_	dis	.
ACa07	5	89989950	.	G	T	.	PASS	GPR98	exoi	NM_	.	missens
ACa07	5	93809079	.	G	A	.	PASS	KIAA08:	exoi	NM_	.	missens
ACa07	5	94936743	rs7633968	C	T	.	PASS	ARSK	exoi	NM_	.	missens
ACa07	5	99401916	.	C	T	.	PASS	.	inte	NR_	dis	.
ACa07	5	1.02E+08	.	A	C	.	PASS	PAM	exoi	NM_	.	missens
ACa07	5	1.02E+08	rs1114874	C	T	.	PASS	PAM	intrc	NM_	.	.
ACa07	5	1.1E+08	.	G	T	.	PASS	.	ups	NM_	.	.
ACa07	5	1.12E+08	.	A	G	.	PASS	EPB41L	exoi	NM_	.	synonyr
ACa07	5	1.16E+08	.	C	T	.	PASS	SEMA6	exoi	NM_	.	synonyr
ACa07	5	1.19E+08	.	G	T	.	PASS	TNFAIP	intrc	NM_	.	.
ACa07	5	1.32E+08	rs9346631	G	A	.	PASS	SOWAH	exoi	NM_	.	unknow
ACa07	5	1.32E+08	.	A	T	.	PASS	AFF4	intrc	NM_	.	.
ACa07	5	1.33E+08	rs7465490	C	T	.	PASS	FSTL4	exoi	NM_	.	synonyr
ACa07	5	1.34E+08	.	C	T	.	PASS	.	ups	NM_	.	.
ACa07	5	1.34E+08	rs7470418	T	C	.	PASS	DDX46	intrc	NM_	.	.
ACa07	5	1.38E+08	rs7665720	C	T	.	PASS	CDC25(	exoi	NM_	.	missens
ACa07	5	1.38E+08	.	A	G	.	PASS	CDC25(	exoi	NM_	.	missens
ACa07	5	1.38E+08	.	A	G	.	PASS	EGR1	exoi	NM_	.	missens
ACa07	5	1.38E+08	rs7721589	G	A	.	PASS	EGR1	exoi	NM_	.	missens
ACa07	5	1.4E+08	.	T	C	.	PASS	ANKHD	exoi	NM_	.	missens
ACa07	5	1.4E+08	.	G	A	.	PASS	PCDHA	exoi	NM_	.	synonyr
ACa07	5	1.4E+08	.	C	T	.	PASS	PCDHA	exoi	NM_	.	synonyr
ACa07	5	1.4E+08	rs7823690	G	A	.	PASS	PCDHA	exoi	NM_	.	synonyr
ACa07	5	1.41E+08	.	G	A	.	PASS	PCDHB	exoi	NM_	.	missens
ACa07	5	1.41E+08	.	C	T	.	PASS	PCDHB	exoi	NM_	.	missens
ACa07	5	1.41E+08	.	C	T	.	PASS	PCDHG	exoi	NM_	.	synonyr
ACa07	5	1.41E+08	rs9814581	C	T	.	PASS	PCDHG	exoi	NM_	.	missens
ACa07	5	1.41E+08	rs5446331	C	T	.	PASS	PCDHG	exoi	NM_	.	synonyr
ACa07	5	1.41E+08	rs3769527	C	T	.	PASS	PCDHG	exoi	NM_	.	synonyr
ACa07	5	1.41E+08	rs7719463	G	A	.	PASS	PCDHG	exoi	NM_	.	missens

ACa07	5	1.41E+08	rs2018026	G	A	.	PASS	KIAA01	exo	NM_	.	missens
ACa07	5	1.47E+08	rs1000063	A	G	.	PASS	JAKMIP	UTF	NM_	NM	.
ACa07	5	1.49E+08	.	T	C	.	PASS	CSNK1	intro	NM_	.	.
ACa07	5	1.49E+08	.	A	T	.	PASS	PDE6A	intro	NM_	.	.
ACa07	5	1.5E+08	.	G	A	.	PASS	SYNPO	exo	NM_	.	missens
ACa07	5	1.51E+08	.	C	A	.	PASS	G3BP1	exo	NM_	.	missens
ACa07	5	1.57E+08	rs5492040	G	A	.	PASS	CYFIP2	intro	NM_	.	.
ACa07	5	1.59E+08	rs5611223	C	T	.	PASS	RNF14	exo	NM_	.	missens
ACa07	5	1.67E+08	.	G	A	.	PASS	TENM2	exo	NM_	.	missens
ACa07	5	1.68E+08	.	C	A	.	PASS	TENM2	exo	NM_	.	missens
ACa07	5	1.68E+08	rs1491693	C	T	.	PASS	WWC1	exo	NM_	.	missens
ACa07	5	1.68E+08	rs7577825	C	T	.	PASS	RARS	exo	NM_	.	missens
ACa07	5	1.68E+08	.	G	T	.	PASS	SLIT3	intro	NM_	.	.
ACa07	5	1.72E+08	.	T	C	.	PASS	SH3PXI	exo	NM_	.	missens
ACa07	5	1.72E+08	rs1822272	C	T	.	PASS	NEURL	intro	NM_	.	.
ACa07	5	1.73E+08	.	C	A	.	PASS	CREBR	intro	NM_	.	.
ACa07	5	1.76E+08	.	T	C	.	PASS	FAF2	intro	NM_	.	.
ACa07	5	1.77E+08	.	G	A	.	PASS	FGFR4	intro	NM_	.	.
ACa07	5	1.77E+08	rs4551119	G	A	.	PASS	FGFR4	intro	NM_	.	.
ACa07	5	1.77E+08	rs5582208	C	T	.	PASS	FGFR4	exo	NM_	.	missens
ACa07	5	1.77E+08	.	C	A	.	PASS	LOC202	ncR	NR_	.	.
ACa07	5	1.77E+08	.	C	T	.	PASS	FAM15	intro	NM_	.	.
ACa07	5	1.78E+08	.	G	A	.	PASS	GRM6	UTF	NM_	NM	.
ACa07	5	1.79E+08	rs1025870	G	A	.	PASS	.	inte	NM_	dis	.
ACa07	5	1.79E+08	.	G	A	.	PASS	MGAT4	UTF	NM_	NM	.
ACa07	5	1.8E+08	.	G	T	.	PASS	GFPT2	intro	NM_	.	.
ACa07	6	4996642	rs7651293	G	A	.	PASS	RPP40	exo	NM_	.	missens
ACa07	6	5001178	rs1023580	A	T	.	PASS	RPP40	intro	NM_	.	.
ACa07	6	7269296	rs1864505	G	A	.	PASS	.	inte	NM_	dis	.
ACa07	6	7481383	.	A	G	.	PASS	.	inte	NM_	dis	.
ACa07	6	7883534	rs2004302	C	T	.	PASS	BLOC1	ncR	NR_	.	.
ACa07	6	10084993	.	T	A	.	PASS	.	inte	NR_	dis	.
ACa07	6	10557615	rs3692600	G	A	.	PASS	GCNT2	intro	NM_	.	.
ACa07	6	12122025	rs7468362	G	A	.	PASS	HIVEP1	exo	NM_	.	missens
ACa07	6	15523454	rs7674897	C	T	.	PASS	DTNBP	splic	NM_	NM	.
ACa07	6	22147112	.	T	G	.	PASS	CASC1	ncR	NR_	.	.
ACa07	6	23854521	.	C	T	.	PASS	.	inte	NM_	dis	.
ACa07	6	25023988	.	G	A	.	PASS	FAM65	intro	NM_	.	.
ACa07	6	26865005	.	T	C	.	PASS	GUSBP	ncR	NR_	.	.
ACa07	6	27179321	.	G	A	.	PASS	.	inte	NR_	dis	.
ACa07	6	28058830	.	C	T	.	PASS	ZSCAN	ncR	NR_	.	.
ACa07	6	28321550	.	G	A	.	PASS	ZKSCAI	intro	NM_	.	.
ACa07	6	28569767	.	T	C	.	PASS	.	inte	NM_	dis	.
ACa07	6	29643881	rs7653588	G	A	.	PASS	ZFP57	intro	NM_	.	.
ACa07	6	29856132	.	A	G	.	PASS	HLA-H	ncR	NR_	.	.
ACa07	6	30530100	rs7511377	C	T	.	PASS	PRR3	intro	NM_	.	.
ACa07	6	30679176	.	G	T	.	PASS	MDC1	intro	NM_	.	.
ACa07	6	31429977	.	G	A	.	PASS	.	ups	NR_	.	.
ACa07	6	31487246	.	A	G	.	PASS	.	inte	NM_	dis	.
ACa07	6	31594834	rs7792598	C	T	.	PASS	PRRC2	exo	NM_	.	synonyr
ACa07	6	31750447	.	G	A	.	PASS	VARS	intro	NM_	.	.
ACa07	6	32412629	.	T	C	.	PASS	HLA-DF	UTF	NM_	NM	.
ACa07	6	33144803	rs1119219	C	T	.	PASS	COL11	exo	NM_	.	missens
ACa07	6	33147259	rs7759216	C	T	.	PASS	COL11	exo	NM_	.	synonyr

ACa07	6	33658820	.	G	A	.	PASS	ITPR3	exoı	NM_	.	missens
ACa07	6	34498074	.	G	T	.	PASS	PACSIN	exoı	NM_	.	missens
ACa07	6	35378846	rs7740360	C	T	.	PASS	PPARD	UTF	NM_	NM	.
ACa07	6	35387747	.	C	T	.	PASS	PPARD	intrc	NM_	.	.
ACa07	6	35392381	rs7715433	C	T	.	PASS	PPARD	exoı	NM_	.	synonyr
ACa07	6	35436310	.	G	A	.	PASS	RPL10A	intrc	NM_	.	.
ACa07	6	35927609	.	G	C	.	PASS	SLC26A	intrc	NM_	.	.
ACa07	6	36710079	rs1007644	G	A	.	PASS	CPNE5	exoı	NM_	.	missens
ACa07	6	39843088	.	A	G	.	PASS	DAAM2	intrc	NM_	.	.
ACa07	6	41217331	rs3751257	C	T	.	PASS	.	dow	NR_	.	.
ACa07	6	41617431	rs7778285	G	A	.	PASS	MDFI	exoı	NM_	.	missens
ACa07	6	41738534	.	C	A	.	PASS	FRS3	exoı	NM_	.	missens
ACa07	6	42153585	rs8008274	C	A	.	PASS	GUCA1	intrc	NM_	.	.
ACa07	6	42227276	rs7806884	C	T	.	PASS	TRERF	exoı	NM_	.	unknow
ACa07	6	42689635	.	G	T	.	PASS	PRPH2	exoı	NM_	.	synonyr
ACa07	6	43100271	rs1434417	C	T	.	PASS	PTK7	exoı	NM_	.	synonyr
ACa07	6	43126633	.	C	T	.	PASS	PTK7	exoı	NM_	.	missens
ACa07	6	43325659	.	G	T	.	PASS	ZNF318	intrc	NM_	.	.
ACa07	6	43484278	.	A	G	.	PASS	YIPF3	intrc	NM_	.	.
ACa07	6	43499264	rs1496900	G	A	.	PASS	XPO5	exoı	NM_	.	synonyr
ACa07	6	44255388	rs1435325	G	A	.	PASS	TCTE1	exoı	NM_	.	missens
ACa07	6	52715113	.	G	A	.	PASS	.	inteı	NM_	dis	.
ACa07	6	56323987	.	C	A	.	PASS	DST	exoı	NM_	.	missens
ACa07	6	63939195	.	C	G	.	PASS	.	inteı	NM_	dis	.
ACa07	6	70890361	.	A	G	.	PASS	COL19A	exoı	NM_	.	synonyr
ACa07	6	71571705	rs2021606	A	G	.	PASS	SMAP1	UTF	NM_	NM	.
ACa07	6	74073122	.	A	T	.	PASS	KHDC3I	intrc	NM_	.	.
ACa07	6	74432967	.	C	A	.	PASS	CD109	intrc	NM_	.	.
ACa07	6	79651030	rs7761622	C	T	.	PASS	PHIP	exoı	NM_	.	missens
ACa07	6	80878661	rs1497660	C	T	.	PASS	BCKDH	exoı	NM_	.	missens
ACa07	6	84629075	rs1002475	T	C	.	PASS	CYB5R	intrc	NM_	.	.
ACa07	6	88218841	rs7619979	G	A	.	PASS	SLC35A	exoı	NM_	.	synonyr
ACa07	6	90497830	rs1015787	G	A	.	PASS	MDN1	intrc	NM_	.	.
ACa07	6	90605889	.	C	T	.	PASS	.	dow	NM_	.	.
ACa07	6	99283939	.	T	C	.	PASS	POU3F	exoı	NM_	.	missens
ACa07	6	1.07E+08	rs5581466	A	C	.	PASS	LOC10C	ncR	NR_	.	.
ACa07	6	1.09E+08	.	T	C	.	PASS	NR2E1	intrc	NM_	.	.
ACa07	6	1.09E+08	.	T	C	.	PASS	SESN1	intrc	NM_	.	.
ACa07	6	1.1E+08	.	T	C	.	PASS	PPIL6	UTF	NM_	NM	.
ACa07	6	1.1E+08	.	G	T	.	PASS	MICAL1	intrc	NM_	.	.
ACa07	6	1.12E+08	.	G	T	.	PASS	REV3L	exoı	NM_	.	missens
ACa07	6	1.22E+08	.	C	T	.	PASS	.	inteı	NM_	dis	.
ACa07	6	1.22E+08	.	G	A	.	PASS	.	inteı	NM_	dis	.
ACa07	6	1.26E+08	.	T	C	.	PASS	TRMT1	intrc	NM_	.	.
ACa07	6	1.3E+08	rs3754749	C	T	.	PASS	LAMA2	intrc	NM_	.	.
ACa07	6	1.3E+08	.	A	C	.	PASS	LAMA2	intrc	NM_	.	.
ACa07	6	1.34E+08	rs7606036	G	A	.	PASS	EYA4	exoı	NM_	.	missens
ACa07	6	1.35E+08	rs5279151	G	A	.	PASS	ALDH8A	intrc	NM_	.	.
ACa07	6	1.37E+08	rs1006659	T	C	.	PASS	IL20RA	intrc	NM_	.	.
ACa07	6	1.39E+08	.	T	C	.	PASS	KIAA12	intrc	NM_	.	.
ACa07	6	1.39E+08	.	C	T	.	PASS	KIAA12	intrc	NM_	.	.
ACa07	6	1.43E+08	rs1910958	G	A	.	PASS	GPR12(	intrc	NM_	.	.
ACa07	6	1.45E+08	rs1401901	G	A	.	PASS	STX11	exoı	NM_	.	missens
ACa07	6	1.47E+08	.	A	G	.	PASS	ADGB	exoı	NM_	.	missens

ACa07	6	1.49E+08	rs7737449	C	T	.	PASS	UST	exoı	NM_	.	missens
ACa07	6	1.5E+08	rs7774557	A	G	.	PASS	GINM1	exoı	NM_	.	missens
ACa07	6	1.5E+08	.	T	C	.	PASS	KATNA1	exoı	NM_	.	missens
ACa07	6	1.5E+08	.	T	C	.	PASS	RAET1E	ncR	NR_	.	.
ACa07	6	1.52E+08	.	C	T	.	PASS	AKAP12	intrc	NM_	.	.
ACa07	6	1.52E+08	.	C	T	.	PASS	SYNE1	intrc	NM_	.	.
ACa07	6	1.53E+08	rs5290225	G	A	.	PASS	SYNE1	intrc	NM_	.	.
ACa07	6	1.53E+08	.	T	C	.	PASS	SYNE1	intrc	NM_	.	.
ACa07	6	1.55E+08	.	T	C	.	PASS	IPCEF1	exoı	NM_	.	missens
ACa07	6	1.55E+08	.	G	A	.	PASS	SCAF8	exoı	NM_	.	missens
ACa07	6	1.58E+08	.	C	T	.	PASS	ARID1B	exoı	NM_	.	stopgaiı
ACa07	6	1.59E+08	rs1015839	G	A	.	PASS	TMEM1	intrc	NM_	.	.
ACa07	6	1.6E+08	.	C	T	.	PASS	SOD2	exoı	NM_	.	missens
ACa07	6	1.6E+08	.	G	A	.	PASS	.	inteı	NM_	dis	.
ACa07	6	1.61E+08	.	C	T	.	PASS	LOC729	ncR	NR_	.	.
ACa07	6	1.61E+08	.	C	T	.	PASS	LPA	intrc	NM_	.	.
ACa07	6	1.61E+08	.	C	T	.	PASS	MAP3K4	intrc	NM_	.	.
ACa07	6	1.62E+08	.	T	C	.	PASS	PARK2	exoı	NM_	.	missens
ACa07	6	1.64E+08	rs7739962	C	T	.	PASS	QKI	exoı	NM_	.	synonyr
ACa07	6	1.66E+08	rs2008105	G	A	.	PASS	C6orf11	exoı	NM_	.	missens
ACa07	6	1.68E+08	rs7558449	C	T	.	PASS	TCP10	intrc	NM_	.	.
ACa07	6	1.7E+08	.	A	G	.	PASS	WDR27	UTF	NM_	NM	.
ACa07	7	296714	.	T	A	.	PASS	FAM20C	exoı	NM_	.	missens
ACa07	7	1458899	.	C	T	.	PASS	.	inteı	NM_	dis	.
ACa07	7	1482145	.	G	T	.	PASS	MICALL	intrc	NM_	.	.
ACa07	7	1515597	.	C	T	.	PASS	INTS1	exoı	NM_	.	missens
ACa07	7	1519120	.	C	T	.	PASS	INTS1	exoı	NM_	.	missens
ACa07	7	1526597	rs7786995	G	A	.	PASS	INTS1	exoı	NM_	.	synonyr
ACa07	7	1589844	.	A	G	.	PASS	TMEM1	intrc	NM_	.	.
ACa07	7	2282801	.	G	A	.	PASS	NUDT1	intrc	NM_	.	.
ACa07	7	2472403	rs5612232	G	A	.	PASS	CHST12	exoı	NM_	.	synonyr
ACa07	7	2958116	.	C	T	.	PASS	CARD11	intrc	NM_	.	.
ACa07	7	4009076	rs3699321	C	T	.	PASS	SDK1	intrc	NM_	.	.
ACa07	7	4150245	.	C	A	.	PASS	SDK1	intrc	NM_	.	.
ACa07	7	4822805	rs5413152	C	T	.	PASS	AP5Z1	intrc	NM_	.	.
ACa07	7	5963539	.	C	T	.	PASS	CCZ1	exoı	NM_	.	missens
ACa07	7	6205433	.	C	A	.	PASS	CYTH3	exoı	NM_	.	missens
ACa07	7	6464401	rs7747652	C	A	.	PASS	DAGLB	exoı	NM_	.	missens
ACa07	7	6548610	.	G	T	.	PASS	GRID2II	exoı	NM_	.	synonyr
ACa07	7	6639930	.	C	T	.	PASS	C7orf26	exoı	NM_	.	missens
ACa07	7	6693528	.	C	T	.	PASS	ZNF316	exoı	NM_	.	synonyr
ACa07	7	6845627	rs7800175	G	A	.	PASS	CCZ1B	intrc	NM_	.	.
ACa07	7	6862125	rs8790524	A	G	.	PASS	CCZ1B	intrc	NM_	.	.
ACa07	7	6862133	rs3726603	G	A	.	PASS	CCZ1B	intrc	NM_	.	.
ACa07	7	7398413	.	T	C	.	PASS	COL28A	exoı	NM_	.	missens
ACa07	7	7612747	.	G	A	.	PASS	MIOS	exoı	NM_	.	missens
ACa07	7	10516161	.	G	A	.	PASS	.	inteı	NR_	dis	.
ACa07	7	11675991	.	C	A	.	PASS	THSD7A	exoı	NM_	.	missens
ACa07	7	20762859	.	T	C	.	PASS	ABCB5	intrc	NM_	.	.
ACa07	7	21788267	rs3710375	C	T	.	PASS	DNAH11	exoı	NM_	.	synonyr
ACa07	7	22459357	.	T	C	.	PASS	STEAP1	UTF	NM_	NM	.
ACa07	7	23651273	.	A	T	.	PASS	CCDC1	intrc	NM_	.	.
ACa07	7	27689124	rs1427054	C	T	.	PASS	HIBADH	exoı	NM_	.	missens
ACa07	7	30475623	.	T	C	.	PASS	NOD1	exoı	NM_	.	missens

ACa07	7	32511099	.	C	T	.	PASS	.	inte	NR_	dis	.
ACa07	7	32660665	.	G	T	.	PASS	DPY19L	ncR	NR_	.	.
ACa07	7	33014358	rs7507081	C	T	.	PASS	FKBP9	exo	NM_	.	synonyr
ACa07	7	33644928	.	C	T	.	PASS	BBS9	UTF	NM_	NM	.
ACa07	7	35006340	.	G	A	.	PASS	DPY19L	intrc	NM_	.	.
ACa07	7	35130022	rs7696767	C	T	.	PASS	DPY19L	ncR	NR_	.	.
ACa07	7	38289110	.	G	A	.	PASS	.	inte	NM_	dis	.
ACa07	7	43484829	rs7755650	G	A	.	PASS	HECW1	exo	NM_	.	synonyr
ACa07	7	43508353	.	G	A	.	PASS	HECW1	intrc	NM_	.	.
ACa07	7	43918365	.	G	A	.	PASS	URGCP	exo	NM_	.	unknow
ACa07	7	44178934	rs1113701	G	A	.	PASS	MYL7	intrc	NM_	.	.
ACa07	7	44619296	.	G	A	.	PASS	TMED4	intrc	NM_	.	.
ACa07	7	44747033	.	G	T	.	PASS	OGDH	intrc	NM_	.	.
ACa07	7	45701804	rs7476235	C	T	.	PASS	ADCY1	exo	NM_	.	synonyr
ACa07	7	45954730	rs1024757	G	A	.	PASS	IGFBP3	intrc	NM_	.	.
ACa07	7	47385996	.	A	C	.	PASS	TNS3	intrc	NM_	.	.
ACa07	7	47853147	.	T	C	.	PASS	C7orf69	exo	NM_	.	unknow
ACa07	7	56183779	.	G	A	.	PASS	NUPR1l	exo	NM_	.	missens
ACa07	7	56439590	.	C	T	.	PASS	.	inte	NM_	dis	.
ACa07	7	56754394	.	G	T	.	PASS	.	inte	NR_	dis	.
ACa07	7	56884079	.	C	T	.	PASS	.	inte	NR_	dis	.
ACa07	7	56886881	rs3749762	T	G	.	PASS	.	inte	NR_	dis	.
ACa07	7	56888099	.	C	T	.	PASS	.	inte	NR_	dis	.
ACa07	7	56888183	rs8799601	C	T	.	PASS	.	inte	NR_	dis	.
ACa07	7	57082082	.	G	A	.	PASS	.	inte	NR_	dis	.
ACa07	7	63022876	.	C	T	.	PASS	.	inte	NR_	dis	.
ACa07	7	63022930	.	C	T	.	PASS	.	inte	NR_	dis	.
ACa07	7	63219077	.	G	T	.	PASS	.	inte	NR_	dis	.
ACa07	7	63219280	.	G	A	.	PASS	.	inte	NR_	dis	.
ACa07	7	64639495	rs6156381	C	T	.	PASS	.	ncR	NR_	.	.
ACa07	7	65222986	.	G	T	.	PASS	CCT6P1	ncR	NR_	.	.
ACa07	7	65223001	.	G	A	.	PASS	CCT6P1	ncR	NR_	.	.
ACa07	7	66520742	rs5480693	T	A	.	PASS	TYW1	intrc	NM_	.	.
ACa07	7	71175845	rs7706902	C	T	.	PASS	WBSCF	exo	NM_	.	missens
ACa07	7	72336710	rs5574064	C	T	.	PASS	SPDYE	ncR	NR_	.	.
ACa07	7	72640092	.	T	C	.	PASS	NCF1B	ncR	NR_	.	.
ACa07	7	72742252	.	G	A	.	PASS	FKBP6	UTF	NM_	NM	.
ACa07	7	72849745	.	C	T	.	PASS	FZD9	exo	NM_	.	missens
ACa07	7	72856782	rs7822351	C	T	.	PASS	BAZ1B	exo	NM_	.	missens
ACa07	7	73151034	.	G	T	.	PASS	ABHD1	intrc	NM_	.	.
ACa07	7	73634643	.	G	A	.	PASS	LAT2	intrc	NM_	.	.
ACa07	7	73935595	rs7822129	G	A	.	PASS	GTF2IR	exo	NM_	.	missens
ACa07	7	74193774	rs3705131	T	C	.	PASS	NCF1	intrc	NM_	.	.
ACa07	7	74608537	rs8799812	T	C	.	PASS	GTF2IP	ncR	NR_	.	.
ACa07	7	76099560	rs1493360	T	C	.	PASS	FDPSP	ncR	NR_	.	.
ACa07	7	76133994	rs1403890	T	A	.	PASS	DTX2	intrc	NM_	.	.
ACa07	7	81613821	.	G	A	.	PASS	CACNA	intrc	NM_	.	.
ACa07	7	82219933	.	A	G	.	PASS	.	inte	NM_	dis	.
ACa07	7	82457265	.	C	A	.	PASS	PCLO	exo	NM_	.	missens
ACa07	7	90747485	rs1413343	G	A	.	PASS	CDK14	exo	NM_	.	missens
ACa07	7	93633731	rs9879024	G	T	.	PASS	.	ups	NM_	.	.
ACa07	7	94039078	rs7641741	G	A	.	PASS	COL1A	exo	NM_	.	missens
ACa07	7	94268607	.	T	G	.	PASS	SGCE	intrc	NM_	.	.
ACa07	7	95166840	.	G	A	.	PASS	ASB4	intrc	NM_	.	.

ACa07	7	97820925	.	G	T	.	PASS	LMTK2	splic	NM_	NM	.
ACa07	7	98551092	rs7821462	C	T	.	PASS	TRRAP	intrc	NM_	.	.
ACa07	7	98563456	rs7752142	C	T	.	PASS	TRRAP	exoı	NM_	.	missens
ACa07	7	98877074	.	C	T	.	PASS	MYH16	ncR	NR_	.	.
ACa07	7	98877268	rs9034524	G	A	.	PASS	MYH16	ncR	NR_	.	.
ACa07	7	1E+08	rs5767320	G	A	.	PASS	PCOLC	exoı	NM_	.	missens
ACa07	7	1E+08	.	C	T	.	PASS	ACTL6E	exoı	NM_	.	synonyr
ACa07	7	1.01E+08	rs7641678	C	T	.	PASS	MUC12	exoı	NM_	.	stopgaiı
ACa07	7	1.01E+08	.	G	A	.	PASS	MOGAT	intrc	NM_	.	.
ACa07	7	1.02E+08	.	G	A	.	PASS	CUX1	exoı	NM_	.	missens
ACa07	7	1.02E+08	rs7590335	C	T	.	PASS	RASA4E	exoı	NM_	.	synonyr
ACa07	7	1.02E+08	rs4729845	T	C	.	PASS	SPDYE:	intrc	NM_	.	.
ACa07	7	1.03E+08	.	C	T	.	PASS	PSMC2	exoı	NM_	.	missens
ACa07	7	1.04E+08	.	G	A	.	PASS	ORC5	exoı	NM_	.	missens
ACa07	7	1.11E+08	.	T	C	.	PASS	LRRN3	exoı	NM_	.	synonyr
ACa07	7	1.12E+08	.	G	A	.	PASS	IFRD1	intrc	NM_	.	.
ACa07	7	1.17E+08	.	G	A	.	PASS	ASZ1	intrc	NM_	.	.
ACa07	7	1.22E+08	rs1457073	C	T	.	PASS	AASS	intrc	NM_	.	.
ACa07	7	1.24E+08	.	G	A	.	PASS	.	inteı	NR_	dis	.
ACa07	7	1.24E+08	rs3755434	C	T	.	PASS	GPR37	exoı	NM_	.	missens
ACa07	7	1.28E+08	.	T	A	.	PASS	.	inteı	NM_	dis	.
ACa07	7	1.28E+08	.	G	T	.	PASS	FLNC	exoı	NM_	.	missens
ACa07	7	1.29E+08	rs2006779	C	T	.	PASS	SMO	exoı	NM_	.	synonyr
ACa07	7	1.29E+08	.	G	T	.	PASS	.	ups	NM_	.	.
ACa07	7	1.32E+08	rs3679590	G	A	.	PASS	PLXNA4	exoı	NM_	.	missens
ACa07	7	1.35E+08	.	C	A	.	PASS	.	inteı	NM_	dis	.
ACa07	7	1.37E+08	.	G	A	.	PASS	DGKI	intrc	NM_	.	.
ACa07	7	1.37E+08	.	A	G	.	PASS	DGKI	exoı	NM_	.	missens
ACa07	7	1.38E+08	.	C	T	.	PASS	CREB3I	exoı	NM_	.	missens
ACa07	7	1.38E+08	rs1825415	G	A	.	PASS	ATP6V0	intrc	NM_	.	.
ACa07	7	1.4E+08	.	T	C	.	PASS	SLC37A	intrc	NM_	.	.
ACa07	7	1.41E+08	.	C	T	.	PASS	.	inteı	NM_	dis	.
ACa07	7	1.41E+08	.	C	T	.	PASS	AGK	intrc	NM_	.	.
ACa07	7	1.42E+08	rs7825497	C	T	.	PASS	MGAM	exoı	NM_	.	missens
ACa07	7	1.42E+08	.	C	T	.	PASS	.	exoı	NM_	.	unknow
ACa07	7	1.42E+08	.	C	A	.	PASS	.	inteı	NR_	dis	.
ACa07	7	1.42E+08	.	C	T	.	PASS	.	inteı	NR_	dis	.
ACa07	7	1.42E+08	.	C	T	.	PASS	.	inteı	NR_	dis	.
ACa07	7	1.42E+08	rs7663697	C	T	.	PASS	.	inteı	NR_	dis	.
ACa07	7	1.43E+08	.	G	A	.	PASS	GSTK1	intrc	NM_	.	.
ACa07	7	1.43E+08	rs3733853	G	A	.	PASS	FAM131	intrc	NM_	.	.
ACa07	7	1.48E+08	rs7598872	C	T	.	PASS	CNTNA	exoı	NM_	.	synonyr
ACa07	7	1.49E+08	rs8019707	C	T	.	PASS	KRBA1	intrc	NM_	.	.
ACa07	7	1.5E+08	rs1447568	G	A	.	PASS	SSPO	exoı	NM_	.	missens
ACa07	7	1.5E+08	.	T	G	.	PASS	ZNF775	UTF	NM_	NM	.
ACa07	7	1.51E+08	.	A	G	.	PASS	NOS3	intrc	NM_	.	.
ACa07	7	1.51E+08	.	C	T	.	PASS	ATG9B	exoı	NM_	.	unknow
ACa07	7	1.51E+08	.	C	T	.	PASS	CDK5	exoı	NM_	.	missens
ACa07	7	1.51E+08	.	C	T	.	PASS	TMUB1	exoı	NM_	.	missens
ACa07	7	1.51E+08	.	C	T	.	PASS	PRKAG	intrc	NM_	.	.
ACa07	7	1.52E+08	.	C	T	.	PASS	PRKAG	intrc	NM_	.	.
ACa07	7	1.52E+08	rs3761181	C	T	.	PASS	KMT2C	exoı	NM_	.	missens
ACa07	7	1.52E+08	.	G	C	.	PASS	ACTR3I	intrc	NM_	.	.
ACa07	7	1.54E+08	rs9367654	G	A	.	PASS	DPP6	intrc	NM_	.	.

ACa07	7	1.57E+08	.	G	T	.	PASS	UBE3C	intro	NM_	.	.
ACa07	7	1.58E+08	rs1056749	G	A	.	PASS	PTPRN2	intro	NM_	.	.
ACa07	7	1.58E+08	rs3696336	T	A	.	PASS	PTPRN2	intro	NM_	.	.
ACa07	7	1.59E+08	.	C	T	.	PASS	.	inter	NM_	dis	.
ACa07	8	328564	.	C	T	.	PASS	FAM87A	ncR	NR_	.	.
ACa07	8	1728466	.	T	A	.	PASS	CLN8	exon	NM_	.	synonyn
ACa07	8	6668771	rs1995647	G	A	.	PASS	XKR5	exon	NM_	.	missens
ACa07	8	6794236	.	C	A	.	PASS	DEFA4	intro	NM_	.	.
ACa07	8	7191342	rs7692119	T	C	.	PASS	USP17L	exon	NM_	.	synonyn
ACa07	8	7915727	rs2946428	C	T	.	PASS	.	inter	NR_	dis	.
ACa07	8	8560334	rs7810476	C	T	.	PASS	CLDN23	exon	NM_	.	synonyn
ACa07	8	10469012	rs7606573	G	A	.	PASS	RP1L1	exon	NM_	.	missens
ACa07	8	10530224	.	G	A	.	PASS	C8orf74	splic	NM_	NM	.
ACa07	8	10584255	rs7511375	C	T	.	PASS	SOX7	intro	NM_	.	.
ACa07	8	11189479	rs7626017	C	T	.	PASS	SLC35G	exon	NM_	.	synonyn
ACa07	8	11204044	.	C	T	.	PASS	TDH	ncR	NR_	.	.
ACa07	8	12237303	.	C	T	.	PASS	FAM66A	ncR	NR_	.	.
ACa07	8	12293333	rs3988723	A	G	.	PASS	FAM86B	intro	NM_	.	.
ACa07	8	12459464	rs3713666	A	T	.	PASS	LOC729	ncR	NR_	.	.
ACa07	8	12944087	rs1036449	G	A	.	PASS	DLC1	intro	NM_	.	.
ACa07	8	15480640	.	T	C	.	PASS	TUSC3	exon	NM_	.	missens
ACa07	8	20231256	rs1470216	A	G	.	PASS	.	inter	NR_	dis	.
ACa07	8	20231262	rs1394112	G	A	.	PASS	.	inter	NR_	dis	.
ACa07	8	21981390	.	C	T	.	PASS	HR	intro	NM_	.	.
ACa07	8	23049182	.	G	T	.	PASS	TNFRSF	UTF	NM_	NM	.
ACa07	8	23152173	.	C	A	.	PASS	R3HCC	intro	NM_	.	.
ACa07	8	23560117	.	G	T	.	PASS	NKX2-6	exon	NM_	.	synonyn
ACa07	8	25267590	rs7690910	C	T	.	PASS	DOCK5	intro	NM_	.	.
ACa07	8	25293068	rs2013688	G	A	.	PASS	KCTD9	intro	NM_	.	.
ACa07	8	26363068	.	G	A	.	PASS	PNMA2	UTF	NM_	NM	.
ACa07	8	27369366	rs7247582	C	T	.	PASS	EPHX2	exon	NM_	.	missens
ACa07	8	28929051	.	G	A	.	PASS	KIF13B	intro	NM_	.	.
ACa07	8	30703725	rs1419780	G	A	.	PASS	TEX15	exon	NM_	.	stopgai
ACa07	8	33451130	rs1198970	G	A	.	PASS	DUSP26	exon	NM_	.	synonyn
ACa07	8	36768413	rs5320288	C	T	.	PASS	KCNU1	intro	NM_	.	.
ACa07	8	37598127	.	C	T	.	PASS	ERLIN2	intro	NM_	.	.
ACa07	8	38272450	rs7612284	G	A	.	PASS	FGFR1	intro	NM_	.	.
ACa07	8	38287473	.	C	T	.	PASS	FGFR1	intro	NM_	.	.
ACa07	8	38684698	rs7608102	G	A	.	PASS	TACC1	exon	NM_	.	missens
ACa07	8	39464012	.	T	C	.	PASS	ADAM1	intro	NM_	.	.
ACa07	8	41507533	.	C	T	.	PASS	.	inter	NM_	dis	.
ACa07	8	41814697	rs3705628	G	A	.	PASS	KAT6A	intro	NM_	.	.
ACa07	8	42227706	rs7605197	G	A	.	PASS	POLB	intro	NM_	.	.
ACa07	8	43821054	.	G	A	.	PASS	.	inter	NM_	dis	.
ACa07	8	43821150	rs3733322	T	G	.	PASS	.	inter	NM_	dis	.
ACa07	8	43822425	rs2003364	T	C	.	PASS	.	inter	NM_	dis	.
ACa07	8	43831173	.	A	C	.	PASS	.	inter	NM_	dis	.
ACa07	8	46844208	rs4498566	A	C	.	PASS	.	inter	NON	dis	.
ACa07	8	46844794	.	G	A	.	PASS	.	inter	NON	dis	.
ACa07	8	46849434	rs4401873	C	G	.	PASS	.	inter	NON	dis	.
ACa07	8	49641620	.	G	T	.	PASS	EFCAB1	exon	NM_	.	missens
ACa07	8	51415308	.	T	C	.	PASS	SNTG1	intro	NM_	.	.
ACa07	8	53853631	.	A	G	.	PASS	.	dow	NM_	.	.
ACa07	8	55591073	rs1871956	C	T	.	PASS	.	inter	NM_	dis	.

ACa07	8	55638925	.	G	A	.	PASS	.	inte	NM_	dis	.	
ACa07	8	59548131	.	T	A	.	PASS	NSMAF	intro	NM_	.	.	
ACa07	8	61178562	rs7793713	G	A	.	PASS	CA8	exo	NM_	.	synonyr	
ACa07	8	61761171	rs1513224	C	T	.	PASS	CHD7	intro	NM_	.	.	
ACa07	8	66695246	rs8897899	G	A	.	PASS	PDE7A	intro	NM_	.	.	
ACa07	8	68070822	.	A	G	.	PASS	CSPP1	exo	NM_	.	synonyr	
ACa07	8	68105600	.	G	T	.	PASS	CSPP1	intro	NM_	.	.	
ACa07	8	69032380	rs7454376	G	A	.	PASS	PREX2	intro	NM_	.	.	
ACa07	8	74169239	.	G	A	.	PASS	C8orf89	exo	NM_	.	missens	
ACa07	8	76577776	.	C	T	.	PASS	.	inte	NM_	dis	.	
ACa07	8	82392876	.	T	C	.	PASS	FABP4	intro	NM_	.	.	
ACa07	8	87670124	.	T	C	.	PASS	CNGB3	intro	NM_	.	.	
ACa07	8	87671445	.	G	A	.	PASS	CNGB3	intro	NM_	.	.	
ACa07	8	88395019	rs1030919	C	T	.	PASS	.	dow	NM_	.	.	
ACa07	8	91057147	rs7559645	G	A	.	PASS	DECR1	exo	NM_	.	missens	
ACa07	8	91057203	.	G	A	.	PASS	DECR1	exo	NM_	.	missens	
ACa07	8	93074699	.	T	C	.	PASS	RUNX1	intro	NM_	.	.	
ACa07	8	95365674	.	G	A	.	PASS	.	inte	NM_	dis	.	
ACa07	8	96087740	rs1000299	T	C	.	PASS	.	inte	NR_	dis	.	
ACa07	8	98954359	.	T	A	.	PASS	MATN2	intro	NM_	.	.	
ACa07	8	1.02E+08	.	G	A	.	PASS	.	ups	NR_	.	.	
ACa07	8	1.04E+08	.	G	A	.	PASS	.	inte	NM_	dis	.	
ACa07	8	1.08E+08	.	C	T	.	PASS	OXR1	UTF	NM_	NM	.	
ACa07	8	1.11E+08	.	C	T	.	PASS	KCNV1	exo	NM_	.	missens	
ACa07	8	1.24E+08	rs1493710	C	T	.	PASS	ATAD2	exo	NM_	.	missens	
ACa07	8	1.25E+08	.	A	G	.	PASS	FER1L6	exo	NM_	.	synonyr	
ACa07	8	1.26E+08	rs7626895	C	T	.	PASS	.	inte	NM_	dis	.	
ACa07	8	1.28E+08	.	C	T	.	PASS	PRNCR	ncR	NR_	.	.	
ACa07	8	1.34E+08	rs3595615	G	A	.	PASS	LRRC6	exo	NM_	.	synonyr	
ACa07	8	1.34E+08	rs1689333	G	A	.	PASS	TG	exo	NM_	.	missens	
ACa07	8	1.41E+08	.	G	T	.	PASS	KCNK9	UTF	NM_	NM	.	
ACa07	8	1.41E+08	.	C	T	.	PASS	TRAPP	intro	NM_	.	.	
ACa07	8	1.42E+08	.	C	A	.	PASS	DENND	exo	NM_	.	synonyr	
ACa07	8	1.42E+08	.	C	A	.	PASS	MROH5	intro	NM_	.	.	
ACa07	8	1.44E+08	.	C	T	.	PASS	BAI1	intro	NM_	.	.	
ACa07	8	1.44E+08	.	G	T	.	PASS	LY6H	intro	NM_	.	.	
ACa07	8	1.44E+08	rs7598006	C	T	.	PASS	ZFP41	exo	NM_	.	missens	
ACa07	8	1.45E+08	rs5644139	G	A	.	PASS	FAM83H	exo	NM_	.	synonyr	
ACa07	8	1.45E+08	.	G	T	.	PASS	FAM83H	exo	NM_	.	missens	
ACa07	8	1.45E+08	rs7819090	C	T	.	PASS	SCRIB	exo	NM_	.	missens	
ACa07	8	1.45E+08	.	C	T	.	PASS	NRBP2	exo	NM_	.	missens	
ACa07	8	1.45E+08	.	G	A	.	PASS	EPPK1	exo	NM_	.	missens	
ACa07	8	1.45E+08	rs2000607	G	A	.	PASS	PLEC	exo	NM_	.	missens	
ACa07	8	1.46E+08	rs5565117	C	T	.	PASS	HSF1	intro	NM_	.	.	
ACa07	8	1.46E+08	.	C	T	.	PASS	HSF1	intro	NM_	.	.	
ACa07	8	1.46E+08	.	G	A	.	PASS	CYHR1	exo	NM_	.	missens	
ACa07	8	1.46E+08	rs7468927	G	A	.	PASS	KIFC2	exo	NM_	.	missens	
ACa07	9	15883	rs7123526	A	G	.	PASS	WASH1	intro	NM_	.	.	
ACa07	9	4576210	rs8987908	C	T	.	PASS	SLC1A1	intro	NM_	.	.	
ACa07	9	13143465	.	C	A	.	PASS	MPDZ	exo	NM_	.	missens	
ACa07	9	14125770	rs7591887	G	A	.	PASS	NFIB	intro	NM_	.	.	
ACa07	9	14819391	.	C	T	.	PASS	FREM1	exo	NM_	.	missens	
ACa07	9	14851329	rs7554261	C	T	.	PASS	FREM1	exo	NM_	.	missens	
ACa07	9	15214070	.	C	T	.	PASS	TTC39E	intro	NM_	.	.	

ACa07	9	16436706	.	G	A	.	PASS	BNC2	exoı	NM_	.	missens
ACa07	9	17143320	rs7717080	G	A	.	PASS	CNTLN	exoı	NM_	.	missens
ACa07	9	19528050	.	G	A	.	PASS	SLC24A	exoı	NM_	.	synonyr
ACa07	9	20944746	rs7552126	C	T	.	PASS	FOCAD	exoı	NM_	.	unknow
ACa07	9	21011377	rs8986502	G	A	.	PASS	PTPLAI	intrc	NM_	.	.
ACa07	9	32567140	rs7703822	C	T	.	PASS	NDUFB	intrc	NM_	.	.
ACa07	9	34398403	.	G	A	.	PASS	FAM219	UTF	NM_	NM	.
ACa07	9	35608407	.	C	T	.	PASS	TESK1	exoı	NM_	.	missens
ACa07	9	37541389	.	G	A	.	PASS	FBXO1(	exoı	NM_	.	missens
ACa07	9	37746388	.	G	A	.	PASS	FRMPD	exoı	NM_	.	stopgaiı
ACa07	9	39078288	rs1616872	A	G	.	PASS	CNTNA	intrc	NM_	.	.
ACa07	9	43915558	rs7820531	G	C	.	PASS	CNTNA	exoı	NM_	.	missens
ACa07	9	47236563	rs6255281	C	T	.	PASS	.	inteı	NR_	dis	.
ACa07	9	65602736	.	C	T	.	PASS	.	inteı	NM_	dis	.
ACa07	9	66553866	rs2003121	C	T	.	PASS	.	ncR	NR_	.	.
ACa07	9	67867765	rs2016275	G	A	.	PASS	.	inteı	NR_	dis	.
ACa07	9	68315458	rs2013763	G	C	.	PASS	.	inteı	NM_	dis	.
ACa07	9	68371780	rs3764322	A	G	.	PASS	.	inteı	NM_	dis	.
ACa07	9	68433386	.	A	C	.	PASS	LOC642	ncR	NR_	.	.
ACa07	9	70177694	.	G	A	.	PASS	FOXD4I	exoı	NM_	.	missens
ACa07	9	70177946	rs7506128	G	A	.	PASS	FOXD4I	exoı	NM_	.	missens
ACa07	9	73946728	.	G	T	.	PASS	.	inteı	NM_	dis	.
ACa07	9	74365113	.	G	T	.	PASS	TMEM2	exoı	NM_	.	synonyr
ACa07	9	77692282	.	C	A	.	PASS	NMRK1	intrc	NM_	.	.
ACa07	9	79239583	rs9660777	T	C	.	PASS	PRUNE	intrc	NM_	.	.
ACa07	9	82006224	rs5294239	G	A	.	PASS	.	inteı	NR_	dis	.
ACa07	9	82465778	.	A	G	.	PASS	.	ncR	NR_	.	.
ACa07	9	84608680	rs5696558	G	A	.	PASS	SPATA3	exoı	NM_	.	missens
ACa07	9	84676646	.	G	A	.	PASS	.	inteı	NM_	dis	.
ACa07	9	86153164	rs7749802	A	G	.	PASS	FRMD3	UTF	NM_	NM	.
ACa07	9	87635048	.	G	A	.	PASS	NTRK2	intrc	NM_	.	.
ACa07	9	88923463	.	C	T	.	PASS	ZCCHC	exoı	NM_	.	missens
ACa07	9	90473759	rs9367277	C	T	.	PASS	LOC392	ncR	NR_	.	.
ACa07	9	91686190	rs1447929	C	T	.	PASS	SHC3	intrc	NM_	.	.
ACa07	9	95033689	.	C	T	.	PASS	IARS	intrc	NM_	.	.
ACa07	9	95874687	.	A	G	.	PASS	C9orf89	intrc	NM_	.	.
ACa07	9	96080176	rs5352400	G	A	.	PASS	WNK2	exoı	NM_	.	missens
ACa07	9	96434042	.	A	G	.	PASS	PHF2	intrc	NM_	.	.
ACa07	9	96441396	.	A	G	.	PASS	PHF2	UTF	NM_	NM	.
ACa07	9	99150692	.	C	T	.	PASS	ZNF367	exoı	NM_	.	missens
ACa07	9	99327874	rs5750212	C	T	.	PASS	CDC14I	intrc	NM_	.	.
ACa07	9	99580381	rs5664919	C	T	.	PASS	ZNF782	exoı	NM_	.	missens
ACa07	9	1.01E+08	rs5346016	G	A	.	PASS	TBC1D2	intrc	NM_	.	.
ACa07	9	1.08E+08	rs5275568	G	A	.	PASS	FSD1L	intrc	NM_	.	.
ACa07	9	1.13E+08	.	A	G	.	PASS	SVEP1	intrc	NM_	.	.
ACa07	9	1.15E+08	rs9418280	C	T	.	PASS	KIAA19	exoı	NM_	.	missens
ACa07	9	1.15E+08	rs7534529	C	T	.	PASS	KIAA19	exoı	NM_	.	missens
ACa07	9	1.16E+08	.	G	T	.	PASS	.	dow	NM_	.	.
ACa07	9	1.16E+08	.	G	T	.	PASS	ALAD	intrc	NM_	.	.
ACa07	9	1.17E+08	rs1017044	C	T	.	PASS	COL27A	intrc	NM_	.	.
ACa07	9	1.17E+08	.	C	T	.	PASS	COL27A	intrc	NM_	.	.
ACa07	9	1.17E+08	rs5296296	C	T	.	PASS	COL27A	intrc	NM_	.	.
ACa07	9	1.17E+08	.	C	A	.	PASS	AKNA	intrc	NM_	.	.
ACa07	9	1.19E+08	rs5393503	G	A	.	PASS	PAPPA	exoı	NM_	.	missens

ACa07	9	1.23E+08	rs5770416	C	T	.	PASS	CDK5R	intrc	NM_	.	.
ACa07	9	1.24E+08	rs7765588	C	T	.	PASS	PHF19	intrc	NM_	.	.
ACa07	9	1.24E+08	.	A	G	.	PASS	PHF19	intrc	NM_	.	.
ACa07	9	1.25E+08	rs7817163	C	T	.	PASS	DAB2IP	exo	NM_	.	synonyr
ACa07	9	1.25E+08	.	G	T	.	PASS	DAB2IP	exo	NM_	.	missens
ACa07	9	1.26E+08	.	C	A	.	PASS	STRBP	intrc	NM_	.	.
ACa07	9	1.27E+08	rs7671231	C	T	.	PASS	NEK6	intrc	NM_	.	.
ACa07	9	1.28E+08	.	T	C	.	PASS	MAPKA	intrc	NM_	.	.
ACa07	9	1.3E+08	.	C	T	.	PASS	RALGP	intrc	NM_	.	.
ACa07	9	1.3E+08	rs7704105	C	T	.	PASS	LRSAM	exo	NM_	.	synonyr
ACa07	9	1.3E+08	rs7514371	G	A	.	PASS	STXBP	exo	NM_	.	missens
ACa07	9	1.3E+08	.	C	A	.	PASS	TTC16	intrc	NM_	.	.
ACa07	9	1.31E+08	rs7724303	C	T	.	PASS	FPGS	exo	NM_	.	missens
ACa07	9	1.31E+08	rs3755590	C	T	.	PASS	ENG	intrc	NM_	.	.
ACa07	9	1.31E+08	rs7704197	C	T	.	PASS	ODF2	exo	NM_	.	synonyr
ACa07	9	1.31E+08	rs3700623	G	A	.	PASS	SPTAN	exo	NM_	.	missens
ACa07	9	1.31E+08	rs1020109	C	T	.	PASS	SPTAN	exo	NM_	.	missens
ACa07	9	1.31E+08	.	T	C	.	PASS	SPTAN	intrc	NM_	.	.
ACa07	9	1.31E+08	.	C	T	.	PASS	SPTAN	intrc	NM_	.	.
ACa07	9	1.32E+08	rs1200306	C	T	.	PASS	ZER1	intrc	NM_	.	.
ACa07	9	1.32E+08	rs4837317	T	C	.	PASS	CCBL1	intrc	NM_	.	.
ACa07	9	1.32E+08	rs4837318	C	T	.	PASS	CCBL1	intrc	NM_	.	.
ACa07	9	1.32E+08	rs7690702	G	A	.	PASS	DOLPP	exo	NM_	.	missens
ACa07	9	1.32E+08	.	G	A	.	PASS	CRAT	intrc	NM_	.	.
ACa07	9	1.32E+08	.	G	A	.	PASS	CRAT	exo	NM_	.	missens
ACa07	9	1.34E+08	rs9375934	C	T	.	PASS	RAPGE	exo	NM_	.	synonyr
ACa07	9	1.35E+08	.	C	T	.	PASS	NTNG2	intrc	NM_	.	.
ACa07	9	1.35E+08	.	C	T	.	PASS	SETX	exo	NM_	.	missens
ACa07	9	1.36E+08	rs4498679	G	A	.	PASS	DDX31	intrc	NM_	.	.
ACa07	9	1.36E+08	.	G	A	.	PASS	GTF3C	UTF	NM_	NM	.
ACa07	9	1.36E+08	.	C	T	.	PASS	RALGD	exo	NM_	.	missens
ACa07	9	1.36E+08	.	A	G	.	PASS	GBGT1	exo	NM_	.	missens
ACa07	9	1.36E+08	.	G	T	.	PASS	SURF4	intrc	NM_	.	.
ACa07	9	1.36E+08	rs7823594	G	A	.	PASS	STKLD1	exo	NM_	.	missens
ACa07	9	1.36E+08	rs7806069	C	T	.	PASS	TMEM8	exo	NM_	.	missens
ACa07	9	1.37E+08	.	G	T	.	PASS	DBH	exo	NM_	.	missens
ACa07	9	1.38E+08	.	C	T	.	PASS	COL5A	exo	NM_	.	missens
ACa07	9	1.38E+08	rs6173790	C	T	.	PASS	COL5A	exo	NM_	.	synonyr
ACa07	9	1.38E+08	.	G	A	.	PASS	COL5A	intrc	NM_	.	.
ACa07	9	1.39E+08	rs7478355	C	T	.	PASS	CAMSA	exo	NM_	.	missens
ACa07	9	1.39E+08	.	C	A	.	PASS	GPSM1	intrc	NM_	.	.
ACa07	9	1.39E+08	rs9942197	C	T	.	PASS	NOTCH	intrc	NM_	.	.
ACa07	9	1.39E+08	.	C	A	.	PASS	NOTCH	exo	NM_	.	missens
ACa07	9	1.39E+08	.	C	T	.	PASS	NOTCH	intrc	NM_	.	.
ACa07	9	1.4E+08	.	G	A	.	PASS	FAM69E	exo	NM_	.	missens
ACa07	9	1.4E+08	.	T	C	.	PASS	CCDC1	ncR	NR_	.	.
ACa07	9	1.4E+08	rs1399152	C	T	.	PASS	TRAF2	exo	NM_	.	synonyr
ACa07	9	1.4E+08	rs5651517	C	T	.	PASS	MAN1B	intrc	NM_	.	.
ACa07	9	1.4E+08	rs7788028	G	A	.	PASS	SLC34A	intrc	NM_	.	.
ACa07	9	1.4E+08	.	C	A	.	PASS	FAM16	exo	NM_	.	missens
ACa07	9	1.4E+08	rs7776402	G	A	.	PASS	TOR4A	exo	NM_	.	synonyr
ACa07	9	1.4E+08	rs1126228	C	T	.	PASS	PNPLA	intrc	NM_	.	.
ACa07	9	1.41E+08	.	G	A	.	PASS	EHMT1	intrc	NM_	.	.
ACa07	9	1.41E+08	rs3707879	C	T	.	PASS	TUBBP	ncR	NR_	.	.

ACa07	10	1053091	rs7547454	C	T	.	PASS	GTPBP4	intrc	NM_	.	.
ACa07	10	1599286	.	C	A	.	PASS	ADARB	intrc	NM_	.	.
ACa07	10	3193659	rs5680149	C	T	.	PASS	PITRM1	intrc	NM_	.	.
ACa07	10	5927584	.	A	T	.	PASS	ANKRD	intrc	NM_	.	.
ACa07	10	7262397	rs2019140	G	A	.	PASS	SFMBT	exo	NM_	.	missens
ACa07	10	7608771	rs7961144	G	A	.	PASS	ITIH5	intrc	NM_	.	.
ACa07	10	7621760	rs3708927	C	T	.	PASS	ITIH5	exo	NM_	.	missens
ACa07	10	8007776	.	C	A	.	PASS	TAF3	intrc	NM_	.	.
ACa07	10	8055842	.	G	A	.	PASS	TAF3	intrc	NM_	.	.
ACa07	10	9444396	.	C	A	.	PASS	.	inte	NR_	dis	.
ACa07	10	12110841	.	C	A	.	PASS	.	ups	NM_	.	.
ACa07	10	15027024	rs1919845	C	T	.	PASS	.	inte	NM_	dis	.
ACa07	10	17086956	rs7658069	G	A	.	PASS	CUBN	intrc	NM_	.	.
ACa07	10	17142108	.	G	T	.	PASS	CUBN	exo	NM_	.	missens
ACa07	10	20436609	.	C	T	.	PASS	PLXDC	intrc	NM_	.	.
ACa07	10	22193634	rs9652901	T	C	.	PASS	DNAJC	intrc	NM_	.	.
ACa07	10	24832764	.	A	T	.	PASS	KIAA12	exo	NM_	.	missens
ACa07	10	24874959	rs7647799	T	C	.	PASS	ARHGA	exo	NM_	.	missens
ACa07	10	24884596	rs1999030	A	G	.	PASS	ARHGA	intrc	NM_	.	.
ACa07	10	26482239	.	A	G	.	PASS	MYO3A	exo	NM_	.	missens
ACa07	10	26851385	.	A	G	.	PASS	APBB1I	intrc	NM_	.	.
ACa07	10	27324093	rs3678495	G	A	.	PASS	ANKRD	exo	NM_	.	missens
ACa07	10	27326015	.	G	T	.	PASS	ANKRD	intrc	NM_	.	.
ACa07	10	27539503	.	G	A	.	PASS	LRRC37	ncR	NR_	.	.
ACa07	10	29801633	rs7766630	G	A	.	PASS	SVIL	intrc	NM_	.	.
ACa07	10	30915215	rs5733337	C	T	.	PASS	LYZL2	intrc	NM_	.	.
ACa07	10	35819163	.	G	A	.	PASS	CCNY	exo	NM_	.	missens
ACa07	10	44283476	.	G	A	.	PASS	HNRNP	ncR	NR_	.	.
ACa07	10	46204293	.	G	A	.	PASS	.	inte	NM_	dis	.
ACa07	10	47714165	.	A	G	.	PASS	.	inte	NM_	dis	.
ACa07	10	48919803	.	C	T	.	PASS	.	inte	NR_	dis	.
ACa07	10	48930140	.	G	A	.	PASS	BMS1P5	ncR	NR_	.	.
ACa07	10	49999086	.	G	A	.	PASS	WDFY4	intrc	NM_	.	.
ACa07	10	50028381	rs9040575	G	A	.	PASS	WDFY4	exo	NM_	.	missens
ACa07	10	50824107	rs3745238	G	A	.	PASS	CHAT	exo	NM_	.	synonyr
ACa07	10	52444579	.	C	A	.	PASS	.	inte	NR_	dis	.
ACa07	10	55664967	rs7512474	G	A	.	PASS	PCDH15	intrc	NM_	.	.
ACa07	10	58583274	.	C	A	.	PASS	.	inte	NM_	dis	.
ACa07	10	61028449	rs1389134	G	A	.	PASS	FAM13C	exo	NM_	.	missens
ACa07	10	61612621	rs5737945	A	G	.	PASS	CCDC6	intrc	NM_	.	.
ACa07	10	70056519	.	G	A	.	PASS	PBLD	intrc	NM_	.	.
ACa07	10	70958717	.	A	G	.	PASS	SUPV3L	intrc	NM_	.	.
ACa07	10	71690164	rs7548920	C	T	.	PASS	COL13A	exo	NM_	.	synonyr
ACa07	10	71880912	.	C	T	.	PASS	AIFM2	exo	NM_	.	missens
ACa07	10	72358965	rs3744653	G	A	.	PASS	PRF1	intrc	NM_	.	.
ACa07	10	73574769	rs2017279	C	T	.	PASS	CDH23	exo	NM_	.	missens
ACa07	10	74790029	.	C	A	.	PASS	P4HA1	exo	NM_	.	missens
ACa07	10	74994864	.	C	A	.	PASS	FAM149	intrc	NM_	.	.
ACa07	10	75238410	rs7759506	A	G	.	PASS	PPP3CB	intrc	NM_	.	.
ACa07	10	75451768	rs7802380	A	G	.	PASS	AGAP5	intrc	NM_	.	.
ACa07	10	75464614	rs8789174	A	G	.	PASS	BMS1P4	ncR	NR_	.	.
ACa07	10	75480330	.	C	T	.	PASS	BMS1P4	ncR	NR_	.	.
ACa07	10	78708754	.	A	G	.	PASS	KCNMA	ncR	NR_	.	.
ACa07	10	81701987	.	A	G	.	PASS	SFTPD	intrc	NM_	.	.

ACa07	10	82348450	rs5681324	C	T	.	PASS	SH2D4E	exoı	NM_	.	missens
ACa07	10	85965536	.	C	T	.	PASS	CDHR1	intrc	NM_	.	.
ACa07	10	85997052	.	C	T	.	PASS	LRIT1	exoı	NM_	.	synonyr
ACa07	10	87898527	.	C	T	.	PASS	GRID1	intrc	NM_	.	.
ACa07	10	93380017	rs5296587	C	T	.	PASS	.	inteı	NR_	dis	.
ACa07	10	94821012	.	G	A	.	PASS	.	upsı	NM_	.	.
ACa07	10	95093021	rs3696055	G	A	.	PASS	MYOF	exoı	NM_	.	stopgair
ACa07	10	95276988	rs7535868	G	A	.	PASS	CEP55	exoı	NM_	.	missens
ACa07	10	96824534	.	G	T	.	PASS	CYP2C8	intrc	NM_	.	.
ACa07	10	97712313	rs9047130	T	G	.	PASS	ENTPD:	ncR	NR_	.	.
ACa07	10	97987175	rs7826084	C	T	.	PASS	BLNK	exoı	NM_	NM	missens
ACa07	10	98510597	.	G	A	.	PASS	.	inteı	NM_	dis	.
ACa07	10	99514368	rs3769880	G	A	.	PASS	ZFYVE2	intrc	NM_	.	.
ACa07	10	99661203	rs9945308	C	T	.	PASS	CRTAC	intrc	NM_	.	.
ACa07	10	1.01E+08	rs5392081	G	A	.	PASS	HPSE2	exoı	NM_	.	missens
ACa07	10	1.02E+08	rs3692674	C	T	.	PASS	CUTC	exoı	NM_	.	missens
ACa07	10	1.02E+08	rs9441434	C	T	.	PASS	SEC31E	intrc	NM_	.	.
ACa07	10	1.04E+08	.	G	A	.	PASS	PPRC1	exoı	NM_	.	missens
ACa07	10	1.04E+08	.	A	G	.	PASS	PSD	intrc	NM_	.	.
ACa07	10	1.04E+08	.	C	T	.	PASS	TRIM8	intrc	NM_	.	.
ACa07	10	1.06E+08	.	C	A	.	PASS	COL17A	intrc	NM_	.	.
ACa07	10	1.06E+08	.	G	A	.	PASS	CFAP58	intrc	NM_	.	.
ACa07	10	1.07E+08	.	T	G	.	PASS	SORCS	intrc	NM_	.	.
ACa07	10	1.18E+08	.	C	T	.	PASS	GFRA1	intrc	NM_	.	.
ACa07	10	1.19E+08	.	C	T	.	PASS	PDZD8	exoı	NM_	.	missens
ACa07	10	1.21E+08	.	T	A	.	PASS	TIAL1	intrc	NM_	.	.
ACa07	10	1.24E+08	.	A	G	.	PASS	BTBD16	intrc	NM_	.	.
ACa07	10	1.27E+08	rs7503782	G	A	.	PASS	FAM175	exoı	NM_	.	synonyr
ACa07	10	1.29E+08	.	C	T	.	PASS	DOCK1	exoı	NM_	.	unknow
ACa07	10	1.29E+08	rs3765225	C	T	.	PASS	DOCK1	intrc	NM_	.	.
ACa07	10	1.3E+08	.	C	T	.	PASS	FOXI2	exoı	NM_	.	missens
ACa07	10	1.34E+08	rs5455183	G	A	.	PASS	PPP2R2	intrc	NM_	.	.
ACa07	10	1.35E+08	.	G	T	.	PASS	NKX6-2	exoı	NM_	.	synonyr
ACa07	10	1.35E+08	.	C	T	.	PASS	CFAP46	intrc	NM_	.	.
ACa07	10	1.35E+08	rs5396027	C	T	.	PASS	CFAP46	intrc	NM_	.	.
ACa07	10	1.35E+08	.	G	T	.	PASS	PRAP1	exoı	NM_	.	missens
ACa07	10	1.35E+08	.	G	A	.	PASS	PAOX	exoı	NM_	.	synonyr
ACa07	10	1.35E+08	rs7793478	G	A	.	PASS	PAOX	intrc	NM_	.	.
ACa07	10	1.35E+08	.	G	A	.	PASS	PAOX	intrc	NM_	.	.
ACa07	10	1.35E+08	.	T	C	.	PASS	MTG1	intrc	NM_	.	.
ACa07	11	130618	.	G	T	.	PASS	LINC01	ncR	NR_	.	.
ACa07	11	196075	rs2003509	C	A	.	PASS	.	upsı	NM_	.	.
ACa07	11	237236	.	G	T	.	PASS	PSMD1	intrc	NM_	.	.
ACa07	11	500049	rs1008718	G	A	.	PASS	RNH1	intrc	NM_	.	.
ACa07	11	551871	rs7793525	C	T	.	PASS	LRRC56	intrc	NM_	.	.
ACa07	11	556906	rs3741463	G	A	.	PASS	LMNTD	exoı	NM_	.	missens
ACa07	11	605299	.	A	G	.	PASS	PHRF1	exoı	NM_	.	missens
ACa07	11	793447	.	G	T	.	PASS	SLC25A	intrc	NM_	.	.
ACa07	11	994418	rs8960121	G	A	.	PASS	AP2A2	intrc	NM_	.	.
ACa07	11	1220701	rs7582346	G	A	.	PASS	.	inteı	NM_	dis	.
ACa07	11	1244666	rs7514641	G	A	.	PASS	MUC5B	intrc	NM_	.	.
ACa07	11	1251120	.	T	C	.	PASS	MUC5B	intrc	NM_	.	.
ACa07	11	1255481	.	G	A	.	PASS	MUC5B	exoı	NM_	.	synonyr
ACa07	11	1257694	.	C	T	.	PASS	MUC5B	exoı	NM_	.	missens

ACa07	11	1281905	rs3689865	C	T	.	PASS	MUC5B	exo	NM_	.	synonyr
ACa07	11	2191119	.	A	G	.	PASS	TH	intrc	NM_	.	.
ACa07	11	2194374	rs1019902	C	T	.	PASS	.	dow	NR_	.	.
ACa07	11	2407268	rs7724812	G	A	.	PASS	CD81	intrc	NM_	.	.
ACa07	11	2431892	.	G	T	.	PASS	TRPM5	intrc	NM_	.	.
ACa07	11	2436853	.	G	A	.	PASS	TRPM5	intrc	NM_	.	.
ACa07	11	4254500	rs5592355	T	C	.	PASS	.	inte	NR_	dis	.
ACa07	11	4389369	.	T	C	.	PASS	OR52B4	exo	NM_	.	missens
ACa07	11	5142343	.	G	A	.	PASS	.	inte	NM_	dis	.
ACa07	11	5655758	rs8669468	C	T	.	PASS	TRIM6-T	intrc	NM_	.	.
ACa07	11	6340358	.	G	A	.	PASS	PRKCD	UTF	NM_	NM	.
ACa07	11	6417299	rs7289628	C	T	.	PASS	APBB1	intrc	NM_	.	.
ACa07	11	6644893	.	G	A	.	PASS	DCHS1	exo	NM_	.	stopgai
ACa07	11	6650772	.	C	T	.	PASS	DCHS1	exo	NM_	.	missens
ACa07	11	12315642	rs7595225	C	T	.	PASS	MICALC	exo	NM_	.	missens
ACa07	11	13408180	rs7811107	C	T	.	PASS	ARNTL	exo	NM_	.	unknow
ACa07	11	14277316	.	G	A	.	PASS	SPON1	exo	NM_	.	unknow
ACa07	11	14316397	rs7824579	C	T	.	PASS	RRAS2	exo	NM_	.	missens
ACa07	11	14865528	.	C	A	.	PASS	PDE3B	exo	NM_	.	missens
ACa07	11	14913494	.	G	A	.	PASS	CYP2R1	intrc	NM_	.	.
ACa07	11	16838236	.	G	A	.	PASS	PLEKHA	intrc	NM_	.	.
ACa07	11	16847770	rs7785451	G	A	.	PASS	PLEKHA	exo	NM_	.	missens
ACa07	11	17598431	.	G	A	.	PASS	OTOG	exo	NM_	.	missens
ACa07	11	18062208	.	G	T	.	PASS	TPH1	exo	NM_	.	synonyr
ACa07	11	18210492	.	C	T	.	PASS	.	inte	NM_	dis	.
ACa07	11	18291106	.	G	T	.	PASS	SAA1	intrc	NM_	.	.
ACa07	11	18426449	.	C	A	.	PASS	LDHA	intrc	NM_	.	.
ACa07	11	20652143	.	A	C	.	PASS	SLC6A5	intrc	NM_	.	.
ACa07	11	21250898	rs1146385	G	A	.	PASS	NELL1	exo	NM_	.	missens
ACa07	11	22777485	.	A	G	.	PASS	GAS2	exo	NM_	.	missens
ACa07	11	23427305	.	A	T	.	PASS	.	inte	NM_	dis	.
ACa07	11	33743791	.	C	A	.	PASS	CD59	intrc	NM_	.	.
ACa07	11	34194650	.	C	T	.	PASS	ABTB2	intrc	NM_	.	.
ACa07	11	43876386	.	C	T	.	PASS	HSD17B	exo	NM_	.	missens
ACa07	11	46726773	.	G	A	.	PASS	ZNF408	exo	NM_	.	missens
ACa07	11	46765806	.	G	A	.	PASS	CKAP5	exo	NM_	.	stopgai
ACa07	11	47193789	rs7731977	C	T	.	PASS	ARFGA	intrc	NM_	.	.
ACa07	11	55135745	.	T	C	.	PASS	OR4A15	exo	NM_	.	missens
ACa07	11	55371921	rs7783566	T	C	.	PASS	.	ups	NM_	.	.
ACa07	11	57314032	.	T	G	.	PASS	SMTNL1	exo	NM_	.	missens
ACa07	11	57983012	rs7556226	G	A	.	PASS	OR1S1	exo	NM_	.	missens
ACa07	11	57995755	rs3705153	C	T	.	PASS	OR10Q1	exo	NM_	.	missens
ACa07	11	59420105	rs7461600	C	T	.	PASS	PATL1	intrc	NM_	.	.
ACa07	11	61249827	rs7613895	G	A	.	PASS	PPP1R3	exo	NM_	.	missens
ACa07	11	61253132	.	C	A	.	PASS	PPP1R3	intrc	NM_	.	.
ACa07	11	61578304	.	G	T	.	PASS	FADS1	exo	NM_	.	synonyr
ACa07	11	62291713	rs7506613	G	A	.	PASS	AHNAK	exo	NM_	.	synonyr
ACa07	11	62361821	rs3688014	C	T	.	PASS	MTA2	exo	NM_	.	missens
ACa07	11	62416338	rs3734262	C	T	.	PASS	INTS5	exo	NM_	.	missens
ACa07	11	62566179	.	T	G	.	PASS	NXF1	UTF	NM_	NM	.
ACa07	11	63721515	rs7551956	C	T	.	PASS	NAA40	exo	NM_	.	missens
ACa07	11	63763957	rs320149	G	A	.	PASS	OTUB1	intrc	NM_	.	.
ACa07	11	64039354	.	T	A	.	PASS	BAD	intrc	NM_	.	.
ACa07	11	64521078	rs7694797	C	T	.	PASS	PYGM	exo	NM_	.	missens

ACa07	11	64899776	rs7599871	G	A	.	PASS	SYVN1	exoı	NM_	.	synonyr
ACa07	11	64937741	rs2009796	C	T	.	PASS	SPDYC	intrc	NM_	.	.
ACa07	11	65036017	.	G	A	.	PASS	POLA2	intrc	NM_	.	.
ACa07	11	65360827	.	G	A	.	PASS	KCNK7	exoı	NM_	.	missens
ACa07	11	65381195	.	G	A	.	PASS	MAP3K	exoı	NM_	.	synonyr
ACa07	11	65389786	rs3731576	G	A	.	PASS	PCNXL	exoı	NM_	.	missens
ACa07	11	65414066	.	G	A	.	PASS	SIPA1	exoı	NM_	.	missens
ACa07	11	65414716	.	T	G	.	PASS	SIPA1	intrc	NM_	.	.
ACa07	11	65487375	.	G	A	.	PASS	RNASE	intrc	NM_	.	.
ACa07	11	65810662	.	C	T	.	PASS	GAL3S1	exoı	NM_	.	synonyr
ACa07	11	66033554	.	C	T	.	PASS	KLC2	intrc	NM_	.	.
ACa07	11	66082361	.	C	T	.	PASS	CD248	exoı	NM_	.	missens
ACa07	11	66108304	.	T	C	.	PASS	BRMS1	exoı	NM_	.	missens
ACa07	11	67166750	.	G	A	.	PASS	PPP1CA	intrc	NM_	.	.
ACa07	11	67378750	rs8917540	C	T	.	PASS	NDUFV	intrc	NM_	.	.
ACa07	11	67815194	rs1432324	C	T	.	PASS	TCIRG1	exoı	NM_	.	synonyr
ACa07	11	68822725	rs7621382	C	T	.	PASS	TPCN2	exoı	NM_	.	missens
ACa07	11	68851602	.	G	A	.	PASS	TPCN2	intrc	NM_	.	.
ACa07	11	70194192	.	A	G	.	PASS	PPFIA1	intrc	NM_	.	.
ACa07	11	71149990	rs7726393	C	T	.	PASS	DHCR7	exoı	NM_	.	missens
ACa07	11	71820226	rs1917587	G	A	.	PASS	LRTOM	UTF	NM_	NM	.
ACa07	11	71931865	.	G	C	.	PASS	FOLR2	intrc	NM_	.	.
ACa07	11	72040728	.	C	A	.	PASS	CLPB	intrc	NM_	.	.
ACa07	11	72070285	.	A	G	.	PASS	CLPB	intrc	NM_	.	.
ACa07	11	72396522	.	C	A	.	PASS	ARAP1	UTF	NM_	NM	.
ACa07	11	72651947	.	T	C	.	PASS	FCHSD	intrc	NM_	.	.
ACa07	11	73964648	.	G	A	.	PASS	PPME1	UTF	NM_	NM	.
ACa07	11	74706128	.	C	T	.	PASS	NEU3	intrc	NM_	.	.
ACa07	11	74873801	rs7806415	G	A	.	PASS	SLCO2I	exoı	NM_	.	missens
ACa07	11	75316988	rs7805688	G	A	.	PASS	MAP6	exoı	NM_	.	missens
ACa07	11	75694576	rs7453149	C	T	.	PASS	UVRAG	intrc	NM_	.	.
ACa07	11	77918543	.	G	A	.	PASS	USP35	intrc	NM_	.	.
ACa07	11	80037592	rs3722049	G	C	.	PASS	.	inteı	NM_	dis	.
ACa07	11	83501466	.	G	A	.	PASS	DLG2	intrc	NM_	.	.
ACa07	11	88300512	.	G	A	.	PASS	GRM5	exoı	NM_	.	missens
ACa07	11	89402770	.	G	A	.	PASS	FOLH1E	intrc	NM_	.	.
ACa07	11	93148372	rs7912478	G	A	.	PASS	CCDC6	intrc	NM_	.	.
ACa07	11	1.03E+08	rs1913813	G	A	.	PASS	DYNC2I	exoı	NM_	.	missens
ACa07	11	1.07E+08	rs7548668	G	A	.	PASS	GUCY1	intrc	NM_	.	.
ACa07	11	1.08E+08	rs7639430	C	T	.	PASS	ELMOD	exoı	NM_	.	missens
ACa07	11	1.08E+08	.	A	G	.	PASS	CUL5	intrc	NM_	.	.
ACa07	11	1.08E+08	.	T	C	.	PASS	ACAT1	exoı	NM_	.	synonyr
ACa07	11	1.08E+08	.	A	G	.	PASS	ATM	intrc	NM_	.	.
ACa07	11	1.11E+08	rs5746434	C	T	.	PASS	C11orf5	UTF	NM_	NM	.
ACa07	11	1.11E+08	rs1042921	G	A	.	PASS	COLCA	ncR	NR_	.	.
ACa07	11	1.12E+08	rs9738553	C	T	.	PASS	SIK2	intrc	NM_	.	.
ACa07	11	1.14E+08	rs7711773	G	A	.	PASS	ZW10	exoı	NM_	.	synonyr
ACa07	11	1.15E+08	rs7553780	T	G	.	PASS	CADM1	intrc	NM_	.	.
ACa07	11	1.17E+08	.	C	T	.	PASS	SIK3	exoı	NM_	.	synonyr
ACa07	11	1.18E+08	rs3706451	C	A	.	PASS	TMPRS	exoı	NM_	.	synonyr
ACa07	11	1.18E+08	.	C	T	.	PASS	SCN2B	exoı	NM_	.	missens
ACa07	11	1.18E+08	.	G	T	.	PASS	MPZL3	intrc	NM_	.	.
ACa07	11	1.18E+08	.	C	A	.	PASS	ARCN1	intrc	NM_	.	.
ACa07	11	1.19E+08	.	G	A	.	PASS	DDX6	exoı	NM_	.	synonyr

ACa07	11	1.2E+08	rs5761141	T	C	.	PASS	ARHGE	exoı	NM_	.	synonyr
ACa07	11	1.2E+08	.	C	A	.	PASS	ARHGE	exoı	NM_	.	missens
ACa07	11	1.21E+08	rs1424863	C	T	.	PASS	TECTA	exoı	NM_	.	missens
ACa07	11	1.24E+08	rs1464053	C	T	.	PASS	OR10G	exoı	NM_	.	missens
ACa07	11	1.24E+08	rs1866274	C	T	.	PASS	OR8G2	exoı	NM_	.	unknow
ACa07	11	1.25E+08	.	C	A	.	PASS	ROBO4	intro	NM_	.	.
ACa07	11	1.25E+08	.	C	T	.	PASS	PKNOX	exoı	NM_	.	missens
ACa07	11	1.26E+08	rs1036245	G	A	.	PASS	FOXRE	exoı	NM_	.	missens
ACa07	11	1.29E+08	rs1499597	C	T	.	PASS	TP53AI	intro	NM_	.	.
ACa07	11	1.3E+08	rs1160026	G	T	.	PASS	PRDM1	exoı	NM_	.	synonyr
ACa07	11	1.3E+08	rs7501981	G	A	.	PASS	ADAMT	exoı	NM_	.	missens
ACa07	11	1.33E+08	.	G	T	.	PASS	OPCML	exoı	NM_	.	missens
ACa07	11	1.34E+08	rs9612999	G	A	.	PASS	.	inteı	NM_	dis	.
ACa07	11	1.34E+08	.	G	A	.	PASS	GLB1L3	intro	NM_	.	.
ACa07	12	542634	rs1492134	C	T	.	PASS	CCDC7	intro	NM_	.	.
ACa07	12	569498	.	G	A	.	PASS	.	ups	NM_	.	.
ACa07	12	663146	rs7477402	G	A	.	PASS	B4GALI	exoı	NM_	.	missens
ACa07	12	923046	.	G	T	.	PASS	WNK1	intro	NM_	.	.
ACa07	12	2711100	.	C	T	.	PASS	CACNA	exoı	NM_	.	synonyr
ACa07	12	2719715	rs2020589	C	T	.	PASS	CACNA	exoı	NM_	.	synonyr
ACa07	12	2750942	rs1044570	G	A	.	PASS	CACNA	intro	NM_	.	.
ACa07	12	3765346	.	C	T	.	PASS	CRACR	intro	NM_	.	.
ACa07	12	4398171	.	C	A	.	PASS	CCND2	intro	NM_	.	.
ACa07	12	4648013	.	G	T	.	PASS	RAD51/	UTF	NM_	NM	.
ACa07	12	4919704	.	A	G	.	PASS	KCNA6	exoı	NM_	.	missens
ACa07	12	6438497	.	G	A	.	PASS	TNFRSI	exoı	NM_	.	missens
ACa07	12	6781789	rs7522876	C	T	.	PASS	ZNF384	intro	NM_	.	.
ACa07	12	6923187	.	C	T	.	PASS	CD4	intro	NM_	.	.
ACa07	12	7286388	.	C	A	.	PASS	CLSTN3	intro	NM_	.	.
ACa07	12	7635360	.	T	C	.	PASS	CD163	splic	NM_	NM	.
ACa07	12	8618152	.	T	A	.	PASS	CLEC6/	exoı	NM_	.	missens
ACa07	12	8807044	.	G	A	.	PASS	MFAP5	exoı	NM_	.	unknow
ACa07	12	9709589	.	A	G	.	PASS	.	inteı	NR_	dis	.
ACa07	12	9713438	rs7607645	T	C	.	PASS	.	inteı	NR_	dis	.
ACa07	12	10131539	.	C	A	.	PASS	CLEC12	intro	NM_	.	.
ACa07	12	11036605	.	G	A	.	PASS	PRH1-F	ncR	NR_	.	.
ACa07	12	11905325	.	C	A	.	PASS	ETV6	intro	NM_	.	.
ACa07	12	12514214	rs7520863	C	T	.	PASS	LOH12(	exoı	NM_	.	unknow
ACa07	12	12514275	.	T	C	.	PASS	LOH12(	exoı	NM_	.	unknow
ACa07	12	13028428	rs7558438	C	T	.	PASS	RPL13A	ncR	NR_	.	.
ACa07	12	18644464	.	C	T	.	PASS	PIK3C2	exoı	NM_	.	missens
ACa07	12	18656280	.	G	T	.	PASS	PIK3C2	exoı	NM_	.	stopgaiı
ACa07	12	19260709	rs9411424	A	C	.	PASS	.	inteı	NM_	dis	.
ACa07	12	21377691	.	A	G	.	PASS	SLCO1I	exoı	NM_	.	missens
ACa07	12	27840344	.	A	G	.	PASS	PPFIBP	exoı	NM_	.	synonyr
ACa07	12	29493982	.	C	T	.	PASS	ERGIC2	UTF	NM_	NM	.
ACa07	12	31116725	rs3715564	C	T	.	PASS	TSPAN	intro	NM_	.	.
ACa07	12	31144980	.	C	T	.	PASS	TSPAN	UTF	NM_	NM	.
ACa07	12	33139537	.	G	A	.	PASS	.	inteı	NM_	dis	.
ACa07	12	38596398	.	C	T	.	PASS	.	inteı	NON	dis	.
ACa07	12	40858460	.	C	T	.	PASS	MUC19	intro	NM_	.	.
ACa07	12	40894116	.	C	T	.	PASS	MUC19	intro	NM_	.	.
ACa07	12	44132686	.	A	T	.	PASS	PUS7L	intro	NM_	.	.
ACa07	12	48888693	.	A	C	.	PASS	C12orf5	exoı	NM_	.	missens

ACa07	12	49165248	.	C	T	.	PASS	ADCY6	intrc	NM_	.	.
ACa07	12	49725168	.	T	C	.	PASS	TROAP	exo	NM_	.	missens
ACa07	12	50026472	rs5557374	G	A	.	PASS	PRPF4	intrc	NM_	.	.
ACa07	12	50036070	rs7778437	G	A	.	PASS	PRPF4	exo	NM_	.	missens
ACa07	12	50195577	.	A	G	.	PASS	NCKAP	intrc	NM_	.	.
ACa07	12	50471384	rs7903986	G	A	.	PASS	ASIC1	intrc	NM_	.	.
ACa07	12	50854873	.	G	A	.	PASS	LARP4	intrc	NM_	.	.
ACa07	12	51512439	.	T	C	.	PASS	TFCP2	exo	NM_	.	missens
ACa07	12	52599942	.	A	G	.	PASS	C12orf8	exo	NM_	.	missens
ACa07	12	52946871	.	G	T	.	PASS	KRT71	UTF	NM_	NM	.
ACa07	12	53002235	rs3777629	C	T	.	PASS	KRT73	exo	NM_	.	synonyr
ACa07	12	53045563	rs5301800	G	A	.	PASS	KRT2	exo	NM_	.	missens
ACa07	12	53201610	.	C	T	.	PASS	KRT4	exo	NM_	.	synonyr
ACa07	12	53459846	.	G	A	.	PASS	SPRYD	intrc	NM_	.	.
ACa07	12	53593996	.	C	T	.	PASS	ITGB7	intrc	NM_	.	.
ACa07	12	53877615	rs9039120	G	A	.	PASS	MAP3K	intrc	NM_	.	.
ACa07	12	54426698	.	G	T	.	PASS	HOXC4	intrc	NM_	.	.
ACa07	12	56645817	rs3688610	C	T	.	PASS	ANKRD	exo	NM_	.	missens
ACa07	12	57432280	rs7688629	C	T	.	PASS	MYO1A	exo	NM_	.	missens
ACa07	12	57458056	rs1929543	T	A	.	PASS	TMEM1	intrc	NM_	.	.
ACa07	12	57523017	rs9453696	G	C	.	PASS	LRP1	intrc	NM_	.	.
ACa07	12	57567098	.	G	A	.	PASS	LRP1	exo	NM_	.	missens
ACa07	12	57577325	rs3702872	G	A	.	PASS	LRP1	intrc	NM_	.	.
ACa07	12	57584906	.	T	C	.	PASS	LRP1	intrc	NM_	.	.
ACa07	12	57628071	rs5363943	G	A	.	PASS	SHMT2	exo	NM_	.	missens
ACa07	12	63954420	.	T	G	.	PASS	DPY19L	exo	NM_	.	missens
ACa07	12	64812712	rs1809265	C	T	.	PASS	XPOT	exo	NM_	.	synonyr
ACa07	12	64825337	.	A	G	.	PASS	XPOT	exo	NM_	.	missens
ACa07	12	69764543	.	A	G	.	PASS	YEATS	exo	NM_	.	unknow
ACa07	12	69995116	.	G	A	.	PASS	CCT2	UTF	NM_	NM	.
ACa07	12	70037457	rs5319845	C	T	.	PASS	BEST3	UTF	NM_	NM	.
ACa07	12	72080578	.	A	C	.	PASS	TMEM1	exo	NM_	.	missens
ACa07	12	76752585	rs3675929	C	T	.	PASS	OSBPL	exo	NM_	.	missens
ACa07	12	77260115	.	G	T	.	PASS	CSRP2	intrc	NM_	.	.
ACa07	12	78582518	.	C	A	.	PASS	NAV3	exo	NM_	.	missens
ACa07	12	78593259	.	G	T	.	PASS	NAV3	exo	NM_	.	missens
ACa07	12	80672808	rs7672581	C	T	.	PASS	OTOGL	exo	NM_	.	synonyr
ACa07	12	80760258	rs9382701	A	G	.	PASS	OTOGL	intrc	NM_	.	.
ACa07	12	82828501	.	G	T	.	PASS	METTL	exo	NM_	.	missens
ACa07	12	94034649	.	C	T	.	PASS	.	inte	NM_	dis	.
ACa07	12	95918387	.	G	A	.	PASS	USP44	intrc	NM_	.	.
ACa07	12	96717860	rs5750458	G	A	.	PASS	CDK17	exo	NM_	.	missens
ACa07	12	97078469	rs1491955	G	A	.	PASS	.	inte	NM_	dis	.
ACa07	12	97222775	.	T	C	.	PASS	.	inte	NM_	dis	.
ACa07	12	1.01E+08	rs7594794	C	T	.	PASS	NR1H4	exo	NM_	.	missens
ACa07	12	1.01E+08	.	C	T	.	PASS	ANO4	intrc	NM_	.	.
ACa07	12	1.02E+08	.	A	G	.	PASS	ARL1	intrc	NM_	.	.
ACa07	12	1.02E+08	.	G	T	.	PASS	MYBPC	intrc	NM_	.	.
ACa07	12	1.04E+08	.	G	T	.	PASS	STAB2	exo	NM_	.	missens
ACa07	12	1.04E+08	.	G	A	.	PASS	TDG	intrc	NM_	.	.
ACa07	12	1.09E+08	.	C	T	.	PASS	WSCD2	intrc	NM_	.	.
ACa07	12	1.09E+08	.	C	T	.	PASS	SART3	exo	NM_	.	missens
ACa07	12	1.1E+08	.	T	C	.	PASS	ALKBH	intrc	NM_	.	.
ACa07	12	1.1E+08	.	C	T	.	PASS	MYO1H	intrc	NM_	.	.

ACa07	12	1.12E+08	.	G	T	.	PASS	.	inte	NM_	dis	.	
ACa07	12	1.12E+08	.	C	A	.	PASS	BRAP	UTF	NM_	NM	.	
ACa07	12	1.13E+08	rs3699442	G	A	.	PASS	HECTD	exo	NM_	.		synonyr
ACa07	12	1.16E+08	rs7648934	C	T	.	PASS	MED13I	intrc	NM_	.		.
ACa07	12	1.18E+08	.	T	G	.	PASS	NOS1	intrc	NM_	.		.
ACa07	12	1.19E+08	.	A	G	.	PASS	TAOK3	intrc	NM_	.		.
ACa07	12	1.2E+08	.	G	T	.	PASS	TMEM2	UTF	NM_	NM	.	
ACa07	12	1.21E+08	rs9094398	C	T	.	PASS	MLEC	UTF	NM_	NM	.	
ACa07	12	1.21E+08	rs7629138	C	T	.	PASS	SPPL3	UTF	NM_	NM	.	
ACa07	12	1.21E+08	.	A	T	.	PASS	OASL	exo	NM_	.		missens
ACa07	12	1.22E+08	rs1000013	G	A	.	PASS	P2RX4	intrc	NM_	.		.
ACa07	12	1.22E+08	.	C	T	.	PASS	KDM2B	intrc	NM_	.		.
ACa07	12	1.23E+08	.	G	T	.	PASS	CLIP1	intrc	NM_	.		.
ACa07	12	1.23E+08	.	G	A	.	PASS	KNTC1	intrc	NM_	.		.
ACa07	12	1.23E+08	rs5485859	G	A	.	PASS	HIP1R	intrc	NM_	.		.
ACa07	12	1.24E+08	.	C	A	.	PASS	SBNO1	intrc	NM_	.		.
ACa07	12	1.24E+08	.	G	A	.	PASS	SBNO1	intrc	NM_	.		.
ACa07	12	1.24E+08	.	T	C	.	PASS	SETD8	intrc	NM_	.		.
ACa07	12	1.24E+08	rs3682772	C	T	.	PASS	DNAH1(	intrc	NM_	.		.
ACa07	12	1.24E+08	.	T	C	.	PASS	DNAH1(	exo	NM_	.		synonyr
ACa07	12	1.24E+08	rs1861196	G	A	.	PASS	DNAH1(	intrc	NM_	.		.
ACa07	12	1.26E+08	.	T	G	.	PASS	TMEM1	intrc	NM_	.		.
ACa07	12	1.29E+08	.	C	T	.	PASS	.	inte	NR_	dis	.	
ACa07	12	1.29E+08	rs7758291	C	T	.	PASS	TMEM1	exo	NM_	.		synonyr
ACa07	12	1.29E+08	rs8685914	G	A	.	PASS	GLT1D1	intrc	NM_	.		.
ACa07	12	1.32E+08	.	G	T	.	PASS	EP400	intrc	NM_	.		.
ACa07	12	1.33E+08	.	T	C	.	PASS	DDX51	intrc	NM_	.		.
ACa07	12	1.33E+08	.	C	T	.	PASS	GALNT!	intrc	NM_	.		.
ACa07	12	1.33E+08	.	C	T	.	PASS	CHFR	exo	NM_	.		missens
ACa07	13	20356757	.	C	T	.	PASS	PSPC1	exo	NM_	.		synonyr
ACa07	13	20656960	.	G	T	.	PASS	ZMYM2	exo	NM_	.		missens
ACa07	13	21974513	rs7650516	G	A	.	PASS	ZDHHC	exo	NM_	.		missens
ACa07	13	23808767	.	G	A	.	PASS	SGCG	exo	NM_	.		synonyr
ACa07	13	27260004	rs7609687	G	A	.	PASS	WASF3	UTF	NM_	NM	.	
ACa07	13	29233178	.	T	C	.	PASS	POMP	UTF	NM_	NM	.	
ACa07	13	32813486	.	G	T	.	PASS	FRY	exo	NM_	.		missens
ACa07	13	34398037	.	T	G	.	PASS	RFC3	intrc	NM_	.		.
ACa07	13	36445225	.	C	T	.	PASS	MIR548	ncR	NR_	.		.
ACa07	13	36827965	.	C	T	.	PASS	CCDC1	exo	NM_	.		missens
ACa07	13	37427588	.	T	C	.	PASS	SMAD9	exo	NM_	.		missens
ACa07	13	41797402	rs1133145	C	T	.	PASS	MTRF1	exo	NM_	.		missens
ACa07	13	43134008	rs9269682	G	A	.	PASS	.	inte	NM_	dis	.	
ACa07	13	46127182	.	G	A	.	PASS	ERICH6	intrc	NM_	.		.
ACa07	13	46154301	rs5302300	C	T	.	PASS	ERICH6	intrc	NM_	.		.
ACa07	13	47361123	rs7755432	C	T	.	PASS	ESD	intrc	NM_	.		.
ACa07	13	71872859	rs5354767	C	T	.	PASS	.	inte	NR_	dis	.	
ACa07	13	72038778	.	A	C	.	PASS	DACH1	intrc	NM_	.		.
ACa07	13	75911047	.	G	A	.	PASS	TBC1D	intrc	NM_	.		.
ACa07	13	90883912	rs5371791	C	T	.	PASS	.	dow	NR_	.		.
ACa07	13	96240011	.	C	T	.	PASS	DZIP1	intrc	NM_	.		.
ACa07	13	97999160	rs1492005	G	A	.	PASS	MBNL2	exo	NM_	.		missens
ACa07	13	1E+08	rs3733851	G	A	.	PASS	TM9SF	exo	NM_	.		synonyr
ACa07	13	1.01E+08	rs3731740	C	T	.	PASS	PCCA	intrc	NM_	.		.
ACa07	13	1.09E+08	.	T	C	.	PASS	ABHD1	exo	NM_	.		missens

ACa07	13	1.09E+08	rs7567578	G	A	.	PASS	MYO16	exoi	NM_	.	missens
ACa07	13	1.11E+08	rs1488011	G	A	.	PASS	COL4A1	exoi	NM_	.	missens
ACa07	13	1.11E+08	rs7563035	G	A	.	PASS	CARS2	exoi	NM_	.	synonyr
ACa07	13	1.11E+08	.	G	A	.	PASS	.	ups	NM_	.	.
ACa07	13	1.12E+08	rs1000744	C	T	.	PASS	ANKRD	UTF	NM_	NM	.
ACa07	13	1.14E+08	rs7522048	G	A	.	PASS	.	inte	NM_	dis	.
ACa07	13	1.14E+08	rs7502995	G	A	.	PASS	MCF2L	exoi	NM_	.	missens
ACa07	13	1.15E+08	rs7709821	C	T	.	PASS	GAS6	exoi	NM_	.	missens
ACa07	14	19501000	rs8791117	C	T	.	PASS	.	inte	NR_	dis	.
ACa07	14	19502175	.	G	T	.	PASS	.	inte	NR_	dis	.
ACa07	14	19720452	rs7445485	C	T	.	PASS	.	inte	NR_	dis	.
ACa07	14	20470724	.	A	G	.	PASS	.	inte	NM_	dis	.
ACa07	14	20869728	rs1453568	C	A	.	PASS	TEP1	exoi	NM_	.	missens
ACa07	14	20897523	rs5497679	C	T	.	PASS	KLHL33	exoi	NM_	.	missens
ACa07	14	21851762	.	C	T	.	PASS	SUPT16	intrc	NM_	.	.
ACa07	14	22748944	.	T	C	.	PASS	.	inte	NM_	dis	.
ACa07	14	23244794	rs3745810	G	A	.	PASS	SLC7A7	intrc	NM_	.	.
ACa07	14	23417206	rs7576169	G	A	.	PASS	HAUS4	exoi	NM_	.	synonyr
ACa07	14	24032908	.	A	G	.	PASS	LOC102	ncR	NR_	.	.
ACa07	14	35557135	rs7777607	G	A	.	PASS	LOC101	ncR	NR_	.	.
ACa07	14	35564107	rs9241538	C	T	.	PASS	LOC101	ncR	NR_	.	.
ACa07	14	36123534	.	T	A	.	PASS	RALGAI	ncR	NR_	.	.
ACa07	14	36207787	.	C	A	.	PASS	RALGAI	exoi	NM_	.	missens
ACa07	14	36645564	.	C	A	.	PASS	PTCSC	ncR	NR_	.	.
ACa07	14	36840760	rs5673369	G	A	.	PASS	.	inte	NM_	dis	.
ACa07	14	38170628	.	C	T	.	PASS	.	inte	NM_	dis	.
ACa07	14	42361109	rs8659367	G	A	.	PASS	LRFN5	exoi	NM_	.	missens
ACa07	14	45650729	.	T	C	.	PASS	FANCM	splic	NM_	NM	.
ACa07	14	50459445	.	G	T	.	PASS	C14orf1	intrc	NM_	.	.
ACa07	14	52536381	.	G	T	.	PASS	.	ups	NM_	.	.
ACa07	14	52782046	rs7462395	G	A	.	PASS	PTGER	exoi	NM_	.	synonyr
ACa07	14	58690314	.	A	T	.	PASS	ACTR1(	intrc	NM_	.	.
ACa07	14	59757839	.	T	C	.	PASS	DAAM1	intrc	NM_	.	.
ACa07	14	59792389	.	G	T	.	PASS	DAAM1	exoi	NM_	.	missens
ACa07	14	59942751	.	T	C	.	PASS	L3HYPD	intrc	NM_	.	.
ACa07	14	60619792	rs1401803	C	T	.	PASS	DHRS7	exoi	NM_	.	synonyr
ACa07	14	64060705	.	A	G	.	PASS	.	inte	NM_	dis	.
ACa07	14	64905889	rs5401543	C	T	.	PASS	MTHFD	exoi	NM_	.	missens
ACa07	14	66723885	rs1404421	C	T	.	PASS	.	inte	NM_	dis	.
ACa07	14	67432208	.	A	G	.	PASS	GPHN	intrc	NM_	.	.
ACa07	14	69345661	.	C	T	.	PASS	ACTN1	intrc	NM_	.	.
ACa07	14	74225682	.	C	T	.	PASS	ELMSAI	intrc	NM_	.	.
ACa07	14	74371771	rs7545827	C	T	.	PASS	ZNF410	exoi	NM_	.	missens
ACa07	14	74387833	rs3764591	C	T	.	PASS	ZNF410	intrc	NM_	.	.
ACa07	14	75019057	.	A	G	.	PASS	LTBP2	exoi	NM_	.	missens
ACa07	14	75231119	.	C	T	.	PASS	YLPM1	intrc	NM_	.	.
ACa07	14	75320856	.	G	T	.	PASS	PROX2	UTF	NM_	NM	.
ACa07	14	75373816	rs1413435	G	A	.	PASS	RPS6KI	exoi	NM_	.	synonyr
ACa07	14	90459861	.	C	A	.	PASS	TDP1	intrc	NM_	.	.
ACa07	14	90759404	.	G	T	.	PASS	NRDE2	intrc	NM_	.	.
ACa07	14	91161849	rs7511076	G	A	.	PASS	TTC7B	exoi	NM_	.	stopgaii
ACa07	14	91701455	rs7632995	G	A	.	PASS	GPR68	UTF	NM_	NM	.
ACa07	14	91752050	.	G	A	.	PASS	CCDC8	intrc	NM_	.	.
ACa07	14	94471264	.	G	A	.	PASS	LINC00!	ncR	NR_	.	.

ACa07	14	94581845	.	C	T	.	PASS	IFI27	intrc	NM_	.	.
ACa07	14	95560026	.	G	A	.	PASS	DICER1	intrc	NM_	.	.
ACa07	14	99641990	.	A	G	.	PASS	BCL11E	exoı	NM_	.	missens
ACa07	14	99697789	.	G	T	.	PASS	BCL11E	exoı	NM_	.	missens
ACa07	14	1.01E+08	.	C	T	.	PASS	DLK1	exoı	NM_	.	synonyr
ACa07	14	1.01E+08	.	T	C	.	PASS	MIR411	ncR	NR_	.	.
ACa07	14	1.02E+08	.	G	A	.	PASS	MIR381	ncR	NR_	.	.
ACa07	14	1.03E+08	rs7571156	G	A	.	PASS	MOK	exoı	NM_	.	synonyr
ACa07	14	1.03E+08	rs9721368	C	T	.	PASS	CDC42I	intrc	NM_	.	.
ACa07	14	1.04E+08	rs1043142	C	T	.	PASS	TRMT6:	intrc	NM_	.	.
ACa07	14	1.05E+08	.	C	T	.	PASS	KIF26A	exoı	NM_	.	missens
ACa07	14	1.05E+08	.	G	T	.	PASS	TMEM1	exoı	NM_	.	missens
ACa07	14	1.05E+08	.	C	T	.	PASS	SIVA1	intrc	NM_	.	.
ACa07	14	1.05E+08	rs7564163	C	T	.	PASS	AKT1	intrc	NM_	.	.
ACa07	14	1.05E+08	.	G	T	.	PASS	CEP17C	intrc	NM_	.	.
ACa07	14	1.06E+08	rs7689001	G	A	.	PASS	JAG2	intrc	NM_	.	.
ACa07	14	1.06E+08	.	C	A	.	PASS	BRF1	intrc	NM_	.	.
ACa07	14	1.06E+08	rs7822187	G	A	.	PASS	PACS2	exoı	NM_	.	missens
ACa07	14	1.06E+08	rs7820214	C	T	.	PASS	.	inteı	NR_	dis	.
ACa07	14	1.06E+08	.	G	A	.	PASS	ADAM6	ncR	NR_	.	.
ACa07	14	1.07E+08	.	C	T	.	PASS	.	inteı	NR_	dis	.
ACa07	15	20511974	rs2005484	G	A	.	PASS	.	inteı	NR_	dis	.
ACa07	15	21071136	.	C	T	.	PASS	POTEB.	intrc	NM_	.	.
ACa07	15	21324830	.	A	G	.	PASS	.	inteı	NR_	dis	.
ACa07	15	22297633	.	A	G	.	PASS	LOC101	ncR	NR_	.	.
ACa07	15	22956666	.	C	T	.	PASS	CYFIP1	intrc	NM_	.	.
ACa07	15	23099573	.	G	A	.	PASS	LOC283	ncR	NR_	.	.
ACa07	15	23419634	.	G	A	.	PASS	.	intrc	NM_	.	.
ACa07	15	23467145	.	C	T	.	PASS	.	intrc	NM_	.	.
ACa07	15	23686244	rs7605191	G	A	.	PASS	.	inteı	NR_	dis	.
ACa07	15	25337269	rs9195421	G	A	.	PASS	.	dow	NR_	.	.
ACa07	15	25431213	.	G	A	.	PASS	.	dow	NR_	.	.
ACa07	15	25444465	rs7485099	T	C	.	PASS	.	ups	NR_	.	.
ACa07	15	25477642	.	A	G	.	PASS	.	dow	NR_	.	.
ACa07	15	26107216	rs9209119	G	A	.	PASS	ATP10A	intrc	NM_	.	.
ACa07	15	28358433	rs3718584	C	T	.	PASS	HERC2	splic	NM_	NM	.
ACa07	15	28478423	.	A	G	.	PASS	HERC2	exoı	NM_	.	missens
ACa07	15	28949022	rs2021672	C	T	.	PASS	GOLGA	exoı	NM_	.	synonyr
ACa07	15	29526740	.	G	T	.	PASS	FAM18	intrc	NM_	.	.
ACa07	15	30382020	.	G	A	.	PASS	GOLGA	exoı	NM_	.	missens
ACa07	15	30663208	.	C	T	.	PASS	CHRFA	intrc	NM_	.	.
ACa07	15	30846509	rs1995696	A	G	.	PASS	.	inteı	NM_	dis	.
ACa07	15	30874998	.	T	C	.	PASS	ULK4P1	ncR	NR_	.	.
ACa07	15	31776610	.	C	T	.	PASS	OTUD7.	exoı	NM_	.	synonyr
ACa07	15	32635938	rs1127716	A	T	.	PASS	.	inteı	NM_	dis	.
ACa07	15	34040248	.	C	T	.	PASS	RYR3	intrc	NM_	.	.
ACa07	15	35385280	.	G	A	.	PASS	.	inteı	NM_	dis	.
ACa07	15	40568144	.	G	A	.	PASS	PAK6	exoı	NM_	.	missens
ACa07	15	40589993	.	G	T	.	PASS	PLCB2	intrc	NM_	.	.
ACa07	15	42158532	rs3704110	C	T	.	PASS	SPTBN!	intrc	NM_	.	.
ACa07	15	42170858	.	G	A	.	PASS	SPTBN!	intrc	NM_	.	.
ACa07	15	43109245	rs5310947	G	A	.	PASS	TTBK2	exoı	NM_	.	synonyr
ACa07	15	43678043	rs7551745	C	T	.	PASS	TUBGC	exoı	NM_	.	stopgaiı
ACa07	15	43891133	rs5360360	C	T	.	PASS	CKMT1I	exoı	NM_	.	stopgaiı

ACa07	15	43901655	.	G	A	.	PASS	STRC	intrc	NM_	.	.
ACa07	15	44003289	rs5322560	G	A	.	PASS	.	inter	NM_	dis	.
ACa07	15	45328596	.	G	A	.	PASS	SORD	intrc	NM_	.	.
ACa07	15	45455872	.	G	T	.	PASS	DUOX1	exon	NM_	.	missens
ACa07	15	48054299	.	A	G	.	PASS	SEMA6I	intrc	NM_	.	.
ACa07	15	48720650	rs7737859	G	A	.	PASS	FBN1	exon	NM_	.	missens
ACa07	15	48782203	rs1409544	C	T	.	PASS	FBN1	exon	NM_	.	missens
ACa07	15	49264890	.	G	T	.	PASS	.	inter	NM_	dis	.
ACa07	15	49869190	.	A	G	.	PASS	FAM227	intrc	NM_	.	.
ACa07	15	50331173	.	G	T	.	PASS	ATP8B4	intrc	NM_	.	.
ACa07	15	50835793	.	A	G	.	PASS	USP50	splic	NM_	NM	.
ACa07	15	52571126	rs3686733	G	A	.	PASS	MYO5C	exon	NM_	.	synonyr
ACa07	15	52680198	.	G	T	.	PASS	MYO5A	intrc	NM_	.	.
ACa07	15	58465302	.	T	C	.	PASS	AQP9	exon	NM_	.	missens
ACa07	15	63132757	rs7793599	G	A	.	PASS	TLN2	exon	NM_	.	missens
ACa07	15	65308790	rs7518805	C	T	.	PASS	MTFMT	exon	NM_	.	missens
ACa07	15	65678326	.	C	G	.	PASS	IGDCC4	exon	NM_	.	missens
ACa07	15	65686840	rs5369412	C	T	.	PASS	IGDCC4	exon	NM_	.	synonyr
ACa07	15	66223005	.	G	A	.	PASS	MEGF1	intrc	NM_	.	.
ACa07	15	67286477	.	T	C	.	PASS	.	inter	NM_	dis	.
ACa07	15	67652167	.	T	C	.	PASS	IQCH	intrc	NM_	.	.
ACa07	15	68502136	rs7568129	C	T	.	PASS	CLN6	intrc	NM_	.	.
ACa07	15	68624562	.	C	T	.	PASS	ITGA11	intrc	NM_	.	.
ACa07	15	72039013	rs5460720	C	T	.	PASS	THSD4	intrc	NM_	.	.
ACa07	15	72069450	.	C	A	.	PASS	THSD4	intrc	NM_	.	.
ACa07	15	72608191	.	G	A	.	PASS	CELF6	exon	NM_	.	missens
ACa07	15	72978799	rs1043159	C	A	.	PASS	BBS4	intrc	NM_	.	.
ACa07	15	74640093	.	G	A	.	PASS	CYP11A	intrc	NM_	.	.
ACa07	15	75551094	.	C	T	.	PASS	GOLGA	intrc	NM_	.	.
ACa07	15	75551112	.	C	T	.	PASS	GOLGA	intrc	NM_	.	.
ACa07	15	76643759	rs9744245	G	A	.	PASS	SCAPEI	intrc	NM_	.	.
ACa07	15	76643778	rs1047518	C	T	.	PASS	SCAPEI	intrc	NM_	.	.
ACa07	15	78217296	rs8789048	A	G	.	PASS	LOC645	ncR	NR_	.	.
ACa07	15	78276624	.	G	T	.	PASS	.	inter	NR_	dis	.
ACa07	15	79065578	.	C	T	.	PASS	ADAMT	intrc	NM_	.	.
ACa07	15	79750037	rs1471333	C	T	.	PASS	KIAA10	exon	NM_	.	synonyr
ACa07	15	80445547	.	G	A	.	PASS	FAH	intrc	NM_	.	.
ACa07	15	81179994	rs5292517	G	A	.	PASS	CEMIP	intrc	NM_	.	.
ACa07	15	81427559	.	T	C	.	PASS	C15orf2	intrc	NM_	.	.
ACa07	15	84611197	rs9410184	C	T	.	PASS	ADAMT	intrc	NM_	.	.
ACa07	15	84908921	rs7920451	A	G	.	PASS	GOLGA	exon	NM_	.	missens
ACa07	15	86284204	.	G	A	.	PASS	AKAP13	intrc	NM_	.	.
ACa07	15	86311985	rs3718116	C	T	.	PASS	KLHL25	exon	NM_	.	missens
ACa07	15	89876682	.	G	A	.	PASS	POLG	exon	NM_	.	missens
ACa07	15	90029782	.	A	T	.	PASS	RHCG	intrc	NM_	.	.
ACa07	15	90188260	rs2012804	G	A	.	PASS	KIF7	exon	NM_	.	synonyr
ACa07	15	90192513	.	C	T	.	PASS	KIF7	exon	NM_	.	synonyr
ACa07	15	90610456	rs1475111	C	T	.	PASS	ZNF710	exon	NM_	.	unknow
ACa07	15	1E+08	rs5584446	T	C	.	PASS	.	ups	NM_	.	.
ACa07	15	1.01E+08	.	T	C	.	PASS	ADAMT	exon	NM_	.	synonyr
ACa07	15	1.01E+08	.	C	T	.	PASS	PRKXP	ncR	NR_	.	.
ACa07	15	1.02E+08	.	G	T	.	PASS	LRRK1	intrc	NM_	.	.
ACa07	15	1.02E+08	rs7764234	G	A	.	PASS	PCSK6	exon	NM_	.	unknow
ACa07	15	1.02E+08	.	C	A	.	PASS	TARSL2	intrc	NM_	.	.

ACa07	15	1.02E+08	.	G	A	.	PASS	.	inte	NM_	dis	.		
ACa07	15	1.02E+08	rs3682087	G	A	.	PASS	.	inte	NM_	dis	.		
ACa07	15	1.02E+08	.	C	T	.	PASS	.	inte	NM_	dis	.		
ACa07	15	1.03E+08	.	A	G	.	PASS	DDX11L	ncR	NR_	.	.		
ACa07	16	150424	rs7613665	G	A	.	PASS	NPRL3	exo	NM_	.	unknow		
ACa07	16	314957	.	G	T	.	PASS	ITFG3	exo	NM_	.	missens		
ACa07	16	324844	.	G	A	.	PASS	RGS11	intro	NM_	.	.		
ACa07	16	544795	rs8927038	G	C	.	PASS	RAB11F	intro	NM_	.	.		
ACa07	16	682922	rs1998911	C	T	.	PASS	WFIKKN	exo	NM_	.	missens		
ACa07	16	721815	rs2010028	C	T	.	PASS	RHOT2	intro	NM_	.	.		
ACa07	16	734141	rs7767345	G	A	.	PASS	JMJD8	exo	NM_	.	missens		
ACa07	16	746934	rs1466306	C	T	.	PASS	FBXL16	exo	NM_	.	missens		
ACa07	16	780555	rs3681933	C	T	.	PASS	NARFL	exo	NM_	.	synonyr		
ACa07	16	847840	.	G	T	.	PASS	CHTF18	exo	NM_	.	synonyr		
ACa07	16	960853	.	C	A	.	PASS	LMF1	intro	NM_	.	.		
ACa07	16	1129064	rs1506415	C	T	.	PASS	SSTR5	exo	NM_	.	synonyr		
ACa07	16	1365820	rs9971019	G	A	.	PASS	UBE2I	intro	NM_	.	.		
ACa07	16	1388641	.	A	T	.	PASS	BAIAP3	exo	NM_	.	missens		
ACa07	16	1390640	rs3710800	C	T	.	PASS	BAIAP3	intro	NM_	.	.		
ACa07	16	1391397	.	G	A	.	PASS	BAIAP3	exo	NM_	.	missens		
ACa07	16	1393987	rs7592188	C	T	.	PASS	BAIAP3	intro	NM_	.	.		
ACa07	16	1397792	.	G	A	.	PASS	BAIAP3	exo	NM_	.	missens		
ACa07	16	1479447	rs5365360	G	A	.	PASS	.	inte	NM_	dis	.		
ACa07	16	1497508	rs7601714	C	T	.	PASS	CLCN7	exo	NM_	.	missens		
ACa07	16	1741777	.	C	T	.	PASS	HN1L	intro	NM_	.	.		
ACa07	16	1748934	rs1450874	C	T	.	PASS	HN1L	exo	NM_	.	missens		
ACa07	16	1798630	.	T	A	.	PASS	MAPK8I	exo	NM_	.	synonyr		
ACa07	16	2004033	rs3749279	G	A	.	PASS	RPL3L	exo	NM_	.	synonyr		
ACa07	16	2034966	.	C	A	.	PASS	GFER	intro	NM_	.	.		
ACa07	16	2049733	rs7634894	G	A	.	PASS	ZNF598	exo	NM_	.	missens		
ACa07	16	2215749	.	G	A	.	PASS	TRAF7	intro	NM_	.	.		
ACa07	16	2523602	.	T	C	.	PASS	NTN3	intro	NM_	.	.		
ACa07	16	3078076	.	G	A	.	PASS	CCDC6	UTF	NM_	NM	.		
ACa07	16	3445074	rs1416929	C	T	.	PASS	ZSCAN	intro	NM_	.	.		
ACa07	16	3487003	.	A	G	.	PASS	ZNF597	exo	NM_	.	synonyr		
ACa07	16	3580692	rs7525617	C	T	.	PASS	CLUAP	intro	NM_	.	.		
ACa07	16	3646372	rs5345285	G	A	.	PASS	SLX4	exo	NM_	.	missens		
ACa07	16	3820858	.	C	A	.	PASS	CREBB	exo	NM_	.	missens		
ACa07	16	3821078	rs5502446	T	A	.	PASS	CREBB	intro	NM_	.	.		
ACa07	16	3843244	rs1894802	G	A	.	PASS	CREBB	intro	NM_	.	.		
ACa07	16	4016487	rs3711593	C	T	.	PASS	ADCY9	exo	NM_	.	synonyr		
ACa07	16	4432151	rs2013575	C	T	.	PASS	VASN	exo	NM_	.	missens		
ACa07	16	4935715	.	G	T	.	PASS	PPL	exo	NM_	.	missens		
ACa07	16	10996015	rs5632991	G	A	.	PASS	CIITA	exo	NM_	.	missens		
ACa07	16	11008860	rs7679436	T	C	.	PASS	CIITA	intro	NM_	.	.		
ACa07	16	11554808	rs9184234	G	A	.	PASS	.	inte	NM_	dis	.		
ACa07	16	14743630	rs7800288	A	G	.	PASS	BFAR	intro	NM_	.	.		
ACa07	16	15104125	rs5668064	G	A	.	PASS	PDXDC	intro	NM_	.	.		
ACa07	16	15474800	rs7507758	G	A	.	PASS	.	inte	NM_	dis	.		
ACa07	16	15733133	.	C	T	.	PASS	KIAA04	UTF	NM_	NM	.		
ACa07	16	15787881	.	C	T	.	PASS	NDE1	intro	NM_	.	.		
ACa07	16	16065490	.	G	A	.	PASS	ABCC1	intro	NM_	.	.		
ACa07	16	16459820	.	T	C	.	PASS	.	inte	NR_	dis	.		
ACa07	16	18887501	rs1784261	A	T	.	PASS	SMG1	exo	NM_	.	missens		

ACa07	16	19194909	rs7575963	G	A	.	PASS	SYT17	exo	NM_	.	missens
ACa07	16	20352562	rs7647908	G	A	.	PASS	UMOD	exo	NM_	.	synonyr
ACa07	16	21208820	.	C	A	.	PASS	ZP2	exo	NM_	.	unknow
ACa07	16	22268950	rs1829590	C	T	.	PASS	EEF2K	intrc	NM_	.	.
ACa07	16	23494295	.	G	A	.	PASS	GGA2	exo	NM_	.	missens
ACa07	16	23703312	.	C	T	.	PASS	ERN2	exo	NM_	.	missens
ACa07	16	25180293	rs9722593	C	T	.	PASS	LCMT1	intrc	NM_	.	.
ACa07	16	26922879	.	G	T	.	PASS	.	inte	NM_	dis	.
ACa07	16	27549562	.	G	A	.	PASS	GTF3C	exo	NM_	.	missens
ACa07	16	27732801	.	C	T	.	PASS	KIAA05	intrc	NM_	.	.
ACa07	16	27761369	rs1412530	G	A	.	PASS	KIAA05	exo	NM_	.	missens
ACa07	16	28167926	rs1909424	A	T	.	PASS	XPO6	intrc	NM_	.	.
ACa07	16	28659481	.	G	A	.	PASS	.	inte	NM_	dis	.
ACa07	16	28846949	rs7491998	C	T	.	PASS	ATXN2L	exo	NM_	.	missens
ACa07	16	28857656	.	T	C	.	PASS	TUFM	UTF	NM_	NM	.
ACa07	16	28912047	rs1820846	G	A	.	PASS	ATP2A1	exo	NM_	.	missens
ACa07	16	29140948	.	G	T	.	PASS	.	inte	NR_	dis	.
ACa07	16	29910712	.	G	T	.	PASS	.	ups	NM_	.	.
ACa07	16	29918302	rs7813295	C	T	.	PASS	KCTD1	exo	NM_	.	missens
ACa07	16	31122275	rs9659529	G	A	.	PASS	BCKDK	intrc	NM_	.	.
ACa07	16	31160723	.	T	C	.	PASS	PRSS3	intrc	NM_	.	.
ACa07	16	31374102	.	G	T	.	PASS	ITGAX	intrc	NM_	.	.
ACa07	16	31895845	.	C	A	.	PASS	ZNF267	exo	NM_	.	synonyr
ACa07	16	32487025	rs7477221	C	T	.	PASS	.	inte	NR_	dis	.
ACa07	16	33106981	rs460256	A	G	.	PASS	.	inte	NR_	dis	.
ACa07	16	33379986	.	A	G	.	PASS	.	inte	NR_	dis	.
ACa07	16	33393640	.	A	G	.	PASS	.	inte	NR_	dis	.
ACa07	16	33406888	rs3744640	G	C	.	PASS	.	inte	NR_	dis	.
ACa07	16	33516019	.	C	A	.	PASS	RNU6-7	ncR	NR_	.	.
ACa07	16	33738959	.	A	C	.	PASS	.	inte	NR_	dis	.
ACa07	16	34303978	.	C	G	.	PASS	.	inte	NR_	dis	.
ACa07	16	46386672	rs1333894	A	C	.	PASS	.	inte	NON	dis	.
ACa07	16	46387429	rs2885478	C	T	.	PASS	.	inte	NON	dis	.
ACa07	16	46391232	rs4249087	T	G	.	PASS	.	inte	NON	dis	.
ACa07	16	46394845	rs5591122	C	T	.	PASS	.	inte	NON	dis	.
ACa07	16	46395166	rs4246405	G	C	.	PASS	.	inte	NON	dis	.
ACa07	16	46395251	rs4292070	G	C	.	PASS	.	inte	NON	dis	.
ACa07	16	46399518	rs8799648	A	G	.	PASS	.	inte	NON	dis	.
ACa07	16	46401894	rs8799977	A	C	.	PASS	.	inte	NON	dis	.
ACa07	16	46405469	rs4291933	A	G	.	PASS	.	inte	NON	dis	.
ACa07	16	46406401	rs4375682	A	G	.	PASS	.	inte	NON	dis	.
ACa07	16	46406551	rs7200104	T	G	.	PASS	.	inte	NON	dis	.
ACa07	16	46406785	rs7188643	A	G	.	PASS	.	inte	NON	dis	.
ACa07	16	46406828	rs9328642	G	C	.	PASS	.	inte	NON	dis	.
ACa07	16	46406854	rs7205231	T	A	.	PASS	.	inte	NON	dis	.
ACa07	16	46407028	rs4445923	A	T	.	PASS	.	inte	NON	dis	.
ACa07	16	46407752	rs2887500	T	G	.	PASS	.	inte	NON	dis	.
ACa07	16	46408061	rs2888543	G	T	.	PASS	.	inte	NON	dis	.
ACa07	16	46420322	rs7664258	G	A	.	PASS	.	inte	NON	dis	.
ACa07	16	46421742	rs4249076	T	C	.	PASS	.	inte	NON	dis	.
ACa07	16	46428673	.	T	C	.	PASS	.	inte	NON	dis	.
ACa07	16	46781878	rs3683497	G	A	.	PASS	MYLK3	exo	NM_	.	synonyr
ACa07	16	47537111	.	C	A	.	PASS	PHKB	intrc	NM_	.	.
ACa07	16	56660201	.	C	A	.	PASS	MT1E	intrc	NM_	.	.

ACa07	16	56864520	.	G	T	.	PASS	NUP93	exo	NM_	.	missens
ACa07	16	56871737	.	A	C	.	PASS	NUP93	intrc	NM_	.	.
ACa07	16	57003671	.	G	A	.	PASS	CETP	intrc	NM_	.	.
ACa07	16	57180194	rs1471990	C	T	.	PASS	CPNE2	exo	NM_	.	synonyr
ACa07	16	57760660	.	T	C	.	PASS	DRC7	intrc	NM_	.	.
ACa07	16	58019162	.	G	T	.	PASS	TEPP	exo	NM_	.	missens
ACa07	16	58313479	.	A	G	.	PASS	CCDC1	intrc	NM_	.	.
ACa07	16	64597212	rs1499903	A	G	.	PASS	.	inte	NON	dis	.
ACa07	16	67218882	.	C	T	.	PASS	EXOC3l	exo	NM_	.	missens
ACa07	16	67223187	rs1054074	A	G	.	PASS	EXOC3l	intrc	NM_	.	.
ACa07	16	67236430	rs7715372	C	T	.	PASS	ELMO3	exo	NM_	.	synonyr
ACa07	16	67298343	rs5618721	G	A	.	PASS	SLC9A5	exo	NM_	.	missens
ACa07	16	67324912	.	G	T	.	PASS	KCTD1	intrc	NM_	.	.
ACa07	16	67404993	rs7545456	C	T	.	PASS	LRRC3	exo	NM_	.	missens
ACa07	16	67432256	rs3696380	G	A	.	PASS	ZDHHC	intrc	NM_	.	.
ACa07	16	67654782	.	C	A	.	PASS	CTCF	intrc	NM_	.	.
ACa07	16	67964672	rs7584347	C	T	.	PASS	CTRL	exo	NM_	.	missens
ACa07	16	67979591	.	C	A	.	PASS	SLC12A	intrc	NM_	.	.
ACa07	16	68056074	.	G	A	.	PASS	DDX28	exo	NM_	.	synonyr
ACa07	16	69418494	.	C	T	.	PASS	TERF2	exo	NM_	.	missens
ACa07	16	70363143	rs8670174	A	G	.	PASS	LOC10	ncR	NR_	.	.
ACa07	16	71464866	rs9278778	C	T	.	PASS	.	inte	NM_	dis	.
ACa07	16	71465222	rs1485518	C	T	.	PASS	.	inte	NM_	dis	.
ACa07	16	71976819	rs3776990	G	A	.	PASS	PKD1L3	exo	NM_	.	unknow
ACa07	16	74446298	rs7502787	G	T	.	PASS	CLEC18	intrc	NM_	.	.
ACa07	16	74709365	.	C	T	.	PASS	MLKL	intrc	NM_	.	.
ACa07	16	75300714	.	C	T	.	PASS	BCAR1	intrc	NM_	.	.
ACa07	16	79781104	rs1469198	A	G	.	PASS	LOC102	ncR	NR_	.	.
ACa07	16	81157276	rs7733922	G	A	.	PASS	PKD1L2	exo	NM_	.	unknow
ACa07	16	81931297	.	T	C	.	PASS	PLCG2	intrc	NM_	.	.
ACa07	16	83949765	rs7483034	C	T	.	PASS	MLYCD	UTF	NM_	NM	.
ACa07	16	84193350	rs1407517	G	A	.	PASS	DNAAF	exo	NM_	.	missens
ACa07	16	84213850	rs7647118	G	A	.	PASS	TAF1C	exo	NM_	.	missens
ACa07	16	85690052	rs9336865	C	T	.	PASS	GSE1	exo	NM_	.	missens
ACa07	16	86601246	.	T	A	.	PASS	FOXC2	exo	NM_	.	missens
ACa07	16	87367554	rs3681305	C	T	.	PASS	FBXO3	exo	NM_	.	synonyr
ACa07	16	87638598	rs9665470	T	C	.	PASS	JPH3	intrc	NM_	.	.
ACa07	16	88500698	rs5606502	G	A	.	PASS	ZNF469	exo	NM_	.	missens
ACa07	16	88721965	rs9646181	C	T	.	PASS	MVD	intrc	NM_	.	.
ACa07	16	88945815	.	C	T	.	PASS	CBFA2T	exo	NM_	.	missens
ACa07	16	89758073	.	C	A	.	PASS	CDK10	intrc	NM_	.	.
ACa07	16	89799935	rs2017315	G	A	.	PASS	ZNF276	exo	NM_	.	synonyr
ACa07	16	89805198	rs7530482	G	A	.	PASS	ZNF276	UTF	NM_	NM	.
ACa07	17	1375434	rs7627705	T	C	.	PASS	MYO1C	exo	NM_	.	missens
ACa07	17	1562751	.	C	T	.	PASS	PRPF8	exo	NM_	.	missens
ACa07	17	1610814	.	A	G	.	PASS	TLCD2	UTF	NM_	NM	.
ACa07	17	1961007	.	T	C	.	PASS	HIC1	exo	NM_	.	synonyr
ACa07	17	1988923	.	C	A	.	PASS	SMG6	intrc	NM_	.	.
ACa07	17	2203721	.	G	T	.	PASS	SMG6	exo	NM_	.	missens
ACa07	17	2290401	rs7803486	C	T	.	PASS	MNT	exo	NM_	.	missens
ACa07	17	2323768	.	A	G	.	PASS	METTL	exo	NM_	.	synonyr
ACa07	17	3765488	.	T	C	.	PASS	CAMKK	UTF	NM_	NM	.
ACa07	17	4145515	.	A	C	.	PASS	ANKFY	intrc	NM_	.	.
ACa07	17	4192509	rs5506079	C	T	.	PASS	UBE2G	intrc	NM_	.	.

ACa07	17	4575748	rs3764321	C	T	.	PASS	PELP1	exo	NM_	.	synonyr
ACa07	17	4926810	rs2008227	G	A	.	PASS	KIF1C	exo	NM_	.	synonyr
ACa07	17	5085969	rs2013516	C	T	.	PASS	ZNF594	exo	NM_	.	missens
ACa07	17	5367081	.	C	T	.	PASS	DHX33	intrc	NM_	.	.
ACa07	17	6493860	.	T	C	.	PASS	KIAA07!	exo	NM_	.	missens
ACa07	17	6599019	.	C	T	.	PASS	SLC13A	intrc	NM_	.	.
ACa07	17	6980456	.	A	G	.	PASS	CLEC1(	intrc	NM_	.	.
ACa07	17	7226101	rs2000463	G	A	.	PASS	NEURL	exo	NM_	.	missens
ACa07	17	7401521	rs7455911	G	A	.	PASS	POLR2/	exo	NM_	.	missens
ACa07	17	8638873	rs1473707	G	A	.	PASS	CCDC4	exo	NM_	.	synonyr
ACa07	17	8732222	.	C	T	.	PASS	PIK3R6	exo	NM_	.	unknow
ACa07	17	9873083	.	C	T	.	PASS	GAS7	splic	NM_	NM	.
ACa07	17	11556040	rs7534759	A	G	.	PASS	DNAH9	intrc	NM_	.	.
ACa07	17	11835436	.	G	T	.	PASS	DNAH9	exo	NM_	.	stopgai
ACa07	17	14205156	.	C	T	.	PASS	HS3ST3	exo	NM_	.	synonyr
ACa07	17	15207385	rs7633797	C	T	.	PASS	TEKT3	exo	NM_	.	synonyr
ACa07	17	15638786	rs6207237	G	A	.	PASS	TBC1D2	intrc	NM_	.	.
ACa07	17	15938128	.	G	A	.	PASS	NCOR1	exo	NM_	.	synonyr
ACa07	17	15961112	.	T	C	.	PASS	NCOR1	intrc	NM_	.	.
ACa07	17	17030234	.	C	T	.	PASS	MPRIP	intrc	NM_	.	.
ACa07	17	17034866	.	G	A	.	PASS	MPRIP	intrc	NM_	.	.
ACa07	17	18058365	rs1157965	G	A	.	PASS	MYO15/	intrc	NM_	.	.
ACa07	17	18060466	.	A	G	.	PASS	MYO15/	splic	NM_	NM	.
ACa07	17	18286697	.	G	A	.	PASS	EVPLL	intrc	NM_	.	.
ACa07	17	20361613	.	C	A	.	PASS	LGALS!	exo	NM_	.	synonyr
ACa07	17	20370796	rs2869918	T	C	.	PASS	LGALS!	UTF	NM_	NM	.
ACa07	17	20916264	rs7529523	G	A	.	PASS	USP22	intrc	NM_	.	.
ACa07	17	21207962	.	G	A	.	PASS	MAP2K	intrc	NM_	.	.
ACa07	17	26084555	.	C	T	.	PASS	NOS2	intrc	NM_	.	.
ACa07	17	26816533	.	A	T	.	PASS	SLC13A	intrc	NM_	.	.
ACa07	17	26820830	.	G	T	.	PASS	SLC13A	intrc	NM_	.	.
ACa07	17	26850910	.	C	T	.	PASS	.	ups	NM_	.	.
ACa07	17	26932168	.	C	T	.	PASS	SPAG5-	ncR	NR_	.	.
ACa07	17	26940926	rs1051245	C	T	.	PASS	SPAG5-	ncR	NR_	.	.
ACa07	17	26962672	.	A	G	.	PASS	KIAA01(	intrc	NM_	.	.
ACa07	17	27308994	.	C	T	.	PASS	SEZ6	exo	NM_	.	missens
ACa07	17	27436897	.	C	T	.	PASS	MYO18/	exo	NM_	.	missens
ACa07	17	28557113	rs7968757	A	G	.	PASS	SLC6A4	intrc	NM_	.	.
ACa07	17	29645931	rs5324904	C	T	.	PASS	EVI2A	exo	NM_	.	missens
ACa07	17	29848139	.	G	T	.	PASS	RAB11F	intrc	NM_	.	.
ACa07	17	30192707	.	A	G	.	PASS	UTP6	intrc	NM_	.	.
ACa07	17	30211542	.	G	T	.	PASS	UTP6	intrc	NM_	.	.
ACa07	17	32905761	.	G	T	.	PASS	C17orf1	intrc	NM_	.	.
ACa07	17	33462347	.	G	A	.	PASS	NLE1	exo	NM_	.	missens
ACa07	17	33679688	rs7610872	C	T	.	PASS	SLFN11	exo	NM_	.	missens
ACa07	17	33818178	.	G	T	.	PASS	.	inte	NM_	dis	.
ACa07	17	34036160	.	C	A	.	PASS	AP2B1	intrc	NM_	.	.
ACa07	17	34797287	rs3702689	T	G	.	PASS	TBC1D3	UTF	NM_	NM	.
ACa07	17	36349473	rs3720623	T	C	.	PASS	.	ups	NM_	.	.
ACa07	17	36646848	.	A	G	.	PASS	ARHGA	intrc	NM_	.	.
ACa07	17	36895087	rs7545869	C	T	.	PASS	PCGF2	exo	NM_	.	missens
ACa07	17	37243776	.	G	T	.	PASS	PLXDC	intrc	NM_	.	.
ACa07	17	37351190	rs7807350	G	A	.	PASS	CACNB	exo	NM_	.	missens
ACa07	17	37359163	.	C	T	.	PASS	RPL19	intrc	NM_	.	.

ACa07	17	37371354	.	C	T	.	PASS	STAC2	intrc	NM_	.	.
ACa07	17	37881332	rs1057519	G	A	.	PASS	ERBB2	exoı	NM_	.	missens
ACa07	17	38711777	rs1996216	C	T	.	PASS	CCR7	exoı	NM_	.	synonyr
ACa07	17	39535402	rs1488746	C	T	.	PASS	KRT34	exoı	NM_	.	synonyr
ACa07	17	39959567	rs7824576	C	T	.	PASS	LEPREL	exoı	NM_	.	synonyr
ACa07	17	40094785	rs7827568	G	A	.	PASS	TTC25	intrc	NM_	.	.
ACa07	17	40107195	.	A	G	.	PASS	TTC25	intrc	NM_	.	.
ACa07	17	40477116	.	G	T	.	PASS	STAT3	intrc	NM_	.	.
ACa07	17	40485855	rs9065729	G	A	.	PASS	STAT3	intrc	NM_	.	.
ACa07	17	40491207	rs5769204	G	A	.	PASS	STAT3	intrc	NM_	.	.
ACa07	17	40715885	.	G	T	.	PASS	COASY	intrc	NM_	.	.
ACa07	17	40940950	rs1042659	C	T	.	PASS	WNK4	intrc	NM_	.	.
ACa07	17	41296281	rs9984123	C	T	.	PASS	NBR2	ncR	NR_	.	.
ACa07	17	41341298	.	A	G	.	PASS	NBR1	intrc	NM_	.	.
ACa07	17	41455004	.	G	A	.	PASS	LINC00!	ncR	NR_	.	.
ACa07	17	41893443	.	G	T	.	PASS	MPP3	exoı	NM_	.	synonyr
ACa07	17	41893978	.	C	A	.	PASS	MPP3	intrc	NM_	.	.
ACa07	17	41898415	rs7617924	G	A	.	PASS	MPP3	exoı	NM_	.	synonyr
ACa07	17	42745490	.	T	G	.	PASS	C17orf1	exoı	NM_	.	synonyr
ACa07	17	43004248	rs9135110	C	T	.	PASS	KIF18B	intrc	NM_	.	.
ACa07	17	43215415	.	G	T	.	PASS	ACBD4	intrc	NM_	.	.
ACa07	17	43322422	rs7805429	C	T	.	PASS	FMNL1	exoı	NM_	.	missens
ACa07	17	43531652	.	C	T	.	PASS	PLEKHI	intrc	NM_	.	.
ACa07	17	43585815	.	C	T	.	PASS	LRRC3	ncR	NR_	.	.
ACa07	17	44363438	.	G	A	.	PASS	.	inteı	NR_	dis	.
ACa07	17	44806355	rs1002396	G	A	.	PASS	NSF	intrc	NM_	.	.
ACa07	17	45128939	rs1056060	A	G	.	PASS	.	inteı	NM_	dis	.
ACa07	17	45664593	rs2644320	C	T	.	PASS	NPEPP:	splic	NM_	NM	.
ACa07	17	45886991	.	A	G	.	PASS	OSBPL	intrc	NM_	.	.
ACa07	17	47294019	rs3749157	G	A	.	PASS	ABI3	exoı	NM_	.	missens
ACa07	17	47376105	rs9379553	G	A	.	PASS	ZNF652	exoı	NM_	.	synonyr
ACa07	17	48051439	.	G	T	.	PASS	DLX4	UTF	NM_	NM	.
ACa07	17	48195592	rs7784106	C	T	.	PASS	SAMD1	exoı	NM_	.	missens
ACa07	17	48218827	.	C	A	.	PASS	PPP1R!	intrc	NM_	.	.
ACa07	17	48227017	.	G	A	.	PASS	PPP1R!	exoı	NM_	.	unknow
ACa07	17	48267799	rs1029268	C	T	.	PASS	COL1A	intrc	NM_	.	.
ACa07	17	48432555	.	G	T	.	PASS	XYLT2	intrc	NM_	.	.
ACa07	17	48602100	rs3894278	A	T	.	PASS	MYCBP	intrc	NM_	.	.
ACa07	17	53344988	.	C	T	.	PASS	HLF	intrc	NM_	.	.
ACa07	17	56770941	.	G	A	.	PASS	RAD51(	intrc	NM_	.	.
ACa07	17	58733825	rs3677870	A	G	.	PASS	PPM1D	intrc	NM_	.	.
ACa07	17	61994969	.	G	T	.	PASS	GH1	intrc	NM_	.	.
ACa07	17	62045564	rs7637250	C	T	.	PASS	SCN4A	exoı	NM_	.	synonyr
ACa07	17	62796399	.	C	A	.	PASS	PLEKHI	ncR	NR_	.	.
ACa07	17	65141955	.	C	T	.	PASS	HELZ	exoı	NM_	.	synonyr
ACa07	17	67133640	.	T	C	.	PASS	ABCA6	exoı	NM_	.	missens
ACa07	17	72206149	.	C	T	.	PASS	MGC16	ncR	NR_	.	.
ACa07	17	72352246	.	C	T	.	PASS	.	dow	NM_	.	.
ACa07	17	72859354	rs5610655	C	T	.	PASS	FDXR	exoı	NM_	.	missens
ACa07	17	73486703	rs1018051	G	C	.	PASS	KIAA01!	intrc	NM_	.	.
ACa07	17	73501559	rs3777274	G	A	.	PASS	CASKIN	intrc	NM_	.	.
ACa07	17	73503825	.	C	T	.	PASS	CASKIN	intrc	NM_	.	.
ACa07	17	73611950	.	G	T	.	PASS	MYO15I	ncR	NR_	.	.
ACa07	17	73624543	.	G	T	.	PASS	RECQL	intrc	NM_	.	.

ACa07	17	73625597	.	G	C	.	PASS	RECQL	intrc	NM_	.	.
ACa07	17	74527640	rs3697050	C	T	.	PASS	CYGB	exoı	NM_	.	missens
ACa07	17	74559126	.	A	G	.	PASS	SNHG1	ncR	NR_	.	.
ACa07	17	74899523	.	C	A	.	PASS	MGAT5	intrc	NM_	.	.
ACa07	17	75191119	rs7670774	G	A	.	PASS	SEC14L	intrc	NM_	.	.
ACa07	17	76454501	.	C	A	.	PASS	DNAH1	intrc	NM_	.	.
ACa07	17	76499024	rs7521502	C	T	.	PASS	DNAH1	exoı	NM_	.	missens
ACa07	17	77100166	.	G	A	.	PASS	RBFOX	exoı	NM_	.	missens
ACa07	17	77808213	.	G	A	.	PASS	CBX4	exoı	NM_	.	missens
ACa07	17	78032382	rs5354610	G	A	.	PASS	CCDC4	exoı	NM_	.	missens
ACa07	17	78156663	rs7737952	G	A	.	PASS	CARD1	intrc	NM_	.	.
ACa07	17	78179400	rs3676544	C	T	.	PASS	CARD1	exoı	NM_	.	synonyr
ACa07	17	78324327	.	G	A	.	PASS	RNF213	intrc	NM_	.	.
ACa07	17	78937995	.	G	A	.	PASS	RPTOR	intrc	NM_	.	.
ACa07	17	79027499	.	G	A	.	PASS	BAIAP2	exoı	NM_	.	missens
ACa07	17	79180303	.	C	A	.	PASS	CEP131	intrc	NM_	.	.
ACa07	17	79279159	rs3678800	C	T	.	PASS	LINC00	ncR	NR_	.	.
ACa07	17	79390607	.	G	A	.	PASS	BAHCC	intrc	NM_	.	.
ACa07	17	79632417	.	C	T	.	PASS	OXLD1	exoı	NM_	.	synonyr
ACa07	17	79916537	.	C	A	.	PASS	NOTUM	intrc	NM_	.	.
ACa07	17	79974405	.	T	C	.	PASS	ASPSCI	splic	NM_	NM	.
ACa07	17	80041663	.	G	A	.	PASS	FASN	exoı	NM_	.	missens
ACa07	17	80047200	rs7594547	G	A	.	PASS	FASN	exoı	NM_	.	missens
ACa07	17	80085557	.	C	T	.	PASS	CCDC5	intrc	NM_	.	.
ACa07	17	80575231	rs1452537	G	A	.	PASS	WDR45	exoı	NM_	.	synonyr
ACa07	17	80789716	.	G	A	.	PASS	ZNF750	exoı	NM_	.	synonyr
ACa07	18	909466	.	G	A	.	PASS	ADCYA	exoı	NM_	.	missens
ACa07	18	2578185	.	A	G	.	PASS	NDC80	intrc	NM_	.	.
ACa07	18	12353187	.	A	G	.	PASS	AFG3L2	intrc	NM_	.	.
ACa07	18	13024458	.	G	A	.	PASS	CEP192	intrc	NM_	.	.
ACa07	18	13885013	.	A	T	.	PASS	MC2R	exoı	NM_	.	missens
ACa07	18	14890007	.	C	A	.	PASS	.	inteı	NM_	dis	.
ACa07	18	15004074	.	G	A	.	PASS	.	inteı	NR_	dis	.
ACa07	18	19093947	.	G	T	.	PASS	GREB1I	exoı	NM_	.	missens
ACa07	18	19115949	rs1390032	C	T	.	PASS	ESCO1	intrc	NM_	.	.
ACa07	18	20948481	.	G	T	.	PASS	TMEM2	intrc	NM_	.	.
ACa07	18	21402267	rs5453420	C	T	.	PASS	LAMA3	exoı	NM_	.	missens
ACa07	18	29847791	rs7793765	C	T	.	PASS	GAREM	UTF	NM_	NM	.
ACa07	18	33719560	.	A	G	.	PASS	ELP2	exoı	NM_	.	synonyr
ACa07	18	43495988	.	A	G	.	PASS	EPG5	exoı	NM_	.	missens
ACa07	18	44656708	rs7789211	G	A	.	PASS	HDHD2	intrc	NM_	.	.
ACa07	18	44656742	.	A	G	.	PASS	HDHD2	intrc	NM_	.	.
ACa07	18	47421391	rs7710491	G	A	.	PASS	MYO5B	exoı	NM_	.	missens
ACa07	18	47778202	.	C	T	.	PASS	CFAP53	intrc	NM_	.	.
ACa07	18	57677257	.	G	A	.	PASS	.	inteı	NM_	dis	.
ACa07	18	60225951	.	G	T	.	PASS	ZCCHC	exoı	NM_	.	missens
ACa07	18	61234143	rs1417032	C	T	.	PASS	SERPIN	exoı	NM_	.	stopgaiı
ACa07	18	61309094	.	T	C	.	PASS	SERPIN	exoı	NM_	.	missens
ACa07	18	67871371	.	A	G	.	PASS	RTTN	exoı	NM_	.	synonyr
ACa07	18	72346490	.	C	A	.	PASS	ZNF407	exoı	NM_	.	missens
ACa07	18	77067295	.	G	T	.	PASS	ATP9B	intrc	NM_	.	.
ACa07	19	312348	rs1020066	C	T	.	PASS	MIER2	intrc	NM_	.	.
ACa07	19	572542	.	C	T	.	PASS	BSG	UTF	NM_	NM	.
ACa07	19	619791	rs3749392	C	T	.	PASS	POLRM	intrc	NM_	.	.

ACa07	19	629937	rs7789396	G	A	.	PASS	POLRM	exo	NM_	.	missens
ACa07	19	736057	.	G	A	.	PASS	PALM	exo	NM_	.	missens
ACa07	19	805209	rs3777310	C	T	.	PASS	PTBP1	intro	NM_	.	.
ACa07	19	1012501	.	G	T	.	PASS	TMEM2	exo	NM_	.	missens
ACa07	19	1079821	rs9456811	C	T	.	PASS	HMHA1	exo	NM_	.	synonyr
ACa07	19	1091690	.	C	T	.	PASS	POLR2I	intro	NM_	.	.
ACa07	19	1105690	rs7620114	G	A	.	PASS	GPX4	exo	NM_	.	unknow
ACa07	19	1109709	rs2022226	C	T	.	PASS	SBNO2	exo	NM_	.	synonyr
ACa07	19	1112702	.	C	T	.	PASS	SBNO2	intro	NM_	.	.
ACa07	19	1122606	rs7799382	G	A	.	PASS	SBNO2	intro	NM_	.	.
ACa07	19	1149369	.	G	A	.	PASS	SBNO2	exo	NM_	.	missens
ACa07	19	1255409	.	C	A	.	PASS	MIDN	intro	NM_	.	.
ACa07	19	1271445	rs3753640	C	T	.	PASS	CIRBP	exo	NM_	.	missens
ACa07	19	1407859	.	G	A	.	PASS	DAZAP	intro	NM_	.	.
ACa07	19	1434018	.	G	A	.	PASS	DAZAP	intro	NM_	.	.
ACa07	19	1531834	rs1007918	C	T	.	PASS	PLK5	exo	NM_	.	synonyr
ACa07	19	2211189	rs3694032	G	A	.	PASS	DOT1L	exo	NM_	.	synonyr
ACa07	19	2341270	rs7522031	G	A	.	PASS	SPPL2E	intro	NM_	.	.
ACa07	19	2757710	.	G	T	.	PASS	SGTA	exo	NM_	.	missens
ACa07	19	2767039	.	C	A	.	PASS	SGTA	intro	NM_	.	.
ACa07	19	3179096	rs3763485	C	T	.	PASS	S1PR4	exo	NM_	.	synonyr
ACa07	19	3457098	rs7773785	C	T	.	PASS	NFIC	intro	NM_	.	.
ACa07	19	3816988	.	G	A	.	PASS	ZFR2	intro	NM_	.	.
ACa07	19	4210478	.	C	T	.	PASS	ANKRD	intro	NM_	.	.
ACa07	19	4217198	rs7591374	G	A	.	PASS	ANKRD	exo	NM_	.	missens
ACa07	19	4362368	.	G	A	.	PASS	SH3GL	exo	NM_	.	stopgai
ACa07	19	4538218	rs1503658	G	A	.	PASS	LRG1	exo	NM_	.	missens
ACa07	19	4544276	.	G	A	.	PASS	SEMA6I	exo	NM_	.	synonyr
ACa07	19	5039983	.	T	C	.	PASS	KDM4B	exo	NM_	.	missens
ACa07	19	5641967	.	C	T	.	PASS	SAFB	intro	NM_	.	.
ACa07	19	5785310	rs1018214	G	A	.	PASS	DUS3L	intro	NM_	.	.
ACa07	19	6477127	rs7632840	G	A	.	PASS	DENND	exo	NM_	.	unknow
ACa07	19	6826394	.	T	C	.	PASS	VAV1	intro	NM_	.	.
ACa07	19	7527157	rs1390681	C	T	.	PASS	ARHGE	exo	NM_	.	missens
ACa07	19	7607845	rs3770694	C	T	.	PASS	PNPLA	intro	NM_	.	.
ACa07	19	7687484	rs3745730	G	A	.	PASS	XAB2	exo	NM_	.	missens
ACa07	19	7688095	rs7516930	C	T	.	PASS	XAB2	exo	NM_	.	synonyr
ACa07	19	7704641	rs1417170	G	A	.	PASS	STXBP	exo	NM_	.	missens
ACa07	19	7806726	.	G	T	.	PASS	CD209	UTF	NM_	NM	.
ACa07	19	7808878	.	C	A	.	PASS	CD209	intro	NM_	.	.
ACa07	19	7920858	rs7595385	C	T	.	PASS	EVI5L	intro	NM_	.	.
ACa07	19	8201121	rs3527749	C	T	.	PASS	FBN3	exo	NM_	.	missens
ACa07	19	8367325	rs7615602	C	T	.	PASS	CD320	UTF	NM_	NM	.
ACa07	19	8503622	rs5308100	T	G	.	PASS	2-Mar	UTF	NM_	NM	.
ACa07	19	8808742	.	C	T	.	PASS	ACTL9	exo	NM_	.	missens
ACa07	19	8893064	rs3694972	C	T	.	PASS	.	inte	NM_	dis	.
ACa07	19	8893178	rs3751712	C	G	.	PASS	.	inte	NM_	dis	.
ACa07	19	9014701	rs5686926	G	A	.	PASS	MUC16	exo	NM_	.	synonyr
ACa07	19	9063844	rs7801357	C	T	.	PASS	MUC16	exo	NM_	.	missens
ACa07	19	9064124	.	C	T	.	PASS	MUC16	exo	NM_	.	synonyr
ACa07	19	9922558	.	A	G	.	PASS	FBXL12	intro	NM_	.	.
ACa07	19	9949144	rs7564749	G	A	.	PASS	PIN1	exo	NM_	.	missens
ACa07	19	9965347	rs7814402	G	A	.	PASS	OLFM2	exo	NM_	.	missens
ACa07	19	10201938	.	C	T	.	PASS	C19orf6	exo	NM_	.	missens

ACa07	19	10218498	.	G	A	.	PASS	PPAN-F	exo	NM_	.	missens
ACa07	19	10225883	.	G	A	.	PASS	P2RY11	UTF	NM_	NM	.
ACa07	19	10394233	.	G	T	.	PASS	ICAM1	exo	NM_	.	missens
ACa07	19	10405808	.	G	A	.	PASS	ICAM5	intro	NM_	.	.
ACa07	19	10475409	rs1383503	G	A	.	PASS	TYK2	exo	NM_	.	synonyr
ACa07	19	10577703	rs2009930	G	A	.	PASS	PDE4A	exo	NM_	.	synonyr
ACa07	19	10662385	.	A	G	.	PASS	ATG4D	intro	NM_	.	.
ACa07	19	10676669	.	C	T	.	PASS	KRI1	exo	NM_	.	missens
ACa07	19	11224018	rs7634492	C	T	.	PASS	LDLR	exo	NM_	.	synonyr
ACa07	19	11240383	rs3737274	G	A	.	PASS	LDLR	intro	NM_	.	.
ACa07	19	11348936	rs3721772	C	T	.	PASS	DOCK6	exo	NM_	.	missens
ACa07	19	11541352	.	A	G	.	PASS	CCDC1	intro	NM_	.	.
ACa07	19	11660011	rs5385135	A	T	.	PASS	CNN1	intro	NM_	.	.
ACa07	19	12766435	.	G	T	.	PASS	MAN2B	intro	NM_	.	.
ACa07	19	13084339	rs3710318	G	A	.	PASS	DAND5	exo	NM_	.	missens
ACa07	19	14076450	.	G	A	.	PASS	RFX1	exo	NM_	.	missens
ACa07	19	14208252	.	A	C	.	PASS	PRKAC	exo	NM_	.	missens
ACa07	19	15540664	.	C	G	.	PASS	WIZ	intro	NM_	.	.
ACa07	19	15839060	.	C	T	.	PASS	OR10H	exo	NM_	.	synonyr
ACa07	19	16437736	.	G	A	.	PASS	KLF2	exo	NM_	.	missens
ACa07	19	16495865	rs1015670	G	A	.	PASS	EPS15L	intro	NM_	.	.
ACa07	19	17108172	.	C	T	.	PASS	CPAMD	intro	NM_	.	.
ACa07	19	17278658	rs1138422	G	A	.	PASS	MYO9B	intro	NM_	.	.
ACa07	19	17338512	.	T	C	.	PASS	OCEL1	intro	NM_	.	.
ACa07	19	17366376	rs9290443	G	A	.	PASS	USHBP	exo	NM_	.	missens
ACa07	19	17452078	rs7698270	G	A	.	PASS	GTPBP	exo	NM_	.	synonyr
ACa07	19	17767088	rs1043222	C	T	.	PASS	UNC13	exo	NM_	.	missens
ACa07	19	17783446	.	T	C	.	PASS	UNC13	intro	NM_	.	.
ACa07	19	18258237	.	G	T	.	PASS	MAST3	intro	NM_	.	.
ACa07	19	18333024	.	C	T	.	PASS	PDE4C	exo	NM_	.	missens
ACa07	19	18967718	rs3719294	C	T	.	PASS	UPF1	exo	NM_	.	synonyr
ACa07	19	19452210	rs2012753	C	T	.	PASS	MAU2	exo	NM_	.	synonyr
ACa07	19	20229551	.	C	A	.	PASS	ZNF90	exo	NM_	.	synonyr
ACa07	19	21838755	.	T	C	.	PASS	.	inte	NM_	dis	.
ACa07	19	22836286	.	T	C	.	PASS	ZNF492	intro	NM_	.	.
ACa07	19	30164554	.	C	T	.	PASS	PLEKHI	intro	NM_	.	.
ACa07	19	31767776	rs7540203	C	T	.	PASS	TSHZ3	exo	NM_	.	missens
ACa07	19	34811007	rs3754283	C	T	.	PASS	KIAA03	exo	NM_	.	missens
ACa07	19	34868527	.	C	G	.	PASS	GPI	intro	NM_	.	.
ACa07	19	35836331	.	A	C	.	PASS	CD22	intro	NM_	.	.
ACa07	19	36124878	rs7656023	G	A	.	PASS	RBM42	exo	NM_	.	missens
ACa07	19	36581331	.	G	A	.	PASS	WDR62	intro	NM_	.	.
ACa07	19	36727209	.	T	C	.	PASS	ZNF146	UTF	NM_	NM	.
ACa07	19	37037627	rs5581240	C	T	.	PASS	ZNF529	UTF	NM_	NM	.
ACa07	19	37780303	.	C	T	.	PASS	.	inte	NR_	dis	.
ACa07	19	37784554	rs4806419	C	A	.	PASS	.	inte	NR_	dis	.
ACa07	19	37785301	rs7125437	C	G	.	PASS	.	inte	NR_	dis	.
ACa07	19	37785451	.	C	T	.	PASS	.	inte	NR_	dis	.
ACa07	19	38375409	rs7728313	G	A	.	PASS	.	dow	NM_	.	.
ACa07	19	38684347	.	G	T	.	PASS	SIPA1L	exo	NM_	.	unknow
ACa07	19	38861397	rs1476199	C	T	.	PASS	CATSPI	exo	NM_	.	missens
ACa07	19	38904308	.	C	T	.	PASS	RASGR	intro	NM_	.	.
ACa07	19	39003154	rs3756003	G	A	.	PASS	RYR1	intro	NM_	.	.
ACa07	19	39051696	rs5371432	A	G	.	PASS	RYR1	intro	NM_	.	.

ACa07	19	39098907	.	G	A	.	PASS	MAP4K:	intrc	NM_	.	.
ACa07	19	39329428	.	C	A	.	PASS	HNRNP	intrc	NM_	.	.
ACa07	19	39381353	rs5721273	A	G	.	PASS	SIRT2	intrc	NM_	.	.
ACa07	19	39739143	rs8917290	G	A	.	PASS	IFNL4	exoı	NM_	.	unknow
ACa07	19	40025388	.	T	C	.	PASS	.	inteı	NM_	dis	.
ACa07	19	40221984	rs7564368	G	A	.	PASS	CLC	UTF	NM_	NM	.
ACa07	19	40316434	rs7738579	G	A	.	PASS	DYRK1I	exoı	NM_	.	unknow
ACa07	19	40376264	.	G	A	.	PASS	FCGBP	intrc	NM_	.	.
ACa07	19	41083493	rs7469641	G	A	.	PASS	SHKBP:	exoı	NM_	.	missen:
ACa07	19	41083673	.	C	T	.	PASS	SHKBP:	intrc	NM_	.	.
ACa07	19	41099064	.	G	A	.	PASS	.	ups1	NM_	.	.
ACa07	19	41119079	.	C	T	.	PASS	LTBP4	exoı	NM_	.	unknow
ACa07	19	41119905	.	C	T	.	PASS	LTBP4	exoı	NM_	.	unknow
ACa07	19	41193028	.	C	A	.	PASS	NUMBL	intrc	NM_	.	.
ACa07	19	41255546	rs1045293	G	C	.	PASS	C19orf5	exoı	NM_	.	missen:
ACa07	19	41808836	.	G	T	.	PASS	HNRNP	exoı	NM_	.	unknow
ACa07	19	41854288	.	G	T	.	PASS	TGFB1	exoı	NM_	.	missen:
ACa07	19	42398329	.	C	T	.	PASS	ARHGE	exoı	NM_	.	missen:
ACa07	19	42880770	.	C	A	.	PASS	MEGF8	exoı	NM_	.	missen:
ACa07	19	43097765	.	C	A	.	PASS	CEACA	exoı	NM_	.	stopgaiı
ACa07	19	43244381	.	T	C	.	PASS	PSG3	intrc	NM_	.	.
ACa07	19	43290727	.	C	T	.	PASS	.	inteı	NM_	dis	.
ACa07	19	44096856	.	G	A	.	PASS	IRGQ	exoı	NM_	.	synonyı
ACa07	19	44492772	.	T	G	.	PASS	ZNF155	intrc	NM_	.	.
ACa07	19	44892038	.	G	T	.	PASS	ZNF285	exoı	NM_	.	missen:
ACa07	19	44932620	rs7737032	C	T	.	PASS	ZNF229	exoı	NM_	.	unknow
ACa07	19	45121644	rs9075637	C	T	.	PASS	IGSF23	intrc	NM_	.	.
ACa07	19	45183512	.	C	T	.	PASS	CEACA	intrc	NM_	.	.
ACa07	19	45207659	rs5632226	C	T	.	PASS	CEACA	intrc	NM_	.	.
ACa07	19	45254635	.	C	T	.	PASS	BCL3	exoı	NM_	.	synonyı
ACa07	19	45315761	.	C	A	.	PASS	BCAM	exoı	NM_	.	missen:
ACa07	19	45395763	.	T	C	.	PASS	TOMM4	intrc	NM_	.	.
ACa07	19	46094263	.	G	T	.	PASS	GPR4	exoı	NM_	.	missen:
ACa07	19	47207795	rs1026645	C	T	.	PASS	PRKD2	exoı	NM_	.	missen:
ACa07	19	47236438	rs3697415	C	T	.	PASS	STRN4	exoı	NM_	.	missen:
ACa07	19	47940535	.	G	A	.	PASS	SLC8A2	intrc	NM_	.	.
ACa07	19	48325475	.	G	A	.	PASS	CRX	intrc	NM_	.	.
ACa07	19	48364154	.	C	T	.	PASS	.	inteı	NM_	dis	.
ACa07	19	48423499	rs6213000	C	G	.	PASS	.	inteı	NR_	dis	.
ACa07	19	49132107	.	G	A	.	PASS	SPHK2	exoı	NM_	.	missen:
ACa07	19	49355406	rs1012379	G	A	.	PASS	PLEKH/	intrc	NM_	.	.
ACa07	19	49601618	.	G	A	.	PASS	SNRNP	intrc	NM_	.	.
ACa07	19	49605507	.	A	G	.	PASS	SNRNP	intrc	NM_	.	.
ACa07	19	49867782	rs5626072	C	T	.	PASS	DKKL1	intrc	NM_	.	.
ACa07	19	49918087	.	C	T	.	PASS	CCDC1	intrc	NM_	.	.
ACa07	19	50046324	.	G	T	.	PASS	RCN3	intrc	NM_	.	.
ACa07	19	50208142	rs7508901	C	T	.	PASS	CPT1C	intrc	NM_	.	.
ACa07	19	50775820	rs3749564	G	A	.	PASS	MYH14	exoı	NM_	.	missen:
ACa07	19	50880852	rs2001122	G	A	.	PASS	NR1H2	UTF	NM_	NM	.
ACa07	19	50910470	.	G	A	.	PASS	POLD1	intrc	NM_	.	.
ACa07	19	50915997	rs5643823	T	G	.	PASS	POLD1	intrc	NM_	.	.
ACa07	19	50920365	rs3695357	C	T	.	PASS	POLD1	intrc	NM_	.	.
ACa07	19	50922148	.	G	A	.	PASS	.	ups1	NM_	.	.
ACa07	19	50944153	rs7738602	G	A	.	PASS	MYBPC	exoı	NM_	.	missen:

ACa07	19	50969451	.	G	A	.	PASS	MYBPC	splic	NM_	NM	.
ACa07	19	51273989	.	A	G	.	PASS	GPR32	exoı	NM_	.	synonyr
ACa07	19	53453763	.	T	C	.	PASS	ZNF816	exoı	NM_	.	missens
ACa07	19	53553611	.	A	G	.	PASS	ERVV-2	exoı	NM_	.	synonyr
ACa07	19	53770304	rs1420234	T	G	.	PASS	VN1R4	exoı	NM_	.	synonyr
ACa07	19	54257306	.	G	A	.	PASS	MIR527	ncR	NR_	.	.
ACa07	19	54483198	.	T	G	.	PASS	CACNG	exoı	NM_	.	missens
ACa07	19	54634642	.	T	C	.	PASS	PRPF31	intrc	NM_	.	.
ACa07	19	54660450	.	G	A	.	PASS	LENG1	intrc	NM_	.	.
ACa07	19	54745439	rs3704562	G	A	.	PASS	LILRA6,	intrc	NM_	.	.
ACa07	19	54941633	.	C	A	.	PASS	TTYH1	intrc	NM_	.	.
ACa07	19	55045145	rs7529171	G	A	.	PASS	KIR3DX	ncR	NR_	.	.
ACa07	19	55685191	.	G	T	.	PASS	SYT5	intrc	NM_	.	.
ACa07	19	55889387	rs3707427	C	T	.	PASS	TMEM1	exoı	NM_	.	missens
ACa07	19	56424219	rs2000514	G	A	.	PASS	NLRP13	exoı	NM_	.	missens
ACa07	19	57327563	rs7704358	C	T	.	PASS	PEG3	exoı	NM_	.	synonyr
ACa07	19	58011532	.	G	A	.	PASS	ZNF773	intrc	NM_	.	.
ACa07	19	58099851	.	G	A	.	PASS	ZIK1	intrc	NM_	.	.
ACa07	19	58499615	rs1474253	C	T	.	PASS	ZNF606	exoı	NM_	.	synonyr
ACa07	19	58571417	.	C	T	.	PASS	ZNF135	UTF	NM_	NM	.
ACa07	19	58858404	rs3733248	C	T	.	PASS	A1BG	intrc	NM_	.	.
ACa07	20	1903097	rs1021538	C	T	.	PASS	SIRPA	exoı	NM_	.	missens
ACa07	20	2413101	rs7627114	T	C	.	PASS	TGM6	intrc	NM_	.	.
ACa07	20	2635601	.	G	A	.	PASS	NOP56	intrc	NM_	.	.
ACa07	20	3245239	rs8672082	C	T	.	PASS	C20orf1	intrc	NM_	.	.
ACa07	20	3642095	.	C	T	.	PASS	GFRA4	intrc	NM_	.	.
ACa07	20	3651693	rs7638624	G	A	.	PASS	ADAM33	exoı	NM_	.	stopgaiı
ACa07	20	4705860	.	C	T	.	PASS	PRND	UTF	NM_	NM	.
ACa07	20	11009666	rs1032256	G	A	.	PASS	.	inteı	NR_	dis	.
ACa07	20	16385687	.	T	C	.	PASS	KIF16B	intrc	NM_	.	.
ACa07	20	18139741	.	A	G	.	PASS	CSRP2I	exoı	NM_	.	missens
ACa07	20	18362090	.	T	C	.	PASS	LINC00	ncR	NR_	.	.
ACa07	20	23669411	rs1509219	G	A	.	PASS	CST4	exoı	NM_	.	missens
ACa07	20	25289014	.	T	C	.	PASS	ABHD12	intrc	NM_	.	.
ACa07	20	25745477	.	C	T	.	PASS	FAM182	ncR	NR_	.	.
ACa07	20	29612803	.	C	T	.	PASS	FRG1B	ncR	NR_	.	.
ACa07	20	29612810	.	G	A	.	PASS	FRG1B	ncR	NR_	.	.
ACa07	20	31043958	rs1845841	C	T	.	PASS	NOL4L	exoı	NM_	.	missens
ACa07	20	31654608	.	C	A	.	PASS	BPIFB3	intrc	NM_	.	.
ACa07	20	32354938	.	G	T	.	PASS	ZNF341	intrc	NM_	.	.
ACa07	20	33247126	.	G	A	.	PASS	PIGU	intrc	NM_	.	.
ACa07	20	33657227	rs7460506	A	T	.	PASS	TRPC4	intrc	NM_	.	.
ACa07	20	34099484	.	A	C	.	PASS	CEP250	UTF	NM_	NM	.
ACa07	20	34263091	rs1480471	C	T	.	PASS	NFS1	exoı	NM_	.	missens
ACa07	20	34329931	rs5431099	T	C	.	PASS	RBM39	UTF	NM_	NM	.
ACa07	20	34806760	.	G	A	.	PASS	EPB41L	intrc	NM_	.	.
ACa07	20	35064896	.	G	A	.	PASS	DLGAP	intrc	NM_	.	.
ACa07	20	35374485	rs8798536	G	A	.	PASS	NDRG3	UTF	NM_	NM	.
ACa07	20	35736905	.	G	T	.	PASS	MROH8	intrc	NM_	.	.
ACa07	20	36718044	rs9848856	C	T	.	PASS	RPRD1I	intrc	NM_	.	.
ACa07	20	37591014	rs9460302	G	A	.	PASS	DHX35	exoı	NM_	.	missens
ACa07	20	37667223	rs3711048	G	A	.	PASS	DHX35	UTF	NM_	NM	.
ACa07	20	39713106	.	G	A	.	PASS	TOP1	exoı	NM_	.	missens
ACa07	20	39794835	.	C	T	.	PASS	PLCG1	exoı	NM_	.	missens

ACa07	20	41100948	.	G	A	.	PASS	PTPRT	exo	NM_	.	stopgai
ACa07	20	41101120	rs3761522	G	A	.	PASS	PTPRT	exo	NM_	.	synonyr
ACa07	20	43349104	.	T	C	.	PASS	WISP2	intrc	NM_	.	.
ACa07	20	43752926	.	G	T	.	PASS	WFDC1	intrc	NM_	.	.
ACa07	20	44666942	.	C	A	.	PASS	SLC12A	intrc	NM_	.	.
ACa07	20	45004077	.	G	T	.	PASS	ELMO2	intrc	NM_	.	.
ACa07	20	45212380	.	G	T	.	PASS	SLC13A	intrc	NM_	.	.
ACa07	20	45905430	.	C	T	.	PASS	ZMYND	exo	NM_	.	missens
ACa07	20	49621024	.	G	A	.	PASS	KCNG1	exo	NM_	.	missens
ACa07	20	50234189	.	C	T	.	PASS	ATP9A	intrc	NM_	.	.
ACa07	20	50721612	.	G	A	.	PASS	ZFP64	intrc	NM_	.	.
ACa07	20	52193647	rs8891302	C	T	.	PASS	ZNF217	exo	NM_	.	synonyr
ACa07	20	52454373	rs8669651	T	C	.	PASS	.	inte	NM_	dis	.
ACa07	20	57429095	.	G	A	.	PASS	GNAS	exo	NM_	.	missens
ACa07	20	57429831	.	C	T	.	PASS	GNAS	exo	NM_	.	missens
ACa07	20	58486765	.	G	A	.	PASS	SYCP2	intrc	NM_	.	.
ACa07	20	60705624	rs7702464	C	T	.	PASS	LSM14E	exo	NM_	.	missens
ACa07	20	61468410	rs7787907	C	T	.	PASS	COL9A	intrc	NM_	.	.
ACa07	20	61468733	rs1813756	G	A	.	PASS	COL9A	intrc	NM_	.	.
ACa07	20	61909505	rs1435421	G	A	.	PASS	ARFGA	exo	NM_	.	synonyr
ACa07	20	62153550	.	C	T	.	PASS	.	dow	NM_	.	.
ACa07	20	62174036	.	A	G	.	PASS	SRMS	intrc	NM_	.	.
ACa07	20	62493372	rs5462318	G	A	.	PASS	ABHD1	exo	NM_	.	missens
ACa07	21	9755905	rs7962225	A	G	.	PASS	.	inte	NON	dis	.
ACa07	21	9826731	.	C	T	.	PASS	.	dow	NR_	.	.
ACa07	21	10087796	.	G	A	.	PASS	.	inte	NR_	dis	.
ACa07	21	10139755	.	C	A	.	PASS	.	inte	NR_	dis	.
ACa07	21	10535504	rs8680295	G	A	.	PASS	.	inte	NR_	dis	.
ACa07	21	10535526	rs3711086	C	A	.	PASS	.	inte	NR_	dis	.
ACa07	21	10614100	rs3775054	C	T	.	PASS	.	inte	NR_	dis	.
ACa07	21	10969870	.	G	A	.	PASS	TPTE	intrc	NM_	.	.
ACa07	21	10970029	.	C	A	.	PASS	TPTE	exo	NM_	.	unknow
ACa07	21	11058391	rs5282430	G	C	.	PASS	BAGE5,	intrc	NM_	.	.
ACa07	21	11107313	.	C	T	.	PASS	.	inte	NM_	dis	.
ACa07	21	11116113	rs665796	T	C	.	PASS	.	inte	NM_	dis	.
ACa07	21	11116122	rs6582845	C	T	.	PASS	.	inte	NM_	dis	.
ACa07	21	11181417	rs915519	G	C	.	PASS	.	inte	NM_	dis	.
ACa07	21	14424309	.	C	T	.	PASS	ANKRD	ncR	NR_	.	.
ACa07	21	14721665	.	T	C	.	PASS	.	inte	NR_	dis	.
ACa07	21	22643653	.	A	G	.	PASS	NCAM2	intrc	NM_	.	.
ACa07	21	27083858	.	C	G	.	PASS	JAM2	intrc	NM_	.	.
ACa07	21	27348250	.	A	G	.	PASS	APP	intrc	NM_	.	.
ACa07	21	28792752	rs9172671	C	T	.	PASS	MIR500	ncR	NR_	.	.
ACa07	21	31720848	.	C	T	.	PASS	KRTAP	exo	NM_	.	missens
ACa07	21	33330766	.	A	G	.	PASS	HUNK	intrc	NM_	.	.
ACa07	21	33340425	.	G	T	.	PASS	HUNK	intrc	NM_	.	.
ACa07	21	34017987	rs1034062	C	T	.	PASS	SYNJ1	exo	NM_	.	synonyr
ACa07	21	34443159	.	C	T	.	PASS	OLIG1	exo	NM_	.	synonyr
ACa07	21	34713283	rs7587939	A	G	.	PASS	IFNAR1	intrc	NM_	.	.
ACa07	21	35190531	.	C	T	.	PASS	ITSN1	intrc	NM_	.	.
ACa07	21	37443165	.	T	G	.	PASS	LOC10C	ncR	NR_	.	.
ACa07	21	38367015	.	C	T	.	PASS	.	inte	NM_	dis	.
ACa07	21	38470307	rs5585229	C	T	.	PASS	TTC3	intrc	NM_	.	.
ACa07	21	40555352	rs7591033	G	A	.	PASS	PSMG1	UTF	NM_	NM	.

ACa07	21	40627700	rs7585989	C	T	.	PASS	BRWD1	exoı	NM_	.	missens
ACa07	21	41165725	.	C	A	.	PASS	IGSF5	intrc	NM_	.	.
ACa07	21	41414479	.	C	A	.	PASS	DSCAM	exoı	NM_	.	synonyr
ACa07	21	41416012	.	T	C	.	PASS	DSCAM	exoı	NM_	.	synonyr
ACa07	21	41452118	.	G	A	.	PASS	DSCAM	exoı	NM_	.	missens
ACa07	21	41710096	.	T	C	.	PASS	DSCAM	exoı	NM_	.	missens
ACa07	21	42716350	.	C	T	.	PASS	FAM3B	intrc	NM_	.	.
ACa07	21	43230856	.	T	C	.	PASS	PRDM1	intrc	NM_	.	.
ACa07	21	43325674	rs9464977	G	A	.	PASS	C2CD2	intrc	NM_	.	.
ACa07	21	43411857	.	G	A	.	PASS	ZBTB21	exoı	NM_	.	missens
ACa07	21	43959597	.	T	C	.	PASS	SLC37A	intrc	NM_	.	.
ACa07	21	44514959	rs1014361	C	A	.	PASS	U2AF1	intrc	NM_	.	.
ACa07	21	45220931	rs7806447	G	A	.	PASS	RRP1	intrc	NM_	.	.
ACa07	21	45547974	rs5662670	C	T	.	PASS	PWP2	exoı	NM_	.	missens
ACa07	21	45856886	rs9852755	C	T	.	PASS	TRPM2	intrc	NM_	.	.
ACa07	21	46320365	rs7565454	G	A	.	PASS	ITGB2	exoı	NM_	.	missens
ACa07	21	46935755	rs7582548	C	T	.	PASS	SLC19A	exoı	NM_	.	synonyr
ACa07	21	47409750	rs3771590	C	T	.	PASS	COL6A1	intrc	NM_	.	.
ACa07	21	47572651	.	G	A	.	PASS	FTCD	intrc	NM_	.	.
ACa07	21	47722647	.	G	A	.	PASS	C21orf5	intrc	NM_	.	.
ACa07	21	47817386	rs7517144	G	A	.	PASS	PCNT	exoı	NM_	.	missens
ACa07	21	47965145	.	G	A	.	PASS	DIP2A	exoı	NM_	.	missens
ACa07	22	16229834	rs2845112	A	G	.	PASS	.	inteı	NR_	dis	.
ACa07	22	16256800	rs8790424	C	G	.	PASS	POTEH	intrc	NM_	.	.
ACa07	22	16287159	.	A	G	.	PASS	POTEH	intrc	NM_	.	.
ACa07	22	16347994	rs1248470	C	A	.	PASS	.	inteı	NM_	dis	.
ACa07	22	17445828	.	G	A	.	PASS	GAB4	intrc	NM_	.	.
ACa07	22	19028635	rs5555078	G	A	.	PASS	DGCR2	exoı	NM_	.	synonyr
ACa07	22	19109530	.	T	A	.	PASS	DGCR2	intrc	NM_	.	.
ACa07	22	20709550	rs1899538	G	A	.	PASS	.	inteı	NM_	dis	.
ACa07	22	20711723	.	C	A	.	PASS	.	inteı	NM_	dis	.
ACa07	22	20795769	.	G	A	.	PASS	.	dow	NM_	.	.
ACa07	22	21066804	rs3714923	G	A	.	PASS	PI4KA	exoı	NM_	.	synonyr
ACa07	22	21800936	rs7724279	G	A	.	PASS	HIC2	exoı	NM_	.	synonyr
ACa07	22	22681696	.	G	A	.	PASS	.	inteı	NR_	dis	.
ACa07	22	23264876	rs3777336	G	A	.	PASS	.	inteı	NM_	dis	.
ACa07	22	24145443	rs3681295	C	T	.	PASS	SMARC	intrc	NM_	.	.
ACa07	22	24584287	.	G	T	.	PASS	SUSD2	exoı	NM_	.	missens
ACa07	22	24837284	.	C	T	.	PASS	ADORA	exoı	NM_	.	missens
ACa07	22	24911361	.	G	T	.	PASS	UPB1	intrc	NM_	.	.
ACa07	22	25019124	rs7723316	G	A	.	PASS	GGT1	exoı	NM_	.	missens
ACa07	22	25041478	.	C	T	.	PASS	POM121	ncR	NR_	.	.
ACa07	22	27282917	.	C	T	.	PASS	.	inteı	NR_	dis	.
ACa07	22	29628179	rs1925641	C	T	.	PASS	EMID1	intrc	NM_	.	.
ACa07	22	30885889	.	T	C	.	PASS	SEC14L	UTF	NM_	NM	.
ACa07	22	31287084	.	G	A	.	PASS	OSBP2	intrc	NM_	.	.
ACa07	22	31489439	rs5710568	G	A	.	PASS	SMTN	intrc	NM_	.	.
ACa07	22	31685923	rs1015431	C	T	.	PASS	PIK3IP1	intrc	NM_	.	.
ACa07	22	32003934	.	G	A	.	PASS	SFI1	exoı	NM_	.	synonyr
ACa07	22	32532909	.	T	C	.	PASS	.	inteı	NR_	dis	.
ACa07	22	32795806	.	T	C	.	PASS	RTCB	intrc	NM_	.	.
ACa07	22	33672966	.	G	A	.	PASS	LARGE	intrc	NM_	.	.
ACa07	22	35812391	rs1379196	C	T	.	PASS	MCM5	exoı	NM_	.	synonyr
ACa07	22	36678570	.	C	T	.	PASS	MYH9	UTF	NM_	NM	.

ACa07	22	37326821	rs3682304	G	A	.	PASS	CSF2R	exon	NM_	.	missens
ACa07	22	37400169	rs5367410	C	T	.	PASS	TEX33	intro	NM_	.	.
ACa07	22	38511635	.	G	A	.	PASS	PLA2G	exon	NM_	.	stopgai
ACa07	22	38610282	.	C	A	.	PASS	MAFF	intro	NM_	.	.
ACa07	22	38622889	rs7523833	A	G	.	PASS	TMEM1	intro	NM_	.	.
ACa07	22	38823720	.	G	A	.	PASS	KCNJ4	exon	NM_	.	missens
ACa07	22	38824095	.	G	A	.	PASS	KCNJ4	exon	NM_	.	missens
ACa07	22	39126505	rs5714435	C	T	.	PASS	GTPBP	intro	NM_	.	.
ACa07	22	39448270	.	C	T	.	PASS	APOBE	exon	NM_	.	synony
ACa07	22	40053731	rs3719126	C	T	.	PASS	CACNA	intro	NM_	.	.
ACa07	22	40708930	.	C	T	.	PASS	TNRC6	exon	NM_	.	missens
ACa07	22	41075625	rs2016059	G	A	.	PASS	MCHR1	exon	NM_	.	missens
ACa07	22	41926891	.	C	T	.	PASS	POLR3	exon	NM_	.	missens
ACa07	22	41928740	rs1838128	C	T	.	PASS	POLR3	exon	NM_	.	missens
ACa07	22	42335257	.	C	A	.	PASS	CENPM	intro	NM_	.	.
ACa07	22	42910976	.	G	A	.	PASS	RRP7A	intro	NM_	.	.
ACa07	22	44178231	rs1916812	C	T	.	PASS	EFCAB	intro	NM_	.	.
ACa07	22	44341889	.	G	A	.	PASS	PNPLA	intro	NM_	.	.
ACa07	22	45597019	rs5584935	C	T	.	PASS	KIAA09	intro	NM_	.	.
ACa07	22	45724214	rs1305515	C	T	.	PASS	FAM11	intro	NM_	.	.
ACa07	22	45809290	.	C	T	.	PASS	SMC1B	intro	NM_	.	.
ACa07	22	45931221	.	A	G	.	PASS	FBLN1	splic	NM_	NM	.
ACa07	22	46130150	.	T	C	.	PASS	ATXN1	intro	NM_	.	.
ACa07	22	46657939	.	G	A	.	PASS	PKDRE	exon	NM_	.	synony
ACa07	22	46760606	.	G	A	.	PASS	CELSR	exon	NM_	.	missens
ACa07	22	50436009	.	C	A	.	PASS	IL17RE	intro	NM_	.	.
ACa07	22	50553197	.	C	A	.	PASS	MOV10	intro	NM_	.	.
ACa07	22	50591485	rs5595729	G	A	.	PASS	MOV10	exon	NM_	.	synony
ACa07	22	50615116	.	G	A	.	PASS	PANX2	intro	NM_	.	.
ACa07	22	50615684	.	G	A	.	PASS	PANX2	exon	NM_	.	synony
ACa07	22	50724661	rs3705843	C	T	.	PASS	PLXNB	exon	NM_	.	synony
ACa07	22	50876480	.	G	A	.	PASS	PPP6R	intro	NM_	.	.
ACa07	22	50961677	.	C	T	.	PASS	NCAPH	exon	NM_	.	missens
ACa07	22	51048083	rs9805300	C	T	.	PASS	MAPK8	intro	NM_	.	.
ACa07	X	1743038	.	A	G	.	PASS	ASMT	intro	NM_	.	.
ACa07	X	2659015	.	T	G	.	PASS	CD99	UTF	NM_	NM	.
ACa07	X	2856352	.	A	C	.	PASS	ARSE	intro	NM_	.	.
ACa07	X	5810797	.	T	C	.	PASS	NLGN4	UTF	NM_	NM	.
ACa07	X	9935829	.	T	C	.	PASS	LOC10	exon	NM_	.	synony
ACa07	X	12737015	.	G	T	.	PASS	FRMPD	intro	NM_	.	.
ACa07	X	13607566	.	C	T	.	PASS	EGFL6	intro	NM_	.	.
ACa07	X	17750225	.	C	T	.	PASS	NHS	exon	NM_	.	missens
ACa07	X	21393119	.	G	A	.	PASS	CNKSR	intro	NM_	.	.
ACa07	X	23876770	rs3730298	C	T	.	PASS	APOO	exon	NM_	.	missens
ACa07	X	25031167	.	C	T	.	PASS	ARX	exon	NM_	.	synony
ACa07	X	27650692	.	T	C	.	PASS	.	inte	NR_	dis	.
ACa07	X	27830696	.	C	T	.	PASS	MAGEB	intro	NM_	.	.
ACa07	X	29414654	.	T	A	.	PASS	IL1RAP	intro	NM_	.	.
ACa07	X	30268937	.	C	A	.	PASS	MAGEB	exon	NM_	.	synony
ACa07	X	30671533	.	G	A	.	PASS	GK	UTF	NM_	NM	.
ACa07	X	30714907	.	T	C	.	PASS	GK	intro	NM_	.	.
ACa07	X	32659702	.	A	C	.	PASS	DMD	intro	NM_	.	.
ACa07	X	35077818	.	A	C	.	PASS	.	inte	NM_	dis	.
ACa07	X	38037524	.	A	C	.	PASS	SRPX	intro	NM_	.	.

ACa07	X	40506680	.	G	A	.	PASS	CXorf38	exoı	NM_	.	synonyr
ACa07	X	44169469	.	G	A	.	PASS	EFHC2	intrc	NM_	.	.
ACa07	X	47663162	.	C	T	.	PASS	.	inteı	NR_	dis	.
ACa07	X	48047195	.	G	T	.	PASS	SSX5	intrc	NM_	.	.
ACa07	X	48760101	rs7825839	C	T	.	PASS	PQBP1	intrc	NM_	.	.
ACa07	X	48781133	rs3679315	G	A	.	PASS	OTUD5	exoı	NM_	.	missens
ACa07	X	48935556	rs1871040	C	T	.	PASS	WDR45	exoı	NM_	.	missens
ACa07	X	49021149	.	G	T	.	PASS	MAGIX	intrc	NM_	.	.
ACa07	X	49034293	.	G	A	.	PASS	PRICKL	intrc	NM_	.	.
ACa07	X	49061747	rs7822715	C	T	.	PASS	CACNA	exoı	NM_	.	synonyr
ACa07	X	49962195	.	C	T	.	PASS	AKAP4	exoı	NM_	.	synonyr
ACa07	X	51638837	.	G	A	.	PASS	MAGED	exoı	NM_	.	missens
ACa07	X	52632804	.	G	A	.	PASS	.	inteı	NM_	dis	.
ACa07	X	53222424	rs7825371	C	T	.	PASS	KDM5C	exoı	NM_	.	missens
ACa07	X	53270791	.	G	T	.	PASS	IQSEC2	intrc	NM_	.	.
ACa07	X	53430792	.	A	C	.	PASS	SMC1A	exoı	NM_	.	missens
ACa07	X	53575150	.	G	T	.	PASS	HUWE1	exoı	NM_	.	missens
ACa07	X	54186095	.	T	C	.	PASS	FAM12(	intrc	NM_	.	.
ACa07	X	54495310	.	C	T	.	PASS	FGD1	splic	NM_	NM	.
ACa07	X	54580986	.	G	A	.	PASS	GNL3L	intrc	NM_	.	.
ACa07	X	54800884	rs9617788	A	G	.	PASS	ITIH6	intrc	NM_	.	.
ACa07	X	61685261	.	T	G	.	PASS	.	inteı	NON	dis	.
ACa07	X	61687523	.	G	C	.	PASS	.	inteı	NON	dis	.
ACa07	X	61691656	.	A	G	.	PASS	.	inteı	NON	dis	.
ACa07	X	61691693	rs7124105	C	A	.	PASS	.	inteı	NON	dis	.
ACa07	X	61710635	.	A	G	.	PASS	.	inteı	NON	dis	.
ACa07	X	61712534	rs3765099	T	G	.	PASS	.	inteı	NON	dis	.
ACa07	X	61717326	.	T	C	.	PASS	.	inteı	NON	dis	.
ACa07	X	61717473	.	T	G	.	PASS	.	inteı	NON	dis	.
ACa07	X	61717952	.	A	C	.	PASS	.	inteı	NON	dis	.
ACa07	X	61719733	.	G	A	.	PASS	.	inteı	NON	dis	.
ACa07	X	63653978	.	C	A	.	PASS	.	inteı	NM_	dis	.
ACa07	X	67944001	.	C	A	.	PASS	STARD	intrc	NM_	.	.
ACa07	X	67991777	rs7687527	G	A	.	PASS	.	inteı	NM_	dis	.
ACa07	X	69497114	.	G	C	.	PASS	ARR3	intrc	NM_	.	.
ACa07	X	70345997	rs7507300	C	T	.	PASS	MED12	exoı	NM_	.	missens
ACa07	X	70443876	rs8632249	C	T	.	PASS	GJB1	exoı	NM_	.	missens
ACa07	X	70613114	rs7685308	G	A	.	PASS	BCYRN	ncR	NR_	.	.
ACa07	X	70628064	.	C	A	.	PASS	BCYRN	ncR	NR_	.	.
ACa07	X	71792574	.	G	A	.	PASS	HDAC8	exoı	NM_	.	missens
ACa07	X	71932644	.	C	T	.	PASS	PHKA1	exoı	NM_	.	missens
ACa07	X	72674385	.	A	G	.	PASS	CDX4	exoı	NM_	.	synonyr
ACa07	X	72989914	.	G	A	.	PASS	.	inteı	NM_	dis	.
ACa07	X	73619356	.	G	T	.	PASS	.	inteı	NM_	dis	.
ACa07	X	73963938	.	C	A	.	PASS	KIAA20	exoı	NM_	.	stopgaiı
ACa07	X	79816293	.	A	G	.	PASS	.	inteı	NM_	dis	.
ACa07	X	80064940	.	C	T	.	PASS	BRWD3	exoı	NM_	.	missens
ACa07	X	91131885	.	G	A	.	PASS	PCDH1	exoı	NM_	.	missens
ACa07	X	91134064	.	C	A	.	PASS	PCDH1	exoı	NM_	.	missens
ACa07	X	91714781	.	C	T	.	PASS	PCDH1	intrc	NM_	.	.
ACa07	X	91751081	.	T	C	.	PASS	PCDH1	intrc	NM_	.	.
ACa07	X	95788407	rs9672698	G	A	.	PASS	.	inteı	NR_	dis	.
ACa07	X	1E+08	.	G	A	.	PASS	NOX1	exoı	NM_	.	missens
ACa07	X	1.01E+08	.	C	A	.	PASS	NXF5	exoı	NM_	.	missens

ACa07	X	1.01E+08	.	C	T	.	PASS	TCEAL2	intro	NM_	.	.
ACa07	X	1.04E+08	rs1507941	C	T	.	PASS	IL1RAP	intro	NM_	.	.
ACa07	X	1.09E+08	.	G	A	.	PASS	ACSL4	UTF	NM_	NM	.
ACa07	X	1.18E+08	.	C	A	.	PASS	LINC01	ncR	NR_	.	.
ACa07	X	1.18E+08	rs8664003	T	C	.	PASS	.	inte	NR_	dis	.
ACa07	X	1.19E+08	.	C	T	.	PASS	SLC25A	ncR	NR_	.	.
ACa07	X	1.2E+08	.	C	T	.	PASS	CUL4B	intro	NM_	.	.
ACa07	X	1.24E+08	.	G	A	.	PASS	.	inte	NM_	dis	.
ACa07	X	1.27E+08	rs1387255	C	T	.	PASS	ACTRT	exo	NM_	.	missens
ACa07	X	1.29E+08	.	A	G	.	PASS	ZNF280	intro	NM_	.	.
ACa07	X	1.33E+08	.	C	A	.	PASS	GPC3	intro	NM_	.	.
ACa07	X	1.35E+08	.	C	T	.	PASS	SLC9A6	intro	NM_	.	.
ACa07	X	1.35E+08	.	G	T	.	PASS	GPR112	intro	NM_	.	.
ACa07	X	1.39E+08	.	A	G	.	PASS	ATP11C	exo	NM_	.	synonyr
ACa07	X	1.4E+08	.	G	A	.	PASS	LDOC1,	inte	NM_	dis	.
ACa07	X	1.49E+08	.	C	T	.	PASS	MAGEA	exo	NM_	.	synonyr
ACa07	X	1.49E+08	rs7823749	C	T	.	PASS	CXorf40	UTF	NM_	NM	.
ACa07	X	1.51E+08	.	G	A	.	PASS	CNGA2	exo	NM_	.	missens
ACa07	X	1.53E+08	rs3712627	C	T	.	PASS	.	inte	NM_	dis	.
ACa07	X	1.53E+08	.	G	T	.	PASS	BGN	exo	NM_	.	missens
ACa07	X	1.53E+08	.	G	A	.	PASS	ATP2B3	intro	NM_	.	.
ACa07	X	1.53E+08	rs7825278	G	A	.	PASS	SSR4	intro	NM_	.	.
ACa07	X	1.53E+08	.	G	A	.	PASS	L1CAM	intro	NM_	.	.
ACa07	X	1.53E+08	.	G	A	.	PASS	L1CAM	intro	NM_	.	.
ACa07	X	1.53E+08	.	G	A	.	PASS	RENBP	splic	NM_	NM	.
ACa07	X	1.53E+08	.	C	T	.	PASS	HCFC1	intro	NM_	.	.
ACa07	X	1.54E+08	.	G	A	.	PASS	SLC10A	exo	NM_	.	synonyr
ACa07	X	1.54E+08	rs7819487	C	T	.	PASS	G6PD	exo	NM_	.	missens
ACa07	X	1.54E+08	.	C	T	.	PASS	DKC1	intro	NM_	.	.
ACa07	X	1.54E+08	.	G	A	.	PASS	MPP1	UTF	NM_	NM	.
ACa07	X	1.54E+08	.	G	A	.	PASS	SMIM9	UTF	NM_	NM	.
ACa07	X	1.55E+08	.	A	G	.	PASS	TMLHE	exo	NM_	.	missens
ACa07	Y	13323970	.	C	T	.	PASS	.	inte	NON	dis	.
ACa07	MT	9459	rs8791767	C	T	.	PASS	.	inte	NON	dis	.
ACa07	MT	11376	.	A	T	.	PASS	.	inte	NON	dis	.
ACa07	MT	15312	.	T	C	.	PASS	.	inte	NON	dis	.
ACaP01	1	16778593	rs5750552	C	T	.	PASS	NECAP	intro	NM_	.	.
ACaP01	1	16899877	rs4101081	C	T	.	PASS	NBPF1	intro	NM_	.	.
ACaP01	1	16914515	rs7629862	A	C	.	PASS	NBPF1	intro	NM_	.	.
ACaP01	1	16960070	.	T	C	.	PASS	.	inte	NR_	dis	.
ACaP01	1	21324110	.	C	A	.	PASS	EIF4G3	exo	NM_	.	missens
ACaP01	1	1.43E+08	rs3772379	G	C	.	PASS	.	inte	NR_	dis	.
ACaP01	1	1.43E+08	rs3720400	G	T	.	PASS	.	inte	NR_	dis	.
ACaP01	1	1.45E+08	rs6181337	T	C	.	PASS	NBPF25	ncR	NR_	.	.
ACaP01	1	2.48E+08	.	G	T	.	PASS	.	ups	NM_	.	.
ACaP01	2	85596991	.	G	T	.	PASS	ELMOD	intro	NM_	.	.
ACaP01	2	91692533	rs1649505	G	C	.	PASS	.	inte	NON	dis	.
ACaP01	3	97311535	rs7933313	G	A	.	PASS	EPHA6	exo	NM_	.	synonyr
ACaP01	3	1.3E+08	rs7480655	C	T	.	PASS	COL6A6	exo	NM_	.	synonyr
ACaP01	3	1.33E+08	.	C	A	.	PASS	TOPBP	exo	NM_	.	missens
ACaP01	4	9232841	.	C	T	.	PASS	.	inte	NM_	dis	.
ACaP01	5	734072	rs671688	A	G	.	PASS	.	inte	NM_	dis	.
ACaP01	5	34179550	rs8681576	G	T	.	PASS	.	inte	NR_	dis	.
ACaP01	5	1.78E+08	.	T	C	.	PASS	CLK4	exo	NM_	.	synonyr

ACaP01	6	1.49E+08	.	A	G	.	PASS	SASH1	intrc	NM_	.	.
ACaP01	7	38503768	.	T	C	.	PASS	AMPH	intrc	NM_	.	.
ACaP01	7	44060096	.	C	T	.	PASS	.	inte	NR_	dis	.
ACaP01	7	1.16E+08	.	C	T	.	PASS	TFEC	splic	NM_	NM	.
ACaP01	7	1.5E+08	rs1471823	G	A	.	PASS	GIMAP5	exon	NM_	.	missens
ACaP01	8	21840110	rs7496607	G	A	.	PASS	XPO7	intrc	NM_	.	.
ACaP01	8	22168690	.	C	T	.	PASS	PIWIL2	exon	NM_	.	synonyr
ACaP01	9	69449944	rs1482733	G	T	.	PASS	.	inte	NM_	dis	.
ACaP01	9	1.31E+08	.	G	T	.	PASS	WDR34	intrc	NM_	.	.
ACaP01	10	47207813	rs2020143	T	C	.	PASS	AGAP9	exon	NM_	.	missens
ACaP01	10	1E+08	.	A	C	.	PASS	HPSE2	exon	NM_	.	missens
ACaP01	11	46797908	rs7731324	G	A	.	PASS	CKAP5	exon	NM_	.	missens
ACaP01	11	57157532	.	A	T	.	PASS	PRG2	intrc	NM_	.	.
ACaP01	12	49433687	rs7734351	G	A	.	PASS	KMT2D	exon	NM_	.	synonyr
ACaP01	12	52642506	rs7769669	C	T	.	PASS	KRT7	exon	NM_	.	missens
ACaP01	14	1.06E+08	.	G	A	.	PASS	JAG2	exon	NM_	.	synonyr
ACaP01	14	1.06E+08	rs7157981	C	T	.	PASS	.	inte	NR_	dis	.
ACaP01	15	20740075	rs2019940	T	G	.	PASS	GOLGA	exon	NM_	.	missens
ACaP01	15	20740088	rs3731461	A	G	.	PASS	GOLGA	exon	NM_	.	synonyr
ACaP01	15	25416142	.	C	A	.	PASS	.	dow	NR_	.	.
ACaP01	15	30771144	.	A	G	.	PASS	.	inte	NM_	dis	.
ACaP01	15	69706957	.	C	A	.	PASS	KIF23	intrc	NM_	.	.
ACaP01	16	27457419	.	C	T	.	PASS	IL21R	intrc	NM_	.	.
ACaP01	17	5328662	rs7781206	T	C	.	PASS	RPAIN	intrc	NM_	.	.
ACaP01	17	36333536	rs3725436	A	C	.	PASS	.	inte	NM_	dis	.
ACaP01	19	33490585	rs7861545	G	A	.	PASS	RHPN2	exon	NM_	.	stopgai
ACaP01	19	40411685	.	C	T	.	PASS	FCGBP	exon	NM_	.	missens
ACaP01	19	41763678	.	C	G	.	PASS	AXL	intrc	NM_	.	.
ACaP01	20	35152507	.	A	T	.	PASS	RP5-97	ncR	NR_	.	.
ACaP01	21	11114515	rs9266067	A	G	.	PASS	.	inte	NM_	dis	.
ACaP01	X	32380995	rs7663121	G	A	.	PASS	DMD	exon	NM_	.	synonyr
ACaP01	X	70636506	rs3397828	A	G	.	PASS	BCYRN	ncR	NR_	.	.
ACaP02	1	16265634	.	G	A	.	PASS	SPEN	intrc	NM_	.	.
ACaP02	1	16931257	rs436293	C	T	.	PASS	NBPF1	intrc	NM_	.	.
ACaP02	1	32128301	.	G	T	.	PASS	COL16A	intrc	NM_	.	.
ACaP02	1	32670897	.	G	C	.	PASS	CCDC2	UTF	NM_	NM	.
ACaP02	1	1.01E+08	rs3721234	C	T	.	PASS	SASS6	intrc	NM_	.	.
ACaP02	1	1.09E+08	.	G	A	.	PASS	FNDC7	intrc	NM_	.	.
ACaP02	1	1.45E+08	rs2007574	G	C	.	PASS	NBPF20	intrc	NM_	.	.
ACaP02	1	1.45E+08	rs9424724	G	C	.	PASS	NBPF25	ncR	NR_	.	.
ACaP02	1	1.52E+08	.	T	G	.	PASS	.	inte	NM_	dis	.
ACaP02	1	1.56E+08	.	G	A	.	PASS	TMEM7	exon	NM_	.	synonyr
ACaP02	1	1.56E+08	.	G	C	.	PASS	C1orf85	exon	NM_	.	missens
ACaP02	1	1.59E+08	.	C	G	.	PASS	SPTA1	intrc	NM_	.	.
ACaP02	1	1.59E+08	.	G	C	.	PASS	DARC	UTF	NM_	NM	.
ACaP02	1	1.6E+08	.	C	T	.	PASS	DCAF8	exon	NM_	.	missens
ACaP02	1	1.62E+08	.	G	C	.	PASS	FCRLB	intrc	NM_	.	.
ACaP02	1	1.83E+08	.	C	T	.	PASS	SHCBP	exon	NM_	.	missens
ACaP02	1	1.97E+08	rs9662955	G	A	.	PASS	CFHR5	intrc	NM_	.	.
ACaP02	1	2.28E+08	rs7570105	G	A	.	PASS	WNT3A	exon	NM_	.	missens
ACaP02	1	2.33E+08	.	C	G	.	PASS	KIAA180	exon	NM_	.	synonyr
ACaP02	1	2.37E+08	.	G	A	.	PASS	MTR	intrc	NM_	.	.
ACaP02	1	2.41E+08	.	C	T	.	PASS	FMN2	UTF	NM_	NM	.
ACaP02	2	38925391	.	C	T	.	PASS	GALM	intrc	NM_	.	.

ACaP02	2	44053534	rs1420196	G	A	.	PASS	ABCG5	exoı	NM_	.	missens
ACaP02	2	1.5E+08	.	T	C	.	PASS	LYPD6E	intrc	NM_	.	.
ACaP02	2	1.6E+08	.	C	T	.	PASS	TANC1	exoı	NM_	.	missens
ACaP02	2	1.97E+08	.	C	G	.	PASS	SLC39A	exoı	NM_	.	missens
ACaP02	2	1.98E+08	rs1857895	C	T	.	PASS	HSPD1	intrc	NM_	.	.
ACaP02	2	2.08E+08	.	G	C	.	PASS	KLF7	intrc	NM_	.	.
ACaP02	2	2.25E+08	.	G	T	.	PASS	WDFY1	intrc	NM_	.	.
ACaP02	2	2.35E+08	.	G	C	.	PASS	MROH2	exoı	NM_	.	missens
ACaP02	2	2.42E+08	rs7784676	G	A	.	PASS	STK25	exoı	NM_	.	missens
ACaP02	3	33434142	.	G	C	.	PASS	UBP1	intrc	NM_	.	.
ACaP02	3	97652428	.	A	G	.	PASS	CRYBG	intrc	NM_	.	.
ACaP02	3	1.34E+08	.	G	T	.	PASS	CEP63	exoı	NM_	.	missens
ACaP02	3	1.34E+08	.	G	A	.	PASS	CEP63	exoı	NM_	.	missens
ACaP02	3	1.38E+08	.	G	C	.	PASS	ARMC8	intrc	NM_	.	.
ACaP02	3	1.68E+08	rs7462149	G	A	.	PASS	GOLIM4	exoı	NM_	.	stopgaiı
ACaP02	3	1.72E+08	.	G	C	.	PASS	TNFSF1	UTF	NM_	NM	.
ACaP02	3	1.79E+08	rs1048860	G	A	.	PASS	PIK3CA	exoı	NM_	.	missens
ACaP02	3	1.84E+08	.	A	C	.	PASS	YEATS2	exoı	NM_	.	missens
ACaP02	4	944346	.	C	T	.	PASS	TMEM1	intrc	NM_	.	.
ACaP02	4	95173994	.	G	A	.	PASS	SMARC	exoı	NM_	.	missens
ACaP02	4	1.09E+08	.	C	T	.	PASS	CYP2U1	intrc	NM_	.	.
ACaP02	4	1.4E+08	.	C	T	.	PASS	NAA15	exoı	NM_	.	missens
ACaP02	4	1.43E+08	.	C	G	.	PASS	INPP4B	exoı	NM_	.	missens
ACaP02	4	1.72E+08	.	C	T	.	PASS	.	dow	NR_	.	.
ACaP02	5	33969172	rs1876921	G	C	.	PASS	SLC45A	intrc	NM_	.	.
ACaP02	5	63665546	.	G	C	.	PASS	RNF180	exoı	NM_	.	missens
ACaP02	5	76029074	.	C	T	.	PASS	F2R	exoı	NM_	.	missens
ACaP02	5	1.34E+08	rs7956159	C	T	.	PASS	SEC24A	exoı	NM_	.	missens
ACaP02	5	1.57E+08	.	C	G	.	PASS	FAM71E	exoı	NM_	.	missens
ACaP02	5	1.63E+08	.	G	A	.	PASS	HMMR	exoı	NM_	.	synonyr
ACaP02	5	1.7E+08	.	G	A	.	PASS	KCNIP1	exoı	NM_	.	missens
ACaP02	6	2692903	.	C	A	.	PASS	MYLK4	intrc	NM_	.	.
ACaP02	6	10894173	.	A	G	.	PASS	SYCP2L	exoı	NM_	.	missens
ACaP02	6	26501806	.	G	A	.	PASS	BTN1A1	intrc	NM_	.	.
ACaP02	6	29911685	rs1787910	C	T	.	PASS	HLA-A	intrc	NM_	.	.
ACaP02	6	30231416	rs5364899	A	G	.	PASS	HLA-L	ncR	NR_	.	.
ACaP02	6	64694337	.	C	G	.	PASS	EYS	exoı	NM_	.	missens
ACaP02	6	65301337	.	C	T	.	PASS	EYS	exoı	NM_	.	missens
ACaP02	6	1.49E+08	.	C	T	.	PASS	.	inteı	NM_	dis	.
ACaP02	6	1.68E+08	.	C	G	.	PASS	KIF25	intrc	NM_	.	.
ACaP02	7	5347198	.	G	C	.	PASS	TNRC18	UTF	NM_	NM	.
ACaP02	7	63210111	.	C	A	.	PASS	.	inteı	NR_	dis	.
ACaP02	7	88956826	.	C	A	.	PASS	ZNF804	intrc	NM_	.	.
ACaP02	7	1.31E+08	.	C	T	.	PASS	.	dow	NR_	.	.
ACaP02	7	1.38E+08	rs3685111	G	C	.	PASS	CREB3I	intrc	NM_	.	.
ACaP02	8	29120432	.	G	A	.	PASS	KIF13B	intrc	NM_	.	.
ACaP02	8	69009261	.	A	T	.	PASS	PREX2	exoı	NM_	.	missens
ACaP02	8	1.22E+08	.	G	A	.	PASS	SNTB1	UTF	NM_	NM	.
ACaP02	8	1.45E+08	.	C	T	.	PASS	NAPRT	exoı	NM_	.	missens
ACaP02	8	1.45E+08	rs3743103	C	T	.	PASS	PLEC	exoı	NM_	.	missens
ACaP02	9	68415292	rs4928838	C	T	.	PASS	.	inteı	NM_	dis	.
ACaP02	9	89876064	.	G	A	.	PASS	.	inteı	NM_	dis	.
ACaP02	9	96398757	.	G	A	.	PASS	PHF2	exoı	NM_	.	synonyr
ACaP02	9	1.17E+08	.	C	T	.	PASS	AKNA	intrc	NM_	.	.

ACaP02	9	1.36E+08	.	T	A	.	PASS	DDX31	intrc	NM_	.	.
ACaP02	10	323462	.	C	T	.	PASS	DIP2C	exo	NM_	.	missens
ACaP02	10	3150111	.	G	A	.	PASS	PFKP	intrc	NM_	.	.
ACaP02	10	88988731	rs3128226	C	G	.	PASS	NUTM2	intrc	NM_	.	.
ACaP02	10	1.35E+08	.	G	C	.	PASS	KNDC1	intrc	NM_	.	.
ACaP02	11	4744416	.	C	T	.	PASS	.	inte	NM_	dis	.
ACaP02	11	19864378	.	C	G	.	PASS	NAV2	intrc	NM_	.	.
ACaP02	11	45948953	.	C	T	.	PASS	GYLTL1	exo	NM_	.	synonyr
ACaP02	11	61735932	.	G	A	.	PASS	.	ups	NM_	.	.
ACaP02	11	78399235	.	G	A	.	PASS	TENM4	exo	NM_	.	synonyr
ACaP02	11	1.17E+08	.	G	A	.	PASS	SIK3	intrc	NM_	.	.
ACaP02	11	1.23E+08	.	G	A	.	PASS	.	ups	NM_	.	.
ACaP02	12	68657	rs5382343	T	C	.	PASS	.	inte	NON	dis	.
ACaP02	12	14650790	.	A	G	.	PASS	ATF7IP	exo	NM_	.	missens
ACaP02	12	27641424	.	C	G	.	PASS	SMCO2	exo	NM_	.	missens
ACaP02	12	64838770	rs8658204	T	A	.	PASS	XPOT	intrc	NM_	.	.
ACaP02	12	80840657	.	C	T	.	PASS	PTPRQ	intrc	NM_	.	.
ACaP02	12	1.13E+08	.	G	A	.	PASS	NAA25	exo	NM_	.	synonyr
ACaP02	12	1.18E+08	rs7707492	G	A	.	PASS	NOS1	exo	NM_	.	missens
ACaP02	13	30353182	.	T	A	.	PASS	UBL3	intrc	NM_	.	.
ACaP02	13	32811934	rs3702294	G	A	.	PASS	FRY	exo	NM_	.	missens
ACaP02	13	39263609	rs4129275	C	T	.	PASS	FREM2	exo	NM_	.	missens
ACaP02	13	39264019	.	C	G	.	PASS	FREM2	exo	NM_	.	missens
ACaP02	13	61987109	.	C	T	.	PASS	PCDH2	exo	NM_	.	missens
ACaP02	13	78337257	.	C	G	.	PASS	SLAIN1	intrc	NM_	.	.
ACaP02	13	78337320	.	C	G	.	PASS	SLAIN1	exo	NM_	.	missens
ACaP02	13	1.03E+08	.	G	A	.	PASS	CCDC1	exo	NM_	.	missens
ACaP02	14	22265543	rs3699116	C	T	.	PASS	.	inte	NM_	dis	.
ACaP02	14	36240320	rs7660001	C	T	.	PASS	RALGA	ncR	NR_	.	.
ACaP02	14	93762356	.	C	T	.	PASS	BTBD7	exo	NM_	.	synonyr
ACaP02	14	95677219	.	G	A	.	PASS	CLMN	splic	NM_	NM	.
ACaP02	14	1.05E+08	rs1214345	C	T	.	PASS	AKT1	exo	NM_	.	missens
ACaP02	14	1.07E+08	.	G	A	.	PASS	.	inte	NR_	dis	.
ACaP02	15	20644362	rs4932010	G	A	.	PASS	HERC2	ncR	NR_	.	.
ACaP02	15	21036908	rs7494426	C	G	.	PASS	.	inte	NR_	dis	.
ACaP02	15	22016259	rs1079463	T	C	.	PASS	CXADR	ncR	NR_	.	.
ACaP02	15	22058425	rs1823548	G	C	.	PASS	LOC33	intrc	NM_	.	.
ACaP02	15	25313171	.	G	A	.	PASS	.	dow	NR_	.	.
ACaP02	15	40681418	rs1861770	C	T	.	PASS	KNSTR	intrc	NM_	.	.
ACaP02	15	44962225	rs9953164	G	A	.	PASS	PATL2	exo	NM_	.	missens
ACaP02	15	45839258	.	G	C	.	PASS	HMGN2	ncR	NR_	.	.
ACaP02	15	72461122	.	G	A	.	PASS	GRAMD	intrc	NM_	.	.
ACaP02	15	78501642	.	G	A	.	PASS	ACSBG	intrc	NM_	.	.
ACaP02	15	81410105	.	C	T	.	PASS	.	inte	NM_	dis	.
ACaP02	16	3081097	.	C	T	.	PASS	CCDC6	exo	NM_	.	missens
ACaP02	16	32214205	.	G	A	.	PASS	.	inte	NR_	dis	.
ACaP02	16	56485604	rs7759203	C	T	.	PASS	OGFOD	exo	NM_	.	missens
ACaP02	16	70802734	.	C	T	.	PASS	VAC14-	ncR	NR_	.	.
ACaP02	17	4803555	.	G	C	.	PASS	C17orf1	exo	NM_	.	missens
ACaP02	17	36486118	rs7810711	C	T	.	PASS	GPR17	exo	NM_	.	missens
ACaP02	17	42828228	rs1509595	G	A	.	PASS	DBF4B	exo	NM_	.	synonyr
ACaP02	17	46691740	.	G	A	.	PASS	HOXB8	exo	NM_	.	synonyr
ACaP02	17	76166892	rs3742496	C	T	.	PASS	SYNGR	intrc	NM_	.	.
ACaP02	17	78356929	rs5611679	C	T	.	PASS	LOC10	ncR	NR_	.	.

ACaP02	17	79917411	rs5502840	G	A	.	PASS	NOTUM	exoı	NM_	.	synonyr
ACaP02	18	14186389	rs1996954	C	T	.	PASS	ANKRD	ncR	NR_	.	.
ACaP02	18	56149006	.	G	C	.	PASS	ALPK2	UTF	NM_	NM	.
ACaP02	19	9376928	.	C	G	.	PASS	.	inteı	NM_	dis	.
ACaP02	19	18426987	.	G	A	.	PASS	LSM4	intrc	NM_	.	.
ACaP02	19	19389565	.	G	T	.	PASS	SUGP1	exoı	NM_	.	missens
ACaP02	19	19906197	.	C	T	.	PASS	ZNF506	exoı	NM_	.	missens
ACaP02	19	36135341	.	C	T	.	PASS	ETV2	intrc	NM_	.	.
ACaP02	19	37441599	.	C	G	.	PASS	ZNF568	exoı	NM_	.	stopgaiı
ACaP02	19	40724034	.	G	A	.	PASS	TTC9B	exoı	NM_	.	synonyr
ACaP02	19	43576796	.	G	C	.	PASS	PSG2	intrc	NM_	.	.
ACaP02	19	55556685	.	C	G	.	PASS	RDH13	intrc	NM_	.	.
ACaP02	20	1162284	.	G	C	.	PASS	TMEM7	intrc	NM_	.	.
ACaP02	20	2411210	.	G	A	.	PASS	TGM6	exoı	NM_	.	synonyr
ACaP02	20	5170828	rs1438597	G	A	.	PASS	CDS2	exoı	NM_	.	missens
ACaP02	20	30534404	.	G	C	.	PASS	PDRG1	intrc	NM_	.	.
ACaP02	20	36759941	.	T	C	.	PASS	TGM2	intrc	NM_	.	.
ACaP02	21	11026667	rs1170118	T	C	.	PASS	BAGE4,	intrc	NM_	.	.
ACaP02	21	17214771	.	C	G	.	PASS	USP25	exoı	NM_	.	stopgaiı
ACaP02	22	19959033	.	G	C	.	PASS	ARVCF	intrc	NM_	.	.
ACaP02	22	31090253	.	C	T	.	PASS	OSBP2	exoı	NM_	.	missens
ACaP02	22	38271958	.	C	A	.	PASS	EIF3L	exoı	NM_	.	synonyr
ACaP02	X	3021841	rs7509188	G	A	.	PASS	ARSF	exoı	NM_	.	missens
ACaP02	X	25031637	.	G	A	.	PASS	ARX	exoı	NM_	.	missens
ACaP02	X	46299761	.	T	A	.	PASS	.	inteı	NR_	dis	.
ACaP02	X	69573529	.	G	A	.	PASS	KIF4A	exoı	NM_	.	missens
ACaP02	X	1.19E+08	.	G	C	.	PASS	NKAP	exoı	NM_	.	missens
ACaP02	X	1.39E+08	.	T	A	.	PASS	SRD5A:	ncR	NR_	.	.
ACaP02	X	1.39E+08	.	T	A	.	PASS	SRD5A:	ncR	NR_	.	.
ACaP02	X	1.51E+08	.	G	C	.	PASS	GABRA	intrc	NM_	.	.
ACaP02	X	1.53E+08	.	C	T	.	PASS	IRAK1	UTF	NM_	NM	.
ACaP02	MT	13324	.	T	C	.	PASS	.	inteı	NON	dis	.
ACaP03	1	1666303	rs1149286	T	C	.	PASS	SLC35E	intrc	NM_	.	.
ACaP03	1	25784928	.	C	A	.	PASS	TMEM5	exoı	NM_	.	missens
ACaP03	1	74835045	.	C	T	.	PASS	FPGT-T	intrc	NM_	.	.
ACaP03	1	86482866	.	G	A	.	PASS	COL24A	exoı	NM_	.	missens
ACaP03	1	1.21E+08	rs1998147	T	C	.	PASS	FCGR1I	UTF	NM_	NM	.
ACaP03	1	1.43E+08	rs8662584	G	T	.	PASS	.	inteı	NR_	dis	.
ACaP03	2	54850869	.	T	A	.	PASS	SPTBN:	intrc	NM_	.	.
ACaP03	2	67630477	.	G	A	.	PASS	ETAA1	exoı	NM_	.	missens
ACaP03	2	85133304	.	C	G	.	PASS	TMSB1(	intrc	NM_	.	.
ACaP03	2	1.22E+08	rs3708851	C	T	.	PASS	GLI2	exoı	NM_	.	synonyr
ACaP03	2	1.36E+08	rs2008806	T	A	.	PASS	R3HDM	intrc	NM_	.	.
ACaP03	2	1.69E+08	rs9042902	C	T	.	PASS	STK39	intrc	NM_	.	.
ACaP03	2	2.18E+08	rs3702772	C	T	.	PASS	IGFBP5	intrc	NM_	.	.
ACaP03	2	2.35E+08	rs5574456	G	A	.	PASS	TRPM8	intrc	NM_	.	.
ACaP03	3	23499994	rs1446637	C	T	.	PASS	MIR548	ncR	NR_	.	.
ACaP03	3	37018036	.	G	T	.	PASS	.	inteı	NM_	dis	.
ACaP03	3	1.14E+08	.	C	T	.	PASS	GRAMD	exoı	NM_	.	synonyr
ACaP03	3	1.14E+08	rs2015548	C	T	.	PASS	DRD3	exoı	NM_	.	missens
ACaP03	4	13475825	rs1163261	C	T	.	PASS	RAB28	intrc	NM_	.	.
ACaP03	4	49165624	rs4695484	C	G	.	PASS	.	inteı	NM_	dis	.
ACaP03	4	49165647	rs4695485	G	T	.	PASS	.	inteı	NM_	dis	.
ACaP03	4	71255517	rs7496227	C	T	.	PASS	SMR3B	exoı	NM_	.	synonyr

ACaP03	4	1.86E+08	rs7758415	G	A	.	PASS	PDLIM3	exo	NM_	.	missens
ACaP03	5	1.4E+08	.	C	T	.	PASS	PCDHA	exo	NM_	.	synonyr
ACaP03	6	31850912	rs1161907	C	T	.	PASS	EHMT2	intrc	NM_	.	.
ACaP03	6	76423203	.	G	T	.	PASS	SENP6	intrc	NM_	.	.
ACaP03	6	80778603	.	T	A	.	PASS	.	inte	NM_	dis	.
ACaP03	6	1.11E+08	rs7577542	C	T	.	PASS	CDK19	UTF	NM_	NM	.
ACaP03	7	5112643	.	G	A	.	PASS	RBAKD	ncR	NR_	.	.
ACaP03	7	87145947	.	C	A	.	PASS	ABCB1	exo	NM_	.	missens
ACaP03	7	1.02E+08	.	T	C	.	PASS	FAM18	intrc	NM_	.	.
ACaP03	8	17849058	rs7722351	C	T	.	PASS	PCM1	exo	NM_	.	synonyr
ACaP03	8	1.42E+08	.	G	T	.	PASS	PTK2	intrc	NM_	.	.
ACaP03	9	1.4E+08	rs7507213	G	A	.	PASS	TRAF2	exo	NM_	.	missens
ACaP03	10	10989346	.	T	A	.	PASS	LINC00	ncR	NR_	.	.
ACaP03	10	15649742	rs7571508	G	A	.	PASS	ITGA8	exo	NM_	.	synonyr
ACaP03	10	38896297	rs5655237	C	T	.	PASS	.	inte	NR_	dis	.
ACaP03	10	74452957	rs7905448	T	A	.	PASS	MCU	intrc	NM_	.	.
ACaP03	10	91597898	.	G	A	.	PASS	LINC00	ncR	NR_	.	.
ACaP03	10	1.04E+08	rs9706750	C	T	.	PASS	LOC10	ncR	NR_	.	.
ACaP03	10	1.04E+08	rs7723659	A	G	.	PASS	SFXN2	intrc	NM_	.	.
ACaP03	10	1.05E+08	.	G	A	.	PASS	INA	exo	NM_	.	synonyr
ACaP03	10	1.23E+08	rs1219134	A	G	.	PASS	FGFR2	exo	NM_	.	missens
ACaP03	11	688411	.	G	A	.	PASS	DEAF1	exo	NM_	.	missens
ACaP03	11	17517143	rs5453728	G	A	.	PASS	USH1C	exo	NM_	.	synonyr
ACaP03	11	17565822	.	A	G	.	PASS	USH1C	exo	NM_	.	synonyr
ACaP03	11	1.2E+08	.	T	C	.	PASS	ARHGE	intrc	NM_	.	.
ACaP03	12	1.13E+08	rs1030864	T	C	.	PASS	OAS1	exo	NM_	.	missens
ACaP03	13	49027224	.	G	A	.	PASS	RB1	exo	NM_	.	synonyr
ACaP03	13	78236783	rs9145408	C	T	.	PASS	.	inte	NM_	dis	.
ACaP03	13	79233446	rs9816818	A	G	.	PASS	.	ups	NM_	.	.
ACaP03	14	23066954	.	G	T	.	PASS	.	ups	NM_	.	.
ACaP03	14	33525230	.	C	A	.	PASS	NPAS3	intrc	NM_	.	.
ACaP03	14	45571879	rs5634662	G	A	.	PASS	PRPF3	exo	NM_	.	synonyr
ACaP03	14	53130502	.	G	A	.	PASS	ERO1L	intrc	NM_	.	.
ACaP03	14	89151457	.	G	A	.	PASS	EML5	exo	NM_	.	missens
ACaP03	14	1E+08	.	C	A	.	PASS	HHIPL1	exo	NM_	.	missens
ACaP03	15	20481082	.	G	T	.	PASS	.	inte	NON	dis	.
ACaP03	15	23388408	rs7780894	G	A	.	PASS	.	inte	NR_	dis	.
ACaP03	15	42042562	.	T	C	.	PASS	MGA	exo	NM_	.	missens
ACaP03	15	43075621	.	C	A	.	PASS	TTBK2	exo	NM_	.	missens
ACaP03	16	67304885	.	C	T	.	PASS	SLC9A5	exo	NM_	.	synonyr
ACaP03	17	8638951	.	A	T	.	PASS	CCDC4	intrc	NM_	.	.
ACaP03	17	21905146	rs5864828	G	C	.	PASS	FLJ360	ncR	NR_	.	.
ACaP03	17	46874382	rs7798230	G	A	.	PASS	TTLL6	intrc	NM_	.	.
ACaP03	18	5395522	.	C	A	.	PASS	EPB41L	intrc	NM_	.	.
ACaP03	19	13418632	rs7477304	G	A	.	PASS	CACNA	exo	NM_	.	synonyr
ACaP03	19	14952045	.	C	A	.	PASS	OR7A1	exo	NM_	.	synonyr
ACaP03	19	35226977	rs9472013	T	C	.	PASS	ZNF181	intrc	NM_	.	.
ACaP03	19	36368719	.	C	T	.	PASS	APLP1	exo	NM_	.	missens
ACaP03	19	44272930	.	C	A	.	PASS	KCNN4	intrc	NM_	.	.
ACaP03	19	51525990	rs3718581	C	T	.	PASS	KLK11	intrc	NM_	.	.
ACaP03	19	53802903	rs1056285	C	T	.	PASS	.	inte	NM_	dis	.
ACaP03	20	2552806	rs5584781	T	A	.	PASS	TMC2	intrc	NM_	.	.
ACaP03	20	25900162	rs6037279	C	A	.	PASS	.	inte	NR_	dis	.
ACaP03	20	34061552	.	G	C	.	PASS	CEP250	intrc	NM_	.	.

ACaP03	20	44657935	.	G	A	.	PASS	SLC12A	UTF	NM_	NM	.
ACaP03	21	10920253	rs2013315	C	T	.	PASS	TPTE	intrc	NM_	.	.
ACaP03	21	10920295	rs2001788	G	A	.	PASS	TPTE	intrc	NM_	.	.
ACaP03	22	46655190	.	G	A	.	PASS	PKDRE.	exoi	NM_	.	missens
ACaP03	X	1.53E+08	.	G	T	.	PASS	IRAK1	exoi	NM_	.	stopgaiı
ACaP03	MT	8177	.	G	A	.	PASS	.	intei	NON	dis	.
ACaP03	MT	15761	.	G	A	.	PASS	.	intei	NON	dis	.
ACaP04	1	237272	rs6263527	T	A	.	PASS	.	intei	NR_	dis	.
ACaP04	1	53540057	.	C	A	.	PASS	PODN	intrc	NM_	.	.
ACaP04	1	1.43E+08	rs7457471	C	G	.	PASS	.	intei	NR_	dis	.
ACaP04	1	1.67E+08	.	T	C	.	PASS	POU2F:	intrc	NM_	.	.
ACaP04	1	2.36E+08	.	C	A	.	PASS	NID1	intrc	NM_	.	.
ACaP04	2	87580821	.	G	C	.	PASS	.	intei	NR_	dis	.
ACaP04	2	90465003	rs3711404	G	A	.	PASS	.	intei	NR_	dis	.
ACaP04	2	1.82E+08	.	A	T	.	PASS	ITGA4	intrc	NM_	.	.
ACaP04	3	1.96E+08	rs7758373	T	C	.	PASS	SDHAP	ncR	NR_	.	.
ACaP04	4	2251695	.	T	A	.	PASS	MXD4	UTF	NM_	NM	.
ACaP04	4	49211467	rs4694947	T	G	.	PASS	.	intei	NM_	dis	.
ACaP04	9	73191428	.	T	C	.	PASS	TRPM3	intrc	NM_	.	.
ACaP04	10	38939588	rs4070334	G	A	.	PASS	.	intei	NR_	dis	.
ACaP04	12	76854256	rs9213947	C	A	.	PASS	OSBPL8	intrc	NM_	.	.
ACaP04	14	50623669	rs1125033	G	A	.	PASS	SOS2	intrc	NM_	.	.
ACaP04	14	75474251	.	A	C	.	PASS	EIF2B2	intrc	NM_	.	.
ACaP04	15	20739825	rs2859043	G	A	.	PASS	GOLGA	exoi	NM_	.	missens
ACaP04	15	23097357	.	G	T	.	PASS	LOC283	ncR	NR_	.	.
ACaP04	18	31463134	.	G	A	.	PASS	NOL4	intrc	NM_	.	.
ACaP05	1	1.43E+08	.	A	C	.	PASS	.	intei	NR_	dis	.
ACaP05	1	1.49E+08	rs2787778	G	T	.	PASS	.	intei	NR_	dis	.
ACaP05	1	1.49E+08	rs2787777	C	T	.	PASS	.	intei	NR_	dis	.
ACaP05	2	90465003	rs3711404	G	A	.	PASS	.	intei	NR_	dis	.
ACaP05	5	21751735	rs1997973	G	T	.	PASS	CDH12	UTF	NM_	NM	.
ACaP05	7	62956286	rs2018986	T	C	.	PASS	.	intei	NR_	dis	.
ACaP05	7	63219077	.	G	T	.	PASS	.	intei	NR_	dis	.
ACaP05	7	1.02E+08	rs1390499	G	C	.	PASS	POLR2.	exoi	NM_	.	missens
ACaP05	8	7386635	rs2077351	A	C	.	PASS	.	intei	NM_	dis	.
ACaP05	9	66500662	rs1136487	C	A	.	PASS	PTGER	ncR	NR_	.	.
ACaP05	9	69083093	rs7648362	A	G	.	PASS	PGM5P	ncR	NR_	.	.
ACaP05	9	74979191	.	G	C	.	PASS	ZFAND!	intrc	NM_	.	.
ACaP05	10	51853633	rs1995206	C	T	.	PASS	FAM21/	exoi	NM_	.	missens
ACaP05	11	135046	rs3713518	C	G	.	PASS	.	intei	NR_	dis	.
ACaP05	11	45927418	rs8790394	A	T	.	PASS	MAPK8I	UTF	NM_	NM	.
ACaP05	17	21906860	rs1155611	C	A	.	PASS	FLJ360(	ncR	NR_	.	.
ACaP05	18	11610531	rs2018600	G	A	.	PASS	SLC35C	exoi	NM_	.	missens
ACaP05	21	10139726	.	G	A	.	PASS	.	intei	NR_	dis	.
ACaP05	21	10139851	rs8792017	A	G	.	PASS	.	intei	NR_	dis	.
ACaP05	21	11097497	rs1043983	A	T	.	PASS	BAGE2,	intrc	NM_	.	.
ACaP05	X	1.39E+08	rs1444677	G	C	.	PASS	.	intei	NM_	dis	.
ACaP06	1	803964	.	G	A	.	PASS	FAM41(	ncR	NR_	.	.
ACaP06	1	5924273	.	A	G	.	PASS	NPHP4	intrc	NM_	.	.
ACaP06	1	5993482	.	C	A	.	PASS	NPHP4	intrc	NM_	.	.
ACaP06	1	9662320	.	C	T	.	PASS	TMEM2	exoi	NM_	.	missens
ACaP06	1	12888733	.	C	T	.	PASS	PRAME	intrc	NM_	.	.
ACaP06	1	12888735	.	G	T	.	PASS	PRAME	intrc	NM_	.	.
ACaP06	1	1E+08	.	T	G	.	PASS	PALMD	UTF	NM_	NM	.

ACaP06	1	1.43E+08	rs3894786	T	C	.	PASS	.	inte	NR_	dis	.
ACaP06	1	1.43E+08	rs1112760	G	A	.	PASS	.	inte	NR_	dis	.
ACaP06	1	1.44E+08	rs2017985	G	A	.	PASS	NBPF2(	intrc	NM_	.	.
ACaP06	1	1.45E+08	rs3713084	T	C	.	PASS	NBPF2(	intrc	NM_	.	.
ACaP06	1	1.45E+08	rs8799710	G	C	.	PASS	NBPF8,	intrc	NM_	.	.
ACaP06	1	1.46E+08	rs6699421	A	G	.	PASS	NBPF25	ncR	NR_	.	.
ACaP06	1	1.49E+08	rs8799669	C	A	.	PASS	NBPF25	ncR	NR_	.	.
ACaP06	1	1.5E+08	.	C	G	.	PASS	OTUD7I	exo	NM_	.	missens
ACaP06	1	1.54E+08	.	C	G	.	PASS	C1orf18	intrc	NM_	.	.
ACaP06	1	1.56E+08	rs4833528	C	T	.	PASS	RIT1	exo	NM_	.	missens
ACaP06	1	1.75E+08	rs8940723	G	A	.	PASS	RABGA	intrc	NM_	.	.
ACaP06	1	1.82E+08	rs7731705	C	T	.	PASS	CACNA	intrc	NM_	.	.
ACaP06	2	88272597	.	G	C	.	PASS	RGPD1	intrc	NM_	.	.
ACaP06	2	89185488	rs7781962	G	T	.	PASS	.	inte	NR_	dis	.
ACaP06	2	99861764	rs2018517	G	A	.	PASS	LYG2	exo	NM_	.	synonyr
ACaP06	2	1.03E+08	.	A	C	.	PASS	SLC9A2	exo	NM_	.	missens
ACaP06	2	1.77E+08	rs7685255	G	A	.	PASS	EVX2	exo	NM_	.	missens
ACaP06	2	1.81E+08	rs7551300	C	T	.	PASS	CWC22	exo	NM_	.	missens
ACaP06	3	39453515	rs6224264	A	G	.	PASS	RPSA	exo	NM_	.	synonyr
ACaP06	3	1.83E+08	.	C	G	.	PASS	ATP11E	intrc	NM_	.	.
ACaP06	3	1.84E+08	.	G	A	.	PASS	ABCC5	exo	NM_	.	synonyr
ACaP06	3	1.84E+08	.	G	A	.	PASS	HTR3C	exo	NM_	.	missens
ACaP06	3	1.85E+08	.	T	G	.	PASS	VPS8	intrc	NM_	.	.
ACaP06	4	1.09E+08	.	C	G	.	PASS	LEF1	intrc	NM_	.	.
ACaP06	4	1.18E+08	rs5742217	T	C	.	PASS	.	inte	NM_	dis	.
ACaP06	4	1.78E+08	rs5549088	G	A	.	PASS	VEGFC	exo	NM_	.	missens
ACaP06	5	34180243	.	G	A	.	PASS	.	inte	NR_	dis	.
ACaP06	5	69206624	rs3759258	G	T	.	PASS	.	inte	NR_	dis	.
ACaP06	5	71648514	.	C	T	.	PASS	PTCD2	exo	NM_	.	missens
ACaP06	5	1.31E+08	.	G	C	.	PASS	ACSL6	intrc	NM_	.	.
ACaP06	5	1.32E+08	.	G	A	.	PASS	LEAP2	intrc	NM_	.	.
ACaP06	5	1.76E+08	.	C	T	.	PASS	EIF4E1I	intrc	NM_	.	.
ACaP06	5	1.76E+08	.	T	G	.	PASS	UNC5A	intrc	NM_	.	.
ACaP06	6	13792301	rs7580301	C	T	.	PASS	MCUR1	intrc	NM_	.	.
ACaP06	6	15504951	rs7598672	G	A	.	PASS	JARID2	intrc	NM_	.	.
ACaP06	6	24667377	.	G	A	.	PASS	ACOT1:	UTF	NM_	NM	.
ACaP06	6	29797317	.	C	A	.	PASS	HLA-G	exo	NM_	.	missens
ACaP06	6	31721280	.	G	C	.	PASS	MSH5-S	ncR	NR_	.	.
ACaP06	6	33137752	.	C	T	.	PASS	COL11A	intrc	NM_	.	.
ACaP06	6	43196698	.	C	T	.	PASS	DNPH1	intrc	NM_	.	.
ACaP06	6	62407056	.	A	G	.	PASS	KHDRB	intrc	NM_	.	.
ACaP06	6	66013918	.	A	T	.	PASS	LOC441	exo	NM_	.	missens
ACaP06	6	1.58E+08	.	G	T	.	PASS	SYNJ2	intrc	NM_	.	.
ACaP06	7	1588922	.	G	A	.	PASS	TMEM1	intrc	NM_	.	.
ACaP06	7	62956286	rs2018986	T	C	.	PASS	.	inte	NR_	dis	.
ACaP06	7	87594053	rs9803140	C	G	.	PASS	ADAM2	intrc	NM_	.	.
ACaP06	7	1.44E+08	rs1208357	G	A	.	PASS	.	inte	NM_	dis	.
ACaP06	7	1.44E+08	rs6248378	G	A	.	PASS	.	inte	NM_	dis	.
ACaP06	7	1.5E+08	.	G	A	.	PASS	GIMAP1	exo	NM_	.	missens
ACaP06	7	1.52E+08	.	G	A	.	PASS	.	inte	NR_	dis	.
ACaP06	8	28695333	.	G	A	.	PASS	INTS9	intrc	NM_	.	.
ACaP06	8	43822531	rs2022368	A	G	.	PASS	.	inte	NM_	dis	.
ACaP06	8	1.45E+08	.	T	G	.	PASS	SCRIB	intrc	NM_	.	.
ACaP06	9	85863034	.	G	C	.	PASS	FRMD3	exo	NM_	.	synonyr

ACaP06	9	94518550	.	C	G	.	PASS	ROR2	intrc	NM_	.	.	
ACaP06	9	98908654	.	T	C	.	PASS	.	inte	NM_	dis	.	
ACaP06	9	1.4E+08	rs7729568	G	A	.	PASS	MAN1B	exo	NM_	.	synonyr	
ACaP06	10	44869797	rs5354201	C	T	.	PASS	CXCL12	intrc	NM_	.	.	
ACaP06	10	1.06E+08	.	A	T	.	PASS	.	ups	NM_	.	.	
ACaP06	10	1.06E+08	.	G	T	.	PASS	CCDC1	exo	NM_	.	stopgai	
ACaP06	10	1.19E+08	.	C	T	.	PASS	KCNK18	exo	NM_	.	missens	
ACaP06	11	802749	rs1474222	G	A	.	PASS	PIDD	exo	NM_	.	synonyr	
ACaP06	11	3394898	.	G	C	.	PASS	ZNF195	UTF	NM_	NM	.	
ACaP06	11	6453188	.	G	A	.	PASS	HPX	exo	NM_	.	missens	
ACaP06	11	10440314	.	G	A	.	PASS	CAND1.	ncR	NR_	.	.	
ACaP06	11	17409674	rs7776543	C	T	.	PASS	KCNJ11	UTF	NM_	NM	.	
ACaP06	11	44956486	rs7802181	G	A	.	PASS	TP53I11	exo	NM_	.	synonyr	
ACaP06	11	58962592	.	C	G	.	PASS	DTX4	intrc	NM_	.	.	
ACaP06	11	65143804	.	C	T	.	PASS	SLC25A	UTF	NM_	NM	.	
ACaP06	11	1.2E+08	rs1009016	G	A	.	PASS	TRIM29	intrc	NM_	.	.	
ACaP06	11	1.26E+08	.	G	A	.	PASS	DDX25	intrc	NM_	.	.	
ACaP06	12	57114257	.	G	C	.	PASS	NACA	exo	NM_	.	missens	
ACaP06	12	1.13E+08	rs8683709	T	C	.	PASS	HECTD	intrc	NM_	.	.	
ACaP06	12	1.22E+08	.	G	T	.	PASS	KDM2B	intrc	NM_	.	.	
ACaP06	12	1.22E+08	.	G	T	.	PASS	HPD	intrc	NM_	.	.	
ACaP06	13	19956998	.	G	A	.	PASS	.	inte	NR_	dis	.	
ACaP06	13	19957240	.	G	A	.	PASS	.	inte	NR_	dis	.	
ACaP06	13	25373510	rs7603455	G	A	.	PASS	RNF17	intrc	NM_	.	.	
ACaP06	13	25826058	.	G	A	.	PASS	MTMR6	exo	NM_	.	missens	
ACaP06	13	36167439	.	A	T	.	PASS	MIR548	ncR	NR_	.	.	
ACaP06	13	39450585	.	A	T	.	PASS	FREM2	intrc	NM_	.	.	
ACaP06	13	75876520	rs1847802	G	A	.	PASS	TBC1D	exo	NM_	.	stopgai	
ACaP06	14	20145911	rs8674109	G	T	.	PASS	.	inte	NM_	dis	.	
ACaP06	14	30095729	.	C	T	.	PASS	PRKD1	exo	NM_	.	missens	
ACaP06	14	35183710	.	C	T	.	PASS	CFL2	UTF	NM_	NM	.	
ACaP06	14	45583374	.	C	G	.	PASS	PRPF39	intrc	NM_	.	.	
ACaP06	14	57949844	rs7590859	C	T	.	PASS	C14orf1	exo	NM_	.	missens	
ACaP06	14	63848931	.	G	A	.	PASS	PPP2R5	intrc	NM_	.	.	
ACaP06	14	70714300	rs1126070	A	G	.	PASS	ADAM2	ncR	NR_	.	.	
ACaP06	14	1.04E+08	.	C	G	.	PASS	TDRD9	intrc	NM_	.	.	
ACaP06	14	1.04E+08	.	C	T	.	PASS	TDRD9	intrc	NM_	.	.	
ACaP06	14	1.04E+08	.	C	T	.	PASS	TDRD9	splic	NM_	NM	.	
ACaP06	15	62547818	.	C	T	.	PASS	.	inte	NM_	dis	.	
ACaP06	15	71433874	.	C	A	.	PASS	THSD4	exo	NM_	.	missens	
ACaP06	16	1394469	.	G	A	.	PASS	BAIAP3	splic	NM_	NM	.	
ACaP06	16	1440797	.	G	A	.	PASS	UNKL	intrc	NM_	.	.	
ACaP06	16	4787945	rs7488576	G	A	.	PASS	C16orf7	exo	NM_	.	missens	
ACaP06	16	5080595	.	G	C	.	PASS	NAGPA	intrc	NM_	.	.	
ACaP06	16	20432607	.	G	A	.	PASS	ACSM5	exo	NM_	.	missens	
ACaP06	16	28123150	.	G	A	.	PASS	XPO6	exo	NM_	.	missens	
ACaP06	16	30080816	.	C	A	.	PASS	ALDOA	splic	NM_	NM	.	
ACaP06	16	46388298	rs7501178	C	T	.	PASS	.	inte	NON	dis	.	
ACaP06	16	46393135	rs2128450	T	A	.	PASS	.	inte	NON	dis	.	
ACaP06	16	46407013	rs4436803	T	G	.	PASS	.	inte	NON	dis	.	
ACaP06	16	46407752	rs2887500	T	G	.	PASS	.	inte	NON	dis	.	
ACaP06	16	70867025	.	C	A	.	PASS	HYDIN	intrc	NM_	.	.	
ACaP06	16	89789052	.	T	C	.	PASS	ZNF276	exo	NM_	.	missens	
ACaP06	17	25936412	.	G	C	.	PASS	KSR1	intrc	NM_	.	.	

ACaP06	17	28419118	.	C	G	.	PASS	EFCAB!	exo	NM_	.	missens
ACaP06	17	38065416	.	G	A	.	PASS	GSDMB	intrc	NM_	.	.
ACaP06	17	40125572	rs1996882	G	T	.	PASS	CNP	exo	NM_	.	missens
ACaP06	17	40870998	.	C	G	.	PASS	EZH1	intrc	NM_	.	.
ACaP06	17	42945543	rs1022498	A	T	.	PASS	EFTUD:	intrc	NM_	.	.
ACaP06	17	62122809	.	C	T	.	PASS	ERN1	exo	NM_	.	missens
ACaP06	17	65021082	rs3720728	C	T	.	PASS	CACNG	exo	NM_	.	synonyr
ACaP06	17	80534231	.	G	A	.	PASS	FOXK2	intrc	NM_	.	.
ACaP06	18	10532779	.	G	A	.	PASS	NAPG	exo	NM_	.	missens
ACaP06	18	12254798	.	C	T	.	PASS	CIDEA	intrc	NM_	.	.
ACaP06	18	14999128	.	G	C	.	PASS	.	inte	NR_	dis	.
ACaP06	18	51888195	rs7558958	C	T	.	PASS	C18orf5	exo	NM_	.	missens
ACaP06	19	38573136	rs9952243	G	A	.	PASS	SIPA1L:	exo	NM_	.	missens
ACaP06	19	49100155	.	C	T	.	PASS	SULT2E	exo	NM_	.	missens
ACaP06	20	13201803	.	G	T	.	PASS	.	ups	NM_	.	.
ACaP06	20	36952898	.	T	G	.	PASS	BPI	intrc	NM_	.	.
ACaP06	21	10204134	rs3697472	C	A	.	PASS	.	inte	NR_	dis	.
ACaP06	21	10204188	rs7969899	C	T	.	PASS	.	inte	NR_	dis	.
ACaP06	21	44579674	.	C	T	.	PASS	.	inte	NM_	dis	.
ACaP06	21	45479544	.	C	G	.	PASS	TRAPP(	intrc	NM_	.	.
ACaP06	22	16277923	rs2212142	T	G	.	PASS	POTEH	intrc	NM_	.	.
ACaP06	22	20194161	.	G	T	.	PASS	LINC00	ncR	NR_	.	.
ACaP06	22	20715963	rs2015076	G	C	.	PASS	.	inte	NM_	dis	.
ACaP06	22	37771552	rs7611606	G	A	.	PASS	ELFN2	exo	NM_	.	missens
ACaP06	22	38469859	.	T	A	.	PASS	PICK1	intrc	NM_	.	.
ACaP06	X	70636506	rs3397828	A	G	.	PASS	BCYRN	ncR	NR_	.	.
ACaP06	X	88008631	.	C	T	.	PASS	CPXCR	exo	NM_	.	synonyr
ACaP06	MT	215	rs8792192	A	G	.	PASS	.	inte	NON	dis	.
ACaP07	1	16767275	.	G	A	.	PASS	NECAP	exo	NM_	.	missens
ACaP07	1	16894795	rs1996890	T	C	.	PASS	NBPF1	intrc	NM_	.	.
ACaP07	1	35370332	.	C	A	.	PASS	DLGAP:	exo	NM_	.	missens
ACaP07	1	1.1E+08	.	A	G	.	PASS	GNAI3	exo	NM_	.	missens
ACaP07	1	1.43E+08	.	T	C	.	PASS	.	inte	NR_	dis	.
ACaP07	1	1.43E+08	rs1694653	C	G	.	PASS	.	inte	NR_	dis	.
ACaP07	1	1.45E+08	rs9441133	T	G	.	PASS	NBPF9,	exo	NM_	.	unknow
ACaP07	1	1.45E+08	rs7819708	C	G	.	PASS	NBPF9,	exo	NM_	.	unknow
ACaP07	1	1.48E+08	rs2022445	T	A	.	PASS	NBPF8	intrc	NM_	.	.
ACaP07	1	1.5E+08	.	C	T	.	PASS	.	inte	NM_	dis	.
ACaP07	1	1.51E+08	.	C	T	.	PASS	ANXA9	intrc	NM_	.	.
ACaP07	1	1.58E+08	.	C	G	.	PASS	.	inte	NM_	dis	.
ACaP07	1	1.83E+08	rs1494980	T	C	.	PASS	RNASE	exo	NM_	.	missens
ACaP07	1	1.86E+08	.	C	A	.	PASS	HMCN1	exo	NM_	.	missens
ACaP07	1	2.15E+08	rs7630457	C	T	.	PASS	CENPF	exo	NM_	.	missens
ACaP07	1	2.33E+08	rs3725088	G	T	.	PASS	KIAA18(	intrc	NM_	.	.
ACaP07	2	42588301	.	T	C	.	PASS	COX7A:	exo	NM_	.	missens
ACaP07	2	91894137	rs6214568	A	G	.	PASS	.	inte	NR_	dis	.
ACaP07	2	1.12E+08	.	C	G	.	PASS	ACOXL	intrc	NM_	.	.
ACaP07	2	1.13E+08	rs1476846	C	T	.	PASS	ANAPC	exo	NM_	.	missens
ACaP07	2	2.31E+08	.	C	G	.	PASS	SP140L	intrc	NM_	.	.
ACaP07	3	42907168	.	G	A	.	PASS	ACKR2	UTF	NM_	NM	.
ACaP07	3	48692539	.	C	A	.	PASS	CELSR:	exo	NM_	.	missens
ACaP07	3	1.09E+08	.	G	T	.	PASS	DPPA2	intrc	NM_	.	.
ACaP07	3	1.13E+08	rs1428044	G	A	.	PASS	SPICE1	exo	NM_	.	synonyr
ACaP07	3	1.55E+08	.	T	C	.	PASS	MME	intrc	NM_	.	.

ACaP07	4	49846	rs4970287	C	T	.	PASS	.	inte	NON	dis	.
ACaP07	4	1.02E+08	.	T	C	.	PASS	PPP3CA	splic	NM_	NM	.
ACaP07	4	1.06E+08	.	G	A	.	PASS	TET2	exon	NM_	.	missens
ACaP07	5	36976324	.	C	T	.	PASS	NIPBL	exon	NM_	.	stopgai
ACaP07	5	1.4E+08	.	C	A	.	PASS	SLC4A9	intro	NM_	.	.
ACaP07	7	37961124	rs9642790	C	T	.	PASS	EPDR1	intro	NM_	.	.
ACaP07	7	56884371	rs6246184	G	A	.	PASS	.	inte	NR_	dis	.
ACaP07	7	56887911	.	C	A	.	PASS	.	inte	NR_	dis	.
ACaP07	7	1E+08	.	C	T	.	PASS	LRCH4,	exon	NM_	.	missens
ACaP07	7	1.22E+08	.	C	A	.	PASS	PTPRZ1	exon	NM_	.	missens
ACaP07	7	1.49E+08	rs8915428	C	T	.	PASS	SSPO	intro	NM_	.	.
ACaP07	7	1.55E+08	.	A	T	.	PASS	PAXIP1	ncR	NR_	.	.
ACaP07	8	28670032	.	G	A	.	PASS	INTS9	intro	NM_	.	.
ACaP07	8	37555820	.	C	G	.	PASS	ZNF703	exon	NM_	.	missens
ACaP07	8	67590502	.	G	A	.	PASS	C8orf44	intro	NM_	.	.
ACaP07	9	5041997	.	G	A	.	PASS	JAK2	intro	NM_	.	.
ACaP07	9	1.4E+08	.	G	A	.	PASS	NDOR1	intro	NM_	.	.
ACaP07	10	75563305	.	C	G	.	PASS	NDST2	intro	NM_	.	.
ACaP07	10	87898673	.	G	C	.	PASS	GRID1	exon	NM_	.	missens
ACaP07	10	1.04E+08	.	G	T	.	PASS	NOLC1	exon	NM_	.	synonyr
ACaP07	10	1.05E+08	.	G	C	.	PASS	PDCD11	intro	NM_	.	.
ACaP07	10	1.35E+08	.	G	A	.	PASS	KNDC1	splic	NM_	NM	.
ACaP07	11	5686420	.	C	A	.	PASS	TRIM5	exon	NM_	.	missens
ACaP07	11	7981551	.	T	C	.	PASS	NLRP10	exon	NM_	.	synonyr
ACaP07	11	30953503	.	C	T	.	PASS	DCDC5	splic	NM_	NM	.
ACaP07	11	36511665	.	C	T	.	PASS	TRAF6	exon	NM_	.	missens
ACaP07	11	94703114	.	T	C	.	PASS	CWC15	intro	NM_	.	.
ACaP07	11	1.17E+08	rs7580622	C	T	.	PASS	DSCAM	exon	NM_	.	synonyr
ACaP07	12	7639196	.	A	G	.	PASS	CD163	exon	NM_	.	missens
ACaP07	12	49725662	.	G	A	.	PASS	.	dow	NM_	.	.
ACaP07	12	50498477	.	C	G	.	PASS	GPD1	exon	NM_	.	missens
ACaP07	12	54757465	.	C	T	.	PASS	GPR84	exon	NM_	.	synonyr
ACaP07	12	58014656	.	G	C	.	PASS	SLC26A	intro	NM_	.	.
ACaP07	12	1.23E+08	.	C	A	.	PASS	HCAR3	exon	NM_	.	missens
ACaP07	13	93518634	.	C	A	.	PASS	GPC5	exon	NM_	.	missens
ACaP07	14	20134633	rs7149806	A	G	.	PASS	.	inte	NM_	dis	.
ACaP07	14	1.04E+08	.	G	T	.	PASS	.	inte	NR_	dis	.
ACaP07	15	23931763	.	T	G	.	PASS	NDN	exon	NM_	.	missens
ACaP07	15	30846509	rs1995696	A	G	.	PASS	.	inte	NM_	dis	.
ACaP07	15	33359832	.	G	T	.	PASS	FMN1	exon	NM_	.	missens
ACaP07	15	57953573	.	C	G	.	PASS	GCOM1	intro	NM_	.	.
ACaP07	15	72534134	.	C	A	.	PASS	PARP6	intro	NM_	.	.
ACaP07	15	90346511	.	G	C	.	PASS	ANPEP	exon	NM_	.	missens
ACaP07	16	6069942	.	G	A	.	PASS	RBFOX	UTF	NM_	NM	.
ACaP07	16	75538587	.	G	T	.	PASS	.	inte	NM_	dis	.
ACaP07	17	10205074	.	G	C	.	PASS	MYH13	intro	NM_	.	.
ACaP07	17	58092326	.	T	C	.	PASS	TBC1D3	ncR	NR_	.	.
ACaP07	18	14629932	rs8790150	G	A	.	PASS	.	inte	NM_	dis	.
ACaP07	19	3811459	rs8673654	T	C	.	PASS	ZFR2	intro	NM_	.	.
ACaP07	19	12776320	rs7668108	G	A	.	PASS	MAN2B	exon	NM_	.	synonyr
ACaP07	19	18707522	rs7683037	G	A	.	PASS	CRLF1	exon	NM_	.	missens
ACaP07	19	22362334	.	T	A	.	PASS	ZNF676	UTF	NM_	NM	.
ACaP07	19	37383903	.	T	G	.	PASS	ZNF829	intro	NM_	.	.
ACaP07	19	39098806	.	C	T	.	PASS	MAP4K1	exon	NM_	.	synonyr

ACaP07	19	46320344	.	T	G	.	PASS	SYMPK	intrc	NM_	.	.	
ACaP07	19	47996617	.	G	C	.	PASS	NAPA-A	ncR	NR_	.	.	
ACaP07	19	49400716	.	C	G	.	PASS	TULP2	intrc	NM_	.	.	
ACaP07	19	49400795	.	G	A	.	PASS	TULP2	intrc	NM_	.	.	
ACaP07	19	51107811	.	A	G	.	PASS	.	ups	NR_	.	.	
ACaP07	19	56244717	.	G	A	.	PASS	NLRP9	exoı	NM_	.	synonyr	
ACaP07	20	42249604	.	G	C	.	PASS	IFT52	intrc	NM_	.	.	
ACaP07	20	50407956	.	A	G	.	PASS	SALL4	exoı	NM_	.	missens	
ACaP07	21	11186426	rs1118412	G	A	.	PASS	.	inteı	NM_	dis	.	
ACaP07	21	28315813	.	C	A	.	PASS	ADAMT	exoı	NM_	.	missens	
ACaP07	21	33746030	.	C	T	.	PASS	URB1	exoı	NM_	.	missens	
ACaP07	22	23029846	rs2006656	T	C	.	PASS	.	inteı	NM_	dis	.	
ACaP07	22	44496060	rs9392199	G	A	.	PASS	PARVB	intrc	NM_	.	.	
ACaP07	X	46320101	rs734038	T	G	.	PASS	KRBOX	intrc	NM_	.	.	
ACaP07	X	89368426	rs1826904	G	C	.	PASS	.	inteı	NM_	dis	.	
ACaP07	X	1.29E+08	.	T	A	.	PASS	OCRL	exoı	NM_	.	missens	
ACaP07	X	1.35E+08	.	C	T	.	PASS	GPR11	exoı	NM_	.	missens	
ACaP07	X	1.45E+08	.	C	A	.	PASS	SLITRK	exoı	NM_	.	missens	
ACaP07	X	1.54E+08	.	G	A	.	PASS	F8	exoı	NM_	.	missens	
ACaP07	MT	13597	.	G	A	.	PASS	.	inteı	NON	dis	.	
ACaP07	MT	15976	.	T	C	.	PASS	.	inteı	NON	dis	.	
ACaP08	1	16984574	rs1810443	G	A	.	PASS	.	inteı	NR_	dis	.	
ACaP08	1	1.43E+08	rs3698413	G	A	.	PASS	.	inteı	NR_	dis	.	
ACaP08	1	1.43E+08	rs3774960	G	A	.	PASS	.	inteı	NR_	dis	.	
ACaP08	1	1.45E+08	rs2596327	T	A	.	PASS	NBPF9,	intrc	NM_	.	.	
ACaP08	1	1.48E+08	rs2020368	T	A	.	PASS	NBPF8	intrc	NM_	.	.	
ACaP08	1	1.49E+08	.	C	A	.	PASS	NBPF25	ncR	NR_	.	.	
ACaP08	2	84816179	.	G	C	.	PASS	DNAH6	intrc	NM_	.	.	
ACaP08	2	91777902	rs2647769	C	T	.	PASS	.	inteı	NON	dis	.	
ACaP08	2	1.05E+08	.	C	T	.	PASS	.	dow	NM_	.	.	
ACaP08	2	1.13E+08	.	C	A	.	PASS	FBLN7	intrc	NM_	.	.	
ACaP08	2	1.13E+08	.	C	G	.	PASS	FBLN7	intrc	NM_	.	.	
ACaP08	2	1.31E+08	rs6216494	T	A	.	PASS	POTEJ	intrc	NM_	.	.	
ACaP08	3	1.42E+08	.	G	T	.	PASS	TFDP2	intrc	NM_	.	.	
ACaP08	4	5020763	.	C	A	.	PASS	CYTL1	intrc	NM_	.	.	
ACaP08	4	69981142	rs1429725	C	T	.	PASS	.	inteı	NM_	dis	.	
ACaP08	5	21491760	rs2871612	T	C	.	PASS	GUSBP	ncR	NR_	.	.	
ACaP08	7	56898132	rs6246206	T	C	.	PASS	.	inteı	NR_	dis	.	
ACaP08	7	65228263	rs3680944	T	C	.	PASS	CCT6P1	ncR	NR_	.	.	
ACaP08	8	58124209	rs1499905	G	C	.	PASS	.	inteı	NM_	dis	.	
ACaP08	10	73570873	.	T	A	.	PASS	CDH23	intrc	NM_	.	.	
ACaP08	10	77732782	rs7710236	G	A	.	PASS	C10orf1	intrc	NM_	.	.	
ACaP08	10	81317406	rs1788433	A	G	.	PASS	SFTPA2	intrc	NM_	.	.	
ACaP08	10	1.24E+08	.	T	G	.	PASS	DMBT1	intrc	NM_	.	.	
ACaP08	11	1.25E+08	.	T	A	.	PASS	PKNOX	intrc	NM_	.	.	
ACaP08	12	9549738	rs1063873	T	G	.	PASS	.	inteı	NR_	dis	.	
ACaP08	14	19509337	rs4247513	T	C	.	PASS	.	inteı	NR_	dis	.	
ACaP08	14	19720452	rs7445485	C	T	.	PASS	.	inteı	NR_	dis	.	
ACaP08	14	20107085	rs8678279	G	A	.	PASS	.	inteı	NM_	dis	.	
ACaP08	14	23292000	.	A	T	.	PASS	.	inteı	NM_	dis	.	
ACaP08	17	36349252	rs3930170	G	T	.	PASS	.	ups	NM_	.	.	
ACaP08	17	48602100	rs3894278	A	T	.	PASS	MYCBP	intrc	NM_	.	.	
ACaP08	21	9873564	rs1963041	G	A	.	PASS	.	inteı	NR_	dis	.	
ACaP08	22	16346025	rs3716554	G	T	.	PASS	.	inteı	NM_	dis	.	

ACaP08	22	20711723	.	C	A	.	PASS	.	inte	NM_	dis	.	
ACaP08	X	1.35E+08	rs7819787	C	A	.	PASS	CT45A1	UTF	NM_	NM	.	
ACaP09	1	1453062	.	T	G	.	PASS	ATAD3A	intrc	NM_	.	.	
ACaP09	1	16899903	rs453347	G	C	.	PASS	NBPF1	intrc	NM_	.	.	
ACaP09	1	45278512	.	G	T	.	PASS	BTBD19	intrc	NM_	.	.	
ACaP09	1	1.43E+08	rs1812127	C	G	.	PASS	.	inte	NR_	dis	.	
ACaP09	1	1.45E+08	.	C	G	.	PASS	NOTCH	intrc	NM_	.	.	
ACaP09	1	1.45E+08	rs8799607	G	T	.	PASS	NBPF25	ncR	NR_	.	.	
ACaP09	1	1.48E+08	rs1451754	C	A	.	PASS	NBPF8	intrc	NM_	.	.	
ACaP09	1	1.48E+08	rs2003945	G	T	.	PASS	NBPF8	intrc	NM_	.	.	
ACaP09	1	1.48E+08	rs7970322	T	G	.	PASS	NBPF8	intrc	NM_	.	.	
ACaP09	1	2.06E+08	.	G	T	.	PASS	NUCKS	intrc	NM_	.	.	
ACaP09	2	2.43E+08	rs1996199	C	T	.	PASS	NEU4	intrc	NM_	.	.	
ACaP09	3	48327615	.	G	T	.	PASS	.	inte	NM_	dis	.	
ACaP09	3	1.7E+08	rs1873413	C	T	.	PASS	PRKCI	intrc	NM_	.	.	
ACaP09	3	1.95E+08	rs3723871	A	G	.	PASS	.	inte	NM_	dis	.	
ACaP09	3	1.95E+08	.	T	C	.	PASS	MUC20	intrc	NM_	.	.	
ACaP09	3	1.96E+08	rs2006820	G	A	.	PASS	SDHAP	ncR	NR_	.	.	
ACaP09	4	9245235	.	T	A	.	PASS	.	ups	NM_	.	.	
ACaP09	5	1.34E+08	.	C	A	.	PASS	CAMLG	UTF	NM_	NM	.	
ACaP09	6	1.6E+08	.	C	T	.	PASS	.	inte	NM_	dis	.	
ACaP09	6	1.61E+08	rs7746421	C	G	.	PASS	LPA	intrc	NM_	.	.	
ACaP09	6	1.71E+08	rs3759780	G	C	.	PASS	.	inte	NM_	dis	.	
ACaP09	7	63215333	.	C	G	.	PASS	.	inte	NR_	dis	.	
ACaP09	7	63215356	.	T	A	.	PASS	.	inte	NR_	dis	.	
ACaP09	7	70229814	.	T	G	.	PASS	AUTS2	exo	NM_	.	missens	
ACaP09	9	44990618	rs2803685	G	T	.	PASS	FAM27(	ncR	NR_	.	.	
ACaP09	10	47736243	rs1996770	C	T	.	PASS	.	inte	NM_	dis	.	
ACaP09	10	47736338	rs1456597	C	A	.	PASS	.	inte	NM_	dis	.	
ACaP09	11	6459067	.	A	C	.	PASS	HPX	intrc	NM_	.	.	
ACaP09	11	57281775	.	T	C	.	PASS	SLC43A	intrc	NM_	.	.	
ACaP09	12	88227	rs3718339	T	G	.	PASS	LOC10(	ncR	NR_	.	.	
ACaP09	12	89912	rs3767721	C	T	.	PASS	LOC10(	ncR	NR_	.	.	
ACaP09	12	64838771	rs5604999	A	T	.	PASS	XPOT	intrc	NM_	.	.	
ACaP09	14	19560932	rs7755744	G	A	.	PASS	POTEG	intrc	NM_	.	.	
ACaP09	14	20097348	rs1018908	C	T	.	PASS	.	inte	NM_	dis	.	
ACaP09	15	23436112	.	G	A	.	PASS	GOLGA	ncR	NR_	.	.	
ACaP09	15	35162992	rs7568595	T	C	.	PASS	AQR	exo	NM_	.	synonyr	
ACaP09	16	22497030	rs3739171	C	T	.	PASS	SMG1P	ncR	NR_	.	.	
ACaP09	16	55803763	rs7137409	G	A	.	PASS	CES1P1	ncR	NR_	.	.	
ACaP09	17	18402736	rs7124761	G	A	.	PASS	.	inte	NM_	dis	.	
ACaP09	17	21906848	rs6149181	G	T	.	PASS	FLJ360(	ncR	NR_	.	.	
ACaP09	17	58288284	rs2870408	T	C	.	PASS	USP32	intrc	NM_	.	.	
ACaP09	17	58288310	rs2848380	C	T	.	PASS	USP32	intrc	NM_	.	.	
ACaP09	17	79977953	.	G	C	.	PASS	STRA13	intrc	NM_	.	.	
ACaP09	18	61637184	.	C	A	.	PASS	.	ups	NM_	.	.	
ACaP09	19	9043665	.	A	G	.	PASS	MUC16	intrc	NM_	.	.	
ACaP09	21	9842077	.	G	T	.	PASS	.	inte	NR_	dis	.	
ACaP09	22	21538112	.	A	T	.	PASS	FAM23(	ncR	NR_	.	.	
ACaP09	22	42538399	rs3609392	C	T	.	PASS	CYP2D7	ncR	NR_	.	.	
ACaP09	X	57475295	.	C	A	.	PASS	FAAH2	intrc	NM_	.	.	
ACaP10	1	1290218	rs7558529	C	T	.	PASS	MXRA8	exo	NM_	.	missens	
ACaP10	1	1407731	.	C	T	.	PASS	ATAD3E	intrc	NM_	.	.	
ACaP10	1	36228102	.	G	C	.	PASS	CLSPN	intrc	NM_	.	.	

ACaP10	1	36291134	.	G	C	.	PASS	AGO4	intrc	NM_	.	.
ACaP10	1	38186581	.	C	T	.	PASS	EPHA1(	intrc	NM_	.	.
ACaP10	1	41827744	.	G	A	.	PASS	.	inte	NM_	dis	.
ACaP10	1	41827857	.	G	T	.	PASS	.	inte	NM_	dis	.
ACaP10	1	87329181	.	C	T	.	PASS	15-Sep	exo	NM_	.	unknow
ACaP10	1	90049401	.	C	T	.	PASS	LRRC8I	exo	NM_	.	stopgai
ACaP10	1	1.15E+08	rs1430207	T	C	.	PASS	SYCP1	intrc	NM_	.	.
ACaP10	1	1.21E+08	.	G	A	.	PASS	NOTCH	intrc	NM_	.	.
ACaP10	1	1.43E+08	rs1996428	C	T	.	PASS	.	inte	NR_	dis	.
ACaP10	1	1.45E+08	.	G	C	.	PASS	NBPF9,	intrc	NM_	.	.
ACaP10	1	1.45E+08	rs3755029	A	T	.	PASS	NBPF2!	ncR	NR_	.	.
ACaP10	1	1.48E+08	rs2849511	G	A	.	PASS	LINC01:	ncR	NR_	.	.
ACaP10	1	1.48E+08	rs7824596	A	C	.	PASS	NBPF8	intrc	NM_	.	.
ACaP10	1	1.48E+08	rs3765099	T	G	.	PASS	NBPF8	intrc	NM_	.	.
ACaP10	1	1.52E+08	rs9099303	C	T	.	PASS	TCHH	exo	NM_	.	missen
ACaP10	1	1.54E+08	.	G	C	.	PASS	ATP8B2	intrc	NM_	.	.
ACaP10	1	1.78E+08	.	G	A	.	PASS	RASAL2	intrc	NM_	.	.
ACaP10	1	2.04E+08	.	A	G	.	PASS	PLEKH/	intrc	NM_	.	.
ACaP10	1	2.07E+08	.	C	T	.	PASS	C4BPB	intrc	NM_	.	.
ACaP10	2	1.79E+08	.	G	T	.	PASS	TTN	splic	NM_	NM	.
ACaP10	2	2.19E+08	.	G	C	.	PASS	TNS1	intrc	NM_	.	.
ACaP10	3	4836817	rs7793139	G	A	.	PASS	ITPR1	exo	NM_	.	missen
ACaP10	3	8983527	.	C	T	.	PASS	RAD18	intrc	NM_	.	.
ACaP10	3	13678138	.	G	C	.	PASS	FBLN2	intrc	NM_	.	.
ACaP10	3	52529448	.	C	T	.	PASS	STAB1	exo	NM_	.	missen
ACaP10	4	1986605	rs7785731	C	T	.	PASS	NELFA	exo	NM_	.	synonyr
ACaP10	4	88901517	rs7749410	G	A	.	PASS	SPP1	intrc	NM_	.	.
ACaP10	5	14652335	rs2382048	A	G	.	PASS	.	inte	NM_	dis	.
ACaP10	5	79933788	rs7817278	C	G	.	PASS	DHFR	exo	NM_	.	missen
ACaP10	5	1.15E+08	.	A	G	.	PASS	COMMI	UTF	NM_	NM	.
ACaP10	5	1.21E+08	rs1140023	G	A	.	PASS	ZNF474	exo	NM_	.	missen
ACaP10	5	1.51E+08	.	C	T	.	PASS	SLC36A	intrc	NM_	.	.
ACaP10	5	1.61E+08	.	G	A	.	PASS	GABRB	exo	NM_	.	stopgai
ACaP10	6	2784638	.	G	C	.	PASS	WRNIP:	splic	NM_	NM	.
ACaP10	6	31833291	.	C	T	.	PASS	SLC44A	exo	NM_	.	missen
ACaP10	6	47220921	.	T	C	.	PASS	TNFRSI	intrc	NM_	.	.
ACaP10	6	1.29E+08	rs9907394	C	T	.	PASS	LAMA2	intrc	NM_	.	.
ACaP10	6	1.58E+08	.	G	A	.	PASS	ARID1B	exo	NM_	.	missen
ACaP10	6	1.68E+08	.	G	A	.	PASS	MLLT4	exo	NM_	.	missen
ACaP10	6	1.71E+08	.	G	A	.	PASS	TBP	intrc	NM_	.	.
ACaP10	7	2701347	.	C	G	.	PASS	TTYH3	exo	NM_	.	missen
ACaP10	7	6045510	.	C	G	.	PASS	PMS2	intrc	NM_	.	.
ACaP10	7	16255796	rs7468373	T	C	.	PASS	ISPD	exo	NM_	.	synonyr
ACaP10	7	43540300	.	A	T	.	PASS	HECW1	exo	NM_	.	missen
ACaP10	7	47622236	.	G	A	.	PASS	.	ups	NM_	.	.
ACaP10	7	1.44E+08	rs5705914	T	G	.	PASS	.	inte	NM_	dis	.
ACaP10	7	1.5E+08	rs1481871	C	T	.	PASS	GIMAP6	exo	NM_	.	missen
ACaP10	8	1624682	rs7588073	G	A	.	PASS	DLGAP:	intrc	NM_	.	.
ACaP10	8	11403517	rs7479925	G	A	.	PASS	BLK	intrc	NM_	.	.
ACaP10	8	43828041	.	T	G	.	PASS	.	inte	NM_	dis	.
ACaP10	8	1.12E+08	rs5327262	C	T	.	PASS	.	inte	NM_	dis	.
ACaP10	8	1.45E+08	rs5696070	G	A	.	PASS	CYC1	intrc	NM_	.	.
ACaP10	9	80144019	rs3682437	G	A	.	PASS	GNA14	exo	NM_	.	missen
ACaP10	9	1.07E+08	rs3746457	G	A	.	PASS	OR13C:	exo	NM_	.	synonyr

ACaP10	9	1.31E+08	rs7528554	G	A	.	PASS	ZER1	intrc	NM_	.	.
ACaP10	9	1.4E+08	rs5640471	G	A	.	PASS	PHPT1	exoı	NM_	.	missens
ACaP10	9	1.41E+08	rs5505690	C	T	.	PASS	CACNA	intrc	NM_	.	.
ACaP10	10	1.13E+08	.	G	A	.	PASS	RBM20	exoı	NM_	.	missens
ACaP10	11	28332552	rs7615769	A	T	.	PASS	METTL:	intrc	NM_	.	.
ACaP10	11	30953473	.	C	T	.	PASS	DCDC5	exoı	NM_	.	synonyı
ACaP10	11	46369302	rs7817387	C	G	.	PASS	DGKZ	exoı	NM_	.	synonyı
ACaP10	11	62414303	.	T	C	.	PASS	.	ups	NM_	.	.
ACaP10	11	63581174	rs7525419	G	A	.	PASS	C11orf8	exoı	NM_	.	missens
ACaP10	11	64976886	.	G	C	.	PASS	CAPN1	intrc	NM_	.	.
ACaP10	11	1.34E+08	.	G	A	.	PASS	GLB1L2	intrc	NM_	.	.
ACaP10	12	1.14E+08	.	C	G	.	PASS	RASAL:	exoı	NM_	.	missens
ACaP10	12	1.22E+08	rs1409770	G	A	.	PASS	WDR66	exoı	NM_	.	missens
ACaP10	13	37541814	.	T	C	.	PASS	ALG5	intrc	NM_	.	.
ACaP10	13	76445122	.	G	T	.	PASS	.	ups	NM_	.	.
ACaP10	14	19425348	.	C	G	.	PASS	.	inteı	NR_	dis	.
ACaP10	14	52508802	.	C	A	.	PASS	NID2	intrc	NM_	.	.
ACaP10	14	68257498	.	A	C	.	PASS	ZFYVE2	intrc	NM_	.	.
ACaP10	14	74375999	.	C	T	.	PASS	ZNF410	intrc	NM_	.	.
ACaP10	14	74376028	.	C	T	.	PASS	ZNF410	intrc	NM_	.	.
ACaP10	16	312533	rs7663922	C	T	.	PASS	ITFG3	exoı	NM_	.	missens
ACaP10	16	843324	rs5750779	C	T	.	PASS	CHTF18	intrc	NM_	.	.
ACaP10	16	5046192	.	C	T	.	PASS	SEC14L	intrc	NM_	.	.
ACaP10	16	23445193	.	T	C	.	PASS	COG7	intrc	NM_	.	.
ACaP10	16	33544403	rs7569695	A	G	.	PASS	RNU6-7	ncR	NR_	.	.
ACaP10	16	33544406	rs7520038	G	A	.	PASS	RNU6-7	ncR	NR_	.	.
ACaP10	17	29171141	.	T	C	.	PASS	ATAD5	intrc	NM_	.	.
ACaP10	17	34432681	rs3713755	C	T	.	PASS	CCL4	exoı	NM_	.	synonyı
ACaP10	17	38857404	rs7744485	C	T	.	PASS	KRT24	exoı	NM_	.	synonyı
ACaP10	17	48538137	rs7460185	G	A	.	PASS	ACSF2	exoı	NM_	.	synonyı
ACaP10	17	57719367	rs8668152	T	G	.	PASS	CLTC	intrc	NM_	.	.
ACaP10	17	60345769	rs8665784	T	G	.	PASS	TBC1D3	ncR	NR_	.	.
ACaP10	17	75471673	.	G	C	.	PASS	9-Sep	exoı	NM_	.	missens
ACaP10	17	77758598	rs1995663	G	A	.	PASS	CBX2	exoı	NM_	.	synonyı
ACaP10	17	80709957	.	G	T	.	PASS	TBCD	UTF	NM_	NM	.
ACaP10	19	37784499	rs3865492	C	G	.	PASS	.	inteı	NR_	dis	.
ACaP10	19	37794112	.	T	C	.	PASS	.	inteı	NR_	dis	.
ACaP10	19	42546812	rs7571526	G	A	.	PASS	GRIK5	exoı	NM_	.	synonyı
ACaP10	19	45448463	rs7565559	G	A	.	PASS	APOC4	exoı	NM_	.	synonyı
ACaP10	20	1559096	.	G	A	.	PASS	SIRPB1	exoı	NM_	.	synonyı
ACaP10	20	30231173	.	C	T	.	PASS	COX4I2	intrc	NM_	.	.
ACaP10	20	35783518	rs3774092	G	A	.	PASS	MROH8	exoı	NM_	.	unknow
ACaP10	21	46841517	.	C	A	.	PASS	COL18A	ncR	NR_	.	.
ACaP10	22	16290842	rs6197100	A	G	.	PASS	.	inteı	NM_	dis	.
ACaP10	22	21537446	.	A	G	.	PASS	FAM230	ncR	NR_	.	.
ACaP10	22	25045815	rs5662423	G	C	.	PASS	POM12	ncR	NR_	.	.
ACaP10	22	42761191	.	C	G	.	PASS	.	inteı	NR_	dis	.
ACaP10	22	45603891	rs1007675	G	A	.	PASS	KIAA09	intrc	NM_	.	.
ACaP10	X	48675681	.	G	C	.	PASS	HDAC6	intrc	NM_	.	.
ACaP10	X	48976038	.	G	A	.	PASS	GPKOW	intrc	NM_	.	.
ACaP10	X	49853406	rs7970448	C	T	.	PASS	CLCN5	exoı	NM_	.	stopgaiı
ACaP10	X	54824610	.	A	G	.	PASS	ITIH6	exoı	NM_	.	missens
ACaP10	X	1.52E+08	.	C	T	.	PASS	CSAG1	UTF	NM_	NM	.
ACaP11	1	17092158	rs4634900	A	G	.	PASS	.	inteı	NM_	dis	.

ACaP11	1	34238488	.	G	A	.	PASS	CSMD2	intrc	NM_	.	.
ACaP11	1	1.43E+08	rs7450506	G	T	.	PASS	.	inte	NR_	dis	.
ACaP11	1	1.43E+08	rs2016919	T	A	.	PASS	.	inte	NR_	dis	.
ACaP11	1	1.43E+08	.	A	T	.	PASS	.	inte	NR_	dis	.
ACaP11	1	1.43E+08	rs8662584	G	T	.	PASS	.	inte	NR_	dis	.
ACaP11	1	1.45E+08	rs4143495	G	A	.	PASS	NBPF25	ncR	NR_	.	.
ACaP11	1	1.48E+08	rs6181077	C	T	.	PASS	NBPF8	intrc	NM_	.	.
ACaP11	1	1.58E+08	.	G	T	.	PASS	CD1D	UTF	NM_	NM	.
ACaP11	1	2.06E+08	rs2003501	G	A	.	PASS	SLC26A	intrc	NM_	.	.
ACaP11	2	61468669	.	G	A	.	PASS	USP34	intrc	NM_	.	.
ACaP11	2	71776472	rs1834895	C	T	.	PASS	DYSF	intrc	NM_	.	.
ACaP11	2	71909724	rs2009908	C	T	.	PASS	DYSF	exo	NM_	.	missens
ACaP11	2	74687101	.	G	C	.	PASS	WBP1	exo	NM_	.	missens
ACaP11	2	98444405	rs9528	T	C	.	PASS	TMEM1	intrc	NM_	.	.
ACaP11	2	1.5E+08	.	A	G	.	PASS	EPC2	UTF	NM_	NM	.
ACaP11	2	2.41E+08	rs3739748	G	A	.	PASS	GPC1	exo	NM_	.	missens
ACaP11	3	27161284	rs5683253	C	T	.	PASS	.	inte	NM_	dis	.
ACaP11	3	50224219	rs1474450	C	T	.	PASS	SEMA3I	intrc	NM_	.	.
ACaP11	3	52014898	rs2004359	G	A	.	PASS	ABHD14	exo	NM_	.	missens
ACaP11	3	1.19E+08	.	C	T	.	PASS	ARHGA	exo	NM_	.	stopgai
ACaP11	4	3516405	.	T	G	.	PASS	LRPAP1	intrc	NM_	.	.
ACaP11	4	6080805	.	T	C	.	PASS	JAKMIP	intrc	NM_	.	.
ACaP11	4	8230040	.	G	A	.	PASS	SH3TC1	exo	NM_	.	synonyr
ACaP11	4	41631469	.	G	A	.	PASS	LIMCH1	intrc	NM_	.	.
ACaP11	4	49165445	rs2932367	A	T	.	PASS	.	inte	NM_	dis	.
ACaP11	4	49235403	.	T	C	.	PASS	.	inte	NM_	dis	.
ACaP11	4	53463959	.	G	A	.	PASS	USP46	intrc	NM_	.	.
ACaP11	4	80328488	rs7669673	C	T	.	PASS	GK2	exo	NM_	.	synonyr
ACaP11	5	60188960	.	C	T	.	PASS	ERCC8	intrc	NM_	.	.
ACaP11	5	60628079	.	C	A	.	PASS	.	ups	NM_	.	.
ACaP11	5	1.02E+08	.	G	A	.	PASS	SLCO4C	exo	NM_	.	missens
ACaP11	5	1.44E+08	.	G	A	.	PASS	YIPF5	UTF	NM_	NM	.
ACaP11	5	1.49E+08	rs5319092	G	A	.	PASS	ABLIM3	UTF	NM_	NM	.
ACaP11	6	10926514	rs5661365	A	T	.	PASS	SYCP2I	intrc	NM_	.	.
ACaP11	6	29797493	.	C	T	.	PASS	HLA-G	intrc	NM_	.	.
ACaP11	6	32053837	.	C	A	.	PASS	TNXB	exo	NM_	.	synonyr
ACaP11	7	1539158	rs1814105	G	A	.	PASS	INTS1	exo	NM_	.	synonyr
ACaP11	7	56870145	rs7827655	G	A	.	PASS	.	inte	NR_	dis	.
ACaP11	7	87323300	rs1484992	G	A	.	PASS	RUNDC	intrc	NM_	.	.
ACaP11	7	1E+08	rs5523938	A	T	.	PASS	EPHB4	intrc	NM_	.	.
ACaP11	7	1.37E+08	.	G	T	.	PASS	DGKI	exo	NM_	.	synonyr
ACaP11	7	1.37E+08	rs3737114	G	A	.	PASS	DGKI	intrc	NM_	.	.
ACaP11	7	1.44E+08	.	C	T	.	PASS	OR2A25	exo	NM_	.	synonyr
ACaP11	8	51675009	rs9982298	G	A	.	PASS	SNTG1	intrc	NM_	.	.
ACaP11	8	92092953	.	C	T	.	PASS	OTUD6I	exo	NM_	.	missens
ACaP11	9	3937164	.	G	A	.	PASS	GLIS3	exo	NM_	.	missens
ACaP11	9	31254421	.	G	A	.	PASS	.	inte	NR_	dis	.
ACaP11	9	67334841	rs9330412	T	C	.	PASS	.	inte	NR_	dis	.
ACaP11	9	95858738	.	G	A	.	PASS	C9orf89	intrc	NM_	.	.
ACaP11	9	1.16E+08	.	C	A	.	PASS	.	inte	NM_	dis	.
ACaP11	9	1.39E+08	rs9459874	G	A	.	PASS	GPSM1	exo	NM_	.	missens
ACaP11	9	1.41E+08	rs9494422	C	T	.	PASS	CACNA	intrc	NM_	.	.
ACaP11	10	25465126	.	C	G	.	PASS	GPR158	exo	NM_	.	missens
ACaP11	10	38344467	.	T	A	.	PASS	ZNF33A	exo	NM_	.	missens

ACaP11	10	49200349	rs2996811	G	A	.	PASS	.	inte	NR_	dis	.
ACaP11	10	51754214	.	G	C	.	PASS	AGAP6	intro	NM_	.	.
ACaP11	10	97725811	.	G	C	.	PASS	ENTPD	ncR	NR_	.	.
ACaP11	10	99426911	rs7792060	G	A	.	PASS	PI4K2A	intro	NM_	.	.
ACaP11	11	12539367	.	T	A	.	PASS	PARVA	intro	NM_	.	.
ACaP11	11	1.08E+08	.	C	A	.	PASS	ATM	intro	NM_	.	.
ACaP11	11	1.19E+08	.	T	G	.	PASS	HYOU1	exo	NM_	.	missens
ACaP11	11	1.34E+08	.	T	C	.	PASS	GLB1L2	intro	NM_	.	.
ACaP11	12	7894149	.	T	C	.	PASS	CLEC4(	intro	NM_	.	.
ACaP11	12	12038881	.	G	A	.	PASS	ETV6	exo	NM_	.	missens
ACaP11	12	25398284	rs1219135	C	T	.	PASS	KRAS	exo	NM_	.	missens
ACaP11	12	57975211	rs3522560	G	A	.	PASS	KIF5A	exo	NM_	.	synonyr
ACaP11	12	75435988	.	T	A	.	PASS	KCNC2	UTF	NM_	NM	.
ACaP11	12	1.04E+08	rs5396169	G	A	.	PASS	GNN	ncR	NR_	.	.
ACaP11	13	84480908	.	G	A	.	PASS	.	inte	NM_	dis	.
ACaP11	14	54907897	.	C	T	.	PASS	CNIH1	intro	NM_	.	.
ACaP11	14	99439761	rs8791962	G	A	.	PASS	.	inte	NM_	dis	.
ACaP11	15	22319705	.	G	A	.	PASS	LOC727	ncR	NR_	.	.
ACaP11	15	23931877	.	A	G	.	PASS	NDN	exo	NM_	.	missens
ACaP11	15	27128535	.	C	T	.	PASS	GABRA	exo	NM_	.	missens
ACaP11	15	32778129	rs7831577	A	C	.	PASS	.	inte	NM_	dis	.
ACaP11	15	84839208	rs2010114	T	A	.	PASS	.	inte	NR_	dis	.
ACaP11	16	25266886	.	G	A	.	PASS	ZKSCAI	intro	NM_	.	.
ACaP11	16	33500640	.	T	C	.	PASS	RNU6-7	ncR	NR_	.	.
ACaP11	16	49313530	.	C	A	.	PASS	CBLN1	intro	NM_	.	.
ACaP11	16	70013113	rs2001829	A	C	.	PASS	PDXDC	ncR	NR_	.	.
ACaP11	16	81941457	.	C	A	.	PASS	PLCG2	intro	NM_	.	.
ACaP11	17	33353396	.	C	T	.	PASS	RFFL	exo	NM_	.	synonyr
ACaP11	17	36349253	.	C	T	.	PASS	.	ups	NM_	.	.
ACaP11	17	37395078	.	C	A	.	PASS	.	inte	NM_	dis	.
ACaP11	17	79424399	.	C	G	.	PASS	BAHCC	intro	NM_	.	.
ACaP11	18	61323506	.	T	A	.	PASS	SERPIN	intro	NM_	.	.
ACaP11	19	3811459	rs8673654	T	C	.	PASS	ZFR2	intro	NM_	.	.
ACaP11	19	7935901	.	A	T	.	PASS	FLJ2218	exo	NM_	.	unknow
ACaP11	19	11319376	rs7680686	C	T	.	PASS	DOCK6	exo	NM_	.	missens
ACaP11	19	36431190	.	A	T	.	PASS	LRFN3	exo	NM_	.	missens
ACaP11	19	40025280	.	G	A	.	PASS	.	inte	NM_	dis	.
ACaP11	19	44635910	.	C	T	.	PASS	ZNF225	exo	NM_	.	synonyr
ACaP11	19	54818898	.	G	A	.	PASS	LILRA5	intro	NM_	.	.
ACaP11	19	55481747	.	G	A	.	PASS	NLRP2	intro	NM_	.	.
ACaP11	19	58385069	rs7705940	G	A	.	PASS	ZNF814	exo	NM_	.	synonyr
ACaP11	20	2083840	.	G	A	.	PASS	STK35	exo	NM_	.	missens
ACaP11	20	23432566	rs2002254	G	A	.	PASS	CST11	intro	NM_	.	.
ACaP11	20	37581121	.	G	T	.	PASS	FAM83I	exo	NM_	.	missens
ACaP11	20	53968515	.	C	T	.	PASS	.	inte	NM_	dis	.
ACaP11	21	22881344	.	T	G	.	PASS	NCAM2	exo	NM_	.	missens
ACaP11	22	33402489	.	G	A	.	PASS	SYN3	exo	NM_	.	synonyr
ACaP11	X	48136636	.	C	G	.	PASS	.	inte	NM_	dis	.
ACaP11	X	1.01E+08	.	G	A	.	PASS	ARMCX	exo	NM_	.	synonyr
ACaP11	X	1.04E+08	.	C	T	.	PASS	TEX13A	exo	NM_	.	missens
ACaP11	X	1.05E+08	rs7533837	C	T	.	PASS	NRK	exo	NM_	.	synonyr
ACaP11	X	1.23E+08	.	C	T	.	PASS	SH2D1A	intro	NM_	.	.
ACaP11	X	1.35E+08	rs7767123	C	T	.	PASS	MAP7D	intro	NM_	.	.
ACaP11	X	1.45E+08	.	C	A	.	PASS	.	inte	NM_	dis	.

ACaP11	X	1.54E+08	rs3981235	G	A	.	PASS	G6PD	exo	NM_	.	missens
ACaP11	MT	12641	.	A	G	.	PASS	.	inte	NON	dis	.
ACaP12	1	10478868	.	C	T	.	PASS	PGD	intrc	NM_	.	.
ACaP12	1	16954895	rs1015792	T	C	.	PASS	CROCC	ncR	NR_	.	.
ACaP12	1	19176042	.	C	T	.	PASS	TAS1R2	exo	NM_	.	synonyr
ACaP12	1	21945544	rs1453424	G	A	.	PASS	RAP1G/	exo	NM_	.	missens
ACaP12	1	92595302	.	G	T	.	PASS	BTBD8	exo	NM_	.	stopgai
ACaP12	1	1.43E+08	rs7704066	T	C	.	PASS	.	inte	NR_	dis	.
ACaP12	1	1.43E+08	rs1415172	A	G	.	PASS	.	inte	NR_	dis	.
ACaP12	1	1.43E+08	rs1256214	A	G	.	PASS	.	inte	NR_	dis	.
ACaP12	1	1.46E+08	rs3721624	C	T	.	PASS	NBPF25	ncR	NR_	.	.
ACaP12	1	1.46E+08	rs5876752	C	T	.	PASS	NBPF25	ncR	NR_	.	.
ACaP12	1	1.49E+08	.	A	G	.	PASS	NBPF25	ncR	NR_	.	.
ACaP12	1	1.5E+08	rs3730063	C	T	.	PASS	CA14	exo	NM_	.	synonyr
ACaP12	1	1.84E+08	.	G	C	.	PASS	NCF2	intrc	NM_	.	.
ACaP12	1	2.11E+08	.	C	G	.	PASS	KCNH1	exo	NM_	.	missens
ACaP12	2	47748923	.	G	A	.	PASS	KCNK12	exo	NM_	.	missens
ACaP12	2	90154277	rs7540104	G	A	.	PASS	.	inte	NR_	dis	.
ACaP12	2	1.55E+08	.	G	A	.	PASS	GALNT	intrc	NM_	.	.
ACaP12	2	1.79E+08	.	C	A	.	PASS	MIR548	ncR	NR_	.	.
ACaP12	2	1.98E+08	.	G	T	.	PASS	HSPD1	intrc	NM_	.	.
ACaP12	2	2.36E+08	rs9825331	G	A	.	PASS	SH3BP4	intrc	NM_	.	.
ACaP12	3	4459126	.	A	G	.	PASS	SUMF1	intrc	NM_	.	.
ACaP12	3	5025541	rs1309395	A	G	.	PASS	BHLHE4	UTF	NM_	NM	.
ACaP12	3	32493464	.	A	C	.	PASS	CMTM7	intrc	NM_	.	.
ACaP12	3	39142000	rs3710999	G	A	.	PASS	GORAS	intrc	NM_	.	.
ACaP12	3	40232079	.	G	T	.	PASS	EIF1B-A	ncR	NR_	.	.
ACaP12	3	49094476	.	G	A	.	PASS	QRICH1	exo	NM_	.	missens
ACaP12	3	49695410	.	G	A	.	PASS	BSN	exo	NM_	.	synonyr
ACaP12	3	49695411	.	G	T	.	PASS	BSN	exo	NM_	.	missens
ACaP12	3	50402763	.	C	T	.	PASS	CACNA	intrc	NM_	.	.
ACaP12	3	1.14E+08	.	T	C	.	PASS	.	inte	NM_	dis	.
ACaP12	3	1.26E+08	rs5729372	T	C	.	PASS	ALDH1L	ncR	NR_	.	.
ACaP12	4	49846	rs4970287	C	T	.	PASS	.	inte	NON	dis	.
ACaP12	4	72675845	rs1456513	C	T	.	PASS	.	inte	NM_	dis	.
ACaP12	4	88056744	.	G	A	.	PASS	AFF1	exo	NM_	.	missens
ACaP12	4	1.19E+08	.	A	G	.	PASS	SNHG8	ncR	NR_	.	.
ACaP12	4	1.26E+08	rs3727861	C	T	.	PASS	FAT4	exo	NM_	.	missens
ACaP12	4	1.74E+08	.	C	T	.	PASS	GALNT	exo	NM_	.	missens
ACaP12	4	1.89E+08	.	A	G	.	PASS	TRIML2	intrc	NM_	.	.
ACaP12	5	1.49E+08	.	G	C	.	PASS	ABLIM3	UTF	NM_	NM	.
ACaP12	5	1.56E+08	.	C	A	.	PASS	HAVCR	intrc	NM_	.	.
ACaP12	6	29640133	.	C	G	.	PASS	MOG	UTF	NM_	NM	.
ACaP12	6	38704724	.	C	G	.	PASS	DNAH8	intrc	NM_	.	.
ACaP12	6	98472367	rs9010911	C	T	.	PASS	.	ups	NR_	.	.
ACaP12	6	1.19E+08	.	A	T	.	PASS	CEP85L	intrc	NM_	.	.
ACaP12	7	5160948	rs1544467	G	C	.	PASS	ZNF890	ncR	NR_	.	.
ACaP12	7	6194972	.	G	C	.	PASS	USP42	intrc	NM_	.	.
ACaP12	7	23775541	.	C	G	.	PASS	STK31	intrc	NM_	.	.
ACaP12	7	27211839	.	C	A	.	PASS	HOXA1(	ncR	NR_	.	.
ACaP12	7	45145116	.	T	C	.	PASS	TBRG4	exo	NM_	.	missens
ACaP12	7	48886013	rs1929995	G	A	.	PASS	.	inte	NM_	dis	.
ACaP12	7	66532216	.	T	G	.	PASS	TYW1	intrc	NM_	.	.
ACaP12	7	75192365	.	G	A	.	PASS	HIP1	exo	NM_	.	synonyr

ACaP12	7	95040962	.	T	A	.	PASS	PON2	splic	NM_	NM	.
ACaP12	7	98552820	.	G	C	.	PASS	TRRAP	exo	NM_	.	missens
ACaP12	7	1.33E+08	rs5753233	C	T	.	PASS	CHCHD	intrc	NM_	.	.
ACaP12	7	1.35E+08	.	G	A	.	PASS	NUP205	intrc	NM_	.	.
ACaP12	8	47697282	.	G	A	.	PASS	.	inte	NON	dis	.
ACaP12	8	71581321	.	G	C	.	PASS	LACTB2	exo	NM_	.	missens
ACaP12	8	73150309	rs5472481	T	C	.	PASS	LOC392	ncR	NR_	.	.
ACaP12	8	87111441	.	G	C	.	PASS	ATP6V0	intrc	NM_	.	.
ACaP12	9	28149024	rs3746546	C	T	.	PASS	LINGO2	intrc	NM_	.	.
ACaP12	9	34372554	.	C	T	.	PASS	KIAA11	exo	NM_	.	unknow
ACaP12	9	72131055	.	C	T	.	PASS	APBA1	exo	NM_	.	missens
ACaP12	9	78686669	.	C	T	.	PASS	PCSK5	exo	NM_	.	missens
ACaP12	9	1.16E+08	rs2868900	G	A	.	PASS	.	inte	NM_	dis	.
ACaP12	9	1.17E+08	rs3719801	C	T	.	PASS	MIR455	ncR	NR_	.	.
ACaP12	9	1.39E+08	rs9976753	G	A	.	PASS	CARD9	intrc	NM_	.	.
ACaP12	9	1.39E+08	rs3763504	C	T	.	PASS	SEC16A	exo	NM_	.	missens
ACaP12	10	3184061	rs9266937	C	T	.	PASS	PITRM1	ncR	NR_	.	.
ACaP12	10	34624952	.	A	T	.	PASS	PARD3	intrc	NM_	.	.
ACaP12	10	46968906	.	A	G	.	PASS	SYT15	intrc	NM_	.	.
ACaP12	10	75290252	.	T	A	.	PASS	USP54	exo	NM_	.	stopgai
ACaP12	10	94274858	.	T	G	.	PASS	IDE	intrc	NM_	.	.
ACaP12	10	1.29E+08	.	A	C	.	PASS	DOCK1	intrc	NM_	.	.
ACaP12	11	5021198	.	A	C	.	PASS	.	dow	NM_	.	.
ACaP12	11	18057716	.	C	T	.	PASS	TPH1	intrc	NM_	.	.
ACaP12	11	18333062	.	T	A	.	PASS	HPS5	intrc	NM_	.	.
ACaP12	11	55234524	.	C	T	.	PASS	.	inte	NM_	dis	.
ACaP12	11	57184188	rs3696017	C	T	.	PASS	SLC43A	intrc	NM_	.	.
ACaP12	11	66743255	.	C	T	.	PASS	C11orf8	intrc	NM_	.	.
ACaP12	11	1.07E+08	.	G	A	.	PASS	CWF19	exo	NM_	.	stopgai
ACaP12	11	1.19E+08	.	G	A	.	PASS	USP2-A	ncR	NR_	.	.
ACaP12	12	48387872	.	A	C	.	PASS	COL2A1	intrc	NM_	.	.
ACaP12	12	1.01E+08	.	G	C	.	PASS	DEPDC	exo	NM_	.	missens
ACaP12	12	1.13E+08	rs8683709	T	C	.	PASS	HECTD	intrc	NM_	.	.
ACaP12	12	1.17E+08	.	G	A	.	PASS	MED13I	intrc	NM_	.	.
ACaP12	12	1.21E+08	rs5681239	C	T	.	PASS	HNF1A	exo	NM_	.	missens
ACaP12	13	99554177	rs7528858	C	T	.	PASS	DOCK9	intrc	NM_	.	.
ACaP12	14	23394231	.	T	C	.	PASS	PRMT5	exo	NM_	.	missens
ACaP12	14	23574113	rs1288039	A	G	.	PASS	.	inte	NM_	dis	.
ACaP12	14	1.02E+08	rs5316143	G	A	.	PASS	.	ups	NR_	.	.
ACaP12	15	23931748	.	C	G	.	PASS	NDN	exo	NM_	.	missens
ACaP12	15	30019030	rs7540721	C	T	.	PASS	TJP1	exo	NM_	.	missens
ACaP12	15	30675581	rs2015691	A	C	.	PASS	CHRFA	UTF	NM_	NM	.
ACaP12	15	30875177	rs7122570	C	G	.	PASS	ULK4P1	ncR	NR_	.	.
ACaP12	15	34649590	.	G	A	.	PASS	NUTM1	exo	NM_	.	synonyr
ACaP12	15	44828896	.	C	T	.	PASS	EIF3J-A	ncR	NR_	.	.
ACaP12	15	78217260	rs1869226	T	A	.	PASS	LOC645	ncR	NR_	.	.
ACaP12	15	78217296	rs8789048	A	G	.	PASS	LOC645	ncR	NR_	.	.
ACaP12	15	78369712	.	C	T	.	PASS	TBC1D2	exo	NM_	.	missens
ACaP12	15	83242000	.	A	T	.	PASS	CPEB1	intrc	NM_	.	.
ACaP12	16	2024786	rs7655357	C	T	.	PASS	TBL3	exo	NM_	.	synonyr
ACaP12	16	3594311	rs2015706	G	A	.	PASS	NLRC3	exo	NM_	.	synonyr
ACaP12	16	5097836	.	T	C	.	PASS	C16orf8	intrc	NM_	.	.
ACaP12	16	71571633	.	C	T	.	PASS	CHST4	exo	NM_	.	synonyr
ACaP12	17	1703092	.	C	G	.	PASS	SMYD4	intrc	NM_	.	.

ACaP12	17	3704387	.	G	A	.	PASS	ITGAE	intrc	NM_	.	.
ACaP12	17	5328662	rs7781206	T	C	.	PASS	RPAIN	intrc	NM_	.	.
ACaP12	17	54403491	.	C	T	.	PASS	ANKFN:	intrc	NM_	.	.
ACaP12	17	72860511	rs1134430	C	T	.	PASS	FDXR	intrc	NM_	.	.
ACaP12	17	73748472	.	G	A	.	PASS	ITGB4	intrc	NM_	.	.
ACaP12	17	78897302	rs7648089	G	A	.	PASS	RPTOR	exoı	NM_	.	synonyr
ACaP12	17	79804058	.	C	T	.	PASS	P4HB	intrc	NM_	.	.
ACaP12	17	80275317	rs3729739	C	T	.	PASS	CD7	exoı	NM_	.	missens
ACaP12	17	80923509	.	C	G	.	PASS	B3GNTI	intrc	NM_	.	.
ACaP12	17	80923732	.	C	G	.	PASS	B3GNTI	intrc	NM_	.	.
ACaP12	18	671490	.	C	T	.	PASS	ENOSF	UTF	NM_	NM	.
ACaP12	18	671523	.	C	T	.	PASS	ENOSF	UTF	NM_	NM	.
ACaP12	18	32919698	.	A	C	.	PASS	ZNF24	intrc	NM_	.	.
ACaP12	19	3456537	rs5282381	C	T	.	PASS	NFIC	intrc	NM_	.	.
ACaP12	19	3456645	.	G	A	.	PASS	NFIC	intrc	NM_	.	.
ACaP12	19	3456958	rs9075262	G	A	.	PASS	NFIC	intrc	NM_	.	.
ACaP12	19	3456966	.	G	T	.	PASS	NFIC	intrc	NM_	.	.
ACaP12	19	9722191	.	C	A	.	PASS	ZNF561	intrc	NM_	.	.
ACaP12	19	13925374	.	C	T	.	PASS	ZSWIM	intrc	NM_	.	.
ACaP12	19	38230186	.	A	C	.	PASS	ZNF573	exoı	NM_	.	missens
ACaP12	19	44770644	.	C	A	.	PASS	ZNF233	intrc	NM_	.	.
ACaP12	19	49247486	.	G	C	.	PASS	IZUMO1	intrc	NM_	.	.
ACaP12	19	49254265	.	G	A	.	PASS	FUT1	exoı	NM_	.	stopgaiı
ACaP12	19	49254480	.	G	A	.	PASS	FUT1	exoı	NM_	.	missens
ACaP12	19	51228430	.	C	T	.	PASS	CLEC11	exoı	NM_	.	synonyr
ACaP12	19	53321368	.	C	A	.	PASS	ZNF28	intrc	NM_	.	.
ACaP12	19	54418876	.	C	A	.	PASS	CACNG	intrc	NM_	.	.
ACaP12	20	39987411	rs7457076	C	T	.	PASS	LPIN3	exoı	NM_	.	missens
ACaP12	21	9826941	.	G	A	.	PASS	.	dow	NR_	.	.
ACaP12	21	10139851	rs8792017	A	G	.	PASS	.	inteı	NR_	dis	.
ACaP12	21	10935163	.	A	C	.	PASS	TPTE	intrc	NM_	.	.
ACaP12	22	18121731	rs1167407	C	T	.	PASS	BCL2L1	intrc	NM_	.	.
ACaP12	22	18898908	rs3760330	A	G	.	PASS	DGCR6	intrc	NM_	.	.
ACaP12	22	31487231	.	C	T	.	PASS	SMTN	exoı	NM_	.	stopgaiı
ACaP12	X	73811619	.	C	T	.	PASS	RLIM	exoı	NM_	.	missens
ACaP12	X	1.39E+08	.	G	T	.	PASS	MCF2	exoı	NM_	.	missens
ACaP12	X	1.41E+08	.	T	A	.	PASS	MAGEC	UTF	NM_	NM	.
ACaP13	1	1416370	rs1153096	T	C	.	PASS	ATAD3I	intrc	NM_	.	.
ACaP13	1	1960807	.	T	G	.	PASS	GABRD	intrc	NM_	.	.
ACaP13	1	38095397	.	G	A	.	PASS	RSPO1	UTF	NM_	NM	.
ACaP13	1	46088944	rs5317072	C	T	.	PASS	CCDC1	exoı	NM_	.	synonyr
ACaP13	1	52761662	.	T	A	.	PASS	ZFYVE	intrc	NM_	.	.
ACaP13	1	57756744	rs7787296	C	T	.	PASS	DAB1	UTF	NM_	NM	.
ACaP13	1	1E+08	.	C	T	.	PASS	AGL	exoı	NM_	.	synonyr
ACaP13	1	1.43E+08	rs7736232	A	T	.	PASS	.	inteı	NR_	dis	.
ACaP13	1	1.43E+08	rs1712174	A	C	.	PASS	.	inteı	NR_	dis	.
ACaP13	1	1.53E+08	.	C	A	.	PASS	LCE2D	UTF	NM_	NM	.
ACaP13	1	1.54E+08	.	C	T	.	PASS	UBAP2I	intrc	NM_	.	.
ACaP13	1	1.61E+08	.	C	T	.	PASS	NIT1	intrc	NM_	.	.
ACaP13	1	1.83E+08	.	T	G	.	PASS	LAMC2	intrc	NM_	.	.
ACaP13	1	1.96E+08	.	G	T	.	PASS	KCNT2	intrc	NM_	.	.
ACaP13	1	1.98E+08	.	A	T	.	PASS	C1orf53	UTF	NM_	NM	.
ACaP13	1	2.03E+08	.	G	A	.	PASS	KDM5B	exoı	NM_	.	missens
ACaP13	1	2.44E+08	.	G	A	.	PASS	SDCCA	intrc	NM_	.	.

ACaP13	2	3391528	.	C	T	.	PASS	TRAPP(	exo	NM_	.	missens
ACaP13	2	3391529	.	C	T	.	PASS	TRAPP(	exo	NM_	.	synonyr
ACaP13	2	15467935	.	C	A	.	PASS	NBAS	exo	NM_	.	missens
ACaP13	2	32474956	.	G	T	.	PASS	NLRC4	exo	NM_	.	missens
ACaP13	2	69300244	.	G	A	.	PASS	ANTXR:	intrc	NM_	.	.
ACaP13	2	95858692	rs2015850	T	C	.	PASS	.	inte	NM_	dis	.
ACaP13	2	98351736	.	A	T	.	PASS	ZAP70	exo	NM_	.	missens
ACaP13	2	1.07E+08	rs2008974	G	T	.	PASS	RGPD3	intrc	NM_	.	.
ACaP13	2	1.19E+08	rs7249504	G	T	.	PASS	INSIG2	intrc	NM_	.	.
ACaP13	2	1.19E+08	.	A	T	.	PASS	INSIG2	intrc	NM_	.	.
ACaP13	2	1.79E+08	.	G	A	.	PASS	TTN	exo	NM_	.	synonyr
ACaP13	2	2.04E+08	.	C	A	.	PASS	ICA1L	UTF	NM_	NM	.
ACaP13	2	2.04E+08	rs5520376	C	T	.	PASS	ABI2	intrc	NM_	.	.
ACaP13	2	2.04E+08	.	C	G	.	PASS	ABI2	intrc	NM_	.	.
ACaP13	2	2.17E+08	rs1816528	G	A	.	PASS	LINC00(	ncR	NR_	.	.
ACaP13	2	2.33E+08	.	G	A	.	PASS	PRSS5(	exo	NM_	.	missens
ACaP13	3	44816983	.	C	G	.	PASS	KIF15	intrc	NM_	.	.
ACaP13	3	46940375	rs9185110	C	G	.	PASS	PTH1R	intrc	NM_	.	.
ACaP13	3	52551451	rs3735896	C	T	.	PASS	STAB1	intrc	NM_	.	.
ACaP13	3	98530296	.	T	C	.	PASS	DCBLD:	intrc	NM_	.	.
ACaP13	3	98531184	.	A	G	.	PASS	DCBLD:	exo	NM_	.	missens
ACaP13	3	1.01E+08	.	T	G	.	PASS	RPL24	intrc	NM_	.	.
ACaP13	3	1.22E+08	.	G	A	.	PASS	ILDR1	UTF	NM_	NM	.
ACaP13	3	1.3E+08	rs1456223	C	T	.	PASS	COL6A(	exo	NM_	.	synonyr
ACaP13	3	1.79E+08	rs1048860	G	A	.	PASS	PIK3CA	exo	NM_	.	missens
ACaP13	4	3516405	.	T	G	.	PASS	LRPAP:	intrc	NM_	.	.
ACaP13	4	4158786	rs4018318	C	T	.	PASS	.	inte	NR_	dis	.
ACaP13	4	42466856	.	C	T	.	PASS	ATP8A1	intrc	NM_	.	.
ACaP13	4	42626770	.	G	A	.	PASS	ATP8A1	intrc	NM_	.	.
ACaP13	4	74810030	.	G	T	.	PASS	.	inte	NM_	dis	.
ACaP13	4	1.41E+08	.	G	C	.	PASS	SCOC	intrc	NM_	.	.
ACaP13	4	1.91E+08	.	C	G	.	PASS	.	inte	NM_	dis	.
ACaP13	5	21461937	.	C	T	.	PASS	GUSBP	ncR	NR_	.	.
ACaP13	5	54696250	.	G	A	.	PASS	SKIV2L:	splic	NM_	NM	.
ACaP13	5	1.2E+08	.	G	A	.	PASS	PRR16	UTF	NM_	NM	.
ACaP13	5	1.31E+08	.	A	T	.	PASS	RAPGE	intrc	NM_	.	.
ACaP13	5	1.41E+08	rs7735798	C	T	.	PASS	PCDHG	exo	NM_	.	missens
ACaP13	5	1.71E+08	.	C	T	.	PASS	FGF18	exo	NM_	.	synonyr
ACaP13	5	1.79E+08	.	G	A	.	PASS	TBC1D!	exo	NM_	.	stopgai
ACaP13	5	1.8E+08	rs3761176	C	T	.	PASS	BTNL9	exo	NM_	.	missens
ACaP13	6	25279438	.	C	T	.	PASS	.	ups	NM_	.	.
ACaP13	6	30621054	rs1419237	T	G	.	PASS	DHX16	exo	NM_	.	missens
ACaP13	6	1.12E+08	rs3687466	C	T	.	PASS	LAMA4	splic	NM_	NM	.
ACaP13	6	1.5E+08	.	G	A	.	PASS	RAET1(	exo	NM_	.	synonyr
ACaP13	6	1.54E+08	.	G	A	.	PASS	OPRM1	exo	NM_	.	synonyr
ACaP13	6	1.56E+08	rs2014763	G	A	.	PASS	TIAM2	exo	NM_	.	missens
ACaP13	6	1.71E+08	rs3759780	G	C	.	PASS	.	inte	NM_	dis	.
ACaP13	7	44524932	rs7676901	A	G	.	PASS	NUDCD	intrc	NM_	.	.
ACaP13	7	44609630	.	T	C	.	PASS	DDX56	exo	NM_	.	missens
ACaP13	7	56884371	rs6246184	G	A	.	PASS	.	inte	NR_	dis	.
ACaP13	7	56893786	rs2013722	C	T	.	PASS	.	inte	NR_	dis	.
ACaP13	7	66458446	.	C	G	.	PASS	SBDS	intrc	NM_	.	.
ACaP13	7	72440173	.	C	T	.	PASS	LOC541	ncR	NR_	.	.
ACaP13	7	1.01E+08	rs7611895	G	A	.	PASS	MUC12	exo	NM_	.	missens

ACaP13	7	1.41E+08	.	A	C	.	PASS	KIAA11	intrc	NM_	.	.
ACaP13	7	1.52E+08	rs1419939	A	T	.	PASS	KMT2C	exol	NM_	.	synonyr
ACaP13	7	1.56E+08	.	G	A	.	PASS	.	intel	NM_	dis	.
ACaP13	8	9593033	.	G	A	.	PASS	TNKS	intrc	NM_	.	.
ACaP13	8	12453566	rs7484521	C	A	.	PASS	LOC72	ncR	NR_	.	.
ACaP13	8	21859882	.	A	C	.	PASS	XPO7	intrc	NM_	.	.
ACaP13	8	22426860	.	T	C	.	PASS	SORBS	intrc	NM_	.	.
ACaP13	8	89131069	.	G	A	.	PASS	MMP16	exol	NM_	.	missen
ACaP13	8	1.05E+08	rs5352633	G	A	.	PASS	RIMS2	intrc	NM_	.	.
ACaP13	8	1.13E+08	rs5594625	A	T	.	PASS	CSMD3	intrc	NM_	.	.
ACaP13	8	1.25E+08	rs3766154	C	T	.	PASS	ANXA1	exol	NM_	.	missen
ACaP13	8	1.45E+08	.	G	A	.	PASS	CCDC1	intrc	NM_	.	.
ACaP13	8	1.45E+08	.	G	A	.	PASS	OPLAH	exol	NM_	.	missen
ACaP13	9	15468835	.	G	T	.	PASS	PSIP1	exol	NM_	.	synonyr
ACaP13	9	17463175	.	T	C	.	PASS	CNTLN	intrc	NM_	.	.
ACaP13	9	44990618	rs2803685	G	T	.	PASS	FAM27	ncR	NR_	.	.
ACaP13	9	90749743	rs7553291	G	A	.	PASS	SPATA	exol	NM_	.	synonyr
ACaP13	9	1.28E+08	.	A	G	.	PASS	GAPVD	exol	NM_	.	missen
ACaP13	10	3190592	rs1901417	G	A	.	PASS	PITRM1	ncR	NR_	.	.
ACaP13	10	33196033	.	G	T	.	PASS	ITGB1	exol	NM_	.	missen
ACaP13	10	38939588	rs4070334	G	A	.	PASS	.	intel	NR_	dis	.
ACaP13	10	81319598	.	C	T	.	PASS	SFTPA	intrc	NM_	.	.
ACaP13	10	89622388	.	G	C	.	PASS	KLLN	UTF	NM_	NM	.
ACaP13	10	97824204	.	G	A	.	PASS	ENTPD	ncR	NR_	.	.
ACaP13	11	1024083	rs7488284	G	A	.	PASS	MUC6	exol	NM_	.	synonyr
ACaP13	11	11952236	.	C	T	.	PASS	USP47	intrc	NM_	.	.
ACaP13	11	17416893	.	G	A	.	PASS	ABCC8	intrc	NM_	.	.
ACaP13	11	18428563	.	T	A	.	PASS	LDHA	intrc	NM_	.	.
ACaP13	11	26559175	.	C	T	.	PASS	ANO3	intrc	NM_	.	.
ACaP13	11	28332512	rs1470918	G	T	.	PASS	METTL	intrc	NM_	.	.
ACaP13	11	62557597	.	A	T	.	PASS	TMEM1	UTF	NM_	NM	.
ACaP13	11	63531795	.	G	A	.	PASS	C11orf9	exol	NM_	.	missen
ACaP13	11	89510434	rs2010003	C	A	.	PASS	.	intel	NM_	dis	.
ACaP13	11	1.03E+08	.	A	T	.	PASS	DYNC2I	intrc	NM_	.	.
ACaP13	12	45270544	.	G	C	.	PASS	NELL2	UTF	NM_	NM	.
ACaP13	12	51890927	rs7761498	A	G	.	PASS	SLC4A	intrc	NM_	.	.
ACaP13	12	56716439	.	G	A	.	PASS	PAN2	exol	NM_	.	missen
ACaP13	12	99145327	.	C	G	.	PASS	ANKS1	intrc	NM_	.	.
ACaP13	12	1.1E+08	.	G	A	.	PASS	MMAB	splic	NM_	NM	.
ACaP13	13	44770472	.	C	G	.	PASS	.	ups	NR_	.	.
ACaP13	13	60849382	.	C	A	.	PASS	.	intel	NM_	dis	.
ACaP13	13	91924856	.	G	A	.	PASS	.	intel	NR_	dis	.
ACaP13	14	19806361	rs2259899	C	A	.	PASS	.	intel	NR_	dis	.
ACaP13	14	20146058	rs7966403	C	A	.	PASS	.	intel	NM_	dis	.
ACaP13	14	23315028	rs7511166	C	T	.	PASS	MMP14	exol	NM_	.	missen
ACaP13	14	23874795	rs1127835	C	T	.	PASS	MYH6	intrc	NM_	.	.
ACaP13	14	36157747	.	C	A	.	PASS	RALGA	splic	NM_	NM	.
ACaP13	14	1.03E+08	.	T	G	.	PASS	CDC42	exol	NM_	.	missen
ACaP13	15	20487853	rs7146667	T	C	.	PASS	.	ups	NR_	.	.
ACaP13	15	25601103	.	T	C	.	PASS	UBE3A	exol	NM_	.	missen
ACaP13	15	29347059	.	G	A	.	PASS	APBA2	intrc	NM_	.	.
ACaP13	15	40468780	.	G	A	.	PASS	BUB1B	exol	NM_	.	missen
ACaP13	15	76073327	.	G	T	.	PASS	.	intel	NR_	dis	.
ACaP13	16	11051861	.	T	C	.	PASS	CLEC1	intrc	NM_	.	.

ACaP13	16	17292440	.	A	T	.	PASS	XYLT1	intrc	NM_	.	.
ACaP13	16	29675597	.	C	T	.	PASS	SPN	exoı	NM_	.	missens
ACaP13	16	31374294	rs1450253	C	T	.	PASS	ITGAX	exoı	NM_	.	synonyr
ACaP13	16	67995561	.	C	G	.	PASS	SLC12A	exoı	NM_	.	missens
ACaP13	16	69966926	rs1135006	G	A	.	PASS	WWP2	intrc	NM_	.	.
ACaP13	17	2282453	rs7673846	G	A	.	PASS	SGSM2	exoı	NM_	.	missens
ACaP13	17	9757917	rs7606360	C	T	.	PASS	GLP2R	exoı	NM_	.	stopgaiı
ACaP13	17	10212819	.	G	A	.	PASS	MYH13	intrc	NM_	.	.
ACaP13	17	27031537	rs5549220	G	A	.	PASS	PROCA	intrc	NM_	.	.
ACaP13	17	42988034	.	A	G	.	PASS	GFAP	intrc	NM_	.	.
ACaP13	17	67075243	.	G	T	.	PASS	ABCA6	intrc	NM_	.	.
ACaP13	17	80861278	rs7400170	C	T	.	PASS	TBCD	splic	NM_	NM	.
ACaP13	18	14183901	rs7837587	T	A	.	PASS	ANKRD	ncR	NR_	.	.
ACaP13	18	43328304	.	C	G	.	PASS	SLC14A	intrc	NM_	.	.
ACaP13	18	51795778	.	G	A	.	PASS	.	ups	NM_	.	.
ACaP13	18	57677517	rs1466769	G	A	.	PASS	.	inteı	NM_	dis	.
ACaP13	18	65945459	rs5634889	C	T	.	PASS	.	inteı	NR_	dis	.
ACaP13	19	1048907	.	C	T	.	PASS	ABCA7	exoı	NM_	.	synonyr
ACaP13	19	8809037	.	G	A	.	PASS	ACTL9	exoı	NM_	.	synonyr
ACaP13	19	12057823	.	A	G	.	PASS	ZNF700	intrc	NM_	.	.
ACaP13	19	14749271	.	G	A	.	PASS	EMR3	intrc	NM_	.	.
ACaP13	19	19971642	.	C	G	.	PASS	.	inteı	NM_	dis	.
ACaP13	19	22778387	.	G	A	.	PASS	.	ups	NR_	.	.
ACaP13	19	22836947	.	A	C	.	PASS	ZNF492	intrc	NM_	.	.
ACaP13	19	36387520	.	C	T	.	PASS	NFKBID	intrc	NM_	.	.
ACaP13	19	37785301	rs7125437	C	G	.	PASS	.	inteı	NR_	dis	.
ACaP13	19	51646206	rs1820521	G	A	.	PASS	SIGLEC	intrc	NM_	.	.
ACaP13	20	3052800	.	C	T	.	PASS	OXT	exoı	NM_	.	synonyr
ACaP13	20	3641469	.	G	A	.	PASS	GFRA4	exoı	NM_	.	stopgaiı
ACaP13	20	8696907	.	T	A	.	PASS	PLCB1	splic	NM_	NM	.
ACaP13	20	61050353	.	G	A	.	PASS	GATA5	exoı	NM_	.	synonyr
ACaP13	21	9873828	rs7964453	C	G	.	PASS	.	inteı	NR_	dis	.
ACaP13	21	11020702	rs3735529	C	A	.	PASS	.	dow	NM_	.	.
ACaP13	21	11181987	rs7280383	T	C	.	PASS	.	inteı	NM_	dis	.
ACaP13	21	22791110	.	G	T	.	PASS	NCAM2	intrc	NM_	.	.
ACaP13	21	36080332	rs7738143	C	T	.	PASS	CLIC6	exoı	NM_	.	synonyr
ACaP13	21	45432440	rs5510648	C	T	.	PASS	TRAPP(	exoı	NM_	.	synonyr
ACaP13	21	47320950	.	G	A	.	PASS	PCBP3	exoı	NM_	.	missens
ACaP13	21	47419581	.	G	A	.	PASS	COL6A1	exoı	NM_	.	missens
ACaP13	22	16230100	rs1262847	G	A	.	PASS	.	inteı	NR_	dis	.
ACaP13	22	22973384	rs86008	A	G	.	PASS	.	dow	NR_	.	.
ACaP13	22	30783120	.	C	A	.	PASS	RNF215	exoı	NM_	.	synonyr
ACaP13	X	10181697	.	T	C	.	PASS	CLCN4	intrc	NM_	.	.
ACaP13	X	18189250	rs7787173	C	T	.	PASS	BEND2	exoı	NM_	.	missens
ACaP13	X	49962162	.	C	T	.	PASS	AKAP4	intrc	NM_	.	.
ACaP13	X	65719566	.	C	T	.	PASS	.	inteı	NM_	dis	.
ACaP13	X	69898849	.	T	C	.	PASS	TEX11	intrc	NM_	.	.
ACaP13	X	70764432	.	C	T	.	PASS	OGT	exoı	NM_	.	missens
ACaP13	X	1.01E+08	.	G	A	.	PASS	TCEAL6	intrc	NM_	.	.
ACaP13	X	1.01E+08	.	C	T	.	PASS	.	inteı	NM_	dis	.
ACaP13	X	1.24E+08	rs9176062	C	T	.	PASS	.	inteı	NM_	dis	.
ACaP13	X	1.51E+08	rs9279180	G	A	.	PASS	.	inteı	NM_	dis	.
ACaP13	X	1.53E+08	.	C	T	.	PASS	IRAK1	exoı	NM_	.	missens
ACaP14	1	18149614	rs3718451	G	A	.	PASS	ACTL8	exoı	NM_	.	synonyr

ACaP14	1	27618712	.	T	A	.	PASS	WDTC1	exo	NM_	.	stopgai
ACaP14	1	34684254	rs7678484	C	T	.	PASS	C1orf94	intro	NM_	.	.
ACaP14	1	35260564	rs7603609	G	A	.	PASS	GJA4	exo	NM_	.	synonyr
ACaP14	1	44323919	.	G	A	.	PASS	ST3GAI	intro	NM_	.	.
ACaP14	1	45811623	.	G	T	.	PASS	TESK2	exo	NM_	.	missens
ACaP14	1	64515280	.	G	C	.	PASS	ROR1	intro	NM_	.	.
ACaP14	1	65332484	.	G	T	.	PASS	JAK1	intro	NM_	.	.
ACaP14	1	1.1E+08	.	G	A	.	PASS	AMPD2	exo	NM_	.	missens
ACaP14	1	1.12E+08	rs7547967	T	G	.	PASS	PIFO	intro	NM_	.	.
ACaP14	1	1.43E+08	rs7528408	C	T	.	PASS	.	inte	NR_	dis	.
ACaP14	1	1.45E+08	rs2009307	C	T	.	PASS	NBPF2(	intro	NM_	.	.
ACaP14	1	1.45E+08	rs3726923	A	G	.	PASS	NBPF25	ncR	NR_	.	.
ACaP14	1	1.45E+08	rs8799825	G	T	.	PASS	NBPF25	ncR	NR_	.	.
ACaP14	1	2.02E+08	.	T	G	.	PASS	PTPN7	intro	NM_	.	.
ACaP14	1	2.28E+08	rs7756172	C	T	.	PASS	OBSCN	intro	NM_	.	.
ACaP14	2	1491745	rs7587514	C	T	.	PASS	TPO	exo	NM_	.	missens
ACaP14	2	1.7E+08	.	G	A	.	PASS	LRP2	UTF	NM_	NM	.
ACaP14	2	1.8E+08	.	A	C	.	PASS	MIR548	ncR	NR_	.	.
ACaP14	2	1.97E+08	rs1411611	C	T	.	PASS	STK17E	intro	NM_	.	.
ACaP14	3	48677416	rs1156481	C	T	.	PASS	CELSR3	exo	NM_	.	missens
ACaP14	3	1.12E+08	.	A	G	.	PASS	SLC9C1	intro	NM_	.	.
ACaP14	3	1.43E+08	.	C	A	.	PASS	PCOLC	intro	NM_	.	.
ACaP14	3	1.84E+08	.	C	T	.	PASS	ABCC5	exo	NM_	.	missens
ACaP14	4	628481	.	T	A	.	PASS	PDE6B	exo	NM_	.	missens
ACaP14	4	875824	.	C	T	.	PASS	GAK	exo	NM_	.	missens
ACaP14	4	2357633	rs7307061	T	C	.	PASS	ZFYVE2	intro	NM_	.	.
ACaP14	4	9245302	rs8796734	A	G	.	PASS	.	ups	NM_	.	.
ACaP14	4	94128702	rs7690288	C	G	.	PASS	GRID2	intro	NM_	.	.
ACaP14	4	1.6E+08	.	T	G	.	PASS	RXFP1	intro	NM_	.	.
ACaP14	4	1.68E+08	rs1003755	G	A	.	PASS	.	inte	NM_	dis	.
ACaP14	5	9136477	.	G	C	.	PASS	SEMA5	intro	NM_	.	.
ACaP14	5	80256656	.	G	A	.	PASS	RASGR	exo	NM_	.	synonyr
ACaP14	5	1.12E+08	.	G	A	.	PASS	APC	exo	NM_	.	missens
ACaP14	5	1.41E+08	.	G	C	.	PASS	PCDHG	ncR	NR_	.	.
ACaP14	5	1.59E+08	.	C	A	.	PASS	EBF1	intro	NM_	.	.
ACaP14	5	1.68E+08	.	T	A	.	PASS	WWC1	exo	NM_	.	synonyr
ACaP14	6	28295175	.	G	T	.	PASS	ZSCAN	exo	NM_	.	missens
ACaP14	6	31138300	rs7589117	C	T	.	PASS	POU5F	exo	NM_	.	missens
ACaP14	6	57393271	.	T	A	.	PASS	PRIM2	intro	NM_	.	.
ACaP14	6	85472394	.	G	A	.	PASS	TBX18	exo	NM_	.	missens
ACaP14	6	1.68E+08	rs441907	T	C	.	PASS	.	inte	NM_	dis	.
ACaP14	7	83675581	.	C	G	.	PASS	SEMA3	intro	NM_	.	.
ACaP14	7	93116217	.	G	C	.	PASS	CALCR	intro	NM_	.	.
ACaP14	7	1.13E+08	.	C	T	.	PASS	C7orf60	exo	NM_	.	synonyr
ACaP14	8	7215692	.	A	T	.	PASS	ZNF705	exo	NM_	.	missens
ACaP14	8	1.13E+08	.	T	C	.	PASS	CSMD3	exo	NM_	.	missens
ACaP14	8	1.34E+08	.	T	C	.	PASS	NDRG1	intro	NM_	.	.
ACaP14	9	14116261	.	G	A	.	PASS	NFIB	exo	NM_	.	stopgai
ACaP14	9	1.08E+08	.	C	T	.	PASS	FKTN	exo	NM_	.	stopgai
ACaP14	9	1.26E+08	.	C	T	.	PASS	OR1L6	exo	NM_	.	synonyr
ACaP14	10	29770662	.	A	C	.	PASS	SVIL	intro	NM_	.	.
ACaP14	10	49200211	rs3125109	G	A	.	PASS	.	inte	NR_	dis	.
ACaP14	10	72531001	.	G	A	.	PASS	TBATA	UTF	NM_	NM	.
ACaP14	10	74452957	rs7905448	T	A	.	PASS	MCU	intro	NM_	.	.

ACaP14	10	92678882	.	C	A	.	PASS	ANKRD	intro	NM_	.	.
ACaP14	10	1.12E+08	rs7646688	A	G	.	PASS	MXI1	exo	NM_	.	missens
ACaP14	10	1.17E+08	.	G	A	.	PASS	ATRNL	intro	NM_	.	.
ACaP14	11	6588138	.	C	T	.	PASS	DNHD1	exo	NM_	.	missens
ACaP14	11	51459427	.	C	A	.	PASS	.	inte	NM_	dis	.
ACaP14	11	76750853	rs3775202	C	T	.	PASS	B3GNT	exo	NM_	.	unknow
ACaP14	11	89701788	.	C	T	.	PASS	TRIM64	exo	NM_	.	synonyr
ACaP14	11	1.08E+08	rs7690752	G	A	.	PASS	.	inte	NM_	dis	.
ACaP14	11	1.17E+08	rs7585846	G	A	.	PASS	SIDT2	exo	NM_	.	missens
ACaP14	12	26809244	.	C	T	.	PASS	ITPR2	exo	NM_	.	synonyr
ACaP14	12	49391553	.	T	G	.	PASS	DDN	exo	NM_	.	missens
ACaP14	12	54974891	.	A	G	.	PASS	PPP1R	intro	NM_	.	.
ACaP14	13	28543079	.	C	T	.	PASS	CDX2	exo	NM_	.	missens
ACaP14	14	19806361	rs2259899	C	A	.	PASS	.	inte	NR_	dis	.
ACaP14	14	20132225	rs2014187	G	T	.	PASS	.	inte	NM_	dis	.
ACaP14	14	69259663	.	G	A	.	PASS	ZFP36L	exo	NM_	.	missens
ACaP14	15	54793046	.	C	G	.	PASS	UNC13	exo	NM_	.	missens
ACaP14	16	1270525	rs7764738	C	T	.	PASS	CACNA	exo	NM_	.	missens
ACaP14	16	23702864	.	G	C	.	PASS	ERN2	intro	NM_	.	.
ACaP14	16	74422830	rs2013540	C	A	.	PASS	.	inte	NR_	dis	.
ACaP14	18	12197920	rs7502623	A	C	.	PASS	.	inte	NM_	dis	.
ACaP14	19	6963763	rs7589667	C	T	.	PASS	EMR4P	ncR	NR_	.	.
ACaP14	19	10116382	.	C	A	.	PASS	COL5A	exo	NM_	.	missens
ACaP14	19	41285709	.	A	T	.	PASS	MIA-RA	ncR	NR_	.	.
ACaP14	19	46521260	.	G	T	.	PASS	CCDC6	intro	NM_	.	.
ACaP14	19	46811870	.	G	A	.	PASS	HIF3A	intro	NM_	.	.
ACaP14	19	56052730	rs9019919	G	A	.	PASS	SBK3	exo	NM_	.	missens
ACaP14	20	10036169	.	C	G	.	PASS	ANKEF	exo	NM_	.	missens
ACaP14	20	10036179	rs7770803	G	C	.	PASS	ANKEF	exo	NM_	.	missens
ACaP14	20	34827993	rs7792821	C	T	.	PASS	AAR2	exo	NM_	.	missens
ACaP14	21	34960922	.	G	A	.	PASS	DONSC	exo	NM_	.	missens
ACaP14	22	30689936	.	C	T	.	PASS	TBC1D	exo	NM_	.	missens
ACaP14	X	20152233	.	G	A	.	PASS	EIF1AX	intro	NM_	.	.
ACaP14	X	25048192	.	G	T	.	PASS	.	inte	NM_	dis	.
ACaP14	X	47426131	.	C	T	.	PASS	ARAF	exo	NM_	.	synonyr
ACaP14	X	54011864	.	C	A	.	PASS	PHF8	intro	NM_	.	.
ACaP14	X	1.02E+08	.	C	G	.	PASS	BEX1	intro	NM_	.	.
ACaP14	X	1.07E+08	.	C	T	.	PASS	TSC22D	UTF	NM_	NM	.
ACaP14	X	1.35E+08	.	G	C	.	PASS	DDX26E	intro	NM_	.	.
ACaP14	X	1.37E+08	.	C	T	.	PASS	ZIC3	exo	NM_	.	synonyr
ACaP14	X	1.54E+08	.	A	G	.	PASS	F8	intro	NM_	.	.
ACaP14	X	1.55E+08	rs4013617	G	A	.	PASS	.	inte	NM_	dis	.
ACaP14	MT	11126	.	G	A	.	PASS	.	inte	NON	dis	.

AACha	Genecode:	wgRna	cytc	targ	tfbs	genomicSu	Repe	avsnp150	co	clinva	gwasCa	1000g2(	1000g2(
.	ENST000C	.	1p3	.	.	Score=0.99	.	rs6264213	.	.	.	.	.
.	ENST000C	.	1p3	.	.	Score=0.99	.	rs8798212	ID	.	.	.	.
.	ENST000C	.	1p3	.	.	.	.	.	.	.	.	.	.
.	ENST000C	.	1p3	.	.	.	Score	rs2011489	.	.	.	.	.
PTCHD	ENST000C	.	1p3	.	Scc	.	.	rs7706792	ID	.	.	.	.
.	ENST000C	.	1p3	.	.	.	.	.	.	.	.	.	.
.	ENST000C	.	1p3	.	.	Score=0.99	.	rs7573447	ID	.	.	.	.
.	ENST000C	.	1p3	.	.	Score=0.96	.	.	.	.	.	.	.
.	ENST000C	.	1p3	.	.	Score=0.96	.	rs643011	.	.	.	.	.
.	.	.	1p3	.	.	Score=0.96	.	rs1891369	ID	.	.	.	.
.	ENST000C	.	1p3	.	.	.	Score	rs7967920	ID	.	.	.	.
.	ENST000C	.	1p3	.	.	.	.	.	.	.	.	.	.
.	ENST000C	.	1p3	.	.	.	.	rs3699237	.	.	.	.	.
.	ENST000C	.	1p3	.	.	.	.	.	.	.	.	.	.
.	ENST000C	.	1p3	.	.	.	.	.	.	.	.	.	.
.	ENST000C	.	1p1	.	Scc	.	.	.	.	.	.	.	.
.	.	.	1p1	.	.	.	Score	.	.	.	.	.	.
.	ENST000C	.	1q2	.	.	Score=0.97	.	rs1809861	ID	.	.	.	.
.	.	.	1q2	.	.	Score=0.98	.	rs3742168	.	.	.	.	.
.	ENST000C	.	1q2	.	.	Score=0.98	Score	rs1409433	.	.	.	.	.
.	ENST000C	.	1q2	.	.	Score=0.97	.	rs7967822	ID	.	.	.	.
.	ENST000C	.	1q2	.	.	Score=0.97	.	rs7763460	ID	.	.	.	.
.	.	.	1q2	.	.	Score=0.98	.	.	.	.	.	.	.
.	ENST000C	.	1q2	.	.	Score=0.98	.	rs1694653	.	.	.	.	.
.	ENST000C	.	1q2	.	.	Score=0.98	.	rs1129555	ID	.	.	.	.
.	.	.	1q2	.	.	Score=0.99	Score	rs3732644	.	.	.	.	0.023
PDE4D	ENST000C	.	1q2	.	Scc	Score=0.92	.	.	.	.	.	.	.
.	ENST000C	.	1q2	.	.	Score=0.99	Score	rs2691756	.	.	.	.	.
.	ENST000C	.	1q2	.	.	Score=0.98	.	.	.	.	.	.	.
.	ENST000C	.	1q2	.	.	Score=0.98	.	.	ID	.	.	.	.
.	ENST000C	.	1q2	.	.	Score=0.99	.	.	ID	.	.	.	.
.	ENST000C	.	1q2	.	.	.	.	rs1932337	.	.	.	.	.
.	ENST000C	.	1q2	.	.	Score=0.98	.	.	.	.	.	.	.
.	ENST000C	.	1q2	.	.	Score=0.98	.	rs2837929	ID	.	.	.	.
.	ENST000C	.	1q2	.	.	Score=0.99	.	rs2983273	ID	.	.	.	0.002
PGLYR	ENST000C	.	1q2	.	.	.	.	rs3740307	ID	.	.	.	.
.	ENST000C	.	1q2	.	.	Score=0.97	Score	rs1674785	ID	.	.	.	0.672
FCGR2	ENST000C	.	1q2	.	.	Score=0.98	.	rs1485348	.	.	.	.	.
POU2F	ENST000C	.	1q2	.	Scc	.	.	rs7598068	.	.	.	.	.
.	ENST000C	.	1q3	.	.	.	.	.	.	.	.	.	.
.	ENST000C	.	1q4	.	.	.	Score	.	.	.	.	.	.
.	ENST000C	.	1q4	.	.	.	.	.	.	.	.	.	.
CEP17(	ENST000C	.	1q4	.	Scc	Score=0.98	.	rs2006447	ID	.	.	.	.
.	.	.	1q4	.	.	Score=0.98	.	rs3739868	ID	.	.	.	.
.	.	.	1q4	.	.	Score=0.98	.	rs1782199	ID	.	.	.	.
.	.	.	2p1	.	.	.	Score	rs3461159	ID	.	.	.	0.005
.	.	.	2p1	.	.	Score=0.98	Score	rs8689209	ID	.	.	.	.
.	ENST000C	.	2p1	.	.	Score=0.99	Score	.	.	.	.	.	.
IL1R1:N	ENST000C	.	2q1	.	.	.	.	.	.	.	.	.	.
.	ENST000C	.	2q1	.	.	Score=0.98	Score	rs3877497	ID	.	.	.	.

.	ENST0000	.	2q1	.	.	.	Score	rs1037863	.	.	.	.	.
POTEF	ENST0000	.	2q2	.	.	Score=0.9	.	rs1839843	ID	.	.	.	0.688
POTEF	ENST0000	.	2q2	.	.	Score=0.9	.	.	ID	.	.	.	.
.	ENST0000	.	2q2	.	.	.	.	.	.	.	.	.	.
ZEB2:N	ENST0000	.	2q2	.	Scc	.	.	.	.	.	.	.	.
.	ENST0000	.	2q3	.	.	.	.	.	.	.	.	.	.
MARS2	ENST0000	.	2q3	.	.	.	.	.	.	.	.	.	.
PLEKH	ENST0000	.	2q3	.	Scc	.	.	.	.	.	.	.	.
.	ENST0000	.	2q3	.	.	.	.	rs7812258	.	.	.	.	.
.	ENST0000	.	2q3	.	.	.	.	rs1010785	ID	.	.	.	.
.	ENST0000	.	2q3	.	.	.	.	rs7526847	.	.	.	.	.
.	ENST0000	.	3p2	.	.	.	.	.	.	.	.	.	.
.	ENST0000	.	3p2	.	.	.	.	.	.	.	.	.	.
KAT2B	ENST0000	.	3p2	.	.	.	.	.	.	.	.	.	.
.	ENST0000	.	3p2	.	.	.	.	.	.	.	.	.	.
SCN10	ENST0000	.	3p2	.	.	.	.	rs1450320	.	Uncer	.	.	.
SCN10	ENST0000	.	3p2	.	.	.	.	rs1398610	.	Uncer	.	.	.
PDZRN	ENST0000	.	3p1	.	Scc	.	.	.	ID	.	.	.	.
OR5H1	ENST0000	.	3q1	.	Scc	Score=0.8	.	rs1126455	ID	.	.	.	0.001
.	ENST0000	.	3q1	.	.	.	.	.	.	.	.	.	.
.	ENST0000	.	3q1	.	.	.	.	rs7541975	.	.	.	.	.
IFT57:N	ENST0000	.	3q1	.	.	.	.	rs3757741	.	.	.	.	.
.	ENST0000	.	3q2	.	.	.	.	.	ID	.	.	.	.
.	ENST0000	.	3q2	.	.	Score=0.9	.	rs6228513	ID	.	.	.	.
.	.	.	3q2	.	.	Score=0.9	Score	.	.	.	.	.	.
.	ENST0000	.	4p1	.	.	.	.	rs4970287	ID	.	.	.	.
.	ENST0000	.	4q2	.	.	.	.	.	.	.	.	.	.
.	.	.	4q2	.	.	.	Score	rs3764069	.	.	.	.	.
.	.	.	4q2	.	.	Score=0.9	Score	rs7963572	.	.	.	.	.
FREM3	ENST0000	.	4q3	.	.	.	.	rs9542709	ID	.	.	.	.
.	ENST0000	.	4q3	.	.	.	.	rs3721454	.	.	.	.	.
TRIML1	ENST0000	.	4q3	.	.	.	.	.	ID	.	.	.	.
.	ENST0000	.	5p1	.	.	Score=0.9	Score	rs649883	.	.	.	.	.
.	ENST0000	.	5p1	.	.	Score=0.9	Score	rs1467072	.	.	.	.	.
.	ENST0000	.	5p1	.	.	.	.	.	.	.	.	.	.
.	ENST0000	.	5p1	.	.	Score=0.9	Score	rs1500138	ID	.	.	.	.
.	ENST0000	.	5p1	.	.	.	.	.	.	.	.	.	.
.	ENST0000	.	5q1	.	.	Score=0.9	.	rs7962294	ID	.	.	.	.
.	ENST0000	.	5q1	.	.	Score=0.9	.	.	.	.	.	.	.
.	ENST0000	.	5q2	.	.	.	.	.	.	.	.	.	.
CAMK4	ENST0000	.	5q2	.	.	.	.	rs7609290	.	.	.	.	.
.	ENST0000	.	5q3	.	.	.	.	.	.	.	.	.	.
PCDHG	ENST0000	.	5q3	.	.	.	.	rs7647428	.	.	.	.	.
.	ENST0000	.	5q3	.	.	Score=0.9	Score	rs1407436	ID	.	.	.	.
.	ENST0000	.	5q3	.	.	Score=0.9	.	rs7380674	.	.	.	.	.
.	ENST0000	.	6p2	.	.	.	.	rs5568001	.	.	.	.	0.001
GSTA1	ENST0000	.	6p1	.	.	Score=0.9	.	rs3977792	.	.	.	.	.
.	ENST0000	.	6p1	.	.	Score=0.9	.	.	.	.	.	.	.
PHIP:N	ENST0000	.	6q1	.	.	.	.	.	.	.	.	.	.
.	ENST0000	.	6q1	.	.	Score=0.9	.	rs9844362	.	.	.	.	.
.	ENST0000	.	6q1	.	.	Score=0.9	.	.	.	.	.	.	.
.	ENST0000	.	6q2	.	.	.	Score	rs9992807	.	.	.	.	.
ARMC2	ENST0000	.	6q2	.	.	.	.	.	.	.	.	.	.
SLC2A	ENST0000	.	6q2	.	.	.	.	rs7554849	.	.	.	.	.

.	ENST0000C	.	6q2	.	.	.	.	.	.	.	.	.	.
ADGB:I	ENST0000C	.	6q2	.	.	.	.	rs7747387	.	.	.	.	.
.	ENST0000C	.	6q2	.	.	Score=0.92	Score	rs1467177	ID	.	.	.	.
.	ENST0000C	.	7p2	.	.	.	.	.	.	.	.	.	.
.	ENST0000C	.	7p2	.	.	.	Score	rs7308136	ID	.	.	.	.
.	ENST0000C	.	7p1	.	.	.	.	.	.	.	.	.	.
.	.	.	7p1	.	.	Score=0.98	.	.	.	.	.	.	.
.	ENST0000C	.	7p1	.	.	Score=0.99	.	rs7911953	.	.	.	.	0.091
.	ENST0000C	.	7p1	.	.	Score=0.99	.	.	ID	.	.	.	0.045
.	ENST0000C	.	7p1	.	.	Score=0.99	.	.	.	.	.	.	.
ZNF71(	ENST0000C	.	7p1	.	.	Score=0.89	.	.	.	.	.	.	.
.	.	.	7q1	.	.	.	Score	rs4311554	ID	.	.	.	.
.	ENST0000C	.	7q1	.	.	Score=0.99	.	.	.	.	.	.	.
.	ENST0000C	.	7q1	.	.	Score=0.99	.	.	.	.	.	.	.
.	.	.	7q1	.	.	Score=0.99	.	rs5467260	.	.	.	.	0.022
.	.	.	7q1	.	.	Score=0.99	.	.	ID	.	.	.	.
.	.	.	7q1	.	.	Score=0.99	.	rs8688020	ID	.	.	.	.
.	ENST0000C	.	7q1	.	.	Score=0.99	Score	rs8799757	ID	.	.	.	.
.	ENST0000C	.	7q2	.	.	.	.	rs7686126	.	.	.	.	.
.	ENST0000C	.	7q2	.	.	Score=0.99	.	rs3727203	ID	.	.	.	.
POT1:N	ENST0000C	.	7q3	.	Scc	.	.	rs7809364	.	.	.	.	.
SSMEN	ENST0000C	.	7q3	.	.	.	.	.	ID	.	.	.	.
KMT2C	ENST0000C	.	7q3	.	.	Score=0.96	.	rs7754267	ID	.	.	.	.
KMT2C	ENST0000C	.	7q3	.	Scc	Score=0.96	.	rs1499922	ID	.	.	.	.
.	ENST0000C	.	7q3	.	.	Score=0.96	Score	.	.	.	.	.	.
.	.	.	7q3	.	.	.	Score	rs1407758	ID	.	.	.	.
.	.	.	7q3	.	.	.	Score	rs1482637	ID	.	.	.	.
.	ENST0000C	.	8p2	.	.	Score=0.98	.	.	.	.	.	.	.
.	ENST0000C	.	8p2	.	.	.	.	.	.	.	.	.	.
.	ENST0000C	.	8p2	.	.	.	.	.	.	.	.	.	.
KCTD9	ENST0000C	.	8p2	.	Scc	.	.	.	.	.	.	.	.
.	.	.	8p1	.	.	.	Score	rs1813428	.	.	.	.	.
.	.	.	8p1	.	.	.	Score	rs2022368	ID	.	.	.	.
.	ENST0000C	.	8q1	.	.	.	.	.	.	.	.	.	.
.	ENST0000C	.	8q2	.	.	.	.	rs1837720	ID	.	.	.	.
.	ENST0000C	.	9p1	.	.	Score=0.98	.	rs1329860	ID	.	.	.	.
.	ENST0000C	.	9q1	.	.	Score=0.99	.	rs2954515	.	.	.	.	.
.	.	.	9q1	.	.	Score=0.99	.	rs1968548	.	.	.	.	.
.	.	.	9q1	.	.	Score=0.99	.	rs2019611	ID	.	.	.	.
.	.	.	9q1	.	.	Score=0.89	Score	rs4928839	.	.	.	.	.
.	ENST0000C	.	9q2	.	.	.	.	.	.	.	.	.	.
.	.	.	9q3	.	.	Score=0.98	.	rs2868900	.	.	.	.	.
ENG:N	ENST0000C	.	9q3	.	.	.	.	.	.	.	.	.	.
FNBP1	ENST0000C	.	9q3	.	.	.	.	.	.	.	.	.	.
.	ENST0000C	.	9q3	.	.	.	.	.	.	.	.	.	.
DBH:N	ENST0000C	.	9q3	.	.	.	.	rs7694907	.	.	.	.	.
HSPA1	ENST0000C	.	10p	.	.	.	.	.	.	.	.	.	.
.	ENST0000C	.	10p	.	.	.	Score	rs1051909	.	.	.	.	.
MYO3A	ENST0000C	.	10p	.	.	.	.	.	ID	.	.	.	.
MYO3A	ENST0000C	.	10p	.	Scc	.	.	.	.	.	.	.	.
UNKNC	ENST0000C	.	10p	.	.	Score=0.93	.	rs7531795	ID	.	.	.	.
SVIL:N	ENST0000C	.	10p	.	.	.	.	rs5566342	.	.	.	.	.
.	ENST0000C	.	10p	.	.	Score=0.96	.	rs1759036	ID	.	.	.	.
.	.	.	10p	.	.	Score=0.92	.	.	.	.	.	.	.

.	ENST0000	.	10p	.	.	Score=0.9	.	rs1494650	ID	.	.	.	.
.	ENST0000	.	10p	.	.	Score=0.9	.	rs5524339	.	.	.	.	0.001
.	ENST0000	.	10q	.	.	Score=0.9	.	rs7827248	ID	.	.	.	.
RBP3:	ENST0000	.	10q	.	.	.	.	rs7821206	.	.	.	.	.
.	ENST0000	.	10q	.	.	.	.	.	.	.	.	.	.
ATOH7	ENST0000	.	10q	.	.	.	.	.	.	.	.	.	.
TET1:	ENST0000	.	10q	.	.	.	.	rs1810812	ID	.	.	.	.
STOX1	ENST0000	.	10q	.	.	.	.	rs1852770	.	.	.	.	.
.	ENST0000	.	10q	.	.	Score=0.9	.	.	.	.	.	.	.
.	ENST0000	.	10q	.	Scc	.	.	.	.	.	.	.	.
.	ENST0000	.	10q	.	.	.	.	rs9002476	ID	.	.	.	.
.	ENST0000	.	10q	.	.	.	Score	.	.	.	.	.	.
.	ENST0000	.	10q	.	.	.	.	rs1028763	.	.	.	.	.
.	ENST0000	.	11p	.	.	.	.	.	.	.	.	.	.
.	ENST0000	.	11p	.	.	.	Score	rs7615769	ID	.	.	.	.
.	ENST0000	.	11p	.	.	.	Score	.	.	.	.	.	.
.	.	.	11p	.	.	Score=0.9	.	.	.	.	.	.	.
.	ENST0000	.	11q	.	.	.	.	.	.	.	.	.	.
PRKRI	ENST0000	.	11q	.	.	Score=0.9	.	.	ID	.	.	.	.
.	ENST0000	.	11q	.	.	Score=0.9	.	.	.	.	.	.	.
.	ENST0000	.	11q	.	.	Score=0.9	.	.	.	.	.	.	.
USP35	ENST0000	.	11q	.	.	.	.	rs7614089	.	.	.	.	.
HEPHL	ENST0000	.	11q	.	Scc	.	.	rs1438612	ID	.	.	.	.
SIK2:N	ENST0000	.	11q	.	.	.	.	.	.	.	.	.	.
OR10S	ENST0000	.	11q	.	.	.	.	rs7757345	ID	.	.	.	.
.	ENST0000	.	11q	.	.	.	.	rs2512286	.	.	.	.	0.164
.	ENST0000	.	12p	.	.	Score=0.9	.	rs5382343	ID	.	.	.	0.375
.	ENST0000	.	12p	.	.	Score=0.9	.	rs8799828	.	.	.	.	.
SLC6A	ENST0000	.	12p	.	.	.	.	.	.	.	.	.	.
.	ENST0000	.	12p	.	.	.	Score	rs8681434	ID	.	.	.	.
.	ENST0000	.	12p	.	.	Score=0.9	.	rs2575282	.	.	.	.	.
.	ENST0000	.	12q	.	.	.	.	.	.	.	.	.	.
.	ENST0000	.	12q	.	.	.	Score	.	.	.	.	.	.
BIN2:N	ENST0000	.	12q	.	.	.	.	.	.	.	.	.	.
.	ENST0000	.	12q	.	.	Score=0.9	.	.	.	.	.	.	.
.	ENST0000	.	12q	.	.	Score=0.8	.	.	.	.	.	.	.
CHST1	ENST0000	.	12q	.	Scc	.	.	rs1043870	ID	.	.	.	.
.	ENST0000	.	12q	.	.	.	Score	.	.	.	.	.	.
.	ENST0000	.	12q	.	.	.	.	.	.	.	.	.	.
PCDH1	ENST0000	.	13q	.	.	.	.	.	.	.	.	.	.
.	ENST0000	.	14q	.	.	Score=0.9	.	.	ID	.	.	.	.
.	ENST0000	.	14q	.	.	Score=0.9	.	rs2014705	ID	.	.	.	.
.	.	.	14q	.	.	Score=0.9	.	rs8794601	ID	.	.	.	.
.	ENST0000	.	14q	.	.	Score=0.9	.	.	.	.	.	.	.
.	ENST0000	.	14q	.	.	Score=0.9	.	rs8674109	.	.	.	.	.
KLHL3	ENST0000	.	14q	.	Scc	.	.	.	.	.	.	.	.
.	.	.	14q	.	.	.	.	.	.	.	.	.	.
.	.	.	14q	.	.	.	.	.	.	.	.	.	.
.	ENST0000	.	14q	.	.	.	.	.	.	.	.	.	.
.	.	.	14q	.	.	.	.	.	.	.	.	.	.
.	ENST0000	.	14q	.	.	.	.	.	.	.	.	.	.
SYNE2	ENST0000	.	14q	.	.	.	.	rs7732738	ID	.	.	.	.
.	ENST0000	.	14q	.	.	Score=0.9	.	.	.	.	.	.	.
.	.	.	14q	.	.	Score=0.9	Score	rs2897118	.	.	.	.	.

.	ENST000C	.	14q	.	.	Score=0.9	.	rs3681255	ID	.	.	.	.
.	.	.	14q	.	.	Score=0.9	.	rs6199969	ID	.	.	.	.
.	ENST000C	.	15q	.	.	Score=0.9	.	.	.	.	.	.	.
.	ENST000C	.	15q	.	.	Score=0.9	Score	.	.	.	.	.	.
.	.	.	15q	.	.	Score=0.9	.	rs3749240	ID	.	.	.	.
.	ENST000C	.	15q	.	.	Score=0.9	.	.	.	.	.	.	.
.	ENST000C	.	15q	.	.	Score=0.9	.	.	.	.	.	.	.
.	ENST000C	.	15q	.	.	Score=0.9	.	.	ID	.	.	.	.
.	.	.	15q	.	.	Score=0.9	.	rs7964394	.	.	.	.	.
.	.	.	15q	.	.	Score=0.9	.	.	.	.	.	.	.
.	.	.	15q	.	.	Score=0.9	.	rs7126232	.	.	.	.	0.384
NPAP1	ENST000C	.	15q	.	.	.	.	.	ID	.	.	.	.
ATP10	ENST000C	.	15q	.	Scc	.	.	.	.	.	.	.	.
GOLGA	ENST000C	.	15q	.	.	Score=0.9	.	rs7695754	ID	.	.	.	.
APBA2	ENST000C	.	15q	.	Scc	.	.	.	.	.	.	.	.
.	ENST000C	.	15q	.	.	Score=0.9	.	.	ID	.	.	.	.
.	ENST000C	.	15q	.	.	Score=0.9	.	.	.	.	.	.	.
.	ENST000C	.	15q	.	.	Score=0.9	.	rs8658676	.	.	.	.	.
.	ENST000C	.	15q	.	.	Score=0.9	.	.	.	.	.	.	.
.	ENST000C	.	15q	.	.	.	Score	rs3683923	ID	.	.	.	.
.	ENST000C	.	15q	.	.	.	.	rs3741666	.	.	.	.	.
.	ENST000C	.	15q	.	.	Score=0.9	.	.	.	.	.	.	.
.	ENST000C	.	15q	.	.	Score=0.9	.	rs5743805	.	.	.	.	0.002
GOLGA	ENST000C	.	15q	.	.	Score=0.9	.	rs1998088	ID	.	.	.	.
GOLGA	ENST000C	.	15q	.	.	Score=0.9	.	rs3775649	ID	.	.	.	.
.	ENST000C	.	15q	.	.	Score=0.9	.	rs6202866	ID	.	.	.	.
.	ENST000C	.	16p	.	.	.	.	rs7561426	.	.	.	.	.
.	ENST000C	.	16p	.	.	.	Score	.	ID	.	.	.	.
CORO	ENST000C	.	16p	.	Scc	.	.	rs7464587	.	.	.	.	.
ZNF64	ENST000C	.	16p	.	.	.	.	.	.	.	.	.	.
.	ENST000C	.	16p	.	.	Score=0.9	.	rs2018870	ID	.	.	.	.
.	.	.	16q	.	.	.	Score	rs4643347	.	.	.	.	.
.	.	.	16q	.	.	.	Score	rs4535017	.	.	.	.	.
.	.	.	16q	.	.	.	Score	rs2128471	.	.	.	.	.
.	.	.	16q	.	.	.	Score	rs2888787	.	.	.	.	.
.	.	.	16q	.	.	.	Score	rs4355081	.	.	.	.	.
.	.	.	16q	.	.	.	Score	rs4249097	.	.	.	.	.
.	.	.	16q	.	.	.	Score	rs4232920	ID	.	.	.	.
.	.	.	16q	.	.	.	Score	rs7970299	.	.	.	.	.
.	.	.	16q	.	.	.	Score	rs1483332	.	.	.	.	.
.	.	.	16q	.	.	.	Score	rs9972801	.	.	.	.	.
.	.	.	16q	.	.	.	Score	rs2878607	.	.	.	.	.
.	.	.	16q	.	.	.	Score	rs4249050	.	.	.	.	.
.	.	.	16q	.	.	.	Score	rs4250282	.	.	.	.	.
.	.	.	16q	.	.	.	Score	rs7198709	.	.	.	.	.
.	.	.	16q	.	.	.	Score	rs4445923	.	.	.	.	.
.	.	.	16q	.	.	.	Score	rs4450416	ID	.	.	.	.
.	.	.	16q	.	.	.	Score	.	.	.	.	.	.
.	.	.	16q	.	.	.	Score	rs8056404	.	.	.	.	.
.	.	.	16q	.	.	.	Score	rs8056672	ID	.	.	.	.
.	.	.	16q	.	.	.	Score	rs6152895	.	.	.	.	.
.	.	.	16q	.	.	.	Score	rs2884801	.	.	.	.	.
.	ENST000C	.	16q	.	.	.	.	.	.	.	.	.	.
CBFB:I	ENST000C	.	16q	.	Scc	.	.	.	ID	.	.	.	.

.	.	.	16q	.	.	.	Score	.	ID	.	.	.	.
ZNF46	ENST000C	.	16q	.	Scc	.	.	.	.	.	.	.	.
PRPF8	ENST000C	.	17p	.	.	.	.	rs7801100	.	.	.	.	.
SPATA	ENST000C	.	17p	.	Scc	.	.	.	.	.	.	.	.
CHD3:I	ENST000C	.	17p	.	Scc	.	.	rs3687584	.	.	.	.	.
.	ENST000C	.	17p	.	.	.	.	.	.	.	.	.	.
.	ENST000C	.	17p	.	.	Score=0.98	.	rs1845094	.	.	.	.	.
.	ENST000C	.	17p	.	.	.	.	.	.	.	.	.	.
.	ENST000C	.	17p	.	.	Score=0.99	.	.	.	.	.	.	.
.	.	.	17p	.	.	Score=0.99	Score	.	.	.	.	.	.
.	ENST000C	.	17p	.	.	Score=0.99	.	rs4503851	.	.	.	.	.
.	.	.	17p	.	.	.	Score	rs1462263	ID	.	.	.	.
.	.	.	17p	.	.	.	Score	rs4362423	ID	.	.	.	.
.	.	.	17p	.	.	.	Score	rs6565430	.	.	.	.	.
.	.	.	17p	.	.	.	Score	rs1434103	.	.	.	.	.
.	.	.	17p	.	.	.	Score	.	.	.	.	.	.
.	ENST000C	.	17q	.	.	.	Score	rs8916657	.	.	.	.	.
.	ENST000C	.	17q	.	.	.	Score	rs1167082	.	.	.	.	0.001
.	ENST000C	.	17q	.	.	.	.	.	.	.	.	.	.
.	ENST000C	.	17q	.	.	.	.	rs1459625	ID	.	.	.	.
.	ENST000C	.	17q	.	.	Score=0.98	.	rs1724395	.	.	.	.	.
.	ENST000C	.	17q	.	.	.	.	.	.	.	.	.	.
.	ENST000C	.	17q	.	.	.	Score	.	.	.	.	.	.
.	ENST000C	.	17q	.	.	.	.	.	.	.	.	.	.
EPB41I	ENST000C	.	18p	.	.	.	.	.	.	.	.	.	.
.	ENST000C	.	18p	.	.	Score=0.98	.	rs5374020	ID	.	.	.	.
.	ENST000C	.	18p	.	.	Score=0.97	.	rs2012257	ID	.	.	.	.
.	.	.	19q	.	.	Score=0.99	Score	.	.	.	.	.	.
.	.	.	19q	.	.	Score=0.98	Score	rs7125437	.	.	.	.	.
.	.	.	19q	.	.	Score=0.98	Score	.	.	.	.	.	.
.	ENST000C	.	19q	.	.	Score=0.94	Score	.	ID	.	.	.	.
.	ENST000C	.	19q	.	.	Score=0.95	.	.	.	.	.	.	.
.	ENST000C	.	19q	.	.	.	.	.	.	.	.	.	.
.	ENST000C	.	19q	.	.	.	.	.	.	.	.	.	.
.	ENST000C	.	19q	.	.	.	.	.	.	.	.	.	.
NLRP5	ENST000C	.	19q	.	.	.	.	.	.	.	.	.	.
.	.	.	19q	.	.	.	Score	rs8677343	.	.	.	.	.
.	ENST000C	.	20q	.	.	.	.	.	.	.	.	.	.
.	ENST000C	.	20q	.	.	.	.	.	.	.	.	.	.
.	ENST000C	.	21p	.	.	Score=0.99	Score	rs8795581	.	.	.	.	.
.	ENST000C	.	21p	.	.	Score=0.98	.	rs8661396	ID	.	.	.	.
.	.	.	21p	.	.	Score=0.97	.	.	.	.	.	.	.
.	ENST000C	.	21p	.	.	Score=0.96	Score	rs7532385	ID	.	.	.	.
.	ENST000C	.	21p	.	.	Score=0.96	Score	rs3737231	.	.	.	.	.
.	.	.	21p	.	.	Score=0.98	.	.	.	.	.	.	.
.	ENST000C	.	22q	.	.	Score=0.99	.	rs2003402	.	.	.	.	.
.	ENST000C	.	22q	.	.	Score=0.99	.	rs2818474	ID	.	.	.	0.103
UNKN(	ENST000C	.	22q	.	.	.	.	rs5464921	ID	.	.	.	.
.	ENST000C	.	22q	.	.	.	.	rs9350299	.	.	.	.	.
.	ENST000C	.	22q	.	.	Score=0.97	.	rs4050109	.	.	.	.	.
.	ENST000C	.	22q	.	.	Score=0.97	.	.	.	.	.	.	.
.	ENST000C	.	22q	.	.	Score=0.98	.	.	.	.	.	.	.
.	ENST000C	.	22q	.	.	.	.	.	.	.	.	.	.
.	ENST000C	.	22q	.	.	.	.	.	.	.	.	.	.

.	ENST0000	.	22q	.	.	.	.	.	.	.	.	.	.
.	ENST0000	.	22q	.	.	Score=0.96	.	rs1425675	.	.	.	.	0.006
.	ENST0000	.	22q	.	.	.	.	rs1054069	.	.	.	.	.
.	ENST0000	.	22q	.	.	.	.	.	.	.	.	.	.
.	ENST0000	.	Xp2	.	.	Score=0.94	.	rs7661994	.	.	.	.	.
.	ENST0000	.	Xp2	.	.	Score=0.95	.	.	.	.	.	.	.
.	.	.	Xp2	.	.	.	Score	.	.	.	.	.	.
TFE3:N	ENST0000	.	Xp1	.	.	.	.	.	.	.	.	.	.
.	ENST0000	.	Xp1	.	.	.	.	.	.	.	.	.	.
.	ENST0000	.	Xp1	.	.	.	.	.	.	.	.	.	.
.	.	.	Xp1	.	Scc	.	.	rs7822715	.	.	.	.	.
TSPYL	ENST0000	.	Xp1	.	.	.	.	rs7821690	.	.	.	.	.
.	.	.	Xq1	.	.	.	Score	.	.	.	.	.	.
.	ENST0000	.	Xq1	.	.	.	.	.	.	.	.	.	.
.	ENST0000	.	Xq2	.	.	.	.	.	.	.	.	.	.
.	ENST0000	.	Xq2	.	.	.	.	.	.	.	.	.	.
.	ENST0000	.	Xq2	.	.	Score=0.89	.	.	.	.	.	.	.
CSTF2	ENST0000	.	Xq2	.	.	.	.	.	.	.	.	.	.
ZCCHC	ENST0000	.	Xq2	.	.	.	.	rs7818697	.	.	.	.	.
HS6ST	ENST0000	.	Xq2	.	Scc	.	.	.	ID	.	.	.	.
MCF2:I	ENST0000	.	Xq2	.	Scc	.	.	.	.	.	.	.	.
.	ENST0000	.	Xq2	.	.	.	.	.	.	.	.	.	.
.	.	.	.	.	.	.	.	rs8788971	ID	.	.	.	.
MTOR:	ENST0000	.	1p3	.	.	.	.	.	.	.	.	.	.
MTOR:	ENST0000	.	1p3	.	Scc	.	.	.	.	.	.	.	.
.	ENST0000	.	1p3	.	.	Score=0.94	.	rs5506852	ID	.	.	.	0.003
.	ENST0000	.	1p3	.	.	Score=0.99	.	rs2994093	.	.	.	.	.
.	ENST0000	.	1p3	.	.	Score=0.99	.	.	ID	.	.	.	.
.	ENST0000	.	1p3	.	.	Score=0.95	.	rs1390932	ID	.	.	.	0.158
.	ENST0000	.	1p3	.	.	.	.	.	.	.	.	.	.
ORC1:I	ENST0000	.	1p3	.	.	.	.	rs3686746	.	.	.	.	.
.	ENST0000	.	1p3	.	.	.	.	.	.	.	.	.	.
.	ENST0000	.	1p2	.	.	.	.	.	.	.	.	.	.
KCNA2	ENST0000	.	1p1	.	.	.	.	.	.	.	.	.	.
.	.	.	1q1	.	.	.	Score	rs2010827	ID	.	.	.	0.217
.	.	.	1q2	.	.	Score=0.96	Score	.	.	.	.	.	.
.	ENST0000	.	1q2	.	.	Score=0.97	.	rs2018849	.	.	.	.	.
.	ENST0000	.	1q2	.	.	Score=0.98	.	rs3724010	.	.	.	.	.
.	ENST0000	.	1q2	.	.	Score=0.98	.	.	.	.	.	.	.
.	.	.	1q2	.	.	Score=0.98	.	rs3772379	.	.	.	.	.
.	ENST0000	.	1q2	.	.	Score=0.98	.	rs8799699	.	.	.	.	.
.	ENST0000	.	1q2	.	.	Score=0.98	.	.	.	.	.	.	.
.	ENST0000	.	1q2	.	.	Score=0.98	.	.	.	.	.	.	.
.	ENST0000	.	1q2	.	.	Score=0.99	Score	rs4649849	.	.	.	.	.
.	ENST0000	.	1q2	.	.	Score=0.98	.	rs8799650	.	.	.	.	.
.	ENST0000	.	1q2	.	.	Score=0.98	.	rs7668601	ID	.	.	.	.
.	ENST0000	.	1q2	.	.	Score=0.98	.	rs7823737	.	.	.	.	.
.	ENST0000	.	1q2	.	.	Score=0.96	.	.	.	.	.	.	.
.	ENST0000	.	1q2	.	.	Score=0.98	.	rs2003436	ID	.	.	.	.
.	.	.	1q2	.	.	Score=0.99	.	rs6181174	ID	.	.	.	.
.	.	.	1q2	.	.	Score=0.99	Score	rs6181174	.	.	.	.	.
.	ENST0000	.	1q2	.	.	.	.	.	.	.	.	.	.
TNR:NI	ENST0000	.	1q2	.	.	.	.	rs7494818	ID	.	.	.	.
.	ENST0000	.	1q2	.	.	.	.	.	.	.	.	.	.

.	ENST000C	.	1q2	.	.	.	.	rs8790889	.	.	.	.	.
.	ENST000C	.	1q4	.	.	.	.	.	.	.	.	.	.
.	ENST000C	.	1q4	.	.	Score=0.9(	Score	.	.	.	.	.	.
.	.	.	1q4	.	.	Score=0.9'	.	.	.	.	.	.	.
.	ENST000C	.	1q4	.	.	.	.	.	.	.	.	.	.
.	ENST000C	.	2p2	.	.	.	.	.	.	.	.	.	.
.	.	.	2p1	.	.	Score=0.9{	Score	rs1020943	ID	.	.	.	.
.	ENST000C	.	2p1	.	.	Score=0.9!	Score	.	.	.	.	.	.
.	ENST000C	.	2p1	.	.	Score=0.9!	Score	rs2004118	ID	.	.	.	.
.	ENST000C	.	2q1	.	.	Score=0.9!	.	rs2021466	ID	.	.	.	.
.	ENST000C	.	2q1	.	.	Score=0.9{	.	rs3694799	ID	.	.	.	.
RGPD4	ENST000C	.	2q1	.	.	Score=0.9!	.	rs5416674	ID	.	.	.	0.002
.	ENST000C	.	2q1	.	.	Score=0.9{	.	rs1053772	.	.	.	.	.
.	ENST000C	.	2q1	.	.	Score=0.9{	.	.	ID	.	.	.	.
.	.	.	2q2	.	.	Score=0.9(	.	rs5382277	.	.	.	.	0.001
.	ENST000C	.	2q2	.	.	.	.	.	.	.	.	.	.
.	ENST000C	.	2q3	.	.	.	.	rs1875087	.	.	.	.	0.003
TTN:NI	ENST000C	.	2q3	.	.	.	.	.	.	.	.	.	.
.	ENST000C	.	2q3	.	.	.	.	.	.	.	.	.	.
.	ENST000C	.	2q3	.	.	.	.	.	.	.	.	.	.
COL4A	ENST000C	.	2q3	.	.	.	.	rs1509794	.	Benig	.	.	0.026
NGEF:I	ENST000C	.	2q3	.	.	.	.	.	ID	.	.	.	.
.	.	.	2q3	.	.	Score=0.9!	.	rs7970263	.	.	.	.	.
.	ENST000C	.	3p2	.	.	.	.	.	.	.	.	.	.
.	ENST000C	.	3p2	.	.	.	.	.	.	.	.	.	.
MST1:N	ENST000C	.	3p2	.	.	Score=0.9'	.	rs6777426	ID	.	.	.	.
.	ENST000C	.	3p2	.	.	Score=0.9'	.	rs6779963	ID	.	.	.	.
UNKNC	ENST000C	.	3p1	.	.	.	.	rs5472981	.	.	.	.	.
.	ENST000C	.	3p1	.	.	.	.	.	.	.	.	.	.
.	ENST000C	.	3q2	.	.	Score=0.9‹	.	.	.	.	.	.	.
PIK3CA	ENST000C	.	3q2	.	.	Score=0.9'	.	rs1219132	ID	Patho	.	.	.
.	ENST000C	.	3q2	.	.	.	.	.	.	.	.	.	.
GMNC:	ENST000C	.	3q2	.	.	.	.	.	.	.	.	.	.
.	ENST000C	.	3q2	.	.	.	Score	.	.	.	.	.	.
.	ENST000C	.	3q2	.	.	Score=0.9!	.	rs4857535	.	.	.	.	.
.	ENST000C	.	4p1	.	.	.	.	.	.	.	.	.	.
USP17	ENST000C	.	4p1	.	.	Score=0.9{	.	.	.	.	.	.	.
.	ENST000C	.	4p1	.	.	Score=0.9!	.	.	.	.	.	.	.
.	.	.	4p1	.	.	Score=0.9!	.	rs7961122	.	.	.	.	.
.	ENST000C	.	4p1	.	.	.	.	.	.	.	.	.	.
.	.	.	4p1	.	.	Score=0.9'	Score	.	ID	.	.	.	.
.	ENST000C	.	4q1	.	.	.	.	.	.	.	.	.	.
PRDM8	ENST000C	.	4q2	.	.	.	Score	rs3705413	.	.	.	.	.
UNKNC	ENST000C	.	4q2	.	.	.	.	.	.	.	.	.	.
.	ENST000C	.	4q2	.	.	.	.	.	.	.	.	.	.
.	ENST000C	.	4q2	.	.	.	.	.	.	.	.	.	.
GYPB:I	ENST000C	.	4q3	.	.	Score=0.9(	.	rs2016625	ID	.	.	.	.
.	ENST000C	.	4q3	.	.	.	.	.	.	.	.	.	.
ZFP42:	ENST000C	.	4q3	.	.	.	.	rs7619626	.	.	.	.	.
.	ENST000C	.	5p1	.	.	Score=0.9'	.	rs8684061	ID	.	.	.	.
.	ENST000C	.	5p1	.	.	.	.	.	.	.	.	.	.
.	.	.	5p1	.	.	Score=0.9!	Score	.	.	.	.	.	.
.	ENST000C	.	5p1	.	.	Score=0.9{	Score	.	.	.	.	.	.
.	.	.	5q1	.	.	.	Score	rs8659304	ID	.	.	.	.

.	.	.	5q1	.	.	.	Score	rs1335780	ID	.	.	.	.
.	.	.	5q1	.	.	.	Score	rs1483787	ID	.	.	.	0.018
.	ENST0000	.	5q1	.	Scc	.	Score	.	.	.	.	.	.
.	ENST0000	.	5q1	.	.	.	Score	rs1416874	.	.	.	.	0.425
.	ENST0000	.	5q3	.	.	.	.	.	.	.	.	.	.
.	ENST0000	.	5q3	.	.	.	Score	rs7284112	.	.	.	.	.
.	ENST0000	.	5q3	.	.	.	.	rs1829546	.	.	.	.	.
STK19:	ENST0000	.	6p2	.	Scc	Score=0.9	.	rs7743469	ID	.	.	.	0.027
.	ENST0000	.	6p2	.	.	Score=0.9	.	rs1403232	.	.	.	.	0.149
.	ENST0000	.	6p2	.	.	.	Score	rs7596914	.	.	.	.	.
.	.	.	6p1	.	.	.	Score	rs4928651	ID	.	.	.	.
DSE:NI	ENST0000	.	6q2	.	Scc	Score=0.9	.	.	.	.	.	.	.
.	ENST0000	.	7p2	.	.	.	.	.	.	.	.	.	.
.	ENST0000	.	7p1	.	.	.	.	rs7467237	.	.	.	.	.
NACAD	ENST0000	.	7p1	.	.	.	.	rs7762925	ID	.	.	.	.
.	.	.	7p1	.	.	Score=0.9	Score	.	.	.	.	.	.
.	ENST0000	.	7p1	.	.	Score=0.9	.	.	.	.	.	.	.
.	ENST0000	.	7p1	.	.	Score=0.9	.	rs1422600	.	.	.	.	.
.	.	.	7q1	.	.	.	Score	rs4460255	.	.	.	.	.
.	.	.	7q1	.	.	Score=0.9	.	.	.	.	.	.	.
.	.	.	7q1	.	.	Score=0.9	.	rs3740384	.	.	.	.	.
.	.	.	7q1	.	.	Score=0.9	.	rs8672068	.	.	.	.	.
.	.	.	7q1	.	.	Score=0.9	.	.	.	.	.	.	.
.	.	.	7q1	.	.	Score=0.9	.	.	.	.	.	.	.
.	.	.	7q1	.	.	Score=0.9	.	.	.	.	.	.	.
.	ENST0000	.	7q1	.	.	Score=0.9	Score	rs5541523	.	.	.	.	0.011
.	ENST0000	.	7q2	.	.	Score=0.9	Score	rs3757000	.	.	.	.	.
GPR85	ENST0000	.	7q3	.	Scc	.	.	.	.	.	.	.	.
.	ENST0000	.	7q3	.	.	.	Score	.	.	.	.	.	.
.	ENST0000	.	7q3	.	.	.	.	.	.	.	.	.	.
.	ENST0000	.	7q3	.	.	.	.	.	.	.	.	.	.
.	ENST0000	.	7q3	.	.	.	.	rs8681384	ID	.	.	.	.
.	ENST0000	.	8p2	.	.	.	.	.	.	.	.	.	.
.	.	.	8p2	.	.	Score=0.9	Score	rs3989791	ID	.	.	.	.
.	.	.	8p2	.	.	Score=0.9	.	.	.	.	.	.	.
RP1L1:	ENST0000	.	8p2	.	.	.	.	rs7469289	ID	.	.	.	.
.	.	.	8p2	.	.	Score=0.9	.	rs7595500	.	.	.	.	.
.	ENST0000	.	8p2	.	.	.	.	.	.	.	.	.	.
.	ENST0000	.	8p2	.	.	.	.	.	.	.	.	.	.
.	.	.	8q1	.	.	.	Score	.	.	.	.	.	.
.	.	.	8q1	.	.	.	Score	rs4440644	.	.	.	.	.
.	ENST0000	.	8q2	.	.	.	Score	.	.	.	.	.	.
.	ENST0000	.	8q2	.	.	.	.	.	.	.	.	.	.
.	ENST0000	.	8q2	.	.	.	.	.	.	.	.	.	.
.	ENST0000	.	9p2	.	.	.	.	rs5356927	.	.	.	.	.
.	ENST0000	.	9p1	.	.	.	.	rs5620843	.	.	.	.	.
CNTNA	ENST0000	.	9p1	.	Scc	Score=0.9	.	rs1412360	ID	.	.	.	.
.	ENST0000	.	9p1	.	.	Score=0.9	.	rs3755065	ID	.	.	.	.
.	.	.	9p1	.	.	Score=0.9	Score	rs5368523	.	.	.	.	0.002
.	ENST0000	.	9q1	.	.	Score=0.9	.	rs7792676	.	.	.	.	.
.	.	.	9q1	.	.	Score=0.9	.	rs1968548	.	.	.	.	.
.	ENST0000	.	9q1	.	Scc	Score=0.9	.	rs6254382	ID	.	.	.	0.37
.	ENST0000	.	9q2	.	.	Score=0.9	.	.	ID	.	.	.	.
.	.	.	9q3	.	.	.	.	rs5781745	.	.	.	.	.

.	ENST0000C	.	9q3	.	.	.	.	.	.	.	.	.	.
CACNA	ENST0000C	.	9q3	.	.	.	.	.	.	.	.	.	.
CUBN:I	ENST0000C	.	10p	.	.	Score=0.9:	.	rs7660547	.	.	.	.	.
.	ENST0000C	.	10p	.	.	.	Score	rs1930137	.	.	.	.	.
.	ENST0000C	.	10q	.	.	Score=0.9!	.	.	.	.	.	.	.
.	ENST0000C	.	10q	.	.	Score=0.9!	.	rs7819664	ID	.	.	.	.
.	ENST0000C	.	10q	.	.	Score=0.9!	.	rs6185008	ID	.	.	.	0.453
.	ENST0000C	.	10q	.	.	Score=0.9	.	.	ID	.	.	.	.
.	ENST0000C	.	10q	.	.	Score=0.9!	.	.	.	.	.	.	.
.	ENST0000C	.	10q	.	.	.	.	.	.	.	.	.	.
.	ENST0000C	.	10q	.	.	.	Score	rs2928119	ID	.	.	.	0.416
.	.	.	10q	.	.	.	Score	.	.	.	.	.	.
.	ENST0000C	.	10q	.	.	.	.	rs1021891	.	.	.	.	.
MUC2:I	ENST0000C	.	11p	.	.	.	.	rs6263724	ID	.	.	.	.
.	ENST0000C	.	11p	.	.	.	.	rs1494932	.	.	.	.	0.004
.	ENST0000C	.	11p	.	.	.	Score	.	.	.	.	.	.
.	ENST0000C	.	11p	.	.	.	.	.	.	.	.	.	.
.	ENST0000C	.	11p	.	.	.	Score	.	.	.	.	.	.
.	ENST0000C	.	11q	.	Scc	.	.	.	.	.	.	.	.
.	ENST0000C	.	11q	.	.	.	.	rs9265024	.	.	.	.	.
.	ENST0000C	.	11q	.	.	.	Score	.	.	.	.	.	.
.	ENST0000C	.	11q	.	.	.	.	.	.	.	.	.	.
.	ENST0000C	.	11q	.	.	.	Score	.	.	.	.	.	.
ARAP1	ENST0000C	.	11q	.	.	.	.	rs3679844	.	.	.	.	.
ARAP1	ENST0000C	.	11q	.	.	.	.	.	.	.	.	.	.
.	ENST0000C	.	11q	.	.	.	.	.	.	.	.	.	.
.	ENST0000C	.	11q	.	.	.	.	rs3831451	ID	.	.	.	.
.	ENST0000C	.	11q	.	.	.	Score	.	.	.	.	.	.
.	ENST0000C	.	11q	.	.	Score=0.9:	.	.	.	.	.	.	.
.	ENST0000C	.	11q	.	.	.	Score	rs1441056	.	.	.	.	.
OR8B8	ENST0000C	.	11q	.	.	.	.	rs5379167	.	.	.	.	.
.	.	.	11q	.	.	.	Score	.	ID	.	.	.	.
.	ENST0000C	.	12p	.	.	Score=0.9	.	rs1482401	ID	.	.	.	.
TULP3:	ENST0000C	.	12p	.	.	.	.	.	.	.	.	.	.
.	ENST0000C	.	12p	.	.	Score=0.9	.	rs2005141	ID	.	.	.	.
ASUN:I	ENST0000C	.	12p	.	Scc	.	.	.	.	.	.	.	.
BAZ2A	ENST0000C	.	12q	.	Scc	.	.	.	.	.	.	.	.
ZBTB3!	ENST0000C	.	12q	.	.	.	.	rs3737629	.	.	.	.	.
.	ENST0000C	.	12q	.	.	.	.	.	.	.	.	.	.
PPFIA2	ENST0000C	.	12q	.	Scc	.	.	.	.	.	.	.	.
.	ENST0000C	.	12q	.	.	.	.	.	.	.	.	.	.
BTBD1	ENST0000C	.	12q	.	.	.	.	.	.	.	.	.	.
.	ENST0000C	.	12q	.	.	.	Score	.	.	.	.	.	.
.	ENST0000C	.	12q	.	.	.	Score	rs3495666	ID	.	.	.	.
.	ENST0000C	.	12q	.	.	.	Score	.	.	.	.	.	.
NCOR2	ENST0000C	.	12q	.	Scc	.	.	.	.	.	.	.	.
.	ENST0000C	.	13q	.	.	Score=0.9!	.	.	.	.	.	.	.
.	ENST0000C	.	13q	.	.	.	.	.	.	.	.	.	.
.	.	.	13q	.	.	.	Score	rs3772091	.	.	.	.	.
.	.	.	13q	.	.	Score=0.9(	.	.	.	.	.	.	.
.	ENST0000C	.	13q	.	.	.	.	.	.	.	.	.	.
.	.	.	14q	.	.	Score=0.9	.	rs2259899	ID	.	.	.	.
OR4K1	ENST0000C	.	14q	.	.	Score=0.9!	.	rs7532438	ID	.	.	.	.
.	ENST0000C	.	14q	.	.	.	.	.	.	.	.	.	.

.	ENST0000	.	14q	.	Sco	.	.	.	.	.	.	.	.
C14orf	ENST0000	.	14q	.	.	.	.	.	.	.	.	.	.
RBM25	ENST0000	.	14q	.	.	.	.	.	.	.	.	.	.
.	ENST0000	.	14q	.	.	.	.	.	.	.	.	.	.
.	ENST0000	.	14q	.	.	.	Score	.	.	.	.	.	.
.	ENST0000	.	14q	.	.	.	.	rs9310230	.	.	.	.	.
.	ENST0000	.	14q	.	.	.	.	.	.	.	.	.	.
.	ENST0000	.	15q	.	.	Score=0.9	.	rs2259724	.	.	.	.	.
.	ENST0000	.	15q	.	.	Score=0.9	.	rs2648130	.	.	.	.	.
.	ENST0000	.	15q	.	.	Score=0.9	.	rs2015085	.	.	.	.	.
.	ENST0000	.	15q	.	.	Score=0.9	.	rs2007357	ID	.	.	.	.
UNKN	ENST0000	.	15q	.	Sco	.	.	.	.	.	.	.	.
VPS18	ENST0000	.	15q	.	Sco	.	.	.	.	.	.	.	.
.	ENST0000	.	15q	.	.	.	Score	.	.	.	.	.	.
.	ENST0000	.	15q	.	.	.	.	.	.	.	.	.	.
.	ENST0000	.	15q	.	.	.	.	.	.	.	.	.	.
.	ENST0000	.	15q	.	.	.	Score	.	.	.	.	.	.
.	ENST0000	.	15q	.	.	Score=0.9	.	.	ID	.	.	.	.
.	ENST0000	.	15q	.	.	Score=0.9	.	rs1401448	ID	.	.	.	.
.	ENST0000	.	15q	.	.	.	Score	rs8788873	.	.	.	.	.
.	ENST0000	.	15q	.	.	Score=0.9	.	rs2870850	ID	.	.	.	.
.	ENST0000	.	15q	.	.	.	.	.	.	.	.	.	.
ADAMT	ENST0000	.	15q	.	.	.	.	rs7724699	.	.	.	.	.
.	ENST0000	.	15q	.	.	Score=0.9	.	.	ID	.	.	.	.
.	ENST0000	.	16p	.	.	Score=0.9	.	rs7760391	ID	.	.	.	.
.	ENST0000	.	16p	.	Sco	.	.	.	.	.	.	.	.
.	ENST0000	.	16p	.	Sco	.	.	.	.	.	.	.	.
SRCA	ENST0000	.	16p	.	Sco	.	.	.	.	.	.	.	.
.	ENST0000	.	16p	.	.	Score=0.9	.	rs2111615	.	.	.	.	.
.	ENST0000	.	16p	.	.	Score=0.9	.	rs1123661	.	.	.	.	.
.	ENST0000	.	16p	.	.	.	.	.	.	.	.	.	.
.	ENST0000	.	16p	.	.	.	.	rs5547762	.	.	.	.	.
.	.	.	16p	.	.	.	Score	rs3718897	.	.	.	.	.
.	.	.	16q	.	.	.	Score	rs4967312	ID	.	.	.	.
.	.	.	16q	.	.	.	Score	rs4270195	ID	.	.	.	.
.	.	.	16q	.	.	.	Score	rs4347658	.	.	.	.	.
.	.	.	16q	.	.	.	Score	rs4246399	.	.	.	.	.
.	.	.	16q	.	.	.	Score	rs5908263	.	.	.	.	.
.	.	.	16q	.	.	.	Score	rs4249045	.	.	.	.	.
.	.	.	16q	.	.	.	Score	rs7188365	ID	.	.	.	.
.	.	.	16q	.	.	.	Score	rs4249050	.	.	.	.	.
.	.	.	16q	.	.	.	Score	rs4505355	.	.	.	.	.
.	.	.	16q	.	.	.	Score	rs1244825	.	.	.	.	.
.	.	.	16q	.	.	.	.	rs1393031	ID	.	.	.	.
.	.	.	16q	.	.	.	.	rs5614462	.	.	.	.	.
.	.	.	16q	.	.	.	Score	rs4967197	.	.	.	.	.
.	.	.	16q	.	.	.	Score	rs4450416	ID	.	.	.	.
.	.	.	16q	.	.	.	Score	rs8056404	.	.	.	.	.
.	.	.	16q	.	.	.	Score	rs4246356	.	.	.	.	.
.	.	.	16q	.	.	.	Score	rs8052624	.	.	.	.	.
.	.	.	16q	.	.	.	Score	rs4249654	.	.	.	.	.
.	.	.	16q	.	.	.	Score	rs2885782	.	.	.	.	.
.	.	.	16q	.	.	.	Score	rs6153388	.	.	.	.	.
.	.	.	16q	.	.	.	Score	.	.	.	.	.	.

.	.	.	16q	.	.	.	Score	rs2875315	.	.	.	.	.
.	.	.	16q	.	.	.	Score	.	.	.	.	.	.
.	.	.	16q	.	.	.	Score	rs1479181	.	.	.	.	.
.	.	.	16q	.	.	.	Score	rs8677676	.	.	.	.	.
.	ENST000C	.	16q	.	.	Score=0.9	Score	.	.	.	.	.	.
HP:NM	ENST000C	.	16q	.	.	Score=0.9	.	rs1891151	.	.	.	.	0.003
.	ENST000C	.	16q	.	.	Score=0.9	.	rs1291890	ID	.	.	.	0.259
.	ENST000C	.	16q	.	.	.	.	.	.	.	.	.	.
ACSF3	ENST000C	.	16q	.	.	.	.	.	.	.	.	.	.
ACSF3	ENST000C	.	16q	.	.	.	.	rs7697160	.	.	.	.	.
.	ENST000C	.	16q	.	.	Score=0.9	.	rs7945920	.	.	.	.	.
.	ENST000C	.	16q	.	.	Score=0.9	.	rs3697515	ID	.	.	.	.
.	ENST000C	.	16q	.	.	Score=0.9	.	rs5596034	ID	.	.	.	0.752
XAF1:N	ENST000C	.	17p	.	.	.	.	.	.	.	.	.	.
.	ENST000C	.	17p	.	.	.	.	.	.	.	.	.	.
.	ENST000C	.	17p	.	.	.	.	.	.	.	.	.	.
.	ENST000C	.	17p	.	.	.	.	.	.	.	.	.	.
.	ENST000C	.	17p	.	.	.	.	rs9806119	.	.	.	.	.
.	ENST000C	.	17p	.	.	.	.	rs7571703	.	.	.	.	.
.	ENST000C	.	17p	.	.	Score=0.9	.	rs1460005	ID	.	.	.	.
.	ENST000C	.	17p	.	.	Score=0.9	.	rs2168782	ID	.	.	.	0.171
.	.	.	17p	.	.	Score=0.9	Score	.	.	.	.	.	.
.	.	.	17p	.	.	.	Score	rs4362423	ID	.	.	.	.
.	.	.	17p	.	.	.	Score	rs4283255	ID	.	.	.	.
.	.	.	17p	.	.	.	Score	rs1403689	.	.	.	.	.
.	.	.	17p	.	.	.	Score	rs1405621	.	.	.	.	.
.	ENST000C	.	17q	.	.	Score=0.9	.	.	.	.	.	.	.
.	ENST000C	.	17q	.	.	Score=0.9	.	rs7126069	.	.	.	.	.
.	ENST000C	.	17q	.	.	Score=0.9	.	rs573849	.	.	.	.	.
.	ENST000C	.	17q	.	.	Score=0.9	.	rs9693530	.	.	.	.	.
.	ENST000C	.	17q	.	.	Score=0.9	Score	.	.	.	.	.	.
.	ENST000C	.	17q	.	.	.	.	.	.	.	.	.	.
.	ENST000C	.	17q	.	.	.	Score	.	.	.	.	.	.
.	ENST000C	.	17q	.	.	Score=0.9	.	rs7516213	.	.	.	.	.
AXIN2:	ENST000C	.	17q	.	Scc	.	.	rs7607356	.	Likely	.	.	.
SEC14	ENST000C	.	17q	.	.	.	.	.	.	.	.	.	.
.	ENST000C	.	17q	.	.	.	Score	.	.	.	.	.	.
.	ENST000C	.	17q	.	.	.	.	rs1138327	.	.	.	.	.
.	ENST000C	.	17q	.	.	.	Score	rs8791354	.	.	.	.	.
.	.	.	18p	.	.	Score=0.9	.	.	.	.	.	.	.
.	ENST000C	.	18p	.	.	Score=0.9	.	rs8658406	ID	.	.	.	.
.	ENST000C	.	18p	.	.	Score=0.9	.	rs8676712	.	.	.	.	.
.	ENST000C	.	19p	.	.	.	.	.	.	.	.	.	.
.	.	.	19p	.	.	.	.	rs3756184	.	.	.	.	.
.	ENST000C	.	19p	.	.	.	.	.	.	.	.	.	.
.	ENST000C	.	19p	.	.	.	.	.	.	.	.	.	.
PNPLA	ENST000C	.	19p	.	.	.	.	rs5460066	.	.	.	.	.
.	ENST000C	.	19p	.	.	.	.	.	.	.	.	.	.
.	ENST000C	.	19q	.	.	.	.	.	.	.	.	.	.
.	ENST000C	.	19q	.	.	.	.	.	.	.	.	.	.
.	.	.	19q	.	.	Score=0.9	Score	rs2385185	.	.	.	.	.
.	.	.	19q	.	.	Score=0.9	Score	rs7125437	.	.	.	.	.
.	ENST000C	.	19q	.	.	.	.	.	.	.	.	.	.
.	ENST000C	.	19q	.	.	Score=0.8	.	rs1390553	.	.	.	.	0.204

FPR1:N	ENST0000	.	19q	.	.	.	.	rs7714460	.	.	.	.	.
.	ENST0000	.	19q	.	.	Score=0.92	.	rs1785512	ID	.	.	.	0.601
.	.	.	19q	.	.	.	.	rs9207323	.	.	.	.	.
RRBP1	ENST0000	.	20p	.	Scc	.	.	.	.	.	.	.	.
.	ENST0000	.	20p	.	.	.	Score	.	.	.	.	.	.
ACSS1	ENST0000	.	20p	.	Scc	.	.	rs7787265	ID	.	.	.	.
.	ENST0000	.	20p	.	.	.	.	rs3775467	.	.	.	.	.
.	ENST0000	.	20q	.	.	Score=0.96	.	.	ID	.	.	.	.
.	ENST0000	.	20q	.	.	.	.	.	.	.	.	.	.
.	ENST0000	.	20q	.	.	.	Score	.	.	.	.	.	.
BIRC7:	ENST0000	.	20q	.	.	.	.	.	.	.	.	.	.
.	.	.	21p	.	.	Score=0.97	Score	rs3747993	.	.	.	.	.
.	.	.	21p	.	.	Score=0.98	Score	rs3730978	.	.	.	.	.
.	.	.	21p	.	.	Score=0.99	Score	rs2927801	ID	.	.	.	.
.	ENST0000	.	21p	.	.	Score=0.98	.	rs1996169	.	.	.	.	.
.	ENST0000	.	21p	.	.	Score=0.96	Score	rs8789866	ID	.	.	.	.
.	.	.	21p	.	.	Score=0.97	.	rs4358221	.	.	.	.	.
.	.	.	21p	.	.	Score=0.98	.	.	.	.	.	.	.
.	.	.	21q	.	.	.	Score	rs8664333	.	.	.	.	.
.	ENST0000	.	21q	.	.	.	Score	rs7967544	ID	.	.	.	.
TRAPP	ENST0000	.	21q	.	Scc	.	.	.	.	.	.	.	.
.	ENST0000	.	21q	.	.	.	.	.	.	.	.	.	.
.	.	.	21q	.	.	Score=0.98	.	.	.	.	.	.	.
TBX1:N	ENST0000	.	22q	.	Scc	.	Score	.	.	.	.	.	.
.	ENST0000	.	22q	.	.	Score=0.99	.	rs3702204	.	.	.	.	.
.	ENST0000	.	22q	.	.	Score=0.99	.	rs1119554	.	.	.	.	0.134
.	ENST0000	.	22q	.	.	Score=0.97	.	rs1880252	.	.	.	.	0.018
.	ENST0000	.	22q	.	.	Score=0.99	.	.	ID	.	.	.	.
VCX:NI	ENST0000	.	Xp2	.	.	Score=0.94	.	rs1637792	ID	.	.	.	.
.	ENST0000	.	Xp2	.	.	.	.	.	.	.	.	.	.
DCAF8	ENST0000	.	Xp2	.	.	Score=0.90	.	.	.	.	.	.	.
.	.	.	Xp2	.	.	.	.	.	.	.	.	.	.
FAM47	ENST0000	.	Xp2	.	.	.	.	rs7740495	ID	.	.	.	.
.	ENST0000	.	Xp1	.	.	.	.	.	.	.	.	.	.
.	ENST0000	.	Xp1	.	.	.	.	.	.	.	.	.	.
.	.	.	Xq1	.	.	.	Score	.	.	.	.	.	.
.	.	.	Xq1	.	.	.	Score	rs8686279	.	.	.	.	.
.	.	.	Xq1	.	.	.	Score	.	.	.	.	.	.
.	.	.	Xq1	.	.	.	Score	.	ID	.	.	.	.
MED12	ENST0000	.	Xq1	.	.	.	.	.	.	.	.	.	.
GPRAS	ENST0000	.	Xq2	.	.	.	.	.	.	.	.	.	.
.	.	.	Xq2	.	.	.	Score	.	.	.	.	.	.
.	ENST0000	.	Xq2	.	.	.	.	.	.	.	.	.	.
.	ENST0000	.	Xq2	.	.	.	.	.	.	.	.	.	.
.	ENST0000	.	Xq2	.	.	.	.	.	.	.	.	.	.
.	ENST0000	.	Xq2	.	.	.	.	rs9883463	.	.	.	.	.
.	ENST0000	.	Xq2	.	.	.	Score	.	.	.	.	.	.
.	ENST0000	.	Xq2	.	.	.	.	rs7821231	ID	.	.	.	.
.	ENST0000	.	Xq2	.	.	.	.	.	.	.	.	.	.
.	.	.	Yq1	.	.	Score=0.96	.	rs3769023	ID	.	.	.	.
.	.	.	.	.	.	.	.	.	.	.	.	.	.
.	.	.	.	.	.	.	.	rs1057516	.	Uncer	.	.	.
.	ENST0000	.	1p3	.	.	Score=0.99	.	.	ID	.	.	.	.
.	ENST0000	.	1p3	.	.	Score=0.99	Score	.	.	.	.	.	.

TP73:N	ENST0000C	.	1p3	.	Scc	.	.	.	.	.	.	.	.
.	ENST0000C	.	1p3	.	.	.	.	rs7579432	.	.	.	.	.
.	ENST0000C	.	1p3	.	.	Score=0.9(	.	rs5565927	.	.	.	.	0.002
.	ENST0000C	.	1p3	.	.	Score=0.9(	.	rs7619917	ID	.	.	.	.
.	ENST0000C	.	1p3	.	.	Score=0.9(	.	rs663828	.	.	.	.	.
.	ENST0000C	.	1p3	.	.	Score=0.9(	.	rs646524	ID	.	.	.	.
.	ENST0000C	.	1p3	.	.	Score=0.9!	.	rs4596945	.	.	.	.	0.247
.	ENST0000C	.	1p3	.	.	Score=0.9:	.	rs2296161	ID	.	.	.	.
EMC1:I	ENST0000C	.	1p3	.	.	.	.	rs5616387	.	.	.	.	.
.	ENST0000C	.	1p3	.	.	.	.	.	.	.	.	.	.
.	ENST0000C	.	1p3	.	.	.	Score	rs9490484	.	.	.	.	.
.	ENST0000C	.	1p3	.	.	.	.	.	.	.	.	.	.
ERICH:	ENST0000C	.	1p3	.	.	.	.	.	.	.	.	.	.
.	ENST0000C	.	1p3	.	.	.	.	.	.	.	.	.	.
NHLH2	ENST0000C	.	1p1	.	.	.	.	.	.	.	.	.	.
.	ENST0000C	.	1q2	.	.	Score=0.9{	Score	rs1409433	.	.	.	.	.
.	ENST0000C	.	1q2	.	.	Score=0.9{	Score	rs2022010	.	.	.	.	.
.	ENST0000C	.	1q2	.	.	Score=0.9'	.	rs7763460	ID	.	.	.	.
.	.	.	1q2	.	.	Score=0.9{	.	rs1457064	.	.	.	.	.
.	ENST0000C	.	1q2	.	.	Score=0.9{	.	.	.	.	.	.	.
.	.	.	1q2	.	.	Score=0.9{	.	rs1148852	ID	.	.	.	.
.	ENST0000C	.	1q2	.	.	.	.	rs3746294	.	.	.	.	0.051
.	ENST0000C	.	1q2	.	.	Score=0.9{	.	rs3675661	ID	.	.	.	.
.	ENST0000C	.	1q2	.	.	Score=0.9{	.	rs7986155	ID	.	.	.	.
MEX3A	ENST0000C	.	1q2	.	.	.	.	rs7593611	ID	.	.	.	.
OR6P1	ENST0000C	.	1q2	.	Scc	.	.	.	.	.	.	.	.
.	ENST0000C	.	1q3	.	.	.	.	.	.	.	.	.	.
PPFIA4	ENST0000C	.	1q3	.	.	.	.	rs7508890	.	.	.	.	.
LEFTY:	ENST0000C	.	1q4	.	.	Score=0.9(	.	rs1454313	ID	.	.	.	0.216
OR6F1	ENST0000C	.	1q4	.	.	.	.	rs3720052	ID	.	.	.	.
.	.	.	1q4	.	.	Score=0.9'	.	rs8789457	ID	.	.	.	.
VSNL1	ENST0000C	.	2p2	.	.	.	.	.	.	.	.	.	.
.	ENST0000C	.	2p1	.	.	.	.	.	.	.	.	.	.
LRRTM	ENST0000C	.	2p1	.	.	.	.	.	.	.	.	.	.
.	ENST0000C	.	2p1	.	Scc	Score=0.9{	.	rs2013675	ID	.	.	.	.
.	ENST0000C	.	2p1	.	.	Score=0.9!	.	.	.	.	.	.	.
.	ENST0000C	.	2q2	.	.	.	Score	.	.	.	.	.	.
.	ENST0000C	.	2q3	.	.	.	Score	rs1473449	ID	.	.	.	.
ITGA4:	ENST0000C	.	2q3	.	.	.	.	.	.	.	.	.	.
.	ENST0000C	.	2q3	.	.	.	.	.	.	.	.	.	.
.	.	.	2q3	.	.	Score=0.9!	Score	.	.	.	.	.	.
.	ENST0000C	.	3p2	.	.	.	Score	rs1050893	ID	.	.	.	.
.	ENST0000C	.	3p2	.	.	.	Score	.	.	.	.	.	.
.	ENST0000C	.	3p1	.	.	.	.	rs1178604	.	.	.	.	0.006
.	.	.	3p1	.	.	.	Score	rs3744125	ID	.	.	.	.
.	ENST0000C	.	3p1	.	.	.	Score	rs1451899	.	.	.	.	0.081
.	ENST0000C	.	3q2	.	.	.	.	.	.	.	.	.	.
.	ENST0000C	.	3q2	.	.	.	.	.	.	.	.	.	.
.	ENST0000C	.	3q2	.	.	.	.	.	.	.	.	.	.
ARMC8	ENST0000C	.	3q2	.	Scc	.	.	.	.	.	.	.	.
PIK3CA	ENST0000C	.	3q2	.	.	.	.	rs1057519	ID	Likely	.	.	.
PIK3CA	ENST0000C	.	3q2	.	.	Score=0.9'	.	rs1048860	ID	Patho	.	.	.
.	ENST0000C	.	3q2	.	.	.	.	.	.	.	.	.	.
ZFYVE	ENST0000C	.	4p1	.	.	.	.	rs3725657	ID	.	.	.	.

.	ENST0000	.	4p1	.	.	.	.	.	.	.	.	.	.
.	.	.	4p1	.	.	Score=0.99	.	rs8796734	.	.	.	.	.
.	.	.	4p1	.	.	Score=0.98	Score	rs5941177	.	.	.	.	.
NPFFR	ENST0000	.	4q1	.	.	.	.	.	.	.	.	.	.
.	ENST0000	.	4q3	.	.	.	Score	.	.	.	.	.	.
.	ENST0000	.	5p1	.	.	Score=0.98	Score	rs4288119	ID	.	.	.	.
.	ENST0000	.	5p1	.	.	Score=0.98	Score	rs8669155	ID	.	.	.	.
JADE2:	ENST0000	.	5q3	.	.	.	.	rs7765625	.	.	.	.	.
.	.	.	5q3	.	.	.	Score	rs5593800	ID	.	.	.	0.391
.	ENST0000	.	5q3	.	.	Score=0.99	.	.	.	.	.	.	.
.	ENST0000	.	6p2	.	.	.	Score	.	.	.	.	.	.
HLA-B:	ENST0000	.	6p2	.	.	Score=0.89	.	rs1513411	.	.	.	.	.
EHMT2	ENST0000	.	6p2	.	Scc	.	.	.	.	.	.	.	.
PPARD	ENST0000	.	6p2	.	.	.	.	rs7668272	.	.	.	.	.
.	ENST0000	.	6p1	.	.	.	.	.	.	.	.	.	.
.	ENST0000	.	6p1	.	.	Score=0.94	.	.	.	.	.	.	.
.	ENST0000	.	6q1	.	.	.	.	.	.	.	.	.	.
.	ENST0000	.	7p2	.	.	Score=0.89	.	.	.	.	.	.	.
.	ENST0000	.	7p1	.	.	.	.	.	.	.	.	.	.
HECW:	ENST0000	.	7p1	.	.	.	.	.	ID	.	.	.	.
.	ENST0000	.	7p1	.	.	.	.	.	.	.	.	.	.
.	.	.	7p1	.	.	Score=0.98	.	.	.	.	.	.	.
.	.	.	7p1	.	.	Score=0.99	.	rs1439657	.	.	.	.	0.189
.	ENST0000	.	7p1	.	.	Score=0.99	.	.	.	.	.	.	.
.	ENST0000	.	7p1	.	.	Score=0.99	.	rs8799634	ID	.	.	.	.
.	ENST0000	.	7p1	.	.	Score=0.99	.	.	.	.	.	.	.
.	ENST0000	.	7p1	.	.	Score=0.99	.	rs2002309	ID	.	.	.	.
.	ENST0000	.	7p1	.	.	Score=0.99	.	.	.	.	.	.	.
.	ENST0000	.	7q1	.	.	Score=0.99	.	rs2018986	ID	.	.	.	.
.	ENST0000	.	7q1	.	.	Score=0.98	.	.	.	.	.	.	.
.	ENST0000	.	7q1	.	.	Score=0.98	.	.	.	.	.	.	.
.	ENST0000	.	7q1	.	.	Score=0.98	Score	.	ID	.	.	.	.
GNAI1:	ENST0000	.	7q2	.	.	.	.	.	.	.	.	.	.
SAMD9	ENST0000	.	7q2	.	.	.	.	.	.	.	.	.	.
PDK4:N	ENST0000	.	7q2	.	.	.	.	rs2676016	.	.	.	.	.
.	ENST0000	.	7q2	.	.	.	.	rs1826826	.	.	.	.	.
.	ENST0000	.	7q3	.	.	.	Score	rs1409779	.	.	.	.	.
.	ENST0000	.	7q3	.	Scc	.	Score	.	.	.	.	.	.
.	ENST0000	.	7q3	.	.	Score=0.96	.	.	.	.	.	.	.
.	.	.	7q3	.	.	.	Score	rs7962502	.	.	.	.	.
ANGPT	ENST0000	.	8p2	.	.	.	.	.	.	.	.	.	.
.	.	.	8p2	.	.	Score=0.99	.	rs3763515	ID	.	.	.	.
.	ENST0000	.	8p2	.	.	Score=0.98	.	rs5673338	.	.	.	.	0.203
.	ENST0000	.	8p2	.	.	.	.	.	.	.	.	.	.
.	ENST0000	.	8q2	.	.	.	.	.	.	.	.	.	.
.	ENST0000	.	8q2	.	.	.	.	.	.	.	.	.	.
.	ENST0000	.	8q2	.	.	.	Score	rs5609552	ID	.	.	.	0.027
.	ENST0000	.	9p2	.	.	.	.	.	.	.	.	.	.
.	ENST0000	.	9p1	.	.	.	Score	rs1463382	ID	.	.	.	0.003
.	.	.	9p1	.	.	Score=0.99	Score	rs7495042	ID	.	.	.	.
.	ENST0000	.	9q1	.	.	Score=0.99	.	.	.	.	.	.	.
.	ENST0000	.	9q1	.	.	Score=0.99	.	rs7714176	.	.	.	.	.
.	ENST0000	.	9q1	.	.	Score=0.89	Score	rs4452915	ID	.	.	.	.
.	ENST0000	.	9q1	.	.	Score=0.89	Score	rs7492487	ID	.	.	.	.

.	ENST000C	.	9q2	.	.	Score=0.9	.	rs391727	.	.	.	.	0.32
.	.	.	9q2	.	.	.	.	.	.	.	.	.	.
.	ENST000C	.	9q2	.	.	.	.	.	.	.	.	.	.
.	ENST000C	.	9q3	.	.	Score=0.9	Score	rs2855049	ID	.	.	.	.
NOTCH	ENST000C	.	9q3	.	.	.	.	rs7771119		Likely	.	.	.
CAMK1	ENST000C	.	10p	.	.	.	.	.	.	.	.	.	.
.	ENST000C	.	10p	.	.	.	.	.	.	.	.	.	.
.	ENST000C	.	10p	.	.	.	.	rs7747854	.	.	.	.	.
.	ENST000C	.	10p	.	.	Score=0.9	.	rs1759035	ID	.	.	.	.
.	.	.	10q	.	.	Score=0.9	.	rs3125109	ID	.	.	.	.
.	ENST000C	.	10q	.	.	.	Score	.	.	.	.	.	.
VCL:NI	ENST000C	.	10q	.	Scc	.	.	rs2018088	.	.	.	.	.
.	ENST000C	.	10q	.	.	Score=0.9	.	.	.	.	.	.	.
.	ENST000C	.	10q	.	.	Score=0.9	.	rs3128226	.	.	.	.	0.524
.	ENST000C	.	10q	.	.	.	.	.	.	.	.	.	.
.	ENST000C	.	10q	.	.	.	Score	.	.	.	.	.	.
.	ENST000C	.	10q	.	Scc	.	.	rs5495128	.	.	.	.	0.001
CPXM2	ENST000C	.	10q	.	Scc	.	.	.	.	.	.	.	.
.	ENST000C	.	11p	.	.	.	.	.	.	.	.	.	.
.	ENST000C	.	11q	.	Scc	.	.	.	.	.	.	.	.
.	ENST000C	.	11q	.	.	Score=0.9	Score	.	.	.	.	.	.
.	ENST000C	.	12p	.	.	Score=0.9	.	rs7827727	ID	.	.	.	.
.	ENST000C	.	12p	.	.	.	.	.	.	.	.	.	.
CHD4:I	ENST000C	.	12p	.	Scc	.	.	.	.	.	.	.	.
.	ENST000C	.	12p	.	.	.	.	.	.	.	.	.	.
.	ENST000C	.	12p	.	.	Score=0.9	.	.	.	.	.	.	.
KRAS:I	ENST000C	.	12p	.	Scc	.	.	rs1219135	ID	Patho	.	.	.
ITPR2:I	ENST000C	.	12p	.	.	.	.	rs3731379	.	.	.	.	.
.	ENST000C	.	12p	.	.	Score=0.9	.	rs4031316	ID	.	.	.	.
PUS7L	ENST000C	.	12q	.	.	.	.	rs5661873	ID	.	.	.	.
FAM18	ENST000C	.	12q	.	.	.	.	rs9815586	ID	.	.	.	.
.	ENST000C	.	12q	.	.	.	.	rs1409167	.	.	.	.	0.006
.	ENST000C	.	12q	.	.	.	.	.	.	.	.	.	.
.	ENST000C	.	12q	.	.	.	.	.	.	.	.	.	.
.	.	.	12q	.	.	.	Score	rs1707911	.	.	.	.	0.268
DDX51	ENST000C	.	12q	.	.	.	.	rs7662808	.	.	.	.	.
.	ENST000C	.	13q	.	.	Score=0.9	.	rs9079318	.	.	.	.	.
.	ENST000C	.	13q	.	.	.	.	.	.	.	.	.	.
FLT3:N	ENST000C	.	13q	.	Scc	.	.	rs7666774	.	.	.	.	.
DCLK1	ENST000C	.	13q	.	.	.	.	.	ID	.	.	.	.
.	ENST000C	.	13q	.	.	Score=0.9	.	.	.	.	.	.	.
.	ENST000C	.	14q	.	.	Score=0.9	.	rs2018046	ID	.	.	.	.
.	ENST000C	.	14q	.	.	Score=0.9	.	rs4982820	.	.	.	.	.
.	.	.	14q	.	.	Score=0.9	Score	rs2005137	.	.	.	.	.
.	ENST000C	.	14q	.	.	Score=0.9	.	.	.	.	.	.	.
.	ENST000C	.	14q	.	.	Score=0.9	Score	.	.	.	.	.	.
.	ENST000C	.	14q	.	.	Score=0.9	.	rs3763109	.	.	.	.	.
.	ENST000C	.	14q	.	.	.	.	.	.	.	.	.	.
.	ENST000C	.	14q	.	.	.	.	rs7592315	.	.	.	.	.
.	.	.	14q	.	.	Score=0.9	Score	.	.	.	.	.	.
.	ENST000C	.	14q	.	.	Score=0.9	.	rs7473295	ID	.	.	.	.
.	.	.	15q	.	.	Score=0.9	.	rs5686085	.	.	.	.	0.001
.	ENST000C	.	15q	.	.	Score=0.9	Score	rs5761409	ID	.	.	.	.
.	.	.	15q	.	.	Score=0.9	Score	rs3684417	.	.	.	.	.

.	ENST0000	.	15q	.	.	Score=0.9	Score	rs1130343	.	.	.	.	.
.	ENST0000	.	15q	.	.	Score=0.9	.	rs1995519	.	.	.	.	.
.	ENST0000	.	15q	.	.	Score=0.9	.	rs1836337	.	.	.	.	.
.	.	.	15q	.	.	Score=0.9	Score	rs3700524	.	.	.	.	.
.	ENST0000	.	15q	.	.	Score=0.9	.	rs3766397	.	.	.	.	.
.	ENST0000	.	15q	.	.	Score=0.9	.	.	.	.	.	.	.
SPINT1	ENST0000	.	15q	.	Scc	.	Score	rs5573362	.	.	.	.	.
.	ENST0000	.	15q	.	.	.	Score	.	.	.	.	.	.
GOLGA	ENST0000	.	15q	.	.	Score=0.9	.	rs7443087	ID	.	.	.	.
.	ENST0000	.	15q	.	.	Score=0.9	.	.	.	.	.	.	.
IFT140	ENST0000	.	16p	.	.	.	.	.	.	.	.	.	.
IL32:N	ENST0000	.	16p	.	.	.	.	.	.	.	.	.	.
.	ENST0000	.	16p	.	.	.	.	.	.	.	.	.	.
.	ENST0000	.	16p	.	.	Score=0.9	.	.	.	.	.	.	.
.	.	.	16q	.	.	.	Score	rs2887413	ID	.	.	.	.
.	.	.	16q	.	.	.	Score	rs4966607	.	.	.	.	.
.	.	.	16q	.	.	.	Score	rs4359628	.	.	.	.	.
.	.	.	16q	.	.	.	Score	rs4249500	ID	.	.	.	.
.	.	.	16q	.	.	.	Score	rs4372866	ID	.	.	.	.
.	.	.	16q	.	.	.	Score	rs4396558	ID	.	.	.	.
.	.	.	16q	.	.	.	Score	rs4249160	.	.	.	.	.
.	.	.	16q	.	.	.	Score	rs4967780	ID	.	.	.	.
.	.	.	16q	.	.	.	Score	rs7203681	.	.	.	.	.
.	.	.	16q	.	.	.	Score	rs7826866	.	.	.	.	.
.	.	.	16q	.	.	.	Score	rs4249045	.	.	.	.	.
.	.	.	16q	.	.	.	Score	rs7188365	ID	.	.	.	.
.	.	.	16q	.	.	.	Score	rs9972801	.	.	.	.	.
.	.	.	16q	.	.	.	Score	rs4249048	.	.	.	.	.
.	.	.	16q	.	.	.	Score	rs7823469	ID	.	.	.	.
.	.	.	16q	.	.	.	.	rs1393031	ID	.	.	.	.
.	.	.	16q	.	.	.	Score	rs4090116	.	.	.	.	.
.	.	.	16q	.	.	.	Score	rs4090115	ID	.	.	.	.
.	.	.	16q	.	.	.	Score	rs4459567	.	.	.	.	.
.	.	.	16q	.	.	.	Score	rs6133865	.	.	.	.	.
.	.	.	16q	.	.	.	Score	rs4341908	.	.	.	.	.
.	.	.	16q	.	.	.	Score	rs4451975	.	.	.	.	.
.	.	.	16q	.	.	.	Score	rs7187636	ID	.	.	.	.
.	ENST0000	.	16q	.	.	Score=0.9	.	.	.	.	.	.	.
PDPR:I	ENST0000	.	16q	.	.	Score=0.9	.	rs2004697	ID	not pr	.	.	.
.	ENST0000	.	16q	.	Scc	.	Score	.	.	.	.	.	.
FANCA	ENST0000	.	16q	.	.	.	.	.	.	.	.	.	.
.	ENST0000	.	16q	.	.	.	Score	.	.	.	.	.	.
.	ENST0000	.	17p	.	.	.	Score	.	.	.	.	.	.
.	ENST0000	.	17p	.	.	.	.	.	.	.	.	.	.
.	ENST0000	.	17p	.	.	.	.	.	.	.	.	.	.
.	.	.	17p	.	.	.	Score	rs1440233	ID	.	.	.	.
.	.	.	17p	.	.	.	Score	rs4362423	ID	.	.	.	.
.	.	.	17q	.	.	Score=0.9	.	rs1409172	ID	.	.	.	0.223
.	.	.	17q	.	.	Score=0.9	.	rs3737234	.	.	.	.	0.212
ARHGA	ENST0000	.	17q	.	.	Score=0.9	.	.	ID	.	.	.	.
.	ENST0000	.	17q	.	.	Score=0.9	.	.	ID	.	.	.	.
.	ENST0000	.	17q	.	.	.	Score	rs8679073	ID	.	.	.	.
.	.	.	17q	.	.	.	Score	.	.	.	.	.	.
.	ENST0000	.	17q	.	.	.	Score	.	.	.	.	.	.

CACNC	ENST0000C	.	17q	.	.	.	.	rs7651139	ID	.	.	.	.
.	ENST0000C	.	17q	.	.	.	.	rs7754213	.	.	.	.	.
.	ENST0000C	.	17q	.	.	.	.	.	.	.	.	.	.
.	ENST0000C	.	17q	.	.	.	.	rs5408783	.	.	.	.	.
CCDC1	ENST0000C	.	17q	.	.	.	.	.	.	.	.	.	.
.	ENST0000C	.	17q	.	.	.	.	.	ID	.	.	.	.
.	ENST0000C	.	17q	.	.	.	.	rs3732207	.	.	.	.	.
CIDEA:	ENST0000C	.	18p	.	.	.	.	.	.	.	.	.	.
SEMA6	ENST0000C	.	19p	.	.	.	.	rs7575217	.	.	.	.	.
.	ENST0000C	.	19p	.	.	.	.	.	.	.	.	.	.
.	ENST0000C	.	19p	.	.	.	.	.	.	.	.	.	.
.	ENST0000C	.	19p	.	.	.	.	.	.	.	.	.	.
.	ENST0000C	.	19p	.	.	.	.	.	.	.	.	.	.
.	.	.	19p	.	.	Score=0.98	Score	rs1492924	ID	.	.	.	.
.	ENST0000C	.	19q	.	.	.	Score	rs1999730	ID	.	.	.	.
.	.	.	19q	.	.	Score=0.98	Score	rs3762437	.	.	.	.	.
.	.	.	19q	.	.	Score=0.98	Score	rs7125437	.	.	.	.	.
SPTBN	ENST0000C	.	19q	.	.	.	Score	.	.	.	.	.	.
.	ENST0000C	.	19q	.	.	.	Score	.	.	.	.	.	.
.	ENST0000C	.	19q	.	.	Score=0.95	.	.	ID	.	.	.	.
PLA2G	ENST0000C	.	19q	.	.	.	.	.	.	.	.	.	.
.	ENST0000C	.	19q	.	.	Score=0.95	.	rs6212787	ID	.	.	.	.
.	ENST0000C	.	19q	.	.	.	Score	.	.	.	.	.	.
.	ENST0000C	.	19q	.	.	Score=0.92	.	rs5492748	.	.	.	.	.
.	.	.	19q	.	.	.	.	.	ID	.	.	.	.
PRKCG	ENST0000C	.	19q	.	.	.	.	rs7600928	ID	.	.	.	.
.	ENST0000C	.	19q	.	.	.	.	.	.	.	.	.	.
ZNF814	ENST0000C	.	19q	.	.	Score=0.94	.	rs7704038	.	.	.	.	.
.	ENST0000C	.	19q	.	.	.	.	.	.	.	.	.	.
.	ENST0000C	.	20p	.	Scc	.	.	.	.	.	.	.	.
.	ENST0000C	.	20p	.	.	.	.	.	.	.	.	.	.
.	ENST0000C	.	20p	.	.	Score=0.96	.	.	.	.	.	.	.
.	ENST0000C	.	20q	.	.	Score=0.96	.	rs9569348	ID	.	.	.	.
.	ENST0000C	.	20q	.	.	.	.	.	.	.	.	.	.
.	ENST0000C	.	21p	.	.	Score=0.97	Score	rs3772733	.	.	.	.	.
.	ENST0000C	.	21p	.	.	Score=0.96	.	rs9184726	ID	.	.	.	.
.	.	.	21p	.	.	Score=0.97	.	rs9266067	.	.	.	.	.
.	.	.	21p	.	.	Score=0.98	Score	.	.	.	.	.	.
.	ENST0000C	.	21q	.	.	.	.	.	.	.	.	.	.
.	ENST0000C	.	21q	.	.	.	Score	rs7967544	ID	.	.	.	.
.	ENST0000C	.	22q	.	.	Score=0.99	.	rs2019683	.	.	.	.	.
.	ENST0000C	.	22q	.	.	.	.	rs7543647	.	.	.	.	.
.	ENST0000C	.	22q	.	.	.	.	rs5711604	.	.	.	.	0.005
.	ENST0000C	.	22q	.	.	Score=0.97	.	rs1856371	ID	.	.	.	0.016
MCM5:	ENST0000C	.	22q	.	Scc	.	.	rs3713772	ID	.	.	.	.
.	ENST0000C	.	22q	.	.	.	.	.	.	.	.	.	.
.	ENST0000C	.	22q	.	.	.	.	.	.	.	.	.	.
.	ENST0000C	.	22q	.	.	.	.	.	.	.	.	.	.
.	.	.	Xp2	.	.	Score=0.99	Score	rs6644312	.	.	.	.	.
.	ENST0000C	.	Xp2	.	.	.	Score	rs2010857	.	.	.	.	0.028
WWC3	ENST0000C	.	Xp2	.	.	.	.	.	ID	.	.	.	.
SRPX:N	ENST0000C	.	Xp1	.	.	.	.	rs7457127	ID	.	.	.	.
.	ENST0000C	.	Xp1	.	.	.	.	.	.	.	.	.	.
CACNA	ENST0000C	.	Xp1	.	.	.	.	rs7825308	ID	.	.	.	.

.	.	.	Xq1	.	.	.	Score	.	.	.	.	.	.
.	.	.	Xq1	.	.	.	Score	.	ID	.	.	.	.
.	.	.	Xq1	.	.	.	Score	rs7756689	.	.	.	.	.
.	ENST000C	.	Xq1	.	.	.	.	rs2486782	ID	.	.	.	0.005
AMOT:	ENST000C	.	Xq2	.	.	.	.	.	ID	.	.	.	.
C1GAL	ENST000C	.	Xq2	.	Scc	.	.	.	.	.	.	.	.
.	.	.	Xq2	.	.	.	.	.	.	.	.	.	.
.	ENST000C	.	Xq2	.	.	.	.	rs1016097	.	.	.	.	.
.	ENST000C	.	Xq2	.	.	.	.	.	.	.	.	.	.
.	ENST000C	.	Xq2	.	.	Score=0.9	.	rs7802961	ID	.	.	.	.
.	.	.	.	.	.	.	.	.	.	.	.	.	.
.	.	.	.	.	.	.	.	rs3868289	.	.	.	.	.
.	.	.	.	.	.	.	.	.	.	.	.	.	.
ATAD3	ENST000C	.	1p3	.	Scc	.	.	rs7466465	.	.	.	.	.
.	ENST000C	.	1p3	.	.	Score=0.9	Score	.	.	.	.	.	.
FBXO2	ENST000C	.	1p3	.	Scc	.	.	rs1454709	.	.	.	.	.
MAD2L	ENST000C	.	1p3	.	Scc	.	.	.	.	.	.	.	.
.	ENST000C	.	1p3	.	.	Score=0.9	.	rs1440990	.	.	.	.	.
.	.	.	1p3	.	.	Score=0.9	Score	rs1157686	ID	.	.	.	0.096
.	.	.	1p3	.	.	Score=0.9	.	.	.	.	.	.	.
.	ENST000C	.	1p3	.	.	Score=0.9	.	rs7511577	ID	.	.	.	.
SH2D5	ENST000C	.	1p3	.	.	.	.	.	ID	.	.	.	.
.	ENST000C	.	1p3	.	.	.	Score	rs5551428	.	.	.	.	.
GRHL3	ENST000C	.	1p3	.	.	.	.	rs1422489	ID	.	.	.	0.001
.	ENST000C	.	1p3	.	.	Score=0.9	Score	.	.	.	.	.	.
.	ENST000C	.	1p3	.	Scc	.	.	.	.	.	.	.	.
UNKNC	ENST000C	.	1p3	.	.	.	.	rs7753177	ID	.	.	.	.
SNIP1:	ENST000C	.	1p3	.	.	.	.	.	.	.	.	.	.
MACF1	ENST000C	.	1p3	.	.	.	.	.	.	.	.	.	.
.	ENST000C	.	1p3	.	.	.	.	.	.	.	.	.	.
.	ENST000C	.	1p3	.	.	.	.	.	.	.	.	.	.
.	ENST000C	.	1p3	.	.	.	.	.	.	.	.	.	.
.	ENST000C	.	1p3	.	.	.	Score	.	ID	.	.	.	.
KLF17:	ENST000C	.	1p3	.	Scc	Score=0.9	.	rs7580073	.	.	.	.	.
.	.	.	1p3	.	.	Score=0.9	Score	rs8662033	ID	.	.	.	.
.	ENST000C	.	1p3	.	.	.	Score	.	.	.	.	.	.
.	ENST000C	.	1p3	.	.	.	.	.	.	.	.	.	.
.	ENST000C	.	1p3	.	.	.	.	.	.	.	.	.	.
.	ENST000C	.	1p3	.	.	.	Score	.	.	.	.	.	.
.	ENST000C	.	1p2	.	.	.	.	.	.	.	.	.	.
.	ENST000C	.	1p2	.	.	.	.	.	.	.	.	.	.
.	ENST000C	.	1p2	.	.	.	.	.	.	.	.	.	.
.	ENST000C	.	1p1	.	.	.	.	rs7598590	.	.	.	.	.
LOC64	ENST000C	.	1p1	.	.	.	.	.	.	.	.	.	.
.	ENST000C	.	1p1	.	.	Score=0.9	.	rs2004173	ID	.	.	.	.
.	ENST000C	.	1q2	.	.	Score=0.9	.	rs1043712	.	.	.	.	.
.	.	.	1q2	.	.	Score=0.9	.	.	.	.	.	.	.
.	ENST000C	.	1q2	.	.	Score=0.9	Score	rs2002777	.	.	.	.	.
.	ENST000C	.	1q2	.	.	Score=0.9	.	rs7506054	.	.	.	.	.
.	ENST000C	.	1q2	.	.	Score=0.9	.	rs1694653	.	.	.	.	.
.	ENST000C	.	1q2	.	.	Score=0.9	.	rs4125366	ID	.	.	.	.
.	ENST000C	.	1q2	.	.	Score=0.9	.	.	.	.	.	.	.
.	ENST000C	.	1q2	.	.	Score=0.9	.	.	.	.	.	.	.
.	ENST000C	.	1q2	.	.	Score=0.9	.	rs6181199	ID	.	.	.	.

.	.	.	1q2	.	.	Score=0.98	.	rs8799948	.	.	.	.	.
.	ENST0000	.	1q2	.	.	Score=0.98	.	rs482979	.	.	.	.	.
.	ENST0000	.	1q2	.	.	Score=0.99	.	rs2021032	.	.	.	.	.
.	ENST0000	.	1q2	.	.	Score=0.99	.	rs2009700	ID	.	.	.	.
.	ENST0000	.	1q2	.	.	Score=0.99	Score	rs2003739	.	.	.	.	.
.	ENST0000	.	1q2	.	.	Score=0.99	Score	rs3705433	.	.	.	.	.
.	ENST0000	.	1q2	.	.	Score=0.99	.	rs226753	ID	.	.	.	.
.	ENST0000	.	1q2	.	.	Score=0.92	.	.	.	.	.	.	.
.	ENST0000	.	1q2	.	.	Score=0.98	.	rs2787777	ID	.	.	.	0.325
.	.	.	1q2	.	.	Score=0.96	.	rs3750007	.	.	.	.	.
.	ENST0000	.	1q2	.	.	.	.	.	.	.	.	.	.
.	ENST0000	.	1q2	.	.	.	.	.	.	.	.	.	.
.	ENST0000	.	1q2	.	.	.	.	.	.	.	.	.	.
SLC50A	ENST0000	.	1q2	.	.	.	.	.	.	.	.	.	.
.	ENST0000	.	1q2	.	.	.	.	.	.	.	.	.	.
.	ENST0000	.	1q2	.	.	.	.	.	.	.	.	.	.
PEAR1	ENST0000	.	1q2	.	Scc	.	.	.	.	.	.	.	.
.	ENST0000	.	1q2	.	.	.	.	.	.	.	.	.	.
ASPM:I	ENST0000	.	1q3	.	.	.	.	.	.	.	.	.	.
.	ENST0000	.	1q3	.	.	.	Score	rs1997462	.	.	.	.	.
PLEKH	ENST0000	.	1q3	.	.	.	.	rs3692540	.	.	.	.	.
C1orf1	ENST0000	.	1q4	.	.	.	.	.	.	.	.	.	.
CAPN2	ENST0000	.	1q4	.	.	.	.	.	.	.	.	.	.
.	ENST0000	.	1q4	.	.	Score=0.98	.	.	.	.	.	.	.
.	ENST0000	.	1q4	.	.	.	.	rs7793949	.	.	.	.	.
.	ENST0000	.	2p2	.	.	.	Score	.	.	.	.	.	.
.	ENST0000	.	2p2	.	.	.	.	.	.	.	.	.	.
SF3B6	ENST0000	.	2p2	.	.	.	.	.	.	.	.	.	.
.	ENST0000	.	2p2	.	.	.	.	.	.	.	.	.	.
.	ENST0000	.	2p2	.	.	.	Score	.	.	.	.	.	.
SLC3A	ENST0000	.	2p2	.	.	.	.	.	.	.	.	.	.
.	ENST0000	.	2p2	.	.	.	.	.	.	.	.	.	.
.	ENST0000	.	2p2	.	.	.	.	.	.	.	.	.	.
.	ENST0000	.	2p1	.	.	.	.	.	.	.	.	.	.
.	ENST0000	.	2p1	.	.	.	.	.	.	.	.	.	.
.	ENST0000	.	2p1	.	.	.	.	.	ID	.	.	.	.
.	ENST0000	.	2p1	.	.	Score=0.94	.	rs3764475	.	.	.	.	.
PTCD3	ENST0000	.	2p1	.	.	.	.	rs1425097	.	.	.	.	.
.	ENST0000	.	2p1	.	.	.	.	.	.	.	.	.	.
.	ENST0000	.	2p1	.	.	.	.	.	.	.	.	.	.
.	ENST0000	.	2p1	.	Scc	Score=0.99	.	rs8671368	ID	.	.	.	.
.	ENST0000	.	2p1	.	.	Score=0.99	.	rs8674281	ID	.	.	.	.
.	ENST0000	.	2p1	.	.	Score=0.99	.	.	.	.	.	.	.
.	.	.	2p1	.	.	Score=0.95	Score	rs1210470	ID	.	.	.	.
.	.	.	2q1	.	.	.	Score	rs2015850	ID	.	.	.	.
SLC9A	ENST0000	.	2q1	.	.	.	.	.	ID	.	.	.	.
.	ENST0000	.	2q1	.	.	.	.	.	.	.	.	.	.
.	ENST0000	.	2q1	.	.	.	.	.	.	.	.	.	.
.	ENST0000	.	2q1	.	.	.	.	rs6216365	.	.	.	.	0.378
.	ENST0000	.	2q1	.	Scc	.	Score	.	.	.	.	.	.
.	ENST0000	.	2q2	.	.	Score=0.98	.	.	.	.	.	.	.
POTEF	ENST0000	.	2q2	.	.	Score=0.98	.	rs1839843	ID	.	.	.	0.688
.	ENST0000	.	2q2	.	.	Score=0.98	Score	rs2001821	.	.	.	.	.
.	ENST0000	.	2q2	.	.	Score=0.96	.	.	.	.	.	.	.

.	ENST0000	.	2q2	.	.	.	.	.	.	.	.	.	.
.	ENST0000	.	2q2	.	.	.	.	.	.	.	.	.	.
.	ENST0000	.	2q3	.	.	.	.	.	.	.	.	.	.
CCDC1	ENST0000	.	2q3	.	.	.	.	.	ID	.	.	.	.
.	ENST0000	.	2q3	.	.	.	.	.	.	.	.	.	.
.	ENST0000	.	2q3	.	.	.	Score	.	.	.	.	.	.
.	ENST0000	.	2q3	.	.	.	.	.	.	.	.	.	.
SP140:	ENST0000	.	2q3	.	.	.	.	.	.	.	.	.	.
.	ENST0000	.	2q3	.	.	.	.	.	.	.	.	.	.
.	ENST0000	.	2q3	.	.	.	.	.	.	.	.	.	.
.	ENST0000	.	3p2	.	.	Score=0.8	Score	.	.	.	.	.	.
.	ENST0000	.	3p2	.	.	.	.	.	.	.	.	.	.
.	ENST0000	.	3p2	.	.	.	.	.	.	.	.	.	.
.	ENST0000	.	3p2	.	.	.	.	.	.	.	.	.	.
DYNC1	ENST0000	.	3p2	.	Scc	.	.	.	.	.	.	.	.
.	ENST0000	.	3p2	.	.	.	.	.	.	.	.	.	.
.	ENST0000	.	3p2	.	.	.	.	.	.	.	.	.	.
NDUFA	ENST0000	.	3p2	.	.	.	.	.	.	.	.	.	.
NPRL2	ENST0000	.	3p2	.	Scc	.	.	.	.	.	.	.	.
.	ENST0000	.	3p2	.	Scc	.	.	.	.	.	.	.	.
.	ENST0000	.	3p2	.	.	.	.	rs7801345	.	.	.	.	.
ITIH3:N	ENST0000	.	3p2	.	.	.	.	rs3677973	.	.	.	.	.
.	ENST0000	.	3p1	.	.	.	.	rs7454520	.	.	.	.	.
.	ENST0000	.	3q1	.	.	.	.	.	.	.	.	.	.
.	ENST0000	.	3q1	.	.	.	.	rs1911937	.	.	.	.	.
ZNF80:	ENST0000	.	3q1	.	.	.	.	.	.	.	.	.	.
GPR15	ENST0000	.	3q1	.	.	.	.	.	.	.	.	.	.
.	ENST0000	.	3q2	.	.	.	.	.	.	.	.	.	.
H1FX:N	ENST0000	.	3q2	.	.	.	.	.	.	.	.	.	.
.	ENST0000	.	3q2	.	.	.	.	.	.	.	.	.	.
MBD4:I	ENST0000	.	3q2	.	.	.	.	.	.	.	.	.	.
.	ENST0000	.	3q2	.	.	.	.	.	.	.	.	.	.
COL6A	ENST0000	.	3q2	.	.	.	.	.	.	.	.	.	.
ATP2C	ENST0000	.	3q2	.	.	.	.	.	.	.	.	.	.
.	ENST0000	.	3q2	.	.	.	.	rs1469630	.	.	.	.	.
FOXL2	ENST0000	.	3q2	.	Scc	.	.	.	.	.	.	.	.
GRK7:I	ENST0000	.	3q2	.	.	.	.	.	ID	.	.	.	.
.	ENST0000	.	3q2	.	.	.	.	.	.	.	.	.	.
.	ENST0000	.	3q2	.	.	.	.	rs7613736	.	.	.	.	.
.	ENST0000	.	3q2	.	.	.	.	.	.	.	.	.	.
.	ENST0000	.	3q2	.	.	.	.	.	.	.	.	.	.
SLC2A	ENST0000	.	3q2	.	.	.	.	.	.	.	.	.	.
FNDC3	ENST0000	.	3q2	.	.	.	.	.	.	.	.	.	.
HTR3E	ENST0000	.	3q2	.	.	.	.	.	.	.	.	.	.
.	ENST0000	.	3q2	.	.	.	Score	.	.	.	.	.	.
.	ENST0000	.	3q2	.	.	.	.	.	.	.	.	.	.
.	ENST0000	.	3q2	.	.	.	.	.	.	.	.	.	.
.	.	.	3q2	.	.	Score=0.9	Score	rs8795450	.	.	.	.	.
.	.	.	3q2	.	.	Score=0.9	Score	.	.	.	.	.	.
.	ENST0000	.	3q2	.	.	Score=0.9	.	rs4927838	ID	.	.	.	.
.	ENST0000	.	3q2	.	Scc	Score=0.9	.	rs1739998	ID	.	.	.	.
.	ENST0000	.	4p1	.	.	.	Score	rs4022005	ID	.	.	.	.
SLC26	ENST0000	.	4p1	.	.	.	.	rs7518089	.	.	.	.	.
PPARG	ENST0000	.	4p1	.	.	.	.	rs7529426	.	.	.	.	.

.	.	.	4p1	.	.	Score=0.9	.	.	.	.	.	.	.
.	ENST0000	.	4p1	.	.	.	.	.	.	.	.	.	.
LIMCH	ENST0000	.	4p1	.	.	.	.	.	.	.	.	.	.
.	.	.	4p1	.	.	Score=0.9	.	rs7541707	ID	.	.	.	.
.	ENST0000	.	4q1	.	.	.	.	.	.	.	.	.	.
.	ENST0000	.	4q1	.	.	.	.	rs5423520	.	.	.	.	0.001
.	ENST0000	.	4q2	.	.	.	.	.	.	.	.	.	.
.	ENST0000	.	4q2	.	.	Score=0.9	.	rs8789415	ID	.	.	.	.
.	ENST0000	.	4q2	.	.	.	.	rs8900187	.	.	.	.	.
.	ENST0000	.	4q2	.	.	.	.	.	.	.	.	.	.
.	ENST0000	.	4q2	.	.	.	Score	rs1378897	.	.	.	.	0.245
PCDH1	ENST0000	.	4q2	.	Scc	.	.	.	.	.	.	.	.
.	ENST0000	.	4q3	.	.	Score=0.9	.	rs3676041	.	.	.	.	.
.	ENST0000	.	4q3	.	.	.	.	.	.	.	.	.	.
.	ENST0000	.	4q3	.	.	.	.	.	.	.	.	.	.
.	.	.	4q3	.	.	.	Score	.	ID	.	.	.	.
PDCD6	ENST0000	.	5p1	.	.	.	.	.	.	.	.	.	.
.	ENST0000	.	5p1	.	.	.	.	.	.	.	.	.	.
.	ENST0000	.	5p1	.	.	Score=0.9	.	.	.	.	.	.	.
.	ENST0000	.	5q1	.	.	.	.	.	.	.	.	.	.
.	ENST0000	.	5q1	.	.	.	.	.	.	.	.	.	.
.	ENST0000	.	5q1	.	.	.	Score	.	.	.	.	.	.
.	.	.	5q1	.	.	.	Score	.	.	.	.	.	.
.	ENST0000	.	5q1	.	.	Score=0.9	.	.	.	.	.	.	.
.	ENST0000	.	5q1	.	.	Score=0.9	Score	rs2848141	ID	.	.	.	.
.	ENST0000	.	5q1	.	Scc	.	.	.	.	.	.	.	.
HEXB:	ENST0000	.	5q1	.	.	.	.	.	.	.	.	.	.
HSD17	ENST0000	.	5q2	.	Scc	.	.	.	.	.	.	.	.
.	ENST0000	.	5q3	.	.	.	.	.	.	.	.	.	.
.	ENST0000	.	5q3	.	.	.	.	.	.	.	.	.	.
.	ENST0000	.	5q3	.	.	.	Score	rs1024566	.	.	.	.	.
.	ENST0000	.	5q3	.	.	.	.	.	.	.	.	.	.
.	ENST0000	.	5q3	.	.	.	Score	.	.	.	.	.	.
.	ENST0000	.	5q3	.	.	.	.	.	.	.	.	.	.
.	ENST0000	.	5q3	.	.	.	.	rs5341551	.	.	.	.	.
.	ENST0000	.	5q3	.	.	.	.	.	.	.	.	.	.
CBY3:	ENST0000	.	5q3	.	.	.	.	.	.	.	.	.	.
DUSP2	ENST0000	.	6p2	.	.	.	.	rs2013466	.	.	.	.	.
.	ENST0000	.	6p2	.	.	.	Score	.	.	.	.	.	.
CDYL:	ENST0000	.	6p2	.	.	.	.	.	.	.	.	.	.
HIST1H	ENST0000	.	6p2	.	.	.	.	rs7489137	.	.	.	.	.
POM12	ENST0000	.	6p2	.	.	.	.	.	.	.	.	.	.
POM12	ENST0000	.	6p2	.	.	.	.	.	.	.	.	.	.
.	ENST0000	.	6p2	.	.	.	.	rs9402071	.	.	.	.	.
DHX16	ENST0000	.	6p2	.	.	.	.	.	.	.	.	.	.
.	ENST0000	.	6p2	.	.	.	.	.	.	.	.	.	.
.	ENST0000	.	6p2	.	.	Score=0.8	.	.	.	.	.	.	.
.	ENST0000	.	6p2	.	.	.	.	.	.	.	.	.	.
ZBTB1	ENST0000	.	6p2	.	.	Score=0.9	.	.	.	.	.	.	.
STK19:	ENST0000	.	6p2	.	Scc	Score=0.9	.	.	.	.	.	.	.
.	ENST0000	.	6p2	.	.	Score=0.9	.	rs5421901	.	.	.	.	.
UNKN	ENST0000	.	6p2	.	Scc	Score=0.9	.	.	.	.	.	.	.
.	ENST0000	.	6p2	.	.	Score=0.9	.	.	.	.	.	.	.
ETV7:	ENST0000	.	6p2	.	.	.	.	.	.	.	.	.	.

.	ENST0000	.	6p2	.	.	.	.	.	.	.	.	.	.
.	ENST0000	.	6p2	.	.	.	.	rs3746609	.	.	.	.	.
GPR11	ENST0000	.	6p1	.	.	Score=0.95	.	rs5482384	.	.	.	.	.
DEFB1	ENST0000	.	6p1	.	.	.	.	rs1442276	.	.	.	.	.
.	ENST0000	.	6q1	.	.	.	.	rs9008212	.	.	.	.	.
.	ENST0000	.	6q1	.	.	.	.	.	.	.	.	.	.
.	ENST0000	.	6q2	.	.	.	.	rs7466151	.	.	.	.	.
CEP85	ENST0000	.	6q2	.	Scc	.	.	.	.	.	.	.	.
.	.	.	6q2	.	.	.	Score	rs5411063	.	.	.	.	0.001
.	ENST0000	.	6q2	.	.	.	.	.	.	.	.	.	.
.	ENST0000	.	7p2	.	.	.	.	rs3695415	.	.	.	.	.
.	ENST0000	.	7p2	.	.	.	.	rs9183664	.	.	.	.	.
.	.	.	7p2	.	.	.	Score	.	.	.	.	.	.
.	ENST0000	.	7p1	.	.	Score=0.94	Score	.	.	.	.	.	.
.	ENST0000	.	7p1	.	.	.	.	rs1485954	.	.	.	.	.
.	ENST0000	.	7p1	.	.	Score=0.99	.	rs3677059	.	.	.	.	.
.	ENST0000	.	7p1	.	.	Score=0.99	.	.	ID	.	.	.	.
.	ENST0000	.	7p1	.	.	Score=0.99	.	.	.	.	.	.	.
.	ENST0000	.	7p1	.	.	Score=0.99	.	.	.	.	.	.	.
.	ENST0000	.	7q1	.	.	Score=0.99	.	rs2018986	ID	.	.	.	.
.	ENST0000	.	7q1	.	.	Score=0.99	Score	rs1860492	ID	.	.	.	0.355
.	.	.	7q1	.	.	Score=0.99	.	.	ID	.	.	.	.
.	.	.	7q1	.	.	Score=0.99	.	.	.	.	.	.	.
.	ENST0000	.	7q1	.	.	Score=0.97	.	rs2687038	.	.	.	.	.
.	ENST0000	.	7q1	.	.	.	.	.	.	.	.	.	.
.	ENST0000	.	7q1	.	Scc	.	.	.	.	.	.	.	.
.	.	.	7q2	.	.	Score=0.95	.	.	.	.	.	.	.
.	ENST0000	.	7q2	.	.	.	.	rs3730249	.	.	.	.	.
RBM28	ENST0000	.	7q3	.	.	.	.	rs3721622	.	.	.	.	.
RBM28	ENST0000	.	7q3	.	.	.	.	.	.	.	.	.	.
.	ENST0000	.	7q3	.	.	.	.	rs1030263	.	.	.	.	.
.	ENST0000	.	7q3	.	.	Score=0.91	.	.	.	.	.	.	.
.	ENST0000	.	7q3	.	.	.	.	.	.	.	.	.	.
.	ENST0000	.	7q3	.	.	Score=0.96	.	rs8678229	.	.	.	.	.
.	ENST0000	.	8p2	.	.	Score=0.98	.	.	.	.	.	.	.
.	.	.	8p2	.	.	Score=0.99	.	rs6264068	.	.	.	.	.
.	ENST0000	.	8p2	.	.	.	Score	.	.	.	.	.	.
.	ENST0000	.	8p2	.	.	Score=0.99	Score	.	.	.	.	.	.
.	ENST0000	.	8p2	.	.	Score=0.99	.	.	.	.	.	.	.
CHMP7	ENST0000	.	8p2	.	.	.	.	rs7607907	.	.	.	.	.
NRG1:I	ENST0000	.	8p1	.	.	.	.	.	.	.	.	.	.
RAB11	ENST0000	.	8p1	.	.	.	.	rs3736343	ID	.	.	.	.
.	ENST0000	.	8q1	.	.	.	.	.	.	.	.	.	.
TERF1	ENST0000	.	8q2	.	.	.	.	.	.	.	.	.	.
.	ENST0000	.	8q2	.	.	.	.	rs9872967	.	.	.	.	.
.	ENST0000	.	8q2	.	.	.	.	.	.	.	.	.	.
.	ENST0000	.	8q2	.	.	.	.	.	.	.	.	.	.
.	ENST0000	.	8q2	.	.	.	Score	.	.	.	.	.	.
.	ENST0000	.	8q2	.	.	.	.	.	.	.	.	.	.
.	ENST0000	.	8q2	.	.	.	.	.	ID	.	.	.	.
TAF2:N	ENST0000	.	8q2	.	.	.	.	.	.	.	.	.	.
.	ENST0000	.	8q2	.	.	.	.	.	.	.	.	.	.
KIAA01	ENST0000	.	8q2	.	.	.	.	rs1436548	.	.	.	.	.
.	ENST0000	.	8q2	.	Scc	.	.	.	.	.	.	.	.

.	ENST0000	.	8q2	.	.	.	.	.	.	.	.	.	.
.	ENST0000	.	9p2	.	.	Score=0.9	.	.	.	.	.	.	.
HAUS6	ENST0000	.	9p2	.	.	.	.	rs7682516	.	.	.	.	.
IFNB1:	ENST0000	.	9p2	.	.	.	.	.	.	.	.	.	.
UBAP2	ENST0000	.	9p1	.	.	.	.	.	.	.	.	.	.
.	ENST0000	.	9p1	.	.	Score=0.9	.	rs3764507	ID	.	.	.	.
.	ENST0000	.	9p1	.	.	Score=0.9	.	.	.	.	.	.	.
.	ENST0000	.	9q1	.	.	Score=0.9	.	.	.	.	.	.	.
.	ENST0000	.	9q2	.	.	Score=0.9	.	.	.	.	.	.	.
.	ENST0000	.	9q2	.	.	.	.	.	.	.	.	.	.
.	ENST0000	.	9q2	.	.	.	.	.	.	.	.	.	.
.	ENST0000	.	9q2	.	.	.	.	.	.	.	.	.	.
ERCC6	ENST0000	.	9q2	.	Scc	.	.	.	.	.	.	.	.
.	ENST0000	.	9q3	.	.	.	.	rs9619494	.	.	.	.	.
DAB2IF	ENST0000	.	9q3	.	.	.	.	.	.	.	.	.	.
LMX1B	ENST0000	.	9q3	.	.	.	.	.	.	.	.	.	.
.	ENST0000	.	9q3	.	.	.	.	.	.	.	.	.	.
.	ENST0000	.	9q3	.	.	.	.	rs7589091	.	.	.	.	.
.	ENST0000	.	9q3	.	.	.	.	.	.	.	.	.	.
.	ENST0000	.	9q3	.	.	.	.	.	.	.	.	.	.
.	ENST0000	.	9q3	.	.	.	.	.	.	.	.	.	.
UNKNC	ENST0000	.	9q3	.	.	.	.	.	.	.	.	.	.
UPF2:N	ENST0000	.	10p	.	.	.	.	.	.	.	.	.	.
SKIDA1	ENST0000	.	10p	.	.	.	Score	rs2018361	ID	.	.	.	.
.	ENST0000	.	10q	.	.	.	.	.	.	.	.	.	.
.	ENST0000	.	10q	.	.	.	.	.	.	.	.	.	.
.	ENST0000	.	10q	.	.	.	.	.	.	.	.	.	.
HKDC1	ENST0000	.	10q	.	.	.	.	rs3760330	.	.	.	.	.
.	ENST0000	.	10q	.	.	.	.	.	.	.	.	.	.
.	ENST0000	.	10q	.	.	.	.	.	.	.	.	.	.
UNC5B	ENST0000	.	10q	.	Scc	.	.	rs7593379	.	.	.	.	.
CDH23	ENST0000	.	10q	.	.	.	.	rs7698966	ID	.	.	.	.
.	.	.	10q	.	.	Score=0.9	Score	.	ID	.	.	.	.
.	.	.	10q	.	.	Score=0.9	Score	.	ID	.	.	.	.
.	ENST0000	.	10q	.	.	.	.	.	.	.	.	.	.
.	ENST0000	.	10q	.	.	.	.	.	.	.	.	.	.
.	ENST0000	.	10q	.	.	.	.	.	.	.	.	.	.
.	ENST0000	.	10q	.	.	Score=0.9	.	rs7966467	.	.	.	.	.
MUC6:	ENST0000	.	11p	.	.	.	.	rs7657851	ID	.	.	.	.
.	ENST0000	.	11p	.	.	Score=0.9	.	rs8667539	ID	.	.	.	.
SBF2:N	ENST0000	.	11p	.	.	.	.	.	.	.	.	.	.
LUZP2	ENST0000	.	11p	.	.	.	.	.	.	.	.	.	.
WT1:N	ENST0000	.	11p	.	Scc	.	.	.	.	.	.	.	.
DGKZ:	ENST0000	.	11p	.	Scc	.	.	.	.	.	.	.	.
.	ENST0000	.	11p	.	.	.	.	.	.	.	.	.	.
.	ENST0000	.	11q	.	.	.	.	.	.	.	.	.	.
PPP1R	ENST0000	.	11q	.	.	.	.	rs7463740	ID	.	.	.	.
.	ENST0000	.	11q	.	Scc	.	Score	.	.	.	.	.	.
.	ENST0000	.	11q	.	Scc	.	.	.	.	.	.	.	.
RCOR2	ENST0000	.	11q	.	Scc	.	.	rs3704854	ID	.	.	.	.
.	ENST0000	.	11q	.	.	.	Score	.	.	.	.	.	.
NUDT2	ENST0000	.	11q	.	.	.	.	.	.	.	.	.	.
.	ENST0000	.	11q	.	.	.	.	.	.	.	.	.	.
FKBP2	ENST0000	.	11q	.	Scc	.	.	.	.	.	.	.	.

.	ENST0000C	.	11q	.	.	.	.	.	.	.	.	.	.
.	ENST0000C	.	11q	.	.	.	.	.	.	.	.	.	.
.	ENST0000C	.	11q	.	.	.	.	rs7459229	.	.	.	.	.
P4HA3	ENST0000C	.	11q	.	.	.	.	.	.	.	.	.	.
.	ENST0000C	.	11q	.	.	.	.	.	.	.	.	.	.
.	ENST0000C	.	11q	.	.	Score=0.9	.	rs3521580	.	.	.	.	.
.	ENST0000C	.	11q	.	.	Score=0.9	.	rs3580670	ID	.	.	.	.
.	ENST0000C	.	11q	.	.	.	.	rs9203240	.	.	.	.	.
ZBTB4	ENST0000C	.	11q	.	Scc	.	.	.	.	.	.	.	.
.	ENST0000C	.	12p	.	.	.	.	.	.	.	.	.	.
.	ENST0000C	.	12p	.	.	.	.	.	.	.	.	.	.
.	ENST0000C	.	12p	.	.	.	.	.	.	.	.	.	.
DDX11	ENST0000C	.	12p	.	Scc	Score=0.9	.	rs7123030	ID	.	.	.	0.131
KIAA15	ENST0000C	.	12p	.	.	.	.	.	.	.	.	.	.
.	ENST0000C	.	12q	.	.	.	.	.	.	.	.	.	.
SCAF1	ENST0000C	.	12q	.	.	.	.	.	.	.	.	.	.
CYP27	ENST0000C	.	12q	.	.	.	.	.	.	.	.	.	.
.	ENST0000C	.	12q	.	.	.	.	.	.	.	.	.	.
.	ENST0000C	.	12q	.	.	.	Score	rs8674713	.	.	.	.	.
.	ENST0000C	.	12q	.	.	.	.	rs9109827	.	.	.	.	.
.	ENST0000C	.	12q	.	.	.	Score	rs9642294	.	.	.	.	.
.	ENST0000C	.	12q	.	.	.	.	.	.	.	.	.	.
.	ENST0000C	.	12q	.	.	.	.	.	.	.	.	.	.
MYBPC	ENST0000C	.	12q	.	.	.	.	.	.	.	.	.	.
.	ENST0000C	.	12q	.	.	.	Score	.	.	.	.	.	.
MED13	ENST0000C	.	12q	.	.	.	.	.	.	.	.	.	.
.	ENST0000C	.	12q	.	.	.	.	.	.	.	.	.	.
.	ENST0000C	.	12q	.	.	.	.	rs7540482	.	.	.	.	.
.	ENST0000C	.	12q	.	.	.	.	.	.	.	.	.	.
.	ENST0000C	.	12q	.	.	.	.	.	.	.	.	.	.
.	ENST0000C	.	13q	.	.	.	.	.	.	.	.	.	.
NBEA:I	ENST0000C	.	13q	.	.	.	.	.	.	.	.	.	.
EPSTI1	ENST0000C	.	13q	.	Scc	.	.	.	.	.	.	.	.
.	ENST0000C	.	13q	.	.	.	.	.	.	.	.	.	.
FGF14	ENST0000C	.	13q	.	.	.	.	rs7720033	.	.	.	.	.
.	ENST0000C	.	14q	.	.	Score=0.9	.	rs1450473	.	.	.	.	.
.	.	.	14q	.	.	.	.	.	.	.	.	.	.
.	ENST0000C	.	14q	.	.	Score=0.9	.	.	.	.	.	.	.
.	ENST0000C	.	14q	.	.	Score=0.9	.	.	.	.	.	.	.
.	ENST0000C	.	14q	.	.	.	.	.	.	.	.	.	.
.	.	.	14q	.	.	.	Score	.	.	.	.	.	.
GCH1:I	ENST0000C	.	14q	.	.	.	.	rs1501582	.	Likely	.	.	.
.	ENST0000C	.	14q	.	.	.	.	.	.	.	.	.	.
.	ENST0000C	.	14q	.	.	.	Score	.	.	.	.	.	.
.	ENST0000C	.	14q	.	.	.	.	.	.	.	.	.	.
SMOC	ENST0000C	.	14q	.	.	.	.	rs7676169	.	.	.	.	.
PAPLN	ENST0000C	.	14q	.	.	.	.	.	.	.	.	.	.
.	ENST0000C	.	14q	.	.	.	.	.	.	.	.	.	.
.	ENST0000C	.	14q	.	.	.	.	.	.	.	.	.	.
.	ENST0000C	.	14q	.	.	.	.	.	.	.	.	.	.
.	ENST0000C	.	14q	.	.	.	.	rs3756754	.	.	.	.	.
.	ENST0000C	.	14q	.	.	.	.	.	ID	.	.	.	.
.	ENST0000C	.	14q	.	.	.	.	rs5537870	.	.	.	.	.
.	.	.	14q	.	.	Score=0.9	.	rs1289410	ID	.	.	.	.

.	ENST0000C	.	15q	.	.	Score=0.99	.	.	.	.	.	.	.
.	ENST0000C	.	15q	.	.	Score=0.98	Score	.	.	.	.	.	.
HERC2	ENST0000C	.	15q	.	Scc	.	.	rs5409237	.	.	.	.	.
.	ENST0000C	.	15q	.	.	Score=0.99	.	.	.	.	.	.	.
.	ENST0000C	.	15q	.	.	Score=0.98	.	.	.	.	.	.	.
.	ENST0000C	.	15q	.	.	Score=0.99	.	.	ID	.	.	.	.
.	ENST0000C	.	15q	.	.	Score=0.95	.	rs2015085	.	.	.	.	.
.	ENST0000C	.	15q	.	.	.	.	rs7481072	.	.	.	.	.
.	ENST0000C	.	15q	.	.	.	.	.	.	.	.	.	.
RPAP1	ENST0000C	.	15q	.	.	.	.	.	.	.	.	.	.
SPTBN	ENST0000C	.	15q	.	.	.	.	rs7754448	.	.	.	.	.
.	ENST0000C	.	15q	.	.	Score=0.98	.	.	.	.	.	.	.
.	ENST0000C	.	15q	.	.	.	.	rs1922871	.	.	.	.	.
.	ENST0000C	.	15q	.	.	.	.	.	.	.	.	.	.
.	ENST0000C	.	15q	.	.	Score=0.97	.	rs6202600	.	.	.	.	0.422
SPATA	ENST0000C	.	15q	.	Scc	.	.	.	ID	.	.	.	.
.	ENST0000C	.	15q	.	Scc	.	.	.	.	.	.	.	.
.	ENST0000C	.	15q	.	.	.	.	.	.	.	.	.	.
.	ENST0000C	.	15q	.	.	.	.	rs5478938	.	.	.	.	.
.	ENST0000C	.	15q	.	.	.	.	.	.	.	.	.	.
.	ENST0000C	.	15q	.	.	Score=0.92	.	.	.	.	.	.	.
.	ENST0000C	.	15q	.	.	.	.	.	.	.	.	.	.
.	.	.	15q	.	.	Score=0.93	.	.	.	.	.	.	.
CHRNE	ENST0000C	.	15q	.	Scc	.	.	.	.	.	.	.	.
ANKRD	ENST0000C	.	15q	.	.	.	.	.	.	.	.	.	.
.	ENST0000C	.	15q	.	.	Score=0.96	.	rs2401418	.	.	.	.	.
.	ENST0000C	.	15q	.	.	.	.	.	.	.	.	.	.
VPS33I	ENST0000C	.	15q	.	.	.	.	.	.	.	.	.	.
.	ENST0000C	.	15q	.	.	.	.	.	.	.	.	.	.
.	ENST0000C	.	15q	.	.	.	.	.	.	.	.	.	.
TARSL	ENST0000C	.	15q	.	.	.	.	rs7510253	ID	.	.	.	.
UNKNC	ENST0000C	.	16p	.	Scc	.	.	rs7504825	.	.	.	.	.
.	ENST0000C	.	16p	.	.	.	.	.	.	.	.	.	.
.	ENST0000C	.	16p	.	.	.	.	.	.	.	.	.	.
TEKT5:	ENST0000C	.	16p	.	.	.	.	.	.	.	.	.	.
TNFRS	ENST0000C	.	16p	.	.	.	.	.	.	.	.	.	.
.	ENST0000C	.	16p	.	.	Score=0.99	.	.	.	.	.	.	.
.	ENST0000C	.	16p	.	.	Score=0.99	.	rs5522924	.	.	.	.	0.034
.	ENST0000C	.	16p	.	.	Score=0.99	.	rs6203288	ID	.	.	.	0.04
SMG1:I	ENST0000C	.	16p	.	.	.	.	rs7654396	.	.	.	.	.
.	ENST0000C	.	16p	.	.	.	Score	.	.	.	.	.	.
.	ENST0000C	.	16p	.	.	.	Score	.	.	.	.	.	.
.	ENST0000C	.	16p	.	Scc	Score=0.99	.	.	.	.	.	.	.
XPO6:N	ENST0000C	.	16p	.	.	.	.	.	.	.	.	.	.
.	ENST0000C	.	16p	.	.	.	.	.	.	.	.	.	.
.	ENST0000C	.	16p	.	.	Score=0.98	.	.	.	.	.	.	.
.	ENST0000C	.	16p	.	.	.	.	.	.	.	.	.	.
.	ENST0000C	.	16p	.	.	.	.	.	.	.	.	.	.
.	ENST0000C	.	16p	.	.	Score=0.96	Score	rs7896651	.	.	.	.	.
.	ENST0000C	.	16q	.	.	.	.	rs7515102	.	.	.	.	.
CNOT1	ENST0000C	.	16q	.	.	.	.	.	.	.	.	.	.
CTCF:N	ENST0000C	.	16q	.	.	.	.	.	.	.	.	.	.
.	ENST0000C	.	16q	.	.	.	.	.	.	.	.	.	.
.	ENST0000C	.	16q	.	.	Score=0.97	.	rs2911030	ID	.	.	.	.

.	ENST0000	.	16q	.	.	.	Score	.	.	.	.	.	.
.	ENST0000	.	16q	.	.	Score=0.99	.	.	ID	.	.	.	.
.	ENST0000	.	17p	.	.	.	.	rs7805194	.	.	.	.	.
.	ENST0000	.	17p	.	.	.	.	.	.	.	.	.	.
TNFSF	ENST0000	.	17p	.	Scc	.	.	.	.	.	.	.	.
DNAH2	ENST0000	.	17p	.	Scc	.	.	.	.	.	.	.	.
MYH13	ENST0000	.	17p	.	.	.	.	.	.	.	.	.	.
DNAH9	ENST0000	.	17p	.	Scc	.	.	.	ID	.	.	.	.
.	ENST0000	.	17p	.	.	.	.	rs5595610	.	.	.	.	.
.	ENST0000	.	17p	.	.	.	.	.	.	.	.	.	.
.	ENST0000	.	17p	.	.	Score=0.98	.	.	.	.	.	.	.
.	ENST0000	.	17p	.	.	Score=0.98	.	.	.	.	.	.	.
.	ENST0000	.	17p	.	.	Score=0.99	.	.	.	.	.	.	.
.	ENST0000	.	17p	.	.	Score=0.99	.	rs7841783	.	.	.	.	.
.	ENST0000	.	17q	.	.	.	.	.	.	.	.	.	.
.	ENST0000	.	17q	.	.	.	.	.	.	.	.	.	.
.	ENST0000	.	17q	.	.	.	.	.	.	.	.	.	.
SUZ12	ENST0000	.	17q	.	.	Score=0.97	.	.	.	.	.	.	.
.	ENST0000	.	17q	.	.	Score=0.97	.	.	.	.	.	.	.
.	ENST0000	.	17q	.	.	.	.	.	.	.	.	.	.
.	ENST0000	.	17q	.	.	Score=0.99	.	.	.	.	.	.	.
.	ENST0000	.	17q	.	.	Score=0.99	.	rs8795183	.	.	.	.	.
.	ENST0000	.	17q	.	.	.	.	.	.	.	.	.	.
.	ENST0000	.	17q	.	.	.	.	.	.	.	.	.	.
.	ENST0000	.	17q	.	.	.	.	.	.	.	.	.	.
.	ENST0000	.	17q	.	.	Score=0.99	.	.	.	.	.	.	.
MED1:I	ENST0000	.	17q	.	.	.	.	.	.	.	.	.	.
.	ENST0000	.	17q	.	.	.	.	rs1035039	.	.	.	.	.
.	ENST0000	.	17q	.	.	.	.	.	.	.	.	.	.
.	ENST0000	.	17q	.	.	.	.	.	.	.	.	.	.
.	ENST0000	.	17q	.	.	.	.	.	.	.	.	.	.
.	ENST0000	.	17q	.	.	.	.	.	.	.	.	.	.
.	ENST0000	.	17q	.	.	.	.	.	.	.	.	.	.
DDX42	ENST0000	.	17q	.	.	.	.	.	.	.	.	.	.
DDX42	ENST0000	.	17q	.	.	.	.	.	.	.	.	.	.
.	ENST0000	.	17q	.	.	.	Score	.	.	.	.	.	.
.	ENST0000	.	17q	.	.	.	.	.	.	.	.	.	.
SLC26	ENST0000	.	17q	.	.	.	.	rs1405528	.	.	.	.	.
BAIAP2	ENST0000	.	17q	.	.	.	.	.	.	.	.	.	.
.	ENST0000	.	17q	.	.	Score=0.99	.	rs3677917	.	.	.	.	.
.	ENST0000	.	18p	.	.	.	.	.	.	.	.	.	.
LRRC3	ENST0000	.	18p	.	Scc	.	.	.	.	.	.	.	.
PPP4R	ENST0000	.	18p	.	.	.	.	.	.	.	.	.	.
.	ENST0000	.	18p	.	.	.	.	.	.	.	.	.	.
PIEZO2	ENST0000	.	18p	.	Scc	.	.	.	.	.	.	.	.
RNMT	ENST0000	.	18p	.	Scc	.	.	.	.	.	.	.	.
.	ENST0000	.	18p	.	.	Score=0.96	Score	.	ID	.	.	.	.
.	ENST0000	.	18q	.	.	.	Score	.	.	.	.	.	.
SKOR2	ENST0000	.	18q	.	.	.	Score	.	.	.	.	.	.
.	ENST0000	.	18q	.	.	.	.	.	.	.	.	.	.
.	ENST0000	.	18q	.	.	.	Score	.	.	.	.	.	.
.	ENST0000	.	18q	.	.	.	.	rs7468202	.	.	.	.	.
CCDC1	ENST0000	.	18q	.	.	.	.	.	.	.	.	.	.
.	ENST0000	.	18q	.	.	.	.	.	.	.	.	.	.

.	.	.	19p	.	.	.	.	rs9545783	.	.	.	.	.
.	ENST0000	.	19p	.	.	.	.	.	.	.	.	.	.
.	ENST0000	.	19p	.	.	.	.	.	.	.	.	.	.
.	ENST0000	.	19p	.	.	.	.	.	.	.	.	.	.
.	ENST0000	.	19p	.	.	.	.	rs3742787	.	.	.	.	.
.	ENST0000	.	19p	.	.	.	Score	rs1418930	.	.	.	.	0.106
THOP1	ENST0000	.	19p	.	.	.	.	.	.	.	.	.	.
.	ENST0000	.	19p	.	.	.	.	rs7627736	.	.	.	.	.
.	ENST0000	.	19p	.	.	.	Score	.	.	.	.	.	.
.	ENST0000	.	19p	.	.	.	.	.	.	.	.	.	.
.	ENST0000	.	19p	.	.	.	.	.	.	.	.	.	.
.	ENST0000	.	19p	.	.	.	.	.	.	.	.	.	.
.	ENST0000	.	19p	.	.	.	Score	.	.	.	.	.	.
.	ENST0000	.	19p	.	.	.	Score	.	.	.	.	.	.
.	ENST0000	.	19p	.	.	.	.	.	.	.	.	.	.
MUC16	ENST0000	.	19p	.	.	.	.	.	.	.	.	.	.
MUC16	ENST0000	.	19p	.	.	.	.	.	.	.	.	.	.
MUC16	ENST0000	.	19p	.	.	.	.	.	ID	.	.	.	.
MUC16	ENST0000	.	19p	.	.	.	.	rs7741875	.	.	.	.	.
.	ENST0000	.	19p	.	.	.	Score	.	.	.	.	.	.
ZNF44	ENST0000	.	19p	.	.	.	.	.	.	.	.	.	.
.	ENST0000	.	19p	.	.	.	.	.	.	.	.	.	.
.	ENST0000	.	19p	.	.	.	.	.	.	.	.	.	.
NWD1	ENST0000	.	19p	.	.	.	.	.	.	.	.	.	.
FAM12	ENST0000	.	19p	.	.	.	.	rs7797020	.	.	.	.	.
.	ENST0000	.	19p	.	.	.	Score	.	.	.	.	.	.
.	ENST0000	.	19p	.	.	.	.	.	.	.	.	.	.
ZNF25	ENST0000	.	19p	.	.	.	.	.	ID	.	.	.	.
.	ENST0000	.	19p	.	.	Score=0.9	.	.	.	.	.	.	.
.	ENST0000	.	19q	.	.	.	.	.	.	.	.	.	.
.	ENST0000	.	19q	.	.	.	.	.	.	.	.	.	.
.	ENST0000	.	19q	.	.	.	.	.	.	.	.	.	.
.	.	.	19q	.	.	Score=0.9	Score	rs8683521	.	.	.	.	.
.	.	.	19q	.	.	Score=0.9	Score	rs8793125	.	.	.	.	.
ZNF85	ENST0000	.	19q	.	.	.	.	rs7653982	ID	.	.	.	.
.	ENST0000	.	19q	.	.	.	Score	rs9896621	.	.	.	.	.
.	ENST0000	.	19q	.	.	Score=0.9	.	.	.	.	.	.	.
.	ENST0000	.	19q	.	.	.	.	rs1408143	.	.	.	.	0.001
.	ENST0000	.	19q	.	.	.	.	.	.	.	.	.	.
.	ENST0000	.	19q	.	.	.	Score	rs1444115	.	.	.	.	.
.	ENST0000	.	19q	.	.	Score=0.9	.	.	.	.	.	.	.
.	ENST0000	.	19q	.	.	.	Score	.	.	.	.	.	.
PLAUR	ENST0000	.	19q	.	.	.	.	rs4251912	.	.	.	.	.
NANOS	ENST0000	.	19q	.	.	.	.	.	.	.	.	.	.
.	ENST0000	.	19q	.	.	.	.	.	.	.	.	.	.
.	ENST0000	.	19q	.	.	.	.	.	.	.	.	.	.
ZNF61	ENST0000	.	19q	.	.	.	.	.	.	.	.	.	.
.	ENST0000	.	19q	.	.	Score=0.9	.	.	.	.	.	.	.
.	.	.	19q	.	.	Score=0.9	.	.	.	.	.	.	.
NCR1:I	ENST0000	.	19q	.	.	.	.	.	.	.	.	.	.
USP29	ENST0000	.	19q	.	.	.	.	.	.	.	.	.	.
USP29	ENST0000	.	19q	.	.	.	.	.	.	.	.	.	.
.	ENST0000	.	20p	.	.	.	.	.	.	.	.	.	.
.	ENST0000	.	20p	.	.	.	.	.	.	.	.	.	.

.	ENST0000	.	20p	.	.	.	.	.	.	.	.	.	.
SIGLEC	ENST0000	.	20p	.	.	.	.	.	.	.	.	.	.
PLCB1	ENST0000	.	20p	.	.	.	.	rs7610831	.	.	.	.	.
.	ENST0000	.	20p	.	.	.	.	.	.	.	.	.	.
.	ENST0000	HBI-43	20p	.	.	.	.	.	.	.	.	.	.
.	ENST0000	.	20q	.	.	Score=0.96	.	rs8120738	ID	.	.	.	.
.	ENST0000	.	20q	.	.	Score=0.96	.	rs7791580	ID	.	.	.	0.001
.	ENST0000	.	20q	.	.	.	.	.	.	.	.	.	.
.	ENST0000	.	20q	.	.	.	.	.	.	.	.	.	.
NCOA6	ENST0000	.	20q	.	Scc	.	.	rs1904867	.	.	.	.	0.001
RNF114	ENST0000	.	20q	.	Scc	.	.	.	.	.	.	.	.
UNKNC	ENST0000	.	20q	.	Scc	.	.	.	.	.	.	.	.
ZNF831	ENST0000	.	20q	.	.	.	.	.	.	.	.	.	.
.	ENST0000	.	20q	.	.	.	Score	.	.	.	.	.	.
.	ENST0000	.	20q	.	.	.	.	.	.	.	.	.	.
.	ENST0000	.	20q	.	.	.	Score	rs1874434	.	.	.	.	.
.	.	.	21p	.	.	Score=0.98	.	.	.	.	.	.	.
.	ENST0000	.	21p	.	.	Score=0.98	.	.	.	.	.	.	.
.	.	.	21p	.	.	Score=0.99	Score	.	.	.	.	.	.
.	.	.	21p	.	.	Score=0.97	.	rs3707796	ID	.	.	.	.
.	ENST0000	.	21p	.	.	Score=0.96	.	rs7469658	ID	.	.	.	.
.	.	.	21p	.	.	Score=0.98	Score	rs4638882	.	.	.	.	.
.	ENST0000	.	21q	.	.	.	.	.	.	.	.	.	.
.	ENST0000	.	21q	.	.	.	.	.	.	.	.	.	.
.	ENST0000	.	21q	.	.	.	.	.	.	.	.	.	.
CBS:NI	ENST0000	.	21q	.	Scc	.	.	.	.	.	.	.	.
.	ENST0000	.	21q	.	.	.	.	rs7276130	ID	.	.	.	.
.	ENST0000	.	21q	.	.	.	.	.	.	.	.	.	.
.	ENST0000	.	22q	.	.	Score=0.99	.	.	.	.	.	.	.
.	ENST0000	.	22q	.	.	Score=0.99	.	.	.	.	.	.	.
.	ENST0000	.	22q	.	.	Score=0.99	Score	.	.	.	.	.	.
.	ENST0000	.	22q	.	.	Score=0.98	.	rs3769174	.	.	.	.	.
PRODH	ENST0000	.	22q	.	.	Score=0.95	.	rs2870983	ID	.	.	.	0.032
.	ENST0000	.	22q	.	.	.	.	.	.	.	.	.	.
.	.	.	22q	.	.	Score=0.99	Score	rs1389774	.	.	.	.	0.067
.	ENST0000	.	22q	.	.	Score=0.98	.	rs3731564	ID	.	.	.	.
.	ENST0000	.	22q	.	.	Score=0.97	.	rs1856371	ID	.	.	.	0.016
.	ENST0000	.	22q	.	.	.	.	.	.	.	.	.	.
.	ENST0000	.	22q	.	.	.	.	rs5669283	.	.	.	.	.
.	ENST0000	.	22q	.	.	.	.	.	.	.	.	.	.
.	ENST0000	.	22q	.	.	.	.	rs7606577	.	.	.	.	.
.	ENST0000	.	22q	.	.	Score=0.94	.	.	.	.	.	.	.
SYNGR	ENST0000	.	22q	.	.	.	.	.	.	.	.	.	.
XPNPE	ENST0000	.	22q	.	.	.	.	rs1409901	.	.	.	.	.
EP300:	ENST0000	.	22q	.	.	.	.	.	.	.	.	.	.
SREBF	ENST0000	.	22q	.	.	.	.	rs3731131	.	.	.	.	.
.	ENST0000	.	22q	.	.	.	Score	.	.	.	.	.	.
.	ENST0000	.	22q	.	.	.	Score	.	.	.	.	.	.
.	ENST0000	.	22q	.	.	.	Score	.	.	.	.	.	.
PLXNB	ENST0000	.	22q	.	.	.	.	rs7711497	ID	.	.	.	.
.	ENST0000	.	Xp2	.	.	Score=0.99	.	.	.	.	.	.	.
.	.	.	Xp2	.	.	Score=0.99	Score	rs8673094	.	.	.	.	.
.	ENST0000	.	Xp2	.	.	Score=0.93	.	rs7643909	.	.	.	.	.
.	ENST0000	.	Xp2	.	.	Score=0.95	.	rs6640384	.	.	.	.	.

TLR7:N	ENST0000	.	Xp2	.	.	.	.	.	.	.	.	.	.
TLR8:N	ENST0000	.	Xp2	.	.	.	.	.	.	.	.	.	.
.	ENST0000	.	Xp2	.	Sco	.	.	.	.	.	.	.	.
MAOA:	ENST0000	.	Xp1	.	.	.	.	.	.	.	.	.	.
.	ENST0000	.	Xp1	.	.	Score=0.95	.	.	.	.	.	.	.
PCSK1	ENST0000	.	Xp1	.	.	.	Score	.	.	.	.	.	.
.	ENST0000	.	Xp1	.	.	Score=0.93	.	.	.	.	.	.	.
.	ENST0000	.	Xp1	.	.	.	.	.	.	.	.	.	.
.	ENST0000	.	Xp1	.	.	.	.	.	.	.	.	.	.
GNL3L	ENST0000	.	Xp1	.	.	.	.	.	.	.	.	.	.
.	ENST0000	.	Xp1	.	.	Score=0.93	.	.	.	.	.	.	.
.	ENST0000	.	Xq1	.	.	.	Score	.	.	.	.	.	.
MED12	ENST0000	.	Xq1	.	.	.	.	.	.	.	.	.	.
NLGN3	ENST0000	.	Xq1	.	Sco	.	.	.	.	.	.	.	.
.	ENST0000	.	Xq1	.	.	Score=0.97	.	.	.	.	.	.	.
.	ENST0000	.	Xq2	.	.	.	.	.	.	.	.	.	.
SASH3	ENST0000	.	Xq2	.	.	.	.	.	.	.	.	.	.
UTP14	ENST0000	.	Xq2	.	.	.	Score	.	ID	.	.	.	.
BCORL	ENST0000	.	Xq2	.	.	.	.	.	.	.	.	.	.
.	ENST0000	.	Xq2	.	.	.	.	.	.	.	.	.	.
ARHGE	ENST0000	.	Xq2	.	Sco	.	.	.	.	.	.	.	.
UNKNO	ENST0000	.	Xq2	.	.	Score=0.92	Score	.	.	.	.	.	.
TREX2	ENST0000	.	Xq2	.	Sco	.	.	rs7824625	.	.	.	.	.
.	ENST0000	.	Xq2	.	.	.	Score	.	.	.	.	.	.
.	ENST0000	.	1p3	.	.	Score=0.99	.	.	.	.	.	.	.
.	ENST0000	.	1p3	.	.	Score=0.99	Score	rs7810569	ID	.	.	.	.
.	ENST0000	.	1p3	.	.	Score=0.90	.	rs1149269	.	.	.	.	.
FAM21	ENST0000	.	1p3	.	.	.	.	.	.	.	.	.	.
.	ENST0000	.	1p3	.	.	.	.	.	.	.	.	.	.
.	ENST0000	.	1p3	.	.	.	.	.	.	.	.	.	.
.	ENST0000	.	1p3	.	.	Score=0.90	.	rs2008876	ID	.	.	.	.
.	ENST0000	.	1p3	.	.	Score=0.96	.	rs5565927	.	.	.	.	0.002
.	ENST0000	.	1p3	.	.	Score=0.96	.	rs6177234	.	.	.	.	.
.	ENST0000	.	1p3	.	.	Score=0.93	.	rs1008195	ID	.	.	.	.
.	ENST0000	.	1p3	.	.	Score=0.95	.	rs5388409	ID	.	.	.	0.025
CROCC	ENST0000	.	1p3	.	Sco	Score=0.93	.	rs6669627	ID	.	.	.	.
.	ENST0000	.	1p3	.	.	.	.	.	.	.	.	.	.
.	ENST0000	.	1p3	.	.	.	.	rs7661411	ID	.	.	.	.
HIVEP3	ENST0000	.	1p3	.	.	.	.	rs7802118	ID	.	.	.	.
.	.	.	1p3	.	.	Score=0.99	Score	rs8664767	ID	.	.	.	.
.	.	.	1p3	.	.	.	Score	.	.	.	.	.	.
.	ENST0000	.	1q2	.	.	Score=0.97	.	rs7581372	.	.	.	.	.
.	ENST0000	.	1q2	.	.	Score=0.97	.	rs7697846	.	.	.	.	.
.	.	.	1q2	.	.	Score=0.98	.	rs2016919	.	.	.	.	.
.	ENST0000	.	1q2	.	.	Score=0.98	.	.	ID	.	.	.	.
.	ENST0000	.	1q2	.	.	Score=0.99	.	rs3703375	.	.	.	.	.
.	ENST0000	.	1q2	.	.	Score=0.98	.	rs1996515	ID	.	.	.	.
.	.	.	1q2	.	.	Score=0.98	.	.	.	.	.	.	.
.	.	.	1q2	.	.	Score=0.98	.	.	.	.	.	.	.
.	ENST0000	.	1q2	.	.	Score=0.99	.	.	ID	.	.	.	.
.	ENST0000	.	1q2	.	.	Score=0.99	Score	.	.	.	.	.	.
.	ENST0000	.	1q2	.	.	Score=0.98	.	.	ID	.	.	.	.
.	ENST0000	.	1q2	.	.	Score=0.98	.	rs8799960	ID	.	.	.	.
.	ENST0000	.	1q2	.	.	Score=0.99	Score	rs2003739	.	.	.	.	.

.	ENST0000	.	1q2	.	.	Score=0.99	Score	rs3705433	.	.	.	.	.
.	ENST0000	.	1q2	.	.	.	.	.	.	.	.	.	.
.	ENST0000	.	1q4	.	.	Score=0.97	.	rs1839036	.	.	.	.	0.404
.	ENST0000	.	1q4	.	.	Score=0.98	Score	.	.	.	.	.	.
.	ENST0000	.	1q4	.	.	Score=0.98	Score	.	.	.	.	.	.
.	ENST0000	.	1q4	.	.	.	.	.	.	.	.	.	.
.	.	.	1q4	.	.	Score=0.97	.	rs1828179	.	.	.	.	.
.	.	.	2p1	.	.	Score=0.98	.	rs4096246	ID	.	.	.	0.026
.	ENST0000	.	2p1	.	.	Score=0.99	.	.	.	.	.	.	.
.	.	.	2p1	.	.	Score=0.95	.	.	.	.	.	.	.
.	ENST0000	.	2p1	.	.	Score=0.99	.	.	.	.	.	.	.
ANAPC	ENST0000	.	2q1	.	.	Score=0.97	.	rs2011280	ID	.	.	.	.
.	.	.	2q1	.	.	Score=0.98	.	rs8685337	ID	.	.	.	.
.	ENST0000	.	2q1	.	.	Score=0.98	.	rs2747968	ID	.	.	.	.
.	ENST0000	.	2q1	.	.	.	Score	rs1029282	.	.	.	.	.
.	ENST0000	.	2q2	.	.	Score=0.98	Score	rs2001821	.	.	.	.	.
.	ENST0000	.	2q2	.	.	Score=0.97	.	rs7800646	ID	.	.	.	.
.	ENST0000	.	2q3	.	.	.	.	.	.	.	.	.	.
.	ENST0000	.	2q3	.	.	.	.	.	.	.	.	.	.
.	ENST0000	.	2q3	.	.	.	.	rs1875561	ID	.	.	.	0.006
.	ENST0000	.	2q3	.	.	.	Score	rs8671576	ID	.	.	.	.
.	ENST0000	.	3p2	.	.	.	.	.	.	.	.	.	.
AGTR1	ENST0000	.	3q2	.	Scc	.	.	.	.	.	.	.	.
.	ENST0000	.	3q2	.	.	.	.	.	.	.	.	.	.
.	ENST0000	.	3q2	.	.	.	.	.	.	.	.	.	.
.	.	.	3q2	.	.	Score=0.97	Score	rs3760511	ID	.	.	.	.
.	ENST0000	.	3q2	.	.	.	.	rs8677455	.	.	.	.	.
.	ENST0000	.	3q2	.	.	Score=0.97	.	rs1128840	ID	.	.	.	.
.	ENST0000	.	4p1	.	.	.	.	.	.	.	.	.	.
.	ENST0000	.	4p1	.	.	.	.	rs1443656	ID	.	.	.	.
.	ENST0000	.	5p1	.	.	.	.	.	.	.	.	.	.
.	ENST0000	.	5p1	.	.	Score=0.98	Score	.	.	.	.	.	.
.	ENST0000	.	5q1	.	.	Score=0.99	Score	rs3759258	.	.	.	.	0.772
.	ENST0000	.	5q1	.	.	Score=0.97	Score	rs8790446	ID	.	.	.	.
.	ENST0000	.	5q2	.	.	Score=0.98	.	.	ID	.	.	.	.
.	ENST0000	.	5q3	.	.	.	.	.	.	.	.	.	.
.	ENST0000	.	5q3	.	.	.	.	.	.	.	.	.	.
COL23	ENST0000	.	5q3	.	.	.	.	.	.	.	.	.	.
.	ENST0000	.	5q3	.	.	.	.	.	.	.	.	.	.
.	.	.	6p2	.	.	.	.	rs3719775	.	.	.	.	.
.	.	.	6p2	.	.	.	.	rs9202904	.	.	.	.	.
.	.	.	6p1	.	.	.	Score	rs4292554	ID	.	.	.	.
.	.	.	6p1	.	.	.	Score	rs4437505	ID	.	.	.	.
.	ENST0000	.	6q2	.	.	.	.	rs7508774	.	.	.	.	.
.	ENST0000	.	6q2	.	.	.	.	.	.	.	.	.	.
.	ENST0000	.	6q2	.	.	Score=0.98	.	rs1436754	.	.	.	.	.
.	ENST0000	.	6q2	.	.	.	Score	.	.	.	.	.	.
.	ENST0000	.	7p2	.	.	.	.	.	.	.	.	.	.
.	ENST0000	.	7p2	.	.	Score=0.99	Score	rs2016650	.	.	.	.	.
.	ENST0000	.	7p1	.	.	.	Score	.	.	.	.	.	.
.	ENST0000	.	7p1	.	.	Score=0.99	.	.	.	.	.	.	.
.	ENST0000	.	7p1	.	.	Score=0.99	.	.	ID	.	.	.	.
.	ENST0000	.	7p1	.	.	Score=0.99	.	.	.	.	.	.	.
.	ENST0000	.	7p1	.	.	Score=0.99	.	.	.	.	.	.	.

.	ENST000C	.	7p1	.	.	Score=0.9	.	.	.	.	.	.	.
.	ENST000C	.	7p1	.	.	Score=0.9	.	.	.	.	.	.	.
.	ENST000C	.	7p1	.	.	Score=0.9	Score	.	.	.	.	.	.
.	.	.	7q1	.	.	.	Score	rs4596537	.	.	.	.	.
.	.	.	7q1	.	.	.	Score	rs2167825	ID	.	.	.	.
.	ENST000C	.	7q1	.	.	Score=0.9	.	rs586765	ID	.	.	.	.
.	.	.	7q1	.	.	Score=0.9	.	rs8688020	ID	.	.	.	.
.	ENST000C	.	7q1	.	.	Score=0.9	.	rs2462259	ID	.	.	.	.
.	ENST000C	.	7q2	.	.	Score=0.9	.	rs6248349	.	.	.	.	.
.	ENST000C	.	7q3	.	.	Score=0.9	.	rs3741264	.	.	.	.	.
.	ENST000C	.	7q3	.	.	Score=0.9	.	.	.	.	.	.	.
.	.	.	8p2	.	.	Score=0.9	.	rs2077351	ID	.	.	.	.
.	ENST000C	.	8p1	.	.	.	.	.	.	.	.	.	.
.	.	.	8p1	.	.	.	Score	rs7503847	.	.	.	.	.
.	.	.	8p1	.	.	.	Score	rs7967153	ID	.	.	.	.
.	.	.	8p1	.	.	.	Score	.	ID	.	.	.	.
.	.	.	8q1	.	.	.	Score	rs4363208	ID	.	.	.	.
.	.	.	8q1	.	.	.	Score	rs3740584	.	.	.	.	.
.	ENST000C	.	8q2	.	.	.	Score	.	.	.	.	.	.
ARC:NI	ENST000C	.	8q2	.	Scc	.	.	.	.	.	.	.	.
.	ENST000C	.	8q2	.	.	.	Score	rs7821104	ID	.	.	.	.
.	.	.	9p1	.	.	Score=0.9	Score	rs7495042	ID	.	.	.	.
.	.	.	9q1	.	.	Score=0.8	Score	rs4928839	.	.	.	.	.
.	ENST000C	.	9q1	.	Scc	Score=0.9	.	rs4568707	.	.	.	.	.
.	ENST000C	.	9q2	.	.	Score=0.9	.	rs1435617	ID	.	.	.	.
.	ENST000C	.	9q2	.	.	Score=0.9	.	rs3773733	.	.	.	.	.
.	ENST000C	.	9q2	.	.	.	Score	.	.	.	.	.	.
.	ENST000C	.	9q3	.	.	.	.	.	.	.	.	.	.
.	ENST000C	.	9q3	.	.	.	Score	.	.	.	.	.	.
.	.	.	10p	.	.	.	Score	rs1397322	ID	.	.	.	.
.	ENST000C	hsa-mir-60	10p	.	.	.	Score	.	.	.	.	.	.
.	ENST000C	.	10p	.	.	.	Score	rs9631413	.	.	.	.	.
.	ENST000C	.	10p	.	.	.	.	.	.	.	.	.	.
.	ENST000C	.	10q	.	.	Score=0.9	Score	rs694157	.	.	.	.	.
.	ENST000C	.	10q	.	.	Score=0.8	.	rs5275172	.	.	.	.	.
.	ENST000C	.	10q	.	.	.	.	rs1888127	.	.	.	.	.
.	.	.	10q	.	.	Score=0.9	.	rs2573327	ID	.	.	.	.
.	ENST000C	.	10q	.	.	Score=0.9	.	rs3128226	.	.	.	.	0.524
.	ENST000C	.	11p	.	.	Score=0.9	.	.	ID	.	.	.	.
.	ENST000C	.	11p	.	.	Score=0.9	.	.	ID	.	.	.	.
.	.	.	11q	.	.	.	Score	rs9035709	ID	.	.	.	.
.	.	.	11q	.	.	Score=0.9	.	rs1874489	ID	.	.	.	.
PRKRII	ENST000C	.	11q	.	.	Score=0.9	.	rs9666739	.	.	.	.	.
.	ENST000C	.	11q	.	.	.	Score	rs5688517	.	.	.	.	.
OR8G5	ENST000C	.	11q	.	.	.	.	.	.	.	.	.	.
.	ENST000C	.	11q	.	.	.	.	.	.	.	.	.	.
.	ENST000C	.	12q	.	.	Score=0.8	.	rs7606061	.	.	.	.	.
ERBB3	ENST000C	.	12q	.	.	.	.	.	ID	.	.	.	.
.	ENST000C	.	12q	.	.	.	.	rs7597010	ID	.	.	.	.
.	ENST000C	.	13q	.	.	Score=0.9	.	rs2004528	.	.	.	.	.
MYO16	ENST000C	.	13q	.	Scc	.	.	rs1510074	ID	.	.	.	.
.	ENST000C	.	14q	.	.	Score=0.9	.	rs7446578	ID	.	.	.	.
.	ENST000C	.	14q	.	.	Score=0.9	.	.	.	.	.	.	.
.	ENST000C	.	14q	.	.	Score=0.9	.	rs2006543	.	.	.	.	.

.	ENST0000	.	14q	.	.	Score=0.9	.	rs2020426	.	.	.	.	.
.	ENST0000	.	14q	.	.	Score=0.9	.	rs7704695	ID	.	.	.	.
UNKNC	ENST0000	.	14q	.	.	.	.	.	.	.	.	.	.
.	ENST0000	.	14q	.	.	.	.	.	.	.	.	.	.
.	ENST0000	.	14q	.	.	Score=0.9	.	.	ID	.	.	.	.
.	.	.	15q	.	.	Score=0.9	Score	rs6200967	ID	.	.	.	0.345
.	ENST0000	.	15q	.	.	Score=0.9	.	.	.	.	.	.	.
.	ENST0000	.	15q	.	.	.	.	.	.	.	.	.	.
.	ENST0000	.	15q	.	.	Score=0.9	.	rs7751825	.	.	.	.	.
.	ENST0000	.	15q	.	.	Score=0.9	Score	rs2449652	ID	.	.	.	0.026
.	ENST0000	.	15q	.	.	Score=0.9	Score	rs2449653	ID	.	.	.	0.167
.	ENST0000	.	15q	.	.	Score=0.9	.	.	.	.	.	.	.
.	ENST0000	.	15q	.	.	Score=0.9	.	.	.	.	.	.	.
.	ENST0000	.	15q	.	.	Score=0.9	.	rs6202600	.	.	.	.	0.422
.	ENST0000	.	15q	.	.	.	.	.	.	.	.	.	.
.	ENST0000	.	15q	.	.	.	.	.	.	.	.	.	.
GOLGA	ENST0000	.	15q	.	.	Score=0.9	.	.	.	.	.	.	.
.	.	.	15q	.	.	Score=0.9	.	rs3743150	.	.	.	.	0.446
.	ENST0000	.	15q	.	.	Score=0.9	.	rs9700260	.	.	.	.	.
.	ENST0000	.	15q	.	.	Score=0.9	.	rs364085	.	.	.	.	.
.	ENST0000	.	16p	.	.	Score=0.9	.	rs2009400	.	.	.	.	.
.	ENST0000	.	16p	.	.	Score=0.9	.	rs2016432	.	.	.	.	.
.	ENST0000	.	16p	.	.	Score=0.9	.	.	ID	.	.	.	.
.	.	.	16p	.	.	Score=0.9	.	rs5667377	.	.	.	.	0.001
.	ENST0000	.	16p	.	.	Score=0.9	.	rs5618411	ID	.	.	.	.
.	.	.	16p	.	.	Score=0.9	Score	rs8792106	.	.	.	.	.
.	.	.	16q	.	.	.	Score	rs4319791	ID	.	.	.	.
.	.	.	16q	.	.	.	Score	rs4349321	.	.	.	.	.
.	.	.	16q	.	.	.	Score	rs4246344	.	.	.	.	.
.	.	.	16q	.	.	.	.	.	.	.	.	.	.
.	.	.	16q	.	.	.	Score	rs8799648	.	.	.	.	.
.	.	.	16q	.	.	.	Score	rs9972801	.	.	.	.	.
.	.	.	16q	.	.	.	Score	rs4324300	ID	.	.	.	.
.	.	.	16q	.	.	.	Score	rs9328621	ID	.	.	.	.
.	.	.	16q	.	.	.	Score	rs4380209	ID	.	.	.	.
.	.	.	16q	.	.	.	Score	rs4291933	.	.	.	.	.
.	.	.	16q	.	.	.	.	rs4011858	ID	.	.	.	.
.	.	.	16q	.	.	.	Score	.	.	.	.	.	.
.	.	.	16q	.	.	.	Score	rs7205231	.	.	.	.	.
.	.	.	16q	.	.	.	Score	rs4093117	.	.	.	.	.
.	.	.	16q	.	.	.	Score	rs4249029	.	.	.	.	.
.	.	.	16q	.	.	.	Score	.	.	.	.	.	.
.	ENST0000	.	16q	.	.	Score=0.9	.	rs6205366	.	.	.	.	.
.	ENST0000	.	17p	.	.	Score=0.9	.	rs2543804	ID	.	.	.	.
.	ENST0000	.	17p	.	.	Score=0.9	.	rs7126379	ID	.	.	.	.
.	ENST0000	.	17p	.	.	Score=0.9	.	rs7126379	ID	.	.	.	.
.	ENST0000	.	17p	.	.	Score=0.9	.	rs7841783	.	.	.	.	.
.	.	.	17p	.	.	.	Score	.	.	.	.	.	.
.	.	.	17p	.	.	.	Score	rs1437650	.	.	.	.	.
.	.	.	17p	.	.	.	Score	.	.	.	.	.	.
.	ENST0000	.	17q	.	.	Score=0.9	.	rs6207957	.	.	.	.	.
KRTAP	ENST0000	.	17q	.	.	Score=0.9	.	rs7482024	ID	.	.	.	.
.	ENST0000	.	17q	.	.	.	.	rs1459625	ID	.	.	.	.
.	ENST0000	.	17q	.	.	Score=0.9	.	.	.	.	.	.	.

.	ENST000C	.	18p	.	.	Score=0.9	.	rs2012257	ID	.	.	.	.
.	ENST000C	.	18p	.	.	Score=0.9	.	rs3734996	.	.	.	.	.
.	ENST000C	.	18q	.	.	.	.	rs7520640	.	.	.	.	.
.	ENST000C	.	19p	.	.	.	.	.	.	.	.	.	.
.	ENST000C	.	19q	.	.	.	.	.	.	.	.	.	.
.	.	.	19q	.	.	Score=0.9	Score	rs7641371	.	.	.	.	.
.	.	.	19q	.	.	Score=0.9	Score	.	.	.	.	.	.
.	ENST000C	.	19q	.	.	Score=0.9	.	.	.	.	.	.	.
.	ENST000C	.	19q	.	.	.	.	.	.	.	.	.	.
.	ENST000C	.	19q	.	.	.	.	rs3499068	ID	.	.	.	.
.	ENST000C	.	19q	.	.	.	.	rs5615613	ID	.	.	.	.
.	ENST000C	.	19q	.	.	Score=0.9	.	rs1334652	ID	.	.	.	.
.	ENST000C	.	19q	.	.	.	.	rs3751555	ID	.	.	.	.
NCOA	ENST000C	.	20q	.	.	.	Score	.	ID	.	.	.	.
.	ENST000C	.	21p	.	.	Score=0.9	.	rs7984422	ID	.	.	.	.
.	.	.	21p	.	.	Score=0.9	Score	rs2017188	ID	.	.	.	.
.	ENST000C	.	22q	.	.	Score=0.9	.	rs3753995	ID	.	.	.	.
.	ENST000C	.	22q	.	.	Score=0.9	.	.	.	.	.	.	.
.	ENST000C	.	22q	.	.	Score=0.9	.	rs2021509	ID	.	.	.	.
.	ENST000C	.	22q	.	.	Score=0.9	.	.	.	.	.	.	.
.	ENST000C	.	22q	.	.	Score=0.9	.	rs6221896	ID	.	.	.	.
.	ENST000C	.	22q	.	.	Score=0.9	.	rs2016329	ID	.	.	.	0.114
.	ENST000C	.	22q	.	.	Score=0.9	.	rs6223125	ID	.	.	.	.
.	ENST000C	.	22q	.	.	Score=0.9	.	rs367416	ID	.	.	.	.
.	ENST000C	.	22q	.	.	Score=0.9	.	rs3609392	.	.	.	.	0.302
.	ENST000C	.	22q	.	.	Score=0.9	.	.	.	.	.	.	.
.	ENST000C	.	22q	.	.	.	.	.	.	.	.	.	.
.	ENST000C	.	Xp1	.	.	.	.	.	.	.	.	.	.
.	.	.	Xq1	.	.	.	Score	.	.	.	.	.	.
.	.	.	Xq1	.	.	.	Score	.	ID	.	.	.	.
.	.	.	Xq1	.	.	.	Score	rs7520049	.	.	.	.	.
.	.	.	Xq1	.	.	.	Score	rs7756689	.	.	.	.	.
.	.	.	Xq1	.	.	.	Score	.	.	.	.	.	.
SNX12	ENST000C	.	Xq1	.	Scc	.	.	rs1048883	ID	.	.	.	.
.	ENST000C	.	Xq1	.	.	.	.	.	.	.	.	.	.
NHSL2	ENST000C	.	Xq1	.	.	.	.	.	.	.	.	.	.
.	ENST000C	.	Xq2	.	.	Score=0.9	.	rs1423249	ID	.	.	.	.
.	.	.	Xq2	.	.	.	.	.	.	.	.	.	.
.	ENST000C	.	1p3	.	.	Score=0.9	.	rs3744991	ID	.	.	.	0.092
.	ENST000C	.	1p3	.	.	.	.	.	.	.	.	.	.
KLHL1	ENST000C	.	1p3	.	Scc	.	.	rs1159856	.	.	.	.	.
UNKN	ENST000C	.	1p3	.	.	.	.	.	.	.	.	.	.
ISG15:	ENST000C	.	1p3	.	.	.	.	.	.	.	.	.	.
.	ENST000C	.	1p3	.	.	.	.	rs5413521	.	.	.	.	.
.	ENST000C	.	1p3	.	.	.	.	rs7680451	ID	.	.	.	.
.	ENST000C	.	1p3	.	.	.	.	.	.	.	.	.	.
MXRA	ENST000C	.	1p3	.	Scc	.	.	.	.	.	.	.	.
.	ENST000C	.	1p3	.	.	.	.	rs7800811	.	.	.	.	.
.	ENST000C	.	1p3	.	.	.	.	.	.	.	.	.	.
.	ENST000C	.	1p3	.	.	Score=0.9	.	rs2014492	.	.	.	.	0.001
UNKN	ENST000C	.	1p3	.	Scc	Score=0.9	.	rs7463384	.	.	.	.	.
.	ENST000C	.	1p3	.	.	Score=0.9	Score	.	.	.	.	.	.
.	ENST000C	.	1p3	.	.	Score=0.9	Score	.	.	.	.	.	.
GABR	ENST000C	.	1p3	.	.	.	.	.	.	.	.	.	.

.	ENST0000C	.	1p3	.	.	.	.	.	.	.	.	.	.
.	ENST0000C	.	1p3	.	.	.	.	.	.	.	.	.	.
MEGF6	ENST0000C	.	1p3	.	.	.	.	.	.	.	.	.	.
.	ENST0000C	.	1p3	.	.	.	Score	.	.	.	.	.	.
UNKNC	ENST0000C	.	1p3	.	Scc	.	.	.	.	.	.	.	.
.	ENST0000C	.	1p3	.	.	.	.	.	.	.	.	.	.
.	ENST0000C	.	1p3	.	.	.	.	rs9430027	.	.	.	.	.
CHD5:I	ENST0000C	.	1p3	.	Scc	.	.	.	ID	.	.	.	.
CHD5:I	ENST0000C	.	1p3	.	Scc	.	.	.	.	.	.	.	.
.	ENST0000C	.	1p3	.	.	.	.	rs9864160	.	.	.	.	.
.	ENST0000C	.	1p3	.	.	.	.	rs7562596	.	.	.	.	.
CAMTA	ENST0000C	.	1p3	.	.	.	.	rs1431018	.	.	.	.	.
.	ENST0000C	.	1p3	.	.	.	Score	.	.	.	.	.	.
CAMTA	ENST0000C	.	1p3	.	.	.	.	rs7748471	.	.	.	.	.
.	ENST0000C	.	1p3	.	.	.	.	rs9446685	.	.	.	.	.
GPR15	ENST0000C	.	1p3	.	Scc	.	.	rs1476580	.	.	.	.	.
.	ENST0000C	.	1p3	.	.	.	Score	.	.	.	.	.	.
.	ENST0000C	.	1p3	.	.	.	.	.	.	.	.	.	.
.	ENST0000C	.	1p3	.	.	.	.	.	.	.	.	.	.
.	ENST0000C	.	1p3	.	.	.	.	rs7934532	.	Uncer	.	.	.
.	ENST0000C	.	1p3	.	.	.	.	.	.	.	.	.	.
.	ENST0000C	.	1p3	.	.	.	Score	.	.	.	.	.	.
VPS13I	ENST0000C	.	1p3	.	.	.	.	.	.	.	.	.	.
.	ENST0000C	.	1p3	.	.	Score=0.99	.	rs2021163	ID	.	.	.	.
.	ENST0000C	.	1p3	.	.	Score=0.99	.	rs5876978	ID	.	.	.	0.307
CASP9	ENST0000C	.	1p3	.	.	.	.	rs4646008	.	.	.	.	.
.	ENST0000C	.	1p3	.	.	.	.	rs3745685	.	.	.	.	.
ARHGE	ENST0000C	.	1p3	.	.	.	.	.	.	.	.	.	.
UNKNC	ENST0000C	.	1p3	.	.	Score=0.96	.	rs3734377	ID	.	.	.	.
.	ENST0000C	.	1p3	.	.	Score=0.96	.	rs6177234	.	.	.	.	.
.	ENST0000C	.	1p3	.	.	Score=0.96	.	rs4112521	.	.	.	.	.
.	ENST0000C	.	1p3	.	.	Score=0.96	.	rs1213433	ID	.	.	.	.
.	.	.	1p3	.	.	Score=0.96	.	rs3930814	ID	.	.	.	.
.	ENST0000C	.	1p3	.	.	.	.	rs5780915	.	.	.	.	0.019
IGSF21	ENST0000C	.	1p3	.	.	.	.	rs3774826	.	.	.	.	.
TAS1R	ENST0000C	.	1p3	.	.	.	.	rs3766882	.	.	.	.	.
UBR4:I	ENST0000C	.	1p3	.	Scc	.	.	rs3471023	.	.	.	.	.
UBR4:I	ENST0000C	.	1p3	.	Scc	.	.	.	.	.	.	.	.
.	ENST0000C	.	1p3	.	.	.	.	.	.	.	.	.	.
.	ENST0000C	.	1p3	.	.	.	.	rs3712996	.	.	.	.	.
.	ENST0000C	.	1p3	.	.	.	Score	.	.	.	.	.	.
.	ENST0000C	.	1p3	.	.	.	.	rs1001195	.	.	.	.	.
.	ENST0000C	.	1p3	.	.	.	.	.	.	.	.	.	.
UNKNC	ENST0000C	.	1p3	.	.	.	.	.	.	.	.	.	.
.	.	.	1p3	.	.	Score=0.96	.	rs7584776	ID	.	.	.	.
CELA3	ENST0000C	.	1p3	.	.	Score=0.96	.	.	.	.	.	.	.
.	ENST0000C	.	1p3	.	.	.	.	.	.	.	.	.	.
.	ENST0000C	.	1p3	.	.	.	.	.	.	.	.	.	.
.	ENST0000C	.	1p3	.	.	.	.	rs1391690	.	.	.	.	.
RUNX3	ENST0000C	.	1p3	.	.	.	.	rs7518873	ID	.	.	.	.
.	ENST0000C	.	1p3	.	.	Score=0.97	.	.	.	.	.	.	.
.	ENST0000C	.	1p3	.	.	.	.	.	.	.	.	.	.
.	ENST0000C	.	1p3	.	.	.	.	.	.	.	.	.	.
FAM11	ENST0000C	.	1p3	.	Scc	.	.	.	.	.	.	.	.

CNKSF	ENST0000	.	1p3	.	.	.	.	rs7737670	.	.	.	.	.
UBXN1	ENST0000	.	1p3	.	Scc	.	.	rs7767332	.	.	.	.	.
RPS6K	ENST0000	.	1p3	.	.	.	.	.	.	.	.	.	.
ARID1/	ENST0000	.	1p3	.	.	.	.	rs8792552	ID	Patho	.	.	.
ZDHHC	ENST0000	.	1p3	.	Scc	.	.	.	.	.	.	.	.
.	ENST0000	.	1p3	.	.	.	.	rs1003250	.	.	.	.	.
.	ENST0000	.	1p3	.	Scc	.	.	rs7625790	.	.	.	.	.
.	ENST0000	.	1p3	.	.	.	.	rs9468364	.	.	.	.	.
.	ENST0000	.	1p3	.	.	Score=0.9	.	.	.	.	.	.	.
.	ENST0000	.	1p3	.	.	.	.	.	ID	.	.	.	.
.	ENST0000	.	1p3	.	.	.	.	.	.	.	.	.	.
FABP3	ENST0000	.	1p3	.	.	.	.	rs7596455	ID	.	.	.	.
SPOCD	ENST0000	.	1p3	.	.	.	.	rs9232513	.	.	.	.	.
.	ENST0000	.	1p3	.	.	.	.	.	.	.	.	.	.
LCK:NI	ENST0000	.	1p3	.	.	.	.	.	.	.	.	.	.
.	.	.	1p3	.	.	.	Score	.	.	.	.	.	.
.	ENST0000	.	1p3	.	.	.	Score	.	.	.	.	.	.
.	ENST0000	.	1p3	.	.	.	.	rs9651423	.	.	.	.	.
RSPO1	ENST0000	.	1p3	.	.	.	.	rs7723940	.	.	.	.	.
.	ENST0000	.	1p3	.	.	.	Score	.	.	.	.	.	.
.	ENST0000	.	1p3	.	.	.	.	rs1814745	.	.	.	.	0.009
.	ENST0000	.	1p3	.	.	.	Score	.	.	.	.	.	.
.	ENST0000	.	1p3	.	.	.	.	.	.	.	.	.	.
.	ENST0000	.	1p3	.	.	.	.	.	.	.	.	.	.
.	ENST0000	.	1p3	.	.	Score=0.9	.	rs5523408	.	.	.	.	.
.	ENST0000	.	1p3	.	.	.	.	.	.	.	.	.	.
PPT1:N	ENST0000	.	1p3	.	Scc	.	.	.	.	.	.	.	.
RLF:NN	ENST0000	.	1p3	.	.	.	.	.	.	.	.	.	.
.	ENST0000	.	1p3	.	Scc	.	.	.	.	.	.	.	.
.	ENST0000	.	1p3	.	.	.	.	rs9093579	.	.	.	.	.
.	ENST0000	.	1p3	.	.	.	.	.	.	.	.	.	.
.	ENST0000	.	1p3	.	.	.	.	rs1179985	.	.	.	.	.
YBX1:N	ENST0000	.	1p3	.	.	.	.	rs7456140	.	.	.	.	.
.	ENST0000	.	1p3	.	.	.	.	.	.	.	.	.	.
.	ENST0000	.	1p3	.	.	.	Score	.	.	.	.	.	.
.	ENST0000	.	1p3	.	.	.	.	.	.	.	.	.	.
CCDC1	ENST0000	.	1p3	.	.	.	.	.	.	.	.	.	.
.	ENST0000	.	1p3	.	.	.	.	rs7290104	.	.	.	.	0.009
.	ENST0000	.	1p3	.	.	.	.	.	.	.	.	.	.
MKNK1	ENST0000	.	1p3	.	Scc	.	.	rs5508458	.	.	.	.	.
.	ENST0000	.	1p3	.	.	Score=0.9	.	.	.	.	.	.	.
.	ENST0000	.	1p3	.	.	.	.	.	.	.	.	.	.
.	ENST0000	.	1p3	.	.	.	Score	rs7570389	.	.	.	.	.
.	ENST0000	.	1p3	.	.	.	.	rs9512571	.	.	.	.	.
.	ENST0000	.	1p3	.	.	.	Score	rs7759188	.	.	.	.	.
CYB5R	ENST0000	.	1p3	.	Scc	.	.	.	.	.	.	.	.
PCSK9	ENST0000	.	1p3	.	.	.	.	rs3769455	.	.	.	.	.
PRKAA	ENST0000	.	1p3	.	.	.	.	rs7815069	ID	.	.	.	.
.	ENST0000	.	1p3	.	.	.	.	.	.	.	.	.	.
.	ENST0000	.	1p3	.	.	.	.	.	.	.	.	.	.
.	ENST0000	.	1p3	.	.	.	.	.	.	.	.	.	.
.	ENST0000	.	1p3	.	.	.	.	.	.	.	.	.	.
.	ENST0000	.	1p3	.	.	.	.	.	.	.	.	.	.
ELTD1	ENST0000	.	1p3	.	.	.	.	rs1785229	.	.	.	.	.

.	ENST000C	.	1p3	.	.	Score=0.9(	.	.	.	.	.	.	.
LPHN2	ENST000C	.	1p3	.	.	.	.	rs1394863	ID	.	.	.	.
LPAR3	ENST000C	.	1p2	.	Scc	.	.	rs7636045	.	.	.	.	.
.	ENST000C	.	1p2	.	.	.	.	.	.	.	.	.	.
.	ENST000C	.	1p2	.	.	.	.	.	.	.	.	.	.
ABCA4	ENST000C	.	1p2	.	.	.	.	.	.	.	.	.	.
ABCA4	ENST000C	.	1p2	.	.	.	.	rs6174943	ID	not pr	.	.	.
.	ENST000C	.	1p2	.	.	.	.	.	.	.	.	.	.
.	ENST000C	.	1p2	.	.	.	.	.	.	.	.	.	.
PRPF3	ENST000C	.	1p1	.	.	.	.	.	.	.	.	.	.
.	ENST000C	.	1p1	.	.	.	.	rs9363214	.	.	.	.	.
.	ENST000C	.	1p1	.	.	.	Score	rs7774328	.	.	.	.	.
RBM15	ENST000C	.	1p1	.	.	.	.	.	.	.	.	.	.
.	ENST000C	.	1p1	.	.	.	Score	.	.	.	.	.	.
UNKNC	ENST000C	.	1p1	.	.	.	.	.	ID	.	.	.	.
.	ENST000C	.	1p1	.	.	.	Score	.	.	.	.	.	.
MOV1C	ENST000C	.	1p1	.	Scc	.	.	rs7556469	ID	.	.	.	.
RHOC:	ENST000C	.	1p1	.	.	.	.	.	.	.	.	.	.
MAGI3:	ENST000C	.	1p1	.	.	.	.	.	.	.	.	.	.
.	ENST000C	.	1p1	.	.	.	.	.	.	.	.	.	.
AMPD1	ENST000C	.	1p1	.	.	.	.	rs3724512	.	.	.	.	.
.	ENST000C	.	1p1	.	.	.	.	.	ID	.	.	.	.
.	ENST000C	.	1p1	.	.	.	.	.	.	.	.	.	.
.	ENST000C	.	1p1	.	.	Score=0.9:	.	.	.	.	.	.	.
.	ENST000C	.	1p1	.	.	.	.	rs1041798	.	.	.	.	.
.	ENST000C	.	1p1	.	.	.	.	.	.	.	.	.	.
.	ENST000C	.	1q2	.	.	Score=0.98	.	.	.	.	.	.	.
UNKNC	ENST000C	.	1q2	.	.	Score=0.98	.	rs9441133	ID	.	.	.	.
.	ENST000C	.	1q2	.	.	Score=0.98	.	rs4067693	ID	.	.	.	.
UNKNC	ENST000C	.	1q2	.	.	Score=0.98	.	.	.	.	.	.	.
UNKNC	ENST000C	.	1q2	.	Scc	.	.	rs7819390	ID	.	.	.	.
.	ENST000C	.	1q2	.	.	Score=0.98	.	rs7515424	.	.	.	.	.
.	.	.	1q2	.	.	Score=0.98	.	.	.	.	.	.	.
POLR3	ENST000C	.	1q2	.	.	.	.	.	.	.	.	.	.
.	ENST000C	.	1q2	.	.	Score=0.97	.	.	.	.	.	.	.
.	ENST000C	.	1q2	.	.	Score=0.99	.	.	.	.	.	.	.
.	ENST000C	.	1q2	.	.	Score=0.92	.	.	.	.	.	.	.
.	ENST000C	.	1q2	.	.	.	.	.	.	.	.	.	.
PI4KB:I	ENST000C	.	1q2	.	.	.	.	.	.	.	.	.	.
POGZ:I	ENST000C	.	1q2	.	Scc	.	.	.	.	.	.	.	.
FLG2:N	ENST000C	.	1q2	.	.	.	.	.	.	.	.	.	.
.	ENST000C	.	1q2	.	.	.	.	rs9479104	.	.	.	.	.
.	ENST000C	.	1q2	.	.	.	.	.	.	.	.	.	.
.	ENST000C	.	1q2	.	.	.	.	.	.	.	.	.	.
DENNI	ENST000C	.	1q2	.	.	.	.	rs7704251	ID	.	.	.	.
.	ENST000C	.	1q2	.	Scc	.	.	.	.	.	.	.	.
.	ENST000C	.	1q2	.	.	.	.	.	.	.	.	.	.
ASH1L	ENST000C	.	1q2	.	.	.	.	.	.	.	.	.	.
.	ENST000C	.	1q2	.	.	.	.	.	.	.	.	.	.
.	ENST000C	.	1q2	.	.	.	.	rs9321901	.	.	.	.	.
.	ENST000C	.	1q2	.	.	.	.	.	.	.	.	.	.
ETV3L:	ENST000C	.	1q2	.	.	.	.	rs3718046	.	.	.	.	.
.	.	.	1q2	.	.	.	Score	rs2000983	ID	.	.	.	.
CD1B:I	ENST000C	.	1q2	.	.	.	.	rs7734794	.	.	.	.	.

.	ENST0000	.	1q2	.	.	.	.	rs7622475	.	.	.	.	.
ADAMT	ENST0000	.	1q2	.	Scc	.	.	.	.	.	.	.	.
C1orf2	ENST0000	.	1q2	.	.	.	.	rs2010794	.	.	.	.	.
RGS4:I	ENST0000	.	1q2	.	.	.	.	.	.	.	.	.	.
PBX1:N	ENST0000	.	1q2	.	Scc	.	.	.	.	.	.	.	.
POU2F	ENST0000	.	1q2	.	Scc	.	.	.	ID	.	.	.	.
.	ENST0000	.	1q2	.	.	.	.	.	.	.	.	.	.
.	ENST0000	.	1q2	.	.	.	.	.	.	.	.	.	.
.	ENST0000	.	1q2	.	.	.	.	.	.	.	.	.	.
.	ENST0000	.	1q2	.	.	.	.	.	.	.	.	.	.
.	ENST0000	.	1q2	.	.	.	.	.	.	.	.	.	.
ASTN1	ENST0000	.	1q2	.	Scc	.	.	rs7763360	ID	.	.	.	.
ABL2:N	ENST0000	.	1q2	.	.	.	.	.	.	.	.	.	.
.	ENST0000	.	1q2	.	.	.	.	rs1014592	.	.	.	.	.
.	ENST0000	.	1q2	.	.	.	Score	.	ID	.	.	.	.
CACNA	ENST0000	.	1q2	.	Scc	.	.	rs7733795	.	.	.	.	.
ZNF64	ENST0000	.	1q2	.	.	.	.	rs7640679	.	.	.	.	.
TEDDM	ENST0000	.	1q2	.	.	.	.	.	.	.	.	.	.
NPL:NI	ENST0000	.	1q2	.	.	.	.	rs7702036	.	.	.	.	.
TPR:NI	ENST0000	.	1q3	.	.	.	.	.	.	.	.	.	.
.	ENST0000	.	1q3	.	.	.	.	.	.	.	.	.	.
.	ENST0000	.	1q3	.	.	.	.	.	.	.	.	.	.
KIF21B	ENST0000	.	1q3	.	Scc	.	.	rs3712350	.	.	.	.	.
.	ENST0000	.	1q3	.	.	.	.	rs3723002	.	.	.	.	.
NAV1:N	ENST0000	.	1q3	.	.	.	.	rs3694010	ID	.	.	.	.
.	ENST0000	.	1q3	.	.	.	.	.	.	.	.	.	.
.	ENST0000	.	1q3	.	.	.	.	rs1044866	.	.	.	.	.
.	ENST0000	.	1q3	.	.	.	.	.	.	.	.	.	.
C4BPA	ENST0000	.	1q3	.	.	.	.	.	.	.	.	.	.
.	ENST0000	.	1q3	.	.	Score=0.9	.	.	.	.	.	.	.
.	ENST0000	.	1q3	.	.	.	.	.	.	.	.	.	.
.	ENST0000	.	1q3	.	.	.	.	.	.	.	.	.	.
.	ENST0000	.	1q3	.	.	.	.	.	.	.	.	.	.
.	ENST0000	.	1q3	.	.	.	.	rs9188348	.	.	.	.	.
FLVCR	ENST0000	.	1q3	.	.	.	.	.	.	.	.	.	.
RPS6K	ENST0000	.	1q3	.	Scc	.	.	.	.	.	.	.	.
.	ENST0000	.	1q4	.	.	.	.	.	.	.	.	.	.
.	ENST0000	.	1q4	.	.	.	.	.	.	.	.	.	.
USH2A	ENST0000	.	1q4	.	.	.	.	rs9106912	.	.	.	.	.
.	ENST0000	.	1q4	.	.	.	.	.	.	.	.	.	.
USH2A	ENST0000	.	1q4	.	.	.	.	rs7564262	ID	.	.	.	.
.	ENST0000	.	1q4	.	.	.	Score	rs1909372	.	.	.	.	0.002
.	ENST0000	.	1q4	.	Scc	.	.	.	.	.	.	.	.
IARS2:	ENST0000	.	1q4	.	.	.	.	.	.	.	.	.	.
IARS2:	ENST0000	.	1q4	.	.	.	.	rs7799500	ID	.	.	.	.
MARC2	ENST0000	.	1q4	.	Scc	.	.	.	.	.	.	.	.
DISP1:	ENST0000	.	1q4	.	.	.	.	.	.	.	.	.	.
.	ENST0000	.	1q4	.	.	.	Score	.	.	.	.	.	.
.	ENST0000	.	1q4	.	.	.	.	.	.	.	.	.	.
LEFTY	ENST0000	.	1q4	.	Scc	Score=0.9	.	.	.	.	.	.	.
.	.	.	1q4	.	.	.	Score	rs9303868	.	.	.	.	.
.	ENST0000	.	1q4	.	.	.	.	.	.	.	.	.	.
OBSCN	ENST0000	.	1q4	.	Scc	.	.	.	.	.	.	.	.
TRIM6	ENST0000	.	1q4	.	.	.	.	.	.	.	.	.	.

.	ENST000C	.	1q4	.	.	.	.	.	.	.	.	.	.
KCNK1	ENST000C	.	1q4	.	Scc	.	.	rs1006991	ID	.	.	.	.
.	ENST000C	.	1q4	.	.	.	.	rs3676265	ID	.	.	.	.
.	ENST000C	.	1q4	.	.	Score=0.9(	.	.	.	.	.	.	.
.	ENST000C	.	1q4	.	.	.	.	.	.	.	.	.	.
.	.	.	1q4	.	.	.	Score	rs1000082	.	.	.	.	.
.	ENST000C	.	1q4	.	.	.	.	rs1825931	.	.	.	.	.
ZNF69:	ENST000C	.	1q4	.	.	.	.	rs7580154	.	.	.	.	.
.	ENST000C	.	2p2	.	.	.	.	rs3724090	.	.	.	.	.
.	ENST000C	.	2p2	.	.	.	.	rs7778730	.	.	.	.	.
TRAPP	ENST000C	.	2p2	.	.	.	.	.	.	.	.	.	.
TRAPP	ENST000C	.	2p2	.	.	.	.	rs5690508	.	.	.	.	0.001
.	.	.	2p2	.	.	.	.	.	.	.	.	.	.
MBOA1	ENST000C	.	2p2	.	.	.	.	rs7490370	.	.	.	.	.
ADAM1	ENST000C	.	2p2	.	.	.	.	rs3691590	.	.	.	.	.
.	ENST000C	.	2p2	.	.	.	Score	rs9653906	.	.	.	.	.
.	ENST000C	.	2p2	.	Scc	.	Score	.	.	.	.	.	.
.	ENST000C	.	2p2	.	.	.	.	rs3765736	.	.	.	.	.
VSNL1	ENST000C	.	2p2	.	Scc	.	.	rs3680831	.	.	.	.	.
KCNS3	ENST000C	.	2p2	.	.	.	.	rs7556987	.	.	.	.	.
KLHL2!	ENST000C	.	2p2	.	Scc	.	.	.	ID	.	.	.	.
.	ENST000C	.	2p2	.	.	.	.	rs5650507	.	.	.	.	.
NCOA1	ENST000C	.	2p2	.	Scc	.	.	rs7736455	.	.	.	.	.
EMILIN	ENST000C	.	2p2	.	.	.	.	rs1479767	.	.	.	.	.
CAD:NI	ENST000C	.	2p2	.	.	.	.	.	.	.	.	.	.
CAD:NI	ENST000C	.	2p2	.	.	.	.	.	.	.	.	.	.
.	ENST000C	.	2p2	.	.	.	.	.	.	.	.	.	.
.	ENST000C	.	2p2	.	.	.	.	.	.	.	.	.	.
BIRC6:	ENST000C	.	2p2	.	.	.	.	rs7664313	ID	.	.	.	.
.	ENST000C	.	2p2	.	.	.	Score	rs1047800	.	.	.	.	.
.	ENST000C	.	2p2	.	.	.	Score	.	.	.	.	.	.
STRN:I	ENST000C	.	2p2	.	.	.	.	.	.	.	.	.	.
.	ENST000C	.	2p2	.	.	.	Score	rs7725290	ID	.	.	.	.
.	ENST000C	.	2p2	.	.	.	.	.	.	.	.	.	.
.	ENST000C	.	2p1	.	.	.	.	.	.	.	.	.	.
.	ENST000C	.	2p1	.	.	.	.	.	.	.	.	.	.
.	ENST000C	.	2p1	.	.	.	.	.	.	.	.	.	.
SPTBN	ENST000C	.	2p1	.	Scc	.	.	.	.	.	.	.	.
.	ENST000C	.	2p1	.	Scc	.	.	.	.	.	.	.	.
.	ENST000C	.	2p1	.	.	.	Score	rs7787019	ID	.	.	.	.
CCDC8	ENST000C	.	2p1	.	.	.	.	rs7608139	.	.	.	.	.
.	ENST000C	.	2p1	.	Scc	.	.	.	.	.	.	.	.
.	ENST000C	.	2p1	.	.	.	.	.	.	.	.	.	.
.	ENST000C	.	2p1	.	.	.	.	.	.	.	.	.	.
.	ENST000C	.	2p1	.	.	.	.	.	.	.	.	.	.
ARHG/	ENST000C	.	2p1	.	.	.	.	.	ID	.	.	.	.
.	ENST000C	.	2p1	.	.	.	.	.	.	.	.	.	.
.	ENST000C	.	2p1	.	.	.	.	.	.	.	.	.	.
.	ENST000C	.	2p1	.	.	.	.	.	.	.	.	.	.
.	ENST000C	.	2p1	.	.	.	.	.	.	.	.	.	.
EVA1A	ENST000C	.	2p1	.	.	.	.	rs7670256	ID	.	.	.	.
DNAH6	ENST000C	.	2p1	.	.	.	.	.	.	.	.	.	.
.	ENST000C	.	2p1	.	.	.	.	rs1883943	.	.	.	.	.
.	ENST000C	.	2p1	.	.	.	.	.	.	.	.	.	.

GGCX:	ENST0000	.	2p1	.	.	.	.	.	.	.	.	.	.
.	ENST0000	.	2p1	.	.	.	.	.	.	.	.	.	.
.	ENST0000	.	2p1	.	.	Score=0.98	.	.	.	.	.	.	.
.	ENST0000	.	2p1	.	.	Score=0.98	.	.	.	.	.	.	.
.	ENST0000	.	2p1	.	.	Score=0.98	.	rs1915368	ID	.	.	.	.
.	.	.	2p1	.	.	Score=0.98	.	rs7778874	.	.	.	.	.
.	.	.	2p1	.	.	Score=0.98	Score	rs2923588	.	.	.	.	.
.	ENST0000	.	2p1	.	.	Score=0.97	.	rs3763447	.	.	.	.	.
.	ENST0000	.	2p1	.	.	Score=0.98	.	.	.	.	.	.	.
.	.	.	2p1	.	.	Score=0.99	.	.	.	.	.	.	.
.	.	.	2p1	.	.	Score=0.98	Score	rs8689209	ID	.	.	.	.
.	.	.	2p1	.	.	Score=0.98	.	rs3759446	ID	.	.	.	.
.	ENST0000	.	2p1	.	.	Score=0.94	.	rs1210521	ID	.	.	.	.
.	ENST0000	.	2q1	.	.	Score=0.97	.	rs2931782	.	.	.	.	.
.	ENST0000	.	2q1	.	.	Score=0.99	.	.	.	.	.	.	.
ASTL:N	ENST0000	.	2q1	.	Scc	.	.	rs7784367	.	.	.	.	.
SNRNF	ENST0000	.	2q1	.	.	.	.	.	.	.	.	.	.
.	ENST0000	.	2q1	.	.	.	Score	rs5704884	.	.	.	.	.
.	ENST0000	.	2q1	.	.	.	.	.	.	.	.	.	.
ANKRD	ENST0000	.	2q1	.	.	Score=0.95	.	rs5571075	.	.	.	.	0.001
.	ENST0000	.	2q1	.	.	Score=0.98	.	.	.	.	.	.	.
TMEM1	ENST0000	.	2q1	.	Scc	.	.	.	.	.	.	.	.
AFF3:N	ENST0000	.	2q1	.	.	.	.	rs7682782	ID	.	.	.	.
IL18R1	ENST0000	.	2q1	.	.	.	.	.	.	.	.	.	.
TGFBR	ENST0000	.	2q1	.	.	.	.	rs7516119	.	.	.	.	.
RGPD3	ENST0000	.	2q1	.	.	Score=0.99	.	rs2018112	.	.	.	.	.
BUB1:N	ENST0000	.	2q1	.	.	.	.	rs7589510	.	.	.	.	.
.	ENST0000	.	2q1	.	.	.	.	.	.	.	.	.	.
.	.	.	2q1	.	.	.	.	.	.	.	.	.	.
IL36A:N	ENST0000	.	2q1	.	.	.	.	.	.	.	.	.	.
.	ENST0000	.	2q1	.	.	Score=0.97	.	.	ID	.	.	.	.
.	ENST0000	.	2q1	.	.	.	.	.	.	.	.	.	.
RALB:N	ENST0000	.	2q1	.	.	.	.	rs7642264	.	.	.	.	.
.	ENST0000	.	2q1	.	.	.	.	.	.	.	.	.	.
.	ENST0000	.	2q1	.	.	.	.	.	.	.	.	.	.
.	ENST0000	.	2q2	.	.	Score=0.96	.	rs7817298	.	.	.	.	.
.	ENST0000	.	2q2	.	.	Score=0.96	.	rs1340756	ID	.	.	.	.
.	ENST0000	.	2q2	.	.	Score=0.97	.	rs1820004	.	.	.	.	.
.	ENST0000	.	2q2	.	.	.	.	.	.	.	.	.	.
.	ENST0000	.	2q2	.	.	.	Score	.	.	.	.	.	.
.	ENST0000	.	2q2	.	.	.	.	.	.	.	.	.	.
NEB:NI	ENST0000	.	2q2	.	.	.	.	.	.	.	.	.	.
.	ENST0000	.	2q2	.	.	.	.	.	.	.	.	.	.
.	ENST0000	.	2q2	.	.	.	.	.	.	.	.	.	.
.	ENST0000	.	2q2	.	.	.	.	rs1012824	.	.	.	.	.
.	ENST0000	.	2q2	.	.	.	Score	rs9859742	.	.	.	.	.
TANC1	ENST0000	.	2q2	.	.	.	.	rs2003571	ID	.	.	.	.
SLC4A	ENST0000	.	2q2	.	Scc	.	.	rs3721419	.	.	.	.	.
.	ENST0000	.	2q2	.	.	.	.	.	.	.	.	.	.
.	ENST0000	.	2q2	.	.	.	.	rs1029714	.	.	.	.	.
.	ENST0000	.	2q2	.	.	.	.	.	.	.	.	.	.
.	ENST0000	.	2q3	.	.	.	Score	.	.	.	.	.	.
.	ENST0000	.	2q3	.	.	.	.	.	.	.	.	.	.
.	ENST0000	.	2q3	.	.	.	.	.	.	.	.	.	.

GORAS	ENST0000	.	2q3	.	.	.	.	rs9215367	.	.	.	.	.
.	ENST0000	.	2q3	.	.	.	Score	.	.	.	.	.	.
.	ENST0000	.	2q3	.	.	.	.	rs9638533	.	.	.	.	.
.	ENST0000	.	2q3	.	.	.	.	.	.	.	.	.	.
.	ENST0000	.	2q3	.	.	.	.	.	.	.	.	.	.
.	ENST0000	.	2q3	.	.	.	Score	.	.	.	.	.	.
.	ENST0000	.	2q3	.	.	.	.	.	.	.	.	.	.
.	ENST0000	.	2q3	.	.	.	Score	.	ID	.	.	.	.
.	ENST0000	.	2q3	.	.	.	.	rs9603080	.	.	.	.	.
DNAJC	ENST0000	.	2q3	.	.	.	.	rs3772721	.	.	.	.	.
FSIP2:I	ENST0000	.	2q3	.	.	.	.	rs3766275	.	.	.	.	.
FSIP2:I	ENST0000	.	2q3	.	.	.	.	.	.	.	.	.	.
.	ENST0000	.	2q3	.	.	.	.	.	.	.	.	.	.
.	ENST0000	.	2q3	.	.	.	.	rs3768239	.	.	.	.	.
.	ENST0000	.	2q3	.	.	.	.	rs7715235	.	.	.	.	.
.	ENST0000	.	2q3	.	Scc	.	.	.	.	.	.	.	.
SDPR:I	ENST0000	.	2q3	.	.	.	.	.	.	.	.	.	.
STK17I	ENST0000	.	2q3	.	.	.	.	.	.	.	.	.	.
KCTD1	ENST0000	.	2q3	.	Scc	.	.	.	.	.	.	.	.
CLK1:N	ENST0000	.	2q3	.	Scc	.	.	.	.	.	.	.	.
KIAA20	ENST0000	.	2q3	.	.	.	.	rs5481928	.	.	.	.	.
.	ENST0000	.	2q3	.	.	.	.	rs1901033	.	.	.	.	0.009
RAPH1	ENST0000	.	2q3	.	.	.	Score	rs7778048	.	.	.	.	.
EEF1B	ENST0000	.	2q3	.	.	.	.	rs1477917	ID	.	.	.	.
.	ENST0000	.	2q3	.	Scc	.	.	rs5379398	.	.	.	.	0.001
CRYGI	ENST0000	.	2q3	.	Scc	.	.	.	.	.	.	.	.
.	ENST0000	.	2q3	.	.	.	.	.	.	.	.	.	.
UNC80	ENST0000	.	2q3	.	.	.	.	.	.	.	.	.	.
.	ENST0000	.	2q3	.	.	.	.	.	.	.	.	.	.
.	ENST0000	.	2q3	.	.	.	.	.	.	.	.	.	.
.	ENST0000	.	2q3	.	.	.	.	.	.	.	.	.	.
.	ENST0000	.	2q3	.	.	.	.	.	.	.	.	.	.
.	ENST0000	.	2q3	.	.	.	.	.	.	.	.	.	.
.	ENST0000	.	2q3	.	.	.	.	.	.	.	.	.	.
.	ENST0000	.	2q3	.	.	.	.	.	.	.	.	.	.
.	ENST0000	.	2q3	.	.	.	.	rs5756405	.	.	.	.	.
.	ENST0000	.	2q3	.	.	.	.	.	.	.	.	.	.
.	ENST0000	.	2q3	.	.	.	.	rs3732211	.	.	.	.	.
DNAJB	ENST0000	.	2q3	.	.	.	.	.	.	.	.	.	.
.	ENST0000	.	2q3	.	.	.	.	rs7469670	.	.	.	.	.
.	ENST0000	.	2q3	.	Scc	.	.	rs5290584	.	.	.	.	.
EPHA4	ENST0000	.	2q3	.	.	.	.	.	.	.	.	.	.
IRS1:N	ENST0000	.	2q3	.	.	.	.	.	.	.	.	.	.
.	ENST0000	.	2q3	.	.	.	.	.	.	.	.	.	.
.	ENST0000	.	2q3	.	.	.	.	rs499449	.	.	.	.	0.426
NMUR	ENST0000	.	2q3	.	.	.	.	rs7533248	.	.	.	.	.
.	ENST0000	.	2q3	.	.	Score=0.9	.	.	.	.	.	.	.
.	ENST0000	.	2q3	.	.	Score=0.9	.	.	.	.	.	.	.
.	ENST0000	.	2q3	.	.	Score=0.9	.	.	.	.	.	.	.
UNKNO	ENST0000	.	2q3	.	Scc	.	.	rs2020267	.	.	.	.	.
.	ENST0000	.	2q3	.	.	.	.	rs1053770	.	.	.	.	.
MROH	ENST0000	.	2q3	.	.	.	.	.	.	.	.	.	.
.	ENST0000	.	2q3	.	.	.	.	.	.	.	.	.	.
PER2:N	ENST0000	.	2q3	.	.	.	.	rs7483373	.	.	.	.	.

.	ENST0000	.	2q3	.	.	.	.	.	.	.	.	.	.
KIF1A:I	ENST0000	.	2q3	.	.	.	.	rs3776284	.	.	.	.	.
.	ENST0000	.	2q3	.	.	.	.	rs1500761	.	.	.	.	0.001
.	ENST0000	.	2q3	.	.	.	.	.	.	.	.	.	.
.	ENST0000	.	2q3	.	.	.	.	.	.	.	.	.	.
.	ENST0000	.	2q3	.	.	.	.	.	.	.	.	.	.
CHL1:N	ENST0000	.	3p2	.	.	.	.	rs3731564	ID	.	.	.	.
.	ENST0000	.	3p2	.	.	.	Score	rs5694312	.	.	.	.	.
.	ENST0000	.	3p2	.	.	.	.	.	.	.	.	.	.
.	ENST0000	.	3p2	.	.	.	Score	.	.	.	.	.	.
.	ENST0000	.	3p2	.	.	.	Score	rs7502023	.	.	.	.	.
.	ENST0000	.	3p2	.	.	.	.	.	.	.	.	.	.
CRELD	ENST0000	.	3p2	.	Scc	.	.	rs7567892	.	.	.	.	.
.	ENST0000	.	3p2	.	.	Score=0.9	.	.	.	.	.	.	.
SLC6A	ENST0000	.	3p2	.	Scc	.	.	.	.	.	.	.	.
.	ENST0000	.	3p2	.	.	.	.	.	.	.	.	.	.
.	.	.	3p2	.	.	.	.	.	.	.	.	.	.
.	ENST0000	.	3p2	.	.	.	Score	rs9925095	.	.	.	.	.
.	ENST0000	.	3p2	.	.	.	.	.	.	.	.	.	.
.	ENST0000	.	3p2	.	.	.	.	.	.	.	.	.	.
CAPN7	ENST0000	.	3p2	.	.	.	.	.	.	.	.	.	.
.	ENST0000	.	3p2	.	.	.	.	.	.	.	.	.	.
.	ENST0000	.	3p2	.	.	.	.	.	.	.	.	.	.
NR1D2	ENST0000	.	3p2	.	.	.	.	.	.	.	.	.	.
.	ENST0000	.	3p2	.	.	.	.	.	.	.	.	.	.
.	.	.	3p2	.	.	.	Score	.	.	.	.	.	.
.	ENST0000	.	3p2	.	.	.	Score	rs8972401	ID	.	.	.	.
TGFBR	ENST0000	.	3p2	.	.	.	.	rs1124655	.	.	.	.	.
.	ENST0000	hsa-mir-46	3p2	.	.	.	Score	.	.	.	.	.	.
UNKNC	ENST0000	.	3p2	.	.	.	.	rs7802104	.	.	.	.	.
.	ENST0000	.	3p2	.	.	.	.	.	.	.	.	.	.
DCLK3	ENST0000	.	3p2	.	.	.	.	rs5407838	.	.	.	.	0.001
.	ENST0000	.	3p2	.	.	.	.	rs5599017	.	.	.	.	.
GOLGA	ENST0000	.	3p2	.	.	.	.	.	.	.	.	.	.
.	ENST0000	.	3p2	.	Scc	.	.	rs3732183	.	.	.	.	.
.	ENST0000	.	3p2	.	.	.	Score	.	.	.	.	.	.
SCN10	ENST0000	.	3p2	.	.	.	.	rs7485706	ID	.	.	.	.
.	ENST0000	.	3p2	.	.	.	.	.	.	.	.	.	.
CCR9:I	ENST0000	.	3p2	.	.	.	.	rs7745693	.	.	.	.	.
.	ENST0000	.	3p2	.	.	.	Score	.	.	.	.	.	.
.	ENST0000	.	3p2	.	.	.	.	.	.	.	.	.	.
CCDC5	ENST0000	.	3p2	.	.	.	.	rs7512571	.	.	.	.	.
.	ENST0000	.	3p2	.	.	.	.	rs5304720	.	.	.	.	.
NCKIPS	ENST0000	.	3p2	.	.	.	.	rs3713830	.	.	.	.	.
WDR6:	ENST0000	.	3p2	.	.	.	.	.	.	.	.	.	.
QRICH	ENST0000	.	3p2	.	.	.	.	rs2001906	.	.	.	.	.
USP4:N	ENST0000	.	3p2	.	.	.	.	.	.	.	.	.	.
BSN:NI	ENST0000	.	3p2	.	.	.	.	.	.	.	.	.	.
.	ENST0000	.	3p2	.	.	.	.	.	.	.	.	.	.
UNKNC	ENST0000	.	3p2	.	Scc	.	.	rs7596904	.	.	.	.	.
.	ENST0000	.	3p2	.	.	.	.	rs1821535	.	.	.	.	.
.	ENST0000	.	3p2	.	.	.	.	.	.	.	.	.	.
PCBP4	ENST0000	.	3p2	.	.	.	.	rs7780907	.	.	.	.	.
.	ENST0000	.	3p2	.	.	.	.	.	.	.	.	.	.

.	ENST0000	.	3p2	.	.	.	.	.	.	.	.	.	.
.	ENST0000	.	3p2	.	.	.	Score	.	.	.	.	.	.
.	ENST0000	.	3p2	.	.	.	.	.	.	.	.	.	.
.	ENST0000	.	3p2	.	.	.	.	rs9624625	.	.	.	.	.
.	ENST0000	.	3p2	.	.	.	.	.	.	.	.	.	.
PBRM1	ENST0000	.	3p2	.	.	.	.	.	.	.	.	.	.
CACNA	ENST0000	.	3p2	.	Scc	.	.	.	.	.	.	.	.
.	ENST0000	.	3p2	.	.	.	.	rs2000990	ID	.	.	.	.
.	ENST0000	.	3p1	.	.	.	Score	rs9858893	.	.	.	.	.
.	ENST0000	.	3p1	.	.	.	Score	rs1042360	.	.	.	.	.
.	ENST0000	.	3p1	.	.	.	.	.	.	.	.	.	.
.	ENST0000	.	3p1	.	.	.	.	.	.	.	.	.	.
.	ENST0000	.	3p1	.	.	.	.	.	.	.	.	.	.
.	ENST0000	.	3p1	.	.	.	.	.	.	.	.	.	.
ADAMT	ENST0000	.	3p1	.	.	.	.	rs7458693	.	.	.	.	.
LMOD3	ENST0000	.	3p1	.	.	.	.	.	.	.	.	.	.
MITF:N	ENST0000	.	3p1	.	.	.	.	.	.	.	.	.	.
.	ENST0000	.	3p1	.	.	Score=0.9	.	rs7794528	ID	.	.	.	.
MINA:N	ENST0000	.	3q1	.	.	.	.	.	.	.	.	.	.
.	.	.	3q1	.	.	.	.	.	.	.	.	.	.
.	ENST0000	.	3q1	.	.	.	.	.	.	.	.	.	.
.	ENST0000	.	3q1	.	.	.	.	.	.	.	.	.	.
CFAP4	ENST0000	.	3q1	.	.	.	.	.	.	.	.	.	.
.	ENST0000	.	3q1	.	Scc	.	.	.	.	.	.	.	.
.	ENST0000	.	3q1	.	.	.	.	.	.	.	.	.	.
STXBP	ENST0000	.	3q1	.	.	.	.	rs3684939	.	.	.	.	.
.	ENST0000	.	3q2	.	.	.	.	rs1001693	ID	.	.	.	.
.	ENST0000	.	3q2	.	.	Score=0.9	.	rs1465223	.	.	.	.	.
.	ENST0000	.	3q2	.	.	.	.	.	.	.	.	.	.
PLXNA	ENST0000	.	3q2	.	.	.	.	rs5528272	.	.	.	.	0.001
TPRA1	ENST0000	.	3q2	.	Scc	.	.	rs7511092	.	.	.	.	.
.	ENST0000	.	3q2	.	.	.	.	.	.	.	.	.	.
.	ENST0000	.	3q2	.	.	Score=0.9	.	rs5631647	.	.	.	.	.
.	ENST0000	.	3q2	.	.	Score=0.9	.	.	.	.	.	.	.
NUDT1	ENST0000	.	3q2	.	.	.	.	.	.	.	.	.	.
.	ENST0000	.	3q2	.	.	.	.	.	.	.	.	.	.
PRR23	ENST0000	.	3q2	.	.	Score=0.9	.	.	.	.	.	.	.
PRR23	ENST0000	.	3q2	.	.	Score=0.9	.	.	.	.	.	.	.
CLSTN	ENST0000	.	3q2	.	Scc	.	.	rs1408440	ID	.	.	.	.
RASA2	ENST0000	.	3q2	.	.	.	.	.	.	.	.	.	.
.	ENST0000	.	3q2	.	.	.	.	.	.	.	.	.	.
.	ENST0000	.	3q2	.	.	.	.	rs7685217	ID	Likely	.	.	0.002
PCOLC	ENST0000	.	3q2	.	.	.	.	rs7705095	.	.	.	.	.
.	ENST0000	.	3q2	.	.	.	.	.	ID	.	.	.	.
.	ENST0000	.	3q2	.	.	.	.	.	.	.	.	.	.
EIF2A:I	ENST0000	.	3q2	.	.	.	.	rs7605662	.	.	.	.	.
.	ENST0000	.	3q2	.	.	.	.	.	.	.	.	.	.
.	ENST0000	hsa-mir-16	3q2	.	Scc	.	.	.	.	.	.	.	.
.	ENST0000	.	3q2	.	.	.	.	.	.	.	.	.	.
.	.	.	3q2	.	.	.	Score	.	.	.	.	.	.
SKIL:N	ENST0000	.	3q2	.	Scc	.	.	rs5388049	.	.	.	.	.
.	.	.	3q2	.	.	.	Score	rs9368630	.	.	.	.	.
.	ENST0000	.	3q2	.	.	Score=0.9	.	.	ID	.	.	.	.
USP13	ENST0000	.	3q2	.	Scc	.	.	rs7652687	.	.	.	.	.

.	ENST000C	.	3q2	.	.	.	.	rs5714458	ID	.	.	.	0.001
MCCC:	ENST000C	.	3q2	.	.	.	.	.	.	.	.	.	.
KLHL6:	ENST000C	.	3q2	.	.	.	.	.	.	.	.	.	.
.	ENST000C	.	3q2	.	.	.	.	rs9015268	.	.	.	.	.
.	ENST000C	.	3q2	.	.	.	Score	.	.	.	.	.	.
ECE2:N	ENST000C	.	3q2	.	.	.	.	rs3748943	ID	.	.	.	.
.	ENST000C	.	3q2	.	.	.	.	.	.	.	.	.	.
LPP:NM	ENST000C	.	3q2	.	Scc	.	.	rs7531929	.	.	.	.	.
.	ENST000C	.	3q2	.	.	.	.	rs7777314	.	.	.	.	.
.	.	.	3q2	.	.	Score=0.9	.	.	.	.	.	.	.
.	.	.	3q2	.	.	Score=0.9	Score	.	.	.	.	.	.
.	ENST000C	.	3q2	.	.	.	.	.	.	.	.	.	.
.	ENST000C	.	3q2	.	.	.	Score	.	.	.	.	.	.
.	ENST000C	.	3q2	.	.	.	Score	rs8866852	.	.	.	.	.
.	ENST000C	.	3q2	.	.	Score=0.9	.	rs7637750	ID	.	.	.	.
.	ENST000C	.	4p1	.	.	.	Score	.	.	.	.	.	.
UNKNC	ENST000C	.	4p1	.	Scc	.	.	rs7620115	.	.	.	.	.
.	ENST000C	.	4p1	.	.	.	.	.	.	.	.	.	.
.	ENST000C	.	4p1	.	.	.	.	rs9125004	.	.	.	.	.
.	ENST000C	.	4p1	.	.	.	.	.	.	.	.	.	.
.	ENST000C	.	4p1	.	.	.	Score	.	.	.	.	.	.
.	ENST000C	.	4p1	.	.	.	.	rs5508099	.	.	.	.	.
.	ENST000C	.	4p1	.	.	.	.	.	.	.	.	.	.
.	ENST000C	.	4p1	.	.	.	Score	.	.	.	.	.	.
.	.	.	4p1	.	.	.	.	rs9333127	.	.	.	.	.
ADRA2	ENST000C	.	4p1	.	.	.	Score	.	.	.	.	.	.
WFS1:I	ENST000C	.	4p1	.	.	.	.	rs5603533	.	other|	.	.	0.001
.	ENST000C	.	4p1	.	.	.	.	.	.	.	.	.	.
.	ENST000C	.	4p1	.	.	.	.	.	ID	.	.	.	.
AFAP1	ENST000C	.	4p1	.	.	.	.	rs3763511	.	.	.	.	.
.	ENST000C	.	4p1	.	.	.	.	rs2006520	.	.	.	.	0.008
SH3TC	ENST000C	.	4p1	.	.	.	.	rs7521101	.	.	.	.	.
ZNF51	ENST000C	.	4p1	.	.	.	.	rs7804064	ID	.	.	.	.
BOD1L	ENST000C	.	4p1	.	.	.	Score	.	.	.	.	.	.
CC2D2	ENST000C	.	4p1	.	Scc	.	.	.	.	.	.	.	.
.	ENST000C	.	4p1	.	.	.	.	.	.	.	.	.	.
CCDC1	ENST000C	.	4p1	.	.	.	.	.	.	.	.	.	.
.	.	.	4p1	.	.	.	Score	rs1420745	.	.	.	.	0.01
.	ENST000C	.	4p1	.	.	.	.	rs5344165	.	.	.	.	.
.	ENST000C	.	4p1	.	.	Score=0.9	.	.	.	.	.	.	.
.	ENST000C	.	4p1	.	.	Score=0.9	.	.	.	.	.	.	.
RHOH:	ENST000C	.	4p1	.	.	.	.	rs7759148	ID	.	.	.	.
NSUN7	ENST000C	.	4p1	.	Scc	.	.	.	.	.	.	.	.
.	ENST000C	.	4p1	.	.	.	Score	rs2856467	ID	.	.	.	.
.	ENST000C	.	4p1	.	.	Score=0.9	.	rs1451863	ID	.	.	.	.
.	.	.	4p1	.	.	Score=0.9	Score	rs5941177	.	.	.	.	.
LRRC6	ENST000C	.	4q1	.	.	.	.	.	ID	.	.	.	.
.	ENST000C	.	4q1	.	Scc	.	.	.	.	.	.	.	.
.	ENST000C	.	4q1	.	.	.	.	.	.	.	.	.	.
.	ENST000C	.	4q1	.	.	.	.	.	.	.	.	.	.
.	ENST000C	.	4q1	.	.	.	.	.	.	.	.	.	.
UGT2B	ENST000C	.	4q1	.	.	.	.	.	ID	.	.	.	.
.	ENST000C	.	4q1	.	.	.	.	.	.	.	.	.	.
.	ENST000C	.	4q1	.	.	.	.	rs7619680	.	.	.	.	.

.	ENST0000	.	4q1	.	Scc	.	.	.	.	.	.	.	.
.	ENST0000	.	4q1	.	.	.	Score	rs1194702	ID	.	.	.	0.126
.	ENST0000	.	4q1	.	.	.	.	.	.	.	.	.	.
PPEF2	ENST0000	.	4q2	.	.	.	.	rs3691209	.	.	.	.	.
CCDC1	ENST0000	.	4q2	.	.	.	.	.	.	.	.	.	.
SHRO(	ENST0000	.	4q2	.	.	.	.	rs7609961	.	.	.	.	.
FRAS1	ENST0000	.	4q2	.	Scc	.	.	.	.	.	.	.	.
.	ENST0000	.	4q2	.	.	.	.	.	.	.	.	.	.
.	ENST0000	.	4q2	.	.	.	.	rs8867538	.	.	.	.	.
FAM13	ENST0000	.	4q2	.	Scc	.	.	.	.	.	.	.	.
.	ENST0000	.	4q2	.	Scc	.	.	.	.	.	.	.	.
TET2:N	ENST0000	.	4q2	.	.	.	.	.	.	.	.	.	.
UNKN(	ENST0000	.	4q2	.	Scc	.	.	rs3719859	.	.	.	.	.
ENPEP	ENST0000	.	4q2	.	.	Score=0.92	.	.	ID	.	.	.	.
.	ENST0000	.	4q2	.	.	Score=0.96	.	.	.	.	.	.	.
.	ENST0000	.	4q2	.	.	Score=0.89	.	.	.	.	.	.	.
.	ENST0000	.	4q2	.	.	Score=0.98	.	.	.	.	.	.	.
METTL	ENST0000	.	4q2	.	Scc	.	.	.	.	.	.	.	.
NDNF:I	ENST0000	.	4q2	.	.	.	.	.	.	.	.	.	.
ANKRD	ENST0000	.	4q2	.	.	.	.	rs7744530	.	.	.	.	.
C4orf29	ENST0000	.	4q2	.	.	.	.	.	.	.	.	.	.
C4orf29	ENST0000	.	4q2	.	.	.	.	rs7741072	.	.	.	.	.
.	.	.	4q2	.	.	Score=0.97	Score	.	ID	.	.	.	.
.	ENST0000	.	4q2	.	.	.	.	.	.	.	.	.	.
TBC1D	ENST0000	.	4q3	.	Scc	.	.	.	.	.	.	.	.
TBC1D	ENST0000	.	4q3	.	.	.	.	.	ID	.	.	.	.
IL15:NM	ENST0000	.	4q3	.	.	.	.	.	.	.	.	.	.
.	ENST0000	.	4q3	.	.	Score=0.96	.	.	.	.	.	.	.
.	ENST0000	.	4q3	.	.	.	Score	rs8661228	.	.	.	.	.
.	ENST0000	.	4q3	.	.	Score=0.97	Score	rs4095550	ID	.	.	.	.
.	ENST0000	.	4q3	.	.	.	Score	.	.	.	.	.	.
TENM3	ENST0000	.	4q3	.	Scc	.	.	.	.	.	.	.	.
.	ENST0000	.	4q3	.	.	.	.	rs9420962	.	.	.	.	.
SORBS	ENST0000	.	4q3	.	.	.	.	.	ID	.	.	.	.
.	ENST0000	.	4q3	.	.	.	.	rs7781796	.	.	.	.	.
.	ENST0000	.	5p1	.	Scc	.	.	.	.	.	.	.	.
CEP72	ENST0000	.	5p1	.	.	.	.	.	.	.	.	.	.
.	ENST0000	.	5p1	.	.	Score=0.97	.	rs412221	ID	.	.	.	.
.	ENST0000	.	5p1	.	.	.	.	rs1006578	ID	.	.	.	.
IRX4:N	ENST0000	.	5p1	.	.	.	Score	.	.	.	.	.	.
.	.	.	5p1	.	.	.	Score	rs9030458	.	.	.	.	.
ICE1:N	ENST0000	.	5p1	.	.	.	.	rs7491497	.	.	.	.	.
UBE2Q	ENST0000	.	5p1	.	Scc	.	.	.	.	.	.	.	.
PAPD7	ENST0000	.	5p1	.	.	.	.	.	ID	.	.	.	.
CTNND	ENST0000	.	5p1	.	Scc	.	.	rs1497666	ID	.	.	.	.
.	ENST0000	.	5p1	.	.	.	Score	rs6554817	.	Benig	.	.	0.548
.	ENST0000	.	5p1	.	.	.	.	rs9140889	.	.	.	.	.
.	ENST0000	.	5p1	.	.	.	.	rs7661668	.	.	.	.	.
.	ENST0000	.	5p1	.	.	Score=0.95	.	rs9251299	.	.	.	.	.
.	ENST0000	.	5p1	.	.	.	.	rs5697598	.	.	.	.	.
.	ENST0000	.	5p1	.	.	Score=0.98	Score	rs8799835	.	.	.	.	.
.	ENST0000	.	5p1	.	.	Score=0.98	Score	rs9765292	ID	.	.	.	.
.	ENST0000	.	5p1	.	.	Score=0.98	Score	rs9765293	.	.	.	.	.
.	ENST0000	.	5p1	.	.	.	.	.	.	.	.	.	.

.	ENST000C	.	5p1	.	Scc	.	.	.	.	.	.	.	.
.	ENST000C	.	5p1	.	.	.	.	.	.	.	.	.	.
.	ENST000C	.	5p1	.	.	.	.	.	.	.	.	.	.
.	.	.	5q1	.	.	.	Score	.	.	.	.	.	.
.	.	.	5q1	.	.	.	Score	rs4327635	.	.	.	.	.
.	.	.	5q1	.	.	.	Score	.	.	.	.	.	.
ACTBL	ENST000C	.	5q1	.	.	.	.	rs2005764	.	.	.	.	.
PDE4D	ENST000C	.	5q1	.	Scc	.	.	.	.	.	.	.	.
.	.	.	5q1	.	.	Score=0.9	.	rs5582099	.	.	.	.	.
.	ENST000C	.	5q1	.	Scc	.	.	.	.	.	.	.	.
.	ENST000C	.	5q1	.	.	.	.	.	.	.	.	.	.
.	ENST000C	.	5q1	.	.	.	.	rs5672287	.	.	.	.	.
MAST4	ENST000C	.	5q1	.	Scc	.	.	.	.	.	.	.	.
MAST4	ENST000C	.	5q1	.	Scc	.	.	rs2016310	.	.	.	.	.
PIK3R1	ENST000C	.	5q1	.	.	.	.	.	.	.	.	.	.
.	ENST000C	.	5q1	.	.	.	Score	.	.	.	.	.	.
.	ENST000C	.	5q1	.	.	Score=0.9	.	.	.	.	.	.	.
.	ENST000C	.	5q1	.	.	.	.	.	.	.	.	.	.
.	ENST000C	.	5q1	.	.	.	Score	.	.	.	.	.	.
.	ENST000C	.	5q1	.	.	.	.	.	.	.	.	.	.
ACOT1	ENST000C	.	5q1	.	.	.	.	.	.	.	.	.	.
.	ENST000C	.	5q1	.	.	Score=0.8	.	.	.	.	.	.	.
.	ENST000C	.	5q1	.	.	.	.	rs3717547	ID	.	.	.	.
.	.	.	5q1	.	.	.	Score	rs7815826	.	.	.	.	.
GPR98	ENST000C	.	5q1	.	.	.	.	.	.	.	.	.	.
KIAA08	ENST000C	.	5q1	.	.	.	.	.	.	.	.	.	.
ARSK:I	ENST000C	.	5q1	.	.	.	.	rs7633968	ID	.	.	.	.
.	ENST000C	.	5q2	.	.	Score=0.9	.	.	.	.	.	.	.
PAM:N	ENST000C	.	5q2	.	Scc	.	.	.	.	.	.	.	.
.	ENST000C	.	5q2	.	.	.	.	rs1114874	.	.	.	.	.
.	ENST000C	.	5q2	.	.	.	.	.	.	.	.	.	.
EPB41I	ENST000C	.	5q2	.	.	.	.	.	.	.	.	.	.
SEMA6	ENST000C	.	5q2	.	.	.	.	.	.	.	.	.	.
.	ENST000C	.	5q2	.	.	.	.	.	.	.	.	.	.
UNKNC	ENST000C	.	5q3	.	Scc	.	.	rs9346631	.	.	.	.	.
.	ENST000C	.	5q3	.	.	.	.	.	.	.	.	.	.
FSTL4:	ENST000C	.	5q3	.	Scc	.	.	rs7465490	.	.	.	.	.
.	.	.	5q3	.	.	.	.	.	.	.	.	.	.
.	ENST000C	.	5q3	.	.	.	.	rs7470418	.	.	.	.	.
CDC25	ENST000C	.	5q3	.	.	.	.	rs7665720	ID	.	.	.	.
CDC25	ENST000C	.	5q3	.	.	.	.	.	.	.	.	.	.
EGR1:I	ENST000C	.	5q3	.	.	.	Score	.	.	.	.	.	.
EGR1:I	ENST000C	.	5q3	.	.	.	Score	rs7721589	.	.	.	.	.
ANKHD	ENST000C	.	5q3	.	Scc	.	.	.	.	.	.	.	.
PCDHA	ENST000C	.	5q3	.	.	Score=0.9	.	.	.	.	.	.	.
PCDHA	ENST000C	.	5q3	.	.	.	.	.	ID	.	.	.	.
PCDHA	ENST000C	.	5q3	.	Scc	.	.	rs7823690	ID	.	.	.	.
PCDHE	ENST000C	.	5q3	.	.	Score=0.9	.	.	ID	.	.	.	.
PCDHE	ENST000C	.	5q3	.	.	Score=0.9	.	.	.	.	.	.	.
PCDHC	ENST000C	.	5q3	.	.	.	.	.	.	.	.	.	.
PCDHC	ENST000C	.	5q3	.	.	.	.	rs9814581	.	.	.	.	.
PCDHC	ENST000C	.	5q3	.	Scc	.	.	rs5446331	ID	.	.	.	.
PCDHC	ENST000C	.	5q3	.	.	.	.	rs3769527	ID	.	.	.	.
PCDHC	ENST000C	.	5q3	.	Scc	.	.	rs7719463	.	.	.	.	.

KIAA01	ENST000C	.	5q3	.	Scc	.	.	rs2018026	ID	.	.	.	0.004
.	ENST000C	.	5q3	.	.	.	Score	rs1000063	.	.	.	.	.
.	ENST000C	.	5q3	.	.	.	.	.	.	.	.	.	.
.	ENST000C	.	5q3	.	.	.	.	.	.	.	.	.	.
SYNPC	ENST000C	.	5q3	.	.	.	.	.	.	.	.	.	.
G3BP1	ENST000C	.	5q3	.	Scc	Score=0.9	.	.	.	.	.	.	.
.	ENST000C	.	5q3	.	.	.	.	rs5492040	.	.	.	.	0.002
RNF14	ENST000C	.	5q3	.	.	.	.	rs5611223	.	.	.	.	.
TENM2	ENST000C	.	5q3	.	Scc	.	.	.	.	.	.	.	.
TENM2	ENST000C	.	5q3	.	.	.	.	.	.	.	.	.	.
WWC1	ENST000C	.	5q3	.	.	.	.	rs1491693	.	.	.	.	.
RARS:I	ENST000C	.	5q3	.	.	.	.	rs7577825	ID	.	.	.	.
.	ENST000C	.	5q3	.	.	.	.	.	.	.	.	.	.
SH3PX	ENST000C	.	5q3	.	.	.	.	.	.	.	.	.	.
.	ENST000C	.	5q3	.	.	.	.	rs1822272	.	.	.	.	.
.	ENST000C	.	5q3	.	.	.	.	.	.	.	.	.	.
.	ENST000C	.	5q3	.	Scc	.	.	.	.	.	.	.	.
.	ENST000C	.	5q3	.	.	.	.	.	.	.	.	.	.
.	ENST000C	.	5q3	.	.	.	.	rs4551119	.	.	.	.	.
FGFR4	ENST000C	.	5q3	.	Scc	.	.	rs5582208	.	.	.	.	.
.	ENST000C	.	5q3	.	.	Score=0.9	.	.	.	.	.	.	.
.	ENST000C	.	5q3	.	.	Score=0.9	.	.	.	.	.	.	.
.	ENST000C	.	5q3	.	.	.	.	.	.	.	.	.	.
.	ENST000C	.	5q3	.	.	Score=0.9	.	rs1025870	.	.	.	.	.
.	ENST000C	.	5q3	.	Scc	.	Score	.	.	.	.	.	.
.	ENST000C	.	5q3	.	.	.	Score	.	.	.	.	.	.
RPP40	ENST000C	.	6p2	.	.	.	.	rs7651293	.	.	.	.	.
.	ENST000C	.	6p2	.	.	.	Score	rs1023580	.	.	.	.	.
.	ENST000C	.	6p2	.	.	.	.	rs1864505	.	.	.	.	.
.	ENST000C	.	6p2	.	.	Score=0.9	.	.	.	.	.	.	.
.	ENST000C	.	6p2	.	.	.	.	rs2004302	.	.	.	.	.
.	ENST000C	.	6p2	.	.	.	.	.	.	.	.	.	.
.	ENST000C	.	6p2	.	.	.	.	rs3692600	ID	.	.	.	.
HIVEP	ENST000C	.	6p2	.	.	.	.	rs7468362	.	.	.	.	.
.	ENST000C	.	6p2	.	.	.	.	rs7674897	.	.	.	.	.
.	ENST000C	.	6p2	.	.	.	.	.	.	.	.	.	.
.	.	.	6p2	.	.	Score=0.9	.	.	.	.	.	.	.
.	ENST000C	.	6p2	.	.	Score=0.9	.	.	.	.	.	.	.
.	ENST000C	.	6p2	.	.	Score=0.9	.	.	.	.	.	.	.
.	ENST000C	.	6p2	.	.	.	.	.	.	.	.	.	.
.	.	.	6p2	.	.	.	.	.	.	.	.	.	.
.	ENST000C	.	6p2	.	.	.	Score	.	.	.	.	.	.
.	ENST000C	.	6p2	.	.	.	.	.	.	.	.	.	.
.	ENST000C	.	6p2	.	.	.	Score	rs7653588	ID	.	.	.	.
.	ENST000C	.	6p2	.	.	Score=0.9	.	.	.	.	.	.	.
.	ENST000C	.	6p2	.	.	.	.	rs7511377	.	.	.	.	.
.	ENST000C	.	6p2	.	.	.	.	.	.	.	.	.	.
.	ENST000C	.	6p2	.	.	.	.	.	.	.	.	.	.
.	.	.	6p2	.	.	.	.	.	.	.	.	.	.
PRRC2	ENST000C	.	6p2	.	.	.	.	rs7792598	.	.	.	.	.
.	ENST000C	.	6p2	.	.	.	.	.	.	.	.	.	.
.	ENST000C	.	6p2	.	.	.	.	.	.	.	.	.	.
COL11	ENST000C	.	6p2	.	.	.	.	rs1119219	.	.	.	.	.
COL11	ENST000C	.	6p2	.	Scc	.	.	rs7759216	.	.	.	.	.

ITPR3:I	ENST0000C	.	6p2	.	Scc	.	.	.	.	.	.	.	.
PACSIN	ENST0000C	.	6p2	.	.	.	.	.	.	.	.	.	.
.	ENST0000C	.	6p2	.	Scc	.	.	rs7740360	.	.	.	.	.
.	ENST0000C	.	6p2	.	.	.	Score	.	.	.	.	.	.
PPARD	ENST0000C	.	6p2	.	.	.	.	rs7715433	.	.	.	.	.
.	ENST0000C	.	6p2	.	.	.	.	.	.	.	.	.	.
.	ENST0000C	.	6p2	.	.	.	.	.	.	.	.	.	.
CPNE5	ENST0000C	.	6p2	.	.	.	.	rs1007644	.	.	.	.	.
.	ENST0000C	.	6p2	.	.	.	.	.	.	.	.	.	.
.	ENST0000C	.	6p2	.	Scc	Score=0.9(	.	rs3751257	.	.	.	.	.
MDFI:N	ENST0000C	.	6p2	.	.	.	.	rs7778285	ID	.	.	.	.
FRS3:N	ENST0000C	.	6p2	.	Scc	.	.	.	ID	.	.	.	.
.	ENST0000C	.	6p2	.	.	.	.	rs8008274	.	.	.	.	0.023
UNKNC	ENST0000C	.	6p2	.	Scc	.	.	rs7806884	ID	.	.	.	.
PRPH2	ENST0000C	.	6p2	.	.	.	.	.	.	.	.	.	.
PTK7:N	ENST0000C	.	6p2	.	.	.	.	rs1434417	ID	.	.	.	.
PTK7:N	ENST0000C	.	6p2	.	Scc	.	.	.	.	.	.	.	.
.	ENST0000C	.	6p2	.	.	.	.	.	.	.	.	.	.
.	ENST0000C	.	6p2	.	.	.	.	.	.	.	.	.	.
XPO5:N	ENST0000C	.	6p2	.	.	.	.	rs1496900	.	.	.	.	.
TCTE1	ENST0000C	.	6p2	.	.	.	.	rs1435325	.	.	.	.	.
.	ENST0000C	.	6p1	.	.	.	.	.	.	.	.	.	.
DST:NI	ENST0000C	.	6p1	.	.	.	.	.	.	.	.	.	.
.	ENST0000C	.	6q1	.	.	Score=0.9!	.	.	.	.	.	.	.
COL19.	ENST0000C	.	6q1	.	.	.	.	.	.	.	.	.	.
.	ENST0000C	.	6q1	.	.	.	.	rs2021606	ID	.	.	.	0.004
.	ENST0000C	.	6q1	.	.	.	.	.	.	.	.	.	.
.	ENST0000C	.	6q1	.	Scc	.	.	.	.	.	.	.	.
PHIP:N	ENST0000C	.	6q1	.	.	.	.	rs7761622	.	.	.	.	.
BCKDH	ENST0000C	.	6q1	.	Scc	.	.	rs1497660	.	Likely	.	.	.
.	ENST0000C	.	6q1	.	.	.	.	rs1002475	.	.	.	.	.
SLC35/	ENST0000C	.	6q1	.	.	.	.	rs7619979	.	.	.	.	.
.	ENST0000C	.	6q1	.	.	.	.	rs1015787	.	.	.	.	.
.	ENST0000C	.	6q1	.	.	.	Score	.	.	.	.	.	.
POU3F	ENST0000C	.	6q1	.	.	.	.	.	.	.	.	.	.
.	ENST0000C	.	6q2	.	.	.	.	rs5581466	ID	.	.	.	.
.	ENST0000C	.	6q2	.	.	.	.	.	.	.	.	.	.
.	ENST0000C	.	6q2	.	.	.	.	.	.	.	.	.	.
.	ENST0000C	.	6q2	.	Scc	.	.	.	.	.	.	.	.
.	ENST0000C	.	6q2	.	.	.	.	.	.	.	.	.	.
REV3L	ENST0000C	.	6q2	.	Scc	.	.	.	.	.	.	.	.
.	.	.	6q2	.	.	.	.	.	ID	.	.	.	.
.	ENST0000C	.	6q2	.	.	.	.	.	.	.	.	.	.
.	ENST0000C	.	6q2	.	.	.	.	.	.	.	.	.	.
.	ENST0000C	.	6q2	.	.	.	.	rs3754749	.	.	.	.	.
.	ENST0000C	.	6q2	.	.	.	.	.	.	.	.	.	.
EYA4:N	ENST0000C	.	6q2	.	.	.	.	rs7606036	.	.	.	.	.
.	ENST0000C	.	6q2	.	.	.	Score	rs5279151	.	.	.	.	.
.	ENST0000C	.	6q2	.	.	.	Score	rs1006659	.	.	.	.	.
.	ENST0000C	.	6q2	.	.	.	.	.	.	.	.	.	.
.	ENST0000C	.	6q2	.	.	.	.	.	.	.	.	.	.
.	ENST0000C	.	6q2	.	.	.	.	rs1910958	.	.	.	.	0.005
STX11:	ENST0000C	.	6q2	.	Scc	.	.	rs1401901	.	.	.	.	.
ADGB:I	ENST0000C	.	6q2	.	.	.	.	.	.	.	.	.	.

UST:NI	ENST000C	.	6q2	.	Scc	.	.	rs7737449	ID	.	.	.	.
GINM1	ENST000C	.	6q2	.	Scc	.	.	rs7774557	.	.	.	.	.
KATNA	ENST000C	.	6q2	.	Scc	.	.	.	.	.	.	.	.
.	ENST000C	.	6q2	.	.	Score=0.9(	.	.	.	.	.	.	.
.	ENST000C	.	6q2	.	.	.	.	.	.	.	.	.	.
.	ENST000C	.	6q2	.	.	.	.	.	.	.	.	.	.
.	ENST000C	.	6q2	.	.	.	.	rs5290225	.	.	.	.	.
.	ENST000C	.	6q2	.	.	.	.	.	ID	.	.	.	.
IPCEF1	ENST000C	.	6q2	.	.	.	.	.	.	.	.	.	.
SCAF8	ENST000C	.	6q2	.	.	.	.	.	.	.	.	.	.
ARID1E	ENST000C	.	6q2	.	.	.	.	.	ID	.	.	.	.
.	ENST000C	.	6q2	.	.	.	.	rs1015839	.	.	.	.	.
SOD2:I	ENST000C	.	6q2	.	Scc	.	.	.	.	.	.	.	.
.	ENST000C	.	6q2	.	.	Score=0.9:	.	.	.	.	.	.	.
.	ENST000C	.	6q2	.	.	.	Score	.	.	.	.	.	.
.	ENST000C	.	6q2	.	.	Score=0.9!	.	.	.	.	.	.	.
.	ENST000C	.	6q2	.	.	.	.	.	.	.	.	.	.
PARK2	ENST000C	.	6q2	.	.	.	.	.	.	.	.	.	.
QKI:NM	ENST000C	.	6q2	.	Scc	.	.	rs7739962	.	.	.	.	.
C6orf1:	ENST000C	.	6q2	.	.	.	.	rs2008105	ID	.	.	.	.
.	ENST000C	.	6q2	.	.	Score=0.9{	.	rs7558449	.	.	.	.	.
.	ENST000C	.	6q2	.	.	.	.	.	.	.	.	.	.
FAM20	ENST000C	.	7p2	.	.	.	.	.	.	.	.	.	.
.	.	.	7p2	.	.	.	.	.	.	.	.	.	.
.	ENST000C	.	7p2	.	.	.	.	.	.	.	.	.	.
INTS1:I	ENST000C	.	7p2	.	.	.	.	.	.	.	.	.	.
INTS1:I	ENST000C	.	7p2	.	.	.	.	.	.	.	.	.	.
INTS1:I	ENST000C	.	7p2	.	Scc	.	.	rs7786995	.	.	.	.	.
.	ENST000C	.	7p2	.	.	.	.	.	.	.	.	.	.
.	ENST000C	.	7p2	.	.	.	Score	.	.	.	.	.	.
CHST1	ENST000C	.	7p2	.	.	.	.	rs5612232	.	.	.	.	.
.	ENST000C	.	7p2	.	.	.	.	.	.	.	.	.	.
.	ENST000C	.	7p2	.	.	.	.	rs3699321	.	.	.	.	.
.	ENST000C	.	7p2	.	.	.	.	.	.	.	.	.	.
.	ENST000C	.	7p2	.	.	.	.	rs5413152	.	.	.	.	.
CCZ1:N	ENST000C	.	7p2	.	.	Score=0.9!	.	.	.	.	.	.	.
CYTH3	ENST000C	.	7p2	.	.	.	.	.	.	.	.	.	.
DAGLB	ENST000C	.	7p2	.	.	.	.	rs7747652	.	.	.	.	.
GRID2I	ENST000C	.	7p2	.	Scc	.	.	.	.	.	.	.	.
C7orf2(	ENST000C	.	7p2	.	.	.	.	.	.	.	.	.	.
ZNF31(	ENST000C	.	7p2	.	Scc	.	.	.	.	.	.	.	.
.	ENST000C	.	7p2	.	.	Score=0.9!	.	rs7800175	ID	.	.	.	.
.	ENST000C	.	7p2	.	.	Score=0.9!	.	rs8790524	.	.	.	.	.
.	ENST000C	.	7p2	.	.	Score=0.9!	.	rs3726603	ID	.	.	.	.
COL28.	ENST000C	.	7p2	.	.	.	.	.	.	.	.	.	.
MIOS:N	ENST000C	.	7p2	.	.	.	.	.	ID	.	.	.	.
.	ENST000C	.	7p2	.	.	.	Score	.	.	.	.	.	.
THSD7	ENST000C	.	7p2	.	.	.	.	.	.	.	.	.	.
.	ENST000C	.	7p2	.	.	.	.	.	.	.	.	.	.
DNAH1	ENST000C	.	7p1	.	.	.	.	rs3710375	.	.	.	.	.
.	ENST000C	.	7p1	.	.	.	Score	.	.	.	.	.	.
.	ENST000C	.	7p1	.	.	.	.	.	.	.	.	.	.
HIBADI	ENST000C	.	7p1	.	.	.	.	rs1427054	.	.	.	.	.
NOD1:I	ENST000C	.	7p1	.	.	.	.	.	.	.	.	.	.

.	.	.	7p1	.	.	.	.	.	.	.	.	.	.
.	ENST0000	.	7p1	.	Sco	Score=0.94	.	.	.	.	.	.	.
FKBP9	ENST0000	.	7p1	.	.	Score=0.97	.	rs7507081	.	.	.	.	.
.	ENST0000	.	7p1	.	.	.	.	.	.	.	.	.	.
.	ENST0000	.	7p1	.	.	Score=0.94	.	.	.	.	.	.	.
.	ENST0000	.	7p1	.	.	Score=0.97	.	rs7696767	ID	.	.	.	.
.	ENST0000	.	7p1	.	Sco	Score=0.94	.	.	.	.	.	.	.
HECW	ENST0000	.	7p1	.	.	.	.	rs7755650	ID	.	.	.	.
.	ENST0000	.	7p1	.	.	.	.	.	.	.	.	.	.
UNKNO	ENST0000	.	7p1	.	.	.	.	.	.	.	.	.	.
.	ENST0000	.	7p1	.	.	.	Score	rs1113701	.	.	.	.	.
.	ENST0000	.	7p1	.	.	.	.	.	.	.	.	.	.
.	ENST0000	.	7p1	.	.	.	.	.	.	.	.	.	.
ADCY1	ENST0000	.	7p1	.	.	.	.	rs7476235	.	.	.	.	.
.	ENST0000	.	7p1	.	.	.	.	rs1024757	.	.	.	.	.
.	ENST0000	.	7p1	.	.	.	.	.	.	.	.	.	.
UNKNO	ENST0000	.	7p1	.	.	.	.	.	.	.	.	.	.
NUPR1	ENST0000	.	7p1	.	.	.	.	.	.	.	.	.	.
.	.	.	7p1	.	.	Score=0.98	.	.	.	.	.	.	.
.	ENST0000	.	7p1	.	.	Score=0.92	.	.	.	.	.	.	.
.	ENST0000	.	7p1	.	.	Score=0.99	.	.	.	.	.	.	.
.	ENST0000	.	7p1	.	.	Score=0.99	.	rs3749762	.	.	.	.	.
.	ENST0000	.	7p1	.	.	Score=0.99	.	.	.	.	.	.	.
.	ENST0000	.	7p1	.	.	Score=0.99	.	rs8799601	ID	.	.	.	.
.	ENST0000	.	7p1	.	.	Score=0.99	.	.	.	.	.	.	.
.	ENST0000	.	7q1	.	.	Score=0.99	.	.	.	.	.	.	.
.	ENST0000	.	7q1	.	.	Score=0.99	.	.	.	.	.	.	.
.	.	.	7q1	.	.	Score=0.99	.	.	.	.	.	.	.
.	.	.	7q1	.	.	Score=0.99	.	.	.	.	.	.	.
.	ENST0000	.	7q1	.	.	Score=0.99	Score	rs6156381	.	.	.	.	.
.	ENST0000	.	7q1	.	.	Score=0.98	.	.	ID	.	.	.	.
.	ENST0000	.	7q1	.	.	Score=0.98	.	.	.	.	.	.	.
.	ENST0000	.	7q1	.	.	Score=0.96	.	rs5480693	ID	.	.	.	0.015
WBSC	ENST0000	.	7q1	.	Sco	.	.	rs7706902	.	.	.	.	.
.	ENST0000	.	7q1	.	.	Score=0.98	Score	rs5574064	.	.	.	.	.
.	ENST0000	.	7q1	.	.	Score=0.99	.	.	.	.	.	.	.
.	ENST0000	.	7q1	.	.	Score=0.96	.	.	.	.	.	.	.
FZD9:N	ENST0000	.	7q1	.	.	.	.	.	.	.	.	.	.
BAZ1B	ENST0000	.	7q1	.	.	.	.	rs7822351	.	.	.	.	.
.	ENST0000	.	7q1	.	.	.	.	.	.	.	.	.	.
.	ENST0000	.	7q1	.	.	.	.	.	.	.	.	.	.
GTF2I	ENST0000	.	7q1	.	.	.	.	rs7822129	.	.	.	.	.
.	ENST0000	.	7q1	.	.	Score=0.99	.	rs3705131	.	.	.	.	.
.	ENST0000	.	7q1	.	.	Score=0.99	.	rs8799812	.	.	.	.	.
.	ENST0000	.	7q1	.	.	Score=0.97	.	rs1493360	.	.	.	.	0.569
.	ENST0000	.	7q1	.	.	Score=0.97	.	rs1403890	.	.	.	.	.
.	ENST0000	.	7q2	.	.	.	.	.	.	.	.	.	.
.	ENST0000	.	7q2	.	.	.	.	.	.	.	.	.	.
PCLO:I	ENST0000	.	7q2	.	.	.	.	.	.	.	.	.	.
CDK14	ENST0000	.	7q2	.	.	.	.	rs1413343	.	.	.	.	.
.	ENST0000	.	7q2	.	.	.	.	rs9879024	.	.	.	.	.
COL1A	ENST0000	.	7q2	.	Sco	.	.	rs7641741	ID	.	.	.	.
.	ENST0000	.	7q2	.	.	.	.	.	.	.	.	.	.
.	ENST0000	.	7q2	.	.	.	.	.	.	.	.	.	.

.	ENST0000	.	7q2	.	.	.	.	.	ID	.	.	.	.
.	ENST0000	.	7q2	.	.	.	.	rs7821462	.	.	.	.	.
TRRAF	ENST0000	.	7q2	.	Scc	.	.	rs7752142	ID	.	.	.	.
.	ENST0000	.	7q2	.	.	.	.	.	.	.	.	.	.
.	ENST0000	.	7q2	.	.	.	.	rs9034524	ID	.	.	.	.
PCOLC	ENST0000	.	7q2	.	Scc	.	.	rs5767320	.	.	.	.	.
ACTL6I	ENST0000	.	7q2	.	Scc	.	.	.	.	.	.	.	.
MUC12	ENST0000	.	7q2	.	.	Score=0.9!	.	rs7641678	.	.	.	.	.
.	ENST0000	.	7q2	.	.	.	.	.	.	.	.	.	.
CUX1:I	ENST0000	.	7q2	.	.	.	.	.	.	.	.	.	.
RASA4	ENST0000	.	7q2	.	.	Score=0.9!	.	rs7590335	.	.	.	.	.
.	ENST0000	.	7q2	.	.	Score=0.9!	.	rs4729845	ID	.	.	.	.
PSMC2	ENST0000	.	7q2	.	.	.	.	.	.	.	.	.	.
ORC5:I	ENST0000	.	7q2	.	Scc	.	.	.	.	.	.	.	.
LRRN3	ENST0000	.	7q3	.	Scc	.	.	.	.	.	.	.	.
.	ENST0000	.	7q3	.	.	.	.	.	.	.	.	.	.
.	ENST0000	.	7q3	.	.	.	.	.	.	.	.	.	.
.	ENST0000	.	7q3	.	.	.	.	rs1457073	.	.	.	.	.
.	ENST0000	.	7q3	.	.	.	.	.	.	.	.	.	.
GPR37	ENST0000	.	7q3	.	.	.	.	rs3755434	.	.	.	.	.
.	ENST0000	.	7q3	.	.	Score=0.9	.	.	.	.	.	.	.
FLNC:I	ENST0000	.	7q3	.	.	Score=0.9(	.	.	.	.	.	.	.
SMO:N	ENST0000	.	7q3	.	.	.	.	rs2006779	.	.	.	.	.
.	.	.	7q3	.	.	.	.	.	.	.	.	.	.
PLXNA	ENST0000	.	7q3	.	.	.	.	rs3679590	.	.	.	.	.
.	.	.	7q3	.	Scc	.	.	.	.	.	.	.	.
.	ENST0000	.	7q3	.	.	.	.	.	.	.	.	.	.
DGKI:I	ENST0000	.	7q3	.	.	.	.	.	.	.	.	.	.
CREB3	ENST0000	.	7q3	.	.	.	.	.	.	.	.	.	.
.	ENST0000	.	7q3	.	.	.	.	rs1825415	.	.	.	.	0.007
.	ENST0000	.	7q3	.	.	.	.	.	.	.	.	.	.
.	.	.	7q3	.	.	.	.	.	.	.	.	.	.
.	ENST0000	.	7q3	.	.	.	.	.	.	.	.	.	.
MGAM:	ENST0000	.	7q3	.	.	.	.	rs7825497	ID	.	.	.	.
UNKNC	ENST0000	.	7q3	.	.	.	.	.	.	.	.	.	.
.	ENST0000	.	7q3	.	.	.	.	.	.	.	.	.	.
.	ENST0000	.	7q3	.	.	.	.	.	.	.	.	.	.
.	ENST0000	.	7q3	.	.	Score=0.9(	.	.	.	.	.	.	.
.	.	.	7q3	.	.	.	.	rs7663697	.	.	.	.	.
.	ENST0000	.	7q3	.	.	.	.	.	.	.	.	.	.
.	ENST0000	.	7q3	.	.	.	.	rs3733853	ID	.	.	.	.
CNTNA	ENST0000	.	7q3	.	.	.	.	rs7598872	.	.	.	.	.
.	ENST0000	.	7q3	.	.	.	.	rs8019707	.	.	.	.	0.013
SSPO:I	ENST0000	.	7q3	.	.	.	.	rs1447568	.	.	.	.	.
.	ENST0000	.	7q3	.	.	.	.	.	.	.	.	.	.
.	ENST0000	.	7q3	.	.	.	.	.	.	.	.	.	.
UNKNC	ENST0000	.	7q3	.	.	.	.	.	.	.	.	.	.
CDK5:I	ENST0000	.	7q3	.	Scc	.	.	.	.	.	.	.	.
TMUB1	ENST0000	.	7q3	.	Scc	.	.	.	.	.	.	.	.
.	ENST0000	.	7q3	.	.	.	.	.	.	.	.	.	.
.	ENST0000	.	7q3	.	.	.	.	.	.	.	.	.	.
KMT2C	ENST0000	.	7q3	.	.	.	.	rs3761181	.	.	.	.	.
.	ENST0000	.	7q3	.	.	Score=0.9	.	.	.	.	.	.	.
.	ENST0000	.	7q3	.	.	Score=0.9	.	rs9367654	.	.	.	.	.

.	ENST000C	.	7q3	.	.	.	.	.	.	.	.	.	.
.	ENST000C	.	7q3	.	.	.	Score	rs1056749	.	.	.	.	.
.	ENST000C	.	7q3	.	.	.	.	rs3696336	.	.	.	.	.
.	ENST000C	.	7q3	.	.	.	.	.	.	.	.	.	.
.	ENST000C	.	8p2	.	.	.	.	.	.	.	.	.	.
CLN8:N	ENST000C	.	8p2	.	Scc	.	.	.	.	.	.	.	.
XKR5:N	ENST000C	.	8p2	.	.	.	.	rs1995647	.	.	.	.	.
.	ENST000C	.	8p2	.	.	.	.	.	.	.	.	.	.
USP17	ENST000C	.	8p2	.	.	Score=0.9(	.	rs7692119	.	.	.	.	.
.	.	.	8p2	.	.	Score=0.9	Score	rs2946428	ID	.	.	.	.
CLDN2	ENST000C	.	8p2	.	.	.	.	rs7810476	.	.	.	.	.
RP1L1:	ENST000C	.	8p2	.	.	.	.	rs7606573	.	.	.	.	.
.	ENST000C	.	8p2	.	.	.	.	.	.	.	.	.	.
.	ENST000C	.	8p2	.	.	.	Score	rs7511375	.	.	.	.	.
SLC35(	ENST000C	.	8p2	.	.	Score=0.9(	.	rs7626017	.	.	.	.	.
.	ENST000C	.	8p2	.	.	.	.	.	.	.	.	.	.
.	ENST000C	.	8p2	.	.	Score=0.9	.	.	.	.	.	.	.
.	ENST000C	.	8p2	.	.	Score=0.9	.	rs3988723	.	.	.	.	0.425
.	ENST000C	.	8p2	.	.	Score=0.9	Score	rs3713666	.	.	.	.	.
.	ENST000C	.	8p2	.	.	.	.	rs1036449	.	.	.	.	.
TUSC3	ENST000C	.	8p2	.	Scc	.	.	.	.	.	.	.	.
.	.	.	8p2	.	.	.	Score	rs1470216	ID	.	.	.	.
.	.	.	8p2	.	.	.	Score	rs1394112	.	.	.	.	.
.	ENST000C	.	8p2	.	.	.	.	.	.	.	.	.	.
.	ENST000C	.	8p2	.	.	.	.	.	.	.	.	.	.
.	ENST000C	.	8p2	.	.	.	.	.	.	.	.	.	.
NKX2-6	ENST000C	.	8p2	.	.	.	.	.	.	.	.	.	.
.	ENST000C	.	8p2	.	.	.	.	rs7690910	.	.	.	.	.
.	ENST000C	.	8p2	.	.	.	.	rs2013688	.	.	.	.	0.001
.	ENST000C	.	8p2	.	.	.	.	.	.	.	.	.	.
EPHX2	ENST000C	.	8p2	.	.	.	.	rs7247582	.	.	.	.	.
.	ENST000C	.	8p1	.	.	.	.	.	.	.	.	.	.
TEX15:	ENST000C	.	8p1	.	.	.	.	rs1419780	ID	.	.	.	.
DUSP2	ENST000C	.	8p1	.	.	.	.	rs1198970	.	.	.	.	.
.	ENST000C	.	8p1	.	.	.	.	rs5320288	.	.	.	.	.
.	ENST000C	.	8p1	.	.	.	Score	.	.	.	.	.	.
.	ENST000C	.	8p1	.	.	.	Score	rs7612284	.	.	.	.	.
.	ENST000C	.	8p1	.	.	.	.	.	.	.	.	.	.
TACC1	ENST000C	.	8p1	.	.	.	.	rs7608102	.	.	.	.	.
.	ENST000C	.	8p1	.	.	.	.	.	.	.	.	.	.
.	ENST000C	.	8p1	.	.	.	.	.	.	.	.	.	.
.	ENST000C	.	8p1	.	.	.	.	rs3705628	.	.	.	.	.
.	ENST000C	.	8p1	.	.	.	.	rs7605197	.	.	.	.	.
.	.	.	8p1	.	.	.	Score	.	.	.	.	.	.
.	.	.	8p1	.	.	.	Score	rs3733322	.	.	.	.	.
.	.	.	8p1	.	.	.	Score	rs2003364	ID	.	.	.	.
.	.	.	8p1	.	.	.	Score	.	.	.	.	.	.
.	.	.	8q1	.	.	.	Score	rs4498566	.	.	.	.	.
.	.	.	8q1	.	.	.	Score	.	.	.	.	.	.
.	.	.	8q1	.	.	.	Score	rs4401873	.	.	.	.	.
EFCAB	ENST000C	.	8q1	.	.	.	.	.	.	.	.	.	.
.	ENST000C	.	8q1	.	.	.	.	.	.	.	.	.	.
.	ENST000C	.	8q1	.	.	.	.	.	.	.	.	.	.
.	ENST000C	.	8q1	.	Scc	.	.	rs1871956	.	.	.	.	.

.	ENST0000	.	8q1	.	Scc	.	.	.	.	.	.	.	.
.	ENST0000	.	8q1	.	.	.	.	.	.	.	.	.	.
CA8:NI	ENST0000	.	8q1	.	.	.	.	rs7793713	.	.	.	.	.
.	ENST0000	.	8q1	.	.	.	.	rs1513224	.	Likely	.	.	.
.	ENST0000	.	8q1	.	.	.	Score	rs8897899	.	.	.	.	.
CSPP1	ENST0000	.	8q1	.	.	.	Score	.	.	.	.	.	.
.	ENST0000	.	8q1	.	.	.	Score	.	.	.	.	.	.
.	ENST0000	.	8q1	.	.	.	.	rs7454376	.	.	.	.	.
C8orf8	ENST0000	.	8q2	.	.	.	.	.	.	.	.	.	.
.	.	.	8q2	.	.	.	Score	.	ID	.	.	.	.
.	ENST0000	.	8q2	.	.	.	.	.	.	.	.	.	.
.	ENST0000	.	8q2	.	.	.	.	.	.	.	.	.	.
.	ENST0000	.	8q2	.	.	.	.	.	.	.	.	.	.
.	ENST0000	.	8q2	.	.	.	.	rs1030919	.	.	.	.	.
DECR1	ENST0000	.	8q2	.	Scc	.	.	rs7559645	ID	.	.	.	.
DECR1	ENST0000	.	8q2	.	Scc	.	.	.	.	.	.	.	.
.	ENST0000	.	8q2	.	Scc	.	.	.	.	.	.	.	.
.	ENST0000	.	8q2	.	.	.	.	.	.	.	.	.	.
.	ENST0000	.	8q2	.	.	.	.	rs1000299	.	.	.	.	.
.	ENST0000	.	8q2	.	.	.	.	.	.	.	.	.	.
.	ENST0000	.	8q2	.	.	.	.	.	.	.	.	.	.
.	ENST0000	.	8q2	.	.	.	.	.	.	.	.	.	.
.	ENST0000	.	8q2	.	.	.	.	.	.	.	.	.	.
KCNV1	ENST0000	.	8q2	.	.	.	.	.	.	.	.	.	.
ATAD2	ENST0000	.	8q2	.	Scc	.	.	rs1493710	.	.	.	.	.
FER1L	ENST0000	.	8q2	.	.	.	.	.	ID	.	.	.	.
.	ENST0000	.	8q2	.	.	.	Score	rs7626895	.	.	.	.	.
.	ENST0000	.	8q2	.	.	.	.	.	.	.	.	.	.
LRRC6	ENST0000	.	8q2	.	.	.	.	rs3595615	.	Benig	.	.	.
TG:NM	ENST0000	.	8q2	.	Scc	.	.	rs1689333	.	.	.	.	.
.	ENST0000	.	8q2	.	.	.	.	.	.	.	.	.	.
.	ENST0000	.	8q2	.	.	.	.	.	.	.	.	.	.
DENND	ENST0000	.	8q2	.	Scc	.	.	.	.	.	.	.	.
.	ENST0000	.	8q2	.	.	.	Score	.	.	.	.	.	.
.	ENST0000	.	8q2	.	.	.	.	.	.	.	.	.	.
.	ENST0000	.	8q2	.	.	.	.	.	.	.	.	.	.
ZFP41	ENST0000	.	8q2	.	.	.	.	rs7598006	ID	.	.	.	.
FAM83	ENST0000	.	8q2	.	.	.	.	rs5644139	ID	.	.	.	0.001
FAM83	ENST0000	.	8q2	.	.	.	.	.	.	.	.	.	.
SCRIB	ENST0000	.	8q2	.	.	.	.	rs7819090	ID	.	.	.	.
NRBP2	ENST0000	.	8q2	.	.	.	.	.	ID	.	.	.	.
EPPK1	ENST0000	.	8q2	.	.	.	.	.	.	.	.	.	.
PLEC:N	ENST0000	.	8q2	.	.	.	.	rs2000607	.	Likely	.	.	.
.	ENST0000	.	8q2	.	.	.	.	rs5565117	.	.	.	.	.
.	ENST0000	.	8q2	.	Scc	.	.	.	.	.	.	.	.
CYHR1	ENST0000	.	8q2	.	.	.	.	.	.	.	.	.	.
KIFC2:	ENST0000	.	8q2	.	.	.	.	rs7468927	.	.	.	.	.
.	ENST0000	.	9p2	.	.	Score=0.9	.	rs7123526	ID	.	.	.	0.208
.	ENST0000	.	9p2	.	.	.	.	rs8987908	.	.	.	.	.
MPDZ:	ENST0000	.	9p2	.	Scc	.	.	.	.	.	.	.	.
.	ENST0000	.	9p2	.	.	.	.	rs7591887	.	.	.	.	.
FREM1	ENST0000	.	9p2	.	.	.	.	.	.	.	.	.	.
FREM1	ENST0000	.	9p2	.	.	.	.	rs7554261	ID	.	.	.	.
.	ENST0000	.	9p2	.	.	.	.	.	.	.	.	.	.

BNC2:	ENST0000	.	9p2	.	Scc	.	.	.	.	.	.	.	.
CNTLN	ENST0000	.	9p2	.	Scc	.	.	rs7717080	.	.	.	.	.
SLC24	ENST0000	.	9p2	.	.	.	.	.	.	.	.	.	.
UNKNC	ENST0000	.	9p2	.	Scc	.	.	rs7552126	ID	.	.	.	.
.	ENST0000	.	9p2	.	.	.	Score	rs8986502	.	.	.	.	.
.	ENST0000	.	9p2	.	.	.	.	rs7703822	.	.	.	.	.
.	ENST0000	.	9p1	.	.	.	.	.	.	.	.	.	.
TESK1	ENST0000	.	9p1	.	.	.	.	.	ID	.	.	.	.
FBXO1	ENST0000	.	9p1	.	Scc	.	.	.	.	.	.	.	.
FRMPD	ENST0000	.	9p1	.	.	.	.	.	ID	.	.	.	.
.	ENST0000	.	9p1	.	.	Score=0.98	.	rs1616872	ID	.	.	.	.
CNTNA	ENST0000	.	9p1	.	.	Score=0.99	Score	rs7820531	ID	.	.	.	.
.	.	.	9p1	.	.	Score=0.99	Score	rs6255281	.	.	.	.	.
.	ENST0000	.	9q1	.	.	Score=0.99	.	.	.	.	.	.	.
.	ENST0000	.	9q1	.	.	Score=0.98	.	rs2003121	ID	.	.	.	.
.	.	.	9q1	.	.	Score=0.99	Score	rs2016275	.	.	.	.	.
.	.	.	9q1	.	.	Score=0.98	.	rs2013763	.	.	.	.	.
.	ENST0000	.	9q1	.	.	Score=0.98	.	rs3764322	ID	.	.	.	.
.	ENST0000	.	9q1	.	.	Score=0.96	.	.	.	.	.	.	.
FOXD4	ENST0000	.	9q2	.	.	Score=0.99	.	.	.	.	.	.	.
FOXD4	ENST0000	.	9q2	.	.	Score=0.99	.	rs7506128	.	.	.	.	.
.	ENST0000	.	9q2	.	.	.	Score	.	.	.	.	.	.
TMEM2	ENST0000	.	9q2	.	Scc	.	.	.	.	.	.	.	.
.	ENST0000	.	9q2	.	.	.	.	.	.	.	.	.	.
.	ENST0000	.	9q2	.	.	.	.	rs9660777	.	.	.	.	.
.	ENST0000	.	9q2	.	.	.	.	rs5294239	.	.	.	.	.
.	.	.	9q2	.	.	.	.	.	.	.	.	.	.
SPATA	ENST0000	.	9q2	.	.	Score=0.92	.	rs5696558	ID	.	.	.	.
.	ENST0000	.	9q2	.	.	Score=0.93	.	.	.	.	.	.	.
.	ENST0000	.	9q2	.	.	.	.	rs7749802	.	.	.	.	.
.	ENST0000	.	9q2	.	.	.	.	.	.	.	.	.	.
ZCCHC	ENST0000	.	9q2	.	.	.	.	.	.	.	.	.	.
.	ENST0000	.	9q2	.	.	Score=0.90	.	rs9367277	.	.	.	.	.
.	ENST0000	.	9q2	.	.	.	.	rs1447929	.	.	.	.	.
.	ENST0000	.	9q2	.	.	.	.	.	.	.	.	.	.
.	ENST0000	.	9q2	.	.	.	Score	.	.	.	.	.	.
WNK2:	ENST0000	.	9q2	.	.	.	.	rs5352400	.	.	.	.	.
.	ENST0000	.	9q2	.	.	.	.	.	ID	.	.	.	.
.	ENST0000	.	9q2	.	Scc	.	.	.	.	.	.	.	.
ZNF36	ENST0000	.	9q2	.	.	.	.	.	.	.	.	.	.
.	ENST0000	.	9q2	.	.	.	.	rs5750212	.	.	.	.	.
ZNF78	ENST0000	.	9q2	.	.	.	.	rs5664919	ID	.	.	.	.
.	ENST0000	.	9q2	.	.	.	.	rs5346016	.	.	.	.	0.001
.	ENST0000	.	9q3	.	.	.	Score	rs5275568	.	.	.	.	.
.	ENST0000	.	9q3	.	.	.	.	.	.	.	.	.	.
KIAA19	ENST0000	.	9q3	.	Scc	.	.	rs9418280	.	.	.	.	.
KIAA19	ENST0000	.	9q3	.	Scc	.	.	rs7534529	.	.	.	.	.
.	ENST0000	.	9q3	.	.	.	Score	.	.	.	.	.	.
.	ENST0000	.	9q3	.	.	.	.	.	.	.	.	.	.
.	ENST0000	.	9q3	.	.	.	.	rs1017044	.	.	.	.	.
.	ENST0000	.	9q3	.	.	.	.	.	.	.	.	.	.
.	ENST0000	.	9q3	.	.	.	.	rs5296296	.	.	.	.	.
.	ENST0000	.	9q3	.	.	.	Score	.	.	.	.	.	.
PAPPA	ENST0000	.	9q3	.	Scc	.	.	rs5393503	ID	.	.	.	.

.	ENST0000C	.	9q3	.	.	.	.	rs5770416	.	.	.	.	.
.	ENST0000C	.	9q3	.	.	.	.	rs7765588	.	.	.	.	.
.	ENST0000C	.	9q3	.	.	.	.	.	.	.	.	.	.
DAB2IF	ENST0000C	.	9q3	.	Scc	.	.	rs7817163	.	.	.	.	.
DAB2IF	ENST0000C	.	9q3	.	Scc	.	.	.	.	.	.	.	.
.	ENST0000C	.	9q3	.	.	.	.	.	.	.	.	.	.
.	ENST0000C	.	9q3	.	.	.	Score	rs7671231	.	.	.	.	.
.	ENST0000C	.	9q3	.	.	.	.	.	.	.	.	.	.
.	ENST0000C	.	9q3	.	.	.	.	.	.	.	.	.	.
LRSAM	ENST0000C	.	9q3	.	.	.	.	rs7704105	.	.	.	.	.
STXBP	ENST0000C	.	9q3	.	.	.	.	rs7514371	.	.	.	.	.
.	ENST0000C	.	9q3	.	.	.	.	.	.	.	.	.	.
FPGS:I	ENST0000C	.	9q3	.	.	.	.	rs7724303	ID	.	.	.	.
.	ENST0000C	.	9q3	.	.	.	.	rs3755590	.	.	.	.	.
ODF2:I	ENST0000C	.	9q3	.	Scc	.	.	rs7704197	.	.	.	.	.
SPTAN	ENST0000C	.	9q3	.	.	.	.	rs3700623	.	Likely	.	.	.
SPTAN	ENST0000C	.	9q3	.	Scc	.	.	rs1020109	.	.	.	.	.
.	ENST0000C	.	9q3	.	.	.	.	.	.	.	.	.	.
.	ENST0000C	.	9q3	.	.	.	.	.	.	.	.	.	.
.	ENST0000C	.	9q3	.	.	.	.	rs1200306	.	.	.	.	.
.	ENST0000C	.	9q3	.	.	.	Score	rs4837317	ID	.	.	.	.
.	ENST0000C	.	9q3	.	.	.	Score	rs4837318	ID	.	.	.	.
DOLPF	ENST0000C	.	9q3	.	.	.	.	rs7690702	.	.	.	.	.
.	ENST0000C	.	9q3	.	.	.	.	.	.	.	.	.	.
CRAT:I	ENST0000C	.	9q3	.	Scc	.	.	.	ID	.	.	.	.
RAPGE	ENST0000C	.	9q3	.	.	.	.	rs9375934	ID	.	.	.	.
.	ENST0000C	.	9q3	.	.	.	.	.	.	.	.	.	.
SETX:I	ENST0000C	.	9q3	.	.	.	.	.	.	.	.	.	.
.	ENST0000C	.	9q3	.	.	.	.	rs4498679	.	.	.	.	0.038
.	ENST0000C	.	9q3	.	.	.	.	.	.	.	.	.	.
RALGD	ENST0000C	.	9q3	.	.	.	Score	.	.	.	.	.	.
GBGT1	ENST0000C	.	9q3	.	.	.	.	.	.	.	.	.	.
.	ENST0000C	.	9q3	.	.	.	.	.	.	.	.	.	.
STKLD	ENST0000C	.	9q3	.	.	.	.	rs7823594	ID	.	.	.	.
TMEM8	ENST0000C	.	9q3	.	.	.	.	rs7806069	.	.	.	.	.
DBH:NI	ENST0000C	.	9q3	.	.	.	.	.	.	.	.	.	.
COL5A	ENST0000C	.	9q3	.	.	.	.	.	.	.	.	.	.
COL5A	ENST0000C	.	9q3	.	.	.	.	rs6173790	.	Benig	.	.	.
.	ENST0000C	.	9q3	.	.	.	.	.	.	.	.	.	.
CAMSA	ENST0000C	.	9q3	.	.	.	.	rs7478355	.	.	.	.	.
.	ENST0000C	.	9q3	.	.	.	.	.	.	.	.	.	.
.	ENST0000C	.	9q3	.	.	.	.	rs9942197	ID	.	.	.	.
NOTCH	ENST0000C	.	9q3	.	.	.	.	.	.	.	.	.	.
.	ENST0000C	.	9q3	.	.	.	.	.	.	.	.	.	.
FAM69	ENST0000C	.	9q3	.	.	.	.	.	.	.	.	.	.
.	ENST0000C	.	9q3	.	.	.	Score	.	.	.	.	.	.
TRAF2	ENST0000C	.	9q3	.	.	.	.	rs1399152	.	.	.	.	.
.	ENST0000C	.	9q3	.	.	.	.	rs5651517	.	.	.	.	.
.	ENST0000C	.	9q3	.	.	.	.	rs7788028	.	.	.	.	.
FAM16	ENST0000C	.	9q3	.	.	.	.	.	.	.	.	.	.
TOR4A	ENST0000C	.	9q3	.	.	.	.	rs7776402	ID	.	.	.	.
.	ENST0000C	.	9q3	.	.	.	.	rs1126228	.	.	.	.	0.001
.	ENST0000C	.	9q3	.	.	.	.	.	.	.	.	.	.
.	ENST0000C	.	9q3	.	.	Score=0.9	.	rs3707879	.	.	.	.	.

.	ENST0000	.	10p	.	.	.	.	rs7547454	.	.	.	.	.
.	ENST0000	.	10p	.	.	.	Score	.	.	.	.	.	.
.	ENST0000	.	10p	.	.	.	.	rs5680149	.	.	.	.	0.001
.	ENST0000	.	10p	.	.	.	Score	.	.	.	.	.	.
SFMBT	ENST0000	.	10p	.	.	.	.	rs2019140	ID	.	.	.	.
.	ENST0000	.	10p	.	.	.	Score	rs7961144	.	.	.	.	.
ITIH5:N	ENST0000	.	10p	.	.	.	.	rs3708927	ID	.	.	.	.
.	ENST0000	.	10p	.	.	.	.	.	.	.	.	.	.
.	ENST0000	.	10p	.	.	.	.	.	.	.	.	.	.
.	.	.	10p	.	.	.	Score	.	.	.	.	.	.
.	.	.	10p	.	.	.	.	.	.	.	.	.	.
.	ENST0000	.	10p	.	.	Score=0.9	Score	rs1919845	.	.	.	.	.
.	ENST0000	.	10p	.	.	.	.	rs7658069	ID	.	.	.	0.03
CUBN:	ENST0000	.	10p	.	.	.	.	.	.	.	.	.	.
.	ENST0000	.	10p	.	.	.	.	.	.	.	.	.	.
.	ENST0000	.	10p	.	.	.	.	rs9652901	.	.	.	.	.
KIAA12	ENST0000	.	10p	.	.	.	.	.	.	.	.	.	.
ARHGA	ENST0000	.	10p	.	Scc	Score=0.9	.	rs7647799	.	.	.	.	.
.	ENST0000	.	10p	.	.	.	.	rs1999030	.	.	.	.	.
MYO3A	ENST0000	.	10p	.	.	.	.	.	.	.	.	.	.
.	ENST0000	.	10p	.	.	.	.	.	ID	.	.	.	.
ANKRD	ENST0000	.	10p	.	.	.	.	rs3678495	ID	Uncer	.	.	.
.	ENST0000	.	10p	.	.	.	.	.	.	.	.	.	.
.	ENST0000	.	10p	.	.	Score=0.9	.	.	ID	.	.	.	.
.	ENST0000	.	10p	.	.	Score=0.9	.	rs7766630	.	.	.	.	.
.	ENST0000	.	10p	.	.	Score=0.9	.	rs5733337	.	.	.	.	.
CCNY:	ENST0000	.	10p	.	Scc	.	.	.	ID	.	.	.	.
.	.	.	10q	.	.	Score=0.9	.	.	.	.	.	.	.
.	ENST0000	.	10q	.	.	Score=0.9	.	.	ID	.	.	.	.
.	ENST0000	.	10q	.	.	Score=0.9	.	.	.	.	.	.	.
.	ENST0000	.	10q	.	.	Score=0.9	.	.	.	.	.	.	.
.	ENST0000	.	10q	.	.	Score=0.9	.	.	.	.	.	.	.
.	ENST0000	.	10q	.	.	.	.	.	.	.	.	.	.
WDFY4	ENST0000	.	10q	.	.	.	.	rs9040575	ID	.	.	.	.
CHAT:	ENST0000	.	10q	.	.	.	.	rs3745238	.	.	.	.	.
.	ENST0000	.	10q	.	.	Score=0.9	.	.	.	.	.	.	.
.	ENST0000	.	10q	.	.	.	.	rs7512474	.	.	.	.	.
.	.	.	10q	.	.	.	Score	.	.	.	.	.	.
FAM13	ENST0000	.	10q	.	.	.	.	rs1389134	ID	.	.	.	.
.	ENST0000	.	10q	.	.	.	.	rs5737945	.	.	.	.	.
.	ENST0000	.	10q	.	.	.	Score	.	.	.	.	.	.
.	ENST0000	.	10q	.	.	.	.	.	.	.	.	.	.
COL13	ENST0000	.	10q	.	.	.	.	rs7548920	ID	.	.	.	.
AIFM2:	ENST0000	.	10q	.	Scc	.	.	.	.	.	.	.	.
.	ENST0000	.	10q	.	.	.	.	rs3744653	.	.	.	.	.
CDH23	ENST0000	.	10q	.	Scc	.	.	rs2017279	ID	Likely	.	.	.
P4HA1	ENST0000	.	10q	.	Scc	.	.	.	.	.	.	.	.
.	ENST0000	.	10q	.	.	.	.	.	.	.	.	.	.
.	ENST0000	.	10q	.	.	.	.	rs7759506	.	.	.	.	.
.	ENST0000	.	10q	.	.	Score=0.9	.	rs7802380	ID	.	.	.	.
.	ENST0000	.	10q	.	.	Score=0.9	Score	rs8789174	.	.	.	.	.
.	ENST0000	.	10q	.	.	Score=0.9	.	.	.	.	.	.	.
.	ENST0000	.	10q	.	.	.	.	.	.	.	.	.	.
.	ENST0000	.	10q	.	.	.	.	.	.	.	.	.	.

SH2D4	ENST000C	.	10q	.	Scc	.	.	rs5681324	.	.	.	.	0.001
.	ENST000C	.	10q	.	.	.	.	.	.	.	.	.	.
LRIT1:I	ENST000C	.	10q	.	.	.	.	.	.	.	.	.	.
.	ENST000C	.	10q	.	.	.	.	.	.	.	.	.	.
.	.	.	10q	.	.	.	Score	rs5296587	.	.	.	.	.
.	ENST000C	.	10q	.	.	.	.	.	.	.	.	.	.
MYOF:	ENST000C	.	10q	.	.	.	.	rs3696055	.	.	.	.	.
CEP55	ENST000C	.	10q	.	.	.	.	rs7535868	.	.	.	.	.
.	ENST000C	.	10q	.	.	.	.	.	.	.	.	.	.
.	ENST000C	.	10q	.	.	.	Score	rs9047130	.	.	.	.	.
BLNK:N	ENST000C	.	10q	.	.	.	.	rs7826084	.	.	.	.	.
.	ENST000C	.	10q	.	.	.	.	.	.	.	.	.	.
.	ENST000C	.	10q	.	.	.	Score	rs3769880	.	.	.	.	.
.	ENST000C	.	10q	.	.	.	.	rs9945308	.	.	.	.	.
HPSE2	ENST000C	.	10q	.	.	.	.	rs5392081	ID	.	.	.	.
CUTC:I	ENST000C	.	10q	.	Scc	.	.	rs3692674	.	.	.	.	.
.	ENST000C	.	10q	.	.	.	.	rs9441434	.	.	.	.	.
PPRC1	ENST000C	.	10q	.	.	.	.	.	.	.	.	.	.
.	ENST000C	.	10q	.	.	.	.	.	.	.	.	.	.
.	ENST000C	.	10q	.	.	.	.	.	.	.	.	.	.
.	ENST000C	.	10q	.	.	.	.	.	.	.	.	.	.
.	ENST000C	.	10q	.	.	.	.	.	.	.	.	.	.
.	ENST000C	.	10q	.	.	.	.	.	.	.	.	.	.
.	ENST000C	.	10q	.	.	.	Score	.	.	.	.	.	.
PDZD8	ENST000C	.	10q	.	.	.	.	.	.	.	.	.	.
.	ENST000C	.	10q	.	.	.	Score	.	.	.	.	.	.
.	ENST000C	.	10q	.	.	.	.	.	.	.	.	.	.
FAM17	ENST000C	.	10q	.	.	.	.	rs7503782	ID	.	.	.	.
UNKNC	ENST000C	.	10q	.	.	.	.	.	.	.	.	.	.
.	ENST000C	.	10q	.	.	.	.	rs3765225	.	.	.	.	.
FOXI2:	ENST000C	.	10q	.	.	.	.	.	ID	.	.	.	.
.	ENST000C	.	10q	.	.	.	.	rs5455183	.	.	.	.	.
NKX6-2	ENST000C	.	10q	.	.	.	.	.	.	.	.	.	.
.	ENST000C	.	10q	.	.	.	.	.	.	.	.	.	.
.	ENST000C	.	10q	.	.	.	.	rs5396027	.	.	.	.	.
PRAP1	ENST000C	.	10q	.	Scc	.	.	.	.	.	.	.	.
PAOX:I	ENST000C	.	10q	.	Scc	.	.	.	.	.	.	.	.
.	ENST000C	.	10q	.	.	.	.	rs7793478	ID	.	.	.	.
.	ENST000C	.	10q	.	.	.	.	.	.	.	.	.	.
.	ENST000C	.	10q	.	.	.	.	.	.	.	.	.	.
.	ENST000C	.	11p	.	.	Score=0.9!	.	.	.	.	.	.	.
.	ENST000C	.	11p	.	.	.	.	rs2003509	ID	.	.	.	.
.	ENST000C	.	11p	.	.	.	.	.	.	.	.	.	.
.	ENST000C	.	11p	.	.	.	.	rs1008718	.	.	.	.	.
.	ENST000C	.	11p	.	.	.	.	rs7793525	.	.	.	.	.
LMNTD	ENST000C	.	11p	.	Scc	.	.	rs3741463	.	.	.	.	.
PHRF1	ENST000C	.	11p	.	.	.	.	.	.	.	.	.	.
.	ENST000C	.	11p	.	.	.	.	.	.	.	.	.	.
.	ENST000C	.	11p	.	.	.	.	rs8960121	.	.	.	.	.
.	ENST000C	.	11p	.	.	.	.	rs7582346	.	.	.	.	.
.	ENST000C	.	11p	.	.	.	.	rs7514641	.	.	.	.	.
.	ENST000C	.	11p	.	.	.	.	.	.	.	.	.	.
MUC5E	ENST000C	.	11p	.	.	.	.	.	ID	.	.	.	.
MUC5E	ENST000C	.	11p	.	Scc	.	.	.	ID	.	.	.	.

MUC5E	ENST0000	.	11p	.	.	.	.	rs3689865	ID	Uncer	.	.	.
.	ENST0000	.	11p	.	.	.	.	.	.	.	.	.	.
.	.	.	11p	.	.	.	.	rs1019902	.	.	.	.	.
.	ENST0000	.	11p	.	.	.	Score	rs7724812	.	.	.	.	.
.	ENST0000	.	11p	.	.	.	.	.	.	.	.	.	.
.	ENST0000	.	11p	.	.	.	.	.	.	.	.	.	.
.	.	.	11p	.	.	Score=0.9	.	rs5592355	ID	.	.	.	0.115
OR52B	ENST0000	.	11p	.	.	.	.	.	.	.	.	.	.
.	ENST0000	.	11p	.	.	.	.	.	ID	.	.	.	.
.	ENST0000	.	11p	.	.	.	Score	rs8669468	ID	.	.	.	.
.	ENST0000	.	11p	.	.	.	.	.	.	.	.	.	.
.	ENST0000	.	11p	.	.	.	.	rs7289628	.	.	.	.	0.001
DCHS1	ENST0000	.	11p	.	.	.	.	.	.	.	.	.	.
DCHS1	ENST0000	.	11p	.	.	.	.	.	.	.	.	.	.
MICAL	ENST0000	.	11p	.	.	.	.	rs7595225	.	.	.	.	.
UNKN	ENST0000	.	11p	.	Scc	.	.	rs7811107	.	.	.	.	.
UNKN	ENST0000	.	11p	.	.	.	.	.	ID	.	.	.	.
RRAS2	ENST0000	.	11p	.	.	.	.	rs7824579	ID	.	.	.	.
PDE3B	ENST0000	.	11p	.	Scc	.	.	.	.	.	.	.	.
.	ENST0000	.	11p	.	.	.	.	.	.	.	.	.	.
.	ENST0000	.	11p	.	.	.	Score	.	.	.	.	.	.
PLEKH	ENST0000	.	11p	.	.	.	.	rs7785451	.	.	.	.	.
OTOG:	ENST0000	.	11p	.	Scc	.	.	.	.	.	.	.	.
TPH1:N	ENST0000	.	11p	.	Scc	.	.	.	.	.	.	.	.
.	ENST0000	.	11p	.	.	.	.	.	.	.	.	.	.
.	ENST0000	.	11p	.	.	Score=0.9	.	.	.	.	.	.	.
.	ENST0000	.	11p	.	.	.	.	.	.	.	.	.	.
.	ENST0000	.	11p	.	.	.	.	.	.	.	.	.	.
NELL1:	ENST0000	.	11p	.	Scc	.	.	rs1146385	.	.	.	.	.
GAS2:N	ENST0000	.	11p	.	.	.	.	.	.	.	.	.	.
.	ENST0000	.	11p	.	.	Score=0.9	.	.	.	.	.	.	.
.	ENST0000	.	11p	.	.	.	.	.	.	.	.	.	.
.	ENST0000	.	11p	.	.	.	.	.	.	.	.	.	.
HSD17	ENST0000	.	11p	.	.	Score=0.9	.	.	.	.	.	.	.
ZNF40	ENST0000	.	11p	.	Scc	.	.	.	.	.	.	.	.
CKAP5	ENST0000	.	11p	.	Scc	.	.	.	.	.	.	.	.
.	ENST0000	.	11p	.	.	.	Score	rs7731977	.	.	.	.	.
OR4A1	ENST0000	.	11q	.	Scc	Score=0.9	.	.	ID	.	.	.	.
.	ENST0000	.	11q	.	.	.	.	rs7783566	.	.	.	.	.
SMTNL	ENST0000	.	11q	.	.	.	.	.	.	.	.	.	.
OR1S1	ENST0000	.	11q	.	.	Score=0.8	.	rs7556226	.	.	.	.	.
OR10Q	ENST0000	.	11q	.	.	.	.	rs3705153	.	.	.	.	.
.	ENST0000	.	11q	.	.	.	.	rs7461600	.	.	.	.	.
PPP1R	ENST0000	.	11q	.	.	.	.	rs7613895	.	.	.	.	.
.	ENST0000	.	11q	.	.	.	.	.	.	.	.	.	.
FADS1	ENST0000	.	11q	.	.	.	.	.	.	.	.	.	.
AHNAK	ENST0000	.	11q	.	.	.	.	rs7506613	.	.	.	.	.
MTA2:N	ENST0000	.	11q	.	.	.	.	rs3688014	.	.	.	.	.
INTS5:I	ENST0000	.	11q	.	.	.	.	rs3734262	.	.	.	.	.
.	ENST0000	.	11q	.	.	.	.	.	.	.	.	.	.
NAA40	ENST0000	.	11q	.	.	.	.	rs7551956	.	.	.	.	.
.	ENST0000	.	11q	.	.	.	.	rs320149	ID	.	.	.	.
.	ENST0000	.	11q	.	.	.	.	.	.	.	.	.	.
PYGM:	ENST0000	.	11q	.	Scc	.	.	rs7694797	.	.	.	.	.

SYVN1	ENST0000C	.	11q	.	Scc	.	.	rs7599871	.	.	.	.	.
.	ENST0000C	.	11q	.	.	.	.	rs2009796	ID	.	.	.	.
.	ENST0000C	.	11q	.	.	.	Score	.	.	.	.	.	.
KCNK7	ENST0000C	.	11q	.	.	.	.	.	.	.	.	.	.
MAP3K	ENST0000C	.	11q	.	Scc	.	.	.	.	.	.	.	.
PCNXL	ENST0000C	.	11q	.	.	.	.	rs3731576	.	.	.	.	.
SIPA1:I	ENST0000C	.	11q	.	.	.	.	.	.	.	.	.	.
.	ENST0000C	.	11q	.	.	.	.	.	.	.	.	.	.
.	ENST0000C	.	11q	.	.	.	.	.	.	.	.	.	.
GAL3S	ENST0000C	.	11q	.	Scc	.	.	.	.	.	.	.	.
.	ENST0000C	.	11q	.	.	.	.	.	.	.	.	.	.
CD248:	ENST0000C	.	11q	.	.	.	.	.	.	.	.	.	.
BRMS1	ENST0000C	.	11q	.	Scc	.	.	.	.	.	.	.	.
.	ENST0000C	.	11q	.	.	.	.	.	.	.	.	.	.
.	ENST0000C	.	11q	.	.	.	.	rs8917540	.	.	.	.	.
TCIRG:	ENST0000C	.	11q	.	.	.	.	rs1432324	.	.	.	.	.
TPCN2	ENST0000C	.	11q	.	.	.	.	rs7621382	.	.	.	.	.
.	ENST0000C	.	11q	.	.	.	.	.	.	.	.	.	.
.	ENST0000C	.	11q	.	.	.	.	.	.	.	.	.	.
DHCR7	ENST0000C	.	11q	.	Scc	.	.	rs7726393	ID	.	.	.	.
.	ENST0000C	.	11q	.	.	.	Score	rs1917587	.	Uncer	.	.	.
.	ENST0000C	.	11q	.	.	.	.	.	.	.	.	.	.
.	ENST0000C	.	11q	.	.	.	.	.	.	.	.	.	.
.	ENST0000C	.	11q	.	.	.	Score	.	.	.	.	.	.
.	ENST0000C	.	11q	.	.	.	.	.	.	.	.	.	.
.	ENST0000C	.	11q	.	.	.	.	.	.	.	.	.	.
.	ENST0000C	.	11q	.	Scc	.	.	.	.	.	.	.	.
.	ENST0000C	.	11q	.	.	.	.	.	.	.	.	.	.
SLCO2	ENST0000C	.	11q	.	.	.	.	rs7806415	.	.	.	.	.
MAP6:N	ENST0000C	.	11q	.	Scc	.	.	rs7805688	.	.	.	.	.
.	ENST0000C	.	11q	.	.	.	.	rs7453149	.	.	.	.	.
.	ENST0000C	.	11q	.	.	.	.	.	.	.	.	.	.
.	ENST0000C	.	11q	.	.	.	Score	rs3722049	.	.	.	.	.
.	ENST0000C	.	11q	.	.	Score=0.9:	.	.	.	.	.	.	.
GRM5:	ENST0000C	.	11q	.	Scc	.	.	.	ID	.	.	.	.
.	ENST0000C	.	11q	.	.	Score=0.9'	.	.	.	.	.	.	.
.	ENST0000C	.	11q	.	.	.	.	rs7912478	.	.	.	.	.
DYNC2	ENST0000C	.	11q	.	.	.	.	rs1913813	.	Uncer	.	.	.
.	ENST0000C	.	11q	.	.	.	.	rs7548668	.	.	.	.	.
ELMOD	ENST0000C	.	11q	.	Scc	.	.	rs7639430	.	.	.	.	.
.	ENST0000C	.	11q	.	.	.	.	.	.	.	.	.	.
ACAT1	ENST0000C	.	11q	.	.	.	.	.	.	.	.	.	.
.	ENST0000C	.	11q	.	.	.	.	.	.	.	.	.	.
.	ENST0000C	.	11q	.	.	.	.	rs5746434	.	.	.	.	.
.	ENST0000C	.	11q	.	Scc	.	.	rs1042921	.	.	.	.	.
.	ENST0000C	.	11q	.	.	.	.	rs9738553	.	.	.	.	.
ZW10:N	ENST0000C	.	11q	.	.	.	.	rs7711773	.	.	.	.	.
.	ENST0000C	.	11q	.	.	.	Score	rs7553780	ID	.	.	.	.
SIK3:N	ENST0000C	.	11q	.	.	.	.	.	.	.	.	.	.
TMPRS	ENST0000C	.	11q	.	.	.	.	rs3706451	.	.	.	.	.
SCN2B	ENST0000C	.	11q	.	.	.	.	.	.	.	.	.	.
.	ENST0000C	.	11q	.	.	.	.	.	.	.	.	.	.
.	ENST0000C	.	11q	.	.	.	.	.	.	.	.	.	.
DDX6:N	ENST0000C	.	11q	.	Scc	.	.	.	.	.	.	.	.

ARHGE	ENST000C	.	11q	.	.	.	.	rs5761141	.	.	.	.	.
ARHGE	ENST000C	.	11q	.	.	.	.	.	.	.	.	.	.
TECTA	ENST000C	.	11q	.	.	.	.	rs1424863	ID	other;	.	.	0.001
OR10G	ENST000C	.	11q	.	Scc	.	.	rs1464053	.	.	.	.	.
UNKNC	ENST000C	.	11q	.	.	.	.	rs1866274	ID	.	.	.	0.008
.	ENST000C	.	11q	.	.	.	.	.	.	.	.	.	.
PKNOX	ENST000C	.	11q	.	.	.	.	.	.	.	.	.	.
FOXRE	ENST000C	.	11q	.	.	.	.	rs1036245	.	.	.	.	.
.	ENST000C	.	11q	.	.	.	Score	rs1499597	.	.	.	.	.
PRDM1	ENST000C	.	11q	.	.	.	.	rs1160026	.	.	.	.	.
ADAMT	ENST000C	.	11q	.	Scc	.	.	rs7501981	.	.	.	.	.
OPCMI	ENST000C	.	11q	.	.	.	.	.	.	.	.	.	.
.	.	.	11q	.	.	.	Score	rs9612999	.	.	.	.	.
.	ENST000C	.	11q	.	.	.	.	.	.	.	.	.	.
.	ENST000C	.	12p	.	.	.	.	rs1492134	.	.	.	.	.
.	.	.	12p	.	.	.	Score	.	.	.	.	.	.
B4GAL	ENST000C	.	12p	.	.	.	.	rs7477402	.	.	.	.	.
.	ENST000C	.	12p	.	.	.	.	.	.	.	.	.	.
CACNA	ENST000C	.	12p	.	Scc	.	.	.	ID	.	.	.	.
CACNA	ENST000C	.	12p	.	.	.	.	rs2020589	.	Likely	.	.	0.001
.	ENST000C	.	12p	.	.	.	.	rs1044570	.	.	.	.	.
.	ENST000C	.	12p	.	.	.	.	.	.	.	.	.	.
.	ENST000C	.	12p	.	.	.	.	.	.	.	.	.	.
.	ENST000C	.	12p	.	.	.	.	.	.	.	.	.	.
KCNA6	ENST000C	.	12p	.	.	.	.	.	.	.	.	.	.
TNFRS	ENST000C	.	12p	.	.	.	Score	.	.	.	.	.	.
.	ENST000C	.	12p	.	.	.	.	rs7522876	.	.	.	.	.
.	ENST000C	.	12p	.	.	.	.	.	.	.	.	.	.
.	ENST000C	.	12p	.	.	.	.	.	.	.	.	.	.
.	ENST000C	.	12p	.	.	.	.	.	.	.	.	.	.
CLEC6	ENST000C	.	12p	.	.	.	.	.	.	.	.	.	.
UNKNC	ENST000C	.	12p	.	.	.	.	.	.	.	.	.	.
.	ENST000C	.	12p	.	.	Score=0.9	.	.	.	.	.	.	.
.	ENST000C	.	12p	.	.	Score=0.9	.	rs7607645	ID	.	.	.	.
.	ENST000C	.	12p	.	.	.	.	.	.	.	.	.	.
.	ENST000C	.	12p	.	.	Score=0.9	.	.	.	.	.	.	.
.	ENST000C	.	12p	.	.	.	.	.	.	.	.	.	.
UNKNC	ENST000C	.	12p	.	Scc	.	.	rs7520863	.	.	.	.	.
UNKNC	ENST000C	.	12p	.	.	.	.	.	ID	.	.	.	.
.	.	.	12p	.	.	.	.	rs7558438	.	.	.	.	.
PIK3C2	ENST000C	.	12p	.	.	.	.	.	.	.	.	.	.
PIK3C2	ENST000C	.	12p	.	.	.	.	.	.	.	.	.	.
.	.	.	12p	.	.	.	.	rs9411424	.	.	.	.	.
SLCO1	ENST000C	.	12p	.	.	.	.	.	.	.	.	.	.
PPFIBF	ENST000C	.	12p	.	.	.	.	.	.	.	.	.	.
.	ENST000C	.	12p	.	.	.	.	.	.	.	.	.	.
.	ENST000C	.	12p	.	.	.	.	rs3715564	.	.	.	.	.
.	ENST000C	.	12p	.	.	.	.	.	.	.	.	.	.
.	ENST000C	.	12p	.	.	Score=0.9(	.	.	.	.	.	.	.
.	ENST000C	.	12q	.	.	Score=0.9	.	.	.	.	.	.	.
.	ENST000C	.	12q	.	.	.	.	.	.	.	.	.	.
.	ENST000C	.	12q	.	.	.	.	.	.	.	.	.	.
.	ENST000C	.	12q	.	.	.	.	.	.	.	.	.	.
C12orf5	ENST000C	.	12q	.	.	.	.	.	.	.	.	.	.

.	ENST0000C	.	12q	.	.	.	.	.	.	.	.	.	.
TROAF	ENST0000C	.	12q	.	.	.	.	.	.	.	.	.	.
.	ENST0000C	.	12q	.	.	.	.	rs5557374	.	.	.	.	0.001
PRPF4	ENST0000C	.	12q	.	.	.	.	rs7778437	ID	.	.	.	.
.	ENST0000C	.	12q	.	.	.	.	.	.	.	.	.	.
.	ENST0000C	.	12q	.	.	.	.	rs7903986	.	.	.	.	.
.	ENST0000C	.	12q	.	.	.	.	.	.	.	.	.	.
TFCP2	ENST0000C	.	12q	.	.	.	.	.	.	.	.	.	.
C12orf8	ENST0000C	.	12q	.	.	.	.	.	.	.	.	.	.
.	ENST0000C	.	12q	.	.	.	.	.	.	.	.	.	.
KRT73	ENST0000C	.	12q	.	.	.	.	rs3777629	.	.	.	.	.
KRT2:N	ENST0000C	.	12q	.	.	.	Score	rs5301800	ID	.	.	.	.
KRT4:N	ENST0000C	.	12q	.	Scc	.	.	.	.	.	.	.	.
.	ENST0000C	.	12q	.	.	.	.	.	.	.	.	.	.
.	ENST0000C	.	12q	.	.	.	Score	.	.	.	.	.	.
.	ENST0000C	.	12q	.	.	.	.	rs9039120	.	.	.	.	.
.	ENST0000C	.	12q	.	Scc	.	.	.	.	.	.	.	.
ANKRD	ENST0000C	.	12q	.	.	.	.	rs3688610	.	.	.	.	.
MYO1A	ENST0000C	.	12q	.	Scc	.	.	rs7688629	.	.	.	.	.
.	ENST0000C	.	12q	.	.	.	.	rs1929543	ID	.	.	.	.
.	ENST0000C	.	12q	.	.	.	.	rs9453696	.	.	.	.	.
LRP1:N	ENST0000C	.	12q	.	.	.	.	.	.	.	.	.	.
.	ENST0000C	.	12q	.	.	.	.	rs3702872	.	.	.	.	.
.	ENST0000C	.	12q	.	.	.	.	.	.	.	.	.	.
SHMT2	ENST0000C	.	12q	.	Scc	.	.	rs5363943	ID	.	.	.	.
DPY19I	ENST0000C	.	12q	.	.	Score=0.9	.	.	.	.	.	.	.
XPOT:I	ENST0000C	.	12q	.	.	.	.	rs1809265	.	.	.	.	.
XPOT:I	ENST0000C	.	12q	.	.	.	.	.	.	.	.	.	.
UNKNC	ENST0000C	.	12q	.	.	.	.	.	.	.	.	.	.
.	ENST0000C	.	12q	.	Scc	.	.	.	.	.	.	.	.
.	ENST0000C	.	12q	.	.	.	Score	rs5319845	.	.	.	.	.
TMEM1	ENST0000C	.	12q	.	.	.	.	.	.	.	.	.	.
OSBPL	ENST0000C	.	12q	.	.	.	.	rs3675929	.	.	.	.	.
.	ENST0000C	.	12q	.	.	.	.	.	.	.	.	.	.
NAV3:N	ENST0000C	.	12q	.	.	.	.	.	.	.	.	.	.
NAV3:N	ENST0000C	.	12q	.	Scc	.	.	.	.	.	.	.	.
OTOGI	ENST0000C	.	12q	.	Scc	.	.	rs7672581	.	.	.	.	.
.	ENST0000C	.	12q	.	.	.	.	rs9382701	.	.	.	.	.
METTL	ENST0000C	.	12q	.	Scc	.	.	.	.	.	.	.	.
.	ENST0000C	.	12q	.	.	Score=0.9	.	.	.	.	.	.	.
.	ENST0000C	.	12q	.	.	.	.	.	.	.	.	.	.
CDK17	ENST0000C	.	12q	.	.	.	.	rs5750458	.	.	.	.	.
.	ENST0000C	.	12q	.	.	.	.	rs1491955	ID	.	.	.	.
.	ENST0000C	.	12q	.	.	.	.	.	.	.	.	.	.
NR1H4	ENST0000C	.	12q	.	.	.	.	rs7594794	.	.	.	.	.
.	ENST0000C	.	12q	.	.	.	.	.	.	.	.	.	.
.	ENST0000C	.	12q	.	.	.	.	.	.	.	.	.	.
.	ENST0000C	.	12q	.	.	.	.	.	.	.	.	.	.
STAB2	ENST0000C	.	12q	.	.	.	.	.	.	.	.	.	.
.	ENST0000C	.	12q	.	.	.	.	.	.	.	.	.	.
.	ENST0000C	.	12q	.	.	.	.	.	.	.	.	.	.
SART3	ENST0000C	.	12q	.	Scc	.	.	.	.	.	.	.	.
.	ENST0000C	.	12q	.	.	.	.	.	.	.	.	.	.
.	ENST0000C	.	12q	.	.	.	.	.	.	.	.	.	.

.	ENST000C	.	12q	.	.	.	.	.	.	.	.	.	.
.	ENST000C	.	12q	.	.	.	.	.	.	.	.	.	.
HECTD	ENST000C	.	12q	.	Scc	.	.	rs3699442	.	.	.	.	.
.	ENST000C	.	12q	.	.	.	.	rs7648934	.	.	.	.	.
.	ENST000C	.	12q	.	.	.	.	.	.	.	.	.	.
.	ENST000C	.	12q	.	.	.	.	.	.	.	.	.	.
.	ENST000C	.	12q	.	.	.	.	.	.	.	.	.	.
.	ENST000C	.	12q	.	Scc	.	.	rs9094398	.	.	.	.	.
.	ENST000C	.	12q	.	.	.	.	rs7629138	.	.	.	.	.
OASL:N	ENST000C	.	12q	.	.	.	.	.	.	.	.	.	.
.	ENST000C	.	12q	.	.	.	Score	rs1000013	ID	.	.	.	.
.	ENST000C	.	12q	.	.	.	.	.	.	.	.	.	.
.	ENST000C	.	12q	.	.	.	.	.	.	.	.	.	.
.	ENST000C	.	12q	.	.	.	Score	.	.	.	.	.	.
.	ENST000C	.	12q	.	.	.	.	rs5485859	.	.	.	.	.
.	ENST000C	.	12q	.	.	.	.	.	.	.	.	.	.
.	ENST000C	.	12q	.	.	.	Score	.	.	.	.	.	.
.	ENST000C	.	12q	.	Scc	.	.	.	.	.	.	.	.
.	ENST000C	.	12q	.	.	.	.	rs3682772	.	.	.	.	.
DNAH1	ENST000C	.	12q	.	.	.	.	.	.	.	.	.	.
.	ENST000C	.	12q	.	.	.	.	rs1861196	.	.	.	.	0.005
.	ENST000C	.	12q	.	.	.	.	.	.	.	.	.	.
.	.	.	12q	.	.	.	Score	.	.	.	.	.	.
TMEM1	ENST000C	.	12q	.	.	.	.	rs7758291	ID	.	.	.	.
.	ENST000C	.	12q	.	.	.	.	rs8685914	.	.	.	.	.
.	ENST000C	.	12q	.	.	.	.	.	.	.	.	.	.
.	ENST000C	.	12q	.	.	.	.	.	.	.	.	.	.
.	ENST000C	.	12q	.	.	.	.	.	.	.	.	.	.
CHFR:I	ENST000C	.	12q	.	Scc	.	.	.	.	.	.	.	.
PSPC1	ENST000C	.	13q	.	.	.	.	.	.	.	.	.	.
ZMYM2	ENST000C	.	13q	.	.	.	.	.	.	.	.	.	.
ZDHHC	ENST000C	.	13q	.	.	.	.	rs7650516	ID	.	.	.	.
SGCG:	ENST000C	.	13q	.	.	.	.	.	.	.	.	.	.
.	ENST000C	.	13q	.	.	.	.	rs7609687	.	.	.	.	.
.	.	.	13q	.	.	.	.	.	.	.	.	.	.
FRY:NI	ENST000C	.	13q	.	.	.	.	.	.	.	.	.	.
.	ENST000C	.	13q	.	.	.	.	.	.	.	.	.	.
.	ENST000C	.	13q	.	Scc	.	.	.	.	.	.	.	.
CCDC1	ENST000C	.	13q	.	.	.	.	.	.	.	.	.	.
SMAD9	ENST000C	.	13q	.	Scc	.	.	.	.	.	.	.	.
MTRF1	ENST000C	.	13q	.	.	.	.	rs1133145	.	.	.	.	.
.	.	.	13q	.	.	.	Score	rs9269682	.	.	.	.	.
.	ENST000C	.	13q	.	.	.	.	.	.	.	.	.	.
.	ENST000C	.	13q	.	.	.	.	rs5302300	.	.	.	.	0.003
.	ENST000C	.	13q	.	.	.	.	rs7755432	.	.	.	.	.
.	.	.	13q	.	.	.	Score	rs5354767	.	.	.	.	.
.	ENST000C	.	13q	.	.	.	.	.	.	.	.	.	.
.	ENST000C	.	13q	.	.	.	.	.	.	.	.	.	.
.	ENST000C	.	13q	.	.	.	.	rs5371791	.	.	.	.	.
.	ENST000C	.	13q	.	.	.	.	.	.	.	.	.	.
MBNL2	ENST000C	.	13q	.	Scc	.	.	rs1492005	.	.	.	.	.
TM9SF	ENST000C	.	13q	.	.	.	.	rs3733851	.	.	.	.	.
.	ENST000C	.	13q	.	.	.	.	rs3731740	.	.	.	.	.
ABHD1	ENST000C	.	13q	.	.	.	.	.	.	.	.	.	.

MYO16	ENST0000	.	13q	.	Scc	.	.	rs7567578	.	.	.	.	.
COL4A	ENST0000	.	13q	.	.	.	.	rs1488011	ID	.	.	.	.
CARS2	ENST0000	.	13q	.	.	.	.	rs7563035	.	.	.	.	.
.	ENST0000	.	13q	.	.	.	.	.	.	.	.	.	.
.	ENST0000	.	13q	.	.	.	.	rs1000744	.	.	.	.	.
.	ENST0000	.	13q	.	.	.	.	rs7522048	ID	.	.	.	.
MCF2L	ENST0000	.	13q	.	.	.	.	rs7502995	.	.	.	.	.
GAS6:	ENST0000	.	13q	.	.	.	.	rs7709821	.	.	.	.	.
.	ENST0000	.	14q	.	.	Score=0.9	.	rs8791117	.	.	.	.	.
.	ENST0000	.	14q	.	.	Score=0.9	.	.	.	.	.	.	.
.	ENST0000	.	14q	.	.	Score=0.9	.	rs7445485	.	.	.	.	.
.	ENST0000	.	14q	.	.	.	.	.	.	.	.	.	.
TEP1:	ENST0000	.	14q	.	.	.	.	rs1453568	.	.	.	.	.
KLHL3	ENST0000	.	14q	.	.	.	.	rs5497679	.	.	.	.	.
.	ENST0000	.	14q	.	.	.	.	.	.	.	.	.	.
.	.	.	14q	.	.	.	.	.	.	.	.	.	.
.	ENST0000	.	14q	.	.	.	.	rs3745810	.	.	.	.	.
HAUS4	ENST0000	.	14q	.	.	.	.	rs7576169	ID	.	.	.	.
.	ENST0000	.	14q	.	.	.	.	.	.	.	.	.	.
.	ENST0000	.	14q	.	.	.	.	rs7777607	.	.	.	.	.
.	ENST0000	.	14q	.	.	.	Score	rs9241538	.	.	.	.	.
.	ENST0000	.	14q	.	.	.	.	.	.	.	.	.	.
RALGA	ENST0000	.	14q	.	.	.	.	.	.	.	.	.	.
.	ENST0000	.	14q	.	.	.	.	.	.	.	.	.	.
.	ENST0000	.	14q	.	.	Score=0.9	.	rs5673369	.	.	.	.	.
.	ENST0000	.	14q	.	.	.	.	.	.	.	.	.	.
LRFN5	ENST0000	.	14q	.	.	.	.	rs8659367	ID	.	.	.	.
.	ENST0000	.	14q	.	.	.	.	.	.	.	.	.	.
.	ENST0000	.	14q	.	.	.	.	.	.	.	.	.	.
.	.	.	14q	.	.	.	.	.	.	.	.	.	.
PTGER	ENST0000	.	14q	.	Scc	.	.	rs7462395	.	.	.	.	.
.	ENST0000	.	14q	.	.	.	.	.	.	.	.	.	.
.	ENST0000	.	14q	.	.	.	.	.	.	.	.	.	.
DAAM1	ENST0000	.	14q	.	.	.	.	.	.	.	.	.	.
.	ENST0000	.	14q	.	.	.	.	.	.	.	.	.	.
DHRS7	ENST0000	.	14q	.	.	.	.	rs1401803	.	.	.	.	.
.	.	.	14q	.	.	.	.	.	.	.	.	.	.
MTHFD	ENST0000	.	14q	.	.	.	.	rs5401543	.	.	.	.	.
.	ENST0000	.	14q	.	.	.	Score	rs1404421	.	.	.	.	0.001
.	ENST0000	.	14q	.	.	.	.	.	.	.	.	.	.
.	ENST0000	.	14q	.	.	.	.	.	.	.	.	.	.
.	ENST0000	.	14q	.	.	.	.	.	.	.	.	.	.
ZNF41	ENST0000	.	14q	.	Scc	.	.	rs7545827	.	.	.	.	.
.	ENST0000	.	14q	.	.	.	.	rs3764591	.	.	.	.	.
LTBP2:	ENST0000	.	14q	.	Scc	.	.	.	.	.	.	.	.
.	ENST0000	.	14q	.	.	.	.	.	.	.	.	.	.
.	ENST0000	.	14q	.	.	.	Score	.	.	.	.	.	.
RPS6K	ENST0000	.	14q	.	.	.	.	rs1413435	.	.	.	.	.
.	ENST0000	.	14q	.	.	.	.	.	.	.	.	.	.
.	ENST0000	.	14q	.	.	.	.	.	.	.	.	.	.
TTC7B	ENST0000	.	14q	.	.	.	.	rs7511076	.	.	.	.	.
.	ENST0000	.	14q	.	.	.	.	rs7632995	.	.	.	.	.
.	ENST0000	.	14q	.	.	.	.	.	.	.	.	.	.
.	ENST0000	.	14q	.	.	.	.	.	.	.	.	.	.

.	ENST000C	.	14q	.	.	.	Score	.	.	.	.	.	.
.	ENST000C	.	14q	.	.	.	.	.	.	.	.	.	.
BCL11I	ENST000C	.	14q	.	.	.	.	.	.	.	.	.	.
BCL11I	ENST000C	.	14q	.	Scc	.	.	.	.	.	.	.	.
DLK1:N	ENST000C	.	14q	.	.	.	.	.	.	.	.	.	.
.	ENST000C	hsa-mir-41	14q	.	Scc	.	.	.	.	.	.	.	.
.	ENST000C	.	14q	.	.	.	.	.	.	.	.	.	.
MOK:N	ENST000C	.	14q	.	Scc	.	.	rs7571156	.	.	.	.	.
.	ENST000C	.	14q	.	.	.	.	rs9721368	.	.	.	.	.
.	ENST000C	.	14q	.	.	.	.	rs1043142	.	.	.	.	.
KIF26A	ENST000C	.	14q	.	.	.	.	.	.	.	.	.	.
TMEM1	ENST000C	.	14q	.	Scc	.	.	.	.	.	.	.	.
.	ENST000C	.	14q	.	.	.	.	.	.	.	.	.	.
.	ENST000C	.	14q	.	.	.	.	rs7564163	.	.	.	.	.
.	ENST000C	.	14q	.	.	.	.	.	.	.	.	.	.
.	ENST000C	.	14q	.	.	.	.	rs7689001	.	.	.	.	.
.	ENST000C	.	14q	.	.	.	.	.	.	.	.	.	.
PACS2	ENST000C	.	14q	.	.	.	.	rs7822187	.	.	.	.	.
.	ENST000C	.	14q	.	.	.	.	rs7820214	.	.	.	.	.
.	ENST000C	.	14q	.	.	Score=0.96	.	.	.	.	.	.	.
.	.	.	14q	.	.	Score=0.89	.	.	.	.	.	.	.
.	.	.	15q	.	.	Score=0.96	Score	rs2005484	ID	.	.	.	.
.	ENST000C	.	15q	.	.	Score=0.99	.	.	.	.	.	.	.
.	.	.	15q	.	.	Score=0.99	Score	.	.	.	.	.	.
.	ENST000C	.	15q	.	.	Score=0.95	.	.	.	.	.	.	.
.	ENST000C	.	15q	.	.	.	.	.	.	.	.	.	.
.	ENST000C	.	15q	.	.	Score=0.98	.	.	.	.	.	.	.
.	ENST000C	.	15q	.	.	Score=0.99	Score	.	.	.	.	.	.
.	ENST000C	.	15q	.	.	Score=0.99	.	.	.	.	.	.	.
.	ENST000C	.	15q	.	.	Score=0.96	Score	rs7605191	.	.	.	.	.
.	ENST000C	.	15q	.	.	.	.	rs9195421	.	.	.	.	.
.	ENST000C	.	15q	.	.	.	.	.	.	.	.	.	.
.	ENST000C	.	15q	.	.	.	.	rs7485099	.	.	.	.	.
.	ENST000C	.	15q	.	.	.	.	.	.	.	.	.	.
.	ENST000C	.	15q	.	.	.	.	rs9209119	.	.	.	.	.
.	ENST000C	.	15q	.	.	.	.	rs3718584	ID	.	.	.	.
HERC2	ENST000C	.	15q	.	.	Score=0.97	.	.	.	.	.	.	.
GOLGA	ENST000C	.	15q	.	.	Score=0.99	.	rs2021672	ID	.	.	.	.
.	ENST000C	.	15q	.	.	.	.	.	.	.	.	.	.
GOLGA	ENST000C	.	15q	.	.	Score=0.99	.	.	.	.	.	.	.
.	ENST000C	.	15q	.	.	Score=0.99	.	.	.	.	.	.	.
.	ENST000C	.	15q	.	.	Score=0.99	Score	rs1995696	.	.	.	.	.
.	ENST000C	.	15q	.	.	Score=0.99	.	.	ID	.	.	.	.
OTUD7	ENST000C	.	15q	.	.	.	.	.	.	.	.	.	.
.	.	.	15q	.	.	Score=0.99	.	rs1127716	.	.	.	.	.
.	ENST000C	.	15q	.	.	.	.	.	.	.	.	.	.
.	ENST000C	.	15q	.	.	.	.	.	.	.	.	.	.
PAK6:N	ENST000C	.	15q	.	Scc	.	.	.	.	.	.	.	.
.	ENST000C	.	15q	.	.	.	.	.	.	.	.	.	.
.	ENST000C	.	15q	.	.	.	.	rs3704110	.	.	.	.	.
.	ENST000C	.	15q	.	.	.	.	.	.	.	.	.	.
TTBK2:	ENST000C	.	15q	.	Scc	.	.	rs5310947	ID	.	.	.	.
TUBGC	ENST000C	.	15q	.	.	.	.	rs7551745	ID	.	.	.	.
CKMT1	ENST000C	.	15q	.	.	Score=0.98	.	rs5360360	.	.	.	.	.

.	ENST0000C	.	15q	.	.	Score=0.9	.	.	.	.	.	.	.
.	ENST0000C	.	15q	.	.	Score=0.9	.	rs5322560	.	.	.	.	.
.	ENST0000C	.	15q	.	.	Score=0.9	.	.	.	.	.	.	.
DUOX1	ENST0000C	.	15q	.	Scc	.	.	.	.	.	.	.	.
.	ENST0000C	.	15q	.	.	.	.	.	.	.	.	.	.
FBN1:N	ENST0000C	.	15q	.	.	.	.	rs7737859	.	.	.	.	.
FBN1:N	ENST0000C	.	15q	.	Scc	.	.	rs1409544	ID	Uncer	.	.	0.001
.	ENST0000C	.	15q	.	.	.	.	.	.	.	.	.	.
.	ENST0000C	.	15q	.	.	.	.	.	.	.	.	.	.
.	ENST0000C	.	15q	.	.	.	.	.	.	.	.	.	.
.	ENST0000C	.	15q	.	.	.	.	.	.	.	.	.	.
MYO5C	ENST0000C	.	15q	.	Scc	.	.	rs3686733	ID	.	.	.	.
.	ENST0000C	.	15q	.	.	.	.	.	.	.	.	.	.
AQP9:N	ENST0000C	.	15q	.	Scc	.	.	.	.	.	.	.	.
TLN2:N	ENST0000C	.	15q	.	.	.	.	rs7793599	.	.	.	.	.
MTFMT	ENST0000C	.	15q	.	.	.	.	rs7518805	.	.	.	.	.
IGDCC	ENST0000C	.	15q	.	Scc	.	.	.	.	.	.	.	.
IGDCC	ENST0000C	.	15q	.	Scc	.	.	rs5369412	.	.	.	.	0.001
.	ENST0000C	.	15q	.	.	.	Score	.	.	.	.	.	.
.	ENST0000C	.	15q	.	.	.	.	.	.	.	.	.	.
.	ENST0000C	.	15q	.	.	.	.	.	.	.	.	.	.
.	ENST0000C	.	15q	.	.	.	.	rs7568129	ID	.	.	.	.
.	ENST0000C	.	15q	.	.	.	.	.	.	.	.	.	.
.	ENST0000C	.	15q	.	.	.	Score	rs5460720	.	.	.	.	0.001
.	ENST0000C	.	15q	.	.	.	.	.	.	.	.	.	.
CELF6	ENST0000C	.	15q	.	.	.	.	.	.	.	.	.	.
.	ENST0000C	.	15q	.	.	.	.	rs1043159	.	.	.	.	.
.	ENST0000C	.	15q	.	.	.	.	.	.	.	.	.	.
.	ENST0000C	.	15q	.	.	Score=0.9	.	.	.	.	.	.	.
.	ENST0000C	.	15q	.	.	Score=0.9	.	.	.	.	.	.	.
.	ENST0000C	.	15q	.	.	.	.	rs9744245	.	.	.	.	.
.	ENST0000C	.	15q	.	.	.	.	rs1047518	.	.	.	.	.
.	ENST0000C	.	15q	.	.	Score=0.9	.	rs8789048	ID	.	.	.	.
.	ENST0000C	.	15q	.	.	Score=0.9	.	.	.	.	.	.	.
.	ENST0000C	.	15q	.	.	Score=0.9	.	.	.	.	.	.	.
KIAA1C	ENST0000C	.	15q	.	.	.	.	rs1471333	ID	.	.	.	.
.	ENST0000C	.	15q	.	.	.	Score	.	.	.	.	.	.
.	ENST0000C	.	15q	.	.	.	.	rs5292517	.	.	.	.	.
.	ENST0000C	.	15q	.	.	.	.	.	.	.	.	.	.
.	ENST0000C	.	15q	.	.	.	.	rs9410184	.	.	.	.	.
GOLGA	ENST0000C	.	15q	.	.	Score=0.9	.	rs7920451	ID	.	.	.	.
.	ENST0000C	.	15q	.	.	.	.	.	.	.	.	.	.
KLHL25	ENST0000C	.	15q	.	.	.	.	rs3718116	.	.	.	.	.
POLG:N	ENST0000C	.	15q	.	.	.	.	.	.	.	.	.	.
.	ENST0000C	.	15q	.	.	.	Score	.	.	.	.	.	.
KIF7:NI	ENST0000C	.	15q	.	Scc	.	.	rs2012804	.	.	.	.	.
KIF7:NI	ENST0000C	.	15q	.	.	.	.	.	ID	.	.	.	.
UNKNC	ENST0000C	.	15q	.	.	.	.	rs1475111	.	.	.	.	.
.	.	.	15q	.	.	.	Score	rs5584446	.	.	.	.	0.001
ADAMT	ENST0000C	.	15q	.	.	.	.	.	.	.	.	.	.
.	ENST0000C	.	15q	.	.	.	.	.	.	.	.	.	.
.	ENST0000C	.	15q	.	.	.	Score	.	.	.	.	.	.
UNKNC	ENST0000C	.	15q	.	Scc	.	.	rs7764234	ID	.	.	.	.
.	ENST0000C	.	15q	.	.	.	.	.	.	.	.	.	.

.	ENST0000	.	15q	.	.	Score=0.9	.	.	.	.	.	.	.
.	ENST0000	.	15q	.	.	Score=0.9	.	rs3682087	.	.	.	.	.
.	ENST0000	.	15q	.	.	Score=0.9	.	.	.	.	.	.	.
.	ENST0000	.	15q	.	.	Score=0.9	.	.	.	.	.	.	.
UNKN	ENST0000	.	16p	.	.	.	.	rs7613665	.	.	.	.	.
ITFG3:	ENST0000	.	16p	.	.	.	.	.	.	.	.	.	.
.	ENST0000	.	16p	.	.	.	.	.	.	.	.	.	.
.	ENST0000	.	16p	.	.	.	Score	rs8927038	.	.	.	.	.
WFIKK	ENST0000	.	16p	.	.	.	.	rs1998911	.	.	.	.	.
.	ENST0000	.	16p	.	.	.	Score	rs2010028	ID	.	.	.	.
JMJD8:	ENST0000	.	16p	.	.	.	Score	rs7767345	ID	.	.	.	.
FBXL1	ENST0000	.	16p	.	.	.	.	rs1466306	.	.	.	.	.
NARFL	ENST0000	.	16p	.	.	.	.	rs3681933	.	.	.	.	.
CHTF1	ENST0000	.	16p	.	.	.	.	.	.	.	.	.	.
.	ENST0000	.	16p	.	.	.	.	.	.	.	.	.	.
SSTR5	ENST0000	.	16p	.	.	.	.	rs1506415	.	.	.	.	.
.	ENST0000	.	16p	.	.	.	.	rs9971019	.	.	.	.	.
BAIAP	ENST0000	.	16p	.	.	.	.	.	.	.	.	.	.
.	ENST0000	.	16p	.	.	.	.	rs3710800	.	.	.	.	.
BAIAP	ENST0000	.	16p	.	.	.	.	.	.	.	.	.	.
.	ENST0000	.	16p	.	.	.	.	rs7592188	.	.	.	.	.
BAIAP	ENST0000	.	16p	.	.	.	.	.	.	.	.	.	.
.	.	.	16p	.	.	.	.	rs5365360	.	.	.	.	0.002
CLCN7	ENST0000	.	16p	.	Sc	.	.	rs7601714	.	.	.	.	.
.	ENST0000	.	16p	.	.	.	.	.	.	.	.	.	.
HN1L:	ENST0000	.	16p	.	.	.	.	rs1450874	ID	.	.	.	.
MAPK	ENST0000	.	16p	.	.	.	.	.	.	.	.	.	.
RPL3L:	ENST0000	.	16p	.	.	.	.	rs3749279	.	.	.	.	.
.	ENST0000	.	16p	.	.	.	.	.	.	.	.	.	.
ZNF59	ENST0000	.	16p	.	.	.	.	rs7634894	.	.	.	.	.
.	ENST0000	.	16p	.	.	.	.	.	.	.	.	.	.
.	ENST0000	.	16p	.	.	.	.	.	.	.	.	.	.
.	ENST0000	.	16p	.	.	.	.	.	.	.	.	.	.
.	ENST0000	.	16p	.	.	.	Score	rs1416929	.	.	.	.	0.015
ZNF59	ENST0000	.	16p	.	Sc	.	.	.	.	.	.	.	.
.	ENST0000	.	16p	.	.	.	.	rs7525617	.	.	.	.	.
SLX4:	ENST0000	.	16p	.	.	.	.	rs5345285	.	Likely	.	.	0.008
CREB	ENST0000	.	16p	.	Sc	.	.	.	.	.	.	.	.
.	ENST0000	.	16p	.	.	.	Score	rs5502446	.	.	.	.	0.001
.	ENST0000	.	16p	.	.	.	.	rs1894802	.	.	.	.	.
ADCY9	ENST0000	.	16p	.	.	.	.	rs3711593	ID	.	.	.	.
VASN:	ENST0000	.	16p	.	.	.	.	rs2013575	.	.	.	.	.
PPL:N	ENST0000	.	16p	.	.	.	.	.	.	.	.	.	.
CIITA:	ENST0000	.	16p	.	.	.	.	rs5632991	ID	.	.	.	0.001
.	ENST0000	.	16p	.	.	.	.	rs7679436	.	.	.	.	.
.	ENST0000	.	16p	.	.	.	.	rs9184234	.	.	.	.	.
.	ENST0000	.	16p	.	.	.	.	rs7800288	.	.	.	.	.
.	ENST0000	.	16p	.	.	Score=0.9	Score	rs5668064	.	.	.	.	.
.	ENST0000	.	16p	.	.	Score=0.9	.	rs7507758	.	.	.	.	.
.	ENST0000	.	16p	.	.	.	.	.	.	.	.	.	.
.	ENST0000	.	16p	.	.	.	.	.	.	.	.	.	.
.	ENST0000	.	16p	.	.	.	Score	.	.	.	.	.	.
.	ENST0000	.	16p	.	Sc	Score=0.9	.	.	.	.	.	.	.
SMG1:	ENST0000	.	16p	.	.	Score=0.9	.	rs1784261	ID	.	.	.	.

SYT17:	ENST000C	.	16p	.	.	.	.	rs7575963	.	.	.	.	.
UMOD:	ENST000C	.	16p	.	.	.	.	rs7647908	.	.	.	.	.
UNKNC	ENST000C	.	16p	.	.	.	.	.	.	.	.	.	.
.	ENST000C	.	16p	.	.	.	.	rs1829590	.	.	.	.	.
GGA2:I	ENST000C	.	16p	.	Scc	.	.	.	.	.	.	.	.
ERN2:I	ENST000C	.	16p	.	.	.	.	.	.	.	.	.	.
.	ENST000C	.	16p	.	.	.	Score	rs9722593	.	.	.	.	.
.	.	.	16p	.	.	.	.	.	.	.	.	.	.
GTF3C	ENST000C	.	16p	.	.	.	.	.	.	.	.	.	.
.	ENST000C	.	16p	.	.	.	.	.	.	.	.	.	.
KIAA05	ENST000C	.	16p	.	.	.	.	rs1412530	.	.	.	.	.
.	ENST000C	.	16p	.	.	.	.	rs1909424	.	.	.	.	0.02
.	ENST000C	.	16p	.	.	Score=0.9	.	.	.	.	.	.	.
ATXN2	ENST000C	.	16p	.	Scc	.	.	rs7491998	.	.	.	.	.
.	ENST000C	.	16p	.	.	.	.	.	.	.	.	.	.
ATP2A	ENST000C	.	16p	.	.	.	.	rs1820846	.	.	.	.	.
.	ENST000C	.	16p	.	.	Score=0.9	.	.	.	.	.	.	.
.	ENST000C	.	16p	.	Scc	.	.	.	.	.	.	.	.
KCTD1	ENST000C	.	16p	.	Scc	.	.	rs7813295	.	.	.	.	.
.	ENST000C	.	16p	.	.	.	Score	rs9659529	.	.	.	.	.
.	ENST000C	.	16p	.	.	.	.	.	.	.	.	.	.
.	ENST000C	.	16p	.	.	Score=0.9	.	.	.	.	.	.	.
ZNF26	ENST000C	.	16p	.	.	.	.	.	.	.	.	.	.
.	ENST000C	.	16p	.	.	Score=0.9	.	rs7477221	.	.	.	.	.
.	ENST000C	.	16p	.	.	Score=0.9	.	rs460256	ID	.	.	.	0.19
.	ENST000C	.	16p	.	.	Score=0.9	.	.	.	.	.	.	.
.	ENST000C	.	16p	.	.	Score=0.9	Score	.	.	.	.	.	.
.	ENST000C	.	16p	.	.	Score=0.9	Score	rs3744640	ID	.	.	.	0.1
.	.	.	16p	.	.	Score=0.9	.	.	.	.	.	.	.
.	ENST000C	.	16p	.	Scc	Score=0.9	.	.	.	.	.	.	.
.	ENST000C	.	16p	.	.	.	Score	.	.	.	.	.	.
.	.	.	16q	.	.	.	Score	rs1333894	.	.	.	.	.
.	.	.	16q	.	.	.	Score	rs2885478	ID	.	.	.	.
.	.	.	16q	.	.	.	Score	rs4249087	ID	.	.	.	.
.	.	.	16q	.	.	.	Score	rs5591122	.	.	.	.	.
.	.	.	16q	.	.	.	Score	rs4246405	.	.	.	.	.
.	.	.	16q	.	.	.	Score	rs4292070	ID	.	.	.	.
.	.	.	16q	.	.	.	Score	rs8799648	.	.	.	.	.
.	.	.	16q	.	.	.	Score	rs8799977	.	.	.	.	.
.	.	.	16q	.	.	.	Score	rs4291933	.	.	.	.	.
.	.	.	16q	.	.	.	Score	rs4375682	.	.	.	.	.
.	.	.	16q	.	.	.	Score	rs7200104	ID	.	.	.	.
.	.	.	16q	.	.	.	Score	rs7188643	.	.	.	.	.
.	.	.	16q	.	.	.	Score	rs9328642	.	.	.	.	.
.	.	.	16q	.	.	.	Score	rs7205231	.	.	.	.	.
.	.	.	16q	.	.	.	Score	rs4445923	.	.	.	.	.
.	.	.	16q	.	.	.	Score	rs2887500	ID	.	.	.	.
.	.	.	16q	.	.	.	Score	rs2888543	.	.	.	.	.
.	.	.	16q	.	.	.	Score	rs7664258	.	.	.	.	.
.	.	.	16q	.	.	.	Score	rs4249076	.	.	.	.	.
.	.	.	16q	.	.	.	Score	.	.	.	.	.	.
MYLK3	ENST000C	.	16q	.	.	.	.	rs3683497	.	.	.	.	.
.	ENST000C	.	16q	.	.	.	.	.	.	.	.	.	.
.	ENST000C	.	16q	.	.	.	.	.	.	.	.	.	.

NUP93	ENST0000C	.	16q	.	.	.	.	.	.	.	.	.	.
.	ENST0000C	.	16q	.	.	.	Score	.	.	.	.	.	.
.	ENST0000C	.	16q	.	.	.	.	.	.	.	.	.	.
CPNE2	ENST0000C	.	16q	.	.	.	.	rs1471990	.	.	.	.	.
.	ENST0000C	.	16q	.	.	.	Score	.	.	.	.	.	.
TEPP:N	ENST0000C	.	16q	.	.	.	.	.	.	.	.	.	.
.	ENST0000C	.	16q	.	.	.	.	.	.	.	.	.	.
.	ENST0000C	.	16q	.	.	.	Score	rs1499903	.	.	.	.	.
EXOC3	ENST0000C	.	16q	.	.	.	.	.	.	.	.	.	.
.	ENST0000C	.	16q	.	.	.	Score	rs1054074	.	.	.	.	.
ELMO3	ENST0000C	.	16q	.	.	.	.	rs7715372	.	.	.	.	.
SLC9A	ENST0000C	.	16q	.	.	.	.	rs5618721	.	.	.	.	.
.	ENST0000C	.	16q	.	.	.	.	.	.	.	.	.	.
LRRC3	ENST0000C	.	16q	.	Scc	.	.	rs7545456	ID	.	.	.	.
.	ENST0000C	.	16q	.	.	.	.	rs3696380	.	.	.	.	.
.	ENST0000C	.	16q	.	.	.	.	.	.	.	.	.	.
CTRL:N	ENST0000C	.	16q	.	Scc	.	.	rs7584347	.	.	.	.	.
.	ENST0000C	.	16q	.	.	.	.	.	.	.	.	.	.
DDX28	ENST0000C	.	16q	.	.	.	.	.	.	.	.	.	.
TERF2	ENST0000C	.	16q	.	.	.	.	.	.	.	.	.	.
.	ENST0000C	.	16q	.	.	.	Score	rs8670174	.	.	.	.	.
.	ENST0000C	.	16q	.	.	.	.	rs9278778	.	.	.	.	.
.	ENST0000C	.	16q	.	.	.	.	rs1485518	.	.	.	.	.
UNKNC	ENST0000C	.	16q	.	.	.	.	rs3776990	.	.	.	.	.
.	ENST0000C	.	16q	.	.	Score=0.9	.	rs7502787	.	.	.	.	.
.	ENST0000C	.	16q	.	.	.	.	.	.	.	.	.	.
.	ENST0000C	.	16q	.	.	.	.	.	.	.	.	.	.
.	ENST0000C	.	16q	.	.	.	Score	rs1469198	.	.	.	.	0.029
UNKNC	ENST0000C	.	16q	.	Scc	.	.	rs7733922	ID	.	.	.	.
.	ENST0000C	.	16q	.	.	.	Score	.	.	.	.	.	.
.	ENST0000C	.	16q	.	Scc	.	.	rs7483034		Likely	.	.	0.104
DNAAF	ENST0000C	.	16q	.	.	.	.	rs1407517	.	.	.	.	.
TAF1C	ENST0000C	.	16q	.	.	.	.	rs7647118	ID	.	.	.	.
GSE1:N	ENST0000C	.	16q	.	.	.	Score	rs9336865	ID	.	.	.	.
FOXC2	ENST0000C	.	16q	.	.	.	.	.	.	.	.	.	.
FBXO3	ENST0000C	.	16q	.	.	.	.	rs3681305	.	.	.	.	0.001
.	ENST0000C	.	16q	.	.	.	.	rs9665470	.	.	.	.	.
ZNF46	ENST0000C	.	16q	.	.	.	.	rs5606502	.	.	.	.	.
.	ENST0000C	.	16q	.	.	.	.	rs9646181	.	.	.	.	.
CBFA2	ENST0000C	.	16q	.	Scc	.	.	.	.	.	.	.	.
.	ENST0000C	.	16q	.	.	.	.	.	.	.	.	.	.
ZNF27	ENST0000C	.	16q	.	.	.	.	rs2017315	.	.	.	.	0.001
.	ENST0000C	.	16q	.	.	.	.	rs7530482	.	.	.	.	.
MYO1C	ENST0000C	.	17p	.	.	.	.	rs7627705	.	.	.	.	.
PRPF8	ENST0000C	.	17p	.	Scc	.	.	.	.	.	.	.	.
.	ENST0000C	.	17p	.	.	.	.	.	.	.	.	.	.
HIC1:N	ENST0000C	.	17p	.	Scc	.	.	.	.	.	.	.	.
.	ENST0000C	.	17p	.	.	.	.	.	.	.	.	.	.
SMG6:I	ENST0000C	.	17p	.	.	.	.	.	.	.	.	.	.
MNT:N	ENST0000C	.	17p	.	.	.	.	rs7803486	.	.	.	.	.
METTL	ENST0000C	.	17p	.	.	.	.	.	.	.	.	.	.
.	ENST0000C	.	17p	.	.	.	.	.	.	.	.	.	.
.	ENST0000C	.	17p	.	.	.	.	.	.	.	.	.	.
.	ENST0000C	.	17p	.	.	.	.	rs5506079	.	.	.	.	0.001

PELP1:	ENST0000C	.	17p	.	Scc	.	.	rs3764321	.	.	.	.	.
KIF1C:I	ENST0000C	.	17p	.	Scc	Score=0.9	.	rs2008227	ID	.	.	.	.
ZNF59	ENST0000C	.	17p	.	.	Score=0.9	.	rs2013516	ID	.	.	.	.
.	ENST0000C	.	17p	.	.	.	Score	.	.	.	.	.	.
KIAA07	ENST0000C	.	17p	.	.	.	.	.	.	.	.	.	.
.	ENST0000C	.	17p	.	.	.	.	.	.	.	.	.	.
.	ENST0000C	.	17p	.	.	.	Score	.	.	.	.	.	.
NEURL	ENST0000C	.	17p	.	Scc	.	.	rs2000463	.	.	.	.	.
POLR2	ENST0000C	.	17p	.	.	.	.	rs7455911	.	.	.	.	.
CCDC4	ENST0000C	.	17p	.	Scc	.	.	rs1473707	.	.	.	.	.
UNKNC	ENST0000C	.	17p	.	.	.	.	.	.	.	.	.	.
.	ENST0000C	.	17p	.	.	.	.	.	.	.	.	.	.
.	ENST0000C	.	17p	.	.	.	.	rs7534759	.	.	.	.	.
DNAH9	ENST0000C	.	17p	.	.	.	.	.	.	.	.	.	.
HS3ST	ENST0000C	.	17p	.	.	.	.	.	.	.	.	.	.
TEKT3:	ENST0000C	.	17p	.	.	.	.	rs7633797	.	.	.	.	.
.	ENST0000C	.	17p	.	.	Score=0.9	.	rs6207237	.	.	.	.	.
NCOR1	ENST0000C	.	17p	.	Scc	.	.	.	.	.	.	.	.
.	ENST0000C	.	17p	.	.	.	.	.	.	.	.	.	.
.	ENST0000C	.	17p	.	.	.	.	.	.	.	.	.	.
.	ENST0000C	.	17p	.	.	.	.	.	.	.	.	.	.
.	ENST0000C	.	17p	.	.	.	.	rs1157965	.	.	.	.	.
.	ENST0000C	.	17p	.	.	.	.	.	.	.	.	.	.
.	ENST0000C	.	17p	.	.	Score=0.9	.	.	.	.	.	.	.
LGALS	ENST0000C	.	17p	.	Scc	Score=0.9	.	.	.	.	.	.	.
.	ENST0000C	.	17p	.	.	Score=0.9	.	rs2869918	ID	.	.	.	0.654
.	ENST0000C	.	17p	.	.	.	.	rs7529523	.	.	.	.	.
.	ENST0000C	.	17p	.	.	.	.	.	.	.	.	.	.
.	ENST0000C	.	17q	.	.	Score=0.9	.	.	.	.	.	.	.
.	ENST0000C	.	17q	.	.	.	.	.	.	.	.	.	.
.	ENST0000C	.	17q	.	.	.	.	.	.	.	.	.	.
.	ENST0000C	.	17q	.	.	.	.	.	.	.	.	.	.
.	ENST0000C	.	17q	.	.	.	Score	.	.	.	.	.	.
.	ENST0000C	.	17q	.	.	.	.	rs1051245	.	.	.	.	.
.	ENST0000C	.	17q	.	.	.	.	.	.	.	.	.	.
SEZ6:N	ENST0000C	.	17q	.	.	.	.	.	.	.	.	.	.
MYO18	ENST0000C	.	17q	.	Scc	.	.	.	.	.	.	.	.
.	ENST0000C	.	17q	.	.	.	Score	rs7968757	ID	.	.	.	.
EVI2A:I	ENST0000C	.	17q	.	Scc	.	.	rs5324904	.	.	.	.	.
.	ENST0000C	.	17q	.	.	.	.	.	.	.	.	.	.
.	ENST0000C	.	17q	.	.	.	.	.	.	.	.	.	.
.	ENST0000C	.	17q	.	.	.	.	.	.	.	.	.	.
.	ENST0000C	.	17q	.	.	.	.	.	.	.	.	.	.
NLE1:N	ENST0000C	.	17q	.	Scc	.	.	.	.	.	.	.	.
SLFN1	ENST0000C	.	17q	.	.	Score=0.8	.	rs7610872	ID	.	.	.	.
.	ENST0000C	.	17q	.	.	Score=0.9	.	.	.	.	.	.	.
.	ENST0000C	.	17q	.	.	.	.	.	.	.	.	.	.
.	ENST0000C	.	17q	.	.	Score=0.9	.	rs3702689	.	.	.	.	.
.	ENST0000C	.	17q	.	.	Score=0.9	Score	rs3720623	.	.	.	.	.
.	ENST0000C	.	17q	.	.	Score=0.9	.	.	ID	.	.	.	.
PCGF2	ENST0000C	.	17q	.	.	.	.	rs7545869	.	.	.	.	.
.	ENST0000C	.	17q	.	.	.	Score	.	.	.	.	.	.
CACNE	ENST0000C	.	17q	.	.	.	.	rs7807350	.	.	.	.	.
.	ENST0000C	.	17q	.	.	.	.	.	.	.	.	.	.

.	ENST0000C	.	17q	.	.	.	.	.	.	.	.	.	.
ERBB2	ENST0000C	.	17q	.	.	.	.	rs1057519	ID	Likely	.	.	.
CCR7:I	ENST0000C	.	17q	.	.	.	.	rs1996216	.	.	.	.	.
KRT34:	ENST0000C	.	17q	.	.	.	.	rs1488746	ID	.	.	.	.
LEPRE	ENST0000C	.	17q	.	Scc	.	.	rs7824576	.	.	.	.	.
.	ENST0000C	.	17q	.	.	.	.	rs7827568	.	.	.	.	.
.	ENST0000C	.	17q	.	.	.	.	.	.	.	.	.	.
.	ENST0000C	.	17q	.	.	.	.	.	.	.	.	.	.
.	ENST0000C	.	17q	.	.	.	.	rs9065729	.	.	.	.	.
.	ENST0000C	.	17q	.	.	.	Score	rs5769204	.	.	.	.	0.003
.	ENST0000C	.	17q	.	.	.	.	.	.	.	.	.	.
.	ENST0000C	.	17q	.	.	.	.	rs1042659	.	.	.	.	.
.	ENST0000C	.	17q	.	.	Score=0.9(	.	rs9984123	.	.	.	.	.
.	ENST0000C	.	17q	.	.	.	.	.	.	.	.	.	.
.	ENST0000C	.	17q	.	.	.	.	.	.	.	.	.	.
MPP3:I	ENST0000C	.	17q	.	Scc	.	.	.	.	.	.	.	.
.	ENST0000C	.	17q	.	.	.	.	.	.	.	.	.	.
MPP3:I	ENST0000C	.	17q	.	Scc	.	.	rs7617924	.	.	.	.	.
C17orf1	ENST0000C	.	17q	.	Scc	.	.	.	.	.	.	.	.
.	ENST0000C	.	17q	.	.	.	.	rs9135110	.	.	.	.	.
.	ENST0000C	.	17q	.	.	.	.	.	.	.	.	.	.
FMNL1	ENST0000C	.	17q	.	Scc	.	.	rs7805429	.	.	.	.	.
.	ENST0000C	.	17q	.	.	Score=0.9(	.	.	.	.	.	.	.
.	ENST0000C	.	17q	.	.	Score=0.9ℓ	.	.	.	.	.	.	.
.	ENST0000C	.	17q	.	.	Score=0.9	.	.	.	.	.	.	.
.	ENST0000C	.	17q	.	.	.	.	rs1002396	.	.	.	.	.
.	ENST0000C	.	17q	.	.	Score=0.9ℓ	.	rs1056060	.	.	.	.	0.304
.	ENST0000C	.	17q	.	.	Score=0.9ℓ	.	rs2644320	ID	.	.	.	.
.	ENST0000C	.	17q	.	.	.	.	.	.	.	.	.	.
ABI3:N	ENST0000C	.	17q	.	.	.	.	rs3749157	.	.	.	.	.
ZNF65:	ENST0000C	.	17q	.	.	.	.	rs9379553	ID	.	.	.	.
.	ENST0000C	.	17q	.	.	.	.	.	.	.	.	.	.
SAMD1	ENST0000C	.	17q	.	Scc	.	.	rs7784106	.	.	.	.	.
.	ENST0000C	.	17q	.	.	.	Score	.	.	.	.	.	.
UNKNC	ENST0000C	.	17q	.	.	.	.	.	.	.	.	.	.
.	ENST0000C	.	17q	.	.	.	.	rs1029268	.	.	.	.	.
.	ENST0000C	.	17q	.	.	.	.	.	.	.	.	.	.
.	ENST0000C	.	17q	.	.	.	Score	rs3894278	ID	.	.	.	.
.	ENST0000C	.	17q	.	.	.	.	.	.	.	.	.	.
.	ENST0000C	.	17q	.	.	.	.	.	.	.	.	.	.
.	ENST0000C	.	17q	.	.	.	Score	rs3677870	.	.	.	.	.
.	ENST0000C	.	17q	.	.	Score=0.9	.	.	.	.	.	.	.
SCN4A	ENST0000C	.	17q	.	Scc	.	.	rs7637250	ID	Likely	.	.	.
.	ENST0000C	.	17q	.	.	Score=0.9(	.	.	.	.	.	.	.
HELZ:N	ENST0000C	.	17q	.	.	.	.	.	.	.	.	.	.
ABCA6	ENST0000C	.	17q	.	.	.	.	.	.	.	.	.	.
.	ENST0000C	.	17q	.	.	.	Score	.	.	.	.	.	.
.	.	.	17q	.	Scc	.	.	.	.	.	.	.	.
FDXR:I	ENST0000C	.	17q	.	Scc	.	.	rs5610655	.	.	.	.	.
.	ENST0000C	.	17q	.	.	.	.	rs1018051	.	.	.	.	.
.	ENST0000C	.	17q	.	.	.	Score	rs3777274	.	.	.	.	.
.	ENST0000C	.	17q	.	.	.	.	.	.	.	.	.	.
.	ENST0000C	.	17q	.	.	.	.	.	.	.	.	.	.
.	ENST0000C	.	17q	.	.	.	.	.	.	.	.	.	.

.	ENST0000	.	17q	.	.	.	.	.	.	.	.	.	.
CYGB:I	ENST0000	.	17q	.	Scc	.	.	rs3697050	.	.	.	.	.
.	ENST0000	.	17q	.	.	.	.	.	.	.	.	.	.
.	ENST0000	.	17q	.	.	.	.	.	.	.	.	.	.
.	ENST0000	.	17q	.	.	.	.	rs7670774	.	.	.	.	.
.	ENST0000	.	17q	.	.	.	.	.	.	.	.	.	.
DNAH1	ENST0000	.	17q	.	Scc	.	.	rs7521502	.	.	.	.	.
RBFOX	ENST0000	.	17q	.	.	.	.	.	.	.	.	.	.
CBX4:N	ENST0000	.	17q	.	Scc	.	.	.	.	.	.	.	.
CCDC4	ENST0000	.	17q	.	.	.	.	rs5354610	ID	.	.	.	.
.	ENST0000	.	17q	.	.	.	.	rs7737952	.	.	.	.	.
CARD1	ENST0000	.	17q	.	.	.	.	rs3676544	.	.	.	.	.
.	ENST0000	.	17q	.	.	.	.	.	.	.	.	.	.
.	ENST0000	.	17q	.	.	.	.	.	.	.	.	.	.
BAIAP2	ENST0000	.	17q	.	.	.	.	.	.	.	.	.	.
.	ENST0000	.	17q	.	.	.	.	.	.	.	.	.	.
.	ENST0000	.	17q	.	.	.	.	rs3678800	.	.	.	.	.
.	ENST0000	.	17q	.	.	.	.	.	.	.	.	.	.
OXLD1	ENST0000	.	17q	.	.	.	.	.	ID	.	.	.	.
.	ENST0000	.	17q	.	.	.	.	.	.	.	.	.	.
.	ENST0000	.	17q	.	.	.	.	.	.	.	.	.	.
FASN:N	ENST0000	.	17q	.	.	.	.	.	.	.	.	.	.
FASN:N	ENST0000	.	17q	.	.	.	.	rs7594547	ID	.	.	.	.
.	ENST0000	.	17q	.	.	.	.	.	.	.	.	.	.
WDR45	ENST0000	.	17q	.	Scc	.	.	rs1452537	ID	.	.	.	.
ZNF75(	ENST0000	.	17q	.	.	.	.	.	.	.	.	.	.
ADCYA	ENST0000	.	18p	.	.	.	.	.	.	.	.	.	.
.	ENST0000	.	18p	.	.	.	.	.	.	.	.	.	.
.	ENST0000	.	18p	.	.	.	.	.	.	.	.	.	.
.	ENST0000	.	18p	.	.	.	Score	.	ID	.	.	.	.
MC2R:I	ENST0000	.	18p	.	.	.	.	.	.	.	.	.	.
.	ENST0000	.	18p	.	.	Score=0.9	.	.	.	.	.	.	.
.	ENST0000	.	18p	.	.	Score=0.9	.	.	ID	.	.	.	.
GREB1	ENST0000	.	18q	.	.	.	.	.	.	.	.	.	.
.	ENST0000	.	18q	.	.	.	.	rs1390032	.	.	.	.	.
.	ENST0000	.	18q	.	.	.	Score	.	.	.	.	.	.
LAMA3	ENST0000	.	18q	.	.	.	.	rs5453420	.	.	.	.	0.001
.	ENST0000	.	18q	.	.	.	.	rs7793765	.	.	.	.	.
ELP2:N	ENST0000	.	18q	.	.	.	.	.	.	.	.	.	.
EPG5:N	ENST0000	.	18q	.	.	.	.	.	.	.	.	.	.
.	ENST0000	.	18q	.	.	.	.	rs7789211	.	.	.	.	.
.	ENST0000	.	18q	.	.	.	.	.	.	.	.	.	.
MYO5E	ENST0000	.	18q	.	.	.	.	rs7710491	.	.	.	.	.
.	ENST0000	.	18q	.	.	.	.	.	.	.	.	.	.
.	ENST0000	.	18q	.	.	Score=0.9:	.	.	.	.	.	.	.
ZCCHC	ENST0000	.	18q	.	.	.	.	.	.	.	.	.	.
SERPII	ENST0000	.	18q	.	.	.	.	rs1417032	.	.	.	.	.
SERPII	ENST0000	.	18q	.	.	Score=0.9:	.	.	.	.	.	.	.
RTTN:I	ENST0000	.	18q	.	.	.	.	.	ID	.	.	.	.
ZNF40	ENST0000	.	18q	.	.	.	.	.	.	.	.	.	.
.	ENST0000	.	18q	.	.	.	.	.	.	.	.	.	.
.	ENST0000	.	19p	.	.	.	.	rs1020066	.	.	.	.	.
.	ENST0000	.	19p	.	.	.	.	.	ID	.	.	.	.
.	ENST0000	.	19p	.	.	.	.	rs3749392	.	.	.	.	.

POLRM	ENST0000	.	19p	.	.	.	.	rs7789396	.	.	.	.	.
PALM:I	ENST0000	.	19p	.	.	.	.	.	.	.	.	.	.
.	ENST0000	.	19p	.	.	.	.	rs3777310	.	.	.	.	0.001
TMEM2	ENST0000	.	19p	.	Scc	.	.	.	.	.	.	.	.
HMHA1	ENST0000	.	19p	.	.	.	.	rs9456811	.	.	.	.	.
.	ENST0000	.	19p	.	.	.	.	.	.	.	.	.	.
UNKNC	ENST0000	.	19p	.	.	.	.	rs7620114	ID	.	.	.	.
SBNO2	ENST0000	.	19p	.	Scc	.	.	rs2022226	.	.	.	.	.
.	ENST0000	.	19p	.	.	.	.	.	.	.	.	.	.
.	ENST0000	.	19p	.	.	.	Score	rs7799382	.	.	.	.	.
SBNO2	ENST0000	.	19p	.	.	.	.	.	.	.	.	.	.
.	ENST0000	.	19p	.	.	.	.	.	.	.	.	.	.
CIRBP:	ENST0000	.	19p	.	.	.	.	rs3753640	.	.	.	.	.
.	ENST0000	.	19p	.	.	.	Score	.	.	.	.	.	.
.	ENST0000	.	19p	.	.	.	.	.	.	.	.	.	.
PLK5:N	ENST0000	.	19p	.	.	.	.	rs1007918	.	.	.	.	.
DOT1L	ENST0000	.	19p	.	Scc	.	.	rs3694032	.	.	.	.	.
.	ENST0000	.	19p	.	.	.	.	rs7522031	ID	.	.	.	.
SGTA:I	ENST0000	.	19p	.	.	.	.	.	ID	.	.	.	.
.	ENST0000	.	19p	.	.	.	.	.	.	.	.	.	.
S1PR4	ENST0000	.	19p	.	Scc	.	.	rs3763485	.	.	.	.	.
.	ENST0000	.	19p	.	.	.	.	rs7773785	.	.	.	.	.
.	ENST0000	.	19p	.	.	.	Score	.	.	.	.	.	.
.	ENST0000	.	19p	.	.	.	Score	.	.	.	.	.	.
ANKRD	ENST0000	.	19p	.	.	.	.	rs7591374	.	.	.	.	.
SH3GL	ENST0000	.	19p	.	.	.	.	.	ID	.	.	.	.
LRG1:N	ENST0000	.	19p	.	.	.	.	rs1503658	.	.	.	.	.
SEMA6	ENST0000	.	19p	.	.	.	Score	.	.	.	.	.	.
KDM4E	ENST0000	.	19p	.	.	.	.	.	.	.	.	.	.
.	ENST0000	.	19p	.	.	.	.	.	.	.	.	.	.
.	ENST0000	.	19p	.	.	.	.	rs1018214	.	.	.	.	.
UNKNC	ENST0000	.	19p	.	.	.	.	rs7632840	ID	.	.	.	.
.	ENST0000	.	19p	.	.	.	Score	.	.	.	.	.	.
ARHGE	ENST0000	.	19p	.	.	.	.	rs1390681	.	.	.	.	.
.	ENST0000	.	19p	.	.	.	.	rs3770694	.	.	.	.	.
XAB2:N	ENST0000	.	19p	.	.	.	.	rs3745730	.	.	.	.	.
XAB2:N	ENST0000	.	19p	.	.	.	.	rs7516930	.	.	.	.	.
STXBP	ENST0000	.	19p	.	.	.	.	rs1417170	ID	.	.	.	.
.	ENST0000	.	19p	.	.	Score=0.8	.	.	.	.	.	.	.
.	ENST0000	.	19p	.	.	.	.	.	.	.	.	.	.
.	ENST0000	.	19p	.	.	.	.	rs7595385	.	.	.	.	.
FBN3:N	ENST0000	.	19p	.	.	.	.	rs3527749	ID	.	.	.	0.005
.	ENST0000	.	19p	.	.	.	.	rs7615602	.	.	.	.	.
.	ENST0000	.	19p	.	.	.	.	rs5308100	.	.	.	.	0.007
ACTL9	ENST0000	.	19p	.	.	.	.	.	ID	.	.	.	.
.	.	.	19p	.	.	Score=0.9	Score	rs3694972	.	.	.	.	.
.	.	.	19p	.	.	Score=0.9	Score	rs3751712	.	.	.	.	.
MUC16	ENST0000	.	19p	.	.	Score=0.9	.	rs5686926	.	.	.	.	0.001
MUC16	ENST0000	.	19p	.	.	.	.	rs7801357	.	.	.	.	.
MUC16	ENST0000	.	19p	.	.	.	.	.	.	.	.	.	.
.	ENST0000	.	19p	.	.	.	.	.	.	.	.	.	.
PIN1:N	ENST0000	.	19p	.	Scc	.	.	rs7564749	.	.	.	.	.
OLFM2	ENST0000	.	19p	.	Scc	.	.	rs7814402	ID	.	.	.	.
C19orf	ENST0000	.	19p	.	.	.	.	.	.	.	.	.	.

PPAN-I	ENST0000C	.	19p	.	Scc	.	.	.	.	.	.	.	.
.	ENST0000C	.	19p	.	.	.	.	.	.	.	.	.	.
ICAM1:	ENST0000C	.	19p	.	.	.	.	.	.	.	.	.	.
.	ENST0000C	.	19p	.	.	.	.	.	.	.	.	.	.
TYK2:N	ENST0000C	.	19p	.	Scc	.	.	rs1383503	ID	.	.	.	.
PDE4A	ENST0000C	.	19p	.	.	.	.	rs2009930	.	.	.	.	.
.	ENST0000C	.	19p	.	.	.	Score	.	.	.	.	.	.
KRI1:N	ENST0000C	.	19p	.	.	.	.	.	.	.	.	.	.
LDLR:N	ENST0000C	.	19p	.	Scc	.	.	rs7634492	ID	.	.	.	.
.	ENST0000C	.	19p	.	.	.	.	rs3737274	ID	.	.	.	.
DOCK6	ENST0000C	.	19p	.	.	.	.	rs3721772	.	.	.	.	.
.	ENST0000C	.	19p	.	.	.	.	.	.	.	.	.	.
.	ENST0000C	.	19p	.	.	.	Score	rs5385135	ID	.	.	.	.
.	ENST0000C	.	19p	.	.	.	Score	.	.	.	.	.	.
DAND5	ENST0000C	.	19p	.	Scc	.	.	rs3710318	.	.	.	.	.
RFX1:N	ENST0000C	.	19p	.	Scc	.	.	.	ID	.	.	.	.
PRKAC	ENST0000C	.	19p	.	Scc	.	.	.	.	.	.	.	.
.	ENST0000C	.	19p	.	Scc	.	Score	.	.	.	.	.	.
OR10H	ENST0000C	.	19p	.	.	Score=0.9	.	.	ID	.	.	.	.
KLF2:N	ENST0000C	.	19p	.	.	.	.	.	.	.	.	.	.
.	ENST0000C	.	19p	.	.	.	.	rs1015670	.	.	.	.	.
.	ENST0000C	.	19p	.	.	.	.	.	.	.	.	.	.
.	ENST0000C	.	19p	.	.	.	Score	rs1138422	.	.	.	.	.
.	ENST0000C	.	19p	.	.	.	Score	.	.	.	.	.	.
USHBP	ENST0000C	.	19p	.	.	.	.	rs9290443	ID	.	.	.	.
GTPBP	ENST0000C	.	19p	.	.	.	.	rs7698270	.	.	.	.	.
UNC13	ENST0000C	.	19p	.	.	.	.	rs1043222	.	.	.	.	.
.	ENST0000C	.	19p	.	.	.	Score	.	.	.	.	.	.
.	ENST0000C	.	19p	.	.	.	.	.	.	.	.	.	.
PDE4C	ENST0000C	.	19p	.	.	.	.	.	.	.	.	.	.
UPF1:N	ENST0000C	.	19p	.	.	.	.	rs3719294	.	.	.	.	.
MAU2:I	ENST0000C	.	19p	.	.	.	.	rs2012753	.	.	.	.	.
ZNF90:	ENST0000C	.	19p	.	.	.	.	.	.	.	.	.	.
.	ENST0000C	.	19p	.	.	Score=0.9	.	.	.	.	.	.	.
.	ENST0000C	.	19p	.	.	Score=0.9	.	.	.	.	.	.	.
.	ENST0000C	.	19q	.	.	.	Score	.	.	.	.	.	.
TSHZ3	ENST0000C	.	19q	.	Scc	.	.	rs7540203	ID	.	.	.	.
KIAA03	ENST0000C	.	19q	.	Scc	.	.	rs3754283	.	.	.	.	.
.	ENST0000C	.	19q	.	.	.	.	.	.	.	.	.	.
.	ENST0000C	.	19q	.	.	.	Score	.	.	.	.	.	.
RBM42	ENST0000C	.	19q	.	Scc	.	.	rs7656023	.	.	.	.	.
.	ENST0000C	.	19q	.	.	.	.	.	.	.	.	.	.
.	ENST0000C	.	19q	.	.	.	.	.	.	.	.	.	.
.	ENST0000C	.	19q	.	.	.	Score	rs5581240	.	.	.	.	.
.	.	.	19q	.	.	Score=0.9	Score	.	.	.	.	.	.
.	.	.	19q	.	.	Score=0.9	.	rs4806419	.	.	.	.	.
.	.	.	19q	.	.	Score=0.9	Score	rs7125437	.	.	.	.	.
.	.	.	19q	.	.	Score=0.9	Score	.	.	.	.	.	.
.	.	.	19q	.	.	.	Score	rs7728313	.	.	.	.	.
UNKNO	ENST0000C	.	19q	.	Scc	.	.	.	.	.	.	.	.
CATSP	ENST0000C	.	19q	.	.	.	.	rs1476199	ID	.	.	.	.
.	ENST0000C	.	19q	.	.	.	Score	.	.	.	.	.	.
.	ENST0000C	.	19q	.	.	.	.	rs3756003	.	.	.	.	0.001
.	ENST0000C	.	19q	.	.	.	Score	rs5371432	.	.	.	.	0.003

.	ENST0000	.	19q	.	.	.	.	.	.	.	.	.	.
.	ENST0000	.	19q	.	.	.	.	.	.	.	.	.	.
.	ENST0000	.	19q	.	.	.	Score	rs5721273	.	.	.	.	0.008
UNKNO	ENST0000	.	19q	.	.	Score=0.9	.	rs8917290	.	.	.	.	.
.	ENST0000	.	19q	.	.	Score=0.9	.	.	.	.	.	.	.
.	ENST0000	.	19q	.	.	.	.	rs7564368	ID	.	.	.	.
UNKNO	ENST0000	.	19q	.	Scc	.	.	rs7738579	ID	.	.	.	.
.	ENST0000	.	19q	.	.	Score=0.9	.	.	.	.	.	.	.
SHKBP	ENST0000	.	19q	.	Scc	.	.	rs7469641	.	.	.	.	.
.	ENST0000	.	19q	.	.	.	Score	.	.	.	.	.	.
.	ENST0000	.	19q	.	.	.	.	.	.	.	.	.	.
UNKNO	ENST0000	.	19q	.	.	.	.	.	.	.	.	.	.
UNKNO	ENST0000	.	19q	.	Scc	.	.	.	.	.	.	.	.
.	ENST0000	.	19q	.	.	.	.	.	.	.	.	.	.
C19orf5	ENST0000	.	19q	.	.	.	.	rs1045293	.	.	.	.	.
UNKNO	ENST0000	.	19q	.	.	.	.	.	.	.	.	.	.
TGFB1	ENST0000	.	19q	.	.	.	.	.	.	.	.	.	.
ARHGE	ENST0000	.	19q	.	.	.	.	.	.	.	.	.	.
MEGF8	ENST0000	.	19q	.	Scc	.	.	.	.	.	.	.	.
CEACA	ENST0000	.	19q	.	.	.	.	.	.	.	.	.	.
.	ENST0000	.	19q	.	.	Score=0.9	.	.	.	.	.	.	.
.	ENST0000	.	19q	.	.	Score=0.9	.	.	.	.	.	.	.
IRGQ:N	ENST0000	.	19q	.	.	.	.	.	.	.	.	.	.
.	ENST0000	.	19q	.	.	.	.	.	.	.	.	.	.
ZNF28	ENST0000	.	19q	.	.	Score=0.9	.	.	.	.	.	.	.
UNKNO	ENST0000	.	19q	.	.	.	.	rs7737032	ID	.	.	.	.
.	ENST0000	.	19q	.	.	.	.	rs9075637	.	.	.	.	.
.	ENST0000	.	19q	.	.	.	.	.	.	.	.	.	.
.	ENST0000	.	19q	.	.	.	Score	rs5632226	.	.	.	.	.
BCL3:N	ENST0000	.	19q	.	.	.	.	.	.	.	.	.	.
BCAM:	ENST0000	.	19q	.	.	.	.	.	.	.	.	.	.
.	ENST0000	.	19q	.	.	.	.	.	.	.	.	.	.
GPR4:N	ENST0000	.	19q	.	Scc	.	.	.	.	.	.	.	.
PRKD2	ENST0000	.	19q	.	.	.	.	rs1026645	.	.	.	.	.
STRN4	ENST0000	.	19q	.	Scc	.	.	rs3697415	ID	.	.	.	.
.	ENST0000	.	19q	.	.	.	Score	.	.	.	.	.	.
.	ENST0000	.	19q	.	.	.	.	.	.	.	.	.	.
.	ENST0000	.	19q	.	.	.	.	.	.	.	.	.	.
.	.	.	19q	.	.	Score=0.9	Score	rs6213000	.	.	.	.	.
SPHK2	ENST0000	.	19q	.	.	.	.	.	.	.	.	.	.
.	ENST0000	.	19q	.	.	.	.	rs1012379	ID	.	.	.	.
.	ENST0000	.	19q	.	.	.	.	.	.	.	.	.	.
.	ENST0000	.	19q	.	.	.	.	.	.	.	.	.	.
.	ENST0000	.	19q	.	.	.	.	rs5626072	.	.	.	.	.
.	ENST0000	.	19q	.	.	.	.	.	.	.	.	.	.
.	ENST0000	.	19q	.	.	.	.	.	.	.	.	.	.
.	ENST0000	.	19q	.	.	.	.	rs7508901	.	.	.	.	.
MYH14	ENST0000	.	19q	.	.	.	.	rs3749564	ID	.	.	.	.
.	ENST0000	.	19q	.	.	.	.	rs2001122	.	.	.	.	0.001
.	ENST0000	.	19q	.	.	.	.	.	.	.	.	.	.
.	ENST0000	.	19q	.	.	.	.	rs5643823	.	.	.	.	.
.	ENST0000	.	19q	.	.	.	.	rs3695357	.	Likely	.	.	.
.	ENST0000	.	19q	.	.	.	.	.	.	.	.	.	.
MYBPC	ENST0000	.	19q	.	.	.	.	rs7738602	ID	.	.	.	.

.	ENST0000	.	19q	.	.	.	.	.	.	.	.	.	.
GPR32	ENST0000	.	19q	.	.	Score=0.9	.	.	.	.	.	.	.
ZNF81	ENST0000	.	19q	.	.	.	.	.	.	.	.	.	.
ERVV-	ENST0000	.	19q	.	.	.	Score	.	.	.	.	.	.
VN1R4	ENST0000	.	19q	.	.	.	.	rs1420234	ID	.	.	.	.
.	ENST0000	hsa-mir-52	19q	.	.	.	.	.	.	.	.	.	.
CACN	ENST0000	.	19q	.	.	.	.	.	.	.	.	.	.
.	ENST0000	.	19q	.	.	.	.	.	.	.	.	.	.
.	ENST0000	.	19q	.	.	.	.	.	.	.	.	.	.
.	ENST0000	.	19q	.	.	Score=0.9	.	rs3704562	.	.	.	.	.
.	ENST0000	.	19q	.	.	.	.	.	.	.	.	.	.
.	ENST0000	.	19q	.	.	.	.	rs7529171	.	.	.	.	.
.	ENST0000	.	19q	.	.	.	.	.	.	.	.	.	.
TMEM1	ENST0000	.	19q	.	.	.	.	rs3707427	.	.	.	.	.
NLRP1	ENST0000	.	19q	.	.	.	.	rs2000514	.	.	.	.	.
PEG3:	ENST0000	.	19q	.	.	.	.	rs7704358	ID	.	.	.	.
.	ENST0000	.	19q	.	.	Score=0.9	.	.	.	.	.	.	.
.	ENST0000	.	19q	.	.	.	.	.	.	.	.	.	.
ZNF60	ENST0000	.	19q	.	.	.	.	rs1474253	.	.	.	.	.
.	ENST0000	.	19q	.	.	.	.	.	.	.	.	.	.
.	ENST0000	.	19q	.	.	.	.	rs3733248	.	.	.	.	.
SIRPA:	ENST0000	.	20p	.	.	Score=0.9	.	rs1021538	ID	.	.	.	.
.	ENST0000	.	20p	.	.	.	.	rs7627114	.	.	.	.	.
.	ENST0000	.	20p	.	.	.	.	.	.	.	.	.	.
.	ENST0000	.	20p	.	.	.	Score	rs8672082	.	.	.	.	.
.	ENST0000	.	20p	.	.	.	.	.	.	.	.	.	.
ADAM3	ENST0000	.	20p	.	.	.	.	rs7638624	.	.	.	.	.
.	ENST0000	.	20p	.	.	.	.	.	.	.	.	.	.
.	ENST0000	.	20p	.	.	.	Score	rs1032256	.	.	.	.	.
.	ENST0000	.	20p	.	.	.	.	.	.	.	.	.	.
CSRP2	ENST0000	.	20p	.	.	.	.	.	.	.	.	.	.
.	ENST0000	.	20p	.	.	.	Score	.	.	.	.	.	.
CST4:	ENST0000	.	20p	.	.	Score=0.9	.	rs1509219	ID	.	.	.	.
.	ENST0000	.	20p	.	.	.	.	.	.	.	.	.	.
.	ENST0000	.	20p	.	.	Score=0.9	.	.	ID	.	.	.	.
.	ENST0000	.	20q	.	.	Score=0.9	.	.	ID	.	.	.	.
.	ENST0000	.	20q	.	.	Score=0.9	.	.	ID	.	.	.	.
NOL4L	ENST0000	.	20q	.	.	.	.	rs1845841	ID	.	.	.	0.001
.	ENST0000	.	20q	.	.	.	.	.	.	.	.	.	.
.	ENST0000	.	20q	.	.	.	.	.	.	.	.	.	.
.	ENST0000	.	20q	.	.	.	Score	.	.	.	.	.	.
.	ENST0000	.	20q	.	.	.	.	rs7460506	ID	.	.	.	0.001
.	ENST0000	.	20q	.	.	.	.	.	.	.	.	.	.
NFS1:	ENST0000	.	20q	.	Scc	.	.	rs1480471	.	.	.	.	.
.	ENST0000	.	20q	.	.	.	Score	rs5431099	ID	.	.	.	.
.	ENST0000	.	20q	.	.	.	.	.	.	.	.	.	.
.	ENST0000	.	20q	.	.	.	.	.	.	.	.	.	.
.	ENST0000	.	20q	.	Scc	.	Score	rs8798536	.	.	.	.	.
.	ENST0000	.	20q	.	.	.	.	.	.	.	.	.	.
.	ENST0000	.	20q	.	.	.	.	rs9848856	.	.	.	.	.
DHX35	ENST0000	.	20q	.	Scc	.	.	rs9460302	.	.	.	.	.
.	ENST0000	.	20q	.	.	.	.	rs3711048	.	.	.	.	0.001
TOP1:	ENST0000	.	20q	.	.	.	.	.	.	.	.	.	.
PLCG1	ENST0000	.	20q	.	Scc	.	.	.	.	.	.	.	.

PTPRT	ENST000C	.	20q	.	.	.	.	.	.	.	.	.	.
PTPRT	ENST000C	.	20q	.	.	.	.	rs3761522	ID	.	.	.	.
.	ENST000C	.	20q	.	.	.	.	.	.	.	.	.	.
.	ENST000C	.	20q	.	.	.	.	.	.	.	.	.	.
.	ENST000C	.	20q	.	.	.	Score	.	.	.	.	.	.
.	ENST000C	.	20q	.	.	.	.	.	.	.	.	.	.
.	ENST000C	.	20q	.	.	.	.	.	.	.	.	.	.
ZMYND	ENST000C	.	20q	.	Scc	.	.	.	ID	.	.	.	.
KCNG1	ENST000C	.	20q	.	.	.	.	.	ID	.	.	.	.
.	ENST000C	.	20q	.	.	.	.	.	.	.	.	.	.
.	ENST000C	.	20q	.	.	.	.	.	.	.	.	.	.
ZNF21	ENST000C	.	20q	.	.	.	.	rs8891302	.	.	.	.	.
.	.	.	20q	.	.	.	Score	rs8669651	.	.	.	.	.
GNAS:I	ENST000C	.	20q	.	.	.	.	.	.	.	.	.	.
GNAS:I	ENST000C	.	20q	.	.	.	.	.	.	.	.	.	.
.	ENST000C	.	20q	.	.	.	.	.	.	.	.	.	.
LSM14	ENST000C	.	20q	.	.	.	.	rs7702464	.	.	.	.	.
.	ENST000C	.	20q	.	.	.	.	rs7787907	.	.	.	.	.
.	ENST000C	.	20q	.	.	.	.	rs1813756	.	.	.	.	0.002
ARFGA	ENST000C	.	20q	.	Scc	.	.	rs1435421	.	.	.	.	.
.	ENST000C	.	20q	.	.	.	.	.	.	.	.	.	.
.	ENST000C	.	20q	.	.	.	.	.	.	.	.	.	.
ABHD1	ENST000C	.	20q	.	.	.	.	rs5462318	ID	.	.	.	.
.	ENST000C	.	21p	.	.	Score=0.9	.	rs7962225	.	.	.	.	.
.	.	.	21p	.	.	Score=0.9	.	.	.	.	.	.	.
.	.	.	21p	.	.	Score=0.9	Score	.	.	.	.	.	.
.	.	.	21p	.	.	Score=0.9	Score	.	.	.	.	.	.
.	ENST000C	.	21p	.	.	Score=0.9	Score	rs8680295	.	.	.	.	.
.	ENST000C	.	21p	.	.	Score=0.9	Score	rs3711086	.	.	.	.	.
.	.	.	21p	.	.	Score=0.9	.	rs3775054	ID	.	.	.	.
.	ENST000C	.	21p	.	.	Score=0.9	Score	.	.	.	.	.	.
UNKNC	ENST000C	.	21p	.	.	Score=0.9	.	.	.	.	.	.	.
.	ENST000C	.	21p	.	.	Score=0.9	.	rs5282430	.	.	.	.	.
.	.	.	21p	.	.	Score=0.9	Score	.	.	.	.	.	.
.	.	.	21p	.	.	Score=0.9	Score	rs665796	.	.	.	.	.
.	.	.	21p	.	.	Score=0.9	Score	rs6582845	ID	.	.	.	.
.	ENST000C	.	21p	.	.	Score=0.9	.	rs915519	ID	.	.	.	.
.	ENST000C	.	21q	.	.	Score=0.9	Score	.	.	.	.	.	.
.	ENST000C	.	21q	.	.	Score=0.9	.	.	ID	.	.	.	.
.	ENST000C	.	21q	.	.	.	Score	.	.	.	.	.	.
.	ENST000C	.	21q	.	.	.	.	.	.	.	.	.	.
.	ENST000C	.	21q	.	.	.	.	.	.	.	.	.	.
.	ENST000C	.	21q	.	.	.	.	rs9172671	.	.	.	.	.
KRTAP	ENST000C	.	21q	.	.	.	.	.	.	.	.	.	.
.	ENST000C	.	21q	.	.	.	Score	.	.	.	.	.	.
.	ENST000C	.	21q	.	.	.	.	.	.	.	.	.	.
SYNJ1:	ENST000C	.	21q	.	.	.	.	rs1034062	.	.	.	.	.
OLIG1:	ENST000C	.	21q	.	Scc	.	Score	.	.	.	.	.	.
.	ENST000C	.	21q	.	.	.	.	rs7587939	.	.	.	.	.
.	ENST000C	.	21q	.	.	.	.	.	.	.	.	.	.
.	ENST000C	.	21q	.	.	.	Score	.	.	.	.	.	.
.	ENST000C	.	21q	.	.	.	.	.	.	.	.	.	.
.	ENST000C	.	21q	.	Scc	.	Score	rs5585229	.	.	.	.	.
.	ENST000C	.	21q	.	.	.	.	rs7591033	.	.	.	.	.

BRWD	ENST000C	.	21q	.	.	.	.	rs7585989	ID	.	.	.	.
.	ENST000C	.	21q	.	.	.	.	.	.	.	.	.	.
DSCAN	ENST000C	.	21q	.	Scc	.	.	.	.	.	.	.	.
DSCAN	ENST000C	.	21q	.	Scc	.	.	.	.	.	.	.	.
DSCAN	ENST000C	.	21q	.	.	.	.	.	ID	.	.	.	.
DSCAN	ENST000C	.	21q	.	.	.	.	.	.	.	.	.	.
.	ENST000C	.	21q	.	.	.	.	.	.	.	.	.	.
.	ENST000C	.	21q	.	.	.	.	.	.	.	.	.	.
.	ENST000C	.	21q	.	.	.	Score	rs9464977	.	.	.	.	.
ZBTB2	ENST000C	.	21q	.	Scc	.	.	.	.	.	.	.	.
.	ENST000C	.	21q	.	.	.	.	.	.	.	.	.	.
.	ENST000C	.	21q	.	.	.	.	rs1014361	.	.	.	.	.
.	ENST000C	.	21q	.	.	.	.	rs7806447	.	.	.	.	.
PWP2:	ENST000C	.	21q	.	Scc	.	.	rs5662670	.	.	.	.	.
.	ENST000C	.	21q	.	.	.	.	rs9852755	.	.	.	.	.
ITGB2:	ENST000C	.	21q	.	Scc	.	.	rs7565454	.	.	.	.	.
SLC19	ENST000C	.	21q	.	.	.	.	rs7582548	ID	.	.	.	.
.	ENST000C	.	21q	.	.	.	.	rs3771590	.	.	.	.	.
.	ENST000C	.	21q	.	.	.	.	.	.	.	.	.	.
.	ENST000C	.	21q	.	.	.	.	.	.	.	.	.	.
PCNT:	ENST000C	.	21q	.	.	.	.	rs7517144	.	.	.	.	.
DIP2A:	ENST000C	.	21q	.	.	.	.	.	.	.	.	.	.
.	ENST000C	.	22q	.	.	Score=0.9	Score	rs2845112	ID	.	.	.	.
.	ENST000C	.	22q	.	.	Score=0.9	.	rs8790424	.	.	.	.	.
.	ENST000C	.	22q	.	.	Score=0.9	.	.	.	.	.	.	.
.	ENST000C	.	22q	.	.	Score=0.9	.	rs1248470	ID	.	.	.	.
.	ENST000C	.	22q	.	.	.	.	.	.	.	.	.	.
DGCR	ENST000C	.	22q	.	.	.	.	rs5555078	.	.	.	.	.
.	ENST000C	.	22q	.	.	.	Score	.	.	.	.	.	.
.	ENST000C	.	22q	.	.	Score=0.9	.	rs1899538	.	.	.	.	.
.	ENST000C	.	22q	.	.	Score=0.9	.	.	.	.	.	.	.
.	ENST000C	.	22q	.	.	.	.	.	.	.	.	.	.
PI4KA:	ENST000C	.	22q	.	Scc	Score=0.9	.	rs3714923	.	.	.	.	.
HIC2:N	ENST000C	.	22q	.	.	.	.	rs7724279	.	.	.	.	.
.	ENST000C	.	22q	.	.	Score=0.9	.	.	.	.	.	.	.
.	ENST000C	.	22q	.	.	.	.	rs3777336	.	.	.	.	.
.	ENST000C	.	22q	.	.	.	.	rs3681295	.	.	.	.	0.001
SUSD2	ENST000C	.	22q	.	.	.	.	.	.	.	.	.	.
ADOR	ENST000C	.	22q	.	.	.	.	.	.	.	.	.	.
.	ENST000C	.	22q	.	.	.	.	.	.	.	.	.	.
GGT1:	ENST000C	.	22q	.	.	Score=0.9	.	rs7723316	.	.	.	.	.
.	ENST000C	.	22q	.	.	Score=0.9	.	.	.	.	.	.	.
.	ENST000C	.	22q	.	.	.	.	.	.	.	.	.	.
.	ENST000C	.	22q	.	.	.	.	rs1925641	.	.	.	.	.
.	ENST000C	.	22q	.	.	.	.	.	.	.	.	.	.
.	ENST000C	.	22q	.	.	.	.	.	.	.	.	.	.
.	ENST000C	.	22q	.	.	.	.	rs5710568	.	.	.	.	.
.	ENST000C	.	22q	.	.	.	.	rs1015431	.	.	.	.	.
SFI1:N	ENST000C	.	22q	.	.	.	.	.	.	.	.	.	.
.	.	.	22q	.	.	Score=0.9	.	.	.	.	.	.	.
.	ENST000C	.	22q	.	.	.	.	.	.	.	.	.	.
.	ENST000C	.	22q	.	.	.	.	.	.	.	.	.	.
MCM5:	ENST000C	.	22q	.	.	.	.	rs1379196	ID	.	.	.	.
.	ENST000C	.	22q	.	.	.	.	.	.	.	.	.	.

CSF2R	ENST0000	.	22q	.	.	.	.	rs3682304	.	.	.	.	0.001
.	ENST0000	.	22q	.	.	.	.	rs5367410	.	.	.	.	.
PLA2G	ENST0000	.	22q	.	Scc	.	.	.	.	.	.	.	.
.	ENST0000	.	22q	.	.	.	.	.	.	.	.	.	.
.	ENST0000	.	22q	.	.	.	.	rs7523833	.	.	.	.	.
KCNJ4	ENST0000	.	22q	.	Scc	.	.	.	ID	.	.	.	.
KCNJ4	ENST0000	.	22q	.	Scc	.	.	.	ID	.	.	.	.
.	ENST0000	.	22q	.	.	.	.	rs5714435	.	.	.	.	0.001
APOBE	ENST0000	.	22q	.	.	Score=0.9	.	.	.	.	.	.	.
.	ENST0000	.	22q	.	.	.	Score	rs3719126	.	.	.	.	0.005
TNRC6	ENST0000	.	22q	.	.	.	.	.	.	.	.	.	.
MCHR1	ENST0000	.	22q	.	.	.	.	rs2016059	.	.	.	.	.
POLR3	ENST0000	.	22q	.	Scc	.	.	.	.	.	.	.	.
POLR3	ENST0000	.	22q	.	.	.	.	rs1838128	.	.	.	.	.
.	ENST0000	.	22q	.	.	.	.	.	.	.	.	.	.
.	ENST0000	.	22q	.	.	Score=0.9	.	.	.	.	.	.	.
.	ENST0000	.	22q	.	.	.	.	rs1916812	ID	.	.	.	.
.	ENST0000	.	22q	.	.	.	.	.	.	.	.	.	.
.	ENST0000	.	22q	.	Scc	.	.	rs5584935	.	.	.	.	0.001
.	ENST0000	.	22q	.	.	.	.	rs1305515	ID	.	.	.	.
.	ENST0000	.	22q	.	.	.	.	.	.	.	.	.	.
.	ENST0000	.	22q	.	.	.	.	.	.	.	.	.	.
.	ENST0000	.	22q	.	.	.	.	.	.	.	.	.	.
PKDRE	ENST0000	.	22q	.	.	.	.	.	.	.	.	.	.
CELSR	ENST0000	.	22q	.	.	.	.	.	.	.	.	.	.
.	ENST0000	.	22q	.	.	.	.	.	.	.	.	.	.
.	ENST0000	.	22q	.	.	.	.	.	.	.	.	.	.
MOV10	ENST0000	.	22q	.	Scc	.	.	rs5595729	.	.	.	.	.
.	ENST0000	.	22q	.	.	.	.	.	.	.	.	.	.
PANX2	ENST0000	.	22q	.	Scc	.	.	.	.	.	.	.	.
PLXNB	ENST0000	.	22q	.	.	.	.	rs3705843	.	.	.	.	0.001
.	ENST0000	.	22q	.	.	Score=0.8	.	.	.	.	.	.	.
NCAPH	ENST0000	.	22q	.	Scc	.	.	.	ID	.	.	.	.
.	ENST0000	.	22q	.	.	.	.	rs9805300	.	.	.	.	.
.	ENST0000	.	Xp2	.	.	Score=0.9	.	.	.	.	.	.	.
.	ENST0000	.	Xp2	.	.	Score=0.9	.	.	.	.	.	.	.
.	ENST0000	.	Xp2	.	.	Score=0.9	.	.	.	.	.	.	.
.	ENST0000	.	Xp2	.	.	.	Score	.	.	.	.	.	.
LOC10	ENST0000	.	Xp2	.	.	.	.	.	.	.	.	.	.
.	ENST0000	.	Xp2	.	.	.	.	.	.	.	.	.	.
.	ENST0000	.	Xp2	.	.	.	.	.	.	.	.	.	.
NHS:N	ENST0000	.	Xp2	.	Scc	.	.	.	.	.	.	.	.
.	ENST0000	.	Xp2	.	Scc	.	.	.	.	.	.	.	.
APOO:	ENST0000	.	Xp2	.	.	.	.	rs3730298	.	.	.	.	.
ARX:N	ENST0000	.	Xp2	.	.	.	.	.	.	.	.	.	.
.	ENST0000	.	Xp2	.	.	.	Score	.	.	.	.	.	.
.	ENST0000	.	Xp2	.	.	.	.	.	.	.	.	.	.
.	ENST0000	.	Xp2	.	.	.	.	.	.	.	.	.	.
MAGEE	ENST0000	.	Xp2	.	.	.	.	.	.	.	.	.	.
.	ENST0000	.	Xp2	.	.	.	.	.	.	.	.	.	.
.	ENST0000	.	Xp2	.	.	.	.	.	.	.	.	.	.
.	ENST0000	.	Xp2	.	.	.	.	.	.	.	.	.	.
.	ENST0000	.	Xp2	.	.	.	.	.	.	.	.	.	.
.	ENST0000	.	Xp1	.	.	.	.	.	.	.	.	.	.

CXorf3	ENST000C	.	Xp1	.	Scc	.	.	.	.	.	.	.	.
.	ENST000C	.	Xp1	.	.	.	.	.	.	.	.	.	.
.	ENST000C	.	Xp1	.	.	.	.	.	.	.	.	.	.
.	ENST000C	.	Xp1	.	.	Score=0.9	.	.	.	.	.	.	.
.	ENST000C	.	Xp1	.	.	.	.	rs7825839	.	.	.	.	.
OTUD5	ENST000C	.	Xp1	.	.	.	.	rs3679315	ID	.	.	.	.
WDR45	ENST000C	.	Xp1	.	Scc	.	.	rs1871040	ID	.	.	.	.
.	ENST000C	.	Xp1	.	.	.	.	.	.	.	.	.	.
.	ENST000C	.	Xp1	.	.	.	.	.	.	.	.	.	.
CACNA	ENST000C	.	Xp1	.	.	.	.	rs7822715	.	.	.	.	.
AKAP4	ENST000C	.	Xp1	.	.	.	.	.	.	.	.	.	.
MAGEI	ENST000C	.	Xp1	.	Scc	.	.	.	.	.	.	.	.
.	.	.	Xp1	.	.	Score=0.9	.	.	.	.	.	.	.
KDM5C	ENST000C	.	Xp1	.	.	.	.	rs7825371	.	.	.	.	.
.	ENST000C	.	Xp1	.	.	.	Score	.	.	.	.	.	.
SMC1A	ENST000C	.	Xp1	.	.	.	.	.	.	.	.	.	.
HUWE	ENST000C	.	Xp1	.	.	.	.	.	.	.	.	.	.
.	ENST000C	.	Xp1	.	.	.	.	.	.	.	.	.	.
.	ENST000C	.	Xp1	.	.	.	.	.	.	.	.	.	.
.	ENST000C	.	Xp1	.	.	.	.	.	.	.	.	.	.
.	ENST000C	.	Xp1	.	.	Score=0.9	.	rs9617788	.	.	.	.	.
.	.	.	Xq1	.	.	.	Score	.	.	.	.	.	.
.	.	.	Xq1	.	.	.	Score	.	.	.	.	.	.
.	.	.	Xq1	.	.	.	Score	.	.	.	.	.	.
.	.	.	Xq1	.	.	.	Score	rs7124105	.	.	.	.	.
.	.	.	Xq1	.	.	.	Score	.	.	.	.	.	.
.	.	.	Xq1	.	.	.	Score	rs3765099	ID	.	.	.	.
.	.	.	Xq1	.	.	.	Score	.	.	.	.	.	.
.	.	.	Xq1	.	.	.	Score	.	.	.	.	.	.
.	.	.	Xq1	.	.	.	Score	.	ID	.	.	.	.
.	.	.	Xq1	.	.	.	Score	.	.	.	.	.	.
.	ENST000C	.	Xq1	.	.	Score=0.9	.	.	.	.	.	.	.
.	ENST000C	.	Xq1	.	.	.	.	.	.	.	.	.	.
.	ENST000C	.	Xq1	.	.	.	.	rs7687527	.	.	.	.	0.001
.	ENST000C	.	Xq1	.	.	.	.	.	.	.	.	.	.
MED12	ENST000C	.	Xq1	.	Scc	.	.	rs7507300	.	.	.	.	.
GJB1:N	ENST000C	.	Xq1	.	.	.	.	rs8632249	.	Patho	.	.	.
.	ENST000C	.	Xq1	.	.	.	Score	rs7685308	ID	.	.	.	.
.	ENST000C	.	Xq1	.	.	.	.	.	.	.	.	.	.
HDAC8	ENST000C	.	Xq1	.	.	.	.	.	.	.	.	.	.
PHKA1	ENST000C	.	Xq1	.	Scc	.	.	.	.	.	.	.	.
CDX4:N	ENST000C	.	Xq1	.	.	.	.	.	.	.	.	.	.
.	ENST000C	.	Xq1	.	.	Score=0.9	.	.	.	.	.	.	.
.	ENST000C	.	Xq1	.	.	Score=0.9	.	.	.	.	.	.	.
KIAA2C	ENST000C	.	Xq1	.	.	.	.	.	.	.	.	.	.
.	ENST000C	.	Xq2	.	.	.	.	.	.	.	.	.	.
BRWD	ENST000C	.	Xq2	.	.	.	.	.	ID	.	.	.	.
PCDH1	ENST000C	.	Xq2	.	Scc	Score=0.9	.	.	.	.	.	.	.
PCDH1	ENST000C	.	Xq2	.	.	Score=0.9	.	.	.	.	.	.	.
.	ENST000C	.	Xq2	.	.	Score=0.9	.	.	.	.	.	.	.
.	ENST000C	.	Xq2	.	.	Score=0.9	.	.	.	.	.	.	.
.	.	.	Xq2	.	.	.	Score	rs9672698	.	.	.	.	.
NOX1:I	ENST000C	.	Xq2	.	.	.	.	.	.	.	.	.	.
NXF5:N	ENST000C	.	Xq2	.	.	Score=0.9	.	.	.	.	.	.	.

.	ENST0000	.	Xq2	.	.	Score=0.9	.	.	.	.	.	.	.
.	ENST0000	.	Xq2	.	.	.	.	rs1507941	.	.	.	.	.
.	ENST0000	.	Xq2	.	.	.	.	.	.	.	.	.	.
.	ENST0000	.	Xq2	.	.	.	.	.	.	.	.	.	.
.	ENST0000	.	Xq2	.	.	.	Score	rs8664003	.	.	.	.	.
.	ENST0000	.	Xq2	.	.	.	.	.	.	.	.	.	.
.	ENST0000	.	Xq2	.	.	.	Score	.	.	.	.	.	.
.	ENST0000	.	Xq2	.	.	.	.	.	.	.	.	.	.
ACTRT	ENST0000	.	Xq2	.	.	.	.	rs1387255	ID	.	.	.	.
.	ENST0000	.	Xq2	.	.	.	.	.	.	.	.	.	.
.	ENST0000	.	Xq2	.	.	.	.	.	.	.	.	.	.
.	ENST0000	.	Xq2	.	.	.	.	.	.	.	.	.	.
.	ENST0000	.	Xq2	.	.	.	.	.	.	.	.	.	.
ATP11	ENST0000	.	Xq2	.	.	.	.	.	.	.	.	.	.
.	ENST0000	.	Xq2	.	.	Score=0.9	Score	.	ID	.	.	.	.
MAGE	ENST0000	.	Xq2	.	.	.	.	.	.	.	.	.	.
.	ENST0000	.	Xq2	.	.	Score=0.9	.	rs7823749	ID	.	.	.	.
CNGA	ENST0000	.	Xq2	.	.	.	.	.	.	.	.	.	.
.	ENST0000	.	Xq2	.	.	.	.	rs3712627	.	.	.	.	.
BGN:N	ENST0000	.	Xq2	.	Sco	.	.	.	.	.	.	.	.
.	ENST0000	.	Xq2	.	.	.	.	.	.	.	.	.	.
.	ENST0000	.	Xq2	.	.	.	.	rs7825278	.	.	.	.	.
.	ENST0000	.	Xq2	.	.	.	.	.	.	.	.	.	.
.	ENST0000	.	Xq2	.	.	.	.	.	.	.	.	.	.
.	ENST0000	.	Xq2	.	.	.	.	.	.	.	.	.	.
.	ENST0000	.	Xq2	.	.	.	.	.	.	.	.	.	.
SLC10	ENST0000	.	Xq2	.	.	.	.	.	.	.	.	.	.
G6PD:I	ENST0000	.	Xq2	.	.	.	.	rs7819487	ID	.	.	.	.
.	ENST0000	.	Xq2	.	.	.	.	.	.	.	.	.	.
.	ENST0000	.	Xq2	.	.	.	.	.	.	.	.	.	.
.	ENST0000	.	Xq2	.	.	.	.	.	.	.	.	.	.
TMLHE	ENST0000	.	Xq2	.	.	.	.	.	.	.	.	.	.
.	.	.	Yq1	.	.	Score=0.9	.	.	.	.	.	.	.
.	.	.	.	.	.	.	.	rs8791767	.	.	.	.	.
.	.	.	.	.	.	.	.	.	.	.	.	.	.
.	.	.	.	.	.	.	.	.	.	.	.	.	.
.	ENST0000	.	1p3	.	.	.	.	rs5750552	.	.	.	0	.
.	ENST0000	.	1p3	.	.	Score=0.9	.	rs4101081	.	.	.	.	.
.	ENST0000	.	1p3	.	.	Score=0.9	.	rs7629862	ID	.	.	.	.
.	ENST0000	.	1p3	.	.	Score=0.9	Score	.	.	.	.	.	.
EIF4G	ENST0000	.	1p3	.	Sco	.	.	.	.	.	.	.	.
.	.	.	1q2	.	.	Score=0.9	.	rs3772379	.	.	.	.	.
.	ENST0000	.	1q2	.	.	Score=0.9	Score	rs3720400	.	.	.	.	.
.	ENST0000	.	1q2	.	.	Score=0.9	Score	rs6181337	.	.	.	.	.
.	ENST0000	.	1q4	.	.	.	.	.	.	.	.	.	.
.	ENST0000	.	2p1	.	.	.	.	.	.	.	.	.	.
.	.	.	2p1	.	.	Score=0.9	Score	rs1649505	.	.	.	.	.
EPHA6	ENST0000	.	3q1	.	.	.	.	rs7933313	.	.	.	0.002	0.001
COL6A	ENST0000	.	3q2	.	.	.	.	rs7480655	ID	.	.	.	.
TOPBF	ENST0000	.	3q2	.	.	.	.	.	.	.	.	.	.
.	ENST0000	.	4p1	.	.	Score=0.9	.	.	.	.	.	.	.
.	ENST0000	.	5p1	.	.	Score=0.9	.	rs671688	.	.	.	.	.
.	ENST0000	.	5p1	.	.	Score=0.9	Score	rs8681576	ID	.	.	.	.
CLK4:N	ENST0000	.	5q3	.	.	.	.	.	.	.	.	.	.

.	ENST0000C	.	6q2	.	.	.	.	.	.	.	.	.	.
.	ENST0000C	.	7p1	.	.	.	.	.	.	.	.	.	.
.	.	.	7p1	.	.	Score=0.9{	Score	.	.	.	.	.	.
.	ENST0000C	.	7q3	.	.	.	.	.	ID	.	.	.	.
GIMAP	ENST0000C	.	7q3	.	.	.	.	rs1471823	.	.	.	0	.
.	ENST0000C	.	8p2	.	.	.	.	rs7496607	.	.	.	.	.
PIWIL2	ENST0000C	.	8p2	.	.	.	.	.	ID	.	.	.	.
.	.	.	9q2	.	.	Score=0.9{	.	rs1482733	ID	.	.	.	.
.	ENST0000C	.	9q3	.	.	.	.	.	.	.	.	.	.
AGAP9	ENST0000C	.	10q	.	.	Score=0.9{	.	rs2020143	ID	.	.	.	.
HPSE2	ENST0000C	.	10q	.	.	.	.	.	.	.	.	.	.
CKAP5	ENST0000C	.	11p	.	Scc	.	.	rs7731324	ID	.	.	.	.
.	ENST0000C	.	11q	.	.	.	.	.	.	.	.	.	.
KMT2D	ENST0000C	.	12q	.	Scc	.	.	rs7734351	.	.	.	.	.
KRT7:N	ENST0000C	.	12q	.	.	.	.	rs7769669	.	.	.	.	.
JAG2:N	ENST0000C	.	14q	.	Scc	.	.	.	.	.	.	.	.
.	.	.	14q	.	.	Score=0.9!	Score	rs7157981	ID	.	.	.	.
GOLGA	ENST0000C	.	15q	.	.	Score=0.9{	.	rs2019940	ID	.	.	0.206	0.211
GOLGA	ENST0000C	.	15q	.	.	Score=0.9{	.	rs3731461	ID	.	.	.	.
.	ENST0000C	.	15q	.	.	.	.	.	.	.	.	.	.
.	ENST0000C	.	15q	.	.	Score=0.9{	.	.	.	.	.	.	.
.	ENST0000C	.	15q	.	.	.	.	.	.	.	.	.	.
.	ENST0000C	.	16p	.	.	.	.	.	.	.	.	.	.
.	ENST0000C	.	17p	.	.	.	Score	rs7781206	ID	.	.	.	.
.	.	.	17q	.	.	Score=0.9{	.	rs3725436	ID	.	.	.	.
RHPN2	ENST0000C	.	19q	.	.	Score=0.9	.	rs7861545	ID	.	.	.	.
FCGBF	.	.	19q	.	.	Score=0.9	.	.	.	.	.	.	.
.	ENST0000C	.	19q	.	.	.	.	.	.	.	.	.	.
.	ENST0000C	.	20q	.	.	.	.	.	.	.	.	.	.
.	.	.	21p	.	.	Score=0.9	.	rs9266067	.	.	.	.	.
DMD:N	ENST0000C	.	Xp2	.	.	.	.	rs7663121	ID	.	.	.	.
.	ENST0000C	.	Xq1	.	.	Score=0.9{	.	rs3397828	ID	.	.	0.374	0.348
.	ENST0000C	.	1p3	.	.	.	.	.	.	.	.	.	.
.	ENST0000C	.	1p3	.	.	Score=0.9!	.	rs436293	ID	.	.	.	.
.	ENST0000C	.	1p3	.	.	.	.	.	.	.	.	.	.
.	ENST0000C	.	1p3	Scc	.	.	.	.	.	.	.	.	.
.	ENST0000C	.	1p2	.	.	.	.	rs3721234	.	.	.	.	.
.	ENST0000C	.	1p1	.	.	.	.	.	.	.	.	.	.
.	.	.	1q2	.	.	Score=0.9{	Score	rs2007574	ID	.	.	.	.
.	ENST0000C	.	1q2	.	.	Score=0.9{	.	rs9424724	ID	.	.	.	.
.	.	.	1q2	.	.	Score=0.9!	.	.	.	.	.	.	.
TMEM7	ENST0000C	.	1q2	.	.	.	.	.	.	.	.	.	.
C1orf8!	ENST0000C	.	1q2	.	Scc	.	.	.	.	.	.	.	.
.	ENST0000C	.	1q2	.	.	.	.	.	.	.	.	.	.
.	ENST0000C	.	1q2	.	.	.	.	.	.	.	.	.	.
DCAF8	ENST0000C	.	1q2	.	Scc	.	.	.	.	.	.	.	.
.	ENST0000C	.	1q2	.	.	.	.	.	.	.	.	.	.
SHCBF	ENST0000C	.	1q2	.	.	.	.	.	ID	.	.	.	.
.	ENST0000C	.	1q3	.	.	.	.	rs9662955	.	.	.	.	.
WNT3A	ENST0000C	.	1q4	.	.	.	.	rs7570105	.	.	.	.	.
KIAA18	ENST0000C	.	1q4	.	Scc	.	.	.	.	.	.	.	.
.	ENST0000C	.	1q4	.	.	.	.	.	.	.	.	.	.
.	ENST0000C	.	1q4	.	Scc	.	.	.	.	.	.	.	.
.	ENST0000C	.	2p2	.	.	Score=0.9	.	.	.	.	.	.	.

ABCG5	ENST0000C	.	2p2	.	.	.	.	rs1420196	.	.	.	.	.
.	ENST0000C	.	2q2	.	.	.	.	.	.	.	.	.	.
TANC1	ENST0000C	.	2q2	.	.	.	.	.	.	.	.	.	.
SLC39/	ENST0000C	.	2q3	.	Scc	.	.	.	.	.	.	.	.
.	ENST0000C	.	2q3	.	.	Score=0.9	Score	rs1857895	.	.	.	0	0.003
.	ENST0000C	.	2q3	.	.	.	.	.	.	.	.	.	.
.	ENST0000C	.	2q3	.	.	.	.	.	.	.	.	.	.
MROH2	ENST0000C	.	2q3	.	.	.	.	.	.	.	.	.	.
STK25:	ENST0000C	.	2q3	.	.	.	.	rs7784676	.	.	.	.	.
.	ENST0000C	.	3p2	.	.	.	.	.	.	.	.	.	.
.	ENST0000C	.	3q1	.	.	.	.	.	.	.	.	.	.
CEP63	ENST0000C	.	3q2	.	.	.	.	.	.	.	.	.	.
CEP63	ENST0000C	.	3q2	.	Scc	.	.	.	.	.	.	.	.
.	ENST0000C	.	3q2	.	.	.	.	.	.	.	.	.	.
GOLIM	ENST0000C	.	3q2	.	.	.	.	rs7462149	ID	.	.	.	.
.	ENST0000C	.	3q2	.	.	.	.	.	.	.	.	.	.
PIK3C/	ENST0000C	.	3q2	.	.	Score=0.9	.	rs1048860	ID	Patho	.	.	.
YEATS	ENST0000C	.	3q2	.	Scc	.	.	.	.	.	.	.	.
.	ENST0000C	.	4p1	.	.	.	.	.	.	.	.	.	.
SMARC	ENST0000C	.	4q2	.	.	.	.	.	.	.	.	.	.
.	ENST0000C	.	4q2	.	.	.	Score	.	.	.	.	.	.
NAA15	ENST0000C	.	4q3	.	Scc	.	.	.	.	.	.	.	.
INPP4E	ENST0000C	.	4q3	.	.	.	.	.	.	.	.	.	.
.	.	.	4q3	.	.	.	.	.	.	.	.	.	.
.	ENST0000C	.	5p1	.	.	.	Score	rs1876921	ID	.	.	.	.
RNF18	ENST0000C	.	5q1	.	Scc	.	.	.	.	.	.	.	.
F2R:NI	ENST0000C	.	5q1	.	.	.	.	.	.	.	.	.	.
SEC24	ENST0000C	.	5q3	.	.	.	.	rs7956159	ID	.	.	0.033	0.031
FAM71	ENST0000C	.	5q3	.	.	.	.	.	.	.	.	.	.
HMMR:	ENST0000C	.	5q3	.	.	.	.	.	.	.	.	.	.
KCNIP	ENST0000C	.	5q3	.	.	.	.	.	ID	.	.	.	.
.	ENST0000C	.	6p2	.	.	.	.	.	.	.	.	.	.
SYCP2	ENST0000C	.	6p2	.	.	.	.	.	.	.	.	.	.
.	ENST0000C	.	6p2	.	.	.	.	.	.	.	.	.	.
.	ENST0000C	.	6p2	.	.	Score=0.9	.	rs1787910	.	.	.	0.309	0.277
.	ENST0000C	.	6p2	.	.	.	.	rs5364899	.	.	.	0.003	0.003
EYS:NI	ENST0000C	.	6q1	.	.	.	.	.	.	.	.	.	.
EYS:NI	ENST0000C	.	6q1	.	Scc	.	.	.	.	.	.	.	.
.	ENST0000C	.	6q2	.	.	.	.	.	.	.	.	.	.
.	ENST0000C	.	6q2	.	.	.	.	.	.	.	.	.	.
.	ENST0000C	.	7p2	.	.	Score=0.9	.	.	.	.	.	.	.
.	.	.	7q1	.	.	Score=0.9	.	.	.	.	.	.	.
.	ENST0000C	.	7q2	.	.	.	.	.	.	.	.	.	.
.	ENST0000C	.	7q3	.	.	.	.	.	.	.	.	.	.
.	ENST0000C	.	7q3	.	.	.	.	rs3685111	.	.	.	.	.
.	ENST0000C	.	8p1	.	.	.	.	.	.	.	.	.	.
PREX2	ENST0000C	.	8q1	.	.	.	.	.	.	.	.	.	.
.	ENST0000C	.	8q2	Scc	.	.	.	.	.	.	.	.	.
NAPRT	ENST0000C	.	8q2	.	.	.	.	.	.	.	.	.	.
PLEC:I	ENST0000C	.	8q2	.	Scc	.	.	rs3743103	ID	.	.	.	.
.	.	.	9q1	.	.	Score=0.8	Score	rs4928838	ID	.	.	.	.
.	.	.	9q2	.	.	.	.	.	.	.	.	.	.
PHF2:I	ENST0000C	.	9q2	.	.	.	.	.	.	.	.	.	.
.	ENST0000C	.	9q3	.	.	.	.	.	.	.	.	.	.

.	ENST0000	.	9q3	.	.	.	.	.	.	.	.	.	.
DIP2C:	ENST0000	.	10p	.	.	.	.	.	.	.	.	.	.
.	ENST0000	.	10p	.	.	.	.	.	.	.	.	.	.
.	ENST0000	.	10q	.	.	Score=0.9	.	rs3128226	.	.	.	0.527	0.524
.	ENST0000	.	10q	.	.	.	.	.	.	.	.	.	.
.	ENST0000	.	11p	.	.	Score=0.9	.	.	.	.	.	.	.
.	ENST0000	.	11p	.	.	.	.	.	.	.	.	.	.
GYLTL	ENST0000	.	11p	.	.	.	.	.	.	.	.	.	.
.	ENST0000	.	11q	.	.	.	.	.	.	.	.	.	.
TENM4	ENST0000	.	11q	.	.	.	.	.	.	.	.	.	.
.	ENST0000	.	11q	.	.	.	.	.	.	.	.	.	.
.	ENST0000	.	11q	.	.	.	.	.	.	.	.	.	.
.	ENST0000	.	12p	.	.	Score=0.9	.	rs5382343	ID	.	.	0.402	0.375
ATF7IP	ENST0000	.	12p	.	Scc	.	.	.	.	.	.	.	.
SMCO2	ENST0000	.	12p	.	.	.	.	.	.	.	.	.	.
.	ENST0000	.	12q	.	.	.	.	rs8658204	ID	.	.	.	.
.	ENST0000	.	12q	.	.	.	Score	.	.	.	.	.	.
NAA25	ENST0000	.	12q	.	.	.	.	.	.	.	.	.	.
NOS1:I	ENST0000	.	12q	.	.	.	.	rs7707492	.	.	.	.	.
.	ENST0000	.	13q	.	.	.	.	.	.	.	.	.	.
FRY:NI	ENST0000	.	13q	.	.	.	.	rs3702294	.	.	.	0	.
FREM2	ENST0000	.	13q	.	.	.	.	rs4129275	ID	Uncer	.	0	.
FREM2	ENST0000	.	13q	.	.	.	.	.	.	.	.	.	.
PCDH2	ENST0000	.	13q	.	.	.	.		ID	.	.	.	.
.	ENST0000	.	13q	.	.	.	.	.	.	.	.	.	.
SLAIN1	ENST0000	.	13q	.	.	.	.	.	.	.	.	.	.
CCDC1	ENST0000	.	13q	.	.	.	.	.	.	.	.	.	.
.	ENST0000	.	14q	.	.	.	.	rs3699116	.	.	.	.	.
.	ENST0000	.	14q	.	.	.	Score	rs7660001	.	.	.	.	.
BTBD7	ENST0000	.	14q	.	Scc	.	.	.	.	.	.	.	.
.	ENST0000	.	14q	.	.	.	.	.	.	.	.	.	.
AKT1:N	ENST0000	.	14q	.	.	.	.	rs1214345	ID	Patho	.	.	.
.	ENST0000	.	14q	.	.	Score=0.9	.	.	.	.	.	.	.
.	ENST0000	.	15q	.	.	Score=0.9	.	rs4932010	ID	.	.	.	.
.	.	.	15q	.	.	Score=0.9	Score	rs7494426	.	.	.	.	.
.	ENST0000	.	15q	.	.	Score=0.9	.	rs1079463	ID	.	.	.	.
.	ENST0000	.	15q	.	.	Score=0.9	Score	rs1823548	.	.	.	0.086	0.088
.	ENST0000	.	15q	.	.	.	.	.	.	.	.	.	.
.	ENST0000	.	15q	.	.	.	Score	rs1861770	.	.	.	0.003	0.002
PATL2:	ENST0000	.	15q	.	.	.	.	rs9953164	ID	.	.	.	.
.	ENST0000	.	15q	.	.	.	Score	.	.	.	.	.	.
.	ENST0000	.	15q	.	.	.	.	.	.	.	.	.	.
.	ENST0000	.	15q	.	.	.	.	.	.	.	.	.	.
.	ENST0000	.	15q	.	.	Score=0.8	Score	.	.	.	.	.	.
CCDC6	ENST0000	.	16p	.	.	.	.		ID	.	.	.	.
.	.	.	16p	.	.	Score=0.9	.	.	.	.	.	.	.
OGFOI	ENST0000	.	16q	.	.	.	.	rs7759203	.	.	.	.	.
.	ENST0000	.	16q	.	.	.	Score	.	.	.	.	.	.
C17orf1	ENST0000	.	17p	.	.	.	.	.	.	.	.	.	.
GPR17	ENST0000	.	17q	.	.	.	.	rs7810711	ID	.	.	.	.
DBF4B	ENST0000	.	17q	.	.	.	.	rs1509595	.	.	.	0	.
HOXB8	ENST0000	.	17q	.	.	.	.	.	.	.	.	.	.
.	ENST0000	.	17q	.	.	.	.	rs3742496	.	.	.	0.002	0.002
.	ENST0000	.	17q	.	.	.	.	rs5611679	.	.	.	.	.

NOTUM	ENST000C	.	17q	.	Scc	.	.	rs5502840	.	.	.	0	.
.	ENST000C	.	18p	.	.	Score=0.9	Score	rs1996954	ID	.	.	.	.
.	ENST000C	.	18q	.	.	.	.	.	.	.	.	.	.
.	ENST000C	.	19p	.	.	.	.	.	.	.	.	.	.
.	ENST000C	.	19p	.	.	.	.	.	.	.	.	.	.
SUGP1	ENST000C	.	19p	.	Scc	.	.	.	.	.	.	.	.
ZNF50	ENST000C	.	19p	.	.	Score=0.8	.	.	.	.	.	.	.
.	ENST000C	.	19q	.	.	.	.	.	.	.	.	.	.
ZNF56	ENST000C	.	19q	.	.	.	.	.	.	.	.	.	.
TTC9B	ENST000C	.	19q	.	.	.	.	.	ID	.	.	.	.
.	ENST000C	.	19q	.	.	Score=0.9	.	.	.	.	.	.	.
.	ENST000C	.	19q	.	.	.	.	.	.	.	.	.	.
.	ENST000C	.	20p	.	.	.	.	.	.	.	.	.	.
TGM6:I	ENST000C	.	20p	.	.	.	.	.	.	.	.	.	.
CDS2:N	ENST000C	.	20p	.	Scc	.	.	rs1438597	.	.	.	.	.
.	ENST000C	.	20q	.	.	.	.	.	.	.	.	.	.
.	ENST000C	.	20q	.	.	.	Score	.	.	.	.	.	.
.	ENST000C	.	21p	.	.	Score=0.9	.	rs1170118	ID	.	.	.	.
USP25	ENST000C	.	21q	.	.	.	.	.	.	.	.	.	.
.	ENST000C	.	22q	.	.	.	.	.	.	.	.	.	.
OSBP2	ENST000C	.	22q	.	.	.	.	.	.	.	.	.	.
EIF3L:N	ENST000C	.	22q	.	Scc	.	.	.	.	.	.	.	.
ARSF:N	ENST000C	.	Xp2	.	.	.	.	rs7509188	ID	.	.	.	.
ARX:NI	ENST000C	.	Xp2	.	Scc	.	.	.	.	.	.	.	.
.	ENST000C	.	Xp1	.	.	Score=0.9	.	.	.	.	.	.	.
KIF4A:I	ENST000C	.	Xq1	.	.	.	.	.	.	.	.	.	.
NKAP:N	ENST000C	.	Xq2	.	Scc	.	.	.	.	.	.	.	.
.	.	.	Xq2	.	.	.	.	.	.	.	.	.	.
.	.	.	Xq2	.	.	.	.	.	.	.	.	.	.
.	ENST000C	.	Xq2	.	Scc	.	.	.	.	.	.	.	.
.	ENST000C	.	Xq2	.	.	.	.	.	.	.	.	.	.
.	.	.	.	.	.	.	.	.	.	.	.	.	.
.	ENST000C	.	1p3	.	.	Score=0.9	.	rs1149286	.	.	.	.	.
TMEM	ENST000C	.	1p3	.	Scc	.	.	.	.	.	.	.	.
.	ENST000C	.	1p3	.	.	.	.	.	.	.	.	.	.
COL24	ENST000C	.	1p2	.	Scc	.	.	.	.	.	.	.	.
.	ENST000C	.	1p1	.	.	Score=0.9	.	rs1998147	.	.	.	.	.
.	ENST000C	.	1q2	.	.	Score=0.9	.	rs8662584	ID	.	.	.	.
.	ENST000C	.	2p1	.	.	.	.	.	.	.	.	.	.
ETAA1	ENST000C	.	2p1	.	.	.	.	.	.	.	.	.	.
.	ENST000C	.	2p1	.	.	.	.	.	.	.	.	.	.
GLI2:N	ENST000C	.	2q1	.	.	.	.	rs3708851	.	.	.	.	.
.	ENST000C	.	2q2	.	.	.	Score	rs2008806	ID	.	.	0	.
.	ENST000C	.	2q2	.	.	.	.	rs9042902	.	.	.	.	.
.	ENST000C	.	2q3	.	.	.	.	rs3702772	.	.	.	.	.
.	ENST000C	.	2q3	.	.	.	.	rs5574456	.	.	.	0	.
.	ENST000C	.	3p2	.	.	.	Score	rs1446637	.	.	.	0.078	0.081
.	ENST000C	.	3p2	.	.	.	.	.	.	.	.	.	.
GRAMI	ENST000C	.	3q1	.	.	.	.	.	.	.	.	.	.
DRD3:N	ENST000C	.	3q1	.	.	.	.	rs2015548	ID	.	.	.	.
.	ENST000C	.	4p1	.	.	.	.	rs1163261	.	.	.	0	.
.	.	.	4p1	.	.	Score=0.9	.	rs4695484	ID	.	.	.	.
.	.	.	4p1	.	.	Score=0.9	Score	rs4695485	ID	.	.	.	.
SMR3E	ENST000C	.	4q1	.	.	.	.	rs7496227	ID	.	.	.	.

PDLIM:	ENST000C	.	4q3	.	.	.	.	rs7758415	ID	.	.	.	.
PCDHA	ENST000C	.	5q3	.	.	Score=0.9:	.	.	ID	.	.	.	.
.	ENST000C	.	6p2	.	.	.	.	rs1161907	.	.	.	.	.
.	ENST000C	.	6q1	.	.	.	.	.	.	.	.	.	.
.	ENST000C	.	6q1	.	.	Score=0.98	.	.	.	.	.	.	.
.	ENST000C	.	6q2	.	.	.	.	rs7577542	.	.	.	.	.
.	ENST000C	.	7p2	.	.	.	.	.	.	.	.	.	.
ABCB1	ENST000C	.	7q2	.	.	.	.	.	.	.	.	.	.
.	ENST000C	.	7q2	.	.	Score=0.98	.	.	.	.	.	.	.
PCM1:I	ENST000C	.	8p2	.	Scc	.	.	rs7722351	.	.	.	.	.
.	ENST000C	.	8q2	.	.	.	.	.	.	.	.	.	.
TRAF2	ENST000C	.	9q3	.	.	.	.	rs7507213	ID	.	.	.	.
.	ENST000C	.	10p	.	Scc	.	.	.	.	.	.	.	.
ITGA8:	ENST000C	.	10p	.	.	.	.	rs7571508	ID	.	.	.	.
.	ENST000C	.	10p	.	.	Score=0.9(	.	rs5655237	.	.	.	0	0.001
.	ENST000C	.	10q	.	.	.	Score	rs7905448	ID	.	.	0.347	0.355
.	ENST000C	.	10q	.	.	.	.	.	.	.	.	.	.
.	ENST000C	.	10q	.	.	.	.	rs9706750	.	.	.	.	.
.	ENST000C	.	10q	.	.	.	.	rs7723659	.	.	.	.	.
INA:NM	ENST000C	.	10q	.	.	.	.	.	ID	.	.	.	.
FGFR2	ENST000C	.	10q	.	.	.	.	rs1219134	ID	Likely	.	.	.
DEAF1	ENST000C	.	11p	.	.	.	.	.	.	.	.	.	.
USH1C	ENST000C	.	11p	.	.	.	.	rs5453728	.	.	.	0	.
USH1C	ENST000C	.	11p	.	Scc	.	.	.	.	.	.	.	.
.	ENST000C	.	11q	.	.	.	.	.	.	.	.	.	.
OAS1:N	ENST000C	.	12q	.	.	.	.	rs1030864	.	.	.	.	.
RB1:NM	ENST000C	.	13q	.	.	.	.	.	.	.	.	.	.
.	.	.	13q	.	.	Score=0.9(	.	rs9145408	.	.	.	.	.
.	.	.	13q	.	.	.	Score	rs9816818	.	.	.	.	.
.	ENST000C	.	14q	.	.	.	.	.	.	.	.	.	.
.	ENST000C	.	14q	.	Scc	.	.	.	.	.	.	.	.
PRPF3	ENST000C	.	14q	.	Scc	.	.	rs5634662	ID	.	.	0	.
.	ENST000C	.	14q	.	.	.	.	.	.	.	.	.	.
EML5:N	ENST000C	.	14q	.	.	.	.	.	.	.	.	.	.
HHIPL1	ENST000C	.	14q	.	.	.	.	.	.	.	.	.	.
.	ENST000C	.	15q	.	.	Score=0.9(	.	.	.	.	.	.	.
.	ENST000C	.	15q	.	.	Score=0.9!	.	rs7780894	.	.	.	.	.
MGA:N	ENST000C	.	15q	.	.	.	.	.	.	.	.	.	.
TTBK2:	ENST000C	.	15q	.	.	.	Score	.	.	.	.	.	.
SLC9A	ENST000C	.	16q	.	.	.	.	.	.	.	.	.	.
.	ENST000C	.	17p	.	.	.	.	.	.	.	.	.	.
.	.	.	17p	.	.	Score=0.9!	.	rs5864828	.	.	.	.	.
.	ENST000C	.	17q	.	.	.	.	rs7798230	.	.	.	.	.
.	ENST000C	.	18p	.	.	.	.	.	.	.	.	.	.
CACNA	ENST000C	.	19p	.	Scc	.	.	rs7477304	ID	.	.	.	.
OR7A1	ENST000C	.	19p	.	.	.	.	.	.	.	.	.	.
.	ENST000C	.	19q	.	.	Score=0.9(	Score	rs9472013	.	.	.	.	.
APLP1:	ENST000C	.	19q	.	.	.	.	.	.	.	.	.	.
.	ENST000C	.	19q	.	.	.	.	.	.	.	.	.	.
.	ENST000C	.	19q	.	.	.	.	rs3718581	.	.	.	.	.
.	ENST000C	.	19q	.	.	.	.	rs1056285	.	.	.	.	.
.	ENST000C	.	20p	.	.	.	.	rs5584781	ID	.	.	0	.
.	ENST000C	.	20p	.	.	Score=0.9!	.	rs6037279	ID	.	.	.	.
.	ENST000C	.	20q	.	.	.	.	.	.	.	.	.	.

.	ENST0000	.	20q	.	Scc	.	.	.	.	.	.	.	.
.	ENST0000	.	21p	.	.	Score=0.96	.	rs2013315	ID	.	.	.	.
.	ENST0000	.	21p	.	.	Score=0.96	Score	rs2001788	ID	.	.	.	.
PKDRE	ENST0000	.	22q	.	.	.	.	.	ID	.	.	.	.
IRAK1:	ENST0000	.	Xq2	.	.	.	.	.	.	.	.	.	.
.	.	.	.	.	.	.	.	.	.	.	.	.	.
.	.	.	.	.	.	.	.	.	.	.	.	.	.
.	ENST0000	.	1p3	.	.	Score=0.99	Score	rs6263527	ID	.	.	.	.
.	ENST0000	.	1p3	.	.	.	.	.	.	.	.	.	.
.	ENST0000	.	1q2	.	.	Score=0.98	.	rs7457471	ID	.	.	.	.
.	ENST0000	.	1q2	.	Scc	.	.	.	.	.	.	.	.
.	ENST0000	.	1q4	.	.	.	.	.	.	.	.	.	.
.	.	.	2p1	.	.	Score=0.98	.	.	ID	.	.	.	.
.	.	.	2p1	.	.	Score=0.95	.	rs3711404	ID	.	.	.	.
.	ENST0000	.	2q3	.	Scc	.	.	.	.	.	.	.	.
.	ENST0000	.	3q2	.	.	Score=0.97	.	rs7758373	ID	.	.	.	.
.	ENST0000	.	4p1	.	.	.	.	.	.	.	.	.	.
.	.	.	4p1	.	.	Score=0.97	Score	rs4694947	.	.	.	.	.
.	ENST0000	.	9q2	.	.	.	Score	.	ID	.	.	.	.
.	ENST0000	.	10p	.	.	Score=0.97	.	rs4070334	ID	.	.	.	.
.	ENST0000	.	12q	.	.	.	Score	rs9213947	.	.	.	.	.
.	ENST0000	.	14q	.	.	.	.	rs1125033	ID	.	.	.	.
.	ENST0000	.	14q	.	.	.	Score	.	.	.	.	.	.
GOLGA	ENST0000	.	15q	.	.	Score=0.99	Score	rs2859043	ID	Benig	.	.	.
.	ENST0000	.	15q	.	.	Score=0.98	.	.	.	.	.	.	.
.	ENST0000	.	18q	.	.	.	Score	.	ID	.	.	.	.
.	ENST0000	.	1q2	.	.	Score=0.98	.	.	.	.	.	.	.
.	ENST0000	.	1q2	.	.	Score=0.98	.	rs2787778	ID	.	.	.	.
.	ENST0000	.	1q2	.	.	Score=0.98	.	rs2787777	ID	.	.	0.319	0.325
.	.	.	2p1	.	.	Score=0.95	.	rs3711404	ID	.	.	.	.
.	ENST0000	.	5p1	.	.	.	Score	rs1997973	.	.	.	.	.
.	ENST0000	.	7q1	.	.	Score=0.99	.	rs2018986	ID	.	.	.	.
.	.	.	7q1	.	.	Score=0.99	.	.	.	.	.	.	.
POLR2	ENST0000	.	7q2	.	.	Score=0.99	.	rs1390499	ID	.	.	.	.
.	.	.	8p2	.	.	Score=0.99	.	rs2077351	ID	.	.	.	.
.	ENST0000	.	9q1	.	.	Score=0.99	.	rs1136487	ID	.	.	.	.
.	ENST0000	.	9q2	.	.	Score=0.99	.	rs7648362	ID	.	.	.	.
.	ENST0000	.	9q2	.	.	.	.	.	.	.	.	.	.
FAM21	ENST0000	.	10q	.	Scc	Score=0.99	.	rs1995206	ID	.	.	.	.
.	ENST0000	.	11p	.	.	Score=0.99	Score	rs3713518	ID	.	.	.	.
.	ENST0000	.	11p	.	.	.	.	rs8790394	.	.	.	.	.
.	ENST0000	.	17p	.	.	Score=0.99	.	rs1155611	ID	.	.	.	.
SLC35	ENST0000	.	18p	.	.	Score=0.97	.	rs2018600	ID	.	.	.	.
.	.	.	21p	.	.	Score=0.99	Score	.	ID	.	.	.	.
.	.	.	21p	.	.	Score=0.99	Score	rs8792017	ID	.	.	.	.
.	ENST0000	.	21p	.	.	.	Score	rs1043983	ID	.	.	.	.
.	ENST0000	.	Xq2	.	.	Score=0.97	.	rs1444677	.	.	.	0.107	0.101
.	ENST0000	.	1p3	.	.	Score=0.96	.	.	.	.	.	.	.
.	ENST0000	.	1p3	.	.	.	.	.	.	.	.	.	.
.	ENST0000	.	1p3	.	.	.	.	.	.	.	.	.	.
TMEM2	ENST0000	.	1p3	.	Scc	.	.	.	.	.	.	.	.
.	ENST0000	.	1p3	.	.	Score=0.94	.	.	.	.	.	.	.
.	ENST0000	.	1p3	.	.	Score=0.94	.	.	.	.	.	.	.
.	ENST0000	.	1p2	.	Scc	.	.	.	.	.	.	.	.

.	ENST0000	.	1q2	.	.	Score=0.9	.	rs3894786	ID	.	.	.	.
.	ENST0000	.	1q2	.	.	Score=0.9	.	rs1112760	ID	.	.	.	.
.	ENST0000	.	1q2	.	.	Score=0.9	.	rs2017985	ID	.	.	.	.
.	ENST0000	.	1q2	.	.	Score=0.9	.	rs3713084	ID	.	.	.	.
.	ENST0000	.	1q2	.	.	Score=0.9	.	rs8799710	.	.	.	.	.
.	ENST0000	.	1q2	.	.	Score=0.9	.	rs6699421	ID	.	.	.	.
.	ENST0000	.	1q2	.	.	Score=0.9	.	rs8799669	.	.	.	.	.
OTUD7	ENST0000	.	1q2	.	.	.	.	.	.	.	.	.	.
.	ENST0000	.	1q2	.	.	.	.	.	.	.	.	.	.
RIT1:N	ENST0000	.	1q2	.	Scc	.	.	rs4833528	ID	not pr	.	.	.
.	ENST0000	.	1q2	.	.	.	.	rs8940723	.	.	.	.	.
.	ENST0000	.	1q2	.	.	.	.	rs7731705	.	.	.	.	.
.	ENST0000	.	2p1	.	.	Score=0.9	.	.	.	.	.	.	.
.	ENST0000	.	2p1	.	.	.	.	rs7781962	.	.	.	.	.
LYG2:N	ENST0000	.	2q1	.	Scc	.	.	rs2018517	ID	.	.	0	0.001
SLC9A	ENST0000	.	2q1	.	Scc	.	.	.	.	.	.	.	.
EVX2:N	ENST0000	.	2q3	.	.	.	.	rs7685255	.	.	.	.	.
CWC22	ENST0000	.	2q3	.	Scc	.	.	rs7551300	.	.	.	.	.
RPSA:N	ENST0000	.	3p2	.	.	.	.	rs6224264	ID	.	.	.	.
.	ENST0000	.	3q2	.	.	.	.	.	.	.	.	.	.
ABCC5	ENST0000	.	3q2	.	Scc	.	.	.	.	.	.	.	.
HTR3C	ENST0000	.	3q2	.	.	.	.	.	.	.	.	.	.
.	ENST0000	.	3q2	.	.	.	.	.	.	.	.	.	.
.	ENST0000	.	4q2	.	.	.	.	.	.	.	.	.	.
.	ENST0000	.	4q2	.	.	.	.	rs5742217	.	.	.	0	.
VEGFC	ENST0000	.	4q3	.	.	.	.	rs5549088	ID	.	.	0	.
.	ENST0000	.	5p1	.	.	Score=0.9	Score	.	.	.	.	.	.
.	ENST0000	.	5q1	.	.	Score=0.9	Score	rs3759258	.	.	.	0.777	0.772
PTCD2	ENST0000	.	5q1	.	.	.	.	.	.	.	.	.	.
.	ENST0000	.	5q3	.	.	.	.	.	.	.	.	.	.
.	ENST0000	.	5q3	.	Scc	.	.	.	.	.	.	.	.
.	ENST0000	.	5q3	.	.	.	.	.	.	.	.	.	.
.	ENST0000	.	5q3	.	.	.	Score	.	.	.	.	.	.
.	ENST0000	.	6p2	.	.	.	Score	rs7580301	.	.	.	.	.
.	ENST0000	.	6p2	.	.	.	.	rs7598672	.	.	.	.	.
.	ENST0000	.	6p2	.	.	.	.	.	.	.	.	.	.
HLA-G:	ENST0000	.	6p2	.	.	Score=0.8	.	.	.	.	.	.	.
.	ENST0000	.	6p2	.	.	.	.	.	.	.	.	.	.
.	ENST0000	.	6p2	.	.	.	.	.	.	.	.	.	.
.	ENST0000	.	6p2	.	.	.	.	.	.	.	.	.	.
.	ENST0000	.	6q1	.	.	.	.	.	.	.	.	.	.
LOC44	ENST0000	.	6q1	.	.	Score=0.9	.	.	.	.	.	.	.
.	ENST0000	.	6q2	.	.	.	Score	.	.	.	.	.	.
.	ENST0000	.	7p2	.	.	.	.	.	.	.	.	.	.
.	ENST0000	.	7q1	.	.	Score=0.9	.	rs2018986	ID	.	.	.	.
.	ENST0000	.	7q2	.	.	.	.	rs9803140	.	.	.	.	.
.	ENST0000	.	7q3	.	.	Score=0.9	Score	rs1208357	ID	.	.	.	.
.	ENST0000	.	7q3	.	.	Score=0.9	Score	rs6248378	ID	.	.	.	.
GIMAP	ENST0000	.	7q3	.	.	.	.	.	.	.	.	.	.
.	ENST0000	.	7q3	.	.	Score=0.9	.	.	.	.	.	.	.
.	ENST0000	.	8p2	.	.	.	.	.	.	.	.	.	.
.	.	.	8p1	.	.	.	Score	rs2022368	ID	.	.	.	.
.	ENST0000	.	8q2	.	.	.	.	.	.	.	.	.	.
FRMD3	ENST0000	.	9q2	.	Scc	.	.	.	.	.	.	.	.

.	ENST0000	.	9q2	.	.	.	.	.	.	.	.	.	.
.	ENST0000	.	9q2	.	.	Score=0.9	.	.	.	.	.	.	.
MAN1E	ENST0000	.	9q3	.	.	.	.	rs7729568	.	.	.	.	.
.	ENST0000	.	10q	.	.	.	.	rs5354201	.	.	.	0	.
.	ENST0000	.	10q	.	.	.	.	.	.	.	.	.	.
CCDC1	ENST0000	.	10q	.	Scc	.	.	.	ID	.	.	.	.
KCNK1	ENST0000	.	10q	.	.	.	.	.	.	.	.	.	.
PIDD:N	ENST0000	.	11p	.	.	.	.	rs1474222	.	.	.	0	0.001
.	ENST0000	.	11p	.	.	.	.	.	.	.	.	.	.
HPX:NI	ENST0000	.	11p	.	Scc	.	.	.	.	.	.	.	.
.	ENST0000	.	11p	.	.	.	Score	.	.	.	.	.	.
.	ENST0000	.	11p	.	.	.	.	rs7776543	.	.	.	.	.
TP53I1	ENST0000	.	11p	.	.	.	.	rs7802181	.	.	.	.	.
.	ENST0000	.	11q	.	.	.	.	.	.	.	.	.	.
.	ENST0000	.	11q	.	.	.	.	.	.	.	.	.	.
.	ENST0000	.	11q	.	.	.	.	rs1009016	.	.	.	.	.
.	ENST0000	.	11q	.	.	.	.	.	.	.	.	.	.
NACA:I	ENST0000	.	12q	.	.	.	.	.	.	.	.	.	.
.	ENST0000	.	12q	.	.	.	Score	rs8683709	ID	.	.	.	.
.	ENST0000	.	12q	.	.	.	.	.	.	.	.	.	.
.	ENST0000	.	12q	.	.	.	.	.	.	.	.	.	.
.	ENST0000	.	13q	.	.	Score=0.9	.	.	.	.	.	.	.
.	ENST0000	.	13q	.	.	Score=0.9	.	.	ID	.	.	.	.
.	ENST0000	.	13q	.	.	.	.	rs7603455	.	.	.	.	.
MTMR	ENST0000	.	13q	.	.	.	.	.	.	.	.	.	.
.	ENST0000	.	13q	.	.	.	.	.	.	.	.	.	.
.	ENST0000	.	13q	.	.	.	.	.	ID	.	.	.	.
TBC1D	ENST0000	.	13q	.	.	.	.	rs1847802	.	.	.	0	.
.	ENST0000	.	14q	.	.	Score=0.9	.	rs8674109	.	.	.	.	.
PRKD1	ENST0000	.	14q	.	Scc	.	.	.	.	.	.	.	.
.	ENST0000	.	14q	.	.	.	.	.	.	.	.	.	.
.	ENST0000	.	14q	.	.	.	.	.	.	.	.	.	.
C14orf	ENST0000	.	14q	.	.	.	.	rs7590859	.	.	.	.	.
.	ENST0000	.	14q	.	.	.	.	.	.	.	.	.	.
.	ENST0000	.	14q	.	.	Score=0.9	.	rs1126070	ID	.	.	.	.
.	ENST0000	.	14q	.	.	.	.	.	.	.	.	.	.
.	ENST0000	.	14q	.	.	.	.	.	.	.	.	.	.
.	ENST0000	.	14q	.	.	.	.	.	.	.	.	.	.
.	ENST0000	.	15q	.	.	.	.	.	.	.	.	.	.
THSD4	ENST0000	.	15q	.	.	.	.	.	.	.	.	.	.
.	ENST0000	.	16p	.	Scc	.	.	.	.	.	.	.	.
.	ENST0000	.	16p	.	.	.	Score	.	.	.	.	.	.
C16orf	ENST0000	.	16p	.	.	.	.	rs7488576	.	.	.	.	.
.	ENST0000	.	16p	.	.	.	Score	.	.	.	.	.	.
ACSM	ENST0000	.	16p	.	.	Score=0.9	.	.	.	.	.	.	.
XPO6:N	ENST0000	.	16p	.	Scc	.	.	.	.	.	.	.	.
.	ENST0000	.	16p	.	.	.	.	.	.	.	.	.	.
.	.	.	16q	.	.	.	Score	rs7501178	.	.	.	.	.
.	.	.	16q	.	.	.	Score	rs2128450	.	.	.	.	.
.	.	.	16q	.	.	.	Score	rs4436803	ID	.	.	.	.
.	.	.	16q	.	.	.	Score	rs2887500	ID	.	.	.	.
.	ENST0000	.	16q	.	.	Score=0.9	.	.	.	.	.	.	.
ZNF27	ENST0000	.	16q	.	.	.	.	.	.	.	.	.	.
.	ENST0000	.	17q	.	.	.	.	.	.	.	.	.	.

EFCAB	ENST0000	.	17q	.	Scc	.	.	.	.	.	.	.	.
.	ENST0000	.	17q	.	.	.	Score	.	.	.	.	.	.
CNP:N	ENST0000	.	17q	.	.	.	.	rs1996882	.	.	.	.	.
.	ENST0000	.	17q	.	.	.	.	.	.	.	.	.	.
.	ENST0000	.	17q	.	.	.	.	rs1022498	.	.	.	.	.
ERN1:N	ENST0000	.	17q	.	.	.	.	.	.	.	.	.	.
CACNG	ENST0000	.	17q	.	.	.	.	rs3720728	.	.	.	.	.
.	ENST0000	.	17q	.	.	.	.	.	.	.	.	.	.
NAPG:	ENST0000	.	18p	.	.	.	.	.	.	.	.	.	.
.	ENST0000	.	18p	.	.	.	.	.	.	.	.	.	.
.	.	.	18p	.	.	Score=0.9	.	.	.	.	.	.	.
C18orf5	ENST0000	.	18q	.	.	.	.	rs7558958	.	.	.	.	.
SIPA1L	ENST0000	.	19q	.	.	.	.	rs9952243	.	.	.	.	.
SULT2	ENST0000	.	19q	.	Scc	.	.	.	.	.	.	.	.
.	.	.	20p	.	.	.	.	.	.	.	.	.	.
.	ENST0000	.	20q	.	.	.	Score	.	.	.	.	.	.
.	ENST0000	.	21p	.	.	Score=0.9	.	rs3697472	.	.	.	.	.
.	ENST0000	.	21p	.	.	Score=0.9	.	rs7969899	ID	.	.	.	.
.	ENST0000	.	21q	.	.	.	.	.	.	.	.	.	.
.	ENST0000	.	21q	.	.	.	.	.	.	.	.	.	.
.	ENST0000	.	22q	.	.	Score=0.9	.	rs2212142	ID	.	.	.	.
.	ENST0000	.	22q	.	.	.	.	.	.	.	.	.	.
.	ENST0000	.	22q	.	.	Score=0.9	.	rs2015076	.	.	.	.	.
ELFN2	ENST0000	.	22q	.	.	.	.	rs7611606	.	.	.	.	.
.	ENST0000	.	22q	.	.	.	.	.	.	.	.	.	.
.	ENST0000	.	Xq1	.	.	Score=0.9	.	rs3397828	ID	.	.	0.374	0.348
CPXCR	ENST0000	.	Xq2	.	.	.	.	.	ID	.	.	.	.
.	.	.	.	.	.	.	.	rs8792192	.	.	.	.	.
NECAP	ENST0000	.	1p3	.	.	.	.	.	.	.	.	.	.
.	ENST0000	.	1p3	.	.	Score=0.9	.	rs1996890	.	.	.	.	.
DLGAP	ENST0000	.	1p3	.	.	.	.	.	.	.	.	.	.
GNAI3	ENST0000	.	1p1	.	Scc	.	.	.	.	.	.	.	.
.	ENST0000	.	1q2	.	.	Score=0.9	.	.	.	.	.	.	.
.	ENST0000	.	1q2	.	.	Score=0.9	.	rs1694653	.	.	.	.	.
UNKNO	ENST0000	.	1q2	.	.	Score=0.9	.	rs9441133	ID	.	.	.	.
UNKNO	ENST0000	.	1q2	.	.	Score=0.9	.	rs7819708	.	.	.	.	.
.	ENST0000	.	1q2	.	.	Score=0.9	.	rs2022445	ID	.	.	.	.
.	.	.	1q2	.	.	.	.	.	.	.	.	.	.
.	ENST0000	.	1q2	.	.	.	.	.	ID	.	.	.	.
.	ENST0000	.	1q2	.	.	Score=0.9	.	.	.	.	.	.	.
RNASE	ENST0000	.	1q2	.	.	.	.	rs1494980	.	.	.	.	.
HMCN1	ENST0000	.	1q3	.	.	.	.	.	.	.	.	.	.
CENPF	ENST0000	.	1q4	.	.	.	.	rs7630457	.	.	.	.	.
.	ENST0000	.	1q4	.	.	.	.	rs3725088	.	.	.	.	.
COX7A	ENST0000	.	2p2	.	.	.	.	.	.	.	.	.	.
.	ENST0000	.	2p1	.	.	Score=0.9	.	rs6214568	ID	.	.	.	.
.	ENST0000	.	2q1	.	.	.	.	.	.	.	.	.	.
ANAPC	ENST0000	.	2q1	.	.	Score=0.9	.	rs1476846	ID	.	.	.	.
.	ENST0000	.	2q3	.	.	Score=0.9	.	.	.	.	.	.	.
.	ENST0000	.	3p2	.	.	.	.	.	.	.	.	.	.
CELSR	ENST0000	.	3p2	.	.	.	.	.	.	.	.	.	.
.	ENST0000	.	3q1	.	.	.	.	.	.	.	.	.	.
SPICE1	ENST0000	.	3q1	.	.	.	.	rs1428044	.	.	.	.	.
.	ENST0000	.	3q2	.	.	.	.	.	.	.	.	.	.

.	ENST000C	.	4p1	.	.	.	.	rs4970287	ID	.	.	.	.
.	ENST000C	.	4q2	.	.	.	.	.	.	.	.	.	.
TET2:N	ENST000C	.	4q2	.	.	.	.	.	ID	.	.	.	.
NIPBL:	ENST000C	.	5p1	.	.	.	.	.	.	.	.	.	.
.	ENST000C	.	5q3	.	.	.	Score	.	.	.	.	.	.
.	ENST000C	.	7p1	.	.	.	.	rs9642790	.	.	.	.	.
.	ENST000C	.	7p1	.	.	Score=0.9	.	rs6246184	ID	.	.	.	.
.	ENST000C	.	7p1	.	.	Score=0.9	.	.	.	.	.	.	.
LRCH4	ENST000C	.	7q2	.	Scc	.	.	.	.	.	.	.	.
PTPRZ	ENST000C	.	7q3	.	.	.	.	.	ID	.	.	.	.
.	ENST000C	.	7q3	.	.	.	.	rs8915428	.	.	.	.	.
.	ENST000C	.	7q3	.	.	.	.	.	.	.	.	.	.
.	ENST000C	.	8p2	.	.	.	.	.	.	.	.	.	.
ZNF70	ENST000C	.	8p1	.	.	.	.	.	.	.	.	.	.
.	ENST000C	.	8q1	.	.	.	.	.	.	.	.	.	.
.	ENST000C	.	9p2	.	.	.	.	.	.	.	.	.	.
.	ENST000C	.	9q3	.	.	.	.	.	.	.	.	.	.
.	ENST000C	.	10q	.	.	.	.	.	.	.	.	.	.
GRID1:	ENST000C	.	10q	.	.	.	.	.	.	.	.	.	.
NOLC1	ENST000C	.	10q	.	.	.	.	.	.	.	.	.	.
.	ENST000C	.	10q	.	.	.	.	.	.	.	.	.	.
.	ENST000C	.	10q	.	.	.	.	.	.	.	.	.	.
TRIM5:	ENST000C	.	11p	.	.	.	.	.	.	.	.	.	.
NLRP1	ENST000C	.	11p	.	.	.	.	.	ID	.	.	.	.
.	ENST000C	.	11p	.	.	.	.	.	.	.	.	.	.
TRAF6	ENST000C	.	11p	.	Scc	Score=0.9	.	.	.	.	.	.	.
.	ENST000C	.	11q	.	.	.	.	.	.	.	.	.	.
DSCAM	ENST000C	.	11q	.	.	.	.	rs7580622	ID	.	.	.	.
CD163	ENST000C	.	12p	.	.	.	.	.	.	.	.	.	.
.	.	.	12q	.	.	.	.	.	.	.	.	.	.
GPD1:N	ENST000C	.	12q	.	.	.	.	.	.	.	.	.	.
GPR84	ENST000C	.	12q	.	.	.	.	.	.	.	.	.	.
.	ENST000C	.	12q	.	.	.	.	.	.	.	.	.	.
HCAR3	ENST000C	.	12q	.	.	Score=0.9	.	.	.	.	.	.	.
GPC5:N	ENST000C	.	13q	.	.	.	.	.	.	.	.	.	.
.	ENST000C	.	14q	.	.	Score=0.9	.	rs7149806	ID	.	.	.	.
.	.	.	14q	.	.	.	Score	.	.	.	.	.	.
NDN:N	ENST000C	.	15q	.	.	Score=0.8	.	.	.	.	.	.	.
.	ENST000C	.	15q	.	.	Score=0.9	Score	rs1995696	.	.	.	.	.
FMN1:N	ENST000C	.	15q	.	.	.	.	.	.	.	.	.	.
.	ENST000C	.	15q	.	.	.	.	.	.	.	.	.	.
.	ENST000C	.	15q	.	.	.	.	.	.	.	.	.	.
ANPEP	ENST000C	.	15q	.	.	.	.	.	.	.	.	.	.
.	ENST000C	.	16p	.	.	.	.	.	.	.	.	.	.
.	ENST000C	.	16q	.	.	.	.	.	.	.	.	.	.
.	ENST000C	.	17p	.	.	.	.	.	.	.	.	.	.
.	ENST000C	.	17q	.	.	Score=0.9	.	.	.	.	.	.	.
.	ENST000C	.	18p	.	.	Score=0.9	.	rs8790150	ID	.	.	.	.
.	ENST000C	.	19p	.	.	.	Score	rs8673654	ID	.	.	.	.
MAN2B	ENST000C	.	19p	.	.	.	.	rs7668108	.	.	.	.	.
CRLF1	ENST000C	.	19p	.	Scc	.	.	rs7683037	.	.	.	.	.
.	ENST000C	.	19p	.	.	Score=0.9	.	.	.	.	.	.	.
.	ENST000C	.	19q	.	.	.	.	.	.	.	.	.	.
MAP4K	ENST000C	.	19q	.	.	.	.	.	.	.	.	.	.

.	ENST0000	.	19q	.	.	.	.	.	.	.	.	.	.
.	ENST0000	.	19q	.	.	.	.	.	.	.	.	.	.
.	ENST0000	.	19q	.	.	.	.	.	.	.	.	.	.
.	ENST0000	.	19q	.	.	.	.	.	.	.	.	.	.
.	.	.	19q	.	.	Score=0.94	.	.	.	.	.	.	.
NLRP9	ENST0000	.	19q	.	.	Score=0.90	.	.	.	.	.	.	.
.	ENST0000	.	20q	.	.	.	.	.	.	.	.	.	.
SALL4:	ENST0000	.	20q	.	.	.	.	.	.	.	.	.	.
.	.	.	21p	.	.	Score=0.98	Score	rs1118412	.	.	.	.	.
ADAMT	ENST0000	.	21q	.	.	.	.	.	.	.	.	.	.
URB1:N	ENST0000	.	21q	.	Scc	.	.	.	.	.	.	.	.
.	.	.	22q	.	.	Score=0.89	.	rs2006656	ID	.	.	.	.
.	ENST0000	.	22q	.	.	.	.	rs9392199	.	.	.	.	.
.	ENST0000	.	Xp1	.	.	.	Score	rs734038	.	.	.	0.207	0.192
.	ENST0000	.	Xq2	.	.	Score=0.98	.	rs1826904	.	.	.	0	.
OCRL:I	ENST0000	.	Xq2	.	Scc	.	.	.	.	.	.	.	.
GPR11	ENST0000	.	Xq2	.	.	.	.	.	ID	.	.	.	.
SLITRK	ENST0000	.	Xq2	.	.	.	.	.	.	.	.	.	.
F8:NM_	ENST0000	.	Xq2	.	.	.	.	.	.	.	.	.	.
.	.	.	.	.	.	.	.	.	.	.	.	.	.
.	.	.	.	.	.	.	.	.	.	.	.	.	.
.	ENST0000	.	1p3	.	.	Score=0.98	Score	rs1810443	ID	.	.	.	.
.	.	.	1q2	.	.	Score=0.98	Score	rs3698413	.	.	.	.	.
.	ENST0000	.	1q2	.	.	Score=0.97	.	rs3774960	.	.	.	.	.
.	ENST0000	.	1q2	.	.	.	.	rs2596327	ID	.	.	.	.
.	ENST0000	.	1q2	.	.	Score=0.98	.	rs2020368	.	.	.	.	.
.	.	.	1q2	.	.	Score=0.99	.	.	.	.	.	.	.
.	ENST0000	.	2p1	.	.	.	Score	.	.	.	.	.	.
.	ENST0000	.	2p1	.	.	Score=0.94	.	rs2647769	ID	.	.	.	.
.	ENST0000	.	2q1	.	.	.	Score	.	.	.	.	.	.
.	ENST0000	.	2q1	.	.	.	Score	.	.	.	.	.	.
.	ENST0000	.	2q1	.	.	.	Score	.	.	.	.	.	.
.	ENST0000	.	2q2	.	.	Score=0.99	Score	rs6216494	.	.	.	.	.
.	ENST0000	.	3q2	.	.	.	Score	.	.	.	.	.	.
.	ENST0000	.	4p1	.	.	.	.	.	.	.	.	.	.
.	.	.	4q1	.	.	.	Score	rs1429725	.	.	.	0.684	0.681
.	ENST0000	.	5p1	.	.	Score=0.98	.	rs2871612	ID	.	.	.	.
.	ENST0000	.	7p1	.	.	Score=0.99	.	rs6246206	ID	.	.	.	.
.	ENST0000	.	7q1	.	.	Score=0.98	.	rs3680944	ID	.	.	.	.
.	ENST0000	.	8q1	.	.	Score=0.97	Score	rs1499905	ID	.	.	.	.
.	ENST0000	.	10q	.	.	.	.	.	.	.	.	.	.
.	ENST0000	.	10q	.	.	.	Score	rs7710236	ID	.	.	.	.
.	ENST0000	.	10q	.	.	.	.	rs1788433	ID	.	.	.	.
.	ENST0000	.	10q	.	.	.	.	.	.	.	.	.	.
.	ENST0000	.	11q	.	.	.	Score	.	.	.	.	.	.
.	ENST0000	.	12p	.	.	Score=0.98	.	rs1063873	.	.	.	0.269	0.267
.	ENST0000	.	14q	.	.	Score=0.99	Score	rs4247513	.	.	.	.	.
.	ENST0000	.	14q	.	.	Score=0.99	.	rs7445485	.	.	.	.	.
.	ENST0000	.	14q	.	.	Score=0.99	.	rs8678279	.	.	.	.	.
.	ENST0000	.	14q	.	.	.	.	.	.	.	.	.	.
.	ENST0000	.	17q	.	.	Score=0.99	.	rs3930170	ID	.	.	.	.
.	ENST0000	.	17q	.	.	.	Score	rs3894278	ID	.	.	.	.
.	.	.	21p	.	.	Score=0.98	.	rs1963041	ID	.	.	.	.
.	ENST0000	.	22q	.	.	Score=0.99	.	rs3716554	.	.	.	.	.

.	ENST000C	.	22q	.	Score=0.9	.	.	.	.	.	.	.
.	ENST000C	.	Xq	.	Score=0.9	.	rs7819787	.	.	.	0.254	0.249
.	ENST000C	.	1p3	.	Score=0.9	.	.	.	.	.	.	.
.	ENST000C	.	1p3	.	Score=0.9	.	rs453347	ID	.	.	0.45	0.437
.	ENST000C	.	1p3	.	.	.	.	.	.	.	.	.
.	ENST000C	.	1q2	.	Score=0.9	.	rs1812127	.	.	.	.	.
.	ENST000C	.	1q2	.	Score=0.9	Score	.	.	.	.	.	.
.	ENST000C	.	1q2	.	Score=0.9	.	rs8799607	.	.	.	.	.
.	ENST000C	.	1q2	.	Score=0.9	.	rs1451754	ID	.	.	.	.
.	ENST000C	.	1q2	.	Score=0.9	.	rs2003945	ID	.	.	.	.
.	ENST000C	.	1q2	.	Score=0.9	.	rs7970322	.	.	.	.	.
.	ENST000C	.	1q3	.	.	.	.	.	.	.	.	.
.	ENST000C	.	2q3	.	.	.	rs1996199	ID	.	.	.	.
.	ENST000C	.	3p2	.	.	Score	.	.	.	.	.	.
.	ENST000C	.	3q2	.	.	.	rs1873413	.	.	.	0.063	0.055
.	.	.	3q2	.	Score=0.9	Score	rs3723871	.	.	.	.	.
.	ENST000C	.	3q2	.	.	Score	.	.	.	.	.	.
.	ENST000C	.	3q2	.	Score=0.9	.	rs2006820	.	.	.	.	.
.	.	.	4p1	.	Score=0.9	.	.	.	.	.	.	.
.	ENST000C	.	5q3	.	.	.	.	.	.	.	.	.
.	.	.	6q2	.	.	Score	.	.	.	.	.	.
.	ENST000C	.	6q2	.	Score=0.9	.	rs7746421	ID	.	.	0.359	0.355
.	.	.	6q2	.	Score=0.9	.	rs3759780	.	.	.	.	.
.	.	.	7q1	.	Score=0.9	.	.	ID	.	.	.	.
.	.	.	7q1	.	Score=0.9	.	.	.	.	.	.	.
AUTS2	ENST000C	.	7q1	Scc	.	Score	.	.	.	.	.	.
.	ENST000C	.	9p1	.	Score=0.9	Score	rs2803685	ID	.	.	.	.
.	.	.	10q	.	Score=0.9	.	rs1996770	.	.	.	.	.
.	.	.	10q	.	Score=0.9	Score	rs1456597	ID	.	.	.	.
.	ENST000C	.	11p	.	.	Score	.	.	.	.	.	.
.	ENST000C	.	11q	Scc	.	.	.	.	.	.	.	.
.	ENST000C	.	12p	.	Score=0.9	.	rs3718339	ID	.	.	.	.
.	ENST000C	.	12p	.	Score=0.9	.	rs3767721	ID	.	.	.	.
.	ENST000C	.	12q	.	.	.	rs5604999	ID	.	.	.	.
.	ENST000C	.	14q	.	Score=0.9	Score	rs7755744	ID	.	.	.	.
.	ENST000C	.	14q	.	Score=0.9	.	rs1018908	ID	.	.	.	.
.	ENST000C	.	15q	.	Score=0.9	.	.	.	.	.	.	.
AQR:N	ENST000C	.	15q	.	.	.	rs7568595	.	.	.	.	.
.	ENST000C	.	16p	.	Score=0.9	Score	rs3739171	.	.	.	.	.
.	ENST000C	.	16q	.	Score=0.9	.	rs7137409	.	.	.	0.251	0.262
.	ENST000C	.	17p	.	Score=0.9	.	rs7124761	.	.	.	.	.
.	ENST000C	.	17p	.	Score=0.9	.	rs6149181	ID	.	.	.	.
.	ENST000C	.	17q	.	Score=0.9	.	rs2870408	ID	.	.	.	.
.	ENST000C	.	17q	.	Score=0.9	.	rs2848380	ID	.	.	.	.
.	ENST000C	.	17q	.	.	.	.	.	.	.	.	.
.	ENST000C	.	18q	.	.	.	.	.	.	.	.	.
.	ENST000C	.	19p	.	.	Score	.	.	.	.	.	.
.	ENST000C	.	21p	.	Score=0.9	.	.	.	.	.	.	.
.	ENST000C	.	22q	.	Score=0.9	.	.	ID	.	.	.	.
.	ENST000C	.	22q	.	Score=0.9	.	rs3609392	.	.	.	0.266	0.302
.	ENST000C	.	Xp1	.	.	.	.	.	.	.	.	.
MXRA	ENST000C	.	1p3	.	.	.	rs7558529	.	.	.	.	.
.	ENST000C	.	1p3	.	Score=0.9	.	.	.	.	.	.	.
.	ENST000C	.	1p3	.	.	.	.	.	.	.	.	.

.	ENST0000	.	1p3	.	.	.	.	.	.	.	.	.	.
.	ENST0000	.	1p3	.	.	.	.	.	.	.	.	.	.
.	ENST0000	.	1p3	.	Scc	.	.	.	.	.	.	.	.
.	ENST0000	.	1p3	.	Scc	.	.	.	.	.	.	.	.
UNKN	ENST0000	.	1p2	.	.	.	.	.	.	.	.	.	.
LRRC8	ENST0000	.	1p2	.	Scc	Score=0.9	.	.	.	.	.	.	.
.	ENST0000	.	1p1	.	Scc	.	.	rs1430207	.	.	.	0.002	0.001
.	ENST0000	.	1p1	.	.	Score=0.9	Score	.	.	.	.	.	.
.	ENST0000	.	1q2	.	.	Score=0.9	.	rs1996428	ID	.	.	.	.
.	ENST0000	.	1q2	.	.	Score=0.9	Score	.	ID	.	.	.	.
.	ENST0000	.	1q2	.	.	Score=0.9	.	rs3755029	.	.	.	.	.
.	ENST0000	.	1q2	.	.	Score=0.9	.	rs2849511	.	.	.	.	.
.	ENST0000	.	1q2	.	.	Score=0.9	.	rs7824596	.	.	.	.	.
.	ENST0000	.	1q2	.	.	Score=0.9	.	rs3765099	ID	.	.	.	.
TCHH:	ENST0000	.	1q2	.	.	.	.	rs9099303	ID	.	.	.	.
.	ENST0000	.	1q2	.	.	.	.	.	.	.	.	.	.
.	ENST0000	.	1q2	.	.	.	.	.	.	.	.	.	.
.	ENST0000	.	1q3	.	.	.	.	.	.	.	.	.	.
.	ENST0000	.	1q3	.	.	.	.	.	.	.	.	.	.
.	ENST0000	.	2q3	.	.	.	.	.	.	.	.	.	.
.	ENST0000	.	2q3	.	.	.	.	.	.	.	.	.	.
ITPR1:	ENST0000	.	3p2	.	.	.	.	rs7793139	ID	.	.	.	.
.	ENST0000	.	3p2	.	.	.	.	.	.	.	.	.	.
.	ENST0000	.	3p2	.	.	.	.	.	.	.	.	.	.
STAB1	ENST0000	.	3p2	.	Scc	.	.	.	.	.	.	.	.
NELFA	ENST0000	.	4p1	.	.	.	.	rs7785731	.	.	.	.	.
.	ENST0000	.	4q2	.	.	.	.	rs7749410	.	.	.	.	.
.	ENST0000	.	5p1	.	.	Score=0.9	.	rs2382048	ID	.	.	.	.
DHFR:	ENST0000	.	5q1	.	.	.	.	rs7817278	.	.	.	.	.
.	ENST0000	.	5q2	.	.	.	.	.	.	.	.	.	.
ZNF47	ENST0000	.	5q2	.	.	.	.	rs1140023	ID	.	.	0	.
.	ENST0000	.	5q3	.	.	.	.	.	.	.	.	.	.
GABRE	ENST0000	.	5q3	.	Scc	.	.	.	.	.	.	.	.
.	ENST0000	.	6p2	.	.	.	.	.	.	.	.	.	.
SLC44	ENST0000	.	6p2	.	.	.	.	.	.	.	.	.	.
.	ENST0000	.	6p1	.	.	.	.	.	.	.	.	.	.
.	ENST0000	.	6q2	.	.	Score=0.9	.	rs9907394	.	.	.	.	.
ARID1	ENST0000	.	6q2	.	.	.	.	.	ID	.	.	.	.
MLLT4	ENST0000	.	6q2	.	.	.	.	.	.	.	.	.	.
.	ENST0000	.	6q2	.	.	.	.	.	.	.	.	.	.
TTYH3	ENST0000	.	7p2	.	Scc	.	.	.	.	.	.	.	.
.	ENST0000	.	7p2	.	.	Score=0.9	.	.	.	.	.	.	.
ISPD:N	ENST0000	.	7p2	.	.	.	.	rs7468373	.	.	.	.	.
HECW	ENST0000	.	7p1	.	.	.	.	.	.	.	.	.	.
.	.	.	7p1	.	.	.	Score	.	.	.	.	.	.
.	ENST0000	.	7q3	.	.	.	.	rs5705914	.	.	.	0	.
GIMAP	ENST0000	.	7q3	.	Scc	.	.	rs1481871	ID	.	.	.	.
.	ENST0000	.	8p2	.	.	.	.	rs7588073	.	.	.	.	.
.	ENST0000	.	8p2	.	.	.	.	rs7479925	.	.	.	.	.
.	.	.	8p1	.	.	.	Score	.	.	.	.	.	.
.	ENST0000	.	8q2	.	.	.	Score	rs5327262	.	.	.	.	.
.	ENST0000	.	8q2	.	.	.	.	rs5696070	.	.	.	0	.
GNA14	ENST0000	.	9q2	.	.	.	.	rs3682437	.	.	.	0	.
OR13C	ENST0000	.	9q3	.	.	.	.	rs3746457	.	.	.	0.003	0.003

.	ENST0000	.	9q3	.	.	.	.	rs7528554	.	.	.	.	.
PHPT1	ENST0000	.	9q3	.	.	.	.	rs5640471	ID	.	.	.	.
.	ENST0000	.	9q3	.	.	.	.	rs5505690	.	.	.	0	.
RBM20	ENST0000	.	10q	.	.	.	.	.	.	.	.	.	.
.	ENST0000	.	11p	.	.	.	Score	rs7615769	ID	.	.	.	.
DCDC5	ENST0000	.	11p	.	.	.	.	.	.	.	.	.	.
DGKZ:I	ENST0000	.	11p	.	Scc	.	.	rs7817387	ID	.	.	.	.
.	.	.	11q	.	.	.	.	.	.	.	.	.	.
C11orf8	ENST0000	.	11q	.	.	.	.	rs7525419	.	.	.	.	.
.	ENST0000	.	11q	.	.	.	.	.	.	.	.	.	.
.	ENST0000	.	11q	.	.	.	.	.	.	.	.	.	.
RASAL	ENST0000	.	12q	.	Scc	.	.	.	.	.	.	.	.
WDR66	ENST0000	.	12q	.	.	.	.	rs1409770	ID	.	.	0	.
.	ENST0000	.	13q	.	.	.	.	.	.	.	.	.	.
.	.	.	13q	.	.	.	.	.	.	.	.	.	.
.	ENST0000	.	14q	.	.	Score=0.9	Score	.	ID	.	.	.	.
.	ENST0000	.	14q	.	.	.	.	.	.	.	.	.	.
.	ENST0000	.	14q	.	.	.	.	.	.	.	.	.	.
.	ENST0000	.	14q	.	.	.	.	.	.	.	.	.	.
.	ENST0000	.	14q	.	.	.	.	.	.	.	.	.	.
ITFG3:I	ENST0000	.	16p	.	.	.	.	rs7663922	.	.	.	.	.
.	ENST0000	.	16p	.	.	.	.	rs5750779	.	.	.	0	.
.	ENST0000	.	16p	.	.	.	.	.	.	.	.	.	.
.	ENST0000	.	16p	.	.	.	.	.	.	.	.	.	.
.	ENST0000	.	16p	.	.	Score=0.9	.	rs7569695	.	.	.	.	.
.	ENST0000	.	16p	.	.	Score=0.9	.	rs7520038	.	.	.	.	.
.	ENST0000	.	17q	.	.	.	Score	.	.	.	.	.	.
CCL4:N	ENST0000	.	17q	.	Scc	Score=0.9	.	rs3713755	.	.	.	0	.
KRT24:	ENST0000	.	17q	.	.	.	.	rs7744485	.	.	.	.	.
ACSF2	ENST0000	.	17q	.	.	.	.	rs7460185	ID	.	.	.	.
.	ENST0000	.	17q	.	.	.	Score	rs8668152	.	.	.	.	.
.	ENST0000	.	17q	.	.	Score=0.9	.	rs8665784	ID	.	.	.	.
SEPT9	ENST0000	.	17q	.	.	.	.	.	.	.	.	.	.
CBX2:N	ENST0000	.	17q	.	.	.	.	rs1995663	.	.	.	0	.
.	ENST0000	.	17q	.	.	.	.	.	.	.	.	.	.
.	.	.	19q	.	.	Score=0.9	.	rs3865492	ID	.	.	0.174	0.216
.	.	.	19q	.	.	Score=0.9	.	.	.	.	.	.	.
GRIK5:	ENST0000	.	19q	.	Scc	.	.	rs7571526	ID	.	.	.	.
APOC4	ENST0000	.	19q	.	.	.	.	rs7565559	ID	.	.	.	.
SIRPB1	ENST0000	.	20p	.	.	.	.	.	.	.	.	.	.
.	ENST0000	.	20q	.	.	.	.	.	.	.	.	.	.
UNKNO	ENST0000	.	20q	.	.	.	.	rs3774092	.	.	.	.	.
.	ENST0000	.	21q	.	.	.	.	.	.	.	.	.	.
.	.	.	22q	.	.	Score=0.9	.	rs6197100	.	.	.	0.168	0.168
.	ENST0000	.	22q	.	.	Score=0.9	.	.	.	.	.	.	.
.	ENST0000	.	22q	.	.	Score=0.9	.	rs5662423	ID	.	.	.	.
.	ENST0000	.	22q	.	.	.	.	.	.	.	.	.	.
.	ENST0000	.	22q	.	.	.	.	rs1007675	.	.	.	.	.
.	ENST0000	.	Xp1	.	.	.	Score	.	.	.	.	.	.
.	ENST0000	.	Xp1	.	.	.	.	.	.	.	.	.	.
CLCN5	ENST0000	.	Xp1	.	.	.	.	rs7970448	.	Patho	.	.	.
ITIH6:N	ENST0000	.	Xp1	.	.	.	.	.	.	.	.	.	.
.	ENST0000	.	Xq2	.	.	Score=0.9	.	.	.	.	.	.	.
.	ENST0000	.	1p3	.	.	Score=0.9	Score	rs4634900	ID	.	.	.	.

.	ENST000C	.	1p3	.	.	.	.	.	.	.	.	.	.
.	.	.	1q2	.	.	Score=0.9	Score	rs7450506	ID	.	.	.	.
.	.	.	1q2	.	.	Score=0.9	.	rs2016919	.	.	.	.	.
.	.	.	1q2	.	.	Score=0.9	.	.	.	.	.	.	.
.	ENST000C	.	1q2	.	.	Score=0.9	.	rs8662584	ID	.	.	.	.
.	ENST000C	.	1q2	.	.	Score=0.9	.	rs4143495	ID	.	.	.	.
.	ENST000C	.	1q2	.	.	Score=0.9	.	rs6181077	ID	.	.	.	.
.	ENST000C	.	1q2	.	.	.	.	.	.	.	.	.	.
.	ENST000C	.	1q3	.	.	.	.	rs2003501	.	.	.	0	0.001
.	ENST000C	.	2p1	.	.	.	.	.	.	.	.	.	.
.	ENST000C	.	2p1	.	Scc	.	.	rs1834895	.	Uncer	.	0	.
DYSF:N	ENST000C	.	2p1	.	.	.	.	rs2009908	ID	Uncer	.	.	.
WBP1:	ENST000C	.	2p1	.	.	.	.	.	.	.	.	.	.
.	ENST000C	.	2q1	.	.	Score=0.9	.	rs9528	ID	.	.	.	.
.	ENST000C	.	2q2	.	Scc	.	.	.	.	.	.	.	.
GPC1:N	ENST000C	.	2q3	.	.	.	.	rs3739748	.	.	.	.	.
.	ENST000C	.	3p2	.	.	.	.	rs5683253	.	.	.	.	.
.	ENST000C	.	3p2	.	.	.	.	rs1474450	.	.	.	0	.
ABHD1	ENST000C	.	3p2	.	Scc	.	.	rs2004359	.	.	.	.	.
ARHGA	ENST000C	.	3q1	.	.	.	.	.	.	.	.	.	.
.	ENST000C	.	4p1	.	.	.	.	.	.	.	.	.	.
.	ENST000C	.	4p1	.	.	.	.	.	.	.	.	.	.
SH3TC	ENST000C	.	4p1	.	Scc	.	.	.	.	.	.	.	.
.	ENST000C	.	4p1	.	Scc	.	.	.	.	.	.	.	.
.	.	.	4p1	.	.	Score=0.9	.	rs2932367	ID	.	.	.	.
.	ENST000C	.	4p1	.	.	Score=0.9	.	.	ID	.	.	.	.
.	ENST000C	.	4q1	.	.	.	.	.	.	.	.	.	.
GK2:NI	ENST000C	.	4q2	.	.	.	.	rs7669673	ID	.	.	.	.
.	ENST000C	.	5q1	.	.	.	.	.	.	.	.	.	.
.	.	.	5q1	.	Scc	.	.	.	.	.	.	.	.
SLCO4	ENST000C	.	5q2	.	Scc	.	.	.	.	.	.	.	.
.	ENST000C	.	5q3	.	Scc	.	.	.	ID	.	.	.	.
.	ENST000C	.	5q3	.	.	.	.	rs5319092	.	.	.	0	.
.	ENST000C	.	6p2	.	.	.	Score	rs5661365	.	.	.	0	.
.	ENST000C	.	6p2	.	.	Score=0.8	.	.	.	.	.	.	.
TNXB:N	ENST000C	.	6p2	.	Scc	.	.	.	.	.	.	.	.
INTS1:I	ENST000C	.	7p2	.	.	.	.	rs1814105	.	.	.	0	.
.	.	.	7p1	.	.	Score=0.9	.	rs7827655	.	.	.	.	.
.	ENST000C	.	7q2	.	.	.	.	rs1484992	.	.	.	0.003	0.002
.	ENST000C	.	7q2	.	.	.	.	rs5523938	.	.	.	0.005	0.004
DGKI:N	ENST000C	.	7q3	.	Scc	.	.	.	ID	.	.	.	.
.	ENST000C	.	7q3	.	.	.	.	rs3737114	.	.	.	.	.
OR2A2	ENST000C	.	7q3	.	Scc	.	.	.	.	.	.	.	.
.	ENST000C	.	8q1	.	.	Score=0.9	.	rs9982298	.	.	.	.	.
OTUD6	ENST000C	.	8q2	.	.	.	.	.	.	.	.	.	.
GLIS3:I	ENST000C	.	9p2	.	.	.	.	.	.	.	.	.	.
.	ENST000C	.	9p2	.	.	.	.	.	.	.	.	.	.
.	ENST000C	.	9q1	.	.	Score=0.9	.	rs9330412	ID	.	.	.	.
.	ENST000C	.	9q2	.	.	.	.	.	.	.	.	.	.
.	.	.	9q3	.	.	Score=0.9	.	.	.	.	.	.	.
GPSM1	ENST000C	.	9q3	.	Scc	.	.	rs9459874	.	.	.	.	.
.	ENST000C	.	9q3	.	.	.	.	rs9494422	.	.	.	.	.
GPR15	ENST000C	.	10p	.	Scc	.	.	.	.	.	.	.	.
ZNF33/	ENST000C	.	10p	.	.	Score=0.9	.	.	.	.	.	.	.

.	.	.	10q	.	.	Score=0.9	.	rs2996811	.	.	.	.	.
.	ENST0000	.	10q	.	.	Score=0.9	.	.	.	.	.	.	.
.	ENST0000	.	10q	.	.	.	.	.	.	.	.	.	.
.	ENST0000	.	10q	.	.	.	.	rs7792060	.	.	.	.	.
.	ENST0000	.	11p	.	.	.	.	.	.	.	.	.	.
.	ENST0000	.	11q	.	.	.	.	.	.	.	.	.	.
HYOU1	ENST0000	.	11q	.	Scc	.	.	.	.	.	.	.	.
.	ENST0000	.	11q	.	.	.	.	.	.	.	.	.	.
.	ENST0000	.	12p	.	.	.	.	.	.	.	.	.	.
ETV6:N	ENST0000	.	12p	.	.	.	.	.	ID	.	.	.	.
KRAS:N	ENST0000	.	12p	.	Scc	.	.	rs1219135	ID	Patho	.	.	.
KIF5A:N	ENST0000	.	12q	.	.	.	.	rs3522560	.	Benig	.	0	.
.	ENST0000	.	12q	.	.	.	.	.	.	.	.	.	.
.	ENST0000	.	12q	.	.	.	.	rs5396169	.	.	.	0	.
.	.	.	13q	.	.	Score=0.9	.	.	.	.	.	.	.
.	ENST0000	.	14q	.	.	.	Score	.	.	.	.	.	.
.	ENST0000	.	14q	.	.	.	.	rs8791962	.	.	.	.	.
.	ENST0000	.	15q	.	.	Score=0.9	.	.	.	.	.	.	.
NDN:N	ENST0000	.	15q	.	Scc	Score=0.8	.	.	.	.	.	.	.
GABRA	ENST0000	.	15q	.	.	.	.	.	ID	.	.	.	.
.	ENST0000	.	15q	.	.	Score=0.9	.	rs7831577	.	.	.	0.095	0.082
.	.	.	15q	.	.	Score=0.9	.	rs2010114	ID	.	.	.	.
.	ENST0000	.	16p	.	.	.	.	.	.	.	.	.	.
.	ENST0000	.	16p	.	.	Score=0.9	.	.	.	.	.	.	.
.	ENST0000	.	16q	.	.	.	.	.	.	.	.	.	.
.	ENST0000	.	16q	.	.	Score=0.9	.	rs2001829	ID	.	.	.	.
.	ENST0000	.	16q	.	.	.	.	.	.	.	.	.	.
RFFL:N	ENST0000	.	17q	.	.	.	.	.	.	.	.	.	.
.	ENST0000	.	17q	.	.	Score=0.9	.	.	.	.	.	.	.
.	ENST0000	.	17q	.	.	.	.	.	.	.	.	.	.
.	ENST0000	.	17q	.	.	.	.	.	.	.	.	.	.
.	ENST0000	.	18q	.	.	Score=0.9	Score	.	.	.	.	.	.
.	ENST0000	.	19p	.	.	.	Score	rs8673654	ID	.	.	.	.
UNKN	ENST0000	.	19p	.	.	.	.	.	.	.	.	.	.
DOCK6	ENST0000	.	19p	.	Scc	.	.	rs7680686	.	.	.	.	.
LRFN3	ENST0000	.	19q	.	.	.	.	.	.	.	.	.	.
.	ENST0000	.	19q	.	.	Score=0.9	.	.	.	.	.	.	.
ZNF22	ENST0000	.	19q	.	.	.	.	.	.	.	.	.	.
.	ENST0000	.	19q	.	.	.	.	.	.	.	.	.	.
.	ENST0000	.	19q	.	.	.	Score	.	.	.	.	.	.
ZNF81	ENST0000	.	19q	.	.	Score=0.9	.	rs7705940	.	.	.	.	.
STK35	ENST0000	.	20p	.	Scc	.	.	.	.	.	.	.	.
.	ENST0000	.	20p	.	.	.	.	rs2002254	.	.	.	0	.
FAM83	ENST0000	.	20q	.	.	.	.	.	.	.	.	.	.
.	.	.	20q	.	.	.	Score	.	.	.	.	.	.
NCAM2	ENST0000	.	21q	.	Scc	.	.	.	.	.	.	.	.
SYN3:N	ENST0000	.	22q	.	.	.	.	.	.	.	.	.	.
.	ENST0000	.	Xp1	.	.	Score=0.9	.	.	.	.	.	.	.
ARMC	ENST0000	.	Xq2	.	.	.	.	.	.	.	.	.	.
TEX13	ENST0000	.	Xq2	.	.	.	.	.	ID	.	.	.	.
NRK:N	ENST0000	.	Xq2	.	.	.	.	rs7533837	.	.	.	0.002	0.001
.	ENST0000	.	Xq2	.	.	.	.	.	.	.	.	.	.
.	ENST0000	.	Xq2	.	.	.	.	rs7767123	.	.	.	.	.
.	ENST0000	.	Xq2	.	.	.	.	.	.	.	.	.	.

G6PD:I	ENST0000C	.	Xq2	.	.	.	.	rs3981235	.	Patho	.	0.002	0.001
.	.	.	.	.	.	.	.	.	.	.	.	.	.
.	ENST0000C	.	1p3	.	.	.	.	.	.	.	.	.	.
.	ENST0000C	.	1p3	.	.	Score=0.9	.	rs1015792	ID	.	.	.	.
TAS1R	ENST0000C	.	1p3	.	.	.	.	.	.	.	.	.	.
RAP1G	ENST0000C	.	1p3	.	.	.	.	rs1453424	.	.	.	.	.
BTBD8	ENST0000C	.	1p2	.	Scc	.	.	.	.	.	.	.	.
.	ENST0000C	.	1q2	.	.	Score=0.9	.	rs7704066	.	.	.	.	.
.	ENST0000C	.	1q2	.	.	Score=0.9	.	rs1415172	ID	.	.	.	.
.	ENST0000C	.	1q2	.	.	Score=0.9	.	rs1256214	ID	.	.	.	.
.	ENST0000C	.	1q2	.	.	Score=0.9	.	rs3721624	.	.	.	.	.
.	ENST0000C	.	1q2	.	.	Score=0.9	.	rs5876752	ID	.	.	0.269	0.314
.	ENST0000C	.	1q2	.	.	Score=0.9	.	.	.	.	.	.	.
CA14:N	ENST0000C	.	1q2	.	Scc	.	.	rs3730063	.	.	.	.	.
.	ENST0000C	.	1q2	.	.	.	.	.	.	.	.	.	.
KCNH1	ENST0000C	.	1q3	.	.	.	.	.	.	.	.	.	.
KCNK1	ENST0000C	.	2p2	.	Scc	.	.	.	.	.	.	.	.
.	.	.	2p1	.	.	Score=0.9	.	rs7540104	.	.	.	.	.
.	ENST0000C	.	2q2	.	.	.	.	.	.	.	.	.	.
.	ENST0000C	.	2q3	.	.	.	.	.	.	.	.	.	.
.	ENST0000C	.	2q3	.	.	.	.	.	.	.	.	.	.
.	ENST0000C	.	2q3	.	Scc	.	.	rs9825331	.	.	.	.	.
.	ENST0000C	.	3p2	.	.	.	.	.	.	.	.	.	.
.	ENST0000C	.	3p2	.	.	.	Score	rs1309395	.	.	.	.	.
.	ENST0000C	.	3p2	.	.	.	.	.	.	.	.	.	.
.	ENST0000C	.	3p2	.	.	.	.	rs3710999	.	.	.	.	.
.	ENST0000C	.	3p2	.	.	.	Score	.	.	.	.	.	.
QRICH	ENST0000C	.	3p2	.	.	.	.	.	.	.	.	.	.
BSN:NI	ENST0000C	.	3p2	.	Scc	.	.	.	.	.	.	.	.
BSN:NI	ENST0000C	.	3p2	.	Scc	.	.	.	.	.	.	.	.
.	ENST0000C	.	3p2	.	.	.	.	.	.	.	.	.	.
.	ENST0000C	.	3q1	.	.	.	.	.	.	.	.	.	.
.	ENST0000C	.	3q2	.	.	.	.	rs5729372	.	.	.	0	.
.	ENST0000C	.	4p1	.	.	.	.	rs4970287	ID	.	.	.	.
.	.	.	4q1	.	.	.	Score	rs1456513	.	.	.	0.002	0.001
AFF1:N	ENST0000C	.	4q2	.	.	.	.	.	.	.	.	.	.
.	ENST0000C	.	4q2	.	.	.	.	.	.	.	.	.	.
FAT4:N	ENST0000C	.	4q2	.	.	.	.	rs3727861	ID	.	.	.	.
GALNT	ENST0000C	.	4q3	.	Scc	.	.	.	.	.	.	.	.
.	ENST0000C	.	4q3	.	.	.	Score	.	.	.	.	.	.
.	ENST0000C	.	5q3	.	.	.	.	.	.	.	.	.	.
.	ENST0000C	.	5q3	.	.	.	.	.	.	.	.	.	.
.	ENST0000C	.	6p2	.	.	.	.	.	.	.	.	.	.
.	ENST0000C	.	6p2	.	.	.	.	.	.	.	.	.	.
.	ENST0000C	.	6q1	.	.	.	.	rs9010911	.	.	.	.	.
.	ENST0000C	.	6q2	.	.	.	.	.	.	.	.	.	.
.	ENST0000C	.	7p2	.	.	Score=0.9	.	rs1544467	ID	.	.	.	.
.	ENST0000C	.	7p2	.	.	.	.	.	.	.	.	.	.
.	ENST0000C	.	7p1	.	.	.	.	.	.	.	.	.	.
.	ENST0000C	.	7p1	.	.	.	.	.	.	.	.	.	.
TBRG4	ENST0000C	.	7p1	.	.	.	.	.	.	.	.	.	.
.	.	.	7p1	.	.	Score=0.9	.	rs1929995	.	.	.	0.002	0.001
.	ENST0000C	.	7q1	.	.	Score=0.9	.	.	.	.	.	.	.
HIP1:N	ENST0000C	.	7q1	.	Scc	.	.	.	.	.	.	.	.

.	ENST000C	.	7q2	.	.	.	.	.	.	.	.	.	.
TRRAF	ENST000C	.	7q2	.	.	.	.	.	.	.	.	.	.
.	ENST000C	.	7q3	.	.	.	.	rs5753233	.	.	.	0	.
.	ENST000C	.	7q3	.	Scc	.	.	.	.	.	.	.	.
.	ENST000C	.	8q1	.	.	.	.	.	.	.	.	.	.
LACTB	ENST000C	.	8q1	.	.	.	.	.	.	.	.	.	.
.	ENST000C	.	8q1	.	.	.	.	rs5472481	.	.	.	0	.
.	ENST000C	.	8q2	.	.	.	.	.	.	.	.	.	.
.	ENST000C	.	9p2	.	.	Score=0.9	.	rs3746546	ID	.	.	.	.
UNKNC	ENST000C	.	9p1	.	Scc	.	.	.	ID	.	.	.	.
APBA1	ENST000C	.	9q2	.	.	.	.	.	.	.	.	.	.
PCSK5	ENST000C	.	9q2	.	Scc	.	.	.	.	.	.	.	.
.	.	.	9q3	.	.	Score=0.9	.	rs2868900	.	.	.	.	.
.	ENST000C	hsa-mir-45	9q3	.	.	.	.	rs3719801	.	.	.	.	.
.	ENST000C	.	9q3	.	.	.	.	rs9976753	.	.	.	.	.
SEC16	ENST000C	.	9q3	.	.	.	.	rs3763504	.	.	.	.	.
.	ENST000C	.	10p	.	.	.	.	rs9266937	.	.	.	.	.
.	ENST000C	.	10p	.	.	.	.	.	.	.	.	.	.
.	ENST000C	.	10q	.	.	.	Score	.	.	.	.	.	.
USP54	ENST000C	.	10q	.	Scc	.	.	.	.	.	.	.	.
.	ENST000C	.	10q	.	.	.	.	.	.	.	.	.	.
.	ENST000C	.	10q	.	.	.	.	.	.	.	.	.	.
.	ENST000C	.	11p	.	.	.	.	.	.	.	.	.	.
.	ENST000C	.	11p	.	.	.	.	.	.	.	.	.	.
.	ENST000C	.	11p	.	.	.	Score	.	ID	.	.	.	.
.	ENST000C	.	11q	.	.	.	.	.	.	.	.	.	.
.	ENST000C	.	11q	.	.	.	.	rs3696017	.	.	.	.	.
.	ENST000C	.	11q	.	.	.	Score	.	.	.	.	.	.
CWF19	ENST000C	.	11q	.	.	.	.	.	.	.	.	.	.
.	ENST000C	.	11q	.	.	.	.	.	.	.	.	.	.
.	ENST000C	.	12q	.	.	.	.	.	.	.	.	.	.
DEPDC	ENST000C	.	12q	.	.	.	.	.	.	.	.	.	.
.	ENST000C	.	12q	.	.	.	Score	rs8683709	ID	.	.	.	.
.	ENST000C	.	12q	.	.	.	.	.	.	.	.	.	.
HNF1A	ENST000C	.	12q	.	Scc	.	.	rs5681239	.	.	.	0.002	0.001
.	ENST000C	.	13q	.	.	.	.	rs7528858	.	.	.	.	.
PRMT5	ENST000C	.	14q	.	.	.	.	.	.	.	.	.	.
.	ENST000C	.	14q	.	.	.	Score	rs1288039	ID	.	.	.	.
.	.	.	14q	.	.	.	.	rs5316143	.	.	.	0	.
NDN:N	ENST000C	.	15q	.	.	Score=0.8	.	.	.	.	.	.	.
TJP1:N	ENST000C	.	15q	.	.	.	.	rs7540721	.	.	.	.	.
.	ENST000C	.	15q	.	.	Score=0.9	.	rs2015691	.	.	.	.	.
.	ENST000C	.	15q	.	.	Score=0.9	.	rs7122570	.	.	.	.	.
NUTM1	ENST000C	.	15q	.	.	.	.	.	.	.	.	.	.
.	ENST000C	.	15q	.	.	.	.	.	.	.	.	.	.
.	ENST000C	.	15q	.	.	Score=0.9	.	rs1869226	ID	.	.	0.043	0.047
.	ENST000C	.	15q	.	.	Score=0.9	.	rs8789048	ID	.	.	.	.
TBC1D	ENST000C	.	15q	.	.	.	.	.	.	.	.	.	.
.	ENST000C	.	15q	.	.	.	.	.	.	.	.	.	.
TBL3:N	ENST000C	.	16p	.	.	.	.	rs7655357	ID	.	.	.	.
NLRC3	ENST000C	.	16p	.	Scc	.	.	rs2015706	.	.	.	0.003	0.002
.	ENST000C	.	16p	.	.	.	.	.	.	.	.	.	.
CHST4	ENST000C	.	16q	.	.	.	.	.	ID	.	.	.	.
.	ENST000C	.	17p	.	.	.	.	.	.	.	.	.	.

.	ENST000C	.	17p	.	.	.	.	.	.	.	.	.	.
.	ENST000C	.	17p	.	.	.	Score	rs7781206	ID	.	.	.	.
.	ENST000C	.	17q	.	.	.	.	.	.	.	.	.	.
.	ENST000C	.	17q	.	.	.	.	rs1134430	.	.	.	0	.
.	ENST000C	.	17q	.	.	.	.	.	.	.	.	.	.
RPTOF	ENST000C	.	17q	.	.	.	.	rs7648089	.	.	.	.	.
.	ENST000C	.	17q	.	.	.	.	.	.	.	.	.	.
CD7:NI	ENST000C	.	17q	.	Scc	.	.	rs3729739	.	.	.	0	.
.	ENST000C	.	17q	.	.	.	.	.	.	.	.	.	.
.	ENST000C	.	17q	.	.	.	.	.	.	.	.	.	.
.	ENST000C	.	18p	.	.	.	.	.	.	.	.	.	.
.	ENST000C	.	18p	.	.	.	.	.	.	.	.	.	.
.	ENST000C	.	18q	.	.	.	.	.	.	.	.	.	.
.	ENST000C	.	19p	.	.	.	.	rs5282381	.	.	.	0	.
.	ENST000C	.	19p	.	Scc	.	.	.	.	.	.	.	.
.	ENST000C	.	19p	.	.	.	Score	rs9075262	.	.	.	.	.
.	ENST000C	.	19p	.	.	.	Score	.	.	.	.	.	.
.	ENST000C	.	19p	.	.	.	Score	.	.	.	.	.	.
.	ENST000C	.	19p	.	.	.	.	.	.	.	.	.	.
ZNF57	ENST000C	.	19q	.	.	.	.	.	.	.	.	.	.
.	ENST000C	.	19q	.	.	.	.	.	.	.	.	.	.
.	ENST000C	.	19q	.	.	.	.	.	.	.	.	.	.
FUT1:N	ENST000C	.	19q	.	Scc	.	.	.	.	.	.	.	.
FUT1:N	ENST000C	.	19q	.	.	.	.	.	.	.	.	.	.
CLEC1	ENST000C	.	19q	.	.	.	.	.	.	.	.	.	.
.	ENST000C	.	19q	.	.	Score=0.9	.	.	.	.	.	.	.
.	ENST000C	.	19q	.	.	.	Score	.	.	.	.	.	.
LPIN3:I	ENST000C	.	20q	.	.	.	.	rs7457076	ID	.	.	.	.
.	.	.	21p	.	.	Score=0.9	Score	.	.	.	.	.	.
.	.	.	21p	.	.	Score=0.9	Score	rs8792017	ID	.	.	.	.
.	ENST000C	.	21p	.	.	Score=0.9	.	.	.	.	.	.	.
.	ENST000C	.	22q	.	.	.	.	rs1167407	.	.	.	0	.
.	ENST000C	.	22q	.	.	Score=0.9	.	rs3760330	ID	.	.	.	.
SMTN:I	ENST000C	.	22q	.	.	.	.	.	.	.	.	.	.
RLIM:N	ENST000C	.	Xq1	.	.	Score=0.9	.	.	.	.	.	.	.
MCF2:I	ENST000C	.	Xq2	.	.	.	.	.	.	.	.	.	.
.	ENST000C	.	Xq2	.	.	.	.	.	.	.	.	.	.
.	ENST000C	.	1p3	.	.	Score=0.9	.	rs1153096	ID	.	.	0.596	0.581
.	ENST000C	.	1p3	.	.	.	.	.	.	.	.	.	.
.	ENST000C	.	1p3	.	.	.	.	.	.	.	.	.	.
CCDC1	ENST000C	.	1p3	.	.	.	.	rs5317072	ID	.	.	0	.
.	ENST000C	.	1p3	.	.	.	.	.	.	.	.	.	.
.	ENST000C	.	1p3	.	Scc	.	.	rs7787296	.	.	.	.	.
AGL:NI	ENST000C	.	1p2	.	.	.	.	.	.	.	.	.	.
.	ENST000C	.	1q2	.	.	Score=0.9	.	rs7736232	.	.	.	0.571	0.554
.	ENST000C	.	1q2	.	.	Score=0.9	.	rs1712174	ID	.	.	.	.
.	ENST000C	.	1q2	.	.	.	.	.	.	.	.	.	.
.	ENST000C	.	1q2	.	.	.	.	.	.	.	.	.	.
.	ENST000C	.	1q2	.	.	.	.	.	ID	.	.	.	.
.	ENST000C	.	1q2	.	.	.	.	.	.	.	.	.	.
.	ENST000C	.	1q3	.	.	.	.	.	.	.	.	.	.
.	.	.	1q3	.	.	.	.	.	.	.	.	.	.
KDM5E	ENST000C	.	1q3	.	.	.	.	.	.	.	.	.	.
.	ENST000C	.	1q4	.	.	.	.	.	.	.	.	.	.

TRAPP	ENST0000	.	2p2	.	.	.	.	.	.	.	.	.	.
TRAPP	ENST0000	.	2p2	.	.	.	.	.	.	.	.	.	.
NBAS:	ENST0000	.	2p2	.	.	.	.	.	.	.	.	.	.
NLRC4	ENST0000	.	2p2	.	.	.	.	.	.	.	.	.	.
.	ENST0000	.	2p1	.	.	.	.	.	.	.	.	.	.
.	.	.	2q1	.	.	.	Score	rs2015850	ID	.	.	.	.
ZAP70:	ENST0000	.	2q1	.	Scc	.	.	.	.	.	.	.	.
.	ENST0000	.	2q1	.	.	Score=0.9	.	rs2008974	.	.	.	.	.
.	ENST0000	.	2q1	.	.	.	.	rs7249504	ID	.	.	0.042	0.042
.	ENST0000	.	2q1	.	.	.	.	.	.	.	.	.	.
TTN:N	ENST0000	.	2q3	.	.	.	.	.	ID	.	.	.	.
.	ENST0000	.	2q3	.	.	.	.	.	.	.	.	.	.
.	ENST0000	.	2q3	.	.	.	.	rs5520376	.	.	.	.	.
.	ENST0000	.	2q3	.	.	.	.	.	.	.	.	.	.
.	ENST0000	.	2q3	.	.	.	Score	rs1816528	.	.	.	0.002	0.001
PRSS5	ENST0000	.	2q3	.	Scc	.	.	.	.	.	.	.	.
.	ENST0000	.	3p2	.	.	.	Score	.	.	.	.	.	.
.	ENST0000	.	3p2	.	.	.	.	rs9185110	.	.	.	.	.
.	ENST0000	.	3p2	.	.	.	.	rs3735896	.	.	.	.	.
.	ENST0000	.	3q1	.	.	.	.	.	.	.	.	.	.
DCBLD	ENST0000	.	3q1	.	.	.	.	.	.	.	.	.	.
.	ENST0000	.	3q1	.	.	.	.	.	.	.	.	.	.
.	ENST0000	.	3q1	.	.	.	.	.	.	.	.	.	.
COL6A	ENST0000	.	3q2	.	.	.	.	rs1456223	ID	.	.	0	.
PIK3C	ENST0000	.	3q2	.	.	Score=0.9	.	rs1048860	ID	Patho	.	.	.
.	ENST0000	.	4p1	.	.	.	.	.	.	.	.	.	.
.	ENST0000	.	4p1	.	.	Score=0.9	.	rs4018318	.	.	.	.	.
.	ENST0000	.	4p1	.	.	.	.	.	.	.	.	.	.
.	ENST0000	.	4p1	.	.	.	.	.	.	.	.	.	.
.	ENST0000	.	4q1	.	.	.	.	.	.	.	.	.	.
.	ENST0000	.	4q3	.	.	.	.	.	.	.	.	.	.
.	.	.	4q3	.	.	Score=0.9	Score	.	.	.	.	.	.
.	ENST0000	.	5p1	.	.	Score=0.9	.	.	.	.	.	.	.
.	ENST0000	.	5q1	.	.	.	.	.	.	.	.	.	.
.	ENST0000	.	5q2	.	.	.	.	.	.	.	.	.	.
.	ENST0000	.	5q3	.	.	.	.	.	.	.	.	.	.
PCDH	ENST0000	.	5q3	.	.	.	.	rs7735798	ID	.	.	.	.
FGF18:	ENST0000	.	5q3	.	.	.	.	.	ID	.	.	.	.
TBC1D	ENST0000	.	5q3	.	.	.	.	.	.	.	.	.	.
BTNL9:	ENST0000	.	5q3	.	.	.	.	rs3761176	ID	.	.	.	.
.	ENST0000	.	6p2	.	Scc	.	.	.	.	.	.	.	.
DHX16	ENST0000	.	6p2	.	Scc	.	.	rs1419237	ID	.	.	.	.
.	ENST0000	.	6q2	.	.	.	.	rs3687466	.	.	.	.	.
RAET1	ENST0000	.	6q2	.	.	Score=0.9	.	.	.	.	.	.	.
OPRM:	ENST0000	.	6q2	.	.	.	.	.	.	.	.	.	.
TIAM2:	ENST0000	.	6q2	.	.	.	.	rs2014763	.	.	.	.	.
.	.	.	6q2	.	.	Score=0.9	.	rs3759780	.	.	.	.	.
.	ENST0000	.	7p1	.	.	.	.	rs7676901	.	.	.	.	.
DDX56	ENST0000	.	7p1	.	.	.	.	.	.	.	.	.	.
.	ENST0000	.	7p1	.	.	Score=0.9	.	rs6246184	ID	.	.	.	.
.	ENST0000	.	7p1	.	.	Score=0.9	.	rs2013722	ID	.	.	.	.
.	ENST0000	.	7q1	.	.	Score=0.9	.	.	.	.	.	.	.
.	.	.	7q1	.	.	Score=0.9	.	.	.	.	.	.	.
MUC12	ENST0000	.	7q2	.	.	Score=0.9	.	rs7611895	.	.	.	.	.

.	ENST0000	.	7q3	.	.	.	.	.	.	.	.	.	.
KMT2C	ENST0000	.	7q3	.	.	Score=0.9	.	rs1419939	ID	.	.	.	.
.	ENST0000	.	7q3	.	.	.	.	.	.	.	.	.	.
.	ENST0000	.	8p2	.	.	.	Score	.	.	.	.	.	.
.	ENST0000	.	8p2	.	.	Score=0.9	.	rs7484521	ID	.	.	.	.
.	ENST0000	.	8p2	.	.	.	.	.	.	.	.	.	.
.	ENST0000	.	8p2	.	.	.	Score	.	ID	.	.	.	.
MMP16	ENST0000	.	8q2	.	Scc	.	.	.	.	.	.	.	.
.	ENST0000	.	8q2	.	.	.	.	rs5352633	.	.	.	0	0.001
.	ENST0000	.	8q2	.	.	.	Score	rs5594625	ID	.	.	0	0.001
ANXA1	ENST0000	.	8q2	.	.	.	.	rs3766154	.	.	.	.	.
.	ENST0000	.	8q2	.	.	.	.	.	.	.	.	.	.
OPLAH	ENST0000	.	8q2	.	.	.	.	.	.	.	.	.	.
PSIP1:I	ENST0000	.	9p2	.	.	.	.	.	.	.	.	.	.
.	ENST0000	.	9p2	.	Scc	.	.	.	.	.	.	.	.
.	ENST0000	.	9p1	.	.	Score=0.9	Score	rs2803685	ID	.	.	.	.
SPATA	ENST0000	.	9q2	.	.	Score=0.9	.	rs7553291	.	.	.	.	.
GAPVD	ENST0000	.	9q3	.	.	.	.	.	.	.	.	.	.
.	ENST0000	.	10p	.	.	.	.	rs1901417	.	.	.	0	.
ITGB1:	ENST0000	.	10p	.	Scc	.	.	.	.	.	.	.	.
.	ENST0000	.	10p	.	.	Score=0.9	.	rs4070334	ID	.	.	.	.
.	ENST0000	.	10q	.	.	.	Score	.	.	.	.	.	.
.	ENST0000	.	10q	.	.	.	.	.	.	.	.	.	.
.	ENST0000	.	10q	.	.	.	.	.	.	.	.	.	.
MUC6:I	ENST0000	.	11p	.	Scc	.	.	rs7488284	.	.	.	.	.
.	ENST0000	.	11p	.	.	.	.	.	.	.	.	.	.
.	ENST0000	.	11p	.	.	.	.	.	.	.	.	.	.
.	ENST0000	.	11p	.	.	.	Score	.	ID	.	.	.	.
.	ENST0000	.	11p	.	.	.	.	.	.	.	.	.	.
.	ENST0000	.	11p	.	.	.	Score	rs1470918	.	.	.	.	.
.	ENST0000	.	11q	.	Scc	.	.	.	.	.	.	.	.
C11orf9	ENST0000	.	11q	.	Scc	.	.	.	.	.	.	.	.
.	ENST0000	.	11q	.	.	Score=0.9	.	rs2010003	ID	.	.	.	.
.	ENST0000	.	11q	.	.	.	.	.	.	.	.	.	.
.	ENST0000	.	12q	.	.	.	.	.	.	.	.	.	.
.	ENST0000	.	12q	.	.	.	.	rs7761498	.	.	.	.	.
PAN2:N	ENST0000	.	12q	.	.	.	.	.	.	.	.	.	.
.	ENST0000	.	12q	.	.	.	.	.	.	.	.	.	.
.	ENST0000	.	12q	.	.	.	.	.	.	.	.	.	.
.	ENST0000	.	13q	.	.	.	.	.	.	.	.	.	.
.	ENST0000	.	13q	.	.	Score=0.9	.	.	.	.	.	.	.
.	.	.	13q	.	.	.	.	.	.	.	.	.	.
.	.	.	14q	.	.	Score=0.9	.	rs2259899	ID	.	.	.	.
.	ENST0000	.	14q	.	.	Score=0.9	.	rs7966403	ID	.	.	.	.
MMP14	ENST0000	.	14q	.	Scc	.	.	rs7511166	ID	.	.	.	.
.	ENST0000	.	14q	.	.	.	.	rs1127835	.	.	.	.	.
.	ENST0000	.	14q	.	.	.	.	.	.	.	.	.	.
CDC42	ENST0000	.	14q	.	Scc	.	.	.	.	.	.	.	.
.	.	.	15q	.	.	Score=0.9	.	rs7146667	ID	.	.	.	.
UBE3A	ENST0000	.	15q	.	Scc	.	.	.	.	.	.	.	.
.	ENST0000	.	15q	.	.	Score=0.9	.	.	.	.	.	.	.
BUB1B	ENST0000	.	15q	.	.	.	.	.	.	.	.	.	.
.	ENST0000	.	15q	.	.	Score=0.9	.	.	.	.	.	.	.
.	ENST0000	.	16p	.	.	.	.	.	.	.	.	.	.

.	ENST0000	.	16p	.	.	.	.	.	.	.	.	.	.
SPN:NI	ENST0000	.	16p	.	.	.	.	.	.	.	.	.	.
ITGAX:	ENST0000	.	16p	.	.	Score=0.9:	.	rs1450253	ID	.	.	.	.
SLC12/	ENST0000	.	16q	.	.	.	.	.	.	.	.	.	.
.	ENST0000	.	16q	.	.	.	.	rs1135006	ID	.	.	.	.
SGSM2	ENST0000	.	17p	.	Scc	.	.	rs7673846	.	.	.	.	.
GLP2R	ENST0000	.	17p	.	Scc	.	.	rs7606360	.	.	.	.	.
.	ENST0000	.	17p	.	.	.	.	.	.	.	.	.	.
.	ENST0000	.	17q	.	.	.	.	rs5549220	.	.	.	0	.
.	ENST0000	.	17q	.	.	.	.	.	.	.	.	.	.
.	ENST0000	.	17q	.	.	.	.	.	.	.	.	.	.
.	ENST0000	.	17q	.	.	.	.	rs7400170	.	.	.	0	.
.	ENST0000	.	18p	.	.	Score=0.9(	.	rs7837587	ID	.	.	.	.
.	ENST0000	.	18q	.	.	.	.	.	.	.	.	.	.
.	ENST0000	.	18q	.	.	.	.	.	.	.	.	.	.
.	ENST0000	.	18q	.	.	Score=0.9:	.	rs1466769	.	.	.	0.013	0.011
.	ENST0000	.	18q	.	.	.	.	rs5634889	.	.	.	0	.
ABCA7	ENST0000	.	19p	.	.	.	.	.	.	.	.	.	.
ACTL9:	ENST0000	.	19p	.	.	.	.	.	.	.	.	.	.
.	ENST0000	.	19p	.	.	.	Score	.	.	.	.	.	.
.	ENST0000	.	19p	.	.	.	.	.	.	.	.	.	.
.	.	.	19p	.	.	.	.	.	.	.	.	.	.
.	ENST0000	.	19p	.	.	.	.	.	.	.	.	.	.
.	ENST0000	.	19p	.	.	Score=0.9!	.	.	.	.	.	.	.
.	ENST0000	.	19q	.	.	.	Score	.	.	.	.	.	.
.	.	.	19q	.	.	Score=0.9{	Score	rs7125437	.	.	.	.	.
.	ENST0000	.	19q	.	.	.	.	rs1820521	.	.	.	0	.
OXT:NI	ENST0000	.	20p	.	.	.	.	.	.	.	.	.	.
GFRA4	ENST0000	.	20p	.	Scc	.	.	.	.	.	.	.	.
.	ENST0000	.	20p	.	.	.	.	.	.	.	.	.	.
GATA5	ENST0000	.	20q	.	.	.	.	.	.	.	.	.	.
.	.	.	21p	.	.	Score=0.9{	.	rs7964453	.	.	.	.	.
.	ENST0000	.	21p	.	.	Score=0.9{	.	rs3735529	ID	.	.	0.061	0.062
.	ENST0000	.	21p	.	.	Score=0.9{	Score	rs7280383	ID	.	.	.	.
.	ENST0000	.	21q	.	.	.	Score	.	.	.	.	.	.
CLIC6:I	ENST0000	.	21q	.	.	.	.	rs7738143	.	.	.	.	.
TRAPP	ENST0000	.	21q	.	Scc	.	.	rs5510648	.	.	.	0	.
PCBP3	ENST0000	.	21q	.	Scc	.	.	.	.	.	.	.	.
COL6A	ENST0000	.	21q	.	Scc	.	.	.	.	.	.	.	.
.	ENST0000	.	22q	.	.	Score=0.9!	.	rs1262847	.	.	.	.	.
.	ENST0000	.	22q	.	.	Score=0.9{	.	rs86008	ID	.	.	.	.
RNF21:	ENST0000	.	22q	.	.	.	.	.	.	.	.	.	.
.	ENST0000	.	Xp2	.	.	.	.	.	.	.	.	.	.
BEND2	ENST0000	.	Xp2	.	.	.	.	rs7787173	.	.	.	.	.
.	ENST0000	.	Xp1	.	.	.	.	.	.	.	.	.	.
.	.	.	Xq1	.	.	.	.	.	.	.	.	.	.
.	ENST0000	.	Xq1	.	.	.	Score	.	.	.	.	.	.
OGT:N	ENST0000	.	Xq1	.	Scc	.	.	.	ID	.	.	.	.
.	ENST0000	.	Xq2	.	.	Score=0.9:	.	.	.	.	.	.	.
.	ENST0000	.	Xq2	.	.	Score=0.9:	.	.	.	.	.	.	.
.	ENST0000	.	Xq2	.	.	.	.	rs9176062	.	.	.	.	.
.	.	.	Xq2	.	.	.	Score	rs9279180	.	.	.	.	.
IRAK1:	ENST0000	.	Xq2	.	.	.	.	.	.	.	.	.	.
ACTL8:	ENST0000	.	1p3	.	.	.	.	rs3718451	ID	.	.	.	.

WDTC:	ENST000C	.	1p3	.	Scc	.	.	.	.	.	.	.	.
.	ENST000C	.	1p3	.	.	.	.	rs7678484	.	.	.	.	.
GJA4:N	ENST000C	.	1p3	.	.	.	.	rs7603609	ID	.	.	.	.
.	ENST000C	.	1p3	.	.	.	.	.	.	.	.	.	.
TESK2	ENST000C	.	1p3	.	Scc	.	.	.	.	.	.	.	.
.	ENST000C	.	1p3	.	.	.	.	.	.	.	.	.	.
.	ENST000C	.	1p3	.	.	.	.	.	.	.	.	.	.
AMPD2	ENST000C	.	1p1	.	.	.	.	.	.	.	.	.	.
.	ENST000C	.	1p1	.	.	.	Score	rs7547967	ID	.	.	.	.
.	ENST000C	.	1q2	.	.	Score=0.9	Score	rs7528408	.	.	.	.	.
.	ENST000C	.	1q2	.	.	Score=0.9	Score	rs2009307	ID	.	.	.	.
.	ENST000C	.	1q2	.	.	Score=0.9	.	rs3726923	ID	.	.	.	.
.	ENST000C	.	1q2	.	.	Score=0.9	Score	rs8799825	.	.	.	.	.
.	ENST000C	.	1q3	.	.	.	.	.	.	.	.	.	.
.	ENST000C	.	1q4	.	.	.	.	rs7756172	.	.	.	.	.
TPO:NI	ENST000C	.	2p2	.	.	.	.	rs7587514	.	.	.	.	.
.	ENST000C	.	2q3	.	Scc	.	.	.	.	.	.	.	.
.	ENST000C	.	2q3	.	.	.	.	.	.	.	.	.	.
.	ENST000C	.	2q3	.	.	.	.	rs1411611	.	.	.	.	.
CELSR	ENST000C	.	3p2	.	.	.	.	rs1156481	ID	.	.	0	.
.	ENST000C	.	3q1	.	.	Score=0.9	.	.	.	.	.	.	.
.	ENST000C	.	3q2	.	.	.	.	.	.	.	.	.	.
ABCC5	ENST000C	.	3q2	.	.	.	.	.	.	.	.	.	.
PDE6B	ENST000C	.	4p1	.	Scc	.	.	.	.	.	.	.	.
GAK:NI	ENST000C	.	4p1	.	.	.	.	.	.	.	.	.	.
.	ENST000C	.	4p1	.	.	.	.	rs7307061	.	.	.	0	.
.	.	.	4p1	.	.	Score=0.9	.	rs8796734	.	.	.	.	.
.	ENST000C	.	4q2	.	.	.	Score	rs7690288	.	.	.	.	.
.	ENST000C	.	4q3	.	.	.	.	.	.	.	.	.	.
.	.	.	4q3	.	.	.	Score	rs1003755	.	.	.	.	.
.	ENST000C	.	5p1	.	.	.	.	.	.	.	.	.	.
RASGF	ENST000C	.	5q1	.	Scc	.	.	.	.	.	.	.	.
APC:NI	ENST000C	.	5q2	.	.	.	.	.	.	.	.	.	.
.	ENST000C	.	5q3	.	.	.	.	.	.	.	.	.	.
.	ENST000C	.	5q3	.	.	.	.	.	.	.	.	.	.
WWC1	ENST000C	.	5q3	.	Scc	.	.	.	.	.	.	.	.
ZSCAN	ENST000C	.	6p2	.	.	.	.	.	.	.	.	.	.
POU5F	ENST000C	.	6p2	.	.	.	.	rs7589117	.	.	.	.	.
.	ENST000C	.	6p1	.	.	.	.	.	.	.	.	.	.
TBX18:	ENST000C	.	6q1	.	Scc	.	.	.	ID	.	.	.	.
.	.	.	6q2	.	.	.	.	rs441907	ID	.	.	.	.
.	ENST000C	.	7q2	.	.	.	.	.	.	.	.	.	.
.	ENST000C	.	7q2	.	.	.	.	.	.	.	.	.	.
C7orf6(	ENST000C	.	7q3	.	.	.	.	.	ID	.	.	.	.
ZNF70!	ENST000C	.	8p2	.	.	Score=0.9	.	.	.	.	.	.	.
CSMD3	ENST000C	.	8q2	.	Scc	.	.	.	.	.	.	.	.
.	ENST000C	.	8q2	.	.	.	Score	.	.	.	.	.	.
NFIB:N	ENST000C	.	9p2	.	Scc	.	.	.	.	.	.	.	.
FKTN:N	ENST000C	.	9q3	.	.	.	.	.	.	.	.	.	.
OR1L6	ENST000C	.	9q3	.	.	.	.	.	.	.	.	.	.
.	ENST000C	.	10p	.	.	Score=0.9	.	.	.	.	.	.	.
.	.	.	10q	.	.	Score=0.9	.	rs3125109	ID	.	.	.	.
.	ENST000C	.	10q	.	.	.	.	.	.	.	.	.	.
.	ENST000C	.	10q	.	.	.	Score	rs7905448	ID	.	.	0.347	0.355

.	ENST0000	.	10q	.	.	.	.	.	.	.	.	.	.
MXI1:N	ENST0000	.	10q	.	.	.	.	rs7646688	ID	.	.	.	.
.	ENST0000	.	10q	.	.	.	.	.	.	.	.	.	.
DNHD1	ENST0000	.	11p	.	.	.	.	.	.	.	.	.	.
.	ENST0000	.	11p	.	.	Score=0.9!	.	.	.	.	.	.	.
UNKN(	ENST0000	.	11q	.	.	.	.	rs3775202	.	.	.	.	.
TRIM6	ENST0000	.	11q	.	.	Score=0.9!	.	.	.	.	.	.	.
.	.	.	11q	.	.	.	Score	rs7690752	.	.	.	.	.
SIDT2:I	ENST0000	.	11q	.	Scc	.	.	rs7585846	ID	.	.	.	.
ITPR2:I	ENST0000	.	12p	.	.	.	.	.	.	.	.	.	.
DDN:N	ENST0000	.	12q	.	.	.	.	.	.	.	.	.	.
.	ENST0000	.	12q	.	.	.	.	.	.	.	.	.	.
CDX2:I	ENST0000	.	13q	.	.	.	.	.	.	.	.	.	.
.	.	.	14q	.	.	Score=0.9	.	rs2259899	ID	.	.	.	.
.	.	.	14q	.	.	Score=0.9!	.	rs2014187	.	.	.	.	.
ZFP36I	ENST0000	.	14q	.	Scc	.	.	.	.	.	.	.	.
UNC13	ENST0000	.	15q	.	.	.	.	.	.	.	.	.	.
CACN/	ENST0000	.	16p	.	.	.	.	rs7764738	.	.	.	.	.
.	ENST0000	.	16p	.	.	.	.	.	.	.	.	.	.
.	ENST0000	.	16q	.	.	Score=0.9	.	rs2013540	ID	.	.	0.324	0.285
.	.	.	18p	.	.	Score=0.9	.	rs7502623	ID	.	.	0.623	0.62
.	ENST0000	.	19p	.	.	.	.	rs7589667	ID	.	.	.	.
COL5A	ENST0000	.	19p	.	.	.	.	.	.	.	.	.	.
.	ENST0000	.	19q	.	.	.	Score	.	.	.	.	.	.
.	ENST0000	.	19q	.	.	.	.	.	.	.	.	.	.
.	ENST0000	.	19q	.	.	.	.	.	.	.	.	.	.
SBK3:I	ENST0000	.	19q	.	Scc	.	.	rs9019919	.	.	.	.	.
ANKEF	ENST0000	.	20p	.	.	.	.	.	.	.	.	.	.
ANKEF	ENST0000	.	20p	.	.	.	.	rs7770803	.	.	.	.	.
AAR2:I	ENST0000	.	20q	.	.	.	.	rs7792821	.	.	.	.	.
DONS(	ENST0000	.	21q	.	.	.	.	.	.	.	.	.	.
TBC1D	ENST0000	.	22q	.	Scc	.	.	.	.	.	.	.	.
.	ENST0000	.	Xp2	.	.	.	Score	.	.	.	.	.	.
.	ENST0000	.	Xp2	.	.	Score=0.9(	.	.	.	.	.	.	.
ARAF:I	ENST0000	.	Xp1	.	Scc	.	.	.	.	.	.	.	.
.	ENST0000	.	Xp1	.	.	.	.	.	.	.	.	.	.
.	ENST0000	.	Xq2	.	.	.	.	.	.	.	.	.	.
.	ENST0000	.	Xq2	.	.	.	.	.	.	.	.	.	.
.	ENST0000	.	Xq2	.	.	.	.	.	.	.	.	.	.
ZIC3:N	ENST0000	.	Xq2	.	.	.	.	.	ID	.	.	.	.
.	ENST0000	.	Xq2	.	.	.	.	.	.	.	.	.	.
.	ENST0000	.	Xq2	.	.	Score=0.9!	.	rs4013617	.	.	.	.	.
.	.	.	.	.	.	.	.	.	ID	.	.	.	.

1000g2(	esp	ExA	ExA	SIF	Polyp	Polyp	Muta	gerp	CAD	Nov	Nov	INFO	FOR	ACa	ACa01T	Ori_REF	Ori_ALT
.	.	.	.	.	.	.	.	.	.	0	0.2	SON	GT:/	0:8,(	0/1:11,3:3	T	C
.	.	.	.	.	.	.	.	.	.	.	.	SON	GT:/	0:11	0/1:7,4:37:	A	G
.	.	.	.	.	.	.	.	.	.	.	.	SON	GT:/	0:50	0/1:83,12:3	C	T
.	.	.	.	.	.	.	.	.	.	.	.	SON	GT:/	0:13	0/1:19,3:37	C	T
.	.	##	0	0.9	0.001	0.001	1.00	.	.	.	.	SON	GT:/	0:71	0/1:135,26	G	A
.	.	.	.	.	.	.	.	.	.	.	.	SON	GT:/	0:21	0/1:35,13:3	C	T
.	.	.	.	.	.	.	.	.	.	0	.	SON	GT:/	0:8,(	0/1:3,2:37:	A	G
.	.	.	.	.	.	.	.	.	.	0	.	SON	GT:/	0:14	0/1:13,3:28	G	T
.	.	.	.	.	.	.	.	.	.	0	0	SON	GT:/	0:30	0/1:69,5:31	C	G
.	.	.	.	.	.	.	.	.	.	0	0	SON	GT:/	0:17	0/1:26,4:37	T	C
.	.	.	.	.	.	.	.	.	.	0	.	SON	GT:/	0:25	0/1:37,3:37	G	A
.	.	.	.	.	.	.	.	.	.	.	.	SON	GT:/	0:11	0/1:20,6:2(	A	C
.	0	##	0	.	.	.	.	3.3	4.54	.	.	SON	GT:/	0:19	0/1:54,14:3	G	A
.	.	.	.	.	.	.	.	.	.	.	.	SON	GT:/	0:49	0/1:83,13:3	G	A
.	.	.	.	.	.	.	.	.	.	.	.	SON	GT:/	0:14	0/1:26,14:3	A	C
.	.	.	.	.	.	.	.	.	.	.	.	SON	GT:/	0:13	0/1:20,7:37	G	A
.	.	.	.	.	.	.	.	.	.	.	.	SON	GT:/	0:10	0/1:21,3:37	A	T
.	.	.	.	.	.	.	.	.	.	.	.	DB;S	GT:/	0:23	0/1:27,3:37	G	A
.	.	.	.	.	.	.	.	.	.	0	0	SON	GT:/	0:18	0/1:24,3:37	A	C
.	.	.	.	.	.	.	.	.	.	0	.	SON	GT:/	0:9,(	0/1:16,4:37	T	C
.	.	.	.	.	.	.	.	.	.	0	0.1	SON	GT:/	0:17	0/1:18,4:37	G	A
.	.	.	.	.	.	.	.	.	.	0	0.3	SON	GT:/	0:12	0/1:14,3:37	C	G
.	.	.	.	.	.	.	.	.	.	0	.	SON	GT:/	0:42	0/1:52,4:34	A	T
.	.	.	.	.	.	.	.	.	.	0	0.1	SON	GT:/	0:30	0/1:46,6:37	C	G
.	.	.	.	.	.	.	.	.	.	.	.	SON	GT:/	0:43	0/1:74,6:37	G	A
0.005	.	.	.	.	.	.	.	.	.	0	0	SON	GT:/	0:8,(	0/1:10,4:37	C	T
.	.	.	.	.	.	.	.	4.8	4.02	.	.	SON	GT:/	0:11	0/1:244,50	T	C
.	.	.	.	.	.	.	.	.	.	0.1	0.5	SON	GT:/	0:8,(	0/1:4,2:37:	T	C
.	.	.	.	.	.	.	.	.	.	0	.	SON	GT:/	0:15	0/1:37,5:34	A	G
.	.	.	.	.	.	.	.	.	.	.	.	SON	GT:/	0:13	0/1:166,6:3	G	T
.	.	.	.	.	.	.	.	.	.	0	.	SON	GT:/	0:37	0/1:64,4:37	G	A
.	.	.	.	.	.	.	.	.	.	.	.	SON	GT:/	0:36	0/1:48,31:3	C	T
.	.	.	.	.	.	.	.	.	.	.	.	SON	GT:/	0:57	0/1:65,5:31	C	G
.	.	.	.	.	.	.	.	.	.	0	0.4	SON	GT:/	0:14	0/1:17,9:37	G	A
0.014	.	.	.	.	.	.	.	.	.	0	.	DB;S	GT:/	0:40	0/1:89,4:37	A	C
.	.	##	0	.	.	.	.	.	.	.	.	SON	GT:/	0:12	0/1:160,70	C	T
0.759	.	.	.	.	.	.	.	.	.	0.2	0.7	SON	GT:/	0:9,(	0/1:18,4:37	C	T
.	0	0	0	0.2	0.006	0.001	1,N	.	.	0	0	SON	GT:/	0:21	0/1:33,3:37	T	G
.	.	##	.	0.1	0.002	0.003	1,D	3.5	3.54	0	.	SON	GT:/	0:15	0/1:190,11	G	A
.	.	.	.	.	.	.	.	.	.	.	.	SON	GT:/	0:24	0/1:45,21:3	G	T
.	.	.	.	.	.	.	.	.	1.82	0	.	SON	GT:/	0:17	0/1:31,9:15	A	C
.	.	.	.	.	.	.	.	.	.	.	.	SON	GT:/	0:49	0/1:64,22:3	A	C
.	.	0	0	.	.	.	.	.	.	0	0.1	SON	GT:/	0:54	0/1:111,5:3	A	G
.	.	.	.	.	.	.	.	.	.	0.2	0.1	DB;S	GT:/	0:64	0/1:130,6:3	A	G
.	.	.	.	.	.	.	.	.	.	0.1	0.1	DB;S	GT:/	0:28	0/1:62,7:37	G	T
0.066	.	.	.	.	.	.	.	.	.	0	0	DB;S	GT:/	0:24	0/1:21,7:37	C	A
.	.	.	.	.	.	.	.	.	.	0	.	SON	GT:/	0:14	0/1:9,3:37:	G	A
.	.	.	.	.	.	.	.	.	.	.	.	SON	GT:/	0:8,(	0/1:6,2:37:	C	T
.	.	.	.	0.1	0.168	0.087	1,D	3.4	2.35	.	.	SON	GT:/	0:10	0/1:149,65	A	G
.	.	.	.	.	.	.	.	.	.	0.1	0.2	SON	GT:/	0:66	0/1:95,5:37	T	G

.	.	.	.	.	.	.	.	.	.	0	0	SOM	GT:/	0:8,(	0/1:11,4:27	A	G
0.739	.	1	1	.	.	.	.	.	.	0	0	SOM	GT:/	0:25	0/1:19,6:37	T	C
.	.	.	.	0.0(	0.965	0.998	1,N	.	.	.	.	SOM	GT:/	0:12	0/1:186,34	C	A
.	.	.	.	.	.	.	.	.	.	.	.	SOM	GT:/	0:19	0/1:37,3:37	C	T
.	.	.	.	0.0(	0.232	0.347	1.00	5.5	2.28	.	.	SOM	GT:/	0:57	0/1:104,17	A	T
.	.	.	.	.	.	.	.	.	.	.	.	SOM	GT:/	0:20	0/1:35,7:18	T	G
.	.	.	.	0.0(	0.006	0.006	0.81	4.4	2.83	.	.	SOM	GT:/	0:13	0/1:262,16	C	T
.	.	.	.	0.8(	0.004	0.0,B	0.98	2.4	.	.	.	SOM	GT:/	0:38	0/1:50,25:3	T	G
.	.	##	0	.	.	.	.	.	.	.	.	SOM	GT:/	0:54	0/1:93,4:37	C	T
.	.	.	.	.	.	.	.	.	.	.	.	SOM	GT:/	0:44	0/1:78,38:3	C	T
.	.	.	.	.	.	.	.	.	.	0	.	SOM	GT:/	0:32	0/1:67,4:37	A	C
.	.	.	.	.	.	.	.	.	.	0	.	SOM	GT:/	0:29	0/1:53,15:1	T	G
.	.	.	.	.	.	.	.	3.4	.	.	.	SOM	GT:/	0:22	0/1:38,8:14	T	G
.	.	.	.	0.3(	0.12,I	0.167	1.00	5.7	2.23	.	.	SOM	GT:/	0:22	0/1:31,29:3	C	G
.	.	.	.	.	.	.	.	.	.	.	.	SOM	GT:/	0:10	0/1:226,71	G	A
.	0	0	0	0.0.	0.997	1.0,D	1.00	4.9	5.22	.	.	SOM	GT:/	0:57	0/1:145,9:3	C	T
2E-04	0	0	0	0.0(	0.399	0.932	1,N	.	.	.	.	SOM	GT:/	0:11	0/1:304,48	C	T
.	.	.	.	.	.	.	.	.	1.45	.	.	SOM	GT:/	0:44	0/1:68,12:3	G	A
4E-04	.	0	.	.	.	.	.	.	.	0	0	DB;S	GT:/	0:62	0/1:139,10	G	T
.	.	.	.	.	.	.	.	.	.	.	.	SOM	GT:/	0:12	0/1:19,4:37	C	G
.	.	##	0	.	.	.	.	.	.	.	.	SOM	GT:/	0:39	0/1:54,6:32	C	T
.	.	.	.	1.0.	0.129	0.478	1.00	5.9	.	.	.	SOM	GT:/	0:27	0/1:45,20:3	G	A
.	.	.	.	.	.	.	.	.	.	.	.	SOM	GT:/	0:15	0/1:28,3:37	C	T
.	.	.	.	.	.	.	.	.	.	0	0.2	SOM	GT:/	0:16	0/1:10,6:37	T	C
.	.	.	.	.	.	.	.	.	.	0	0	SOM	GT:/	0:16	0/1:29,5:37	C	T
.	.	.	.	.	.	.	.	.	.	0.2	0.2	DB;S	GT:/	0:24	0/1:38,4:37	C	T
.	.	.	.	.	.	.	.	.	.	0	.	SOM	GT:/	0:22	0/1:27,6:17	T	G
.	.	.	.	.	.	.	.	.	.	0	0	SOM	GT:/	0:9,(	0/1:2,3:28:	C	T
.	.	.	.	.	.	.	.	.	.	0	.	SOM	GT:/	0:10	0/1:15,4:37	G	A
.	.	.	.	0.1'	.	.	0.94	.	.	.	.	SOM	GT:/	0:36	0/1:63,20:3	C	T
.	0	##	0	.	.	.	.	.	.	0	.	SOM	GT:/	0:19	0/1:27,13:3	C	T
.	.	.	.	.	.	.	.	.	.	.	.	SOM	GT:/	0:44	0/1:57,7:37	C	T
.	.	.	.	.	.	.	.	.	.	0	0	DB;S	GT:/	0:51	0/1:170,7:3	T	C
.	.	.	.	.	.	.	.	.	.	.	.	SOM	GT:/	0:40	0/1:119,6:3	G	A
.	.	.	.	.	.	.	.	.	.	.	.	SOM	GT:/	0:21	0/1:55,11:3	C	A
.	.	.	.	.	.	.	.	.	.	0	0	SOM	GT:/	0:36	0/1:120,8:3	A	G
.	.	.	.	.	.	.	.	.	2.36	.	.	SOM	GT:/	0:11	0/1:34,5:21	T	G
.	.	.	.	.	.	.	.	.	.	0.1	0.2	SOM	GT:/	0:16	0/1:37,5:37	T	C
.	.	.	.	.	.	.	.	.	.	.	.	SOM	GT:/	0:67	0/1:88,4:37	C	G
.	.	.	.	.	.	.	.	.	.	.	.	SOM	GT:/	0:26	0/1:54,25:3	A	G
.	.	##	0	.	.	.	.	.	.	.	.	SOM	GT:/	0:48	0/1:109,20	C	T
.	.	.	.	.	.	.	.	.	1.72	.	.	SOM	GT:/	0:43	0/1:59,39:3	C	T
.	.	##	0	0.0(	0.998	1.0,D	1,D	5.7	3.22	.	.	SOM	GT:/	0:71	0/1:153,10	C	T
.	.	.	.	.	.	.	.	.	1.61	0.1	0	SOM	GT:/	0:9,(	0/1:12,6:37	A	C
.	.	.	.	.	.	.	.	.	.	.	0	DB;S	GT:/	0:22	0/1:35,5:31	G	C
2E-04	.	.	.	.	.	.	.	.	.	.	.	SOM	GT:/	0:37	0/1:43,15:3	G	A
.	.	.	.	0.4.	0.001	0.0,B	0.99	.	.	.	.	SOM	GT:/	0:51	0/1:40,36:3	T	G
.	.	.	.	.	.	.	.	.	.	.	.	SOM	GT:/	0:15	0/1:19,5:37	T	C
.	.	.	.	0.0:	0.991	0.998	1,D	5.2	4.56	.	.	SOM	GT:/	0:30	0/1:66,5:37	A	G
.	.	.	.	.	.	.	.	.	.	.	.	SOM	GT:/	0:8,(	0/1:13,8:37	C	T
.	.	.	.	.	.	.	.	.	.	.	.	SOM	GT:/	0:87	0/1:114,54	T	A
.	.	.	.	.	.	.	.	.	.	0	0	SOM	GT:/	0:10	0/1:17,6:37	T	C
.	.	.	.	0.1!	0.01,I	0.063	1,N	.	.	.	.	SOM	GT:/	0:22	0/1:38,11:3	C	T
.	.	##	0	.	.	.	.	.	.	.	.	SOM	GT:/	0:81	0/1:132,7:3	G	A

.	.	.	.	.	.	.	.	.	.	.	.	SOM	GT:	0:65	0/1:78,45:3	G	A
0.038	0	0	0	.	.	.	.	.	.	.	.	DB;S	GT:	0:43	0/1:63,8:37	C	T
.	.	.	.	.	.	.	.	.	.	0	0	SOM	GT:	0:23	0/1:42,4:37	C	T
.	.	.	.	.	.	.	.	.	.	.	.	SOM	GT:	0:11	0/1:24,9:13	G	T
4E-04	.	.	.	.	.	.	.	.	.	.	.	DB;S	GT:	0:19	0/1:40,3:37	G	C
.	.	.	.	.	.	.	.	.	.	.	.	SOM	GT:	0:38	0/1:60,27:3	G	A
.	.	.	.	.	.	.	.	.	.	.	.	SOM	GT:	0:23	0/1:176,6:3	C	T
0.125	.	.	.	.	.	.	.	.	.	0	0	SOM	GT:	0:8,	0/1:22,3:37	C	T
0.016	.	.	.	.	.	.	.	.	.	0	0	SOM	GT:	0:10	0/1:20,4:37	G	C
.	.	.	.	.	.	.	.	.	.	.	.	SOM	GT:	0:12	0/1:58,16:3	C	T
.	.	.	.	0.0	0.367	0.883	1,N	.	.	.	.	SOM	GT:	0:10	0/1:143,81	A	C
.	.	.	.	.	.	.	.	.	.	0	.	DB;S	GT:	0:22	0/1:64,5:37	G	T
.	.	.	.	.	.	.	.	.	.	.	.	SOM	GT:	0:52	0/1:66,7:35	G	T
.	.	.	.	.	.	.	.	.	.	.	.	SOM	GT:	0:90	0/1:91,17:3	G	A
0.098	.	.	.	.	.	.	.	.	.	0	.	SOM	GT:	0:28	0/1:41,4:37	G	A
.	.	.	.	.	.	.	.	.	.	0	.	SOM	GT:	0:30	0/1:42,4:37	C	G
.	.	.	.	.	.	.	.	.	.	0	.	SOM	GT:	0:12	0/1:12,6:35	G	A
.	.	.	.	.	.	.	.	.	.	.	.	SOM	GT:	0:34	0/1:70,5:37	G	T
.	.	##	0	.	.	.	.	.	.	.	.	SOM	GT:	0:41	0/1:74,7:37	G	A
.	0	0	0	.	.	.	.	.	.	0.1	0.1	SOM	GT:	0:39	0/1:92,7:37	G	C
.	.	##	0	0.0	0.994	1.0,D	1,D	4.6	3.91	.	.	SOM	GT:	0:49	0/1:81,12:3	G	A
.	.	.	.	.	.	.	.	2.4	.	.	.	SOM	GT:	0:69	0/1:70,35:3	G	A
.	.	0	0	.	.	.	.	.	.	0	0	SOM	GT:	0:88	0/1:131,7:3	A	G
.	.	0	0	0.0	1.0,D	1.0,D	1.00	4.9	4.26	0	0	SOM	GT:	0:10	0/1:177,10	C	T
.	.	.	.	.	.	.	.	.	2.11	.	.	SOM	GT:	0:21	0/1:29,3:37	C	T
.	.	.	.	.	.	.	.	.	.	0.3	0.4	SOM	GT:	0:11	0/1:8,3:28:	C	T
.	.	.	.	.	.	.	.	.	.	0.3	0.4	SOM	GT:	0:12	0/1:7,3:33:	T	C
.	.	.	.	.	.	.	.	.	.	.	.	SOM	GT:	0:64	0/1:1256,3	C	A
.	.	.	.	.	.	.	.	.	.	.	.	SOM	GT:	0:10	0/1:10,5:37	A	G
.	.	.	.	.	.	.	.	.	.	0	.	SOM	GT:	0:15	0/1:21,6:17	A	C
.	.	.	.	0.0	0.833	0.699	1,D	5.6	4.01	.	.	SOM	GT:	0:12	0/1:18,7:18	T	G
4E-04	.	.	.	.	.	.	.	.	.	.	.	SOM	GT:	0:10	0/1:16,11:3	G	A
.	.	.	.	.	.	.	.	.	.	0	.	SOM	GT:	0:14	0/1:67,5:37	A	G
.	.	.	.	.	.	.	.	.	.	.	.	SOM	GT:	0:16	0/1:15,8:33	T	C
0.001	0	0	0	.	.	.	.	.	.	0	0	SOM	GT:	0:35	0/1:50,8:37	A	T
.	.	0	0	.	.	.	.	.	.	.	.	DB;S	GT:	0:38	0/1:115,5:3	T	C
.	.	.	.	.	.	.	.	.	.	0	0	DB;S	GT:	0:55	0/1:89,4:37	T	C
.	.	.	.	.	.	.	.	.	.	0.1	0.2	SOM	GT:	0:12	0/1:21,6:37	C	A
.	.	.	.	.	.	.	.	.	.	0	0.1	SOM	GT:	0:9,	0/1:10,3:37	G	A
.	.	0	0	.	.	.	.	.	.	0	0	DB;S	GT:	0:50	0/1:119,6:3	A	C
.	.	.	.	.	.	.	.	.	.	.	.	SOM	GT:	0:17	0/1:226,10	G	A
.	.	.	.	.	.	.	.	.	.	0	0.1	SOM	GT:	0:9,	0/1:13,7:37	G	A
.	.	.	.	.	.	.	.	3.7	.	.	.	SOM	GT:	0:85	0/1:98,58:3	C	T
.	.	.	.	.	.	.	.	5.6	2.81	.	.	SOM	GT:	0:39	0/1:61,10:1	T	G
.	.	##	0	.	.	.	.	.	.	.	.	SOM	GT:	0:85	0/1:115,53	A	G
.	.	##	0	.	.	.	.	.	.	.	.	SOM	GT:	0:11	0/1:171,53	C	T
.	.	.	.	0.0	1.0,D	1.0,D	1,D	5.7	5.41	.	.	SOM	GT:	0:55	0/1:70,32:3	G	A
.	.	.	.	.	.	.	.	.	.	.	.	DB;S	GT:	0:37	0/1:72,5:37	T	G
.	.	.	.	0.1	0.022	0.02,I	1,N	.	.	.	.	SOM	GT:	0:31	0/1:29,5:37	C	T
.	.	.	.	0.0	0.996	1.0,D	1,D	5.3	4.51	.	.	SOM	GT:	0:18	0/1:27,13:3	G	A
.	.	##	0	0.1	0.001	0.002	1,N	.	.	0	.	SOM	GT:	0:67	0/1:84,37:3	G	A
.	.	##	0	.	.	.	.	.	.	.	.	SOM	GT:	0:12	0/1:189,82	C	T
.	.	0	0	.	.	.	.	.	.	0	.	DB;S	GT:	0:45	0/1:74,4:37	G	T
.	.	.	.	.	.	.	.	.	.	.	.	SOM	GT:	0:24	0/1:35,14:3	C	T

.	.	.	.	.	.	.	.	.	.	0	0.4	SON	GT:/	0:8,(	0/1:3,4:37:	A	C
2E-04	.	.	.	.	.	.	.	.	.	0	.	SON	GT:/	0:54	0/1:83,14:3	C	T
.	.	0	0	.	.	.	.	.	.	0	.	SON	GT:/	0:39	0/1:59,5:37	T	A
.	.	##	0	.	.	.	.	5.4	2.57	.	.	SON	GT:/	0:94	0/1:144,42	C	T
.	.	.	.	.	.	.	.	.	.	.	.	SON	GT:/	0:9,(	0/1:3,3:24:	C	A
.	.	.	.	.	.	.	.	2.7	.	.	.	SON	GT:/	0:90	0/1:133,26	C	T
2E-04	0	##	0	.	.	.	.	.	.	.	.	SON	GT:/	0:12	0/1:191,27	C	T
2E-04	##	##	0	0.1(	0.056	0.685	1,N	4.4	.	.	.	SON	GT:/	0:44	0/1:55,19:3	G	A
.	.	.	.	.	.	.	.	.	.	.	.	SON	GT:/	0:17	0/1:20,4:37	G	T
.	.	.	.	.	.	.	.	2.1	.	.	.	SON	GT:/	0:51	0/1:53,7:2(	A	C
.	.	.	.	.	.	.	.	.	.	0	0	SON	GT:/	0:38	0/1:72,5:37	C	T
.	.	.	.	.	.	.	.	.	.	.	.	SON	GT:/	0:20	0/1:9,3:28:	A	G
.	.	.	.	.	.	.	.	.	.	.	.	SON	GT:/	0:22	0/1:37,24:3	G	A
.	.	.	.	.	.	.	.	.	.	.	.	SON	GT:/	0:9,(	0/1:14,6:37	G	T
.	.	.	.	.	.	.	.	.	.	0	0	SON	GT:/	0:11	0/1:19,3:37	A	T
.	.	.	.	.	.	.	.	.	.	.	.	SON	GT:/	0:8,(	0/1:18,9:35	G	A
.	.	.	.	.	.	.	.	.	.	.	.	SON	GT:/	0:8,(	0/1:16,3:37	T	G
.	.	.	.	.	.	.	.	.	.	.	.	SON	GT:/	0:84	0/1:141,48	G	A
.	.	.	.	0.0	0.295	0.594	0.99	.	2.32	0	.	SON	GT:/	0:43	0/1:79,5:37	T	A
.	.	.	.	.	.	.	.	.	.	0	.	SON	GT:/	0:20	0/1:33,13:1	T	G
.	.	.	.	.	.	.	.	2.4	.	0	.	SON	GT:/	0:20	0/1:33,13:1	T	G
.	.	##	0	.	.	.	.	.	2.22	.	.	SON	GT:/	0:10	0/1:137,62	C	T
0.001	0	0	0	0.0(	0.029	0.595	0.98	5.2	2.47	0	.	SON	GT:/	0:43	0/1:87,23:3	C	T
.	.	.	.	.	.	.	.	.	.	.	.	SON	GT:/	0:13	0/1:251,18	G	T
.	.	##	.	0.1(	0.001	0.002	1,N	.	.	0	.	SON	GT:/	0:89	0/1:175,16	C	T
0.155	.	.	.	.	.	.	.	.	.	0.1	0.1	DB;S	GT:/	0:47	0/1:94,5:34	T	C
0.205	.	.	.	.	.	.	.	.	.	0.1	0	SON	GT:/	0:19	0/1:56,4:37	T	C
.	.	.	.	.	.	.	.	.	.	0	.	SON	GT:/	0:66	0/1:77,9:37	C	G
.	.	.	.	0.0	0.949	0.994	1,D	5.2	4.41	.	.	SON	GT:/	0:46	0/1:36,41:3	A	C
.	.	.	.	.	.	.	.	.	.	0	0.1	SON	GT:/	0:9,(	0/1:5,2:37:	T	C
.	.	.	.	.	.	.	.	.	.	0	.	SON	GT:/	0:8,(	0/1:17,3:37	A	G
.	.	.	.	.	.	.	.	.	.	.	.	SON	GT:/	0:25	0/1:28,14:3	G	A
.	.	.	.	.	.	.	.	.	1.67	.	.	SON	GT:/	0:22	0/1:42,22:3	G	C
.	.	.	.	.	.	.	.	3.6	.	.	.	SON	GT:/	0:47	0/1:71,33:3	C	T
.	.	.	.	.	.	.	.	.	1.42	0	.	SON	GT:/	0:55	0/1:116,5:3	A	C
.	.	.	.	.	.	.	.	.	.	.	.	SON	GT:/	0:21	0/1:36,5:37	G	A
.	.	.	.	.	.	.	.	.	.	.	.	SON	GT:/	0:15	0/1:349,13	C	T
.	.	.	.	.	.	.	.	.	.	.	.	SON	GT:/	0:15	0/1:53,4:37	C	T
.	.	.	.	.	.	.	.	3.2	1.26	.	.	SON	GT:/	0:42	0/1:65,27:3	C	T
.	.	.	.	0.0	0.967	0.993	0.99	3.4	1.91	.	.	SON	GT:/	0:91	0/1:163,16	C	A
.	.	.	.	.	.	.	.	.	.	0	0	SON	GT:/	0:22	0/1:37,3:37	G	A
.	.	.	.	.	.	.	.	.	.	0	0	SON	GT:/	0:16	0/1:22,3:33	C	T
.	.	.	.	.	.	.	.	.	.	0	.	SON	GT:/	0:8,(	0/1:5,2:37:	G	A
.	.	.	.	.	.	.	.	.	.		0	SON	GT:/	0:8,(	0/1:7,3:37:	C	T
.	.	.	.	.	.	.	.	.	.	0	0	SON	GT:/	0:11	0/1:13,3:37	G	T
.	.	.	.	0.0(	0.131	0.164	0.82	3.6	2.01	.	.	SON	GT:/	0:88	0/1:106,76	C	T
.	.	.	.	.	.	.	.	3.6	.	0	.	SON	GT:/	0:11	0/1:10,6:2(	A	C
.	.	.	.	.	.	.	.	.	.	0	.	SON	GT:/	0:11	0/1:11,5:19	A	C
.	.	.	.	.	.	.	.	.	.	.	.	SON	GT:/	0:33	0/1:44,32:3	G	A
.	.	.	.	.	.	.	.	2.8	1.36	.	.	SON	GT:/	0:18	0/1:33,13:3	G	A
.	.	.	.	.	.	.	.	.	.	.	.	SON	GT:/	0:13	0/1:15,11:3	T	G
.	.	##	0	0.0(	0.352	0.934	1,D	4.3	1.26	.	.	SON	GT:/	0:89	0/1:100,51	G	A
.	.	.	.	.	.	.	.	.	1.47	.	.	SON	GT:/	0:24	0/1:47,3:33	G	T
.	.	.	.	.	.	.	.	.	.	0.1	0.3	SON	GT:/	0:8,(	0/1:2,3:33:	C	T

.	.	.	.	.	.	.	.	.	.	0.1	0	SOM	GT:/	0:11	0/1:14,6:3	T	A
.	.	.	.	.	.	.	.	.	.	0	0.4	DB;S	GT:/	0:22	0/1:22,3:3	T	C
.	.	.	.	.	.	.	.	.	1.48	.	.	SOM	GT:/	0:47	0/1:1228,6	C	A
.	.	.	.	.	.	.	.	.	.	0	0	SOM	GT:/	0:10	0/1:28,3:3	T	G
.	.	.	.	.	.	.	.	.	.	0	0.1	SOM	GT:/	0:42	0/1:87,4:3	G	A
.	.	.	.	.	.	.	.	.	.	0	0	SOM	GT:/	0:40	0/1:75,12:	C	T
.	.	.	.	.	.	.	.	.	.	.	.	SOM	GT:/	0:36	0/1:58,5:3	C	T
.	.	.	.	.	.	.	.	.	.	.	.	SOM	GT:/	0:70	0/1:179,8:	C	T
.	.	.	.	.	.	.	.	.	.	.	.	SOM	GT:/	0:26	0/1:34,4:3	T	C
.	.	.	.	.	.	.	.	.	.	.	.	SOM	GT:/	0:18	0/1:52,10:	A	G
0.379	.	.	.	.	.	.	.	.	.	0	0.2	SOM	GT:/	0:8,(	0/1:12,5:3	C	T
.	.	.	.	.	.	.	.	.	1.74	.	.	SOM	GT:/	0:99	0/1:351,12	G	A
.	.	.	.	.	.	.	.	.	.	.	.	SOM	GT:/	0:20	0/1:638,22	G	A
.	.	##	0	0.0	.	.	1,N	.	.	.	.	SOM	GT:/	0:11	0/1:379,32	C	T
.	.	.	.	0.0	0.993	1.0,D	1.00	5.1	4.80	.	.	SOM	GT:/	0:71	0/1:181,49	G	A
.	.	.	.	.	.	.	.	.	.	.	.	SOM	GT:/	0:51	0/1:123,5:	T	C
.	.	.	.	.	.	.	.	.	.	0	.	SOM	GT:/	0:41	0/1:50,4:3	C	A
.	.	.	.	.	.	.	.	.	.	.	.	SOM	GT:/	0:49	0/1:57,4:3	G	A
.	.	.	.	.	.	.	.	.	.	0	.	SOM	GT:/	0:93	0/1:126,16	G	T
.	.	.	.	.	.	.	.	2.5	.	0	.	SOM	GT:/	0:8,(	0/1:54,4:3	G	A
.	.	.	.	.	.	.	.	.	.	.	.	SOM	GT:/	0:16	0/1:56,11:	G	A
.	.	.	.	.	.	.	.	.	.	.	.	SOM	GT:/	0:49	0/1:155,10	C	T
4E-04	.	.	.	.	.	.	.	.	.	0	.	SOM	GT:/	0:11	0/1:309,29	G	A
.	.	.	.	.	.	.	.	.	.	0	.	SOM	GT:/	0:10	0/1:14,4:3	A	G
.	.	0	0	0.3	.	.	1,N	.	.	.	.	SOM	GT:/	0:24	0/1:42,3:3	A	G
.	.	0	0	.	.	.	.	.	.	.	.	SOM	GT:/	0:14	0/1:69,9:3	A	G
.	.	.	.	.	.	.	.	.	.	.	.	SOM	GT:/	0:56	0/1:149,39	G	A
.	.	.	.	.	.	.	.	.	.	.	.	SOM	GT:/	0:23	0/1:70,7:3	C	T
.	.	##	0	.	.	.	.	.	1.60	.	0	SOM	GT:/	0:89	0/1:235,14	C	T
.	.	.	.	.	.	.	.	3.8	.	.	.	SOM	GT:/	0:10	0/1:219,69	G	A
8E-04	.	.	.	.	.	.	.	.	.	0	0	SOM	GT:/	0:26	0/1:79,4:3	C	T
.	.	.	.	.	.	.	.	.	.	.	.	DB;S	GT:/	0:24	0/1:36,3:3	T	C
.	.	.	.	.	.	.	.	.	.	0	.	DB;S	GT:/	0:34	0/1:42,3:3	T	C
.	.	.	.	.	.	.	.	.	.	.	.	DB;S	GT:/	0:23	0/1:31,3:3	T	C
.	.	.	.	.	.	.	.	.	.	.	.	DB;S	GT:/	0:56	0/1:107,5:	C	G
.	.	.	.	.	.	.	.	.	.	0	.	DB;S	GT:/	0:84	0/1:189,7:	T	A
.	.	.	.	.	.	.	.	.	.	.	.	DB;S	GT:/	0:31	0/1:25,5:3	T	C
.	.	.	.	.	.	.	.	.	.	0	.	SOM	GT:/	0:42	0/1:131,5:	C	T
.	.	.	.	.	.	.	.	.	.	0	.	SOM	GT:/	0:25	0/1:50,4:3	T	G
.	.	.	.	.	.	.	.	.	.	.	.	SOM	GT:/	0:40	0/1:80,4:3	A	T
.	.	.	.	.	.	.	.	.	.	0	.	DB;S	GT:/	0:66	0/1:155,8:	A	G
.	.	.	.	.	.	.	.	.	.	.	.	DB;S	GT:/	0:49	0/1:97,5:3	A	T
.	.	.	.	.	.	.	.	.	.	0	.	DB;S	GT:/	0:36	0/1:56,4:3	C	G
.	.	.	.	.	.	.	.	.	.	0	.	DB;S	GT:/	0:47	0/1:73,5:3	T	C
.	.	.	.	.	.	.	.	.	.	0	.	DB;S	GT:/	0:36	0/1:49,8:3	T	C
.	.	.	.	.	.	.	.	.	.	0	.	DB;S	GT:/	0:44	0/1:102,5:	A	T
.	.	.	.	.	.	.	.	.	.	0	.	SOM	GT:/	0:20	0/1:37,4:3	A	G
.	.	.	.	.	.	.	.	.	.	.	.	DB;S	GT:/	0:20	0/1:29,3:3	G	A
.	.	.	.	.	.	.	.	.	.	0	.	DB;S	GT:/	0:36	0/1:68,4:3	C	G
.	.	.	.	.	.	.	.	.	.	0	.	DB;S	GT:/	0:33	0/1:60,4:3	A	C
.	.	.	.	.	.	.	.	.	.	0	.	SOM	GT:/	0:20	0/1:41,3:3	G	A
.	.	.	.	.	.	.	.	.	.	.	.	SOM	GT:/	0:13	0/1:31,3:3	T	C
.	.	.	.	.	.	.	.	.	.	.	.	SOM	GT:/	0:9,(	0/1:17,7:1	A	C
.	.	.	.	.	.	.	1,A	5	5.87	.	.	SOM	GT:/	0:76	0/1:74,21:	C	T

.	.	.	.	.	.	.	.	.	.	0	0	SON	GT:/	0:9,(	0/1:5,4:24:	C	G
.	.	.	.	0.0(	0.586	0.998	1,N	2.8	.	.	.	SON	GT:/	0:49	0/1:47,40:3	C	T
.	.	##	0	.	.	.	.	.	1.43	.	0	SON	GT:/	0:74	0/1:115,48	G	A
.	.	.	.	0.2(	0.058	0.082	1,D	3.4	3.15	.	.	SON	GT:/	0:25	0/1:40,19:3	T	C
4E-04	0	##	0	0.5	0.007	0.093	0.99	.	.	.	.	SON	GT:/	0:93	0/1:133,64	G	A
.	.	.	.	.	.	.	.	.	.	.	.	SON	GT:/	0:21	0/1:30,9:12	A	C
0.006	.	0	0	.	.	.	.	.	.	.	.	SON	GT:/	0:13	0/1:217,10	C	T
.	.	.	.	.	.	.	.	.	.	.	.	SON	GT:/	0:89	0/1:141,71	G	A
.	.	.	.	.	.	.	.	.	.	.	.	SON	GT:/	0:63	0/1:114,5:3	G	T
.	.	.	.	.	.	.	.	.	.	0	.	SON	GT:/	0:28	0/1:55,4:37	C	G
.	.	.	.	.	.	.	.	.	.	0	0	DB;S	GT:/	0:26	0/1:20,5:37	G	T
.	.	.	.	.	.	.	.	.	.	0	.	SON	GT:/	0:9,(	0/1:12,3:37	C	A
.	.	.	.	.	.	.	.	.	.	0	0	SON	GT:/	0:33	0/1:53,4:37	T	G
.	.	.	.	.	.	.	.	.	.	0	0	DB;S	GT:/	0:34	0/1:55,4:37	T	G
.	.	.	.	.	.	.	.	.	.	0	.	SON	GT:/	0:34	0/1:55,5:37	G	A
.	.	.	.	.	.	.	.	.	.	.	.	SON	GT:/	0:10	0/1:5,2:37:	G	C
.	.	.	.	.	.	.	.	.	.	.	.	SON	GT:/	0:16	0/1:34,17:3	T	A
0.021	.	.	.	.	.	.	.	.	.	0	0	DB;S	GT:/	0:37	0/1:58,13:3	A	T
.	.	.	.	.	.	.	.	2.3	.	0	.	SON	GT:/	0:44	0/1:84,16:1	T	G
.	.	.	.	.	.	.	.	.	.	0	0.2	SON	GT:/	0:34	0/1:82,5:37	G	A
.	.	.	.	.	.	.	.	.	.	0	0.1	SON	GT:/	0:77	0/1:132,5:3	T	C
.	.	.	.	.	.	.	.	2.6	.	.	.	SON	GT:/	0:54	0/1:65,30:3	G	A
.	.	.	.	.	.	.	.	.	.	.	.	SON	GT:/	0:37	0/1:39,9:14	T	G
.	.	.	.	.	.	.	.	.	.	.	.	SON	GT:/	0:43	0/1:95,29:1	A	C
.	.	.	.	.	.	.	.	5.5	2.32	.	.	SON	GT:/	0:45	0/1:82,40:3	C	T
.	.	.	.	.	.	.	.	.	.	0	.	SON	GT:/	0:8,(	0/1:1,2:37:	T	C
.	.	.	.	.	.	.	.	.	.	0	.	SON	GT:/	0:10	0/1:143,6:3	C	G
.	.	.	.	.	.	.	.	.	.	.	.	SON	GT:/	0:13	0/1:39,4:37	G	A
.	.	.	.	.	.	.	.	.	.	0	.	SON	GT:/	0:20	0/1:54,4:37	C	G
.	.	.	.	.	.	.	.	.	.	0	.	SON	GT:/	0:34	0/1:73,4:34	C	T
.	.	.	.	.	.	.	.	.	.	0	.	SON	GT:/	0:10	0/1:18,4:37	A	G
.	.	.	.	.	.	.	.	.	.	.	.	SON	GT:/	0:19	0/1:57,6:37	G	T
.	.	.	.	.	.	.	.	3.2	.	.	.	SON	GT:/	0:43	0/1:71,28:3	G	A
.	.	.	.	.	.	.	.	.	.	.	.	SON	GT:/	0:10	0/1:29,11:3	G	A
.	.	.	.	.	.	.	.	.	.	.	.	SON	GT:/	0:48	0/1:86,4:37	G	T
.	.	.	.	0.8	0.0,B	0.0,B	1,N	.	.	0	.	SON	GT:/	0:10	0/1:213,39	G	A
.	.	.	.	.	.	.	.	.	.	.	.	SON	GT:/	0:10	0/1:18,3:37	T	C
.	.	.	.	.	.	.	.	.	.	0	.	DB;S	GT:/	0:20	0/1:33,26:3	G	A
.	.	.	.	.	.	.	.	.	.	.	.	SON	GT:/	0:14	0/1:29,11:3	C	T
.	.	.	.	.	.	.	.	.	.	0	.	SON	GT:/	0:8,(	0/1:7,3:37:	C	T
.	.	.	.	.	.	.	.	.	.	0.1	0.1	SON	GT:/	0:28	0/1:20,7:35	G	A
.	.	.	.	.	.	.	.	.	.	0	0	SON	GT:/	0:8,(	0/1:13,3:37	G	T
.	.	.	.	.	.	.	.	.	.	0	0	SON	GT:/	0:10	0/1:11,4:34	G	A
.	.	.	.	.	.	.	.	.	.	0	0	SON	GT:/	0:14	0/1:30,5:37	A	T
.	.	.	.	.	.	.	.	.	.	.	.	SON	GT:/	0:28	0/1:33,10:3	G	A
.	.	.	.	.	.	.	.	.	.	0	0	SON	GT:/	0:11	0/1:3,2:37:	C	A
0.24	.	.	.	.	.	.	.	.	.	0	0	SON	GT:/	0:10	0/1:15,3:37	G	A
2E-04	.	##	0	.	.	.	.	.	.	.	.	SON	GT:/	0:53	0/1:136,13	C	T
.	.	.	.	.	.	.	.	.	.	.	.	SON	GT:/	0:17	0/1:23,8:37	A	G
0.065	.	.	.	.	.	.	.	.	.	0	.	DB;S	GT:/	0:36	0/1:81,5:37	C	G
.	.	.	.	.	.	.	.	.	.	0	.	SON	GT:/	0:36	0/1:81,5:37	T	G
.	.	.	.	.	.	.	.	.	.		0	SON	GT:/	0:56	0/1:76,8:37	A	C
.	.	.	.	.	.	.	.	3	.	.	.	SON	GT:/	0:26	0/1:39,16:3	C	T
.	.	.	.	.	.	.	.	3.3	3.32	.	.	SON	GT:/	0:23	0/1:41,8:18	A	C

.	.	.	.	.	.	.	.	.	.	.	.	SOM	GT:/	0:32	0/1:72,29:3	G	A
0.031	.	.	.	.	.	.	.	.	.	0	.	SOM	GT:/	0:15	0/1:29,3:33	C	T
.	.	.	.	.	.	.	.	.	.	.	.	SOM	GT:/	0:45	0/1:67,29:3	G	A
.	.	.	.	.	.	.	.	.	.	.	.	SOM	GT:/	0:37	0/1:45,22:3	G	A
.	.	##	0	.	.	.	.	.	.	.	.	SOM	GT:/	0:17	0/1:34,5:31	C	T
.	.	.	.	.	.	.	.	.	.	.	0	SOM	GT:/	0:8,(	0/1:5,2:37:	C	T
.	.	.	.	.	.	.	.	.	.	0	0	SOM	GT:/	0:8,(	0/1:10,4:24	T	C
.	.	.	.	0.0(	0.505	0.857	0.99	4.6	4.05	.	.	SOM	GT:/	0:12	0/1:140,84	G	A
.	.	.	.	.	.	.	.	.	1.53	.	.	DB;S	GT:/	0:20	0/1:41,21:3	G	A
.	.	.	.	.	.	.	.	.	.	.	.	SOM	GT:/	0:26	0/1:23,3:37	C	T
.	.	0	0	.	.	.	.	.	.	.	.	SOM	GT:/	0:45	0/1:90,17:3	C	T
.	.	##	0	.	.	.	.	.	.	.	.	SOM	GT:/	0:92	0/1:135,52	C	T
.	.	.	.	.	.	.	.	.	.	0	.	SOM	GT:/	0:8,(	0/1:20,4:37	A	T
.	.	.	.	.	.	.	.	.	.	.	.	SOM	GT:/	0:12	0/1:9,4:37:	T	G
.	.	.	.	.	.	.	.	4.4	.	.	.	SOM	GT:/	0:26	0/1:40,12:3	C	T
.	.	.	.	.	.	.	.	3.1	1.56	.	.	SOM	GT:/	0:22	0/1:44,4:37	C	T
.	.	.	.	.	.	.	.	.	.	.	.	SOM	GT:/	0:37	0/1:49,28:3	C	T
.	.	.	.	.	.	.	.	3.6	1.91	.	.	SOM	GT:/	0:15	0/1:232,91	G	A
.	.	##	0	.	0.995	1.0,D	1,N	.	.	.	.	SOM	GT:/	0:79	0/1:132,44	G	A
.	.	.	.	0.0(	0.04,I	0.48,I	0.99	.	1.45	.	.	SOM	GT:/	0:14	0/1:294,15	C	T
.	.	.	.	0.6	0.004	0.005	0.99	.	.	.	.	SOM	GT:/	0:40	0/1:83,7:33	C	T
.	.	.	.	.	.	.	.	.	.	.	.	SOM	GT:/	0:12	0/1:180,58	C	T
.	.	.	.	.	.	.	.	.	.	.	.	SOM	GT:/	0:32	0/1:32,121	G	A
.	.	.	.	0.0(	0.433	0.884	1,D	5.2	3.32	.	.	SOM	GT:/	0:90	0/1:146,78	C	T
.	.	.	.	0.1:	0.039	0.107	1.00	5.3	4.84	.	.	SOM	GT:/	0:64	0/1:122,64	C	T
0.002	.	.	.	.	.	.	.	.	.	0	0	SOM	GT:/	0:21	0/1:9,3:28:	G	A
.	.	0	0	.	.	.	.	.	.	0	0.2	DB;S	GT:/	0:21	0/1:35,4:37	G	A
.	.	.	.	.	.	.	.	.	.	.	.	SOM	GT:/	0:22	0/1:29,4:37	C	T
0.034	.	.	.	.	.	.	.	.	1.65	0.1	0.2	SOM	GT:/	0:9,(	0/1:20,4:37	C	T
.	.	.	.	.	.	.	.	.	.	.	.	SOM	GT:/	0:10	0/1:20,9:37	A	C
4E-04	##	##	0	.	.	.	.	2.3	.	.	.	SOM	GT:/	0:36	0/1:40,41:3	G	A
.	.	.	.	.	.	.	.	.	.	.	.	SOM	GT:/	0:30	0/1:46,47:3	T	C
.	.	.	.	.	.	.	.	.	.	.	.	SOM	GT:/	0:17	0/1:25,26:3	T	C
.	.	.	.	.	.	.	.	2.9	.	.	.	SOM	GT:/	0:55	0/1:80,66:3	C	T
0.248	.	.	.	.	.	.	.	.	.	0	0.2	SOM	GT:/	0:12	0/1:5,3:37:	G	C
.	.	.	.	.	.	.	.	.	.	0	0	SOM	GT:/	0:15	0/1:30,5:37	C	A
.	.	.	.	.	.	.	.	.	.	0	0	SOM	GT:/	0:8,(	0/1:17,3:37	T	G
.	.	.	.	.	.	.	.	.	.	0	.	SOM	GT:/	0:33	0/1:63,4:34	A	T
.	.	.	.	.	.	.	.	.	.	.	.	SOM	GT:/	0:22	0/1:61,5:37	T	C
.	.	.	.	.	.	.	.	.	.	0	0	SOM	GT:/	0:29	0/1:78,5:37	G	C
.	.	.	.	.	.	.	.	.	.	.	.	SOM	GT:/	0:9,(	0/1:15,5:37	G	A
.	.	.	.	.	.	.	.	.	.	.	.	SOM	GT:/	0:35	0/1:106,6:3	C	A
.	.	.	.	.	.	.	.	.	.	.	.	SOM	GT:/	0:35	0/1:107,6:3	A	C
.	.	.	.	.	.	.	.	.	.	0	0.3	SOM	GT:/	0:8,(	0/1:5,3:37:	A	G
.	.	.	.	.	.	.	.	.	.	0	.	SOM	GT:/	0:21	0/1:58,8:37	A	C
.	.	.	.	.	.	.	.	.	.	0	0	SOM	GT:/	0:23	0/1:30,4:37	C	G
.	.	.	.	.	.	.	.	.	.	0	0	SOM	GT:/	0:23	0/1:30,4:37	C	T
.	.	.	.	.	.	.	.	.	.	.	.	SOM	GT:/	0:8,(	0/1:18,14:3	C	T
.	.	.	.	.	.	.	.	.	.	0.1	0.2	SOM	GT:/	0:24	0/1:54,5:31	G	A
.	.	.	.	.	.	.	.	.	.	0	0.5	SOM	GT:/	0:8,(	0/1:6,5:37:	G	A
.	.	.	.	.	.	.	.	.	.	0	0.5	SOM	GT:/	0:10	0/1:7,5:37:	G	A
.	.	.	.	.	.	.	.	.	.	.	.	SOM	GT:/	0:17	0/1:37,5:21	A	C
.	.	##	0	0.0'	0.997	1.0,D	1,D	5.7	5.14	0	.	SOM	GT:/	0:65	0/1:121,19	C	T
.	.	.	.	.	.	.	.	.	.	.	.	SOM	GT:/	0:23	0/1:34,15:3	C	T

.	.	.	.	.	.	.	.	.	.	.	.	SOM	GT:/	0:19	0/1:38,7:17	C	A
.	.	.	.	.	.	.	.	.	.	.	.	SOM	GT:/	0:28	0/1:40,36:3	G	T
.	.	.	.	.	.	.	.	.	.	.	.	SOM	GT:/	0:19	0/1:43,24:3	G	A
.	.	.	.	.	.	.	.	.	.	.	.	SOM	GT:/	0:24	0/1:40,5:37	A	G
.	.	.	.	.	.	.	.	3.7	2.73	.	.	SOM	GT:/	0:58	0/1:72,42:3	C	A
.	.	.	.	.	.	.	.	.	.	.	.	SOM	GT:/	0:39	0/1:67,65:3	A	G
.	.	.	.	.	.	.	.	.	.	.	.	SOM	GT:/	0:11	0/1:36,4:37	C	A
.	.	.	.	.	.	.	.	.	.	.	0	SOM	GT:/	0:8,(	0/1:12,4:37	C	T
.	.	.	.	.	.	.	.	.	.	0	0.2	SOM	GT:/	0:8,(	0/1:14,5:37	A	T
.	.	.	.	.	.	.	.	.	.	0.1	0	SOM	GT:/	0:13	0/1:47,19:3	A	T
.	.	.	.	.	.	.	.	.	.	0.1	0	SOM	GT:/	0:36	0/1:111,6:3	T	C
4E-04	.	##	0	0.2	0.001	0.008	1.00	2.3	.	0	.	SOM	GT:/	0:97	0/1:132,12	G	A
.	.	.	.	.	.	.	.	.	.	0	.	DB;S	GT:/	0:62	0/1:125,15	A	C
.	.	.	.	.	.	.	.	.	.	0	.	SOM	GT:/	0:23	0/1:32,3:37	C	T
2E-04	.	.	.	.	.	.	.	.	.	0	0	SOM	GT:/	0:8,(	0/1:6,9:34	C	T
.	.	.	.	.	.	.	.	3	1.31	.	.	SOM	GT:/	0:14	0/1:23,8:37	G	C
6E-04	.	.	.	.	.	.	.	.	.	.	0	SOM	GT:/	0:11	0/1:17,19:3	G	A
.	.	.	.	0.0	0.054	0.102	1,N	3.6	.	.	.	SOM	GT:/	0:52	0/1:62,50:3	G	T
.	.	.	.	.	.	.	.	.	.	.	.	SOM	GT:/	0:8,(	0/1:15,9:35	A	G
.	.	.	.	.	.	.	.	.	.	.	.	SOM	GT:/	0:45	0/1:10,58:3	T	C
0.005	0	0	0	0.4	0.004	0.007	1,N	.	.	0	0	SOM	GT:/	0:79	0/1:66,33:3	C	T
.	.	.	.	0.0	0.961	0.999	1,D	5.1	5.69	.	.	SOM	GT:/	0:42	0/1:88,8:37	C	T
.	.	.	.	.	.	.	.	.	.	0	0.1	SOM	GT:/	0:8,(	0/1:0,2:37	T	C
.	.	.	.	.	.	.	1,D	5.2	4.50	.	.	SOM	GT:/	0:13	0/1:31,21:3	G	A
.	.	.	.	.	.	.	.	5.2	1.86	.	.	SOM	GT:/	0:9,(	0/1:10,13:3	G	A
.	.	0	0	0.2	0.002	0.001	1,D	4.8	4.41	0	0	DB;S	GT:/	0:77	0/1:159,6:3	G	C
.	.	.	.	.	.	.	.	.	.	0.2	0.3	DB;S	GT:/	0:27	0/1:78,4:37	G	A
.	.	##	0	.	.	.	.	.	.	0	.	SOM	GT:/	0:34	0/1:36,36:3	G	A
.	.	.	.	.	.	.	.	4.1	.	.	.	SOM	GT:/	0:54	0/1:81,41:3	C	T
.	.	.	.	.	.	.	.	.	.	.	.	SOM	GT:/	0:28	0/1:26,18:3	C	G
.	.	.	.	0.0	0.912	0.995	1,D	5.8	5.36	.	.	DB;S	GT:/	0:19	0/1:62,173	G	A
.	.	.	.	.	.	.	.	.	.	.	.	SOM	GT:/	0:29	0/1:85,213	C	G
.	.	.	.	.	.	.	.	.	.	.	.	SOM	GT:/	0:55	0/1:397,59	A	G
.	.	.	.	.	.	.	.	.	.	.	.	SOM	GT:/	0:8,(	0/1:93,35:3	G	T
.	.	.	.	.	.	.	.	.	.	0	0.3	DB;S	GT:/	0:31	0/1:244,8:3	A	G
.	.	.	.	.	.	.	.	.	.	.	.	SOM	GT:/	0:11	0/1:115,11	C	T
.	.	.	.	0.0	.	.	0.73	.	2.06	.	.	SOM	GT:/	0:60	0/1:71,9:37	G	T
.	.	.	.	.	.	.	.	.	.	.	.	SOM	GT:/	0:30	0/1:412,11	G	T
.	.	.	.	.	.	.	.	.	.	0	.	SOM	GT:/	0:57	0/1:95,7:37	C	G
.	.	.	.	.	.	.	.	.	.	.	.	SOM	GT:/	0:18	0/1:10,14:3	C	T
.	.	.	.	.	.	.	.	.	.	0	.	SOM	GT:/	0:8,(	0/1:17,3:37	A	G
.	.	.	.	.	.	.	.	.	.	.	.	SOM	GT:/	0:15	0/1:21,15:3	G	A
.	.	##	.	.	.	.	.	2.4	.	0	.	SOM	GT:/	0:11	0/1:313,25	C	T
.	.	.	.	.	0.965	0.999	.	4	5.22	.	.	SOM	GT:/	0:22	0/1:565,40	C	T
.	.	.	.	.	.	.	.	.	.	.	.	SOM	GT:/	0:12	0/1:22,16:3	T	C
.	.	.	.	.	.	.	.	.	2.00	.	.	SOM	GT:/	0:9,(	0/1:31,5:37	G	A
.	.	0	0	0.4	0.002	0.004	1,N	.	.	0	0	SOM	GT:/	0:60	0/1:142,9:3	T	G
.	.	.	.	.	.	.	.	.	.	.	.	SOM	GT:/	0:16	0/1:16,12:3	G	A
.	.	##	0	.	.	.	.	.	.	.	.	SOM	GT:/	0:11	0/1:140,13	C	T
.	.	.	.	.	.	.	.	.	.	0	.	SOM	GT:/	0:11	0/1:21,4:37	C	G
.	.	.	.	.	.	.	.	.	1.84	.	.	DB;S	GT:/	0:33	0/1:55,43:3	C	T
.	.	.	.	.	.	.	.	.	.	.	.	SOM	GT:/	0:16	0/1:20,3:37	G	A
.	.	.	.	.	.	.	.	.	.	0	0	SOM	GT:/	0:20	0/1:48,4:37	G	A
.	.	.	.	.	.	.	.	.	.	0	.	SOM	GT:/	0:13	0/1:14,9:34	A	G

.	.	.	.	.	.	.	.	.	.	0	.	SON	GT:/	0:12	0/1:18,3:37	A	C
0.038	.	.	.	.	.	.	.	.	.	0	0	SON	GT:/	0:12	0/1:34,6:37	G	C
.	.	.	.	.	.	.	.	3.5	2.17	.	.	SON	GT:/	0:39	0/1:52,65:3	C	A
0.237	.	.	.	.	.	.	.	.	.	0.2	0.4	SON	GT:/	0:8,(	0/1:7,11:35	T	G
.	.	.	.	.	.	.	.	.	.	.	.	SON	GT:/	0:20	0/1:27,18:3	G	A
.	.	##	0	.	.	.	.	.	.	0	0	DB;S	GT:/	0:44	0/1:90,4:37	G	A
4E-04	0	0	0	.	.	.	.	.	.	.	.	SON	GT:/	0:23	0/1:50,49:3	C	A
0.026	.	0	0	.	.	.	.	.	.	0	0	DB;S	GT:/	0:90	0/1:227,7:3	A	G
0.047	.	0	0	.	.	.	.	.	.	0.1	0.2	SON	GT:/	0:69	0/1:26,21:3	T	C
.	.	.	.	.	.	.	.	.	.	.	.	SON	GT:/	0:33	0/1:58,4:37	C	T
.	.	.	.	.	.	.	.	.	.	0	0	DB;S	GT:/	0:62	0/1:265,9:3	T	A
.	.	.	.	.	.	.	1,D	6	5.89	.	.	SON	GT:/	0:41	0/1:68,62:3	G	T
.	.	.	.	.	.	.	.	.	.	.	.	SON	GT:/	0:15	0/1:32,6:20	A	C
.	.	##	0	.	.	.	.	.	.	.	.	SON	GT:/	0:36	0/1:55,53:3	A	G
.	.	0	0	0.0	0.0,B	0.0,B	1,N	.	.	.	.	SON	GT:/	0:55	0/1:79,5:37	C	T
.	.	.	.	.	.	.	.	.	.	0	.	SON	GT:/	0:12	0/1:18,7:37	G	T
.	.	.	.	.	.	.	.	.	.	0	.	SON	GT:/	0:8,(	0/1:23,5:37	A	G
.	.	.	.	.	.	.	.	.	.	.	.	SON	GT:/	0:11	0/1:0,2:37:	C	T
.	.	.	.	.	.	.	.	.	.	.	.	DB;S	GT:/	0:76	0/1:166,10	G	C
.	.	.	.	.	.	.	.	.	.	0	.	SON	GT:/	0:38	0/1:93,7:35	C	A
.	.	.	.	.	.	.	.	.	.	0	.	SON	GT:/	0:20	0/1:47,4:34	G	A
.	.	.	.	.	.	.	.	.	.	.	.	SON	GT:/	0:74	0/1:100,5:3	G	A
.	.	.	.	.	.	.	.	.	.	.	.	SON	GT:/	0:75	0/1:70,4:34	C	G
.	.	.	.	.	.	.	.	.	.	.	.	DB;S	GT:/	0:10	0/1:224,7:3	G	A
.	.	.	.	.	.	.	.	.	.	.	.	SON	GT:/	0:21	0/1:36,6:37	G	T
0.002	.	.	.	.	.	.	.	.	.	0	0	SON	GT:/	0:8,(	0/1:13,3:37	C	T
.	.	.	.	.	.	.	.	.	.	0	0	SON	GT:/	0:8,(	0/1:6,2:37:	T	C
.	.	.	.	.	.	.	.	4.9	.	.	.	SON	GT:/	0:57	0/1:93,71:3	A	G
.	.	.	.	.	.	.	.	.	.	0	0	SON	GT:/	0:15	0/1:12,5:21	T	G
.	.	.	.	.	.	.	.	.	.	0	.	SON	GT:/	0:14	0/1:28,6:17	T	G
.	.	.	.	.	.	.	.	.	.	0	.	SON	GT:/	0:14	0/1:30,6:18	T	G
.	.	.	.	.	.	.	.	.	.	0	0	SON	GT:/	0:8,(	0/1:2,2:37:	G	C
.	.	.	.	.	.	.	.	.	.	.	.	SON	GT:/	0:8,(	0/1:16,11:3	C	T
.	.	.	.	.	.	.	.	.	.	0	0	SON	GT:/	0:10	0/1:21,3:37	C	G
.	.	.	.	.	.	.	.	.	.	0	0	SON	GT:/	0:12	0/1:24,7:33	G	A
.	.	##	0	.	.	.	.	.	.	0	.	SON	GT:/	0:18	0/1:274,20	G	A
.	.	.	.	.	.	.	.	.	.	0.1	0.1	SON	GT:/	0:8,(	0/1:22,5:37	C	G
.	.	.	.	.	.	.	.	.	.	.	.	SON	GT:/	0:8,(	0/1:28,3:37	G	A
.	.	.	.	.	.	.	.	.	.	.	.	SON	GT:/	0:20	0/1:33,17:3	G	T
.	.	.	.	.	.	.	.	.	.	.	.	SON	GT:/	0:23	0/1:59,5:37	G	C
.	.	.	.	.	.	.	.	.	.	.	.	DB;S	GT:/	0:43	0/1:104,5:3	C	T
.	.	.	.	.	.	.	.	.	.	.	.	SON	GT:/	0:8,(	0/1:7,7:37:	G	T
.	.	.	.	.	.	.	.	.	.	.	.	SON	GT:/	0:80	0/1:109,10	G	A
.	.	.	.	.	.	.	.	2.2	.	.	.	SON	GT:/	0:17	0/1:35,20:3	C	T
6E-04	.	.	.	.	.	.	.	.	.	0	.	SON	GT:/	0:19	0/1:74,6:33	G	A
2E-04	.	.	.	.	.	.	.	.	.	.	.	SON	GT:/	0:37	0/1:75,17:3	C	T
.	0	0	0	1.0	0.013	0.004	1.00	.	.	0	0.3	DB;S	GT:/	0:20	0/1:46,3:37	T	C
.	.	.	.	.	.	.	.	.	.	0	0	SON	GT:/	0:12	0/1:28,6:32	C	A
4E-04	.	.	.	.	.	.	.	.	.	0	0	SON	GT:/	0:13	0/1:22,6:37	T	A
.	.	.	.	.	.	.	.	.	.	0	0	SON	GT:/	0:34	0/1:67,5:37	G	T
.	.	.	.	.	.	.	.	.	.	0.1	0.2	SON	GT:/	0:8,(	0/1:26,5:37	C	A
0.321	.	.	.	.	.	.	.	.	.	0.1	0.2	DB;S	GT:/	0:29	0/1:63,5:34	A	G
.	.	.	.	.	.	.	.	.	.	0	.	SON	GT:/	0:22	0/1:55,5:34	G	T
6E-04	.	.	.	.	.	.	.	.	.	.	.	SON	GT:/	0:14	0/1:17,15:3	G	A

.	.	.	.	.	.	.	.	.	2.31	.	.	SOM	GT:/	0:82	0/1:169,7:3	G	A
.	.	.	.	0.0	1.0,D	1.0,D	1,D	4.9	4.64	.	.	SOM	GT:/	0:99	0/1:134,80	G	A
.	.	##	0	.	.	.	.	.	.	.	.	SOM	GT:/	0:50	0/1:66,77:3	C	T
.	.	.	.	.	.	.	.	.	.	0	0	SOM	GT:/	0:15	0/1:10,5:31	C	T
.	.	.	.	.	.	.	.	.	.	.	.	SOM	GT:/	0:47	0/1:63,50:3	T	G
.	.	.	.	.	.	.	.	.	.	0.1	0.3	SOM	GT:/	0:8,(	0/1:15,4:34	T	A
0.41	.	.	.	.	.	.	.	.	.	.	.	SOM	GT:/	0:8,(	0/1:24,11:3	G	C
.	.	.	.	.	.	.	.	.	.	.	.	SOM	GT:/	0:43	0/1:120,8:3	T	A
.	.	.	.	.	.	.	.	.	.	.	.	SOM	GT:/	0:30	0/1:32,12:3	G	A
.	.	.	.	.	.	.	.	.	.	.	.	SOM	GT:/	0:52	0/1:73,50:3	C	T
0.156	.	.	.	.	.	.	.	2.7	.	0.4	0.5	DB;S	GT:/	0:31	0/1:50,49:3	C	G
.	.	.	.	.	.	.	.	.	.	.	.	SOM	GT:/	0:16	0/1:3,2:37:	C	G
.	.	.	.	.	.	.	.	.	.	.	.	SOM	GT:/	0:18	0/1:228,15	C	T
.	.	.	.	.	.	.	.	.	.	0.1	0	SOM	GT:/	0:9,(	0/1:3,2:37:	A	C
0.021	.	.	.	.	.	.	.	.	.	0	0	SOM	GT:/	0:39	0/1:98,6:32	T	C
.	.	.	.	.	.	.	.	.	.	.	.	SOM	GT:/	0:11	0/1:40,7:19	T	G
.	.	.	.	.	.	.	.	3.5	.	.	.	SOM	GT:/	0:38	0/1:137,5:3	G	A
.	.	.	.	.	.	.	.	.	1.64	.	.	SOM	GT:/	0:52	0/1:89,9:37	G	A
.	.	.	.	.	.	.	.	.	1.75	.	.	SOM	GT:/	0:22	0/1:37,21:3	G	A
.	.	.	.	.	.	.	.	2.9	.	.	.	SOM	GT:/	0:27	0/1:17,25:3	G	C
.	.	.	.	.	.	.	.	.	.	.	.	SOM	GT:/	0:12	0/1:21,19:3	T	C
.	.	.	.	0.0	0.12,I	0.912	1,N	2.8	.	.	.	SOM	GT:/	0:90	0/1:192,7:3	C	T
.	.	.	.	.	.	.	.	.	.	.	.	SOM	GT:/	0:11	0/1:8,15:33	G	A
.	.	##	0	.	.	.	.	.	.	.	.	SOM	GT:/	0:53	0/1:65,56:3	G	A
.	.	.	.	.	.	.	.	.	.	.	.	SOM	GT:/	0:79	0/1:125,99	G	A
.	.	.	.	.	.	.	.	.	.	.	.	SOM	GT:/	0:21	0/1:28,7:35	C	A
.	.	.	.	.	.	.	.	.	.	0	0.2	DB;S	GT:/	0:35	0/1:65,4:37	A	G
.	.	.	.	.	.	.	.	.	.	.	.	SOM	GT:/	0:13	0/1:14,15:3	A	T
.	.	.	.	.	.	.	.	.	.	.	.	SOM	GT:/	0:52	0/1:122,20	C	T
.	.	.	.	.	.	.	.	.	.	.	.	SOM	GT:/	0:8,(	0/1:11,5:37	C	T
2E-04	.	##	0	0.0	0.993	1.0,D	0.96	2.9	3.63	.	.	SOM	GT:/	0:33	0/1:58,43:3	G	T
.	.	.	.	.	.	.	.	.	.	.	0	SOM	GT:/	0:13	0/1:13,6:32	T	C
.	.	.	.	.	.	.	.	.	.	0.1	0.3	SOM	GT:/	0:61	0/1:120,8:3	C	G
.	.	.	.	0.0(	0.999	1.0,D	1.00	5.2	3.95	.	.	SOM	GT:/	0:60	0/1:75,67:3	T	A
.	.	.	.	.	.	.	.	3.7	2.31	0	.	SOM	GT:/	0:11	0/1:31,4:37	T	C
.	.	.	.	.	.	.	.	2.9	.	.	.	SOM	GT:/	0:64	0/1:58,47:3	G	A
.	.	.	.	.	.	.	.	5.7	1.53	.	.	SOM	GT:/	0:53	0/1:61,42:3	T	C
.	##	##	0	0.2	0.002	0.009	0.68	4.3	2.22	.	.	SOM	GT:/	0:63	0/1:84,77:3	G	A
.	.	.	.	.	.	.	.	4.3	1.40	.	.	SOM	GT:/	0:19	0/1:22,15:3	C	T
.	.	.	.	.	.	.	1,A	.	5.99	.	.	SOM	GT:/	0:59	0/1:80,70:3	G	A
.	.	.	.	.	.	.	.	.	.	.	.	SOM	GT:/	0:23	0/1:48,17:3	C	G
.	.	.	.	0.0!	0.949	0.997	1.00	4.1	4.28	.	.	SOM	GT:/	0:51	0/1:94,69:3	C	T
.	.	.	.	.	.	.	.	.	.	0	.	SOM	GT:/	0:15	0/1:62,6:37	G	A
.	.	.	.	.	.	.	.	.	.	0	.	SOM	GT:/	0:17	0/1:48,5:37	A	G
.	.	.	.	.	.	.	.	.	.	.	.	SOM	GT:/	0:37	0/1:59,41:3	G	C
.	.	.	.	0.0	0.997	1.0,D	1.00	3.9	3.50	.	.	SOM	GT:/	0:40	0/1:56,37:3	C	G
.	.	.	.	.	.	.	.	6	1.44	.	.	SOM	GT:/	0:37	0/1:69,6:37	A	G
.	.	.	.	.	.	.	.	.	1.48	.	.	SOM	GT:/	0:24	0/1:39,24:3	A	C
.	.	.	.	.	.	.	.	.	.	0	0	SOM	GT:/	0:8,(	0/1:8,3:33:	T	C
.	.	.	.	.	.	.	.	.	.	.	.	SOM	GT:/	0:91	0/1:134,86	G	T
.	.	.	.	.	.	.	.	.	.	.	.	SOM	GT:/	0:16	0/1:34,31:3	C	G
.	.	.	.	.	.	.	.	.	.	0.1	0.2	SOM	GT:/	0:13	0/1:29,7:37	C	A
.	.	##	0	0.0(	0.419	0.949	0.98	3.8	4.06	.	.	SOM	GT:/	0:10	0/1:152,12	G	A
.	.	.	.	.	.	.	.	4.4	1.49	.	.	SOM	GT:/	0:37	0/1:119,6:3	T	A

.	.	.	.	.	.	.	.	4.6	1.64	.	.	SOM	GT:/	0:84	0/1:110,66	G	T
.	.	.	.	.	0.934	0.998	1,N	2.2	.	.	.	SOM	GT:/	0:22	0/1:26,20:3	T	C
.	.	.	.	0.2(	0.068	0.396	0.99	5.8	3.14	0	.	SOM	GT:/	0:17	0/1:32,9:12	G	C
.	.	.	.	.	.	.	.	.	.	.	.	SOM	GT:/	0:75	0/1:104,63	G	A
.	.	.	.	.	.	.	.	.	.	0	.	SOM	GT:/	0:16	0/1:27,8:16	A	C
.	.	.	.	.	.	.	.	.	.	.	.	SOM	GT:/	0:52	0/1:82,56:3	C	T
.	.	.	.	.	.	.	.	.	.	.	.	SOM	GT:/	0:16	0/1:13,20:3	C	T
.	.	.	.	.	.	.	.	.	.	.	0	SOM	GT:/	0:15	0/1:29,3:37	A	G
.	.	.	.	.	.	.	.	.	.	.	.	SOM	GT:/	0:55	0/1:169,12	G	T
.	.	.	.	.	.	.	.	.	.	0	0	SOM	GT:/	0:66	0/1:175,8:3	C	A
.	.	.	.	.	.	.	.	.	.	0	0	SOM	GT:/	0:70	0/1:123,5:3	A	G
.	.	.	.	.	.	.	.	.	1.30	.	.	SOM	GT:/	0:46	0/1:87,50:3	C	A
.	.	.	.	.	.	.	.	3	.	.	.	SOM	GT:/	0:11	0/1:164,13	A	G
.	.	.	.	.	.	.	.	.	.	.	.	SOM	GT:/	0:35	0/1:72,8:33	G	A
.	.	.	.	.	.	.	.	2.7	.	.	.	SOM	GT:/	0:68	0/1:75,67:3	C	T
.	.	.	.	.	.	.	.	.	.	.	.	SOM	GT:/	0:44	0/1:62,62:3	C	T
.	.	.	.	.	.	.	.	.	.	.	.	SOM	GT:/	0:12	0/1:15,15:3	C	A
.	.	.	.	.	.	.	.	.	.	0	.	SOM	GT:/	0:37	0/1:34,10:3	C	T
.	.	.	.	.	.	.	.	.	.	0	0	SOM	GT:/	0:10	0/1:31,3:37	T	C
.	.	.	.	.	.	.	.	.	.	.	.	SOM	GT:/	0:9,(	0/1:10,4:21	C	A
.	.	.	.	.	.	.	.	.	.	0	0	SOM	GT:/	0:47	0/1:100,8:3	G	A
.	.	.	.	.	.	.	.	.	.	.	.	SOM	GT:/	0:44	0/1:101,6:3	C	T
.	.	##	0	.	.	.	.	5.4	2.61	.	.	SOM	GT:/	0:48	0/1:119,9:3	C	T
.	.	.	.	.	.	.	.	.	.	.	.	SOM	GT:/	0:58	0/1:70,4:34	G	A
.	.	0	0	.	.	.	.	.	.	0	0	SOM	GT:/	0:21	0/1:36,6:37	C	T
.	.	.	.	.	.	.	.	3.2	.	.	.	SOM	GT:/	0:15	0/1:19,6:37	C	G
.	.	.	.	.	.	.	.	3.9	.	.	.	SOM	GT:/	0:12	0/1:167,12	G	A
.	.	.	.	0.0	0.004	0.001	0.99	2.8	.	.	.	SOM	GT:/	0:67	0/1:110,76	C	T
.	.	.	.	.	.	.	.	.	.	0	.	SOM	GT:/	0:11	0/1:39,4:37	A	G
.	.	.	.	.	.	.	.	.	.	0	.	SOM	GT:/	0:20	0/1:43,5:37	T	C
.	.	.	.	.	.	.	.	.	.	.	.	SOM	GT:/	0:12	0/1:20,16:3	C	A
2E-04	.	.	.	.	.	.	.	.	.	.	.	SOM	GT:/	0:17	0/1:27,3:37	C	T
.	.	.	.	.	.	.	.	.	.	.	.	SOM	GT:/	0:32	0/1:56,42:3	C	T
.	.	.	.	.	.	.	.	.	.	0	.	SOM	GT:/	0:11	0/1:42,7:37	G	T
.	.	.	.	.	.	.	.	.	.	0	.	DB;S	GT:/	0:51	0/1:245,10	G	C
.	.	.	.	.	.	.	.	.	.	0	.	DB;S	GT:/	0:38	0/1:195,8:3	A	T
.	.	.	.	.	.	.	.	.	.	0	.	DB;S	GT:/	0:37	0/1:100,6:3	T	C
.	.	.	.	.	.	.	.	.	.	.	.	DB;S	GT:/	0:36	0/1:106,5:3	A	G
.	.	.	.	.	.	.	.	.	.	0	.	DB;S	GT:/	0:62	0/1:183,7:3	T	C
.	.	.	.	.	.	.	.	.	.	.	.	DB;S	GT:/	0:78	0/1:202,8:3	G	T
.	.	.	.	.	.	.	.	.	.	0	.	DB;S	GT:/	0:49	0/1:121,6:3	C	G
.	.	.	.	.	.	.	.	.	.	0	.	DB;S	GT:/	0:46	0/1:127,6:3	C	T
.	.	.	.	.	.	.	.	.	.	0	.	DB;S	GT:/	0:35	0/1:86,8:33	T	G
.	.	.	.	.	.	.	.	.	.	0	.	SOM	GT:/	0:50	0/1:117,8:3	A	G
.	.	.	.	.	.	.	.	.	.	.	.	DB;S	GT:/	0:52	0/1:125,6:3	A	C
.	.	.	.	.	.	.	.	.	.	0	.	DB;S	GT:/	0:36	0/1:149,7:3	T	G
.	.	.	.	.	.	.	.	.	.	0	.	SOM	GT:/	0:34	0/1:71,7:37	A	G
.	.	.	.	.	.	.	.	.	.	0	.	DB;S	GT:/	0:55	0/1:180,7:3	C	G
.	.	.	.	.	.	.	.	.	.	.	.	DB;S	GT:/	0:80	0/1:275,14	C	T
.	.	.	.	.	.	.	.	.	.	.	.	DB;S	GT:/	0:70	0/1:207,7:3	G	T
.	.	.	.	.	.	.	.	.	.	.	.	DB;S	GT:/	0:66	0/1:161,11	A	T
.	.	.	.	.	.	.	.	.	.	.	.	SOM	GT:/	0:18	0/1:43,3:37	G	T
.	.	.	.	.	.	.	.	.	.	.	.	DB;S	GT:/	0:19	0/1:41,4:37	C	T
.	.	.	.	.	.	.	.	.	.	.	.	SOM	GT:/	0:17	0/1:40,3:37	G	C

.	.	.	.	.	.	.	.	.	.	0	.	DB;S	GT:/	0:44	0/1:147,10	T	C
.	.	.	.	.	.	.	.	.	.	.	.	SOM	GT:/	0:11	0/1:45,3:37	G	A
.	.	.	.	.	.	.	.	.	.	0	.	SOM	GT:/	0:14	0/1:42,4:37	C	G
.	.	.	.	.	.	.	.	.	.	.	.	SOM	GT:/	0:14	0/1:34,3:37	C	T
.	.	.	.	.	.	.	.	.	.	.	.	SOM	GT:/	0:28	0/1:39,25:3	C	G
1E-03	.	0	0	0.0	0.086	0.044	1,D	5.2	.	0	0	SOM	GT:/	0:12	0/1:307,18	G	C
0.317	.	.	.	.	.	.	.	.	.	0	0.1	DB;S	GT:/	0:39	0/1:83,4:37	T	C
.	.	.	.	.	.	.	.	.	.	.	.	SOM	GT:/	0:15	0/1:10,11:3	G	T
.	.	.	.	0.0	0.942	0.988	1,D	5.3	3.11	.	.	SOM	GT:/	0:15	0/1:81,200	C	A
.	.	##	.	0.0	0.885	0.933	1,D	5.3	3.92	.	.	SOM	GT:/	0:15	0/1:58,203	C	T
.	.	.	.	.	.	.	.	.	.	0	0	SOM	GT:/	0:18	0/1:51,4:37	C	A
.	.	.	.	.	.	.	.	.	.	0	0.1	SOM	GT:/	0:9,(	0/1:23,4:37	C	A
0.533	.	.	.	.	.	.	.	.	.	0	0.2	SOM	GT:/	0:9,(	0/1:20,4:37	A	G
.	.	.	.	0.7	0.047	0.042	1,N	.	.	.	.	SOM	GT:/	0:35	0/1:50,22:3	G	A
.	.	.	.	.	.	.	.	.	.	0	0	SOM	GT:/	0:17	0/1:18,6:22	A	C
.	.	.	.	.	.	.	.	.	.	.	.	SOM	GT:/	0:94	0/1:129,91	C	T
.	.	.	.	.	.	.	.	.	2.31	0	0	SOM	GT:/	0:36	0/1:50,39:3	G	A
.	.	.	.	.	.	.	.	.	.	.	.	SOM	GT:/	0:11	0/1:13,16:3	C	T
.	.	.	.	.	.	.	.	.	.	.	.	SOM	GT:/	0:16	0/1:29,16:3	C	T
.	.	.	.	.	.	.	.	.	.	0.1	0.1	SOM	GT:/	0:16	0/1:29,7:37	T	A
0.134	.	.	.	.	.	.	.	.	.	0.2	0.2	SOM	GT:/	0:10	0/1:31,14:3	A	G
.	.	.	.	.	.	.	.	.	.	0	.	SOM	GT:/	0:51	0/1:139,5:3	C	G
.	.	.	.	.	.	.	.	.	.	0	0	SOM	GT:/	0:37	0/1:45,7:37	T	G
.	.	.	.	.	.	.	.	.	.	0	.	DB;S	GT:/	0:37	0/1:55,5:37	A	G
.	.	.	.	.	.	.	.	.	.	0	.	SOM	GT:/	0:10	0/1:48,9:37	T	G
.	.	.	.	.	.	.	.	.	.	.	.	SOM	GT:/	0:9,(	0/1:41,3:37	C	T
.	.	.	.	.	.	.	.	.	.	.	.	SOM	GT:/	0:45	0/1:64,5:34	G	C
.	.	.	.	.	.	.	.	.	.	.	.	SOM	GT:/	0:45	0/1:82,4:34	C	T
.	.	.	.	.	.	.	.	.	.	0.1	0	DB;S	GT:/	0:38	0/1:117,8:3	T	C
.	.	.	.	.	.	.	.	.	.	0	0	SOM	GT:/	0:9,(	0/1:17,4:37	T	A
.	.	.	.	.	.	.	.	.	.	0	0	SOM	GT:/	0:8,(	0/1:16,3:37	T	A
.	.	.	.	.	.	.	.	.	2.77	.	.	SOM	GT:/	0:59	0/1:70,63:3	C	G
.	.	.	.	.	.	.	.	2.7	2.69	.	.	SOM	GT:/	0:28	0/1:61,4:37	G	A
.	.	.	.	.	.	.	.	.	.	0	0	SOM	GT:/	0:96	0/1:186,6:3	G	T
.	.	##	0	.	.	.	.	.	.	0	.	SOM	GT:/	0:96	0/1:107,11	G	A
.	.	.	.	0.6	0.098	0.066	1,D	5.1	2.42	.	.	SOM	GT:/	0:53	0/1:80,58:3	C	T
.	.	.	.	.	.	.	.	.	.	0	.	SOM	GT:/	0:8,(	0/1:8,8:34	G	A
0.053	.	.	.	.	.	.	.	.	.	.	.	DB;S	GT:/	0:26	0/1:57,27:3	C	T
.	.	.	.	.	.	.	.	.	.	0	0	SOM	GT:/	0:15	0/1:36,4:37	A	T
.	.	.	.	.	.	.	.	.	.	0	0	SOM	GT:/	0:8,(	0/1:6,5:37	G	C
.	.	.	.	.	.	.	.	.	.	0	0	SOM	GT:/	0:11	0/1:25,3:37	G	A
.	.	.	.	.	.	.	.	.	.	0	0	SOM	GT:/	0:10	0/1:25,3:37	C	T
.	.	.	.	.	.	.	.	.	.	.	.	SOM	GT:/	0:42	0/1:85,16:3	C	A
.	##	##	0	.	.	.	.	.	.	.	.	SOM	GT:/	0:10	0/1:146,12	G	A
.	.	.	.	.	.	.	.	.	.	.	.	SOM	GT:/	0:54	0/1:66,38:3	G	A
.	.	.	.	.	.	.	.	.	.	.	.	SOM	GT:/	0:56	0/1:65,38:3	G	T
2E-04	.	.	.	0.1	0.806	0.985	0.64	5.1	2.59	.	.	SOM	GT:/	0:15	0/1:215,16	G	A
.	.	.	.	.	.	.	.	.	.	.	.	SOM	GT:/	0:56	0/1:99,61:3	T	C
.	.	.	.	.	.	.	.	4.1	1.24	.	.	SOM	GT:/	0:56	0/1:70,51:3	C	T
.	.	.	.	.	.	.	.	.	.	.	.	SOM	GT:/	0:51	0/1:73,44:3	G	A
.	.	.	.	.	.	.	.	.	.	.	.	SOM	GT:/	0:11	0/1:15,3:37	G	C
.	.	.	.	.	.	.	.	.	.	0	.	SOM	GT:/	0:18	0/1:34,3:37	C	G
.	.	.	.	.	.	.	.	.	.	.	.	SOM	GT:/	0:90	0/1:129,10	G	C
0.13	.	.	.	.	.	.	.	.	.	0.2	0.2	SOM	GT:/	0:9,(	0/1:15,15:3	T	A

.	.	##	0	0.0(	0.773	0.928	1,N	.	2.55	.	.	SON	GT:/	0:85	0/1:136,11	G	A
0.722	.	.	.	.	.	.	.	.	.	0.3	0.5	DB;S	GT:/	0:48	0/1:124,8:3	T	C
.	.	.	.	.	.	.	.	.	.	.	.	SON	GT:/	0:8,(	0/1:10,6:32	G	A
.	.	.	.	0.0	0.793	0.976	1.00	5.8	4.40	.	.	SON	GT:/	0:61	0/1:70,66:3	T	G
.	.	.	.	.	.	.	.	.	.	.	.	SON	GT:/	0:46	0/1:60,48:3	G	A
.	.	##	0	.	.	.	.	.	.	.	.	SON	GT:/	0:58	0/1:88,43:3	G	A
.	##	##	0	.	.	.	.	.	.	.	.	SON	GT:/	0:45	0/1:75,52:3	G	A
.	.	.	.	.	.	.	.	.	.	0	.	SON	GT:/	0:12	0/1:31,5:34	C	T
.	.	.	.	.	.	.	.	3.1	1.79	.	.	SON	GT:/	0:25	0/1:35,21:3	C	A
.	.	.	.	.	.	.	.	.	.	.	.	SON	GT:/	0:32	0/1:43,29:3	G	A
.	.	.	.	0.2	0.974	0.998	0.99	3.9	1.92	.	.	SON	GT:/	0:55	0/1:93,63:3	G	A
.	.	.	.	.	.	.	.	.	.	0	0.2	SON	GT:/	0:8,(	0/1:15,5:37	C	G
.	.	.	.	.	.	.	.	.	.	0	0.1	SON	GT:/	0:8,(	0/1:2,4:37:	G	A
.	.	.	.	.	.	.	.	.	.	0.1	0	SON	GT:/	0:48	0/1:91,4:37	G	A
.	.	.	.	.	.	.	.	.	.	0	0.1	SON	GT:/	0:21	0/1:48,10:3	T	C
.	.	.	.	.	.	.	.	.	.	0	0	SON	GT:/	0:27	0/1:76,4:37	G	T
.	.	.	.	.	.	.	.	.	.	0	0	DB;S	GT:/	0:32	0/1:57,6:37	G	A
.	.	.	.	.	.	.	.	.	.	0	0	SON	GT:/	0:8,(	0/1:10,3:33	T	A
.	.	.	.	.	.	.	.	.	.	.	.	SON	GT:/	0:9,(	0/1:4,6:30:	A	C
.	.	.	.	.	.	.	.	.	.	.	.	SON	GT:/	0:11	0/1:13,3:37	T	G
.	.	.	.	.	.	.	.	4.5	.	.	.	SON	GT:/	0:70	0/1:99,93:3	G	A
.	.	.	.	.	.	.	.	2.9	2.16	.	.	SON	GT:/	0:34	0/1:70,52:3	G	A
.	.	.	.	.	.	.	.	.	.	.	.	SON	GT:/	0:14	0/1:6,2:37:	C	T
.	.	.	.	0.0(	0.012	0.313	0.99	2	2.54	.	.	SON	GT:/	0:18	0/1:4,14:35	G	A
.	.	.	.	.	.	.	.	.	.	0	0.1	SON	GT:/	0:10	0/1:1,4:34:	C	T
0.078	.	.	.	.	.	.	.	.	.	.	.	SON	GT:/	0:22	0/1:5,6:37:	G	T
0.029	.	0	0	.	.	.	.	.	.	0	0	SON	GT:/	0:55	0/1:80,4:37	C	T
.	.	.	.	.	.	.	.	.	.	.	.	SON	GT:/	0:31	0/1:20,4:27	G	A
.	0	0	0	0.5	0.0,B	0.002	1,D	.	.	0	.	DB;S	GT:/	0:34	0/1:46,5:37	G	C
.	.	.	.	.	.	.	.	.	.	.	.	SON	GT:/	0:18	0/1:47,21:3	G	A
.	.	.	.	.	0.926	0.986	0.99	3.6	2.28	.	.	SON	GT:/	0:11	0/1:131,10	G	A
.	.	.	.	.	.	.	.	.	.	.	.	SON	GT:/	0:8,(	0/1:5,2:37:	C	A
5E-04	.	##	0	.	.	.	.	.	.	.	.	SON	GT:/	0:12	0/1:191,15	G	A
.	.	.	.	.	.	.	.	.	.	.	.	SON	GT:/	0:21	0/1:36,10:3	G	C
.	.	.	.	.	.	.	.	.	.	.	.	SON	GT:/	0:23	0/1:47,10:3	G	T
.	.	.	.	.	.	.	.	.	.	.	.	SON	GT:/	0:12	0/1:30,3:37	G	T
.	.	.	.	.	.	.	.	.	.	.	.	SON	GT:/	0:9,(	0/1:5,2:37:	A	C
.	.	.	.	.	.	.	.	.	.	.	.	SON	GT:/	0:15	0/1:44,5:34	C	T
.	.	.	.	.	.	.	.	.	.	.	.	SON	GT:/	0:21	0/1:47,5:37	G	C
.	.	.	.	.	.	.	1,A	4.3	12.6	.	.	SON	GT:/	0:58	0/1:68,54:3	C	T
.	.	.	.	.	.	.	.	.	1.66	.	.	SON	GT:/	0:47	0/1:130,5:3	T	A
.	.	.	.	.	.	.	.	.	.	.	.	SON	GT:/	0:11	0/1:13,4:34	G	A
.	.	.	.	.	.	.	.	.	.	.	.	SON	GT:/	0:52	0/1:131,17	A	G
.	.	.	.	.	.	.	.	.	1.46	.	.	SON	GT:/	0:39	0/1:66,52:3	C	A
.	.	.	.	.	.	.	.	.	.	.	.	SON	GT:/	0:49	0/1:69,60:3	C	T
.	.	.	.	.	.	.	.	.	.	.	.	SON	GT:/	0:36	0/1:81,15:1	T	G
.	.	.	.	.	.	.	.	.	.	0	0	SON	GT:/	0:13	0/1:9,4:37:	G	C
.	.	.	.	.	.	.	.	.	.	0	.	SON	GT:/	0:29	0/1:70,5:37	G	A
.	.	.	.	.	.	.	.	.	.	.	.	SON	GT:/	0:41	0/1:79,60:3	G	A
.	.	.	.	.	.	.	.	.	.	.	.	SON	GT:/	0:11	0/1:19,3:37	C	A
.	.	.	.	.	.	.	.	.	.	.	.	SON	GT:/	0:56	0/1:406,14	C	T
.	.	.	.	.	.	.	.	.	.	.	.	SON	GT:/	0:30	0/1:290,27	C	T
.	.	.	.	.	.	.	.	.	.	0	0	SON	GT:/	0:8,(	0/1:11,3:37	A	G
.	.	.	.	.	.	.	.	.	.	.	0	SON	GT:/	0:33	0/1:61,4:34	G	A

.	.	.	.	0.0	0.99	1.0,D	1,D	4.6	5.20	.	.	SOM	GT:/	0:90	0/1:190,12	C	T
.	.	##	0	.	.	.	.	.	.	.	.	SOM	GT:/	0:41	0/1:95,5:37	C	T
0.001	.	.	.	.	.	.	.	.	.	0	.	SOM	GT:/	0:62	0/1:117,5:3	T	C
.	.	.	.	.	.	.	.	.	.	0.1	0.1	SOM	GT:/	0:47	0/1:100,11	T	C
.	.	.	.	.	.	.	.	.	.	0	0	DB;S	GT:/	0:43	0/1:100,6:3	T	C
.	.	.	.	.	.	.	.	.	.	0.1	0.2	DB;S	GT:/	0:19	0/1:34,5:37	T	G
0.192	.	.	.	.	.	.	.	.	.	0.1	0.2	DB;S	GT:/	0:19	0/1:37,6:37	G	C
.	.	.	.	.	.	.	.	.	.	0.3	0.3	DB;S	GT:/	0:63	0/1:108,5:3	C	T
4E-04	.	##	0	0.2	0.376	0.966	1.00	5.8	3.60	0	.	SOM	GT:/	0:12	0/1:164,12	C	T
.	.	.	.	.	.	.	.	.	.	.	.	SOM	GT:/	0:49	0/1:92,7:33	G	A
.	.	.	.	.	.	.	.	.	1.42	.	.	SOM	GT:/	0:38	0/1:97,11:3	G	T
.	.	.	.	.	.	.	.	2.7	.	0	.	SOM	GT:/	0:19	0/1:16,9:14	T	G
.	.	.	.	0.0	0.826	0.992	1,N	4.5	1.58	.	.	SOM	GT:/	0:40	0/1:65,7:37	G	T
.	.	.	.	.	.	.	.	.	.	.	.	SOM	GT:/	0:66	0/1:114,8:3	C	T
.	.	.	.	0.0	0.997	1.0,D	1,D	.	2.95	.	.	SOM	GT:/	0:66	0/1:119,9:3	G	A
.	.	.	.	.	.	.	.	.	.	0	.	SOM	GT:/	0:15	0/1:18,4:37	T	C
.	.	.	.	.	.	.	.	.	.	0	.	SOM	GT:/	0:8,(	0/1:9,3:37:	G	A
.	.	.	.	.	.	.	.	.	.	0	0.3	SOM	GT:/	0:14	0/1:17,3:37	C	G
.	.	.	.	.	.	.	.	.	.	0	0	SOM	GT:/	0:70	0/1:112,6:3	G	A
.	.	.	.	.	.	.	.	.	.	.	.	SOM	GT:/	0:30	0/1:45,5:37	C	G
.	.	.	.	.	.	.	.	.	.	0.1	0	DB;S	GT:/	0:20	0/1:18,3:37	G	C
0.114	.	.	.	.	.	.	.	.	.	0	0	SOM	GT:/	0:8,(	0/1:11,6:37	A	G
.	.	.	.	.	.	.	.	.	.	.	.	SOM	GT:/	0:27	0/1:45,4:37	C	G
.	.	.	.	.	.	.	.	.	.	0	0	DB;S	GT:/	0:34	0/1:66,4:37	T	A
.	.	##	0	.	.	.	.	.	.	.	.	SOM	GT:/	0:60	0/1:99,14:3	G	A
.	.	.	.	0.0	0.927	0.998	0.92	2.1	2.54	.	.	SOM	GT:/	0:56	0/1:93,5:29	G	T
.	.	.	.	.	.	.	.	.	.	.	.	SOM	GT:/	0:20	0/1:46,3:33	G	C
.	.	##	0	0.0	1.0,D	1.0,D	1,D	5	5.82	.	.	SOM	GT:/	0:81	0/1:144,11	G	A
0.128	0	0	0	1.0	0.0,B	0.0,B	1.00	3.3	.	0	0.1	SOM	GT:/	0:24	0/1:37,4:37	A	G
.	##	##	0	0.0	0.01,I	0.047	1,N	.	1.51	.	.	SOM	GT:/	0:27	0/1:71,11:3	C	T
.	.	0	0	.	.	.	.	.	.	0	0	SOM	GT:/	0:40	0/1:99,11:3	A	G
.	.	.	.	1.0	0.001	0.0,B	1,D	5.4	.	.	.	SOM	GT:/	0:34	0/1:60,7:37	C	A
.	.	.	.	.	.	.	.	.	1.35	.	.	SOM	GT:/	0:54	0/1:92,9:37	T	A
.	.	.	.	.	.	.	.	.	.	.	.	SOM	GT:/	0:11	0/1:188,18	G	A
.	.	.	.	.	.	.	.	3.2	1.43	0	0	SOM	GT:/	0:11	0/1:19,4:37	G	A
.	.	.	.	.	.	.	.	.	.	.	.	SOM	GT:/	0:51	0/1:39,4:37	G	C
.	.	.	.	.	.	.	.	.	.	.	.	SOM	GT:/	0:16	0/1:15,4:27	T	C
.	.	.	.	.	.	.	.	.	.	0	0	SOM	GT:/	0:12	0/1:15,3:37	G	T
.	.	.	.	0.0	0.707	0.763	1.00	4.2	2.72	.	.	SOM	GT:/	0:13	0/1:189,12	C	T
.	.	.	.	.	.	.	.	3.3	.	.	.	SOM	GT:/	0:41	0/1:82,6:37	C	T
.	.	.	.	.	.	.	.	.	.	.	.	SOM	GT:/	0:9,(	0/1:5,2:37:	C	A
.	.	.	.	.	.	.	.	.	.	0	.	SOM	GT:/	0:10	0/1:7,3:37:	G	C
.	.	.	.	.	.	.	.	.	.	0	.	SOM	GT:/	0:10	0/1:7,3:33:	C	T
0.002	.	0	0	.	.	.	.	.	.	0	0	DB;S	GT:/	0:55	0/1:98,7:37	G	A
.	.	.	.	.	.	.	.	.	1.95	0	0	SOM	GT:/	0:16	0/1:18,3:37	G	A
0.105	.	.	.	.	.	.	.	.	.	.	.	SOM	GT:/	0:14	0/1:20,4:27	C	T
.	.	.	.	.	.	.	.	.	.	.	.	SOM	GT:/	0:96	0/1:141,13	G	A
.	.	.	.	.	.	.	.	.	1.31	.	.	SOM	GT:/	0:22	0/1:47,10:1	A	C
.	.	.	.	.	.	.	.	.	.	.	.	SOM	GT:/	0:27	0/1:25,14:1	A	C
.	.	.	.	.	.	.	.	.	.	.	.	SOM	GT:/	0:38	0/1:71,8:37	T	G
.	.	.	.	0.1	0.655	0.936	1,D	5.6	5.16	.	.	SOM	GT:/	0:42	0/1:66,7:37	G	A
.	.	##	0	0.0	0.909	0.991	1,D	5.8	5.70	.	.	DB;S	GT:/	0:26	0/1:38,6:37	G	A
.	.	.	.	.	.	.	.	.	1.41	.	.	SOM	GT:/	0:52	0/1:79,6:35	C	A
.	.	0	0	0.1	0.565	0.981	0.99	4.2	3.28	.	.	SOM	GT:/	0:20	0/1:339,26	G	A

.	.	.	.	.	.	.	.	.	.	.	.	SOM	GT:/	0:10	0/1:163,14	A	G
.	.	.	.	.	.	.	.	.	.	0	.	SOM	GT:/	0:51	0/1:85,7:35	A	G
.	.	.	.	.	.	.	.	.	.	0	.	SOM	GT:/	0:8,(	0/1:4,3:37:	C	T
.	.	.	.	0.2	0.002	0.002	1,N	2.2	1.28	.	.	SOM	GT:/	0:69	0/1:119,5:3	C	T
.	.	.	.	.	.	.	.	.	.	.	.	SOM	GT:/	0:26	0/1:49,7:37	A	C
.	.	.	.	.	.	.	.	.	.	0.1	0.2	SOM	GT:/	0:13	0/1:27,3:37	G	A
.	.	.	.	.	.	.	.	.	.	0	0	SOM	GT:/	0:42	0/1:54,5:37	C	T
.	.	##	0	0.2	0.085	0.804	1,D	2.4	1.31	.	.	SOM	GT:/	0:17	0/1:280,26	G	A
0.381	.	.	.	.	.	.	.	.	.	0.1	0.2	DB;S	GT:/	0:19	0/1:17,3:33	C	T
.	.	.	.	.	.	.	.	.	.	.	.	SOM	GT:/	0:17	0/1:25,3:37	G	A
.	.	.	.	.	.	.	.	.	.	.	.	SOM	GT:/	0:15	0/1:21,8:14	A	C
.	.	.	.	0.0	0.999	1.0,D	0.91	3.2	.	.	.	SOM	GT:/	0:19	0/1:89,9:35	G	C
.	.	.	.	0.0	0.91,	0.99,	0.86	3.5	3.17	.	.	SOM	GT:/	0:75	0/1:104,13	C	A
.	.	##	0	0.0	0.469	0.99,	0.99	3.7	2.54	.	.	SOM	GT:/	0:77	0/1:160,11	C	T
.	.	.	.	.	.	.	.	.	.	.	.	SOM	GT:/	0:30	0/1:57,4:37	G	A
.	.	.	.	.	.	.	.	.	.	.	0	SOM	GT:/	0:53	0/1:82,9:32	T	A
.	.	.	.	.	.	.	.	.	.	.	.	SOM	GT:/	0:59	0/1:88,6:37	T	G
.	.	.	.	.	.	.	.	.	.	.	.	SOM	GT:/	0:27	0/1:46,3:37	C	T
.	.	.	.	.	.	.	.	.	.	.	.	SOM	GT:/	0:36	0/1:63,5:37	C	T
.	.	.	.	0.0	0.997	1.0,D	1,D	5.6	5.94	.	.	SOM	GT:/	0:79	0/1:141,10	G	A
.	.	.	.	.	.	.	.	.	.	0	.	SOM	GT:/	0:46	0/1:90,13:1	T	G
.	.	.	.	.	.	.	.	.	.	0	.	SOM	GT:/	0:73	0/1:128,5:3	C	T
0.069	.	.	.	.	.	.	.	.	.	0.2	0.2	SOM	GT:/	0:9,(	0/1:8,11:37	G	A
.	.	.	.	.	.	.	.	.	.	.	.	SOM	GT:/	0:52	0/1:30,6:28	C	T
.	.	.	.	.	.	.	.	.	.	.	.	SOM	GT:/	0:31	0/1:22,4:37	G	T
.	.	.	.	.	.	.	.	.	.	.	.	SOM	GT:/	0:12	0/1:4,2:37:	A	G
.	.	.	.	.	.	.	.	.	.	0	0	SOM	GT:/	0:9,(	0/1:8,3:28:	C	G
.	.	.	.	.	.	.	.	.	.	.	.	SOM	GT:/	0:21	0/1:10,6:37	C	A
.	.	.	.	.	.	.	.	.	.	0	0	SOM	GT:/	0:23	0/1:15,3:37	T	C
.	.	.	.	.	.	.	.	.	.	.	0	SOM	GT:/	0:70	0/1:94,6:35	C	T
.	.	.	.	.	.	.	.	.	.	.	0	SOM	GT:/	0:85	0/1:113,6:3	C	T
.	.	.	.	.	.	.	.	.	.	0	0	SOM	GT:/	0:11	0/1:15,3:28	G	A
.	.	.	.	0.0	0.041	0.006	1,D	6.1	4.63	.	.	SOM	GT:/	0:37	0/1:61,6:37	G	A
.	.	.	.	0.0	0.681	0.989	0.99	3.7	.	.	.	SOM	GT:/	0:52	0/1:83,16:3	C	T
.	.	.	.	0.0	1.0,D	1.0,D	1,D	5.3	5.07	.	.	SOM	GT:/	0:40	0/1:87,4:37	C	T
0.008	.	.	.	.	.	.	.	.	.	.	.	SOM	GT:/	0:39	0/1:73,4:34	G	A
0.005	.	.	.	.	.	.	.	.	.	0	.	SOM	GT:/	0:32	0/1:68,6:37	C	T
.	.	.	.	.	.	.	.	.	1.34	.	.	SOM	GT:/	0:10	0/1:16,5:19	A	C
.	.	.	.	.	.	.	.	.	.	.	.	SOM	GT:/	0:28	0/1:54,4:37	G	C
.	.	.	.	.	.	.	.	.	.	.	.	SOM	GT:/	0:8,(	0/1:5,3:33:	C	T
.	.	.	.	.	.	.	.	.	.	.	.	SOM	GT:/	0:53	0/1:66,5:37	G	C
.	.	.	.	.	.	.	.	.	.	0	.	SOM	GT:/	0:19	0/1:27,3:37	G	A
0.148	.	.	.	.	.	.	.	.	.	.	.	SOM	GT:/	0:90	0/1:91,4:37	T	A
.	.	.	.	.	.	.	.	.	.	.	.	SOM	GT:/	0:10	0/1:15,3:37	C	G
.	.	.	.	.	.	.	.	3.2	2.52	.	.	SOM	GT:/	0:19	0/1:45,3:33	C	T
.	.	.	.	.	.	.	.	.	.	0	.	SOM	GT:/	0:22	0/1:32,5:37	G	A
0.039	.	.	.	.	.	.	.	.	.	0	0	SOM	GT:/	0:12	0/1:11,3:33	T	C
.	.	.	.	.	.	.	.	.	.	.	.	SOM	GT:/	0:46	0/1:75,5:37	G	A
6E-04	.	.	.	.	.	.	.	.	.	0	.	SOM	GT:/	0:37	0/1:73,5:37	C	T
.	.	.	.	.	.	.	.	.	.	.	.	SOM	GT:/	0:37	0/1:50,10:3	G	A
.	.	.	.	.	.	.	.	.	.	.	.	SOM	GT:/	0:94	0/1:158,7:3	C	T
.	.	.	.	.	.	.	.	.	.	.	.	SOM	GT:/	0:15	0/1:11,5:37	G	A
.	.	0	0	.	.	.	.	.	.	0.1	0	DB;S	GT:/	0:47	0/1:72,7:37	C	T
.	.	0	0	.	.	.	.	.	.	0	.	SOM	GT:/	0:47	0/1:72,5:37	T	C

0.371	0	0	0	.	.	.	.	.	.	0	0.1	SOM	GT:/	0:23	0/1:27,3:37	G	C
.	.	.	.	.	.	.	.	.	.	.	.	SOM	GT:/	0:69	0/1:94,8:35	G	A
.	.	.	.	.	.	.	.	.	.	.	.	SOM	GT:/	0:17	0/1:20,3:33	G	T
.	.	.	.	.	.	.	.	.	.	0	0	DB;S	GT:/	0:33	0/1:57,4:37	T	C
.	.	##	0	.	.	.	.	.	.	.	.	SOM	GT:/	0:12	0/1:233,19	G	A
.	.	.	.	0.0	0.999	1.0,D	1,D	5.7	4.61	.	.	SOM	GT:/	0:78	0/1:119,7:3	G	A
.	.	.	.	.	.	.	.	.	.	.	.	SOM	GT:/	0:21	0/1:14,3:37	G	A
.	.	##	0	.	.	.	.	.	.	.	.	SOM	GT:/	0:62	0/1:122,12	G	A
.	.	.	.	.	.	.	.	.	.	0	.	DB;S	GT:/	0:22	0/1:36,5:37	A	G
.	.	.	.	.	.	.	.	.	.	0.3	0.2	SOM	GT:/	0:10	0/1:24,4:37	G	A
.	.	.	.	.	.	.	.	2.9	.	.	.	SOM	GT:/	0:27	0/1:36,8:37	G	A
.	0	##	0	0.0	0.979	0.999	1,D	5.8	5.60	.	.	SOM	GT:/	0:58	0/1:158,6:3	G	A
.	.	.	.	.	.	.	.	.	.	.	.	SOM	GT:/	0:22	0/1:30,4:37	A	G
0.433	.	1	1	.	.	.	.	.	.	0.1	0.5	SOM	GT:/	0:48	0/1:90,5:37	C	G
.	.	.	.	.	.	.	.	.	.	.	.	SOM	GT:/	0:19	0/1:31,4:37	G	A
.	.	.	.	.	.	.	.	.	.	.	.	SOM	GT:/	0:18	0/1:35,9:15	C	G
4E-04	.	.	.	.	.	.	.	.	.	.	.	SOM	GT:/	0:12	0/1:21,7:37	C	T
.	.	.	.	0.3	0.019	0.049	1,D	5.2	2.12	.	.	SOM	GT:/	0:10	0/1:198,14	C	A
.	.	.	.	.	.	.	.	.	1.70	.	.	SOM	GT:/	0:39	0/1:85,6:37	G	T
.	.	.	.	.	.	.	.	.	.	.	.	SOM	GT:/	0:12	0/1:30,4:37	C	T
.	.	.	.	.	.	.	.	.	.	.	.	SOM	GT:/	0:9,(	0/1:7,5:32:	A	T
.	.	0	0	.	.	.	.	2.1	.	.	.	SOM	GT:/	0:12	0/1:14,3:37	T	C
.	.	.	.	.	.	.	.	.	.	.	.	SOM	GT:/	0:11	0/1:198,16	C	A
.	.	.	.	.	.	.	.	.	.	.	.	SOM	GT:/	0:93	0/1:181,20	G	C
.	.	.	.	.	.	.	.	2.9	.	.	.	SOM	GT:/	0:31	0/1:32,4:37	G	C
.	.	.	.	.	.	.	.	.	.	0	.	SOM	GT:/	0:16	0/1:41,8:35	C	A
.	.	##	0	0.0	0.361	0.517	1,D	5.7	4.40	0	.	DB;S	GT:/	0:17	0/1:46,4:37	C	T
.	##	##	0	0.4	0.052	0.375	0.51	4	2.41	.	.	SOM	GT:/	0:49	0/1:70,7:37	C	T
.		0	0	.	.	.	.	.	.	.	.	DB;S	GT:/	0:11	0/1:243,8:3	T	C
0.001	.	0	0	0.1	0.03,I	0.075	1.00	.	1.87	.	.	SOM	GT:/	0:45	0/1:58,8:37	C	T
.	.	.	.	.	.	.	1,A	.	3.73	.	.	SOM	GT:/	0:51	0/1:70,12:3	G	A
0.001	.	.	.	.	.	.	.	.	.	0	0	SOM	GT:/	0:37	0/1:68,5:37	G	A
.	.	.	.	.	.	.	.	2.6	.	.	.	SOM	GT:/	0:15	0/1:36,4:37	C	A
.	.	.	.	.	.	.	.	.	.	.	.	SOM	GT:/	0:52	0/1:89,11:3	G	A
0.22	.	.	.	.	.	.	.	.	.	0.1	0.3	SOM	GT:/	0:8,(	0/1:6,9:37:	T	C
.	.	##	0	0.0	1.0,D	1.0,D	1,D	5	4.51	.	.	SOM	GT:/	0:84	0/1:155,9:3	G	A
.	.	.	.	.	.	.	.	.	.	.	.	SOM	GT:/	0:9,(	0/1:25,4:37	C	T
.	.	.	.	.	.	.	.	.	.	.	.	SOM	GT:/	0:31	0/1:72,10:1	A	C
.	.	##	0	.	.	.	1,A	4.9	5.89	.	.	SOM	GT:/	0:17	0/1:45,3:37	G	A
.	.	.	.	0.0	0.997	1.0,D	1,D	6.2	4.86	.	.	SOM	GT:/	0:31	0/1:79,4:37	C	T
.	.	.	.	.	.	.	.	.	.	.	.	SOM	GT:/	0:83	0/1:149,14	C	T
.	.	.	.	.	.	.	.	.	.	0	.	SOM	GT:/	0:33	0/1:44,5:37	T	G
.	.	.	.	.	.	.	.	.	.	0	0	SOM	GT:/	0:9,(	0/1:12,3:37	G	A
.	.	.	.	.	.	.	.	.	.	0	.	SOM	GT:/	0:9,(	0/1:9,3:37:	C	T
.	.	.	.	.	.	.	.	.	.	0	0	SOM	GT:/	0:8,(	0/1:4,3:37:	T	G
.	.	.	.	.	.	.	.	.	.	.	.	SOM	GT:/	0:10	0/1:20,3:37	T	C
.	.	.	.	.	.	.	.	.	.	0	0	SOM	GT:/	0:10	0/1:198,7:3	C	T
.	.	.	.	.	.	.	.	.	3.49	.	.	SOM	GT:/	0:43	0/1:69,8:34	G	T
.	.	##	0	.	.	.	.	.	.	.	.	SOM	GT:/	0:10	0/1:210,12	G	A
.	.	.	.	.	.	.	.	.	.	0	.	SOM	GT:/	0:23	0/1:30,3:37	T	A
.	.	.	.	.	.	.	.	.	.	0	0.3	DB;S	GT:/	0:44	0/1:67,4:34	G	A
2E-04	.	.	.	.	.	.	.	.	.	0	0	SOM	GT:/	0:8,(	0/1:17,3:37	C	T
.	.	.	.	.	.	.	.	.	.	0.2	0.2	DB;S	GT:/	0:22	0/1:51,8:37	G	C
.	.	.	.	.	.	.	.	.	.	.	.	SOM	GT:/	0:8,(	0/1:5,2:37:	G	T

.	.	.	.	.	.	.	.	.	.	0	0.1	SON	GT:/	0:10	0/1:7,8:35:	A	C
.	.	.	.	.	.	.	.	.	.	0	0	SON	GT:/	0:10	0/1:15,6:37	C	G
.	.	.	.	.	.	.	.	.	.	0	0.1	SON	GT:/	0:9,(	0/1:18,3:33	A	G
.	.	.	.	.	.	.	.	.	.	0	0.2	SON	GT:/	0:8,(	0/1:18,4:37	G	A
.	.	0	0	.	.	.	.	.	.	0	0	SON	GT:/	0:74	0/1:153,8:3	G	A
.	.	.	.	.	.	.	.	2.8	2.38	.	.	SON	GT:/	0:22	0/1:312,17	G	A
2E-04	.	##	0	.	.	.	.	.	.	.	.	SON	GT:/	0:68	0/1:145,10	C	T
.	.	.	.	.	.	.	.	.	.	.	.	SON	GT:/	0:20	0/1:27,3:37	C	T
.	.	0	0	1.0	0.0,B	0.0,B	1.00	.	.	0	0	SON	GT:/	0:42	0/1:72,4:37	A	G
.	.	.	.	.	.	.	.	.	.	.	.	SON	GT:/	0:9,(	0/1:1,2:37:	G	A
.	.	.	.	0.5	0.001	0.0,B	1,N	.	.	.	.	SON	GT:/	0:99	0/1:163,14	T	C
.	.	.	.	.	.	.	.	.	.	.	.	SON	GT:/	0:18	0/1:304,14	A	T
.	.	.	.	.	.	.	.	.	.	.	.	SON	GT:/	0:38	0/1:71,4:37	C	T
.	.	.	.	.	.	.	.	.	.	.	.	SON	GT:/	0:15	0/1:20,3:33	G	A
.	.	.	.	.	.	.	.	.	.	0	.	DB;S	GT:/	0:23	0/1:75,11:3	C	G
.	.	.	.	.	.	.	.	.	.	0	.	DB;S	GT:/	0:25	0/1:49,4:37	C	T
.	.	.	.	.	.	.	.	.	.	.	.	SON	GT:/	0:35	0/1:123,6:3	T	G
.	.	.	.	.	.	.	.	.	.	0	.	DB;S	GT:/	0:42	0/1:132,9:3	G	A
.	.	.	.	.	.	.	.	.	.	0	.	DB;S	GT:/	0:59	0/1:235,13	C	T
.	.	.	.	.	.	.	.	.	.	0	.	DB;S	GT:/	0:65	0/1:265,14	T	A
.	.	.	.	.	.	.	.	.	.	0	.	DB;S	GT:/	0:36	0/1:135,6:3	C	T
.	.	.	.	.	.	.	.	.	.	0	.	DB;S	GT:/	0:35	0/1:138,6:3	G	C
.	.	.	.	.	.	.	.	.	.	.	.	DB;S	GT:/	0:43	0/1:70,4:37	C	T
.	.	.	.	.	.	.	.	.	.	0	.	SON	GT:/	0:8,(	0/1:55,4:37	G	A
.	.	.	.	.	.	.	.	.	.	0	.	DB;S	GT:/	0:22	0/1:103,8:3	T	C
.	.	.	.	.	.	.	.	.	.	.	.	DB;S	GT:/	0:34	0/1:116,6:3	G	T
.	.	.	.	.	.	.	.	.	.	0	.	DB;S	GT:/	0:38	0/1:156,6:3	A	G
.	.	.	.	.	.	.	.	.	.	0	.	DB;S	GT:/	0:39	0/1:155,7:3	G	C
.	.	.	.	.	.	.	.	.	.	0	0	SON	GT:/	0:8,(	0/1:25,3:37	C	A
.	.	.	.	.	.	.	.	.	.	0	.	SON	GT:/	0:22	0/1:71,4:37	A	G
.	.	.	.	.	.	.	.	.	.	.	.	DB;S	GT:/	0:43	0/1:107,5:3	C	T
.	.	.	.	.	.	.	.	.	.	0	.	DB;S	GT:/	0:43	0/1:184,7:3	C	T
.	.	.	.	.	.	.	.	.	.	.	.	DB;S	GT:/	0:30	0/1:112,5:3	C	T
.	.	.	.	.	.	.	.	.	.	.	.	SON	GT:/	0:14	0/1:38,3:37	A	T
.	.	.	.	.	.	.	.	.	.	.	.	DB;S	GT:/	0:35	0/1:167,11	A	C
.	.	.	.	.	.	.	.	.	.	0	.	DB;S	GT:/	0:27	0/1:117,8:3	A	C
.	.	.	.	.	.	.	.	.	.	0	.	DB;S	GT:/	0:21	0/1:82,4:34	T	C
.	.	.	.	.	.	.	.	.	.	.	.	SON	GT:/	0:33	0/1:61,8:34	C	A
.	.	0	0	0.6	0.0,B	0.0,B	1,N	.	.	0	0	SON	GT:/	0:79	0/1:161,7:3	A	G
.	.	.	.	.	.	.	.	.	.	0	0	SON	GT:/	0:14	0/1:20,4:24	A	C
.	.	.	.	0.1(	0.002	0.0,B	1,N	.	1.72	.	.	SON	GT:/	0:46	0/1:69,8:37	C	G
.	.	.	.	.	.	.	.	.	.	.	.	SON	GT:/	0:35	0/1:85,5:31	C	A
.	.	.	.	.	.	.	.	.	.	.	.	SON	GT:/	0:19	0/1:29,5:21	T	G
.	.	.	.	.	.	.	.	3.2	.	.	.	SON	GT:/	0:58	0/1:122,7:3	G	A
.	.	.	.	.	.	.	.	.	.	.	.	SON	GT:/	0:79	0/1:146,11	G	T
.	.	.	.	.	.	.	.	.	.	.	.	SON	GT:/	0:14	0/1:28,3:37	G	A
.	.	.	.	.	.	.	.	.	.	0	0	SON	GT:/	0:16	0/1:31,4:37	T	G
0.344	.	.	.	.	.	.	.	.	.	0	0.2	SON	GT:/	0:11	0/1:9,4:37:	A	G
0.177	.	.	.	.	.	.	.	.	.	0	0.2	SON	GT:/	0:13	0/1:10,3:37	C	A
.	.	.	.	.	0.137	0.458	1.00	4.9	3.16	0	.	SON	GT:/	0:25	0/1:30,4:37	C	T
.	.	.	.	.	.	.	.	.	.	.	.	SON	GT:/	0:37	0/1:59,4:37	A	G
.	.	.	.	.	.	.	.	.	.	0	0	DB;S	GT:/	0:29	0/1:39,4:37	A	T
.	.	.	.	.	.	.	.	.	.	.	.	SON	GT:/	0:18	0/1:0,2:37:	G	A
.	.	.	.	.	.	.	.	.	.	.	.	SON	GT:/	0:33	0/1:42,4:34	G	A

.	.	##	.	0.0(	0.985	1.0,D	1.00	3.4	4.41	.	.	SON	GT:/	0:13	0/1:225,21	C	T
.	.	0	0	.	.	.	.	2.5	.	.	.	SON	GT:/	0:88	0/1:170,17	G	A
.	.	.	.	.	.	.	.	.	.	.	.	SON	GT:/	0:93	0/1:159,7:3	G	A
4E-04	.	.	.	.	.	.	.	.	.	.	.	SON	GT:/	0:21	0/1:29,5:26	C	T
.	.	.	.	.	.	.	.	4	.	.	.	SON	GT:/	0:55	0/1:104,7:3	G	A
.	.	.	.	.	.	.	1,N	.	.	.	.	SON	GT:/	0:10	0/1:165,13	C	T
.	##	##	0	.	.	.	.	.	.	0	.	SON	GT:/	0:87	0/1:143,9:3	C	T
.	.	.	.	.	.	.	.	.	1.42	.	.	SON	GT:/	0:14	0/1:255,23	C	T
.	.	0	.	.	.	.	.	.	.	0	.	SON	GT:/	0:17	0/1:13,3:37	C	T
.	.	.	.	.	.	.	.	.	.	.	.	SON	GT:/	0:32	0/1:36,3:37	G	A
.	.	.	.	.	.	.	.	.	.	.	.	SON	GT:/	0:65	0/1:130,5:3	G	T
.	.	.	.	.	.	.	.	.	.	.	.	SON	GT:/	0:11	0/1:23,5:37	G	T
.	.	.	.	.	.	.	.	2.4	1.26	.	.	SON	GT:/	0:10	0/1:18,3:37	C	A
.	.	.	.	.	.	.	.	.	.	.	.	SON	GT:/	0:11	0/1:12,3:37	G	A
.	.	.	.	.	.	.	.	.	.	0	0.1	SON	GT:/	0:8,(	0/1:6,4:34:	T	C
.	.	.	.	.	.	.	.	.	.	0	0	SON	GT:/	0:26	0/1:35,3:37	G	T
.	.	.	.	.	.	.	.	.	.	0	.	SON	GT:/	0:38	0/1:68,4:37	C	G
.	.	.	.	.	.	.	.	3.1	.	.	.	SON	GT:/	0:24	0/1:40,7:37	C	T
.	.	.	.	.	.	.	.	.	.	0	.	SON	GT:/	0:22	0/1:50,11:1	A	C
.	.	.	.	.	.	.	.	.	.	.	.	SON	GT:/	0:19	0/1:28,3:37	C	G
.	.	.	.	0.0(	0.809	0.975	0.81	.	3.51	.	.	DB;S	GT:/	0:37	0/1:55,5:37	C	A
.	.	.	.	.	.	.	.	.	.	0	.	DB;S	GT:/	0:34	0/1:75,4:34	C	A
.	.	.	.	.	.	.	.	.	.	.	.	SON	GT:/	0:17	0/1:27,7:16	A	C
2E-04	.	.	.	.	.	.	.	.	.	0	0	SON	GT:/	0:11	0/1:33,3:37	T	C
.	.	.	.	.	.	.	.	.	.	0	.	SON	GT:/	0:85	0/1:173,6:3	T	C
.	.	##	0	.	.	.	.	.	.	.	.	SON	GT:/	0:75	0/1:156,15	G	A
.	.	.	.	.	.	.	.	.	.	.	.	SON	GT:/	0:39	0/1:76,8:37	G	T
.	.	##	0	0.0:	0.961	0.998	0.76	2.6	3.22	.	.	SON	GT:/	0:17	0/1:294,23	T	G
.	.	.	.	.	.	.	.	2.9	.	.	.	SON	GT:/	0:42	0/1:75,4:37	C	T
.	.	.	.	.	.	.	.	.	.	.	.	SON	GT:/	0:17	0/1:41,7:33	C	T
.	.	.	.	.	.	.	.	.	.	.	.	SON	GT:/	0:21	0/1:33,3:37	G	T
.	.	.	.	.	.	.	.	.	.	.	.	SON	GT:/	0:87	0/1:157,7:3	C	A
.	.	.	.	.	.	.	.	.	.	.	0	SON	GT:/	0:23	0/1:27,3:37	A	G
.	.	.	.	.	.	.	.	.	.	.	.	SON	GT:/	0:41	0/1:71,9:34	C	A
.	.	.	.	.	.	.	.	.	.	0.1	0.2	SON	GT:/	0:10	0/1:17,4:37	C	T
.	.	.	.	.	.	.	.	.	.	0	0	SON	GT:/	0:41	0/1:41,3:37	C	T
.	.	.	.	.	.	.	.	.	.	0	.	SON	GT:/	0:54	0/1:104,5:3	A	G
.	.	.	.	.	.	.	.	.	.	.	.	SON	GT:/	0:9,(	0/1:14,3:37	G	A
.	.	.	.	.	.	.	.	.	.	.	.	SON	GT:/	0:29	0/1:63,4:37	C	T
.	.	.	.	.	.	.	.	.	.	.	.	SON	GT:/	0:12	0/1:9,3:37:	T	G
.	.	.	.	.	.	.	.	.	.	.	.	SON	GT:/	0:56	0/1:101,5:3	C	T
.	.	.	.	.	.	.	.	.	.	.	.	SON	GT:/	0:20	0/1:50,4:37	C	T
1E-03	.	0	0	.	.	.	.	.	.	0	0	SON	GT:/	0:18	0/1:330,16	G	A
0.03	.	.	.	.	.	.	.	.	.	0	0	SON	GT:/	0:22	0/1:40,3:37	C	T
.	##	##	0	.	.	.	.	.	.	.	.	SON	GT:/	0:63	0/1:126,13	C	T
.	.	.	.	.	.	.	.	3.5	.	.	.	SON	GT:/	0:56	0/1:87,9:35	G	A
.	.	.	.	.	.	.	.	.	2.38	.	.	SON	GT:/	0:25	0/1:31,3:37	G	A
.	.	.	.	.	.	.	.	.	.	.	.	SON	GT:/	0:26	0/1:63,4:37	C	A
.	.	.	.	.	.	.	.	.	.	0	0.1	SON	GT:/	0:31	0/1:19,4:37	G	T
0.034	.	.	.	.	.	.	.	.	.	.	.	SON	GT:/	0:11	0/1:17,4:37	T	C
.	.	.	.	0.0(	0.754	0.898	0.97	5	3.18	.	.	SON	GT:/	0:11	0/1:219,22	C	T
.	.	##	0	0.0·	0.805	0.998	1,D	5.1	3.90	.	.	SON	GT:/	0:48	0/1:99,6:35	C	T
.	.	.	.	.	.	.	.	2.6	.	.	.	SON	GT:/	0:93	0/1:181,16	G	A
.	.	0	0	0.0.	0.999	1.0,D	1,D	4.9	3.75	.	.	SON	GT:/	0:43	0/1:81,4:37	G	A

.	.	.	.	.	.	.	.	.	.	.		.	SON	GT:/	0:26	0/1:116,5:3	G	C
.	.	.	.	.	.	.	.	.	.		0	.	SON	GT:/	0:13	0/1:43,6:37	T	C
.	.	.	.	.	.	.	.	.	.		0	.	SON	GT:/	0:13	0/1:44,4:37	A	G
0.048	0	.	.	.	.	.	.	.	.		0	0	DB;S	GT:/	0:60	0/1:107,9:3	A	T
.	.	.	.	0.0	0.416	0.974	0.93	4.8	.	.		.	SON	GT:/	0:71	0/1:166,13	G	A
.	.	.	.	0.4	0.001	0.0,B	1.00	.	.	.		.	SON	GT:/	0:89	0/1:195,17	T	C
.	.	.	.	.	.	.	.	.	.	.		.	SON	GT:/	0:65	0/1:150,10	C	T
.	.	.	.	.	.	.	.	.	.	.		.	SON	GT:/	0:17	0/1:35,5:37	C	A
.	.	.	.	.	.	.	.	.	.	.		.	SON	GT:/	0:20	0/1:37,6:37	G	A
.	.	0	0	.	.	.	.	.	3.39	.		.	SON	GT:/	0:17	0/1:15,3:37	G	A
.	.	.	.	.	.	.	.	.	.	.		.	SON	GT:/	0:26	0/1:99,6:35	T	C
.	.	.	.	.	.	.	.	.	.	.		.	SON	GT:/	0:29	0/1:107,5:3	G	A
.	.	.	.	.	.	.	.	.	.	.		.	SON	GT:/	0:22	0/1:68,10:3	C	A
.	.	##	0	.	.	.	.	.	.	.		.	SON	GT:/	0:95	0/1:120,52	C	T
.	.	.	.	.	.	.	.	.	.	.		.	SON	GT:/	0:40	0/1:83,12:3	C	G
2E-04	0	0	0	0.8	0.062	0.877	0.99	4.2	2.67	.		.	SON	GT:/	0:92	0/1:141,9:3	C	T
.	.	.	.	0.0	0.38,l	0.273	1,D	5.4	4.81	.		.	SON	GT:/	0:14	0/1:192,47	C	T
.	.	0	0	.	.	.	.	.	.		0	0	SON	GT:/	0:17	0/1:26,6:33	G	A
0.081	.	.	.	.	.	.	.	.	.		0	0.4	SON	GT:/	0:8,(	0/1:3,2:37:	G	C
.	.	.	.	.	.	.	.	.	.	.		.	SON	GT:/	0:31	0/1:31,3:37	T	A
.	.	.	.	.	.	.	.	.	.		0	0	SON	GT:/	0:8,(	0/1:15,5:37	C	T
.	.	.	.	0.0	0.052	0.103	1,N	3	2.65	.		.	SON	GT:/	0:40	0/1:54,13:3	G	C
2E-04	.	.	.	.	.	.	.	.	.	.		.	SON	GT:/	0:13	0/1:11,4:37	C	T
0.001	0	0	0	0.2	0.005	0.055	1,D	5	3.03		0	0	SON	GT:/	0:46	0/1:49,18:3	G	A
.	.	.	.	.	.	.	.	.	.	.		.	SON	GT:/	0:13	0/1:17,5:37	G	A
.	.	.	.	.	.	.	.	2.3	1.84	.		.	SON	GT:/	0:86	0/1:102,35	G	A
.	.	##	0	0.0	0.001	0.002	1,D	3.2	2.14	.		.	SON	GT:/	0:93	0/1:97,26:3	G	A
.	.	.	.	0.0	0.18,l	0.236	1.00	6.2	2.89	.		.	SON	GT:/	0:39	0/1:67,4:37	C	T
.	.	.	.	0.0	0.998	1.0,D	1,D	5.9	5.31	.		.	SON	GT:/	0:30	0/1:50,5:37	G	C
.	.	.	.	.	.	.	.	2.3	2.51	.		.	SON	GT:/	0:37	0/1:78,13:3	G	T
.	.	.	.	.	.	.	.	.	.	.		.	SON	GT:/	0:25	0/1:60,7:37	G	T
.	.	.	.	.	.	.	.	.	.	.		.	SON	GT:/	0:12	0/1:26,7:37	G	C
.	.	.	.	.	.	.	.	.	.		0	.	SON	GT:/	0:18	0/1:36,3:37	A	G
.	.	##	0	0.0	0.002	0.029	1,N	.	1.80	.		.	SON	GT:/	0:23	0/1:325,84	C	T
.	.	.	.	.	.	.	.	.	.		0	0.1	SON	GT:/	0:8,(	0/1:6,2:37:	C	A
.	.	.	.	.	.	.	.	2.2	1.31	.		.	SON	GT:/	0:41	0/1:57,23:3	C	T
.	.	.	.	.	.	.	.	.	.	.		.	SON	GT:/	0:28	0/1:37,14:3	C	G
.	.	.	.	.	.	.	.	.	.	.		.	SON	GT:/	0:20	0/1:38,4:37	G	A
.	.	.	.	.	.	.	.	.	.	.		.	SON	GT:/	0:10	0/1:15,5:37	G	A
.	.	.	.	.	.	.	.	.	.	.		.	SON	GT:/	0:11	0/1:19,4:37	G	A
.	.	.	.	.	.	.	.	.	.	.		.	SON	GT:/	0:8,(	0/1:6,2:37:	C	A
.	.	.	.	.	.	.	.	.	.	.		.	SON	GT:/	0:31	0/1:52,14:3	G	A
.	.	.	.	.	.	.	.	.	1.48	.		.	SON	GT:/	0:56	0/1:66,10:3	G	A
.	.	.	.	.	.	.	.	.	.	.		.	SON	GT:/	0:10	0/1:146,43	G	A
.	.	0	0	.	.	.	.	.	.		0	0.3	SON	GT:/	0:11	0/1:24,7:37	G	A
.	.	.	.	.	.	.	.	.	.		0	0	SON	GT:/	0:12	0/1:12,4:37	C	T
.	.	.	.	.	.	.	.	.	.	.		.	SON	GT:/	0:9,(	0/1:6,2:37:	C	T
.	.	.	.	.	.	.	.	.	.		0	0	SON	GT:/	0:13	0/1:12,3:33	G	A
.	.	.	.	.	.	.	.	.	.		0	0	SON	GT:/	0:41	0/1:80,4:37	A	G
.	.	.	.	.	.	.	.	.	.		0	0.1	SON	GT:/	0:20	0/1:37,6:37	C	G
.	.	.	.	.	.	.	.	.	.		0	.	DB;S	GT:/	0:52	0/1:60,8:37	A	T
.	.	.	.	.	.	.	.	.	.	.		.	SON	GT:/	0:68	0/1:130,5:3	T	G
.	.	.	.	.	.	.	.	.	.	.		.	SON	GT:/	0:9,(	0/1:6,2:37:	A	G
.	.	.	.	.	.	.	.	.	.	.		.	SON	GT:/	0:8,(	0/1:14,3:33	G	A

.	.	.	.	.	.	.	.	.	.	.	.	SOM	GT:/	0:19	0/1:32,4:37	C	A
.	.	.	.	.	.	.	.	.	.	0	.	DB;S	GT:/	0:27	0/1:36,4:37	C	T
.	.	.	.	.	.	.	.	.	.	.	.	SOM	GT:/	0:16	0/1:191,8:3	T	G
.	.	.	.	.	.	.	.	.	.	.	.	SOM	GT:/	0:18	0/1:169,15	C	G
.	.	.	.	.	.	.	.	.	.	0	0	SOM	GT:/	0:12	0/1:12,4:37	G	A
.	.	.	.	.	.	.	.	.	.	0	0	SOM	GT:/	0:12	0/1:12,4:37	G	A
.	.	.	.	.	.	.	.	.	1.97	0	0	SOM	GT:/	0:11	0/1:12,3:37	G	C
.	.	.	.	.	.	.	.	.	.	.	.	SOM	GT:/	0:9,(	0/1:8,16:36	G	A
0.398	.	.	.	.	.	.	.	.	.	0	0.2	SOM	GT:/	0:24	0/1:41,7:37	C	T
.	.	.	.	.	.	.	.	.	.	0	.	SOM	GT:/	0:14	0/1:17,4:37	T	A
.	.	.	.	.	.	.	.	.	1.76	.	.	SOM	GT:/	0:18	0/1:40,9:37	C	T
.	.	.	.	.	.	.	.	.	.	.	.	SOM	GT:/	0:62	0/1:92,28:3	C	G
.	.	.	.	.	.	.	.	.	.	.	.	SOM	GT:/	0:31	0/1:37,10:3	C	A
.	.	.	.	.	.	.	.	.	.	.	.	SOM	GT:/	0:69	0/1:120,29	A	T
.	.	.	.	.	.	.	.	.	.	.	.	SOM	GT:/	0:19	0/1:32,12:2	G	T
.	.	.	.	.	.	.	.	3.3	1.34	.	.	SOM	GT:/	0:51	0/1:74,5:37	C	T
.	.	.	.	.	.	.	.	2.8	1.23	.	.	SOM	GT:/	0:85	0/1:183,14	C	T
.	.	.	.	.	.	.	.	2	.	.	.	SOM	GT:/	0:42	0/1:58,4:37	C	T
.	.	.	.	0.3	0.082	0.11,I	1,N	.	.	.	.	SOM	GT:/	0:58	0/1:77,28:3	C	G
2E-04	0	0	0	.	.	.	.	.	.	.	.	SOM	GT:/	0:12	0/1:20,3:37	G	A
.	##	0	0	0.0(	0.333	0.988	1.00	5.6	3.54	.	.	SOM	GT:/	0:52	0/1:81,26:3	G	A
.	.	.	.	0.1	0.582	0.994	0.99	2.6	3.80	.	.	SOM	GT:/	0:25	0/1:60,5:37	G	A
.	.	.	.	0.0.	1.0,D	1.0,D	1,D	5.7	5.03	0	.	SOM	GT:/	0:75	0/1:121,27	C	T
.	.	.	.	.	.	.	.	.	.	.	.	SOM	GT:/	0:13	0/1:16,3:37	G	A
.	.	##	0	.	.	.	.	.	.	.	.	SOM	GT:/	0:38	0/1:64,14:3	G	A
.	.	.	.	.	.	.	.	.	1.87	.	.	SOM	GT:/	0:28	0/1:44,12:3	C	T
.	.	.	.	.	.	.	.	.	.	.	.	SOM	GT:/	0:9,(	0/1:20,8:33	C	G
.	.	.	.	.	.	.	.	3.4	.	.	.	DB;S	GT:/	0:40	0/1:76,25:3	G	C
.	.	.	.	.	.	.	.	.	.	.	.	SOM	GT:/	0:31	0/1:49,16:3	G	C
.	.	.	.	.	.	.	.	.	.	0	.	SOM	GT:/	0:9,(	0/1:5,2:37:	C	A
.	.	.	.	.	.	.	.	4.1	.	.	.	SOM	GT:/	0:69	0/1:81,25:3	C	T
.	.	.	.	.	.	.	.	3.9	.	.	.	SOM	GT:/	0:13	0/1:28,3:37	C	T
.	.	.	.	.	.	.	.	.	.	.	.	SOM	GT:/	0:36	0/1:58,19:3	A	G
.	.	.	.	.	.	.	.	.	1.61	.	.	SOM	GT:/	0:17	0/1:21,3:37	C	G
.	.	.	.	.	.	.	.	.	.	.	.	SOM	GT:/	0:12	0/1:10,3:37	C	G
.	.	.	.	.	.	.	.	.	.	0	.	SOM	GT:/	0:27	0/1:37,12:3	C	T
.	.	.	.	.	.	.	.	2.6	.		0	SOM	GT:/	0:31	0/1:58,4:37	C	T
.	##	##	0	.	.	.	.	.	.	.	.	SOM	GT:/	0:59	0/1:87,17:3	C	T
.	.	.	.	.	.	.	.	.	.	.	.	SOM	GT:/	0:77	0/1:107,20	C	T
.	.	.	.	.	.	.	.	.	1.57	.	.	SOM	GT:/	0:39	0/1:48,7:33	G	A
.	.	.	.	.	.	.	.	.	.	0	.	SOM	GT:/	0:17	0/1:23,3:37	A	G
.	.	.	.	.	.	.	.	.	.	0	.	SOM	GT:/	0:18	0/1:24,3:37	C	A
.	.	.	.	.	.	.	.	.	1.90	.	.	SOM	GT:/	0:27	0/1:30,5:31	C	T
.	.	.	.	.	.	.	.	.	.	0.1	0.5	SOM	GT:/	0:8,(	0/1:10,5:37	G	A
.	.	.	.	.	.	.	.	.	.	0	0.1	SOM	GT:/	0:8,(	0/1:13,3:37	T	C
.	.	.	.	.	.	.	.	3.8	1.39	.	.	SOM	GT:/	0:90	0/1:123,26	C	T
.	.	.	.	.	.	.	.	.	.	.	.	SOM	GT:/	0:29	0/1:37,15:3	G	A
.	.	.	.	.	.	.	.	.	1.92	.	.	SOM	GT:/	0:31	0/1:35,12:3	G	C
0.408	.	.	.	.	.	.	.	.	.	0	0	DB;S	GT:/	0:28	0/1:62,5:37	A	T
.	.	.	.	.	.	.	.	5.4	3.63	.	.	SOM	GT:/	0:10	0/1:180,7:3	C	T
.	.	.	.	.	.	.	.	.	.	.	.	SOM	GT:/	0:16	0/1:27,3:37	C	T
0.739	.	1	1	.	.	.	.	.	.	0	0	SOM	GT:/	0:17	0/1:40,3:37	T	C
.	.	.	.	.	.	.	.	.	.	0	.	SOM	GT:/	0:9,(	0/1:16,3:37	A	G
.	.	.	.	.	.	.	.	.	.	.	.	SOM	GT:/	0:18	0/1:139,15	C	T

.	.	.	.	.	.	.	.	.	.	.	.	SON	GT:/	0:17	0/1:26,7:35	G	T
.	.	.	.	.	.	.	.	.	.	.	.	SON	GT:/	0:22	0/1:40,3:37	G	C
.	.	.	.	.	.	.	.	.	.	.	.	SON	GT:/	0:14	0/1:21,7:14	T	G
.	.	.	.	0.1	0.104	0.291	0.94	2.4	2.90	.	.	SON	GT:/	0:37	0/1:64,18:3	C	T
.	.	.	.	.	.	.	.	.	.	.	.	SON	GT:/	0:15	0/1:10,6:15	T	G
.	.	.	.	.	.	.	.	.	.	.	.	SON	GT:/	0:9,(	0/1:16,4:30	T	C
.	.	.	.	.	.	.	.	.	.	.	.	SON	GT:/	0:33	0/1:41,12:3	C	T
.	.	.	.	0.3	0.05,I	0.118	1,N	.	.	.	.	SON	GT:/	0:26	0/1:47,11:3	G	A
.	.	.	.	.	.	.	.	.	.	.	.	SON	GT:/	0:13	0/1:30,9:37	G	A
.	.	.	.	.	.	.	.	.	.	.	.	SON	GT:/	0:25	0/1:20,5:37	C	T
.	.	.	.	.	.	.	.	.	.	.	.	SON	GT:/	0:67	0/1:114,5:3	G	A
.	.	.	.	.	.	.	.	.	.	.	.	SON	GT:/	0:45	0/1:89,22:3	G	A
.	.	.	.	.	.	.	.	.	.	.	.	SON	GT:/	0:16	0/1:23,4:37	G	A
.	.	.	.	.	.	.	.	.	.	.	.	SON	GT:/	0:10	0/1:14,3:37	G	T
.	.	.	.	0.0	0.166	0.146	1,D	5.5	4.87	.	.	SON	GT:/	0:37	0/1:47,18:3	C	G
.	.	.	.	.	.	.	.	.	.	.	.	SON	GT:/	0:55	0/1:95,22:3	C	T
.	.	.	.	.	.	.	.	.	.	.	.	SON	GT:/	0:15	0/1:29,6:37	G	A
.	.	.	.	.	.	.	.	4.6	2.01	.	.	SON	GT:/	0:98	0/1:168,47	C	T
.	.	.	.	0.1	0.376	0.722	1,D	5.4	4.72	.	.	SON	GT:/	0:10	0/1:179,35	C	T
.	.	.	.	.	.	.	.	.	.	.	.	SON	GT:/	0:16	0/1:271,65	G	A
.	.	##	.	.	.	.	.	.	1.27	0	.	SON	GT:/	0:17	0/1:225,71	C	T
.	##	.	.	0.1	0.67,I	0.939	1.00	5.4	4.66	.	.	SON	GT:/	0:10	0/1:165,17	G	A
.	.	##	0	.	.	.	.	.	.	.	.	SON	GT:/	0:52	0/1:100,24	G	A
.	.	.	.	.	.	.	.	.	.	.	.	SON	GT:/	0:33	0/1:50,21:3	A	T
6E-04	0	0	0	.	.	.	.	3.1	.	.	.	SON	GT:/	0:17	0/1:22,10:3	G	A
.	.	.	.	0.1	0.005	0.011	1,N	.	.	.	.	SON	GT:/	0:79	0/1:289,9:3	C	T
.	.	.	.	0.1	0.203	0.437	1,N	3.6	3.56	.	.	SON	GT:/	0:68	0/1:221,12	C	G
.	.	.	.	.	.	.	.	.	.	.	.	SON	GT:/	0:43	0/1:107,15	C	T
.	.	.	.	0.0	0.993	0.999	1.00	3.5	3.61	.	.	SON	GT:/	0:11	0/1:373,11	C	T
.	.	.	.	.	.	.	.	.	.	.	.	SON	GT:/	0:21	0/1:61,14:3	C	T
.	.	.	.	0.1	0.599	0.873	1,N	4.2	1.92	.	.	SON	GT:/	0:68	0/1:207,25	C	T
.	.	.	.	.	.	.	.	.	.	.	.	SON	GT:/	0:41	0/1:145,25	C	T
.	.	.	.	.	.	.	.	2.9	.	.	.	SON	GT:/	0:71	0/1:209,23	G	A
.	.	.	.	.	.	.	.	.	.	.	.	SON	GT:/	0:32	0/1:121,28	C	T
0.002	0	0	0	.	.	.	.	.	.	.	.	SON	GT:/	0:26	0/1:60,64:3	G	A
.	.	.	.	.	.	.	1,D	3.4	8.37	.	.	SON	GT:/	0:59	0/1:178,17	C	A
.	.	.	.	.	.	.	.	.	.	.	.	SON	GT:/	0:11	0/1:361,52	G	A
.	.	.	.	.	.	.	.	.	.	.	.	SON	GT:/	0:13	0/1:44,5:37	T	G
.	.	.	.	.	.	.	.	2.8	.	.	.	SON	GT:/	0:9,(	0/1:25,6:37	G	C
.	.	.	.	.	.	.	.	.	2.10	.	.	SON	GT:/	0:18	0/1:41,20:3	C	T
.	.	.	.	.	.	.	.	.	.	.	.	SON	GT:/	0:25	0/1:77,12:3	G	A
.	.	.	.	.	.	.	.	.	.	.	.	SON	GT:/	0:40	0/1:116,40	G	C
.	.	.	.	.	.	.	.	5.6	.	.	.	SON	GT:/	0:70	0/1:245,30	C	T
.	.	.	.	0.4	1.0,D	1.0,D	1,N	.	.	.	.	SON	GT:/	0:93	0/1:154,19	C	G
.	.	.	.	.	.	.	.	.	.	.	.	SON	GT:/	0:22	0/1:68,9:34	G	A
.	.	.	.	.	.	.	.	.	.	.	.	SON	GT:/	0:24	0/1:46,11:3	G	A
.	.	.	.	.	.	.	.	.	.	.	.	SON	GT:/	0:8,(	0/1:18,7:37	C	T
.	.	.	.	.	.	.	.	.	.	0	.	SON	GT:/	0:17	0/1:54,5:37	C	T
.	.	.	.	.	.	.	.	.	.	0	0	SON	GT:/	0:10	0/1:28,5:34	C	T
.	.	.	.	.	.	.	.	.	.	0	0	SON	GT:/	0:11	0/1:36,5:31	G	A
.	.	.	.	.	.	.	.	.	.	0	0	SON	GT:/	0:14	0/1:45,5:37	C	T
.	.	.	.	.	.	.	.	.	.	0.4	0.4	DB;S	GT:/	0:41	0/1:72,10:3	C	T
.	.	0	0	.	.	.	.	2.1	.	.	.	SON	GT:/	0:84	0/1:131,43	C	T
.	.	##	0	0.0	0.0,B	0.0,B	1.00	3	2.06	0	.	SON	GT:/	0:42	0/1:75,20:3	G	A

.	.	.	.	.	.	.	.	.	.	.	.	SON	GT:/	0:10	0/1:21,3:37	G	A
.	.	.	.	.	.	.	.	4	1.61	.	.	SON	GT:/	0:21	0/1:31,10:3	G	A
.	.	.	.	0.0(	0.979	0.99,I	1,D	5.6	4.79	.	.	SON	GT:/	0:57	0/1:113,29	C	G
.	.	.	.	.	.	.	.	.	.	0	0	DB;S	GT:/	0:20	0/1:14,5:34	G	T
.	.	.	.	.	.	.	.	.	.	.	.	SON	GT:/	0:19	0/1:41,5:34	G	A
2E-04	.	.	.	.	.	.	.	.	.	.	.	SON	GT:/	0:38	0/1:77,27:3	C	T
.	.	.	.	.	.	.	.	.	.	.	.	SON	GT:/	0:14	0/1:15,7:37	G	C
.	.	.	.	.	.	.	.	.	.	0	0.1	SON	GT:/	0:9,(	0/1:22,3:37	C	A
.	.	.	.	.	.	.	.	.	.	.	.	SON	GT:/	0:18	0/1:35,11:3	C	T
.	.	.	.	.	.	.	.	.	.	.	.	SON	GT:/	0:28	0/1:50,13:3	G	A
0.108	.	.	.	.	.	.	.	.	1.30	0	0	SON	GT:/	0:9,(	0/1:11,3:33	A	T
.	.	.	.	0.0	1.0,D	1.0,D	1,D	4.7	4.00	.	.	SON	GT:/	0:17	0/1:265,68	G	C
.	.	.	.	.	.	.	.	.	.	0	.	SON	GT:/	0:11	0/1:21,4:37	A	G
.	.	.	.	.	.	.	.	3.1	.	.	.	SON	GT:/	0:26	0/1:33,7:18	T	G
.	.	.	.	.	.	.	.	.	.	.	.	SON	GT:/	0:10	0/1:34,5:37	T	C
.	.	.	.	.	.	.	.	.	.	0	0	SON	GT:/	0:10	0/1:18,3:37	G	C
.	.	.	.	0.5	0.003	0.001	1,D	5.8	.	.	.	SON	GT:/	0:17	0/1:322,15	G	A
.	.	.	.	.	.	.	.	.	.	.	.	SON	GT:/	0:10	0/1:21,3:37	C	T
.	.	.	.	.	.	.	.	.	.	.	.	SON	GT:/	0:14	0/1:26,13:3	G	A
.	.	.	.	.	.	.	.	.	.	.	.	SON	GT:/	0:14	0/1:22,8:12	A	C
.	.	.	.	.	.	.	.	.	.	.	.	SON	GT:/	0:14	0/1:38,7:37	G	A
.	.	.	.	.	.	.	.	2.5	.	.	.	SON	GT:/	0:11	0/1:20,5:37	T	A
.	.	.	.	.	.	.	.	.	.	.	.	SON	GT:/	0:11	0/1:13,6:37	C	T
.	.	.	.	.	.	.	.	.	.	.	.	SON	GT:/	0:29	0/1:48,8:37	G	C
.	.	.	.	.	.	.	.	.	.	0	0	SON	GT:/	0:22	0/1:49,15:3	C	T
.	.	.	.	.	.	.	.	.	.	.	.	SON	GT:/	0:26	0/1:43,14:3	C	T
.	.	.	.	.	.	.	1,A	.	7.72	.	.	SON	GT:/	0:37	0/1:58,14:3	C	T
.	.	.	.	0.0	0.992	1.0,D	1,D	5.8	5.12	.	.	SON	GT:/	0:69	0/1:110,28	C	T
.	.	.	.	.	.	.	.	2.1	1.99	.	.	SON	GT:/	0:35	0/1:36,12:3	C	A
.	.	.	.	.	.	.	.	.	2.08	.	.	SON	GT:/	0:12	0/1:21,6:30	C	A
.	.	.	.	.	.	.	.	.	.	.	.	SON	GT:/	0:40	0/1:70,15:3	A	C
.	.	.	.	.	.	.	.	.	.	.	.	SON	GT:/	0:24	0/1:45,12:3	C	T
.	.	.	.	.	.	.	.	.	.	.	.	SON	GT:/	0:10	0/1:23,3:37	G	C
.	.	.	.	.	.	.	.	.	.	.	.	SON	GT:/	0:31	0/1:63,4:37	G	A
1E-03	.	.	.	.	.	.	.	.	1.83	.	0	SON	GT:/	0:12	0/1:35,3:37	G	A
.	.	.	.	.	.	.	.	.	3.63	.	.	SON	GT:/	0:83	0/1:80,26:3	C	T
.	.	.	.	.	.	.	.	3.2	.	.	.	SON	GT:/	0:13	0/1:209,7:3	G	C
.	.	##	.	0.4	0.474	0.986	1.00	5.9	2.50	.	.	SON	GT:/	0:31	0/1:64,5:37	C	G
.	.	.	.	.	.	.	.	.	.	.	.	SON	GT:/	0:12	0/1:37,5:37	C	G
.	.	.	.	0.0	0.045	0.088	0.99	5.6	2.43	.	.	SON	GT:/	0:68	0/1:114,21	C	G
.	.	##	0	.	.	.	.	5.2	1.69	.	.	SON	GT:/	0:24	0/1:411,94	G	A
.	.	.	.	0.2	0.007	0.017	1,N	.	.	.	.	SON	GT:/	0:76	0/1:130,30	C	T
.	.	.	.	0.0	0.106	0.358	1,N	.	.	.	.	SON	GT:/	0:92	0/1:170,45	C	G
.	.	.	.	.	.	.	.	.	.	.	.	SON	GT:/	0:12	0/1:14,5:37	C	T
.	.	.	.	.	.	.	.	4	.	.	.	SON	GT:/	0:49	0/1:97,12:3	G	C
.	.	.	.	.	.	.	.	.	.	.	.	SON	GT:/	0:15	0/1:31,10:3	G	C
.	.	.	.	.	.	.	.	.	.	.	.	SON	GT:/	0:34	0/1:63,9:37	G	A
.	.	.	.	.	.	.	.	.	.	.	.	SON	GT:/	0:15	0/1:33,9:35	G	A
.	.	.	.	.	.	.	.	.	.	.	.	SON	GT:/	0:12	0/1:237,42	G	A
.	.	.	.	0.0	0.794	0.988	1.00	3.8	3.27	.	.	SON	GT:/	0:97	0/1:156,49	C	T
2E-04	.	0	0	.	.	.	.	.	.	.	.	SON	GT:/	0:59	0/1:115,7:3	C	T
.	.	.	.	.	.	.	.	2.4	1.28	.	.	SON	GT:/	0:18	0/1:332,10	C	T
.	.	.	.	.	.	.	.	.	.	.	.	SON	GT:/	0:13	0/1:286,10	C	T
.	.	.	.	.	.	.	.	.	.	.	.	SON	GT:/	0:37	0/1:50,18:3	C	A

.	.	.	.	.	.	.	.	.	.	.	.	SOM	GT:/	0:24	0/1:35,8:17	T	G
.	##	.	.	.	.	.	.	.	4.09	0	.	SOM	GT:/	0:13	0/1:23,8:17	A	C
2E-04	.	##	0	0.0.	0.826	0.997	1.00	5.4	3.50	0	.	SOM	GT:/	0:14	0/1:195,46	C	T
0.003	0	0	0	0.0·	0.0,B	0.0,B	1,N	.	.	.	.	SOM	GT:/	0:42	0/1:74,15:3	G	A
.	.	.	.	.	.	.	.	.	.	.	.	SOM	GT:/	0:48	0/1:103,5:3	C	G
.	.	.	.	.	.	.	.	.	.	.	.	SOM	GT:/	0:15	0/1:27,8:37	C	T
.	.	##	0	.	.	.	.	.	1.60	.	.	SOM	GT:/	0:21	0/1:36,6:37	C	T
.	.	.	.	0.0(	0.998	1.0,D	1.00	6.2	5.48	.	.	SOM	GT:/	0:70	0/1:131,26	C	T
8E-04	.	.	.	.	.	.	.	.	.	.	.	SOM	GT:/	0:8,(	0/1:7,3:37:	G	A
.	.	.	.	.	.	.	.	.	.	.	.	SOM	GT:/	0:98	0/1:146,39	G	C
.	0	##	0	.	.	.	.	.	.	.	.	SOM	GT:/	0:57	0/1:80,23:3	G	A
.	.	.	.	.	.	.	.	.	.	.	.	SOM	GT:/	0:59	0/1:97,21:3	C	T
.	.	.	.	.	.	.	.	.	.	.	.	SOM	GT:/	0:26	0/1:32,4:37	G	T
.	.	.	.	.	.	.	.	.	.	.	.	SOM	GT:/	0:14	0/1:19,7:37	C	T
0.008	0	0	0	.	.	.	.	.	.	0	.	SOM	GT:/	0:50	0/1:70,22:3	C	T
.	.	.	.	.	.	.	.	.	.	.	.	SOM	GT:/	0:21	0/1:20,5:34	G	A
.	.	.	.	.	.	.	.	.	.	.	.	SOM	GT:/	0:69	0/1:48,5:34	C	G
.	.	.	.	.	.	.	.	.	.	.	.	SOM	GT:/	0:24	0/1:43,4:37	A	T
.	.	.	.	.	.	.	.	.	.	.	.	SOM	GT:/	0:25	0/1:37,3:37	T	C
.	.	.	.	.	.	.	.	.	.	0	0	SOM	GT:/	0:10	0/1:24,5:34	T	C
0.359	.	.	.	.	.	.	.	.	.	0.1	0.4	SOM	GT:/	0:8,(	0/1:2,3:37:	C	T
.	.	.	.	.	.	.	.	.	.	.	.	SOM	GT:/	0:34	0/1:12,3:29	C	A
.	.	.	.	.	.	.	.	.	.	0	.	SOM	GT:/	0:20	0/1:31,6:37	G	C
.	.	##	0	.	.	.	.	2.1	.	0	.	SOM	GT:/	0:11	0/1:164,6:3	T	C
.	.	.	.	.	.	.	.	.	.	0	.	SOM	GT:/	0:12	0/1:35,10:1	A	C
.	.	.	.	0.1'	.	.	0.51	2.6	.	.	.	SOM	GT:/	0:68	0/1:104,26	G	T
.	.	.	.	.	.	.	.	.	.	0	0	SOM	GT:/	0:13	0/1:32,4:37	A	G
.	##	0	0	.	.	.	.	.	.	.	.	SOM	GT:/	0:14	0/1:191,49	G	A
2E-04	.	##	0	0.0(	0.736	0.974	0.77	4.5	4.43	.	.	SOM	GT:/	0:75	0/1:164,8:3	G	A
.	.	.	.	0.4(	0.044	0.014	1,D	5.9	2.69	.	.	SOM	GT:/	0:59	0/1:113,7:3	G	C
.	.	.	.	.	.	.	.	3.9	.	.	.	SOM	GT:/	0:48	0/1:81,16:3	C	T
.	.	.	.	.	.	.	.	.	.	.	.	SOM	GT:/	0:41	0/1:68,16:3	C	T
.	.	.	.	.	.	.	.	.	.	.	.	SOM	GT:/	0:64	0/1:93,21:3	G	C
.	.	.	.	.	.	.	.	.	.	0	0	SOM	GT:/	0:9,(	0/1:19,3:37	G	T
.	.	.	.	.	.	.	.	.	.	.	.	SOM	GT:/	0:48	0/1:84,8:37	G	A
.	.	.	.	.	.	.	.	.	.	.	.	SOM	GT:/	0:16	0/1:16,3:37	C	T
.	.	.	.	.	.	.	.	.	.	.	.	SOM	GT:/	0:60	0/1:66,15:3	G	A
.	.	.	.	.	.	.	.	.	.	.	.	SOM	GT:/	0:36	0/1:41,3:37	A	G
.	.	.	.	.	.	.	.	.	.	.	.	SOM	GT:/	0:19	0/1:20,5:37	G	A
.	.	0	0	0.0!	0.781	0.982	1,D	5.8	3.58	.	.	SOM	GT:/	0:36	0/1:50,15:3	C	T
.	.	.	.	0.3:	0.42,I	0.961	1,N	4.9	3.65	.	.	SOM	GT:/	0:11	0/1:181,7:3	G	A
.	0	##	0	0.1·	0.021	0.178	0.99	3.2	.	.	.	SOM	GT:/	0:51	0/1:76,24:3	G	A
.	.	.	.	.	.	.	.	.	.	.	.	SOM	GT:/	0:29	0/1:37,11:3	C	T
.	.	.	.	.	.	.	.	2.8	.	.	.	SOM	GT:/	0:35	0/1:51,18:3	G	A
.	.	.	.	.	.	.	.	.	.	.	.	SOM	GT:/	0:39	0/1:54,4:37	T	A
.	.	.	.	.	.	.	.	.	.	0	0	SOM	GT:/	0:38	0/1:53,9:37	G	A
.	.	.	.	.	.	.	.	.	.	.	.	SOM	GT:/	0:15	0/1:26,7:37	G	A
.	.	.	.	.	.	.	.	.	.	.	.	SOM	GT:/	0:11	0/1:10,3:28	T	A
.	.	.	.	.	.	.	.	.	.	.	.	SOM	GT:/	0:31	0/1:56,16:3	G	C
.	.	.	.	.	.	.	.	.	.	.	.	SOM	GT:/	0:85	0/1:127,28	G	A
.	.	.	.	0.1:	0.721	0.972	1.00	2.2	3.43	.	.	SOM	GT:/	0:32	0/1:52,18:3	G	T
.	.	.	.	.	.	.	.	.	.	.	.	SOM	GT:/	0:25	0/1:22,7:35	G	A
.	.	.	.	0.1:	0.986	0.991	1,D	4.8	2.47	.	.	SOM	GT:/	0:56	0/1:73,25:3	G	C
.	.	.	.	.	.	.	.	.	.	.	.	SOM	GT:/	0:12	0/1:22,7:37	G	C

.	.	.	.	.	.	.	.	.	.	.	.	SOM	GT:/	0:25	0/1:50,13:3	G	A
.	.	.	.	.	.	.	.	.	.	.	.	SOM	GT:/	0:26	0/1:31,8:37	C	T
.	.	##	0	0.1	0.015	0.008	0.99	.	2.58	.	.	SOM	GT:/	0:17	0/1:30,6:37	C	T
.	.	.	.	0.8	0.004	0.025	1,N	.	.	.	.	SOM	GT:/	0:46	0/1:93,4:37	C	T
.	.	.	.	.	.	.	1,A	5.9	8.08	.	.	SOM	GT:/	0:46	0/1:61,9:37	G	C
.	.	.	.	.	.	.	.	.	.	0	0.1	SOM	GT:/	0:10	0/1:14,5:37	T	C
.	.	.	.	.	.	.	.	.	.	.	.	SOM	GT:/	0:40	0/1:72,7:37	C	G
.	.	.	.	.	.	.	.	.	.	.	.	SOM	GT:/	0:26	0/1:30,3:37	G	C
.	.	.	.	.	.	.	.	.	.	.	.	SOM	GT:/	0:9,(	0/1:12,5:37	G	A
.	.	.	.	.	.	.	.	2.4	.	.	.	SOM	GT:/	0:11	0/1:21,9:37	C	G
.	.	.	.	.	.	.	.	.	.	.	.	SOM	GT:/	0:16	0/1:23,6:35	C	G
.	.	.	.	.	.	.	.	.	.	.	.	SOM	GT:/	0:54	0/1:95,30:3	T	G
.	.	.	.	0.0	0.396	0.673	0.57	6	2.71	.	.	SOM	GT:/	0:22	0/1:48,11:3	G	C
.	.	.	.	.	.	.	.	3	1.95	.	.	SOM	GT:/	0:20	0/1:36,11:3	C	T
.	.	.	.	.	.	.	.	5.1	1.57	.	.	SOM	GT:/	0:65	0/1:110,32	C	T
.	.	.	.	.	.	.	.	3	1.29	.	.	SOM	GT:/	0:98	0/1:123,30	G	A
.	.	.	.	.	.	.	.	.	.	.	.	SOM	GT:/	0:17	0/1:230,62	G	A
.	.	##	0	.	.	.	.	.	2.08	.	.	SOM	GT:/	0:30	0/1:39,5:34	C	T
.	.	.	.	.	.	.	.	.	.	.	.	SOM	GT:/	0:24	0/1:60,4:37	C	A
.	.	.	.	.	.	.	.	.	.	.	.	SOM	GT:/	0:20	0/1:37,3:37	G	T
.	.	.	.	.	.	.	.	.	.	.	.	SOM	GT:/	0:24	0/1:40,12:3	C	T
.	.	.	.	.	.	.	.	.	.	.	.	SOM	GT:/	0:78	0/1:105,20	C	T
.	.	.	.	.	.	.	1,A	5.2	10.2	.	.	SOM	GT:/	0:32	0/1:54,19:3	C	A
.	.	0	0	.	.	.	.	.	.	0	0	DB;S	GT:/	0:45	0/1:86,10:3	T	C
.	.	.	.	.	.	.	.	.	.	.	.	SOM	GT:/	0:16	0/1:16,7:22	T	G
.	.	.	.	.	.	.	.	.	.	.	.	SOM	GT:/	0:15	0/1:16,7:25	T	G
.	.	.	.	.	.	.	.	2	.	.	.	SOM	GT:/	0:29	0/1:29,5:34	G	T
.	##	##	0	.	.	.	.	.	.	.	.	SOM	GT:/	0:72	0/1:118,34	G	A
.	.	.	.	.	.	.	.	.	2.53	.	.	SOM	GT:/	0:14	0/1:23,6:32	G	A
.	.	.	.	.	.	.	.	.	.	.	.	SOM	GT:/	0:48	0/1:77,15:3	G	C
.	.	##	0	0.0	0.542	0.996	1.00	4.8	4.62	.	.	SOM	GT:/	0:11	0/1:165,36	G	A
.	.	##	0	.	.	.	.	4.3	2.58	.	.	SOM	GT:/	0:68	0/1:123,20	G	A
.	.	.	.	.	.	.	.	.	.	0	.	SOM	GT:/	0:8,(	0/1:16,3:37	C	T
.	.	.	.	.	.	.	.	.	.	0	.	SOM	GT:/	0:8,(	0/1:15,4:37	A	G
.	.	.	.	.	.	.	.	.	.	.	.	SOM	GT:/	0:12	0/1:20,3:37	C	G
.	.	.	.	.	.	.	.	.	1.39	.	.	SOM	GT:/	0:11	0/1:19,5:37	G	A
.	.	.	.	.	.	.	.	.	.	.	.	SOM	GT:/	0:24	0/1:18,9:35	G	A
.	.	.	.	.	.	.	.	.	.	0.1	0.1	SOM	GT:/	0:21	0/1:36,3:37	A	G
.	.	.	.	.	.	.	.	.	.	.	.	SOM	GT:/	0:93	0/1:128,45	G	A
.	.	.	.	.	.	.	.	.	.	.	.	SOM	GT:/	0:26	0/1:47,4:37	C	G
.	.	.	.	0.0	0.991	1.0,D	1,D	5	4.63	.	.	SOM	GT:/	0:28	0/1:54,12:3	G	A
.	.	.	.	.	.	.	1,A	4.6	3.58	.	.	SOM	GT:/	0:71	0/1:118,32	C	T
.	.	.	.	0.0	0.154	0.463	.	2.3	4.08	.	.	SOM	GT:/	0:22	0/1:42,3:37	C	T
.	.	.	.	0.0	0.684	0.933	1,D	4.2	3.80	.	.	SOM	GT:/	0:77	0/1:122,13	A	G
.	.	.	.	.	.	.	.	2.2	.	.	.	SOM	GT:/	0:65	0/1:99,21:3	G	T
.	.	.	.	.	.	.	.	.	.	.	.	SOM	GT:/	0:11	0/1:20,14:3	C	A
.	.	##	0	0.6	0.996	1.0,D	0.83	4	3.94	.	.	SOM	GT:/	0:77	0/1:125,26	C	T
.	.	.	.	.	.	.	.	.	2.32	.	.	SOM	GT:/	0:43	0/1:96,17:1	T	G
.	.	.	.	.	.	.	.	.	.	.	.	SOM	GT:/	0:29	0/1:39,8:37	C	T
.	0	##	0	0.9	0.001	0.016	0.98	3.8	.	.	.	SOM	GT:/	0:53	0/1:87,21:3	C	T
.	.	.	.	.	.	.	.	.	1.36	.	.	SOM	GT:/	0:22	0/1:25,12:3	C	G
.	.	.	.	0.0	0.962	0.999	1,D	3.1	2.73	.	.	SOM	GT:/	0:14	0/1:238,53	C	G
.	.	.	.	.	.	.	.	3	1.80	.	.	SOM	GT:/	0:94	0/1:135,45	G	T
.	.	.	.	0.0	0.425	0.697	1,D	4	4.83	.	.	SOM	GT:/	0:33	0/1:46,13:3	G	A

.	.	.	.	.	.	.	.	2.3	.	.	.	SON	GT:/	0:24	0/1:40,12:3	G	A
.	.	.	.	.	.	.	.	.	.	.	.	SON	GT:/	0:51	0/1:84,21:3	C	T
.	.	##	0	.	.	.	.	.	.	.	.	SON	GT:/	0:93	0/1:196,11	C	T
.	.	.	.	0.6	0.01,I	0.017	0.92	5.6	1.76	.	.	SON	GT:/	0:28	0/1:55,5:37	C	G
.	.	.	.	.	.	.	.	3.4	3.20	.	.	SON	GT:/	0:11	0/1:19,5:37	G	A
.	.	.	.	.	.	.	.	.	.	0	0	SON	GT:/	0:82	0/1:125,7:3	T	C
.	.	.	.	.	.	.	.	.	.	0	0	SON	GT:/	0:89	0/1:129,7:3	C	T
.	.	.	.	.	.	.	.	.	.	0	0	SON	GT:/	0:15	0/1:38,3:33	C	T
.	.	.	.	0.3	0.989	1.0,D	1,D	6.2	5.79	.	.	SON	GT:/	0:35	0/1:48,18:3	C	T
.	.	.	.	.	.	.	.	.	2.83	.	.	SON	GT:/	0:79	0/1:117,32	C	G
.	.	.	.	.	.	.	.	.	.	.	.	SON	GT:/	0:47	0/1:66,16:3	C	T
.	.	.	.	.	.	.	.	2.7	1.91	.	.	SON	GT:/	0:65	0/1:86,17:3	C	T
0.104	.	##	0	0.0	0.99,I	1.0,D	1,D	4	3.20	0	.	SON	GT:/	0:9,(	0/1:17,3:33	G	A
.	.	.	.	0.0	0.135	0.347	1,N	.	2.37	.	.	SON	GT:/	0:37	0/1:56,15:3	G	A
.	.	.	.	.	.	.	.	.	.	.	.	SON	GT:/	0:17	0/1:23,16:3	G	C
.	.	.	.	0.0	0.59,I	0.96,I	1,N	3.9	.	.	.	SON	GT:/	0:38	0/1:94,5:37	C	G
.	.	.	.	0.3	0.003	0.002	1,N	4.3	.	.	.	SON	GT:/	0:15	0/1:227,60	C	T
.	.	.	.	.	.	.	.	3.2	.	.	.	SON	GT:/	0:15	0/1:47,13:3	C	G
.	.	.	.	.	.	.	.	.	.	.	.	SON	GT:/	0:8,(	0/1:19,4:37	G	A
.	.	.	.	.	.	.	.	.	.	.	.	SON	GT:/	0:12	0/1:20,8:37	C	T
.	.	.	.	.	.	.	.	.	2.04	.	.	SON	GT:/	0:18	0/1:29,12:3	C	T
.	.	.	.	.	.	.	.	.	.	.	.	SON	GT:/	0:24	0/1:42,4:27	T	G
.	.	.	.	0.0	.	.	1,N	.	.	.	.	SON	GT:/	0:36	0/1:81,6:37	C	A
.	.	.	.	0.0	0.999	1.0,D	1,D	6	4.89	.	.	SON	GT:/	0:45	0/1:97,29:3	G	C
.	.	.	.	.	.	.	.	.	.	.	.	SON	GT:/	0:10	0/1:6,2:37:	C	T
.	.	.	.	.	.	.	.	.	.	.	.	SON	GT:/	0:43	0/1:76,21:3	C	T
.	.	.	.	.	.	.	.	.	.	.	.	SON	GT:/	0:21	0/1:15,7:37	G	A
.	.	##	0	.	.	.	.	.	.	.	.	SON	GT:/	0:47	0/1:91,6:30	G	A
.	.	.	.	.	.	.	.	.	.	.	.	SON	GT:/	0:37	0/1:66,17:3	G	A
.	.	.	.	.	.	.	.	.	.	.	.	SON	GT:/	0:45	0/1:51,10:3	C	G
.	.	.	.	.	.	.	.	2.7	2.30	.	.	SON	GT:/	0:15	0/1:17,5:37	A	C
.	.	.	.	0.0	0.991	0.998	1.00	5.2	4.92	.	.	SON	GT:/	0:25	0/1:36,8:37	G	C
.	.	.	.	.	.	.	1.00	3.6	1.82	.	.	SON	GT:/	0:65	0/1:103,28	G	C
.	.	.	.	.	.	.	.	.	.	.	.	SON	GT:/	0:32	0/1:31,3:37	C	A
.	.	0	0	.	.	.	.	5.1	1.83	.	.	SON	GT:/	0:99	0/1:145,51	G	T
.	.	.	.	.	.	.	.	.	.	0	0.3	SON	GT:/	0:8,(	0/1:7,4:37:	C	T
.	.	.	.	.	.	.	.	.	.	.	.	SON	GT:/	0:16	0/1:19,4:24	A	C
.	.	.	.	.	.	.	.	2.2	.	.	.	SON	GT:/	0:53	0/1:87,16:3	G	A
.	.	.	.	.	.	.	.	.	.	.	.	SON	GT:/	0:16	0/1:20,5:37	G	A
.	.	.	.	.	.	.	.	.	.	.	.	SON	GT:/	0:16	0/1:28,5:37	C	T
.	.	.	.	.	.	.	.	.	.	.	.	SON	GT:/	0:9,(	0/1:19,5:37	G	C
2E-04	0	0	0	.	.	.	.	.	.	.	.	SON	GT:/	0:14	0/1:47,11:3	C	T
.	.	.	.	.	.	.	.	.	.	.	.	SON	GT:/	0:23	0/1:45,3:37	C	A
.	.	.	.	.	.	.	.	.	.	.	.	SON	GT:/	0:22	0/1:47,9:37	C	A
.	.	.	.	.	.	.	.	.	.	.	.	SON	GT:/	0:41	0/1:75,18:3	C	A
.	.	.	.	0.0	0.579	0.994	0.66	4.2	3.13	.	.	SON	GT:/	0:53	0/1:96,4:37	G	A
.	.	.	.	.	.	.	.	.	.	.	.	SON	GT:/	0:14	0/1:233,12	C	T
.	.	.	.	.	.	.	.	.	.	.	.	SON	GT:/	0:39	0/1:44,15:3	G	A
.	.	.	.	.	.	.	.	.	.	.	.	SON	GT:/	0:37	0/1:59,9:37	G	C
.	.	.	.	.	.	.	.	.	.	0	.	SON	GT:/	0:67	0/1:93,19:3	G	A
.	##	.	.	.	.	.	.	.	1.75	.	.	SON	GT:/	0:53	0/1:69,14:3	G	A
.	.	.	.	.	.	.	.	.	.	0	0	SON	GT:/	0:14	0/1:33,7:20	T	G
2E-04	.	.	.	.	.	.	.	.	.	.	.	SON	GT:/	0:58	0/1:61,27:3	G	A
.	.	.	.	.	.	.	.	.	.	0	0.1	DB;S	GT:/	0:31	0/1:61,4:37	G	A

.	.	.	.	.	.	.	.	.	.	.	.	SOM	GT:/	0:20	0/1:33,3:37	C	T
.	.	.	.	.	.	.	.	.	.	.	.	SOM	GT:/	0:9,(	0/1:32,13:3	C	A
2E-04	.	##	0	.	.	.	.	4.2	1.36	.	.	SOM	GT:/	0:13	0/1:261,42	C	T
.	.	.	.	.	.	.	.	.	.	.	.	SOM	GT:/	0:13	0/1:16,6:15	T	G
.	.	.	.	.	.	.	.	.	1.97	.	.	SOM	GT:/	0:24	0/1:44,5:21	T	G
.	.	.	.	.	.	.	.	.	.	.	.	SOM	GT:/	0:48	0/1:80,4:37	T	C
.	.	.	.	.	.	.	.	.	.	0	0	SOM	GT:/	0:60	0/1:171,8:3	C	A
.	.	.	.	.	.	.	.	.	1.94	.	.	SOM	GT:/	0:87	0/1:112,28	C	T
.	.	.	.	.	.	.	.	.	3.36	.	.	SOM	GT:/	0:41	0/1:91,33:3	C	T
.	.	.	.	0.0	0.973	1.0,D	1,D	5.8	4.85	.	.	SOM	GT:/	0:76	0/1:101,26	C	G
.	.	##	0	.	.	.	.	.	.	.	.	SOM	GT:/	0:77	0/1:114,39	G	A
.	.	.	.	.	.	.	.	.	.	.	.	SOM	GT:/	0:16	0/1:27,5:37	G	T
2E-04	.	.	.	.	.	.	.	.	.	.	.	SOM	GT:/	0:29	0/1:78,16:3	C	T
.	.	.	.	.	.	.	.	.	.	.	.	SOM	GT:/	0:33	0/1:59,14:3	C	T
0.324	.	.	.	.	.	.	.	.	.	0	0	SOM	GT:/	0:9,(	0/1:19,3:37	G	A
.	.	.	.	.	.	.	.	.	.	.	.	SOM	GT:/	0:16	0/1:34,7:37	G	A
.	.	.	.	.	.	.	.	3	1.84	.	.	SOM	GT:/	0:23	0/1:31,3:37	G	A
.	.	.	.	.	.	.	.	.	.	.	.	SOM	GT:/	0:23	0/1:33,3:37	C	T
4E-04	.	.	.	.	.	.	.	.	.	0	.	SOM	GT:/	0:56	0/1:147,25	C	T
.	.	.	.	.	.	.	.	.	.	.	.	SOM	GT:/	0:52	0/1:89,12:3	C	A
.	.	.	.	.	.	.	.	.	.	.	.	SOM	GT:/	0:22	0/1:35,6:37	G	C
.	.	.	.	.	.	.	.	.	.	.	.	SOM	GT:/	0:29	0/1:57,4:37	G	A
.	.	.	.	.	.	.	.	.	.	.	.	SOM	GT:/	0:11	0/1:20,7:37	C	T
.	.	.	.	0.0	0.996	0.999	1,D	4	4.10	.	.	SOM	GT:/	0:97	0/1:254,8:3	C	T
.	.	.	.	0.0	0.037	0.214	1,N	.	.	.	.	SOM	GT:/	0:98	0/1:165,29	C	G
.	.	.	.	.	.	.	.	.	.	0	0	DB;S	GT:/	0:22	0/1:35,4:34	T	C
.	.	.	.	.	.	.	.	.	.	.	.	SOM	GT:/	0:27	0/1:85,12:3	C	T
.	.	.	.	0.2	0.146	0.587	1,D	5.5	2.88	.	.	SOM	GT:/	0:55	0/1:115,22	C	G
.	.	.	.	.	.	.	.	.	.	.	.	SOM	GT:/	0:10	0/1:22,4:37	C	G
.	.	.	.	.	.	.	.	.	.	0	.	SOM	GT:/	0:14	0/1:38,6:37	C	T
.	.	##	0	0.8	0.005	0.102	1,N	.	.	0	.	SOM	GT:/	0:31	0/1:65,12:3	C	T
.	.	##	0	.	0.978	0.999	1,D	5.7	3.83	.	.	SOM	GT:/	0:89	0/1:144,35	G	A
.	.	.	.	.	.	.	.	.	.	.	.	SOM	GT:/	0:28	0/1:51,15:3	C	T
.	.	.	.	.	.	.	.	.	.	.	.	SOM	GT:/	0:44	0/1:58,10:3	G	A
.	.	.	.	.	.	.	1,A	5.1	5.03	.	.	SOM	GT:/	0:67	0/1:88,6:37	G	A
.	.	.	.	0.0	0.213	0.759	0.67	4.4	4.04	.	.	SOM	GT:/	0:57	0/1:120,7:3	C	T
.	.	.	.	.	.	.	.	.	.	.	.	SOM	GT:/	0:21	0/1:24,13:3	G	A
0.021	.	.	.	.	.	.	.	.	.	0	.	SOM	GT:/	0:11	0/1:14,6:35	C	T
0.04	.	##	0	.	.	.	.	.	.	0	0	SOM	GT:/	0:10	0/1:6,2:37:	T	C
.	.	##	0	0.1	0.968	0.997	1.00	5.9	4.60	.	.	SOM	GT:/	0:46	0/1:83,22:3	C	T
.	.	.	.	.	.	.	.	.	.	.	.	SOM	GT:/	0:45	0/1:65,22:3	G	A
.	.	.	.	.	.	.	.	.	.	.	.	SOM	GT:/	0:8,(	0/1:23,6:37	C	T
.	.	.	.	.	.	.	.	.	.	.	.	SOM	GT:/	0:62	0/1:104,26	A	G
.	.	.	.	0.1	0.974	0.999	1,D	5.8	5.29	.	.	SOM	GT:/	0:72	0/1:119,25	C	G
.	.	.	.	.	.	.	.	.	.	.	.	SOM	GT:/	0:10	0/1:26,6:37	G	A
.	.	.	.	.	.	.	.	.	.	.	.	SOM	GT:/	0:65	0/1:101,21	G	A
.	.	.	.	.	.	.	.	.	.	.	.	SOM	GT:/	0:89	0/1:114,76	T	A
.	.	.	.	.	.	.	.	2.1	.	.	.	SOM	GT:/	0:12	0/1:14,6:37	C	T
.	.	.	.	.	.	.	.	.	.	0	.	DB;S	GT:/	0:32	0/1:60,6:37	A	C
.	.	##	0	.	.	.	.	.	.	.	.	SOM	GT:/	0:11	0/1:28,4:34	C	G
.	.	.	.	0.0	0.928	0.993	1,D	5.9	5.60	.	.	SOM	GT:/	0:67	0/1:96,27:3	C	T
.	.	.	.	0.0	0.996	0.999	1,D	5	4.69	.	.	SOM	GT:/	0:88	0/1:156,47	C	G
.	.	.	.	.	.	.	.	2.8	2.61	.	.	SOM	GT:/	0:10	0/1:17,9:37	T	A
.	.	.	.	.	.	.	.	.	.	0.1	.	SOM	GT:/	0:22	0/1:31,6:37	A	G

.	.	.	.	.	.	.	.	.	.	.	.	SON	GT:/	0:27	0/1:47,7:37	G	A
.	.	.	.	.	.	.	.	.	.	.	.	SON	GT:/	0:13	0/1:20,3:37	C	T
.	.	##	0	.	.	.	.	.	.	.	.	SON	GT:/	0:12	0/1:204,59	C	T
.	.	.	.	.	.	.	.	.	.	.	.	SON	GT:/	0:45	0/1:99,6:37	C	T
.	.	.	.	0.0(	0.098	0.332	0.91	.	2.91	.	.	SON	GT:/	0:42	0/1:58,14:3	C	G
.	.	.	.	.	.	.	.	4.3	.	.	.	SON	GT:/	0:30	0/1:56,4:37	C	T
.	.	.	.	0.0(	0.992	0.996	1.00	3.9	4.25	.	.	SON	GT:/	0:52	0/1:88,17:3	A	G
.	.	.	.	0.7	0.106	0.652	1,N	.	.	.	.	SON	GT:/	0:23	0/1:41,13:3	G	C
2E-04	.	.	.	.	.	.	.	.	.	.	.	SON	GT:/	0:23	0/1:42,17:3	G	A
.	.	.	.	.	.	.	.	.	.	.	.	SON	GT:/	0:53	0/1:60,23:3	C	G
.	.	.	.	.	.	.	.	.	.	.	.	SON	GT:/	0:70	0/1:104,27	G	C
.	.	.	.	.	.	.	.	.	.	.	.	SON	GT:/	0:34	0/1:62,11:3	C	T
.	.	.	.	.	.	.	.	.	.	.	.	SON	GT:/	0:13	0/1:228,26	G	A
.	.	.	.	.	.	.	.	.	.	0	.	SON	GT:/	0:35	0/1:59,4:37	G	A
.	.	.	.	.	.	.	.	.	.	.	.	SON	GT:/	0:26	0/1:33,8:35	C	T
.	.	.	.	.	.	.	.	.	2.06	.	.	SON	GT:/	0:70	0/1:142,26	G	C
.	.	.	.	.	.	.	.	4.3	2.33	.	.	SON	GT:/	0:10	0/1:19,5:37	G	A
.	.	.	.	0.0	0.634	0.991	1,D	4.8	2.26	.	.	SON	GT:/	0:24	0/1:34,6:37	C	G
.	.	.	.	.	.	.	.	4.7	.	.	.	SON	GT:/	0:17	0/1:28,5:34	C	A
.	.	.	.	.	.	.	.	.	.	.	.	SON	GT:/	0:13	0/1:33,4:37	G	C
.	.	.	.	.	.	.	.	.	.	.	.	SON	GT:/	0:16	0/1:25,4:37	G	T
.	.	.	.	.	.	.	.	.	.	.	.	SON	GT:/	0:49	0/1:59,7:37	C	T
.	.	.	.	.	.	.	.	.	1.26	.	.	SON	GT:/	0:10	0/1:142,43	G	A
.	.	.	.	.	.	.	.	4.3	3.84	.	.	SON	GT:/	0:27	0/1:40,8:37	G	C
.	.	.	.	.	.	.	.	3.4	2.30	.	.	SON	GT:/	0:50	0/1:72,21:3	G	A
.	.	.	.	.	.	.	.	.	.	0	.	SON	GT:/	0:15	0/1:35,4:30	C	A
.	.	.	.	0.0(	0.219	0.412	1,D	5.4	2.54	.	.	SON	GT:/	0:73	0/1:136,8:3	G	C
.	.	.	.	.	.	.	.	2	.	.	.	SON	GT:/	0:33	0/1:76,14:3	C	T
.	.	.	.	.	.	.	.	2.3	.	.	.	SON	GT:/	0:24	0/1:37,10:3	G	A
.	.	.	.	.	.	.	.	.	.	.	.	SON	GT:/	0:36	0/1:57,14:3	C	T
.	.	.	.	.	.	.	.	.	1.24	.	.	SON	GT:/	0:45	0/1:87,26:3	C	T
.	.	.	.	.	.	.	.	.	.	.	.	SON	GT:/	0:33	0/1:46,17:3	C	T
.	.	.	.	.	.	.	.	4.4	1.99	.	.	SON	GT:/	0:17	0/1:338,10	C	G
.	.	.	.	0.0!	0.003	0.012	0.99	6	2.11	.	.	SON	GT:/	0:24	0/1:34,5:37	G	C
.	.	.	.	0.0!	0.013	0.029	0.98	3.9	1.98	.	.	SON	GT:/	0:24	0/1:35,5:34	G	T
.	.	.	.	.	.	.	.	.	.	.	.	SON	GT:/	0:36	0/1:37,9:17	T	G
.	.	.	.	.	.	.	.	.	.	.	.	SON	GT:/	0:11	0/1:178,27	G	C
1E-03	0	0	0	.	.	.	.	2.4	.	.	.	SON	GT:/	0:49	0/1:99,27:3	C	T
.	.	.	.	.	.	.	.	5.3	1.40	.	.	SON	GT:/	0:11	0/1:151,47	C	T
.	.	.	.	.	.	.	.	.	.	0.1	0	SON	GT:/	0:8,(	0/1:15,3:37	A	G
.	.	.	.	.	.	.	.	.	.	.	.	SON	GT:/	0:19	0/1:30,6:37	G	C
.	.	.	.	.	.	.	.	.	.	.	.	SON	GT:/	0:11	0/1:212,8:3	G	A
.	.	.	.	0.0	0.096	0.053	0.86	3.3	2.65	.	.	SON	GT:/	0:42	0/1:73,17:3	C	G
.	.	.	.	.	.	.	.	2.3	.	.	.	SON	GT:/	0:14	0/1:32,4:37	G	C
.	.	.	.	0.0!	.	.	1,D	5.1	4.06	.	.	SON	GT:/	0:30	0/1:62,12:3	C	G
.	.	.	.	1.0	0.002	0.001	1,D	.	.	.	.	SON	GT:/	0:37	0/1:61,12:3	G	A
.	.	.	.	.	.	.	.	.	.	0	0	SON	GT:/	0:20	0/1:39,3:37	C	T
.	.	.	.	.	.	.	.	.	.	.	.	SON	GT:/	0:11	0/1:14,3:29	G	T
.	.	.	.	0.0(	.	.	1,D	3.8	3.39	.	.	SON	GT:/	0:85	0/1:150,27	C	T
.	.	.	.	.	.	.	.	2.3	.	.	.	SON	GT:/	0:11	0/1:12,5:37	G	A
.	.	.	.	.	.	.	.	.	.	.	.	SON	GT:/	0:13	0/1:38,3:37	C	T
.	.	.	.	.	.	.	.	.	.	.	.	SON	GT:/	0:34	0/1:50,7:37	C	T
.	.	.	.	.	.	.	.	3.6	.	.	.	SON	GT:/	0:38	0/1:67,19:3	G	A
.	.	.	.	.	.	.	.	.	.	.	.	SON	GT:/	0:29	0/1:44,11:3	A	G

.	.	.	.	.	.	.	.	.	.	.	.	SOM	GT:/	0:18	0/1:28,5:37	C	T
.	.	.	.	.	.	.	.	.	.	.	.	SOM	GT:/	0:59	0/1:83,22:3	G	A
.	.	.	.	.	.	.	.	.	.	.	.	SOM	GT:/	0:12	0/1:16,11:3	G	C
.	.	.	.	.	.	.	.	.	.	.	.	SOM	GT:/	0:38	0/1:71,19:3	G	T
6E-04	0	0	0	.	.	.	.	.	.	.	.	SOM	GT:/	0:50	0/1:70,17:3	G	A
0.023	.	.	.	.	.	.	.	.	.	0	0.1	SOM	GT:/	0:9,(	0/1:10,3:37	A	T
.	.	.	.	.	.	.	1,A	.	5.18	.	.	SOM	GT:/	0:58	0/1:109,5:3	G	T
.	.	.	.	.	.	.	.	.	.	.	.	SOM	GT:/	0:36	0/1:66,5:37	G	A
.	.	.	.	.	.	.	.	.	1.54	.	.	SOM	GT:/	0:13	0/1:22,8:37	C	T
.	.	.	.	.	.	.	.	.	.	.	.	SOM	GT:/	0:13	0/1:25,8:37	G	C
.	.	.	.	.	.	.	.	.	.	.	.	SOM	GT:/	0:8,(	0/1:10,3:28	G	A
.	.	.	.	.	.	.	.	.	.	.	.	SOM	GT:/	0:69	0/1:104,27	T	A
.	.	.	.	.	.	.	.	.	.	.	.	SOM	GT:/	0:9,(	0/1:5,2:37:	A	G
.	.	.	.	.	.	.	.	.	.	.	.	SOM	GT:/	0:13	0/1:20,3:37	G	C
.	.	.	.	.	.	.	.	.	.	.	.	SOM	GT:/	0:39	0/1:65,22:3	C	G
.	.	.	.	.	.	.	1,A	2.1	49.9	.	.	SOM	GT:/	0:95	0/1:157,29	G	T
.	.	.	.	0.0(	0.206	0.376	1,N	.	.	.	.	SOM	GT:/	0:89	0/1:154,26	G	A
.	.	.	.	.	.	.	.	.	.	.	.	SOM	GT:/	0:62	0/1:86,35:3	G	A
.	.	##	0	0.0.	0.609	0.7,P	1,N	.	1.70	.	.	SOM	GT:/	0:10	0/1:167,42	G	A
.	.	.	.	.	.	.	.	.	.	.	.	SOM	GT:/	0:57	0/1:81,17:3	C	T
.	.	.	.	0.0(	0.999	1.0,D	0.82	.	2.14	.	.	SOM	GT:/	0:89	0/1:163,39	G	C
.	.	.	.	.	.	.	.	.	2.25	.	.	SOM	GT:/	0:90	0/1:147,42	G	A
.	.	.	.	.	.	.	.	.	1.68	.	.	SOM	GT:/	0:71	0/1:82,29:3	G	T
.	.	.	.	0.1(	0.682	0.958	1,N	.	.	.	.	SOM	GT:/	0:31	0/1:70,15:3	C	G
.	.	0	0	0.1!	0.006	0.013	1,N	.	.	.	.	SOM	GT:/	0:44	0/1:97,15:3	G	A
.	.	.	.	.	.	.	.	.	.	.	.	SOM	GT:/	0:9,(	0/1:9,3:37:	G	A
.	.	.	.	.	.	.	.	.	.	.	.	SOM	GT:/	0:97	0/1:154,44	A	T
.	.	.	.	.	0.007	0.015	1,N	.	.	.	.	SOM	GT:/	0:18	0/1:323,67	C	T
.	.	.	.	.	.	.	.	.	.	.	.	SOM	GT:/	0:10	0/1:194,51	G	A
.	.	.	.	.	.	.	.	.	.	.	.	SOM	GT:/	0:39	0/1:53,11:3	C	G
.	.	.	.	0.0'	0.975	0.998	0.99	6	4.03	.	.	SOM	GT:/	0:70	0/1:129,26	G	A
.	.	.	.	.	.	.	.	.	3.43	.	.	SOM	GT:/	0:41	0/1:71,9:37	G	C
.	.	.	.	.	.	.	.	.	.	.	.	SOM	GT:/	0:11	0/1:24,3:37	T	G
.	.	.	.	.	.	.	.	.	.	.	.	SOM	GT:/	0:11	0/1:25,3:37	T	C
.	.	##	0	.	0.708	0.999	1,D	.	.	.	.	SOM	GT:/	0:12	0/1:251,47	C	T
.	.	.	.	.	.	.	.	.	.	.	.	SOM	GT:/	0:32	0/1:47,9:37	C	T
.	.	.	.	.	.	.	.	.	3.20	.	.	SOM	GT:/	0:12	0/1:13,3:37	G	A
4E-04	.	.	.	.	.	.	.	.	.	0	0	SOM	GT:/	0:11	0/1:164,65	C	T
.	.	.	.	.	.	.	.	5.4	1.35	.	.	SOM	GT:/	0:50	0/1:82,18:3	G	C
1E-03	.	.	.	.	.	.	.	.	.	.	.	SOM	GT:/	0:68	0/1:77,29:3	C	T
.	.	.	.	.	.	.	.	.	.	0	.	SOM	GT:/	0:33	0/1:55,15:3	G	A
.	.	.	.	.	.	.	.	.	.	.	.	SOM	GT:/	0:8,(	0/1:14,3:37	G	C
0.002	0	0	0	.	.	.	.	.	.	.	0	DB;S	GT:/	0:52	0/1:78,22:3	G	A
.	.	.	.	0.5(	0.023	0.089	1,N	2.3	.	.	.	SOM	GT:/	0:11	0/1:200,52	C	G
.	.	.	.	.	.	.	.	.	.	.	.	SOM	GT:/	0:35	0/1:40,13:3	G	T
.	.	.	.	.	.	.	.	.	.	.	.	SOM	GT:/	0:13	0/1:34,5:37	C	A
.	.	.	.	.	.	.	.	.	.	.	.	SOM	GT:/	0:79	0/1:103,30	G	A
.	.	.	.	.	.	.	.	.	.	0	.	SOM	GT:/	0:8,(	0/1:19,3:37	T	G
.	.	.	.	.	.	.	.	.	.	.	.	SOM	GT:/	0:34	0/1:29,13:3	G	A
.	.	.	.	0.4!	0.353	0.824	1,N	3.2	.	.	.	SOM	GT:/	0:55	0/1:96,17:3	G	C
.	.	.	.	0.0(	0.993	1.0,D	0.99	.	1.34	.	.	SOM	GT:/	0:49	0/1:84,14:3	G	C
.	.	.	.	0.0(	0.006	0.026	1.00	.	.	.	.	SOM	GT:/	0:44	0/1:75,14:3	G	C
.	.	.	.	.	.	.	.	.	.	.	.	SOM	GT:/	0:10	0/1:24,9:37	C	T
.	.	.	.	.	.	.	.	.	.	.	.	SOM	GT:/	0:20	0/1:27,4:24	T	G

.	.	.	.	.	.	.	.	.	.	.	.	SON	GT:/	0:14	0/1:26,10:3	G	A
.	.	.	.	0.0(	0.806	0.994	1,N	.	1.83	.	.	SON	GT:/	0:80	0/1:139,28	G	A
.	.	##	0	0.0'	0.89(	0.99,I	0.99	.	3.67	.	.	SON	GT:/	0:23	0/1:58,10:3	C	G
.	.	.	.	.	.	.	.	2	.	.	.	SON	GT:/	0:32	0/1:81,5:37	C	G
.	.	.	.	.	.	.	.	5.2	3.13	.	.	SON	GT:/	0:32	0/1:73,21:3	G	A
.	.	.	.	.	.	.	.	.	.	0.1	0.2	SON	GT:/	0:21	0/1:52,9:37	C	G
6E-04	.	0	0	0.0:	0.548	0.526	0.99	.	2.37	0.1	0.3	DB;S	GT:/	0:22	0/1:59,6:32	G	A
.	.	.	.	.	.	.	.	.	.	.	.	SON	GT:/	0:30	0/1:33,13:3	C	T
.	.	.	.	.	.	.	.	.	1.29	.	.	SON	GT:/	0:91	0/1:183,43	C	G
2E-04	##	##	0	0.0(	0.0,B	0.0,B	0.99	2.7	1.92	.	.	SON	GT:/	0:63	0/1:96,24:3	C	T
.	.	.	.	.	.	.	1,A	4	4.42	.	.	SON	GT:/	0:64	0/1:97,21:3	C	T
.	.	.	.	.	.	.	1,A	5.5	8.81	.	.	SON	GT:/	0:20	0/1:56,5:37	C	T
.	.	.	.	.	.	.	.	4.1	.	.	.	SON	GT:/	0:23	0/1:536,30	C	T
.	.	.	.	.	.	.	.	.	.	.	.	SON	GT:/	0:15	0/1:15,3:28	G	T
.	.	.	.	.	.	.	.	.	.	.	.	SON	GT:/	0:42	0/1:79,16:3	G	A
.	.	.	.	.	.	.	.	.	.	0	0	SON	GT:/	0:11	0/1:17,3:37	G	A
.	.	.	.	.	.	.	.	.	.	0	0.1	SON	GT:/	0:11	0/1:17,3:37	C	T
.	.	.	.	.	.	.	.	.	.	0	0	DB;S	GT:/	0:22	0/1:22,5:37	A	C
.	.	.	.	.	.	.	.	.	.	0	0	SON	GT:/	0:23	0/1:54,7:37	T	G
.	.	.	.	.	.	.	.	.	.	0.1	0.1	SON	GT:/	0:15	0/1:30,9:37	G	A
.	.	.	.	.	.	.	.	.	1.41	.	.	DB;S	GT:/	0:54	0/1:150,6:3	C	T
.	.	.	.	.	.	.	.	.	.	0	0	SON	GT:/	0:9,(	0/1:12,3:28	C	A
.	.	.	.	.	.	.	.	.	.	.	.	SON	GT:/	0:38	0/1:40,12:3	T	C
.	.	.	.	.	.	.	.	.	.	.	.	SON	GT:/	0:32	0/1:26,9:34	G	A
.	.	.	.	.	.	.	.	.	.	.	.	SON	GT:/	0:75	0/1:111,20	G	A
.	.	.	.	0.0(	1.0,D	1.0,D	1.00	3.7	3.81	.	.	DB;S	GT:/	0:87	0/1:69,62:3	G	C
.	.	.	.	.	.	.	.	.	.	0.1	0.4	DB;S	GT:/	0:32	0/1:45,6:37	G	A
.	.	.	.	.	.	.	.	.	.	.	.	SON	GT:/	0:42	0/1:46,15:3	C	G
.	.	.	.	.	.	.	.	.	.	.	.	SON	GT:/	0:70	0/1:122,11	C	T
.	.	.	.	.	.	.	.	.	.	.	.	SON	GT:/	0:13	0/1:28,6:35	T	A
.	.	.	.	.	.	.	.	.	.	0	0	SON	GT:/	0:11	0/1:28,4:37	G	C
.	.	.	.	.	.	.	.	.	.	0	0	SON	GT:/	0:8,(	0/1:12,4:37	G	T
0.04	0	0	0	0.5:	0.011	0.026	0.99	.	.	0	0	DB;S	GT:/	0:60	0/1:105,5:3	C	T
.	.	.	.	.	.	.	.	.	.	.	.	SON	GT:/	0:30	0/1:28,7:37	G	A
0.148	.	.	.	.	.	.	.	.	.	.	0	SON	GT:/	0:8,(	0/1:7,3:37:	C	G
.	.	.	.	.	.	.	.	.	.	0.2	0.4	SON	GT:/	0:14	0/1:31,11:3	G	C
0.03	.	.	.	.	.	.	.	.	.	0	0	SON	GT:/	0:11	0/1:47,3:37	C	T
.	.	.	.	.	.	.	.	3.3	.	.	.	SON	GT:/	0:32	0/1:39,9:37	C	G
.	.	.	.	.	.	.	.	.	.	.	.	SON	GT:/	0:12	0/1:181,61	G	A
.	.	.	.	.	.	.	.	.	1.37	.	.	SON	GT:/	0:17	0/1:28,9:32	G	T
.	.	##	0	.	.	.	.	.	2.00	.	.	SON	GT:/	0:12	0/1:151,41	G	A
.	.	.	.	.	.	.	.	.	.	.	.	SON	GT:/	0:14	0/1:15,17:3	G	C
.	.	.	.	0.0:	0.04,I	0.026	1,N	.	1.58	.	.	SON	GT:/	0:70	0/1:159,7:3	G	C
.	##	##	0	0.0:	0.931	1.0,D	1.00	4.7	3.81	.	.	SON	GT:/	0:46	0/1:79,4:37	C	T
.	.	.	.	0.0(	0.005	0.016	0.82	4.5	1.39	.	.	SON	GT:/	0:11	0/1:190,52	G	C
.	##	##	0	.	.	.	.	.	.	.	.	SON	GT:/	0:88	0/1:131,45	G	A
.	.	.	.	.	.	.	.	.	.	.	.	SON	GT:/	0:30	0/1:37,13:3	C	G
.	.	.	.	.	.	.	.	.	.	.	.	SON	GT:/	0:16	0/1:274,68	C	T
.	.	.	.	.	.	.	.	.	.	.	.	SON	GT:/	0:9,(	0/1:15,8:37	G	A
.	.	##	0	.	.	.	.	.	.	.	.	SON	GT:/	0:12	0/1:159,30	G	A
.	.	.	.	.	.	.	.	.	.	.	.	SON	GT:/	0:16	0/1:26,3:37	C	T
.	.	.	.	.	.	.	.	.	.	.	.	SON	GT:/	0:9,(	0/1:3,2:37:	G	A
.	.	##	0	.	.	.	.	.	.	.	.	SON	GT:/	0:13	0/1:21,5:37	T	C
.	.	.	.	.	.	.	.	.	.	0	0	SON	GT:/	0:19	0/1:13,4:37	G	A

.	.	.	.	0.0!	0.005	0.005	1,N	3.9	.	.	.	SON	GT:/	0:47	0/1:88,27:3	C	G
.	.	.	.	.	.	.	1,D	5.6	5.60	.	.	SON	GT:/	0:67	0/1:117,8:3	C	A
.	.	.	.	.	.	.	.	.	.	.	.	SON	GT:/	0:27	0/1:54,13:3	G	T
.	.	.	.	0.0:	0.185	0.645	1,D	5.9	4.00	.	.	SON	GT:/	0:60	0/1:73,16:3	G	A
.	.	.	.	.	.	.	.	.	.	.	.	SON	GT:/	0:53	0/1:72,18:3	G	A
.	.	.	.	.	.	.	.	.	.	.	.	SON	GT:/	0:20	0/1:33,9:37	G	A
.	.	.	.	.	.	.	.	.	2.04	.	.	SON	GT:/	0:32	0/1:41,12:3	C	G
.	.	.	.	.	.	.	.	5.1	1.74	.	.	SON	GT:/	0:64	0/1:84,15:3	G	A
.	.	.	.	.	.	.	.	2.6	2.43	.	.	SON	GT:/	0:34	0/1:62,21:3	A	G
.	.	.	.	.	.	.	.	.	.	.	.	SON	GT:/	0:53	0/1:102,37	G	A
.	.	.	.	.	.	.	.	.	.	.	.	SON	GT:/	0:32	0/1:66,18:3	C	T
.	.	.	.	.	.	.	.	.	.	0	0	SON	GT:/	0:14	0/1:24,4:37	T	C
.	.	.	.	.	.	.	1,A	4.7	9.66	.	.	SON	GT:/	0:62	0/1:108,29	C	G
.	.	.	.	.	.	.	1,A	4.4	6.48	.	.	SON	GT:/	0:48	0/1:75,21:3	G	A
.	.	.	.	.	.	.	.	.	.	.	.	SON	GT:/	0:23	0/1:47,8:37	C	G
.	.	.	.	.	.	.	.	.	.	.	.	SON	GT:/	0:48	0/1:57,17:3	G	A
.	.	.	.	.	.	.	1,A	5.2	5.59	.	.	SON	GT:/	0:38	0/1:63,22:3	C	T
.	.	.	.	0.0:	0.771	0.935	1,N	.	1.24	.	.	SON	GT:/	0:22	0/1:47,11:3	G	C
.	.	.	.	0.0:	0.577	0.879	1.00	4.9	3.19	.	.	SON	GT:/	0:74	0/1:98,23:3	G	A
.	.	.	.	.	.	.	.	.	.	.	.	SON	GT:/	0:24	0/1:26,14:3	G	A
.	.	.	.	0.4	0.002	0.001	1.00	4.3	1.36	.	.	SON	GT:/	0:78	0/1:144,35	C	T
.	.	.	.	0.2(	0.002	0.008	0.79	4.1	.	.	.	SON	GT:/	0:20	0/1:59,9:37	G	A
.	.	0	0	0.6'	0.86,I	0.998	0.83	4.1	.	.	.	SON	GT:/	0:63	0/1:98,25:3	G	A
.	.	.	.	.	.	.	.	.	.	0	.	SON	GT:/	0:15	0/1:23,8:14	A	C
.	.	.	.	.	.	.	.	.	.	0	0	SON	GT:/	0/1:1	0:11,0:.:11	T	C
.	.	.	.	.	.	.	.	.	.	0	0	SON	GT:/	0/1:1	0:13,0:.:13	G	A
.	.	.	.	.	.	.	.	.	.	0	0	SON	GT:/	0/1:5	0:17,0:.:17	C	T
.	.	.	.	.	.	.	.	.	.	.	.	SON	GT:/	0/1:3	0:19,0:.:19	G	T
.	.	.	.	.	.	.	.	.	.	.	.	SON	GT:/	0/1:4	0:24,0:.:24	G	A
.	.	.	.	.	.	.	.	.	.	0	.	SON	GT:/	0/1:3	0:20,0:.:20	A	C
.	.	.	.	.	.	.	.	.	.	0.1	0.4	DB;S	GT:/	0/1:4	0:31,0:.:31	C	T
0.001	.	.	.	.	.	.	.	.	.	0	.	SON	GT:/	0/1:7	0:40,1:.:41	T	C
.	.	.	.	.	.	.	.	.	.	0	0.1	SON	GT:/	0/1:1	0:15,0:.:10	C	A
.	.	.	.	.	.	.	.	.	.	0.3	0.4	DB;S	GT:/	0/1:4	0:20,0:.:20	C	G
0.005	.	.	.	.	.	.	.	.	.	0	0	SON	GT:/	0/1:1	0:10,0:.:10	G	A
.	.	0	0	0.0(	1.0,D	1.0,D	1.00	4.5	3.37	.	.	DB;S	GT:/	0/1:7	0:46,1:.:47	G	C
.	.	.	.	.	.	.	.	.	.	0	.	SON	GT:/	0/1:8	0:45,1:.:46	A	C
.	.	##	0	.	.	.	.	.	.	.	.	SON	GT:/	0/1:1	0:93,0:.:93	C	T
.	.	##	0	0.0!	0.98:	1.0,D	1.00	4.8	3.42	.	.	SON	GT:/	0/1:1	0:70,1:.:71	C	T
.	.	.	.	.	.	.	.	.	.	0	0	SON	GT:/	0/1:2	0:8,0:.:9:0.	C	T
.	.	.	.	.	.	.	.	.	.	.	.	SON	GT:/	0/1:9	0:45,1:.:46	G	C
.	.	.	.	.	.	.	.	.	.	0.3	0.5	DB;S	GT:/	0/1:1	0:19,0:.:17	T	C
.	.	.	.	.	.	.	.	.	.	0.3	0.4	SON	GT:/	0/1:1	0:17,0:.:15	A	C
.	.	.	.	.	.	.	.	.	.	0	0.1	SON	GT:/	0/1:9	0:8,0:.:9:0.	T	A
.	.	.	.	.	.	.	.	.	.	0	0	SON	GT:/	0/1:4	0:26,0:.:26	C	A
.	.	.	.	.	.	.	.	.	.	0	0	SON	GT:/	0/1:1	0:9,0:.:7:0.	C	A
.	.	.	.	.	.	.	.	.	.	.	.	SON	GT:/	0/1:9	0:53,1:.:47	T	A
.	.	.	.	.	.	.	.	.	.	0	.	SON	GT:/	0/1:4	0:28,0:.:28	A	T
.	.	.	.	.	.	.	.	.	.	0	.	SON	GT:/	0/1:6	0:45,0:.:44	C	G
.	.	.	.	.	.	.	.	.	.	0	.	SON	GT:/	0/1:2	0:10,0:.:10	G	A
.	.	.	.	.	.	.	.	.	.	0	.	SON	GT:/	0/1:1	0:8,0:.:8:0.	T	A
.	.	.	.	.	.	.	.	.	.	.	.	SON	GT:/	0/1:6	0:43,0:.:40	G	T
.	.	.	.	.	.	.	.	.	.	0	.	SON	GT:/	0/1:1	0:8,0:.:6:0.	G	C
.	.	.	.	.	.	.	.	.	.	0	0	SON	GT:/	0/1:1	0:11,0:.:11	G	A

.	.	.	.	.	.	.	.	.	.	0	0	SOM	GT:	0/1:1	0:12,0:..:12	G	A
.	.	.	.	.	.	.	.	.	.	0	.	SOM	GT:	0/1:9	0:43,0:..:43	T	G
0.351	.	.	.	.	.	.	.	.	.	0	0.2	SOM	GT:	0/1:1	0:12,0:..:7:0	T	A
.	.	.	.	.	.	.	.	.	.	0	0	SOM	GT:	0/1:2	0:10,0:..:9:0	T	C
.	.	.	.	.	.	.	.	.	.	0	0	SOM	GT:	0/1:2	0:10,0:..:9:0	A	G
.	.	.	.	.	.	.	.	.	.	.	.	SOM	GT:	0/1:3	0:23,0:..:23	A	C
.	.	.	.	.	.	.	.	.	.	0	0.1	SOM	GT:	0/1:2	0:15,0:..:16	T	C
0.052	.	.	.	.	.	.	.	.	.	0.2	0.5	SOM	GT:	0/1:1	0:9,0:..:9:0.	A	C
.	.	.	.	.	.	.	.	.	.	.	.	SOM	GT:	0/1:3	0:52,0:..:22	G	C
.	.	.	.	.	.	.	.	.	.	.	.	SOM	GT:	0/1:4	0:8,0:..:6:0.	G	A
.	.	.	.	.	.	.	.	.	.	.	.	SOM	GT:	0/1:9	0:69,1:..:68	G	T
.	.	0	0	0.0	0.107	0.431	1,N	4.8	2.17	0.1	.	SOM	GT:	0/1:2	0:9,0:..:9:0.	G	A
.	.	.	.	.	.	.	.	.	.	0	.	SOM	GT:	0/1:7	0:33,1:..:33	G	A
.	.	.	.	.	.	.	.	.	.	0	0	SOM	GT:	0/1:7	0:37,0:..:37	A	G
.	.	.	.	.	.	.	.	.	.	.	.	SOM	GT:	0/1:7	0:52,1:..:52	G	T
.	.	.	.	.	.	.	.	.	.	0	.	SOM	GT:	0/1:1	0:14,1:..:10	A	G
.	.	.	.	.	.	.	.	.	.	.	.	SOM	GT:	0/1:1	0:70,0:..:70	C	T
.	.	.	.	.	.	.	.	.	.	.	.	SOM	GT:	0/1:1	0:10,0:..:10	T	C
.	.	.	.	.	.	.	.	.	.	.	.	SOM	GT:	0/1:4	0:25,0:..:25	A	T
0.002	.	.	.	.	.	.	.	.	.	0	0	SOM	GT:	0/1:4	0:8,0:..:8:0.	C	T
.	.	.	.	.	.	.	.	3.9	.	0	.	SOM	GT:	0/1:1	0:12,0:..:12	C	G
.	.	.	.	.	.	.	.	.	1.88	0	.	SOM	GT:	0/1:2	0:20,0:..:20	T	G
.	.	.	.	.	.	.	.	2.5	.	.	.	SOM	GT:	0/1:7	0:25,0:..:25	G	A
.	.	.	.	.	.	.	.	.	.	0	.	SOM	GT:	0/1:2	0:24,0:..:24	T	G
.	.	.	.	.	.	.	.	.	.	0	.	SOM	GT:	0/1:2	0:23,0:..:23	T	G
.	.	.	.	.	.	.	.	.	.	0	0.2	SOM	GT:	0/1:1	0:14,0:..:14	G	A
.	.	.	.	.	.	.	.	.	.	0	0.1	SOM	GT:	0/1:3	0:10,0:..:11	A	G
.	.	.	.	.	.	.	.	.	.	.	.	SOM	GT:	0/1:9	0:34,0:..:33	A	G
.	.	.	.	.	.	.	.	.	.	.	.	SOM	GT:	0/1:1	0:57,1:..:59	C	T
4E-04	0	0	0	.	.	.	.	.	.	0	.	SOM	GT:	0/1:3	0:27,0:..:27	G	A
.	.	.	.	.	.	.	.	.	.	.	.	SOM	GT:	0/1:6	0:32,0:..:32	G	A
.	.	.	.	.	.	.	.	.	.	0	0	SOM	GT:	0/1:4	0:23,0:..:20	C	T
0.477	.	.	.	.	.	.	.	.	.	0.1	0.4	SOM	GT:	0/1:1	0:9,0:..:7:0.	G	T
.	.	.	.	.	.	.	.	.	.	0	.	SOM	GT:	0/1:3	0:20,0:..:20	T	C
.	.	.	.	.	.	.	.	.	.	0	.	SOM	GT:	0/1:1	0:59,1:..:60	G	A
.	.	.	.	.	.	.	.	.	.	0	.	SOM	GT:	0/1:1	0:18,0:..:18	A	C
.	.	.	.	.	.	.	.	.	.	.	.	SOM	GT:	0/1:2	0:15,0:..:15	A	G
.	.	.	.	0.1(	0.127	0.05,I	0.69	.	1.65	.	.	SOM	GT:	0/1:1	0:74,0:..:74	C	A
.	.	.	.	.	.	.	.	.	.	.	.	SOM	GT:	0/1:9	0:45,0:..:45	C	A
.	##	##	0	.	.	.	.	2.1	.	.	.	SOM	GT:	0/1:1	0:75,0:..:75	T	C
.	.	.	.	.	.	.	.	.	.	0	.	SOM	GT:	0/1:5	0:35,0:..:35	C	G
.	.	.	.	.	.	.	.	.	.	0	0	DB;S	GT:	0/1:1	0:59,1:..:59	A	G
.	.	.	.	.	.	.	.	.	.	0	.	DB;S	GT:	0/1:1	0:43,1:..:35	G	T
.	.	##	0	.	.	.	.	.	.	.	.	SOM	GT:	0/1:1	0:77,0:..:77	C	T
.	.	.	.	.	.	.	.	.	.	.	.	SOM	GT:	0/1:4	0:24,0:..:24	A	C
.	.	.	.	.	.	.	.	.	.	0	.	SOM	GT:	0/1:1	0:9,0:..:9:0.	G	A
.	.	.	.	.	.	.	.	.	.	.	.	SOM	GT:	0/1:5	0:8,0:..:6:0.	A	G
.	.	.	.	.	.	.	.	.	.	0	.	SOM	GT:	0/1:4	0:20,1:..:21	A	C
.	.	.	.	.	.	.	.	.	.	0	0	SOM	GT:	0/1:9	0:12,0:..:9:0	T	A
.	.	.	.	.	.	.	.	.	.	.	.	SOM	GT:	0/1:1	0:8,0:..:8:0.	T	G
.	.	.	.	.	.	.	.	.	.	.	.	SOM	GT:	0/1:6	0:46,0:..:45	C	G
.	.	.	.	.	.	.	.	.	.	.	.	SOM	GT:	0/1:4	0:27,0:..:27	C	G
.	.	.	.	.	.	.	.	.	.	.	.	SOM	GT:	0/1:2	0:18,0:..:5:0	C	G
.	.	.	.	.	.	.	.	.	.	.	.	SOM	GT:	0/1:2	0:10,0:..:9:0	C	A

.	.	.	.	.	.	.	.	.	.	.	.	SOM	GT:/	0/1:3	0:20,0:..:13	G	T
.	.	.	.	.	.	.	.	.	.	.	.	SOM	GT:/	0/1:3	0:23,0:..:13	C	T
.	.	.	.	.	.	.	.	.	.	.	.	SOM	GT:/	0/1:3	0:23,0:..:23	A	C
.	.	.	.	.	.	.	.	.	.	.	.	DB;S	GT:/	0/1:6	0:39,0:..:14	C	A
.	.	.	.	.	.	.	.	.	.	0	.	DB;S	GT:/	0/1:3	0:22,0:..:14	A	C
.	.	.	.	.	.	.	.	.	.	0	0.4	SOM	GT:/	0/1:6	0:8,0:..:4:0.	G	T
.	.	.	.	.	.	.	.	.	.	0	.	SOM	GT:/	0/1:3	0:9,0:..:7:0.	G	A
.	.	.	.	.	.	.	.	.	.	0	0	DB;S	GT:/	0/1:4	0:23,0:..:22	C	T
.	.	.	.	.	.	.	.	.	.	0	.	SOM	GT:/	0/1:1	0:9,0:..:4:0.	G	A
.	.	.	.	.	.	.	.	.	.	0	0.1	SOM	GT:/	0/1:2	0:14,0:..:14	C	G
.	.	.	.	.	.	.	.	4.2	3.16	.	.	SOM	GT:/	0/1:1	0:87,1:..:71	T	C
.	.	.	.	.	.	.	.	.	.	0.2	0.3	SOM	GT:/	0/1:2	0:24,0:..:24	A	C
.	.	.	.	.	.	.	.	.	.	.	.	SOM	GT:/	0/1:6	0:25,0:..:25	T	G
.	.	.	.	.	.	.	.	.	.	.	.	SOM	GT:/	0/1:9	0:34,0:..:27	T	G
.	.	.	.	.	.	.	.	.	.	.	.	SOM	GT:/	0/1:2	0:18,0:..:7:0	G	A
.	.	.	.	.	.	.	.	.	.	.	.	SOM	GT:/	0/1:4	0:20,0:..:12	A	G
.	.	.	.	.	.	.	.	.	.	.	.	SOM	GT:/	0/1:5	0:50,0:..:19	C	G
.	.	.	.	.	.	.	.	.	.	0	.	SOM	GT:/	0/1:8	0:37,1:..:23	G	C
.	.	.	.	.	.	.	.	.	.	0	.	SOM	GT:/	0/1:2	0:9,0:..:9:0.	C	T
.	.	.	.	0.0(	0.998	0.999	1.00	4.8	4.21	.	.	DB;S	GT:/	0/1:2	0:88,0:..:88	G	A
.	.	0	0	.	.	.	.	.	.	0	0	SOM	GT:/	0/1:1	0:14,1:..:15	T	G
.	.	.	.	.	.	.	.	.	.	.	.	SOM	GT:/	0/1:3	0:18,1:..:19	G	A
.	.	0	0	.	.	.	.	.	.	0	0	DB;S	GT:/	0/1:8	0:48,1:..:46	A	C
.	.	.	.	.	.	.	.	.	.	0.1	0.2	DB;S	GT:/	0/1:7	0:38,1:..:39	G	A
.	.	.	.	.	.	.	.	.	.	0	0.1	SOM	GT:/	0/1:1	0:8,0:..:8:0.	T	C
.	.	.	.	.	.	.	.	.	.	0	0	SOM	GT:/	0/1:9	0:57,0:..:57	T	C
.	.	.	.	.	.	.	.	.	1.65	0	.	SOM	GT:/	0/1:3	0:26,1:..:27	C	G
.	.	.	.	.	.	.	.	.	.	.	.	SOM	GT:/	0/1:6	0:37,0:..:37	T	G
.	.	.	.	.	.	.	.	.	.	0	0	SOM	GT:/	0/1:9	0:8,0:..:7:0.	C	T
.	.	.	.	.	.	.	.	.	.	0	0.2	SOM	GT:/	0/1:6	0:8,0:..:8:0.	T	C
.	.	.	.	.	.	.	.	.	.	0	.	SOM	GT:/	0/1:2	0:16,0:..:16	G	T
.	.	.	.	.	.	.	.	.	.	0	.	SOM	GT:/	0/1:1	0:13,0:..:13	T	C
.	.	.	.	.	.	.	.	2.5	.	.	.	SOM	GT:/	0/1:1	0:50,1:..:51	T	C
.	.	.	.	.	.	.	.	.	.	0	0.1	SOM	GT:/	0/1:1	0:12,0:..:12	A	G
1E-03	.	.	.	.	.	.	.	.	.	.	.	SOM	GT:/	0/1:7	0:11,0:..:11	G	A
6E-04	0	0	0	.	.	.	.	.	2.28	.	.	SOM	GT:/	0/1:9	0:38,0:..:38	G	A
.	.	.	.	.	.	.	.	.	.	0	0.1	SOM	GT:/	0/1:2	0:12,0:..:12	T	C
0.433	.	1	1	.	.	.	.	.	.	0.1	0.5	SOM	GT:/	0/1:7	0:41,0:..:29	C	G
.	.	.	.	.	.	.	.	.	.	0	.	SOM	GT:/	0/1:4	0:23,0:..:23	T	C
.	.	.	.	.	.	.	.	.	.	0	.	SOM	GT:/	0/1:4	0:24,0:..:24	G	A
.	.	.	.	.	.	.	.	.	.	0	.	SOM	GT:/	0/1:1	0:8,0:..:8:0.	C	T
.	.	.	.	.	.	.	.	.	.	0	0	SOM	GT:/	0/1:3	0:20,0:..:20	T	C
.	.	.	.	0.0.	0.037	0.053	1,D	5.1	3.04	.	.	DB;S	GT:/	0/1:6	0:39,0:..:39	G	T
4E-04	.	.	.	.	.	.	.	.	.	0	0	SOM	GT:/	0/1:6	0:10,0:..:10	G	A
.	.	.	.	.	.	.	.	4.2	2.69	0	.	SOM	GT:/	0/1:1	0:67,1:..:68	G	A
.	.	.	.	.	.	.	.	.	.	.	.	SOM	GT:/	0/1:6	0:36,0:..:36	T	G
.	.	.	.	.	.	.	.	.	.	0	.	SOM	GT:/	0/1:3	0:12,0:..:12	T	C
.	.	.	.	0.0(	0.26,l	0.612	1,D	4.6	3.17	.	.	SOM	GT:/	0/1:1	0:70,1:..:71	T	C
.	.	.	.	.	.	.	.	3.9	1.70	0	0	SOM	GT:/	0/1:5	0:40,1:..:41	C	T
.	.	.	.	.	.	.	.	.	.	.	.	SOM	GT:/	0/1:7	0:30,0:..:30	C	T
4E-04	##	##	0	.	.	.	.	.	.	.	.	SOM	GT:/	0/1:3	0:176,1:..:1	G	A
.	.	.	.	.	.	.	.	.	.	0	0	DB;S	GT:/	0/1:7	0:49,1:..:50	T	C
.	.	.	.	.	.	.	.	.	.	.	.	SOM	GT:/	0/1:4	0:8,0:..:4:0.	C	T
.	.	.	.	.	.	.	.	.	.	0	0	SOM	GT:/	0/1:2	0:10,0:..:9:0	C	T

.	.	.	.	.	.	.	.	.	.	0	0	SOM	GT:A	0/1:2	0:10,0:..:9:0	T	C
.	.	.	.	.	.	.	.	.	.	.	.	SOM	GT:A	0/1:1	0:19,0:..:9:0	G	T
.	.	.	.	0.5	0.559	0.99,I	1,D	3.9	2.76	.	.	SOM	GT:A	0/1:1	0:92,0:..:92	G	A
.	.	.	.	.	.	.	.	.	.	0	.	SOM	GT:A	0/1:1	0:20,0:..:22	A	T
.	.	.	.	.	.	.	.	.	.	.	.	SOM	GT:A	0/1:6	0:29,0:..:29	C	T
0.258	.	.	.	.	.	.	.	.	.	0	0.1	SOM	GT:A	0/1:6	0:9,0:..:9:0.	C	T
.	.	.	.	.	.	.	.	.	.	.	.	SOM	GT:A	0/1:5	0:22,0:..:14	C	T
.	.	.	.	.	.	.	.	.	.	0	.	SOM	GT:A	0/1:4	0:27,1:..:28	A	C
.	.	.	.	.	.	.	.	.	.	.	.	SOM	GT:A	0/1:1	0:75,1:..:72	G	C
0.192	.	.	.	.	.	.	.	.	.	0	0	DB;S	GT:A	0/1:7	0:38,0:..:38	T	C
0.485	.	.	.	.	.	.	.	.	.	0.1	0.1	DB;S	GT:A	0/1:8	0:41,0:..:41	T	C
.	.	.	.	.	.	.	.	.	.	.	.	SOM	GT:A	0/1:4	0:38,0:..:25	G	A
.	.	.	.	.	.	.	.	.	.	.	.	SOM	GT:A	0/1:7	0:11,0:..:6:0	G	A
0.324	.	.	.	.	.	.	.	.	.	0	0	SOM	GT:A	0/1:1	0:11,0:..:11	G	A
.	.	.	.	.	.	.	.	2.1	.	.	.	SOM	GT:A	0/1:6	0:39,0:..:39	A	C
.	.	.	.	.	.	.	.	.	2.12	.	.	SOM	GT:A	0/1:8	0:44,1:..:45	T	A
.	.	.	.	0.0(	.	.	0.99	.	1.82	.	.	SOM	GT:A	0/1:2	0:20,0:..:13	C	T
0.327	.	.	.	.	.	.	.	.	.	0	0.5	SOM	GT:A	0/1:1	0:12,0:..:12	T	G
.	.	.	.	.	.	.	.	.	.	.	.	SOM	GT:A	0/1:1	0:10,0:..:10	C	G
8E-04	.	.	.	.	.	.	.	.	.	.	.	DB;S	GT:A	0/1:6	0:34,1:..:35	T	C
.	.	.	.	.	.	.	.	.	.	0	0	SOM	GT:A	0/1:2	0:13,0:..:14	G	A
.	.	.	.	.	.	.	.	.	.	0	.	SOM	GT:A	0/1:1	0:8,0:..:8:0.	C	T
.	.	.	.	.	.	.	.	.	.	0	.	SOM	GT:A	0/1:1	0:11,0:..:9:0	C	T
4E-04	.	.	.	.	.	.	.	.	.	.	.	SOM	GT:A	0/1:1	0:12,0:..:7:0	T	C
.	.	0	0	.	.	.	.	.	.	0	.	SOM	GT:A	0/1:4	0:18,0:..:18	T	C
.	.	.	.	.	.	.	.	.	.	0	.	SOM	GT:A	0/1:1	0:16,0:..:16	A	T
.	.	.	.	.	.	.	.	.	.	0	.	DB;S	GT:A	0/1:1	0:54,0:..:53	C	T
.	.	.	.	.	.	.	.	.	.	0	.	DB;S	GT:A	0/1:2	0:58,1:..:58	G	A
.	.	.	.	.	.	.	.	.	.	.	.	SOM	GT:A	0/1:2	0:20,0:..:15	G	A
.	.	.	.	.	.	.	.	.	.	.	.	SOM	GT:A	0/1:4	0:18,0:..:10	A	T
.	.	.	.	.	.	.	.	.	.	.	.	SOM	GT:A	0/1:9	0:30,0:..:25	A	G
.	.	.	.	.	.	.	.	.	.	0	.	DB;S	GT:A	0/1:1	0:35,1:..:29	A	G
.	.	.	.	.	.	.	.	.	.	0	.	DB;S	GT:A	0/1:1	0:46,1:..:47	G	A
.	.	.	.	.	.	.	.	.	.	0	0.1	DB;S	GT:A	0/1:8	0:35,0:..:32	T	C
.	.	.	.	.	.	.	.	.	.	0	0.2	DB;S	GT:A	0/1:8	0:35,1:..:32	C	T
.	.	.	.	.	.	.	.	.	.	0	0	DB;S	GT:A	0/1:7	0:30,0:..:16	A	G
.	.	.	.	.	.	.	.	.	.	0	.	DB;S	GT:A	0/1:4	0:27,0:..:12	G	C
.	.	.	.	.	.	.	.	.	.	.	.	DB;S	GT:A	0/1:6	0:33,1:..:21	G	C
.	.	.	.	.	.	.	.	.	.	0	.	DB;S	GT:A	0/1:1	0:46,1:..:42	T	A
.	.	.	.	.	.	.	.	.	.	0	.	SOM	GT:A	0/1:6	0:39,0:..:23	C	A
.	.	.	.	.	.	.	.	.	.	0	.	DB;S	GT:A	0/1:1	0:50,0:..:40	G	A
.	.	.	.	.	.	.	.	.	.	.	.	SOM	GT:A	0/1:5	0:11,0:..:2:0	C	T
.	.	.	.	.	.	.	.	.	.	0	.	SOM	GT:A	0/1:1	0:9,0:..:8:0.	G	A
.	.	.	.	.	.	.	.	.	.	.	.	SOM	GT:A	0/1:9	0:48,1:..:49	G	A
.	.	.	.	.	.	.	.	.	.	.	.	SOM	GT:A	0/1:8	0:38,1:..:39	T	C
.	.	.	.	.	.	.	.	.	.	0	.	DB;S	GT:A	0/1:6	0:38,1:..:40	G	A
.	.	.	.	.	.	.	.	.	.	0	.	SOM	GT:A	0/1:8	0:34,0:..:29	G	A
.	.	.	.	.	.	.	.	.	.	.	.	SOM	GT:A	0/1:4	0:10,0:..:7:0	T	A
.	.	.	.	.	.	.	.	.	.	0	.	SOM	GT:A	0/1:2	0:13,0:..:12	T	G
.	.	.	.	.	.	.	.	.	.	.	.	SOM	GT:A	0/1:1	0:9,0:..:8:0.	G	A
.	.	.	.	.	.	.	.	.	.	0	.	SOM	GT:A	0/1:4	0:13,0:..:12	C	T
.	.	##	0	.	.	.	.	.	.	.	.	SOM	GT:A	0/1:8	0:46,0:..:46	G	T
.	.	.	.	.	.	.	.	.	.	0	0.2	SOM	GT:A	0/1:5	0:28,0:..:24	G	A
.	.	.	.	.	.	.	.	.	.	0	.	SOM	GT:A	0/1:3	0:23,0:..:23	A	T

.	.	.	.	.	.	.	.	.	.	0	.	SON	GT:/	0/1:8	0:62,0:..:50	C		G
.	.	.	.	.	.	.	.	.	.	.	.	SON	GT:/	0/1:7	0:67,1:..:53	A		T
.	.	0	0	.	.	.	.	.	.	.	.	SON	GT:/	0/1:3	0:27,0:..:28	G		A
.	.	.	.	.	.	.	.	.	.	.	.	SON	GT:/	0/1:4	0:27,0:..:27	A		C
.	.	.	.	.	.	.	.	2.1	.	.	.	DB;S	GT:/	0/1:5	0:44,1:..:45	T		A
.	.	.	.	.	.	.	.	.	.	0	0	SON	GT:/	0/1:3	0:10,0:..:10	G		T
.	.	.	.	.	.	.	.	.	.	0	.	SON	GT:/	0/1:3	0:17,0:..:17	G		A
.	.	.	.	.	.	.	.	.	.	0	.	SON	GT:/	0/1:2	0:16,0:..:16	C		A
.	.	.	.	.	.	.	.	.	.	0	.	SON	GT:/	0/1:1	0:13,1:..:14	T		A
.	.	0	0	.	.	.	.	.	.	0	0	SON	GT:/	0/1:9	0:49,1:..:50	A		G
.	.	0	0	.	.	.	.	.	1.77	0	0	DB;S	GT:/	0/1:7	0:37,1:..:38	C		T
.	.	0	0	.	.	.	.	.	.	0	0	DB;S	GT:/	0/1:7	0:27,1:..:28	G		A
.	.	0	0	.	.	.	.	.	.	0	0	SON	GT:/	0/1:4	0:38,0:..:33	G		A
.	.	0	0	.	.	.	.	.	.	.	.	SON	GT:/	0/1:8	0:58,1:..:59	C		T
.	.	.	.	.	.	.	.	.	.	0.3	0.4	DB;S	GT:/	0/1:3	0:23,0:..:23	A		G
.	.	.	.	.	.	.	.	.	.	0	0	DB;S	GT:/	0/1:3	0:43,0:..:42	T		C
.	.	.	.	.	.	.	.	.	.	0.1	0.2	SON	GT:/	0/1:1	0:11,0:..:6:0	G		A
.	.	.	.	.	.	.	.	.	.	.	.	SON	GT:/	0/1:1	0:9,0:..:5:0.	A		T
.	.	.	.	.	.	.	.	.	.	0	0	SON	GT:/	0/1:8	0:8,0:..:6:0.	A		G
.	.	.	.	.	.	.	.	.	.	.	.	SON	GT:/	0/1:5	0:39,1:..:35	T		G
.	.	.	.	.	.	.	.	.	.	0	0	DB;S	GT:/	0/1:6	0:33,0:..:33	C		T
0.24	.	.	.	.	.	.	.	.	.	0	0.1	SON	GT:/	0/1:1	0:8,0:..:8:0.	G		T
.	.	.	.	.	.	.	.	.	.	0	0	DB;S	GT:/	0/1:6	0:33,0:..:31	C		T
.	.	.	.	.	.	.	.	.	.	0	0	DB;S	GT:/	0/1:4	0:22,0:..:22	T		C
0.458	.	.	.	.	.	.	.	.	.	0.1	0.3	DB;S	GT:/	0/1:6	0:36,0:..:36	C		T
.	.	.	.	.	.	.	.	.	.	0	0	SON	GT:/	0/1:7	0:35,0:..:35	C		A
.	.	.	.	.	.	.	.	.	.	.	.	SON	GT:/	0/1:6	0:24,0:..:24	G		A
.	.	.	.	.	.	.	.	3.1	1.73	.	.	SON	GT:/	0/1:8	0:39,1:..:40	C		T
.	.	.	.	.	.	.	.	.	.	.	.	SON	GT:/	0/1:1	0:51,0:..:46	G		C
.	.	.	.	.	.	.	.	.	.	.	.	SON	GT:/	0/1:6	0:39,0:..:27	C		T
.	.	.	.	.	.	.	.	.	.	.	.	SON	GT:/	0/1:6	0:38,1:..:27	T		C
.	.	.	.	.	.	.	.	.	.	0	.	SON	GT:/	0/1:5	0:35,0:..:26	A		G
.	.	.	.	.	.	.	.	.	.	.	.	SON	GT:/	0/1:2	0:15,0:..:10	A		C
.	.	.	.	0.2	0.0,B	0.0,B	1.00	5.3	.	.	.	SON	GT:/	0/1:8	0:38,1:..:39	C		T
.	.	.	.	.	.	.	.	.	.	.	.	SON	GT:/	0/1:3	0:23,1:..:24	T		A
.	.	.	.	0.1	0.002	0.001	1,N	.	.	.	.	SON	GT:/	0/1:1	0:71,0:..:71	G		A
.	.	.	.	.	.	.	.	.	.	0	0	SON	GT:/	0/1:5	0:23,0:..:23	G		A
.	.	.	.	.	.	.	.	.	.	.	.	SON	GT:/	0/1:3	0:16,0:..:16	C		T
0.039	.	.	.	.	.	.	.	.	.	0	0.1	SON	GT:/	0:11	0/1:15,8:37	C		T
.	.	.	.	.	.	.	.	.	.	.	.	SON	GT:/	0:43	0/1:72,8:35	C		T
0.007	0	0	0	.	.	.	.	.	1.30	.	.	DB;S	GT:/	0:22	0/1:424,23	C		T
.	.	.	.	0.4	0.042	0.089	1,N	.	1.28	.	.	SON	GT:/	0:15	0/1:304,38	G		A
.	.	.	.	0.0	0.983	1.0,D	1.00	.	1.51	.	.	SON	GT:/	0:16	0/1:267,58	C		T
2E-04	.	.	.	.	.	.	.	.	.	.	.	SON	GT:/	0:46	0/1:92,14:3	C		T
.	.	##	0	.	.	.	.	.	.	.	.	SON	GT:/	0:69	0/1:112,19	C		T
.	.	.	.	.	.	.	.	.	.	.	.	SON	GT:/	0:79	0/1:127,28	G		A
.	.	.	.	0.2	0.062	0.84,I	1,N	2.7	1.91	.	.	SON	GT:/	0:29	0/1:63,6:37	C		T
.	.	##	0	.	.	.	.	.	.	.	.	SON	GT:/	0:51	0/1:93,15:3	C		T
.	.	.	.	.	.	.	.	.	.	.	.	SON	GT:/	0:95	0/1:137,24	G		A
2E-04	##	##	0	.	.	.	.	.	.	0	.	SON	GT:/	0:12	0/1:206,32	C		T
.	.	##	0	.	.	.	.	.	.	.	.	SON	GT:/	0:31	0/1:505,66	G		A
.	.	.	.	.	.	.	.	.	.	.	.	SON	GT:/	0:39	0/1:68,4:37	C		T
.	.	.	.	.	.	.	.	.	.	.	.	SON	GT:/	0:19	0/1:28,5:37	C		T
.	.	.	.	.	.	.	.	.	.	.	.	SON	GT:/	0:65	0/1:153,15	C		A

.	.	.	.	.	.	.	.	.	.	.	.	SON	GT:/	0:23	0/1:37,4:37	G	A
.	.	.	.	.	.	.	.	.	.	.	.	SON	GT:/	0:77	0/1:111,25	G	A
.	.	.	.	.	.	.	.	.	.	.	.	SON	GT:/	0:13	0/1:238,7:3	A	G
.	.	.	.	.	.	.	.	.	.	.	.	SON	GT:/	0:28	0/1:62,11:3	C	A
.	.	.	.	0.1	0.988	1.0,D	0.99	5.5	4.01	.	.	SON	GT:/	0:17	0/1:293,62	G	T
.	.	.	.	.	.	.	.	.	.	.	.	SON	GT:/	0:29	0/1:47,6:37	G	A
.	.	.	.	.	.	.	.	.	.	.	.	SON	GT:/	0:37	0/1:54,5:37	C	T
.	.	.	.	0.0	0.988	1.0,D	1.00	4.5	6.11	.	.	SON	GT:/	0:98	0/1:190,30	C	T
.	.	.	.	0.0	0.996	0.997	1,D	4.8	4.48	.	.	SON	GT:/	0:57	0/1:87,15:3	C	T
.	.	.	.	.	.	.	.	.	.	.	.	SON	GT:/	0:22	0/1:50,11:3	G	A
.	.	##	0	.	.	.	.	.	2.54	.	.	SON	GT:/	0:12	0/1:179,36	C	T
4E-04	##	0	0	.	.	.	.	.	.	.	.	SON	GT:/	0:22	0/1:340,60	C	T
.	.	.	.	.	.	.	.	.	.	.	.	SON	GT:/	0:16	0/1:27,5:37	T	A
.	.	##	0	.	.	.	.	2	1.90	.	.	SON	GT:/	0:69	0/1:85,22:3	C	T
.	.	.	.	.	.	.	.	.	.	.	.	SON	GT:/	0:29	0/1:34,11:3	C	T
.	##	##	0	0.1	0.594	0.975	1,N	.	1.35	.	.	SON	GT:/	0:64	0/1:138,23	G	A
.	.	.	.	.	.	.	.	.	.	.	.	SON	GT:/	0:42	0/1:56,8:37	C	T
.	.	.	.	.	.	.	.	.	.	.	.	SON	GT:/	0:22	0/1:37,11:3	C	T
.	.	.	.	.	.	.	.	.	.	.	.	SON	GT:/	0:17	0/1:35,6:37	A	G
0.007	0	0	0	.	.	.	.	.	.	.	.	DB;S	GT:/	0:11	0/1:203,38	C	T
.	.	.	.	.	.	.	.	3	1.88	.	.	SON	GT:/	0:33	0/1:58,15:3	C	T
.	.	.	.	.	.	.	.	.	3.36	.	.	SON	GT:/	0:11	0/1:6,5:37:	G	T
.	.	.	.	.	0.899	0.994	1.00	6.1	4.05	.	.	SON	GT:/	0:35	0/1:66,8:3	C	T
.	.	.	.	.	.	.	.	.	.	0	0	SON	GT:/	0:65	0/1:90,7:37	G	C
0.259	.	0	0	.	.	.	.	.	.	0	.	SON	GT:/	0:9,(	0/1:12,4:37	C	T
.	.	##	0	0.3	0.338	0.975	1,N	.	2.01	.	.	DB;S	GT:/	0:77	0/1:148,23	G	A
.	##	##	0	.	.	.	.	.	.	.	.	SON	GT:/	0:10	0/1:163,30	C	T
.	.	.	.	0.0	0.763	0.998	0.96	2.2	4.05	.	.	SON	GT:/	0:45	0/1:111,20	G	A
.	0	0	0	.	.	.	.	.	.	0	0	SON	GT:/	0:22	0/1:333,26	G	A
.	.	.	.	.	.	.	.	.	.	0	0.1	SON	GT:/	0:22	0/1:39,3:37	C	A
.	.	.	.	.	.	.	.	.	.	0.1	0.2	DB;S	GT:/	0:23	0/1:30,3:37	T	C
.	.	.	.	.	.	.	.	.	.	0.2	0.3	DB;S	GT:/	0:56	0/1:81,11:3	A	T
.	.	.	.	.	.	.	.	.	.	.	.	DB;S	GT:/	0:24	0/1:35,3:37	G	A
0.005	.	.	.	.	.	.	.	.	.	0	0	DB;S	GT:/	0:44	0/1:71,15:3	G	A
.	.	##	.	0.0	0.023	0.065	1.00	4	3.56	.	0	SON	GT:/	0:49	0/1:92,19:3	C	T
.	##	##	0	0.0	0.535	0.98,I	0.99	4.7	2.17	.	.	SON	GT:/	0:11	0/1:183,42	C	T
0.005	0	0	0	.	.	.	.	.	.	.	.	DB;S	GT:/	0:47	0/1:80,16:3	G	A
.	.	.	.	.	.	.	.	.	1.26	.	.	SON	GT:/	0:16	0/1:284,59	G	A
.	.	.	.	.	.	.	.	.	.	.	.	SON	GT:/	0:46	0/1:74,8:37	A	G
.	##	##	0	.	.	.	.	4.6	1.47	.	.	SON	GT:/	0:36	0/1:64,12:3	C	T
.	.	.	.	.	.	.	.	2.2	.	.	.	SON	GT:/	0:82	0/1:134,19	G	A
.	.	.	.	.	.	.	.	.	.	.	.	SON	GT:/	0:26	0/1:58,11:3	G	A
.	.	.	.	.	.	.	.	.	.	.	.	SON	GT:/	0:68	0/1:105,17	C	T
.	.	.	.	.	.	.	.	.	.	.	.	SON	GT:/	0:78	0/1:106,15	A	G
.	.	.	.	.	.	.	.	.	.	0	.	SON	GT:/	0:32	0/1:54,4:37	C	T
.	.	.	.	0.5	0.044	0.055	1,N	.	.	.	.	SON	GT:/	0:23	0/1:48,15:3	C	A
.	.	.	.	.	.	.	.	3	.	.	.	SON	GT:/	0:10	0/1:28,6:37	G	A
.	.	.	.	.	.	.	.	.	1.26	.	.	SON	GT:/	0:49	0/1:64,21:3	G	T
0.002	.	.	.	.	.	.	.	.	.	.	.	SON	GT:/	0:8,(	0/1:5,3:37:	C	T
.	.	##	0	.	.	.	.	.	.	.	.	SON	GT:/	0:40	0/1:62,15:3	G	A
.	.	.	.	.	.	.	.	.	1.51	.	.	SON	GT:/	0:17	0/1:43,8:37	G	T
.	.	.	.	.	.	.	.	.	1.49	.	.	SON	GT:/	0:37	0/1:83,15:3	G	A
.	.	.	.	.	.	.	.	2.5	.	.	.	SON	GT:/	0:13	0/1:38,4:37	G	A
.	.	.	.	.	.	.	.	2.4	.	.	.	SON	GT:/	0:16	0/1:234,63	C	T

.	.	##	0	0.0	0.001	0.006	1,N	3.5	1.43	.	.	SOM	GT:/	0:14	0/1:220,38	C	T
.	.	##	0	0.0	1.0,D	1.0,D	1.00	5.5	4.84	.	.	SOM	GT:/	0:75	0/1:127,32	C	T
.	.	.	.	0.5	0.206	0.937	1,D	5.1	4.66	.	0	SOM	GT:/	0:74	0/1:92,22:3	G	A
.	.	.	.	.	.	.	1,D	5	10.3	.	.	SOM	GT:/	0:12	0/1:221,42	C	T
.	.	.	.	0.0	0.001	0.008	1.00	2.2	1.43	.	.	SOM	GT:/	0:92	0/1:127,33	C	T
.	.	.	.	.	.	.	.	.	.	.	.	SOM	GT:/	0:10	0/1:6,2:37	G	A
.	.	.	.	.	.	.	.	.	3.14	.	.	SOM	GT:/	0:67	0/1:129,12	C	T
.	.	.	.	.	.	.	.	.	2.64	.	.	SOM	GT:/	0:17	0/1:29,5:37	G	A
.	.	.	.	.	.	.	.	.	.	0	.	SOM	GT:/	0:58	0/1:69,16:3	C	T
.	.	.	.	.	.	.	.	.	.	.	.	SOM	GT:/	0:67	0/1:68,18:3	G	A
.	.	.	.	.	.	.	.	2.3	.	.	.	SOM	GT:/	0:10	0/1:23,3:37	G	C
.	.	##	0	.	.	.	.	2.3	1.55	0	.	SOM	GT:/	0:78	0/1:144,31	G	A
.	.	.	.	0.0	0.237	0.947	1,N	.	1.35	0	.	SOM	GT:/	0:79	0/1:129,24	G	A
.	.	.	.	.	.	.	.	3.2	2.43	.	.	SOM	GT:/	0:86	0/1:130,25	G	A
.	.	.	.	0.0	0.714	0.772	1.00	.	3.12	.	.	SOM	GT:/	0:57	0/1:75,12:3	C	A
.	.	.	.	.	.	.	.	.	.	.	.	SOM	GT:/	0:15	0/1:12,3:37	G	A
.	.	.	.	.	.	.	.	.	.	.	.	SOM	GT:/	0:22	0/1:37,6:37	C	T
.	.	.	.	.	.	.	.	2.3	.	.	.	SOM	GT:/	0:20	0/1:35,8:37	A	G
.	.	##	0	0.1	0.079	0.161	1.00	3.8	2.32	.	.	SOM	GT:/	0:59	0/1:109,27	G	A
.	.	.	.	.	.	.	.	.	.	.	.	SOM	GT:/	0:29	0/1:46,13:3	G	A
0.002	.	0	0	.	.	.	.	.	.	0	0	SOM	GT:/	0:70	0/1:100,26	C	T
.	.	.	.	.	.	.	.	.	.	.	.	SOM	GT:/	0:20	0/1:39,3:37	C	T
.	.	.	.	.	.	.	.	5.8	4.31	.	.	SOM	GT:/	0:53	0/1:61,18:3	G	A
.	.	.	.	.	.	.	.	5.6	1.81	.	.	SOM	GT:/	0:30	0/1:59,9:37	A	C
4E-04	.	.	.	.	.	.	.	.	.	.	.	SOM	GT:/	0:46	0/1:71,14:3	C	T
.	.	.	.	.	.	.	.	.	.	.	.	SOM	GT:/	0:73	0/1:118,20	G	T
.	.	.	.	0.9	0.999	1.0,D	1,D	4.4	5.13	.	.	SOM	GT:/	0:13	0/1:232,50	G	A
.	.	.	.	.	.	.	.	3.1	.	.	.	SOM	GT:/	0:63	0/1:97,18:3	T	C
.	.	.	.	.	.	.	.	5.4	1.33	.	.	SOM	GT:/	0:30	0/1:38,8:35	G	A
.	.	.	.	.	.	.	.	4.8	2.89	.	.	SOM	GT:/	0:54	0/1:60,23:3	G	T
.	.	.	.	.	.	.	.	.	.	.	.	SOM	GT:/	0:43	0/1:87,15:1	T	A
0.01	0	0	0	.	.	.	.	.	.	.	.	DB;S	GT:/	0:33	0/1:37,5:37	C	T
.	.	##	0	0.0	0.007	0.029	1,D	5.4	3.98	.	.	SOM	GT:/	0:12	0/1:215,32	G	A
.	.	.	.	.	.	.	.	.	.	.	.	SOM	GT:/	0:52	0/1:87,17:3	G	A
.	.	.	.	.	.	.	.	.	1.80	.	.	SOM	GT:/	0:15	0/1:21,3:37	C	T
.	.	.	.	.	.	.	.	.	.	.	.	SOM	GT:/	0:69	0/1:118,24	C	T
.	.	.	.	0.0	0.955	0.998	1,N	.	3.39	.	.	SOM	GT:/	0:13	0/1:227,40	C	A
0.013	.	.	.	.	.	.	.	.	.	0	0	DB;S	GT:/	0:32	0/1:36,8:37	C	T
.	.	.	.	.	.	.	.	3.1	.	.	.	SOM	GT:/	0:47	0/1:71,7:35	A	G
2E-04	.	##	0	.	.	.	.	.	.	.	.	SOM	GT:/	0:54	0/1:94,26:3	C	T
.	.	.	.	.	.	.	.	.	.	.	.	SOM	GT:/	0:66	0/1:54,4:37	C	T
.	.	.	.	.	.	.	.	.	.	.	.	SOM	GT:/	0:27	0/1:36,10:3	G	T
.	.	.	.	.	.	.	.	2.3	.	.	.	SOM	GT:/	0:48	0/1:89,12:3	C	T
.	.	.	.	.	.	.	.	.	.	.	.	SOM	GT:/	0:8,(	0/1:15,3:37	C	T
.	.	.	.	.	.	.	.	.	.	.	.	SOM	GT:/	0:22	0/1:46,9:37	C	T
.	.	.	.	0.4	0.779	0.98,I	1,D	5.2	4.82	.	.	SOM	GT:/	0:48	0/1:71,7:37	G	A
.	0	##	0	0.0	0.124	0.729	1,D	3.9	4.67	0	.	SOM	GT:/	0:51	0/1:87,12:3	G	A
.	.	##	0	0.0	0.966	1.0,D	1,D	2.8	3.49	0	.	SOM	GT:/	0:48	0/1:74,7:37	C	T
.	.	.	.	.	.	.	.	.	.	.	.	SOM	GT:/	0:11	0/1:143,34	C	T
.	.	.	.	.	.	.	.	.	.	.	.	SOM	GT:/	0:32	0/1:52,12:3	T	A
.	.	.	.	.	.	.	.	.	.	.	.	SOM	GT:/	0:43	0/1:110,21	C	T
.	.	.	.	.	.	.	.	.	.	.	.	SOM	GT:/	0:12	0/1:14,3:37	C	A
.	.	.	.	.	.	.	.	.	.	.	.	SOM	GT:/	0:33	0/1:39,10:3	A	G
.	.	##	0	.	.	.	.	.	.	.	.	DB;S	GT:/	0:40	0/1:65,12:3	G	A

.	.	.	.	.	.	.	.	.	.	.	.	SON	GT:/	0:16	0/1:17,5:34	G	A
.	.	##	.	.	.	.	.	.	.	.	.	SON	GT:/	0:54	0/1:87,11:3	C	T
.	.	##	0	0.1	0.001	0.001	0.99	.	1.97	.	.	SON	GT:/	0:89	0/1:151,15	C	T
.	.	.	.	.	.	.	.	.	.	.	.	SON	GT:/	0:24	0/1:42,9:37	C	A
.	.	.	.	.	.	.	.	.	.	.	.	SON	GT:/	0:26	0/1:41,4:37	G	T
.	.	.	.	.	.	.	.	3.5	.	.	.	SON	GT:/	0:71	0/1:105,26	G	C
.	.	##	0	.	.	.	.	.	1.40	.	.	DB;S	GT:/	0:48	0/1:93,18:3	G	A
.	.	.	.	.	.	.	.	.	.	.	.	SON	GT:/	0:15	0/1:19,3:37	T	A
.	.	.	.	.	.	.	.	.	.	.	.	SON	GT:/	0:18	0/1:29,5:37	A	T
.	.	.	.	0.0	0.979	0.995	1,D	5.6	1.99	.	.	SON	GT:/	0:48	0/1:79,11:3	A	C
.	.	.	.	.	.	.	.	.	.	.	.	SON	GT:/	0:10	0/1:194,33	C	T
.	.	.	.	.	.	.	.	.	.	0	.	SON	GT:/	0:31	0/1:32,9:37	G	A
.	.	.	.	0.0	0.996	1.0,D	1,D	4.9	4.51	.	.	SON	GT:/	0:11	0/1:197,36	C	T
.	.	.	.	.	.	.	.	.	.	.	.	SON	GT:/	0:56	0/1:85,4:37	T	C
.	.	.	.	.	.	.	1,A	.	3.27	.	.	SON	GT:/	0:19	0/1:47,10:3	C	T
.	.	.	.	.	.	.	.	.	.	.	.	SON	GT:/	0:10	0/1:125,25	T	C
.	.	##	0	0.0	0.676	0.997	1.00	4.1	3.60	.	.	SON	GT:/	0:96	0/1:146,38	C	T
.	.	.	.	.	.	.	.	5.4	2.78	.	.	SON	GT:/	0:10	0/1:164,31	G	A
.	.	.	.	0.0	1.0,D	1.0,D	1,D	5.5	4.45	.	.	SON	GT:/	0:78	0/1:85,17:3	G	T
.	.	.	.	.	.	.	.	.	.	.	.	SON	GT:/	0:76	0/1:115,24	T	G
.	##	##	0	0.0	0.999	1.0,D	1,D	5.5	5.31	.	.	SON	GT:/	0:27	0/1:38,5:37	C	T
.	.	.	.	.	.	.	.	.	.	0	.	SON	GT:/	0:60	0/1:72,10:3	G	A
.	.	.	.	.	.	.	.	.	.	.	.	SON	GT:/	0:14	0/1:20,3:37	G	T
.	.	.	.	.	.	.	.	.	.	.	.	SON	GT:/	0:17	0/1:238,44	C	T
.	.	.	.	.	.	.	.	.	.	.	.	SON	GT:/	0:11	0/1:11,3:37	C	T
.	.	.	.	.	.	.	.	2.2	2.04	.	.	SON	GT:/	0:34	0/1:59,8:35	A	G
.	.	.	.	.	.	.	.	.	.	.	.	SON	GT:/	0:17	0/1:25,3:37	T	G
.	.	0	0	.	.	.	.	.	.	0	0.1	DB;S	GT:/	0:72	0/1:142,8:3	T	G
.	.	.	.	.	.	.	.	.	.	.	0	DB;S	GT:/	0:46	0/1:68,5:37	G	C
.	.	.	.	.	.	.	.	.	.	.	.	SON	GT:/	0:12	0/1:205,37	A	G
.	.	##	0	.	.	.	.	6.2	3.22	.	.	SON	GT:/	0:11	0/1:214,10	C	T
.	.	.	.	.	.	.	.	.	.	.	0.1	DB;S	GT:/	0:69	0/1:101,5:3	T	A
.	.	.	.	.	.	.	.	.	.	.	.	SON	GT:/	0:48	0/1:35,6:37	C	T
.	.	.	.	.	.	.	.	4.9	3.57	.	.	SON	GT:/	0:51	0/1:80,16:3	C	T
.	.	.	.	.	.	.	.	.	.	.	.	SON	GT:/	0:13	0/1:140,36	G	A
.	.	.	.	.	.	.	.	.	.	.	.	SON	GT:/	0:33	0/1:383,15	G	A
.	.	.	.	.	.	.	.	.	.	.	.	SON	GT:/	0:88	0/1:153,23	C	T
.	.	.	.	.	.	.	.	.	.	.	.	DB;S	GT:/	0:90	0/1:151,29	C	T
.	.	.	.	.	.	.	1,D	5.5	10.0	.	.	SON	GT:/	0:62	0/1:111,13	G	A
.	.	.	.	0.0	0.997	0.999	1.00	6	3.34	.	.	SON	GT:/	0:66	0/1:75,19:3	T	C
.	.	.	.	.	.	.	.	.	.	.	.	SON	GT:/	0:34	0/1:530,10	G	A
.	.	.	.	.	.	.	.	.	.	.	.	SON	GT:/	0:32	0/1:37,3:37	C	T
.	.	.	.	.	.	.	.	.	.	.	.	SON	GT:/	0:54	0/1:74,21:3	C	T
.	.	.	.	.	.	.	.	.	.	.	.	SON	GT:/	0:33	0/1:46,11:3	A	G
.	.	##	0	0.0	0.249	0.978	0.99	3.8	3.72	.	.	SON	GT:/	0:53	0/1:113,21	G	A
.	.	.	.	.	.	.	1,D	4.3	3.54	.	.	SON	GT:/	0:10	0/1:150,34	C	A
.	.	.	.	.	.	.	.	.	1.74	.	.	SON	GT:/	0:60	0/1:114,20	C	T
.	.	.	.	0.0	0.0,B	0.0,B	0.99	4.2	2.12	.	.	SON	GT:/	0:57	0/1:87,18:3	C	A
.	.	.	.	.	.	.	.	.	1.62	.	.	SON	GT:/	0:23	0/1:36,5:37	C	A
.	.	.	.	.	.	.	.	.	.	.	.	SON	GT:/	0:20	0/1:22,8:37	C	T
.	.	.	.	.	.	.	.	.	.	.	.	SON	GT:/	0:37	0/1:40,11:3	T	C
.	.	##	0	0.3	0.004	0.007	1,N	.	.	.	0	SON	GT:/	0:70	0/1:130,27	C	T
.	.	.	.	.	.	.	.	.	.	0	0	SON	GT:/	0:12	0/1:21,3:33	A	G
.	.	##	0	.	.	.	.	.	.	.	.	SON	GT:/	0:42	0/1:56,6:37	G	A

.	.	##	0	.	.	.	.	.	.	.	.	SON	GT:/	0:30	0/1:47,11:3	C	T
.	.	.	.	0.0	0.948	1.0,D	0.79	3.9	3.05	.	.	SON	GT:/	0:11	0/1:168,31	G	A
8E-04	.	##	0	.	.	.	.	2.1	.	.	.	SON	GT:/	0:16	0/1:252,55	C	T
.	.	.	.	0.1	0.642	0.989	1,N	2.9	1.79	.	.	SON	GT:/	0:80	0/1:114,21	C	A
.	.	.	.	0.1	0.776	0.958	1,D	6.1	3.94	.	.	SON	GT:/	0:72	0/1:118,5:3	A	G
.	.	.	.	.	.	.	.	.	.	.	.	SON	GT:/	0:12	0/1:259,38	C	T
.	.	.	.	.	.	.	.	.	.	.	.	SON	GT:/	0:15	0/1:32,3:37	G	A
.	.	.	.	.	.	.	.	.	.	.	.	SON	GT:/	0:19	0/1:28,5:37	A	G
.	.	.	.	.	.	.	.	.	.	.	.	SON	GT:/	0:25	0/1:23,7:37	C	T
.	.	.	.	.	.	.	.	.	.	.	.	SON	GT:/	0:11	0/1:25,6:37	G	A
.	.	.	.	.	.	.	.	.	.	.	.	SON	GT:/	0:34	0/1:33,10:3	T	G
.	.	##	0	.	.	.	.	.	.	.	.	SON	GT:/	0:61	0/1:111,22	C	T
.	.	.	.	.	.	.	1,D	5.4	3.73	.	.	SON	GT:/	0:10	0/1:187,39	C	A
.	.	.	.	.	.	.	.	.	.	.	.	SON	GT:/	0:22	0/1:30,8:37	C	T
.	.	.	.	.	.	.	.	.	.	.	.	DB;S	GT:/	0:36	0/1:45,6:32	C	T
.	.	##	.	0.0	0.829	0.996	1,D	4.2	4.18	.	.	SON	GT:/	0:45	0/1:79,20:3	C	A
.	.	##	0	0.5	0.004	0.013	1,N	.	.	.	.	SON	GT:/	0:10	0/1:170,28	G	A
.	.	.	.	0.0	0.998	1.0,D	0.96	4.8	4.07	.	.	SON	GT:/	0:69	0/1:103,16	G	A
.	.	##	0	0.0	0.998	1.0,D	1,D	5.6	4.70	.	.	DB;S	GT:/	0:70	0/1:102,24	C	T
.	.	.	.	0.0	0.645	0.976	1.00	5.1	2.99	.	.	SON	GT:/	0:27	0/1:35,9:37	C	A
.	.	.	.	.	.	.	.	.	.	.	.	SON	GT:/	0:22	0/1:46,4:37	C	T
.	.	.	.	.	.	.	.	.	.	.	.	SON	GT:/	0:39	0/1:42,8:37	A	G
.	##	##	0	0.0	0.999	1.0,D	1.00	4	4.09	0	.	SON	GT:/	0:44	0/1:84,15:3	G	A
.	##	##	.	.	.	.	.	3.4	.	.	.	SON	GT:/	0:60	0/1:101,10	C	T
.	##	##	0	1.0	0.011	0.027	0.97	2.8	.	.	.	SON	GT:/	0:19	0/1:299,47	G	A
.	.	.	.	.	.	.	.	.	2.55	.	.	SON	GT:/	0:20	0/1:32,4:37	G	T
.	.	.	.	.	.	.	.	.	.	.	.	SON	GT:/	0:16	0/1:23,4:37	C	T
.	.	.	.	.	.	.	.	.	.	.	.	SON	GT:/	0:53	0/1:84,17:3	C	T
.	.	.	.	.	.	.	.	.	.	.	.	SON	GT:/	0:30	0/1:51,14:3	T	G
.	.	.	.	.	.	.	.	.	.	.	.	SON	GT:/	0:11	0/1:14,4:34	G	A
.	.	.	.	.	.	.	.	.	.	.	.	SON	GT:/	0:60	0/1:72,16:3	T	C
.	.	.	.	.	.	.	.	.	.	.	.	SON	GT:/	0:20	0/1:46,6:37	C	G
.	.	.	.	.	.	.	.	.	.	.	.	SON	GT:/	0:26	0/1:39,8:37	T	C
.	.	.	.	.	.	.	.	.	.	.	.	SON	GT:/	0:61	0/1:130,17	C	T
.	.	.	.	0.2	0.012	0.102	1,N	.	3.19	.	.	SON	GT:/	0:12	0/1:178,29	C	T
.	.	.	.	0.0	1.0,D	1.0,D	1,D	6	3.50	.	.	SON	GT:/	0:32	0/1:52,10:3	G	T
.	.	.	.	.	.	.	.	2.3	.	.	.	SON	GT:/	0:57	0/1:99,22:3	A	G
.	.	.	.	.	.	.	.	.	.	.	.	SON	GT:/	0:38	0/1:66,8:37	G	A
.	.	.	.	0.0	0.691	0.976	0.98	3.2	1.82	.	.	SON	GT:/	0:13	0/1:207,37	T	C
.	.	.	.	.	.	.	.	.	.	.	.	SON	GT:/	0:57	0/1:67,9:37	G	T
.	.	##	0	0.2	0.021	0.021	0.99	.	2.30	.	.	SON	GT:/	0:73	0/1:87,20:3	C	T
8E-04	.	0	0	.	.	.	.	.	.	.	0	SON	GT:/	0:18	0/1:41,8:37	C	T
.	.	.	.	.	.	.	1,D	6.1	4.56	.	.	SON	GT:/	0:54	0/1:70,11:3	C	T
.	.	.	.	.	.	.	.	2.6	.	.	.	SON	GT:/	0:88	0/1:125,23	C	T
.	.	##	0	.	0.13,I	0.48,I	1.00	4.9	2.72	0	.	SON	GT:/	0:40	0/1:74,8:37	C	T
.	.	.	.	0.1	0.593	0.767	1.00	2.9	3.54	.	.	SON	GT:/	0:31	0/1:61,12:3	C	A
.	.	.	.	0.0	0.27,I	0.274	1.00	5.8	3.31	.	.	SON	GT:/	0:52	0/1:91,14:3	T	G
.	.	.	.	.	.	.	.	.	.	.	.	SON	GT:/	0:33	0/1:34,3:37	C	T
.	.	.	.	.	.	.	.	.	.	.	.	SON	GT:/	0:60	0/1:104,21	G	A
.	.	.	.	0.0	0.828	0.999	1,N	.	2.96	.	.	SON	GT:/	0:19	0/1:250,46	G	A
.	.	.	.	.	.	.	.	.	.	.	.	SON	GT:/	0:13	0/1:37,3:37	C	T
.	.	.	.	.	.	.	.	.	.	.	.	SON	GT:/	0:68	0/1:106,24	G	A
.	.	.	.	0.0	0.071	0.223	0.96	3.9	4.16	.	.	SON	GT:/	0:16	0/1:33,6:17	A	T
.	.	.	.	0.0	1.0,D	1.0,D	1,D	5.3	4.11	.	.	SON	GT:/	0:48	0/1:94,18:3	G	A

.	.	.	.	.	.	.	.	.	.	0	.	SOM	GT:	0:26	0/1:25,6:37	C	T
.	.	.	.	.	.	.	.	.	.	.	0	SOM	GT:	0:16	0/1:244,60	C	T
0.001	0	0	0	.	.	.	.	.	.	.	.	SOM	GT:	0:46	0/1:94,14:3	C	T
.	.	.	.	.	.	.	.	.	.	.	.	SOM	GT:	0:34	0/1:51,9:37	C	T
.	.	.	.	.	.	.	1,D	6.1	4.95	.	.	SOM	GT:	0:30	0/1:38,13:3	C	T
.	.	.	.	.	.	.	.	.	.	.	.	SOM	GT:	0:53	0/1:65,28:3	G	A
0.001	0	0	0	.	.	.	.	.	.	.	.	SOM	GT:	0:67	0/1:134,15	C	T
.	.	##	0	0.4	0.992	1.0,D	1.00	3.6	3.57	0	.	SOM	GT:	0:47	0/1:71,12:3	C	T
.	0	0	0	.	.	.	.	.	.	.	.	SOM	GT:	0:30	0/1:55,9:37	G	A
.	.	##	0	.	.	.	.	.	.	.	.	SOM	GT:	0:18	0/1:323,62	G	A
.	.	.	.	0.0	0.271	0.617	1.00	3.9	3.19	.	.	SOM	GT:	0:18	0/1:318,62	A	G
2E-04	.	##	0	0.0	0.938	0.999	1,D	4.7	4.09	0	.	SOM	GT:	0:54	0/1:98,17:3	G	A
.	.	.	.	.	.	.	.	.	.	.	.	SOM	GT:	0:73	0/1:87,20:3	T	C
.	.	##	0	0.0	0.999	1.0,D	1,D	4.7	4.78	.	.	SOM	GT:	0:33	0/1:57,6:37	G	A
.	##	##	0	0.0	0.897	0.998	1,D	5.5	3.85	.	.	SOM	GT:	0:59	0/1:106,16	C	T
.	.	.	.	.	.	.	.	.	.	.	.	SOM	GT:	0:46	0/1:55,20:3	G	A
.	.	.	.	.	.	.	.	2.1	1.37	.	.	SOM	GT:	0:17	0/1:22,5:37	C	T
2E-04	0	##	0	.	.	.	.	3.1	.	.	.	SOM	GT:	0:28	0/1:57,6:37	C	T
.	##	##	0	.	.	.	.	.	.	.	.	SOM	GT:	0:95	0/1:153,26	C	T
.	.	##	0	0.0	0.999	1.0,D	1,D	5.6	3.16	.	.	SOM	GT:	0:72	0/1:149,24	C	T
.	.	.	.	0.0	0.997	1.0,D	1,D	5.2	6.08	.	.	SOM	GT:	0:92	0/1:168,27	G	A
2E-04	.	.	.	.	.	.	.	.	.	.	.	SOM	GT:	0:34	0/1:70,11:3	G	A
.	.	##	0	.	.	.	.	.	.	.	.	SOM	GT:	0:48	0/1:95,26:3	G	A
2E-04	0	##	0	0.2	0.001	0.002	0.98	.	.	.	.	SOM	GT:	0:71	0/1:98,20:3	G	A
.	.	.	.	0.0	0.008	0.002	0.77	.	2.96	.	.	SOM	GT:	0:57	0/1:116,22	G	A
.	.	.	.	1.0	0.197	0.645	1,D	4.7	.	.	.	SOM	GT:	0:87	0/1:110,13	A	T
.	.	.	.	.	.	.	.	3	.	.	.	SOM	GT:	0:11	0/1:13,3:37	G	A
.	.	.	.	.	.	.	.	.	.	.	.	SOM	GT:	0:23	0/1:37,7:37	G	A
.	.	##	0	0.0	0.987	0.999	1,D	5.6	5.41	0	.	SOM	GT:	0:34	0/1:64,11:3	G	A
.	.	.	.	.	.	.	.	.	.	.	.	SOM	GT:	0:17	0/1:25,4:37	G	A
.	.	.	.	.	.	.	.	.	.	.	.	SOM	GT:	0:19	0/1:32,9:37	A	G
.	.	.	.	0.1	0.022	0.005	1,D	5.3	3.98	.	.	SOM	GT:	0:50	0/1:71,14:3	C	T
.	.	0	0	.	.	.	.	.	.	.	.	DB;S	GT:	0:46	0/1:73,13:3	C	T
.	.	.	.	.	.	.	.	2.4	.	.	.	SOM	GT:	0:11	0/1:16,5:37	T	G
.	.	.	.	.	.	.	.	3.2	.	.	.	SOM	GT:	0:41	0/1:67,8:37	T	C
.	.	.	.	.	.	.	.	.	.	.	.	SOM	GT:	0:34	0/1:30,6:37	C	A
.	.	.	.	.	.	.	.	.	.	.	.	SOM	GT:	0:27	0/1:41,9:37	C	T
.	.	.	.	0.6	0.018	0.02,I	0.99	5.6	1.44	.	.	SOM	GT:	0:81	0/1:122,27	C	A
.	.	.	.	.	.	.	.	5.2	2.70	.	.	SOM	GT:	0:39	0/1:53,13:3	T	C
.	.	##	.	.	.	.	.	.	.	.	.	SOM	GT:	0:16	0/1:29,5:37	T	C
.	.	##	0	.	.	.	.	.	.	.	.	SOM	GT:	0:29	0/1:38,10:3	C	T
.	.	.	.	.	.	.	.	2.3	1.51	.	.	SOM	GT:	0:21	0/1:24,5:37	T	C
.	.	.	.	.	.	.	.	.	.	.	.	SOM	GT:	0:24	0/1:42,8:37	C	T
.	.	.	.	.	.	.	.	.	.	.	.	SOM	GT:	0:16	0/1:37,4:37	A	T
.	.	.	.	.	.	.	.	.	1.28	.	.	SOM	GT:	0:18	0/1:43,9:37	A	C
.	.	.	.	0.2	0.036	0.094	0.93	4	.	.	.	SOM	GT:	0:48	0/1:88,9:37	C	T
.	.	.	.	.	.	.	.	.	.	.	.	SOM	GT:	0:22	0/1:34,8:37	A	G
.	.	.	.	.	.	.	.	.	.	.	.	SOM	GT:	0:52	0/1:78,15:3	C	A
.	.	.	.	.	.	.	.	.	.	.	.	SOM	GT:	0:72	0/1:135,23	C	T
.	.	.	.	.	.	.	.	.	.	.	.	SOM	GT:	0:58	0/1:95,15:3	C	A
.	.	##	0	.	.	.	.	.	.	.	.	SOM	GT:	0:95	0/1:123,31	C	T
.	.	.	.	0.8	0.163	0.571	0.95	4.5	.	.	.	SOM	GT:	0:30	0/1:53,10:3	A	G
2E-04	.	.	.	.	.	.	.	.	.	.	.	SOM	GT:	0:25	0/1:43,3:37	G	A
.	.	.	.	.	.	.	.	2.2	.	.	.	SOM	GT:	0:55	0/1:71,17:3	G	A

.	.	.	.	.	.	.	.	5	.	.	.	SON	GT:/	0:34	0/1:70,11:3	G	T
.	.	.	.	.	.	.	.	.	.	.	.	SON	GT:/	0:49	0/1:67,11:3	T	C
.	.	.	.	.	.	.	.	.	.	.	.	SON	GT:/	0:28	0/1:69,4:37	C	T
.	.	.	.	.	.	.	.	.	1.67	.	.	SON	GT:/	0:25	0/1:28,6:37	A	C
0.004	.	.	.	.	.	.	.	.	.	0	.	SON	GT:/	0:24	0/1:22,3:37	G	A
.	.	.	.	.	.	.	.	.	.	0.2	0.5	SON	GT:/	0:8,(	0/1:23,12:3	G	A
.	.	.	.	.	.	.	.	.	.	0.3	0.5	SON	GT:/	0:9,(	0/1:30,13:3	G	A
.	0	##	0	.	.	.	.	2.1	3.65	.	.	SON	GT:/	0:16	0/1:287,50	C	T
.	.	.	.	.	.	.	.	.	1.49	.	.	SON	GT:/	0:18	0/1:240,52	T	G
.	.	.	.	.	.	.	.	.	.	.	.	SON	GT:/	0:18	0/1:27,7:35	T	C
.	.	.	.	.	.	.	.	.	.	0	.	SON	GT:/	0:8,(	0/1:17,4:30	G	A
.	.	.	.	.	.	.	.	.	.	0	.	SON	GT:/	0:10	0/1:17,4:37	C	A
.	.	.	.	.	.	.	.	.	.	0.1	0.1	DB;S	GT:/	0:38	0/1:71,8:37	G	A
2E-04	.	0	0	.	.	.	.	2.3	1.39	.	.	SON	GT:/	0:63	0/1:65,7:37	G	A
.	.	.	.	.	.	.	.	.	.	.	.	SON	GT:/	0:61	0/1:102,22	A	G
.	.	##	0	.	.	.	.	.	.	.	.	SON	GT:/	0:76	0/1:115,24	C	T
.	.	.	.	.	.	.	.	3.4	1.32	.	0	SON	GT:/	0:43	0/1:60,17:3	C	T
.	.	.	.	.	.	.	.	.	.	.	.	SON	GT:/	0:45	0/1:84,14:3	G	A
.	.	.	.	.	.	.	.	.	.	.	.	SON	GT:/	0:70	0/1:128,23	T	C
2E-04	.	0	0	.	.	.	.	.	.	.	.	SON	GT:/	0:14	0/1:206,34	G	A
.	.	.	.	.	.	.	.	.	.	.	.	SON	GT:/	0:46	0/1:51,5:37	T	C
.	.	.	.	.	.	.	.	.	.	.	.	SON	GT:/	0:30	0/1:35,8:37	C	A
.	.	##	0	0.0(	0.997	1.0,D	1,D	5.7	5.60	.	.	SON	GT:/	0:68	0/1:117,16	C	T
.	.	.	.	0.7	0.013	0.003	1,N	.	.	.	.	SON	GT:/	0:29	0/1:43,4:37	G	A
.	.	##	0	.	.	.	.	.	.	.	.	SON	GT:/	0:60	0/1:92,17:3	G	A
.	0	0	0	1.0	0.0,B	0.002	1,N	2.3	.	.	.	SON	GT:/	0:27	0/1:381,68	C	T
.	.	##	0	0.0	0.588	0.993	0.85	4	2.75	.	.	SON	GT:/	0:42	0/1:41,12:3	C	T
.	.	.	.	.	.	.	.	.	.	.	.	SON	GT:/	0:46	0/1:83,12:3	G	A
.	.	.	.	.	.	.	.	.	.	.	.	SON	GT:/	0:18	0/1:22,5:37	A	G
.	.	.	.	0.0(	0.996	0.999	1.00	4.5	3.86	.	.	SON	GT:/	0:58	0/1:82,20:3	A	G
.	.	.	.	.	.	.	.	.	.	.	.	SON	GT:/	0:91	0/1:172,36	G	A
.	.	.	.	.	.	.	.	.	.	.	.	SON	GT:/	0:68	0/1:77,12:3	T	C
.	.	##	0	.	.	.	.	.	.	.	0	SON	GT:/	0:37	0/1:70,14:3	C	T
.	.	.	.	.	.	.	.	.	.	0	.	SON	GT:/	0:35	0/1:38,13:1	T	A
.	.	.	.	.	.	.	.	.	.	.	.	SON	GT:/	0:24	0/1:49,14:2	G	A
.	.	0	0	.	.	.	.	.	.	.	.	SON	GT:/	0:62	0/1:82,12:3	C	T
.	.	.	.	.	.	.	.	.	.	0.2	0	DB;S	GT:/	0:30	0/1:40,4:37	G	C
8E-04	.	.	.	.	.	.	.	.	.	0	.	SON	GT:/	0:19	0/1:38,12:3	C	T
.	.	.	.	.	.	.	.	.	.	.	.	SON	GT:/	0:9,(	0/1:7,3:37:	A	G
.	.	.	.	.	.	.	.	.	.	.	.	SON	GT:/	0:24	0/1:28,4:37	C	T
.	.	.	.	.	.	.	.	.	.	.	.	SON	GT:/	0:14	0/1:28,3:37	C	A
.	.	.	.	0.6	0.474	0.976	0.99	5.4	.	.	.	SON	GT:/	0:43	0/1:87,17:3	G	A
.	.	.	.	.	.	.	.	.	1.25	.	.	SON	GT:/	0:10	0/1:176,28	G	T
.	.	.	.	.	.	.	.	.	.	.	.	DB;S	GT:/	0:44	0/1:63,10:3	G	A
.	.	.	.	.	.	.	.	.	.	0	.	SON	GT:/	0:27	0/1:41,8:35	C	T
.	.	.	.	.	.	.	.	3	.	.	.	SON	GT:/	0:10	0/1:15,6:24	A	C
.	.	##	0	0.0	1.0,D	1.0,D	1,D	5.1	4.07	.	.	SON	GT:/	0:56	0/1:85,14:3	C	T
.	##	##	0	0.1(	0.031	0.157	0.99	5.6	3.15	.	.	SON	GT:/	0:27	0/1:44,6:37	G	A
.	.	.	.	.	.	.	.	.	3.88	.	.	SON	GT:/	0:33	0/1:46,3:37	G	T
.	.	.	.	.	.	.	.	.	1.43	.	.	SON	GT:/	0:29	0/1:29,10:3	C	T
.	.	.	.	.	.	.	.	.	.	.	.	SON	GT:/	0:56	0/1:127,8:3	G	A
.	.	.	.	.	.	.	.	.	.	.	.	SON	GT:/	0:29	0/1:41,5:37	T	G
.	.	.	.	.	.	.	.	.	.	.	.	SON	GT:/	0:86	0/1:155,15	G	T
.	.	.	.	.	.	.	.	3.5	1.73	.	.	SON	GT:/	0:35	0/1:55,13:3	A	G

.	.	.	.	.	.	.	1,A	3.6	5.01	.	.	SON	GT:/	0:39	0/1:51,10:3	C	T
.	.	.	.	.	.	.	.	.	.	.	.	SON	GT:/	0:21	0/1:30,4:37	T	A
.	.	.	.	.	.	.	.	.	.	.	.	SON	GT:/	0:9,(	0/1:14,4:37	G	A
.	.	.	.	.	.	.	.	3.5	1.85	.	.	SON	GT:/	0:32	0/1:36,6:37	T	C
.	.	.	.	.	.	.	.	.	3.87	.	.	SON	GT:/	0:25	0/1:44,6:37	T	C
.	.	.	.	.	.	.	.	.	.	.	.	SON	GT:/	0:13	0/1:11,5:37	G	A
.	.	.	.	.	.	.	.	.	.	.	.	SON	GT:/	0:10	0/1:8,3:37:	A	T
.	.	.	.	.	.	.	.	.	.	.	.	SON	GT:/	0:8,(	0/1:3,2:37:	G	A
.	.	0	0	.	.	.	.	.	.	.	.	SON	GT:/	0:21	0/1:22,9:37	T	A
.	##	.	.	0.0	0.876	0.998	1,D	6	4.95	.	.	SON	GT:/	0:77	0/1:107,16	C	T
.	.	.	.	1.0	.	.	1,N	.	.	.	.	SON	GT:/	0:39	0/1:62,19:3	T	C
.	.	.	.	0.1	.	.	1,N	2.8	.	.	.	SON	GT:/	0:60	0/1:87,20:3	A	G
.	.	.	.	.	.	.	.	.	3.07	.	.	SON	GT:/	0:32	0/1:59,10:3	T	C
.	.	##	.	.	.	.	.	.	.	.	.	SON	GT:/	0:23	0/1:42,4:34	G	A
.	.	##	0	.	.	.	.	.	.	.	.	SON	GT:/	0:80	0/1:112,20	C	T
.	.	.	.	.	.	.	.	5.2	4.01	.	.	SON	GT:/	0:35	0/1:53,5:37	G	A
.	.	.	.	0.0	0.045	0.075	1,N	.	2.39	.	.	SON	GT:/	0:10	0/1:152,22	G	A
.	.	.	.	0.0	1.0,D	1.0,D	1,D	.	4.12	.	.	DB;S	GT:/	0:21	0/1:40,10:3	T	A
.	.	.	.	0.0	0.999	1.0,D	0.95	5.5	5.36	.	.	SON	GT:/	0:81	0/1:98,22:3	C	T
.	.	.	.	0.0	0.985	0.986	1.00	5.4	4.37	.	.	SON	GT:/	0:66	0/1:78,11:3	T	C
.	.	##	0	.	.	.	.	.	.	.	.	SON	GT:/	0:10	0/1:156,40	G	A
0.007	.	.	.	.	.	.	.	.	.	0	0	SON	GT:/	0:12	0/1:27,6:37	T	A
.	.	##	0	.	.	.	.	.	.	.	.	SON	GT:/	0:74	0/1:169,37	A	G
0.002	0	0	0	.	.	.	.	.	.	.	.	SON	GT:/	0:67	0/1:100,17	G	A
0.002	.	0	0	0.0	.	.	1,D	5.8	5.44	.	.	SON	GT:/	0:64	0/1:129,22	G	A
.	.	.	.	0.2	0.024	0.001	1.00	.	.	.	.	SON	GT:/	0:99	0/1:152,27	G	C
.	.	.	.	.	.	.	.	.	.	.	.	SON	GT:/	0:18	0/1:31,9:37	C	A
.	.	.	.	0.0	0.999	1.0,D	1.00	5.4	4.90	.	.	SON	GT:/	0:49	0/1:117,18	G	A
.	.	.	.	.	.	.	.	.	.	.	.	SON	GT:/	0:87	0/1:145,22	C	T
.	.	.	.	.	.	.	.	.	.	.	.	SON	GT:/	0:57	0/1:113,13	A	G
.	.	.	.	.	.	.	.	.	.	.	.	SON	GT:/	0:19	0/1:22,3:37	T	C
.	.	.	.	.	.	.	.	.	.	.	.	SON	GT:/	0:35	0/1:40,15:3	G	T
.	.	.	.	0.0	.	.	.	4.8	4.78	.	.	SON	GT:/	0:46	0/1:89,19:3	A	T
.	.	.	.	.	.	.	.	2.9	.	.	.	SON	GT:/	0:32	0/1:55,7:37	C	A
.	.	.	.	.	.	.	.	.	.	.	.	SON	GT:/	0:48	0/1:77,23:3	G	T
2E-04	.	0	0	0.0	0.0,B	0.0,B	1,N	.	.	.	.	SON	GT:/	0:98	0/1:179,29	G	A
.	.	.	.	.	.	.	.	2.9	.	.	.	SON	GT:/	0:16	0/1:25,7:37	G	T
.	##	##	0	.	.	.	.	.	.	.	.	SON	GT:/	0:66	0/1:96,27:3	G	A
.	.	.	.	.	.	.	.	2.7	.	.	.	SON	GT:/	0:61	0/1:106,17	G	A
.	.	##	0	.	.	.	.	.	.	.	.	SON	GT:/	0:67	0/1:125,18	C	T
0.003	.	0	0	.	.	.	.	.	.	.	0	SON	GT:/	0:80	0/1:159,27	C	T
.	.	.	.	0.0	0.211	0.171	1,D	6.2	2.79	.	.	SON	GT:/	0:66	0/1:147,23	T	A
.	.	.	.	.	.	.	.	.	.	.	.	SON	GT:/	0:87	0/1:142,28	C	T
.	.	.	.	.	.	.	.	.	.	.	.	DB;S	GT:/	0:33	0/1:27,3:37	A	T
0.284	0	0	0	.	.	.	.	.	2.16	0.4	0.4	DB;S	GT:/	0:53	0/1:78,12:3	C	T
.	.	##	0	0.0	0.993	1.0,D	0.97	3.3	2.52	.	.	SON	GT:/	0:13	0/1:218,36	G	A
.	.	.	.	.	.	.	.	5.3	2.55	.	.	SON	GT:/	0:18	0/1:43,3:37	C	A
.	.	.	.	.	.	.	.	.	.	.	.	SON	GT:/	0:15	0/1:25,7:37	G	T
.	.	.	.	.	.	.	.	.	.	.	.	SON	GT:/	0:56	0/1:102,18	G	A
.	##	##	0	0.0	0.988	0.995	0.98	5.5	3.13	.	.	SON	GT:/	0:51	0/1:91,22:3	G	A
.	.	.	.	.	.	.	.	.	.	.	.	SON	GT:/	0:8,(	0/1:12,4:37	C	T
.	.	.	.	.	.	.	.	.	.	.	.	SON	GT:/	0:63	0/1:136,25	C	T
.	.	.	.	.	.	.	.	2.8	.	.	.	SON	GT:/	0:37	0/1:50,14:3	C	T
.	.	.	.	0.0	0.997	1.0,D	1,D	4.4	3.71	.	.	SON	GT:/	0:51	0/1:92,19:3	C	T

.	.	.	.	.	.	.	.	.	.	.	.	SOM	GT:/	0:18	0/1:34,7:37	C	T
.	##	##	0	.	.	.	.	.	.	.	.	SOM	GT:/	0:30	0/1:65,9:34	G	A
0.007	0	0	0	.	.	.	.	.	.	0	0	SOM	GT:/	0:10	0/1:179,40	C	T
.	.	.	.	.	.	.	.	.	.	.	.	SOM	GT:/	0:15	0/1:9,3:37:	G	T
.	.	.	.	.	.	.	.	.	.	.	.	SOM	GT:/	0:20	0/1:29,6:37	C	T
.	.	.	.	.	.	.	.	4.6	2.74	.	.	SOM	GT:/	0:49	0/1:91,17:3	G	A
.	##	##	0	.	.	.	.	4.8	1.24	.	.	SOM	GT:/	0:29	0/1:45,7:37	C	T
0.002	.	.	.	.	.	.	.	.	.	.	.	SOM	GT:/	0:8,(	0/1:14,5:37	C	T
.	.	.	.	.	.	.	.	.	2.21	.	.	SOM	GT:/	0:41	0/1:53,16:3	C	T
.	.	.	.	.	.	.	.	2.5	1.28	.	.	SOM	GT:/	0:14	0/1:32,4:37	G	A
.	.	.	.	.	.	.	.	.	.	.	.	SOM	GT:/	0:19	0/1:38,11:3	C	T
.	.	.	.	.	.	.	.	4.1	2.41	.	.	SOM	GT:/	0:48	0/1:85,10:3	T	C
.	.	##	0	0.0	0.966	1.0,D	0.99	5.2	3.90	0	.	SOM	GT:/	0:27	0/1:50,6:37	C	T
.	.	.	.	.	.	.	.	.	2.23	.	.	SOM	GT:/	0:21	0/1:41,4:37	C	T
.	.	.	.	0.0	0.993	1.0,D	1,D	4.8	4.39	.	.	SOM	GT:/	0:36	0/1:61,11:3	C	T
.	.	.	.	.	.	.	.	.	.	.	.	SOM	GT:/	0:19	0/1:38,8:37	G	A
.	.	.	.	.	.	.	.	.	.	.	.	SOM	GT:/	0:13	0/1:8,3:37:	G	A
.	.	.	.	.	.	.	.	.	.	0	0	SOM	GT:/	0:8,(	0/1:15,3:37	C	T
.	.	.	.	.	.	.	.	.	.	.	.	SOM	GT:/	0:77	0/1:139,18	G	T
.	.	.	.	.	.	.	.	.	.	.	.	SOM	GT:/	0:16	0/1:22,3:37	C	A
.	.	.	.	0.0	0.998	1.0,D	1.00	2.3	3.38	.	.	SOM	GT:/	0:16	0/1:23,9:37	T	A
.	.	.	.	.	.	.	.	.	.	.	.	SOM	GT:/	0:31	0/1:35,13:3	T	C
.	.	.	.	.	.	.	.	3.8	1.28	.	.	SOM	GT:/	0:26	0/1:44,5:37	G	T
.	.	.	.	.	.	.	.	2.1	.	.	.	SOM	GT:/	0:40	0/1:71,10:3	G	A
.	.	.	.	.	.	.	.	.	1.31	.	.	SOM	GT:/	0:39	0/1:69,8:37	C	T
.	.	.	.	.	.	.	.	.	.	.	.	SOM	GT:/	0:20	0/1:36,9:37	A	G
.	.	##	0	.	.	.	.	.	.	.	.	SOM	GT:/	0:22	0/1:33,11:3	A	G
2E-04	0	##	0	0.3	0.003	0.003	1.00	5.4	1.35	.	.	DB;S	GT:/	0:74	0/1:115,25	G	A
.	.	.	.	.	.	.	.	.	.	.	.	SOM	GT:/	0:26	0/1:42,9:37	C	T
.	.	##	0	.	.	.	.	.	.	0	.	SOM	GT:/	0:62	0/1:78,22:3	C	T
.	.	.	.	.	.	.	.	.	.	.	.	SOM	GT:/	0:45	0/1:59,13:3	G	T
4E-04	.	##	0	0.5	0.001	0.002	1,N	.	.	0	0	SOM	GT:/	0:17	0/1:303,57	G	A
8E-04	.	.	.	.	.	.	.	.	.	.	.	SOM	GT:/	0:56	0/1:64,12:3	G	A
.	.	.	.	.	.	.	.	.	.	.	.	SOM	GT:/	0:9,(	0/1:28,4:37	T	C
.	0	##	0	.	.	.	.	.	.	.	.	SOM	GT:/	0:36	0/1:72,11:3	G	A
.	.	.	.	.	.	.	.	2.3	.	.	.	SOM	GT:/	0:30	0/1:59,12:3	C	T
.	.	##	0	.	.	.	.	.	.	.	.	SOM	GT:/	0:63	0/1:88,14:3	G	A
.	.	.	.	.	.	.	.	.	.	.	.	SOM	GT:/	0:49	0/1:71,13:3	G	A
.	.	##	0	0.1	0.951	0.995	1,D	5	3.50	.	.	SOM	GT:/	0:10	0/1:184,41	G	A
.	.	.	.	.	.	.	.	.	.	0	.	SOM	GT:/	0:21	0/1:31,6:35	G	A
.	.	.	.	.	.	.	.	2	.	.	.	SOM	GT:/	0:47	0/1:51,8:35	G	A
.	.	0	0	0.0	0.007	0.022	0.99	2.9	2.38	.	.	SOM	GT:/	0:15	0/1:236,46	C	T
.	.	##	0	.	.	.	.	.	.	0	.	SOM	GT:/	0:16	0/1:280,51	G	A
.	##	##	0	1.0	0.09,I	0.608	0.98	3.6	.	.	.	SOM	GT:/	0:64	0/1:113,24	C	T
.	.	.	.	0.0	1.0,D	1.0,D	1.00	5.3	3.33	.	.	SOM	GT:/	0:10	0/1:164,33	C	T
2E-04	.	##	0	.	.	.	.	.	.		0	SOM	GT:/	0:12	0/1:241,50	C	T
.	.	.	.	0.0	0.816	0.999	0.99	2.3	4.32	.	.	SOM	GT:/	0:10	0/1:141,28	G	A
.	.	.	.	.	.	.	1,A	.	6.60	.	.	SOM	GT:/	0:12	0/1:187,42	C	T
.	.	.	.	.	.	.	.	2.8	.	.	.	SOM	GT:/	0:31	0/1:55,7:37	G	T
.	.	##	0	0.1	0.666	0.985	1.00	5.1	4.02	.	.	SOM	GT:/	0:15	0/1:257,46	C	T
6E-04	0	0	0	.	.	.	.	.	.	.	.	SOM	GT:/	0:72	0/1:85,17:3	G	A
.	.	.	.	.	.	.	.	.	.	.	.	SOM	GT:/	0:52	0/1:90,15:3	A	G
.	.	##	0	0.0	1.0,D	1.0,D	1,D	4.4	4.28	.	.	SOM	GT:/	0:82	0/1:127,27	G	A
.	.	.	.	.	.	.	1,D	4.5	3.79	.	.	DB;S	GT:/	0:26	0/1:76,7:37	A	G

.	.	.	.	.	.	.	.	.	.	.	.	SOM	GT:/	0:38	0/1:50,8:37	G	A
.	.	.	.	.	.	.	.	.	2.42	.	.	SOM	GT:/	0:20	0/1:27,4:37	A	G
.	.	.	.	.	.	.	.	.	.	.	.	SOM	GT:/	0:12	0/1:230,43	C	T
.	.	.	.	.	.	.	.	.	.	.	.	SOM	GT:/	0:27	0/1:41,9:37	C	T
.	.	.	.	.	.	.	.	.	.	.	.	SOM	GT:/	0:24	0/1:26,8:37	A	T
.	.	.	.	0.1	0.997	0.999	1.00	5.6	5.03	.	.	SOM	GT:/	0:66	0/1:105,19	T	G
.	.	.	.	0.9	0.373	0.58,I	0.99	5.4	1.88	.	.	SOM	GT:/	0:55	0/1:95,25:3	C	T
.	.	.	.	.	.	.	.	.	.	.	.	DB;S	GT:/	0:20	0/1:29,8:35	C	T
.	.	.	.	.	.	.	.	.	.	.	.	DB;S	GT:/	0:36	0/1:47,5:26	T	C
.	.	.	.	.	.	.	.	.	.	.	.	SOM	GT:/	0:36	0/1:47,5:27	T	C
.	.	.	.	.	.	.	.	.	.	.	.	SOM	GT:/	0:13	0/1:30,6:37	G	A
.	.	.	.	.	.	.	.	.	1.48	.	.	SOM	GT:/	0:67	0/1:79,14:3	C	A
.	.	.	.	.	.	.	.	.	.	.	.	SOM	GT:/	0:61	0/1:81,20:3	G	T
.	.	.	.	.	.	.	.	4.2	2.40	.	.	SOM	GT:/	0:54	0/1:55,12:3	G	T
.	.	##	0	.	.	.	.	.	2.23	.	.	SOM	GT:/	0:98	0/1:153,34	C	T
.	.	.	.	0.5	0.001	0.0,B	1,N	.	.	.	.	SOM	GT:/	0:10	0/1:143,26	A	G
.	.	.	.	0.0	0.962	0.998	1.00	5.9	3.83	.	.	SOM	GT:/	0:76	0/1:89,22:3	C	T
.	.	##	0	.	.	.	.	2	.	.	.	SOM	GT:/	0:29	0/1:37,8:33	C	T
.	.	.	.	0.3	0.156	0.575	0.90	5	.	.	.	SOM	GT:/	0:25	0/1:46,9:37	G	A
.	.	.	.	.	.	.	.	.	.	.	.	SOM	GT:/	0:20	0/1:26,6:37	A	G
.	.	.	.	.	.	.	.	.	.	.	.	SOM	GT:/	0:46	0/1:76,15:3	G	T
.	.	.	.	.	.	.	.	2.2	4.04	0	.	SOM	GT:/	0:24	0/1:37,5:37	C	T
.	.	.	.	.	.	.	.	2.5	.	.	.	SOM	GT:/	0:28	0/1:41,6:35	G	T
.	.	.	.	.	.	.	.	.	.	.	.	SOM	GT:/	0:44	0/1:92,13:3	A	C
.	.	.	.	.	.	.	.	.	.	.	.	SOM	GT:/	0:31	0/1:50,10:3	C	A
.	##	##	0	0.0	0.572	0.996	1.00	5.5	4.81	.	.	SOM	GT:/	0:28	0/1:59,18:3	G	A
.	.	.	.	.	.	.	.	2.7	.	.	.	SOM	GT:/	0:20	0/1:47,10:3	G	A
.	.	.	.	.	.	.	.	.	.	.	.	SOM	GT:/	0:25	0/1:60,6:37	G	A
.	.	.	.	.	.	.	.	3.2	.	.	.	SOM	GT:/	0:79	0/1:121,35	C	A
4E-04	.	0	0	.	.	.	.	.	.	0	0	SOM	GT:/	0:55	0/1:86,13:3	G	A
.	.	##	0	.	.	.	.	2.6	1.42	.	.	SOM	GT:/	0:12	0/1:200,54	G	A
.	.	.	.	.	.	.	.	.	.	.	.	SOM	GT:/	0:26	0/1:79,15:3	A	G
.	.	.	.	.	.	.	.	.	.	.	.	SOM	GT:/	0:40	0/1:51,11:3	C	T
.	.	.	.	.	.	.	.	.	3.01	.	.	SOM	GT:/	0:25	0/1:45,9:37	A	G
.	.	.	.	0.0	0.931	0.999	1,D	3.7	4.27	.	.	SOM	GT:/	0:74	0/1:110,20	G	A
.	.	.	.	.	.	.	.	.	.	.	.	SOM	GT:/	0:58	0/1:98,17:3	C	A
.	.	.	.	0.0	0.932	0.998	1,N	.	1.53	.	.	SOM	GT:/	0:35	0/1:53,8:33	G	A
.	.	.	.	.	.	.	.	.	.	.	.	SOM	GT:/	0:94	0/1:153,24	C	T
8E-04	##	##	0	.	.	.	.	.	.	.	.	SOM	GT:/	0:31	0/1:51,6:33	C	T
.	.	.	.	.	.	.	.	4.6	.	.	.	SOM	GT:/	0:56	0/1:54,12:3	G	A
.	.	.	.	.	.	.	.	.	.	.	.	SOM	GT:/	0:10	0/1:14,4:37	C	T
0.048	0	0	0	.	.	.	.	.	.	0	0	DB;S	GT:/	0:20	0/1:22,4:37	A	T
.	.	##	0	0.1	0.021	0.102	1.00	3.9	3.82	.	.	SOM	GT:/	0:31	0/1:60,8:37	C	T
.	.	.	.	.	.	.	.	.	.	.	.	SOM	GT:/	0:46	0/1:60,9:34	G	A
.	.	.	.	.	.	.	.	.	.	.	.	SOM	GT:/	0:42	0/1:48,8:37	C	T
.	.	##	0	.	.	.	.	3.2	.	0	.	SOM	GT:/	0:62	0/1:94,12:3	C	T
.	.	.	.	.	.	.	.	.	.	.	.	SOM	GT:/	0:9,(	0/1:16,4:37	C	A
.	.	.	.	.	.	.	.	6.1	2.79	.	.	SOM	GT:/	0:22	0/1:54,8:37	G	A
.	.	.	.	.	.	.	.	.	.	.	.	SOM	GT:/	0:98	0/1:136,21	G	A
.	.	.	.	.	.	.	.	.	.	.	.	SOM	GT:/	0:9,(	0/1:14,4:37	G	C
2E-04	.	##	0	0.0	0.999	1.0,D	1.00	6.1	4.63	.	.	SOM	GT:/	0:38	0/1:51,4:37	C	T
.	.	.	.	.	.	.	.	.	.	.	.	SOM	GT:/	0:9,(	0/1:12,6:37	G	A
.	.	.	.	.	.	.	.	.	1.54	.	.	SOM	GT:/	0:34	0/1:49,4:37	G	T
.	.	##	0	.	.	.	.	.	.	.	.	SOM	GT:/	0:47	0/1:100,23	C	T

2E-04	.	.	.	.	.	.	.	.	.	.		0	SON	GT:/	0:27	0/1:50,11:3	C	T
.	.	.	.	.	.	.	.	5.6	.	.		.	SON	GT:/	0:82	0/1:125,19	T	G
.	.	.	.	0.2	0.455	0.836	1.00	5.7	4.22	.		.	SON	GT:/	0:10	0/1:166,23	C	T
.	.	.	.	.	.	.	.	.	.	.		.	SON	GT:/	0:59	0/1:83,15:3	C	T
.	.	.	.	.	.	.	.	.	.	.		.	SON	GT:/	0:60	0/1:84,18:3	T	C
.	##	##	0	0.8	0.024	0.083	1,D	5.1	.	.		.	SON	GT:/	0:63	0/1:66,16:3	G	A
.	.	.	.	.	.	.	.	.	.	.		.	SON	GT:/	0:39	0/1:63,9:34	T	A
.	.	##	0	.	.	.	.	.	1.48	.		.	SON	GT:/	0:54	0/1:65,16:3	C	T
.	.	##	0	.	.	.	.	2.5	.	.		.	SON	GT:/	0:33	0/1:51,11:3	G	A
.	.	.	.	.	.	.	.	.	.	.		.	SON	GT:/	0:24	0/1:57,6:37	C	T
.	.	.	.	.	.	.	.	.	.	.	0	0	SON	GT:/	0:10	0/1:19,4:37	C	T
.	.	.	.	.	.	.	.	3.1	.	.		.	SON	GT:/	0:43	0/1:58,12:3	C	T
.	.	.	.	.	.	.	.	.	.	.		.	SON	GT:/	0:51	0/1:76,24:3	C	T
.	.	.	.	.	.	.	.	.	.	.	0	.	SON	GT:/	0:9,(	0/1:11,7:37	G	A
.	.	.	.	.	.	.	.	.	.	.	0	0	DB;S	GT:/	0:75	0/1:132,7:3	A	G
.	.	.	.	.	.	.	.	.	.	.		.	SON	GT:/	0:8,(	0/1:14,4:30	G	T
.	.	##	0	0.0	0.931	1.0,D	0.79	3.3	1.95		0	0	SON	GT:/	0:80	0/1:175,35	G	A
.	.	.	.	.	.	.	.	.	.	.		.	SON	GT:/	0:79	0/1:179,6:3	C	A
.	.	.	.	.	.	.	.	.	.	.		.	SON	GT:/	0:45	0/1:66,16:3	C	T
.	.	.	.	.	.	.	.	.	.	.	0	.	SON	GT:/	0:43	0/1:67,5:37	C	T
.	.	.	.	.	.	.	.	.	.	.		.	SON	GT:/	0:70	0/1:90,18:3	G	A
2E-04	.	.	.	.	.	.	.	.	.	.		.	SON	GT:/	0:19	0/1:23,5:37	G	A
.	.	.	.	.	.	.	.	.	.	.		.	SON	GT:/	0:68	0/1:85,30:3	T	C
.	.	.	.	.	.	.	.	.	.	.		.	SON	GT:/	0:16	0/1:14,5:37	G	A
.	.	.	.	.	.	.	.	.	.	.		.	SON	GT:/	0:10	0/1:176,29	G	A
.	.	.	.	.	.	.	1,D	.	3.49	.		.	SON	GT:/	0:9,(	0/1:25,8:35	C	T
4E-04	0	0	0	.	.	.	.	.	.	.		.	DB;S	GT:/	0:14	0/1:283,24	C	T
.	.	.	.	.	.	.	.	.	.	.		.	SON	GT:/	0:14	0/1:24,3:37	G	A
.	.	.	.	.	.	.	.	.	1.69	.		.	SON	GT:/	0:72	0/1:111,17	G	A
.	.	##	0	0.2	0.002	0.004	1,D	4.1	1.85	.		.	SON	GT:/	0:35	0/1:62,7:37	G	A
0.002	.	0	0	.	.	.	.	.	.		0	0	SON	GT:/	0:36	0/1:70,10:3	C	T
.	.	##	0	.	.	.	.	.	.	.		.	SON	GT:/	0:12	0/1:211,38	C	T
.	.	.	.	0.0	0.997	1.0,D	0.99	6.2	4.57	.		.	SON	GT:/	0:62	0/1:111,19	G	A
.	.	.	.	0.0	0.565	0.959	0.68	3.7	1.42	.		.	SON	GT:/	0:60	0/1:51,5:37	C	A
.	.	.	.	0.0	1.0,D	1.0,D	1,D	5.4	4.96	.		.	SON	GT:/	0:59	0/1:88,12:3	G	A
.	.	.	.	.	.	.	.	.	.	.		.	SON	GT:/	0:31	0/1:43,6:32	A	T
.	.	.	.	0.1	0.988	0.994	0.87	5.6	3.33	.		.	SON	GT:/	0:40	0/1:55,16:3	T	C
0.003	.	.	.	.	.	.	.	.	.		0	0	SON	GT:/	0:8,(	0/1:6,7:37	A	G
2E-04	.	##	0	.	.	.	.	2.2	2.78	.		.	SON	GT:/	0:20	0/1:27,9:37	C	T
.	.	.	.	.	.	.	.	.	.	.		.	SON	GT:/	0:55	0/1:85,15:3	C	T
.	.	.	.	.	.	.	.	.	1.70	.		.	SON	GT:/	0:27	0/1:49,6:37	G	T
.	.	##	0	0.0	0.988	0.998	1,D	5.9	5.07	.		.	SON	GT:/	0:67	0/1:120,26	G	A
.	.	.	.	0.2	0.159	0.475	0.99	4.1	2.31	.		.	SON	GT:/	0:56	0/1:64,12:3	A	T
.	.	.	.	.	.	.	.	.	.	.		.	DB;S	GT:/	0:37	0/1:53,12:3	C	T
.	.	.	.	.	.	.	.	.	.	.	0.1	0.2	SON	GT:/	0:20	0/1:26,4:37	C	T
.	.	.	.	.	.	.	.	.	.	.	0	.	SON	GT:/	0:8,(	0/1:6,4:37	C	T
.	.	.	.	0.1	0.008	0.127	1,N	.	1.50	.		.	SON	GT:/	0:14	0/1:224,37	C	T
.	.	.	.	.	.	.	.	.	1.36	.		.	SON	GT:/	0:13	0/1:23,4:37	C	T
.	.	.	.	.	.	.	.	.	.	.		.	SON	GT:/	0:69	0/1:88,14:3	G	C
.	.	.	.	.	.	.	.	.	.	.		.	SON	GT:/	0:9,(	0/1:14,4:37	C	A
.	.	.	.	.	.	.	.	.	.	.		.	SON	GT:/	0:18	0/1:41,8:37	G	A
.	.	.	.	.	.	.	1,A	.	5.21	.		.	SON	GT:/	0:17	0/1:254,36	C	T
.	.	.	.	.	.	.	.	3.8	.	.		.	SON	GT:/	0:30	0/1:40,4:37	A	T
.	.	##	0	.	.	.	.	.	.	.		.	SON	GT:/	0:27	0/1:32,4:37	C	T

.	.	.	.	.	.	.	1,D	5.6	4.33	.	.	SOM	GT:/	0:44	0/1:79,16:3	C	T
0.126	.	0	0	.	.	.	.	.	.	0	0	DB;S	GT:/	0:20	0/1:47,15:3	T	A
.	.	.	.	.	.	.	.	.	.	.	.	SOM	GT:/	0:14	0/1:12,3:37	G	T
.	##	0	0	0.0	1.0,D	1.0,D	1.00	3.9	3.90	.	.	SOM	GT:/	0:49	0/1:74,10:3	C	T
.	.	.	.	.	.	.	.	4.7	1.51	.	.	SOM	GT:/	0:73	0/1:131,18	G	T
.	.	##	0	0.0	0.998	1.0,D	1.00	5.7	5.10	.	.	SOM	GT:/	0:53	0/1:87,13:3	C	T
.	.	.	.	.	.	.	1,A	.	8.91	.	.	SOM	GT:/	0:34	0/1:74,8:35	G	T
.	.	.	.	.	.	.	.	.	1.24	.	.	SOM	GT:/	0:8,(	0/1:14,4:37	A	G
.	.	.	.	.	.	.	.	.	.	.	.	SOM	GT:/	0:30	0/1:35,4:37	T	C
.	.	.	.	0.0:	0.999	1.0,D	1,D	5.6	3.37	.	.	SOM	GT:/	0:12	0/1:231,37	G	T
.	.	.	.	.	.	.	.	3.9	3.01	.	.	SOM	GT:/	0:24	0/1:38,4:37	C	A
.	.	.	.	0.0	1.0,D	1.0,D	1,D	5.5	5.97	.	.	SOM	GT:/	0:86	0/1:109,16	G	A
.	##	##	0	0.0	0.999	1.0,D	1,D	5.4	5.22	.	.	SOM	GT:/	0:96	0/1:131,18	C	T
.	.	.	.	.	.	.	.	.	.	.	.	SOM	GT:/	0:84	0/1:180,41	C	T
.	.	.	.	.	.	.	.	.	.	.	.	SOM	GT:/	0:15	0/1:19,3:37	A	G
.	.	.	.	.	.	.	.	.	.	.	.	SOM	GT:/	0:44	0/1:46,12:3	A	G
.	.	.	.	.	.	.	.	.	.	.	.	DB;S	GT:/	0:28	0/1:21,5:37	A	G
.	.	.	.	.	.	.	1,A	5.8	7.24	.	.	SOM	GT:/	0:55	0/1:55,12:3	G	T
.	.	.	.	0.0	0.327	0.627	1,D	3.9	2.49	.	.	SOM	GT:/	0:34	0/1:67,17:3	C	A
.	.	##	0	0.2	0.014	0.006	0.64	4.2	1.72	.	.	SOM	GT:/	0:67	0/1:118,23	T	C
.	.	.	.	0.0	0.999	1.0,D	1,D	6	4.97	.	.	SOM	GT:/	0:48	0/1:54,8:37	G	T
.	.	##	.	0.1	0.574	0.819	1.00	5.1	4.00	.	.	SOM	GT:/	0:33	0/1:80,14:3	A	G
.	.	.	.	.	.	.	.	.	.	0	0	SOM	GT:/	0:10	0/1:21,4:37	C	A
.	.	.	.	.	.	.	.	.	.	.	0	SOM	GT:/	0:99	0/1:155,29	G	A
.	.	.	.	.	.	.	.	3.2	.	.	.	SOM	GT:/	0:47	0/1:115,27	G	T
.	.	.	.	.	.	.	.	.	1.27	.	.	SOM	GT:/	0:95	0/1:133,17	G	A
.	.	.	.	.	.	.	.	3.1	.	.	.	SOM	GT:/	0:34	0/1:66,10:3	A	G
.	.	.	.	.	.	.	.	.	.	.	.	SOM	GT:/	0:36	0/1:57,10:3	C	A
.	.	.	.	.	.	.	.	.	.	0	0	SOM	GT:/	0:9,(	0/1:7,3:37:	T	C
.	.	0	0	.	.	.	.	.	.	0.1	0	SOM	GT:/	0:15	0/1:26,5:37	G	A
.	.	.	.	.	.	.	.	.	.	.	.	SOM	GT:/	0:12	0/1:27,6:37	G	A
.	.	.	.	0.0	0.641	0.909	1,D	3.7	4.04	.	.	SOM	GT:/	0:53	0/1:76,21:3	A	G
.	.	.	.	.	.	.	.	.	.	.	.	SOM	GT:/	0:27	0/1:27,6:37	C	T
.	.	.	.	.	.	.	.	.	.	.	.	SOM	GT:/	0:58	0/1:67,12:3	G	A
.	.	##	0	.	.	.	.	.	.	.	.	SOM	GT:/	0:58	0/1:107,21	G	A
.	.	.	.	.	.	.	.	.	.	.	.	SOM	GT:/	0:32	0/1:47,12:3	A	G
.	.	.	.	0.0	0.596	0.938	1,N	2.5	.	.	.	SOM	GT:/	0:83	0/1:132,22	G	A
.	.	.	.	.	.	.	.	.	.	0	0.1	DB;S	GT:/	0:45	0/1:71,4:37	G	C
.	.	.	.	.	.	.	.	.	.	.	.	SOM	GT:/	0:45	0/1:55,7:37	C	T
.	.	.	.	0.5	0.0,B	0.0,B	1,N	.	.	.	.	SOM	GT:/	0:38	0/1:751,26	C	A
.	.	.	.	.	.	.	.	.	.	0	.	SOM	GT:/	0:39	0/1:44,3:37	G	A
.	.	##	0	0.1	0.33,I	0.728	1,N	4.9	3.90	.	.	SOM	GT:/	0:57	0/1:71,20:3	G	A
.	.	.	.	0.0	0.998	1.0,D	1,D	5	3.70	.	.	SOM	GT:/	0:11	0/1:26,7:37	G	T
.	.	.	.	0.0	0.152	0.569	1.00	5.4	2.84	.	.	SOM	GT:/	0:68	0/1:97,26:3	A	G
2E-04	0	0	0	.	.	.	.	.	.	.	.	SOM	GT:/	0:70	0/1:119,25	G	A
0.261	0	0	1	.	.	.	.	.	.	0.5	0.5	DB;S	GT:/	0:52	0/1:74,16:3	G	A
.	.	.	.	.	.	.	.	.	.	.	.	SOM	GT:/	0:10	0/1:13,4:37	G	C
.	.	0	0	.	.	.	.	.	.	.	.	SOM	GT:/	0:83	0/1:163,26	G	A
.	.	.	.	.	.	.	.	.	.	.	.	SOM	GT:/	0:40	0/1:72,19:3	G	A
2E-04	.	##	0	.	.	.	.	.	.	.	.	SOM	GT:/	0:73	0/1:115,17	G	A
.	.	.	.	.	.	.	.	.	.	.	.	SOM	GT:/	0:35	0/1:83,4:37	G	C
.	.	.	.	.	.	.	.	.	.	.	.	SOM	GT:/	0:36	0/1:87,4:37	G	T
.	.	.	.	.	.	.	.	.	.	.	.	DB;S	GT:/	0:35	0/1:82,6:37	C	T
.	.	.	.	.	.	.	.	5.5	2.43	.	.	SOM	GT:/	0:49	0/1:77,5:37	C	A

.	.	.	.	.	.	.	.	.	.	.	.	SOM	GT:/	0:18	0/1:23,3:37	T		A
.	.	.	.	.	.	.	.	.	.	.	.	SOM	GT:/	0:37	0/1:41,3:37	A		G
.	.	.	.	.	.	.	.	.	.	.	.	SOM	GT:/	0:46	0/1:65,10:3	G		T
.	.	.	.	.	.	.	.	.	.	.	.	SOM	GT:/	0:8,(	0/1:17,3:37	T		C
.	.	.	.	.	.	.	.	.	.	0	.	SOM	GT:/	0:8,(	0/1:22,4:37	T		C
.	.	.	.	.	.	.	.	.	.	.	.	SOM	GT:/	0:8,(	0/1:22,4:37	A		G
.	##	##	.	.	0.267	0.507	1.00	4.8	1.30	.	.	SOM	GT:/	0:10	0/1:220,37	C		T
.	.	.	.	0.4	0.14,I	0.931	1,D	4.3	1.68	.	.	SOM	GT:/	0:45	0/1:81,14:3	C		T
2E-04	.	.	.	.	.	.	.	.	.	.	.	SOM	GT:/	0:13	0/1:209,40	C		T
.	.	.	.	.	.	.	1,D	5.3	2.11	.	.	SOM	GT:/	0:39	0/1:61,7:37	C		A
.	.	.	.	.	.	.	.	.	2.24	.	.	SOM	GT:/	0:40	0/1:63,8:37	G		A
6E-04	.	.	.	.	.	.	.	2.2	.	0	0	SOM	GT:/	0:20	0/1:29,7:37	G		A
.	.	.	.	0.4	0.001	0.0,B	1,N	.	1.81	.	.	SOM	GT:/	0:12	0/1:192,41	A		G
0.001	0	0	0	0.0	0.436	0.988	1,N	.	2.82	.	.	SOM	GT:/	0:11	0/1:255,13	C		T
.	.	.	.	.	.	.	.	5.2	.	.	.	SOM	GT:/	0:53	0/1:70,7:35	C		T
.	.	.	.	.	.	.	.	.	.	.	.	SOM	GT:/	0:8,(	0/1:15,5:37	A		G
.	.	.	.	.	.	.	.	.	.	0	.	SOM	GT:/	0:24	0/1:41,5:37	C		G
.	.	.	.	.	.	.	.	2.9	1.25	.	.	SOM	GT:/	0:34	0/1:55,9:37	C		A
.	.	.	.	.	.	.	.	.	.	.	.	SOM	GT:/	0:12	0/1:12,3:37	A		G
.	.	.	.	.	.	.	.	.	3.64	.	.	SOM	GT:/	0:19	0/1:29,5:37	C		T
.	.	.	.	.	.	.	.	3.8	.	.	.	SOM	GT:/	0:47	0/1:70,14:3	G		A
.	.	.	.	.	.	.	.	3.8	.	.	.	SOM	GT:/	0:15	0/1:13,5:37	G		A
.	##	##	0	.	.	.	.	.	.	.	.	SOM	GT:/	0:20	0/1:28,8:30	C		T
.	.	.	.	.	.	.	.	.	.	0	.	SOM	GT:/	0:23	0/1:32,7:37	C		T
.	.	.	.	0.5	0.013	0.04,I	1,N	2.1	.	.	.	SOM	GT:/	0:10	0/1:154,38	G		T
.	.	.	.	0.6	0.002	0.0,B	1,N	.	.	.	.	SOM	GT:/	0:53	0/1:92,18:3	G		A
.	.	##	0	0.0	0.136	0.588	0.87	5.8	2.16	0	.	SOM	GT:/	0:32	0/1:53,10:3	C		T
.	.	.	.	.	.	.	.	.	.	.	.	SOM	GT:/	0:25	0/1:43,5:37	C		T
.	.	.	.	0.6	0.311	0.745	1,D	3.6	2.38	.	.	DB;S	GT:/	0:29	0/1:48,8:37	A		C
8E-04	.	.	.	.	.	.	.	.	.	.	.	DB;S	GT:/	0:30	0/1:21,4:37	C		T
.	.	.	.	.	.	.	.	.	.	.	.	SOM	GT:/	0:16	0/1:36,10:3	G		T
.	.	.	.	.	.	.	.	.	.	.	.	SOM	GT:/	0:96	0/1:149,25	A		G
.	.	.	.	0.0	0.601	0.832	1,D	5.9	5.19	.	.	SOM	GT:/	0:43	0/1:69,8:37	C		T
.	.	.	.	.	.	.	.	.	.	.	.	SOM	GT:/	0:13	0/1:12,4:37	G		T
.	.	.	.	0.0	0.972	1.0,D	0.61	4.2	4.51	.	.	SOM	GT:/	0:13	0/1:19,7:31	G		A
.	.	.	.	.	.	.	.	4.9	.	.	.	SOM	GT:/	0:23	0/1:30,11:3	A		T
.	.	##	0	.	.	.	.	.	1.26	.	.	SOM	GT:/	0:57	0/1:84,17:3	C		T
.	.	.	.	.	.	.	.	.	2.16	.	.	SOM	GT:/	0:16	0/1:21,4:37	C		T
.	.	##	0	.	.	.	.	.	.	.	.	SOM	GT:/	0:21	0/1:28,8:37	T		C
.	.	##	0	0.0	0.99,I	1.0,D	0.97	5.4	3.82	0	.	SOM	GT:/	0:51	0/1:66,15:3	C		T
.	.	.	.	0.0	0.994	0.999	1.00	4.7	3.65	.	.	SOM	GT:/	0:36	0/1:69,17:3	A		G
.	.	.	.	1.0	0.0,B	0.0,B	1,N	.	.	.	.	DB;S	GT:/	0:10	0/1:204,38	A		G
.	.	##	0	0.1	0.001	0.003	1.00	3.6	1.87	.	.	SOM	GT:/	0:10	0/1:205,41	G		A
.	.	.	.	0.4	0.035	0.044	0.99	4.2	1.79	.	.	SOM	GT:/	0:70	0/1:103,20	T		C
.	.	.	.	.	.	.	.	.	.	.	.	SOM	GT:/	0:11	0/1:184,34	G		A
.	.	.	.	.	.	.	.	.	.	.	.	SOM	GT:/	0:16	0/1:262,49	C		T
.	.	##	0	.	.	.	.	.	.	0	.	SOM	GT:/	0:55	0/1:84,21:3	G		A
.	.	.	.	0.0	1.0,D	1.0,D	0.81	4.2	3.64	.	.	SOM	GT:/	0:14	0/1:241,42	G		A
.	.	.	.	0.0	0.941	0.994	1.00	4.2	2.91	.	.	SOM	GT:/	0:12	0/1:195,40	C		T
.	.	.	.	.	.	.	.	.	.	.	.	SOM	GT:/	0:65	0/1:111,27	C		T
.	.	.	.	0.0	0.272	0.479	1,N	3.4	2.43	.	.	SOM	GT:/	0:76	0/1:141,32	C		T
0.002	.	##	0	.	.	.	.	.	.	0	.	SOM	GT:/	0:89	0/1:153,7:3	C		T
.	##	##	0	.	.	.	.	.	.	.	.	SOM	GT:/	0:20	0/1:353,57	C		T
.	.	##	0	0.0	0.875	0.998	1,D	4	3.02	.	.	SOM	GT:/	0:10	0/1:160,30	G		A

8E-04	.	##	0	0.0(	0.001	0.003	1,N	.	.	0	0	SON	GT:/	0:70	0/1:140,30	G	A
.	.	.	.	.	.	.	.	.	.	0	.	SON	GT:/	0:19	0/1:43,10:3	A	G
.	.	.	.	.	.	.	.	2.7	.	.	.	SON	GT:/	0:24	0/1:25,4:37	T	C
.	.	.	.	.	.	.	.	.	.	.	.	SON	GT:/	0:23	0/1:55,15:3	A	T
.	.	.	.	0.0:	0.647	0.978	0.86	5.5	3.81	.	.	SON	GT:/	0:11	0/1:214,52	G	A
.	.	.	.	0.0.	0.879	0.998	1,D	5.4	3.63	.	.	SON	GT:/	0:63	0/1:100,18	C	A
4E-04	.	.	.	.	.	.	.	.	.	0	0	SON	GT:/	0:16	0/1:28,6:37	G	A
2E-04	.	0	0	0.1:	0.514	0.96,I	1.00	5.7	3.45	.	.	SON	GT:/	0:86	0/1:146,25	C	T
.	.	.	.	0.0:	0.537	0.984	0.99	5.2	3.64	.	.	SON	GT:/	0:29	0/1:75,12:3	G	A
.	.	.	.	0.0.	0.986	0.997	1.00	5.8	4.50	.	.	SON	GT:/	0:91	0/1:142,19	C	A
.	0	##	0	0.1'	0.281	0.981	0.99	4.3	.	.	.	SON	GT:/	0:72	0/1:133,19	C	T
.	.	##	0	0.0.	0.999	1.0,D	1,D	5.6	3.40	0	.	SON	GT:/	0:48	0/1:65,5:34	C	T
.	.	.	.	.	.	.	.	5.1	.	.	.	SON	GT:/	0:49	0/1:94,19:3	G	T
.	.	.	.	0.0(	0.075	0.181	1,D	5.8	3.16	.	.	SON	GT:/	0:14	0/1:259,53	T	C
2E-04	.	.	.	.	.	.	.	.	.	0	.	SON	GT:/	0:14	0/1:11,3:37	C	T
.	.	.	.	.	.	.	.	.	.	.	.	SON	GT:/	0:28	0/1:50,7:37	C	A
.	.	.	.	.	.	.	.	5.1	3.89	.	.	SON	GT:/	0:27	0/1:47,4:37	T	C
.	.	.	.	.	.	.	.	.	.	.	.	SON	GT:/	0:12	0/1:213,42	G	A
0.003	.	0	0	.	.	.	.	.	.	0	.	DB;S	GT:/	0:88	0/1:180,36	G	A
4E-04	.	##	0	0.0(	0.996	1.0,D	1,D	3.6	3.58	.	.	SON	GT:/	0:22	0/1:367,69	C	T
.	.	.	.	.	.	.	.	3.2	3.75	.	.	SON	GT:/	0:47	0/1:75,9:37	C	A
.	.	.	.	.	.	.	.	.	.	.	.	SON	GT:/	0:33	0/1:36,13:3	C	T
.	.	.	.	.	.	.	.	.	.	.	.	SON	GT:/	0:21	0/1:38,5:37	G	A
.	.	.	.	.	.	.	.	.	.	0	0	SON	GT:/	0:21	0/1:40,13:3	G	A
.	.	.	.	.	.	.	.	3.7	3.15	.	.	SON	GT:/	0:25	0/1:44,7:37	G	A
.	.	.	.	.	.	.	.	.	.	.	.	SON	GT:/	0:8,(	0/1:9,4:37:	G	T
.	.	##	0	0.1.	0.017	0.14,I	0.73	5.2	1.40	0	.	SON	GT:/	0:83	0/1:120,21	G	A
.	.	.	.	.	.	.	.	.	.	.	.	SON	GT:/	0:15	0/1:19,6:37	A	T
0.002	.	.	.	.	.	.	.	.	.	.	0	SON	GT:/	0:33	0/1:54,7:37	G	A
.	.	.	.	.	.	.	.	.	.	.	.	SON	GT:/	0:37	0/1:49,8:37	A	G
.	.	##	0	.	.	.	.	.	.	.	.	SON	GT:/	0:57	0/1:80,13:3	C	T
.	.	.	.	.	.	.	.	.	.	.	.	SON	GT:/	0:16	0/1:12,3:37	T	A
.	##	##	0	.	.	.	.	.	.	0	.	DB;S	GT:/	0:58	0/1:83,7:37	G	A
.	.	##	0	0.0:	0.951	1.0,D	1,D	5.9	6.16	.	.	SON	GT:/	0:90	0/1:144,22	G	A
.	.	##	0	.	.	.	.	.	.	.	.	SON	GT:/	0:43	0/1:77,16:3	C	T
.	.	.	.	.	.	.	.	.	.	.	.	SON	GT:/	0:28	0/1:29,7:37	T	G
.	.	.	.	.	.	.	.	.	.	.	.	SON	GT:/	0:17	0/1:50,6:37	C	T
.	.	.	.	.	.	.	.	.	.	.	.	SON	GT:/	0:12	0/1:23,3:37	G	A
.	.	.	.	.	.	.	.	.	.	.	.	SON	GT:/	0:42	0/1:65,14:3	T	C
.	.	.	.	.	.	.	.	.	.	.	.	SON	GT:/	0:19	0/1:30,5:34	G	A
.	.	.	.	.	.	.	.	.	.	.	.	SON	GT:/	0:11	0/1:17,3:37	C	T
.	.	.	.	.	.	.	.	.	2.64	.	.	SON	GT:/	0:18	0/1:20,3:33	G	A
.	.	.	.	.	.	.	.	.	.	.	.	SON	GT:/	0:20	0/1:50,5:37	T	C
.	.	0	0	.	.	.	.	.	.	.	.	DB;S	GT:/	0:20	0/1:34,3:37	G	A
.	.	.	.	.	.	.	.	.	.	.	.	SON	GT:/	0:52	0/1:85,7:35	A	G
.	.	.	.	.	.	.	.	.	.	.	.	SON	GT:/	0:57	0/1:91,15:3	C	T
.	.	.	.	.	.	.	.	.	.	.	.	SON	GT:/	0:57	0/1:85,17:3	G	T
.	.	.	.	.	.	.	.	.	.	.	.	SON	GT:/	0:59	0/1:98,10:3	G	A
.	.	.	.	.	.	.	.	.	.	.	.	SON	GT:/	0:29	0/1:62,11:3	A	G
.	.	##	0	.	.	.	.	.	.	.	0	SON	GT:/	0:81	0/1:117,22	C	T
.	.	.	.	.	.	.	.	.	.	.	.	SON	GT:/	0:10	0/1:180,28	G	A
.	.	.	.	.	.	.	.	.	.	.	.	SON	GT:/	0:15	0/1:15,3:37	T	C
.	.	.	.	0.0.	1.0,D	1.0,D	1,D	4.2	4.48	.	.	DB;S	GT:/	0:63	0/1:97,19:3	C	T
.	.	0	0	.	.	.	.	.	.	0	.	SON	GT:/	0:99	0/1:169,26	C	T

.	.	.	.	0.0	1.0,D	1.0,D	1,D	5.7	5.41	.	.	SON	GT:/	0:64	0/1:116,13	G	A
.	.	.	.	0.0	0.973	0.996	1,D	3.6	2.38	.	.	SON	GT:/	0:68	0/1:129,23	G	T
.	.	##	0	.	.	.	.	.	1.66	.	.	SON	GT:/	0:51	0/1:76,11:3	C	T
.	.	.	.	.	.	.	.	3.3	.	.	.	SON	GT:/	0:12	0/1:16,4:37	C	T
.	.	##	0	.	.	.	.	.	.	0	.	SON	GT:/	0:11	0/1:159,30	C	T
.	.	.	.	.	.	.	.	.	1.32	.	.	SON	GT:/	0:40	0/1:48,8:37	G	A
.	.	.	.	.	.	.	.	2.9	2.80	.	.	SON	GT:/	0:28	0/1:37,10:1	G	C
.	.	.	.	0.0	0.124	0.865	1,D	4	2.29	.	.	SON	GT:/	0:60	0/1:93,23:3	G	A
.	.	.	.	.	.	.	.	.	.	.	.	SON	GT:/	0:84	0/1:145,25	A	G
.	.	.	.	.	.	.	.	2.1	.	.	.	SON	GT:/	0:26	0/1:40,4:37	C	T
.	.	##	0	0.9	0.006	0.014	1,N	.	.	.	.	SON	GT:/	0:83	0/1:132,36	G	A
.	.	.	.	0.0	0.687	0.991	0.70	4.9	1.80	.	.	SON	GT:/	0:96	0/1:146,30	C	A
0.005	.	0	0	.	.	.	.	2.2	.	0	0	DB;S	GT:/	0:71	0/1:132,10	C	A
.	.	##	0	.	.	.	.	.	1.37	0	.	SON	GT:/	0:60	0/1:86,16:3	C	T
.	.	.	.	.	.	.	.	.	.	.	.	SON	GT:/	0:84	0/1:152,29	G	T
2E-04	0	##	0	.	.	.	.	.	.	0	.	SON	GT:/	0:67	0/1:123,24	C	T
.	.	.	.	0.0	1.0,D	1.0,D	1,D	5.9	4.56	.	.	SON	GT:/	0:73	0/1:107,27	C	T
.	.	.	.	.	.	.	.	.	.	.	.	SON	GT:/	0:23	0/1:28,5:37	G	T
.	.	.	.	.	.	.	.	.	.	.	.	SON	GT:/	0:60	0/1:111,23	A	G
4E-04	0	0	0	.	.	.	.	.	.	.	.	SON	GT:/	0:40	0/1:66,11:3	G	A
.	0	0	0	0.0	0.451	0.99,I	1,N	2.6	2.98	.	.	SON	GT:/	0:10	0/1:175,38	G	A
.	.	.	.	.	.	.	.	.	.	.	.	SON	GT:/	0:39	0/1:68,13:3	G	A
.	.	.	.	0.0	0.997	1.0,D	.	6	3.85	.	.	SON	GT:/	0:73	0/1:118,19	C	A
.	.	.	.	.	.	.	.	.	.	.	.	SON	GT:/	0:10	0/1:200,24	C	G
.	.	.	.	.	.	.	.	3.4	.	.	.	SON	GT:/	0:34	0/1:39,7:37	A	G
0.001	.	0	0	.	.	.	.	.	.	0	0	SON	GT:/	0:13	0/1:19,7:31	A	G
.	.	.	.	.	.	.	.	.	.	0	.	SON	GT:/	0:24	0/1:32,10:1	A	T
.	.	.	.	.	.	.	.	2.9	.	.	.	SON	GT:/	0:45	0/1:58,11:3	C	A
.	.	##	0	0.0	0.001	0.007	0.65	4.8	2.89	.	.	SON	GT:/	0:66	0/1:76,13:3	C	T
2E-04	0	##	0	0.0	1.0,D	1.0,D	1,D	.	3.70	.	.	SON	GT:/	0:82	0/1:116,30	C	T
.	.	.	.	.	.	.	.	.	.	.	.	SON	GT:/	0:21	0/1:38,8:37	T	C
.	.	##	0	.	.	.	.	.	1.51	.	.	SON	GT:/	0:32	0/1:52,5:37	G	A
.	.	.	.	.	.	.	.	.	.	.	.	SON	GT:/	0:12	0/1:9,3:37:	G	A
.	.	.	.	.	.	.	.	2.4	1.98	.	.	SON	GT:/	0:8,(	0/1:13,4:37	C	T
.	.	.	.	0.0	0.997	0.999	1,D	4.5	3.86	.	.	SON	GT:/	0:16	0/1:242,48	T	C
2E-04	.	.	.	.	.	.	.	.	.	0	.	SON	GT:/	0:65	0/1:103,20	A	C
.	.	.	.	.	.	.	.	5.9	3.72	.	.	SON	GT:/	0:41	0/1:47,9:37	T	C
.	.	.	.	.	.	.	.	.	.	.	.	SON	GT:/	0:28	0/1:43,6:37	T	C
.	.	.	.	.	.	.	.	2.5	2.14	.	.	SON	GT:/	0:14	0/1:22,5:37	T	C
.	.	.	.	.	.	.	.	.	.	.	.	SON	GT:/	0:35	0/1:31,3:37	G	T
.	.	.	.	0.0	0.972	1.0,D	0.99	5.8	3.48	.	.	SON	GT:/	0:70	0/1:83,13:3	G	T
.	.	.	.	.	.	.	.	.	.	.	.	SON	GT:/	0:21	0/1:30,3:33	C	T
.	.	.	.	.	.	.	.	.	.	.	.	SON	GT:/	0:26	0/1:41,6:37	G	A
.	.	.	.	.	.	.	.	.	.	.	.	SON	GT:/	0:23	0/1:29,10:3	T	C
2E-04	0	##	0	.	.	.	.	.	.	0	.	SON	GT:/	0:54	0/1:96,13:3	C	T
.	.	.	.	.	.	.	.	.	.	.	.	SON	GT:/	0:30	0/1:65,4:37	A	C
.	.	##	0	0.0	0.998	1.0,D	1,D	5.8	4.37	.	.	SON	GT:/	0:28	0/1:54,16:3	G	A
.	.	.	.	.	.	.	.	.	.	0	.	SON	GT:/	0:16	0/1:30,8:37	G	A
.	.	.	.	.	.	.	.	.	.	.	.	SON	GT:/	0:22	0/1:19,7:37	T	C
.	.	.	.	.	.	.	.	.	.	.	.	SON	GT:/	0:56	0/1:93,20:3	T	C
.	.	.	.	.	.	.	.	2.9	.	.	.	SON	GT:/	0:73	0/1:116,24	C	T
1E-03	0	0	0	.	.	.	.	2.9	2.40	0	0	SON	GT:/	0:22	0/1:36,10:3	G	A
.	##	##	0	0.0	0.362	0.935	1,D	5.2	4.87	.	.	SON	GT:/	0:22	0/1:384,83	G	A
.	.	.	.	0.3	0.002	0.01,I	1,N	.	.	.	.	SON	GT:/	0:96	0/1:119,20	A	G

.	.	##	0	0.0(	0.986	1.0,D	1,D	5.6	4.88	.	.	SON	GT:/	0:51	0/1:89,17:3	C	T
.	.	##	0	0.1:	0.003	0.004	0.98	3.1	2.10	.	.	SON	GT:/	0:34	0/1:48,10:3	A	G
.	.	.	.	0.0(	0.997	1.0,D	1,D	5.8	4.44	.	.	SON	GT:/	0:48	0/1:67,9:35	T	C
.	.	.	.	.	.	.	.	.	.	.	.	SON	GT:/	0:36	0/1:54,8:37	T	C
.	.	.	.	.	.	.	.	.	.	.	.	SON	GT:/	0:29	0/1:46,9:37	C	T
.	.	.	.	.	.	.	.	2.6	2.16	.	.	SON	GT:/	0:20	0/1:49,6:37	C	T
.	.	##	0	.	.	.	.	.	.	.	.	SON	GT:/	0:82	0/1:118,17	G	A
.	.	.	.	.	.	.	.	.	.	.	.	SON	GT:/	0:71	0/1:116,16	T	C
.	.	.	.	0.1:	0.115	0.525	1,D	4.6	1.33	.	.	SON	GT:/	0:24	0/1:39,11:3	T	C
.	.	.	.	0.0	0.998	1.0,D	1,D	4.6	5.20	.	.	SON	GT:/	0:53	0/1:68,5:34	G	A
.	.	.	.	.	.	.	1,A	4.9	8.66	.	.	SON	GT:/	0:74	0/1:120,18	C	T
.	.	.	.	.	.	.	.	.	.	.	.	SON	GT:/	0:10	0/1:173,43	G	A
.	.	.	.	0.0	1.0,D	1.0,D	1,D	5.8	4.61	.	.	SON	GT:/	0:50	0/1:86,11:3	C	T
.	.	.	.	.	.	.	.	.	.	0	.	SON	GT:/	0:92	0/1:148,21	G	A
.	.	.	.	.	.	.	.	.	.	.	.	SON	GT:/	0:70	0/1:142,15	C	T
.	.	.	.	.	.	.	.	.	.	.	.	SON	GT:/	0:69	0/1:46,7:37	C	T
.	.	.	.	.	.	.	.	2.9	1.44	.	.	SON	GT:/	0:11	0/1:15,8:37	C	T
.	.	.	.	0.3(	0.968	0.993	1,D	4.7	.	.	.	SON	GT:/	0:57	0/1:74,17:3	T	C
.	.	##	0	.	.	.	.	5.7	.	.	.	SON	GT:/	0:30	0/1:61,12:3	C	T
4E-04	##	##	0	0.0(	0.04,I	0.231	1,N	.	3.09	.	.	SON	GT:/	0:12	0/1:199,43	G	A
.	.	##	0	.	.	.	.	.	.	0	.	SON	GT:/	0:14	0/1:224,24	C	T
.	.	.	.	.	.	.	.	.	.	.	.	SON	GT:/	0:27	0/1:43,7:37	A	G
.	.	.	.	0.0(	0.959	0.996	1,D	5.1	3.79	.	.	SON	GT:/	0:10	0/1:155,27	T	A
.	.	.	.	.	.	.	.	.	.	.	.	SON	GT:/	0:11	0/1:16,3:37	C	T
.	.	.	.	.	.	.	.	3	.	.	.	SON	GT:/	0:97	0/1:184,36	G	T
.	.	.	.	0.3	0.039	0.041	0.96	4.4	1.40	.	.	SON	GT:/	0:54	0/1:103,19	C	T
.	.	.	.	0.0:	0.097	0.167	1.00	5	3.97	.	.	SON	GT:/	0:51	0/1:88,17:3	C	T
.	.	##	0	.	.	.	.	.	.	.	.	SON	GT:/	0:90	0/1:134,22	G	A
.	.	.	.	.	.	.	.	.	.	.	.	SON	GT:/	0:99	0/1:188,25	A	G
.	.	.	.	.	.	.	.	.	.	.	.	SON	GT:/	0:14	0/1:254,51	G	A
2E-04	.	##	0	.	.	.	.	.	.	.	.	SON	GT:/	0:18	0/1:298,64	G	A
.	.	.	.	.	.	.	.	.	.	.	.	SON	GT:/	0:52	0/1:100,5:3	C	T
.	##	.	.	.	.	.	.	.	.	.	.	SON	GT:/	0:68	0/1:89,18:3	C	T
.	.	.	.	.	.	.	.	2.5	.	.	.	SON	GT:/	0:9,(	0/1:7,7:37:	C	A
.	.	.	.	.	.	.	.	.	1.40	.	.	SON	GT:/	0:42	0/1:61,18:3	C	T
.	.	.	.	0.0	1.0,D	1.0,D	1,D	3.6	3.67	.	.	SON	GT:/	0:78	0/1:96,11:3	C	T
.	.	.	.	0.1	0.002	0.0,B	1.00	4.5	3.16	.	.	SON	GT:/	0:93	0/1:136,30	C	A
.	.	##	0	0.0	0.624	0.966	1.00	.	3.84	.	.	SON	GT:/	0:54	0/1:70,10:3	C	A
.	.	.	.	.	.	.	.	.	.	.	.	SON	GT:/	0:17	0/1:47,11:3	G	T
.	.	.	.	0.0	0.999	1.0,D	1.00	3.2	3.89	.	.	SON	GT:/	0:16	0/1:255,56	C	T
.	.	.	.	.	.	.	.	.	2.05	.	.	SON	GT:/	0:29	0/1:51,10:3	C	T
.	.	##	0	.	.	.	.	.	.	.	.	SON	GT:/	0:13	0/1:147,15	G	A
.	.	.	.	.	.	.	.	.	.	0	0	SON	GT:/	0:13	0/1:35,4:37	A	G
.	.	.	.	.	.	.	.	.	.	0	.	SON	GT:/	0:12	0/1:33,3:37	G	A
.	.	.	.	0.5	0.021	0.011	1,N	.	.	.	.	SON	GT:/	0:32	0/1:63,15:3	T	C
.	.	.	.	0.0	0.998	1.0,D	1,D	5.7	4.60	.	.	SON	GT:/	0:54	0/1:82,10:3	G	A
.	.	.	.	.	.	.	.	.	.	.	.	SON	GT:/	0:35	0/1:57,12:3	G	A
.	.	.	.	0.0(	1.0,D	1.0,D	1,D	5.6	3.42	.	.	SON	GT:/	0:14	0/1:257,48	C	A
.	.	.	.	.	.	.	.	.	2.47	.	.	SON	GT:/	0:40	0/1:48,11:3	T	C
.	.	##	0	.	.	.	.	5.1	1.42	.	.	SON	GT:/	0:57	0/1:94,21:3	C	T
.	.	.	.	.	.	.	.	.	.	.	.	SON	GT:/	0:35	0/1:47,10:3	T	C
.	.	.	.	.	.	.	.	.	.	.	.	SON	GT:/	0:28	0/1:59,8:37	A	T
.	##	##	0	0.1:	0.095	0.122	1.00	5.9	3.45	.	.	SON	GT:/	0:29	0/1:43,9:37	C	T
.	.	.	.	0.0:	0.006	0.013	1,N	.	.	.	.	SON	GT:/	0:60	0/1:100,25	T	C

.	.	.	.	.	.	.	.	.	.	.	.	SOM	GT:/	0:15	0/1:19,3:37	C	T
.	.	.	.	.	.	.	.	.	.	.	.	SOM	GT:/	0:31	0/1:32,6:37	G	T
.	.	##	0	.	.	.	.	.	.	0	0	SOM	GT:/	0:69	0/1:96,30:3	C	T
.	.	.	.	.	.	.	.	3.1	.	.	.	SOM	GT:/	0:31	0/1:44,10:3	C	T
.	.	.	.	.	.	.	.	.	.	.	.	SOM	GT:/	0:19	0/1:16,6:35	G	A
.	.	##	0	.	.	.	.	4.1	1.68	.	0	SOM	GT:/	0:58	0/1:86,18:3	C	T
.	.	.	.	.	.	.	.	.	.	.	.	SOM	GT:/	0:11	0/1:142,30	G	A
.	.	##	0	.	.	.	.	.	.	.	.	SOM	GT:/	0:15	0/1:244,53	G	A
.	.	.	.	.	.	.	.	.	.	.	.	SOM	GT:/	0:17	0/1:55,12:3	G	A
.	.	.	.	0.0	0.993	1.0,D	0.96	.	3.31	.	.	SOM	GT:/	0:17	0/1:278,43	G	A
2E-04	.	.	.	.	.	.	.	.	.	.	.	DB;S	GT:/	0:44	0/1:71,16:3	G	A
.	.	.	.	.	.	.	.	.	.	.	.	SOM	GT:/	0:46	0/1:104,11	G	A
.	.	.	.	.	.	.	.	.	.	.	.	SOM	GT:/	0:11	0/1:153,40	G	T
.	.	##	0	.	.	.	.	.	.	0	.	SOM	GT:/	0:21	0/1:42,7:37	C	T
.	.	.	.	.	.	.	.	.	.	.	.	SOM	GT:/	0:28	0/1:66,8:34	G	A
.	.	.	.	.	.	.	.	.	.	.	.	SOM	GT:/	0:42	0/1:73,15:3	A	C
.	.	.	.	.	.	.	.	.	.	.	.	SOM	GT:/	0:33	0/1:71,19:3	T	C
.	.	.	.	0.4	0.025	0.011	0.98	.	.	.	.	SOM	GT:/	0:54	0/1:59,19:3	G	A
.	.	.	.	.	.	.	.	.	.	0	.	SOM	GT:/	0:49	0/1:101,5:3	C	T
.	.	.	.	.	.	.	.	.	.	.	.	SOM	GT:/	0:91	0/1:140,28	G	T
.	.	.	.	.	.	.	.	.	.	.	.	SOM	GT:/	0:37	0/1:40,3:37	C	T
.	.	.	.	.	.	.	.	.	.	.	.	SOM	GT:/	0:34	0/1:81,8:37	T	G
.	.	.	.	.	.	.	.	.	.	.	.	SOM	GT:/	0:64	0/1:87,6:35	C	T
.	.	.	.	.	.	.	.	.	.	.	.	SOM	GT:/	0:52	0/1:46,5:37	C	T
.	.	.	.	.	.	.	.	.	.	.	.	SOM	GT:/	0:22	0/1:37,3:37	G	A
.	.	.	.	.	.	.	.	.	.	.	.	SOM	GT:/	0:69	0/1:136,8:3	C	T
.	.	.	.	.	.	.	.	.	.	.	.	SOM	GT:/	0:47	0/1:97,12:3	C	T
.	.	.	.	.	.	.	.	.	.	0	.	SOM	GT:/	0:33	0/1:42,8:33	G	T
.	.	.	.	.	.	.	.	.	.	.	.	SOM	GT:/	0:41	0/1:41,3:33	G	A
.	.	.	.	.	.	.	.	.	.	0.1	0.5	SOM	GT:/	0:9,(	0/1:6,2:37:	C	T
.	.	.	.	.	.	.	.	2.1	.	0	0	SOM	GT:/	0:18	0/1:30,3:37	G	T
.	.	.	.	.	.	.	.	2.1	.	.	.	SOM	GT:/	0:18	0/1:29,3:37	G	A
0.019	.	.	.	.	.	.	.	.	.	0	0	SOM	GT:/	0:17	0/1:40,3:37	T	A
.	.	##	0	0.0	0.924	0.994	0.97	5.3	3.78	.	.	SOM	GT:/	0:43	0/1:76,16:3	C	T
0.004	.	.	.	.	.	.	.	.	.	.	0	SOM	GT:/	0:9,(	0/1:26,5:37	C	T
.	.	.	.	.	.	.	.	.	.	.	.	SOM	GT:/	0:75	0/1:105,6:3	T	C
.	.	.	.	.	.	.	.	.	.	.	.	SOM	GT:/	0:12	0/1:159,34	G	A
.	.	.	.	0.0	0.978	1.0,D	1.00	3.5	3.09	.	.	SOM	GT:/	0:20	0/1:354,61	C	T
.	.	##	0	0.2	0.781	0.994	0.99	4.8	2.69	.	.	SOM	GT:/	0:96	0/1:165,21	C	T
.	.	.	.	.	.	.	.	3	.	.	.	SOM	GT:/	0:49	0/1:94,4:37	G	T
.	.	.	.	.	.	.	.	.	.	0	.	SOM	GT:/	0:40	0/1:51,9:37	G	A
.	.	##	0	0.1	0.996	1.0,D	1.00	4.7	5.21	.	.	SOM	GT:/	0:11	0/1:152,35	G	A
.	##	.	.	.	.	.	.	3.5	.	.	.	SOM	GT:/	0:17	0/1:306,11	T	C
.	.	.	.	.	.	.	.	.	.	.	.	SOM	GT:/	0:41	0/1:60,9:37	T	C
0.598	.	.	.	.	.	.	.	.	.	0.2	0.1	SOM	GT:/	0:18	0/1:16,10:3	T	C
.	.	.	.	.	.	.	.	.	.	0	0	SOM	GT:/	0:13	0/1:11,3:37	T	A
.	.	.	.	.	.	.	.	5.8	3.77	.	.	SOM	GT:/	0:41	0/1:48,14:3	G	A
.	.	.	.	.	.	.	.	.	.	.	.	SOM	GT:/	0:26	0/1:56,15:3	A	G
.	.	.	.	0.0	0.998	1.0,D	1.00	5.1	2.61	.	.	SOM	GT:/	0:39	0/1:46,8:37	C	A
6E-04	0	##	0	0.0	0.916	0.981	0.99	5.9	3.17	.	.	SOM	GT:/	0:35	0/1:34,4:37	G	A
.	.	.	.	.	.	.	.	.	.	.	.	SOM	GT:/	0:10	0/1:14,6:37	G	T
.	.	##	0	0.0	0.997	1.0,D	1,D	5.6	4.80	.	.	SOM	GT:/	0:87	0/1:124,22	G	A
.	.	.	.	.	.	.	.	.	.	.	.	SOM	GT:/	0:68	0/1:102,31	T	G
.	.	.	.	.	.	.	.	.	.	.	.	SOM	GT:/	0:52	0/1:76,8:35	G	A

.	.	.	.	.	.	.	1,D	5.5	4.26	.	.	SON	GT:/	0:98	0/1:166,34	G	T
.	.	##	0	.	.	.	.	.	.	.	.	SON	GT:/	0:42	0/1:65,8:37	C	T
.	.	##	0	0.0	0.701	0.998	1,D	6	4.93	0	.	SON	GT:/	0:33	0/1:61,8:37	C	T
.	.	.	.	.	.	.	.	.	.	.	.	SON	GT:/	0:30	0/1:45,9:37	C	T
.	.	.	.	.	.	.	.	.	.	.	.	SON	GT:/	0:44	0/1:66,11:3	G	A
.	.	##	.	0.0	0.61,I	0.975	1.00	3.1	4.07	.	.	SON	GT:/	0:90	0/1:147,24	G	A
.	.	.	.	.	.	.	.	3.1	.	.	.	SON	GT:/	0:11	0/1:178,46	C	T
.	.	0	0	.	.	.	1,A	.	13.8	.	.	SON	GT:/	0:89	0/1:921,27	C	T
.	.	.	.	.	.	.	.	.	.	.	.	SON	GT:/	0:10	0/1:24,3:37	G	A
.	.	.	.	0.0	0.996	1.0,D	1,D	4.4	4.77	.	.	SON	GT:/	0:13	0/1:202,34	G	A
.	.	0	0	.	.	.	.	.	1.45	.	.	SON	GT:/	0:97	0/1:64,10:3	C	T
.	.	.	.	.	.	.	.	.	.	.	.	SON	GT:/	0:41	0/1:43,3:37	T	C
.	.	.	.	0.0	1.0,D	1.0,D	1,D	3.7	3.79	.	.	SON	GT:/	0:34	0/1:74,14:3	C	T
.	.	.	.	0.0	0.747	0.999	1.00	5.7	3.34	.	.	SON	GT:/	0:43	0/1:59,8:37	G	A
.	.	.	.	.	.	.	.	.	.	.	.	SON	GT:/	0:75	0/1:121,24	T	C
.	.	.	.	.	.	.	.	.	.	.	.	SON	GT:/	0:23	0/1:39,5:31	G	A
.	.	.	.	.	.	.	.	.	.	.	.	SON	GT:/	0:25	0/1:37,11:3	G	A
0.001	.	.	.	.	.	.	.	.	.	.	.	SON	GT:/	0:32	0/1:37,4:37	C	T
.	.	.	.	.	.	.	.	.	.	.	.	SON	GT:/	0:48	0/1:72,12:3	G	A
.	##	##	0	0.0	0.861	0.989	0.99	5.5	4.90	.	.	SON	GT:/	0:10	0/1:151,46	C	T
.	.	.	.	.	.	.	.	2.8	2.65	.	.	SON	GT:/	0:16	0/1:19,5:34	T	A
.	.	.	.	0.0	0.977	0.998	1.00	4.9	3.01	.	.	SON	GT:/	0:74	0/1:119,22	G	T
2E-04	.	##	0	.	.	.	.	.	1.31	.	.	SON	GT:/	0:14	0/1:235,36	C	T
.	.	.	.	.	.	.	.	.	.	.	.	SON	GT:/	0:64	0/1:98,12:3	G	T
.	##	##	0	0.0	0.846	0.997	0.98	4	3.66	.	.	SON	GT:/	0:91	0/1:174,27	G	A
.	.	.	.	.	.	.	.	.	.	.	.	SON	GT:/	0:52	0/1:97,13:3	C	A
.	.	.	.	.	.	.	.	.	.	.	.	DB;S	GT:/	0:51	0/1:61,10:2	G	A
.	.	.	.	1.0	0.004	0.001	0.99	.	.	.	.	SON	GT:/	0:52	0/1:102,16	A	G
.	.	.	.	0.6	0.002	0.001	1,N	.	.	.	.	SON	GT:/	0:16	0/1:40,7:37	C	T
0.002	.	##	0	.	.	.	.	.	.	.	.	SON	GT:/	0:18	0/1:27,10:3	G	A
.	.	.	.	.	.	.	.	2.3	3.13	.	.	SON	GT:/	0:29	0/1:43,7:37	T	C
.	.	.	.	.	.	.	.	.	.	.	.	SON	GT:/	0:64	0/1:88,15:3	C	T
.	.	.	.	.	.	.	.	.	1.64	.	.	SON	GT:/	0:40	0/1:80,14:3	C	T
.	.	##	0	0.0	0.217	0.938	1.00	5.6	3.26	.	.	SON	GT:/	0:32	0/1:46,6:37	C	T
.	.	.	.	0.1	.	.	.	5	.	.	.	SON	GT:/	0:34	0/1:60,10:3	C	T
.	.	.	.	.	.	.	.	.	.	.	.	SON	GT:/	0:20	0/1:387,61	C	A
.	.	.	.	.	.	.	.	.	.	.	.	SON	GT:/	0:15	0/1:235,47	C	T
.	.	.	.	.	.	.	.	.	.	.	.	SON	GT:/	0:12	0/1:191,10	C	T
.	.	0	0	.	.	.	.	.	.	.	.	SON	GT:/	0:13	0/1:16,4:37	C	T
.	.	.	.	.	.	.	.	.	.	.	.	SON	GT:/	0:26	0/1:66,16:3	G	A
.	0	0	0	.	.	.	.	.	.	0	.	SON	GT:/	0:51	0/1:75,19:3	G	A
.	.	##	0	.	.	.	.	.	.	.	.	SON	GT:/	0:57	0/1:84,11:3	C	T
0.003	.	.	.	.	.	.	.	.	.	0	0	DB;S	GT:/	0:27	0/1:60,16:3	C	T
4E-04	0	0	0	.	0.999	1.0,D	.	3.7	.	.	.	SON	GT:/	0:11	0/1:190,37	G	A
.	.	.	.	.	.	.	.	.	.	.	.	SON	GT:/	0:62	0/1:100,12	T	G
.	.	.	.	.	.	.	.	.	.	.	.	SON	GT:/	0:68	0/1:100,18	A	G
.	.	.	.	.	0.283	0.68,I	.	5.3	1.29	.	.	SON	GT:/	0:65	0/1:104,16	C	T
.	.	.	.	0.0	0.997	1.0,D	1,D	4.6	5.06	.	.	SON	GT:/	0:99	0/1:151,36	C	T
.	.	.	.	0.1	0.015	0.297	0.99	4.8	4.97	.	.	SON	GT:/	0:24	0/1:41,6:32	C	T
.	.	.	.	.	.	.	.	.	.	.	.	SON	GT:/	0:35	0/1:60,7:29	C	T
.	.	.	.	.	.	.	.	.	.	.	.	SON	GT:/	0:18	0/1:41,6:37	C	T
.	##	##	0	0.4	0.002	0.003	1,N	.	1.38	.	.	SON	GT:/	0:36	0/1:65,5:37	C	T
.	.	.	.	.	.	.	.	.	.	.	.	SON	GT:/	0:35	0/1:47,6:35	G	C
.	.	.	.	.	.	.	.	.	.	.	.	SON	GT:/	0:21	0/1:35,4:37	G	A

.	.	.	.	.	.	.	.	.	.	.	.	SON	GT:/	0:37	0/1:47,5:34	G	T
.	.	.	.	.	.	.	.	.	.	.	.	SON	GT:/	0:8,(	0/1:19,4:37	G	A
.	.	.	.	.	.	.	.	.	.	.	.	SON	GT:/	0:11	0/1:5,2:37:	T	A
.	.	.	.	.	.	.	.	.	1.63	.	.	SON	GT:/	0:47	0/1:80,10:3	C	T
.	.	.	.	.	.	.	.	.	.	.	.	SON	GT:/	0:10	0/1:143,29	C	T
.	.	.	.	.	.	.	.	.	1.65	.	.	SON	GT:/	0:84	0/1:127,25	T	A
.	0	0	0	.	.	.	.	.	.	.	.	SON	GT:/	0:12	0/1:173,36	G	A
.	.	.	.	.	.	.	.	.	.	.	.	SON	GT:/	0:57	0/1:72,22:3	C	A
.	.	0	0	.	.	.	.	.	.	.	.	SON	GT:/	0:16	0/1:252,36	T	C
.	.	.	.	.	.	.	.	.	.	0	.	SON	GT:/	0:14	0/1:20,4:37	C	T
.	.	##	0	.	.	.	.	3.3	.	.	.	SON	GT:/	0:10	0/1:176,26	C	T
.	.	##	0	0.0(	0.054	0.487	1,N	.	3.36	.	.	SON	GT:/	0:68	0/1:107,11	G	A
.	.	.	.	.	.	.	1,D	4.7	.	.	.	SON	GT:/	0:90	0/1:148,34	G	A
.	.	.	.	.	.	.	.	.	.	.	.	SON	GT:/	0:39	0/1:59,7:37	C	T
.	.	##	0	.	.	.	.	.	.	0	.	SON	GT:/	0:92	0/1:111,13	C	T
.	.	.	.	.	.	.	.	.	.	.	.	SON	GT:/	0:25	0/1:31,10:3	C	T
.	.	.	.	.	.	.	.	.	.	.	.	SON	GT:/	0:53	0/1:85,36:3	C	T
0.244	.	.	.	.	.	.	.	.	.	0.3	0.5	SON	GT:/	0:9,(	0/1:5,9:37:	A	G
.	.	.	.	.	.	.	.	.	.	.	.	SON	GT:/	0:21	0/1:27,3:37	A	T
.	.	.	.	.	.	.	.	.	.	.	.	SON	GT:/	0:12	0/1:28,3:37	G	A
.	.	.	.	0.9(	0.972	0.981	1.00	5.6	2.25	.	.	SON	GT:/	0:40	0/1:65,9:37	T	C
.	.	.	.	.	.	.	.	.	.	.	.	SON	GT:/	0:11	0/1:8,5:37:	A	G
.	.	.	.	.	.	.	.	.	.	0	0	SON	GT:/	0:11	0/1:9,5:32:	G	A
.	.	.	.	.	.	.	.	.	.	.	.	SON	GT:/	0:47	0/1:70,17:3	C	T
.	.	.	.	.	.	.	.	.	.	.	.	SON	GT:/	0:43	0/1:83,18:3	G	T
.	.	.	.	.	.	.	.	4	2.52	.	.	SON	GT:/	0:17	0/1:40,7:37	C	A
.	.	.	.	.	.	.	.	.	.	.	.	SON	GT:/	0:91	0/1:182,30	G	T
.	.	0	0	.	.	.	.	.	.	.	.	SON	GT:/	0:85	0/1:157,26	C	T
2E-04	.	0	0	.	.	.	.	.	.	.	.	SON	GT:/	0:43	0/1:58,9:37	G	A
.	.	.	.	.	.	.	.	.	.	0	.	SON	GT:/	0:23	0/1:32,9:37	G	A
.	##	0	0	0.2(	0.001	0.002	1,N	.	.	.	.	DB;S	GT:/	0:85	0/1:185,32	C	T
.	.	.	.	.	.	.	.	.	.	.	.	SON	GT:/	0:68	0/1:119,24	G	A
.	0	##	0	.	.	.	1,A	.	5.79	.	.	SON	GT:/	0:64	0/1:101,19	G	A
0.031	0	0	0	.	.	.	.	.	.	.	.	DB;S	GT:/	0:76	0/1:140,29	G	A
.	.	##	0	.	.	.	.	.	.	.	0	SON	GT:/	0:18	0/1:48,7:37	C	T
.	.	.	.	.	.	.	.	.	.	.	.	SON	GT:/	0:20	0/1:13,4:37	C	T
.	.	##	0	.	.	.	.	.	.	.	.	SON	GT:/	0:44	0/1:47,10:3	G	A
.	.	.	.	.	.	.	.	5.6	1.92	.	.	SON	GT:/	0:57	0/1:94,14:3	C	T
.	.	##	0	0.0(	0.994	0.999	1,D	3.7	3.34	.	.	SON	GT:/	0:36	0/1:65,10:3	G	A
.	.	.	.	.	.	.	.	3.3	.	.	.	SON	GT:/	0:27	0/1:18,6:37	T	C
.	.	.	.	.	.	.	.	.	.	.	.	SON	GT:/	0:22	0/1:30,5:37	C	T
.	0	##	0	0.0(	.	.	1.00	.	2.48	.	.	SON	GT:/	0:51	0/1:99,16:3	G	A
.	.	0	0	.	.	.	.	.	.	.	0	SON	GT:/	0:23	0/1:34,8:35	G	A
.	.	.	.	.	.	.	.	.	.	.	.	SON	GT:/	0:12	0/1:58,4:37	G	A
.	.	.	.	.	.	.	.	.	.	.	.	SON	GT:/	0:12	0/1:87,4:37	T	G
.	.	.	.	.	.	.	.	.	.	0	.	SON	GT:/	0:13	0/1:94,10:3	T	C
.	.	.	.	.	.	.	.	.	.	.	.	SON	GT:/	0:12	0/1:98,5:37	A	C
.	.	.	.	.	.	.	.	.	.	.	.	SON	GT:/	0:27	0/1:377,12	A	C
.	.	.	.	.	.	.	.	.	.	.	.	SON	GT:/	0:14	0/1:62,5:37	G	A
.	.	.	.	.	.	.	.	.	.	0	.	DB;S	GT:/	0:35	0/1:267,13	C	G
.	.	.	.	0.7!	0.061	0.097	0.99	5.2	.	.	.	SON	GT:/	0:35	0/1:62,5:34	G	T
.	.	.	.	.	.	.	.	2.5	.	.	.	SON	GT:/	0:30	0/1:38,7:37	T	C
.	.	.	.	.	.	.	.	.	.	.	.	SON	GT:/	0:15	0/1:23,4:37	A	G
.	.	.	.	.	.	.	.	.	.	.	.	SON	GT:/	0:23	0/1:40,4:37	C	T

.	.	.	.	.	.	.	.	4.9	4.20	.	.	SOM	GT:/	0:36	0/1:52,6:37	G	A
.	.	.	.	.	.	.	.	.	1.75	.	.	SOM	GT:/	0:16	0/1:20,3:37	T	A
.	.	##	0	.	.	.	.	.	.	.	.	SOM	GT:/	0:26	0/1:40,6:37	G	A
4E-04	0	0	0	.	.	.	.	2.3	.	.	0	SOM	GT:/	0:55	0/1:86,19:3	C	T
.	.	.	.	.	.	.	.	.	.	.	.	SOM	GT:/	0:10	0/1:18,4:34	G	A
.	.	.	.	.	.	.	.	2.7	.	.	.	SOM	GT:/	0:30	0/1:62,10:3	A	G
.	.	.	.	.	.	.	.	.	.	.	.	SOM	GT:/	0:24	0/1:51,10:3	G	T
.	.	##	0	.	.	.	.	.	.	.	.	SOM	GT:/	0:25	0/1:50,6:37	G	A
.	.	.	.	.	.	.	.	4.1	.	.	.	SOM	GT:/	0:27	0/1:52,9:37	G	A
.	.	.	.	.	.	.	.	.	.	.	.	SOM	GT:/	0:8,(	0/1:12,3:37	C	T
.	.	.	.	.	.	.	.	.	.	.	.	SOM	GT:/	0:21	0/1:32,3:37	T	C
.	.	.	.	.	.	.	.	.	.	.	.	SOM	GT:/	0:81	0/1:117,12	T	C
.	.	.	.	.	.	.	.	.	.	.	.	SOM	GT:/	0:33	0/1:64,16:3	G	A
.	.	.	.	.	.	.	.	.	.	.	.	SOM	GT:/	0:62	0/1:82,11:3	C	T
.	.	##	0	0.0	1.0,D	1.0,D	1,D	5	5.17	.	.	SOM	GT:/	0:78	0/1:127,19	G	A
.	.	.	.	0.0	0.941	0.997	1,D	5.9	5.77	.	.	SOM	GT:/	0:69	0/1:114,5:3	G	A
.	.	.	.	.	.	.	.	.	.	.	.	SOM	GT:/	0:36	0/1:70,8:37	T	C
.	.	.	.	.	.	.	.	.	.	0	.	SOM	GT:/	0:17	0/1:21,4:37	G	A
.	.	.	.	.	.	.	.	.	.	.	.	SOM	GT:/	0:36	0/1:48,6:37	T	C
.	.	.	.	.	.	.	.	.	.	.	.	SOM	GT:/	0:12	0/1:15,4:37	T	A
.	.	.	.	.	.	.	.	.	.	.	.	SOM	GT:/	0:31	0/1:38,7:37	G	A
.	.	.	.	.	.	.	.	.	.	.	.	SOM	GT:/	0:32	0/1:38,5:37	G	A
.	.	.	.	.	.	.	.	2.6	1.45	.	.	SOM	GT:/	0:19	0/1:18,6:37	C	T
.	.	.	.	0.0	0.255	0.898	0.68	4.8	3.48	.	.	SOM	GT:/	0:10	0/1:157,39	C	T
6E-04	0	0	0	0.1	0.037	0.108	1.00	3.1	1.59	0	.	SOM	GT:/	0:61	0/1:111,25	C	T
.	.	.	.	.	.	.	.	.	.	.	.	SOM	GT:/	0:50	0/1:67,11:3	A	G
.	.	.	.	.	.	.	.	.	.	.	.	SOM	GT:/	0:8,(	0/1:11,3:37	C	T
.	.	.	.	.	.	.	.	.	.	.	.	SOM	GT:/	0:42	0/1:57,13:3	C	T
0.017	0	0	0	.	.	.	.	.	.	.	.	DB;S	GT:/	0:65	0/1:79,15:3	G	A
0.011	.	0	.	0.1	0.002	0.014	1,P	.	1.51	0	.	DB;S	GT:/	0:10	0/1:152,20	G	A
.	.	.	.	.	.	.	.	.	.	.	.	SOM	GT:/	0:22	0/1:42,5:37	G	T
.	.	.	.	.	.	.	.	.	.	.	.	SOM	GT:/	0:19	0/1:30,5:37	C	T
.	.	.	.	.	.	.	.	.	1.36	.	.	SOM	GT:/	0:71	0/1:84,5:34	C	A
.	.	.	.	.	.	.	.	.	.	.	.	SOM	GT:/	0:29	0/1:39,7:35	C	A
.	.	.	.	.	.	.	.	.	.	.	0	SOM	GT:/	0:31	0/1:53,12:3	C	T
.	.	.	.	.	.	.	.	.	.	.	.	SOM	GT:/	0:60	0/1:131,5:3	G	T
.	.	##	0	.	0.008	0.166	1,N	.	.	.	.	SOM	GT:/	0:87	0/1:123,10	C	T
2E-04	.	##	0	.	.	.	.	.	.	.	.	SOM	GT:/	0:48	0/1:62,21:3	G	A
.	.	.	.	0.0	0.034	0.278	0.98	4	1.69	.	.	SOM	GT:/	0:68	0/1:119,14	G	T
.	.	##	0	1.0	0.001	0.004	1.00	3.3	.	.	.	SOM	GT:/	0:21	0/1:36,8:29	C	T
.	.	.	.	0.4	0.862	0.998	1.00	3.8	3.69	.	.	SOM	GT:/	0:67	0/1:145,22	C	T
.	.	.	.	.	0.119	0.389	1.00	.	.	.	.	SOM	GT:/	0:18	0/1:328,65	G	A
1E-03	0	0	0	0.3	0.001	0.0,B	0.99	.	1.25	.	.	SOM	GT:/	0:15	0/1:238,43	G	A
0.002	.	.	.	.	.	.	.	.	.	0	.	SOM	GT:/	0:17	0/1:26,7:37	C	T
.	.	.	.	.	.	.	1,D	3.6	2.41	.	.	SOM	GT:/	0:75	0/1:172,31	C	T
.	.	.	.	0.0	0.981	1.0,D	1.00	5.2	3.52	.	.	SOM	GT:/	0:42	0/1:66,9:37	G	A
.	.	0	0	0.2	0.001	0.002	1,N	.	1.50	.	.	SOM	GT:/	0:70	0/1:150,29	G	A
0.278	.	0	0	.	.	.	.	.	.	0	0	SOM	GT:/	0:9,(	0/1:17,4:37	A	G
.	.	.	.	.	.	.	.	.	.	.	.	SOM	GT:/	0:19	0/1:31,3:37	C	T
.	.	.	.	0.0	0.075	0.101	1,D	6.2	3.93	.	.	SOM	GT:/	0:84	0/1:161,22	C	A
.	.	##	0	.	.	.	.	.	.	.	.	SOM	GT:/	0:58	0/1:65,18:3	G	A
.	.	.	.	0.1	0.269	0.57,I	0.95	3.1	2.96	.	.	SOM	GT:/	0:62	0/1:108,15	C	T
.	.	##	0	0.0	0.93,I	1.0,D	1.00	6	5.23	.	.	SOM	GT:/	0:60	0/1:87,11:3	C	T
.	.	.	.	.	.	.	.	.	.	.	.	SOM	GT:/	0:18	0/1:36,5:37	C	T

.	.	.	.	0.0	1.0,D	1.0,D	1,D	5.9	3.74	.	.	SOM	GT:/	0:63	0/1:121,20	G	A
.	.	##	0	0.0	0.988	1.0,D	0.99	5.2	4.81	.	.	SOM	GT:/	0:29	0/1:45,8:37	G	A
.	.	.	.	.	.	.	.	.	.	.	.	SOM	GT:/	0:26	0/1:48,8:35	G	A
.	.	##	0	.	.	.	.	.	1.41	.	.	SOM	GT:/	0:71	0/1:96,20:3	C	T
.	.	.	.	.	.	.	.	.	.	.	.	SOM	GT:/	0:26	0/1:38,5:34	G	A
.	.	0	0	.	.	.	.	.	.	0	.	SOM	GT:/	0:63	0/1:117,22	C	T
.	.	.	.	.	.	.	.	.	.	.	.	SOM	GT:/	0:23	0/1:50,9:37	G	A
.	.	.	.	0.0	1.0,D	1.0,D	1,D	5.3	4.47	.	.	SOM	GT:/	0:12	0/1:197,31	C	T
.	.	.	.	0.1	0.022	0.024	1,D	5.9	2.74	.	.	SOM	GT:/	0:15	0/1:216,42	G	A
.	.	.	.	.	.	.	1,D	5.5	13.0	.	.	SOM	GT:/	0:95	0/1:178,38	G	A
.	.	.	.	.	.	.	.	.	.	0	.	SOM	GT:/	0:12	0/1:29,5:37	A	G
.	.	.	.	.	.	.	.	.	.	.	.	SOM	GT:/	0:10	0/1:2,6:37:	G	C
.	.	.	.	.	.	.	.	.	.	.	.	SOM	GT:/	0:8,(	0/1:2,3:37:	C	T
.	.	.	.	.	.	.	.	.	.	.	.	SOM	GT:/	0:65	0/1:137,16	C	T
.	.	0	0	.	.	.	.	.	.	0	0	SOM	GT:/	0:13	0/1:32,3:37	C	T
.	.	.	.	.	.	.	.	.	.	0	0.2	SOM	GT:/	0:9,(	0/1:12,3:37	G	A
.	.	.	.	.	.	.	.	.	.	.	.	SOM	GT:/	0:9,(	0/1:11,3:37	G	C
.	.	.	.	.	.	.	.	.	.	0	.	SOM	GT:/	0:11	0/1:31,3:37	A	G
.	.	.	.	.	.	.	.	.	.	.	.	SOM	GT:/	0:36	0/1:46,8:37	A	C
.	.	.	.	0.0	0.041	0.745	1,N	.	.	.	.	SOM	GT:/	0:13	0/1:176,17	G	A
.	.	##	0	0.0	0.001	0.003	1.00	.	.	.	.	SOM	GT:/	0:25	0/1:23,4:37	G	A
.	.	.	.	.	.	.	.	.	.	.	.	SOM	GT:/	0:10	0/1:10,4:37	G	T
.	.	.	.	.	.	.	.	3.5	.	.	.	SOM	GT:/	0:93	0/1:155,27	G	T
.	.	.	.	.	.	.	.	3.7	.	.	.	SOM	GT:/	0:23	0/1:19,5:31	C	A
.	.	.	.	.	.	.	.	.	.	.	.	SOM	GT:/	0:36	0/1:52,8:37	T	C
.	.	.	.	.	.	.	.	.	1.82	.	.	SOM	GT:/	0:70	0/1:126,28	G	A
.	.	.	.	.	.	.	.	.	.	.	.	SOM	GT:/	0:17	0/1:30,6:37	A	G
2E-04	.	##	0	1.0	0.002	0.019	1,N	.	.	.	.	SOM	GT:/	0:10	0/1:126,23	G	A
.	.	.	.	.	.	.	.	.	.	.	.	SOM	GT:/	0:26	0/1:53,10:3	G	A
.	.	0	0	.	.	.	.	4.6	.	.	.	SOM	GT:/	0:26	0/1:54,17:3	A	G
.	.	.	.	.	.	.	.	.	.	.	.	SOM	GT:/	0:31	0/1:62,10:3	G	A
.	.	.	.	0.0	1.0,D	1.0,D	1,D	5.7	4.59	.	.	SOM	GT:/	0:46	0/1:99,11:3	C	T
.	.	.	.	.	.	.	.	.	.	.	.	SOM	GT:/	0:13	0/1:202,38	C	T
8E-04	0	0	0	.	.	.	.	.	.	.	.	SOM	GT:/	0:63	0/1:106,22	C	T
.	.	.	.	.	.	.	.	.	.	.	.	SOM	GT:/	0:19	0/1:24,9:37	C	T
.	.	.	.	.	.	.	.	.	.	.	.	SOM	GT:/	0:36	0/1:72,16:3	A	G
4E-04	.	0	0	0.3	0.0,B	0.006	1,N	.	1.43	.	.	SOM	GT:/	0:14	0/1:262,46	G	A
.	.	.	.	.	.	.	.	.	.	0	0	SOM	GT:/	0:12	0/1:17,3:37	A	G
.	.	.	.	.	.	.	.	6.1	2.42	.	.	SOM	GT:/	0:23	0/1:33,4:37	A	G
.	.	.	.	0.4	0.001	0.0,B	1,N	4.4	.	.	.	SOM	GT:/	0:52	0/1:83,9:37	C	T
6E-04	.	.	.	.	.	.	.	.	.	0	0	SOM	GT:/	0:35	0/1:47,10:3	C	T
2E-04	.	##	0	0.0	0.487	0.977	0.89	2.2	3.05	.	.	SOM	GT:/	0:94	0/1:177,11	C	T
6E-04	.	.	.	.	.	.	.	.	.	0	0	SOM	GT:/	0:78	0/1:123,27	G	A
.	.	.	.	.	.	.	.	.	.	.	.	SOM	GT:/	0:11	0/1:18,4:37	G	A
.	.	.	.	.	.	.	.	.	1.27	.	.	SOM	GT:/	0:24	0/1:39,8:37	A	G
.	.	.	.	0.0	0.264	0.918	0.99	5.1	3.26	.	.	SOM	GT:/	0:86	0/1:144,35	C	T
.	.	##	0	0.3	0.018	0.023	0.99	5.8	.	.	.	SOM	GT:/	0:10	0/1:173,32	C	T
.	.	.	.	.	.	.	.	.	.	.	.	SOM	GT:/	0:12	0/1:24,4:34	G	T
.	.	.	.	.	.	.	.	.	.	.	.	SOM	GT:/	0:69	0/1:173,13	G	T
.	.	.	.	.	.	.	.	2.8	.	.	.	SOM	GT:/	0:82	0/1:151,29	C	T
.	.	.	.	.	.	.	.	2.8	.	.	.	SOM	GT:/	0:73	0/1:102,14	C	T
4E-04	.	.	.	.	.	.	.	.	.	0	0	SOM	GT:/	0:90	0/1:137,26	C	T
.	.	.	.	.	.	.	.	.	.	.	.	SOM	GT:/	0:19	0/1:19,4:37	C	A
.	.	.	.	0.1	0.008	0.02,I	1.00	.	.	.	.	SOM	GT:/	0:48	0/1:105,16	G	A

1E-03	.	.	.	.	.	.	.	.	.	0	.	SON	GT:/	0:17	0/1:23,9:37	C	T
.	.	##	0	.	.	.	.	.	.	.	.	SON	GT:/	0:35	0/1:61,13:3	C	T
.	.	.	.	.	.	.	.	.	.	.	.	SON	GT:/	0:16	0/1:25,4:37	A	G
.	.	##	0	.	.	.	.	.	.	.	.	SON	GT:/	0:56	0/1:118,16	C	T
.	.	.	.	0.0!	0.982	0.995	1,D	5.2	4.96	.	.	SON	GT:/	0:69	0/1:114,15	G	T
.	.	.	.	.	.	.	.	.	.	.	.	SON	GT:/	0:36	0/1:56,9:37	C	A
.	.	.	.	.	.	.	.	.	.	.	.	SON	GT:/	0:8,(	0/1:13,5:37	C	T
.	.	.	.	.	.	.	.	.	.	.	.	SON	GT:/	0:69	0/1:113,19	T	C
.	.	.	.	.	.	.	.	.	.	.	.	SON	GT:/	0:64	0/1:137,5:3	C	T
.	.	##	0	.	.	.	.	.	.	0	0	SON	GT:/	0:67	0/1:120,5:3	C	T
.	.	##	0	0.7(	0.386	0.86,I	1,D	5.5	3.10	0	.	SON	GT:/	0:59	0/1:105,25	G	A
.	.	.	.	.	.	.	.	.	.	.	.	SON	GT:/	0:68	0/1:120,9:3	C	A
.	.	##	0	0.2!	0.008	0.006	1.00	2.8	.	0	.	SON	GT:/	0:11	0/1:186,30	C	T
.	.	.	.	.	.	.	.	.	.	.	.	SON	GT:/	0:52	0/1:103,20	C	T
.	.	##	0	.	.	.	.	4.8	.	.	.	SON	GT:/	0:63	0/1:125,25	C	T
.	##	##	0	0.0	0.991	0.999	1,D	6	4.62	0	.	SON	GT:/	0:57	0/1:100,15	G	A
.	.	.	.	0.0	0.985	1.0,D	1,D	4.9	4.69	.	.	SON	GT:/	0:82	0/1:117,19	C	T
.	.	.	.	.	.	.	.	2.4	2.14	.	.	SON	GT:/	0:94	0/1:163,31	T	C
.	.	.	.	.	.	.	.	.	.	.	.	SON	GT:/	0:98	0/1:152,32	C	T
0.005	.	.	.	.	.	.	.	.	.	0	.	DB;S	GT:/	0:41	0/1:57,8:35	C	T
.	.	.	.	.	.	.	.	.	.	0	0	DB;S	GT:/	0:23	0/1:28,6:37	T	C
.	.	.	.	.	.	.	.	.	.	0	0	DB;S	GT:/	0:21	0/1:27,5:29	C	T
.	.	##	0	0.7!	0.004	0.005	1.00	.	2.06	.	.	SON	GT:/	0:56	0/1:117,24	G	A
.	.	.	.	.	.	.	.	.	.	.	.	SON	GT:/	0:64	0/1:103,25	G	A
.	.	.	.	0.1!	0.018	0.032	1.00	.	.	.	.	SON	GT:/	0:96	0/1:133,42	G	A
.	.	.	.	.	.	.	.	.	.	.	.	SON	GT:/	0:85	0/1:150,24	C	T
.	.	.	.	.	.	.	.	.	.	.	.	SON	GT:/	0:80	0/1:171,29	C	T
.	.	.	.	0.1(	0.018	0.041	1,N	3	3.01	.	.	SON	GT:/	0:10	0/1:210,10	C	T
0.087	0	.	.	.	.	.	.	.	.	0.1	0.1	DB;S	GT:/	0:35	0/1:34,8:37	G	A
.	.	.	.	.	.	.	.	.	.	.	.	SON	GT:/	0:47	0/1:85,13:3	G	A
.	.	.	.	0.4!	0.6,P	0.94,I	1,N	.	3.02	.	.	SON	GT:/	0:46	0/1:73,22:3	C	T
.	.	.	.	0.3(	0.013	0.047	1,N	2.7	.	.	.	SON	GT:/	0:18	0/1:16,6:32	A	G
.	.	.	.	.	.	.	.	.	.	.	.	SON	GT:/	0:58	0/1:112,14	G	T
.	.	##	0	0.8	0.008	0.016	1,D	.	.	0	.	SON	GT:/	0:10	0/1:178,31	G	A
.	.	0	0	0.9	0.005	0.007	0.88	3.7	.	.	.	SON	GT:/	0:71	0/1:139,23	C	T
.	.	.	.	0.1(	0.477	0.389	1,N	.	.	.	.	SON	GT:/	0:68	0/1:111,18	G	T
.	.	.	.	0.0	0.985	1.0,D	0.98	4.5	1.94	.	.	SON	GT:/	0:15	0/1:200,41	C	T
2E-04	0	0	0	.	.	.	.	.	.	.	.	DB;S	GT:/	0:67	0/1:125,26	C	T
.	.	.	.	.	.	.	.	.	.	.	.	SON	GT:/	0:61	0/1:123,20	G	A
.	.	##	0	0.0(	0.975	0.999	0.99	4	3.94	.	.	SON	GT:/	0:43	0/1:106,18	C	T
.	.	.	.	.	.	.	.	.	.	.	.	SON	GT:/	0:63	0/1:136,24	C	A
.	.	.	.	.	.	.	.	.	1.39	.	.	DB;S	GT:/	0:56	0/1:86,8:37	C	T
.	.	.	.	0.0	0.998	1.0,D	1,D	4.5	4.54	.	.	SON	GT:/	0:35	0/1:63,12:3	C	A
.	.	.	.	.	.	.	.	2.9	.	.	.	SON	GT:/	0:43	0/1:76,10:3	C	T
.	.	##	0	0.4!	0.0,B	0.001	1.00	3.5	.	.	.	SON	GT:/	0:70	0/1:130,17	G	A
.	.	.	.	.	.	.	.	.	.	.	.	SON	GT:/	0:10	0/1:21,5:37	T	C
.	##	##	0	.	.	.	.	.	.	.	.	SON	GT:/	0:12	0/1:190,26	C	T
2E-04	.	.	.	.	.	.	.	.	.	0	.	SON	GT:/	0:18	0/1:24,6:37	C	T
.	.	.	.	.	.	.	.	2.1	.	.	.	SON	GT:/	0:87	0/1:155,24	G	A
.	.	.	.	0.0!	0.966	0.999	0.53	.	3.45	.	.	SON	GT:/	0:60	0/1:101,37	C	A
.	.	.	.	.	.	.	.	.	.	.	.	SON	GT:/	0:10	0/1:204,41	G	A
8E-04	.	.	.	.	.	.	.	.	.	0	.	DB;S	GT:/	0:51	0/1:99,18:3	C	T
.	.	.	.	.	.	.	.	.	.	.	.	SON	GT:/	0:11	0/1:182,44	G	A
.	.	.	.	.	.	.	.	.	.	.	.	SON	GT:/	0:12	0/1:168,45	C	T

.	.	##	0	.	.	.	.	.	.	.		.	SON	GT:/	0:21	0/1:18,4:37	C	T
.	.	.	.	.	.	.	.	.	.	.		.	SON	GT:/	0:21	0/1:44,6:37	C	A
2E-04	.	.	.	.	.	.	.	.	.	.		.	SON	GT:/	0:14	0/1:28,5:37	C	T
.	.	.	.	.	.	.	.	.	.	.		.	SON	GT:/	0:23	0/1:27,5:31	A	T
4E-04	.	0	0	0.0	0.932	0.999	0.99	.	2.73	.		0	SON	GT:/	0:43	0/1:64,11:3	G	A
.	.	.	.	.	.	.	.	.	.	.		.	SON	GT:/	0:10	0/1:1,2:37:	G	A
.	##	##	0	0.0	1.0,D	1.0,D	0.99	4.3	5.25	.		.	SON	GT:/	0:28	0/1:481,73	C	T
.	.	.	.	.	.	.	.	3.2	.	.		.	SON	GT:/	0:57	0/1:75,22:3	C	A
.	.	.	.	.	.	.	.	.	.	.		.	SON	GT:/	0:35	0/1:68,9:37	G	A
.	.	.	.	.	.	.	.	.	.	.		.	SON	GT:/	0:17	0/1:16,5:37	C	A
.	.	.	.	.	.	.	.	.	.	.		.	SON	GT:/	0:15	0/1:23,6:28	C	A
6E-04	.	.	.	.	.	.	.	.	.	.		.	SON	GT:/	0:15	0/1:21,3:37	C	T
0.007	0	0	0	.	.	.	.	.	.		0	0.1	DB;S	GT:/	0:37	0/1:65,10:3	G	A
.	.	.	.	0.0	0.999	1.0,D	1.00	4.6	2.46	.		.	SON	GT:/	0:42	0/1:73,11:3	G	T
.	.	.	.	.	.	.	.	.	.	.		.	SON	GT:/	0:21	0/1:27,3:37	C	T
.	.	.	.	.	.	.	.	3	2.07	.		.	SON	GT:/	0:26	0/1:35,4:37	T	C
.	.	.	.	0.7	0.236	0.804	1,N	.	2.47	.		.	SON	GT:/	0:53	0/1:54,15:3	A	T
.	.	##	0	0.0	0.998	1.0,D	1,D	5	5.02	.		.	SON	GT:/	0:38	0/1:50,11:3	T	C
0.003	0	0	0	.	.	.	.	.	.	.		.	SON	GT:/	0:33	0/1:54,7:37	A	G
.	.	.	.	0.0	0.994	0.999	1,D	5.8	4.12	.		.	SON	GT:/	0:33	0/1:104,8:3	A	G
.	.	.	.	.	.	.	.	.	.	.		.	SON	GT:/	0:59	0/1:92,18:3	A	G
.	##	0	0	0.1	0.787	0.99,I	1,N	.	3.04	.		.	SON	GT:/	0:62	0/1:94,11:3	G	A
.	.	.	.	.	.	.	.	.	.	.		.	SON	GT:/	0:11	0/1:12,3:37	G	T
.	.	.	.	.	.	.	.	.	.		0	.	SON	GT:/	0:56	0/1:69,14:3	G	A
.	.	##	0	.	.	.	.	.	1.99	.		.	SON	GT:/	0:27	0/1:61,11:3	G	A
2E-04	.	##	0	.	.	.	.	.	.	.		.	SON	GT:/	0:25	0/1:411,67	C	T
.	.	.	.	0.7	0.999	1.0,D	1.00	5.9	3.84	.		.	SON	GT:/	0:27	0/1:32,4:34	G	A
.	.	.	.	.	.	.	.	.	.	.		.	SON	GT:/	0:9,(	0/1:9,3:37:	G	A
.	.	.	.	.	.	.	.	.	.	.		.	SON	GT:/	0:19	0/1:27,3:37	G	A
.	.	.	.	.	.	.	.	.	.	.		.	SON	GT:/	0:65	0/1:74,4:37	A	G
.	.	.	.	.	.	.	.	.	.	.		.	SON	GT:/	0:13	0/1:179,23	C	T
.	.	.	.	.	.	.	.	.	.	.		.	SON	GT:/	0:13	0/1:28,3:37	G	A
.	.	.	.	.	.	.	.	.	.		0	.	SON	GT:/	0:40	0/1:64,6:37	G	A
.	.	.	.	0.1	0.011	0.025	1,N	.	.		0	.	SON	GT:/	0:46	0/1:97,20:3	G	A
.	##	##	0	.	.	.	.	.	.	.		.	SON	GT:/	0:47	0/1:79,12:3	G	A
.	.	.	.	.	.	.	.	.	.	.		.	SON	GT:/	0:22	0/1:39,14:3	C	A
.	.	.	.	.	.	.	.	.	.	.		.	SON	GT:/	0:53	0/1:117,14	G	A
.	.	.	.	.	.	.	.	.	.	.		.	SON	GT:/	0:12	0/1:11,5:37	C	A
6E-04	0	0	0	0.3	0.736	0.996	1,D	6	2.56		0	.	SON	GT:/	0:28	0/1:46,5:37	G	A
8E-04	.	.	.	.	.	.	.	3.6	1.58	.		.	SON	GT:/	0:8,(	0/1:7,3:37:	A	G
.	.	.	.	.	.	.	.	.	.		0	.	SON	GT:/	0:11	0/1:26,6:37	G	A
.	.	.	.	.	.	.	.	.	.	.		.	SON	GT:/	0:13	0/1:17,3:37	A	G
.	.	##	0	.	.	.	.	.	.		0	0	SON	GT:/	0:56	0/1:89,13:3	C	T
.	.	.	.	0.0	0.983	0.998	1,D	6.2	5.20	.		.	SON	GT:/	0:48	0/1:99,7:37	C	T
.	.	.	.	.	.	.	.	.	.	.		.	SON	GT:/	0:63	0/1:96,24:3	G	A
2E-04	0	##	0	0.0	0.996	1.0,D	1,D	5.2	4.17	.		.	SON	GT:/	0:10	0/1:177,36	C	T
.	.	.	.	0.0	0.995	1.0,D	1,D	4.4	3.97	.		.	SON	GT:/	0:37	0/1:46,5:37	C	A
.	.	.	.	.	.	.	.	.	.	.		.	SON	GT:/	0:31	0/1:74,9:37	C	A
.	.	##	0	.	.	.	.	2.1	1.45	.		.	SON	GT:/	0:28	0/1:40,5:37	A	G
.	.	0	0	.	.	.	.	.	.	.		.	SON	GT:/	0:70	0/1:95,4:37	A	G
.	.	.	.	.	.	.	.	.	.	.		.	SON	GT:/	0:21	0/1:28,6:28	A	G
.	.	.	.	.	.	.	.	.	.	.		.	SON	GT:/	0:14	0/1:19,4:37	C	T
.	.	.	.	.	.	.	.	.	2.57	.		.	SON	GT:/	0:38	0/1:69,6:37	A	G
.	.	.	.	.	.	.	.	.	.	.		.	SON	GT:/	0:39	0/1:57,7:37	A	G

2E-04	.	##	0	0.0:	0.998	1.0,D	1.00	5.2	4.83	0	.	SOM	GT:/	0:43	0/1:86,18:3	C	T
.	.	.	.	.	.	.	.	4.4	1.39	.	.	SOM	GT:/	0:18	0/1:38,10:3	C	T
.	.	.	.	.	.	.	.	.	1.28	.	.	SOM	GT:/	0:43	0/1:88,20:3	C	T
.	.	.	.	.	.	.	.	.	.	.	.	SOM	GT:/	0:17	0/1:50,4:37	C	T
.	.	.	.	.	.	.	.	.	.	.	.	SOM	GT:/	0:8,(	0/1:12,4:37	C	T
.	.	.	.	.	.	.	.	.	1.59	.	.	SOM	GT:/	0:10	0/1:148,34	G	A
.	##	##	.	.	.	.	1,A	3.9	12.2	.	0	SOM	GT:/	0:48	0/1:56,16:3	G	A
.	.	##	0	0.1:	0.006	0.066	0.72	3.9	3.01	.	.	SOM	GT:/	0:21	0/1:53,13:3	G	A
.	.	.	.	.	.	.	.	.	.	.	.	SOM	GT:/	0:40	0/1:39,9:37	G	T
.	.	.	.	.	.	.	.	.	2.29	.	.	SOM	GT:/	0:17	0/1:12,4:37	T	G
.	.	##	0	0.0	0.336	0.912	0.99	5.8	3.59	.	.	SOM	GT:/	0:52	0/1:104,22	C	T
.	.	.	.	.	.	.	.	.	.	.	.	SOM	GT:/	0:11	0/1:42,4:37	G	A
.	.	.	.	.	.	.	.	.	.	.	.	SOM	GT:/	0:38	0/1:48,19:3	G	A
.	.	.	.	.	.	.	.	.	.	.	.	SOM	GT:/	0:37	0/1:41,11:3	C	T
.	.	##	0	0.0	0.828	0.999	1,D	4.8	4.65	.	.	SOM	GT:/	0:65	0/1:85,17:3	G	A
.	##	##	0	0.0	1.0,D	1.0,D	1,D	4.9	4.09	.	.	SOM	GT:/	0:47	0/1:77,13:3	C	T
.	.	.	.	.	.	.	.	.	.	.	.	SOM	GT:/	0:56	0/1:86,18:3	C	T
.	.	.	.	0.0	0.93,I	1.0,D	0.99	5.6	4.37	.	.	SOM	GT:/	0:12	0/1:188,47	G	A
.	.	.	.	.	.	.	.	3	2.85	.	.	SOM	GT:/	0:60	0/1:96,21:3	A	G
.	.	.	.	.	.	.	.	.	.	.	0	SOM	GT:/	0:15	0/1:229,49	C	T
.	.	.	.	.	.	.	.	.	.	.	.	SOM	GT:/	0:36	0/1:65,18:3	C	A
.	.	.	.	.	.	.	.	.	.	.	.	SOM	GT:/	0:17	0/1:12,6:37	G	A
.	.	.	.	.	.	.	.	.	.	.	.	SOM	GT:/	0:17	0/1:26,8:37	T	G
.	.	.	.	.	.	.	.	.	.	.	.	SOM	GT:/	0:18	0/1:30,5:34	C	T
.	.	.	.	0.0:	0.455	0.822	0.95	3.9	1.76	.	.	SOM	GT:/	0:70	0/1:109,20	C	T
.	.	.	.	.	.	.	.	.	.	.	.	SOM	GT:/	0:9,(	0/1:7,6:35:	T	A
.	.	.	.	.	.	.	.	.	.	.	.	SOM	GT:/	0:42	0/1:60,12:3	A	G
.	.	##	0	.	.	.	.	.	.	0	.	SOM	GT:/	0:55	0/1:73,15:3	G	A
.	.	.	.	.	.	.	1,A	5	8.40	.	.	SOM	GT:/	0:67	0/1:130,21	C	T
.	0	0	0	.	.	.	.	.	1.65	.	.	SOM	GT:/	0:31	0/1:53,12:3	C	T
.	.	.	.	0.0(	0.993	1.0,D	1,D	3.8	4.93	.	.	SOM	GT:/	0:53	0/1:91,28:3	C	T
2E-04	.	.	.	.	.	.	.	.	1.50	.	.	SOM	GT:/	0:63	0/1:109,22	G	A
.	.	.	.	.	.	.	.	.	1.46	.	.	SOM	GT:/	0:57	0/1:123,7:3	G	T
.	.	.	.	.	.	.	.	.	.	.	.	SOM	GT:/	0:24	0/1:55,5:37	C	T
2E-04	.	0	0	.	.	.	.	.	.	.	.	SOM	GT:/	0:36	0/1:77,4:37	C	T
.	.	.	.	0.6	0.002	0.001	1,N	.	.	.	.	SOM	GT:/	0:81	0/1:153,17	G	T
.	.	.	.	.	.	.	.	2.8	.	.	.	SOM	GT:/	0:15	0/1:251,58	G	A
.	.	##	0	.	.	.	.	.	.	.	.	SOM	GT:/	0:37	0/1:62,12:3	G	A
.	.	.	.	.	.	.	.	.	.	.	.	SOM	GT:/	0:19	0/1:292,52	G	A
.	.	.	.	.	.	.	.	.	.	.	.	SOM	GT:/	0:44	0/1:55,15:3	T	C
.	.	.	.	.	.	.	.	.	.	.	.	SOM	GT:/	0:21	0/1:33,6:33	G	T
.	.	.	.	.	.	.	.	.	.	.	.	SOM	GT:/	0:12	0/1:4,2:37:	C	A
.	.	.	.	.	.	.	.	3.2	2.43	.	.	SOM	GT:/	0:29	0/1:47,7:35	G	T
.	.	.	.	.	.	.	.	.	.	.	.	SOM	GT:/	0:19	0/1:36,8:37	G	A
.	.	##	0	.	.	.	.	.	.	.	.	SOM	GT:/	0:16	0/1:295,76	C	T
.	##	##	0	0.1:	0.002	0.008	1,N	.	.	.	.	SOM	GT:/	0:46	0/1:86,21:3	G	A
.	.	.	.	0.1	0.257	0.687	1.00	4.7	1.29	.	.	SOM	GT:/	0:56	0/1:89,21:3	A	G
.	.	.	.	.	.	.	.	.	.	.	.	SOM	GT:/	0:86	0/1:139,30	G	T
.	.	.	.	.	.	.	.	.	.	.	.	SOM	GT:/	0:24	0/1:52,13:3	G	A
.	.	0	0	.	.	.	.	.	.	.	.	SOM	GT:/	0:51	0/1:96,24:3	G	A
.	.	0	0	.	.	.	.	.	.	.	.	SOM	GT:/	0:75	0/1:103,26	G	A
.	.	.	.	.	.	.	.	.	.	.	.	SOM	GT:/	0:93	0/1:131,25	T	C
.	.	.	.	.	.	.	.	.	.	.	.	SOM	GT:/	0:28	0/1:40,6:37	G	A
.	.	.	.	0.0	0.993	1.0,D	1.00	3.2	1.94	.	.	SOM	GT:/	0:95	0/1:164,33	C	T

.	0	##	0	.	.	.	.	.	.	0	.	SOM	GT:/	0:23	0/1:394,61	C	T
.	.	.	.	.	.	.	.	.	.	.	.	SOM	GT:/	0:10	0/1:23,5:37	A	G
.	.	.	.	.	.	.	.	.	.	.	.	SOM	GT:/	0:48	0/1:90,25:3	C	T
.	.	.	.	.	.	.	.	.	.	.	.	SOM	GT:/	0:51	0/1:91,14:3	G	A
.	.	.	.	.	.	.	.	.	.	.	.	SOM	GT:/	0:72	0/1:99,24:3	G	T
.	.	.	.	.	.	.	.	.	.	.	.	SOM	GT:/	0:19	0/1:32,11:3	G	A
0.18	.	.	.	.	.	.	.	.	.	.	.	SOM	GT:/	0:8,(	0/1:6,2:37:	T	C
.	.	.	.	0.1	0.015	0.027	1,N	4.8	.	.	.	SOM	GT:/	0:83	0/1:116,18	T	C
.	.	.	.	.	.	.	.	.	.	.	.	SOM	GT:/	0:39	0/1:55,7:37	G	A
.	.	.	.	.	.	.	.	.	.	.	.	SOM	GT:/	0:26	0/1:27,3:37	C	T
.	.	.	.	.	.	.	.	3.4	1.62	.	.	SOM	GT:/	0:65	0/1:130,6:3	G	A
0.01	0	0	0	.	.	.	.	3.9	.	0	0	DB;S	GT:/	0:10	0/1:199,43	C	T
.	.	.	.	.	.	.	1,D	5.4	16.1	.	.	SOM	GT:/	0:83	0/1:137,20	G	A
.	.	.	.	0.7	0.052	0.024	0.99	5.2	.	.	.	SOM	GT:/	0:13	0/1:219,44	C	T
0.003	0	0	0	0.1	0.001	0.008	1,N	.	1.50	0	.	DB;S	GT:/	0:73	0/1:127,23	C	T
.	.	##	0	.	.	.	.	.	.	0	0	SOM	GT:/	0:37	0/1:43,5:37	C	T
.	.	.	.	.	0.966	0.999	.	5.7	5.19	.	.	SOM	GT:/	0:79	0/1:102,15	G	A
.	.	##	0	0.0	0.992	1.0,D	1,D	4.7	5.02	.	.	SOM	GT:/	0:20	0/1:37,8:37	C	T
.	.	.	.	0.0	1.0,D	1.0,D	1,D	4.9	4.04	.	.	SOM	GT:/	0:47	0/1:86,17:3	C	A
.	.	.	.	.	.	.	.	3.1	3.01	.	.	SOM	GT:/	0:21	0/1:39,8:37	G	A
.	.	.	.	.	.	.	.	.	.	.	.	SOM	GT:/	0:10	0/1:35,6:37	G	A
.	.	##	0	0.0	0.151	0.549	1,D	3.2	2.63	.	.	SOM	GT:/	0:14	0/1:263,48	G	A
.	.	.	.	0.3	.	.	0.99	2.7	1.67	.	.	SOM	GT:/	0:64	0/1:94,15:3	G	A
.	.	.	.	.	.	.	.	.	.	.	.	SOM	GT:/	0:21	0/1:25,6:37	G	T
.	.	.	.	.	.	.	.	.	.	.	.	SOM	GT:/	0:55	0/1:106,17	C	T
.	.	.	.	.	.	.	.	.	.	.	.	SOM	GT:/	0:41	0/1:51,9:35	G	T
.	.	.	.	.	.	.	.	.	.	.	.	SOM	GT:/	0:35	0/1:59,6:37	C	A
.	.	.	.	.	.	.	.	.	.	.	.	SOM	GT:/	0:59	0/1:75,8:37	A	C
0.005	0	0	0	0.0	0.978	1.0,D	1,D	5.8	5.04	.	.	DB;S	GT:/	0:96	0/1:137,19	G	A
.	.	.	.	0.4	0.032	0.002	1.00	5.6	2.60	.	.	SOM	GT:/	0:31	0/1:44,7:37	A	G
.	.	.	.	.	.	.	.	.	.	.	.	SOM	GT:/	0:16	0/1:34,4:37	A	T
.	.	.	.	.	.	.	.	.	1.90	.	.	SOM	GT:/	0:19	0/1:47,9:37	C	A
.	.	.	.	.	.	.	.	.	.	.	.	SOM	GT:/	0:69	0/1:96,21:3	C	T
.	.	.	.	0.0	0.995	1.0,D	1,D	5.4	4.86	.	.	SOM	GT:/	0:52	0/1:62,20:3	C	T
.	.	.	.	0.1	0.029	0.703	0.63	2.3	2.96	.	.	SOM	GT:/	0:11	0/1:203,46	G	A
.	.	.	.	.	.	.	1,D	3.5	11.1	.	.	SOM	GT:/	0:64	0/1:107,22	G	A
.	.	##	.	.	.	.	.	.	.	.	.	SOM	GT:/	0:75	0/1:129,25	C	T
.	.	.	.	0.0	0.977	0.999	1.00	3.5	.	.	.	SOM	GT:/	0:22	0/1:54,9:34	T	C
.	.	.	.	.	.	.	.	.	.	.	.	SOM	GT:/	0:45	0/1:59,8:37	T	C
.	.	.	.	0.0	1.0,D	1.0,D	1.00	4.4	4.35	.	.	SOM	GT:/	0:53	0/1:115,7:3	T	G
.	.	##	0	0.0	1.0,D	1.0,D	0.90	3.2	.	.	.	SOM	GT:/	0:16	0/1:247,51	G	A
.	##	.	.	1.0	0.004	0.006	1,N	.	.	.	.	SOM	GT:/	0:77	0/1:102,16	C	T
.	.	##	0	.	.	.	.	.	.	0	.	SOM	GT:/	0:35	0/1:45,5:37	C	T
.	.	##	0	0.0	1.0,D	1.0,D	1.00	5	4.11	.	.	SOM	GT:/	0:98	0/1:165,21	G	A
.	.	.	.	.	.	.	.	.	.	.	.	SOM	GT:/	0:17	0/1:24,7:37	C	A
.	.	.	.	.	.	.	.	5.1	.	.	.	SOM	GT:/	0:14	0/1:250,37	G	T
.	.	##	0	.	.	.	.	.	.	.	0	SOM	GT:/	0:68	0/1:88,17:3	G	A
.	##	##	0	0.0	0.288	0.928	1.00	5	3.02	.	.	SOM	GT:/	0:67	0/1:119,13	C	T
.	##	##	0	0.0	0.005	0.006	0.75	3.7	1.63	.	.	SOM	GT:/	0:92	0/1:179,31	C	T
.	.	.	.	.	.	.	.	4.7	.	.	.	SOM	GT:/	0:15	0/1:37,8:34	T	G
.	.	##	0	0.3	0.812	0.997	1,D	5.3	3.80	.	.	SOM	GT:/	0:34	0/1:60,10:3	C	T
0.023	0	0	0	.	.	.	.	.	.	.	.	DB;S	GT:/	0:40	0/1:96,17:3	G	A
.	.	.	.	.	.	.	.	.	.	.	.	SOM	GT:/	0:32	0/1:70,11:3	T	A
.	.	##	0	0.0	0.069	0.218	1.00	5	3.71	.	.	SOM	GT:/	0:45	0/1:93,19:3	C	T

.	.	##	0	.	.	.	.	.	1.45	.	.	SOM	GT:/	0:86	0/1:157,30	G	A
2E-04	0	0	0	.	.	.	.	.	.	.	.	SOM	GT:/	0:25	0/1:42,6:37	C	T
.	.	.	.	.	.	.	.	.	.	.	.	SOM	GT:/	0:15	0/1:22,5:37	G	A
.	.	.	.	0.2	0.0,B	0.0,B	1,N	.	.	.	.	SOM	GT:/	0:86	0/1:178,38	G	A
.	.	.	.	.	.	.	.	3.1	.	.	.	SOM	GT:/	0:49	0/1:95,22:3	G	A
.	##	##	0	0.1	0.662	0.998	0.82	2.3	4.16	.	.	SOM	GT:/	0:59	0/1:131,22	G	A
.	.	.	.	0.0	0.718	0.991	0.96	3.8	3.80	.	.	SOM	GT:/	0:45	0/1:96,23:3	G	A
.	.	.	.	.	.	.	.	.	.	.	.	SOM	GT:/	0:12	0/1:26,4:34	T	G
.	.	.	.	.	.	.	.	.	.	.	.	SOM	GT:/	0:16	0/1:230,32	G	A
.	.	.	.	.	.	.	.	2.6	.	.	.	SOM	GT:/	0:13	0/1:196,44	C	T
.	.	.	.	.	.	.	.	2.1	.	.	.	SOM	GT:/	0:61	0/1:105,18	C	T
.	.	.	.	0.1	0.979	0.995	0.53	2.3	2.09	.	.	SOM	GT:/	0:59	0/1:123,24	C	T
.	.	.	.	0.0	0.992	0.999	1.00	5	4.68	.	.	SOM	GT:/	0:67	0/1:151,23	T	C
.	.	.	.	.	.	.	.	.	.	.	.	SOM	GT:/	0:23	0/1:30,6:37	G	A
.	.	.	.	.	.	.	.	.	.	0	.	SOM	GT:/	0:52	0/1:52,8:32	C	T
.	##	##	0	.	.	.	.	.	3.28	.	.	SOM	GT:/	0:24	0/1:466,80	C	T
.	.	##	0	0.0	0.998	1.0,D	1,D	4.1	3.12	.	.	SOM	GT:/	0:27	0/1:427,85	C	T
.	.	.	.	.	.	.	.	.	.	.	.	SOM	GT:/	0:25	0/1:21,6:37	G	A
.	.	.	.	.	.	.	.	.	.	.	.	SOM	GT:/	0:28	0/1:33,7:33	A	G
.	.	##	0	0.0	0.959	1.0,D	1.00	4.8	4.03	.	.	SOM	GT:/	0:74	0/1:116,29	C	T
0.002	.	.	.	.	.	.	.	2.5	.	.	.	SOM	GT:/	0:59	0/1:120,15	G	A
.	.	.	.	.	.	.	.	.	.	.	.	SOM	GT:/	0:28	0/1:34,3:37	G	C
.	.	.	.	.	.	.	.	2.4	.	.	.	SOM	GT:/	0:39	0/1:55,8:37	C	A
.	.	.	.	.	.	.	.	.	.	.	.	SOM	GT:/	0:38	0/1:59,9:37	A	G
.	.	.	.	.	.	.	.	.	.	.	.	SOM	GT:/	0:19	0/1:20,6:37	C	A
.	.	.	.	.	.	.	.	.	.	.	.	SOM	GT:/	0:22	0/1:22,4:37	T	C
.	.	.	.	.	.	.	.	4.4	.	.	.	SOM	GT:/	0:36	0/1:50,17:3	G	A
.	.	.	.	.	.	.	.	.	.	.	.	SOM	GT:/	0:19	0/1:33,9:37	C	T
.	.	##	0	0.2	0.05,I	0.652	1,N	.	.	.	.	SOM	GT:/	0:75	0/1:97,17:3	G	A
.	.	##	0	0.1	0.004	0.096	1,N	.	1.64	.	.	SOM	GT:/	0:59	0/1:93,15:3	G	A
.	.	##	0	.	.	.	.	.	.	.	.	SOM	GT:/	0:29	0/1:59,13:3	C	T
.	.	.	.	.	.	.	.	.	.	.	.	SOM	GT:/	0:11	0/1:220,40	G	A
.	.	.	.	.	.	.	.	.	.	0	0.1	SOM	GT:/	0:10	0/1:2,2:37:	G	C
.	.	.	.	.	.	.	.	.	.	.	.	SOM	GT:/	0:44	0/1:61,9:35	G	A
.	.	.	.	0.0	0.999	1.0,D	1,D	5.6	4.14	.	.	SOM	GT:/	0:50	0/1:100,29	G	A
.	.	.	.	.	.	.	.	.	.	.	.	SOM	GT:/	0:20	0/1:27,8:37	G	A
0.007	.	0	0	.	.	.	.	.	.	.	.	DB;S	GT:/	0:30	0/1:41,9:35	G	A
0.001	0	0	0	0.0	0.233	0.647	1,D	5.6	3.66	.	.	SOM	GT:/	0:44	0/1:65,15:3	G	A
.	.	.	.	.	.	.	.	.	.	.	.	SOM	GT:/	0:20	0/1:26,6:37	G	A
.	.	##	0	0.0	0.204	0.284	1,D	5.3	3.64	.	.	SOM	GT:/	0:68	0/1:90,23:3	C	T
.	.	.	.	.	.	.	.	3.9	1.89	.	.	SOM	GT:/	0:28	0/1:44,9:35	A	G
.	.	.	.	.	.	.	.	.	1.33	.	.	SOM	GT:/	0:48	0/1:62,14:3	T	C
.	.	.	.	.	.	.	.	.	1.75	.	.	SOM	GT:/	0:99	0/1:150,24	A	G
4E-04	.	.	.	.	.	.	.	.	.	.	.	SOM	GT:/	0:30	0/1:69,7:37	C	T
.	.	.	.	.	.	.	.	5.8	5.38	.	.	SOM	GT:/	0:16	0/1:35,12:3	G	A
.	.	.	.	.	.	.	.	.	.	.	.	SOM	GT:/	0:63	0/1:89,17:3	C	T
.	.	##	0	.	.	.	.	.	.	.	.	SOM	GT:/	0:35	0/1:50,13:3	G	A
.	.	.	.	.	.	.	.	.	.	.	0	SOM	GT:/	0:26	0/1:22,15:3	T	G
.	.	.	.	.	.	.	.	4.8	.	.	.	SOM	GT:/	0:83	0/1:110,16	C	T
.	.	.	.	.	.	.	.	2.7	.	.	.	SOM	GT:/	0:75	0/1:111,21	C	A
.	.	.	.	0.1	0.211	0.673	0.98	4.9	3.03	.	.	SOM	GT:/	0:70	0/1:130,27	C	T
.	.	.	.	.	.	.	.	.	.	.	.	SOM	GT:/	0:21	0/1:31,6:37	G	T
.	.	.	.	.	.	.	.	.	2.81	.	.	SOM	GT:/	0:78	0/1:149,35	C	A
.	.	.	.	.	.	.	.	4.7	.	.	.	SOM	GT:/	0:43	0/1:65,11:3	G	A

.	.	##	0	.	.	.	.	.	.	.	.	SOM	GT:/	0:38	0/1:65,13:3	T	C
.	.	.	.	0.0(	1.0,D	1.0,D	1,D	4.7	4.88	.	.	SOM	GT:/	0:42	0/1:68,5:37	C	A
0.002	0	0	0	0.0:	0.977	1.0,D	0.83	4.3	3.69	0	.	SOM	GT:/	0:10	0/1:200,36	C	T
0.003	0	0	0	0.0(	0.77,I	0.819	1,N	.	1.29	.	.	SOM	GT:/	0:25	0/1:403,73	C	T
0.002	.	0	0	.	.	.	.	.	.	0	.	SOM	GT:/	0:14	0/1:176,31	C	T
.	.	.	.	.	.	.	.	2.9	.	.	.	SOM	GT:/	0:58	0/1:88,15:3	C	A
.	.	.	.	0.0	0.998	0.998	1,D	5.5	3.68	.	.	SOM	GT:/	0:48	0/1:61,11:3	C	T
.	.	.	.	0.0	1.0,D	1.0,D	1,D	5.4	5.19	.	.	SOM	GT:/	0:78	0/1:124,28	G	A
4E-04	.	0	0	.	.	.	.	.	.	.	.	SOM	GT:/	0:36	0/1:74,10:3	C	T
.	.	##	.	.	.	.	.	.	.	.	.	DB;S	GT:/	0:21	0/1:398,69	G	T
.	.	##	0	0.1	1.0,D	1.0,D	1,D	5.9	4.06	.	.	SOM	GT:/	0:72	0/1:108,27	G	A
.	.	.	.	0.0!	0.61,I	0.566	0.97	.	3.26	.	.	SOM	GT:/	0:87	0/1:139,26	G	T
.	.	.	.	.	.	.	.	.	.	.	.	SOM	GT:/	0:14	0/1:17,3:37	G	A
.	.	.	.	.	.	.	.	.	.	.	.	SOM	GT:/	0:9,(	0/1:17,3:37	G	A
.	.	.	.	.	.	.	.	.	.	.	.	SOM	GT:/	0:22	0/1:31,4:27	C	T
.	.	.	.	.	.	.	.	2	.	.	.	SOM	GT:/	0:9,(	0/1:45,11:3	G	A
.	.	##	0	0.5(	0.168	0.642	0.85	4.9	3.51	.	.	SOM	GT:/	0:45	0/1:77,9:32	G	A
.	.	.	.	.	.	.	.	.	.	.	.	SOM	GT:/	0:17	0/1:31,5:37	G	T
.	.	.	.	.	.	.	.	3.7	1.29	.	.	SOM	GT:/	0:14	0/1:220,39	C	T
4E-04	##	##	0	.	.	.	.	.	.	0	.	SOM	GT:/	0:13	0/1:175,37	C	T
.	.	.	.	.	.	.	.	.	.	0	0	SOM	GT:/	0:43	0/1:69,12:3	G	A
.	.	.	.	.	.	.	.	.	.	.	0	SOM	GT:/	0:17	0/1:21,3:37	C	T
.	.	.	.	.	.	.	.	.	.	.	.	SOM	GT:/	0:91	0/1:144,29	C	A
.	.	.	.	.	.	.	.	2.3	.	.	.	SOM	GT:/	0:28	0/1:48,9:37	G	T
.	.	.	.	0.0:	0.928	0.997	1.00	4.9	3.64	.	.	SOM	GT:/	0:12	0/1:226,36	A	G
.	.	.	.	0.1:	0.005	0.04,I	1,N	.	2.00	.	.	SOM	GT:/	0:83	0/1:127,22	G	A
.	.	.	.	.	.	.	.	.	3.44	.	.	SOM	GT:/	0:19	0/1:42,11:3	C	T
.	.	.	.	.	.	.	.	.	.	.	.	SOM	GT:/	0:8,(	0/1:10,3:37	C	T
.	.	.	.	.	.	.	.	.	.	.	.	SOM	GT:/	0:87	0/1:130,32	C	A
.	.	.	.	.	.	.	1.00	2.9	1.47	.	.	SOM	GT:/	0:35	0/1:68,13:3	T	C
.	.	.	.	0.1:	0.001	0.0,B	1,N	.	.	.	.	SOM	GT:/	0:57	0/1:91,18:3	T	A
.	.	.	.	0.1!	0.666	0.885	1,D	3.1	2.21	.	.	SOM	GT:/	0:35	0/1:58,10:3	G	A
.	.	.	.	.	.	.	.	2.2	1.34	.	.	SOM	GT:/	0:8,(	0/1:17,3:37	A	G
.	.	.	.	.	.	.	.	.	.	0	.	DB;S	GT:/	0:21	0/1:28,3:37	T	C
.	.	.	.	.	.	.	.	.	.	.	.	SOM	GT:/	0:64	0/1:90,16:3	C	A
.	.	.	.	.	.	.	.	.	.	.	.	SOM	GT:/	0:69	0/1:131,21	G	A
.	.	.	.	.	.	.	.	.	2.01	.	.	SOM	GT:/	0:50	0/1:83,15:3	C	A
.	.	##	0	0.0	0.99,I	1.0,D	1,D	.	4.09	.	.	SOM	GT:/	0:46	0/1:79,14:3	C	T
.	.	.	.	0.0(	0.93,I	0.996	1,D	6	3.76	.	.	SOM	GT:/	0:51	0/1:76,12:3	T	C
.	.	##	0	.	.	.	.	.	.	.	.	SOM	GT:/	0:17	0/1:17,3:37	C	T
.	.	.	.	0.0(	0.999	1.0,D	1.00	4	4.05	.	.	SOM	GT:/	0:39	0/1:61,6:37	C	T
.	.	.	.	.	.	.	1,A	3.9	7.41	.	.	SOM	GT:/	0:25	0/1:43,6:37	G	T
.	.	.	.	.	.	.	.	.	.	.	.	SOM	GT:/	0:8,(	0/1:27,8:37	A	C
.	.	.	.	0.0	0.865	0.988	0.96	3.7	1.94	.	.	SOM	GT:/	0:27	0/1:28,5:37	A	G
.	.	.	.	.	.	.	.	.	.	.	.	SOM	GT:/	0:44	0/1:61,11:3	A	G
.	.	.	.	.	.	.	.	3.1	2.96	.	.	SOM	GT:/	0:36	0/1:51,7:35	C	T
.	0	##	0	.	.	.	.	.	.	.	.	SOM	GT:/	0:62	0/1:111,17	C	T
.	.	.	.	.	.	.	.	.	.	.	.	SOM	GT:/	0:36	0/1:65,11:3	C	T
.	.	.	.	.	.	.	.	.	.	.	.	SOM	GT:/	0:36	0/1:66,7:37	G	A
.	.	.	.	.	.	.	.	.	.	.	.	SOM	GT:/	0:10	0/1:15,7:33	C	T
.	.	.	.	.	.	.	.	.	.	.	.	SOM	GT:/	0:9,(	0/1:13,3:37	C	T
.	.	.	.	.	.	.	.	.	.	.	.	SOM	GT:/	0:13	0/1:42,4:37	C	T
.	.	.	.	.	.	.	.	.	.	.	.	SOM	GT:/	0:27	0/1:29,5:37	A	T
.	.	.	.	0.0!	0.069	0.246	1,N	.	.	.	.	SOM	GT:/	0:55	0/1:90,18:3	A	C

.	.	.	.	.	.	.	.	.	.	.	.	SON	GT:/	0:36	0/1:61,8:37	C	T
.	.	.	.	0.0	0.808	0.98,I	1,N	.	2.20	.	.	SON	GT:/	0:15	0/1:281,45	T	C
2E-04	.	.	.	.	.	.	.	.	1.67	.	.	SON	GT:/	0:66	0/1:126,36	G	A
.	.	##	0	0.0	0.996	1.0,D	1,D	4.6	5.03	.	.	SON	GT:/	0:56	0/1:92,24:3	G	A
.	.	.	.	.	.	.	.	.	.	.	.	SON	GT:/	0:40	0/1:68,15:3	A	G
0.035	.	.	.	.	.	.	.	2.5	3.31	.	.	DB;S	GT:/	0:24	0/1:42,8:37	G	A
.	.	.	.	.	.	.	.	.	.	.	.	SON	GT:/	0:77	0/1:95,25:3	G	A
.	.	.	.	0.0	0.987	0.996	1,D	5.7	5.20	.	.	SON	GT:/	0:34	0/1:55,7:37	T	C
.	.	.	.	.	.	.	.	.	.	.	.	SON	GT:/	0:58	0/1:102,11	A	G
.	.	.	.	.	.	.	.	.	.	.	.	SON	GT:/	0:45	0/1:73,21:3	G	T
.	##	##	.	.	.	.	.	.	.	0	.	SON	GT:/	0:20	0/1:38,4:37	C	T
2E-04	.	##	0	0.1	0.446	0.995	0.96	.	.	.	.	SON	GT:/	0:63	0/1:99,19:3	G	A
.	.	.	.	.	.	.	.	4.6	.	.	.	SON	GT:/	0:69	0/1:117,29	C	T
.	.	.	.	.	.	.	.	.	.	.	.	SON	GT:/	0:38	0/1:62,24:3	G	A
.	.	.	.	.	.	.	.	.	2.01	.	.	SON	GT:/	0:49	0/1:114,5:3	C	T
.	.	.	.	.	.	.	.	.	.	.	.	SON	GT:/	0:36	0/1:75,13:3	G	A
.	.	.	.	.	.	.	.	5.3	2.00	.	.	SON	GT:/	0:14	0/1:23,5:26	G	T
.	##	##	0	0.0	0.871	0.991	0.99	4.1	4.54	.	.	SON	GT:/	0:41	0/1:48,5:37	C	T
.	.	##	0	0.0	0.98,I	1.0,D	1.00	4.9	4.81	.	.	SON	GT:/	0:82	0/1:111,22	C	T
0.021	.	.	.	.	.	.	.	.	.	0	.	DB;S	GT:/	0:34	0/1:39,7:37	T	A
.	.	.	.	.	.	.	.	.	.	.	.	SON	GT:/	0:14	0/1:25,4:37	G	C
.	.	.	.	0.0	0.998	0.999	1,D	5	4.83	.	.	SON	GT:/	0:95	0/1:155,32	G	A
.	##	##	0	.	.	.	.	.	.	0	.	SON	GT:/	0:38	0/1:70,12:3	G	A
.	.	.	.	.	.	.	.	.	.	.	.	SON	GT:/	0:26	0/1:34,3:37	T	C
2E-04	.	0	0	0.0	0.014	0.068	1.00	.	3.03	.	.	SON	GT:/	0:10	0/1:180,30	G	A
.	.	.	.	0.1	0.272	0.565	0.99	3	2.43	.	.	SON	GT:/	0:68	0/1:107,29	T	G
2E-04	.	0	0	.	.	.	.	3.9	.	.	.	SON	GT:/	0:45	0/1:54,11:3	C	T
.	.	.	.	0.0	0.549	0.956	1,D	5.2	4.98	.	.	SON	GT:/	0:56	0/1:74,10:3	A	G
.	.	.	.	0.2	0.012	0.019	1,D	4.9	2.97	.	.	SON	GT:/	0:58	0/1:76,15:3	A	G
.	.	.	.	0.0	0.001	0.003	1,N	.	.	.	.	SON	GT:/	0:55	0/1:88,17:3	G	A
.	.	##	0	.	.	.	.	2.9	.	.	.	SON	GT:/	0:50	0/1:93,14:3	C	T
.	.	.	.	0.0	0.946	0.999	1.00	5.5	4.78	.	.	SON	GT:/	0:35	0/1:59,6:37	A	C
.	##	##	0	0.4	0.195	0.736	1.00	5.9	2.50	.	.	SON	GT:/	0:30	0/1:38,7:37	C	T
.	.	.	.	.	.	.	.	.	1.50	.	.	SON	GT:/	0:58	0/1:78,12:3	G	T
.	.	.	.	0.2	0.976	0.999	1,D	5.1	3.90	.	.	SON	GT:/	0:37	0/1:64,11:3	C	A
.	.	.	.	0.0	0.999	1.0,D	1,D	5.7	4.55	.	.	SON	GT:/	0:50	0/1:79,8:37	G	T
.	.	##	0	.	.	.	.	3.1	1.36	0	.	SON	GT:/	0:59	0/1:61,13:3	C	T
.	.	.	.	.	.	.	.	.	.	0	.	SON	GT:/	0:38	0/1:39,10:3	A	G
.	.	.	.	0.0	0.864	0.997	0.99	3.3	2.82	.	.	SON	GT:/	0:32	0/1:64,9:37	G	T
.	.	.	.	.	.	.	.	.	.	.	.	SON	GT:/	0:72	0/1:112,20	C	T
.	.	.	.	.	.	.	.	.	.	.	.	SON	GT:/	0:30	0/1:38,6:37	G	A
2E-04	.	##	0	0.0	0.266	0.923	1,D	5.7	3.88	.	.	SON	GT:/	0:58	0/1:84,12:3	G	A
2E-04	.	##	.	.	.	.	.	.	.	.	.	SON	GT:/	0:26	0/1:54,8:37	G	A
.	.	.	.	.	.	.	.	.	.	.	.	SON	GT:/	0:27	0/1:35,5:37	T	C
.	.	##	0	0.5	0.002	0.002	1,N	4.1	.	.	.	SON	GT:/	0:75	0/1:130,30	C	T
.	.	.	.	.	.	.	.	.	.	.	.	SON	GT:/	0:53	0/1:87,9:35	C	T
.	.	.	.	.	.	.	.	.	.	.	.	SON	GT:/	0:29	0/1:29,10:3	A	G
.	.	.	.	.	.	.	.	4.4	.	.	.	SON	GT:/	0:23	0/1:43,9:35	G	T
.	.	.	.	0.0	0.999	1.0,D	1,D	5.4	4.00	.	.	SON	GT:/	0:71	0/1:96,20:3	G	T
.	.	.	.	.	.	.	.	.	.	.	.	SON	GT:/	0:48	0/1:80,13:3	G	A
.	.	.	.	.	.	.	.	.	.	.	.	SON	GT:/	0:75	0/1:132,24	C	T
.	.	.	.	0.2	1.0,D	1.0,D	1,D	6	2.22	.	.	SON	GT:/	0:23	0/1:57,7:37	C	T
.	.	.	.	.	.	.	.	.	.	.	.	SON	GT:/	0:13	0/1:14,4:37	T	C
.	.	.	.	.	.	.	.	.	.	.	.	SON	GT:/	0:19	0/1:36,6:37	C	T

.	.	.	.	.	.	.	.	.	.	.	.	SON	GT:/	0:9,(	0/1:9,3:37:	G	T
.	.	.	.	.	.	.	.	3.7	.	.	.	SON	GT:/	0:22	0/1:25,4:37	C	A
.	##	##	0	.	.	.	.	.	.	.	.	SON	GT:/	0:59	0/1:87,15:3	G	A
.	.	##	0	.	.	.	.	.	.	.	.	SON	GT:/	0:52	0/1:82,9:34	C	T
.	.	.	.	.	.	.	.	.	.	.	.	SON	GT:/	0:62	0/1:95,4:37	T	G
.	.	.	.	.	.	.	.	.	.	.	.	SON	GT:/	0:16	0/1:27,7:35	A	G
.	.	.	.	.	.	.	.	.	3.27	.	.	SON	GT:/	0:83	0/1:124,28	G	T
.	.	.	.	.	.	.	.	3.6	2.68	.	.	SON	GT:/	0:18	0/1:28,5:37	C	T
.	.	0	0	.	.	.	.	.	2.56	.	.	SON	GT:/	0:38	0/1:55,7:37	C	T
.	.	.	.	0.7	0.001	0.001	1,N	.	.	.	.	SON	GT:/	0:68	0/1:82,20:3	A	T
.	.	.	.	.	.	.	.	.	.	.	.	SON	GT:/	0:9,(	0/1:9,3:37:	G	A
.	.	.	.	.	.	.	.	.	.	.	.	SON	GT:/	0:40	0/1:59,14:3	C	T
.	.	.	.	.	.	.	.	.	.	.	.	SON	GT:/	0:15	0/1:23,4:37	G	T
.	.	.	.	.	.	.	.	.	.	.	.	SON	GT:/	0:12	0/1:21,3:37	G	A
2E-04	.	.	.	.	.	.	.	.	.	.	.	SON	GT:/	0:57	0/1:94,11:3	G	A
.	.	.	.	.	.	.	.	.	.	.	.	SON	GT:/	0:18	0/1:32,7:37	C	A
.	.	.	.	.	.	.	.	.	.	.	.	SON	GT:/	0:14	0/1:19,3:37	G	A
.	.	.	.	.	.	.	.	.	2.11	.	.	SON	GT:/	0:22	0/1:32,4:37	T	C
.	##	##	0	.	.	.	.	.	.	.	.	SON	GT:/	0:28	0/1:45,12:3	C	T
.	.	.	.	.	.	.	.	.	.	.	.	SON	GT:/	0:36	0/1:58,10:3	T	C
0.001	.	0	0	.	.	.	.	.	.	0	0	SON	GT:/	0:11	0/1:165,45	G	A
.	.	.	.	.	.	.	.	5.5	4.12	.	.	SON	GT:/	0:18	0/1:23,5:37	T	G
.	.	.	.	.	.	.	.	.	.	.	.	SON	GT:/	0:42	0/1:74,14:3	C	T
.	.	##	0	.	.	.	.	.	.	.	.	SON	GT:/	0:17	0/1:257,62	C	T
.	.	.	.	0.3	0.814	0.999	1,N	4.4	1.24	0	0	SON	GT:/	0:40	0/1:75,16:3	G	A
.	.	.	.	.	.	.	.	.	.	.	.	SON	GT:/	0:39	0/1:63,12:3	G	T
.	.	.	.	.	.	.	.	.	.	.	.	SON	GT:/	0:13	0/1:227,35	T	C
.	.	.	.	.	.	.	.	.	.	.	.	SON	GT:/	0:46	0/1:61,11:3	C	T
.	.	.	.	0.0	0.976	0.999	1,D	5.8	5.60	.	.	SON	GT:/	0:57	0/1:98,18:3	C	T
.	.	.	.	.	.	.	.	.	.	.	.	SON	GT:/	0:65	0/1:142,5:3	C	T
.	.	.	.	.	0.77,I	0.99,I	1.00	5.2	4.36	.	.	SON	GT:/	0:34	0/1:40,8:37	G	T
.	.	##	0	0.0	0.743	0.958	1,D	4.9	4.00	0	.	SON	GT:/	0:26	0/1:37,6:37	G	A
.	.	.	.	.	.	.	.	.	.	.	.	SON	GT:/	0:29	0/1:50,8:37	G	A
.	.	##	0	.	.	.	.	.	.	.	.	SON	GT:/	0:57	0/1:80,19:3	G	A
.	.	.	.	.	.	.	.	4.7	1.34	.	.	SON	GT:/	0:18	0/1:27,7:37	T	C
.	.	.	.	.	0.338	0.352	1,D	5.9	3.74	.	.	SON	GT:/	0:27	0/1:60,6:37	G	T
.	.	.	.	.	.	.	.	.	.	.	.	SON	GT:/	0:29	0/1:34,6:37	T	G
.	.	.	.	.	.	.	.	.	.	.	.	SON	GT:/	0:36	0/1:70,20:3	C	T
.	.	.	.	0.0	0.005	0.013	1,N	2.1	3.35	.	.	SON	GT:/	0:57	0/1:75,14:3	C	T
.	.	.	.	0.0	0.963	0.998	1,D	5.5	2.69	.	.	SON	GT:/	0:54	0/1:82,9:37	T	C
.	.	##	.	1.0	0.116	0.84,I	0.99	4.1	1.69	.	.	DB;S	GT:/	0:26	0/1:37,7:37	C	T
.	.	.	.	.	.	.	.	.	.	.	.	SON	GT:/	0:9,(	0/1:13,4:37	G	A
.	.	.	.	.	.	.	.	.	.	.	.	SON	GT:/	0:9,(	0/1:13,4:37	G	A
6E-04	.	.	.	.	.	.	.	.	.	0	0	SON	GT:/	0:43	0/1:53,11:3	C	T
.	.	##	0	.	.	.	.	.	.	.	.	SON	GT:/	0:24	0/1:37,5:34	C	T
2E-04	.	.	.	.	.	.	.	.	.	.	.	SON	GT:/	0:8,(	0/1:5,2:37:	C	T
.	.	.	.	.	.	.	.	4.7	3.36	.	.	SON	GT:/	0:41	0/1:53,8:37	A	C
.	.	.	.	.	.	.	.	.	.	.	.	SON	GT:/	0:39	0/1:58,8:37	G	A
4E-04	.	.	.	.	.	.	.	.	.	.	0	SON	GT:/	0:9,(	0/1:11,5:34	C	T
.	.	.	.	.	.	.	.	.	.	.	.	SON	GT:/	0:71	0/1:113,17	C	T
.	##	##	0	0.0	0.997	1.0,D	1,D	5.9	5.76	.	.	SON	GT:/	0:11	0/1:173,27	G	A
.	##	0	0	.	.	.	.	.	.	.	.	SON	GT:/	0:15	0/1:33,6:37	G	A
.	##	##	0	.	.	.	.	.	.	.	.	SON	GT:/	0:21	0/1:32,5:37	C	T
.	.	.	.	0.5	0.001	0.004	0.99	5.9	.	.	.	SON	GT:/	0:56	0/1:105,10	T	C

.	.	##	0	0.0(	0.997	1.0,D	1.00	5.4	5.22	.	.	SON	GT:/	0:63	0/1:131,21	G	A
.	##	0	0	0.0(	0.995	1.0,D	1.00	3.8	3.80	.	.	SON	GT:/	0:58	0/1:89,14:3	G	A
.	.	##	0	.	.	.	.	.	.	.	.	SON	GT:/	0:13	0/1:256,97	G	A
.	.	.	.	.	.	.	.	.	.	.	.	SON	GT:/	0:38	0/1:66,9:35	G	A
.	.	.	.	.	.	.	.	.	.	.	.	SON	GT:/	0:72	0/1:119,22	C	T
.	.	.	.	.	.	.	.	.	.	.	.	SON	GT:/	0:26	0/1:27,5:37	G	A
.	.	##	0	0.0	0.995	1.0,D	1.00	4.3	3.99	.	.	SON	GT:/	0:33	0/1:56,7:37	G	A
.	.	##	0	0.7	0.001	0.003	1.00	.	.	.	.	SON	GT:/	0:82	0/1:124,26	C	T
.	.	.	.	.	.	.	.	.	.	.	.	SON	GT:/	0:9,(	0/1:7,3:37	C	T
.	.	.	.	.	.	.	.	.	.	.	.	SON	GT:/	0:33	0/1:76,4:37	G	T
.	.	.	.	.	.	.	.	.	.	0	0	SON	GT:/	0:12	0/1:12,4:37	C	T
.	.	.	.	.	.	.	.	2.8	1.66	.	.	SON	GT:/	0:15	0/1:267,12	A	G
.	.	##	.	0.1	0.001	0.0,B	1,N	3	.	0	0	SON	GT:/	0:77	0/1:106,12	C	A
8E-04	.	0	0	0.0	0.168	0.545	0.99	5.5	3.45	.	.	SON	GT:/	0:82	0/1:152,22	C	T
.	.	.	.	.	0.007	0.049	1,N	.	1.36	.	.	SON	GT:/	0:30	0/1:63,16:3	C	T
.	.	.	.	.	.	.	.	3.1	.	.	.	SON	GT:/	0:28	0/1:34,4:37	T	C
.	##	.	.	.	.	.	.	.	.	.	.	SON	GT:/	0:30	0/1:67,6:37	G	A
.	.	##	0	.	.	.	.	.	.	.	.	SON	GT:/	0:86	0/1:149,23	G	A
.	.	.	.	.	.	.	.	.	.	.	.	SON	GT:/	0:13	0/1:206,40	A	G
.	.	##	0	.	.	.	.	.	.	.	.	SON	GT:/	0:17	0/1:40,6:37	G	A
.	.	.	.	.	.	.	.	.	.	.	.	SON	GT:/	0:9,(	0/1:10,5:37	C	T
.	.	.	.	.	.	.	.	.	.	.	.	SON	GT:/	0:9,(	0/1:12,4:37	T	A
.	.	.	.	0.0	0.068	0.033	1,N	.	.	.	.	SON	GT:/	0:18	0/1:58,7:37	C	A
.	.	.	.	.	.	.	.	.	.	.	.	SON	GT:/	0:38	0/1:60,16:3	C	A
0.006	.	0	0	.	.	.	.	.	.	.	.	SON	GT:/	0:14	0/1:252,32	G	A
.	.	.	.	.	.	.	.	4.5	6.74	.	.	SON	GT:/	0:40	0/1:82,18:3	C	T
.	.	.	.	0.0	0.142	0.955	1,D	4.8	1.42	.	.	SON	GT:/	0:36	0/1:59,10:3	G	A
.	.	.	.	.	.	.	1,D	5.3	3.23	.	.	SON	GT:/	0:44	0/1:68,11:3	T	C
.	.	.	.	.	.	.	.	.	.	.	.	SON	GT:/	0:33	0/1:47,7:35	G	T
.	.	.	.	.	.	.	.	2.1	.	.	.	SON	GT:/	0:37	0/1:59,12:3	G	T
.	.	##	0	.	.	.	.	.	1.31	.	.	SON	GT:/	0:15	0/1:279,44	G	A
.	.	.	.	.	.	.	.	3.5	1.56	.	.	SON	GT:/	0:67	0/1:111,22	A	T
.	.	.	.	.	.	.	.	.	.	.	.	SON	GT:/	0:15	0/1:14,6:35	T	C
.	.	.	.	0.0	0.999	1.0,D	1,D	5.4	3.88	.	.	SON	GT:/	0:32	0/1:64,10:3	G	T
.	.	.	.	.	.	.	.	2.1	.	.	.	SON	GT:/	0:46	0/1:73,12:3	T	C
.	##	##	0	.	.	.	.	.	.	.	.	SON	GT:/	0:53	0/1:89,13:3	C	T
.	.	.	.	.	.	.	.	.	.	.	.	SON	GT:/	0:26	0/1:20,3:37	A	G
2E-04	.	##	0	0.0	0.091	0.417	1,D	3.1	2.69	.	.	SON	GT:/	0:59	0/1:88,7:37	C	T
2E-04	.	.	.	.	.	.	.	.	.	0	.	SON	GT:/	0:15	0/1:40,9:37	C	T
.	.	.	.	.	.	.	.	.	.	.	.	SON	GT:/	0:16	0/1:23,5:37	A	G
.	.	.	.	.	.	.	.	2.6	1.79	.	.	SON	GT:/	0:10	0/1:185,33	C	T
.	.	.	.	.	.	.	.	.	.	.	.	SON	GT:/	0:21	0/1:30,8:33	C	T
.	.	##	0	0.1	0.816	0.998	1,D	2.7	2.21	.	.	SON	GT:/	0:18	0/1:35,15:3	C	T
.	##	##	0	.	.	.	.	.	1.98	.	.	SON	GT:/	0:27	0/1:45,4:37	C	T
.	.	.	.	0.5	0.549	0.979	0.91	4.9	3.01	.	.	SON	GT:/	0:92	0/1:149,19	A	G
.	.	.	.	.	.	.	.	2	.	.	.	SON	GT:/	0:65	0/1:118,13	C	T
.	.	.	.	.	.	.	.	.	.	.	.	SON	GT:/	0:21	0/1:39,9:35	G	T
6E-04	##	0	0	.	.	.	.	.	.	.	.	SON	GT:/	0:95	0/1:149,15	G	A
.	.	.	.	.	.	.	.	.	.	.	.	SON	GT:/	0:14	0/1:22,3:37	C	A
.	.	.	.	.	.	.	.	.	.	.	.	SON	GT:/	0:9,(	0/1:17,6:37	G	T
.	.	##	0	.	.	.	1,A	5.7	5.57	.	.	SON	GT:/	0:49	0/1:69,12:3	G	A
.	.	0	0	.	.	.	.	.	1.26	.	.	SON	GT:/	0:34	0/1:54,9:37	G	A
.	.	.	.	.	.	.	.	.	.	.	.	SON	GT:/	0:38	0/1:53,11:3	G	A
.	.	.	.	.	.	.	.	.	.	.	.	SON	GT:/	0:98	0/1:163,34	G	A

.	.	.	.	.	.	.	.	.	.	.	.	SON	GT:/	0:15	0/1:20,4:37	C	T
.	.	.	.	.	.	.	.	.	.	.	.	SON	GT:/	0:12	0/1:10,3:37	G	A
.	.	.	.	0.0(	0.853	0.984	0.99	4.3	3.06	.	.	SON	GT:/	0:56	0/1:85,15:3	A	G
.	.	.	.	0.2(	0.993	0.999	1,D	5.4	3.15	.	.	SON	GT:/	0:38	0/1:62,8:37	G	T
.	.	.	.	.	.	.	.	3.5	.	.	.	SON	GT:/	0:21	0/1:358,73	C	T
.	.	.	.	.	.	.	.	4.2	2.86	.	.	SON	GT:/	0:25	0/1:75,11:3	T	C
.	.	.	.	.	.	.	.	.	.	.	.	SON	GT:/	0:20	0/1:28,5:37	G	A
.	.	##	0	.	.	.	.	.	.	.	.	SON	GT:/	0:31	0/1:54,6:37	G	A
.	.	.	.	.	.	.	.	.	.	.	.	SON	GT:/	0:15	0/1:21,6:37	C	T
.	.	.	.	.	.	.	.	.	.	.	.	SON	GT:/	0:81	0/1:150,23	C	T
.	.	.	.	0.2:	0.002	0.02,I	1,D	.	.	.	.	SON	GT:/	0:13	0/1:32,10:3	C	T
.	.	.	.	0.3:	.	.	1.00	3.7	1.88	.	.	SON	GT:/	0:60	0/1:125,18	G	T
.	.	.	.	.	.	.	.	.	.	.	.	SON	GT:/	0:12	0/1:19,3:37	C	T
.	.	##	0	.	.	.	.	.	.	0	0	SON	GT:/	0:66	0/1:129,16	C	T
.	.	.	.	.	.	.	.	.	.	.	.	SON	GT:/	0:20	0/1:43,5:37	G	T
.	.	##	0	.	.	.	.	.	.	.	.	SON	GT:/	0:13	0/1:209,32	G	A
.	.	.	.	.	.	.	.	.	.	.	.	SON	GT:/	0:31	0/1:54,15:3	C	A
.	.	##	0	0.3	0.024	0.104	0.99	2.3	1.34	.	.	SON	GT:/	0:99	0/1:171,33	G	A
.	.	##	0	.	.	.	.	.	.	.	.	SON	GT:/	0:84	0/1:137,21	C	T
.	.	.	.	.	.	.	.	.	.	.	.	SON	GT:/	0:22	0/1:42,8:37	G	A
.	.	.	.	.	.	.	.	.	.	.	.	SON	GT:/	0:38	0/1:48,12:3	C	T
.	.	.	.	.	.	.	.	.	.	0.1	0.2	SON	GT:/	0:8,(	0/1:9,3:37:	G	A
.	.	.	.	.	.	.	.	.	.	.	.	SON	GT:/	0:12	0/1:212,15	C	T
.	.	.	.	.	.	.	.	.	.	.	.	SON	GT:/	0:24	0/1:29,4:37	A	G
.	.	.	.	.	.	.	.	.	.	.	.	SON	GT:/	0:96	0/1:167,17	A	G
.	.	.	.	.	.	.	.	.	.	.	.	SON	GT:/	0:28	0/1:23,3:37	C	T
.	.	.	.	.	.	.	.	.	.	.	.	SON	GT:/	0:46	0/1:73,10:3	G	A
.	.	.	.	.	.	.	.	.	.	.	.	SON	GT:/	0:40	0/1:51,9:37	G	A
.	.	.	.	.	.	.	.	.	.	0	.	SON	GT:/	0:60	0/1:93,9:37	C	T
.	.	0	0	0.1(	.	.	1,N	.	.	0	.	SON	GT:/	0:78	0/1:155,26	G	A
.	.	.	.	.	.	.	.	.	.	.	.	SON	GT:/	0:75	0/1:125,24	G	A
.	.	.	.	.	.	.	.	.	.	.	.	SON	GT:/	0:11	0/1:7,3:37:	G	A
.	.	.	.	.	.	.	.	.	.	.	.	SON	GT:/	0:25	0/1:37,7:37	T	C
.	.	.	.	.	.	.	.	.	.	.	.	SON	GT:/	0:13	0/1:187,29	A	G
.	.	.	.	.	.	.	.	.	.	.	.	SON	GT:/	0:24	0/1:48,6:37	G	A
.	0	##	0	.	.	.	.	.	.	0	.	SON	GT:/	0:70	0/1:113,19	C	T
.	.	.	.	0.0	0.91,I	0.967	1,D	4	4.18	.	.	SON	GT:/	0:11	0/1:67,16:3	A	G
.	.	##	0	.	.	.	.	.	.	0	.	SON	GT:/	0:55	0/1:35,4:34	C	T
.	.	.	.	.	.	.	.	.	.	.	.	SON	GT:/	0:24	0/1:26,3:37	G	T
.	.	.	.	0.1!	.	.	1,N	.	.	0	.	SON	GT:/	0:14	0/1:238,9:3	G	A
.	.	.	.	.	.	.	.	.	.	0	.	SON	GT:/	0:40	0/1:66,9:32	C	T
.	.	.	.	.	.	.	.	.	.	0.2	0.3	SON	GT:/	0:10	0/1:14,6:33	A	G
.	.	.	.	.	.	.	.	.	.	.	.	SON	GT:/	0:45	0/1:67,8:37	T	C
.	.	.	.	.	.	.	.	.	.	.	.	SON	GT:/	0:16	0/1:331,67	C	T
.	.	.	.	.	.	.	.	.	.	.	.	SON	GT:/	0:19	0/1:46,4:37	A	T
.	.	.	.	.	.	.	.	.	.	.	.	SON	GT:/	0:29	0/1:31,9:37	C	T
.	.	.	.	.	.	.	.	2.3	.	.	.	SON	GT:/	0:11	0/1:30,7:37	G	A
.	.	.	.	0.1!	0.924	1.0,D	1.00	4.5	2.61	.	.	SON	GT:/	0:76	0/1:142,32	G	A
.	.	.	.	.	.	.	.	.	2.07	.	.	SON	GT:/	0:39	0/1:48,7:37	G	T
0.001	0	0	0	.	.	.	.	.	.	.	.	SON	GT:/	0:86	0/1:175,42	C	T
.	.	.	.	.	.	.	.	.	.	.	.	SON	GT:/	0:87	0/1:117,24	G	A
.	.	##	0	.	.	.	.	.	.	.	.	SON	GT:/	0:60	0/1:93,12:3	G	A
.	.	##	0	.	.	.	1,A	3.8	5.48	.	.	SON	GT:/	0:55	0/1:101,14	C	T
2E-04	.	##	0	.	.	.	1,D	4.3	7.65	.	.	SON	GT:/	0:12	0/1:186,22	C	T

.	.	.	.	.	.	.	.	.	.	.	.	SOM	GT:/	0:30	0/1:37,5:37	G	A
2E-04	.	.	.	.	.	.	.	.	.	.	.	SOM	GT:/	0:16	0/1:198,35	G	A
.	.	.	.	.	.	.	.	.	.	.	.	SOM	GT:/	0:48	0/1:60,10:3	G	A
.	.	.	.	0.0	0.99,	1.0,D	1,D	4.4	3.92	.	.	SOM	GT:/	0:10	0/1:215,30	G	T
.	.	.	.	.	.	.	.	.	.	.	.	SOM	GT:/	0:16	0/1:31,3:37	A	G
.	.	##	0	0.0	0.474	0.969	0.96	5.8	4.26	.	.	DB;S	GT:/	0:60	0/1:90,18:3	G	A
4E-04	0	0	0	0.0	0.997	1.0,D	1,D	5.7	5.61	.	.	SOM	GT:/	0:10	0/1:186,27	C	T
.	.	.	.	.	.	.	.	.	.	.	.	SOM	GT:/	0:39	0/1:69,14:3	G	T
.	.	.	.	.	.	.	.	.	.	.	.	SOM	GT:/	0:15	0/1:27,3:37	A	G
.	.	.	.	.	.	.	.	.	.	.	.	SOM	GT:/	0:42	0/1:66,8:37	G	T
.	.	.	.	.	.	.	1,D	6	3.25	.	.	SOM	GT:/	0:27	0/1:37,6:37	A	G
.	.	##	0	.	.	.	.	.	.	.	.	SOM	GT:/	0:64	0/1:106,21	G	A
.	.	.	.	.	.	.	.	.	.	.	.	SOM	GT:/	0:17	0/1:21,5:34	G	T
.	.	.	.	0.0	0.999	1.0,D	1,D	5.5	3.28	.	.	SOM	GT:/	0:67	0/1:110,14	T	C
.	.	##	0	0.0	0.999	1.0,D	1,D	5.6	5.38	.	.	SOM	GT:/	0:42	0/1:59,9:37	G	A
.	.	##	0	0.0	0.984	1.0,D	0.98	2.8	3.48	0	0	SOM	GT:/	0:42	0/1:62,13:3	C	T
.	.	.	.	0.1	0.005	0.001	1,N	2.8	.	.	.	SOM	GT:/	0:32	0/1:37,4:34	C	G
2E-04	.	##	0	.	.	.	.	3.7	.	.	.	SOM	GT:/	0:71	0/1:122,18	C	T
.	.	.	.	.	.	.	.	.	.	.	.	SOM	GT:/	0:15	0/1:21,6:37	G	A
.	.	.	.	.	.	.	.	.	.	.	.	SOM	GT:/	0:27	0/1:48,8:33	T	C
.	.	.	.	.	.	.	.	3.3	3.65	.	.	SOM	GT:/	0:54	0/1:62,13:3	T	C
.	.	##	0	.	.	.	.	.	.	.	.	SOM	GT:/	0:32	0/1:64,16:3	C	T
.	.	.	.	.	.	.	.	.	.	.	.	SOM	GT:/	0:61	0/1:105,10	C	T
2E-04	.	.	.	.	.	.	.	.	.	0	0	SOM	GT:/	0:23	0/1:35,11:3	C	T
.	.	.	.	.	.	.	.	.	.	.	.	SOM	GT:/	0:34	0/1:44,12:3	C	A
.	.	.	.	0.1	0.998	1.0,D	1,D	6	4.74	.	.	SOM	GT:/	0:55	0/1:73,10:3	G	A
.	.	.	.	.	.	.	.	.	.	.	.	SOM	GT:/	0:76	0/1:143,25	C	A
.	.	.	.	.	.	.	.	.	.	.	.	SOM	GT:/	0:8,(	0/1:11,6:37	G	A
.	.	.	.	.	.	.	.	.	.	0	.	SOM	GT:/	0:69	0/1:70,5:37	C	T
.	.	.	.	.	.	.	.	.	.	0	.	SOM	GT:/	0:64	0/1:70,5:37	C	T
.	.	.	.	.	.	.	.	.	.	.	.	SOM	GT:/	0:14	0/1:20,6:32	G	A
.	.	.	.	.	.	.	.	.	.	.	.	SOM	GT:/	0:12	0/1:19,6:37	C	T
.	.	.	.	.	.	.	.	.	.	0	0	SOM	GT:/	0:46	0/1:110,6:3	A	G
.	.	.	.	.	.	.	.	.	.	.	.	SOM	GT:/	0:41	0/1:61,15:3	G	T
.	.	.	.	.	.	.	.	2.5	1.34	.	.	SOM	GT:/	0:35	0/1:527,98	C	T
.	0	##	0	.	.	.	.	.	.	.	.	SOM	GT:/	0:10	0/1:159,25	C	T
.	.	.	.	.	.	.	.	.	.	.	.	SOM	GT:/	0:15	0/1:290,14	G	A
2E-04	.	##	0	.	.	.	.	.	.	0	.	SOM	GT:/	0:58	0/1:108,20	G	A
.	.	.	.	.	.	.	.	.	.	.	.	SOM	GT:/	0:14	0/1:20,7:37	T	C
.	.	.	.	.	.	.	.	.	.	.	.	SOM	GT:/	0:9,(	0/1:17,3:37	C	T
.	.	0	.	1.0	.	.	1,N	.	.	.	.	SOM	GT:/	0:29	0/1:38,6:28	A	G
.	.	.	.	.	.	.	.	.	.	.	.	SOM	GT:/	0:23	0/1:39,4:37	G	A
.	##	##	0	0.0	0.458	0.901	1.00	5.4	3.98	0	.	SOM	GT:/	0:14	0/1:233,40	C	T
.	.	.	.	0.0	0.791	0.999	1,D	5.1	4.69	.	.	SOM	GT:/	0:86	0/1:149,23	G	A
.	.	.	.	.	.	.	.	.	.	.	.	SOM	GT:/	0:27	0/1:72,18:3	A	T
.	.	##	.	.	.	.	.	.	.	0	.	SOM	GT:/	0:10	0/1:171,36	G	A
.	.	.	.	.	.	.	.	.	1.71	.	.	SOM	GT:/	0:46	0/1:66,10:3	C	T
.	0	##	0	.	.	.	.	.	.	.	.	SOM	GT:/	0:10	0/1:200,28	C	T
1E-03	.	.	.	.	.	.	.	.	.	0	.	SOM	GT:/	0:18	0/1:38,7:37	T	C
.	.	.	.	.	.	.	.	.	.	.	.	SOM	GT:/	0:80	0/1:164,33	T	C
.	.	.	.	.	.	.	.	.	.	.	.	SOM	GT:/	0:35	0/1:54,5:37	C	T
.	.	.	.	.	.	.	.	.	.	.	.	SOM	GT:/	0:45	0/1:59,6:32	G	T
.	.	##	0	0.0	0.998	1.0,D	1.00	5.7	3.91	.	.	SOM	GT:/	0:40	0/1:68,15:3	G	A
.	.	.	.	.	.	.	.	3.4	.	.	.	SOM	GT:/	0:23	0/1:37,6:37	C	A

.	.	.	.	.	.	.	.	.	.	.	.	SON	GT:/	0:59	0/1:612,25	G	A
.	.	.	.	.	.	.	.	.	.	.	.	SON	GT:/	0:73	0/1:768,52	G	A
.	.	.	.	.	.	.	.	.	.	.	.	SON	GT:/	0:17	0/1:248,63	C	T
.	.	.	.	.	.	.	.	.	.	.	.	SON	GT:/	0:29	0/1:428,14	A	G
.	.	##	0	.	0.002	0.001	0.77	3.5	.	.	.	SON	GT:/	0:68	0/1:112,25	G	A
.	.	.	.	0.1(	0.143	0.302	1,N	.	.	.	.	SON	GT:/	0:11	0/1:208,32	G	T
.	.	.	.	.	.	.	.	.	.	.	.	SON	GT:/	0:12	0/1:216,42	G	A
.	.	.	.	.	.	.	.	.	.	.	.	SON	GT:/	0:9,(	0/1:5,2:37:	G	C
2E-04	.	##	0	0.0:	0.024	0.491	1.00	.	.	.	.	SON	GT:/	0:79	0/1:128,17	C	T
.	.	0	0	.	.	.	.	.	.	0	.	DB;S	GT:/	0:66	0/1:123,8:3	C	T
.	.	##	0	0.2	0.001	0.006	1,N	.	1.60	.	.	SON	GT:/	0:46	0/1:74,11:3	G	A
2E-04	##	##	0	0.0	0.862	0.999	1,D	4.2	3.34	.	.	SON	GT:/	0:17	0/1:298,68	C	T
.	##	##	0	.	.	.	.	.	.	0	.	SON	GT:/	0:25	0/1:458,78	C	T
.	.	.	.	.	.	.	.	3	.	.	.	SON	GT:/	0:31	0/1:36,6:32	G	T
.	.	.	.	.	.	.	.	.	.	.	.	SON	GT:/	0:8,(	0/1:14,6:37	C	A
.	##	.	.	.	.	.	.	.	.	.	.	SON	GT:/	0:97	0/1:178,29	C	T
.	.	.	.	.	.	.	.	.	.	.	.	SON	GT:/	0:28	0/1:63,8:37	G	A
.	.	.	.	0.0	0.608	0.958	1,N	2.8	2.07	.	.	SON	GT:/	0:44	0/1:42,13:3	A	T
2E-04	.	.	.	.	.	.	.	.	.	.	.	SON	GT:/	0:11	0/1:13,4:37	C	T
.	.	.	.	0.3:	0.499	0.833	0.98	2.6	1.55	.	.	SON	GT:/	0:65	0/1:140,28	G	A
.	.	##	0	.	.	.	.	.	.	.	.	SON	GT:/	0:43	0/1:90,19:3	C	T
.	.	.	.	0.0	0.999	1.0,D	1,D	5.2	5.25	.	.	SON	GT:/	0:12	0/1:214,40	G	A
4E-04	.	.	.	.	.	.	.	.	.	0	.	SON	GT:/	0:83	0/1:118,23	G	A
.	.	0	0	0.0	0.999	1.0,D	1,D	4.8	4.71	.	.	SON	GT:/	0:69	0/1:120,23	C	T
.	.	.	.	.	.	.	.	.	.	0	.	SON	GT:/	0:22	0/1:26,11:3	C	T
.	##	##	0	0.0(	0.999	1.0,D	1.00	.	3.51	.	.	SON	GT:/	0:20	0/1:326,55	C	T
.	.	.	.	.	.	.	.	.	1.74	.	.	SON	GT:/	0:99	0/1:138,33	T	A
4E-04	##	##	0	.	.	.	.	.	.	.	0	SON	GT:/	0:45	0/1:87,24:3	G	A
.	.	.	.	.	.	.	.	.	.	.	.	SON	GT:/	0:30	0/1:50,12:3	C	A
.	.	##	0	0.4	0.0,B	0.001	1,N	.	.	0	.	SON	GT:/	0:10	0/1:188,35	G	A
.	.	.	.	.	.	.	.	.	.	.	.	SON	GT:/	0:43	0/1:93,15:3	G	A
.	.	.	.	.	.	.	.	.	.	.	.	SON	GT:/	0:32	0/1:55,8:37	T	C
.	.	.	.	.	.	.	.	.	.	.	.	SON	GT:/	0:66	0/1:92,15:3	G	A
0.003	.	.	.	.	.	.	.	.	.	0	0	SON	GT:/	0:30	0/1:40,4:37	C	T
.	.	.	.	.	.	.	.	.	.	.	.	SON	GT:/	0:48	0/1:76,11:3	A	G
.	.	##	0	.	.	.	.	.	.	.	.	SON	GT:/	0:36	0/1:59,8:37	C	T
0.002	.	0	0	0.3!	0.0,B	0.0,B	1,N	.	.	0	0	SON	GT:/	0:52	0/1:92,27:3	G	A
.	.	.	.	0.0!	0.891	0.997	1.00	5.7	3.29	.	.	SON	GT:/	0:61	0/1:90,18:3	C	A
4E-04	.	.	.	.	.	.	.	.	.	.	.	SON	GT:/	0:14	0/1:23,3:37	T	A
2E-04	.	.	.	.	.	.	.	.	.	.	.	SON	GT:/	0:28	0/1:38,9:37	G	A
.	##	##	0	.	.	.	.	.	.	.	.	SON	GT:/	0:22	0/1:408,78	C	T
.	0	0	0	0.0	0.0,B	0.003	1,N	.	.	0	.	SON	GT:/	0:90	0/1:140,39	C	T
.	.	.	.	0.0!	0.997	0.999	0.96	4.8	2.03	.	.	SON	GT:/	0:10	0/1:173,41	G	T
2E-04	.	##	0	0.2	0.0,B	0.0,B	1,N	.	.	0	.	SON	GT:/	0:12	0/1:193,34	G	A
.	.	.	.	.	.	.	.	.	.	.	.	SON	GT:/	0:37	0/1:55,11:3	T	C
.	.	.	.	.	.	.	.	.	.	.	.	SON	GT:/	0:28	0/1:55,8:37	G	A
.	.	##	0	.	.	.	.	.	2.57	.	.	SON	GT:/	0:27	0/1:38,8:37	A	G
2E-04	.	.	.	.	.	.	.	.	2.86	.	.	SON	GT:/	0:31	0/1:53,7:37	G	A
.	.	0	0	.	.	.	.	.	.	.	.	SON	GT:/	0:81	0/1:61,13:3	G	A
.	.	.	.	.	.	.	.	5.6	2.61	.	.	SON	GT:/	0:14	0/1:29,8:35	C	T
.	.	.	.	.	.	.	.	.	.	.	.	SON	GT:/	0:16	0/1:17,5:37	C	T
.	.	.	.	.	.	.	.	.	.	.	.	SON	GT:/	0:22	0/1:20,5:37	G	A
.	.	.	.	.	.	.	.	2.1	.	.	.	SON	GT:/	0:10	0/1:150,17	T	C
.	.	0	0	0.0	0.0,B	0.0,B	1.00	4.2	2.45	0	.	DB;S	GT:/	0:91	0/1:157,7:3	A	T

.	.	0	0	0.6	0.002	0.004	1,N	2.2	.		0	.	SON	GT:/	0:18	0/1:304,52	G	A
.	.	##	0	.	.	.	.	.	.	.		.	SON	GT:/	0:72	0/1:101,26	G	A
.	.	.	.	.	0.997	1.0,D	1.00	3.2	2.86	.		.	SON	GT:/	0:50	0/1:81,6:37	C	A
.	##	##	.	.	.	.	.	.	.	.		.	SON	GT:/	0:82	0/1:155,28	C	T
.	.	.	.	0.0	0.999	1.0,D	1,D	5.7	4.84	.		.	SON	GT:/	0:60	0/1:80,16:3	G	A
.	.	.	.	0.3	0.755	0.998	0.99	4.6	2.75	.		.	SON	GT:/	0:77	0/1:126,24	C	T
.	.	.	.	.	.	.	.	.	.	.		.	SON	GT:/	0:22	0/1:29,4:37	C	T
.	.	.	.	.	.	.	.	.	.	.		.	SON	GT:/	0:19	0/1:33,3:37	G	T
.	.	.	.	0.0	0.999	1.0,D	0.73	3.6	4.00	.		.	SON	GT:/	0:55	0/1:93,17:3	G	A
.	.	.	.	.	.	.	.	.	.	.		.	SON	GT:/	0:55	0/1:74,12:3	C	T
2E-04	##	##	0	0.0	0.99,I	1.0,D	1.00	5.2	3.22	.		.	SON	GT:/	0:11	0/1:157,33	G	A
0.006	.	.	.	.	.	.	.	.	.		0	0	SON	GT:/	0:25	0/1:26,7:35	A	T
.	.	.	.	0.4	.	.	1,N	.	.	.		.	SON	GT:/	0:27	0/1:20,5:37	G	A
.	.	.	.	0.0	0.997	1.0,D	1,D	5.8	3.66	.		.	SON	GT:/	0:11	0/1:179,38	C	T
.	.	.	.	.	.	.	.	.	1.23	.		.	SON	GT:/	0:52	0/1:91,5:37	T	C
2E-04	.	##	0	0.0	0.923	1.0,D	0.99	3.4	4.90	.		.	SON	GT:/	0:13	0/1:223,31	G	A
.	.	.	.	.	.	.	.	.	.	.		.	SON	GT:/	0:68	0/1:96,14:3	G	T
.	.	.	.	.	.	.	.	.	.	.		.	SON	GT:/	0:8,(	0/1:6,2:37:	G	T
.	.	##	0	0.2	0.469	0.99,I	0.89	4.5	.		0	0	SON	GT:/	0:13	0/1:198,45	C	T
.	.	.	.	.	.	.	.	.	.	.		.	SON	GT:/	0:37	0/1:57,9:37	G	A
.	.	.	.	.	.	.	.	3.4	2.32	.		.	SON	GT:/	0:67	0/1:106,21	T	C
.	.	.	.	.	.	.	.	.	.	.		.	SON	GT:/	0:43	0/1:82,17:3	G	T
.	.	.	.	.	.	.	.	.	.	.		.	SON	GT:/	0:10	0/1:203,22	C	A
2E-04	.	.	.	.	.	.	.	.	.		0	0	DB;S	GT:/	0:48	0/1:62,4:37	C	T
0.215	.	.	.	.	.	.	.	.	.		0	.	SON	GT:/	0:24	0/1:29,10:3	A	G
.	.	.	.	.	.	.	.	2.7	.	.		.	SON	GT:/	0:23	0/1:350,28	A	G
.	.	.	.	.	.	.	.	.	.		0	.	SON	GT:/	0:49	0/1:83,6:37	A	G
0.02	.	.	.	.	.	.	.	.	.		0	0.1	SON	GT:/	0:8,(	0/1:3,3:37:	G	C
.	.	.	.	.	.	.	.	.	.	.		.	SON	GT:/	0:9,(	0/1:5,2:37:	C	A
.	.	.	.	.	.	.	.	.	.	.		.	SON	GT:/	0:21	0/1:36,3:37	A	C
.	.	.	.	.	.	.	.	.	.	.		.	SON	GT:/	0:9,(	0/1:13,3:37	C	G
.	.	.	.	.	.	.	.	.	.		0	.	DB;S	GT:/	0:19	0/1:112,5:3	A	C
.	.	.	.	.	.	.	.	.	.		0	.	DB;S	GT:/	0:19	0/1:193,8:3	C	T
.	.	.	.	.	.	.	.	.	.		0	.	DB;S	GT:/	0:22	0/1:245,9:3	T	G
.	.	.	.	.	.	.	.	.	.	.		.	DB;S	GT:/	0:19	0/1:171,7:3	C	T
.	.	.	.	.	.	.	.	.	.		0	.	DB;S	GT:/	0:28	0/1:424,22	G	C
.	.	.	.	.	.	.	.	.	.		0	.	DB;S	GT:/	0:39	0/1:679,33	G	C
.	.	.	.	.	.	.	.	.	.	.		.	SON	GT:/	0:23	0/1:292,12	A	G
.	.	.	.	.	.	.	.	.	.		0	.	SON	GT:/	0:30	0/1:265,9:3	A	C
.	.	.	.	.	.	.	.	.	.		0	0	DB;S	GT:/	0:24	0/1:213,8:3	A	G
.	.	.	.	.	.	.	.	.	.	.		.	DB;S	GT:/	0:29	0/1:233,11	A	G
.	.	.	.	.	.	.	.	.	.		0	.	DB;S	GT:/	0:34	0/1:228,8:3	T	G
.	.	.	.	.	.	.	.	.	.	.		.	DB;S	GT:/	0:35	0/1:364,12	A	G
.	.	.	.	.	.	.	.	.	.		0	.	DB;S	GT:/	0:34	0/1:446,15	G	C
.	.	.	.	.	.	.	.	.	.		0	.	DB;S	GT:/	0:37	0/1:502,15	T	A
.	.	.	.	.	.	.	.	.	.		0	.	DB;S	GT:/	0:23	0/1:363,12	A	T
.	.	.	.	.	.	.	.	.	.		0	.	DB;S	GT:/	0:27	0/1:221,16	T	G
.	.	.	.	.	.	.	.	.	.		0	.	SON	GT:/	0:10	0/1:60,5:37	G	T
.	.	.	.	.	.	.	.	.	.		0	.	SON	GT:/	0:13	0/1:142,6:3	G	A
.	.	.	.	.	.	.	.	.	.	.		.	SON	GT:/	0:10	0/1:99,7:37	T	C
.	.	.	.	.	.	.	.	.	.	.		.	SON	GT:/	0:10	0/1:24,3:37	T	C
.	0	##	0	.	.	.	.	.	.	.		.	SON	GT:/	0:15	0/1:237,60	G	A
.	.	.	.	.	.	.	.	.	.	.		.	SON	GT:/	0:24	0/1:60,4:37	C	A
.	.	.	.	.	.	.	.	.	.	.		.	SON	GT:/	0:49	0/1:90,20:3	C	A

.	.	.	.	0.1!	0.006	0.002	1.00	.	1.43	.	.	SON	GT:/	0:50	0/1:91,17:3	G	T
.	.	.	.	.	.	.	.	.	.	.	.	SON	GT:/	0:25	0/1:29,12:3	A	C
.	.	.	.	.	.	.	.	.	.	.	.	SON	GT:/	0:47	0/1:66,12:3	G	A
4E-04	0	0	.	.	.	.	.	.	.	.	.	SON	GT:/	0:20	0/1:328,59	C	T
.	.	.	.	.	.	.	.	.	.	.	.	SON	GT:/	0:53	0/1:79,18:3	T	C
.	.	.	.	0.0!	0.742	0.986	0.67	3.1	3.79	.	.	SON	GT:/	0:61	0/1:80,13:3	G	T
.	.	.	.	.	.	.	.	.	1.73	.	.	SON	GT:/	0:17	0/1:31,6:37	A	G
.	.	.	.	.	.	.	.	.	.	0	.	SON	GT:/	0:8,(	0/1:9,3:37:	A	G
.	.	.	.	0.1'	0.821	0.996	1,N	.	1.95	.	.	SON	GT:/	0:93	0/1:195,41	C	T
.	.	.	.	.	.	.	.	.	.	.	.	SON	GT:/	0:30	0/1:50,5:37	A	G
.	.	##	0	.	.	.	.	.	.	.	0	SON	GT:/	0:13	0/1:180,35	C	T
2E-04	.	##	0	0.0:	0.996	1.0,D	1.00	5.3	3.77	.	.	SON	GT:/	0:89	0/1:125,24	G	A
.	.	.	.	.	.	.	.	2.2	1.30	.	.	SON	GT:/	0:60	0/1:95,9:34	G	T
.	.	##	0	0.0(	0.819	0.996	1.00	.	3.55	.	.	SON	GT:/	0:48	0/1:98,10:3	C	T
.	0	##	0	.	.	.	.	.	.	0	.	SON	GT:/	0:32	0/1:53,9:37	G	A
.	.	.	.	.	.	.	.	.	.	.	.	SON	GT:/	0:19	0/1:38,6:32	C	A
.	.	##	0	.	0.03,I	0.124	0.98	.	1.80	.	.	SON	GT:/	0:65	0/1:141,5:3	C	T
.	.	.	.	.	.	.	.	.	.	.	.	SON	GT:/	0:41	0/1:53,6:37	C	A
.	.	.	.	.	.	.	.	.	.	.	.	SON	GT:/	0:10	0/1:197,38	G	A
.	.	.	.	0.5:	0.894	0.931	0.99	5.5	3.13	.	.	SON	GT:/	0:31	0/1:77,10:3	C	T
.	.	.	.	.	.	.	.	.	.	0	0	SON	GT:/	0:38	0/1:50,12:2	A	G
.	.	.	.	.	.	.	.	.	.	.	.	SON	GT:/	0:25	0/1:34,7:33	C	T
1E-03	.	.	.	.	.	.	.	.	.	.	.	SON	GT:/	0:38	0/1:71,13:3	C	T
.	0	##	0	.	0.715	0.996	.	4.1	.	.	.	SON	GT:/	0:25	0/1:44,9:37	G	A
.	.	0	0	.	.	.	.	.	.	0	0	SON	GT:/	0:10	0/1:21,4:37	G	T
.	.	.	.	.	.	.	.	.	.	.	.	SON	GT:/	0:46	0/1:56,4:37	C	T
.	.	.	.	.	.	.	.	.	.	.	.	SON	GT:/	0:38	0/1:55,10:3	C	T
0.01	.	.	.	.	.	.	.	.	.	0	0	SON	GT:/	0:10	0/1:8,6:37:	A	G
.	.	0	0	.	0.081	0.205	.	.	.	0	.	SON	GT:/	0:38	0/1:40,7:35	G	A
.	.	.	.	.	.	.	.	.	.	.	.	SON	GT:/	0:14	0/1:14,3:37	T	C
0.063	.	.	.	.	.	.	.	.	.	0.1	0	DB;S	GT:/	0:34	0/1:56,4:37	C	T
6E-04	0	##	0	0.0:	0.846	0.984	0.98	5.1	2.66	.	0	SON	GT:/	0:38	0/1:51,15:3	G	A
.	.	##	0	0.0(	0.707	0.993	1.00	4	2.25	.	.	SON	GT:/	0:64	0/1:101,24	G	A
.	.	.	.	0.0(	0.833	0.997	1.00	.	2.53	.	.	SON	GT:/	0:24	0/1:43,10:3	C	T
.	.	.	.	0.0	1.0,D	1.0,D	1,D	4.7	4.17	.	.	SON	GT:/	0:14	0/1:224,47	T	A
2E-04	##	##	0	.	.	.	.	.	.	0	.	SON	GT:/	0:11	0/1:226,39	C	T
.	.	.	.	.	.	.	.	.	.	.	.	SON	GT:/	0:58	0/1:96,17:3	T	C
2E-04	.	##	0	0.1!	0.011	0.025	1,N	.	.	.	.	SON	GT:/	0:11	0/1:167,25	G	A
.	.	.	.	.	.	.	.	.	.	.	.	SON	GT:/	0:20	0/1:32,6:37	C	T
.	.	.	.	0.0(	0.969	1.0,D	1,D	4.7	4.08	.	.	SON	GT:/	0:13	0/1:272,45	C	T
.	.	.	.	.	.	.	.	.	.	.	.	SON	GT:/	0:70	0/1:107,13	C	A
2E-04	.	##	0	.	.	.	.	.	.	.	.	SON	GT:/	0:62	0/1:110,24	G	A
.	.	##	0	.	.	.	.	.	.	.	.	SON	GT:/	0:11	0/1:195,35	G	A
.	.	##	0	0.0	0.96,I	0.994	1.00	4.9	4.09	0	.	SON	GT:/	0:81	0/1:155,26	T	C
.	.	.	.	0.6:	0.001	0.001	1,D	5.6	3.09	.	.	SON	GT:/	0:73	0/1:90,15:3	C	T
.	.	.	.	.	.	.	.	2.2	.	.	.	SON	GT:/	0:10	0/1:18,6:37	A	G
.	.	.	.	.	.	.	.	.	.	.	.	SON	GT:/	0:63	0/1:122,24	T	C
.	.	.	.	.	.	.	.	.	.	.	.	SON	GT:/	0:13	0/1:25,5:37	C	A
.	.	.	.	0.2(	0.03,I	0.142	0.90	5.5	.	.	.	SON	GT:/	0:82	0/1:128,7:3	G	T
.	.	##	0	0.0(	0.035	0.492	0.97	3.9	4.11	.	.	SON	GT:/	0:40	0/1:41,6:37	C	T
.	.	.	.	.	.	.	.	.	.	.	.	SON	GT:/	0:80	0/1:122,14	A	G
.	.	.	.	.	.	.	.	3.4	2.72	.	.	SON	GT:/	0:62	0/1:97,16:3	T	C
.	.	.	.	.	.	.	.	3.4	.	.	.	SON	GT:/	0:11	0/1:13,4:37	A	C
4E-04	.	##	0	.	.	.	.	3.2	.	.	.	SON	GT:/	0:32	0/1:57,7:37	C	T

.	##	0	0	.	.	.	.	.	.	.	.	SON	GT:/	0:58	0/1:71,15:3	C	T
2E-04	.	0	0	.	.	.	.	.	.	.	.	SON	GT:/	0:65	0/1:87,15:3	G	A
.	.	0	0	0.5	0.0,B	0.002	1,N	.	.	.	.	SON	GT:/	0:15	0/1:265,64	C	T
.	.	.	.	.	.	.	.	.	.	.	.	SON	GT:/	0:8,(	0/1:15,4:37	C	T
.	.	.	.	0.1	0.01,	0.019	0.98	.	.	.	.	SON	GT:/	0:26	0/1:29,12:3	T	C
.	.	.	.	.	.	.	.	.	.	.	0	SON	GT:/	0:61	0/1:89,16:3	C	T
.	.	.	.	.	.	.	.	.	.	.	.	SON	GT:/	0:15	0/1:27,13:3	A	G
1E-03	0	0	0	0.3	0.311	0.921	0.99	5.4	3.35	.	.	SON	GT:/	0:74	0/1:123,23	G	A
.	.	##	0	.	1.0,D	1.0,D	1,D	5.6	5.52	.	.	SON	GT:/	0:68	0/1:96,16:3	G	A
.	0	0	0	.	.	.	.	.	.	.	.	SON	GT:/	0:10	0/1:184,27	G	A
.	.	.	.	.	.	.	.	.	.	.	.	SON	GT:/	0:89	0/1:126,20	C	T
.	.	.	.	.	.	.	.	.	1.68	.	.	SON	GT:/	0:64	0/1:118,21	C	T
.	.	##	0	.	.	.	.	.	2.87	.	.	SON	GT:/	0:14	0/1:8,3:37:	A	G
.	.	.	.	.	.	.	1,A	4.1	19.1	.	.	SON	GT:/	0:48	0/1:75,8:37	G	T
.	.	.	.	.	.	.	.	3.5	.	.	.	SON	GT:/	0:90	0/1:117,21	C	T
.	.	##	0	.	.	.	.	.	.	.	.	SON	GT:/	0:74	0/1:139,29	C	T
.	.	0	0	.	.	.	.	.	.	.	.	SON	GT:/	0:19	0/1:150,41	G	A
.	.	.	.	.	.	.	.	3.2	.	.	.	SON	GT:/	0:33	0/1:59,8:37	G	A
.	.	.	.	.	.	.	.	.	.	.	.	SON	GT:/	0:32	0/1:34,9:37	T	C
.	.	.	.	.	.	.	.	.	.	.	.	SON	GT:/	0:68	0/1:126,29	C	T
.	.	.	.	.	.	.	.	.	.	.	.	SON	GT:/	0:31	0/1:46,10:3	G	A
0.002	.	.	.	.	.	.	.	.	.	.	.	DB;S	GT:/	0:67	0/1:102,19	G	A
.	.	.	.	.	.	.	.	.	.	.	.	SON	GT:/	0:84	0/1:148,36	A	G
.	.	.	.	.	.	.	.	.	.	.	.	SON	GT:/	0:19	0/1:32,6:35	G	A
.	.	.	.	.	.	.	.	.	.	.	.	SON	GT:/	0:16	0/1:244,43	C	A
0.875	.	1	0	.	.	.	.	2.3	1.54	0.1	0.2	DB;S	GT:/	0:58	0/1:127,6:3	T	C
.	.	##	0	.	.	.	.	.	.	.	.	SON	GT:/	0:50	0/1:91,16:3	G	A
.	.	.	.	.	.	.	.	.	.	.	.	SON	GT:/	0:16	0/1:36,6:37	G	A
.	.	.	.	.	.	.	.	.	.	.	.	SON	GT:/	0:16	0/1:31,7:37	C	T
.	.	.	.	.	.	.	.	.	.	0	.	SON	GT:/	0:17	0/1:17,10:1	A	T
.	.	.	.	.	.	.	1,N	.	.	.	.	SON	GT:/	0:66	0/1:82,14:3	G	T
.	.	.	.	.	.	.	.	.	.	.	.	SON	GT:/	0:86	0/1:124,18	C	T
.	.	.	.	.	.	.	.	.	.	.	.	SON	GT:/	0:26	0/1:33,7:37	C	T
.	.	.	.	.	.	.	.	.	.	0	.	SON	GT:/	0:59	0/1:90,18:3	C	T
.	.	.	.	.	.	.	.	.	.	.	.	SON	GT:/	0:69	0/1:118,21	A	G
.	.	.	.	0.0	0.116	0.22,	0.94	2.9	2.39	.	.	SON	GT:/	0:96	0/1:159,15	C	T
.	.	.	.	0.0	0.917	0.99,	1.00	4.9	5.48	.	.	SON	GT:/	0:48	0/1:87,16:3	C	T
.	.	.	.	.	.	.	.	.	.	0	0	SON	GT:/	0:12	0/1:15,8:35	A	G
2E-04	.	##	0	1.0	0.0,B	0.0,B	1,N	.	.	.	.	SON	GT:/	0:67	0/1:91,12:3	C	T
.	.	.	.	.	.	.	.	.	.	.	.	SON	GT:/	0:53	0/1:86,15:3	G	T
.	.	.	.	.	.	.	.	2.5	2.71	.	.	SON	GT:/	0:14	0/1:15,5:37	A	G
.	.	.	.	.	.	.	.	.	.	.	.	SON	GT:/	0:16	0/1:36,3:37	G	T
.	.	.	.	.	.	.	.	.	.	.	.	SON	GT:/	0:58	0/1:60,9:35	G	T
.	.	.	.	0.5	0.503	0.627	1.00	4.1	2.37	.	.	SON	GT:/	0:63	0/1:96,17:3	G	A
.	.	##	0	0.5	0.013	0.248	1,N	.	.	.	.	SON	GT:/	0:99	0/1:142,30	C	T
.	.	.	.	.	.	.	.	.	.	.	.	SON	GT:/	0:16	0/1:25,7:37	G	T
.	.	.	.	.	.	.	.	.	.	.	.	SON	GT:/	0:19	0/1:29,6:37	C	A
.	.	.	.	.	.	.	.	.	.	0.1	0.2	SON	GT:/	0:42	0/1:86,6:37	T	G
.	.	.	.	.	.	.	.	.	.	0	0	SON	GT:/	0:12	0/1:27,3:37	T	C
.	.	.	.	.	.	.	.	.	.	.	.	SON	GT:/	0:36	0/1:55,4:37	A	G
.	.	##	0	.	0.824	0.977	1.00	4.1	1.52	.	.	SON	GT:/	0:44	0/1:70,15:3	C	T
.	.	.	.	.	.	.	.	.	.	.	.	SON	GT:/	0:26	0/1:43,5:37	G	T
.	.	##	0	0.0	0.999	1.0,D	1.00	2.6	4.46	.	.	SON	GT:/	0:62	0/1:100,14	G	A
.	.	.	.	.	.	.	.	.	.	.	.	SON	GT:/	0:37	0/1:65,9:37	C	T

.	.	.	.	.	.	.	.	.	.	.	.	SON	GT:/	0:20	0/1:365,74	C	T
.	.	.	.	0.0(	0.926	0.999	1,D	5.1	3.24	.	.	SON	GT:/	0:13	0/1:208,37	G	A
8E-04	##	##	0	.	.	.	.	.	.	.	0	SON	GT:/	0:59	0/1:96,25:3	C	T
2E-04	0	0	0	.	.	.	.	.	.	.	.	SON	GT:/	0:15	0/1:293,46	C	T
.	.	##	0	.	.	.	.	.	.	.	.	SON	GT:/	0:13	0/1:195,45	C	T
.	.	##	0	.	.	.	.	.	.	.	.	SON	GT:/	0:57	0/1:70,18:3	G	A
.	.	.	.	.	.	.	.	.	.	.	.	SON	GT:/	0:64	0/1:114,22	A	G
.	.	.	.	.	.	.	.	.	1.44	.	.	SON	GT:/	0:57	0/1:93,4:37	G	T
.	.	.	.	.	.	.	.	.	.	.	.	SON	GT:/	0:49	0/1:128,20	G	A
6E-04	.	.	.	.	.	.	.	.	.	0	0	SON	GT:/	0:14	0/1:37,7:35	G	A
.	.	.	.	.	.	.	.	.	.	.	.	SON	GT:/	0:39	0/1:41,13:3	G	T
.	.	.	.	.	.	.	.	.	.	.	.	SON	GT:/	0:10	0/1:17,3:37	C	T
.	.	.	.	.	.	.	.	.	.	.	.	SON	GT:/	0:22	0/1:25,9:34	C	T
.	.	.	.	.	.	.	.	.	.	.	.	SON	GT:/	0:41	0/1:78,18:3	A	G
.	.	.	.	.	.	.	.	.	.	.	.	SON	GT:/	0:87	0/1:150,21	G	A
.	.	.	.	.	.	.	.	2.1	.	.	.	SON	GT:/	0:66	0/1:106,18	G	T
.	.	.	.	.	.	.	.	.	.	.	.	SON	GT:/	0:65	0/1:98,14:3	C	A
.	.	##	0	.	.	.	.	.	.	0	.	SON	GT:/	0:62	0/1:98,24:3	G	A
.	.	.	.	.	.	.	.	2.8	.	.	.	SON	GT:/	0:62	0/1:64,14:3	T	G
.	.	.	.	.	.	.	.	.	.	.	.	SON	GT:/	0:35	0/1:49,6:37	C	T
.	.	.	.	.	.	.	.	.	.	.	.	SON	GT:/	0:19	0/1:37,8:33	G	T
.	.	##	0	0.1	0.925	0.997	1,D	4.5	4.18	.	.	SON	GT:/	0:11	0/1:177,31	C	T
.	.	.	.	.	.	.	.	.	3.18	.	.	SON	GT:/	0:80	0/1:108,22	C	T
.	.	.	.	.	.	.	.	.	.	.	.	SON	GT:/	0:59	0/1:112,23	C	T
.	.	.	.	.	.	.	.	.	.	.	.	SON	GT:/	0:83	0/1:119,22	G	A
.	.	.	.	.	.	.	.	.	.	.	.	SON	GT:/	0:23	0/1:41,9:33	G	A
0.402	.	.	.	.	.	.	.	.	.	0.2	0.3	SON	GT:/	0:9,(	0/1:7,13:37	A	G
.	.	0	0	.	.	.	.	3.4	.	.	.	SON	GT:/	0:46	0/1:89,10:3	C	T
.	.	.	.	.	.	.	.	.	.	.	.	SON	GT:/	0:20	0/1:34,5:37	A	G
.	##	##	0	0.0'	0.062	0.473	1,N	.	2.27	.	.	SON	GT:/	0:55	0/1:94,14:3	G	A
.	.	.	.	.	.	.	.	.	.	.	.	SON	GT:/	0:14	0/1:217,33	G	A
.	.	.	.	.	.	.	.	3.7	.	.	.	SON	GT:/	0:21	0/1:29,7:35	G	T
.	.	##	0	0.0	0.996	1.0,D	1.00	5.3	5.31	.	.	SON	GT:/	0:41	0/1:85,13:3	C	T
.	.	.	.	.	.	.	.	.	1.41	.	.	SON	GT:/	0:50	0/1:69,16:3	C	A
.	.	.	.	.	0.098	0.51,I	.	3.5	2.51	.	.	SON	GT:/	0:83	0/1:130,27	G	A
.	.	.	.	.	.	.	.	.	.	.	.	SON	GT:/	0:63	0/1:139,17	C	T
.	.	.	.	.	.	.	.	.	.	.	.	SON	GT:/	0:16	0/1:25,7:35	G	T
.	.	.	.	.	.	.	.	.	.	0	.	DB;S	GT:/	0:31	0/1:39,5:29	A	T
.	.	.	.	.	.	.	.	5.5	3.34	.	.	SON	GT:/	0:10	0/1:19,4:37	C	T
.	.	.	.	.	.	.	.	.	.	.	.	SON	GT:/	0:8,(	0/1:9,4:37:	G	A
1E-03	.	.	.	.	.	.	.	.	.	.	.	SON	GT:/	0:26	0/1:39,7:37	A	G
.	.	.	.	.	.	.	.	.	.	.	.	SON	GT:/	0:84	0/1:124,11	G	T
.	.	##	0	.	.	.	.	.	.	.	.	SON	GT:/	0:10	0/1:224,35	C	T
.	.	.	.	.	.	.	.	2.8	.	.	.	SON	GT:/	0:21	0/1:241,46	C	A
.	.	.	.	.	.	.	.	.	.	.	.	SON	GT:/	0:50	0/1:67,12:3	C	T
.	.	.	.	0.0(	0.997	1.0,D	0.97	4.8	2.38	.	.	SON	GT:/	0:38	0/1:41,11:3	T	C
.	.	.	.	.	.	.	.	.	.	.	.	SON	GT:/	0:29	0/1:27,9:37	C	T
.	.	.	.	.	.	.	.	.	.	.	.	SON	GT:/	0:41	0/1:68,6:37	C	T
2E-04	.	##	0	0.0	1.0,D	1.0,D	1,D	3.9	5.02	.	.	SON	GT:/	0:11	0/1:170,26	C	T
.	.	.	.	.	.	.	.	3.2	1.45	.	.	SON	GT:/	0:41	0/1:76,17:3	G	C
.	.	##	0	.	.	.	.	.	.	.	.	SON	GT:/	0:41	0/1:66,18:3	G	A
.	.	.	.	.	.	.	.	4.7	.	.	.	SON	GT:/	0:57	0/1:74,15:3	C	T
.	.	.	.	.	.	.	.	.	.	.	.	SON	GT:/	0:63	0/1:86,6:37	G	T
.	.	.	.	.	.	.	.	.	.	.	.	SON	GT:/	0:36	0/1:69,10:3	G	T

.	.	.	.	.	.	.	.	.	.	.	.	SON	GT:/	0:55	0/1:97,10:3	G	C
.	##	##	0	0.0(	0.956	1.0,D	1.00	3.4	4.53	.	.	SON	GT:/	0:13	0/1:215,37	C	T
.	.	.	.	.	.	.	.	.	.	.	.	SON	GT:/	0:34	0/1:62,9:37	A	G
.	.	.	.	.	.	.	.	2.6	1.73	.	.	SON	GT:/	0:31	0/1:54,5:34	C	A
.	.	0	0	.	.	.	.	.	.	.	.	SON	GT:/	0:43	0/1:58,16:3	G	A
.	.	.	.	.	.	.	.	.	.	.	.	SON	GT:/	0:8,(	0/1:15,4:37	C	A
.	.	0	0	.	0.393	0.981	0.98	5.5	2.78	0	0	SON	GT:/	0:70	0/1:114,22	C	T
.	.	.	.	.	0.998	1.0,D	1,D	4.6	2.18	.	.	SON	GT:/	0:83	0/1:128,24	G	A
.	.	.	.	0.0(	0.996	1.0,D	1.00	4.1	3.94	.	.	SON	GT:/	0:24	0/1:32,10:3	G	A
2E-04	.	##	0	0.0(	0.966	1.0,D	1,N	.	1.46	0	.	SON	GT:/	0:65	0/1:107,15	G	A
.	.	.	.	.	.	.	.	.	.	.	.	SON	GT:/	0:62	0/1:106,23	G	A
.	##	##	0	.	.	.	.	.	.	.	.	SON	GT:/	0:41	0/1:80,6:37	C	T
.	.	.	.	.	.	.	.	.	.	.	.	SON	GT:/	0:20	0/1:31,3:33	G	A
.	.	.	.	.	.	.	.	.	.	.	.	SON	GT:/	0:32	0/1:35,6:37	G	A
.	.	.	.	0.0(	0.997	1.0,D	1,D	5.3	5.05	.	.	SON	GT:/	0:80	0/1:126,14	G	A
.	.	.	.	.	.	.	.	.	.	.	.	SON	GT:/	0:22	0/1:21,5:37	C	A
.	.	##	.	.	.	.	.	.	.	0	0	SON	GT:/	0:28	0/1:35,8:37	C	T
.	.	.	.	.	.	.	.	.	.	.	.	SON	GT:/	0:24	0/1:55,12:3	G	A
.	.	.	.	.	.	.	.	.	.	.	.	SON	GT:/	0:66	0/1:123,17	C	T
.	.	.	.	.	.	.	.	.	.	.	.	SON	GT:/	0:26	0/1:54,20:3	C	A
.	.	.	.	.	.	.	1,D	3.8	.	.	.	SON	GT:/	0:18	0/1:38,6:37	T	C
.	.	.	.	0.1:	0.824	0.994	0.96	4.5	2.47	.	.	SON	GT:/	0:10	0/1:176,38	G	A
.	.	##	0	0.0(	0.923	1.0,D	1.00	3.3	3.15	.	.	SON	GT:/	0:12	0/1:188,34	G	A
.	.	.	.	.	.	.	.	.	1.54	.	.	SON	GT:/	0:89	0/1:160,23	C	T
.	0	0	0	.	.	.	.	2.5	.	0	0	SON	GT:/	0:47	0/1:89,18:3	G	A
.	.	.	.	.	.	.	.	2.6	.	.	.	SON	GT:/	0:10	0/1:173,25	G	A
.	.	.	.	0.3	0.085	0.716	0.99	3.1	.	.	.	SON	GT:/	0:15	0/1:35,5:34	G	A
.	.	.	.	.	.	.	.	2.2	.	.	.	SON	GT:/	0:45	0/1:70,16:3	A	G
.	.	.	.	.	.	.	.	2.3	.	.	.	SON	GT:/	0:41	0/1:50,8:37	A	G
.	.	.	.	.	.	.	.	.	.	0	.	SON	GT:/	0:27	0/1:53,12:3	G	A
.	.	.	.	0.3(	0.038	0.131	0.64	4.1	2.86	.	.	SON	GT:/	0:17	0/1:260,57	A	T
.	.	.	.	.	.	.	.	.	.	.	.	SON	GT:/	0:20	0/1:40,3:37	C	A
.	.	.	.	.	.	.	.	.	.	.	.	SON	GT:/	0:11	0/1:187,36	G	A
.	.	.	.	0.0'	0.837	0.983	1,D	5.9	3.34	.	.	SON	GT:/	0:22	0/1:36,7:37	G	T
2E-04	.	.	.	.	.	.	.	.	.	.	.	SON	GT:/	0:39	0/1:48,10:3	C	T
.	.	.	.	.	.	.	.	.	.	.	.	SON	GT:/	0:27	0/1:52,13:3	G	T
2E-04	.	##	0	0.0	0.042	0.313	0.99	4.8	1.42	.	.	SON	GT:/	0:81	0/1:129,30	C	T
.	.	##	0	.	.	.	.	4	.	.	.	SON	GT:/	0:35	0/1:39,8:33	C	T
.	.	.	.	.	.	.	.	.	.	.	.	SON	GT:/	0:49	0/1:75,19:3	A	G
.	.	.	.	0.0(	0.999	1.0,D	1,D	5.9	3.90	.	.	SON	GT:/	0:36	0/1:68,7:37	A	G
.	.	##	0	.	.	.	.	.	.	0	.	SON	GT:/	0:34	0/1:41,8:37	G	A
.	.	.	.	.	.	.	.	3.1	.	.	.	SON	GT:/	0:28	0/1:33,6:37	A	G
.	.	##	0	0.0	0.77,I	0.999	1.00	4.8	4.35	.	.	SON	GT:/	0:85	0/1:143,25	G	A
.	.	.	.	.	.	.	.	.	.	.	.	SON	GT:/	0:18	0/1:50,10:3	C	T
.	.	.	.	.	.	.	.	.	.	.	.	SON	GT:/	0:14	0/1:22,3:37	G	A
.	.	.	.	0.0(	0.994	0.999	0.99	.	3.46	.	.	SON	GT:/	0:28	0/1:29,5:37	G	T
4E-04	##	0	0	.	.	.	1,D	.	.	.	.	SON	GT:/	0:41	0/1:59,17:3	C	T
.	.	.	.	0.0	0.443	0.529	1,N	3.8	2.23	.	.	SON	GT:/	0:60	0/1:66,8:37	T	C
.	.	.	.	.	.	.	.	.	1.56	.	.	SON	GT:/	0:25	0/1:40,6:37	A	G
.	.	.	.	0.0'	0.8,P	0.993	1,N	.	.	.	.	SON	GT:/	0:50	0/1:68,15:3	C	A
.	.	.	.	.	.	.	.	.	.	.	.	SON	GT:/	0:42	0/1:49,13:3	G	T
.	.	.	.	.	.	.	.	.	1.76	.	.	SON	GT:/	0:56	0/1:78,17:3	C	T
.	.	.	.	.	.	.	.	2.7	3.73	.	.	SON	GT:/	0:17	0/1:39,7:33	C	T
.	##	##	0	.	.	.	.	.	.	.	.	SON	GT:/	0:30	0/1:59,11:3	C	T

.	.	##	0	.	0.0,B	0.0,B	1,N	.	.	.	.	SON	GT:/	0:90	0/1:123,22	G	A
.	.	.	.	0.3	0.22,	0.839	0.86	2.8	.	.	.	SON	GT:/	0:16	0/1:284,54	G	A
2E-04	##	##	0	.	.	.	.	.	.	.	0	SON	GT:/	0:62	0/1:111,19	C	T
.	.	.	.	0.0	0.997	1.0,D	0.99	.	2.19	.	.	SON	GT:/	0:78	0/1:157,33	G	T
.	.	.	.	.	.	.	.	.	.	.	.	SON	GT:/	0:12	0/1:192,38	C	T
.	.	.	.	.	.	.	.	.	.	.	.	SON	GT:/	0:13	0/1:10,3:37	C	T
2E-04	0	0	0	0.0	0.246	0.546	1,D	3.5	2.62	.	.	DB;S	GT:/	0:53	0/1:121,25	G	A
4E-04	0	##	0	.	.	.	.	.	.	.	.	SON	GT:/	0:46	0/1:61,19:3	C	T
.	.	.	.	.	.	.	.	.	.	.	.	SON	GT:/	0:22	0/1:33,10:3	C	T
.	.	0	0	.	.	.	.	.	.	0	.	SON	GT:/	0:14	0/1:23,4:37	G	A
.	.	.	.	0.0	0.12,	0.875	0.57	.	.	.	.	SON	GT:/	0:45	0/1:87,17:3	G	A
.	.	.	.	.	.	.	.	.	1.24	.	.	SON	GT:/	0:56	0/1:98,29:3	C	A
.	##	##	0	0.1	0.924	1.0,D	1,D	2.1	3.16	.	.	SON	GT:/	0:11	0/1:173,40	C	T
.	.	.	.	.	.	.	.	.	2.19	.	.	SON	GT:/	0:11	0/1:11,4:37	G	A
.	.	.	.	.	.	.	.	.	.	.	.	SON	GT:/	0:18	0/1:37,8:37	G	A
.	.	.	.	.	.	.	.	.	.	0	.	SON	GT:/	0:16	0/1:252,47	C	T
4E-04	##	##	0	.	.	.	.	.	1.68	.	.	SON	GT:/	0:19	0/1:281,80	G	A
.	.	0	0	.	.	.	.	.	.	.	.	SON	GT:/	0:11	0/1:211,34	G	A
.	.	.	.	0.5	0.128	0.199	0.99	4.7	1.33	.	.	SON	GT:/	0:11	0/1:176,28	G	T
.	.	.	.	.	.	.	.	.	.	.	.	SON	GT:/	0:33	0/1:72,11:3	C	A
.	##	##	0	.	.	.	.	.	1.31	.	.	SON	GT:/	0:14	0/1:256,45	C	T
.	.	.	.	.	.	.	.	.	.	.	.	SON	GT:/	0:9,(	0/1:26,5:37	C	T
.	.	.	.	.	.	.	.	.	.	.	.	SON	GT:/	0:25	0/1:23,9:37	G	A
.	.	.	.	.	.	.	.	.	.	.	.	SON	GT:/	0:27	0/1:43,9:35	C	T
.	.	##	0	0.0	0.387	0.815	1,N	.	2.00	.	.	SON	GT:/	0:82	0/1:130,23	G	A
.	.	.	.	.	.	.	1,D	3.7	6.30	.	.	SON	GT:/	0:66	0/1:99,20:3	G	A
6E-04	0	0	0	0.0	0.802	0.999	1,N	.	4.56	.	.	SON	GT:/	0:53	0/1:112,12	G	A
.	.	.	.	.	.	.	.	.	.	.	.	SON	GT:/	0:29	0/1:64,9:35	G	A
.	.	.	.	0.0	0.338	0.416	1,D	4	4.18	.	.	SON	GT:/	0:10	0/1:174,46	T	C
.	.	.	.	.	.	.	.	.	1.51	.	.	SON	GT:/	0:63	0/1:102,12	C	T
.	.	.	.	.	.	.	.	.	.	0	.	SON	GT:/	0:94	0/1:169,30	G	A
.	.	##	0	.	.	.	.	.	1.52	.	.	SON	GT:/	0:97	0/1:154,30	G	A
.	.	.	.	.	.	.	.	.	.	.	.	SON	GT:/	0:31	0/1:53,5:37	T	C
6E-04	##	##	0	0.0	0.14,	0.534	1.00	5.3	3.72	.	.	SON	GT:/	0:92	0/1:161,32	C	T
.	##	0	0	.	.	.	.	.	.	0	.	SON	GT:/	0:10	0/1:168,29	C	T
.	##	##	0	0.0	0.072	0.146	1,D	4.1	2.79	.	.	SON	GT:/	0:19	0/1:314,56	G	A
.	.	##	0	.	.	.	.	.	.	.	.	SON	GT:/	0:76	0/1:107,24	C	T
2E-04	##	##	0	0.0	0.997	1.0,D	1.00	5.4	5.41	0	.	SON	GT:/	0:39	0/1:90,15:3	G	A
.	.	.	.	.	.	.	.	.	1.28	.	.	SON	GT:/	0:10	0/1:22,5:37	G	T
.	.	.	.	.	.	.	.	.	.	.	.	SON	GT:/	0:10	0/1:143,22	C	A
.	.	##	0	.	.	.	.	.	.	.	.	SON	GT:/	0:85	0/1:137,25	C	T
0.015	0	0	0	0.5	0.013	0.084	1,N	.	.	0	0	DB;S	GT:/	0:16	0/1:333,59	C	T
.	.	##	0	.	.	.	.	.	.	.	.	SON	GT:/	0:19	0/1:318,58	C	T
0.002	.	.	.	.	.	.	.	2.2	.	.	.	SON	GT:/	0:16	0/1:15,3:37	T	G
.	.	.	.	0.1	0.003	0.004	1,N	.	.	0	.	SON	GT:/	0:78	0/1:118,19	C	T
.	.	.	.	.	.	.	.	.	.	.	.	SON	GT:/	0:8,(	0/1:6,2:37:	C	T
.	.	.	.	.	.	.	.	.	.	.	.	SON	GT:/	0:11	0/1:12,3:37	C	G
6E-04	.	0	0	.	.	.	.	.	.	.	0	SON	GT:/	0:12	0/1:108,29	G	A
.	.	##	0	0.3	0.0,B	0.0,B	1,N	.	.	.	.	SON	GT:/	0:11	0/1:166,37	C	T
.	.	.	.	.	.	.	.	.	.	.	.	SON	GT:/	0:11	0/1:213,39	C	T
.	.	.	.	.	.	.	.	.	.	.	.	SON	GT:/	0:11	0/1:21,6:37	A	G
.	.	.	.	0.1	0.593	0.953	1.00	4.3	2.96	0	.	SON	GT:/	0:37	0/1:52,11:3	G	A
.	.	##	0	0.0	0.959	1.0,D	1.00	4.8	2.99	.	.	SON	GT:/	0:12	0/1:196,40	G	A
.	.	.	.	0.0	0.725	0.994	0.67	2.8	3.44	.	.	SON	GT:/	0:61	0/1:97,18:3	C	T

.	.	.	.	1.0	0.191	0.327	0.91	5.5	.	.	.	SOM	GT:/	0:75	0/1:111,21	G	A
.	.	.	.	.	.	.	.	.	.	.	.	SOM	GT:/	0:31	0/1:68,4:37	G	A
.	.	.	.	0.2	0.249	0.514	1.00	.	.	0	.	SOM	GT:/	0:47	0/1:81,16:3	G	T
.	.	.	.	.	.	.	.	.	.	.	.	SOM	GT:/	0:10	0/1:18,3:37	G	A
2E-04	.	##	0	.	.	.	.	.	1.42	.	.	SOM	GT:/	0:13	0/1:232,40	G	A
.	.	##	0	.	.	.	.	.	.	.	.	SOM	GT:/	0:16	0/1:241,46	G	A
.	.	.	.	.	.	.	.	.	.	.	.	SOM	GT:/	0:9,(	0/1:19,5:37	A	G
.	.	.	.	0.0	0.978	0.993	1,D	4.4	4.77	.	.	SOM	GT:/	0:70	0/1:115,16	C	T
.	.	##	0	.	.	.	.	.	.	.	.	SOM	GT:/	0:25	0/1:50,21:3	C	T
.	##	##	0	.	.	.	.	.	.	.	.	SOM	GT:/	0:52	0/1:70,18:3	G	A
.	##	##	0	0.0	0.998	1.0,D	1,D	4.8	5.30	.	.	SOM	GT:/	0:49	0/1:86,14:3	C	T
.	.	.	.	.	.	.	.	.	.	.	.	SOM	GT:/	0:11	0/1:38,7:37	A	G
8E-04	.	.	.	.	.	.	.	.	.	0	0	SOM	GT:/	0:31	0/1:45,7:37	A	T
.	.	.	.	.	.	.	.	.	.	.	.	SOM	GT:/	0:39	0/1:59,6:35	G	T
.	0	##	0	0.2	0.007	0.012	1,N	.	1.25	0	.	SOM	GT:/	0:13	0/1:222,31	G	A
.	.	.	.	0.0	0.918	0.998	1.00	3	3.50	.	.	SOM	GT:/	0:55	0/1:87,21:3	G	A
.	.	.	.	0.0	0.889	0.99,I	1,D	4.5	4.51	.	.	SOM	GT:/	0:53	0/1:109,18	A	C
.	.	.	.	0.2	0.216	0.641	1,N	2.4	.	.	.	SOM	GT:/	0:11	0/1:162,25	C	G
.	.	.	.	.	.	.	.	.	.	.	.	SOM	GT:/	0:34	0/1:637,41	C	T
.	.	.	.	0.0	0.999	1.0,D	1.00	2.2	3.41	.	.	SOM	GT:/	0:94	0/1:173,35	G	A
.	.	.	.	.	.	.	.	.	.	.	.	SOM	GT:/	0:38	0/1:49,10:3	G	A
.	.	.	.	.	.	.	.	.	.	.	.	SOM	GT:/	0:71	0/1:115,20	C	T
4E-04	.	.	.	.	.	.	.	.	.	.	.	DB;S	GT:/	0:46	0/1:71,16:3	G	A
.	.	.	.	.	.	.	.	.	.	.	.	SOM	GT:/	0:32	0/1:27,6:37	T	C
.	.	.	.	0.0	0.908	1.0,D	1,N	.	1.71	.	.	SOM	GT:/	0:80	0/1:117,25	G	A
.	.	##	0	.	.	.	.	.	.	.	.	SOM	GT:/	0:59	0/1:106,16	G	A
.	.	.	.	0.3	0.113	0.804	0.98	4.7	2.67	.	.	SOM	GT:/	0:90	0/1:170,35	C	T
.	.	.	.	.	.	.	.	.	.	.	.	SOM	GT:/	0:28	0/1:46,7:37	T	C
.	.	.	.	.	.	.	.	.	.	.	.	SOM	GT:/	0:98	0/1:157,35	G	T
.	.	.	.	0.0	0.997	1.0,D	0.99	4.4	4.50	.	.	SOM	GT:/	0:50	0/1:83,13:3	C	T
.	##	##	0	.	.	.	.	.	.	0	.	SOM	GT:/	0:24	0/1:70,12:3	C	T
2E-04	.	.	.	.	.	.	.	.	.	.	.	SOM	GT:/	0:56	0/1:72,12:3	C	T
.	.	.	.	.	.	.	.	.	.	.	.	SOM	GT:/	0:21	0/1:302,60	C	A
.	.	.	.	.	.	.	.	.	.	0	.	SOM	GT:/	0:17	0/1:271,23	T	C
.	.	.	.	.	.	.	.	.	.	.	.	SOM	GT:/	0:11	0/1:200,22	T	C
.	.	.	.	.	.	.	.	.	.	.	.	SOM	GT:/	0:34	0/1:50,4:37	C	T
.	.	##	0	0.4	0.959	0.997	1,D	5.8	2.48	.	.	SOM	GT:/	0:72	0/1:99,16:3	C	T
.	##	##	0	0.0	0.011	0.022	1.00	2.1	2.96	.	.	SOM	GT:/	0:23	0/1:49,14:3	C	T
.	.	.	.	.	.	.	.	.	.	.	.	SOM	GT:/	0:84	0/1:159,23	C	G
.	.	.	.	.	.	.	.	.	.	.	.	SOM	GT:/	0:8,(	0/1:13,3:37	A	C
.	.	##	0	0.0	0.011	0.168	1,D	4.9	3.90	.	.	SOM	GT:/	0:58	0/1:87,16:3	G	A
.	.	.	.	.	.	.	.	.	.	.	.	SOM	GT:/	0:69	0/1:120,19	G	A
.	.	.	.	.	.	.	.	2.4	.	.	.	SOM	GT:/	0:20	0/1:32,8:37	T	C
2E-04	.	.	.	.	.	.	.	.	.	0	.	SOM	GT:/	0:12	0/1:20,3:37	C	T
.	.	.	.	.	.	.	.	.	.	.	.	SOM	GT:/	0:9,(	0/1:25,3:37	C	T
.	.	.	.	.	.	.	.	.	.	0	0	DB;S	GT:/	0:19	0/1:43,4:37	C	A
.	.	.	.	.	.	.	.	.	.	0	.	SOM	GT:/	0:42	0/1:87,4:37	C	G
.	.	.	.	.	.	.	.	.	.	0	.	SOM	GT:/	0:35	0/1:86,5:37	C	T
.	.	.	.	.	.	.	.	.	.	.	.	SOM	GT:/	0:14	0/1:32,3:37	G	A
.	.	.	.	0.2	0.031	0.021	0.93	3.2	1.73	.	.	SOM	GT:/	0:32	0/1:69,11:3	G	T
6E-04	0	0	0	0.2	0.058	0.103	1,N	.	2.00	.	.	SOM	GT:/	0:62	0/1:94,22:3	C	T
.	.	.	.	.	.	.	.	2.2	.	.	.	SOM	GT:/	0:13	0/1:19,5:37	C	T
2E-04	.	##	0	.	.	.	.	.	.	0	.	SOM	GT:/	0:10	0/1:187,29	G	A
0.004	.	.	.	.	.	.	.	.	.	0	0	SOM	GT:/	0:45	0/1:55,5:34	A	G

.	.	.	.	.	.	.	.	.	.	.	.	SON	GT:/	0:19	0/1:34,7:3	G	A
.	.	.	.	.	.	.	.	.	.	.	.	SON	GT:/	0:21	0/1:47,5:2	C	A
0.009	.	.	.	.	.	.	.	.	.	.	.	SON	GT:/	0:38	0/1:76,11:	A	G
.	.	.	.	.	.	.	.	2.5	3.60	.	.	SON	GT:/	0:72	0/1:139,21	G	A
.	.	.	.	.	.	.	.	.	1.35	.	.	SON	GT:/	0:15	0/1:23,3:37	T	C
.	.	##	0	.	.	.	.	.	.	.	0	SON	GT:/	0:12	0/1:15,5:37	G	A
.	.	##	0	0.1	0.246	0.968	0.99	4.2	3.28	.	.	SON	GT:/	0:40	0/1:71,17:	G	A
.	.	.	.	.	.	.	.	2.4	.	.	.	SON	GT:/	0:62	0/1:73,25:	G	A
.	.	##	0	.	0.794	0.986	1.00	4.8	3.90	.	.	SON	GT:/	0:13	0/1:251,41	G	A
.	.	.	.	.	.	.	.	.	.	.	.	SON	GT:/	0:44	0/1:78,10:	C	T
.	.	.	.	.	.	.	.	2.4	.	.	.	SON	GT:/	0:26	0/1:59,7:37	G	A
.	.	.	.	0.0	0.07,I	0.503	1,N	3	2.64	.	.	SON	GT:/	0:14	0/1:244,52	C	T
.	.	.	.	0.4	0.042	0.009	0.55	3.6	2.75	.	.	SON	GT:/	0:65	0/1:152,27	C	T
.	.	.	.	.	.	.	.	.	2.41	.	.	SON	GT:/	0:9,	0/1:15,4:37	C	A
.	.	.	.	0.0	0.237	0.748	1.00	3.5	2.25	.	.	SON	GT:/	0:42	0/1:87,19:	G	C
.	.	.	.	.	.	.	1,A	4.6	7.89	.	.	SON	GT:/	0:31	0/1:80,19:	G	T
.	.	.	.	0.2	0.831	0.991	0.98	4.8	2.51	.	.	SON	GT:/	0:69	0/1:139,5:	G	T
.	.	.	.	0.0	0.02,I	0.149	0.88	2.9	2.50	.	.	SON	GT:/	0:41	0/1:77,13:	C	T
.	.	.	.	0.0	0.999	1.0,D	1,D	4.2	3.92	.	.	SON	GT:/	0:65	0/1:129,22	C	A
.	.	.	.	.	.	.	1,A	.	4.13	.	.	SON	GT:/	0:91	0/1:123,29	C	A
.	.	.	.	.	.	.	.	.	.	0	0	SON	GT:/	0:73	0/1:149,9:	T	C
.	.	.	.	.	.	.	.	.	.	.	.	SON	GT:/	0:24	0/1:31,3:37	C	T
.	.	.	.	.	.	.	.	.	.	.	.	SON	GT:/	0:19	0/1:382,65	G	A
.	.	.	.	.	.	.	.	.	.	.	.	SON	GT:/	0:41	0/1:61,9:37	T	G
.	.	.	.	.	0.004	0.012	1,N	.	2.49	.	.	SON	GT:/	0:59	0/1:89,13:	G	T
.	.	##	0	.	0.015	0.316	1,N	.	1.91	0	.	SON	GT:/	0:10	0/1:171,32	C	T
.	.	.	.	.	.	.	.	.	.	.	.	SON	GT:/	0:14	0/1:18,4:37	C	T
.	.	.	.	.	.	.	.	.	.	.	.	SON	GT:/	0:59	0/1:73,12:	C	T
2E-04	.	.	.	.	.	.	.	.	.	.	.	SON	GT:/	0:27	0/1:41,10:	C	T
.	.	.	.	.	.	.	.	.	2.03	.	.	SON	GT:/	0:10	0/1:198,47	C	T
.	.	.	.	0.0	0.999	1.0,D	0.98	3.8	2.47	.	.	SON	GT:/	0:10	0/1:189,36	C	A
.	.	.	.	.	.	.	.	.	1.42	.	.	SON	GT:/	0:9,	0/1:24,5:37	T	C
.	.	.	.	0.0	0.534	0.712	1,D	2.4	3.32	.	.	SON	GT:/	0:12	0/1:196,25	G	T
.	.	.	.	0.0	0.84,I	0.995	0.99	5.3	2.86	.	.	SON	GT:/	0:10	0/1:201,37	C	T
.	##	##	0	0.0	0.969	0.997	1,D	4.7	3.93	0	0	SON	GT:/	0:14	0/1:190,36	C	T
.	.	.	.	.	.	.	.	.	.	.	.	SON	GT:/	0:34	0/1:41,7:35	G	A
.	.	.	.	.	.	.	.	.	.	.	.	SON	GT:/	0:27	0/1:25,5:37	G	A
.	.	.	.	.	.	.	.	.	.	.	.	SON	GT:/	0:66	0/1:104,25	C	T
.	.	.	.	.	.	.	.	.	.	.	.	SON	GT:/	0:9,	0/1:22,3:37	C	G
.	.	.	.	0.0	0.228	0.957	1,D	4.1	3.36	.	.	SON	GT:/	0:20	0/1:306,67	G	A
.	.	.	.	.	.	.	.	.	.	.	.	SON	GT:/	0:51	0/1:109,23	G	A
.	.	.	.	.	.	.	.	.	.	.	.	SON	GT:/	0:46	0/1:70,5:37	G	A
.	.	.	.	.	.	.	.	4.4	2.17	.	.	SON	GT:/	0:47	0/1:57,10:	A	G
4E-04	.	.	.	.	.	.	.	.	.	.	.	SON	GT:/	0:17	0/1:29,12:	C	T
.	.	.	.	.	.	.	.	.	.	.	.	SON	GT:/	0:23	0/1:32,9:34	C	T
.	.	.	.	.	.	.	.	2.5	1.43	.	.	SON	GT:/	0:64	0/1:129,21	G	T
.	.	.	.	.	.	.	.	.	.	.	.	SON	GT:/	0:63	0/1:103,15	C	T
.	0	0	0	0.0	0.974	1.0,D	1.00	3.1	4.07	.	.	SON	GT:/	0:17	0/1:50,6:37	G	A
6E-04	0	0	0	.	.	.	.	4.4	1.63	.	.	SON	GT:/	0:10	0/1:151,29	G	A
.	.	.	.	.	.	.	.	.	.	.	.	SON	GT:/	0:17	0/1:273,59	G	A
0.015	.	.	.	.	.	.	.	.	.	0	0	DB;	GT:/	0:20	0/1:35,6:37	T	G
.	##	##	0	.	.	.	.	.	.	.	.	SON	GT:/	0:40	0/1:77,13:	C	T
.	.	.	.	.	.	.	.	.	.	.	.	SON	GT:/	0:21	0/1:20,6:37	G	A
.	.	0	0	0.1	0.004	0.001	0.97	.	1.31	.	.	SON	GT:/	0:66	0/1:97,20:	G	A

.	.	.	.	.	.	.	1,D	2.8	1.87	.	.	SOM	GT:/	0:96	0/1:161,28	G	A
.	.	.	.	.	.	.	.	.	.	.	.	SOM	GT:/	0:15	0/1:241,45	A	G
.	.	.	.	0.0:	1.0,D	1.0,D	1,D	.	2.92	.	.	SOM	GT:/	0:13	0/1:197,38	T	C
.	.	.	.	.	.	.	.	.	.	.	.	SOM	GT:/	0:49	0/1:59,13:3	A	G
.	0	0	0	.	.	.	.	.	.	0	0	SOM	GT:/	0:66	0/1:107,5:3	T	G
.	.	.	.	.	.	.	.	.	.	.	.	SOM	GT:/	0:90	0/1:190,9:3	G	A
.	.	.	.	0.3:	0.57,I	0.782	.	4.8	3.58	.	.	SOM	GT:/	0:17	0/1:264,36	T	G
.	.	.	.	.	.	.	.	.	.	.	.	SOM	GT:/	0:11	0/1:25,7:37	T	C
.	.	.	.	.	.	.	.	.	.	.	.	SOM	GT:/	0:22	0/1:42,5:37	G	A
.	0	##	0	.	.	.	.	.	.	.	.	SOM	GT:/	0:96	0/1:141,7:3	G	A
.	.	.	.	.	.	.	.	2.9	1.43	.	.	SOM	GT:/	0:88	0/1:139,27	C	A
.	.	##	.	.	.	.	1,N	.	2.84	.	.	SOM	GT:/	0:99	0/1:143,30	G	A
.	.	.	.	.	.	.	.	.	.	.	.	SOM	GT:/	0:10	0/1:15,4:34	G	T
.	.	##	0	0.1:	0.078	0.864	1,N	.	2.24	0	.	SOM	GT:/	0:11	0/1:146,44	C	T
.	##	0	0	0.0:	0.37,I	0.529	1,N	.	.	0	.	SOM	GT:/	0:47	0/1:83,13:3	G	A
.	.	##	0	.	.	.	.	.	.	0	.	SOM	GT:/	0:63	0/1:89,11:3	C	T
.	.	.	.	.	.	.	.	.	1.27	.	.	SOM	GT:/	0:20	0/1:26,7:35	G	A
.	.	.	.	.	.	.	.	.	.	.	.	SOM	GT:/	0:37	0/1:52,12:3	G	A
.	0	0	0	.	.	.	.	.	.	.	.	SOM	GT:/	0:32	0/1:64,4:37	C	T
.	.	.	.	.	.	.	.	.	.	.	.	SOM	GT:/	0:13	0/1:254,45	C	T
.	##	##	0	.	.	.	.	.	.	.	.	SOM	GT:/	0:89	0/1:162,25	C	T
.	.	.	.	0.2:	0.122	0.555	1,N	.	.	.	.	SOM	GT:/	0:20	0/1:348,55	C	T
.	.	##	0	.	.	.	.	3.8	3.57	0	.	SOM	GT:/	0:17	0/1:31,4:34	T	C
.	.	.	.	.	.	.	.	.	1.56	.	.	SOM	GT:/	0:85	0/1:139,22	G	A
.	.	.	.	.	.	.	.	.	.	0	.	SOM	GT:/	0:24	0/1:35,6:37	C	T
.	.	.	.	.	.	.	.	.	.	.	.	SOM	GT:/	0:21	0/1:42,5:37	C	T
.	.	0	0	.	.	.	1,A	3.4	8.95	.	.	SOM	GT:/	0:37	0/1:50,8:37	G	A
.	.	.	.	.	.	.	.	.	.	.	.	SOM	GT:/	0:12	0/1:16,6:37	C	T
.	.	.	.	.	.	.	.	.	.	.	.	SOM	GT:/	0:42	0/1:56,17:3	G	A
.	.	.	.	.	.	.	.	.	.	.	.	SOM	GT:/	0:14	0/1:35,9:37	T	C
.	.	.	.	0.0:	0.98,I	0.99,I	1,D	5.6	4.31	.	.	SOM	GT:/	0:55	0/1:89,18:3	A	G
.	.	.	.	.	.	.	.	.	.	.	.	SOM	GT:/	0:30	0/1:46,4:37	T	C
2E-04	0	##	0	0.0:	0.583	0.928	1,N	.	1.82	0	.	SOM	GT:/	0:48	0/1:108,16	G	A
.	.	.	.	.	.	.	.	.	.	.	.	SOM	GT:/	0:10	0/1:15,4:37	T	C
.	.	.	.	.	.	.	.	.	.	0	.	SOM	GT:/	0:9,(	0/1:27,4:37	C	T
.	.	.	.	.	.	.	.	.	.	0	.	SOM	GT:/	0:15	0/1:39,3:37	C	T
.	.	.	.	.	.	.	.	.	.	0	.	SOM	GT:/	0:16	0/1:41,3:37	G	A
2E-04	.	##	0	0.3:	0.023	0.142	1.00	5	2.85	.	0	SOM	GT:/	0:55	0/1:113,11	C	T
.	.	.	.	.	.	.	.	.	.	.	.	SOM	GT:/	0:84	0/1:128,14	C	A
.	.	.	.	.	.	.	.	2.6	.	.	.	SOM	GT:/	0:14	0/1:24,5:37	G	T
.	.	.	.	.	.	.	.	.	.	0	0	SOM	GT:/	0:10	0/1:6,2:37:	G	A
0.022	0	0	0	.	.	.	.	.	.	0	0	DB;S	GT:/	0:40	0/1:86,6:37	A	T
.	.	.	.	.	.	.	.	.	.	.	.	SOM	GT:/	0:14	0/1:29,6:37	A	C
.	0	##	0	0.0.	0.992	1.0,D	1,D	5	5.37	.	.	SOM	GT:/	0:52	0/1:100,16	C	T
0.001	.	.	.	.	.	.	.	.	.	0	.	SOM	GT:/	0:20	0/1:40,12:3	T	C
.	.	.	.	.	.	.	.	.	1.86	.	.	SOM	GT:/	0:73	0/1:141,5:3	G	A
.	.	.	.	.	.	.	.	.	.	.	.	SOM	GT:/	0:44	0/1:60,8:35	G	A
.	.	.	.	.	.	.	.	.	2.54	0	0	SOM	GT:/	0:9,(	0/1:25,3:37	G	A
.	.	.	.	.	.	.	.	.	.	.	.	SOM	GT:/	0:15	0/1:29,7:37	G	T
.	.	.	.	.	.	.	.	.	.	.	.	SOM	GT:/	0:31	0/1:27,6:37	C	T
.	.	.	.	0.0.	0.987	0.996	1.00	4.4	5.38	.	.	SOM	GT:/	0:11	0/1:184,35	G	A
2E-04	##	##	0	.	.	.	.	3.8	3.08	0	0	SOM	GT:/	0:53	0/1:73,10:3	G	A
.	.	.	.	0.6(	0.012	0.02,I	0.75	3.1	.	.	.	SOM	GT:/	0:29	0/1:35,7:37	G	A
.	.	.	.	0.0.	0.999	1.0,D	1,D	4.5	2.75	.	.	SOM	GT:/	0:79	0/1:162,15	C	T

.	.	.	.	.	.	.	1,A	4.3	8.44	.	.	SOM	GT:	0:14	0/1:261,38	G	A
.	0	##	0	.	.	.	.	.	.	.	.	SOM	GT:	0:78	0/1:129,24	G	A
.	.	.	.	.	.	.	.	2.5	.	.	.	SOM	GT:	0:21	0/1:26,5:37	T	C
.	.	.	.	.	.	.	.	.	.	.	.	SOM	GT:	0:81	0/1:108,15	G	T
.	.	.	.	.	.	.	1,N	.	.	.	.	SOM	GT:	0:27	0/1:48,12:3	C	A
.	.	.	.	.	.	.	.	.	.	.	.	SOM	GT:	0:29	0/1:43,8:37	G	T
.	.	.	.	.	.	.	.	.	.	.	.	SOM	GT:	0:63	0/1:112,20	G	T
.	.	.	.	0.0	0.99	1.0,D	1.00	5.6	4.89	.	.	SOM	GT:	0:61	0/1:94,18:3	C	T
.	.	.	.	0.0	0.994	1.0,D	1,D	5.2	4.06	.	.	SOM	GT:	0:96	0/1:158,25	G	A
.	.	.	.	.	.	.	.	.	.	.	.	SOM	GT:	0:40	0/1:66,11:3	C	T
.	.	.	.	.	.	.	.	.	.	.	.	SOM	GT:	0:45	0/1:49,17:3	G	A
.	.	.	.	.	.	.	.	.	.	.	.	SOM	GT:	0:82	0/1:124,29	C	T
.	.	.	.	.	.	.	.	.	.	0	0	SOM	GT:	0:9,(	0/1:10,4:37	T	C
.	.	.	.	0.0	0.008	0.213	1,N	.	.	.	.	SOM	GT:	0:21	0/1:374,82	G	A
.	.	.	.	0.0	0.032	0.437	1,N	.	2.41	.	.	SOM	GT:	0:64	0/1:105,15	C	T
.	.	.	.	.	.	.	.	.	.	.	.	SOM	GT:	0:16	0/1:14,6:37	G	A
.	.	##	0	0.0	0.995	1.0,D	1.00	3.6	2.21	.	.	SOM	GT:	0:57	0/1:89,13:3	C	T
.	.	##	0	.	.	.	.	.	.	.	.	SOM	GT:	0:72	0/1:118,16	C	T
4E-04	.	.	.	.	.	.	.	.	.	0	.	SOM	GT:	0:31	0/1:75,11:3	G	A
0.002	0	0	0	.	.	.	.	.	3.33	.	.	SOM	GT:	0:17	0/1:267,33	G	A
.	.	.	.	.	.	.	.	.	.	0	.	SOM	GT:	0:13	0/1:12,3:37	C	T
.	.	.	.	.	.	.	.	.	.	.	.	SOM	GT:	0:12	0/1:20,3:37	A	G
2E-04	.	##	0	0.0	0.997	1.0,D	1.00	4.6	3.93	.	.	SOM	GT:	0:11	0/1:197,39	G	A
.	.	.	.	.	.	.	.	.	.	.	.	SOM	GT:	0:59	0/1:37,11:3	A	G
.	.	.	.	.	.	.	.	.	.	.	.	SOM	GT:	0:8,(	0/1:15,3:28	C	T
.	.	.	.	.	.	.	.	.	.	0.1	0.1	SOM	GT:	0:38	0/1:58,4:37	G	A
.	.	.	.	.	.	.	.	.	.	0	0	SOM	GT:	0:58	0/1:108,7:3	C	A
.	.	.	.	.	.	.	.	.	.	0	0.1	SOM	GT:	0:9,(	0/1:15,5:37	G	A
.	.	.	.	.	.	.	.	.	.	0	0.1	SOM	GT:	0:8,(	0/1:17,3:37	C	A
.	.	.	.	.	.	.	.	.	.	0.2	0.4	SOM	GT:	0:14	0/1:19,8:37	C	T
.	.	.	.	.	.	.	.	.	.	.	.	SOM	GT:	0:78	0/1:115,14	G	A
.	.	.	.	.	.	.	.	.	.	.	.	SOM	GT:	0:11	0/1:203,8:3	C	A
2E-04	.	.	.	.	.	.	.	.	.	.	.	SOM	GT:	0:70	0/1:104,6:3	G	C
.	.	.	.	.	.	.	.	.	.	.	.	SOM	GT:	0:9,(	0/1:1,5:34:	C	T
.	.	.	.	.	.	.	.	.	.	0.3	0.6	SOM	GT:	0:9,(	0/1:14,4:37	T	C
.	.	.	.	.	.	.	.	.	.	0.3	0.5	SOM	GT:	0:9,(	0/1:14,3:37	C	T
.	.	.	.	.	.	.	.	.	.	0.1	0.2	DB;S	GT:	0:33	0/1:55,4:37	G	C
.	.	.	.	.	.	.	.	.	.	.	.	SOM	GT:	0:30	0/1:63,5:37	C	T
.	.	.	.	.	.	.	.	.	.	.	.	SOM	GT:	0:31	0/1:30,7:37	T	C
.	.	.	.	.	.	.	.	.	.	.	.	SOM	GT:	0:33	0/1:50,9:37	A	G
.	.	.	.	.	.	.	.	2.5	.	.	.	SOM	GT:	0:24	0/1:36,5:37	C	G
.	.	.	.	.	.	.	.	.	.	.	.	SOM	GT:	0:75	0/1:100,20	A	G
.	.	.	.	.	.	.	.	.	.	.	.	SOM	GT:	0:18	0/1:26,10:3	C	T
.	.	.	.	0.5	0.106	0.245	1,N	.	.	.	.	SOM	GT:	0:11	0/1:181,31	C	T
.	.	.	.	.	.	.	.	.	.	.	.	SOM	GT:	0:14	0/1:23,8:37	A	G
.	.	.	.	.	.	.	.	.	.	.	.	SOM	GT:	0:21	0/1:32,9:37	G	T
.	.	.	.	.	.	.	.	.	.	.	.	SOM	GT:	0:37	0/1:52,5:37	C	T
.	.	.	.	.	.	.	.	3.8	.	.	.	SOM	GT:	0:28	0/1:39,7:37	C	T
.	.	##	0	.	.	.	.	.	1.49	.	.	SOM	GT:	0:22	0/1:33,11:3	A	G
.	.	.	.	.	.	.	.	.	.	0	.	SOM	GT:	0:47	0/1:67,11:3	C	T
.	.	.	.	.	.	.	.	.	.	.	.	SOM	GT:	0:12	0/1:8,3:37:	T	G
.	.	.	.	.	.	.	.	.	.	.	.	SOM	GT:	0:10	0/1:24,4:37	C	T
.	.	.	.	.	.	.	.	.	1.71	.	.	SOM	GT:	0:22	0/1:36,5:37	C	T
.	.	##	0	.	.	.	.	.	.	.	.	SOM	GT:	0:51	0/1:96,22:3	G	A

.	.	##	0	0.0(	0.993	1.0,D	1.00	4.8	4.76	.	.	SON	GT:/	0:30	0/1:57,17:3	C	T
.	.	.	.	.	.	.	.	.	.	.	.	SON	GT:/	0:28	0/1:46,6:37	C	A
.	.	.	.	.	.	.	.	.	.	.	.	SON	GT:/	0:93	0/1:133,34	C	A
.	.	.	.	.	.	.	.	.	.	.	.	SON	GT:/	0:42	0/1:73,17:3	T	C
.	.	.	.	0.0(	0.998	1.0,D	1.00	5	2.65	.	.	SON	GT:/	0:36	0/1:69,14:3	G	A
.	.	.	.	0.0'	0.996	1.0,D	1.00	5.3	3.79	.	.	SON	GT:/	0:44	0/1:81,20:3	T	C
.	.	.	.	.	.	.	.	.	.	.	.	SON	GT:/	0:29	0/1:39,9:37	C	T
.	.	.	.	.	.	.	.	.	.	.	.	SON	GT:/	0:16	0/1:30,5:37	T	C
.	.	.	.	.	.	.	.	.	.	.	.	SON	GT:/	0:34	0/1:46,11:3	G	A
.	.	.	.	0.0·	0.987	1.0,D	1.00	5.3	4.28	.	.	SON	GT:/	0:73	0/1:109,13	G	A
.	.	.	.	.	.	.	.	.	.	.	.	SON	GT:/	0:23	0/1:44,5:37	T	C
.	.	.	.	.	.	.	.	.	.	.	.	SON	GT:/	0:12	0/1:13,6:35	C	A
.	.	.	.	.	.	.	.	.	.	.	.	SON	GT:/	0:53	0/1:88,13:3	G	A
2E-04	.	##	.	0.0.	0.96,I	1.0,D	0.99	.	2.81	.	.	SON	GT:/	0:10	0/1:135,30	C	T
.	.	.	.	.	.	.	.	.	.	.	.	SON	GT:/	0:18	0/1:41,8:35	C	T
.	.	##	0	0.0(	0.998	1.0,D	1,D	5	4.64	.	.	SON	GT:/	0:72	0/1:98,18:3	G	A
.	.	##	0	.	.	.	.	.	.	.	.	SON	GT:/	0:14	0/1:240,38	C	T
.	0	.	.	.	.	.	.	.	.	.	.	SON	GT:/	0:35	0/1:94,26:3	C	T
.	.	.	.	.	.	.	.	.	.	.	.	SON	GT:/	0:15	0/1:32,3:37	G	A
.	.	.	.	.	.	.	.	.	.	.	.	SON	GT:/	0:20	0/1:35,4:34	G	A
.	.	##	0	0.0(	0.997	1.0,D	0.99	5.5	3.51	.	.	SON	GT:/	0:90	0/1:131,28	G	A
.	.	.	.	0.4:	0.329	0.837	0.99	4.5	1.36	.	.	SON	GT:/	0:36	0/1:72,11:3	G	A
.	.	.	.	.	.	.	.	.	.	0.1	0.3	SON	GT:/	0:17	0/1:36,4:37	A	G
.	.	.	.	.	.	.	.	.	.	0	0.1	SON	GT:/	0:10	0/1:11,6:35	C	G
.	.	.	.	.	.	.	.	.	.	.	.	SON	GT:/	0:23	0/1:28,7:37	A	G
.	.	.	.	.	.	.	.	.	.	0.2	0.3	DB;S	GT:/	0:19	0/1:36,5:37	C	A
.	.	.	.	.	.	.	.	.	.	.	.	SON	GT:/	0:49	0/1:90,15:3	G	A
4E-04	.	##	0	.	.	.	.	.	.	.	.	SON	GT:/	0:16	0/1:284,46	G	A
.	.	.	.	.	.	.	.	.	2.51	0	.	SON	GT:/	0:8,(	0/1:19,5:19	T	A
0.002	.	0	0	.	.	.	.	.	.	.	.	SON	GT:/	0:59	0/1:61,6:37	G	A
.	.	.	.	.	.	.	.	.	.	0	0	SON	GT:/	0:61	0/1:90,5:37	C	A
.	.	.	.	.	.	.	.	.	.	.	.	SON	GT:/	0:24	0/1:39,3:37	G	A
.	.	##	0	.	.	.	.	.	.	.	.	SON	GT:/	0:14	0/1:213,40	G	A
.	.	##	0	.	.	.	.	.	.	.	.	SON	GT:/	0:12	0/1:188,41	G	A
.	.	.	.	.	.	.	.	.	.	.	.	SON	GT:/	0:11	0/1:166,24	G	A
.	##	##	0	.	.	.	.	.	.	.	.	SON	GT:/	0:52	0/1:63,14:3	G	A
2E-04	##	##	0	.	.	.	.	.	.	.	.	SON	GT:/	0:59	0/1:120,25	C	T
.	.	.	.	0.0:	0.265	0.745	1,N	.	1.99	.	.	SON	GT:/	0:12	0/1:216,34	G	T
.	.	.	.	0.0(	0.002	0.009	0.76	3.3	2.16	.	.	SON	GT:/	0:11	0/1:175,39	C	T
.	.	.	.	.	.	.	.	2.3	.	.	.	SON	GT:/	0:44	0/1:70,11:3	G	T
.	.	##	0	0.2'	0.019	0.127	0.99	3.7	1.77	.	.	SON	GT:/	0:10	0/1:14,5:37	G	A
.	.	.	.	.	.	.	.	.	.	.	.	SON	GT:/	0:44	0/1:89,6:37	C	T
.	.	.	.	.	.	.	.	.	.	.	.	SON	GT:/	0:22	0/1:33,9:37	C	T
4E-04	.	.	.	.	.	.	.	.	.	.	.	SON	GT:/	0:45	0/1:73,16:3	C	T
.	.	.	.	.	.	.	.	.	.	.	.	SON	GT:/	0:50	0/1:85,13:3	T	C
.	.	.	.	.	.	.	.	.	.	.	.	SON	GT:/	0:20	0/1:44,10:3	G	A
1E-03	.	0	0	.	.	.	.	.	.	.	.	SON	GT:/	0:15	0/1:33,4:37	G	A
.	.	.	.	.	.	.	.	2.2	.	.	.	SON	GT:/	0:27	0/1:40,6:37	C	T
.	.	.	.	.	.	.	.	4	2.97	.	.	SON	GT:/	0:81	0/1:137,28	G	A
.	.	.	.	.	.	.	.	.	.	.	.	SON	GT:/	0:16	0/1:24,5:34	T	C
.	.	.	.	.	.	.	.	.	.	.	.	SON	GT:/	0:18	0/1:45,8:37	T	C
.	.	.	.	.	.	.	.	.	.	.	.	SON	GT:/	0:49	0/1:81,13:3	G	A
.	##	##	0	.	.	.	.	.	.	.	.	SON	GT:/	0:72	0/1:109,16	C	T
.	.	.	.	.	.	.	.	.	.	.	.	SON	GT:/	0:42	0/1:67,13:3	C	T

2E-04	##	0	0	0.2	0.205	0.863	1,N	.	2.01	0	0	SOM	GT:/	0:66	0/1:153,27	G	A
2E-04	.	.	.	.	.	.	.	.	.	.	.	SOM	GT:/	0:47	0/1:64,6:37	C	T
.	.	.	.	.	.	.	1,A	4.5	7.78	.	.	SOM	GT:/	0:53	0/1:83,15:3	G	A
.	.	.	.	.	.	.	.	.	.	.	.	SOM	GT:/	0:10	0/1:30,5:37	C	A
.	.	##	0	.	.	.	.	.	.	.	.	SOM	GT:/	0:75	0/1:137,28	A	G
.	.	.	.	0.0	1.0,D	1.0,D	1,D	4.9	4.46	.	.	SOM	GT:/	0:17	0/1:297,53	G	A
.	.	.	.	0.0	0.781	0.999	0.99	4.4	3.54	.	.	SOM	GT:/	0:14	0/1:285,62	G	A
2E-04	.	.	.	.	.	.	.	.	.	.	.	SOM	GT:/	0:82	0/1:164,27	C	T
.	.	.	.	.	.	.	.	.	.	.	.	SOM	GT:/	0:26	0/1:429,80	C	T
1E-03	.	.	.	.	.	.	.	.	.	0	.	SOM	GT:/	0:49	0/1:90,18:3	C	T
.	.	.	.	0.2	0.975	0.999	0.99	5.6	4.50	.	.	SOM	GT:/	0:54	0/1:89,19:3	C	T
.	.	##	0	0.4	0.002	0.035	1,N	.	.	.	.	SOM	GT:/	0:12	0/1:154,35	G	A
.	.	.	.	0.0	0.721	0.948	1,D	5.4	5.33	.	.	SOM	GT:/	0:86	0/1:154,8:3	C	T
2E-04	.	##	0	0.0	0.998	1.0,D	1,D	4.4	3.92	.	.	SOM	GT:/	0:69	0/1:138,20	C	T
.	.	.	.	.	.	.	.	.	.	.	.	SOM	GT:/	0:26	0/1:52,6:37	C	A
.	.	.	.	.	.	.	.	.	.	.	.	SOM	GT:/	0:36	0/1:30,12:3	G	A
2E-04	.	##	0	.	.	.	.	.	.	.	.	SOM	GT:/	0:82	0/1:120,20	C	T
.	.	.	.	.	.	.	.	.	.	.	.	SOM	GT:/	0:22	0/1:28,5:37	G	A
2E-04	.	.	.	.	.	.	.	.	.	0	0	SOM	GT:/	0:13	0/1:13,3:37	C	T
.	.	.	.	.	.	.	.	.	.	0	0	DB;S	GT:/	0:37	0/1:79,4:34	C	T
.	.	.	.	.	.	.	.	2.2	1.77	.	.	SOM	GT:/	0:26	0/1:51,12:3	C	T
.	.	.	.	.	.	.	.	5.5	2.28	.	.	SOM	GT:/	0:30	0/1:54,10:3	A	G
.	.	.	.	.	.	.	.	.	.	.	.	SOM	GT:/	0:16	0/1:32,5:37	T	C
.	.	.	.	.	.	.	.	.	.	.	.	SOM	GT:/	0:65	0/1:127,33	G	A
.	.	.	.	0.0	0.218	0.937	1,N	2.7	1.74	.	.	SOM	GT:/	0:12	0/1:160,42	G	A
.	.	.	.	.	.	.	.	.	.	.	.	SOM	GT:/	0:75	0/1:115,22	C	A
.	.	.	.	.	.	.	.	.	.	.	.	SOM	GT:/	0:15	0/1:31,3:37	C	A
.	.	.	.	.	.	.	.	.	.	.	.	SOM	GT:/	0:52	0/1:100,5:3	G	A
.	.	.	.	.	.	.	.	.	.	.	.	SOM	GT:/	0:8,	0/1:7,3:37:	G	A
.	.	.	.	.	.	.	.	3.7	.	.	.	SOM	GT:/	0:21	0/1:341,55	G	A
2E-04	##	##	0	.	.	.	.	.	.	.	.	SOM	GT:/	0:54	0/1:122,21	C	T
.	.	.	.	.	.	.	.	.	.	.	.	SOM	GT:/	0:53	0/1:69,12:3	G	A
.	.	.	.	0.0	0.999	1.0,D	1.00	4.8	4.78	.	.	SOM	GT:/	0:96	0/1:212,25	C	T
.	.	.	.	.	.	.	.	.	.	.	.	SOM	GT:/	0:20	0/1:37,11:3	C	T
.	.	.	.	.	.	.	.	.	.	.	.	SOM	GT:/	0:19	0/1:27,11:3	A	G
.	.	.	.	.	.	.	.	.	.	.	.	SOM	GT:/	0:22	0/1:35,10:3	T	G
.	.	.	.	.	.	.	.	.	.	.	.	SOM	GT:/	0:50	0/1:87,12:3	A	C
.	.	.	.	.	.	.	.	.	.	.	.	SOM	GT:/	0:70	0/1:130,19	T	C
.	.	.	.	.	.	.	.	.	.	.	.	SOM	GT:/	0:51	0/1:90,17:3	T	C
.	.	.	.	.	.	.	.	2.1	.	.	.	SOM	GT:/	0:37	0/1:50,13:3	G	T
.	.	.	.	.	.	.	.	.	.	.	.	SOM	GT:/	0:17	0/1:14,5:37	C	T
.	.	.	.	0.0	1.0,D	1.0,D	1,D	5.6	4.37	.	.	SOM	GT:/	0:11	0/1:183,32	C	T
.	.	.	.	.	.	.	.	.	1.86	.	.	SOM	GT:/	0:71	0/1:111,28	G	A
.	.	##	0	0.0	0.456	0.916	1,N	.	2.58	.	.	SOM	GT:/	0:61	0/1:96,13:3	C	T
.	.	.	.	.	.	.	.	.	.	.	.	SOM	GT:/	0:71	0/1:99,18:3	C	T
.	.	.	.	.	.	.	.	.	.	.	.	SOM	GT:/	0:94	0/1:125,34	T	C
.	.	.	.	.	.	.	.	.	.	.	.	SOM	GT:/	0:15	0/1:31,8:37	C	T
.	.	.	.	.	.	.	.	.	.	.	.	SOM	GT:/	0:58	0/1:52,17:3	T	A
.	.	.	.	.	.	.	.	.	.	.	.	SOM	GT:/	0:64	0/1:132,10	C	A
.	.	.	.	.	.	.	.	3	4.36	.	.	SOM	GT:/	0:12	0/1:20,6:37	G	A
.	.	.	.	.	.	.	.	.	.	.	.	SOM	GT:/	0:43	0/1:40,5:31	T	C
.	.	.	.	.	.	.	.	.	.	.	.	SOM	GT:/	0:29	0/1:49,7:35	A	C
.	.	.	.	.	.	.	.	.	.	.	.	SOM	GT:/	0:45	0/1:73,9:35	A	C
.	.	.	.	.	.	.	.	5.2	3.80	.	.	SOM	GT:/	0:30	0/1:50,4:37	A	C

.	.	.	.	.	.	.	.	.	1.99	.	.	SON	GT:/	0:12	0/1:176,35	G	A
.	.	.	.	.	.	.	.	.	.	.	.	SON	GT:/	0:42	0/1:60,6:37	G	A
.	.	.	.	.	.	.	.	.	.	.	.	SON	GT:/	0:99	0/1:134,15	C	T
.	.	.	.	.	.	.	.	.	.	.	.	SON	GT:/	0:86	0/1:170,13	G	T
.	.	0	0	.	.	.	.	.	.	.	.	SON	GT:/	0:21	0/1:308,65	C	T
.	##	##	0	0.3:	0.034	0.507	1,D	3.3	3.27	.	.	SON	GT:/	0:14	0/1:234,47	G	A
.	.	##	0	0.1·	0.843	0.999	1,D	4	4.03	.	.	SON	GT:/	0:12	0/1:191,37	C	T
.	.	.	.	.	.	.	.	.	.	.	.	SON	GT:/	0:14	0/1:259,34	G	T
.	.	.	.	.	.	.	.	2.7	1.77	.	.	SON	GT:/	0:38	0/1:59,18:3	G	A
.	.	##	0	.	.	.	.	.	.	.	.	SON	GT:/	0:89	0/1:156,34	C	T
.	.	.	.	.	.	.	.	4.2	1.57	.	.	SON	GT:/	0:65	0/1:100,12	C	T
.	.	.	.	0.0!	0.104	0.703	0.96	2.4	1.63	.	.	SON	GT:/	0:63	0/1:138,25	G	A
.	.	.	.	.	.	.	.	.	.	.	.	SON	GT:/	0:14	0/1:13,4:37	G	A
.	.	##	0	0.3(	0.039	0.585	1,D	3.6	1.69	.	.	SON	GT:/	0:11	0/1:192,25	C	T
.	.	.	.	.	.	.	.	.	.	.	.	SON	GT:/	0:42	0/1:58,12:3	G	T
.	.	.	.	0.3!	0.079	0.357	1,D	4.6	3.14	.	.	SON	GT:/	0:74	0/1:102,19	A	C
.	.	.	.	0.0:	0.124	0.206	0.98	5.8	1.66	.	.	SON	GT:/	0:10	0/1:171,22	G	T
.	.	.	.	.	.	.	.	.	.	.	.	SON	GT:/	0:14	0/1:22,8:37	T	C
.	.	.	.	.	.	.	1,D	5.1	3.90	.	.	SON	GT:/	0:11	0/1:228,8:3	C	T
.	.	.	.	.	.	.	.	.	.	.	.	SON	GT:/	0:94	0/1:147,23	G	A
.	.	.	.	.	.	.	.	2.4	.	.	.	SON	GT:/	0:22	0/1:48,8:35	A	G
.	.	.	.	.	.	.	.	.	.	.	.	SON	GT:/	0:8,(	0/1:33,3:37	T	G
.	.	.	.	.	.	.	.	.	.	.	.	SON	GT:/	0:11	0/1:221,8:3	G	C
.	.	.	.	.	.	.	.	.	.	.	.	SON	GT:/	0:29	0/1:327,12	A	G
.	.	.	.	.	.	.	.	.	.	.	.	DB;S	GT:/	0:30	0/1:344,10	C	A
.	.	.	.	.	.	.	.	.	.	.	.	SON	GT:/	0:8,(	0/1:44,4:34	A	G
.	.	.	.	.	.	.	.	.	.	.	.	SON	GT:/	0:10	0/1:69,7:37	T	G
.	.	.	.	.	.	.	.	.	.	.	.	SON	GT:/	0:10	0/1:36,16:3	T	C
.	.	.	.	.	.	.	.	.	.	.	.	SON	GT:/	0:10	0/1:47,4:34	T	G
.	.	.	.	.	.	.	.	.	.	.	.	SON	GT:/	0:8,(	0/1:157,8:3	A	C
.	.	.	.	.	.	.	.	.	.	0	.	SON	GT:/	0:17	0/1:143,6:3	G	A
.	.	.	.	.	.	.	.	.	.	.	.	SON	GT:/	0:9,(	0/1:20,4:37	C	A
.	.	.	.	.	.	.	.	.	.	.	.	SON	GT:/	0:14	0/1:181,34	C	A
3E-04	.	.	.	.	.	.	.	.	.	.	.	SON	GT:/	0:29	0/1:30,6:35	G	A
.	.	.	.	.	.	.	.	.	.	.	.	SON	GT:/	0:60	0/1:80,16:3	G	C
.	.	##	0	0.0(	0.977	0.998	1,D	4.6	3.56	.	.	SON	GT:/	0:96	0/1:199,41	C	T
.	.	.	.	0.0(	0.892	0.999	0.98	2.9	1.95	.	.	SON	GT:/	0:11	0/1:216,42	C	T
.	.	0	0	.	.	.	.	.	.	.	.	SON	GT:/	0:16	0/1:18,4:37	G	A
.	.	.	.	.	.	.	.	.	.	.	.	SON	GT:/	0:99	0/1:132,26	C	A
.	.	.	.	0.0(	0.019	0.288	1.00	3.8	1.91	.	.	SON	GT:/	0:93	0/1:150,21	G	A
.	.	.	.	0.1:	0.997	1.0,D	1,D	4.5	4.69	.	.	SON	GT:/	0:30	0/1:46,9:35	C	T
.	.	.	.	.	.	.	.	.	.	.	.	SON	GT:/	0:41	0/1:50,10:3	A	G
.	.	.	.	.	.	.	.	.	.	.	.	SON	GT:/	0:77	0/1:120,20	G	A
.	.	.	.	.	.	.	.	.	.	.	.	SON	GT:/	0:11	0/1:6,4:37:	G	T
.	.	.	.	.	.	.	1,A	6.1	9.05	.	.	SON	GT:/	0:79	0/1:120,34	C	A
.	.	.	.	.	.	.	.	2.8	.	.	.	SON	GT:/	0:8,(	0/1:24,3:37	A	G
.	.	.	.	0.0(	0.581	0.963	0.99	5	4.16	.	.	SON	GT:/	0:40	0/1:62,13:3	C	T
.	.	.	.	0.1	0.993	1.0,D	1.00	4.7	2.63	.	.	SON	GT:/	0:66	0/1:104,21	G	A
.	.	.	.	0.0:	0.93,I	0.996	0.99	5.2	3.37	.	.	SON	GT:/	0:23	0/1:344,68	C	A
.	.	.	.	.	.	.	.	.	1.55	.	.	SON	GT:/	0:24	0/1:25,9:37	C	T
.	.	.	.	.	.	.	.	.	.	.	.	SON	GT:/	0:12	0/1:23,4:37	T	C
.	.	.	.	.	.	.	.	.	.	.	.	SON	GT:/	0:8,(	0/1:14,5:37	G	A
.	.	.	.	0.0(	0.999	1.0,D	1,D	3.9	4.36	.	.	SON	GT:/	0:85	0/1:134,21	G	A
.	.	.	.	0.0(	0.025	0.032	1.00	.	.	.	.	SON	GT:/	0:85	0/1:85,15:3	C	A

.	.	.	.	.	.	.	.	.	.	.	.	SOM	GT:/	0:21	0/1:51,5:37	C	T
0.013	0	0	0	.	.	.	.	.	.	.	.	SOM	GT:/	0:82	0/1:147,17	C	T
.	.	.	.	.	.	.	.	3.4	.	.	.	SOM	GT:/	0:99	0/1:176,46	G	A
.	.	.	.	.	.	.	.	.	.	.	.	SOM	GT:/	0:50	0/1:77,16:3	C	A
.	.	.	.	.	.	.	.	.	.	.	.	SOM	GT:/	0:15	0/1:5,2:37:	T	C
.	.	.	.	.	.	.	.	2.4	2.35	.	.	SOM	GT:/	0:12	0/1:209,36	C	T
.	.	.	.	.	.	.	.	.	.	.	.	SOM	GT:/	0:41	0/1:82,11:3	C	T
.	.	.	.	.	.	.	.	.	.	.	.	SOM	GT:/	0:10	0/1:36,9:34	G	A
.	##	0	0	1.0.	0.032	0.075	0.92	2.9	.	0	.	SOM	GT:/	0:64	0/1:102,19	C	T
.	.	.	.	.	.	.	.	.	.	.	.	SOM	GT:/	0:9,(	0/1:11,4:37	A	G
.	.	.	.	.	.	.	.	.	.	.	.	SOM	GT:/	0:13	0/1:28,7:35	C	A
.	.	.	.	.	.	.	.	.	.	.	.	SOM	GT:/	0:57	0/1:78,14:3	C	T
.	.	.	.	.	.	.	.	.	.	.	.	SOM	GT:/	0:32	0/1:47,8:37	G	T
.	.	.	.	.	.	.	.	.	1.46	.	.	SOM	GT:/	0:29	0/1:35,3:37	A	G
.	.	.	.	.	.	.	.	.	.	.	.	SOM	GT:/	0:16	0/1:21,9:35	G	A
.	.	.	.	.	.	.	.	.	.	.	.	SOM	GT:/	0:69	0/1:87,16:3	C	T
.	.	0	0	.	.	.	.	.	.	0	0	SOM	GT:/	0:69	0/1:121,10	C	T
.	.	.	.	0.0!	0.698	0.955	1.00	5.3	2.25	.	.	SOM	GT:/	0:61	0/1:120,25	G	A
.	.	##	0	.	.	.	.	.	.	.	.	SOM	GT:/	0:10	0/1:205,29	C	T
.	.	.	.	0.0'	0.897	0.992	1.00	4.8	4.69	.	.	SOM	GT:/	0:93	0/1:158,13	G	T
.	.	.	.	.	.	.	.	.	.	.	.	SOM	GT:/	0:36	0/1:73,18:3	G	A
.	.	##	0	.	.	.	.	.	.	.	.	SOM	GT:/	0:75	0/1:108,36	G	A
.	.	.	.	.	.	.	.	.	.	.	0	SOM	GT:/	0:46	0/1:65,14:3	G	A
.	.	.	.	.	.	.	.	.	1.53	.	.	SOM	GT:/	0:15	0/1:8,3:37:	G	A
.	.	.	.	.	.	.	.	4	1.79	.	.	SOM	GT:/	0:86	0/1:182,15	G	A
.	.	.	.	.	.	.	.	.	.	.	0	SOM	GT:/	0:14	0/1:25,6:37	C	T
.	.	.	.	.	.	.	.	5.5	.	.	.	SOM	GT:/	0:18	0/1:260,52	G	A
.	.	##	0	0.1!	0.128	0.6,P	1,D	4.7	3.31	.	.	SOM	GT:/	0:56	0/1:97,22:3	C	T
.	.	.	.	.	.	.	.	.	.	.	.	SOM	GT:/	0:36	0/1:92,19:3	C	T
.	.	.	.	.	.	.	.	.	1.48	.	.	SOM	GT:/	0:27	0/1:54,9:37	G	A
.	.	.	.	.	.	.	.	.	.	.	.	SOM	GT:/	0:37	0/1:54,12:3	G	A
.	.	.	.	0.0(	0.982	1.0,D	1,D	4.2	2.12	.	.	SOM	GT:/	0:52	0/1:64,20:3	A	G
.	.	.	.	.	.	.	.	.	.	.	.	SOM	GT:/	0:9,(	0/1:6,3:37:	C	T
.	.	.	.	.	.	.	.	.	.	.	.	SOM	GT:/	0:34	0/1:411,49	C	T
.	.	.	.	.	.	.	.	.	.	.	.	SOM	GT:/	0:27	0/1:325,56	A	T
.	.	.	.	.	.	.	.	.	.	.	.	SOM	GT:/	0:19	0/1:148,50	T	C
2E-04	.	.	.	.	.	.	.	.	.	.	.	SOM	GT:/	0/1:(	0:41,0:.:41	C	T
.	.	.	.	.	.	.	.	.	.	0	0.1	SOM	GT:/	0/1:1	0:9,0:.:6:0.	C	T
.	.	.	.	.	.	.	.	.	.	0	0	SOM	GT:/	0/1:5	0:17,0:.:16	A	C
.	.	.	.	.	.	.	.	.	.	0	0	SOM	GT:/	0/1:1	0:9,0:.:10:(	T	C
.	.	.	.	.	.	.	1,D	5.6	4.73	.	.	SOM	GT:/	0/1:3	0:18,0:.:18	C	A
.	.	.	.	.	.	.	.	.	.	0	0	SOM	GT:/	0/1:3	0:13,0:.:16	G	C
.	.	.	.	.	.	.	.	.	.	0	0.1	SOM	GT:/	0/1:9	0:8,0:.:8:0.	G	T
.	.	.	.	.	.	.	.	.	.	0	.	SOM	GT:/	0/1:2	0:11,0:.:11	T	C
.	.	.	.	.	.	.	.	.	.	.	.	SOM	GT:/	0/1:2	0:82,0:.:82	G	T
.	.	.	.	.	.	.	.	.	.	.	.	SOM	GT:/	0/1:5	0:28,0:.:28	G	T
.	.	.	.	.	.	.	.	.	.	0	0	SOM	GT:/	0/1:1	0:13,0:.:13	G	C
4E-04	##	0	0	.	.	.	.	.	1.33	0	0	DB;S	GT:/	0/1:1	0:49,0:.:49	G	A
.	.	##	0	.	.	.	.	.	.	.	.	SOM	GT:/	0/1:8	0:25,0:.:25	C	T
.	.	.	.	0.0(	1.0,D	1.0,D	1,D	5.8	4.66	.	.	SOM	GT:/	0/1:4	0:21,0:.:21	C	A
.	.	.	.	.	.	.	.	.	.	.	.	SOM	GT:/	0/1:2	0:180,0:.:1	C	T
.	.	.	.	.	.	.	.	.	.	0	0	DB;S	GT:/	0/1:5	0:35,0:.:35	A	G
.	.	.	.	.	.	.	.	.	.	0	0	SOM	GT:/	0/1:3	0:13,0:.:10	G	T
.	.	.	.	.	.	.	.	3.4	.	.	.	SOM	GT:/	0/1:8	0:58,0:.:58	T	C

.	.	.	.	.	.	.	.	.	.	.	.	SOM	GT:/	0/1:1	0:9,0:.:9:0.	A	G
.	.	.	.	.	.	.	.	.	.	.	.	SOM	GT:/	0/1:4	0:24,0:.:24	T	C
.	.	.	.	.	.	.	.	.	.	.	.	SOM	GT:/	0/1:4	0:15,0:.:15	C	T
.	.	.	.	.	.	.	1,D	5.2	4.11	.	.	SOM	GT:/	0/1:4	0:25,0:.:25	C	T
4E-04	0	##	0	0.5	0.005	0.007	1,N	.	.	.	.	SOM	GT:/	0/1:3	0:200,0:.:2	G	A
.	.	##	0	.	.	.	.	2.6	2.20	.	.	SOM	GT:/	0/1:1	0:51,0:.:51	G	A
.	.	.	.	.	.	.	.	.	1.24	.	.	SOM	GT:/	0/1:3	0:20,0:.:20	C	T
.	.	.	.	.	.	.	.	.	.	0	0	SOM	GT:/	0/1:7	0:9,0:.:8:0.	G	T
.	.	.	.	.	.	.	.	.	.	.	.	SOM	GT:/	0/1:2	0:12,0:.:12	G	T
.	.	0	0	.	.	.	.	.	.	.	.	SOM	GT:/	0/1:4	0:38,1:.:26	T	C
.	.	.	.	0.5	0.001	0.001	1,N	2.8	.	.	.	SOM	GT:/	0/1:3	0:30,0:.:30	A	C
.	.	##	0	0.0	0.02,|	0.056	1.00	5.5	3.45	.	.	SOM	GT:/	0/1:3	0:24,0:.:24	G	A
.	.	.	.	.	.	.	.	.	1.63	.	.	SOM	GT:/	0/1:2	0:8,0:.:8:0.	A	T
.	.	##	0	.	.	.	.	.	.	0	.	SOM	GT:/	0/1:2	0:128,0:.:1	G	A
.	.	##	0	0.0	0.353	0.978	1,N	.	1.80	0	0	SOM	GT:/	0/1:9	0:44,0:.:44	C	T
.	.	.	.	.	.	.	.	.	.	.	.	SOM	GT:/	0/1:5	0:29,0:.:29	G	A
.	.	.	.	.	.	.	.	.	.	0.1	0.2	DB;S	GT:/	0/1:4	0:35,0:.:34	C	T
0.075	.	0	0	.	0.006	0.013	1,N	.	.	0.2	0.2	SOM	GT:/	0/1:5	0:40,0:.:30	T	G
.	.	0	0	.	.	.	.	.	.	0.3	0.2	SOM	GT:/	0/1:5	0:42,0:.:31	A	G
.	.	.	.	.	.	.	.	.	.	.	.	SOM	GT:/	0/1:1	0:99,0:.:99	C	A
.	.	.	.	.	.	.	.	.	.	0.1	0.1	SOM	GT:/	0/1:1	0:11,0:.:6:0	A	G
.	.	.	.	.	.	.	.	.	1.81	.	.	SOM	GT:/	0/1:1	0:9,0:.:9:0.	C	A
.	.	.	.	.	.	.	.	.	.	.	.	SOM	GT:/	0/1:7	0:51,0:.:51	C	T
.	.	0	0	.	.	.	.	.	.	.	.	SOM	GT:/	0/1:2	0:9,0:.:9:0.	T	C
.	.	.	.	.	.	.	.	.	.	0	0.4	SOM	GT:/	0/1:2	0:13,0:.:11	A	C
.	.	0	0	.	.	.	1,A	4.7	8.33	0.1	0	DB;S	GT:/	0/1:1	0:112,1:.:1	G	A
.	.	.	.	.	.	.	.	4.4	1.68	.	.	SOM	GT:/	0/1:2	0:183,0:.:1	C	T
.	.	.	.	.	.	.	.	3.6	.	.	.	SOM	GT:/	0/1:4	0:22,0:.:22	C	G
.	.	.	.	.	.	.	.	.	.	.	.	SOM	GT:/	0/1:4	0:26,0:.:26	A	T
.	.	.	.	.	.	.	.	.	.	0	.	SOM	GT:/	0/1:1	0:39,1:.:40	A	G
.	.	##	0	.	.	.	.	.	.	0	.	SOM	GT:/	0/1:6	0:26,0:.:26	G	A
0.144	.	0	0	.	.	.	.	.	.	0.1	0.1	SOM	GT:/	0/1:3	0:12,0:.:12	A	G
.	.	.	.	.	.	.	.	.	.	.	.	SOM	GT:/	0/1:4	0:12,0:.:12	G	A
.	.	.	.	.	.	.	.	.	.	0.3	0.4	DB;S	GT:/	0/1:4	0:33,1:.:34	C	T
.	.	.	.	.	.	.	.	3.1	1.70	.	.	SOM	GT:/	0/1:4	0:26,0:.:26	G	T
.	.	.	.	.	.	.	.	2.4	2.84	.	.	SOM	GT:/	0/1:1	0:84,1:.:86	G	C
.	##	##	0	.	.	.	.	.	.	.	.	SOM	GT:/	0/1:4	0:14,0:.:14	C	T
.	.	.	.	.	.	.	.	2.6	2.07	.	.	SOM	GT:/	0/1:3	0:16,0:.:16	G	A
.	.	.	.	.	.	.	.	.	.	0	0.1	SOM	GT:/	0/1:1	0:9,0:.:9:0.	G	C
.	.	.	.	.	.	.	.	.	.	0	0.2	SOM	GT:/	0/1:3	0:11,0:.:11	G	C
.	.	.	.	.	.	.	.	.	.	.	.	SOM	GT:/	0/1:9	0:56,1:.:57	T	G
.	.	.	.	.	.	.	1,D	.	1.30	.	.	SOM	GT:/	0/1:3	0:213,0:.:2	G	A
.	.	.	.	0.1	0.018	0.008	1.00	4.4	1.76	.	.	SOM	GT:/	0/1:2	0:89,0:.:90	G	C
.	.	.	.	.	.	.	.	.	.	.	.	SOM	GT:/	0/1:5	0:19,0:.:20	C	G
.	.	.	.	.	.	.	.	4	.	.	.	SOM	GT:/	0/1:1	0:85,0:.:85	G	C
.	.	.	.	0.0	0.99,|	0.998	1,D	5.7	5.56	.	.	SOM	GT:/	0/1:6	0:20,0:.:20	C	T
.	.	.	.	.	.	.	.	.	.	.	.	SOM	GT:/	0/1:7	0:30,0:.:30	G	C
.	.	.	.	0.1	0.392	0.738	0.98	5	2.81	.	.	SOM	GT:/	0/1:2	0:26,0:.:26	C	T
.	.	.	.	.	.	.	.	.	.	.	.	SOM	GT:/	0/1:1	0:9,0:.:9:0.	G	A
.	.	##	0	0.0	0.995	1.0,D	1,D	4.8	5.21	.	.	SOM	GT:/	0/1:1	0:102,0:.:1	G	A
.	.	.	.	.	.	.	.	2.5	1.40	.	.	SOM	GT:/	0/1:4	0:35,0:.:36	C	G
.	.	.	.	.	.	.	.	.	.	.	.	SOM	GT:/	0/1:4	0:23,0:.:23	G	A
.	.	.	.	.	.	.	.	.	1.24	.	.	SOM	GT:/	0/1:3	0:12,0:.:12	C	T
.	.	.	.	.	.	.	.	2.5	.	.	.	SOM	GT:/	0/1:5	0:23,0:.:23	C	T

.	.	##	.	0.0	0.998	1.0,D	1,D	5.6	4.51	.	.	SOM	GT:A	0/1:5	0:17,0:..:18	G	A
.	.	.	.	.	.	.	.	.	.	.	.	SOM	GT:A	0/1:9	0:52,0:..:52	T	C
.	.	.	.	0.0	0.748	0.986	1.00	5.9	5.01	.	.	SOM	GT:A	0/1:5	0:31,0:..:31	C	T
.	.	.	.	0.0	0.991	0.999	1,D	4.9	3.98	.	.	SOM	GT:A	0/1:8	0:47,0:..:47	C	G
6E-04	.	.	.	.	.	.	.	.	.	.	.	SOM	GT:A	0/1:1	0:12,0:..:12	C	T
.	.	.	.	.	.	.	.	.	.	.	.	SOM	GT:A	0/1:6	0:37,0:..:37	G	C
.	.	.	.	.	.	.	.	3	1.75	.	.	SOM	GT:A	0/1:3	0:22,0:..:22	G	T
.	.	.	.	0.0	.	.	0.99	2.2	.	.	.	SOM	GT:A	0/1:2	0:173,0:..:1	G	C
.	.	##	0	0.0	0.432	0.962	1,D	5.2	3.79	.	.	SOM	GT:A	0/1:4	0:23,0:..:23	G	A
.	.	.	.	.	.	.	.	.	.	.	.	SOM	GT:A	0/1:4	0:31,0:..:31	G	C
.	.	.	.	.	.	.	.	.	.	.	.	SOM	GT:A	0/1:5	0:36,0:..:36	A	G
.	.	.	.	0.0	0.924	0.994	1,D	3.2	3.19	.	.	SOM	GT:A	0/1:9	0:54,0:..:54	G	T
.	.	.	.	0.4	0.015	0.026	1,D	4.2	.	.	.	SOM	GT:A	0/1:9	0:54,0:..:55	G	A
.	.	.	.	.	.	.	.	.	.	.	.	SOM	GT:A	0/1:3	0:10,0:..:10	G	C
.	.	##	0	.	.	.	0.99	2.3	8.58	.	.	SOM	GT:A	0/1:1	0:61,0:..:61	G	A
.	.	.	.	.	.	.	.	.	.	.	.	SOM	GT:A	0/1:1	0:55,0:..:55	G	C
.	.	##	0	0.0	0.909	0.991	1,D	5.8	5.70	.	.	DB;S	GT:A	0/1:3	0:25,0:..:25	G	A
.	.	.	.	0.0	0.002	0.002	0.81	.	3.23	.	.	SOM	GT:A	0/1:8	0:32,0:..:32	A	C
.	.	.	.	.	.	.	.	.	.	.	.	SOM	GT:A	0/1:6	0:33,0:..:33	C	T
.	.	.	.	0.0	0.394	0.817	1.00	5.6	2.56	.	.	SOM	GT:A	0/1:5	0:39,0:..:39	G	A
.	.	.	.	.	.	.	.	.	.	.	.	SOM	GT:A	0/1:3	0:19,0:..:19	C	T
.	.	.	.	0.0	0.721	0.941	1,D	5.3	5.17	.	.	SOM	GT:A	0/1:3	0:20,0:..:21	C	T
.	.	.	.	0.0	0.996	1.0,D	1,D	6.2	4.90	.	.	SOM	GT:A	0/1:5	0:17,0:..:17	C	G
.	.	.	.	.	.	.	.	.	.	.	.	SOM	GT:A	0/1:3	0:13,0:..:13	C	T
.	.	.	.	.	.	.	.	.	.	.	0	SOM	GT:A	0/1:1	0:14,0:..:14	G	C
.	.	.	.	0.0	0.996	1.0,D	1.00	5.6	4.57	.	.	SOM	GT:A	0/1:4	0:24,0:..:24	G	C
.	.	.	.	0.1	0.001	0.0,B	1,N	.	.	.	.	SOM	GT:A	0/1:8	0:36,0:..:37	C	T
0.007	.	0	0	0.1	0.153	0.768	0.63	3.9	1.85	0	0	DB;S	GT:A	0/1:6	0:37,0:..:37	C	T
.	.	.	.	0.0	0.383	0.974	1.00	4.5	2.94	.	.	SOM	GT:A	0/1:1	0:77,0:..:77	C	G
.	.	.	.	.	.	.	.	.	.	.	.	SOM	GT:A	0/1:5	0:30,0:..:31	G	A
.	.	.	.	0.6	0.006	0.056	1,D	4.2	2.60	.	.	SOM	GT:A	0/1:1	0:141,0:..:1	G	A
.	.	.	.	.	.	.	.	.	.	.	.	SOM	GT:A	0/1:1	0:8,0:..:8:0.	C	A
.	.	.	.	0.3	0.0,B	0.001	1,N	.	.	.	.	SOM	GT:A	0/1:4	0:21,0:..:21	A	G
.	.	.	.	.	.	.	.	.	.	.	.	SOM	GT:A	0/1:1	0:146,0:..:1	G	A
0.213	.	.	.	.	.	.	.	.	.	0.2	0.3	DB;S	GT:A	0/1:7	0:39,1:..:40	C	T
6E-04	.	.	.	.	.	.	.	.	.	0	0	SOM	GT:A	0/1:1	0:8,0:..:8:0.	A	G
.	.	.	.	0.0	0.993	1.0,D	0.99	5.2	2.21	.	.	SOM	GT:A	0/1:3	0:17,0:..:17	C	G
.	.	.	.	0.0	0.036	0.037	1.00	2.5	.	.	.	SOM	GT:A	0/1:5	0:30,0:..:30	C	T
.	.	.	.	.	.	.	.	.	.	.	.	SOM	GT:A	0/1:2	0:12,0:..:12	C	T
.	.	.	.	.	.	.	.	.	.	.	.	SOM	GT:A	0/1:1	0:72,0:..:73	C	G
.	.	.	.	.	.	.	.	3.7	2.89	.	.	SOM	GT:A	0/1:4	0:27,0:..:27	G	C
.	.	.	.	.	.	.	.	.	.	0	.	SOM	GT:A	0/1:1	0:29,0:..:28	C	A
.	.	.	.	.	.	.	.	.	.	.	.	SOM	GT:A	0/1:6	0:45,0:..:45	C	A
.	.	.	.	.	.	.	.	4.8	1.97	.	.	SOM	GT:A	0/1:8	0:54,0:..:54	C	T
.	.	##	0	.	.	.	.	.	.	.	.	SOM	GT:A	0/1:8	0:44,0:..:44	G	C
.	.	.	.	.	.	.	.	.	.	.	.	SOM	GT:A	0/1:1	0:153,0:..:1	G	A
.	.	.	.	0.0	0.138	0.202	1,D	6.2	3.33	.	.	SOM	GT:A	0/1:4	0:18,0:..:18	A	T
.	.	.	.	.	.	.	.	4	1.65	.	.	SOM	GT:A	0/1:4	0:30,0:..:30	G	A
.	.	.	.	0.2	0.012	0.004	1,N	.	1.51	.	.	SOM	GT:A	0/1:4	0:39,0:..:39	C	T
.	##	##	0	0.0	0.674	0.992	0.98	3.8	1.87	0	.	SOM	GT:A	0/1:1	0:125,0:..:1	C	T
.	.	0	0	.	.	.	.	.	.	0.1	0	DB;S	GT:A	0/1:5	0:43,1:..:38	C	T
.	.	.	.	.	.	.	.	.	.	.	.	SOM	GT:A	0/1:3	0:22,0:..:22	G	A
.	.	.	.	.	.	.	.	4.9	1.37	.	.	SOM	GT:A	0/1:1	0:122,0:..:1	G	A
.	.	.	.	.	.	.	.	.	.	.	.	SOM	GT:A	0/1:1	0:130,0:..:1	C	T

.	.	.	.	.	.	.	.	.	.	.	.	SON	GT:	0/1:1	0:69,0:..:70	T	A
.	.	.	.	0.0(	0.978	0.999	1,D	6	2.81	.	.	SON	GT:	0/1:2	0:126,0:..:1	C	T
.	.	.	.	.	.	.	.	.	.	.	.	SON	GT:	0/1:1	0:81,0:..:81	G	A
0.433	.	1	1	.	.	.	.	.	.	0.1	0.5	SON	GT:	0/1:3	0:44,1:..:26	C	G
.	.	.	.	.	.	.	.	.	.	.	.	SON	GT:	0/1:6	0:87,0:..:88	G	C
.	.	.	.	.	.	.	.	.	.	.	.	SON	GT:	0/1:1	0:114,0:..:1	C	T
.	.	.	.	.	.	.	.	.	1.76	.	.	SON	GT:	0/1:4	0:33,0:..:33	C	G
.	.	.	.	.	.	.	.	.	.	.	.	SON	GT:	0/1:2	0:130,0:..:1	C	T
.	.	.	.	.	.	.	.	.	.	.	.	SON	GT:	0/1:2	0:163,0:..:1	G	A
.	.	.	.	.	.	.	.	3.9	1.41	.	.	SON	GT:	0/1:6	0:43,0:..:43	G	A
.	.	.	.	.	.	.	.	.	.	.	.	SON	GT:	0/1:6	0:51,0:..:51	G	A
.	.	.	.	.	.	.	.	.	.	.	.	SON	GT:	0/1:3	0:35,0:..:35	G	A
0.205	.	.	.	.	.	.	.	.	.	0.1	0	SON	GT:	0/1:2	0:15,0:..:13	T	C
.	.	.	.	0.0	0.998	1.0,D	1,D	6.1	3.62	.	.	SON	GT:	0/1:1	0:44,0:..:45	A	G
.	.	.	.	0.0	0.453	0.849	1.00	2.8	.	.	.	SON	GT:	0/1:3	0:24,0:..:24	C	G
.	.	.	.	.	.	.	.	.	.	0	0	SON	GT:	0/1:2	0:15,0:..:15	T	A
.	.	.	.	.	.	.	.	.	.	.	.	SON	GT:	0/1:3	0:16,0:..:17	C	T
.	.	.	.	.	.	.	.	2.5	.	.	.	SON	GT:	0/1:5	0:29,0:..:29	G	A
.	.	.	.	0.0	1.0,D	1.0,D	1,D	5	5.03	.	.	DB;S	GT:	0/1:1	0:52,0:..:52	G	A
.	.	.	.	.	.	.	.	.	.	.	.	SON	GT:	0/1:3	0:14,0:..:14	T	A
4E-04	##	##	0	.	0.947	0.997	1,D	6	5.36	.	.	SON	GT:	0/1:1	0:42,0:..:42	G	A
4E-04	0	0	0	0.0	0.676	0.997	1.00	5.1	3.59	.	.	DB;S	GT:	0/1:2	0:104,0:..:1	C	T
.	.	.	.	0.0	0.344	0.356	0.94	3.2	1.48	.	.	SON	GT:	0/1:9	0:46,0:..:46	C	G
.	.	.	.	0.1	0.031	0.064	0.99	5.9	1.36	.	.	SON	GT:	0/1:4	0:31,0:..:31	C	T
.	.	.	.	.	.	.	.	.	.	.	.	SON	GT:	0/1:6	0:28,0:..:28	C	G
.	.	.	.	0.1	0.419	0.881	1.00	5.7	2.53	.	.	SON	GT:	0/1:7	0:37,0:..:37	C	G
.	.	.	.	.	0.095	0.27,I	1,N	.	2.37	.	.	SON	GT:	0/1:6	0:49,0:..:49	G	A
.	0	0	0	.	.	.	.	.	.	.	.	SON	GT:	0/1:3	0:27,0:..:27	C	T
.	.	.	.	.	.	.	.	.	.	.	.	SON	GT:	0/1:4	0:14,0:..:14	C	T
.	.	.	.	.	.	.	.	.	.	.	.	SON	GT:	0/1:3	0:8,0:..:9:0.	C	T
.	.	.	.	.	.	.	.	4.1	.	.	.	SON	GT:	0/1:5	0:43,0:..:43	G	A
.	.	##	0	0.0	1.0,D	1.0,D	1,D	4.6	4.45	.	.	DB;S	GT:	0/1:7	0:81,0:..:82	C	T
.	.	.	.	.	.	.	.	.	.	.	.	SON	GT:	0/1:7	0:35,0:..:35	G	A
.	.	0	0	.	.	.	.	.	.	0	.	DB;S	GT:	0/1:4	0:58,1:..:57	G	A
.	.	.	.	.	.	.	.	.	.	0	0	SON	GT:	0/1:8	0:9,0:..:8:0.	C	G
.	.	0	0	.	.	.	.	.	.	0.4	0.3	SON	GT:	0/1:4	0:33,1:..:30	T	C
0.073	.	.	.	.	.	.	.	.	.	0	0.1	SON	GT:	0/1:4	0:17,0:..:17	G	C
.	.	.	.	.	.	.	.	.	.	.	.	SON	GT:	0/1:9	0:58,1:..:59	G	A
4E-04	.	0	0	.	.	.	.	.	.	0	0	SON	GT:	0/1:8	0:49,0:..:49	C	T
.	.	.	.	0.0(	0.803	0.992	1,N	.	1.98	.	.	SON	GT:	0/1:1	0:81,0:..:81	G	A
.	.	.	.	.	.	.	.	.	.	.	.	SON	GT:	0/1:1	0:60,0:..:60	G	C
.	.	.	.	.	.	.	.	4.6	.	.	.	SON	GT:	0/1:4	0:14,0:..:14	G	A
.	.	.	.	.	.	.	.	.	.	.	.	SON	GT:	0/1:8	0:47,0:..:47	G	A
.	.	.	.	.	.	.	.	.	.	.	.	SON	GT:	0/1:9	0:76,0:..:77	C	T
.	.	.	.	0.0(	0.963	1.0,D	1,D	5.4	3.69	.	.	SON	GT:	0/1:1	0:105,0:..:1	C	T
.	.	.	.	.	.	.	.	.	.	.	.	SON	GT:	0/1:1	0:18,0:..:18	G	A
.	.	##	0	0.0(	0.594	0.987	1.00	5.8	5.62	.	.	SON	GT:	0/1:1	0:50,0:..:50	C	T
.	.	.	.	.	.	.	.	.	.	.	.	SON	GT:	0/1:7	0:44,0:..:44	C	T
.	.	.	.	0.0(	0.568	0.899	1.00	2.6	2.10	.	.	SON	GT:	0/1:1	0:90,0:..:90	G	C
.	.	##	0	.	0.002	0.001	1,N	.	.	.	.	SON	GT:	0/1:2	0:247,0:..:2	C	T
0.001	0	0	0	.	.	.	.	.	.	.	.	SON	GT:	0/1:2	0:178,0:..:1	G	A
.	.	.	.	.	.	.	.	3	.	.	.	SON	GT:	0/1:2	0:202,0:..:2	G	A
4E-04	.	0	0	.	.	.	.	.	.	.	.	SON	GT:	0/1:3	0:199,0:..:1	C	T
.	.	.	.	.	.	.	.	.	.	0	.	SON	GT:	0/1:3	0:25,0:..:25	C	T

2E-04	.	.	.	.	.	.	.	.	2.32	.	.	SON	GT:/	0/1:3	0:32,0:..:32	G	A
.	.	0	0	.	.	.	.	.	.	0	0	SON	GT:/	0/1:2	0:14,0:..:14	C	T
.	.	.	.	.	.	.	.	.	.	.	.	SON	GT:/	0/1:2	0:22,0:..:23	G	C
.	.	.	.	.	.	.	.	2.8	.	.	.	SON	GT:/	0/1:7	0:24,0:..:24	C	G
.	.	.	.	.	.	.	.	.	.	.	.	SON	GT:/	0/1:4	0:13,0:..:13	G	A
.	.	.	.	0.0	0.998	0.999	1.00	3.1	3.49	.	.	SON	GT:/	0/1:7	0:57,0:..:57	G	T
.	.	.	.	0.0	0.112	0.43,I	1,N	.	.	.	.	SON	GT:/	0/1:1	0:65,0:..:65	C	T
.	.	.	.	.	.	.	.	.	.	.	.	SON	GT:/	0/1:3	0:32,0:..:32	C	T
.	.	.	.	.	.	.	1,D	4	6.43	.	.	SON	GT:/	0/1:6	0:37,0:..:37	C	G
.	.	.	.	.	.	.	.	.	1.59	.	0	SON	GT:/	0/1:5	0:62,0:..:62	G	A
.	.	.	.	.	.	.	.	.	.	.	.	SON	GT:/	0/1:1	0:76,0:..:76	G	C
.	.	.	.	.	.	.	.	.	.	.	.	SON	GT:/	0/1:1	0:118,0:..:1	C	G
.	.	.	.	.	.	.	.	.	.	.	.	SON	GT:/	0/1:9	0:48,0:..:48	G	C
.	.	.	.	.	.	.	.	5.9	.	.	.	SON	GT:/	0/1:4	0:11,0:..:11	G	A
.	##	##	0	0.4	0.002	0.005	0.98	.	1.70	0	0	SON	GT:/	0/1:1	0:44,0:..:44	G	A
.	.	.	.	.	.	.	.	.	.	.	.	SON	GT:/	0/1:6	0:17,0:..:17	G	C
.	.	.	.	.	.	.	.	.	.	.	.	SON	GT:/	0/1:1	0:8,0:..:8:0.	T	C
.	.	.	.	.	.	.	.	.	.	0	0	DB;S	GT:/	0/1:4	0:28,0:..:27	T	C
.	.	.	.	.	.	.	1,A	5.5	10.7	.	.	SON	GT:/	0/1:5	0:27,0:..:27	C	G
.	.	.	.	.	.	.	.	.	.	.	.	DB;S	GT:/	0/1:1	0:20,0:..:20	G	C
.	.	.	.	0.3	0.107	0.487	1,N	.	2.74	.	.	SON	GT:/	0/1:2	0:157,0:..:1	C	T
.	.	.	.	.	.	.	.	4.4	.	.	.	SON	GT:/	0/1:4	0:24,0:..:24	C	A
.	.	0	0	0.0	0.935	0.371	1.00	3.5	2.43	.	.	SON	GT:/	0/1:4	0:42,0:..:42	G	A
.	.	.	.	0.0	0.795	0.995	0.97	2.7	2.50	.	.	SON	GT:/	0/1:6	0:16,0:..:16	G	A
.	.	.	.	.	.	.	.	.	.	.	.	SON	GT:/	0/1:8	0:9,0:..:9:0.	T	A
.	.	.	.	0.3	0.906	0.999	1,D	4.9	4.26	.	.	SON	GT:/	0/1:7	0:34,0:..:34	G	A
.	.	.	.	0.0	0.058	0.07,I	1,N	.	.	.	.	SON	GT:/	0/1:1	0:157,0:..:1	G	C
.	.	.	.	.	.	.	.	.	.	0	.	SON	GT:/	0/1:6	0:10,0:..:10	T	A
.	.	.	.	.	.	.	.	.	.	.	.	SON	GT:/	0/1:1	0:15,0:..:15	T	A
.	.	.	.	.	.	.	.	.	.	.	.	SON	GT:/	0/1:4	0:16,0:..:16	G	C
.	.	.	.	.	.	.	.	.	.	.	.	SON	GT:/	0/1:6	0:52,0:..:52	C	T
.	.	.	.	.	.	.	.	.	.	.	.	SON	GT:/	0/1:1	0:45,0:..:45	T	C
.	.	0	0	.	.	.	.	.	.	0	0	DB;S	GT:/	0/1:1	0:37,1:..:35	T	C
.	.	.	.	1.0	0.059	0.048	1,D	4.9	.	.	.	SON	GT:/	0/1:7	0:48,0:..:48	C	A
.	.	.	.	.	.	.	.	.	.	.	.	SON	GT:/	0/1:1	0:93,0:..:93	C	T
.	.	.	.	0.4	0.016	0.007	0.98	4.1	1.44	.	.	SON	GT:/	0/1:5	0:31,0:..:31	G	A
.	.	.	.	.	.	.	.	.	1.47	0	0.1	SON	GT:/	0/1:2	0:9,0:..:9:0.	T	C
.	.	.	.	.	.	.	.	.	.	0.2	0.2	SON	GT:/	0/1:1	0:13,0:..:13	G	T
.	.	.	.	.	.	.	.	.	1.67	.	.	SON	GT:/	0/1:1	0:49,0:..:49	T	A
.	.	.	.	0.5	0.003	0.001	1,N	.	.	.	.	SON	GT:/	0/1:1	0:72,0:..:74	G	A
.	.	.	.	.	.	.	.	3.3	.	.	.	SON	GT:/	0/1:3	0:185,1:..:1	C	G
.	##	##	0	.	.	.	.	.	.	.	.	SON	GT:/	0/1:8	0:85,0:..:85	C	T
0.007	.	0	0	.	.	.	.	.	.	0	0	SON	GT:/	0/1:1	0:61,0:..:61	T	A
.	.	.	.	.	.	.	.	.	.	.	.	SON	GT:/	0/1:4	0:27,0:..:27	C	T
.	0	##	0	.	.	.	.	.	.	.	.	SON	GT:/	0/1:1	0:127,0:..:1	C	T
.	.	0	0	.	.	.	.	.	.	0	.	SON	GT:/	0/1:1	0:105,0:..:1	G	A
0.102	.	.	.	.	.	.	.	.	.	0	0.1	SON	GT:/	0/1:1	0:9,0:..:9:0.	C	T
.	.	.	.	.	.	.	.	.	.	.	.	SON	GT:/	0/1:4	0:22,0:..:22	G	T
.	.	.	.	.	.	.	.	4.8	.	.	.	SON	GT:/	0/1:6	0:40,0:..:40	C	T
.	##	##	0	0.4	0.001	0.002	1,N	2	1.66	.	.	SON	GT:/	0/1:8	0:64,0:..:64	C	T
0.014	.	.	.	.	.	.	.	.	.	0	0	DB;S	GT:/	0/1:1	0:72,0:..:72	C	T
.	.	.	.	.	.	.	.	.	.	0.4	0.5	DB;S	GT:/	0/1:4	0:52,1:..:48	C	G
.	.	.	.	.	.	.	.	.	.	0.4	0.4	DB;S	GT:/	0/1:3	0:40,1:..:37	G	T
.	.	##	0	.	.	.	.	.	.	.	.	SON	GT:/	0/1:3	0:127,0:..:1	C	T

.	.	##	0	0.0	0.391	0.84,l	1,D	2.7	2.81	.	.	SON	GT:/	0/1:1	0:68,0::68	G	A
.	.	.	.	.	.	.	.	.	.	.	.	SON	GT:/	0/1:2	0:337,0::3	C	T
.	.	.	.	.	.	.	.	.	.	.	.	DB;S	GT:/	0/1:4	0:33,0::33	C	T
.	.	.	.	.	.	.	.	.	.	.	.	SON	GT:/	0/1:6	0:24,0::24	G	T
.	.	.	.	.	.	.	.	.	.	.	.	SON	GT:/	0/1:1	0:74,0::74	T	A
.	.	##	0	.	.	.	.	.	.	.	.	SON	GT:/	0/1:1	0:46,0::46	C	T
.	.	.	.	0.0	0.14,l	0.557	.	.	1.91	.	.	SON	GT:/	0/1:4	0:44,0::44	G	A
.	.	.	.	1.0	0.004	0.0,B	1.00	4	.	.	.	SON	GT:/	0/1:4	0:23,0::23	C	A
.	.	.	.	.	.	.	.	.	.	.	.	SON	GT:/	0/1:3	0:23,0::20	T	C
.	.	##	0	.	.	.	.	.	.	.	.	SON	GT:/	0/1:8	0:31,0::31	C	T
.	.	.	.	.	.	.	.	.	.	.	.	SON	GT:/	0/1:5	0:21,0::21	G	T
.	.	##	0	0.4	0.294	0.866	0.99	4.5	2.15	.	.	SON	GT:/	0/1:9	0:81,0::82	G	A
.	.	.	.	.	.	.	.	3.9	2.86	.	.	SON	GT:/	0/1:5	0:24,0::24	T	A
.	.	##	0	.	.	.	.	.	.	.	.	SON	GT:/	0/1:4	0:27,0::27	G	A
0.002	.	.	.	.	.	.	.	.	.	0	.	SON	GT:/	0/1:3	0:25,0::25	C	T
0.327	.	.	.	.	.	.	.	.	.	0	.	DB;S	GT:/	0/1:4	0:19,0::19	T	A
.	.	.	.	.	.	.	.	.	.	.	.	SON	GT:/	0/1:4	0:32,0::32	G	A
.	.	.	.	.	.	.	.	.	.	.	.	SON	GT:/	0/1:9	0:63,0::63	C	T
.	.	##	0	.	.	.	.	.	.	.	.	SON	GT:/	0/1:1	0:68,1::69	A	G
.	.	.	.	.	.	.	.	.	1.46	.	.	SON	GT:/	0/1:6	0:113,0::1	G	A
.	.	.	.	0.0	0.637	0.887	1,D	5.8	4.15	.	.	DB;S	GT:/	0/1:7	0:46,0::46	A	G
.	.	.	.	0.0	0.994	1.0,D	1,D	4	4.84	.	.	SON	GT:/	0/1:1	0:107,0::1	G	A
2E-04	.	##	0	.	.	.	.	.	.	0	.	SON	GT:/	0/1:4	0:38,0::38	G	A
.	.	.	.	.	.	.	.	.	1.62	.	.	SON	GT:/	0/1:1	0:101,0::1	A	G
.	.	.	.	.	.	.	.	2.4	.	.	.	SON	GT:/	0/1:1	0:51,0::51	T	C
.	.	.	.	0.2	1.0,D	1.0,D	1,D	4.8	3.46	.	.	SON	GT:/	0/1:8	0:55,0::56	T	C
.	.	.	.	.	.	.	.	.	.	.	.	SON	GT:/	0/1:8	0:44,0::44	G	A
.	.	.	.	.	.	.	.	.	.	.	.	SON	GT:/	0/1:1	0:19,0::19	C	T
.	.	.	.	.	.	.	.	.	.	0	.	SON	GT:/	0/1:4	0:21,0::21	A	G
.	.	.	.	.	.	.	.	.	1.45	.	.	SON	GT:/	0/1:2	0:123,0::1	G	T
.	.	.	.	.	.	.	.	5.7	4.01	.	.	SON	GT:/	0/1:4	0:37,0::37	C	A
2E-04	.	##	0	.	.	.	.	.	.	.	.	SON	GT:/	0/1:5	0:22,0::22	G	A
.	.	.	.	.	.	.	.	.	.	.	.	SON	GT:/	0/1:6	0:45,0::45	G	A
.	.	.	.	0.0	1.0,D	1.0,D	1,D	5	4.65	.	.	SON	GT:/	0/1:4	0:23,0::23	G	A
.	.	.	.	0.0	0.945	0.994	1.00	5.1	4.41	.	.	SON	GT:/	0/1:1	0:66,0::66	C	A
.	.	.	.	.	.	.	.	.	.	0	0	SON	GT:/	0/1:2	0:9,0::9:0.	G	T
.	.	.	.	.	.	.	.	.	.	0	.	SON	GT:/	0/1:4	0:17,0::15	G	A
.	.	.	.	0.1	0.039	0.104	1.00	3.8	.	.	.	SON	GT:/	0/1:7	0:48,0::48	T	C
.	.	.	.	0.7	0.003	0.0,B	0.99	2.5	.	.	.	SON	GT:/	0/1:3	0:26,0::26	C	A
.	.	.	.	.	.	.	.	.	.	.	.	SON	GT:/	0/1:2	0:196,0::1	C	T
.	.	.	.	.	.	.	.	4.1	1.54	.	.	SON	GT:/	0/1:1	0:82,1::83	A	T
.	.	.	.	.	.	.	.	.	.	0	0	SON	GT:/	0/1:7	0:15,0::12	G	C
.	.	0	0	.	.	.	.	.	.	.	.	SON	GT:/	0/1:5	0:24,0::24	G	A
.	.	.	.	.	.	.	.	.	.	.	.	SON	GT:/	0/1:8	0:27,0::27	C	A
.	.	##	0	.	.	.	.	.	.	.	.	SON	GT:/	0/1:7	0:25,0::26	G	A
.	.	.	.	.	.	.	.	.	.	.	.	SON	GT:/	0/1:6	0:46,0::46	C	A
.	.	.	.	.	.	.	.	.	.	.	.	SON	GT:/	0/1:9	0:74,0::74	T	C
.	.	.	.	0.0	0.66,l	0.955	0.75	4.4	3.62	.	.	SON	GT:/	0/1:4	0:13,0::13	C	T
.	.	.	.	.	.	.	.	.	.	0	.	SON	GT:/	0/1:5	0:8,0::8:0.	C	A
.	##	##	0	.	.	.	.	.	.	.	.	SON	GT:/	0/1:3	0:9,0::9:0.	C	T
.	.	.	.	.	.	.	.	.	.	.	.	SON	GT:/	0/1:1	0:120,0::1	C	T
0.002	.	0	0	.	.	.	.	.	.	.	.	SON	GT:/	0/1:6	0:26,0::26	T	A
.	.	.	.	.	.	.	.	.	.	0.2	0.2	DB;S	GT:/	0/1:5	0:38,0::37	C	A
.	.	.	.	.	.	.	.	.	.	.	.	SON	GT:/	0/1:1	0:45,0::45	G	C

.	.	.	.	.	.	.	.	.	2.11	.	.	SOM	GT:/	0/1:5	0:36,0:..:36	G	A
.	.	.	.	.	.	.	.	.	.	0.1	0.2	SOM	GT:/	0/1:1	0:19,0:..:19	C	T
.	.	.	.	.	.	.	.	.	.	0.1	0.2	SOM	GT:/	0/1:1	0:16,0:..:16	G	A
.	.	.	.	0.2	0.008	0.014	1,N	.	.	.	.	SOM	GT:/	0/1:1	0:57,0:..:57	G	A
.	.	.	.	.	.	.	1,A	.	4.71	.	.	SOM	GT:/	0/1:1	0:98,0:..:98	G	T
.	.	.	.	.	.	.	.	.	.	.	.	SOM	GT:/	0/1:5	0:9,0:..:9:0.	G	A
.	.	.	.	.	.	.	.	.	.	.	.	SOM	GT:/	0/1:4	0:8,0:..:8:0.	G	A
.	.	.	.	.	.	.	.	.	.	0	0.1	SOM	GT:/	0/1:8	0:9,0:..:8:0.	T	A
.	.	.	.	.	.	.	.	3.3	.	.	.	SOM	GT:/	0/1:3	0:24,0:..:24	C	A
.	.	.	.	.	.	.	.	.	.	0	0.1	SOM	GT:/	0/1:1	0:72,1:..:70	C	G
.	.	.	.	.	.	.	.	.	.	.	.	SOM	GT:/	0/1:3	0:13,0:..:13	T	C
.	.	.	.	.	.	.	.	.	.	.	.	SOM	GT:/	0/1:3	0:31,0:..:31	C	A
.	.	.	.	.	.	.	.	.	.	0	0	SOM	GT:/	0/1:1	0:14,0:..:9:0	G	C
.	.	.	.	.	.	.	.	.	.	0	0	SOM	GT:/	0/1:1	0:8,0:..:8:0.	G	A
.	.	.	.	.	.	.	.	5.1	4.13	.	.	SOM	GT:/	0/1:5	0:15,0:..:15	A	T
.	.	.	.	.	.	.	.	.	1.24	0	0.1	SOM	GT:/	0/1:3	0:21,0:..:14	T	C
.	.	.	.	.	.	.	.	.	.	0	.	SOM	GT:/	0/1:1	0:12,0:..:13	T	A
.	.	.	.	.	.	.	.	.	.	0.1	0	SOM	GT:/	0/1:1	0:11,0:..:9:0	T	G
.	.	.	.	.	.	.	.	.	.	.	.	SOM	GT:/	0/1:1	0:9,0:..:9:0.	T	C
.	.	.	.	.	.	.	.	.	.	0.1	0.3	DB;S	GT:/	0/1:2	0:35,1:..:34	G	A
.	.	.	.	.	.	.	.	.	.	0	.	SOM	GT:/	0/1:1	0:17,0:..:17	C	A
.	.	0	0	.	.	.	.	.	.	0	.	DB;S	GT:/	0/1:3	0:45,1:..:46	G	A
.	.	.	.	.	.	.	.	.	.	.	.	SOM	GT:/	0/1:4	0:20,0:..:20	A	C
.	.	0	0	.	0.851	0.997	1,N	.	.	0	0	DB;S	GT:/	0/1:5	0:35,0:..:38	G	A
.	.	.	.	.	.	.	.	.	.	.	.	SOM	GT:/	0/1:1	0:9,0:..:9:0.	G	T
.	.	.	.	.	.	.	.	.	.	0	.	SOM	GT:/	0/1:2	0:9,0:..:9:0.	G	A
.	.	.	.	.	.	.	.	.	.	.	.	SOM	GT:/	0:12	0/1:26,5:31	A	C
.	.	.	.	.	.	.	.	.	.	0	0.1	SOM	GT:/	0:36	0/1:51,6:28	G	T
0.398	.	.	.	.	.	.	.	.	.	0	0.2	SOM	GT:/	0:35	0/1:53,6:30	C	T
.	.	.	.	.	.	.	.	.	.	0	0	SOM	GT:/	0:14	0/1:14,4:37	G	A
.	.	.	.	.	.	.	.	3.1	.	.	.	SOM	GT:/	0:12	0/1:23,4:34	G	T
.	.	.	.	.	.	.	.	.	.	0	0	SOM	GT:/	0:15	0/1:11,6:37	T	C
.	.	.	.	.	.	.	.	.	.	0	.	SOM	GT:/	0:17	0/1:26,14:3	G	T
.	0	0	0	0.0	0.647	0.998	1,D	.	3.26	0	0.1	SOM	GT:/	0:22	0/1:44,4:37	G	C
.	.	.	.	.	.	.	.	.	.	0.2	0.3	SOM	GT:/	0:12	0/1:12,4:30	A	C
.	.	.	.	.	.	.	.	.	.	0.1	0.2	DB;S	GT:/	0:32	0/1:74,9:35	C	A
.	.	.	.	.	.	.	.	.	.	0	.	SOM	GT:/	0:33	0/1:30,13:3	A	G
.	.	.	.	.	.	.	.	.	.	0	.	SOM	GT:/	0:8,0	0/1:22,5:13	G	C
.	.	0	0	0.1	0.016	0.239	1,N	.	.	0.1	0.1	SOM	GT:/	0:10	0/1:27,4:37	C	T
.	.	.	.	.	.	.	.	.	.	0	0.1	SOM	GT:/	0:19	0/1:23,5:37	C	G
.	.	.	.	.	.	.	.	.	.	0	.	SOM	GT:/	0:8,0	0/1:11,5:11	A	T
.	.	.	.	.	.	.	.	.	.	0	.	DB;S	GT:/	0:23	0/1:36,8:30	C	A
.	.	0	0	.	.	.	.	.	1.95	.	.	SOM	GT:/	0:42	0/1:42,6:35	G	A
.	.	.	.	.	.	.	.	.	.	0.1	0	SOM	GT:/	0:37	0/1:74,11:3	G	A
.	.	.	.	.	.	.	.	.	.	0.2	0	SOM	GT:/	0:36	0/1:59,12:3	A	G
.	.	0	0	.	.	.	.	.	.	0.1	0.4	DB;S	GT:/	0:26	0/1:33,6:37	A	T
0.042	.	.	.	.	.	.	.	.	.	0.1	0	SOM	GT:/	0:14	0/1:28,4:37	G	C
.	.	.	.	.	.	.	.	.	.	.	.	SOM	GT:/	0/1:6	0:48,0:..:41	G	A
.	.	.	.	.	.	.	.	.	.	.	.	SOM	GT:/	0/1:5	0:58,0:..:58	A	G
.	.	.	.	.	.	.	.	3.5	.	.	.	SOM	GT:/	0/1:3	0:9,0:..:9:0.	C	A
.	.	.	.	0.0	0.993	1.0,D	0.99	4.8	3.80	.	.	SOM	GT:/	0/1:5	0:47,0:..:47	C	T
.	.	.	.	.	.	.	.	.	.	0	.	SOM	GT:/	0/1:4	0:25,0:..:25	C	T
.	.	.	.	.	.	.	.	.	.	0	.	SOM	GT:/	0/1:4	0:25,0:..:25	G	T
.	.	.	.	.	.	.	.	5.9	3.35	.	.	SOM	GT:/	0/1:2	0:8,0:..:8:0.	T	G

.	.	.	.	.	.	.	.	.	.	0.1	0.2	DB;S	GT:/	0/1:3	0:20,0:..:20	T	C
.	.	.	.	.	.	.	.	.	.	0.1	0.3	SOM	GT:/	0/1:3	0:21,0:..:21	G	A
.	.	.	.	.	.	.	.	.	.	0.1	0.1	SOM	GT:/	0/1:4	0:18,0:..:16	G	A
.	.	.	.	.	.	.	.	.	.	.	0	SOM	GT:/	0/1:7	0:33,1:..:26	T	C
.	.	.	.	.	.	.	.	.	.	0	0	SOM	GT:/	0/1:7	0:38,1:..:29	G	C
.	.	.	.	.	.	.	.	.	1.23	0.1	0.2	SOM	GT:/	0/1:4	0:17,0:..:11	A	G
.	.	.	.	.	.	.	.	.	.	0	0.2	SOM	GT:/	0/1:1	0:12,0:..:6:0	C	A
.	.	.	.	1.0	0.023	0.004	1.00	3.2	1.46	.	.	SOM	GT:/	0/1:1	0:34,1:..:35	C	G
.	.	.	.	.	.	.	.	.	.	.	.	SOM	GT:/	0/1:5	0:17,0:..:17	C	G
.	.	.	.	0.1	0.998	0.999	1,D	5.8	4.66	.	.	SOM	GT:/	0/1:7	0:22,0:..:23	C	T
.	.	.	.	.	.	.	.	5.4	.	.	.	SOM	GT:/	0/1:5	0:28,0:..:28	G	A
.	.	.	.	.	.	.	.	.	.	0	0	SOM	GT:/	0/1:9	0:22,0:..:22	C	T
.	.	.	.	.	.	.	.	.	.	.	.	SOM	GT:/	0/1:5	0:50,1:..:37	G	C
.	.	##	.	.	.	.	.	4.5	2.39	.	.	SOM	GT:/	0/1:1	0:98,0:..:98	G	T
4E-04	##	0	0	.	.	.	.	.	.	0	.	SOM	GT:/	0/1:1	0:28,0:..:28	G	A
.	.	.	.	0.2	0.001	0.001	0.99	4.8	1.44	.	.	SOM	GT:/	0/1:2	0:136,0:..:1	A	C
.	.	##	0	0.0	0.07,I	0.298	0.92	4.9	2.12	.	.	SOM	GT:/	0/1:2	0:215,0:..:2	G	A
.	.	##	0	0.2	0.001	0.001	1,N	.	.	.	.	SOM	GT:/	0/1:1	0:50,0:..:50	C	T
.	.	0	0	.	.	.	.	2.7	.	.	.	DB;S	GT:/	0/1:1	0:29,0:..:29	A	G
.	.	.	.	.	.	.	.	.	.	.	.	SOM	GT:/	0/1:5	0:12,0:..:12	C	G
.	.	.	.	.	.	.	.	5.6	1.28	.	.	SOM	GT:/	0/1:2	0:100,1:..:1	G	A
.	.	.	.	0.4	0.848	0.991	1.00	2.5	2.33	.	.	SOM	GT:/	0/1:2	0:58,1:..:59	G	A
.	.	.	.	.	.	.	.	2.8	.	.	.	SOM	GT:/	0/1:2	0:12,0:..:12	T	G
.	.	.	.	.	.	.	.	.	.	.	.	SOM	GT:/	0/1:2	0:11,0:..:11	C	G
2E-04	.	.	.	.	.	.	.	.	.	.	.	SOM	GT:/	0/1:4	0:20,0:..:20	T	C
4E-04	.	##	0	.	0.99,I	1.0,D	1,D	5.7	4.68	.	.	SOM	GT:/	0/1:1	0:43,1:..:45	G	A
.	.	.	.	.	.	.	.	.	.	0	0	SOM	GT:/	0/1:3	0:8,0:..:8:0.	G	A
0.477	.	.	.	.	.	.	.	.	.	0.1	0.4	SOM	GT:/	0/1:1	0:15,0:..:9:0	G	T
.	.	.	.	1.0	0.002	0.001	1,N	.	.	.	.	SOM	GT:/	0/1:3	0:11,0:..:11	C	T
.	.	.	.	.	.	.	.	.	.	.	.	SOM	GT:/	0/1:1	0:116,0:..:1	G	C
.	.	.	.	.	.	.	.	.	.	.	.	SOM	GT:/	0/1:8	0:42,0:..:42	G	A
.	.	.	.	.	.	.	.	.	2.48	.	.	SOM	GT:/	0/1:1	0:132,0:..:1	C	T
.	.	.	.	.	.	.	.	.	.	.	.	SOM	GT:/	0/1:4	0:35,0:..:35	T	G
.	.	.	.	.	.	.	.	.	.	.	.	SOM	GT:/	0/1:7	0:30,0:..:30	C	T
.	.	.	.	.	.	.	.	.	.	.	.	SOM	GT:/	0/1:6	0:46,0:..:46	G	A
.	.	.	.	.	.	.	.	2.4	1.48	.	.	SOM	GT:/	0/1:8	0:68,0:..:68	G	A
.	.	.	.	0.0	0.986	0.996	1.00	.	.	.	.	SOM	GT:/	0/1:4	0:293,1:..:2	C	A
.	.	.	.	.	.	.	.	3.9	.	.	.	SOM	GT:/	0/1:1	0:48,0:..:48	G	C
.	.	.	.	.	.	.	.	.	.	.	.	SOM	GT:/	0/1:1	0:86,1:..:87	C	T
.	.	.	.	.	.	.	.	2.7	1.88	.	.	SOM	GT:/	0/1:1	0:85,0:..:85	C	T
.	.	.	.	.	.	.	.	5.3	4.40	.	.	SOM	GT:/	0/1:4	0:24,0:..:24	A	G
.	.	.	.	.	.	.	.	.	.	.	.	SOM	GT:/	0/1:1	0:88,0:..:75	A	T
.	.	.	.	.	.	.	.	.	.	.	.	SOM	GT:/	0/1:1	0:108,0:..:1	G	T
.	.	.	.	.	.	.	.	.	.	.	.	SOM	GT:/	0/1:5	0:42,0:..:42	G	A
.	.	.	.	.	.	.	.	.	.	0	0	SOM	GT:/	0/1:1	0:18,0:..:9:0	T	C
.	.	.	.	.	.	.	.	.	1.59	.	.	SOM	GT:/	0/1:3	0:31,0:..:31	C	G
.	.	.	.	.	.	.	.	.	.	0.1	0	SOM	GT:/	0/1:2	0:16,0:..:16	G	A
.	.	.	.	.	.	.	.	.	.	0.1	0	SOM	GT:/	0/1:2	0:13,0:..:13	G	A
.	.	.	.	0.0	0.477	0.988	1.00	.	3.03	.	.	SOM	GT:/	0/1:8	0:59,1:..:60	G	A
.	.	.	.	.	.	.	.	3.7	.	.	.	SOM	GT:/	0/1:2	0:152,1:..:1	G	A
.	.	.	.	.	.	.	.	.	1.91	.	.	SOM	GT:/	0/1:5	0:17,0:..:17	G	A
.	.	.	.	.	.	.	.	.	.	0	.	SOM	GT:/	0/1:2	0:10,0:..:10	A	G
.	.	.	.	.	.	.	.	.	.	.	0	SOM	GT:/	0/1:7	0:50,0:..:50	T	G
.	.	.	.	.	.	.	.	2.9	.	.	.	SOM	GT:/	0/1:9	0:39,0:..:39	G	C

.	.	.	.	.	.	.	.	2.2	.	.	.	SOM	GT:/	0/1:8	0:40,0:..:40	C	G
.	.	.	.	.	.	.	.	.	.	.	.	SOM	GT:/	0/1:3	0:13,0:..:13	T	C
.	.	##	0	.	.	.	.	.	.	.	.	SOM	GT:/	0/1:8	0:38,1:..:39	G	A
2E-04	.	.	.	.	.	.	.	.	.	.	0	SOM	GT:/	0/1:1	0:150,0:..:1	C	T
.	.	.	.	.	.	.	.	.	.	.	.	SOM	GT:/	0/1:7	0:40,1:..:41	A	T
.	.	.	.	.	.	.	1,A	6	7.18	.	.	SOM	GT:/	0/1:5	0:21,0:..:21	G	T
.	.	.	.	0.5	0.001	0.002	1,N	2.9	.	.	.	SOM	GT:/	0/1:2	0:83,0:..:83	C	T
8E-04	0	0	0	.	.	.	.	.	.	.	.	SOM	GT:/	0/1:1	0:86,0:..:86	G	A
.	.	.	.	.	.	.	.	.	.	.	.	SOM	GT:/	0/1:3	0:23,0:..:23	G	C
.	.	.	.	0.0	0.972	1.0,D	0.96	5.7	3.84	.	.	SOM	GT:/	0/1:1	0:77,1:..:78	G	A
.	.	.	.	.	.	.	.	.	.	.	.	SOM	GT:/	0/1:2	0:17,0:..:17	G	A
.	.	##	0	.	.	.	.	.	.	.	.	SOM	GT:/	0/1:1	0:126,1:..:1	C	T
.	.	##	0	.	.	.	.	2.1	.	.	.	SOM	GT:/	0/1:2	0:161,0:..:1	G	A
.	.	.	.	.	.	.	.	.	.	.	.	SOM	GT:/	0/1:4	0:18,0:..:18	C	G
.	.	.	.	.	.	.	.	.	.	.	.	SOM	GT:/	0/1:1	0:119,1:..:1	C	T
.	.	.	.	.	.	.	.	.	.	.	.	SOM	GT:/	0/1:3	0:33,0:..:33	G	A
.	.	.	.	0.0	.	.	1,N	.	.	.	.	SOM	GT:/	0/1:1	0:150,1:..:1	G	A
.	.	.	.	0.9	0.019	0.053	1,N	.	1.98	.	.	SOM	GT:/	0/1:6	0:28,0:..:28	G	C
.	.	.	.	.	.	.	.	.	.	.	.	SOM	GT:/	0/1:5	0:15,0:..:15	T	C
.	.	.	.	.	.	.	.	.	.	0	.	DB;S	GT:/	0/1:2	0:19,0:..:23	G	T
.	.	.	.	.	.	.	.	.	.	.	.	SOM	GT:/	0/1:2	0:102,1:..:1	G	T
.	.	.	.	.	.	.	.	.	.	.	.	SOM	GT:/	0/1:6	0:56,0:..:56	G	A
.	.	.	.	.	.	.	.	.	.	.	.	SOM	GT:/	0/1:4	0:30,0:..:30	G	A
.	.	##	0	.	.	.	.	.	.	.	.	SOM	GT:/	0/1:1	0:13,0:..:13	G	A
.	.	.	.	0.0	0.999	0.999	1,D	4.7	4.37	.	.	SOM	GT:/	0/1:8	0:42,0:..:42	G	A
.	.	.	.	.	.	.	.	.	.	.	.	SOM	GT:/	0/1:9	0:57,1:..:58	A	T
.	.	.	.	.	.	.	.	.	.	.	.	SOM	GT:/	0/1:3	0:20,0:..:20	A	T
2E-04	.	##	0	.	.	.	1,A	4.1	26.1	.	.	SOM	GT:/	0/1:4	0:15,0:..:15	G	A
.	.	.	.	.	.	.	.	.	.	0	0	SOM	GT:/	0/1:3	0:34,1:..:27	G	T
.	.	.	.	0.1	0.817	0.975	1,D	5.5	5.27	.	.	SOM	GT:/	0/1:4	0:24,0:..:24	C	T
.	.	.	.	.	.	.	.	.	1.25	.	.	SOM	GT:/	0/1:1	0:65,0:..:65	C	T
.	.	.	.	.	.	.	.	.	.	.	.	SOM	GT:/	0/1:2	0:17,0:..:17	C	G
.	.	##	0	1.0	0.006	0.015	1,D	3.8	.	.	.	SOM	GT:/	0/1:1	0:39,0:..:39	C	T
.	.	.	.	.	.	.	.	.	1.47	.	.	SOM	GT:/	0/1:5	0:22,0:..:22	G	A
.	.	.	.	.	.	.	.	.	.	0.1	0	DB;S	GT:/	0/1:1	0:41,0:..:41	A	G
.	.	.	.	.	.	.	.	.	.	.	.	SOM	GT:/	0/1:3	0:10,0:..:10	C	G
.	.	.	.	.	.	.	.	.	.	.	.	SOM	GT:/	0/1:3	0:11,0:..:11	C	T
.	.	.	.	.	.	.	.	.	.	.	.	SOM	GT:/	0/1:3	0:8,0:..:9:0.	C	T
.	.	.	.	.	.	.	.	.	.	.	.	SOM	GT:/	0/1:9	0:91,0:..:91	C	T
.	.	.	.	0.0	0.548	0.948	1,N	4.3	2.60	.	.	SOM	GT:/	0/1:2	0:14,0:..:14	C	A
.	.	.	.	.	.	.	1,D	4.2	3.22	.	.	SOM	GT:/	0/1:1	0:119,0:..:1	G	A
.	.	.	.	.	.	.	.	.	.	.	.	SOM	GT:/	0/1:4	0:31,0:..:31	G	A
.	.	##	0	0.0	0.083	0.507	1.00	.	3.78	.	.	SOM	GT:/	0/1:7	0:47,0:..:47	G	A
.	.	.	.	.	.	.	.	.	.	.	.	SOM	GT:/	0/1:8	0:63,0:..:63	G	C
.	.	.	.	0.1	0.0,B	0.0,B	1,N	.	.	.	.	SOM	GT:/	0/1:8	0:46,0:..:46	G	A
.	.	.	.	0.7	0.002	0.0,B	0.71	4.8	.	.	.	SOM	GT:/	0/1:3	0:243,1:..:2	G	A
.	.	.	.	.	.	.	.	3	.	.	.	SOM	GT:/	0/1:5	0:434,0:..:4	C	A
.	.	.	.	.	.	.	.	.	.	0	.	DB;S	GT:/	0/1:5	0:19,0:..:14	C	T
.	.	.	.	.	.	.	.	.	.	0	0	DB;S	GT:/	0/1:3	0:22,0:..:20	T	A
.	.	.	.	.	.	.	.	.	.	0.1	0	DB;S	GT:/	0/1:9	0:35,1:..:34	T	G
.	.	.	.	.	.	.	.	.	.	0	.	DB;S	GT:/	0/1:7	0:36,1:..:34	T	G
.	.	.	.	.	.	.	.	.	.	.	.	SOM	GT:/	0/1:4	0:20,0:..:20	C	A
.	.	.	.	0.4	0.002	0.001	1,N	.	.	.	.	SOM	GT:/	0/1:2	0:228,1:..:2	T	C
.	.	.	.	.	.	.	.	.	.	.	.	SOM	GT:/	0/1:2	0:139,0:..:1	G	C

.	.	.	.	0.8	0.05,I	0.049	1,D	5.8	1.77	.	.	SON	GT:/	0/1:3	0:25,0:.:25	C	G
.	.	.	.	.	.	.	.	.	.	.	.	SON	GT:/	0/1:9	0:40,0:.:40	G	A
.	##	##	.	0.0	0.429	0.801	1,D	.	2.66	.	.	SON	GT:/	0/1:3	0:201,1:.:2	G	T
.	.	.	.	.	.	.	.	2.9	.	.	.	SON	GT:/	0/1:3	0:15,0:.:15	C	G
.	.	.	.	.	.	.	.	.	.	.	.	SON	GT:/	0/1:1	0:9,0:.:9:0.	A	T
.	.	.	.	0.0	0.048	0.028	1,D	5.3	4.86	.	.	SON	GT:/	0/1:2	0:171,1:.:1	C	T
.	##	##	0	.	.	.	.	.	1.52	0	.	SON	GT:/	0/1:2	0:158,0:.:1	C	T
.	.	.	.	.	.	.	.	.	1.30	.	.	SON	GT:/	0/1:1	0:11,0:.:11	G	A
.	.	.	.	1.0	0.08,I	0.358	1,D	5.9	2.88	.	.	SON	GT:/	0/1:7	0:29,0:.:29	G	A
.	.	.	.	.	.	.	.	.	.	.	.	SON	GT:/	0/1:9	0:66,1:.:67	C	T
.	.	.	.	.	.	.	.	.	.	.	.	SON	GT:/	0/1:3	0:24,0:.:24	G	C
.	.	##	0	0.1	0.0,B	0.0,B	1,N	.	.	.	.	SON	GT:/	0/1:6	0:30,0:.:31	C	T
.	.	.	.	0.0	0.049	0.661	0.97	4.4	2.60	.	.	SON	GT:/	0/1:2	0:184,0:.:1	G	A
.	.	.	.	0.0	0.779	0.999	1,N	3.3	2.81	.	.	SON	GT:/	0/1:8	0:49,0:.:49	C	T
.	.	.	.	.	.	.	.	.	.	.	.	SON	GT:/	0/1:1	0:8,0:.:8:0.	G	T
.	.	.	.	.	.	.	.	.	.	.	.	SON	GT:/	0/1:2	0:9,0:.:9:0.	T	G
.	.	.	.	.	.	.	.	.	.	0.1	0.4	SON	GT:/	0/1:4	0:26,0:.:18	C	A
.	.	.	.	.	.	.	.	.	.	0	0.1	SON	GT:/	0/1:4	0:24,0:.:16	C	T
.	.	.	.	.	.	.	.	.	.	.	.	SON	GT:/	0/1:1	0:86,1:.:88	C	T
.	.	.	.	.	.	.	.	.	.	.	.	SON	GT:/	0/1:7	0:22,0:.:22	C	G
.	.	0	0	.	.	.	.	.	.	.	.	SON	GT:/	0/1:9	0:55,0:.:52	T	G
.	.	.	.	.	.	.	.	.	.	.	.	SON	GT:/	0/1:1	0:83,0:.:83	G	T
.	.	.	.	.	.	.	.	.	.	0.1	0.1	SON	GT:/	0/1:3	0:33,1:.:33	G	C
.	.	0	0	0.6	0.002	0.014	0.64	4.5	.	.	.	SON	GT:/	0/1:7	0:41,0:.:41	G	A
.	.	.	.	.	.	.	.	.	.	.	.	SON	GT:/	0/1:2	0:178,1:.:1	T	A
0.144	.	0	0	.	.	.	.	.	.	0.1	0.1	SON	GT:/	0/1:2	0:8,0:.:8:0.	A	G
.	.	.	.	.	.	.	.	.	.	.	.	SON	GT:/	0/1:6	0:31,0:.:31	C	T
.	.	.	.	.	.	.	.	.	.	0	.	SON	GT:/	0/1:4	0:14,0:.:14	A	G
.	.	.	.	0.0	0.739	0.985	1,D	5.7	4.01	.	.	SON	GT:/	0/1:1	0:58,0:.:58	G	A
.	.	.	.	.	.	.	.	.	.	0	0	SON	GT:/	0/1:3	0:26,0:.:26	T	C
.	.	.	.	0.0	0.916	0.997	1,D	4.6	1.92	.	.	SON	GT:/	0/1:1	0:93,0:.:93	C	A
.	.	.	.	0.0	0.933	0.995	1,D	5.4	4.16	.	.	SON	GT:/	0/1:4	0:15,0:.:15	A	G
.	.	.	.	.	.	.	.	.	.	0	0	SON	GT:/	0/1:5	0:21,0:.:21	T	C
.	.	.	.	.	.	.	.	.	.	0	0.1	SON	GT:/	0/1:1	0:17,0:.:17	C	G
.	.	0	0	.	.	.	.	.	.	0	0.1	DB;S	GT:/	0/1:9	0:60,1:.:60	T	G
.	.	0	0	.	.	.	.	.	1.48	.	.	SON	GT:/	0/1:6	0:51,0:.:38	C	G
.	.	.	.	.	.	.	.	.	.	0	0	SON	GT:/	0/1:2	0:26,0:.:21	T	A
.	.	.	.	.	.	.	.	.	.	.	.	SON	GT:/	0/1:7	0:47,0:.:47	C	T
.	.	.	.	.	.	.	.	.	.		0	SON	GT:/	0/1:7	0:44,0:.:44	C	T
.	.	.	.	.	.	.	.	.	.	.	.	SON	GT:/	0/1:1	0:17,0:.:17	C	G
.	##	##	0	0.4	0.005	0.002	1,N	.	.	.	.	SON	GT:/	0/1:1	0:87,0:.:88	T	C
.	.	.	.	0.0	0.998	0.999	0.98	5.5	4.27	.	.	SON	GT:/	0/1:5	0:42,0:.:42	C	A
.	.	##	0	0.0	0.98,I	1.0,D	0.98	5.6	5.56	.	.	SON	GT:/	0/1:1	0:80,0:.:80	C	T
.	.	.	.	.	.	.	.	.	.	.	.	SON	GT:/	0/1:6	0:39,0:.:39	G	T
.	.	.	.	0.0	0.031	0.034	1,D	5.3	3.35	.	.	SON	GT:/	0/1:2	0:168,0:.:1	T	C
.	.	.	.	.	.	.	.	.	.	0.1	0.1	SON	GT:/	0/1:6	0:42,1:.:38	A	G
.	.	.	.	.	.	.	.	.	.	.	.	SON	GT:/	0/1:2	0:13,0:.:13	C	G
.	.	0	0	0.1	0.02,I	0.108	0.88	4.6	3.30	0	0	SON	GT:/	0/1:2	0:10,0:.:10	C	T
.	.	.	.	.	.	.	.	.	.	.	.	SON	GT:/	0/1:3	0:22,0:.:22	C	G
.	.	.	.	.	.	.	.	.	.	.	.	SON	GT:/	0/1:3	0:10,0:.:10	G	A
.	.	.	.	0.0	0.58,I	0.896	0.99	4.8	2.62	.	.	SON	GT:/	0/1:3	0:191,0:.:1	C	A
.	.	.	.	.	.	.	.	.	.	.	.	SON	GT:/	0/1:3	0:15,0:.:15	G	T
.	0	##	0	.	.	.	.	3.4	.	.	.	SON	GT:/	0/1:7	0:48,0:.:48	G	A
.	.	.	.	.	.	.	.	.	.	.	.	SON	GT:/	0/1:5	0:38,0:.:39	T	C

.	.	.	.	.	.	.	.	.	.	0.2	0.2	DB;S	GT:/	0/1:2	0:19,0:..:19	C	T
.	.	.	.	.	.	.	.	4.3	2.02	.	.	SON	GT:/	0/1:5	0:47,0:..:47	T	C
.	.	.	.	0.6	0.001	0.024	1,N	.	.	.	.	SON	GT:/	0/1:9	0:59,0:..:60	G	A
.	.	.	.	.	.	.	1,A	5.6	8.61	.	.	SON	GT:/	0/1:8	0:59,0:..:59	C	T
.	.	.	.	.	.	.	.	.	.	.	.	SON	GT:/	0/1:6	0:28,0:..:28	C	A
.	.	.	.	.	.	.	.	.	.	.	.	SON	GT:/	0/1:8	0:45,0:..:45	C	T
.	.	.	.	.	.	.	.	.	.	0.1	0	SON	GT:/	0/1:1	0:11,0:..:8:0	G	A
.	.	.	.	.	.	.	.	.	.	0	.	SON	GT:/	0/1:8	0:8,0:..:3:0.	C	A
.	.	.	.	.	.	.	.	3.9	4.59	.	.	SON	GT:/	0/1:1	0:108,0:..:1	C	T
.	.	.	.	0.0	0.998	1.0,D	1.00	.	4.03	.	.	SON	GT:/	0/1:5	0:31,0:..:31	C	A
.	.	##	0	.	.	.	.	.	.	0	.	SON	GT:/	0/1:8	0:60,1:..:61	C	T
.	.	.	.	.	.	.	.	.	.	.	.	SON	GT:/	0/1:3	0:24,0:..:24	A	T
.	.	.	.	.	.	.	.	.	.	.	.	SON	GT:/	0/1:1	0:18,0:..:19	G	A
.	.	.	.	0.0	0.995	1.0,D	1,D	2.2	2.54	.	.	SON	GT:/	0/1:9	0:105,0:..:1	C	G
.	.	.	.	.	.	.	.	.	.	.	.	SON	GT:/	0/1:5	0:29,0:..:30	G	A
.	.	.	.	.	.	.	.	.	.	.	.	SON	GT:/	0/1:1	0:68,1:..:69	G	A
.	.	.	.	.	.	.	.	.	.	.	.	SON	GT:/	0/1:8	0:71,0:..:72	G	A
.	.	.	.	.	.	.	.	3.3	.	.	.	SON	GT:/	0/1:3	0:18,0:..:18	C	G
.	.	.	.	0.6	0.015	0.014	0.94	5.1	2.33	.	.	SON	GT:/	0/1:1	0:82,0:..:82	G	C
.	.	.	.	.	.	.	.	5.3	.	.	.	SON	GT:/	0/1:1	0:92,0:..:92	G	T
.	.	.	.	.	.	.	.	.	.	.	.	SON	GT:/	0/1:1	0:54,0:..:56	G	C
.	.	.	.	.	.	.	1,D	4	3.48	.	.	SON	GT:/	0/1:6	0:35,0:..:35	G	A
.	.	.	.	0.2	0.02,I	0.009	1,N	.	.	.	.	SON	GT:/	0/1:8	0:40,0:..:40	C	A
.	.	.	.	.	.	.	.	.	.	.	.	SON	GT:/	0/1:5	0:43,0:..:43	T	C
.	.	.	.	.	.	.	.	3.5	.	.	.	SON	GT:/	0/1:3	0:21,0:..:21	C	T
.	.	.	.	0.0	0.743	0.983	1,D	5.5	4.92	.	.	SON	GT:/	0/1:9	0:89,0:..:90	C	T
.	.	.	.	.	.	.	.	.	.	.	.	SON	GT:/	0/1:5	0:35,0:..:35	T	C
.	.	##	0	.	.	.	.	.	.	.	.	SON	GT:/	0/1:5	0:40,0:..:40	C	T
.	.	.	.	0.0	0.08,I	0.22,I	1,N	5.3	1.44	.	.	SON	GT:/	0/1:1	0:98,0:..:98	A	G
.	.	.	.	.	.	.	.	.	.	.	.	SON	GT:/	0/1:1	0:16,0:..:16	G	A
.	.	.	.	0.0	1.0,D	1.0,D	1,D	4.8	3.94	.	.	SON	GT:/	0/1:1	0:88,0:..:88	C	G
.	.	.	.	.	.	.	.	3.9	.	.	.	SON	GT:/	0/1:2	0:165,0:..:1	C	T
.	.	.	.	.	.	.	.	.	.	.	.	SON	GT:/	0/1:1	0:144,0:..:1	G	C
.	.	.	.	0.2	0.68,I	0.846	1,D	2.3	.	.	.	SON	GT:/	0/1:2	0:122,0:..:1	C	A
.	.	.	.	0.0	0.617	0.934	1,N	4	.	.	.	SON	GT:/	0/1:7	0:48,0:..:48	C	A
.	.	.	.	.	.	.	.	.	.	0.1	0.1	DB;S	GT:/	0/1:5	0:24,0:..:24	A	G
.	.	.	.	.	.	.	.	.	.	.	.	SON	GT:/	0/1:4	0:24,0:..:24	G	T
.	.	.	.	0.0	0.988	0.999	0.99	3.2	3.49	.	.	SON	GT:/	0/1:1	0:120,0:..:1	T	G
.	.	.	.	.	.	.	.	.	.	0.2	0.3	SON	GT:/	0/1:7	0:8,0:..:8:0.	A	G
.	.	.	.	0.0	0.281	0.867	.	3	.	.	.	SON	GT:/	0/1:1	0:79,0:..:79	G	T
.	.	.	.	.	.	.	.	.	.	.	.	SON	GT:/	0/1:3	0:24,0:..:24	C	G
.	.	.	.	.	.	.	.	.	.	.	.	SON	GT:/	0/1:5	0:31,0:..:31	C	A
.	.	.	.	0.6	0.004	0.002	1,N	.	.	.	.	SON	GT:/	0/1:1	0:80,0:..:80	G	C
.	.	.	.	.	.	.	.	.	.	.	.	SON	GT:/	0/1:3	0:25,0:..:25	G	A
.	.	.	.	.	.	.	.	.	.	.	.	SON	GT:/	0/1:5	0:48,0:..:43	G	T
.	.	.	.	.	.	.	.	.	.	.	.	SON	GT:/	0/1:1	0:110,0:..:1	G	C
.	.	.	.	.	.	.	.	.	.	.	.	SON	GT:/	0/1:1	0:10,0:..:10	T	C
.	.	.	.	.	.	.	.	.	.	0	.	SON	GT:/	0/1:1	0:15,0:..:14	G	A
.	.	.	.	.	.	.	.	.	.	.	.	SON	GT:/	0/1:3	0:16,0:..:16	T	C
.	.	##	0	.	.	.	1.00	.	.	.	.	SON	GT:/	0/1:1	0:83,0:..:83	G	A
.	.	##	0	0.0	0.998	1.0,D	1,D	5	4.32	.	.	SON	GT:/	0/1:2	0:128,0:..:1	G	A
.	.	.	.	.	.	.	.	.	.	.	.	SON	GT:/	0/1:8	0:65,0:..:65	T	A
.	.	.	.	.	.	.	.	.	.	.	.	SON	GT:/	0/1:1	0:151,0:..:1	T	G
.	.	.	.	.	.	.	.	2.9	.	.	.	SON	GT:/	0/1:3	0:21,0:..:21	C	T

.	.	.	.	.	.	.	.	.	.	0	.	SOM	GT:/	0/1:3	0:29,0:.:29	T	G
.	.	.	.	.	.	.	.	.	.	.	.	SOM	GT:/	0/1:1	0:100,0:.:1	G	C
.	.	.	.	.	.	.	.	.	.	.	.	SOM	GT:/	0/1:7	0:52,0:.:52	C	G
.	.	.	.	.	.	.	.	.	.	.	.	SOM	GT:/	0/1:3	0:37,0:.:37	G	A
.	.	.	.	.	.	.	.	.	.	.	.	SOM	GT:/	0/1:5	0:27,0:.:27	A	G
.	.	.	.	.	.	.	.	.	.	.	.	SOM	GT:/	0/1:6	0:29,0:.:29	G	A
.	.	.	.	.	.	.	.	.	.	.	.	SOM	GT:/	0/1:3	0:11,0:.:11	G	C
.	.	.	.	0.0	0.422	0.938	1,D	2.9	1.62	.	.	SOM	GT:/	0/1:1	0:94,0:.:94	A	G
.	.	.	.	.	.	.	.	.	.	0	0	DB;S	GT:/	0/1:4	0:33,1:.:35	G	A
.	.	.	.	0.0	1.0,D	1.0,D	1,D	5.4	4.70	.	.	SOM	GT:/	0/1:3	0:27,0:.:27	C	A
.	.	.	.	0.0	0.776	0.97,I	0.99	5.9	3.43	.	.	SOM	GT:/	0/1:7	0:52,0:.:52	C	T
.	.	.	.	.	.	.	.	.	.	0.2	0	SOM	GT:/	0/1:3	0:28,0:.:28	T	C
.	.	.	.	.	.	.	.	3.1	4.26	.	.	SOM	GT:/	0/1:8	0:65,0:.:65	G	A
0.243	.	.	.	.	.	.	.	.	.	0	0	SOM	GT:/	0/1:5	0:23,0:.:24	T	G
5E-04	.	.	.	.	.	.	.	.	.	.	.	SOM	GT:/	0/1:4	0:12,0:.:12	G	C
.	.	.	.	0.1	0.483	0.919	1.00	5.7	2.46	.	.	SOM	GT:/	0/1:1	0:47,0:.:47	T	A
.	.	.	.	0.0	0.992	1.0,D	1.00	2.3	1.66	.	.	SOM	GT:/	0/1:7	0:45,0:.:45	C	T
.	.	.	.	0.0	0.868	0.993	1.00	5.8	2.89	.	.	SOM	GT:/	0/1:6	0:48,0:.:48	C	A
.	.	.	.	0.0	0.991	1.0,D	1,D	4.5	4.02	.	.	SOM	GT:/	0/1:8	0:40,0:.:40	G	A
.	.	.	.	.	.	.	.	.	.	.	.	SOM	GT:/	0/1:1	0:60,0:.:60	G	A
.	.	.	.	.	.	.	.	.	.	.	.	SOM	GT:/	0/1:1	0:56,0:.:57	T	C
.	.	.	.	.	.	.	.	.	.	0	.	SOM	GT:/	0/1:1	0:11,0:.:9:0	G	A
.	.	.	.	.	.	.	.	.	.	0	0	SOM	GT:/	0/1:3	0:10,0:.:10	G	A
.	.	.	.	.	.	.	.	.	.	0.1	0.4	SOM	GT:/	0/1:2	0:31,0:.:22	G	A
.	.	.	.	.	.	.	.	.	.	0.5	0.5	DB;S	GT:/	0/1:3	0:24,0:.:24	T	A
.	.	.	.	.	.	.	.	.	.	.	0	SOM	GT:/	0/1:1	0:16,0:.:14	T	A
.	.	.	.	.	.	.	.	.	.	0	.	SOM	GT:/	0/1:5	0:37,1:.:25	C	A
.	.	.	.	.	.	.	.	.	1.25	0	0	SOM	GT:/	0/1:1	0:10,0:.:10	G	C
.	.	.	.	.	.	.	.	.	1.90	0.1	0	DB;S	GT:/	0/1:6	0:37,1:.:38	C	T
.	.	.	.	.	.	.	.	.	.	0	.	SOM	GT:/	0/1:1	0:8,0:.:8:0.	C	T
.	.	.	.	.	.	.	.	.	.	.	.	SOM	GT:/	0/1:1	0:8,0:.:8:0.	C	A
.	.	.	.	.	.	.	.	.	.	.	.	SOM	GT:/	0/1:1	0:8,0:.:8:0.	C	G
.	.	.	.	.	.	.	.	.	.	0	0.4	SOM	GT:/	0/1:9	0:9,0:.:8:0.	T	A
.	.	.	.	.	.	.	.	.	.	.	.	SOM	GT:/	0/1:4	0:29,0:.:29	G	T
.	.	.	.	.	.	.	.	.	.	.	.	SOM	GT:/	0/1:1	0:8,0:.:8:0.	C	A
0.468	.	.	.	.	.	.	.	.	.	0.2	0.6	SOM	GT:/	0/1:9	0:9,0:.:9:0.	C	T
.	.	.	.	.	.	.	.	.	.	0	0	SOM	GT:/	0/1:6	0:33,0:.:33	T	C
.	.	.	.	.	.	.	.	.	.	0	0	DB;S	GT:/	0/1:4	0:25,0:.:25	T	C
.	.	.	.	.	.	.	.	2.1	.	0.1	0	SOM	GT:/	0/1:5	0:44,1:.:45	T	C
.	.	.	.	.	.	.	.	.	.	0	.	SOM	GT:/	0/1:1	0:10,0:.:10	G	C
.	.	.	.	.	.	.	.	.	.	0	.	SOM	GT:/	0/1:3	0:12,0:.:12	T	A
.	.	.	.	.	.	.	.	.	.	0	.	SOM	GT:/	0/1:1	0:10,0:.:10	G	A
.	.	.	.	.	.	.	.	.	.	0	0	DB;S	GT:/	0/1:5	0:25,0:.:26	A	G
.	.	.	.	.	.	.	.	.	.	.	.	SOM	GT:/	0/1:1	0:36,1:.:37	T	G
.	.	.	.	.	.	.	.	.	.	.	.	SOM	GT:/	0/1:3	0:10,0:.:11	T	A
0.134	.	.	.	.	.	.	.	.	.	0.2	0.2	SOM	GT:/	0/1:8	0:8,0:.:8:0.	T	G
.	.	.	.	.	.	.	.	.	.	0.1	0.3	SOM	GT:/	0/1:1	0:10,0:.:9:0	T	C
.	.	.	.	.	.	.	.	.	.	0	0	SOM	GT:/	0/1:4	0:22,0:.:20	C	T
.	.	.	.	.	.	.	.	.	.	0	0	SOM	GT:/	0/1:4	0:37,1:.:26	G	A
.	.	.	.	.	.	.	.	.	.	0	.	SOM	GT:/	0/1:2	0:9,0:.:9:0.	A	T
.	.	.	.	.	.	.	.	.	.	0	0.1	DB;S	GT:/	0/1:4	0:26,0:.:26	G	T
.	.	.	.	.	.	.	.	.	.	0	.	DB;S	GT:/	0/1:6	0:40,0:.:40	A	T
.	.	.	.	.	.	.	.	.	.	0	0	DB;S	GT:/	0/1:2	0:19,0:.:17	G	A
.	.	.	.	.	.	.	.	.	.	0	0	SOM	GT:/	0/1:3	0:12,0:.:12	G	T

.	.	.	.	.	.	.	.	.	.	0	0	SOM	GT:/	0/1:4	0:22,0:..:21	C	A
0.268	.	.	.	.	.	.	.	.	.	.	0	SOM	GT:/	0/1:1	0:131,1:..:7	C	A
.	.	.	.	.	.	.	.	.	.	.	.	SOM	GT:/	0:87	0/1:106,27	T	G
0.444	.	.	.	.	.	.	.	.	.	0.2	0.5	DB;S	GT:/	0:22	0/1:23,4:37	G	C
.	.	.	.	.	.	.	.	.	.	.	.	SOM	GT:/	0:16	0/1:28,7:17	G	T
.	.	.	.	.	.	.	.	.	.	0.1	0.4	SOM	GT:/	0:12	0/1:17,5:37	C	G
.	.	.	.	.	.	.	.	.	.	0	0	SOM	GT:/	0:13	0/1:25,4:37	C	G
.	.	.	.	.	.	.	.	.	.	0	.	SOM	GT:/	0:22	0/1:59,7:35	G	T
.	.	.	.	.	.	.	.	.	.	0.2	0.5	SOM	GT:/	0:14	0/1:29,17:3	C	A
.	.	.	.	.	.	.	.	.	.	0	0	SOM	GT:/	0:11	0/1:25,5:34	G	T
.	.	.	.	.	.	.	.	.	.	.	.	SOM	GT:/	0:37	0/1:36,6:37	T	G
.	.	.	.	.	.	.	.	.	.	.	.	SOM	GT:/	0:8,(	0/1:34,6:37	G	T
.	.	.	.	.	.	.	.	.	.	0	0.1	SOM	GT:/	0:18	0/1:40,8:21	C	T
.	.	.	.	.	.	.	.	.	.	.	.	SOM	GT:/	0:13	0/1:20,8:14	G	T
0.011	.	.	.	.	.	.	.	.	.	0.1	0	SOM	GT:/	0:15	0/1:29,24:3	C	T
.	.	.	.	.	.	.	.	.	.	0.1	0.4	SOM	GT:/	0:8,(	0/1:11,8:33	A	G
.	.	.	.	.	.	.	.	.	.	.	.	SOM	GT:/	0:9,(	0/1:26,4:37	T	C
.	.	.	.	.	.	.	.	.	.	0.1	0.3	SOM	GT:/	0:18	0/1:16,5:34	G	A
.	.	.	.	.	.	.	.	.	.	.	.	SOM	GT:/	0:10	0/1:30,6:37	T	A
.	.	.	.	.	.	.	.	.	.	.	.	SOM	GT:/	0:13	0/1:39,5:29	C	A
.	.	.	.	.	.	.	.	.	.	.	.	SOM	GT:/	0:20	0/1:25,4:37	C	T
0.336	.	.	.	.	.	.	.	.	.	0.1	0.3	DB;S	GT:/	0:51	0/1:125,17	C	G
.	.	.	.	.	.	.	.	.	.	0.1	0.1	SOM	GT:/	0:12	0/1:11,7:33	G	C
.	.	.	.	.	.	.	.	.	.	0	.	SOM	GT:/	0:41	0/1:117,18	C	G
.	.	.	.	.	.	.	.	.	.	0	.	SOM	GT:/	0:46	0/1:117,17	T	A
.	.	.	.	0.0:	0.173	0.763	1.00	5.2	2.74	0	.	SOM	GT:/	0:58	0/1:134,13	T	G
.	.	.	.	.	.	.	.	.	.	0	.	SOM	GT:/	0:8,(	0/1:9,5:37:	G	T
.	.	.	.	.	.	.	.	.	.	0	0	SOM	GT:/	0:8,(	0/1:5,6:37:	C	T
.	.	.	.	.	.	.	.	.	.	0	0.1	SOM	GT:/	0:8,(	0/1:9,5:37:	C	A
.	.	.	.	.	.	.	.	2.7	.	.	.	SOM	GT:/	0:15	0/1:57,7:20	A	C
.	.	.	.	.	.	.	.	.	1.80	.	.	SOM	GT:/	0:12	0/1:44,6:37	T	C
.	.	.	.	.	.	.	.	.	.	0	0	SOM	GT:/	0:35	0/1:72,9:37	T	G
.	.	.	.	.	.	.	.	.	.	0	0.1	SOM	GT:/	0:17	0/1:35,4:34	C	T
.	.	.	.	.	.	.	.	.	.	.	.	SOM	GT:/	0:11	0/1:34,4:30	A	T
.	.	.	.	.	.	.	.	.	.	0	0.1	SOM	GT:/	0:10	0/1:20,7:35	G	A
.	.	.	.	.	.	.	.	.	.	0	0.1	SOM	GT:/	0:14	0/1:43,4:37	C	T
.	.	.	.	.	.	.	.	.	.	0	.	SOM	GT:/	0:16	0/1:34,6:37	G	A
.	.	##	0	.	.	.	.	.	1.26	0	.	SOM	GT:/	0:12	0/1:19,36:3	T	C
.	.	.	.	.	.	.	.	.	.	0	0	SOM	GT:/	0:13	0/1:7,4:37:	C	T
0.49	.	.	.	.	.	.	.	.	.	0	0.2	DB;S	GT:/	0:37	0/1:72,8:37	G	A
.	.	.	.	.	.	.	.	.	.	0	0	SOM	GT:/	0:18	0/1:17,8:35	G	A
.	.	.	.	.	.	.	.	.	.	0	0.1	DB;S	GT:/	0:20	0/1:84,10:3	G	T
.	.	.	.	.	.	.	.	.	.	0	0.1	DB;S	GT:/	0:34	0/1:55,9:33	T	C
.	.	.	.	.	.	.	.	2.2	.	0	0.1	DB;S	GT:/	0:40	0/1:70,10:3	C	T
.	.	.	.	.	.	.	.	.	.	.	.	SOM	GT:/	0:16	0/1:44,4:30	G	C
.	.	.	.	.	.	.	.	.	.	.	.	SOM	GT:/	0:11	0/1:38,4:37	C	A
.	.	.	.	.	.	.	.	.	.	.	.	SOM	GT:/	0:12	0/1:8,4:37:	A	G
.	.	.	.	.	.	.	.	.	.	0	0	SOM	GT:/	0:9,(	0/1:13,6:33	G	T
.	.	.	.	.	.	.	.	.	.	.	.	SOM	GT:/	0:44	0/1:143,17	A	T
0.458	.	.	.	.	.	.	.	.	.	0.1	0.3	DB;S	GT:/	0:40	0/1:118,13	C	T
.	.	.	.	.	.	.	.	.	.	.	.	SOM	GT:/	0:9,(	0/1:23,4:37	C	A
.	.	##	0	0.1:	0.987	1.0,D	1.00	4.2	2.42	.	.	SOM	GT:/	0/1:9	0:104,0:..:1	C	T
.	.	.	.	.	.	.	.	2.3	.	.	.	SOM	GT:/	0/1:5	0:47,0:..:44	C	T
.	.	.	.	.	.	.	.	2.9	.	.	.	SOM	GT:/	0/1:4	0:33,0:..:34	G	C

.	.	.	.	.	.	.	.	2.6	.	.	.	SOM	GT:/	0/1:2	0:11,0:..11	G	C
.	.	.	.	.	.	.	.	2.3	.	.	.	SOM	GT:/	0/1:1	0:108,0:..1	C	T
.	.	.	.	0.0	.	.	0.99	3.2	2.83	.	.	SOM	GT:/	0/1:8	0:85,0:..85	G	A
.	.	.	.	.	.	.	.	2.1	1.40	.	.	SOM	GT:/	0/1:1	0:139,0:..1	G	T
.	.	.	.	.	.	.	.	.	.	.	.	SOM	GT:/	0/1:3	0:12,0:..12	C	T
.	.	.	.	.	.	.	1,A	5.3	7.23	.	.	SOM	GT:/	0/1:3	0:30,0:..30	C	T
0.015	.	.	.	.	.	.	.	.	.	0	0	SOM	GT:/	0/1:2	0:8,0:..8:0.	T	C
.	.	.	.	.	.	.	.	.	.	.	0	SOM	GT:/	0/1:2	0:11,0:..11	G	A
.	.	.	.	.	.	.	.	.	.	0.1	0.4	SOM	GT:/	0/1:1	0:13,0:..12	C	T
.	.	.	.	.	.	.	.	.	.	0	0	SOM	GT:/	0/1:1	0:9,0:..9:0.	G	C
.	.	.	.	.	.	.	.	.	.	0	.	SOM	GT:/	0/1:8	0:20,0:..19	A	T
.	.	.	.	.	.	.	.	.	.	.	0	SOM	GT:/	0/1:3	0:57,1:..43	G	A
.	.	.	.	.	.	.	.	.	.	.	.	SOM	GT:/	0/1:4	0:17,0:..17	A	C
.	.	.	.	.	.	.	.	.	.	0	0.1	SOM	GT:/	0/1:3	0:16,0:..16	T	G
.	.	.	.	0.0	0.912	0.996	0.96	4.3	3.06	0	.	SOM	GT:/	0/1:3	0:384,0:..3	C	T
.	.	.	.	.	.	.	.	5.4	1.82	.	.	SOM	GT:/	0/1:1	0:105,0:..1	G	C
.	.	.	.	.	.	.	.	.	.	.	.	SOM	GT:/	0/1:1	0:108,0:..1	G	A
.	.	.	.	.	.	.	.	.	.	.	.	SOM	GT:/	0/1:1	0:106,0:..1	A	G
.	.	.	.	.	.	.	.	.	.	.	.	SOM	GT:/	0/1:1	0:102,0:..1	C	T
.	.	.	.	.	.	.	.	.	.	.	.	SOM	GT:/	0/1:3	0:30,0:..30	G	T
.	.	.	.	.	.	.	.	3	.	.	.	SOM	GT:/	0/1:6	0:47,0:..47	G	C
.	.	##	0	0.4	0.179	0.796	1,D	5.4	3.42	.	.	SOM	GT:/	0/1:1	0:143,0:..1	G	A
.	.	.	.	.	.	.	.	2.3	.	.	.	SOM	GT:/	0/1:3	0:17,0:..17	C	T
.	.	.	.	.	.	.	.	.	.	.	.	SOM	GT:/	0/1:1	0:110,0:..1	G	C
.	.	.	.	0.1	0.495	0.899	1,N	3.3	1.89	.	.	SOM	GT:/	0/1:1	0:145,0:..1	C	T
.	.	##	0	.	.	.	.	.	1.59	.	.	SOM	GT:/	0/1:1	0:120,0:..1	C	T
.	.	##	0	.	.	.	.	.	.	.	.	SOM	GT:/	0/1:2	0:17,0:..17	G	A
.	.	.	.	.	.	.	.	.	.	0.1	0	SOM	GT:/	0/1:5	0:14,0:..14	A	G
.	.	##	0	0.0	0.653	0.918	0.97	4.9	3.84	.	.	SOM	GT:/	0/1:2	0:23,0:..23	C	G
.	.	.	.	.	.	.	.	.	.	.	.	SOM	GT:/	0/1:1	0:155,0:..1	A	G
0.001	0	0	0	0.2	0.18,I	0.791	0.94	3.4	.	.	.	DB;S	GT:/	0/1:2	0:109,0:..1	G	A
.	.	.	.	.	.	.	.	.	.	.	.	SOM	GT:/	0/1:6	0:14,0:..14	C	T
.	.	.	.	.	.	.	1,D	2.9	6.40	.	.	SOM	GT:/	0/1:1	0:67,0:..67	G	A
.	.	.	.	.	.	.	1,D	5.1	4.27	.	.	SOM	GT:/	0/1:1	0:55,0:..55	G	C
.	.	.	.	0.0	0.819	0.919	1.00	4.4	4.65	.	.	SOM	GT:/	0/1:1	0:148,0:..1	C	T
.	.	.	.	.	.	.	.	.	.	.	.	SOM	GT:/	0/1:1	0:125,0:..1	T	C
.	.	.	.	.	.	.	.	.	.	.	.	SOM	GT:/	0/1:8	0:23,0:..23	C	T
.	.	.	.	0.0	0.178	0.693	1.00	5.1	2.94	0	.	SOM	GT:/	0/1:3	0:311,1:..3	G	A
.	.	.	.	0.0	0.029	0.004	0.85	3.7	2.36	.	.	SOM	GT:/	0/1:2	0:106,1:..1	G	A
.	.	.	.	.	.	.	.	.	.	.	.	SOM	GT:/	0/1:2	0:21,0:..21	G	A
.	.	.	.	0.0	0.266	0.891	0.75	4.5	3.26	.	.	SOM	GT:/	0/1:1	0:141,1:..1	C	G
.	.	.	.	.	.	.	.	2.6	2.09	.	.	SOM	GT:/	0/1:3	0:17,0:..17	C	G
.	.	##	0	.	.	.	.	.	.	.	.	SOM	GT:/	0/1:4	0:26,0:..26	T	C
.	.	.	.	0.0	0.616	0.915	1,D	5.1	3.61	.	.	SOM	GT:/	0/1:7	0:25,0:..25	A	T
.	.	.	.	.	.	.	.	.	.	.	.	SOM	GT:/	0/1:6	0:61,0:..61	G	A
2E-04	.	0	0	.	.	.	.	4.6	2.69	.	.	SOM	GT:/	0/1:4	0:161,0:..1	T	G
.	0	##	0	0.0	0.881	0.999	1,N	3.2	1.45	.	.	SOM	GT:/	0/1:3	0:310,1:..3	C	T
.	.	##	0	.	.	.	.	2.4	.	.	.	SOM	GT:/	0/1:2	0:185,0:..1	G	A
.	.	##	0	.	.	.	.	.	.	0	.	SOM	GT:/	0/1:8	0:59,0:..59	G	A
.	.	.	.	.	.	.	.	.	.	.	.	SOM	GT:/	0/1:4	0:8,0:..7:0.	T	G
.	.	.	.	.	.	.	.	.	.	.	.	SOM	GT:/	0/1:2	0:17,0:..17	C	T
2E-04	.	##	0	.	.	.	.	2.9	2.55	.	.	SOM	GT:/	0/1:1	0:109,0:..1	G	A
2E-04	##	##	0	0.1	0.041	0.064	0.75	4.1	2.41	.	.	SOM	GT:/	0/1:1	0:28,0:..28	G	A
6E-04	.	0	0	.	.	.	.	.	.	0	0	SOM	GT:/	0/1:5	0:29,0:..29	G	A

.	.	##	0	.	.	.	.	.	.	.	.	SOM	GT:/	0/1:8	0:27,0:..:27	G	A
.	.	##	0	0.1	0.557	0.853	0.98	2.8	3.03	0	.	SOM	GT:/	0/1:2	0:178,0:..:1	G	A
2E-04	.	.	.	.	.	.	.	.	.	.	.	SOM	GT:/	0/1:1	0:10,0:..:10	C	T
.	.	.	.	0.0	0.999	1.0,D	1,D	5.2	5.53	.	.	SOM	GT:/	0/1:4	0:25,0:..:25	G	A
.	.	.	.	.	.	.	.	.	.	0	0	SOM	GT:/	0/1:1	0:10,0:..:10	A	T
.	.	.	.	.	.	.	.	.	.	.	.	SOM	GT:/	0/1:2	0:24,0:..:25	C	T
.	.	0	0	.	.	.	.	3.4	1.86	0	0	SOM	GT:/	0/1:9	0:10,0:..:10	C	G
.	.	.	.	.	.	.	.	.	2.44	.	.	SOM	GT:/	0/1:3	0:41,0:..:41	T	C
.	.	##	0	0.0	0.002	0.012	0.99	.	3.22	.	.	SOM	GT:/	0/1:2	0:32,0:..:32	G	A
.	.	.	.	.	.	.	.	.	.	.	.	SOM	GT:/	0/1:1	0:90,0:..:90	G	C
.	.	.	.	.	.	.	.	.	.	.	.	SOM	GT:/	0/1:1	0:97,0:..:97	G	A
.	.	.	.	0.6	0.999	1.0,D	1,D	4.7	4.05	.	.	SOM	GT:/	0/1:1	0:141,1:..:1	C	G
0.001	0	0	0	0.1	0.277	0.881	0.88	4.4	3.43	.	.	SOM	GT:/	0/1:1	0:109,0:..:1	G	A
.	.	.	.	.	.	.	.	3.9	1.92	.	.	SOM	GT:/	0/1:2	0:21,0:..:21	T	C
.	.	.	.	.	.	.	.	.	2.06	.	.	SOM	GT:/	0/1:9	0:29,0:..:29	G	T
.	.	.	.	.	.	.	.	.	.	0.1	0.1	SOM	GT:/	0/1:2	0:8,0:..:8:0.	C	G
.	.	.	.	.	.	.	.	.	.	.	.	SOM	GT:/	0/1:1	0:120,0:..:1	C	A
.	.	.	.	.	.	.	.	.	.	.	.	SOM	GT:/	0/1:1	0:88,0:..:88	A	C
.	.	.	.	.	.	.	.	.	2.69	.	.	SOM	GT:/	0/1:3	0:21,0:..:21	C	T
.	.	.	.	.	.	.	.	.	4.15	.	.	SOM	GT:/	0/1:4	0:30,0:..:30	C	T
.	.	##	0	0.0	0.541	0.948	1,N	.	2.41	.	.	SOM	GT:/	0/1:6	0:55,1:..:56	C	T
2E-04	.	##	0	.	.	.	.	.	.	.	.	SOM	GT:/	0/1:1	0:15,0:..:15	C	T
.	.	.	.	.	.	.	.	.	.	.	.	SOM	GT:/	0/1:9	0:41,0:..:41	C	T
.	.	.	.	.	.	.	.	.	.	.	.	SOM	GT:/	0/1:2	0:9,0:..:9:0.	T	C
.	.	.	.	.	.	.	.	.	.	0	0	SOM	GT:/	0/1:5	0:53,1:..:53	A	G
.	.	.	.	.	.	.	.	.	.	0	0	SOM	GT:/	0/1:6	0:50,1:..:50	G	A
.	.	.	.	.	.	.	.	.	.	.	.	SOM	GT:/	0/1:5	0:23,0:..:24	T	C
2E-04	.	##	0	.	.	.	1.00	.	.	.	.	SOM	GT:/	0/1:1	0:84,0:..:84	C	T
.	.	0	0	.	.	.	.	3.8	.	.	.	SOM	GT:/	0/1:1	0:66,0:..:66	C	T
.	.	##	0	.	.	.	.	.	.	.	.	SOM	GT:/	0/1:1	0:149,0:..:1	G	A
.	.	.	.	.	.	.	.	.	.	.	.	SOM	GT:/	0/1:2	0:13,0:..:13	T	G
.	.	.	.	.	.	.	.	.	.	0	0	SOM	GT:/	0/1:1	0:14,0:..:14	T	G
.	.	.	.	0.0	0.0,B	0.0,B	1,N	.	.	.	.	SOM	GT:/	0/1:8	0:56,0:..:56	G	C
1E-03	0	0	0	.	.	.	.	.	.	0	0	SOM	GT:/	0/1:1	0:162,0:..:1	G	A
.	.	.	.	.	.	.	.	.	.	.	.	SOM	GT:/	0/1:2	0:27,0:..:27	G	T
0.348	.	.	.	.	.	.	.	.	.	0.1	0.2	DB;S	GT:/	0/1:1	0:19,0:..:19	C	G
.	.	.	.	.	.	.	.	.	.	0	.	SOM	GT:/	0/1:1	0:9,0:..:9:0.	T	C
.	.	##	.	.	.	.	.	.	1.49	0	.	SOM	GT:/	0/1:1	0:126,0:..:1	G	A
.	.	##	0	.	.	.	.	.	.	.	.	SOM	GT:/	0/1:2	0:165,1:..:1	G	A
.	.	.	.	.	.	.	.	2.4	.	.	.	SOM	GT:/	0/1:2	0:164,0:..:1	G	A
.	.	.	.	.	.	.	.	4	3.19	.	.	SOM	GT:/	0/1:7	0:38,0:..:38	C	T
.	##	##	0	0.0	0.942	1.0,D	1.00	4.6	1.33	.	.	SOM	GT:/	0/1:1	0:38,0:..:38	G	A
.	.	.	.	.	.	.	.	.	.	.	.	SOM	GT:/	0/1:1	0:95,1:..:96	C	A
0.086	.	.	.	.	.	.	.	.	.	0	0.1	SOM	GT:/	0/1:1	0:11,0:..:10	A	G
.	.	.	.	.	.	.	.	.	.	.	.	SOM	GT:/	0/1:5	0:83,0:..:35	A	G
.	.	0	0	.	.	.	.	.	.	0	0	SOM	GT:/	0/1:4	0:40,1:..:39	G	C
.	.	.	.	.	.	.	.	2.3	.	.	.	SOM	GT:/	0/1:8	0:60,1:..:61	C	G
.	.	.	.	.	.	.	.	.	.	.	.	SOM	GT:/	0/1:3	0:25,0:..:25	G	A
.	.	.	.	.	.	.	.	.	.	.	.	SOM	GT:/	0/1:4	0:30,0:..:30	G	C
.	.	.	.	.	.	.	.	.	.	.	.	SOM	GT:/	0/1:2	0:110,0:..:1	G	A
.	.	.	.	.	.	.	1,A	4.6	8.20	.	.	SOM	GT:/	0/1:1	0:70,0:..:70	C	T
.	.	.	.	0.1	0.007	0.02,I	1,N	.	.	.	.	SOM	GT:/	0/1:9	0:37,0:..:37	A	G
.	.	.	.	.	.	.	.	.	.	.	.	SOM	GT:/	0/1:1	0:112,1:..:1	C	T
.	.	.	.	.	.	.	.	.	.	0	0	SOM	GT:/	0/1:2	0:16,0:..:15	A	G

.	.	.	.	.	.	.	.	.	.	0	.	SOM	GT:	0/1:5	0:36,0:..:37	G	A
.	.	.	.	.	.	.	.	.	.	0.1	0.3	SOM	GT:	0/1:1	0:8,0:..:7:0.	G	T
.	.	.	.	.	.	.	.	.	.	0	0.1	SOM	GT:	0/1:1	0:9,0:..:9:0.	T	A
.	.	.	.	.	.	.	.	.	.	0	.	SOM	GT:	0/1:4	0:54,0:..:53	A	T
.	.	.	.	.	.	.	.	.	.	0.2	0.2	SOM	GT:	0/1:1	0:9,0:..:8:0.	G	T
.	.	0	0	.	.	.	.	.	.	0.2	0.4	DB;S	GT:	0/1:6	0:39,1:..:40	G	A
.	.	.	.	.	.	.	.	.	.	.	.	SOM	GT:	0/1:3	0:40,1:..:34	C	T
.	.	.	.	.	.	.	.	.	.	.	.	SOM	GT:	0/1:7	0:49,0:..:49	G	T
2E-04	.	##	0	.	.	.	.	.	.	.	.	SOM	GT:	0/1:6	0:50,0:..:51	G	A
.	.	.	.	.	.	.	.	3.7	1.32	.	.	SOM	GT:	0/1:2	0:21,0:..:21	G	A
2E-04	.	##	0	.	.	.	.	3.4	2.15	0	0	SOM	GT:	0/1:1	0:110,1:..:1	C	T
.	.	##	0	0.0	1.0,D	1.0,D	1.00	3.1	4.05	0	.	SOM	GT:	0/1:2	0:199,1:..:2	C	T
.	.	.	.	0.0	0.996	0.999	0.99	4.6	3.60	.	.	SOM	GT:	0/1:4	0:274,0:..:2	G	C
.	.	.	.	.	.	.	.	.	.	0	0	SOM	GT:	0/1:2	0:15,0:..:13	T	C
.	.	.	.	.	.	.	.	3.3	.	.	.	SOM	GT:	0/1:4	0:38,0:..:38	A	G
.	##	##	0	0.0	0.12,I	0.086	0.58	.	2.62	.	.	SOM	GT:	0/1:8	0:58,0:..:58	G	A
.	.	0	0	0.4	0.002	0.002	1,N	.	.	0	.	SOM	GT:	0/1:4	0:34,0:..:36	C	T
2E-04	0	0	0	.	.	.	.	2.2	.	.	.	SOM	GT:	0/1:1	0:84,0:..:84	C	T
.	0	0	0	0.6	0.003	0.009	1,N	.	2.04	.	.	SOM	GT:	0/1:1	0:80,0:..:80	G	A
.	.	.	.	.	.	.	1,D	4.1	9.70	.	.	SOM	GT:	0/1:2	0:148,0:..:1	C	T
.	.	.	.	.	.	.	.	.	.	.	.	SOM	GT:	0/1:7	0:47,0:..:47	T	G
.	.	.	.	.	.	.	.	.	.	.	.	SOM	GT:	0/1:9	0:69,1:..:70	T	C
.	.	.	.	.	.	.	.	.	.	.	.	SOM	GT:	0/1:9	0:55,0:..:55	G	A
.	.	.	.	.	.	.	.	.	.	.	.	SOM	GT:	0/1:6	0:47,0:..:48	G	A
.	.	.	.	.	.	.	.	.	.	0.2	0.2	DB;S	GT:	0/1:3	0:26,0:..:23	A	T
.	.	.	.	.	.	.	.	.	.	0	0	SOM	GT:	0/1:1	0:9,0:..:9:0.	T	C
.	.	.	.	.	.	.	.	.	.	.	.	SOM	GT:	0/1:2	0:25,0:..:25	G	A
.	.	##	0	.	.	.	.	.	.	.	.	SOM	GT:	0/1:1	0:92,0:..:93	C	T
.	.	.	.	.	.	.	.	.	.	.	.	SOM	GT:	0/1:5	0:28,0:..:28	C	T
.	.	.	.	.	.	.	.	2.2	1.64	.	.	SOM	GT:	0/1:3	0:16,0:..:16	C	A
.	.	.	.	0.2	0.002	0.005	0.99	4.3	1.98	.	.	SOM	GT:	0/1:2	0:130,1:..:1	G	A
.	.	.	.	.	.	.	.	2.7	.	.	.	SOM	GT:	0/1:6	0:59,0:..:59	G	A
2E-04	.	##	0	.	.	.	.	.	.	0	.	SOM	GT:	0/1:8	0:55,0:..:55	G	A
2E-04	.	.	.	.	.	.	.	2.3	.	0	.	SOM	GT:	0/1:3	0:21,0:..:21	A	T
.	.	.	.	.	.	.	.	.	.	.	.	SOM	GT:	0/1:1	0:146,1:..:1	C	T
.	.	.	.	.	.	.	.	.	.	.	.	SOM	GT:	0/1:7	0:47,0:..:47	C	A
4E-04	0	0	0	.	.	.	.	.	.	.	.	SOM	GT:	0/1:9	0:58,1:..:60	G	A
.	.	.	.	.	.	.	.	.	.	0	0.1	SOM	GT:	0/1:7	0:8,0:..:7:0.	G	A
4E-04	.	##	0	.	.	.	.	.	3.53	0	.	SOM	GT:	0/1:6	0:43,0:..:43	G	A
0.003	.	.	.	.	.	.	.	.	.	.	0	SOM	GT:	0/1:1	0:58,1:..:59	A	T
.	.	.	.	.	.	.	.	5.4	1.35	.	.	SOM	GT:	0/1:2	0:153,0:..:1	G	T
.	##	##	0	.	.	.	.	.	.	0	.	SOM	GT:	0/1:5	0:25,0:..:25	G	A
.	.	.	.	.	.	.	.	.	.	.	.	SOM	GT:	0/1:2	0:251,1:..:2	C	T
.	.	.	.	.	.	.	.	.	.	.	.	SOM	GT:	0/1:9	0:8,0:..:8:0.	G	A
.	.	.	.	0.0	0.996	1.0,D	1,D	5.8	4.25	.	.	SOM	GT:	0/1:8	0:79,0:..:80	C	T
.	.	.	.	0.0	0.999	1.0,D	1,D	5.9	4.98	.	.	SOM	GT:	0/1:1	0:100,1:..:1	G	A
.	.	.	.	.	.	.	.	.	.	0	.	SOM	GT:	0/1:6	0:56,1:..:57	G	A
.	.	.	.	.	.	.	.	.	.	0.1	0.3	DB;S	GT:	0/1:2	0:22,0:..:22	T	C
.	.	.	.	.	.	.	.	.	.	.	.	SOM	GT:	0/1:1	0:11,0:..:11	G	A
.	.	.	.	.	.	.	.	.	.	.	.	SOM	GT:	0/1:1	0:9,0:..:9:0.	C	A
.	.	.	.	0.0	0.544	0.9,P	1.00	2.7	3.35	.	.	SOM	GT:	0/1:1	0:85,0:..:85	G	A
.	.	.	.	.	.	.	.	4.9	4.07	.	.	SOM	GT:	0/1:4	0:25,0:..:25	C	T
.	.	.	.	0.1	0.2,B	0.598	0.99	2.9	2.50	.	.	SOM	GT:	0/1:1	0:74,1:..:76	C	G
.	.	.	.	0.1	0.559	0.831	1,N	.	.	.	.	SOM	GT:	0/1:7	0:65,0:..:66	T	A

.	.	.	.	.	.	.	.	.	.	0	0	SOM	GT:/	0/1:1	0:8,0:.:8:0.	G	A
.	.	.	.	.	.	.	.	.	.	.	.	SOM	GT:/	0/1:7	0:43,0:.:44	G	C
.	.	.	.	.	.	.	.	.	.	.	.	SOM	GT:/	0/1:3	0:28,0:.:28	G	C
.	.	##	0	.	.	.	.	.	.	.	.	SOM	GT:/	0/1:5	0:52,0:.:52	G	A
.	.	.	.	.	.	.	.	.	.	.	.	SOM	GT:/	0/1:9	0:93,0:.:93	T	A
.	.	.	.	.	.	.	.	.	.	.	.	SOM	GT:/	0/1:3	0:20,0:.:20	C	A
.	.	.	.	0.0	1.0,D	1.0,D	1,D	.	4.09	.	.	SOM	GT:/	0/1:8	0:81,1:.:82	T	G
.	.	.	.	.	.	.	.	.	.	.	.	SOM	GT:/	0/1:1	0:55,0:.:56	T	C
.	.	.	.	.	.	.	.	.	.	.	.	SOM	GT:/	0/1:3	0:14,0:.:14	T	C
.	.	.	.	0.0	1.0,D	1.0,D	1,D	5	3.25	.	.	SOM	GT:/	0/1:1	0:63,0:.:63	G	A
.	.	##	0	0.0	0.361	0.517	1,D	5.7	4.40	0	.	DB;S	GT:/	0/1:2	0:19,0:.:19	C	T
8E-04	0	0	0	.	.	.	.	.	.	.	.	DB;S	GT:/	0/1:2	0:154,0:.:1	G	A
.	.	.	.	.	.	.	.	.	.	.	.	SOM	GT:/	0/1:1	0:86,1:.:88	T	A
2E-04	.	.	.	.	.	.	.	.	.	.	.	SOM	GT:/	0/1:1	0:62,0:.:62	G	A
.	.	.	.	.	.	.	.	.	.	.	.	SOM	GT:/	0/1:5	0:33,0:.:33	G	A
.	.	.	.	.	.	.	.	.	1.37	.	.	SOM	GT:/	0/1:4	0:47,1:.:48	C	T
.	.	.	.	.	.	.	.	.	1.50	.	0	SOM	GT:/	0/1:5	0:36,0:.:36	G	A
.	.	.	.	.	.	.	.	.	.	0	.	SOM	GT:/	0/1:3	0:31,0:.:31	G	A
.	.	.	.	0.1	0.687	0.623	0.88	.	2.43	.	.	SOM	GT:/	0/1:1	0:146,1:.:1	A	G
.	.	.	.	0.0	0.67,	0.978	1,D	4.4	3.66	.	.	SOM	GT:/	0/1:1	0:110,0:.:1	C	T
0.146	.	.	.	.	.	.	.	.	.	0	0	SOM	GT:/	0/1:1	0:10,0:.:9:0	A	C
.	.	.	.	.	.	.	.	.	.	0	.	SOM	GT:/	0/1:5	0:41,0:.:34	T	A
.	.	.	.	.	.	.	.	.	.	.	.	SOM	GT:/	0/1:1	0:13,0:.:13	G	A
.	.	.	.	.	.	.	.	2.3	2.10	.	.	SOM	GT:/	0/1:1	0:70,0:.:70	T	C
.	.	.	.	.	.	.	.	.	.	.	.	SOM	GT:/	0/1:1	0:98,1:.:99	C	A
.	.	.	.	.	.	.	.	.	.	0	.	SOM	GT:/	0/1:6	0:37,1:.:37	A	C
.	.	.	.	.	.	.	.	.	.	.	.	SOM	GT:/	0/1:4	0:30,0:.:30	C	A
.	.	.	.	.	.	.	.	2.2	1.33	.	.	SOM	GT:/	0/1:5	0:40,0:.:40	C	T
.	.	.	.	.	.	.	.	.	.	0	.	SOM	GT:/	0/1:1	0:53,0:.:53	C	T
.	.	.	.	.	.	.	.	3	1.38	.	.	SOM	GT:/	0/1:1	0:66,0:.:66	C	A
.	.	.	.	.	.	.	.	.	.	.	.	SOM	GT:/	0/1:1	0:86,0:.:86	C	G
.	.	.	.	.	.	.	.	.	.	.	.	SOM	GT:/	0/1:1	0:18,0:.:18	T	A
.	.	.	.	.	.	.	.	.	.	.	.	SOM	GT:/	0/1:3	0:13,0:.:13	T	C
.	.	.	.	.	.	.	.	.	.	.	.	SOM	GT:/	0/1:2	0:9,0:.:10:0	A	T
.	.	##	0	0.7	0.001	0.0,B	0.98	.	.	.	.	SOM	GT:/	0/1:2	0:103,0:.:1	C	T
.	.	.	.	0.0	0.996	1.0,D	1.00	4.9	3.96	.	.	SOM	GT:/	0/1:9	0:55,0:.:55	A	T
.	.	.	.	.	.	.	.	.	.	.	.	SOM	GT:/	0/1:2	0:19,0:.:19	G	A
.	.	.	.	.	.	.	.	.	1.73	.	.	SOM	GT:/	0/1:1	0:75,0:.:75	C	T
.	.	.	.	.	.	.	.	.	.	.	.	SOM	GT:/	0/1:1	0:72,0:.:72	G	A
.	.	.	.	.	.	.	.	.	.	.	.	SOM	GT:/	0/1:4	0:29,0:.:29	G	A
.	.	##	0	.	.	.	.	.	.	.	.	SOM	GT:/	0/1:1	0:125,1:.:1	G	A
.	.	.	.	0.0	0.999	1.0,D	1.00	4	5.08	.	.	SOM	GT:/	0/1:1	0:150,1:.:1	G	A
2E-04	0	##	0	.	.	.	.	.	.	.	.	SOM	GT:/	0/1:1	0:52,0:.:52	G	A
.	.	.	.	0.0	0.011	0.014	1.00	.	3.33	.	.	SOM	GT:/	0/1:8	0:56,0:.:56	G	T
.	.	.	.	.	.	.	.	.	.	.	.	SOM	GT:/	0/1:3	0:26,0:.:26	C	T
.	.	.	.	0.0	0.462	0.93,	1.00	.	3.58	.	.	SOM	GT:/	0/1:2	0:19,0:.:19	T	G
.	.	.	.	.	.	.	.	.	.	.	.	SOM	GT:/	0/1:1	0:125,0:.:1	G	A
.	.	.	.	.	.	.	.	.	.	.	.	SOM	GT:/	0/1:1	0:72,0:.:72	C	G
.	.	.	.	.	.	.	.	.	.	.	.	SOM	GT:/	0/1:5	0:373,0:.:3	G	A
.	.	.	.	.	0.937	0.999	1,N	2.4	1.61	.	.	SOM	GT:/	0/1:1	0:110,1:.:1	C	T
3E-04	.	0	0	.	.	.	.	.	1.38	.	0	SOM	GT:/	0/1:9	0:65,1:.:66	C	T
.	.	.	.	.	.	.	.	.	.	.	.	SOM	GT:/	0/1:1	0:104,0:.:1	C	T
.	.	##	0	.	.	.	.	.	.	.	.	SOM	GT:/	0/1:1	0:52,0:.:52	C	T
.	.	.	.	.	.	.	.	.	.	.	.	SOM	GT:/	0/1:4	0:23,0:.:23	C	A

5E-04	.	0	0	0.0	0.998	1.0,D	1,D	4.8	3.91	.	.	SOM	GT:/	0/1:2	0:100,0:..:1	G	A
.	.	.	.	.	.	.	.	.	.	.	.	SOM	GT:/	0/1:2	0:15,0:..:15	A	G
.	.	.	.	.	.	.	.	.	.	.	.	SOM	GT:/	0:39	0/1:32,4:37	C	T
.	.	.	.	.	.	.	.	.	.	0.3	0.4	DB;S	GT:/	0:34	0/1:83,16:3	T	C
.	.	.	.	.	.	.	.	.	.	0	.	SOM	GT:/	0:86	0/1:81,11:3	C	T
.	##	##	0	0.0	0.999	1.0,D	1.00	2.9	3.92	.	.	SOM	GT:/	0:35	0/1:85,32:3	G	A
.	.	.	.	.	.	.	1,A	5.9	5.29	.	.	SOM	GT:/	0:30	0/1:63,13:3	G	T
.	.	.	.	.	.	.	.	.	.	0.1	0.1	DB;S	GT:/	0:33	0/1:73,8:37	T	C
.	.	.	.	.	.	.	.	.	.	0	0.1	SOM	GT:/	0:8,0	0/1:9,6:37:	A	G
.	.	.	.	.	.	.	.	.	.	0	0	SOM	GT:/	0:8,0	0/1:22,8:37	A	G
.	.	0	0	.	.	.	.	.	.	0	0	SOM	GT:/	0:19	0/1:29,7:35	C	T
0.245	.	0	0	.	.	.	.	.	.	0.1	0	SOM	GT:/	0:35	0/1:38,11:3	C	T
.	.	.	.	.	.	.	.	.	.	0	.	SOM	GT:/	0:42	0/1:107,38	A	G
.	##	##	0	.	.	.	.	.	.	.	.	SOM	GT:/	0:12	0/1:122,18	C	T
.	.	.	.	.	.	.	.	.	.	.	.	SOM	GT:/	0:95	0/1:99,12:3	G	C
.	.	.	.	0.0	0.915	0.992	1,D	3.6	3.92	.	.	SOM	GT:/	0:12	0/1:138,18	C	G
.	.	.	.	0.0	1.0,D	1.0,D	1,D	4.4	4.19	.	.	SOM	GT:/	0:78	0/1:282,65	G	A
.	.	0	0	.	.	.	.	.	.	.	.	SOM	GT:/	0:13	0/1:93,15:3	G	A
.	.	.	.	.	.	.	.	.	.	.	.	SOM	GT:/	0:41	0/1:28,7:37	G	A
.	.	.	.	.	.	.	.	.	2.12	.	.	SOM	GT:/	0:37	0/1:68,13:3	C	A
.	.	.	.	.	.	.	.	.	.	.	.	SOM	GT:/	0:11	0/1:26,4:37	G	T
.	.	.	.	.	.	.	.	.	.	.	.	SOM	GT:/	0:34	0/1:57,20:3	G	A
.	.	.	.	.	.	.	.	.	.	.	.	SOM	GT:/	0:37	0/1:31,4:37	A	G
.	.	.	.	.	.	.	.	.	.	0	.	DB;S	GT:/	0:55	0/1:38,6:37	A	G
.	.	.	.	.	.	.	.	.	.	.	.	SOM	GT:/	0:53	0/1:32,10:3	A	C
.	##	##	0	.	.	.	.	.	1.89	.	.	SOM	GT:/	0:98	0/1:45,7:37	G	A
.	.	.	.	.	.	.	.	.	2.87	.	.	SOM	GT:/	0:49	0/1:46,8:37	G	T
.	.	.	.	0.0	0.452	0.9,P	1,D	6.2	4.76	.	.	SOM	GT:/	0:12	0/1:89,20:3	G	A
.	.	.	.	.	.	.	.	5.7	.	.	.	SOM	GT:/	0:20	0/1:249,51	G	A
.	.	.	.	0.0	0.87,	0.998	1.00	5.7	1.95	.	.	SOM	GT:/	0:20	0/1:250,51	G	T
.	.	.	.	.	.	.	.	.	.	.	.	SOM	GT:/	0:91	0/1:193,29	C	T
.	.	.	.	.	.	.	.	.	.	.	.	SOM	GT:/	0:10	0/1:150,42	T	C
2E-04	.	.	.	.	.	.	.	.	.	.	.	SOM	GT:/	0:52	0/1:85,17:3	T	C
.	.	.	.	.	.	.	.	.	.	0.2	0.2	DB;S	GT:/	0:36	0/1:29,4:37	C	T
2E-04	.	.	.	.	.	.	.	.	.	0	0	SOM	GT:/	0:8,0	0/1:13,13:3	C	T
.	.	.	.	0.5	0.0,B	0.0,B	0.99	2.9	.	.	.	SOM	GT:/	0:55	0/1:86,13:3	G	A
.	.	.	.	.	.	.	.	2.2	.	.	.	SOM	GT:/	0:33	0/1:70,7:35	A	G
.	##	##	0	0.2	0.97,	0.999	1.00	5.7	4.26	.	.	SOM	GT:/	0:52	0/1:117,20	C	T
.	.	.	.	0.0	1.0,D	1.0,D	1,D	5.8	4.93	.	.	SOM	GT:/	0:33	0/1:59,11:3	C	T
.	.	.	.	.	.	.	.	.	.	.	.	SOM	GT:/	0:39	0/1:23,5:37	A	G
.	.	.	.	.	.	.	.	2.5	.	.	.	SOM	GT:/	0:34	0/1:37,5:37	G	C
.	.	.	.	.	.	.	.	.	.	.	.	SOM	GT:/	0:25	0/1:15,4:37	C	A
.	.	.	.	.	.	.	.	.	.	.	.	SOM	GT:/	0:31	0/1:17,12:3	C	G
.	.	.	.	.	.	.	.	.	.	.	.	SOM	GT:/	0:8,0	0/1:13,10:3	C	G
.	.	.	.	.	.	.	.	4.9	4.04	.	.	SOM	GT:/	0:19	0/1:42,38:3	C	T
.	.	.	.	.	.	.	.	.	.	.	.	SOM	GT:/	0:34	0/1:25,19:3	A	T
.	.	.	.	.	.	.	.	.	.	0.1	0	SOM	GT:/	0:8,0	0/1:11,10:3	G	C
.	.	.	.	.	.	.	.	.	.	.	.	SOM	GT:/	0:12	0/1:21,5:31	G	C
.	.	.	.	.	.	.	.	.	.	.	.	SOM	GT:/	0:17	0/1:39,8:32	C	G
.	.	.	.	.	.	.	.	.	.	.	.	SOM	GT:/	0:11	0/1:64,15:3	C	A
.	.	.	.	0.1	0.431	0.876	1.00	5.7	2.17	.	.	SOM	GT:/	0:21	0/1:174,27	T	C
2E-04	.	.	.	.	.	.	.	.	.	.	.	SOM	GT:/	0:54	0/1:78,22:3	G	A
.	.	.	.	.	.	.	.	2.3	.	.	.	SOM	GT:/	0:23	0/1:50,8:37	T	G
.	.	.	.	.	.	.	.	4.6	1.39	.	.	SOM	GT:/	0:10	0/1:81,15:3	G	A

.	.	.	.	.	.	.	.	.	.	.	.	SON	GT:/	0:26	0/1:61,17:3	T	A
.	.	.	.	0.0(	0.992	1.0,D	1,D	4.7	4.84	.	.	SON	GT:/	0:15	0/1:173,45	G	C
2E-04	.	.	.	.	.	.	.	.	.	0	.	SON	GT:/	0:16	0/1:38,8:37	C	T
.	.	.	.	.	.	.	.	5	.	.	.	SON	GT:/	0:27	0/1:85,24:3	G	A
.	.	.	.	.	.	.	.	.	.	.	.	SON	GT:/	0:43	0/1:65,18:3	G	A
.	.	.	.	0.0(	0.992	1.0,D	1,D	3.8	5.83	.	.	SON	GT:/	0:11	0/1:223,24	G	C
2E-04	.	.	.	.	.	.	.	.	.	.	.	SON	GT:/	0:21	0/1:40,13:3	T	C
.	.	.	.	.	.	.	.	.	.	.	.	SON	GT:/	0:36	0/1:41,8:34	G	C
.	.	.	.	.	.	.	.	.	.	.	.	SON	GT:/	0:14	0/1:95,34:3	C	T
.	.	.	.	.	0.052	0.509	0.93	5.8	2.26	.	.	SON	GT:/	0:59	0/1:185,30	C	T
.	.	.	.	0.0:	0.225	0.957	1,D	5.9	5.10	.	.	SON	GT:/	0:21	0/1:453,10	C	T
.	.	.	.	0.0(	1.0,D	1.0,D	1.00	5.7	5.01	.	.	SON	GT:/	0:14	0/1:92,15:3	C	T
.	.	.	.	.	.	.	.	.	.	0	0.1	SON	GT:/	0:8,(	0/1:33,5:37	G	A
.	##	##	0	.	.	.	.	4.5	2.74	.	.	SON	GT:/	0:82	0/1:81,17:3	C	T
.	.	.	.	.	.	.	.	.	.	.	.	SON	GT:/	0:10	0/1:219,40	G	A
.	##	##	0	0.0:	0.951	1.0,D	1.00	5.6	4.24	.	.	SON	GT:/	0:19	0/1:267,45	C	T
.	.	.	.	.	.	.	.	.	.	.	.	SON	GT:/	0:29	0/1:11,7:37	C	T
.	.	.	.	.	.	.	.	4.6	.	.	.	SON	GT:/	0:15	0/1:37,7:35	A	T
.	.	.	.	.	.	.	.	.	.	.	.	SON	GT:/	0:17	0/1:23,6:24	A	G
.	.	.	.	.	.	.	1,A	5.5	6.00	.	.	SON	GT:/	0:28	0/1:44,13:3	T	A
.	.	.	.	.	.	.	.	.	.	.	.	SON	GT:/	0:30	0/1:34,5:37	T	G
.	.	.	.	.	.	.	.	.	.	0	.	SON	GT:/	0:9,(	0/1:20,5:22	A	C
.	.	.	.	.	.	.	.	.	.	0	.	SON	GT:/	0:25	0/1:25,6:20	A	C
.	.	.	.	.	.	.	.	.	.	.	.	SON	GT:/	0:19	0/1:25,10:3	C	T
.	.	.	.	.	.	.	.	.	.	.	.	SON	GT:/	0:31	0/1:24,8:33	T	A
.	.	.	.	.	.	.	.	.	.	.	.	SON	GT:/	0:9,(	0/1:6,5:37:	C	T
.	##	##	0	.	.	.	.	.	.	.	.	SON	GT:/	0:58	0/1:49,7:37	C	T
.	.	.	.	.	.	.	.	.	.	.	.	SON	GT:/	0:21	0/1:18,7:35	C	T
.	.	.	.	.	.	.	1,D	5.6	6.86	.	.	SON	GT:/	0:38	0/1:80,21:3	G	A
.	.	.	.	.	.	.	.	.	.	.	.	SON	GT:/	0:59	0/1:36,10:3	G	A
.	.	.	.	.	.	.	.	.	.	.	.	SON	GT:/	0:76	0/1:40,8:37	A	C
.	.	.	.	0.0:	0.998	1.0,D	0.95	4.9	3.23	.	.	SON	GT:/	0:40	0/1:88,27:3	G	C
.	.	.	.	.	.	.	.	.	.	.	.	SON	GT:/	0:42	0/1:29,7:35	T	C
.	.	.	.	.	.	.	.	.	1.39	.	.	SON	GT:/	0:17	0/1:23,4:37	G	A
2E-04	.	##	0	0.0(	0.754	0.988	1,D	2.5	3.43	0	.	SON	GT:/	0:59	0/1:166,43	C	T
.	.	##	0	.	.	.	.	.	.	.	.	SON	GT:/	0:29	0/1:57,12:3	C	T
.	.	.	.	0.3	0.079	0.059	0.99	4.9	1.32	.	.	SON	GT:/	0:63	0/1:34,10:3	T	C
.	.	0	0	.	.	.	.	4	1.94	0.1	0	DB;S	GT:/	0:43	0/1:38,4:37	A	G
2E-04	.	.	.	.	.	.	.	.	.	0	.	SON	GT:/	0:57	0/1:47,6:37	G	A
.	.	.	.	0.1	0.444	0.392	0.86	3.2	2.29	.	.	SON	GT:/	0:15	0/1:315,41	C	G
.	.	##	0	0.0	0.978	0.999	1,D	5.8	4.07	0	.	SON	GT:/	0:40	0/1:71,8:35	C	T
.	.	0	0	.	.	.	.	.	1.62	.	.	SON	GT:/	0:16	0/1:30,4:37	A	C
.	.	0	0	.	.	.	.	.	.	0	.	SON	GT:/	0:43	0/1:101,18	C	G
.	.	.	.	.	.	.	.	.	.	.	.	SON	GT:/	0:18	0/1:123,29	G	A
.	.	.	.	.	.	.	.	.	.	.	.	SON	GT:/	0:15	0/1:287,60	C	T
0.04	.	.	.	.	.	.	.	.	.	0	.	SON	GT:/	0:39	0/1:66,7:37	T	A
.	.	.	.	.	.	.	.	.	.	0	0	SON	GT:/	0:38	0/1:70,8:37	A	G
.	.	.	.	0.1	0.811	0.996	0.99	2.8	2.64	.	.	SON	GT:/	0:75	0/1:179,31	C	T
.	.	.	.	.	.	.	.	.	.	.	0	SON	GT:/	0:37	0/1:39,4:37	A	T
.	.	##	0	.	.	.	.	.	.	.	.	SON	GT:/	0:78	0/1:217,38	C	T
4E-04	##	##	0	.	.	.	.	.	.	0	0	SON	GT:/	0:10	0/1:63,11:3	G	A
.	.	.	.	.	.	.	.	.	1.69	.	.	SON	GT:/	0:39	0/1:42,10:3	T	C
.	.	.	.	.	.	.	.	.	.	0	.	SON	GT:/	0:90	0/1:59,13:3	C	T
.	.	.	.	.	.	.	.	2	1.99	.	.	SON	GT:/	0:19	0/1:24,4:37	C	G

.	.	.	.	.	.	.	.	.	.	.	.	SOM	GT:/	0:11	0/1:140,32	G	A
.	.	0	0	.	.	.	.	.	.	.	.	SOM	GT:/	0:14	0/1:33,4:37	T	C
.	.	.	.	.	.	.	.	.	.	.	.	SOM	GT:/	0:34	0/1:73,18:3	C	T
4E-04	0	##	0	.	.	.	.	.	.	.	.	DB;S	GT:/	0:21	0/1:442,88	C	T
.	.	.	.	.	.	.	.	.	.	.	.	SOM	GT:/	0:11	0/1:282,54	G	A
.	.	##	0	.	.	.	.	.	.	.	.	SOM	GT:/	0:19	0/1:314,13	G	A
.	.	.	.	.	.	.	.	.	.	.	.	SOM	GT:/	0:14	0/1:27,4:37	C	T
2E-04	##	##	0	0.7	0.058	0.596	1,N	.	.	.	.	SOM	GT:/	0:25	0/1:102,12	C	T
.	.	.	.	.	.	.	.	.	.	.	.	SOM	GT:/	0:45	0/1:50,19:3	C	G
.	.	.	.	.	.	.	.	.	.	.	.	SOM	GT:/	0:44	0/1:37,12:3	C	G
.	.	.	.	.	.	.	.	.	.	.	.	SOM	GT:/	0:49	0/1:56,15:3	C	T
.	.	.	.	.	.	.	.	.	.	.	.	SOM	GT:/	0:47	0/1:49,11:3	C	T
.	.	.	.	.	.	.	.	.	.	.	.	SOM	GT:/	0:28	0/1:20,4:37	A	C
2E-04	.	.	.	.	.	.	.	3.5	2.39	0	.	SOM	GT:/	0:65	0/1:109,29	C	T
.	.	.	.	.	.	.	.	3.8	2.10	.	.	SOM	GT:/	0:44	0/1:91,21:3	G	A
.	.	.	.	.	.	.	.	.	.	.	.	SOM	GT:/	0:66	0/1:93,18:3	G	A
.	.	.	.	.	.	.	.	.	.	.	.	SOM	GT:/	0:62	0/1:90,16:3	G	T
.	.	.	.	.	.	.	.	.	.	.	.	SOM	GT:/	0:8,(	0/1:11,4:37	C	A
.	.	.	.	.	0.969	1.0,D	1,D	4.3	3.43	.	.	SOM	GT:/	0:36	0/1:60,24:2	C	T
.	.	.	.	0.1	0.018	0.018	1,N	.	1.37	.	.	SOM	GT:/	0:60	0/1:104,25	A	C
.	.	.	.	.	.	.	.	.	.	.	.	SOM	GT:/	0:20	0/1:26,4:34	C	A
.	.	.	.	.	.	.	.	.	.	.	.	SOM	GT:/	0:35	0/1:18,6:37	G	C
.	.	.	.	.	.	.	1,D	4.7	11.6	.	.	SOM	GT:/	0:83	0/1:143,24	G	A
.	.	.	.	0.0	0.723	0.977	1,N	5.1	3.83	.	.	SOM	GT:/	0:92	0/1:71,20:3	G	A
.	.	.	.	.	.	.	1,D	.	.	.	.	SOM	GT:/	0:11	0/1:256,35	C	T
.	.	.	.	.	.	.	.	.	.	.	.	SOM	GT:/	0:36	0/1:36,4:37	C	A
.	.	.	.	.	.	.	.	.	.	.	.	SOM	GT:/	0:52	0/1:29,6:35	C	A
.	.	0	0	0.0	0.931	0.999	1.00	3	2.89	.	0	SOM	GT:/	0:96	0/1:79,17:3	C	T
.	.	.	.	.	.	.	.	.	.	0	.	SOM	GT:/	0:9,(	0/1:62,14:3	G	A
.	.	.	.	.	.	.	.	.	.	0.2	0	SOM	GT:/	0:37	0/1:70,9:37	A	G
.	.	.	.	.	.	.	.	.	.	0	0	SOM	GT:/	0:12	0/1:25,9:37	A	C
0.004	.	.	.	.	.	.	.	.	.	.	.	DB;S	GT:/	0:30	0/1:78,17:3	C	T
.	.	.	.	.	.	.	.	.	.	0.4	0.4	DB;S	GT:/	0:52	0/1:105,12	A	G
.	.	.	.	.	.	.	1,A	3.1	4.59	.	.	SOM	GT:/	0:17	0/1:245,63	C	T
.	.	.	.	0.1	0.036	0.104	0.94	5.4	2.06	.	.	SOM	GT:/	0:83	0/1:85,21:3	C	T
.	.	.	.	0.0	1.0,D	1.0,D	0.96	3.8	2.70	.	.	SOM	GT:/	0:44	0/1:72,18:3	G	T
.	.	.	.	.	.	.	.	.	.	.	.	SOM	GT:/	0:16	0/1:29,9:37	T	A
0.437	.	.	.	.	.	.	.	.	.	0.5	0.5	DB;S	GT:/	0:53	0/1:16,7:37	T	C
.	.	.	.	.	.	.	.	.	.	.	.	SOM	GT:/	0:39	0/1:11,38:3	T	G
.	.	.	.	.	.	.	.	3	.	0	.	SOM	GT:/	0:11	0/1:237,77	G	A
0.004	.	0	0	.	.	.	.	.	.	.	.	SOM	GT:/	0:15	0/1:252,22	C	T
.	.	.	.	.	.	.	.	4.6	.	.	.	SOM	GT:/	0:15	0/1:40,7:37	T	A
.	.	##	0	.	.	.	.	.	.	.	.	SOM	GT:/	0:22	0/1:61,16:3	C	T
.	.	.	.	.	.	.	.	5.6	.	.	.	SOM	GT:/	0:23	0/1:39,16:3	C	T
0.317	.	.	.	.	.	.	.	.	.	0	0	SOM	GT:/	0:51	0/1:86,10:3	A	T
.	.	.	.	.	.	.	.	.	.	0.1	0.1	SOM	GT:/	0:22	0/1:28,5:34	A	C
.	.	.	.	.	.	.	.	.	.	.	.	SOM	GT:/	0:94	0/1:123,96	C	A
.	.	.	.	.	.	.	.	.	.	.	.	SOM	GT:/	0:11	0/1:25,4:37	C	T
.	.	.	.	.	.	.	.	.	.	.	.	SOM	GT:/	0:50	0/1:35,21:3	C	T
.	.	.	.	.	.	.	.	.	.	.	.	SOM	GT:/	0:67	0/1:84,60:3	T	G
.	.	.	.	.	.	.	.	.	.	.	.	SOM	GT:/	0:11	0/1:12,8:35	G	T
.	.	.	.	.	.	.	.	.	.	.	.	SOM	GT:/	0:26	0/1:25,22:3	A	T
.	.	.	.	0.0	0.857	0.993	1.00	5.4	4.17	.	.	SOM	GT:/	0:36	0/1:28,21:3	G	A
.	.	.	.	.	.	.	.	.	.	.	.	SOM	GT:/	0:54	0/1:68,38:3	G	A

.	.	.	.	0.0(	0.988	1.0,D	1.00	5.1	3.49	.	.	SON	GT:/	0:22	0/1:341,19	C	T
.	.	.	.	.	.	.	.	.	.	.	.	SON	GT:/	0:22	0/1:341,19	C	T
.	.	.	.	0.0(	0.999	1.0,D	1.00	3.1	3.33	.	.	SON	GT:/	0:48	0/1:43,29:3	C	A
.	.	.	.	0.1'	0.003	0.002	.	2.1	1.30	.	.	SON	GT:/	0:56	0/1:59,25:3	G	T
.	.	.	.	.	.	.	.	.	.	.	.	SON	GT:/	0:37	0/1:35,14:3	G	A
.	.	.	.	.	.	.	.	.	.	0	0.1	SON	GT:/	0:9,(	0/1:17,9:37	T	C
.	.	.	.	0.0	0.985	1.0,D	1,D	5.5	4.92	.	.	SON	GT:/	0:95	0/1:179,16	A	T
.	.	.	.	.	.	.	.	.	.	0	0.1	SON	GT:/	0:21	0/1:34,6:37	G	T
0.057	.	.	.	.	.	.	.	.	.	0	0	DB;S	GT:/	0:45	0/1:16,17:2	G	T
.	.	.	.	.	.	.	.	.	1.59	.	.	SON	GT:/	0:12	0/1:8,5:29:	A	T
.	.	.	.	.	.	.	.	.	.	.	.	SON	GT:/	0:46	0/1:34,37:3	G	A
.	.	.	.	.	.	.	.	3.5	2.23	.	.	SON	GT:/	0:28	0/1:35,28:3	C	A
.	.	.	.	.	.	.	.	.	.	.	.	SON	GT:/	0:68	0/1:56,28:3	C	T
.	.	.	.	.	.	.	.	.	.	.	.	SON	GT:/	0:43	0/1:25,13:3	C	G
2E-04	.	.	.	.	.	.	.	.	.	0	0	SON	GT:/	0:12	0/1:11,6:37	G	A
.	.	.	.	.	.	.	.	4.7	3.12	.	.	SON	GT:/	0:17	0/1:185,13	G	A
.	.	.	.	.	.	.	.	.	.	.	.	SON	GT:/	0:8,(	0/1:11,6:35	C	G
.	.	.	.	.	.	.	.	.	.	.	.	SON	GT:/	0:12	0/1:129,11	C	G
.	0	##	0	.	.	.	.	.	.	.	.	SON	GT:/	0:96	0/1:181,44	C	T
.	.	.	.	.	.	.	.	.	2.91	.	.	SON	GT:/	0:80	0/1:93,69:3	T	C
.	.	.	.	0.4!	0.005	0.002	1,D	4.7	1.64	.	.	SON	GT:/	0:60	0/1:66,43:3	A	G
.	.	.	.	.	.	.	.	.	1.87	.	.	SON	GT:/	0:31	0/1:42,9:35	T	G
.	.	.	.	.	.	.	.	.	1.92	.	.	SON	GT:/	0:15	0/1:29,16:3	G	A
0.004	0	0	0	.	.	.	.	4.6	.	0	.	SON	GT:/	0:24	0/1:415,20	C	T
.	.	##	0	0.0(	0.909	0.991	1,D	5.8	5.70	.	.	DB;S	GT:/	0:28	0/1:33,92:3	G	A
.	.	.	.	.	.	.	.	.	.	.	.	SON	GT:/	0:79	0/1:128,27	T	G
.	.	.	.	.	.	.	.	.	.	.	0	DB;S	GT:/	0:55	0/1:94,60:3	C	T
.	.	.	.	.	.	.	.	4.1	.	.	.	SON	GT:/	0:30	0/1:35,33:3	C	T
.	.	.	.	.	.	.	.	.	.	0	.	SON	GT:/	0:63	0/1:77,40:3	G	A
.	.	.	.	.	.	.	.	.	.	.	.	SON	GT:/	0:19	0/1:16,11:3	G	T
.	.	.	.	.	.	.	.	.	.	.	.	SON	GT:/	0:15	0/1:193,56	G	C
.	.	.	.	.	.	.	.	.	.	.	.	SON	GT:/	0:12	0/1:22,5:37	C	G
.	.	.	.	.	.	.	.	.	.	.	.	SON	GT:/	0:76	0/1:53,31:3	C	T
.	.	.	.	.	.	.	1,D	5.3	4.66	.	.	SON	GT:/	0:16	0/1:28,18:3	G	A
.	.	.	.	.	.	.	.	.	1.74	.	.	SON	GT:/	0:91	0/1:77,44:3	G	A
.	.	.	.	.	.	.	.	.	.	.	.	SON	GT:/	0:17	0/1:23,4:37	A	T
.	.	##	0	0.0	0.999	1.0,D	1,N	3.2	1.65	.	.	SON	GT:/	0:25	0/1:278,17	C	T
.	.	.	.	.	.	.	.	.	.	.	.	SON	GT:/	0:68	0/1:72,39:3	C	T
.	.	.	.	.	.	.	1,A	.	5.99	.	.	SON	GT:/	0:79	0/1:65,72:3	G	A
.	##	##	0	0.0(	0.953	1.0,D	0.87	5	3.10	.	.	SON	GT:/	0:12	0/1:176,11	C	T
.	.	.	.	.	.	.	.	.	.	.	.	SON	GT:/	0:20	0/1:18,18:3	C	T
.	.	0	0	0.0:	0.873	0.997	0.99	5.5	3.85	.	.	SON	GT:/	0:27	0/1:39,21:3	T	G
.	##	##	0	.	.	.	.	.	.	.	.	SON	GT:/	0:33	0/1:49,30:3	C	T
.	.	.	.	.	.	.	.	.	.	.	.	SON	GT:/	0:73	0/1:72,44:3	G	A
.	.	.	.	.	.	.	.	6	4.00	.	.	SON	GT:/	0:62	0/1:68,48:3	G	A
.	0	0	0	0.0(	1.0,D	1.0,D	1.00	6	5.21	.	.	SON	GT:/	0:23	0/1:247,61	G	A
.	.	.	.	.	.	.	.	.	.	0.1	0.1	SON	GT:/	0:12	0/1:5,7:37:	G	C
.	.	##	0	.	.	.	.	.	.	.	.	SON	GT:/	0:30	0/1:40,21:3	A	G
.	.	.	.	0.3(	0.035	0.006	0.99	.	1.83	.	.	SON	GT:/	0:12	0/1:148,12	T	C
.	.	.	.	.	.	.	.	.	.	0.1	0	SON	GT:/	0:13	0/1:20,6:37	G	A
.	.	.	.	.	.	.	.	.	.	0	0	SON	GT:/	0:9,(	0/1:29,6:33	C	T
.	.	.	.	.	.	.	.	.	.	.	.	SON	GT:/	0:20	0/1:15,16:3	C	G
.	.	.	.	.	.	.	.	.	.	.	.	SON	GT:/	0:86	0/1:95,49:3	C	T
.	.	0	0	0.1:	.	.	1,N	.	.	0	.	SON	GT:/	0:25	0/1:488,15	G	A

.	.	.	.	.	.	.	.	.	.	.	.	SON	GT:/	0:10	0/1:23,13:3	A	C
.	0	0	0	.	.	.	.	2.3	1.34	0	0	SON	GT:/	0:89	0/1:153,17	A	T
.	.	.	.	.	.	.	.	.	.	.	.	SON	GT:/	0:16	0/1:222,14	G	A
.	.	.	.	.	.	.	.	.	.	.	.	SON	GT:/	0:23	0/1:30,19:3	G	A
.	.	0	0	.	.	.	.	.	.	0	0.2	SON	GT:/	0:15	0/1:27,4:37	C	A
.	.	.	.	.	.	.	.	.	.	0	.	SON	GT:/	0:13	0/1:22,14:1	A	C
.	.	.	.	.	.	.	.	.	.	.	.	SON	GT:/	0:16	0/1:37,4:37	T	C
.	.	.	.	0.0(	0.997	1.0,D	1,D	5.3	5.19	.	.	SON	GT:/	0:15	0/1:26,21:3	G	A
2E-04	.	.	.	.	.	.	.	.	.	.	.	SON	GT:/	0:9,(	0/1:7,6:35:	G	A
0.002	.	.	.	.	.	.	.	.	.	.	0	SON	GT:/	0:11	0/1:16,20:3	A	T
.	##	##	0	0.0(	0.998	1.0,D	1.00	4.8	4.42	.	.	SON	GT:/	0:61	0/1:67,7:37	C	T
.	.	.	.	.	.	.	.	.	.	.	.	SON	GT:/	0:23	0/1:9,17:34	G	A
.	.	.	.	.	1.0,D	1.0,D	.	4.4	1.95	.	.	SON	GT:/	0:58	0/1:25,67:3	G	A
.	.	.	.	.	.	.	.	5.6	.	.	.	SON	GT:/	0:21	0/1:39,18:3	G	T
.	.	.	.	.	.	.	.	.	1.54	.	.	SON	GT:/	0:8,(	0/1:13,14:3	T	C
.	.	.	.	.	.	.	.	.	.	0	.	SON	GT:/	0:10	0/1:5,6:37:	G	T
.	.	##	0	.	.	.	.	.	.	.	.	SON	GT:/	0:37	0/1:38,11:3	G	A
.	.	.	.	0.4(	0.836	0.826	1,D	6.1	3.43	.	.	SON	GT:/	0:17	0/1:29,13:3	A	G
2E-04	.	.	.	.	.	.	.	.	.	.	.	SON	GT:/	0:27	0/1:32,32:3	G	A
.	.	.	.	0.0·	0.017	0.002	1,D	6	4.31	.	.	SON	GT:/	0:45	0/1:26,18:3	G	T
.	.	.	.	.	.	.	.	.	.	0.1	0.3	DB;S	GT:/	0:26	0/1:9,4:37:	G	A
.	.	.	.	.	.	.	.	.	.	.	.	SON	GT:/	0:12	0/1:172,11	C	T
.	.	.	.	.	.	.	.	.	1.27	.	.	SON	GT:/	0:28	0/1:38,13:3	G	C
.	.	.	.	.	.	.	.	.	.	.	.	SON	GT:/	0:11	0/1:11,9:37	G	A
.	.	##	0	.	.	.	.	.	.	.	0	SON	GT:/	0:90	0/1:142,86	G	A
.	.	.	.	.	.	.	.	2.4	.	.	.	SON	GT:/	0:41	0/1:30,23:3	C	T
.	.	.	.	.	.	.	.	.	.	.	.	SON	GT:/	0:68	0/1:47,48:3	G	A
.	.	.	.	.	.	.	.	.	.	0	.	SON	GT:/	0:23	0/1:19,5:37	T	A
.	.	.	.	.	.	.	.	.	.	.	.	SON	GT:/	0:8,(	0/1:11,8:37	C	T
.	.	.	.	.	.	.	.	.	.	0	0	SON	GT:/	0:19	0/1:8,5:37:	G	T
.	.	.	.	.	.	.	.	.	.	0	.	SON	GT:/	0:8,(	0/1:28,6:13	A	T
.	.	.	.	.	0.999	1.0,D	.	3.3	2.24	.	.	SON	GT:/	0:11	0/1:25,29:3	G	A
.	.	.	.	.	.	.	.	.	.	0	.	SON	GT:/	0:46	0/1:58,6:37	C	A
.	.	.	.	.	.	.	.	3.8	1.25	.	.	SON	GT:/	0:30	0/1:59,6:37	A	T
.	.	.	.	.	.	.	.	2.3	1.46	.	.	SON	GT:/	0:68	0/1:38,35:3	G	C
.	.	##	.	.	.	.	.	.	1.76	.	0	SON	GT:/	0:31	0/1:23,20:3	A	G
.	.	.	.	0.0(	0.597	0.594	0.99	4.3	3.78	.	.	SON	GT:/	0:39	0/1:96,16:3	G	A
.	.	.	.	.	.	.	.	.	.	.	.	SON	GT:/	0:31	0/1:18,19:3	C	G
.	.	.	.	.	.	.	.	2.7	.	.	.	SON	GT:/	0:51	0/1:50,41:3	G	A
.	.	.	.	.	.	.	.	.	.	.	.	SON	GT:/	0:20	0/1:19,10:3	C	G
.	.	.	.	.	.	.	.	.	.	.	.	SON	GT:/	0:9,(	0/1:9,8:37:	C	A
.	.	.	.	.	.	.	.	.	.	.	.	SON	GT:/	0:44	0/1:53,98:3	G	A
.	.	.	.	.	.	.	.	.	.	0.1	0.2	SON	GT:/	0:34	0/1:66,8:37	C	A
.	.	.	.	.	.	.	.	.	.	0	0	SON	GT:/	0:48	0/1:39,7:37	C	A
.	.	##	0	0.0'	0.002	0.003	1,N	4.5	1.47	.	.	SON	GT:/	0:19	0/1:175,10	C	T
.	.	##	.	.	.	.	.	.	.	.	.	DB;S	GT:/	0:93	0/1:95,73:3	C	T
.	.	.	.	.	.	.	1,D	6	3.52	.	.	SON	GT:/	0:51	0/1:43,39:3	C	A
.	.	.	.	0.0	1.0,D	1.0,D	1,D	5.5	3.84	.	.	SON	GT:/	0:89	0/1:186,46	T	G
.	.	.	.	.	.	.	.	.	.	0	0.1	DB;S	GT:/	0:20	0/1:24,7:33	T	C
.	.	.	.	0.6	0.007	0.071	1.00	5.8	1.48	.	.	SON	GT:/	0:34	0/1:31,28:3	T	C
.	.	.	.	.	.	.	.	.	.	.	.	SON	GT:/	0:70	0/1:96,55:3	G	A
.	.	.	.	0.0(	1.0,D	1.0,D	1,D	5.6	5.71	.	.	SON	GT:/	0:44	0/1:36,26:3	G	A
.	.	.	.	.	.	.	.	.	.	.	.	SON	GT:/	0:91	0/1:102,54	G	T
.	.	.	.	.	.	.	.	.	1.33	.	.	SON	GT:/	0:8,(	0/1:13,4:37	T	C

.	.	.	.	.	.	.	.	.	.	.	.	SON	GT:/	0:26	0/1:39,14:3	A	T
.	.	.	.	0.0'	0.017	0.006	1,N	.	1.70	.	.	SON	GT:/	0:30	0/1:295,22	C	T
.	0	##	0	.	.	.	.	.	.	.	.	SON	GT:/	0:74	0/1:85,19:3	C	T
.	.	.	.	0.0(	0.526	0.73,I	1,D	5.8	5.49	.	.	SON	GT:/	0:23	0/1:249,56	C	G
.	.	.	.	.	.	.	.	.	4.49	0	0	DB;S	GT:/	0:63	0/1:50,30:3	G	A
.	.	##	0	0.0(	0.982	0.999	0.99	5.6	4.76	0	.	SON	GT:/	0:21	0/1:248,17	G	A
.	.	##	0	.	.	.	1,A	.	5.58	.	.	SON	GT:/	0:56	0/1:59,32:3	C	T
.	.	.	.	.	.	.	.	.	.	.	.	SON	GT:/	0:13	0/1:103,78	G	A
4E-04	.	##	0	.	.	.	.	.	.	0	.	SON	GT:/	0:51	0/1:55,34:3	G	A
.	.	.	.	.	.	.	.	3.9	1.88	.	.	SON	GT:/	0:46	0/1:57,33:3	A	G
.	.	.	.	.	.	.	.	.	.	.	.	SON	GT:/	0:45	0/1:50,47:3	G	T
0.011	0	0	0	.	.	.	.	2.5	.	.	.	DB;S	GT:/	0:12	0/1:211,14	C	T
.	.	##	0	.	.	.	.	.	.	0.1	0.2	DB;S	GT:/	0:22	0/1:57,8:37	T	A
.	.	.	.	.	.	.	.	.	.	.	.	SON	GT:/	0:29	0/1:9,37:35	C	G
.	.	.	.	.	.	.	.	.	.	.	.	SON	GT:/	0:50	0/1:6,31:37	G	A
0.021	.	.	.	.	.	.	.	.	.	0	0	SON	GT:/	0:13	0/1:11,4:37	G	A
2E-04	.	.	.	.	.	.	.	.	.	.	.	SON	GT:/	0:10	0/1:5,6:37:	C	T
.	.	.	.	.	.	.	.	2.9	.	.	.	SON	GT:/	0:46	0/1:56,30:3	C	T
.	.	.	.	.	.	.	.	.	.	.	.	SON	GT:/	0:54	0/1:60,45:3	G	A
.	.	.	.	.	.	.	.	.	.	.	.	SON	GT:/	0:21	0/1:30,6:37	A	G
.	.	.	.	.	.	.	.	.	.	.	.	SON	GT:/	0:35	0/1:38,31:3	G	A
.	.	.	.	.	.	.	.	.	.	.	.	SON	GT:/	0:66	0/1:56,37:3	C	G
.	.	.	.	.	.	.	.	.	.	.	.	SON	GT:/	0:21	0/1:20,20:3	G	A
.	.	.	.	.	.	.	.	.	.	.	.	SON	GT:/	0:36	0/1:52,13:3	A	C
.	.	.	.	.	.	.	.	.	.	.	.	SON	GT:/	0:38	0/1:50,24:3	C	T
.	.	.	.	.	.	.	.	.	.	0	.	SON	GT:/	0:23	0/1:27,4:37	C	G
0.002	.	.	.	.	.	.	.	.	.	.	.	SON	GT:/	0:61	0/1:61,41:3	G	A
.	.	.	.	.	.	.	.	.	1.50	.	.	SON	GT:/	0:53	0/1:69,53:3	C	T
.	.	.	.	.	.	.	1,A	.	.	.	.	SON	GT:/	0:26	0/1:53,17:3	G	A
.	.	.	.	.	.	.	.	.	1.84	.	.	DB;S	GT:/	0:21	0/1:29,22:3	T	A
.	.	.	.	.	.	.	.	.	.	.	.	SON	GT:/	0:43	0/1:91,38:3	G	A
.	.	.	.	.	.	.	.	.	.	0	0.1	SON	GT:/	0:14	0/1:23,4:37	C	G
0.071	.	.	.	.	.	.	.	.	.	0.1	0.1	SON	GT:/	0:12	0/1:17,11:3	C	A
.	.	.	.	.	.	.	.	.	.	0	0	SON	GT:/	0:17	0/1:33,5:37	T	C
.	.	.	.	.	.	.	.	.	.	.	.	SON	GT:/	0:8,(	0/1:9,4:37:	G	T
.	.	##	0	.	.	.	.	.	1.27	.	.	SON	GT:/	0:34	0/1:22,24:3	C	T
2E-04	.	.	.	.	.	.	.	3.3	3.26	.	.	SON	GT:/	0:90	0/1:116,73	C	T
.	.	.	.	0.0(	0.999	0.999	1,D	4.9	5.51	.	.	SON	GT:/	0:89	0/1:116,50	G	A
.	.	.	.	0.0(	0.999	1.0,D	1,D	4.2	3.76	.	.	SON	GT:/	0:10	0/1:102,68	G	A
.	.	.	.	.	.	.	.	.	.	0.1	0.2	SON	GT:/	0:8,(	0/1:24,6:37	G	A
.	.	.	.	.	.	.	.	.	.	0	0.1	SON	GT:/	0:34	0/1:41,5:37	A	G
.	.	.	.	.	.	.	.	.	.	.	.	SON	GT:/	0:50	0/1:33,19:3	C	A
.	.	.	.	.	.	.	.	.	.	.	.	SON	GT:/	0:80	0/1:95,74:3	T	C
.	.	##	0	0.0(	0.186	0.958	1,N	3.7	1.71	.	.	SON	GT:/	0:63	0/1:75,46:3	C	T
.	.	.	.	.	.	.	.	.	.	.	.	SON	GT:/	0:42	0/1:41,25:3	C	T
.	.	.	.	.	.	.	.	.	.	.	.	SON	GT:/	0:16	0/1:30,19:3	C	T
.	.	.	.	.	.	.	.	.	.	0	0	SON	GT:/	0:20	0/1:35,4:37	T	C
.	.	.	.	0.0:	1.0,D	1.0,D	1,D	5.2	4.19	.	.	SON	GT:/	0:48	0/1:44,36:3	C	T
.	.	.	.	.	.	.	.	.	.	.	.	SON	GT:/	0:45	0/1:26,15:3	G	A
.	.	.	.	.	.	.	.	.	.	.	.	SON	GT:/	0:26	0/1:28,35:3	C	T
.	.	.	.	.	.	.	.	.	.	.	.	SON	GT:/	0:46	0/1:55,33:3	C	T
.	.	.	.	.	.	.	.	.	.	0	.	SON	GT:/	0:10	0/1:10,7:37	G	A
.	.	.	.	0.1:	0.928	0.999	1,N	4	3.15	.	.	SON	GT:/	0:15	0/1:248,55	C	T
.	0	##	0	.	.	.	.	.	.	0	.	SON	GT:/	0:14	0/1:298,62	G	A

.	.	.	.	.	.	.	1,A	4.8	8.04	.	.	SOM	GT:/	0:32	0/1:83,25:3	T	A
.	.	##	0	.	.	.	.	.	1.66	.	.	SOM	GT:/	0:18	0/1:47,8:37	C	T
.	.	##	0	.	.	.	.	.	.	.	.	SOM	GT:/	0:29	0/1:304,33	G	A
.	.	.	.	.	.	.	.	.	1.72	.	.	SOM	GT:/	0:16	0/1:31,10:3	G	A
.	.	.	.	0.6	0.005	0.001	0.98	3.8	1.70	.	.	SOM	GT:/	0:33	0/1:44,9:37	G	T
.	.	.	.	.	.	.	.	.	.	.	.	SOM	GT:/	0:24	0/1:65,19:3	G	C
.	.	.	.	.	.	.	.	.	.	.	.	SOM	GT:/	0:34	0/1:40,23:3	G	T
.	.	.	.	0.0	0.982	0.999	1,D	4.2	5.24	.	.	SOM	GT:/	0:22	0/1:279,77	G	A
.	.	.	.	.	.	.	.	.	.	0	0	DB;S	GT:/	0:25	0/1:30,5:37	T	G
.	.	.	.	.	.	.	.	.	.	0	0	SOM	GT:/	0:27	0/1:44,5:37	C	T
.	.	.	.	.	.	.	.	.	.	0.1	0.3	SOM	GT:/	0:9,(	0/1:32,4:37	C	T
.	.	.	.	.	.	.	.	.	.	0	0	SOM	GT:/	0:10	0/1:30,5:37	A	G
.	.	.	.	.	.	.	.	.	.	.	.	SOM	GT:/	0:35	0/1:47,8:33	G	T
.	.	.	.	.	.	.	.	.	1.68	0	.	SOM	GT:/	0:22	0/1:70,11:1	T	G
.	.	##	0	.	.	.	.	.	.	.	.	SOM	GT:/	0:31	0/1:518,47	C	T
.	.	##	0	0.0	1.0,D	1.0,D	1,D	2.7	3.85	.	.	SOM	GT:/	0:23	0/1:42,10:3	C	T
.	.	.	.	.	.	.	.	4.5	.	.	.	SOM	GT:/	0:12	0/1:130,17	G	A
.	.	.	.	.	.	.	.	.	1.48	.	.	SOM	GT:/	0:78	0/1:99,17:3	A	C
.	.	##	0	.	.	.	.	.	.	.	.	SOM	GT:/	0:23	0/1:26,11:3	C	T
0.01	0	0	0	0.0(	0.17,I	0.899	0.99	4	3.11	0	.	DB;S	GT:/	0:16	0/1:125,40	C	T
.	.	.	.	.	.	.	.	.	.	.	.	SOM	GT:/	0:35	0/1:53,5:37	A	G
.	.	.	.	.	.	.	.	.	.	.	.	SOM	GT:/	0:41	0/1:72,20:3	C	A
.	.	.	.	0.0(	0.996	1.0,D	1,D	5.7	5.49	.	.	SOM	GT:/	0:34	0/1:107,13	C	T
.	.	.	.	0.2	0.005	0.001	1.00	.	.	.	.	SOM	GT:/	0:56	0/1:49,7:37	T	A
.	.	.	.	0.0	0.999	1.0,D	1,D	5.8	4.83	.	.	SOM	GT:/	0:17	0/1:120,57	C	T
0.016	.	.	.	.	.	.	.	.	.	.	.	DB;S	GT:/	0:25	0/1:25,7:37	T	C
.	.	.	.	.	.	.	.	.	.	0	.	SOM	GT:/	0:27	0/1:34,5:31	A	G
.	.	.	.	.	.	.	.	3.4	.	.	.	SOM	GT:/	0:26	0/1:30,6:37	C	G
.	.	.	.	.	.	.	.	.	.	.	.	SOM	GT:/	0:18	0/1:21,7:17	T	G
.	.	.	.	.	.	.	.	.	.	0	0.1	SOM	GT:/	0:9,(	0/1:13,4:34	G	A
.	.	.	.	.	.	.	.	.	.	.	.	SOM	GT:/	0:25	0/1:82,16:3	G	C
.	.	.	.	.	.	.	.	.	2.50	.	.	SOM	GT:/	0:11	0/1:130,18	G	A
.	.	.	.	0.0	0.936	0.997	1.00	5.9	4.12	.	.	SOM	GT:/	0:62	0/1:73,26:3	G	A
.	.	.	.	.	.	.	.	4.1	.	.	.	SOM	GT:/	0:45	0/1:56,14:3	G	C
.	.	.	.	.	.	.	.	2.7	2.12	.	.	SOM	GT:/	0:79	0/1:85,20:3	C	A
.	.	.	.	.	.	.	.	2.5	.	.	.	SOM	GT:/	0:12	0/1:223,76	T	A
.	.	.	.	1.0	0.0,B	0.0,B	1,N	.	.	.	.	SOM	GT:/	0:31	0/1:55,20:3	G	T
.	.	##	0	0.0	0.004	0.313	0.99	2.5	2.21	.	.	SOM	GT:/	0:14	0/1:150,34	C	T
.	.	.	.	.	.	.	.	2.3	1.83	.	.	SOM	GT:/	0:28	0/1:25,5:34	T	A
.	.	.	.	0.0(	0.054	0.665	1.00	4.9	1.86	.	.	SOM	GT:/	0:14	0/1:112,15	G	A
.	.	.	.	.	.	.	.	.	.	0	0	DB;S	GT:/	0:26	0/1:18,5:37	T	C
.	.	.	.	.	.	.	.	.	.	.	.	SOM	GT:/	0:40	0/1:36,10:3	C	G
.	.	.	.	.	.	.	.	.	.	.	.	SOM	GT:/	0:31	0/1:35,5:31	G	C
.	.	.	.	.	.	.	.	5	.	.	.	SOM	GT:/	0:16	0/1:107,56	C	T
.	.	.	.	0.0	.	.	0.98	.	2.98	.	.	SOM	GT:/	0:14	0/1:163,70	A	T
.	.	.	.	0.9	0.99,I	0.999	1.00	5.8	1.57	.	.	SOM	GT:/	0:76	0/1:86,17:3	T	C
.	.	.	.	.	.	.	.	.	.	.	.	SOM	GT:/	0:70	0/1:154,57	T	C
.	.	.	.	.	.	.	1,A	5.9	13.3	.	.	SOM	GT:/	0:43	0/1:94,22:3	G	A
.	.	.	.	.	.	.	1,D	.	2.38	.	.	SOM	GT:/	0:48	0/1:60,6:37	C	T
.	.	.	.	.	.	.	.	.	.	.	.	SOM	GT:/	0:53	0/1:100,10	C	T
.	.	.	.	.	.	.	.	.	1.26	0	.	SOM	GT:/	0:10	0/1:21,7:11	A	C
.	.	.	.	.	.	.	.	.	.	0.3	0.2	SOM	GT:/	0:8,(	0/1:20,8:35	G	A
.	.	.	.	.	.	.	.	2.1	.	.	.	SOM	GT:/	0:20	0/1:77,7:37	G	A
0.327	.	.	.	.	.	.	.	.	.	0	.	DB;S	GT:/	0:19	0/1:26,5:37	T	A

.	.	.	.	.	.	.	.	.	.	.	.	SOM	GT:/	0:35	0/1:38,10:3	C	A
.	.	##	0	0.9	0.008	0.025	0.97	4.8	.	.	.	SOM	GT:/	0:48	0/1:70,25:3	A	G
.	.	.	.	.	.	.	.	.	.	.	.	SOM	GT:/	0:27	0/1:36,10:3	G	A
.	.	##	0	0.1	0.003	0.019	1,N	.	.	.	.	SOM	GT:/	0:52	0/1:132,13	C	T
.	.	.	.	.	.	.	.	.	.	.	.	SOM	GT:/	0:53	0/1:54,11:3	C	A
.	.	.	.	.	.	.	.	.	3.00	.	.	SOM	GT:/	0:11	0/1:78,15:3	C	T
.	.	.	.	.	.	.	.	.	.	.	.	SOM	GT:/	0:81	0/1:91,33:3	C	T
.	.	.	.	.	.	.	.	.	.	.	.	SOM	GT:/	0:22	0/1:43,25:3	G	A
.	.	##	0	0.1	0.105	0.205	1.00	5.1	3.30	.	.	SOM	GT:/	0:57	0/1:90,13:3	G	A
.	.	.	.	.	.	.	.	3.7	.	.	.	SOM	GT:/	0:14	0/1:364,68	C	T
.	.	.	.	0.2	0.087	0.358	1,N	.	.	.	.	SOM	GT:/	0:17	0/1:152,46	T	G
.	.	.	.	.	.	.	.	.	.	.	.	SOM	GT:/	0:19	0/1:25,7:35	A	G
.	.	.	.	0.0	0.872	0.983	0.88	4.2	3.66	.	.	SOM	GT:/	0:11	0/1:91,26:3	C	T
.	.	.	.	.	.	.	.	.	.	0.1	0.2	SOM	GT:/	0:37	0/1:30,7:37	C	A
.	.	.	.	.	.	.	.	.	.	0	0.1	SOM	GT:/	0:15	0/1:8,5:37:	G	T
.	.	.	.	0.0	.	.	1,D	4.6	1.63	.	.	SOM	GT:/	0:58	0/1:96,19:3	G	A
.	.	.	.	0.0	0.554	0.927	1.00	5.1	4.23	.	.	SOM	GT:/	0:32	0/1:52,6:37	C	G
.	.	##	0	0.2	0.004	0.012	1,N	.	.	.	.	SOM	GT:/	0:19	0/1:111,33	C	T
.	.	.	.	.	.	.	.	.	.	.	.	SOM	GT:/	0:44	0/1:75,22:3	G	C
0.318	.	.	.	.	.	.	.	.	.	0.1	0.2	SOM	GT:/	0:23	0/1:27,6:37	C	A
0.291	.	.	.	.	.	.	.	.	.	0.3	0.6	SOM	GT:/	0:9,(	0/1:14,4:37	A	C
.	.	0	0	.	.	.	.	3.2	2.71	.	.	SOM	GT:/	0:28	0/1:106,13	C	T
.	.	.	.	0.0	0.993	0.999	0.99	4.6	2.87	.	.	SOM	GT:/	0:11	0/1:176,34	C	A
.	.	.	.	.	.	.	.	.	.	.	.	SOM	GT:/	0:9,(	0/1:16,5:37	A	T
.	.	.	.	.	.	.	.	.	3.11	.	.	SOM	GT:/	0:28	0/1:40,14:3	G	T
.	.	.	.	.	.	.	.	.	.	.	.	SOM	GT:/	0:29	0/1:16,6:37	G	A
.	.	.	.	.	.	.	.	.	3.37	.	.	SOM	GT:/	0:10	0/1:86,28:3	G	A
.	.	.	.	0.0	0.499	0.883	1.00	4.7	4.04	.	.	SOM	GT:/	0:38	0/1:52,17:3	C	G
.	.	##	.	0.3	0.002	0.002	0.97	.	1.77	.	.	SOM	GT:/	0:40	0/1:53,17:3	G	C
.	.	##	0	0.1	0.014	0.087	0.98	4.1	1.73	.	.	SOM	GT:/	0:83	0/1:189,17	C	T
.	.	.	.	0.0	0.996	0.999	1.00	4.1	4.31	.	.	SOM	GT:/	0:13	0/1:9,4:30:	G	A
.	.	.	.	0.0	0.999	1.0,D	1,D	4.8	5.03	.	.	SOM	GT:/	0:20	0/1:243,39	C	T
.	.	.	.	.	.	.	.	.	.	.	.	SOM	GT:/	0:9,(	0/1:23,8:37	G	A
.	.	.	.	.	.	.	.	.	.	.	.	SOM	GT:/	0:22	0/1:48,17:3	G	T
.	.	.	.	.	.	.	.	.	.	0	.	SOM	GT:/	0:12	0/1:147,35	C	T
.	.	.	.	.	.	.	.	.	.	.	.	SOM	GT:/	0:13	0/1:16,7:37	C	A
.	.	.	.	.	.	.	.	2	.	.	.	SOM	GT:/	0:23	0/1:13,6:37	C	G
.	.	.	.	.	.	.	.	2.5	2.83	.	.	SOM	GT:/	0:11	0/1:82,23:3	C	T
.	.	.	.	.	.	.	.	.	.	.	.	SOM	GT:/	0:28	0/1:15,10:3	G	C
.	.	.	.	.	.	.	.	2.9	.	.	.	SOM	GT:/	0:21	0/1:182,17	C	T
.	.	.	.	.	.	.	.	.	.	.	.	SOM	GT:/	0:33	0/1:51,12:3	A	G
.	.	.	.	.	.	.	.	.	1.29	0.5	0.5	SOM	GT:/	0:13	0/1:29,10:3	G	A
.	.	.	.	.	.	.	.	.	.	.	.	SOM	GT:/	0:55	0/1:82,117	G	A

shared_ho	shared_he	OMIM	GWA	HGM	GO_	GO_	GO_M	KEG	PID	BIO	REACTOME_PATHWAY
0	0	.	NA	.	.	.	.	.	.	.	.
0	0	.	NA	.	.	.	.	.	.	.	.
0	0	.	NA	.	.	.	.	.	.	.	.
0	0	.	NA	.	.	.	.	.	.	.	.
0	0	.	NA	.	.	.	.	.	.	.	.
0	0	Homocysti	NA	.	CAR	.	OXID	KEG	.	.	REACTOME_METABOLISM_OF_
0	0	.	NA	.	.	.	.	.	.	.	.
0	0	.	NA	.	.	.	.	.	.	.	.
0	0	.	NA	.	.	.	.	.	.	.	.
0	0	.	NA	.	.	.	.	.	.	.	.
0	0	Rhabdomy	NA	.	CELI	.	TRAN	.	.	.	.
0	0	.	NA	.	.	NUC	RNA_	KEG	PID	.	REACTOME_RNA_POL_II_TRAN
0	0	.	NA	.	RNA	ORG	RNA_	KEG	.	.	REACTOME_PROCESSING_OF_
0	0	.	NA	.	.	.	.	.	.	.	.
0	0	.	NA	.	CAR	.	OXID	KEG	.	.	REACTOME_BIOLOGICAL_OXID
0	0	.	NA	.	LIPID	INTR	TRAN	.	.	.	REACTOME_PHOSPHOLIPID_M
0	0	.	NA	.	.	.	.	.	.	.	.
0	0	.	NA	.	.	.	.	.	.	.	.
0	0	.	NA	.	.	.	.	.	.	.	.
0	0	.	NA	.	.	.	.	.	.	.	.
0	0	.	NA	.	.	.	.	.	.	.	.
0	0	.	NA	.	.	.	.	.	.	.	.
0	0	.	NA	.	.	.	.	.	.	.	.
0	0	.	NA	.	.	.	.	.	.	.	.
0	0	.	NA	.	.	.	.	.	.	.	.
0	0	.	NA	.	.	.	.	.	.	.	.
0	0	.	NA	.	.	.	.	.	.	.	.
0	0	.	NA	.	.	.	.	.	.	.	.
0	0	.	NA	.	.	.	.	.	.	.	.
0	0	.	NA	.	.	.	.	.	.	.	.
0	0	.	NA	.	.	.	.	.	.	.	.
0	0	.	NA	.	.	.	.	.	.	.	.
0	0	.	NA	.	.	.	.	.	.	.	.
0	0	.	NA	.	.	.	.	.	.	.	.
0	0	.	NA	.	.	.	.	.	.	.	.
0	0	.	NA	.	MUL	.	RECE	.	.	.	.
0	0	.	NA	.	.	.	.	.	.	.	.
0	0	{Malaria, re	NA	.	SIGN	.	.	KEG	PID	BIO	REACTOME_IMMUNOREGULAT
0	0	.	NA	.	NEG	NUC	TRAN	.	PID	.	REACTOME_RNA_POL_III_TRA
0	0	.	NA	.	.	.	.	.	.	.	.
0	0	HEM skele	NA	.	.	ORG	DNA_	.	.	.	REACTOME_METABOLISM_OF_
0	0	.	NA	.	.	.	.	.	.	.	.
0	0	.	NA	.	.	.	.	.	.	.	.
0	0	.	NA	.	.	.	.	.	.	.	.
0	0	.	NA	.	.	.	.	.	.	.	.
0	0	.	NA	.	.	.	.	.	.	.	.
0	0	.	NA	.	.	.	.	.	.	.	.
0	0	.	NA	.	.	.	.	.	.	.	.
0	0	.	NA	.	.	INTR	INTEI	KEG	PID	BIO	REACTOME_SIGNALING_BY_IL
0	0	.	NA	.	.	.	.	.	.	.	.

0	0	.	NA	.	.	.	.	.	.	.	.
0	0	.	NA	.	.	.	.	.	.	.	.
0	0	.	NA	.	.	.	.	.	.	.	.
0	0	.	NA	.	.	.	.	KE(	.	.	REACTOME_METABOLISM_OF_
0	0	Mowat-Wil	NA	.	.	.	.	.	.	.	.
0	0	.	NA	.	CELI	NUC	.	.	PID	.	.
0	0	.	NA	.	RNA	ORG	LIGAS	KE(	.	.	REACTOME_MITOCHONDRIAL_
0	0	.	NA	.	.	.	.	.	.	.	.
0	0	.	NA	.	.	INTR	G_PR	KE(	.	.	REACTOME_SIGNALING_BY_G
0	0	Spinal mus	NA	.	PRO	.	UNFC	.	.	.	.
0	0	.	NA	.	.	.	.	.	.	.	.
0	0	.	NA	.	RNA	.	TRAN	.	PID	.	.
0	0	.	NA	.	.	.	.	.	.	.	.
0	0	.	NA	.	N_TI	NUC	HIST(	KE(	PID	BIO(	REACTOME_PRE_NOTCH_TRA
0	0	Nail disord	NA	.	MEM	CYT	LIPAS	KE(	.	.	.
0	0	.	NA	.	EST/	.	.	.	.	.	.
0	0	.	NA	CM1	EST/	.	.	.	.	.	.
0	0	.	NA	.	.	.	.	.	.	.	.
0	0	.	NA	.	.	.	.	.	.	.	.
0	0	.	NA	.	.	.	.	.	.	.	.
0	0	.	NA	.	.	.	.	.	.	.	.
0	0	.	NA	.	REG	.	.	KE(	.	.	.
0	0	.	NA	.	.	.	.	.	.	.	.
0	0	.	NA	.	.	.	.	.	.	.	.
0	0	.	NA	.	.	.	.	.	.	.	.
0	0	.	NA	.	.	.	.	.	.	.	.
0	0	.	NA	.	.	.	.	.	PID	.	.
0	0	.	NA	.	.	.	.	.	.	.	.
0	0	.	NA	.	.	.	.	.	.	.	.
0	0	.	NA	.	.	.	.	.	.	.	.
0	0	.	NA	.	.	.	.	.	.	.	.
0	0	.	NA	.	.	.	.	.	.	.	.
0	0	.	NA	.	.	.	.	.	.	.	.
0	0	.	NA	.	.	.	.	.	.	.	.
0	0	.	NA	.	.	.	.	.	.	.	.
0	0	.	NA	.	.	.	.	.	.	.	.
0	0	.	NA	.	.	.	.	.	.	.	REACTOME_METABOLISM_OF_
0	0	.	NA	.	.	.	.	KE(	.	.	.
0	0	.	NA	.	.	.	.	.	.	.	.
0	0	.	NA	.	.	.	.	.	.	.	.
0	0	.	NA	.	BIOF	.	.	KE(	PID	BIO(	REACTOME_SIGNALLING_BY_I
0	0	.	NA	.	CELI	PRO	PROT	KE(	PID	BIO(	REACTOME_SIGNALING_BY_W
0	0	.	NA	.	.	.	.	.	.	.	.
0	0	.	NA	.	.	.	.	.	.	.	.
0	0	.	NA	.	.	.	.	.	.	.	.
0	0	.	NA	.	.	.	.	.	.	.	.
0	0	.	NA	.	COF	.	.	KE(	.	.	REACTOME_BIOLOGICAL_OXII
0	0	.	NA	.	.	.	.	.	.	.	.
0	0	.	NA	.	.	.	.	.	.	.	.
0	0	.	NA	.	.	.	.	.	.	.	.
0	0	.	NA	.	.	.	.	.	.	.	.
0	0	Coenzyme	NA	.	ISOF	.	TRAN	KE(	.	.	.
0	0	.	NA	.	.	.	.	.	.	.	.
0	0	.	NA	.	.	.	.	.	.	.	REACTOME_TRANSMEMBRANI

0	0	.	NA	.	MUS	MEM	.	.	.	BIO(	.
0	0	.	NA	.	.	.	.	.	.	.	.
0	0	.	NA	.	.	.	.	KE(	.	.	.
0	0	.	NA	.	MAC	EUK	TRAN	.	.	.	REACTOME_TRANSLATION;RE
0	0	.	NA	.	.	.	.	.	.	.	.
0	0	.	NA	.	MUS	.	EXOF	.	.	.	.
0	0	.	NA	.	.	.	.	.	.	.	.
0	0	.	NA	.	.	.	.	.	.	.	.
0	0	.	NA	.	.	.	.	.	.	.	.
0	0	.	NA	.	.	.	.	.	.	.	.
0	0	.	NA	.	.	.	.	.	.	.	.
0	0	.	NA	.	.	.	.	.	.	.	.
0	0	.	NA	.	.	.	.	.	.	.	.
0	0	.	NA	.	.	.	.	.	.	.	.
0	0	.	NA	.	.	.	.	.	.	.	.
0	0	.	NA	.	.	.	.	.	.	.	.
0	0	.	NA	.	.	.	.	.	.	.	.
0	0	.	NA	.	.	.	.	.	.	.	.
0	0	.	NA	.	.	.	.	.	.	.	.
0	0	.	NA	.	.	.	.	KE(	.	.	.
0	0	.	NA	.	DNA	.	SING	.	PID	.	REACTOME_MEIOSIS;REACTO
0	0	.	NA	.	.	.	.	.	.	.	.
0	0	.	NA	.	.	.	.	.	.	.	.
0	0	.	NA	.	.	.	.	.	.	.	.
0	0	.	NA	.	.	.	.	.	.	.	.
0	0	.	NA	.	.	.	.	.	.	.	.
0	0	.	NA	.	.	.	.	.	.	.	.
0	0	.	NA	.	.	.	.	.	.	.	.
0	0	.	NA	.	.	.	.	.	.	.	.
0	0	.	NA	.	EST/	ORG	.	.	.	.	.
0	0	.	NA	.	.	.	.	.	.	.	.
0	0	.	NA	.	.	.	.	.	.	.	.
0	0	.	NA	.	.	.	.	.	.	.	.
0	0	.	NA	.	.	.	.	.	.	.	.
0	0	.	NA	.	PHO	CYT(	PHOS	KE(	.	.	.
0	0	.	NA	.	.	.	.	.	.	.	.
0	0	.	NA	.	.	.	.	.	.	.	.
0	0	.	NA	.	.	.	.	.	.	.	.
0	0	.	NA	.	.	.	.	.	.	.	.
0	0	.	NA	.	.	.	.	.	.	.	.
0	0	.	NA	.	BIOF	.	NUCL	.	.	.	.
0	0	.	NA	.	.	.	.	.	.	.	.
0	0	Telangiect	NA	.	REG	INTF	.	.	PID	.	.
0	0	.	NA	.	.	.	IDEN	.	.	.	.
0	0	Thrombotic	NA	.	BIOF	PRO	INTE(	.	.	.	.
0	0	Dopamine	NA	.	CELI	CYT(	.	KE(	.	BIO(	REACTOME_METABOLISM_OF_
0	0	.	NA	.	.	.	.	.	.	.	.
0	0	.	NA	.	BIOF	.	.	.	.	.	REACTOME_MEMBRANE_BIND
0	0	Deafness,	NA	.	PRO	ORG	HYDF	.	.	.	.
0	0	Deafness,	NA	.	PRO	ORG	HYDF	.	.	.	.
0	0	.	NA	.	.	.	.	.	.	.	.
0	0	.	NA	.	MUS	CYT(	ACTII	.	PID	.	.
0	0	.	NA	.	.	.	.	.	.	.	.
0	0	.	NA	.	.	.	.	.	.	.	.

0	0	.	NA	.	.	.	.	.	.	.	.
0	0	.	NA	.	.	.	.	.	.	.	.
0	0	.	NA	.	.	.	.	.	.	.	.
0	0	?Retinitis p	NA	.	LIPIL	.	.	.	.	.	.
0	0	.	NA	.	.	.	.	.	.	.	.
0	0	.	NA	.	.	.	.	.	.	.	.
0	0	.	NA	.	.	.	.	.	.	.	.
0	0	Preeclamp	NA	.	.	.	.	.	.	.	.
0	0	.	NA	.	.	.	.	.	.	.	.
0	0	Cholestery	NA	.	GLY(	CYT(	.	KE(	.	.	.
0	0	.	NA	.	.	.	.	.	.	.	.
0	0	Epidermoly	NA	.	SYS	INTF	.	.	PID	.	REACTOME_CELL_CELL_COMI
0	0	.	NA	.	.	.	.	.	.	.	.
0	0	.	NA	.	.	.	.	.	.	.	.
0	0	.	NA	.	.	.	.	.	.	.	.
0	0	.	NA	.	PRO	.	ACID	KE(	PID	BIO(	REACTOME_SIGNALLING_BY_I
0	0	.	NA	.	.	.	.	.	.	.	.
0	0	.	NA	.	.	.	.	.	.	.	.
0	0	.	NA	.	SIGN	.	.	.	.	.	.
0	0	.	NA	.	RNA	NUC	.	.	.	.	.
0	0	.	NA	.	RNA	NUC	.	.	.	.	.
0	0	.	NA	.	.	.	.	.	.	.	.
0	0	.	NA	.	.	.	.	.	.	.	.
0	0	.	NA	.	ENZ	CYT(	NUCL	.	PID	.	.
0	0	.	NA	.	.	.	.	KE(	.	.	REACTOME_SIGNALING_BY_G
0	0	.	NA	.	.	.	.	.	.	.	.
0	0	.	NA	.	.	.	.	.	.	.	.
0	0	.	NA	.	.	.	.	.	.	.	.
0	0	.	NA	.	.	.	.	.	.	.	.
0	0	.	NA	.	REG	SYN	.	.	PID	.	.
0	0	.	NA	.	.	.	.	.	.	.	.
0	0	.	NA	.	.	.	.	.	.	.	.
0	0	.	NA	.	.	.	.	.	.	.	REACTOME_TRANSMISSION_A
0	0	.	NA	.	.	.	.	.	.	.	.
0	0	.	NA	.	.	.	.	.	.	.	.
0	0	.	NA	.	.	.	.	.	.	.	.
0	0	.	NA	.	SULI	.	SULF	KE(	.	.	REACTOME_CHONDROITIN_SU
0	0	.	NA	.	.	.	.	.	.	.	.
0	0	.	NA	.	SYS	.	PHOS	.	PID	.	.
0	0	.	NA	.	.	.	.	.	.	.	.
0	0	.	NA	.	.	.	.	.	.	.	.
0	0	.	NA	.	.	.	.	.	.	.	.
0	0	.	NA	.	.	.	.	.	.	.	.
0	0	.	NA	.	.	.	.	.	.	.	.
0	0	.	NA	.	.	.	.	.	.	.	.
0	0	.	NA	.	.	.	.	.	.	.	.
0	0	.	NA	.	.	.	.	.	.	.	.
0	0	.	NA	.	.	.	.	.	.	.	.
0	0	.	NA	.	.	.	.	.	.	.	.
0	0	.	NA	.	.	.	.	.	.	.	.
0	0	.	NA	.	.	.	.	.	.	.	.
0	0	Emery-Dre	NA	.	.	.	.	.	.	.	REACTOME_MEIOSIS;REACTO
0	0	.	NA	.	.	.	.	.	.	.	.
0	0	.	NA	.	.	.	.	.	.	.	.

0	0	.	NA	.	.	.	.	.	.	.	.
0	0	.	NA	.	.	.	.	.	.	.	.
0	0	.	NA	.	.	.	.	.	.	.	.
0	0	.	NA	.	.	.	.	.	.	.	.
0	0	.	NA	.	.	.	.	.	.	.	.
0	0	.	NA	.	.	.	.	.	.	.	.
0	0	.	NA	.	.	.	.	.	.	.	.
0	0	.	NA	.	.	.	.	.	.	.	.
0	0	.	NA	.	.	.	.	.	.	.	.
0	0	.	NA	.	.	.	.	.	.	.	.
0	0	.	NA	.	.	.	.	.	.	.	.
0	0	.	NA	.	.	.	.	.	.	.	.
0	0	.	NA	.	.	.	.	.	.	.	REACTOME_TRANSMEMBRANI
0	0	.	NA	.	.	.	.	.	.	.	.
0	0	.	NA	.	SYS	.	.	.	.	.	.
0	0	.	NA	.	.	.	.	.	.	.	.
0	0	.	NA	.	.	.	.	.	.	.	.
0	0	.	NA	.	.	.	.	.	.	.	.
0	0	.	NA	.	.	.	.	.	.	.	.
0	0	.	NA	.	.	.	.	.	.	.	.
0	0	.	NA	.	.	.	.	.	.	.	.
0	0	.	NA	.	.	.	.	.	.	.	.
0	0	.	NA	.	.	.	.	.	.	.	.
0	0	.	NA	.	.	.	.	.	.	.	.
0	0	.	NA	.	.	.	.	.	.	.	.
0	0	.	NA	.	.	.	.	.	.	.	.
0	0	.	NA	.	SIGN	.	GTPA	.	.	.	REACTOME_SIGNALING_BY_G
0	0	Deafness,	NA	.	.	.	.	.	.	.	.
0	0	Immunode	NA	.	.	ORG	STRU	.	.	.	.
0	0	.	NA	.	.	.	.	.	.	.	.
0	0	.	NA	.	.	.	.	.	.	.	.
0	0	.	NA	.	.	.	.	.	.	.	.
0	0	.	NA	.	.	.	.	.	.	.	.
0	0	.	NA	.	.	.	.	.	.	.	.
0	0	.	NA	.	.	.	.	.	.	.	.
0	0	.	NA	.	.	.	.	.	.	.	.
0	0	.	NA	.	.	.	.	.	.	.	.
0	0	.	NA	.	.	.	.	.	.	.	.
0	0	.	NA	.	.	.	.	.	.	.	.
0	0	.	NA	.	.	.	.	.	.	.	.
0	0	.	NA	.	.	.	.	.	.	.	.
0	0	.	NA	.	.	.	.	.	.	.	.
0	0	.	NA	.	.	.	.	.	.	.	.
0	0	.	NA	.	.	.	.	.	.	.	.
0	0	.	NA	.	.	.	.	.	.	.	.
0	0	.	NA	.	.	.	.	.	.	.	.
0	0	.	NA	.	.	.	.	.	.	.	.
0	0	.	NA	.	.	.	.	.	.	.	.
0	0	.	NA	.	.	.	.	.	.	.	.
0	0	.	NA	.	.	.	.	.	.	.	.
0	0	.	NA	.	.	.	.	.	.	.	.
0	0	.	NA	.	.	.	.	.	.	.	.
0	0	Growth ret	NA	.	PAT	.	.	.	.	.	.
0	0	Myeloid leu	NA	.	RNA	.	RNA_	.	PID	.	.

0	0	.	NA	.	.	.	.	.	.	.	.
0	0	Brittle corn	NA	.	.	.	.	.	.	.	.
0	0	Retinitis pi	NA	.	RNA	ORG	RNA_	KEG	.	.	REACTOME_PROCESSING_OF
0	0	.	NA	.	.	.	.	.	.	.	.
0	0	.	NA	.	RNA	.	HYDR	.	PID	.	.
0	0	.	NA	.	GAM	ORG	.	.	.	.	.
0	0	.	NA	.	.	.	.	.	.	.	.
0	0	Deafness,	NA	.	SEN	.	.	.	.	.	.
0	0	.	NA	.	.	.	.	.	.	.	.
0	0	.	NA	.	.	.	.	.	.	.	.
0	0	.	NA	.	.	.	.	.	.	.	.
0	0	.	NA	.	.	.	.	.	.	.	.
0	0	.	NA	.	.	.	.	.	.	.	.
0	0	.	NA	.	.	.	.	.	.	.	.
0	0	.	NA	.	.	.	.	.	.	.	.
0	0	.	NA	.	.	.	.	.	.	.	.
0	0	.	NA	.	.	.	.	.	.	.	.
0	0	.	NA	.	.	.	.	.	.	.	.
0	0	.	NA	.	CELL	.	HYDR	.	.	.	.
0	0	Cortical dy	NA	.	MICR	ORG	STRU	.	PID	.	REACTOME_CELL_CYCLE;REA
0	0	.	NA	.	.	.	.	.	.	.	.
0	0	.	NA	.	.	.	.	.	.	.	.
0	0	Amelogene	NA	.	RNA	.	ENZY	KEG	PID	BIO	REACTOME_SIGNALLING_BY_N
0	0	.	NA	.	.	.	.	.	.	.	.
0	0	.	NA	.	.	CELL	.	KEG	.	.	.
0	0	.	NA	.	.	.	.	.	.	.	.
0	0	.	NA	.	.	.	.	.	.	.	.
0	0	.	NA	.	.	.	.	.	.	.	.
0	0	.	NA	.	.	.	.	.	.	.	.
0	0	.	NA	.	.	.	.	.	.	.	.
0	0	.	NA	.	.	.	OXY	KEG	.	.	REACTOME_BIOLOGICAL_OXID
0	0	.	NA	.	.	.	.	.	.	.	.
0	0	.	NA	.	RNA	.	TRAN	.	PID	.	.
0	0	.	NA	.	.	.	.	.	.	.	.
0	0	.	NA	.	CAR	.	ELEC	KEG	.	.	.
0	0	.	NA	.	.	ORG	.	.	.	.	.
0	0	.	NA	.	.	.	.	.	.	.	.
0	0	.	NA	.	.	.	.	.	.	.	.
0	0	.	NA	.	REG	.	TRAN	KEG	PID	.	.
0	0	.	NA	.	.	.	.	.	.	.	.
0	0	.	NA	.	.	.	.	.	.	.	.
0	0	.	NA	.	.	.	.	.	.	.	.
0	0	.	NA	.	.	.	.	.	.	.	.
0	0	.	NA	.	.	.	.	.	.	.	.
0	0	.	NA	.	.	.	.	.	.	.	.
0	0	.	NA	.	.	.	.	.	.	.	.
0	0	.	NA	.	.	.	.	.	.	.	.
0	0	.	NA	.	DNA	NUC	.	.	.	.	.
0	0	.	NA	.	CAR	INTR	TRAN	KEG	.	.	REACTOME_BIOLOGICAL_OXID
0	0	.	NA	.	.	.	.	.	.	.	.
0	0	.	NA	.	.	.	.	.	.	.	.
0	0	.	NA	.	.	.	.	.	.	.	.
0	0	.	NA	.	.	ORG	.	.	.	.	.
0	0	.	NA	.	.	.	.	.	.	.	.

0	0	.	NA	.	.	.	.	.	.	.	.
0	0	.	NA	.	.	.	.	.	.	.	.
0	0	.	NA	.	SYS	INTR	.	.	.	.	.
0	0	.	NA	.	SYS	INTR	.	.	.	.	.
0	0	.	NA	.	GAM	ORG	CHR	.	.	.	.
0	0	.	NA	.	.	.	.	.	.	.	.
0	0	.	NA	.	.	.	.	.	.	.	.
0	0	Renal cell	NA	.	.	.	.	.	PID	.	.
0	0	Renal cell	NA	.	.	.	.	.	PID	.	.
0	0	Aland Islar	NA	.	DETI	INTR	CATI	KEG	.	.	.
0	0	.	NA	.	.	.	.	.	.	.	.
0	0	.	NA	.	REG	ORG	.	.	.	.	REACTOME_DIABETES_PATHW
0	0	.	NA	.	.	.	.	.	.	.	.
0	0	Androgen i	NA	.	EST	CYT	LIGAI	KEG	PID	.	REACTOME_GENERIC_TRANS
0	0	.	NA	.	.	.	.	.	.	.	.
0	0	Mental reta	NA	.	.	.	.	.	.	.	.
0	0	.	NA	.	.	.	.	.	.	.	.
0	0	.	NA	.	RNA	NUC	RNA_	.	.	.	REACTOME_PROCESSING_OF
0	0	.	NA	.	.	.	.	.	.	.	.
0	0	.	NA	.	.	.	.	KEG	.	.	REACTOME_HS_GAG_BIOSYN
0	0	.	NA	.	.	CYT	.	.	PID	.	REACTOME_SIGNALING_BY_R
0	0	.	NA	.	.	.	.	.	.	.	.
0	0	.	NA	.	.	.	.	.	.	.	.
0	0	.	NA	.	REG	MEM	KINA	KEG	PID	BIO	REACTOME_SIGNALLING_BY_
0	0	.	NA	.	REG	MEM	KINA	KEG	PID	BIO	REACTOME_SIGNALLING_BY_
0	0	.	NA	.	.	.	.	.	.	.	.
0	0	.	NA	.	.	.	.	.	.	.	.
0	0	.	NA	.	.	.	.	.	.	.	.
0	0	.	NA	.	.	.	.	.	.	.	.
0	0	.	NA	.	.	.	.	.	.	.	.
0	0	Meier-Gorl	NA	.	DNA	NUC	DNA_	KEG	PID	BIO	REACTOME_ACTIVATION_OF_
0	0	.	NA	.	.	.	.	.	.	.	.
0	0	Myopia 21,	NA	.	.	.	.	.	.	.	.
0	0	.	NA	.	EST	INTR	CATI	.	.	.	REACTOME_NEURONAL_SYST
0	0	.	NA	.	.	.	.	.	.	.	.
0	0	.	NA	.	.	.	.	.	.	.	.
0	0	.	NA	.	.	.	.	.	.	.	.
0	0	.	NA	.	.	.	.	.	.	.	.
0	0	.	NA	.	.	.	.	.	.	.	.
0	0	.	NA	.	.	.	.	.	.	.	.
0	0	.	NA	.	.	.	.	.	.	.	.
0	0	.	NA	.	.	.	.	.	.	.	.
0	0	.	NA	.	.	.	.	.	.	.	.
0	0	.	NA	.	.	.	.	.	.	.	.
0	0	.	NA	.	.	.	.	.	.	.	.
0	0	.	NA	.	.	.	.	.	.	.	.
0	0	.	NA	.	.	.	.	.	.	.	.
0	0	.	NA	.	.	.	.	.	.	.	.
0	0	.	NA	.	.	.	.	.	.	.	.
0	0	.	NA	.	.	.	.	.	.	.	.
0	0	.	NA	.	.	.	.	.	.	.	.
0	0	.	NA	.	.	.	.	.	.	.	.
0	0	.	NA	.	.	.	.	KEG	.	.	.
0	0	.	NA	.	STEI	CYT	TRAN	KEG	.	.	.

0	0	.	NA	.	.	.	.	.	.	.	.
0	0	.	NA	.	.	.	.	.	.	.	.
0	0	.	NA	.	.	.	.	.	.	.	.
0	0	.	NA	.	.	.	.	.	.	.	.
0	0	.	NA	.	.	.	.	.	.	.	REACTOME_SIGNALING_BY_G
0	0	.	NA	.	.	NUC	PROT	.	PID	BIO(	REACTOME_DEVELOPMENTAL
0	0	.	NA	.	.	.	.	.	.	.	.
0	0	.	NA	.	.	.	.	.	.	.	.
0	0	.	NA	.	.	.	.	.	.	.	.
0	0	.	NA	.	.	.	.	.	.	.	.
0	0	.	NA	.	.	.	.	.	.	.	.
0	0	.	NA	.	.	.	.	.	.	.	.
0	0	.	NA	.	AGIN	.	.	.	PID	.	REACTOME_CELL_CELL_COMI
0	0	.	NA	.	.	.	.	.	.	.	.
0	0	.	NA	.	.	.	.	.	.	.	.
0	0	.	NA	.	.	.	.	.	.	.	.
0	0	Bardet-Bie	NA	.	.	.	.	.	.	.	.
0	0	Cardiomyo	NA	.	MUS	ORG	NUCL	KEG	.	.	.
0	0	.	NA	.	.	.	.	.	.	.	.
0	0	AICA-ribos	NA	.	NUC	.	HYDF	KEG	.	.	REACTOME_METABOLISM_OF_
0	0	Alport sync	NA	.	REG	PRO	STRU	KEG	PID	BIO(	REACTOME_DEVELOPMENTAL
0	0	.	NA	.	.	.	.	KEG	PID	.	REACTOME_SIGNALING_BY_R
0	0	.	NA	.	.	.	.	.	.	.	.
0	0	Spinocerel	NA	.	.	INTF	CATI(	KEG	.	.	.
0	0	.	NA	.	.	.	.	.	.	.	.
0	0	T-cell immu	NA	.	.	.	.	.	PID	.	.
0	0	T-cell immu	NA	.	.	.	.	.	PID	.	.
0	0	.	NA	.	.	.	.	.	.	.	.
0	0	Tietz albini	NA	.	.	.	TRAN	KEG	PID	.	.
0	0	.	NA	.	.	.	.	.	.	.	.
0	0	Breast can	NA	.	.	.	PHOS	KEG	PID	BIO(	REACTOME_SIGNALLING_BY_N
0	0	.	NA	.	.	.	.	.	.	.	REACTOME_FACTORS_INVOLV
0	0	.	NA	.	.	.	.	.	.	.	.
0	0	.	NA	.	.	.	.	.	.	.	.
0	0	.	NA	.	.	.	.	.	.	.	.
0	0	.	NA	.	VIRA	.	TRAN	KEG	PID	BIO(	.
0	0	.	NA	.	.	.	.	.	.	.	.
0	0	.	NA	.	.	.	.	.	.	.	.
0	0	.	NA	.	.	.	.	.	.	.	.
0	0	.	NA	.	.	.	.	.	.	.	.
0	0	.	NA	.	.	.	.	.	.	.	.
0	0	.	NA	.	.	.	.	.	.	.	.
0	0	.	NA	.	.	.	.	.	.	.	.
0	0	{Alcohol de	NA	.	.	.	.	KEG	.	.	REACTOME_BIOLOGICAL_OXID
0	0	.	NA	.	.	.	.	.	.	.	.
0	0	Cardiac arr	NA	.	.	.	.	.	.	.	.
0	0	[Blood grou	NA	.	.	INTF	.	.	.	.	.
0	0	.	NA	.	.	.	.	.	.	.	.
0	0	.	NA	.	.	.	.	.	.	.	.
0	0	.	NA	.	.	.	.	.	.	.	.
0	0	.	NA	.	.	.	.	.	.	.	.
0	0	.	NA	.	.	.	.	.	.	.	.
0	0	.	NA	.	.	.	.	.	.	.	.
0	0	.	NA	.	.	.	.	.	.	.	.

0	0	.	NA	.	.	.	.	.	.	.	.
0	0	.	NA	.	.	.	.	.	.	.	.
0	0	.	NA	.	.	.	.	.	.	.	.
0	0	.	NA	.	.	.	.	.	.	.	.
0	0	Charcot-Ma	NA	.	.	.	.	.	.	.	.
0	0	.	NA	.	ORG	.	RAS_	KE(	PID	.	REACTOME_FACTORS_INVOLV
0	0	.	NA	.	RNA	NUC	TRAN	KE(	PID	.	REACTOME_PRE_NOTCH_TRA
0	0	.	NA	.	BIOF	NUC	PHOS	.	.	.	.
0	0	.	NA	.	.	.	.	.	.	.	.
0	0	.	NA	.	EST/	.	CATI(	.	.	.	REACTOME_NEURONAL_SYST
0	0	.	NA	.	.	.	.	.	.	.	.
0	0	.	NA	.	.	CYT(	ISOM	KE(	.	.	REACTOME_CHONDROITIN_SU
0	0	.	NA	.	RNA	NUC	.	.	.	.	.
0	0	Ciliary dysk	NA	.	.	.	.	.	.	.	.
0	0	.	NA	.	.	.	.	.	.	.	.
0	0	.	NA	.	.	.	.	.	.	.	.
0	0	.	NA	.	.	.	.	.	.	.	.
0	0	.	NA	.	.	.	.	.	.	.	.
0	0	.	NA	.	.	.	.	.	.	.	.
0	0	.	NA	.	.	.	.	.	.	.	.
0	0	.	NA	.	.	.	.	.	.	.	.
0	0	.	NA	.	.	.	.	.	.	.	.
0	0	.	NA	.	.	.	.	.	.	.	.
0	0	.	NA	.	.	.	.	.	.	.	.
0	0	.	NA	.	.	.	.	.	.	.	.
0	0	.	NA	.	.	.	.	.	.	.	.
0	0	.	NA	.	.	.	.	.	.	.	.
0	0	.	NA	.	SIGN	.	.	.	.	.	.
0	0	.	NA	.	EST/	CELI	ANIO	.	.	.	REACTOME_TRANSMEMBRANI
0	0	.	NA	.	EST/	ORG	NUCL	.	.	.	.
0	0	.	NA	.	EST/	ORG	NUCL	.	.	.	.
0	0	.	NA	.	BIOF	INTF	PROT	KE(	.	.	.
0	0	.	NA	.	.	.	.	.	.	.	.
0	0	.	NA	.	.	.	.	.	.	.	.
0	0	.	NA	.	.	.	.	.	.	.	.
0	0	Occult mac	NA	.	.	.	.	.	.	.	.
0	0	.	NA	.	.	.	.	.	.	.	.
0	0	.	NA	.	.	.	.	.	.	.	.
0	0	Spastic pa	NA	.	.	.	.	KE(	.	.	REACTOME_MEMBRANE_TRAF
0	0	.	NA	.	.	.	.	.	.	.	.
0	0	.	NA	.	.	.	.	.	.	.	.
0	0	.	NA	.	.	.	.	.	.	.	.
0	0	.	NA	.	.	.	.	.	.	.	.
0	0	.	NA	.	SYS'	.	.	.	.	.	.
0	0	.	NA	.	.	.	.	.	PID	.	REACTOME_TRANSMEMBRANI
0	0	Lymphopla	NA	.	RNA	.	.	.	PID	.	.
0	0	.	NA	.	.	.	.	.	.	.	.
0	0	.	NA	.	.	.	.	.	.	.	.
0	0	.	NA	.	.	.	.	.	.	.	.
0	0	.	NA	.	.	.	.	.	.	.	.
0	0	.	NA	.	.	.	.	.	.	.	.
0	0	.	NA	.	.	.	.	.	.	.	.
0	0	.	NA	.	.	.	.	.	.	.	.
0	0	.	NA	.	.	.	.	.	.	.	.

0	0	.	NA	.	.	.	GTPA	KEG	PID	BIO	REACTOME_SIGNALLING_BY_I
0	0	.	NA	.	CELI	MEM	CATI	KEG	.	.	REACTOME_TRANSMISSION_A
0	0	Megalobla	NA	.	ESTA	MEM	PROT	.	.	.	REACTOME_METABOLISM_OF_
0	0	.	NA	.	.	.	.	.	.	.	.
0	0	.	NA	.	.	.	.	.	.	.	.
0	0	.	NA	.	.	.	.	.	.	.	.
0	0	.	NA	.	.	.	.	.	.	.	REACTOME_MITOCHONDRIAL_
0	0	.	NA	.	.	.	.	.	.	.	.
0	0	.	NA	.	.	.	.	.	.	.	.
0	0	.	NA	.	ESTA	.	CYTO	.	.	.	.
0	0	.	NA	.	.	.	.	KEG	.	.	REACTOME_TRANSMEMBRANI
0	0	.	NA	.	.	.	.	.	.	.	.
0	0	.	NA	.	.	.	.	.	.	.	.
0	0	.	NA	.	DIGE	PRO	STRU	KEG	.	.	REACTOME_O_LINKED_GLYCO
0	0	{Pulmonary	NA	.	.	.	.	.	.	.	REACTOME_O_LINKED_GLYCO
0	0	.	NA	.	.	.	.	.	.	.	.
0	0	.	NA	.	SIGN	ORG	RAS_	.	.	.	.
0	0	Exostoses,	NA	.	SYS	ORG	.	KEG	.	.	REACTOME_HS_GAG_BIOSYN
0	0	.	NA	.	.	.	.	.	.	.	.
0	0	.	NA	.	POS	.	.	KEG	PID	.	REACTOME_VEGF_LIGAND_RE
0	0	.	NA	.	BIOF	CYT	ACID	KEG	.	.	REACTOME_DIABETES_PATHW
0	0	.	NA	.	.	ORG	.	.	.	.	REACTOME_MHC_CLASS_II_AI
0	0	.	NA	.	.	.	.	.	.	.	.
0	0	.	NA	.	REG	CYT	RAS_	KEG	PID	.	REACTOME_SIGNALING_BY_R
0	0	.	NA	.	REG	CYT	RAS_	KEG	PID	.	REACTOME_SIGNALING_BY_R
0	0	.	NA	.	.	.	.	.	.	.	.
0	0	Deafness,	NA	.	LYSO	ORG	CALM	.	.	.	.
0	0	.	NA	.	RNA	NUC	.	.	.	.	.
0	0	.	NA	.	.	.	.	.	.	.	.
0	0	.	NA	.	GLY	INTR	EXOF	KEG	.	.	.
0	0	.	NA	.	.	.	.	KEG	.	.	REACTOME_SIGNALING_BY_G
0	0	.	NA	.	.	.	.	.	.	.	.
0	0	.	NA	.	.	.	.	.	.	.	.
0	0	.	NA	.	.	.	.	.	.	.	.
0	0	.	NA	.	.	.	.	.	.	.	.
0	0	.	NA	.	.	.	.	.	.	.	.
0	0	.	NA	.	.	.	.	.	.	.	.
0	0	.	NA	.	.	.	.	.	.	.	.
0	0	Deafness,	NA	.	.	.	.	.	.	.	.
0	0	.	NA	.	CELI	CYT	.	.	.	.	.
0	0	.	NA	.	.	.	.	.	.	.	.
0	0	.	NA	.	.	.	.	.	.	.	.
0	0	.	NA	.	.	.	.	.	.	.	.
0	0	.	NA	.	.	.	.	.	.	.	.
0	0	Spermatog	NA	.	SYS	CELI	OXID	KEG	.	BIO	REACTOME_LATENT_INFECTIO
0	0	.	NA	.	.	NUC	TRAN	KEG	PID	BIO	REACTOME_DEVELOPMENTAL
0	0	.	NA	.	DNA	NON	TRAN	KEG	PID	BIO	REACTOME_TRIF_MEDIATED_
0	0	.	NA	.	.	.	.	.	.	.	.
0	0	.	NA	.	.	.	.	.	.	.	.
0	0	.	NA	.	.	.	.	.	.	.	.
0	0	.	NA	.	PRO	CYT	SERI	.	.	.	REACTOME_IMMUNE_SYSTEM
0	0	.	NA	.	.	.	.	.	.	.	.
0	0	.	NA	.	.	.	.	KEG	.	.	REACTOME_SIGNALING_BY_G
0	0	.	NA	.	.	.	.	.	.	.	.

0	0	.	NA	.	.	.	.	.	.	.	.
0	0	.	NA	.	.	.	.	.	.	.	.
0	0	.	NA	.	.	.	.	KE(	.	.	.
0	0	Glaucoma	NA	.	EST/	PRO	.	.	.	.	.
0	0	.	NA	.	.	.	.	.	.	.	.
0	0	.	NA	.	.	.	.	.	.	.	.
0	0	.	NA	.	.	.	.	.	.	.	.
0	0	.	NA	.	.	.	.	.	.	.	.
0	0	.	NA	.	.	.	.	.	.	.	.
0	0	.	NA	.	.	.	.	.	.	.	.
0	0	.	NA	.	.	.	.	.	.	.	.
0	0	.	NA	.	EST/	.	CATI(	KE(	.	.	.
0	0	.	NA	.	.	.	.	.	.	.	.
0	0	Bartter syn	NA	.	.	MEM	CATI(	.	.	.	REACTOME_TRANSMEMBRANI
0	0	.	NA	.	.	.	.	.	.	.	.
0	0	.	NA	.	.	.	.	.	.	.	.
0	0	.	NA	.	.	INTF	.	KE(	.	.	REACTOME_DEVELOPMENTAL
0	0	.	NA	.	.	.	.	.	.	.	.
0	0	.	NA	.	.	.	.	.	.	.	.
0	0	.	NA	.	.	.	.	.	.	.	.
0	0	.	NA	.	.	.	.	.	.	.	.
0	0	.	NA	.	.	.	.	.	.	.	REACTOME_TRANSMEMBRANI
0	0	Weill-Marc	NA	.	.	.	.	.	.	.	.
0	0	.	NA	.	.	.	.	.	.	.	.
0	0	.	NA	.	.	.	.	.	.	.	.
0	0	Lissencepl	NA	.	CEN	ORG	MICR	.	.	.	.
0	0	.	NA	.	.	.	.	.	.	.	.
0	0	Floating-Ha	NA	.	RNA	.	TRAN	.	.	.	.
0	0	.	NA	.	.	.	.	.	.	.	.
0	0	.	NA	.	.	.	.	.	.	.	.
0	0	.	NA	.	.	.	.	.	.	.	.
0	0	.	NA	.	.	.	.	.	.	.	.
0	0	.	NA	.	.	.	.	.	.	.	.
0	0	.	NA	.	.	.	.	.	.	.	.
0	0	.	NA	.	.	.	.	.	.	.	.
0	0	.	NA	.	.	.	.	.	.	.	.
0	0	.	NA	.	.	.	.	.	.	.	.
0	0	.	NA	.	.	.	.	.	.	.	.
0	0	.	NA	.	.	.	.	.	.	.	.
0	0	.	NA	.	.	.	.	.	.	.	.
0	0	.	NA	.	.	.	.	.	.	.	.
0	0	.	NA	.	.	.	.	.	.	.	.
0	0	.	NA	.	.	.	.	.	.	.	.
0	0	.	NA	.	.	.	.	.	.	.	.
0	0	.	NA	.	.	.	.	.	.	.	.
0	0	.	NA	.	.	.	.	.	.	.	.
0	0	.	NA	.	.	.	.	.	.	.	.
0	0	.	NA	.	.	.	.	.	.	.	.
0	0	.	NA	.	.	.	.	.	.	.	.
0	0	.	NA	.	.	.	.	.	.	.	.
0	0	.	NA	.	.	.	.	.	.	.	.
0	0	.	NA	.	.	.	.	.	.	.	.
0	0	.	NA	.	.	.	.	.	.	.	.
0	0	.	NA	.	.	.	.	.	.	.	.

0	0	.	NA	.	.	.	.	.	.	.	.	
0	0	.	NA	.	.	.	.	.	.	.	.	
0	0	.	NA	.	.	.	.	.	.	.	.	
0	0	.	NA	.	.	.	.	.	.	.	.	
0	0	Ciliary dysk	NA	.	.	.	.	.	.	.	.	
0	0	[Anhaptogl	NA	.	DEFI	.	.	.	.	.	.	
0	0	.	NA	.	.	.	.	.	.	.	.	
0	0	.	NA	.	.	.	.	.	.	.	.	
0	0	Combined	NA	.	.	.	.	.	.	.	.	
0	0	Combined	NA	.	.	.	.	.	.	.	.	
0	0	.	NA	.	.	.	.	.	.	.	.	
0	0	.	NA	.	.	.	.	.	.	.	.	
0	0	.	NA	.	.	.	.	.	.	.	.	
0	0	.	NA	.	.	.	.	.	.	.	REACTOME_INTERFERON_ALF	
0	0	.	NA	.	CAL(	CELI	IDEN	KE(	.	.	REACTOME_CELL_CELL_COMI	
0	0	.	NA	.	.	.	.	.	.	.	.	
0	0	.	NA	.	.	.	MOT(	KE(	.	.	.	
0	0	.	NA	.	GAM	ORG	.	.	.	.	.	
0	0	.	NA	.	GAM	ORG	.	.	.	.	.	
0	0	.	NA	.	.	.	.	.	.	.	.	
0	0	.	NA	.	.	.	.	.	.	.	.	
0	0	.	NA	.	.	.	.	.	.	.	.	
0	0	.	NA	.	.	.	.	.	.	.	.	
0	0	.	NA	.	.	.	.	.	.	.	.	
0	0	.	NA	.	.	.	.	.	.	.	.	
0	0	.	NA	.	.	.	.	.	.	.	.	
0	0	.	NA	.	.	.	.	.	.	.	.	
0	0	.	NA	.	.	.	.	.	.	.	.	
0	0	.	NA	.	.	.	.	.	.	.	.	
0	0	.	NA	.	.	.	.	.	.	.	.	
0	0	.	NA	.	.	.	.	.	.	.	.	
0	0	Night blind	NA	.	.	.	.	.	.	.	.	
0	0	.	NA	.	.	.	.	.	.	.	.	
0	0	.	NA	.	.	.	.	.	.	.	.	
0	0	Colorectal	NA	.	SIGN	NUC	.	KE(	PID_	.	.	
0	0	.	NA	.	.	CYT(	.	.	.	.	.	
0	0	.	NA	.	.	.	.	.	.	.	.	
0	0	.	NA	.	.	.	.	.	.	.	.	
0	0	.	NA	.	PRO	ORG	CHAF	.	.	.	REACTOME_PROTEIN_FOLDIN	
0	0	.	NA	.	.	.	.	.	.	.	.	
0	0	.	NA	.	.	.	.	.	.	.	.	
0	0	.	NA	.	.	.	.	.	.	.	.	
0	0	Cerebral cr	NA	.	BIOS	.	METH	KE(	.	.	REACTOME_METABOLISM_OF_	
0	0	.	NA	.	.	.	.	.	.	.	.	
0	0	.	NA	.	.	.	META	KE(	.	.	.	
0	0	.	NA	.	.	.	META	KE(	.	.	.	
0	0	Spastic par	NA	.	.	.	.	.	.	.	.	
0	0	.	NA	.	ESTA	INTR	ACTI	.	.	.	REACTOME_GLUTAMATE_NEU	
0	0	.	NA	.	REG	NUC	SEQU	.	.	.	.	
0	0	.	NA	.	.	.	.	.	.	.	.	
0	0	.	NA	.	.	.	.	.	.	.	.	
0	0	.	NA	.	.	.	.	.	.	.	.	
0	0	Central cor	NA	.	ESTA	INTR	CATI(	KE(	.	.	.	
0	0	.	NA	.	.	.	.	.	.	.	.	

0	0	.	NA	.	REG	INTF	PEPT	KE(	PID	BIO(	REACTOME_SIGNALING_BY_G
0	0	.	NA	.	.	.	.	.	.	.	.
0	0	.	NA	.	.	.	.	.	.	.	.
0	0	.	NA	.	BIOS	RIBC	RECE	.	.	.	.
0	0	.	NA	.	.	.	.	.	.	.	.
0	0	.	NA	.	.	.	.	KE(	PID	.	REACTOME_BIOLOGICAL_OXII
0	0	.	NA	.	.	.	.	KE(	PID	.	REACTOME_BIOLOGICAL_OXII
0	0	.	NA	.	.	.	.	.	.	.	.
0	0	.	NA	.	.	.	.	.	.	.	.
0	0	.	NA	.	.	.	.	.	.	.	.
0	0	.	NA	.	.	.	.	.	.	.	.
0	0	.	NA	.	.	.	.	.	.	.	.
0	0	.	NA	.	.	.	.	.	.	.	.
0	0	.	NA	.	.	.	.	.	.	.	.
0	0	.	NA	.	.	.	.	.	.	.	.
0	0	.	NA	.	.	.	.	.	.	.	.
0	0	.	NA	.	.	.	.	.	.	.	.
0	0	.	NA	.	.	.	.	.	.	.	.
0	0	.	NA	.	.	.	.	.	.	.	.
0	0	.	NA	.	.	.	.	.	.	.	.
0	0	.	NA	.	EST/	INTF	CATI(	.	.	.	.
0	0	.	NA	.	.	.	.	.	.	.	.
0	0	.	NA	.	.	.	.	.	.	.	.
0	0	Conotrunca	NA	.	SYS	NUC	PROT	.	.	.	.
0	0	.	NA	.	.	.	.	.	.	.	.
0	0	.	NA	.	.	.	.	.	.	.	.
0	0	.	NA	.	.	.	.	.	.	.	.
0	0	.	NA	.	.	.	.	.	.	.	.
0	0	.	NA	.	GAM	ORG	CHR(	.	.	.	.
0	0	Opitz GBB	NA	.	PAT	ORG	.	KE(	.	.	.
0	0	.	NA	.	.	.	.	.	.	.	.
0	0	.	NA	.	.	.	.	.	.	.	.
0	0	.	NA	.	.	.	.	.	.	.	.
0	0	.	NA	.	BIOF	.	.	.	.	.	.
0	0	.	NA	.	BIOF	.	.	.	.	.	.
0	0	.	NA	.	.	.	.	.	.	.	.
0	0	.	NA	.	.	.	.	.	.	.	.
0	0	.	NA	.	.	.	.	.	.	.	.
0	0	.	NA	.	.	.	.	.	.	.	.
0	0	Lujan-Fryn	NA	.	RNA	NUC	RNA_	.	PID	.	REACTOME_DEVELOPMENTAL
0	0	.	NA	.	.	.	.	.	.	.	.
0	0	.	NA	.	.	.	.	.	.	.	.
0	0	Lissenceph	NA	.	SYS	ORG	MICR	.	PID	.	REACTOME_DEVELOPMENTAL
0	0	Dent disea	NA	.	LIPIE	ORG	PHOS	KE(	.	.	REACTOME_SIGNALING_BY_R
0	0	Hypothyroi	NA	.	SIGN	INTF	PROT	.	.	.	.
0	0	.	NA	.	.	.	.	.	.	.	.
0	0	.	NA	.	SYS	CYT(	ENZY	KE(	.	.	.
0	0	.	NA	.	.	.	.	.	.	.	.
0	0	Cardiac va	NA	.	NUC	CYT(	ACTII	KE(	PID	.	.
0	0	.	NA	.	.	.	.	.	.	.	.
0	0	.	NA	.	.	.	.	.	.	.	.
0	0	.	NA	.	.	.	.	.	.	.	.
0	0	.	NA	.	.	.	.	.	.	.	.
0	0	.	NA	.	REG	NUC	NUCL	.	PID	BIO(	.

0	0	?Neurobla	NA	.	DNA	.	TRAN	KE	PID	BIO	.
0	0	.	NA	.	.	.	.	.	.	.	.
0	0	.	NA	.	.	.	.	.	.	.	.
0	0	.	NA	.	.	.	.	.	.	.	.
0	0	.	NA	.	.	.	.	.	.	.	.
0	0	.	NA	.	.	.	.	.	.	.	.
0	0	.	NA	.	.	.	.	.	.	.	.
0	0	.	NA	.	.	.	.	.	.	.	.
0	0	.	NA	.	.	.	.	.	.	.	.
0	0	.	NA	.	.	.	.	.	.	.	.
0	0	.	NA	.	.	.	.	.	.	.	REACTOME_IMMUNE_SYSTEM
0	0	Hypobetali	NA	.	REG	EXTI	INTE	.	PID	.	.
0	0	.	NA	.	.	.	.	.	.	.	.
0	0	.	NA	.	SEN	.	.	.	PID	.	.
0	0	.	NA	.	SYS	.	.	.	.	.	.
0	0	.	NA	.	.	.	.	.	.	.	.
0	0	.	NA	.	.	.	.	.	.	.	.
0	0	.	NA	.	.	.	.	.	.	.	.
0	0	.	NA	.	.	.	.	.	.	.	.
0	0	.	NA	.	.	.	.	.	.	.	.
0	0	.	NA	.	.	.	.	.	.	.	.
0	0	.	NA	.	.	.	.	.	.	.	.
0	0	.	NA	.	.	.	.	.	.	.	.
0	0	.	NA	.	.	.	.	.	.	.	.
0	0	.	NA	.	.	.	.	.	.	.	.
0	0	.	NA	.	.	.	.	.	.	.	.
0	0	.	NA	.	.	.	STRU	.	.	.	.
0	0	.	NA	.	.	.	.	.	.	.	.
0	0	.	NA	.	ENZ	.	.	KE	.	.	REACTOME_DEVELOPMENTAL
0	0	.	NA	.	.	.	.	KE	.	.	REACTOME_SIGNALING_BY_G
0	0	.	NA	.	.	.	.	.	.	.	.
0	0	.	NA	.	.	.	.	.	.	.	.
0	0	.	NA	.	.	.	.	.	.	.	.
0	0	.	NA	.	.	.	.	.	.	.	.
0	0	.	NA	.	.	.	.	.	.	.	.
0	0	.	NA	.	.	.	.	.	.	.	.
0	0	.	NA	.	.	.	.	.	.	.	.
0	0	.	NA	.	BIOF	.	ACID	.	.	.	.
0	0	.	NA	.	.	.	IDEN	KE	PID	BIO	.
0	0	AICA-ribos	NA	.	NUC	.	HYDF	KE	.	.	REACTOME_METABOLISM_OF_
0	0	.	NA	.	.	.	.	.	.	.	.
0	0	.	NA	.	.	.	.	.	.	.	REACTOME_FACTORS_INVOLV
0	0	.	NA	.	.	.	.	.	.	.	REACTOME_FACTORS_INVOLV
0	0	.	NA	.	.	.	.	KE	.	.	REACTOME_TRANSMISSION_A
0	0	.	NA	.	.	.	.	.	.	.	.
0	0	Vesicouret	NA	.	SYS	CEL	IDEN	KE	.	.	REACTOME_DEVELOPMENTAL
0	0	.	NA	.	DEFI	.	LIPAS	KE	.	.	REACTOME_PHOSPHOLIPID_M
0	0	.	NA	.	.	.	.	.	.	.	.
0	0	.	NA	.	.	.	.	KE	.	.	.
0	0	.	NA	.	.	.	.	.	.	.	.
0	0	Breast can	NA	.	.	.	PHOS	KE	PID	BIO	REACTOME_SIGNALLING_BY_
0	0	Breast can	NA	CM1	.	.	PHOS	KE	PID	BIO	REACTOME_SIGNALLING_BY_
0	0	.	NA	.	.	.	.	.	.	.	.
0	0	.	NA	.	.	.	.	.	PID	.	.

0	0	.	NA	.	.	RIBC	RNA_	.	.	.	.
0	0	.	NA	.	.	.	.	.	.	.	.
0	0	.	NA	.	.	.	.	.	.	.	.
0	0	.	NA	.	DETI	INTR	.	KE(	.	.	REACTOME_GASTRIN_CREB_S
0	0	.	NA	.	.	.	.	.	.	.	.
0	0	.	NA	.	.	.	.	.	.	.	.
0	0	.	NA	.	.	.	.	.	.	.	.
0	0	.	NA	.	.	.	.	.	.	.	.
0	0	.	NA	.	.	.	.	.	.	.	.
0	0	.	NA	.	.	.	.	.	.	.	.
0	0	.	NA	.	RES	CYT(	.	.	.	.	.
0	0	{Abacavir h	NA	.	DEFI	CELI	.	KE(	.	.	REACTOME_ANTIGEN_PROCE
0	0	.	NA	.	.	.	.	KE(	PID_	.	REACTOME_RNA_POL_I_TRAN
0	0	.	NA	.	REG	.	LIGAI	KE(	PID_	BIO(	REACTOME_GENERIC_TRANS(
0	0	.	NA	.	.	.	.	KE(	.	.	REACTOME_PPARA_ACTIVATE
0	0	.	NA	.	.	.	TRAN	KE(	.	.	REACTOME_BIOLOGICAL_OXID
0	0	.	NA	.	SYS	.	.	.	PID_	.	REACTOME_EXTRACELLULAR_
0	0	.	NA	.	.	.	.	.	.	.	.
0	0	.	NA	.	.	.	.	.	.	.	.
0	0	.	NA	.	.	.	.	.	.	.	.
0	0	Diabetes m	NA	.	REG	.	CARE	KE(	PID_	BIO(	REACTOME_DEVELOPMENTAL
0	0	.	NA	.	.	.	.	.	.	.	.
0	0	.	NA	.	.	.	.	.	.	.	.
0	0	.	NA	.	.	.	.	.	.	.	.
0	0	.	NA	.	.	.	.	.	.	.	.
0	0	.	NA	.	.	.	.	.	.	.	.
0	0	.	NA	.	.	.	.	.	.	.	.
0	0	.	NA	.	.	.	.	.	.	.	.
0	0	.	NA	.	.	.	.	.	.	.	.
0	0	.	NA	.	.	.	.	.	.	.	.
0	0	.	NA	.	.	.	.	.	.	.	.
0	0	.	NA	.	.	.	.	.	.	.	.
0	0	.	NA	.	.	.	.	.	.	.	.
0	0	.	NA	.	.	EXTI	HYDF	KE(	PID_	BIO(	REACTOME_TRANSMISSION_A
0	0	Tumoral ca	NA	.	.	.	.	.	.	.	.
0	0	.	NA	.	GLU	CYT(	PHOS	.	.	.	REACTOME_PYRUVATE_META
0	0	.	NA	.	.	.	.	.	.	.	.
0	0	.	NA	.	.	.	.	.	.	.	.
0	0	Premature	NA	.	.	.	.	.	.	.	.
0	0	.	NA	.	.	.	.	.	.	.	.
0	0	.	NA	.	.	.	.	.	.	.	.
0	0	.	NA	.	SIGN	EXTI	RECE	.	PID_	.	REACTOME_CELL_SURFACE_I
0	0	.	NA	.	.	.	.	.	.	.	.
0	0	.	NA	.	.	.	.	.	.	.	.
0	0	.	NA	.	.	.	.	.	.	.	.
0	0	.	NA	.	.	.	.	.	.	.	REACTOME_TRANSMEMBRANI
0	0	Cohen syn	NA	.	.	.	.	.	.	.	.
0	0	Seizures, b	NA	.	EST/	INTR	CATI(	.	.	.	REACTOME_DEVELOPMENTAL
0	0	.	NA	.	.	.	.	.	.	.	.
0	0	Craniosync	NA	.	.	INTR	.	KE(	.	.	.
0	0	.	NA	.	.	.	.	.	.	.	.
0	0	.	NA	.	.	.	.	.	.	.	.
0	0	.	NA	.	.	.	.	.	.	.	.
0	0	.	NA	.	.	.	.	.	.	.	.
0	0	.	NA	.	.	.	.	.	.	.	.

0	0	.	NA	.	.	ADH	STRU	.	.	.	.
0	0	.	NA	.	.	.	.	.	.	.	.
0	0	.	NA	.	.	.	.	.	.	.	REACTOME_TRANSMEMBRAN
0	0	Maturity-or	NA	.	REG	CYT	.	KE	.	.	.
0	0	Aortic valv	NA	.	MUS	NUC	.	KE	PID	BIO	.
0	0	.	NA	.	.	MAC	.	.	.	.	.
0	0	Brugada sy	NA	.	MUS	MEM	CATI	KE	.	.	REACTOME_DEVELOPMENTAL
0	0	.	NA	.	.	NUC	.	.	.	.	.
0	0	.	NA	.	.	.	.	.	.	.	.
0	0	.	NA	.	.	.	.	.	.	.	.
0	0	.	NA	.	.	.	.	.	.	.	.
0	0	Cardiomyo	NA	.	MAC	ADH	OXID	KE	PID	BIO	REACTOME_RESPONSE_TO_E
0	0	.	NA	.	.	.	.	.	.	.	.
0	0	.	NA	.	.	.	.	.	.	.	.
0	0	.	NA	.	.	.	.	.	.	.	.
0	0	.	NA	.	.	.	.	KE	.	.	REACTOME_TRANSMEMBRAN
0	0	.	NA	.	.	.	.	.	.	.	.
0	0	.	NA	.	.	.	.	.	.	.	.
0	0	.	NA	.	.	.	.	.	.	.	.
0	0	.	NA	.	.	.	.	.	.	.	.
0	0	.	NA	.	.	.	.	.	.	.	.
0	0	.	NA	.	.	.	.	.	.	.	.
0	0	.	NA	.	EST	.	KINAS	.	.	.	.
0	0	.	NA	.	RNA	.	HYDR	.	PID	.	.
0	0	.	NA	.	SYS	.	.	.	.	.	.
0	0	.	NA	.	.	.	.	.	.	.	.
0	0	Bladder ca	NA	CM0	.	.	.	KE	PID	BIO	REACTOME_SIGNALLING_BY_
0	0	.	NA	.	SIGN	CYT	CATI	KE	.	.	REACTOME_SIGNALLING_BY_
0	0	Warsaw br	NA	.	POS	ORG	.	.	.	.	REACTOME_DIABETES_PATHW
0	0	.	NA	.	.	.	.	.	.	.	.
0	0	.	NA	.	.	.	.	.	.	.	.
0	0	.	NA	.	.	.	.	.	.	.	.
0	0	.	NA	.	.	.	.	.	.	.	REACTOME_MEMBRANE_TRAF
0	0	.	NA	.	.	.	.	.	.	.	.
0	0	.	NA	.	.	.	.	.	.	.	.
0	0	.	NA	.	.	.	.	.	.	.	.
0	0	.	NA	.	.	.	.	.	.	.	.
0	0	.	NA	.	.	.	.	.	.	.	.
0	0	Leukemia,	NA	.	ENZ	INTR	TRAN	KE	.	BIO	.
0	0	.	NA	.	SYS	INTR	RECE	.	.	.	.
0	0	.	NA	.	.	.	.	.	.	.	.
0	0	.	NA	.	.	.	.	.	.	.	.
0	0	.	NA	.	.	.	.	.	.	.	.
0	0	.	NA	.	.	.	.	.	.	.	.
0	0	.	NA	.	.	.	.	.	.	.	.
0	0	.	NA	.	.	.	.	.	.	.	.
0	0	.	NA	.	.	.	.	.	.	.	.
0	0	.	NA	.	.	.	.	.	.	.	.
0	0	.	NA	.	.	.	.	.	.	.	.
0	0	.	NA	.	.	.	.	.	.	.	.
0	0	.	NA	.	.	.	.	.	.	.	.
0	0	.	NA	.	.	.	.	.	.	.	.
0	0	.	NA	.	.	.	.	.	.	.	.
0	0	.	NA	.	.	.	.	.	.	.	.

0	0	.	NA	.	.	.	.	.	.	.	.
0	0	.	NA	.	.	.	.	.	.	.	.
0	0	.	NA	.	.	.	.	.	.	.	.
0	0	.	NA	.	.	.	.	.	.	.	.
0	0	.	NA	.	.	.	.	.	.	.	.
0	0	.	NA	.	.	.	.	.	.	.	REACTOME_TRANSMISSION_A
0	0	.	NA	.	.	EXTI	PROT	.	.	.	.
0	0	.	NA	.	.	.	.	.	.	.	.
0	0	.	NA	.	.	.	.	.	.	.	.
0	0	.	NA	.	.	.	.	.	.	.	.
0	0	Mainzer-Sa	NA	.	.	.	.	.	.	.	.
0	0	.	NA	.	DEFI	EXTI	.	.	.	.	.
0	0	.	NA	.	.	.	.	.	.	.	.
0	0	.	NA	.	.	.	.	.	.	.	.
0	0	.	NA	.	.	.	.	.	.	.	.
0	0	.	NA	.	.	.	.	.	.	.	.
0	0	.	NA	.	.	.	.	.	.	.	.
0	0	.	NA	.	.	.	.	.	.	.	.
0	0	.	NA	.	.	.	.	.	.	.	.
0	0	.	NA	.	.	.	.	.	.	.	.
0	0	.	NA	.	.	.	.	.	.	.	.
0	0	.	NA	.	.	.	.	.	.	.	.
0	0	.	NA	.	.	.	.	.	.	.	.
0	0	.	NA	.	.	.	.	.	.	.	.
0	0	.	NA	.	.	.	.	.	.	.	.
0	0	.	NA	.	.	.	.	.	.	.	.
0	0	.	NA	.	.	.	.	.	.	.	.
0	0	.	NA	.	.	.	.	.	.	.	.
0	0	.	NA	.	.	.	.	.	.	.	.
0	0	.	NA	.	.	.	.	.	.	.	.
0	0	.	NA	.	.	.	.	.	.	.	.
0	0	.	NA	.	.	.	.	.	.	.	.
0	0	.	NA	.	.	.	.	.	.	.	.
0	0	.	NA	.	.	.	.	.	.	.	.
0	0	.	NA	.	.	.	.	.	.	.	.
0	0	.	NA	.	.	.	.	.	.	.	.
0	0	.	NA	.	.	.	.	.	.	.	.
0	0	.	NA	.	.	.	.	.	.	.	.
0	0	.	NA	.	.	.	.	.	.	.	.
0	0	.	NA	.	.	.	.	.	.	.	REACTOME_PYRUVATE_META
0	0	.	NA	.	.	.	.	.	.	.	.
0	0	Fanconi ar	NA	.	DNA	CYT	.	.	PID	.	REACTOME_FANCONI_ANEMIA
0	0	.	NA	.	.	.	.	KEG	.	.	.
0	0	.	NA	.	.	.	.	.	.	.	.
0	0	.	NA	.	NEG	.	TRAN	.	.	.	.
0	0	Mitochondi	NA	.	.	.	.	.	.	.	.
0	0	.	NA	.	.	.	.	.	.	.	.
0	0	.	NA	.	.	.	.	.	.	.	.
0	0	.	NA	.	.	.	.	.	.	.	.
0	0	.	NA	.	.	.	.	.	.	.	.
0	0	.	NA	.	.	.	.	.	.	.	.
0	0	.	NA	.	.	.	.	.	.	.	.
0	0	.	NA	.	.	.	.	.	.	.	.
0	0	.	NA	.	.	.	.	.	.	.	.
0	0	Koolen-De	NA	.	.	.	.	.	.	.	.

0	0	.	NA	.	.	.	CATI	KE(	.	.	.
0	0	.	NA	.	.	.	.	.	.	.	.
0	0	.	NA	.	.	.	.	KE(	.	.	.
0	0	Amyotroph	NA	.	PRO	ORG	HYDF	.	.	.	.
0	0	.	NA	.	.	.	.	.	.	.	.
0	0	{Diabetes r	NA	.	REG	INTF	G_PF	KE(	.	.	REACTOME_GASTRIN_CREB_S
0	0	.	NA	.	.	.	.	KE(	PID	BIO(	REACTOME_VITAMIN_B5_PANT
0	0	.	NA	.	DNA	CYT(	PROT	.	.	.	.
0	0	.	NA	.	.	.	.	KE(	.	.	.
0	0	.	NA	.	RNA	.	RNA_	.	.	.	REACTOME_DIABETES_PATHW
0	0	.	NA	.	DNA	.	.	KE(	.	.	REACTOME_TRANSCRIPTION_
0	0	.	NA	.	EST/	ORG	.	.	.	.	REACTOME_MITOCHONDRIAL_
0	0	.	NA	.	.	.	.	.	.	.	.
0	0	.	NA	.	.	.	.	.	.	.	.
0	0	.	NA	.	.	.	.	.	.	.	.
0	0	.	NA	.	.	.	.	.	.	.	.
0	0	.	NA	.	.	.	.	.	.	.	.
0	0	.	NA	.	EST/	NUC	ACTI	.	.	.	REACTOME_DEVELOPMENTAL
0	0	.	NA	.	.	.	.	KE(	.	.	REACTOME_DIABETES_PATHW
0	0	.	NA	.	.	.	.	.	.	.	.
0	0	.	NA	.	LIPIC	CYT(	LIPAS	.	.	.	REACTOME_ACYL_CHAIN_REM
0	0	.	NA	.	.	.	.	.	.	.	REACTOME_METABOLISM_OF_
0	0	.	NA	.	.	.	.	.	.	.	.
0	0	.	NA	.	.	.	.	.	.	.	.
0	0	.	NA	.	.	.	.	.	.	.	.
0	0	Spinocerel	NA	.	DNA	.	PHOS	KE(	PID	BIO(	REACTOME_SIGNALLING_BY_I
0	0	.	NA	.	.	.	.	.	.	.	REACTOME_GENERIC_TRANS(
0	0	.	NA	.	.	.	.	.	.	.	.
0	0	.	NA	.	.	.	.	.	.	.	.
0	0	Spinocerel	NA	.	.	.	.	.	.	.	.
0	0	.	NA	.	.	.	.	.	.	.	.
0	0	.	NA	.	.	.	.	.	.	.	.
0	0	.	NA	.	.	.	.	.	.	.	.
0	0	.	NA	.	GAM	EXTI	.	.	.	.	.
0	0	.	NA	.	.	.	.	.	.	.	.
0	0	.	NA	.	.	.	.	.	.	.	.
0	0	.	NA	.	.	.	.	.	.	.	.
0	0	.	NA	.	.	.	.	.	.	.	.
0	0	.	NA	.	.	.	.	.	.	.	.
0	0	.	NA	.	.	.	.	.	.	.	.
0	0	.	NA	.	.	.	.	.	.	.	.
0	0	.	NA	.	ANA	.	TRAN	.	.	.	.
0	0	.	NA	.	.	.	.	.	.	.	.
0	0	.	NA	.	.	.	.	.	.	.	.
0	0	.	NA	.	DNA	NUC	.	KE(	.	BIO(	REACTOME_ACTIVATION_OF_
0	0	.	NA	.	.	.	.	.	.	.	.
0	0	.	NA	.	.	.	.	.	.	.	.
0	0	.	NA	.	.	.	.	.	.	.	.
0	0	.	NA	.	.	.	.	.	.	.	.
0	0	.	NA	.	ACTI	ORG	CATI	.	.	.	.
0	0	.	NA	.	.	.	.	.	.	.	.
0	0	.	NA	.	.	CELI	.	.	.	.	.
0	0	Neutropeni	NA	.	REG	NON	GTPA	KE(	PID	.	REACTOME_TCR_SIGNALING;F
0	0	Aland Islar	NA	.	DETI	INTF	CATI	KE(	.	.	.

0	0	.	NA	.	.	.	.	.	.	.	.
0	0	.	NA	.	.	.	.	.	.	.	.
0	0	.	NA	.	.	.	.	.	.	.	.
0	0	.	NA	.	.	.	.	.	.	.	.
0	0	.	NA	.	SYS	ORG	.	.	.	.	REACTOME_SIGNALING_BY_H
0	0	Tn polyagg	NA	.	.	.	.	KEC	.	.	REACTOME_O_LINKED_GLYCC
0	0	.	NA	.	.	.	.	.	.	.	.
0	0	.	NA	.	.	CYT	.	.	PID	.	REACTOME_SIGNALING_BY_R
0	0	CHILD syn	NA	.	BIOS	CYT	STEF	KEC	.	.	REACTOME_METABOLISM_OF_
0	0	.	NA	.	.	.	.	.	.	.	.
0	0	.	NA	.	.	.	.	.	.	.	.
0	0	.	NA	.	.	.	.	.	.	.	.
0	0	.	NA	.	.	.	.	.	.	.	.
0	0	.	NA	.	.	.	.	.	.	.	.
0	0	.	NA	.	REG	CYT	NUCL	.	PID	BIO	.
0	0	.	NA	.	BIOF	.	ACID	KEC	.	.	REACTOME_IMMUNE_SYSTEM
0	0	.	NA	.	MITC	.	.	KEC	.	.	REACTOME_DNA_REPAIR
0	0	.	NA	.	.	.	.	.	.	.	.
0	0	.	NA	.	.	.	.	.	.	.	.
0	0	.	NA	.	.	.	.	.	.	.	.
0	0	.	NA	.	.	.	.	.	.	.	.
0	0	.	NA	.	.	.	.	.	.	.	.
0	0	.	NA	.	.	.	.	.	.	.	.
0	0	.	NA	.	.	.	.	.	.	.	.
0	0	[Rh-negati	NA	.	.	INTR	.	.	.	.	.
0	0	.	NA	.	MUL	PRO	INTE	.	PID	.	REACTOME_EXTRACELLULAR_
0	0	.	NA	.	.	.	.	.	.	.	.
0	0	Psychomo	NA	.	.	.	.	.	PID	.	.
0	0	.	NA	.	.	CYT	.	.	.	.	.
0	0	.	NA	.	.	CYT	.	.	.	.	.
0	0	.	NA	.	.	CYT	.	.	.	.	.
0	0	.	NA	.	.	CYT	.	.	.	.	.
0	0	.	NA	.	NUC	.	TRAN	.	.	.	.
0	0	.	NA	.	.	.	.	.	.	.	.
0	0	.	NA	.	.	.	.	.	.	.	.
0	0	.	NA	.	.	.	.	.	.	.	.
0	0	.	NA	.	.	.	.	KEC	.	.	REACTOME_CELL_CELL_COMI
0	0	.	NA	.	.	.	.	.	.	.	.
0	0	.	NA	.	.	.	.	.	.	.	.
0	0	.	NA	.	MUL	.	.	.	PID	.	.
0	0	Cone-rod c	NA	.	EST	CELI	NUCL	KEC	.	.	REACTOME_ABC_FAMILY_PRC
0	0	.	NA	.	.	.	.	.	.	.	.
0	0	.	NA	.	.	MEM	.	.	.	.	.
0	0	.	NA	.	.	.	.	.	.	.	.
0	0	.	NA	.	.	.	.	.	.	.	REACTOME_CROSS_PRESENT
0	0	.	NA	.	.	.	.	.	.	.	.
0	0	.	NA	.	.	.	.	.	.	.	.
0	0	.	NA	.	.	.	.	.	.	.	.
0	0	.	NA	.	.	.	.	.	.	.	.
0	0	.	NA	.	.	.	.	.	.	.	.
0	0	.	NA	.	.	.	.	.	.	.	.
0	0	.	NA	.	.	.	.	.	.	.	.
0	0	.	NA	.	.	.	.	.	.	.	.
0	0	.	NA	.	.	.	.	.	.	.	.

0	0	.	NA	.	.	.	.	.	.	.	.
0	0	.	NA	.	.	.	.	.	.	.	.
0	0	.	NA	.	.	.	.	.	.	.	.
0	0	.	NA	.	.	.	.	.	.	.	.
0	0	.	NA	.	.	.	.	.	.	.	.
0	0	.	NA	.	.	.	.	.	.	.	.
0	0	.	NA	.	.	.	.	.	.	.	.
0	0	.	NA	.	.	.	.	.	.	.	.
0	0	.	NA	.	.	.	.	.	.	.	.
0	0	.	NA	.	.	.	.	.	.	.	.
0	0	.	NA	.	.	.	.	.	.	.	.
0	0	.	NA	.	RNA	NUC	.	.	.	.	.
0	0	.	NA	.	.	.	.	.	.	.	.
0	0	.	NA	.	REG	.	.	.	.	.	.
0	0	.	NA	.	ACTI	ORG	RAS_	KE(	PID	BIO(	REACTOME_SIGNALING_BY_R
0	0	.	NA	.	.	.	.	.	.	.	.
0	0	.	NA	.	.	.	.	.	.	.	.
0	0	{Myocardia	NA	.	POS	INTF	.	KE(	.	.	.
0	0	Microcepha	NA	.	.	.	.	.	.	.	.
0	0	.	NA	.	.	.	.	.	.	.	.
0	0	.	NA	.	.	.	.	.	.	.	.
0	0	.	NA	.	.	.	.	.	.	.	.
0	0	.	NA	.	.	.	.	KE(	PID	BIO(	.
0	0	.	NA	.	.	.	.	.	.	.	.
0	0	.	NA	.	.	.	.	.	PID	.	REACTOME_SIGNALING_BY_R
0	0	.	NA	.	.	.	.	KE(	PID	BIO(	.
0	0	.	NA	.	.	.	.	.	.	.	REACTOME_RNA_POL_I_TRAN
0	0	.	NA	.	.	.	.	.	.	.	.
0	0	.	NA	.	.	.	LIGA	KE(	PID	.	REACTOME_METABOLISM_OF_
0	0	.	NA	.	.	.	.	.	.	.	.
0	0	Cystinuria	NA	.	EST	INTF	ACTI	.	.	.	REACTOME_AMINO_ACID_TRA
0	0	Cystinuria	NA	.	EST	INTF	ACTI	.	.	.	REACTOME_AMINO_ACID_TRA
0	0	.	NA	.	.	.	.	KE(	PID	.	REACTOME_SIGNALING_BY_R
0	0	.	NA	.	.	.	.	.	.	.	.
0	0	.	NA	.	.	.	.	.	.	.	REACTOME_SIGNALING_BY_R
0	0	Miyoshi mu	NA	.	SYS	MEM	.	.	.	.	.
0	0	.	NA	.	ISOF	ORG	OXID	KE(	.	.	.
0	0	.	NA	.	.	.	.	.	.	.	.
0	0	.	NA	.	.	.	.	.	.	.	.
0	0	.	NA	.	.	.	.	.	.	.	.
0	0	.	NA	.	.	.	.	.	.	.	.
0	0	.	NA	.	.	.	.	.	.	.	.
0	0	.	NA	.	.	.	.	.	.	.	.
0	0	.	NA	.	.	.	.	.	.	.	.
0	0	.	NA	.	.	.	.	.	.	.	.
0	0	.	NA	.	.	INTF	CATI	.	.	.	REACTOME_TRANSMEMBRANI
0	0	.	NA	.	.	.	.	.	.	.	.
0	0	.	NA	.	.	.	.	KE(	.	BIO(	REACTOME_RNA_POL_I_TRAN
0	0	.	NA	.	.	.	.	.	.	.	.
0	0	.	NA	.	.	.	.	.	.	.	.
0	0	.	NA	.	.	.	.	.	.	.	.
0	0	.	NA	.	.	.	.	.	.	.	.
0	0	.	NA	.	.	.	.	.	.	.	.
0	0	.	NA	.	.	.	.	.	.	.	.

0	0	.	NA	.	NEG	CYT	.	KEG	.	.	.
0	0	.	NA	.	.	.	.	.	.	.	.
0	0	.	NA	.	.	.	.	.	.	.	.
0	0	.	NA	.	.	.	.	.	.	.	.
0	0	.	NA	.	.	NUC	.	.	.	.	REACTOME_RNA_POL_III_TRA
0	0	Carbamoyl	NA	.	.	CYT	LIGAS	KEG	.	.	REACTOME_METABOLISM_OF_
0	0	.	NA	.	.	.	.	.	.	.	.
0	0	.	NA	.	DEFI	NUC	TRAN	.	.	.	.
0	0	.	NA	.	DEFI	NUC	TRAN	.	.	.	.
0	0	Brachydac	NA	.	RNA	NUC	TRAN	.	PID	BIO	REACTOME_NOTCH1_INTRACE
0	0	.	NA	.	.	.	.	.	.	.	.
0	0	.	NA	.	.	.	.	KEG	.	.	REACTOME_FACTORS_INVOLV
0	0	.	NA	.	.	.	.	.	.	.	.
0	0	.	NA	.	.	.	.	.	.	.	.
0	0	.	NA	.	.	.	.	KEG	.	.	.
0	0	Gray platel	NA	.	.	.	.	.	.	.	.
0	0	.	NA	.	.	.	.	.	.	.	.
0	0	Mitochondr	NA	.	.	.	.	.	.	.	.
0	0	.	NA	.	.	.	.	.	.	.	.
0	0	.	NA	.	.	.	.	KEG	.	.	.
0	0	.	NA	.	SYS	INTR	LIPO	.	.	.	.
0	0	.	NA	.	.	.	.	.	.	.	.
0	0	Robinow s	NA	.	SIGN	EXTI	RECE	KEG	PID	.	REACTOME_SIGNALING_BY_G
0	0	.	NA	.	.	.	.	KEG	PID	.	.
0	0	.	NA	.	.	.	.	.	.	.	.
0	0	.	NA	.	.	.	.	.	.	.	.
0	0	.	NA	.	.	.	.	KEG	.	.	.
0	0	.	NA	.	.	.	.	KEG	.	.	.
0	0	.	NA	.	.	.	.	.	.	.	.
0	0	.	NA	.	.	.	.	.	.	.	.
0	0	.	NA	.	.	NUC	ENDO	KEG	.	.	REACTOME_BASE_EXCISION_
0	0	.	NA	.	.	NUC	ENDO	KEG	.	.	REACTOME_BASE_EXCISION_
0	0	.	NA	.	.	.	.	.	.	.	.
0	0	Hailey-Hail	NA	.	REG	ORG	CATI	.	.	.	REACTOME_TRANSMEMBRANE
0	0	.	NA	.	.	.	.	.	.	.	.
0	0	Blepharopl	NA	.	DNA	NUC	ENZY	.	.	.	.
0	0	.	NA	.	.	.	.	KEG	PID	.	.
0	0	Cutaneous	NA	.	DNA	.	PROT	KEG	PID	BIO	REACTOME_MEIOSIS;REACTO
0	0	Bleeding d	NA	.	.	.	.	.	.	.	REACTOME_SIGNALING_BY_G
0	0	Leukemia,	NA	.	SYS	CYT	PROT	.	.	.	.
0	0	.	NA	.	.	.	.	.	.	.	.
0	0	Fanconi-Bi	NA	.	EST	INTR	SUGA	KEG	PID	.	REACTOME_DEVELOPMENTAL
0	0	.	NA	.	.	.	.	.	.	.	.
0	0	.	NA	.	.	.	.	.	.	.	.
0	0	.	NA	.	.	ORG	SMAL	KEG	.	.	.
0	0	.	NA	.	DNA	ORG	.	KEG	PID	.	REACTOME_CELL_CYCLE;REA
0	0	.	NA	.	.	.	.	.	.	.	.
0	0	.	NA	.	.	.	.	.	.	.	.
0	0	.	NA	.	.	.	.	.	.	.	.
0	0	.	NA	.	.	.	.	.	.	.	.
0	0	.	NA	.	.	.	.	.	.	.	.
0	0	.	NA	.	.	.	.	.	.	.	REACTOME_GENERIC_TRANS
0	0	.	NA	.	.	INTR	.	.	.	.	REACTOME_TRANSMEMBRANE
0	0	.	NA	.	REG	NUC	RNA_	KEG	PID	BIO	REACTOME_DEVELOPMENTAL

0	0	.	NA	.	.	.	.	.	.	.	.
0	0	.	NA	.	.	.	NUCL	.	.	.	.
0	0	.	NA	.	.	.	.	.	.	.	.
0	0	.	NA	.	.	.	.	.	.	.	.
0	0	.	NA	.	.	.	.	KEC	.	.	.
0	0	.	NA	.	.	.	.	KEC	.	.	.
0	0	.	NA	.	SIGN	CYT	.	.	.	.	.
0	0	Wolfram sy	NA	.	.	MAC	.	.	.	.	.
0	0	.	NA	.	.	EXTI	CARE	.	.	.	REACTOME_EXTRACELLULAR_
0	0	.	NA	.	.	.	.	.	.	.	.
0	0	Ceroid lipo	NA	.	.	.	.	KEC	.	.	.
0	0	.	NA	.	.	.	.	.	.	.	.
0	0	[Blood grou	NA	.	.	INTR	.	.	.	.	.
0	0	.	NA	.	SIGN	CYT	PHOS	KEC	.	.	REACTOME_NITRIC_OXIDE_ST
0	0	Short rib-p	NA	.	.	CYT	.	.	.	.	.
0	0	.	NA	.	.	.	.	.	.	.	.
0	0	.	NA	.	CELI	CYT	CATI	.	.	.	.
0	0	.	NA	.	.	.	.	.	.	.	.
0	0	Increased	NA	.	SYS	INTR	RECE	KEC	PID	BIO	REACTOME_GROWTH_HORMC
0	0	.	NA	.	.	.	.	.	.	.	.
0	0	.	NA	.	.	.	.	.	.	.	.
0	0	.	NA	.	.	.	.	.	.	.	.
0	0	.	NA	.	.	.	.	.	.	.	.
0	0	.	NA	.	.	.	.	.	.	.	REACTOME_RNA_POL_I_TRAN
0	0	.	NA	.	.	.	.	.	.	.	.
0	0	Sandhoff d	NA	.	.	.	PROT	KEC	.	.	REACTOME_GLYCOSPHINGOL
0	0	Sandhoff d	NA	.	.	.	PROT	KEC	.	.	REACTOME_GLYCOSPHINGOL
0	0	D-bifunctio	NA	.	.	CYT	LIPID	KEC	.	BIO	REACTOME_BILE_ACID_AND_E
0	0	Usher sync	NA	.	.	.	.	KEC	.	.	REACTOME_CYTOSOLIC_TRNA
0	0	Usher sync	NA	.	.	.	.	KEC	.	.	REACTOME_CYTOSOLIC_TRNA
0	0	.	NA	.	SIGN	NUC	HYDF	.	.	.	.
0	0	Hyperekple	NA	.	REG	INTR	EXCI	KEC	.	.	REACTOME_TRANSMEMBRANI
0	0	.	NA	.	.	.	.	.	.	.	.
0	0	.	NA	.	.	.	.	.	.	.	REACTOME_DEVELOPMENTAL
0	0	.	NA	.	.	.	.	.	.	.	.
0	0	.	NA	.	.	.	.	KEC	.	.	.
0	0	.	NA	.	.	.	.	.	.	.	.
0	0	.	NA	.	CELI	.	.	.	.	.	.
0	0	.	NA	.	RES	CYT	.	.	.	.	.
0	0	.	NA	.	GAM	.	.	.	.	.	.
0	0	.	NA	.	.	.	.	KEC	.	.	REACTOME_MEIOSIS;REACTO
0	0	.	NA	.	.	.	.	.	.	.	.
0	0	.	NA	.	.	.	.	.	.	.	.
0	0	.	NA	.	.	.	.	.	.	.	.
0	0	.	NA	.	RNA	NUC	RNA_	KEC	.	.	.
0	0	.	NA	.	RNA	NUC	RNA_	KEC	.	.	.
0	0	.	NA	.	.	.	.	.	.	.	.
0	0	{Asthma, s	NA	.	RNA	CELI	RECE	KEC	PID	BIO	REACTOME_EXTRINSIC_PATH
0	0	.	NA	.	.	.	.	.	.	.	.
0	0	.	NA	.	BIOF	NUC	PHOS	.	.	.	.
0	0	.	NA	.	.	.	.	.	.	.	.
0	0	Adrenal hy	NA	.	.	.	.	KEC	.	.	REACTOME_METABOLISM_OF_
0	0	.	NA	.	.	.	.	.	.	.	.
0	0	.	NA	.	RNA	NUC	SPEC	KEC	.	.	.

0	0	.	NA	.	.	.	.	.	.	.	.	
0	0	Combined	NA	.	.	.	.	KEG	.	.	REACTOME_MITOCHONDRIAL_	
0	0	.	NA	.	.	INTR	G_PR	.	.	.	.	
0	0	.	NA	.	.	.	.	.	.	.	REACTOME_DEFENSINS;REAC	
0	0	.	NA	.	MITO	ORG	PHOS	KEG	.	.	.	
0	0	.	NA	.	REG	CYT	ENZY	.	.	.	.	
0	0	.	NA	.	.	.	.	.	.	.	.	
0	0	.	NA	.	.	.	.	.	.	.	.	
0	0	.	NA	.	.	.	.	.	.	.	.	
0	0	.	NA	.	.	.	.	.	.	.	.	
0	0	.	NA	.	RNA	NUC	.	.	.	.	.	
0	0	Spastic pa	NA	.	.	.	.	.	.	.	.	
0	0	.	NA	.	.	.	.	.	.	.	.	
0	0	.	NA	.	.	.	.	.	.	.	.	
0	0	.	NA	.	.	.	.	.	.	.	.	
0	0	.	NA	.	.	.	.	.	.	.	.	
0	0	.	NA	.	.	.	.	.	.	.	.	
0	0	.	NA	.	.	.	.	.	.	.	.	
0	0	.	NA	.	.	.	.	.	.	.	.	
0	0	.	NA	.	.	.	.	.	.	.	.	
0	0	.	NA	.	.	.	.	.	.	.	.	
0	0	.	NA	.	.	.	.	.	.	.	.	
0	0	.	NA	.	.	.	.	.	.	.	.	
0	0	.	NA	.	.	.	.	.	.	.	.	
0	0	.	NA	.	.	.	.	KEG	.	.	REACTOME_O_LINKED_GLYCO	
0	0	{Prostate c	NA	.	REG	CYT	STRU	KEG	PID	.	.	
0	0	.	NA	.	.	.	.	.	.	.	.	
0	0	.	NA	.	.	.	.	.	.	.	.	
0	0	Alopecia, r	NA	.	.	.	.	.	.	.	.	
0	0	Alopecia, r	NA	.	.	.	.	.	.	.	.	
0	0	.	NA	.	.	.	.	.	.	.	.	
0	0	.	NA	.	.	.	.	.	.	.	.	
0	0	Weaver sy	NA	.	RNA	NUC	HIST	.	.	.	.	
0	0	.	NA	.	.	.	.	.	.	.	.	
0	0	.	NA	.	.	.	.	.	.	.	.	
0	0	.	NA	.	.	.	.	.	.	.	.	
0	0	Maturity-or	NA	.	SIGN	.	.	.	PID	.	REACTOME_ANTIGEN_ACTIVA	
0	0	.	NA	.	.	.	.	.	.	.	.	
0	0	.	NA	.	.	.	.	.	.	.	.	
0	0	.	NA	.	.	.	.	.	.	.	REACTOME_MEMBRANE_TRAF	
0	0	{?Schizoph	NA	.	NEG	.	TRAN	KEG	PID	BIO	REACTOME_SIGNALING_BY_E	
0	0	.	NA	.	.	.	.	KEG	.	.	.	
0	0	.	NA	.	EST	.	.	KEG	.	.	REACTOME_INSULIN_RECEPT	
0	0	.	NA	.	.	NUC	IDEN	.	PID	BIO	REACTOME_MEIOSIS;REACTO	
0	0	.	NA	.	SYS	.	TRAN	.	PID	.	REACTOME_NOTCH1_INTRACE	
0	0	.	NA	.	.	.	.	.	.	.	.	
0	0	.	NA	.	.	.	.	.	.	.	.	
0	0	.	NA	.	.	.	.	.	.	.	.	
0	0	.	NA	.	.	.	.	.	.	.	.	
0	0	.	NA	.	.	.	.	.	.	.	.	
0	0	.	NA	.	RNA	NUC	RNA_	KEG	.	.	.	
0	0	.	NA	.	.	.	.	.	.	.	.	
0	0	Spastic pa	NA	.	.	.	.	.	.	.	.	
0	0	Spastic pa	NA	.	.	.	.	.	.	.	.	

0	0	Mental reta	NA	.	.	.	.	.	.	.	.
0	0	.	NA	.	.	.	.	.	.	.	.
0	0	.	NA	.	.	.	.	.	.	.	.
0	0	.	NA	.	REG	.	.	KE(	PID	BIO(	REACTOME_TRAF6_MEDIATED
0	0	.	NA	.	.	.	.	.	.	.	.
0	0	.	NA	.	.	.	.	.	.	.	.
0	0	.	NA	.	.	.	.	.	.	.	.
0	0	.	NA	.	.	.	.	.	.	.	.
0	0	.	NA	.	.	.	.	.	.	.	.
0	0	.	NA	.	.	.	.	.	.	.	.
0	0	Choreoaca	NA	.	.	.	.	.	.	.	.
0	0	.	NA	.	.	.	.	.	PID	.	REACTOME_NOTCH1_INTRACE
0	0	.	NA	.	.	.	.	.	.	.	.
0	0	.	NA	.	.	.	.	.	.	.	.
0	0	.	NA	.	.	.	.	.	PID	.	.
0	0	Nail-patella	NA	.	RNA	NUC	TRAN	.	.	.	.
0	0	.	NA	.	CELI	.	.	.	.	.	.
0	0	.	NA	.	.	.	.	.	.	.	.
0	0	.	NA	.	.	.	.	.	.	.	.
0	0	.	NA	.	ENZ	.	.	KE(	PID	BIO(	REACTOME_SIGNALLING_BY_I
0	0	.	NA	.	.	.	.	.	.	.	.
0	0	.	NA	.	.	.	.	.	.	.	.
0	0	.	NA	.	BIOF	CYT	IDEN	.	.	.	REACTOME_METABOLISM_OF_
0	0	.	NA	.	.	.	.	.	.	.	.
0	0	.	NA	.	.	.	.	.	.	.	.
0	0	.	NA	.	.	.	.	.	.	.	.
0	0	.	NA	.	.	.	.	.	.	.	.
0	0	.	NA	.	.	.	.	.	.	.	.
0	0	.	NA	.	.	.	.	.	.	.	.
0	0	.	NA	.	.	.	.	KE(	PID	.	REACTOME_DEVELOPMENTAL
0	0	.	NA	.	.	.	.	KE(	PID	.	REACTOME_DEVELOPMENTAL
0	0	Deafness,	NA	.	REG	NON	.	.	.	.	.
0	0	.	NA	.	.	.	.	.	.	.	.
0	0	.	NA	.	.	.	.	.	.	.	.
0	0	Microceph	NA	.	M_P	ORG	MICR	.	.	.	REACTOME_MHC_CLASS_II_AI
0	0	.	NA	.	.	NUC	TRAN	.	.	.	.
0	0	{Age-relate	NA	.	.	.	LIPID	.	PID	.	.
0	0	.	NA	.	.	.	.	.	.	.	.
0	0	.	NA	.	.	.	.	.	.	.	REACTOME_O_LINKED_GLYCO
0	0	.	NA	.	.	.	.	.	.	.	.
0	0	Charcot-M	NA	.	.	MEM	.	.	.	.	.
0	0	.	NA	.	.	.	.	.	.	.	.
0	0	Denys-Dra	NA	.	.	.	.	.	PID	BIO(	.
0	0	.	NA	.	.	NUC	NUCL	KE(	.	.	.
0	0	.	NA	.	.	.	.	KE(	.	.	.
0	0	.	NA	.	.	.	.	.	.	.	.
0	0	.	NA	.	.	.	.	.	.	.	.
0	0	.	NA	.	.	.	.	.	.	.	.
0	0	.	NA	.	.	.	.	.	.	.	.
0	0	.	NA	.	.	.	.	.	.	.	.
0	0	.	NA	.	.	.	.	.	.	.	.
0	0	.	NA	.	.	.	.	.	.	.	.
0	0	.	NA	.	POS	.	.	KE(	PID	.	REACTOME_VEGF_LIGAND_RE
0	0	.	NA	.	.	CYT	DRU(	.	.	.	.

0	0	.	NA	.	.	CYT	DRU	.	.	.	.
0	0	.	NA	.	.	.	.	.	.	.	.
0	0	Glaucoma	NA	.	.	.	.	.	.	.	.
0	0	.	NA	.	.	.	.	KE	.	.	.
0	0	.	NA	.	.	INTR	.	KE	PID	.	REACTOME_DEVELOPMENTAL
0	0	.	NA	.	.	ORG	PEPT	.	.	.	.
0	0	.	NA	.	.	ORG	PEPT	.	.	.	.
0	0	Megalence	NA	.	.	.	.	.	.	.	.
0	0	.	NA	.	.	.	.	.	.	.	.
0	0	.	NA	.	.	.	HYDR	KE	.	.	REACTOME_GLYCOLYSIS;REA
0	0	.	NA	.	.	.	HYDR	KE	.	.	REACTOME_GLYCOLYSIS;REA
0	0	.	NA	.	.	.	.	.	.	.	REACTOME_MEMBRANE_TRAF
0	0	Warsaw br	NA	.	POS	ORG	.	.	.	.	REACTOME_DIABETES_PATHW
0	0	.	NA	.	.	.	.	.	.	.	.
0	0	.	NA	.	.	.	.	.	.	.	.
0	0	.	NA	.	RNA	NUC	RNA_	.	.	.	.
0	0	Vitamin D-	NA	.	BIOS	.	.	KE	.	.	REACTOME_METABOLISM_OF_
0	0	Vitamin D-	NA	.	BIOS	.	.	KE	.	.	REACTOME_METABOLISM_OF_
0	0	.	NA	.	.	.	.	.	.	.	.
0	0	.	NA	.	.	.	.	.	.	.	.
0	0	Deafness,	NA	.	.	.	.	.	.	.	.
0	0	.	NA	.	.	.	.	.	.	.	.
0	0	Mental reta	NA	.	CELI	.	.	.	PID	BIO	.
0	0	Arthrogryp	NA	.	.	CYT	STRU	.	.	.	REACTOME_STRIATED_MUSCL
0	0	.	NA	.	.	.	.	.	.	.	.
0	0	Transposit	NA	.	.	.	.	.	.	.	REACTOME_DEVELOPMENTAL
0	0	.	NA	.	.	.	.	.	.	.	.
0	0	.	NA	.	ACTI	.	LIPAS	KE	PID	.	REACTOME_ACYL_CHAIN_REM
0	0	.	NA	.	.	.	.	.	.	.	.
0	0	.	NA	.	.	.	.	.	.	.	.
0	0	.	NA	.	EST	.	.	.	PID	.	.
0	0	.	NA	.	.	ORG	.	.	.	.	.
0	0	.	NA	.	.	.	.	.	.	.	.
0	0	.	NA	.	.	.	.	.	PID	.	REACTOME_FACTORS_INVOLV
0	0	Spinocereb	NA	.	SYS	.	RECE	KE	.	.	.
0	0	.	NA	.	.	.	.	.	.	.	.
0	0	.	NA	.	.	.	.	.	.	.	.
0	0	.	NA	.	.	.	.	.	.	.	.
0	0	.	NA	.	.	.	.	.	.	.	.
0	0	.	NA	.	.	.	.	.	.	.	.
0	0	.	NA	.	.	.	.	.	.	.	.
0	0	Dystonia, D	NA	.	REG	.	HYDR	KE	.	.	REACTOME_TETRAHYDROBIO
0	0	.	NA	.	.	.	.	.	.	.	.
0	0	{Pheochror	NA	.	RNA	.	TRAN	KE	PID	BIO	REACTOME_CELL_CYCLE;REA
0	0	{Pheochror	NA	.	RNA	.	TRAN	KE	PID	BIO	REACTOME_CELL_CYCLE;REA
0	0	Microphtha	NA	.	.	.	.	.	.	.	.
0	0	.	NA	.	.	.	.	.	.	.	.
0	0	.	NA	.	.	.	.	.	.	.	.
0	0	.	NA	.	.	.	.	.	.	.	.
0	0	.	NA	.	.	.	CYST	KE	.	.	REACTOME_METABOLISM_OF_
0	0	.	NA	.	.	.	.	.	.	.	.
0	0	.	NA	.	SIGN	.	.	.	PID	BIO	.
0	0	.	NA	.	.	.	.	.	.	.	.
0	0	.	NA	.	.	.	.	.	.	.	.

0	0	.	NA	.	.	.	.	.	.	.	.
0	0	.	NA	.	.	.	.	.	.	.	.
0	0	[Skin/hair/e	NA	.	.	.	.	KE(	.	.	REACTOME_IMMUNE_SYSTEM
0	0	.	NA	.	.	.	.	.	.	.	.
0	0	.	NA	.	.	.	.	.	.	.	.
0	0	.	NA	.	.	.	.	.	.	.	.
0	0	.	NA	.	.	.	.	.	.	.	.
0	0	.	NA	.	.	.	.	.	.	.	.
0	0	.	NA	.	.	.	.	.	.	.	.
0	0	.	NA	.	.	.	.	.	.	.	.
0	0	.	NA	.	.	.	.	.	.	.	REACTOME_DEVELOPMENTAL
0	0	.	NA	.	.	.	.	.	.	.	.
0	0	.	NA	.	.	.	.	.	.	.	.
0	0	.	NA	.	MAC	EUK.	TRAN	.	.	.	REACTOME_TRANSLATION;RE
0	0	.	NA	.	.	.	.	.	.	.	.
0	0	.	NA	.	.	.	.	.	.	.	.
0	0	.	NA	.	.	.	.	.	.	.	.
0	0	.	NA	.	SYS	MEM	ENZY	.	.	.	.
0	0	.	NA	.	.	.	.	.	.	.	.
0	0	Cardiofacic	NA	.	SIGN	.	PHOS	KE(	PID	BIO(	REACTOME_SIGNALLING_BY_I
0	0	.	NA	.	SIGN	.	.	KE(	PID	BIO(	REACTOME_SIGNALLING_BY_I
0	0	.	NA	.	.	.	.	.	.	.	.
0	0	.	NA	.	.	.	.	.	.	.	.
0	0	.	NA	.	EST/	INTF	CATI(	KE(	.	.	REACTOME_TRANSMISSION_A
0	0	.	NA	.	.	.	.	.	.	.	.
0	0	.	NA	.	.	.	.	.	.	.	.
0	0	Fanconi ar	NA	.	.	.	.	.	PID	.	.
0	0	Arthrogryp	NA	.	EST/	.	.	.	.	.	.
0	0	.	NA	.	.	INTF	LIPID	.	.	.	.
0	0	{Coronary	NA	.	MUS	NUC	.	.	PID	BIO(	REACTOME_SIGNALLING_BY_I
0	0	.	NA	.	.	.	.	KE(	.	.	.
0	0	.	NA	.	.	.	.	.	.	.	.
0	0	Osteopetrc	NA	.	.	.	.	.	.	.	.
0	0	.	NA	.	.	ORG	PROT	.	.	.	.
0	0	.	NA	.	.	.	.	.	.	.	.
0	0	.	NA	.	SIGN	INTF	RECE	KE(	.	BIO(	.
0	0	.	NA	.	.	.	.	.	.	.	.
0	0	.	NA	.	.	.	.	.	.	.	.
0	0	.	NA	.	.	.	.	.	.	.	.
0	0	.	NA	.	BIOF	CYT(	PHOS	.	.	.	REACTOME_METABOLISM_OF_
0	0	.	NA	.	BIOF	CYT(	TRAN	.	PID	BIO(	REACTOME_INSULIN_RECEPT(
0	0	.	NA	.	.	.	.	.	.	.	REACTOME_RNA_POL_III_TRA
0	0	.	NA	.	.	.	.	.	.	.	.
0	0	.	NA	.	EST/	.	PROT	.	.	.	.
0	0	.	NA	.	EST/	.	PROT	.	.	.	.
0	0	.	NA	.	.	.	.	.	.	.	.
0	0	.	NA	.	.	.	.	.	.	.	.
0	0	{Systemic l	NA	.	.	INTF	.	KE(	PID	BIO(	REACTOME_CELL_SURFACE_I
0	0	.	NA	.	.	.	.	.	.	.	.
0	0	.	NA	.	.	CYT(	.	.	.	.	REACTOME_ASSOCIATION_OF
0	0	.	NA	.	.	.	.	KE(	.	.	.
0	0	.	NA	.	RNA	NUC	CATI(	.	.	.	.
0	0	.	NA	.	.	.	.	.	.	.	.
0	0	.	NA	.	.	.	.	.	.	.	.

0	0	.	NA	.	.	.	.	.	.	.	.
0	0	.	NA	.	.	.	.	.	.	.	.
0	0	Bleeding d	NA	.	EST	INTR	CATI	KEG	.	.	REACTOME_ELEVATION_OF_C
0	0	.	NA	.	.	.	.	.	.	.	.
0	0	.	NA	.	SYS	INTR	.	KEG	.	BIO	.
0	0	.	NA	.	.	.	.	KEG	.	.	.
0	0	.	NA	.	.	.	MOT	KEG	.	.	.
0	0	.	NA	.	GAM	ORG	.	.	.	.	.
0	0	Mitochondr	NA	.	.	.	.	.	.	.	.
0	0	.	NA	.	RNA	ORG	RNA_	KEG	PID	.	REACTOME_DEVELOPMENTAL
0	0	.	NA	.	.	.	.	.	.	.	.
0	0	.	NA	.	.	.	.	.	.	.	.
0	0	.	NA	.	.	.	.	.	.	.	.
0	0	.	NA	.	.	.	.	.	.	.	.
0	0	.	NA	.	.	.	.	.	.	.	.
0	0	.	NA	.	.	.	.	.	.	.	.
0	0	.	NA	.	.	.	.	.	.	.	.
0	0	.	NA	.	.	NUC	HIST	.	.	.	.
0	0	.	NA	.	.	NUC	HIST	.	.	.	.
0	0	.	NA	.	REG	ORG	.	.	.	.	REACTOME_SIGNALING_BY_R
0	0	.	NA	.	.	.	.	.	.	.	.
0	0	.	NA	.	.	.	.	.	.	.	.
0	0	.	NA	.	.	.	.	.	.	.	.
0	0	Acetyl-CoA	NA	.	.	.	LIGA	KEG	.	BIO	REACTOME_TRIGLYCERIDE_B
0	0	.	NA	.	EST	ORG	.	.	.	.	.
0	0	.	NA	.	.	.	.	.	.	.	.
0	0	.	NA	.	RNA	NUC	RNA_	.	PID	BIO	REACTOME_DEVELOPMENTAL
0	0	.	NA	.	.	.	.	.	.	.	.
0	0	.	NA	.	.	.	.	.	.	.	.
0	0	Ovalocytos	NA	.	REG	INTR	ANIO	.	.	.	REACTOME_TRANSMEMBRAN
0	0	.	NA	.	.	.	.	KEG	.	.	.
0	0	.	NA	.	.	MEM	CATI	KEG	PID	.	REACTOME_DEVELOPMENTAL
0	0	.	NA	.	.	CYT	RECE	.	.	.	.
0	0	.	NA	.	.	.	.	KEG	.	.	.
0	0	.	NA	.	.	.	.	KEG	.	.	.
0	0	.	NA	.	.	.	.	.	.	.	.
0	0	Hemophag	NA	.	.	.	.	.	.	.	.
0	0	.	NA	.	.	.	.	.	.	.	.
0	0	.	NA	.	SYS	MEM	MOLE	KEG	PID	BIO	.
0	0	.	NA	.	.	.	.	.	.	.	.
0	0	.	NA	.	REG	INTR	.	.	.	.	.
0	0	.	NA	.	.	.	.	.	.	.	.
0	0	.	NA	.	.	.	.	.	.	.	.
0	0	.	NA	.	.	CYT	OXID	.	.	.	.
0	0	.	NA	.	.	.	.	.	.	.	.
0	0	.	NA	.	RNA	NUC	S_AD	.	.	.	REACTOME_RNA_POL_II_TRAN
0	0	.	NA	.	.	.	.	.	.	.	.
0	0	Scalp-ear-	NA	.	.	.	.	.	.	.	.
0	0	.	NA	.	.	.	.	.	.	.	.
0	0	Pitt-Hopkin	NA	.	RNA	NUC	TRAN	.	PID	.	REACTOME_DEVELOPMENTAL
0	0	.	NA	.	.	.	.	.	.	.	.
0	0	Osteolysis,	NA	.	POS	.	RECE	KEG	.	BIO	.
0	0	.	NA	.	.	.	.	.	.	.	.
0	0	.	NA	.	.	.	.	.	.	.	REACTOME_TRANSMEMBRAN

0	0	.	NA	.	.	.	.	.	.	.	.
0	0	.	NA	.	.	.	.	.	.	.	REACTOME_SIGNALING_BY_R
0	0	.	NA	.	.	.	.	.	.	.	REACTOME_SIGNALING_BY_R
0	0	Leukemia,	NA	.	RNA	.	PROT	.	PID	.	REACTOME_DEVELOPMENTAL
0	0	Leukemia,	NA	.	RNA	.	PROT	.	PID	.	REACTOME_DEVELOPMENTAL
0	0	.	NA	.	.	.	.	.	.	.	.
0	0	.	NA	.	.	.	META	KEG	.	.	.
0	0	.	NA	.	.	.	META	KEG	.	.	.
0	0	.	NA	.	RNA	.	TRAN	.	PID	.	.
0	0	.	NA	.	CHR	NUC	CHRO	.	.	.	.
0	0	.	NA	.	.	.	.	.	.	.	.
0	0	Hemophag	NA	.	.	.	.	.	.	.	.
0	0	{Reduced t	NA	.	REG	EXTI	ENZY	KEG	.	.	REACTOME_DEVELOPMENTAL
0	0	.	NA	.	.	.	.	.	.	.	.
0	0	.	NA	.	.	.	.	.	.	.	.
0	0	.	NA	.	.	MEM	.	.	.	.	REACTOME_O_LINKED_GLYCO
0	0	.	NA	.	.	MEM	.	.	.	.	REACTOME_O_LINKED_GLYCO
0	0	.	NA	.	.	MEM	.	.	.	.	REACTOME_O_LINKED_GLYCO
0	0	.	NA	.	.	MEM	.	.	.	.	REACTOME_O_LINKED_GLYCO
0	0	.	NA	.	.	.	TRAN	.	.	.	.
0	0	.	NA	.	.	.	.	.	.	.	REACTOME_GENERIC_TRANS
0	0	.	NA	.	ESTA	.	.	.	.	.	.
0	0	.	NA	.	RNA	ORG	RNA_	.	PID	BIO	REACTOME_SMAD2_SMAD3_S
0	0	.	NA	.	.	.	.	.	.	.	.
0	0	.	NA	.	.	.	.	.	.	.	.
0	0	Thyroid dys	NA	.	.	INTR	INOR	.	.	.	REACTOME_METABOLISM_OF_
0	0	.	NA	.	.	.	.	.	.	.	.
0	0	.	NA	.	.	.	.	.	.	.	.
0	0	.	NA	.	.	.	.	.	.	.	.
0	0	.	NA	.	.	.	.	.	.	.	.
0	0	.	NA	.	.	.	.	.	.	.	.
0	0	.	NA	.	.	.	.	.	.	.	.
0	0	.	NA	.	.	.	.	.	.	.	.
0	0	.	NA	.	.	.	.	.	.	.	.
0	0	.	NA	.	.	.	.	.	.	.	.
0	0	.	NA	.	.	.	.	.	.	.	.
0	0	.	NA	.	.	.	.	.	.	.	.
0	0	.	NA	.	CELL	MEM	RAS_	KEG	PID	BIO	REACTOME_SIGNALLING_BY_N
0	0	.	NA	.	.	.	GLUT	KEG	.	.	REACTOME_TRANSMISSION_A
0	0	.	NA	.	.	.	GLUT	KEG	.	.	REACTOME_TRANSMISSION_A
0	0	.	NA	.	.	.	.	.	.	.	.
0	0	.	NA	.	BEH	CELL	ENZY	KEG	PID	.	REACTOME_METABOLISM_OF_
0	0	.	NA	.	BEH	CELL	ENZY	KEG	PID	.	REACTOME_METABOLISM_OF_
0	0	.	NA	.	.	.	.	.	.	.	.
0	0	.	NA	.	SYS	.	TRAN	.	.	.	.
0	0	.	NA	.	.	.	.	.	.	.	.
0	0	.	NA	.	.	.	.	.	.	.	REACTOME_GENERIC_TRANS
0	0	.	NA	.	.	.	.	.	.	.	.
0	0	.	NA	.	.	.	.	.	.	.	.
0	0	.	NA	.	REG	NUC	RECE	KEG	.	.	.
0	0	.	NA	.	.	.	SMAL	.	.	.	.
0	0	.	NA	.	.	.	SMAL	.	.	.	.
0	0	.	NA	.	SIGN	INTR	.	.	.	.	REACTOME_CELL_CELL_COMM
0	0	Spinocereb	NA	.	.	.	.	.	.	.	.

0	0	.	NA	.	.	.	.	.	.	.	.
0	0	.	NA	.	.	.	.	KEG	.	.	.
0	0	Epileptic e	NA	.	.	CYT	.	KEG	PID	BIO	REACTOME_GASTRIN_CREB_S
0	0	.	NA	.	.	.	.	.	.	.	.
0	0	.	NA	.	.	.	.	.	.	.	.
0	0	.	NA	.	.	.	.	.	.	.	.
0	0	.	NA	.	.	.	.	.	.	.	.
0	0	.	NA	.	SYS	.	.	KEG	PID	BIO	REACTOME_SIGNALING_BY_IL
0	0	.	NA	.	RNA	NUC	TRAN	.	PID	.	REACTOME_DEVELOPMENTAL
0	0	.	NA	.	RNA	NUC	TRAN	.	PID	.	REACTOME_DEVELOPMENTAL
0	0	.	NA	.	.	.	.	.	.	.	.
0	0	.	NA	.	.	.	.	.	PID	.	.
0	0	.	NA	.	.	.	.	.	.	.	.
0	0	.	NA	.	.	.	.	.	.	.	.
0	0	.	NA	.	.	.	.	.	.	.	.
0	0	.	NA	.	.	.	.	.	.	BIO	.
0	0	.	NA	.	.	.	.	.	.	.	.
0	0	.	NA	.	.	.	.	.	.	.	.
0	0	.	NA	.	.	.	.	.	.	.	.
0	0	.	NA	.	.	.	.	.	.	.	.
0	0	.	NA	.	.	.	.	.	.	.	.
0	0	.	NA	.	.	.	.	.	.	.	.
0	0	.	NA	.	.	.	.	.	.	.	.
0	0	.	NA	.	.	.	.	.	.	.	.
0	0	.	NA	.	.	CYT	.	.	.	.	.
0	0	Homocysti	NA	CM9	.	CYT	HYDF	KEG	.	.	REACTOME_SULFUR_AMINO_A
0	0	.	NA	.	.	.	.	.	.	.	.
0	0	Knobloch s	NA	.	SYS	PRO	.	.	PID	.	.
0	0	.	NA	.	.	.	.	.	.	.	.
0	0	.	NA	.	.	.	.	.	.	.	.
0	0	.	NA	.	.	.	.	.	.	.	.
0	0	.	NA	.	.	.	.	.	.	.	.
0	0	Hyperprolir	NA	CM0	.	.	OXID	KEG	.	.	REACTOME_METABOLISM_OF_
0	0	.	NA	.	.	.	.	.	.	.	.
0	0	.	NA	.	.	.	.	.	.	.	.
0	0	.	NA	.	.	.	.	.	.	.	.
0	0	.	NA	.	.	.	.	.	.	.	.
0	0	.	NA	.	.	.	.	.	.	.	.
0	0	.	NA	.	ANA	.	.	.	.	.	.
0	0	.	NA	.	EST	CYT	.	.	PID	.	.
0	0	.	NA	.	PAT	EXTI	.	KEG	.	.	REACTOME_PRE_NOTCH_EXP
0	0	.	NA	.	.	.	.	.	.	.	.
0	0	.	NA	.	.	INTR	.	.	.	.	.
0	0	Nephronop	NA	.	.	.	.	.	.	.	.
0	0	Colorectal	NA	.	REG	NUC	TRAN	KEG	PID	BIO	REACTOME_DEVELOPMENTAL
0	0	.	NA	.	RNA	.	RNA_	.	.	.	REACTOME_PPARA_ACTIVATE
0	0	.	NA	.	.	.	.	KEG	.	.	REACTOME_MEIOSIS;REACTO
0	0	.	NA	.	.	.	.	.	.	.	.
0	0	.	NA	.	CELI	.	NUCL	.	.	.	.
0	0	.	NA	.	.	.	.	KEG	.	.	.
0	0	.	NA	.	.	.	.	KEG	.	.	.
0	0	.	NA	.	.	.	.	.	.	.	.
0	0	.	NA	.	GAM	ORG	.	.	.	.	.
0	0	.	NA	.	.	.	.	.	.	.	.

0	0	.	NA	.	POS	.	RNA_	KE(	.	BIO(	REACTOME_TRAFFICKING_AN
0	0	.	NA	.	POS	.	RNA_	KE(	.	.	REACTOME_TRAFFICKING_AN
0	0	Becker mu	NA	.	BIOS	PRO	ACTII	KE(	.	BIO(	REACTOME_STRIATED_MUSCI
0	0	Brunner sy	NA	.	BEH.	.	.	KE(	.	.	REACTOME_BIOLOGICAL_OXII
0	0	?Sarcoma,	NA	.	.	NUC	TRAN	.	.	.	.
0	0	.	NA	.	.	EXTI	RECE	.	.	.	.
0	0	.	NA	.	.	.	.	.	.	.	.
0	0	.	NA	.	.	.	.	.	.	.	.
0	0	.	NA	.	.	.	.	.	.	.	.
0	0	.	NA	.	.	.	.	.	.	.	.
0	0	.	NA	.	.	.	.	.	.	.	REACTOME_TCA_CYCLE_AND_
0	0	.	NA	.	.	.	.	.	.	.	.
0	0	Lujan-Fryn	NA	.	RNA	NUC	RNA_	.	PID	.	REACTOME_DEVELOPMENTAL
0	0	{Asperger :	NA	.	.	.	.	KE(	.	.	.
0	0	.	NA	.	.	.	.	.	.	.	.
0	0	.	NA	.	.	.	.	.	.	.	.
0	0	.	NA	.	.	.	.	.	.	.	.
0	0	.	NA	.	.	.	.	.	.	.	.
0	0	.	NA	.	.	.	.	.	.	.	.
0	0	.	NA	.	.	.	.	.	.	.	.
0	0	Mental reta	NA	.	.	.	.	KE(	PID	BIO(	REACTOME_SIGNALING_BY_R
0	0	.	NA	.	MUL	.	DNA_	.	.	.	.
0	0	.	NA	.	DNA	.	HYDF	.	.	.	.
0	0	.	NA	.	.	INTF	.	.	.	.	REACTOME_APOPTOTIC_CLEA
0	0	.	NA	.	.	.	.	.	.	.	.
0	0	.	NA	.	.	.	.	.	.	.	.
0	0	.	NA	.	.	.	.	.	.	.	.
0	0	.	NA	.	.	.	.	.	.	.	.
0	0	.	NA	.	.	.	.	.	.	.	.
0	0	.	NA	.	.	.	.	.	.	.	.
0	0	.	NA	.	.	.	.	.	.	.	.
0	0	.	NA	.	.	.	.	.	.	.	.
0	0	.	NA	.	.	.	.	.	.	.	.
0	0	.	NA	.	.	.	.	.	.	.	.
0	0	.	NA	.	.	.	.	.	.	.	.
0	0	.	NA	.	CEN	ORG	STRU	.	.	.	.
0	0	Rhabdomy	NA	.	CELI	.	TRAN	.	.	.	.
0	0	.	NA	.	REG	CYT(	.	.	.	.	REACTOME_INTERFERON_ALF
0	0	.	NA	.	NUC	.	TRAN	.	.	.	.
0	0	.	NA	.	.	.	.	.	.	.	.
0	0	.	NA	.	.	.	.	.	.	.	.
0	0	.	NA	.	.	.	.	.	.	.	.
0	0	.	NA	.	.	.	.	.	.	.	.
0	0	.	NA	.	.	.	.	.	.	.	.
0	0	.	NA	.	.	.	.	.	.	.	.
0	0	.	NA	.	.	.	.	.	.	.	.
0	0	.	NA	.	.	.	.	.	.	.	.
0	0	.	NA	.	.	.	.	.	.	.	.
0	0	.	NA	.	.	.	.	.	.	.	.
0	0	.	NA	.	.	.	.	.	.	.	.
0	0	.	NA	.	.	.	.	.	.	.	.
0	0	.	NA	.	.	.	.	.	.	.	.
0	0	.	NA	.	.	.	.	.	.	.	.
0	0	.	NA	.	.	.	.	.	.	.	.

0	0	.	NA	.	.	.	.	.	.	.	.
0	0	.	NA	.	.	.	.	.	.	.	.
0	0	.	NA	.	.	.	.	.	.	.	.
0	0	.	NA	.	.	.	.	.	.	.	.
0	0	.	NA	.	.	.	.	.	.	.	.
0	0	.	NA	.	.	.	.	KE(	PID	.	REACTOME_DEVELOPMENTAL
0	0	.	NA	.	.	.	.	.	.	.	.
0	0	.	NA	.	.	.	.	.	.	.	.
0	0	.	NA	.	.	.	.	.	.	.	.
0	0	.	NA	.	.	.	.	.	.	.	.
0	0	.	NA	.	.	.	.	.	.	.	.
0	0	.	NA	.	.	.	.	KE(	.	.	REACTOME_INHIBITION_OF_TI
0	0	.	NA	.	.	.	.	.	.	.	.
0	0	.	NA	.	.	.	.	.	.	.	.
0	0	.	NA	.	.	.	.	.	.	.	.
0	0	.	NA	.	.	.	.	.	.	.	.
0	0	.	NA	.	.	.	.	.	.	.	.
0	0	.	NA	.	BIOS	ORG	STEF	KE(	.	.	.
0	0	Hypomyeli	NA	.	EST/	CYT(	ACTI'	.	.	.	REACTOME_MITOCHONDRIAL_
0	0	.	NA	.	.	.	.	KE(	.	.	REACTOME_IMMUNE_SYSTEM
0	0	.	NA	.	.	.	.	.	.	.	.
0	0	.	NA	.	.	.	.	.	.	.	.
0	0	Hypertensi	NA	.	REG	INTF	.	KE(	PID	BIO(	REACTOME_GASTRIN_CREB_
0	0	Cerebellar	NA	.	REG	EXTI	OXID	KE(	PID	.	REACTOME_TRANSMEMBRANI
0	0	Cerebellar	NA	.	REG	EXTI	OXID	KE(	PID	.	REACTOME_TRANSMEMBRANI
0	0	.	NA	.	.	.	.	.	.	.	.
0	0	.	NA	.	.	.	.	.	.	.	.
0	0	.	NA	.	.	.	.	.	.	.	.
0	0	.	NA	.	.	.	.	.	.	.	REACTOME_GENERIC_TRANS(
0	0	.	NA	.	AXO	EXTI	RECE	KE(	PID	.	REACTOME_DEVELOPMENTAL
0	0	Neuropathy	NA	.	.	.	.	.	.	.	.
0	0	.	NA	.	.	.	.	.	.	.	.
0	0	.	NA	.	.	.	.	.	.	.	.
0	0	.	NA	.	.	.	.	.	.	.	.
0	0	.	NA	.	.	.	.	.	.	.	.
0	0	Hyperglyci	NA	.	.	.	.	.	.	.	REACTOME_AMINO_ACID_TRA
0	0	.	NA	.	REG	.	.	KE(	PID	.	.
0	0	.	NA	.	.	.	.	.	.	.	REACTOME_EXTRACELLULAR_
0	0	Ehlers-Dar	NA	.	.	.	META	.	.	.	REACTOME_EXTRACELLULAR_
0	0	.	NA	.	.	.	.	.	.	.	.
0	0	.	NA	.	.	.	.	.	.	.	.
0	0	.	NA	.	.	.	.	.	.	.	.
0	0	.	NA	.	.	.	.	.	.	.	.
0	0	.	NA	.	.	.	.	.	.	.	.
0	0	Emery-Dre	NA	.	ORG	SYN.	ACTI	.	.	.	REACTOME_MEIOSIS;REACTO
0	0	.	NA	.	.	.	.	.	.	.	.
0	0	.	NA	.	.	.	.	.	.	.	.
0	0	.	NA	.	.	.	.	.	.	.	.
0	0	.	NA	.	.	.	.	.	.	.	.
0	0	.	NA	.	.	.	.	.	.	.	.
0	0	.	NA	.	.	.	.	.	.	.	.
0	0	.	NA	.	.	.	.	.	.	.	.
0	0	.	NA	.	.	.	.	.	.	.	.
0	0	.	NA	.	.	.	.	.	.	.	.

0	0	.	NA	.	.	.	.	.	.	.	.
0	0	.	NA	.	.	.	.	.	.	.	.
0	0	.	NA	.	.	.	.	.	.	.	.
0	0	.	NA	.	.	.	.	.	.	.	.
0	0	.	NA	.	.	.	.	.	.	.	.
0	0	.	NA	.	.	.	.	.	.	.	.
0	0	.	NA	.	.	.	.	.	.	.	.
0	0	.	NA	.	.	.	.	KEG	.	.	REACTOME_ACTIVATED_NOTC
0	0	.	NA	.	.	.	.	KEG	.	.	.
0	0	.	NA	.	.	.	.	.	.	.	.
0	0	.	NA	.	.	.	.	.	.	.	.
0	0	.	NA	.	.	.	.	.	.	.	.
0	0	.	NA	.	.	.	.	.	.	.	.
0	0	.	NA	.	.	.	.	.	.	.	.
0	0	.	NA	.	.	.	.	.	.	.	.
0	0	.	NA	.	.	.	.	.	.	.	.
0	0	.	NA	.	.	.	.	.	.	.	.
0	0	.	NA	.	.	.	.	.	.	.	.
0	0	.	NA	.	.	.	.	.	.	.	.
0	0	Myoclonus	NA	.	.	.	.	.	.	.	.
0	0	5-oxoprolir	NA	.	.	.	.	KEG	.	.	REACTOME_BIOLOGICAL_OXID
0	0	.	NA	.	.	.	.	.	.	.	.
0	0	.	NA	.	.	.	.	.	.	.	.
0	0	.	NA	.	.	.	.	.	.	.	.
0	0	.	NA	.	.	.	.	.	.	.	.
0	0	.	NA	.	.	.	.	.	.	.	.
0	0	.	NA	.	.	.	.	.	.	.	.
0	0	.	NA	.	.	.	.	.	.	.	.
0	0	.	NA	.	.	.	.	.	PID	.	REACTOME_SIGNALLING_BY_I
0	0	.	NA	.	.	.	.	.	.	.	.
0	0	.	NA	.	.	.	.	.	.	.	.
0	0	.	NA	.	.	.	.	.	.	.	.
0	0	.	NA	.	RNA	.	.	.	PID	BIO(	.
0	0	.	NA	.	.	.	.	.	.	.	.
0	0	.	NA	.	.	.	.	.	.	.	.
0	0	.	NA	.	NEG	CYT(	.	.	.	.	.
0	0	.	NA	.	.	.	.	.	.	.	.
0	0	.	NA	.	.	.	.	.	.	.	.
0	0	.	NA	.	.	.	.	.	.	.	.
0	0	.	NA	.	.	.	.	.	.	.	.
0	0	.	NA	.	.	.	.	.	.	.	.
0	0	.	NA	.	.	.	.	.	.	.	.
0	0	.	NA	.	SIGN	.	.	.	.	.	.
0	0	.	NA	.	.	.	.	.	.	.	.
0	0	.	NA	.	.	.	.	KEG	.	.	.
0	0	.	NA	.	CELI	.	.	.	.	.	.
0	0	Pachyonyc	NA	.	SYS	ORG	.	.	.	.	.
0	0	Lethal con(	NA	.	ENZ	INTF	TRAN	KEG	PID	BIO(	REACTOME_SIGNALING_BY_EI
0	0	.	NA	.	DNA	NUC	DAM/	KEG	.	.	REACTOME_BASE_EXCISION_I
0	0	.	NA	.	.	.	.	.	.	.	.
0	0	.	NA	.	SYS	NUC	ACTI	.	.	.	.
0	0	.	NA	.	.	.	.	.	.	.	.
0	0	.	NA	.	.	.	.	.	.	.	.
0	0	.	NA	.	.	.	.	.	.	.	.

0	0	.	NA	.	.	.	.	.	.	.	.
0	0	.	NA	.	.	.	.	.	.	.	.
0	0	{Cerebral i	NA	.	BIOF	.	PHOS	KEG	PID	BIO	REACTOME_GASTRIN_CREB_S
0	0	Tyrosinemi	NA	.	CAR	CYT	OXID	KEG	.	.	REACTOME_METABOLISM_OF_
0	0	.	NA	.	.	.	.	.	.	.	.
0	0	.	NA	.	.	.	.	.	.	.	.
0	0	.	NA	.	.	.	.	.	.	.	.
0	0	.	NA	.	.	.	.	.	.	.	.
0	0	.	NA	.	.	.	.	.	.	.	.
0	0	.	NA	.	.	.	.	.	.	.	.
0	0	.	NA	.	.	.	.	.	.	.	.
0	0	.	NA	.	.	.	.	.	.	.	.
0	0	.	NA	.	.	.	.	.	.	.	.
0	0	.	NA	.	.	.	.	.	.	.	.
0	0	.	NA	.	CAR	ORG	LIGAS	KEG	.	.	REACTOME_BILE_ACID_AND_B
0	0	.	NA	.	EST/	CYT	.	.	PID	.	.
0	0	.	NA	.	.	.	.	.	.	.	.
0	0	.	NA	.	.	.	.	.	.	.	.
0	0	.	NA	.	.	.	.	.	.	.	.
0	0	.	NA	.	.	.	.	.	.	.	.
0	0	.	NA	.	.	.	.	.	.	.	.
0	0	.	NA	.	.	.	.	.	.	.	.
0	0	.	NA	.	.	.	.	.	.	.	.
0	0	.	NA	.	.	.	.	.	.	.	.
0	0	.	NA	.	.	.	.	.	.	.	.
0	0	.	NA	.	.	.	.	.	.	.	.
0	0	.	NA	.	.	.	.	.	.	.	.
0	0	.	NA	.	.	.	.	.	.	.	.
0	0	.	NA	.	.	.	.	.	.	.	.
0	0	.	NA	.	.	.	.	.	.	.	.
0	0	.	NA	.	.	.	.	.	.	.	.
0	0	.	NA	.	.	.	.	.	.	.	.
0	0	.	NA	.	.	.	.	.	.	.	.
0	0	.	NA	.	.	.	.	.	.	.	.
0	0	.	NA	.	.	.	.	.	.	.	.
0	0	.	NA	.	.	.	.	.	.	.	.
0	0	.	NA	.	.	.	.	.	.	.	.
0	0	.	NA	.	.	.	.	.	.	.	.
0	0	.	NA	.	.	.	.	.	.	.	.
0	0	.	NA	.	.	.	.	.	.	.	.
0	0	.	NA	.	.	.	.	.	.	.	.
0	0	.	NA	.	.	.	.	.	.	.	.
0	0	.	NA	.	.	.	.	.	.	.	.
0	0	.	NA	.	.	.	.	.	.	.	.
0	0	.	NA	.	.	.	.	.	.	.	.
0	0	.	NA	.	.	.	.	.	.	.	.
0	0	.	NA	.	.	.	.	.	.	.	.
0	0	.	NA	.	.	.	.	.	.	.	.
0	0	.	NA	.	.	.	.	.	.	.	.
0	0	.	NA	.	.	.	.	.	.	.	.
0	0	.	NA	.	.	.	.	.	.	.	.
0	0	.	NA	.	.	.	.	.	.	.	.
0	0	Cortical dy	NA	.	MICF	ORG	STRU	.	PID	.	REACTOME_CELL_CYCLE;REA
0	0	.	NA	.	.	.	.	.	.	.	.

0	0	.	NA	.	.	.	.	.	.	.	.
0	0	.	NA	.	.	.	.	.	.	.	.
0	0	.	NA	.	RES	.	.	.	.	.	.
0	0	.	NA	.	.	.	.	.	.	.	.
0	0	.	NA	.	IMMU	INTR	.	KEG	PID	.	.
0	0	.	NA	.	.	.	.	.	.	.	.
0	0	.	NA	.	.	.	.	.	.	.	.
0	0	.	NA	.	.	.	.	.	.	.	.
0	0	.	NA	.	SEN	ORG	.	.	.	.	.
0	0	.	NA	.	.	.	.	.	.	.	.
0	0	.	NA	.	.	.	.	.	.	.	.
0	0	.	NA	.	SIGN	INTR	RECE	KEG	.	.	REACTOME_IMMUNOREGULAT
0	0	.	NA	.	.	.	.	.	.	.	.
0	0	.	NA	.	RNA	NUC	TRAN	.	PID	.	REACTOME_DEVELOPMENTAL
0	0	.	NA	.	.	.	.	.	.	.	.
0	0	.	NA	.	.	.	.	.	.	.	.
0	0	.	NA	.	.	.	.	.	.	.	.
0	0	.	NA	.	.	.	.	.	.	.	.
0	0	.	NA	.	.	.	.	.	.	.	.
0	0	.	NA	.	.	.	.	.	.	.	.
0	0	.	NA	.	.	.	.	.	.	.	.
0	0	.	NA	.	.	.	.	.	.	.	.
0	0	.	NA	.	.	.	.	.	.	.	.
0	0	.	NA	.	.	.	.	.	.	.	.
0	0	.	NA	.	.	.	.	.	.	.	.
0	0	.	NA	.	.	.	.	.	.	.	.
0	0	.	NA	.	CELL	NON	ACTI	KEG	PID	.	.
0	0	Mental reta	NA	.	.	.	.	.	.	.	.
0	0	.	NA	.	.	.	.	.	.	.	.
0	0	.	NA	.	.	.	.	.	.	.	.
0	0	.	NA	.	.	.	.	.	.	.	.
0	0	.	NA	.	.	.	.	.	.	.	.
0	0	.	NA	.	.	.	.	.	.	.	.
0	0	.	NA	.	.	.	.	.	.	.	.
0	0	.	NA	.	.	.	.	.	.	.	.
0	0	.	NA	.	.	.	.	.	.	.	.
0	0	.	NA	.	.	.	.	.	.	.	.
0	0	.	NA	.	.	.	.	.	.	.	.
0	0	.	NA	.	.	.	.	.	.	.	.
0	0	.	NA	.	.	.	.	.	PID	.	.
0	0	.	NA	.	.	.	.	.	.	.	.
0	0	.	NA	.	.	.	.	.	.	.	.
0	0	.	NA	.	BIOF	.	.	KEG	.	.	REACTOME_ANTIVIRAL_MECH
0	0	.	NA	.	.	.	.	.	.	.	.
0	0	.	NA	.	.	.	.	KEG	.	.	REACTOME_IMMUNE_SYSTEM
0	0	.	NA	.	.	.	.	.	.	.	.
0	0	.	NA	.	.	.	.	.	.	.	.
0	0	.	NA	.	.	.	.	.	.	.	.
0	0	.	NA	.	POS	.	.	.	.	.	REACTOME_ACTIVATED_NOTC
0	0	.	NA	.	REG	CYT	NUCL	.	PID	BIO	.
0	0	.	NA	.	REG	CYT	NUCL	.	PID	BIO	.
0	0	.	NA	.	REG	CYT	NUCL	.	PID	BIO	.
0	0	.	NA	.	REG	CYT	NUCL	.	PID	BIO	.
0	0	{Epilepsy,	NA	.	SIGN	INTR	GABA	KEG	.	.	.

0	0	.	NA	.	CELI	MEM	PHOS	KE(	PID	BIO(	REACTOME_TGF_BETA_RECEI
0	0	Peroxisom	NA	.	EST/	CYT(	.	KE(	.	.	.
0	0	.	NA	.	.	.	.	.	.	.	.
0	0	.	NA	.	DNA	CYT(	HYDF	KE(	PID	BIO(	REACTOME_APOPTOSIS_INDU
0	0	Nephronop	NA	.	.	.	.	.	.	.	REACTOME_SIGNALING_BY_H
0	0	Nephronop	NA	.	.	.	.	.	.	.	REACTOME_SIGNALING_BY_H
0	0	.	NA	.	.	.	.	.	.	.	.
0	0	.	NA	.	.	.	.	.	.	.	.
0	0	.	NA	.	.	.	.	.	.	.	.
0	0	.	NA	.	.	.	.	.	.	.	.
0	0	.	NA	.	.	.	TRAN	.	.	.	.
0	0	Cerebellar	NA	.	.	.	.	.	.	.	.
0	0	Cerebellar	NA	.	.	.	.	.	.	.	.
0	0	Cerebellar	NA	.	.	.	.	.	.	.	.
0	0	.	NA	.	.	.	.	.	.	.	REACTOME_TRANSMEMBRANI
0	0	.	NA	.	.	.	.	.	.	.	.
0	0	.	NA	.	.	.	.	.	.	.	.
0	0	.	NA	.	.	.	.	.	.	.	.
0	0	.	NA	.	.	.	.	.	.	.	.
0	0	Ehlers-Dar	NA	.	SYS	.	PROT	KE(	.	.	REACTOME_EXTRACELLULAR_
0	0	.	NA	.	POS	.	RECE	KE(	.	.	.
0	0	.	NA	.	.	.	.	.	.	.	.
0	0	.	NA	.	.	.	.	.	.	.	.
0	0	.	NA	.	.	.	.	.	.	.	.
0	0	.	NA	.	.	.	.	.	.	.	.
0	0	.	NA	.	REG	.	ENZY	KE(	PID	BIO(	REACTOME_SIGNALLING_BY_I
0	0	Cataract 6,	NA	.	SIGN	INTR	.	KE(	PID	.	.
0	0	.	NA	.	.	.	.	.	.	BIO(	.
0	0	.	NA	.	.	.	.	.	.	.	.
0	0	.	NA	.	.	.	.	.	.	.	.
0	0	.	NA	.	.	.	.	.	.	.	.
0	0	.	NA	.	.	.	.	.	.	.	.
0	0	.	NA	.	.	.	.	.	.	.	.
0	0	Parkinson	NA	.	.	.	.	.	.	.	.
0	0	.	NA	.	.	.	.	.	.	.	.
0	0	.	NA	.	DETI	.	TAST	KE(	.	.	REACTOME_SIGNALING_BY_G
0	0	.	NA	.	.	.	.	.	.	.	REACTOME_IMMUNE_SYSTEM
0	0	.	NA	.	.	.	.	.	.	.	REACTOME_IMMUNE_SYSTEM
0	0	.	NA	.	.	.	.	.	.	.	.
0	0	.	NA	.	.	ORG	ACTI	.	.	.	.
0	0	.	NA	.	.	.	.	.	.	.	.
0	0	.	NA	.	SIGN	CYT(	GTPA	.	.	.	REACTOME_RAP1_SIGNALLING
0	0	Dyssegme	NA	.	.	.	.	KE(	.	.	REACTOME_HS_GAG_DEGRAD
0	0	Dyssegme	NA	.	.	.	.	KE(	.	.	REACTOME_HS_GAG_DEGRAD
0	0	.	NA	.	.	.	.	.	.	.	.
0	0	.	NA	.	STEI	.	SERI	.	.	.	.
0	0	.	NA	.	.	.	.	.	.	.	.
0	0	Galactose	NA	.	.	.	.	KE(	.	.	REACTOME_METABOLISM_OF_
0	0	HMG-CoA	NA	.	.	.	.	KE(	.	.	REACTOME_METABOLISM_OF_
0	0	.	NA	.	RNA	.	TRAN	.	PID	.	.
0	0	Rh-null dis	NA	.	.	INTR	.	.	.	.	.
0	0	.	NA	.	GLY(	ORG	CATI(	KE(	.	.	REACTOME_METABOLISM_OF_
0	0	.	NA	.	SIGN	.	TUBU	KE(	PID	.	.
0	0	.	NA	.	.	.	.	.	.	.	.

0	0	.	NA	.	ENZ	CELL	PROT	.	PID	.	.
0	0	.	NA	.	.	.	.	.	.	.	.
0	0	.	NA	.	SIGN	.	PHOS	KEG	PID	BIO	REACTOME_SIGNALLING_BY_N
0	0	Mental reta	NA	.	RNA	NUC	TRAN	.	.	.	.
0	0	.	NA	.	.	.	.	.	.	.	.
0	0	.	NA	.	REG	INTR	CATI	KEG	PID	.	REACTOME_HYALURONAN_UP
0	0	.	NA	.	REG	INTR	CATI	KEG	PID	.	REACTOME_HYALURONAN_UP
0	0	.	NA	.	.	.	.	.	.	.	.
0	0	.	NA	.	.	.	.	.	.	.	.
0	0	.	NA	.	ANA	.	.	.	.	.	.
0	0	.	NA	.	IMMU	.	.	.	.	.	.
0	0	.	NA	.	NEG	.	.	KEG	.	.	.
0	0	.	NA	.	.	.	.	.	.	.	.
0	0	.	NA	.	.	.	.	.	.	.	.
0	0	SCID due t	NA	.	REG	ORG	PROT	KEG	PID	BIO	REACTOME_SIGNALING_BY_S
0	0	.	NA	.	.	.	.	.	.	.	.
0	0	.	NA	.	.	NUC	.	.	.	.	.
0	0	.	NA	.	MUL	.	DNA_	.	.	.	.
0	0	Palmoplan	NA	.	.	.	.	.	PID	.	.
0	0	.	NA	.	.	.	.	.	.	.	.
0	0	.	NA	.	.	.	.	.	.	.	.
0	0	.	NA	.	.	CYT	.	.	.	.	.
0	0	.	NA	.	.	CYT	.	.	.	.	.
0	0	.	NA	.	.	CYT	.	.	.	.	.
0	0	.	NA	.	.	.	.	.	.	.	.
0	0	.	NA	.	REG	CYT	MRN	.	.	.	.
0	0	Ceroid lipo	NA	.	REG	AXO	THIO	KEG	.	.	.
0	0	.	NA	.	.	.	CATI	.	.	.	.
0	0	.	NA	.	.	.	CATI	.	.	.	.
0	0	Deafness,	NA	.	EST	.	.	.	.	.	REACTOME_NEURONAL_SYST
0	0	Deafness,	NA	.	EST	.	.	.	.	.	REACTOME_NEURONAL_SYST
0	0	.	NA	.	.	.	.	.	.	.	.
0	0	.	NA	.	RNA	.	SING	.	PID	.	REACTOME_PROCESSING_OF_
0	0	.	NA	.	.	NUC	.	.	.	.	REACTOME_DEVELOPMENTAL
0	0	.	NA	.	.	.	.	.	.	.	.
0	0	.	NA	.	BIOF	.	PHOS	.	PID	.	.
0	0	.	NA	.	.	.	.	.	.	.	.
0	0	Adenocarc	NA	.	DNA	NUC	.	KEG	.	.	.
0	0	Spastic pa	NA	.	CAR	INSC	HYDF	.	.	.	.
0	0	.	NA	.	BIOF	.	NUCL	KEG	PID	BIO	REACTOME_SPRY_REGULATIC
0	0	.	NA	.	.	.	.	.	.	.	.
0	0	.	NA	.	.	.	.	.	.	.	.
0	0	.	NA	.	.	.	.	.	.	.	.
0	0	{Myocardia	NA	.	EST	.	RECE	.	PID	.	REACTOME_PLATELET_HOME
0	0	.	NA	.	RNA	NUC	SPEC	.	.	.	.
0	0	.	NA	.	.	.	.	.	.	.	.
0	0	Hyperchole	NA	.	REG	EXTI	SERI	.	.	.	.
0	0	.	NA	.	BIOF	.	.	KEG	PID	BIO	REACTOME_ACTIVATED_AMPK
0	0	Ciliary dysk	NA	.	.	.	.	.	PID	.	.
0	0	.	NA	.	.	.	.	KEG	PID	BIO	REACTOME_IL_7_SIGNALING;F
0	0	.	NA	.	.	.	.	.	.	.	.
0	0	.	NA	.	CELL	CYT	.	KEG	.	.	.
0	0	.	NA	.	PRO	ORG	LIPID	KEG	.	.	REACTOME_METABOLISM_OF_
0	0	.	NA	.	.	INTR	G_PR	.	.	.	.

0	0	.	NA	.	.	.	.	.	.	.	.	
0	0	.	NA	.	.	INTF	G_PF	.	.	.	.	
0	0	.	NA	.	REG	INTF	LIPID	KEG	PID	BIO(	REACTOME_GASTRIN_CREB_S
0	0	.	NA	.	.	.	.	.	.	.	.	
0	0	.	NA	.	ENZ	INTF	CARE	.	PID	BIO(	.	
0	0	Cone-rod c	NA	.	ESTA	CELI	NUCL	KEG	.	.	REACTOME_ABC_FAMILY_PRO
0	0	Cone-rod c	NA	.	ESTA	CELI	NUCL	KEG	.	.	REACTOME_ABC_FAMILY_PRO
0	0	.	NA	.	.	.	.	.	.	.	.	
0	0	Fibrochonc	NA	.	SYS	PRO	.	KEG	PID	.	REACTOME_EXTRACELLULAR_
0	0	.	NA	.	.	.	.	KEG	.	.	.	
0	0	.	NA	.	.	.	.	.	.	.	.	
0	0	.	NA	.	.	.	.	.	.	.	.	
0	0	Megakaryc	NA	.	.	.	.	.	.	.	.	
0	0	.	NA	.	AMIN	.	CARE	KEG	.	.	.	
0	0	.	NA	.	.	.	.	.	.	.	.	
0	0	.	NA	.	.	.	.	.	.	.	.	
0	0	.	NA	.	.	.	.	.	.	.	REACTOME_PRE_NOTCH_TRA
0	0	.	NA	.	POS	.	HYDF	.	PID	.	REACTOME_SIGNALING_BY_R
0	0	.	NA	.	.	.	.	KEG	PID	.	.	
0	0	Myoadenyl	NA	.	.	.	HYDF	KEG	.	.	REACTOME_METABOLISM_OF_
0	0	Myoadenyl	NA	.	.	.	HYDF	KEG	.	.	REACTOME_METABOLISM_OF_
0	0	Bamforth-L	NA	.	RNA	NUC	HYDF	.	.	.	.	
0	0	.	NA	.	.	NUC	.	.	.	.	.	
0	0	.	NA	.	.	.	.	.	.	.	.	
0	0	Alagille syr	NA	.	REG	INTF	SPEC	KEG	PID	.	REACTOME_PRE_NOTCH_TRA
0	0	Alagille syr	NA	.	REG	INTF	SPEC	KEG	PID	.	REACTOME_PRE_NOTCH_TRA
0	0	.	NA	.	.	.	.	.	.	.	.	
0	0	.	NA	.	.	.	.	.	.	.	.	
0	0	.	NA	.	.	.	.	.	.	.	.	
0	0	.	NA	.	.	.	.	.	.	.	.	
0	0	.	NA	.	ESTA	.	.	KEG	.	.	.	
0	0	.	NA	.	.	.	.	.	.	.	.	
0	0	.	NA	.	.	.	.	.	.	.	.	
0	0	.	NA	.	RNA	NUC	NUCL	KEG	.	.	REACTOME_RNA_POL_III_TRA
0	0	.	NA	.	.	.	.	.	.	.	.	
0	0	.	NA	.	.	.	.	.	.	.	.	
0	0	.	NA	.	.	.	.	.	.	.	.	
0	0	.	NA	.	.	.	.	KEG	.	.	REACTOME_MITOCHONDRIAL_
0	0	.	NA	.	PHO	CYT(	PHOS	KEG	PID	.	REACTOME_SYNTHESIS_OF_F
0	0	.	NA	.	.	.	.	.	.	.	.	
0	0	.	NA	.	.	.	.	.	.	.	.	
0	0	.	NA	.	MUL	.	RECE	.	.	.	.	
0	0	.	NA	.	.	.	.	.	.	.	.	
0	0	.	NA	.	RNA	NUC	.	.	.	.	.	
0	0	.	NA	.	.	.	.	.	.	.	.	
0	0	.	NA	.	.	.	.	KEG	.	.	REACTOME_IMMUNE_SYSTEM
0	0	.	NA	.	.	INTF	.	.	.	.	REACTOME_MITOCHONDRIAL_
0	0	.	NA	.	RNA	CELI	RNA_	KEG	.	.	.	
0	0	.	NA	.	.	.	.	.	.	.	.	
0	0	.	NA	.	RNA	NUC	TRAN	.	.	.	REACTOME_CELL_CYCLE;REA
0	0	.	NA	.	RNA	.	RAS_	KEG	PID	BIO(	REACTOME_SIGNALING_BY_R
0	0	.	NA	.	.	.	.	.	.	.	.	
0	0	.	NA	.	.	.	.	.	.	.	.	
0	0	.	NA	.	.	INTF	.	KEG	.	.	.	

0	0	.	NA	.	DEFI	.	.	.	PID	.	REACTOME_INNATE_IMMUNE_
0	0	.	NA	.	SYS	.	.	.	.	.	.
0	0	.	NA	.	.	.	.	.	.	.	.
0	0	.	NA	.	REG	.	GTPA	.	.	.	REACTOME_SIGNALING_BY_G
0	0	Leukemia,	NA	.	RNA	NUC	.	.	PID	.	.
0	0	.	NA	.	NEG	NUC	TRAN	.	PID	.	REACTOME_RNA_POL_III_TRA
0	0	.	NA	.	.	.	.	.	.	.	.
0	0	Platelet alp	NA	.	.	ORG	.	KEC	PID	BIO	REACTOME_CELL_SURFACE_I
0	0	.	NA	.	.	CYT	.	.	.	.	.
0	0	.	NA	.	.	.	.	.	.	.	.
0	0	.	NA	.	PRO	.	META	.	.	.	REACTOME_DIABETES_PATHW
0	0	.	NA	.	.	.	.	.	.	.	.
0	0	Leukemia,	NA	.	BIOF	.	CATI	KEC	.	.	REACTOME_DEVELOPMENTAL
0	0	Nephrotic s	NA	.	ESTA	INTR	.	.	PID	.	.
0	0	.	NA	.	.	.	.	.	.	.	.
0	0	.	NA	.	CELI	MEM	CATI	KEC	.	.	.
0	0	.	NA	.	.	.	.	.	.	.	.
0	0	.	NA	.	.	.	.	.	.	.	.
0	0	.	NA	.	.	.	.	KEC	.	.	.
0	0	.	NA	.	NUC	ORG	.	KEC	.	.	REACTOME_METABOLISM_OF_
0	0	Leber cong	NA	.	ANA	.	.	.	.	.	.
0	0	.	NA	.	.	.	.	.	.	.	.
0	0	.	NA	.	.	.	.	.	.	.	.
0	0	Hypokalem	NA	.	SYS	MEM	CATI	KEC	.	.	REACTOME_DEVELOPMENTAL
0	0	.	NA	.	.	.	.	.	.	.	.
0	0	.	NA	.	ESTA	ORG	.	.	.	.	REACTOME_MITOCHONDRIAL_
0	0	.	NA	.	.	.	.	KEC	.	.	REACTOME_PHOSPHOLIPID_M
0	0	?Epilepsy,	NA	.	.	INTR	.	KEC	.	.	REACTOME_DEVELOPMENTAL
0	0	.	NA	.	.	.	.	KEC	PID	.	REACTOME_REGULATION_OF_
0	0	.	NA	.	.	.	.	.	.	.	.
0	0	.	NA	.	.	.	.	KEC	.	.	REACTOME_DEVELOPMENTAL
0	0	Cortisone r	NA	.	.	.	.	KEC	.	.	REACTOME_METABOLISM_OF_
0	0	.	NA	.	.	.	.	.	.	.	.
0	0	.	NA	.	.	.	.	.	.	.	.
0	0	Ataxia, pos	NA	.	ESTA	INTR	.	.	.	.	REACTOME_TRANSMEMBRANI
0	0	.	NA	.	SIGN	.	PHOS	.	.	.	.
0	0	Choanal at	NA	.	BIOF	.	PROT	.	.	.	.
0	0	Choanal at	NA	.	BIOF	.	PROT	.	.	.	.
0	0	Retinitis pi	NA	.	REG	PRO	PROT	.	.	.	.
0	0	Retinitis pi	NA	.	REG	PRO	PROT	.	.	.	.
0	0	Retinitis pi	NA	.	REG	PRO	PROT	.	.	.	.
0	0	.	NA	.	RNA	.	PROT	.	.	.	REACTOME_GENERIC_TRANS
0	0	Hypermang	NA	.	.	.	.	.	.	.	.
0	0	.	NA	.	.	.	.	KEC	.	.	REACTOME_MITOCHONDRIAL_
0	0	.	NA	.	.	.	.	KEC	.	.	REACTOME_MITOCHONDRIAL_
0	0	.	NA	.	.	.	.	.	.	.	.
0	0	.	NA	.	.	.	.	.	.	.	.
0	0	.	NA	.	.	NUC	NUCL	.	.	.	.
0	0	.	NA	.	.	.	.	.	.	.	.
0	0	Left-right a	NA	.	ENZ	.	.	KEC	.	.	REACTOME_DEVELOPMENTAL
0	0	.	NA	.	.	.	.	.	.	.	.
0	0	Coenzyme	NA	.	.	.	.	.	.	.	.
0	0	.	NA	.	.	.	.	.	PID	.	REACTOME_SIGNALING_BY_R
0	0	.	NA	.	.	.	.	.	.	.	.

0	0	.	NA	.	PRO	.	PROT	.	.	.	.
0	0	.	NA	.	EST	INTR	CATI	.	.	.	REACTOME_NEURONAL_SYST
0	0	Arrhythmo	NA	.	REG	MEM	CATI	KEG	.	BIO	.
0	0	.	NA	.	.	.	.	.	.	.	.
0	0	Megalence	NA	.	BIOF	.	.	KEG	PID	.	REACTOME_SIGNALLING_BY_N
0	0	.	NA	.	.	.	.	.	.	.	.
0	0	.	NA	.	.	.	.	.	.	.	REACTOME_GENERIC_TRANS
0	0	.	NA	.	.	.	.	.	.	.	.
0	0	Thromboc	NA	.	.	INTR	.	KEG	.	.	REACTOME_METABOLISM_OF_
0	0	.	NA	.	.	.	.	.	.	.	.
0	0	.	NA	.	.	.	.	.	.	.	.
0	0	.	NA	.	.	.	.	.	.	.	.
0	0	.	NA	.	.	.	.	.	.	.	.
0	0	.	NA	.	.	.	.	KEG	.	.	REACTOME_ACYL_CHAIN_REM
0	0	Inflammato	NA	.	CELL	INTR	.	KEG	PID	BIO	REACTOME_SIGNALLING_BY_N
0	0	.	NA	.	.	.	.	.	.	.	.
0	0	.	NA	.	.	.	.	.	.	.	.
0	0	.	NA	.	.	.	.	.	.	.	.
0	0	.	NA	.	.	.	.	.	.	.	.
0	0	.	NA	.	EST	.	CATI	.	.	.	REACTOME_NEURONAL_SYST
0	0	.	NA	.	.	.	.	.	.	.	.
0	0	.	NA	.	.	.	MOLE	.	PID	.	.
0	0	.	NA	.	.	NUC	PROT	.	PID	BIO	REACTOME_DEVELOPMENTAL
0	0	.	NA	.	.	.	.	.	.	.	.
0	0	.	NA	.	.	.	LIGA	KEG	PID	.	REACTOME_METABOLISM_OF_
0	0	.	NA	.	.	.	LIGA	KEG	PID	.	REACTOME_METABOLISM_OF_
0	0	.	NA	.	.	.	.	.	.	.	.
0	0	{Neuroblas	NA	.	SYS	INTR	TRAN	.	.	.	.
0	0	.	NA	.	CELL	.	.	KEG	.	.	.
0	0	.	NA	.	.	.	.	.	.	.	.
0	0	.	NA	.	.	.	.	.	.	.	.
0	0	.	NA	.	.	.	.	.	PID	.	.
0	0	.	NA	.	EST	INTR	CATI	KEG	.	.	REACTOME_TRANSMEMBRANE
0	0	Leigh synd	NA	.	EST	ORG	MICR	.	.	.	.
0	0	.	NA	.	.	.	.	.	.	.	.
0	0	.	NA	.	.	.	.	.	.	.	.
0	0	.	NA	.	.	ORG	ACTI	.	PID	.	REACTOME_DEVELOPMENTAL
0	0	.	NA	.	.	ORG	ACTI	.	PID	.	REACTOME_DEVELOPMENTAL
0	0	.	NA	.	SYS	ORG	.	.	PID	.	REACTOME_SIGNALLING_BY_N
0	0	Doyne hon	NA	.	SEN	PRO	.	.	.	.	.
0	0	.	NA	.	.	.	.	.	.	.	.
0	0	.	NA	.	.	.	.	.	.	.	.
0	0	.	NA	.	.	.	.	.	.	.	.
0	0	.	NA	.	GLU	.	NUCL	KEG	.	.	REACTOME_BIOLOGICAL_OXID
0	0	.	NA	.	.	.	.	.	.	.	REACTOME_SIGNALING_BY_IL
0	0	.	NA	.	.	.	.	.	.	.	REACTOME_SIGNALING_BY_R
0	0	GAPO syn	NA	.	.	.	.	.	PID	.	.
0	0	.	NA	.	.	.	ACTI	.	.	.	.
0	0	.	NA	.	AMIN	.	.	KEG	.	.	.
0	0	Miyoshi mu	NA	.	SYS	MEM	.	.	.	.	.
0	0	.	NA	.	.	.	.	.	.	.	.
0	0	.	NA	.	.	.	.	.	.	.	.
0	0	.	NA	.	.	.	.	.	.	.	.
0	0	.	NA	.	ISOF	ORG	OXID	KEG	.	.	.

0	0	Pseudoxar	NA	.	REG	INTF	LYAS	.	.	.	REACTOME_PTM_GAMMA_CAF
0	0	.	NA	.	MUS	MEM	.	KE(	.	.	.
0	0	.	NA	.	.	.	.	.	.	.	.
0	0	.	NA	.	.	.	.	.	.	.	.
0	0	.	NA	.	.	.	.	.	.	.	.
0	0	.	NA	.	.	.	.	.	.	.	.
0	0	.	NA	.	.	.	.	.	.	.	.
0	0	.	NA	.	.	.	.	.	.	.	.
0	0	.	NA	.	.	.	.	.	.	.	.
0	0	.	NA	.	.	.	.	.	.	.	.
0	0	.	NA	.	.	.	.	.	.	.	.
0	0	.	NA	.	.	.	.	.	.	.	.
0	0	.	NA	.	.	.	.	.	.	.	.
0	0	.	NA	.	.	.	.	.	.	.	.
0	0	.	NA	.	.	.	.	.	.	.	.
0	0	.	NA	.	PRO	.	.	.	.	.	.
0	0	Retinitis pi(	NA	.	RNA	ORG	HYDF	KE(	.	.	REACTOME_PROCESSING_OF_
0	0	.	NA	.	.	.	.	.	.	.	.
0	0	.	NA	.	.	.	.	.	.	.	.
0	0	.	NA	.	.	.	.	.	.	.	.
0	0	.	NA	.	.	.	.	.	.	.	.
0	0	.	NA	.	.	.	.	.	.	.	.
0	0	.	NA	.	MUL	NUC	.	.	.	.	.
0	0	.	NA	.	SIGN	MEM	.	KE(	PID	BIO(	.
0	0	.	NA	.	ENZ'	MEM	SMAI	.	PID	.	.
0	0	.	NA	.	.	.	.	.	.	.	.
0	0	Colorectal	NA	.	MIT(	ORG	.	KE(	PID	.	REACTOME_CELL_CYCLE;REA
0	0	.	NA	.	.	.	.	.	.	.	.
0	0	.	NA	.	.	.	.	.	.	.	.
0	0	.	NA	.	.	.	.	.	.	.	.
0	0	.	NA	.	.	.	.	.	.	.	.
0	0	.	NA	.	.	CELI	.	.	.	.	.
0	0	.	NA	.	SIGN	.	GTP_	KE(	PID	.	REACTOME_SIGNALLING_BY_I
0	0	Thrombopl	NA	.	REG	.	SERI	KE(	PID	BIO(	REACTOME_PTM_GAMMA_CAF
0	0	.	NA	.	.	.	.	.	.	.	.
0	0	.	NA	.	LIPI	ORG	LIPAS	KE(	.	.	REACTOME_GLYCOSPHINGOL
0	0	.	NA	.	.	.	.	.	.	.	.
0	0	.	NA	.	.	.	.	.	.	.	.
0	0	.	NA	.	SIGN	INTF	.	.	.	.	REACTOME_SIGNALING_BY_G
0	0	.	NA	.	.	.	.	.	.	.	.
0	0	.	NA	.	EST/	.	.	.	.	.	.
0	0	Nemaline r	NA	.	.	SAR	STRU	.	.	.	REACTOME_STRIATED_MUSCI
0	0	.	NA	.	.	.	.	KE(	PID	.	REACTOME_MEMBRANE_TRAF
0	0	.	NA	.	.	.	.	.	.	.	.
0	0	.	NA	.	.	.	.	KE(	.	.	.
0	0	.	NA	.	.	.	.	.	.	.	.
0	0	.	NA	.	.	.	.	.	.	.	.
0	0	.	NA	.	.	.	.	.	.	.	.
0	0	.	NA	.	IMMU	.	SERI	.	.	.	REACTOME_INTEGRATION_OF
0	0	.	NA	.	IMMU	.	SERI	.	.	.	REACTOME_INTEGRATION_OF
0	0	.	NA	.	.	.	.	.	.	.	.
0	0	Cholestasi	NA	.	EST/	INTF	CATI(	KE(	.	BIO(	REACTOME_BILE_ACID_AND_E
0	0	Bardet-Bie	NA	.	.	.	.	.	.	.	.
0	0	.	NA	.	.	.	.	.	.	.	.

0	0	.	NA	.	.	.	.	.	.	.	.
0	0	.	NA	.	.	.	.	KEG	.	.	REACTOME_CELL_CYCLE;REA
0	0	.	NA	.	SIGN	CELI	GTPA	KEG	.	.	REACTOME_INTEGRIN_CELL_S
0	0	Wiskott-Al	NA	.	MAC	NON	ACTI	.	PID	.	.
0	0	Multiple pte	NA	.	REG	INTF	CATI	KEG	PID	BIO	REACTOME_TRANSMISSION_A
0	0	.	NA	.	.	.	.	.	.	.	.
0	0	Rhizomelic	NA	.	BIOS	CYT	TRAN	KEG	.	.	REACTOME_PEROXISOMAL_LI
0	0	Pigmented	NA	.	SIGN	.	.	KEG	.	.	REACTOME_SIGNALING_BY_G
0	0	Cardiomyo	NA	.	MUS	ORG	NUCL	KEG	.	.	.
0	0	.	NA	.	.	.	.	.	.	.	.
0	0	.	NA	.	.	.	.	.	.	.	.
0	0	.	NA	.	.	.	.	.	.	.	.
0	0	.	NA	.	.	.	.	KEG	PID	BIO	REACTOME_DEVELOPMENTAL
0	0	Ehlers-Dar	NA	.	SYS	PRO	.	KEG	PID	.	REACTOME_DEVELOPMENTAL
0	0	.	NA	.	.	.	.	.	.	.	.
0	0	Candidiasi	NA	.	RNA	CYT	RECE	KEG	PID	BIO	REACTOME_SIGNALING_BY_S
0	0	.	NA	.	.	CYT	LIPID	.	.	.	.
0	0	.	NA	.	CELI	NUC	NUCL	.	.	.	.
0	0	.	NA	.	.	.	.	.	.	.	.
0	0	3MC syndr	NA	.	CELI	.	PHOS	.	.	.	.
0	0	.	NA	.	.	.	.	.	.	.	.
0	0	.	NA	.	.	.	.	.	.	.	.
0	0	.	NA	.	.	.	.	.	.	.	.
0	0	.	NA	.	.	.	.	.	.	.	REACTOME_TRANSLATION;RE
0	0	.	NA	.	SYS	INTF	INTE	.	.	.	.
0	0	Cataract 4,	NA	.	SEN	.	.	.	.	.	.
0	0	.	NA	.	.	.	.	.	.	.	.
0	0	.	NA	.	.	.	.	.	.	.	.
0	0	.	NA	.	.	.	.	.	.	.	.
0	0	.	NA	.	.	.	.	.	.	.	.
0	0	Carbamoyl	NA	.	.	CYT	LIGA	KEG	.	.	REACTOME_METABOLISM_OF_
0	0	.	NA	.	.	.	.	.	PID	BIO	.
0	0	.	NA	.	.	.	.	.	.	.	.
0	0	.	NA	.	.	.	.	.	.	.	.
0	0	{Buruli ulc	NA	.	MUL	INTF	.	KEG	PID	.	REACTOME_LATENT_INFECTI
0	0	Cholestasi	NA	.	MAC	.	.	.	.	.	.
0	0	.	NA	.	.	.	.	.	.	.	.
0	0	.	NA	.	.	.	.	.	.	.	.
0	0	Spinal mus	NA	.	PRO	.	UNF	.	.	.	.
0	0	?Muscular	NA	.	ORG	SAR	STRU	KEG	PID	.	REACTOME_STRIATED_MUSCI
0	0	.	NA	.	MUS	NUC	.	.	.	.	.
0	0	.	NA	.	SIGN	INTF	.	KEG	PID	BIO	.
0	0	{Coronary	NA	.	ENZ	VESI	PROT	KEG	PID	BIO	REACTOME_SIGNALLING_BY_N
0	0	.	NA	.	.	.	.	.	.	.	.
0	0	.	NA	.	.	.	.	.	.	.	.
0	0	.	NA	.	REG	INTF	NEUF	KEG	.	.	REACTOME_GASTRIN_CREB_S
0	0	Perlman sy	NA	.	.	.	.	.	.	.	.
0	0	Perlman sy	NA	.	.	.	.	.	.	.	.
0	0	.	NA	.	.	.	.	KEG	.	.	.
0	0	.	NA	.	SIGN	.	PHOS	KEG	PID	.	.
0	0	.	NA	.	.	.	.	.	.	.	.
0	0	.	NA	.	.	.	.	.	.	.	.
0	0	.	NA	.	.	.	.	.	.	.	.
0	0	Advanced	NA	.	RHY	.	.	KEG	PID	.	REACTOME_BMAL1_CLOCK_NI

0	0	.	NA	.	.	.	.	.	.	.	.
0	0	Mental reta	NA	.	EST/	.	.	.	.	.	.
0	0	Mental reta	NA	.	EST/	.	.	.	.	.	.
0	0	.	NA	.	.	.	.	.	.	.	.
0	0	.	NA	.	.	.	.	.	.	.	.
0	0	.	NA	.	.	.	.	.	.	.	.
0	0	.	NA	.	SIGN	INTR	.	.	.	.	.
0	0	.	NA	.	SYS	.	.	.	PID	.	REACTOME_DEVELOPMENTAL
0	0	.	NA	.	.	.	.	.	.	.	.
0	0	.	NA	.	.	.	.	.	.	.	.
0	0	.	NA	.	.	.	.	.	.	.	.
0	0	.	NA	.	.	.	.	.	.	.	.
0	0	Atrioventric	NA	.	.	.	.	.	.	.	.
0	0	Fanconi ar	NA	.	.	.	.	.	PID	BIO(	REACTOME_REGULATION_OF_
0	0	.	NA	.	.	.	.	.	.	.	REACTOME_TRANSMISSION_A
0	0	.	NA	.	EST/	ORG	.	.	.	.	.
0	0	.	NA	.	.	.	.	.	.	.	.
0	0	Pontocereb	NA	.	.	.	.	.	.	.	.
0	0	LEOPARD	NA	.	CELI	ORG	.	KE(	PID	BIO(	REACTOME_SIGNALLING_BY_N
0	0	.	NA	.	.	.	.	KE(	PID	.	.
0	0	.	NA	.	.	.	.	.	.	.	.
0	0	.	NA	.	.	.	.	.	.	.	.
0	0	.	NA	.	.	.	.	.	.	.	REACTOME_NEURONAL_SYST
0	0	.	NA	.	RNA	NUC	.	.	.	.	REACTOME_GENERIC_TRANS(
0	0	.	NA	.	.	NUC	KINA:	.	.	BIO(	.
0	0	.	NA	.	.	.	.	.	.	.	.
0	0	.	NA	.	.	.	.	.	.	.	REACTOME_TRANSMEMBRANE
0	0	Colorectal	NA	.	POS	INTR	TRAN	KE(	PID	BIO(	REACTOME_TGF_BETA_RECEI
0	0	.	NA	.	.	.	.	.	.	.	.
0	0	.	NA	.	GLY(	ORG	TRAN	KE(	.	.	.
0	0	.	NA	.	.	CELI	.	.	.	.	.
0	0	.	NA	.	.	.	.	.	.	.	.
0	0	Colorectal	NA	.	DNA	.	SING	KE(	PID	.	REACTOME_MEIOSIS;REACTO
0	0	.	NA	.	EST/	ORG	.	.	.	.	.
0	0	Esophagea	NA	.	NEG	CYT(	.	.	.	.	.
0	0	.	NA	.	.	.	.	KE(	.	.	.
0	0	.	NA	.	EST/	.	.	.	.	.	.
0	0	.	NA	.	.	.	.	.	.	.	.
0	0	.	NA	.	REG	INTR	.	KE(	.	.	REACTOME_SIGNALING_BY_G
0	0	.	NA	.	.	.	.	.	.	.	.
0	0	.	NA	.	.	.	.	KE(	.	.	.
0	0	.	NA	.	.	.	.	.	.	.	.
0	0	EBD invers	NA	.	SYS	PRO	.	.	PID	.	REACTOME_EXTRACELLULAR_
0	0	.	NA	.	NUC	.	.	.	.	.	.
0	0	.	NA	.	.	.	.	.	.	.	.
0	0	.	NA	.	.	.	.	.	.	.	.
0	0	.	NA	.	.	CYT(	CYST	.	.	.	.
0	0	.	NA	.	CELI	NUC	.	.	.	.	.
0	0	.	NA	.	.	.	.	.	.	.	.
0	0	.	NA	.	.	.	.	KE(	.	.	.
0	0	.	NA	.	.	.	.	.	.	.	.
0	0	.	NA	.	MIT(	.	RNA_	.	PID	.	.
0	0	.	NA	.	MIT(	.	RNA_	.	PID	.	.
0	0	.	NA	.	COF	.	.	KE(	PID	BIO(	REACTOME_PPARA_ACTIVATE

0	0	.	NA	.	.	.	.	KE(	.	.	.
0	0	.	NA	.	.	.	.	KE(	.	.	.
0	0	.	NA	.	.	.	.	KE(	.	.	.
0	0	.	NA	.	.	.	.	KE(	.	.	.
0	0	.	NA	.	CHR	.	.	.	.	.	.
0	0	.	NA	.	CHR	.	.	.	.	.	.
0	0	Sinoatrial r	NA	.	EST/	MEM	CATI(	KE(	.	.	.
0	0	.	NA	.	.	.	.	.	.	.	.
0	0	.	NA	.	.	.	.	KE(	.	.	REACTOME_TRANSMISSION_A
0	0	.	NA	.	.	.	.	KE(	.	.	REACTOME_TRANSMISSION_A
0	0	Hypogonac	NA	.	.	.	.	.	PID	.	REACTOME_SIGNALING_BY_F(
0	0	.	NA	.	SIGN	NUC	.	KE(	PID	.	REACTOME_REGULATION_OF_
0	0	.	NA	.	.	.	.	.	.	.	.
0	0	.	NA	.	BIOF	CYT(	NUCL	.	.	.	.
0	0	.	NA	.	GLY(	PRO	.	.	.	.	.
0	0	.	NA	.	.	.	.	.	.	.	.
0	0	Tietz albini	NA	.	.	.	TRAN	KE(	PID	.	.
0	0	.	NA	.	.	.	.	.	.	.	.
0	0	.	NA	.	.	.	.	.	PID	.	.
0	0	.	NA	.	.	.	.	.	.	.	.
0	0	.	NA	.	.	.	.	.	.	.	.
0	0	.	NA	.	GAM	NUC	.	.	.	.	.
0	0	.	NA	.	.	.	.	.	.	.	.
0	0	.	NA	.	.	.	.	.	.	.	.
0	0	.	NA	.	.	.	.	.	.	.	.
0	0	.	NA	.	.	.	.	.	.	.	.
0	0	.	NA	.	.	.	.	KE(	.	.	.
0	0	.	NA	.	.	.	.	.	.	.	.
0	0	Urocanase	NA	.	.	.	.	KE(	.	.	REACTOME_METABOLISM_OF_
0	0	.	NA	.	MUL	.	RECE	KE(	.	.	REACTOME_DEVELOPMENTAL
0	0	.	NA	.	LIPIE	INTR	G_PF	.	.	.	.
0	0	.	NA	.	.	.	.	.	.	.	.
0	0	.	NA	.	.	.	.	.	.	.	.
0	0	.	NA	.	.	.	.	.	.	.	.
0	0	.	NA	.	.	.	.	.	.	.	.
0	0	.	NA	.	.	.	.	KE(	.	.	.
0	0	.	NA	.	.	.	.	.	.	.	.
0	0	.	NA	.	.	.	.	.	.	.	.
0	0	.	NA	.	.	.	.	.	.	.	.
0	0	.	NA	.	.	.	RAS_	KE(	PID	.	.
0	0	.	NA	.	.	.	TRAN	KE(	PID	.	.
0	0	Cutaneous	NA	.	DNA	.	PROT	KE(	PID	BIO(	REACTOME_MEIOSIS;REACTO
0	0	.	NA	.	.	.	.	.	.	.	REACTOME_EXTRACELLULAR_
0	0	.	NA	.	.	.	.	.	.	.	.
0	0	.	NA	.	.	.	.	.	.	.	.
0	0	.	NA	.	MAC	CYT(	TRAN	.	PID	.	.
0	0	Combined	NA	.	.	.	.	.	.	.	.
0	0	.	NA	.	.	.	.	.	.	.	.
0	0	.	NA	.	.	.	.	.	.	.	.
0	0	.	NA	.	.	.	.	.	.	.	.
0	0	.	NA	.	RNA	.	TRAN	.	PID	BIO(	REACTOME_TRANSCRIPTIONA
0	0	.	NA	.	.	.	.	.	.	.	.
0	0	.	NA	.	REG	INTR	CATI(	KE(	.	.	REACTOME_NEURONAL_SYST
0	0	.	NA	.	.	.	CYST	.	.	.	.

0	0	.	NA	.	.	.	.	.	.	.	.
0	0	3-Methylcr	NA	.	.	.	.	KEG	.	.	REACTOME_METABOLISM_OF_
0	0	.	NA	.	.	.	.	.	.	.	.
0	0	.	NA	.	.	.	.	.	.	.	.
0	0	.	NA	.	.	.	.	.	.	.	.
0	0	.	NA	.	SYST	ORG	CATIO	.	.	.	.
0	0	.	NA	.	CELL	EXTR	RECE	.	.	.	REACTOME_SIGNALING_BY_G
0	0	Leukemia,	NA	.	.	.	.	.	PID	.	.
0	0	.	NA	.	.	.	.	.	.	.	.
0	0	.	NA	.	.	.	.	.	.	.	.
0	0	.	NA	.	.	.	.	.	.	.	.
0	0	.	NA	.	.	INTR	RECE	.	.	.	REACTOME_O_LINKED_GLYCO
0	0	.	NA	.	.	.	.	.	.	.	.
0	0	.	NA	.	.	.	.	.	.	.	.
0	0	.	NA	.	.	.	.	.	.	.	.
0	0	.	NA	.	.	.	.	.	.	.	.
0	0	.	NA	.	.	.	.	.	.	.	.
0	0	.	NA	.	VIRA	.	TRAN	KEG	PID	BIOC	.
0	0	.	NA	.	.	.	.	.	.	.	.
0	0	{?Bladder (	NA	.	.	.	.	.	PID	.	.
0	0	.	NA	.	SIGN	INTR	CATIO	.	.	.	.
0	0	.	NA	.	ANAT	.	.	KEG	.	.	.
0	0	.	NA	.	RNA_	.	TRAN	.	PID	.	.
0	0	Fetal akine	NA	.	.	.	.	.	.	.	.
0	0	.	NA	.	.	.	.	.	.	.	.
0	0	{Congestiv	NA	.	REG	INTR	AMIN	KEG	.	.	.
0	0	Deafness,	NA	.	SYST	INTR	.	.	.	.	REACTOME_DIABETES_PATHW
0	0	.	NA	.	.	.	.	KEG	.	BIOC	.
0	0	.	NA	.	.	.	.	.	.	.	.
0	0	.	NA	.	.	.	.	.	.	.	.
0	0	.	NA	.	.	NON	.	KEG	.	.	.
0	0	.	NA	.	.	.	.	.	.	.	.
0	0	.	NA	.	.	.	.	.	.	.	.
0	0	.	NA	.	.	.	.	.	.	.	.
0	0	COACH sy	NA	.	.	.	.	.	.	.	.
0	0	.	NA	.	.	.	.	.	.	.	.
0	0	.	NA	.	.	.	.	.	.	.	.
0	0	.	NA	.	.	.	.	.	.	.	.
0	0	.	NA	.	BIOP	.	ACID	KEG	.	.	REACTOME_NEGATIVE_REGUL
0	0	.	NA	.	BIOP	.	ACID	KEG	.	.	REACTOME_NEGATIVE_REGUL
0	0	.	NA	.	.	.	.	.	.	.	.
0	0	.	NA	.	SIGN	CYTO	GTP_	KEG	.	.	REACTOME_SIGNALING_BY_R
0	0	.	NA	.	.	.	.	.	.	.	.
0	0	.	NA	.	SIGN	INTR	GABA	KEG	.	.	REACTOME_TRANSMISSION_A
0	0	.	NA	.	.	.	.	.	.	.	.
0	0	.	NA	.	.	.	.	.	.	.	.
0	0	.	NA	.	.	.	.	.	.	.	.
0	0	.	NA	.	.	.	.	.	.	.	.
0	0	.	NA	.	.	.	.	.	PID	.	REACTOME_DIABETES_PATHW
0	0	.	NA	.	.	.	.	.	.	.	.
0	0	.	NA	.	RES	INTR	PEPT	.	.	.	.
0	0	.	NA	.	LIPI	.	.	KEG	.	.	REACTOME_BIOLOGICAL_OXID
0	0	.	NA	.	.	.	.	.	.	.	.
0	0	Renal tubu	NA	.	.	INTR	CATIO	KEG	.	.	REACTOME_TRANSMEMBRAN

0	0	.	NA	.	.	.	.	.	.	.	REACTOME_EXTRACELLULAR_
0	0	.	NA	.	.	EXTI	.	.	.	.	.
0	0	.	NA	.	.	.	.	.	.	.	.
0	0	.	NA	.	BIOF	.	PHOS	.	.	.	.
0	0	.	NA	.	.	.	.	.	.	.	.
0	0	.	NA	.	PAT	ADH	ACTII	.	.	.	.
0	0	Fraser syn	NA	.	.	.	.	.	.	.	.
0	0	.	NA	.	.	.	.	.	.	.	REACTOME_G0_AND_EARLY_(
0	0	.	NA	.	.	.	.	.	.	.	.
0	0	.	NA	.	.	.	.	.	.	.	REACTOME_SIGNALING_BY_R
0	0	.	NA	.	RNA	CYT(	TRAN	KE(	PID	BIO(	.
0	0	Myelodysp	NA	.	.	.	.	.	.	.	.
0	0	.	NA	.	.	.	.	.	.	.	.
0	0	.	NA	.	CELI	INTF	META	KE(	.	.	.
0	0	.	NA	.	.	.	.	.	.	.	.
0	0	.	NA	.	.	.	.	KE(	.	.	REACTOME_HS_GAG_BIOSYN
0	0	.	NA	.	.	.	.	.	.	.	.
0	0	.	NA	.	.	.	.	.	.	.	.
0	0	.	NA	.	.	.	.	.	.	.	.
0	0	.	NA	.	.	.	.	.	.	.	.
0	0	.	NA	.	.	.	.	.	.	.	.
0	0	.	NA	.	.	.	.	.	.	.	.
0	0	.	NA	.	.	.	.	.	.	.	.
0	0	.	NA	.	.	.	.	.	.	.	.
0	0	.	NA	.	.	.	.	.	.	.	.
0	0	.	NA	.	.	.	.	.	.	.	.
0	0	.	NA	.	POS	INTF	.	KE(	.	BIO(	.
0	0	[Blood grou	NA	.	.	INTF	.	.	.	.	.
0	0	.	NA	.	.	CYT(	.	.	.	.	.
0	0	.	NA	.	EST/	NON	.	.	.	.	.
0	0	.	NA	.	.	.	.	KE(	.	.	REACTOME_O_LINKED_GLYCO
0	0	.	NA	.	.	.	.	.	.	.	.
0	0	.	NA	.	.	.	.	.	.	.	.
0	0	.	NA	.	.	NON	MOLE	.	.	.	.
0	0	.	NA	.	.	.	.	.	.	.	.
0	0	.	NA	.	.	.	CATI(	KE(	PID	.	REACTOME_TRANSMEMBRANI
0	0	.	NA	.	.	.	.	.	.	.	REACTOME_CELL_CYCLE;REA
0	0	.	NA	.	.	.	.	.	.	.	.
0	0	.	NA	.	.	INTF	CATI(	.	.	.	REACTOME_TRANSMEMBRANI
0	0	.	NA	.	SYS	.	.	.	.	.	.
0	0	.	NA	.	.	.	.	.	.	.	.
0	0	.	NA	.	.	.	.	.	.	.	.
0	0	.	NA	.	.	.	.	KE(	.	.	.
0	0	.	NA	.	ORG	NUC	.	KE(	.	.	.
0	0	Mental reta	NA	.	SIGN	CYT(	.	.	.	.	.
0	0	Ciliary dysl	NA	.	.	.	.	.	.	.	.
0	0	.	NA	.	ENZ	.	PHOS	.	PID	BIO(	REACTOME_SIGNALING_BY_R
0	0	.	NA	.	ENZ	.	PHOS	.	PID	BIO(	REACTOME_SIGNALING_BY_R
0	0	.	NA	.	.	.	.	.	.	.	.
0	0	.	NA	.	.	.	.	.	.	.	.
0	0	.	NA	.	.	.	.	.	.	.	.
0	0	.	NA	.	.	.	.	.	.	.	.
0	0	.	NA	.	.	.	.	.	.	.	.
0	0	.	NA	.	.	.	.	.	.	.	.

0	0	.	NA	.	BIOF	.	.	KEC	PID	BIO	REACTOME_ACTIVATED_AMPK
0	0	Succinyl C	NA	.	.	CYT	TRAN	KEC	.	.	REACTOME_METABOLISM_OF_
0	0	.	NA	.	.	CYT	TRAN	KEC	PID	.	REACTOME_PPARA_ACTIVATE
0	0	.	NA	.	.	.	.	.	.	.	.
0	0	.	NA	.	.	.	.	.	.	.	.
0	0	.	NA	.	.	.	.	.	.	.	.
0	0	.	NA	.	.	.	.	.	.	.	.
0	0	Acrodysost	NA	.	.	INSC	HYDF	KEC	.	.	REACTOME_SIGNALING_BY_G
0	0	.	NA	.	.	.	.	.	.	.	.
0	0	.	NA	.	.	.	.	.	.	.	REACTOME_CELL_CYCLE;REA
0	0	.	NA	.	ENZ	PRO	INTE	KEC	.	.	.
0	0	.	NA	.	.	.	.	.	.	.	.
0	0	.	NA	.	.	.	.	.	.	.	.
0	0	.	NA	.	.	.	.	.	.	.	.
0	0	Agammagl	NA	.	ENZ	CYT	HORI	KEC	PID	BIO	REACTOME_SIGNALLING_BY_I
0	0	.	NA	.	DNA	NUC	.	.	PID	BIO	REACTOME_CELL_CYCLE;REA
0	0	.	NA	.	.	.	.	.	.	.	.
0	0	.	NA	.	.	ORG	.	.	PID	.	.
0	0	.	NA	.	.	.	.	.	.	.	.
0	0	.	NA	.	G_PI	INTR	.	.	.	.	.
0	0	.	NA	.	COF	CYT	THIO	KEC	.	.	.
0	0	.	NA	.	.	.	.	.	.	.	.
0	0	Wagner sy	NA	.	MUL	PRO	CARE	KEC	PID	.	REACTOME_CS_DS_DEGRADA
0	0	.	NA	.	.	.	.	.	.	.	.
0	0	Febrile sei	NA	.	REG	CELI	CATI	.	.	.	.
0	0	.	NA	.	.	.	.	.	.	.	.
0	0	.	NA	.	.	.	ARYL	.	.	.	REACTOME_PTM_GAMMA_CAR
0	0	.	NA	.	.	.	.	.	.	.	.
0	0	.	NA	.	BIOF	ORG	CHRO	.	.	.	.
0	0	.	NA	.	BIOF	ORG	CHRO	.	.	.	.
0	0	.	NA	.	.	.	.	.	.	.	.
0	0	.	NA	.	.	.	.	.	.	.	.
0	0	.	NA	.	.	.	.	KEC	.	.	REACTOME_DEVELOPMENTAL
0	0	.	NA	.	CELI	CYT	ENZY	.	.	.	.
0	0	.	NA	.	.	.	.	.	.	.	.
0	0	.	NA	.	RNA	.	TRAN	.	.	.	.
0	0	.	NA	.	.	.	.	.	.	.	.
0	0	.	NA	.	.	.	.	.	.	.	.
0	0	.	NA	.	.	.	.	KEC	.	.	.
0	0	.	NA	.	REG	.	.	KEC	PID	BIO	REACTOME_CELL_CYCLE;REA
0	0	.	NA	.	REG	.	.	KEC	PID	BIO	REACTOME_CELL_CYCLE;REA
0	0	.	NA	.	NUC	NUC	TRAN	KEC	PID	BIO	REACTOME_INTERFERON_ALF
0	0	.	NA	.	NUC	NUC	TRAN	KEC	PID	BIO	REACTOME_INTERFERON_ALF
0	0	.	NA	.	.	.	.	.	.	.	.
0	0	.	NA	.	SYS	INTR	.	.	.	.	.
0	0	.	NA	.	.	.	.	.	.	.	.
0	0	.	NA	.	SYS	INTR	.	.	.	.	.
0	0	.	NA	.	SYS	INTR	.	.	.	.	.
0	0	.	NA	.	.	.	.	.	.	.	.
0	0	.	NA	.	.	.	.	.	.	.	.
0	0	.	NA	.	.	.	.	.	.	.	.
0	0	.	NA	.	.	.	.	.	.	.	.
0	0	.	NA	.	.	.	.	.	.	.	.
0	0	.	NA	.	.	.	.	.	.	.	.

0	0	.	NA	.	.	CYT	.	.	.	.	.
0	0	.	NA	.	.	.	.	.	.	.	.
0	0	.	NA	.	BIOF	.	PHOS	KEG	PID	BIOC	REACTOME_SIGNALING_BY_W
0	0	Retinitis pi	NA	.	SEN	.	.	KEG	PID	.	REACTOME_CGMP_EFFECTS;F
0	0	.	NA	.	.	CELI	ACTII	.	.	.	.
0	0	.	NA	.	SIGN	NUC	HYDF	.	.	.	.
0	0	.	NA	.	CELI	CYT	.	KEG	PID	.	.
0	0	.	NA	.	.	.	.	.	.	.	.
0	0	.	NA	.	.	.	.	.	.	.	.
0	0	.	NA	.	.	.	.	.	.	.	.
0	0	[Memory, e	NA	.	.	.	.	.	.	.	REACTOME_SIGNALING_BY_H
0	0	.	NA	.	RNA	CYT	.	KEG	.	.	REACTOME_CYTOSOLIC_TRNA
0	0	.	NA	.	SYS	EXTI	.	KEG	.	.	REACTOME_DEVELOPMENTAL
0	0	Frank-ter H	NA	.	.	.	.	.	.	.	.
0	0	.	NA	.	.	.	.	.	.	.	.
0	0	.	NA	.	.	.	.	.	.	.	.
0	0	.	NA	.	.	.	.	.	.	.	.
0	0	{Cancer pr	NA	.	ENZ	INTF	TRAN	KEG	PID	.	REACTOME_NEGATIVE_REGUI
0	0	{Cancer pr	NA	.	ENZ	INTF	TRAN	KEG	PID	.	REACTOME_NEGATIVE_REGUI
0	0	{Cancer pr	NA	.	ENZ	INTF	TRAN	KEG	PID	.	REACTOME_NEGATIVE_REGUI
0	0	.	NA	.	.	.	.	.	.	.	.
0	0	.	NA	.	.	.	.	.	.	.	REACTOME_TRANSLATION;RE
0	0	Night blind	NA	.	GLU	INTF	GLUT	KEG	.	.	REACTOME_SIGNALING_BY_G
0	0	.	NA	.	.	.	.	.	.	.	.
0	0	.	NA	.	GLY	.	TRAN	KEG	.	.	REACTOME_METABOLISM_OF_
0	0	.	NA	.	ENE	.	.	KEG	.	.	REACTOME_METABOLISM_OF_
0	0	.	NA	.	.	ORG	HYDF	.	.	.	.
0	0	.	NA	.	.	ORG	HYDF	.	.	.	.
0	0	.	NA	.	.	.	.	.	.	.	.
0	0	.	NA	.	.	.	.	.	.	.	.
0	0	.	NA	.	.	.	.	.	.	.	.
0	0	.	NA	.	.	.	.	.	.	.	.
0	0	Adult i phe	NA	.	BIOS	CELI	TRAN	KEG	.	.	.
0	0	.	NA	.	.	NUC	DNA_	.	.	.	.
0	0	Hermansky	NA	.	ORG	AXO	IDEN	.	.	.	REACTOME_MEMBRANE_TRAF
0	0	.	NA	.	.	.	.	.	.	.	.
0	0	.	NA	.	.	.	.	.	.	.	.
0	0	.	NA	.	.	.	.	.	.	.	.
0	0	.	NA	.	.	.	.	.	.	.	.
0	0	.	NA	.	.	.	.	.	.	.	.
0	0	.	NA	.	.	.	.	.	.	.	.
0	0	.	NA	.	.	.	.	.	.	.	REACTOME_GENERIC_TRANS
0	0	.	NA	.	.	.	.	.	.	.	.
0	0	Diabetes m	NA	.	.	.	.	.	.	.	.
0	0	Hemochroi	NA	.	.	.	.	.	.	.	.
0	0	.	NA	.	.	.	.	.	.	.	.
0	0	.	NA	.	.	NUC	.	.	PID	.	REACTOME_HOMOLOGOUS_R
0	0	.	NA	.	.	.	.	.	.	.	.
0	0	.	NA	.	.	.	.	.	.	.	.
0	0	.	NA	.	.	.	.	.	.	.	.
0	0	.	NA	.	.	.	LIGAS	KEG	.	.	REACTOME_CYTOSOLIC_TRNA
0	0	.	NA	.	.	CYT	.	KEG	PID	BIOC	.
0	0	Deafness,	NA	.	.	.	.	KEG	PID	.	REACTOME_EXTRACELLULAR_
0	0	Deafness,	NA	.	.	.	.	KEG	PID	.	REACTOME_EXTRACELLULAR_

0	0	{Diabetes,	NA	.	SIGN	BRU	CATI	KEG	.	.	REACTOME_SIGNALLING_BY_N
0	0	.	NA	.	.	.	.	.	.	.	.
0	0	.	NA	.	REG	.	LIGAI	KEG	PID	BIO	REACTOME_GENERIC_TRANS
0	0	.	NA	.	REG	.	LIGAI	KEG	PID	BIO	REACTOME_GENERIC_TRANS
0	0	.	NA	.	REG	.	LIGAI	KEG	PID	BIO	REACTOME_GENERIC_TRANS
0	0	.	NA	.	ANA	.	.	KEG	.	.	REACTOME_TRANSLATION;RE
0	0	Spermatog	NA	.	.	.	.	.	.	.	.
0	0	.	NA	.	.	.	.	.	.	.	.
0	0	.	NA	.	.	.	.	KEG	.	.	.
0	0	.	NA	.	.	.	.	.	.	.	.
0	0	.	NA	.	RNA	CYT	TRAN	.	.	.	.
0	0	.	NA	.	ENZ	.	.	.	PID	.	REACTOME_SIGNALING_BY_F
0	0	Retinitis pi	NA	.	REG	.	ENZY	KEG	PID	.	.
0	0	.	NA	.	RNA	NUC	TRAN	.	.	.	.
0	0	Choriodal (	NA	.	SEN	INTR	.	KEG	.	.	.
0	0	.	NA	.	SIGN	INTR	TRAN	.	.	.	.
0	0	.	NA	.	SIGN	INTR	TRAN	.	.	.	.
0	0	.	NA	.	.	.	.	.	PID	.	.
0	0	.	NA	.	.	MEM	.	.	.	.	.
0	0	.	NA	.	.	.	.	.	.	.	REACTOME_MICRORNA_MIRN
0	0	.	NA	.	.	.	.	.	.	.	.
0	0	.	NA	.	.	.	.	.	.	.	.
0	0	Neuropath	NA	.	ORG	PRO	INTE	.	PID	.	.
0	0	.	NA	.	.	.	.	.	.	.	.
0	0	.	NA	.	SYS	.	.	.	.	.	REACTOME_EXTRACELLULAR_
0	0	.	NA	.	.	.	.	KEG	.	.	.
0	0	Hydatidifor	NA	.	.	.	.	.	.	.	.
0	0	.	NA	.	.	.	.	.	.	.	.
0	0	.	NA	.	.	.	.	.	.	.	.
0	0	Maple syru	NA	CM0	CAR	ORG	LYAS	KEG	.	.	REACTOME_METABOLISM_OF_
0	0	.	NA	.	SEN	CYT	ELEC	.	.	.	.
0	0	Congenital	NA	.	EST	INTR	SUBS	.	.	.	REACTOME_TRANSMEMBRANI
0	0	.	NA	.	.	.	.	.	.	.	.
0	0	.	NA	.	.	.	.	.	.	.	.
0	0	.	NA	.	POS	.	IDEN	.	.	.	.
0	0	.	NA	.	.	.	.	.	.	.	.
0	0	.	NA	.	SYS	.	LIGAI	.	.	.	REACTOME_GENERIC_TRANS
0	0	.	NA	.	RES	NUC	.	KEG	PID	.	.
0	0	.	NA	.	.	.	.	.	.	.	.
0	0	.	NA	.	.	CYT	PROT	.	.	.	.
0	0	.	NA	.	DNA	.	NUCL	.	.	.	REACTOME_DNA_REPAIR
0	0	.	NA	.	.	.	.	.	.	.	.
0	0	.	NA	.	.	.	.	.	.	.	.
0	0	.	NA	.	.	.	.	KEG	.	.	.
0	0	Muscular d	NA	.	MUS	PRO	STRU	KEG	PID	BIO	REACTOME_INTEGRIN_CELL_
0	0	Muscular d	NA	.	MUS	PRO	STRU	KEG	PID	BIO	REACTOME_INTEGRIN_CELL_
0	0	Cardiomyo	NA	.	ANA	.	.	.	.	.	.
0	0	.	NA	.	ISOF	.	OXID	.	.	.	.
0	0	.	NA	.	.	.	.	KEG	.	.	.
0	0	.	NA	.	.	.	.	.	.	.	.
0	0	.	NA	.	.	.	.	.	.	.	.
0	0	.	NA	.	.	INTR	G_PF	.	.	.	.
0	0	Hemophag	NA	.	MEM	.	.	KEG	.	.	REACTOME_BOTULINUM_NEU
0	0	.	NA	.	.	.	.	.	.	.	.

0	0	.	NA	.	BIOF	INTF	SULF	KEC	.	.	REACTOME_CHONDROITIN_SU
0	0	.	NA	.	.	.	.	.	.	.	.
0	0	.	NA	.	INTE	ORG	HYDF	.	PID	.	.
0	0	.	NA	.	.	.	.	.	.	.	.
0	0	.	NA	.	SIGN	CYT	KINA	.	.	.	.
0	0	Emery-Dre	NA	.	ORG	SYN	ACTII	.	.	.	REACTOME_MEIOSIS;REACTO
0	0	Emery-Dre	NA	.	ORG	SYN	ACTII	.	.	.	REACTOME_MEIOSIS;REACTO
0	0	Emery-Dre	NA	.	ORG	SYN	ACTII	.	.	.	REACTOME_MEIOSIS;REACTO
0	0	.	NA	.	RES	.	OXID	.	PID	.	.
0	0	.	NA	.	.	.	.	.	.	.	.
0	0	Mental reta	NA	.	.	NUC	DNA_	.	.	.	.
0	0	.	NA	.	.	.	.	.	.	.	.
0	0	{Microvasc	NA	.	RNA	CYT	OXID	KEC	PID	BIO	.
0	0	.	NA	.	.	.	.	.	.	.	.
0	0	.	NA	.	.	.	.	.	.	.	.
0	0	[LPA defici	NA	.	SYS	.	PROT	.	PID	.	REACTOME_METABOLISM_OF_
0	0	.	NA	.	REG	.	PHOS	KEC	PID	BIO	.
0	0	Adenocarc	NA	.	BIOF	CYT	ACID	KEC	PID	BIO	REACTOME_IMMUNE_SYSTEM
0	0	.	NA	.	.	.	.	.	.	.	.
0	0	.	NA	.	.	.	.	.	.	.	.
0	0	.	NA	.	.	CYT	.	.	.	.	.
0	0	.	NA	.	.	.	.	.	.	.	.
0	0	Raine sync	NA	.	.	.	.	.	.	.	.
0	0	.	NA	.	.	.	.	.	.	.	.
0	0	.	NA	.	.	.	.	.	.	.	.
0	0	.	NA	.	RNA	NUC	.	.	.	.	.
0	0	.	NA	.	RNA	NUC	.	.	.	.	.
0	0	.	NA	.	RNA	NUC	.	.	.	.	.
0	0	.	NA	.	.	.	.	.	.	.	.
0	0	.	NA	.	RES	.	HYDF	.	.	.	.
0	0	.	NA	.	SULI	.	SULF	KEC	.	.	REACTOME_CHONDROITIN_SU
0	0	Immunode	NA	.	REG	MEM	PROT	KEC	PID	.	REACTOME_DOWNSTREAM_S
0	0	.	NA	.	.	.	.	.	.	.	.
0	0	.	NA	.	.	.	.	.	.	.	.
0	0	Spastic pa	NA	.	.	.	.	.	.	.	.
0	0	.	NA	.	.	.	.	.	.	.	.
0	0	.	NA	.	EST	MEM	LIPID	.	PID	BIO	.
0	0	.	NA	.	.	.	.	.	PID	.	REACTOME_GASTRIN_CREB_S
0	0	.	NA	.	.	.	.	.	.	.	.
0	0	.	NA	.	.	.	.	.	.	.	.
0	0	.	NA	.	.	.	.	.	.	.	.
0	0	.	NA	.	.	.	.	.	.	.	.
0	0	.	NA	.	.	.	.	.	.	.	.
0	0	.	NA	.	.	.	.	.	.	.	.
0	0	.	NA	.	.	.	.	.	.	.	REACTOME_EXTRACELLULAR_
0	0	.	NA	.	.	.	.	.	.	.	.
0	0	.	NA	.	.	.	.	.	.	.	.
0	0	.	NA	.	.	.	.	.	.	.	.
0	0	.	NA	.	.	.	.	KEC	.	.	REACTOME_ABC_FAMILY_PRC
0	0	Ciliary dysl	NA	.	.	.	.	.	.	.	.
0	0	.	NA	.	.	.	.	.	.	.	.
0	0	.	NA	.	.	.	.	.	.	.	.
0	0	.	NA	.	.	.	.	KEC	.	.	REACTOME_METABOLISM_OF_
0	0	.	NA	.	PRO	CYT	ENZY	KEC	.	.	REACTOME_TRIF_MEDIATED_

0	0	.	NA	.	.	.	.	.	.	.	.
0	0	.	NA	.	.	.	.	.	.	.	.
0	0	.	NA	.	PRO	CYT	CATI	.	.	.	REACTOME_ASSOCIATION_OF
0	0	Bardet-Bie	NA	.	.	.	.	.	.	.	.
0	0	.	NA	.	.	.	.	.	.	.	.
0	0	.	NA	.	.	.	.	.	.	.	.
0	0	.	NA	.	.	.	.	.	.	.	.
0	0	.	NA	.	.	.	.	.	.	.	.
0	0	.	NA	.	.	.	.	.	.	.	.
0	0	.	NA	.	.	.	.	.	.	.	.
0	0	.	NA	.	.	.	.	KE	.	BIO	.
0	0	.	NA	.	POS	.	.	.	.	.	.
0	0	Alpha-keto	NA	.	GEN	ORG	OXID	KE	.	BIO	REACTOME_PYRUVATE_META
0	0	.	NA	.	.	.	.	KE	PID	BIO	REACTOME_SIGNALLING_BY_
0	0	.	NA	.	MUS	NUC	ENZY	KE	PID	BIO	REACTOME_DIABETES_PATHW
0	0	.	NA	.	.	.	.	.	.	.	.
0	0	.	NA	.	.	.	.	.	.	.	.
0	0	.	NA	.	.	.	.	.	.	.	.
0	0	.	NA	.	.	.	.	.	.	.	.
0	0	.	NA	.	.	.	.	.	.	.	.
0	0	.	NA	.	.	.	.	.	.	.	.
0	0	.	NA	.	.	.	.	.	.	.	.
0	0	.	NA	.	.	.	.	.	.	.	.
0	0	.	NA	.	.	.	.	.	.	.	.
0	0	.	NA	.	.	.	.	.	.	.	.
0	0	.	NA	.	.	.	.	.	.	.	.
0	0	.	NA	.	.	.	.	.	.	.	.
0	0	.	NA	.	.	.	.	.	.	.	.
0	0	.	NA	.	.	.	.	.	.	.	.
0	0	.	NA	.	.	.	.	.	.	.	.
0	0	.	NA	.	.	.	.	.	.	.	.
0	0	.	NA	.	.	.	.	.	.	.	.
0	0	.	NA	.	.	.	.	.	.	.	.
0	0	.	NA	.	.	.	.	KE	.	.	REACTOME_O_LINKED_GLYCO
0	0	.	NA	.	.	.	.	.	.	.	.
0	0	.	NA	.	.	.	.	.	.	.	.
0	0	.	NA	.	PRO	.	DRU	.	.	.	REACTOME_MEIOSIS;REACTO
0	0	.	NA	.	SYS	INTR	RECE	KE	PID	.	REACTOME_SIGNALING_BY_G
0	0	.	NA	.	.	.	.	.	.	.	.
0	0	.	NA	.	.	.	.	.	.	.	.
0	0	.	NA	.	POS	MEM	PROT	.	PID	.	.
0	0	.	NA	.	.	.	.	KE	.	.	.
0	0	Chronic gr	NA	.	DEFI	CYT	HYDF	KE	PID	.	.
0	0	.	NA	.	.	.	.	.	.	.	.
0	0	.	NA	.	.	.	.	.	.	.	.
0	0	.	NA	.	.	.	.	KE	.	.	REACTOME_ACTIVATED_NOTC
0	0	.	NA	.	ESTA	MEM	CATI	KE	.	.	.
0	0	.	NA	.	.	.	.	.	.	.	.
0	0	.	NA	.	ORG	SYN	LIPID	.	.	.	.
0	0	.	NA	.	.	CYT	.	.	.	.	.
0	0	.	NA	.	.	.	.	.	.	.	.
0	0	Ehlers-Dar	NA	.	SYS	PRO	.	KE	PID	.	REACTOME_DEVELOPMENTAL
0	0	Dystonia-1	NA	.	MUS	PRO	.	.	.	.	.
0	0	.	NA	.	.	.	.	.	.	.	REACTOME_IMMUNE_SYSTEM

0	0	.	NA	.	PRO	INTF	PHOS	.	.	.	.
0	0	.	NA	.	SIGN	NUC	TRAN	.	PID	BIO	.
0	0	.	NA	.	SIGN	NUC	TRAN	.	PID	BIO	.
0	0	.	NA	.	.	.	.	.	.	.	.
0	0	.	NA	.	.	.	.	.	.	.	.
0	0	.	NA	.	MUL	.	.	.	.	.	REACTOME_EXTRACELLULAR_
0	0	.	NA	.	.	NUC	STRU	.	.	.	.
0	0	.	NA	.	.	.	.	.	.	.	REACTOME_O_LINKED_GLYCO
0	0	.	NA	.	.	.	.	.	.	.	.
0	0	.	NA	.	RNA	.	RNA_	.	.	.	REACTOME_SIGNALING_BY_F
0	0	.	NA	.	.	.	.	.	.	.	.
0	0	.	NA	.	.	.	.	KEG	.	.	.
0	0	.	NA	.	.	CYT	.	KEG	.	BIO	REACTOME_SIGNALING_BY_W
0	0	.	NA	.	DNA	NUC	NUCL	KEG	.	BIO	REACTOME_ACTIVATION_OF_
0	0	.	NA	.	.	.	.	.	.	.	.
0	0	.	NA	.	MUS	.	.	.	.	.	.
0	0	.	NA	.	.	CYT	.	.	.	.	.
0	0	Hyperlysin	NA	.	PRO	CYT	.	KEG	.	.	REACTOME_METABOLISM_OF_
0	0	.	NA	.	.	.	.	.	.	.	.
0	0	.	NA	.	SIGN	INTF	.	KEG	.	BIO	.
0	0	.	NA	.	.	.	.	.	.	.	.
0	0	Myopathy,	NA	.	.	.	.	KEG	.	.	REACTOME_CELL_CELL_COMI
0	0	Basal cell	NA	.	.	INTF	RECE	KEG	PID	BIO	REACTOME_SIGNALING_BY_G
0	0	.	NA	.	.	.	.	.	.	.	.
0	0	.	NA	.	.	.	.	.	.	.	.
0	0	.	NA	.	.	.	.	.	.	.	.
0	0	.	NA	.	.	CYT	PHOS	KEG	.	.	REACTOME_GASTRIN_CREB_S
0	0	.	NA	.	.	CYT	PHOS	KEG	.	.	REACTOME_GASTRIN_CREB_S
0	0	.	NA	.	.	.	.	KEG	.	.	.
0	0	Renal tubu	NA	.	SYS	CELI	.	KEG	.	.	REACTOME_INSULIN_RECEPT
0	0	.	NA	.	.	.	.	.	.	.	.
0	0	.	NA	.	.	.	.	.	.	.	.
0	0	Cataract 3	NA	.	.	.	.	KEG	.	.	.
0	0	.	NA	.	BIOF	.	.	KEG	.	.	REACTOME_METABOLISM_OF_
0	0	.	NA	.	.	.	.	.	.	.	.
0	0	.	NA	.	.	.	.	.	.	.	.
0	0	.	NA	.	.	.	.	.	.	.	.
0	0	.	NA	.	.	.	.	.	.	.	.
0	0	.	NA	.	.	.	.	.	.	.	.
0	0	.	NA	.	.	.	IDEN	KEG	.	.	.
0	0	.	NA	.	.	.	.	.	.	.	.
0	0	Cortical dy	NA	.	.	.	.	KEG	.	.	.
0	0	.	NA	.	.	.	.	.	.	.	.
0	0	.	NA	.	.	.	.	.	PID	.	.
0	0	.	NA	.	.	.	.	.	.	.	.
0	0	{Alzheimer	NA	.	.	CYT	OXID	KEG	PID	BIO	REACTOME_TETRAHYDROBIO
0	0	.	NA	.	ORG	.	.	.	.	.	.
0	0	.	NA	.	SYS	AXO	PHOS	KEG	PID	BIO	REACTOME_DEVELOPMENTAL
0	0	.	NA	.	.	.	.	.	.	.	.
0	0	Cardiomyo	NA	.	.	.	.	KEG	.	BIO	REACTOME_ACTIVATED_AMPK
0	0	Cardiomyo	NA	.	.	.	.	KEG	.	BIO	REACTOME_ACTIVATED_AMPK
0	0	.	NA	.	.	.	.	.	.	.	.
0	0	.	NA	.	.	.	.	.	.	.	.
0	0	Ventricular	NA	.	.	.	.	.	.	.	.

0	0	.	NA	.	PRO	.	ACID	KE	.	.	REACTOME_IMMUNE_SYSTEM
0	0	.	NA	.	BIOF	INTF	PROT	KE	.	.	.
0	0	.	NA	.	BIOF	INTF	PROT	KE	.	.	.
0	0	.	NA	.	.	.	.	.	.	.	.
0	0	.	NA	.	.	.	.	.	.	.	.
0	0	Ceroid lipo	NA	.	SYS	CYT	.	.	.	.	.
0	0	.	NA	.	.	.	.	.	.	.	.
0	0	.	NA	.	.	.	.	.	.	.	REACTOME_DEFENSINS;REAC
0	0	.	NA	.	.	.	.	.	.	.	.
0	0	.	NA	.	.	.	.	.	.	.	.
0	0	.	NA	.	CAL	CELI	IDEN	KE	.	.	.
0	0	Occult mac	NA	.	.	.	.	.	.	.	.
0	0	.	NA	.	.	.	.	.	.	.	.
0	0	.	NA	.	.	.	.	.	.	.	.
0	0	.	NA	.	.	.	.	.	.	.	.
0	0	.	NA	.	.	.	.	.	.	.	.
0	0	.	NA	.	.	.	.	.	.	.	.
0	0	.	NA	.	.	.	.	.	.	.	.
0	0	.	NA	.	.	.	.	.	.	.	.
0	0	Colorectal	NA	.	REG	.	GTPA	.	PID	.	REACTOME_SIGNALING_BY_R
0	0	Mental reta	NA	.	GLY	ORG	TRAN	KE	.	.	REACTOME_METABOLISM_OF_
0	0	.	NA	.	.	.	.	.	.	.	.
0	0	.	NA	.	.	.	.	.	.	.	.
0	0	Alopecia ur	NA	.	.	.	.	.	.	.	.
0	0	.	NA	.	SIGN	.	RECE	KE	PID	BIO	.
0	0	.	NA	.	.	.	.	.	.	.	.
0	0	Persistent	NA	.	SYS	.	TRAN	.	.	.	.
0	0	.	NA	.	.	.	.	.	.	.	REACTOME_FACTORS_INVOLV
0	0	.	NA	.	.	.	.	.	.	.	.
0	0	.	NA	.	.	.	.	.	.	.	.
0	0	{Hyperchol	NA	.	.	.	.	KE	.	.	.
0	0	.	NA	.	EST	CYT	KINA	.	PID	.	.
0	0	.	NA	.	.	.	.	.	.	.	REACTOME_MEIOSIS;REACTO
0	0	.	NA	.	.	.	.	.	.	.	.
0	0	.	NA	.	.	.	.	.	.	.	.
0	0	Spastic pa	NA	.	.	ORG	.	.	.	.	.
0	0	Hypogonac	NA	.	SYS	INTF	TRAN	KE	PID	.	REACTOME_DEVELOPMENTAL
0	0	Hypogonac	NA	.	SYS	INTF	TRAN	KE	PID	.	REACTOME_DEVELOPMENTAL
0	0	.	NA	.	.	.	.	.	PID	.	.
0	0	.	NA	.	GAM	CELI	.	.	.	.	.
0	0	.	NA	.	.	.	.	.	.	.	.
0	0	.	NA	.	NEG	NUC	TRAN	.	.	.	.
0	0	.	NA	.	DNA	ORG	MICR	KE	.	.	REACTOME_BASE_EXCISION_
0	0	.	NA	.	.	.	.	.	.	.	.
0	0	.	NA	.	.	.	.	.	.	.	.
0	0	.	NA	.	.	.	.	.	.	.	.
0	0	.	NA	.	.	.	.	.	.	.	.
0	0	.	NA	.	.	.	.	.	.	.	.
0	0	.	NA	.	.	.	.	.	.	.	.
0	0	.	NA	.	.	.	.	.	.	.	.
0	0	.	NA	.	.	.	.	.	.	.	.
0	0	.	NA	.	.	PRO	.	.	.	.	.
0	0	.	NA	.	.	.	.	.	.	.	.
0	0	.	NA	.	.	.	.	.	.	.	.

0	0	.	NA	.	.	.	.	.	.	.	.
0	0	.	NA	.	MEM	CYT	RECE	.	PID	BIO	.
0	0	Cerebellar	NA	.	.	.	HYDF	KEC	.	.	.
0	0	CHARGE	NA	.	.	.	.	.	PID	.	.
0	0	.	NA	.	.	.	HYDF	KEC	.	.	REACTOME_SIGNALING_BY_G
0	0	.	NA	.	.	.	.	.	.	.	.
0	0	.	NA	.	.	.	.	.	.	.	.
0	0	.	NA	.	SIGN	.	RAS_	.	PID	.	.
0	0	.	NA	.	.	.	.	.	.	.	.
0	0	.	NA	.	.	.	.	.	.	.	.
0	0	.	NA	.	.	CYT	LIPID	KEC	PID	.	REACTOME_DEVELOPMENTAL
0	0	Achromato	NA	.	SEN	.	.	.	PID	.	.
0	0	Achromato	NA	.	SEN	.	.	.	PID	.	.
0	0	.	NA	.	.	.	.	.	.	.	.
0	0	?DECR de	NA	.	.	CYT	OXID	.	.	.	REACTOME_MITOCHONDRIAL_
0	0	?DECR de	NA	.	.	CYT	OXID	.	.	.	REACTOME_MITOCHONDRIAL_
0	0	.	NA	.	GEN	.	TRAN	KEC	.	.	.
0	0	.	NA	.	.	.	.	.	.	.	.
0	0	.	NA	.	.	.	.	.	.	.	.
0	0	.	NA	.	.	.	.	.	.	.	.
0	0	.	NA	.	.	.	.	.	.	.	.
0	0	.	NA	.	.	.	.	.	.	.	.
0	0	.	NA	.	.	.	.	.	.	.	.
0	0	.	NA	.	EST	.	AUXI	.	.	.	REACTOME_NEURONAL_SYST
0	0	.	NA	.	.	.	.	.	.	.	.
0	0	.	NA	.	.	.	.	.	.	.	.
0	0	.	NA	.	.	.	.	.	.	.	.
0	0	.	NA	.	.	.	.	.	.	.	.
0	0	Ciliary dysl	NA	.	.	.	.	.	.	.	.
0	0	Thyroid dy	NA	.	.	.	.	KEC	.	.	.
0	0	Birk-Barel	NA	.	.	.	.	.	.	.	REACTOME_NEURONAL_SYST
0	0	Mental reta	NA	.	.	.	.	.	.	.	.
0	0	.	NA	.	.	.	.	.	.	.	.
0	0	.	NA	.	.	.	.	.	.	.	.
0	0	.	NA	.	SYS	INTF	G_PF	KEC	.	.	.
0	0	.	NA	.	SYS	.	.	.	.	.	.
0	0	.	NA	.	.	.	.	.	.	.	.
0	0	Amelogene	NA	.	.	.	.	.	.	.	.
0	0	Amelogene	NA	.	.	.	.	.	.	.	.
0	0	.	NA	.	.	.	.	.	.	.	.
0	0	.	NA	.	.	.	.	.	.	.	.
0	0	.	NA	.	.	.	.	.	.	.	.
0	0	Epidermoly	NA	.	.	.	STRU	.	.	.	REACTOME_APOPTOTIC_CLEA
0	0	.	NA	.	.	.	TRAN	.	PID	.	.
0	0	.	NA	.	.	.	TRAN	.	PID	.	.
0	0	.	NA	.	.	.	.	.	.	.	.
0	0	.	NA	.	.	.	.	.	.	.	.
0	0	.	NA	.	.	.	.	.	.	.	.
0	0	{?Schizoph	NA	.	EST	INTF	ACTI	.	.	.	REACTOME_GLUTAMATE_NEU
0	0	Hydroceph	NA	.	.	.	.	KEC	.	.	.
0	0	.	NA	.	.	NUC	TRAN	.	PID	.	REACTOME_RNA_POL_III_TRA
0	0	Bifid nose	NA	.	.	.	.	.	.	.	.
0	0	Bifid nose	NA	.	.	.	.	.	.	.	.
0	0	.	NA	.	.	.	.	.	.	.	.

0	0	.	NA	.	.	.	.	.	.	.	.
0	0	.	NA	.	.	.	.	.	.	.	.
0	0	.	NA	.	.	.	.	.	PID_	.	REACTOME_TRANSMEMBRANI
0	0	.	NA	.	.	.	.	.	.	.	.
0	0	.	NA	.	.	.	.	.	.	.	.
0	0	.	NA	.	.	ORG	.	KE(	.	.	REACTOME_TCA_CYCLE_AND_
0	0	.	NA	.	.	.	.	.	.	.	.
0	0	.	NA	.	GAM	.	PHOS	.	.	.	REACTOME_CELL_CELL_COMI
0	0	.	NA	.	.	.	.	.	.	.	.
0	0	.	NA	.	.	.	.	.	.	.	.
0	0	.	NA	.	.	.	.	.	.	.	.
0	0	.	NA	.	.	.	.	.	.	.	.
0	0	.	NA	.	.	.	.	.	.	.	.
0	0	.	NA	.	.	.	.	.	.	.	.
0	0	.	NA	.	.	.	.	.	.	.	.
0	0	.	NA	.	.	.	.	.	.	.	.
0	0	.	NA	.	.	.	.	.	.	.	.
0	0	.	NA	.	.	.	.	.	.	.	.
0	0	.	NA	.	.	.	.	.	.	.	.
0	0	.	NA	.	.	.	.	.	.	.	.
0	0	.	NA	.	.	.	.	.	.	.	.
0	0	.	NA	.	.	.	.	.	.	.	.
0	0	.	NA	.	.	INTF	.	.	.	.	.
0	0	.	NA	.	.	.	.	.	.	.	.
0	0	.	NA	.	CELI	.	.	.	.	.	.
0	0	.	NA	.	.	.	.	.	.	.	.
0	0	.	NA	.	.	.	.	.	.	.	.
0	0	.	NA	.	.	.	.	.	.	.	.
0	0	.	NA	.	.	.	.	.	.	.	.
0	0	.	NA	.	.	.	.	.	.	.	.
0	0	Obesity, hy	NA	.	ENZ'	INTF	GRO\	KE(	PID_	.	REACTOME_SIGNALLING_BY_I
0	0	.	NA	.	.	.	.	.	.	.	.
0	0	.	NA	.	.	.	.	.	.	.	.
0	0	.	NA	.	SYS'	.	.	KE(	PID_	.	REACTOME_SIGNALLING_BY_I
0	0	.	NA	.	.	CYT(	.	KE(	.	.	REACTOME_CYTOSOLIC_TRN/
0	0	.	NA	.	.	.	.	.	.	.	.
0	0	.	NA	.	BIOF	.	NUCL	.	.	.	.
0	0	.	NA	.	.	NUC	.	.	.	.	.
0	0	.	NA	.	.	NUC	.	.	.	.	.
0	0	.	NA	.	RNA	NUC	TRAN	.	.	.	.
0	0	.	NA	.	.	NUC	PROT	KE(	PID_	.	.
0	0	.	NA	.	.	.	.	.	.	.	.
0	0	.	NA	.	.	.	.	.	.	.	.
0	0	.	NA	.	.	.	.	.	.	.	.
0	0	.	NA	.	.	.	.	.	.	.	.
0	0	.	NA	.	.	.	.	.	.	.	.
0	0	.	NA	.	.	.	.	.	.	.	.
0	0	.	NA	.	.	.	.	.	.	.	.
0	0	Porphyria,	NA	.	COF	.	HYDF	KE(	.	BIO(	REACTOME_METABOLISM_OF_
0	0	.	NA	.	.	.	.	.	.	.	REACTOME_EXTRACELLULAR_
0	0	.	NA	.	.	.	.	.	.	.	REACTOME_EXTRACELLULAR_
0	0	.	NA	.	.	.	.	.	.	.	REACTOME_EXTRACELLULAR_
0	0	.	NA	.	.	.	.	.	.	.	.
0	0	.	NA	.	.	EXTI	META	.	.	.	REACTOME_DIABETES_PATHW

0	0	Microcepha	NA	.	.	ORG	KINA	.	.	.	REACTOME_CELL_CYCLE;REA
0	0	.	NA	.	.	.	.	.	.	.	.
0	0	.	NA	.	.	.	.	.	.	.	.
0	0	.	NA	.	.	.	.	.	PID	.	.
0	0	.	NA	.	.	.	.	.	PID	.	.
0	0	.	NA	.	.	NUC	.	.	.	.	.
0	0	.	NA	.	POS	CYT	NUCL	.	.	.	.
0	0	.	NA	.	.	.	.	.	PID	.	REACTOME_SIGNALLING_BY_I
0	0	.	NA	.	.	.	.	.	.	.	.
0	0	Charcot-M	NA	.	.	.	.	.	.	.	REACTOME_IMMUNE_SYSTEM
0	0	Epileptic e	NA	.	.	.	.	.	.	.	REACTOME_GLUTAMATE_NEU
0	0	.	NA	.	.	.	.	.	.	.	.
0	0	.	NA	.	NUC	CYT	ACID	KEG	.	.	REACTOME_METABOLISM_OF_
0	0	Telangiecta	NA	.	REG	INTR	.	.	PID	.	.
0	0	.	NA	.	.	.	STRU	.	PID	.	.
0	0	Epileptic e	NA	.	.	ORG	ACTI	KEG	PID	BIO	REACTOME_APOPTOTIC_CLEA
0	0	Epileptic e	NA	.	.	ORG	ACTI	KEG	PID	BIO	REACTOME_APOPTOTIC_CLEA
0	0	Epileptic e	NA	.	.	ORG	ACTI	KEG	PID	BIO	REACTOME_APOPTOTIC_CLEA
0	0	Epileptic e	NA	.	.	ORG	ACTI	KEG	PID	BIO	REACTOME_APOPTOTIC_CLEA
0	0	.	NA	.	REG	UBIQ	ACID	.	.	.	.
0	0	.	NA	.	AMIN	CYT	.	.	.	.	REACTOME_METABOLISM_OF_
0	0	.	NA	.	AMIN	CYT	.	.	.	.	REACTOME_METABOLISM_OF_
0	0	.	NA	.	GLY	ORG	HYDF	KEG	.	.	REACTOME_METABOLISM_OF_
0	0	?Carnitine	NA	.	GEN	.	.	KEG	.	.	REACTOME_PEROXISOMAL_LI
0	0	?Carnitine	NA	.	GEN	.	.	KEG	.	.	REACTOME_PEROXISOMAL_LI
0	0	.	NA	.	ENZ	.	.	KEG	PID	BIO	REACTOME_SIGNALLING_BY_I
0	0	.	NA	.	SYS	INTR	.	.	.	.	.
0	0	Amyotroph	NA	.	DNA	NUC	DNA_	.	.	.	.
0	0	.	NA	.	.	.	.	.	.	.	.
0	0	.	NA	.	.	NUC	.	.	.	.	REACTOME_RNA_POL_III_TRA
0	0	.	NA	.	.	.	GTPA	KEG	PID	BIO	REACTOME_SIGNALLING_BY_I
0	0	.	NA	.	GLY	.	.	KEG	.	.	.
0	0	.	NA	.	.	CYT	.	.	.	.	.
0	0	.	NA	.	.	.	.	.	.	.	.
0	0	.	NA	.	.	.	.	.	.	.	.
0	0	Dopamine	NA	.	CELI	CYT	.	KEG	.	BIO	REACTOME_METABOLISM_OF_
0	0	Ehlers-Dar	NA	.	.	PRO	.	KEG	PID	.	REACTOME_DEVELOPMENTAL
0	0	Ehlers-Dar	NA	.	.	PRO	.	KEG	PID	.	REACTOME_DEVELOPMENTAL
0	0	Ehlers-Dar	NA	.	.	PRO	.	KEG	PID	.	REACTOME_DEVELOPMENTAL
0	0	.	NA	.	.	.	.	.	.	.	.
0	0	.	NA	.	.	.	.	.	.	.	.
0	0	Aortic valv	NA	.	MUS	NUC	.	KEG	PID	BIO	.
0	0	Aortic valv	NA	.	MUS	NUC	.	KEG	PID	BIO	.
0	0	Aortic valv	NA	.	MUS	NUC	.	KEG	PID	BIO	.
0	0	.	NA	.	.	.	.	.	.	.	.
0	0	.	NA	.	.	.	.	.	.	.	.
0	0	.	NA	.	POS	.	.	KEG	PID	BIO	REACTOME_EXTRINSIC_PATHY
0	0	Mental reta	NA	.	OLIG	INTR	CATI	KEG	.	.	REACTOME_METABOLISM_OF_
0	0	Hypophosp	NA	.	REG	MEM	CATI	.	.	.	REACTOME_TRANSMEMBRANI
0	0	.	NA	.	.	.	.	.	.	.	.
0	0	.	NA	.	.	.	.	.	.	.	.
0	0	.	NA	.	.	.	.	.	.	.	.
0	0	Kleefstra s	NA	.	ONE	.	HIST	KEG	.	.	.
0	0	.	NA	.	.	.	.	.	.	.	.

0	0	.	NA	.	REG	CYT(	HYDF	.	.	.	.
0	0	.	NA	.	.	.	HYDF	.	.	.	.
0	0	.	NA	.	PRO	ORG	META	.	.	.	.
0	0	.	NA	.	.	.	.	.	.	.	.
0	0	.	NA	.	.	.	.	.	.	.	.
0	0	.	NA	.	.	.	.	.	.	.	.
0	0	.	NA	.	.	.	.	.	.	.	.
0	0	.	NA	.	MAIN	NUC	.	.	.	.	.
0	0	.	NA	.	MAIN	NUC	.	.	.	.	.
0	0	.	NA	.	.	.	.	.	.	.	.
0	0	.	NA	.	.	.	.	.	.	.	.
0	0	.	NA	.	.	.	.	.	.	.	.
0	0	Megaloblas	NA	.	EST/	MEM	PROT	.	.	.	REACTOME_METABOLISM_OF_
0	0	Megaloblas	NA	.	EST/	MEM	PROT	.	.	.	REACTOME_METABOLISM_OF_
0	0	.	NA	.	.	.	.	.	.	.	.
0	0	.	NA	.	NEG	CYT(	ENZY	.	.	.	.
0	0	.	NA	.	SYS	CYT(	.	.	.	.	.
0	0	.	NA	.	.	.	.	.	.	.	.
0	0	.	NA	.	.	.	.	.	.	.	.
0	0	Deafness,	NA	.	PRO	ORG	HYDF	.	.	.	.
0	0	.	NA	.	.	.	.	.	.	.	REACTOME_INTEGRIN_CELL_S
0	0	Thromboc	NA	.	.	.	.	.	.	.	.
0	0	Thromboc	NA	.	.	.	.	.	.	.	.
0	0	.	NA	.	.	.	.	.	.	.	.
0	0	.	NA	.	MUS	CYT(	ACTII	.	PID	.	.
0	0	.	NA	.	.	.	.	.	.	.	.
0	0	.	NA	.	.	NUC	.	.	.	.	.
0	0	.	NA	.	.	.	.	.	.	.	.
0	0	.	NA	.	.	.	.	.	.	.	.
0	0	.	NA	.	.	.	.	.	.	.	.
0	0	.	NA	.	.	.	.	.	.	.	.
0	0	.	NA	.	.	.	.	.	.	.	.
0	0	.	NA	.	.	.	.	.	.	.	.
0	0	.	NA	.	.	.	.	.	.	.	.
0	0	Myasthenic	NA	.	.	CYT(	.	KEC	.	.	REACTOME_PHOSPHOLIPID_M
0	0	.	NA	.	.	.	.	.	.	.	.
0	0	Deafness,	NA	.	REG	NON	.	.	.	.	.
0	0	.	NA	.	.	.	.	.	.	.	.
0	0	.	NA	.	.	.	.	.	.	.	.
0	0	Thyroid pa	NA	.	.	.	STRU	KEC	.	.	.
0	0	.	NA	.	.	.	.	.	.	.	.
0	0	.	NA	.	DNA	ORG	DNA_	.	.	.	.
0	0	.	NA	.	SYS	PRO	CARE	.	PID	.	REACTOME_EXTRACELLULAR_
0	0	.	NA	.	CELI	ORG	NUCL	.	PID	.	.
0	0	Hemophag	NA	.	MUL	.	.	KEC	PID	BIO(	.
0	0	Deafness,	NA	.	REG	NON	.	.	.	.	.
0	0	.	NA	.	.	CYT(	OXID	KEC	.	.	.
0	0	.	NA	.	.	.	.	.	.	.	.
0	0	.	NA	.	.	PRO	CATI(	KEC	PID	BIO(	REACTOME_SIGNALING_BY_G
0	0	.	NA	.	.	.	.	.	.	.	.
0	0	.	NA	.	.	.	.	.	.	.	.
0	0	.	NA	.	.	.	.	.	.	.	.
0	0	.	NA	.	.	.	.	.	.	.	.
0	0	.	NA	.	REG	MEM	CARE	.	PID	.	.

0	0	.	NA	.	.	.	.	.	.	.	.
0	0	Cone-rod c	NA	.	.	.	.	.	.	.	.
0	0	.	NA	.	.	ORG	.	.	.	.	.
0	0	.	NA	.	.	.	.	KEC	.	.	.
0	0	.	NA	.	.	.	.	.	.	.	.
0	0	.	NA	.	.	.	.	.	.	.	.
0	0	.	NA	.	SYS	MEM	.	.	PID	.	.
0	0	.	NA	.	.	.	.	.	.	.	.
0	0	Rhabdomy	NA	.	.	.	.	KEC	.	.	REACTOME_BIOLOGICAL_OXID
0	0	.	NA	.	.	.	.	.	.	.	.
0	0	Agammagl	NA	.	SYS	.	MOLE	KEC	PID	BIO	REACTOME_ANTIGEN_ACTIVA
0	0	.	NA	.	.	.	.	.	.	.	.
0	0	Spastic pa	NA	.	.	.	.	.	.	.	.
0	0	.	NA	.	.	.	.	.	.	.	.
0	0	Urofacial s	NA	.	.	.	HYDF	KEC	.	.	REACTOME_HS_GAG_DEGRAD
0	0	.	NA	.	.	.	.	.	.	.	.
0	0	.	NA	.	.	.	.	.	.	.	.
0	0	.	NA	.	.	.	.	.	.	.	.
0	0	.	NA	.	.	.	.	KEC	.	.	.
0	0	.	NA	.	.	.	.	.	.	.	.
0	0	Epidermoly	NA	.	SYS	INTR	.	.	PID	.	REACTOME_CELL_CELL_COMI
0	0	.	NA	.	.	.	.	.	.	.	.
0	0	.	NA	.	.	.	.	.	.	.	REACTOME_SIGNALLING_BY_I
0	0	.	NA	.	.	.	RECE	.	PID	.	REACTOME_DEVELOPMENTAL
0	0	.	NA	.	.	.	.	.	.	.	.
0	0	.	NA	.	RNA	CYT	RNA_	.	.	.	.
0	0	.	NA	.	.	.	.	.	.	.	.
0	0	.	NA	.	.	.	.	.	.	.	.
0	0	.	NA	.	CELI	CYT	GTPA	KEC	PID	BIO	REACTOME_DEVELOPMENTAL
0	0	.	NA	.	CELI	CYT	GTPA	KEC	PID	BIO	REACTOME_DEVELOPMENTAL
0	0	.	NA	.	.	.	.	.	.	.	.
0	0	.	NA	.	.	.	.	KEC	.	BIO	.
0	0	.	NA	.	.	.	.	.	.	.	.
0	0	.	NA	.	.	.	.	.	.	.	.
0	0	.	NA	.	.	.	.	.	.	.	.
0	0	.	NA	.	.	.	.	.	.	.	.
0	0	.	NA	.	.	.	.	KEC	PID	.	REACTOME_METABOLISM_OF_
0	0	.	NA	.	.	.	.	KEC	PID	.	REACTOME_METABOLISM_OF_
0	0	.	NA	.	.	.	.	KEC	PID	.	REACTOME_METABOLISM_OF_
0	0	.	NA	.	.	.	.	.	.	.	.
0	0	.	NA	.	.	.	.	.	.	.	.
0	0	.	NA	.	.	.	.	.	.	.	.
0	0	.	NA	.	.	PRO	.	KEC	.	.	REACTOME_SIGNALING_BY_W
0	0	.	NA	.	SYS	EXTI	ENZY	.	.	.	.
0	0	.	NA	.	.	.	.	.	.	.	.
0	0	.	NA	.	.	.	.	.	.	.	.
0	0	.	NA	.	.	.	.	.	.	.	.
0	0	Epileptic e	NA	.	EST/	ORG	ACTI	.	.	.	.
0	0	.	NA	.	.	.	.	KEC	.	.	REACTOME_SIGNALLING_BY_I
0	0	.	NA	.	.	.	.	.	.	.	.
0	0	{Pulmonar	NA	.	.	.	.	.	.	.	REACTOME_O_LINKED_GLYCC
0	0	{Pulmonar	NA	.	.	.	.	.	.	.	REACTOME_O_LINKED_GLYCC
0	0	{Pulmonar	NA	.	.	.	.	.	.	.	REACTOME_O_LINKED_GLYCC
0	0	{Pulmonar	NA	.	.	.	.	.	.	.	REACTOME_O_LINKED_GLYCC

0	0	{Pulmonar	NA	.	.	.	.	.	.	.	REACTOME_O_LINKED_GLYCC
0	0	Segawa sy	NA	.	ANA	.	OXID	KEG	PID	BIO	REACTOME_METABOLISM_OF_
0	0	.	NA	.	.	.	.	.	.	.	.
0	0	Immunode	NA	.	PHO	INTR	.	KEG	PID	.	REACTOME_IMMUNOREGULAT
0	0	.	NA	.	.	INTR	SUBS	KEG	.	.	.
0	0	.	NA	.	.	INTR	SUBS	KEG	.	.	.
0	0	.	NA	.	.	.	.	.	.	.	.
0	0	.	NA	.	.	.	.	KEG	.	.	REACTOME_SIGNALING_BY_G
0	0	.	NA	.	.	.	.	.	.	.	.
0	0	.	NA	.	.	.	.	.	.	.	.
0	0	.	NA	.	.	.	.	.	.	.	.
0	0	.	NA	.	REG	NUC	TRAN	KEG	PID	.	.
0	0	.	NA	.	.	.	.	.	.	.	.
0	0	.	NA	.	.	.	.	.	.	.	.
0	0	.	NA	.	.	.	.	.	.	.	.
0	0	.	NA	.	RNA	NUC	.	KEG	PID	.	REACTOME_BMAL1_CLOCK_NI
0	0	.	NA	.	.	.	.	.	.	.	.
0	0	Ovarian ca	NA	.	.	.	HYDF	KEG	.	.	.
0	0	.	NA	.	.	.	HYDF	KEG	.	BIO	REACTOME_INSULIN_RECEPT
0	0	Rickets du	NA	.	.	.	.	.	.	.	REACTOME_METABOLISM_OF_
0	0	.	NA	.	.	.	.	.	PID	.	.
0	0	.	NA	.	.	.	.	.	PID	.	.
0	0	Deafness,	NA	.	.	.	.	.	.	.	.
0	0	.	NA	.	.	.	.	KEG	.	BIO	REACTOME_METABOLISM_OF_
0	0	.	NA	.	.	.	.	.	.	.	.
0	0	.	NA	.	REG	.	G_PF	.	PID	.	REACTOME_TRIF_MEDIATED_
0	0	Glycogen s	NA	.	.	CYT	OXID	KEG	PID	BIO	REACTOME_PYRUVATE_META
0	0	Hyperekple	NA	.	CELI	.	CATI	.	.	.	REACTOME_TRANSMEMBRANI
0	0	.	NA	.	SYS	.	.	.	.	.	.
0	0	.	NA	.	CELI	ORG	.	.	PID	BIO	REACTOME_APOPTOTIC_CLEA
0	0	.	NA	.	.	.	.	.	.	.	.
0	0	Hemolytic	NA	.	REG	CELI	.	KEG	.	.	.
0	0	.	NA	.	.	.	.	.	.	.	.
0	0	.	NA	.	.	.	.	KEG	.	.	REACTOME_TRIGLYCERIDE_B
0	0	.	NA	.	.	.	IDEN	.	.	.	.
0	0	.	NA	.	EST/	ORG	.	.	PID	.	REACTOME_CELL_CYCLE;REA
0	0	.	NA	.	.	.	.	KEG	.	.	.
0	0	.	NA	.	.	.	.	KEG	.	.	REACTOME_SIGNALING_BY_G
0	0	.	NA	.	.	.	.	.	.	.	.
0	0	.	NA	.	.	.	.	.	.	.	.
0	0	.	NA	.	.	.	.	KEG	.	.	REACTOME_SIGNALING_BY_G
0	0	.	NA	.	.	.	.	KEG	.	.	REACTOME_SIGNALING_BY_G
0	0	.	NA	.	.	.	.	KEG	.	.	REACTOME_MRNA_DECAY_BY
0	0	.	NA	.	.	.	.	.	.	.	.
0	0	.	NA	.	.	.	.	.	.	.	.
0	0	.	NA	.	PHO	.	OXID	KEG	.	.	REACTOME_PPARA_ACTIVATE
0	0	.	NA	.	.	.	.	.	.	.	.
0	0	.	NA	.	ORG	.	.	.	PID	.	.
0	0	.	NA	.	RNA	NUC	.	.	.	.	.
0	0	.	NA	.	.	.	.	.	.	.	REACTOME_PROCESSING_OF_
0	0	.	NA	.	.	.	.	.	.	.	.
0	0	.	NA	.	.	.	.	.	.	.	.
0	0	.	NA	.	CELI	.	.	KEG	PID	BIO	REACTOME_SIGNALLING_BY_I
0	0	McArdle di	NA	.	GLU	.	TRAN	KEG	.	BIO	REACTOME_GLYCOGEN_BREA

0	0	.	NA	.	BIOF	CYT	ACID	KEC	.	.	REACTOME_DIABETES_PATHV
0	0	.	NA	.	.	NUC	KINA	KEC	.	.	.
0	0	.	NA	.	.	.	.	KEC	.	.	REACTOME_ACTIVATION_OF_
0	0	.	NA	.	EST	.	CATI	.	.	.	REACTOME_NEURONAL_SYST
0	0	.	NA	.	PRO	ORG	PHOS	KEC	PID	BIO	.
0	0	.	NA	.	.	.	.	.	.	.	.
0	0	Metastasis	NA	.	CELI	.	GTPA	KEC	PID	.	REACTOME_RAP1_SIGNALLIN
0	0	Metastasis	NA	.	CELI	.	GTPA	KEC	PID	.	REACTOME_RAP1_SIGNALLIN
0	0	Aicardi-Go	NA	.	.	.	.	KEC	.	.	.
0	0	.	NA	.	.	.	.	.	.	.	.
0	0	.	NA	.	.	ORG	.	.	.	.	REACTOME_MHC_CLASS_II_AI
0	0	.	NA	.	.	PRO	.	.	.	.	.
0	0	.	NA	.	.	.	.	.	.	.	.
0	0	.	NA	.	BIOF	.	PHOS	KEC	PID	BIO	REACTOME_DOWNREGULATIC
0	0	Mitochondi	NA	.	.	ORG	.	KEC	.	.	REACTOME_TCA_CYCLE_AND_
0	0	Osteopetrc	NA	.	POS	INTR	.	KEC	.	.	REACTOME_INSULIN_RECEPT
0	0	[Skin/hair/e	NA	.	.	.	.	.	.	.	.
0	0	[Skin/hair/e	NA	.	.	.	.	.	.	.	.
0	0	.	NA	.	SIGN	CYT	.	.	.	.	.
0	0	Smith-Lem	NA	.	BIOS	ORG	OXID	KEC	.	.	REACTOME_METABOLISM_OF_
0	0	Deafness,	NA	.	.	.	.	.	.	.	.
0	0	.	NA	.	EST	CELI	VITAI	.	.	.	.
0	0	.	NA	.	.	.	.	.	.	.	.
0	0	.	NA	.	.	.	.	.	.	.	.
0	0	.	NA	.	REG	CYT	RAS_	KEC	PID	.	REACTOME_SIGNALING_BY_R
0	0	.	NA	.	.	.	.	.	.	.	.
0	0	.	NA	.	BIOF	.	PROT	.	.	.	.
0	0	.	NA	.	LIPIE	INTR	.	KEC	.	.	REACTOME_GLYCOSPHINGOL
0	0	.	NA	.	.	.	.	.	.	.	REACTOME_TRANSMEMBRANI
0	0	.	NA	.	.	.	.	.	.	.	.
0	0	.	NA	.	DNA	CYT	.	.	.	.	.
0	0	.	NA	.	.	.	.	.	.	.	.
0	0	.	NA	.	.	.	.	.	.	.	.
0	0	.	NA	.	.	MEM	PHOS	.	.	.	.
0	0	.	NA	.	G_PI	INTR	GLUT	KEC	.	.	.
0	0	.	NA	.	.	.	.	.	.	.	.
0	0	.	NA	.	.	.	.	.	.	.	.
0	0	Asphyxiatir	NA	.	ORG	CYT	.	KEC	.	.	REACTOME_MHC_CLASS_II_AI
0	0	.	NA	.	.	.	PHOS	KEC	.	.	REACTOME_NITRIC_OXIDE_ST
0	0	.	NA	.	.	.	.	.	.	.	.
0	0	.	NA	.	CELI	.	CATI	KEC	.	.	REACTOME_SIGNALING_BY_EI
0	0	Alpha-metl	NA	.	.	CYT	TRAN	KEC	.	.	REACTOME_METABOLISM_OF_
0	0	Ataxia-tela	NA	.	DNA	.	PHOS	KEC	PID	BIO	REACTOME_MEIOSIS;REACTO
0	0	.	NA	.	.	.	.	.	.	.	.
0	0	.	NA	.	.	.	.	.	.	.	.
0	0	.	NA	.	ENZ	CYT	NUCL	.	PID	.	.
0	0	.	NA	.	MITC	ORG	SEQU	.	.	.	REACTOME_CELL_CYCLE;REA
0	0	.	NA	.	POS	CELI	PROT	KEC	.	.	REACTOME_CELL_CELL_COMI
0	0	.	NA	.	BIOF	.	NUCL	.	PID	.	.
0	0	.	NA	.	.	.	.	.	.	.	.
0	0	Atrial fibrilla	NA	.	EST	INTR	CATI	.	.	.	REACTOME_DEVELOPMENTAL
0	0	.	NA	.	.	.	.	.	.	.	.
0	0	.	NA	.	EST	ORG	.	.	.	.	REACTOME_COPI_MEDIATED_
0	0	.	NA	.	.	RIBC	RNA_	KEC	.	.	REACTOME_MRNA_DECAY_BY

0	0	Leukemia,	NA	.	.	.	.	KE(	PID	BIO(	REACTOME_SIGNALING_BY_R
0	0	Leukemia,	NA	.	.	.	.	KE(	PID	BIO(	REACTOME_SIGNALING_BY_R
0	0	Deafness,	NA	.	SEN	.	.	.	.	.	.
0	0	.	NA	.	.	.	.	KE(	.	.	REACTOME_SIGNALING_BY_G
0	0	.	NA	.	.	.	.	KE(	.	.	.
0	0	.	NA	.	SYS	INTF	RECE	.	.	.	.
0	0	.	NA	.	.	.	.	.	.	.	.
0	0	Leigh synd	NA	.	.	CYT(	.	.	.	.	.
0	0	.	NA	.	CELI	CYT(	.	KE(	PID	.	.
0	0	.	NA	.	.	.	.	.	.	.	.
0	0	.	NA	.	.	.	.	.	.	.	.
0	0	{Ovarian ca	NA	.	CELI	INTF	PEPT	.	.	.	.
0	0	.	NA	.	.	.	.	.	.	.	.
0	0	.	NA	.	.	.	.	.	.	.	.
0	0	.	NA	.	.	.	.	.	.	.	.
0	0	.	NA	.	.	.	.	.	.	.	.
0	0	.	NA	.	.	.	.	.	.	.	.
0	0	Neuropath	NA	.	EST/	CYT(	NUCL	.	.	.	.
0	0	Brugada sy	NA	.	REG	ORG	CATI(	KE(	.	.	.
0	0	Brugada sy	NA	.	REG	ORG	CATI(	KE(	.	.	.
0	0	Brugada sy	NA	.	REG	ORG	CATI(	KE(	.	.	.
0	0	.	NA	.	.	.	.	.	.	.	.
0	0	.	NA	.	POS	MAC	.	KE(	PID	BIO(	REACTOME_CELL_CYCLE;REA
0	0	.	NA	.	.	.	SING	.	.	.	.
0	0	.	NA	.	EST/	INTF	CATI(	.	.	.	REACTOME_NEURONAL_SYST
0	0	Periodic fe	NA	.	RNA	INTF	RECE	KE(	PID	BIO(	REACTOME_EXTRINSIC_PATH
0	0	.	NA	.	SYS	.	.	.	.	.	.
0	0	OKT4 epitc	NA	.	SYS	MEN	KINA:	KE(	PID	BIO(	REACTOME_NEF_MEDIATES_D
0	0	.	NA	.	.	.	.	.	.	.	.
0	0	.	NA	.	.	INTF	.	.	.	.	.
0	0	.	NA	.	.	.	.	.	.	.	.
0	0	.	NA	.	.	.	STRU	.	PID	.	.
0	0	.	NA	.	.	.	.	.	.	.	.
0	0	.	NA	.	.	.	.	.	.	.	.
0	0	.	NA	.	.	.	.	.	.	.	.
0	0	.	NA	.	.	.	.	.	.	.	.
0	0	Leukemia,	NA	.	.	.	TRAN	KE(	.	.	.
0	0	.	NA	.	.	.	.	.	.	.	.
0	0	.	NA	.	.	.	.	.	.	.	.
0	0	.	NA	.	.	.	.	.	.	.	.
0	0	.	NA	.	.	.	.	KE(	.	BIO(	REACTOME_SYNTHESIS_OF_F
0	0	.	NA	.	.	.	.	KE(	.	BIO(	REACTOME_SYNTHESIS_OF_F
0	0	.	NA	.	.	.	.	.	.	.	.
0	0	Hyperbiliru	NA	.	EST/	INTF	ACTI'	.	.	.	REACTOME_BILE_ACID_AND_E
0	0	.	NA	.	.	.	.	.	.	.	.
0	0	.	NA	.	EST/	CYT(	.	.	.	.	.
0	0	.	NA	.	.	.	.	.	.	.	.
0	0	.	NA	.	.	.	.	.	.	.	.
0	0	.	NA	.	.	.	.	.	.	.	.
0	0	.	NA	.	.	.	.	.	.	.	.
0	0	.	NA	.	.	.	.	.	.	.	REACTOME_O_LINKED_GLYCC
0	0	.	NA	.	.	.	.	.	.	.	REACTOME_O_LINKED_GLYCC
0	0	.	NA	.	.	.	.	.	.	.	.
0	0	.	NA	.	.	.	.	.	.	.	.

0	0	.	NA	.	.	.	.	KE(	PID	.	REACTOME_SIGNALLING_BY_I
0	0	.	NA	.	.	CYT(	.	.	.	.	.
0	0	.	NA	.	.	.	.	KE(	.	.	.
0	0	.	NA	.	.	.	.	KE(	.	.	.
0	0	.	NA	.	.	.	.	.	.	.	.
0	0	.	NA	.	.	.	.	.	.	.	.
0	0	.	NA	.	.	.	.	.	.	.	.
0	0	.	NA	.	RNA	.	TRAN	.	PID	.	.
0	0	.	NA	.	.	.	.	.	.	.	.
0	0	.	NA	.	.	.	.	.	.	.	.
0	0	.	NA	.	.	.	.	.	.	.	.
0	0	Ichthyosis	NA	.	SYS	ORG	STRU	.	.	.	.
0	0	White spor	NA	.	ANA	.	.	.	.	.	.
0	0	.	NA	.	.	.	.	.	.	.	.
0	0	.	NA	.	.	INTR	IDEN	KE(	PID	.	REACTOME_IMMUNOREGULAT
0	0	.	NA	.	PRO	CYT(	KINA	KE(	PID	BIO(	.
0	0	.	NA	.	.	.	.	.	.	.	.
0	0	.	NA	.	.	.	.	.	.	.	.
0	0	Deafness,	NA	.	SEN	ORG	.	.	.	.	.
0	0	.	NA	.	.	.	.	.	.	.	.
0	0	.	NA	.	LIPIL	INTR	CATI(	KE(	PID	.	.
0	0	.	NA	.	LIPIL	INTR	CATI(	KE(	PID	.	.
0	0	.	NA	.	LIPIL	INTR	CATI(	KE(	PID	.	.
0	0	.	NA	.	LIPIL	INTR	CATI(	KE(	PID	.	.
0	0	.	NA	.	.	CYT(	.	KE(	.	.	.
0	0	Spermatog	NA	.	.	.	.	.	.	.	.
0	0	.	NA	.	.	.	RNA_	.	.	.	.
0	0	.	NA	.	.	.	RNA_	.	.	.	.
0	0	.	NA	.	.	NUC	TRAN	.	PID	.	.
0	0	.	NA	.	.	.	.	.	.	.	REACTOME_ASSOCIATION_OF
0	0	.	NA	.	.	.	.	.	.	.	.
0	0	.	NA	.	.	.	.	.	.	.	.
0	0	.	NA	.	.	.	.	.	.	.	.
0	0	.	NA	.	.	.	.	.	PID	.	.
0	0	.	NA	.	.	.	.	.	.	.	.
0	0	.	NA	.	.	.	.	.	.	.	.
0	0	Deafness,	NA	.	.	.	.	.	.	.	.
0	0	Deafness,	NA	.	.	.	.	.	.	.	.
0	0	.	NA	.	.	.	.	.	.	.	.
0	0	.	NA	.	.	.	.	.	.	.	.
0	0	.	NA	.	.	NUC	.	.	.	.	.
0	0	.	NA	.	BIOF	.	.	.	.	.	.
0	0	.	NA	.	.	.	.	.	.	.	.
0	0	.	NA	.	.	.	.	.	.	.	.
0	0	.	NA	.	SIGN	.	PROT	.	PID	BIO(	.
0	0	.	NA	.	.	.	.	.	.	.	.
0	0	.	NA	.	.	CYT(	HYDF	.	.	.	.
0	0	Arthrogryp	NA	.	.	CYT(	STRU	.	.	.	REACTOME_STRIATED_MUSCI
0	0	.	NA	.	MUL	INTR	CARE	.	.	.	REACTOME_HYALURONAN_UF
0	0	.	NA	.	DNA	NUC	DAM/	KE(	.	.	REACTOME_BASE_EXCISION_I
0	0	.	NA	.	.	.	.	.	.	.	.
0	0	Porokerato	NA	.	.	.	.	.	.	.	.
0	0	.	NA	.	.	.	DAM/	.	.	.	REACTOME_DNA_REPAIR
0	0	.	NA	.	.	.	.	.	.	.	.

0	0	.	NA	.	.	.	.	.	.	.	.
0	0	.	NA	.	SIGN	CYT(	ACID	.	.	.	.
0	0	.	NA	.	.	.	.	.	.	.	.
0	0	Transposit	NA	.	.	.	.	.	.	.	REACTOME_DEVELOPMENTAL
0	0	Spermatog	NA	.	SYST	CELI	OXID	KEG	.	BIO(	REACTOME_LATENT_INFECTIO
0	0	.	NA	.	PRO	.	PHOS	KEG	PID	.	.
0	0	.	NA	.	.	.	.	.	.	.	.
0	0	.	NA	.	.	.	.	.	.	.	REACTOME_METABOLISM_OF_
0	0	.	NA	.	.	.	.	.	.	.	.
0	0	.	NA	.	.	ORG	RNA_	.	.	.	REACTOME_INTERFERON_GAI
0	0	.	NA	.	SIGN	INTR	CATI(	KEG	.	.	.
0	0	.	NA	.	.	.	.	.	.	.	.
0	0	.	NA	.	M_P	ORG	PROT	.	PID	.	REACTOME_CELL_CYCLE;REA
0	0	.	NA	.	MITC	ORG	.	.	.	.	REACTOME_CELL_CYCLE;REA
0	0	.	NA	.	.	.	.	.	.	.	REACTOME_MEMBRANE_TRAF
0	0	.	NA	.	.	.	.	.	.	.	.
0	0	.	NA	.	.	.	.	.	.	.	.
0	0	.	NA	.	.	.	.	KEG	PID	.	.
0	0	.	NA	.	.	.	.	.	.	.	.
0	0	.	NA	.	.	.	.	.	.	.	.
0	0	.	NA	.	.	.	.	.	.	.	.
0	0	.	NA	.	.	.	.	.	.	.	.
0	0	.	NA	.	.	.	.	.	.	.	.
0	0	.	NA	.	.	.	.	.	.	.	.
0	0	.	NA	.	.	.	.	.	.	.	.
0	0	.	NA	.	.	.	.	.	.	.	.
0	0	.	NA	.	.	.	.	.	.	.	.
0	0	.	NA	.	.	.	.	KEG	.	.	REACTOME_O_LINKED_GLYCO
0	0	.	NA	.	MITC	.	.	.	.	.	.
0	0	.	NA	.	.	.	.	.	.	.	.
0	0	.	NA	.	.	.	.	.	.	.	REACTOME_SIGNALING_BY_F(
0	0	.	NA	.	.	.	.	.	.	.	.
0	0	Muscular d	NA	.	MUS	PRO	.	KEG	.	.	.
0	0	.	NA	.	MAC	.	.	KEG	.	BIO(	.
0	0	Keratosis li	NA	.	.	.	.	KEG	.	.	.
0	0	.	NA	.	.	.	.	.	.	.	.
0	0	.	NA	.	DNA	ORG	HYDF	KEG	PID	.	REACTOME_CELL_CYCLE;REA
0	0	.	NA	.	.	.	.	.	.	.	.
0	0	.	NA	.	.	.	.	.	.	.	.
0	0	Pulmonary	NA	.	.	.	.	KEG	PID	.	.
0	0	.	NA	.	BIOS	CYT(	TRAN	.	.	.	.
0	0	.	NA	.	.	.	.	.	.	.	.
0	0	.	NA	.	.	.	.	.	.	.	.
0	0	.	NA	.	.	.	.	.	.	.	.
0	0	.	NA	.	.	.	.	.	.	.	.
0	0	.	NA	.	.	.	.	.	.	.	.
0	0	.	NA	.	.	.	.	.	.	.	.
0	0	.	NA	.	.	.	.	.	PID	.	.
0	0	.	NA	.	.	.	.	.	.	.	.
0	0	.	NA	.	.	CYT(	.	.	.	.	.
0	0	.	NA	.	.	.	.	.	.	.	.
0	0	.	NA	.	EST/	INTR	.	.	.	.	.
0	0	Propionica	NA	.	.	.	LIGA!	KEG	.	.	REACTOME_MITOCHONDRIAL_
0	0	.	NA	.	.	.	.	.	.	.	.

0	0	.	NA	.	SYS	NUC	ACTI	.	.	.	.
0	0	Angiopathy	NA	.	.	.	.	KE(	PID	BIO(	REACTOME_DEVELOPMENTAL
0	0	.	NA	.	.	.	.	KE(	.	.	REACTOME_MITOCHONDRIAL_
0	0	.	NA	.	.	.	.	.	.	.	.
0	0	.	NA	.	.	.	.	.	.	.	.
0	0	.	NA	.	.	.	.	.	.	.	.
0	0	.	NA	.	.	.	.	.	PID	.	.
0	0	.	NA	.	SIGN	.	RECE	.	.	.	.
0	0	.	NA	.	.	.	.	.	.	.	.
0	0	.	NA	.	.	.	.	.	.	.	.
0	0	.	NA	.	.	.	.	.	.	.	.
0	0	.	NA	.	.	.	.	.	.	.	.
0	0	.	NA	.	DNA	ORG	NUCL	.	.	BIO(	.
0	0	.	NA	.	.	.	.	.	.	.	.
0	0	.	NA	.	RNA	.	TRAN	.	.	.	REACTOME_RNA_POL_II_TRAN
0	0	.	NA	.	.	.	.	.	.	.	.
0	0	Lysinuric p	NA	.	EST/	INTR	.	.	.	.	REACTOME_CELL_SURFACE_I
0	0	.	NA	.	.	.	.	.	.	.	.
0	0	.	NA	.	.	.	.	.	.	.	.
0	0	.	NA	.	.	.	.	.	.	.	.
0	0	.	NA	.	.	.	.	.	.	.	.
0	0	.	NA	.	.	.	.	.	.	.	.
0	0	.	NA	.	.	.	.	.	.	.	.
0	0	.	NA	.	.	.	.	.	.	.	.
0	0	.	NA	.	.	.	.	.	.	.	.
0	0	.	NA	.	.	.	.	.	.	.	.
0	0	.	NA	.	.	.	.	.	.	.	.
0	0	Fanconi ar	NA	.	.	.	.	.	PID	.	REACTOME_FANCONI_ANEMIA
0	0	.	NA	.	.	.	.	.	.	.	.
0	0	.	NA	.	.	.	.	.	.	.	.
0	0	{Asthma, a	NA	.	SIGN	INTR	RHOI	KE(	.	.	REACTOME_SIGNALING_BY_G
0	0	.	NA	.	.	.	.	.	.	.	.
0	0	.	NA	.	.	.	.	KE(	PID	.	.
0	0	.	NA	.	.	.	.	KE(	PID	.	.
0	0	.	NA	.	.	.	.	.	.	.	.
0	0	.	NA	.	.	.	.	.	.	.	.
0	0	.	NA	.	.	.	.	.	.	.	.
0	0	.	NA	.	.	CYT(	LIGA:	KE(	.	.	REACTOME_METABOLISM_OF_
0	0	.	NA	.	.	.	.	.	.	.	.
0	0	Molybdenu	NA	.	.	.	.	.	.	BIO(	REACTOME_METABOLISM_OF_
0	0	Bleeding d	NA	.	FOC	ADH	INTE(	KE(	PID	BIO(	REACTOME_CELL_CELL_COMI
0	0	.	NA	.	.	.	.	.	.	.	.
0	0	.	NA	.	.	.	.	.	.	.	.
0	0	.	NA	.	.	.	.	.	.	.	.
0	0	Glaucoma	NA	.	EST/	PRO	.	.	.	.	.
0	0	.	NA	.	.	.	.	.	.	.	.
0	0	.	NA	.	.	.	.	.	.	.	.
0	0	.	NA	.	.	.	.	.	.	.	.
0	0	Spinocerel	NA	.	.	.	.	.	.	.	REACTOME_DOUBLE_STRAND
0	0	.	NA	.	.	.	.	.	.	.	.
0	0	.	NA	.	.	.	.	.	.	.	.
0	0	.	NA	.	SIGN	INTR	.	.	.	.	REACTOME_GASTRIN_CREB_S
0	0	Hydroceph	NA	.	REG	INSC	PROT	.	.	.	.
0	0	.	NA	.	.	.	.	.	.	.	.

0	0	.	NA	.	.	.	.	.	.	.	REACTOME_INTERFERON_ALF
0	0	Goiter, mul	NA	.	MAC	.	HYDF	.	PID	.	REACTOME_MICRORNA_MIRN
0	0	.	NA	.	.	.	.	.	.	.	.
0	0	.	NA	.	.	.	.	.	.	.	.
0	0	.	NA	.	MUL	EXTI	.	.	PID	.	REACTOME_ACTIVATED_NOTC
0	0	.	NA	.	.	.	.	.	.	.	.
0	0	.	NA	.	.	.	.	.	.	.	.
0	0	.	NA	.	.	.	.	.	.	.	.
0	0	.	NA	.	BIOF	CELI	NUCL	.	.	.	.
0	0	.	NA	.	.	.	.	.	.	.	.
0	0	.	NA	.	.	.	.	.	.	.	REACTOME_MHC_CLASS_II_AI
0	0	.	NA	.	.	.	.	.	.	.	.
0	0	.	NA	.	CELI	.	RECE	.	.	.	.
0	0	Breast can	NA	.	REG	MEM	PHOS	KEC	PID	BIO(	REACTOME_SIGNALLING_BY_I
0	0	.	NA	.	.	.	.	.	.	.	.
0	0	.	NA	.	SYS	INTF	RECE	KEC	PID	.	REACTOME_ACTIVATED_NOTC
0	0	.	NA	.	RNA	.	.	.	.	.	REACTOME_RNA_POL_III_TRA
0	0	.	NA	.	.	.	.	.	.	.	.
0	0	.	NA	.	.	.	.	.	.	.	.
0	0	.	NA	.	.	.	.	.	.	.	.
0	0	.	NA	.	.	.	.	.	.	.	.
0	0	.	NA	.	.	.	.	.	.	.	.
0	0	.	NA	.	.	.	.	.	.	.	.
0	0	.	NA	.	.	.	.	.	.	.	.
0	0	.	NA	.	.	.	.	.	.	.	.
0	0	.	NA	.	SYS	CYT	RAS_	KEC	.	.	.
0	0	.	NA	.	.	.	.	.	.	.	.
0	0	.	NA	.	.	.	.	.	.	.	.
0	0	.	NA	.	.	.	.	.	.	.	.
0	0	.	NA	.	.	.	.	.	.	.	.
0	0	.	NA	.	.	.	.	.	.	.	.
0	0	.	NA	.	.	.	.	.	.	.	.
0	0	.	NA	.	.	.	.	.	.	.	.
0	0	.	NA	.	.	.	.	.	.	.	.
0	0	.	NA	.	.	.	.	.	.	.	REACTOME_TRANSMEMBRANI
0	0	[Skin/hair/e	NA	.	.	.	.	KEC	.	.	REACTOME_IMMUNE_SYSTEM
0	0	[Skin/hair/e	NA	.	.	.	.	KEC	.	.	REACTOME_IMMUNE_SYSTEM
0	0	.	NA	.	.	.	.	.	.	.	.
0	0	.	NA	.	.	.	.	.	.	.	.
0	0	.	NA	.	.	.	.	.	.	.	.
0	0	.	NA	.	.	.	.	.	.	.	REACTOME_TRANSMISSION_A
0	0	.	NA	.	.	.	.	.	.	.	.
0	0	.	NA	.	.	.	.	.	.	.	.
0	0	.	NA	.	.	.	.	.	.	.	.
0	0	.	NA	.	.	.	.	.	.	.	.
0	0	.	NA	.	EST/	.	CATI	KEC	.	.	.
0	0	.	NA	.	.	.	.	.	.	.	.
0	0	.	NA	.	.	.	.	KEC	.	BIO(	REACTOME_DEVELOPMENTAL
0	0	Platelet PL	NA	.	REG	.	LIPAS	KEC	PID	.	REACTOME_GASTRIN_CREB_S
0	0	.	NA	.	.	.	.	.	.	.	REACTOME_DEVELOPMENTAL
0	0	.	NA	.	.	.	.	.	.	.	REACTOME_DEVELOPMENTAL
0	0	Spinocerel	NA	.	.	.	.	.	.	.	.
0	0	.	NA	.	.	ORG	.	.	.	.	.
0	0	.	NA	.	.	.	.	KEC	.	.	REACTOME_METABOLISM_OF_

0	0	Deafness,	NA	.	.	.	.	.	.	.	.
0	0	.	NA	.	.	.	.	.	.	.	.
0	0	?Cataract,	NA	.	SEN	.	.	KEG	.	.	.
0	0	.	NA	.	SIGN	.	.	.	.	.	.
0	0	.	NA	.	.	.	.	KEG	.	.	REACTOME_DEVELOPMENTAL
0	0	Acromicric	NA	.	SYS	PRO	CATI	.	PID	.	REACTOME_INTEGRIN_CELL_S
0	0	Acromicric	NA	CM0	SYS	PRO	CATI	.	PID	.	REACTOME_INTEGRIN_CELL_S
0	0	.	NA	.	.	.	.	.	.	.	.
0	0	.	NA	.	.	.	.	.	.	.	.
0	0	.	NA	.	.	.	.	.	.	.	REACTOME_TRANSMEMBRANE
0	0	.	NA	.	.	.	.	.	.	.	.
0	0	.	NA	.	.	.	.	.	.	.	.
0	0	Griscelli sy	NA	.	.	CYT	.	.	.	.	REACTOME_DIABETES_PATHW
0	0	.	NA	.	EST	INTR	ACTI	.	.	.	REACTOME_TRANSMEMBRANE
0	0	.	NA	.	MAC	.	.	KEG	.	.	.
0	0	Combined	NA	.	.	.	.	KEG	.	.	.
0	0	.	NA	.	.	.	.	.	.	.	.
0	0	.	NA	.	.	.	.	.	.	.	.
0	0	.	NA	.	.	.	.	.	.	.	.
0	0	.	NA	.	.	.	.	.	.	.	.
0	0	.	NA	.	.	.	.	.	.	.	.
0	0	Ceroid lipo	NA	.	REG	ORG	PROT	.	.	.	.
0	0	.	NA	.	MUS	INTR	COLL	KEG	PID	.	REACTOME_INTEGRIN_CELL_S
0	0	.	NA	.	.	.	.	.	.	.	.
0	0	.	NA	.	.	.	.	.	.	.	.
0	0	.	NA	.	.	.	.	.	.	.	.
0	0	Bardet-Bie	NA	.	CEN	ORG	MICR	.	.	.	.
0	0	Adrenal ins	NA	.	BIOS	.	OXYG	KEG	.	.	REACTOME_METABOLISM_OF_
0	0	.	NA	.	.	.	.	.	.	.	.
0	0	.	NA	.	.	.	.	.	.	.	.
0	0	.	NA	.	.	.	.	.	.	.	.
0	0	.	NA	.	.	.	.	.	.	.	.
0	0	.	NA	.	.	.	.	.	.	.	.
0	0	.	NA	.	.	.	.	.	.	.	.
0	0	.	NA	.	.	.	.	.	.	.	.
0	0	.	NA	.	.	.	.	.	.	.	.
0	0	Tyrosinemi	NA	.	CAR	.	.	KEG	.	.	REACTOME_METABOLISM_OF_
0	0	.	NA	.	.	.	.	.	.	.	.
0	0	.	NA	.	.	.	.	.	.	.	.
0	0	.	NA	.	.	.	.	.	.	.	.
0	0	.	NA	.	.	.	.	.	.	.	.
0	0	.	NA	.	.	CELL	.	.	PID	BIO	REACTOME_SIGNALING_BY_R
0	0	.	NA	.	.	CYT	.	.	.	.	.
0	0	Mitochond	NA	.	DNA	CYT	NUCL	.	.	.	.
0	0	.	NA	.	REG	INTR	CATI	.	.	.	REACTOME_TRANSMEMBRANE
0	0	Acrocallosa	NA	.	.	.	.	.	.	.	.
0	0	Acrocallosa	NA	.	.	.	.	.	.	.	.
0	0	.	NA	.	.	.	.	.	.	.	.
0	0	.	NA	.	.	.	.	.	.	.	.
0	0	Weill-Marc	NA	.	.	.	.	.	.	.	.
0	0	.	NA	.	.	.	.	.	.	.	.
0	0	.	NA	.	.	.	.	.	.	.	.
0	0	.	NA	.	CELL	.	SERI	.	.	.	REACTOME_SIGNALLING_BY_N
0	0	.	NA	.	.	.	.	KEG	.	.	.

0	0	.	NA	.	.	.	.	.	.	.	.
0	0	.	NA	.	.	.	.	.	.	.	.
0	0	.	NA	.	.	.	.	.	.	.	.
0	0	.	NA	.	.	.	.	.	.	.	.
0	0	.	NA	.	.	.	.	.	.	.	.
0	0	.	NA	.	.	.	.	.	.	.	.
0	0	.	NA	.	SIGN	.	GTPA	.	.	.	REACTOME_SIGNALING_BY_G
0	0	.	NA	.	.	.	.	KEC	PID	.	.
0	0	.	NA	.	.	.	.	.	.	.	.
0	0	.	NA	.	REG	ORG	.	.	.	.	REACTOME_SIGNALING_BY_R
0	0	.	NA	.	.	.	.	.	.	.	.
0	0	.	NA	.	.	.	.	.	.	.	.
0	0	.	NA	.	REG	.	.	.	.	.	.
0	0	.	NA	.	.	.	.	.	.	.	.
0	0	Lipase defi	NA	.	.	.	.	.	.	.	.
0	0	Somatosta	NA	.	SIGN	INTR	NEUR	KEC	.	.	REACTOME_SIGNALING_BY_G
0	0	.	NA	.	BIOF	NUC	.	KEC	PID	.	REACTOME_MEIOSIS;REACTO
0	0	.	NA	.	REG	.	PROT	.	.	.	.
0	0	.	NA	.	REG	.	PROT	.	.	.	.
0	0	.	NA	.	REG	.	PROT	.	.	.	.
0	0	.	NA	.	REG	.	PROT	.	.	.	.
0	0	.	NA	.	REG	.	PROT	.	.	.	.
0	0	.	NA	.	.	.	.	.	.	.	.
0	0	Osteopetrc	NA	.	.	.	.	.	.	.	.
0	0	.	NA	.	.	.	.	.	.	.	.
0	0	.	NA	.	.	.	.	.	.	.	.
0	0	.	NA	.	EST	CYT	KINAS	KEC	PID	.	.
0	0	.	NA	.	BIOS	RIBC	STRU	KEC	.	.	REACTOME_TRANSLATION;RE
0	0	Myopathy,	NA	.	.	.	.	.	.	.	REACTOME_MITOCHONDRIAL_
0	0	.	NA	.	.	.	.	.	.	.	.
0	0	.	NA	.	POS	UBIQ	ACID	.	.	.	.
0	0	.	NA	.	.	.	.	KEC	.	.	REACTOME_DEVELOPMENTAL
0	0	.	NA	.	.	.	.	.	.	.	.
0	0	.	NA	.	.	.	.	.	.	.	.
0	0	.	NA	.	.	.	.	.	.	.	REACTOME_GENERIC_TRANS
0	0	.	NA	.	.	.	.	.	.	.	.
0	0	Fanconi ar	NA	.	.	.	.	.	.	.	.
0	0	Rubinstein	NA	.	RNA	CYT	TRAN	KEC	PID	BIO	REACTOME_DEVELOPMENTAL
0	0	Rubinstein	NA	.	RNA	CYT	TRAN	KEC	PID	BIO	REACTOME_DEVELOPMENTAL
0	0	Rubinstein	NA	.	RNA	CYT	TRAN	KEC	PID	BIO	REACTOME_DEVELOPMENTAL
0	0	.	NA	.	.	INTR	PHOS	KEC	PID	.	REACTOME_SIGNALLING_BY_N
0	0	.	NA	.	.	.	.	.	.	.	.
0	0	.	NA	.	.	NON	STRU	.	.	.	.
0	0	.	NA	.	IMMU	.	RNA_	KEC	.	.	REACTOME_INTERFERON_GAI
0	0	.	NA	.	IMMU	.	RNA_	KEC	.	.	REACTOME_INTERFERON_GAI
0	0	.	NA	.	.	.	.	.	.	.	.
0	0	.	NA	.	CELL	INTR	STRU	.	.	.	.
0	0	.	NA	.	.	.	.	.	.	.	.
0	0	.	NA	.	.	.	.	.	.	.	.
0	0	.	NA	.	.	.	.	.	.	.	.
0	0	Lissenceph	NA	.	CEN	ORG	MICR	.	.	.	.
0	0	.	NA	.	EST	INTR	HYDR	KEC	PID	.	REACTOME_ABC_FAMILY_PRO
0	0	.	NA	.	.	.	.	.	.	.	.
0	0	.	NA	.	BIOF	CYT	PHOS	.	.	.	REACTOME_METABOLISM_OF_

0	0	.	NA	.	.	.	.	.	.	.	.
0	0	Glomerulo	NA	.	DEFI	MEM	.	.	.	.	.
0	0	.	NA	.	REP	.	RECE	.	.	.	.
0	0	.	NA	.	BIOF	CYT	TRAN	.	PID	BIO	REACTOME_INSULIN_RECEPT
0	0	.	NA	.	.	ORG	SMAL	KEG	.	.	.
0	0	.	NA	.	BIOF	.	NUCL	.	.	.	.
0	0	.	NA	.	ONE	.	PROT	KEG	.	.	.
0	0	.	NA	.	.	.	.	.	.	.	.
0	0	.	NA	.	.	NUC	.	.	.	.	.
0	0	.	NA	.	.	.	.	.	.	.	.
0	0	.	NA	.	.	.	.	.	.	.	.
0	0	.	NA	.	ESTA	.	PROT	.	.	.	.
0	0	.	NA	.	.	.	.	.	.	.	.
0	0	.	NA	.	.	.	.	.	.	.	.
0	0	Combined	NA	.	BIOF	CYT	TRAN	.	.	.	.
0	0	Brody myo	NA	.	ESTA	.	CATI	KEG	.	.	REACTOME_PRE_NOTCH_EXP
0	0	.	NA	.	.	.	.	.	.	.	.
0	0	.	NA	.	.	.	.	.	.	.	.
0	0	.	NA	.	DNA	.	.	.	.	.	.
0	0	Branched-	NA	.	CAR	ORG	NUCL	.	.	.	.
0	0	.	NA	.	.	CYT	SERI	.	.	.	.
0	0	.	NA	.	SYS	INTR	RECE	KEG	PID	BIO	REACTOME_CELL_SURFACE_I
0	0	.	NA	.	MUL	.	.	.	.	.	REACTOME_GENERIC_TRANS
0	0	.	NA	.	.	.	.	.	.	.	.
0	0	.	NA	.	.	.	.	.	.	.	.
0	0	.	NA	.	.	.	.	.	.	.	.
0	0	.	NA	.	.	.	.	.	.	.	.
0	0	.	NA	.	.	.	.	.	.	.	.
0	0	.	NA	.	.	.	.	.	.	.	.
0	0	.	NA	.	.	.	.	.	.	.	.
0	0	.	NA	.	.	.	.	.	.	.	.
0	0	.	NA	.	.	.	.	.	.	.	.
0	0	.	NA	.	.	.	.	.	.	.	.
0	0	.	NA	.	.	.	.	.	.	.	.
0	0	.	NA	.	.	.	.	.	.	.	.
0	0	.	NA	.	.	.	.	.	.	.	.
0	0	.	NA	.	.	.	.	.	.	.	.
0	0	.	NA	.	.	.	.	.	.	.	.
0	0	.	NA	.	.	.	.	.	.	.	.
0	0	.	NA	.	.	.	.	.	.	.	.
0	0	.	NA	.	.	.	.	.	.	.	.
0	0	.	NA	.	.	.	.	.	.	.	.
0	0	.	NA	.	.	.	.	.	.	.	.
0	0	.	NA	.	.	.	.	.	.	.	.
0	0	.	NA	.	.	.	.	.	.	.	.
0	0	.	NA	.	.	.	.	.	.	.	.
0	0	.	NA	.	.	.	.	.	.	.	.
0	0	.	NA	.	.	.	.	.	.	.	.
0	0	.	NA	.	.	.	.	.	.	.	.
0	0	.	NA	.	.	.	.	.	.	.	.
0	0	.	NA	.	.	.	.	.	.	.	.
0	0	.	NA	.	.	.	.	KEG	.	.	.
0	0	Phosphory	NA	.	GEN	.	.	KEG	.	.	REACTOME_GLYCOGEN_BREA
0	0	.	NA	.	.	.	.	.	.	.	.

0	0	.	NA	.	.	.	.	.	.	.	REACTOME_METABOLISM_OF_
0	0	.	NA	.	.	.	.	.	.	.	REACTOME_METABOLISM_OF_
0	0	Hyperalpha	NA	.	REG	EXTI	LIPID	.	.	.	REACTOME_HDL_MEDIATED_L
0	0	.	NA	.	.	.	.	.	.	.	.
0	0	.	NA	.	.	.	.	.	.	.	.
0	0	.	NA	.	.	.	.	.	.	.	.
0	0	.	NA	.	.	MAC	.	.	.	.	.
0	0	.	NA	.	.	.	.	.	.	.	.
0	0	.	NA	.	.	.	.	.	.	.	.
0	0	.	NA	.	.	.	.	.	.	.	.
0	0	.	NA	.	.	.	.	.	.	.	.
0	0	.	NA	.	.	INTF	CATI(	.	.	.	REACTOME_TRANSMEMBRANI
0	0	.	NA	.	.	.	.	.	.	.	.
0	0	.	NA	.	.	.	.	.	.	.	.
0	0	.	NA	.	.	.	.	.	.	.	.
0	0	.	NA	.	RNA	NUC	CATI(	.	.	.	.
0	0	.	NA	.	PRO	EXTI	.	.	.	.	.
0	0	.	NA	.	REG	INTF	CATI(	.	.	.	REACTOME_TRANSMEMBRANI
0	0	.	NA	.	.	.	.	.	.	.	.
0	0	.	NA	.	DNA	ORG	SING	.	PID	.	REACTOME_MEIOSIS;REACTO
0	0	.	NA	.	.	.	.	.	.	.	.
0	0	.	NA	.	.	.	.	.	.	.	.
0	0	.	NA	.	.	.	.	.	.	.	.
0	0	.	NA	.	.	.	.	.	.	.	.
0	0	.	NA	.	.	.	.	.	.	.	.
0	0	.	NA	.	.	.	.	.	.	.	.
0	0	.	NA	.	REG	ADH	KINA:	KE(	PID	BIO(	REACTOME_INTEGRIN_CELL_S
0	0	.	NA	.	.	.	.	.	.	.	.
0	0	.	NA	.	.	.	.	.	.	.	.
0	0	Autoinflam	NA	.	.	.	.	KE(	PID	.	REACTOME_ANTIGEN_ACTIVA
0	0	Malonyl-Co	NA	.	COF	CYT(	LYAS	KE(	.	.	.
0	0	.	NA	.	.	.	.	.	.	.	.
0	0	.	NA	.	RNA	.	RNA_	.	.	.	REACTOME_RNA_POL_I_TRAN
0	0	.	NA	.	.	.	.	.	.	.	.
0	0	Lymphedei	NA	.	SYS	NUC	TRAN	.	.	.	.
0	0	.	NA	.	.	.	.	.	.	.	.
0	0	Huntington	NA	.	.	.	.	.	.	.	.
0	0	Brittle corn	NA	.	.	.	.	.	.	.	.
0	0	.	NA	.	.	.	LYAS	KE(	.	.	REACTOME_METABOLISM_OF_
0	0	.	NA	.	CELI	.	.	.	PID	.	.
0	0	.	NA	.	INTE	.	PHOS	.	.	.	.
0	0	.	NA	.	.	.	.	.	.	.	.
0	0	.	NA	.	.	.	.	.	.	.	.
0	0	Glomerulos	NA	.	.	ORG	.	.	.	.	.
0	0	Retinitis pi	NA	.	RNA	ORG	RNA_	KE(	.	.	REACTOME_PROCESSING_OF_
0	0	.	NA	.	.	.	.	.	.	.	.
0	0	.	NA	.	RNA	.	TRAN	.	PID	BIO(	.
0	0	.	NA	.	DNA	ORG	TELC	.	PID	.	REACTOME_METABOLISM_OF_
0	0	.	NA	.	DNA	ORG	TELC	.	PID	.	REACTOME_METABOLISM_OF_
0	0	.	NA	.	RNA	.	TRAN	.	.	.	.
0	0	.	NA	.	.	.	.	.	.	.	.
0	0	.	NA	.	.	.	.	KE(	.	BIO(	REACTOME_TRANSMISSION_A
0	0	.	NA	.	.	ORG	.	.	.	.	.
0	0	.	NA	.	BIOF	.	.	KE(	.	BIO(	REACTOME_IMMUNE_SYSTEM

0	0	.	NA	.	.	.	.	.	PID	BIO(	.
0	0	.	NA	.	EST/	CYT(	.	.	.	.	.
0	0	.	NA	.	.	.	.	.	.	.	.
0	0	.	NA	.	.	.	.	.	.	.	.
0	0	.	NA	.	.	.	.	.	.	.	.
0	0	.	NA	.	.	.	.	.	.	.	REACTOME_TRANSMEMBRANI
0	0	.	NA	.	.	MEM	CARE	.	.	.	.
0	0	.	NA	.	.	.	.	.	.	.	.
0	0	.	NA	.	.	NUC	.	KE(	.	BIO(	REACTOME_MICRORNA_MIRN/
0	0	.	NA	.	.	.	.	.	.	.	.
0	0	.	NA	.	.	.	.	.	PID	.	REACTOME_PHOSPHOLIPID_M
0	0	.	NA	.	NEG	.	TRAN	.	.	.	.
0	0	.	NA	.	GAM	ORG	.	.	.	.	.
0	0	.	NA	.	GAM	ORG	.	.	.	.	.
0	0	.	NA	.	SULI	INTR	SULF	KE(	.	.	REACTOME_HS_GAG_BIOSYN
0	0	.	NA	.	.	.	.	.	.	.	.
0	0	.	NA	.	.	.	.	.	.	.	.
0	0	.	NA	.	RNA	.	TRAN	.	PID	BIO(	REACTOME_DEVELOPMENTAL
0	0	.	NA	.	RNA	.	TRAN	.	PID	BIO(	REACTOME_DEVELOPMENTAL
0	0	.	NA	.	.	.	.	.	.	.	.
0	0	.	NA	.	.	.	.	.	.	.	.
0	0	Deafness,	NA	.	SEN	.	.	.	.	.	.
0	0	Deafness,	NA	.	SEN	.	.	.	.	.	.
0	0	.	NA	.	.	.	.	.	.	.	.
0	0	.	NA	.	.	.	.	.	.	.	.
0	0	.	NA	.	.	.	.	.	.	.	.
0	0	.	NA	.	.	.	.	.	.	.	.
0	0	.	NA	.	RNA	.	PHOS	KE(	PID	BIO(	REACTOME_TRIF_MEDIATED_
0	0	{Hypertens	NA	.	MUL	CYT(	OXID	KE(	PID	BIO(	REACTOME_LATENT_INFECTIO
0	0	.	NA	.	.	INTR	CATI(	.	.	.	REACTOME_TRANSMEMBRANI
0	0	.	NA	.	.	INTR	CATI(	.	.	.	REACTOME_TRANSMEMBRANI
0	0	.	NA	.	.	.	.	.	.	.	.
0	0	.	NA	.	.	.	.	.	.	.	.
0	0	.	NA	.	.	.	.	.	.	.	.
0	0	.	NA	.	.	.	.	.	.	.	.
0	0	.	NA	.	.	.	.	.	.	.	.
0	0	.	NA	.	CELI	.	.	.	.	.	REACTOME_SIGNALING_BY_F(
0	0	{Anxiety-re	NA	.	REG	INTR	ACTI	.	.	.	.
0	0	.	NA	.	.	.	.	.	.	.	.
0	0	.	NA	.	.	.	.	KE(	.	.	.
0	0	.	NA	.	.	.	.	.	.	.	.
0	0	.	NA	.	.	.	.	.	.	.	.
0	0	.	NA	.	.	.	.	.	.	.	.
0	0	.	NA	.	.	.	.	.	.	.	.
0	0	.	NA	.	.	NUC	.	.	.	.	.
0	0	.	NA	.	.	.	.	.	.	.	.
0	0	.	NA	.	.	.	.	KE(	.	.	REACTOME_SIGNALLING_BY_I
0	0	.	NA	.	.	.	.	.	.	.	.
0	0	.	NA	.	.	.	.	.	.	.	.
0	0	.	NA	.	.	.	.	.	.	.	.
0	0	.	NA	.	.	.	TRAN	.	.	.	.
0	0	.	NA	.	.	.	.	.	.	.	.
0	0	.	NA	.	.	MEM	CATI(	KE(	.	.	REACTOME_DEVELOPMENTAL
0	0	.	NA	.	BIOS	.	STRU	KE(	.	.	REACTOME_TRANSLATION;RE

0	0	.	NA	.	.	.	.	.	.	.	.
0	0	Adenocarc	NA	.	SYS	EXTI	PHOS	KEG	PID	.	REACTOME_DEVELOPMENTAL
0	0	.	NA	.	REG	INTR	.	KEG	.	BIO	REACTOME_SIGNALING_BY_G
0	0	.	NA	.	SYS	.	.	.	.	.	.
0	0	.	NA	.	MEIO	ORG	.	.	.	.	.
0	0	.	NA	.	.	CYTO	.	.	.	.	.
0	0	.	NA	.	.	CYTO	.	.	.	.	.
0	0	Hyper-IgE	NA	.	RNA	CYTO	RECE	KEG	PID	BIO	REACTOME_SIGNALLING_BY_N
0	0	Hyper-IgE	NA	.	RNA	CYTO	RECE	KEG	PID	BIO	REACTOME_SIGNALLING_BY_N
0	0	Hyper-IgE	NA	.	RNA	CYTO	RECE	KEG	PID	BIO	REACTOME_SIGNALLING_BY_N
0	0	.	NA	.	COF	.	PHOS	KEG	.	.	REACTOME_VITAMIN_B5_PAN
0	0	Pseudohyp	NA	.	ESTA	CELL	NUCL	.	.	.	.
0	0	.	NA	.	.	.	.	.	.	.	.
0	0	.	NA	.	.	.	.	.	.	.	.
0	0	.	NA	.	.	.	.	.	.	.	.
0	0	.	NA	.	SIGN	INTR	PHOS	.	.	.	.
0	0	.	NA	.	SIGN	INTR	PHOS	.	.	.	.
0	0	.	NA	.	SIGN	INTR	PHOS	.	.	.	.
0	0	.	NA	.	.	.	.	.	.	.	.
0	0	.	NA	.	.	.	.	.	.	.	.
0	0	.	NA	.	.	.	.	.	.	.	.
0	0	.	NA	.	.	.	.	.	.	.	.
0	0	Osteopetro	NA	.	.	.	.	.	.	.	.
0	0	.	NA	.	.	.	.	.	.	.	.
0	0	.	NA	.	.	.	.	.	.	.	.
0	0	.	NA	.	.	.	.	KEG	.	.	REACTOME_TRANSMISSION_A
0	0	.	NA	.	.	.	.	.	.	.	.
0	0	.	NA	.	.	CYTO	EXOF	.	.	.	REACTOME_IMMUNE_SYSTEM
0	0	.	NA	.	.	.	.	.	.	.	.
0	0	.	NA	.	BIOF	LAM	.	.	.	.	.
0	0	.	NA	.	.	.	.	.	.	.	.
0	0	.	NA	.	.	.	.	.	.	.	.
0	0	.	NA	.	.	.	.	.	.	.	.
0	0	.	NA	.	REG	NUC	.	.	.	.	.
0	0	.	NA	.	REG	NUC	.	.	.	.	.
0	0	Caffey dise	NA	.	SYS	.	.	KEG	PID	.	REACTOME_DEVELOPMENTAL
0	0	{Pseudoxa	NA	.	.	.	.	KEG	.	.	.
0	0	.	NA	.	GAM	CYTO	.	.	.	.	.
0	0	.	NA	.	RNA	NUC	DOUI	.	.	.	.
0	0	Fanconi ar	NA	.	DNA	NUC	DNA_	KEG	.	.	REACTOME_MEIOSIS;REACTO
0	0	Breast can	NA	.	BIOF	NUC	PHOS	KEG	PID	.	.
0	0	Growth hor	NA	.	ESTA	.	HEMA	KEG	.	BIO	REACTOME_GROWTH_HORMO
0	0	Hyperkaler	NA	.	ESTA	.	CATI	.	.	.	REACTOME_DEVELOPMENTAL
0	0	.	NA	.	.	.	.	.	.	.	.
0	0	.	NA	.	.	.	.	.	.	.	.
0	0	.	NA	.	.	.	.	KEG	.	.	REACTOME_ABCA_TRANSPOR
0	0	.	NA	.	.	.	.	.	.	.	.
0	0	.	NA	.	.	.	.	.	.	.	.
0	0	.	NA	.	BIOS	CYTO	ELEC	.	PID	.	.
0	0	.	NA	.	.	.	.	.	.	.	.
0	0	.	NA	.	.	CYTO	.	.	.	.	.
0	0	.	NA	.	.	CYTO	.	.	.	.	.
0	0	.	NA	.	.	.	.	.	.	.	.
0	0	.	NA	.	DNA	.	.	.	.	.	.

0	0 .	NA	.	DNA	.	.	.	.	.	.	
0	0 .	NA	.	RES	.	OXID	.	.	.	.	
0	0 .	NA	.	.	.	.	.	.	.	.	
0	0 .	NA	.	.	.	.	KE(	.	.	.	
0	0 .	NA	.	.	CYT(	.	.	.	.	.	
0	0 .	NA	.	.	.	.	.	.	.	.	
0	0 .	NA	.	.	.	.	.	.	.	.	
0	0 .	NA	.	.	.	.	.	.	.	.	
0	0 .	NA	.	CELI	NUC	TRAN	.	PID	.	.	
0	0 Ciliary dysk	NA	.	.	.	.	.	.	.	.	
0	0 Pityriasis ru	NA	.	POS	CYT(	PROT	.	.	.	.	
0	0 Pityriasis ru	NA	.	POS	CYT(	PROT	.	.	.	.	
0	0 {Moyamoya	NA	.	.	.	.	.	.	.	.	
0	0 .	NA	.	.	.	.	KE(	PID	.	REACTOME_INSULIN_RECEPT(	
0	0 .	NA	.	SYS	MEN	MOLE	KE(	PID	BIO(	.	
0	0 .	NA	.	.	.	.	.	.	.	.	
0	0 .	NA	.	.	.	.	.	.	.	.	
0	0 .	NA	.	.	.	.	.	.	.	.	
0	0 .	NA	.	.	.	.	.	.	.	.	
0	0 .	NA	.	.	.	.	.	.	.	.	
0	0 Alveolar sc	NA	.	.	.	.	.	.	.	.	
0	0 .	NA	.	.	.	.	KE(	PID	BIO(	REACTOME_VITAMIN_B5_PAN	
0	0 .	NA	.	.	.	.	KE(	PID	BIO(	REACTOME_VITAMIN_B5_PAN	
0	0 .	NA	.	.	.	.	.	.	.	.	
0	0 .	NA	.	.	.	.	.	.	.	.	
0	0 Seborrhea	NA	.	.	.	.	.	.	.	.	
0	0 .	NA	.	MUL	CELI	NEUF	.	.	.	REACTOME_SIGNALLING_BY_I	
0	0 .	NA	.	M_P	ORG	.	.	PID	.	REACTOME_CELL_CYCLE;REA	
0	0 Ataxia, spa	NA	.	.	CYT(	UNF(	.	.	.	.	
0	0 .	NA	.	.	.	.	.	.	.	REACTOME_CELL_CYCLE;REA	
0	0 Glucocortic	NA	.	SIGN	INTF	NEUF	KE(	.	.	REACTOME_SIGNALING_BY_G	
0	0 .	NA	.	.	.	.	.	.	.	.	
0	0 .	NA	.	.	.	.	.	.	.	.	
0	0 .	NA	.	.	.	.	.	.	.	.	
0	0 .	NA	.	.	.	.	.	.	.	.	
0	0 .	NA	.	.	.	.	.	.	.	.	
0	0 Cardiomyo	NA	.	SYS	PRO	.	KE(	PID	BIO(	REACTOME_CELL_CELL_COMI	
0	0 .	NA	.	.	.	.	.	.	.	.	
0	0 .	NA	.	RNA	NUC	DNA_	.	.	.	.	
0	0 Vici syndro	NA	.	.	.	.	.	.	.	.	
0	0 .	NA	.	.	.	.	.	.	.	.	
0	0 .	NA	.	.	.	.	.	.	.	.	
0	0 Microvillus	NA	.	.	.	.	.	.	.	REACTOME_TRANSMEMBRANI	
0	0 .	NA	.	.	.	.	.	.	.	.	
0	0 .	NA	.	.	.	.	.	.	.	.	
0	0 .	NA	.	.	CYT(	.	.	.	.	.	
0	0 .	NA	.	.	.	PROT	.	.	.	.	
0	0 .	NA	.	.	.	.	.	.	.	.	
0	0 Polymicrog	NA	.	.	.	.	.	.	.	.	
0	0 .	NA	.	.	.	.	.	.	.	.	
0	0 .	NA	.	.	.	.	.	.	.	REACTOME_TRANSMEMBRANI	
0	0 .	NA	.	.	.	.	.	.	.	.	
0	0 [Blood grou	NA	.	SIGN	.	.	.	PID	.	REACTOME_PYRUVATE_META	
0	0 .	NA	.	RNA	CYT(	NUCI	.	.	.	REACTOME_TRANSCRIPTION;I	

0	0	.	NA	.	RNA	CYT	NUCL	.	.	.	REACTOME_TRANSCRIPTION;I
0	0	.	NA	.	.	INTR	.	.	.	.	.
0	0	.	NA	.	RNA	NUC	RNA_	.	.	.	REACTOME_PROCESSING_OF_
0	0	.	NA	.	.	.	.	.	.	.	.
0	0	.	NA	.	.	.	.	.	.	.	REACTOME_SIGNALING_BY_R
0	0	.	NA	.	RNA	NUC	NUCL	KEG	.	.	REACTOME_RNA_POL_III_TRA
0	0	.	NA	.	MEM	CYT	OXID	KEG	.	.	.
0	0	.	NA	.	.	.	.	.	.	.	.
0	0	.	NA	.	.	.	.	.	.	.	.
0	0	.	NA	.	.	.	.	.	.	.	.
0	0	.	NA	.	.	.	.	.	.	.	.
0	0	.	NA	.	.	.	.	.	.	.	.
0	0	.	NA	.	RES	.	RNA_	.	.	.	.
0	0	.	NA	.	GAM	.	RNA_	.	.	.	.
0	0	.	NA	.	GAM	.	RNA_	.	.	.	.
0	0	.	NA	.	.	.	.	.	.	.	.
0	0	.	NA	.	.	.	.	KEG	.	.	.
0	0	.	NA	.	.	.	.	.	.	.	.
0	0	.	NA	.	.	CYT	.	.	.	.	.
0	0	.	NA	.	.	CYT	.	.	.	.	.
0	0	.	NA	.	REG	INTR	LIPID	KEG	PID	.	REACTOME_SIGNALING_BY_G
0	0	.	NA	.	RNA	.	TRAN	.	PID	.	.
0	0	.	NA	.	.	.	.	.	.	.	.
0	0	.	NA	.	.	.	.	.	.	.	.
0	0	.	NA	.	.	.	.	.	.	.	.
0	0	Leukemia,	NA	.	SYS	.	.	KEG	PID	.	.
0	0	.	NA	.	.	.	.	.	.	.	.
0	0	.	NA	.	.	.	.	KEG	.	.	.
0	0	.	NA	.	.	.	.	.	.	.	.
0	0	.	NA	.	ORG	NUC	DOUI	.	PID	.	.
0	0	.	NA	.	.	.	.	.	.	.	.
0	0	.	NA	.	.	.	.	.	.	.	.
0	0	.	NA	.	.	.	TRAN	KEG	PID	BIO	REACTOME_SIGNALING_BY_R
0	0	.	NA	.	.	.	.	.	PID	BIO	REACTOME_SIGNALING_BY_R
0	0	Spastic pa	NA	.	.	.	.	.	.	.	.
0	0	.	NA	.	DNA	.	.	KEG	.	.	REACTOME_TRANSCRIPTION_
0	0	.	NA	.	DNA	.	.	KEG	.	.	REACTOME_TRANSCRIPTION_
0	0	Hemophag	NA	.	.	.	.	.	.	.	.
0	0	{Dengue fe	NA	.	MUL	.	CARE	.	.	.	.
0	0	{Dengue fe	NA	.	MUL	.	CARE	.	.	.	.
0	0	.	NA	.	.	.	.	.	.	.	.
0	0	.	NA	.	.	.	.	.	.	.	.
0	0	Methylmal	NA	.	.	CYT	.	.	.	.	.
0	0	.	NA	.	.	.	.	.	.	.	.
0	0	.	NA	.	.	.	.	.	.	.	.
0	0	.	NA	.	.	.	.	.	.	.	.
0	0	.	NA	.	.	.	.	.	.	.	.
0	0	.	NA	.	.	MEM	.	.	.	.	REACTOME_O_LINKED_GLYCO
0	0	.	NA	.	.	MEM	.	.	.	.	REACTOME_O_LINKED_GLYCO
0	0	.	NA	.	.	MEM	.	.	.	.	REACTOME_O_LINKED_GLYCO
0	0	.	NA	.	.	.	.	.	.	.	.
0	0	.	NA	.	M_P	NUC	.	KEG	PID	BIO	REACTOME_ANTIVIRAL_MECH
0	0	.	NA	.	.	.	.	.	.	.	.
0	0	.	NA	.	.	.	.	.	.	.	.

0	0	.	NA	.	RNA	NUC	.	.	.	.	.
0	0	.	NA	.	G_PI	INTR	RECE	KEG	.	.	REACTOME_GASTRIN_CREB_S
0	0	{Malaria, c	NA	.	.	INTR	RECE	KEG	PID	BIO	REACTOME_IMMUNOREGULAT
0	0	.	NA	.	.	INTR	.	.	.	.	.
0	0	Tyrosine ki	NA	.	BIOF	.	PHOS	KEG	PID	BIO	REACTOME_SIGNALING_BY_IL
0	0	.	NA	.	SIGN	INSO	HYDR	KEG	.	.	.
0	0	.	NA	.	EST/	ORG	MICR	KEG	.	.	.
0	0	.	NA	.	.	.	.	.	.	.	.
0	0	Hyperchole	NA	.	GLY	INTR	LIPOI	KEG	.	.	REACTOME_METABOLISM_OF_
0	0	Hyperchole	NA	.	GLY	INTR	LIPOI	KEG	.	.	REACTOME_METABOLISM_OF_
0	0	Adams-Oli	NA	.	.	.	.	.	PID	.	REACTOME_FACTORS_INVOLV
0	0	.	NA	.	.	.	.	.	.	.	.
0	0	.	NA	.	REG	.	.	.	.	.	.
0	0	Mannosido	NA	.	GLY	.	HYDR	KEG	.	.	.
0	0	.	NA	.	.	.	.	.	.	.	.
0	0	.	NA	.	IMMU	.	RNA_	.	.	.	.
0	0	.	NA	.	.	.	.	KEG	PID	BIO	REACTOME_SIGNALLING_BY_N
0	0	.	NA	.	.	.	.	.	.	.	.
0	0	.	NA	.	.	.	.	KEG	.	.	REACTOME_SIGNALING_BY_G
0	0	.	NA	.	.	.	.	.	.	.	.
0	0	.	NA	.	.	.	.	.	.	.	REACTOME_SIGNALING_BY_E
0	0	.	NA	.	.	.	.	.	.	.	.
0	0	{Celiac dis	NA	.	EST/	ORG	HYDR	.	PID	.	REACTOME_SIGNALING_BY_R
0	0	.	NA	.	.	.	.	.	.	.	.
0	0	.	NA	.	.	.	PROT	.	.	.	.
0	0	.	NA	.	.	.	.	.	.	.	.
0	0	.	NA	.	.	.	.	.	.	.	.
0	0	.	NA	.	.	.	.	.	.	.	.
0	0	.	NA	.	.	.	.	.	.	.	.
0	0	.	NA	.	.	.	HYDR	KEG	.	.	REACTOME_SIGNALING_BY_G
0	0	.	NA	.	DNA	ORG	HYDR	.	.	.	.
0	0	.	NA	.	.	.	.	.	.	.	.
0	0	.	NA	.	.	.	.	.	.	.	.
0	0	.	NA	.	.	.	.	.	.	.	.
0	0	.	NA	.	.	.	.	.	.	.	REACTOME_GENERIC_TRANS
0	0	.	NA	.	.	.	.	.	.	.	.
0	0	.	NA	.	.	.	.	.	.	.	.
0	0	.	NA	.	.	.	.	.	.	.	.
0	0	Hemolytic	NA	.	REG	.	.	KEG	.	BIO	REACTOME_GLYCOLYSIS;REA
0	0	.	NA	.	IMMU	INTR	.	KEG	PID	.	.
0	0	.	NA	.	.	.	.	.	.	.	.
0	0	Microceph	NA	.	.	.	.	.	.	.	.
0	0	.	NA	.	RNA	NUC	CARE	.	.	.	.
0	0	.	NA	.	.	.	.	.	.	.	REACTOME_GENERIC_TRANS
0	0	.	NA	.	.	.	.	.	.	.	.
0	0	.	NA	.	.	.	.	.	.	.	.
0	0	.	NA	.	.	.	.	.	.	.	.
0	0	.	NA	.	.	.	.	.	.	.	.
0	0	.	NA	.	.	.	.	.	.	.	.
0	0	.	NA	.	.	.	.	.	.	.	.
0	0	.	NA	.	.	.	.	.	.	.	.
0	0	.	NA	.	REG	MEM	LIPID	KEG	PID	.	.
0	0	Central co	NA	.	EST/	INTR	CATI	KEG	.	.	.
0	0	Central co	NA	.	EST/	INTR	CATI	KEG	.	.	.

0	0	.	NA	.	SYS	.	NUCL	KEG	PID	BIO	.
0	0	.	NA	.	.	NUC	RNA_	.	.	.	REACTOME_PROCESSING_OF_
0	0	.	NA	.	MUS	ORG	CATI	.	PID	.	.
0	0	.	NA	.	.	.	.	.	.	.	.
0	0	.	NA	.	.	.	.	.	.	.	.
0	0	Cold-induc	NA	.	MUL	.	.	.	.	.	.
0	0	.	NA	.	RNA	NUC	TRAN	.	.	BIO	.
0	0	.	NA	.	.	.	.	.	.	.	.
0	0	.	NA	.	.	.	.	.	.	.	.
0	0	.	NA	.	.	.	.	.	.	.	.
0	0	.	NA	.	.	.	.	.	.	.	.
0	0	Cutis laxa,	NA	.	REG	PRO	CATI	.	.	.	.
0	0	Cutis laxa,	NA	.	REG	PRO	CATI	.	.	.	.
0	0	.	NA	.	SYS	.	.	KEG	.	.	.
0	0	.	NA	.	.	.	.	.	.	.	.
0	0	.	NA	.	RNA	ORG	RNA_	.	.	.	REACTOME_PROCESSING_OF_
0	0	Camurati-E	NA	.	REG	PRO	TRAN	KEG	PID	BIO	REACTOME_DEVELOPMENTAL
0	0	.	NA	.	CELL	MEM	RAS_	KEG	PID	BIO	REACTOME_SIGNALLING_BY_I
0	0	Carpenter	NA	.	.	.	.	.	.	.	.
0	0	.	NA	.	IMMU	INTR	.	.	.	.	.
0	0	.	NA	.	MUL	.	.	.	.	.	.
0	0	.	NA	.	.	.	.	.	.	.	.
0	0	.	NA	.	.	.	.	.	.	.	.
0	0	.	NA	.	.	.	TRAN	.	.	.	REACTOME_GENERIC_TRANS
0	0	.	NA	.	.	.	.	.	.	.	REACTOME_GENERIC_TRANS
0	0	.	NA	.	.	.	.	.	.	.	.
0	0	.	NA	.	.	.	.	.	.	.	.
0	0	.	NA	.	.	.	.	.	.	.	.
0	0	Deafness,	NA	.	.	.	.	.	.	.	.
0	0	Leukemia/I	NA	.	POS	CYT	PROT	.	PID	.	.
0	0	[Blood grou	NA	.	SIGN	INTR	RECE	.	.	.	.
0	0	.	NA	.	.	.	.	KEG	.	.	REACTOME_MITOCHONDRIAL_
0	0	.	NA	.	SIGN	INTR	.	.	.	.	REACTOME_GASTRIN_CREB_S
0	0	.	NA	.	.	.	.	.	.	.	.
0	0	.	NA	.	SIGN	CYT	STRU	.	.	.	.
0	0	.	NA	.	.	.	.	KEG	.	.	REACTOME_TRANSMEMBRANI
0	0	Cone-rod r	NA	.	SYS	.	.	.	.	.	.
0	0	.	NA	.	.	.	.	.	.	.	.
0	0	.	NA	.	.	.	.	.	.	.	.
0	0	.	NA	.	.	CYT	.	KEG	PID	.	REACTOME_SPHINGOLIPID_DE
0	0	.	NA	.	.	.	.	.	.	.	.
0	0	.	NA	.	RNA	ORG	RNA_	KEG	.	.	REACTOME_PROCESSING_OF_
0	0	.	NA	.	RNA	ORG	RNA_	KEG	.	.	REACTOME_PROCESSING_OF_
0	0	.	NA	.	ANA	EXTI	.	.	.	.	.
0	0	.	NA	.	.	.	.	.	.	.	.
0	0	.	NA	.	.	.	.	.	.	.	.
0	0	.	NA	.	.	.	.	KEG	PID	.	.
0	0	Deafness,	NA	.	.	.	.	KEG	.	.	REACTOME_DEVELOPMENTAL
0	0	.	NA	.	NEG	CYT	.	.	PID	.	REACTOME_GENERIC_TRANS
0	0	Mandibular	NA	.	DNA	NUC	NUCL	KEG	.	.	REACTOME_BASE_EXCISION_I
0	0	Mandibular	NA	.	DNA	NUC	NUCL	KEG	.	.	REACTOME_BASE_EXCISION_I
0	0	Mandibular	NA	.	DNA	NUC	NUCL	KEG	.	.	REACTOME_BASE_EXCISION_I
0	0	.	NA	.	.	.	.	.	.	.	.
0	0	.	NA	.	.	.	STRU	.	.	.	REACTOME_STRIATED_MUSCL

0	0	.	NA	.	.	.	STRU	.	.	.	REACTOME_STRIATED_MUSCL
0	0	.	NA	.	SIGN	INTR	.	.	.	.	.
0	0	.	NA	.	.	.	.	.	.	.	.
0	0	.	NA	.	.	.	.	.	.	.	.
0	0	.	NA	.	.	.	.	.	.	.	.
0	0	.	NA	.	.	.	.	.	.	.	.
0	0	.	NA	.	.	.	.	KEG	.	.	REACTOME_TRANSMISSION_A
0	0	Retinitis pig	NA	.	RNA	NUC	RIBO	KEG	.	.	.
0	0	.	NA	.	.	.	.	.	.	.	.
0	0	.	NA	.	SIGN	INTR	RECE	KEG	.	.	REACTOME_IMMUNOREGULAT
0	0	.	NA	.	.	INTR	.	.	.	.	.
0	0	.	NA	.	.	.	.	.	.	.	.
0	0	.	NA	.	CELL	.	.	.	.	.	REACTOME_INTEGRATION_OF
0	0	.	NA	.	.	.	.	.	.	.	.
0	0	.	NA	.	.	.	.	.	.	.	.
0	0	.	NA	.	.	.	.	.	.	.	.
0	0	.	NA	.	.	.	.	.	.	.	REACTOME_GENERIC_TRANS
0	0	.	NA	.	.	.	.	.	.	.	REACTOME_GENERIC_TRANS
0	0	.	NA	.	.	.	.	.	.	.	REACTOME_GENERIC_TRANS
0	0	.	NA	.	.	.	.	.	.	.	.
0	0	.	NA	.	.	EXTI	.	.	.	.	.
0	0	.	NA	.	.	MEM	.	.	.	.	REACTOME_CELL_CELL_COM
0	0	Spinocereb	NA	.	.	.	.	.	.	.	.
0	0	Spinocereb	NA	.	RNA	ORG	RNA_	.	.	.	REACTOME_ASSOCIATION_OF
0	0	.	NA	.	.	.	.	.	.	.	.
0	0	.	NA	.	.	.	.	.	.	.	REACTOME_DEVELOPMENTAL
0	0	.	NA	.	.	.	.	.	.	.	.
0	0	.	NA	.	.	.	.	.	.	.	.
0	0	.	NA	.	.	.	.	.	.	.	.
0	0	.	NA	.	.	.	.	.	.	.	.
0	0	.	NA	.	.	NUC	.	.	.	.	.
0	0	.	NA	.	.	.	.	.	.	.	.
0	0	.	NA	.	.	.	.	.	.	.	.
0	0	Polyneurop	NA	.	.	.	.	.	.	.	.
0	0	.	NA	.	.	.	.	.	.	.	.
0	0	.	NA	.	.	.	.	.	.	.	.
0	0	.	NA	.	.	.	.	.	.	.	.
0	0	.	NA	.	.	.	.	.	.	.	.
0	0	.	NA	.	.	CYT	.	.	.	.	.
0	0	.	NA	.	.	.	.	.	.	.	.
0	0	.	NA	.	PHO	ORG	LIPID	KEG	.	.	REACTOME_METABOLISM_OF_
0	0	.	NA	.	EST/	ORG	PROT	.	.	.	.
0	0	.	NA	.	CEN	ORG	KINAS	.	.	.	REACTOME_CELL_CYCLE;REA
0	0	.	NA	.	BIOF	ORG	PROT	.	.	.	REACTOME_METABOLISM_OF_
0	0	.	NA	.	.	NUC	.	.	.	.	.
0	0	Mental reta	NA	.	.	.	.	KEG	PID	.	REACTOME_TRANSMISSION_A
0	0	.	NA	.	.	.	.	.	.	.	.
0	0	.	NA	.	.	CYT	.	.	.	.	.
0	0	.	NA	.	.	.	.	.	.	.	.
0	0	.	NA	.	.	.	.	.	.	.	.
0	0	.	NA	.	.	.	.	.	.	.	.
0	0	.	NA	.	.	.	.	.	.	.	.
0	0	DNA topois	NA	.	RES	NUC	CHRO	.	PID	.	.
0	0	.	NA	.	.	CYT	.	KEG	PID	BIO	REACTOME_SIGNALLING_BY_I

0	0	.	NA	.	ENZ'	INTF	PROT	.	.	.	.
0	0	.	NA	.	ENZ'	INTF	PROT	.	.	.	.
0	0	.	NA	.	SIGN	CELI	.	.	.	.	.
0	0	.	NA	.	MUL	EXTI	.	.	.	.	.
0	0	.	NA	.	.	.	.	.	.	.	REACTOME_TRANSMEMBRANI
0	0	.	NA	.	.	.	.	.	PID	.	.
0	0	.	NA	.	.	.	.	.	.	.	REACTOME_TRANSMEMBRANI
0	0	.	NA	.	.	.	.	.	.	.	.
0	0	.	NA	.	.	.	.	.	.	.	REACTOME_NEURONAL_SYST
0	0	.	NA	.	.	.	.	.	.	.	REACTOME_TRANSMEMBRANI
0	0	.	NA	.	.	.	.	.	.	.	.
0	0	.	NA	.	.	.	TRAN	.	.	.	.
0	0	.	NA	.	.	.	.	.	.	.	.
0	0	ACTH-inde	NA	.	REG	EXTI	HYDF	KEC	.	BIO(	REACTOME_SIGNALING_BY_G
0	0	ACTH-inde	NA	.	REG	EXTI	HYDF	KEC	.	BIO(	REACTOME_SIGNALING_BY_G
0	0	.	NA	.	.	.	.	.	.	.	REACTOME_MEIOSIS;REACTO
0	0	.	NA	.	.	.	.	.	.	.	.
0	0	Epiphyseal	NA	.	.	PRO	.	.	PID	.	REACTOME_DEVELOPMENTAL
0	0	Epiphyseal	NA	.	.	PRO	.	.	PID	.	REACTOME_DEVELOPMENTAL
0	0	.	NA	.	.	.	.	KEC	PID	BIO(	REACTOME_COPI_MEDIATED_
0	0	.	NA	.	.	.	.	.	.	.	.
0	0	.	NA	.	.	.	.	.	.	.	.
0	0	.	NA	.	.	.	.	.	.	.	.
0	0	.	NA	.	.	.	.	.	.	.	.
0	0	.	NA	.	.	.	.	.	.	.	.
0	0	.	NA	.	.	.	.	.	.	.	.
0	0	.	NA	.	.	.	.	.	.	.	.
0	0	.	NA	.	.	.	.	.	.	.	.
0	0	.	NA	.	.	.	.	.	.	.	.
0	0	.	NA	.	.	.	.	.	.	.	.
0	0	.	NA	.	BIOF	INTF	PROT	.	.	.	.
0	0	.	NA	.	BIOF	INTF	PROT	.	.	.	.
0	0	.	NA	.	.	.	.	.	.	.	.
0	0	.	NA	.	.	.	.	.	.	.	.
0	0	.	NA	.	.	.	.	.	.	.	.
0	0	.	NA	.	.	.	.	.	.	.	.
0	0	.	NA	.	.	.	.	.	.	.	.
0	0	.	NA	.	.	.	.	.	.	.	.
0	0	.	NA	.	.	.	.	.	.	.	.
0	0	.	NA	.	CELI	INTF	.	KEC	.	.	.
0	0	.	NA	.	.	.	.	KEC	PID	.	REACTOME_CELL_SURFACE_I
0	0	Alzheimer	NA	.	REG	INTF	PROT	KEC	PID	BIO(	REACTOME_TRIF_MEDIATED_
0	0	.	NA	.	.	.	.	.	.	.	.
0	0	.	NA	.	.	.	.	.	.	.	.
0	0	.	NA	.	SIGN	.	PHOS	.	.	.	.
0	0	.	NA	.	SIGN	.	PHOS	.	.	.	.
0	0	.	NA	.	EST/	.	PHOS	KEC	PID	BIO(	REACTOME_PHOSPHOLIPID_M
0	0	.	NA	.	.	.	.	.	.	.	.
0	0	.	NA	.	RES	INTF	RECE	KEC	PID	BIO(	REACTOME_INTERFERON_ALF
0	0	.	NA	.	EST/	.	.	.	PID	.	REACTOME_SIGNALING_BY_R
0	0	.	NA	.	.	.	.	.	.	.	.
0	0	.	NA	.	.	.	.	.	.	.	.
0	0	.	NA	.	.	.	.	.	.	.	.
0	0	.	NA	.	.	INTF	.	.	.	.	.

0	0	.	NA	.	.	NUC	.	.	.	.	.	
0	0	.	NA	.	.	.	.	KEG	.	.	.	
0	0	.	NA	.	SYS	INTR	.	.	PID	.	REACTOME_CELL_CELL_COMI	
0	0	.	NA	.	SYS	INTR	.	.	PID	.	REACTOME_CELL_CELL_COMI	
0	0	.	NA	.	SYS	INTR	.	.	PID	.	REACTOME_CELL_CELL_COMI	
0	0	.	NA	.	SYS	INTR	.	.	PID	.	REACTOME_CELL_CELL_COMI	
0	0	.	NA	.	EST	.	.	.	.	.	.	
0	0	.	NA	.	.	.	.	.	PID	.	.	
0	0	.	NA	.	.	CYT	.	.	.	.	.	
0	0	.	NA	.	.	.	.	.	.	.	.	
0	0	.	NA	.	.	.	.	.	.	.	.	
0	0	.	NA	.	RNA	NUC	.	KEG	.	.	REACTOME_PROCESSING_OF_	
0	0	.	NA	.	.	ORG	.	.	.	.	.	
0	0	.	NA	.	SIGN	.	.	.	.	.	.	
0	0	.	NA	.	EST	INTR	CATI	.	.	.	.	
0	0	Leukocyte	NA	.	BIOF	.	KINA	KEG	PID	BIO	REACTOME_IMMUNOREGULAT	
0	0	.	NA	.	EST	INTR	COFA	.	.	.	REACTOME_METABOLISM_OF_	
0	0	Bethlem m	NA	.	.	.	.	KEG	PID	.	REACTOME_DEVELOPMENTAL	
0	0	Glutamate	NA	.	COF	CYT	.	KEG	.	.	REACTOME_METABOLISM_OF_	
0	0	.	NA	.	.	.	.	.	.	.	.	
0	0	Microcepha	NA	.	ANA	.	.	.	.	BIO	REACTOME_CELL_CYCLE;REA	
0	0	.	NA	.	.	.	.	.	.	.	.	
0	0	.	NA	.	.	.	.	.	.	.	.	
0	0	.	NA	.	.	.	.	.	.	.	.	
0	0	.	NA	.	.	.	.	.	.	.	.	
0	0	.	NA	.	.	.	.	.	.	.	.	
0	0	.	NA	.	.	.	.	.	.	.	.	
0	0	DiGeorge s	NA	.	SYS	.	.	.	.	.	.	
0	0	DiGeorge s	NA	.	SYS	.	.	.	.	.	.	
0	0	.	NA	.	.	.	.	.	.	.	.	
0	0	.	NA	.	.	.	.	.	.	.	.	
0	0	.	NA	.	.	.	.	.	.	.	.	
0	0	.	NA	.	PHO	ORG	PHOS	KEG	PID	.	REACTOME_SYNTHESIS_OF_P	
0	0	.	NA	.	.	NUC	PROT	.	.	.	.	
0	0	.	NA	.	.	.	.	.	.	.	.	
0	0	.	NA	.	.	.	.	.	.	.	.	
0	0	Mental reta	NA	.	DNA	NUC	.	.	PID	.	.	
0	0	.	NA	.	.	.	.	.	.	.	.	
0	0	.	NA	.	REG	INTR	.	KEG	PID	.	REACTOME_SIGNALLING_BY_I	
0	0	Beta-ureid	NA	.	.	.	HYDR	KEG	.	.	REACTOME_PYRIMIDINE_CATA	
0	0	Glutathioni	NA	.	CAR	.	.	KEG	.	.	REACTOME_BIOLOGICAL_OXID	
0	0	.	NA	.	.	.	.	.	.	.	.	
0	0	.	NA	.	.	.	.	.	.	.	.	
0	0	.	NA	.	.	.	.	.	.	.	.	
0	0	.	NA	.	.	.	.	.	.	.	.	
0	0	.	NA	.	.	.	.	.	.	.	.	
0	0	.	NA	.	MUS	NON	ACTI	.	.	.	.	
0	0	.	NA	.	.	.	.	.	.	.	.	
0	0	.	NA	.	.	.	.	.	.	.	.	
0	0	.	NA	.	.	.	.	.	.	.	.	
0	0	.	NA	.	.	.	.	.	.	.	.	
0	0	Muscular d	NA	.	REG	ORG	TRAN	.	.	.	.	
0	0	.	NA	.	DNA	NUC	.	KEG	.	BIO	REACTOME_ACTIVATION_OF_	
0	0	Deafness,	NA	.	REG	ORG	HYDR	KEG	.	.	REACTOME_DEVELOPMENTAL	

0	0	Surfactant	NA	.	RES	INTF	INTEI	KEC	PID	BIO(	REACTOME_SIGNALING_BY_IL
0	0	.	NA	.	.	.	.	.	.	.	.
0	0	Infantile ne	NA	CM1	MEM	.	LIPAS	KEC	.	.	REACTOME_ACYL_CHAIN_REM
0	0	.	NA	.	RNA	.	.	.	.	BIO(	REACTOME_FACTORS_INVOLV
0	0	.	NA	.	.	.	.	.	.	.	.
0	0	.	NA	.	EST/	INTF	CATI(	.	.	.	REACTOME_TRANSMISSION_A
0	0	.	NA	.	EST/	INTF	CATI(	.	.	.	REACTOME_TRANSMISSION_A
0	0	.	NA	.	SIGN	.	GTP_	.	.	.	.
0	0	.	NA	.	RNA	ORG	CATI(	.	.	.	.
0	0	.	NA	.	SIGN	.	.	KEC	.	.	REACTOME_DEVELOPMENTAL
0	0	.	NA	.	.	.	.	.	.	.	REACTOME_PRE_NOTCH_TRA
0	0	.	NA	.	REG	INTF	NEUF	KEC	.	.	REACTOME_GASTRIN_CREB_S
0	0	.	NA	.	RNA	NUC	NUCL	KEC	.	.	REACTOME_RNA_POL_III_TRA
0	0	.	NA	.	RNA	NUC	NUCL	KEC	.	.	REACTOME_RNA_POL_III_TRA
0	0	.	NA	.	.	.	.	.	.	.	REACTOME_CELL_CYCLE;REA
0	0	.	NA	.	.	.	.	.	.	.	.
0	0	.	NA	.	.	.	.	.	.	.	.
0	0	.	NA	.	LIPIL	INTF	TRAN	KEC	.	.	REACTOME_PHOSPHOLIPID_M
0	0	.	NA	.	.	.	.	.	.	.	.
0	0	.	NA	.	.	.	.	.	.	.	.
0	0	.	NA	.	.	.	.	KEC	.	.	REACTOME_MEIOSIS;REACTO
0	0	Synpolydac	NA	.	.	PRO	STRU	.	.	.	.
0	0	Spinocereb	NA	.	SYS	CYT(	.	.	.	.	.
0	0	.	NA	.	EST/	MEM	.	.	.	.	.
0	0	.	NA	.	SYS	INTF	.	.	.	.	.
0	0	.	NA	.	.	.	.	.	.	.	.
0	0	.	NA	.	CELL	.	NUCL	.	.	.	.
0	0	.	NA	.	CELL	.	NUCL	.	.	.	.
0	0	.	NA	.	.	.	.	.	.	.	REACTOME_NEURONAL_SYST
0	0	.	NA	.	.	.	.	.	.	.	REACTOME_NEURONAL_SYST
0	0	.	NA	.	.	.	.	KEC	.	.	.
0	0	.	NA	.	.	.	.	.	.	.	.
0	0	.	NA	.	.	.	.	.	.	.	.
0	0	.	NA	.	ENZ	CYT(	KINA:	KEC	.	.	.
0	0	.	NA	.	BIOS	.	.	KEC	.	.	.
0	0	.	NA	.	.	INTF	.	KEC	.	.	.
0	0	Chondrody	NA	.	SYS	.	ARYL	.	.	.	REACTOME_PTM_GAMMA_CAF
0	0	.	NA	.	.	.	.	KEC	.	.	.
0	0	.	NA	.	.	.	.	.	.	.	.
0	0	.	NA	.	.	.	.	.	.	.	.
0	0	.	NA	.	CELL	EXTI	INTE(	.	.	.	.
0	0	Cataract 4(	NA	.	.	.	.	.	.	.	.
0	0	.	NA	.	.	.	.	.	.	.	.
0	0	.	NA	.	.	.	.	.	.	.	.
0	0	Epileptic er	NA	.	.	.	.	.	.	.	.
0	0	.	NA	.	.	.	.	.	.	.	.
0	0	.	NA	.	.	.	.	.	.	.	.
0	0	Mental reta	NA	.	BEH.	.	.	.	.	.	.
0	0	.	NA	.	.	.	.	.	.	.	.
0	0	Glycerol kii	NA	.	.	.	.	KEC	.	.	REACTOME_TRIGLYCERIDE_B
0	0	Glycerol kii	NA	.	.	.	.	KEC	.	.	REACTOME_TRIGLYCERIDE_B
0	0	Becker mu	NA	.	BIOS	PRO	ACTII	KEC	.	BIO(	REACTOME_STRIATED_MUSCL
0	0	.	NA	.	.	.	.	.	.	.	.
0	0	.	NA	.	.	CELI	.	.	.	.	.

0	0	.	NA	.	.	.	.	.	.	.	.
0	0	.	NA	.	.	.	.	.	.	.	.
0	0	.	NA	.	.	.	.	.	.	.	.
0	0	.	NA	.	.	.	.	.	.	.	.
0	0	Renpennin	NA	.	RNA	NUC	TRAN	KE(	.	.	.
0	0	.	NA	.	.	.	.	KE(	.	.	REACTOME_NEGATIVE_REGUI
0	0	Neurodege	NA	.	.	.	.	.	.	.	.
0	0	.	NA	.	.	.	.	.	.	.	.
0	0	.	NA	.	.	.	.	.	.	.	.
0	0	Aland Islar	NA	.	DETI	INTF	CATI(	KE(	.	.	.
0	0	.	NA	.	SIGN	NON	KINA:	.	.	.	.
0	0	.	NA	.	CELI	MAC	.	KE(	PID	.	REACTOME_SIGNALLING_BY_I
0	0	.	NA	.	.	.	.	.	.	.	.
0	0	Mental reta	NA	.	.	.	.	.	.	.	.
0	0	Mental reta	NA	.	.	.	.	KE(	.	.	.
0	0	.	NA	.	DNA	ORG	PROT	KE(	PID	.	REACTOME_MEIOSIS;REACTO
0	0	Mental reta	NA	.	PRO	NUC	ACID	KE(	PID	.	REACTOME_IMMUNE_SYSTEM
0	0	.	NA	.	.	.	.	.	.	.	.
0	0	Aarskog-S	NA	.	REG	CYT(	GTPA	KE(	PID	.	REACTOME_SIGNALING_BY_R
0	0	.	NA	.	.	.	.	.	.	.	.
0	0	.	NA	.	.	.	.	.	.	.	.
0	0	.	NA	.	.	.	.	.	.	.	.
0	0	.	NA	.	.	.	.	.	.	.	.
0	0	.	NA	.	.	.	.	.	.	.	.
0	0	.	NA	.	.	.	.	.	.	.	.
0	0	.	NA	.	.	.	.	.	.	.	.
0	0	.	NA	.	.	.	.	.	.	.	.
0	0	.	NA	.	.	.	.	.	.	.	.
0	0	.	NA	.	.	.	.	.	.	.	.
0	0	.	NA	.	.	.	.	.	.	.	.
0	0	.	NA	.	.	.	.	.	.	.	.
0	0	.	NA	.	.	.	.	.	.	.	.
0	0	.	NA	.	.	.	.	.	.	.	REACTOME_SIGNALING_BY_R
0	0	.	NA	.	.	.	.	.	.	.	.
0	0	.	NA	.	SIGN	CYT(	.	.	PID	.	.
0	0	Lujan-Fryn	NA	.	RNA	NUC	RNA_	.	PID	.	REACTOME_DEVELOPMENTAL
0	0	Charcot-M	NA	CM9	SYS	CELI	.	.	.	.	REACTOME_MEMBRANE_TRAI
0	0	.	NA	.	.	.	.	.	.	.	.
0	0	.	NA	.	.	.	.	.	.	.	.
0	0	Cornelia de	NA	.	RNA	NUC	HYDF	.	PID	BIO(	REACTOME_NOTCH1_INTRACI
0	0	Muscle gly	NA	.	GEN	.	PHOS	KE(	.	.	REACTOME_GLYCOGEN_BREA
0	0	.	NA	.	.	.	.	.	PID	.	.
0	0	.	NA	.	.	.	.	.	.	.	.
0	0	.	NA	.	.	.	.	.	.	.	.
0	0	?Mental re	NA	.	.	.	.	.	.	.	.
0	0	.	NA	.	.	.	.	.	.	.	.
0	0	Mental reta	NA	.	.	.	.	.	.	.	.
0	0	.	NA	.	.	INTF	.	.	.	.	.
0	0	.	NA	.	.	INTF	.	.	.	.	.
0	0	.	NA	.	.	INTF	.	.	.	.	.
0	0	.	NA	.	.	INTF	.	.	.	.	.
0	0	.	NA	.	.	.	.	.	.	.	.
0	0	.	NA	.	ESTA	.	VOLT	KE(	PID	BIO(	.
0	0	.	NA	.	ESTA	CYT(	RNA_	.	.	.	.

0	0	.	NA	.	.	.	.	.	.	.	.
0	0	.	NA	.	SYS`	.	.	.	.	.	.
0	0	Mental reta	NA	.	BEH.	.	LIGA!	KE(	.	.	REACTOME_TRIGLYCERIDE_B
0	0	.	NA	.	.	.	.	.	.	.	.
0	0	.	NA	.	.	.	.	.	.	.	.
0	0	.	NA	.	.	.	.	.	.	.	.
0	0	Mental reta	NA	.	.	.	.	KE(	.	.	.
0	0	.	NA	.	.	.	.	.	.	.	.
0	0	.	NA	.	.	.	.	.	.	.	.
0	0	.	NA	.	.	.	.	.	.	.	.
0	0	Simpson-C	NA	.	ANA	INTF	.	.	.	.	REACTOME_HS_GAG_DEGRAD
0	0	Mental reta	NA	.	.	ORG	CATI(	KE(	.	.	REACTOME_TRANSMEMBRANI
0	0	.	NA	.	.	INTF	G_PF	.	.	.	.
0	0	.	NA	.	.	.	.	.	.	.	REACTOME_TRANSMEMBRANI
0	0	.	NA	.	NEG	NUC	.	.	.	.	.
0	0	.	NA	.	.	.	.	.	.	.	.
0	0	.	NA	.	.	.	.	.	.	.	.
0	0	.	NA	.	.	.	.	.	.	.	.
0	0	.	NA	.	.	.	.	.	.	.	.
0	0	.	NA	.	.	MEM	.	.	PID	.	REACTOME_CS_DS_DEGRADA
0	0	Spinocerel	NA	.	.	MEM	CATI(	KE(	.	.	REACTOME_TRANSMEMBRANI
0	0	.	NA	.	.	.	.	.	.	.	REACTOME_TRANSLATION;RE
0	0	CRASH sy	NA	.	SYS`	.	.	KE(	PID	BIO(	REACTOME_DEVELOPMENTAL
0	0	CRASH sy	NA	.	SYS`	.	.	KE(	PID	BIO(	REACTOME_DEVELOPMENTAL
0	0	.	NA	.	REG	.	PROT	KE(	.	.	.
0	0	Mental reta	NA	.	RNA	CYT(	IDEN`	.	.	.	.
0	0	.	NA	.	.	.	.	.	.	.	.
0	0	Favism;He	NA	.	GLU	CYT(	.	KE(	.	.	REACTOME_METABOLISM_OF_
0	0	Dyskeratos	NA	.	DNA	NUC	NUCL	.	PID	.	REACTOME_CELL_CYCLE;REA
0	0	.	NA	.	SIGN	INTF	PHOS	.	.	.	.
0	0	.	NA	.	.	.	.	.	.	.	.
0	0	Epsilon-trir	NA	.	.	.	.	KE(	.	.	REACTOME_METABOLISM_OF_
0	0	.	NA	.	.	.	.	.	.	.	.
0	0	.	NA	.	.	.	.	.	.	.	.
0	0	.	NA	.	.	.	.	.	.	.	.
0	0	.	NA	.	.	.	.	.	.	.	.
0	0	.	NA	.	EST/	ORG	.	.	.	.	.
0	0	.	NA	.	.	.	.	.	.	.	.
0	0	.	NA	.	.	.	.	.	.	.	.
0	0	.	NA	.	.	.	.	.	.	.	.
0	0	.	NA	.	MAC	CYT(	RNA_	KE(	.	BIO(	REACTOME_ANTIVIRAL_MECH
0	0	.	NA	.	.	.	.	.	.	.	.
0	0	.	NA	.	.	.	.	.	.	.	.
0	0	.	NA	.	.	.	.	.	.	.	.
0	0	.	NA	.	.	.	.	.	.	.	.
0	0	?Deafness	NA	.	.	.	.	.	.	.	.
0	0	.	NA	.	.	.	.	.	.	.	.
0	0	.	NA	.	.	.	.	KE(	PID	.	.
0	0	.	NA	.	.	.	.	KE(	.	.	.
0	0	.	NA	.	RES	NUC	PROT	.	PID	.	.
0	0	.	NA	.	.	.	.	.	.	.	.
0	0	.	NA	.	.	.	.	.	.	.	.
0	0	.	NA	.	.	.	.	.	.	.	.
0	0	.	NA	.	.	.	.	.	.	.	.

0	0	.	NA	.	.	.	.	.	.	.	.
0	0	.	NA	.	EST/	COA	.	KEC	PID	BIO(	.
0	0	.	NA	.	.	.	.	.	.	.	.
0	0	.	NA	.	.	.	TRAN	.	PID	.	.
0	0	.	NA	.	.	.	.	.	.	.	.
0	0	.	NA	.	EST/	ORG	.	.	.	.	.
0	0	.	NA	.	.	.	.	KEC	.	.	.
0	0	.	NA	.	.	.	.	.	.	.	.
0	0	.	NA	.	.	.	.	.	.	.	.
0	0	.	NA	.	.	.	.	.	.	.	.
0	0	Urofacial s	NA	.	.	.	HYDF	KEC	.	.	REACTOME_HS_GAG_DEGRAD
0	0	.	NA	.	EST/	ORG	.	.	PID	.	REACTOME_CELL_CYCLE;REA
0	0	.	NA	.	.	EXTI	CARE	KEC	.	.	.
0	0	.	NA	.	.	.	.	.	.	.	.
0	0	.	NA	.	DNA	CYT(	.	.	.	.	.
0	0	.	NA	.	SYS	INTF	RECE	KEC	PID	.	REACTOME_ACTIVATED_NOTC
0	0	.	NA	.	.	.	.	.	.	.	.
0	0	.	NA	.	.	.	.	.	.	.	.
0	0	.	NA	.	.	.	.	.	.	.	.
0	0	.	NA	.	.	.	.	.	.	.	.
0	0	.	NA	.	.	.	.	.	.	.	.
0	0	.	NA	.	MICF	ORG	MICR	.	PID	.	REACTOME_CELL_CYCLE;REA
0	0	Immunode	NA	.	.	.	.	KEC	.	.	.
0	0	.	NA	.	DNA	NUC	PROT	.	.	.	.
0	0	.	NA	.	.	.	.	.	.	.	.
0	0	.	NA	.	.	.	.	.	.	.	.
0	0	.	NA	.	.	.	.	.	.	.	.
0	0	.	NA	.	SIGN	INTF	TRAN	.	PID	.	.
0	0	.	NA	.	.	.	.	.	.	.	.
0	0	.	NA	.	.	.	.	.	.	.	.
0	0	Becker mu	NA	.	BIOS	PRO	ACTII	KEC	.	BIO(	REACTOME_STRIATED_MUSCL
0	0	.	NA	.	.	.	.	.	.	.	.
0	0	Megakaryc	NA	.	.	.	.	.	PID	BIO(	.
0	0	.	NA	.	.	.	.	.	.	.	.
0	0	.	NA	.	MUL	PRO	INTE(	.	PID	.	REACTOME_EXTRACELLULAR_
0	0	{Bardet-Bie	NA	.	.	.	.	.	.	.	.
0	0	.	NA	.	CEN	ORG	.	.	.	.	.
0	0	.	NA	.	.	.	.	.	.	.	.
0	0	.	NA	.	.	.	.	.	.	.	.
0	0	.	NA	.	.	.	.	.	.	.	.
0	0	.	NA	.	.	.	.	.	.	.	.
0	0	.	NA	.	.	.	.	.	.	.	.
0	0	.	NA	.	.	.	.	.	.	.	.
0	0	Elliptocytos	NA	.	ACTI	ORG	ACTII	.	.	.	REACTOME_DEVELOPMENTAL
0	0	[Blood grou	NA	.	.	MEM	.	.	.	.	REACTOME_SIGNALING_BY_G
0	0	.	NA	.	.	.	.	.	.	.	.
0	0	.	NA	.	.	.	.	.	.	.	.
0	0	.	NA	.	.	.	.	.	.	.	.
0	0	Nephropatl	NA	.	.	.	.	.	.	.	.
0	0	.	NA	.	.	.	.	KEC	PID	.	REACTOME_SIGNALING_BY_G
0	0	.	NA	.	PRO	.	PROT	.	.	.	.
0	0	Homocystii	NA	.	.	.	.	KEC	.	.	REACTOME_SULFUR_AMINO_/
0	0	.	NA	.	.	.	.	KEC	.	.	.
0	0	.	NA	.	.	.	.	KEC	.	.	.

0	0	Sitosterole	NA	.	.	.	PROT	KEG	.	.	REACTOME_ABCA_TRANSPOR
0	0	.	NA	.	.	.	.	.	.	.	.
0	0	.	NA	.	.	.	.	.	.	.	.
0	0	.	NA	.	.	.	.	.	.	.	REACTOME_TRANSMEMBRANI
0	0	Leukodystr	NA	.	CELI	ORG	CHAF	KEG	PID	.	REACTOME_MITOCHONDRIAL_
0	0	.	NA	.	RNA	.	CATI	.	.	.	.
0	0	.	NA	.	.	CYT	LIPID	.	.	.	.
0	0	.	NA	.	.	.	.	.	.	.	.
0	0	.	NA	.	SIGN	.	IDEN	.	.	.	.
0	0	.	NA	.	RNA	NUC	SPEC	.	.	.	.
0	0	.	NA	.	.	.	.	.	.	.	.
0	0	Seckel syn	NA	.	.	ORG	.	.	.	.	REACTOME_CELL_CYCLE;REA
0	0	Seckel syn	NA	.	.	ORG	.	.	.	.	REACTOME_CELL_CYCLE;REA
0	0	.	NA	.	.	.	.	.	.	.	.
0	0	.	NA	.	.	ORG	.	.	.	.	.
0	0	.	NA	.	POS	INTF	.	KEG	PID	BIO	REACTOME_EXTRINSIC_PATHY
0	0	Breast can	NA	CM1	.	.	PHOS	KEG	PID	BIO	REACTOME_SIGNALLING_BY_I
0	0	.	NA	.	.	.	.	.	.	.	.
0	0	.	NA	.	.	.	.	.	.	.	.
0	0	Adermatog	NA	.	DNA	.	.	.	.	.	.
0	0	Spastic pa	NA	.	.	.	.	KEG	.	.	REACTOME_BIOLOGICAL_OXID
0	0	.	NA	.	RNA	NUC	TRAN	.	.	.	.
0	0	.	NA	.	SIGN	.	PHOS	KEG	.	.	REACTOME_SYNTHESIS_OF_F
0	0	.	NA	.	.	.	.	.	.	.	.
0	0	Albinism, c	NA	.	.	.	.	.	.	.	.
0	0	.	NA	.	.	.	.	.	.	.	.
0	0	.	NA	.	REG	INTF	PEPT	KEG	PID	BIO	REACTOME_GASTRIN_CREB_S
0	0	.	NA	.	.	.	.	.	.	.	.
0	0	.	NA	.	.	.	.	.	.	.	.
0	0	{Breast ca	NA	.	.	.	.	KEG	.	.	REACTOME_HYALURONAN_UF
0	0	.	NA	.	SIGN	.	.	.	.	.	.
0	0	.	NA	.	.	.	.	.	.	.	.
0	0	.	NA	.	.	.	.	.	.	.	.
0	0	.	NA	.	.	INTF	RECE	.	.	.	.
0	0	{Hypersens	NA	.	.	.	.	KEG	PID	BIO	REACTOME_NEF_MEDIATED_D
0	0	.	NA	.	.	.	.	.	.	.	.
0	0	Retinitis pi	NA	.	.	.	.	.	.	.	.
0	0	Retinitis pi	NA	.	.	.	.	.	.	.	.
0	0	.	NA	.	.	.	.	.	.	.	.
0	0	.	NA	.	M_P	ORG	.	.	.	.	.
0	0	.	NA	.	.	.	.	.	.	.	.
0	0	.	NA	.	.	.	.	.	.	.	.
0	0	.	NA	.	.	.	.	.	.	.	.
0	0	.	NA	.	.	.	.	.	.	.	.
0	0	.	NA	.	.	.	.	KEG	.	.	.
0	0	.	NA	.	EST	CYT	KINA	.	PID	.	.
0	0	.	NA	.	SIGN	.	RAS_	.	PID	.	.
0	0	.	NA	.	SYS	PRO	.	.	.	.	.
0	0	.	NA	.	.	.	.	.	.	.	.
0	0	Epidermoly	NA	.	.	.	STRU	.	.	.	REACTOME_APOPTOTIC_CLEA
0	0	.	NA	.	.	.	.	.	.	.	.
0	0	.	NA	.	.	.	.	.	.	.	.
0	0	.	NA	.	.	NUC	.	.	.	.	.
0	0	.	NA	.	.	.	.	.	.	.	.

0	0	.	NA	.	.	.	.	.	.	.	.
0	0	.	NA	.	.	.	.	.	.	.	.
0	0	.	NA	.	.	.	PHOS	KE	.	BIO	REACTOME_GLYCOLYSIS;REA
0	0	.	NA	.	.	.	.	.	.	.	.
0	0	.	NA	.	.	.	.	.	.	.	.
0	0	.	NA	.	.	.	.	.	.	.	.
0	0	.	NA	.	.	.	.	.	.	.	.
0	0	.	NA	.	REG	.	.	.	.	.	.
0	0	.	NA	.	.	.	.	.	.	.	.
0	0	.	NA	.	.	.	.	.	.	.	.
0	0	.	NA	.	BIOF	.	NUCL	.	PID	.	.
0	0	.	NA	.	.	.	.	.	.	.	.
0	0	.	NA	.	.	.	.	.	.	.	.
0	0	.	NA	.	DNA	NUC	.	.	.	.	.
0	0	.	NA	.	.	.	.	.	.	.	.
0	0	.	NA	.	.	.	RNA_	.	.	.	.
0	0	Deafness,	NA	.	.	.	.	.	.	.	.
0	0	.	NA	.	.	.	.	.	.	.	.
0	0	Spermatog	NA	.	SYS	CELL	OXID	KE	.	BIO	REACTOME_LATENT_INFECTIO
0	0	.	NA	.	.	.	.	.	.	.	.
0	0	.	NA	.	.	.	.	.	.	.	.
0	0	Fraser syn	NA	.	.	.	.	.	.	.	.
0	0	Fraser syn	NA	.	.	.	.	.	.	.	.
0	0	.	NA	.	.	.	.	.	.	.	.
0	0	.	NA	.	.	.	.	.	.	.	.
0	0	.	NA	.	.	.	.	.	.	.	.
0	0	.	NA	.	.	.	.	.	.	.	.
0	0	.	NA	.	.	.	.	.	.	.	.
0	0	.	NA	.	.	.	.	.	.	.	.
0	0	.	NA	.	.	.	.	.	.	.	.
0	0	.	NA	.	.	.	.	.	.	.	.
0	0	Breast can	NA	.	REG	MEM	PHOS	KE	PID	BIO	REACTOME_SIGNALLING_BY_
0	0	.	NA	.	.	.	.	.	.	.	.
0	0	.	NA	.	.	.	.	.	.	.	.
0	0	.	NA	.	.	.	.	.	.	.	.
0	0	.	NA	.	.	.	.	.	.	.	.
0	0	.	NA	.	.	.	.	.	.	.	.
0	0	.	NA	.	.	.	.	.	.	.	.
0	0	.	NA	.	.	.	.	.	.	.	.
0	0	.	NA	.	.	.	.	.	.	.	.
0	0	.	NA	.	.	.	.	.	.	.	.
0	0	.	NA	.	.	.	.	.	.	.	.
0	0	.	NA	.	.	.	.	.	.	.	.
0	0	.	NA	.	.	.	.	.	.	.	.
0	0	.	NA	.	.	.	.	.	.	.	.
0	0	.	NA	.	.	.	.	.	.	.	.
0	0	.	NA	.	.	.	.	.	.	.	.
0	0	.	NA	.	.	.	.	.	.	.	.
0	0	.	NA	.	.	.	.	.	.	.	.
0	0	Night blind	NA	.	.	.	.	.	.	.	.
0	0	.	NA	.	.	.	.	.	.	.	.
0	0	.	NA	.	.	.	.	.	.	.	.
0	0	.	NA	.	.	INTR	.	.	.	.	.
0	0	.	NA	.	.	.	.	.	.	.	.

0	0	.	NA	.	.	.	.	.	.	.	.
0	0	.	NA	.	.	.	.	.	.	.	.
0	0	.	NA	.	.	.	.	.	.	.	.
0	0	.	NA	.	.	.	.	.	.	.	.
0	0	.	NA	.	RNA	ORG	.	KEC	.	.	REACTOME_MRNA_DECAY_BY
0	0	.	NA	.	.	.	.	.	.	.	REACTOME_PROCESSING_OF
0	0	.	NA	.	.	ORG	.	.	.	.	REACTOME_GENERIC_TRANS
0	0	.	NA	.	.	.	.	.	.	.	.
0	0	.	NA	.	.	.	.	.	.	.	.
0	0	.	NA	.	.	.	.	.	.	.	.
0	0	.	NA	.	.	.	.	.	.	.	.
0	0	.	NA	.	.	.	.	.	.	.	.
0	0	.	NA	.	.	.	.	.	.	.	.
0	0	Spinocereb	NA	.	.	.	.	.	.	.	.
0	0	.	NA	.	.	.	.	KEC	.	.	REACTOME_PHOSPHOLIPID_M
0	0	.	NA	.	.	.	.	.	.	.	.
0	0	.	NA	.	REG	.	TRAN	KEC	PID	.	.
0	0	.	NA	.	.	.	.	.	.	.	.
0	0	.	NA	.	BIOF	.	.	.	.	.	.
0	0	.	NA	.	MUL	.	.	.	.	.	.
0	0	.	NA	.	.	.	.	.	.	.	.
0	0	.	NA	.	.	.	.	.	.	.	.
0	0	.	NA	.	.	.	ARYL	.	.	.	REACTOME_PTM_GAMMA_CAF
0	0	Epileptic er	NA	.	.	.	.	.	.	.	.
0	0	.	NA	.	.	.	.	.	.	.	.
0	0	.	NA	.	EST/	ORG	MICR	.	.	.	REACTOME_DEVELOPMENTAL
0	0	.	NA	.	.	.	.	.	.	.	.
0	0	.	NA	.	.	.	.	.	.	.	.
0	0	.	NA	.	.	.	.	.	.	.	.
0	0	.	NA	.	SIGN	INTF	NEUF	KEC	.	BIOC	REACTOME_TRANSMISSION_A
0	0	.	NA	.	PRO	.	KINA:	KEC	PID	BIOC	REACTOME_SIGNALLING_BY_I
0	0	.	NA	.	.	.	.	.	.	.	.
0	0	.	NA	.	.	.	.	.	.	.	.
0	0	.	NA	.	.	.	.	.	.	.	.
0	0	.	NA	.	.	.	.	.	.	.	.
0	0	.	NA	.	.	.	.	.	PID	.	REACTOME_EXTRACELLULAR_
0	0	.	NA	.	.	.	.	.	.	.	REACTOME_CROSS_PRESENT
0	0	.	NA	.	.	.	.	.	.	.	.
0	0	.	NA	.	.	ORG	ACTII	.	PID	.	REACTOME_DEVELOPMENTAL
0	0	.	NA	.	.	.	.	.	.	.	.
0	0	.	NA	.	.	.	.	.	.	.	.
0	0	Holoprosen	NA	.	DNA	.	CATIC	KEC	PID	BIOC	.
0	0	.	NA	.	.	.	.	.	.	.	.
0	0	.	NA	.	.	.	.	.	PID	.	.
0	0	.	NA	.	.	.	.	.	.	.	REACTOME_DIABETES_PATHW
0	0	.	NA	.	.	.	.	.	.	.	.
0	0	.	NA	.	.	.	.	.	.	.	.
0	0	.	NA	.	.	.	.	.	.	.	.
0	0	.	NA	.	.	.	.	.	.	.	.
0	0	{Essential t	NA	.	SIGN	INTF	AMIN	KEC	.	.	REACTOME_SIGNALING_BY_G
0	0	Cone-rod c	NA	.	.	.	HYDF	.	.	.	.
0	0	.	NA	.	.	.	.	.	.	.	.
0	0	.	NA	.	.	.	.	.	.	.	.
0	0	.	NA	.	.	EXTI	.	.	.	.	.

0	0	.	NA	.	.	.	.	.	.	.	.
0	0	.	NA	.	SYS	INTR	.	.	.	.	.
0	0	.	NA	.	.	.	.	KEG	PID	.	REACTOME_RNA_POL_I_TRAN
0	0	.	NA	.	.	.	.	.	.	.	.
0	0	.	NA	.	.	.	.	.	.	.	.
0	0	.	NA	.	.	.	.	.	.	.	REACTOME_DEVELOPMENTAL
0	0	.	NA	.	.	.	.	.	.	.	.
0	0	{Colchicine	NA	.	ESTA	CELL	NUCL	KEG	PID	BIO	REACTOME_ABACAVIR_TRANS
0	0	.	NA	.	.	.	.	.	.	.	.
0	0	Thyroid ca	NA	.	.	ORG	.	.	.	.	REACTOME_CELL_CYCLE;REA
0	0	.	NA	.	SIGN	NON	PROT	KEG	PID	BIO	REACTOME_APOPTOTIC_CLEA
0	0	.	NA	.	POS	.	.	KEG	PID	BIO	REACTOME_EXTRINSIC_PATHY
0	0	.	NA	.	.	.	.	.	.	.	.
0	0	.	NA	.	.	INTR	.	KEG	PID	.	REACTOME_INTEGRIN_CELL_S
0	0	.	NA	.	.	.	.	.	.	.	.
0	0	.	NA	.	.	.	.	.	.	.	.
0	0	.	NA	.	.	.	.	.	.	.	.
0	0	.	NA	.	.	.	.	.	.	.	.
0	0	.	NA	.	.	.	.	.	.	.	.
0	0	.	NA	.	.	ORG	STRU	.	.	.	.
0	0	Antley-Bixle	NA	.	.	CELL	.	KEG	PID	.	REACTOME_NEGATIVE_REGUL
0	0	.	NA	.	RNA	NUC	.	.	.	.	.
0	0	Deafness,	NA	.	REG	CYTO	.	.	.	BIO	.
0	0	Deafness,	NA	.	REG	CYTO	.	.	.	BIO	.
0	0	Leukemia,	NA	.	.	.	.	KEG	PID	BIO	REACTOME_SIGNALING_BY_R
0	0	{Diabetes r	NA	.	NUC	CYTO	.	.	.	.	REACTOME_INTERFERON_GAI
0	0	Bladder ca	NA	.	REG	NUC	KINA	KEG	PID	BIO	REACTOME_CELL_CYCLE;REA
0	0	.	NA	.	.	.	.	.	.	.	.
0	0	.	NA	.	.	.	.	.	.	.	.
0	0	.	NA	.	.	.	.	.	.	.	.
0	0	.	NA	.	.	.	.	.	.	.	.
0	0	.	NA	.	.	.	.	.	.	.	.
0	0	.	NA	.	PRO	VESI	.	KEG	.	.	REACTOME_DIABETES_PATHW
0	0	.	NA	.	.	.	.	.	.	.	.
0	0	.	NA	.	.	.	.	.	.	.	.
0	0	.	NA	.	.	.	.	.	.	.	.
0	0	.	NA	.	.	.	.	.	.	.	.
0	0	.	NA	.	.	.	.	.	.	.	.
0	0	Spinocereb	NA	.	.	.	.	.	.	.	.
0	0	.	NA	.	.	INTR	CATI	.	.	.	REACTOME_TRANSMEMBRANE
0	0	.	NA	.	.	.	.	.	.	.	.
0	0	.	NA	.	.	.	.	.	.	.	.
0	0	.	NA	.	.	.	.	.	.	.	.
0	0	.	NA	.	.	CELL	.	KEG	.	.	.
0	0	Episodic at	NA	.	REG	INTR	CATI	KEG	.	.	REACTOME_TRANSMISSION_A
0	0	.	NA	.	.	.	.	KEG	.	.	REACTOME_SIGNALING_BY_G
0	0	.	NA	.	.	.	.	.	.	.	.
0	0	.	NA	.	SYS	PRO	.	.	.	.	.
0	0	.	NA	.	ESTA	INTR	CATI	.	.	.	REACTOME_NEURONAL_SYST
0	0	.	NA	.	.	.	.	.	.	.	.
0	0	.	NA	.	.	.	.	.	.	.	.
0	0	.	NA	.	.	.	.	.	.	.	.
0	0	.	NA	.	.	.	.	.	.	.	.
0	0	.	NA	.	CEN	ORG	KINA	.	.	.	REACTOME_CELL_CYCLE;REA

0	0	.	NA	.	.	.	.	.	.	.	REACTOME_TRANSMEMBRANI
0	0	.	NA	.	BIOF	INTF	PROT	.	.	.	.
0	0	.	NA	.	BIOF	INTF	PROT	.	.	.	.
0	0	.	NA	.	EST/	MEM	.	.	.	.	.
0	0	.	NA	.	PRO	.	KINA:	KEC	PID	BIO(	REACTOME_SIGNALLING_BY_I
0	0	.	NA	.	.	.	.	.	.	.	.
0	0	.	NA	.	.	.	.	.	.	.	.
0	0	.	NA	.	.	.	.	.	.	.	.
0	0	.	NA	.	.	.	.	.	.	.	.
0	0	.	NA	.	.	.	.	.	.	.	.
0	0	.	NA	.	NEG	NUC	TRAN	.	PID	.	REACTOME_RNA_POL_III_TRA
0	0	.	NA	.	.	.	.	.	PID	.	.
0	0	.	NA	.	.	.	.	.	.	.	.
0	0	.	NA	.	.	.	.	.	.	.	.
0	0	.	NA	.	.	.	IDEN	KEC	PID	BIO(	.
0	0	.	NA	.	.	.	.	.	.	.	.
0	0	.	NA	.	RNA	.	TRAN	.	PID	.	.
0	0	.	NA	.	.	.	.	.	.	.	.
0	0	.	NA	.	EST/	.	.	.	.	.	.
0	0	.	NA	.	.	.	.	.	.	.	.
0	0	.	NA	.	.	.	.	.	.	.	.
0	0	.	NA	.	.	.	.	KEC	PID	.	REACTOME_SIGNALING_BY_R
0	0	Leukoence	NA	.	REG	CYT(	TRAN	.	.	BIO(	REACTOME_TRANSLATION;RE
0	0	.	NA	.	.	.	.	.	.	.	.
0	0	.	NA	.	.	.	.	.	.	.	.
0	0	.	NA	.	.	ORG	RNA_	.	.	.	.
0	0	.	NA	.	.	.	.	.	.	.	.
0	0	.	NA	.	.	.	.	.	.	.	.
0	0	.	NA	.	.	.	.	.	.	.	.
0	0	.	NA	.	.	.	.	.	.	.	.
0	0	.	NA	.	.	.	.	.	.	.	REACTOME_CELL_CELL_COMI
0	0	.	NA	.	.	.	.	.	.	.	.
0	0	.	NA	.	.	.	.	.	.	.	.
0	0	.	NA	.	.	.	.	KEC	.	.	.
0	0	.	NA	.	.	.	.	.	.	.	.
0	0	.	NA	.	.	.	.	.	.	.	.
0	0	.	NA	.	.	.	.	.	.	.	.
0	0	.	NA	.	.	.	.	.	PID	.	.
0	0	.	NA	.	.	.	.	.	.	.	.
0	0	.	NA	.	.	.	.	.	.	.	.
0	0	{Diabetes r	NA	.	EST/	CYT(	KINA:	KEC	PID	.	.
0	0	.	NA	.	.	.	.	.	.	.	.
0	0	.	NA	.	.	.	.	.	.	.	.
0	0	.	NA	.	.	.	.	.	.	.	.
0	0	.	NA	.	.	.	.	.	.	.	.
0	0	.	NA	.	.	.	.	.	.	.	.
0	0	.	NA	.	.	.	.	.	.	.	.
0	0	.	NA	.	.	.	.	.	.	.	.
0	0	Nephronop	NA	.	.	.	.	.	.	.	REACTOME_SIGNALING_BY_H
0	0	Nephronop	NA	.	.	.	.	.	.	.	REACTOME_SIGNALING_BY_H
0	0	.	NA	.	.	.	.	.	.	.	.
0	0	.	NA	.	.	.	.	.	.	.	.
0	0	.	NA	.	.	.	.	.	.	.	.
0	0	.	NA	.	.	.	.	.	.	.	.

0	0	.	NA	.	.	.	.	.	.	.	.
0	0	.	NA	.	.	.	.	.	.	.	.
0	0	.	NA	.	.	.	.	.	.	.	.
0	0	.	NA	.	.	.	.	.	.	.	.
0	0	.	NA	.	.	.	.	.	.	.	.
0	0	.	NA	.	.	.	.	.	.	.	.
0	0	.	NA	.	.	.	.	.	.	.	.
0	0	.	NA	.	SIGN	CYT(	.	.	.	.	.
0	0	.	NA	.	.	.	.	.	.	.	.
0	0	Noonan sy	NA	CM1	SIGN	MEM	GTP_	.	PID	.	REACTOME_SIGNALLING_BY_I
0	0	.	NA	.	.	.	.	.	.	.	.
0	0	.	NA	.	CELI	MEM	CATI(	KE(	.	.	.
0	0	.	NA	.	.	.	.	.	.	.	.
0	0	.	NA	.	.	.	.	.	.	.	.
0	0	.	NA	.	.	.	.	.	.	.	.
0	0	.	NA	.	.	INTF	CATI(	.	.	.	REACTOME_TRANSMEMBRANI
0	0	.	NA	.	.	.	.	.	.	.	.
0	0	.	NA	.	.	ORG	RNA_	.	.	.	.
0	0	.	NA	.	.	CYT(	RECE	KE(	.	.	REACTOME_TRANSLATION;RE
0	0	.	NA	.	EST/	.	HYDF	.	.	.	REACTOME_TRANSMEMBRANI
0	0	.	NA	.	EST/	INTF	NUCL	KE(	.	.	REACTOME_HYALURONAN_ME
0	0	.	NA	.	.	.	.	.	.	.	REACTOME_TRANSMEMBRANI
0	0	.	NA	.	.	.	.	.	.	.	.
0	0	Sebaceous	NA	.	.	.	SEQU	KE(	PID	BIO(	.
0	0	.	NA	.	.	.	.	.	.	.	.
0	0	.	NA	.	SIGN	.	.	KE(	PID	.	REACTOME_VEGF_LIGAND_RE
0	0	.	NA	.	.	.	.	.	.	.	.
0	0	.	NA	.	.	.	.	.	.	.	.
0	0	.	NA	.	.	.	.	.	.	.	.
0	0	Myelodysp	NA	.	.	.	.	KE(	.	.	REACTOME_TRIGLYCERIDE_B
0	0	.	NA	.	.	.	.	.	.	.	.
0	0	.	NA	.	.	.	.	KE(	.	.	.
0	0	.	NA	.	.	.	.	KE(	PID	.	REACTOME_DEVELOPMENTAL
0	0	.	NA	.	.	.	.	.	.	.	.
0	0	.	NA	.	SYS'	.	.	.	.	.	.
0	0	.	NA	.	.	.	.	.	.	.	.
0	0	{Asthma, s	NA	.	DEFI	.	RECE	KE(	.	.	REACTOME_ANTIGEN_PROCE
0	0	.	NA	.	.	.	.	.	.	.	.
0	0	Deafness,	NA	.	.	.	.	KE(	PID	.	REACTOME_EXTRACELLULAR_
0	0	.	NA	.	.	.	.	.	.	.	.
0	0	.	NA	.	.	.	.	.	.	.	.
0	0	.	NA	.	.	.	.	.	.	.	.
0	0	.	NA	.	.	.	.	KE(	.	BIO(	REACTOME_PHOSPHOLIPID_M
0	0	.	NA	.	.	.	.	.	.	.	.
0	0	.	NA	.	.	.	.	.	.	.	.
0	0	.	NA	.	SYS'	.	.	.	.	.	.
0	0	.	NA	.	.	.	.	.	.	.	.
0	0	.	NA	.	.	.	.	.	.	.	.
0	0	.	NA	.	.	.	.	.	.	.	.
0	0	.	NA	.	.	.	.	.	.	.	.
0	0	.	NA	.	RNA	NUC	.	.	.	.	.
0	0	.	NA	.	.	.	.	.	.	.	.
0	0	.	NA	.	.	.	.	.	.	.	.
0	0	.	NA	.	.	.	.	.	.	.	.

0	0	Brachydac	NA	.	SIGN	INTR	TRAN	.	PID	.	.
0	0	.	NA	.	.	.	.	.	.	.	.
0	0	Mental reta	NA	.	OLIG	INTR	CATI	KEG	.	.	REACTOME_METABOLISM_OF_
0	0	{AIDS, resi	NA	.	REG	.	G_PR	KEG	PID	BIO	REACTOME_SIGNALING_BY_EI
0	0	.	NA	.	.	.	.	.	.	.	.
0	0	.	NA	.	.	.	.	.	.	.	.
0	0	{Migraine,	NA	.	.	.	.	.	.	.	REACTOME_NEURONAL_SYST
0	0	.	NA	.	SIGN	.	RECE	KEG	PID	.	.
0	0	.	NA	.	.	.	.	.	.	.	.
0	0	.	NA	.	REG	EXTI	COFA	.	.	.	.
0	0	.	NA	.	.	.	.	.	.	.	.
0	0	Diabetes m	NA	.	ESTA	INTR	.	KEG	PID	.	REACTOME_NEURONAL_SYST
0	0	.	NA	.	NEG	.	.	.	.	.	.
0	0	.	NA	.	.	.	.	KEG	.	.	REACTOME_ACTIVATED_NOTC
0	0	.	NA	.	.	.	.	.	.	.	.
0	0	.	NA	.	RNA	.	TRAN	.	.	.	.
0	0	.	NA	.	CELI	RIBO	HYDR	.	.	.	.
0	0	.	NA	.	BIOS	CYTO	.	.	PID	.	.
0	0	.	NA	.	.	.	.	.	.	.	.
0	0	.	NA	.	.	.	.	.	.	.	.
0	0	Hawkinsinu	NA	.	CAR	.	.	KEG	.	.	REACTOME_METABOLISM_OF_
0	0	.	NA	.	.	.	.	.	.	.	.
0	0	.	NA	.	.	.	.	.	.	.	.
0	0	.	NA	.	.	.	.	.	.	.	.
0	0	.	NA	.	.	CYTO	.	KEG	.	.	REACTOME_PHOSPHOLIPID_M
0	0	.	NA	.	.	.	.	.	.	.	.
0	0	Fraser syn	NA	.	.	.	.	.	.	.	.
0	0	.	NA	.	.	.	.	.	PID	.	.
0	0	.	NA	.	.	.	.	.	.	.	.
0	0	.	NA	.	BIOF	INTR	PHOS	.	PID	.	REACTOME_SPHINGOLIPID_DE
0	0	Nemaline r	NA	.	.	.	.	KEG	PID	.	.
0	0	.	NA	.	.	.	.	.	.	.	.
0	0	.	NA	.	.	.	.	.	.	.	.
0	0	.	NA	.	.	CYTO	PROT	KEG	.	BIO	REACTOME_SIGNALING_BY_W
0	0	.	NA	.	.	.	.	.	.	.	.
0	0	.	NA	.	.	.	.	.	.	.	.
0	0	.	NA	.	.	.	.	.	.	.	.
0	0	.	NA	.	.	.	.	.	.	.	.
0	0	.	NA	.	.	.	.	.	.	.	.
0	0	.	NA	.	.	.	.	.	.	.	.
0	0	.	NA	.	REG	.	PROT	.	.	.	.
0	0	.	NA	.	.	.	.	.	.	.	.
0	0	.	NA	.	.	.	.	.	.	.	.
0	0	.	NA	.	ESTA	INTR	HYDR	KEG	.	.	.
0	0	.	NA	.	.	.	.	KEG	.	.	.
0	0	.	NA	.	ESTA	.	PROT	.	.	.	.
0	0	Glycogen s	NA	.	GLU	.	LYAS	KEG	PID	BIO	REACTOME_RESPONSE_TO_E
0	0	.	NA	.	.	.	.	.	.	.	.
0	0	.	NA	.	.	.	.	.	.	.	.
0	0	.	NA	.	.	.	.	.	.	.	.
0	0	.	NA	.	.	.	.	.	.	.	.
0	0	Ciliary dysk	NA	.	.	.	.	.	.	.	.
0	0	.	NA	.	.	.	.	.	.	.	.
0	0	.	NA	.	.	.	.	.	PID	.	.

0	0	.	NA	.	.	.	.	.	.	.	.
0	0	.	NA	.	.	.	.	.	.	.	.
0	0	.	NA	.	CELI	.	HYDF	.	.	.	.
0	0	Weaver sy	NA	.	ANA	.	CHR(	.	.	.	.
0	0	Mandibulof	NA	.	RNA	ORG	HYDF	KE(	.	.	REACTOME_PROCESSING_OF_
0	0	.	NA	.	REG	ORG	NUCL	KE(	.	.	REACTOME_DIABETES_PATHW
0	0	.	NA	.	.	INTF	CATI(	KE(	.	.	REACTOME_TRANSMISSION_A
0	0	.	NA	.	RNA	.	RNA_	.	.	.	.
0	0	.	NA	.	EST	CYT(	.	.	.	.	.
0	0	.	NA	.	DNA	CYT(	PROT	.	.	.	.
0	0	.	NA	.	.	.	.	.	.	.	.
0	0	.	NA	.	.	.	.	.	.	.	.
0	0	.	NA	.	.	.	.	.	.	.	.
0	0	.	NA	.	STEI	CYT(	SULF	KE(	.	.	REACTOME_BIOLOGICAL_OXID
0	0	.	NA	.	.	.	.	.	.	.	.
0	0	.	NA	.	.	INTF	.	.	.	.	.
0	0	.	NA	.	.	.	.	.	.	.	.
0	0	.	NA	.	.	.	.	.	.	.	.
0	0	.	NA	.	.	.	.	.	.	.	.
0	0	.	NA	.	EST	INTF	CATI(	.	.	.	.
0	0	.	NA	.	.	.	.	.	.	.	.
0	0	.	NA	.	.	.	.	.	.	.	.
0	0	.	NA	.	.	.	.	.	.	.	.
0	0	.	NA	.	.	.	.	.	.	.	.
0	0	.	NA	.	DNA	SYN	HYDF	.	.	.	REACTOME_TRANSMISSION_A
0	0	.	NA	.	.	.	.	.	.	.	.
0	0	.	NA	.	.	.	.	.	.	.	.
0	0	.	NA	.	.	.	.	.	.	.	.
0	0	.	NA	.	EST	ORG	.	.	.	.	.
0	0	.	NA	.	.	.	.	.	.	.	.
0	0	.	NA	.	.	.	.	.	.	.	.
0	0	Auriculocor	NA	.	REG	.	HYDF	KE(	PID	.	REACTOME_TRANSMISSION_A
0	0	.	NA	.	.	.	.	.	.	.	.
0	0	.	NA	.	.	.	.	.	.	.	.
0	0	.	NA	.	.	.	.	.	.	.	.
0	0	.	NA	.	.	.	.	.	.	.	.
0	0	.	NA	.	.	.	.	.	.	.	.
0	0	.	NA	.	.	.	.	.	.	.	.
0	0	.	NA	.	CELI	CELI	LIPID	.	.	.	.
0	0	.	NA	.	.	.	.	.	.	.	.
0	0	Prostate ca	NA	.	.	.	RNA_	.	.	.	REACTOME_INTERFERON_ALF
0	0	{Macular d	NA	.	.	.	.	.	.	.	.
0	0	.	NA	.	MUS	ORG	PROT	.	PID	.	.
0	0	.	NA	.	PRO	.	PROT	.	.	.	.
0	0	.	NA	.	.	.	CATI(	KE(	.	.	REACTOME_TCA_CYCLE_AND_
0	0	.	NA	.	.	.	.	.	.	.	.
0	0	.	NA	.	.	.	.	.	.	.	.
0	0	.	NA	.	.	.	.	KE(	.	.	REACTOME_INHIBITION_OF_TI
0	0	.	NA	.	.	.	.	.	.	.	.
0	0	.	NA	.	.	.	.	.	.	.	.
0	0	.	NA	.	.	INTF	G_PF	.	.	.	.
0	0	.	NA	.	.	.	.	.	.	.	.
0	0	.	NA	.	.	.	.	.	.	.	.
0	0	Membrano	NA	.	CELI	INTF	META	KE(	.	.	.

0	0	.	NA	.	.	.	.	.	.	.	.
0	0	.	NA	.	.	.	.	KE(	PID_	BIO(	REACTOME_SIGNALING_BY_G
0	0	Myelodysp	NA	.	.	.	.	.	.	.	.
0	0	Cornelia de	NA	.	.	.	.	.	.	.	.
0	0	.	NA	.	.	.	.	.	.	.	REACTOME_TRANSMEMBRANI
0	0	.	NA	.	.	.	.	.	.	.	.
0	0	.	NA	.	.	.	.	.	.	.	.
0	0	.	NA	.	.	.	.	.	.	.	.
0	0	Cardiomyo	NA	.	SYS	NUC	.	.	.	.	.
0	0	{H. pylori ir	NA	.	SYS	INTR	PROT	KE(	.	.	.
0	0	.	NA	.	.	.	.	.	PID_	.	.
0	0	.	NA	.	.	.	.	.	.	.	.
0	0	.	NA	.	RNA_	NUC	.	.	.	.	.
0	0	.	NA	.	.	.	.	.	.	.	.
0	0	.	NA	.	.	.	.	.	.	.	.
0	0	Erythrocyto	NA	.	SYS	.	PROT	KE(	PID_	BIO(	REACTOME_SIGNALING_BY_S(
0	0	.	NA	.	GEN	CYT(	NUCL	.	.	.	.
0	0	.	NA	.	.	INTR	.	KE(	.	.	REACTOME_HS_GAG_BIOSYN
0	0	.	NA	.	.	.	.	KE(	.	.	.
0	0	.	NA	.	RNA_	ORG	.	.	.	.	.
0	0	.	NA	.	.	NUC	TRAN	.	.	.	.
0	0	.	NA	.	.	.	.	.	.	.	.
0	0	.	NA	.	.	.	.	.	.	.	.
0	0	.	NA	.	.	.	.	.	.	.	.
0	0	.	NA	.	.	.	.	.	.	.	.
0	0	.	NA	.	PRO	.	ACID_	KE(	PID_	BIO(	REACTOME_SIGNALLING_BY_I
0	0	.	NA	.	RNA_	NUC	RNA_	KE(	.	.	.
0	0	.	NA	.	SYS	CELI	PROT	.	.	.	REACTOME_CELL_CELL_COMI
0	0	.	NA	.	.	INTR	.	.	.	.	.
0	0	.	NA	.	.	.	.	.	.	.	.
0	0	Hypertrigly	NA	.	.	.	OXID	KE(	.	.	REACTOME_TRIGLYCERIDE_B
0	0	.	NA	.	.	.	.	.	.	.	.
0	0	.	NA	.	.	.	.	.	.	.	.
0	0	.	NA	.	SIGN	INTR	.	.	.	.	REACTOME_SIGNALING_BY_G
0	0	.	NA	.	.	INTR	.	.	.	.	REACTOME_HS_GAG_DEGRAD
0	0	.	NA	.	.	.	.	.	.	.	.
0	0	.	NA	.	.	.	.	.	.	.	.
0	0	Prader-Wil	NA	.	NEG	.	.	.	PID_	.	.
0	0	.	NA	.	.	.	.	.	.	.	.
0	0	.	NA	.	.	.	.	.	PID_	.	.
0	0	.	NA	.	.	.	.	.	.	.	.
0	0	.	NA	.	.	.	.	.	.	.	.
0	0	.	NA	.	.	INTR	META	KE(	PID_	BIO(	.
0	0	.	NA	.	.	ORG	PROT	.	.	.	.
0	0	.	NA	.	.	.	.	.	.	.	.
0	0	.	NA	.	.	.	MOT(	KE(	.	.	.
0	0	.	NA	.	.	.	.	.	.	.	.
0	0	.	NA	.	.	.	.	.	.	.	.
0	0	.	NA	.	.	.	.	.	.	.	.
0	0	Mannosido	NA	.	GLY(	.	HYDF	KE(	.	.	.
0	0	Cold-induc	NA	.	.	EXTI	RECE	.	.	.	.
0	0	.	NA	.	.	.	.	.	.	.	.
0	0	.	NA	.	.	.	.	.	.	.	.
0	0	.	NA	.	SYS	.	NUCL	KE(	PID_	BIO(	.

0	0	.	NA	.	.	NUC	.	KE(	.	.	.
0	0	.	NA	.	.	.	.	.	.	.	.
0	0	.	NA	.	SEN	.	.	.	.	.	.
0	0	.	NA	.	SEN	.	.	.	.	.	.
0	0	.	NA	.	.	.	.	.	.	.	.
0	0	.	NA	.	.	.	.	.	.	.	.
0	0	.	NA	.	.	.	.	.	.	.	.
0	0	Duane-rad	NA	.	.	.	.	.	PID	.	.
0	0	.	NA	.	.	.	.	.	.	.	.
0	0	.	NA	.	PRO	PRO	INTE(	.	.	.	.
0	0	.	NA	.	.	.	.	.	.	.	.
0	0	.	NA	.	.	.	.	.	.	.	.
0	0	.	NA	.	.	.	.	KE(	PID	.	REACTOME_CELL_CELL_COMI
0	0	.	NA	.	.	.	.	.	.	.	.
0	0	.	NA	.	.	.	.	.	.	.	.
0	0	Dent disea	NA	.	LIPIE	ORG	PHOS	KE(	.	.	REACTOME_SIGNALING_BY_R
0	0	.	NA	.	.	INTR	G_PF	.	.	.	.
0	0	.	NA	.	.	.	.	.	.	.	.
0	0	Hemophilia	NA	CM0	REG	.	.	KE(	.	BIO(	REACTOME_RESPONSE_TO_E
0	0	.	NA	.	.	.	.	.	.	.	.
0	0	.	NA	.	.	.	.	.	.	.	.
0	0	.	NA	.	.	.	.	.	.	.	.
0	0	.	NA	.	.	.	.	.	.	.	.
0	0	.	NA	.	.	.	.	.	.	.	.
0	0	.	NA	.	EST/	.	.	KE(	.	.	.
0	0	.	NA	.	.	.	.	.	.	.	.
0	0	.	NA	.	.	.	.	.	.	.	.
0	0	.	NA	.	.	.	.	.	.	.	.
0	0	.	NA	.	.	.	.	.	.	.	.
0	0	.	NA	.	.	.	.	.	.	.	.
0	0	.	NA	.	.	.	.	.	.	.	.
0	0	.	NA	.	.	.	.	.	.	.	.
0	0	.	NA	.	.	.	.	.	.	.	.
0	0	.	NA	.	.	.	TRAN	KE(	PID	.	.
0	0	.	NA	.	SIGN	EXTI	RECE	.	.	.	.
0	0	.	NA	.	.	.	.	.	.	.	.
0	0	.	NA	.	.	.	.	.	.	.	.
0	0	.	NA	.	.	.	.	.	.	.	.
0	0	.	NA	.	.	.	.	.	.	.	.
0	0	.	NA	.	.	.	.	.	.	.	.
0	0	Deafness,	NA	.	REG	NON	.	.	.	.	.
0	0	Albinism, c	NA	.	.	.	.	.	.	.	.
0	0	Pulmonary	NA	.	.	.	.	.	PID	.	.
0	0	.	NA	.	MUL	ORG	RECE	.	.	.	.
0	0	.	NA	.	.	.	.	.	.	.	.
0	0	.	NA	.	.	.	.	.	.	.	.
0	0	.	NA	.	.	.	.	.	.	.	.
0	0	.	NA	.	.	.	.	.	.	.	.
0	0	.	NA	.	.	.	.	.	.	.	.
0	0	.	NA	.	.	.	.	.	.	.	.
0	0	.	NA	.	.	.	.	.	.	.	.
0	0	.	NA	.	GAM	CYT(	.	.	.	.	.
0	0	.	NA	.	.	.	.	.	.	.	.
0	0	.	NA	.	.	.	.	.	.	.	.

0	0	.	NA	.	.	.	.	.	.	.	.
0	0	.	NA	.	.	.	.	.	.	.	.
0	0	.	NA	.	.	.	.	.	.	.	.
0	0	.	NA	.	.	.	.	.	.	.	.
0	0	.	NA	.	.	.	.	.	.	.	.
0	0	.	NA	.	.	.	.	.	.	.	.
0	0	.	NA	.	.	.	.	.	.	.	.
0	0	.	NA	.	.	.	.	.	.	.	.
0	0	.	NA	.	.	.	.	.	.	.	.
0	0	.	NA	.	.	.	.	.	.	.	.
0	0	.	NA	.	.	.	.	.	.	.	.
0	0	.	NA	.	.	.	.	.	.	.	.
0	0	.	NA	.	.	ORG	HYDR	KEG	.	.	REACTOME_GLYCOSPHINGOL
0	0	.	NA	.	.	.	.	.	.	.	.
0	0	.	NA	.	ESTA	CYTO	NUCL	KEG	PID	.	REACTOME_SIGNALLING_BY_I
0	0	.	NA	.	.	.	.	.	.	.	.
0	0	.	NA	.	PRO	MEM	.	.	PID	.	.
0	0	.	NA	.	.	.	.	.	.	.	.
0	0	.	NA	.	.	.	.	.	.	.	.
0	0	.	NA	.	DEFI	CYTO	.	.	.	.	.
0	0	.	NA	.	.	.	.	.	.	.	.
0	0	[LPA defici	NA	.	SYS	.	PROT	.	PID	.	REACTOME_METABOLISM_OF_
0	0	.	NA	.	.	.	.	.	.	.	.
0	0	.	NA	.	.	.	.	.	.	.	.
0	0	.	NA	.	.	.	.	.	.	.	.
0	0	.	NA	.	.	.	.	.	.	.	.
0	0	.	NA	.	.	.	.	.	.	.	.
0	0	.	NA	.	.	.	.	.	.	.	.
0	0	.	NA	.	.	.	.	.	.	.	.
0	0	.	NA	.	REG	EXTI	COFA	.	.	.	.
0	0	.	NA	.	ESTA	INTR	ACTI	.	.	.	REACTOME_AMINO_ACID_TRA
0	0	.	NA	.	.	.	.	.	.	.	.
0	0	.	NA	.	.	.	.	.	.	.	.
0	0	.	NA	.	.	.	RNA_	.	.	.	.
0	0	.	NA	.	.	.	.	.	.	.	.
0	0	.	NA	.	.	.	.	.	.	.	.
0	0	.	NA	.	.	.	.	.	.	.	.
0	0	.	NA	.	.	.	.	KEG	.	.	.
0	0	.	NA	.	.	.	.	.	.	.	.
0	0	.	NA	.	ANA	CYTO	HYDR	.	PID	.	.
0	0	.	NA	.	.	.	.	.	.	.	.
0	0	.	NA	.	.	.	.	.	.	.	.
0	0	.	NA	.	.	.	.	.	.	.	.
0	0	.	NA	.	.	.	.	.	.	.	.
0	0	.	NA	.	.	.	.	.	.	.	.
0	0	.	NA	.	.	.	.	.	.	.	.
0	0	.	NA	.	.	MEM	.	.	.	.	REACTOME_O_LINKED_GLYCO
0	0	.	NA	.	.	.	.	.	.	.	.
0	0	.	NA	.	.	.	.	.	.	.	.
0	0	.	NA	.	.	.	.	.	.	.	.
0	0	.	NA	.	.	.	.	.	.	.	.
0	0	.	NA	.	.	.	.	.	.	.	.
0	0	.	NA	.	.	.	.	.	.	.	.
0	0	.	NA	.	.	.	.	PID	.	.	.

0	0	.	NA	.	.	.	.	.	.	.	.
0	0	.	NA	.	.	.	.	.	.	.	.
0	0	.	NA	.	.	.	.	.	.	.	.
0	0	.	NA	.	.	.	.	.	.	.	.
0	0	.	NA	.	PRO	ORG	.	.	.	.	.
0	0	.	NA	.	.	.	.	.	.	.	.
0	0	.	NA	.	DNA	.	DNA_	.	.	.	REACTOME_MEIOSIS;REACTO
0	0	Alagille syr	NA	.	REG	INTF	SPEC	KEC	PID	.	REACTOME_PRE_NOTCH_TRA
0	0	.	NA	.	.	.	.	.	.	.	.
0	0	.	NA	.	.	.	.	.	.	.	.
0	0	.	NA	.	.	.	.	.	.	.	.
0	0	.	NA	.	.	.	.	.	.	.	.
0	0	.	NA	.	.	.	.	.	.	.	.
0	0	.	NA	.	.	.	.	.	.	.	.
0	0	.	NA	.	.	.	CATI(	.	.	.	.
0	0	.	NA	.	.	.	.	.	.	.	.
0	0	.	NA	.	SIGN	.	RAS_	.	.	.	.
0	0	.	NA	.	.	.	.	.	.	.	.
0	0	.	NA	.	REG	.	.	KEC	PID	.	REACTOME_REGULATION_OF_
0	0	Cardiomyo	NA	.	MUS	ORG	NUCL	KEC	.	.	.
0	0	.	NA	.	.	.	.	.	PID	BIO(	.
0	0	Spinocerel	NA	.	.	INTF	CATI(	KEC	.	.	.
0	0	.	NA	.	.	.	.	.	.	.	.
0	0	.	NA	.	.	PRO	CATI(	.	.	.	.
0	0	.	NA	.	SYS	INTF	LIPOI	.	.	.	.
0	0	.	NA	.	.	.	.	.	.	.	.
0	0	.	NA	.	.	.	.	KEC	PID	.	REACTOME_INTEGRIN_CELL_S
0	0	.	NA	.	.	.	.	.	.	.	.
0	0	Megaloblas	NA	.	.	.	.	KEC	PID	BIO(	REACTOME_TETRAHYDROBIO
0	0	.	NA	.	.	.	.	.	.	.	.
0	0	.	NA	.	.	.	.	.	.	.	.
0	0	.	NA	.	.	.	.	.	.	.	.
0	0	.	NA	.	SIGN	INTF	GABA	KEC	.	.	REACTOME_TRANSMISSION_A
0	0	.	NA	.	DNA	NUC	HYDF	.	.	.	.
0	0	.	NA	.	.	.	.	.	.	.	REACTOME_PHOSPHOLIPID_M
0	0	.	NA	.	.	.	.	KEC	.	.	REACTOME_PPARA_ACTIVATE
0	0	Muscular d	NA	.	MUS	PRO	STRU	KEC	PID	BIO(	REACTOME_INTEGRIN_CELL_S
0	0	Mental reta	NA	.	.	NUC	DNA_	.	.	.	.
0	0	.	NA	.	SIGN	CELL	PROT	KEC	PID	.	.
0	0	Spinocerel	NA	.	RNA	NUC	RNA_	KEC	PID	BIO(	REACTOME_RNA_POL_III_TRA
0	0	.	NA	.	.	.	.	.	.	.	.
0	0	Colorectal	NA	.	DNA	.	SING	KEC	PID	.	.
0	0	Muscular d	NA	.	.	.	.	.	.	.	.
0	0	.	NA	.	.	.	.	.	.	.	.
0	0	.	NA	.	.	.	.	.	.	.	.
0	0	.	NA	.	.	.	.	.	.	.	.
0	0	.	NA	.	.	.	.	.	.	.	.
0	0	.	NA	.	.	.	.	.	.	.	.
0	0	Maturity-or	NA	.	SIGN	.	.	.	PID	.	REACTOME_ANTIGEN_ACTIVA
0	0	.	NA	.	.	.	.	.	.	.	.
0	0	.	NA	.	.	.	.	.	.	.	.
0	0	.	NA	.	.	CYT(	ELEC	KEC	.	.	REACTOME_TCA_CYCLE_AND_
0	0	.	NA	.	SIGN	EXTI	HYDF	KEC	PID	.	REACTOME_GASTRIN_CREB_S
0	0	.	NA	.	.	.	.	KEC	.	.	.

0	0	.	NA	.	REG	UBIQ	ACID	.	.	.	.
0	0	.	NA	.	.	CYT	.	KEG	.	.	.
0	0	.	NA	.	CELI	MEM	CATI	KEG	.	.	REACTOME_TRANSMISSION_A
0	0	Cardiomyo	NA	.	.	.	.	.	.	.	.
0	0	.	NA	.	.	.	.	.	.	.	.
0	0	.	NA	.	.	.	.	.	.	.	.
0	0	.	NA	.	.	NUC	NUCL	KEG	.	.	.
0	0	.	NA	.	.	.	.	.	.	.	.
0	0	.	NA	.	.	.	.	.	.	.	.
0	0	.	NA	.	POS	.	.	KEG	PID	BIO	.
0	0	.	NA	.	.	.	.	.	.	.	.
0	0	.	NA	.	.	.	RAS_	.	PID	.	.
0	0	.	NA	.	.	.	.	.	.	.	.
0	0	.	NA	.	GLY	.	TRAN	KEG	.	.	REACTOME_METABOLISM_OF_
0	0	.	NA	.	.	.	.	.	.	.	.
0	0	.	NA	.	.	.	.	.	.	.	.
0	0	.	NA	.	.	PRO	COLL	.	.	.	.
0	0	Spastic pa	NA	.	.	.	.	.	.	.	.
0	0	.	NA	.	.	.	.	.	.	.	.
0	0	.	NA	.	.	.	.	.	.	.	.
0	0	.	NA	.	.	.	.	.	.	.	.
0	0	.	NA	.	.	.	.	.	.	.	.
0	0	.	NA	.	.	.	.	.	.	.	.
0	0	Congenital	NA	.	REG	ORG	.	.	.	.	.
0	0	.	NA	.	.	.	.	.	.	.	.
0	0	.	NA	.	.	.	.	.	.	.	.
0	0	.	NA	.	.	INTR	.	.	.	.	.
0	0	.	NA	.	RES	EXTI	G_PR	KEG	PID	BIO	REACTOME_SIGNALING_BY_G
0	0	.	NA	.	.	.	.	.	.	.	.
0	0	.	NA	.	.	.	.	.	.	.	.
0	0	.	NA	.	.	.	.	KEG	PID	.	REACTOME_GAP_JUNCTION_D
0	0	.	NA	.	.	.	.	.	.	.	.
0	0	Amyotroph	NA	.	PRO	ORG	HYDR	.	.	.	.
0	0	46XY sex r	NA	.	.	.	.	.	.	.	.
0	0	.	NA	.	PRO	ORG	CHAP	.	.	.	REACTOME_PROTEIN_FOLDIN
0	0	.	NA	.	.	.	.	.	.	.	.
0	0	.	NA	.	.	.	.	.	.	.	.
0	0	.	NA	.	.	.	GLUT	KEG	.	.	REACTOME_TRANSMISSION_A
0	0	.	NA	.	LIPID	.	SUBS	.	.	.	.
0	0	.	NA	.	SIGN	INTR	.	.	.	.	REACTOME_CELL_CELL_COMM
0	0	Exocrine p	NA	.	.	.	CATI	KEG	.	.	.
0	0	.	NA	.	.	.	.	.	.	.	.
0	0	.	NA	.	.	.	.	.	.	.	.
0	0	.	NA	.	.	.	.	.	.	.	.
0	0	.	NA	.	.	.	.	.	.	.	.
0	0	.	NA	.	.	.	.	.	.	.	.
0	0	.	NA	.	.	.	.	.	.	.	.
0	0	.	NA	.	.	.	.	.	.	.	.
0	0	Chondrody	NA	.	BIOP	NUC	HYDR	.	PID	BIO	REACTOME_NOTCH1_INTRACE
0	0	.	NA	.	.	.	.	.	.	.	.
0	0	Dent disea	NA	CM0	EST	INTR	.	.	.	.	.
0	0	.	NA	.	.	.	.	.	.	.	.
0	0	.	NA	.	.	.	.	.	.	.	.
0	0	.	NA	.	.	.	.	.	.	.	.

0	0	.	NA	.	.	.	.	.	.	.	.
0	0	.	NA	.	.	.	.	.	.	.	.
0	0	.	NA	.	.	.	.	.	.	.	.
0	0	.	NA	.	.	.	.	.	.	.	.
0	0	.	NA	.	.	.	.	.	.	.	.
0	0	.	NA	.	.	.	.	.	.	.	.
0	0	.	NA	.	.	.	.	.	.	.	.
0	0	.	NA	.	POS	INTR	LIPID	KEG	.	.	.
0	0	.	NA	.	.	.	.	.	.	.	REACTOME_TRANSMEMBRAN
0	0	.	NA	.	.	.	.	.	.	.	.
0	0	Miyoshi mu	NA	.	SYS	MEM	.	.	.	.	.
0	0	Miyoshi mu	NA	.	SYS	MEM	.	.	.	.	.
0	0	.	NA	.	.	.	.	.	.	.	.
0	0	.	NA	.	.	.	.	.	.	.	.
0	0	.	NA	.	.	.	.	.	.	.	.
0	0	.	NA	.	.	INTR	.	.	PID	.	REACTOME_DEVELOPMENTAL
0	0	.	NA	.	.	.	.	.	.	.	.
0	0	.	NA	.	.	EXTI	.	KEG	.	.	.
0	0	.	NA	.	.	.	.	.	.	.	.
0	0	Adams-Oli	NA	.	.	.	.	.	.	.	REACTOME_SIGNALING_BY_R
0	0	Myopia 23,	NA	.	PRO	INTR	CARE	.	PID	.	.
0	0	.	NA	.	.	RIBO	RNA_	.	.	.	.
0	0	.	NA	.	.	.	.	.	.	.	.
0	0	.	NA	.	.	.	.	.	.	.	.
0	0	.	NA	.	.	.	.	.	.	.	.
0	0	.	NA	.	.	.	.	.	.	.	.
0	0	.	NA	.	.	.	.	.	.	.	.
0	0	.	NA	.	.	.	.	KEG	.	.	.
0	0	Cockayne	NA	.	DNA	ORG	HYDF	KEG	.	.	REACTOME_TRANSCRIPTION_
0	0	.	NA	.	.	.	.	.	.	.	.
0	0	.	NA	.	.	.	.	.	.	.	REACTOME_TRANSMEMBRAN
0	0	.	NA	.	.	.	.	.	.	.	.
0	0	.	NA	.	.	.	.	KEG	.	.	REACTOME_DEVELOPMENTAL
0	0	.	NA	.	.	.	.	.	.	.	.
0	0	{Asthma, s	NA	.	DEFI	.	RECE	KEG	.	.	REACTOME_ANTIGEN_PROCE
0	0	Ehlers-Dar	NA	.	ORG	PRO	INTE	KEG	.	.	.
0	0	.	NA	.	RNA	NUC	.	.	.	.	.
0	0	.	NA	.	.	.	.	.	.	.	.
0	0	{Colchicine	NA	.	EST	CELI	NUCL	KEG	PID	BIO	REACTOME_ABACAVIR_TRANS
0	0	.	NA	.	SYS	INTR	.	KEG	PID	.	.
0	0	.	NA	.	.	CYT	PHOS	KEG	.	.	REACTOME_GASTRIN_CREB_
0	0	.	NA	.	.	CYT	PHOS	KEG	.	.	REACTOME_GASTRIN_CREB_
0	0	.	NA	.	.	.	.	KEG	.	.	REACTOME_SIGNALING_BY_G
0	0	.	NA	.	.	PRO	.	.	.	.	.
0	0	.	NA	.	.	.	.	.	.	.	.
0	0	Diabetes m	NA	.	RNA	NUC	SPEC	.	.	.	.
0	0	.	NA	.	.	.	.	.	.	.	.
0	0	.	NA	.	.	.	.	.	.	.	.
0	0	.	NA	.	.	.	.	.	.	.	.
0	0	.	NA	.	.	.	.	.	.	.	.
0	0	.	NA	.	.	.	.	.	.	.	.
0	0	.	NA	.	CELI	MEM	CATI	KEG	.	.	REACTOME_TRANSMISSION_A
0	0	.	NA	.	.	.	.	.	.	.	.
0	0	.	NA	.	.	.	.	.	.	.	REACTOME_GENERIC_TRANS

0	0	.	NA	.	.	.	.	.	.	.	.
0	0	.	NA	.	.	.	.	.	.	.	.
0	0	.	NA	.	.	.	.	.	.	.	.
0	0	.	NA	.	PHO	INTR	PHOS	.	PID	.	REACTOME_SYNTHESIS_OF_P
0	0	.	NA	.	.	.	.	KEG	PID	.	REACTOME_CELL_CELL_COMM
0	0	Ataxia-tela	NA	.	DNA	.	PHOS	KEG	PID	BIOC	REACTOME_MEIOSIS;REACTO
0	0	.	NA	.	.	CYTO	.	.	.	.	REACTOME_DIABETES_PATHW
0	0	.	NA	.	.	.	.	.	.	.	.
0	0	.	NA	.	.	.	.	.	.	.	.
0	0	Leukemia,	NA	.	.	.	TRAN	KEG	.	.	.
0	0	Bladder ca	NA	CM0	.	.	.	KEG	PID	BIOC	REACTOME_SIGNALLING_BY_N
0	0	Spastic pa	NA	.	ESTA	ORG	.	.	.	.	REACTOME_MHC_CLASS_II_AN
0	0	.	NA	.	.	.	.	.	.	.	REACTOME_NEURONAL_SYST
0	0	.	NA	.	.	.	.	.	.	.	.
0	0	.	NA	.	.	.	.	.	.	.	.
0	0	.	NA	.	.	.	.	.	.	.	.
0	0	.	NA	.	.	.	.	.	.	.	.
0	0	.	NA	.	.	.	.	.	.	.	.
0	0	Prader-Wil	NA	.	NEG	.	.	.	PID	.	.
0	0	.	NA	.	SIGN	INTR	.	KEG	.	BIOC	REACTOME_TRANSMISSION_A
0	0	.	NA	.	.	.	.	.	.	.	.
0	0	.	NA	.	.	.	.	.	.	.	.
0	0	.	NA	.	.	.	.	.	.	.	.
0	0	.	NA	.	.	.	.	.	.	.	.
0	0	.	NA	.	SYST	.	.	.	.	.	.
0	0	.	NA	.	.	.	.	.	.	.	.
0	0	Autoinflam	NA	.	.	.	.	KEG	PID	.	REACTOME_ANTIGEN_ACTIVA
0	0	.	NA	.	.	.	.	.	PID	.	.
0	0	.	NA	.	.	.	.	.	.	.	.
0	0	.	NA	.	.	.	.	.	.	.	.
0	0	.	NA	.	.	.	.	.	.	.	.
0	0	.	NA	.	.	.	PROT	.	.	.	.
0	0	.	NA	.	.	.	.	.	.	.	.
0	0	.	NA	.	.	.	.	.	.	.	.
0	0	Adams-Oli	NA	.	.	.	.	.	PID	.	REACTOME_FACTORS_INVOLV
0	0	.	NA	.	.	.	.	.	.	.	.
0	0	.	NA	.	.	.	.	.	.	.	.
0	0	.	NA	.	.	.	.	.	.	.	REACTOME_GENERIC_TRANS
0	0	.	NA	.	.	.	.	.	.	.	.
0	0	.	NA	.	REG	CYTO	PROT	.	.	.	.
0	0	.	NA	.	.	.	.	.	.	.	.
0	0	.	NA	.	.	.	.	.	.	.	.
0	0	.	NA	.	.	.	.	.	.	.	.
0	0	.	NA	.	.	.	.	.	.	.	.
0	0	.	NA	.	.	.	.	.	.	.	.
0	0	.	NA	.	CELL	INTR	.	KEG	.	.	.
0	0	.	NA	.	REG	COA	.	.	.	.	REACTOME_TRANSMISSION_A
0	0	.	NA	.	.	.	.	.	.	.	.
0	0	.	NA	.	.	.	.	.	.	.	.
0	0	.	NA	.	.	.	.	.	.	.	.
0	0	.	NA	.	.	.	.	.	PID	.	.
0	0	Lymphopro	NA	.	CELL	CYTO	.	KEG	PID	.	.
0	0	.	NA	.	.	.	.	.	.	.	.
0	0	.	NA	.	.	.	.	.	.	.	.

0	0	Favism;He	NA	CM9	GLU	CYT	.	KEC	.	.	REACTOME_METABOLISM_OF_
0	0	.	NA	.	.	.	.	.	.	.	.
0	0	.	NA	.	NUC	.	.	KEC	.	.	REACTOME_METABOLISM_OF_
0	0	.	NA	.	.	.	.	.	.	.	.
0	0	.	NA	.	DETI	.	TAST	KEC	.	.	REACTOME_SIGNALING_BY_G
0	0	.	NA	.	SIGN	CYT	GTPA	.	.	.	REACTOME_RAP1_SIGNALLING
0	0	.	NA	.	.	.	.	.	.	.	.
0	0	.	NA	.	.	.	.	.	.	.	.
0	0	.	NA	.	.	.	.	.	.	.	.
0	0	.	NA	.	.	.	.	.	.	.	.
0	0	.	NA	.	.	.	.	.	.	.	.
0	0	.	NA	.	.	.	.	.	.	.	.
0	0	.	NA	.	.	.	.	.	.	.	.
0	0	.	NA	.	.	INTR	HYDR	KEC	.	.	REACTOME_REVERSIBLE_HYD
0	0	Chronic gr	NA	.	DEFI	.	ELEC	KEC	PID	BIO	REACTOME_ANTIGEN_PROCE
0	0	.	NA	.	MUS	INTR	CATI	.	.	.	REACTOME_NEURONAL_SYST
0	0	.	NA	.	.	.	.	.	.	.	.
0	0	.	NA	.	.	.	.	.	.	.	.
0	0	.	NA	.	.	.	.	KEC	.	.	REACTOME_O_LINKED_GLYCO
0	0	.	NA	.	.	.	.	.	.	.	.
0	0	Leukodystr	NA	.	CELI	ORG	CHAR	KEC	PID	.	REACTOME_MITOCHONDRIAL_
0	0	.	NA	.	ESTA	NUC	.	.	.	.	.
0	0	Multiple su	NA	.	.	.	.	KEC	.	.	REACTOME_PTM_GAMMA_CAR
0	0	.	NA	.	.	.	.	KEC	PID	.	REACTOME_BMAL1_CLOCK_N
0	0	.	NA	.	.	.	.	.	.	.	.
0	0	.	NA	.	.	.	.	.	PID	.	REACTOME_CELL_CYCLE;REA
0	0	.	NA	.	.	.	.	.	.	.	.
0	0	.	NA	.	.	.	.	.	.	.	.
0	0	.	NA	.	CELI	NUC	.	.	.	.	.
0	0	.	NA	.	CELI	NUC	.	.	.	.	.
0	0	.	NA	.	.	.	.	KEC	.	.	.
0	0	.	NA	.	.	.	.	.	.	.	.
0	0	.	NA	.	.	.	.	.	.	.	.
0	0	.	NA	.	.	.	.	.	.	.	.
0	0	.	NA	.	.	.	.	.	.	.	.
0	0	.	NA	.	.	.	TRAN	.	.	.	.
0	0	.	NA	.	.	.	.	.	.	.	.
0	0	.	NA	.	.	.	.	.	.	.	.
0	0	.	NA	.	GLY	.	TRAN	KEC	.	.	REACTOME_O_LINKED_GLYCO
0	0	.	NA	.	.	.	.	.	.	.	.
0	0	.	NA	.	.	.	.	KEC	.	.	REACTOME_DEVELOPMENTAL
0	0	{Atopy, res	NA	.	.	.	.	.	.	.	.
0	0	Narcolepsy	NA	.	SYS	.	.	.	.	.	.
0	0	.	NA	.	.	.	.	.	.	.	.
0	0	.	NA	.	.	.	.	.	.	.	.
0	0	.	NA	.	.	.	.	.	.	.	.
0	0	.	NA	.	.	.	.	.	.	.	.
0	0	.	NA	.	.	.	.	.	.	.	.
0	0	.	NA	.	.	.	.	.	.	.	.
0	0	.	NA	.	.	.	.	.	.	.	.
0	0	.	NA	.	POS	CYT	.	.	.	.	.
0	0	.	NA	.	.	.	.	.	.	.	.
0	0	.	NA	.	.	.	.	.	.	.	.
0	0	{Prostate c	NA	.	REG	CYT	STRU	KEC	PID	.	.

0	0	{Coronary	NA	.	.	.	HYDF	.	.	.	.
0	0	.	NA	.	SIGN	NUC	TRAN	.	PID	BIO	.
0	0	.	NA	.	.	CYT	.	.	.	.	.
0	0	.	NA	.	NUC	.	.	.	.	.	REACTOME_METABOLISM_OF_
0	0	.	NA	.	.	.	.	.	.	.	.
0	0	.	NA	.	.	.	.	.	.	.	.
0	0	.	NA	.	.	.	.	.	.	.	.
0	0	.	NA	.	.	.	.	KEC	.	.	REACTOME_INSULIN_RECEPT
0	0	.	NA	.	.	.	.	.	.	.	.
0	0	.	NA	.	.	.	.	.	.	.	.
0	0	.	NA	.	SYS	COA	.	.	.	.	.
0	0	.	NA	.	BIOF	EXTI	.	.	.	.	REACTOME_SIGNALLING_BY_I
0	0	.	NA	.	.	.	.	.	.	.	.
0	0	.	NA	.	.	.	.	.	.	.	.
0	0	Candidiasi	NA	.	POS	CYT	PROT	KEC	.	.	REACTOME_INNATE_IMMUNE_
0	0	.	NA	.	.	.	.	.	.	.	.
0	0	.	NA	.	.	.	.	.	.	.	.
0	0	.	NA	.	SYS	CELI	.	KEC	.	.	REACTOME_CELL_CELL_COMI
0	0	.	NA	.	.	.	.	.	.	.	.
0	0	.	NA	.	.	.	.	.	.	.	.
0	0	.	NA	.	REP	EXTI	META	KEC	.	.	.
0	0	.	NA	.	CELI	CYT	GTPA	KEC	PID	BIO	REACTOME_DEVELOPMENTAL
0	0	.	NA	.	.	.	.	.	.	.	.
0	0	.	NA	.	.	.	.	KEC	.	BIO	REACTOME_METABOLISM_OF_
0	0	Hermansky	NA	.	.	.	.	.	.	.	.
0	0	.	NA	.	.	.	.	.	.	.	.
0	0	.	NA	.	.	.	.	.	.	.	.
0	0	.	NA	.	.	.	.	.	.	.	.
0	0	.	NA	.	.	.	.	.	.	.	.
0	0	.	NA	.	.	.	.	.	.	.	.
0	0	Achondrog	NA	.	SYS	.	.	KEC	PID	.	REACTOME_DEVELOPMENTAL
0	0	.	NA	.	.	.	.	.	.	.	.
0	0	.	NA	.	.	.	.	.	.	.	.
0	0	Transposit	NA	.	.	.	.	.	.	.	REACTOME_DEVELOPMENTAL
0	0	Diabetes m	NA	CM0	REG	MAC	TRAN	KEC	PID	BIO	REACTOME_DEVELOPMENTAL
0	0	.	NA	.	.	.	.	.	PID	.	REACTOME_FACTORS_INVOLV
0	0	.	NA	.	ONE	.	METH	.	PID	.	REACTOME_METABOLISM_OF_
0	0	.	NA	.	.	.	.	.	.	.	.
0	0	.	NA	.	.	.	.	.	.	.	.
0	0	Prader-Wil	NA	.	NEG	.	.	.	PID	.	.
0	0	.	NA	.	MAC	CELI	.	KEC	PID	.	REACTOME_APOPTOTIC_CLEA
0	0	.	NA	.	.	.	.	.	.	.	REACTOME_TRANSMISSION_A
0	0	.	NA	.	.	.	.	.	.	.	.
0	0	.	NA	.	.	.	.	.	.	.	.
0	0	.	NA	.	.	.	.	.	.	.	.
0	0	.	NA	.	.	.	.	.	.	.	.
0	0	.	NA	.	.	.	.	.	.	.	.
0	0	.	NA	.	.	.	.	.	.	.	.
0	0	.	NA	.	.	.	.	KEC	PID	.	.
0	0	.	NA	.	SIGN	.	RECE	.	.	.	.
0	0	.	NA	.	REG	CYT	.	.	.	.	.
0	0	.	NA	.	.	.	.	.	.	.	.
0	0	.	NA	.	AMIN	ORG	SULF	KEC	.	.	.
0	0	.	NA	.	.	.	.	.	.	.	.

0	0	.	NA	.	.	INTF	.	KEC	PID	.	REACTOME_INTEGRIN_CELL_S
0	0	.	NA	.	DNA	NUC	PROT	.	.	.	.
0	0	.	NA	.	.	.	.	.	.	.	.
0	0	.	NA	.	BIOS	CYT	ELEC	.	PID	.	.
0	0	Epidermoly	NA	.	.	INTF	.	KEC	PID	.	REACTOME_CELL_CELL_COMI
0	0	.	NA	.	.	.	.	KEC	PID	.	REACTOME_INSULIN_RECEPT
0	0	.	NA	.	BIOF	CELI	OXID	.	.	BIO	REACTOME_EXTRACELLULAR_
0	0	.	NA	.	CELI	INTF	RECE	KEC	.	BIO	.
0	0	.	NA	.	.	.	.	.	.	.	REACTOME_O_LINKED_GLYCC
0	0	.	NA	.	.	.	.	.	.	.	REACTOME_O_LINKED_GLYCC
0	0	.	NA	.	.	.	.	.	.	.	.
0	0	.	NA	.	.	.	.	.	.	.	.
0	0	.	NA	.	NEG	.	TRAN	.	.	.	.
0	0	.	NA	.	RNA	.	TRAN	.	PID	.	.
0	0	.	NA	.	RNA	.	TRAN	.	PID	.	.
0	0	.	NA	.	RNA	.	TRAN	.	PID	.	.
0	0	.	NA	.	RNA	.	TRAN	.	PID	.	.
0	0	.	NA	.	.	.	.	.	.	.	REACTOME_GENERIC_TRANS
0	0	.	NA	.	.	.	.	.	.	.	.
0	0	.	NA	.	.	.	.	.	.	.	.
0	0	.	NA	.	.	.	.	.	.	.	.
0	0	.	NA	.	REP	INTF	.	.	.	.	.
0	0	[Bombay p	NA	.	GLY	INTF	TRAN	KEC	.	.	.
0	0	[Bombay p	NA	.	GLY	INTF	TRAN	KEC	.	.	.
0	0	.	NA	.	POS	EXTI	RECE	.	.	.	.
0	0	.	NA	.	.	.	.	.	.	.	REACTOME_GENERIC_TRANS
0	0	.	NA	.	.	.	.	KEC	.	.	.
0	0	.	NA	.	.	.	.	.	.	.	REACTOME_TRIGLYCERIDE_B
0	0	.	NA	.	.	.	.	.	.	.	.
0	0	.	NA	.	.	.	.	.	.	.	.
0	0	.	NA	.	BIOF	INTF	PROT	.	.	.	.
0	0	.	NA	.	.	INTF	.	.	.	.	.
0	0	.	NA	.	SYS	PRO	.	.	.	.	.
0	0	.	NA	.	MUS	NON	ACTI	.	.	.	.
0	0	.	NA	.	.	.	.	.	.	.	.
0	0	.	NA	.	.	CYT	.	.	PID	.	REACTOME_SIGNALING_BY_R
0	0	.	NA	.	.	.	.	.	.	.	.
0	0	.	NA	.	.	.	.	.	.	.	.
0	0	{Epilepsy,	NA	.	SIGN	INTF	GABA	KEC	.	.	.
0	0	Palmoplan	NA	.	.	.	.	.	PID	.	.
0	0	.	NA	.	.	.	.	.	.	.	.
0	0	.	NA	.	NUC	.	.	KEC	PID	BIO	REACTOME_DOWNREGULATIC
0	0	Ciliary dysl	NA	.	.	.	.	.	PID	.	.
0	0	Glycogen s	NA	.	.	MAC	.	KEC	.	.	REACTOME_GLYCOGEN_BREA
0	0	.	NA	.	.	.	.	.	.	.	.
0	0	.	NA	.	.	.	.	.	.	.	.
0	0	.	NA	.	.	.	.	.	.	.	.
0	0	.	NA	.	.	.	.	.	.	.	.
0	0	.	NA	.	.	.	HYDF	.	.	.	.
0	0	Epidermoly	NA	.	SYS	.	.	KEC	PID	.	REACTOME_CELL_CELL_COMI
0	0	.	NA	.	.	.	.	.	.	.	.
0	0	.	NA	.	.	.	.	.	.	.	.
0	0	.	NA	.	RNA	NUC	TRAN	.	.	.	.
0	0	Senior-Lok	NA	.	BIOF	.	.	KEC	PID	.	REACTOME_CELL_CYCLE;REA

0	0	.	NA	.	.	.	.	.	.	.	.
0	0	.	NA	.	.	.	.	.	.	.	.
0	0	Short statu	NA	.	.	.	.	.	.	.	.
0	0	.	NA	.	REG	.	NUCL	KEG	PID	.	REACTOME_INNATE_IMMUNE_
0	0	GAPO syn	NA	.	.	.	.	.	PID	.	.
0	0	.	NA	.	.	.	.	.	.	.	.
0	0	Selective T	NA	.	POS	MEM	.	KEG	PID	BIOC	REACTOME_TCR_SIGNALING;F
0	0	.	NA	.	.	.	.	.	.	.	.
0	0	.	NA	.	.	.	.	.	.	.	.
0	0	.	NA	.	.	.	.	.	.	.	.
0	0	Cardiomyo	NA	.	MUS	ORG	NUCL	KEG	.	.	.
0	0	.	NA	.	.	.	.	.	.	.	.
0	0	.	NA	.	BIOF	CYT	PROT	KEG	PID	BIOC	.
0	0	.	NA	.	BIOF	CYT	PROT	KEG	PID	BIOC	.
0	0	.	NA	.	.	.	.	.	.	.	.
0	0	Microphtha	NA	.	.	.	.	.	.	.	.
0	0	.	NA	.	M_P	ORG	DNA_	.	.	BIOC	REACTOME_MHC_CLASS_II_AI
0	0	.	NA	.	SYS	INTR	G_PF	KEG	.	.	REACTOME_SIGNALING_BY_G
0	0	.	NA	.	SYS	INTR	LIPOI	.	.	.	.
0	0	.	NA	.	REG	INTR	.	.	.	.	.
0	0	.	NA	.	REG	INTR	.	.	.	.	.
0	0	.	NA	.	BIOS	.	STRU	KEG	.	.	REACTOME_TRANSLATION;RE
0	0	Deafness,	NA	.	.	.	.	.	.	.	.
0	0	.	NA	.	.	.	.	KEG	.	.	.
0	0	Breast can	NA	CM1	.	.	PHOS	KEG	PID	BIOC	REACTOME_SIGNALLING_BY_I
0	0	Myopia 23,	NA	.	PRO	INTR	CARE	.	PID	.	.
0	0	.	NA	.	.	.	.	.	.	.	.
0	0	.	NA	.	.	.	.	.	.	.	REACTOME_TRANSMEMBRANI
0	0	.	NA	.	.	.	.	.	.	.	REACTOME_TRANSMEMBRANI
0	0	.	NA	.	.	.	.	.	.	.	.
0	0	.	NA	.	.	.	.	.	.	.	.
0	0	.	NA	.	.	.	.	.	.	.	.
0	0	.	NA	.	.	.	.	.	.	.	.
0	0	.	NA	.	.	.	.	KEG	.	.	.
0	0	.	NA	.	.	.	.	.	.	.	.
0	0	.	NA	.	.	MEM	RAS_	.	.	.	.
0	0	.	NA	.	.	.	.	.	.	.	.
0	0	.	NA	.	POS	ORG	RECE	KEG	PID	.	REACTOME_NEGATIVE_REGUI
0	0	.	NA	.	.	.	.	.	.	.	.
0	0	.	NA	.	.	.	.	.	.	.	.
0	0	.	NA	.	.	.	.	.	.	.	.
0	0	.	NA	.	RNA	NUC	RNA_	KEG	.	.	.
0	0	Cardiomyo	NA	.	.	PRO	STRU	KEG	PID	BIOC	.
0	0	.	NA	.	.	.	.	KEG	.	.	.
0	0	.	NA	.	SIGN	INTR	PEPT	KEG	PID	.	REACTOME_SIGNALING_BY_G
0	0	.	NA	.	.	.	GTPA	KEG	PID	.	REACTOME_SIGNALING_BY_R
0	0	.	NA	.	.	.	.	.	.	.	.
0	0	.	NA	.	.	.	.	.	.	.	.
0	0	.	NA	.	.	ORG	HYDF	.	.	.	.
0	0	.	NA	.	.	.	.	.	.	.	.
0	0	.	NA	.	.	.	.	.	.	.	.
0	0	Shwachma	NA	.	.	.	.	.	.	.	.
0	0	.	NA	.	.	.	.	.	.	.	.
0	0	.	NA	.	.	.	.	.	.	.	REACTOME_O_LINKED_GLYCC

0	0	.	NA	.	.	.	.	.	.	.	.
0	0	.	NA	.	.	.	.	.	.	.	.
0	0	.	NA	.	.	.	.	.	.	.	.
0	0	.	NA	.	BIOF	ORG	TRAN	.	PID	BIO(	.
0	0	.	NA	.	.	.	.	.	.	.	.
0	0	.	NA	.	EST/	ORG	.	.	.	.	.
0	0	.	NA	.	ACTI	ADH	STRU	.	.	.	REACTOME_MUSCLE_CONTRA
0	0	.	NA	.	.	INTR	CATI(	.	.	.	REACTOME_DEGRADATION_O
0	0	.	NA	.	.	.	.	.	.	.	.
0	0	.	NA	.	.	.	.	.	.	.	.
0	0	.	NA	.	.	.	.	.	.	.	.
0	0	.	NA	.	.	.	.	.	.	.	.
0	0	5-oxoprolin	NA	.	.	.	.	KE(	.	.	REACTOME_BIOLOGICAL_OXID
0	0	.	NA	.	.	.	.	.	.	.	REACTOME_HIV_INFECTION;R
0	0	.	NA	.	.	.	.	.	.	.	.
0	0	.	NA	.	.	.	.	.	.	.	.
0	0	.	NA	.	.	.	.	.	.	.	.
0	0	.	NA	.	.	.	.	.	.	.	.
0	0	.	NA	.	.	.	.	.	.	.	.
0	0	.	NA	.	HOM	CYT(	IDEN	KE(	PID	BIO(	REACTOME_DEVELOPMENTAL
0	0	.	NA	.	.	.	.	.	.	.	.
0	0	Pulmonary	NA	.	.	.	.	.	PID	.	.
0	0	Cowden sy	NA	.	.	.	.	.	.	.	.
0	0	.	NA	.	.	.	.	.	.	.	.
0	0	.	NA	.	.	.	.	.	.	.	REACTOME_O_LINKED_GLYCO
0	0	.	NA	.	.	.	.	.	.	.	.
0	0	Diabetes m	NA	.	EST/	.	CATI(	KE(	PID	.	REACTOME_NEURONAL_SYST
0	0	Glycogen s	NA	.	.	CYT(	OXID	KE(	PID	BIO(	REACTOME_PYRUVATE_META
0	0	Dystonia 2	NA	.	.	.	.	.	.	.	.
0	0	.	NA	.	.	.	.	.	.	.	.
0	0	.	NA	.	.	.	.	.	.	.	.
0	0	.	NA	.	.	.	.	.	.	.	.
0	0	.	NA	.	.	.	.	.	.	.	.
0	0	Asphyxiatin	NA	.	ORG	CYT(	.	KE(	.	.	REACTOME_MHC_CLASS_II_AI
0	0	.	NA	.	.	.	.	.	.	.	.
0	0	.	NA	.	.	.	.	.	.	.	REACTOME_TRANSMEMBRANI
0	0	.	NA	.	.	.	.	.	.	.	.
0	0	.	NA	.	.	.	.	.	.	.	.
0	0	Methylmalo	NA	.	.	.	.	KE(	.	.	.
0	0	.	NA	.	.	.	.	.	.	.	.
0	0	.	NA	.	.	.	.	.	.	.	.
0	0	.	NA	.	.	.	.	.	.	.	.
0	0	.	NA	.	.	.	.	.	.	.	.
0	0	.	NA	.	.	.	.	.	.	.	.
0	0	.	NA	.	PRO	INTR	CATI(	KE(	PID	.	REACTOME_DEGRADATION_O
0	0	Atrial septa	NA	.	REG	ORG	HYDF	KE(	.	.	REACTOME_STRIATED_MUSCI
0	0	.	NA	.	.	.	.	.	.	.	.
0	0	.	NA	.	BIOF	CELI	NUCL	.	.	.	.
0	0	.	NA	.	.	.	.	.	.	.	.
0	0	Angelman	NA	.	BIOF	.	.	KE(	PID	BIO(	REACTOME_IMMUNE_SYSTEM
0	0	.	NA	.	SYS	.	.	.	.	.	.
0	0	Colorectal	NA	.	MIT(	ORG	.	KE(	PID	.	REACTOME_INHIBITION_OF_TI
0	0	.	NA	.	.	.	.	.	.	.	.
0	0	.	NA	.	.	.	.	.	.	.	.

0	0	{Pseudoxa	NA	.	BIOS	.	UDP_	KEG	.	.	.
0	0	.	NA	.	POS	INTR	RECE	KEG	.	.	REACTOME_CELL_SURFACE_I
0	0	.	NA	.	SYS	INTR	RECE	KEG	PID	BIO	REACTOME_CELL_SURFACE_I
0	0	.	NA	.	REG	INTR	CATI	.	.	.	REACTOME_TRANSMEMBRAN
0	0	.	NA	.	MUL	UBIQ	ACID	KEG	.	.	.
0	0	.	NA	.	.	.	.	.	.	.	.
0	0	.	NA	.	POS	INTR	G_PR	KEG	.	.	REACTOME_SIGNALING_BY_G
0	0	.	NA	.	.	.	MOT	KEG	.	.	.
0	0	.	NA	.	.	.	.	.	.	.	.
0	0	Alexander	NA	.	.	ORG	STRU	.	.	.	REACTOME_SIGNALING_BY_E
0	0	.	NA	.	.	.	.	KEG	.	.	REACTOME_ABCA_TRANSPOR
0	0	.	NA	.	PRO	ORG	CHAP	.	.	.	REACTOME_PROTEIN_FOLDIN
0	0	.	NA	.	.	.	.	.	.	.	.
0	0	[Blood grou	NA	.	EST	INTR	SUBS	.	.	.	REACTOME_TRANSMEMBRAN
0	0	.	NA	.	.	.	.	.	.	.	.
0	0	.	NA	.	.	.	.	.	.	.	.
0	0	.	NA	.	.	.	.	.	.	.	.
0	0	.	NA	.	.	INTR	NUCL	KEG	.	.	REACTOME_ABCA_TRANSPOR
0	0	.	NA	.	.	.	.	.	.	.	.
0	0	.	NA	.	.	.	.	.	.	.	.
0	0	.	NA	.	.	INTR	G_PR	.	.	.	REACTOME_SIGNALING_BY_G
0	0	.	NA	.	.	.	.	.	.	.	.
0	0	.	NA	.	.	.	.	.	.	.	.
0	0	.	NA	.	.	.	.	.	.	.	REACTOME_GENERIC_TRANS
0	0	.	NA	.	.	.	.	.	.	.	.
0	0	.	NA	.	.	.	.	.	.	.	.
0	0	.	NA	.	.	INTR	CARE	.	.	.	.
0	0	.	NA	.	SIGN	.	.	.	.	.	REACTOME_GASTRIN_CREB_S
0	0	.	NA	.	.	.	.	.	.	.	REACTOME_DEVELOPMENTAL
0	0	Epileptic e	NA	.	.	CYT	.	KEG	PID	BIO	REACTOME_GASTRIN_CREB_S
0	0	.	NA	.	.	.	.	.	.	.	REACTOME_FACTORS_INVOLV
0	0	.	NA	.	.	.	.	.	.	.	.
0	0	.	NA	.	.	.	.	.	.	.	.
0	0	.	NA	.	.	.	.	.	.	.	.
0	0	.	NA	.	CELL	INTR	.	KEG	.	.	.
0	0	.	NA	.	.	.	.	.	.	.	.
0	0	.	NA	.	EST	INTR	CATI	.	.	.	.
0	0	.	NA	.	.	CYT	.	.	.	.	.
0	0	Bethlem m	NA	.	.	.	.	KEG	PID	.	REACTOME_DEVELOPMENTAL
0	0	.	NA	.	.	.	.	.	.	.	.
0	0	.	NA	.	.	.	.	.	.	.	.
0	0	.	NA	.	.	.	.	.	.	.	.
0	0	.	NA	.	INOR	.	VOLT	.	.	.	.
0	0	.	NA	.	.	.	.	.	.	.	.
0	0	.	NA	.	SIGN	NON	KINA	.	.	.	.
0	0	.	NA	.	.	.	.	.	.	.	.
0	0	.	NA	.	.	.	.	.	.	.	.
0	0	.	NA	.	GLY	CYT	TRAN	.	.	.	.
0	0	.	NA	.	.	.	.	.	.	.	.
0	0	.	NA	.	.	.	.	.	.	.	.
0	0	.	NA	.	.	.	.	.	.	.	.
0	0	.	NA	.	.	.	.	.	.	.	.
0	0	.	NA	.	PRO	.	KINA	KEG	PID	BIO	REACTOME_SIGNALLING_BY_N
0	0	.	NA	.	.	.	.	.	.	.	.

0	0	.	NA	.	.	.	.	.	.	.	.
0	0	.	NA	.	.	.	.	.	.	.	.
0	0	.	NA	.	EST	INTR	.	.	.	.	REACTOME_MEMBRANE_TRAF
0	0	Epileptic en	NA	.	.	.	TRAN	KEG	.	.	REACTOME_O_LINKED_GLYCO
0	0	.	NA	.	BIOF	NUC	PHOS	.	.	.	.
0	0	.	NA	.	ENZ	INTR	TRAN	.	.	.	.
0	0	.	NA	.	.	.	.	KEG	PID	BIO	REACTOME_IL_7_SIGNALING;F
0	0	.	NA	.	.	.	.	KEG	.	.	REACTOME_METABOLISM_OF_
0	0	.	NA	.	.	.	.	.	.	.	.
0	0	.	NA	.	.	.	.	.	.	.	.
0	0	.	NA	.	.	.	.	.	.	.	.
0	0	.	NA	.	.	.	.	.	.	.	.
0	0	.	NA	.	.	.	.	.	.	.	.
0	0	.	NA	.	BIOF	CYT	PROT	KEG	PID	BIO	.
0	0	.	NA	.	.	.	.	.	PID	.	REACTOME_SIGNALING_BY_R
0	0	Thromboc	NA	.	.	INTR	.	KEG	.	.	REACTOME_METABOLISM_OF_
0	0	Donnai-Ba	NA	.	GLY	CYT	.	KEG	PID	.	REACTOME_METABOLISM_OF_
0	0	.	NA	.	.	.	.	.	.	.	.
0	0	.	NA	.	CELL	NUC	NUCL	.	.	.	.
0	0	.	NA	.	.	INTR	G_PR	.	.	.	.
0	0	.	NA	.	.	.	.	.	.	.	.
0	0	.	NA	.	.	.	.	.	.	.	REACTOME_EXTRACELLULAR_
0	0	.	NA	.	EST	INTR	NUCL	KEG	.	.	REACTOME_HYALURONAN_ME
0	0	Night blind	NA	.	DETI	.	.	KEG	PID	.	REACTOME_CGMP_EFFECTS;F
0	0	.	NA	.	.	.	PHOS	.	.	.	REACTOME_MEMBRANE_TRAF
0	0	.	NA	.	.	.	.	.	PID	.	.
0	0	.	NA	.	.	.	.	.	.	.	.
0	0	.	NA	.	GLU	INTR	.	KEG	.	.	.
0	0	.	NA	.	.	.	.	KEG	.	.	REACTOME_SIGNALING_BY_G
0	0	.	NA	.	.	.	.	.	.	.	.
0	0	.	NA	.	SYS	.	.	KEG	.	.	REACTOME_DEVELOPMENTAL
0	0	.	NA	.	.	.	.	KEG	PID	.	REACTOME_SIGNALING_BY_R
0	0	Adenoma,	NA	.	NEG	ORG	KINA	KEG	PID	BIO	REACTOME_APOPTOTIC_CLEA
0	0	.	NA	.	.	.	.	.	.	.	.
0	0	.	NA	.	.	.	.	.	.	.	REACTOME_DEVELOPMENTAL
0	0	[Memory, e	NA	.	.	.	.	.	.	.	REACTOME_SIGNALING_BY_H
0	0	.	NA	.	.	.	.	.	.	.	.
0	0	.	NA	.	RNA	.	TRAN	.	PID	.	.
0	0	.	NA	.	DNA	.	NUCL	KEG	.	.	REACTOME_ACTIVATION_OF_
0	0	.	NA	.	.	.	.	.	.	.	.
0	0	.	NA	.	.	.	.	.	.	.	.
0	0	{Hypogona	NA	.	.	EXTI	.	KEG	.	.	REACTOME_DEVELOPMENTAL
0	0	{Osteopor	NA	.	REG	.	HORI	KEG	.	.	.
0	0	.	NA	.	.	.	.	.	.	.	.
0	0	.	NA	.	.	.	.	.	.	.	.
0	0	.	NA	.	.	.	.	.	.	.	.
0	0	Charcot-M	NA	.	RES	.	.	.	PID	.	.
0	0	.	NA	.	.	NUC	TRAN	.	PID	.	REACTOME_RNA_POL_III_TRA
0	0	Cardiomyo	NA	.	MUS	EXTI	.	.	.	.	.
0	0	.	NA	.	.	.	.	KEG	.	.	REACTOME_SIGNALING_BY_G
0	0	.	NA	.	MUS	CYT	ACTI	.	PID	.	.
0	0	.	NA	.	.	.	.	.	.	.	.
0	0	.	NA	.	.	.	.	.	.	.	.
0	0	.	NA	.	.	.	.	.	.	.	.

0	0	.	NA	.	SIGN	NUC	DNA_	.	.	.	REACTOME_PPARA_ACTIVATE
0	0	Neurofibro:	NA	.	NUC	NUC	TRAN	.	.	.	.
0	0	.	NA	.	.	.	.	.	.	.	.
0	0	.	NA	.	.	.	.	.	.	.	.
0	0	.	NA	.	.	.	.	.	.	.	.
0	0	.	NA	.	.	.	TRAN	KEG	.	.	REACTOME_O_LINKED_GLYCO
0	0	.	NA	.	.	.	.	.	.	.	.
0	0	.	NA	.	.	.	.	.	.	.	.
0	0	.	NA	.	.	.	.	.	.	.	.
0	0	.	NA	.	SIGN	CYT	CATI	KEG	.	.	REACTOME_SIGNALLING_BY_N
0	0	.	NA	.	.	.	.	.	.	.	.
0	0	.	NA	.	.	.	PHOS	KEG	.	.	.
0	0	.	NA	.	RNA	.	TRAN	.	.	.	REACTOME_INTEGRATION_OF
0	0	.	NA	.	.	.	.	.	.	.	.
0	0	.	NA	.	.	.	.	.	.	.	.
0	0	.	NA	.	.	.	MRNA	.	PID	.	REACTOME_METABOLISM_OF_
0	0	.	NA	.	.	.	.	.	.	.	.
0	0	{Epilepsy, (	NA	.	MUS	MEM	CATI	KEG	.	.	REACTOME_DEVELOPMENTAL
0	0	.	NA	.	BIOF	.	NUCL	.	.	.	.
0	0	.	NA	.	.	.	.	.	.	.	.
0	0	.	NA	.	.	.	.	.	.	.	.
0	0	.	NA	.	.	INTF	G_PF	.	.	.	.
0	0	.	NA	.	MUS	PRO	.	KEG	.	.	REACTOME_EXTRACELLULAR_
0	0	.	NA	.	.	.	.	.	.	.	.
0	0	.	NA	.	.	.	.	.	.	.	.
0	0	.	NA	.	.	.	.	.	PID	.	REACTOME_REGULATION_OF_
0	0	.	NA	.	.	.	.	.	.	.	.
0	0	.	NA	.	.	.	.	.	.	.	.
0	0	.	NA	.	.	.	.	.	.	.	.
0	0	.	NA	.	.	.	.	.	.	.	.
0	0	.	NA	.	.	.	.	.	.	.	.
0	0	.	NA	.	.	CELI	PROT	.	.	.	.
0	0	.	NA	.	MAC	.	.	.	.	BIO	REACTOME_TRANSLATION;RE
0	0	.	NA	.	.	.	.	.	.	.	.
0	0	.	NA	.	BIOF	.	.	KEG	.	BIO	.
0	0	Mental reta	NA	.	.	.	.	.	.	.	.
0	0	.	NA	.	.	.	.	.	PID	.	.
0	0	.	NA	.	RNA	.	TRAN	.	.	.	.
0	0	.	NA	.	.	.	.	.	.	.	.
0	0	Congenital	NA	CI97	PAT	.	.	.	.	.	.
0	0	Hemophilia	NA	.	REG	.	.	KEG	.	BIO	REACTOME_RESPONSE_TO_E
0	0	.	NA	.	.	.	.	.	.	.	.
0	0	.	NA	.	.	.	.	.	.	.	.

_VITAMINS_AND_COFACTORS

NSCRIPTION;REACTOME_RNA_POL_II_TRANSCRIPTION_PRE_INITIATION_AND_PROMOTER_
_CAPPED_INTRON_CONTAINING_PRE_MRNA;REACTOME_MRNA_PROCESSING;REACTOME_

DATIONS;REACTOME_CYTOCHROME_P450_ARRANGED_BY_SUBSTRATE_TYPE;REACTOME_
METABOLISM;REACTOME_SYNTHESIS_OF_PE;REACTOME_SYNTHESIS_OF_PC;REACTOME_C

TORY_INTERACTIONS_BETWEEN_A_LYMPHOID_AND_A_NON_LYMPHOID_CELL;REACTOME_
NSCRIPTION;REACTOME_TRANSCRIPTION;REACTOME_RNA_POL_I_RNA_POL_III_AND_MITC

_LIPIDS_AND_LIPOPROTEINS;REACTOME_CHOLESTEROL_BIOSYNTHESIS

S;REACTOME_IL1_SIGNALING;REACTOME_IMMUNE_SYSTEM;REACTOME_CYTOKINE_SIGNA

_PROTEINS;REACTOME_POST_TRANSLATIONAL_PROTEIN_MODIFICATION;REACTOME_ASP

_TRNA_AMINOACYLATION;REACTOME_TRNA_AMINOACYLATION

PCR;REACTOME_CLASS_B_2_SECRETIN_FAMILY_RECEPTORS;REACTOME_GPCR_DOWNST

NSCRIPTION_AND_TRANSLATION;REACTOME_YAP1_AND_WWTR1_TAZ_STIMULATED_GEN

_NON_CODING_RNA;REACTOME_ANTIVIRAL_MECHANISM_BY_IFN_STIMULATED_GENES;RE

NGF;REACTOME_DAG_AND_IP3_SIGNALING;REACTOME_SIGNALING_BY_ERBB2;REACTOME_
NT;REACTOME_SIGNALLING_BY_NGF;REACTOME_CTNNB1_PHOSPHORYLATION_CASCADE

DATIONS;REACTOME_GLUTATHIONE_CONJUGATION;REACTOME_PHASE_II_CONJUGATION

E_TRANSPORT_OF_SMALL_MOLECULES;REACTOME_SLC_MEDIATED_TRANSMEMBRANE_T

ACTOME_FORMATION_OF_THE_TERNARY_COMPLEX_AND_SUBSEQUENTLY_THE_43S_COM

ME_CELL_CYCLE;REACTOME_CHROMOSOME_MAINTENANCE;REACTOME_MEIOTIC_SYNAP

_AMINO_ACIDS_AND_DERIVATIVES;REACTOME_AMINE_DERIVED_HORMONES

ING_AND_TARGETTING_OF_GAG_PROTEINS;REACTOME_HIV_INFECTION;REACTOME_HIV_

MUNICATION;REACTOME_EXTRACELLULAR_MATRIX_ORGANIZATION;REACTOME_COLLAGE

NGF;REACTOME_TRIF_MEDIATED_TLR3_SIGNALING;REACTOME_TCR_SIGNALING;REACTOM

PCR;REACTOME_OLFACTORY_SIGNALING_PATHWAY;REACTOME_GPCR_DOWNSTREAM_S

ACROSS_CHEMICAL_SYNAPSES;REACTOME_NEURONAL_SYSTEM;REACTOME_AMINO_ACID_

JLFATE_BIOSYNTHESIS;REACTOME_CHONDROITIN_SULFATE_DERMATAN_SULFATE_METAB

ME_CELL_CYCLE;REACTOME_CHROMOSOME_MAINTENANCE;REACTOME_MEIOTIC_SYNAPS

E_TRANSPORT_OF_SMALL_MOLECULES;REACTOME_ION_TRANSPORT_BY_P_TYPE_ATPAS

PCR;REACTOME_GPCR_DOWNSTREAM_SIGNALING;REACTOME_G_ALPHA_I_SIGNALLING_E

_CAPPED_INTRON_CONTAINING_PRE_MRNA;REACTOME_MRNA_PROCESSING;REACTOME_

CTOME_CELL_CYCLE_MITOTIC;REACTOME_RECRUITMENT_OF_MITOTIC_CENTROSOME_PR

NGF;REACTOME_DAG_AND_IP3_SIGNALING;REACTOME_SIGNALING_BY_ERBB2;REACTOME_

DATIONS;REACTOME_XENOBIOTICS;REACTOME_CYTOCHROME_P450_ARRANGED_BY_SUB

DATIONS;REACTOME_GLUTATHIONE_CONJUGATION;REACTOME_PHASE_II_CONJUGATION

VAYS;REACTOME_ACTIVATION_OF_CHAPERONE_GENES_BY_XBP1S;REACTOME_UNFOLDE

CRIPTION_PATHWAY;REACTOME_NUCLEAR_RECEPTOR_TRANSCRIPTION_PATHWAY

_INTRONLESS_PRE_MRNAS;REACTOME_PROCESSING_OF_CAPPED_INTRON_CONTAINING_

THESIS;REACTOME_HEPARAN_SULFATE_HEPARIN_HS_GAG_METABOLISM;REACTOME_GLY

HO_GTPASES;REACTOME_SIGNALLING_BY_NGF;REACTOME_NRAGE_SIGNALS_DEATH_THF

NGF;REACTOME_SIGNALING_BY_SCF_KIT;REACTOME_SIGNALING_BY_ERBB4;REACTOME_S

NGF;REACTOME_SIGNALING_BY_SCF_KIT;REACTOME_SIGNALING_BY_ERBB4;REACTOME_S

THE_PRE_REPLICATIVE_COMPLEX;REACTOME_CELL_CYCLE;REACTOME_ORC1_REMOVAL_

EM;REACTOME_VOLTAGE_GATED_POTASSIUM_CHANNELS;REACTOME_POTASSIUM_CHAN

PCR;REACTOME_GPCR_DOWNSTREAM_SIGNALING;REACTOME_G_ALPHA_I_SIGNALLING_E
L_BIOLOGY;REACTOME_BMAL1_CLOCK_NPAS2_ACTIVATES_CIRCADIAN_EXPRESSION;REAC

MUNICATION;REACTOME_CELL_EXTRACELLULAR_MATRIX_INTERACTIONS;REACTOME_CEL

_NUCLEOTIDES;REACTOME_PURINE_RIBONUCLEOSIDE_MONOPHOSPHATE_BIOSYNTHESIS
L_BIOLOGY;REACTOME_EXTRACELLULAR_MATRIX_ORGANIZATION;REACTOME_COLLAGEN_
HO_GTPASES;REACTOME_SIGNALLING_BY_NGF;REACTOME_NRAGE_SIGNALS_DEATH_THR

NGF;REACTOME_SIGNALING_BY_SCF_KIT;REACTOME_CELL_CELL_COMMUNICATION;REACT
VED_IN_MEGAKARYOCYTE_DEVELOPMENT_AND_PLATELET_PRODUCTION;REACTOME_HEM

DATIONS;REACTOME_PHASE1_FUNCTIONALIZATION_OF_COMPOUNDS;REACTOME_ETHANO

VED_IN_MEGAKARYOCYTE_DEVELOPMENT_AND_PLATELET_PRODUCTION;REACTOME_HEM
NSCRIPTION_AND_TRANSLATION;REACTOME_PRE_NOTCH_EXPRESSION_AND_PROCESSIN

EM;REACTOME_TANDEM_PORE_DOMAIN_POTASSIUM_CHANNELS;REACTOME_POTASSIUM

LFATE_DERMATAN_SULFATE_METABOLISM;REACTOME_GLYCOSAMINOGLYCAN_METABOL

E_TRANSPORT_OF_SMALL_MOLECULES;REACTOME_SLC_MEDIATED_TRANSMEMBRANE_T

FICKING;REACTOME_MEMBRANE_BINDING_AND_TARGETTING_OF_GAG_PROTEINS;REACT

E_TRANSPORT_OF_SMALL_MOLECULES;REACTOME_SLC_MEDIATED_TRANSMEMBRANE_T

NGF;REACTOME_SIGNALLING_TO_RAS;REACTOME_NGF_SIGNALLING_VIA_TRKA_FROM_TH
ACROSS_CHEMICAL_SYNAPSES;REACTOME_NEURONAL_SYSTEM
_STEROID_HORMONES_AND_VITAMINS_A_AND_D;REACTOME_HDL_MEDIATED_LIPID_TRAN

_PROTEIN_IMPORT;REACTOME_METABOLISM_OF_PROTEINS

E_TRANSPORT_OF_SMALL_MOLECULES;REACTOME_AQUAPORIN_MEDIATED_TRANSPORT;

OSYLATION_OF_MUCINS;REACTOME_TERMINATION_OF_O_GLYCAN_BIOSYNTHESIS;REACT
OSYLATION_OF_MUCINS;REACTOME_TERMINATION_OF_O_GLYCAN_BIOSYNTHESIS;REACT

THESIS;REACTOME_HEPARAN_SULFATE_HEPARIN_HS_GAG_METABOLISM;REACTOME_GLY

ECEPTOR_INTERACTIONS;REACTOME_RESPONSE_TO_ELEVATED_PLATELET_CYTOSOLIC_
WAYS;REACTOME_ACTIVATION_OF_CHAPERONE_GENES_BY_XBP1S;REACTOME_UNFOLDE
NTIGEN_PRESENTATION;REACTOME_FACTORS_INVOLVED_IN_MEGAKARYOCYTE_DEVELO

HO_GTPASES
HO_GTPASES

PCR;REACTOME_OLFACTORY_SIGNALING_PATHWAY;REACTOME_GPCR_DOWNSTREAM_S

ON_OF_HOMO_SAPIENS_WITH_MYCOBACTERIUM_TUBERCULOSIS;REACTOME_NITRIC_OXI
_BIOLOGY;REACTOME_NOTCH1_INTRACELLULAR_DOMAIN_REGULATES_TRANSCRIPTION;
TLR3_SIGNALING;REACTOME_RIP_MEDIATED_NFKB_ACTIVATION_VIA_DAI;REACTOME_APO

;REACTOME_ADAPTIVE_IMMUNE_SYSTEM;REACTOME_CLASS_I_MHC_MEDIATED_ANTIGEN

PCR;REACTOME_OLFACTORY_SIGNALING_PATHWAY;REACTOME_GPCR_DOWNSTREAM_S

E_TRANSPORT_OF_SMALL_MOLECULES;REACTOME_SLC_MEDIATED_TRANSMEMBRANE_T

._BIOLOGY;REACTOME_AXON_GUIDANCE;REACTOME_MYOGENESIS;REACTOME_NETRIN1_

E_TRANSPORT_OF_SMALL_MOLECULES;REACTOME_SLC_MEDIATED_TRANSMEMBRANE_T

PHA_BETA_SIGNALING;REACTOME_INTERFERON_SIGNALING;REACTOME_IMMUNE_SYSTEM
MUNICATION;REACTOME_CELL_CELL_JUNCTION_ORGANIZATION;REACTOME_TIGHT_JUNC

G;REACTOME_POST_CHAPERONIN_TUBULIN_FOLDING_PATHWAY;REACTOME_METABOLIS

_AMINO_ACIDS_AND_DERIVATIVES

IROTRANSMITTER_RELEASE_CYCLE;REACTOME_TRANSMISSION_ACROSS_CHEMICAL_SYN

PCR;REACTOME_PEPTIDE_LIGAND_BINDING_RECEPTORS;REACTOME_CLASS_A1_RHODOF

DATIONS;REACTOME_PHASE1_FUNCTIONALIZATION_OF_COMPOUNDS;REACTOME_ETHANC
DATIONS;REACTOME_PHASE1_FUNCTIONALIZATION_OF_COMPOUNDS;REACTOME_ETHANC

._BIOLOGY;REACTOME_PPARA_ACTIVATES_GENE_EXPRESSION;REACTOME_GENERIC_TR/

._BIOLOGY;REACTOME_AXON_GUIDANCE;REACTOME_L1CAM_INTERACTIONS
HO_GTPASES;REACTOME_MEMBRANE_TRAFFICKING;REACTOME_TRANS_GOLGI_NETWOR

;REACTOME_ADAPTIVE_IMMUNE_SYSTEM;REACTOME_CLASS_I_MHC_MEDIATED_ANTIGEN

._BIOLOGY;REACTOME_SIGNALING_BY_NODAL
PCR;REACTOME_OLFACTORY_SIGNALING_PATHWAY;REACTOME_GPCR_DOWNSTREAM_S

_NUCLEOTIDES;REACTOME_PURINE_RIBONUCLEOSIDE_MONOPHOSPHATE_BIOSYNTHESIS

/ED_IN_MEGAKARYOCYTE_DEVELOPMENT_AND_PLATELET_PRODUCTION;REACTOME_KINI
/ED_IN_MEGAKARYOCYTE_DEVELOPMENT_AND_PLATELET_PRODUCTION;REACTOME_KINI
ACROSS_CHEMICAL_SYNAPSES;REACTOME_NEURONAL_SYSTEM

._BIOLOGY;REACTOME_AXON_GUIDANCE;REACTOME_SIGNALING_BY_ROBO_RECEPTOR
IETABOLISM;REACTOME_GASTRIN_CREB_SIGNALLING_PATHWAY_VIA_PKC_AND_MAPK;RE/

NGF;REACTOME_SIGNALING_BY_SCF_KIT;REACTOME_CELL_CELL_COMMUNICATION;REACT
NGF;REACTOME_SIGNALING_BY_SCF_KIT;REACTOME_CELL_CELL_COMMUNICATION;REACT

SIGNALLING_PATHWAY_VIA_PKC_AND_MAPK;REACTOME_SIGNALING_BY_GPCR;REACTOME

SSING_CROSS_PRESENTATION;REACTOME_ENDOSOMAL_VACUOLAR_PATHWAY;REACTOM
ISCRIPTION;REACTOME_TRANSCRIPTION;REACTOME_RNA_POL_I_RNA_POL_III_AND_MITO
CRIPTION_PATHWAY;REACTOME_NUCLEAR_RECEPTOR_TRANSCRIPTION_PATHWAY
ES_GENE_EXPRESSION;REACTOME_METABOLISM_OF_LIPIDS_AND_LIPOPROTEINS;REACTO
DATIONS;REACTOME_GLUTATHIONE_CONJUGATION;REACTOME_PHASE_II_CONJUGATION
_MATRIX_ORGANIZATION;REACTOME_COLLAGEN_FORMATION

_BIOLOGY;REACTOME_REGULATION_OF_BETA_CELL_DEVELOPMENT;REACTOME_REGULA

ACROSS_CHEMICAL_SYNAPSES;REACTOME_NEURONAL_SYSTEM;REACTOME_SIGNALING_

BOLISM_AND_CITRIC_ACID_TCA_CYCLE;REACTOME_TCA_CYCLE_AND_RESPIRATORY_ELE

NTERACTIONS_AT_THE_VASCULAR_WALL;REACTOME_TIE2_SIGNALING;REACTOME_HEMO

E_TRANSPORT_OF_SMALL_MOLECULES;REACTOME_SLC_MEDIATED_TRANSMEMBRANE_T

_BIOLOGY;REACTOME_NEURONAL_SYSTEM;REACTOME_AXON_GUIDANCE;REACTOME_L1

E_TRANSPORT_OF_SMALL_MOLECULES;REACTOME_SLC_MEDIATED_TRANSMEMBRANE_T

_BIOLOGY;REACTOME_TRANSMISSION_ACROSS_CHEMICAL_SYNAPSES;REACTOME_NEUR

LEVATED_PLATELET_CYTOSOLIC_CA2_;REACTOME_MUSCLE_CONTRACTION;REACTOME_

E_TRANSPORT_OF_SMALL_MOLECULES;REACTOME_AQUAPORIN_MEDIATED_TRANSPORT;

NGF;REACTOME_SIGNALING_BY_SCF_KIT;REACTOME_DEVELOPMENTAL_BIOLOGY;REACTC
NGF;REACTOME_DAG_AND_IP3_SIGNALING;REACTOME_SIGNALING_BY_ERBB2;REACTOME_
VAYS;REACTOME_ACTIVATION_OF_CHAPERONE_GENES_BY_XBP1S;REACTOME_UNFOLDE

FFICKING;REACTOME_TRANS_GOLGI_NETWORK_VESICLE_BUDDING;REACTOME_GOLGI_AS

ACROSS_CHEMICAL_SYNAPSES;REACTOME_NEURONAL_SYSTEM;REACTOME_NEUROTRAN

BOLISM_AND_CITRIC_ACID_TCA_CYCLE;REACTOME_TCA_CYCLE_AND_RESPIRATORY_ELE

A_PATHWAY;REACTOME_DNA_REPAIR

SIGNALLING_PATHWAY_VIA_PKC_AND_MAPK;REACTOME_SIGNALING_BY_GPCR;REACTOME
TOTHENATE_METABOLISM;REACTOME_METABOLISM_OF_VITAMINS_AND_COFACTORS;REA

VAYS;REACTOME_PERK_REGULATED_GENE_EXPRESSION;REACTOME_ACTIVATION_OF_GE
_COUPLED_NER_TC_NER;REACTOME_NUCLEOTIDE_EXCISION_REPAIR;REACTOME_FORMAT
_PROTEIN_IMPORT;REACTOME_METABOLISM_OF_PROTEINS

_BIOLOGY;REACTOME_AXON_GUIDANCE;REACTOME_NCAM_SIGNALING_FOR_NEURITE_OU
VAYS;REACTOME_PERK_REGULATED_GENE_EXPRESSION;REACTOME_ACTIVATION_OF_GE

MODELLING_OF_PI;REACTOME_ACYL_CHAIN_REMODELLING_OF_PC;REACTOME_PHOSPHO
_AMINO_ACIDS_AND_DERIVATIVES;REACTOME_GLYCOPROTEIN_HORMONES;REACTOME_F

NGF;REACTOME_DAG_AND_IP3_SIGNALING;REACTOME_SIGNALING_BY_ERBB2;REACTOME_
CRIPTION_PATHWAY

THE_PRE_REPLICATIVE_COMPLEX;REACTOME_CELL_CYCLE;REACTOME_ORC1_REMOVAL_

REACTOME_GENERATION_OF_SECOND_MESSENGER_MOLECULES;REACTOME_IMMUNE_SY

IPPO
)SYLATION_OF_MUCINS;REACTOME_METABOLISM_OF_PROTEINS;REACTOME_POST_TRAN

HO_GTPASES;REACTOME_SIGNALLING_BY_NGF;REACTOME_NRAGE_SIGNALS_DEATH_THF
_LIPIDS_AND_LIPOPROTEINS;REACTOME_CHOLESTEROL_BIOSYNTHESIS

;REACTOME_ADAPTIVE_IMMUNE_SYSTEM;REACTOME_CLASS_I_MHC_MEDIATED_ANTIGEN

_MATRIX_ORGANIZATION;REACTOME_COLLAGEN_FORMATION

MUNICATION;REACTOME_CELL_CELL_JUNCTION_ORGANIZATION;REACTOME_TIGHT_JUNC

)TEINS_MEDIATED_TRANSPORT;REACTOME_TRANSMEMBRANE_TRANSPORT_OF_SMALL_M

TATION_OF_SOLUBLE_EXOGENOUS_ANTIGENS_ENDOSOMES;REACTOME_ANTIGEN_PROCE

HO_GTPASES;REACTOME_SIGNALLING_BY_NGF;REACTOME_NRAGE_SIGNALS_DEATH_THR

HO_GTPASES;REACTOME_SIGNALLING_BY_NGF;REACTOME_NRAGE_SIGNALS_DEATH_THR

ISCRIPTION_TERMINATION;REACTOME_RNA_POL_I_TRANSCRIPTION;REACTOME_TRANSCR

_NUCLEOTIDES;REACTOME_PYRIMIDINE_METABOLISM

ANSPORT_ACROSS_THE_PLASMA_MEMBRANE;REACTOME_TRANSMEMBRANE_TRANSPORT

ANSPORT_ACROSS_THE_PLASMA_MEMBRANE;REACTOME_TRANSMEMBRANE_TRANSPORT

HO_GTPASES

HO_GTPASES

E_TRANSPORT_OF_SMALL_MOLECULES;REACTOME_SLC_MEDIATED_TRANSMEMBRANE_TI

ISCRIPTION_TERMINATION;REACTOME_RNA_POL_I_TRANSCRIPTION;REACTOME_TRANSCR

NSCRIPTION_INITIATION_FROM_TYPE_2_PROMOTER;REACTOME_RNA_POL_III_TRANSCRIP
_AMINO_ACIDS_AND_DERIVATIVES

ELLULAR_DOMAIN_REGULATES_TRANSCRIPTION;REACTOME_SIGNALING_BY_NOTCH1;REA

/ED_IN_MEGAKARYOCYTE_DEVELOPMENT_AND_PLATELET_PRODUCTION;REACTOME_HEM

PCR;REACTOME_CLASS_B_2_SECRETIN_FAMILY_RECEPTORS;REACTOME_GPCR_LIGAND_

REPAIR;REACTOME_RESOLUTION_OF_AP_SITES_VIA_THE_MULTIPLE_NUCLEOTIDE_PATCH
REPAIR;REACTOME_RESOLUTION_OF_AP_SITES_VIA_THE_MULTIPLE_NUCLEOTIDE_PATCH

E_TRANSPORT_OF_SMALL_MOLECULES;REACTOME_ION_TRANSPORT_BY_P_TYPE_ATPAS

ME_CELL_CYCLE;REACTOME_CELL_CYCLE_CHECKPOINTS;REACTOME_REGULATION_OF_T
PCR;REACTOME_CLASS_A1_RHODOPSIN_LIKE_RECEPTORS;REACTOME_P2Y_RECEPTORS

_BIOLOGY;REACTOME_REGULATION_OF_BETA_CELL_DEVELOPMENT;REACTOME_REGULA

CTOME_CELL_CYCLE_MITOTIC;REACTOME_CELL_CYCLE_CHECKPOINTS;REACTOME_TRAN

CRIPTION_PATHWAY
E_TRANSPORT_OF_SMALL_MOLECULES;REACTOME_SLC_MEDIATED_TRANSMEMBRANE_TI
_BIOLOGY;REACTOME_BMAL1_CLOCK_NPAS2_ACTIVATES_CIRCADIAN_EXPRESSION;REAC

_MATRIX_ORGANIZATION;REACTOME_COLLAGEN_FORMATION

TIMULATES_GUANYLATE_CYCLASE;REACTOME_PLATELET_HOMEOSTASIS;REACTOME_HEM

ONE_RECEPTOR_SIGNALING;REACTOME_SIGNALING_BY_ERBB4;REACTOME_PROLACTIN_R

ISCRIPTION_TERMINATION;REACTOME_RNA_POL_I_TRANSCRIPTION;REACTOME_RNA_POL

IPID_METABOLISM;REACTOME_PHOSPHOLIPID_METABOLISM;REACTOME_CS_DS_DEGRAD
IPID_METABOLISM;REACTOME_PHOSPHOLIPID_METABOLISM;REACTOME_CS_DS_DEGRAD
BILE_SALT_METABOLISM;REACTOME_SYNTHESIS_OF_BILE_ACIDS_AND_BILE_SALTS_VIA_7
A_AMINOACYLATION;REACTOME_TRNA_AMINOACYLATION
A_AMINOACYLATION;REACTOME_TRNA_AMINOACYLATION

E_TRANSPORT_OF_SMALL_MOLECULES;REACTOME_ION_CHANNEL_TRANSPORT;REACTOM

_BIOLOGY;REACTOME_TRANSCRIPTIONAL_REGULATION_OF_WHITE_ADIPOCYTE_DIFFERE

ME_CELL_CYCLE;REACTOME_RNA_POL_I_TRANSCRIPTION;REACTOME_TRANSCRIPTION;R

WAY_FOR_APOPTOSIS;REACTOME_DEVELOPMENTAL_BIOLOGY;REACTOME_TRANSCRIPTI

_STEROID_HORMONES_AND_VITAMINS_A_AND_D;REACTOME_BIOLOGICAL_OXIDATIONS;R

_TRNA_AMINOACYLATION;REACTOME_TRNA_AMINOACYLATION

TOME_BETA_DEFENSINS;REACTOME_INNATE_IMMUNE_SYSTEM;REACTOME_IMMUNE_SYS

OSYLATION_OF_MUCINS;REACTOME_METABOLISM_OF_PROTEINS;REACTOME_POST_TRAN

TES_B_CELL_RECEPTOR_LEADING_TO_GENERATION_OF_SECOND_MESSENGERS;REACTO

FFICKING;REACTOME_ENDOSOMAL_SORTING_COMPLEX_REQUIRED_FOR_TRANSPORT_ES
RBB4;REACTOME_DOWNREGULATION_OF_ERBB2_ERBB3_SIGNALING;REACTOME_SIGNALI

OR_RECYCLING;REACTOME_NEF_MEDIATES_DOWN_MODULATION_OF_CELL_SURFACE_RE
ME_CELL_CYCLE;REACTOME_CHROMOSOME_MAINTENANCE;REACTOME_MEIOTIC_SYNAP
ELLULAR_DOMAIN_REGULATES_TRANSCRIPTION;REACTOME_SIGNALING_BY_NOTCH1;REA

_IRF7_ACTIVATION;REACTOME_FACTORS_INVOLVED_IN_MEGAKARYOCYTE_DEVELOPME

ELLULAR_DOMAIN_REGULATES_TRANSCRIPTION;REACTOME_SIGNALING_BY_NOTCH1;REA

NGF;REACTOME_ARMS_MEDIATED_ACTIVATION;REACTOME_PROLONGED_ERK_ACTIVATIO

_MRNA;REACTOME_METABOLISM_OF_RNA;REACTOME_NONSENSE_MEDIATED_DECAY_EN

_BIOLOGY;REACTOME_REGULATION_OF_APOPTOSIS;REACTOME_AXON_GUIDANCE;REAC

_BIOLOGY;REACTOME_REGULATION_OF_APOPTOSIS;REACTOME_AXON_GUIDANCE;REAC

NTIGEN_PRESENTATION;REACTOME_FACTORS_INVOLVED_IN_MEGAKARYOCYTE_DEVELO

SYLATION_OF_MUCINS;REACTOME_TERMINATION_OF_O_GLYCAN_BIOSYNTHESIS;REACT

ECEPTOR_INTERACTIONS;REACTOME_RESPONSE_TO_ELEVATED_PLATELET_CYTOSOLIC_

._BIOLOGY;REACTOME_AXON_GUIDANCE;REACTOME_NCAM1_INTERACTIONS;REACTOME_

CTOME_GLUCONEOGENESIS;REACTOME_METABOLISM_OF_CARBOHYDRATES;REACTOME_
CTOME_GLUCONEOGENESIS;REACTOME_METABOLISM_OF_CARBOHYDRATES;REACTOME_
FFICKING;REACTOME_TRANS_GOLGI_NETWORK_VESICLE_BUDDING;REACTOME_GOLGI_AS
VAYS;REACTOME_ACTIVATION_OF_CHAPERONE_GENES_BY_XBP1S;REACTOME_UNFOLDE

_STEROID_HORMONES_AND_VITAMINS_A_AND_D;REACTOME_BIOLOGICAL_OXIDATIONS;RI
_STEROID_HORMONES_AND_VITAMINS_A_AND_D;REACTOME_BIOLOGICAL_OXIDATIONS;RI

LE_CONTRACTION;REACTOME_MUSCLE_CONTRACTION

._BIOLOGY;REACTOME_PPARA_ACTIVATES_GENE_EXPRESSION;REACTOME_METABOLISM_

MODELLING_OF_PI;REACTOME_ACYL_CHAIN_REMODELLING_OF_PC;REACTOME_PHOSPHO

VED_IN_MEGAKARYOCYTE_DEVELOPMENT_AND_PLATELET_PRODUCTION;REACTOME_HEM

PTERIN_BH4_SYNTHESIS_RECYCLING_SALVAGE_AND_REGULATION;REACTOME_ENOS_AC

CTOME_CELL_CYCLE_MITOTIC;REACTOME_CYCLIN_E_ASSOCIATED_EVENTS_DURING_G1_
CTOME_CELL_CYCLE_MITOTIC;REACTOME_CYCLIN_E_ASSOCIATED_EVENTS_DURING_G1_

_STEROID_HORMONES_AND_VITAMINS_A_AND_D;REACTOME_TRAFFICKING_AND_PROCES

;REACTOME_ADAPTIVE_IMMUNE_SYSTEM;REACTOME_CLASS_I_MHC_MEDIATED_ANTIGEN

_BIOLOGY;REACTOME_AXON_GUIDANCE;REACTOME_NCAM_SIGNALING_FOR_NEURITE_O

ACTOME_FORMATION_OF_THE_TERNARY_COMPLEX_AND_SUBSEQUENTLY_THE_43S_CON

NGF;REACTOME_SIGNALING_BY_SCF_KIT;REACTOME_DEVELOPMENTAL_BIOLOGY;REACTC
NGF;REACTOME_NGF_SIGNALLING_VIA_TRKA_FROM_THE_PLASMA_MEMBRANE

ACROSS_CHEMICAL_SYNAPSES;REACTOME_NEURONAL_SYSTEM;REACTOME_NEUROTRAN

NGF;REACTOME_DEVELOPMENTAL_BIOLOGY;REACTOME_TRIF_MEDIATED_TLR3_SIGNALIN

_MRNA;REACTOME_METABOLISM_OF_RNA;REACTOME_NONSENSE_MEDIATED_DECAY_EN
OR_SIGNALLING_CASCADE;REACTOME_PKB_MEDIATED_EVENTS;REACTOME_SIGNALING_E
NSCRIPTION_INITIATION_FROM_TYPE_2_PROMOTER;REACTOME_RNA_POL_III_TRANSCRIP

NTERACTIONS_AT_THE_VASCULAR_WALL;REACTOME_INTEGRIN_CELL_SURFACE_INTERA

_TRIC_CCT_WITH_TARGET_PROTEINS_DURING_BIOSYNTHESIS;REACTOME_PROTEIN_FOL

:YTOSOLIC_CA2_LEVELS;REACTOME_PLATELET_HOMEOSTASIS;REACTOME_PLATELET_CA

._BIOLOGY;REACTOME_PPARA_ACTIVATES_GENE_EXPRESSION;REACTOME_RORA_ACTIVA

HO_GTPASES

IOSYNTHESIS;REACTOME_FATTY_ACYL_COA_BIOSYNTHESIS;REACTOME_INTEGRATION_O

._BIOLOGY;REACTOME_BMAL1_CLOCK_NPAS2_ACTIVATES_CIRCADIAN_EXPRESSION;REAC

E_TRANSPORT_OF_SMALL_MOLECULES;REACTOME_SLC_MEDIATED_TRANSMEMBRANE_TI

._BIOLOGY;REACTOME_AXON_GUIDANCE;REACTOME_NCAM1_INTERACTIONS;REACTOME_

NSCRIPTION;REACTOME_MRNA_CAPPING;REACTOME_MRNA_PROCESSING;REACTOME_TR

._BIOLOGY;REACTOME_MYOGENESIS

E_TRANSPORT_OF_SMALL_MOLECULES;REACTOME_ION_TRANSPORT_BY_P_TYPE_ATPAS

HO_GTPASES
HO_GTPASES
._BIOLOGY;REACTOME_MYOGENESIS
._BIOLOGY;REACTOME_MYOGENESIS

._BIOLOGY;REACTOME_PPARA_ACTIVATES_GENE_EXPRESSION;REACTOME_METABOLISM_

)SYLATION_OF_MUCINS;REACTOME_TERMINATION_OF_O_GLYCAN_BIOSYNTHESIS;REACT(
)SYLATION_OF_MUCINS;REACTOME_TERMINATION_OF_O_GLYCAN_BIOSYNTHESIS;REACT(
)SYLATION_OF_MUCINS;REACTOME_TERMINATION_OF_O_GLYCAN_BIOSYNTHESIS;REACT(
)SYLATION_OF_MUCINS;REACTOME_TERMINATION_OF_O_GLYCAN_BIOSYNTHESIS;REACT(

CRIPTION_PATHWAY

MAD4_HETEROTRIMER_REGULATES_TRANSCRIPTION;REACTOME_TRANSCRIPTIONAL_ACT

_AMINO_ACIDS_AND_DERIVATIVES;REACTOME_AMINE_DERIVED_HORMONES;REACTOME_

NGF;REACTOME_P75_NTR_RECEPTOR_MEDIATED_SIGNALLING;REACTOME_SIGNALING_BY
ACROSS_CHEMICAL_SYNAPSES;REACTOME_NEURONAL_SYSTEM;REACTOME_NEUROTRAN
ACROSS_CHEMICAL_SYNAPSES;REACTOME_NEURONAL_SYSTEM;REACTOME_NEUROTRAN

_PROTEINS;REACTOME_POST_TRANSLATIONAL_MODIFICATION_SYNTHESIS_OF_GPI_ANCH
_PROTEINS;REACTOME_POST_TRANSLATIONAL_MODIFICATION_SYNTHESIS_OF_GPI_ANCH

CRIPTION_PATHWAY

MUNICATION;REACTOME_SIGNAL_REGULATORY_PROTEIN_SIRP_FAMILY_INTERACTIONS

:IGNALLING_PATHWAY_VIA_PKC_AND_MAPK;REACTOME_TRANSMISSION_ACROSS_CHEMI(

.S;REACTOME_REGULATION_OF_SIGNALING_BY_CBL;REACTOME_IL_3_5_AND_GM_CSF_SI(
._BIOLOGY;REACTOME_BMAL1_CLOCK_NPAS2_ACTIVATES_CIRCADIAN_EXPRESSION;REAC
._BIOLOGY;REACTOME_BMAL1_CLOCK_NPAS2_ACTIVATES_CIRCADIAN_EXPRESSION;REAC

ACID_METABOLISM;REACTOME_METABOLISM_OF_AMINO_ACIDS_AND_DERIVATIVES

_AMINO_ACIDS_AND_DERIVATIVES

'RESSION_AND_PROCESSING;REACTOME_PRE_NOTCH_PROCESSING_IN_GOLGI;REACTOM

._BIOLOGY;REACTOME_BMAL1_CLOCK_NPAS2_ACTIVATES_CIRCADIAN_EXPRESSION;REAC
:S_GENE_EXPRESSION;REACTOME_METABOLISM_OF_LIPIDS_AND_LIPOPROTEINS;REACTC
ME_CELL_CYCLE;REACTOME_CHROMOSOME_MAINTENANCE;REACTOME_MEIOTIC_SYNAP

D_PROCESSING_OF_ENDOSOMAL_TLR;REACTOME_TRAF6_MEDIATED_INDUCTION_OF_NFk
D_PROCESSING_OF_ENDOSOMAL_TLR;REACTOME_TRAF6_MEDIATED_INDUCTION_OF_NFk
.E_CONTRACTION;REACTOME_MUSCLE_CONTRACTION
DATIONS;REACTOME_TRANSMISSION_ACROSS_CHEMICAL_SYNAPSES;REACTOME_NEURO

_RESPIRATORY_ELECTRON_TRANSPORT;REACTOME_RESPIRATORY_ELECTRON_TRANSPO

._BIOLOGY;REACTOME_PPARA_ACTIVATES_GENE_EXPRESSION;REACTOME_GENERIC_TRA

HO_GTPASES;REACTOME_SIGNALLING_BY_NGF;REACTOME_CELL_CELL_COMMUNICATION

AVAGE_OF_CELLULAR_PROTEINS;REACTOME_APOPTOSIS;REACTOME_APOPTOTIC_EXECU

PHA_BETA_SIGNALING;REACTOME_INTERFERON_SIGNALING;REACTOME_IMMUNE_SYSTEM

_BIOLOGY;REACTOME_TCR_SIGNALING;REACTOME_GENERATION_OF_SECOND_MESSEN

HE_PROTEOLYTIC_ACTIVITY_OF_APC_C_REQUIRED_FOR_THE_ONSET_OF_ANAPHASE_BY_

_PROTEIN_IMPORT;REACTOME_GLUCONEOGENESIS;REACTOME_METABOLISM_OF_PROTE
;REACTOME_ADAPTIVE_IMMUNE_SYSTEM;REACTOME_CLASS_I_MHC_MEDIATED_ANTIGEN

SIGNALLING_PATHWAY_VIA_PKC_AND_MAPK;REACTOME_SIGNALING_BY_GPCR;REACTOME
E_TRANSPORT_OF_SMALL_MOLECULES;REACTOME_SLC_MEDIATED_TRANSMEMBRANE_T
E_TRANSPORT_OF_SMALL_MOLECULES;REACTOME_SLC_MEDIATED_TRANSMEMBRANE_T

CRIPTION_PATHWAY
_BIOLOGY;REACTOME_AXON_GUIDANCE;REACTOME_ACTIVATION_OF_RAC;REACTOME_SI

NSPORT_ACROSS_THE_PLASMA_MEMBRANE;REACTOME_TRANSMEMBRANE_TRANSPORT

_MATRIX_ORGANIZATION;REACTOME_COLLAGEN_FORMATION
_MATRIX_ORGANIZATION;REACTOME_COLLAGEN_FORMATION

ME_CELL_CYCLE;REACTOME_CHROMOSOME_MAINTENANCE;REACTOME_MEIOTIC_SYNAP

CH1_TRANSMITS_SIGNAL_TO_THE_NUCLEUS;REACTOME_SIGNALING_BY_NOTCH1;REACTO

DATIONS;REACTOME_GLUTATHIONE_CONJUGATION;REACTOME_PHASE_II_CONJUGATION

NGF;REACTOME_SIGNALING_BY_SCF_KIT;REACTOME_SIGNALING_BY_ERBB4;REACTOME_S

RBB4;REACTOME_DOWNREGULATION_OF_ERBB2_ERBB3_SIGNALING;REACTOME_SIGNALI
REPAIR;REACTOME_RESOLUTION_OF_AP_SITES_VIA_THE_MULTIPLE_NUCLEOTIDE_PATCH

SIGNALLING_PATHWAY_VIA_PKC_AND_MAPK;REACTOME_SIGNALING_BY_GPCR;REACTOME
_AMINO_ACIDS_AND_DERIVATIVES

BILE_SALT_METABOLISM;REACTOME_SYNTHESIS_OF_BILE_ACIDS_AND_BILE_SALTS_VIA_7

CTOME_CELL_CYCLE_MITOTIC;REACTOME_RECRUITMENT_OF_MITOTIC_CENTROSOME_P

TORY_INTERACTIONS_BETWEEN_A_LYMPHOID_AND_A_NON_LYMPHOID_CELL;REACTOME_

_BIOLOGY;REACTOME_BMAL1_CLOCK_NPAS2_ACTIVATES_CIRCADIAN_EXPRESSION;REAC

ANISM_BY_IFN_STIMULATED_GENES;REACTOME_INTERFERON_ALPHA_BETA_SIGNALING;F

;REACTOME_ADAPTIVE_IMMUNE_SYSTEM;REACTOME_CLASS_I_MHC_MEDIATED_ANTIGEN

CH1_TRANSMITS_SIGNAL_TO_THE_NUCLEUS;REACTOME_SIGNALING_BY_NOTCH1;REACTO

PTOR_SIGNALING_IN_EMT_EPITHELIAL_TO_MESENCHYMAL_TRANSITION;REACTOME_GPVI_

ICED_DNA_FRAGMENTATION;REACTOME_APOPTOSIS;REACTOME_APOPTOTIC_EXECUTION
IPPO
IPPO

E_TRANSPORT_OF_SMALL_MOLECULES;REACTOME_SLC_MEDIATED_TRANSMEMBRANE_TI

_MATRIX_ORGANIZATION;REACTOME_COLLAGEN_FORMATION

NGF;REACTOME_SIGNALING_BY_SCF_KIT;REACTOME_SIGNALING_BY_ERBB4;REACTOME_S

PCR;REACTOME_CLASS_C_3_METABOTROPIC_GLUTAMATE_PHEROMONE_RECEPTORS;RE
;REACTOME_ADAPTIVE_IMMUNE_SYSTEM;REACTOME_CLASS_I_MHC_MEDIATED_ANTIGEN
;REACTOME_ADAPTIVE_IMMUNE_SYSTEM;REACTOME_CLASS_I_MHC_MEDIATED_ANTIGEN

G;REACTOME_IMMUNE_SYSTEM;REACTOME_ADAPTIVE_IMMUNE_SYSTEM
DATION;REACTOME_CHONDROITIN_SULFATE_DERMATAN_SULFATE_METABOLISM;REACTO
DATION;REACTOME_CHONDROITIN_SULFATE_DERMATAN_SULFATE_METABOLISM;REACTO

_CARBOHYDRATES
_LIPIDS_AND_LIPOPROTEINS;REACTOME_FATTY_ACID_TRIACYLGLYCEROL_AND_KETONE_

_PROTEINS;REACTOME_POST_TRANSLATIONAL_PROTEIN_MODIFICATION;REACTOME_ASP

NGF;REACTOME_DEVELOPMENTAL_BIOLOGY;REACTOME_TRIF_MEDIATED_TLR3_SIGNALIN

PTAKE_AND_DEGRADATION;REACTOME_HYALURONAN_METABOLISM;REACTOME_GLYCOSA
PTAKE_AND_DEGRADATION;REACTOME_HYALURONAN_METABOLISM;REACTOME_GLYCOSA

CF_KIT;REACTOME_REGULATION_OF_KIT_SIGNALING;REACTOME_NEF_MEDIATES_DOWN_

EM;REACTOME_VOLTAGE_GATED_POTASSIUM_CHANNELS;REACTOME_POTASSIUM_CHAN
EM;REACTOME_VOLTAGE_GATED_POTASSIUM_CHANNELS;REACTOME_POTASSIUM_CHAN

_CAPPED_INTRON_CONTAINING_PRE_MRNA;REACTOME_MRNA_PROCESSING;REACTOME_
_BIOLOGY;REACTOME_PPARA_ACTIVATES_GENE_EXPRESSION;REACTOME_GENERIC_TRA

ON_OF_FGF_SIGNALING;REACTOME_NEGATIVE_REGULATION_OF_FGFR_SIGNALING;REACT

OSTASIS;REACTOME_PLATELET_SENSITIZATION_BY_LDL;REACTOME_HEMOSTASIS

K_STIMULATES_FATTY_ACID_OXIDATION_IN_MUSCLE;REACTOME_INSULIN_RECEPTOR_SIG

REACTOME_ANTIVIRAL_MECHANISM_BY_IFN_STIMULATED_GENES;REACTOME_SIGNALING_

_PROTEINS;REACTOME_POST_TRANSLATIONAL_MODIFICATION_SYNTHESIS_OF_GPI_ANCH

SIGNALLING_PATHWAY_VIA_PKC_AND_MAPK;REACTOME_SIGNALING_BY_GPCR;REACTOME

OTEINS_MEDIATED_TRANSPORT;REACTOME_TRANSMEMBRANE_TRANSPORT_OF_SMALL_M
OTEINS_MEDIATED_TRANSPORT;REACTOME_TRANSMEMBRANE_TRANSPORT_OF_SMALL_M

_MATRIX_ORGANIZATION;REACTOME_COLLAGEN_FORMATION

NSCRIPTION_AND_TRANSLATION;REACTOME_PRE_NOTCH_EXPRESSION_AND_PROCESSIN
HO_GTPASES;REACTOME_DEVELOPMENTAL_BIOLOGY;REACTOME_SIGNALING_BY_GPCR;R

_NUCLEOTIDES;REACTOME_PURINE_SALVAGE;REACTOME_PURINE_METABOLISM
_NUCLEOTIDES;REACTOME_PURINE_SALVAGE;REACTOME_PURINE_METABOLISM

NSCRIPTION_AND_TRANSLATION;REACTOME_SIGNALING_BY_NOTCH2;REACTOME_PRE_N
NSCRIPTION_AND_TRANSLATION;REACTOME_SIGNALING_BY_NOTCH2;REACTOME_PRE_N

NSCRIPTION_INITIATION_FROM_TYPE_2_PROMOTER;REACTOME_RNA_POL_III_TRANSCRIP

_TRNA_AMINOACYLATION;REACTOME_TRNA_AMINOACYLATION
PIPS_AT_THE_GOLGI_MEMBRANE;REACTOME_PHOSPHOLIPID_METABOLISM;REACTOME_PI

;REACTOME_ADAPTIVE_IMMUNE_SYSTEM;REACTOME_CLASS_I_MHC_MEDIATED_ANTIGEN
_PROTEIN_IMPORT;REACTOME_METABOLISM_OF_PROTEINS

CTOME_CELL_CYCLE_MITOTIC;REACTOME_MITOTIC_M_M_G1_PHASES;REACTOME_DNA_R
HO_GTPASES;REACTOME_SIGNALLING_BY_NGF;REACTOME_DEVELOPMENTAL_BIOLOGY;R

_SYSTEM;REACTOME_IMMUNE_SYSTEM;REACTOME_COMPLEMENT_CASCADE;REACTOME_

PCR;REACTOME_GPCR_DOWNSTREAM_SIGNALING;REACTOME_G_ALPHA_I_SIGNALLING_E

NSCRIPTION;REACTOME_TRANSCRIPTION;REACTOME_RNA_POL_I_RNA_POL_III_AND_MITC

NTERACTIONS_AT_THE_VASCULAR_WALL;REACTOME_RESPONSE_TO_ELEVATED_PLATEL

VAYS;REACTOME_REGULATION_OF_INSULIN_LIKE_GROWTH_FACTOR_IGF_ACTIVITY_BY_IN

_BIOLOGY;REACTOME_AXON_GUIDANCE;REACTOME_SIGNALING_BY_ROBO_RECEPTOR

_NON_CODING_RNA;REACTOME_ANTIVIRAL_MECHANISM_BY_IFN_STIMULATED_GENES;RE

_BIOLOGY;REACTOME_AXON_GUIDANCE;REACTOME_NCAM1_INTERACTIONS;REACTOME_

_PROTEIN_IMPORT;REACTOME_METABOLISM_OF_PROTEINS
IETABOLISM;REACTOME_SYNTHESIS_OF_PE;REACTOME_GLYCEROPHOSPHOLIPID_BIOSYN
_BIOLOGY;REACTOME_AXON_GUIDANCE;REACTOME_NCAM1_INTERACTIONS;REACTOME_
_COMPLEMENT_CASCADE;REACTOME_INNATE_IMMUNE_SYSTEM;REACTOME_IMMUNE_SYS

_BIOLOGY;REACTOME_AXON_GUIDANCE;REACTOME_CRMPS_IN_SEMA3A_SIGNALING;REA
_STEROID_HORMONES_AND_VITAMINS_A_AND_D;REACTOME_STEROID_HORMONES;REAC

E_TRANSPORT_OF_SMALL_MOLECULES;REACTOME_IRON_UPTAKE_AND_TRANSPORT

CRIPTION_PATHWAY;REACTOME_NUCLEAR_RECEPTOR_TRANSCRIPTION_PATHWAY

_TRNA_AMINOACYLATION;REACTOME_TRNA_AMINOACYLATION
_TRNA_AMINOACYLATION;REACTOME_TRNA_AMINOACYLATION

_BIOLOGY;REACTOME_SIGNALING_BY_NODAL;REACTOME_RESPONSE_TO_ELEVATED_PL

HO_GTPASES;REACTOME_SIGNALLING_BY_NGF;REACTOME_NRAGE_SIGNALS_DEATH_THR

'EM;REACTOME_TANDEM_PORE_DOMAIN_POTASSIUM_CHANNELS;REACTOME_POTASSIUM

NGF;REACTOME_SIGNALING_BY_SCF_KIT;REACTOME_DEVELOPMENTAL_BIOLOGY;REACTC

CRIPTION_PATHWAY

_AMINO_ACIDS_AND_DERIVATIVES;REACTOME_AMINE_DERIVED_HORMONES

MODELLING_OF_PC;REACTOME_PHOSPHOLIPID_METABOLISM;REACTOME_ACYL_CHAIN_RE
NGF;REACTOME_GROWTH_HORMONE_RECEPTOR_SIGNALING;REACTOME_SIGNALING_BY_

'EM;REACTOME_INTEGRATION_OF_ENERGY_METABOLISM;REACTOME_REGULATION_OF_IN

_BIOLOGY;REACTOME_BMAL1_CLOCK_NPAS2_ACTIVATES_CIRCADIAN_EXPRESSION;REAC

_NUCLEOTIDES;REACTOME_PYRIMIDINE_METABOLISM
_NUCLEOTIDES;REACTOME_PYRIMIDINE_METABOLISM

E_TRANSPORT_OF_SMALL_MOLECULES;REACTOME_SLC_MEDIATED_TRANSMEMBRANE_TI

_BIOLOGY;REACTOME_CELL_CELL_COMMUNICATION;REACTOME_AXON_GUIDANCE;REACT
_BIOLOGY;REACTOME_CELL_CELL_COMMUNICATION;REACTOME_AXON_GUIDANCE;REACT
NGF;REACTOME_P75_NTR_RECEPTOR_MEDIATED_SIGNALLING

DATIONS;REACTOME_METABOLISM_OF_CARBOHYDRATES;REACTOME_GLUCURONIDATION
S;REACTOME_IL1_SIGNALING;REACTOME_IRAK1_RECRUITS_IKK_COMPLEX;REACTOME_TF
HO_GTPASES

RBOXYLATION_HYPUSINE_FORMATION_AND_ARYLSULFATASE_ACTIVATION;REACTOME_GA

_CAPPED_INTRON_CONTAINING_PRE_MRNA;REACTOME_MRNA_PROCESSING;REACTOME_

CTOME_CELL_CYCLE_MITOTIC;REACTOME_MITOTIC_M_M_G1_PHASES;REACTOME_DNA_R

NGF;REACTOME_SIGNALLING_TO_RAS;REACTOME_NGF_SIGNALLING_VIA_TRKA_FROM_TH
RBOXYLATION_HYPUSINE_FORMATION_AND_ARYLSULFATASE_ACTIVATION;REACTOME_GA

IPID_METABOLISM;REACTOME_PHOSPHOLIPID_METABOLISM;REACTOME_SPHINGOLIPID_M

PCR;REACTOME_CLASS_A1_RHODOPSIN_LIKE_RECEPTORS;REACTOME_GPCR_LIGAND_BI

LE_CONTRACTION;REACTOME_MUSCLE_CONTRACTION
FFICKING;REACTOME_SIGNALING_BY_EGFR_IN_CANCER;REACTOME_EGFR_DOWNREGULA

_ENERGY_METABOLISM;REACTOME_REGULATION_OF_INSULIN_SECRETION;REACTOME_S
_ENERGY_METABOLISM;REACTOME_REGULATION_OF_INSULIN_SECRETION;REACTOME_S

BILE_SALT_METABOLISM;REACTOME_SYNTHESIS_OF_BILE_ACIDS_AND_BILE_SALTS_VIA_7A

CTOME_MHC_CLASS_II_ANTIGEN_PRESENTATION;REACTOME_CELL_CYCLE_MITOTIC;REA
SURFACE_INTERACTIONS;REACTOME_INTEGRATION_OF_ENERGY_METABOLISM;REACTOM

CROSS_CHEMICAL_SYNAPSES;REACTOME_NEURONAL_SYSTEM;REACTOME_NEUROTRAN

PID_METABOLISM;REACTOME_METABOLISM_OF_LIPIDS_AND_LIPOPROTEINS
PCR;REACTOME_GPCR_DOWNSTREAM_SIGNALING;REACTOME_G_ALPHA_S_SIGNALLING_

_BIOLOGY;REACTOME_ANTIGEN_PROCESSING_CROSS_PRESENTATION;REACTOME_CELL_
_BIOLOGY;REACTOME_EXTRACELLULAR_MATRIX_ORGANIZATION;REACTOME_COLLAGEN_

CF_KIT;REACTOME_GROWTH_HORMONE_RECEPTOR_SIGNALING;REACTOME_ANTIVIRAL_

ACTOME_METABOLISM_OF_PROTEINS

_AMINO_ACIDS_AND_DERIVATIVES

N_OF_HOMO_SAPIENS_WITH_MYCOBACTERIUM_TUBERCULOSIS;REACTOME_TRANSMEM

E_CONTRACTION;REACTOME_MUSCLE_CONTRACTION

NGF;REACTOME_GROWTH_HORMONE_RECEPTOR_SIGNALING;REACTOME_INSULIN_RECE

SIGNALLING_PATHWAY_VIA_PKC_AND_MAPK;REACTOME_SIGNALING_BY_GPCR;REACTOME

PAS2_ACTIVATES_CIRCADIAN_EXPRESSION;REACTOME_CIRCADIAN_CLOCK

_BIOLOGY;REACTOME_AXON_GUIDANCE;REACTOME_L1CAM_INTERACTIONS

_THE_FANCONI_ANEMIA_PATHWAY;REACTOME_FANCONI_ANEMIA_PATHWAY;REACTOME_
ACROSS_CHEMICAL_SYNAPSES;REACTOME_NEURONAL_SYSTEM;REACTOME_NEUROTRAN

NGF;REACTOME_SIGNALING_BY_SCF_KIT;REACTOME_DEVELOPMENTAL_BIOLOGY;REACTC

EM;REACTOME_VOLTAGE_GATED_POTASSIUM_CHANNELS;REACTOME_POTASSIUM_CHAN
CRIPTION_PATHWAY;REACTOME_NUCLEAR_RECEPTOR_TRANSCRIPTION_PATHWAY

E_TRANSPORT_OF_SMALL_MOLECULES;REACTOME_SLC_MEDIATED_TRANSMEMBRANE_T
PTOR_SIGNALING_IN_EMT_EPITHELIAL_TO_MESENCHYMAL_TRANSITION;REACTOME_DOW

ME_MEIOTIC_RECOMBINATION

PCR;REACTOME_PEPTIDE_LIGAND_BINDING_RECEPTORS;REACTOME_CLASS_A1_RHODOF

_MATRIX_ORGANIZATION;REACTOME_COLLAGEN_FORMATION

ES_GENE_EXPRESSION;REACTOME_METABOLISM_OF_LIPIDS_AND_LIPOPROTEINS;REACTC

ACROSS_CHEMICAL_SYNAPSES;REACTOME_NEURONAL_SYSTEM
ACROSS_CHEMICAL_SYNAPSES;REACTOME_NEURONAL_SYSTEM
GFR_IN_DISEASE;REACTOME_SIGNALING_BY_FGFR
_APOPTOSIS;REACTOME_ROLE_OF_DCC_IN_REGULATING_APOPTOSIS;REACTOME_APOPT

_AMINO_ACIDS_AND_DERIVATIVES
_BIOLOGY;REACTOME_AXON_GUIDANCE;REACTOME_CRMPS_IN_SEMA3A_SIGNALING;REA

ME_CELL_CYCLE;REACTOME_CELL_CYCLE_CHECKPOINTS;REACTOME_REGULATION_OF_T
_MATRIX_ORGANIZATION;REACTOME_COLLAGEN_FORMATION

AL_ACTIVITY_OF_SMAD2_SMAD3_SMAD4_HETEROTRIMER;REACTOME_DOWNREGULATION_

EM;REACTOME_CGMP_EFFECTS;REACTOME_NITRIC_OXIDE_STIMULATES_GUANYLATE_CY

_AMINO_ACIDS_AND_DERIVATIVES;REACTOME_BRANCHED_CHAIN_AMINO_ACID_CATABOL

PCR;REACTOME_PEPTIDE_LIGAND_BINDING_RECEPTORS;REACTOME_CLASS_A1_RHODOP

OSYLATION_OF_MUCINS;REACTOME_TERMINATION_OF_O_GLYCAN_BIOSYNTHESIS;REACTO

VAYS;REACTOME_ACTIVATION_OF_CHAPERONE_GENES_BY_XBP1S;REACTOME_UNFOLDE

LATORS_OF_RIG_I_MDA5_SIGNALING;REACTOME_RIG_I_MDA5_MEDIATED_INDUCTION_OF_

LATORS_OF_RIG_I_MDA5_SIGNALING;REACTOME_RIG_I_MDA5_MEDIATED_INDUCTION_OF_

HO_GTPASES

ACROSS_CHEMICAL_SYNAPSES;REACTOME_NEURONAL_SYSTEM;REACTOME_NEUROTRAN

VAYS;REACTOME_INSULIN_SYNTHESIS_AND_PROCESSING

DATIONS;REACTOME_GLUCURONIDATION;REACTOME_PHASE_II_CONJUGATION

E_TRANSPORT_OF_SMALL_MOLECULES;REACTOME_SLC_MEDIATED_TRANSMEMBRANE_TI

_MATRIX_ORGANIZATION;REACTOME_COLLAGEN_FORMATION

G1;REACTOME_CELL_CYCLE;REACTOME_CELL_CYCLE_MITOTIC;REACTOME_MITOTIC_G1_(

HO_GTPASES

THESIS;REACTOME_HEPARAN_SULFATE_HEPARIN_HS_GAG_METABOLISM;REACTOME_GLY

OSYLATION_OF_MUCINS;REACTOME_METABOLISM_OF_PROTEINS;REACTOME_POST_TRAN

E_TRANSPORT_OF_SMALL_MOLECULES;REACTOME_SLC_MEDIATED_TRANSMEMBRANE_T
CTOME_CELL_CYCLE_MITOTIC;REACTOME_RECRUITMENT_OF_MITOTIC_CENTROSOME_P

E_TRANSPORT_OF_SMALL_MOLECULES;REACTOME_SLC_MEDIATED_TRANSMEMBRANE_T

HO_GTPASES;REACTOME_SIGNALLING_BY_NGF;REACTOME_DEVELOPMENTAL_BIOLOGY;R
HO_GTPASES;REACTOME_SIGNALLING_BY_NGF;REACTOME_DEVELOPMENTAL_BIOLOGY;R

K_STIMULATES_FATTY_ACID_OXIDATION_IN_MUSCLE;REACTOME_INSULIN_RECEPTOR_SIG
_LIPIDS_AND_LIPOPROTEINS;REACTOME_FATTY_ACID_TRIACYLGLYCEROL_AND_KETONE_
ES_GENE_EXPRESSION;REACTOME_METABOLISM_OF_LIPIDS_AND_LIPOPROTEINS;REACTO

PCR;REACTOME_OPIOID_SIGNALLING;REACTOME_DARPP_32_EVENTS;REACTOME_GPCR_I

CTOME_CELL_CYCLE_MITOTIC;REACTOME_MITOTIC_M_M_G1_PHASES;REACTOME_CHROM

NGF;REACTOME_SIGNALING_BY_SCF_KIT;REACTOME_CELL_CELL_COMMUNICATION;REACT
CTOME_CELL_CYCLE_CHECKPOINTS;REACTOME_ACTIVATION_OF_ATR_IN_RESPONSE_TO

ATION;REACTOME_CHONDROITIN_SULFATE_BIOSYNTHESIS;REACTOME_CHONDROITIN_SUL

RBOXYLATION_HYPUSINE_FORMATION_AND_ARYLSULFATASE_ACTIVATION;REACTOME_GL

_BIOLOGY;REACTOME_AXON_GUIDANCE;REACTOME_OTHER_SEMAPHORIN_INTERACTION

CTOME_CELL_CYCLE_MITOTIC;REACTOME_CELL_CYCLE_CHECKPOINTS;REACTOME_CYCL
CTOME_CELL_CYCLE_MITOTIC;REACTOME_CELL_CYCLE_CHECKPOINTS;REACTOME_CYCL
PHA_BETA_SIGNALING;REACTOME_INTERFERON_SIGNALING;REACTOME_IMMUNE_SYSTEM
PHA_BETA_SIGNALING;REACTOME_INTERFERON_SIGNALING;REACTOME_IMMUNE_SYSTEM

/NT;REACTOME_CTNNB1_PHOSPHORYLATION_CASCADE
REACTOME_NITRIC_OXIDE_STIMULATES_GUANYLATE_CYCLASE;REACTOME_PLATELET_HC

IPPO
A_AMINOACYLATION;REACTOME_TRNA_AMINOACYLATION
._BIOLOGY;REACTOME_AXON_GUIDANCE;REACTOME_NETRIN1_SIGNALING

LATION_OF_FGFR_SIGNALING;REACTOME_INSULIN_RECEPTOR_SIGNALLING_CASCADE;RE
LATION_OF_FGFR_SIGNALING;REACTOME_INSULIN_RECEPTOR_SIGNALLING_CASCADE;RE
LATION_OF_FGFR_SIGNALING;REACTOME_INSULIN_RECEPTOR_SIGNALLING_CASCADE;RE

ACTOME_SRP_DEPENDENT_COTRANSLATIONAL_PROTEIN_TARGETING_TO_MEMBRANE;RE
PCR;REACTOME_CLASS_C_3_METABOTROPIC_GLUTAMATE_PHEROMONE_RECEPTORS;RE

_PROTEINS;REACTOME_POST_TRANSLATIONAL_PROTEIN_MODIFICATION;REACTOME_ASP
_PROTEINS;REACTOME_POST_TRANSLATIONAL_PROTEIN_MODIFICATION;REACTOME_SYN

FFICKING;REACTOME_TRANS_GOLGI_NETWORK_VESICLE_BUDDING;REACTOME_GOLGI_AS

CRIPTION_PATHWAY

ECOMBINATION_REPAIR_OF_REPLICATION_INDEPENDENT_DOUBLE_STRAND_BREAKS;REA

A_AMINOACYLATION;REACTOME_TRNA_AMINOACYLATION

_MATRIX_ORGANIZATION;REACTOME_COLLAGEN_FORMATION
_MATRIX_ORGANIZATION;REACTOME_COLLAGEN_FORMATION

NGF;REACTOME_DAG_AND_IP3_SIGNALING;REACTOME_SIGNALING_BY_ERBB2;REACTOME_

CRIPTION_PATHWAY;REACTOME_NUCLEAR_RECEPTOR_TRANSCRIPTION_PATHWAY
CRIPTION_PATHWAY;REACTOME_NUCLEAR_RECEPTOR_TRANSCRIPTION_PATHWAY
CRIPTION_PATHWAY;REACTOME_NUCLEAR_RECEPTOR_TRANSCRIPTION_PATHWAY
ACTOME_SRP_DEPENDENT_COTRANSLATIONAL_PROTEIN_TARGETING_TO_MEMBRANE;RE

GFR_IN_DISEASE;REACTOME_FRS2_MEDIATED_CASCADE;REACTOME_DOWNSTREAM_SIGI

A_BIOGENESIS;REACTOME_REGULATORY_RNA_PATHWAYS

_MATRIX_ORGANIZATION;REACTOME_COLLAGEN_FORMATION

_AMINO_ACIDS_AND_DERIVATIVES;REACTOME_BRANCHED_CHAIN_AMINO_ACID_CATABOL

E_TRANSPORT_OF_SMALL_MOLECULES;REACTOME_SLC_MEDIATED_TRANSMEMBRANE_TI

CRIPTION_PATHWAY;REACTOME_NUCLEAR_RECEPTOR_TRANSCRIPTION_PATHWAY

SURFACE_INTERACTIONS
SURFACE_INTERACTIONS

ROTOXICITY;REACTOME_PROTEOLYTIC_CLEAVAGE_OF_SNARE_COMPLEX_PROTEINS;REA

JLFATE_DERMATAN_SULFATE_METABOLISM;REACTOME_GLYCOSAMINOGLYCAN_METABOL

ME_CELL_CYCLE;REACTOME_CHROMOSOME_MAINTENANCE;REACTOME_MEIOTIC_SYNAP
ME_CELL_CYCLE;REACTOME_CHROMOSOME_MAINTENANCE;REACTOME_MEIOTIC_SYNAP
ME_CELL_CYCLE;REACTOME_CHROMOSOME_MAINTENANCE;REACTOME_MEIOTIC_SYNAP

_LIPIDS_AND_LIPOPROTEINS;REACTOME_LIPID_DIGESTION_MOBILIZATION_AND_TRANSPO

;REACTOME_ADAPTIVE_IMMUNE_SYSTEM;REACTOME_CLASS_I_MHC_MEDIATED_ANTIGEN

JLFATE_BIOSYNTHESIS;REACTOME_CHONDROITIN_SULFATE_DERMATAN_SULFATE_METAE
IGNALING_EVENTS_OF_B_CELL_RECEPTOR_BCR;REACTOME_ACTIVATION_OF_NF_KAPPAE

SIGNALLING_PATHWAY_VIA_PKC_AND_MAPK;REACTOME_SIGNALING_BY_GPCR;REACTOME

_MATRIX_ORGANIZATION;REACTOME_COLLAGEN_FORMATION

)TEINS_MEDIATED_TRANSPORT;REACTOME_TRANSMEMBRANE_TRANSPORT_OF_SMALL_M

_AMINO_ACIDS_AND_DERIVATIVES;REACTOME_BRANCHED_CHAIN_AMINO_ACID_CATABOL
TLR3_SIGNALING;REACTOME_TAK1_ACTIVATES_NFKB_BY_PHOSPHORYLATION_AND_ACTIV

_TRIC_CCT_WITH_TARGET_PROTEINS_DURING_BIOSYNTHESIS;REACTOME_PROTEIN_FOL

BOLISM_AND_CITRIC_ACID_TCA_CYCLE;REACTOME_TCA_CYCLE_AND_RESPIRATORY_ELE
NGF;REACTOME_DAG_AND_IP3_SIGNALING;REACTOME_SIGNALING_BY_ERBB2;REACTOME_
VAYS;REACTOME_REGULATION_OF_INSULIN_LIKE_GROWTH_FACTOR_IGF_ACTIVITY_BY_IN

OSYLATION_OF_MUCINS;REACTOME_METABOLISM_OF_PROTEINS;REACTOME_POST_TRAN

ME_CELL_CYCLE;REACTOME_CHROMOSOME_MAINTENANCE;REACTOME_MEIOTIC_SYNAP
PCR;REACTOME_CLASS_B_2_SECRETIN_FAMILY_RECEPTORS;REACTOME_GPCR_LIGAND_

CH1_TRANSMITS_SIGNAL_TO_THE_NUCLEUS;REACTOME_SIGNALING_BY_NOTCH1;REACTO

_BIOLOGY;REACTOME_EXTRACELLULAR_MATRIX_ORGANIZATION;REACTOME_CELL_SURF

;REACTOME_ADAPTIVE_IMMUNE_SYSTEM;REACTOME_CLASS_I_MHC_MEDIATED_ANTIGEN

_MATRIX_ORGANIZATION;REACTOME_COLLAGEN_FORMATION

OSYLATION_OF_MUCINS;REACTOME_TERMINATION_OF_O_GLYCAN_BIOSYNTHESIS;REACTO

GFR_IN_DISEASE;REACTOME_SIGNALING_BY_FGFR1_MUTANTS;REACTOME_SIGNALING_BY

VNT;REACTOME_CROSS_PRESENTATION_OF_SOLUBLE_EXOGENOUS_ANTIGENS_ENDOSOM
THE_PRE_REPLICATIVE_COMPLEX;REACTOME_CELL_CYCLE;REACTOME_ORC1_REMOVAL_

_AMINO_ACIDS_AND_DERIVATIVES

MUNICATION;REACTOME_CELL_EXTRACELLULAR_MATRIX_INTERACTIONS;REACTOME_CEL
PCR;REACTOME_CLASS_B_2_SECRETIN_FAMILY_RECEPTORS;REACTOME_GPCR_LIGAND_

SIGNALLING_PATHWAY_VIA_PKC_AND_MAPK;REACTOME_SIGNALING_BY_GPCR;REACTOME
SIGNALLING_PATHWAY_VIA_PKC_AND_MAPK;REACTOME_SIGNALING_BY_GPCR;REACTOME

OR_RECYCLING;REACTOME_LATENT_INFECTION_OF_HOMO_SAPIENS_WITH_MYCOBACTER

_CARBOHYDRATES;REACTOME_DIGESTION_OF_DIETARY_CARBOHYDRATE

PTERIN_BH4_SYNTHESIS_RECYCLING_SALVAGE_AND_REGULATION;REACTOME_LATENT_I

_BIOLOGY;REACTOME_SIGNALING_BY_GPCR;REACTOME_OPIOID_SIGNALLING;REACTOME

K_STIMULATES_FATTY_ACID_OXIDATION_IN_MUSCLE;REACTOME_INSULIN_RECEPTOR_SIG
K_STIMULATES_FATTY_ACID_OXIDATION_IN_MUSCLE;REACTOME_INSULIN_RECEPTOR_SIG

;REACTOME_ADAPTIVE_IMMUNE_SYSTEM;REACTOME_CLASS_I_MHC_MEDIATED_ANTIGEN

TOME_INNATE_IMMUNE_SYSTEM;REACTOME_IMMUNE_SYSTEM

HO_GTPASES
_PROTEINS;REACTOME_POST_TRANSLATIONAL_PROTEIN_MODIFICATION;REACTOME_ASP

VED_IN_MEGAKARYOCYTE_DEVELOPMENT_AND_PLATELET_PRODUCTION;REACTOME_HEM

ME_MEIOTIC_RECOMBINATION

_BIOLOGY;REACTOME_NEGATIVE_REGULATION_OF_FGFR_SIGNALING;REACTOME_INSULI
_BIOLOGY;REACTOME_NEGATIVE_REGULATION_OF_FGFR_SIGNALING;REACTOME_INSULI

REPAIR;REACTOME_RESOLUTION_OF_AP_SITES_VIA_THE_MULTIPLE_NUCLEOTIDE_PATCH

PCR;REACTOME_GPCR_DOWNSTREAM_SIGNALING;REACTOME_G_ALPHA_S_SIGNALLING_

_BIOLOGY;REACTOME_METABOLISM_OF_LIPIDS_AND_LIPOPROTEINS;REACTOME_TRANSC

_FATTY_ACID_BETA_OXIDATION;REACTOME_METABOLISM_OF_LIPIDS_AND_LIPOPROTEINS

_FATTY_ACID_BETA_OXIDATION;REACTOME_METABOLISM_OF_LIPIDS_AND_LIPOPROTEINS

EM;REACTOME_VOLTAGE_GATED_POTASSIUM_CHANNELS;REACTOME_POTASSIUM_CHAN

EM;REACTOME_TANDEM_PORE_DOMAIN_POTASSIUM_CHANNELS;REACTOME_POTASSIUM

AVAGE_OF_CELLULAR_PROTEINS;REACTOME_CELL_CELL_COMMUNICATION;REACTOME_C

IROTRANSMITTER_RELEASE_CYCLE;REACTOME_TRANSMISSION_ACROSS_CHEMICAL_SYN

NSCRIPTION;REACTOME_TRANSCRIPTION;REACTOME_RNA_POL_I_RNA_POL_III_AND_MITC

E_TRANSPORT_OF_SMALL_MOLECULES;REACTOME_SLC_MEDIATED_TRANSMEMBRANE_TI

_RESPIRATORY_ELECTRON_TRANSPORT;REACTOME_RESPIRATORY_ELECTRON_TRANSPO

MUNICATION;REACTOME_CELL_EXTRACELLULAR_MATRIX_INTERACTIONS;REACTOME_CEL

NGF;REACTOME_NGF_SIGNALLING_VIA_TRKA_FROM_THE_PLASMA_MEMBRANE

NGF;REACTOME_INSULIN_RECEPTOR_SIGNALLING_CASCADE;REACTOME_SIGNALLING_TO_

A_AMINOACYLATION;REACTOME_TRNA_AMINOACYLATION

_PORPHYRINS

_MATRIX_ORGANIZATION;REACTOME_COLLAGEN_FORMATION

_MATRIX_ORGANIZATION;REACTOME_COLLAGEN_FORMATION

_MATRIX_ORGANIZATION;REACTOME_COLLAGEN_FORMATION

VAYS;REACTOME_REGULATION_OF_INSULIN_LIKE_GROWTH_FACTOR_IGF_ACTIVITY_BY_IN

CTOME_CELL_CYCLE_MITOTIC;REACTOME_RECRUITMENT_OF_MITOTIC_CENTROSOME_PI

NGF;REACTOME_SIGNALING_BY_SCF_KIT;REACTOME_SIGNALING_BY_ERBB4;REACTOME_S

;REACTOME_ADAPTIVE_IMMUNE_SYSTEM;REACTOME_CLASS_I_MHC_MEDIATED_ANTIGEN
IROTRANSMITTER_RELEASE_CYCLE;REACTOME_TRANSMISSION_ACROSS_CHEMICAL_SYN

_VITAMINS_AND_COFACTORS

AVAGE_OF_CELLULAR_PROTEINS;REACTOME_DEVELOPMENTAL_BIOLOGY;REACTOME_CEL
AVAGE_OF_CELLULAR_PROTEINS;REACTOME_DEVELOPMENTAL_BIOLOGY;REACTOME_CEL
AVAGE_OF_CELLULAR_PROTEINS;REACTOME_DEVELOPMENTAL_BIOLOGY;REACTOME_CEL
AVAGE_OF_CELLULAR_PROTEINS;REACTOME_DEVELOPMENTAL_BIOLOGY;REACTOME_CEL

_AMINO_ACIDS_AND_DERIVATIVES;REACTOME_AMINO_ACID_SYNTHESIS_AND_INTERCONV
_AMINO_ACIDS_AND_DERIVATIVES;REACTOME_AMINO_ACID_SYNTHESIS_AND_INTERCONV
_PROTEINS;REACTOME_POST_TRANSLATIONAL_PROTEIN_MODIFICATION;REACTOME_SYN
PID_METABOLISM;REACTOME_METABOLISM_OF_LIPIDS_AND_LIPOPROTEINS
PID_METABOLISM;REACTOME_METABOLISM_OF_LIPIDS_AND_LIPOPROTEINS
NGF;REACTOME_ARMS_MEDIATED_ACTIVATION;REACTOME_PROLONGED_ERK_ACTIVATIO

NSCRIPTION_INITIATION_FROM_TYPE_2_PROMOTER;REACTOME_RNA_POL_III_TRANSCRIP
NGF;REACTOME_SIGNALLING_TO_RAS;REACTOME_NGF_SIGNALLING_VIA_TRKA_FROM_TH

_AMINO_ACIDS_AND_DERIVATIVES;REACTOME_AMINE_DERIVED_HORMONES
_BIOLOGY;REACTOME_EXTRACELLULAR_MATRIX_ORGANIZATION;REACTOME_COLLAGEN_
_BIOLOGY;REACTOME_EXTRACELLULAR_MATRIX_ORGANIZATION;REACTOME_COLLAGEN_
_BIOLOGY;REACTOME_EXTRACELLULAR_MATRIX_ORGANIZATION;REACTOME_COLLAGEN_

WAY_FOR_APOPTOSIS;REACTOME_TRAF6_MEDIATED_IRF7_ACTIVATION;REACTOME_TRAF
_PROTEINS;REACTOME_POST_TRANSLATIONAL_PROTEIN_MODIFICATION;REACTOME_ASP
E_TRANSPORT_OF_SMALL_MOLECULES;REACTOME_SLC_MEDIATED_TRANSMEMBRANE_TI

_STEROID_HORMONES_AND_VITAMINS_A_AND_D;REACTOME_HDL_MEDIATED_LIPID_TRAN
_STEROID_HORMONES_AND_VITAMINS_A_AND_D;REACTOME_HDL_MEDIATED_LIPID_TRAN

SURFACE_INTERACTIONS;REACTOME_P130CAS_LINKAGE_TO_MAPK_SIGNALING_FOR_INTE

IETABOLISM;REACTOME_SYNTHESIS_OF_PC;REACTOME_GLYCEROPHOSPHOLIPID_BIOSYN

_MATRIX_ORGANIZATION;REACTOME_COLLAGEN_FORMATION

PCR;REACTOME_OPIOID_SIGNALLING;REACTOME_DARPP_32_EVENTS

DATIONS;REACTOME_XENOBIOTICS;REACTOME_CYTOCHROME_P450_ARRANGED_BY_SUB

TES_B_CELL_RECEPTOR_LEADING_TO_GENERATION_OF_SECOND_MESSENGERS;REACTO

DATION;REACTOME_HEPARAN_SULFATE_HEPARIN_HS_GAG_METABOLISM;REACTOME_GLY

MUNICATION;REACTOME_EXTRACELLULAR_MATRIX_ORGANIZATION;REACTOME_COLLAGE

NGF;REACTOME_P75_NTR_RECEPTOR_MEDIATED_SIGNALLING
_BIOLOGY;REACTOME_AXON_GUIDANCE;REACTOME_NCAM1_INTERACTIONS;REACTOME_

_BIOLOGY;REACTOME_AXON_GUIDANCE;REACTOME_NETRIN1_SIGNALING;REACTOME_DC
_BIOLOGY;REACTOME_AXON_GUIDANCE;REACTOME_NETRIN1_SIGNALING;REACTOME_DC

_AMINO_ACIDS_AND_DERIVATIVES;REACTOME_BIOLOGICAL_OXIDATIONS;REACTOME_PHA
_AMINO_ACIDS_AND_DERIVATIVES;REACTOME_BIOLOGICAL_OXIDATIONS;REACTOME_PHA
_AMINO_ACIDS_AND_DERIVATIVES;REACTOME_BIOLOGICAL_OXIDATIONS;REACTOME_PHA

NT;REACTOME_CROSS_PRESENTATION_OF_SOLUBLE_EXOGENOUS_ANTIGENS_ENDOSON

NGF;REACTOME_DEVELOPMENTAL_BIOLOGY;REACTOME_NEF_MEDIATES_DOWN_MODULA

OSYLATION_OF_MUCINS;REACTOME_TERMINATION_OF_O_GLYCAN_BIOSYNTHESIS;REACT
OSYLATION_OF_MUCINS;REACTOME_TERMINATION_OF_O_GLYCAN_BIOSYNTHESIS;REACT
OSYLATION_OF_MUCINS;REACTOME_TERMINATION_OF_O_GLYCAN_BIOSYNTHESIS;REACT
OSYLATION_OF_MUCINS;REACTOME_TERMINATION_OF_O_GLYCAN_BIOSYNTHESIS;REACT

)SYLATION_OF_MUCINS;REACTOME_TERMINATION_OF_O_GLYCAN_BIOSYNTHESIS;REACT(
_AMINO_ACIDS_AND_DERIVATIVES;REACTOME_AMINE_DERIVED_HORMONES

'ORY_INTERACTIONS_BETWEEN_A_LYMPHOID_AND_A_NON_LYMPHOID_CELL;REACTOME_

PCR;REACTOME_OLFACTORY_SIGNALING_PATHWAY;REACTOME_GPCR_DOWNSTREAM_S

PAS2_ACTIVATES_CIRCADIAN_EXPRESSION;REACTOME_PPARA_ACTIVATES_GENE_EXPRE

OR_SIGNALLING_CASCADE;REACTOME_SIGNALING_BY_GPCR;REACTOME_GPCR_DOWNST
_STEROID_HORMONES_AND_VITAMINS_A_AND_D;REACTOME_BIOLOGICAL_OXIDATIONS;RI

_AMINO_ACIDS_AND_DERIVATIVES;REACTOME_AMINE_DERIVED_HORMONES

TLR3_SIGNALING;REACTOME_RIP_MEDIATED_NFKB_ACTIVATION_VIA_DAI;REACTOME_GAS
BOLISM_AND_CITRIC_ACID_TCA_CYCLE;REACTOME_TCA_CYCLE_AND_RESPIRATORY_ELE
E_TRANSPORT_OF_SMALL_MOLECULES;REACTOME_SLC_MEDIATED_TRANSMEMBRANE_TI

VAGE_OF_CELLULAR_PROTEINS;REACTOME_CASPASE_MEDIATED_CLEAVAGE_OF_CYTOS

IOSYNTHESIS;REACTOME_FATTY_ACYL_COA_BIOSYNTHESIS;REACTOME_METABOLISM_OF

CTOME_CELL_CYCLE_MITOTIC;REACTOME_RECRUITMENT_OF_MITOTIC_CENTROSOME_PI

PCR;REACTOME_OLFACTORY_SIGNALING_PATHWAY;REACTOME_GPCR_DOWNSTREAM_S

PCR;REACTOME_OLFACTORY_SIGNALING_PATHWAY;REACTOME_GPCR_DOWNSTREAM_S
PCR;REACTOME_OLFACTORY_SIGNALING_PATHWAY;REACTOME_GPCR_DOWNSTREAM_S
'_5_TO_3_EXORIBONUCLEASE;REACTOME_METABOLISM_OF_MRNA;REACTOME_DEADENYL

S_GENE_EXPRESSION;REACTOME_ALPHA_LINOLENIC_ACID_ALA_METABOLISM;REACTOME

_CAPPED_INTRON_CONTAINING_PRE_MRNA;REACTOME_TRANSPORT_OF_MATURE_TRAN

NGF;REACTOME_SIGNALING_BY_SCF_KIT;REACTOME_SIGNALING_BY_ERBB4;REACTOME_
KDOWN_GLYCOGENOLYSIS;REACTOME_METABOLISM_OF_CARBOHYDRATES;REACTOME_

VAYS;REACTOME_ACTIVATION_OF_CHAPERONE_GENES_BY_XBP1S;REACTOME_UNFOLDE

THE_PRE_REPLICATIVE_COMPLEX;REACTOME_CELL_CYCLE;REACTOME_PROCESSIVE_SY
EM;REACTOME_TANDEM_PORE_DOMAIN_POTASSIUM_CHANNELS;REACTOME_POTASSIUM

;REACTOME_IMMUNE_SYSTEM;REACTOME_ADAPTIVE_IMMUNE_SYSTEM
;REACTOME_IMMUNE_SYSTEM;REACTOME_ADAPTIVE_IMMUNE_SYSTEM

NTIGEN_PRESENTATION;REACTOME_FACTORS_INVOLVED_IN_MEGAKARYOCYTE_DEVELOP

ON_OF_TGF_BETA_RECEPTOR_SIGNALING;REACTOME_TGF_BETA_RECEPTOR_SIGNALING_
_RESPIRATORY_ELECTRON_TRANSPORT;REACTOME_RESPIRATORY_ELECTRON_TRANSPO
OR_RECYCLING;REACTOME_LATENT_INFECTION_OF_HOMO_SAPIENS_WITH_MYCOBACTE

_LIPIDS_AND_LIPOPROTEINS;REACTOME_CHOLESTEROL_BIOSYNTHESIS

HO_GTPASES

IPID_METABOLISM;REACTOME_PHOSPHOLIPID_METABOLISM;REACTOME_SPHINGOLIPID_M
E_TRANSPORT_OF_SMALL_MOLECULES;REACTOME_SLC_MEDIATED_TRANSMEMBRANE_T

NTIGEN_PRESENTATION;REACTOME_IMMUNE_SYSTEM;REACTOME_ADAPTIVE_IMMUNE_SY
TIMULATES_GUANYLATE_CYCLASE;REACTOME_PLATELET_HOMEOSTASIS;REACTOME_HEM

RBB2;REACTOME_HIV_INFECTION;REACTOME_HOST_INTERACTIONS_OF_HIV_FACTORS;RE
_AMINO_ACIDS_AND_DERIVATIVES;REACTOME_BRANCHED_CHAIN_AMINO_ACID_CATABOL
ME_CELL_CYCLE;REACTOME_P53_INDEPENDENT_G1_S_DNA_DAMAGE_CHECKPOINT;REAC

CTOME_CELL_CYCLE_MITOTIC;REACTOME_MITOTIC_M_M_G1_PHASES;REACTOME_DNA_R
MUNICATION;REACTOME_ADHERENS_JUNCTIONS_INTERACTIONS;REACTOME_CELL_CELL_

_BIOLOGY;REACTOME_AXON_GUIDANCE;REACTOME_L1CAM_INTERACTIONS;REACTOME_

TRANSPORT;REACTOME_MEMBRANE_TRAFFICKING
_5_TO_3_EXORIBONUCLEASE;REACTOME_METABOLISM_OF_MRNA;REACTOME_DEADENYL

HO_GTPASES;REACTOME_SIGNALLING_BY_NGF;REACTOME_DEVELOPMENTAL_BIOLOGY;R
HO_GTPASES;REACTOME_SIGNALLING_BY_NGF;REACTOME_DEVELOPMENTAL_BIOLOGY;R

PCR;REACTOME_OLFACTORY_SIGNALING_PATHWAY;REACTOME_GPCR_DOWNSTREAM_S

CTOME_CELL_CYCLE_MITOTIC;REACTOME_G1_PHASE;REACTOME_MITOTIC_G1_G1_S_PHA

EM;REACTOME_VOLTAGE_GATED_POTASSIUM_CHANNELS;REACTOME_POTASSIUM_CHAN
WAY_FOR_APOPTOSIS;REACTOME_APOPTOSIS

OWN_MODULATION_OF_CELL_SURFACE_RECEPTORS_BY_RECRUITING_THEM_TO_CLATH

IPS_AT_THE_GOLGI_MEMBRANE;REACTOME_PHOSPHOLIPID_METABOLISM;REACTOME_SY
IPS_AT_THE_GOLGI_MEMBRANE;REACTOME_PHOSPHOLIPID_METABOLISM;REACTOME_SY

ILE_SALT_METABOLISM;REACTOME_RECYCLING_OF_BILE_ACIDS_AND_SALTS;REACTOME

SYLATION_OF_MUCINS;REACTOME_TERMINATION_OF_O_GLYCAN_BIOSYNTHESIS;REACT
SYLATION_OF_MUCINS;REACTOME_TERMINATION_OF_O_GLYCAN_BIOSYNTHESIS;REACT

NGF;REACTOME_DAG_AND_IP3_SIGNALING;REACTOME_SIGNALING_BY_ERBB2;REACTOME_

TORY_INTERACTIONS_BETWEEN_A_LYMPHOID_AND_A_NON_LYMPHOID_CELL;REACTOME_

E_TRIC_CCT_WITH_TARGET_PROTEINS_DURING_BIOSYNTHESIS;REACTOME_PREFOLDIN_M

LE_CONTRACTION;REACTOME_MUSCLE_CONTRACTION
PTAKE_AND_DEGRADATION;REACTOME_HYALURONAN_METABOLISM;REACTOME_GLYCOSA
REPAIR;REACTOME_RESOLUTION_OF_AP_SITES_VIA_THE_MULTIPLE_NUCLEOTIDE_PATCH

._BIOLOGY;REACTOME_PPARA_ACTIVATES_GENE_EXPRESSION;REACTOME_METABOLISM_
ON_OF_HOMO_SAPIENS_WITH_MYCOBACTERIUM_TUBERCULOSIS;REACTOME_NITRIC_OXI

_PROTEINS;REACTOME_POST_TRANSLATIONAL_PROTEIN_MODIFICATION;REACTOME_ASP

MMA_SIGNALING;REACTOME_INTERFERON_ALPHA_BETA_SIGNALING;REACTOME_INTERFE

CTOME_CELL_CYCLE_MITOTIC;REACTOME_MITOTIC_M_M_G1_PHASES;REACTOME_DNA_R
CTOME_CELL_CYCLE_MITOTIC;REACTOME_MITOTIC_M_M_G1_PHASES;REACTOME_DNA_R
FFICKING;REACTOME_TRANS_GOLGI_NETWORK_VESICLE_BUDDING;REACTOME_GOLGI_AS

OSYLATION_OF_MUCINS;REACTOME_METABOLISM_OF_PROTEINS;REACTOME_POST_TRAN

GFR_IN_DISEASE;REACTOME_SIGNALING_BY_FGFR1_MUTANTS;REACTOME_SIGNALING_BY

CTOME_CELL_CYCLE_MITOTIC;REACTOME_CELL_CYCLE_CHECKPOINTS;REACTOME_TRAN

_FATTY_ACID_BETA_OXIDATION;REACTOME_METABOLISM_OF_LIPIDS_AND_LIPOPROTEINS

_BIOLOGY;REACTOME_EXTRACELLULAR_MATRIX_ORGANIZATION;REACTOME_COLLAGEN_
_TRNA_AMINOACYLATION;REACTOME_TRNA_AMINOACYLATION

NSCRIPTION;REACTOME_TRANSCRIPTION;REACTOME_FORMATION_OF_RNA_POL_II_ELONG

NTERACTIONS_AT_THE_VASCULAR_WALL;REACTOME_BASIGIN_INTERACTIONS;REACTOMI

_PATHWAY;REACTOME_DNA_REPAIR

PCR;REACTOME_CLASS_A1_RHODOPSIN_LIKE_RECEPTORS;REACTOME_EICOSANOID_LIG/

_VITAMINS_AND_COFACTORS

_VITAMINS_AND_COFACTORS
MUNICATION;REACTOME_RESPONSE_TO_ELEVATED_PLATELET_CYTOSOLIC_CA2_;REACTC

_BREAK_REPAIR;REACTOME_DNA_REPAIR

SIGNALLING_PATHWAY_VIA_PKC_AND_MAPK;REACTOME_SIGNALING_BY_GPCR;REACTOME

PHA_BETA_SIGNALING;REACTOME_INTERFERON_SIGNALING;REACTOME_IMMUNE_SYSTEM
A_BIOGENESIS;REACTOME_REGULATORY_RNA_PATHWAYS

CH1_TRANSMITS_SIGNAL_TO_THE_NUCLEUS;REACTOME_SIGNALING_BY_NOTCH1;REACTO

NTIGEN_PRESENTATION;REACTOME_FACTORS_INVOLVED_IN_MEGAKARYOCYTE_DEVELOP

NGF;REACTOME_SIGNALING_BY_SCF_KIT;REACTOME_DEVELOPMENTAL_BIOLOGY;REACTO

CH1_TRANSMITS_SIGNAL_TO_THE_NUCLEUS;REACTOME_SIGNALING_BY_NOTCH4;REACTO
NSCRIPTION_INITIATION_FROM_TYPE_2_PROMOTER;REACTOME_RNA_POL_III_TRANSCRIP

E_TRANSPORT_OF_SMALL_MOLECULES;REACTOME_ION_TRANSPORT_BY_P_TYPE_ATPAS
;REACTOME_ADAPTIVE_IMMUNE_SYSTEM;REACTOME_CLASS_I_MHC_MEDIATED_ANTIGEN
;REACTOME_ADAPTIVE_IMMUNE_SYSTEM;REACTOME_CLASS_I_MHC_MEDIATED_ANTIGEN

ACROSS_CHEMICAL_SYNAPSES;REACTOME_NEURONAL_SYSTEM;REACTOME_NEUROTRAN

_BIOLOGY;REACTOME_AXON_GUIDANCE;REACTOME_ACTIVATION_OF_RAC;REACTOME_SI
SIGNALLING_PATHWAY_VIA_PKC_AND_MAPK;REACTOME_TRANSMISSION_ACROSS_CHEMI
_BIOLOGY;REACTOME_AXON_GUIDANCE;REACTOME_NCAM_SIGNALING_FOR_NEURITE_OU
_BIOLOGY;REACTOME_AXON_GUIDANCE;REACTOME_NCAM_SIGNALING_FOR_NEURITE_OU

_AMINO_ACIDS_AND_DERIVATIVES

_BIOLOGY;REACTOME_METABOLISM_OF_PROTEINS;REACTOME_AXON_GUIDANCE;REACT
SURFACE_INTERACTIONS
SURFACE_INTERACTIONS

E_TRANSPORT_OF_SMALL_MOLECULES;REACTOME_ION_TRANSPORT_BY_P_TYPE_ATPAS

VAYS;REACTOME_INSULIN_SYNTHESIS_AND_PROCESSING
E_TRANSPORT_OF_SMALL_MOLECULES;REACTOME_PASSIVE_TRANSPORT_BY_AQUAPORI

SURFACE_INTERACTIONS

_STEROID_HORMONES_AND_VITAMINS_A_AND_D;REACTOME_BIOLOGICAL_OXIDATIONS;RI

_AMINO_ACIDS_AND_DERIVATIVES

HO_GTPASES;REACTOME_SIGNALLING_BY_NGF;REACTOME_NRAGE_SIGNALS_DEATH_THF

E_TRANSPORT_OF_SMALL_MOLECULES;REACTOME_SLC_MEDIATED_TRANSMEMBRANE_TI

NGF;REACTOME_DEVELOPMENTAL_BIOLOGY;REACTOME_SIGNALING_BY_NODAL

PCR;REACTOME_GPCR_DOWNSTREAM_SIGNALING;REACTOME_G_ALPHA_I_SIGNALLING_E

HO_GTPASES

PCR;REACTOME_PEPTIDE_LIGAND_BINDING_RECEPTORS;REACTOME_CLASS_A1_RHODOF
ME_CELL_CYCLE;REACTOME_CHROMOSOME_MAINTENANCE;REACTOME_MEIOTIC_SYNAP

ACTOME_SRP_DEPENDENT_COTRANSLATIONAL_PROTEIN_TARGETING_TO_MEMBRANE;RE
_PROTEIN_IMPORT;REACTOME_METABOLISM_OF_PROTEINS

_BIOLOGY;REACTOME_MYOGENESIS

CRIPTION_PATHWAY

_BIOLOGY;REACTOME_BMAL1_CLOCK_NPAS2_ACTIVATES_CIRCADIAN_EXPRESSION;REAC
_BIOLOGY;REACTOME_BMAL1_CLOCK_NPAS2_ACTIVATES_CIRCADIAN_EXPRESSION;REAC
_BIOLOGY;REACTOME_BMAL1_CLOCK_NPAS2_ACTIVATES_CIRCADIAN_EXPRESSION;REAC
NGF;REACTOME_DAG_AND_IP3_SIGNALING;REACTOME_SIGNALING_BY_ERBB2;REACTOME_

MMA_SIGNALING;REACTOME_INTERFERON_SIGNALING;REACTOME_IMMUNE_SYSTEM;REA
MMA_SIGNALING;REACTOME_INTERFERON_SIGNALING;REACTOME_IMMUNE_SYSTEM;REA

TEINS_MEDIATED_TRANSPORT;REACTOME_TRANSMEMBRANE_TRANSPORT_OF_SMALL_M

_MRNA;REACTOME_METABOLISM_OF_RNA;REACTOME_NONSENSE_MEDIATED_DECAY_EN

OR_SIGNALLING_CASCADE;REACTOME_PKB_MEDIATED_EVENTS;REACTOME_SIGNALING_E

RESSION_AND_PROCESSING;REACTOME_PRE_NOTCH_PROCESSING_IN_GOLGI;REACTOM

NTERACTIONS_AT_THE_VASCULAR_WALL;REACTOME_INTEGRIN_CELL_SURFACE_INTERA(
CRIPTION_PATHWAY

AKDOWN_GLYCOGENOLYSIS;REACTOME_METABOLISM_OF_CARBOHYDRATES;REACTOME_

_NON_CODING_RNA;REACTOME_ANTIVIRAL_MECHANISM_BY_IFN_STIMULATED_GENES;RE
_NON_CODING_RNA;REACTOME_ANTIVIRAL_MECHANISM_BY_IFN_STIMULATED_GENES;RE
.IPID_TRANSPORT;REACTOME_METABOLISM_OF_LIPIDS_AND_LIPOPROTEINS;REACTOME_I

E_TRANSPORT_OF_SMALL_MOLECULES;REACTOME_SLC_MEDIATED_TRANSMEMBRANE_TI

E_TRANSPORT_OF_SMALL_MOLECULES;REACTOME_SLC_MEDIATED_TRANSMEMBRANE_TI

ME_CELL_CYCLE;REACTOME_CHROMOSOME_MAINTENANCE;REACTOME_MEIOTIC_SYNAP

SURFACE_INTERACTIONS;REACTOME_P130CAS_LINKAGE_TO_MAPK_SIGNALING_FOR_INTE

TES_B_CELL_RECEPTOR_LEADING_TO_GENERATION_OF_SECOND_MESSENGERS;REACTC

ISCRIPTION_TERMINATION;REACTOME_RNA_POL_I_TRANSCRIPTION;REACTOME_TRANSCF

_LIPIDS_AND_LIPOPROTEINS;REACTOME_CHOLESTEROL_BIOSYNTHESIS

_CAPPED_INTRON_CONTAINING_PRE_MRNA;REACTOME_MRNA_PROCESSING;REACTOME_

_MRNA;REACTOME_METABOLISM_OF_RNA;REACTOME_NONSENSE_MEDIATED_DECAY_ENI
_MRNA;REACTOME_METABOLISM_OF_RNA;REACTOME_NONSENSE_MEDIATED_DECAY_ENI

ACROSS_CHEMICAL_SYNAPSES;REACTOME_NEURONAL_SYSTEM;REACTOME_NEUROTRAN

;REACTOME_ADAPTIVE_IMMUNE_SYSTEM;REACTOME_CLASS_I_MHC_MEDIATED_ANTIGEN

E_TRANSPORT_OF_SMALL_MOLECULES;REACTOME_SLC_MEDIATED_TRANSMEMBRANE_T

A_BIOGENESIS;REACTOME_REGULATORY_RNA_PATHWAYS;REACTOME_PROCESSING_OF_

IETABOLISM;REACTOME_SYNTHESIS_OF_PIPS_AT_THE_PLASMA_MEMBRANE;REACTOME_

THESIS;REACTOME_HEPARAN_SULFATE_HEPARIN_HS_GAG_METABOLISM;REACTOME_GLY

_BIOLOGY;REACTOME_BMAL1_CLOCK_NPAS2_ACTIVATES_CIRCADIAN_EXPRESSION;REAC

_BIOLOGY;REACTOME_BMAL1_CLOCK_NPAS2_ACTIVATES_CIRCADIAN_EXPRESSION;REAC

TLR3_SIGNALING;REACTOME_MAP_KINASE_ACTIVATION_IN_TLR_CASCADE;REACTOME_AC

ON_OF_HOMO_SAPIENS_WITH_MYCOBACTERIUM_TUBERCULOSIS;REACTOME_NITRIC_OXI

E_TRANSPORT_OF_SMALL_MOLECULES;REACTOME_SLC_MEDIATED_TRANSMEMBRANE_T

E_TRANSPORT_OF_SMALL_MOLECULES;REACTOME_SLC_MEDIATED_TRANSMEMBRANE_T

GFR_IN_DISEASE;REACTOME_SIGNALING_BY_FGFR1_MUTANTS;REACTOME_SIGNALING_BY

NGF;REACTOME_DEVELOPMENTAL_BIOLOGY;REACTOME_NEF_MEDIATES_DOWN_MODULA

_BIOLOGY;REACTOME_TRANSMISSION_ACROSS_CHEMICAL_SYNAPSES;REACTOME_NEUR

ACTOME_SRP_DEPENDENT_COTRANSLATIONAL_PROTEIN_TARGETING_TO_MEMBRANE;RE

_BIOLOGY;REACTOME_DOWNREGULATION_OF_ERBB2_ERBB3_SIGNALING;REACTOME_SIG
PCR;REACTOME_PEPTIDE_LIGAND_BINDING_RECEPTORS;REACTOME_CLASS_A1_RHODOF

NGF;REACTOME_SIGNALING_BY_SCF_KIT;REACTOME_GROWTH_HORMONE_RECEPTOR_SI
NGF;REACTOME_SIGNALING_BY_SCF_KIT;REACTOME_GROWTH_HORMONE_RECEPTOR_SI
NGF;REACTOME_SIGNALING_BY_SCF_KIT;REACTOME_GROWTH_HORMONE_RECEPTOR_SI
TOTHENATE_METABOLISM;REACTOME_METABOLISM_OF_VITAMINS_AND_COFACTORS

ACROSS_CHEMICAL_SYNAPSES;REACTOME_NEURONAL_SYSTEM;REACTOME_NEUROTRAN

;REACTOME_ADAPTIVE_IMMUNE_SYSTEM;REACTOME_CLASS_I_MHC_MEDIATED_ANTIGEN

_BIOLOGY;REACTOME_EXTRACELLULAR_MATRIX_ORGANIZATION;REACTOME_CELL_SURF

ME_FACTORS_INVOLVED_IN_MEGAKARYOCYTE_DEVELOPMENT_AND_PLATELET_PRODUC

ONE_RECEPTOR_SIGNALING;REACTOME_SIGNALING_BY_ERBB4;REACTOME_PROLACTIN_R
_BIOLOGY;REACTOME_AXON_GUIDANCE;REACTOME_L1CAM_INTERACTIONS;REACTOME_I

RTERS_IN_LIPID_HOMEOSTASIS;REACTOME_ABC_FAMILY_PROTEINS_MEDIATED_TRANSPO

OR_SIGNALLING_CASCADE;REACTOME_REGULATION_OF_AMPK_ACTIVITY_VIA_LKB1;REAC

TOTHENATE_METABOLISM;REACTOME_METABOLISM_OF_VITAMINS_AND_COFACTORS;REA
TOTHENATE_METABOLISM;REACTOME_METABOLISM_OF_VITAMINS_AND_COFACTORS;REA

NGF;REACTOME_NGF_SIGNALLING_VIA_TRKA_FROM_THE_PLASMA_MEMBRANE;REACTOMI
CTOME_CELL_CYCLE_MITOTIC;REACTOME_MITOTIC_M_M_G1_PHASES;REACTOME_DNA_R

CTOME_CELL_CYCLE_MITOTIC;REACTOME_RECRUITMENT_OF_MITOTIC_CENTROSOME_PI
PCR;REACTOME_PEPTIDE_LIGAND_BINDING_RECEPTORS;REACTOME_CLASS_A1_RHODOF

MUNICATION;REACTOME_CELL_JUNCTION_ORGANIZATION

E_TRANSPORT_OF_SMALL_MOLECULES;REACTOME_AQUAPORIN_MEDIATED_TRANSPORT;

E_TRANSPORT_OF_SMALL_MOLECULES;REACTOME_ION_TRANSPORT_BY_P_TYPE_ATPAS

BOLISM_AND_CITRIC_ACID_TCA_CYCLE;REACTOME_TCA_CYCLE_AND_RESPIRATORY_ELE
REACTOME_RNA_POL_I_RNA_POL_III_AND_MITOCHONDRIAL_TRANSCRIPTION

REACTOME_RNA_POL_I_RNA_POL_III_AND_MITOCHONDRIAL_TRANSCRIPTION

_CAPPED_INTRON_CONTAINING_PRE_MRNA;REACTOME_MRNA_PROCESSING;REACTOME_

HO_GTPASES
NSCRIPTION_INITIATION_FROM_TYPE_2_PROMOTER;REACTOME_MICRORNA_MIRNA_BIOG

PCR;REACTOME_CLASS_A1_RHODOPSIN_LIKE_RECEPTORS;REACTOME_GPCR_DOWNSTR

HO_GTPASES;REACTOME_SIGNALLING_BY_NGF;REACTOME_SIGNALING_BY_SCF_KIT;REAC
HO_GTPASES;REACTOME_SIGNALLING_BY_NGF;REACTOME_TGF_BETA_RECEPTOR_SIGNA

_COUPLED_NER_TC_NER;REACTOME_NUCLEOTIDE_EXCISION_REPAIR;REACTOME_FORMA
_COUPLED_NER_TC_NER;REACTOME_NUCLEOTIDE_EXCISION_REPAIR;REACTOME_FORMA

OSYLATION_OF_MUCINS;REACTOME_TERMINATION_OF_O_GLYCAN_BIOSYNTHESIS;REACT
OSYLATION_OF_MUCINS;REACTOME_TERMINATION_OF_O_GLYCAN_BIOSYNTHESIS;REACT
OSYLATION_OF_MUCINS;REACTOME_TERMINATION_OF_O_GLYCAN_BIOSYNTHESIS;REACT

ANISM_BY_IFN_STIMULATED_GENES;REACTOME_INTERFERON_SIGNALING;REACTOME_NE

SIGNALLING_PATHWAY_VIA_PKC_AND_MAPK;REACTOME_SIGNALING_BY_GPCR;REACTOME
TORY_INTERACTIONS_BETWEEN_A_LYMPHOID_AND_A_NON_LYMPHOID_CELL;REACTOME_

S;REACTOME_INTERFERON_ALPHA_BETA_SIGNALING;REACTOME_REGULATION_OF_IFNA_

_LIPIDS_AND_LIPOPROTEINS;REACTOME_LIPID_DIGESTION_MOBILIZATION_AND_TRANSPO
_LIPIDS_AND_LIPOPROTEINS;REACTOME_LIPID_DIGESTION_MOBILIZATION_AND_TRANSPO
VED_IN_MEGAKARYOCYTE_DEVELOPMENT_AND_PLATELET_PRODUCTION;REACTOME_HEM

NGF;REACTOME_DAG_AND_IP3_SIGNALING;REACTOME_CELL_CYCLE;REACTOME_SIGNALIN

PCR;REACTOME_OLFACTORY_SIGNALING_PATHWAY;REACTOME_GPCR_DOWNSTREAM_S

GFR_IN_CANCER;REACTOME_EGFR_DOWNREGULATION

HO_GTPASES

PCR;REACTOME_OPIOID_SIGNALLING;REACTOME_DARPP_32_EVENTS;REACTOME_GPCR_

CRIPTION_PATHWAY

CTOME_GLUCONEOGENESIS;REACTOME_METABOLISM_OF_CARBOHYDRATES;REACTOME_

CRIPTION_PATHWAY

_CAPPED_INTRON_CONTAINING_PRE_MRNA;REACTOME_MRNA_PROCESSING;REACTOME_

_CAPPED_INTRON_CONTAINING_PRE_MRNA;REACTOME_MRNA_PROCESSING;REACTOME_
_BIOLOGY;REACTOME_TGF_BETA_RECEPTOR_SIGNALING_IN_EMT_EPITHELIAL_TO_MESE
NGF;REACTOME_P75_NTR_RECEPTOR_MEDIATED_SIGNALLING;REACTOME_SIGNALING_BY

CRIPTION_PATHWAY
CRIPTION_PATHWAY

_PROTEIN_IMPORT;REACTOME_METABOLISM_OF_PROTEINS
SIGNALLING_PATHWAY_VIA_PKC_AND_MAPK;REACTOME_SIGNALING_BY_GPCR;REACTOME

E_TRANSPORT_OF_SMALL_MOLECULES;REACTOME_SLC_MEDIATED_TRANSMEMBRANE_T

E_NOVO_BIOSYNTHESIS;REACTOME_PHOSPHOLIPID_METABOLISM;REACTOME_SPHINGOLI

_CAPPED_INTRON_CONTAINING_PRE_MRNA;REACTOME_MRNA_PROCESSING;REACTOME_
_CAPPED_INTRON_CONTAINING_PRE_MRNA;REACTOME_MRNA_PROCESSING;REACTOME_

_BIOLOGY;REACTOME_AXON_GUIDANCE;REACTOME_SEMA4D_IN_SEMAPHORIN_SIGNALIN
CRIPTION_PATHWAY;REACTOME_NUCLEAR_RECEPTOR_TRANSCRIPTION_PATHWAY
REPAIR;REACTOME_RESOLUTION_OF_AP_SITES_VIA_THE_MULTIPLE_NUCLEOTIDE_PATCH
REPAIR;REACTOME_RESOLUTION_OF_AP_SITES_VIA_THE_MULTIPLE_NUCLEOTIDE_PATCH
REPAIR;REACTOME_RESOLUTION_OF_AP_SITES_VIA_THE_MULTIPLE_NUCLEOTIDE_PATCH

LE_CONTRACTION;REACTOME_MUSCLE_CONTRACTION

.E_CONTRACTION;REACTOME_MUSCLE_CONTRACTION

ACROSS_CHEMICAL_SYNAPSES;REACTOME_NEURONAL_SYSTEM;REACTOME_NEUROTRAN

TORY_INTERACTIONS_BETWEEN_A_LYMPHOID_AND_A_NON_LYMPHOID_CELL;REACTOME_

E_ENERGY_METABOLISM;REACTOME_REGULATION_OF_INSULIN_SECRETION

CRIPTION_PATHWAY
CRIPTION_PATHWAY
CRIPTION_PATHWAY

MUNICATION;REACTOME_CELL_SURFACE_INTERACTIONS_AT_THE_VASCULAR_WALL;REAC

E_TRIC_CCT_WITH_TARGET_PROTEINS_DURING_BIOSYNTHESIS;REACTOME_PROTEIN_FOL

L_BIOLOGY;REACTOME_AXON_GUIDANCE;REACTOME_NCAM1_INTERACTIONS;REACTOME_

_PROTEINS;REACTOME_POST_TRANSLATIONAL_MODIFICATION_SYNTHESIS_OF_GPI_ANCH

ACTOME_CELL_CYCLE_MITOTIC;REACTOME_RECRUITMENT_OF_MITOTIC_CENTROSOME_PI
_VITAMINS_AND_COFACTORS

ACROSS_CHEMICAL_SYNAPSES;REACTOME_NEURONAL_SYSTEM;REACTOME_NEUROTRAN

NGF;REACTOME_DEVELOPMENTAL_BIOLOGY;REACTOME_DAG_AND_IP3_SIGNALING;REACT

E_TRANSPORT_OF_SMALL_MOLECULES;REACTOME_SLC_MEDIATED_TRANSMEMBRANE_TI

E_TRANSPORT_OF_SMALL_MOLECULES;REACTOME_SLC_MEDIATED_TRANSMEMBRANE_TI

EM;REACTOME_VOLTAGE_GATED_POTASSIUM_CHANNELS;REACTOME_POTASSIUM_CHAN
E_TRANSPORT_OF_SMALL_MOLECULES;REACTOME_ION_TRANSPORT_BY_P_TYPE_ATPAS

PCR;REACTOME_INTEGRATION_OF_ENERGY_METABOLISM;REACTOME_TRANSMEMBRANE
PCR;REACTOME_INTEGRATION_OF_ENERGY_METABOLISM;REACTOME_TRANSMEMBRANE
ME_CELL_CYCLE;REACTOME_CHROMOSOME_MAINTENANCE;REACTOME_MEIOTIC_SYNAP

_BIOLOGY;REACTOME_EXTRACELLULAR_MATRIX_ORGANIZATION;REACTOME_COLLAGEN_
_BIOLOGY;REACTOME_EXTRACELLULAR_MATRIX_ORGANIZATION;REACTOME_COLLAGEN_
TRANSPORT;REACTOME_MEMBRANE_TRAFFICKING;REACTOME_DIABETES_PATHWAYS;RE

NTERACTIONS_AT_THE_VASCULAR_WALL;REACTOME_INTEGRIN_CELL_SURFACE_INTERA
TLR3_SIGNALING;REACTOME_RIP_MEDIATED_NFKB_ACTIVATION_VIA_DAI;REACTOME_GAS

IETABOLISM;REACTOME_SYNTHESIS_OF_PIPS_AT_THE_PLASMA_MEMBRANE;REACTOME_

PHA_BETA_SIGNALING;REACTOME_REGULATION_OF_IFNA_SIGNALING;REACTOME_INTERF
HO_GTPASES;REACTOME_SIGNALLING_BY_NGF;REACTOME_NRAGE_SIGNALS_DEATH_THI

MUNICATION;REACTOME_DSCAM_INTERACTIONS
MUNICATION;REACTOME_DSCAM_INTERACTIONS
MUNICATION;REACTOME_DSCAM_INTERACTIONS
MUNICATION;REACTOME_DSCAM_INTERACTIONS

_CAPPED_INTRON_CONTAINING_PRE_MRNA;REACTOME_TRANSPORT_OF_MATURE_TRANS

TORY_INTERACTIONS_BETWEEN_A_LYMPHOID_AND_A_NON_LYMPHOID_CELL;REACTOME_
_VITAMINS_AND_COFACTORS
_BIOLOGY;REACTOME_EXTRACELLULAR_MATRIX_ORGANIZATION;REACTOME_COLLAGEN_
_AMINO_ACIDS_AND_DERIVATIVES

CTOME_CELL_CYCLE_MITOTIC;REACTOME_RECRUITMENT_OF_MITOTIC_CENTROSOME_PI

PIPS_AT_THE_GOLGI_MEMBRANE;REACTOME_PHOSPHOLIPID_METABOLISM;REACTOME_PI_

NGF;REACTOME_NGF_SIGNALLING_VIA_TRKA_FROM_THE_PLASMA_MEMBRANE;REACTOMI
ABOLISM;REACTOME_METABOLISM_OF_NUCLEOTIDES;REACTOME_PYRIMIDINE_METABOLIS
DATIONS;REACTOME_GLUTATHIONE_CONJUGATION;REACTOME_PHASE_II_CONJUGATION

THE_PRE_REPLICATIVE_COMPLEX;REACTOME_CELL_CYCLE;REACTOME_ORC1_REMOVAL_
_BIOLOGY;REACTOME_AXON_GUIDANCE;REACTOME_SEMA4D_IN_SEMAPHORIN_SIGNALIN

S;REACTOME_IL_3_5_AND_GM_CSF_SIGNALING;REACTOME_IL_RECEPTOR_SHC_SIGNALIN

/ODELLING_OF_PC;REACTOME_PHOSPHOLIPID_METABOLISM;REACTOME_ACYL_CHAIN_RE
/ED_IN_MEGAKARYOCYTE_DEVELOPMENT_AND_PLATELET_PRODUCTION;REACTOME_HEN

CROSS_CHEMICAL_SYNAPSES;REACTOME_NEURONAL_SYSTEM;REACTOME_NEUROTRAN
CROSS_CHEMICAL_SYNAPSES;REACTOME_NEURONAL_SYSTEM;REACTOME_NEUROTRAN

_BIOLOGY;REACTOME_AXON_GUIDANCE;REACTOME_NCAM1_INTERACTIONS;REACTOME_
NSCRIPTION_AND_TRANSLATION;REACTOME_PRE_NOTCH_EXPRESSION_AND_PROCESSII
SIGNALLING_PATHWAY_VIA_PKC_AND_MAPK;REACTOME_SIGNALING_BY_GPCR;REACTOME
NSCRIPTION_INITIATION_FROM_TYPE_2_PROMOTER;REACTOME_RNA_POL_III_TRANSCRIP
NSCRIPTION_INITIATION_FROM_TYPE_2_PROMOTER;REACTOME_RNA_POL_III_TRANSCRIP
CTOME_CELL_CYCLE_MITOTIC;REACTOME_MITOTIC_M_M_G1_PHASES;REACTOME_DNA_R

IETABOLISM;REACTOME_GLYCEROPHOSPHOLIPID_BIOSYNTHESIS;REACTOME_METABOLIS

ME_CELL_CYCLE;REACTOME_CHROMOSOME_MAINTENANCE;REACTOME_MEIOTIC_SYNAP

EM
EM

RBOXYLATION_HYPUSINE_FORMATION_AND_ARYLSULFATASE_ACTIVATION;REACTOME_GL

IOSYNTHESIS;REACTOME_METABOLISM_OF_LIPIDS_AND_LIPOPROTEINS;REACTOME_FATT
IOSYNTHESIS;REACTOME_METABOLISM_OF_LIPIDS_AND_LIPOPROTEINS;REACTOME_FATT
E_CONTRACTION;REACTOME_MUSCLE_CONTRACTION

LATORS_OF_RIG_I_MDA5_SIGNALING;REACTOME_RIG_I_MDA5_MEDIATED_INDUCTION_OF_

NGF;REACTOME_NRAGE_SIGNALS_DEATH_THROUGH_JNK;REACTOME_REGULATION_OF_A

ME_CELL_CYCLE;REACTOME_PROCESSING_OF_CAPPED_INTRON_CONTAINING_PRE_MRN
;REACTOME_ADAPTIVE_IMMUNE_SYSTEM;REACTOME_CLASS_I_MHC_MEDIATED_ANTIGEN

HO_GTPASES;REACTOME_SIGNALLING_BY_NGF;REACTOME_NRAGE_SIGNALS_DEATH_THR

HO_GTPASES

_BIOLOGY;REACTOME_PPARA_ACTIVATES_GENE_EXPRESSION;REACTOME_GENERIC_TR
FICKING;REACTOME_GAP_JUNCTION_TRAFFICKING;REACTOME_GAP_JUNCTION_ASSEMB

ELLULAR_DOMAIN_REGULATES_TRANSCRIPTION;REACTOME_SIGNALING_BY_NOTCH1;REA
KDOWN_GLYCOGENOLYSIS;REACTOME_METABOLISM_OF_CARBOHYDRATES;REACTOME_

IOSYNTHESIS;REACTOME_FATTY_ACYL_COA_BIOSYNTHESIS;REACTOME_METABOLISM_OF

DATION;REACTOME_CHONDROITIN_SULFATE_DERMATAN_SULFATE_METABOLISM;REACTO
E_TRANSPORT_OF_SMALL_MOLECULES;REACTOME_SLC_MEDIATED_TRANSMEMBRANE_TI

E_TRANSPORT_OF_SMALL_MOLECULES;REACTOME_ION_TRANSPORT_BY_P_TYPE_ATPAS

ATION;REACTOME_CHONDROITIN_SULFATE_BIOSYNTHESIS;REACTOME_CHONDROITIN_SUL
E_TRANSPORT_OF_SMALL_MOLECULES;REACTOME_PLATELET_HOMEOSTASIS;REACTOME
ACTOME_SRP_DEPENDENT_COTRANSLATIONAL_PROTEIN_TARGETING_TO_MEMBRANE;RE
_BIOLOGY;REACTOME_CELL_SURFACE_INTERACTIONS_AT_THE_VASCULAR_WALL;REACT
_BIOLOGY;REACTOME_CELL_SURFACE_INTERACTIONS_AT_THE_VASCULAR_WALL;REACT

_CARBOHYDRATES
CTOME_CHROMOSOME_MAINTENANCE;REACTOME_TELOMERE_MAINTENANCE;REACTOME

_AMINO_ACIDS_AND_DERIVATIVES

ANISM_BY_IFN_STIMULATED_GENES;REACTOME_INTERFERON_SIGNALING;REACTOME_IM

DATION;REACTOME_HEPARAN_SULFATE_HEPARIN_HS_GAG_METABOLISM;REACTOME_GLY
CTOME_CELL_CYCLE_MITOTIC;REACTOME_RECRUITMENT_OF_MITOTIC_CENTROSOME_P

H1_TRANSMITS_SIGNAL_TO_THE_NUCLEUS;REACTOME_SIGNALING_BY_NOTCH4;REACTO

CTOME_MHC_CLASS_II_ANTIGEN_PRESENTATION;REACTOME_CELL_CYCLE_MITOTIC;REA

E_CONTRACTION;REACTOME_MUSCLE_CONTRACTION

_MATRIX_ORGANIZATION;REACTOME_COLLAGEN_FORMATION

_BIOLOGY;REACTOME_AXON_GUIDANCE;REACTOME_NCAM_SIGNALING_FOR_NEURITE_O
PCR;REACTOME_PEPTIDE_LIGAND_BINDING_RECEPTORS;REACTOME_CLASS_A1_RHODO

PCR;REACTOME_CLASS_B_2_SECRETIN_FAMILY_RECEPTORS;REACTOME_GPCR_LIGAND_

ACID_METABOLISM;REACTOME_METABOLISM_OF_AMINO_ACIDS_AND_DERIVATIVES;REAC

RTERS_IN_LIPID_HOMEOSTASIS;REACTOME_ABC_FAMILY_PROTEINS_MEDIATED_TRANSPO

E_TRANSPORT_OF_SMALL_MOLECULES;REACTOME_SLC_MEDIATED_TRANSMEMBRANE_TI
_PROTEIN_IMPORT;REACTOME_METABOLISM_OF_PROTEINS

CTOME_CELL_CYCLE_MITOTIC;REACTOME_RECRUITMENT_OF_MITOTIC_CENTROSOME_PI
CTOME_CELL_CYCLE_MITOTIC;REACTOME_RECRUITMENT_OF_MITOTIC_CENTROSOME_PI

WAY_FOR_APOPTOSIS;REACTOME_APOPTOSIS
NGF;REACTOME_SIGNALING_BY_SCF_KIT;REACTOME_CELL_CELL_COMMUNICATION;REACT

DATIONS;REACTOME_CYTOCHROME_P450_ARRANGED_BY_SUBSTRATE_TYPE;REACTOME_

PIPS_AT_THE_EARLY_ENDOSOME_MEMBRANE;REACTOME_PHOSPHOLIPID_METABOLISM;RI

SIGNALLING_PATHWAY_VIA_PKC_AND_MAPK;REACTOME_SIGNALING_BY_GPCR;REACTOME

TAKE_AND_DEGRADATION;REACTOME_HYALURONAN_METABOLISM;REACTOME_GLYCOSA

DOWNREGULATION_OF_MHC_CLASS_I_COMPLEX_CELL_SURFACE_EXPRESSION;REACTOM

AVAGE_OF_CELLULAR_PROTEINS;REACTOME_CELL_CELL_COMMUNICATION;REACTOME_C

CTOME_METABOLISM_OF_CARBOHYDRATES;REACTOME_GLUCOSE_METABOLISM

ON_OF_HOMO_SAPIENS_WITH_MYCOBACTERIUM_TUBERCULOSIS;REACTOME_NITRIC_OXI

NGF;REACTOME_SIGNALING_BY_SCF_KIT;REACTOME_DEVELOPMENTAL_BIOLOGY;REACTC

'_5_TO_3_EXORIBONUCLEASE;REACTOME_METABOLISM_OF_MRNA;REACTOME_DEADENYL
_CAPPED_INTRON_CONTAINING_PRE_MRNA;REACTOME_MRNA_PROCESSING;REACTOME_
CRIPTION_PATHWAY

IETABOLISM;REACTOME_GLYCEROPHOSPHOLIPID_BIOSYNTHESIS;REACTOME_METABOLIS

RBOXYLATION_HYPUSINE_FORMATION_AND_ARYLSULFATASE_ACTIVATION;REACTOME_GL

_BIOLOGY;REACTOME_MHC_CLASS_II_ANTIGEN_PRESENTATION;REACTOME_AXON_GUIDA

ACROSS_CHEMICAL_SYNAPSES;REACTOME_NEURONAL_SYSTEM;REACTOME_NEUROTRAN
NGF;REACTOME_TRIF_MEDIATED_TLR3_SIGNALING;REACTOME_P75NTR_RECRUITS_SIGNA

_MATRIX_ORGANIZATION;REACTOME_COLLAGEN_FORMATION
TATION_OF_SOLUBLE_EXOGENOUS_ANTIGENS_ENDOSOMES;REACTOME_ANTIGEN_PROCE

_BIOLOGY;REACTOME_CELL_CELL_COMMUNICATION;REACTOME_AXON_GUIDANCE;REACT

VAYS;REACTOME_REGULATION_OF_INSULIN_LIKE_GROWTH_FACTOR_IGF_ACTIVITY_BY_IN

PCR;REACTOME_CLASS_A1_RHODOPSIN_LIKE_RECEPTORS;REACTOME_AMINE_LIGAND_B

ISCRIPTION;REACTOME_TRANSCRIPTION;REACTOME_RNA_POL_I_RNA_POL_III_AND_MITO(

._BIOLOGY;REACTOME_PPARA_ACTIVATES_GENE_EXPRESSION;REACTOME_METABOLISM_

SPORT_AND_METABOLISM;REACTOME_ABC_FAMILY_PROTEINS_MEDIATED_TRANSPORT;RI

.CTOME_CELL_CYCLE_MITOTIC;REACTOME_RECRUITMENT_OF_MITOTIC_CENTROSOME_PI
AVAGE_OF_CELLULAR_PROTEINS;REACTOME_DEVELOPMENTAL_BIOLOGY;REACTOME_CEL
WAY_FOR_APOPTOSIS;REACTOME_TRAF6_MEDIATED_IRF7_ACTIVATION;REACTOME_TRAF

SURFACE_INTERACTIONS

LATION_OF_FGFR_SIGNALING;REACTOME_INSULIN_RECEPTOR_SIGNALLING_CASCADE;RE

HO_GTPASES;REACTOME_SIGNALLING_BY_NGF;REACTOME_DEVELOPMENTAL_BIOLOGY;R
MMA_SIGNALING;REACTOME_INTERFERON_ALPHA_BETA_SIGNALING;REACTOME_INTERFE
.CTOME_ORC1_REMOVAL_FROM_CHROMATIN;REACTOME_CELL_CYCLE_MITOTIC;REACTOM

VAYS;REACTOME_INSULIN_SYNTHESIS_AND_PROCESSING

E_TRANSPORT_OF_SMALL_MOLECULES;REACTOME_SLC_MEDIATED_TRANSMEMBRANE_TI

ACROSS_CHEMICAL_SYNAPSES;REACTOME_NEURONAL_SYSTEM;REACTOME_INTEGRATIOI
PCR;REACTOME_OLFACTORY_SIGNALING_PATHWAY;REACTOME_GPCR_DOWNSTREAM_S

TEM;REACTOME_POTASSIUM_CHANNELS

.CTOME_CELL_CYCLE_MITOTIC;REACTOME_RECRUITMENT_OF_MITOTIC_CENTROSOME_PI

E_TRANSPORT_OF_SMALL_MOLECULES;REACTOME_SLC_MEDIATED_TRANSMEMBRANE_TI

NGF;REACTOME_TRIF_MEDIATED_TLR3_SIGNALING;REACTOME_P75NTR_RECRUITS_SIGNA

NSCRIPTION;REACTOME_TRANSCRIPTION;REACTOME_RNA_POL_I_RNA_POL_III_AND_MITC

HO_GTPASES;REACTOME_SIGNALLING_BY_NGF;REACTOME_DEVELOPMENTAL_BIOLOGY;R
ACTOME_METABOLISM_OF_PROTEINS

MUNICATION;REACTOME_ADHERENS_JUNCTIONS_INTERACTIONS;REACTOME_CELL_CELL_

IPPO
IPPO

NGF;REACTOME_NGF_SIGNALLING_VIA_TRKA_FROM_THE_PLASMA_MEMBRANE;REACTOM

E_TRANSPORT_OF_SMALL_MOLECULES;REACTOME_SLC_MEDIATED_TRANSMEMBRANE_T

ACTOME_FORMATION_OF_THE_TERNARY_COMPLEX_AND_SUBSEQUENTLY_THE_43S_CON
E_TRANSPORT_OF_SMALL_MOLECULES;REACTOME_ION_TRANSPORT_BY_P_TYPE_ATPAS
ETABOLISM;REACTOME_GLYCOSAMINOGLYCAN_METABOLISM;REACTOME_ABC_FAMILY_PF
E_TRANSPORT_OF_SMALL_MOLECULES;REACTOME_ION_CHANNEL_TRANSPORT;REACTON

ECEPTOR_INTERACTIONS;REACTOME_RESPONSE_TO_ELEVATED_PLATELET_CYTOSOLIC_

IOSYNTHESIS;REACTOME_FATTY_ACYL_COA_BIOSYNTHESIS;REACTOME_METABOLISM_OF

_BIOLOGY;REACTOME_REGULATION_OF_APOPTOSIS;REACTOME_AXON_GUIDANCE;REAC

SSING_CROSS_PRESENTATION;REACTOME_ENDOSOMAL_VACUOLAR_PATHWAY;REACTON

_MATRIX_ORGANIZATION;REACTOME_COLLAGEN_FORMATION

METABOLISM;REACTOME_SYNTHESIS_OF_PIPS_AT_THE_PLASMA_MEMBRANE;REACTOME_

_PROTEINS;REACTOME_POST_TRANSLATIONAL_PROTEIN_MODIFICATION;REACTOME_ASP
RBB4;REACTOME_NUCLEAR_SIGNALING_BY_ERBB4;REACTOME_SIGNALING_BY_GPCR;REA

EM;REACTOME_TANDEM_PORE_DOMAIN_POTASSIUM_CHANNELS;REACTOME_POTASSIUM

EM;REACTOME_INTEGRATION_OF_ENERGY_METABOLISM;REACTOME_REGULATION_OF_IN

H1_TRANSMITS_SIGNAL_TO_THE_NUCLEUS;REACTOME_SIGNALING_BY_NOTCH1;REACTO

_AMINO_ACIDS_AND_DERIVATIVES

IETABOLISM;REACTOME_SYNTHESIS_OF_PIPS_AT_THE_PLASMA_MEMBRANE;REACTOME_

E_NOVO_BIOSYNTHESIS;REACTOME_PHOSPHOLIPID_METABOLISM;REACTOME_SPHINGOLI

NT;REACTOME_CTNNB1_PHOSPHORYLATION_CASCADE;REACTOME_CELL_CYCLE;REACTO

LEVATED_PLATELET_CYTOSOLIC_CA2_;REACTOME_GLYCOLYSIS;REACTOME_GLUCONEO

_CAPPED_INTRON_CONTAINING_PRE_MRNA;REACTOME_MRNA_PROCESSING;REACTOME_
VAYS;REACTOME_UNFOLDED_PROTEIN_RESPONSE
.CROSS_CHEMICAL_SYNAPSES;REACTOME_NEURONAL_SYSTEM;REACTOME_NEUROTRAN

DATIONS;REACTOME_CYTOSOLIC_SULFONATION_OF_SMALL_MOLECULES;REACTOME_PHA

.CROSS_CHEMICAL_SYNAPSES;REACTOME_NEURONAL_SYSTEM;REACTOME_NEUROTRAN

.CROSS_CHEMICAL_SYNAPSES;REACTOME_NEURONAL_SYSTEM;REACTOME_SIGNALING_E

PHA_BETA_SIGNALING;REACTOME_INTERFERON_SIGNALING;REACTOME_IMMUNE_SYSTEM

_RESPIRATORY_ELECTRON_TRANSPORT;REACTOME_RESPIRATORY_ELECTRON_TRANSPO

HE_PROTEOLYTIC_ACTIVITY_OF_APC_C_REQUIRED_FOR_THE_ONSET_OF_ANAPHASE_BY_

PCR;REACTOME_OPIOID_SIGNALLING;REACTOME_DARPP_32_EVENTS

E_TRANSPORT_OF_SMALL_MOLECULES;REACTOME_SLC_MEDIATED_TRANSMEMBRANE_T

CF_KIT;REACTOME_GROWTH_HORMONE_RECEPTOR_SIGNALING;REACTOME_SIGNALING_

THESIS;REACTOME_HEPARAN_SULFATE_HEPARIN_HS_GAG_METABOLISM;REACTOME_GLY

NGF;REACTOME_TRIF_MEDIATED_TLR3_SIGNALING;REACTOME_TCR_SIGNALING;REACTOM

MUNICATION;REACTOME_DSCAM_INTERACTIONS

IOSYNTHESIS;REACTOME_PHOSPHOLIPID_METABOLISM;REACTOME_SYNTHESIS_OF_PA;R

PCR;REACTOME_CLASS_A1_RHODOPSIN_LIKE_RECEPTORS;REACTOME_GPCR_DOWNSTR
DATION;REACTOME_CHONDROITIN_SULFATE_DERMATAN_SULFATE_METABOLISM;REACTO

MUNICATION;REACTOME_CELL_EXTRACELLULAR_MATRIX_INTERACTIONS;REACTOME_CEL

HO_GTPASES;REACTOME_MEMBRANE_TRAFFICKING;REACTOME_TRANS_GOLGI_NETWOR

LEVATED_PLATELET_CYTOSOLIC_CA2_;REACTOME_FORMATION_OF_FIBRIN_CLOT_CLOTT

IPID_METABOLISM;REACTOME_PHOSPHOLIPID_METABOLISM;REACTOME_SPHINGOLIPID_M

NGF;REACTOME_CELL_CELL_COMMUNICATION;REACTOME_P75NTR_RECRUITS_SIGNALLIN

_LIPIDS_AND_LIPOPROTEINS;REACTOME_LIPID_DIGESTION_MOBILIZATION_AND_TRANSPO

NSPORT_ACROSS_THE_PLASMA_MEMBRANE;REACTOME_TRANSMEMBRANE_TRANSPORT

SYLATION_OF_MUCINS;REACTOME_TERMINATION_OF_O_GLYCAN_BIOSYNTHESIS;REACT

ME_CELL_CYCLE;REACTOME_CHROMOSOME_MAINTENANCE;REACTOME_MEIOTIC_SYNAP
NSCRIPTION_AND_TRANSLATION;REACTOME_SIGNALING_BY_NOTCH2;REACTOME_PRE_N

_COMPLEMENT_CASCADE;REACTOME_INNATE_IMMUNE_SYSTEM;REACTOME_IMMUNE_SY

SURFACE_INTERACTIONS;REACTOME_SIGNALING_BY_PDGF

PTERIN_BH4_SYNTHESIS_RECYCLING_SALVAGE_AND_REGULATION;REACTOME_METABOL

CROSS_CHEMICAL_SYNAPSES;REACTOME_NEURONAL_SYSTEM;REACTOME_NEUROTRAN

IETABOLISM;REACTOME_SYNTHESIS_OF_PC;REACTOME_GLYCEROPHOSPHOLIPID_BIOSYN
S_GENE_EXPRESSION;REACTOME_METABOLISM_OF_LIPIDS_AND_LIPOPROTEINS;REACTO
SURFACE_INTERACTIONS

NSCRIPTION_INITIATION_FROM_TYPE_2_PROMOTER;REACTOME_RNA_POL_II_TRANSCRIP

TES_B_CELL_RECEPTOR_LEADING_TO_GENERATION_OF_SECOND_MESSENGERS;REACTO

_RESPIRATORY_ELECTRON_TRANSPORT;REACTOME_MITOCHONDRIAL_PROTEIN_IMPORT;
SIGNALLING_PATHWAY_VIA_PKC_AND_MAPK;REACTOME_SIGNALING_BY_GPCR;REACTOME

CROSS_CHEMICAL_SYNAPSES;REACTOME_NEURONAL_SYSTEM

_PROTEINS;REACTOME_POST_TRANSLATIONAL_PROTEIN_MODIFICATION;REACTOME_SYN

PCR;REACTOME_PEPTIDE_LIGAND_BINDING_RECEPTORS;REACTOME_CLASS_A1_RHODO

DEGRADATION;REACTOME_SIGNALLING_BY_NGF;REACTOME_DEVELOPMENTAL_BIOLOGY;

G;REACTOME_POST_CHAPERONIN_TUBULIN_FOLDING_PATHWAY;REACTOME_METABOLIS

CROSS_CHEMICAL_SYNAPSES;REACTOME_NEURONAL_SYSTEM;REACTOME_NEUROTRAN

MUNICATION;REACTOME_SIGNAL_REGULATORY_PROTEIN_SIRP_FAMILY_INTERACTIONS

ELLULAR_DOMAIN_REGULATES_TRANSCRIPTION;REACTOME_SIGNALING_BY_NOTCH1;REA

E_TRANSPORT_OF_SMALL_MOLECULES;REACTOME_SLC_MEDIATED_TRANSMEMBRANE_T

_BIOLOGY;REACTOME_HS_GAG_DEGRADATION;REACTOME_CHONDROITIN_SULFATE_DER

HO_GTPASES

COUPLED_NER_TC_NER;REACTOME_NUCLEOTIDE_EXCISION_REPAIR;REACTOME_FORMA

E_TRANSPORT_OF_SMALL_MOLECULES;REACTOME_SLC_MEDIATED_TRANSMEMBRANE_T

_BIOLOGY;REACTOME_AXON_GUIDANCE;REACTOME_NETRIN1_SIGNALING;REACTOME_DC

SSING_CROSS_PRESENTATION;REACTOME_ENDOSOMAL_VACUOLAR_PATHWAY;REACTOM

SPORT_AND_METABOLISM;REACTOME_ABC_FAMILY_PROTEINS_MEDIATED_TRANSPORT;R

SIGNALLING_PATHWAY_VIA_PKC_AND_MAPK;REACTOME_SIGNALING_BY_GPCR;REACTOME
SIGNALLING_PATHWAY_VIA_PKC_AND_MAPK;REACTOME_SIGNALING_BY_GPCR;REACTOME
PCR;REACTOME_OLFACTORY_SIGNALING_PATHWAY;REACTOME_GPCR_DOWNSTREAM_S

ACROSS_CHEMICAL_SYNAPSES;REACTOME_NEURONAL_SYSTEM

CRIPTION_PATHWAY

'IPS_AT_THE_EARLY_ENDOSOME_MEMBRANE;REACTOME_SYNTHESIS_OF_PIPS_AT_THE_(
MUNICATION;REACTOME_CELL_EXTRACELLULAR_MATRIX_INTERACTIONS;REACTOME_CEL
ME_CELL_CYCLE;REACTOME_P53_INDEPENDENT_G1_S_DNA_DAMAGE_CHECKPOINT;REAC
VAYS;REACTOME_ACTIVATION_OF_CHAPERONE_GENES_BY_XBP1S;REACTOME_UNFOLDE

NGF;REACTOME_SIGNALING_BY_SCF_KIT;REACTOME_DEVELOPMENTAL_BIOLOGY;REACTC
NTIGEN_PRESENTATION;REACTOME_FACTORS_INVOLVED_IN_MEGAKARYOCYTE_DEVELOF
EM;REACTOME_INTEGRATION_OF_ENERGY_METABOLISM;REACTOME_REGULATION_OF_IN

ACROSS_CHEMICAL_SYNAPSES;REACTOME_NEURONAL_SYSTEM;REACTOME_NEUROTRAN

TES_B_CELL_RECEPTOR_LEADING_TO_GENERATION_OF_SECOND_MESSENGERS;REACTC

VED_IN_MEGAKARYOCYTE_DEVELOPMENT_AND_PLATELET_PRODUCTION;REACTOME_HEM

CRIPTION_PATHWAY

ACROSS_CHEMICAL_SYNAPSES;REACTOME_NEURONAL_SYSTEM;REACTOME_NEUROTRAN

_CARBOHYDRATES

_CARBOHYDRATES

PCR;REACTOME_CLASS_C_3_METABOTROPIC_GLUTAMATE_PHEROMONE_RECEPTORS;RE
G;REACTOME_IMMUNE_SYSTEM;REACTOME_ADAPTIVE_IMMUNE_SYSTEM

DRATION_OF_CARBON_DIOXIDE
SSING_CROSS_PRESENTATION;REACTOME_LATENT_INFECTION_OF_HOMO_SAPIENS_WITI
EM;REACTOME_VOLTAGE_GATED_POTASSIUM_CHANNELS;REACTOME_POTASSIUM_CHAN

OSYLATION_OF_MUCINS;REACTOME_METABOLISM_OF_PROTEINS;REACTOME_POST_TRAN

_PROTEIN_IMPORT;REACTOME_METABOLISM_OF_PROTEINS

RBOXYLATION_HYPUSINE_FORMATION_AND_ARYLSULFATASE_ACTIVATION;REACTOME_GL
PAS2_ACTIVATES_CIRCADIAN_EXPRESSION;REACTOME_CIRCADIAN_CLOCK

CTOME_CELL_CYCLE_MITOTIC;REACTOME_MITOTIC_M_M_G1_PHASES;REACTOME_DNA_R

OSYLATION_OF_MUCINS;REACTOME_METABOLISM_OF_PROTEINS;REACTOME_POST_TRAN

_BIOLOGY;REACTOME_AXON_GUIDANCE;REACTOME_NETRIN1_SIGNALING;REACTOME_DC

_NON_CODING_RNA;REACTOME_ANTIVIRAL_MECHANISM_BY_IFN_STIMULATED_GENES;RE

OR_RECYCLING;REACTOME_LATENT_INFECTION_OF_HOMO_SAPIENS_WITH_MYCOBACTEI

NGF

_SYSTEM;REACTOME_IMMUNE_SYSTEM;REACTOME_NOD1_2_SIGNALING_PATHWAY;REACT

MUNICATION;REACTOME_TGF_BETA_RECEPTOR_SIGNALING_IN_EMT_EPITHELIAL_TO_MES

._BIOLOGY;REACTOME_AXON_GUIDANCE;REACTOME_NETRIN1_SIGNALING;REACTOME_DC

_AMINO_ACIDS_AND_DERIVATIVES;REACTOME_AMINE_DERIVED_HORMONES

._BIOLOGY;REACTOME_EXTRACELLULAR_MATRIX_ORGANIZATION;REACTOME_COLLAGEN_

._BIOLOGY;REACTOME_PPARA_ACTIVATES_GENE_EXPRESSION;REACTOME_METABOLISM_
._BIOLOGY;REACTOME_REGULATION_OF_BETA_CELL_DEVELOPMENT;REACTOME_REGULA
/ED_IN_MEGAKARYOCYTE_DEVELOPMENT_AND_PLATELET_PRODUCTION;REACTOME_HEM
_NON_CODING_RNA;REACTOME_METABOLISM_OF_RNA

AVAGE_OF_CELLULAR_PROTEINS;REACTOME_MEMBRANE_TRAFFICKING;REACTOME_SIGN
ACROSS_CHEMICAL_SYNAPSES;REACTOME_NEURONAL_SYSTEM;REACTOME_NEUROTRAN

SURFACE_INTERACTIONS

MUNICATION;REACTOME_INTEGRIN_CELL_SURFACE_INTERACTIONS;REACTOME_CELL_JUN
OR_SIGNALLING_CASCADE;REACTOME_REGULATION_OF_AMPK_ACTIVITY_VIA_LKB1;REAC
_MATRIX_ORGANIZATION;REACTOME_COLLAGEN_FORMATION;REACTOME_METABOLISM_

OSYLATION_OF_MUCINS;REACTOME_METABOLISM_OF_PROTEINS;REACTOME_POST_TRAN
OSYLATION_OF_MUCINS;REACTOME_METABOLISM_OF_PROTEINS;REACTOME_POST_TRAN

CRIPTION_PATHWAY

CRIPTION_PATHWAY

IOSYNTHESIS;REACTOME_PHOSPHOLIPID_METABOLISM;REACTOME_SYNTHESIS_OF_PE;R

HO_GTPASES;REACTOME_SIGNALLING_BY_NGF;REACTOME_NRAGE_SIGNALS_DEATH_TH

ON_OF_TGF_BETA_RECEPTOR_SIGNALING;REACTOME_TGF_BETA_RECEPTOR_SIGNALING_

AKDOWN_GLYCOGENOLYSIS;REACTOME_METABOLISM_OF_CARBOHYDRATES;REACTOME_

MUNICATION;REACTOME_CELL_JUNCTION_ORGANIZATION

CTOME_CELL_CYCLE_MITOTIC;REACTOME_RECRUITMENT_OF_MITOTIC_CENTROSOME_P

_SYSTEM;REACTOME_IMMUNE_SYSTEM;REACTOME_INFLAMMASOMES;REACTOME_NUCLE(

REACTOME_TRANSLOCATION_OF_ZAP_70_TO_IMMUNOLOGICAL_SYNAPSE;REACTOME_GEI

NTIGEN_PRESENTATION;REACTOME_FACTORS_INVOLVED_IN_MEGAKARYOCYTE_DEVELOF
PCR;REACTOME_CLASS_B_2_SECRETIN_FAMILY_RECEPTORS;REACTOME_GPCR_DOWNST

ACTOME_SRP_DEPENDENT_COTRANSLATIONAL_PROTEIN_TARGETING_TO_MEMBRANE;RE

NGF;REACTOME_SIGNALING_BY_SCF_KIT;REACTOME_CELL_CELL_COMMUNICATION;REACT

E_TRANSPORT_OF_SMALL_MOLECULES;REACTOME_ION_TRANSPORT_BY_P_TYPE_ATPAS
E_TRANSPORT_OF_SMALL_MOLECULES;REACTOME_ION_TRANSPORT_BY_P_TYPE_ATPAS

LATION_OF_FGFR_SIGNALING;REACTOME_INSULIN_RECEPTOR_SIGNALLING_CASCADE;RE

PCR;REACTOME_PEPTIDE_LIGAND_BINDING_RECEPTORS;REACTOME_CLASS_A1_RHODOF
HO_GTPASES;REACTOME_SIGNALLING_BY_NGF;REACTOME_PPARA_ACTIVATES_GENE_EX

OSYLATION_OF_MUCINS;REACTOME_TERMINATION_OF_O_GLYCAN_BIOSYNTHESIS;REACT(

ACTION;REACTOME_SMOOTH_MUSCLE_CONTRACTION
F_THE_EXTRACELLULAR_MATRIX;REACTOME_EXTRACELLULAR_MATRIX_ORGANIZATION

DATIONS;REACTOME_GLUTATHIONE_CONJUGATION;REACTOME_PHASE_II_CONJUGATION
EACTOME_HIV_LIFE_CYCLE;REACTOME_EARLY_PHASE_OF_HIV_LIFE_CYCLE;REACTOME_H

_BIOLOGY;REACTOME_CELL_CELL_COMMUNICATION;REACTOME_IMMUNOREGULATORY_I

OSYLATION_OF_MUCINS;REACTOME_TERMINATION_OF_O_GLYCAN_BIOSYNTHESIS;REACTO

EM;REACTOME_INTEGRATION_OF_ENERGY_METABOLISM;REACTOME_ABC_FAMILY_PROTE
BOLISM_AND_CITRIC_ACID_TCA_CYCLE;REACTOME_TCA_CYCLE_AND_RESPIRATORY_ELE

NTIGEN_PRESENTATION;REACTOME_IMMUNE_SYSTEM;REACTOME_ADAPTIVE_IMMUNE_SY

E_TRANSPORT_OF_SMALL_MOLECULES;REACTOME_SLC_MEDIATED_TRANSMEMBRANE_TI

F_THE_EXTRACELLULAR_MATRIX;REACTOME_EXTRACELLULAR_MATRIX_ORGANIZATION
LE_CONTRACTION;REACTOME_MUSCLE_CONTRACTION

;REACTOME_ADAPTIVE_IMMUNE_SYSTEM;REACTOME_CLASS_I_MHC_MEDIATED_ANTIGEN

HE_PROTEOLYTIC_ACTIVITY_OF_APC_C_REQUIRED_FOR_THE_ONSET_OF_ANAPHASE_BY_

NTERACTIONS_AT_THE_VASCULAR_WALL;REACTOME_BASIGIN_INTERACTIONS;REACTOMI
NTERACTIONS_AT_THE_VASCULAR_WALL;REACTOME_INTEGRIN_CELL_SURFACE_INTERA(
E_TRANSPORT_OF_SMALL_MOLECULES;REACTOME_SLC_MEDIATED_TRANSMEMBRANE_TI

PCR;REACTOME_CLASS_B_2_SECRETIN_FAMILY_RECEPTORS;REACTOME_GLUCAGON_TYI

RBB4;REACTOME_NUCLEAR_SIGNALING_BY_ERBB4
RTERS_IN_LIPID_HOMEOSTASIS;REACTOME_ABC_FAMILY_PROTEINS_MEDIATED_TRANSPO
G;REACTOME_POST_CHAPERONIN_TUBULIN_FOLDING_PATHWAY;REACTOME_METABOLIS

E_TRANSPORT_OF_SMALL_MOLECULES;REACTOME_SLC_MEDIATED_TRANSMEMBRANE_TI

RTERS_IN_LIPID_HOMEOSTASIS;REACTOME_ABC_FAMILY_PROTEINS_MEDIATED_TRANSPO

PCR;REACTOME_CLASS_B_2_SECRETIN_FAMILY_RECEPTORS;REACTOME_GPCR_LIGAND_

CRIPTION_PATHWAY

SIGNALLING_PATHWAY_VIA_PKC_AND_MAPK;REACTOME_SIGNALING_BY_GPCR;REACTOME
_BIOLOGY;REACTOME_AXON_GUIDANCE;REACTOME_NCAM1_INTERACTIONS;REACTOME_
SIGNALLING_PATHWAY_VIA_PKC_AND_MAPK;REACTOME_TRANSMISSION_ACROSS_CHEMI(
VED_IN_MEGAKARYOCYTE_DEVELOPMENT_AND_PLATELET_PRODUCTION;REACTOME_HEM

_BIOLOGY;REACTOME_EXTRACELLULAR_MATRIX_ORGANIZATION;REACTOME_COLLAGEN_

NGF;REACTOME_TRIF_MEDIATED_TLR3_SIGNALING;REACTOME_P75NTR_RECRUITS_SIGNA

FFICKING;REACTOME_GAP_JUNCTION_TRAFFICKING;REACTOME_GAP_JUNCTION_ASSEMBL
OSYLATION_OF_MUCINS;REACTOME_TERMINATION_OF_O_GLYCAN_BIOSYNTHESIS;REACTO

REACTOME_ANTIVIRAL_MECHANISM_BY_IFN_STIMULATED_GENES;REACTOME_SIGNALING_
_NUCLEOTIDES;REACTOME_PURINE_SALVAGE;REACTOME_PURINE_METABOLISM

HO_GTPASES;REACTOME_SIGNALLING_BY_NGF;REACTOME_NRAGE_SIGNALS_DEATH_THR
_AMINO_ACIDS_AND_DERIVATIVES;REACTOME_AMINE_DERIVED_HORMONES
_STEROID_HORMONES_AND_VITAMINS_A_AND_D;REACTOME_STEROID_HORMONES;REAC

_MATRIX_ORGANIZATION;REACTOME_COLLAGEN_FORMATION
ETABOLISM;REACTOME_GLYCOSAMINOGLYCAN_METABOLISM;REACTOME_ABC_FAMILY_PR
REACTOME_NITRIC_OXIDE_STIMULATES_GUANYLATE_CYCLASE;REACTOME_PLATELET_HO
FFICKING;REACTOME_TRANS_GOLGI_NETWORK_VESICLE_BUDDING;REACTOME_GOLGI_AS

PCR;REACTOME_PEPTIDE_LIGAND_BINDING_RECEPTORS;REACTOME_CLASS_A1_RHODOP

_BIOLOGY;REACTOME_AXON_GUIDANCE;REACTOME_OTHER_SEMAPHORIN_INTERACTION
HO_GTPASES;REACTOME_SIGNALLING_BY_NGF;REACTOME_TRANSMISSION_ACROSS_CHE
AVAGE_OF_CELLULAR_PROTEINS;REACTOME_SIGNALING_BY_WNT;REACTOME_CTNNB1_P

_BIOLOGY;REACTOME_TRANSCRIPTIONAL_REGULATION_OF_WHITE_ADIPOCYTE_DIFFERE
IPPO

THE_PRE_REPLICATIVE_COMPLEX;REACTOME_CELL_CYCLE;REACTOME_PROCESSIVE_SYI

_BIOLOGY;REACTOME_AXON_GUIDANCE;REACTOME_CRMPS_IN_SEMA3A_SIGNALING;REA

NSCRIPTION;REACTOME_TRANSCRIPTION;REACTOME_RNA_POL_I_RNA_POL_III_AND_MITO

PCR;REACTOME_OLFACTORY_SIGNALING_PATHWAY;REACTOME_GPCR_DOWNSTREAM_S

ES_GENE_EXPRESSION;REACTOME_METABOLISM_OF_LIPIDS_AND_LIPOPROTEINS;REACTC

OSYLATION_OF_MUCINS;REACTOME_METABOLISM_OF_PROTEINS;REACTOME_POST_TRAN

NGF;REACTOME_DAG_AND_IP3_SIGNALING;REACTOME_SIGNALING_BY_ERBB2;REACTOME_

_ENERGY_METABOLISM;REACTOME_REGULATION_OF_INSULIN_SECRETION;REACTOME_II

_MRNA;REACTOME_METABOLISM_OF_RNA;REACTOME_DESTABILIZATION_OF_MRNA_BY_B

_BIOLOGY;REACTOME_AXON_GUIDANCE;REACTOME_NCAM1_INTERACTIONS;REACTOME_

_MATRIX_ORGANIZATION;REACTOME_COLLAGEN_FORMATION

_HYPOXIA_INDUCIBLE_FACTOR_HIF_BY_OXYGEN;REACTOME_OXYGEN_DEPENDENT_PROL

ACTOME_FORMATION_OF_THE_TERNARY_COMPLEX_AND_SUBSEQUENTLY_THE_43S_COM

LEVATED_PLATELET_CYTOSOLIC_CA2_;REACTOME_FORMATION_OF_FIBRIN_CLOT_CLOTT

OPENING;REACTOME_TRANSCRIPTION;REACTOME_RNA_POL_II_PRE_TRANSCRIPTION_EVI
_MRNA_SPLICING

_PHASE1_FUNCTIONALIZATION_OF_COMPOUNDS
GLYCEROPHOSPHOLIPID_BIOSYNTHESIS;REACTOME_METABOLISM_OF_LIPIDS_AND_LIPOPI

_IMMUNE_SYSTEM;REACTOME_ADAPTIVE_IMMUNE_SYSTEM
OCHONDRIAL_TRANSCRIPTION;REACTOME_RNA_POL_III_TRANSCRIPTION_INITIATION_FROI

ALING_IN_IMMUNE_SYSTEM

ARAGINE_N_LINKED_GLYCOSYLATION;REACTOME_TRANSPORT_TO_THE_GOLGI_AND_SUE

TREAM_SIGNALING;REACTOME_G_ALPHA_S_SIGNALLING_EVENTS;REACTOME_GPCR_LIGA

E_EXPRESSION;REACTOME_PRE_NOTCH_EXPRESSION_AND_PROCESSING;REACTOME_NC

ACTOME_PROCESSING_OF_CAPPED_INTRON_CONTAINING_PRE_MRNA;REACTOME_TRANS

_SIGNALING_BY_EGFR_IN_CANCER;REACTOME_NGF_SIGNALLING_VIA_TRKA_FROM_THE_I
E;REACTOME_SPRY_REGULATION_OF_FGF_SIGNALING;REACTOME_TRIF_MEDIATED_TLR3_

RANSPORT;REACTOME_TRANSPORT_OF_GLUCOSE_AND_OTHER_SUGARS_BILE_SALTS_AI

MPLEX;REACTOME_ACTIVATION_OF_THE_MRNA_UPON_BINDING_OF_THE_CAP_BINDING_C

SIS;REACTOME_PACKAGING_OF_TELOMERE_ENDS;REACTOME_TELOMERE_MAINTENANCE

LIFE_CYCLE;REACTOME_LATE_PHASE_OF_HIV_LIFE_CYCLE

N_FORMATION;REACTOME_CELL_JUNCTION_ORGANIZATION

ME_DOWNSTREAM_TCR_SIGNALING;REACTOME_P75NTR_RECRUITS_SIGNALLING_COMPLE

IGNALING

_TRANSPORT_ACROSS_THE_PLASMA_MEMBRANE;REACTOME_TRANSMEMBRANE_TRANSF

BOLISM;REACTOME_GLYCOSAMINOGLYCAN_METABOLISM;REACTOME_METABOLISM_OF_C

SIS

ES;REACTOME_ION_CHANNEL_TRANSPORT

EVENTS

_MRNA_SPLICING;REACTOME_MRNA_SPLICING_MINOR_PATHWAY

ROTEINS_AND_COMPLEXES;REACTOME_LOSS_OF_NLP_FROM_MITOTIC_CENTROSOMES;R

_SIGNALING_BY_EGFR_IN_CANCER;REACTOME_NGF_SIGNALLING_VIA_TRKA_FROM_THE_I

STRATE_TYPE;REACTOME_PHASE1_FUNCTIONALIZATION_OF_COMPOUNDS

D_PROTEIN_RESPONSE

_PRE_MRNA;REACTOME_RNA_POL_II_TRANSCRIPTION;REACTOME_MRNA_PROCESSING;RE

'COSAMINOGLYCAN_METABOLISM;REACTOME_METABOLISM_OF_CARBOHYDRATES
ROUGH_JNK;REACTOME_CELL_DEATH_SIGNALLING_VIA_NRAGE_NRIF_AND_NADE;REACTO

SIGNALING_BY_ERBB2;REACTOME_SIGNALING_BY_EGFR_IN_CANCER;REACTOME_PI3K_EVI
SIGNALING_BY_ERBB2;REACTOME_SIGNALING_BY_EGFR_IN_CANCER;REACTOME_PI3K_EVI

_FROM_CHROMATIN;REACTOME_ASSOCIATION_OF_LICENSING_FACTORS_WITH_THE_PRE_

INELS

EVENTS
CTOME_PPARA_ACTIVATES_GENE_EXPRESSION;REACTOME_RORA_ACTIVATES_CIRCADIAN

L_JUNCTION_ORGANIZATION

S;REACTOME_PURINE_METABOLISM
_FORMATION;REACTOME_INTEGRIN_CELL_SURFACE_INTERACTIONS;REACTOME_SIGNALIN
ROUGH_JNK;REACTOME_CELL_DEATH_SIGNALLING_VIA_NRAGE_NRIF_AND_NADE;REACTO

TOME_SIGNALING_BY_ERBB4;REACTOME_SIGNALING_BY_ERBB2;REACTOME_SIGNALING_E
MOSTASIS

OL_OXIDATION

/IOSTASIS;REACTOME_HIV_INFECTION;REACTOME_HOST_INTERACTIONS_OF_HIV_FACTOR
NG;REACTOME_NOTCH1_INTRACELLULAR_DOMAIN_REGULATES_TRANSCRIPTION;REACTO

I_CHANNELS

LISM;REACTOME_METABOLISM_OF_CARBOHYDRATES

RANSPORT;REACTOME_TRANSPORT_OF_INORGANIC_CATIONS_ANIONS_AND_AMINO_ACID

TOME_ENDOSOMAL_SORTING_COMPLEX_REQUIRED_FOR_TRANSPORT_ESCRT;REACTOME

RANSPORT;REACTOME_TRANSPORT_OF_INORGANIC_CATIONS_ANIONS_AND_AMINO_ACID

E_PLASMA_MEMBRANE;REACTOME_SIGNALLING_TO_ERKS;REACTOME_P38MAPK_EVENTS

SPORT;REACTOME_STEROID_HORMONES;REACTOME_METABOLISM_OF_LIPIDS_AND_LIPO

REACTOME_REGULATION_OF_WATER_BALANCE_BY_RENAL_AQUAPORINS

OME_METABOLISM_OF_PROTEINS;REACTOME_POST_TRANSLATIONAL_PROTEIN_MODIFICA
OME_METABOLISM_OF_PROTEINS;REACTOME_POST_TRANSLATIONAL_PROTEIN_MODIFICA

COSAMINOGLYCAN_METABOLISM;REACTOME_METABOLISM_OF_CARBOHYDRATES

CA2_;REACTOME_HEMOSTASIS;REACTOME_PLATELET_ACTIVATION_SIGNALING_AND_AGG
D_PROTEIN_RESPONSE
PMENT_AND_PLATELET_PRODUCTION;REACTOME_KINESINS;REACTOME_HEMOSTASIS;REA

IGNALING

DE_STIMULATES_GUANYLATE_CYCLASE;REACTOME_PLATELET_HOMEOSTASIS;REACTOME
REACTOME_SIGNALING_BY_NOTCH1;REACTOME_TRANSCRIPTIONAL_ACTIVITY_OF_SMAD2
PTOSIS_INDUCED_DNA_FRAGMENTATION;REACTOME_TAK1_ACTIVATES_NFKB_BY_PHOSP

_PROCESSING_PRESENTATION;REACTOME_ANTIGEN_PROCESSING_UBIQUITINATION_PRO

IGNALING

RANSPORT;REACTOME_TRANSPORT_OF_INORGANIC_CATIONS_ANIONS_AND_AMINO_ACID

SIGNALING

RANSPORT;REACTOME_TRANSPORT_OF_VITAMINS_NUCLEOSIDES_AND_RELATED_MOLEC

I;REACTOME_CYTOKINE_SIGNALING_IN_IMMUNE_SYSTEM
TION_INTERACTIONS;REACTOME_CELL_JUNCTION_ORGANIZATION

M_OF_PROTEINS

IAPSES;REACTOME_NEURONAL_SYSTEM;REACTOME_NEUROTRANSMITTER_RELEASE_CYC

PSIN_LIKE_RECEPTORS;REACTOME_GPCR_DOWNSTREAM_SIGNALING;REACTOME_G_ALPH

OL_OXIDATION
OL_OXIDATION

ANSCRIPTION_PATHWAY;REACTOME_METABOLISM_OF_LIPIDS_AND_LIPOPROTEINS;REACT

K_VESICLE_BUDDING;REACTOME_SYNTHESIS_OF_PIPS_AT_THE_GOLGI_MEMBRANE;REAC

_PROCESSING_PRESENTATION;REACTOME_ANTIGEN_PROCESSING_UBIQUITINATION_PRC

IGNALING

;;REACTOME_PURINE_METABOLISM

ESINS;REACTOME_HEMOSTASIS
ESINS;REACTOME_HEMOSTASIS

ACTOME_GLYCEROPHOSPHOLIPID_BIOSYNTHESIS;REACTOME_SIGNALING_BY_GPCR;REA(

TOME_SIGNALING_BY_ERBB4;REACTOME_SIGNALING_BY_ERBB2;REACTOME_SIGNALING_E
TOME_SIGNALING_BY_ERBB4;REACTOME_SIGNALING_BY_ERBB2;REACTOME_SIGNALING_E

E_PEPTIDE_LIGAND_BINDING_RECEPTORS;REACTOME_CLASS_A1_RHODOPSIN_LIKE_RECE

ME_ER_PHAGOSOME_PATHWAY;REACTOME_IMMUNOREGULATORY_INTERACTIONS_BETWE
CHONDRIAL_TRANSCRIPTION;REACTOME_RNA_POL_I_TRANSCRIPTION_INITIATION

OME_FATTY_ACID_TRIACYLGLYCEROL_AND_KETONE_BODY_METABOLISM

ATION_OF_GENE_EXPRESSION_IN_BETA_CELLS;REACTOME_TRANSMEMBRANE_TRANSPOR

BY_GPCR;REACTOME_INTEGRATION_OF_ENERGY_METABOLISM;REACTOME_OPIOID_SIGNA

CTRON_TRANSPORT;REACTOME_REGULATION_OF_PYRUVATE_DEHYDROGENASE_PDH_C

STASIS

RANSPORT;REACTOME_TRANSPORT_OF_INORGANIC_CATIONS_ANIONS_AND_AMINO_ACID

CAM_INTERACTIONS;REACTOME_INTERACTION_BETWEEN_L1_AND_ANKYRINS;REACTOME_

RANSPORT;REACTOME_TRANSPORT_OF_VITAMINS_NUCLEOSIDES_AND_RELATED_MOLEC

[illegible]ONAL_SYSTEM;REACTOME_INTEGRATION_OF_ENERGY_METABOLISM;REACTOME_AXON_[illegible]

[illegible]SMOOTH_MUSCLE_CONTRACTION;REACTOME_HEMOSTASIS;REACTOME_PLATELET_ACTIV

REACTOME_REGULATION_OF_WATER_BALANCE_BY_RENAL_AQUAPORINS

[illegible]ME_SIGNALING_BY_ERBB4;REACTOME_SIGNALING_BY_ERBB2;REACTOME_SIGNALING_BY_
_SIGNALING_BY_EGFR_IN_CANCER;REACTOME_ANTIGEN_ACTIVATES_B_CELL_RECEPTOR
D_PROTEIN_RESPONSE

[illegible]SSOCIATED_VESICLE_BIOGENESIS

SMITTER_RECEPTOR_BINDING_AND_DOWNSTREAM_TRANSMISSION_IN_THE_POSTSYNAP

CTRON_TRANSPORT;REACTOME_REGULATION_OF_PYRUVATE_DEHYDROGENASE_PDH_C

E_INTEGRATION_OF_ENERGY_METABOLISM;REACTOME_GLUCAGON_SIGNALING_IN_METAI
ACTOME_TRIGLYCERIDE_BIOSYNTHESIS;REACTOME_FATTY_ACYL_COA_BIOSYNTHESIS;RE

ENES_BY_ATF4;REACTOME_UNFOLDED_PROTEIN_RESPONSE;REACTOME_METABOLISM_O
TION_OF_TRANSCRIPTION_COUPLED_NER_TC_NER_REPAIR_COMPLEX;REACTOME_DNA_F

UT_GROWTH;REACTOME_L1CAM_INTERACTIONS;REACTOME_INTERACTION_BETWEEN_L1_
ENES_BY_ATF4;REACTOME_UNFOLDED_PROTEIN_RESPONSE;REACTOME_METABOLISM_O

LIPID_METABOLISM;REACTOME_ACYL_CHAIN_REMODELLING_OF_PE;REACTOME_GLYCERO
PEPTIDE_HORMONE_BIOSYNTHESIS

_SIGNALING_BY_EGFR_IN_CANCER;REACTOME_NGF_SIGNALLING_VIA_TRKA_FROM_THE_I

_FROM_CHROMATIN;REACTOME_CELL_CYCLE_MITOTIC;REACTOME_CELL_CYCLE_CHECKP

YSTEM;REACTOME_ADAPTIVE_IMMUNE_SYSTEM

SLATIONAL_PROTEIN_MODIFICATION

ROUGH_JNK;REACTOME_CELL_DEATH_SIGNALLING_VIA_NRAGE_NRIF_AND_NADE;REACTO

_PROCESSING_PRESENTATION;REACTOME_ANTIGEN_PROCESSING_UBIQUITINATION_PRO

TION_INTERACTIONS;REACTOME_CELL_JUNCTION_ORGANIZATION

MOLECULES

ESSING_CROSS_PRESENTATION;REACTOME_INTERFERON_GAMMA_SIGNALING;REACTOME

ROUGH_JNK;REACTOME_CELL_DEATH_SIGNALLING_VIA_NRAGE_NRIF_AND_NADE;REACTO

ROUGH_JNK;REACTOME_CELL_DEATH_SIGNALLING_VIA_NRAGE_NRIF_AND_NADE;REACTO

RIPTION;REACTOME_RNA_POL_I_RNA_POL_III_AND_MITOCHONDRIAL_TRANSCRIPTION;REA

_OF_SMALL_MOLECULES;REACTOME_SLC_MEDIATED_TRANSMEMBRANE_TRANSPORT;RE

_OF_SMALL_MOLECULES;REACTOME_SLC_MEDIATED_TRANSMEMBRANE_TRANSPORT;RE

RANSPORT;REACTOME_TRANSPORT_OF_INORGANIC_CATIONS_ANIONS_AND_AMINO_ACI

RIPTION;REACTOME_RNA_POL_I_RNA_POL_III_AND_MITOCHONDRIAL_TRANSCRIPTION;REA

TION;REACTOME_TRANSCRIPTION;REACTOME_RNA_POL_I_RNA_POL_III_AND_MITOCHOND

CTOME_SIGNALING_BY_NOTCH

MOSTASIS;REACTOME_INNATE_IMMUNE_SYSTEM;REACTOME_IMMUNE_SYSTEM;REACTOME

BINDING

_REPLACEMENT_PATHWAY;REACTOME_BASE_FREE_SUGAR_PHOSPHATE_REMOVAL_VIA_
_REPLACEMENT_PATHWAY;REACTOME_BASE_FREE_SUGAR_PHOSPHATE_REMOVAL_VIA_

ES;REACTOME_ION_CHANNEL_TRANSPORT

THE_FANCONI_ANEMIA_PATHWAY;REACTOME_FANCONI_ANEMIA_PATHWAY;REACTOME_D
;REACTOME_NUCLEOTIDE_LIKE_PURINERGIC_RECEPTORS;REACTOME_GPCR_DOWNSTRE

TION_OF_GENE_EXPRESSION_IN_BETA_CELLS;REACTOME_INTEGRATION_OF_ENERGY_M

ISCRIPTION_COUPLED_NER_TC_NER;REACTOME_POL_SWITCHING;REACTOME_NUCLEOTI

RANSPORT;REACTOME_TRANSPORT_OF_INORGANIC_CATIONS_ANIONS_AND_AMINO_ACID
TOME_PPARA_ACTIVATES_GENE_EXPRESSION;REACTOME_RORA_ACTIVATES_CIRCADIAN

IOSTASIS

ECEPTOR_SIGNALING;REACTOME_NUCLEAR_SIGNALING_BY_ERBB4;REACTOME_IMMUNE_

_II_TRANSCRIPTION;REACTOME_MRNA_CAPPING;REACTOME_TRANSCRIPTION_COUPLED_

ATION;REACTOME_HYALURONAN_UPTAKE_AND_DEGRADATION;REACTOME_HYALURONAN
ATION;REACTOME_HYALURONAN_UPTAKE_AND_DEGRADATION;REACTOME_HYALURONAN
ALPHA_HYDROXYCHOLESTEROL;REACTOME_SYNTHESIS_OF_BILE_ACIDS_AND_BILE_SALT

ME_LIGAND_GATED_ION_CHANNEL_TRANSPORT

ENTIATION

EACTOME_RNA_POL_I_RNA_POL_III_AND_MITOCHONDRIAL_TRANSCRIPTION;REACTOME_C

ONAL_REGULATION_OF_WHITE_ADIPOCYTE_DIFFERENTIATION;REACTOME_APOPTOSIS

EACTOME_CYTOCHROME_P450_ARRANGED_BY_SUBSTRATE_TYPE;REACTOME_PHASE1_F

;TEM

SLATIONAL_PROTEIN_MODIFICATION

OME_SIGNALING_BY_THE_B_CELL_RECEPTOR_BCR;REACTOME_IMMUNE_SYSTEM;REACTO

CRT
NG_BY_ERBB2;REACTOME_GRB2_EVENTS_IN_ERBB2_SIGNALING;REACTOME_PI3K_EVENTS

ECEPTORS_BY_RECRUITING_THEM_TO_CLATHRIN_ADAPTERS;REACTOME_LATENT_INFECT
SIS;REACTOME_PACKAGING_OF_TELOMERE_ENDS;REACTOME_TELOMERE_MAINTENANCE
CTOME_SIGNALING_BY_NOTCH

IT_AND_PLATELET_PRODUCTION;REACTOME_TRAF3_DEPENDENT_IRF_ACTIVATION_PATH

CTOME_SIGNALING_BY_NOTCH

N_EVENTS;REACTOME_NGF_SIGNALLING_VIA_TRKA_FROM_THE_PLASMA_MEMBRANE;REA

HANCED_BY_THE_EXON_JUNCTION_COMPLEX

TOME_ROLE_OF_DCC_IN_REGULATING_APOPTOSIS;REACTOME_NETRIN1_SIGNALING;REA
TOME_ROLE_OF_DCC_IN_REGULATING_APOPTOSIS;REACTOME_NETRIN1_SIGNALING;REA

PMENT_AND_PLATELET_PRODUCTION;REACTOME_KINESINS;REACTOME_HEMOSTASIS;REA

OME_METABOLISM_OF_PROTEINS;REACTOME_POST_TRANSLATIONAL_PROTEIN_MODIFICA

CA2_;REACTOME_HEMOSTASIS;REACTOME_PLATELET_ACTIVATION_SIGNALING_AND_AGG

NCAM_SIGNALING_FOR_NEURITE_OUT_GROWTH;REACTOME_L1CAM_INTERACTIONS;REA(

_GLUCOSE_METABOLISM
_GLUCOSE_METABOLISM
SSOCIATED_VESICLE_BIOGENESIS
D_PROTEIN_RESPONSE

EACTOME_CYTOCHROME_P450_ARRANGED_BY_SUBSTRATE_TYPE;REACTOME_PHASE1_F
EACTOME_CYTOCHROME_P450_ARRANGED_BY_SUBSTRATE_TYPE;REACTOME_PHASE1_F

_OF_LIPIDS_AND_LIPOPROTEINS;REACTOME_FATTY_ACID_TRIACYLGLYCEROL_AND_KETO

LIPID_METABOLISM;REACTOME_SYNTHESIS_OF_PA;REACTOME_ACYL_CHAIN_REMODELLI

MOSTASIS

TIVATION_AND_REGULATION

_S_TRANSITION_;REACTOME_G1_S_TRANSITION;REACTOME_MITOTIC_G1_G1_S_PHASES;R
_S_TRANSITION_;REACTOME_G1_S_TRANSITION;REACTOME_MITOTIC_G1_G1_S_PHASES;R

SSING_OF_ENDOSOMAL_TLR;REACTOME_MHC_CLASS_II_ANTIGEN_PRESENTATION;REACT(

_PROCESSING_PRESENTATION;REACTOME_ANTIGEN_PROCESSING_UBIQUITINATION_PRO

UT_GROWTH;REACTOME_L1CAM_INTERACTIONS;REACTOME_INTERACTION_BETWEEN_L1_

MPLEX;REACTOME_ACTIVATION_OF_THE_MRNA_UPON_BINDING_OF_THE_CAP_BINDING_C

OME_TRIF_MEDIATED_TLR3_SIGNALING;REACTOME_SIGNALING_BY_ERBB4;REACTOME_SIG

SMITTER_RECEPTOR_BINDING_AND_DOWNSTREAM_TRANSMISSION_IN_THE_POSTSYNAP

G;REACTOME_NGF_SIGNALLING_VIA_TRKA_FROM_THE_PLASMA_MEMBRANE;REACTOME_

HANCED_BY_THE_EXON_JUNCTION_COMPLEX
BY_INSULIN_RECEPTOR;REACTOME_MTORC1_MEDIATED_SIGNALLING;REACTOME_PI3K_CA
TION;REACTOME_TRANSCRIPTION;REACTOME_RNA_POL_I_RNA_POL_III_AND_MITOCHOND

CTIONS;REACTOME_HEMOSTASIS

DING;REACTOME_METABOLISM_OF_PROTEINS

LCIUM_HOMEOSTASIS;REACTOME_HEMOSTASIS

ATES_CIRCADIAN_EXPRESSION;REACTOME_METABOLISM_OF_LIPIDS_AND_LIPOPROTEINS;

F_ENERGY_METABOLISM;REACTOME_METABOLISM_OF_LIPIDS_AND_LIPOPROTEINS;REAC

TOME_PPARA_ACTIVATES_GENE_EXPRESSION;REACTOME_RORA_ACTIVATES_CIRCADIAN

RANSPORT;REACTOME_TRANSPORT_OF_INORGANIC_CATIONS_ANIONS_AND_AMINO_ACID

NCAM_SIGNALING_FOR_NEURITE_OUT_GROWTH

ANSCRIPTION;REACTOME_HIV_INFECTION;REACTOME_HIV_LIFE_CYCLE;REACTOME_LATE_

ES;REACTOME_ION_CHANNEL_TRANSPORT

_OF_LIPIDS_AND_LIPOPROTEINS;REACTOME_FATTY_ACID_TRIACYLGLYCEROL_AND_KETO

OME_METABOLISM_OF_PROTEINS;REACTOME_POST_TRANSLATIONAL_PROTEIN_MODIFICA
OME_METABOLISM_OF_PROTEINS;REACTOME_POST_TRANSLATIONAL_PROTEIN_MODIFICA
OME_METABOLISM_OF_PROTEINS;REACTOME_POST_TRANSLATIONAL_PROTEIN_MODIFICA
OME_METABOLISM_OF_PROTEINS;REACTOME_POST_TRANSLATIONAL_PROTEIN_MODIFICA

TIVITY_OF_SMAD2_SMAD3_SMAD4_HETEROTRIMER;REACTOME_GENERIC_TRANSCRIPTION

TRANSMEMBRANE_TRANSPORT_OF_SMALL_MOLECULES;REACTOME_SLC_MEDIATED_TRA

_GPCR;REACTOME_G_ALPHA1213_SIGNALLING_EVENTS;REACTOME_GPCR_DOWNSTREAM
SMITTER_RECEPTOR_BINDING_AND_DOWNSTREAM_TRANSMISSION_IN_THE_POSTSYNAP
SMITTER_RECEPTOR_BINDING_AND_DOWNSTREAM_TRANSMISSION_IN_THE_POSTSYNAP

HORED_PROTEINS;REACTOME_POST_TRANSLATIONAL_PROTEIN_MODIFICATION;REACTOM
HORED_PROTEINS;REACTOME_POST_TRANSLATIONAL_PROTEIN_MODIFICATION;REACTOM

CAL_SYNAPSES;REACTOME_NEURONAL_SYSTEM;REACTOME_SIGNALING_BY_GPCR;REAC

GNALING;REACTOME_HIV_INFECTION;REACTOME_HOST_INTERACTIONS_OF_HIV_FACTORS
CTOME_PPARA_ACTIVATES_GENE_EXPRESSION;REACTOME_RORA_ACTIVATES_CIRCADIAN
CTOME_PPARA_ACTIVATES_GENE_EXPRESSION;REACTOME_RORA_ACTIVATES_CIRCADIAN

E_SIGNALING_BY_NOTCH

CTOME_PPARA_ACTIVATES_GENE_EXPRESSION;REACTOME_PRE_NOTCH_TRANSCRIPTION
OME_FATTY_ACID_TRIACYLGLYCEROL_AND_KETONE_BODY_METABOLISM
SIS

KB_AND_MAP_KINASES_UPON_TLR7_8_OR_9_ACTIVATION;REACTOME_TRAF6_MEDIATED_I
KB_AND_MAP_KINASES_UPON_TLR7_8_OR_9_ACTIVATION;REACTOME_TRAF6_MEDIATED_I

NAL_SYSTEM;REACTOME_PHASE1_FUNCTIONALIZATION_OF_COMPOUNDS;REACTOME_NE

ORT;REACTOME_RESPIRATORY_ELECTRON_TRANSPORT_ATP_SYNTHESIS_BY_CHEMIOSM

ANSCRIPTION_PATHWAY;REACTOME_METABOLISM_OF_LIPIDS_AND_LIPOPROTEINS;REACT

I;REACTOME_NRAGE_SIGNALS_DEATH_THROUGH_JNK;REACTOME_CELL_DEATH_SIGNALL

ITION_PHASE

I;REACTOME_CYTOKINE_SIGNALING_IN_IMMUNE_SYSTEM

3ER_MOLECULES;REACTOME_AXON_GUIDANCE;REACTOME_SIGNALING_BY_ROBO_RECEP

_MITOTIC_SPINDLE_CHECKPOINT_COMPONENTS;REACTOME_CELL_CYCLE;REACTOME_CE

INS;REACTOME_METABOLISM_OF_CARBOHYDRATES;REACTOME_GLUCOSE_METABOLISM
_PROCESSING_PRESENTATION;REACTOME_ANTIGEN_PROCESSING_UBIQUITINATION_PRC

E_PEPTIDE_LIGAND_BINDING_RECEPTORS;REACTOME_CLASS_A1_RHODOPSIN_LIKE_RECE
RANSPORT;REACTOME_TRANSPORT_OF_GLUCOSE_AND_OTHER_SUGARS_BILE_SALTS_AI
RANSPORT;REACTOME_TRANSPORT_OF_GLUCOSE_AND_OTHER_SUGARS_BILE_SALTS_AI

GNALING_BY_ROBO_RECEPTOR;REACTOME_NETRIN1_SIGNALING

_OF_SMALL_MOLECULES;REACTOME_SLC_MEDIATED_TRANSMEMBRANE_TRANSPORT;RE.

SIS

ME_SIGNALING_BY_NOTCH

IGNALING_BY_ERBB2;REACTOME_SIGNALING_BY_EGFR_IN_CANCER;REACTOME_PI3K_EV

NG_BY_ERBB2;REACTOME_PI3K_EVENTS_IN_ERBB2_SIGNALING
_REPLACEMENT_PATHWAY;REACTOME_BASE_FREE_SUGAR_PHOSPHATE_REMOVAL_VIA_

E_G_ALPHA_Q_SIGNALLING_EVENTS;REACTOME_GPCR_DOWNSTREAM_SIGNALING;REACT

ALPHA_HYDROXYCHOLESTEROL;REACTOME_SYNTHESIS_OF_BILE_ACIDS_AND_BILE_SALT

ROTEINS_AND_COMPLEXES;REACTOME_LOSS_OF_NLP_FROM_MITOTIC_CENTROSOMES;R

IMMUNE_SYSTEM;REACTOME_ADAPTIVE_IMMUNE_SYSTEM

TOME_PPARA_ACTIVATES_GENE_EXPRESSION;REACTOME_RORA_ACTIVATES_CIRCADIAN

REACTOME_INTERFERON_SIGNALING;REACTOME_NEGATIVE_REGULATORS_OF_RIG_I_MDA

_PROCESSING_PRESENTATION;REACTOME_ANTIGEN_PROCESSING_UBIQUITINATION_PRO

ME_SIGNALING_BY_NOTCH

_MEDIATED_ACTIVATION_CASCADE;REACTOME_HEMOSTASIS;REACTOME_SIGNALING_BY_

_PHASE

RANSPORT;REACTOME_TRANSPORT_OF_GLUCOSE_AND_OTHER_SUGARS_BILE_SALTS_A

IGNALING_BY_ERBB2;REACTOME_SIGNALING_BY_EGFR_IN_CANCER;REACTOME_PI3K_EV

ACTOME_GPCR_LIGAND_BINDING
_PROCESSING_PRESENTATION;REACTOME_ANTIGEN_PROCESSING_UBIQUITINATION_PRO
_PROCESSING_PRESENTATION;REACTOME_ANTIGEN_PROCESSING_UBIQUITINATION_PRO

ME_HS_GAG_BIOSYNTHESIS;REACTOME_HEPARAN_SULFATE_HEPARIN_HS_GAG_METABO
ME_HS_GAG_BIOSYNTHESIS;REACTOME_HEPARAN_SULFATE_HEPARIN_HS_GAG_METABO

BODY_METABOLISM

ARAGINE_N_LINKED_GLYCOSYLATION;REACTOME_TRANSPORT_TO_THE_GOLGI_AND_SU

G;REACTOME_NGF_SIGNALLING_VIA_TRKA_FROM_THE_PLASMA_MEMBRANE;REACTOME_

AMINOGLYCAN_METABOLISM;REACTOME_TRANSMEMBRANE_TRANSPORT_OF_SMALL_MOL
AMINOGLYCAN_METABOLISM;REACTOME_TRANSMEMBRANE_TRANSPORT_OF_SMALL_MOL

_MODULATION_OF_CELL_SURFACE_RECEPTORS_BY_RECRUITING_THEM_TO_CLATHRIN_AI

INELS
INELS

_MRNA_SPLICING
ANSCRIPTION_PATHWAY;REACTOME_METABOLISM_OF_LIPIDS_AND_LIPOPROTEINS;REACT

TOME_SIGNALING_BY_FGFR_IN_DISEASE;REACTOME_SIGNALING_BY_FGFR

SNALLING_CASCADE;REACTOME_INTEGRATION_OF_ENERGY_METABOLISM;REACTOME_RE

_BY_ILS;REACTOME_IL_3_5_AND_GM_CSF_SIGNALING;REACTOME_IL_RECEPTOR_SHC_SIG

HORED_PROTEINS;REACTOME_POST_TRANSLATIONAL_PROTEIN_MODIFICATION

E_CLASS_A1_RHODOPSIN_LIKE_RECEPTORS;REACTOME_G_ALPHA_Q_SIGNALLING_EVENT

MOLECULES
MOLECULES

NG;REACTOME_SIGNALING_BY_NOTCH
REACTOME_AXON_GUIDANCE;REACTOME_G_ALPHA1213_SIGNALLING_EVENTS;REACTOME

OTCH_EXPRESSION_AND_PROCESSING;REACTOME_PRE_NOTCH_PROCESSING_IN_GOLGI
OTCH_EXPRESSION_AND_PROCESSING;REACTOME_PRE_NOTCH_PROCESSING_IN_GOLGI

TION;REACTOME_TRANSCRIPTION;REACTOME_RNA_POL_I_RNA_POL_III_AND_MITOCHOND

_METABOLISM;REACTOME_METABOLISM_OF_LIPIDS_AND_LIPOPROTEINS

_PROCESSING_PRESENTATION;REACTOME_ANTIGEN_PROCESSING_UBIQUITINATION_PRO

REPLICATION;REACTOME_MITOTIC_PROMETAPHASE
REACTOME_NRAGE_SIGNALS_DEATH_THROUGH_JNK;REACTOME_CELL_DEATH_SIGNALLIN

_CREATION_OF_C4_AND_C2_ACTIVATORS;REACTOME_INITIAL_TRIGGERING_OF_COMPLEM

EVENTS;REACTOME_G_ALPHA_Z_SIGNALLING_EVENTS

OCHONDRIAL_TRANSCRIPTION;REACTOME_RNA_POL_III_TRANSCRIPTION_INITIATION_FROM

ET_CYTOSOLIC_CA2_;REACTOME_HEMOSTASIS;REACTOME_PLATELET_ACTIVATION_SIGN

NSULIN_LIKE_GROWTH_FACTOR_BINDING_PROTEINS_IGFBPS

ACTOME_PROCESSING_OF_CAPPED_INTRON_CONTAINING_PRE_MRNA;REACTOME_TRANS

NCAM_SIGNALING_FOR_NEURITE_OUT_GROWTH

NTHESIS;REACTOME_METABOLISM_OF_LIPIDS_AND_LIPOPROTEINS
NCAM_SIGNALING_FOR_NEURITE_OUT_GROWTH;REACTOME_L1CAM_INTERACTIONS
STEM;REACTOME_COMPLEMENT_CASCADE

CTOME_OTHER_SEMAPHORIN_INTERACTIONS;REACTOME_SEMA3A_PAK_DEPENDENT_AX
TOME_METABOLISM_OF_LIPIDS_AND_LIPOPROTEINS

ATELET_CYTOSOLIC_CA2_;REACTOME_HEMOSTASIS;REACTOME_PLATELET_ACTIVATION_S

ROUGH_JNK;REACTOME_CELL_DEATH_SIGNALLING_VIA_NRAGE_NRIF_AND_NADE;REACTO

l_CHANNELS

OME_SIGNALING_BY_ERBB4;REACTOME_DOWNREGULATION_OF_ERBB2_ERBB3_SIGNALING

EMODELLING_OF_PE;REACTOME_GLYCEROPHOSPHOLIPID_BIOSYNTHESIS;REACTOME_MET
_ERBB4;REACTOME_SIGNALING_BY_EGFR_IN_CANCER;REACTOME_NUCLEAR_SIGNALING_

NSULIN_SECRETION_BY_GLUCAGON_LIKE_PEPTIDE1;REACTOME_REGULATION_OF_INSULI

TOME_PPARA_ACTIVATES_GENE_EXPRESSION;REACTOME_RORA_ACTIVATES_CIRCADIAN

RANSPORT;REACTOME_TRANSPORT_OF_INORGANIC_CATIONS_ANIONS_AND_AMINO_ACID

TOME_NCAM_SIGNALING_FOR_NEURITE_OUT_GROWTH;REACTOME_L1CAM_INTERACTION
TOME_NCAM_SIGNALING_FOR_NEURITE_OUT_GROWTH;REACTOME_L1CAM_INTERACTION

l;REACTOME_PHASE_II_CONJUGATION;REACTOME_GLUCOSE_METABOLISM
RAF6_MEDIATED_INDUCTION_OF_NFKB_AND_MAP_KINASES_UPON_TLR7_8_OR_9_ACTIVAT

\MMA_CARBOXYLATION_TRANSPORT_AND_AMINO_TERMINAL_CLEAVAGE_OF_PROTEINS;F

_MRNA_SPLICING;REACTOME_MRNA_SPLICING_MINOR_PATHWAY

REPLICATION;REACTOME_MITOTIC_PROMETAPHASE

E_PLASMA_MEMBRANE;REACTOME_SIGNALLING_TO_ERKS;REACTOME_P38MAPK_EVENTS
\MMA_CARBOXYLATION_TRANSPORT_AND_AMINO_TERMINAL_CLEAVAGE_OF_PROTEINS;F

METABOLISM;REACTOME_METABOLISM_OF_LIPIDS_AND_LIPOPROTEINS

NDING

TION;REACTOME_ENDOSOMAL_SORTING_COMPLEX_REQUIRED_FOR_TRANSPORT_ESCRT

YNTHESIS_SECRETION_AND_INACTIVATION_OF_GIP;REACTOME_INCRETIN_SYNTHESIS_SE
YNTHESIS_SECRETION_AND_INACTIVATION_OF_GIP;REACTOME_INCRETIN_SYNTHESIS_SE

ALPHA_HYDROXYCHOLESTEROL;REACTOME_RECYCLING_OF_BILE_ACIDS_AND_SALTS;RE

CTOME_RECRUITMENT_OF_MITOTIC_CENTROSOME_PROTEINS_AND_COMPLEXES;REACTC
IE_INTEGRIN_ALPHAIIB_BETA3_SIGNALING;REACTOME_REGULATION_OF_INSULIN_SECRET

SMITTER_RECEPTOR_BINDING_AND_DOWNSTREAM_TRANSMISSION_IN_THE_POSTSYNAP

EVENTS;REACTOME_CGMP_EFFECTS;REACTOME_NITRIC_OXIDE_STIMULATES_GUANYLAT

_SURFACE_INTERACTIONS_AT_THE_VASCULAR_WALL;REACTOME_PECAM1_INTERACTION
_FORMATION;REACTOME_SIGNALING_BY_PDGF;REACTOME_AXON_GUIDANCE;REACTOME_

MECHANISM_BY_IFN_STIMULATED_GENES;REACTOME_SIGNALING_BY_FGFR_IN_DISEASE;F

BRANE_TRANSPORT_OF_SMALL_MOLECULES;REACTOME_SLC_MEDIATED_TRANSMEMBRA

PTOR_SIGNALLING_CASCADE;REACTOME_NGF_SIGNALLING_VIA_TRKA_FROM_THE_PLASM

E_PEPTIDE_LIGAND_BINDING_RECEPTORS;REACTOME_CLASS_A1_RHODOPSIN_LIKE_RECE

DNA_REPAIR
SMITTER_RELEASE_CYCLE;REACTOME_TRANSMEMBRANE_TRANSPORT_OF_SMALL_MOLE

OME_SIGNALING_BY_ERBB4;REACTOME_SIGNALING_BY_ERBB2;REACTOME_GRB2_EVENTS_

INELS

RANSPORT;REACTOME_TRANSPORT_OF_INORGANIC_CATIONS_ANIONS_AND_AMINO_ACID
NREGULATION_OF_TGF_BETA_RECEPTOR_SIGNALING;REACTOME_TGF_BETA_RECEPTOR

PSIN_LIKE_RECEPTORS;REACTOME_CHEMOKINE_RECEPTORS_BIND_CHEMOKINES;REACTO

OME_FATTY_ACID_TRIACYLGLYCEROL_AND_KETONE_BODY_METABOLISM;REACTOME_MET

OSIS

CTOME_OTHER_SEMAPHORIN_INTERACTIONS;REACTOME_SEMA3A_PAK_DEPENDENT_AX

THE_FANCONI_ANEMIA_PATHWAY;REACTOME_FANCONI_ANEMIA_PATHWAY;REACTOME_D

_OF_SMAD2_3_SMAD4_TRANSCRIPTIONAL_ACTIVITY;REACTOME_GENERIC_TRANSCRIPTIO

CLASE;REACTOME_PLATELET_HOMEOSTASIS;REACTOME_HEMOSTASIS;REACTOME_POTA

ISM

PSIN_LIKE_RECEPTORS;REACTOME_GPCR_DOWNSTREAM_SIGNALING;REACTOME_G_ALPH

OME_METABOLISM_OF_PROTEINS;REACTOME_POST_TRANSLATIONAL_PROTEIN_MODIFICA

D_PROTEIN_RESPONSE

_IFN_ALPHA_BETA_PATHWAYS;REACTOME_INNATE_IMMUNE_SYSTEM;REACTOME_IMMUNE
_IFN_ALPHA_BETA_PATHWAYS;REACTOME_INNATE_IMMUNE_SYSTEM;REACTOME_IMMUNE

SMITTER_RECEPTOR_BINDING_AND_DOWNSTREAM_TRANSMISSION_IN_THE_POSTSYNAP

RANSPORT;REACTOME_TRANSPORT_OF_INORGANIC_CATIONS_ANIONS_AND_AMINO_ACID

G1_S_PHASES

YCOSAMINOGLYCAN_METABOLISM;REACTOME_METABOLISM_OF_CARBOHYDRATES

SLATIONAL_PROTEIN_MODIFICATION

RANSPORT;REACTOME_TRANSPORT_OF_INORGANIC_CATIONS_ANIONS_AND_AMINO_ACID
ROTEINS_AND_COMPLEXES;REACTOME_LOSS_OF_NLP_FROM_MITOTIC_CENTROSOMES;R

RANSPORT;REACTOME_TRANSPORT_OF_INORGANIC_CATIONS_ANIONS_AND_AMINO_ACID

REACTOME_GASTRIN_CREB_SIGNALLING_PATHWAY_VIA_PKC_AND_MAPK;REACTOME_NRA
REACTOME_GASTRIN_CREB_SIGNALLING_PATHWAY_VIA_PKC_AND_MAPK;REACTOME_NRA

NALLING_CASCADE;REACTOME_REGULATION_OF_AMPK_ACTIVITY_VIA_LKB1;REACTOME_
BODY_METABOLISM
ME_FATTY_ACID_TRIACYLGLYCEROL_AND_KETONE_BODY_METABOLISM;REACTOME_CHC

DOWNSTREAM_SIGNALING;REACTOME_G_ALPHA_S_SIGNALLING_EVENTS

MOSOME_MAINTENANCE;REACTOME_DEPOSITION_OF_NEW_CENPA_CONTAINING_NUCLEC

TOME_IL_7_SIGNALING;REACTOME_SIGNALING_BY_ERBB4;REACTOME_SIGNALING_BY_ERE
_REPLICATION_STRESS;REACTOME_G2_M_CHECKPOINTS

FATE_DERMATAN_SULFATE_METABOLISM;REACTOME_HEPARAN_SULFATE_HEPARIN_HS_

YCOSPHINGOLIPID_METABOLISM;REACTOME_PHOSPHOLIPID_METABOLISM;REACTOME_T

S;REACTOME_SEMAPHORIN_INTERACTIONS

IN_A_B1_ASSOCIATED_EVENTS_DURING_G2_M_TRANSITION;REACTOME_MITOTIC_G2_G2_
IN_A_B1_ASSOCIATED_EVENTS_DURING_G2_M_TRANSITION;REACTOME_MITOTIC_G2_G2_
I;REACTOME_CYTOKINE_SIGNALING_IN_IMMUNE_SYSTEM
I;REACTOME_CYTOKINE_SIGNALING_IN_IMMUNE_SYSTEM

OMEOSTASIS;REACTOME_HEMOSTASIS

ACTOME_SIGNALING_BY_FGFR_IN_DISEASE;REACTOME_SIGNALING_BY_FGFR_MUTANTS;F
ACTOME_SIGNALING_BY_FGFR_IN_DISEASE;REACTOME_SIGNALING_BY_FGFR_MUTANTS;F
ACTOME_SIGNALING_BY_FGFR_IN_DISEASE;REACTOME_SIGNALING_BY_FGFR_MUTANTS;F

EACTOME_PEPTIDE_CHAIN_ELONGATION;REACTOME_METABOLISM_OF_PROTEINS;REACTO
EACTOME_GPCR_LIGAND_BINDING

ARAGINE_N_LINKED_GLYCOSYLATION;REACTOME_TRANSPORT_TO_THE_GOLGI_AND_SUB
THESIS_OF_SUBSTRATES_IN_N_GLYCAN_BIOSYTHESIS;REACTOME_ASPARAGINE_N_LINKE

SSOCIATED_VESICLE_BIOGENESIS

ACTOME_DOUBLE_STRAND_BREAK_REPAIR;REACTOME_DNA_REPAIR

_SIGNALING_BY_EGFR_IN_CANCER;REACTOME_ANTIGEN_ACTIVATES_B_CELL_RECEPTOR

EACTOME_PEPTIDE_CHAIN_ELONGATION;REACTOME_METABOLISM_OF_PROTEINS;REACT(

NALING_OF_ACTIVATED_FGFR;REACTOME_SIGNALING_BY_FGFR

ISM

RANSPORT;REACTOME_TRANSPORT_OF_VITAMINS_NUCLEOSIDES_AND_RELATED_MOLEC

CTOME_NEURONAL_SYSTEM

LISM;REACTOME_METABOLISM_OF_CARBOHYDRATES

SIS
SIS
SIS

RT;REACTOME_LIPOPROTEIN_METABOLISM

_PROCESSING_PRESENTATION;REACTOME_ANTIGEN_PROCESSING_UBIQUITINATION_PRO

BOLISM;REACTOME_GLYCOSAMINOGLYCAN_METABOLISM;REACTOME_METABOLISM_OF_C
B_IN_B_CELLS;REACTOME_SIGNALING_BY_THE_B_CELL_RECEPTOR_BCR;REACTOME_TCR_

E_G_ALPHA_Q_SIGNALLING_EVENTS;REACTOME_GPCR_DOWNSTREAM_SIGNALING;REACT

MOLECULES

ISM
VATION_OF_IKKS_COMPLEX;REACTOME_MAP_KINASE_ACTIVATION_IN_TLR_CASCADE;REA

LDING;REACTOME_METABOLISM_OF_PROTEINS

CTRON_TRANSPORT;REACTOME_METABOLISM_OF_AMINO_ACIDS_AND_DERIVATIVES;REA
_SIGNALING_BY_EGFR_IN_CANCER;REACTOME_NGF_SIGNALLING_VIA_TRKA_FROM_THE_I
NSULIN_LIKE_GROWTH_FACTOR_BINDING_PROTEINS_IGFBPS

SLATIONAL_PROTEIN_MODIFICATION

SIS
BINDING

OME_SIGNALING_BY_NOTCH

ACE_INTERACTIONS_AT_THE_VASCULAR_WALL;REACTOME_COLLAGEN_FORMATION;REA

_PROCESSING_PRESENTATION;REACTOME_ANTIGEN_PROCESSING_UBIQUITINATION_PRO

OME_METABOLISM_OF_PROTEINS;REACTOME_POST_TRANSLATIONAL_PROTEIN_MODIFICA

Y_FGFR1_FUSION_MUTANTS;REACTOME_SIGNALING_BY_FGFR_MUTANTS

MES;REACTOME_ANTIGEN_PROCESSING_CROSS_PRESENTATION;REACTOME_ER_PHAGOS
_FROM_CHROMATIN;REACTOME_ASSOCIATION_OF_LICENSING_FACTORS_WITH_THE_PRE_

L_JUNCTION_ORGANIZATION
_BINDING

E_G_ALPHA_Q_SIGNALLING_EVENTS;REACTOME_GPCR_DOWNSTREAM_SIGNALING;REACT
E_G_ALPHA_Q_SIGNALLING_EVENTS;REACTOME_GPCR_DOWNSTREAM_SIGNALING;REACT

RIUM_TUBERCULOSIS;REACTOME_TRANSMEMBRANE_TRANSPORT_OF_SMALL_MOLECULE

NFECTION_OF_HOMO_SAPIENS_WITH_MYCOBACTERIUM_TUBERCULOSIS;REACTOME_ENC

_DARPP_32_EVENTS;REACTOME_AXON_GUIDANCE;REACTOME_CRMPS_IN_SEMA3A_SIGNA

SNALLING_CASCADE;REACTOME_INTEGRATION_OF_ENERGY_METABOLISM;REACTOME_RE
SNALLING_CASCADE;REACTOME_INTEGRATION_OF_ENERGY_METABOLISM;REACTOME_RE

_PROCESSING_PRESENTATION;REACTOME_ANTIGEN_PROCESSING_UBIQUITINATION_PRC

ARAGINE_N_LINKED_GLYCOSYLATION

MOSTASIS

N_RECEPTOR_SIGNALLING_CASCADE;REACTOME_SIGNALING_BY_FGFR_IN_DISEASE;REAC
N_RECEPTOR_SIGNALLING_CASCADE;REACTOME_SIGNALING_BY_FGFR_IN_DISEASE;REAC

I_REPLACEMENT_PATHWAY;REACTOME_BASE_FREE_SUGAR_PHOSPHATE_REMOVAL_VIA_

EVENTS

:RIPTIONAL_REGULATION_OF_WHITE_ADIPOCYTE_DIFFERENTIATION;REACTOME_HORMON

;;REACTOME_FATTY_ACID_TRIACYLGLYCEROL_AND_KETONE_BODY_METABOLISM
;;REACTOME_FATTY_ACID_TRIACYLGLYCEROL_AND_KETONE_BODY_METABOLISM

INELS

I_CHANNELS

ASPASE_MEDIATED_CLEAVAGE_OF_CYTOSKELETAL_PROTEINS;REACTOME_CELL_JUNCTI

IAPSES;REACTOME_NEURONAL_SYSTEM;REACTOME_NEUROTRANSMITTER_RELEASE_CYC

OCHONDRIAL_TRANSCRIPTION;REACTOME_RNA_POL_III_TRANSCRIPTION_TERMINATION

RANSPORT;REACTOME_TRANSPORT_OF_INORGANIC_CATIONS_ANIONS_AND_AMINO_ACID

ORT;REACTOME_RESPIRATORY_ELECTRON_TRANSPORT_ATP_SYNTHESIS_BY_CHEMIOSM

L_JUNCTION_ORGANIZATION

_RAS;REACTOME_NGF_SIGNALLING_VIA_TRKA_FROM_THE_PLASMA_MEMBRANE;REACTOM

NSULIN_LIKE_GROWTH_FACTOR_BINDING_PROTEINS_IGFBPS

ROTEINS_AND_COMPLEXES;REACTOME_LOSS_OF_NLP_FROM_MITOTIC_CENTROSOMES;R

SIGNALING_BY_ERBB2;REACTOME_SIGNALING_BY_EGFR_IN_CANCER;REACTOME_PI3K_EV

_PROCESSING_PRESENTATION;REACTOME_ANTIGEN_PROCESSING_UBIQUITINATION_PRO
APSES;REACTOME_NEURONAL_SYSTEM;REACTOME_NEUROTRANSMITTER_RELEASE_CYC

L_CELL_COMMUNICATION;REACTOME_CASPASE_MEDIATED_CLEAVAGE_OF_CYTOSKELET
L_CELL_COMMUNICATION;REACTOME_CASPASE_MEDIATED_CLEAVAGE_OF_CYTOSKELET
L_CELL_COMMUNICATION;REACTOME_CASPASE_MEDIATED_CLEAVAGE_OF_CYTOSKELET
L_CELL_COMMUNICATION;REACTOME_CASPASE_MEDIATED_CLEAVAGE_OF_CYTOSKELET

VERSION_TRANSAMINATION;REACTOME_TRYPTOPHAN_CATABOLISM
VERSION_TRANSAMINATION;REACTOME_TRYPTOPHAN_CATABOLISM
THESIS_OF_SUBSTRATES_IN_N_GLYCAN_BIOSYTHESIS;REACTOME_ASPARAGINE_N_LINKE

N_EVENTS;REACTOME_NGF_SIGNALLING_VIA_TRKA_FROM_THE_PLASMA_MEMBRANE;REA

TION;REACTOME_TRANSCRIPTION;REACTOME_RNA_POL_I_RNA_POL_III_AND_MITOCHOND
E_PLASMA_MEMBRANE;REACTOME_SIGNALLING_TO_ERKS;REACTOME_P38MAPK_EVENTS

_FORMATION;REACTOME_SIGNALING_BY_PDGF;REACTOME_AXON_GUIDANCE;REACTOME_
_FORMATION;REACTOME_SIGNALING_BY_PDGF;REACTOME_AXON_GUIDANCE;REACTOME_
_FORMATION;REACTOME_SIGNALING_BY_PDGF;REACTOME_AXON_GUIDANCE;REACTOME_

F6_MEDIATED_NFKB_ACTIVATION;REACTOME_RIG_I_MDA5_MEDIATED_INDUCTION_OF_IFN_
ARAGINE_N_LINKED_GLYCOSYLATION;REACTOME_CALNEXIN_CALRETICULIN_CYCLE;REAC
RANSPORT;REACTOME_TRANSPORT_OF_INORGANIC_CATIONS_ANIONS_AND_AMINO_ACID

SPORT;REACTOME_STEROID_HORMONES;REACTOME_METABOLISM_OF_LIPIDS_AND_LIPO
SPORT;REACTOME_STEROID_HORMONES;REACTOME_METABOLISM_OF_LIPIDS_AND_LIPO

EGRINS;REACTOME_GRB2_SOS_PROVIDES_LINKAGE_TO_MAPK_SIGNALING_FOR_INTERGR

NTHESIS;REACTOME_TRANSMISSION_ACROSS_CHEMICAL_SYNAPSES;REACTOME_NEURON

STRATE_TYPE;REACTOME_PHASE1_FUNCTIONALIZATION_OF_COMPOUNDS

OME_SIGNALING_BY_THE_B_CELL_RECEPTOR_BCR;REACTOME_SIGNALING_BY_ILS;REACT

'COSAMINOGLYCAN_METABOLISM;REACTOME_METABOLISM_OF_CARBOHYDRATES

N_FORMATION;REACTOME_CELL_JUNCTION_ORGANIZATION

NCAM_SIGNALING_FOR_NEURITE_OUT_GROWTH

C_MEDIATED_ATTRACTIVE_SIGNALING;REACTOME_FACTORS_INVOLVED_IN_MEGAKARYO

C_MEDIATED_ATTRACTIVE_SIGNALING;REACTOME_FACTORS_INVOLVED_IN_MEGAKARYO

SE1_FUNCTIONALIZATION_OF_COMPOUNDS;REACTOME_METABOLISM_OF_POLYAMINES

SE1_FUNCTIONALIZATION_OF_COMPOUNDS;REACTOME_METABOLISM_OF_POLYAMINES

SE1_FUNCTIONALIZATION_OF_COMPOUNDS;REACTOME_METABOLISM_OF_POLYAMINES

MES;REACTOME_ANTIGEN_PROCESSING_CROSS_PRESENTATION;REACTOME_ER_PHAGOS

TION_OF_CELL_SURFACE_RECEPTORS_BY_RECRUITING_THEM_TO_CLATHRIN_ADAPTERS

OME_METABOLISM_OF_PROTEINS;REACTOME_POST_TRANSLATIONAL_PROTEIN_MODIFIC

OME_METABOLISM_OF_PROTEINS;REACTOME_POST_TRANSLATIONAL_PROTEIN_MODIFIC

OME_METABOLISM_OF_PROTEINS;REACTOME_POST_TRANSLATIONAL_PROTEIN_MODIFIC

OME_METABOLISM_OF_PROTEINS;REACTOME_POST_TRANSLATIONAL_PROTEIN_MODIFIC

OME_METABOLISM_OF_PROTEINS;REACTOME_POST_TRANSLATIONAL_PROTEIN_MODIFICA

IMMUNE_SYSTEM;REACTOME_ADAPTIVE_IMMUNE_SYSTEM

IGNALING

SSION;REACTOME_RORA_ACTIVATES_CIRCADIAN_EXPRESSION;REACTOME_CIRCADIAN_R

TREAM_SIGNALING;REACTOME_G_ALPHA_S_SIGNALLING_EVENTS;REACTOME_CGMP_EFFE
EACTOME_CYTOCHROME_P450_ARRANGED_BY_SUBSTRATE_TYPE;REACTOME_PHASE1_F

TRIN_CREB_SIGNALLING_PATHWAY_VIA_PKC_AND_MAPK;REACTOME_SIGNALING_BY_GPC
CTRON_TRANSPORT;REACTOME_PYRUVATE_METABOLISM
RANSPORT;REACTOME_TRANSPORT_OF_GLUCOSE_AND_OTHER_SUGARS_BILE_SALTS_A

SKELETAL_PROTEINS;REACTOME_APOPTOSIS;REACTOME_APOPTOTIC_EXECUTION_PHAS

_LIPIDS_AND_LIPOPROTEINS;REACTOME_FATTY_ACID_TRIACYLGLYCEROL_AND_KETONE_

ROTEINS_AND_COMPLEXES;REACTOME_LOSS_OF_NLP_FROM_MITOTIC_CENTROSOMES;R

IGNALING

IGNALING
IGNALING
ATION_DEPENDENT_MRNA_DECAY;REACTOME_METABOLISM_OF_RNA

_METABOLISM_OF_LIPIDS_AND_LIPOPROTEINS;REACTOME_FATTY_ACID_TRIACYLGLYCE

SCRIPT_TO_CYTOPLASM;REACTOME_MRNA_PROCESSING;REACTOME_TRANSPORT_OF_M

SIGNALING_BY_ERBB2;REACTOME_SIGNALING_BY_EGFR_IN_CANCER;REACTOME_PI3K_EV
GLUCOSE_METABOLISM

D_PROTEIN_RESPONSE

NTHESIS_ON_THE_LAGGING_STRAND;REACTOME_CELL_CYCLE_MITOTIC;REACTOME_M_G
I_CHANNELS

PMENT_AND_PLATELET_PRODUCTION;REACTOME_KINESINS;REACTOME_HEMOSTASIS;REA

_ACTIVATES_SMADS;REACTOME_SIGNALING_BY_GPCR;REACTOME_OPIOID_SIGNALLING;R
ORT;REACTOME_RESPIRATORY_ELECTRON_TRANSPORT_ATP_SYNTHESIS_BY_CHEMIOSM
RIUM_TUBERCULOSIS;REACTOME_TRANSMEMBRANE_TRANSPORT_OF_SMALL_MOLECULE

METABOLISM;REACTOME_METABOLISM_OF_LIPIDS_AND_LIPOPROTEINS
RANSPORT;REACTOME_TRANSPORT_OF_VITAMINS_NUCLEOSIDES_AND_RELATED_MOLEC

STEM
MOSTASIS

EACTOME_IMMUNE_SYSTEM;REACTOME_ADAPTIVE_IMMUNE_SYSTEM;REACTOME_CLASS_
ISM;REACTOME_METABOLISM_OF_LIPIDS_AND_LIPOPROTEINS;REACTOME_FATTY_ACID_T
CTOME_CELL_CYCLE_CHECKPOINTS;REACTOME_HOMOLOGOUS_RECOMBINATION_REPAIR

REPLICATION;REACTOME_MITOTIC_PROMETAPHASE
_JUNCTION_ORGANIZATION;REACTOME_CELL_JUNCTION_ORGANIZATION

NTERACTION_BETWEEN_L1_AND_ANKYRINS

LATION_DEPENDENT_MRNA_DECAY;REACTOME_METABOLISM_OF_RNA

REACTOME_NRAGE_SIGNALS_DEATH_THROUGH_JNK;REACTOME_CELL_DEATH_SIGNALLIN
REACTOME_NRAGE_SIGNALS_DEATH_THROUGH_JNK;REACTOME_CELL_DEATH_SIGNALLIN

IGNALING

ASES

INELS

IRIN_ADAPTERS;REACTOME_DEFENSINS;REACTOME_TCR_SIGNALING;REACTOME_DOWNS

YNTHESIS_OF_PIPS_AT_THE_PLASMA_MEMBRANE;REACTOME_PI_METABOLISM;REACTOME
YNTHESIS_OF_PIPS_AT_THE_PLASMA_MEMBRANE;REACTOME_PI_METABOLISM;REACTOME

_TRANSMEMBRANE_TRANSPORT_OF_SMALL_MOLECULES;REACTOME_SLC_MEDIATED_TR

OME_METABOLISM_OF_PROTEINS;REACTOME_POST_TRANSLATIONAL_PROTEIN_MODIFICA
OME_METABOLISM_OF_PROTEINS;REACTOME_POST_TRANSLATIONAL_PROTEIN_MODIFICA

_SIGNALING_BY_EGFR_IN_CANCER;REACTOME_NGF_SIGNALLING_VIA_TRKA_FROM_THE_[illegible]

[illegible]INTEGRIN_CELL_SURFACE_INTERACTIONS;REACTOME_IMMUNE_SYSTEM;REACTOME_ADA[illegible]

[illegible]MEDIATED_TRANSFER_OF_SUBSTRATE_TO_CCT_TRIC;REACTOME_PROTEIN_FOLDING;REA[illegible]

[illegible]AMINOGLYCAN_METABOLISM;REACTOME_METABOLISM_OF_CARBOHYDRATES
[illegible]_REPLACEMENT_PATHWAY;REACTOME_BASE_FREE_SUGAR_PHOSPHATE_REMOVAL_VIA_[illegible]

_OF_LIPIDS_AND_LIPOPROTEINS;REACTOME_FATTY_ACID_TRIACYLGLYCEROL_AND_KETO
DE_STIMULATES_GUANYLATE_CYCLASE;REACTOME_PLATELET_HOMEOSTASIS;REACTOME

ARAGINE_N_LINKED_GLYCOSYLATION;REACTOME_N_GLYCAN_TRIMMING_IN_THE_ER_AND

RON_SIGNALING;REACTOME_IMMUNE_SYSTEM;REACTOME_CYTOKINE_SIGNALING_IN_IMM

EPLICATION;REACTOME_MITOTIC_PROMETAPHASE
EPLICATION;REACTOME_MITOTIC_PROMETAPHASE
SOCIATED_VESICLE_BIOGENESIS

SLATIONAL_PROTEIN_MODIFICATION

_FGFR1_FUSION_MUTANTS;REACTOME_SIGNALING_BY_FGFR_MUTANTS

NSCRIPTION_COUPLED_NER_TC_NER;REACTOME_POL_SWITCHING;REACTOME_NUCLEOTI

;REACTOME_FATTY_ACID_TRIACYLGLYCEROL_AND_KETONE_BODY_METABOLISM

_FORMATION;REACTOME_INTEGRIN_CELL_SURFACE_INTERACTIONS;REACTOME_SIGNALI

GATION_COMPLEX_;REACTOME_ELONGATION_ARREST_AND_RECOVERY;REACTOME_RNA_

E_AMINO_ACID_TRANSPORT_ACROSS_THE_PLASMA_MEMBRANE;REACTOME_TRANSMEM

AND_BINDING_RECEPTORS;REACTOME_PROSTANOID_LIGAND_RECEPTORS;REACTOME_G

OME_CELL_EXTRACELLULAR_MATRIX_INTERACTIONS;REACTOME_CELL_JUNCTION_ORGA

E_CLASS_A1_RHODOPSIN_LIKE_RECEPTORS;REACTOME_G_ALPHA_Q_SIGNALLING_EVENT

I;REACTOME_CYTOKINE_SIGNALING_IN_IMMUNE_SYSTEM

)ME_SIGNALING_BY_NOTCH

PMENT_AND_PLATELET_PRODUCTION;REACTOME_KINESINS;REACTOME_HEMOSTASIS;REA

)ME_TETRAHYDROBIOPTERIN_BH4_SYNTHESIS_RECYCLING_SALVAGE_AND_REGULATION;

)ME_SIGNALING_BY_NOTCH2;REACTOME_SIGNALING_BY_NOTCH1;REACTOME_SIGNALING_
TION;REACTOME_TRANSCRIPTION;REACTOME_RNA_POL_I_RNA_POL_III_AND_MITOCHONE

ES;REACTOME_ION_CHANNEL_TRANSPORT
_PROCESSING_PRESENTATION;REACTOME_ANTIGEN_PROCESSING_UBIQUITINATION_PRO
_PROCESSING_PRESENTATION;REACTOME_ANTIGEN_PROCESSING_UBIQUITINATION_PRO

SMITTER_RECEPTOR_BINDING_AND_DOWNSTREAM_TRANSMISSION_IN_THE_POSTSYNAP

GNALING_BY_ROBO_RECEPTOR
CAL_SYNAPSES;REACTOME_NEURONAL_SYSTEM;REACTOME_SIGNALING_BY_GPCR;REAC
UT_GROWTH;REACTOME_L1CAM_INTERACTIONS;REACTOME_INTERACTION_BETWEEN_L1_
UT_GROWTH;REACTOME_L1CAM_INTERACTIONS;REACTOME_INTERACTION_BETWEEN_L1_

OME_POST_TRANSLATIONAL_MODIFICATION_SYNTHESIS_OF_GPI_ANCHORED_PROTEINS;I

ES;REACTOME_ION_CHANNEL_TRANSPORT

NS;REACTOME_AQUAPORIN_MEDIATED_TRANSPORT

EACTOME_CYTOCHROME_P450_ARRANGED_BY_SUBSTRATE_TYPE;REACTOME_PHASE1_F

ROUGH_JNK;REACTOME_CELL_DEATH_SIGNALLING_VIA_NRAGE_NRIF_AND_NADE;REACTO

RANSPORT;REACTOME_TRANSPORT_OF_GLUCOSE_AND_OTHER_SUGARS_BILE_SALTS_AI

EVENTS

PSIN_LIKE_RECEPTORS;REACTOME_GPCR_DOWNSTREAM_SIGNALING;REACTOME_G_ALPH
SIS

EACTOME_PEPTIDE_CHAIN_ELONGATION;REACTOME_METABOLISM_OF_PROTEINS;REACTO

CTOME_PPARA_ACTIVATES_GENE_EXPRESSION;REACTOME_PRE_NOTCH_TRANSCRIPTION
CTOME_PPARA_ACTIVATES_GENE_EXPRESSION;REACTOME_PRE_NOTCH_TRANSCRIPTION
CTOME_PPARA_ACTIVATES_GENE_EXPRESSION;REACTOME_PRE_NOTCH_TRANSCRIPTION
_SIGNALING_BY_EGFR_IN_CANCER;REACTOME_NGF_SIGNALLING_VIA_TRKA_FROM_THE_

CTOME_CYTOKINE_SIGNALING_IN_IMMUNE_SYSTEM
CTOME_CYTOKINE_SIGNALING_IN_IMMUNE_SYSTEM

MOLECULES

HANCED_BY_THE_EXON_JUNCTION_COMPLEX

3Y_INSULIN_RECEPTOR;REACTOME_MTORC1_MEDIATED_SIGNALLING;REACTOME_PI3K_CA

E_TRANSMEMBRANE_TRANSPORT_OF_SMALL_MOLECULES;REACTOME_PLATELET_HOME

CTIONS;REACTOME_HEMOSTASIS

_GLUCOSE_METABOLISM

ACTOME_PROCESSING_OF_CAPPED_INTRON_CONTAINING_PRE_MRNA;REACTOME_TRANS
ACTOME_PROCESSING_OF_CAPPED_INTRON_CONTAINING_PRE_MRNA;REACTOME_TRANS
LIPID_DIGESTION_MOBILIZATION_AND_TRANSPORT;REACTOME_LIPOPROTEIN_METABOLIS

RANSPORT;REACTOME_TRANSPORT_OF_INORGANIC_CATIONS_ANIONS_AND_AMINO_ACID

RANSPORT;REACTOME_TRANSPORT_OF_INORGANIC_CATIONS_ANIONS_AND_AMINO_ACID

SIS;REACTOME_PACKAGING_OF_TELOMERE_ENDS;REACTOME_TELOMERE_MAINTENANCE

EGRINS;REACTOME_INTEGRIN_ALPHAIIB_BETA3_SIGNALING;REACTOME_SIGNALING_BY_PD

OME_SIGNALING_BY_THE_B_CELL_RECEPTOR_BCR;REACTOME_GPVI_MEDIATED_ACTIVATI

RIPTION;REACTOME_RNA_POL_I_RNA_POL_III_AND_MITOCHONDRIAL_TRANSCRIPTION;REA

_MRNA_SPLICING;REACTOME_MRNA_SPLICING_MINOR_PATHWAY

HANCED_BY_THE_EXON_JUNCTION_COMPLEX
HANCED_BY_THE_EXON_JUNCTION_COMPLEX

SMITTER_RECEPTOR_BINDING_AND_DOWNSTREAM_TRANSMISSION_IN_THE_POSTSYNAP

_PROCESSING_PRESENTATION;REACTOME_ANTIGEN_PROCESSING_UBIQUITINATION_PRO

RANSPORT;REACTOME_TRANSPORT_OF_GLUCOSE_AND_OTHER_SUGARS_BILE_SALTS_AI

_CAPPED_INTRON_CONTAINING_PRE_MRNA;REACTOME_RNA_POL_II_TRANSCRIPTION;REA

PI_METABOLISM;REACTOME_SIGNALING_BY_GPCR;REACTOME_GPVI_MEDIATED_ACTIVATI

'COSAMINOGLYCAN_METABOLISM;REACTOME_METABOLISM_OF_CARBOHYDRATES

TOME_SIGNALING_BY_ERBB4;REACTOME_NUCLEAR_SIGNALING_BY_ERBB4;REACTOME_P

TOME_SIGNALING_BY_ERBB4;REACTOME_NUCLEAR_SIGNALING_BY_ERBB4;REACTOME_P

TIVATED_TAK1_MEDIATES_P38_MAPK_ACTIVATION;REACTOME_TRAF6_MEDIATED_INDUCT

DE_STIMULATES_GUANYLATE_CYCLASE;REACTOME_PLATELET_HOMEOSTASIS;REACTOME

RANSPORT;REACTOME_TRANSPORT_OF_GLUCOSE_AND_OTHER_SUGARS_BILE_SALTS_AI

RANSPORT;REACTOME_TRANSPORT_OF_GLUCOSE_AND_OTHER_SUGARS_BILE_SALTS_AI

'_FGFR1_FUSION_MUTANTS;REACTOME_SIGNALING_BY_FGFR_MUTANTS

TION_OF_CELL_SURFACE_RECEPTORS_BY_RECRUITING_THEM_TO_CLATHRIN_ADAPTERS

ONAL_SYSTEM;REACTOME_AXON_GUIDANCE;REACTOME_NCAM1_INTERACTIONS;REACTO

EACTOME_PEPTIDE_CHAIN_ELONGATION;REACTOME_METABOLISM_OF_PROTEINS;REACT

GNALING_BY_ERBB2;REACTOME_GRB2_EVENTS_IN_ERBB2_SIGNALING;REACTOME_PI3K_EV
PSIN_LIKE_RECEPTORS;REACTOME_CHEMOKINE_RECEPTORS_BIND_CHEMOKINES;REACTO

GNALING;REACTOME_NGF_SIGNALLING_VIA_TRKA_FROM_THE_PLASMA_MEMBRANE;REAC
GNALING;REACTOME_NGF_SIGNALLING_VIA_TRKA_FROM_THE_PLASMA_MEMBRANE;REAC
GNALING;REACTOME_NGF_SIGNALLING_VIA_TRKA_FROM_THE_PLASMA_MEMBRANE;REAC

SMITTER_RECEPTOR_BINDING_AND_DOWNSTREAM_TRANSMISSION_IN_THE_POSTSYNAPT

_PROCESSING_PRESENTATION;REACTOME_ANTIGEN_PROCESSING_UBIQUITINATION_PRO

FACE_INTERACTIONS_AT_THE_VASCULAR_WALL;REACTOME_COLLAGEN_FORMATION;REAC

TION;REACTOME_MEIOTIC_RECOMBINATION;REACTOME_HEMOSTASIS

RECEPTOR_SIGNALING;REACTOME_NUCLEAR_SIGNALING_BY_ERBB4;REACTOME_DIABETES
NTERACTION_BETWEEN_L1_AND_ANKYRINS

RT;REACTOME_TRANSMEMBRANE_TRANSPORT_OF_SMALL_MOLECULES

TOME_ENERGY_DEPENDENT_REGULATION_OF_MTOR_BY_LKB1_AMPK;REACTOME_PKB_M

ACTOME_TRIGLYCERIDE_BIOSYNTHESIS;REACTOME_FATTY_ACYL_COA_BIOSYNTHESIS;REA
ACTOME_TRIGLYCERIDE_BIOSYNTHESIS;REACTOME_FATTY_ACYL_COA_BIOSYNTHESIS;REA

E_SIGNALING_BY_GPCR;REACTOME_CLASS_B_2_SECRETIN_FAMILY_RECEPTORS;REACTO
REPLICATION;REACTOME_MITOTIC_PROMETAPHASE

ROTEINS_AND_COMPLEXES;REACTOME_LOSS_OF_NLP_FROM_MITOTIC_CENTROSOMES;R
PSIN_LIKE_RECEPTORS;REACTOME_GPCR_DOWNSTREAM_SIGNALING;REACTOME_G_ALPH

REACTOME_REGULATION_OF_WATER_BALANCE_BY_RENAL_AQUAPORINS

ES;REACTOME_ION_CHANNEL_TRANSPORT

CTRON_TRANSPORT;REACTOME_CELL_SURFACE_INTERACTIONS_AT_THE_VASCULAR_WA

_MRNA_SPLICING

ENESIS;REACTOME_REGULATORY_RNA_PATHWAYS;REACTOME_PROCESSING_OF_CAPPE

EAM_SIGNALING;REACTOME_G_ALPHA_I_SIGNALLING_EVENTS;REACTOME_GPCR_LIGAND

CTOME_ANTIGEN_ACTIVATES_B_CELL_RECEPTOR_LEADING_TO_GENERATION_OF_SECON
ALING_IN_EMT_EPITHELIAL_TO_MESENCHYMAL_TRANSITION;REACTOME_NRAGE_SIGNALS_

TION_OF_TRANSCRIPTION_COUPLED_NER_TC_NER_REPAIR_COMPLEX;REACTOME_DNA_F
TION_OF_TRANSCRIPTION_COUPLED_NER_TC_NER_REPAIR_COMPLEX;REACTOME_DNA_F

OME_METABOLISM_OF_PROTEINS;REACTOME_POST_TRANSLATIONAL_PROTEIN_MODIFICA
OME_METABOLISM_OF_PROTEINS;REACTOME_POST_TRANSLATIONAL_PROTEIN_MODIFICA
OME_METABOLISM_OF_PROTEINS;REACTOME_POST_TRANSLATIONAL_PROTEIN_MODIFICA

EGATIVE_REGULATORS_OF_RIG_I_MDA5_SIGNALING;REACTOME_RIG_I_MDA5_MEDIATED_I

E_CLASS_A1_RHODOPSIN_LIKE_RECEPTORS;REACTOME_G_ALPHA_Q_SIGNALLING_EVENT
_INTEGRIN_CELL_SURFACE_INTERACTIONS;REACTOME_INTERFERON_GAMMA_SIGNALING;

_SIGNALING;REACTOME_INTERFERON_SIGNALING;REACTOME_IL_6_SIGNALING;REACTOME

RT;REACTOME_LIPOPROTEIN_METABOLISM;REACTOME_CHYLOMICRON_MEDIATED_LIPID_
RT;REACTOME_LIPOPROTEIN_METABOLISM;REACTOME_CHYLOMICRON_MEDIATED_LIPID_
MOSTASIS

NG_BY_ERBB2;REACTOME_SIGNALING_BY_EGFR_IN_CANCER;REACTOME_NGF_SIGNALLIN

IGNALING

DOWNSTREAM_SIGNALING;REACTOME_G_ALPHA_S_SIGNALLING_EVENTS

_GLUCOSE_METABOLISM

_MRNA_SPLICING

_MRNA_SPLICING
NCHYMAL_TRANSITION;REACTOME_DOWNREGULATION_OF_TGF_BETA_RECEPTOR_SIGNA
_GPCR;REACTOME_G_ALPHA1213_SIGNALLING_EVENTS;REACTOME_GPCR_DOWNSTREAM

E_CLASS_A1_RHODOPSIN_LIKE_RECEPTORS;REACTOME_G_ALPHA_Q_SIGNALLING_EVENT

RANSPORT;REACTOME_TRANSPORT_OF_INORGANIC_CATIONS_ANIONS_AND_AMINO_ACID

PID_METABOLISM;REACTOME_METABOLISM_OF_LIPIDS_AND_LIPOPROTEINS

_MRNA_SPLICING
_MRNA_SPLICING

G;REACTOME_SEMAPHORIN_INTERACTIONS;REACTOME_SEMA4D_INDUCED_CELL_MIGRA

_REPLACEMENT_PATHWAY;REACTOME_CELL_CYCLE;REACTOME_PROCESSIVE_SYNTHES
_REPLACEMENT_PATHWAY;REACTOME_CELL_CYCLE;REACTOME_PROCESSIVE_SYNTHES
_REPLACEMENT_PATHWAY;REACTOME_CELL_CYCLE;REACTOME_PROCESSIVE_SYNTHES

SMITTER_RECEPTOR_BINDING_AND_DOWNSTREAM_TRANSMISSION_IN_THE_POSTSYNAP

IMMUNE_SYSTEM;REACTOME_ADAPTIVE_IMMUNE_SYSTEM

CTOME_SIGNAL_REGULATORY_PROTEIN_SIRP_FAMILY_INTERACTIONS;REACTOME_HEMOS

DING;REACTOME_METABOLISM_OF_PROTEINS

NCAM_SIGNALING_FOR_NEURITE_OUT_GROWTH

HORED_PROTEINS;REACTOME_POST_TRANSLATIONAL_PROTEIN_MODIFICATION

ROTEINS_AND_COMPLEXES;REACTOME_LOSS_OF_NLP_FROM_MITOTIC_CENTROSOMES;R

SMITTER_RECEPTOR_BINDING_AND_DOWNSTREAM_TRANSMISSION_IN_THE_POSTSYNAP

TOME_ANTIVIRAL_MECHANISM_BY_IFN_STIMULATED_GENES;REACTOME_SIGNALING_BY_E

RANSPORT;REACTOME_TRANSPORT_OF_INORGANIC_CATIONS_ANIONS_AND_AMINO_ACID

RANSPORT;REACTOME_TRANSPORT_OF_GLUCOSE_AND_OTHER_SUGARS_BILE_SALTS_AI

INELS
ES;REACTOME_ION_CHANNEL_TRANSPORT

_TRANSPORT_OF_SMALL_MOLECULES;REACTOME_GLUCAGON_SIGNALING_IN_METABOLI
_TRANSPORT_OF_SMALL_MOLECULES;REACTOME_GLUCAGON_SIGNALING_IN_METABOLI
SIS

_FORMATION;REACTOME_SIGNALING_BY_PDGF;REACTOME_AXON_GUIDANCE;REACTOME_
_FORMATION;REACTOME_SIGNALING_BY_PDGF;REACTOME_AXON_GUIDANCE;REACTOME_
EACTOME_ACTIVATION_OF_CHAPERONE_GENES_BY_XBP1S;REACTOME_UNFOLDED_PROT

CTIONS;REACTOME_HEMOSTASIS
STRIN_CREB_SIGNALLING_PATHWAY_VIA_PKC_AND_MAPK;REACTOME_RESPONSE_TO_ELE

PI_METABOLISM;REACTOME_METABOLISM_OF_LIPIDS_AND_LIPOPROTEINS

ERON_SIGNALING;REACTOME_IMMUNE_SYSTEM;REACTOME_CYTOKINE_SIGNALING_IN_IM
ROUGH_JNK;REACTOME_CELL_DEATH_SIGNALLING_VIA_NRAGE_NRIF_AND_NADE;REACTO

SCRIPT_TO_CYTOPLASM;REACTOME_RNA_POL_II_TRANSCRIPTION;REACTOME_MRNA_PRO

_CELL_SURFACE_INTERACTIONS_AT_THE_VASCULAR_WALL;REACTOME_INTEGRIN_CELL_S

_FORMATION;REACTOME_SIGNALING_BY_PDGF;REACTOME_AXON_GUIDANCE;REACTOME_

ROTEINS_AND_COMPLEXES;REACTOME_LOSS_OF_NLP_FROM_MITOTIC_CENTROSOMES;R

_METABOLISM;REACTOME_METABOLISM_OF_LIPIDS_AND_LIPOPROTEINS

E_SIGNALING_BY_GPCR;REACTOME_CLASS_A1_RHODOPSIN_LIKE_RECEPTORS;REACTOMI
SM

_FROM_CHROMATIN;REACTOME_CELL_CYCLE_MITOTIC;REACTOME_CELL_CYCLE_CHECKP
G;REACTOME_SEMAPHORIN_INTERACTIONS;REACTOME_SEMA4D_INDUCED_CELL_MIGRAT

G;REACTOME_IL_2_SIGNALING;REACTOME_IMMUNE_SYSTEM;REACTOME_CYTOKINE_SIGN

EMODELLING_OF_PE;REACTOME_GLYCEROPHOSPHOLIPID_BIOSYNTHESIS;REACTOME_MET
MOSTASIS

SMITTER_RECEPTOR_BINDING_AND_DOWNSTREAM_TRANSMISSION_IN_THE_POSTSYNAP
SMITTER_RECEPTOR_BINDING_AND_DOWNSTREAM_TRANSMISSION_IN_THE_POSTSYNAP

NCAM_SIGNALING_FOR_NEURITE_OUT_GROWTH
NG;REACTOME_REGULATORY_RNA_PATHWAYS;REACTOME_SIGNALING_BY_NOTCH
E_PEPTIDE_LIGAND_BINDING_RECEPTORS;REACTOME_CLASS_A1_RHODOPSIN_LIKE_RECE
TION;REACTOME_TRANSCRIPTION;REACTOME_RNA_POL_I_RNA_POL_III_AND_MITOCHONE
TION;REACTOME_TRANSCRIPTION;REACTOME_RNA_POL_I_RNA_POL_III_AND_MITOCHONE
EPLICATION;REACTOME_MITOTIC_PROMETAPHASE

M_OF_LIPIDS_AND_LIPOPROTEINS

SIS

YCOSPHINGOLIPID_METABOLISM;REACTOME_PHOSPHOLIPID_METABOLISM;REACTOME_T

Y_ACID_TRIACYLGLYCEROL_AND_KETONE_BODY_METABOLISM
Y_ACID_TRIACYLGLYCEROL_AND_KETONE_BODY_METABOLISM

_IFN_ALPHA_BETA_PATHWAYS;REACTOME_INNATE_IMMUNE_SYSTEM;REACTOME_IMMUNE

APOPTOSIS;REACTOME_CELL_DEATH_SIGNALLING_VIA_NRAGE_NRIF_AND_NADE;REACTOM

A;REACTOME_CELL_CYCLE_MITOTIC;REACTOME_MRNA_PROCESSING;REACTOME_MRNA_
_PROCESSING_PRESENTATION;REACTOME_ANTIGEN_PROCESSING_UBIQUITINATION_PRO

ROUGH_JNK;REACTOME_CELL_DEATH_SIGNALLING_VIA_NRAGE_NRIF_AND_NADE;REACTO

ANSCRIPTION_PATHWAY;REACTOME_METABOLISM_OF_LIPIDS_AND_LIPOPROTEINS;REACT
LY

CTOME_SIGNALING_BY_NOTCH
_GLUCOSE_METABOLISM

F_LIPIDS_AND_LIPOPROTEINS;REACTOME_FATTY_ACID_TRIACYLGLYCEROL_AND_KETONE_

ME_HS_GAG_BIOSYNTHESIS;REACTOME_HEPARAN_SULFATE_HEPARIN_HS_GAG_METABO
RANSPORT;REACTOME_TRANSPORT_OF_INORGANIC_CATIONS_ANIONS_AND_AMINO_ACID

ES;REACTOME_ION_CHANNEL_TRANSPORT

LFATE_DERMATAN_SULFATE_METABOLISM;REACTOME_HEPARAN_SULFATE_HEPARIN_HS_
E_PLATELET_CALCIUM_HOMEOSTASIS;REACTOME_ION_TRANSPORT_BY_P_TYPE_ATPASES
EACTOME_METABOLISM_OF_PROTEINS
OME_BASIGIN_INTERACTIONS;REACTOME_AXON_GUIDANCE;REACTOME_L1CAM_INTERACT
OME_BASIGIN_INTERACTIONS;REACTOME_AXON_GUIDANCE;REACTOME_L1CAM_INTERACT

E_EXTENSION_OF_TELOMERES

MUNE_SYSTEM;REACTOME_CYTOKINE_SIGNALING_IN_IMMUNE_SYSTEM

YCOSAMINOGLYCAN_METABOLISM;REACTOME_METABOLISM_OF_CARBOHYDRATES
ROTEINS_AND_COMPLEXES;REACTOME_LOSS_OF_NLP_FROM_MITOTIC_CENTROSOMES;R

OME_SIGNALING_BY_NOTCH2;REACTOME_SIGNALING_BY_NOTCH1;REACTOME_SIGNALING_

CTOME_MITOTIC_M_M_G1_PHASES;REACTOME_FACTORS_INVOLVED_IN_MEGAKARYOCYTI

UT_GROWTH;REACTOME_L1CAM_INTERACTIONS;REACTOME_INTERACTION_BETWEEN_L1_
PSIN_LIKE_RECEPTORS;REACTOME_GPCR_LIGAND_BINDING

_BINDING

TOME_BIOLOGICAL_OXIDATIONS;REACTOME_PHASE_II_CONJUGATION

RT;REACTOME_TRANSMEMBRANE_TRANSPORT_OF_SMALL_MOLECULES;REACTOME_MET

RANSPORT;REACTOME_TRANSPORT_OF_GLUCOSE_AND_OTHER_SUGARS_BILE_SALTS_A

ROTEINS_AND_COMPLEXES;REACTOME_LOSS_OF_NLP_FROM_MITOTIC_CENTROSOMES;R

ROTEINS_AND_COMPLEXES;REACTOME_LOSS_OF_NLP_FROM_MITOTIC_CENTROSOMES;R

TOME_SIGNALING_BY_ERBB4;REACTOME_SIGNALING_BY_ERBB2;REACTOME_SIGNALING_

_PHASE1_FUNCTIONALIZATION_OF_COMPOUNDS

EACTOME_SYNTHESIS_OF_PIPS_AT_THE_PLASMA_MEMBRANE;REACTOME_PI_METABOLIS

E_PEPTIDE_LIGAND_BINDING_RECEPTORS;REACTOME_CLASS_A1_RHODOPSIN_LIKE_RECE

AMINOGLYCAN_METABOLISM;REACTOME_METABOLISM_OF_CARBOHYDRATES

E_ANTIGEN_PROCESSING_CROSS_PRESENTATION;REACTOME_ENDOSOMAL_VACUOLAR_

ASPASE_MEDIATED_CLEAVAGE_OF_CYTOSKELETAL_PROTEINS;REACTOME_CELL_JUNCTI

)DE_STIMULATES_GUANYLATE_CYCLASE;REACTOME_PLATELET_HOMEOSTASIS;REACTOME

)ME_TETRAHYDROBIOPTERIN_BH4_SYNTHESIS_RECYCLING_SALVAGE_AND_REGULATION;

LATION_DEPENDENT_MRNA_DECAY;REACTOME_METABOLISM_OF_RNA
_MRNA_SPLICING

M_OF_LIPIDS_AND_LIPOPROTEINS

LYCOSPHINGOLIPID_METABOLISM;REACTOME_PHOSPHOLIPID_METABOLISM;REACTOME_T

ANCE;REACTOME_L1CAM_INTERACTIONS;REACTOME_RECYCLING_PATHWAY_OF_L1;REAC

SMITTER_RECEPTOR_BINDING_AND_DOWNSTREAM_TRANSMISSION_IN_THE_POSTSYNAP
ALLING_COMPLEXES;REACTOME_P75NTR_SIGNALS_VIA_NFKB;REACTOME_NFKB_IS_ACTIV

ESSING_CROSS_PRESENTATION;REACTOME_INTERFERON_GAMMA_SIGNALING;REACTOME

TOME_NCAM_SIGNALING_FOR_NEURITE_OUT_GROWTH;REACTOME_L1CAM_INTERACTION

NSULIN_LIKE_GROWTH_FACTOR_BINDING_PROTEINS_IGFBPS

INDING_RECEPTORS;REACTOME_GPCR_DOWNSTREAM_SIGNALING;REACTOME_G_ALPHA_

:HONDRIAL_TRANSCRIPTION;REACTOME_RNA_POL_I_TRANSCRIPTION_INITIATION

_OF_LIPIDS_AND_LIPOPROTEINS;REACTOME_FATTY_ACID_TRIACYLGLYCEROL_AND_KETO

EACTOME_TRANSMEMBRANE_TRANSPORT_OF_SMALL_MOLECULES

ROTEINS_AND_COMPLEXES;REACTOME_LOSS_OF_NLP_FROM_MITOTIC_CENTROSOMES;R
L_CELL_COMMUNICATION;REACTOME_INTEGRIN_CELL_SURFACE_INTERACTIONS;REACTC
F6_MEDIATED_NFKB_ACTIVATION;REACTOME_RIG_I_MDA5_MEDIATED_INDUCTION_OF_IFN_

ACTOME_SIGNALING_BY_FGFR_IN_DISEASE;REACTOME_ACTIVATED_POINT_MUTANTS_OF

REACTOME_NRAGE_SIGNALS_DEATH_THROUGH_JNK;REACTOME_CELL_DEATH_SIGNALLIN
RON_SIGNALING;REACTOME_IMMUNE_SYSTEM;REACTOME_CYTOKINE_SIGNALING_IN_IMM
ME_CYCLIN_E_ASSOCIATED_EVENTS_DURING_G1_S_TRANSITION_;REACTOME_G1_PHASE;

RANSPORT;REACTOME_TRANSPORT_OF_INORGANIC_CATIONS_ANIONS_AND_AMINO_ACID

N_OF_ENERGY_METABOLISM;REACTOME_REGULATION_OF_INSULIN_SECRETION
IGNALING

ROTEINS_AND_COMPLEXES;REACTOME_LOSS_OF_NLP_FROM_MITOTIC_CENTROSOMES;R

RANSPORT;REACTOME_TRANSPORT_OF_INORGANIC_CATIONS_ANIONS_AND_AMINO_ACID

LLING_COMPLEXES;REACTOME_P75NTR_SIGNALS_VIA_NFKB;REACTOME_NFKB_IS_ACTIVA

CHONDRIAL_TRANSCRIPTION;REACTOME_RNA_POL_III_TRANSCRIPTION_INITIATION_FROM

REACTOME_NRAGE_SIGNALS_DEATH_THROUGH_JNK;REACTOME_CELL_DEATH_SIGNALLIN

_JUNCTION_ORGANIZATION;REACTOME_CELL_JUNCTION_ORGANIZATION

E_SIGNALLING_TO_ERKS;REACTOME_SIGNALLING_TO_P38_VIA_RIT_AND_RIN

RANSPORT;REACTOME_TRANSPORT_OF_INORGANIC_CATIONS_ANIONS_AND_AMINO_ACID

MPLEX;REACTOME_SRP_DEPENDENT_COTRANSLATIONAL_PROTEIN_TARGETING_TO_MEMI
ES;REACTOME_ION_CHANNEL_TRANSPORT
ROTEINS_MEDIATED_TRANSPORT;REACTOME_TRANSMEMBRANE_TRANSPORT_OF_SMALL_
ME_LIGAND_GATED_ION_CHANNEL_TRANSPORT

CA2_;REACTOME_HEMOSTASIS;REACTOME_PLATELET_ACTIVATION_SIGNALING_AND_AGG

F_LIPIDS_AND_LIPOPROTEINS;REACTOME_FATTY_ACID_TRIACYLGLYCEROL_AND_KETONE_

TOME_ROLE_OF_DCC_IN_REGULATING_APOPTOSIS;REACTOME_NETRIN1_SIGNALING;REA(

ME_ER_PHAGOSOME_PATHWAY;REACTOME_IMMUNOREGULATORY_INTERACTIONS_BETWI

PI_METABOLISM;REACTOME_METABOLISM_OF_LIPIDS_AND_LIPOPROTEINS

ARAGINE_N_LINKED_GLYCOSYLATION;REACTOME_CALNEXIN_CALRETICULIN_CYCLE;REAC
CTOME_PEPTIDE_LIGAND_BINDING_RECEPTORS;REACTOME_CLASS_A1_RHODOPSIN_LIKE

_CHANNELS

NSULIN_SECRETION;REACTOME_POTASSIUM_CHANNELS;REACTOME_INWARDLY_RECTIFYI

ME_SIGNALING_BY_NOTCH

PI_METABOLISM;REACTOME_METABOLISM_OF_LIPIDS_AND_LIPOPROTEINS

PID_METABOLISM;REACTOME_METABOLISM_OF_LIPIDS_AND_LIPOPROTEINS

OME_CELL_CYCLE_MITOTIC;REACTOME_COSTIMULATION_BY_THE_CD28_FAMILY;REACTOM

GENESIS;REACTOME_METABOLISM_OF_CARBOHYDRATES;REACTOME_HEMOSTASIS;REAC

_MRNA_SPLICING;REACTOME_MRNA_SPLICING_MINOR_PATHWAY

SMITTER_RECEPTOR_BINDING_AND_DOWNSTREAM_TRANSMISSION_IN_THE_POSTSYNAP

ASE_II_CONJUGATION

SMITTER_RECEPTOR_BINDING_AND_DOWNSTREAM_TRANSMISSION_IN_THE_POSTSYNAP

BY_GPCR;REACTOME_OPIOID_SIGNALLING;REACTOME_ADENYLATE_CYCLASE_INHIBITORY_

I;REACTOME_CYTOKINE_SIGNALING_IN_IMMUNE_SYSTEM

ORT;REACTOME_RESPIRATORY_ELECTRON_TRANSPORT_ATP_SYNTHESIS_BY_CHEMIOSM

_MITOTIC_SPINDLE_CHECKPOINT_COMPONENTS;REACTOME_CELL_CYCLE;REACTOME_CE

RANSPORT;REACTOME_TRANSPORT_OF_INORGANIC_CATIONS_ANIONS_AND_AMINO_ACID

BY_ERBB4;REACTOME_PROLACTIN_RECEPTOR_SIGNALING;REACTOME_NUCLEAR_SIGNAL

'COSAMINOGLYCAN_METABOLISM;REACTOME_METABOLISM_OF_CARBOHYDRATES

/IE_DOWNSTREAM_TCR_SIGNALING;REACTOME_P75NTR_RECRUITS_SIGNALLING_COMPLE

EACTOME_GLYCEROPHOSPHOLIPID_BIOSYNTHESIS;REACTOME_METABOLISM_OF_LIPIDS_

EAM_SIGNALING;REACTOME_G_ALPHA_I_SIGNALLING_EVENTS;REACTOME_GPCR_LIGAND
ME_HS_GAG_BIOSYNTHESIS;REACTOME_HEPARAN_SULFATE_HEPARIN_HS_GAG_METABO

.L_JUNCTION_ORGANIZATION

K_VESICLE_BUDDING;REACTOME_SYNTHESIS_OF_PIPS_AT_THE_GOLGI_MEMBRANE;REAC

TING_CASCADE;REACTOME_INTRINSIC_PATHWAY;REACTOME_HEMOSTASIS;REACTOME_PL

IETABOLISM;REACTOME_METABOLISM_OF_LIPIDS_AND_LIPOPROTEINS

G_COMPLEXES;REACTOME_P75NTR_SIGNALS_VIA_NFKB;REACTOME_P75_NTR_RECEPTOF

RT;REACTOME_LIPOPROTEIN_METABOLISM

_OF_SMALL_MOLECULES;REACTOME_SLC_MEDIATED_TRANSMEMBRANE_TRANSPORT;RE

OME_METABOLISM_OF_PROTEINS;REACTOME_POST_TRANSLATIONAL_PROTEIN_MODIFICA

SIS
OTCH_EXPRESSION_AND_PROCESSING;REACTOME_PRE_NOTCH_PROCESSING_IN_GOLGI

STEM;REACTOME_COMPLEMENT_CASCADE

LISM_OF_VITAMINS_AND_COFACTORS;REACTOME_CELL_CYCLE;REACTOME_ENOS_ACTIVA

SMITTER_RECEPTOR_BINDING_AND_DOWNSTREAM_TRANSMISSION_IN_THE_POSTSYNAP

NTHESIS;REACTOME_TRANSMEMBRANE_TRANSPORT_OF_SMALL_MOLECULES;REACTOME_
OME_FATTY_ACID_TRIACYLGLYCEROL_AND_KETONE_BODY_METABOLISM

TION;REACTOME_RNA_POL_III_TRANSCRIPTION;REACTOME_RNA_POL_II_TRANSCRIPTION_

OME_SIGNALING_BY_THE_B_CELL_RECEPTOR_BCR;REACTOME_IMMUNE_SYSTEM;REACTO

;REACTOME_METABOLISM_OF_PROTEINS;REACTOME_RESPIRATORY_ELECTRON_TRANSP
E_INTEGRATION_OF_ENERGY_METABOLISM;REACTOME_G_ALPHA_Q_SIGNALLING_EVENTS

THESIS_OF_SUBSTRATES_IN_N_GLYCAN_BIOSYTHESIS;REACTOME_ASPARAGINE_N_LINKE

PSIN_LIKE_RECEPTORS;REACTOME_CHEMOKINE_RECEPTORS_BIND_CHEMOKINES;REACTO

REACTOME_MEMBRANE_TRAFFICKING;REACTOME_TRANS_GOLGI_NETWORK_VESICLE_BU

M_OF_PROTEINS

SMITTER_RECEPTOR_BINDING_AND_DOWNSTREAM_TRANSMISSION_IN_THE_POSTSYNAP

CTOME_SIGNALING_BY_NOTCH

RANSPORT;REACTOME_TRANSPORT_OF_INORGANIC_CATIONS_ANIONS_AND_AMINO_ACID

:MATAN_SULFATE_METABOLISM;REACTOME_HS_GAG_BIOSYNTHESIS;REACTOME_HEPARA

TION_OF_TRANSCRIPTION_COUPLED_NER_TC_NER_REPAIR_COMPLEX;REACTOME_DNA_F

RANSPORT;REACTOME_TRANSPORT_OF_VITAMINS_NUCLEOSIDES_AND_RELATED_MOLEC

:C_MEDIATED_ATTRACTIVE_SIGNALING

/IE_ER_PHAGOSOME_PATHWAY;REACTOME_IMMUNOREGULATORY_INTERACTIONS_BETWI

EACTOME_TRANSMEMBRANE_TRANSPORT_OF_SMALL_MOLECULES

E_G_ALPHA_Q_SIGNALLING_EVENTS;REACTOME_GPCR_DOWNSTREAM_SIGNALING;REACT
E_G_ALPHA_Q_SIGNALLING_EVENTS;REACTOME_GPCR_DOWNSTREAM_SIGNALING;REACT
IGNALING

GOLGI_MEMBRANE;REACTOME_PHOSPHOLIPID_METABOLISM;REACTOME_SYNTHESIS_OF_
L_JUNCTION_ORGANIZATION
CTOME_CELL_CYCLE_CHECKPOINTS;REACTOME_HOMOLOGOUS_RECOMBINATION_REPAIR
D_PROTEIN_RESPONSE

OME_SIGNALING_BY_ERBB4;REACTOME_SIGNALING_BY_ERBB2;REACTOME_SIGNALING_BY_
PMENT_AND_PLATELET_PRODUCTION;REACTOME_KINESINS;REACTOME_HEMOSTASIS;REA
NSULIN_SECRETION_BY_GLUCAGON_LIKE_PEPTIDE1;REACTOME_REGULATION_OF_INSULI

SMITTER_RECEPTOR_BINDING_AND_DOWNSTREAM_TRANSMISSION_IN_THE_POSTSYNAP

OME_SIGNALING_BY_THE_B_CELL_RECEPTOR_BCR;REACTOME_GPVI_MEDIATED_ACTIVATI

MOSTASIS

SMITTER_RELEASE_CYCLE;REACTOME_DOPAMINE_NEUROTRANSMITTER_RELEASE_CYCL

:EACTOME_GPCR_LIGAND_BINDING

H_MYCOBACTERIUM_TUBERCULOSIS;REACTOME_IMMUNE_SYSTEM;REACTOME_ADAPTIVE
INELS

SLATIONAL_PROTEIN_MODIFICATION

LYCOSPHINGOLIPID_METABOLISM;REACTOME_PHOSPHOLIPID_METABOLISM;REACTOME_T

REPLICATION

SLATIONAL_PROTEIN_MODIFICATION

C_MEDIATED_ATTRACTIVE_SIGNALING

ACTOME_PROCESSING_OF_CAPPED_INTRON_CONTAINING_PRE_MRNA;REACTOME_TRANS

RIUM_TUBERCULOSIS;REACTOME_TRANSMEMBRANE_TRANSPORT_OF_SMALL_MOLECULE

TOME_NUCLEOTIDE_BINDING_DOMAIN_LEUCINE_RICH_REPEAT_CONTAINING_RECEPTOR_I

SENCHYMAL_TRANSITION;REACTOME_CELL_CELL_JUNCTION_ORGANIZATION;REACTOME_T

C_MEDIATED_ATTRACTIVE_SIGNALING;REACTOME_FACTORS_INVOLVED_IN_MEGAKARYO

_FORMATION;REACTOME_INTEGRIN_CELL_SURFACE_INTERACTIONS;REACTOME_SIGNALIN

_OF_LIPIDS_AND_LIPOPROTEINS;REACTOME_FATTY_ACID_TRIACYLGLYCEROL_AND_KETO
ATION_OF_GENE_EXPRESSION_IN_BETA_CELLS
MOSTASIS

ALING_BY_HIPPO;REACTOME_APOPTOTIC_CLEAVAGE_OF_CELL_ADHESION_PROTEINS;RE
SMITTER_RECEPTOR_BINDING_AND_DOWNSTREAM_TRANSMISSION_IN_THE_POSTSYNAP

NCTION_ORGANIZATION
TOME_ENERGY_DEPENDENT_REGULATION_OF_MTOR_BY_LKB1_AMPK;REACTOME_PKB_M
OF_LIPIDS_AND_LIPOPROTEINS;REACTOME_LIPID_DIGESTION_MOBILIZATION_AND_TRANSF

SLATIONAL_PROTEIN_MODIFICATION
SLATIONAL_PROTEIN_MODIFICATION

EACTOME_SYNTHESIS_OF_PC;REACTOME_GLYCEROPHOSPHOLIPID_BIOSYNTHESIS;REACT

ROUGH_JNK;REACTOME_CELL_DEATH_SIGNALLING_VIA_NRAGE_NRIF_AND_NADE;REACTO

_ACTIVATES_SMADS;REACTOME_SIGNALING_BY_TGF_BETA_RECEPTOR_COMPLEX

_GLUCOSE_METABOLISM

ROTEINS_AND_COMPLEXES;REACTOME_LOSS_OF_NLP_FROM_MITOTIC_CENTROSOMES;R

OTIDE_BINDING_DOMAIN_LEUCINE_RICH_REPEAT_CONTAINING_RECEPTOR_NLR_SIGNALIN

NERATION_OF_SECOND_MESSENGER_MOLECULES;REACTOME_IMMUNE_SYSTEM;REACTO

PMENT_AND_PLATELET_PRODUCTION;REACTOME_KINESINS;REACTOME_HEMOSTASIS;REA
TREAM_SIGNALING;REACTOME_G_ALPHA_S_SIGNALLING_EVENTS;REACTOME_GPCR_LIGA

EACTOME_PEPTIDE_CHAIN_ELONGATION;REACTOME_METABOLISM_OF_PROTEINS;REACTO

TOME_SIGNALING_BY_ERBB4;REACTOME_SIGNALING_BY_ERBB2;REACTOME_SIGNALING_E

ES;REACTOME_ION_CHANNEL_TRANSPORT
ES;REACTOME_ION_CHANNEL_TRANSPORT

ACTOME_SIGNALING_BY_FGFR_IN_DISEASE;REACTOME_ACTIVATED_POINT_MUTANTS_OF

PSIN_LIKE_RECEPTORS;REACTOME_OPIOID_SIGNALLING;REACTOME_G_PROTEIN_ACTIVAT
PRESSION;REACTOME_NRAGE_SIGNALS_DEATH_THROUGH_JNK;REACTOME_CELL_DEATH

OME_METABOLISM_OF_PROTEINS;REACTOME_POST_TRANSLATIONAL_PROTEIN_MODIFICA

HOST_INTERACTIONS_OF_HIV_FACTORS;REACTOME_INTERACTIONS_OF_VPR_WITH_HOST

NTERACTIONS_BETWEEN_A_LYMPHOID_AND_A_NON_LYMPHOID_CELL;REACTOME_CELL_

OME_METABOLISM_OF_PROTEINS;REACTOME_POST_TRANSLATIONAL_PROTEIN_MODIFICA

EINS_MEDIATED_TRANSPORT;REACTOME_TRANSMEMBRANE_TRANSPORT_OF_SMALL_MO
CTRON_TRANSPORT;REACTOME_PYRUVATE_METABOLISM

STEM

RANSPORT;REACTOME_TRANSPORT_OF_INORGANIC_CATIONS_ANIONS_AND_AMINO_ACI

_PROCESSING_PRESENTATION;REACTOME_ANTIGEN_PROCESSING_UBIQUITINATION_PRO

_MITOTIC_SPINDLE_CHECKPOINT_COMPONENTS;REACTOME_CELL_CYCLE;REACTOME_CE

E_HEMOSTASIS
CTIONS;REACTOME_HEMOSTASIS
RANSPORT;REACTOME_TRANSPORT_OF_INORGANIC_CATIONS_ANIONS_AND_AMINO_ACID

PE_LIGAND_RECEPTORS;REACTOME_GPCR_DOWNSTREAM_SIGNALING;REACTOME_G_ALI

RT;REACTOME_TRANSMEMBRANE_TRANSPORT_OF_SMALL_MOLECULES
M_OF_PROTEINS

RANSPORT;REACTOME_TRANSPORT_OF_GLUCOSE_AND_OTHER_SUGARS_BILE_SALTS_AI

RT;REACTOME_TRANSMEMBRANE_TRANSPORT_OF_SMALL_MOLECULES

_BINDING

E_PEPTIDE_LIGAND_BINDING_RECEPTORS;REACTOME_CLASS_A1_RHODOPSIN_LIKE_RECE
NCAM_SIGNALING_FOR_NEURITE_OUT_GROWTH
CAL_SYNAPSES;REACTOME_NEURONAL_SYSTEM;REACTOME_SIGNALING_BY_GPCR;REACT
MOSTASIS

_FORMATION;REACTOME_SIGNALING_BY_PDGF;REACTOME_AXON_GUIDANCE;REACTOME_

ALLING_COMPLEXES;REACTOME_P75NTR_SIGNALS_VIA_NFKB;REACTOME_NFKB_IS_ACTIVA

LY
OME_PRE_NOTCH_EXPRESSION_AND_PROCESSING;REACTOME_PRE_NOTCH_PROCESSIN

_BY_ILS;REACTOME_IL_3_5_AND_GM_CSF_SIGNALING;REACTOME_IL_RECEPTOR_SHC_SIG

ROUGH_JNK;REACTOME_CELL_DEATH_SIGNALLING_VIA_NRAGE_NRIF_AND_NADE;REACTO

TOME_METABOLISM_OF_LIPIDS_AND_LIPOPROTEINS

ROTEINS_MEDIATED_TRANSPORT;REACTOME_TRANSMEMBRANE_TRANSPORT_OF_SMALL_
OMEOSTASIS;REACTOME_HEMOSTASIS
SSOCIATED_VESICLE_BIOGENESIS

PSIN_LIKE_RECEPTORS;REACTOME_GPCR_DOWNSTREAM_SIGNALING;REACTOME_G_ALPH

IS;REACTOME_SEMAPHORIN_INTERACTIONS
EMICAL_SYNAPSES;REACTOME_NRAGE_SIGNALS_DEATH_THROUGH_JNK;REACTOME_NEU
HOSPHORYLATION_CASCADE;REACTOME_APOPTOSIS;REACTOME_APOPTOTIC_EXECUTIO

ENTIATION

NTHESIS_ON_THE_LAGGING_STRAND;REACTOME_CELL_CYCLE_MITOTIC;REACTOME_M_G

CTOME_SEMA3A_PAK_DEPENDENT_AXON_REPULSION;REACTOME_SEMAPHORIN_INTERA

OCHONDRIAL_TRANSCRIPTION;REACTOME_RNA_POL_III_TRANSCRIPTION_TERMINATION

IGNALING

ME_FATTY_ACID_TRIACYLGLYCEROL_AND_KETONE_BODY_METABOLISM

SLATIONAL_PROTEIN_MODIFICATION

_SIGNALING_BY_EGFR_IN_CANCER;REACTOME_ANTIGEN_ACTIVATES_B_CELL_RECEPTOR

NCRETIN_SYNTHESIS_SECRETION_AND_INACTIVATION;REACTOME_SYNTHESIS_SECRETIO

RF1;REACTOME_REGULATION_OF_MRNA_STABILITY_BY_PROTEINS_THAT_BIND_AU_RICH_

NCAM_SIGNALING_FOR_NEURITE_OUT_GROWTH

INE_HYDROXYLATION_OF_HYPOXIA_INDUCIBLE_FACTOR_ALPHA

MPLEX;REACTOME_ACTIVATION_OF_THE_MRNA_UPON_BINDING_OF_THE_CAP_BINDING_C

ING_CASCADE;REACTOME_INTRINSIC_PATHWAY;REACTOME_HEMOSTASIS;REACTOME_PL

ENTS;REACTOME_HIV_INFECTION;REACTOME_HIV_LIFE_CYCLE;REACTOME_LATE_PHASE_

ROTEINS

M_TYPE_3_PROMOTER

3SEQUENT_MODIFICATION;REACTOME_N_GLYCAN_ANTENNAE_ELONGATION;REACTOME_N

ND_BINDING

OTCH1_INTRACELLULAR_DOMAIN_REGULATES_TRANSCRIPTION;REACTOME_SIGNALING_BY

SPORT_OF_MATURE_TRANSCRIPT_TO_CYTOPLASM;REACTOME_TRANSMEMBRANE_TRANS

PLASMA_MEMBRANE;REACTOME_SIGNALING_BY_FGFR_IN_DISEASE;REACTOME_TRANSMIS
_SIGNALING;REACTOME_NEGATIVE_REGULATION_OF_FGFR_SIGNALING;REACTOME_CELL_

ND_ORGANIC_ACIDS_METAL_IONS_AND_AMINE_COMPOUNDS;REACTOME_FACILITATIVE_N

OMPLEX_AND_EIFS_AND_SUBSEQUENT_BINDING_TO_43S;REACTOME_METABOLISM_OF_P

E

XES;REACTOME_REGULATED_PROTEOLYSIS_OF_P75NTR;REACTOME_P75NTR_SIGNALS_V

PORT_OF_SMALL_MOLECULES;REACTOME_SLC_MEDIATED_TRANSMEMBRANE_TRANSPOR

ARBOHYDRATES

EACTOME_RECRUITMENT_OF_NUMA_TO_MITOTIC_CENTROSOMES;REACTOME_MITOTIC_[illegible]

PLASMA_MEMBRANE;REACTOME_SIGNALING_BY_FGFR_IN_DISEASE;REACTOME_SIGNALIN[illegible]

EACTOME_MRNA_SPLICING;REACTOME_PROCESSING_OF_CAPPED_INTRONLESS_PRE_MRI

ME_P75_NTR_RECEPTOR_MEDIATED_SIGNALLING;REACTOME_SIGNALING_BY_GPCR;REAC

ENTS_IN_ERBB4_SIGNALING;REACTOME_PI3K_EVENTS_IN_ERBB2_SIGNALING;REACTOME_
ENTS_IN_ERBB4_SIGNALING;REACTOME_PI3K_EVENTS_IN_ERBB2_SIGNALING;REACTOME_

_REPLICATIVE_COMPLEX;REACTOME_E2F_ENABLED_INHIBITION_OF_PRE_REPLICATION_C

I_EXPRESSION;REACTOME_YAP1_AND_WWTR1_TAZ_STIMULATED_GENE_EXPRESSION;RE

NG_BY_PDGF;REACTOME_AXON_GUIDANCE;REACTOME_NCAM1_INTERACTIONS;REACTOMI
ME_P75_NTR_RECEPTOR_MEDIATED_SIGNALLING;REACTOME_SIGNALING_BY_GPCR;REAC

3Y_CONSTITUTIVELY_ACTIVE_EGFR;REACTOME_SIGNALING_BY_EGFR_IN_CANCER;REACT

:S;REACTOME_THE_ROLE_OF_NEF_IN_HIV1_REPLICATION_AND_DISEASE_PATHOGENESIS
ME_SIGNALING_BY_NOTCH1;REACTOME_GENERIC_TRANSCRIPTION_PATHWAY;REACTOMI

)S_OLIGOPEPTIDES

E_HIV_INFECTION;REACTOME_HIV_LIFE_CYCLE;REACTOME_LATE_PHASE_OF_HIV_LIFE_CY

)S_OLIGOPEPTIDES

;

)PROTEINS;REACTOME_LIPID_DIGESTION_MOBILIZATION_AND_TRANSPORT;REACTOME_LI

ATION
ATION

REGATION

ACTOME_IMMUNE_SYSTEM;REACTOME_ADAPTIVE_IMMUNE_SYSTEM

E_HEMOSTASIS
_SMAD3_SMAD4_HETEROTRIMER;REACTOME_DOWNREGULATION_OF_SMAD2_3_SMAD4_T
PHORYLATION_AND_ACTIVATION_OF_IKKS_COMPLEX;REACTOME_TRAF6_MEDIATED_NFKB_

)TEASOME_DEGRADATION

)S_OLIGOPEPTIDES

:ULES;REACTOME_TRANSPORT_OF_ORGANIC_ANIONS

CLE;REACTOME_TRANSMEMBRANE_TRANSPORT_OF_SMALL_MOLECULES;REACTOME_SLC

HA_I_SIGNALLING_EVENTS;REACTOME_GPCR_LIGAND_BINDING

TOME_FATTY_ACID_TRIACYLGLYCEROL_AND_KETONE_BODY_METABOLISM;REACTOME_TR

CTOME_PHOSPHOLIPID_METABOLISM;REACTOME_PI_METABOLISM;REACTOME_GOLGI_ASS

)TEASOME_DEGRADATION

CTOME_G_ALPHA_Q_SIGNALLING_EVENTS;REACTOME_GPCR_DOWNSTREAM_SIGNALING;F

3Y_CONSTITUTIVELY_ACTIVE_EGFR;REACTOME_SIGNALING_BY_EGFR_IN_CANCER;REACT(

3Y_CONSTITUTIVELY_ACTIVE_EGFR;REACTOME_SIGNALING_BY_EGFR_IN_CANCER;REACT(

EPTORS;REACTOME_G_ALPHA_Q_SIGNALLING_EVENTS;REACTOME_GPCR_DOWNSTREAM_

EEN_A_LYMPHOID_AND_A_NON_LYMPHOID_CELL;REACTOME_INTERFERON_GAMMA_SIGNA

RT_OF_SMALL_MOLECULES;REACTOME_SLC_MEDIATED_TRANSMEMBRANE_TRANSPORT;R

ALLING;REACTOME_ADENYLATE_CYCLASE_INHIBITORY_PATHWAY;REACTOME_NEUROTRA

OMPLEX;REACTOME_PYRUVATE_METABOLISM

OS_OLIGOPEPTIDES

_VOLTAGE_GATED_POTASSIUM_CHANNELS;REACTOME_POTASSIUM_CHANNELS

:ULES

;UIDANCE;REACTOME_NCAM1_INTERACTIONS;REACTOME_REGULATION_OF_INSULIN_SEC

ATION_SIGNALING_AND_AGGREGATION

_CONSTITUTIVELY_ACTIVE_EGFR;REACTOME_GRB2_EVENTS_IN_ERBB2_SIGNALING;REAC
_LEADING_TO_GENERATION_OF_SECOND_MESSENGERS;REACTOME_SIGNALING_BY_THE

TIC_CELL;REACTOME_ACETYLCHOLINE_BINDING_AND_DOWNSTREAM_EVENTS;REACTOME

OMPLEX;REACTOME_PYRUVATE_METABOLISM

3OLIC_REGULATION;REACTOME_G_ALPHA_Q_SIGNALLING_EVENTS;REACTOME_CLASS_B_
ACTOME_INTEGRATION_OF_ENERGY_METABOLISM;REACTOME_METABOLISM_OF_LIPIDS_/

F_MRNA;REACTOME_METABOLISM_OF_RNA;REACTOME_REGULATION_OF_MRNA_STABILIT
REPAIR

_AND_ANKYRINS
F_MRNA;REACTOME_MRNA_DECAY_BY_3_TO_5_EXORIBONUCLEASE;REACTOME_DEADENY

OPHOSPHOLIPID_BIOSYNTHESIS;REACTOME_METABOLISM_OF_LIPIDS_AND_LIPOPROTEINS

PLASMA_MEMBRANE;REACTOME_SIGNALING_BY_FGFR_IN_DISEASE;REACTOME_RESPONS

OINTS;REACTOME_M_G1_TRANSITION;REACTOME_G1_S_TRANSITION;REACTOME_SYNTHE

ME_P75_NTR_RECEPTOR_MEDIATED_SIGNALLING;REACTOME_SIGNALING_BY_GPCR;REAC

)TEASOME_DEGRADATION

E_INTERFERON_SIGNALING;REACTOME_IMMUNE_SYSTEM;REACTOME_ADAPTIVE_IMMUNE_

ME_P75_NTR_RECEPTOR_MEDIATED_SIGNALLING;REACTOME_SIGNALING_BY_GPCR;REAC

ME_P75_NTR_RECEPTOR_MEDIATED_SIGNALLING;REACTOME_SIGNALING_BY_GPCR;REAC

CTOME_RNA_POL_I_TRANSCRIPTION_INITIATION

ACTOME_TRANSPORT_OF_INORGANIC_CATIONS_ANIONS_AND_AMINO_ACIDS_OLIGOPEPT
ACTOME_TRANSPORT_OF_INORGANIC_CATIONS_ANIONS_AND_AMINO_ACIDS_OLIGOPEPT

DS_OLIGOPEPTIDES

CTOME_RNA_POL_I_TRANSCRIPTION_INITIATION

)RIAL_TRANSCRIPTION

E_TOLL_RECEPTOR_CASCADES

_THE_SINGLE_NUCLEOTIDE_REPLACEMENT_PATHWAY;REACTOME_DNA_REPAIR;REACTOM
_THE_SINGLE_NUCLEOTIDE_REPLACEMENT_PATHWAY;REACTOME_DNA_REPAIR;REACTOM

NA_REPAIR;REACTOME_CHROMOSOME_MAINTENANCE;REACTOME_ACTIVATION_OF_ATR_
EAM_SIGNALING;REACTOME_G_ALPHA_I_SIGNALLING_EVENTS;REACTOME_SIGNAL_AMPLIF

ETABOLISM;REACTOME_TRANSMEMBRANE_TRANSPORT_OF_SMALL_MOLECULES;REACTO

DE_EXCISION_REPAIR;REACTOME_REPAIR_SYNTHESIS_FOR_GAP_FILLING_BY_DNA_POL_I

)S_OLIGOPEPTIDES
I_EXPRESSION;REACTOME_CIRCADIAN_REPRESSION_OF_EXPRESSION_BY_REV_ERBA;RE/

_SYSTEM;REACTOME_CYTOKINE_SIGNALING_IN_IMMUNE_SYSTEM

_NER_TC_NER;REACTOME_RNA_POL_II_TRANSCRIPTION_PRE_INITIATION_AND_PROMOTER

_METABOLISM;REACTOME_CHONDROITIN_SULFATE_DERMATAN_SULFATE_METABOLISM;R
_METABOLISM;REACTOME_CHONDROITIN_SULFATE_DERMATAN_SULFATE_METABOLISM;R
S;REACTOME_ALPHA_LINOLENIC_ACID_ALA_METABOLISM;REACTOME_PEROXISOMAL_LIPI

CHROMOSOME_MAINTENANCE;REACTOME_DEPOSITION_OF_NEW_CENPA_CONTAINING_NU

UNCTIONALIZATION_OF_COMPOUNDS;REACTOME_ENDOGENOUS_STEROLS;REACTOME_S

ME_ADAPTIVE_IMMUNE_SYSTEM

S_IN_ERBB4_SIGNALING;REACTOME_SHC1_EVENTS_IN_ERBB4_SIGNALING;REACTOME_PI3

TION_OF_HOMO_SAPIENS_WITH_MYCOBACTERIUM_TUBERCULOSIS;REACTOME_TRANSME
[illegible]

WAY;REACTOME_INTERFERON_ALPHA_BETA_SIGNALING;REACTOME_REGULATION_OF_IFN

ACTOME_SIGNALLING_TO_ERKS;REACTOME_SIGNALING_BY_PDGF;REACTOME_DOWNSTRE

CTOME_APOPTOSIS
CTOME_APOPTOSIS

ACTOME_IMMUNE_SYSTEM;REACTOME_ADAPTIVE_IMMUNE_SYSTEM

ATION

REGATION

CTOME_SIGNAL_TRANSDUCTION_BY_L1;REACTOME_INTERFERON_GAMMA_SIGNALING;REA

UNCTIONALIZATION_OF_COMPOUNDS;REACTOME_STEROID_HORMONES;REACTOME_MET.
UNCTIONALIZATION_OF_COMPOUNDS;REACTOME_STEROID_HORMONES;REACTOME_MET.

NE_BODY_METABOLISM;REACTOME_TRANSCRIPTIONAL_REGULATION_OF_WHITE_ADIPOC

NG_OF_PG;REACTOME_ACYL_CHAIN_REMODELLING_OF_PE;REACTOME_ACYL_CHAIN_REM

EACTOME_S_PHASE
EACTOME_S_PHASE

OME_STEROID_HORMONES;REACTOME_METABOLISM_OF_LIPIDS_AND_LIPOPROTEINS;RE

)TEASOME_DEGRADATION

_AND_ANKYRINS

OMPLEX_AND_EIFS_AND_SUBSEQUENT_BINDING_TO_43S;REACTOME_METABOLISM_OF_PI

SNALING_BY_ERBB2;REACTOME_GRB2_EVENTS_IN_ERBB2_SIGNALING;REACTOME_SIGNAL

TIC_CELL;REACTOME_ACETYLCHOLINE_BINDING_AND_DOWNSTREAM_EVENTS;REACTOME

NUCLEAR_EVENTS_KINASE_AND_TRANSCRIPTION_FACTOR_ACTIVATION;REACTOME_ERK_

ASCADE
)RIAL_TRANSCRIPTION;REACTOME_RNA_POL_III_TRANSCRIPTION_INITIATION_FROM_TYPE

REACTOME_FATTY_ACID_TRIACYLGLYCEROL_AND_KETONE_BODY_METABOLISM;REACTO

TOME_FATTY_ACID_TRIACYLGLYCEROL_AND_KETONE_BODY_METABOLISM

I_EXPRESSION;REACTOME_YAP1_AND_WWTR1_TAZ_STIMULATED_GENE_EXPRESSION;RE

DS_OLIGOPEPTIDES

_PHASE_OF_HIV_LIFE_CYCLE

NE_BODY_METABOLISM;REACTOME_TRANSCRIPTIONAL_REGULATION_OF_WHITE_ADIPOC

ATION
ATION
ATION
ATION

I_PATHWAY;REACTOME_SIGNALING_BY_TGF_BETA_RECEPTOR_COMPLEX

NSMEMBRANE_TRANSPORT;REACTOME_TRANSPORT_OF_INORGANIC_CATIONS_ANIONS_

M_SIGNALING
TIC_CELL;REACTOME_ACTIVATION_OF_KAINATE_RECEPTORS_UPON_GLUTAMATE_BINDIN
TIC_CELL;REACTOME_ACTIVATION_OF_KAINATE_RECEPTORS_UPON_GLUTAMATE_BINDIN

IE_HEMOSTASIS
IE_HEMOSTASIS

TOME_INTEGRATION_OF_ENERGY_METABOLISM;REACTOME_OPIOID_SIGNALLING;REACTO

;;REACTOME_THE_ROLE_OF_NEF_IN_HIV1_REPLICATION_AND_DISEASE_PATHOGENESIS;R
I_EXPRESSION;REACTOME_YAP1_AND_WWTR1_TAZ_STIMULATED_GENE_EXPRESSION;RE
I_EXPRESSION;REACTOME_YAP1_AND_WWTR1_TAZ_STIMULATED_GENE_EXPRESSION;RE

_AND_TRANSLATION;REACTOME_RORA_ACTIVATES_CIRCADIAN_EXPRESSION;REACTOME

RF7_ACTIVATION_IN_TLR7_8_OR_9_SIGNALING;REACTOME_INNATE_IMMUNE_SYSTEM;REA
RF7_ACTIVATION_IN_TLR7_8_OR_9_SIGNALING;REACTOME_INNATE_IMMUNE_SYSTEM;REA

UROTRANSMITTER_RELEASE_CYCLE;REACTOME_NOREPINEPHRINE_NEUROTRANSMITTER

OTIC_COUPLING_AND_HEAT_PRODUCTION_BY_UNCOUPLING_PROTEINS_

TOME_FATTY_ACID_TRIACYLGLYCEROL_AND_KETONE_BODY_METABOLISM;REACTOME_TR

ING_VIA_NRAGE_NRIF_AND_NADE;REACTOME_P75_NTR_RECEPTOR_MEDIATED_SIGNALLI

TOR;REACTOME_IMMUNE_SYSTEM;REACTOME_ADAPTIVE_IMMUNE_SYSTEM

LL_CYCLE_MITOTIC;REACTOME_CELL_CYCLE_CHECKPOINTS;REACTOME_REGULATION_OF

)TEASOME_DEGRADATION

EPTORS;REACTOME_G_ALPHA_Q_SIGNALLING_EVENTS;REACTOME_GPCR_DOWNSTREAM_
ND_ORGANIC_ACIDS_METAL_IONS_AND_AMINE_COMPOUNDS;REACTOME_METAL_ION_SLC
ND_ORGANIC_ACIDS_METAL_IONS_AND_AMINE_COMPOUNDS;REACTOME_METAL_ION_SLC

ACTOME_TRANSPORT_OF_INORGANIC_CATIONS_ANIONS_AND_AMINO_ACIDS_OLIGOPEPT

ENTS_IN_ERBB4_SIGNALING;REACTOME_PI3K_EVENTS_IN_ERBB2_SIGNALING;REACTOME_

_THE_SINGLE_NUCLEOTIDE_REPLACEMENT_PATHWAY;REACTOME_DNA_REPAIR;REACTOM

OME_G_ALPHA_Z_SIGNALLING_EVENTS;REACTOME_EFFECTS_OF_PIP2_HYDROLYSIS;REA

S_VIA_24_HYDROXYCHOLESTEROL;REACTOME_SYNTHESIS_OF_BILE_ACIDS_AND_BILE_SA

EACTOME_RECRUITMENT_OF_NUMA_TO_MITOTIC_CENTROSOMES;REACTOME_MITOTIC_

I_EXPRESSION;REACTOME_YAP1_AND_WWTR1_TAZ_STIMULATED_GENE_EXPRESSION;REA

A5_SIGNALING;REACTOME_RIG_I_MDA5_MEDIATED_INDUCTION_OF_IFN_ALPHA_BETA_PATI

OTEASOME_DEGRADATION

TGF_BETA_RECEPTOR_COMPLEX;REACTOME_PLATELET_ACTIVATION_SIGNALING_AND_A

ND_ORGANIC_ACIDS_METAL_IONS_AND_AMINE_COMPOUNDS;REACTOME_FACILITATIVE_N

ENTS_IN_ERBB4_SIGNALING;REACTOME_PI3K_EVENTS_IN_ERBB2_SIGNALING;REACTOME_

TEASOME_DEGRADATION
TEASOME_DEGRADATION

LISM;REACTOME_GLYCOSAMINOGLYCAN_METABOLISM;REACTOME_A_TETRASACCHARIDE
LISM;REACTOME_GLYCOSAMINOGLYCAN_METABOLISM;REACTOME_A_TETRASACCHARIDE

SEQUENT_MODIFICATION

GASTRIN_CREB_SIGNALLING_PATHWAY_VIA_PKC_AND_MAPK;REACTOME_NUCLEAR_EVEN

.ECULES;REACTOME_SLC_MEDIATED_TRANSMEMBRANE_TRANSPORT;REACTOME_TRANSI
.ECULES;REACTOME_SLC_MEDIATED_TRANSMEMBRANE_TRANSPORT;REACTOME_TRANSI

DAPTERS;REACTOME_CELL_SURFACE_INTERACTIONS_AT_THE_VASCULAR_WALL;REACTO

'OME_FATTY_ACID_TRIACYLGLYCEROL_AND_KETONE_BODY_METABOLISM;REACTOME_TR

GULATION_OF_AMPK_ACTIVITY_VIA_LKB1;REACTOME_ENERGY_DEPENDENT_REGULATION

NALING;REACTOME_REGULATION_OF_IFNG_SIGNALING;REACTOME_INTERFERON_GAMMA

S;REACTOME_GPCR_DOWNSTREAM_SIGNALING;REACTOME_G_ALPHA_I_SIGNALLING_EVE

_GPCR_DOWNSTREAM_SIGNALING;REACTOME_SEMA4D_IN_SEMAPHORIN_SIGNALING;REA

;REACTOME_GENERIC_TRANSCRIPTION_PATHWAY;REACTOME_NOTCH_HLH_TRANSCRIPT

;REACTOME_GENERIC_TRANSCRIPTION_PATHWAY;REACTOME_NOTCH_HLH_TRANSCRIPT

RIAL_TRANSCRIPTION;REACTOME_RNA_POL_III_TRANSCRIPTION_INITIATION_FROM_TYPE

TEASOME_DEGRADATION

G_VIA_NRAGE_NRIF_AND_NADE;REACTOME_P75_NTR_RECEPTOR_MEDIATED_SIGNALLIN

ENT

M_TYPE_3_PROMOTER

ALING_AND_AGGREGATION

SPORT_OF_MATURE_TRANSCRIPT_TO_CYTOPLASM;REACTOME_TRANSMEMBRANE_TRANS

ON_REPULSION;REACTOME_SEMAPHORIN_INTERACTIONS;REACTOME_SEMA3A_PLEXIN_R

SIGNALING_AND_AGGREGATION

ME_P75_NTR_RECEPTOR_MEDIATED_SIGNALLING;REACTOME_SIGNALING_BY_GPCR;REAC

G;REACTOME_SIGNALING_BY_ERBB2;REACTOME_SIGNALING_BY_EGFR_IN_CANCER;REACT

TABOLISM_OF_LIPIDS_AND_LIPOPROTEINS
_BY_ERBB4;REACTOME_ACTIVATED_NOTCH1_TRANSMITS_SIGNAL_TO_THE_NUCLEUS;REA

N_SECRETION;REACTOME_VOLTAGE_GATED_POTASSIUM_CHANNELS;REACTOME_POTASS

I_EXPRESSION;REACTOME_YAP1_AND_WWTR1_TAZ_STIMULATED_GENE_EXPRESSION;RE

OS_OLIGOPEPTIDES;REACTOME_PLATELET_HOMEOSTASIS;REACTOME_PLATELET_CALCIU

S;REACTOME_INTERACTION_BETWEEN_L1_AND_ANKYRINS;REACTOME_NEPHRIN_INTERA
S;REACTOME_INTERACTION_BETWEEN_L1_AND_ANKYRINS;REACTOME_NEPHRIN_INTERA

ION;REACTOME_MYD88_MAL_CASCADE_INITIATED_ON_PLASMA_MEMBRANE;REACTOME_I

REACTOME_METABOLISM_OF_PROTEINS;REACTOME_POST_TRANSLATIONAL_PROTEIN_MC

REACTOME_CELL_SURFACE_INTERACTIONS_AT_THE_VASCULAR_WALL;REACTOME_COMM

-

ECRETION_AND_INACTIVATION;REACTOME_SYNTHESIS_SECRETION_AND_INACTIVATION_(
ECRETION_AND_INACTIVATION;REACTOME_SYNTHESIS_SECRETION_AND_INACTIVATION_(

ACTOME_SYNTHESIS_OF_BILE_ACIDS_AND_BILE_SALTS;REACTOME_METABOLISM_OF_LIPI

OME_LOSS_OF_NLP_FROM_MITOTIC_CENTROSOMES;REACTOME_MITOTIC_G2_G2_M_PHAS
ION_BY_GLUCAGON_LIKE_PEPTIDE1;REACTOME_REGULATION_OF_INSULIN_SECRETION;R

TIC_CELL;REACTOME_ACETYLCHOLINE_BINDING_AND_DOWNSTREAM_EVENTS;REACTOME

E_CYCLASE;REACTOME_PLATELET_HOMEOSTASIS;REACTOME_HEMOSTASIS

S;REACTOME_INTEGRIN_CELL_SURFACE_INTERACTIONS;REACTOME_AXON_GUIDANCE;RE
_NCAM1_INTERACTIONS;REACTOME_NCAM_SIGNALING_FOR_NEURITE_OUT_GROWTH

REACTOME_SIGNALING_BY_FGFR1_MUTANTS;REACTOME_SIGNALING_BY_FGFR1_FUSION_

ANE_TRANSPORT;REACTOME_TRANSPORT_OF_GLUCOSE_AND_OTHER_SUGARS_BILE_SAL

IA_MEMBRANE;REACTOME_PI3K_AKT_ACTIVATION;REACTOME_SIGNALING_BY_INSULIN_RE

EPTORS;REACTOME_G_ALPHA_Q_SIGNALLING_EVENTS;REACTOME_GPCR_DOWNSTREAM_

ECULES;REACTOME_SLC_MEDIATED_TRANSMEMBRANE_TRANSPORT;REACTOME_TRANSPO

_IN_ERBB2_SIGNALING;REACTOME_SIGNALING_BY_EGFR_IN_CANCER;REACTOME_SHC1_E

OS_OLIGOPEPTIDES
_SIGNALING_ACTIVATES_SMADS;REACTOME_SIGNALING_BY_TGF_BETA_RECEPTOR_COM

OME_GPCR_DOWNSTREAM_SIGNALING;REACTOME_G_ALPHA_I_SIGNALLING_EVENTS;REA

TABOLISM_OF_PORPHYRINS

ON_REPULSION;REACTOME_SEMAPHORIN_INTERACTIONS;REACTOME_SEMA3A_PLEXIN_R

NA_REPAIR;REACTOME_CHROMOSOME_MAINTENANCE;REACTOME_ACTIVATION_OF_ATR_

N_PATHWAY;REACTOME_SIGNALING_BY_TGF_BETA_RECEPTOR_COMPLEX

ASSIUM_CHANNELS

HA_I_SIGNALLING_EVENTS;REACTOME_GPCR_LIGAND_BINDING

ATION

_SYSTEM;REACTOME_ADAPTIVE_IMMUNE_SYSTEM;REACTOME_CLASS_I_MHC_MEDIATED_
_SYSTEM;REACTOME_ADAPTIVE_IMMUNE_SYSTEM;REACTOME_CLASS_I_MHC_MEDIATED_

TIC_CELL;REACTOME_TRANSMEMBRANE_TRANSPORT_OF_SMALL_MOLECULES;REACTOMI

DS_OLIGOPEPTIDES

)S_OLIGOPEPTIDES
EACTOME_MITOTIC_G2_G2_M_PHASES

)S_OLIGOPEPTIDES

.GE_SIGNALS_DEATH_THROUGH_JNK;REACTOME_CELL_DEATH_SIGNALLING_VIA_NRAGE_I
.GE_SIGNALS_DEATH_THROUGH_JNK;REACTOME_CELL_DEATH_SIGNALLING_VIA_NRAGE_I

ENERGY_DEPENDENT_REGULATION_OF_MTOR_BY_LKB1_AMPK;REACTOME_REGULATION_

[illegible]LESTEROL_BIOSYNTHESIS

[illegible]SOMES_AT_THE_CENTROMERE;REACTOME_DNA_REPLICATION;REACTOME_MITOTIC_PR[illegible]

[illegible]B2;REACTOME_SIGNALING_BY_CONSTITUTIVELY_ACTIVE_EGFR;REACTOME_SIGNALING_[illegible]

_GAG_METABOLISM;REACTOME_GLYCOSAMINOGLYCAN_METABOLISM;REACTOME_A_TETR[illegible]

HE_ACTIVATION_OF_ARYLSULFATASES;REACTOME_METABOLISM_OF_PROTEINS;REACTO[illegible]

_M_PHASES;REACTOME_ACTIVATION_OF_ATR_IN_RESPONSE_TO_REPLICATION_STRESS;[illegible]
_M_PHASES;REACTOME_ACTIVATION_OF_ATR_IN_RESPONSE_TO_REPLICATION_STRESS;[illegible]

REACTOME_FRS2_MEDIATED_CASCADE;REACTOME_PI_3K_CASCADE;REACTOME_DOWNST
REACTOME_FRS2_MEDIATED_CASCADE;REACTOME_PI_3K_CASCADE;REACTOME_DOWNST
REACTOME_FRS2_MEDIATED_CASCADE;REACTOME_PI_3K_CASCADE;REACTOME_DOWNST

OME_3_UTR_MEDIATED_TRANSLATIONAL_REGULATION;REACTOME_METABOLISM_OF_MRN

BSEQUENT_MODIFICATION;REACTOME_N_GLYCAN_ANTENNAE_ELONGATION;REACTOME_N
ED_GLYCOSYLATION;REACTOME_BIOSYNTHESIS_OF_THE_N_GLYCAN_PRECURSOR_DOLIC

_LEADING_TO_GENERATION_OF_SECOND_MESSENGERS;REACTOME_SIGNALING_BY_THE_

OME_3_UTR_MEDIATED_TRANSLATIONAL_REGULATION;REACTOME_METABOLISM_OF_MRN

ULES

OTEASOME_DEGRADATION

ARBOHYDRATES
_SIGNALING;REACTOME_DOWNSTREAM_TCR_SIGNALING;REACTOME_IMMUNE_SYSTEM;RE

OME_EFFECTS_OF_PIP2_HYDROLYSIS;REACTOME_HEMOSTASIS;REACTOME_PLATELET_A

CTOME_JNK_C_JUN_KINASES_PHOSPHORYLATION_AND_ACTIVATION_MEDIATED_BY_ACTI

.CTOME_CITRIC_ACID_CYCLE_TCA_CYCLE
PLASMA_MEMBRANE;REACTOME_SIGNALING_BY_FGFR_IN_DISEASE;REACTOME_TRANSMIS

CTOME_PLATELET_ADHESION_TO_EXPOSED_COLLAGEN;REACTOME_INTEGRIN_CELL_SUR

OTEASOME_DEGRADATION

ATION

SOME_PATHWAY;REACTOME_CELL_CYCLE;REACTOME_ORC1_REMOVAL_FROM_CHROMAT
_REPLICATIVE_COMPLEX;REACTOME_E2F_ENABLED_INHIBITION_OF_PRE_REPLICATION_C

OME_EFFECTS_OF_PIP2_HYDROLYSIS;REACTOME_HEMOSTASIS;REACTOME_PLATELET_A
OME_EFFECTS_OF_PIP2_HYDROLYSIS;REACTOME_HEMOSTASIS;REACTOME_PLATELET_A

S;REACTOME_IRON_UPTAKE_AND_TRANSPORT;REACTOME_TRANSFERRIN_ENDOCYTOSIS

OS_ACTIVATION_AND_REGULATION;REACTOME_NITRIC_OXIDE_STIMULATES_GUANYLATE_

ALING;REACTOME_SEMAPHORIN_INTERACTIONS;REACTOME_FACTORS_INVOLVED_IN_MEC

GULATION_OF_AMPK_ACTIVITY_VIA_LKB1;REACTOME_ENERGY_DEPENDENT_REGULATION
GULATION_OF_AMPK_ACTIVITY_VIA_LKB1;REACTOME_ENERGY_DEPENDENT_REGULATION

)TEASOME_DEGRADATION

CTOME_SIGNALING_BY_FGFR1_MUTANTS;REACTOME_SIGNALING_BY_ACTIVATED_POINT_M
CTOME_SIGNALING_BY_FGFR1_MUTANTS;REACTOME_SIGNALING_BY_ACTIVATED_POINT_M

_THE_SINGLE_NUCLEOTIDE_REPLACEMENT_PATHWAY;REACTOME_DNA_REPAIR;REACTOM

NE_SENSITIVE_LIPASE_HSL_MEDIATED_TRIACYLGLYCEROL_HYDROLYSIS;REACTOME_LIPID

ON_ORGANIZATION;REACTOME_APOPTOSIS;REACTOME_APOPTOTIC_EXECUTION_PHASE

CLE;REACTOME_TRANSMEMBRANE_TRANSPORT_OF_SMALL_MOLECULES;REACTOME_SLC

)S_OLIGOPEPTIDES

OTIC_COUPLING_AND_HEAT_PRODUCTION_BY_UNCOUPLING_PROTEINS_

ME_SIGNALLING_TO_ERKS;REACTOME_SIGNALING_BY_INSULIN_RECEPTOR;REACTOME_SI

EACTOME_MITOTIC_G2_G2_M_PHASES

ENTS_IN_ERBB4_SIGNALING;REACTOME_PI3K_EVENTS_IN_ERBB2_SIGNALING;REACTOME_

OTEASOME_DEGRADATION
CLE;REACTOME_INTEGRATION_OF_ENERGY_METABOLISM;REACTOME_DOPAMINE_NEURO

TAL_PROTEINS;REACTOME_AXON_GUIDANCE;REACTOME_NCAM_SIGNALING_FOR_NEURITE
TAL_PROTEINS;REACTOME_AXON_GUIDANCE;REACTOME_NCAM_SIGNALING_FOR_NEURITE
TAL_PROTEINS;REACTOME_AXON_GUIDANCE;REACTOME_NCAM_SIGNALING_FOR_NEURITE
TAL_PROTEINS;REACTOME_AXON_GUIDANCE;REACTOME_NCAM_SIGNALING_FOR_NEURITE

ED_GLYCOSYLATION;REACTOME_BIOSYNTHESIS_OF_THE_N_GLYCAN_PRECURSOR_DOLIC

ACTOME_SIGNALLING_TO_ERKS;REACTOME_SIGNALING_BY_PDGF;REACTOME_DOWNSTRE

ORIAL_TRANSCRIPTION

_NCAM1_INTERACTIONS;REACTOME_NCAM_SIGNALING_FOR_NEURITE_OUT_GROWTH
_NCAM1_INTERACTIONS;REACTOME_NCAM_SIGNALING_FOR_NEURITE_OUT_GROWTH
_NCAM1_INTERACTIONS;REACTOME_NCAM_SIGNALING_FOR_NEURITE_OUT_GROWTH

_ALPHA_BETA_PATHWAYS;REACTOME_APOPTOSIS;REACTOME_INNATE_IMMUNE_SYSTEM;
CTOME_N_GLYCAN_TRIMMING_IN_THE_ER_AND_CALNEXIN_CALRETICULIN_CYCLE
OS_OLIGOPEPTIDES

)PROTEINS;REACTOME_LIPID_DIGESTION_MOBILIZATION_AND_TRANSPORT;REACTOME_LI
)PROTEINS;REACTOME_LIPID_DIGESTION_MOBILIZATION_AND_TRANSPORT;REACTOME_LI

.INS_;REACTOME_INTEGRIN_ALPHAIIB_BETA3_SIGNALING;REACTOME_PLATELET_AGGREG

NAL_SYSTEM;REACTOME_NEUROTRANSMITTER_RELEASE_CYCLE;REACTOME_ACETYLCHC

OME_REGULATION_OF_SIGNALING_BY_CBL;REACTOME_IL_3_5_AND_GM_CSF_SIGNALING;[illegible]

CYTE_DEVELOPMENT_AND_PLATELET_PRODUCTION;REACTOME_HEMOSTASIS
CYTE_DEVELOPMENT_AND_PLATELET_PRODUCTION;REACTOME_HEMOSTASIS

SOME_PATHWAY;REACTOME_CELL_CYCLE;REACTOME_ORC1_REMOVAL_FROM_CHROMAT

S;REACTOME_SIGNALING_BY_EGFR_IN_CANCER;REACTOME_NGF_SIGNALLING_VIA_TRKA_

ATION
ATION
ATION
ATION

ATION

REPRESSION_OF_EXPRESSION_BY_REV_ERBA;REACTOME_METABOLISM_OF_LIPIDS_AND_L

ECTS;REACTOME_NITRIC_OXIDE_STIMULATES_GUANYLATE_CYCLASE;REACTOME_PLATELE
UNCTIONALIZATION_OF_COMPOUNDS;REACTOME_STEROID_HORMONES;REACTOME_METAB

CR;REACTOME_PEPTIDE_LIGAND_BINDING_RECEPTORS;REACTOME_CLASS_A1_RHODOPSI

ND_ORGANIC_ACIDS_METAL_IONS_AND_AMINE_COMPOUNDS;REACTOME_NA_CL_DEPEND

E

_BODY_METABOLISM;REACTOME_SYNTHESIS_OF_VERY_LONG_CHAIN_FATTY_ACYL_COAS

EACTOME_MITOTIC_M_M_G1_PHASES;REACTOME_MITOTIC_G2_G2_M_PHASES;REACTOME

ROL_AND_KETONE_BODY_METABOLISM

ATURE_MRNA_DERIVED_FROM_AN_INTRONLESS_TRANSCRIPT

ENTS_IN_ERBB4_SIGNALING;REACTOME_PI3K_EVENTS_IN_ERBB2_SIGNALING;REACTOME_

1_TRANSITION;REACTOME_G1_S_TRANSITION;REACTOME_POL_SWITCHING;REACTOME_S

ACTOME_IMMUNE_SYSTEM;REACTOME_ADAPTIVE_IMMUNE_SYSTEM

EACTOME_DARPP_32_EVENTS;REACTOME_METABOLISM_OF_LIPIDS_AND_LIPOPROTEINS;I
OTIC_COUPLING_AND_HEAT_PRODUCTION_BY_UNCOUPLING_PROTEINS_
S;REACTOME_IRON_UPTAKE_AND_TRANSPORT;REACTOME_TRANSFERRIN_ENDOCYTOSIS

ULES;REACTOME_TRANSPORT_OF_ORGANIC_ANIONS

_MHC_MEDIATED_ANTIGEN_PROCESSING_PRESENTATION;REACTOME_ANTIGEN_PROCES
RIACYLGLYCEROL_AND_KETONE_BODY_METABOLISM
_OF_REPLICATION_INDEPENDENT_DOUBLE_STRAND_BREAKS;REACTOME_P53_DEPENDEN

G_VIA_NRAGE_NRIF_AND_NADE;REACTOME_P75_NTR_RECEPTOR_MEDIATED_SIGNALLING
G_VIA_NRAGE_NRIF_AND_NADE;REACTOME_P75_NTR_RECEPTOR_MEDIATED_SIGNALLING

TREAM_TCR_SIGNALING;REACTOME_PHOSPHORYLATION_OF_CD3_AND_TCR_ZETA_CHAIN

E_METABOLISM_OF_LIPIDS_AND_LIPOPROTEINS
E_METABOLISM_OF_LIPIDS_AND_LIPOPROTEINS

ANSMEMBRANE_TRANSPORT;REACTOME_METABOLISM_OF_LIPIDS_AND_LIPOPROTEINS;R

ATION
ATION

PLASMA_MEMBRANE;REACTOME_SIGNALING_BY_FGFR_IN_DISEASE;REACTOME_TRANSMIS

PTIVE_IMMUNE_SYSTEM

CTOME_FORMATION_OF_TUBULIN_FOLDING_INTERMEDIATES_BY_CCT_TRIC;REACTOME_N

THE_SINGLE_NUCLEOTIDE_REPLACEMENT_PATHWAY;REACTOME_DNA_REPAIR;REACTOM

NE_BODY_METABOLISM;REACTOME_TRANSCRIPTIONAL_REGULATION_OF_WHITE_ADIPOC
E_HEMOSTASIS

)_CALNEXIN_CALRETICULIN_CYCLE

MUNE_SYSTEM

DE_EXCISION_REPAIR;REACTOME_REPAIR_SYNTHESIS_FOR_GAP_FILLING_BY_DNA_POL_I

NG_BY_PDGF;REACTOME_AXON_GUIDANCE;REACTOME_NCAM1_INTERACTIONS;REACTOMI

_POL_II_PRE_TRANSCRIPTION_EVENTS;REACTOME_HIV_INFECTION;REACTOME_HIV_LIFE_

RANE_TRANSPORT_OF_SMALL_MOLECULES;REACTOME_SLC_MEDIATED_TRANSMEMBRAI

PCR_DOWNSTREAM_SIGNALING;REACTOME_G_ALPHA_S_SIGNALLING_EVENTS;REACTOMI

NIZATION;REACTOME_NEPHRIN_INTERACTIONS;REACTOME_HEMOSTASIS;REACTOME_PLA

S;REACTOME_GPCR_DOWNSTREAM_SIGNALING;REACTOME_GPCR_LIGAND_BINDING

ACTOME_IMMUNE_SYSTEM;REACTOME_ADAPTIVE_IMMUNE_SYSTEM

REACTOME_SIGNALING_BY_ERBB4;REACTOME_DOWNREGULATION_OF_ERBB2_ERBB3_SI

_BY_NOTCH3;REACTOME_RECEPTOR_LIGAND_BINDING_INITIATES_THE_SECOND_PROTEO
ORIAL_TRANSCRIPTION

OTEASOME_DEGRADATION
OTEASOME_DEGRADATION

TIC_CELL;REACTOME_ACETYLCHOLINE_BINDING_AND_DOWNSTREAM_EVENTS;REACTOME

TOME_INTEGRATION_OF_ENERGY_METABOLISM;REACTOME_OPIOID_SIGNALLING;REACTO
_AND_ANKYRINS
_AND_ANKYRINS

REACTOME_OTHER_SEMAPHORIN_INTERACTIONS;REACTOME_SEMAPHORIN_INTERACTION

UNCTIONALIZATION_OF_COMPOUNDS;REACTOME_ENDOGENOUS_STEROLS;REACTOME_S

ME_P75_NTR_RECEPTOR_MEDIATED_SIGNALLING;REACTOME_SIGNALING_BY_GPCR;REAC

ND_ORGANIC_ACIDS_METAL_IONS_AND_AMINE_COMPOUNDS;REACTOME_AMINE_COMPOU

HA_I_SIGNALLING_EVENTS;REACTOME_GPCR_LIGAND_BINDING

OME_3_UTR_MEDIATED_TRANSLATIONAL_REGULATION;REACTOME_METABOLISM_OF_MRN

_AND_TRANSLATION;REACTOME_RORA_ACTIVATES_CIRCADIAN_EXPRESSION;REACTOME_
_AND_TRANSLATION;REACTOME_RORA_ACTIVATES_CIRCADIAN_EXPRESSION;REACTOME_
_AND_TRANSLATION;REACTOME_RORA_ACTIVATES_CIRCADIAN_EXPRESSION;REACTOME_
PLASMA_MEMBRANE;REACTOME_SIGNALING_BY_FGFR_IN_DISEASE;REACTOME_TRANSMIS

ASCADE

OSTASIS;REACTOME_PLATELET_CALCIUM_HOMEOSTASIS;REACTOME_ION_TRANSPORT_B

SPORT_OF_MATURE_TRANSCRIPT_TO_CYTOPLASM;REACTOME_TRANSMEMBRANE_TRANS
SPORT_OF_MATURE_TRANSCRIPT_TO_CYTOPLASM;REACTOME_TRANSMEMBRANE_TRANS
M

DS_OLIGOPEPTIDES

DS_OLIGOPEPTIDES

E

DGF;REACTOME_DOWNSTREAM_SIGNAL_TRANSDUCTION;REACTOME_PLATELET_AGGREGA

ON_CASCADE;REACTOME_HEMOSTASIS;REACTOME_INNATE_IMMUNE_SYSTEM;REACTOMI

CTOME_RNA_POL_I_TRANSCRIPTION_INITIATION

TIC_CELL;REACTOME_ACTIVATION_OF_NMDA_RECEPTOR_UPON_GLUTAMATE_BINDING_AI

DTEASOME_DEGRADATION

ND_ORGANIC_ACIDS_METAL_IONS_AND_AMINE_COMPOUNDS;REACTOME_BILE_SALT_AND_

CTOME_MRNA_CAPPING;REACTOME_TRANSCRIPTION_COUPLED_NER_TC_NER;REACTOM

ON_CASCADE;REACTOME_GPCR_DOWNSTREAM_SIGNALING;REACTOME_G_BETA_GAMMA_

PARA_ACTIVATES_GENE_EXPRESSION;REACTOME_RORA_ACTIVATES_CIRCADIAN_EXPRES
PARA_ACTIVATES_GENE_EXPRESSION;REACTOME_RORA_ACTIVATES_CIRCADIAN_EXPRES

TION_OF_NFKB_AND_MAP_KINASES_UPON_TLR7_8_OR_9_ACTIVATION;REACTOME_NFKB_A
E_HEMOSTASIS
ND_ORGANIC_ACIDS_METAL_IONS_AND_AMINE_COMPOUNDS;REACTOME_BILE_SALT_AND_
ND_ORGANIC_ACIDS_METAL_IONS_AND_AMINE_COMPOUNDS;REACTOME_BILE_SALT_AND_

S;REACTOME_SIGNALING_BY_EGFR_IN_CANCER;REACTOME_NGF_SIGNALLING_VIA_TRKA_

OME_NCAM_SIGNALING_FOR_NEURITE_OUT_GROWTH
OME_3_UTR_MEDIATED_TRANSLATIONAL_REGULATION;REACTOME_METABOLISM_OF_MRN

VENTS_IN_ERBB2_SIGNALING;REACTOME_AXON_GUIDANCE;REACTOME_SEMA4D_IN_SEMA
OME_GPCR_DOWNSTREAM_SIGNALING;REACTOME_G_ALPHA_I_SIGNALLING_EVENTS;REA

CTOME_SIGNALING_BY_FGFR_IN_DISEASE;REACTOME_SIGNALING_BY_FGFR1_MUTANTS;RI
CTOME_SIGNALING_BY_FGFR_IN_DISEASE;REACTOME_SIGNALING_BY_FGFR1_MUTANTS;RI
CTOME_SIGNALING_BY_FGFR_IN_DISEASE;REACTOME_SIGNALING_BY_FGFR1_MUTANTS;RI

TIC_CELL;REACTOME_TRAFFICKING_OF_AMPA_RECEPTORS;REACTOME_TRAFFICKING_OF

OTEASOME_DEGRADATION

CTOME_PLATELET_ADHESION_TO_EXPOSED_COLLAGEN;REACTOME_INTEGRIN_CELL_SUR

S_PATHWAYS;REACTOME_SYNTHESIS_SECRETION_AND_DEACYLATION_OF_GHRELIN;REA

IEDIATED_EVENTS;REACTOME_SIGNALING_BY_INSULIN_RECEPTOR;REACTOME_MTORC1_I

ACTOME_INTEGRATION_OF_ENERGY_METABOLISM;REACTOME_METABOLISM_OF_LIPIDS_A
ACTOME_INTEGRATION_OF_ENERGY_METABOLISM;REACTOME_METABOLISM_OF_LIPIDS_A

ME_GLUCAGON_TYPE_LIGAND_RECEPTORS;REACTOME_GPCR_DOWNSTREAM_SIGNALING

EACTOME_MITOTIC_G2_G2_M_PHASES
HA_S_SIGNALLING_EVENTS;REACTOME_GPCR_LIGAND_BINDING

ALL;REACTOME_BASIGIN_INTERACTIONS;REACTOME_INTEGRIN_CELL_SURFACE_INTERACT

D_INTRON_CONTAINING_PRE_MRNA;REACTOME_RNA_POL_II_TRANSCRIPTION;REACTOME

_BINDING

D_MESSENGERS;REACTOME_SIGNALING_BY_THE_B_CELL_RECEPTOR_BCR;REACTOME_N
_DEATH_THROUGH_JNK;REACTOME_CELL_DEATH_SIGNALLING_VIA_NRAGE_NRIF_AND_NA

REPAIR
REPAIR

ATION
ATION
ATION

NDUCTION_OF_IFN_ALPHA_BETA_PATHWAYS;REACTOME_INNATE_IMMUNE_SYSTEM;REAC

S;REACTOME_P2Y_RECEPTORS;REACTOME_NUCLEOTIDE_LIKE_PURINERGIC_RECEPTORS
REACTOME_INTERFERON_SIGNALING;REACTOME_IMMUNE_SYSTEM;REACTOME_ADAPTIVE

_IMMUNE_SYSTEM;REACTOME_CYTOKINE_SIGNALING_IN_IMMUNE_SYSTEM

_TRANSPORT
_TRANSPORT

G_VIA_TRKA_FROM_THE_PLASMA_MEMBRANE;REACTOME_SIGNALING_BY_FGFR_IN_DISEA

ALING;REACTOME_TGF_BETA_RECEPTOR_SIGNALING_ACTIVATES_SMADS;REACTOME_RES
M_SIGNALING

S;REACTOME_GPCR_DOWNSTREAM_SIGNALING;REACTOME_GPCR_LIGAND_BINDING

OS_OLIGOPEPTIDES;REACTOME_PLATELET_HOMEOSTASIS;REACTOME_PLATELET_CALCIUI

TION_AND_GROWTH_CONE_COLLAPSE

IS_ON_THE_LAGGING_STRAND;REACTOME_CELL_CYCLE_MITOTIC;REACTOME_TRANSCRIF
IS_ON_THE_LAGGING_STRAND;REACTOME_CELL_CYCLE_MITOTIC;REACTOME_TRANSCRIF
IS_ON_THE_LAGGING_STRAND;REACTOME_CELL_CYCLE_MITOTIC;REACTOME_TRANSCRIF

TIC_CELL;REACTOME_TRAFFICKING_OF_AMPA_RECEPTORS

;TASIS

EACTOME_MITOTIC_G2_G2_M_PHASES

TIC_CELL;REACTOME_TRAFFICKING_OF_AMPA_RECEPTORS

:RBB2;REACTOME_SIGNALING_BY_CONSTITUTIVELY_ACTIVE_EGFR;REACTOME_SIGNALINC

DS_OLIGOPEPTIDES

ND_ORGANIC_ACIDS_METAL_IONS_AND_AMINE_COMPOUNDS;REACTOME_BILE_SALT_AND_

C_REGULATION;REACTOME_REGULATION_OF_INSULIN_SECRETION_BY_GLUCAGON_LIKE_
C_REGULATION;REACTOME_REGULATION_OF_INSULIN_SECRETION_BY_GLUCAGON_LIKE_

_NCAM1_INTERACTIONS;REACTOME_NCAM_SIGNALING_FOR_NEURITE_OUT_GROWTH
_NCAM1_INTERACTIONS;REACTOME_NCAM_SIGNALING_FOR_NEURITE_OUT_GROWTH
EIN_RESPONSE

EVATED_PLATELET_CYTOSOLIC_CA2_;REACTOME_SIGNALING_BY_GPCR;REACTOME_PEPT

MUNE_SYSTEM
ME_P75_NTR_RECEPTOR_MEDIATED_SIGNALLING;REACTOME_SIGNALING_BY_GPCR;REAC

OCESSING;REACTOME_MRNA_SPLICING;REACTOME_TRANSCRIPTION;REACTOME_MRNA_3

SURFACE_INTERACTIONS;REACTOME_HEMOSTASIS;REACTOME_IMMUNE_SYSTEM;REACTO

_NCAM1_INTERACTIONS;REACTOME_NCAM_SIGNALING_FOR_NEURITE_OUT_GROWTH

EACTOME_MITOTIC_G2_G2_M_PHASES

E_NUCLEOTIDE_LIKE_PURINERGIC_RECEPTORS;REACTOME_GPCR_DOWNSTREAM_SIGNA

OINTS;REACTOME_M_G1_TRANSITION;REACTOME_G1_S_TRANSITION;REACTOME_SYNTHE
TION_AND_GROWTH_CONE_COLLAPSE

IALING_IN_IMMUNE_SYSTEM

TABOLISM_OF_LIPIDS_AND_LIPOPROTEINS

TIC_CELL;REACTOME_INHIBITION_OF_VOLTAGE_GATED_CA2_CHANNELS_VIA_GBETA_GAM
TIC_CELL;REACTOME_INHIBITION_OF_VOLTAGE_GATED_CA2_CHANNELS_VIA_GBETA_GAM

EPTORS;REACTOME_G_ALPHA_Q_SIGNALLING_EVENTS;REACTOME_GPCR_DOWNSTREAM_
ORIAL_TRANSCRIPTION;REACTOME_RNA_POL_III_TRANSCRIPTION_INITIATION_FROM_TYPE
ORIAL_TRANSCRIPTION;REACTOME_RNA_POL_III_TRANSCRIPTION_INITIATION_FROM_TYPE

HE_ACTIVATION_OF_ARYLSULFATASES;REACTOME_METABOLISM_OF_PROTEINS;REACTOM

E_SYSTEM

ME_P75_NTR_RECEPTOR_MEDIATED_SIGNALLING;REACTOME_ROLE_OF_DCC_IN_REGULAT

SPLICING;REACTOME_MITOTIC_M_M_G1_PHASES;REACTOME_CHROMOSOME_MAINTENAN
OTEASOME_DEGRADATION

ME_P75_NTR_RECEPTOR_MEDIATED_SIGNALLING;REACTOME_SIGNALING_BY_GPCR;REAC

TOME_FATTY_ACID_TRIACYLGLYCEROL_AND_KETONE_BODY_METABOLISM;REACTOME_TR

_BODY_METABOLISM;REACTOME_SYNTHESIS_OF_VERY_LONG_CHAIN_FATTY_ACYL_COAS

LISM;REACTOME_GLYCOSAMINOGLYCAN_METABOLISM;REACTOME_A_TETRASACCHARIDE
OS_OLIGOPEPTIDES

_GAG_METABOLISM;REACTOME_GLYCOSAMINOGLYCAN_METABOLISM;REACTOME_A_TETR
;REACTOME_ION_CHANNEL_TRANSPORT;REACTOME_HEMOSTASIS

TIONS;REACTOME_INTERACTION_BETWEEN_L1_AND_ANKYRINS;REACTOME_SIGNAL_TRAN
TIONS;REACTOME_INTERACTION_BETWEEN_L1_AND_ANKYRINS;REACTOME_SIGNAL_TRAN

EACTOME_MITOTIC_M_M_G1_PHASES;REACTOME_MITOTIC_G2_G2_M_PHASES;REACTOME

_BY_NOTCH3;REACTOME_RECEPTOR_LIGAND_BINDING_INITIATES_THE_SECOND_PROTEO

E_DEVELOPMENT_AND_PLATELET_PRODUCTION;REACTOME_KINESINS;REACTOME_DNA_F

_AND_ANKYRINS

ABOLISM_OF_LIPIDS_AND_LIPOPROTEINS;REACTOME_LIPID_DIGESTION_MOBILIZATION_AN

ND_ORGANIC_ACIDS_METAL_IONS_AND_AMINE_COMPOUNDS;REACTOME_ZINC_TRANSPOI

EACTOME_MITOTIC_G2_G2_M_PHASES
EACTOME_MITOTIC_G2_G2_M_PHASES

3Y_CONSTITUTIVELY_ACTIVE_EGFR;REACTOME_SIGNALING_BY_EGFR_IN_CANCER;REACT(

M;REACTOME_METABOLISM_OF_LIPIDS_AND_LIPOPROTEINS

EPTORS;REACTOME_G_ALPHA_Q_SIGNALLING_EVENTS;REACTOME_GPCR_DOWNSTREAM_

PATHWAY;REACTOME_ER_PHAGOSOME_PATHWAY;REACTOME_NEF_MEDIATES_DOWN_M

ON_ORGANIZATION;REACTOME_APOPTOSIS;REACTOME_APOPTOTIC_EXECUTION_PHASE

E_HEMOSTASIS

REACTOME_SIGNALING_BY_ERBB4;REACTOME_DOWNREGULATION_OF_ERBB2_ERBB3_SI(

HE_ACTIVATION_OF_ARYLSULFATASES;REACTOME_METABOLISM_OF_PROTEINS;REACTOM

TOME_FACTORS_INVOLVED_IN_MEGAKARYOCYTE_DEVELOPMENT_AND_PLATELET_PROD

TIC_CELL;REACTOME_TRANSMEMBRANE_TRANSPORT_OF_SMALL_MOLECULES;REACTOM
ATED_AND_SIGNALS_SURVIVAL;REACTOME_P75_NTR_RECEPTOR_MEDIATED_SIGNALLING;

E_INTERFERON_SIGNALING;REACTOME_IMMUNE_SYSTEM;REACTOME_ADAPTIVE_IMMUNE_

S;REACTOME_INTERACTION_BETWEEN_L1_AND_ANKYRINS;REACTOME_NEPHRIN_INTERAC

_I_SIGNALLING_EVENTS;REACTOME_GPCR_LIGAND_BINDING

NE_BODY_METABOLISM;REACTOME_TRANSCRIPTIONAL_REGULATION_OF_WHITE_ADIPOC

EACTOME_MITOTIC_G2_G2_M_PHASES
OME_P130CAS_LINKAGE_TO_MAPK_SIGNALING_FOR_INTEGRINS;REACTOME_GRB2_SOS_P
_ALPHA_BETA_PATHWAYS;REACTOME_APOPTOSIS;REACTOME_INNATE_IMMUNE_SYSTEM;

_FGFR2;REACTOME_SIGNALING_BY_FGFR_MUTANTS;REACTOME_FRS2_MEDIATED_CASCA

G_VIA_NRAGE_NRIF_AND_NADE;REACTOME_P75_NTR_RECEPTOR_MEDIATED_SIGNALLING
MUNE_SYSTEM
;REACTOME_G1_S_TRANSITION;REACTOME_SYNTHESIS_OF_DNA;REACTOME_MITOTIC_G1

OS_OLIGOPEPTIDES

EACTOME_MITOTIC_G2_G2_M_PHASES

)S_OLIGOPEPTIDES

ATED_AND_SIGNALS_SURVIVAL;REACTOME_P75_NTR_RECEPTOR_MEDIATED_SIGNALLING;

M_TYPE_3_PROMOTER

G_VIA_NRAGE_NRIF_AND_NADE;REACTOME_P75_NTR_RECEPTOR_MEDIATED_SIGNALLING

)S_OLIGOPEPTIDES

BRANE;REACTOME_ACTIVATION_OF_THE_MRNA_UPON_BINDING_OF_THE_CAP_BINDING_C

_MOLECULES;REACTOME_METABOLISM_OF_CARBOHYDRATES

REGATION

_BODY_METABOLISM;REACTOME_SYNTHESIS_OF_VERY_LONG_CHAIN_FATTY_ACYL_COAS

CTOME_APOPTOSIS

EEN_A_LYMPHOID_AND_A_NON_LYMPHOID_CELL;REACTOME_INTERFERON_GAMMA_SIGN/

CTOME_N_GLYCAN_TRIMMING_IN_THE_ER_AND_CALNEXIN_CALRETICULIN_CYCLE
E_RECEPTORS;REACTOME_CHEMOKINE_RECEPTORS_BIND_CHEMOKINES;REACTOME_GP(

ING_K_CHANNELS

ME_CTLA4_INHIBITORY_SIGNALING;REACTOME_MITOTIC_M_M_G1_PHASES;REACTOME_PL/

TOME_GLUCOSE_METABOLISM;REACTOME_PLATELET_ACTIVATION_SIGNALING_AND_AGG

TIC_CELL;REACTOME_TRAFFICKING_OF_AMPA_RECEPTORS

TIC_CELL;REACTOME_TRAFFICKING_OF_AMPA_RECEPTORS;REACTOME_TRAFFICKING_OF

_PATHWAY;REACTOME_NEUROTRANSMITTER_RECEPTOR_BINDING_AND_DOWNSTREAM_

OTIC_COUPLING_AND_HEAT_PRODUCTION_BY_UNCOUPLING_PROTEINS_

LL_CYCLE_MITOTIC;REACTOME_CELL_CYCLE_CHECKPOINTS;REACTOME_REGULATION_OF

DS_OLIGOPEPTIDES

LING_BY_ERBB4;REACTOME_SIGNALING_BY_ILS;REACTOME_IL_3_5_AND_GM_CSF_SIGNALI

XES;REACTOME_REGULATED_PROTEOLYSIS_OF_P75NTR;REACTOME_P75NTR_SIGNALS_V

AND_LIPOPROTEINS;REACTOME_FATTY_ACID_TRIACYLGLYCEROL_AND_KETONE_BODY_M

_BINDING
LISM;REACTOME_GLYCOSAMINOGLYCAN_METABOLISM;REACTOME_A_TETRASACCHARIDE

:TOME_PHOSPHOLIPID_METABOLISM;REACTOME_PI_METABOLISM;REACTOME_GOLGI_ASS

.ATELET_ACTIVATION_SIGNALING_AND_AGGREGATION

R_MEDIATED_SIGNALLING;REACTOME_CELL_CELL_JUNCTION_ORGANIZATION;REACTOME_

ACTOME_TRANSPORT_OF_INORGANIC_CATIONS_ANIONS_AND_AMINO_ACIDS_OLIGOPEPT

ATION

;REACTOME_GENERIC_TRANSCRIPTION_PATHWAY;REACTOME_NOTCH_HLH_TRANSCRIPT

TION_AND_REGULATION;REACTOME_CELL_CYCLE_MITOTIC;REACTOME_G1_S_TRANSITIO

TIC_CELL;REACTOME_TRANSMEMBRANE_TRANSPORT_OF_SMALL_MOLECULES;REACTOM

_SLC_MEDIATED_TRANSMEMBRANE_TRANSPORT;REACTOME_TRANSPORT_OF_GLUCOSE_

_PRE_INITIATION_AND_PROMOTER_OPENING;REACTOME_TRANSCRIPTION;REACTOME_RN

ME_ADAPTIVE_IMMUNE_SYSTEM

ORT;REACTOME_RESPIRATORY_ELECTRON_TRANSPORT_ATP_SYNTHESIS_BY_CHEMIOSM
;REACTOME_REGULATION_OF_INSULIN_SECRETION;REACTOME_REGULATION_OF_INSULI

ED_GLYCOSYLATION;REACTOME_BIOSYNTHESIS_OF_THE_N_GLYCAN_PRECURSOR_DOLIC

OME_GPCR_LIGAND_BINDING

UDDING;REACTOME_SIGNALING_BY_EGFR_IN_CANCER;REACTOME_NGF_SIGNALLING_VIA_

TIC_CELL;REACTOME_ACTIVATION_OF_KAINATE_RECEPTORS_UPON_GLUTAMATE_BINDIN

)S_OLIGOPEPTIDES

\N_SULFATE_HEPARIN_HS_GAG_METABOLISM;REACTOME_GLYCOSAMINOGLYCAN_METAB(

REPAIR

:ULES;REACTOME_TRANSPORT_OF_ORGANIC_ANIONS

EEN_A_LYMPHOID_AND_A_NON_LYMPHOID_CELL;REACTOME_INTERFERON_GAMMA_SIGN/

'OME_EFFECTS_OF_PIP2_HYDROLYSIS;REACTOME_HEMOSTASIS;REACTOME_PLATELET_A
'OME_EFFECTS_OF_PIP2_HYDROLYSIS;REACTOME_HEMOSTASIS;REACTOME_PLATELET_A

_PIPS_AT_THE_PLASMA_MEMBRANE;REACTOME_PI_METABOLISM;REACTOME_METABOLISM

R_OF_REPLICATION_INDEPENDENT_DOUBLE_STRAND_BREAKS;REACTOME_P53_DEPENDEN

_CONSTITUTIVELY_ACTIVE_EGFR;REACTOME_GRB2_EVENTS_IN_ERBB2_SIGNALING;REAC
ACTOME_IMMUNE_SYSTEM;REACTOME_ADAPTIVE_IMMUNE_SYSTEM
N_SECRETION;REACTOME_VOLTAGE_GATED_POTASSIUM_CHANNELS;REACTOME_POTASS

TIC_CELL;REACTOME_TRANSMEMBRANE_TRANSPORT_OF_SMALL_MOLECULES;REACTOME

ON_CASCADE;REACTOME_HEMOSTASIS;REACTOME_INNATE_IMMUNE_SYSTEM;REACTOME

E

:_IMMUNE_SYSTEM;REACTOME_CLASS_I_MHC_MEDIATED_ANTIGEN_PROCESSING_PRESEN

HE_ACTIVATION_OF_ARYLSULFATASES;REACTOME_METABOLISM_OF_PROTEINS;REACTON

SPORT_OF_MATURE_TRANSCRIPT_TO_CYTOPLASM;REACTOME_TRANSMEMBRANE_TRANS

S;REACTOME_IRON_UPTAKE_AND_TRANSPORT;REACTOME_TRANSFERRIN_ENDOCYTOSIS

NLR_SIGNALING_PATHWAYS

TIGHT_JUNCTION_INTERACTIONS;REACTOME_CELL_JUNCTION_ORGANIZATION;REACTOME

CYTE_DEVELOPMENT_AND_PLATELET_PRODUCTION;REACTOME_HEMOSTASIS

NG_BY_PDGF;REACTOME_AXON_GUIDANCE;REACTOME_NCAM1_INTERACTIONS;REACTOME

NE_BODY_METABOLISM;REACTOME_TRANSCRIPTIONAL_REGULATION_OF_WHITE_ADIPOC

ACTOME_APOPTOSIS;REACTOME_APOPTOTIC_EXECUTION_PHASE
TIC_CELL;REACTOME_ACETYLCHOLINE_BINDING_AND_DOWNSTREAM_EVENTS;REACTOME

IEDIATED_EVENTS;REACTOME_SIGNALING_BY_INSULIN_RECEPTOR;REACTOME_MTORC1_I
PORT;REACTOME_LIPOPROTEIN_METABOLISM;REACTOME_CHYLOMICRON_MEDIATED_LIPI

TOME_METABOLISM_OF_LIPIDS_AND_LIPOPROTEINS;REACTOME_FATTY_ACID_TRIACYLGL

ME_P75_NTR_RECEPTOR_MEDIATED_SIGNALLING;REACTOME_SIGNALING_BY_GPCR;REAC

EACTOME_MITOTIC_G2_G2_M_PHASES;REACTOME_SIGNALLING_BY_NGF;REACTOME_SIGN

G_PATHWAYS

ME_ADAPTIVE_IMMUNE_SYSTEM

CTOME_IMMUNE_SYSTEM;REACTOME_ADAPTIVE_IMMUNE_SYSTEM
ND_BINDING

OME_3_UTR_MEDIATED_TRANSLATIONAL_REGULATION;REACTOME_METABOLISM_OF_MR

Y_CONSTITUTIVELY_ACTIVE_EGFR;REACTOME_SIGNALING_BY_EGFR_IN_CANCER;REACT

_FGFR2;REACTOME_SIGNALING_BY_FGFR3_MUTANTS;REACTOME_SIGNALING_BY_FGFR_

ION;REACTOME_GPCR_DOWNSTREAM_SIGNALING;REACTOME_G_ALPHA_I_SIGNALLING_E
_SIGNALLING_VIA_NRAGE_NRIF_AND_NADE;REACTOME_P75_NTR_RECEPTOR_MEDIATED_

ATION

_CELLULAR_PROTEINS;REACTOME_INTEGRATION_OF_PROVIRUS;REACTOME_APOBEC3G_

SURFACE_INTERACTIONS_AT_THE_VASCULAR_WALL;REACTOME_PLATELET_ADHESION_T

ATION

LECULES;REACTOME_REGULATION_OF_INSULIN_SECRETION;REACTOME_POTASSIUM_CH

S_OLIGOPEPTIDES

TEASOME_DEGRADATION

LL_CYCLE_MITOTIC;REACTOME_CELL_CYCLE_CHECKPOINTS;REACTOME_REGULATION_O

)S_OLIGOPEPTIDES

PHA_S_SIGNALLING_EVENTS;REACTOME_GPCR_LIGAND_BINDING

ND_ORGANIC_ACIDS_METAL_IONS_AND_AMINE_COMPOUNDS;REACTOME_AMINE_COMPOU

EPTORS;REACTOME_G_ALPHA_Q_SIGNALLING_EVENTS;REACTOME_GPCR_DOWNSTREAM_

TOME_INTEGRATION_OF_ENERGY_METABOLISM;REACTOME_OPIOID_SIGNALLING;REACTO

_NCAM1_INTERACTIONS;REACTOME_NCAM_SIGNALING_FOR_NEURITE_OUT_GROWTH

ATED_AND_SIGNALS_SURVIVAL;REACTOME_P75_NTR_RECEPTOR_MEDIATED_SIGNALLING;

G_IN_GOLGI;REACTOME_KERATAN_SULFATE_BIOSYNTHESIS;REACTOME_KERATAN_SULFA

NALING;REACTOME_REGULATION_OF_IFNG_SIGNALING;REACTOME_INTERFERON_GAMMA

ME_P75_NTR_RECEPTOR_MEDIATED_SIGNALLING;REACTOME_SIGNALING_BY_GPCR;REAC

_MOLECULES;REACTOME_METABOLISM_OF_CARBOHYDRATES

HA_S_SIGNALLING_EVENTS;REACTOME_GPCR_LIGAND_BINDING

RONAL_SYSTEM;REACTOME_CELL_DEATH_SIGNALLING_VIA_NRAGE_NRIF_AND_NADE;REA
N_PHASE

1_TRANSITION;REACTOME_G1_S_TRANSITION;REACTOME_POL_SWITCHING;REACTOME_S

CTIONS;REACTOME_SEMA3A_PLEXIN_REPULSION_SIGNALING_BY_INHIBITING_INTEGRIN_A

_LEADING_TO_GENERATION_OF_SECOND_MESSENGERS;REACTOME_SIGNALING_BY_THE_

N_AND_INACTIVATION_OF_GLP1

_ELEMENTS

OMPLEX_AND_EIFS_AND_SUBSEQUENT_BINDING_TO_43S;REACTOME_METABOLISM_OF_PI

LATELET_ACTIVATION_SIGNALING_AND_AGGREGATION

OF_HIV_LIFE_CYCLE

N_GLYCAN_ANTENNAE_ELONGATION_IN_THE_MEDIAL_TRANS_GOLGI

Y_NOTCH1;REACTOME_GENERIC_TRANSCRIPTION_PATHWAY;REACTOME_RNA_POL_I_TRA

SPORT_OF_SMALL_MOLECULES;REACTOME_MRNA_PROCESSING;REACTOME_TRANSPORT

SSION_ACROSS_CHEMICAL_SYNAPSES;REACTOME_NEURONAL_SYSTEM;REACTOME_SIGN
_CYCLE;REACTOME_NGF_SIGNALLING_VIA_TRKA_FROM_THE_PLASMA_MEMBRANE;REACTO

A_INDEPENDENT_GLUCOSE_TRANSPORTERS

ROTEINS;REACTOME_3_UTR_MEDIATED_TRANSLATIONAL_REGULATION

'IA_NFKB;REACTOME_NRIF_SIGNALS_CELL_DEATH_FROM_THE_NUCLEUS;REACTOME_NFK

T;REACTOME_TRANSPORT_OF_INORGANIC_CATIONS_ANIONS_AND_AMINO_ACIDS_OLIGOI

G2_G2_M_PHASES

G_BY_GPCR;REACTOME_INTEGRATION_OF_ENERGY_METABOLISM;REACTOME_OPIOID_SI

NA;REACTOME_TRANSCRIPTION;REACTOME_MRNA_3_END_PROCESSING;REACTOME_CLE

CTOME_G_ALPHA1213_SIGNALLING_EVENTS;REACTOME_GPCR_DOWNSTREAM_SIGNALING

_DOWNSTREAM_SIGNALING_EVENTS_OF_B_CELL_RECEPTOR_BCR;REACTOME_SIGNALING
_DOWNSTREAM_SIGNALING_EVENTS_OF_B_CELL_RECEPTOR_BCR;REACTOME_SIGNALING

OMPLEX_FORMATION;REACTOME_CELL_CYCLE_MITOTIC;REACTOME_CELL_CYCLE_CHECK

ACTOME_CIRCADIAN_REPRESSION_OF_EXPRESSION_BY_REV_ERBA;REACTOME_GENERIC

E_NCAM_SIGNALING_FOR_NEURITE_OUT_GROWTH
CTOME_G_ALPHA1213_SIGNALLING_EVENTS;REACTOME_GPCR_DOWNSTREAM_SIGNALING

OME_PI3K_EVENTS_IN_ERBB4_SIGNALING;REACTOME_PI3K_EVENTS_IN_ERBB2_SIGNALIN

E_NOTCH_HLH_TRANSCRIPTION_PATHWAY;REACTOME_SIGNALING_BY_NOTCH

CLE

POPROTEIN_METABOLISM

TRANSCRIPTIONAL_ACTIVITY;REACTOME_GENERIC_TRANSCRIPTION_PATHWAY;REACTOME
_ACTIVATION;REACTOME_TRAF6_MEDIATED_INDUCTION_OF_NFKB_AND_MAP_KINASES_UP

_MEDIATED_TRANSMEMBRANE_TRANSPORT;REACTOME_TRANSPORT_OF_INORGANIC_CA

ANSCRIPTIONAL_REGULATION_OF_WHITE_ADIPOCYTE_DIFFERENTIATION

OCIATED_VESICLE_BIOGENESIS;REACTOME_METABOLISM_OF_LIPIDS_AND_LIPOPROTEINS

REACTOME_EFFECTS_OF_PIP2_HYDROLYSIS;REACTOME_METABOLISM_OF_LIPIDS_AND_LI

OME_PI3K_EVENTS_IN_ERBB4_SIGNALING;REACTOME_PI3K_EVENTS_IN_ERBB2_SIGNALIN
OME_PI3K_EVENTS_IN_ERBB4_SIGNALING;REACTOME_PI3K_EVENTS_IN_ERBB2_SIGNALIN

_SIGNALING;REACTOME_GPCR_LIGAND_BINDING

ALING;REACTOME_INTERFERON_ALPHA_BETA_SIGNALING;REACTOME_INTERFERON_SIGN/

EACTOME_GLUCOSE_TRANSPORT;REACTOME_METABOLISM_OF_CARBOHYDRATES;REAC

NSMITTER_RECEPTOR_BINDING_AND_DOWNSTREAM_TRANSMISSION_IN_THE_POSTSYNA

:RETION;REACTOME_NCAM_SIGNALING_FOR_NEURITE_OUT_GROWTH;REACTOME_INHIBIT

TOME_SIGNALING_BY_EGFR_IN_CANCER;REACTOME_SHC1_EVENTS_IN_ERBB4_SIGNALIN(
_B_CELL_RECEPTOR_BCR;REACTOME_NGF_SIGNALLING_VIA_TRKA_FROM_THE_PLASMA_I

E_HIGHLY_CALCIUM_PERMEABLE_POSTSYNAPTIC_NICOTINIC_ACETYLCHOLINE_RECEPTOR

2_SECRETIN_FAMILY_RECEPTORS;REACTOME_GLUCAGON_TYPE_LIGAND_RECEPTORS;RE
AND_LIPOPROTEINS;REACTOME_FATTY_ACID_TRIACYLGLYCEROL_AND_KETONE_BODY_M

Y_BY_PROTEINS_THAT_BIND_AU_RICH_ELEMENTS;REACTOME_DESTABILIZATION_OF_MRI

YLATION_DEPENDENT_MRNA_DECAY;REACTOME_METABOLISM_OF_RNA;REACTOME_DEST

S

E_TO_ELEVATED_PLATELET_CYTOSOLIC_CA2_;REACTOME_TRANSMISSION_ACROSS_CHE

ESIS_OF_DNA;REACTOME_MITOTIC_G1_G1_S_PHASES;REACTOME_MITOTIC_M_M_G1_PHAS

:TOME_G_ALPHA1213_SIGNALLING_EVENTS;REACTOME_GPCR_DOWNSTREAM_SIGNALING

_SYSTEM;REACTOME_CYTOKINE_SIGNALING_IN_IMMUNE_SYSTEM;REACTOME_CLASS_I_MI

:TOME_G_ALPHA1213_SIGNALLING_EVENTS;REACTOME_GPCR_DOWNSTREAM_SIGNALING

:TOME_G_ALPHA1213_SIGNALLING_EVENTS;REACTOME_GPCR_DOWNSTREAM_SIGNALING

'IDES;REACTOME_AMINO_ACID_AND_OLIGOPEPTIDE_SLC_TRANSPORTERS
'IDES;REACTOME_AMINO_ACID_AND_OLIGOPEPTIDE_SLC_TRANSPORTERS

ME_RESOLUTION_OF_AP_SITES_VIA_THE_SINGLE_NUCLEOTIDE_REPLACEMENT_PATHWAY
ME_RESOLUTION_OF_AP_SITES_VIA_THE_SINGLE_NUCLEOTIDE_REPLACEMENT_PATHWAY

IN_RESPONSE_TO_REPLICATION_STRESS;REACTOME_MEIOTIC_SYNAPSIS;REACTOME_G2
FICATION;REACTOME_ADP_SIGNALLING_THROUGH_P2RY12;REACTOME_GPCR_LIGAND_BIN

OME_REGULATION_OF_INSULIN_SECRETION;REACTOME_SLC_MEDIATED_TRANSMEMBRANI

N_TC_NER;REACTOME_SYNTHESIS_OF_DNA;REACTOME_DNA_REPAIR;REACTOME_CHROM

ACTOME_METABOLISM_OF_LIPIDS_AND_LIPOPROTEINS;REACTOME_FATTY_ACID_TRIACYL

R_OPENING;REACTOME_MRNA_PROCESSING;REACTOME_TRANSCRIPTION;REACTOME_NU

REACTOME_KERATAN_SULFATE_KERATIN_METABOLISM;REACTOME_KERATAN_SULFATE_D
REACTOME_KERATAN_SULFATE_KERATIN_METABOLISM;REACTOME_KERATAN_SULFATE_D
D_METABOLISM;REACTOME_METABOLISM_OF_LIPIDS_AND_LIPOPROTEINS

JCLEOSOMES_AT_THE_CENTROMERE;REACTOME_RNA_POL_I_PROMOTER_OPENING;REAC

TEROID_HORMONES;REACTOME_METABOLISM_OF_LIPIDS_AND_LIPOPROTEINS

K_EVENTS_IN_ERBB2_SIGNALING;REACTOME_NUCLEAR_SIGNALING_BY_ERBB4

MBRANE_TRANSPORT_OF_SMALL_MOLECULES;REACTOME_IRON_UPTAKE_AND_TRANSPO

NA_SIGNALING;REACTOME_INTERFERON_SIGNALING;REACTOME_RIG_I_MDA5_MEDIATED_

EAM_SIGNAL_TRANSDUCTION;REACTOME_SIGNALING_BY_ILS;REACTOME_REGULATION_OI

ACTOME_INTERFERON_SIGNALING;REACTOME_IMMUNE_SYSTEM;REACTOME_CYTOKINE_S

ABOLISM_OF_LIPIDS_AND_LIPOPROTEINS
ABOLISM_OF_LIPIDS_AND_LIPOPROTEINS

YTE_DIFFERENTIATION

IODELLING_OF_PS;REACTOME_GLYCEROPHOSPHOLIPID_BIOSYNTHESIS;REACTOME_META

ACTOME_INNATE_IMMUNE_SYSTEM;REACTOME_IMMUNE_SYSTEM;REACTOME_TOLL_RECE

ROTEINS;REACTOME_3_UTR_MEDIATED_TRANSLATIONAL_REGULATION

ING_BY_EGFR_IN_CANCER;REACTOME_SHC1_EVENTS_IN_ERBB4_SIGNALING;REACTOME_

E_HIGHLY_CALCIUM_PERMEABLE_POSTSYNAPTIC_NICOTINIC_ACETYLCHOLINE_RECEPTOR

_MAPK_TARGETS;REACTOME_MYOGENESIS;REACTOME_MAP_KINASE_ACTIVATION_IN_TLR

_3_PROMOTER;REACTOME_RNA_POL_III_TRANSCRIPTION_TERMINATION;REACTOME_RNA

ME_CIRCADIAN_CLOCK;REACTOME_TRANSCRIPTIONAL_REGULATION_OF_WHITE_ADIPOC[illegible]

ACTOME_CIRCADIAN_REPRESSION_OF_EXPRESSION_BY_REV_ERBA;REACTOME_GENERI[illegible]

YTE_DIFFERENTIATION

AND_AMINO_ACIDS_OLIGOPEPTIDES;REACTOME_AMINO_ACID_AND_OLIGOPEPTIDE_SLC_T

G;REACTOME_IONOTROPIC_ACTIVITY_OF_KAINATE_RECEPTORS
G;REACTOME_IONOTROPIC_ACTIVITY_OF_KAINATE_RECEPTORS

ME_NEUROTRANSMITTER_RECEPTOR_BINDING_AND_DOWNSTREAM_TRANSMISSION_IN_T

EACTOME_IMMUNE_SYSTEM;REACTOME_CYTOKINE_SIGNALING_IN_IMMUNE_SYSTEM
ACTOME_CIRCADIAN_REPRESSION_OF_EXPRESSION_BY_REV_ERBA;REACTOME_GENERIC
ACTOME_CIRCADIAN_REPRESSION_OF_EXPRESSION_BY_REV_ERBA;REACTOME_GENERIC

_PRE_NOTCH_EXPRESSION_AND_PROCESSING;REACTOME_NOTCH1_INTRACELLULAR_DO

CTOME_IMMUNE_SYSTEM;REACTOME_TOLL_RECEPTOR_CASCADES
CTOME_IMMUNE_SYSTEM;REACTOME_TOLL_RECEPTOR_CASCADES

_RELEASE_CYCLE

ANSCRIPTIONAL_REGULATION_OF_WHITE_ADIPOCYTE_DIFFERENTIATION

NG;REACTOME_SIGNALING_BY_GPCR;REACTOME_G_ALPHA1213_SIGNALLING_EVENTS;RE

F_MITOTIC_CELL_CYCLE;REACTOME_APC_C_CDH1_MEDIATED_DEGRADATION_OF_CDC20_

_SIGNALING;REACTOME_GPCR_LIGAND_BINDING
C_TRANSPORTERS;REACTOME_IRON_UPTAKE_AND_TRANSPORT
C_TRANSPORTERS;REACTOME_IRON_UPTAKE_AND_TRANSPORT

TIDES;REACTOME_AMINO_ACID_AND_OLIGOPEPTIDE_SLC_TRANSPORTERS

_DOWNSTREAM_SIGNALING_EVENTS_OF_B_CELL_RECEPTOR_BCR;REACTOME_SIGNALING

ME_RESOLUTION_OF_AP_SITES_VIA_THE_SINGLE_NUCLEOTIDE_REPLACEMENT_PATHWAY

CTOME_HEMOSTASIS;REACTOME_PLATELET_ACTIVATION_SIGNALING_AND_AGGREGATION

LTS;REACTOME_PEROXISOMAL_LIPID_METABOLISM;REACTOME_METABOLISM_OF_LIPIDS_

G2_G2_M_PHASES

ACTOME_CIRCADIAN_REPRESSION_OF_EXPRESSION_BY_REV_ERBA;REACTOME_GENERIC

HWAYS;REACTOME_INNATE_IMMUNE_SYSTEM;REACTOME_IMMUNE_SYSTEM;REACTOME_

GGREGATION

A_INDEPENDENT_GLUCOSE_TRANSPORTERS

_DOWNSTREAM_SIGNALING_EVENTS_OF_B_CELL_RECEPTOR_BCR;REACTOME_SIGNALING

E_LINKER_SEQUENCE_IS_REQUIRED_FOR_GAG_SYNTHESIS;REACTOME_METABOLISM_OF_
E_LINKER_SEQUENCE_IS_REQUIRED_FOR_GAG_SYNTHESIS;REACTOME_METABOLISM_OF_

ITS_KINASE_AND_TRANSCRIPTION_FACTOR_ACTIVATION;REACTOME_ERK_MAPK_TARGET

PORT_OF_INORGANIC_CATIONS_ANIONS_AND_AMINO_ACIDS_OLIGOPEPTIDES;REACTOME_
PORT_OF_INORGANIC_CATIONS_ANIONS_AND_AMINO_ACIDS_OLIGOPEPTIDES;REACTOME_

ME_PECAM1_INTERACTIONS;REACTOME_TCR_SIGNALING;REACTOME_DOWNSTREAM_TCR

ANSCRIPTIONAL_REGULATION_OF_WHITE_ADIPOCYTE_DIFFERENTIATION

_OF_MTOR_BY_LKB1_AMPK;REACTOME_REGULATION_OF_RHEB_GTPASE_ACTIVITY_BY_A

_SIGNALING;REACTOME_INTERFERON_ALPHA_BETA_SIGNALING;REACTOME_REGULATION

ENTS;REACTOME_GPCR_LIGAND_BINDING

ACTOME_SEMAPHORIN_INTERACTIONS;REACTOME_SEMA4D_INDUCED_CELL_MIGRATION_A

TION_PATHWAY;REACTOME_RECEPTOR_LIGAND_BINDING_INITIATES_THE_SECOND_PROTE
TION_PATHWAY;REACTOME_RECEPTOR_LIGAND_BINDING_INITIATES_THE_SECOND_PROTE

E_3_PROMOTER;REACTOME_RNA_POL_III_TRANSCRIPTION_TERMINATION;REACTOME_RNA

G;REACTOME_SIGNALING_BY_GPCR;REACTOME_AXON_GUIDANCE;REACTOME_G_ALPHA12

SPORT_OF_SMALL_MOLECULES;REACTOME_MRNA_PROCESSING;REACTOME_TRANSPORT

EPULSION_SIGNALING_BY_INHIBITING_INTEGRIN_ADHESION

CTOME_G_ALPHA1213_SIGNALLING_EVENTS;REACTOME_GPCR_DOWNSTREAM_SIGNALING

TOME_PI3K_EVENTS_IN_ERBB4_SIGNALING;REACTOME_PI3K_EVENTS_IN_ERBB2_SIGNALIN

CTOME_SIGNALING_BY_NOTCH1;REACTOME_REGULATED_PROTEOLYSIS_OF_P75NTR;REA

SIUM_CHANNELS

ACTOME_CIRCADIAN_REPRESSION_OF_EXPRESSION_BY_REV_ERBA;REACTOME_GENERIC

M_HOMEOSTASIS;REACTOME_HEMOSTASIS

CTIONS
CTIONS

NNATE_IMMUNE_SYSTEM;REACTOME_ACTIVATED_TLR4_SIGNALLING;REACTOME_IMMUNE_

)DIFICATION

ON_PATHWAY;REACTOME_METABOLISM_OF_PROTEINS;REACTOME_FORMATION_OF_FIBR

)F_GLP1
)F_GLP1

DS_AND_LIPOPROTEINS

;ES;REACTOME_IMMUNE_SYSTEM;REACTOME_ADAPTIVE_IMMUNE_SYSTEM
EACTOME_RAP1_SIGNALLING;REACTOME_PLATELET_AGGREGATION_PLUG_FORMATION;R

E_HIGHLY_CALCIUM_PERMEABLE_POSTSYNAPTIC_NICOTINIC_ACETYLCHOLINE_RECEPTOR

EACTOME_L1CAM_INTERACTIONS;REACTOME_SIGNAL_TRANSDUCTION_BY_L1;REACTOME_

_MUTANTS;REACTOME_SIGNALING_BY_FGFR_MUTANTS;REACTOME_SIGNALING_BY_PDGF;

_TS_AND_ORGANIC_ACIDS_METAL_IONS_AND_AMINE_COMPOUNDS;REACTOME_METAL_IO

ECEPTOR;REACTOME_SIGNAL_ATTENUATION;REACTOME_SOS_MEDIATED_SIGNALLING;REA

_SIGNALING;REACTOME_G_ALPHA_I_SIGNALLING_EVENTS;REACTOME_GPCR_LIGAND_BIN

ORT_OF_GLUCOSE_AND_OTHER_SUGARS_BILE_SALTS_AND_ORGANIC_ACIDS_METAL_ION

EVENTS_IN_ERBB4_SIGNALING;REACTOME_INSULIN_RECEPTOR_SIGNALLING_CASCADE;RE

PLEX

CTOME_GPCR_LIGAND_BINDING

EPULSION_SIGNALING_BY_INHIBITING_INTEGRIN_ADHESION

IN_RESPONSE_TO_REPLICATION_STRESS;REACTOME_MEIOTIC_SYNAPSIS;REACTOME_G2

_ANTIGEN_PROCESSING_PRESENTATION;REACTOME_ANTIGEN_PROCESSING_UBIQUITINA
_ANTIGEN_PROCESSING_PRESENTATION;REACTOME_ANTIGEN_PROCESSING_UBIQUITINA

E_GABA_A_RECEPTOR_ACTIVATION;REACTOME_GABA_RECEPTOR_ACTIVATION;REACTOM

NRIF_AND_NADE;REACTOME_P75_NTR_RECEPTOR_MEDIATED_SIGNALLING;REACTOME_SI
NRIF_AND_NADE;REACTOME_P75_NTR_RECEPTOR_MEDIATED_SIGNALLING;REACTOME_SI

_OF_RHEB_GTPASE_ACTIVITY_BY_AMPK;REACTOME_PKB_MEDIATED_EVENTS;REACTOME_

METAPHASE

Y_EGFR_IN_CANCER;REACTOME_PI3K_EVENTS_IN_ERBB4_SIGNALING;REACTOME_PI3K_E

ASACCHARIDE_LINKER_SEQUENCE_IS_REQUIRED_FOR_GAG_SYNTHESIS;REACTOME_ME

ME_SPHINGOLIPID_METABOLISM;REACTOME_POST_TRANSLATIONAL_PROTEIN_MODIFICAT

EACTOME_G2_M_CHECKPOINTS;REACTOME_G2_M_DNA_DAMAGE_CHECKPOINT
EACTOME_G2_M_CHECKPOINTS;REACTOME_G2_M_DNA_DAMAGE_CHECKPOINT

TREAM_SIGNALING_OF_ACTIVATED_FGFR;REACTOME_PHOSPHOLIPASE_C_MEDIATED_CAS
TREAM_SIGNALING_OF_ACTIVATED_FGFR;REACTOME_PHOSPHOLIPASE_C_MEDIATED_CAS
TREAM_SIGNALING_OF_ACTIVATED_FGFR;REACTOME_PHOSPHOLIPASE_C_MEDIATED_CAS

NA;REACTOME_METABOLISM_OF_RNA;REACTOME_INFLUENZA_LIFE_CYCLE;REACTOME_INI

N_GLYCAN_ANTENNAE_ELONGATION_IN_THE_MEDIAL_TRANS_GOLGI
HOL_LIPID_LINKED_OLIGOSACCHARIDE_LLO_AND_TRANSFER_TO_A_NASCENT_PROTEIN

_B_CELL_RECEPTOR_BCR;REACTOME_NGF_SIGNALLING_VIA_TRKA_FROM_THE_PLASMA_

NA;REACTOME_METABOLISM_OF_RNA;REACTOME_INFLUENZA_LIFE_CYCLE;REACTOME_IN

:EACTOME_ADAPTIVE_IMMUNE_SYSTEM

CTIVATION_SIGNALING_AND_AGGREGATION

VATED_HUMAN_TAK1;REACTOME_ACTIVATED_TAK1_MEDIATES_P38_MAPK_ACTIVATION;RI

SSION_ACROSS_CHEMICAL_SYNAPSES;REACTOME_NEURONAL_SYSTEM;REACTOME_SIGN

RFACE_INTERACTIONS;REACTOME_SIGNALING_BY_PDGF;REACTOME_GPVI_MEDIATED_ACT

IN;REACTOME_DOWNSTREAM_SIGNALING_EVENTS_OF_B_CELL_RECEPTOR_BCR;REACTO
OMPLEX_FORMATION;REACTOME_CELL_CYCLE_MITOTIC;REACTOME_CELL_CYCLE_CHECK

CTIVATION_SIGNALING_AND_AGGREGATION
CTIVATION_SIGNALING_AND_AGGREGATION

;_AND_RECYCLING;REACTOME_SIGNALING_BY_INSULIN_RECEPTOR

CYCLASE;REACTOME_PLATELET_HOMEOSTASIS;REACTOME_HEMOSTASIS

GAKARYOCYTE_DEVELOPMENT_AND_PLATELET_PRODUCTION;REACTOME_HEMOSTASIS

I_OF_MTOR_BY_LKB1_AMPK;REACTOME_REGULATION_OF_RHEB_GTPASE_ACTIVITY_BY_A
I_OF_MTOR_BY_LKB1_AMPK;REACTOME_REGULATION_OF_RHEB_GTPASE_ACTIVITY_BY_A

/IUTANTS_OF_FGFR1;REACTOME_SIGNALING_BY_FGFR_MUTANTS;REACTOME_AXON_GUID
/IUTANTS_OF_FGFR1;REACTOME_SIGNALING_BY_FGFR_MUTANTS;REACTOME_AXON_GUID

/IE_RESOLUTION_OF_AP_SITES_VIA_THE_SINGLE_NUCLEOTIDE_REPLACEMENT_PATHWAY

)_DIGESTION_MOBILIZATION_AND_TRANSPORT

_MEDIATED_TRANSMEMBRANE_TRANSPORT;REACTOME_TRANSPORT_OF_INORGANIC_CA

GNAL_ATTENUATION;REACTOME_SHC_MEDIATED_SIGNALLING;REACTOME_SHC_RELATED

_DOWNSTREAM_SIGNALING_EVENTS_OF_B_CELL_RECEPTOR_BCR;REACTOME_SIGNALING

TRANSMITTER_RELEASE_CYCLE;REACTOME_ACETYLCHOLINE_NEUROTRANSMITTER_RELE

E_OUT_GROWTH;REACTOME_L1CAM_INTERACTIONS;REACTOME_INTERACTION_BETWEEN
E_OUT_GROWTH;REACTOME_L1CAM_INTERACTIONS;REACTOME_INTERACTION_BETWEEN
E_OUT_GROWTH;REACTOME_L1CAM_INTERACTIONS;REACTOME_INTERACTION_BETWEEN
E_OUT_GROWTH;REACTOME_L1CAM_INTERACTIONS;REACTOME_INTERACTION_BETWEEN

HOL_LIPID_LINKED_OLIGOSACCHARIDE_LLO_AND_TRANSFER_TO_A_NASCENT_PROTEIN

EAM_SIGNAL_TRANSDUCTION;REACTOME_SIGNALING_BY_ILS;REACTOME_REGULATION_OI

REACTOME_IMMUNE_SYSTEM

POPROTEIN_METABOLISM
POPROTEIN_METABOLISM

ATION_PLUG_FORMATION;REACTOME_HEMOSTASIS;REACTOME_PLATELET_ACTIVATION_[illegible]

[illegible]LINE_NEUROTRANSMITTER_RELEASE_CYCLE;REACTOME_METABOLISM_OF_LIPIDS_AND_[illegible]

REACTOME_IMMUNE_SYSTEM;REACTOME_ADAPTIVE_IMMUNE_SYSTEM;REACTOME_CYTO

IN;REACTOME_DOWNSTREAM_SIGNALING_EVENTS_OF_B_CELL_RECEPTOR_BCR;REACTO

FROM_THE_PLASMA_MEMBRANE;REACTOME_MHC_CLASS_II_ANTIGEN_PRESENTATION;RE

LIPOPROTEINS;REACTOME_FATTY_ACID_TRIACYLGLYCEROL_AND_KETONE_BODY_METAB(

ET_HOMEOSTASIS;REACTOME_PKB_MEDIATED_EVENTS;REACTOME_SIGNALING_BY_INSUL
ABOLISM_OF_LIPIDS_AND_LIPOPROTEINS

IN_LIKE_RECEPTORS;REACTOME_G_ALPHA_Q_SIGNALLING_EVENTS;REACTOME_GPCR_D(

ENT_NEUROTRANSMITTER_TRANSPORTERS;REACTOME_AMINE_COMPOUND_SLC_TRANS[illegible]

;

E_DNA_REPLICATION;REACTOME_MITOTIC_PROMETAPHASE

_DOWNSTREAM_SIGNALING_EVENTS_OF_B_CELL_RECEPTOR_BCR;REACTOME_SIGNALING

YNTHESIS_OF_DNA;REACTOME_MITOTIC_G1_G1_S_PHASES;REACTOME_MITOTIC_M_M_G1

REACTOME_HORMONE_SENSITIVE_LIPASE_HSL_MEDIATED_TRIACYLGLYCEROL_HYDROLY

S_AND_RECYCLING;REACTOME_SIGNALING_BY_INSULIN_RECEPTOR

SSING_UBIQUITINATION_PROTEASOME_DEGRADATION;REACTOME_VIF_MEDIATED_DEGRAI

NT_G1_DNA_DAMAGE_RESPONSE;REACTOME_REGULATION_OF_THE_FANCONI_ANEMIA_P/

G;REACTOME_SIGNALING_BY_GPCR;REACTOME_AXON_GUIDANCE;REACTOME_G_ALPHA12
G;REACTOME_SIGNALING_BY_GPCR;REACTOME_AXON_GUIDANCE;REACTOME_G_ALPHA12

NS;REACTOME_TRANSLOCATION_OF_ZAP_70_TO_IMMUNOLOGICAL_SYNAPSE;REACTOME_

REACTOME_TRANSPORT_OF_VITAMINS_NUCLEOSIDES_AND_RELATED_MOLECULES;REACT

SSION_ACROSS_CHEMICAL_SYNAPSES;REACTOME_NEURONAL_SYSTEM;REACTOME_SIGN

METABOLISM_OF_PROTEINS

ME_RESOLUTION_OF_AP_SITES_VIA_THE_SINGLE_NUCLEOTIDE_REPLACEMENT_PATHWAY

YTE_DIFFERENTIATION

N_TC_NER;REACTOME_SYNTHESIS_OF_DNA;REACTOME_DNA_REPAIR;REACTOME_CHROM

E_NCAM_SIGNALING_FOR_NEURITE_OUT_GROWTH

CYCLE;REACTOME_LATE_PHASE_OF_HIV_LIFE_CYCLE

NE_TRANSPORT;REACTOME_TRANSPORT_OF_INORGANIC_CATIONS_ANIONS_AND_AMINO_

E_GPCR_LIGAND_BINDING

TELET_ACTIVATION_SIGNALING_AND_AGGREGATION

GNALING;REACTOME_SIGNALING_BY_ERBB2;REACTOME_SIGNALING_BY_EGFR_IN_CANCEI

LYTIC_CLEAVAGE_OF_NOTCH_RECEPTOR;REACTOME_SIGNALING_BY_NOTCH

E_HIGHLY_CALCIUM_PERMEABLE_POSTSYNAPTIC_NICOTINIC_ACETYLCHOLINE_RECEPTOI

ME_NEUROTRANSMITTER_RECEPTOR_BINDING_AND_DOWNSTREAM_TRANSMISSION_IN_T

NS;REACTOME_POST_TRANSLATIONAL_PROTEIN_MODIFICATION;REACTOME_SYNTHESIS_(

TEROID_HORMONES;REACTOME_METABOLISM_OF_LIPIDS_AND_LIPOPROTEINS

CTOME_G_ALPHA1213_SIGNALLING_EVENTS;REACTOME_GPCR_DOWNSTREAM_SIGNALING

JND_SLC_TRANSPORTERS

NA;REACTOME_METABOLISM_OF_RNA;REACTOME_INFLUENZA_LIFE_CYCLE;REACTOME_INI

_YAP1_AND_WWTR1_TAZ_STIMULATED_GENE_EXPRESSION;REACTOME_PRE_NOTCH_EXF
_YAP1_AND_WWTR1_TAZ_STIMULATED_GENE_EXPRESSION;REACTOME_PRE_NOTCH_EXF
_YAP1_AND_WWTR1_TAZ_STIMULATED_GENE_EXPRESSION;REACTOME_PRE_NOTCH_EXF
SSION_ACROSS_CHEMICAL_SYNAPSES;REACTOME_NEURONAL_SYSTEM;REACTOME_SIGN

Y_P_TYPE_ATPASES;REACTOME_ION_CHANNEL_TRANSPORT;REACTOME_SIGNALING_BY_

SPORT_OF_SMALL_MOLECULES;REACTOME_MRNA_PROCESSING;REACTOME_TRANSPORT
SPORT_OF_SMALL_MOLECULES;REACTOME_MRNA_PROCESSING;REACTOME_TRANSPORT

ATION_PLUG_FORMATION;REACTOME_HEMOSTASIS;REACTOME_PLATELET_ACTIVATION_S

E_IMMUNE_SYSTEM;REACTOME_TOLL_RECEPTOR_CASCADES;REACTOME_ADAPTIVE_IMM

ND_POSTSYNAPTIC_EVENTS;REACTOME_POST_NMDA_RECEPTOR_ACTIVATION_EVENTS

_ORGANIC_ANION_SLC_TRANSPORTERS

E_RNA_POL_II_TRANSCRIPTION_PRE_INITIATION_AND_PROMOTER_OPENING;REACTOME_

_SIGNALLING_THROUGH_PI3KGAMMA;REACTOME_G_PROTEIN_BETA_GAMMA_SIGNALLING

SSION;REACTOME_NOTCH1_INTRACELLULAR_DOMAIN_REGULATES_TRANSCRIPTION;REAC

SSION;REACTOME_NOTCH1_INTRACELLULAR_DOMAIN_REGULATES_TRANSCRIPTION;REAC

AND_MAP_KINASES_ACTIVATION_MEDIATED_BY_TLR4_SIGNALING_REPERTOIRE;REACTOMI

_ORGANIC_ANION_SLC_TRANSPORTERS

_ORGANIC_ANION_SLC_TRANSPORTERS

FROM_THE_PLASMA_MEMBRANE;REACTOME_MHC_CLASS_II_ANTIGEN_PRESENTATION;RE

NA;REACTOME_METABOLISM_OF_RNA;REACTOME_INFLUENZA_LIFE_CYCLE;REACTOME_INI

APHORIN_SIGNALING;REACTOME_SEMAPHORIN_INTERACTIONS;REACTOME_SEMA4D_INDU
CTOME_GPCR_LIGAND_BINDING

EACTOME_SIGNALING_BY_FGFR1_FUSION_MUTANTS;REACTOME_SIGNALING_BY_FGFR_MU
EACTOME_SIGNALING_BY_FGFR1_FUSION_MUTANTS;REACTOME_SIGNALING_BY_FGFR_MU
EACTOME_SIGNALING_BY_FGFR1_FUSION_MUTANTS;REACTOME_SIGNALING_BY_FGFR_MU

_GLUR2_CONTAINING_AMPA_RECEPTORS

RFACE_INTERACTIONS;REACTOME_SIGNALING_BY_PDGF;REACTOME_GPVI_MEDIATED_ACT

CTOME_IMMUNE_SYSTEM;REACTOME_CYTOKINE_SIGNALING_IN_IMMUNE_SYSTEM

MEDIATED_SIGNALLING;REACTOME_PI3K_CASCADE

AND_LIPOPROTEINS;REACTOME_FATTY_ACID_TRIACYLGLYCEROL_AND_KETONE_BODY_M
AND_LIPOPROTEINS;REACTOME_FATTY_ACID_TRIACYLGLYCEROL_AND_KETONE_BODY_M

G;REACTOME_G_ALPHA_S_SIGNALLING_EVENTS;REACTOME_GPCR_LIGAND_BINDING

TIONS;REACTOME_PYRUVATE_METABOLISM;REACTOME_HEMOSTASIS

E_RNA_POL_III_TRANSCRIPTION;REACTOME_MRNA_CAPPING;REACTOME_TRANSCRIPTION_

IRAGE_SIGNALS_DEATH_THROUGH_JNK;REACTOME_CELL_DEATH_SIGNALLING_VIA_NRAG
ADE;REACTOME_P75_NTR_RECEPTOR_MEDIATED_SIGNALLING;REACTOME_SIGNALING_BY_

TOME_IMMUNE_SYSTEM;REACTOME_CYTOKINE_SIGNALING_IN_IMMUNE_SYSTEM

;;REACTOME_GPCR_DOWNSTREAM_SIGNALING;REACTOME_G_ALPHA_S_SIGNALLING_EVE
E_IMMUNE_SYSTEM;REACTOME_CYTOKINE_SIGNALING_IN_IMMUNE_SYSTEM

ASE;REACTOME_SIGNALING_BY_GPCR;REACTOME_INTEGRATION_OF_ENERGY_METABOLIS

;PONSE_TO_ELEVATED_PLATELET_CYTOSOLIC_CA2_;REACTOME_TRANSCRIPTIONAL_REG

M_HOMEOSTASIS;REACTOME_HEMOSTASIS

'TION_COUPLED_NER_TC_NER;REACTOME_POL_SWITCHING;REACTOME_NUCLEOTIDE_EX
'TION_COUPLED_NER_TC_NER;REACTOME_POL_SWITCHING;REACTOME_NUCLEOTIDE_EX
'TION_COUPLED_NER_TC_NER;REACTOME_POL_SWITCHING;REACTOME_NUCLEOTIDE_EX

;_BY_EGFR_IN_CANCER;REACTOME_ANTIGEN_ACTIVATES_B_CELL_RECEPTOR_LEADING_

_ORGANIC_ANION_SLC_TRANSPORTERS

PEPTIDE1;REACTOME_REGULATION_OF_INSULIN_SECRETION;REACTOME_CLASS_B_2_SE(

PEPTIDE1;REACTOME_REGULATION_OF_INSULIN_SECRETION;REACTOME_CLASS_B_2_SE(

IDE_LIGAND_BINDING_RECEPTORS;REACTOME_CLASS_A1_RHODOPSIN_LIKE_RECEPTORS

:TOME_G_ALPHA1213_SIGNALLING_EVENTS;REACTOME_GPCR_DOWNSTREAM_SIGNALING

_END_PROCESSING;REACTOME_CLEAVAGE_OF_GROWING_TRANSCRIPT_IN_THE_TERMIN

ME_ADAPTIVE_IMMUNE_SYSTEM

LING;REACTOME_G_ALPHA_S_SIGNALLING_EVENTS;REACTOME_GPCR_LIGAND_BINDING

SIS_OF_DNA;REACTOME_MITOTIC_G1_G1_S_PHASES;REACTOME_MITOTIC_M_M_G1_PHA

IMA_SUBUNITS;REACTOME_GABA_B_RECEPTOR_ACTIVATION;REACTOME_GABA_RECEPTC
IMA_SUBUNITS;REACTOME_GABA_B_RECEPTOR_ACTIVATION;REACTOME_GABA_RECEPTC

_SIGNALING;REACTOME_G_ALPHA_I_SIGNALLING_EVENTS;REACTOME_GPCR_LIGAND_BINI
:_3_PROMOTER;REACTOME_RNA_POL_III_TRANSCRIPTION_TERMINATION;REACTOME_RNA
:_3_PROMOTER;REACTOME_RNA_POL_III_TRANSCRIPTION_TERMINATION;REACTOME_RNA

ME_SPHINGOLIPID_METABOLISM;REACTOME_POST_TRANSLATIONAL_PROTEIN_MODIFICAT

ING_APOPTOSIS;REACTOME_APOPTOSIS

CE;REACTOME_DNA_REPLICATION;REACTOME_MITOTIC_PROMETAPHASE;REACTOME_ME

TOME_G_ALPHA1213_SIGNALLING_EVENTS;REACTOME_GPCR_DOWNSTREAM_SIGNALING

ANSCRIPTIONAL_REGULATION_OF_WHITE_ADIPOCYTE_DIFFERENTIATION

;

E_LINKER_SEQUENCE_IS_REQUIRED_FOR_GAG_SYNTHESIS;REACTOME_METABOLISM_OF_

RASACCHARIDE_LINKER_SEQUENCE_IS_REQUIRED_FOR_GAG_SYNTHESIS;REACTOME_ME

NSDUCTION_BY_L1;REACTOME_RECYCLING_PATHWAY_OF_L1;REACTOME_HEMOSTASIS
NSDUCTION_BY_L1;REACTOME_RECYCLING_PATHWAY_OF_L1;REACTOME_HEMOSTASIS

_DNA_REPLICATION;REACTOME_MITOTIC_PROMETAPHASE

LYTIC_CLEAVAGE_OF_NOTCH_RECEPTOR;REACTOME_SIGNALING_BY_NOTCH

EPLICATION;REACTOME_HEMOSTASIS;REACTOME_IMMUNE_SYSTEM;REACTOME_ADAPTI

ND_TRANSPORT

RTERS;REACTOME_METAL_ION_SLC_TRANSPORTERS

OME_PI3K_EVENTS_IN_ERBB4_SIGNALING;REACTOME_PI3K_EVENTS_IN_ERBB2_SIGNALIN

_SIGNALING;REACTOME_GPCR_LIGAND_BINDING;REACTOME_THROMBIN_SIGNALLING_THR

ODULATION_OF_CELL_SURFACE_RECEPTORS_BY_RECRUITING_THEM_TO_CLATHRIN_ADA

GNALING;REACTOME_SIGNALING_BY_ERBB2;REACTOME_SIGNALING_BY_EGFR_IN_CANCEI

ME_SPHINGOLIPID_METABOLISM;REACTOME_POST_TRANSLATIONAL_PROTEIN_MODIFICAT

UCTION;REACTOME_KINESINS;REACTOME_HEMOSTASIS;REACTOME_IMMUNE_SYSTEM;RE

E_GABA_A_RECEPTOR_ACTIVATION;REACTOME_GABA_RECEPTOR_ACTIVATION;REACTOM
REACTOME_TAK1_ACTIVATES_NFKB_BY_PHOSPHORYLATION_AND_ACTIVATION_OF_IKKS_

_SYSTEM;REACTOME_CYTOKINE_SIGNALING_IN_IMMUNE_SYSTEM;REACTOME_CLASS_I_MI

CTIONS

YTE_DIFFERENTIATION

ROVIDES_LINKAGE_TO_MAPK_SIGNALING_FOR_INTERGRINS_;REACTOME_INTEGRIN_ALPH
REACTOME_IMMUNE_SYSTEM

ADE;REACTOME_PI_3K_CASCADE;REACTOME_DOWNSTREAM_SIGNALING_OF_ACTIVATED_

;REACTOME_SIGNALING_BY_GPCR;REACTOME_AXON_GUIDANCE;REACTOME_G_ALPHA12

_G1_S_PHASES;REACTOME_INHIBITION_OF_REPLICATION_INITIATION_OF_DAMAGED_DNA_

REACTOME_TAK1_ACTIVATES_NFKB_BY_PHOSPHORYLATION_AND_ACTIVATION_OF_IKKS_

;REACTOME_SIGNALING_BY_GPCR;REACTOME_AXON_GUIDANCE;REACTOME_G_ALPHA12

:OMPLEX_AND_EIFS_AND_SUBSEQUENT_BINDING_TO_43S;REACTOME_PEPTIDE_CHAIN_EL

;

ALING;REACTOME_INTERFERON_ALPHA_BETA_SIGNALING;REACTOME_INTERFERON_SIGN/

CR_DOWNSTREAM_SIGNALING;REACTOME_G_ALPHA_I_SIGNALLING_EVENTS;REACTOME_(

ATELET_HOMEOSTASIS;REACTOME_PLATELET_SENSITIZATION_BY_LDL;REACTOME_DNA_F

REGATION

:_GLUR2_CONTAINING_AMPA_RECEPTORS

TRANSMISSION_IN_THE_POSTSYNAPTIC_CELL;REACTOME_PLC_BETA_MEDIATED_EVENTS

F_MITOTIC_CELL_CYCLE;REACTOME_APC_C_CDH1_MEDIATED_DEGRADATION_OF_CDC20_

NG;REACTOME_IL_RECEPTOR_SHC_SIGNALING;REACTOME_FACTORS_INVOLVED_IN_MEG/

/IA_NFKB;REACTOME_NRIF_SIGNALS_CELL_DEATH_FROM_THE_NUCLEUS;REACTOME_NFK

IETABOLISM

E_LINKER_SEQUENCE_IS_REQUIRED_FOR_GAG_SYNTHESIS;REACTOME_METABOLISM_OF_

OCIATED_VESICLE_BIOGENESIS;REACTOME_METABOLISM_OF_LIPIDS_AND_LIPOPROTEINS

TIGHT_JUNCTION_INTERACTIONS;REACTOME_CELL_JUNCTION_ORGANIZATION

IDES;REACTOME_AMINO_ACID_AND_OLIGOPEPTIDE_SLC_TRANSPORTERS

ION_PATHWAY;REACTOME_RECEPTOR_LIGAND_BINDING_INITIATES_THE_SECOND_PROTE

N;REACTOME_MITOTIC_G1_G1_S_PHASES;REACTOME_E2F_MEDIATED_REGULATION_OF_D

E_GABA_A_RECEPTOR_ACTIVATION;REACTOME_GABA_RECEPTOR_ACTIVATION;REACTOM

_AND_OTHER_SUGARS_BILE_SALTS_AND_ORGANIC_ACIDS_METAL_IONS_AND_AMINE_COM

A_POL_I_RNA_POL_III_AND_MITOCHONDRIAL_TRANSCRIPTION;REACTOME_RNA_POL_II_PR

IOTIC_COUPLING_AND_HEAT_PRODUCTION_BY_UNCOUPLING_PROTEINS_
N_SECRETION_BY_ACETYLCHOLINE;REACTOME_ADP_SIGNALLING_THROUGH_P2RY1;REAC

HOL_LIPID_LINKED_OLIGOSACCHARIDE_LLO_AND_TRANSFER_TO_A_NASCENT_PROTEIN

TRKA_FROM_THE_PLASMA_MEMBRANE;REACTOME_MHC_CLASS_II_ANTIGEN_PRESENTATI

G;REACTOME_IONOTROPIC_ACTIVITY_OF_KAINATE_RECEPTORS

OLISM;REACTOME_A_TETRASACCHARIDE_LINKER_SEQUENCE_IS_REQUIRED_FOR_GAG_S

ALING;REACTOME_INTERFERON_ALPHA_BETA_SIGNALING;REACTOME_INTERFERON_SIGN

CTIVATION_SIGNALING_AND_AGGREGATION
CTIVATION_SIGNALING_AND_AGGREGATION

⁄_OF_LIPIDS_AND_LIPOPROTEINS

NT_G1_DNA_DAMAGE_RESPONSE;REACTOME_REGULATION_OF_THE_FANCONI_ANEMIA_P/

TOME_SIGNALING_BY_EGFR_IN_CANCER;REACTOME_SHC1_EVENTS_IN_ERBB4_SIGNALIN(

SIUM_CHANNELS

E_GABA_A_RECEPTOR_ACTIVATION;REACTOME_GABA_RECEPTOR_ACTIVATION;REACTOM

E_IMMUNE_SYSTEM;REACTOME_TOLL_RECEPTOR_CASCADES;REACTOME_ADAPTIVE_IMM

NTATION

ME_SPHINGOLIPID_METABOLISM;REACTOME_POST_TRANSLATIONAL_PROTEIN_MODIFICAT

SPORT_OF_SMALL_MOLECULES;REACTOME_MRNA_PROCESSING;REACTOME_TRANSPORT

S_AND_RECYCLING;REACTOME_SIGNALING_BY_INSULIN_RECEPTOR

E_SIGNALING_BY_TGF_BETA_RECEPTOR_COMPLEX

E_NCAM_SIGNALING_FOR_NEURITE_OUT_GROWTH

YTE_DIFFERENTIATION

E_HIGHLY_CALCIUM_PERMEABLE_POSTSYNAPTIC_NICOTINIC_ACETYLCHOLINE_RECEPTOR

MEDIATED_SIGNALLING;REACTOME_PI3K_CASCADE
D_TRANSPORT

YCEROL_AND_KETONE_BODY_METABOLISM

:TOME_G_ALPHA1213_SIGNALLING_EVENTS;REACTOME_GPCR_DOWNSTREAM_SIGNALING

NALING_BY_SCF_KIT;REACTOME_DEVELOPMENTAL_BIOLOGY;REACTOME_SIGNALING_BY_I

NA;REACTOME_METABOLISM_OF_RNA;REACTOME_INFLUENZA_LIFE_CYCLE;REACTOME_INI

OME_PI3K_EVENTS_IN_ERBB4_SIGNALING;REACTOME_PI3K_EVENTS_IN_ERBB2_SIGNALIN

MUTANTS;REACTOME_FRS2_MEDIATED_CASCADE;REACTOME_PI_3K_CASCADE;REACTOME

VENTS;REACTOME_GPCR_LIGAND_BINDING
_SIGNALLING;REACTOME_SIGNALING_BY_GPCR;REACTOME_G_ALPHA1213_SIGNALLING_E

_MEDIATED_RESISTANCE_TO_HIV1_INFECTION

)_EXPOSED_COLLAGEN;REACTOME_BASIGIN_INTERACTIONS;REACTOME_INTEGRIN_CELL_

ANNELS;REACTOME_INWARDLY_RECTIFYING_K_CHANNELS

=_MITOTIC_CELL_CYCLE;REACTOME_MITOTIC_M_M_G1_PHASES;REACTOME_DNA_REPLICA

JND_SLC_TRANSPORTERS

_SIGNALING;REACTOME_GPCR_LIGAND_BINDING

ME_NEUROTRANSMITTER_RECEPTOR_BINDING_AND_DOWNSTREAM_TRANSMISSION_IN_T

REACTOME_TAK1_ACTIVATES_NFKB_BY_PHOSPHORYLATION_AND_ACTIVATION_OF_IKKS_

ATE_KERATIN_METABOLISM;REACTOME_GLYCOSAMINOGLYCAN_METABOLISM;REACTOME_

_SIGNALING;REACTOME_INTERFERON_ALPHA_BETA_SIGNALING;REACTOME_REGULATION

CTOME_G_ALPHA1213_SIGNALLING_EVENTS;REACTOME_GPCR_DOWNSTREAM_SIGNALING

ACTOME_P75_NTR_RECEPTOR_MEDIATED_SIGNALLING;REACTOME_SIGNALING_BY_GPCR;

YNTHESIS_OF_DNA;REACTOME_MITOTIC_G1_G1_S_PHASES;REACTOME_MITOTIC_M_M_G1

DHESION

_B_CELL_RECEPTOR_BCR;REACTOME_NGF_SIGNALLING_VIA_TRKA_FROM_THE_PLASMA_

ROTEINS;REACTOME_3_UTR_MEDIATED_TRANSLATIONAL_REGULATION

ANSCRIPTION;REACTOME_NOTCH_HLH_TRANSCRIPTION_PATHWAY;REACTOME_TRANSCRI

_OF_MATURE_MRNA_DERIVED_FROM_AN_INTRONLESS_TRANSCRIPT;REACTOME_SLC_ME

ALING_BY_GPCR;REACTOME_OPIOID_SIGNALLING;REACTOME_CA_DEPENDENT_EVENTS;R
OME_SIGNALING_BY_FGFR_IN_DISEASE;REACTOME_NUCLEAR_EVENTS_KINASE_AND_TRA

B_IS_ACTIVATED_AND_SIGNALS_SURVIVAL;REACTOME_CELL_DEATH_SIGNALLING_VIA_NR

PEPTIDES;REACTOME_AMINO_ACID_AND_OLIGOPEPTIDE_SLC_TRANSPORTERS

GNALLING;REACTOME_CA_DEPENDENT_EVENTS;REACTOME_DARPP_32_EVENTS;REACTOM

AVAGE_OF_GROWING_TRANSCRIPT_IN_THE_TERMINATION_REGION_

_BY_THE_B_CELL_RECEPTOR_BCR;REACTOME_INSULIN_RECEPTOR_SIGNALLING_CASCAI
_BY_THE_B_CELL_RECEPTOR_BCR;REACTOME_INSULIN_RECEPTOR_SIGNALLING_CASCAI

POINTS;REACTOME_CDC6_ASSOCIATION_WITH_THE_ORC_ORIGIN_COMPLEX;REACTOME_

C_TRANSCRIPTION_PATHWAY;REACTOME_METABOLISM_OF_LIPIDS_AND_LIPOPROTEINS;R

G;REACTOME_INSULIN_RECEPTOR_SIGNALLING_CASCADE;REACTOME_CELL_SURFACE_IN

E_METABOLISM_OF_LIPIDS_AND_LIPOPROTEINS;REACTOME_FATTY_ACID_TRIACYLGLYCEF
PON_TLR7_8_OR_9_ACTIVATION;REACTOME_ADVANCED_GLYCOSYLATION_ENDPRODUCT_

TIONS_ANIONS_AND_AMINO_ACIDS_OLIGOPEPTIDES;REACTOME_AMINO_ACID_AND_OLIG[illegible]

S

POPROTEINS;REACTOME_HORMONE_SENSITIVE_LIPASE_HSL_MEDIATED_TRIACYLGLYCER

G;REACTOME_INSULIN_RECEPTOR_SIGNALLING_CASCADE;REACTOME_CELL_SURFACE_IN

G;REACTOME_INSULIN_RECEPTOR_SIGNALLING_CASCADE;REACTOME_CELL_SURFACE_IN

ALING;REACTOME_IMMUNE_SYSTEM;REACTOME_ADAPTIVE_IMMUNE_SYSTEM;REACTOME_

TOME_REGULATION_OF_GLUCOKINASE_BY_GLUCOKINASE_REGULATORY_PROTEIN

PTIC_CELL;REACTOME_PLC_BETA_MEDIATED_EVENTS;REACTOME_G_PROTEIN_ACTIVATIC

ION_OF_INSULIN_SECRETION_BY_ADRENALINE_NORADRENALINE

G;REACTOME_DOWNSTREAM_SIGNALING_EVENTS_OF_B_CELL_RECEPTOR_BCR;REACTOM
MEMBRANE;REACTOME_SIGNALING_BY_FGFR_IN_DISEASE;REACTOME_GASTRIN_CREB_SI

RS

EACTOME_GPCR_DOWNSTREAM_SIGNALING;REACTOME_G_ALPHA_S_SIGNALLING_EVENTS
ETABOLISM

NA_BY_KSRP

TABILIZATION_OF_MRNA_BY_BRF1;REACTOME_REGULATION_OF_MRNA_STABILITY_BY_PRO

EMICAL_SYNAPSES;REACTOME_NEURONAL_SYSTEM;REACTOME_SIGNALING_BY_GPCR;REA

SES;REACTOME_ASSEMBLY_OF_THE_PRE_REPLICATIVE_COMPLEX;REACTOME_DNA_REPL

HC_MEDIATED_ANTIGEN_PROCESSING_PRESENTATION

_M_CHECKPOINTS;REACTOME_G2_M_DNA_DAMAGE_CHECKPOINT
NDING;REACTOME_HEMOSTASIS;REACTOME_PLATELET_ACTIVATION_SIGNALING_AND_AG

E_TRANSPORT;REACTOME_TRANSPORT_OF_GLUCOSE_AND_OTHER_SUGARS_BILE_SALTS

MOSOME_MAINTENANCE;REACTOME_GLOBAL_GENOMIC_NER_GG_NER;REACTOME_LAGGI

GLYCEROL_AND_KETONE_BODY_METABOLISM;REACTOME_CIRCADIAN_CLOCK;REACTOME

CLEOTIDE_EXCISION_REPAIR;REACTOME_FORMATION_OF_RNA_POL_II_ELONGATION_COM

DEGRADATION;REACTOME_GLYCOSAMINOGLYCAN_METABOLISM;REACTOME_SPHINGOLIPI

DEGRADATION;REACTOME_GLYCOSAMINOGLYCAN_METABOLISM;REACTOME_SPHINGOLIPI

CTOME_MEIOTIC_RECOMBINATION;REACTOME_MEIOTIC_SYNAPSIS;REACTOME_AMYLOIDS

)RT;REACTOME_TRANSFERRIN_ENDOCYTOSIS_AND_RECYCLING;REACTOME_SIGNALING_E

INDUCTION_OF_IFN_ALPHA_BETA_PATHWAYS;REACTOME_HEMOSTASIS;REACTOME_INNA

F_SIGNALING_BY_CBL;REACTOME_IL_3_5_AND_GM_CSF_SIGNALING;REACTOME_IMMUNE_

SIGNALING_IN_IMMUNE_SYSTEM

ABOLISM_OF_LIPIDS_AND_LIPOPROTEINS

PTOR_CASCADES;REACTOME_ADAPTIVE_IMMUNE_SYSTEM

_INSULIN_RECEPTOR_SIGNALLING_CASCADE;REACTOME_ARMS_MEDIATED_ACTIVATION;RI

RS;REACTOME_PRESYNAPTIC_NICOTINIC_ACETYLCHOLINE_RECEPTORS

R_CASCADE;REACTOME_MAPK_TARGETS_NUCLEAR_EVENTS_MEDIATED_BY_MAP_KINASES

_POL_III_CHAIN_ELONGATION

YTE_DIFFERENTIATION

C_TRANSCRIPTION_PATHWAY;REACTOME_NUCLEAR_RECEPTOR_TRANSCRIPTION_PATHW

TRANSPORTERS

THE_POSTSYNAPTIC_CELL;REACTOME_PLC_BETA_MEDIATED_EVENTS;REACTOME_G_ALPH

C_TRANSCRIPTION_PATHWAY;REACTOME_METABOLISM_OF_LIPIDS_AND_LIPOPROTEINS;R
C_TRANSCRIPTION_PATHWAY;REACTOME_METABOLISM_OF_LIPIDS_AND_LIPOPROTEINS;R

MAIN_REGULATES_TRANSCRIPTION;REACTOME_CIRCADIAN_REPRESSION_OF_EXPRESSIO

ACTOME_GPCR_DOWNSTREAM_SIGNALING;REACTOME_CELL_EXTRACELLULAR_MATRIX_IN

AND_OTHER_APC_C_CDH1_TARGETED_PROTEINS_IN_LATE_MITOSIS_EARLY_G1;REACTON

_BY_THE_B_CELL_RECEPTOR_BCR;REACTOME_NGF_SIGNALLING_VIA_TRKA_FROM_THE_

\N

_AND_LIPOPROTEINS

:_TRANSCRIPTION_PATHWAY;REACTOME_METABOLISM_OF_LIPIDS_AND_LIPOPROTEINS;R

:YTOKINE_SIGNALING_IN_IMMUNE_SYSTEM

_BY_THE_B_CELL_RECEPTOR_BCR;REACTOME_NGF_SIGNALLING_VIA_TRKA_FROM_THE_

_LIPIDS_AND_LIPOPROTEINS;REACTOME_METABOLISM_OF_CARBOHYDRATES;REACTOME_
_LIPIDS_AND_LIPOPROTEINS;REACTOME_METABOLISM_OF_CARBOHYDRATES;REACTOME_

'S;REACTOME_TRANSMISSION_ACROSS_CHEMICAL_SYNAPSES;REACTOME_NEURONAL_SY

_METABOLISM_OF_CARBOHYDRATES
_METABOLISM_OF_CARBOHYDRATES

R_SIGNALING;REACTOME_PHOSPHORYLATION_OF_CD3_AND_TCR_ZETA_CHAINS;REACTOM
[truncated: 71,590 more chars]
