# Supplementary material for: Genomic alterations caused by HPV integration in a cohort of Chinese endocervical adenocarcinomas
Source: Cancer Gene Ther. 2021 Jan 4;28(12):1353–64. doi: 10.1038/s41417-020-00283-4 (PMC8636260; doi:10.1038/s41417-020-00283-4)
Supplement: Supplementary file 8 — Supplementary Table 7 [file 41417_2020_283_MOESM8_ESM.pdf]

Supplementary Table 7

Detail of somatic SNV detected in each case by whole exome sequencing.

Patient	CHRO	POS	ID	REF	ALT	QUAL	FIL	GeneNam	Func	Ger	Gen	Ex	AAChar
ACa01	1	####	rs6264213	T	C	.	PAS	LINC0100	ncRNA_	NR_	.	.	.
ACa01	1	####	rs8798212	A	G	.	PAS	LINC0100	ncRNA_	NR_	.	.	.
ACa01	1	####	.	C	T	.	PAS	HES4	UTR3	NM_	NM_	.	.
ACa01	1	####	rs2011489	C	T	.	PAS	.	intergen	NM_	dist=	.	.
ACa01	1	####	rs7706792	G	A	.	PAS	PTCHD2	exonic	NM_	.	mis	PTCHD
ACa01	1	####	.	C	T	.	PAS	MTHFR	intronic	NM_	.	.	.
ACa01	1	####	rs7573447	A	G	.	PAS	PRAMEF2	intronic	NM_	.	.	.
ACa01	1	####	.	G	T	.	PAS	NBPF1	intronic	NM_	.	.	.
ACa01	1	####	rs643011	C	G	.	PAS	NBPF1	intronic	NM_	.	.	.
ACa01	1	####	rs1891369	T	C	.	PAS	.	upstrea	NR_	.	.	.
ACa01	1	####	rs7967920	G	A	.	PAS	PAX7	intronic	NM_	.	.	.
ACa01	1	####	.	A	C	.	PAS	TAF12	intronic	NM_	.	.	.
ACa01	1	####	rs3699237	G	A	.	PAS	SF3A3	intronic	NM_	.	.	.
ACa01	1	####	.	G	A	.	PAS	.	intergen	NR_	dist=	.	.
ACa01	1	####	.	A	C	.	PAS	CYP2J2	intronic	NM_	.	.	.
ACa01	1	####	.	G	A	.	PAS	CEPT1	intronic	NM_	.	.	.
ACa01	1	####	.	A	T	.	PAS	.	intergen	NR_	dist=	.	.
ACa01	1	####	rs1809861	G	A	.	PAS	.	intergen	NOI	dist=	.	.
ACa01	1	####	rs3742168	A	C	.	PAS	.	intergen	NR_	dist=	.	.
ACa01	1	####	rs1409433	T	C	.	PAS	.	intergen	NR_	dist=	.	.
ACa01	1	####	rs7967822	G	A	.	PAS	.	ncRNA_	NR_	.	.	.
ACa01	1	####	rs7763460	C	G	.	PAS	.	ncRNA_	NR_	.	.	.
ACa01	1	####	.	A	T	.	PAS	.	intergen	NR_	dist=	.	.
ACa01	1	####	rs1694653	C	G	.	PAS	.	intergen	NR_	dist=	.	.
ACa01	1	####	rs1129555	G	A	.	PAS	NBPF8,NE	intronic	NM_	.	.	.
ACa01	1	####	rs3732644	C	T	.	PAS	NBPF8,NE	intronic	NM_	.	.	.
ACa01	1	####	.	T	C	.	PAS	PDE4DIP	exonic	NM_	.	mis	PDE4D
ACa01	1	####	rs2691756	T	C	.	PAS	NBPF9,NE	intronic	NM_	.	.	.
ACa01	1	####	.	A	G	.	PAS	NBPF25P	ncRNA_	NR_	.	.	.
ACa01	1	####	.	G	T	.	PAS	NBPF25P	ncRNA_	NR_	.	.	.
ACa01	1	####	.	G	A	.	PAS	NBPF25P	ncRNA_	NR_	.	.	.
ACa01	1	####	rs1932337	C	T	.	PAS	.	intergen	NR_	dist=	.	.
ACa01	1	####	.	C	G	.	PAS	NBPF8	intronic	NM_	.	.	.
ACa01	1	####	rs2837929	G	A	.	PAS	NBPF8	intronic	NM_	.	.	.
ACa01	1	####	rs2983273	A	C	.	PAS	.	intergen	NR_	dist=	.	.
ACa01	1	####	rs3740307	C	T	.	PAS	PGLYRP3	exonic	NM_	.	syr	PGLYR
ACa01	1	####	rs1674785	C	T	.	PAS	.	UTR3	NM_	NM_	.	.
ACa01	1	####	rs1485348	T	G	.	PAS	FCGR2B	exonic	NM_	.	mis	FCGR2
ACa01	1	####	rs7598068	G	A	.	PAS	POU2F1	exonic	NM_	.	mis	POU2F
ACa01	1	####	.	G	T	.	PAS	DIEXF	intronic	NM_	.	.	.
ACa01	1	####	.	A	C	.	PAS	LBR	intronic	NM_	.	.	.
ACa01	1	####	.	A	C	.	PAS	LOC1001	ncRNA_	NR_	.	.	.
ACa01	1	####	rs2006447	A	G	.	PAS	CEP170	exonic	NM_	.	syr	CEP17
ACa01	1	####	rs3739868	A	G	.	PAS	.	upstrea	NM_	.	.	.
ACa01	1	####	rs1782199	G	T	.	PAS	.	upstrea	NM_	.	.	.
ACa01	2	####	rs3461159	C	A	.	PAS	.	intergen	NR_	dist=	.	.
ACa01	2	####	rs8689209	G	A	.	PAS	.	intergen	NOI	dist=	.	.
ACa01	2	####	.	C	T	.	PAS	LOC6543	ncRNA_	NR_	.	.	.
ACa01	2	####	.	A	G	.	PAS	IL1R1	exonic	NM_	.	mis	IL1R1:N
ACa01	2	####	rs3877497	T	G	.	PAS	FAM138B	ncRNA_	NR_	.	.	.
ACa01	2	####	rs1037863	A	G	.	PAS	CNTNAP5	intronic	NM_	.	.	.

ACa01	2	####	rs1839843	T	C	.	PA!	POTEF	exonic	NM_	.	syr	POTEF
ACa01	2	####	.	C	A	.	PA!	POTEF	exonic	NM_	.	mis	POTEF
ACa01	2	####	.	C	T	.	PA!	MGAT5	intronic	NM_	.	.	.
ACa01	2	####	.	A	T	.	PA!	ZEB2	exonic	NM_	.	mis	ZEB2:N
ACa01	2	####	.	T	G	.	PA!	CDCA7	intronic	NM_	.	.	.
ACa01	2	####	.	C	T	.	PA!	MARS2	exonic	NM_	.	mis	MARS2
ACa01	2	####	.	T	G	.	PA!	PLEKHM3	exonic	NM_	.	mis	PLEKHI
ACa01	2	####	rs7812258	C	T	.	PA!	PTH2R	intronic	NM_	.	.	.
ACa01	2	####	rs1010785	C	T	.	PA!	DNAJB2	intronic	NM_	.	.	.
ACa01	2	####	rs7526847	A	C	.	PA!	.	intergen	NM_	dist=	.	.
ACa01	3	####	.	T	G	.	PA!	NR2C2	intronic	NM_	.	.	.
ACa01	3	####	.	T	G	.	PA!	CAPN7	intronic	NM_	.	.	.
ACa01	3	####	.	C	G	.	PA!	KAT2B	exonic	NM_	.	mis	KAT2B:
ACa01	3	####	.	G	A	.	PA!	PLCD1	intronic	NM_	.	.	.
ACa01	3	####	rs1450320	C	T	.	PA!	SCN10A	exonic	NM_	.	mis	SCN10/
ACa01	3	####	rs1398610	C	T	.	PA!	SCN10A	exonic	NM_	.	mis	SCN10/
ACa01	3	####	.	G	A	.	PA!	PDZRN3	exonic	NM_	.	syr	PDZRN
ACa01	3	####	rs1126455	G	T	.	PA!	OR5H14	exonic	NM_	.	syr	OR5H1
ACa01	3	####	.	C	G	.	PA!	NFKBIZ	intronic	NM_	.	.	.
ACa01	3	####	rs7541975	C	T	.	PA!	NFKBIZ	intronic	NM_	.	.	.
ACa01	3	####	rs3757741	G	A	.	PA!	IFT57	exonic	NM_	.	mis	IFT57:N
ACa01	3	####	.	C	T	.	PA!	.	intergen	NR_	dist=	.	.
ACa01	3	####	rs6228513	T	C	.	PA!	.	intergen	NM_	dist=	.	.
ACa01	3	####	.	C	T	.	PA!	.	intergen	NM_	dist=	.	.
ACa01	4	####	rs4970287	C	T	.	PA!	.	intergen	NOI	dist=	.	.
ACa01	4	####	.	T	G	.	PA!	FAM175A	intronic	NM_	.	.	.
ACa01	4	####	rs3764069	C	T	.	PA!	.	intergen	NR_	dist=	.	.
ACa01	4	####	rs7963572	G	A	.	PA!	.	intergen	NR_	dist=	.	.
ACa01	4	####	rs9542709	C	T	.	PA!	FREM3	exonic	NM_	.	mis	FREM3
ACa01	4	####	rs3721454	C	T	.	PA!	FAM198B	intronic	NM_	.	.	.
ACa01	4	####	.	C	T	.	PA!	TRIML1	exonic	NM_	.	syr	TRIML1
ACa01	5	####	rs649883	T	C	.	PA!	ZDHHC11	intronic	NM_	.	.	.
ACa01	5	####	rs1467072	G	A	.	PA!	SDHAP3	ncRNA_	NR_	.	.	.
ACa01	5	####	.	C	A	.	PA!	.	intergen	NM_	dist=	.	.
ACa01	5	####	rs1500138	A	G	.	PA!	GUSBP1	ncRNA_	NR_	.	.	.
ACa01	5	####	.	T	G	.	PA!	NUP155	intronic	NM_	.	.	.
ACa01	5	####	rs7962294	T	C	.	PA!	NLN	intronic	NM_	.	.	.
ACa01	5	####	.	C	G	.	PA!	LOC4410(	ncRNA_	NR_	.	.	.
ACa01	5	####	.	A	G	.	PA!	PPIP5K2	intronic	NM_	.	.	.
ACa01	5	####	rs7609290	C	T	.	PA!	CAMK4	exonic	NM_	.	syr	CAMK4
ACa01	5	####	.	C	T	.	PA!	PPP2CA	intronic	NM_	.	.	.
ACa01	5	####	rs7647428	C	T	.	PA!	PCDHGC4	exonic	NM_	.	mis	PCDHG
ACa01	5	####	rs1407436	A	C	.	PA!	.	intergen	NR_	dist=	.	.
ACa01	5	####	rs7380674	G	C	.	PA!	.	intergen	NR_	dist=	.	.
ACa01	6	####	rs5568001	G	A	.	PA!	.	intergen	NM_	dist=	.	.
ACa01	6	####	rs3977792	T	G	.	PA!	GSTA1	exonic	NM_	.	mis	GSTA1
ACa01	6	####	.	T	C	.	PA!	.	intergen	NM_	dist=	.	.
ACa01	6	####	.	A	G	.	PA!	PHIP	exonic	NM_	.	mis	PHIP:N
ACa01	6	####	rs9844362	C	T	.	PA!	.	intergen	NM_	dist=	.	.
ACa01	6	####	.	T	A	.	PA!	.	intergen	NM_	dist=	.	.
ACa01	6	####	rs9992807	T	C	.	PA!	PDSS2	intronic	NM_	.	.	.
ACa01	6	####	.	C	T	.	PA!	ARMC2	exonic	NM_	.	mis	ARMC2
ACa01	6	####	rs7554849	G	A	.	PA!	SLC2A12	exonic	NM_	.	syr	SLC2A1
ACa01	6	####	.	G	A	.	PA!	UTRN	intronic	NM_	.	.	.

ACa01	6	####	rs7747387	C	T	.	PA!	ADGB	exonic	NM_	.	syr	ADGB:I
ACa01	6	####	rs1467177	C	T	.	PA!	RAET1L	intronic	NM_	.	.	.
ACa01	7	####	.	G	T	.	PA!	EIF3B	intronic	NM_	.	.	.
ACa01	7	####	rs7308136	G	C	.	PA!	SNX13	intronic	NM_	.	.	.
ACa01	7	####	.	G	A	.	PA!	AEBP1	intronic	NM_	.	.	.
ACa01	7	####	.	C	T	.	PA!	.	intergen	NM_	dist=	.	.
ACa01	7	####	rs7911953	C	T	.	PA!	.	intergen	NR_	dist=	.	.
ACa01	7	####	.	G	C	.	PA!	.	intergen	NR_	dist=	.	.
ACa01	7	####	.	C	T	.	PA!	.	intergen	NR_	dist=	.	.
ACa01	7	####	.	A	C	.	PA!	ZNF716	exonic	NM_	.	mis	ZNF716
ACa01	7	####	rs4311554	G	T	.	PA!	.	intergen	NOI	dist=	.	.
ACa01	7	####	.	G	T	.	PA!	.	intergen	NR_	dist=	.	.
ACa01	7	####	.	G	A	.	PA!	.	intergen	NR_	dist=	.	.
ACa01	7	####	rs5467260	G	A	.	PA!	.	intergen	NR_	dist=	.	.
ACa01	7	####	.	C	G	.	PA!	.	intergen	NR_	dist=	.	.
ACa01	7	####	rs8688020	G	A	.	PA!	.	intergen	NR_	dist=	.	.
ACa01	7	####	rs8799757	G	T	.	PA!	GTF2IRD2	intronic	NM_	.	.	.
ACa01	7	####	rs7686126	G	A	.	PA!	HEPACAN	intronic	NM_	.	.	.
ACa01	7	####	rs3727203	G	C	.	PA!	POLR2J3	intronic	NM_	.	.	.
ACa01	7	####	rs7809364	G	A	.	PA!	POT1	exonic	NM_	.	mis	POT1:N
ACa01	7	####	.	G	A	.	PA!	SSMEM1	exonic	NM_	.	syr	SSMEN
ACa01	7	####	rs7754267	A	G	.	PA!	KMT2C	exonic	NM_	.	syr	KMT2C
ACa01	7	####	rs1499922	C	T	.	PA!	KMT2C	exonic	NM_	.	mis	KMT2C
ACa01	7	####	.	C	T	.	PA!	KMT2C	UTR5	NM_	NM_	.	.
ACa01	7	####	rs1407758	C	T	.	PA!	.	intergen	NM_	dist=	.	.
ACa01	7	####	rs1482637	T	C	.	PA!	.	intergen	NM_	dist=	.	.
ACa01	8	####	.	C	A	.	PA!	.	intergen	NR_	dist=	.	.
ACa01	8	####	.	A	G	.	PA!	MTUS1	intronic	NM_	.	.	.
ACa01	8	####	.	A	C	.	PA!	XPO7	intronic	NM_	.	.	.
ACa01	8	####	.	T	G	.	PA!	KCTD9	exonic	NM_	.	mis	KCTD9
ACa01	8	####	rs1813428	G	A	.	PA!	.	intergen	NM_	dist=	.	.
ACa01	8	####	rs2022368	A	G	.	PA!	.	intergen	NM_	dist=	.	.
ACa01	8	####	.	T	C	.	PA!	LOC3922	ncRNA_	NR_	.	.	.
ACa01	8	####	rs1837720	A	T	.	PA!	IMPA1	intronic	NM_	.	.	.
ACa01	9	####	rs1329860	T	C	.	PA!	CNTNAP3	intronic	NM_	.	.	.
ACa01	9	####	rs2954515	T	C	.	PA!	.	intergen	NM_	dist=	.	.
ACa01	9	####	rs1968548	C	A	.	PA!	PTGER4P	ncRNA_	NR_	.	.	.
ACa01	9	####	rs2019611	G	A	.	PA!	PTGER4P	ncRNA_	NR_	.	.	.
ACa01	9	####	rs4928839	A	C	.	PA!	.	intergen	NM_	dist=	.	.
ACa01	9	####	.	G	A	.	PA!	WNK2	intronic	NM_	.	.	.
ACa01	9	####	rs2868900	G	A	.	PA!	.	intergen	NM_	dist=	.	.
ACa01	9	####	.	C	T	.	PA!	ENG	exonic	NM_	.	syr	ENG:NI
ACa01	9	####	.	T	G	.	PA!	FNBP1	exonic	NM_	.	syr	FNBP1:
ACa01	9	####	.	A	G	.	PA!	ADAMTS1	intronic	NM_	.	.	.
ACa01	9	####	rs7694907	C	T	.	PA!	DBH	exonic	NM_	.	syr	DBH:NI
ACa01	10	####	.	G	A	.	PA!	HSPA14	exonic	NM_	.	mis	HSPA1
ACa01	10	####	rs1051909	T	G	.	PA!	NMT2	intronic	NM_	.	.	.
ACa01	10	####	.	C	T	.	PA!	MYO3A	exonic	NM_	.	mis	MYO3A
ACa01	10	####	.	G	A	.	PA!	MYO3A	exonic	NM_	.	mis	MYO3A
ACa01	10	####	rs7531795	G	A	.	PA!	ARMC4	exonic	NM_	.	unl	UNKNC
ACa01	10	####	rs5566342	C	T	.	PA!	SVIL	exonic	NM_	.	syr	SVIL:NI
ACa01	10	####	rs1759036	G	T	.	PA!	ANKRD30	intronic	NM_	.	.	.
ACa01	10	####	.	C	T	.	PA!	.	intergen	NM_	dist=	.	.
ACa01	10	####	rs1494650	A	C	.	PA!	.	intergen	NR_	dist=	.	.

ACa01	10	####	rs5524339	C	T	.	PA!	ACTR3BP	ncRNA_	NR_	.	.	.	
ACa01	10	####	rs7827248	T	A	.	PA!	AGAP9	intronic	NM_	.	.	.	
ACa01	10	####	rs7821206	C	T	.	PA!	RBP3	exonic	NM_	.	mis	RBP3:N	
ACa01	10	####	.	C	A	.	PA!	.	intergen	NM_	dist=	.	.	
ACa01	10	####	.	C	T	.	PA!	ATOH7	exonic	NM_	.	syr	ATOH7	
ACa01	10	####	rs1810812	C	T	.	PA!	TET1	exonic	NM_	.	syr	TET1:N	
ACa01	10	####	rs1852770	G	A	.	PA!	STOX1	exonic	NM_	.	mis	STOX1	
ACa01	10	####	.	G	T	.	PA!	.	upstrea	NM_	.	.	.	
ACa01	10	####	.	A	C	.	PA!	LIPA	intronic	NM_	.	.	.	
ACa01	10	####	rs9002476	C	T	.	PA!	.	intergen	NM_	dist=	.	.	
ACa01	10	####	.	A	G	.	PA!	COL17A1	intronic	NM_	.	.	.	
ACa01	10	####	rs1028763	G	A	.	PA!	TRUB1	intronic	NM_	.	.	.	
ACa01	11	####	.	G	T	.	PA!	.	intergen	NM_	dist=	.	.	
ACa01	11	####	rs7615769	A	T	.	PA!	METTL15	intronic	NM_	.	.	.	
ACa01	11	####	.	G	A	.	PA!	TRAF6	intronic	NM_	.	.	.	
ACa01	11	####	.	T	G	.	PA!	.	intergen	NM_	dist=	.	.	
ACa01	11	####	.	G	A	.	PA!	MS4A15	intronic	NM_	.	.	.	
ACa01	11	####	.	T	A	.	PA!	PRKRIR	exonic	NM_	.	mis	PRKRIF	
ACa01	11	####	.	T	G	.	PA!	INTS4	intronic	NM_	.	.	.	
ACa01	11	####	.	T	G	.	PA!	INTS4	intronic	NM_	.	.	.	
ACa01	11	####	rs7614089	C	T	.	PA!	USP35	exonic	NM_	.	syr	USP35:	
ACa01	11	####	rs1438612	C	T	.	PA!	HEPHL1	exonic	NM_	.	mis	HEPHL	
ACa01	11	####	.	G	T	.	PA!	SIK2	exonic	NM_	.	syr	SIK2:NI	
ACa01	11	####	rs7757345	C	T	.	PA!	OR10S1	exonic	NM_	.	mis	OR10S	
ACa01	11	####	rs2512286	T	C	.	PA!	.	intergen	NM_	dist=	.	.	
ACa01	12	####	rs5382343	T	C	.	PA!	.	ncRNA_	NR_	.	.	.	
ACa01	12	####	rs8799828	C	G	.	PA!	LOC1002ٍ	ncRNA_	NR_	.	.	.	
ACa01	12	####	.	A	C	.	PA!	SLC6A13	exonic	NM_	.	mis	SLC6A1	
ACa01	12	####	rs8681434	T	C	.	PA!	ERC1	UTR5	NM_	NM_	.	.	
ACa01	12	####	rs2575282	A	G	.	PA!	.	intergen	NM_	dist=	.	.	
ACa01	12	####	.	G	A	.	PA!	PDZRN4	intronic	NM_	.	.	.	
ACa01	12	####	.	G	C	.	PA!	SLC38A1	UTR3	NM_	NM_	.	.	
ACa01	12	####	.	C	T	.	PA!	BIN2	exonic	NM_	.	syr	BIN2:NI	
ACa01	12	####	.	A	C	.	PA!	.	intergen	NR_	dist=	.	.	
ACa01	12	####	.	G	A	.	PA!	CAPS2	intronic	NM_	.	.	.	
ACa01	12	####	rs1043870	C	T	.	PA!	CHST11	exonic	NM_	.	syr	CHST1	
ACa01	12	####	.	C	T	.	PA!	CCDC63	intronic	NM_	.	.	.	
ACa01	12	####	.	C	T	.	PA!	CIT	intronic	NM_	.	.	.	
ACa01	13	####	.	C	A	.	PA!	PCDH17	exonic	NM_	.	mis	PCDH1	
ACa01	14	####	.	G	A	.	PA!	.	intergen	NR_	dist=	.	.	
ACa01	14	####	rs2014705	C	T	.	PA!	.	intergen	NR_	dist=	.	.	
ACa01	14	####	rs8794601	G	A	.	PA!	.	intergen	NR_	dist=	.	.	
ACa01	14	####	.	C	T	.	PA!	.	intergen	NR_	dist=	.	.	
ACa01	14	####	rs8674109	G	T	.	PA!	.	intergen	NM_	dist=	.	.	
ACa01	14	####	.	C	T	.	PA!	KLHL33	exonic	NM_	.	mis	KLHL3	
ACa01	14	####	.	A	C	.	PA!	.	intergen	NM_	dist=	.	.	
ACa01	14	####	.	A	C	.	PA!	.	intergen	NM_	dist=	.	.	
ACa01	14	####	.	G	A	.	PA!	TRAPPC6	intronic	NM_	.	.	.	
ACa01	14	####	.	G	A	.	PA!	.	upstrea	NM_	.	.	.	
ACa01	14	####	.	T	G	.	PA!	KIAA0586	intronic	NM_	.	.	.	
ACa01	14	####	rs7732738	G	A	.	PA!	SYNE2	exonic	NM_	.	mis	SYNE2	
ACa01	14	####	.	G	T	.	PA!	WDR25	intronic	NM_	.	.	.	
ACa01	14	####	rs2897118	C	T	.	PA!	.	intergen	NM_	dist=	.	.	
ACa01	14	####	rs3681255	T	A	.	PA!	.	upstrea	NR_	.	.	.	

ACa01	14	####	rs6199969	T	C	.	PA:	.	intergen	NR_	dist=	.	.
ACa01	15	####	.	C	A	.	PA:	.	intergen	NOI	dist=	.	.
ACa01	15	####	.	T	G	.	PA:	.	intergen	NOI	dist=	.	.
ACa01	15	####	rs3749240	G	A	.	PA:	.	intergen	NR_	dist=	.	.
ACa01	15	####	.	C	T	.	PA:	.	intergen	NR_	dist=	.	.
ACa01	15	####	.	C	T	.	PA:	CXADRP2	ncRNA_	NR_	.	.	.
ACa01	15	####	.	C	T	.	PA:	GOLGA8D	ncRNA_	NR_	.	.	.
ACa01	15	####	rs7964394	T	C	.	PA:	HERC2P2	ncRNA_	NR_	.	.	.
ACa01	15	####	.	A	G	.	PA:	.	intergen	NR_	dist=	.	.
ACa01	15	####	rs7126232	C	T	.	PA:	.	intronic	NM_	.	.	.
ACa01	15	####	.	G	A	.	PA:	NPAP1	exonic	NM_	.	syr	NPAP1
ACa01	15	####	.	G	A	.	PA:	ATP10A	exonic	NM_	.	syr	ATP10/
ACa01	15	####	rs7695754	C	T	.	PA:	GOLGA8N	exonic	NM_	.	mis	GOLGA
ACa01	15	####	.	G	A	.	PA:	APBA2	exonic	NM_	.	mis	APBA2:
ACa01	15	####	.	T	C	.	PA:	ULK4P2,U	ncRNA_	NR_	.	.	.
ACa01	15	####	.	C	A	.	PA:	.	intergen	NR_	dist=	.	.
ACa01	15	####	rs8658676	G	A	.	PA:	GOLGA8C	intronic	NM_	.	.	.
ACa01	15	####	.	G	T	.	PA:	GOLGA8C	intronic	NM_	.	.	.
ACa01	15	####	rs3683923	G	A	.	PA:	DISP2	UTR5	NM_	NM_	.	.
ACa01	15	####	rs3741666	G	A	.	PA:	VPS13C	intronic	NM_	.	.	.
ACa01	15	####	.	C	T	.	PA:	.	intergen	NM_	dist=	.	.
ACa01	15	####	rs5743805	G	A	.	PA:	.	upstreal	NR_	.	.	.
ACa01	15	####	rs1998088	A	G	.	PA:	GOLGA6L	exonic	NM_	.	syr	GOLGA
ACa01	15	####	rs3775649	A	G	.	PA:	GOLGA6L	exonic	NM_	.	mis	GOLGA
ACa01	15	####	rs6202866	A	G	.	PA:	WASH3P	ncRNA_	NR_	.	.	.
ACa01	16	####	rs7561426	G	A	.	PA:	RGS11	UTR3	NM_	NM_	.	.
ACa01	16	####	.	C	T	.	PA:	OTOA	intronic	NM_	.	.	.
ACa01	16	####	rs7464587	C	T	.	PA:	CORO1A	exonic	NM_	.	syr	CORO1
ACa01	16	####	.	G	A	.	PA:	ZNF646	exonic	NM_	.	syr	ZNF646
ACa01	16	####	rs2018870	C	T	.	PA:	.	intergen	NR_	dist=	.	.
ACa01	16	####	rs4643347	T	C	.	PA:	.	intergen	NOI	dist=	.	.
ACa01	16	####	rs4535017	T	C	.	PA:	.	intergen	NOI	dist=	.	.
ACa01	16	####	rs2128471	T	C	.	PA:	.	intergen	NOI	dist=	.	.
ACa01	16	####	rs2888787	C	G	.	PA:	.	intergen	NOI	dist=	.	.
ACa01	16	####	rs4355081	T	A	.	PA:	.	intergen	NOI	dist=	.	.
ACa01	16	####	rs4249097	T	C	.	PA:	.	intergen	NOI	dist=	.	.
ACa01	16	####	rs4232920	C	T	.	PA:	.	intergen	NOI	dist=	.	.
ACa01	16	####	rs7970299	T	G	.	PA:	.	intergen	NOI	dist=	.	.
ACa01	16	####	rs1483332	A	T	.	PA:	.	intergen	NOI	dist=	.	.
ACa01	16	####	rs9972801	A	G	.	PA:	.	intergen	NOI	dist=	.	.
ACa01	16	####	rs2878607	A	T	.	PA:	.	intergen	NOI	dist=	.	.
ACa01	16	####	rs4249050	C	G	.	PA:	.	intergen	NOI	dist=	.	.
ACa01	16	####	rs4250282	T	C	.	PA:	.	intergen	NOI	dist=	.	.
ACa01	16	####	rs7198709	T	C	.	PA:	.	intergen	NOI	dist=	.	.
ACa01	16	####	rs4445923	A	T	.	PA:	.	intergen	NOI	dist=	.	.
ACa01	16	####	rs4450416	A	G	.	PA:	.	intergen	NOI	dist=	.	.
ACa01	16	####	.	G	A	.	PA:	.	intergen	NOI	dist=	.	.
ACa01	16	####	rs8056404	C	G	.	PA:	.	intergen	NOI	dist=	.	.
ACa01	16	####	rs8056672	A	C	.	PA:	.	intergen	NOI	dist=	.	.
ACa01	16	####	rs6152895	G	A	.	PA:	.	intergen	NOI	dist=	.	.
ACa01	16	####	rs2884801	T	C	.	PA:	.	intergen	NOI	dist=	.	.
ACa01	16	####	.	A	C	.	PA:	FTO	intronic	NM_	.	.	.
ACa01	16	####	.	C	T	.	PA:	CBFB	exonic	NM_	.	sto	CBFB:N
ACa01	16	####	.	C	G	.	PA:	.	intergen	NM_	dist=	.	.

ACa01	16	####	.	C	T	.	PA:	ZNF469	exonic	NM_	.	mis	ZNF469
ACa01	17	####	rs7801100	G	A	.	PA:	PRPF8	exonic	NM_	.	syr	PRPF8:
ACa01	17	####	.	T	C	.	PA:	SPATA22	exonic	NM_	.	mis	SPATA:
ACa01	17	####	rs3687584	G	A	.	PA:	CHD3	exonic	NM_	.	mis	CHD3:N
ACa01	17	####	.	A	C	.	PA:	DNAH9	intronic	NM_	.	.	.
ACa01	17	####	rs1845094	C	T	.	PA:	MEIS3P1	ncRNA_	NR_	.	.	.
ACa01	17	####	.	G	A	.	PA:	MYO15A	intronic	NM_	.	.	.
ACa01	17	####	.	G	T	.	PA:	FLJ36000	ncRNA_	NR_	.	.	.
ACa01	17	####	.	C	G	.	PA:	FLJ36000	ncRNA_	NR_	.	.	.
ACa01	17	####	rs4503851	G	T	.	PA:	FLJ36000	ncRNA_	NR_	.	.	.
ACa01	17	####	rs1462263	C	A	.	PA:	.	intergen	NM_	dist=	.	.
ACa01	17	####	rs4362423	T	G	.	PA:	.	intergen	NM_	dist=	.	.
ACa01	17	####	rs6565430	T	G	.	PA:	.	intergen	NM_	dist=	.	.
ACa01	17	####	rs1434103	G	A	.	PA:	.	intergen	NM_	dist=	.	.
ACa01	17	####	.	G	C	.	PA:	.	intergen	NM_	dist=	.	.
ACa01	17	####	rs8916657	T	A	.	PA:	.	intergen	NM_	dist=	.	.
ACa01	17	####	rs1167082	A	T	.	PA:	FBXL20	intronic	NM_	.	.	.
ACa01	17	####	.	T	G	.	PA:	CNP	intronic	NM_	.	.	.
ACa01	17	####	rs1459625	G	A	.	PA:	TUBG1	intronic	NM_	.	.	.
ACa01	17	####	rs1724395	T	C	.	PA:	.	intergen	NR_	dist=	.	.
ACa01	17	####	.	G	A	.	PA:	TEX14	intronic	NM_	.	.	.
ACa01	17	####	.	T	G	.	PA:	FAM20A,F	intronic	NM_	.	.	.
ACa01	17	####	.	A	C	.	PA:	CD300LB	intronic	NM_	.	.	.
ACa01	18	####	.	C	T	.	PA:	EPB41L3	exonic	NM_	.	syr	EPB41L
ACa01	18	####	rs5374020	T	C	.	PA:	.	intergen	NM_	dist=	.	.
ACa01	18	####	rs2012257	C	G	.	PA:	.	intergen	NM_	dist=	.	.
ACa01	19	####	.	G	A	.	PA:	.	intergen	NM_	dist=	.	.
ACa01	19	####	rs7125437	C	G	.	PA:	.	intergen	NR_	dist=	.	.
ACa01	19	####	.	C	T	.	PA:	.	intergen	NR_	dist=	.	.
ACa01	19	####	.	A	G	.	PA:	CYP2F1	intronic	NM_	.	.	.
ACa01	19	####	.	G	T	.	PA:	.	intergen	NR_	dist=	.	.
ACa01	19	####	.	G	A	.	PA:	POU2F2	intronic	NM_	.	.	.
ACa01	19	####	.	G	A	.	PA:	CD177	intronic	NM_	.	.	.
ACa01	19	####	.	G	T	.	PA:	DHDH	intronic	NM_	.	.	.
ACa01	19	####	.	G	A	.	PA:	NLRP5	exonic	NM_	.	mis	NLRP5:
ACa01	19	####	rs8677343	T	C	.	PA:	.	intergen	NR_	dist=	.	.
ACa01	20	####	.	G	A	.	PA:	CNBD2	intronic	NM_	.	.	.
ACa01	20	####	.	C	T	.	PA:	TGM2	intronic	NM_	.	.	.
ACa01	21	####	rs8795581	C	T	.	PA:	.	intergen	NOI	dist=	.	.
ACa01	21	####	rs8661396	G	A	.	PA:	TEKT4P2	ncRNA_	NR_	.	.	.
ACa01	21	####	.	G	T	.	PA:	.	intergen	NR_	dist=	.	.
ACa01	21	####	rs7532385	G	A	.	PA:	BAGE2,B/	intronic	NM_	.	.	.
ACa01	21	####	rs3737231	A	T	.	PA:	BAGE2,B/	intronic	NM_	.	.	.
ACa01	21	####	.	G	A	.	PA:	.	intergen	NM_	dist=	.	.
ACa01	22	####	rs2003402	C	A	.	PA:	POTEH	intronic	NM_	.	.	.
ACa01	22	####	rs2818474	G	A	.	PA:	.	intergen	NM_	dist=	.	.
ACa01	22	####	rs5464921	C	T	.	PA:	CECR2	exonic	NM_	.	unl	UNKNC
ACa01	22	####	rs9350299	A	G	.	PA:	GGT5	intronic	NM_	.	.	.
ACa01	22	####	rs4050109	C	G	.	PA:	.	intergen	NM_	dist=	.	.
ACa01	22	####	.	T	G	.	PA:	.	intergen	NM_	dist=	.	.
ACa01	22	####	.	A	C	.	PA:	POM121L	ncRNA_	NR_	.	.	.
ACa01	22	####	.	C	T	.	PA:	MYO18B	intronic	NM_	.	.	.
ACa01	22	####	.	A	C	.	PA:	THOC5	intronic	NM_	.	.	.
ACa01	22	####	.	G	A	.	PA:	RNF215	intronic	NM_	.	.	.

ACa01	22	####	rs1425675	C	T	.	PA:	CYP2D7	ncRNA_	NR_	.	.	.
ACa01	22	####	rs1054069	G	A	.	PA:	CELSR1	intronic	NM_	.	.	.
ACa01	22	####	.	G	A	.	PA:	CELSR1	intronic	NM_	.	.	.
ACa01	X	####	rs7661994	C	T	.	PA:	VCX	UTR5	NM_	NM_	.	.
ACa01	X	####	.	C	T	.	PA:	.	intergen	NM_	dist=	.	.
ACa01	X	####	.	T	C	.	PA:	.	intergen	NM_	dist=	.	.
ACa01	X	####	.	G	A	.	PA:	TFE3	exonic	NM_	.	mis	TFE3:N
ACa01	X	####	.	G	A	.	PA:	TFE3	intronic	NM_	.	.	.
ACa01	X	####	.	C	T	.	PA:	CACNA1F	intronic	NM_	.	.	.
ACa01	X	####	rs7822715	C	T	.	PA:	.	upstrea	NM_	.	.	.
ACa01	X	####	rs7821690	C	T	.	PA:	TSPYL2	exonic	NM_	.	syr	TSPYL2
ACa01	X	####	.	A	T	.	PA:	.	intergen	NOI	dist=	.	.
ACa01	X	####	.	T	G	.	PA:	AR	intronic	NM_	.	.	.
ACa01	X	####	.	C	T	.	PA:	.	downstr	NM_	.	.	.
ACa01	X	####	.	C	T	.	PA:	BRWD3	intronic	NM_	.	.	.
ACa01	X	####	.	C	T	.	PA:	.	intergen	NM_	dist=	.	.
ACa01	X	####	.	G	A	.	PA:	CSTF2	exonic	NM_	.	syr	CSTF2:
ACa01	X	####	rs7818697	G	A	.	PA:	ZCCHC18	exonic	NM_	.	mis	ZCCHC
ACa01	X	####	.	C	T	.	PA:	HS6ST2	exonic	NM_	.	mis	HS6ST2
ACa01	X	####	.	C	T	.	PA:	MCF2	exonic	NM_	.	mis	MCF2:N
ACa01	X	####	.	C	T	.	PA:	CD99L2	intronic	NM_	.	.	.
ACa01	MT	7830	rs8788971	G	A	.	PA:	.	intergen	NOI	dist=	.	.
ACa02	1	####	.	C	T	.	PA:	MTOR	exonic	NM_	.	mis	MTOR:I
ACa02	1	####	.	C	T	.	PA:	MTOR	exonic	NM_	.	mis	MTOR:I
ACa02	1	####	rs5506852	G	A	.	PA:	.	intergen	NM_	dist=	.	.
ACa02	1	####	rs2994093	G	A	.	PA:	PRAMEF2	UTR3	NM_	NM_	.	.
ACa02	1	####	.	C	T	.	PA:	.	intronic	NM_	.	.	.
ACa02	1	####	rs1390932	C	T	.	PA:	ESPNP	ncRNA_	NR_	.	.	.
ACa02	1	####	.	A	C	.	PA:	KIF17	intronic	NM_	.	.	.
ACa02	1	####	rs3686746	G	A	.	PA:	ORC1	exonic	NM_	.	syr	ORC1:N
ACa02	1	####	.	T	C	.	PA:	LRRC40	intronic	NM_	.	.	.
ACa02	1	####	.	T	C	.	PA:	ZNF644	intronic	NM_	.	.	.
ACa02	1	####	.	C	T	.	PA:	KCNA2	exonic	NM_	.	syr	KCNA2
ACa02	1	####	rs2010827	G	C	.	PA:	.	intergen	NOI	dist=	.	.
ACa02	1	####	.	C	A	.	PA:	.	intergen	NOI	dist=	.	.
ACa02	1	####	rs2018849	T	G	.	PA:	.	intergen	NOI	dist=	.	.
ACa02	1	####	rs3724010	A	T	.	PA:	.	intergen	NR_	dist=	.	.
ACa02	1	####	.	T	C	.	PA:	.	intergen	NR_	dist=	.	.
ACa02	1	####	rs3772379	G	C	.	PA:	.	intergen	NR_	dist=	.	.
ACa02	1	####	rs8799699	G	A	.	PA:	NBPF20	intronic	NM_	.	.	.
ACa02	1	####	.	C	A	.	PA:	NBPF9,NE	intronic	NM_	.	.	.
ACa02	1	####	.	A	C	.	PA:	NBPF8,NE	intronic	NM_	.	.	.
ACa02	1	####	rs4649849	A	G	.	PA:	NBPF9,NE	intronic	NM_	.	.	.
ACa02	1	####	rs8799650	A	C	.	PA:	NBPF25P	ncRNA_	NR_	.	.	.
ACa02	1	####	rs7668601	C	G	.	PA:	NBPF25P	ncRNA_	NR_	.	.	.
ACa02	1	####	rs7823737	C	T	.	PA:	NBPF25P	ncRNA_	NR_	.	.	.
ACa02	1	####	.	C	T	.	PA:	NBPF8	intronic	NM_	.	.	.
ACa02	1	####	rs2003436	G	A	.	PA:	NBPF8	intronic	NM_	.	.	.
ACa02	1	####	rs6181174	G	A	.	PA:	NBPF8	intronic	NM_	.	.	.
ACa02	1	####	rs6181174	G	A	.	PA:	NBPF8	intronic	NM_	.	.	.
ACa02	1	####	.	A	C	.	PA:	LOC1005(	ncRNA_	NR_	.	.	.
ACa02	1	####	rs7494818	C	T	.	PA:	TNR	exonic	NM_	.	mis	TNR:NM
ACa02	1	####	.	C	T	.	PA:	SOAT1	intronic	NM_	.	.	.
ACa02	1	####	rs8790889	C	A	.	PA:	AXDND1	intronic	NM_	.	.	.

ACa02	1	####	.	G	T	.	PA!	RNU5F-1	ncRNA_	NR_	.	.	.	
ACa02	1	####	.	G	A	.	PA!	ZNF678	intronic	NM_	.	.	.	
ACa02	1	####	.	A	G	.	PA!	.	intergen	NM_	dist=	.	.	
ACa02	1	####	.	C	A	.	PA!	RGS7	intronic	NM_	.	.	.	
ACa02	2	####	.	A	G	.	PA!	NCOA1	intronic	NM_	.	.	.	
ACa02	2	####	rs1020943	C	A	.	PA!	.	intergen	NR_	dist=	.	.	
ACa02	2	####	.	C	T	.	PA!	LOC6543	ncRNA_	NR_	.	.	.	
ACa02	2	####	rs2004118	A	T	.	PA!	.	intergen	NR_	dist=	.	.	
ACa02	2	####	rs2021466	A	T	.	PA!	.	intergen	NR_	dist=	.	.	
ACa02	2	####	rs3694799	T	C	.	PA!	ANKRD36	intronic	NM_	.	.	.	
ACa02	2	####	rs5416674	G	A	.	PA!	RGPD4	exonic	NM_	.	mis	RGPD4	
ACa02	2	####	rs1053772	A	C	.	PA!	LIMS1	intronic	NM_	.	.	.	
ACa02	2	####	.	C	T	.	PA!	WASH2P	ncRNA_	NR_	.	.	.	
ACa02	2	####	rs5382277	C	T	.	PA!	RNU6-81F	ncRNA_	NR_	.	.	.	
ACa02	2	####	.	G	C	.	PA!	7-Mar	intronic	NM_	.	.	.	
ACa02	2	####	rs1875087	G	A	.	PA!	BBS5	intronic	NM_	.	.	.	
ACa02	2	####	.	G	T	.	PA!	TTN	exonic	NM_	.	mis	TTN:NM	
ACa02	2	####	.	A	G	.	PA!	CARF	intronic	NM_	.	.	.	
ACa02	2	####	.	T	C	.	PA!	ATIC	UTR3	NM_	NM_	.	.	
ACa02	2	####	rs1509794	C	T	.	PA!	COL4A4	exonic	NM_	.	mis	COL4A	
ACa02	2	####	.	C	T	.	PA!	NGEF	exonic	NM_	.	mis	NGEF:N	
ACa02	2	####	rs7970263	T	C	.	PA!	.	intergen	NR_	dist=	.	.	
ACa02	3	####	.	G	A	.	PA!	ITPR1	splicing	NM_	NM_	.	.	
ACa02	3	####	.	G	A	.	PA!	.	UTR3	NM_	NM_	.	.	
ACa02	3	####	rs6777426	G	C	.	PA!	MST1	exonic	NM_	.	mis	MST1:N	
ACa02	3	####	rs6779963	G	A	.	PA!	MST1	intronic	NM_	.	.	.	
ACa02	3	####	rs5472981	G	A	.	PA!	FRMD4B	exonic	NM_	.	unl	UNKNC	
ACa02	3	####	.	C	T	.	PA!	MITF	intronic	NM_	.	.	.	
ACa02	3	####	.	C	G	.	PA!	.	intergen	NM_	dist=	.	.	
ACa02	3	####	rs1219132	G	A	.	PA!	PIK3CA	exonic	NM_	.	mis	PIK3CA	
ACa02	3	####	.	C	G	.	PA!	MFN1	intronic	NM_	.	.	.	
ACa02	3	####	.	A	G	.	PA!	GMNC	exonic	NM_	.	syr	GMNC:	
ACa02	3	####	.	G	T	.	PA!	.	ncRNA_	NR_	.	.	.	
ACa02	3	####	rs4857535	A	G	.	PA!	LOC22072	ncRNA_	NR_	.	.	.	
ACa02	4	####	.	C	T	.	PA!	CTBP1	intronic	NM_	.	.	.	
ACa02	4	####	.	G	T	.	PA!	USP17L1(	exonic	NM_	.	mis	USP17l	
ACa02	4	####	.	G	T	.	PA!	.	intergen	NM_	dist=	.	.	
ACa02	4	####	rs7961122	C	G	.	PA!	.	upstrea	NM_	.	.	.	
ACa02	4	####	.	C	T	.	PA!	MIR1273H	ncRNA_	NR_	.	.	.	
ACa02	4	####	.	A	G	.	PA!	.	intergen	NM_	dist=	.	.	
ACa02	4	####	.	G	A	.	PA!	.	intergen	NR_	dist=	.	.	
ACa02	4	####	rs3705413	C	T	.	PA!	PRDM8	exonic	NM_	.	syr	PRDM8	
ACa02	4	####	.	C	T	.	PA!	ADH1C	exonic	NM_	.	unl	UNKNC	
ACa02	4	####	.	T	C	.	PA!	SLC9B2	UTR3	NM_	NM_	.	.	
ACa02	4	####	.	G	A	.	PA!	ANK2	intronic	NM_	.	.	.	
ACa02	4	####	rs2016625	T	G	.	PA!	GYPB	exonic	NM_	.	mis	GYPB:N	
ACa02	4	####	.	G	A	.	PA!	WDR17	intronic	NM_	.	.	.	
ACa02	4	####	rs7619626	C	T	.	PA!	ZFP42	exonic	NM_	.	syr	ZFP42:	
ACa02	5	####	rs8684061	C	G	.	PA!	LOC4421	ncRNA_	NR_	.	.	.	
ACa02	5	####	.	C	T	.	PA!	OTULIN	intronic	NM_	.	.	.	
ACa02	5	####	.	G	A	.	PA!	.	intergen	NR_	dist=	.	.	
ACa02	5	####	.	G	A	.	PA!	.	intergen	NR_	dist=	.	.	
ACa02	5	####	rs8659304	A	G	.	PA!	.	intergen	NOI	dist=	.	.	
ACa02	5	####	rs1335780	A	C	.	PA!	.	intergen	NOI	dist=	.	.	

ACa02	5	####	rs1483787	G	C	.	PA!	.	intergen	NOI	dist=	.	.
ACa02	5	####	.	C	A	.	PA!	RGS7BP	UTR5	NM_	NM_	.	.
ACa02	5	####	rs1416874	T	G	.	PA!	.	intergen	NR_	dist=	.	.
ACa02	5	####	.	G	A	.	PA!	SH3TC2	intronic	NM_	.	.	.
ACa02	5	####	rs7284112	G	A	.	PA!	DOCK2	intronic	NM_	.	.	.
ACa02	5	####	rs1829546	C	A	.	PA!	MAML1	intronic	NM_	.	.	.
ACa02	6	####	rs7743469	A	G	.	PA!	STK19	exonic	NM_	.	syr	STK19:
ACa02	6	####	rs1403232	T	C	.	PA!	CYP21A1I	ncRNA_	NR_	.	.	.
ACa02	6	####	rs7596914	C	T	.	PA!	KCNK17	intronic	NM_	.	.	.
ACa02	6	####	rs4928651	T	A	.	PA!	.	intergen	NR_	dist=	.	.
ACa02	6	####	.	G	T	.	PA!	DSE	exonic	NM_	.	sto	DSE:NI
ACa02	7	####	.	A	C	.	PA!	INTS1	intronic	NM_	.	.	.
ACa02	7	####	rs7467237	A	G	.	PA!	NME8	intronic	NM_	.	.	.
ACa02	7	####	rs7762925	C	T	.	PA!	NACAD	exonic	NM_	.	mis	NACAD
ACa02	7	####	.	G	T	.	PA!	.	intergen	NR_	dist=	.	.
ACa02	7	####	.	A	G	.	PA!	.	intergen	NR_	dist=	.	.
ACa02	7	####	rs1422600	C	T	.	PA!	.	intergen	NR_	dist=	.	.
ACa02	7	####	rs4460255	G	C	.	PA!	.	intergen	NOI	dist=	.	.
ACa02	7	####	.	C	A	.	PA!	.	intergen	NR_	dist=	.	.
ACa02	7	####	rs3740384	G	A	.	PA!	.	intergen	NR_	dist=	.	.
ACa02	7	####	rs8672068	G	A	.	PA!	.	intergen	NR_	dist=	.	.
ACa02	7	####	.	C	G	.	PA!	.	intergen	NR_	dist=	.	.
ACa02	7	####	.	G	A	.	PA!	.	intergen	NR_	dist=	.	.
ACa02	7	####	.	G	T	.	PA!	.	intergen	NR_	dist=	.	.
ACa02	7	####	rs5541523	C	T	.	PA!	GTF2IP1,I	ncRNA_	NR_	.	.	.
ACa02	7	####	rs3757000	T	C	.	PA!	.	intergen	NM_	dist=	.	.
ACa02	7	####	.	A	G	.	PA!	GPR85	exonic	NM_	.	syr	GPR85
ACa02	7	####	.	T	G	.	PA!	SLC4A2	intronic	NM_	.	.	.
ACa02	7	####	.	T	G	.	PA!	ABCF2	intronic	NM_	.	.	.
ACa02	7	####	.	T	G	.	PA!	ABCF2	intronic	NM_	.	.	.
ACa02	7	####	rs8681384	G	C	.	PA!	PTPRN2	intronic	NM_	.	.	.
ACa02	8	####	.	C	T	.	PA!	.	intergen	NM_	dist=	.	.
ACa02	8	####	rs3989791	C	G	.	PA!	.	intergen	NR_	dist=	.	.
ACa02	8	####	.	G	A	.	PA!	.	intergen	NR_	dist=	.	.
ACa02	8	####	rs7469289	G	A	.	PA!	RP1L1	exonic	NM_	.	syr	RP1L1:
ACa02	8	####	rs7595500	C	G	.	PA!	FAM90A2!	ncRNA_	NR_	.	.	.
ACa02	8	####	.	G	A	.	PA!	MIR3926-:	ncRNA_	NR_	.	.	.
ACa02	8	####	.	G	T	.	PA!	VPS37A	intronic	NM_	.	.	.
ACa02	8	####	.	G	C	.	PA!	.	intergen	NOI	dist=	.	.
ACa02	8	####	rs4440644	C	T	.	PA!	.	intergen	NOI	dist=	.	.
ACa02	8	####	.	G	T	.	PA!	ZNF704	intronic	NM_	.	.	.
ACa02	8	####	.	G	A	.	PA!	CSMD3	intronic	NM_	.	.	.
ACa02	8	####	.	C	T	.	PA!	LY6H	intronic	NM_	.	.	.
ACa02	9	####	rs5356927	G	A	.	PA!	SLC24A2	intronic	NM_	.	.	.
ACa02	9	####	rs5620843	C	T	.	PA!	PAX5	intronic	NM_	.	.	.
ACa02	9	####	rs1412360	T	C	.	PA!	CNTNAP3	exonic	NM_	.	mis	CNTNA
ACa02	9	####	rs3755065	C	A	.	PA!	GLIDR	ncRNA_	NR_	.	.	.
ACa02	9	####	rs5368523	T	A	.	PA!	.	upstreai	NR_	.	.	.
ACa02	9	####	rs7792676	G	T	.	PA!	PTGER4P	ncRNA_	NR_	.	.	.
ACa02	9	####	rs1968548	C	A	.	PA!	PTGER4P	ncRNA_	NR_	.	.	.
ACa02	9	####	rs6254382	A	G	.	PA!	LOC6422:	ncRNA_	NR_	.	.	.
ACa02	9	####	.	G	T	.	PA!	.	intergen	NR_	dist=	.	.
ACa02	9	####	rs5781745	G	A	.	PA!	.	intergen	NM_	dist=	.	.
ACa02	9	####	.	G	A	.	PA!	RALGDS	intronic	NM_	.	.	.

ACa02	9	####	.	G	A	.	PA!	CACNA1E	exonic	NM_	.	mis	CACNA
ACa02	10	####	rs7660547	C	T	.	PA!	CUBN	exonic	NM_	.	syr	CUBN:I
ACa02	10	####	rs1930137	C	T	.	PA!	MLLT10	intronic	NM_	.	.	.
ACa02	10	####	.	T	G	.	PA!	BMS1	splicing	NM_	NM_	.	.
ACa02	10	####	rs7819664	T	A	.	PA!	FAM35DP	ncRNA_	NR_	.	.	.
ACa02	10	####	rs6185008	G	C	.	PA!	TIMM23B,	intronic	NM_	.	.	.
ACa02	10	####	.	T	A	.	PA!	AGAP5	intronic	NM_	.	.	.
ACa02	10	####	.	G	A	.	PA!	.	intergen	NR_	dist=	.	.
ACa02	10	####	.	C	T	.	PA!	PKD2L1	intronic	NM_	.	.	.
ACa02	10	####	rs2928119	C	G	.	PA!	RAB11FIF	intronic	NM_	.	.	.
ACa02	10	####	.	C	G	.	PA!	.	intergen	NR_	dist=	.	.
ACa02	10	####	rs1021891	C	T	.	PA!	SCART1	ncRNA_	NR_	.	.	.
ACa02	11	####	rs6263724	A	C	.	PA!	MUC2	exonic	NM_	.	syr	MUC2:I
ACa02	11	####	rs1494932	T	C	.	PA!	MUC5B	intronic	NM_	.	.	.
ACa02	11	####	.	T	G	.	PA!	ST5	intronic	NM_	.	.	.
ACa02	11	####	.	G	A	.	PA!	IPO7	intronic	NM_	.	.	.
ACa02	11	####	.	G	A	.	PA!	EXT2	intronic	NM_	.	.	.
ACa02	11	####	.	G	A	.	PA!	SYT7	intronic	NM_	.	.	.
ACa02	11	####	rs9265024	G	C	.	PA!	VEGFB	UTR3	NM_	NM_	.	.
ACa02	11	####	.	T	C	.	PA!	SYVN1	intronic	NM_	.	.	.
ACa02	11	####	.	C	T	.	PA!	KLC2	UTR3	NM_	NM_	.	.
ACa02	11	####	.	G	A	.	PA!	LOC1001	ncRNA_	NR_	.	.	.
ACa02	11	####	rs3679844	G	A	.	PA!	ARAP1	exonic	NM_	.	syr	ARAP1
ACa02	11	####	.	G	A	.	PA!	ARAP1	exonic	NM_	.	syr	ARAP1
ACa02	11	####	.	C	A	.	PA!	GDPD5	intronic	NM_	.	.	.
ACa02	11	####	rs3831451	A	G	.	PA!	MYO7A	intronic	NM_	.	.	.
ACa02	11	####	.	A	T	.	PA!	INTS4	intronic	NM_	.	.	.
ACa02	11	####	.	C	T	.	PA!	.	intergen	NM_	dist=	.	.
ACa02	11	####	rs1441056	C	T	.	PA!	BACE1	intronic	NM_	.	.	.
ACa02	11	####	rs5379167	G	T	.	PA!	OR8B8	exonic	NM_	.	mis	OR8B8
ACa02	11	####	.	T	C	.	PA!	.	intergen	NM_	dist=	.	.
ACa02	12	####	rs1482401	C	G	.	PA!	.	intergen	NR_	dist=	.	.
ACa02	12	####	.	T	A	.	PA!	TULP3	exonic	NM_	.	mis	TULP3:
ACa02	12	####	rs2005141	T	C	.	PA!	.	intergen	NR_	dist=	.	.
ACa02	12	####	.	G	A	.	PA!	ASUN	exonic	NM_	.	syr	ASUN:I
ACa02	12	####	.	T	C	.	PA!	BAZ2A	exonic	NM_	.	syr	BAZ2A:
ACa02	12	####	rs3737629	G	A	.	PA!	ZBTB39	exonic	NM_	.	mis	ZBTB3!
ACa02	12	####	.	C	T	.	PA!	OTOGL	intronic	NM_	.	.	.
ACa02	12	####	.	G	A	.	PA!	PPFIA2	exonic	NM_	.	sto	PPFIA2
ACa02	12	####	.	C	G	.	PA!	.	intergen	NM_	dist=	.	.
ACa02	12	####	.	C	T	.	PA!	BTBD11	exonic	NM_	.	mis	BTBD1:
ACa02	12	####	.	G	A	.	PA!	PPTC7	intronic	NM_	.	.	.
ACa02	12	####	rs3495666	A	G	.	PA!	PPTC7	intronic	NM_	.	.	.
ACa02	12	####	.	G	C	.	PA!	NOS1	intronic	NM_	.	.	.
ACa02	12	####	.	C	G	.	PA!	NCOR2	exonic	NM_	.	mis	NCOR2
ACa02	13	####	.	A	G	.	PA!	HMGB1	UTR3	NM_	NM_	.	.
ACa02	13	####	.	A	C	.	PA!	VWA8	intronic	NM_	.	.	.
ACa02	13	####	rs3772091	T	C	.	PA!	.	intergen	NR_	dist=	.	.
ACa02	13	####	.	G	T	.	PA!	.	intergen	NM_	dist=	.	.
ACa02	13	####	.	C	G	.	PA!	TPP2	intronic	NM_	.	.	.
ACa02	14	####	rs2259899	C	A	.	PA!	.	intergen	NR_	dist=	.	.
ACa02	14	####	rs7532438	G	A	.	PA!	OR4K15	exonic	NM_	.	mis	OR4K1!
ACa02	14	####	.	T	A	.	PA!	.	intergen	NM_	dist=	.	.
ACa02	14	####	.	G	T	.	PA!	.	intergen	NM_	dist=	.	.

ACa02	14	####	.	T	C	.	PA:	C14orf183	exonic	NM_	.	mis	C14orf1
ACa02	14	####	.	G	C	.	PA:	RBM25	exonic	NM_	.	mis	RBM25
ACa02	14	####	.	G	A	.	PA:	LTBP2	intronic	NM_	.	.	.
ACa02	14	####	.	A	C	.	PA:	SLC25A47	intronic	NM_	.	.	.
ACa02	14	####	rs9310230	C	T	.	PA:	MEG3	ncRNA_	NR_	.	.	.
ACa02	14	####	.	C	T	.	PA:	TRMT61A	intronic	NM_	.	.	.
ACa02	15	####	rs2259724	A	G	.	PA:	LINC0119	ncRNA_	NR_	NR_	.	.
ACa02	15	####	rs2648130	G	T	.	PA:	.	intergen	NM_	dist=	.	.
ACa02	15	####	rs2015085	C	A	.	PA:	.	intergen	NR_	dist=	.	.
ACa02	15	####	rs2007357	A	G	.	PA:	.	intergen	NM_	dist=	.	.
ACa02	15	####	.	C	A	.	PA:	RYR3	exonic	NM_	.	unl	UNKNC
ACa02	15	####	.	A	G	.	PA:	VPS18	exonic	NM_	.	syr	VPS18:
ACa02	15	####	.	G	A	.	PA:	SLC12A1	intronic	NM_	.	.	.
ACa02	15	####	.	C	T	.	PA:	RAB8B	UTR5	NM_	NM_	.	.
ACa02	15	####	.	C	T	.	PA:	PKM	intronic	NM_	.	.	.
ACa02	15	####	.	C	A	.	PA:	NEO1	intronic	NM_	.	.	.
ACa02	15	####	.	C	T	.	PA:	GOLGA6C	intronic	NM_	.	.	.
ACa02	15	####	rs1401448	T	C	.	PA:	.	intergen	NR_	dist=	.	.
ACa02	15	####	rs8788873	C	A	.	PA:	SCAPER	intronic	NM_	.	.	.
ACa02	15	####	rs2870850	G	A	.	PA:	.	intergen	NM_	dist=	.	.
ACa02	15	####	.	C	T	.	PA:	SLCO3A1	intronic	NM_	.	.	.
ACa02	15	####	rs7724699	C	T	.	PA:	ADAMTS1	exonic	NM_	.	syr	ADAMT
ACa02	15	####	.	G	A	.	PA:	WASH3P	ncRNA_	NR_	.	.	.
ACa02	16	####	rs7760391	C	T	.	PA:	NPIPA1	intronic	NM_	.	.	.
ACa02	16	####	.	C	G	.	PA:	NDE1	intronic	NM_	.	.	.
ACa02	16	####	.	G	A	.	PA:	.	intergen	NM_	dist=	.	.
ACa02	16	####	.	C	T	.	PA:	SRCAP	exonic	NM_	.	mis	SRCAP
ACa02	16	####	rs2111615	A	G	.	PA:	TP53TG3I	intronic	NM_	.	.	.
ACa02	16	####	rs1123661	T	C	.	PA:	TP53TG3I	intronic	NM_	.	.	.
ACa02	16	####	.	C	A	.	PA:	.	intergen	NR_	dist=	.	.
ACa02	16	####	rs5547762	C	T	.	PA:	.	intergen	NR_	dist=	.	.
ACa02	16	####	rs3718897	C	T	.	PA:	.	intergen	NR_	dist=	.	.
ACa02	16	####	rs4967312	G	T	.	PA:	.	intergen	NOI	dist=	.	.
ACa02	16	####	rs4270195	G	C	.	PA:	.	intergen	NOI	dist=	.	.
ACa02	16	####	rs4347658	A	T	.	PA:	.	intergen	NOI	dist=	.	.
ACa02	16	####	rs4246399	T	C	.	PA:	.	intergen	NOI	dist=	.	.
ACa02	16	####	rs5908263	A	G	.	PA:	.	intergen	NOI	dist=	.	.
ACa02	16	####	rs4249045	T	C	.	PA:	.	intergen	NOI	dist=	.	.
ACa02	16	####	rs7188365	G	T	.	PA:	.	intergen	NOI	dist=	.	.
ACa02	16	####	rs4249050	C	G	.	PA:	.	intergen	NOI	dist=	.	.
ACa02	16	####	rs4505355	C	T	.	PA:	.	intergen	NOI	dist=	.	.
ACa02	16	####	rs1244825	T	G	.	PA:	.	intergen	NOI	dist=	.	.
ACa02	16	####	rs1393031	A	G	.	PA:	.	intergen	NOI	dist=	.	.
ACa02	16	####	rs5614462	A	C	.	PA:	.	intergen	NOI	dist=	.	.
ACa02	16	####	rs4967197	T	G	.	PA:	.	intergen	NOI	dist=	.	.
ACa02	16	####	rs4450416	A	G	.	PA:	.	intergen	NOI	dist=	.	.
ACa02	16	####	rs8056404	C	G	.	PA:	.	intergen	NOI	dist=	.	.
ACa02	16	####	rs4246356	C	T	.	PA:	.	intergen	NOI	dist=	.	.
ACa02	16	####	rs8052624	G	T	.	PA:	.	intergen	NOI	dist=	.	.
ACa02	16	####	rs4249654	A	T	.	PA:	.	intergen	NOI	dist=	.	.
ACa02	16	####	rs2885782	G	T	.	PA:	.	intergen	NOI	dist=	.	.
ACa02	16	####	rs6153388	C	T	.	PA:	.	intergen	NOI	dist=	.	.
ACa02	16	####	.	G	C	.	PA:	.	intergen	NOI	dist=	.	.
ACa02	16	####	rs2875315	T	C	.	PA:	.	intergen	NOI	dist=	.	.

ACa02	16	####	.	G	A	.	PA!	.	intergen	NOI	dist=	.	.
ACa02	16	####	rs1479181	C	G	.	PA!	.	intergen	NOI	dist=	.	.
ACa02	16	####	rs8677676	C	T	.	PA!	.	intergen	NOI	dist=	.	.
ACa02	16	####	.	C	G	.	PA!	HYDIN	intronic	NM_	.	.	.
ACa02	16	####	rs1891151	G	C	.	PA!	HP	exonic	NM_	.	mis	HP:NM_
ACa02	16	####	rs1291890	T	C	.	PA!	HPR	intronic	NM_	.	.	.
ACa02	16	####	.	G	T	.	PA!	CNTNAP4	intronic	NM_	.	.	.
ACa02	16	####	.	C	A	.	PA!	ACSF3	exonic	NM_	.	mis	ACSF3:
ACa02	16	####	rs7697160	C	T	.	PA!	ACSF3	exonic	NM_	.	mis	ACSF3:
ACa02	16	####	rs7945920	C	A	.	PA!	.	ncRNA_	NR_	.	.	.
ACa02	16	####	rs3697515	C	A	.	PA!	.	ncRNA_	NR_	.	.	.
ACa02	16	####	rs5596034	A	G	.	PA!	.	ncRNA_	NR_	.	.	.
ACa02	17	####	.	G	A	.	PA!	XAF1	exonic	NM_	.	mis	XAF1:N
ACa02	17	####	.	A	C	.	PA!	CLDN7	intronic	NM_	.	.	.
ACa02	17	####	.	C	T	.	PA!	LOC2840	ncRNA_	NR_	.	.	.
ACa02	17	####	.	G	A	.	PA!	MYH13	intronic	NM_	.	.	.
ACa02	17	####	rs9806119	C	T	.	PA!	DNAH9	intronic	NM_	.	.	.
ACa02	17	####	rs7571703	C	T	.	PA!	DNAH9	intronic	NM_	.	.	.
ACa02	17	####	rs1460005	T	A	.	PA!	CCDC144	intronic	NM_	.	.	.
ACa02	17	####	rs2168782	A	G	.	PA!	.	intergen	NR_	dist=	.	.
ACa02	17	####	.	C	G	.	PA!	FLJ36000	ncRNA_	NR_	.	.	.
ACa02	17	####	rs4362423	T	G	.	PA!	.	intergen	NM_	dist=	.	.
ACa02	17	####	rs4283255	A	G	.	PA!	.	intergen	NM_	dist=	.	.
ACa02	17	####	rs1403689	T	G	.	PA!	.	intergen	NM_	dist=	.	.
ACa02	17	####	rs1405621	C	T	.	PA!	.	intergen	NM_	dist=	.	.
ACa02	17	####	.	G	C	.	PA!	.	intergen	NM_	dist=	.	.
ACa02	17	####	rs7126069	C	T	.	PA!	TBC1D3F	intronic	NM_	.	.	.
ACa02	17	####	rs573849	T	C	.	PA!	.	upstrea	NM_	.	.	.
ACa02	17	####	rs9693530	T	A	.	PA!	.	intergen	NM_	dist=	.	.
ACa02	17	####	.	T	A	.	PA!	LOC4404	ncRNA_	NR_	.	.	.
ACa02	17	####	.	C	G	.	PA!	GPR179	intronic	NM_	.	.	.
ACa02	17	####	.	G	A	.	PA!	CA10	UTR5	NM_	NM_	.	.
ACa02	17	####	rs7516213	G	T	.	PA!	TBC1D3P	ncRNA_	NR_	.	.	.
ACa02	17	####	rs7607356	G	A	.	PA!	AXIN2	exonic	NM_	.	syr	AXIN2:I
ACa02	17	####	.	C	T	.	PA!	SEC14L1	exonic	NM_	.	mis	SEC14I
ACa02	17	####	.	G	A	.	PA!	RBFOX3	intronic	NM_	.	.	.
ACa02	17	####	rs1138327	C	T	.	PA!	BAIAP2-A	ncRNA_	NR_	.	.	.
ACa02	17	####	rs8791354	A	T	.	PA!	TBCD	intronic	NM_	.	.	.
ACa02	18	####	.	G	C	.	PA!	.	intergen	NR_	dist=	.	.
ACa02	18	####	rs8658406	G	A	.	PA!	.	intergen	NM_	dist=	.	.
ACa02	18	####	rs8676712	C	T	.	PA!	.	intergen	NM_	dist=	.	.
ACa02	19	####	.	C	A	.	PA!	GAMT	intronic	NM_	.	.	.
ACa02	19	####	rs3756184	G	A	.	PA!	.	upstrea	NM_	.	.	.
ACa02	19	####	.	G	A	.	PA!	THOP1	UTR5	NM_	NM_	.	.
ACa02	19	####	.	G	T	.	PA!	THOP1	UTR5	NM_	NM_	.	.
ACa02	19	####	rs5460066	G	A	.	PA!	PNPLA6	exonic	NM_	.	mis	PNPLA
ACa02	19	####	.	T	C	.	PA!	SLC1A6	intronic	NM_	.	.	.
ACa02	19	####	.	C	T	.	PA!	CEBPG	UTR5	NM_	NM_	.	.
ACa02	19	####	.	G	A	.	PA!	ARHGAP	intronic	NM_	.	.	.
ACa02	19	####	rs2385185	G	C	.	PA!	.	intergen	NR_	dist=	.	.
ACa02	19	####	rs7125437	C	G	.	PA!	.	intergen	NR_	dist=	.	.
ACa02	19	####	.	G	C	.	PA!	RYR1	intronic	NM_	.	.	.
ACa02	19	####	rs1390553	T	A	.	PA!	.	intergen	NM_	dist=	.	.
ACa02	19	####	rs7714460	G	A	.	PA!	FPR1	exonic	NM_	.	mis	FPR1:N

ACa02	19	####	rs1785512	T	C	.	PA!	TPM3P9	ncRNA_	NR_	.	.	.
ACa02	19	####	rs9207323	G	A	.	PA!	.	intergen	NM_	dist=	.	.
ACa02	20	####	.	T	G	.	PA!	RRBP1	exonic	NM_	.	mis	RRBP1
ACa02	20	####	.	G	A	.	PA!	.	intergen	NR_	dist=	.	.
ACa02	20	####	rs7787265	G	A	.	PA!	ACSS1	exonic	NM_	.	syr	ACSS1
ACa02	20	####	rs3775467	G	A	.	PA!	ACSS1	intronic	NM_	.	.	.
ACa02	20	####	.	C	T	.	PA!	FRG1B	ncRNA_	NR_	.	.	.
ACa02	20	####	.	C	A	.	PA!	L3MBTL1	intronic	NM_	.	.	.
ACa02	20	####	.	G	A	.	PA!	ZSWIM1	intronic	NM_	.	.	.
ACa02	20	####	.	G	A	.	PA!	BIRC7	exonic	NM_	.	mis	BIRC7:I
ACa02	21	####	rs3747993	C	G	.	PA!	.	intergen	NOI	dist=	.	.
ACa02	21	####	rs3730978	G	A	.	PA!	.	intergen	NOI	dist=	.	.
ACa02	21	####	rs2927801	G	A	.	PA!	.	intergen	NR_	dist=	.	.
ACa02	21	####	rs1996169	T	C	.	PA!	BAGE2,BA	intronic	NM_	.	.	.
ACa02	21	####	rs8789866	G	T	.	PA!	BAGE4,BA	intronic	NM_	.	.	.
ACa02	21	####	rs4358221	G	A	.	PA!	.	intergen	NM_	dist=	.	.
ACa02	21	####	.	T	A	.	PA!	.	intergen	NM_	dist=	.	.
ACa02	21	####	rs8664333	A	C	.	PA!	.	upstrear	NM_	.	.	.
ACa02	21	####	rs7967544	T	G	.	PA!	LOC1001	ncRNA_	NR_	.	.	.
ACa02	21	####	.	G	A	.	PA!	TRAPPC1	exonic	NM_	.	syr	TRAPP
ACa02	21	####	.	G	A	.	PA!	DIP2A	intronic	NM_	.	.	.
ACa02	21	####	.	C	T	.	PA!	.	intergen	NM_	dist=	.	.
ACa02	22	####	.	G	A	.	PA!	TBX1	exonic	NM_	.	mis	TBX1:N
ACa02	22	####	rs3702204	C	T	.	PA!	.	intergen	NM_	dist=	.	.
ACa02	22	####	rs1119554	G	T	.	PA!	.	intergen	NM_	dist=	.	.
ACa02	22	####	rs1880252	C	T	.	PA!	POM121L	ncRNA_	NR_	.	.	.
ACa02	22	####	.	G	A	.	PA!	.	intergen	NR_	dist=	.	.
ACa02	X	####	rs1637792	G	C	.	PA!	VCX	exonic	NM_	.	mis	VCX:NI
ACa02	X	####	.	G	A	.	PA!	MID1	intronic	NM_	.	.	.
ACa02	X	####	.	G	A	.	PA!	DCAF8L2	exonic	NM_	.	mis	DCAF8
ACa02	X	####	.	C	A	.	PA!	.	intergen	NM_	dist=	.	.
ACa02	X	####	rs7740495	G	A	.	PA!	FAM47A	exonic	NM_	.	syr	FAM47
ACa02	X	####	.	G	C	.	PA!	CDK16	intronic	NM_	.	.	.
ACa02	X	####	.	G	T	.	PA!	CDK16	UTR5	NM_	NM_	.	.
ACa02	X	####	.	G	T	.	PA!	.	intergen	NOI	dist=	.	.
ACa02	X	####	rs8686279	A	C	.	PA!	.	intergen	NOI	dist=	.	.
ACa02	X	####	.	C	T	.	PA!	.	intergen	NOI	dist=	.	.
ACa02	X	####	.	G	C	.	PA!	.	intergen	NOI	dist=	.	.
ACa02	X	####	.	C	T	.	PA!	MED12	exonic	NM_	.	sto	MED12
ACa02	X	####	.	T	A	.	PA!	GPRASP1	exonic	NM_	.	syr	GPRAS
ACa02	X	####	.	G	A	.	PA!	.	intergen	NM_	dist=	.	.
ACa02	X	####	.	A	G	.	PA!	DCX	intronic	NM_	.	.	.
ACa02	X	####	.	C	A	.	PA!	OCRL	UTR3	NM_	NM_	.	.
ACa02	X	####	.	C	T	.	PA!	IGSF1	intronic	NM_	.	.	.
ACa02	X	####	rs9883463	T	G	.	PA!	MAP7D3	intronic	NM_	.	.	.
ACa02	X	####	.	G	C	.	PA!	FGF13	intronic	NM_	.	.	.
ACa02	X	####	rs7821231	G	A	.	PA!	.	upstrear	NR_	.	.	.
ACa02	X	####	.	G	A	.	PA!	FLNA	intronic	NM_	.	.	.
ACa02	Y	####	rs3769023	C	A	.	PA!	.	intergen	NOI	dist=	.	.
ACa02	MT	759	.	C	T	.	PA!	.	intergen	NOI	dist=	.	.
ACa02	MT	####	rs1057516	C	T	.	PA!	.	intergen	NOI	dist=	.	.
ACa03	1	####	.	A	G	.	PA!	.	intergen	NR_	dist=	.	.
ACa03	1	####	.	G	A	.	PA!	CDK11A,C	intronic	NM_	.	.	.
ACa03	1	####	.	C	T	.	PA!	TP73	exonic	NM_	.	mis	TP73:N

ACa03	1	####	rs7579432	C	T	.	PA!	VPS13D	intronic	NM_	.	.	.
ACa03	1	####	rs5565927	T	C	.	PA!	NBPF1	intronic	NM_	.	.	.
ACa03	1	####	rs7619917	T	C	.	PA!	NBPF1	intronic	NM_	.	.	.
ACa03	1	####	rs663828	T	C	.	PA!	NBPF1	intronic	NM_	.	.	.
ACa03	1	####	rs646524	T	G	.	PA!	NBPF1	intronic	NM_	.	.	.
ACa03	1	####	rs4596945	G	C	.	PA!	NBPF1	intronic	NM_	.	.	.
ACa03	1	####	rs2296161	C	T	.	PA!	.	upstreal	NR_	.	.	.
ACa03	1	####	rs5616387	C	T	.	PA!	EMC1	exonic	NM_	.	mis	EMC1:N
ACa03	1	####	.	G	A	.	PA!	ZC3H12A	intronic	NM_	.	.	.
ACa03	1	####	rs9490484	G	T	.	PA!	HECTD3	intronic	NM_	.	.	.
ACa03	1	####	.	T	G	.	PA!	ANGPTL3	UTR3	NM_	NM_	.	.
ACa03	1	####	.	G	T	.	PA!	ERICH3	exonic	NM_	.	mis	ERICH3
ACa03	1	####	.	C	T	.	PA!	RABGGTE	intronic	NM_	.	.	.
ACa03	1	####	.	G	A	.	PA!	NHLH2	exonic	NM_	.	mis	NHLH2
ACa03	1	####	rs1409433	T	C	.	PA!	.	intergen	NR_	dist=	.	.
ACa03	1	####	rs2022010	G	A	.	PA!	.	intergen	NR_	dist=	.	.
ACa03	1	####	rs7763460	C	G	.	PA!	.	ncRNA_	NR_	.	.	.
ACa03	1	####	rs1457064	G	A	.	PA!	.	intergen	NR_	dist=	.	.
ACa03	1	####	.	C	G	.	PA!	NBPF20	intronic	NM_	.	.	.
ACa03	1	####	rs1148852	G	C	.	PA!	NBPF9,NE	intronic	NM_	.	.	.
ACa03	1	####	rs3746294	A	G	.	PA!	NBPF20,F	intronic	NM_	.	.	.
ACa03	1	####	rs3675661	C	G	.	PA!	NBPF25P	ncRNA_	NR_	.	.	.
ACa03	1	####	rs7986155	T	A	.	PA!	NBPF8	intronic	NM_	.	.	.
ACa03	1	####	rs7593611	G	A	.	PA!	MEX3A	exonic	NM_	.	syr	MEX3A
ACa03	1	####	.	G	T	.	PA!	OR6P1	exonic	NM_	.	mis	OR6P1
ACa03	1	####	.	G	C	.	PA!	LAD1	intronic	NM_	.	.	.
ACa03	1	####	rs7508890	G	A	.	PA!	PPFIA4	exonic	NM_	.	mis	PPFIA4
ACa03	1	####	rs1454313	A	G	.	PA!	LEFTY1	exonic	NM_	.	mis	LEFTY1
ACa03	1	####	rs3720052	C	T	.	PA!	OR6F1	exonic	NM_	.	mis	OR6F1:
ACa03	1	####	rs8789457	A	G	.	PA!	.	upstreal	NM_	.	.	.
ACa03	2	####	.	C	A	.	PA!	VSNL1	exonic	NM_	.	mis	VSNL1:
ACa03	2	####	.	T	A	.	PA!	LRRTM4	UTR5	NM_	NM_	.	.
ACa03	2	####	.	G	A	.	PA!	LRRTM1	exonic	NM_	.	syr	LRRTM
ACa03	2	####	rs2013675	G	A	.	PA!	.	intergen	NR_	dist=	.	.
ACa03	2	####	.	G	C	.	PA!	RGPD1,R	intronic	NM_	.	.	.
ACa03	2	####	.	T	C	.	PA!	.	intergen	NM_	dist=	.	.
ACa03	2	####	rs1473449	G	T	.	PA!	UBR3	intronic	NM_	.	.	.
ACa03	2	####	.	C	T	.	PA!	ITGA4	exonic	NM_	.	mis	ITGA4:I
ACa03	2	####	.	C	T	.	PA!	ATIC	splicing	NM_	NM_	.	.
ACa03	2	####	.	C	A	.	PA!	.	intergen	NR_	dist=	.	.
ACa03	3	####	rs1050893	G	C	.	PA!	KIF9	intronic	NM_	.	.	.
ACa03	3	####	.	C	T	.	PA!	KIF9	intronic	NM_	.	.	.
ACa03	3	####	rs1178604	G	A	.	PA!	CACNA2D	intronic	NM_	.	.	.
ACa03	3	####	rs3744125	G	A	.	PA!	.	intergen	NM_	dist=	.	.
ACa03	3	####	rs1451899	C	T	.	PA!	ROBO2	intronic	NM_	.	.	.
ACa03	3	####	.	G	A	.	PA!	MGLL	intronic	NM_	.	.	.
ACa03	3	####	.	A	C	.	PA!	NUDT16	UTR3	NM_	NM_	.	.
ACa03	3	####	.	A	C	.	PA!	IL20RB	intronic	NM_	.	.	.
ACa03	3	####	.	T	G	.	PA!	ARMC8	exonic	NM_	.	syr	ARMC8
ACa03	3	####	rs1057519	G	A	.	PA!	PIK3CA	exonic	NM_	.	mis	PIK3CA
ACa03	3	####	rs1048860	G	A	.	PA!	PIK3CA	exonic	NM_	.	mis	PIK3CA
ACa03	3	####	.	C	A	.	PA!	FYTTD1	intronic	NM_	.	.	.
ACa03	4	####	rs3725657	G	A	.	PA!	ZFYVE28	exonic	NM_	.	mis	ZFYVE2
ACa03	4	####	.	A	G	.	PA!	JAKMIP1	splicing	NM_	NM_	.	.

ACa03	4	####	rs8796734	A	G	.	PA!	.	upstrea	NM_	.	.	.
ACa03	4	####	rs5941177	C	T	.	PA!	.	intergen	NM_	dist=	.	.
ACa03	4	####	.	C	T	.	PA!	NPFFR2	exonic	NM_	.	mis	NPFFR
ACa03	4	####	.	A	C	.	PA!	SPOCK3	intronic	NM_	.	.	.
ACa03	5	####	rs4288119	G	A	.	PA!	.	intergen	NR_	dist=	.	.
ACa03	5	####	rs8669155	C	T	.	PA!	.	intergen	NR_	dist=	.	.
ACa03	5	####	rs7765625	G	A	.	PA!	JADE2	exonic	NM_	.	mis	JADE2:
ACa03	5	####	rs5593800	C	T	.	PA!	.	intergen	NM_	dist=	.	.
ACa03	5	####	.	G	A	.	PA!	.	intergen	NR_	dist=	.	.
ACa03	6	####	.	A	C	.	PA!	BPHL	intronic	NM_	.	.	.
ACa03	6	####	rs1513411	G	C	.	PA!	HLA-B	exonic	NM_	.	mis	HLA-B:I
ACa03	6	####	.	C	A	.	PA!	EHMT2	exonic	NM_	.	mis	EHMT2
ACa03	6	####	rs7668272	C	T	.	PA!	PPARD	exonic	NM_	.	mis	PPARD
ACa03	6	####	.	G	A	.	PA!	TNFRSF2	intronic	NM_	.	.	.
ACa03	6	####	.	T	A	.	PA!	GSTA2	intronic	NM_	.	.	.
ACa03	6	####	.	T	G	.	PA!	COL12A1	intronic	NM_	.	.	.
ACa03	7	####	.	C	T	.	PA!	THSD7A	intronic	NM_	.	.	.
ACa03	7	####	.	C	T	.	PA!	C7orf31	intronic	NM_	.	.	.
ACa03	7	####	.	G	A	.	PA!	HECW1	exonic	NM_	.	mis	HECW1
ACa03	7	####	.	T	G	.	PA!	GCK	intronic	NM_	.	.	.
ACa03	7	####	.	C	T	.	PA!	.	intergen	NM_	dist=	.	.
ACa03	7	####	rs1439657	G	A	.	PA!	.	intergen	NR_	dist=	.	.
ACa03	7	####	.	C	T	.	PA!	.	intergen	NR_	dist=	.	.
ACa03	7	####	rs8799634	G	T	.	PA!	.	intergen	NR_	dist=	.	.
ACa03	7	####	.	A	G	.	PA!	.	intergen	NR_	dist=	.	.
ACa03	7	####	rs2002309	C	G	.	PA!	.	intergen	NR_	dist=	.	.
ACa03	7	####	.	C	A	.	PA!	.	intergen	NR_	dist=	.	.
ACa03	7	####	rs2018986	T	C	.	PA!	.	intergen	NR_	dist=	.	.
ACa03	7	####	.	C	T	.	PA!	SPDYE5	intronic	NM_	.	.	.
ACa03	7	####	.	C	T	.	PA!	SPDYE5	intronic	NM_	.	.	.
ACa03	7	####	.	G	A	.	PA!	.	intergen	NM_	dist=	.	.
ACa03	7	####	.	G	A	.	PA!	GNAI1	exonic	NM_	.	mis	GNAI1:
ACa03	7	####	.	C	T	.	PA!	SAMD9	exonic	NM_	.	mis	SAMD9
ACa03	7	####	rs2676016	C	T	.	PA!	PDK4	exonic	NM_	.	mis	PDK4:N
ACa03	7	####	rs1826826	G	A	.	PA!	NYAP1	intronic	NM_	.	.	.
ACa03	7	####	rs1409779	C	T	.	PA!	ST7	intronic	NM_	.	.	.
ACa03	7	####	.	A	C	.	PA!	NOBOX	intronic	NM_	.	.	.
ACa03	7	####	.	G	C	.	PA!	KMT2C	intronic	NM_	.	.	.
ACa03	7	####	rs7962502	C	T	.	PA!	.	intergen	NM_	dist=	.	.
ACa03	8	####	.	G	C	.	PA!	ANGPT2	exonic	NM_	.	syr	ANGPT
ACa03	8	####	rs3763515	G	A	.	PA!	.	intergen	NR_	dist=	.	.
ACa03	8	####	rs5673338	T	A	.	PA!	.	intergen	NR_	dist=	.	.
ACa03	8	####	.	C	G	.	PA!	LOC1019	ncRNA_	NR_	.	.	.
ACa03	8	####	.	C	T	.	PA!	SLC26A7	intronic	NM_	.	.	.
ACa03	8	####	.	G	A	.	PA!	VPS13B	intronic	NM_	.	.	.
ACa03	8	####	rs5609552	T	C	.	PA!	KCNQ3	intronic	NM_	.	.	.
ACa03	9	####	.	G	A	.	PA!	FOCAD	intronic	NM_	.	.	.
ACa03	9	####	rs1463382	C	T	.	PA!	IL11RA	intronic	NM_	.	.	.
ACa03	9	####	rs7495042	G	A	.	PA!	.	upstrea	NR_	.	.	.
ACa03	9	####	.	C	T	.	PA!	.	ncRNA_	NR_	.	.	.
ACa03	9	####	rs7714176	G	A	.	PA!	AQP7P1	ncRNA_	NR_	.	.	.
ACa03	9	####	rs4452915	C	T	.	PA!	.	intergen	NM_	dist=	.	.
ACa03	9	####	rs7492487	T	C	.	PA!	.	intergen	NM_	dist=	.	.
ACa03	9	####	rs391727	G	C	.	PA!	PGM5	intronic	NM_	.	.	.

ACa03	9	####	.	G	A	.	PA!	.	intergen	NM	dist=	.	.
ACa03	9	####	.	G	T	.	PA!	SLC28A3	intronic	NM_	.	.	.
ACa03	9	####	rs2855049	T	C	.	PA!	CEL	intronic	NM_	.	.	.
ACa03	9	####	rs7771119	G	A	.	PA!	NOTCH1	exonic	NM_	.	syr	NOTCH
ACa03	10	####	.	G	A	.	PA!	CAMK1D	exonic	NM_	.	mis	CAMK1
ACa03	10	####	.	G	A	.	PA!	CACNB2	intronic	NM_	.	.	.
ACa03	10	####	rs7747854	G	A	.	PA!	CCNY	UTR3	NM	NM_	.	.
ACa03	10	####	rs1759035	A	G	.	PA!	ANKRD30	intronic	NM_	.	.	.
ACa03	10	####	rs3125109	G	A	.	PA!	.	intergen	NR	dist=	.	.
ACa03	10	####	.	G	A	.	PA!	TBATA	intronic	NM_	.	.	.
ACa03	10	####	rs2018088	G	A	.	PA!	VCL	exonic	NM_	.	mis	VCL:NM
ACa03	10	####	.	A	G	.	PA!	.	intergen	NM	dist=	.	.
ACa03	10	####	rs3128226	C	G	.	PA!	NUTM2A	intronic	NM_	.	.	.
ACa03	10	####	.	G	A	.	PA!	.	intergen	NM	dist=	.	.
ACa03	10	####	.	C	G	.	PA!	RAB11FIF	UTR3	NM	NM_	.	.
ACa03	10	####	rs5495128	C	T	.	PA!	.	intergen	NM	dist=	.	.
ACa03	10	####	.	C	A	.	PA!	CPXM2	exonic	NM_	.	mis	CPXM2
ACa03	11	####	.	G	T	.	PA!	TSPAN18	intronic	NM_	.	.	.
ACa03	11	####	.	C	T	.	PA!	C11orf80	intronic	NM_	.	.	.
ACa03	11	####	.	A	T	.	PA!	MIR5692A	ncRNA_	NR_	.	.	.
ACa03	12	####	rs7827727	T	C	.	PA!	LOC1002	ncRNA_	NR_	.	.	.
ACa03	12	####	.	C	A	.	PA!	AKAP3	UTR5	NM	NM_	.	.
ACa03	12	####	.	G	C	.	PA!	CHD4	exonic	NM_	.	mis	CHD4:N
ACa03	12	####	.	G	C	.	PA!	ZNF384	intronic	NM_	.	.	.
ACa03	12	####	.	C	A	.	PA!	ZNF705A	intronic	NM_	.	.	.
ACa03	12	####	rs1219135	C	T	.	PA!	KRAS	exonic	NM_	.	mis	KRAS:N
ACa03	12	####	rs3731379	C	T	.	PA!	ITPR2	exonic	NM_	.	mis	ITPR2:N
ACa03	12	####	rs4031316	T	C	.	PA!	DDX11	intronic	NM_	.	.	.
ACa03	12	####	rs5661873	C	T	.	PA!	PUS7L	exonic	NM_	.	mis	PUS7L:
ACa03	12	####	rs9815586	G	A	.	PA!	FAM186A	exonic	NM_	.	sto	FAM18
ACa03	12	####	rs1409167	G	A	.	PA!	MMP19	intronic	NM_	.	.	.
ACa03	12	####	.	C	A	.	PA!	HIP1R	intronic	NM_	.	.	.
ACa03	12	####	.	G	A	.	PA!	DNAH10	intronic	NM_	.	.	.
ACa03	12	####	rs1707911	T	C	.	PA!	.	intergen	NM	dist=	.	.
ACa03	12	####	rs7662808	G	A	.	PA!	DDX51	exonic	NM_	.	mis	DDX51:
ACa03	13	####	rs9079318	C	T	.	PA!	.	intergen	NR	dist=	.	.
ACa03	13	####	.	A	C	.	PA!	GSX1	intronic	NM_	.	.	.
ACa03	13	####	rs7666774	G	A	.	PA!	FLT3	exonic	NM_	.	sto	FLT3:N
ACa03	13	####	.	C	T	.	PA!	DCLK1	exonic	NM_	.	mis	DCLK1:
ACa03	13	####	.	C	T	.	PA!	SPACA7	intronic	NM_	.	.	.
ACa03	14	####	rs2018046	T	G	.	PA!	LOC1019	ncRNA_	NR_	.	.	.
ACa03	14	####	rs4982820	G	A	.	PA!	.	intergen	NM	dist=	.	.
ACa03	14	####	rs2005137	C	T	.	PA!	.	intergen	NM	dist=	.	.
ACa03	14	####	.	T	G	.	PA!	.	intergen	NR	dist=	.	.
ACa03	14	####	.	T	C	.	PA!	.	intergen	NM	dist=	.	.
ACa03	14	####	rs3763109	C	T	.	PA!	.	intergen	NM	dist=	.	.
ACa03	14	####	.	G	T	.	PA!	.	intergen	NM	dist=	.	.
ACa03	14	####	rs7592315	G	A	.	PA!	LRRC16B	intronic	NM_	.	.	.
ACa03	14	####	.	T	A	.	PA!	.	intergen	NM	dist=	.	.
ACa03	14	####	rs7473295	G	A	.	PA!	.	intergen	NR	dist=	.	.
ACa03	15	####	rs5686085	C	T	.	PA!	.	intergen	NOI	dist=	.	.
ACa03	15	####	rs5761409	G	C	.	PA!	.	intergen	NOI	dist=	.	.
ACa03	15	####	rs3684417	G	T	.	PA!	.	intergen	NM	dist=	.	.
ACa03	15	####	rs1130343	A	C	.	PA!	.	intergen	NR	dist=	.	.

ACa03	15	####	rs1995519	C	G	.	PA!	.	intergen	NR_	dist=	.	.
ACa03	15	####	rs1836337	A	G	.	PA!	.	upstrea	NR_	.	.	.
ACa03	15	####	rs3700524	G	A	.	PA!	.	intronic	NM_	.	.	.
ACa03	15	####	rs3766397	G	A	.	PA!	GOLGA8S	ncRNA_	NR_	.	.	.
ACa03	15	####	.	G	A	.	PA!	CHRFAM7	intronic	NM_	.	.	.
ACa03	15	####	rs5573362	C	T	.	PA!	SPINT1	exonic	NM_	.	syr	SPINT1
ACa03	15	####	.	C	T	.	PA!	.	downstr	NM_	.	.	.
ACa03	15	####	rs7443087	A	G	.	PA!	GOLGA6E	exonic	NM_	.	mis	GOLGA
ACa03	15	####	.	G	A	.	PA!	GOLGA6L	intronic	NM_	.	.	.
ACa03	16	####	.	T	C	.	PA!	IFT140	exonic	NM_	.	mis	IFT140:
ACa03	16	####	.	A	T	.	PA!	IL32	exonic	NM_	.	syr	IL32:NM
ACa03	16	####	.	C	T	.	PA!	SNX29	intronic	NM_	.	.	.
ACa03	16	####	.	G	A	.	PA!	RRN3P2	ncRNA_	NR_	.	.	.
ACa03	16	####	rs2887413	C	G	.	PA!	.	intergen	NOI	dist=	.	.
ACa03	16	####	rs4966607	C	T	.	PA!	.	intergen	NOI	dist=	.	.
ACa03	16	####	rs4359628	T	G	.	PA!	.	intergen	NOI	dist=	.	.
ACa03	16	####	rs4249500	G	A	.	PA!	.	intergen	NOI	dist=	.	.
ACa03	16	####	rs4372866	C	T	.	PA!	.	intergen	NOI	dist=	.	.
ACa03	16	####	rs4396558	T	A	.	PA!	.	intergen	NOI	dist=	.	.
ACa03	16	####	rs4249160	C	T	.	PA!	.	intergen	NOI	dist=	.	.
ACa03	16	####	rs4967780	G	C	.	PA!	.	intergen	NOI	dist=	.	.
ACa03	16	####	rs7203681	C	T	.	PA!	.	intergen	NOI	dist=	.	.
ACa03	16	####	rs7826866	G	A	.	PA!	.	intergen	NOI	dist=	.	.
ACa03	16	####	rs4249045	T	C	.	PA!	.	intergen	NOI	dist=	.	.
ACa03	16	####	rs7188365	G	T	.	PA!	.	intergen	NOI	dist=	.	.
ACa03	16	####	rs9972801	A	G	.	PA!	.	intergen	NOI	dist=	.	.
ACa03	16	####	rs4249048	G	C	.	PA!	.	intergen	NOI	dist=	.	.
ACa03	16	####	rs7823469	C	A	.	PA!	.	intergen	NOI	dist=	.	.
ACa03	16	####	rs1393031	A	G	.	PA!	.	intergen	NOI	dist=	.	.
ACa03	16	####	rs4090116	C	T	.	PA!	.	intergen	NOI	dist=	.	.
ACa03	16	####	rs4090115	C	T	.	PA!	.	intergen	NOI	dist=	.	.
ACa03	16	####	rs4459567	C	T	.	PA!	.	intergen	NOI	dist=	.	.
ACa03	16	####	rs6133865	A	T	.	PA!	.	intergen	NOI	dist=	.	.
ACa03	16	####	rs4341908	A	C	.	PA!	.	intergen	NOI	dist=	.	.
ACa03	16	####	rs4451975	A	C	.	PA!	.	intergen	NOI	dist=	.	.
ACa03	16	####	rs7187636	T	C	.	PA!	.	intergen	NOI	dist=	.	.
ACa03	16	####	.	C	A	.	PA!	PDXDC2F	ncRNA_	NR_	.	.	.
ACa03	16	####	rs2004697	A	G	.	PA!	PDPR	exonic	NM_	.	mis	PDPR:N
ACa03	16	####	.	A	C	.	PA!	.	upstrea	NM_	.	.	.
ACa03	16	####	.	C	G	.	PA!	FANCA	exonic	NM_	.	mis	FANCA
ACa03	16	####	.	C	A	.	PA!	SPIRE2	intronic	NM_	.	.	.
ACa03	17	####	.	T	G	.	PA!	SMYD4	intronic	NM_	.	.	.
ACa03	17	####	.	G	A	.	PA!	GAS7	UTR5	NM_	NM_	.	.
ACa03	17	####	.	G	T	.	PA!	ATPAF2	UTR5	NM_	NM_	.	.
ACa03	17	####	rs1440233	G	A	.	PA!	.	intergen	NM_	dist=	.	.
ACa03	17	####	rs4362423	T	G	.	PA!	.	intergen	NM_	dist=	.	.
ACa03	17	####	rs1409172	A	G	.	PA!	.	intergen	NM_	dist=	.	.
ACa03	17	####	rs3737234	C	A	.	PA!	.	intergen	NR_	dist=	.	.
ACa03	17	####	.	C	T	.	PA!	ARHGAP2	exonic	NM_	.	mis	ARHGA
ACa03	17	####	.	A	G	.	PA!	ARHGAP2	intronic	NM_	.	.	.
ACa03	17	####	rs8679073	A	T	.	PA!	GSDMA	intronic	NM_	.	.	.
ACa03	17	####	.	G	A	.	PA!	.	intergen	NM_	dist=	.	.
ACa03	17	####	.	G	A	.	PA!	KANSL1	intronic	NM_	.	.	.
ACa03	17	####	rs7651139	C	T	.	PA!	CACNG5	exonic	NM_	.	mis	CACNG

ACa03	17	####	rs7754213	G	A	.	PA!	MYO15B	ncRNA_	NR_	.	.	.
ACa03	17	####	.	G	A	.	PA!	MGAT5B	intronic	NM_	.	.	.
ACa03	17	####	rs5408783	C	T	.	PA!	9-Sep	intronic	NM_	.	.	.
ACa03	17	####	.	G	A	.	PA!	CCDC137	exonic	NM_	.	syr	CCDC1
ACa03	17	####	.	C	T	.	PA!	GCGR	intronic	NM_	.	.	.
ACa03	17	####	rs3732207	C	T	.	PA!	FASN	intronic	NM_	.	.	.
ACa03	18	####	.	C	T	.	PA!	CIDEA	exonic	NM_	.	syr	CIDEA:
ACa03	19	####	rs7575217	C	T	.	PA!	SEMA6B	exonic	NM_	.	syr	SEMA6
ACa03	19	####	.	G	A	.	PA!	KHSRP	intronic	NM_	.	.	.
ACa03	19	####	.	G	T	.	PA!	XAB2	intronic	NM_	.	.	.
ACa03	19	####	.	G	T	.	PA!	TIMM44	intronic	NM_	.	.	.
ACa03	19	####	.	C	A	.	PA!	FBN3	intronic	NM_	.	.	.
ACa03	19	####	rs1492924	G	A	.	PA!	.	intergen	NM_	dist=	.	.
ACa03	19	####	rs1999730	T	C	.	PA!	CEP89	intronic	NM_	.	.	.
ACa03	19	####	rs3762437	G	T	.	PA!	.	intergen	NR_	dist=	.	.
ACa03	19	####	rs7125437	C	G	.	PA!	.	intergen	NR_	dist=	.	.
ACa03	19	####	.	C	T	.	PA!	SPTBN4	exonic	NM_	.	syr	SPTBN
ACa03	19	####	.	A	C	.	PA!	EXOSC5	intronic	NM_	.	.	.
ACa03	19	####	.	C	G	.	PA!	.	intergen	NM_	dist=	.	.
ACa03	19	####	.	C	A	.	PA!	PLA2G4C	exonic	NM_	.	mis	PLA2G4
ACa03	19	####	rs6212787	C	A	.	PA!	CGB8	intronic	NM_	.	.	.
ACa03	19	####	.	A	C	.	PA!	KLK15	intronic	NM_	.	.	.
ACa03	19	####	rs5492748	T	C	.	PA!	TPM3P9	ncRNA_	NR_	.	.	.
ACa03	19	####	.	T	C	.	PA!	.	downstr	NR_	.	.	.
ACa03	19	####	rs7600928	G	A	.	PA!	PRKCG	exonic	NM_	.	syr	PRKCG
ACa03	19	####	.	G	T	.	PA!	ZNF211	intronic	NM_	.	.	.
ACa03	19	####	rs7704038	T	G	.	PA!	ZNF814	exonic	NM_	.	mis	ZNF814
ACa03	19	####	.	C	T	.	PA!	MZF1-AS1	ncRNA_	NR_	.	.	.
ACa03	20	####	.	C	T	.	PA!	TGM6	intronic	NM_	.	.	.
ACa03	20	####	.	G	T	.	PA!	.	intergen	NM_	dist=	.	.
ACa03	20	####	.	C	A	.	PA!	MIR663AH	ncRNA_	NR_	.	.	.
ACa03	20	####	rs9569348	A	G	.	PA!	FRG1B	ncRNA_	NR_	.	.	.
ACa03	20	####	.	C	A	.	PA!	WFDC2	intronic	NM_	.	.	.
ACa03	21	####	rs3772733	C	T	.	PA!	.	intergen	NR_	dist=	.	.
ACa03	21	####	rs9184726	C	T	.	PA!	BAGE,BA(	intronic	NM_	.	.	.
ACa03	21	####	rs9266067	A	G	.	PA!	.	intergen	NM_	dist=	.	.
ACa03	21	####	.	G	A	.	PA!	.	intergen	NM_	dist=	.	.
ACa03	21	####	.	C	T	.	PA!	DONSON	intronic	NM_	.	.	.
ACa03	21	####	rs7967544	T	G	.	PA!	LOC10013	ncRNA_	NR_	.	.	.
ACa03	22	####	rs2019683	C	T	.	PA!	.	intergen	NM_	dist=	.	.
ACa03	22	####	rs7543647	C	T	.	PA!	LZTR1	intronic	NM_	.	.	.
ACa03	22	####	rs5711604	G	A	.	PA!	TTC28-AS	ncRNA_	NR_	.	.	.
ACa03	22	####	rs1856371	C	T	.	PA!	.	intergen	NM_	dist=	.	.
ACa03	22	####	rs3713772	C	T	.	PA!	MCM5	exonic	NM_	.	syr	MCM5:I
ACa03	22	####	.	G	A	.	PA!	TMEM184	intronic	NM_	.	.	.
ACa03	22	####	.	G	A	.	PA!	NPTXR	UTR3	NM_	NM_	.	.
ACa03	22	####	.	C	A	.	PA!	LINC0089	ncRNA_	NR_	.	.	.
ACa03	X	####	rs6644312	G	T	.	PA!	.	intergen	NM_	dist=	.	.
ACa03	X	####	rs2010857	T	C	.	PA!	SHROOM	intronic	NM_	.	.	.
ACa03	X	####	.	C	T	.	PA!	WWC3	exonic	NM_	.	mis	WWC3
ACa03	X	####	rs7457127	C	T	.	PA!	SRPX	exonic	NM_	.	mis	SRPX:N
ACa03	X	####	.	G	A	.	PA!	WAS	intronic	NM_	.	.	.
ACa03	X	####	rs7825308	G	A	.	PA!	CACNA1F	exonic	NM_	.	mis	CACNA
ACa03	X	####	.	G	C	.	PA!	.	intergen	NOI	dist=	.	.

ACa03	X	####	.	T	C	.	PA!	.	intergen	NOI	dist=	.	.	
ACa03	X	####	rs7756689	A	G	.	PA!	.	intergen	NOI	dist=	.	.	
ACa03	X	####	rs2486782	A	T	.	PA!	CHIC1	intronic	NM_	.	.	.	
ACa03	X	####	.	G	A	.	PA!	AMOT	exonic	NM_	.	mis	AMOT:I	
ACa03	X	####	.	T	C	.	PA!	C1GALT1	exonic	NM_	.	mis	C1GAL	
ACa03	X	####	.	C	T	.	PA!	.	upstrea	NM_	.	.	.	
ACa03	X	####	rs1016097	C	A	.	PA!	MCF2	intronic	NM_	.	.	.	
ACa03	X	####	.	G	A	.	PA!	NSDHL	intronic	NM_	.	.	.	
ACa03	X	####	rs7802961	G	A	.	PA!	.	intergen	NM_	dist=	.	.	
ACa03	MT	4451	.	T	C	.	PA!	.	intergen	NOI	dist=	.	.	
ACa03	MT	5777	rs3868289	G	A	.	PA!	.	intergen	NOI	dist=	.	.	
ACa03	MT	####	.	C	A	.	PA!	.	intergen	NOI	dist=	.	.	
ACa04	1	####	rs7466465	C	T	.	PA!	ATAD3C	exonic	NM_	.	syr	ATAD3	
ACa04	1	####	.	C	G	.	PA!	CDK11B	intronic	NM_	.	.	.	
ACa04	1	####	rs1454709	C	T	.	PA!	FBXO2	exonic	NM_	.	mis	FBXO2	
ACa04	1	####	.	C	T	.	PA!	MAD2L2	exonic	NM_	.	mis	MAD2L	
ACa04	1	####	rs1440990	G	A	.	PA!	PRAMEF2	UTR3	NM_	NM_	.	.	
ACa04	1	####	rs1157686	G	C	.	PA!	.	intergen	NM_	dist=	.	.	
ACa04	1	####	.	T	A	.	PA!	.	upstrea	NR_	.	.	.	
ACa04	1	####	rs7511577	C	T	.	PA!	.	intergen	NM_	dist=	.	.	
ACa04	1	####	.	G	C	.	PA!	SH2D5	exonic	NM_	.	mis	SH2D5:	
ACa04	1	####	rs5551428	C	T	.	PA!	TCEA3	intronic	NM_	.	.	.	
ACa04	1	####	rs1422489	G	A	.	PA!	GRHL3	exonic	NM_	.	mis	GRHL3	
ACa04	1	####	.	G	A	.	PA!	RHD	intronic	NM_	.	.	.	
ACa04	1	####	.	G	A	.	PA!	COL16A1	intronic	NM_	.	.	.	
ACa04	1	####	rs7753177	G	A	.	PA!	RNF19B	exonic	NM_	.	unl	UNKNC	
ACa04	1	####	.	C	T	.	PA!	SNIP1	exonic	NM_	.	mis	SNIP1:I	
ACa04	1	####	.	G	C	.	PA!	MACF1	exonic	NM_	.	mis	MACF1	
ACa04	1	####	.	G	T	.	PA!	MACF1	intronic	NM_	.	.	.	
ACa04	1	####	.	G	T	.	PA!	MACF1	intronic	NM_	.	.	.	
ACa04	1	####	.	G	C	.	PA!	MACF1	intronic	NM_	.	.	.	
ACa04	1	####	.	A	G	.	PA!	SCMH1	intronic	NM_	.	.	.	
ACa04	1	####	rs7580073	C	T	.	PA!	KLF17	exonic	NM_	.	mis	KLF17:I	
ACa04	1	####	rs8662033	C	A	.	PA!	.	intergen	NM_	dist=	.	.	
ACa04	1	####	.	C	T	.	PA!	CYB5RL	intronic	NM_	.	.	.	
ACa04	1	####	.	C	G	.	PA!	INADL	intronic	NM_	.	.	.	
ACa04	1	####	.	G	A	.	PA!	.	intergen	NR_	dist=	.	.	
ACa04	1	####	.	G	A	.	PA!	FPGT-TNI	intronic	NM_	.	.	.	
ACa04	1	####	.	G	A	.	PA!	EVI5	intronic	NM_	.	.	.	
ACa04	1	####	.	C	A	.	PA!	ABCA4	intronic	NM_	.	.	.	
ACa04	1	####	.	G	A	.	PA!	SNX7	intronic	NM_	.	.	.	
ACa04	1	####	rs7598590	G	A	.	PA!	CD53	intronic	NM_	.	.	.	
ACa04	1	####	.	G	A	.	PA!	LOC6433	exonic	NM_	.	syr	LOC64	
ACa04	1	####	rs2004173	G	A	.	PA!	FCGR1B	UTR3	NM_	NM_	.	.	
ACa04	1	####	rs1043712	C	T	.	PA!	.	intergen	NOI	dist=	.	.	
ACa04	1	####	.	C	T	.	PA!	.	intergen	NR_	dist=	.	.	
ACa04	1	####	rs2002777	G	A	.	PA!	.	intergen	NR_	dist=	.	.	
ACa04	1	####	rs7506054	A	G	.	PA!	.	intergen	NR_	dist=	.	.	
ACa04	1	####	rs1694653	C	G	.	PA!	.	intergen	NR_	dist=	.	.	
ACa04	1	####	rs4125366	A	T	.	PA!	NBPF20,N	intronic	NM_	.	.	.	
ACa04	1	####	.	T	G	.	PA!	NBPF25P	ncRNA_	NR_	.	.	.	
ACa04	1	####	.	A	G	.	PA!	NBPF25P	ncRNA_	NR_	.	.	.	
ACa04	1	####	rs6181199	G	A	.	PA!	NBPF11,N	intronic	NM_	.	.	.	
ACa04	1	####	rs8799948	C	A	.	PA!	NBPF8	intronic	NM_	.	.	.	

ACa04	1	####	rs482979	C	T	.	PA!	NBPF25P	ncRNA_	NR_	.	.	.
ACa04	1	####	rs2021032	T	G	.	PA!	NBPF25P	ncRNA_	NR_	.	.	.
ACa04	1	####	rs2009700	C	G	.	PA!	NBPF25P	ncRNA_	NR_	.	.	.
ACa04	1	####	rs2003739	G	A	.	PA!	NBPF25P	ncRNA_	NR_	.	.	.
ACa04	1	####	rs3705433	G	A	.	PA!	NBPF25P	ncRNA_	NR_	.	.	.
ACa04	1	####	rs226753	G	C	.	PA!	NBPF25P	ncRNA_	NR_	.	.	.
ACa04	1	####	.	G	A	.	PA!	NBPF25P	ncRNA_	NR_	.	.	.
ACa04	1	####	rs2787777	C	T	.	PA!	.	intergen	NR_	dist=	.	.
ACa04	1	####	rs3750007	T	A	.	PA!	.	downstr	NM_	.	.	.
ACa04	1	####	.	C	T	.	PA!	.	intergen	NM_	dist=	.	.
ACa04	1	####	.	C	G	.	PA!	INTS3	intronic	NM_	.	.	.
ACa04	1	####	.	C	A	.	PA!	SLC27A3	intronic	NM_	.	.	.
ACa04	1	####	.	A	T	.	PA!	SLC50A1	exonic	NM_	.	syr	SLC50/
ACa04	1	####	.	G	T	.	PA!	ARHGEF2	intronic	NM_	.	.	.
ACa04	1	####	.	C	T	.	PA!	PEAR1	intronic	NM_	.	.	.
ACa04	1	####	.	C	T	.	PA!	PEAR1	exonic	NM_	.	syr	PEAR1
ACa04	1	####	.	C	T	.	PA!	TNFSF4	intronic	NM_	.	.	.
ACa04	1	####	.	C	G	.	PA!	ASPM	exonic	NM_	.	mi!	ASPM:!
ACa04	1	####	rs1997462	G	A	.	PA!	KIF21B	intronic	NM_	.	.	.
ACa04	1	####	rs3692540	G	A	.	PA!	PLEKHA6	exonic	NM_	.	mi!	PLEKH/
ACa04	1	####	.	G	A	.	PA!	C1orf115	exonic	NM_	.	mi!	C1orf11
ACa04	1	####	.	C	T	.	PA!	CAPN2	exonic	NM_	.	mi!	CAPN2
ACa04	1	####	.	G	A	.	PA!	.	intergen	NM_	dist=	.	.
ACa04	1	####	rs7793949	G	A	.	PA!	OBSCN	intronic	NM_	.	.	.
ACa04	2	####	.	C	T	.	PA!	ASAP2	UTR5	NM_	NM_	.	.
ACa04	2	####	.	C	G	.	PA!	TAF1B	intronic	NM_	.	.	.
ACa04	2	####	.	G	C	.	PA!	SF3B6	exonic	NM_	.	syr	SF3B6:
ACa04	2	####	.	G	C	.	PA!	CAD	intronic	NM_	.	.	.
ACa04	2	####	.	C	A	.	PA!	.	intergen	NM_	dist=	.	.
ACa04	2	####	.	C	T	.	PA!	SLC3A1	exonic	NM_	.	syr	SLC3A1
ACa04	2	####	.	C	T	.	PA!	SLC3A1,P	UTR3	NM_	NM_	.	.
ACa04	2	####	.	A	G	.	PA!	RHOQ	intronic	NM_	.	.	.
ACa04	2	####	.	C	G	.	PA!	.	intergen	NM_	dist=	.	.
ACa04	2	####	.	C	G	.	PA!	ARHGAP2	intronic	NM_	.	.	.
ACa04	2	####	.	C	T	.	PA!	DYSF	intronic	NM_	.	.	.
ACa04	2	####	rs3764475	C	T	.	PA!	RETSAT	intronic	NM_	.	.	.
ACa04	2	####	rs1425097	C	T	.	PA!	PTCD3	exonic	NM_	.	syr	PTCD3
ACa04	2	####	.	C	T	.	PA!	.	downstr	NM_	.	.	.
ACa04	2	####	.	G	A	.	PA!	RNF103-C	intronic	NM_	.	.	.
ACa04	2	####	rs8671368	A	G	.	PA!	RGPD2,R	intronic	NM_	.	.	.
ACa04	2	####	rs8674281	C	A	.	PA!	RGPD1,R	intronic	NM_	.	.	.
ACa04	2	####	.	C	T	.	PA!	LOC2850	ncRNA_	NR_	.	.	.
ACa04	2	####	rs1210470	G	A	.	PA!	.	intergen	NR_	dist=	.	.
ACa04	2	####	rs2015850	T	C	.	PA!	.	intergen	NM_	dist=	.	.
ACa04	2	####	.	C	T	.	PA!	SLC9A2	exonic	NM_	.	syr	SLC9A2
ACa04	2	####	.	G	A	.	PA!	ACOXL	intronic	NM_	.	.	.
ACa04	2	####	.	G	C	.	PA!	POLR1B	intronic	NM_	.	.	.
ACa04	2	####	rs6216365	A	T	.	PA!	INSIG2	intronic	NM_	.	.	.
ACa04	2	####	.	C	T	.	PA!	IWS1	UTR5	NM_	NM_	.	.
ACa04	2	####	.	C	T	.	PA!	POTEF	intronic	NM_	.	.	.
ACa04	2	####	rs1839843	T	C	.	PA!	POTEF	exonic	NM_	.	syr	POTEF
ACa04	2	####	rs2001821	A	G	.	PA!	POTEE	intronic	NM_	.	.	.
ACa04	2	####	.	C	T	.	PA!	LOC4010	ncRNA_	NR_	.	.	.
ACa04	2	####	.	G	T	.	PA!	RPRM	UTR3	NM_	NM_	.	.

ACa04	2	####	.	G	C	.	PA!	WDSUB1	intronic	NM_	.	.	.
ACa04	2	####	.	T	G	.	PA!	.	intergen	NM_	dist=	.	.
ACa04	2	####	.	C	T	.	PA!	CCDC141	exonic	NM_	.	mis	CCDC1
ACa04	2	####	.	T	G	.	PA!	GTF3C3	intronic	NM_	.	.	.
ACa04	2	####	.	T	C	.	PA!	CPS1	intronic	NM_	.	.	.
ACa04	2	####	.	C	T	.	PA!	TMEM169	intronic	NM_	.	.	.
ACa04	2	####	.	G	A	.	PA!	SP140	exonic	NM_	.	mis	SP140:[illegible]
ACa04	2	####	.	G	A	.	PA!	SP140	intronic	NM_	.	.	.
ACa04	2	####	.	C	T	.	PA!	HDAC4	intronic	NM_	.	.	.
ACa04	3	####	.	G	A	.	PA!	.	intergen	NM_	dist=	.	.
ACa04	3	####	.	G	A	.	PA!	ZFYVE20	intronic	NM_	.	.	.
ACa04	3	####	.	G	A	.	PA!	CAPN7	intronic	NM_	.	.	.
ACa04	3	####	.	G	T	.	PA!	OSBPL10	intronic	NM_	.	.	.
ACa04	3	####	.	C	G	.	PA!	DYNC1LI1	exonic	NM_	.	mis	DYNC1
ACa04	3	####	.	C	T	.	PA!	NBEAL2	intronic	NM_	.	.	.
ACa04	3	####	.	G	A	.	PA!	KIF9-AS1	ncRNA_	NR_	.	.	.
ACa04	3	####	.	C	T	.	PA!	NDUFAF3	exonic	NM_	.	syr	NDUFA
ACa04	3	####	.	C	T	.	PA!	NPRL2	exonic	NM_	.	mis	NPRL2:
ACa04	3	####	.	G	A	.	PA!	CACNA2D	intronic	NM_	.	.	.
ACa04	3	####	rs7801345	C	T	.	PA!	STAB1	intronic	NM_	.	.	.
ACa04	3	####	rs3677973	G	A	.	PA!	ITIH3	exonic	NM_	.	mis	ITIH3:N
ACa04	3	####	rs7454520	G	A	.	PA!	WNT5A	intronic	NM_	.	.	.
ACa04	3	####	.	A	T	.	PA!	EPHA6	intronic	NM_	.	.	.
ACa04	3	####	rs1911937	G	A	.	PA!	MIR548G	ncRNA_	NR_	.	.	.
ACa04	3	####	.	C	T	.	PA!	ZNF80	exonic	NM_	.	mis	ZNF80:
ACa04	3	####	.	C	G	.	PA!	GPR156	exonic	NM_	.	mis	GPR15
ACa04	3	####	.	C	T	.	PA!	SEMA5B	intronic	NM_	.	.	.
ACa04	3	####	.	C	T	.	PA!	H1FX	exonic	NM_	.	mis	H1FX:N
ACa04	3	####	.	C	T	.	PA!	RPL32P3	ncRNA_	NR_	.	.	.
ACa04	3	####	.	C	T	.	PA!	MBD4	exonic	NM_	.	mis	MBD4:N
ACa04	3	####	.	C	T	.	PA!	MBD4	intronic	NM_	.	.	.
ACa04	3	####	.	G	A	.	PA!	COL6A5	exonic	NM_	.	syr	COL6A
ACa04	3	####	.	C	T	.	PA!	ATP2C1	exonic	NM_	.	syr	ATP2C
ACa04	3	####	rs1469630	G	A	.	PA!	NME9	intronic	NM_	.	.	.
ACa04	3	####	.	C	A	.	PA!	FOXL2	exonic	NM_	.	sto	FOXL2:
ACa04	3	####	.	G	A	.	PA!	GRK7	exonic	NM_	.	syr	GRK7:N
ACa04	3	####	.	T	G	.	PA!	ATR	intronic	NM_	.	.	.
ACa04	3	####	rs7613736	G	C	.	PA!	P2RY12,M	intronic	NM_	.	.	.
ACa04	3	####	.	C	T	.	PA!	MLF1	intronic	NM_	.	.	.
ACa04	3	####	.	G	A	.	PA!	ZBBX	intronic	NM_	.	.	.
ACa04	3	####	.	G	C	.	PA!	SLC2A2	exonic	NM_	.	syr	SLC2A
ACa04	3	####	.	C	T	.	PA!	FNDC3B	exonic	NM_	.	syr	FNDC3
ACa04	3	####	.	C	G	.	PA!	HTR3E	exonic	NM_	.	mis	HTR3E
ACa04	3	####	.	G	A	.	PA!	SENP2	intronic	NM_	.	.	.
ACa04	3	####	.	G	A	.	PA!	RFC4	intronic	NM_	.	.	.
ACa04	3	####	.	C	T	.	PA!	HRASLS	intronic	NM_	.	.	.
ACa04	3	####	rs8795450	C	T	.	PA!	.	intergen	NM_	dist=	.	.
ACa04	3	####	.	C	T	.	PA!	.	intergen	NM_	dist=	.	.
ACa04	3	####	rs4927838	G	A	.	PA!	SDHAP1	ncRNA_	NR_	.	.	.
ACa04	3	####	rs1739998	C	T	.	PA!	SDHAP1	ncRNA_	NR_	.	.	.
ACa04	4	####	rs4022005	C	T	.	PA!	ZNF595,Z	intronic	NM_	.	.	.
ACa04	4	####	rs7518089	C	T	.	PA!	SLC26A1	exonic	NM_	.	syr	SLC26
ACa04	4	####	rs7529426	G	A	.	PA!	PPARGC1	exonic	NM_	.	mis	PPARG
ACa04	4	####	.	G	A	.	PA!	.	intergen	NR_	dist=	.	.

ACa04	4	####	.	G	A	.	PA!	N4BP2	splicing	NM_	NM_	.	.
ACa04	4	####	.	C	G	.	PA!	LIMCH1	exonic	NM_	.	mis	LIMCH1
ACa04	4	####	rs7541707	G	T	.	PA!	.	intergen	NM_	dist=	.	.
ACa04	4	####	.	G	A	.	PA!	AASDH	intronic	NM_	.	.	.
ACa04	4	####	rs5423520	C	T	.	PA!	MTHFD2L	intronic	NM_	.	.	.
ACa04	4	####	.	G	C	.	PA!	ANXA3	intronic	NM_	.	.	.
ACa04	4	####	rs8789415	C	A	.	PA!	CISD2	UTR3	NM_	NM_	.	.
ACa04	4	####	rs8900187	C	T	.	PA!	COL25A1	intronic	NM_	.	.	.
ACa04	4	####	.	G	A	.	PA!	SNHG8	ncRNA_	NR_	.	.	.
ACa04	4	####	rs1378897	A	T	.	PA!	MFSD8	intronic	NM_	.	.	.
ACa04	4	####	.	G	C	.	PA!	PCDH10	exonic	NM_	.	mis	PCDH1
ACa04	4	####	rs3676041	A	G	.	PA!	GYPB	intronic	NM_	.	.	.
ACa04	4	####	.	T	G	.	PA!	GUCY1A3	intronic	NM_	.	.	.
ACa04	4	####	.	T	C	.	PA!	NEK1	intronic	NM_	.	.	.
ACa04	4	####	.	G	C	.	PA!	.	upstreal	NR_	.	.	.
ACa04	5	####	.	G	A	.	PA!	PDCD6	exonic	NM_	.	mis	PDCD6
ACa04	5	####	.	C	T	.	PA!	LINC0119	ncRNA_	NR_	.	.	.
ACa04	5	####	.	G	A	.	PA!	GHR	intronic	NM_	.	.	.
ACa04	5	####	.	A	C	.	PA!	ANKRD55	intronic	NM_	.	.	.
ACa04	5	####	.	G	A	.	PA!	.	intergen	NM_	dist=	.	.
ACa04	5	####	.	T	A	.	PA!	MAST4	intronic	NM_	.	.	.
ACa04	5	####	.	C	T	.	PA!	.	intergen	NM_	dist=	.	.
ACa04	5	####	.	G	C	.	PA!	GTF2H2B	ncRNA_	NR_	.	.	.
ACa04	5	####	rs2848141	C	T	.	PA!	.	intergen	NR_	dist=	.	.
ACa04	5	####	.	C	T	.	PA!	HEXB	splicing	NM_	NM_	.	.
ACa04	5	####	.	C	T	.	PA!	HEXB	exonic	NM_	.	sto	HEXB:N
ACa04	5	####	.	C	T	.	PA!	HSD17B4	exonic	NM_	.	mis	HSD17I
ACa04	5	####	.	C	A	.	PA!	HARS	intronic	NM_	.	.	.
ACa04	5	####	.	C	A	.	PA!	HARS	intronic	NM_	.	.	.
ACa04	5	####	rs1024566	A	C	.	PA!	G3BP1	intronic	NM_	.	.	.
ACa04	5	####	.	C	T	.	PA!	GLRA1	intronic	NM_	.	.	.
ACa04	5	####	.	G	C	.	PA!	ADAM19	intronic	NM_	.	.	.
ACa04	5	####	.	G	A	.	PA!	EBF1	intronic	NM_	.	.	.
ACa04	5	####	rs5341551	G	A	.	PA!	TENM2	intronic	NM_	.	.	.
ACa04	5	####	.	C	T	.	PA!	EIF4E1B	intronic	NM_	.	.	.
ACa04	5	####	.	G	C	.	PA!	CBY3	exonic	NM_	.	syr	CBY3:N
ACa04	6	####	rs2013466	C	G	.	PA!	DUSP22	exonic	NM_	.	mis	DUSP2
ACa04	6	####	.	C	G	.	PA!	BPHL	intronic	NM_	.	.	.
ACa04	6	####	.	C	G	.	PA!	CDYL	exonic	NM_	.	mis	CDYL:N
ACa04	6	####	rs7489137	G	A	.	PA!	HIST1H2E	exonic	NM_	.	syr	HIST1H
ACa04	6	####	.	C	T	.	PA!	POM121L	exonic	NM_	.	mis	POM12
ACa04	6	####	.	C	G	.	PA!	POM121L	exonic	NM_	.	mis	POM12
ACa04	6	####	rs9402071	C	T	.	PA!	PGBD1	intronic	NM_	.	.	.
ACa04	6	####	.	G	C	.	PA!	DHX16	exonic	NM_	.	syr	DHX16:
ACa04	6	####	.	G	C	.	PA!	DHX16	intronic	NM_	.	.	.
ACa04	6	####	.	G	A	.	PA!	.	upstreal	NM_	.	.	.
ACa04	6	####	.	G	A	.	PA!	TNF	intronic	NM_	.	.	.
ACa04	6	####	.	G	A	.	PA!	ZBTB12	exonic	NM_	.	syr	ZBTB12
ACa04	6	####	.	C	T	.	PA!	STK19	exonic	NM_	.	mis	STK19:
ACa04	6	####	rs5421901	C	T	.	PA!	CYP21A1I	ncRNA_	NR_	.	.	.
ACa04	6	####	.	C	T	.	PA!	CYP21A2	exonic	NM_	.	unl	UNKNC
ACa04	6	####	.	C	T	.	PA!	CYP21A1I	ncRNA_	NR_	.	.	.
ACa04	6	####	.	C	A	.	PA!	ETV7	exonic	NM_	.	syr	ETV7:N
ACa04	6	####	.	T	G	.	PA!	CMTR1	intronic	NM_	.	.	.

ACa04	6	####	rs3746609	A	C	.	PA!	AARS2	intronic	NM_	.	.	.
ACa04	6	####	rs5482384	C	T	.	PA!	GPR116	exonic	NM_	.	mis	GPR11(
ACa04	6	####	rs1442276	G	A	.	PA!	DEFB112	exonic	NM_	.	mis	DEFB1:
ACa04	6	####	rs9008212	C	G	.	PA!	TTK	intronic	NM_	.	.	.
ACa04	6	####	.	C	T	.	PA!	CASP8AP	intronic	NM_	.	.	.
ACa04	6	####	rs7466151	C	T	.	PA!	AK9	intronic	NM_	.	.	.
ACa04	6	####	.	C	T	.	PA!	CEP85L	exonic	NM_	.	mis	CEP85I
ACa04	6	####	rs5411063	G	A	.	PA!	.	intergen	NM_	dist=	.	.
ACa04	6	####	.	G	C	.	PA!	LOC1001:	ncRNA_	NR_	.	.	.
ACa04	7	####	rs3695415	G	A	.	PA!	INTS1	intronic	NM_	.	.	.
ACa04	7	####	rs9183664	C	T	.	PA!	AP5Z1	intronic	NM_	.	.	.
ACa04	7	####	.	G	T	.	PA!	.	intergen	NM_	dist=	.	.
ACa04	7	####	.	C	T	.	PA!	.	intergen	NR_	dist=	.	.
ACa04	7	####	rs1485954	C	T	.	PA!	MYO1G	intronic	NM_	.	.	.
ACa04	7	####	rs3677059	G	A	.	PA!	.	intergen	NR_	dist=	.	.
ACa04	7	####	.	C	G	.	PA!	.	intergen	NR_	dist=	.	.
ACa04	7	####	.	A	T	.	PA!	.	intergen	NR_	dist=	.	.
ACa04	7	####	.	T	C	.	PA!	.	intergen	NR_	dist=	.	.
ACa04	7	####	rs2018986	T	C	.	PA!	.	intergen	NR_	dist=	.	.
ACa04	7	####	rs1860492	C	T	.	PA!	.	intergen	NR_	dist=	.	.
ACa04	7	####	.	C	A	.	PA!	.	intergen	NR_	dist=	.	.
ACa04	7	####	.	G	C	.	PA!	.	intergen	NR_	dist=	.	.
ACa04	7	####	rs2687038	T	C	.	PA!	TYW1	intronic	NM_	.	.	.
ACa04	7	####	.	A	C	.	PA!	WBSCR1	intronic	NM_	.	.	.
ACa04	7	####	.	G	T	.	PA!	HIP1	intronic	NM_	.	.	.
ACa04	7	####	.	A	G	.	PA!	.	intergen	NM_	dist=	.	.
ACa04	7	####	rs3730249	G	A	.	PA!	SRRT	intronic	NM_	.	.	.
ACa04	7	####	rs3721622	G	A	.	PA!	RBM28	exonic	NM_	.	mis	RBM28
ACa04	7	####	.	G	C	.	PA!	RBM28	exonic	NM_	.	mis	RBM28
ACa04	7	####	rs1030263	C	T	.	PA!	KCP	intronic	NM_	.	.	.
ACa04	7	####	.	C	T	.	PA!	.	intergen	NM_	dist=	.	.
ACa04	7	####	.	G	C	.	PA!	EZH2	intronic	NM_	.	.	.
ACa04	7	####	rs8678229	G	T	.	PA!	KMT2C	intronic	NM_	.	.	.
ACa04	8	####	.	G	A	.	PA!	.	intergen	NR_	dist=	.	.
ACa04	8	####	rs6264068	C	T	.	PA!	.	intergen	NR_	dist=	.	.
ACa04	8	####	.	G	A	.	PA!	BLK	intronic	NM_	.	.	.
ACa04	8	####	.	A	G	.	PA!	FAM86B1	intronic	NM_	.	.	.
ACa04	8	####	.	G	A	.	PA!	FAM86B2	intronic	NM_	.	.	.
ACa04	8	####	rs7607907	C	T	.	PA!	CHMP7	exonic	NM_	.	mis	CHMP7
ACa04	8	####	.	G	A	.	PA!	NRG1	exonic	NM_	.	mis	NRG1:N
ACa04	8	####	rs3736343	G	A	.	PA!	RAB11FIF	exonic	NM_	.	mis	RAB11I
ACa04	8	####	.	C	T	.	PA!	ATP6V1H	intronic	NM_	.	.	.
ACa04	8	####	.	G	A	.	PA!	TERF1	exonic	NM_	.	syr	TERF1:
ACa04	8	####	rs9872967	T	A	.	PA!	HEY1	UTR3	NM_	NM_	.	.
ACa04	8	####	.	G	A	.	PA!	.	intergen	NM_	dist=	.	.
ACa04	8	####	.	G	A	.	PA!	.	intergen	NM_	dist=	.	.
ACa04	8	####	.	T	A	.	PA!	.	intergen	NR_	dist=	.	.
ACa04	8	####	.	G	C	.	PA!	RGS22	intronic	NM_	.	.	.
ACa04	8	####	.	G	A	.	PA!	CSMD3	intronic	NM_	.	.	.
ACa04	8	####	.	G	T	.	PA!	TAF2	exonic	NM_	.	mis	TAF2:N
ACa04	8	####	.	G	A	.	PA!	.	intergen	NR_	dist=	.	.
ACa04	8	####	rs1436548	G	C	.	PA!	KIAA0196	exonic	NM_	.	mis	KIAA01
ACa04	8	####	.	G	C	.	PA!	KIAA0196	intronic	NM_	.	.	.
ACa04	8	####	.	G	A	.	PA!	TRAPPC9	intronic	NM_	.	.	.

ACa04	9	####	.	C	T	.	PA:	LOC3897(	ncRNA_	NR_	.	.	.
ACa04	9	####	rs7682516	C	T	.	PA:	HAUS6	exonic	NM_	.	mis	HAUS6
ACa04	9	####	.	C	T	.	PA:	IFNB1	exonic	NM_	.	mis	IFNB1:N
ACa04	9	####	.	G	C	.	PA:	UBAP2	exonic	NM_	.	sto	UBAP2
ACa04	9	####	rs3764507	T	C	.	PA:	CNTNAP3	intronic	NM_	.	.	.
ACa04	9	####	.	C	G	.	PA:	LOC1027:	intergen	NR_	dist=	.	.
ACa04	9	####	.	G	C	.	PA:	FAM27E3	ncRNA_	NR_	.	.	.
ACa04	9	####	.	G	A	.	PA:	.	intergen	NM_	dist=	.	.
ACa04	9	####	.	C	G	.	PA:	SMC5-AS:	ncRNA_	NR_	.	.	.
ACa04	9	####	.	C	G	.	PA:	VPS13A	intronic	NM_	.	.	.
ACa04	9	####	.	T	G	.	PA:	TLE4	intronic	NM_	.	.	.
ACa04	9	####	.	G	C	.	PA:	ERCC6L2	exonic	NM_	.	mis	ERCC6
ACa04	9	####	rs9619494	C	T	.	PA:	HSDL2	splicing	NM_	NM_	.	.
ACa04	9	####	.	C	T	.	PA:	DAB2IP	exonic	NM_	.	syr	DAB2IF
ACa04	9	####	.	G	A	.	PA:	LMX1B	exonic	NM_	.	syr	LMX1B
ACa04	9	####	.	G	A	.	PA:	CERCAM	intronic	NM_	.	.	.
ACa04	9	####	rs7589091	C	T	.	PA:	IER5L	UTR3	NM_	NM_	.	.
ACa04	9	####	.	C	A	.	PA:	GPR107	splicing	NM_	NM_	.	.
ACa04	9	####	.	G	T	.	PA:	RAPGEF1	intronic	NM_	.	.	.
ACa04	9	####	.	C	T	.	PA:	.	intergen	NM_	dist=	.	.
ACa04	9	####	.	C	T	.	PA:	PNPLA7	exonic	NM_	.	unl	UNKNC
ACa04	10	####	.	C	A	.	PA:	UPF2	exonic	NM_	.	sto	UPF2:N
ACa04	10	####	rs2018361	T	C	.	PA:	SKIDA1	exonic	NM_	.	syr	SKIDA1
ACa04	10	####	.	T	G	.	PA:	RASSF4	intronic	NM_	.	.	.
ACa04	10	####	.	T	G	.	PA:	RASSF4	intronic	NM_	.	.	.
ACa04	10	####	.	G	T	.	PA:	HKDC1	intronic	NM_	.	.	.
ACa04	10	####	rs3760330	G	A	.	PA:	HKDC1	exonic	NM_	.	syr	HKDC1
ACa04	10	####	.	G	A	.	PA:	HKDC1	UTR3	NM_	NM_	.	.
ACa04	10	####	.	G	C	.	PA:	UNC5B	splicing	NM_	NM_	.	.
ACa04	10	####	rs7593379	G	A	.	PA:	UNC5B	exonic	NM_	.	mis	UNC5B
ACa04	10	####	rs7698966	G	A	.	PA:	CDH23	exonic	NM_	.	syr	CDH23
ACa04	10	####	.	C	T	.	PA:	.	intergen	NM_	dist=	.	.
ACa04	10	####	.	A	G	.	PA:	.	intergen	NM_	dist=	.	.
ACa04	10	####	.	C	G	.	PA:	KIF11	intronic	NM_	.	.	.
ACa04	10	####	.	G	A	.	PA:	PDCD11	intronic	NM_	.	.	.
ACa04	10	####	.	G	A	.	PA:	PLEKHA1	intronic	NM_	.	.	.
ACa04	10	####	rs7966467	A	G	.	PA:	.	intergen	NM_	dist=	.	.
ACa04	11	####	rs7657851	G	A	.	PA:	MUC6	exonic	NM_	.	syr	MUC6:N
ACa04	11	####	rs8667539	C	G	.	PA:	.	intergen	NR_	dist=	.	.
ACa04	11	####	.	G	A	.	PA:	SBF2	exonic	NM_	.	mis	SBF2:N
ACa04	11	####	.	C	T	.	PA:	LUZP2	exonic	NM_	.	sto	LUZP2:
ACa04	11	####	.	C	T	.	PA:	WT1	exonic	NM_	.	mis	WT1:NI
ACa04	11	####	.	A	G	.	PA:	DGKZ	exonic	NM_	.	mis	DGKZ:N
ACa04	11	####	.	G	T	.	PA:	ARFGAP2	intronic	NM_	.	.	.
ACa04	11	####	.	C	A	.	PA:	.	downstr	NM_	.	.	.
ACa04	11	####	rs7463740	C	T	.	PA:	PPP1R32	exonic	NM_	.	mis	PPP1R:
ACa04	11	####	.	T	G	.	PA:	RAB3IL1	intronic	NM_	.	.	.
ACa04	11	####	.	C	T	.	PA:	MIR3680-:	ncRNA_	NR_	.	.	.
ACa04	11	####	rs3704854	C	T	.	PA:	RCOR2	exonic	NM_	.	mis	RCOR2
ACa04	11	####	.	C	G	.	PA:	TRPT1	intronic	NM_	.	.	.
ACa04	11	####	.	C	G	.	PA:	NUDT22	exonic	NM_	.	mis	NUDT2
ACa04	11	####	.	G	T	.	PA:	VEGFB	intronic	NM_	.	.	.
ACa04	11	####	.	G	A	.	PA:	FKBP2	exonic	NM_	.	mis	FKBP2:
ACa04	11	####	.	G	A	.	PA:	FKBP2	UTR3	NM_	NM_	.	.

ACa04	11	####	.	C	T	.	PA!	CCDC88B	intronic	NM_	.	.	.
ACa04	11	####	rs7459229	C	T	.	PA!	LTBP3	intronic	NM_	.	.	.
ACa04	11	####	.	C	G	.	PA!	P4HA3	exonic	NM_	.	mis	P4HA3:
ACa04	11	####	.	G	A	.	PA!	NCAM1	intronic	NM_	.	.	.
ACa04	11	####	rs3521580	T	C	.	PA!	PCSK7	intronic	NM_	.	.	.
ACa04	11	####	rs3580670	C	T	.	PA!	PCSK7	intronic	NM_	.	.	.
ACa04	11	####	rs9203240	C	T	.	PA!	HEPACAN	UTR3	NM_	NM_	.	.
ACa04	11	####	.	C	T	.	PA!	ZBTB44	exonic	NM_	.	mis	ZBTB44
ACa04	12	####	.	C	G	.	PA!	ENO2	intronic	NM_	.	.	.
ACa04	12	####	.	C	T	.	PA!	ENO2	intronic	NM_	.	.	.
ACa04	12	####	.	C	T	.	PA!	NECAP1	UTR5	NM_	NM_	.	.
ACa04	12	####	rs7123030	G	A	.	PA!	DDX11	exonic	NM_	.	mis	DDX11:
ACa04	12	####	.	G	A	.	PA!	KIAA1551	exonic	NM_	.	mis	KIAA15
ACa04	12	####	.	G	C	.	PA!	PDZRN4	intronic	NM_	.	.	.
ACa04	12	####	.	C	G	.	PA!	SCAF11	exonic	NM_	.	mis	SCAF1:
ACa04	12	####	.	C	T	.	PA!	CYP27B1	exonic	NM_	.	mis	CYP27I
ACa04	12	####	.	C	G	.	PA!	CYP27B1	intronic	NM_	.	.	.
ACa04	12	####	rs8674713	G	A	.	PA!	PPM1H	intronic	NM_	.	.	.
ACa04	12	####	rs9109827	C	T	.	PA!	MDM1	intronic	NM_	.	.	.
ACa04	12	####	rs9642294	C	T	.	PA!	PTPRQ	intronic	NM_	.	.	.
ACa04	12	####	.	T	G	.	PA!	C12orf29	intronic	NM_	.	.	.
ACa04	12	####	.	C	A	.	PA!	CRADD	intronic	NM_	.	.	.
ACa04	12	####	.	G	C	.	PA!	MYBPC1	exonic	NM_	.	mis	MYBPC
ACa04	12	####	.	C	T	.	PA!	RBM19	intronic	NM_	.	.	.
ACa04	12	####	.	C	T	.	PA!	MED13L	exonic	NM_	.	syr	MED13
ACa04	12	####	.	G	A	.	PA!	GCN1L1	intronic	NM_	.	.	.
ACa04	12	####	rs7540482	G	A	.	PA!	PLA2G1B	intronic	NM_	.	.	.
ACa04	12	####	.	G	A	.	PA!	SBNO1	intronic	NM_	.	.	.
ACa04	12	####	.	C	G	.	PA!	EP400	intronic	NM_	.	.	.
ACa04	13	####	.	A	C	.	PA!	IFT88	intronic	NM_	.	.	.
ACa04	13	####	.	G	C	.	PA!	NBEA	exonic	NM_	.	mis	NBEA:N
ACa04	13	####	.	G	C	.	PA!	EPSTI1	exonic	NM_	.	sto	EPSTI1
ACa04	13	####	.	C	A	.	PA!	DOCK9	intronic	NM_	.	.	.
ACa04	13	####	rs7720033	G	T	.	PA!	FGF14	exonic	NM_	.	syr	FGF14:
ACa04	14	####	rs1450473	C	T	.	PA!	.	intergen	NR_	dist=	.	.
ACa04	14	####	.	A	C	.	PA!	.	intergen	NM_	dist=	.	.
ACa04	14	####	.	G	A	.	PA!	DHRS4-A!	ncRNA_	NR_	.	.	.
ACa04	14	####	.	G	A	.	PA!	DHRS4-A!	ncRNA_	NR_	.	.	.
ACa04	14	####	.	C	T	.	PA!	.	intergen	NM_	dist=	.	.
ACa04	14	####	.	G	C	.	PA!	.	intergen	NR_	dist=	.	.
ACa04	14	####	rs1501582	C	T	.	PA!	GCH1	exonic	NM_	.	syr	GCH1:N
ACa04	14	####	.	C	A	.	PA!	LRRC9	ncRNA_	NR_	.	.	.
ACa04	14	####	.	C	A	.	PA!	MAX,CHU	intronic	NM_	.	.	.
ACa04	14	####	.	C	A	.	PA!	MAX,CHU	intronic	NM_	.	.	.
ACa04	14	####	rs7676169	G	A	.	PA!	SMOC1	exonic	NM_	.	mis	SMOC1
ACa04	14	####	.	C	T	.	PA!	PAPLN	exonic	NM_	.	syr	PAPLN
ACa04	14	####	.	G	A	.	PA!	ANGEL1	intronic	NM_	.	.	.
ACa04	14	####	.	G	C	.	PA!	.	intergen	NM_	dist=	.	.
ACa04	14	####	.	G	A	.	PA!	LGMN	intronic	NM_	.	.	.
ACa04	14	####	rs3756754	G	A	.	PA!	SERPINA:	ncRNA_	NR_	.	.	.
ACa04	14	####	.	T	G	.	PA!	MTA1	intronic	NM_	.	.	.
ACa04	14	####	rs5537870	G	A	.	PA!	.	intergen	NR_	dist=	.	.
ACa04	14	####	rs1289410	G	A	.	PA!	.	intergen	NR_	dist=	.	.
ACa04	15	####	.	C	T	.	PA!	.	intergen	NR_	dist=	.	.

ACa04	15	####	.	C	A	.	PA!	LOC2836{	ncRNA_	NR_	.	.	.
ACa04	15	####	rs5409237	C	T	.	PA!	HERC2	exonic	NM_	.	syr	HERC2
ACa04	15	####	.	T	G	.	PA!	.	intergen	NR_	dist=	.	.
ACa04	15	####	.	T	G	.	PA!	PDCD6IPI	ncRNA_	NR_	.	.	.
ACa04	15	####	.	T	C	.	PA!	ULK4P1,U	ncRNA_	NR_	.	.	.
ACa04	15	####	rs2015085	C	A	.	PA!	.	intergen	NR_	dist=	.	.
ACa04	15	####	rs7481072	C	T	.	PA!	RPAP1	intronic	NM_	.	.	.
ACa04	15	####	.	C	T	.	PA!	RPAP1	intronic	NM_	.	.	.
ACa04	15	####	.	C	G	.	PA!	RPAP1	exonic	NM_	.	mis	RPAP1
ACa04	15	####	rs7754448	G	A	.	PA!	SPTBN5	exonic	NM_	.	syr	SPTBN
ACa04	15	####	.	G	T	.	PA!	CATSPER	intronic	NM_	.	.	.
ACa04	15	####	rs1922871	C	T	.	PA!	MFAP1	intronic	NM_	.	.	.
ACa04	15	####	.	C	T	.	PA!	EIF3J	intronic	NM_	.	.	.
ACa04	15	####	rs6202600	G	A	.	PA!	.	intergen	NM_	dist=	.	.
ACa04	15	####	.	G	A	.	PA!	SPATA5L:	exonic	NM_	.	syr	SPATA!
ACa04	15	####	.	G	A	.	PA!	RFX7	intronic	NM_	.	.	.
ACa04	15	####	.	C	T	.	PA!	ANXA2	intronic	NM_	.	.	.
ACa04	15	####	rs5478938	C	T	.	PA!	RBPMS2	intronic	NM_	.	.	.
ACa04	15	####	.	C	A	.	PA!	MAP2K1	intronic	NM_	.	.	.
ACa04	15	####	.	G	C	.	PA!	MAP2K5	intronic	NM_	.	.	.
ACa04	15	####	.	G	A	.	PA!	ISLR	UTR3	NM_	NM_	.	.
ACa04	15	####	.	C	T	.	PA!	.	intergen	NM_	dist=	.	.
ACa04	15	####	.	C	T	.	PA!	CHRNB4	exonic	NM_	.	mis	CHRNE
ACa04	15	####	.	C	G	.	PA!	ANKRD34	exonic	NM_	.	mis	ANKRD
ACa04	15	####	rs2401418	T	C	.	PA!	.	intergen	NM_	dist=	.	.
ACa04	15	####	.	C	T	.	PA!	FANCI	splicing	NM_	NM_	.	.
ACa04	15	####	.	C	G	.	PA!	VPS33B	exonic	NM_	.	mis	VPS33E
ACa04	15	####	.	C	G	.	PA!	MCTP2	intronic	NM_	.	.	.
ACa04	15	####	.	C	T	.	PA!	MEF2A	intronic	NM_	.	.	.
ACa04	15	####	rs7510253	C	T	.	PA!	TARSL2	exonic	NM_	.	mis	TARSL:
ACa04	16	####	rs7504825	G	A	.	PA!	NPRL3	exonic	NM_	.	unl	UNKNC
ACa04	16	####	.	C	T	.	PA!	CLCN7	intronic	NM_	.	.	.
ACa04	16	####	.	G	A	.	PA!	RBFOX1	intronic	NM_	.	.	.
ACa04	16	####	.	G	A	.	PA!	TEKT5	exonic	NM_	.	sto	TEKT5:
ACa04	16	####	.	C	T	.	PA!	TNFRSF1	exonic	NM_	.	mis	TNFRS
ACa04	16	####	.	G	A	.	PA!	NOMO1	intronic	NM_	.	.	.
ACa04	16	####	rs5522924	C	T	.	PA!	NPIPA5	intronic	NM_	.	.	.
ACa04	16	####	rs6203288	T	C	.	PA!	.	intergen	NR_	dist=	.	.
ACa04	16	####	rs7654396	C	T	.	PA!	SMG1	exonic	NM_	.	mis	SMG1:N
ACa04	16	####	.	G	A	.	PA!	EEF2K	intronic	NM_	.	.	.
ACa04	16	####	.	C	T	.	PA!	POLR3E	intronic	NM_	.	.	.
ACa04	16	####	.	A	G	.	PA!	SMG1P1	ncRNA_	NR_	.	.	.
ACa04	16	####	.	C	G	.	PA!	XPO6	exonic	NM_	.	mis	XPO6:N
ACa04	16	####	.	G	A	.	PA!	XPO6	intronic	NM_	.	.	.
ACa04	16	####	.	G	A	.	PA!	.	intergen	NR_	dist=	.	.
ACa04	16	####	.	T	A	.	PA!	RNF40	intronic	NM_	.	.	.
ACa04	16	####	.	C	T	.	PA!	ITGAM	intronic	NM_	.	.	.
ACa04	16	####	rs7896651	A	C	.	PA!	.	intergen	NR_	dist=	.	.
ACa04	16	####	rs7515102	C	G	.	PA!	LONP2	splicing	NM_	NM_	.	.
ACa04	16	####	.	C	T	.	PA!	CNOT1	exonic	NM_	.	mis	CNOT1
ACa04	16	####	.	C	G	.	PA!	CTCF	exonic	NM_	.	mis	CTCF:N
ACa04	16	####	.	T	A	.	PA!	ZNF821	UTR5	NM_	NM_	.	.
ACa04	16	####	rs2911030	A	G	.	PA!	LOC2839:	ncRNA_	NR_	.	.	.
ACa04	16	####	.	G	A	.	PA!	.	intergen	NM_	dist=	.	.

ACa04	16	####	.	C	T	.	PA!	.	ncRNA_	NR_	.	.	.
ACa04	17	####	rs7805194	C	T	.	PA!	P2RX1	intronic	NM_	.	.	.
ACa04	17	####	.	C	T	.	PA!	ALOX12-A	ncRNA_	NR_	.	.	.
ACa04	17	####	.	C	G	.	PA!	TNFSF12-	exonic	NM_	.	mis	TNFSF1
ACa04	17	####	.	C	T	.	PA!	DNAH2	exonic	NM_	.	syr	DNAH2
ACa04	17	####	.	A	G	.	PA!	MYH13	exonic	NM_	.	mis	MYH13
ACa04	17	####	.	G	C	.	PA!	DNAH9	exonic	NM_	.	mis	DNAH9
ACa04	17	####	rs5595610	G	A	.	PA!	TTC19	intronic	NM_	.	.	.
ACa04	17	####	.	C	G	.	PA!	SREBF1	UTR3	NM_	NM_	.	.
ACa04	17	####	.	G	C	.	PA!	ZNF286B	intronic	NM_	.	.	.
ACa04	17	####	.	C	T	.	PA!	CCDC144	ncRNA_	NR_	.	.	.
ACa04	17	####	.	G	A	.	PA!	LGALS9B	intronic	NM_	.	.	.
ACa04	17	####	rs7841783	G	A	.	PA!	FLJ36000	ncRNA_	NR_	.	.	.
ACa04	17	####	.	C	T	.	PA!	TLCD1	UTR3	NM_	NM_	.	.
ACa04	17	####	.	G	C	.	PA!	ERAL1	intronic	NM_	.	.	.
ACa04	17	####	.	G	A	.	PA!	.	upstrea	NR_	.	.	.
ACa04	17	####	.	C	G	.	PA!	SUZ12	exonic	NM_	.	mis	SUZ12:
ACa04	17	####	.	C	A	.	PA!	SUZ12	intronic	NM_	.	.	.
ACa04	17	####	.	G	C	.	PA!	RHOT1	intronic	NM_	.	.	.
ACa04	17	####	.	G	T	.	PA!	LOC1010(	intronic	NM_	.	.	.
ACa04	17	####	rs8795183	C	T	.	PA!	TBC1D3H	intronic	NM_	.	.	.
ACa04	17	####	.	G	A	.	PA!	MRM1	intronic	NM_	.	.	.
ACa04	17	####	.	G	C	.	PA!	ACACA	intronic	NM_	.	.	.
ACa04	17	####	.	G	A	.	PA!	SYNRG	intronic	NM_	.	.	.
ACa04	17	####	.	C	A	.	PA!	.	upstrea	NM_	.	.	.
ACa04	17	####	.	G	C	.	PA!	MED1	exonic	NM_	.	mis	MED1:N
ACa04	17	####	rs1035039	C	T	.	PA!	LOC1005(	ncRNA_	NR_	.	.	.
ACa04	17	####	.	G	A	.	PA!	ZNF385C	intronic	NM_	.	.	.
ACa04	17	####	.	C	T	.	PA!	SLC4A1	intronic	NM_	.	.	.
ACa04	17	####	.	C	T	.	PA!	EME1	intronic	NM_	.	.	.
ACa04	17	####	.	C	T	.	PA!	CACNA1G	intronic	NM_	.	.	.
ACa04	17	####	.	C	G	.	PA!	BZRAP1	splicing	NM_	NM_	.	.
ACa04	17	####	.	G	C	.	PA!	DDX42	exonic	NM_	.	mis	DDX42:
ACa04	17	####	.	G	T	.	PA!	DDX42	exonic	NM_	.	mis	DDX42:
ACa04	17	####	.	T	G	.	PA!	CEP112	intronic	NM_	.	.	.
ACa04	17	####	.	G	C	.	PA!	UNC13D	intronic	NM_	.	.	.
ACa04	17	####	rs1405528	C	T	.	PA!	SLC26A11	exonic	NM_	.	syr	SLC26A
ACa04	17	####	.	C	T	.	PA!	BAIAP2	exonic	NM_	.	syr	BAIAP2
ACa04	17	####	rs3677917	A	G	.	PA!	FLJ43681	ncRNA_	NR_	.	.	.
ACa04	18	####	.	G	C	.	PA!	L3MBTL4	intronic	NM_	.	.	.
ACa04	18	####	.	G	A	.	PA!	LRRC30	exonic	NM_	.	syr	LRRC3(
ACa04	18	####	.	C	G	.	PA!	PPP4R1	exonic	NM_	.	mis	PPP4R.
ACa04	18	####	.	G	C	.	PA!	TXNDC2	UTR5	NM_	NM_	.	.
ACa04	18	####	.	C	G	.	PA!	PIEZO2	exonic	NM_	.	mis	PIEZO2
ACa04	18	####	.	G	A	.	PA!	RNMT	exonic	NM_	.	mis	RNMT:I
ACa04	18	####	.	C	T	.	PA!	ANKRD20	ncRNA_	NR_	.	.	.
ACa04	18	####	.	G	T	.	PA!	KCTD1	intronic	NM_	.	.	.
ACa04	18	####	.	C	T	.	PA!	SKOR2	exonic	NM_	.	mis	SKOR2
ACa04	18	####	.	G	A	.	PA!	TCF4	intronic	NM_	.	.	.
ACa04	18	####	.	C	T	.	PA!	.	intergen	NR_	dist=	.	.
ACa04	18	####	rs7468202	C	T	.	PA!	TNFRSF1	intronic	NM_	.	.	.
ACa04	18	####	.	G	A	.	PA!	CCDC102	exonic	NM_	.	syr	CCDC1
ACa04	18	####	.	A	G	.	PA!	ATP9B	intronic	NM_	.	.	.
ACa04	19	####	rs9545783	C	T	.	PA!	.	upstrea	NM_	.	.	.

ACa04	19	####	.	G	A	.	PA!	HMHA1	intronic	NM_	.	.	.
ACa04	19	####	.	G	C	.	PA!	HMHA1	intronic	NM_	.	.	.
ACa04	19	####	.	G	T	.	PA!	TCF3	intronic	NM_	.	.	.
ACa04	19	####	rs3742787	G	A	.	PA!	TCF3	intronic	NM_	.	.	.
ACa04	19	####	rs1418930	A	T	.	PA!	SCAMP4	intronic	NM_	.	.	.
ACa04	19	####	.	G	T	.	PA!	THOP1	exonic	NM_	.	sto	THOP1
ACa04	19	####	rs7627736	G	A	.	PA!	THOP1	intronic	NM_	.	.	.
ACa04	19	####	.	C	T	.	PA!	NFIC	UTR5	NM_	NM_	.	.
ACa04	19	####	.	G	C	.	PA!	CHAF1A	intronic	NM_	.	.	.
ACa04	19	####	.	G	A	.	PA!	ACSBG2	intronic	NM_	.	.	.
ACa04	19	####	.	T	A	.	PA!	STXBP2	intronic	NM_	.	.	.
ACa04	19	####	.	A	G	.	PA!	ANGPTL4	intronic	NM_	.	.	.
ACa04	19	####	.	G	C	.	PA!	RAB11B-A	ncRNA_	NR_	.	.	.
ACa04	19	####	.	C	G	.	PA!	MYO1F	intronic	NM_	.	.	.
ACa04	19	####	.	G	T	.	PA!	MUC16	exonic	NM_	.	sto	MUC16
ACa04	19	####	.	G	A	.	PA!	MUC16	exonic	NM_	.	mis	MUC16
ACa04	19	####	.	G	A	.	PA!	MUC16	exonic	NM_	.	syn	MUC16
ACa04	19	####	rs7741875	G	A	.	PA!	MUC16	exonic	NM_	.	mis	MUC16
ACa04	19	####	.	C	T	.	PA!	QTRT1	intronic	NM_	.	.	.
ACa04	19	####	.	G	C	.	PA!	ZNF442	exonic	NM_	.	mis	ZNF442
ACa04	19	####	.	G	A	.	PA!	HOOK2	intronic	NM_	.	.	.
ACa04	19	####	.	G	T	.	PA!	JUNB	UTR5	NM_	NM_	.	.
ACa04	19	####	.	C	G	.	PA!	NWD1	exonic	NM_	.	mis	NWD1:
ACa04	19	####	rs7797020	G	A	.	PA!	FAM129C	exonic	NM_	.	mis	FAM12!
ACa04	19	####	.	G	A	.	PA!	SLC5A5	intronic	NM_	.	.	.
ACa04	19	####	.	A	T	.	PA!	YJEFN3	intronic	NM_	.	.	.
ACa04	19	####	.	C	T	.	PA!	ZNF253	exonic	NM_	.	mis	ZNF253
ACa04	19	####	.	G	A	.	PA!	ZNF676	UTR5	NM_	NM_	.	.
ACa04	19	####	.	C	G	.	PA!	PLEKHF1	intronic	NM_	.	.	.
ACa04	19	####	.	G	A	.	PA!	.	intergen	NM_	dist=	.	.
ACa04	19	####	.	G	C	.	PA!	HAUS5	intronic	NM_	.	.	.
ACa04	19	####	rs8683521	T	G	.	PA!	.	intergen	NM_	dist=	.	.
ACa04	19	####	rs8793125	T	C	.	PA!	.	intergen	NM_	dist=	.	.
ACa04	19	####	rs7653982	C	T	.	PA!	ZNF850	exonic	NM_	.	mis	ZNF850
ACa04	19	####	rs9896621	C	T	.	PA!	SAMD4B	intronic	NM_	.	.	.
ACa04	19	####	.	G	A	.	PA!	FCGBP	intronic	NM_	.	.	.
ACa04	19	####	rs1408143	C	T	.	PA!	ARHGEF1	intronic	NM_	.	.	.
ACa04	19	####	.	G	C	.	PA!	GRIK5	intronic	NM_	.	.	.
ACa04	19	####	rs1444115	C	T	.	PA!	GRIK5	intronic	NM_	.	.	.
ACa04	19	####	.	G	A	.	PA!	PSG10P	ncRNA_	NR_	.	.	.
ACa04	19	####	.	G	C	.	PA!	PLAUR	intronic	NM_	.	.	.
ACa04	19	####	rs4251912	G	A	.	PA!	PLAUR	exonic	NM_	.	syn	PLAUR
ACa04	19	####	.	C	G	.	PA!	NANOS2	exonic	NM_	.	mis	NANOS
ACa04	19	####	.	G	T	.	PA!	NPAS1	intronic	NM_	.	.	.
ACa04	19	####	.	C	A	.	PA!	SCAF1	intronic	NM_	.	.	.
ACa04	19	####	.	G	A	.	PA!	ZNF615	exonic	NM_	.	syn	ZNF615
ACa04	19	####	.	T	G	.	PA!	.	downstr	NM_	.	.	.
ACa04	19	####	.	G	A	.	PA!	.	upstrea	NM_	.	.	.
ACa04	19	####	.	G	C	.	PA!	NCR1	exonic	NM_	.	mis	NCR1:N
ACa04	19	####	.	G	C	.	PA!	USP29	exonic	NM_	.	mis	USP29:
ACa04	19	####	.	G	C	.	PA!	USP29	exonic	NM_	.	mis	USP29:
ACa04	20	####	.	C	T	.	PA!	SIRPB1	intronic	NM_	.	.	.
ACa04	20	####	.	T	G	.	PA!	TGM6	intronic	NM_	.	.	.
ACa04	20	####	.	G	A	.	PA!	TMC2	intronic	NM_	.	.	.

ACa04	20	####	.	G	A	.	PA!	SIGLEC1	exonic	NM_	.	mis	SIGLEC
ACa04	20	####	rs7610831	C	G	.	PA!	PLCB1	exonic	NM_	.	mis	PLCB1:
ACa04	20	####	.	C	G	.	PA!	BANF2	intronic	NM_	.	.	.
ACa04	20	####	.	G	A	.	PA!	SNORD17	ncRNA_	NR_	.	.	.
ACa04	20	####	rs8120738	C	G	.	PA!	FRG1B	ncRNA_	NR_	.	.	.
ACa04	20	####	rs7791580	G	A	.	PA!	FRG1B	ncRNA_	NR_	.	.	.
ACa04	20	####	.	C	T	.	PA!	HCK	UTR5	NM_	NM_	.	.
ACa04	20	####	.	C	G	.	PA!	NCOA6	intronic	NM_	.	.	.
ACa04	20	####	rs1904867	C	T	.	PA!	NCOA6	exonic	NM_	.	mis	NCOA6
ACa04	20	####	.	C	T	.	PA!	RNF114	exonic	NM_	.	sto	RNF114
ACa04	20	####	.	C	T	.	PA!	DOK5	exonic	NM_	.	unl	UNKNC
ACa04	20	####	.	C	T	.	PA!	ZNF831	exonic	NM_	.	syr	ZNF831
ACa04	20	####	.	G	T	.	PA!	OSBPL2	intronic	NM_	.	.	.
ACa04	20	####	.	G	A	.	PA!	COL20A1	intronic	NM_	.	.	.
ACa04	20	####	rs1874434	G	A	.	PA!	MYT1	intronic	NM_	.	.	.
ACa04	21	####	.	C	T	.	PA!	.	intergen	NR_	dist=	.	.
ACa04	21	####	.	A	C	.	PA!	.	intergen	NR_	dist=	.	.
ACa04	21	####	.	T	G	.	PA!	.	intergen	NR_	dist=	.	.
ACa04	21	####	rs3707796	G	A	.	PA!	.	intergen	NR_	dist=	.	.
ACa04	21	####	rs7469658	C	T	.	PA!	BAGE2,BA	intronic	NM_	.	.	.
ACa04	21	####	rs4638882	C	A	.	PA!	.	intergen	NM_	dist=	.	.
ACa04	21	####	.	T	C	.	PA!	CBR3-AS1	ncRNA_	NR_	.	.	.
ACa04	21	####	.	G	A	.	PA!	.	intergen	NM_	dist=	.	.
ACa04	21	####	.	G	A	.	PA!	UBASH3A	intronic	NM_	.	.	.
ACa04	21	####	.	G	C	.	PA!	CBS	exonic	NM_	.	mis	CBS:NM
ACa04	21	####	rs7276130	G	A	.	PA!	FAM207A	intronic	NM_	.	.	.
ACa04	21	####	.	C	G	.	PA!	COL18A1	intronic	NM_	.	.	.
ACa04	22	####	.	C	T	.	PA!	.	intergen	NOI	dist=	.	.
ACa04	22	####	.	T	A	.	PA!	.	intergen	NM_	dist=	.	.
ACa04	22	####	.	G	C	.	PA!	.	intergen	NM_	dist=	.	.
ACa04	22	####	rs3769174	G	T	.	PA!	GGT3P	ncRNA_	NR_	.	.	.
ACa04	22	####	rs2870983	C	T	.	PA!	PRODH	exonic	NM_	.	mis	PRODH
ACa04	22	####	.	G	A	.	PA!	.	intergen	NM_	dist=	.	.
ACa04	22	####	rs1389774	C	G	.	PA!	.	intergen	NR_	dist=	.	.
ACa04	22	####	rs3731564	G	C	.	PA!	POM121L	ncRNA_	NR_	.	.	.
ACa04	22	####	rs1856371	C	T	.	PA!	.	intergen	NM_	dist=	.	.
ACa04	22	####	.	C	G	.	PA!	KIAA1656	ncRNA_	NR_	.	.	.
ACa04	22	####	rs5669283	G	A	.	PA!	PES1	intronic	NM_	.	.	.
ACa04	22	####	.	G	T	.	PA!	TOM1	intronic	NM_	.	.	.
ACa04	22	####	rs7606577	G	A	.	PA!	MFNG	intronic	NM_	.	.	.
ACa04	22	####	.	G	C	.	PA!	APOBEC3	ncRNA_	NR_	.	.	.
ACa04	22	####	.	G	C	.	PA!	SYNGR1	exonic	NM_	.	mis	SYNGR
ACa04	22	####	rs1409901	C	T	.	PA!	XPNPEP3	exonic	NM_	.	mis	XPNPE
ACa04	22	####	.	G	C	.	PA!	EP300	exonic	NM_	.	mis	EP300:
ACa04	22	####	rs3731131	G	A	.	PA!	SREBF2	exonic	NM_	.	syr	SREBF
ACa04	22	####	.	C	G	.	PA!	SMC1B	intronic	NM_	.	.	.
ACa04	22	####	.	C	T	.	PA!	.	intergen	NR_	dist=	.	.
ACa04	22	####	.	G	A	.	PA!	MOV10L1	intronic	NM_	.	.	.
ACa04	22	####	rs7711497	G	A	.	PA!	PLXNB2	exonic	NM_	.	syr	PLXNB
ACa04	X	####	.	C	T	.	PA!	P2RY8	UTR3	NM_	NM_	.	.
ACa04	X	####	rs8673094	G	A	.	PA!	.	intergen	NM_	dist=	.	.
ACa04	X	####	rs7643909	T	C	.	PA!	VCX3B	UTR5	NM_	NM_	.	.
ACa04	X	####	rs6640384	G	A	.	PA!	.	intergen	NM_	dist=	.	.
ACa04	X	####	.	C	G	.	PA!	TLR7	exonic	NM_	.	mis	TLR7:N

ACa04	X	####	.	C	A	.	PA!	TLR8	exonic	NM_	.	sto	TLR8:N
ACa04	X	####	.	G	T	.	PA!	DMD	intronic	NM_	.	.	.
ACa04	X	####	.	G	A	.	PA!	MAOA	exonic	NM_	.	mis	MAOA:I
ACa04	X	####	.	G	A	.	PA!	SSX1	intronic	NM_	.	.	.
ACa04	X	####	.	G	A	.	PA!	PCSK1N	exonic	NM_	.	syr	PCSK1I
ACa04	X	####	.	C	G	.	PA!	USP27X-A	ncRNA_	NR_	.	.	.
ACa04	X	####	.	G	A	.	PA!	FAM120C	UTR3	NM_	NM_	.	.
ACa04	X	####	.	A	G	.	PA!	FAM120C	intronic	NM_	.	.	.
ACa04	X	####	.	G	A	.	PA!	GNL3L	exonic	NM_	.	syr	GNL3L:
ACa04	X	####	.	C	T	.	PA!	UQCRBP1	ncRNA_	NR_	.	.	.
ACa04	X	####	.	T	C	.	PA!	TEX11	intronic	NM_	.	.	.
ACa04	X	####	.	C	G	.	PA!	MED12	exonic	NM_	.	sto	MED12
ACa04	X	####	.	G	A	.	PA!	NLGN3	exonic	NM_	.	sto	NLGN3
ACa04	X	####	.	C	G	.	PA!	BCYRN1	ncRNA_	NR_	.	.	.
ACa04	X	####	.	G	A	.	PA!	.	intergen	NM_	dist=	.	.
ACa04	X	####	.	C	T	.	PA!	SASH3	exonic	NM_	.	sto	SASH3
ACa04	X	####	.	G	C	.	PA!	UTP14A	exonic	NM_	.	mis	UTP14A
ACa04	X	####	.	G	A	.	PA!	BCORL1	exonic	NM_	.	mis	BCORL
ACa04	X	####	.	G	A	.	PA!	FIRRE	ncRNA_	NR_	.	.	.
ACa04	X	####	.	C	T	.	PA!	ARHGEF6	exonic	NM_	.	mis	ARHGE
ACa04	X	####	.	G	A	.	PA!	HMGB3	exonic	NM_	.	unl	UNKNC
ACa04	X	####	rs7824625	G	A	.	PA!	TREX2	exonic	NM_	.	mis	TREX2:
ACa04	X	####	.	A	C	.	PA!	BCAP31	intronic	NM_	.	.	.
ACa06	1	####	.	T	C	.	PA!	.	intergen	NR_	dist=	.	.
ACa06	1	####	rs7810569	G	A	.	PA!	.	intergen	NR_	dist=	.	.
ACa06	1	####	rs1149269	C	T	.	PA!	ATAD3A	intronic	NM_	.	.	.
ACa06	1	####	.	G	T	.	PA!	FAM213B	exonic	NM_	.	syr	FAM21:
ACa06	1	####	.	G	A	.	PA!	MEGF6	intronic	NM_	.	.	.
ACa06	1	####	.	A	C	.	PA!	GPR157	intronic	NM_	.	.	.
ACa06	1	####	rs2008876	C	T	.	PA!	.	intergen	NM_	dist=	.	.
ACa06	1	####	rs5565927	T	C	.	PA!	NBPF1	intronic	NM_	.	.	.
ACa06	1	####	rs6177234	C	A	.	PA!	NBPF1	intronic	NM_	.	.	.
ACa06	1	####	rs1008195	C	G	.	PA!	CROCCP2	ncRNA_	NR_	.	.	.
ACa06	1	####	rs5388409	G	A	.	PA!	ESPNP	ncRNA_	NR_	.	.	.
ACa06	1	####	rs6669627	G	C	.	PA!	CROCC	exonic	NM_	.	mis	CROCC
ACa06	1	####	.	A	C	.	PA!	PAX7	intronic	NM_	.	.	.
ACa06	1	####	rs7661411	C	T	.	PA!	IFI6	intronic	NM_	.	.	.
ACa06	1	####	rs7802118	C	T	.	PA!	HIVEP3	exonic	NM_	.	mis	HIVEP3
ACa06	1	####	rs8664767	C	T	.	PA!	.	intergen	NM_	dist=	.	.
ACa06	1	####	.	G	C	.	PA!	.	intergen	NM_	dist=	.	.
ACa06	1	####	rs7581372	T	C	.	PA!	.	intergen	NOI	dist=	.	.
ACa06	1	####	rs7697846	A	C	.	PA!	.	intergen	NOI	dist=	.	.
ACa06	1	####	rs2016919	T	A	.	PA!	.	intergen	NR_	dist=	.	.
ACa06	1	####	.	C	A	.	PA!	.	intergen	NR_	dist=	.	.
ACa06	1	####	rs3703375	C	A	.	PA!	.	intergen	NR_	dist=	.	.
ACa06	1	####	rs1996515	T	A	.	PA!	.	intergen	NR_	dist=	.	.
ACa06	1	####	.	A	T	.	PA!	.	intergen	NR_	dist=	.	.
ACa06	1	####	.	C	G	.	PA!	.	intergen	NR_	dist=	.	.
ACa06	1	####	.	G	A	.	PA!	LINC0113	ncRNA_	NR_	.	.	.
ACa06	1	####	.	T	A	.	PA!	NBPF9,NC	intronic	NM_	.	.	.
ACa06	1	####	.	G	T	.	PA!	NBPF25P	ncRNA_	NR_	.	.	.
ACa06	1	####	rs8799960	G	C	.	PA!	NBPF8	intronic	NM_	.	.	.
ACa06	1	####	rs2003739	G	A	.	PA!	NBPF25P	ncRNA_	NR_	.	.	.
ACa06	1	####	rs3705433	G	A	.	PA!	NBPF25P	ncRNA_	NR_	.	.	.

ACa06	1	####	.	T	G	.	PA!	FCRL4	intronic	NM_	.	.	.
ACa06	1	####	rs1839036	T	A	.	PA!	.	intergen	NR_	dist=	.	.
ACa06	1	####	.	T	C	.	PA!	.	intergen	NM_	dist=	.	.
ACa06	1	####	.	A	G	.	PA!	.	intergen	NM_	dist=	.	.
ACa06	1	####	.	A	C	.	PA!	ENAH	intronic	NM_	.	.	.
ACa06	1	####	rs1828179	T	C	.	PA!	.	intergen	NM_	dist=	.	.
ACa06	2	####	rs4096246	A	C	.	PA!	.	intergen	NR_	dist=	.	.
ACa06	2	####	.	G	C	.	PA!	RGPD1,R	intronic	NM_	.	.	.
ACa06	2	####	.	G	A	.	PA!	.	intergen	NOI	dist=	.	.
ACa06	2	####	.	G	T	.	PA!	.	intergen	NR_	dist=	.	.
ACa06	2	####	rs2011280	G	A	.	PA!	ANAPC1	exonic	NM_	.	mis	ANAPC
ACa06	2	####	rs8685337	G	A	.	PA!	.	upstreai	NR_	.	.	.
ACa06	2	####	rs2747968	A	G	.	PA!	WASH2P	ncRNA_	NR_	.	.	.
ACa06	2	####	rs1029282	G	T	.	PA!	INSIG2	intronic	NM_	.	.	.
ACa06	2	####	rs2001821	A	G	.	PA!	POTEE	intronic	NM_	.	.	.
ACa06	2	####	rs7800646	C	T	.	PA!	.	intergen	NM_	dist=	.	.
ACa06	2	####	.	T	C	.	PA!	DHRS9	intronic	NM_	.	.	.
ACa06	2	####	.	A	T	.	PA!	SLC25A12	intronic	NM_	.	.	.
ACa06	2	####	rs1875561	C	T	.	PA!	UBE2E3	intronic	NM_	.	.	.
ACa06	2	####	rs8671576	C	G	.	PA!	IKZF2	intronic	NM_	.	.	.
ACa06	3	####	.	T	G	.	PA!	KLHL18	intronic	NM_	.	.	.
ACa06	3	####	.	G	A	.	PA!	AGTR1	exonic	NM_	.	syr	AGTR1
ACa06	3	####	.	T	G	.	PA!	CP	intronic	NM_	.	.	.
ACa06	3	####	.	T	G	.	PA!	CP	intronic	NM_	.	.	.
ACa06	3	####	rs3760511	G	A	.	PA!	.	intergen	NM_	dist=	.	.
ACa06	3	####	rs8677455	A	G	.	PA!	LINC0088	ncRNA_	NR_	.	.	.
ACa06	3	####	rs1128840	A	G	.	PA!	.	intergen	NR_	dist=	.	.
ACa06	4	####	.	C	T	.	PA!	ZNF595,Z	intronic	NM_	.	.	.
ACa06	4	####	rs1443656	G	A	.	PA!	SLIT2	intronic	NM_	.	.	.
ACa06	5	####	.	G	A	.	PA!	FAM134B	intronic	NM_	.	.	.
ACa06	5	####	.	C	T	.	PA!	.	intergen	NR_	dist=	.	.
ACa06	5	####	rs3759258	G	T	.	PA!	.	intergen	NR_	dist=	.	.
ACa06	5	####	rs8790446	T	C	.	PA!	NBPF22P	ncRNA_	NR_	.	.	.
ACa06	5	####	.	G	A	.	PA!	LOC1001	ncRNA_	NR_	.	.	.
ACa06	5	####	.	A	C	.	PA!	SLC36A2	intronic	NM_	.	.	.
ACa06	5	####	.	A	G	.	PA!	CCNG1	intronic	NM_	.	.	.
ACa06	5	####	.	C	A	.	PA!	COL23A1	exonic	NM_	.	mis	COL23/
ACa06	5	####	.	C	A	.	PA!	ADAMTS2	intronic	NM_	.	.	.
ACa06	6	####	rs3719775	T	C	.	PA!	.	upstreai	NM_	.	.	.
ACa06	6	####	rs9202904	C	G	.	PA!	.	upstreai	NM_	.	.	.
ACa06	6	####	rs4292554	A	G	.	PA!	.	intergen	NR_	dist=	.	.
ACa06	6	####	rs4437505	G	T	.	PA!	.	intergen	NR_	dist=	.	.
ACa06	6	####	rs7508774	C	T	.	PA!	KIAA1244	intronic	NM_	.	.	.
ACa06	6	####	.	A	C	.	PA!	SYNE1	intronic	NM_	.	.	.
ACa06	6	####	rs1436754	G	A	.	PA!	TCP10L2	intronic	NM_	.	.	.
ACa06	6	####	.	A	G	.	PA!	WDR27	intronic	NM_	.	.	.
ACa06	7	####	.	A	C	.	PA!	SDK1	intronic	NM_	.	.	.
ACa06	7	####	rs2016650	T	A	.	PA!	RSPH10B	intronic	NM_	.	.	.
ACa06	7	####	.	T	G	.	PA!	.	intergen	NM_	dist=	.	.
ACa06	7	####	.	C	G	.	PA!	.	intergen	NR_	dist=	.	.
ACa06	7	####	.	C	G	.	PA!	.	intergen	NR_	dist=	.	.
ACa06	7	####	.	C	G	.	PA!	.	intergen	NR_	dist=	.	.
ACa06	7	####	.	C	A	.	PA!	.	intergen	NR_	dist=	.	.
ACa06	7	####	.	G	T	.	PA!	.	intergen	NR_	dist=	.	.

ACa06	7	####	.	C	T	.	PA!	.	intergen	NR_	dist=	.	.
ACa06	7	####	.	A	C	.	PA!	.	intergen	NR_	dist=	.	.
ACa06	7	####	rs4596537	C	A	.	PA!	.	intergen	NOI	dist=	.	.
ACa06	7	####	rs2167825	A	C	.	PA!	.	intergen	NOI	dist=	.	.
ACa06	7	####	rs586765	G	T	.	PA!	.	intergen	NR_	dist=	.	.
ACa06	7	####	rs8688020	G	A	.	PA!	.	intergen	NR_	dist=	.	.
ACa06	7	####	rs2462259	C	T	.	PA!	DTX2	intronic	NM_	.	.	.
ACa06	7	####	rs6248349	G	A	.	PA!	POLR2J2	intronic	NM_	.	.	.
ACa06	7	####	rs3741264	C	G	.	PA!	LINC0100	ncRNA_	NR_	.	.	.
ACa06	7	####	.	T	C	.	PA!	KMT2C	intronic	NM_	.	.	.
ACa06	8	####	rs2077351	A	C	.	PA!	.	intergen	NM_	dist=	.	.
ACa06	8	####	.	T	G	.	PA!	PPAPDC1	intronic	NM_	.	.	.
ACa06	8	####	rs7503847	T	G	.	PA!	.	intergen	NM_	dist=	.	.
ACa06	8	####	rs7967153	G	A	.	PA!	.	intergen	NM_	dist=	.	.
ACa06	8	####	.	A	G	.	PA!	.	intergen	NM_	dist=	.	.
ACa06	8	####	rs4363208	C	G	.	PA!	.	intergen	NOI	dist=	.	.
ACa06	8	####	rs3740584	G	C	.	PA!	.	intergen	NOI	dist=	.	.
ACa06	8	####	.	C	T	.	PA!	.	ncRNA_	NR_	.	.	.
ACa06	8	####	.	G	A	.	PA!	ARC	exonic	NM_	.	mis	ARC:NI
ACa06	8	####	rs7821104	T	G	.	PA!	OPLAH	intronic	NM_	.	.	.
ACa06	9	####	rs7495042	G	A	.	PA!	.	upstrea	NR_	.	.	.
ACa06	9	####	rs4928839	A	C	.	PA!	.	intergen	NM_	dist=	.	.
ACa06	9	####	rs4568707	G	A	.	PA!	LOC6422	ncRNA_	NR_	.	.	.
ACa06	9	####	rs1435617	T	C	.	PA!	.	intergen	NM_	dist=	.	.
ACa06	9	####	rs3773733	T	C	.	PA!	.	intergen	NM_	dist=	.	.
ACa06	9	####	.	C	G	.	PA!	NXNL2	intronic	NM_	.	.	.
ACa06	9	####	.	T	G	.	PA!	.	intergen	NM_	dist=	.	.
ACa06	9	####	.	C	T	.	PA!	MAPKAP1	intronic	NM_	.	.	.
ACa06	10	####	rs1397322	T	C	.	PA!	.	intergen	NM_	dist=	.	.
ACa06	10	####	.	G	T	.	PA!	MIR603	ncRNA_	NR_	.	.	.
ACa06	10	####	rs9631413	T	C	.	PA!	.	intergen	NM_	dist=	.	.
ACa06	10	####	.	T	C	.	PA!	CREM	intronic	NM_	.	.	.
ACa06	10	####	rs694157	A	G	.	PA!	.	intergen	NR_	dist=	.	.
ACa06	10	####	rs5275172	G	A	.	PA!	.	intergen	NM_	dist=	.	.
ACa06	10	####	rs1888127	G	A	.	PA!	DLG5	intronic	NM_	.	.	.
ACa06	10	####	rs2573327	T	C	.	PA!	.	intergen	NM_	dist=	.	.
ACa06	10	####	rs3128226	C	G	.	PA!	NUTM2A	intronic	NM_	.	.	.
ACa06	11	####	.	T	C	.	PA!	.	intergen	NR_	dist=	.	.
ACa06	11	####	.	G	A	.	PA!	.	intergen	NR_	dist=	.	.
ACa06	11	####	rs9035709	C	T	.	PA!	.	intergen	NR_	dist=	.	.
ACa06	11	####	rs1874489	T	C	.	PA!	.	intergen	NM_	dist=	.	.
ACa06	11	####	rs9666739	G	T	.	PA!	PRKRIR	exonic	NM_	.	mis	PRKRIF
ACa06	11	####	rs5688517	G	A	.	PA!	.	intergen	NM_	dist=	.	.
ACa06	11	####	.	G	A	.	PA!	OR8G5	exonic	NM_	.	mis	OR8G5
ACa06	11	####	.	T	G	.	PA!	NTM	intronic	NM_	.	.	.
ACa06	12	####	rs7606061	T	C	.	PA!	KRT6A	intronic	NM_	.	.	.
ACa06	12	####	.	T	C	.	PA!	ERBB3	exonic	NM_	.	mis	ERBB3
ACa06	12	####	rs7597010	C	T	.	PA!	TDG	UTR5	NM_	NM_	.	.
ACa06	13	####	rs2004528	C	T	.	PA!	.	intergen	NR_	dist=	.	.
ACa06	13	####	rs1510074	G	A	.	PA!	MYO16	exonic	NM_	.	syr	MYO16
ACa06	14	####	rs7446578	T	C	.	PA!	DUXAP10	ncRNA_	NR_	.	.	.
ACa06	14	####	.	C	T	.	PA!	.	intergen	NR_	dist=	.	.
ACa06	14	####	rs2006543	C	T	.	PA!	.	intergen	NM_	dist=	.	.
ACa06	14	####	rs2020426	T	C	.	PA!	.	intergen	NM_	dist=	.	.

ACa06	14	####	rs7704695	G	T	.	PA!	.	intergen	NM_	dist=	.	.	
ACa06	14	####	.	G	A	.	PA!	PRKCH	exonic	NM_	.	unl	UNKNC
ACa06	14	####	.	A	T	.	PA!	GSTZ1	intronic	NM_	.	.	.
ACa06	14	####	.	C	T	.	PA!	.	intergen	NR_	dist=	.	.
ACa06	15	####	rs6200967	C	T	.	PA!	.	intergen	NOI	dist=	.	.
ACa06	15	####	.	C	T	.	PA!	.	intergen	NR_	dist=	.	.
ACa06	15	####	.	A	C	.	PA!	.	downstr	NR_	.	.	.
ACa06	15	####	rs7751825	G	C	.	PA!	GOLGA8T	ncRNA_	NR_	.	.	.
ACa06	15	####	rs2449652	T	C	.	PA!	LOC1002{	ncRNA_	NR_	.	.	.
ACa06	15	####	rs2449653	T	C	.	PA!	LOC1002{	ncRNA_	NR_	.	.	.
ACa06	15	####	.	G	A	.	PA!	GOLGA8K	intronic	NM_	.	.	.
ACa06	15	####	.	G	A	.	PA!	.	upstrea	NM_	.	.	.
ACa06	15	####	rs6202600	G	A	.	PA!	.	intergen	NM_	dist=	.	.
ACa06	15	####	.	A	C	.	PA!	SLC27A2	intronic	NM_	.	.	.
ACa06	15	####	.	T	A	.	PA!	SNX1	UTR5	NM_	NM_	.	.
ACa06	15	####	.	C	T	.	PA!	GOLGA6L	exonic	NM_	.	mis	GOLGA
ACa06	15	####	rs3743150	T	G	.	PA!	.	intergen	NM_	dist=	.	.
ACa06	15	####	rs9700260	C	G	.	PA!	GOLGA6L	ncRNA_	NR_	.	.	.
ACa06	15	####	rs364085	T	C	.	PA!	.	upstrea	NR_	.	.	.
ACa06	16	####	rs2009400	G	A	.	PA!	.	intergen	NR_	dist=	.	.
ACa06	16	####	rs2016432	C	T	.	PA!	.	intergen	NR_	dist=	.	.
ACa06	16	####	.	C	T	.	PA!	.	intergen	NR_	dist=	.	.
ACa06	16	####	rs5667377	T	C	.	PA!	.	intergen	NR_	dist=	.	.
ACa06	16	####	rs5618411	T	C	.	PA!	RRN3P2	ncRNA_	NR_	.	.	.
ACa06	16	####	rs8792106	A	T	.	PA!	.	downstr	NR_	.	.	.
ACa06	16	####	rs4319791	C	T	.	PA!	.	intergen	NOI	dist=	.	.
ACa06	16	####	rs4349321	G	A	.	PA!	.	intergen	NOI	dist=	.	.
ACa06	16	####	rs4246344	G	A	.	PA!	.	intergen	NOI	dist=	.	.
ACa06	16	####	.	A	T	.	PA!	.	intergen	NOI	dist=	.	.
ACa06	16	####	rs8799648	A	G	.	PA!	.	intergen	NOI	dist=	.	.
ACa06	16	####	rs9972801	A	G	.	PA!	.	intergen	NOI	dist=	.	.
ACa06	16	####	rs4324300	G	A	.	PA!	.	intergen	NOI	dist=	.	.
ACa06	16	####	rs9328621	T	C	.	PA!	.	intergen	NOI	dist=	.	.
ACa06	16	####	rs4380209	C	T	.	PA!	.	intergen	NOI	dist=	.	.
ACa06	16	####	rs4291933	A	G	.	PA!	.	intergen	NOI	dist=	.	.
ACa06	16	####	rs4011858	G	C	.	PA!	.	intergen	NOI	dist=	.	.
ACa06	16	####	.	G	C	.	PA!	.	intergen	NOI	dist=	.	.
ACa06	16	####	rs7205231	T	A	.	PA!	.	intergen	NOI	dist=	.	.
ACa06	16	####	rs4093117	C	A	.	PA!	.	intergen	NOI	dist=	.	.
ACa06	16	####	rs4249029	G	A	.	PA!	.	intergen	NOI	dist=	.	.
ACa06	16	####	.	C	T	.	PA!	.	intergen	NOI	dist=	.	.
ACa06	16	####	rs6205366	G	A	.	PA!	.	ncRNA_	NR_	.	.	.
ACa06	17	####	rs2543804	G	A	.	PA!	CCDC144	ncRNA_	NR_	.	.	.
ACa06	17	####	rs7126379	T	C	.	PA!	.	upstrea	NR_	.	.	.
ACa06	17	####	rs7126379	G	A	.	PA!	.	upstrea	NR_	.	.	.
ACa06	17	####	rs7841783	G	A	.	PA!	FLJ36000	ncRNA_	NR_	.	.	.
ACa06	17	####	.	T	A	.	PA!	.	intergen	NM_	dist=	.	.
ACa06	17	####	rs1437650	T	G	.	PA!	.	intergen	NM_	dist=	.	.
ACa06	17	####	.	G	A	.	PA!	.	intergen	NM_	dist=	.	.
ACa06	17	####	rs6207957	C	T	.	PA!	.	intronic	NM_	.	.	.
ACa06	17	####	rs7482024	G	T	.	PA!	KRTAP9-7	exonic	NM_	.	syr	KRTAP
ACa06	17	####	rs1459625	G	A	.	PA!	TUBG1	intronic	NM_	.	.	.
ACa06	17	####	.	A	T	.	PA!	.	intergen	NR_	dist=	.	.
ACa06	18	####	rs2012257	C	G	.	PA!	.	intergen	NM_	dist=	.	.

ACa06	18	####	rs3734996	A	T	.	PA!	.	intergen	NM_	dist=	.	.	
ACa06	18	####	rs7520640	G	A	.	PA!	SERPINB:	intronic	NM_	.	.	.	
ACa06	19	####	.	A	C	.	PA!	ARRDC5	intronic	NM_	.	.	.	
ACa06	19	####	.	T	A	.	PA!	CD22	intronic	NM_	.	.	.	
ACa06	19	####	rs7641371	G	T	.	PA!	.	intergen	NR_	dist=	.	.	
ACa06	19	####	.	G	A	.	PA!	.	intergen	NR_	dist=	.	.	
ACa06	19	####	.	C	A	.	PA!	.	upstreai	NM_	.	.	.	
ACa06	19	####	.	T	A	.	PA!	EML2	intronic	NM_	.	.	.	
ACa06	19	####	rs3499068	A	G	.	PA!	.	downstr	NR_	.	.	.	
ACa06	19	####	rs5615613	C	T	.	PA!	.	downstr	NR_	.	.	.	
ACa06	19	####	rs1334652	G	A	.	PA!	LILRA6,LI	intronic	NM_	.	.	.	
ACa06	19	####	rs3751555	G	A	.	PA!	.	intergen	NM_	dist=	.	.	
ACa06	20	####	.	C	T	.	PA!	NCOA6	exonic	NM_	.	syr	NCOA6	
ACa06	21	####	rs7984422	A	G	.	PA!	.	intergen	NOI	dist=	.	.	
ACa06	21	####	rs2017188	T	C	.	PA!	.	intergen	NM_	dist=	.	.	
ACa06	22	####	rs3753995	G	A	.	PA!	.	intergen	NM_	dist=	.	.	
ACa06	22	####	.	A	T	.	PA!	.	intergen	NM_	dist=	.	.	
ACa06	22	####	rs2021509	A	G	.	PA!	.	intergen	NM_	dist=	.	.	
ACa06	22	####	.	T	G	.	PA!	DGCR6L,T	intergen	NM_	dist=	.	.	
ACa06	22	####	rs6221896	C	T	.	PA!	.	intergen	NM_	dist=	.	.	
ACa06	22	####	rs2016329	G	T	.	PA!	.	intergen	NR_	dist=	.	.	
ACa06	22	####	rs6223125	C	T	.	PA!	POM121L	ncRNA_	NR_	.	.	.	
ACa06	22	####	rs367416	T	C	.	PA!	.	intergen	NM_	dist=	.	.	
ACa06	22	####	rs3609392	C	T	.	PA!	CYP2D7	ncRNA_	NR_	.	.	.	
ACa06	22	####	.	C	A	.	PA!	RRP7A	intronic	NM_	.	.	.	
ACa06	22	####	.	G	A	.	PA!	PARVG	intronic	NM_	.	.	.	
ACa06	X	####	.	C	T	.	PA!	KDM5C	UTR5	NM_	NM_	.	.	
ACa06	X	####	.	G	C	.	PA!	.	intergen	NOI	dist=	.	.	
ACa06	X	####	.	C	T	.	PA!	.	intergen	NOI	dist=	.	.	
ACa06	X	####	rs7520049	T	C	.	PA!	.	intergen	NOI	dist=	.	.	
ACa06	X	####	rs7756689	A	G	.	PA!	.	intergen	NOI	dist=	.	.	
ACa06	X	####	.	A	C	.	PA!	.	intergen	NOI	dist=	.	.	
ACa06	X	####	rs1048883	C	T	.	PA!	SNX12	exonic	NM_	.	mis	SNX12:	
ACa06	X	####	.	T	A	.	PA!	.	intergen	NM_	dist=	.	.	
ACa06	X	####	.	G	A	.	PA!	NHSL2	exonic	NM_	.	mis	NHSL2:	
ACa06	X	####	rs1423249	G	A	.	PA!	.	intergen	NM_	dist=	.	.	
ACa06	X	####	.	C	T	.	PA!	.	intergen	NM_	dist=	.	.	
ACa07	1	####	rs3744991	C	T	.	PA!	LINC0100	ncRNA_	NR_	.	.	.	
ACa07	1	####	.	C	T	.	PA!	NOC2L	intronic	NM_	.	.	.	
ACa07	1	####	rs1159856	C	T	.	PA!	KLHL17	exonic	NM_	.	syr	KLHL17	
ACa07	1	####	.	G	A	.	PA!	PERM1	exonic	NM_	.	unl	UNKNC	
ACa07	1	####	.	C	T	.	PA!	ISG15	exonic	NM_	.	mis	ISG15:I	
ACa07	1	####	rs5413521	C	T	.	PA!	FAM132A	UTR3	NM_	NM_	.	.	
ACa07	1	####	rs7680451	C	T	.	PA!	UBE2J2	intronic	NM_	.	.	.	
ACa07	1	####	.	G	A	.	PA!	CPTP	intronic	NM_	.	.	.	
ACa07	1	####	.	C	T	.	PA!	MXRA8	exonic	NM_	.	mis	MXRA8	
ACa07	1	####	rs7800811	C	T	.	PA!	CCNL2	intronic	NM_	.	.	.	
ACa07	1	####	.	G	A	.	PA!	MIB2	intronic	NM_	.	.	.	
ACa07	1	####	rs2014492	C	T	.	PA!	CDK11B	intronic	NM_	.	.	.	
ACa07	1	####	rs7463384	G	A	.	PA!	CDK11B	exonic	NM_	.	unl	UNKNC	
ACa07	1	####	.	C	T	.	PA!	CDK11B	intronic	NM_	.	.	.	
ACa07	1	####	.	C	T	.	PA!	CDK11B	intronic	NM_	.	.	.	
ACa07	1	####	.	C	A	.	PA!	GABRD	exonic	NM_	.	syr	GABRD	
ACa07	1	####	.	G	A	.	PA!	PRKCZ	intronic	NM_	.	.	.	

ACa07	1	####	.	G	A	.	PA!	PEX10	intronic	NM_	.	.	.
ACa07	1	####	.	A	G	.	PA!	MEGF6	exonic	NM_	.	syr	MEGF6
ACa07	1	####	.	C	A	.	PA!	DFFB	intronic	NM_	.	.	.
ACa07	1	####	.	G	T	.	PA!	NPHP4	exonic	NM_	.	unl	UNKNC
ACa07	1	####	.	G	A	.	PA!	NPHP4	intronic	NM_	.	.	.
ACa07	1	####	rs9430027	C	T	.	PA!	CHD5	intronic	NM_	.	.	.
ACa07	1	####	.	C	T	.	PA!	CHD5	exonic	NM_	.	mis	CHD5:N
ACa07	1	####	.	C	T	.	PA!	CHD5	exonic	NM_	.	mis	CHD5:N
ACa07	1	####	rs9864160	G	A	.	PA!	RNF207	intronic	NM_	.	.	.
ACa07	1	####	rs7562596	C	T	.	PA!	ZBTB48	intronic	NM_	.	.	.
ACa07	1	####	rs1431018	C	T	.	PA!	CAMTA1	exonic	NM_	.	syr	CAMTA
ACa07	1	####	.	T	A	.	PA!	CAMTA1	intronic	NM_	.	.	.
ACa07	1	####	rs7748471	C	T	.	PA!	CAMTA1	exonic	NM_	.	syr	CAMTA
ACa07	1	####	rs9446685	C	T	.	PA!	SLC2A7	intronic	NM_	.	.	.
ACa07	1	####	rs1476580	G	A	.	PA!	GPR157	exonic	NM_	.	mis	GPR15
ACa07	1	####	.	C	T	.	PA!	SLC25A33	intronic	NM_	.	.	.
ACa07	1	####	.	C	T	.	PA!	CLCN6	intronic	NM_	.	.	.
ACa07	1	####	.	A	G	.	PA!	.	intergen	NM_	dist=	.	.
ACa07	1	####	rs7934532	C	T	.	PA!	PLOD1	intronic	NM_	.	.	.
ACa07	1	####	.	C	T	.	PA!	TNFRSF8	intronic	NM_	.	.	.
ACa07	1	####	.	G	T	.	PA!	VPS13D	intronic	NM_	.	.	.
ACa07	1	####	.	C	T	.	PA!	VPS13D	exonic	NM_	.	mis	VPS13D
ACa07	1	####	rs2021163	G	C	.	PA!	PRAMEF4	intronic	NM_	.	.	.
ACa07	1	####	rs5876978	C	T	.	PA!	PRAMEF1	intronic	NM_	.	.	.
ACa07	1	####	rs4646008	G	A	.	PA!	CASP9	exonic	NM_	.	mis	CASP9
ACa07	1	####	rs3745685	C	T	.	PA!	EPHA2	UTR3	NM_	NM_	.	.
ACa07	1	####	.	G	A	.	PA!	ARHGEF1	exonic	NM_	.	mis	ARHGE
ACa07	1	####	rs3734377	G	A	.	PA!	NBPF1	exonic	NM_	.	unl	UNKNC
ACa07	1	####	rs6177234	C	A	.	PA!	NBPF1	intronic	NM_	.	.	.
ACa07	1	####	rs4112521	T	C	.	PA!	NBPF1	intronic	NM_	.	.	.
ACa07	1	####	rs1213433	A	T	.	PA!	NBPF1	UTR5	NM_	NM_	.	.
ACa07	1	####	rs3930814	G	A	.	PA!	.	upstrea	NR_	.	.	.
ACa07	1	####	rs5780915	G	A	.	PA!	ATP13A2	intronic	NM_	.	.	.
ACa07	1	####	rs3774826	C	T	.	PA!	IGSF21	exonic	NM_	.	mis	IGSF21
ACa07	1	####	rs3766882	C	T	.	PA!	TAS1R2	exonic	NM_	.	mis	TAS1R
ACa07	1	####	rs3471023	G	A	.	PA!	UBR4	exonic	NM_	.	syr	UBR4:N
ACa07	1	####	.	G	A	.	PA!	UBR4	exonic	NM_	.	syr	UBR4:N
ACa07	1	####	.	A	G	.	PA!	MRTO4	intronic	NM_	.	.	.
ACa07	1	####	rs3712996	C	T	.	PA!	CAPZB	UTR3	NM_	NM_	.	.
ACa07	1	####	.	G	A	.	PA!	TMCO4	intronic	NM_	.	.	.
ACa07	1	####	rs1001195	G	A	.	PA!	RAP1GAP	intronic	NM_	.	.	.
ACa07	1	####	.	C	T	.	PA!	HSPG2	intronic	NM_	.	.	.
ACa07	1	####	.	A	G	.	PA!	HSPG2	exonic	NM_	.	unl	UNKNC
ACa07	1	####	rs7584776	C	T	.	PA!	.	upstrea	NM_	.	.	.
ACa07	1	####	.	C	A	.	PA!	CELA3A	exonic	NM_	.	mis	CELA3A
ACa07	1	####	.	G	A	.	PA!	.	intronic	NM_	.	.	.
ACa07	1	####	.	G	T	.	PA!	GALE	UTR3	NM_	NM_	.	.
ACa07	1	####	rs1391690	C	T	.	PA!	HMGCL	intronic	NM_	.	.	.
ACa07	1	####	rs7518873	G	A	.	PA!	RUNX3	exonic	NM_	.	syr	RUNX3
ACa07	1	####	.	G	T	.	PA!	RHCE	intronic	NM_	.	.	.
ACa07	1	####	.	G	A	.	PA!	MAN1C1	intronic	NM_	.	.	.
ACa07	1	####	.	G	A	.	PA!	STMN1	intronic	NM_	.	.	.
ACa07	1	####	.	C	T	.	PA!	FAM110D	exonic	NM_	.	syr	FAM110
ACa07	1	####	rs7737670	C	T	.	PA!	CNKSR1	exonic	NM_	.	mis	CNKSR

ACa07	1	####	rs7767332	C	T	.	PA!	UBXN11	exonic	NM_	.	mis	UBXN1
ACa07	1	####	.	G	A	.	PA!	RPS6KA1	exonic	NM_	.	mis	RPS6K/
ACa07	1	####	rs8792552	C	T	.	PA!	ARID1A	exonic	NM_	.	sto	ARID1A
ACa07	1	####	.	C	T	.	PA!	ZDHHC18	exonic	NM_	.	mis	ZDHHC
ACa07	1	####	rs1003250	G	A	.	PA!	SLC9A1	intronic	NM_	.	.	.
ACa07	1	####	rs7625790	C	T	.	PA!	SLC9A1	intronic	NM_	.	.	.
ACa07	1	####	rs9468364	G	A	.	PA!	WDTC1	intronic	NM_	.	.	.
ACa07	1	####	.	C	T	.	PA!	LOC6449(	ncRNA_	NR_	.	.	.
ACa07	1	####	.	G	A	.	PA!	EYA3	intronic	NM_	.	.	.
ACa07	1	####	.	G	C	.	PA!	YTHDF2	intronic	NM_	.	.	.
ACa07	1	####	rs7596455	G	A	.	PA!	FABP3	exonic	NM_	.	syr	FABP3:
ACa07	1	####	rs9232513	G	A	.	PA!	SPOCD1	exonic	NM_	.	mis	SPOCD
ACa07	1	####	.	G	A	.	PA!	TMEM39E	intronic	NM_	.	.	.
ACa07	1	####	.	C	A	.	PA!	LCK	exonic	NM_	.	mis	LCK:NM
ACa07	1	####	.	G	A	.	PA!	.	intergen	NM_	dist=	.	.
ACa07	1	####	.	C	T	.	PA!	S100PBP	intronic	NM_	.	.	.
ACa07	1	####	rs9651423	A	G	.	PA!	ZMYM4	intronic	NM_	.	.	.
ACa07	1	####	rs7723940	G	A	.	PA!	RSPO1	exonic	NM_	.	mis	RSPO1
ACa07	1	####	.	G	A	.	PA!	C1orf109	intronic	NM_	.	.	.
ACa07	1	####	rs1814745	C	T	.	PA!	EPHA10	intronic	NM_	.	.	.
ACa07	1	####	.	C	T	.	PA!	MACF1	intronic	NM_	.	.	.
ACa07	1	####	.	G	A	.	PA!	MACF1	intronic	NM_	.	.	.
ACa07	1	####	.	A	C	.	PA!	MACF1	UTR3	NM_	NM_	.	.
ACa07	1	####	rs5523408	C	T	.	PA!	PPIEL	ncRNA_	NR_	.	.	.
ACa07	1	####	.	G	T	.	PA!	PABPC4	intronic	NM_	.	.	.
ACa07	1	####	.	G	A	.	PA!	PPT1	exonic	NM_	.	mis	PPT1:N
ACa07	1	####	.	T	C	.	PA!	RLF	exonic	NM_	.	syr	RLF:NM
ACa07	1	####	.	G	A	.	PA!	RLF	UTR3	NM_	NM_	.	.
ACa07	1	####	rs9093579	G	T	.	PA!	KCNQ4	intronic	NM_	.	.	.
ACa07	1	####	.	T	A	.	PA!	KCNQ4	intronic	NM_	.	.	.
ACa07	1	####	rs1179985	C	T	.	PA!	FOXJ3	intronic	NM_	.	.	.
ACa07	1	####	rs7456140	G	A	.	PA!	YBX1	exonic	NM_	.	mis	YBX1:N
ACa07	1	####	.	G	A	.	PA!	MED8	intronic	NM_	.	.	.
ACa07	1	####	.	C	T	.	PA!	KDM4A-A!	ncRNA_	NR_	.	.	.
ACa07	1	####	.	C	T	.	PA!	PLK3	intronic	NM_	.	.	.
ACa07	1	####	.	C	A	.	PA!	CCDC17	exonic	NM_	.	mis	CCDC1
ACa07	1	####	rs7290104	C	T	.	PA!	RAD54L	intronic	NM_	.	.	.
ACa07	1	####	.	A	G	.	PA!	FAAH	intronic	NM_	.	.	.
ACa07	1	####	rs5508458	C	T	.	PA!	MKNK1	exonic	NM_	.	syr	MKNK1
ACa07	1	####	.	C	T	.	PA!	CYP4Z2P	ncRNA_	NR_	.	.	.
ACa07	1	####	.	G	T	.	PA!	AGBL4	intronic	NM_	.	.	.
ACa07	1	####	rs7570389	C	T	.	PA!	TTC39A	intronic	NM_	.	.	.
ACa07	1	####	rs9512571	C	T	.	PA!	LRP8	intronic	NM_	.	.	.
ACa07	1	####	rs7759188	C	T	.	PA!	GLIS1	intronic	NM_	.	.	.
ACa07	1	####	.	G	A	.	PA!	CYB5RL	exonic	NM_	.	mis	CYB5R
ACa07	1	####	rs3769455	G	A	.	PA!	PCSK9	exonic	NM_	.	mis	PCSK9
ACa07	1	####	rs7815069	C	T	.	PA!	PRKAA2	exonic	NM_	.	mis	PRKAA
ACa07	1	####	.	C	T	.	PA!	DAB1	intronic	NM_	.	.	.
ACa07	1	####	.	T	A	.	PA!	JAK1	intronic	NM_	.	.	.
ACa07	1	####	.	C	T	.	PA!	WDR78	intronic	NM_	.	.	.
ACa07	1	####	.	C	A	.	PA!	FPGT,FP(	intronic	NM_	.	.	.
ACa07	1	####	.	A	G	.	PA!	PIGK	intronic	NM_	.	.	.
ACa07	1	####	rs1785229	G	A	.	PA!	ELTD1	exonic	NM_	.	syr	ELTD1:
ACa07	1	####	.	G	A	.	PA!	.	intergen	NM_	dist=	.	.

ACa07	1	####	rs1394863	C	T	.	PA!	LPHN2	exonic	NM_	.	syr	LPHN2:
ACa07	1	####	rs7636045	C	T	.	PA!	LPAR3	exonic	NM_	.	mis	LPAR3:
ACa07	1	####	.	C	A	.	PA!	ZNHIT6	intronic	NM_	.	.	.
ACa07	1	####	.	G	T	.	PA!	TGFBR3	intronic	NM_	.	.	.
ACa07	1	####	.	G	C	.	PA!	ABCA4	exonic	NM_	.	syr	ABCA4
ACa07	1	####	rs6174943	G	A	.	PA!	ABCA4	exonic	NM_	.	syr	ABCA4
ACa07	1	####	.	T	A	.	PA!	LRRC39	intronic	NM_	.	.	.
ACa07	1	####	.	A	T	.	PA!	COL11A1	intronic	NM_	.	.	.
ACa07	1	####	.	A	C	.	PA!	PRPF38B	exonic	NM_	.	mis	PRPF38
ACa07	1	####	rs9363214	C	T	.	PA!	CELSR2	intronic	NM_	.	.	.
ACa07	1	####	rs7774328	G	A	.	PA!	STRIP1	intronic	NM_	.	.	.
ACa07	1	####	.	C	T	.	PA!	RBM15	exonic	NM_	.	mis	RBM15
ACa07	1	####	.	T	C	.	PA!	CHIA	UTR5	NM_	NM_	.	.
ACa07	1	####	.	C	T	.	PA!	C1orf162	exonic	NM_	.	unl	UNKNO
ACa07	1	####	.	T	C	.	PA!	FAM212B	UTR3	NM_	NM_	.	.
ACa07	1	####	rs7556469	C	T	.	PA!	MOV10	exonic	NM_	.	mis	MOV10
ACa07	1	####	.	G	A	.	PA!	RHOC	exonic	NM_	.	syr	RHOC:I
ACa07	1	####	.	G	T	.	PA!	MAGI3	exonic	NM_	.	mis	MAGI3:
ACa07	1	####	.	T	G	.	PA!	AMPD1	intronic	NM_	.	.	.
ACa07	1	####	rs3724512	C	T	.	PA!	AMPD1	exonic	NM_	.	mis	AMPD1
ACa07	1	####	.	G	A	.	PA!	TTF2	intronic	NM_	.	.	.
ACa07	1	####	.	G	T	.	PA!	SPAG17,V	intronic	NM_	.	.	.
ACa07	1	####	.	C	T	.	PA!	NBPF7	intronic	NM_	.	.	.
ACa07	1	####	rs1041798	C	T	.	PA!	NOTCH2	intronic	NM_	.	.	.
ACa07	1	####	.	A	G	.	PA!	NOTCH2	splicing	NM_	NM_	.	.
ACa07	1	####	.	T	G	.	PA!	ANKRD20	ncRNA_	NR_	.	.	.
ACa07	1	####	rs9441133	T	G	.	PA!	NBPF9,NE	exonic	NM_	.	unl	UNKNO
ACa07	1	####	rs4067693	G	C	.	PA!	NBPF20,N	intronic	NM_	.	.	.
ACa07	1	####	.	A	G	.	PA!	NBPF9,NE	exonic	NM_	.	unl	UNKNO
ACa07	1	####	rs7819390	C	T	.	PA!	SEC22B	exonic	NM_	.	unl	UNKNO
ACa07	1	####	rs7515424	T	A	.	PA!	NBPF25P	ncRNA_	NR_	.	.	.
ACa07	1	####	.	C	T	.	PA!	NBPF25P	ncRNA_	NR_	.	.	.
ACa07	1	####	.	C	T	.	PA!	POLR3C	exonic	NM_	.	mis	POLR3
ACa07	1	####	.	G	A	.	PA!	NBPF25P	ncRNA_	NR_	.	.	.
ACa07	1	####	.	G	A	.	PA!	LOC1010	ncRNA_	NR_	.	.	.
ACa07	1	####	.	C	T	.	PA!	NBPF25P	ncRNA_	NR_	.	.	.
ACa07	1	####	.	C	T	.	PA!	TARS2	intronic	NM_	.	.	.
ACa07	1	####	.	G	A	.	PA!	PI4KB	exonic	NM_	.	sto	PI4KB:I
ACa07	1	####	.	T	C	.	PA!	POGZ	exonic	NM_	.	mis	POGZ:I
ACa07	1	####	.	G	A	.	PA!	FLG2	exonic	NM_	.	syr	FLG2:N
ACa07	1	####	rs9479104	C	T	.	PA!	PGLYRP3	intronic	NM_	.	.	.
ACa07	1	####	.	C	T	.	PA!	S100A16	intronic	NM_	.	.	.
ACa07	1	####	.	A	G	.	PA!	INTS3	intronic	NM_	.	.	.
ACa07	1	####	rs7704251	G	A	.	PA!	DENND4E	exonic	NM_	.	mis	DENND
ACa07	1	####	.	C	A	.	PA!	UBE2Q1	splicing	NM_	NM_	.	.
ACa07	1	####	.	C	T	.	PA!	MTX1	intronic	NM_	.	.	.
ACa07	1	####	.	C	A	.	PA!	ASH1L	exonic	NM_	.	mis	ASH1L:
ACa07	1	####	.	C	A	.	PA!	YY1AP1	intronic	NM_	.	.	.
ACa07	1	####	rs9321901	C	T	.	PA!	PMF1	UTR3	NM_	NM_	.	.
ACa07	1	####	.	T	C	.	PA!	ARHGEF1	intronic	NM_	.	.	.
ACa07	1	####	rs3718046	C	T	.	PA!	ETV3L	exonic	NM_	.	mis	ETV3L:
ACa07	1	####	rs2000983	A	G	.	PA!	.	intergen	NM_	dist=	.	.
ACa07	1	####	rs7734794	G	A	.	PA!	CD1B	exonic	NM_	.	syr	CD1B:N
ACa07	1	####	rs7622475	C	T	.	PA!	CRP	intronic	NM_	.	.	.

ACa07	1	####	.	G	A	.	PA:	ADAMTS4	exonic	NM_	.	mis	ADAMT
ACa07	1	####	rs2010794	C	T	.	PA:	C1orf226	exonic	NM_	.	syr	C1orf22
ACa07	1	####	.	C	A	.	PA:	RGS4	exonic	NM_	.	mis	RGS4:N
ACa07	1	####	.	A	G	.	PA:	PBX1	exonic	NM_	.	mis	PBX1:N
ACa07	1	####	.	C	T	.	PA:	POU2F1	exonic	NM_	.	syr	POU2F
ACa07	1	####	.	G	A	.	PA:	CCDC181	intronic	NM_	.	.	.
ACa07	1	####	.	A	G	.	PA:	SELP	intronic	NM_	.	.	.
ACa07	1	####	.	C	T	.	PA:	SCYL3	intronic	NM_	.	.	.
ACa07	1	####	.	G	A	.	PA:	RABGAP1	intronic	NM_	.	.	.
ACa07	1	####	.	T	G	.	PA:	PAPPA2	intronic	NM_	.	.	.
ACa07	1	####	rs7763360	C	T	.	PA:	ASTN1	exonic	NM_	.	syr	ASTN1:
ACa07	1	####	.	C	A	.	PA:	ABL2	exonic	NM_	.	sto	ABL2:N
ACa07	1	####	rs1014592	C	T	.	PA:	NPHS2	UTR5	NM_	NM_	.	.
ACa07	1	####	.	C	T	.	PA:	CEP350	intronic	NM_	.	.	.
ACa07	1	####	rs7733795	C	A	.	PA:	CACNA1E	exonic	NM_	.	mis	CACNA
ACa07	1	####	rs7640679	G	A	.	PA:	ZNF648	exonic	NM_	.	mis	ZNF648
ACa07	1	####	.	G	A	.	PA:	TEDDM1	exonic	NM_	.	mis	TEDDM
ACa07	1	####	rs7702036	C	T	.	PA:	NPL	exonic	NM_	.	mis	NPL:NM
ACa07	1	####	.	C	A	.	PA:	TPR	exonic	NM_	.	mis	TPR:NM
ACa07	1	####	.	C	T	.	PA:	CRB1	intronic	NM_	.	.	.
ACa07	1	####	.	A	G	.	PA:	CAMSAP2	intronic	NM_	.	.	.
ACa07	1	####	rs3712350	G	A	.	PA:	KIF21B	exonic	NM_	.	mis	KIF21B
ACa07	1	####	rs3723002	C	T	.	PA:	CACNA1S	intronic	NM_	.	.	.
ACa07	1	####	rs3694010	G	A	.	PA:	NAV1	exonic	NM_	.	mis	NAV1:N
ACa07	1	####	.	G	T	.	PA:	TIMM17A	intronic	NM_	.	.	.
ACa07	1	####	rs1044866	C	T	.	PA:	ETNK2	intronic	NM_	.	.	.
ACa07	1	####	.	C	T	.	PA:	CNTN2	intronic	NM_	.	.	.
ACa07	1	####	.	T	G	.	PA:	C4BPA	exonic	NM_	.	syr	C4BPA
ACa07	1	####	.	G	A	.	PA:	CR1L	intronic	NM_	.	.	.
ACa07	1	####	.	T	C	.	PA:	PLXNA2	intronic	NM_	.	.	.
ACa07	1	####	.	C	G	.	PA:	HSD11B1	intronic	NM_	.	.	.
ACa07	1	####	.	T	C	.	PA:	RCOR3	intronic	NM_	.	.	.
ACa07	1	####	rs9188348	C	T	.	PA:	TATDN3	intronic	NM_	.	.	.
ACa07	1	####	.	C	T	.	PA:	FLVCR1	exonic	NM_	.	mis	FLVCR
ACa07	1	####	.	G	T	.	PA:	RPS6KC1	exonic	NM_	.	mis	RPS6K
ACa07	1	####	.	A	G	.	PA:	PTPN14	intronic	NM_	.	.	.
ACa07	1	####	.	G	A	.	PA:	PTPN14	intronic	NM_	.	.	.
ACa07	1	####	rs9106912	T	C	.	PA:	USH2A	exonic	NM_	.	mis	USH2A
ACa07	1	####	.	G	T	.	PA:	USH2A	intronic	NM_	.	.	.
ACa07	1	####	rs7564262	C	T	.	PA:	USH2A	exonic	NM_	.	mis	USH2A
ACa07	1	####	rs1909372	C	T	.	PA:	ESRRG	UTR3	NM_	NM_	.	.
ACa07	1	####	.	C	T	.	PA:	SLC30A10	splicing	NM_	NM_	.	.
ACa07	1	####	.	C	T	.	PA:	IARS2	exonic	NM_	.	syr	IARS2:I
ACa07	1	####	rs7799500	C	T	.	PA:	IARS2	exonic	NM_	.	mis	IARS2:I
ACa07	1	####	.	C	A	.	PA:	2-Mar	exonic	NM_	.	mis	MARC2
ACa07	1	####	.	T	G	.	PA:	DISP1	exonic	NM_	.	mis	DISP1:I
ACa07	1	####	.	C	T	.	PA:	NVL	intronic	NM_	.	.	.
ACa07	1	####	.	G	A	.	PA:	TMEM63A	intronic	NM_	.	.	.
ACa07	1	####	.	G	A	.	PA:	LEFTY2	exonic	NM_	.	mis	LEFTY2
ACa07	1	####	rs9303868	C	T	.	PA:	.	intergen	NM_	dist=	.	.
ACa07	1	####	.	G	A	.	PA:	ADCK3	intronic	NM_	.	.	.
ACa07	1	####	.	A	T	.	PA:	OBSCN	exonic	NM_	.	mis	OBSCN
ACa07	1	####	.	G	A	.	PA:	TRIM67	exonic	NM_	.	mis	TRIM67
ACa07	1	####	.	C	T	.	PA:	KIAA1804	intronic	NM_	.	.	.

ACa07	1	####	rs1006991	C	T	.	PA!	KCNK1	exonic	NM_	.	syr	KCNK1
ACa07	1	####	rs3676265	C	T	.	PA!	RYR2	intronic	NM_	.	.	.
ACa07	1	####	.	C	T	.	PA!	PLD5	intronic	NM_	.	.	.
ACa07	1	####	.	C	T	.	PA!	AKT3	splicing	NM_	NM_	.	.
ACa07	1	####	rs1000082	G	A	.	PA!	.	intergen	NM_	dist=	.	.
ACa07	1	####	rs1825931	C	T	.	PA!	ZNF496	intronic	NM_	.	.	.
ACa07	1	####	rs7580154	C	T	.	PA!	ZNF692	exonic	NM_	.	mis	ZNF692
ACa07	2	####	rs3724090	G	A	.	PA!	TPO	intronic	NM_	.	.	.
ACa07	2	####	rs7778730	G	A	.	PA!	TSSC1	intronic	NM_	.	.	.
ACa07	2	####	.	A	G	.	PA!	TRAPPC1	exonic	NM_	.	mis	TRAPP
ACa07	2	####	rs5690508	G	A	.	PA!	TRAPPC1	exonic	NM_	.	mis	TRAPP
ACa07	2	####	.	T	C	.	PA!	.	intergen	NR_	dist=	.	.
ACa07	2	####	rs7490370	G	A	.	PA!	MBOAT2	exonic	NM_	.	mis	MBOAT
ACa07	2	####	rs3691590	C	T	.	PA!	ADAM17	exonic	NM_	.	mis	ADAM1
ACa07	2	####	rs9653906	G	A	.	PA!	PQLC3	intronic	NM_	.	.	.
ACa07	2	####	.	C	T	.	PA!	FAM49A	intronic	NM_	.	.	.
ACa07	2	####	rs3765736	C	T	.	PA!	FAM49A	intronic	NM_	.	.	.
ACa07	2	####	rs3680831	C	T	.	PA!	VSNL1	exonic	NM_	.	syr	VSNL1:
ACa07	2	####	rs7556987	C	T	.	PA!	KCNS3	exonic	NM_	.	mis	KCNS3
ACa07	2	####	.	G	A	.	PA!	KLHL29	exonic	NM_	.	mis	KLHL29
ACa07	2	####	rs5650507	G	A	.	PA!	ITSN2	intronic	NM_	.	.	.
ACa07	2	####	rs7736455	G	A	.	PA!	NCOA1	exonic	NM_	.	syr	NCOA1
ACa07	2	####	rs1479767	G	A	.	PA!	EMILIN1	exonic	NM_	.	mis	EMILIN
ACa07	2	####	.	G	A	.	PA!	CAD	exonic	NM_	.	mis	CAD:NI
ACa07	2	####	.	A	T	.	PA!	CAD	exonic	NM_	.	mis	CAD:NI
ACa07	2	####	.	G	A	.	PA!	FNDC4	intronic	NM_	.	.	.
ACa07	2	####	.	G	A	.	PA!	ALK	intronic	NM_	.	.	.
ACa07	2	####	rs7664313	G	A	.	PA!	BIRC6	exonic	NM_	.	mis	BIRC6:I
ACa07	2	####	rs1047800	G	A	.	PA!	TTC27	intronic	NM_	.	.	.
ACa07	2	####	.	A	G	.	PA!	LINC0048	ncRNA_	NR_	.	.	.
ACa07	2	####	.	C	T	.	PA!	STRN	exonic	NM_	.	mis	STRN:I
ACa07	2	####	rs7725290	C	T	.	PA!	SLC8A1	intronic	NM_	.	.	.
ACa07	2	####	.	T	G	.	PA!	LRPPRC	intronic	NM_	.	.	.
ACa07	2	####	.	T	C	.	PA!	PPP1R21	UTR3	NM_	NM_	.	.
ACa07	2	####	.	C	A	.	PA!	STON1-G	intronic	NM_	.	.	.
ACa07	2	####	.	C	T	.	PA!	SPTBN1	intronic	NM_	.	.	.
ACa07	2	####	.	C	A	.	PA!	SPTBN1	exonic	NM_	.	mis	SPTBN
ACa07	2	####	.	T	C	.	PA!	RTN4	UTR5	NM_	NM_	.	.
ACa07	2	####	rs7787019	T	C	.	PA!	EFEMP1	intronic	NM_	.	.	.
ACa07	2	####	rs7608139	C	T	.	PA!	CCDC85A	exonic	NM_	.	syr	CCDC8
ACa07	2	####	.	T	C	.	PA!	BCL11A	UTR3	NM_	NM_	.	.
ACa07	2	####	.	C	T	.	PA!	WDPCP	intronic	NM_	.	.	.
ACa07	2	####	.	A	T	.	PA!	UGP2	intronic	NM_	.	.	.
ACa07	2	####	.	A	C	.	PA!	PELI1	UTR3	NM_	NM_	.	.
ACa07	2	####	.	C	T	.	PA!	ARHGAP2	exonic	NM_	.	mis	ARHGA
ACa07	2	####	.	A	G	.	PA!	ANTXR1	intronic	NM_	.	.	.
ACa07	2	####	.	C	A	.	PA!	ADD2	intronic	NM_	.	.	.
ACa07	2	####	.	C	T	.	PA!	NAGK	intronic	NM_	.	.	.
ACa07	2	####	.	C	A	.	PA!	DYSF	intronic	NM_	.	.	.
ACa07	2	####	rs7670256	C	T	.	PA!	EVA1A	exonic	NM_	.	syr	EVA1A:
ACa07	2	####	.	A	G	.	PA!	DNAH6	exonic	NM_	.	mis	DNAH6
ACa07	2	####	rs1883943	G	A	.	PA!	KCMF1	intronic	NM_	.	.	.
ACa07	2	####	.	G	A	.	PA!	RETSAT	intronic	NM_	.	.	.
ACa07	2	####	.	G	T	.	PA!	GGCX	exonic	NM_	.	syr	GGCX:I

ACa07	2	####	.	T	C	.	PA!	VAMP5	intronic	NM_	.	.	.	
ACa07	2	####	.	C	T	.	PA!	RGPD2,R	intronic	NM_	.	.	.	
ACa07	2	####	.	A	C	.	PA!	.	intergen	NR_	dist=	.	.	
ACa07	2	####	rs1915368	G	A	.	PA!	.	intergen	NR_	dist=	.	.	
ACa07	2	####	rs7778874	G	A	.	PA!	.	intergen	NR_	dist=	.	.	
ACa07	2	####	rs2923588	G	A	.	PA!	.	intergen	NR_	dist=	.	.	
ACa07	2	####	rs3763447	C	T	.	PA!	.	intergen	NR_	dist=	.	.	
ACa07	2	####	.	T	G	.	PA!	.	intergen	NR_	dist=	.	.	
ACa07	2	####	.	T	C	.	PA!	.	intergen	NR_	dist=	.	.	
ACa07	2	####	rs8689209	G	A	.	PA!	.	intergen	NOI	dist=	.	.	
ACa07	2	####	rs3759446	C	A	.	PA!	.	intergen	NOI	dist=	.	.	
ACa07	2	####	rs1210521	G	A	.	PA!	.	intergen	NOI	dist=	.	.	
ACa07	2	####	rs2931782	G	A	.	PA!	ANKRD20	ncRNA_	NR_	.	.	.	
ACa07	2	####	.	A	G	.	PA!	TRIM43B	intronic	NM_	.	.	.	
ACa07	2	####	rs7784367	C	T	.	PA!	ASTL	exonic	NM_	.	syr	ASTL:N	
ACa07	2	####	.	C	T	.	PA!	SNRNP20	exonic	NM_	.	syr	SNRNP	
ACa07	2	####	rs5704884	G	A	.	PA!	FER1L5	intronic	NM_	.	.	.	
ACa07	2	####	.	T	C	.	PA!	CNNM3	intronic	NM_	.	.	.	
ACa07	2	####	rs5571075	G	A	.	PA!	ANKRD36	exonic	NM_	.	syr	ANKRD	
ACa07	2	####	.	T	C	.	PA!	ANKRD36	intronic	NM_	.	.	.	
ACa07	2	####	.	C	A	.	PA!	TMEM131	exonic	NM_	.	syr	TMEM1	
ACa07	2	####	rs7682782	C	T	.	PA!	AFF3	exonic	NM_	.	mis	AFF3:N	
ACa07	2	####	.	G	A	.	PA!	IL18R1	exonic	NM_	.	mis	IL18R1:	
ACa07	2	####	rs7516119	G	A	.	PA!	TGFBRAF	exonic	NM_	.	syr	TGFBR	
ACa07	2	####	rs2018112	C	T	.	PA!	RGPD3	exonic	NM_	.	mis	RGPD3	
ACa07	2	####	rs7589510	C	T	.	PA!	BUB1	exonic	NM_	.	mis	BUB1:N	
ACa07	2	####	.	G	A	.	PA!	.	intergen	NM_	dist=	.	.	
ACa07	2	####	.	A	G	.	PA!	.	downstr	NM_	.	.	.	
ACa07	2	####	.	A	G	.	PA!	IL36A	exonic	NM_	.	mis	IL36A:N	
ACa07	2	####	.	G	A	.	PA!	.	intergen	NM_	dist=	.	.	
ACa07	2	####	.	T	C	.	PA!	DPP10	intronic	NM_	.	.	.	
ACa07	2	####	rs7642264	C	T	.	PA!	RALB	exonic	NM_	.	syr	RALB:N	
ACa07	2	####	.	T	A	.	PA!	PROC	intronic	NM_	.	.	.	
ACa07	2	####	.	G	A	.	PA!	MYO7B	intronic	NM_	.	.	.	
ACa07	2	####	rs7817298	C	T	.	PA!	SMPD4	intronic	NM_	.	.	.	
ACa07	2	####	rs1340756	G	C	.	PA!	.	intergen	NM_	dist=	.	.	
ACa07	2	####	rs1820004	C	T	.	PA!	.	intergen	NM_	dist=	.	.	
ACa07	2	####	.	A	G	.	PA!	GPR39	intronic	NM_	.	.	.	
ACa07	2	####	.	C	T	.	PA!	MIR7853	ncRNA_	NR_	.	.	.	
ACa07	2	####	.	C	A	.	PA!	LRP1B	intronic	NM_	.	.	.	
ACa07	2	####	.	G	A	.	PA!	NEB	exonic	NM_	.	mis	NEB:NI	
ACa07	2	####	.	G	T	.	PA!	STAM2	intronic	NM_	.	.	.	
ACa07	2	####	.	G	A	.	PA!	FMNL2	intronic	NM_	.	.	.	
ACa07	2	####	rs1012824	C	T	.	PA!	PRPF40A	intronic	NM_	.	.	.	
ACa07	2	####	rs9859742	A	C	.	PA!	ARL6IP6	intronic	NM_	.	.	.	
ACa07	2	####	rs2003571	C	T	.	PA!	TANC1	exonic	NM_	.	mis	TANC1	
ACa07	2	####	rs3721419	G	A	.	PA!	SLC4A10	exonic	NM_	.	mis	SLC4A1	
ACa07	2	####	.	G	T	.	PA!	DPP4	intronic	NM_	.	.	.	
ACa07	2	####	rs1029714	C	T	.	PA!	DPP4	intronic	NM_	.	.	.	
ACa07	2	####	.	G	A	.	PA!	SCN3A	intronic	NM_	.	.	.	
ACa07	2	####	.	T	G	.	PA!	ABCB11	intronic	NM_	.	.	.	
ACa07	2	####	.	G	T	.	PA!	BBS5	UTR5	NM_	NM_	.	.	
ACa07	2	####	.	A	G	.	PA!	LOC1019	ncRNA_	NR_	.	.	.	
ACa07	2	####	rs9215367	C	T	.	PA!	GORASP2	exonic	NM_	.	sto	GORAS	

ACa07	2	####	.	T	A	.	PA!	DYNC1I2	intronic	NM_	.	.	.
ACa07	2	####	rs9638533	G	A	.	PA!	RAPGEF4	intronic	NM_	.	.	.
ACa07	2	####	.	T	C	.	PA!	WIPF1	intronic	NM_	.	.	.
ACa07	2	####	.	T	C	.	PA!	CHRNA1	intronic	NM_	.	.	.
ACa07	2	####	.	G	A	.	PA!	.	intergen	NM_	dist=	.	.
ACa07	2	####	.	A	T	.	PA!	AGPS	intronic	NM_	.	.	.
ACa07	2	####	.	G	A	.	PA!	PDE11A	intronic	NM_	.	.	.
ACa07	2	####	rs9603080	T	A	.	PA!	TTN	intronic	NM_	.	.	.
ACa07	2	####	rs3772721	C	T	.	PA!	DNAJC10	exonic	NM_	.	mis	DNAJC
ACa07	2	####	rs3766275	T	C	.	PA!	FSIP2	exonic	NM_	.	mis	FSIP2:N
ACa07	2	####	.	A	G	.	PA!	FSIP2	exonic	NM_	.	mis	FSIP2:N
ACa07	2	####	.	T	C	.	PA!	ITGAV	intronic	NM_	.	.	.
ACa07	2	####	rs3768239	G	A	.	PA!	COL5A2	intronic	NM_	.	.	.
ACa07	2	####	rs7715235	C	T	.	PA!	WDR75	intronic	NM_	.	.	.
ACa07	2	####	.	G	A	.	PA!	STAT1	intronic	NM_	.	.	.
ACa07	2	####	.	G	A	.	PA!	SDPR	exonic	NM_	.	mis	SDPR:N
ACa07	2	####	.	T	A	.	PA!	STK17B	exonic	NM_	.	mis	STK17B
ACa07	2	####	.	C	T	.	PA!	KCTD18	exonic	NM_	.	mis	KCTD18
ACa07	2	####	.	T	C	.	PA!	CLK1	exonic	NM_	.	mis	CLK1:N
ACa07	2	####	rs5481928	G	A	.	PA!	KIAA2012	exonic	NM_	.	syn	KIAA20
ACa07	2	####	rs1901033	T	A	.	PA!	NBEAL1	intronic	NM_	.	.	.
ACa07	2	####	rs7778048	A	G	.	PA!	RAPH1	exonic	NM_	.	syn	RAPH1
ACa07	2	####	rs1477917	G	A	.	PA!	EEF1B2	exonic	NM_	.	syn	EEF1B2
ACa07	2	####	rs5379398	G	A	.	PA!	ADAM23	intronic	NM_	.	.	.
ACa07	2	####	.	G	C	.	PA!	CRYGD	exonic	NM_	.	mis	CRYGD
ACa07	2	####	.	C	A	.	PA!	.	intergen	NM_	dist=	.	.
ACa07	2	####	.	G	A	.	PA!	UNC80	exonic	NM_	.	mis	UNC80
ACa07	2	####	.	C	T	.	PA!	UNC80	intronic	NM_	.	.	.
ACa07	2	####	.	A	G	.	PA!	UNC80	intronic	NM_	.	.	.
ACa07	2	####	.	T	C	.	PA!	CPS1	intronic	NM_	.	.	.
ACa07	2	####	.	G	T	.	PA!	TNS1	intronic	NM_	.	.	.
ACa07	2	####	.	A	T	.	PA!	.	intergen	NM_	dist=	.	.
ACa07	2	####	.	C	A	.	PA!	CATIP-AS	ncRNA_	NR_	.	.	.
ACa07	2	####	.	G	T	.	PA!	SLC11A1	splicing	NM_	NM_	.	.
ACa07	2	####	rs5756405	G	A	.	PA!	VIL1	intronic	NM_	.	.	.
ACa07	2	####	.	G	T	.	PA!	TTLL4	intronic	NM_	.	.	.
ACa07	2	####	rs3732211	G	A	.	PA!	CCDC108	intronic	NM_	.	.	.
ACa07	2	####	.	G	A	.	PA!	DNAJB2	exonic	NM_	.	syn	DNAJB2
ACa07	2	####	rs7469670	C	T	.	PA!	DES	intronic	NM_	.	.	.
ACa07	2	####	rs5290584	C	T	.	PA!	SPEG	splicing	NM_	NM_	.	.
ACa07	2	####	.	T	A	.	PA!	EPHA4	exonic	NM_	.	mis	EPHA4
ACa07	2	####	.	C	T	.	PA!	IRS1	exonic	NM_	.	syn	IRS1:NI
ACa07	2	####	.	A	T	.	PA!	LOC65484	ncRNA_	NR_	.	.	.
ACa07	2	####	rs499449	C	T	.	PA!	FBXO36	intronic	NM_	.	.	.
ACa07	2	####	rs7533248	G	A	.	PA!	NMUR1	exonic	NM_	.	mis	NMUR1
ACa07	2	####	.	C	A	.	PA!	DIS3L2	splicing	NM_	NM_	.	.
ACa07	2	####	.	G	T	.	PA!	DIS3L2	intronic	NM_	.	.	.
ACa07	2	####	.	G	A	.	PA!	ALPPL2	intronic	NM_	.	.	.
ACa07	2	####	rs2020267	G	A	.	PA!	INPP5D	exonic	NM_	.	unl	UNKNC
ACa07	2	####	rs1053770	C	T	.	PA!	MROH2A	intronic	NM_	.	.	.
ACa07	2	####	.	C	T	.	PA!	MROH2A	exonic	NM_	.	syn	MROH2
ACa07	2	####	.	C	T	.	PA!	IQCA1	intronic	NM_	.	.	.
ACa07	2	####	rs7483373	C	T	.	PA!	PER2	exonic	NM_	.	mis	PER2:N
ACa07	2	####	.	C	T	.	PA!	LOC15093	ncRNA_	NR_	.	.	.

ACa07	2	####	rs3776284	G	A	.	PA!	KIF1A	exonic	NM_	.	syr	KIF1A:N
ACa07	2	####	rs1500761	C	T	.	PA!	KIF1A	intronic	NM_	.	.	.
ACa07	2	####	.	G	T	.	PA!	.	upstreai	NM_	.	.	.
ACa07	2	####	.	C	T	.	PA!	.	intergen	NR_	dist=	.	.
ACa07	2	####	.	G	A	.	PA!	SNED1	intronic	NM_	.	.	.
ACa07	3	####	rs3731564	C	T	.	PA!	CHL1	exonic	NM_	.	syr	CHL1:N
ACa07	3	####	rs5694312	C	T	.	PA!	CNTN6	intronic	NM_	.	.	.
ACa07	3	####	.	C	T	.	PA!	LMCD1	intronic	NM_	.	.	.
ACa07	3	####	.	G	A	.	PA!	LHFPL4	UTR5	NM_	NM_	.	.
ACa07	3	####	rs7502023	C	T	.	PA!	.	intergen	NM_	dist=	.	.
ACa07	3	####	.	T	C	.	PA!	IL17RC	intronic	NM_	.	.	.
ACa07	3	####	rs7567892	C	T	.	PA!	CRELD1	exonic	NM_	.	mis	CRELD
ACa07	3	####	.	C	T	.	PA!	FANCD2	intronic	NM_	.	.	.
ACa07	3	####	.	C	T	.	PA!	SLC6A11	exonic	NM_	.	mis	SLC6A1
ACa07	3	####	.	G	A	.	PA!	TAMM41	intronic	NM_	.	.	.
ACa07	3	####	.	G	A	.	PA!	.	intergen	NM_	dist=	.	.
ACa07	3	####	rs9925095	C	T	.	PA!	TSEN2	intronic	NM_	.	.	.
ACa07	3	####	.	G	T	.	PA!	RAF1	intronic	NM_	.	.	.
ACa07	3	####	.	C	A	.	PA!	IQSEC1	intronic	NM_	.	.	.
ACa07	3	####	.	T	A	.	PA!	CAPN7	exonic	NM_	.	mis	CAPN7
ACa07	3	####	.	T	C	.	PA!	TBC1D5	intronic	NM_	.	.	.
ACa07	3	####	.	G	T	.	PA!	KCNH8	intronic	NM_	.	.	.
ACa07	3	####	.	G	A	.	PA!	NR1D2	exonic	NM_	.	syr	NR1D2
ACa07	3	####	.	C	T	.	PA!	TOP2B	intronic	NM_	.	.	.
ACa07	3	####	.	A	G	.	PA!	.	intergen	NM_	dist=	.	.
ACa07	3	####	rs8972401	A	G	.	PA!	SLC4A7	intronic	NM_	.	.	.
ACa07	3	####	rs1124655	G	A	.	PA!	TGFBR2	exonic	NM_	.	mis	TGFBR
ACa07	3	####	.	C	T	.	PA!	.	intergen	NM_	dist=	.	.
ACa07	3	####	rs7802104	C	T	.	PA!	STT3B	exonic	NM_	.	unl	UNKNC
ACa07	3	####	.	G	T	.	PA!	STAC	intronic	NM_	.	.	.
ACa07	3	####	rs5407838	G	A	.	PA!	DCLK3	exonic	NM_	.	mis	DCLK3:
ACa07	3	####	rs5599017	G	A	.	PA!	MLH1	intronic	NM_	.	.	.
ACa07	3	####	.	T	C	.	PA!	GOLGA4	exonic	NM_	.	syr	GOLGA
ACa07	3	####	rs3732183	G	A	.	PA!	DLEC1	intronic	NM_	.	.	.
ACa07	3	####	.	C	T	.	PA!	EXOG	intronic	NM_	.	.	.
ACa07	3	####	rs7485706	G	A	.	PA!	SCN10A	exonic	NM_	.	syr	SCN10/
ACa07	3	####	.	G	A	.	PA!	ZKSCAN7	intronic	NM_	.	.	.
ACa07	3	####	rs7745693	G	A	.	PA!	CCR9	exonic	NM_	.	mis	CCR9:N
ACa07	3	####	.	G	A	.	PA!	.	upstreai	NM_	.	.	.
ACa07	3	####	.	G	A	.	PA!	SETD2	intronic	NM_	.	.	.
ACa07	3	####	rs7512571	C	T	.	PA!	CCDC51	exonic	NM_	.	mis	CCDC5
ACa07	3	####	rs5304720	G	A	.	PA!	COL7A1	intronic	NM_	.	.	.
ACa07	3	####	rs3713830	C	T	.	PA!	NCKIPSD	exonic	NM_	.	mis	NCKIPS
ACa07	3	####	.	C	T	.	PA!	WDR6	exonic	NM_	.	mis	WDR6:
ACa07	3	####	rs2001906	C	T	.	PA!	QRICH1	exonic	NM_	.	syr	QRICH:
ACa07	3	####	.	G	A	.	PA!	USP4	exonic	NM_	.	mis	USP4:N
ACa07	3	####	.	C	T	.	PA!	BSN	exonic	NM_	.	sto	BSN:NM
ACa07	3	####	.	G	T	.	PA!	CAMKV	intronic	NM_	.	.	.
ACa07	3	####	rs7596904	C	T	.	PA!	CACNA2D	exonic	NM_	.	unl	UNKNC
ACa07	3	####	rs1821535	G	A	.	PA!	IQCF5-AS	ncRNA_	NR_	.	.	.
ACa07	3	####	.	A	G	.	PA!	PCBP4	intronic	NM_	.	.	.
ACa07	3	####	rs7780907	G	A	.	PA!	PCBP4	exonic	NM_	.	mis	PCBP4
ACa07	3	####	.	A	G	.	PA!	ALAS1	splicing	NM_	NM_	.	.
ACa07	3	####	.	G	A	.	PA!	DNAH1	intronic	NM_	.	.	.

ACa07	3	####	.	A	G	.	PA!	DNAH1	intronic	NM_	.	.	.
ACa07	3	####	.	C	T	.	PA!	DNAH1	intronic	NM_	.	.	.
ACa07	3	####	rs9624625	C	T	.	PA!	SEMA3G	intronic	NM_	.	.	.
ACa07	3	####	.	A	T	.	PA!	PBRM1	intronic	NM_	.	.	.
ACa07	3	####	.	T	G	.	PA!	PBRM1	exonic	NM_	.	mis	PBRM1
ACa07	3	####	.	C	T	.	PA!	CACNA1D	exonic	NM_	.	mis	CACNA
ACa07	3	####	rs2000990	C	T	.	PA!	ACTR8	intronic	NM_	.	.	.
ACa07	3	####	rs9858893	T	C	.	PA!	CACNA2D	intronic	NM_	.	.	.
ACa07	3	####	rs1042360	T	C	.	PA!	CACNA2D	intronic	NM_	.	.	.
ACa07	3	####	.	G	A	.	PA!	IL17RD	intronic	NM_	.	.	.
ACa07	3	####	.	C	A	.	PA!	APPL1	intronic	NM_	.	.	.
ACa07	3	####	.	G	T	.	PA!	DENND6A	UTR3	NM_	NM_	.	.
ACa07	3	####	.	G	T	.	PA!	PXK	intronic	NM_	.	.	.
ACa07	3	####	rs7458693	C	T	.	PA!	ADAMTS9	exonic	NM_	.	syn	ADAMT
ACa07	3	####	.	A	G	.	PA!	LMOD3	exonic	NM_	.	mis	LMOD3
ACa07	3	####	.	C	T	.	PA!	MITF	exonic	NM_	.	mis	MITF:N
ACa07	3	####	rs7794528	C	T	.	PA!	FAM86DP	ncRNA_	NR_	.	.	.
ACa07	3	####	.	G	A	.	PA!	MINA	exonic	NM_	.	mis	MINA:N
ACa07	3	####	.	A	G	.	PA!	.	intergen	NR_	dist=	.	.
ACa07	3	####	.	G	T	.	PA!	KIAA1524	intronic	NM_	.	.	.
ACa07	3	####	.	C	T	.	PA!	MORC1	intronic	NM_	.	.	.
ACa07	3	####	.	G	T	.	PA!	CFAP44	exonic	NM_	.	syn	CFAP4
ACa07	3	####	.	A	C	.	PA!	TMEM39A	splicing	NM_	NM_	.	.
ACa07	3	####	.	C	A	.	PA!	TMEM39A	intronic	NM_	.	.	.
ACa07	3	####	rs3684939	G	A	.	PA!	STXBP5L	exonic	NM_	.	mis	STXBP
ACa07	3	####	rs1001693	G	A	.	PA!	SEMA5B	intronic	NM_	.	.	.
ACa07	3	####	rs1465223	G	A	.	PA!	.	intergen	NM_	dist=	.	.
ACa07	3	####	.	C	A	.	PA!	UROC1	intronic	NM_	.	.	.
ACa07	3	####	rs5528272	G	A	.	PA!	PLXNA1	exonic	NM_	.	syn	PLXNA
ACa07	3	####	rs7511092	G	A	.	PA!	TPRA1	exonic	NM_	.	syn	TPRA1
ACa07	3	####	.	A	G	.	PA!	.	intergen	NR_	dist=	.	.
ACa07	3	####	rs5631647	C	T	.	PA!	LOC6537	ncRNA_	NR_	.	.	.
ACa07	3	####	.	A	G	.	PA!	RPL32P3	ncRNA_	NR_	.	.	.
ACa07	3	####	.	G	A	.	PA!	NUDT16	exonic	NM_	.	mis	NUDT1
ACa07	3	####	.	C	A	.	PA!	ACPP	UTR3	NM_	NM_	.	.
ACa07	3	####	.	G	A	.	PA!	PRR23A	exonic	NM_	.	mis	PRR23
ACa07	3	####	.	C	T	.	PA!	PRR23C	exonic	NM_	.	syn	PRR23
ACa07	3	####	rs1408440	C	T	.	PA!	CLSTN2	exonic	NM_	.	syn	CLSTN
ACa07	3	####	.	G	A	.	PA!	RASA2	exonic	NM_	.	syn	RASA2
ACa07	3	####	.	C	T	.	PA!	TFDP2	intronic	NM_	.	.	.
ACa07	3	####	rs7685217	A	T	.	PA!	ATR	intronic	NM_	.	.	.
ACa07	3	####	rs7705095	C	T	.	PA!	PCOLCE2	exonic	NM_	.	mis	PCOLC
ACa07	3	####	.	G	A	.	PA!	.	intergen	NM_	dist=	.	.
ACa07	3	####	.	C	T	.	PA!	.	intergen	NR_	dist=	.	.
ACa07	3	####	rs7605662	C	T	.	PA!	EIF2A	exonic	NM_	.	syn	EIF2A:N
ACa07	3	####	.	C	A	.	PA!	GFM1	intronic	NM_	.	.	.
ACa07	3	####	.	G	A	.	PA!	MIR16-2	ncRNA_	NR_	.	.	.
ACa07	3	####	.	G	A	.	PA!	.	intergen	NM_	dist=	.	.
ACa07	3	####	.	G	C	.	PA!	.	intergen	NM_	dist=	.	.
ACa07	3	####	rs5388049	C	T	.	PA!	SKIL	exonic	NM_	.	mis	SKIL:NI
ACa07	3	####	rs9368630	G	A	.	PA!	.	intergen	NM_	dist=	.	.
ACa07	3	####	.	G	T	.	PA!	KCNMB3	UTR5	NM_	NM_	.	.
ACa07	3	####	rs7652687	C	T	.	PA!	USP13	exonic	NM_	.	syn	USP13
ACa07	3	####	rs5714458	C	T	.	PA!	LOC1019	ncRNA_	NR_	.	.	.

ACa07	3	####	.	T	G	.	PA!	MCCC1	exonic	NM_	.	mis	MCCC1
ACa07	3	####	.	C	T	.	PA!	KLHL6	exonic	NM_	.	mis	KLHL6:
ACa07	3	####	rs9015268	C	T	.	PA!	HTR3D	intronic	NM_	.	.	.
ACa07	3	####	.	T	C	.	PA!	VWA5B2	intronic	NM_	.	.	.
ACa07	3	####	rs3748943	G	A	.	PA!	ECE2	exonic	NM_	.	mis	ECE2:N
ACa07	3	####	.	T	A	.	PA!	SST	intronic	NM_	.	.	.
ACa07	3	####	rs7531929	C	T	.	PA!	LPP	exonic	NM_	.	syr	LPP:NM
ACa07	3	####	rs7777314	G	A	.	PA!	TMEM44	intronic	NM_	.	.	.
ACa07	3	####	.	C	T	.	PA!	.	intergen	NM_	dist=	.	.
ACa07	3	####	.	C	T	.	PA!	.	intergen	NM_	dist=	.	.
ACa07	3	####	.	C	T	.	PA!	MUC4	intronic	NM_	.	.	.
ACa07	3	####	.	C	T	.	PA!	.	intergen	NM_	dist=	.	.
ACa07	3	####	rs8866852	G	A	.	PA!	NRROS	intronic	NM_	.	.	.
ACa07	3	####	rs7637750	A	G	.	PA!	.	intergen	NR_	dist=	.	.
ACa07	4	####	.	G	T	.	PA!	ABCA11P	ncRNA_	NR_	.	.	.
ACa07	4	####	rs7620115	G	A	.	PA!	TMEM175	exonic	NM_	.	unl	UNKNC
ACa07	4	####	.	C	A	.	PA!	CTBP1	intronic	NM_	.	.	.
ACa07	4	####	rs9125004	C	T	.	PA!	FAM53A	intronic	NM_	.	.	.
ACa07	4	####	.	C	T	.	PA!	TACC3	intronic	NM_	.	.	.
ACa07	4	####	.	G	A	.	PA!	LETM1	intronic	NM_	.	.	.
ACa07	4	####	rs5508099	G	A	.	PA!	WHSC1	intronic	NM_	.	.	.
ACa07	4	####	.	T	C	.	PA!	MXD4	intronic	NM_	.	.	.
ACa07	4	####	.	G	A	.	PA!	DOK7	intronic	NM_	.	.	.
ACa07	4	####	rs9333127	G	A	.	PA!	.	intronic	NM_	.	.	.
ACa07	4	####	.	C	T	.	PA!	ADRA2C	exonic	NM_	.	sto	ADRA2
ACa07	4	####	rs5603533	C	T	.	PA!	WFS1	exonic	NM_	.	syr	WFS1:N
ACa07	4	####	.	G	A	.	PA!	PPP2R2C	intronic	NM_	.	.	.
ACa07	4	####	.	G	A	.	PA!	SORCS2	intronic	NM_	.	.	.
ACa07	4	####	rs3763511	G	A	.	PA!	AFAP1	exonic	NM_	.	mis	AFAP1:
ACa07	4	####	rs2006520	C	T	.	PA!	ABLIM2	intronic	NM_	.	.	.
ACa07	4	####	rs7521101	C	T	.	PA!	SH3TC1	exonic	NM_	.	syr	SH3TC
ACa07	4	####	rs7804064	G	A	.	PA!	ZNF518B	exonic	NM_	.	mis	ZNF518
ACa07	4	####	.	C	A	.	PA!	BOD1L1	exonic	NM_	.	mis	BOD1L
ACa07	4	####	.	G	A	.	PA!	CC2D2A	exonic	NM_	.	mis	CC2D2
ACa07	4	####	.	A	T	.	PA!	PACRGL	intronic	NM_	.	.	.
ACa07	4	####	.	T	C	.	PA!	CCDC149	exonic	NM_	.	mis	CCDC1
ACa07	4	####	rs1420745	A	G	.	PA!	.	intergen	NR_	dist=	.	.
ACa07	4	####	rs5344165	C	T	.	PA!	UBE2K	intronic	NM_	.	.	.
ACa07	4	####	.	C	T	.	PA!	UBE2K	intronic	NM_	.	.	.
ACa07	4	####	.	G	T	.	PA!	.	intergen	NM_	dist=	.	.
ACa07	4	####	rs7759148	G	A	.	PA!	RHOH	exonic	NM_	.	mis	RHOH:I
ACa07	4	####	.	A	T	.	PA!	NSUN7	exonic	NM_	.	mis	NSUN7
ACa07	4	####	rs2856467	C	T	.	PA!	GABRB1	intronic	NM_	.	.	.
ACa07	4	####	rs1451863	C	T	.	PA!	.	intergen	NM_	dist=	.	.
ACa07	4	####	rs5941177	C	T	.	PA!	.	intergen	NM_	dist=	.	.
ACa07	4	####	.	C	T	.	PA!	LRRC66	exonic	NM_	.	mis	LRRC6
ACa07	4	####	.	C	T	.	PA!	DANCR	ncRNA_	NR_	.	.	.
ACa07	4	####	.	G	C	.	PA!	EXOC1	intronic	NM_	.	.	.
ACa07	4	####	.	C	A	.	PA!	THEGL	intronic	NM_	.	.	.
ACa07	4	####	.	G	A	.	PA!	TMPRSS1	intronic	NM_	.	.	.
ACa07	4	####	.	C	T	.	PA!	UGT2B4	exonic	NM_	.	sto	UGT2B
ACa07	4	####	.	A	T	.	PA!	CSN1S1	splicing	NM_	NM_	.	.
ACa07	4	####	rs7619680	C	T	.	PA!	SLC4A4	intronic	NM_	.	.	.
ACa07	4	####	.	C	T	.	PA!	ADAMTS3	splicing	NM_	NM_	.	.

ACa07	4	####	rs1194702	T	A	.	PA!	AFM	intronic	NM_	.	.	.
ACa07	4	####	.	G	T	.	PA!	RASSF6	intronic	NM_	.	.	.
ACa07	4	####	rs3691209	C	T	.	PA!	PPEF2	exonic	NM_	.	mis	PPEF2:
ACa07	4	####	.	G	T	.	PA!	CCDC158	exonic	NM_	.	syr	CCDC1
ACa07	4	####	rs7609961	C	T	.	PA!	SHROOM	exonic	NM_	.	mis	SHROC
ACa07	4	####	.	G	T	.	PA!	FRAS1	exonic	NM_	.	sto	FRAS1:
ACa07	4	####	.	A	G	.	PA!	LIN54	UTR5	NM_	NM_	.	.
ACa07	4	####	rs8867538	T	C	.	PA!	WDFY3	intronic	NM_	.	.	.
ACa07	4	####	.	G	T	.	PA!	FAM13A	exonic	NM_	.	mis	FAM13/
ACa07	4	####	.	C	A	.	PA!	NFKB1	intronic	NM_	.	.	.
ACa07	4	####	.	G	A	.	PA!	TET2	exonic	NM_	.	mis	TET2:N
ACa07	4	####	rs3719859	C	T	.	PA!	TBCK	exonic	NM_	.	unl	UNKNC
ACa07	4	####	.	C	T	.	PA!	ENPEP	exonic	NM_	.	syr	ENPEP
ACa07	4	####	.	A	G	.	PA!	.	intergen	NM_	dist=	.	.
ACa07	4	####	.	A	G	.	PA!	NDST3	intronic	NM_	.	.	.
ACa07	4	####	.	A	G	.	PA!	LOC7292	ncRNA_	NR_	.	.	.
ACa07	4	####	.	G	T	.	PA!	METTL14	exonic	NM_	.	sto	METTL
ACa07	4	####	.	C	A	.	PA!	NDNF	exonic	NM_	.	mis	NDNF:N
ACa07	4	####	rs7744530	T	C	.	PA!	ANKRD50	exonic	NM_	.	mis	ANKRD
ACa07	4	####	.	G	T	.	PA!	C4orf29	exonic	NM_	.	mis	C4orf29
ACa07	4	####	rs7741072	A	G	.	PA!	C4orf29	exonic	NM_	.	mis	C4orf29
ACa07	4	####	.	C	A	.	PA!	.	intergen	NR_	dist=	.	.
ACa07	4	####	.	G	A	.	PA!	.	intergen	NR_	dist=	.	.
ACa07	4	####	.	G	T	.	PA!	TBC1D9	exonic	NM_	.	syr	TBC1D
ACa07	4	####	.	G	A	.	PA!	TBC1D9	exonic	NM_	.	syr	TBC1D
ACa07	4	####	.	A	G	.	PA!	IL15	exonic	NM_	.	syr	IL15:NM
ACa07	4	####	.	C	A	.	PA!	GYPB	intronic	NM_	.	.	.
ACa07	4	####	rs8661228	T	C	.	PA!	TRIM2	intronic	NM_	.	.	.
ACa07	4	####	rs4095550	G	A	.	PA!	KLHL2	intronic	NM_	.	.	.
ACa07	4	####	.	G	A	.	PA!	GALNTL6	intronic	NM_	.	.	.
ACa07	4	####	.	A	G	.	PA!	TENM3	exonic	NM_	.	mis	TENM3
ACa07	4	####	rs9420962	C	T	.	PA!	CCDC110	intronic	NM_	.	.	.
ACa07	4	####	.	G	A	.	PA!	SORBS2	exonic	NM_	.	syr	SORBS
ACa07	4	####	rs7781796	G	A	.	PA!	F11-AS1	ncRNA_	NR_	.	.	.
ACa07	5	####	.	A	G	.	PA!	SLC9A3	intronic	NM_	.	.	.
ACa07	5	####	.	G	A	.	PA!	CEP72	exonic	NM_	.	mis	CEP72:
ACa07	5	####	rs412221	G	C	.	PA!	ZDHHC11	intronic	NM_	.	.	.
ACa07	5	####	rs1006578	C	T	.	PA!	SLC12A7	intronic	NM_	.	.	.
ACa07	5	####	.	C	A	.	PA!	IRX4	exonic	NM_	.	mis	IRX4:NI
ACa07	5	####	rs9030458	G	A	.	PA!	.	intergen	NM_	dist=	.	.
ACa07	5	####	rs7491497	G	A	.	PA!	ICE1	exonic	NM_	.	mis	ICE1:NI
ACa07	5	####	.	G	T	.	PA!	UBE2QL1	exonic	NM_	.	mis	UBE2Q
ACa07	5	####	.	A	G	.	PA!	PAPD7	exonic	NM_	.	mis	PAPD7
ACa07	5	####	rs1497666	G	A	.	PA!	CTNND2	exonic	NM_	.	syr	CTNND
ACa07	5	####	rs6554817	G	A	.	PA!	DNAH5	intronic	NM_	.	.	.
ACa07	5	####	rs9140889	G	C	.	PA!	TRIO	intronic	NM_	.	.	.
ACa07	5	####	rs7661668	G	A	.	PA!	TRIO	intronic	NM_	.	.	.
ACa07	5	####	rs9251299	G	A	.	PA!	.	intergen	NM_	dist=	.	.
ACa07	5	####	rs5697598	G	A	.	PA!	ADAMTS1	intronic	NM_	.	.	.
ACa07	5	####	rs8799835	G	C	.	PA!	.	intergen	NR_	dist=	.	.
ACa07	5	####	rs9765292	G	T	.	PA!	.	intergen	NR_	dist=	.	.
ACa07	5	####	rs9765293	C	T	.	PA!	.	intergen	NR_	dist=	.	.
ACa07	5	####	.	C	A	.	PA!	MIR3650,I	ncRNA_	NR_	.	.	.
ACa07	5	####	.	T	A	.	PA!	PRKAA1	UTR3	NM_	NM_	.	.

ACa07	5	####	.	A	G	.	PA:	OXCT1	intronic	NM_	.	.	.
ACa07	5	####	.	G	T	.	PA:	HMGCS1	intronic	NM_	.	.	.
ACa07	5	####	.	T	C	.	PA:	.	intergen	NOI	dist=	.	.
ACa07	5	####	rs4327635	T	C	.	PA:	.	intergen	NOI	dist=	.	.
ACa07	5	####	.	A	G	.	PA:	.	intergen	NOI	dist=	.	.
ACa07	5	####	rs2005764	C	T	.	PA:	ACTBL2	exonic	NM_	.	mis	ACTBL:
ACa07	5	####	.	C	T	.	PA:	PDE4D	exonic	NM_	.	mis	PDE4D
ACa07	5	####	rs5582099	C	T	.	PA:	.	intergen	NM_	dist=	.	.
ACa07	5	####	.	C	A	.	PA:	CENPK	splicing	NM_	NM_	.	.
ACa07	5	####	.	G	A	.	PA:	ERBB2IP	intronic	NM_	.	.	.
ACa07	5	####	rs5672287	G	A	.	PA:	MAST4	intronic	NM_	.	.	.
ACa07	5	####	.	A	G	.	PA:	MAST4	exonic	NM_	.	mis	MAST4
ACa07	5	####	rs2016310	C	T	.	PA:	MAST4	exonic	NM_	.	mis	MAST4
ACa07	5	####	.	C	T	.	PA:	PIK3R1	exonic	NM_	.	syr	PIK3R1
ACa07	5	####	.	A	G	.	PA:	RAD17	intronic	NM_	.	.	.
ACa07	5	####	.	C	G	.	PA:	GUSBP9,(	ncRNA_	NR_	.	.	.
ACa07	5	####	.	C	A	.	PA:	MAP1B	intronic	NM_	.	.	.
ACa07	5	####	.	A	G	.	PA:	ANKDD1B	intronic	NM_	.	.	.
ACa07	5	####	.	C	T	.	PA:	HOMER1	UTR5	NM_	NM_	.	.
ACa07	5	####	.	G	A	.	PA:	ACOT12	exonic	NM_	.	syr	ACOT1
ACa07	5	####	.	G	A	.	PA:	TMEM167	UTR3	NM_	NM_	.	.
ACa07	5	####	rs3717547	C	T	.	PA:	VCAN	intronic	NM_	.	.	.
ACa07	5	####	rs7815826	C	T	.	PA:	.	intergen	NM_	dist=	.	.
ACa07	5	####	.	G	T	.	PA:	GPR98	exonic	NM_	.	mis	GPR98
ACa07	5	####	.	G	A	.	PA:	KIAA0825	exonic	NM_	.	mis	KIAA08
ACa07	5	####	rs7633968	C	T	.	PA:	ARSK	exonic	NM_	.	mis	ARSK:N
ACa07	5	####	.	C	T	.	PA:	.	intergen	NR_	dist=	.	.
ACa07	5	####	.	A	C	.	PA:	PAM	exonic	NM_	.	mis	PAM:NI
ACa07	5	####	rs1114874	C	T	.	PA:	PAM	intronic	NM_	.	.	.
ACa07	5	####	.	G	T	.	PA:	.	upstrea	NM_	.	.	.
ACa07	5	####	.	A	G	.	PA:	EPB41L4A	exonic	NM_	.	syr	EPB41L
ACa07	5	####	.	C	T	.	PA:	SEMA6A	exonic	NM_	.	syr	SEMA6
ACa07	5	####	.	G	T	.	PA:	TNFAIP8	intronic	NM_	.	.	.
ACa07	5	####	rs9346631	G	A	.	PA:	SOWAHA	exonic	NM_	.	unl	UNKNC
ACa07	5	####	.	A	T	.	PA:	AFF4	intronic	NM_	.	.	.
ACa07	5	####	rs7465490	C	T	.	PA:	FSTL4	exonic	NM_	.	syr	FSTL4:
ACa07	5	####	.	C	T	.	PA:	.	upstrea	NM_	.	.	.
ACa07	5	####	rs7470418	T	C	.	PA:	DDX46	intronic	NM_	.	.	.
ACa07	5	####	rs7665720	C	T	.	PA:	CDC25C	exonic	NM_	.	mis	CDC25
ACa07	5	####	.	A	G	.	PA:	CDC25C	exonic	NM_	.	mis	CDC25
ACa07	5	####	.	A	G	.	PA:	EGR1	exonic	NM_	.	mis	EGR1:N
ACa07	5	####	rs7721589	G	A	.	PA:	EGR1	exonic	NM_	.	mis	EGR1:N
ACa07	5	####	.	T	C	.	PA:	ANKHD1-I	exonic	NM_	.	mis	ANKHD
ACa07	5	####	.	G	A	.	PA:	PCDHA4	exonic	NM_	.	syr	PCDHA
ACa07	5	####	.	C	T	.	PA:	PCDHA13	exonic	NM_	.	syr	PCDHA
ACa07	5	####	rs7823690	G	A	.	PA:	PCDHAC1	exonic	NM_	.	syr	PCDHA
ACa07	5	####	.	G	A	.	PA:	PCDHB6	exonic	NM_	.	mis	PCDHB
ACa07	5	####	.	C	T	.	PA:	PCDHB8	exonic	NM_	.	mis	PCDHB
ACa07	5	####	.	C	T	.	PA:	PCDHGA2	exonic	NM_	.	syr	PCDHG
ACa07	5	####	rs9814581	C	T	.	PA:	PCDHGA2	exonic	NM_	.	mis	PCDHG
ACa07	5	####	rs5446331	C	T	.	PA:	PCDHGA3	exonic	NM_	.	syr	PCDHG
ACa07	5	####	rs3769527	C	T	.	PA:	PCDHGB3	exonic	NM_	.	syr	PCDHG
ACa07	5	####	rs7719463	G	A	.	PA:	PCDHGB6	exonic	NM_	.	mis	PCDHG
ACa07	5	####	rs2018026	G	A	.	PA:	KIAA0141	exonic	NM_	.	mis	KIAA01

ACa07	5	####	rs1000063	A	G	.	PA!	JAKMIP2	UTR5	NM_	NM_	.	.	
ACa07	5	####	.	T	C	.	PA!	CSNK1A1	intronic	NM_	.	.	.	
ACa07	5	####	.	A	T	.	PA!	PDE6A	intronic	NM_	.	.	.	
ACa07	5	####	.	G	A	.	PA!	SYNPO	exonic	NM_	.	mis	SYNPO	
ACa07	5	####	.	C	A	.	PA!	G3BP1	exonic	NM_	.	mis	G3BP1:	
ACa07	5	####	rs5492040	G	A	.	PA!	CYFIP2	intronic	NM_	.	.	.	
ACa07	5	####	rs5611223	C	T	.	PA!	RNF145	exonic	NM_	.	mis	RNF14!	
ACa07	5	####	.	G	A	.	PA!	TENM2	exonic	NM_	.	mis	TENM2	
ACa07	5	####	.	C	A	.	PA!	TENM2	exonic	NM_	.	mis	TENM2	
ACa07	5	####	rs1491693	C	T	.	PA!	WWC1	exonic	NM_	.	mis	WWC1	
ACa07	5	####	rs7577825	C	T	.	PA!	RARS	exonic	NM_	.	mis	RARS:N	
ACa07	5	####	.	G	T	.	PA!	SLIT3	intronic	NM_	.	.	.	
ACa07	5	####	.	T	C	.	PA!	SH3PXD2	exonic	NM_	.	mis	SH3PX	
ACa07	5	####	rs1822272	C	T	.	PA!	NEURL1B	intronic	NM_	.	.	.	
ACa07	5	####	.	C	A	.	PA!	CREBRF	intronic	NM_	.	.	.	
ACa07	5	####	.	T	C	.	PA!	FAF2	intronic	NM_	.	.	.	
ACa07	5	####	.	G	A	.	PA!	FGFR4	intronic	NM_	.	.	.	
ACa07	5	####	rs4551119	G	A	.	PA!	FGFR4	intronic	NM_	.	.	.	
ACa07	5	####	rs5582208	C	T	.	PA!	FGFR4	exonic	NM_	.	mis	FGFR4	
ACa07	5	####	.	C	A	.	PA!	LOC2021	ncRNA_	NR_	.	.	.	
ACa07	5	####	.	C	T	.	PA!	FAM153A	intronic	NM_	.	.	.	
ACa07	5	####	.	G	A	.	PA!	GRM6	UTR3	NM_	NM_	.	.	
ACa07	5	####	rs1025870	G	A	.	PA!	.	intergen	NM_	dist=	.	.	
ACa07	5	####	.	G	A	.	PA!	MGAT4B	UTR3	NM_	NM_	.	.	
ACa07	5	####	.	G	T	.	PA!	GFPT2	intronic	NM_	.	.	.	
ACa07	6	####	rs7651293	G	A	.	PA!	RPP40	exonic	NM_	.	mis	RPP40:	
ACa07	6	####	rs1023580	A	T	.	PA!	RPP40	intronic	NM_	.	.	.	
ACa07	6	####	rs1864505	G	A	.	PA!	.	intergen	NM_	dist=	.	.	
ACa07	6	####	.	A	G	.	PA!	.	intergen	NM_	dist=	.	.	
ACa07	6	####	rs2004302	C	T	.	PA!	BLOC1S5	ncRNA_	NR_	.	.	.	
ACa07	6	####	.	T	A	.	PA!	.	intergen	NR_	dist=	.	.	
ACa07	6	####	rs3692600	G	A	.	PA!	GCNT2	intronic	NM_	.	.	.	
ACa07	6	####	rs7468362	G	A	.	PA!	HIVEP1	exonic	NM_	.	mis	HIVEP1	
ACa07	6	####	rs7674897	C	T	.	PA!	DTNBP1	splicing	NM_	NM_	.	.	
ACa07	6	####	.	T	G	.	PA!	CASC15,(	ncRNA_	NR_	.	.	.	
ACa07	6	####	.	C	T	.	PA!	.	intergen	NM_	dist=	.	.	
ACa07	6	####	.	G	A	.	PA!	FAM65B	intronic	NM_	.	.	.	
ACa07	6	####	.	T	C	.	PA!	GUSBP2	ncRNA_	NR_	.	.	.	
ACa07	6	####	.	G	A	.	PA!	.	intergen	NR_	dist=	.	.	
ACa07	6	####	.	C	T	.	PA!	ZSCAN12	ncRNA_	NR_	.	.	.	
ACa07	6	####	.	G	A	.	PA!	ZKSCAN3	intronic	NM_	.	.	.	
ACa07	6	####	.	T	C	.	PA!	.	intergen	NM_	dist=	.	.	
ACa07	6	####	rs7653588	G	A	.	PA!	ZFP57	intronic	NM_	.	.	.	
ACa07	6	####	.	A	G	.	PA!	HLA-H	ncRNA_	NR_	.	.	.	
ACa07	6	####	rs7511377	C	T	.	PA!	PRR3	intronic	NM_	.	.	.	
ACa07	6	####	.	G	T	.	PA!	MDC1	intronic	NM_	.	.	.	
ACa07	6	####	.	G	A	.	PA!	.	upstrea	NR_	.	.	.	
ACa07	6	####	.	A	G	.	PA!	.	intergen	NM_	dist=	.	.	
ACa07	6	####	rs7792598	C	T	.	PA!	PRRC2A	exonic	NM_	.	syr	PRRC2	
ACa07	6	####	.	G	A	.	PA!	VARS	intronic	NM_	.	.	.	
ACa07	6	####	.	T	C	.	PA!	HLA-DRA	UTR3	NM_	NM_	.	.	
ACa07	6	####	rs1119219	C	T	.	PA!	COL11A2	exonic	NM_	.	mis	COL11/	
ACa07	6	####	rs7759216	C	T	.	PA!	COL11A2	exonic	NM_	.	syr	COL11/	
ACa07	6	####	.	G	A	.	PA!	ITPR3	exonic	NM_	.	mis	ITPR3:N	

ACa07	6	####	.	G	T	.	PA!	PACSIN1	exonic	NM_	.	mis	PACSIN
ACa07	6	####	rs7740360	C	T	.	PA!	PPARD	UTR5	NM_	NM_	.	.
ACa07	6	####	.	C	T	.	PA!	PPARD	intronic	NM_	.	.	.
ACa07	6	####	rs7715433	C	T	.	PA!	PPARD	exonic	NM_	.	syr	PPARD
ACa07	6	####	.	G	A	.	PA!	RPL10A	intronic	NM_	.	.	.
ACa07	6	####	.	G	C	.	PA!	SLC26A8	intronic	NM_	.	.	.
ACa07	6	####	rs1007644	G	A	.	PA!	CPNE5	exonic	NM_	.	mis	CPNE5
ACa07	6	####	.	A	G	.	PA!	DAAM2	intronic	NM_	.	.	.
ACa07	6	####	rs3751257	C	T	.	PA!	.	downstr	NR_	.	.	.
ACa07	6	####	rs7778285	G	A	.	PA!	MDFI	exonic	NM_	.	mis	MDFI:N
ACa07	6	####	.	C	A	.	PA!	FRS3	exonic	NM_	.	mis	FRS3:N
ACa07	6	####	rs8008274	C	A	.	PA!	GUCA1B	intronic	NM_	.	.	.
ACa07	6	####	rs7806884	C	T	.	PA!	TRERF1	exonic	NM_	.	unl	UNKNC
ACa07	6	####	.	G	T	.	PA!	PRPH2	exonic	NM_	.	syr	PRPH2
ACa07	6	####	rs1434417	C	T	.	PA!	PTK7	exonic	NM_	.	syr	PTK7:N
ACa07	6	####	.	C	T	.	PA!	PTK7	exonic	NM_	.	mis	PTK7:N
ACa07	6	####	.	G	T	.	PA!	ZNF318	intronic	NM_	.	.	.
ACa07	6	####	.	A	G	.	PA!	YIPF3	intronic	NM_	.	.	.
ACa07	6	####	rs1496900	G	A	.	PA!	XPO5	exonic	NM_	.	syr	XPO5:N
ACa07	6	####	rs1435325	G	A	.	PA!	TCTE1	exonic	NM_	.	mis	TCTE1:
ACa07	6	####	.	G	A	.	PA!	.	intergen	NM_	dist=	.	.
ACa07	6	####	.	C	A	.	PA!	DST	exonic	NM_	.	mis	DST:NM
ACa07	6	####	.	C	G	.	PA!	.	intergen	NM_	dist=	.	.
ACa07	6	####	.	A	G	.	PA!	COL19A1	exonic	NM_	.	syr	COL19A
ACa07	6	####	rs2021606	A	G	.	PA!	SMAP1	UTR3	NM_	NM_	.	.
ACa07	6	####	.	A	T	.	PA!	KHDC3L	intronic	NM_	.	.	.
ACa07	6	####	.	C	A	.	PA!	CD109	intronic	NM_	.	.	.
ACa07	6	####	rs7761622	C	T	.	PA!	PHIP	exonic	NM_	.	mis	PHIP:N
ACa07	6	####	rs1497660	C	T	.	PA!	BCKDHB	exonic	NM_	.	mis	BCKDH
ACa07	6	####	rs1002475	T	C	.	PA!	CYB5R4	intronic	NM_	.	.	.
ACa07	6	####	rs7619979	G	A	.	PA!	SLC35A1	exonic	NM_	.	syr	SLC35A
ACa07	6	####	rs1015787	G	A	.	PA!	MDN1	intronic	NM_	.	.	.
ACa07	6	####	.	C	T	.	PA!	.	downstr	NM_	.	.	.
ACa07	6	####	.	T	C	.	PA!	POU3F2	exonic	NM_	.	mis	POU3F
ACa07	6	####	rs5581466	A	C	.	PA!	LOC10042	ncRNA_	NR_	.	.	.
ACa07	6	####	.	T	C	.	PA!	NR2E1	intronic	NM_	.	.	.
ACa07	6	####	.	T	C	.	PA!	SESN1	intronic	NM_	.	.	.
ACa07	6	####	.	T	C	.	PA!	PPIL6	UTR5	NM_	NM_	.	.
ACa07	6	####	.	G	T	.	PA!	MICAL1	intronic	NM_	.	.	.
ACa07	6	####	.	G	T	.	PA!	REV3L	exonic	NM_	.	mis	REV3L:
ACa07	6	####	.	C	T	.	PA!	.	intergen	NM_	dist=	.	.
ACa07	6	####	.	G	A	.	PA!	.	intergen	NM_	dist=	.	.
ACa07	6	####	.	T	C	.	PA!	TRMT11	intronic	NM_	.	.	.
ACa07	6	####	rs3754749	C	T	.	PA!	LAMA2	intronic	NM_	.	.	.
ACa07	6	####	.	A	C	.	PA!	LAMA2	intronic	NM_	.	.	.
ACa07	6	####	rs7606036	G	A	.	PA!	EYA4	exonic	NM_	.	mis	EYA4:N
ACa07	6	####	rs5279151	G	A	.	PA!	ALDH8A1	intronic	NM_	.	.	.
ACa07	6	####	rs1006659	T	C	.	PA!	IL20RA	intronic	NM_	.	.	.
ACa07	6	####	.	T	C	.	PA!	KIAA1244	intronic	NM_	.	.	.
ACa07	6	####	.	C	T	.	PA!	KIAA1244	intronic	NM_	.	.	.
ACa07	6	####	rs1910958	G	A	.	PA!	GPR126	intronic	NM_	.	.	.
ACa07	6	####	rs1401901	G	A	.	PA!	STX11	exonic	NM_	.	mis	STX11:
ACa07	6	####	.	A	G	.	PA!	ADGB	exonic	NM_	.	mis	ADGB:N
ACa07	6	####	rs7737449	C	T	.	PA!	UST	exonic	NM_	.	mis	UST:NM

ACa07	6	####	rs7774557	A	G	.	PA!	GINM1	exonic	NM_	.	mis	GINM1:
ACa07	6	####	.	T	C	.	PA!	KATNA1	exonic	NM_	.	mis	KATNA
ACa07	6	####	.	T	C	.	PA!	RAET1E-/	ncRNA_	NR_	.	.	.
ACa07	6	####	.	C	T	.	PA!	AKAP12	intronic	NM_	.	.	.
ACa07	6	####	.	C	T	.	PA!	SYNE1	intronic	NM_	.	.	.
ACa07	6	####	rs5290225	G	A	.	PA!	SYNE1	intronic	NM_	.	.	.
ACa07	6	####	.	T	C	.	PA!	SYNE1	intronic	NM_	.	.	.
ACa07	6	####	.	T	C	.	PA!	IPCEF1	exonic	NM_	.	mis	IPCEF1
ACa07	6	####	.	G	A	.	PA!	SCAF8	exonic	NM_	.	mis	SCAF8:
ACa07	6	####	.	C	T	.	PA!	ARID1B	exonic	NM_	.	sto	ARID1E
ACa07	6	####	rs1015839	G	A	.	PA!	TMEM181	intronic	NM_	.	.	.
ACa07	6	####	.	C	T	.	PA!	SOD2	exonic	NM_	.	mis	SOD2:N
ACa07	6	####	.	G	A	.	PA!	.	intergen	NM_	dist=	.	.
ACa07	6	####	.	C	T	.	PA!	LOC7296(	ncRNA_	NR_	.	.	.
ACa07	6	####	.	C	T	.	PA!	LPA	intronic	NM_	.	.	.
ACa07	6	####	.	C	T	.	PA!	MAP3K4	intronic	NM_	.	.	.
ACa07	6	####	.	T	C	.	PA!	PARK2	exonic	NM_	.	mis	PARK2
ACa07	6	####	rs7739962	C	T	.	PA!	QKI	exonic	NM_	.	syr	QKI:NM
ACa07	6	####	rs2008105	G	A	.	PA!	C6orf118	exonic	NM_	.	mis	C6orf11
ACa07	6	####	rs7558449	C	T	.	PA!	TCP10	intronic	NM_	.	.	.
ACa07	6	####	.	A	G	.	PA!	WDR27	UTR5	NM_	NM_	.	.
ACa07	7	####	.	T	A	.	PA!	FAM20C	exonic	NM_	.	mis	FAM20(
ACa07	7	####	.	C	T	.	PA!	.	intergen	NM_	dist=	.	.
ACa07	7	####	.	G	T	.	PA!	MICALL2	intronic	NM_	.	.	.
ACa07	7	####	.	C	T	.	PA!	INTS1	exonic	NM_	.	mis	INTS1:N
ACa07	7	####	.	C	T	.	PA!	INTS1	exonic	NM_	.	mis	INTS1:N
ACa07	7	####	rs7786995	G	A	.	PA!	INTS1	exonic	NM_	.	syr	INTS1:N
ACa07	7	####	.	A	G	.	PA!	TMEM184	intronic	NM_	.	.	.
ACa07	7	####	.	G	A	.	PA!	NUDT1	intronic	NM_	.	.	.
ACa07	7	####	rs5612232	G	A	.	PA!	CHST12	exonic	NM_	.	syr	CHST1:
ACa07	7	####	.	C	T	.	PA!	CARD11	intronic	NM_	.	.	.
ACa07	7	####	rs3699321	C	T	.	PA!	SDK1	intronic	NM_	.	.	.
ACa07	7	####	.	C	A	.	PA!	SDK1	intronic	NM_	.	.	.
ACa07	7	####	rs5413152	C	T	.	PA!	AP5Z1	intronic	NM_	.	.	.
ACa07	7	####	.	C	T	.	PA!	CCZ1	exonic	NM_	.	mis	CCZ1:N
ACa07	7	####	.	C	A	.	PA!	CYTH3	exonic	NM_	.	mis	CYTH3
ACa07	7	####	rs7747652	C	A	.	PA!	DAGLB	exonic	NM_	.	mis	DAGLB
ACa07	7	####	.	G	T	.	PA!	GRID2IP	exonic	NM_	.	syr	GRID2I
ACa07	7	####	.	C	T	.	PA!	C7orf26	exonic	NM_	.	mis	C7orf26
ACa07	7	####	.	C	T	.	PA!	ZNF316	exonic	NM_	.	syr	ZNF316
ACa07	7	####	rs7800175	G	A	.	PA!	CCZ1B	intronic	NM_	.	.	.
ACa07	7	####	rs8790524	A	G	.	PA!	CCZ1B	intronic	NM_	.	.	.
ACa07	7	####	rs3726603	G	A	.	PA!	CCZ1B	intronic	NM_	.	.	.
ACa07	7	####	.	T	C	.	PA!	COL28A1	exonic	NM_	.	mis	COL28/
ACa07	7	####	.	G	A	.	PA!	MIOS	exonic	NM_	.	mis	MIOS:N
ACa07	7	####	.	G	A	.	PA!	.	intergen	NR_	dist=	.	.
ACa07	7	####	.	C	A	.	PA!	THSD7A	exonic	NM_	.	mis	THSD7.
ACa07	7	####	.	T	C	.	PA!	ABCB5	intronic	NM_	.	.	.
ACa07	7	####	rs3710375	C	T	.	PA!	DNAH11	exonic	NM_	.	syr	DNAH1
ACa07	7	####	.	T	C	.	PA!	STEAP1B	UTR3	NM_	NM_	.	.
ACa07	7	####	.	A	T	.	PA!	CCDC126	intronic	NM_	.	.	.
ACa07	7	####	rs1427054	C	T	.	PA!	HIBADH	exonic	NM_	.	mis	HIBADH
ACa07	7	####	.	T	C	.	PA!	NOD1	exonic	NM_	.	mis	NOD1:N
ACa07	7	####	.	C	T	.	PA!	.	intergen	NR_	dist=	.	.

ACa07	7	####	.	G	T	.	PA!	DPY19L1F	ncRNA_	NR_	.	.	.
ACa07	7	####	rs7507081	C	T	.	PA!	FKBP9	exonic	NM_	.	syr	FKBP9:
ACa07	7	####	.	C	T	.	PA!	BBS9	UTR3	NM_	NM_	.	.
ACa07	7	####	.	G	A	.	PA!	DPY19L1	intronic	NM_	.	.	.
ACa07	7	####	rs7696767	C	T	.	PA!	DPY19L2F	ncRNA_	NR_	.	.	.
ACa07	7	####	.	G	A	.	PA!	.	intergen	NM_	dist=	.	.
ACa07	7	####	rs7755650	G	A	.	PA!	HECW1	exonic	NM_	.	syr	HECW1
ACa07	7	####	.	G	A	.	PA!	HECW1	intronic	NM_	.	.	.
ACa07	7	####	.	G	A	.	PA!	URGCP	exonic	NM_	.	unl	UNKNC
ACa07	7	####	rs1113701	G	A	.	PA!	MYL7	intronic	NM_	.	.	.
ACa07	7	####	.	G	A	.	PA!	TMED4	intronic	NM_	.	.	.
ACa07	7	####	.	G	T	.	PA!	OGDH	intronic	NM_	.	.	.
ACa07	7	####	rs7476235	C	T	.	PA!	ADCY1	exonic	NM_	.	syr	ADCY1
ACa07	7	####	rs1024757	G	A	.	PA!	IGFBP3	intronic	NM_	.	.	.
ACa07	7	####	.	A	C	.	PA!	TNS3	intronic	NM_	.	.	.
ACa07	7	####	.	T	C	.	PA!	C7orf69	exonic	NM_	.	unl	UNKNC
ACa07	7	####	.	G	A	.	PA!	NUPR1L	exonic	NM_	.	mis	NUPR1
ACa07	7	####	.	C	T	.	PA!	.	intergen	NM_	dist=	.	.
ACa07	7	####	.	G	T	.	PA!	.	intergen	NR_	dist=	.	.
ACa07	7	####	.	C	T	.	PA!	.	intergen	NR_	dist=	.	.
ACa07	7	####	rs3749762	T	G	.	PA!	.	intergen	NR_	dist=	.	.
ACa07	7	####	.	C	T	.	PA!	.	intergen	NR_	dist=	.	.
ACa07	7	####	rs8799601	C	T	.	PA!	.	intergen	NR_	dist=	.	.
ACa07	7	####	.	G	A	.	PA!	.	intergen	NR_	dist=	.	.
ACa07	7	####	.	C	T	.	PA!	.	intergen	NR_	dist=	.	.
ACa07	7	####	.	C	T	.	PA!	.	intergen	NR_	dist=	.	.
ACa07	7	####	.	G	T	.	PA!	.	intergen	NR_	dist=	.	.
ACa07	7	####	.	G	A	.	PA!	.	intergen	NR_	dist=	.	.
ACa07	7	####	rs6156381	C	T	.	PA!	.	ncRNA_	NR_	.	.	.
ACa07	7	####	.	G	T	.	PA!	CCT6P1	ncRNA_	NR_	.	.	.
ACa07	7	####	.	G	A	.	PA!	CCT6P1	ncRNA_	NR_	.	.	.
ACa07	7	####	rs5480693	T	A	.	PA!	TYW1	intronic	NM_	.	.	.
ACa07	7	####	rs7706902	C	T	.	PA!	WBSCR17	exonic	NM_	.	mis	WBSCF
ACa07	7	####	rs5574064	C	T	.	PA!	SPDYE7P	ncRNA_	NR_	.	.	.
ACa07	7	####	.	T	C	.	PA!	NCF1B	ncRNA_	NR_	.	.	.
ACa07	7	####	.	G	A	.	PA!	FKBP6	UTR5	NM_	NM_	.	.
ACa07	7	####	.	C	T	.	PA!	FZD9	exonic	NM_	.	mis	FZD9:N
ACa07	7	####	rs7822351	C	T	.	PA!	BAZ1B	exonic	NM_	.	mis	BAZ1B:
ACa07	7	####	.	G	T	.	PA!	ABHD11	intronic	NM_	.	.	.
ACa07	7	####	.	G	A	.	PA!	LAT2	intronic	NM_	.	.	.
ACa07	7	####	rs7822129	G	A	.	PA!	GTF2IRD1	exonic	NM_	.	mis	GTF2IF
ACa07	7	####	rs3705131	T	C	.	PA!	NCF1	intronic	NM_	.	.	.
ACa07	7	####	rs8799812	T	C	.	PA!	GTF2IP1,I	ncRNA_	NR_	.	.	.
ACa07	7	####	rs1493360	T	C	.	PA!	FDPSP2	ncRNA_	NR_	.	.	.
ACa07	7	####	rs1403890	T	A	.	PA!	DTX2	intronic	NM_	.	.	.
ACa07	7	####	.	G	A	.	PA!	CACNA2D	intronic	NM_	.	.	.
ACa07	7	####	.	A	G	.	PA!	.	intergen	NM_	dist=	.	.
ACa07	7	####	.	C	A	.	PA!	PCLO	exonic	NM_	.	mis	PCLO:N
ACa07	7	####	rs1413343	G	A	.	PA!	CDK14	exonic	NM_	.	mis	CDK14:
ACa07	7	####	rs9879024	G	T	.	PA!	.	upstrear	NM_	.	.	.
ACa07	7	####	rs7641741	G	A	.	PA!	COL1A2	exonic	NM_	.	mis	COL1A2
ACa07	7	####	.	T	G	.	PA!	SGCE	intronic	NM_	.	.	.
ACa07	7	####	.	G	A	.	PA!	ASB4	intronic	NM_	.	.	.
ACa07	7	####	.	G	T	.	PA!	LMTK2	splicing	NM_	NM_	.	.

ACa07	7	####	rs7821462	C	T	.	PA!	TRRAP	intronic	NM_	.	.	.
ACa07	7	####	rs7752142	C	T	.	PA!	TRRAP	exonic	NM_	.	mis	TRRAP
ACa07	7	####	.	C	T	.	PA!	MYH16	ncRNA_	NR_	.	.	.
ACa07	7	####	rs9034524	G	A	.	PA!	MYH16	ncRNA_	NR_	.	.	.
ACa07	7	####	rs5767320	G	A	.	PA!	PCOLCE	exonic	NM_	.	mis	PCOLC
ACa07	7	####	.	C	T	.	PA!	ACTL6B	exonic	NM_	.	syr	ACTL6I
ACa07	7	####	rs7641678	C	T	.	PA!	MUC12	exonic	NM_	.	sto	MUC12
ACa07	7	####	.	G	A	.	PA!	MOGAT3	intronic	NM_	.	.	.
ACa07	7	####	.	G	A	.	PA!	CUX1	exonic	NM_	.	mis	CUX1:N
ACa07	7	####	rs7590335	C	T	.	PA!	RASA4B	exonic	NM_	.	syr	RASA4I
ACa07	7	####	rs4729845	T	C	.	PA!	SPDYE2B	intronic	NM_	.	.	.
ACa07	7	####	.	C	T	.	PA!	PSMC2	exonic	NM_	.	mis	PSMC2
ACa07	7	####	.	G	A	.	PA!	ORC5	exonic	NM_	.	mis	ORC5:N
ACa07	7	####	.	T	C	.	PA!	LRRN3	exonic	NM_	.	syr	LRRN3
ACa07	7	####	.	G	A	.	PA!	IFRD1	intronic	NM_	.	.	.
ACa07	7	####	.	G	A	.	PA!	ASZ1	intronic	NM_	.	.	.
ACa07	7	####	rs1457073	C	T	.	PA!	AASS	intronic	NM_	.	.	.
ACa07	7	####	.	G	A	.	PA!	.	intergen	NR_	dist=	.	.
ACa07	7	####	rs3755434	C	T	.	PA!	GPR37	exonic	NM_	.	mis	GPR37
ACa07	7	####	.	T	A	.	PA!	.	intergen	NM_	dist=	.	.
ACa07	7	####	.	G	T	.	PA!	FLNC	exonic	NM_	.	mis	FLNC:N
ACa07	7	####	rs2006779	C	T	.	PA!	SMO	exonic	NM_	.	syr	SMO:N
ACa07	7	####	.	G	T	.	PA!	.	upstreai	NM_	.	.	.
ACa07	7	####	rs3679590	G	A	.	PA!	PLXNA4	exonic	NM_	.	mis	PLXNA4
ACa07	7	####	.	C	A	.	PA!	.	intergen	NM_	dist=	.	.
ACa07	7	####	.	G	A	.	PA!	DGKI	intronic	NM_	.	.	.
ACa07	7	####	.	A	G	.	PA!	DGKI	exonic	NM_	.	mis	DGKI:N
ACa07	7	####	.	C	T	.	PA!	CREB3L2	exonic	NM_	.	mis	CREB3
ACa07	7	####	rs1825415	G	A	.	PA!	ATP6V0A4	intronic	NM_	.	.	.
ACa07	7	####	.	T	C	.	PA!	SLC37A3	intronic	NM_	.	.	.
ACa07	7	####	.	C	T	.	PA!	.	intergen	NM_	dist=	.	.
ACa07	7	####	.	C	T	.	PA!	AGK	intronic	NM_	.	.	.
ACa07	7	####	rs7825497	C	T	.	PA!	MGAM	exonic	NM_	.	mis	MGAM:
ACa07	7	####	.	C	T	.	PA!	.	exonic	NM_	.	unl	UNKNC
ACa07	7	####	.	C	A	.	PA!	.	intergen	NR_	dist=	.	.
ACa07	7	####	.	C	T	.	PA!	.	intergen	NR_	dist=	.	.
ACa07	7	####	.	C	T	.	PA!	.	intergen	NR_	dist=	.	.
ACa07	7	####	rs7663697	C	T	.	PA!	.	intergen	NR_	dist=	.	.
ACa07	7	####	.	G	A	.	PA!	GSTK1	intronic	NM_	.	.	.
ACa07	7	####	rs3733853	G	A	.	PA!	FAM131B	intronic	NM_	.	.	.
ACa07	7	####	rs7598872	C	T	.	PA!	CNTNAP2	exonic	NM_	.	syr	CNTNA
ACa07	7	####	rs8019707	C	T	.	PA!	KRBA1	intronic	NM_	.	.	.
ACa07	7	####	rs1447568	G	A	.	PA!	SSPO	exonic	NM_	.	mis	SSPO:N
ACa07	7	####	.	T	G	.	PA!	ZNF775	UTR3	NM_	NM_	.	.
ACa07	7	####	.	A	G	.	PA!	NOS3	intronic	NM_	.	.	.
ACa07	7	####	.	C	T	.	PA!	ATG9B	exonic	NM_	.	unl	UNKNC
ACa07	7	####	.	C	T	.	PA!	CDK5	exonic	NM_	.	mis	CDK5:N
ACa07	7	####	.	C	T	.	PA!	TMUB1	exonic	NM_	.	mis	TMUB1
ACa07	7	####	.	C	T	.	PA!	PRKAG2	intronic	NM_	.	.	.
ACa07	7	####	.	C	T	.	PA!	PRKAG2	intronic	NM_	.	.	.
ACa07	7	####	rs3761181	C	T	.	PA!	KMT2C	exonic	NM_	.	mis	KMT2C
ACa07	7	####	.	G	C	.	PA!	ACTR3B	intronic	NM_	.	.	.
ACa07	7	####	rs9367654	G	A	.	PA!	DPP6	intronic	NM_	.	.	.
ACa07	7	####	.	G	T	.	PA!	UBE3C	intronic	NM_	.	.	.

ACa07	7	####	rs1056749	G	A	.	PA!	PTPRN2	intronic	NM_	.	.	.
ACa07	7	####	rs3696336	T	A	.	PA!	PTPRN2	intronic	NM_	.	.	.
ACa07	7	####	.	C	T	.	PA!	.	intergen	NM_	dist=	.	.
ACa07	8	####	.	C	T	.	PA!	FAM87A	ncRNA_	NR_	.	.	.
ACa07	8	####	.	T	A	.	PA!	CLN8	exonic	NM_	.	syr	CLN8:N
ACa07	8	####	rs1995647	G	A	.	PA!	XKR5	exonic	NM_	.	mis	XKR5:N
ACa07	8	####	.	C	A	.	PA!	DEFA4	intronic	NM_	.	.	.
ACa07	8	####	rs7692119	T	C	.	PA!	USP17L1	exonic	NM_	.	syr	USP17l
ACa07	8	####	rs2946428	C	T	.	PA!	.	intergen	NR_	dist=	.	.
ACa07	8	####	rs7810476	C	T	.	PA!	CLDN23	exonic	NM_	.	syr	CLDN2:
ACa07	8	####	rs7606573	G	A	.	PA!	RP1L1	exonic	NM_	.	mis	RP1L1:
ACa07	8	####	.	G	A	.	PA!	C8orf74	splicing	NM_	NM_	.	.
ACa07	8	####	rs7511375	C	T	.	PA!	SOX7	intronic	NM_	.	.	.
ACa07	8	####	rs7626017	C	T	.	PA!	SLC35G5	exonic	NM_	.	syr	SLC35(
ACa07	8	####	.	C	T	.	PA!	TDH	ncRNA_	NR_	.	.	.
ACa07	8	####	.	C	T	.	PA!	FAM66A	ncRNA_	NR_	.	.	.
ACa07	8	####	rs3988723	A	G	.	PA!	FAM86B2	intronic	NM_	.	.	.
ACa07	8	####	rs3713666	A	T	.	PA!	LOC7297:	ncRNA_	NR_	.	.	.
ACa07	8	####	rs1036449	G	A	.	PA!	DLC1	intronic	NM_	.	.	.
ACa07	8	####	.	T	C	.	PA!	TUSC3	exonic	NM_	.	mis	TUSC3
ACa07	8	####	rs1470216	A	G	.	PA!	.	intergen	NR_	dist=	.	.
ACa07	8	####	rs1394112	G	A	.	PA!	.	intergen	NR_	dist=	.	.
ACa07	8	####	.	C	T	.	PA!	HR	intronic	NM_	.	.	.
ACa07	8	####	.	G	T	.	PA!	TNFRSF1	UTR3	NM_	NM_	.	.
ACa07	8	####	.	C	A	.	PA!	R3HCC1	intronic	NM_	.	.	.
ACa07	8	####	.	G	T	.	PA!	NKX2-6	exonic	NM_	.	syr	NKX2-6
ACa07	8	####	rs7690910	C	T	.	PA!	DOCK5	intronic	NM_	.	.	.
ACa07	8	####	rs2013688	G	A	.	PA!	KCTD9	intronic	NM_	.	.	.
ACa07	8	####	.	G	A	.	PA!	PNMA2	UTR3	NM_	NM_	.	.
ACa07	8	####	rs7247582	C	T	.	PA!	EPHX2	exonic	NM_	.	mis	EPHX2
ACa07	8	####	.	G	A	.	PA!	KIF13B	intronic	NM_	.	.	.
ACa07	8	####	rs1419780	G	A	.	PA!	TEX15	exonic	NM_	.	sto	TEX15:
ACa07	8	####	rs1198970	G	A	.	PA!	DUSP26	exonic	NM_	.	syr	DUSP2
ACa07	8	####	rs5320288	C	T	.	PA!	KCNU1	intronic	NM_	.	.	.
ACa07	8	####	.	C	T	.	PA!	ERLIN2	intronic	NM_	.	.	.
ACa07	8	####	rs7612284	G	A	.	PA!	FGFR1	intronic	NM_	.	.	.
ACa07	8	####	.	C	T	.	PA!	FGFR1	intronic	NM_	.	.	.
ACa07	8	####	rs7608102	G	A	.	PA!	TACC1	exonic	NM_	.	mis	TACC1
ACa07	8	####	.	T	C	.	PA!	ADAM18	intronic	NM_	.	.	.
ACa07	8	####	.	C	T	.	PA!	.	intergen	NM_	dist=	.	.
ACa07	8	####	rs3705628	G	A	.	PA!	KAT6A	intronic	NM_	.	.	.
ACa07	8	####	rs7605197	G	A	.	PA!	POLB	intronic	NM_	.	.	.
ACa07	8	####	.	G	A	.	PA!	.	intergen	NM_	dist=	.	.
ACa07	8	####	rs3733322	T	G	.	PA!	.	intergen	NM_	dist=	.	.
ACa07	8	####	rs2003364	T	C	.	PA!	.	intergen	NM_	dist=	.	.
ACa07	8	####	.	A	C	.	PA!	.	intergen	NM_	dist=	.	.
ACa07	8	####	rs4498566	A	C	.	PA!	.	intergen	NOI	dist=	.	.
ACa07	8	####	.	G	A	.	PA!	.	intergen	NOI	dist=	.	.
ACa07	8	####	rs4401873	C	G	.	PA!	.	intergen	NOI	dist=	.	.
ACa07	8	####	.	G	T	.	PA!	EFCAB1	exonic	NM_	.	mis	EFCAB
ACa07	8	####	.	T	C	.	PA!	SNTG1	intronic	NM_	.	.	.
ACa07	8	####	.	A	G	.	PA!	.	downstr	NM_	.	.	.
ACa07	8	####	rs1871956	C	T	.	PA!	.	intergen	NM_	dist=	.	.
ACa07	8	####	.	G	A	.	PA!	.	intergen	NM_	dist=	.	.

ACa07	8	####	.	T	A	.	PA!	NSMAF	intronic	NM_	.	.	.
ACa07	8	####	rs7793713	G	A	.	PA!	CA8	exonic	NM_	.	syr	CA8:NM
ACa07	8	####	rs1513224	C	T	.	PA!	CHD7	intronic	NM_	.	.	.
ACa07	8	####	rs8897899	G	A	.	PA!	PDE7A	intronic	NM_	.	.	.
ACa07	8	####	.	A	G	.	PA!	CSPP1	exonic	NM_	.	syr	CSPP1
ACa07	8	####	.	G	T	.	PA!	CSPP1	intronic	NM_	.	.	.
ACa07	8	####	rs7454376	G	A	.	PA!	PREX2	intronic	NM_	.	.	.
ACa07	8	####	.	G	A	.	PA!	C8orf89	exonic	NM_	.	mis	C8orf89
ACa07	8	####	.	C	T	.	PA!	.	intergen	NM_	dist=	.	.
ACa07	8	####	.	T	C	.	PA!	FABP4	intronic	NM_	.	.	.
ACa07	8	####	.	T	C	.	PA!	CNGB3	intronic	NM_	.	.	.
ACa07	8	####	.	G	A	.	PA!	CNGB3	intronic	NM_	.	.	.
ACa07	8	####	rs1030919	C	T	.	PA!	.	downstr	NM_	.	.	.
ACa07	8	####	rs7559645	G	A	.	PA!	DECR1	exonic	NM_	.	mis	DECR1
ACa07	8	####	.	G	A	.	PA!	DECR1	exonic	NM_	.	mis	DECR1
ACa07	8	####	.	T	C	.	PA!	RUNX1T1	intronic	NM_	.	.	.
ACa07	8	####	.	G	A	.	PA!	.	intergen	NM_	dist=	.	.
ACa07	8	####	rs1000299	T	C	.	PA!	.	intergen	NR_	dist=	.	.
ACa07	8	####	.	T	A	.	PA!	MATN2	intronic	NM_	.	.	.
ACa07	8	####	.	G	A	.	PA!	.	upstrear	NR_	.	.	.
ACa07	8	####	.	G	A	.	PA!	.	intergen	NM_	dist=	.	.
ACa07	8	####	.	C	T	.	PA!	OXR1	UTR5	NM_	NM_	.	.
ACa07	8	####	.	C	T	.	PA!	KCNV1	exonic	NM_	.	mis	KCNV1
ACa07	8	####	rs1493710	C	T	.	PA!	ATAD2	exonic	NM_	.	mis	ATAD2:
ACa07	8	####	.	A	G	.	PA!	FER1L6	exonic	NM_	.	syr	FER1L6
ACa07	8	####	rs7626895	C	T	.	PA!	.	intergen	NM_	dist=	.	.
ACa07	8	####	.	C	T	.	PA!	PRNCR1	ncRNA_	NR_	.	.	.
ACa07	8	####	rs3595615	G	A	.	PA!	LRRC6	exonic	NM_	.	syr	LRRC6
ACa07	8	####	rs1689333	G	A	.	PA!	TG	exonic	NM_	.	mis	TG:NM_
ACa07	8	####	.	G	T	.	PA!	KCNK9	UTR3	NM_	NM_	.	.
ACa07	8	####	.	C	T	.	PA!	TRAPPC9	intronic	NM_	.	.	.
ACa07	8	####	.	C	A	.	PA!	DENND3	exonic	NM_	.	syr	DENND
ACa07	8	####	.	C	A	.	PA!	MROH5	intronic	NM_	.	.	.
ACa07	8	####	.	C	T	.	PA!	BAI1	intronic	NM_	.	.	.
ACa07	8	####	.	G	T	.	PA!	LY6H	intronic	NM_	.	.	.
ACa07	8	####	rs7598006	C	T	.	PA!	ZFP41	exonic	NM_	.	mis	ZFP41:
ACa07	8	####	rs5644139	G	A	.	PA!	FAM83H	exonic	NM_	.	syr	FAM83I
ACa07	8	####	.	G	T	.	PA!	FAM83H	exonic	NM_	.	mis	FAM83I
ACa07	8	####	rs7819090	C	T	.	PA!	SCRIB	exonic	NM_	.	mis	SCRIB:
ACa07	8	####	.	C	T	.	PA!	NRBP2	exonic	NM_	.	mis	NRBP2
ACa07	8	####	.	G	A	.	PA!	EPPK1	exonic	NM_	.	mis	EPPK1:
ACa07	8	####	rs2000607	G	A	.	PA!	PLEC	exonic	NM_	.	mis	PLEC:N
ACa07	8	####	rs5565117	C	T	.	PA!	HSF1	intronic	NM_	.	.	.
ACa07	8	####	.	C	T	.	PA!	HSF1	intronic	NM_	.	.	.
ACa07	8	####	.	G	A	.	PA!	CYHR1	exonic	NM_	.	mis	CYHR1
ACa07	8	####	rs7468927	G	A	.	PA!	KIFC2	exonic	NM_	.	mis	KIFC2:N
ACa07	9	####	rs7123526	A	G	.	PA!	WASH1	intronic	NM_	.	.	.
ACa07	9	####	rs8987908	C	T	.	PA!	SLC1A1	intronic	NM_	.	.	.
ACa07	9	####	.	C	A	.	PA!	MPDZ	exonic	NM_	.	mis	MPDZ:N
ACa07	9	####	rs7591887	G	A	.	PA!	NFIB	intronic	NM_	.	.	.
ACa07	9	####	.	C	T	.	PA!	FREM1	exonic	NM_	.	mis	FREM1
ACa07	9	####	rs7554261	C	T	.	PA!	FREM1	exonic	NM_	.	mis	FREM1
ACa07	9	####	.	C	T	.	PA!	TTC39B	intronic	NM_	.	.	.
ACa07	9	####	.	G	A	.	PA!	BNC2	exonic	NM_	.	mis	BNC2:N

ACa07	9	####	rs7717080	G	A	.	PA!	CNTLN	exonic	NM_	.	mis	CNTLN
ACa07	9	####	.	G	A	.	PA!	SLC24A2	exonic	NM_	.	syr	SLC24/
ACa07	9	####	rs7552126	C	T	.	PA!	FOCAD	exonic	NM_	.	unl	UNKNC
ACa07	9	####	rs8986502	G	A	.	PA!	PTPLAD2	intronic	NM_	.	.	.
ACa07	9	####	rs7703822	C	T	.	PA!	NDUFB6	intronic	NM_	.	.	.
ACa07	9	####	.	G	A	.	PA!	FAM219A	UTR3	NM_	NM_	.	.
ACa07	9	####	.	C	T	.	PA!	TESK1	exonic	NM_	.	mis	TESK1:
ACa07	9	####	.	G	A	.	PA!	FBXO10	exonic	NM_	.	mis	FBXO1(
ACa07	9	####	.	G	A	.	PA!	FRMPD1	exonic	NM_	.	sto	FRMPD
ACa07	9	####	rs1616872	A	G	.	PA!	CNTNAP3	intronic	NM_	.	.	.
ACa07	9	####	rs7820531	G	C	.	PA!	CNTNAP3	exonic	NM_	.	mis	CNTNA
ACa07	9	####	rs6255281	C	T	.	PA!	.	intergen	NR_	dist=	.	.
ACa07	9	####	.	C	T	.	PA!	.	intergen	NM_	dist=	.	.
ACa07	9	####	rs2003121	C	T	.	PA!	.	ncRNA_	NR_	.	.	.
ACa07	9	####	rs2016275	G	A	.	PA!	.	intergen	NR_	dist=	.	.
ACa07	9	####	rs2013763	G	C	.	PA!	.	intergen	NM_	dist=	.	.
ACa07	9	####	rs3764322	A	G	.	PA!	.	intergen	NM_	dist=	.	.
ACa07	9	####	.	A	C	.	PA!	LOC64223	ncRNA_	NR_	.	.	.
ACa07	9	####	.	G	A	.	PA!	FOXD4L5	exonic	NM_	.	mis	FOXD4
ACa07	9	####	rs7506128	G	A	.	PA!	FOXD4L5	exonic	NM_	.	mis	FOXD4
ACa07	9	####	.	G	T	.	PA!	.	intergen	NM_	dist=	.	.
ACa07	9	####	.	G	T	.	PA!	TMEM2	exonic	NM_	.	syr	TMEM2
ACa07	9	####	.	C	A	.	PA!	NMRK1	intronic	NM_	.	.	.
ACa07	9	####	rs9660777	T	C	.	PA!	PRUNE2	intronic	NM_	.	.	.
ACa07	9	####	rs5294239	G	A	.	PA!	.	intergen	NR_	dist=	.	.
ACa07	9	####	.	A	G	.	PA!	.	ncRNA_	NR_	.	.	.
ACa07	9	####	rs5696558	G	A	.	PA!	SPATA31I	exonic	NM_	.	mis	SPATA:
ACa07	9	####	.	G	A	.	PA!	.	intergen	NM_	dist=	.	.
ACa07	9	####	rs7749802	A	G	.	PA!	FRMD3	UTR5	NM_	NM_	.	.
ACa07	9	####	.	G	A	.	PA!	NTRK2	intronic	NM_	.	.	.
ACa07	9	####	.	C	T	.	PA!	ZCCHC6	exonic	NM_	.	mis	ZCCHC
ACa07	9	####	rs9367277	C	T	.	PA!	LOC39236	ncRNA_	NR_	.	.	.
ACa07	9	####	rs1447929	C	T	.	PA!	SHC3	intronic	NM_	.	.	.
ACa07	9	####	.	C	T	.	PA!	IARS	intronic	NM_	.	.	.
ACa07	9	####	.	A	G	.	PA!	C9orf89	intronic	NM_	.	.	.
ACa07	9	####	rs5352400	G	A	.	PA!	WNK2	exonic	NM_	.	mis	WNK2:I
ACa07	9	####	.	A	G	.	PA!	PHF2	intronic	NM_	.	.	.
ACa07	9	####	.	A	G	.	PA!	PHF2	UTR3	NM_	NM_	.	.
ACa07	9	####	.	C	T	.	PA!	ZNF367	exonic	NM_	.	mis	ZNF367
ACa07	9	####	rs5750212	C	T	.	PA!	CDC14B	intronic	NM_	.	.	.
ACa07	9	####	rs5664919	C	T	.	PA!	ZNF782	exonic	NM_	.	mis	ZNF782
ACa07	9	####	rs5346016	G	A	.	PA!	TBC1D2	intronic	NM_	.	.	.
ACa07	9	####	rs5275568	G	A	.	PA!	FSD1L	intronic	NM_	.	.	.
ACa07	9	####	.	A	G	.	PA!	SVEP1	intronic	NM_	.	.	.
ACa07	9	####	rs9418280	C	T	.	PA!	KIAA1958	exonic	NM_	.	mis	KIAA19
ACa07	9	####	rs7534529	C	T	.	PA!	KIAA1958	exonic	NM_	.	mis	KIAA19
ACa07	9	####	.	G	T	.	PA!	.	downstr	NM_	.	.	.
ACa07	9	####	.	G	T	.	PA!	ALAD	intronic	NM_	.	.	.
ACa07	9	####	rs1017044	C	T	.	PA!	COL27A1	intronic	NM_	.	.	.
ACa07	9	####	.	C	T	.	PA!	COL27A1	intronic	NM_	.	.	.
ACa07	9	####	rs5296296	C	T	.	PA!	COL27A1	intronic	NM_	.	.	.
ACa07	9	####	.	C	A	.	PA!	AKNA	intronic	NM_	.	.	.
ACa07	9	####	rs5393503	G	A	.	PA!	PAPPA	exonic	NM_	.	mis	PAPPA
ACa07	9	####	rs5770416	C	T	.	PA!	CDK5RAF	intronic	NM_	.	.	.

ACa07	9	####	rs7765588	C	T	.	PA!	PHF19	intronic	NM_	.	.	.
ACa07	9	####	.	A	G	.	PA!	PHF19	intronic	NM_	.	.	.
ACa07	9	####	rs7817163	C	T	.	PA!	DAB2IP	exonic	NM_	.	syr	DAB2IF
ACa07	9	####	.	G	T	.	PA!	DAB2IP	exonic	NM_	.	mis	DAB2IF
ACa07	9	####	.	C	A	.	PA!	STRBP	intronic	NM_	.	.	.
ACa07	9	####	rs7671231	C	T	.	PA!	NEK6	intronic	NM_	.	.	.
ACa07	9	####	.	T	C	.	PA!	MAPKAP1	intronic	NM_	.	.	.
ACa07	9	####	.	C	T	.	PA!	RALGPS1	intronic	NM_	.	.	.
ACa07	9	####	rs7704105	C	T	.	PA!	LRSAM1	exonic	NM_	.	syr	LRSAM
ACa07	9	####	rs7514371	G	A	.	PA!	STXBP1	exonic	NM_	.	mis	STXBP
ACa07	9	####	.	C	A	.	PA!	TTC16	intronic	NM_	.	.	.
ACa07	9	####	rs7724303	C	T	.	PA!	FPGS	exonic	NM_	.	mis	FPGS:N
ACa07	9	####	rs3755590	C	T	.	PA!	ENG	intronic	NM_	.	.	.
ACa07	9	####	rs7704197	C	T	.	PA!	ODF2	exonic	NM_	.	syr	ODF2:N
ACa07	9	####	rs3700623	G	A	.	PA!	SPTAN1	exonic	NM_	.	mis	SPTAN
ACa07	9	####	rs1020109	C	T	.	PA!	SPTAN1	exonic	NM_	.	mis	SPTAN
ACa07	9	####	.	T	C	.	PA!	SPTAN1	intronic	NM_	.	.	.
ACa07	9	####	.	C	T	.	PA!	SPTAN1	intronic	NM_	.	.	.
ACa07	9	####	rs1200306	C	T	.	PA!	ZER1	intronic	NM_	.	.	.
ACa07	9	####	rs4837317	T	C	.	PA!	CCBL1	intronic	NM_	.	.	.
ACa07	9	####	rs4837318	C	T	.	PA!	CCBL1	intronic	NM_	.	.	.
ACa07	9	####	rs7690702	G	A	.	PA!	DOLPP1	exonic	NM_	.	mis	DOLPP
ACa07	9	####	.	G	A	.	PA!	CRAT	intronic	NM_	.	.	.
ACa07	9	####	.	G	A	.	PA!	CRAT	exonic	NM_	.	mis	CRAT:N
ACa07	9	####	rs9375934	C	T	.	PA!	RAPGEF1	exonic	NM_	.	syr	RAPGE
ACa07	9	####	.	C	T	.	PA!	NTNG2	intronic	NM_	.	.	.
ACa07	9	####	.	C	T	.	PA!	SETX	exonic	NM_	.	mis	SETX:N
ACa07	9	####	rs4498679	G	A	.	PA!	DDX31	intronic	NM_	.	.	.
ACa07	9	####	.	G	A	.	PA!	GTF3C5	UTR5	NM_	NM_	.	.
ACa07	9	####	.	C	T	.	PA!	RALGDS	exonic	NM_	.	mis	RALGD
ACa07	9	####	.	A	G	.	PA!	GBGT1	exonic	NM_	.	mis	GBGT1
ACa07	9	####	.	G	T	.	PA!	SURF4	intronic	NM_	.	.	.
ACa07	9	####	rs7823594	G	A	.	PA!	STKLD1	exonic	NM_	.	mis	STKLD
ACa07	9	####	rs7806069	C	T	.	PA!	TMEM8C	exonic	NM_	.	mis	TMEM8
ACa07	9	####	.	G	T	.	PA!	DBH	exonic	NM_	.	mis	DBH:NI
ACa07	9	####	.	C	T	.	PA!	COL5A1	exonic	NM_	.	mis	COL5A
ACa07	9	####	rs6173790	C	T	.	PA!	COL5A1	exonic	NM_	.	syr	COL5A
ACa07	9	####	.	G	A	.	PA!	COL5A1	intronic	NM_	.	.	.
ACa07	9	####	rs7478355	C	T	.	PA!	CAMSAP1	exonic	NM_	.	mis	CAMSA
ACa07	9	####	.	C	A	.	PA!	GPSM1	intronic	NM_	.	.	.
ACa07	9	####	rs9942197	C	T	.	PA!	NOTCH1	intronic	NM_	.	.	.
ACa07	9	####	.	C	A	.	PA!	NOTCH1	exonic	NM_	.	mis	NOTCH
ACa07	9	####	.	C	T	.	PA!	NOTCH1	intronic	NM_	.	.	.
ACa07	9	####	.	G	A	.	PA!	FAM69B	exonic	NM_	.	mis	FAM69I
ACa07	9	####	.	T	C	.	PA!	CCDC183	ncRNA_	NR_	.	.	.
ACa07	9	####	rs1399152	C	T	.	PA!	TRAF2	exonic	NM_	.	syr	TRAF2:
ACa07	9	####	rs5651517	C	T	.	PA!	MAN1B1	intronic	NM_	.	.	.
ACa07	9	####	rs7788028	G	A	.	PA!	SLC34A3	intronic	NM_	.	.	.
ACa07	9	####	.	C	A	.	PA!	FAM166A	exonic	NM_	.	mis	FAM16
ACa07	9	####	rs7776402	G	A	.	PA!	TOR4A	exonic	NM_	.	syr	TOR4A
ACa07	9	####	rs1126228	C	T	.	PA!	PNPLA7	intronic	NM_	.	.	.
ACa07	9	####	.	G	A	.	PA!	EHMT1	intronic	NM_	.	.	.
ACa07	9	####	rs3707879	C	T	.	PA!	TUBBP5	ncRNA_	NR_	.	.	.
ACa07	10	####	rs7547454	C	T	.	PA!	GTPBP4	intronic	NM_	.	.	.

ACa07	10	####	.	C	A	.	PA!	ADARB2	intronic	NM_	.	.	.
ACa07	10	####	rs5680149	C	T	.	PA!	PITRM1	intronic	NM_	.	.	.
ACa07	10	####	.	A	T	.	PA!	ANKRD16	intronic	NM_	.	.	.
ACa07	10	####	rs2019140	G	A	.	PA!	SFMBT2	exonic	NM_	.	mis	SFMBT
ACa07	10	####	rs7961144	G	A	.	PA!	ITIH5	intronic	NM_	.	.	.
ACa07	10	####	rs3708927	C	T	.	PA!	ITIH5	exonic	NM_	.	mis	ITIH5:N
ACa07	10	####	.	C	A	.	PA!	TAF3	intronic	NM_	.	.	.
ACa07	10	####	.	G	A	.	PA!	TAF3	intronic	NM_	.	.	.
ACa07	10	####	.	C	A	.	PA!	.	intergen	NR_	dist=	.	.
ACa07	10	####	.	C	A	.	PA!	.	upstrear	NM_	.	.	.
ACa07	10	####	rs1919845	C	T	.	PA!	.	intergen	NM_	dist=	.	.
ACa07	10	####	rs7658069	G	A	.	PA!	CUBN	intronic	NM_	.	.	.
ACa07	10	####	.	G	T	.	PA!	CUBN	exonic	NM_	.	mis	CUBN:I
ACa07	10	####	.	C	T	.	PA!	PLXDC2	intronic	NM_	.	.	.
ACa07	10	####	rs9652901	T	C	.	PA!	DNAJC1	intronic	NM_	.	.	.
ACa07	10	####	.	A	T	.	PA!	KIAA1217	exonic	NM_	.	mis	KIAA12
ACa07	10	####	rs7647799	T	C	.	PA!	ARHGAP2	exonic	NM_	.	mis	ARHGA
ACa07	10	####	rs1999030	A	G	.	PA!	ARHGAP2	intronic	NM_	.	.	.
ACa07	10	####	.	A	G	.	PA!	MYO3A	exonic	NM_	.	mis	MYO3A
ACa07	10	####	.	A	G	.	PA!	APBB1IP	intronic	NM_	.	.	.
ACa07	10	####	rs3678495	G	A	.	PA!	ANKRD26	exonic	NM_	.	mis	ANKRD
ACa07	10	####	.	G	T	.	PA!	ANKRD26	intronic	NM_	.	.	.
ACa07	10	####	.	G	A	.	PA!	LRRC37A	ncRNA_	NR_	.	.	.
ACa07	10	####	rs7766630	G	A	.	PA!	SVIL	intronic	NM_	.	.	.
ACa07	10	####	rs5733337	C	T	.	PA!	LYZL2	intronic	NM_	.	.	.
ACa07	10	####	.	G	A	.	PA!	CCNY	exonic	NM_	.	mis	CCNY:I
ACa07	10	####	.	G	A	.	PA!	HNRNPA3	ncRNA_	NR_	.	.	.
ACa07	10	####	.	G	A	.	PA!	.	intergen	NM_	dist=	.	.
ACa07	10	####	.	A	G	.	PA!	.	intergen	NM_	dist=	.	.
ACa07	10	####	.	C	T	.	PA!	.	intergen	NR_	dist=	.	.
ACa07	10	####	.	G	A	.	PA!	BMS1P5	ncRNA_	NR_	.	.	.
ACa07	10	####	.	G	A	.	PA!	WDFY4	intronic	NM_	.	.	.
ACa07	10	####	rs9040575	G	A	.	PA!	WDFY4	exonic	NM_	.	mis	WDFY4
ACa07	10	####	rs3745238	G	A	.	PA!	CHAT	exonic	NM_	.	syr	CHAT:I
ACa07	10	####	.	C	A	.	PA!	.	intergen	NR_	dist=	.	.
ACa07	10	####	rs7512474	G	A	.	PA!	PCDH15	intronic	NM_	.	.	.
ACa07	10	####	.	C	A	.	PA!	.	intergen	NM_	dist=	.	.
ACa07	10	####	rs1389134	G	A	.	PA!	FAM13C	exonic	NM_	.	mis	FAM13(
ACa07	10	####	rs5737945	A	G	.	PA!	CCDC6	intronic	NM_	.	.	.
ACa07	10	####	.	G	A	.	PA!	PBLD	intronic	NM_	.	.	.
ACa07	10	####	.	A	G	.	PA!	SUPV3L1	intronic	NM_	.	.	.
ACa07	10	####	rs7548920	C	T	.	PA!	COL13A1	exonic	NM_	.	syr	COL13/
ACa07	10	####	.	C	T	.	PA!	AIFM2	exonic	NM_	.	mis	AIFM2:I
ACa07	10	####	rs3744653	G	A	.	PA!	PRF1	intronic	NM_	.	.	.
ACa07	10	####	rs2017279	C	T	.	PA!	CDH23	exonic	NM_	.	mis	CDH23
ACa07	10	####	.	C	A	.	PA!	P4HA1	exonic	NM_	.	mis	P4HA1:
ACa07	10	####	.	C	A	.	PA!	FAM149B	intronic	NM_	.	.	.
ACa07	10	####	rs7759506	A	G	.	PA!	PPP3CB	intronic	NM_	.	.	.
ACa07	10	####	rs7802380	A	G	.	PA!	AGAP5	intronic	NM_	.	.	.
ACa07	10	####	rs8789174	A	G	.	PA!	BMS1P4	ncRNA_	NR_	.	.	.
ACa07	10	####	.	C	T	.	PA!	BMS1P4	ncRNA_	NR_	.	.	.
ACa07	10	####	.	A	G	.	PA!	KCNMA1-	ncRNA_	NR_	.	.	.
ACa07	10	####	.	A	G	.	PA!	SFTPD	intronic	NM_	.	.	.
ACa07	10	####	rs5681324	C	T	.	PA!	SH2D4B	exonic	NM_	.	mis	SH2D4I

ACa07	10	####	.	C	T	.	PA!	CDHR1	intronic	NM_	.	.	.
ACa07	10	####	.	C	T	.	PA!	LRIT1	exonic	NM_	.	syr	LRIT1:N
ACa07	10	####	.	C	T	.	PA!	GRID1	intronic	NM_	.	.	.
ACa07	10	####	rs5296587	C	T	.	PA!	.	intergen	NR_	dist=	.	.
ACa07	10	####	.	G	A	.	PA!	.	upstreaı	NM_	.	.	.
ACa07	10	####	rs3696055	G	A	.	PA!	MYOF	exonic	NM_	.	sto	MYOF:I
ACa07	10	####	rs7535868	G	A	.	PA!	CEP55	exonic	NM_	.	mis	CEP55:
ACa07	10	####	.	G	T	.	PA!	CYP2C8	intronic	NM_	.	.	.
ACa07	10	####	rs9047130	T	G	.	PA!	ENTPD1-/	ncRNA_	NR_	.	.	.
ACa07	10	####	rs7826084	C	T	.	PA!	BLNK	exonic;s	NM_	NM_	mis	BLNK:N
ACa07	10	####	.	G	A	.	PA!	.	intergen	NM_	dist=	.	.
ACa07	10	####	rs3769880	G	A	.	PA!	ZFYVE27	intronic	NM_	.	.	.
ACa07	10	####	rs9945308	C	T	.	PA!	CRTAC1	intronic	NM_	.	.	.
ACa07	10	####	rs5392081	G	A	.	PA!	HPSE2	exonic	NM_	.	mis	HPSE2
ACa07	10	####	rs3692674	C	T	.	PA!	CUTC	exonic	NM_	.	mis	CUTC:N
ACa07	10	####	rs9441434	C	T	.	PA!	SEC31B	intronic	NM_	.	.	.
ACa07	10	####	.	G	A	.	PA!	PPRC1	exonic	NM_	.	mis	PPRC1
ACa07	10	####	.	A	G	.	PA!	PSD	intronic	NM_	.	.	.
ACa07	10	####	.	C	T	.	PA!	TRIM8	intronic	NM_	.	.	.
ACa07	10	####	.	C	A	.	PA!	COL17A1	intronic	NM_	.	.	.
ACa07	10	####	.	G	A	.	PA!	CFAP58	intronic	NM_	.	.	.
ACa07	10	####	.	T	G	.	PA!	SORCS3	intronic	NM_	.	.	.
ACa07	10	####	.	C	T	.	PA!	GFRA1	intronic	NM_	.	.	.
ACa07	10	####	.	C	T	.	PA!	PDZD8	exonic	NM_	.	mis	PDZD8
ACa07	10	####	.	T	A	.	PA!	TIAL1	intronic	NM_	.	.	.
ACa07	10	####	.	A	G	.	PA!	BTBD16	intronic	NM_	.	.	.
ACa07	10	####	rs7503782	G	A	.	PA!	FAM175B	exonic	NM_	.	syr	FAM17!
ACa07	10	####	.	C	T	.	PA!	DOCK1	exonic	NM_	.	unl	UNKNC
ACa07	10	####	rs3765225	C	T	.	PA!	DOCK1	intronic	NM_	.	.	.
ACa07	10	####	.	C	T	.	PA!	FOXI2	exonic	NM_	.	mis	FOXI2:I
ACa07	10	####	rs5455183	G	A	.	PA!	PPP2R2D	intronic	NM_	.	.	.
ACa07	10	####	.	G	T	.	PA!	NKX6-2	exonic	NM_	.	syr	NKX6-2
ACa07	10	####	.	C	T	.	PA!	CFAP46	intronic	NM_	.	.	.
ACa07	10	####	rs5396027	C	T	.	PA!	CFAP46	intronic	NM_	.	.	.
ACa07	10	####	.	G	T	.	PA!	PRAP1	exonic	NM_	.	mis	PRAP1
ACa07	10	####	.	G	A	.	PA!	PAOX	exonic	NM_	.	syr	PAOX:N
ACa07	10	####	rs7793478	G	A	.	PA!	PAOX	intronic	NM_	.	.	.
ACa07	10	####	.	G	A	.	PA!	PAOX	intronic	NM_	.	.	.
ACa07	10	####	.	T	C	.	PA!	MTG1	intronic	NM_	.	.	.
ACa07	11	####	.	G	T	.	PA!	LINC0100	ncRNA_	NR_	.	.	.
ACa07	11	####	rs2003509	C	A	.	PA!	.	upstreaı	NM_	.	.	.
ACa07	11	####	.	G	T	.	PA!	PSMD13	intronic	NM_	.	.	.
ACa07	11	####	rs1008718	G	A	.	PA!	RNH1	intronic	NM_	.	.	.
ACa07	11	####	rs7793525	C	T	.	PA!	LRRC56	intronic	NM_	.	.	.
ACa07	11	####	rs3741463	G	A	.	PA!	LMNTD2	exonic	NM_	.	mis	LMNTD
ACa07	11	####	.	A	G	.	PA!	PHRF1	exonic	NM_	.	mis	PHRF1
ACa07	11	####	.	G	T	.	PA!	SLC25A2:	intronic	NM_	.	.	.
ACa07	11	####	rs8960121	G	A	.	PA!	AP2A2	intronic	NM_	.	.	.
ACa07	11	####	rs7582346	G	A	.	PA!	.	intergen	NM_	dist=	.	.
ACa07	11	####	rs7514641	G	A	.	PA!	MUC5B	intronic	NM_	.	.	.
ACa07	11	####	.	T	C	.	PA!	MUC5B	intronic	NM_	.	.	.
ACa07	11	####	.	G	A	.	PA!	MUC5B	exonic	NM_	.	syr	MUC5B
ACa07	11	####	.	C	T	.	PA!	MUC5B	exonic	NM_	.	mis	MUC5B
ACa07	11	####	rs3689865	C	T	.	PA!	MUC5B	exonic	NM_	.	syr	MUC5B

ACa07	11	####	.	A	G	.	PA!	TH	intronic	NM_	.	.	.
ACa07	11	####	rs1019902	C	T	.	PA!	.	downstr	NR_	.	.	.
ACa07	11	####	rs7724812	G	A	.	PA!	CD81	intronic	NM_	.	.	.
ACa07	11	####	.	G	T	.	PA!	TRPM5	intronic	NM_	.	.	.
ACa07	11	####	.	G	A	.	PA!	TRPM5	intronic	NM_	.	.	.
ACa07	11	####	rs5592355	T	C	.	PA!	.	intergen	NR_	dist=	.	.
ACa07	11	####	.	T	C	.	PA!	OR52B4	exonic	NM_	.	mis	OR52B4
ACa07	11	####	.	G	A	.	PA!	.	intergen	NM_	dist=	.	.
ACa07	11	####	rs8669468	C	T	.	PA!	TRIM6-TR	intronic	NM_	.	.	.
ACa07	11	####	.	G	A	.	PA!	PRKCDBF	UTR3	NM_	NM_	.	.
ACa07	11	####	rs7289628	C	T	.	PA!	APBB1	intronic	NM_	.	.	.
ACa07	11	####	.	G	A	.	PA!	DCHS1	exonic	NM_	.	sto	DCHS1
ACa07	11	####	.	C	T	.	PA!	DCHS1	exonic	NM_	.	mis	DCHS1
ACa07	11	####	rs7595225	C	T	.	PA!	MICALCL	exonic	NM_	.	mis	MICALC
ACa07	11	####	rs7811107	C	T	.	PA!	ARNTL	exonic	NM_	.	unl	UNKNC
ACa07	11	####	.	G	A	.	PA!	SPON1	exonic	NM_	.	unl	UNKNC
ACa07	11	####	rs7824579	C	T	.	PA!	RRAS2	exonic	NM_	.	mis	RRAS2
ACa07	11	####	.	C	A	.	PA!	PDE3B	exonic	NM_	.	mis	PDE3B
ACa07	11	####	.	G	A	.	PA!	CYP2R1	intronic	NM_	.	.	.
ACa07	11	####	.	G	A	.	PA!	PLEKHA7	intronic	NM_	.	.	.
ACa07	11	####	rs7785451	G	A	.	PA!	PLEKHA7	exonic	NM_	.	mis	PLEKH.
ACa07	11	####	.	G	A	.	PA!	OTOG	exonic	NM_	.	mis	OTOG:I
ACa07	11	####	.	G	T	.	PA!	TPH1	exonic	NM_	.	syr	TPH1:N
ACa07	11	####	.	C	T	.	PA!	.	intergen	NM_	dist=	.	.
ACa07	11	####	.	G	T	.	PA!	SAA1	intronic	NM_	.	.	.
ACa07	11	####	.	C	A	.	PA!	LDHA	intronic	NM_	.	.	.
ACa07	11	####	.	A	C	.	PA!	SLC6A5	intronic	NM_	.	.	.
ACa07	11	####	rs1146385	G	A	.	PA!	NELL1	exonic	NM_	.	mis	NELL1:
ACa07	11	####	.	A	G	.	PA!	GAS2	exonic	NM_	.	mis	GAS2:N
ACa07	11	####	.	A	T	.	PA!	.	intergen	NM_	dist=	.	.
ACa07	11	####	.	C	A	.	PA!	CD59	intronic	NM_	.	.	.
ACa07	11	####	.	C	T	.	PA!	ABTB2	intronic	NM_	.	.	.
ACa07	11	####	.	C	T	.	PA!	HSD17B1:	exonic	NM_	.	mis	HSD17I
ACa07	11	####	.	G	A	.	PA!	ZNF408	exonic	NM_	.	mis	ZNF408
ACa07	11	####	.	G	A	.	PA!	CKAP5	exonic	NM_	.	sto	CKAP5
ACa07	11	####	rs7731977	C	T	.	PA!	ARFGAP2	intronic	NM_	.	.	.
ACa07	11	####	.	T	C	.	PA!	OR4A15	exonic	NM_	.	mis	OR4A1!
ACa07	11	####	rs7783566	T	C	.	PA!	.	upstreal	NM_	.	.	.
ACa07	11	####	.	T	G	.	PA!	SMTNL1	exonic	NM_	.	mis	SMTNL
ACa07	11	####	rs7556226	G	A	.	PA!	OR1S1	exonic	NM_	.	mis	OR1S1
ACa07	11	####	rs3705153	C	T	.	PA!	OR10Q1	exonic	NM_	.	mis	OR10Q
ACa07	11	####	rs7461600	C	T	.	PA!	PATL1	intronic	NM_	.	.	.
ACa07	11	####	rs7613895	G	A	.	PA!	PPP1R32	exonic	NM_	.	mis	PPP1R:
ACa07	11	####	.	C	A	.	PA!	PPP1R32	intronic	NM_	.	.	.
ACa07	11	####	.	G	T	.	PA!	FADS1	exonic	NM_	.	syr	FADS1:
ACa07	11	####	rs7506613	G	A	.	PA!	AHNAK	exonic	NM_	.	syr	AHNAK
ACa07	11	####	rs3688014	C	T	.	PA!	MTA2	exonic	NM_	.	mis	MTA2:N
ACa07	11	####	rs3734262	C	T	.	PA!	INTS5	exonic	NM_	.	mis	INTS5:I
ACa07	11	####	.	T	G	.	PA!	NXF1	UTR3	NM_	NM_	.	.
ACa07	11	####	rs7551956	C	T	.	PA!	NAA40	exonic	NM_	.	mis	NAA40:
ACa07	11	####	rs320149	G	A	.	PA!	OTUB1	intronic	NM_	.	.	.
ACa07	11	####	.	T	A	.	PA!	BAD	intronic	NM_	.	.	.
ACa07	11	####	rs7694797	C	T	.	PA!	PYGM	exonic	NM_	.	mis	PYGM:I
ACa07	11	####	rs7599871	G	A	.	PA!	SYVN1	exonic	NM_	.	syr	SYVN1:

ACa07	11	####	rs2009796	C	T	.	PA!	SPDYC	intronic	NM_	.	.	.
ACa07	11	####	.	G	A	.	PA!	POLA2	intronic	NM_	.	.	.
ACa07	11	####	.	G	A	.	PA!	KCNK7	exonic	NM_	.	mis	KCNK7
ACa07	11	####	.	G	A	.	PA!	MAP3K11	exonic	NM_	.	syr	MAP3K
ACa07	11	####	rs3731576	G	A	.	PA!	PCNXL3	exonic	NM_	.	mis	PCNXL
ACa07	11	####	.	G	A	.	PA!	SIPA1	exonic	NM_	.	mis	SIPA1:N
ACa07	11	####	.	T	G	.	PA!	SIPA1	intronic	NM_	.	.	.
ACa07	11	####	.	G	A	.	PA!	RNASEH2	intronic	NM_	.	.	.
ACa07	11	####	.	C	T	.	PA!	GAL3ST3	exonic	NM_	.	syr	GAL3S'
ACa07	11	####	.	C	T	.	PA!	KLC2	intronic	NM_	.	.	.
ACa07	11	####	.	C	T	.	PA!	CD248	exonic	NM_	.	mis	CD248:
ACa07	11	####	.	T	C	.	PA!	BRMS1	exonic	NM_	.	mis	BRMS1
ACa07	11	####	.	G	A	.	PA!	PPP1CA	intronic	NM_	.	.	.
ACa07	11	####	rs8917540	C	T	.	PA!	NDUFV1	intronic	NM_	.	.	.
ACa07	11	####	rs1432324	C	T	.	PA!	TCIRG1	exonic	NM_	.	syr	TCIRG1
ACa07	11	####	rs7621382	C	T	.	PA!	TPCN2	exonic	NM_	.	mis	TPCN2
ACa07	11	####	.	G	A	.	PA!	TPCN2	intronic	NM_	.	.	.
ACa07	11	####	.	A	G	.	PA!	PPFIA1	intronic	NM_	.	.	.
ACa07	11	####	rs7726393	C	T	.	PA!	DHCR7	exonic	NM_	.	mis	DHCR7
ACa07	11	####	rs1917587	G	A	.	PA!	LRTOMT	UTR3	NM_	NM_	.	.
ACa07	11	####	.	G	C	.	PA!	FOLR2	intronic	NM_	.	.	.
ACa07	11	####	.	C	A	.	PA!	CLPB	intronic	NM_	.	.	.
ACa07	11	####	.	A	G	.	PA!	CLPB	intronic	NM_	.	.	.
ACa07	11	####	.	C	A	.	PA!	ARAP1	UTR3	NM_	NM_	.	.
ACa07	11	####	.	T	C	.	PA!	FCHSD2	intronic	NM_	.	.	.
ACa07	11	####	.	G	A	.	PA!	PPME1	UTR3	NM_	NM_	.	.
ACa07	11	####	.	C	T	.	PA!	NEU3	intronic	NM_	.	.	.
ACa07	11	####	rs7806415	G	A	.	PA!	SLCO2B1	exonic	NM_	.	mis	SLCO2
ACa07	11	####	rs7805688	G	A	.	PA!	MAP6	exonic	NM_	.	mis	MAP6:N
ACa07	11	####	rs7453149	C	T	.	PA!	UVRAG	intronic	NM_	.	.	.
ACa07	11	####	.	G	A	.	PA!	USP35	intronic	NM_	.	.	.
ACa07	11	####	rs3722049	G	C	.	PA!	.	intergen	NM_	dist=	.	.
ACa07	11	####	.	G	A	.	PA!	DLG2	intronic	NM_	.	.	.
ACa07	11	####	.	G	A	.	PA!	GRM5	exonic	NM_	.	mis	GRM5:I
ACa07	11	####	.	G	A	.	PA!	FOLH1B	intronic	NM_	.	.	.
ACa07	11	####	rs7912478	G	A	.	PA!	CCDC67	intronic	NM_	.	.	.
ACa07	11	####	rs1913813	G	A	.	PA!	DYNC2H1	exonic	NM_	.	mis	DYNC2
ACa07	11	####	rs7548668	G	A	.	PA!	GUCY1A2	intronic	NM_	.	.	.
ACa07	11	####	rs7639430	C	T	.	PA!	ELMOD1	exonic	NM_	.	mis	ELMOD
ACa07	11	####	.	A	G	.	PA!	CUL5	intronic	NM_	.	.	.
ACa07	11	####	.	T	C	.	PA!	ACAT1	exonic	NM_	.	syr	ACAT1:
ACa07	11	####	.	A	G	.	PA!	ATM	intronic	NM_	.	.	.
ACa07	11	####	rs5746434	C	T	.	PA!	C11orf53	UTR3	NM_	NM_	.	.
ACa07	11	####	rs1042921	G	A	.	PA!	COLCA1	ncRNA_	NR_	.	.	.
ACa07	11	####	rs9738553	C	T	.	PA!	SIK2	intronic	NM_	.	.	.
ACa07	11	####	rs7711773	G	A	.	PA!	ZW10	exonic	NM_	.	syr	ZW10:N
ACa07	11	####	rs7553780	T	G	.	PA!	CADM1	intronic	NM_	.	.	.
ACa07	11	####	.	C	T	.	PA!	SIK3	exonic	NM_	.	syr	SIK3:NI
ACa07	11	####	rs3706451	C	A	.	PA!	TMPRSS1	exonic	NM_	.	syr	TMPRS
ACa07	11	####	.	C	T	.	PA!	SCN2B	exonic	NM_	.	mis	SCN2B
ACa07	11	####	.	G	T	.	PA!	MPZL3	intronic	NM_	.	.	.
ACa07	11	####	.	C	A	.	PA!	ARCN1	intronic	NM_	.	.	.
ACa07	11	####	.	G	A	.	PA!	DDX6	exonic	NM_	.	syr	DDX6:N
ACa07	11	####	rs5761141	T	C	.	PA!	ARHGEF1	exonic	NM_	.	syr	ARHGE

ACa07	11	####	.	C	A	.	PA!	ARHGEF1	exonic	NM_	.	mis	ARHGE
ACa07	11	####	rs1424863	C	T	.	PA!	TECTA	exonic	NM_	.	mis	TECTA
ACa07	11	####	rs1464053	C	T	.	PA!	OR10G7	exonic	NM_	.	mis	OR10G
ACa07	11	####	rs1866274	C	T	.	PA!	OR8G2	exonic	NM_	.	unl	UNKNO
ACa07	11	####	.	C	A	.	PA!	ROBO4	intronic	NM_	.	.	.
ACa07	11	####	.	C	T	.	PA!	PKNOX2	exonic	NM_	.	mis	PKNOX
ACa07	11	####	rs1036245	G	A	.	PA!	FOXRED1	exonic	NM_	.	mis	FOXRE
ACa07	11	####	rs1499597	C	T	.	PA!	TP53AIP1	intronic	NM_	.	.	.
ACa07	11	####	rs1160026	G	T	.	PA!	PRDM10	exonic	NM_	.	syr	PRDM1
ACa07	11	####	rs7501981	G	A	.	PA!	ADAMTS1	exonic	NM_	.	mis	ADAMT
ACa07	11	####	.	G	T	.	PA!	OPCML	exonic	NM_	.	mis	OPCML
ACa07	11	####	rs9612999	G	A	.	PA!	.	intergen	NM_	dist=	.	.
ACa07	11	####	.	G	A	.	PA!	GLB1L3	intronic	NM_	.	.	.
ACa07	12	####	rs1492134	C	T	.	PA!	CCDC77	intronic	NM_	.	.	.
ACa07	12	####	.	G	A	.	PA!	.	upstrear	NM_	.	.	.
ACa07	12	####	rs7477402	G	A	.	PA!	B4GALNT	exonic	NM_	.	mis	B4GALI
ACa07	12	####	.	G	T	.	PA!	WNK1	intronic	NM_	.	.	.
ACa07	12	####	.	C	T	.	PA!	CACNA1C	exonic	NM_	.	syr	CACNA
ACa07	12	####	rs2020589	C	T	.	PA!	CACNA1C	exonic	NM_	.	syr	CACNA
ACa07	12	####	rs1044570	G	A	.	PA!	CACNA1C	intronic	NM_	.	.	.
ACa07	12	####	.	C	T	.	PA!	CRACR2A	intronic	NM_	.	.	.
ACa07	12	####	.	C	A	.	PA!	CCND2	intronic	NM_	.	.	.
ACa07	12	####	.	G	T	.	PA!	RAD51AP	UTR5	NM_	NM_	.	.
ACa07	12	####	.	A	G	.	PA!	KCNA6	exonic	NM_	.	mis	KCNA6
ACa07	12	####	.	G	A	.	PA!	TNFRSF1	exonic	NM_	.	mis	TNFRS
ACa07	12	####	rs7522876	C	T	.	PA!	ZNF384	intronic	NM_	.	.	.
ACa07	12	####	.	C	T	.	PA!	CD4	intronic	NM_	.	.	.
ACa07	12	####	.	C	A	.	PA!	CLSTN3	intronic	NM_	.	.	.
ACa07	12	####	.	T	C	.	PA!	CD163	splicing	NM_	NM_	.	.
ACa07	12	####	.	T	A	.	PA!	CLEC6A	exonic	NM_	.	mis	CLEC6A
ACa07	12	####	.	G	A	.	PA!	MFAP5	exonic	NM_	.	unl	UNKNO
ACa07	12	####	.	A	G	.	PA!	.	intergen	NR_	dist=	.	.
ACa07	12	####	rs7607645	T	C	.	PA!	.	intergen	NR_	dist=	.	.
ACa07	12	####	.	C	A	.	PA!	CLEC12A	intronic	NM_	.	.	.
ACa07	12	####	.	G	A	.	PA!	PRH1-PRI	ncRNA_	NR_	.	.	.
ACa07	12	####	.	C	A	.	PA!	ETV6	intronic	NM_	.	.	.
ACa07	12	####	rs7520863	C	T	.	PA!	LOH12CR	exonic	NM_	.	unl	UNKNO
ACa07	12	####	.	T	C	.	PA!	LOH12CR	exonic	NM_	.	unl	UNKNO
ACa07	12	####	rs7558438	C	T	.	PA!	RPL13AP	ncRNA_	NR_	.	.	.
ACa07	12	####	.	C	T	.	PA!	PIK3C2G	exonic	NM_	.	mis	PIK3C2
ACa07	12	####	.	G	T	.	PA!	PIK3C2G	exonic	NM_	.	sto	PIK3C2
ACa07	12	####	rs9411424	A	C	.	PA!	.	intergen	NM_	dist=	.	.
ACa07	12	####	.	A	G	.	PA!	SLCO1B1	exonic	NM_	.	mis	SLCO1
ACa07	12	####	.	A	G	.	PA!	PPFIBP1	exonic	NM_	.	syr	PPFIBP
ACa07	12	####	.	C	T	.	PA!	ERGIC2	UTR3	NM_	NM_	.	.
ACa07	12	####	rs3715564	C	T	.	PA!	TSPAN11	intronic	NM_	.	.	.
ACa07	12	####	.	C	T	.	PA!	TSPAN11	UTR3	NM_	NM_	.	.
ACa07	12	####	.	G	A	.	PA!	.	intergen	NM_	dist=	.	.
ACa07	12	####	.	C	T	.	PA!	.	intergen	NOI	dist=	.	.
ACa07	12	####	.	C	T	.	PA!	MUC19	intronic	NM_	.	.	.
ACa07	12	####	.	C	T	.	PA!	MUC19	intronic	NM_	.	.	.
ACa07	12	####	.	A	T	.	PA!	PUS7L	intronic	NM_	.	.	.
ACa07	12	####	.	A	C	.	PA!	C12orf54	exonic	NM_	.	mis	C12orf5
ACa07	12	####	.	C	T	.	PA!	ADCY6	intronic	NM_	.	.	.

ACa07	12	####	.	T	C	.	PA!	TROAP	exonic	NM_	.	mis	TROAP
ACa07	12	####	rs5557374	G	A	.	PA!	PRPF40B	intronic	NM_	.	.	.
ACa07	12	####	rs7778437	G	A	.	PA!	PRPF40B	exonic	NM_	.	mis	PRPF4(
ACa07	12	####	.	A	G	.	PA!	NCKAP5L	intronic	NM_	.	.	.
ACa07	12	####	rs7903986	G	A	.	PA!	ASIC1	intronic	NM_	.	.	.
ACa07	12	####	.	G	A	.	PA!	LARP4	intronic	NM_	.	.	.
ACa07	12	####	.	T	C	.	PA!	TFCP2	exonic	NM_	.	mis	TFCP2:
ACa07	12	####	.	A	G	.	PA!	C12orf80	exonic	NM_	.	mis	C12orf8
ACa07	12	####	.	G	T	.	PA!	KRT71	UTR5	NM_	NM_	.	.
ACa07	12	####	rs3777629	C	T	.	PA!	KRT73	exonic	NM_	.	syr	KRT73:
ACa07	12	####	rs5301800	G	A	.	PA!	KRT2	exonic	NM_	.	mis	KRT2:N
ACa07	12	####	.	C	T	.	PA!	KRT4	exonic	NM_	.	syr	KRT4:N
ACa07	12	####	.	G	A	.	PA!	SPRYD3	intronic	NM_	.	.	.
ACa07	12	####	.	C	T	.	PA!	ITGB7	intronic	NM_	.	.	.
ACa07	12	####	rs9039120	G	A	.	PA!	MAP3K12	intronic	NM_	.	.	.
ACa07	12	####	.	G	T	.	PA!	HOXC4	intronic	NM_	.	.	.
ACa07	12	####	rs3688610	C	T	.	PA!	ANKRD52	exonic	NM_	.	mis	ANKRD
ACa07	12	####	rs7688629	C	T	.	PA!	MYO1A	exonic	NM_	.	mis	MYO1A
ACa07	12	####	rs1929543	T	A	.	PA!	TMEM194	intronic	NM_	.	.	.
ACa07	12	####	rs9453696	G	C	.	PA!	LRP1	intronic	NM_	.	.	.
ACa07	12	####	.	G	A	.	PA!	LRP1	exonic	NM_	.	mis	LRP1:N
ACa07	12	####	rs3702872	G	A	.	PA!	LRP1	intronic	NM_	.	.	.
ACa07	12	####	.	T	C	.	PA!	LRP1	intronic	NM_	.	.	.
ACa07	12	####	rs5363943	G	A	.	PA!	SHMT2	exonic	NM_	.	mis	SHMT2
ACa07	12	####	.	T	G	.	PA!	DPY19L2	exonic	NM_	.	mis	DPY19I
ACa07	12	####	rs1809265	C	T	.	PA!	XPOT	exonic	NM_	.	syr	XPOT:N
ACa07	12	####	.	A	G	.	PA!	XPOT	exonic	NM_	.	mis	XPOT:N
ACa07	12	####	.	A	G	.	PA!	YEATS4	exonic	NM_	.	unl	UNKNC
ACa07	12	####	.	G	A	.	PA!	CCT2	UTR3	NM_	NM_	.	.
ACa07	12	####	rs5319845	C	T	.	PA!	BEST3	UTR3	NM_	NM_	.	.
ACa07	12	####	.	A	C	.	PA!	TMEM19	exonic	NM_	.	mis	TMEM1
ACa07	12	####	rs3675929	C	T	.	PA!	OSBPL8	exonic	NM_	.	mis	OSBPL
ACa07	12	####	.	G	T	.	PA!	CSRP2	intronic	NM_	.	.	.
ACa07	12	####	.	C	A	.	PA!	NAV3	exonic	NM_	.	mis	NAV3:N
ACa07	12	####	.	G	T	.	PA!	NAV3	exonic	NM_	.	mis	NAV3:N
ACa07	12	####	rs7672581	C	T	.	PA!	OTOGL	exonic	NM_	.	syr	OTOGL
ACa07	12	####	rs9382701	A	G	.	PA!	OTOGL	intronic	NM_	.	.	.
ACa07	12	####	.	G	T	.	PA!	METTL25	exonic	NM_	.	mis	METTL
ACa07	12	####	.	C	T	.	PA!	.	intergen	NM_	dist=	.	.
ACa07	12	####	.	G	A	.	PA!	USP44	intronic	NM_	.	.	.
ACa07	12	####	rs5750458	G	A	.	PA!	CDK17	exonic	NM_	.	mis	CDK17:
ACa07	12	####	rs1491955	G	A	.	PA!	.	intergen	NM_	dist=	.	.
ACa07	12	####	.	T	C	.	PA!	.	intergen	NM_	dist=	.	.
ACa07	12	####	rs7594794	C	T	.	PA!	NR1H4	exonic	NM_	.	mis	NR1H4
ACa07	12	####	.	C	T	.	PA!	ANO4	intronic	NM_	.	.	.
ACa07	12	####	.	A	G	.	PA!	ARL1	intronic	NM_	.	.	.
ACa07	12	####	.	G	T	.	PA!	MYBPC1	intronic	NM_	.	.	.
ACa07	12	####	.	G	T	.	PA!	STAB2	exonic	NM_	.	mis	STAB2:
ACa07	12	####	.	G	A	.	PA!	TDG	intronic	NM_	.	.	.
ACa07	12	####	.	C	T	.	PA!	WSCD2	intronic	NM_	.	.	.
ACa07	12	####	.	C	T	.	PA!	SART3	exonic	NM_	.	mis	SART3:
ACa07	12	####	.	T	C	.	PA!	ALKBH2	intronic	NM_	.	.	.
ACa07	12	####	.	C	T	.	PA!	MYO1H	intronic	NM_	.	.	.
ACa07	12	####	.	G	T	.	PA!	.	intergen	NM_	dist=	.	.

ACa07	12	####	.	C	A	.	PA!	BRAP	UTR5	NM_	NM_	.	.	
ACa07	12	####	rs3699442	G	A	.	PA!	HECTD4	exonic	NM_	.	syr	HECTD	
ACa07	12	####	rs7648934	C	T	.	PA!	MED13L	intronic	NM_	.	.	.	
ACa07	12	####	.	T	G	.	PA!	NOS1	intronic	NM_	.	.	.	
ACa07	12	####	.	A	G	.	PA!	TAOK3	intronic	NM_	.	.	.	
ACa07	12	####	.	G	T	.	PA!	TMEM233	UTR3	NM_	NM_	.	.	
ACa07	12	####	rs9094398	C	T	.	PA!	MLEC	UTR5	NM_	NM_	.	.	
ACa07	12	####	rs7629138	C	T	.	PA!	SPPL3	UTR3	NM_	NM_	.	.	
ACa07	12	####	.	A	T	.	PA!	OASL	exonic	NM_	.	mis	OASL:N	
ACa07	12	####	rs1000013	G	A	.	PA!	P2RX4	intronic	NM_	.	.	.	
ACa07	12	####	.	C	T	.	PA!	KDM2B	intronic	NM_	.	.	.	
ACa07	12	####	.	G	T	.	PA!	CLIP1	intronic	NM_	.	.	.	
ACa07	12	####	.	G	A	.	PA!	KNTC1	intronic	NM_	.	.	.	
ACa07	12	####	rs5485859	G	A	.	PA!	HIP1R	intronic	NM_	.	.	.	
ACa07	12	####	.	C	A	.	PA!	SBNO1	intronic	NM_	.	.	.	
ACa07	12	####	.	G	A	.	PA!	SBNO1	intronic	NM_	.	.	.	
ACa07	12	####	.	T	C	.	PA!	SETD8	intronic	NM_	.	.	.	
ACa07	12	####	rs3682772	C	T	.	PA!	DNAH10	intronic	NM_	.	.	.	
ACa07	12	####	.	T	C	.	PA!	DNAH10	exonic	NM_	.	syr	DNAH1	
ACa07	12	####	rs1861196	G	A	.	PA!	DNAH10	intronic	NM_	.	.	.	
ACa07	12	####	.	T	G	.	PA!	TMEM132	intronic	NM_	.	.	.	
ACa07	12	####	.	C	T	.	PA!	.	intergen	NR_	dist=	.	.	
ACa07	12	####	rs7758291	C	T	.	PA!	TMEM132	exonic	NM_	.	syr	TMEM1	
ACa07	12	####	rs8685914	G	A	.	PA!	GLT1D1	intronic	NM_	.	.	.	
ACa07	12	####	.	G	T	.	PA!	EP400	intronic	NM_	.	.	.	
ACa07	12	####	.	T	C	.	PA!	DDX51	intronic	NM_	.	.	.	
ACa07	12	####	.	C	T	.	PA!	GALNT9	intronic	NM_	.	.	.	
ACa07	12	####	.	C	T	.	PA!	CHFR	exonic	NM_	.	mis	CHFR:N	
ACa07	13	####	.	C	T	.	PA!	PSPC1	exonic	NM_	.	syr	PSPC1	
ACa07	13	####	.	G	T	.	PA!	ZMYM2	exonic	NM_	.	mis	ZMYM2	
ACa07	13	####	rs7650516	G	A	.	PA!	ZDHHC20	exonic	NM_	.	mis	ZDHHC	
ACa07	13	####	.	G	A	.	PA!	SGCG	exonic	NM_	.	syr	SGCG:I	
ACa07	13	####	rs7609687	G	A	.	PA!	WASF3	UTR3	NM_	NM_	.	.	
ACa07	13	####	.	T	C	.	PA!	POMP	UTR5	NM_	NM_	.	.	
ACa07	13	####	.	G	T	.	PA!	FRY	exonic	NM_	.	mis	FRY:NM	
ACa07	13	####	.	T	G	.	PA!	RFC3	intronic	NM_	.	.	.	
ACa07	13	####	.	C	T	.	PA!	MIR548F5	ncRNA_	NR_	.	.	.	
ACa07	13	####	.	C	T	.	PA!	CCDC169	exonic	NM_	.	mis	CCDC1	
ACa07	13	####	.	T	C	.	PA!	SMAD9	exonic	NM_	.	mis	SMAD9	
ACa07	13	####	rs1133145	C	T	.	PA!	MTRF1	exonic	NM_	.	mis	MTRF1	
ACa07	13	####	rs9269682	G	A	.	PA!	.	intergen	NM_	dist=	.	.	
ACa07	13	####	.	G	A	.	PA!	ERICH6B	intronic	NM_	.	.	.	
ACa07	13	####	rs5302300	C	T	.	PA!	ERICH6B	intronic	NM_	.	.	.	
ACa07	13	####	rs7755432	C	T	.	PA!	ESD	intronic	NM_	.	.	.	
ACa07	13	####	rs5354767	C	T	.	PA!	.	intergen	NR_	dist=	.	.	
ACa07	13	####	.	A	C	.	PA!	DACH1	intronic	NM_	.	.	.	
ACa07	13	####	.	G	A	.	PA!	TBC1D4	intronic	NM_	.	.	.	
ACa07	13	####	rs5371791	C	T	.	PA!	.	downstr	NR_	.	.	.	
ACa07	13	####	.	C	T	.	PA!	DZIP1	intronic	NM_	.	.	.	
ACa07	13	####	rs1492005	G	A	.	PA!	MBNL2	exonic	NM_	.	mis	MBNL2	
ACa07	13	####	rs3733851	G	A	.	PA!	TM9SF2	exonic	NM_	.	syr	TM9SF	
ACa07	13	####	rs3731740	C	T	.	PA!	PCCA	intronic	NM_	.	.	.	
ACa07	13	####	.	T	C	.	PA!	ABHD13	exonic	NM_	.	mis	ABHD1	
ACa07	13	####	rs7567578	G	A	.	PA!	MYO16	exonic	NM_	.	mis	MYO16	

ACa07	13	####	rs1488011	G	A	.	PA!	COL4A1	exonic	NM_	.	mis	COL4A
ACa07	13	####	rs7563035	G	A	.	PA!	CARS2	exonic	NM_	.	syr	CARS2
ACa07	13	####	.	G	A	.	PA!	.	upstreai	NM_	.	.	.
ACa07	13	####	rs1000744	C	T	.	PA!	ANKRD10	UTR3	NM_	NM_	.	.
ACa07	13	####	rs7522048	G	A	.	PA!	.	intergen	NM_	dist=	.	.
ACa07	13	####	rs7502995	G	A	.	PA!	MCF2L	exonic	NM_	.	mis	MCF2L
ACa07	13	####	rs7709821	C	T	.	PA!	GAS6	exonic	NM_	.	mis	GAS6:N
ACa07	14	####	rs8791117	C	T	.	PA!	.	intergen	NR_	dist=	.	.
ACa07	14	####	.	G	T	.	PA!	.	intergen	NR_	dist=	.	.
ACa07	14	####	rs7445485	C	T	.	PA!	.	intergen	NR_	dist=	.	.
ACa07	14	####	.	A	G	.	PA!	.	intergen	NM_	dist=	.	.
ACa07	14	####	rs1453568	C	A	.	PA!	TEP1	exonic	NM_	.	mis	TEP1:N
ACa07	14	####	rs5497679	C	T	.	PA!	KLHL33	exonic	NM_	.	mis	KLHL33
ACa07	14	####	.	C	T	.	PA!	SUPT16H	intronic	NM_	.	.	.
ACa07	14	####	.	T	C	.	PA!	.	intergen	NM_	dist=	.	.
ACa07	14	####	rs3745810	G	A	.	PA!	SLC7A7	intronic	NM_	.	.	.
ACa07	14	####	rs7576169	G	A	.	PA!	HAUS4	exonic	NM_	.	syr	HAUS4
ACa07	14	####	.	A	G	.	PA!	LOC1027	ncRNA_	NR_	.	.	.
ACa07	14	####	rs7777607	G	A	.	PA!	LOC1019	ncRNA_	NR_	.	.	.
ACa07	14	####	rs9241538	C	T	.	PA!	LOC1019	ncRNA_	NR_	.	.	.
ACa07	14	####	.	T	A	.	PA!	RALGAPA	ncRNA_	NR_	.	.	.
ACa07	14	####	.	C	A	.	PA!	RALGAPA	exonic	NM_	.	mis	RALGA
ACa07	14	####	.	C	A	.	PA!	PTCSC3	ncRNA_	NR_	.	.	.
ACa07	14	####	rs5673369	G	A	.	PA!	.	intergen	NM_	dist=	.	.
ACa07	14	####	.	C	T	.	PA!	.	intergen	NM_	dist=	.	.
ACa07	14	####	rs8659367	G	A	.	PA!	LRFN5	exonic	NM_	.	mis	LRFN5:
ACa07	14	####	.	T	C	.	PA!	FANCM	splicing	NM_	NM_	.	.
ACa07	14	####	.	G	T	.	PA!	C14orf182	intronic	NM_	.	.	.
ACa07	14	####	.	G	T	.	PA!	.	upstreai	NM_	.	.	.
ACa07	14	####	rs7462395	G	A	.	PA!	PTGER2	exonic	NM_	.	syr	PTGER
ACa07	14	####	.	A	T	.	PA!	ACTR10	intronic	NM_	.	.	.
ACa07	14	####	.	T	C	.	PA!	DAAM1	intronic	NM_	.	.	.
ACa07	14	####	.	G	T	.	PA!	DAAM1	exonic	NM_	.	mis	DAAM1
ACa07	14	####	.	T	C	.	PA!	L3HYPDH	intronic	NM_	.	.	.
ACa07	14	####	rs1401803	C	T	.	PA!	DHRS7	exonic	NM_	.	syr	DHRS7
ACa07	14	####	.	A	G	.	PA!	.	intergen	NM_	dist=	.	.
ACa07	14	####	rs5401543	C	T	.	PA!	MTHFD1	exonic	NM_	.	mis	MTHFD
ACa07	14	####	rs1404421	C	T	.	PA!	.	intergen	NM_	dist=	.	.
ACa07	14	####	.	A	G	.	PA!	GPHN	intronic	NM_	.	.	.
ACa07	14	####	.	C	T	.	PA!	ACTN1	intronic	NM_	.	.	.
ACa07	14	####	.	C	T	.	PA!	ELMSAN1	intronic	NM_	.	.	.
ACa07	14	####	rs7545827	C	T	.	PA!	ZNF410	exonic	NM_	.	mis	ZNF41(
ACa07	14	####	rs3764591	C	T	.	PA!	ZNF410	intronic	NM_	.	.	.
ACa07	14	####	.	A	G	.	PA!	LTBP2	exonic	NM_	.	mis	LTBP2:
ACa07	14	####	.	C	T	.	PA!	YLPM1	intronic	NM_	.	.	.
ACa07	14	####	.	G	T	.	PA!	PROX2	UTR3	NM_	NM_	.	.
ACa07	14	####	rs1413435	G	A	.	PA!	RPS6KL1	exonic	NM_	.	syr	RPS6KI
ACa07	14	####	.	C	A	.	PA!	TDP1	intronic	NM_	.	.	.
ACa07	14	####	.	G	T	.	PA!	NRDE2	intronic	NM_	.	.	.
ACa07	14	####	rs7511076	G	A	.	PA!	TTC7B	exonic	NM_	.	sto	TTC7B:
ACa07	14	####	rs7632995	G	A	.	PA!	GPR68	UTR5	NM_	NM_	.	.
ACa07	14	####	.	G	A	.	PA!	CCDC88C	intronic	NM_	.	.	.
ACa07	14	####	.	G	A	.	PA!	LINC0052	ncRNA_	NR_	.	.	.
ACa07	14	####	.	C	T	.	PA!	IFI27	intronic	NM_	.	.	.

ACa07	14	####	.	G	A	.	PA!	DICER1	intronic	NM_	.	.	.
ACa07	14	####	.	A	G	.	PA!	BCL11B	exonic	NM_	.	mis	BCL11E
ACa07	14	####	.	G	T	.	PA!	BCL11B	exonic	NM_	.	mis	BCL11E
ACa07	14	####	.	C	T	.	PA!	DLK1	exonic	NM_	.	syr	DLK1:N
ACa07	14	####	.	T	C	.	PA!	MIR411	ncRNA_	NR_	.	.	.
ACa07	14	####	.	G	A	.	PA!	MIR381H(	ncRNA_	NR_	.	.	.
ACa07	14	####	rs7571156	G	A	.	PA!	MOK	exonic	NM_	.	syr	MOK:N
ACa07	14	####	rs9721368	C	T	.	PA!	CDC42BP	intronic	NM_	.	.	.
ACa07	14	####	rs1043142	C	T	.	PA!	TRMT61A	intronic	NM_	.	.	.
ACa07	14	####	.	C	T	.	PA!	KIF26A	exonic	NM_	.	mis	KIF26A
ACa07	14	####	.	G	T	.	PA!	TMEM179	exonic	NM_	.	mis	TMEM1
ACa07	14	####	.	C	T	.	PA!	SIVA1	intronic	NM_	.	.	.
ACa07	14	####	rs7564163	C	T	.	PA!	AKT1	intronic	NM_	.	.	.
ACa07	14	####	.	G	T	.	PA!	CEP170B	intronic	NM_	.	.	.
ACa07	14	####	rs7689001	G	A	.	PA!	JAG2	intronic	NM_	.	.	.
ACa07	14	####	.	C	A	.	PA!	BRF1	intronic	NM_	.	.	.
ACa07	14	####	rs7822187	G	A	.	PA!	PACS2	exonic	NM_	.	mis	PACS2
ACa07	14	####	rs7820214	C	T	.	PA!	.	intergen	NR_	dist=	.	.
ACa07	14	####	.	G	A	.	PA!	ADAM6	ncRNA_	NR_	.	.	.
ACa07	14	####	.	C	T	.	PA!	.	intergen	NR_	dist=	.	.
ACa07	15	####	rs2005484	G	A	.	PA!	.	intergen	NR_	dist=	.	.
ACa07	15	####	.	C	T	.	PA!	POTEB,P(	intronic	NM_	.	.	.
ACa07	15	####	.	A	G	.	PA!	.	intergen	NR_	dist=	.	.
ACa07	15	####	.	A	G	.	PA!	LOC1019z	ncRNA_	NR_	.	.	.
ACa07	15	####	.	C	T	.	PA!	CYFIP1	intronic	NM_	.	.	.
ACa07	15	####	.	G	A	.	PA!	LOC28368	ncRNA_	NR_	.	.	.
ACa07	15	####	.	G	A	.	PA!	.	intronic	NM_	.	.	.
ACa07	15	####	.	C	T	.	PA!	.	intronic	NM_	.	.	.
ACa07	15	####	rs7605191	G	A	.	PA!	.	intergen	NR_	dist=	.	.
ACa07	15	####	rs9195421	G	A	.	PA!	.	downstr	NR_	.	.	.
ACa07	15	####	.	G	A	.	PA!	.	downstr	NR_	.	.	.
ACa07	15	####	rs7485099	T	C	.	PA!	.	upstreal	NR_	.	.	.
ACa07	15	####	.	A	G	.	PA!	.	downstr	NR_	.	.	.
ACa07	15	####	rs9209119	G	A	.	PA!	ATP10A	intronic	NM_	.	.	.
ACa07	15	####	rs3718584	C	T	.	PA!	HERC2	splicing	NM_	NM_	.	.
ACa07	15	####	.	A	G	.	PA!	HERC2	exonic	NM_	.	mis	HERC2
ACa07	15	####	rs2021672	C	T	.	PA!	GOLGA8N	exonic	NM_	.	syr	GOLGA
ACa07	15	####	.	G	T	.	PA!	FAM189A:	intronic	NM_	.	.	.
ACa07	15	####	.	G	A	.	PA!	GOLGA8J	exonic	NM_	.	mis	GOLGA
ACa07	15	####	.	C	T	.	PA!	CHRFAM7	intronic	NM_	.	.	.
ACa07	15	####	rs1995696	A	G	.	PA!	.	intergen	NM_	dist=	.	.
ACa07	15	####	.	T	C	.	PA!	ULK4P1,U	ncRNA_	NR_	.	.	.
ACa07	15	####	.	C	T	.	PA!	OTUD7A	exonic	NM_	.	syr	OTUD7
ACa07	15	####	rs1127716	A	T	.	PA!	.	intergen	NM_	dist=	.	.
ACa07	15	####	.	C	T	.	PA!	RYR3	intronic	NM_	.	.	.
ACa07	15	####	.	G	A	.	PA!	.	intergen	NM_	dist=	.	.
ACa07	15	####	.	G	A	.	PA!	PAK6	exonic	NM_	.	mis	PAK6:N
ACa07	15	####	.	G	T	.	PA!	PLCB2	intronic	NM_	.	.	.
ACa07	15	####	rs3704110	C	T	.	PA!	SPTBN5	intronic	NM_	.	.	.
ACa07	15	####	.	G	A	.	PA!	SPTBN5	intronic	NM_	.	.	.
ACa07	15	####	rs5310947	G	A	.	PA!	TTBK2	exonic	NM_	.	syr	TTBK2:
ACa07	15	####	rs7551745	C	T	.	PA!	TUBGCP4	exonic	NM_	.	sto	TUBGC
ACa07	15	####	rs5360360	C	T	.	PA!	CKMT1B	exonic	NM_	.	sto	CKMT1
ACa07	15	####	.	G	A	.	PA!	STRC	intronic	NM_	.	.	.

ACa07	15	####	rs5322560	G	A	.	PA!	.	intergen	NM_	dist=	.	.
ACa07	15	####	.	G	A	.	PA!	SORD	intronic	NM_	.	.	.
ACa07	15	####	.	G	T	.	PA!	DUOX1	exonic	NM_	.	mis	DUOX1
ACa07	15	####	.	A	G	.	PA!	SEMA6D	intronic	NM_	.	.	.
ACa07	15	####	rs7737859	G	A	.	PA!	FBN1	exonic	NM_	.	mis	FBN1:N
ACa07	15	####	rs1409544	C	T	.	PA!	FBN1	exonic	NM_	.	mis	FBN1:N
ACa07	15	####	.	G	T	.	PA!	.	intergen	NM_	dist=	.	.
ACa07	15	####	.	A	G	.	PA!	FAM227B	intronic	NM_	.	.	.
ACa07	15	####	.	G	T	.	PA!	ATP8B4	intronic	NM_	.	.	.
ACa07	15	####	.	A	G	.	PA!	USP50	splicing	NM_	NM_	.	.
ACa07	15	####	rs3686733	G	A	.	PA!	MYO5C	exonic	NM_	.	syr	MYO5C
ACa07	15	####	.	G	T	.	PA!	MYO5A	intronic	NM_	.	.	.
ACa07	15	####	.	T	C	.	PA!	AQP9	exonic	NM_	.	mis	AQP9:N
ACa07	15	####	rs7793599	G	A	.	PA!	TLN2	exonic	NM_	.	mis	TLN2:N
ACa07	15	####	rs7518805	C	T	.	PA!	MTFMT	exonic	NM_	.	mis	MTFMT
ACa07	15	####	.	C	G	.	PA!	IGDCC4	exonic	NM_	.	mis	IGDCC4
ACa07	15	####	rs5369412	C	T	.	PA!	IGDCC4	exonic	NM_	.	syr	IGDCC4
ACa07	15	####	.	G	A	.	PA!	MEGF11	intronic	NM_	.	.	.
ACa07	15	####	.	T	C	.	PA!	.	intergen	NM_	dist=	.	.
ACa07	15	####	.	T	C	.	PA!	IQCH	intronic	NM_	.	.	.
ACa07	15	####	rs7568129	C	T	.	PA!	CLN6	intronic	NM_	.	.	.
ACa07	15	####	.	C	T	.	PA!	ITGA11	intronic	NM_	.	.	.
ACa07	15	####	rs5460720	C	T	.	PA!	THSD4	intronic	NM_	.	.	.
ACa07	15	####	.	C	A	.	PA!	THSD4	intronic	NM_	.	.	.
ACa07	15	####	.	G	A	.	PA!	CELF6	exonic	NM_	.	mis	CELF6:
ACa07	15	####	rs1043159	C	A	.	PA!	BBS4	intronic	NM_	.	.	.
ACa07	15	####	.	G	A	.	PA!	CYP11A1	intronic	NM_	.	.	.
ACa07	15	####	.	C	T	.	PA!	GOLGA6C	intronic	NM_	.	.	.
ACa07	15	####	.	C	T	.	PA!	GOLGA6C	intronic	NM_	.	.	.
ACa07	15	####	rs9744245	G	A	.	PA!	SCAPER	intronic	NM_	.	.	.
ACa07	15	####	rs1047518	C	T	.	PA!	SCAPER	intronic	NM_	.	.	.
ACa07	15	####	rs8789048	A	G	.	PA!	LOC6457	ncRNA_	NR_	.	.	.
ACa07	15	####	.	G	T	.	PA!	.	intergen	NR_	dist=	.	.
ACa07	15	####	.	C	T	.	PA!	ADAMTS7	intronic	NM_	.	.	.
ACa07	15	####	rs1471333	C	T	.	PA!	KIAA1024	exonic	NM_	.	syr	KIAA10
ACa07	15	####	.	G	A	.	PA!	FAH	intronic	NM_	.	.	.
ACa07	15	####	rs5292517	G	A	.	PA!	CEMIP	intronic	NM_	.	.	.
ACa07	15	####	.	T	C	.	PA!	C15orf26	intronic	NM_	.	.	.
ACa07	15	####	rs9410184	C	T	.	PA!	ADAMTSL	intronic	NM_	.	.	.
ACa07	15	####	rs7920451	A	G	.	PA!	GOLGA6L	exonic	NM_	.	mis	GOLGA
ACa07	15	####	.	G	A	.	PA!	AKAP13	intronic	NM_	.	.	.
ACa07	15	####	rs3718116	C	T	.	PA!	KLHL25	exonic	NM_	.	mis	KLHL25
ACa07	15	####	.	G	A	.	PA!	POLG	exonic	NM_	.	mis	POLG:N
ACa07	15	####	.	A	T	.	PA!	RHCG	intronic	NM_	.	.	.
ACa07	15	####	rs2012804	G	A	.	PA!	KIF7	exonic	NM_	.	syr	KIF7:NM
ACa07	15	####	.	C	T	.	PA!	KIF7	exonic	NM_	.	syr	KIF7:NM
ACa07	15	####	rs1475111	C	T	.	PA!	ZNF710	exonic	NM_	.	unl	UNKNC
ACa07	15	####	rs5584446	T	C	.	PA!	.	upstrea	NM_	.	.	.
ACa07	15	####	.	T	C	.	PA!	ADAMTS1	exonic	NM_	.	syr	ADAMT
ACa07	15	####	.	C	T	.	PA!	PRKXP1	ncRNA_	NR_	.	.	.
ACa07	15	####	.	G	T	.	PA!	LRRK1	intronic	NM_	.	.	.
ACa07	15	####	rs7764234	G	A	.	PA!	PCSK6	exonic	NM_	.	unl	UNKNC
ACa07	15	####	.	C	A	.	PA!	TARSL2	intronic	NM_	.	.	.
ACa07	15	####	.	G	A	.	PA!	.	intergen	NM_	dist=	.	.

ACa07	15	####	rs3682087	G	A	.	PA!	.	intergen	NM_	dist=	.	.
ACa07	15	####	.	C	T	.	PA!	.	intergen	NM_	dist=	.	.
ACa07	15	####	.	A	G	.	PA!	DDX11L9	ncRNA_	NR_	.	.	.
ACa07	16	####	rs7613665	G	A	.	PA!	NPRL3	exonic	NM_	.	unl	UNKNC
ACa07	16	####	.	G	T	.	PA!	ITFG3	exonic	NM_	.	mis	ITFG3:I
ACa07	16	####	.	G	A	.	PA!	RGS11	intronic	NM_	.	.	.
ACa07	16	####	rs8927038	G	C	.	PA!	RAB11FIF	intronic	NM_	.	.	.
ACa07	16	####	rs1998911	C	T	.	PA!	WFIKKN1	exonic	NM_	.	mis	WFIKKI
ACa07	16	####	rs2010028	C	T	.	PA!	RHOT2	intronic	NM_	.	.	.
ACa07	16	####	rs7767345	G	A	.	PA!	JMJD8	exonic	NM_	.	mis	JMJD8:
ACa07	16	####	rs1466306	C	T	.	PA!	FBXL16	exonic	NM_	.	mis	FBXL16
ACa07	16	####	rs3681933	C	T	.	PA!	NARFL	exonic	NM_	.	syr	NARFL
ACa07	16	####	.	G	T	.	PA!	CHTF18	exonic	NM_	.	syr	CHTF18
ACa07	16	####	.	C	A	.	PA!	LMF1	intronic	NM_	.	.	.
ACa07	16	####	rs1506415	C	T	.	PA!	SSTR5	exonic	NM_	.	syr	SSTR5:
ACa07	16	####	rs9971019	G	A	.	PA!	UBE2I	intronic	NM_	.	.	.
ACa07	16	####	.	A	T	.	PA!	BAIAP3	exonic	NM_	.	mis	BAIAP3
ACa07	16	####	rs3710800	C	T	.	PA!	BAIAP3	intronic	NM_	.	.	.
ACa07	16	####	.	G	A	.	PA!	BAIAP3	exonic	NM_	.	mis	BAIAP3
ACa07	16	####	rs7592188	C	T	.	PA!	BAIAP3	intronic	NM_	.	.	.
ACa07	16	####	.	G	A	.	PA!	BAIAP3	exonic	NM_	.	mis	BAIAP3
ACa07	16	####	rs5365360	G	A	.	PA!	.	intergen	NM_	dist=	.	.
ACa07	16	####	rs7601714	C	T	.	PA!	CLCN7	exonic	NM_	.	mis	CLCN7
ACa07	16	####	.	C	T	.	PA!	HN1L	intronic	NM_	.	.	.
ACa07	16	####	rs1450874	C	T	.	PA!	HN1L	exonic	NM_	.	mis	HN1L:N
ACa07	16	####	.	T	A	.	PA!	MAPK8IP3	exonic	NM_	.	syr	MAPK8
ACa07	16	####	rs3749279	G	A	.	PA!	RPL3L	exonic	NM_	.	syr	RPL3L:
ACa07	16	####	.	C	A	.	PA!	GFER	intronic	NM_	.	.	.
ACa07	16	####	rs7634894	G	A	.	PA!	ZNF598	exonic	NM_	.	mis	ZNF598
ACa07	16	####	.	G	A	.	PA!	TRAF7	intronic	NM_	.	.	.
ACa07	16	####	.	T	C	.	PA!	NTN3	intronic	NM_	.	.	.
ACa07	16	####	.	G	A	.	PA!	CCDC64B	UTR3	NM_	NM_	.	.
ACa07	16	####	rs1416929	C	T	.	PA!	ZSCAN32	intronic	NM_	.	.	.
ACa07	16	####	.	A	G	.	PA!	ZNF597	exonic	NM_	.	syr	ZNF597
ACa07	16	####	rs7525617	C	T	.	PA!	CLUAP1	intronic	NM_	.	.	.
ACa07	16	####	rs5345285	G	A	.	PA!	SLX4	exonic	NM_	.	mis	SLX4:N
ACa07	16	####	.	C	A	.	PA!	CREBBP	exonic	NM_	.	mis	CREBB
ACa07	16	####	rs5502446	T	A	.	PA!	CREBBP	intronic	NM_	.	.	.
ACa07	16	####	rs1894802	G	A	.	PA!	CREBBP	intronic	NM_	.	.	.
ACa07	16	####	rs3711593	C	T	.	PA!	ADCY9	exonic	NM_	.	syr	ADCY9
ACa07	16	####	rs2013575	C	T	.	PA!	VASN	exonic	NM_	.	mis	VASN:N
ACa07	16	####	.	G	T	.	PA!	PPL	exonic	NM_	.	mis	PPL:NM
ACa07	16	####	rs5632991	G	A	.	PA!	CIITA	exonic	NM_	.	mis	CIITA:N
ACa07	16	####	rs7679436	T	C	.	PA!	CIITA	intronic	NM_	.	.	.
ACa07	16	####	rs9184234	G	A	.	PA!	.	intergen	NM_	dist=	.	.
ACa07	16	####	rs7800288	A	G	.	PA!	BFAR	intronic	NM_	.	.	.
ACa07	16	####	rs5668064	G	A	.	PA!	PDXDC1	intronic	NM_	.	.	.
ACa07	16	####	rs7507758	G	A	.	PA!	.	intergen	NM_	dist=	.	.
ACa07	16	####	.	C	T	.	PA!	KIAA0430	UTR5	NM_	NM_	.	.
ACa07	16	####	.	C	T	.	PA!	NDE1	intronic	NM_	.	.	.
ACa07	16	####	.	G	A	.	PA!	ABCC1	intronic	NM_	.	.	.
ACa07	16	####	.	T	C	.	PA!	.	intergen	NR_	dist=	.	.
ACa07	16	####	rs1784261	A	T	.	PA!	SMG1	exonic	NM_	.	mis	SMG1:I
ACa07	16	####	rs7575963	G	A	.	PA!	SYT17	exonic	NM_	.	mis	SYT17:

ACa07	16	####	rs7647908	G	A	.	PA!	UMOD	exonic	NM_	.	syr	UMOD:
ACa07	16	####	.	C	A	.	PA!	ZP2	exonic	NM_	.	unl	UNKNC
ACa07	16	####	rs1829590	C	T	.	PA!	EEF2K	intronic	NM_	.	.	.
ACa07	16	####	.	G	A	.	PA!	GGA2	exonic	NM_	.	mis	GGA2:N
ACa07	16	####	.	C	T	.	PA!	ERN2	exonic	NM_	.	mis	ERN2:N
ACa07	16	####	rs9722593	C	T	.	PA!	LCMT1	intronic	NM_	.	.	.
ACa07	16	####	.	G	T	.	PA!	.	intergen	NM_	dist=	.	.
ACa07	16	####	.	G	A	.	PA!	GTF3C1	exonic	NM_	.	mis	GTF3C
ACa07	16	####	.	C	T	.	PA!	KIAA0556	intronic	NM_	.	.	.
ACa07	16	####	rs1412530	G	A	.	PA!	KIAA0556	exonic	NM_	.	mis	KIAA05
ACa07	16	####	rs1909424	A	T	.	PA!	XPO6	intronic	NM_	.	.	.
ACa07	16	####	.	G	A	.	PA!	.	intergen	NM_	dist=	.	.
ACa07	16	####	rs7491998	C	T	.	PA!	ATXN2L	exonic	NM_	.	mis	ATXN2I
ACa07	16	####	.	T	C	.	PA!	TUFM	UTR5	NM_	NM_	.	.
ACa07	16	####	rs1820846	G	A	.	PA!	ATP2A1	exonic	NM_	.	mis	ATP2A1
ACa07	16	####	.	G	T	.	PA!	.	intergen	NR_	dist=	.	.
ACa07	16	####	.	G	T	.	PA!	.	upstreal	NM_	.	.	.
ACa07	16	####	rs7813295	C	T	.	PA!	KCTD13	exonic	NM_	.	mis	KCTD1
ACa07	16	####	rs9659529	G	A	.	PA!	BCKDK	intronic	NM_	.	.	.
ACa07	16	####	.	T	C	.	PA!	PRSS36	intronic	NM_	.	.	.
ACa07	16	####	.	G	T	.	PA!	ITGAX	intronic	NM_	.	.	.
ACa07	16	####	.	C	A	.	PA!	ZNF267	exonic	NM_	.	syr	ZNF267
ACa07	16	####	rs7477221	C	T	.	PA!	.	intergen	NR_	dist=	.	.
ACa07	16	####	rs460256	A	G	.	PA!	.	intergen	NR_	dist=	.	.
ACa07	16	####	.	A	G	.	PA!	.	intergen	NR_	dist=	.	.
ACa07	16	####	.	A	G	.	PA!	.	intergen	NR_	dist=	.	.
ACa07	16	####	rs3744640	G	C	.	PA!	.	intergen	NR_	dist=	.	.
ACa07	16	####	.	C	A	.	PA!	RNU6-76F	ncRNA_	NR_	.	.	.
ACa07	16	####	.	A	C	.	PA!	.	intergen	NR_	dist=	.	.
ACa07	16	####	.	C	G	.	PA!	.	intergen	NR_	dist=	.	.
ACa07	16	####	rs1333894	A	C	.	PA!	.	intergen	NOI	dist=	.	.
ACa07	16	####	rs2885478	C	T	.	PA!	.	intergen	NOI	dist=	.	.
ACa07	16	####	rs4249087	T	G	.	PA!	.	intergen	NOI	dist=	.	.
ACa07	16	####	rs5591122	C	T	.	PA!	.	intergen	NOI	dist=	.	.
ACa07	16	####	rs4246405	G	C	.	PA!	.	intergen	NOI	dist=	.	.
ACa07	16	####	rs4292070	G	C	.	PA!	.	intergen	NOI	dist=	.	.
ACa07	16	####	rs8799648	A	G	.	PA!	.	intergen	NOI	dist=	.	.
ACa07	16	####	rs8799977	A	C	.	PA!	.	intergen	NOI	dist=	.	.
ACa07	16	####	rs4291933	A	G	.	PA!	.	intergen	NOI	dist=	.	.
ACa07	16	####	rs4375682	A	G	.	PA!	.	intergen	NOI	dist=	.	.
ACa07	16	####	rs7200104	T	G	.	PA!	.	intergen	NOI	dist=	.	.
ACa07	16	####	rs7188643	A	G	.	PA!	.	intergen	NOI	dist=	.	.
ACa07	16	####	rs9328642	G	C	.	PA!	.	intergen	NOI	dist=	.	.
ACa07	16	####	rs7205231	T	A	.	PA!	.	intergen	NOI	dist=	.	.
ACa07	16	####	rs4445923	A	T	.	PA!	.	intergen	NOI	dist=	.	.
ACa07	16	####	rs2887500	T	G	.	PA!	.	intergen	NOI	dist=	.	.
ACa07	16	####	rs2888543	G	T	.	PA!	.	intergen	NOI	dist=	.	.
ACa07	16	####	rs7664258	G	A	.	PA!	.	intergen	NOI	dist=	.	.
ACa07	16	####	rs4249076	T	C	.	PA!	.	intergen	NOI	dist=	.	.
ACa07	16	####	.	T	C	.	PA!	.	intergen	NOI	dist=	.	.
ACa07	16	####	rs3683497	G	A	.	PA!	MYLK3	exonic	NM_	.	syr	MYLK3
ACa07	16	####	.	C	A	.	PA!	PHKB	intronic	NM_	.	.	.
ACa07	16	####	.	C	A	.	PA!	MT1E	intronic	NM_	.	.	.
ACa07	16	####	.	G	T	.	PA!	NUP93	exonic	NM_	.	mis	NUP93:

ACa07	16	####	.	A	C	.	PA!	NUP93	intronic	NM_	.	.	.
ACa07	16	####	.	G	A	.	PA!	CETP	intronic	NM_	.	.	.
ACa07	16	####	rs1471990	C	T	.	PA!	CPNE2	exonic	NM_	.	syr	CPNE2
ACa07	16	####	.	T	C	.	PA!	DRC7	intronic	NM_	.	.	.
ACa07	16	####	.	G	T	.	PA!	TEPP	exonic	NM_	.	mis	TEPP:N
ACa07	16	####	.	A	G	.	PA!	CCDC113	intronic	NM_	.	.	.
ACa07	16	####	rs1499903	A	G	.	PA!	.	intergen	NOI	dist=	.	.
ACa07	16	####	.	C	T	.	PA!	EXOC3L1	exonic	NM_	.	mis	EXOC3
ACa07	16	####	rs1054074	A	G	.	PA!	EXOC3L1	intronic	NM_	.	.	.
ACa07	16	####	rs7715372	C	T	.	PA!	ELMO3	exonic	NM_	.	syr	ELMO3
ACa07	16	####	rs5618721	G	A	.	PA!	SLC9A5	exonic	NM_	.	mis	SLC9A5
ACa07	16	####	.	G	T	.	PA!	KCTD19	intronic	NM_	.	.	.
ACa07	16	####	rs7545456	C	T	.	PA!	LRRC36	exonic	NM_	.	mis	LRRC3
ACa07	16	####	rs3696380	G	A	.	PA!	ZDHHC1	intronic	NM_	.	.	.
ACa07	16	####	.	C	A	.	PA!	CTCF	intronic	NM_	.	.	.
ACa07	16	####	rs7584347	C	T	.	PA!	CTRL	exonic	NM_	.	mis	CTRL:N
ACa07	16	####	.	C	A	.	PA!	SLC12A4	intronic	NM_	.	.	.
ACa07	16	####	.	G	A	.	PA!	DDX28	exonic	NM_	.	syr	DDX28:
ACa07	16	####	.	C	T	.	PA!	TERF2	exonic	NM_	.	mis	TERF2:
ACa07	16	####	rs8670174	A	G	.	PA!	LOC1005(	ncRNA_	NR_	.	.	.
ACa07	16	####	rs9278778	C	T	.	PA!	.	intergen	NM_	dist=	.	.
ACa07	16	####	rs1485518	C	T	.	PA!	.	intergen	NM_	dist=	.	.
ACa07	16	####	rs3776990	G	A	.	PA!	PKD1L3	exonic	NM_	.	unl	UNKNC
ACa07	16	####	rs7502787	G	T	.	PA!	CLEC18B	intronic	NM_	.	.	.
ACa07	16	####	.	C	T	.	PA!	MLKL	intronic	NM_	.	.	.
ACa07	16	####	.	C	T	.	PA!	BCAR1	intronic	NM_	.	.	.
ACa07	16	####	rs1469198	A	G	.	PA!	LOC1024(	ncRNA_	NR_	.	.	.
ACa07	16	####	rs7733922	G	A	.	PA!	PKD1L2	exonic	NM_	.	unl	UNKNC
ACa07	16	####	.	T	C	.	PA!	PLCG2	intronic	NM_	.	.	.
ACa07	16	####	rs7483034	C	T	.	PA!	MLYCD	UTR3	NM_	NM_	.	.
ACa07	16	####	rs1407517	G	A	.	PA!	DNAAF1	exonic	NM_	.	mis	DNAAF
ACa07	16	####	rs7647118	G	A	.	PA!	TAF1C	exonic	NM_	.	mis	TAF1C:
ACa07	16	####	rs9336865	C	T	.	PA!	GSE1	exonic	NM_	.	mis	GSE1:N
ACa07	16	####	.	T	A	.	PA!	FOXC2	exonic	NM_	.	mis	FOXC2
ACa07	16	####	rs3681305	C	T	.	PA!	FBXO31	exonic	NM_	.	syr	FBXO3
ACa07	16	####	rs9665470	T	C	.	PA!	JPH3	intronic	NM_	.	.	.
ACa07	16	####	rs5606502	G	A	.	PA!	ZNF469	exonic	NM_	.	mis	ZNF469
ACa07	16	####	rs9646181	C	T	.	PA!	MVD	intronic	NM_	.	.	.
ACa07	16	####	.	C	T	.	PA!	CBFA2T3	exonic	NM_	.	mis	CBFA2T
ACa07	16	####	.	C	A	.	PA!	CDK10	intronic	NM_	.	.	.
ACa07	16	####	rs2017315	G	A	.	PA!	ZNF276	exonic	NM_	.	syr	ZNF276
ACa07	16	####	rs7530482	G	A	.	PA!	ZNF276	UTR3	NM_	NM_	.	.
ACa07	17	####	rs7627705	T	C	.	PA!	MYO1C	exonic	NM_	.	mis	MYO1C
ACa07	17	####	.	C	T	.	PA!	PRPF8	exonic	NM_	.	mis	PRPF8:
ACa07	17	####	.	A	G	.	PA!	TLCD2	UTR3	NM_	NM_	.	.
ACa07	17	####	.	T	C	.	PA!	HIC1	exonic	NM_	.	syr	HIC1:N
ACa07	17	####	.	C	A	.	PA!	SMG6	intronic	NM_	.	.	.
ACa07	17	####	.	G	T	.	PA!	SMG6	exonic	NM_	.	mis	SMG6:N
ACa07	17	####	rs7803486	C	T	.	PA!	MNT	exonic	NM_	.	mis	MNT:NI
ACa07	17	####	.	A	G	.	PA!	METTL16	exonic	NM_	.	syr	METTL
ACa07	17	####	.	T	C	.	PA!	CAMKK1	UTR3	NM_	NM_	.	.
ACa07	17	####	.	A	C	.	PA!	ANKFY1	intronic	NM_	.	.	.
ACa07	17	####	rs5506079	C	T	.	PA!	UBE2G1	intronic	NM_	.	.	.
ACa07	17	####	rs3764321	C	T	.	PA!	PELP1	exonic	NM_	.	syr	PELP1:

ACa07	17	####	rs2008227	G	A	.	PA:	KIF1C	exonic	NM_	.	syr	KIF1C:N
ACa07	17	####	rs2013516	C	T	.	PA:	ZNF594	exonic	NM_	.	mis	ZNF594
ACa07	17	####	.	C	T	.	PA:	DHX33	intronic	NM_	.	.	.
ACa07	17	####	.	T	C	.	PA:	KIAA0753	exonic	NM_	.	mis	KIAA07
ACa07	17	####	.	C	T	.	PA:	SLC13A5	intronic	NM_	.	.	.
ACa07	17	####	.	A	G	.	PA:	CLEC10A	intronic	NM_	.	.	.
ACa07	17	####	rs2000463	G	A	.	PA:	NEURL4	exonic	NM_	.	mis	NEURL
ACa07	17	####	rs7455911	G	A	.	PA:	POLR2A	exonic	NM_	.	mis	POLR2/
ACa07	17	####	rs1473707	G	A	.	PA:	CCDC42	exonic	NM_	.	syr	CCDC4
ACa07	17	####	.	C	T	.	PA:	PIK3R6	exonic	NM_	.	unl	UNKNC
ACa07	17	####	.	C	T	.	PA:	GAS7	splicing	NM_	NM_	.	.
ACa07	17	####	rs7534759	A	G	.	PA:	DNAH9	intronic	NM_	.	.	.
ACa07	17	####	.	G	T	.	PA:	DNAH9	exonic	NM_	.	sto	DNAH9
ACa07	17	####	.	C	T	.	PA:	HS3ST3B	exonic	NM_	.	syr	HS3ST:
ACa07	17	####	rs7633797	C	T	.	PA:	TEKT3	exonic	NM_	.	syr	TEKT3:
ACa07	17	####	rs6207237	G	A	.	PA:	TBC1D26	intronic	NM_	.	.	.
ACa07	17	####	.	G	A	.	PA:	NCOR1	exonic	NM_	.	syr	NCOR1
ACa07	17	####	.	T	C	.	PA:	NCOR1	intronic	NM_	.	.	.
ACa07	17	####	.	C	T	.	PA:	MPRIP	intronic	NM_	.	.	.
ACa07	17	####	.	G	A	.	PA:	MPRIP	intronic	NM_	.	.	.
ACa07	17	####	rs1157965	G	A	.	PA:	MYO15A	intronic	NM_	.	.	.
ACa07	17	####	.	A	G	.	PA:	MYO15A	splicing	NM_	NM_	.	.
ACa07	17	####	.	G	A	.	PA:	EVPLL	intronic	NM_	.	.	.
ACa07	17	####	.	C	A	.	PA:	LGALS9B	exonic	NM_	.	syr	LGALS!
ACa07	17	####	rs2869918	T	C	.	PA:	LGALS9B	UTR5	NM_	NM_	.	.
ACa07	17	####	rs7529523	G	A	.	PA:	USP22	intronic	NM_	.	.	.
ACa07	17	####	.	G	A	.	PA:	MAP2K3	intronic	NM_	.	.	.
ACa07	17	####	.	C	T	.	PA:	NOS2	intronic	NM_	.	.	.
ACa07	17	####	.	A	T	.	PA:	SLC13A2	intronic	NM_	.	.	.
ACa07	17	####	.	G	T	.	PA:	SLC13A2	intronic	NM_	.	.	.
ACa07	17	####	.	C	T	.	PA:	.	upstrea	NM_	.	.	.
ACa07	17	####	.	C	T	.	PA:	SPAG5-A:	ncRNA_	NR_	.	.	.
ACa07	17	####	rs1051245	C	T	.	PA:	SPAG5-A:	ncRNA_	NR_	.	.	.
ACa07	17	####	.	A	G	.	PA:	KIAA0100	intronic	NM_	.	.	.
ACa07	17	####	.	C	T	.	PA:	SEZ6	exonic	NM_	.	mis	SEZ6:N
ACa07	17	####	.	C	T	.	PA:	MYO18A	exonic	NM_	.	mis	MYO18
ACa07	17	####	rs7968757	A	G	.	PA:	SLC6A4	intronic	NM_	.	.	.
ACa07	17	####	rs5324904	C	T	.	PA:	EVI2A	exonic	NM_	.	mis	EVI2A:N
ACa07	17	####	.	G	T	.	PA:	RAB11FIF	intronic	NM_	.	.	.
ACa07	17	####	.	A	G	.	PA:	UTP6	intronic	NM_	.	.	.
ACa07	17	####	.	G	T	.	PA:	UTP6	intronic	NM_	.	.	.
ACa07	17	####	.	G	T	.	PA:	C17orf102	intronic	NM_	.	.	.
ACa07	17	####	.	G	A	.	PA:	NLE1	exonic	NM_	.	mis	NLE1:N
ACa07	17	####	rs7610872	C	T	.	PA:	SLFN11	exonic	NM_	.	mis	SLFN1:
ACa07	17	####	.	G	T	.	PA:	.	intergen	NM_	dist=	.	.
ACa07	17	####	.	C	A	.	PA:	AP2B1	intronic	NM_	.	.	.
ACa07	17	####	rs3702689	T	G	.	PA:	TBC1D3B	UTR3	NM_	NM_	.	.
ACa07	17	####	rs3720623	T	C	.	PA:	.	upstrea	NM_	.	.	.
ACa07	17	####	.	A	G	.	PA:	ARHGAP2	intronic	NM_	.	.	.
ACa07	17	####	rs7545869	C	T	.	PA:	PCGF2	exonic	NM_	.	mis	PCGF2
ACa07	17	####	.	G	T	.	PA:	PLXDC1	intronic	NM_	.	.	.
ACa07	17	####	rs7807350	G	A	.	PA:	CACNB1	exonic	NM_	.	mis	CACNB
ACa07	17	####	.	C	T	.	PA:	RPL19	intronic	NM_	.	.	.
ACa07	17	####	.	C	T	.	PA:	STAC2	intronic	NM_	.	.	.

ACa07	17	####	rs1057519	G	A	.	PA!	ERBB2	exonic	NM_	.	mis	ERBB2
ACa07	17	####	rs1996216	C	T	.	PA!	CCR7	exonic	NM_	.	syr	CCR7:N
ACa07	17	####	rs1488746	C	T	.	PA!	KRT34	exonic	NM_	.	syr	KRT34:
ACa07	17	####	rs7824576	C	T	.	PA!	LEPREL4	exonic	NM_	.	syr	LEPREI
ACa07	17	####	rs7827568	G	A	.	PA!	TTC25	intronic	NM_	.	.	.
ACa07	17	####	.	A	G	.	PA!	TTC25	intronic	NM_	.	.	.
ACa07	17	####	.	G	T	.	PA!	STAT3	intronic	NM_	.	.	.
ACa07	17	####	rs9065729	G	A	.	PA!	STAT3	intronic	NM_	.	.	.
ACa07	17	####	rs5769204	G	A	.	PA!	STAT3	intronic	NM_	.	.	.
ACa07	17	####	.	G	T	.	PA!	COASY	intronic	NM_	.	.	.
ACa07	17	####	rs1042659	C	T	.	PA!	WNK4	intronic	NM_	.	.	.
ACa07	17	####	rs9984123	C	T	.	PA!	NBR2	ncRNA_	NR_	.	.	.
ACa07	17	####	.	A	G	.	PA!	NBR1	intronic	NM_	.	.	.
ACa07	17	####	.	G	A	.	PA!	LINC0091	ncRNA_	NR_	.	.	.
ACa07	17	####	.	G	T	.	PA!	MPP3	exonic	NM_	.	syr	MPP3:N
ACa07	17	####	.	C	A	.	PA!	MPP3	intronic	NM_	.	.	.
ACa07	17	####	rs7617924	G	A	.	PA!	MPP3	exonic	NM_	.	syr	MPP3:N
ACa07	17	####	.	T	G	.	PA!	C17orf104	exonic	NM_	.	syr	C17orf1
ACa07	17	####	rs9135110	C	T	.	PA!	KIF18B	intronic	NM_	.	.	.
ACa07	17	####	.	G	T	.	PA!	ACBD4	intronic	NM_	.	.	.
ACa07	17	####	rs7805429	C	T	.	PA!	FMNL1	exonic	NM_	.	mis	FMNL1
ACa07	17	####	.	C	T	.	PA!	PLEKHM1	intronic	NM_	.	.	.
ACa07	17	####	.	C	T	.	PA!	LRRC37A	ncRNA_	NR_	.	.	.
ACa07	17	####	.	G	A	.	PA!	.	intergen	NR_	dist=	.	.
ACa07	17	####	rs1002396	G	A	.	PA!	NSF	intronic	NM_	.	.	.
ACa07	17	####	rs1056060	A	G	.	PA!	.	intergen	NM_	dist=	.	.
ACa07	17	####	rs2644320	C	T	.	PA!	NPEPPS	splicing	NM_	NM_	.	.
ACa07	17	####	.	A	G	.	PA!	OSBPL7	intronic	NM_	.	.	.
ACa07	17	####	rs3749157	G	A	.	PA!	ABI3	exonic	NM_	.	mis	ABI3:NI
ACa07	17	####	rs9379553	G	A	.	PA!	ZNF652	exonic	NM_	.	syr	ZNF652
ACa07	17	####	.	G	T	.	PA!	DLX4	UTR3	NM_	NM_	.	.
ACa07	17	####	rs7784106	C	T	.	PA!	SAMD14	exonic	NM_	.	mis	SAMD1
ACa07	17	####	.	C	A	.	PA!	PPP1R9B	intronic	NM_	.	.	.
ACa07	17	####	.	G	A	.	PA!	PPP1R9B	exonic	NM_	.	unl	UNKNC
ACa07	17	####	rs1029268	C	T	.	PA!	COL1A1	intronic	NM_	.	.	.
ACa07	17	####	.	G	T	.	PA!	XYLT2	intronic	NM_	.	.	.
ACa07	17	####	rs3894278	A	T	.	PA!	MYCBPAF	intronic	NM_	.	.	.
ACa07	17	####	.	C	T	.	PA!	HLF	intronic	NM_	.	.	.
ACa07	17	####	.	G	A	.	PA!	RAD51C	intronic	NM_	.	.	.
ACa07	17	####	rs3677870	A	G	.	PA!	PPM1D	intronic	NM_	.	.	.
ACa07	17	####	.	G	T	.	PA!	GH1	intronic	NM_	.	.	.
ACa07	17	####	rs7637250	C	T	.	PA!	SCN4A	exonic	NM_	.	syr	SCN4A
ACa07	17	####	.	C	A	.	PA!	PLEKHM1	ncRNA_	NR_	.	.	.
ACa07	17	####	.	C	T	.	PA!	HELZ	exonic	NM_	.	syr	HELZ:N
ACa07	17	####	.	T	C	.	PA!	ABCA6	exonic	NM_	.	mis	ABCA6
ACa07	17	####	.	C	T	.	PA!	MGC1627	ncRNA_	NR_	.	.	.
ACa07	17	####	.	C	T	.	PA!	.	downstr	NM_	.	.	.
ACa07	17	####	rs5610655	C	T	.	PA!	FDXR	exonic	NM_	.	mis	FDXR:N
ACa07	17	####	rs1018051	G	C	.	PA!	KIAA0195	intronic	NM_	.	.	.
ACa07	17	####	rs3777274	G	A	.	PA!	CASKIN2	intronic	NM_	.	.	.
ACa07	17	####	.	C	T	.	PA!	CASKIN2	intronic	NM_	.	.	.
ACa07	17	####	.	G	T	.	PA!	MYO15B	ncRNA_	NR_	.	.	.
ACa07	17	####	.	G	T	.	PA!	RECQL5	intronic	NM_	.	.	.
ACa07	17	####	.	G	C	.	PA!	RECQL5	intronic	NM_	.	.	.

ACa07	17	####	rs3697050	C	T	.	PA!	CYGB	exonic	NM_	.	mis	CYGB:N
ACa07	17	####	.	A	G	.	PA!	SNHG16	ncRNA_	NR_	.	.	.
ACa07	17	####	.	C	A	.	PA!	MGAT5B	intronic	NM_	.	.	.
ACa07	17	####	rs7670774	G	A	.	PA!	SEC14L1	intronic	NM_	.	.	.
ACa07	17	####	.	C	A	.	PA!	DNAH17	intronic	NM_	.	.	.
ACa07	17	####	rs7521502	C	T	.	PA!	DNAH17	exonic	NM_	.	mis	DNAH1
ACa07	17	####	.	G	A	.	PA!	RBFOX3	exonic	NM_	.	mis	RBFOX
ACa07	17	####	.	G	A	.	PA!	CBX4	exonic	NM_	.	mis	CBX4:N
ACa07	17	####	rs5354610	G	A	.	PA!	CCDC40	exonic	NM_	.	mis	CCDC4
ACa07	17	####	rs7737952	G	A	.	PA!	CARD14	intronic	NM_	.	.	.
ACa07	17	####	rs3676544	C	T	.	PA!	CARD14	exonic	NM_	.	syr	CARD1
ACa07	17	####	.	G	A	.	PA!	RNF213	intronic	NM_	.	.	.
ACa07	17	####	.	G	A	.	PA!	RPTOR	intronic	NM_	.	.	.
ACa07	17	####	.	G	A	.	PA!	BAIAP2	exonic	NM_	.	mis	BAIAP2
ACa07	17	####	.	C	A	.	PA!	CEP131	intronic	NM_	.	.	.
ACa07	17	####	rs3678800	C	T	.	PA!	LINC0048	ncRNA_	NR_	.	.	.
ACa07	17	####	.	G	A	.	PA!	BAHCC1	intronic	NM_	.	.	.
ACa07	17	####	.	C	T	.	PA!	OXLD1	exonic	NM_	.	syr	OXLD1
ACa07	17	####	.	C	A	.	PA!	NOTUM	intronic	NM_	.	.	.
ACa07	17	####	.	T	C	.	PA!	ASPSCR1	splicing	NM_	NM_	.	.
ACa07	17	####	.	G	A	.	PA!	FASN	exonic	NM_	.	mis	FASN:N
ACa07	17	####	rs7594547	G	A	.	PA!	FASN	exonic	NM_	.	mis	FASN:N
ACa07	17	####	.	C	T	.	PA!	CCDC57	intronic	NM_	.	.	.
ACa07	17	####	rs1452537	G	A	.	PA!	WDR45B	exonic	NM_	.	syr	WDR45
ACa07	17	####	.	G	A	.	PA!	ZNF750	exonic	NM_	.	syr	ZNF750
ACa07	18	####	.	G	A	.	PA!	ADCYAP1	exonic	NM_	.	mis	ADCYA
ACa07	18	####	.	A	G	.	PA!	NDC80	intronic	NM_	.	.	.
ACa07	18	####	.	A	G	.	PA!	AFG3L2	intronic	NM_	.	.	.
ACa07	18	####	.	G	A	.	PA!	CEP192	intronic	NM_	.	.	.
ACa07	18	####	.	A	T	.	PA!	MC2R	exonic	NM_	.	mis	MC2R:N
ACa07	18	####	.	C	A	.	PA!	.	intergen	NM_	dist=	.	.
ACa07	18	####	.	G	A	.	PA!	.	intergen	NR_	dist=	.	.
ACa07	18	####	.	G	T	.	PA!	GREB1L	exonic	NM_	.	mis	GREB1
ACa07	18	####	rs1390032	C	T	.	PA!	ESCO1	intronic	NM_	.	.	.
ACa07	18	####	.	G	T	.	PA!	TMEM241	intronic	NM_	.	.	.
ACa07	18	####	rs5453420	C	T	.	PA!	LAMA3	exonic	NM_	.	mis	LAMA3
ACa07	18	####	rs7793765	C	T	.	PA!	GAREM	UTR3	NM_	NM_	.	.
ACa07	18	####	.	A	G	.	PA!	ELP2	exonic	NM_	.	syr	ELP2:N
ACa07	18	####	.	A	G	.	PA!	EPG5	exonic	NM_	.	mis	EPG5:N
ACa07	18	####	rs7789211	G	A	.	PA!	HDHD2	intronic	NM_	.	.	.
ACa07	18	####	.	A	G	.	PA!	HDHD2	intronic	NM_	.	.	.
ACa07	18	####	rs7710491	G	A	.	PA!	MYO5B	exonic	NM_	.	mis	MYO5B
ACa07	18	####	.	C	T	.	PA!	CFAP53	intronic	NM_	.	.	.
ACa07	18	####	.	G	A	.	PA!	.	intergen	NM_	dist=	.	.
ACa07	18	####	.	G	T	.	PA!	ZCCHC2	exonic	NM_	.	mis	ZCCHC
ACa07	18	####	rs1417032	C	T	.	PA!	SERPINB!	exonic	NM_	.	sto	SERPIN
ACa07	18	####	.	T	C	.	PA!	SERPINB	exonic	NM_	.	mis	SERPIN
ACa07	18	####	.	A	G	.	PA!	RTTN	exonic	NM_	.	syr	RTTN:N
ACa07	18	####	.	C	A	.	PA!	ZNF407	exonic	NM_	.	mis	ZNF407
ACa07	18	####	.	G	T	.	PA!	ATP9B	intronic	NM_	.	.	.
ACa07	19	####	rs1020066	C	T	.	PA!	MIER2	intronic	NM_	.	.	.
ACa07	19	####	.	C	T	.	PA!	BSG	UTR5	NM_	NM_	.	.
ACa07	19	####	rs3749392	C	T	.	PA!	POLRMT	intronic	NM_	.	.	.
ACa07	19	####	rs7789396	G	A	.	PA!	POLRMT	exonic	NM_	.	mis	POLRM

ACa07	19	####	.	G	A	.	PA!	PALM	exonic	NM_	.	mis	PALM:N
ACa07	19	####	rs3777310	C	T	.	PA!	PTBP1	intronic	NM_	.	.	.
ACa07	19	####	.	G	T	.	PA!	TMEM259	exonic	NM_	.	mis	TMEM2
ACa07	19	####	rs9456811	C	T	.	PA!	HMHA1	exonic	NM_	.	syn	HMHA1
ACa07	19	####	.	C	T	.	PA!	POLR2E	intronic	NM_	.	.	.
ACa07	19	####	rs7620114	G	A	.	PA!	GPX4	exonic	NM_	.	unl	UNKNC
ACa07	19	####	rs2022226	C	T	.	PA!	SBNO2	exonic	NM_	.	syn	SBNO2
ACa07	19	####	.	C	T	.	PA!	SBNO2	intronic	NM_	.	.	.
ACa07	19	####	rs7799382	G	A	.	PA!	SBNO2	intronic	NM_	.	.	.
ACa07	19	####	.	G	A	.	PA!	SBNO2	exonic	NM_	.	mis	SBNO2
ACa07	19	####	.	C	A	.	PA!	MIDN	intronic	NM_	.	.	.
ACa07	19	####	rs3753640	C	T	.	PA!	CIRBP	exonic	NM_	.	mis	CIRBP:
ACa07	19	####	.	G	A	.	PA!	DAZAP1	intronic	NM_	.	.	.
ACa07	19	####	.	G	A	.	PA!	DAZAP1	intronic	NM_	.	.	.
ACa07	19	####	rs1007918	C	T	.	PA!	PLK5	exonic	NM_	.	syn	PLK5:N
ACa07	19	####	rs3694032	G	A	.	PA!	DOT1L	exonic	NM_	.	syn	DOT1L:
ACa07	19	####	rs7522031	G	A	.	PA!	SPPL2B	intronic	NM_	.	.	.
ACa07	19	####	.	G	T	.	PA!	SGTA	exonic	NM_	.	mis	SGTA:N
ACa07	19	####	.	C	A	.	PA!	SGTA	intronic	NM_	.	.	.
ACa07	19	####	rs3763485	C	T	.	PA!	S1PR4	exonic	NM_	.	syn	S1PR4:
ACa07	19	####	rs7773785	C	T	.	PA!	NFIC	intronic	NM_	.	.	.
ACa07	19	####	.	G	A	.	PA!	ZFR2	intronic	NM_	.	.	.
ACa07	19	####	.	C	T	.	PA!	ANKRD24	intronic	NM_	.	.	.
ACa07	19	####	rs7591374	G	A	.	PA!	ANKRD24	exonic	NM_	.	mis	ANKRD
ACa07	19	####	.	G	A	.	PA!	SH3GL1	exonic	NM_	.	sto	SH3GL
ACa07	19	####	rs1503658	G	A	.	PA!	LRG1	exonic	NM_	.	mis	LRG1:N
ACa07	19	####	.	G	A	.	PA!	SEMA6B	exonic	NM_	.	syn	SEMA6
ACa07	19	####	.	T	C	.	PA!	KDM4B	exonic	NM_	.	mis	KDM4B
ACa07	19	####	.	C	T	.	PA!	SAFB	intronic	NM_	.	.	.
ACa07	19	####	rs1018214	G	A	.	PA!	DUS3L	intronic	NM_	.	.	.
ACa07	19	####	rs7632840	G	A	.	PA!	DENND1C	exonic	NM_	.	unl	UNKNC
ACa07	19	####	.	T	C	.	PA!	VAV1	intronic	NM_	.	.	.
ACa07	19	####	rs1390681	C	T	.	PA!	ARHGEF1	exonic	NM_	.	mis	ARHGE
ACa07	19	####	rs3770694	C	T	.	PA!	PNPLA6	intronic	NM_	.	.	.
ACa07	19	####	rs3745730	G	A	.	PA!	XAB2	exonic	NM_	.	mis	XAB2:N
ACa07	19	####	rs7516930	C	T	.	PA!	XAB2	exonic	NM_	.	syn	XAB2:N
ACa07	19	####	rs1417170	G	A	.	PA!	STXBP2	exonic	NM_	.	mis	STXBP
ACa07	19	####	.	G	T	.	PA!	CD209	UTR3	NM_	NM_	.	.
ACa07	19	####	.	C	A	.	PA!	CD209	intronic	NM_	.	.	.
ACa07	19	####	rs7595385	C	T	.	PA!	EVI5L	intronic	NM_	.	.	.
ACa07	19	####	rs3527749	C	T	.	PA!	FBN3	exonic	NM_	.	mis	FBN3:N
ACa07	19	####	rs7615602	C	T	.	PA!	CD320	UTR3	NM_	NM_	.	.
ACa07	19	####	rs5308100	T	G	.	PA!	2-Mar	UTR3	NM_	NM_	.	.
ACa07	19	####	.	C	T	.	PA!	ACTL9	exonic	NM_	.	mis	ACTL9:
ACa07	19	####	rs3694972	C	T	.	PA!	.	intergen	NM_	dist=	.	.
ACa07	19	####	rs3751712	C	G	.	PA!	.	intergen	NM_	dist=	.	.
ACa07	19	####	rs5686926	G	A	.	PA!	MUC16	exonic	NM_	.	syn	MUC16
ACa07	19	####	rs7801357	C	T	.	PA!	MUC16	exonic	NM_	.	mis	MUC16
ACa07	19	####	.	C	T	.	PA!	MUC16	exonic	NM_	.	syn	MUC16
ACa07	19	####	.	A	G	.	PA!	FBXL12	intronic	NM_	.	.	.
ACa07	19	####	rs7564749	G	A	.	PA!	PIN1	exonic	NM_	.	mis	PIN1:NI
ACa07	19	####	rs7814402	G	A	.	PA!	OLFM2	exonic	NM_	.	mis	OLFM2
ACa07	19	####	.	C	T	.	PA!	C19orf66	exonic	NM_	.	mis	C19orf6
ACa07	19	####	.	G	A	.	PA!	PPAN-P2I	exonic	NM_	.	mis	PPAN-I

ACa07	19	####	.	G	A	.	PA!	P2RY11,P	UTR3	NM_	NM_	.	.
ACa07	19	####	.	G	T	.	PA!	ICAM1	exonic	NM_	.	mis	ICAM1:
ACa07	19	####	.	G	A	.	PA!	ICAM5	intronic	NM_	.	.	.
ACa07	19	####	rs1383503	G	A	.	PA!	TYK2	exonic	NM_	.	syr	TYK2:N
ACa07	19	####	rs2009930	G	A	.	PA!	PDE4A	exonic	NM_	.	syr	PDE4A
ACa07	19	####	.	A	G	.	PA!	ATG4D	intronic	NM_	.	.	.
ACa07	19	####	.	C	T	.	PA!	KRI1	exonic	NM_	.	mis	KRI1:NI
ACa07	19	####	rs7634492	C	T	.	PA!	LDLR	exonic	NM_	.	syr	LDLR:N
ACa07	19	####	rs3737274	G	A	.	PA!	LDLR	intronic	NM_	.	.	.
ACa07	19	####	rs3721772	C	T	.	PA!	DOCK6	exonic	NM_	.	mis	DOCK6
ACa07	19	####	.	A	G	.	PA!	CCDC151	intronic	NM_	.	.	.
ACa07	19	####	rs5385135	A	T	.	PA!	CNN1	intronic	NM_	.	.	.
ACa07	19	####	.	G	T	.	PA!	MAN2B1	intronic	NM_	.	.	.
ACa07	19	####	rs3710318	G	A	.	PA!	DAND5	exonic	NM_	.	mis	DAND5
ACa07	19	####	.	G	A	.	PA!	RFX1	exonic	NM_	.	mis	RFX1:N
ACa07	19	####	.	A	C	.	PA!	PRKACA	exonic	NM_	.	mis	PRKAC
ACa07	19	####	.	C	G	.	PA!	WIZ	intronic	NM_	.	.	.
ACa07	19	####	.	C	T	.	PA!	OR10H2	exonic	NM_	.	syr	OR10H
ACa07	19	####	.	G	A	.	PA!	KLF2	exonic	NM_	.	mis	KLF2:N
ACa07	19	####	rs1015670	G	A	.	PA!	EPS15L1	intronic	NM_	.	.	.
ACa07	19	####	.	C	T	.	PA!	CPAMD8	intronic	NM_	.	.	.
ACa07	19	####	rs1138422	G	A	.	PA!	MYO9B	intronic	NM_	.	.	.
ACa07	19	####	.	T	C	.	PA!	OCEL1	intronic	NM_	.	.	.
ACa07	19	####	rs9290443	G	A	.	PA!	USHBP1	exonic	NM_	.	mis	USHBP
ACa07	19	####	rs7698270	G	A	.	PA!	GTPBP3	exonic	NM_	.	syr	GTPBP
ACa07	19	####	rs1043222	C	T	.	PA!	UNC13A	exonic	NM_	.	mis	UNC13
ACa07	19	####	.	T	C	.	PA!	UNC13A	intronic	NM_	.	.	.
ACa07	19	####	.	G	T	.	PA!	MAST3	intronic	NM_	.	.	.
ACa07	19	####	.	C	T	.	PA!	PDE4C	exonic	NM_	.	mis	PDE4C
ACa07	19	####	rs3719294	C	T	.	PA!	UPF1	exonic	NM_	.	syr	UPF1:N
ACa07	19	####	rs2012753	C	T	.	PA!	MAU2	exonic	NM_	.	syr	MAU2:N
ACa07	19	####	.	C	A	.	PA!	ZNF90	exonic	NM_	.	syr	ZNF90:
ACa07	19	####	.	T	C	.	PA!	.	intergen	NM_	dist=	.	.
ACa07	19	####	.	T	C	.	PA!	ZNF492	intronic	NM_	.	.	.
ACa07	19	####	.	C	T	.	PA!	PLEKHF1	intronic	NM_	.	.	.
ACa07	19	####	rs7540203	C	T	.	PA!	TSHZ3	exonic	NM_	.	mis	TSHZ3:
ACa07	19	####	rs3754283	C	T	.	PA!	KIAA0355	exonic	NM_	.	mis	KIAA03
ACa07	19	####	.	C	G	.	PA!	GPI	intronic	NM_	.	.	.
ACa07	19	####	.	A	C	.	PA!	CD22	intronic	NM_	.	.	.
ACa07	19	####	rs7656023	G	A	.	PA!	RBM42	exonic	NM_	.	mis	RBM42
ACa07	19	####	.	G	A	.	PA!	WDR62	intronic	NM_	.	.	.
ACa07	19	####	.	T	C	.	PA!	ZNF146	UTR5	NM_	NM_	.	.
ACa07	19	####	rs5581240	C	T	.	PA!	ZNF529	UTR3	NM_	NM_	.	.
ACa07	19	####	.	C	T	.	PA!	.	intergen	NR_	dist=	.	.
ACa07	19	####	rs4806419	C	A	.	PA!	.	intergen	NR_	dist=	.	.
ACa07	19	####	rs7125437	C	G	.	PA!	.	intergen	NR_	dist=	.	.
ACa07	19	####	.	C	T	.	PA!	.	intergen	NR_	dist=	.	.
ACa07	19	####	rs7728313	G	A	.	PA!	.	downstr	NM_	.	.	.
ACa07	19	####	.	G	T	.	PA!	SIPA1L3	exonic	NM_	.	unl	UNKNC
ACa07	19	####	rs1476199	C	T	.	PA!	CATSPER	exonic	NM_	.	mis	CATSP
ACa07	19	####	.	C	T	.	PA!	RASGRP4	intronic	NM_	.	.	.
ACa07	19	####	rs3756003	G	A	.	PA!	RYR1	intronic	NM_	.	.	.
ACa07	19	####	rs5371432	A	G	.	PA!	RYR1	intronic	NM_	.	.	.
ACa07	19	####	.	G	A	.	PA!	MAP4K1	intronic	NM_	.	.	.

ACa07	19	####	.	C	A	.	PA!	HNRNPL	intronic	NM_	.	.	.
ACa07	19	####	rs5721273	A	G	.	PA!	SIRT2	intronic	NM_	.	.	.
ACa07	19	####	rs8917290	G	A	.	PA!	IFNL4	exonic	NM_	.	unl	UNKNC
ACa07	19	####	.	T	C	.	PA!	.	intergen	NM_	dist=	.	.
ACa07	19	####	rs7564368	G	A	.	PA!	CLC	UTR3	NM_	NM_	.	.
ACa07	19	####	rs7738579	G	A	.	PA!	DYRK1B	exonic	NM_	.	unl	UNKNC
ACa07	19	####	.	G	A	.	PA!	FCGBP	intronic	NM_	.	.	.
ACa07	19	####	rs7469641	G	A	.	PA!	SHKBP1	exonic	NM_	.	mis	SHKBP
ACa07	19	####	.	C	T	.	PA!	SHKBP1	intronic	NM_	.	.	.
ACa07	19	####	.	G	A	.	PA!	.	upstrea	NM_	.	.	.
ACa07	19	####	.	C	T	.	PA!	LTBP4	exonic	NM_	.	unl	UNKNC
ACa07	19	####	.	C	T	.	PA!	LTBP4	exonic	NM_	.	unl	UNKNC
ACa07	19	####	.	C	A	.	PA!	NUMBL	intronic	NM_	.	.	.
ACa07	19	####	rs1045293	G	C	.	PA!	C19orf54	exonic	NM_	.	mis	C19orf5
ACa07	19	####	.	G	T	.	PA!	HNRNPUL	exonic	NM_	.	unl	UNKNC
ACa07	19	####	.	G	T	.	PA!	TGFB1	exonic	NM_	.	mis	TGFB1:
ACa07	19	####	.	C	T	.	PA!	ARHGEF1	exonic	NM_	.	mis	ARHGE
ACa07	19	####	.	C	A	.	PA!	MEGF8	exonic	NM_	.	mis	MEGF8
ACa07	19	####	.	C	A	.	PA!	CEACAM8	exonic	NM_	.	sto	CEACA
ACa07	19	####	.	T	C	.	PA!	PSG3	intronic	NM_	.	.	.
ACa07	19	####	.	C	T	.	PA!	.	intergen	NM_	dist=	.	.
ACa07	19	####	.	G	A	.	PA!	IRGQ	exonic	NM_	.	syr	IRGQ:N
ACa07	19	####	.	T	G	.	PA!	ZNF155	intronic	NM_	.	.	.
ACa07	19	####	.	G	T	.	PA!	ZNF285	exonic	NM_	.	mis	ZNF285
ACa07	19	####	rs7737032	C	T	.	PA!	ZNF229	exonic	NM_	.	unl	UNKNC
ACa07	19	####	rs9075637	C	T	.	PA!	IGSF23	intronic	NM_	.	.	.
ACa07	19	####	.	C	T	.	PA!	CEACAM1	intronic	NM_	.	.	.
ACa07	19	####	rs5632226	C	T	.	PA!	CEACAM1	intronic	NM_	.	.	.
ACa07	19	####	.	C	T	.	PA!	BCL3	exonic	NM_	.	syr	BCL3:N
ACa07	19	####	.	C	A	.	PA!	BCAM	exonic	NM_	.	mis	BCAM:I
ACa07	19	####	.	T	C	.	PA!	TOMM40	intronic	NM_	.	.	.
ACa07	19	####	.	G	T	.	PA!	GPR4	exonic	NM_	.	mis	GPR4:N
ACa07	19	####	rs1026645	C	T	.	PA!	PRKD2	exonic	NM_	.	mis	PRKD2
ACa07	19	####	rs3697415	C	T	.	PA!	STRN4	exonic	NM_	.	mis	STRN4
ACa07	19	####	.	G	A	.	PA!	SLC8A2	intronic	NM_	.	.	.
ACa07	19	####	.	G	A	.	PA!	CRX	intronic	NM_	.	.	.
ACa07	19	####	.	C	T	.	PA!	.	intergen	NM_	dist=	.	.
ACa07	19	####	rs6213000	C	G	.	PA!	.	intergen	NR_	dist=	.	.
ACa07	19	####	.	G	A	.	PA!	SPHK2	exonic	NM_	.	mis	SPHK2
ACa07	19	####	rs1012379	G	A	.	PA!	PLEKHA4	intronic	NM_	.	.	.
ACa07	19	####	.	G	A	.	PA!	SNRNP70	intronic	NM_	.	.	.
ACa07	19	####	.	A	G	.	PA!	SNRNP70	intronic	NM_	.	.	.
ACa07	19	####	rs5626072	C	T	.	PA!	DKKL1	intronic	NM_	.	.	.
ACa07	19	####	.	C	T	.	PA!	CCDC155	intronic	NM_	.	.	.
ACa07	19	####	.	G	T	.	PA!	RCN3	intronic	NM_	.	.	.
ACa07	19	####	rs7508901	C	T	.	PA!	CPT1C	intronic	NM_	.	.	.
ACa07	19	####	rs3749564	G	A	.	PA!	MYH14	exonic	NM_	.	mis	MYH14
ACa07	19	####	rs2001122	G	A	.	PA!	NR1H2	UTR5	NM_	NM_	.	.
ACa07	19	####	.	G	A	.	PA!	POLD1	intronic	NM_	.	.	.
ACa07	19	####	rs5643823	T	G	.	PA!	POLD1	intronic	NM_	.	.	.
ACa07	19	####	rs3695357	C	T	.	PA!	POLD1	intronic	NM_	.	.	.
ACa07	19	####	.	G	A	.	PA!	.	upstrea	NM_	.	.	.
ACa07	19	####	rs7738602	G	A	.	PA!	MYBPC2	exonic	NM_	.	mis	MYBPC
ACa07	19	####	.	G	A	.	PA!	MYBPC2	splicing	NM_	NM_	.	.

ACa07	19	####	.	A	G	.	PA!	GPR32	exonic	NM_	.	syr	GPR32
ACa07	19	####	.	T	C	.	PA!	ZNF816	exonic	NM_	.	mis	ZNF816
ACa07	19	####	.	A	G	.	PA!	ERVV-2	exonic	NM_	.	syr	ERVV-2
ACa07	19	####	rs1420234	T	G	.	PA!	VN1R4	exonic	NM_	.	syr	VN1R4:
ACa07	19	####	.	G	A	.	PA!	MIR527	ncRNA_	NR_	.	.	.
ACa07	19	####	.	T	G	.	PA!	CACNG8	exonic	NM_	.	mis	CACNG
ACa07	19	####	.	T	C	.	PA!	PRPF31	intronic	NM_	.	.	.
ACa07	19	####	.	G	A	.	PA!	LENG1	intronic	NM_	.	.	.
ACa07	19	####	rs3704562	G	A	.	PA!	LILRA6,LI	intronic	NM_	.	.	.
ACa07	19	####	.	C	A	.	PA!	TTYH1	intronic	NM_	.	.	.
ACa07	19	####	rs7529171	G	A	.	PA!	KIR3DX1	ncRNA_	NR_	.	.	.
ACa07	19	####	.	G	T	.	PA!	SYT5	intronic	NM_	.	.	.
ACa07	19	####	rs3707427	C	T	.	PA!	TMEM190	exonic	NM_	.	mis	TMEM1
ACa07	19	####	rs2000514	G	A	.	PA!	NLRP13	exonic	NM_	.	mis	NLRP1:
ACa07	19	####	rs7704358	C	T	.	PA!	PEG3	exonic	NM_	.	syr	PEG3:N
ACa07	19	####	.	G	A	.	PA!	ZNF773	intronic	NM_	.	.	.
ACa07	19	####	.	G	A	.	PA!	ZIK1	intronic	NM_	.	.	.
ACa07	19	####	rs1474253	C	T	.	PA!	ZNF606	exonic	NM_	.	syr	ZNF606
ACa07	19	####	.	C	T	.	PA!	ZNF135	UTR5	NM_	NM_	.	.
ACa07	19	####	rs3733248	C	T	.	PA!	A1BG	intronic	NM_	.	.	.
ACa07	20	####	rs1021538	C	T	.	PA!	SIRPA	exonic	NM_	.	mis	SIRPA:I
ACa07	20	####	rs7627114	T	C	.	PA!	TGM6	intronic	NM_	.	.	.
ACa07	20	####	.	G	A	.	PA!	NOP56	intronic	NM_	.	.	.
ACa07	20	####	rs8672082	C	T	.	PA!	C20orf194	intronic	NM_	.	.	.
ACa07	20	####	.	C	T	.	PA!	GFRA4	intronic	NM_	.	.	.
ACa07	20	####	rs7638624	G	A	.	PA!	ADAM33	exonic	NM_	.	sto	ADAM3
ACa07	20	####	.	C	T	.	PA!	PRND	UTR3	NM_	NM_	.	.
ACa07	20	####	rs1032256	G	A	.	PA!	.	intergen	NR_	dist=	.	.
ACa07	20	####	.	T	C	.	PA!	KIF16B	intronic	NM_	.	.	.
ACa07	20	####	.	A	G	.	PA!	CSRP2BP	exonic	NM_	.	mis	CSRP2
ACa07	20	####	.	T	C	.	PA!	LINC0085	ncRNA_	NR_	.	.	.
ACa07	20	####	rs1509219	G	A	.	PA!	CST4	exonic	NM_	.	mis	CST4:N
ACa07	20	####	.	T	C	.	PA!	ABHD12	intronic	NM_	.	.	.
ACa07	20	####	.	C	T	.	PA!	FAM182B	ncRNA_	NR_	.	.	.
ACa07	20	####	.	C	T	.	PA!	FRG1B	ncRNA_	NR_	.	.	.
ACa07	20	####	.	G	A	.	PA!	FRG1B	ncRNA_	NR_	.	.	.
ACa07	20	####	rs1845841	C	T	.	PA!	NOL4L	exonic	NM_	.	mis	NOL4L:
ACa07	20	####	.	C	A	.	PA!	BPIFB3	intronic	NM_	.	.	.
ACa07	20	####	.	G	T	.	PA!	ZNF341	intronic	NM_	.	.	.
ACa07	20	####	.	G	A	.	PA!	PIGU	intronic	NM_	.	.	.
ACa07	20	####	rs7460506	A	T	.	PA!	TRPC4AP	intronic	NM_	.	.	.
ACa07	20	####	.	A	C	.	PA!	CEP250	UTR3	NM_	NM_	.	.
ACa07	20	####	rs1480471	C	T	.	PA!	NFS1	exonic	NM_	.	mis	NFS1:N
ACa07	20	####	rs5431099	T	C	.	PA!	RBM39	UTR5	NM_	NM_	.	.
ACa07	20	####	.	G	A	.	PA!	EPB41L1	intronic	NM_	.	.	.
ACa07	20	####	.	G	A	.	PA!	DLGAP4	intronic	NM_	.	.	.
ACa07	20	####	rs8798536	G	A	.	PA!	NDRG3	UTR5	NM_	NM_	.	.
ACa07	20	####	.	G	T	.	PA!	MROH8	intronic	NM_	.	.	.
ACa07	20	####	rs9848856	C	T	.	PA!	RPRD1B	intronic	NM_	.	.	.
ACa07	20	####	rs9460302	G	A	.	PA!	DHX35	exonic	NM_	.	mis	DHX35:
ACa07	20	####	rs3711048	G	A	.	PA!	DHX35	UTR3	NM_	NM_	.	.
ACa07	20	####	.	G	A	.	PA!	TOP1	exonic	NM_	.	mis	TOP1:N
ACa07	20	####	.	C	T	.	PA!	PLCG1	exonic	NM_	.	mis	PLCG1
ACa07	20	####	.	G	A	.	PA!	PTPRT	exonic	NM_	.	sto	PTPRT

ACa07	20	####	rs3761522	G	A	.	PA!	PTPRT	exonic	NM_	.	syr	PTPRT
ACa07	20	####	.	T	C	.	PA!	WISP2	intronic	NM_	.	.	.
ACa07	20	####	.	G	T	.	PA!	WFDC12	intronic	NM_	.	.	.
ACa07	20	####	.	C	A	.	PA!	SLC12A5	intronic	NM_	.	.	.
ACa07	20	####	.	G	T	.	PA!	ELMO2	intronic	NM_	.	.	.
ACa07	20	####	.	G	T	.	PA!	SLC13A3	intronic	NM_	.	.	.
ACa07	20	####	.	C	T	.	PA!	ZMYND8	exonic	NM_	.	mis	ZMYND
ACa07	20	####	.	G	A	.	PA!	KCNG1	exonic	NM_	.	mis	KCNG1
ACa07	20	####	.	C	T	.	PA!	ATP9A	intronic	NM_	.	.	.
ACa07	20	####	.	G	A	.	PA!	ZFP64	intronic	NM_	.	.	.
ACa07	20	####	rs8891302	C	T	.	PA!	ZNF217	exonic	NM_	.	syr	ZNF217
ACa07	20	####	rs8669651	T	C	.	PA!	.	intergen	NM_	dist=	.	.
ACa07	20	####	.	G	A	.	PA!	GNAS	exonic	NM_	.	mis	GNAS:I
ACa07	20	####	.	C	T	.	PA!	GNAS	exonic	NM_	.	mis	GNAS:I
ACa07	20	####	.	G	A	.	PA!	SYCP2	intronic	NM_	.	.	.
ACa07	20	####	rs7702464	C	T	.	PA!	LSM14B	exonic	NM_	.	mis	LSM14I
ACa07	20	####	rs7787907	C	T	.	PA!	COL9A3	intronic	NM_	.	.	.
ACa07	20	####	rs1813756	G	A	.	PA!	COL9A3	intronic	NM_	.	.	.
ACa07	20	####	rs1435421	G	A	.	PA!	ARFGAP1	exonic	NM_	.	syr	ARFGA
ACa07	20	####	.	C	T	.	PA!	.	downstr	NM_	.	.	.
ACa07	20	####	.	A	G	.	PA!	SRMS	intronic	NM_	.	.	.
ACa07	20	####	rs5462318	G	A	.	PA!	ABHD16B	exonic	NM_	.	mis	ABHD1
ACa07	21	####	rs7962225	A	G	.	PA!	.	intergen	NOI	dist=	.	.
ACa07	21	####	.	C	T	.	PA!	.	downstr	NR_	.	.	.
ACa07	21	####	.	G	A	.	PA!	.	intergen	NR_	dist=	.	.
ACa07	21	####	.	C	A	.	PA!	.	intergen	NR_	dist=	.	.
ACa07	21	####	rs8680295	G	A	.	PA!	.	intergen	NR_	dist=	.	.
ACa07	21	####	rs3711086	C	A	.	PA!	.	intergen	NR_	dist=	.	.
ACa07	21	####	rs3775054	C	T	.	PA!	.	intergen	NR_	dist=	.	.
ACa07	21	####	.	G	A	.	PA!	TPTE	intronic	NM_	.	.	.
ACa07	21	####	.	C	A	.	PA!	TPTE	exonic	NM_	.	unl	UNKNC
ACa07	21	####	rs5282430	G	C	.	PA!	BAGE5,BA	intronic	NM_	.	.	.
ACa07	21	####	.	C	T	.	PA!	.	intergen	NM_	dist=	.	.
ACa07	21	####	rs665796	T	C	.	PA!	.	intergen	NM_	dist=	.	.
ACa07	21	####	rs6582845	C	T	.	PA!	.	intergen	NM_	dist=	.	.
ACa07	21	####	rs915519	G	C	.	PA!	.	intergen	NM_	dist=	.	.
ACa07	21	####	.	C	T	.	PA!	ANKRD30	ncRNA_	NR_	.	.	.
ACa07	21	####	.	T	C	.	PA!	.	intergen	NR_	dist=	.	.
ACa07	21	####	.	A	G	.	PA!	NCAM2	intronic	NM_	.	.	.
ACa07	21	####	.	C	G	.	PA!	JAM2	intronic	NM_	.	.	.
ACa07	21	####	.	A	G	.	PA!	APP	intronic	NM_	.	.	.
ACa07	21	####	rs9172671	C	T	.	PA!	MIR5009	ncRNA_	NR_	.	.	.
ACa07	21	####	.	C	T	.	PA!	KRTAP23	exonic	NM_	.	mis	KRTAP
ACa07	21	####	.	A	G	.	PA!	HUNK	intronic	NM_	.	.	.
ACa07	21	####	.	G	T	.	PA!	HUNK	intronic	NM_	.	.	.
ACa07	21	####	rs1034062	C	T	.	PA!	SYNJ1	exonic	NM_	.	syr	SYNJ1:
ACa07	21	####	.	C	T	.	PA!	OLIG1	exonic	NM_	.	syr	OLIG1:I
ACa07	21	####	rs7587939	A	G	.	PA!	IFNAR1	intronic	NM_	.	.	.
ACa07	21	####	.	C	T	.	PA!	ITSN1	intronic	NM_	.	.	.
ACa07	21	####	.	T	G	.	PA!	LOC1001	ncRNA_	NR_	.	.	.
ACa07	21	####	.	C	T	.	PA!	.	intergen	NM_	dist=	.	.
ACa07	21	####	rs5585229	C	T	.	PA!	TTC3	intronic	NM_	.	.	.
ACa07	21	####	rs7591033	G	A	.	PA!	PSMG1	UTR5	NM_	NM_	.	.
ACa07	21	####	rs7585989	C	T	.	PA!	BRWD1	exonic	NM_	.	mis	BRWD1

ACa07	21	####	.	C	A	.	PA!	IGSF5	intronic	NM_	.	.	.
ACa07	21	####	.	C	A	.	PA!	DSCAM	exonic	NM_	.	syr	DSCAM
ACa07	21	####	.	T	C	.	PA!	DSCAM	exonic	NM_	.	syr	DSCAM
ACa07	21	####	.	G	A	.	PA!	DSCAM	exonic	NM_	.	mis	DSCAM
ACa07	21	####	.	T	C	.	PA!	DSCAM	exonic	NM_	.	mis	DSCAM
ACa07	21	####	.	C	T	.	PA!	FAM3B	intronic	NM_	.	.	.
ACa07	21	####	.	T	C	.	PA!	PRDM15	intronic	NM_	.	.	.
ACa07	21	####	rs9464977	G	A	.	PA!	C2CD2	intronic	NM_	.	.	.
ACa07	21	####	.	G	A	.	PA!	ZBTB21	exonic	NM_	.	mis	ZBTB21
ACa07	21	####	.	T	C	.	PA!	SLC37A1	intronic	NM_	.	.	.
ACa07	21	####	rs1014361	C	A	.	PA!	U2AF1	intronic	NM_	.	.	.
ACa07	21	####	rs7806447	G	A	.	PA!	RRP1	intronic	NM_	.	.	.
ACa07	21	####	rs5662670	C	T	.	PA!	PWP2	exonic	NM_	.	mis	PWP2:I
ACa07	21	####	rs9852755	C	T	.	PA!	TRPM2	intronic	NM_	.	.	.
ACa07	21	####	rs7565454	G	A	.	PA!	ITGB2	exonic	NM_	.	mis	ITGB2:I
ACa07	21	####	rs7582548	C	T	.	PA!	SLC19A1	exonic	NM_	.	syr	SLC19A
ACa07	21	####	rs3771590	C	T	.	PA!	COL6A1	intronic	NM_	.	.	.
ACa07	21	####	.	G	A	.	PA!	FTCD	intronic	NM_	.	.	.
ACa07	21	####	.	G	A	.	PA!	C21orf58	intronic	NM_	.	.	.
ACa07	21	####	rs7517144	G	A	.	PA!	PCNT	exonic	NM_	.	mis	PCNT:N
ACa07	21	####	.	G	A	.	PA!	DIP2A	exonic	NM_	.	mis	DIP2A:I
ACa07	22	####	rs2845112	A	G	.	PA!	.	intergen	NR_	dist=	.	.
ACa07	22	####	rs8790424	C	G	.	PA!	POTEH	intronic	NM_	.	.	.
ACa07	22	####	.	A	G	.	PA!	POTEH	intronic	NM_	.	.	.
ACa07	22	####	rs1248470	C	A	.	PA!	.	intergen	NM_	dist=	.	.
ACa07	22	####	.	G	A	.	PA!	GAB4	intronic	NM_	.	.	.
ACa07	22	####	rs5555078	G	A	.	PA!	DGCR2	exonic	NM_	.	syr	DGCR2
ACa07	22	####	.	T	A	.	PA!	DGCR2	intronic	NM_	.	.	.
ACa07	22	####	rs1899538	G	A	.	PA!	.	intergen	NM_	dist=	.	.
ACa07	22	####	.	C	A	.	PA!	.	intergen	NM_	dist=	.	.
ACa07	22	####	.	G	A	.	PA!	.	downstr	NM_	.	.	.
ACa07	22	####	rs3714923	G	A	.	PA!	PI4KA	exonic	NM_	.	syr	PI4KA:N
ACa07	22	####	rs7724279	G	A	.	PA!	HIC2	exonic	NM_	.	syr	HIC2:N
ACa07	22	####	.	G	A	.	PA!	.	intergen	NR_	dist=	.	.
ACa07	22	####	rs3777336	G	A	.	PA!	.	intergen	NM_	dist=	.	.
ACa07	22	####	rs3681295	C	T	.	PA!	SMARCB1	intronic	NM_	.	.	.
ACa07	22	####	.	G	T	.	PA!	SUSD2	exonic	NM_	.	mis	SUSD2
ACa07	22	####	.	C	T	.	PA!	ADORA2A	exonic	NM_	.	mis	ADORA
ACa07	22	####	.	G	T	.	PA!	UPB1	intronic	NM_	.	.	.
ACa07	22	####	rs7723316	G	A	.	PA!	GGT1	exonic	NM_	.	mis	GGT1:N
ACa07	22	####	.	C	T	.	PA!	POM121L	ncRNA_	NR_	.	.	.
ACa07	22	####	.	C	T	.	PA!	.	intergen	NR_	dist=	.	.
ACa07	22	####	rs1925641	C	T	.	PA!	EMID1	intronic	NM_	.	.	.
ACa07	22	####	.	T	C	.	PA!	SEC14L4	UTR3	NM_	NM_	.	.
ACa07	22	####	.	G	A	.	PA!	OSBP2	intronic	NM_	.	.	.
ACa07	22	####	rs5710568	G	A	.	PA!	SMTN	intronic	NM_	.	.	.
ACa07	22	####	rs1015431	C	T	.	PA!	PIK3IP1	intronic	NM_	.	.	.
ACa07	22	####	.	G	A	.	PA!	SFI1	exonic	NM_	.	syr	SFI1:NI
ACa07	22	####	.	T	C	.	PA!	.	intergen	NR_	dist=	.	.
ACa07	22	####	.	T	C	.	PA!	RTCB	intronic	NM_	.	.	.
ACa07	22	####	.	G	A	.	PA!	LARGE	intronic	NM_	.	.	.
ACa07	22	####	rs1379196	C	T	.	PA!	MCM5	exonic	NM_	.	syr	MCM5:I
ACa07	22	####	.	C	T	.	PA!	MYH9	UTR3	NM_	NM_	.	.
ACa07	22	####	rs3682304	G	A	.	PA!	CSF2RB	exonic	NM_	.	mis	CSF2R

ACa07	22	####	rs5367410	C	T	.	PA!	TEX33	intronic	NM_	.	.	.
ACa07	22	####	.	G	A	.	PA!	PLA2G6	exonic	NM_	.	sto	PLA2G(
ACa07	22	####	.	C	A	.	PA!	MAFF	intronic	NM_	.	.	.
ACa07	22	####	rs7523833	A	G	.	PA!	TMEM184	intronic	NM_	.	.	.
ACa07	22	####	.	G	A	.	PA!	KCNJ4	exonic	NM_	.	mis	KCNJ4:
ACa07	22	####	.	G	A	.	PA!	KCNJ4	exonic	NM_	.	mis	KCNJ4:
ACa07	22	####	rs5714435	C	T	.	PA!	GTPBP1	intronic	NM_	.	.	.
ACa07	22	####	.	C	T	.	PA!	APOBEC3	exonic	NM_	.	syr	APOBE
ACa07	22	####	rs3719126	C	T	.	PA!	CACNA1I	intronic	NM_	.	.	.
ACa07	22	####	.	C	T	.	PA!	TNRC6B	exonic	NM_	.	mis	TNRC6
ACa07	22	####	rs2016059	G	A	.	PA!	MCHR1	exonic	NM_	.	mis	MCHR1
ACa07	22	####	.	C	T	.	PA!	POLR3H	exonic	NM_	.	mis	POLR3
ACa07	22	####	rs1838128	C	T	.	PA!	POLR3H	exonic	NM_	.	mis	POLR3
ACa07	22	####	.	C	A	.	PA!	CENPM	intronic	NM_	.	.	.
ACa07	22	####	.	G	A	.	PA!	RRP7A	intronic	NM_	.	.	.
ACa07	22	####	rs1916812	C	T	.	PA!	EFCAB6	intronic	NM_	.	.	.
ACa07	22	####	.	G	A	.	PA!	PNPLA3	intronic	NM_	.	.	.
ACa07	22	####	rs5584935	C	T	.	PA!	KIAA0930	intronic	NM_	.	.	.
ACa07	22	####	rs1305515	C	T	.	PA!	FAM118A	intronic	NM_	.	.	.
ACa07	22	####	.	C	T	.	PA!	SMC1B	intronic	NM_	.	.	.
ACa07	22	####	.	A	G	.	PA!	FBLN1	splicing	NM_	NM_	.	.
ACa07	22	####	.	T	C	.	PA!	ATXN10	intronic	NM_	.	.	.
ACa07	22	####	.	G	A	.	PA!	PKDREJ	exonic	NM_	.	syr	PKDRE
ACa07	22	####	.	G	A	.	PA!	CELSR1	exonic	NM_	.	mis	CELSR
ACa07	22	####	.	C	A	.	PA!	IL17REL	intronic	NM_	.	.	.
ACa07	22	####	.	C	A	.	PA!	MOV10L1	intronic	NM_	.	.	.
ACa07	22	####	rs5595729	G	A	.	PA!	MOV10L1	exonic	NM_	.	syr	MOV10
ACa07	22	####	.	G	A	.	PA!	PANX2	intronic	NM_	.	.	.
ACa07	22	####	.	G	A	.	PA!	PANX2	exonic	NM_	.	syr	PANX2
ACa07	22	####	rs3705843	C	T	.	PA!	PLXNB2	exonic	NM_	.	syr	PLXNB:
ACa07	22	####	.	G	A	.	PA!	PPP6R2	intronic	NM_	.	.	.
ACa07	22	####	.	C	T	.	PA!	NCAPH2	exonic	NM_	.	mis	NCAPH
ACa07	22	####	rs9805300	C	T	.	PA!	MAPK8IP2	intronic	NM_	.	.	.
ACa07	X	####	.	A	G	.	PA!	ASMT	intronic	NM_	.	.	.
ACa07	X	####	.	T	G	.	PA!	CD99	UTR3	NM_	NM_	.	.
ACa07	X	####	.	A	C	.	PA!	ARSE	intronic	NM_	.	.	.
ACa07	X	####	.	T	C	.	PA!	NLGN4X	UTR3	NM_	NM_	.	.
ACa07	X	####	.	T	C	.	PA!	LOC10028	exonic	NM_	.	syr	LOC10(
ACa07	X	####	.	G	T	.	PA!	FRMPD4	intronic	NM_	.	.	.
ACa07	X	####	.	C	T	.	PA!	EGFL6	intronic	NM_	.	.	.
ACa07	X	####	.	C	T	.	PA!	NHS	exonic	NM_	.	mis	NHS:NM
ACa07	X	####	.	G	A	.	PA!	CNKSR2	intronic	NM_	.	.	.
ACa07	X	####	rs3730298	C	T	.	PA!	APOO	exonic	NM_	.	mis	APOO:I
ACa07	X	####	.	C	T	.	PA!	ARX	exonic	NM_	.	syr	ARX:NM
ACa07	X	####	.	T	C	.	PA!	.	intergen	NR_	dist=	.	.
ACa07	X	####	.	C	T	.	PA!	MAGEB10	intronic	NM_	.	.	.
ACa07	X	####	.	T	A	.	PA!	IL1RAPL1	intronic	NM_	.	.	.
ACa07	X	####	.	C	A	.	PA!	MAGEB1	exonic	NM_	.	syr	MAGEE
ACa07	X	####	.	G	A	.	PA!	GK	UTR5	NM_	NM_	.	.
ACa07	X	####	.	T	C	.	PA!	GK	intronic	NM_	.	.	.
ACa07	X	####	.	A	C	.	PA!	DMD	intronic	NM_	.	.	.
ACa07	X	####	.	A	C	.	PA!	.	intergen	NM_	dist=	.	.
ACa07	X	####	.	A	C	.	PA!	SRPX	intronic	NM_	.	.	.
ACa07	X	####	.	G	A	.	PA!	CXorf38	exonic	NM_	.	syr	CXorf38

ACa07	X	####	.	G	A	.	PA!	EFHC2	intronic	NM_	.	.	.	
ACa07	X	####	.	C	T	.	PA!	.	intergen	NR_	dist=	.	.	
ACa07	X	####	.	G	T	.	PA!	SSX5	intronic	NM_	.	.	.	
ACa07	X	####	rs7825839	C	T	.	PA!	PQBP1	intronic	NM_	.	.	.	
ACa07	X	####	rs3679315	G	A	.	PA!	OTUD5	exonic	NM_	.	mis	OTUD5	
ACa07	X	####	rs1871040	C	T	.	PA!	WDR45	exonic	NM_	.	mis	WDR45	
ACa07	X	####	.	G	T	.	PA!	MAGIX	intronic	NM_	.	.	.	
ACa07	X	####	.	G	A	.	PA!	PRICKLE3	intronic	NM_	.	.	.	
ACa07	X	####	rs7822715	C	T	.	PA!	CACNA1F	exonic	NM_	.	syr	CACNA	
ACa07	X	####	.	C	T	.	PA!	AKAP4	exonic	NM_	.	syr	AKAP4:	
ACa07	X	####	.	G	A	.	PA!	MAGED1	exonic	NM_	.	mis	MAGED	
ACa07	X	####	.	G	A	.	PA!	.	intergen	NM_	dist=	.	.	
ACa07	X	####	rs7825371	C	T	.	PA!	KDM5C	exonic	NM_	.	mis	KDM5C	
ACa07	X	####	.	G	T	.	PA!	IQSEC2	intronic	NM_	.	.	.	
ACa07	X	####	.	A	C	.	PA!	SMC1A	exonic	NM_	.	mis	SMC1A	
ACa07	X	####	.	G	T	.	PA!	HUWE1	exonic	NM_	.	mis	HUWE1	
ACa07	X	####	.	T	C	.	PA!	FAM120C	intronic	NM_	.	.	.	
ACa07	X	####	.	C	T	.	PA!	FGD1	splicing	NM_	NM_	.	.	
ACa07	X	####	.	G	A	.	PA!	GNL3L	intronic	NM_	.	.	.	
ACa07	X	####	rs9617788	A	G	.	PA!	ITIH6	intronic	NM_	.	.	.	
ACa07	X	####	.	T	G	.	PA!	.	intergen	NOI	dist=	.	.	
ACa07	X	####	.	G	C	.	PA!	.	intergen	NOI	dist=	.	.	
ACa07	X	####	.	A	G	.	PA!	.	intergen	NOI	dist=	.	.	
ACa07	X	####	rs7124105	C	A	.	PA!	.	intergen	NOI	dist=	.	.	
ACa07	X	####	.	A	G	.	PA!	.	intergen	NOI	dist=	.	.	
ACa07	X	####	rs3765099	T	G	.	PA!	.	intergen	NOI	dist=	.	.	
ACa07	X	####	.	T	C	.	PA!	.	intergen	NOI	dist=	.	.	
ACa07	X	####	.	T	G	.	PA!	.	intergen	NOI	dist=	.	.	
ACa07	X	####	.	A	C	.	PA!	.	intergen	NOI	dist=	.	.	
ACa07	X	####	.	G	A	.	PA!	.	intergen	NOI	dist=	.	.	
ACa07	X	####	.	C	A	.	PA!	.	intergen	NM_	dist=	.	.	
ACa07	X	####	.	C	A	.	PA!	STARD8	intronic	NM_	.	.	.	
ACa07	X	####	rs7687527	G	A	.	PA!	.	intergen	NM_	dist=	.	.	
ACa07	X	####	.	G	C	.	PA!	ARR3	intronic	NM_	.	.	.	
ACa07	X	####	rs7507300	C	T	.	PA!	MED12	exonic	NM_	.	mis	MED12	
ACa07	X	####	rs8632249	C	T	.	PA!	GJB1	exonic	NM_	.	mis	GJB1:N	
ACa07	X	####	rs7685308	G	A	.	PA!	BCYRN1	ncRNA_	NR_	.	.	.	
ACa07	X	####	.	C	A	.	PA!	BCYRN1	ncRNA_	NR_	.	.	.	
ACa07	X	####	.	G	A	.	PA!	HDAC8	exonic	NM_	.	mis	HDAC8	
ACa07	X	####	.	C	T	.	PA!	PHKA1	exonic	NM_	.	mis	PHKA1	
ACa07	X	####	.	A	G	.	PA!	CDX4	exonic	NM_	.	syr	CDX4:N	
ACa07	X	####	.	G	A	.	PA!	.	intergen	NM_	dist=	.	.	
ACa07	X	####	.	G	T	.	PA!	.	intergen	NM_	dist=	.	.	
ACa07	X	####	.	C	A	.	PA!	KIAA2022	exonic	NM_	.	sto	KIAA20	
ACa07	X	####	.	A	G	.	PA!	.	intergen	NM_	dist=	.	.	
ACa07	X	####	.	C	T	.	PA!	BRWD3	exonic	NM_	.	mis	BRWD3	
ACa07	X	####	.	G	A	.	PA!	PCDH11X	exonic	NM_	.	mis	PCDH1	
ACa07	X	####	.	C	A	.	PA!	PCDH11X	exonic	NM_	.	mis	PCDH1	
ACa07	X	####	.	C	T	.	PA!	PCDH11X	intronic	NM_	.	.	.	
ACa07	X	####	.	T	C	.	PA!	PCDH11X	intronic	NM_	.	.	.	
ACa07	X	####	rs9672698	G	A	.	PA!	.	intergen	NR_	dist=	.	.	
ACa07	X	####	.	G	A	.	PA!	NOX1	exonic	NM_	.	mis	NOX1:N	
ACa07	X	####	.	C	A	.	PA!	NXF5	exonic	NM_	.	mis	NXF5:N	
ACa07	X	####	.	C	T	.	PA!	TCEAL2	intronic	NM_	.	.	.	

ACa07	X	####	rs1507941	C	T	.	PA!	IL1RAPL2	intronic	NM_	.	.	.
ACa07	X	####	.	G	A	.	PA!	ACSL4	UTR3	NM_	NM_	.	.
ACa07	X	####	.	C	A	.	PA!	LINC0128	ncRNA_	NR_	.	.	.
ACa07	X	####	rs8664003	T	C	.	PA!	.	intergen	NR_	dist=	.	.
ACa07	X	####	.	C	T	.	PA!	SLC25A5-	ncRNA_	NR_	.	.	.
ACa07	X	####	.	C	T	.	PA!	CUL4B	intronic	NM_	.	.	.
ACa07	X	####	.	G	A	.	PA!	.	intergen	NM_	dist=	.	.
ACa07	X	####	rs1387255	C	T	.	PA!	ACTRT1	exonic	NM_	.	mis	ACTRT
ACa07	X	####	.	A	G	.	PA!	ZNF280C	intronic	NM_	.	.	.
ACa07	X	####	.	C	A	.	PA!	GPC3	intronic	NM_	.	.	.
ACa07	X	####	.	C	T	.	PA!	SLC9A6	intronic	NM_	.	.	.
ACa07	X	####	.	G	T	.	PA!	GPR112	intronic	NM_	.	.	.
ACa07	X	####	.	A	G	.	PA!	ATP11C	exonic	NM_	.	syr	ATP11C
ACa07	X	####	.	G	A	.	PA!	LDOC1,SF	intergen	NM_	dist=	.	.
ACa07	X	####	.	C	T	.	PA!	MAGEA11	exonic	NM_	.	syr	MAGEA
ACa07	X	####	rs7823749	C	T	.	PA!	CXorf40B	UTR3	NM_	NM_	.	.
ACa07	X	####	.	G	A	.	PA!	CNGA2	exonic	NM_	.	mis	CNGA2
ACa07	X	####	rs3712627	C	T	.	PA!	.	intergen	NM_	dist=	.	.
ACa07	X	####	.	G	T	.	PA!	BGN	exonic	NM_	.	mis	BGN:NI
ACa07	X	####	.	G	A	.	PA!	ATP2B3	intronic	NM_	.	.	.
ACa07	X	####	rs7825278	G	A	.	PA!	SSR4	intronic	NM_	.	.	.
ACa07	X	####	.	G	A	.	PA!	L1CAM	intronic	NM_	.	.	.
ACa07	X	####	.	G	A	.	PA!	L1CAM	intronic	NM_	.	.	.
ACa07	X	####	.	G	A	.	PA!	RENBP	splicing	NM_	NM_	.	.
ACa07	X	####	.	C	T	.	PA!	HCFC1	intronic	NM_	.	.	.
ACa07	X	####	.	G	A	.	PA!	SLC10A3	exonic	NM_	.	syr	SLC10A
ACa07	X	####	rs7819487	C	T	.	PA!	G6PD	exonic	NM_	.	mis	G6PD:N
ACa07	X	####	.	C	T	.	PA!	DKC1	intronic	NM_	.	.	.
ACa07	X	####	.	G	A	.	PA!	MPP1	UTR5	NM_	NM_	.	.
ACa07	X	####	.	G	A	.	PA!	SMIM9	UTR5	NM_	NM_	.	.
ACa07	X	####	.	A	G	.	PA!	TMLHE	exonic	NM_	.	mis	TMLHE
ACa07	Y	####	.	C	T	.	PA!	.	intergen	NOI	dist=	.	.
ACa07	MT	9459	rs8791767	C	T	.	PA!	.	intergen	NOI	dist=	.	.
ACa07	MT	####	.	A	T	.	PA!	.	intergen	NOI	dist=	.	.
ACa07	MT	####	.	T	C	.	PA!	.	intergen	NOI	dist=	.	.
ACaP01	1	####	rs5750552	C	T	.	PA!	NECAP2	intronic	NM_	.	.	.
ACaP01	1	####	rs4101081	C	T	.	PA!	NBPF1	intronic	NM_	.	.	.
ACaP01	1	####	rs7629862	A	C	.	PA!	NBPF1	intronic	NM_	.	.	.
ACaP01	1	####	.	T	C	.	PA!	.	intergen	NR_	dist=	.	.
ACaP01	1	####	.	C	A	.	PA!	EIF4G3	exonic	NM_	.	mis	EIF4G3
ACaP01	1	####	rs3772379	G	C	.	PA!	.	intergen	NR_	dist=	.	.
ACaP01	1	####	rs3720400	G	T	.	PA!	.	intergen	NR_	dist=	.	.
ACaP01	1	####	rs6181337	T	C	.	PA!	NBPF25P	ncRNA_	NR_	.	.	.
ACaP01	1	####	.	G	T	.	PA!	.	upstreal	NM_	.	.	.
ACaP01	2	####	.	G	T	.	PA!	ELMOD3	intronic	NM_	.	.	.
ACaP01	2	####	rs1649505	G	C	.	PA!	.	intergen	NOI	dist=	.	.
ACaP01	3	####	rs7933313	G	A	.	PA!	EPHA6	exonic	NM_	.	syr	EPHA6
ACaP01	3	####	rs7480655	C	T	.	PA!	COL6A6	exonic	NM_	.	syr	COL6A(
ACaP01	3	####	.	C	A	.	PA!	TOPBP1	exonic	NM_	.	mis	TOPBP
ACaP01	4	####	.	C	T	.	PA!	.	intergen	NM_	dist=	.	.
ACaP01	5	####	rs671688	A	G	.	PA!	.	intergen	NM_	dist=	.	.
ACaP01	5	####	rs8681576	G	T	.	PA!	.	intergen	NR_	dist=	.	.
ACaP01	5	####	.	T	C	.	PA!	CLK4	exonic	NM_	.	syr	CLK4:N
ACaP01	6	####	.	A	G	.	PA!	SASH1	intronic	NM_	.	.	.

ACaP01	7	####	.	T	C	.	PA!	AMPH	intronic	NM_	.	.	.
ACaP01	7	####	.	C	T	.	PA!	.	intergen	NR_	dist=	.	.
ACaP01	7	####	.	C	T	.	PA!	TFEC	splicing	NM_	NM_	.	.
ACaP01	7	####	rs1471823	G	A	.	PA!	GIMAP5,G	exonic	NM_	.	mis	GIMAP!
ACaP01	8	####	rs7496607	G	A	.	PA!	XPO7	intronic	NM_	.	.	.
ACaP01	8	####	.	C	T	.	PA!	PIWIL2	exonic	NM_	.	syr	PIWIL2
ACaP01	9	####	rs1482733	G	T	.	PA!	.	intergen	NM_	dist=	.	.
ACaP01	9	####	.	G	T	.	PA!	WDR34	intronic	NM_	.	.	.
ACaP01	10	####	rs2020143	T	C	.	PA!	AGAP9	exonic	NM_	.	mis	AGAP9
ACaP01	10	####	.	A	C	.	PA!	HPSE2	exonic	NM_	.	mis	HPSE2
ACaP01	11	####	rs7731324	G	A	.	PA!	CKAP5	exonic	NM_	.	mis	CKAP5
ACaP01	11	####	.	A	T	.	PA!	PRG2	intronic	NM_	.	.	.
ACaP01	12	####	rs7734351	G	A	.	PA!	KMT2D	exonic	NM_	.	syr	KMT2D
ACaP01	12	####	rs7769669	C	T	.	PA!	KRT7	exonic	NM_	.	mis	KRT7:N
ACaP01	14	####	.	G	A	.	PA!	JAG2	exonic	NM_	.	syr	JAG2:N
ACaP01	14	####	rs7157981	C	T	.	PA!	.	intergen	NR_	dist=	.	.
ACaP01	15	####	rs2019940	T	G	.	PA!	GOLGA6L	exonic	NM_	.	mis	GOLGA
ACaP01	15	####	rs3731461	A	G	.	PA!	GOLGA6L	exonic	NM_	.	syr	GOLGA
ACaP01	15	####	.	C	A	.	PA!	.	downstr	NR_	.	.	.
ACaP01	15	####	.	A	G	.	PA!	.	intergen	NM_	dist=	.	.
ACaP01	15	####	.	C	A	.	PA!	KIF23	intronic	NM_	.	.	.
ACaP01	16	####	.	C	T	.	PA!	IL21R	intronic	NM_	.	.	.
ACaP01	17	####	rs7781206	T	C	.	PA!	RPAIN	intronic	NM_	.	.	.
ACaP01	17	####	rs3725436	A	C	.	PA!	.	intergen	NM_	dist=	.	.
ACaP01	19	####	rs7861545	G	A	.	PA!	RHPN2	exonic	NM_	.	sto	RHPN2
ACaP01	19	####	.	C	T	.	PA!	FCGBP	exonic	NM_	.	mis	FCGBP
ACaP01	19	####	.	C	G	.	PA!	AXL	intronic	NM_	.	.	.
ACaP01	20	####	.	A	T	.	PA!	RP5-977B	ncRNA_	NR_	.	.	.
ACaP01	21	####	rs9266067	A	G	.	PA!	.	intergen	NM_	dist=	.	.
ACaP01	X	####	rs7663121	G	A	.	PA!	DMD	exonic	NM_	.	syr	DMD:N
ACaP01	X	####	rs3397828	A	G	.	PA!	BCYRN1	ncRNA_	NR_	.	.	.
ACaP02	1	####	.	G	A	.	PA!	SPEN	intronic	NM_	.	.	.
ACaP02	1	####	rs436293	C	T	.	PA!	NBPF1	intronic	NM_	.	.	.
ACaP02	1	####	.	G	T	.	PA!	COL16A1	intronic	NM_	.	.	.
ACaP02	1	####	.	G	C	.	PA!	CCDC28B	UTR3	NM_	NM_	.	.
ACaP02	1	####	rs3721234	C	T	.	PA!	SASS6	intronic	NM_	.	.	.
ACaP02	1	####	.	G	A	.	PA!	FNDC7	intronic	NM_	.	.	.
ACaP02	1	####	rs2007574	G	C	.	PA!	NBPF20,N	intronic	NM_	.	.	.
ACaP02	1	####	rs9424724	G	C	.	PA!	NBPF25P	ncRNA_	NR_	.	.	.
ACaP02	1	####	.	T	G	.	PA!	.	intergen	NM_	dist=	.	.
ACaP02	1	####	.	G	A	.	PA!	TMEM79	exonic	NM_	.	syr	TMEM7
ACaP02	1	####	.	G	C	.	PA!	C1orf85	exonic	NM_	.	mis	C1orf85
ACaP02	1	####	.	C	G	.	PA!	SPTA1	intronic	NM_	.	.	.
ACaP02	1	####	.	G	C	.	PA!	DARC	UTR5	NM_	NM_	.	.
ACaP02	1	####	.	C	T	.	PA!	DCAF8	exonic	NM_	.	mis	DCAF8
ACaP02	1	####	.	G	C	.	PA!	FCRLB	intronic	NM_	.	.	.
ACaP02	1	####	.	C	T	.	PA!	SHCBP1L	exonic	NM_	.	mis	SHCBP
ACaP02	1	####	rs9662955	G	A	.	PA!	CFHR5	intronic	NM_	.	.	.
ACaP02	1	####	rs7570105	G	A	.	PA!	WNT3A	exonic	NM_	.	mis	WNT3A
ACaP02	1	####	.	C	G	.	PA!	KIAA1804	exonic	NM_	.	syr	KIAA18
ACaP02	1	####	.	G	A	.	PA!	MTR	intronic	NM_	.	.	.
ACaP02	1	####	.	C	T	.	PA!	FMN2	UTR3	NM_	NM_	.	.
ACaP02	2	####	.	C	T	.	PA!	GALM	intronic	NM_	.	.	.
ACaP02	2	####	rs1420196	G	A	.	PA!	ABCG5	exonic	NM_	.	mis	ABCG5

ACaP02	2	####	.	T	C	.	PA:	LYPD6B	intronic	NM_	.	.	.
ACaP02	2	####	.	C	T	.	PA:	TANC1	exonic	NM_	.	mis	TANC1
ACaP02	2	####	.	C	G	.	PA:	SLC39A1(	exonic	NM_	.	mis	SLC39/
ACaP02	2	####	rs1857895	C	T	.	PA:	HSPD1	intronic	NM_	.	.	.
ACaP02	2	####	.	G	C	.	PA:	KLF7	intronic	NM_	.	.	.
ACaP02	2	####	.	G	T	.	PA:	WDFY1	intronic	NM_	.	.	.
ACaP02	2	####	.	G	C	.	PA:	MROH2A	exonic	NM_	.	mis	MROH2
ACaP02	2	####	rs7784676	G	A	.	PA:	STK25	exonic	NM_	.	mis	STK25:
ACaP02	3	####	.	G	C	.	PA:	UBP1	intronic	NM_	.	.	.
ACaP02	3	####	.	A	G	.	PA:	CRYBG3	intronic	NM_	.	.	.
ACaP02	3	####	.	G	T	.	PA:	CEP63	exonic	NM_	.	mis	CEP63:
ACaP02	3	####	.	G	A	.	PA:	CEP63	exonic	NM_	.	mis	CEP63:
ACaP02	3	####	.	G	C	.	PA:	ARMC8,N	intronic	NM_	.	.	.
ACaP02	3	####	rs7462149	G	A	.	PA:	GOLIM4	exonic	NM_	.	sto	GOLIM
ACaP02	3	####	.	G	C	.	PA:	TNFSF10	UTR5	NM_	NM_	.	.
ACaP02	3	####	rs1048860	G	A	.	PA:	PIK3CA	exonic	NM_	.	mis	PIK3CA
ACaP02	3	####	.	A	C	.	PA:	YEATS2	exonic	NM_	.	mis	YEATS:
ACaP02	4	####	.	C	T	.	PA:	TMEM175	intronic	NM_	.	.	.
ACaP02	4	####	.	G	A	.	PA:	SMARCAI	exonic	NM_	.	mis	SMARC
ACaP02	4	####	.	C	T	.	PA:	CYP2U1	intronic	NM_	.	.	.
ACaP02	4	####	.	C	T	.	PA:	NAA15	exonic	NM_	.	mis	NAA15:
ACaP02	4	####	.	C	G	.	PA:	INPP4B	exonic	NM_	.	mis	INPP4E
ACaP02	4	####	.	C	T	.	PA:	.	downstr	NR_	.	.	.
ACaP02	5	####	rs1876921	G	C	.	PA:	SLC45A2	intronic	NM_	.	.	.
ACaP02	5	####	.	G	C	.	PA:	RNF180	exonic	NM_	.	mis	RNF18(
ACaP02	5	####	.	C	T	.	PA:	F2R	exonic	NM_	.	mis	F2R:NM
ACaP02	5	####	rs7956159	C	T	.	PA:	SEC24A	exonic	NM_	.	mis	SEC24/
ACaP02	5	####	.	C	G	.	PA:	FAM71B	exonic	NM_	.	mis	FAM71I
ACaP02	5	####	.	G	A	.	PA:	HMMR	exonic	NM_	.	syr	HMMR:
ACaP02	5	####	.	G	A	.	PA:	KCNIP1	exonic	NM_	.	mis	KCNIP1
ACaP02	6	####	.	C	A	.	PA:	MYLK4	intronic	NM_	.	.	.
ACaP02	6	####	.	A	G	.	PA:	SYCP2L	exonic	NM_	.	mis	SYCP2I
ACaP02	6	####	.	G	A	.	PA:	BTN1A1	intronic	NM_	.	.	.
ACaP02	6	####	rs1787910	C	T	.	PA:	HLA-A	intronic	NM_	.	.	.
ACaP02	6	####	rs5364899	A	G	.	PA:	HLA-L	ncRNA_	NR_	.	.	.
ACaP02	6	####	.	C	G	.	PA:	EYS	exonic	NM_	.	mis	EYS:NM
ACaP02	6	####	.	C	T	.	PA:	EYS	exonic	NM_	.	mis	EYS:NM
ACaP02	6	####	.	C	T	.	PA:	.	intergen	NM_	dist=	.	.
ACaP02	6	####	.	C	G	.	PA:	KIF25	intronic	NM_	.	.	.
ACaP02	7	####	.	G	C	.	PA:	TNRC18	UTR3	NM_	NM_	.	.
ACaP02	7	####	.	C	A	.	PA:	.	intergen	NR_	dist=	.	.
ACaP02	7	####	.	C	A	.	PA:	ZNF804B	intronic	NM_	.	.	.
ACaP02	7	####	.	C	T	.	PA:	.	downstr	NR_	.	.	.
ACaP02	7	####	rs3685111	G	C	.	PA:	CREB3L2	intronic	NM_	.	.	.
ACaP02	8	####	.	G	A	.	PA:	KIF13B	intronic	NM_	.	.	.
ACaP02	8	####	.	A	T	.	PA:	PREX2	exonic	NM_	.	mis	PREX2
ACaP02	8	####	.	G	A	.	PA:	SNTB1	UTR3	NM_	NM_	.	.
ACaP02	8	####	.	C	T	.	PA:	NAPRT1	exonic	NM_	.	mis	NAPRT
ACaP02	8	####	rs3743103	C	T	.	PA:	PLEC	exonic	NM_	.	mis	PLEC:N
ACaP02	9	####	rs4928838	C	T	.	PA:	.	intergen	NM_	dist=	.	.
ACaP02	9	####	.	G	A	.	PA:	.	intergen	NM_	dist=	.	.
ACaP02	9	####	.	G	A	.	PA:	PHF2	exonic	NM_	.	syr	PHF2:N
ACaP02	9	####	.	C	T	.	PA:	AKNA	intronic	NM_	.	.	.
ACaP02	9	####	.	T	A	.	PA:	DDX31	intronic	NM_	.	.	.

ACaP02	10	####	.	C	T	.	PAS	DIP2C	exonic	NM_	.	mis	DIP2C:I
ACaP02	10	####	.	G	A	.	PAS	PFKP	intronic	NM_	.	.	.
ACaP02	10	####	rs3128226	C	G	.	PAS	NUTM2A	intronic	NM_	.	.	.
ACaP02	10	####	.	G	C	.	PAS	KNDC1	intronic	NM_	.	.	.
ACaP02	11	####	.	C	T	.	PAS	.	intergen	NM_	dist=	.	.
ACaP02	11	####	.	C	G	.	PAS	NAV2	intronic	NM_	.	.	.
ACaP02	11	####	.	C	T	.	PAS	GYLTL1B	exonic	NM_	.	syr	GYLTL1
ACaP02	11	####	.	G	A	.	PAS	.	upstrear	NM_	.	.	.
ACaP02	11	####	.	G	A	.	PAS	TENM4	exonic	NM_	.	syr	TENM4
ACaP02	11	####	.	G	A	.	PAS	SIK3	intronic	NM_	.	.	.
ACaP02	11	####	.	G	A	.	PAS	.	upstrear	NM_	.	.	.
ACaP02	12	####	rs5382343	T	C	.	PAS	.	intergen	NOI	dist=	.	.
ACaP02	12	####	.	A	G	.	PAS	ATF7IP	exonic	NM_	.	mis	ATF7IP
ACaP02	12	####	.	C	G	.	PAS	SMCO2	exonic	NM_	.	mis	SMCO2
ACaP02	12	####	rs8658204	T	A	.	PAS	XPOT	intronic	NM_	.	.	.
ACaP02	12	####	.	C	T	.	PAS	PTPRQ	intronic	NM_	.	.	.
ACaP02	12	####	.	G	A	.	PAS	NAA25	exonic	NM_	.	syr	NAA25:
ACaP02	12	####	rs7707492	G	A	.	PAS	NOS1	exonic	NM_	.	mis	NOS1:N
ACaP02	13	####	.	T	A	.	PAS	UBL3	intronic	NM_	.	.	.
ACaP02	13	####	rs3702294	G	A	.	PAS	FRY	exonic	NM_	.	mis	FRY:NM
ACaP02	13	####	rs4129275	C	T	.	PAS	FREM2	exonic	NM_	.	mis	FREM2
ACaP02	13	####	.	C	G	.	PAS	FREM2	exonic	NM_	.	mis	FREM2
ACaP02	13	####	.	C	T	.	PAS	PCDH20	exonic	NM_	.	mis	PCDH2
ACaP02	13	####	.	C	G	.	PAS	SLAIN1	intronic	NM_	.	.	.
ACaP02	13	####	.	C	G	.	PAS	SLAIN1	exonic	NM_	.	mis	SLAIN1
ACaP02	13	####	.	G	A	.	PAS	CCDC168	exonic	NM_	.	mis	CCDC1
ACaP02	14	####	rs3699116	C	T	.	PAS	.	intergen	NM_	dist=	.	.
ACaP02	14	####	rs7660001	C	T	.	PAS	RALGAPA	ncRNA_	NR_	.	.	.
ACaP02	14	####	.	C	T	.	PAS	BTBD7	exonic	NM_	.	syr	BTBD7:
ACaP02	14	####	.	G	A	.	PAS	CLMN	splicing	NM_	NM_	.	.
ACaP02	14	####	rs1214345	C	T	.	PAS	AKT1	exonic	NM_	.	mis	AKT1:N
ACaP02	14	####	.	G	A	.	PAS	.	intergen	NR_	dist=	.	.
ACaP02	15	####	rs4932010	G	A	.	PAS	HERC2P3	ncRNA_	NR_	.	.	.
ACaP02	15	####	rs7494426	C	G	.	PAS	.	intergen	NR_	dist=	.	.
ACaP02	15	####	rs1079463	T	C	.	PAS	CXADRP2	ncRNA_	NR_	.	.	.
ACaP02	15	####	rs1823548	G	C	.	PAS	LOC33901	intronic	NM_	.	.	.
ACaP02	15	####	.	G	A	.	PAS	.	downstr	NR_	.	.	.
ACaP02	15	####	rs1861770	C	T	.	PAS	KNSTRN	intronic	NM_	.	.	.
ACaP02	15	####	rs9953164	G	A	.	PAS	PATL2	exonic	NM_	.	mis	PATL2:
ACaP02	15	####	.	G	C	.	PAS	HMGN2P4	ncRNA_	NR_	.	.	.
ACaP02	15	####	.	G	A	.	PAS	GRAMD2	intronic	NM_	.	.	.
ACaP02	15	####	.	G	A	.	PAS	ACSBG1	intronic	NM_	.	.	.
ACaP02	15	####	.	C	T	.	PAS	.	intergen	NM_	dist=	.	.
ACaP02	16	####	.	C	T	.	PAS	CCDC64B	exonic	NM_	.	mis	CCDC6
ACaP02	16	####	.	G	A	.	PAS	.	intergen	NR_	dist=	.	.
ACaP02	16	####	rs7759203	C	T	.	PAS	OGFOD1	exonic	NM_	.	mis	OGFOD
ACaP02	16	####	.	C	T	.	PAS	VAC14-AS	ncRNA_	NR_	.	.	.
ACaP02	17	####	.	G	C	.	PAS	C17orf107	exonic	NM_	.	mis	C17orf1
ACaP02	17	####	rs7810711	C	T	.	PAS	GPR179	exonic	NM_	.	mis	GPR179
ACaP02	17	####	rs1509595	G	A	.	PAS	DBF4B	exonic	NM_	.	syr	DBF4B:
ACaP02	17	####	.	G	A	.	PAS	HOXB8	exonic	NM_	.	syr	HOXB8
ACaP02	17	####	rs3742496	C	T	.	PAS	SYNGR2	intronic	NM_	.	.	.
ACaP02	17	####	rs5611679	C	T	.	PAS	LOC10029	ncRNA_	NR_	.	.	.
ACaP02	17	####	rs5502840	G	A	.	PAS	NOTUM	exonic	NM_	.	syr	NOTUM

ACaP02	18	####	rs1996954	C	T	.	PA:	ANKRD20	ncRNA_	NR_	.	.	.
ACaP02	18	####	.	G	C	.	PA:	ALPK2	UTR3	NM_	NM_	.	.
ACaP02	19	####	.	C	G	.	PA:	.	intergen	NM_	dist=	.	.
ACaP02	19	####	.	G	A	.	PA:	LSM4	intronic	NM_	.	.	.
ACaP02	19	####	.	G	T	.	PA:	SUGP1	exonic	NM_	.	mis	SUGP1
ACaP02	19	####	.	C	T	.	PA:	ZNF506	exonic	NM_	.	mis	ZNF506
ACaP02	19	####	.	C	T	.	PA:	ETV2	intronic	NM_	.	.	.
ACaP02	19	####	.	C	G	.	PA:	ZNF568	exonic	NM_	.	sto	ZNF568
ACaP02	19	####	.	G	A	.	PA:	TTC9B	exonic	NM_	.	syr	TTC9B:
ACaP02	19	####	.	G	C	.	PA:	PSG2	intronic	NM_	.	.	.
ACaP02	19	####	.	C	G	.	PA:	RDH13	intronic	NM_	.	.	.
ACaP02	20	####	.	G	C	.	PA:	TMEM74B	intronic	NM_	.	.	.
ACaP02	20	####	.	G	A	.	PA:	TGM6	exonic	NM_	.	syr	TGM6:N
ACaP02	20	####	rs1438597	G	A	.	PA:	CDS2	exonic	NM_	.	mis	CDS2:N
ACaP02	20	####	.	G	C	.	PA:	PDRG1	intronic	NM_	.	.	.
ACaP02	20	####	.	T	C	.	PA:	TGM2	intronic	NM_	.	.	.
ACaP02	21	####	rs1170118	T	C	.	PA:	BAGE4,BA	intronic	NM_	.	.	.
ACaP02	21	####	.	C	G	.	PA:	USP25	exonic	NM_	.	sto	USP25:
ACaP02	22	####	.	G	C	.	PA:	ARVCF	intronic	NM_	.	.	.
ACaP02	22	####	.	C	T	.	PA:	OSBP2	exonic	NM_	.	mis	OSBP2
ACaP02	22	####	.	C	A	.	PA:	EIF3L	exonic	NM_	.	syr	EIF3L:N
ACaP02	X	####	rs7509188	G	A	.	PA:	ARSF	exonic	NM_	.	mis	ARSF:N
ACaP02	X	####	.	G	A	.	PA:	ARX	exonic	NM_	.	mis	ARX:NM
ACaP02	X	####	.	T	A	.	PA:	.	intergen	NR_	dist=	.	.
ACaP02	X	####	.	G	A	.	PA:	KIF4A	exonic	NM_	.	mis	KIF4A:N
ACaP02	X	####	.	G	C	.	PA:	NKAP	exonic	NM_	.	mis	NKAP:N
ACaP02	X	####	.	T	A	.	PA:	SRD5A1P	ncRNA_	NR_	.	.	.
ACaP02	X	####	.	T	A	.	PA:	SRD5A1P	ncRNA_	NR_	.	.	.
ACaP02	X	####	.	G	C	.	PA:	GABRA3	intronic	NM_	.	.	.
ACaP02	X	####	.	C	T	.	PA:	IRAK1	UTR3	NM_	NM_	.	.
ACaP02	MT	####	.	T	C	.	PA:	.	intergen	NOI	dist=	.	.
ACaP03	1	####	rs1149286	T	C	.	PA:	SLC35E2	intronic	NM_	.	.	.
ACaP03	1	####	.	C	A	.	PA:	TMEM57	exonic	NM_	.	mis	TMEM5
ACaP03	1	####	.	C	T	.	PA:	FPGT-TNI	intronic	NM_	.	.	.
ACaP03	1	####	.	G	A	.	PA:	COL24A1	exonic	NM_	.	mis	COL24A
ACaP03	1	####	rs1998147	T	C	.	PA:	FCGR1B	UTR3	NM_	NM_	.	.
ACaP03	1	####	rs8662584	G	T	.	PA:	.	intergen	NR_	dist=	.	.
ACaP03	2	####	.	T	A	.	PA:	SPTBN1	intronic	NM_	.	.	.
ACaP03	2	####	.	G	A	.	PA:	ETAA1	exonic	NM_	.	mis	ETAA1:
ACaP03	2	####	.	C	G	.	PA:	TMSB10	intronic	NM_	.	.	.
ACaP03	2	####	rs3708851	C	T	.	PA:	GLI2	exonic	NM_	.	syr	GLI2:NI
ACaP03	2	####	rs2008806	T	A	.	PA:	R3HDM1	intronic	NM_	.	.	.
ACaP03	2	####	rs9042902	C	T	.	PA:	STK39	intronic	NM_	.	.	.
ACaP03	2	####	rs3702772	C	T	.	PA:	IGFBP5	intronic	NM_	.	.	.
ACaP03	2	####	rs5574456	G	A	.	PA:	TRPM8	intronic	NM_	.	.	.
ACaP03	3	####	rs1446637	C	T	.	PA:	MIR548AC	ncRNA_	NR_	.	.	.
ACaP03	3	####	.	G	T	.	PA:	.	intergen	NM_	dist=	.	.
ACaP03	3	####	.	C	T	.	PA:	GRAMD1C	exonic	NM_	.	syr	GRAMD
ACaP03	3	####	rs2015548	C	T	.	PA:	DRD3	exonic	NM_	.	mis	DRD3:N
ACaP03	4	####	rs1163261	C	T	.	PA:	RAB28	intronic	NM_	.	.	.
ACaP03	4	####	rs4695484	C	G	.	PA:	.	intergen	NM_	dist=	.	.
ACaP03	4	####	rs4695485	G	T	.	PA:	.	intergen	NM_	dist=	.	.
ACaP03	4	####	rs7496227	C	T	.	PA:	SMR3B	exonic	NM_	.	syr	SMR3B
ACaP03	4	####	rs7758415	G	A	.	PA:	PDLIM3	exonic	NM_	.	mis	PDLIM3

ACaP03	5	####	.	C	T	.	PA:	PCDHA6	exonic	NM_	.	syr	PCDHA
ACaP03	6	####	rs1161907	C	T	.	PA:	EHMT2	intronic	NM_	.	.	.
ACaP03	6	####	.	G	T	.	PA:	SENP6	intronic	NM_	.	.	.
ACaP03	6	####	.	T	A	.	PA:	.	intergen	NM_	dist=	.	.
ACaP03	6	####	rs7577542	C	T	.	PA:	CDK19	UTR3	NM_	NM_	.	.
ACaP03	7	####	.	G	A	.	PA:	RBAKDN	ncRNA_	NR_	.	.	.
ACaP03	7	####	.	C	A	.	PA:	ABCB1	exonic	NM_	.	mis	ABCB1
ACaP03	7	####	.	T	C	.	PA:	FAM185A	intronic	NM_	.	.	.
ACaP03	8	####	rs7722351	C	T	.	PA:	PCM1	exonic	NM_	.	syr	PCM1:N
ACaP03	8	####	.	G	T	.	PA:	PTK2	intronic	NM_	.	.	.
ACaP03	9	####	rs7507213	G	A	.	PA:	TRAF2	exonic	NM_	.	mis	TRAF2:
ACaP03	10	####	.	T	A	.	PA:	LINC0071	ncRNA_	NR_	.	.	.
ACaP03	10	####	rs7571508	G	A	.	PA:	ITGA8	exonic	NM_	.	syr	ITGA8:I
ACaP03	10	####	rs5655237	C	T	.	PA:	.	intergen	NR_	dist=	.	.
ACaP03	10	####	rs7905448	T	A	.	PA:	MCU	intronic	NM_	.	.	.
ACaP03	10	####	.	G	A	.	PA:	LINC0086	ncRNA_	NR_	.	.	.
ACaP03	10	####	rs9706750	C	T	.	PA:	LOC1005(	ncRNA_	NR_	.	.	.
ACaP03	10	####	rs7723659	A	G	.	PA:	SFXN2	intronic	NM_	.	.	.
ACaP03	10	####	.	G	A	.	PA:	INA	exonic	NM_	.	syr	INA:NM
ACaP03	10	####	rs1219134	A	G	.	PA:	FGFR2	exonic	NM_	.	mis	FGFR2
ACaP03	11	####	.	G	A	.	PA:	DEAF1	exonic	NM_	.	mis	DEAF1:
ACaP03	11	####	rs5453728	G	A	.	PA:	USH1C	exonic	NM_	.	syr	USH1C
ACaP03	11	####	.	A	G	.	PA:	USH1C	exonic	NM_	.	syr	USH1C
ACaP03	11	####	.	T	C	.	PA:	ARHGEF1	intronic	NM_	.	.	.
ACaP03	12	####	rs1030864	T	C	.	PA:	OAS1	exonic	NM_	.	mis	OAS1:N
ACaP03	13	####	.	G	A	.	PA:	RB1	exonic	NM_	.	syr	RB1:NM
ACaP03	13	####	rs9145408	C	T	.	PA:	.	intergen	NM_	dist=	.	.
ACaP03	13	####	rs9816818	A	G	.	PA:	.	upstrea	NM_	.	.	.
ACaP03	14	####	.	G	T	.	PA:	.	upstrea	NM_	.	.	.
ACaP03	14	####	.	C	A	.	PA:	NPAS3	intronic	NM_	.	.	.
ACaP03	14	####	rs5634662	G	A	.	PA:	PRPF39	exonic	NM_	.	syr	PRPF3!
ACaP03	14	####	.	G	A	.	PA:	ERO1L	intronic	NM_	.	.	.
ACaP03	14	####	.	G	A	.	PA:	EML5	exonic	NM_	.	mis	EML5:N
ACaP03	14	####	.	C	A	.	PA:	HHIPL1	exonic	NM_	.	mis	HHIPL1
ACaP03	15	####	.	G	T	.	PA:	.	intergen	NOI	dist=	.	.
ACaP03	15	####	rs7780894	G	A	.	PA:	.	intergen	NR_	dist=	.	.
ACaP03	15	####	.	T	C	.	PA:	MGA	exonic	NM_	.	mis	MGA:N
ACaP03	15	####	.	C	A	.	PA:	TTBK2	exonic	NM_	.	mis	TTBK2:
ACaP03	16	####	.	C	T	.	PA:	SLC9A5	exonic	NM_	.	syr	SLC9A!
ACaP03	17	####	.	A	T	.	PA:	CCDC42	intronic	NM_	.	.	.
ACaP03	17	####	rs5864828	G	C	.	PA:	FLJ36000	ncRNA_	NR_	.	.	.
ACaP03	17	####	rs7798230	G	A	.	PA:	TTLL6	intronic	NM_	.	.	.
ACaP03	18	####	.	C	A	.	PA:	EPB41L3	intronic	NM_	.	.	.
ACaP03	19	####	rs7477304	G	A	.	PA:	CACNA1A	exonic	NM_	.	syr	CACNA
ACaP03	19	####	.	C	A	.	PA:	OR7A10	exonic	NM_	.	syr	OR7A1(
ACaP03	19	####	rs9472013	T	C	.	PA:	ZNF181	intronic	NM_	.	.	.
ACaP03	19	####	.	C	T	.	PA:	APLP1	exonic	NM_	.	mis	APLP1:
ACaP03	19	####	.	C	A	.	PA:	KCNN4	intronic	NM_	.	.	.
ACaP03	19	####	rs3718581	C	T	.	PA:	KLK11	intronic	NM_	.	.	.
ACaP03	19	####	rs1056285	C	T	.	PA:	.	intergen	NM_	dist=	.	.
ACaP03	20	####	rs5584781	T	A	.	PA:	TMC2	intronic	NM_	.	.	.
ACaP03	20	####	rs6037279	C	A	.	PA:	.	intergen	NR_	dist=	.	.
ACaP03	20	####	.	G	C	.	PA:	CEP250	intronic	NM_	.	.	.
ACaP03	20	####	.	G	A	.	PA:	SLC12A5	UTR5	NM_	NM_	.	.

ACaP03	21	####	rs2013315	C	T	.	PA	TPTE	intronic	NM_	.	.	.
ACaP03	21	####	rs2001788	G	A	.	PA	TPTE	intronic	NM_	.	.	.
ACaP03	22	####	.	G	A	.	PA	PKDREJ	exonic	NM_	.	mis	PKDRE
ACaP03	X	####	.	G	T	.	PA	IRAK1	exonic	NM_	.	sto	IRAK1:I
ACaP03	MT	8177	.	G	A	.	PA	.	intergen	NOI	dist=	.	.
ACaP03	MT	####	.	G	A	.	PA	.	intergen	NOI	dist=	.	.
ACaP04	1	####	rs6263527	T	A	.	PA	.	intergen	NR_	dist=	.	.
ACaP04	1	####	.	C	A	.	PA	PODN	intronic	NM_	.	.	.
ACaP04	1	####	rs7457471	C	G	.	PA	.	intergen	NR_	dist=	.	.
ACaP04	1	####	.	T	C	.	PA	POU2F1	intronic	NM_	.	.	.
ACaP04	1	####	.	C	A	.	PA	NID1	intronic	NM_	.	.	.
ACaP04	2	####	.	G	C	.	PA	.	intergen	NR_	dist=	.	.
ACaP04	2	####	rs3711404	G	A	.	PA	.	intergen	NR_	dist=	.	.
ACaP04	2	####	.	A	T	.	PA	ITGA4	intronic	NM_	.	.	.
ACaP04	3	####	rs7758373	T	C	.	PA	SDHAP1	ncRNA_	NR_	.	.	.
ACaP04	4	####	.	T	A	.	PA	MXD4	UTR3	NM_	NM_	.	.
ACaP04	4	####	rs4694947	T	G	.	PA	.	intergen	NM_	dist=	.	.
ACaP04	9	####	.	T	C	.	PA	TRPM3	intronic	NM_	.	.	.
ACaP04	10	####	rs4070334	G	A	.	PA	.	intergen	NR_	dist=	.	.
ACaP04	12	####	rs9213947	C	A	.	PA	OSBPL8	intronic	NM_	.	.	.
ACaP04	14	####	rs1125033	G	A	.	PA	SOS2	intronic	NM_	.	.	.
ACaP04	14	####	.	A	C	.	PA	EIF2B2	intronic	NM_	.	.	.
ACaP04	15	####	rs2859043	G	A	.	PA	GOLGA6L	exonic	NM_	.	mis	GOLGA
ACaP04	15	####	.	G	T	.	PA	LOC28368	ncRNA_	NR_	.	.	.
ACaP04	18	####	.	G	A	.	PA	NOL4	intronic	NM_	.	.	.
ACaP05	1	####	.	A	C	.	PA	.	intergen	NR_	dist=	.	.
ACaP05	1	####	rs2787778	G	T	.	PA	.	intergen	NR_	dist=	.	.
ACaP05	1	####	rs2787777	C	T	.	PA	.	intergen	NR_	dist=	.	.
ACaP05	2	####	rs3711404	G	A	.	PA	.	intergen	NR_	dist=	.	.
ACaP05	5	####	rs1997973	G	T	.	PA	CDH12	UTR3	NM_	NM_	.	.
ACaP05	7	####	rs2018986	T	C	.	PA	.	intergen	NR_	dist=	.	.
ACaP05	7	####	.	G	T	.	PA	.	intergen	NR_	dist=	.	.
ACaP05	7	####	rs1390499	G	C	.	PA	POLR2J3	exonic	NM_	.	mis	POLR2
ACaP05	8	####	rs2077351	A	C	.	PA	.	intergen	NM_	dist=	.	.
ACaP05	9	####	rs1136487	C	A	.	PA	PTGER4P	ncRNA_	NR_	.	.	.
ACaP05	9	####	rs7648362	A	G	.	PA	PGM5P2	ncRNA_	NR_	.	.	.
ACaP05	9	####	.	G	C	.	PA	ZFAND5	intronic	NM_	.	.	.
ACaP05	10	####	rs1995206	C	T	.	PA	FAM21A,F	exonic	NM_	.	mis	FAM21I
ACaP05	11	####	rs3713518	C	G	.	PA	.	intergen	NR_	dist=	.	.
ACaP05	11	####	rs8790394	A	T	.	PA	MAPK8IP1	UTR3	NM_	NM_	.	.
ACaP05	17	####	rs1155611	C	A	.	PA	FLJ36000	ncRNA_	NR_	.	.	.
ACaP05	18	####	rs2018600	G	A	.	PA	SLC35G4	exonic	NM_	.	mis	SLC35C
ACaP05	21	####	.	G	A	.	PA	.	intergen	NR_	dist=	.	.
ACaP05	21	####	rs8792017	A	G	.	PA	.	intergen	NR_	dist=	.	.
ACaP05	21	####	rs1043983	A	T	.	PA	BAGE2,BA	intronic	NM_	.	.	.
ACaP05	X	####	rs1444677	G	C	.	PA	.	intergen	NM_	dist=	.	.
ACaP06	1	####	.	G	A	.	PA	FAM41C	ncRNA_	NR_	.	.	.
ACaP06	1	####	.	A	G	.	PA	NPHP4	intronic	NM_	.	.	.
ACaP06	1	####	.	C	A	.	PA	NPHP4	intronic	NM_	.	.	.
ACaP06	1	####	.	C	T	.	PA	TMEM201	exonic	NM_	.	mis	TMEM2
ACaP06	1	####	.	C	T	.	PA	PRAMEF1	intronic	NM_	.	.	.
ACaP06	1	####	.	G	T	.	PA	PRAMEF1	intronic	NM_	.	.	.
ACaP06	1	####	.	T	G	.	PA	PALMD	UTR5	NM_	NM_	.	.
ACaP06	1	####	rs3894786	T	C	.	PA	.	intergen	NR_	dist=	.	.

ACaP06	1	####	rs1112760	G	A	.	PA:	.	intergen	NR_	dist=	.	.
ACaP06	1	####	rs2017985	G	A	.	PA:	NBPF20	intronic	NM_	.	.	.
ACaP06	1	####	rs3713084	T	C	.	PA:	NBPF20,N	intronic	NM_	.	.	.
ACaP06	1	####	rs8799710	G	C	.	PA:	NBPF8,NE	intronic	NM_	.	.	.
ACaP06	1	####	rs6699421	A	G	.	PA:	NBPF25P	ncRNA_	NR_	.	.	.
ACaP06	1	####	rs8799669	C	A	.	PA:	NBPF25P	ncRNA_	NR_	.	.	.
ACaP06	1	####	.	C	G	.	PA:	OTUD7B	exonic	NM_	.	mis	OTUD7
ACaP06	1	####	.	C	G	.	PA:	C1orf189	intronic	NM_	.	.	.
ACaP06	1	####	rs4833528	C	T	.	PA:	RIT1	exonic	NM_	.	mis	RIT1:NI
ACaP06	1	####	rs8940723	G	A	.	PA:	RABGAP1	intronic	NM_	.	.	.
ACaP06	1	####	rs7731705	C	T	.	PA:	CACNA1E	intronic	NM_	.	.	.
ACaP06	2	####	.	G	C	.	PA:	RGPD1	intronic	NM_	.	.	.
ACaP06	2	####	rs7781962	G	T	.	PA:	.	intergen	NR_	dist=	.	.
ACaP06	2	####	rs2018517	G	A	.	PA:	LYG2	exonic	NM_	.	syn	LYG2:N
ACaP06	2	####	.	A	C	.	PA:	SLC9A2	exonic	NM_	.	mis	SLC9A2
ACaP06	2	####	rs7685255	G	A	.	PA:	EVX2	exonic	NM_	.	mis	EVX2:N
ACaP06	2	####	rs7551300	C	T	.	PA:	CWC22	exonic	NM_	.	mis	CWC22
ACaP06	3	####	rs6224264	A	G	.	PA:	RPSA	exonic	NM_	.	syn	RPSA:N
ACaP06	3	####	.	C	G	.	PA:	ATP11B	intronic	NM_	.	.	.
ACaP06	3	####	.	G	A	.	PA:	ABCC5	exonic	NM_	.	syn	ABCC5
ACaP06	3	####	.	G	A	.	PA:	HTR3C	exonic	NM_	.	mis	HTR3C
ACaP06	3	####	.	T	G	.	PA:	VPS8	intronic	NM_	.	.	.
ACaP06	4	####	.	C	G	.	PA:	LEF1	intronic	NM_	.	.	.
ACaP06	4	####	rs5742217	T	C	.	PA:	.	intergen	NM_	dist=	.	.
ACaP06	4	####	rs5549088	G	A	.	PA:	VEGFC	exonic	NM_	.	mis	VEGFC
ACaP06	5	####	.	G	A	.	PA:	.	intergen	NR_	dist=	.	.
ACaP06	5	####	rs3759258	G	T	.	PA:	.	intergen	NR_	dist=	.	.
ACaP06	5	####	.	C	T	.	PA:	PTCD2	exonic	NM_	.	mis	PTCD2
ACaP06	5	####	.	G	C	.	PA:	ACSL6	intronic	NM_	.	.	.
ACaP06	5	####	.	G	A	.	PA:	LEAP2	intronic	NM_	.	.	.
ACaP06	5	####	.	C	T	.	PA:	EIF4E1B	intronic	NM_	.	.	.
ACaP06	5	####	.	T	G	.	PA:	UNC5A	intronic	NM_	.	.	.
ACaP06	6	####	rs7580301	C	T	.	PA:	MCUR1	intronic	NM_	.	.	.
ACaP06	6	####	rs7598672	G	A	.	PA:	JARID2	intronic	NM_	.	.	.
ACaP06	6	####	.	G	A	.	PA:	ACOT13	UTR5	NM_	NM_	.	.
ACaP06	6	####	.	C	A	.	PA:	HLA-G	exonic	NM_	.	mis	HLA-G:
ACaP06	6	####	.	G	C	.	PA:	MSH5-SAI	ncRNA_	NR_	.	.	.
ACaP06	6	####	.	C	T	.	PA:	COL11A2	intronic	NM_	.	.	.
ACaP06	6	####	.	C	T	.	PA:	DNPH1	intronic	NM_	.	.	.
ACaP06	6	####	.	A	G	.	PA:	KHDRBS2	intronic	NM_	.	.	.
ACaP06	6	####	.	A	T	.	PA:	LOC44115	exonic	NM_	.	mis	LOC441
ACaP06	6	####	.	G	T	.	PA:	SYNJ2	intronic	NM_	.	.	.
ACaP06	7	####	.	G	A	.	PA:	TMEM184	intronic	NM_	.	.	.
ACaP06	7	####	rs2018986	T	C	.	PA:	.	intergen	NR_	dist=	.	.
ACaP06	7	####	rs9803140	C	G	.	PA:	ADAM22	intronic	NM_	.	.	.
ACaP06	7	####	rs1208357	G	A	.	PA:	.	intergen	NM_	dist=	.	.
ACaP06	7	####	rs6248378	G	A	.	PA:	.	intergen	NM_	dist=	.	.
ACaP06	7	####	.	G	A	.	PA:	GIMAP1	exonic	NM_	.	mis	GIMAP1
ACaP06	7	####	.	G	A	.	PA:	.	intergen	NR_	dist=	.	.
ACaP06	8	####	.	G	A	.	PA:	INTS9	intronic	NM_	.	.	.
ACaP06	8	####	rs2022368	A	G	.	PA:	.	intergen	NM_	dist=	.	.
ACaP06	8	####	.	T	G	.	PA:	SCRIB	intronic	NM_	.	.	.
ACaP06	9	####	.	G	C	.	PA:	FRMD3	exonic	NM_	.	syn	FRMD3
ACaP06	9	####	.	C	G	.	PA:	ROR2	intronic	NM_	.	.	.

ACaP06	9	####	.	T	C	.	PA!	.	intergen	NM_	dist=	.	.
ACaP06	9	####	rs7729568	G	A	.	PA!	MAN1B1	exonic	NM_	.	syr	MAN1B
ACaP06	10	####	rs5354201	C	T	.	PA!	CXCL12	intronic	NM_	.	.	.
ACaP06	10	####	.	A	T	.	PA!	.	upstrear	NM_	.	.	.
ACaP06	10	####	.	G	T	.	PA!	CCDC147	exonic	NM_	.	sto	CCDC1
ACaP06	10	####	.	C	T	.	PA!	KCNK18	exonic	NM_	.	mis	KCNK1
ACaP06	11	####	rs1474222	G	A	.	PA!	PIDD	exonic	NM_	.	syr	PIDD:N
ACaP06	11	####	.	G	C	.	PA!	ZNF195	UTR5	NM_	NM_	.	.
ACaP06	11	####	.	G	A	.	PA!	HPX	exonic	NM_	.	mis	HPX:NM
ACaP06	11	####	.	G	A	.	PA!	CAND1.11	ncRNA_	NR_	.	.	.
ACaP06	11	####	rs7776543	C	T	.	PA!	KCNJ11	UTR5	NM_	NM_	.	.
ACaP06	11	####	rs7802181	G	A	.	PA!	TP53I11	exonic	NM_	.	syr	TP53I1
ACaP06	11	####	.	C	G	.	PA!	DTX4	intronic	NM_	.	.	.
ACaP06	11	####	.	C	T	.	PA!	SLC25A45	UTR3	NM_	NM_	.	.
ACaP06	11	####	rs1009016	G	A	.	PA!	TRIM29	intronic	NM_	.	.	.
ACaP06	11	####	.	G	A	.	PA!	DDX25	intronic	NM_	.	.	.
ACaP06	12	####	.	G	C	.	PA!	NACA	exonic	NM_	.	mis	NACA:N
ACaP06	12	####	rs8683709	T	C	.	PA!	HECTD4	intronic	NM_	.	.	.
ACaP06	12	####	.	G	T	.	PA!	KDM2B	intronic	NM_	.	.	.
ACaP06	12	####	.	G	T	.	PA!	HPD	intronic	NM_	.	.	.
ACaP06	13	####	.	G	A	.	PA!	.	intergen	NR_	dist=	.	.
ACaP06	13	####	.	G	A	.	PA!	.	intergen	NR_	dist=	.	.
ACaP06	13	####	rs7603455	G	A	.	PA!	RNF17	intronic	NM_	.	.	.
ACaP06	13	####	.	G	A	.	PA!	MTMR6	exonic	NM_	.	mis	MTMR6
ACaP06	13	####	.	A	T	.	PA!	MIR548F5	ncRNA_	NR_	.	.	.
ACaP06	13	####	.	A	T	.	PA!	FREM2	intronic	NM_	.	.	.
ACaP06	13	####	rs1847802	G	A	.	PA!	TBC1D4	exonic	NM_	.	sto	TBC1D4
ACaP06	14	####	rs8674109	G	T	.	PA!	.	intergen	NM_	dist=	.	.
ACaP06	14	####	.	C	T	.	PA!	PRKD1	exonic	NM_	.	mis	PRKD1
ACaP06	14	####	.	C	T	.	PA!	CFL2	UTR5	NM_	NM_	.	.
ACaP06	14	####	.	C	G	.	PA!	PRPF39	intronic	NM_	.	.	.
ACaP06	14	####	rs7590859	C	T	.	PA!	C14orf105	exonic	NM_	.	mis	C14orf1
ACaP06	14	####	.	G	A	.	PA!	PPP2R5E	intronic	NM_	.	.	.
ACaP06	14	####	rs1126070	A	G	.	PA!	ADAM21P	ncRNA_	NR_	.	.	.
ACaP06	14	####	.	C	G	.	PA!	TDRD9	intronic	NM_	.	.	.
ACaP06	14	####	.	C	T	.	PA!	TDRD9	intronic	NM_	.	.	.
ACaP06	14	####	.	C	T	.	PA!	TDRD9	splicing	NM_	NM_	.	.
ACaP06	15	####	.	C	T	.	PA!	.	intergen	NM_	dist=	.	.
ACaP06	15	####	.	C	A	.	PA!	THSD4	exonic	NM_	.	mis	THSD4
ACaP06	16	####	.	G	A	.	PA!	BAIAP3	splicing	NM_	NM_	.	.
ACaP06	16	####	.	G	A	.	PA!	UNKL	intronic	NM_	.	.	.
ACaP06	16	####	rs7488576	G	A	.	PA!	C16orf71	exonic	NM_	.	mis	C16orf7
ACaP06	16	####	.	G	C	.	PA!	NAGPA	intronic	NM_	.	.	.
ACaP06	16	####	.	G	A	.	PA!	ACSM5	exonic	NM_	.	mis	ACSM5
ACaP06	16	####	.	G	A	.	PA!	XPO6	exonic	NM_	.	mis	XPO6:N
ACaP06	16	####	.	C	A	.	PA!	ALDOA	splicing	NM_	NM_	.	.
ACaP06	16	####	rs7501178	C	T	.	PA!	.	intergen	NOI	dist=	.	.
ACaP06	16	####	rs2128450	T	A	.	PA!	.	intergen	NOI	dist=	.	.
ACaP06	16	####	rs4436803	T	G	.	PA!	.	intergen	NOI	dist=	.	.
ACaP06	16	####	rs2887500	T	G	.	PA!	.	intergen	NOI	dist=	.	.
ACaP06	16	####	.	C	A	.	PA!	HYDIN	intronic	NM_	.	.	.
ACaP06	16	####	.	T	C	.	PA!	ZNF276	exonic	NM_	.	mis	ZNF276
ACaP06	17	####	.	G	C	.	PA!	KSR1	intronic	NM_	.	.	.
ACaP06	17	####	.	C	G	.	PA!	EFCAB5	exonic	NM_	.	mis	EFCAB

ACaP06	17	####	.	G	A	.	PA	GSDMB	intronic	NM_	.	.	.
ACaP06	17	####	rs1996882	G	T	.	PA	CNP	exonic	NM_	.	mis	CNP:NI
ACaP06	17	####	.	C	G	.	PA	EZH1	intronic	NM_	.	.	.
ACaP06	17	####	rs1022498	A	T	.	PA	EFTUD2	intronic	NM_	.	.	.
ACaP06	17	####	.	C	T	.	PA	ERN1	exonic	NM_	.	mis	ERN1:N
ACaP06	17	####	rs3720728	C	T	.	PA	CACNG4	exonic	NM_	.	syn	CACNG
ACaP06	17	####	.	G	A	.	PA	FOXK2	intronic	NM_	.	.	.
ACaP06	18	####	.	G	A	.	PA	NAPG	exonic	NM_	.	mis	NAPG:N
ACaP06	18	####	.	C	T	.	PA	CIDEA	intronic	NM_	.	.	.
ACaP06	18	####	.	G	C	.	PA	.	intergen	NR_	dist=	.	.
ACaP06	18	####	rs7558958	C	T	.	PA	C18orf54	exonic	NM_	.	mis	C18orf5
ACaP06	19	####	rs9952243	G	A	.	PA	SIPA1L3	exonic	NM_	.	mis	SIPA1L
ACaP06	19	####	.	C	T	.	PA	SULT2B1	exonic	NM_	.	mis	SULT2B
ACaP06	20	####	.	G	T	.	PA	.	upstrea	NM_	.	.	.
ACaP06	20	####	.	T	G	.	PA	BPI	intronic	NM_	.	.	.
ACaP06	21	####	rs3697472	C	A	.	PA	.	intergen	NR_	dist=	.	.
ACaP06	21	####	rs7969899	C	T	.	PA	.	intergen	NR_	dist=	.	.
ACaP06	21	####	.	C	T	.	PA	.	intergen	NM_	dist=	.	.
ACaP06	21	####	.	C	G	.	PA	TRAPPC1	intronic	NM_	.	.	.
ACaP06	22	####	rs2212142	T	G	.	PA	POTEH	intronic	NM_	.	.	.
ACaP06	22	####	.	G	T	.	PA	LINC0089	ncRNA_	NR_	.	.	.
ACaP06	22	####	rs2015076	G	C	.	PA	.	intergen	NM_	dist=	.	.
ACaP06	22	####	rs7611606	G	A	.	PA	ELFN2	exonic	NM_	.	mis	ELFN2:
ACaP06	22	####	.	T	A	.	PA	PICK1	intronic	NM_	.	.	.
ACaP06	X	####	rs3397828	A	G	.	PA	BCYRN1	ncRNA_	NR_	.	.	.
ACaP06	X	####	.	C	T	.	PA	CPXCR1	exonic	NM_	.	syn	CPXCR
ACaP06	MT	215	rs8792192	A	G	.	PA	.	intergen	NOI	dist=	.	.
ACaP07	1	####	.	G	A	.	PA	NECAP2	exonic	NM_	.	mis	NECAP
ACaP07	1	####	rs1996890	T	C	.	PA	NBPF1	intronic	NM_	.	.	.
ACaP07	1	####	.	C	A	.	PA	DLGAP3	exonic	NM_	.	mis	DLGAP
ACaP07	1	####	.	A	G	.	PA	GNAI3	exonic	NM_	.	mis	GNAI3:
ACaP07	1	####	.	T	C	.	PA	.	intergen	NR_	dist=	.	.
ACaP07	1	####	rs1694653	C	G	.	PA	.	intergen	NR_	dist=	.	.
ACaP07	1	####	rs9441133	T	G	.	PA	NBPF9,NE	exonic	NM_	.	unk	UNKNC
ACaP07	1	####	rs7819708	C	G	.	PA	NBPF9,NE	exonic	NM_	.	unk	UNKNC
ACaP07	1	####	rs2022445	T	A	.	PA	NBPF8	intronic	NM_	.	.	.
ACaP07	1	####	.	C	T	.	PA	.	intergen	NM_	dist=	.	.
ACaP07	1	####	.	C	T	.	PA	ANXA9	intronic	NM_	.	.	.
ACaP07	1	####	.	C	G	.	PA	.	intergen	NM_	dist=	.	.
ACaP07	1	####	rs1494980	T	C	.	PA	RNASEL	exonic	NM_	.	mis	RNASE
ACaP07	1	####	.	C	A	.	PA	HMCN1	exonic	NM_	.	mis	HMCN1
ACaP07	1	####	rs7630457	C	T	.	PA	CENPF	exonic	NM_	.	mis	CENPF
ACaP07	1	####	rs3725088	G	T	.	PA	KIAA1804	intronic	NM_	.	.	.
ACaP07	2	####	.	T	C	.	PA	COX7A2L	exonic	NM_	.	mis	COX7A
ACaP07	2	####	rs6214568	A	G	.	PA	.	intergen	NR_	dist=	.	.
ACaP07	2	####	.	C	G	.	PA	ACOXL	intronic	NM_	.	.	.
ACaP07	2	####	rs1476846	C	T	.	PA	ANAPC1	exonic	NM_	.	mis	ANAPC
ACaP07	2	####	.	C	G	.	PA	SP140L	intronic	NM_	.	.	.
ACaP07	3	####	.	G	A	.	PA	ACKR2	UTR3	NM_	NM_	.	.
ACaP07	3	####	.	C	A	.	PA	CELSR3	exonic	NM_	.	mis	CELSR
ACaP07	3	####	.	G	T	.	PA	DPPA2	intronic	NM_	.	.	.
ACaP07	3	####	rs1428044	G	A	.	PA	SPICE1	exonic	NM_	.	syn	SPICE1
ACaP07	3	####	.	T	C	.	PA	MME	intronic	NM_	.	.	.
ACaP07	4	####	rs4970287	C	T	.	PA	.	intergen	NOI	dist=	.	.

ACaP07	4	####	.	T	C	.	PA!	PPP3CA	splicing	NM_	NM_	.	.	
ACaP07	4	####	.	G	A	.	PA!	TET2	exonic	NM_	.	mis	TET2:N	
ACaP07	5	####	.	C	T	.	PA!	NIPBL	exonic	NM_	.	sto	NIPBL:I	
ACaP07	5	####	.	C	A	.	PA!	SLC4A9	intronic	NM_	.	.	.	
ACaP07	7	####	rs9642790	C	T	.	PA!	EPDR1	intronic	NM_	.	.	.	
ACaP07	7	####	rs6246184	G	A	.	PA!	.	intergen	NR_	dist=	.	.	
ACaP07	7	####	.	C	A	.	PA!	.	intergen	NR_	dist=	.	.	
ACaP07	7	####	.	C	T	.	PA!	LRCH4,ZA	exonic	NM_	.	mis	LRCH4	
ACaP07	7	####	.	C	A	.	PA!	PTPRZ1	exonic	NM_	.	mis	PTPRZ	
ACaP07	7	####	rs8915428	C	T	.	PA!	SSPO	intronic	NM_	.	.	.	
ACaP07	7	####	.	A	T	.	PA!	PAXIP1-A	ncRNA_	NR_	.	.	.	
ACaP07	8	####	.	G	A	.	PA!	INTS9	intronic	NM_	.	.	.	
ACaP07	8	####	.	C	G	.	PA!	ZNF703	exonic	NM_	.	mis	ZNF703	
ACaP07	8	####	.	G	A	.	PA!	C8orf44-S	intronic	NM_	.	.	.	
ACaP07	9	####	.	G	A	.	PA!	JAK2	intronic	NM_	.	.	.	
ACaP07	9	####	.	G	A	.	PA!	NDOR1	intronic	NM_	.	.	.	
ACaP07	10	####	.	C	G	.	PA!	NDST2	intronic	NM_	.	.	.	
ACaP07	10	####	.	G	C	.	PA!	GRID1	exonic	NM_	.	mis	GRID1:	
ACaP07	10	####	.	G	T	.	PA!	NOLC1	exonic	NM_	.	syr	NOLC1	
ACaP07	10	####	.	G	C	.	PA!	PDCD11	intronic	NM_	.	.	.	
ACaP07	10	####	.	G	A	.	PA!	KNDC1	splicing	NM_	NM_	.	.	
ACaP07	11	####	.	C	A	.	PA!	TRIM5	exonic	NM_	.	mis	TRIM5:	
ACaP07	11	####	.	T	C	.	PA!	NLRP10	exonic	NM_	.	syr	NLRP1(	
ACaP07	11	####	.	C	T	.	PA!	DCDC5	splicing	NM_	NM_	.	.	
ACaP07	11	####	.	C	T	.	PA!	TRAF6	exonic	NM_	.	mis	TRAF6:	
ACaP07	11	####	.	T	C	.	PA!	CWC15	intronic	NM_	.	.	.	
ACaP07	11	####	rs7580622	C	T	.	PA!	DSCAML1	exonic	NM_	.	syr	DSCAM	
ACaP07	12	####	.	A	G	.	PA!	CD163	exonic	NM_	.	mis	CD163:	
ACaP07	12	####	.	G	A	.	PA!	.	downstr	NM_	.	.	.	
ACaP07	12	####	.	C	G	.	PA!	GPD1	exonic	NM_	.	mis	GPD1:N	
ACaP07	12	####	.	C	T	.	PA!	GPR84	exonic	NM_	.	syr	GPR84	
ACaP07	12	####	.	G	C	.	PA!	SLC26A1(	intronic	NM_	.	.	.	
ACaP07	12	####	.	C	A	.	PA!	HCAR3	exonic	NM_	.	mis	HCAR3	
ACaP07	13	####	.	C	A	.	PA!	GPC5	exonic	NM_	.	mis	GPC5:N	
ACaP07	14	####	rs7149806	A	G	.	PA!	.	intergen	NM_	dist=	.	.	
ACaP07	14	####	.	G	T	.	PA!	.	intergen	NR_	dist=	.	.	
ACaP07	15	####	.	T	G	.	PA!	NDN	exonic	NM_	.	mis	NDN:NI	
ACaP07	15	####	rs1995696	A	G	.	PA!	.	intergen	NM_	dist=	.	.	
ACaP07	15	####	.	G	T	.	PA!	FMN1	exonic	NM_	.	mis	FMN1:N	
ACaP07	15	####	.	C	G	.	PA!	GCOM1,M	intronic	NM_	.	.	.	
ACaP07	15	####	.	C	A	.	PA!	PARP6	intronic	NM_	.	.	.	
ACaP07	15	####	.	G	C	.	PA!	ANPEP	exonic	NM_	.	mis	ANPEP	
ACaP07	16	####	.	G	A	.	PA!	RBFOX1	UTR5	NM_	NM_	.	.	
ACaP07	16	####	.	G	T	.	PA!	.	intergen	NM_	dist=	.	.	
ACaP07	17	####	.	G	C	.	PA!	MYH13	intronic	NM_	.	.	.	
ACaP07	17	####	.	T	C	.	PA!	TBC1D3P	ncRNA_	NR_	.	.	.	
ACaP07	18	####	rs8790150	G	A	.	PA!	.	intergen	NM_	dist=	.	.	
ACaP07	19	####	rs8673654	T	C	.	PA!	ZFR2	intronic	NM_	.	.	.	
ACaP07	19	####	rs7668108	G	A	.	PA!	MAN2B1	exonic	NM_	.	syr	MAN2B	
ACaP07	19	####	rs7683037	G	A	.	PA!	CRLF1	exonic	NM_	.	mis	CRLF1:	
ACaP07	19	####	.	T	A	.	PA!	ZNF676	UTR3	NM_	NM_	.	.	
ACaP07	19	####	.	T	G	.	PA!	ZNF829	intronic	NM_	.	.	.	
ACaP07	19	####	.	C	T	.	PA!	MAP4K1	exonic	NM_	.	syr	MAP4K	
ACaP07	19	####	.	T	G	.	PA!	SYMPK	intronic	NM_	.	.	.	

ACaP07	19	####	.	G	C	.	PA!	NAPA-AS:	ncRNA_	NR_	.	.	.	
ACaP07	19	####	.	C	G	.	PA!	TULP2	intronic	NM_	.	.	.	
ACaP07	19	####	.	G	A	.	PA!	TULP2	intronic	NM_	.	.	.	
ACaP07	19	####	.	A	G	.	PA!	.	upstreal	NR_	.	.	.	
ACaP07	19	####	.	G	A	.	PA!	NLRP9	exonic	NM_	.	syr	NLRP9:	
ACaP07	20	####	.	G	C	.	PA!	IFT52	intronic	NM_	.	.	.	
ACaP07	20	####	.	A	G	.	PA!	SALL4	exonic	NM_	.	mis	SALL4:I	
ACaP07	21	####	rs1118412	G	A	.	PA!	.	intergen	NM_	dist=	.	.	
ACaP07	21	####	.	C	A	.	PA!	ADAMTS5	exonic	NM_	.	mis	ADAMT	
ACaP07	21	####	.	C	T	.	PA!	URB1	exonic	NM_	.	mis	URB1:N	
ACaP07	22	####	rs2006656	T	C	.	PA!	.	intergen	NM_	dist=	.	.	
ACaP07	22	####	rs9392199	G	A	.	PA!	PARVB	intronic	NM_	.	.	.	
ACaP07	X	####	rs734038	T	G	.	PA!	KRBOX4	intronic	NM_	.	.	.	
ACaP07	X	####	rs1826904	G	C	.	PA!	.	intergen	NM_	dist=	.	.	
ACaP07	X	####	.	T	A	.	PA!	OCRL	exonic	NM_	.	mis	OCRL:N	
ACaP07	X	####	.	C	T	.	PA!	GPR112	exonic	NM_	.	mis	GPR11:	
ACaP07	X	####	.	C	A	.	PA!	SLITRK2	exonic	NM_	.	mis	SLITRK	
ACaP07	X	####	.	G	A	.	PA!	F8	exonic	NM_	.	mis	F8:NM_	
ACaP07	MT	####	.	G	A	.	PA!	.	intergen	NOI	dist=	.	.	
ACaP07	MT	####	.	T	C	.	PA!	.	intergen	NOI	dist=	.	.	
ACaP08	1	####	rs1810443	G	A	.	PA!	.	intergen	NR_	dist=	.	.	
ACaP08	1	####	rs3698413	G	A	.	PA!	.	intergen	NR_	dist=	.	.	
ACaP08	1	####	rs3774960	G	A	.	PA!	.	intergen	NR_	dist=	.	.	
ACaP08	1	####	rs2596327	T	A	.	PA!	NBPF9,SE	intronic	NM_	.	.	.	
ACaP08	1	####	rs2020368	T	A	.	PA!	NBPF8	intronic	NM_	.	.	.	
ACaP08	1	####	.	C	A	.	PA!	NBPF25P	ncRNA_	NR_	.	.	.	
ACaP08	2	####	.	G	C	.	PA!	DNAH6	intronic	NM_	.	.	.	
ACaP08	2	####	rs2647769	C	T	.	PA!	.	intergen	NOI	dist=	.	.	
ACaP08	2	####	.	C	T	.	PA!	.	downstr	NM_	.	.	.	
ACaP08	2	####	.	C	A	.	PA!	FBLN7	intronic	NM_	.	.	.	
ACaP08	2	####	.	C	G	.	PA!	FBLN7	intronic	NM_	.	.	.	
ACaP08	2	####	rs6216494	T	A	.	PA!	POTEJ	intronic	NM_	.	.	.	
ACaP08	3	####	.	G	T	.	PA!	TFDP2	intronic	NM_	.	.	.	
ACaP08	4	####	.	C	A	.	PA!	CYTL1	intronic	NM_	.	.	.	
ACaP08	4	####	rs1429725	C	T	.	PA!	.	intergen	NM_	dist=	.	.	
ACaP08	5	####	rs2871612	T	C	.	PA!	GUSBP1	ncRNA_	NR_	.	.	.	
ACaP08	7	####	rs6246206	T	C	.	PA!	.	intergen	NR_	dist=	.	.	
ACaP08	7	####	rs3680944	T	C	.	PA!	CCT6P1,L	ncRNA_	NR_	.	.	.	
ACaP08	8	####	rs1499905	G	C	.	PA!	.	intergen	NM_	dist=	.	.	
ACaP08	10	####	.	T	A	.	PA!	CDH23	intronic	NM_	.	.	.	
ACaP08	10	####	rs7710236	G	A	.	PA!	C10orf11	intronic	NM_	.	.	.	
ACaP08	10	####	rs1788433	A	G	.	PA!	SFTPA2	intronic	NM_	.	.	.	
ACaP08	10	####	.	T	G	.	PA!	DMBT1	intronic	NM_	.	.	.	
ACaP08	11	####	.	T	A	.	PA!	PKNOX2	intronic	NM_	.	.	.	
ACaP08	12	####	rs1063873	T	G	.	PA!	.	intergen	NR_	dist=	.	.	
ACaP08	14	####	rs4247513	T	C	.	PA!	.	intergen	NR_	dist=	.	.	
ACaP08	14	####	rs7445485	C	T	.	PA!	.	intergen	NR_	dist=	.	.	
ACaP08	14	####	rs8678279	G	A	.	PA!	.	intergen	NM_	dist=	.	.	
ACaP08	14	####	.	A	T	.	PA!	.	intergen	NM_	dist=	.	.	
ACaP08	17	####	rs3930170	G	T	.	PA!	.	upstreal	NM_	.	.	.	
ACaP08	17	####	rs3894278	A	T	.	PA!	MYCBPAF	intronic	NM_	.	.	.	
ACaP08	21	####	rs1963041	G	A	.	PA!	.	intergen	NR_	dist=	.	.	
ACaP08	22	####	rs3716554	G	T	.	PA!	.	intergen	NM_	dist=	.	.	
ACaP08	22	####	.	C	A	.	PA!	.	intergen	NM_	dist=	.	.	

ACaP08	X	####	rs7819787	C	A	.	PA!	CT45A1	UTR3	NM_	NM_	.	.	
ACaP09	1	####	.	T	G	.	PA!	ATAD3A	intronic	NM_	.	.	.	
ACaP09	1	####	rs453347	G	C	.	PA!	NBPF1	intronic	NM_	.	.	.	
ACaP09	1	####	.	G	T	.	PA!	BTBD19	intronic	NM_	.	.	.	
ACaP09	1	####	rs1812127	C	G	.	PA!	.	intergen	NR_	dist=	.	.	
ACaP09	1	####	.	C	G	.	PA!	NOTCH2N	intronic	NM_	.	.	.	
ACaP09	1	####	rs8799607	G	T	.	PA!	NBPF25P	ncRNA_	NR_	.	.	.	
ACaP09	1	####	rs1451754	C	A	.	PA!	NBPF8	intronic	NM_	.	.	.	
ACaP09	1	####	rs2003945	G	T	.	PA!	NBPF8	intronic	NM_	.	.	.	
ACaP09	1	####	rs7970322	T	G	.	PA!	NBPF8	intronic	NM_	.	.	.	
ACaP09	1	####	.	G	T	.	PA!	NUCKS1	intronic	NM_	.	.	.	
ACaP09	2	####	rs1996199	C	T	.	PA!	NEU4	intronic	NM_	.	.	.	
ACaP09	3	####	.	G	T	.	PA!	.	intergen	NM_	dist=	.	.	
ACaP09	3	####	rs1873413	C	T	.	PA!	PRKCI	intronic	NM_	.	.	.	
ACaP09	3	####	rs3723871	A	G	.	PA!	.	intergen	NM_	dist=	.	.	
ACaP09	3	####	.	T	C	.	PA!	MUC20	intronic	NM_	.	.	.	
ACaP09	3	####	rs2006820	G	A	.	PA!	SDHAP1	ncRNA_	NR_	.	.	.	
ACaP09	4	####	.	T	A	.	PA!	.	upstrea	NM_	.	.	.	
ACaP09	5	####	.	C	A	.	PA!	CAMLG	UTR3	NM_	NM_	.	.	
ACaP09	6	####	.	C	T	.	PA!	.	intergen	NM_	dist=	.	.	
ACaP09	6	####	rs7746421	C	G	.	PA!	LPA	intronic	NM_	.	.	.	
ACaP09	6	####	rs3759780	G	C	.	PA!	.	intergen	NM_	dist=	.	.	
ACaP09	7	####	.	C	G	.	PA!	.	intergen	NR_	dist=	.	.	
ACaP09	7	####	.	T	A	.	PA!	.	intergen	NR_	dist=	.	.	
ACaP09	7	####	.	T	G	.	PA!	AUTS2	exonic	NM_	.	mis	AUTS2:	
ACaP09	9	####	rs2803685	G	T	.	PA!	FAM27C	ncRNA_	NR_	.	.	.	
ACaP09	10	####	rs1996770	C	T	.	PA!	.	intergen	NM_	dist=	.	.	
ACaP09	10	####	rs1456597	C	A	.	PA!	.	intergen	NM_	dist=	.	.	
ACaP09	11	####	.	A	C	.	PA!	HPX	intronic	NM_	.	.	.	
ACaP09	11	####	.	T	C	.	PA!	SLC43A1	intronic	NM_	.	.	.	
ACaP09	12	####	rs3718339	T	G	.	PA!	LOC1002	ncRNA_	NR_	.	.	.	
ACaP09	12	####	rs3767721	C	T	.	PA!	LOC1002	ncRNA_	NR_	.	.	.	
ACaP09	12	####	rs5604999	A	T	.	PA!	XPOT	intronic	NM_	.	.	.	
ACaP09	14	####	rs7755744	G	A	.	PA!	POTEG	intronic	NM_	.	.	.	
ACaP09	14	####	rs1018908	C	T	.	PA!	.	intergen	NM_	dist=	.	.	
ACaP09	15	####	.	G	A	.	PA!	GOLGA8E	ncRNA_	NR_	.	.	.	
ACaP09	15	####	rs7568595	T	C	.	PA!	AQR	exonic	NM_	.	syr	AQR:NI	
ACaP09	16	####	rs3739171	C	T	.	PA!	SMG1P1	ncRNA_	NR_	.	.	.	
ACaP09	16	####	rs7137409	G	A	.	PA!	CES1P1	ncRNA_	NR_	.	.	.	
ACaP09	17	####	rs7124761	G	A	.	PA!	.	intergen	NM_	dist=	.	.	
ACaP09	17	####	rs6149181	G	T	.	PA!	FLJ36000	ncRNA_	NR_	.	.	.	
ACaP09	17	####	rs2870408	T	C	.	PA!	USP32	intronic	NM_	.	.	.	
ACaP09	17	####	rs2848380	C	T	.	PA!	USP32	intronic	NM_	.	.	.	
ACaP09	17	####	.	G	C	.	PA!	STRA13	intronic	NM_	.	.	.	
ACaP09	18	####	.	C	A	.	PA!	.	upstrea	NM_	.	.	.	
ACaP09	19	####	.	A	G	.	PA!	MUC16	intronic	NM_	.	.	.	
ACaP09	21	####	.	G	T	.	PA!	.	intergen	NR_	dist=	.	.	
ACaP09	22	####	.	A	T	.	PA!	FAM230B	ncRNA_	NR_	.	.	.	
ACaP09	22	####	rs3609392	C	T	.	PA!	CYP2D7P	ncRNA_	NR_	.	.	.	
ACaP09	X	####	.	C	A	.	PA!	FAAH2	intronic	NM_	.	.	.	
ACaP10	1	####	rs7558529	C	T	.	PA!	MXRA8	exonic	NM_	.	mis	MXRA8	
ACaP10	1	####	.	C	T	.	PA!	ATAD3B	intronic	NM_	.	.	.	
ACaP10	1	####	.	G	C	.	PA!	CLSPN	intronic	NM_	.	.	.	
ACaP10	1	####	.	G	C	.	PA!	AGO4	intronic	NM_	.	.	.	

ACaP10	1	####	.	C	T	.	PA!	EPHA10	intronic	NM_	.	.	.
ACaP10	1	####	.	G	A	.	PA!	.	intergen	NM_	dist=	.	.
ACaP10	1	####	.	G	T	.	PA!	.	intergen	NM_	dist=	.	.
ACaP10	1	####	.	C	T	.	PA!	15-Sep	exonic	NM_	.	unl	UNKNO
ACaP10	1	####	.	C	T	.	PA!	LRRC8B	exonic	NM_	.	sto	LRRC8
ACaP10	1	####	rs1430207	T	C	.	PA!	SYCP1	intronic	NM_	.	.	.
ACaP10	1	####	.	G	A	.	PA!	NOTCH2	intronic	NM_	.	.	.
ACaP10	1	####	rs1996428	C	T	.	PA!	.	intergen	NR_	dist=	.	.
ACaP10	1	####	.	G	C	.	PA!	NBPF9,NO	intronic	NM_	.	.	.
ACaP10	1	####	rs3755029	A	T	.	PA!	NBPF25P	ncRNA_	NR_	.	.	.
ACaP10	1	####	rs2849511	G	A	.	PA!	LINC0113	ncRNA_	NR_	.	.	.
ACaP10	1	####	rs7824596	A	C	.	PA!	NBPF8	intronic	NM_	.	.	.
ACaP10	1	####	rs3765099	T	G	.	PA!	NBPF8	intronic	NM_	.	.	.
ACaP10	1	####	rs9099303	C	T	.	PA!	TCHH	exonic	NM_	.	mis	TCHH:N
ACaP10	1	####	.	G	C	.	PA!	ATP8B2	intronic	NM_	.	.	.
ACaP10	1	####	.	G	A	.	PA!	RASAL2	intronic	NM_	.	.	.
ACaP10	1	####	.	A	G	.	PA!	PLEKHA6	intronic	NM_	.	.	.
ACaP10	1	####	.	C	T	.	PA!	C4BPB	intronic	NM_	.	.	.
ACaP10	2	####	.	G	T	.	PA!	TTN	splicing	NM_	NM_	.	.
ACaP10	2	####	.	G	C	.	PA!	TNS1	intronic	NM_	.	.	.
ACaP10	3	####	rs7793139	G	A	.	PA!	ITPR1	exonic	NM_	.	mis	ITPR1:N
ACaP10	3	####	.	C	T	.	PA!	RAD18	intronic	NM_	.	.	.
ACaP10	3	####	.	G	C	.	PA!	FBLN2	intronic	NM_	.	.	.
ACaP10	3	####	.	C	T	.	PA!	STAB1	exonic	NM_	.	mis	STAB1:
ACaP10	4	####	rs7785731	C	T	.	PA!	NELFA	exonic	NM_	.	syr	NELFA:
ACaP10	4	####	rs7749410	G	A	.	PA!	SPP1	intronic	NM_	.	.	.
ACaP10	5	####	rs2382048	A	G	.	PA!	.	intergen	NM_	dist=	.	.
ACaP10	5	####	rs7817278	C	G	.	PA!	DHFR	exonic	NM_	.	mis	DHFR:N
ACaP10	5	####	.	A	G	.	PA!	COMMD10	UTR5	NM_	NM_	.	.
ACaP10	5	####	rs1140023	G	A	.	PA!	ZNF474	exonic	NM_	.	mis	ZNF474
ACaP10	5	####	.	C	T	.	PA!	SLC36A3	intronic	NM_	.	.	.
ACaP10	5	####	.	G	A	.	PA!	GABRB2	exonic	NM_	.	sto	GABRB
ACaP10	6	####	.	G	C	.	PA!	WRNIP1	splicing	NM_	NM_	.	.
ACaP10	6	####	.	C	T	.	PA!	SLC44A4	exonic	NM_	.	mis	SLC44A
ACaP10	6	####	.	T	C	.	PA!	TNFRSF2	intronic	NM_	.	.	.
ACaP10	6	####	rs9907394	C	T	.	PA!	LAMA2	intronic	NM_	.	.	.
ACaP10	6	####	.	G	A	.	PA!	ARID1B	exonic	NM_	.	mis	ARID1E
ACaP10	6	####	.	G	A	.	PA!	MLLT4	exonic	NM_	.	mis	MLLT4:
ACaP10	6	####	.	G	A	.	PA!	TBP	intronic	NM_	.	.	.
ACaP10	7	####	.	C	G	.	PA!	TTYH3	exonic	NM_	.	mis	TTYH3:
ACaP10	7	####	.	C	G	.	PA!	PMS2	intronic	NM_	.	.	.
ACaP10	7	####	rs7468373	T	C	.	PA!	ISPD	exonic	NM_	.	syr	ISPD:N
ACaP10	7	####	.	A	T	.	PA!	HECW1	exonic	NM_	.	mis	HECW1
ACaP10	7	####	.	G	A	.	PA!	.	upstrean	NM_	.	.	.
ACaP10	7	####	rs5705914	T	G	.	PA!	.	intergen	NM_	dist=	.	.
ACaP10	7	####	rs1481871	C	T	.	PA!	GIMAP6	exonic	NM_	.	mis	GIMAP(
ACaP10	8	####	rs7588073	G	A	.	PA!	DLGAP2	intronic	NM_	.	.	.
ACaP10	8	####	rs7479925	G	A	.	PA!	BLK	intronic	NM_	.	.	.
ACaP10	8	####	.	T	G	.	PA!	.	intergen	NM_	dist=	.	.
ACaP10	8	####	rs5327262	C	T	.	PA!	.	intergen	NM_	dist=	.	.
ACaP10	8	####	rs5696070	G	A	.	PA!	CYC1	intronic	NM_	.	.	.
ACaP10	9	####	rs3682437	G	A	.	PA!	GNA14	exonic	NM_	.	mis	GNA14
ACaP10	9	####	rs3746457	G	A	.	PA!	OR13C3	exonic	NM_	.	syr	OR13C
ACaP10	9	####	rs7528554	G	A	.	PA!	ZER1	intronic	NM_	.	.	.

ACaP10	9	####	rs5640471	G	A	.	PA!	PHPT1	exonic	NM_	.	mis	PHPT1:
ACaP10	9	####	rs5505690	C	T	.	PA!	CACNA1E	intronic	NM_	.	.	.
ACaP10	10	####	.	G	A	.	PA!	RBM20	exonic	NM_	.	mis	RBM20
ACaP10	11	####	rs7615769	A	T	.	PA!	METTL15	intronic	NM_	.	.	.
ACaP10	11	####	.	C	T	.	PA!	DCDC5	exonic	NM_	.	syr	DCDC5
ACaP10	11	####	rs7817387	C	G	.	PA!	DGKZ	exonic	NM_	.	syr	DGKZ:N
ACaP10	11	####	.	T	C	.	PA!	.	upstrear	NM_	.	.	.
ACaP10	11	####	rs7525419	G	A	.	PA!	C11orf84	exonic	NM_	.	mis	C11orf8
ACaP10	11	####	.	G	C	.	PA!	CAPN1	intronic	NM_	.	.	.
ACaP10	11	####	.	G	A	.	PA!	GLB1L2	intronic	NM_	.	.	.
ACaP10	12	####	.	C	G	.	PA!	RASAL1	exonic	NM_	.	mis	RASAL
ACaP10	12	####	rs1409770	G	A	.	PA!	WDR66	exonic	NM_	.	mis	WDR66
ACaP10	13	####	.	T	C	.	PA!	ALG5	intronic	NM_	.	.	.
ACaP10	13	####	.	G	T	.	PA!	.	upstrear	NM_	.	.	.
ACaP10	14	####	.	C	G	.	PA!	.	intergen	NR_	dist=	.	.
ACaP10	14	####	.	C	A	.	PA!	NID2	intronic	NM_	.	.	.
ACaP10	14	####	.	A	C	.	PA!	ZFYVE26	intronic	NM_	.	.	.
ACaP10	14	####	.	C	T	.	PA!	ZNF410	intronic	NM_	.	.	.
ACaP10	14	####	.	C	T	.	PA!	ZNF410	intronic	NM_	.	.	.
ACaP10	16	####	rs7663922	C	T	.	PA!	ITFG3	exonic	NM_	.	mis	ITFG3:N
ACaP10	16	####	rs5750779	C	T	.	PA!	CHTF18	intronic	NM_	.	.	.
ACaP10	16	####	.	C	T	.	PA!	SEC14L5	intronic	NM_	.	.	.
ACaP10	16	####	.	T	C	.	PA!	COG7	intronic	NM_	.	.	.
ACaP10	16	####	rs7569695	A	G	.	PA!	RNU6-76F	ncRNA_	NR_	.	.	.
ACaP10	16	####	rs7520038	G	A	.	PA!	RNU6-76F	ncRNA_	NR_	.	.	.
ACaP10	17	####	.	T	C	.	PA!	ATAD5	intronic	NM_	.	.	.
ACaP10	17	####	rs3713755	C	T	.	PA!	CCL4	exonic	NM_	.	syr	CCL4:N
ACaP10	17	####	rs7744485	C	T	.	PA!	KRT24	exonic	NM_	.	syr	KRT24:
ACaP10	17	####	rs7460185	G	A	.	PA!	ACSF2	exonic	NM_	.	syr	ACSF2:
ACaP10	17	####	rs8668152	T	G	.	PA!	CLTC	intronic	NM_	.	.	.
ACaP10	17	####	rs8665784	T	G	.	PA!	TBC1D3P	ncRNA_	NR_	.	.	.
ACaP10	17	####	.	G	C	.	PA!	9-Sep	exonic	NM_	.	mis	SEPT9:
ACaP10	17	####	rs1995663	G	A	.	PA!	CBX2	exonic	NM_	.	syr	CBX2:N
ACaP10	17	####	.	G	T	.	PA!	TBCD	UTR5	NM_	NM_	.	.
ACaP10	19	####	rs3865492	C	G	.	PA!	.	intergen	NR_	dist=	.	.
ACaP10	19	####	.	T	C	.	PA!	.	intergen	NR_	dist=	.	.
ACaP10	19	####	rs7571526	G	A	.	PA!	GRIK5	exonic	NM_	.	syr	GRIK5:
ACaP10	19	####	rs7565559	G	A	.	PA!	APOC4	exonic	NM_	.	syr	APOC4
ACaP10	20	####	.	G	A	.	PA!	SIRPB1	exonic	NM_	.	syr	SIRPB1
ACaP10	20	####	.	C	T	.	PA!	COX4I2	intronic	NM_	.	.	.
ACaP10	20	####	rs3774092	G	A	.	PA!	MROH8	exonic	NM_	.	unl	UNKNC
ACaP10	21	####	.	C	A	.	PA!	COL18A1-	ncRNA_	NR_	.	.	.
ACaP10	22	####	rs6197100	A	G	.	PA!	.	intergen	NM_	dist=	.	.
ACaP10	22	####	.	A	G	.	PA!	FAM230B	ncRNA_	NR_	.	.	.
ACaP10	22	####	rs5662423	G	C	.	PA!	POM121L	ncRNA_	NR_	.	.	.
ACaP10	22	####	.	C	G	.	PA!	.	intergen	NR_	dist=	.	.
ACaP10	22	####	rs1007675	G	A	.	PA!	KIAA0930	intronic	NM_	.	.	.
ACaP10	X	####	.	G	C	.	PA!	HDAC6	intronic	NM_	.	.	.
ACaP10	X	####	.	G	A	.	PA!	GPKOW	intronic	NM_	.	.	.
ACaP10	X	####	rs7970448	C	T	.	PA!	CLCN5	exonic	NM_	.	sto	CLCN5
ACaP10	X	####	.	A	G	.	PA!	ITIH6	exonic	NM_	.	mis	ITIH6:N
ACaP10	X	####	.	C	T	.	PA!	CSAG1	UTR5	NM_	NM_	.	.
ACaP11	1	####	rs4634900	A	G	.	PA!	.	intergen	NM_	dist=	.	.
ACaP11	1	####	.	G	A	.	PA!	CSMD2	intronic	NM_	.	.	.

ACaP11	1	####	rs7450506	G	T	.	PA!	.	intergen	NR_	dist=	.	.
ACaP11	1	####	rs2016919	T	A	.	PA!	.	intergen	NR_	dist=	.	.
ACaP11	1	####	.	A	T	.	PA!	.	intergen	NR_	dist=	.	.
ACaP11	1	####	rs8662584	G	T	.	PA!	.	intergen	NR_	dist=	.	.
ACaP11	1	####	rs4143495	G	A	.	PA!	NBPF25P	ncRNA_	NR_	.	.	.
ACaP11	1	####	rs6181077	C	T	.	PA!	NBPF8	intronic	NM_	.	.	.
ACaP11	1	####	.	G	T	.	PA!	CD1D	UTR5	NM_	NM_	.	.
ACaP11	1	####	rs2003501	G	A	.	PA!	SLC26A9	intronic	NM_	.	.	.
ACaP11	2	####	.	G	A	.	PA!	USP34	intronic	NM_	.	.	.
ACaP11	2	####	rs1834895	C	T	.	PA!	DYSF	intronic	NM_	.	.	.
ACaP11	2	####	rs2009908	C	T	.	PA!	DYSF	exonic	NM_	.	mis	DYSF:N
ACaP11	2	####	.	G	C	.	PA!	WBP1	exonic	NM_	.	mis	WBP1:I
ACaP11	2	####	rs9528	T	C	.	PA!	TMEM131	intronic	NM_	.	.	.
ACaP11	2	####	.	A	G	.	PA!	EPC2	UTR3	NM_	NM_	.	.
ACaP11	2	####	rs3739748	G	A	.	PA!	GPC1	exonic	NM_	.	mis	GPC1:N
ACaP11	3	####	rs5683253	C	T	.	PA!	.	intergen	NM_	dist=	.	.
ACaP11	3	####	rs1474450	C	T	.	PA!	SEMA3F	intronic	NM_	.	.	.
ACaP11	3	####	rs2004359	G	A	.	PA!	ABHD14A	exonic	NM_	.	mis	ABHD1
ACaP11	3	####	.	C	T	.	PA!	ARHGAP3	exonic	NM_	.	sto	ARHGA
ACaP11	4	####	.	T	G	.	PA!	LRPAP1	intronic	NM_	.	.	.
ACaP11	4	####	.	T	C	.	PA!	JAKMIP1	intronic	NM_	.	.	.
ACaP11	4	####	.	G	A	.	PA!	SH3TC1	exonic	NM_	.	syr	SH3TC
ACaP11	4	####	.	G	A	.	PA!	LIMCH1	intronic	NM_	.	.	.
ACaP11	4	####	rs2932367	A	T	.	PA!	.	intergen	NM_	dist=	.	.
ACaP11	4	####	.	T	C	.	PA!	.	intergen	NM_	dist=	.	.
ACaP11	4	####	.	G	A	.	PA!	USP46	intronic	NM_	.	.	.
ACaP11	4	####	rs7669673	C	T	.	PA!	GK2	exonic	NM_	.	syr	GK2:NN
ACaP11	5	####	.	C	T	.	PA!	ERCC8	intronic	NM_	.	.	.
ACaP11	5	####	.	C	A	.	PA!	.	upstrea	NM_	.	.	.
ACaP11	5	####	.	G	A	.	PA!	SLCO4C1	exonic	NM_	.	mis	SLCO4
ACaP11	5	####	.	G	A	.	PA!	YIPF5	UTR3	NM_	NM_	.	.
ACaP11	5	####	rs5319092	G	A	.	PA!	ABLIM3	UTR3	NM_	NM_	.	.
ACaP11	6	####	rs5661365	A	T	.	PA!	SYCP2L	intronic	NM_	.	.	.
ACaP11	6	####	.	C	T	.	PA!	HLA-G	intronic	NM_	.	.	.
ACaP11	6	####	.	C	A	.	PA!	TNXB	exonic	NM_	.	syr	TNXB:N
ACaP11	7	####	rs1814105	G	A	.	PA!	INTS1	exonic	NM_	.	syr	INTS1:I
ACaP11	7	####	rs7827655	G	A	.	PA!	.	intergen	NR_	dist=	.	.
ACaP11	7	####	rs1484992	G	A	.	PA!	RUNDC3E	intronic	NM_	.	.	.
ACaP11	7	####	rs5523938	A	T	.	PA!	EPHB4	intronic	NM_	.	.	.
ACaP11	7	####	.	G	T	.	PA!	DGKI	exonic	NM_	.	syr	DGKI:N
ACaP11	7	####	rs3737114	G	A	.	PA!	DGKI	intronic	NM_	.	.	.
ACaP11	7	####	.	C	T	.	PA!	OR2A25	exonic	NM_	.	syr	OR2A2
ACaP11	8	####	rs9982298	G	A	.	PA!	SNTG1	intronic	NM_	.	.	.
ACaP11	8	####	.	C	T	.	PA!	OTUD6B	exonic	NM_	.	mis	OTUD6
ACaP11	9	####	.	G	A	.	PA!	GLIS3	exonic	NM_	.	mis	GLIS3:I
ACaP11	9	####	.	G	A	.	PA!	.	intergen	NR_	dist=	.	.
ACaP11	9	####	rs9330412	T	C	.	PA!	.	intergen	NR_	dist=	.	.
ACaP11	9	####	.	G	A	.	PA!	C9orf89	intronic	NM_	.	.	.
ACaP11	9	####	.	C	A	.	PA!	.	intergen	NM_	dist=	.	.
ACaP11	9	####	rs9459874	G	A	.	PA!	GPSM1	exonic	NM_	.	mis	GPSM1
ACaP11	9	####	rs9494422	C	T	.	PA!	CACNA1E	intronic	NM_	.	.	.
ACaP11	10	####	.	C	G	.	PA!	GPR158	exonic	NM_	.	mis	GPR15
ACaP11	10	####	.	T	A	.	PA!	ZNF33A	exonic	NM_	.	mis	ZNF33A
ACaP11	10	####	rs2996811	G	A	.	PA!	.	intergen	NR_	dist=	.	.

ACaP11	10	####	.	G	C	.	PA!	AGAP6	intronic	NM_	.	. .
ACaP11	10	####	.	G	C	.	PA!	ENTPD1-/	ncRNA_	NR_	.	. .
ACaP11	10	####	rs7792060	G	A	.	PA!	PI4K2A	intronic	NM_	.	. .
ACaP11	11	####	.	T	A	.	PA!	PARVA	intronic	NM_	.	. .
ACaP11	11	####	.	C	A	.	PA!	ATM	intronic	NM_	.	. .
ACaP11	11	####	.	T	G	.	PA!	HYOU1	exonic	NM_	.	mis HYOU1
ACaP11	11	####	.	T	C	.	PA!	GLB1L2	intronic	NM_	.	. .
ACaP11	12	####	.	T	C	.	PA!	CLEC4C	intronic	NM_	.	. .
ACaP11	12	####	.	G	A	.	PA!	ETV6	exonic	NM_	.	mis ETV6:N
ACaP11	12	####	rs1219135	C	T	.	PA!	KRAS	exonic	NM_	.	mis KRAS:N
ACaP11	12	####	rs3522560	G	A	.	PA!	KIF5A	exonic	NM_	.	syr KIF5A:N
ACaP11	12	####	.	T	A	.	PA!	KCNC2	UTR3	NM_	NM_	. .
ACaP11	12	####	rs5396169	G	A	.	PA!	GNN	ncRNA_	NR_	.	. .
ACaP11	13	####	.	G	A	.	PA!	.	intergen	NM_	dist=	. .
ACaP11	14	####	.	C	T	.	PA!	CNIH1	intronic	NM_	.	. .
ACaP11	14	####	rs8791962	G	A	.	PA!	.	intergen	NM_	dist=	. .
ACaP11	15	####	.	G	A	.	PA!	LOC72792	ncRNA_	NR_	.	. .
ACaP11	15	####	.	A	G	.	PA!	NDN	exonic	NM_	.	mis NDN:NI
ACaP11	15	####	.	C	T	.	PA!	GABRA5	exonic	NM_	.	mis GABRA
ACaP11	15	####	rs7831577	A	C	.	PA!	.	intergen	NM_	dist=	. .
ACaP11	15	####	rs2010114	T	A	.	PA!	.	intergen	NR_	dist=	. .
ACaP11	16	####	.	G	A	.	PA!	ZKSCAN2	intronic	NM_	.	. .
ACaP11	16	####	.	T	C	.	PA!	RNU6-76F	ncRNA_	NR_	.	. .
ACaP11	16	####	.	C	A	.	PA!	CBLN1	intronic	NM_	.	. .
ACaP11	16	####	rs2001829	A	C	.	PA!	PDXDC2F	ncRNA_	NR_	.	. .
ACaP11	16	####	.	C	A	.	PA!	PLCG2	intronic	NM_	.	. .
ACaP11	17	####	.	C	T	.	PA!	RFFL	exonic	NM_	.	syr RFFL:N
ACaP11	17	####	.	C	T	.	PA!	.	upstreal	NM_	.	. .
ACaP11	17	####	.	C	A	.	PA!	.	intergen	NM_	dist=	. .
ACaP11	17	####	.	C	G	.	PA!	BAHCC1	intronic	NM_	.	. .
ACaP11	18	####	.	T	A	.	PA!	SERPINB:	intronic	NM_	.	. .
ACaP11	19	####	rs8673654	T	C	.	PA!	ZFR2	intronic	NM_	.	. .
ACaP11	19	####	.	A	T	.	PA!	FLJ22184	exonic	NM_	.	unl UNKNC
ACaP11	19	####	rs7680686	C	T	.	PA!	DOCK6	exonic	NM_	.	mis DOCK6
ACaP11	19	####	.	A	T	.	PA!	LRFN3	exonic	NM_	.	mis LRFN3:
ACaP11	19	####	.	G	A	.	PA!	.	intergen	NM_	dist=	. .
ACaP11	19	####	.	C	T	.	PA!	ZNF225	exonic	NM_	.	syr ZNF225
ACaP11	19	####	.	G	A	.	PA!	LILRA5	intronic	NM_	.	. .
ACaP11	19	####	.	G	A	.	PA!	NLRP2	intronic	NM_	.	. .
ACaP11	19	####	rs7705940	G	A	.	PA!	ZNF814	exonic	NM_	.	syr ZNF814
ACaP11	20	####	.	G	A	.	PA!	STK35	exonic	NM_	.	mis STK35:
ACaP11	20	####	rs2002254	G	A	.	PA!	CST11	intronic	NM_	.	. .
ACaP11	20	####	.	G	T	.	PA!	FAM83D	exonic	NM_	.	mis FAM83I
ACaP11	20	####	.	C	T	.	PA!	.	intergen	NM_	dist=	. .
ACaP11	21	####	.	T	G	.	PA!	NCAM2	exonic	NM_	.	mis NCAM2
ACaP11	22	####	.	G	A	.	PA!	SYN3	exonic	NM_	.	syr SYN3:N
ACaP11	X	####	.	C	G	.	PA!	.	intergen	NM_	dist=	. .
ACaP11	X	####	.	G	A	.	PA!	ARMCX2	exonic	NM_	.	syr ARMCX
ACaP11	X	####	.	C	T	.	PA!	TEX13A	exonic	NM_	.	mis TEX13A
ACaP11	X	####	rs7533837	C	T	.	PA!	NRK	exonic	NM_	.	syr NRK:NI
ACaP11	X	####	.	C	T	.	PA!	SH2D1A	intronic	NM_	.	. .
ACaP11	X	####	rs7767123	C	T	.	PA!	MAP7D3	intronic	NM_	.	. .
ACaP11	X	####	.	C	A	.	PA!	.	intergen	NM_	dist=	. .
ACaP11	X	####	rs3981235	G	A	.	PA!	G6PD	exonic	NM_	.	mis G6PD:N

ACaP11	MT	####	.	A	G	.	PA:	.	intergen	NOI	dist=	.	.	
ACaP12	1	####	.	C	T	.	PA:	PGD	intronic	NM_	.	.	.	
ACaP12	1	####	rs1015792	T	C	.	PA:	CROCCP2	ncRNA_	NR_	.	.	.	
ACaP12	1	####	.	C	T	.	PA:	TAS1R2	exonic	NM_	.	syr	TAS1R2	
ACaP12	1	####	rs1453424	G	A	.	PA:	RAP1GAP	exonic	NM_	.	mis	RAP1G	
ACaP12	1	####	.	G	T	.	PA:	BTBD8	exonic	NM_	.	sto	BTBD8:	
ACaP12	1	####	rs7704066	T	C	.	PA:	.	intergen	NR_	dist=	.	.	
ACaP12	1	####	rs1415172	A	G	.	PA:	.	intergen	NR_	dist=	.	.	
ACaP12	1	####	rs1256214	A	G	.	PA:	.	intergen	NR_	dist=	.	.	
ACaP12	1	####	rs3721624	C	T	.	PA:	NBPF25P	ncRNA_	NR_	.	.	.	
ACaP12	1	####	rs5876752	C	T	.	PA:	NBPF25P	ncRNA_	NR_	.	.	.	
ACaP12	1	####	.	A	G	.	PA:	NBPF25P	ncRNA_	NR_	.	.	.	
ACaP12	1	####	rs3730063	C	T	.	PA:	CA14	exonic	NM_	.	syr	CA14:N	
ACaP12	1	####	.	G	C	.	PA:	NCF2	intronic	NM_	.	.	.	
ACaP12	1	####	.	C	G	.	PA:	KCNH1	exonic	NM_	.	mis	KCNH1	
ACaP12	2	####	.	G	A	.	PA:	KCNK12	exonic	NM_	.	mis	KCNK1	
ACaP12	2	####	rs7540104	G	A	.	PA:	.	intergen	NR_	dist=	.	.	
ACaP12	2	####	.	G	A	.	PA:	GALNT13	intronic	NM_	.	.	.	
ACaP12	2	####	.	C	A	.	PA:	MIR548N	ncRNA_	NR_	.	.	.	
ACaP12	2	####	.	G	T	.	PA:	HSPD1	intronic	NM_	.	.	.	
ACaP12	2	####	rs9825331	G	A	.	PA:	SH3BP4	intronic	NM_	.	.	.	
ACaP12	3	####	.	A	G	.	PA:	SUMF1	intronic	NM_	.	.	.	
ACaP12	3	####	rs1309395	A	G	.	PA:	BHLHE40	UTR3	NM_	NM_	.	.	
ACaP12	3	####	.	A	C	.	PA:	CMTM7	intronic	NM_	.	.	.	
ACaP12	3	####	rs3710999	G	A	.	PA:	GORASP1	intronic	NM_	.	.	.	
ACaP12	3	####	.	G	T	.	PA:	EIF1B-AS	ncRNA_	NR_	.	.	.	
ACaP12	3	####	.	G	A	.	PA:	QRICH1	exonic	NM_	.	mis	QRICH1	
ACaP12	3	####	.	G	A	.	PA:	BSN	exonic	NM_	.	syr	BSN:NM	
ACaP12	3	####	.	G	T	.	PA:	BSN	exonic	NM_	.	mis	BSN:NM	
ACaP12	3	####	.	C	T	.	PA:	CACNA2D	intronic	NM_	.	.	.	
ACaP12	3	####	.	T	C	.	PA:	.	intergen	NM_	dist=	.	.	
ACaP12	3	####	rs5729372	T	C	.	PA:	ALDH1L1-	ncRNA_	NR_	.	.	.	
ACaP12	4	####	rs4970287	C	T	.	PA:	.	intergen	NOI	dist=	.	.	
ACaP12	4	####	rs1456513	C	T	.	PA:	.	intergen	NM_	dist=	.	.	
ACaP12	4	####	.	G	A	.	PA:	AFF1	exonic	NM_	.	mis	AFF1:N	
ACaP12	4	####	.	A	G	.	PA:	SNHG8	ncRNA_	NR_	.	.	.	
ACaP12	4	####	rs3727861	C	T	.	PA:	FAT4	exonic	NM_	.	mis	FAT4:N	
ACaP12	4	####	.	C	T	.	PA:	GALNT7	exonic	NM_	.	mis	GALNT	
ACaP12	4	####	.	A	G	.	PA:	TRIML2	intronic	NM_	.	.	.	
ACaP12	5	####	.	G	C	.	PA:	ABLIM3	UTR3	NM_	NM_	.	.	
ACaP12	5	####	.	C	A	.	PA:	HAVCR1	intronic	NM_	.	.	.	
ACaP12	6	####	.	C	G	.	PA:	MOG	UTR3	NM_	NM_	.	.	
ACaP12	6	####	.	C	G	.	PA:	DNAH8	intronic	NM_	.	.	.	
ACaP12	6	####	rs9010911	C	T	.	PA:	.	upstrear	NR_	.	.	.	
ACaP12	6	####	.	A	T	.	PA:	CEP85L	intronic	NM_	.	.	.	
ACaP12	7	####	rs1544467	G	C	.	PA:	ZNF890P	ncRNA_	NR_	.	.	.	
ACaP12	7	####	.	G	C	.	PA:	USP42	intronic	NM_	.	.	.	
ACaP12	7	####	.	C	G	.	PA:	STK31	intronic	NM_	.	.	.	
ACaP12	7	####	.	C	A	.	PA:	HOXA10-I	ncRNA_	NR_	.	.	.	
ACaP12	7	####	.	T	C	.	PA:	TBRG4	exonic	NM_	.	mis	TBRG4	
ACaP12	7	####	rs1929995	G	A	.	PA:	.	intergen	NM_	dist=	.	.	
ACaP12	7	####	.	T	G	.	PA:	TYW1	intronic	NM_	.	.	.	
ACaP12	7	####	.	G	A	.	PA:	HIP1	exonic	NM_	.	syr	HIP1:NI	
ACaP12	7	####	.	T	A	.	PA:	PON2	splicing	NM_	NM_	.	.	

ACaP12	7	####	.	G	C	.	PA:	TRRAP	exonic	NM_	.	mis	TRRAP
ACaP12	7	####	rs5753233	C	T	.	PA:	CHCHD3	intronic	NM_	.	.	.
ACaP12	7	####	.	G	A	.	PA:	NUP205	intronic	NM_	.	.	.
ACaP12	8	####	.	G	A	.	PA:	.	intergen	NOI	dist=	.	.
ACaP12	8	####	.	G	C	.	PA:	LACTB2	exonic	NM_	.	mis	LACTB:
ACaP12	8	####	rs5472481	T	C	.	PA:	LOC3922:	ncRNA_	NR_	.	.	.
ACaP12	8	####	.	G	C	.	PA:	ATP6V0D:	intronic	NM_	.	.	.
ACaP12	9	####	rs3746546	C	T	.	PA:	LINGO2	intronic	NM_	.	.	.
ACaP12	9	####	.	C	T	.	PA:	KIAA1161	exonic	NM_	.	unl	UNKNC
ACaP12	9	####	.	C	T	.	PA:	APBA1	exonic	NM_	.	mis	APBA1:
ACaP12	9	####	.	C	T	.	PA:	PCSK5	exonic	NM_	.	mis	PCSK5
ACaP12	9	####	rs2868900	G	A	.	PA:	.	intergen	NM_	dist=	.	.
ACaP12	9	####	rs3719801	C	T	.	PA:	MIR455	ncRNA_	NR_	.	.	.
ACaP12	9	####	rs9976753	G	A	.	PA:	CARD9	intronic	NM_	.	.	.
ACaP12	9	####	rs3763504	C	T	.	PA:	SEC16A	exonic	NM_	.	mis	SEC16/
ACaP12	10	####	rs9266937	C	T	.	PA:	PITRM1-A	ncRNA_	NR_	.	.	.
ACaP12	10	####	.	A	T	.	PA:	PARD3	intronic	NM_	.	.	.
ACaP12	10	####	.	A	G	.	PA:	SYT15	intronic	NM_	.	.	.
ACaP12	10	####	.	T	A	.	PA:	USP54	exonic	NM_	.	sto	USP54:
ACaP12	10	####	.	T	G	.	PA:	IDE	intronic	NM_	.	.	.
ACaP12	10	####	.	A	C	.	PA:	DOCK1	intronic	NM_	.	.	.
ACaP12	11	####	.	A	C	.	PA:	.	downstr	NM_	.	.	.
ACaP12	11	####	.	C	T	.	PA:	TPH1	intronic	NM_	.	.	.
ACaP12	11	####	.	T	A	.	PA:	HPS5	intronic	NM_	.	.	.
ACaP12	11	####	.	C	T	.	PA:	.	intergen	NM_	dist=	.	.
ACaP12	11	####	rs3696017	C	T	.	PA:	SLC43A3	intronic	NM_	.	.	.
ACaP12	11	####	.	C	T	.	PA:	C11orf86	intronic	NM_	.	.	.
ACaP12	11	####	.	G	A	.	PA:	CWF19L2	exonic	NM_	.	sto	CWF19
ACaP12	11	####	.	G	A	.	PA:	USP2-AS1	ncRNA_	NR_	.	.	.
ACaP12	12	####	.	A	C	.	PA:	COL2A1	intronic	NM_	.	.	.
ACaP12	12	####	.	G	C	.	PA:	DEPDC4	exonic	NM_	.	mis	DEPDC
ACaP12	12	####	rs8683709	T	C	.	PA:	HECTD4	intronic	NM_	.	.	.
ACaP12	12	####	.	G	A	.	PA:	MED13L	intronic	NM_	.	.	.
ACaP12	12	####	rs5681239	C	T	.	PA:	HNF1A	exonic	NM_	.	mis	HNF1A
ACaP12	13	####	rs7528858	C	T	.	PA:	DOCK9	intronic	NM_	.	.	.
ACaP12	14	####	.	T	C	.	PA:	PRMT5	exonic	NM_	.	mis	PRMT5
ACaP12	14	####	rs1288039	A	G	.	PA:	.	intergen	NM_	dist=	.	.
ACaP12	14	####	rs5316143	G	A	.	PA:	.	upstreal	NR_	.	.	.
ACaP12	15	####	.	C	G	.	PA:	NDN	exonic	NM_	.	mis	NDN:NI
ACaP12	15	####	rs7540721	C	T	.	PA:	TJP1	exonic	NM_	.	mis	TJP1:N
ACaP12	15	####	rs2015691	A	C	.	PA:	CHRFAM7	UTR5	NM_	NM_	.	.
ACaP12	15	####	rs7122570	C	G	.	PA:	ULK4P1,U	ncRNA_	NR_	.	.	.
ACaP12	15	####	.	G	A	.	PA:	NUTM1	exonic	NM_	.	syr	NUTM1
ACaP12	15	####	.	C	T	.	PA:	EIF3J-AS1	ncRNA_	NR_	.	.	.
ACaP12	15	####	rs1869226	T	A	.	PA:	LOC6457!	ncRNA_	NR_	.	.	.
ACaP12	15	####	rs8789048	A	G	.	PA:	LOC6457!	ncRNA_	NR_	.	.	.
ACaP12	15	####	.	C	T	.	PA:	TBC1D2B	exonic	NM_	.	mis	TBC1D:
ACaP12	15	####	.	A	T	.	PA:	CPEB1	intronic	NM_	.	.	.
ACaP12	16	####	rs7655357	C	T	.	PA:	TBL3	exonic	NM_	.	syr	TBL3:N
ACaP12	16	####	rs2015706	G	A	.	PA:	NLRC3	exonic	NM_	.	syr	NLRC3
ACaP12	16	####	.	T	C	.	PA:	C16orf89	intronic	NM_	.	.	.
ACaP12	16	####	.	C	T	.	PA:	CHST4	exonic	NM_	.	syr	CHST4
ACaP12	17	####	.	C	G	.	PA:	SMYD4	intronic	NM_	.	.	.
ACaP12	17	####	.	G	A	.	PA:	ITGAE	intronic	NM_	.	.	.

ACaP12	17	####	rs7781206	T	C	.	PA!	RPAIN	intronic	NM_	.	.	.	
ACaP12	17	####	.	C	T	.	PA!	ANKFN1	intronic	NM_	.	.	.	
ACaP12	17	####	rs1134430	C	T	.	PA!	FDXR	intronic	NM_	.	.	.	
ACaP12	17	####	.	G	A	.	PA!	ITGB4	intronic	NM_	.	.	.	
ACaP12	17	####	rs7648089	G	A	.	PA!	RPTOR	exonic	NM_	.	syr	RPTOR	
ACaP12	17	####	.	C	T	.	PA!	P4HB	intronic	NM_	.	.	.	
ACaP12	17	####	rs3729739	C	T	.	PA!	CD7	exonic	NM_	.	mis	CD7:NM	
ACaP12	17	####	.	C	G	.	PA!	B3GNTL1	intronic	NM_	.	.	.	
ACaP12	17	####	.	C	G	.	PA!	B3GNTL1	intronic	NM_	.	.	.	
ACaP12	18	####	.	C	T	.	PA!	ENOSF1	UTR3	NM_	NM_	.	.	
ACaP12	18	####	.	C	T	.	PA!	ENOSF1	UTR3	NM_	NM_	.	.	
ACaP12	18	####	.	A	C	.	PA!	ZNF24	intronic	NM_	.	.	.	
ACaP12	19	####	rs5282381	C	T	.	PA!	NFIC	intronic	NM_	.	.	.	
ACaP12	19	####	.	G	A	.	PA!	NFIC	intronic	NM_	.	.	.	
ACaP12	19	####	rs9075262	G	A	.	PA!	NFIC	intronic	NM_	.	.	.	
ACaP12	19	####	.	G	T	.	PA!	NFIC	intronic	NM_	.	.	.	
ACaP12	19	####	.	C	A	.	PA!	ZNF561	intronic	NM_	.	.	.	
ACaP12	19	####	.	C	T	.	PA!	ZSWIM4	intronic	NM_	.	.	.	
ACaP12	19	####	.	A	C	.	PA!	ZNF573	exonic	NM_	.	mis	ZNF573	
ACaP12	19	####	.	C	A	.	PA!	ZNF233	intronic	NM_	.	.	.	
ACaP12	19	####	.	G	C	.	PA!	IZUMO1	intronic	NM_	.	.	.	
ACaP12	19	####	.	G	A	.	PA!	FUT1	exonic	NM_	.	sto	FUT1:N	
ACaP12	19	####	.	G	A	.	PA!	FUT1	exonic	NM_	.	mis	FUT1:N	
ACaP12	19	####	.	C	T	.	PA!	CLEC11A	exonic	NM_	.	syr	CLEC1:	
ACaP12	19	####	.	C	A	.	PA!	ZNF28	intronic	NM_	.	.	.	
ACaP12	19	####	.	C	A	.	PA!	CACNG7	intronic	NM_	.	.	.	
ACaP12	20	####	rs7457076	C	T	.	PA!	LPIN3	exonic	NM_	.	mis	LPIN3:N	
ACaP12	21	####	.	G	A	.	PA!	.	downstr	NR_	.	.	.	
ACaP12	21	####	rs8792017	A	G	.	PA!	.	intergen	NR_	dist=	.	.	
ACaP12	21	####	.	A	C	.	PA!	TPTE	intronic	NM_	.	.	.	
ACaP12	22	####	rs1167407	C	T	.	PA!	BCL2L13	intronic	NM_	.	.	.	
ACaP12	22	####	rs3760330	A	G	.	PA!	DGCR6	intronic	NM_	.	.	.	
ACaP12	22	####	.	C	T	.	PA!	SMTN	exonic	NM_	.	sto	SMTN:N	
ACaP12	X	####	.	C	T	.	PA!	RLIM	exonic	NM_	.	mis	RLIM:N	
ACaP12	X	####	.	G	T	.	PA!	MCF2	exonic	NM_	.	mis	MCF2:N	
ACaP12	X	####	.	T	A	.	PA!	MAGEC1	UTR3	NM_	NM_	.	.	
ACaP13	1	####	rs1153096	T	C	.	PA!	ATAD3B	intronic	NM_	.	.	.	
ACaP13	1	####	.	T	G	.	PA!	GABRD	intronic	NM_	.	.	.	
ACaP13	1	####	.	G	A	.	PA!	RSPO1	UTR5	NM_	NM_	.	.	
ACaP13	1	####	rs5317072	C	T	.	PA!	CCDC17	exonic	NM_	.	syr	CCDC1	
ACaP13	1	####	.	T	A	.	PA!	ZFYVE9	intronic	NM_	.	.	.	
ACaP13	1	####	rs7787296	C	T	.	PA!	DAB1	UTR5	NM_	NM_	.	.	
ACaP13	1	####	.	C	T	.	PA!	AGL	exonic	NM_	.	syr	AGL:NM	
ACaP13	1	####	rs7736232	A	T	.	PA!	.	intergen	NR_	dist=	.	.	
ACaP13	1	####	rs1712174	A	C	.	PA!	.	intergen	NR_	dist=	.	.	
ACaP13	1	####	.	C	A	.	PA!	LCE2D	UTR3	NM_	NM_	.	.	
ACaP13	1	####	.	C	T	.	PA!	UBAP2L	intronic	NM_	.	.	.	
ACaP13	1	####	.	C	T	.	PA!	NIT1	intronic	NM_	.	.	.	
ACaP13	1	####	.	T	G	.	PA!	LAMC2	intronic	NM_	.	.	.	
ACaP13	1	####	.	G	T	.	PA!	KCNT2	intronic	NM_	.	.	.	
ACaP13	1	####	.	A	T	.	PA!	C1orf53	UTR5	NM_	NM_	.	.	
ACaP13	1	####	.	G	A	.	PA!	KDM5B	exonic	NM_	.	mis	KDM5B	
ACaP13	1	####	.	G	A	.	PA!	SDCCAG8	intronic	NM_	.	.	.	
ACaP13	2	####	.	C	T	.	PA!	TRAPPC1	exonic	NM_	.	mis	TRAPP	

ACaP13	2	####	.	C	T	.	PA:	TRAPPC1	exonic	NM_	.	syr	TRAPP
ACaP13	2	####	.	C	A	.	PA:	NBAS	exonic	NM_	.	mis	NBAS:N
ACaP13	2	####	.	G	T	.	PA:	NLRC4	exonic	NM_	.	mis	NLRC4
ACaP13	2	####	.	G	A	.	PA:	ANTXR1	intronic	NM_	.	.	.
ACaP13	2	####	rs2015850	T	C	.	PA:	.	intergen	NM_	dist=	.	.
ACaP13	2	####	.	A	T	.	PA:	ZAP70	exonic	NM_	.	mis	ZAP70:
ACaP13	2	####	rs2008974	G	T	.	PA:	RGPD3	intronic	NM_	.	.	.
ACaP13	2	####	rs7249504	G	T	.	PA:	INSIG2	intronic	NM_	.	.	.
ACaP13	2	####	.	A	T	.	PA:	INSIG2	intronic	NM_	.	.	.
ACaP13	2	####	.	G	A	.	PA:	TTN	exonic	NM_	.	syr	TTN:NM
ACaP13	2	####	.	C	A	.	PA:	ICA1L	UTR3	NM_	NM_	.	.
ACaP13	2	####	rs5520376	C	T	.	PA:	ABI2	intronic	NM_	.	.	.
ACaP13	2	####	.	C	G	.	PA:	ABI2	intronic	NM_	.	.	.
ACaP13	2	####	rs1816528	G	A	.	PA:	LINC0060	ncRNA_	NR_	.	.	.
ACaP13	2	####	.	G	A	.	PA:	PRSS56	exonic	NM_	.	mis	PRSS5
ACaP13	3	####	.	C	G	.	PA:	KIF15	intronic	NM_	.	.	.
ACaP13	3	####	rs9185110	C	G	.	PA:	PTH1R	intronic	NM_	.	.	.
ACaP13	3	####	rs3735896	C	T	.	PA:	STAB1	intronic	NM_	.	.	.
ACaP13	3	####	.	T	C	.	PA:	DCBLD2	intronic	NM_	.	.	.
ACaP13	3	####	.	A	G	.	PA:	DCBLD2	exonic	NM_	.	mis	DCBLD
ACaP13	3	####	.	T	G	.	PA:	RPL24	intronic	NM_	.	.	.
ACaP13	3	####	.	G	A	.	PA:	ILDR1	UTR3	NM_	NM_	.	.
ACaP13	3	####	rs1456223	C	T	.	PA:	COL6A6	exonic	NM_	.	syr	COL6A
ACaP13	3	####	rs1048860	G	A	.	PA:	PIK3CA	exonic	NM_	.	mis	PIK3CA
ACaP13	4	####	.	T	G	.	PA:	LRPAP1	intronic	NM_	.	.	.
ACaP13	4	####	rs4018318	C	T	.	PA:	.	intergen	NR_	dist=	.	.
ACaP13	4	####	.	C	T	.	PA:	ATP8A1	intronic	NM_	.	.	.
ACaP13	4	####	.	G	A	.	PA:	ATP8A1	intronic	NM_	.	.	.
ACaP13	4	####	.	G	T	.	PA:	.	intergen	NM_	dist=	.	.
ACaP13	4	####	.	G	C	.	PA:	SCOC	intronic	NM_	.	.	.
ACaP13	4	####	.	C	G	.	PA:	.	intergen	NM_	dist=	.	.
ACaP13	5	####	.	C	T	.	PA:	GUSBP1	ncRNA_	NR_	.	.	.
ACaP13	5	####	.	G	A	.	PA:	SKIV2L2	splicing	NM_	NM_	.	.
ACaP13	5	####	.	G	A	.	PA:	PRR16	UTR5	NM_	NM_	.	.
ACaP13	5	####	.	A	T	.	PA:	RAPGEF6	intronic	NM_	.	.	.
ACaP13	5	####	rs7735798	C	T	.	PA:	PCDHGB4	exonic	NM_	.	mis	PCDHG
ACaP13	5	####	.	C	T	.	PA:	FGF18	exonic	NM_	.	syr	FGF18:
ACaP13	5	####	.	G	A	.	PA:	TBC1D9B	exonic	NM_	.	sto	TBC1D
ACaP13	5	####	rs3761176	C	T	.	PA:	BTNL9	exonic	NM_	.	mis	BTNL9:
ACaP13	6	####	.	C	T	.	PA:	.	upstrea	NM_	.	.	.
ACaP13	6	####	rs1419237	T	G	.	PA:	DHX16	exonic	NM_	.	mis	DHX16:
ACaP13	6	####	rs3687466	C	T	.	PA:	LAMA4	splicing	NM_	NM_	.	.
ACaP13	6	####	.	G	A	.	PA:	RAET1G	exonic	NM_	.	syr	RAET1
ACaP13	6	####	.	G	A	.	PA:	OPRM1	exonic	NM_	.	syr	OPRM1
ACaP13	6	####	rs2014763	G	A	.	PA:	TIAM2	exonic	NM_	.	mis	TIAM2:I
ACaP13	6	####	rs3759780	G	C	.	PA:	.	intergen	NM_	dist=	.	.
ACaP13	7	####	rs7676901	A	G	.	PA:	NUDCD3	intronic	NM_	.	.	.
ACaP13	7	####	.	T	C	.	PA:	DDX56	exonic	NM_	.	mis	DDX56:
ACaP13	7	####	rs6246184	G	A	.	PA:	.	intergen	NR_	dist=	.	.
ACaP13	7	####	rs2013722	C	T	.	PA:	.	intergen	NR_	dist=	.	.
ACaP13	7	####	.	C	G	.	PA:	SBDS	intronic	NM_	.	.	.
ACaP13	7	####	.	C	T	.	PA:	LOC54147	ncRNA_	NR_	.	.	.
ACaP13	7	####	rs7611895	G	A	.	PA:	MUC12	exonic	NM_	.	mis	MUC12
ACaP13	7	####	.	A	C	.	PA:	KIAA1147	intronic	NM_	.	.	.

ACaP13	7	####	rs1419939	A	T	.	PA:	KMT2C	exonic	NM_	.	syr	KMT2C
ACaP13	7	####	.	G	A	.	PA:	.	intergen	NM_	dist=	.	.
ACaP13	8	####	.	G	A	.	PA:	TNKS	intronic	NM_	.	.	.
ACaP13	8	####	rs7484521	C	A	.	PA:	LOC7297	ncRNA_	NR_	.	.	.
ACaP13	8	####	.	A	C	.	PA:	XPO7	intronic	NM_	.	.	.
ACaP13	8	####	.	T	C	.	PA:	SORBS3	intronic	NM_	.	.	.
ACaP13	8	####	.	G	A	.	PA:	MMP16	exonic	NM_	.	mis	MMP16
ACaP13	8	####	rs5352633	G	A	.	PA:	RIMS2	intronic	NM_	.	.	.
ACaP13	8	####	rs5594625	A	T	.	PA:	CSMD3	intronic	NM_	.	.	.
ACaP13	8	####	rs3766154	C	T	.	PA:	ANXA13	exonic	NM_	.	mis	ANXA1
ACaP13	8	####	.	G	A	.	PA:	CCDC166	intronic	NM_	.	.	.
ACaP13	8	####	.	G	A	.	PA:	OPLAH	exonic	NM_	.	mis	OPLAH
ACaP13	9	####	.	G	T	.	PA:	PSIP1	exonic	NM_	.	syr	PSIP1:N
ACaP13	9	####	.	T	C	.	PA:	CNTLN	intronic	NM_	.	.	.
ACaP13	9	####	rs2803685	G	T	.	PA:	FAM27C	ncRNA_	NR_	.	.	.
ACaP13	9	####	rs7553291	G	A	.	PA:	SPATA31	exonic	NM_	.	syr	SPATA
ACaP13	9	####	.	A	G	.	PA:	GAPVD1	exonic	NM_	.	mis	GAPVD
ACaP13	10	####	rs1901417	G	A	.	PA:	PITRM1-A	ncRNA_	NR_	.	.	.
ACaP13	10	####	.	G	T	.	PA:	ITGB1	exonic	NM_	.	mis	ITGB1:I
ACaP13	10	####	rs4070334	G	A	.	PA:	.	intergen	NR_	dist=	.	.
ACaP13	10	####	.	C	T	.	PA:	SFTPA2	intronic	NM_	.	.	.
ACaP13	10	####	.	G	C	.	PA:	KLLN	UTR5	NM_	NM_	.	.
ACaP13	10	####	.	G	A	.	PA:	ENTPD1-/	ncRNA_	NR_	.	.	.
ACaP13	11	####	rs7488284	G	A	.	PA:	MUC6	exonic	NM_	.	syr	MUC6:N
ACaP13	11	####	.	C	T	.	PA:	USP47	intronic	NM_	.	.	.
ACaP13	11	####	.	G	A	.	PA:	ABCC8	intronic	NM_	.	.	.
ACaP13	11	####	.	T	A	.	PA:	LDHA	intronic	NM_	.	.	.
ACaP13	11	####	.	C	T	.	PA:	ANO3	intronic	NM_	.	.	.
ACaP13	11	####	rs1470918	G	T	.	PA:	METTL15	intronic	NM_	.	.	.
ACaP13	11	####	.	A	T	.	PA:	TMEM179	UTR3	NM_	NM_	.	.
ACaP13	11	####	.	G	A	.	PA:	C11orf95	exonic	NM_	.	mis	C11orf9
ACaP13	11	####	rs2010003	C	A	.	PA:	.	intergen	NM_	dist=	.	.
ACaP13	11	####	.	A	T	.	PA:	DYNC2H1	intronic	NM_	.	.	.
ACaP13	12	####	.	G	C	.	PA:	NELL2	UTR5	NM_	NM_	.	.
ACaP13	12	####	rs7761498	A	G	.	PA:	SLC4A8	intronic	NM_	.	.	.
ACaP13	12	####	.	G	A	.	PA:	PAN2	exonic	NM_	.	mis	PAN2:N
ACaP13	12	####	.	C	G	.	PA:	ANKS1B	intronic	NM_	.	.	.
ACaP13	12	####	.	G	A	.	PA:	MMAB	splicing	NM_	NM_	.	.
ACaP13	13	####	.	C	G	.	PA:	.	upstreal	NR_	.	.	.
ACaP13	13	####	.	C	A	.	PA:	.	intergen	NM_	dist=	.	.
ACaP13	13	####	.	G	A	.	PA:	.	intergen	NR_	dist=	.	.
ACaP13	14	####	rs2259899	C	A	.	PA:	.	intergen	NR_	dist=	.	.
ACaP13	14	####	rs7966403	C	A	.	PA:	.	intergen	NM_	dist=	.	.
ACaP13	14	####	rs7511166	C	T	.	PA:	MMP14	exonic	NM_	.	mis	MMP14
ACaP13	14	####	rs1127835	C	T	.	PA:	MYH6	intronic	NM_	.	.	.
ACaP13	14	####	.	C	A	.	PA:	RALGAPA	splicing	NM_	NM_	.	.
ACaP13	14	####	.	T	G	.	PA:	CDC42BP	exonic	NM_	.	mis	CDC42
ACaP13	15	####	rs7146667	T	C	.	PA:	.	upstreal	NR_	.	.	.
ACaP13	15	####	.	T	C	.	PA:	UBE3A	exonic	NM_	.	mis	UBE3A
ACaP13	15	####	.	G	A	.	PA:	APBA2	intronic	NM_	.	.	.
ACaP13	15	####	.	G	A	.	PA:	BUB1B	exonic	NM_	.	mis	BUB1B
ACaP13	15	####	.	G	T	.	PA:	.	intergen	NR_	dist=	.	.
ACaP13	16	####	.	T	C	.	PA:	CLEC16A	intronic	NM_	.	.	.
ACaP13	16	####	.	A	T	.	PA:	XYLT1	intronic	NM_	.	.	.

ACaP13	16	####	.	C	T	.	PA!	SPN	exonic	NM_	.	mis	SPN:NM
ACaP13	16	####	rs1450253	C	T	.	PA!	ITGAX	exonic	NM_	.	syr	ITGAX:
ACaP13	16	####	.	C	G	.	PA!	SLC12A4	exonic	NM_	.	mis	SLC12A
ACaP13	16	####	rs1135006	G	A	.	PA!	WWP2	intronic	NM_	.	.	.
ACaP13	17	####	rs7673846	G	A	.	PA!	SGSM2	exonic	NM_	.	mis	SGSM2
ACaP13	17	####	rs7606360	C	T	.	PA!	GLP2R	exonic	NM_	.	sto	GLP2R
ACaP13	17	####	.	G	A	.	PA!	MYH13	intronic	NM_	.	.	.
ACaP13	17	####	rs5549220	G	A	.	PA!	PROCA1	intronic	NM_	.	.	.
ACaP13	17	####	.	A	G	.	PA!	GFAP	intronic	NM_	.	.	.
ACaP13	17	####	.	G	T	.	PA!	ABCA6	intronic	NM_	.	.	.
ACaP13	17	####	rs7400170	C	T	.	PA!	TBCD	splicing	NM_	NM_	.	.
ACaP13	18	####	rs7837587	T	A	.	PA!	ANKRD20	ncRNA_	NR_	.	.	.
ACaP13	18	####	.	C	G	.	PA!	SLC14A1	intronic	NM_	.	.	.
ACaP13	18	####	.	G	A	.	PA!	.	upstrea	NM_	.	.	.
ACaP13	18	####	rs1466769	G	A	.	PA!	.	intergen	NM_	dist=	.	.
ACaP13	18	####	rs5634889	C	T	.	PA!	.	intergen	NR_	dist=	.	.
ACaP13	19	####	.	C	T	.	PA!	ABCA7	exonic	NM_	.	syr	ABCA7
ACaP13	19	####	.	G	A	.	PA!	ACTL9	exonic	NM_	.	syr	ACTL9:
ACaP13	19	####	.	A	G	.	PA!	ZNF700	intronic	NM_	.	.	.
ACaP13	19	####	.	G	A	.	PA!	EMR3	intronic	NM_	.	.	.
ACaP13	19	####	.	C	G	.	PA!	.	intergen	NM_	dist=	.	.
ACaP13	19	####	.	G	A	.	PA!	.	upstrea	NR_	.	.	.
ACaP13	19	####	.	A	C	.	PA!	ZNF492	intronic	NM_	.	.	.
ACaP13	19	####	.	C	T	.	PA!	NFKBID	intronic	NM_	.	.	.
ACaP13	19	####	rs7125437	C	G	.	PA!	.	intergen	NR_	dist=	.	.
ACaP13	19	####	rs1820521	G	A	.	PA!	SIGLEC7	intronic	NM_	.	.	.
ACaP13	20	####	.	C	T	.	PA!	OXT	exonic	NM_	.	syr	OXT:NM
ACaP13	20	####	.	G	A	.	PA!	GFRA4	exonic	NM_	.	sto	GFRA4
ACaP13	20	####	.	T	A	.	PA!	PLCB1	splicing	NM_	NM_	.	.
ACaP13	20	####	.	G	A	.	PA!	GATA5	exonic	NM_	.	syr	GATA5
ACaP13	21	####	rs7964453	C	G	.	PA!	.	intergen	NR_	dist=	.	.
ACaP13	21	####	rs3735529	C	A	.	PA!	.	downstr	NM_	.	.	.
ACaP13	21	####	rs7280383	T	C	.	PA!	.	intergen	NM_	dist=	.	.
ACaP13	21	####	.	G	T	.	PA!	NCAM2	intronic	NM_	.	.	.
ACaP13	21	####	rs7738143	C	T	.	PA!	CLIC6	exonic	NM_	.	syr	CLIC6:N
ACaP13	21	####	rs5510648	C	T	.	PA!	TRAPPC1	exonic	NM_	.	syr	TRAPP
ACaP13	21	####	.	G	A	.	PA!	PCBP3	exonic	NM_	.	mis	PCBP3
ACaP13	21	####	.	G	A	.	PA!	COL6A1	exonic	NM_	.	mis	COL6A
ACaP13	22	####	rs1262847	G	A	.	PA!	.	intergen	NR_	dist=	.	.
ACaP13	22	####	rs86008	A	G	.	PA!	.	downstr	NR_	.	.	.
ACaP13	22	####	.	C	A	.	PA!	RNF215	exonic	NM_	.	syr	RNF215
ACaP13	X	####	.	T	C	.	PA!	CLCN4	intronic	NM_	.	.	.
ACaP13	X	####	rs7787173	C	T	.	PA!	BEND2	exonic	NM_	.	mis	BEND2
ACaP13	X	####	.	C	T	.	PA!	AKAP4	intronic	NM_	.	.	.
ACaP13	X	####	.	C	T	.	PA!	.	intergen	NM_	dist=	.	.
ACaP13	X	####	.	T	C	.	PA!	TEX11	intronic	NM_	.	.	.
ACaP13	X	####	.	C	T	.	PA!	OGT	exonic	NM_	.	mis	OGT:NI
ACaP13	X	####	.	G	A	.	PA!	TCEAL6	intronic	NM_	.	.	.
ACaP13	X	####	.	C	T	.	PA!	.	intergen	NM_	dist=	.	.
ACaP13	X	####	rs9176062	C	T	.	PA!	.	intergen	NM_	dist=	.	.
ACaP13	X	####	rs9279180	G	A	.	PA!	.	intergen	NM_	dist=	.	.
ACaP13	X	####	.	C	T	.	PA!	IRAK1	exonic	NM_	.	mis	IRAK1:I
ACaP14	1	####	rs3718451	G	A	.	PA!	ACTL8	exonic	NM_	.	syr	ACTL8:
ACaP14	1	####	.	T	A	.	PA!	WDTC1	exonic	NM_	.	sto	WDTC1

ACaP14	1	####	rs7678484	C	T	.	PA!	C1orf94	intronic	NM_	.	.	.	
ACaP14	1	####	rs7603609	G	A	.	PA!	GJA4	exonic	NM_	.	syr	GJA4:N	
ACaP14	1	####	.	G	A	.	PA!	ST3GAL3	intronic	NM_	.	.	.	
ACaP14	1	####	.	G	T	.	PA!	TESK2	exonic	NM_	.	mis	TESK2:	
ACaP14	1	####	.	G	C	.	PA!	ROR1	intronic	NM_	.	.	.	
ACaP14	1	####	.	G	T	.	PA!	JAK1	intronic	NM_	.	.	.	
ACaP14	1	####	.	G	A	.	PA!	AMPD2	exonic	NM_	.	mis	AMPD2	
ACaP14	1	####	rs7547967	T	G	.	PA!	PIFO	intronic	NM_	.	.	.	
ACaP14	1	####	rs7528408	C	T	.	PA!	.	intergen	NR_	dist=	.	.	
ACaP14	1	####	rs2009307	C	T	.	PA!	NBPF20,N	intronic	NM_	.	.	.	
ACaP14	1	####	rs3726923	A	G	.	PA!	NBPF25P	ncRNA_	NR_	.	.	.	
ACaP14	1	####	rs8799825	G	T	.	PA!	NBPF25P	ncRNA_	NR_	.	.	.	
ACaP14	1	####	.	T	G	.	PA!	PTPN7	intronic	NM_	.	.	.	
ACaP14	1	####	rs7756172	C	T	.	PA!	OBSCN	intronic	NM_	.	.	.	
ACaP14	2	####	rs7587514	C	T	.	PA!	TPO	exonic	NM_	.	mis	TPO:NM	
ACaP14	2	####	.	G	A	.	PA!	LRP2	UTR5	NM_	NM_	.	.	
ACaP14	2	####	.	A	C	.	PA!	MIR548N	ncRNA_	NR_	.	.	.	
ACaP14	2	####	rs1411611	C	T	.	PA!	STK17B	intronic	NM_	.	.	.	
ACaP14	3	####	rs1156481	C	T	.	PA!	CELSR3	exonic	NM_	.	mis	CELSR	
ACaP14	3	####	.	A	G	.	PA!	SLC9C1	intronic	NM_	.	.	.	
ACaP14	3	####	.	C	A	.	PA!	PCOLCE2	intronic	NM_	.	.	.	
ACaP14	3	####	.	C	T	.	PA!	ABCC5	exonic	NM_	.	mis	ABCC5	
ACaP14	4	####	.	T	A	.	PA!	PDE6B	exonic	NM_	.	mis	PDE6B	
ACaP14	4	####	.	C	T	.	PA!	GAK	exonic	NM_	.	mis	GAK:NM	
ACaP14	4	####	rs7307061	T	C	.	PA!	ZFYVE28	intronic	NM_	.	.	.	
ACaP14	4	####	rs8796734	A	G	.	PA!	.	upstrear	NM_	.	.	.	
ACaP14	4	####	rs7690288	C	G	.	PA!	GRID2	intronic	NM_	.	.	.	
ACaP14	4	####	.	T	G	.	PA!	RXFP1	intronic	NM_	.	.	.	
ACaP14	4	####	rs1003755	G	A	.	PA!	.	intergen	NM_	dist=	.	.	
ACaP14	5	####	.	G	C	.	PA!	SEMA5A	intronic	NM_	.	.	.	
ACaP14	5	####	.	G	A	.	PA!	RASGRF2	exonic	NM_	.	syr	RASGR	
ACaP14	5	####	.	G	A	.	PA!	APC	exonic	NM_	.	mis	APC:NM	
ACaP14	5	####	.	G	C	.	PA!	PCDHGB8	ncRNA_	NR_	.	.	.	
ACaP14	5	####	.	C	A	.	PA!	EBF1	intronic	NM_	.	.	.	
ACaP14	5	####	.	T	A	.	PA!	WWC1	exonic	NM_	.	syr	WWC1	
ACaP14	6	####	.	G	T	.	PA!	ZSCAN31	exonic	NM_	.	mis	ZSCAN	
ACaP14	6	####	rs7589117	C	T	.	PA!	POU5F1	exonic	NM_	.	mis	POU5F	
ACaP14	6	####	.	T	A	.	PA!	PRIM2	intronic	NM_	.	.	.	
ACaP14	6	####	.	G	A	.	PA!	TBX18	exonic	NM_	.	mis	TBX18:	
ACaP14	6	####	rs441907	T	C	.	PA!	.	intergen	NM_	dist=	.	.	
ACaP14	7	####	.	C	G	.	PA!	SEMA3A	intronic	NM_	.	.	.	
ACaP14	7	####	.	G	C	.	PA!	CALCR	intronic	NM_	.	.	.	
ACaP14	7	####	.	C	T	.	PA!	C7orf60	exonic	NM_	.	syr	C7orf60	
ACaP14	8	####	.	A	T	.	PA!	ZNF705G	exonic	NM_	.	mis	ZNF705	
ACaP14	8	####	.	T	C	.	PA!	CSMD3	exonic	NM_	.	mis	CSMD3	
ACaP14	8	####	.	T	C	.	PA!	NDRG1	intronic	NM_	.	.	.	
ACaP14	9	####	.	G	A	.	PA!	NFIB	exonic	NM_	.	sto	NFIB:N	
ACaP14	9	####	.	C	T	.	PA!	FKTN	exonic	NM_	.	sto	FKTN:N	
ACaP14	9	####	.	C	T	.	PA!	OR1L6	exonic	NM_	.	syr	OR1L6:	
ACaP14	10	####	.	A	C	.	PA!	SVIL	intronic	NM_	.	.	.	
ACaP14	10	####	rs3125109	G	A	.	PA!	.	intergen	NR_	dist=	.	.	
ACaP14	10	####	.	G	A	.	PA!	TBATA	UTR3	NM_	NM_	.	.	
ACaP14	10	####	rs7905448	T	A	.	PA!	MCU	intronic	NM_	.	.	.	
ACaP14	10	####	.	C	A	.	PA!	ANKRD1	intronic	NM_	.	.	.	

ACaP14	10	####	rs7646688	A	G	.	PA	MXI1	exonic	NM_	.	mis	MXI1:N
ACaP14	10	####	.	G	A	.	PA	ATRNL1	intronic	NM_	.	.	.
ACaP14	11	####	.	C	T	.	PA	DNHD1	exonic	NM_	.	mis	DNHD1
ACaP14	11	####	.	C	A	.	PA	.	intergen	NM	dist=	.	.
ACaP14	11	####	rs3775202	C	T	.	PA	B3GNT6	exonic	NM_	.	unl	UNKNC
ACaP14	11	####	.	C	T	.	PA	TRIM64	exonic	NM_	.	syr	TRIM64
ACaP14	11	####	rs7690752	G	A	.	PA	.	intergen	NM	dist=	.	.
ACaP14	11	####	rs7585846	G	A	.	PA	SIDT2	exonic	NM_	.	mis	SIDT2:N
ACaP14	12	####	.	C	T	.	PA	ITPR2	exonic	NM_	.	syr	ITPR2:N
ACaP14	12	####	.	T	G	.	PA	DDN	exonic	NM_	.	mis	DDN:NI
ACaP14	12	####	.	A	G	.	PA	PPP1R1A	intronic	NM_	.	.	.
ACaP14	13	####	.	C	T	.	PA	CDX2	exonic	NM_	.	mis	CDX2:N
ACaP14	14	####	rs2259899	C	A	.	PA	.	intergen	NR	dist=	.	.
ACaP14	14	####	rs2014187	G	T	.	PA	.	intergen	NM	dist=	.	.
ACaP14	14	####	.	G	A	.	PA	ZFP36L1	exonic	NM_	.	mis	ZFP36L
ACaP14	15	####	.	C	G	.	PA	UNC13C	exonic	NM_	.	mis	UNC13
ACaP14	16	####	rs7764738	C	T	.	PA	CACNA1H	exonic	NM_	.	mis	CACNA
ACaP14	16	####	.	G	C	.	PA	ERN2	intronic	NM_	.	.	.
ACaP14	16	####	rs2013540	C	A	.	PA	.	intergen	NR	dist=	.	.
ACaP14	18	####	rs7502623	A	C	.	PA	.	intergen	NM	dist=	.	.
ACaP14	19	####	rs7589667	C	T	.	PA	EMR4P	ncRNA_	NR_	.	.	.
ACaP14	19	####	.	C	A	.	PA	COL5A3	exonic	NM_	.	mis	COL5A
ACaP14	19	####	.	A	T	.	PA	MIA-RAB4	ncRNA_	NR_	.	.	.
ACaP14	19	####	.	G	T	.	PA	CCDC61	intronic	NM_	.	.	.
ACaP14	19	####	.	G	A	.	PA	HIF3A	intronic	NM_	.	.	.
ACaP14	19	####	rs9019919	G	A	.	PA	SBK3	exonic	NM_	.	mis	SBK3:N
ACaP14	20	####	.	C	G	.	PA	ANKEF1	exonic	NM_	.	mis	ANKEF
ACaP14	20	####	rs7770803	G	C	.	PA	ANKEF1	exonic	NM_	.	mis	ANKEF
ACaP14	20	####	rs7792821	C	T	.	PA	AAR2	exonic	NM_	.	mis	AAR2:N
ACaP14	21	####	.	G	A	.	PA	DONSON	exonic	NM_	.	mis	DONSC
ACaP14	22	####	.	C	T	.	PA	TBC1D10	exonic	NM_	.	mis	TBC1D
ACaP14	X	####	.	G	A	.	PA	EIF1AX	intronic	NM_	.	.	.
ACaP14	X	####	.	G	T	.	PA	.	intergen	NM	dist=	.	.
ACaP14	X	####	.	C	T	.	PA	ARAF	exonic	NM_	.	syr	ARAF:N
ACaP14	X	####	.	C	A	.	PA	PHF8	intronic	NM_	.	.	.
ACaP14	X	####	.	C	G	.	PA	BEX1	intronic	NM_	.	.	.
ACaP14	X	####	.	C	T	.	PA	TSC22D3	UTR5	NM	NM_	.	.
ACaP14	X	####	.	G	C	.	PA	DDX26B	intronic	NM_	.	.	.
ACaP14	X	####	.	C	T	.	PA	ZIC3	exonic	NM_	.	syr	ZIC3:NI
ACaP14	X	####	.	A	G	.	PA	F8	intronic	NM_	.	.	.
ACaP14	X	####	rs4013617	G	A	.	PA	.	intergen	NM	dist=	.	.
ACaP14	MT	####	.	G	A	.	PA	.	intergen	NOI	dist=	.	.

Genecc	wgRna	cytoBand	targ	tfbs	gen	Repeat	avsnp	cosmic82	cli	gwas(	1000g2(	1000g2(	1000g2(
ENST0	.	1p36.33	.	.	Scc	.	rs626	.	.	.	.	.	.
ENST0	.	1p36.33	.	.	Scc	.	rs879	ID=COSN2	.	.	.	.	.
ENST0	.	1p36.33	.	.	.	.	.	.	.	.	.	.	.
ENST0	.	1p36.23	.	.	.	Score=273	rs201	.	.	.	.	.	.
ENST0	.	1p36.22	.	Scc	.	.	rs770	ID=COSM3	.	.	.	.	.
ENST0	.	1p36.22	.	.	.	.	.	.	.	.	.	.	.
ENST0	.	1p36.21	.	.	Scc	.	rs757	ID=COSN1	.	.	.	.	.
ENST0	.	1p36.13	.	.	Scc	.	.	.	.	.	.	.	.
ENST0	.	1p36.13	.	.	Scc	.	rs643	.	.	.	.	.	.
.	.	1p36.13	.	.	Scc	.	rs189	ID=COSN2	.	.	.	.	.
ENST0	.	1p36.13	.	.	.	Score=981	rs796	ID=COSN2	.	.	.	.	.
ENST0	.	1p35.3	.	.	.	.	.	.	.	.	.	.	.
ENST0	.	1p34.3	.	.	.	.	rs369	.	.	.	.	.	.
ENST0	.	1p32.2	.	.	.	.	.	.	.	.	.	.	.
ENST0	.	1p32.1	.	.	.	.	.	.	.	.	.	.	.
ENST0	.	1p13.3	.	Scc	.	.	.	.	.	.	.	.	.
.	.	1p11.2	.	.	.	Score=140	.	.	.	.	.	.	.
ENST0	.	1q21.1	.	.	Scc	.	rs180	ID=COSN1	.	.	.	.	.
.	.	1q21.1	.	.	Scc	.	rs374	.	.	.	.	.	.
ENST0	.	1q21.1	.	.	Scc	Score=190	rs140	.	.	.	.	.	.
ENST0	.	1q21.1	.	.	Scc	.	rs796	ID=COSN1	.	.	.	.	.
ENST0	.	1q21.1	.	.	Scc	.	rs776	ID=COSN1	.	.	.	.	.
.	.	1q21.1	.	.	Scc	.	.	.	.	.	.	.	.
ENST0	.	1q21.1	.	.	Scc	.	rs169	.	.	.	.	.	.
ENST0	.	1q21.1	.	.	Scc	.	rs112	ID=COSN2	.	.	.	.	.
.	.	1q21.1	.	.	Scc	Score=664	rs373	.	.	.	.	0.023	0.005
ENST0	.	1q21.1	.	Scc	Scc	.	.	.	.	.	.	.	.
ENST0	.	1q21.1	.	.	Scc	Score=636	rs269	.	.	.	.	.	.
ENST0	.	1q21.1	.	.	Scc	.	.	.	.	.	.	.	.
ENST0	.	1q21.1	.	.	Scc	.	.	ID=COSN2	.	.	.	.	.
ENST0	.	1q21.1	.	.	Scc	.	.	ID=COSN1	.	.	.	.	.
ENST0	.	1q21.1	.	.	.	.	rs193	.	.	.	.	.	.
ENST0	.	1q21.2	.	.	Scc	.	.	.	.	.	.	.	.
ENST0	.	1q21.2	.	.	Scc	.	rs283	ID=COSN1	.	.	.	.	.
ENST0	.	1q21.2	.	.	Scc	.	rs298	ID=COSN2	.	.	.	0.002	0.014
ENST0	.	1q21.3	.	.	.	.	rs374	ID=COSM2	.	.	.	.	.
ENST0	.	1q23.3	.	.	Scc	Score=180	rs167	ID=COSN1	.	.	.	0.672	0.759
ENST0	.	1q23.3	.	.	Scc	.	rs148	.	.	.	.	.	.
ENST0	.	1q24.2	.	Scc	.	.	rs759	.	.	.	.	.	.
ENST0	.	1q32.2	.	.	.	.	.	.	.	.	.	.	.
ENST0	.	1q42.12	.	.	.	Score=160	.	.	.	.	.	.	.
ENST0	.	1q43	.	.	.	.	.	.	.	.	.	.	.
ENST0	.	1q43	.	Scc	Scc	.	rs200	ID=COSM4	.	.	.	.	.
.	.	1q44	.	.	Scc	.	rs373	ID=COSN1	.	.	.	.	.
.	.	1q44	.	.	Scc	.	rs178	ID=COSN1	.	.	.	.	.
.	.	2p11.2	.	.	.	Score=240	rs346	ID=COSN1	.	.	.	0.005	0.066
.	.	2p11.1	.	.	Scc	Score=231	rs868	ID=COSN1	.	.	.	.	.
ENST0	.	2p11.1	.	.	Scc	Score=157	.	.	.	.	.	.	.
ENST0	.	2q12.1	.	.	.	.	.	.	.	.	.	.	.
ENST0	.	2q13	.	.	Scc	Score=335	rs387	ID=COSN8	.	.	.	.	.
ENST0	.	2q14.3	.	.	.	Score=130	rs103	.	.	.	.	.	.

ENST0	.	2q21.1	.	.	Scc	.	rs183	ID=COSM3	.	.	.	0.688	0.739
ENST0	.	2q21.1	.	.	Scc	.	.	ID=COSM3	.	.	.	.	.
ENST0	.	2q21.3	.	.	.	.	.	.	.	.	.	.	.
ENST0	.	2q22.3	.	Scc	.	.	.	.	.	.	.	.	.
ENST0	.	2q31.1	.	.	.	.	.	.	.	.	.	.	.
ENST0	.	2q33.1	.	.	.	.	.	.	.	.	.	.	.
ENST0	.	2q33.3	.	Scc	.	.	.	.	.	.	.	.	.
ENST0	.	2q34	.	.	.	.	rs781	.	.	.	.	.	.
ENST0	.	2q35	.	.	.	.	rs101	ID=COSN1	.	.	.	.	.
ENST0	.	2q37.1	.	.	.	.	rs752	.	.	.	.	.	.
ENST0	.	3p25.1	.	.	.	.	.	.	.	.	.	.	.
ENST0	.	3p25.1	.	.	.	.	.	.	.	.	.	.	.
ENST0	.	3p24.3	.	.	.	.	.	.	.	.	.	.	.
ENST0	.	3p22.2	.	.	.	.	.	.	.	.	.	.	.
ENST0	.	3p22.2	.	.	.	.	rs145	.	Ur	.	.	.	.
ENST0	.	3p22.2	.	.	.	.	rs139	.	Ur	.	.	.	2E-04
ENST0	.	3p13	.	Scc	.	.	.	ID=COSM4	.	.	.	.	.
ENST0	.	3q11.2	.	Scc	Scc	.	rs112	ID=COSM6	.	.	.	0.001	4E-04
ENST0	.	3q12.3	.	.	.	.	.	.	.	.	.	.	.
ENST0	.	3q12.3	.	.	.	.	rs754	.	.	.	.	.	.
ENST0	.	3q13.12	.	.	.	.	rs375	.	.	.	.	.	.
ENST0	.	3q28	.	.	.	.	.	ID=COSN6	.	.	.	.	.
ENST0	.	3q29	.	.	Scc	.	rs622	ID=COSN2	.	.	.	.	.
.	.	3q29	.	.	Scc	Score=735	.	.	.	.	.	.	.
ENST0	.	4p16.3	.	.	.	.	rs497	ID=COSN1	.	.	.	.	.
ENST0	.	4q21.23	.	.	.	.	.	.	.	.	.	.	.
.	.	4q26	.	.	.	Score=124	rs376	.	.	.	.	.	.
.	.	4q28.3	.	.	Scc	Score=105	rs796	.	.	.	.	.	.
ENST0	.	4q31.21	.	.	.	.	rs954	ID=COSM4	.	.	.	.	.
ENST0	.	4q32.1	.	.	.	.	rs372	.	.	.	.	.	.
ENST0	.	4q35.2	.	.	.	.	.	ID=COSM1	.	.	.	.	.
ENST0	.	5p15.33	.	.	Scc	Score=337	rs649	.	.	.	.	.	.
ENST0	.	5p15.33	.	.	Scc	Score=148	rs146	.	.	.	.	.	.
ENST0	.	5p14.3	.	.	.	.	.	.	.	.	.	.	.
ENST0	.	5p14.3	.	.	Scc	Score=217	rs150	ID=COSN2	.	.	.	.	.
ENST0	.	5p13.2	.	.	.	.	.	.	.	.	.	.	.
ENST0	.	5q12.3	.	.	Scc	.	rs796	ID=COSN8	.	.	.	.	.
ENST0	.	5q13.2	.	.	Scc	.	.	.	.	.	.	.	.
ENST0	.	5q21.1	.	.	.	.	.	.	.	.	.	.	.
ENST0	.	5q22.1	.	.	.	.	rs760	.	.	.	.	.	.
ENST0	.	5q31.1	.	.	.	.	.	.	.	.	.	.	.
ENST0	.	5q31.3	.	.	.	.	rs764	.	.	.	.	.	.
ENST0	.	5q35.2	.	.	Scc	Score=232	rs140	ID=COSN1	.	.	.	.	.
ENST0	.	5q35.3	.	.	Scc	.	rs738	.	.	.	.	.	.
ENST0	.	6p24.3	.	.	.	.	rs556	.	.	.	.	0.001	2E-04
ENST0	.	6p12.2	.	.	Scc	.	rs397	.	.	.	.	.	.
ENST0	.	6p11.2	.	.	Scc	.	.	.	.	.	.	.	.
ENST0	.	6q14.1	.	.	.	.	.	.	.	.	.	.	.
ENST0	.	6q14.1	.	.	Scc	.	rs984	.	.	.	.	.	.
ENST0	.	6q16.3	.	.	Scc	.	.	.	.	.	.	.	.
ENST0	.	6q21	.	.	.	Score=105	rs999	.	.	.	.	.	.
ENST0	.	6q21	.	.	.	.	.	.	.	.	.	.	.
ENST0	.	6q23.2	.	.	.	.	rs755	.	.	.	.	.	.
ENST0	.	6q24.2	.	.	.	.	.	.	.	.	.	.	.

ENST0	.	6q24.3	.	.	.	.	rs774	.	.	.	.	.	0.038
ENST0	.	6q25.1	.	.	Scc	Score=169	rs146	ID=COSN2	.	.	.	.	.
ENST0	.	7p22.3	.	.	.	.	.	.	.	.	.	.	.
ENST0	.	7p21.1	.	.	.	Score=685	rs730	ID=COSN1	.	.	.	.	4E-04
ENST0	.	7p13	.	.	.	.	.	.	.	.	.	.	.
.	.	7p11.2	.	.	Scc	.	.	.	.	.	.	.	.
ENST0	.	7p11.2	.	.	Scc	.	rs791	.	.	.	.	0.091	0.125
ENST0	.	7p11.2	.	.	Scc	.	.	ID=COSN2	.	.	.	0.045	0.016
ENST0	.	7p11.2	.	.	Scc	.	.	.	.	.	.	.	.
ENST0	.	7p11.2	.	.	Scc	.	.	.	.	.	.	.	.
.	.	7q11.21	.	.	.	Score=133	rs431	ID=COSN1	.	.	.	.	.
ENST0	.	7q11.21	.	.	Scc	.	.	.	.	.	.	.	.
ENST0	.	7q11.21	.	.	Scc	.	.	.	.	.	.	.	.
.	.	7q11.21	.	.	Scc	.	rs546	.	.	.	.	0.022	0.098
.	.	7q11.21	.	.	Scc	.	.	ID=COSN2	.	.	.	.	.
.	.	7q11.21	.	.	Scc	.	rs868	ID=COSN2	.	.	.	.	.
ENST0	.	7q11.23	.	.	Scc	Score=204	rs879	ID=COSN2	.	.	.	.	.
ENST0	.	7q21.3	.	.	.	.	rs768	.	.	.	.	.	.
ENST0	.	7q22.1	.	.	Scc	.	rs372	ID=COSN1	.	.	.	.	.
ENST0	.	7q31.33	.	Scc	.	.	rs780	.	.	.	.	.	.
ENST0	.	7q32.2	.	.	.	.	.	ID=COSM3	.	.	.	.	.
ENST0	.	7q36.1	.	.	Scc	.	rs775	ID=COSM3	.	.	.	.	.
ENST0	.	7q36.1	.	Scc	Scc	.	rs149	ID=COSM1	.	.	.	.	.
ENST0	.	7q36.1	.	.	Scc	Score=231	.	.	.	.	.	.	.
.	.	7q36.3	.	.	.	Score=955	rs140	ID=COSN2	.	.	.	.	.
.	.	7q36.3	.	.	.	Score=955	rs148	ID=COSN2	.	.	.	.	.
ENST0	.	8p23.1	.	.	Scc	.	.	.	.	.	.	.	.
ENST0	.	8p22	.	.	.	.	.	.	.	.	.	.	.
ENST0	.	8p21.3	.	.	.	.	.	.	.	.	.	.	.
ENST0	.	8p21.2	.	Scc	.	.	.	.	.	.	.	.	.
.	.	8p11.21	.	.	.	Score=488	rs181	.	.	.	.	.	4E-04
.	.	8p11.1	.	.	.	Score=126	rs202	ID=COSN2	.	.	.	.	.
ENST0	.	8q13.3	.	.	.	.	.	.	.	.	.	.	.
ENST0	.	8q21.13	.	.	.	.	rs183	ID=COSM6	.	.	.	.	0.001
ENST0	.	9p13.1	.	.	Scc	.	rs132	ID=COSN1	.	.	.	.	.
ENST0	.	9q12	.	.	Scc	.	rs295	.	.	.	.	.	.
.	.	9q13	.	.	Scc	.	rs196	.	.	.	.	.	.
.	.	9q13	.	.	Scc	.	rs201	ID=COSN2	.	.	.	.	.
.	.	9q13	.	.	Scc	Score=540	rs492	.	.	.	.	.	.
ENST0	.	9q22.31	.	.	.	.	.	.	.	.	.	.	.
.	.	9q32	.	.	Scc	.	rs286	.	.	.	.	.	.
ENST0	.	9q34.11	.	.	.	.	.	.	.	.	.	.	.
ENST0	.	9q34.11	.	.	.	.	.	.	.	.	.	.	.
ENST0	.	9q34.2	.	.	.	.	.	.	.	.	.	.	.
ENST0	.	9q34.2	.	.	.	.	rs769	.	.	.	.	.	.
ENST0	.	10p13	.	.	.	.	.	.	.	.	.	.	.
ENST0	.	10p13	.	.	.	Score=288	rs105	.	.	.	.	.	.
ENST0	.	10p12.1	.	.	.	.	.	ID=COSM2	.	.	.	.	.
ENST0	.	10p12.1	.	Scc	.	.	.	.	.	.	.	.	.
ENST0	.	10p12.1	.	.	Scc	.	rs753	ID=COSM3	.	.	.	.	.
ENST0	.	10p11.23	.	.	.	.	rs556	.	.	.	.	.	.
ENST0	.	10p11.21	.	.	Scc	.	rs175	ID=COSN2	.	.	.	.	.
.	.	10p11.1	.	.	Scc	.	.	.	.	.	.	.	.
ENST0	.	10p11.1	.	.	Scc	.	rs149	ID=COSN2	.	.	.	.	.

ENST0	.	10p11.1	.	.	Scc	.	rs552	.	.	.	.	0.001	2E-04
ENST0	.	10q11.22	.	.	Scc	.	rs782	ID=COSN2	.	.	.	.	.
ENST0	.	10q11.22	.	.	.	.	rs782	.	.	.	.	.	.
ENST0	.	10q21.2	.	.	.	.	.	.	.	.	.	.	.
ENST0	.	10q21.3	.	.	.	.	.	.	.	.	.	.	.
ENST0	.	10q21.3	.	.	.	.	rs181	ID=COSM4	.	.	.	.	2E-04
ENST0	.	10q22.1	.	.	.	.	rs185	.	.	.	.	.	2E-04
ENST0	.	10q22.2	.	.	Scc	.	.	.	.	.	.	.	.
ENST0	.	10q23.31	.	Scc	.	.	.	.	.	.	.	.	.
ENST0	.	10q24.1	.	.	.	.	rs900	ID=COSN2	.	.	.	.	.
ENST0	.	10q25.1	.	.	.	Score=963	.	.	.	.	.	.	.
ENST0	.	10q25.3	.	.	.	.	rs102	.	.	.	.	.	.
ENST0	.	11p15.4	.	.	.	.	.	.	.	.	.	.	.
ENST0	.	11p14.1	.	.	.	Score=228	rs761	ID=COSN2	.	.	.	.	.
ENST0	.	11p12	.	.	.	Score=299	.	.	.	.	.	.	.
.	.	11p11.12	.	.	Scc	.	.	.	.	.	.	.	.
ENST0	.	11q12.2	.	.	.	.	.	.	.	.	.	.	.
ENST0	.	11q13.5	.	.	Scc	.	.	ID=COSM2	.	.	.	.	.
ENST0	.	11q14.1	.	.	Scc	.	.	.	.	.	.	.	.
ENST0	.	11q14.1	.	.	Scc	.	.	.	.	.	.	.	.
ENST0	.	11q14.1	.	.	.	.	rs761	.	.	.	.	.	.
ENST0	.	11q21	.	Scc	.	.	rs143	ID=COSM5	.	.	.	.	0.001
ENST0	.	11q23.1	.	.	.	.	.	.	.	.	.	.	.
ENST0	.	11q24.1	.	.	.	.	rs775	ID=COSM4	.	.	.	.	.
ENST0	.	11q24.2	.	.	.	.	rs251	.	.	.	.	0.164	0.155
ENST0	.	12p13.33	.	.	Scc	.	rs538	ID=COSN1	.	.	.	0.375	0.205
ENST0	.	12p13.33	.	.	Scc	.	rs879	.	.	.	.	.	.
ENST0	.	12p13.33	.	.	.	.	.	.	.	.	.	.	.
ENST0	.	12p13.33	.	.	.	Score=286	rs868	ID=COSN1	.	.	.	.	.
ENST0	.	12p11.21	.	.	Scc	.	rs257	.	.	.	.	.	.
ENST0	.	12q12	.	.	.	.	.	.	.	.	.	.	.
ENST0	.	12q13.11	.	.	.	Score=333	.	.	.	.	.	.	.
ENST0	.	12q13.13	.	.	.	.	.	.	.	.	.	.	.
ENST0	.	12q15	.	.	Scc	.	.	.	.	.	.	.	.
ENST0	.	12q21.2	.	.	Scc	.	.	.	.	.	.	.	.
ENST0	.	12q23.3	.	Scc	.	.	rs104	ID=COSM5	.	.	.	.	.
ENST0	.	12q24.11	.	.	.	Score=256	.	.	.	.	.	.	.
ENST0	.	12q24.23	.	.	.	.	.	.	.	.	.	.	.
ENST0	.	13q21.1	.	.	.	.	.	.	.	.	.	.	.
ENST0	.	14q11.2	.	.	Scc	.	.	ID=COSN2	.	.	.	.	.
ENST0	.	14q11.2	.	.	Scc	.	rs201	ID=COSN1	.	.	.	.	.
.	.	14q11.2	.	.	Scc	.	rs879	ID=COSN1	.	.	.	.	.
ENST0	.	14q11.2	.	.	Scc	.	.	.	.	.	.	.	.
ENST0	.	14q11.2	.	.	Scc	.	rs867	.	.	.	.	.	.
ENST0	.	14q11.2	.	Scc	.	.	.	.	.	.	.	.	.
.	.	14q11.2	.	.	.	.	.	.	.	.	.	.	.
.	.	14q11.2	.	.	.	.	.	.	.	.	.	.	.
ENST0	.	14q21.1	.	.	.	.	.	.	.	.	.	.	.
.	.	14q22.1	.	.	.	.	.	.	.	.	.	.	.
ENST0	.	14q23.1	.	.	.	.	.	.	.	.	.	.	.
ENST0	.	14q23.2	.	.	.	.	rs773	ID=COSM4	.	.	.	.	.
ENST0	.	14q32.2	.	.	Scc	.	.	.	.	.	.	.	.
.	.	14q32.33	.	.	Scc	Score=142	rs289	.	.	.	.	.	.
ENST0	.	14q32.33	.	.	Scc	.	rs368	ID=COSN1	.	.	.	.	.

.	.	14q32.33	.	.	Scc	.	rs619	ID=COSN1	.	.	.	.	.
ENST0	.	15q11.1	.	.	Scc	.	.	.	.	.	.	.	.
ENST0	.	15q11.1	.	.	Scc	Score=182	.	.	.	.	.	.	.
.	.	15q11.2	.	.	Scc	.	rs374	ID=COSN2	.	.	.	.	.
ENST0	.	15q11.2	.	.	Scc	.	.	.	.	.	.	.	.
ENST0	.	15q11.2	.	.	Scc	.	.	.	.	.	.	.	.
ENST0	.	15q11.2	.	.	Scc	.	.	ID=COSN2	.	.	.	.	.
.	.	15q11.2	.	.	Scc	.	rs796	.	.	.	.	.	.
.	.	15q11.2	.	.	Scc	.	.	.	.	.	.	.	.
.	.	15q11.2	.	.	Scc	.	rs712	.	.	.	.	0.384	0.379
ENST0	.	15q11.2	.	.	.	.	.	ID=COSM3	.	.	.	.	.
ENST0	.	15q12	.	Scc	.	.	.	.	.	.	.	.	.
ENST0	.	15q13.1	.	.	Scc	.	rs769	ID=COSN1	.	.	.	.	.
ENST0	.	15q13.1	.	Scc	.	.	.	.	.	.	.	.	.
ENST0	.	15q13.2	.	.	Scc	.	.	ID=COSN2	.	.	.	.	.
ENST0	.	15q13.2	.	.	Scc	.	.	.	.	.	.	.	.
ENST0	.	15q13.3	.	.	Scc	.	rs865	.	.	.	.	.	.
ENST0	.	15q13.3	.	.	Scc	.	.	.	.	.	.	.	.
ENST0	.	15q15.1	.	.	.	Score=372	rs368	ID=COSN2	.	.	.	.	.
ENST0	.	15q22.2	.	.	.	.	rs374	.	.	.	.	.	.
ENST0	.	15q25.1	.	.	Scc	.	.	.	.	.	.	.	.
ENST0	.	15q25.2	.	.	Scc	.	rs574	.	.	.	.	0.002	4E-04
ENST0	.	15q25.2	.	.	Scc	.	rs199	ID=COSM5	.	.	.	.	.
ENST0	.	15q25.2	.	.	Scc	.	rs377	ID=COSM9	.	.	.	.	.
ENST0	.	15q26.3	.	.	Scc	.	rs620	ID=COSN2	.	.	.	.	.
ENST0	.	16p13.3	.	.	.	.	rs756	.	.	.	.	.	.
ENST0	.	16p12.2	.	.	.	Score=426	.	ID=COSN1	.	.	.	.	.
ENST0	.	16p11.2	.	Scc	.	.	rs746	.	.	.	.	.	.
ENST0	.	16p11.2	.	.	.	.	.	.	.	.	.	.	.
ENST0	.	16p11.2	.	.	Scc	.	rs201	ID=COSN2	.	.	.	.	8E-04
.	.	16q11.2	.	.	.	Score=108	rs464	.	.	.	.	.	.
.	.	16q11.2	.	.	.	Score=117	rs453	.	.	.	.	.	.
.	.	16q11.2	.	.	.	Score=956	rs212	.	.	.	.	.	.
.	.	16q11.2	.	.	.	Score=111	rs288	.	.	.	.	.	.
.	.	16q11.2	.	.	.	Score=114	rs435	.	.	.	.	.	.
.	.	16q11.2	.	.	.	Score=114	rs424	.	.	.	.	.	.
.	.	16q11.2	.	.	.	Score=989	rs423	ID=COSN2	.	.	.	.	.
.	.	16q11.2	.	.	.	Score=111	rs797	.	.	.	.	.	.
.	.	16q11.2	.	.	.	Score=111	rs148	.	.	.	.	.	.
.	.	16q11.2	.	.	.	Score=111	rs997	.	.	.	.	.	.
.	.	16q11.2	.	.	.	Score=111	rs287	.	.	.	.	.	.
.	.	16q11.2	.	.	.	Score=111	rs424	.	.	.	.	.	.
.	.	16q11.2	.	.	.	Score=112	rs425	.	.	.	.	.	.
.	.	16q11.2	.	.	.	Score=989	rs719	.	.	.	.	.	.
.	.	16q11.2	.	.	.	Score=114	rs444	.	.	.	.	.	.
.	.	16q11.2	.	.	.	Score=114	rs445	ID=COSN1	.	.	.	.	.
.	.	16q11.2	.	.	.	Score=979	.	.	.	.	.	.	.
.	.	16q11.2	.	.	.	Score=979	rs805	.	.	.	.	.	.
.	.	16q11.2	.	.	.	Score=112	rs805	ID=COSN1	.	.	.	.	.
.	.	16q11.2	.	.	.	Score=112	rs615	.	.	.	.	.	.
.	.	16q11.2	.	.	.	Score=112	rs288	.	.	.	.	.	.
ENST0	.	16q12.2	.	.	.	.	.	.	.	.	.	.	.
ENST0	.	16q22.1	.	Scc	.	.	.	ID=COSM5	.	.	.	.	.
.	.	16q22.1	.	.	.	Score=136	.	ID=COSN2	.	.	.	.	.

ENST0	.	16q24.2	.	Scc	.	.	.	.	.	.	.	.	.
ENST0	.	17p13.3	.	.	.	.	rs780	.	.	.	.	.	.
ENST0	.	17p13.2	.	Scc	.	.	.	.	.	.	.	.	.
ENST0	.	17p13.1	.	Scc	.	.	rs368	.	.	.	.	.	4E-04
ENST0	.	17p12	.	.	.	.	.	.	.	.	.	.	.
ENST0	.	17p12	.	.	Scc	.	rs184	.	.	.	.	.	0.006
ENST0	.	17p11.2	.	.	.	.	.	.	.	.	.	.	.
ENST0	.	17p11.2	.	.	Scc	.	.	.	.	.	.	.	.
.	.	17p11.2	.	.	Scc	Score=297	.	.	.	.	.	.	.
ENST0	.	17p11.2	.	.	Scc	.	rs450	.	.	.	.	.	.
.	.	17p11.1	.	.	.	Score=113	rs146	ID=COSN1	.	.	.	.	.
.	.	17p11.1	.	.	.	Score=113	rs436	ID=COSN2	.	.	.	.	.
.	.	17p11.1	.	.	.	Score=113	rs656	.	.	.	.	.	.
.	.	17p11.1	.	.	.	Score=113	rs143	.	.	.	.	.	.
.	.	17p11.1	.	.	.	Score=113	.	.	.	.	.	.	.
ENST0	.	17q11.2	.	.	.	Score=227	rs891	.	.	.	.	.	.
ENST0	.	17q12	.	.	.	Score=224	rs116	.	.	.	.	0.001	0.021
ENST0	.	17q21.2	.	.	.	.	.	.	.	.	.	.	.
ENST0	.	17q21.2	.	.	.	.	rs145	ID=COSN1	.	.	.	.	.
ENST0	.	17q21.31	.	.	Scc	.	rs172	.	.	.	.	.	.
ENST0	.	17q22	.	.	.	.	.	.	.	.	.	.	.
ENST0	.	17q24.2	.	.	.	Score=131	.	.	.	.	.	.	.
ENST0	.	17q25.1	.	.	.	.	.	.	.	.	.	.	.
ENST0	.	18p11.31	.	.	.	.	.	.	.	.	.	.	.
ENST0	.	18p11.21	.	.	Scc	.	rs537	ID=COSM5	.	.	.	.	.
ENST0	.	18p11.21	.	.	Scc	.	rs201	ID=COSN2	.	.	.	.	.
.	.	19q13.12	.	.	Scc	Score=106	.	.	.	.	.	.	.
.	.	19q13.12	.	.	Scc	Score=123	rs712	.	.	.	.	.	.
.	.	19q13.12	.	.	Scc	Score=123	.	.	.	.	.	.	.
ENST0	.	19q13.2	.	.	Scc	Score=204	.	ID=COSN2	.	.	.	.	.
ENST0	.	19q13.2	.	.	Scc	.	.	.	.	.	.	.	.
ENST0	.	19q13.2	.	.	.	.	.	.	.	.	.	.	.
ENST0	.	19q13.31	.	.	.	.	.	.	.	.	.	.	.
ENST0	.	19q13.33	.	.	.	.	.	.	.	.	.	.	.
ENST0	.	19q13.43	.	.	.	.	.	.	.	.	.	.	.
.	.	19q13.43	.	.	.	Score=109	rs867	.	.	.	.	.	.
ENST0	.	20q11.23	.	.	.	.	.	.	.	.	.	.	.
ENST0	.	20q11.23	.	.	.	.	.	.	.	.	.	.	.
ENST0	.	21p11.2	.	.	Scc	Score=165	rs879	.	.	.	.	.	.
ENST0	.	21p11.2	.	.	Scc	.	rs866	ID=COSN2	.	.	.	.	.
.	.	21p11.2	.	.	Scc	.	.	.	.	.	.	.	.
ENST0	.	21p11.1	.	.	Scc	Score=191	rs753	ID=COSN1	.	.	.	.	.
ENST0	.	21p11.1	.	.	Scc	Score=914	rs373	.	.	.	.	.	.
.	.	21p11.1	.	.	Scc	.	.	.	.	.	.	.	.
ENST0	.	22q11.1	.	.	Scc	.	rs200	.	.	.	.	.	.
ENST0	.	22q11.1	.	.	Scc	.	rs281	ID=COSN5	.	.	.	0.103	0.24
ENST0	.	22q11.21	.	.	.	.	rs546	ID=COSM4	.	.	.	.	2E-04
ENST0	.	22q11.23	.	.	.	.	rs935	.	.	.	.	.	.
ENST0	.	22q11.23	.	.	Scc	.	rs405	.	.	.	.	.	0.065
ENST0	.	22q11.23	.	.	Scc	.	.	.	.	.	.	.	.
ENST0	.	22q11.23	.	.	Scc	.	.	.	.	.	.	.	.
ENST0	.	22q12.1	.	.	.	.	.	.	.	.	.	.	.
ENST0	.	22q12.2	.	.	.	.	.	.	.	.	.	.	.
ENST0	.	22q12.2	.	.	.	.	.	.	.	.	.	.	.

ENST0	.	22q13.2	.	.	Scc	.	rs142	.	.	.	.	0.006	0.031
ENST0	.	22q13.31	.	.	.	.	rs105	.	.	.	.	.	.
ENST0	.	22q13.31	.	.	.	.	.	.	.	.	.	.	.
ENST0	.	Xp22.31	.	.	Scc	.	rs766	.	.	.	.	.	.
ENST0	.	Xp22.31	.	.	Scc	.	.	.	.	.	.	.	.
.	.	Xp21.3	.	.	.	Score=114	.	.	.	.	.	.	.
ENST0	.	Xp11.23	.	.	.	.	.	.	.	.	.	.	.
ENST0	.	Xp11.23	.	.	.	.	.	.	.	.	.	.	.
ENST0	.	Xp11.23	.	.	.	.	.	.	.	.	.	.	.
.	.	Xp11.22	.	Scc	.	.	rs782	.	.	.	.	.	.
ENST0	.	Xp11.22	.	.	.	.	rs782	.	.	.	.	.	.
.	.	Xq11.1	.	.	.	Score=120	.	.	.	.	.	.	.
ENST0	.	Xq12	.	.	.	.	.	.	.	.	.	.	.
ENST0	.	Xq21.1	.	.	.	.	.	.	.	.	.	.	.
ENST0	.	Xq21.1	.	.	.	.	.	.	.	.	.	.	.
ENST0	.	Xq21.32	.	.	Scc	.	.	.	.	.	.	.	.
ENST0	.	Xq22.1	.	.	.	.	.	.	.	.	.	.	.
ENST0	.	Xq22.2	.	.	.	.	rs781	.	.	.	.	.	.
ENST0	.	Xq26.2	.	Scc	.	.	.	ID=COSM4	.	.	.	.	.
ENST0	.	Xq27.1	.	Scc	.	.	.	.	.	.	.	.	.
ENST0	.	Xq28	.	.	.	.	.	.	.	.	.	.	.
.	.	.	.	.	.	.	rs878	ID=COSM4	.	.	.	.	.
ENST0	.	1p36.22	.	.	.	.	.	.	.	.	.	.	.
ENST0	.	1p36.22	.	Scc	.	.	.	.	.	.	.	.	.
ENST0	.	1p36.21	.	.	Scc	.	rs550	ID=COSN1	.	.	.	0.003	0.002
ENST0	.	1p36.21	.	.	Scc	.	rs299	.	.	.	.	.	.
ENST0	.	1p36.21	.	.	Scc	.	.	ID=COSN1	.	.	.	.	.
ENST0	.	1p36.13	.	.	Scc	.	rs139	ID=COSN1	.	.	.	0.158	0.034
ENST0	.	1p36.12	.	.	.	.	.	.	.	.	.	.	.
ENST0	.	1p32.3	.	.	.	.	rs368	.	.	.	.	.	4E-04
ENST0	.	1p31.1	.	.	.	.	.	.	.	.	.	.	.
ENST0	.	1p22.2	.	.	.	.	.	.	.	.	.	.	.
ENST0	.	1p13.3	.	.	.	.	.	.	.	.	.	.	.
.	.	1q12	.	.	.	Score=545	rs201	ID=COSN1	.	.	.	0.217	0.248
.	.	1q21.1	.	.	Scc	Score=308	.	.	.	.	.	.	.
ENST0	.	1q21.1	.	.	Scc	.	rs201	.	.	.	.	.	.
ENST0	.	1q21.1	.	.	Scc	.	rs372	.	.	.	.	.	.
ENST0	.	1q21.1	.	.	Scc	.	.	.	.	.	.	.	.
.	.	1q21.1	.	.	Scc	.	rs377	.	.	.	.	.	.
ENST0	.	1q21.1	.	.	Scc	.	rs879	.	.	.	.	.	.
ENST0	.	1q21.1	.	.	Scc	.	.	.	.	.	.	.	.
ENST0	.	1q21.1	.	.	Scc	.	.	.	.	.	.	.	.
ENST0	.	1q21.1	.	.	Scc	Score=277	rs464	.	.	.	.	.	.
ENST0	.	1q21.1	.	.	Scc	.	rs879	.	.	.	.	.	.
ENST0	.	1q21.1	.	.	Scc	.	rs766	ID=COSN2	.	.	.	.	.
ENST0	.	1q21.1	.	.	Scc	.	rs782	.	.	.	.	.	.
ENST0	.	1q21.2	.	.	Scc	.	.	.	.	.	.	.	.
ENST0	.	1q21.2	.	.	Scc	.	rs200	ID=COSN1	.	.	.	.	.
.	.	1q21.2	.	.	Scc	.	rs618	ID=COSN1	.	.	.	.	.
.	.	1q21.2	.	.	Scc	Score=222	rs618	.	.	.	.	.	.
ENST0	.	1q25.1	.	.	.	.	.	.	.	.	.	.	.
ENST0	.	1q25.1	.	.	.	.	rs749	ID=COSM4	.	.	.	.	.
ENST0	.	1q25.2	.	.	.	.	.	.	.	.	.	.	.
ENST0	.	1q25.2	.	.	.	.	rs879	.	.	.	.	.	.

ENST0	.	1q41	.	.	.	.	.	.	.	.	.	.	.
ENST0	.	1q42.13	.	.	Scc	Score=498	.	.	.	.	.	.	.
.	.	1q42.13	.	.	Scc	.	.	.	.	.	.	.	.
ENST0	.	1q43	.	.	.	.	.	.	.	.	.	.	.
ENST0	.	2p23.3	.	.	.	.	.	.	.	.	.	.	.
.	.	2p11.2	.	.	Scc	Score=409	rs102	ID=COSN2	.	.	.	.	.
ENST0	.	2p11.1	.	.	Scc	Score=219	.	.	.	.	.	.	.
ENST0	.	2p11.1	.	.	Scc	Score=223	rs200	ID=COSN2	.	.	.	.	.
ENST0	.	2q11.1	.	.	Scc	.	rs202	ID=COSN1	.	.	.	.	.
ENST0	.	2q11.2	.	.	Scc	.	rs369	ID=COSN1	.	.	.	.	.
ENST0	.	2q12.3	.	.	Scc	.	rs541	ID=COSM1	.	.	.	0.002	4E-04
ENST0	.	2q12.3	.	.	Scc	.	rs105	.	.	.	.	.	.
ENST0	.	2q13	.	.	Scc	.	.	ID=COSN2	.	.	.	.	.
.	.	2q21.1	.	.	Scc	.	rs538	.	.	.	.	0.001	2E-04
ENST0	.	2q24.2	.	.	.	.	.	.	.	.	.	.	.
ENST0	.	2q31.1	.	.	.	.	rs187	.	.	.	.	0.003	6E-04
ENST0	.	2q31.2	.	.	.	.	.	.	.	.	.	.	.
ENST0	.	2q33.2	.	.	.	.	.	.	.	.	.	.	.
ENST0	.	2q35	.	.	.	.	.	.	.	.	.	.	.
ENST0	.	2q36.3	.	.	.	.	rs150	.	Be	.	.	0.026	0.005
ENST0	.	2q37.1	.	.	.	.	.	ID=COSM(	.	.	.	.	.
.	.	2q37.3	.	.	Scc	.	rs797	.	.	.	.	.	.
ENST0	.	3p26.1	.	.	.	.	.	.	.	.	.	.	.
ENST0	.	3p23	.	.	.	.	.	.	.	.	.	.	.
ENST0	.	3p21.31	.	.	Scc	.	rs677	ID=COSM2	.	.	.	.	.
ENST0	.	3p21.31	.	.	Scc	.	rs677	ID=COSN2	.	.	.	.	.
ENST0	.	3p14.1	.	.	.	.	rs547	.	.	.	.	.	.
ENST0	.	3p13	.	.	.	.	.	.	.	.	.	.	.
ENST0	.	3q21.2	.	.	Scc	.	.	.	.	.	.	.	.
ENST0	.	3q26.32	.	.	Scc	.	rs121	ID=COSM1	Pa	.	.	.	.
ENST0	.	3q26.33	.	.	.	.	.	.	.	.	.	.	.
ENST0	.	3q28	.	.	.	.	.	.	.	.	.	.	.
ENST0	.	3q29	.	.	.	Score=234	.	.	.	.	.	.	.
ENST0	.	3q29	.	.	Scc	.	rs485	.	.	.	.	.	.
ENST0	.	4p16.3	.	.	.	.	.	.	.	.	.	.	.
ENST0	.	4p16.1	.	.	Scc	.	.	.	.	.	.	.	.
ENST0	.	4p16.1	.	.	Scc	.	.	.	.	.	.	.	.
.	.	4p16.1	.	.	Scc	.	rs796	.	.	.	.	.	.
ENST0	.	4p14	.	.	.	.	.	.	.	.	.	.	.
.	.	4p11	.	.	Scc	Score=214	.	ID=COSN2	.	.	.	.	.
ENST0	.	4q12	.	.	.	.	.	.	.	.	.	.	.
ENST0	.	4q21.21	.	.	.	Score=219	rs370	.	.	.	.	.	.
ENST0	.	4q23	.	.	.	.	.	.	.	.	.	.	.
ENST0	.	4q24	.	.	.	.	.	.	.	.	.	.	.
ENST0	.	4q26	.	.	.	.	.	.	.	.	.	.	.
ENST0	.	4q31.21	.	.	Scc	.	rs201	ID=COSM1	.	.	.	.	.
ENST0	.	4q34.2	.	.	.	.	.	.	.	.	.	.	.
ENST0	.	4q35.2	.	.	.	.	rs761	.	.	.	.	.	.
ENST0	.	5p15.31	.	.	Scc	.	rs868	ID=COSN8	.	.	.	.	.
ENST0	.	5p15.2	.	.	.	.	.	.	.	.	.	.	.
.	.	5p15.1	.	.	Scc	Score=175	.	.	.	.	.	.	.
ENST0	.	5p13.2	.	.	Scc	Score=142	.	.	.	.	.	.	.
.	.	5q11.1	.	.	.	Score=122	rs865	ID=COSN2	.	.	.	.	.
.	.	5q11.1	.	.	.	Score=121	rs133	ID=COSN2	.	.	.	.	.

.	.	5q11.1	.	.	.	Score=119	rs148	ID=COSN2	.	.	.	0.018	0.038
ENST0	.	5q12.3	.	Scc	.	Score=23;I	.	.	.	.	.	.	.
ENST0	.	5q14.1	.	.	.	Score=789	rs141	.	.	.	.	0.425	0.237
ENST0	.	5q32	.	.	.	.	.	.	.	.	.	.	.
ENST0	.	5q35.1	.	.	.	Score=255	rs728	.	.	.	.	.	.
ENST0	.	5q35.3	.	.	.	.	rs182	.	.	.	.	.	4E-04
ENST0	.	6p21.33	.	Scc	Scc	.	rs774	ID=COSM3	.	.	.	0.027	0.026
ENST0	.	6p21.33	.	.	Scc	.	rs140	.	.	.	.	0.149	0.047
ENST0	.	6p21.2	.	.	.	Score=474	rs759	.	.	.	.	.	.
.	.	6p11.1	.	.	.	Score=118	rs492	ID=COSN1	.	.	.	.	.
ENST0	.	6q22.1	.	Scc	Scc	.	.	.	.	.	.	.	.
ENST0	.	7p22.3	.	.	.	.	.	.	.	.	.	.	.
ENST0	.	7p14.1	.	.	.	.	rs746	.	.	.	.	.	.
ENST0	.	7p13	.	.	.	.	rs776	ID=COSM5	.	.	.	.	.
.	.	7p11.2	.	.	Scc	Score=188	.	.	.	.	.	.	.
ENST0	.	7p11.2	.	.	Scc	.	.	.	.	.	.	.	.
ENST0	.	7p11.2	.	.	Scc	.	rs142	.	.	.	.	.	.
.	.	7q11.21	.	.	.	Score=133	rs446	.	.	.	.	.	.
.	.	7q11.21	.	.	Scc	.	.	.	.	.	.	.	.
.	.	7q11.21	.	.	Scc	.	rs374	.	.	.	.	.	.
.	.	7q11.21	.	.	Scc	.	rs867	.	.	.	.	.	.
.	.	7q11.21	.	.	Scc	.	.	.	.	.	.	.	.
.	.	7q11.21	.	.	Scc	.	.	.	.	.	.	.	.
.	.	7q11.21	.	.	Scc	.	.	.	.	.	.	.	.
ENST0	.	7q11.23	.	.	Scc	Score=254	rs554	.	.	.	.	0.011	0.002
ENST0	.	7q22.1	.	.	Scc	Score=215	rs375	.	.	.	.	.	.
ENST0	.	7q31.1	.	Scc	.	.	.	.	.	.	.	.	.
ENST0	.	7q36.1	.	.	.	Score=216	.	.	.	.	.	.	.
ENST0	.	7q36.1	.	.	.	.	.	.	.	.	.	.	.
ENST0	.	7q36.1	.	.	.	.	.	.	.	.	.	.	.
ENST0	.	7q36.3	.	.	.	.	rs868	ID=COSN1	.	.	.	.	.
ENST0	.	8p23.3	.	.	.	.	.	.	.	.	.	.	.
.	.	8p23.1	.	.	Scc	Score=942	rs398	ID=COSN2	.	.	.	.	.
.	.	8p23.1	.	.	Scc	.	.	.	.	.	.	.	.
ENST0	.	8p23.1	.	.	.	.	rs746	ID=COSM6	.	.	.	.	.
.	.	8p23.1	.	.	Scc	.	rs759	.	.	.	.	.	.
ENST0	.	8p23.1	.	.	.	.	.	.	.	.	.	.	.
ENST0	.	8p22	.	.	.	.	.	.	.	.	.	.	.
.	.	8q11.1	.	.	.	Score=124	.	.	.	.	.	.	.
.	.	8q11.1	.	.	.	Score=124	rs444	.	.	.	.	.	.
ENST0	.	8q21.13	.	.	.	Score=261	.	.	.	.	.	.	.
ENST0	.	8q23.3	.	.	.	.	.	.	.	.	.	.	.
ENST0	.	8q24.3	.	.	.	.	.	.	.	.	.	.	.
ENST0	.	9p22.1	.	.	.	.	rs535	.	.	.	.	.	6E-04
ENST0	.	9p13.2	.	.	.	.	rs562	.	.	.	.	.	2E-04
ENST0	.	9p13.1	.	Scc	Scc	.	rs141	ID=COSM5	.	.	.	.	.
ENST0	.	9p12	.	.	Scc	.	rs375	ID=COSN1	.	.	.	.	.
.	.	9p12	.	.	Scc	Score=288	rs536	.	.	.	.	0.002	4E-04
ENST0	.	9q13	.	.	Scc	.	rs779	.	.	.	.	.	.
.	.	9q13	.	.	Scc	.	rs196	.	.	.	.	.	.
ENST0	.	9q13	.	Scc	Scc	.	rs625	ID=COSN6	.	.	.	0.37	0.321
ENST0	.	9q22.2	.	.	Scc	.	.	ID=COSN2	.	.	.	.	.
.	.	9q33.2	.	.	.	.	rs578	.	.	.	.	.	6E-04
ENST0	.	9q34.2	.	.	.	.	.	.	.	.	.	.	.

ENST0	.	9q34.3	.	.	.	.	.	.	.	.	.	.	.
ENST0	.	10p13	.	.	Scc	.	rs766	.	.	.	.	.	.
ENST0	.	10p12.31	.	.	.	Score=124	rs193	.	.	.	.	.	.
ENST0	.	10q11.21	.	.	Scc	.	.	.	.	.	.	.	.
ENST0	.	10q11.22	.	.	Scc	.	rs781	ID=COSN1	.	.	.	.	.
ENST0	.	10q11.23	.	.	Scc	.	rs618	ID=COSN1	.	.	.	0.453	0.41
ENST0	.	10q22.2	.	.	Scc	.	.	ID=COSN1	.	.	.	.	.
ENST0	.	10q22.3	.	.	Scc	.	.	.	.	.	.	.	.
ENST0	.	10q24.31	.	.	.	.	.	.	.	.	.	.	.
ENST0	.	10q26.11	.	.	.	Score=835	rs292	ID=COSN1	.	.	.	0.416	0.156
.	.	10q26.2	.	.	.	Score=422	.	.	.	.	.	.	.
ENST0	.	10q26.3	.	.	.	.	rs102	.	.	.	.	.	.
ENST0	.	11p15.5	.	.	.	.	rs626	ID=COSM4	.	.	.	.	.
ENST0	.	11p15.5	.	.	.	.	rs149	.	.	.	.	0.004	0.021
ENST0	.	11p15.4	.	.	.	Score=282	.	.	.	.	.	.	.
ENST0	.	11p15.4	.	.	.	.	.	.	.	.	.	.	.
ENST0	.	11p11.2	.	.	.	Score=153	.	.	.	.	.	.	.
ENST0	.	11q12.2	.	Scc	.	.	.	.	.	.	.	.	.
ENST0	.	11q13.1	.	.	.	.	rs926	.	.	.	.	.	.
ENST0	.	11q13.1	.	.	.	Score=252	.	.	.	.	.	.	.
ENST0	.	11q13.2	.	.	.	.	.	.	.	.	.	.	.
ENST0	.	11q13.2	.	.	.	Score=23;I	.	.	.	.	.	.	.
ENST0	.	11q13.4	.	.	.	.	rs367	.	.	.	.	.	.
ENST0	.	11q13.4	.	.	.	.	.	.	.	.	.	.	.
ENST0	.	11q13.4	.	.	.	.	.	.	.	.	.	.	.
ENST0	.	11q13.5	.	.	.	.	rs383	ID=COSN2	.	.	.	.	.
ENST0	.	11q14.1	.	.	.	Score=383	.	.	.	.	.	.	.
ENST0	.	11q22.3	.	.	Scc	.	.	.	.	.	.	.	.
ENST0	.	11q23.3	.	.	.	Score=223	rs144	.	.	.	.	.	.
ENST0	.	11q24.2	.	.	.	.	rs537	.	.	.	.	.	2E-04
.	.	11q25	.	.	.	Score=240	.	ID=COSN2	.	.	.	.	.
ENST0	.	12p13.33	.	.	Scc	.	rs148	ID=COSN7	.	.	.	.	.
ENST0	.	12p13.33	.	.	.	.	.	.	.	.	.	.	.
ENST0	.	12p13.31	.	.	Scc	.	rs200	ID=COSN5	.	.	.	.	.
ENST0	.	12p11.23	.	Scc	.	.	.	.	.	.	.	.	.
ENST0	.	12q13.3	.	Scc	.	.	.	.	.	.	.	.	.
ENST0	.	12q13.3	.	.	.	.	rs373	.	.	.	.	.	.
ENST0	.	12q21.31	.	.	.	.	.	.	.	.	.	.	.
ENST0	.	12q21.31	.	Scc	.	.	.	.	.	.	.	.	.
ENST0	.	12q23.2	.	.	.	.	.	.	.	.	.	.	.
ENST0	.	12q23.3	.	.	.	.	.	.	.	.	.	.	.
ENST0	.	12q24.11	.	.	.	Score=893	.	.	.	.	.	.	.
ENST0	.	12q24.11	.	.	.	Score=459	rs349	ID=COSN1	.	.	.	.	.
ENST0	.	12q24.22	.	.	.	Score=316	.	.	.	.	.	.	.
ENST0	.	12q24.31	.	Scc	.	.	.	.	.	.	.	.	.
ENST0	.	13q12.3	.	.	Scc	.	.	.	.	.	.	.	.
ENST0	.	13q14.11	.	.	.	.	.	.	.	.	.	.	.
.	.	13q14.11	.	.	.	Score=105	rs377	.	.	.	.	.	.
.	.	13q22.3	.	.	Scc	.	.	.	.	.	.	.	.
ENST0	.	13q33.1	.	.	.	.	.	.	.	.	.	.	.
.	.	14q11.2	.	.	Scc	.	rs225	ID=COSN1	.	.	.	.	.
ENST0	.	14q11.2	.	.	Scc	.	rs753	ID=COSM4	.	.	.	.	.
ENST0	.	14q11.2	.	.	.	.	.	.	.	.	.	.	.
ENST0	.	14q11.2	.	Scc	.	.	.	.	.	.	.	.	.

ENST0	.	14q21.3	.	.	.	.	.	.	.	.	.	.	.
ENST0	.	14q24.2	.	.	.	.	.	.	.	.	.	.	.
ENST0	.	14q24.3	.	.	.	.	.	.	.	.	.	.	.
ENST0	.	14q32.2	.	.	.	Score=190	.	.	.	.	.	.	.
ENST0	.	14q32.2	.	.	.	.	rs931	.	.	.	.	.	.
ENST0	.	14q32.32	.	.	.	.	.	.	.	.	.	.	.
ENST0	.	15q11.2	.	.	Scc	.	rs225	.	.	.	.	.	.
ENST0	.	15q13.2	.	.	Scc	.	rs264	.	.	.	.	.	.
ENST0	.	15q13.2	.	.	Scc	.	rs201	.	.	.	.	.	.
ENST0	.	15q13.3	.	.	Scc	.	rs200	ID=COSN2	.	.	.	.	.
ENST0	.	15q14	.	Scc	.	.	.	.	.	.	.	.	.
ENST0	.	15q15.1	.	Scc	.	.	.	.	.	.	.	.	.
ENST0	.	15q21.1	.	.	.	Score=444	.	.	.	.	.	.	.
ENST0	.	15q22.2	.	.	.	.	.	.	.	.	.	.	.
ENST0	.	15q23	.	.	.	.	.	.	.	.	.	.	.
ENST0	.	15q24.1	.	.	.	Score=820	.	.	.	.	.	.	.
ENST0	.	15q24.2	.	.	Scc	.	.	ID=COSN2	.	.	.	.	.
ENST0	.	15q24.2	.	.	Scc	.	rs140	ID=COSN1	.	.	.	.	.
ENST0	.	15q24.3	.	.	.	Score=216	rs878	.	.	.	.	.	.
ENST0	.	15q25.2	.	.	Scc	.	rs287	ID=COSN1	.	.	.	.	.
ENST0	.	15q26.1	.	.	.	.	.	.	.	.	.	.	.
ENST0	.	15q26.3	.	.	.	.	rs772	.	.	.	.	.	.
ENST0	.	15q26.3	.	.	Scc	.	.	ID=COSM4	.	.	.	.	.
ENST0	.	16p13.11	.	.	Scc	.	rs776	ID=COSN2	.	.	.	.	.
ENST0	.	16p13.11	.	Scc	.	.	.	.	.	.	.	.	.
ENST0	.	16p11.2	.	Scc	.	.	.	.	.	.	.	.	.
ENST0	.	16p11.2	.	Scc	.	.	.	.	.	.	.	.	.
ENST0	.	16p11.2	.	.	Scc	.	rs211	.	.	.	.	.	.
ENST0	.	16p11.2	.	.	Scc	.	rs112	.	.	.	.	.	.
ENST0	.	16p11.2	.	.	.	.	.	.	.	.	.	.	.
ENST0	.	16p11.1	.	.	.	.	rs554	.	.	.	.	.	2E-04
.	.	16p11.1	.	.	.	Score=354	rs371	.	.	.	.	.	.
.	.	16q11.2	.	.	.	Score=108	rs496	ID=COSN1	.	.	.	.	.
.	.	16q11.2	.	.	.	Score=117	rs427	ID=COSN1	.	.	.	.	.
.	.	16q11.2	.	.	.	Score=117	rs434	.	.	.	.	.	.
.	.	16q11.2	.	.	.	Score=113	rs424	.	.	.	.	.	.
.	.	16q11.2	.	.	.	Score=110	rs590	.	.	.	.	.	.
.	.	16q11.2	.	.	.	Score=111	rs424	.	.	.	.	.	.
.	.	16q11.2	.	.	.	Score=111	rs718	ID=COSN2	.	.	.	.	.
.	.	16q11.2	.	.	.	Score=111	rs424	.	.	.	.	.	.
.	.	16q11.2	.	.	.	Score=112	rs450	.	.	.	.	.	.
.	.	16q11.2	.	.	.	Score=112	rs124	.	.	.	.	.	.
.	.	16q11.2	.	.	.	.	rs139	ID=COSN1	.	.	.	.	.
.	.	16q11.2	.	.	.	.	rs561	.	.	.	.	.	.
.	.	16q11.2	.	.	.	Score=114	rs496	.	.	.	.	.	.
.	.	16q11.2	.	.	.	Score=114	rs445	ID=COSN1	.	.	.	.	.
.	.	16q11.2	.	.	.	Score=979	rs805	.	.	.	.	.	.
.	.	16q11.2	.	.	.	Score=979	rs424	.	.	.	.	.	.
.	.	16q11.2	.	.	.	Score=979	rs805	.	.	.	.	.	.
.	.	16q11.2	.	.	.	Score=979	rs424	.	.	.	.	.	.
.	.	16q11.2	.	.	.	Score=102	rs288	.	.	.	.	.	.
.	.	16q11.2	.	.	.	Score=102	rs615	.	.	.	.	.	.
.	.	16q11.2	.	.	.	Score=102	.	.	.	.	.	.	.
.	.	16q11.2	.	.	.	Score=112	rs287	.	.	.	.	.	.

.	.	16q11.2	.	.	.	Score=112	.	.	.	.	.	.	.
.	.	16q11.2	.	.	.	Score=110	rs147	.	.	.	.	.	.
.	.	16q11.2	.	.	.	Score=110	rs867	.	.	.	.	.	.
ENST0	.	16q22.2	.	.	Scc	Score=193	.	.	.	.	.	.	.
ENST0	.	16q22.2	.	.	Scc	.	rs189	.	.	.	.	0.003	1E-03
ENST0	.	16q22.2	.	.	Scc	.	rs129	ID=COSN2	.	.	.	0.259	0.317
ENST0	.	16q23.1	.	.	.	.	.	.	.	.	.	.	.
ENST0	.	16q24.3	.	.	.	.	.	.	.	.	.	.	.
ENST0	.	16q24.3	.	.	.	.	rs769	.	.	.	.	.	.
ENST0	.	16q24.3	.	.	Scc	.	rs794	.	.	.	.	.	.
ENST0	.	16q24.3	.	.	Scc	.	rs369	ID=COSN2	.	.	.	.	.
ENST0	.	16q24.3	.	.	Scc	.	rs559	ID=COSN2	.	.	.	0.752	0.533
ENST0	.	17p13.1	.	.	.	.	.	.	.	.	.	.	.
ENST0	.	17p13.1	.	.	.	.	.	.	.	.	.	.	.
ENST0	.	17p13.1	.	.	.	.	.	.	.	.	.	.	.
ENST0	.	17p13.1	.	.	.	.	.	.	.	.	.	.	.
ENST0	.	17p12	.	.	.	.	rs980	.	.	.	.	.	.
ENST0	.	17p12	.	.	.	.	rs757	.	.	.	.	.	.
ENST0	.	17p11.2	.	.	Scc	.	rs146	ID=COSN1	.	.	.	.	.
ENST0	.	17p11.2	.	.	Scc	.	rs216	ID=COSN1	.	.	.	0.171	0.134
.	.	17p11.2	.	.	Scc	Score=297	.	.	.	.	.	.	.
.	.	17p11.1	.	.	.	Score=113	rs436	ID=COSN2	.	.	.	.	.
.	.	17p11.1	.	.	.	Score=113	rs428	ID=COSN2	.	.	.	.	.
.	.	17p11.1	.	.	.	Score=113	rs140	.	.	.	.	.	.
.	.	17p11.1	.	.	.	Score=113	rs140	.	.	.	.	.	.
ENST0	.	17q11.2	.	.	Scc	.	.	.	.	.	.	.	.
ENST0	.	17q12	.	.	Scc	.	rs712	.	.	.	.	.	.
ENST0	.	17q12	.	.	Scc	.	rs573	.	.	.	.	.	.
ENST0	.	17q12	.	.	Scc	.	rs969	.	.	.	.	.	.
ENST0	.	17q12	.	.	Scc	Score=213	.	.	.	.	.	.	.
ENST0	.	17q12	.	.	.	.	.	.	.	.	.	.	.
ENST0	.	17q22	.	.	.	Score=225	.	.	.	.	.	.	.
ENST0	.	17q23.2	.	.	Scc	.	rs751	.	.	.	.	.	.
ENST0	.	17q24.1	.	Scc	.	.	rs760	.	Lik	.	.	.	.
ENST0	.	17q25.2	.	.	.	.	.	.	.	.	.	.	.
ENST0	.	17q25.3	.	.	.	Score=821	.	.	.	.	.	.	.
ENST0	.	17q25.3	.	.	.	.	rs113	.	.	.	.	.	0.053
ENST0	.	17q25.3	.	.	.	Score=821	rs879	.	.	.	.	.	.
.	.	18p11.32	.	.	Scc	.	.	.	.	.	.	.	.
ENST0	.	18p11.21	.	.	Scc	.	rs865	ID=COSN8	.	.	.	.	.
ENST0	.	18p11.21	.	.	Scc	.	rs867	.	.	.	.	.	.
ENST0	.	19p13.3	.	.	.	.	.	.	.	.	.	.	.
.	.	19p13.3	.	.	.	.	rs375	.	.	.	.	.	.
ENST0	.	19p13.3	.	.	.	.	.	.	.	.	.	.	.
ENST0	.	19p13.3	.	.	.	.	.	.	.	.	.	.	.
ENST0	.	19p13.2	.	.	.	.	rs546	.	.	.	.	.	2E-04
ENST0	.	19p13.12	.	.	.	.	.	.	.	.	.	.	.
ENST0	.	19q13.11	.	.	.	.	.	.	.	.	.	.	.
ENST0	.	19q13.12	.	.	.	.	.	.	.	.	.	.	.
.	.	19q13.12	.	.	Scc	Score=349	rs238	.	.	.	.	.	.
.	.	19q13.12	.	.	Scc	Score=123	rs712	.	.	.	.	.	.
ENST0	.	19q13.2	.	.	.	.	.	.	.	.	.	.	.
ENST0	.	19q13.2	.	.	Scc	.	rs139	.	.	.	.	0.204	0.13
ENST0	.	19q13.41	.	.	.	.	rs771	.	.	.	.	.	.

ENST0	.	19q13.42	.	.	Scc	.	rs178	ID=COSM5	.	.	.	0.601	0.722
.	.	19q13.42	.	.	.	.	rs920	.	.	.	.	.	.
ENST0	.	20p12.1	.	Scc	.	.	.	.	.	.	.	.	.
ENST0	.	20p11.21	.	.	.	Score=353	.	.	.	.	.	.	.
ENST0	.	20p11.21	.	Scc	.	.	rs778	ID=COSM1	.	.	.	.	.
ENST0	.	20p11.21	.	.	.	.	rs377	.	.	.	.	.	.
ENST0	.	20q11.21	.	.	Scc	.	.	ID=COSN2	.	.	.	.	.
ENST0	.	20q13.12	.	.	.	.	.	.	.	.	.	.	.
ENST0	.	20q13.12	.	.	.	Score=333	.	.	.	.	.	.	.
ENST0	.	20q13.33	.	.	.	.	.	.	.	.	.	.	.
.	.	21p11.2	.	.	Scc	Score=192	rs374	.	.	.	.	.	.
.	.	21p11.2	.	.	Scc	Score=208	rs373	.	.	.	.	.	.
.	.	21p11.2	.	.	Scc	Score=218	rs292	ID=COSN1	.	.	.	.	.
ENST0	.	21p11.1	.	.	Scc	.	rs199	.	.	.	.	.	.
ENST0	.	21p11.1	.	.	Scc	Score=914	rs878	ID=COSN1	.	.	.	.	.
.	.	21p11.1	.	.	Scc	.	rs435	.	.	.	.	.	.
.	.	21p11.1	.	.	Scc	.	.	.	.	.	.	.	.
.	.	21q22.11	.	.	.	Score=120	rs866	.	.	.	.	.	.
ENST0	.	21q22.12	.	.	.	Score=454	rs796	ID=COSN1	.	.	.	.	.
ENST0	.	21q22.3	.	Scc	.	.	.	.	.	.	.	.	.
ENST0	.	21q22.3	.	.	.	.	.	.	.	.	.	.	.
.	.	21q22.3	.	.	Scc	.	.	.	.	.	.	.	.
ENST0	.	22q11.21	.	Scc	.	Score=208	.	.	.	.	.	.	.
ENST0	.	22q11.21	.	.	Scc	.	rs370	.	.	.	.	.	.
ENST0	.	22q11.21	.	.	Scc	.	rs111	.	.	.	.	0.134	0.078
ENST0	.	22q11.21	.	.	Scc	.	rs188	.	.	.	.	0.018	0.029
ENST0	.	22q11.21	.	.	Scc	.	.	ID=COSN8	.	.	.	.	.
ENST0	.	Xp22.31	.	.	Scc	.	rs163	ID=COSM4	.	.	.	.	.
ENST0	.	Xp22.2	.	.	.	.	.	.	.	.	.	.	.
ENST0	.	Xp21.3	.	.	Scc	.	.	.	.	.	.	.	.
.	.	Xp21.2	.	.	.	.	.	.	.	.	.	.	.
ENST0	.	Xp21.1	.	.	.	.	rs774	ID=COSM2	.	.	.	.	5E-04
ENST0	.	Xp11.23	.	.	.	.	.	.	.	.	.	.	.
ENST0	.	Xp11.23	.	.	.	.	.	.	.	.	.	.	.
.	.	Xq11.1	.	.	.	Score=120	.	.	.	.	.	.	.
.	.	Xq11.1	.	.	.	Score=120	rs868	.	.	.	.	.	.
.	.	Xq11.1	.	.	.	Score=120	.	.	.	.	.	.	.
.	.	Xq11.1	.	.	.	Score=120	.	ID=COSN1	.	.	.	.	.
ENST0	.	Xq13.1	.	.	.	.	.	.	.	.	.	.	.
ENST0	.	Xq22.1	.	.	.	.	.	.	.	.	.	.	.
.	.	Xq22.2	.	.	.	Score=193	.	.	.	.	.	.	.
ENST0	.	Xq23	.	.	.	.	.	.	.	.	.	.	.
ENST0	.	Xq26.1	.	.	.	.	.	.	.	.	.	.	.
ENST0	.	Xq26.2	.	.	.	.	.	.	.	.	.	.	.
ENST0	.	Xq26.3	.	.	.	.	rs988	.	.	.	.	.	.
ENST0	.	Xq27.1	.	.	.	Score=119	.	.	.	.	.	.	.
ENST0	.	Xq27.3	.	.	.	.	rs782	ID=COSN1	.	.	.	.	.
ENST0	.	Xq28	.	.	.	.	.	.	.	.	.	.	.
.	.	Yq12	.	.	Scc	.	rs376	ID=COSN1	.	.	.	.	.
.	.	.	.	.	.	.	.	.	.	.	.	.	.
.	.	.	.	.	.	.	rs105	.	Ur	.	.	.	.
ENST0	.	1p36.33	.	.	Scc	.	.	ID=COSN2	.	.	.	.	.
ENST0	.	1p36.33	.	.	Scc	Score=162	.	.	.	.	.	.	.
ENST0	.	1p36.32	.	Scc	.	.	.	.	.	.	.	.	.

ENST0	.	1p36.22	.	.	.	.	rs757	.	.	.	.	.	.
ENST0	.	1p36.13	.	.	Scc	.	rs556	.	.	.	.	0.002	0.001
ENST0	.	1p36.13	.	.	Scc	.	rs761	ID=COSN1	.	.	.	.	.
ENST0	.	1p36.13	.	.	Scc	.	rs663	.	.	.	.	.	.
ENST0	.	1p36.13	.	.	Scc	.	rs646	ID=COSN1	.	.	.	.	.
ENST0	.	1p36.13	.	.	Scc	.	rs459	.	.	.	.	0.247	0.192
ENST0	.	1p36.13	.	.	Scc	.	rs229	ID=COSN2	.	.	.	.	.
ENST0	.	1p36.13	.	.	.	.	rs561	.	.	.	.	.	4E-04
ENST0	.	1p34.3	.	.	.	.	.	.	.	.	.	.	.
ENST0	.	1p34.1	.	.	.	Score=366	rs949	.	.	.	.	.	.
ENST0	.	1p31.3	.	.	.	.	.	.	.	.	.	.	.
ENST0	.	1p31.1	.	.	.	.	.	.	.	.	.	.	.
ENST0	.	1p31.1	.	.	.	.	.	.	.	.	.	.	.
ENST0	.	1p13.1	.	.	.	.	.	.	.	.	.	.	.
ENST0	.	1q21.1	.	.	Scc	Score=190	rs140	.	.	.	.	.	.
ENST0	.	1q21.1	.	.	Scc	Score=190	rs202	.	.	.	.	.	.
ENST0	.	1q21.1	.	.	Scc	.	rs776	ID=COSN1	.	.	.	.	.
.	.	1q21.1	.	.	Scc	.	rs145	.	.	.	.	.	.
ENST0	.	1q21.1	.	.	Scc	.	.	.	.	.	.	.	.
.	.	1q21.1	.	.	Scc	.	rs114	ID=COSN2	.	.	.	.	.
ENST0	.	1q21.1	.	.	.	.	rs374	.	.	.	.	0.051	0.114
ENST0	.	1q21.1	.	.	Scc	.	rs367	ID=COSN1	.	.	.	.	.
ENST0	.	1q21.2	.	.	Scc	.	rs798	ID=COSN2	.	.	.	.	.
ENST0	.	1q22	.	.	.	.	rs759	ID=COSM4	.	.	.	.	.
ENST0	.	1q23.1	.	Scc	.	.	.	.	.	.	.	.	.
ENST0	.	1q32.1	.	.	.	.	.	.	.	.	.	.	.
ENST0	.	1q32.1	.	.	.	.	rs750	.	.	.	.	.	.
ENST0	.	1q42.12	.	.	Scc	.	rs145	ID=COSM4	.	.	.	0.216	0.128
ENST0	.	1q44	.	.	.	.	rs372	ID=COSM6	.	.	.	.	.
.	.	1q44	.	.	Scc	.	rs878	ID=COSN1	.	.	.	.	.
ENST0	.	2p24.2	.	.	.	.	.	.	.	.	.	.	.
ENST0	.	2p12	.	.	.	.	.	.	.	.	.	.	.
ENST0	.	2p12	.	.	.	.	.	.	.	.	.	.	.
ENST0	.	2p11.2	.	Scc	Scc	.	rs201	ID=COSN1	.	.	.	.	.
ENST0	.	2p11.2	.	.	Scc	.	.	.	.	.	.	.	.
ENST0	.	2q22.1	.	.	.	Score=134	.	.	.	.	.	.	.
ENST0	.	2q31.1	.	.	.	Score=407	rs147	ID=COSN2	.	.	.	.	.
ENST0	.	2q31.3	.	.	.	.	.	.	.	.	.	.	.
ENST0	.	2q35	.	.	.	.	.	.	.	.	.	.	.
.	.	2q37.3	.	.	Scc	Score=225	.	.	.	.	.	.	.
ENST0	.	3p21.31	.	.	.	Score=112	rs105	ID=COSN2	.	.	.	.	.
ENST0	.	3p21.31	.	.	.	Score=112	.	.	.	.	.	.	.
ENST0	.	3p14.3	.	.	.	.	rs117	.	.	.	.	0.006	0.002
.	.	3p12.3	.	.	.	Score=384	rs374	ID=COSN7	.	.	.	.	.
ENST0	.	3p12.3	.	.	.	Score=123	rs145	.	.	.	.	0.081	0.105
ENST0	.	3q21.3	.	.	.	.	.	.	.	.	.	.	.
ENST0	.	3q22.1	.	.	.	.	.	.	.	.	.	.	.
ENST0	.	3q22.3	.	.	.	.	.	.	.	.	.	.	.
ENST0	.	3q22.3	.	Scc	.	.	.	.	.	.	.	.	.
ENST0	.	3q26.32	.	.	.	.	rs105	ID=COSM4	Lik	.	.	.	.
ENST0	.	3q26.32	.	.	Scc	.	rs104	ID=COSM7	Pa	.	.	.	.
ENST0	.	3q29	.	.	.	.	.	.	.	.	.	.	.
ENST0	.	4p16.3	.	.	.	.	rs372	ID=COSM5	.	.	.	.	.
ENST0	.	4p16.1	.	.	.	.	.	.	.	.	.	.	.

.	.	4p16.1	.	.	Scc	.	rs879	.	.	.	.	.	.
.	.	4p11	.	.	Scc	Score=160	rs594	.	.	.	.	.	.
ENST0	.	4q13.3	.	.	.	.	.	.	.	.	.	.	.
ENST0	.	4q32.3	.	.	.	Score=408	.	.	.	.	.	.	.
ENST0	.	5p13.2	.	.	Scc	Score=132	rs428	ID=COSN1	.	.	.	.	.
ENST0	.	5p13.2	.	.	Scc	Score=234	rs866	ID=COSN1	.	.	.	.	.
ENST0	.	5q31.1	.	.	.	.	rs776	.	.	.	.	.	.
.	.	5q35.1	.	.	.	Score=774	rs559	ID=COSN2	.	.	.	0.391	0.381
ENST0	.	5q35.3	.	.	Scc	.	.	.	.	.	.	.	.
ENST0	.	6p25.2	.	.	.	Score=199	.	.	.	.	.	.	.
ENST0	.	6p21.33	.	.	Scc	.	rs151	.	.	.	.	.	.
ENST0	.	6p21.33	.	Scc	.	.	.	.	.	.	.	.	.
ENST0	.	6p21.31	.	.	.	.	rs766	.	.	.	.	.	.
ENST0	.	6p12.3	.	.	.	.	.	.	.	.	.	.	.
ENST0	.	6p12.2	.	.	Scc	.	.	.	.	.	.	.	.
ENST0	.	6q14.1	.	.	.	.	.	.	.	.	.	.	.
ENST0	.	7p21.3	.	.	Scc	.	.	.	.	.	.	.	.
ENST0	.	7p15.3	.	.	.	.	.	.	.	.	.	.	.
ENST0	.	7p13	.	.	.	.	.	ID=COSM4	.	.	.	.	.
ENST0	.	7p13	.	.	.	.	.	.	.	.	.	.	.
.	.	7p11.2	.	.	Scc	.	.	.	.	.	.	.	.
.	.	7p11.2	.	.	Scc	.	rs143	.	.	.	.	0.189	0.069
ENST0	.	7p11.2	.	.	Scc	.	.	.	.	.	.	.	.
ENST0	.	7p11.2	.	.	Scc	.	rs879	ID=COSN1	.	.	.	.	.
ENST0	.	7p11.2	.	.	Scc	.	.	.	.	.	.	.	.
ENST0	.	7p11.2	.	.	Scc	.	rs200	ID=COSN1	.	.	.	.	.
ENST0	.	7p11.2	.	.	Scc	.	.	.	.	.	.	.	.
ENST0	.	7q11.21	.	.	Scc	.	rs201	ID=COSN2	.	.	.	.	.
ENST0	.	7q11.23	.	.	Scc	.	.	.	.	.	.	.	.
ENST0	.	7q11.23	.	.	Scc	.	.	.	.	.	.	.	.
ENST0	.	7q11.23	.	.	Scc	Score=228	.	ID=COSN2	.	.	.	.	.
ENST0	.	7q21.11	.	.	.	.	.	.	.	.	.	.	.
ENST0	.	7q21.2	.	.	.	.	.	.	.	.	.	.	.
ENST0	.	7q21.3	.	.	.	.	rs267	.	.	.	.	.	.
ENST0	.	7q22.1	.	.	.	.	rs182	.	.	.	.	.	0.008
ENST0	.	7q31.2	.	.	.	Score=221	rs140	.	.	.	.	.	0.005
ENST0	.	7q35	.	Scc	.	Score=195	.	.	.	.	.	.	.
ENST0	.	7q36.1	.	.	Scc	.	.	.	.	.	.	.	.
.	.	7q36.3	.	.	.	Score=119	rs796	.	.	.	.	.	.
ENST0	.	8p23.1	.	.	.	.	.	.	.	.	.	.	.
.	.	8p23.1	.	.	Scc	.	rs376	ID=COSN2	.	.	.	.	.
ENST0	.	8p23.1	.	.	Scc	.	rs567	.	.	.	.	0.203	0.148
ENST0	.	8p22	.	.	.	.	.	.	.	.	.	.	.
ENST0	.	8q21.3	.	.	.	.	.	.	.	.	.	.	.
ENST0	.	8q22.2	.	.	.	.	.	.	.	.	.	.	.
ENST0	.	8q24.22	.	.	.	Score=241	rs560	ID=COSN1	.	.	.	0.027	0.039
ENST0	.	9p21.3	.	.	.	.	.	.	.	.	.	.	.
ENST0	.	9p13.3	.	.	.	Score=201	rs146	ID=COSN2	.	.	.	0.003	6E-04
.	.	9p11.2	.	.	Scc	Score=748	rs749	ID=COSN2	.	.	.	.	.
ENST0	.	9q13	.	.	Scc	.	.	.	.	.	.	.	.
ENST0	.	9q13	.	.	Scc	.	rs771	.	.	.	.	.	.
ENST0	.	9q13	.	.	Scc	Score=540	rs445	ID=COSN2	.	.	.	.	.
ENST0	.	9q13	.	.	Scc	Score=540	rs749	ID=COSN2	.	.	.	.	.
ENST0	.	9q21.11	.	.	Scc	.	rs391	.	.	.	.	0.32	0.371

.	.	9q21.13	.	.	.	.	.	.	.	.	.	.	.
ENST0	.	9q21.33	.	.	.	.	.	.	.	.	.	.	.
ENST0	.	9q34.2	.	.	Scc	Score=290	rs285	ID=COSN1	.	.	.	.	.
ENST0	.	9q34.3	.	.	.	.	rs777	.	Lik	.	.	.	.
ENST0	.	10p13	.	.	.	.	.	.	.	.	.	.	.
ENST0	.	10p12.33	.	.	.	.	.	.	.	.	.	.	.
ENST0	.	10p11.21	.	.	.	.	rs774	.	.	.	.	.	.
ENST0	.	10p11.21	.	.	Scc	.	rs175	ID=COSN1	.	.	.	.	.
.	.	10q11.22	.	.	Scc	.	rs312	ID=COSN1	.	.	.	.	.
ENST0	.	10q22.1	.	.	.	Score=350	.	.	.	.	.	.	.
ENST0	.	10q22.2	.	Scc	.	.	rs201	.	.	.	.	.	.
ENST0	.	10q23.1	.	.	Scc	.	.	.	.	.	.	.	.
ENST0	.	10q23.2	.	.	Scc	.	rs312	.	.	.	.	0.524	0.433
ENST0	.	10q24.1	.	.	.	.	.	.	.	.	.	.	.
ENST0	.	10q26.11	.	.	.	Score=209	.	.	.	.	.	.	.
ENST0	.	10q26.13	.	Scc	.	.	rs549	.	.	.	.	0.001	4E-04
ENST0	.	10q26.13	.	Scc	.	.	.	.	.	.	.	.	.
ENST0	.	11p11.2	.	.	.	.	.	.	.	.	.	.	.
ENST0	.	11q13.2	.	Scc	.	.	.	.	.	.	.	.	.
ENST0	.	11q14.3	.	.	Scc	Score=351	.	.	.	.	.	.	.
ENST0	.	12p13.33	.	.	Scc	.	rs782	ID=COSN1	.	.	.	.	.
ENST0	.	12p13.32	.	.	.	.	.	.	.	.	.	.	.
ENST0	.	12p13.31	.	Scc	.	.	.	.	.	.	.	.	.
ENST0	.	12p13.31	.	.	.	.	.	.	.	.	.	.	.
ENST0	.	12p13.31	.	.	Scc	.	.	.	.	.	.	.	.
ENST0	.	12p12.1	.	Scc	.	.	rs121	ID=COSM5	Pa	.	.	.	.
ENST0	.	12p11.23	.	.	.	.	rs373	.	.	.	.	.	.
ENST0	.	12p11.21	.	.	Scc	.	rs403	ID=COSM5	.	.	.	.	.
ENST0	.	12q12	.	.	.	.	rs566	ID=COSM6	.	.	.	.	0.001
ENST0	.	12q13.12	.	.	.	.	rs981	ID=COSM1	.	.	.	.	.
ENST0	.	12q13.2	.	.	.	.	rs140	.	.	.	.	0.006	0.001
ENST0	.	12q24.31	.	.	.	.	.	.	.	.	.	.	.
ENST0	.	12q24.31	.	.	.	.	.	.	.	.	.	.	.
.	.	12q24.33	.	.	.	Score=112	rs170	.	.	.	.	0.268	0.22
ENST0	.	12q24.33	.	.	.	.	rs766	.	.	.	.	.	.
ENST0	.	13q12.11	.	.	Scc	.	rs907	.	.	.	.	.	.
ENST0	.	13q12.2	.	.	.	.	.	.	.	.	.	.	.
ENST0	.	13q12.2	.	Scc	.	.	rs766	.	.	.	.	.	.
ENST0	.	13q13.3	.	.	.	.	.	ID=COSM1	.	.	.	.	.
ENST0	.	13q34	.	.	Scc	.	.	.	.	.	.	.	.
ENST0	.	14q11.2	.	.	Scc	.	rs201	ID=COSN1	.	.	.	.	.
ENST0	.	14q11.2	.	.	Scc	.	rs498	.	.	.	.	.	.
.	.	14q11.2	.	.	Scc	Score=180	rs200	.	.	.	.	.	.
ENST0	.	14q11.2	.	.	Scc	.	.	.	.	.	.	.	.
ENST0	.	14q11.2	.	.	Scc	Score=384	.	.	.	.	.	.	.
ENST0	.	14q11.2	.	.	Scc	.	rs376	.	.	.	.	.	.
ENST0	.	14q11.2	.	.	.	.	.	.	.	.	.	.	.
ENST0	.	14q11.2	.	.	.	.	rs759	.	.	.	.	.	.
.	.	14q32.33	.	.	Scc	Score=132	.	.	.	.	.	.	.
ENST0	.	14q32.33	.	.	Scc	.	rs747	ID=COSN1	.	.	.	.	.
.	.	15q11.1	.	.	Scc	.	rs568	.	.	.	.	0.001	2E-04
ENST0	.	15q11.1	.	.	Scc	Score=203	rs576	ID=COSN2	.	.	.	.	.
.	.	15q11.2	.	.	Scc	Score=749	rs368	.	.	.	.	.	.
ENST0	.	15q11.2	.	.	Scc	Score=278	rs113	.	.	.	.	.	.

ENST0	.	15q11.2	.	.	Scc	.	rs199	.	.	.	.	.	.
ENST0	.	15q11.2	.	.	Scc	.	rs183	.	.	.	.	.	.
.	.	15q11.2	.	.	Scc	Score=275	rs370	.	.	.	.	.	.
ENST0	.	15q11.2	.	.	Scc	.	rs376	.	.	.	.	.	.
ENST0	.	15q13.2	.	.	Scc	.	.	.	.	.	.	.	.
ENST0	.	15q15.1	.	Scc	.	Score=21;I	rs557	.	.	.	.	.	2E-04
ENST0	.	15q15.1	.	.	.	Score=789	.	.	.	.	.	.	.
ENST0	.	15q24.1	.	.	Scc	.	rs744	ID=COSM:	.	.	.	.	.
ENST0	.	15q25.2	.	.	Scc	.	.	.	.	.	.	.	.
ENST0	.	16p13.3	.	.	.	.	.	.	.	.	.	.	.
ENST0	.	16p13.3	.	.	.	.	.	.	.	.	.	.	.
ENST0	.	16p13.13	.	.	.	.	.	.	.	.	.	.	.
ENST0	.	16p11.2	.	.	Scc	.	.	.	.	.	.	.	.
.	.	16q11.2	.	.	.	Score=112	rs288	ID=COSN1	.	.	.	.	.
.	.	16q11.2	.	.	.	Score=108	rs496	.	.	.	.	.	.
.	.	16q11.2	.	.	.	Score=108	rs435	.	.	.	.	.	.
.	.	16q11.2	.	.	.	Score=117	rs424	ID=COSN1	.	.	.	.	.
.	.	16q11.2	.	.	.	Score=117	rs437	ID=COSN1	.	.	.	.	.
.	.	16q11.2	.	.	.	Score=117	rs439	ID=COSN2	.	.	.	.	.
.	.	16q11.2	.	.	.	Score=117	rs424	.	.	.	.	.	.
.	.	16q11.2	.	.	.	Score=111	rs496	ID=COSN2	.	.	.	.	.
.	.	16q11.2	.	.	.	Score=114	rs720	.	.	.	.	.	.
.	.	16q11.2	.	.	.	Score=111	rs782	.	.	.	.	.	.
.	.	16q11.2	.	.	.	Score=111	rs424	.	.	.	.	.	.
.	.	16q11.2	.	.	.	Score=111	rs718	ID=COSN2	.	.	.	.	.
.	.	16q11.2	.	.	.	Score=111	rs997	.	.	.	.	.	.
.	.	16q11.2	.	.	.	Score=111	rs424	.	.	.	.	.	.
.	.	16q11.2	.	.	.	Score=111	rs782	ID=COSN1	.	.	.	.	.
.	.	16q11.2	.	.	.	.	rs139	ID=COSN1	.	.	.	.	.
.	.	16q11.2	.	.	.	Score=114	rs409	.	.	.	.	.	.
.	.	16q11.2	.	.	.	Score=114	rs409	ID=COSN2	.	.	.	.	.
.	.	16q11.2	.	.	.	Score=114	rs445	.	.	.	.	.	.
.	.	16q11.2	.	.	.	Score=114	rs613	.	.	.	.	.	.
.	.	16q11.2	.	.	.	Score=979	rs434	.	.	.	.	.	.
.	.	16q11.2	.	.	.	Score=979	rs445	.	.	.	.	.	.
.	.	16q11.2	.	.	.	Score=112	rs718	ID=COSN1	.	.	.	.	.
ENST0	.	16q22.1	.	.	Scc	.	.	.	.	.	.	.	.
ENST0	.	16q22.1	.	.	Scc	.	rs200	ID=COSM:	no	.	.	.	.
ENST0	.	16q22.1	.	Scc	.	Score=217	.	.	.	.	.	.	.
ENST0	.	16q24.3	.	.	.	.	.	.	.	.	.	.	.
ENST0	.	16q24.3	.	.	.	Score=277	.	.	.	.	.	.	.
ENST0	.	17p13.3	.	.	.	Score=214	.	.	.	.	.	.	.
ENST0	.	17p13.1	.	.	.	.	.	.	.	.	.	.	.
ENST0	.	17p11.2	.	.	.	.	.	.	.	.	.	.	.
.	.	17p11.1	.	.	.	Score=113	rs144	ID=COSN1	.	.	.	.	.
.	.	17p11.1	.	.	.	Score=113	rs436	ID=COSN2	.	.	.	.	.
.	.	17q12	.	.	Scc	.	rs140	ID=COSN1	.	.	.	0.223	0.344
.	.	17q12	.	.	Scc	.	rs373	.	.	.	.	0.212	0.177
ENST0	.	17q12	.	.	Scc	.	.	ID=COSM!	.	.	.	.	.
ENST0	.	17q12	.	.	Scc	.	.	ID=COSN1	.	.	.	.	.
ENST0	.	17q21.1	.	.	.	Score=324	rs867	ID=COSN1	.	.	.	.	.
.	.	17q21.31	.	.	.	Score=876	.	.	.	.	.	.	.
ENST0	.	17q21.31	.	.	.	Score=200	.	.	.	.	.	.	.
ENST0	.	17q24.2	.	.	.	.	rs765	ID=COSM:	.	.	.	.	.

ENST0	.	17q25.1	.	.	.	.	rs775	.	.	.	.	.	.
ENST0	.	17q25.2	.	.	.	.	.	.	.	.	.	.	.
ENST0	.	17q25.3	.	.	.	.	rs540	.	.	.	.	.	4E-04
ENST0	.	17q25.3	.	.	.	.	.	.	.	.	.	.	.
ENST0	.	17q25.3	.	.	.	.	.	ID=COSN2	.	.	.	.	.
ENST0	.	17q25.3	.	.	.	.	rs373	.	.	.	.	.	.
ENST0	.	18p11.21	.	.	.	.	.	.	.	.	.	.	.
ENST0	.	19p13.3	.	.	.	.	rs757	.	.	.	.	.	.
ENST0	.	19p13.3	.	.	.	.	.	.	.	.	.	.	.
ENST0	.	19p13.2	.	.	.	.	.	.	.	.	.	.	.
ENST0	.	19p13.2	.	.	.	.	.	.	.	.	.	.	.
ENST0	.	19p13.2	.	.	.	.	.	.	.	.	.	.	.
.	.	19p13.2	.	.	Scc	Score=182	rs149	ID=COSN2	.	.	.	.	.
ENST0	.	19q13.11	.	.	.	Score=624	rs199	ID=COSN6	.	.	.	.	.
.	.	19q13.12	.	.	Scc	Score=123	rs376	.	.	.	.	.	.
.	.	19q13.12	.	.	Scc	Score=123	rs712	.	.	.	.	.	.
ENST0	.	19q13.2	.	.	.	Score=21;I	.	.	.	.	.	.	.
ENST0	.	19q13.2	.	.	.	Score=160	.	.	.	.	.	.	.
ENST0	.	19q13.31	.	.	Scc	.	.	ID=COSN2	.	.	.	.	.
ENST0	.	19q13.33	.	.	.	.	.	.	.	.	.	.	.
ENST0	.	19q13.33	.	.	Scc	.	rs621	ID=COSN2	.	.	.	.	.
ENST0	.	19q13.33	.	.	.	Score=219	.	.	.	.	.	.	.
ENST0	.	19q13.42	.	.	Scc	.	rs549	.	.	.	.	.	2E-04
.	.	19q13.42	.	.	.	.	.	ID=COSN2	.	.	.	.	.
ENST0	.	19q13.42	.	.	.	.	rs760	ID=COSM2	.	.	.	.	.
ENST0	.	19q13.43	.	.	.	.	.	.	.	.	.	.	.
ENST0	.	19q13.43	.	.	Scc	.	rs770	.	.	.	.	.	.
ENST0	.	19q13.43	.	.	.	.	.	.	.	.	.	.	.
ENST0	.	20p13	.	Scc	.	.	.	.	.	.	.	.	.
ENST0	.	20p12.3	.	.	.	.	.	.	.	.	.	.	.
ENST0	.	20p11.1	.	.	Scc	.	.	.	.	.	.	.	.
ENST0	.	20q11.21	.	.	Scc	.	rs956	ID=COSN1	.	.	.	.	.
ENST0	.	20q13.12	.	.	.	.	.	.	.	.	.	.	.
ENST0	.	21p11.2	.	.	Scc	Score=333	rs377	.	.	.	.	.	.
ENST0	.	21p11.1	.	.	Scc	.	rs918	ID=COSN2	.	.	.	.	.
.	.	21p11.1	.	.	Scc	.	rs926	.	.	.	.	.	.
.	.	21p11.1	.	.	Scc	Score=195	.	.	.	.	.	.	.
ENST0	.	21q22.11	.	.	.	.	.	.	.	.	.	.	.
ENST0	.	21q22.12	.	.	.	Score=454	rs796	ID=COSN1	.	.	.	.	.
ENST0	.	22q11.1	.	.	Scc	.	rs201	.	.	.	.	.	.
ENST0	.	22q11.21	.	.	.	.	rs754	.	.	.	.	.	.
ENST0	.	22q12.1	.	.	.	.	rs571	.	.	.	.	0.005	1E-03
ENST0	.	22q12.2	.	.	Scc	.	rs185	ID=COSN1	.	.	.	0.016	0.03
ENST0	.	22q12.3	.	Scc	.	.	rs371	ID=COSM5	.	.	.	.	.
ENST0	.	22q13.1	.	.	.	.	.	.	.	.	.	.	.
ENST0	.	22q13.1	.	.	.	.	.	.	.	.	.	.	.
ENST0	.	22q13.31	.	.	.	.	.	.	.	.	.	.	.
.	.	Xp22.33	.	.	Scc	Score=187	rs664	.	.	.	.	.	.
ENST0	.	Xp22.2	.	.	.	Score=456	rs201	.	.	.	.	0.028	0.034
ENST0	.	Xp22.2	.	.	.	.	.	ID=COSM2	.	.	.	.	.
ENST0	.	Xp11.4	.	.	.	.	rs745	ID=COSM4	.	.	.	.	.
ENST0	.	Xp11.23	.	.	.	.	.	.	.	.	.	.	.
ENST0	.	Xp11.23	.	.	.	.	rs782	ID=COSM3	.	.	.	.	.
.	.	Xq11.1	.	.	.	Score=120	.	.	.	.	.	.	.

.	.	Xq11.1	.	.	.	Score=120	.	ID=COSN2	.	.	.	.	.
.	.	Xq11.1	.	.	.	Score=120	rs775	.		.	.	.	.
ENST0	.	Xq13.2	.	.	.	.	rs248	ID=COSN1	.	.	.	0.005	0.048
ENST0	.	Xq23	.	.	.	.	.	ID=COSM3	.	.	.	.	.
ENST0	.	Xq24	.	Scc	.	.	.	.	.	.	.	.	.
.	.	Xq25	.	.	.	.	.	.	.	.	.	.	.
ENST0	.	Xq27.1	.	.	.	.	rs101	.	.	.	.	.	.
ENST0	.	Xq28	.	.	.	.	.	.	.	.	.	.	.
ENST0	.	Xq28	.	.	Scc	.	rs780	ID=COSM5	.	.	.	.	.
.	.	.	.	.	.	.	.	.	.	.	.	.	.
.	.	.	.	.	.	.	rs386	.	.	.	.	.	.
.	.	.	.	.	.	.	.	.	.	.	.	.	.
ENST0	.	1p36.33	.	Scc	.	.	rs746	.	.	.	.	.	.
ENST0	.	1p36.33	.	.	Scc	Score=161	.	.	.	.	.	.	.
ENST0	.	1p36.22	.	Scc	.	.	rs145	.	.	.	.	.	2E-04
ENST0	.	1p36.22	.	Scc	.	.	.	.	.	.	.	.	.
ENST0	.	1p36.21	.	.	Scc	.	rs144	.	.	.	.	.	.
.	.	1p36.21	.	.	Scc	Score=207	rs115	ID=COSN6	.	.	.	0.096	0.081
.	.	1p36.13	.	.	Scc	.	.	.	.	.	.	.	.
ENST0	.	1p36.13	.	.	Scc	.	rs751	ID=COSN1	.	.	.	.	.
ENST0	.	1p36.12	.	.	.	.	.	ID=COSM4	.	.	.	.	.
ENST0	.	1p36.12	.	.	.	Score=636	rs555	.	.	.	.	.	2E-04
ENST0	.	1p36.11	.	.	.	.	rs142	ID=COSM1	.	.	.	0.001	0.001
ENST0	.	1p36.11	.	.	Scc	Score=278	.	.	.	.	.	.	.
ENST0	.	1p35.2	.	Scc	.	.	.	.	.	.	.	.	.
ENST0	.	1p35.1	.	.	.	.	rs775	ID=COSM5	.	.	.	.	.
ENST0	.	1p34.3	.	.	.	.	.	.	.	.	.	.	.
ENST0	.	1p34.3	.	.	.	.	.	.	.	.	.	.	.
ENST0	.	1p34.3	.	.	.	.	.	.	.	.	.	.	.
ENST0	.	1p34.3	.	.	.	.	.	.	.	.	.	.	.
ENST0	.	1p34.3	.	.	.	.	.	.	.	.	.	.	.
ENST0	.	1p34.2	.	.	.	Score=809	.	ID=COSN2	.	.	.	.	.
ENST0	.	1p34.1	.	Scc	Scc	.	rs758	.	.	.	.	.	.
.	.	1p34.1	.	.	Scc	Score=104	rs866	ID=COSN1	.	.	.	.	.
ENST0	.	1p32.3	.	.	.	Score=410	.	.	.	.	.	.	.
ENST0	.	1p31.3	.	.	.	.	.	.	.	.	.	.	.
ENST0	.	1p31.1	.	.	.	.	.	.	.	.	.	.	.
ENST0	.	1p31.1	.	.	.	Score=288	.	.	.	.	.	.	.
ENST0	.	1p22.1	.	.	.	.	.	.	.	.	.	.	.
ENST0	.	1p22.1	.	.	.	.	.	.	.	.	.	.	.
ENST0	.	1p21.3	.	.	.	.	.	.	.	.	.	.	.
ENST0	.	1p13.3	.	.	.	.	rs759	.	.	.	.	.	.
ENST0	.	1p13.2	.	.	.	.	.	.	.	.	.	.	.
ENST0	.	1p11.2	.	.	Scc	.	rs200	ID=COSN6	.	.	.	.	.
ENST0	.	1q21.1	.	.	Scc	.	rs104	.	.	.	.	.	.
.	.	1q21.1	.	.	Scc	.	.	.	.	.	.	.	.
ENST0	.	1q21.1	.	.	Scc	Score=169	rs200	.	.	.	.	.	.
ENST0	.	1q21.1	.	.	Scc	.	rs750	.	.	.	.	.	.
ENST0	.	1q21.1	.	.	Scc	.	rs169	.	.	.	.	.	.
ENST0	.	1q21.1	.	.	Scc	.	rs412	ID=COSN1	.	.	.	.	.
ENST0	.	1q21.1	.	.	Scc	.	.	.	.	.	.	.	.
ENST0	.	1q21.1	.	.	Scc	.	.	.	.	.	.	.	.
ENST0	.	1q21.2	.	.	Scc	.	rs618	ID=COSN1	.	.	.	.	.
.	.	1q21.2	.	.	Scc	.	rs879	.	.	.	.	.	.

ENST0	.	1q21.2	.	.	Scc	.	rs482	.	.	.	.	.	.
ENST0	.	1q21.2	.	.	Scc	.	rs202	.	.	.	.	.	.
ENST0	.	1q21.2	.	.	Scc	.	rs200	ID=COSM4	.	.	.	.	.
ENST0	.	1q21.2	.	.	Scc	Score=173	rs200	.	.	.	.	.	.
ENST0	.	1q21.2	.	.	Scc	Score=173	rs370	.	.	.	.	.	.
ENST0	.	1q21.2	.	.	Scc	.	rs226	ID=COSN1	.	.	.	.	.
ENST0	.	1q21.2	.	.	Scc	.	.	.	.	.	.	.	.
ENST0	.	1q21.2	.	.	Scc	.	rs278	ID=COSN1	.	.	.	0.325	0.398
.	.	1q21.3	.	.	Scc	.	rs375	.	.	.	.	.	.
ENST0	.	1q21.3	.	.	.	.	.	.	.	.	.	.	.
ENST0	.	1q21.3	.	.	.	.	.	.	.	.	.	.	.
ENST0	.	1q21.3	.	.	.	.	.	.	.	.	.	.	.
ENST0	.	1q22	.	.	.	.	.	.	.	.	.	.	.
ENST0	.	1q22	.	.	.	.	.	.	.	.	.	.	.
ENST0	.	1q23.1	.	.	.	.	.	.	.	.	.	.	.
ENST0	.	1q23.1	.	Scc	.	.	.	.	.	.	.	.	.
ENST0	.	1q25.1	.	.	.	.	.	.	.	.	.	.	.
ENST0	.	1q31.3	.	.	.	.	.	.	.	.	.	.	.
ENST0	.	1q32.1	.	.	.	Score=252	rs199	.	.	.	.	.	2E-04
ENST0	.	1q32.1	.	.	.	.	rs369	.	.	.	.	.	.
ENST0	.	1q41	.	.	.	.	.	.	.	.	.	.	.
ENST0	.	1q41	.	.	.	.	.	.	.	.	.	.	.
ENST0	.	1q42.11	.	.	Scc	.	.	.	.	.	.	.	.
ENST0	.	1q42.13	.	.	.	.	rs779	.	.	.	.	.	.
ENST0	.	2p25.1	.	.	.	Score=30;I	.	.	.	.	.	.	.
ENST0	.	2p25.1	.	.	.	.	.	.	.	.	.	.	.
ENST0	.	2p23.3	.	.	.	.	.	.	.	.	.	.	.
ENST0	.	2p23.3	.	.	.	.	.	.	.	.	.	.	.
ENST0	.	2p23.3	.	.	.	Score=144	.	.	.	.	.	.	.
ENST0	.	2p21	.	.	.	.	.	.	.	.	.	.	.
ENST0	.	2p21	.	.	.	.	.	.	.	.	.	.	.
ENST0	.	2p21	.	.	.	.	.	.	.	.	.	.	.
ENST0	.	2p14	.	.	.	.	.	.	.	.	.	.	.
ENST0	.	2p13.3	.	.	.	.	.	.	.	.	.	.	.
ENST0	.	2p13.2	.	.	.	.	.	ID=COSN1	.	.	.	.	.
ENST0	.	2p11.2	.	.	Scc	.	rs376	.	.	.	.	.	.
ENST0	.	2p11.2	.	.	.	.	rs142	.	.	.	.	.	.
ENST0	.	2p11.2	.	.	.	.	.	.	.	.	.	.	.
ENST0	.	2p11.2	.	.	.	.	.	.	.	.	.	.	.
ENST0	.	2p11.2	.	Scc	Scc	.	rs867	ID=COSN2	.	.	.	.	.
ENST0	.	2p11.2	.	.	Scc	.	rs867	ID=COSN2	.	.	.	.	.
ENST0	.	2p11.2	.	.	Scc	.	.	.	.	.	.	.	.
.	.	2p11.2	.	.	Scc	Score=184	rs121	ID=COSN2	.	.	.	.	.
.	.	2q11.1	.	.	.	Score=788	rs201	ID=COSN2	.	.	.	.	.
ENST0	.	2q12.1	.	.	.	.	.	ID=COSM5	.	.	.	.	.
ENST0	.	2q13	.	.	.	.	.	.	.	.	.	.	.
ENST0	.	2q13	.	.	.	.	.	.	.	.	.	.	.
ENST0	.	2q14.2	.	.	.	.	rs621	.	.	.	.	0.378	0.408
ENST0	.	2q14.3	.	Scc	.	Score=21;I	.	.	.	.	.	.	.
ENST0	.	2q21.1	.	.	Scc	.	.	.	.	.	.	.	.
ENST0	.	2q21.1	.	.	Scc	.	rs183	ID=COSM3	.	.	.	0.688	0.739
ENST0	.	2q21.1	.	.	Scc	Score=488	rs200	.	.	.	.	.	.
ENST0	.	2q21.1	.	.	Scc	.	.	.	.	.	.	.	.
ENST0	.	2q23.3	.	.	.	.	.	.	.	.	.	.	.

ENST0	.	2q24.2	.	.	.	.	.	.	.	.	.	.	.
ENST0	.	2q31.2	.	.	.	.	.	.	.	.	.	.	.
ENST0	.	2q31.2	.	.	.	.	.	ID=COSM:	.	.	.	.	.
ENST0	.	2q33.1	.	.	.	.	.	.	.	.	.	.	.
ENST0	.	2q34	.	.	.	Score=992	.	.	.	.	.	.	.
ENST0	.	2q35	.	.	.	.	.	.	.	.	.	.	.
ENST0	.	2q37.1	.	.	.	.	.	.	.	.	.	.	.
ENST0	.	2q37.1	.	.	.	.	.	.	.	.	.	.	.
ENST0	.	2q37.3	.	.	.	.	.	.	.	.	.	.	.
ENST0	.	3p25.2	.	.	Scc	Score=105	.	.	.	.	.	.	.
ENST0	.	3p25.1	.	.	.	.	.	.	.	.	.	.	.
ENST0	.	3p25.1	.	.	.	.	.	.	.	.	.	.	.
ENST0	.	3p23	.	.	.	.	.	.	.	.	.	.	.
ENST0	.	3p22.3	.	Scc	.	.	.	.	.	.	.	.	.
ENST0	.	3p21.31	.	.	.	.	.	.	.	.	.	.	.
ENST0	.	3p21.31	.	.	.	.	.	.	.	.	.	.	.
ENST0	.	3p21.31	.	.	.	.	.	.	.	.	.	.	.
ENST0	.	3p21.31	.	Scc	.	.	.	.	.	.	.	.	.
ENST0	.	3p21.31	.	Scc	.	.	.	.	.	.	.	.	.
ENST0	.	3p21.1	.	.	.	.	rs780	.	.	.	.	.	.
ENST0	.	3p21.1	.	.	.	.	rs367	.	.	.	.	.	.
ENST0	.	3p14.3	.	.	.	.	rs745	.	.	.	.	.	.
ENST0	.	3q11.2	.	.	.	.	.	.	.	.	.	.	.
ENST0	.	3q12.1	.	.	.	.	rs191	.	.	.	.	.	6E-04
ENST0	.	3q13.31	.	.	.	.	.	.	.	.	.	.	.
ENST0	.	3q13.33	.	.	.	.	.	.	.	.	.	.	.
ENST0	.	3q21.1	.	.	.	.	.	.	.	.	.	.	.
ENST0	.	3q21.3	.	.	.	.	.	.	.	.	.	.	.
ENST0	.	3q21.3	.	.	.	.	.	.	.	.	.	.	.
ENST0	.	3q21.3	.	.	.	.	.	.	.	.	.	.	.
ENST0	.	3q21.3	.	.	.	.	.	.	.	.	.	.	.
ENST0	.	3q22.1	.	.	.	.	.	.	.	.	.	.	.
ENST0	.	3q22.1	.	.	.	.	.	.	.	.	.	.	.
ENST0	.	3q22.3	.	.	.	.	rs146	.	.	.	.	.	0.002
ENST0	.	3q22.3	.	Scc	.	.	.	.	.	.	.	.	.
ENST0	.	3q23	.	.	.	.	.	ID=COSM:	.	.	.	.	.
ENST0	.	3q23	.	.	.	.	.	.	.	.	.	.	.
ENST0	.	3q25.1	.	.	.	.	rs761	.	.	.	.	.	.
ENST0	.	3q25.32	.	.	.	.	.	.	.	.	.	.	.
ENST0	.	3q26.1	.	.	.	.	.	.	.	.	.	.	.
ENST0	.	3q26.2	.	.	.	.	.	.	.	.	.	.	.
ENST0	.	3q26.31	.	.	.	.	.	.	.	.	.	.	.
ENST0	.	3q27.1	.	.	.	.	.	.	.	.	.	.	.
ENST0	.	3q27.2	.	.	.	Score=303	.	.	.	.	.	.	.
ENST0	.	3q27.3	.	.	.	.	.	.	.	.	.	.	.
ENST0	.	3q29	.	.	.	.	.	.	.	.	.	.	.
.	.	3q29	.	.	Scc	Score=127	rs879	.	.	.	.	.	.
.	.	3q29	.	.	Scc	Score=735	.	.	.	.	.	.	.
ENST0	.	3q29	.	.	Scc	.	rs492	ID=COSN2	.	.	.	.	.
ENST0	.	3q29	.	Scc	Scc	.	rs173	ID=COSN1	.	.	.	.	.
ENST0	.	4p16.3	.	.	.	Score=207	rs402	ID=COSN2	.	.	.	.	.
ENST0	.	4p16.3	.	.	.	.	rs751	.	.	.	.	.	.
ENST0	.	4p15.2	.	.	.	.	rs752	.	.	.	.	.	.
.	.	4p15.1	.	.	Scc	.	.	.	.	.	.	.	.

ENST0	.	4p14	.	.	.	.	.	.	.	.	.	.	.
ENST0	.	4p13	.	.	.	.	.	.	.	.	.	.	.
.	.	4p11	.	.	Scc	.	rs754	ID=COSN1	.	.	.	.	.
ENST0	.	4q12	.	.	.	.	.	.	.	.	.	.	.
ENST0	.	4q13.3	.	.	.	.	rs542	.	.	.	.	0.001	2E-04
ENST0	.	4q21.21	.	.	.	.	.	.	.	.	.	.	.
ENST0	.	4q24	.	.	Scc	.	rs878	ID=COSN2	.	.	.	.	.
ENST0	.	4q25	.	.	.	.	rs890	.	.	.	.	.	.
ENST0	.	4q26	.	.	.	.	.	.	.	.	.	.	.
ENST0	.	4q28.2	.	.	.	Score=234	rs137	.	.	.	.	0.245	0.108
ENST0	.	4q28.3	.	Scc	.	.	.	.	.	.	.	.	.
ENST0	.	4q31.21	.	.	Scc	.	rs367	.	.	.	.	.	.
ENST0	.	4q32.1	.	.	.	.	.	.	.	.	.	.	.
ENST0	.	4q33	.	.	.	.	.	.	.	.	.	.	.
.	.	4q35.2	.	.	.	Score=950	.	ID=COSN2	.	.	.	.	.
ENST0	.	5p15.33	.	.	.	.	.	.	.	.	.	.	.
ENST0	.	5p15.2	.	.	.	.	.	.	.	.	.	.	.
ENST0	.	5p13.1	.	.	Scc	.	.	.	.	.	.	.	.
ENST0	.	5q11.2	.	.	.	.	.	.	.	.	.	.	.
ENST0	.	5q11.2	.	.	.	.	.	.	.	.	.	.	.
ENST0	.	5q12.3	.	.	.	Score=155	.	.	.	.	.	.	.
.	.	5q13.1	.	.	.	Score=221	.	.	.	.	.	.	.
ENST0	.	5q13.2	.	.	Scc	.	.	.	.	.	.	.	.
ENST0	.	5q13.2	.	.	Scc	Score=360	rs284	ID=COSN2	.	.	.	.	.
ENST0	.	5q13.3	.	Scc	.	.	.	.	.	.	.	.	.
ENST0	.	5q13.3	.	.	.	.	.	.	.	.	.	.	.
ENST0	.	5q23.1	.	Scc	.	.	.	.	.	.	.	.	.
ENST0	.	5q31.3	.	.	.	.	.	.	.	.	.	.	.
ENST0	.	5q31.3	.	.	.	.	.	.	.	.	.	.	.
ENST0	.	5q33.1	.	.	.	Score=249	rs102	.	.	.	.	.	.
ENST0	.	5q33.1	.	.	.	.	.	.	.	.	.	.	.
ENST0	.	5q33.3	.	.	.	Score=708	.	.	.	.	.	.	.
ENST0	.	5q33.3	.	.	.	.	.	.	.	.	.	.	.
ENST0	.	5q34	.	.	.	.	rs534	.	.	.	.	.	1E-03
ENST0	.	5q35.2	.	.	.	.	.	.	.	.	.	.	.
ENST0	.	5q35.3	.	.	.	.	.	.	.	.	.	.	.
ENST0	.	6p25.3	.	.	.	.	rs201	.	.	.	.	.	.
ENST0	.	6p25.2	.	.	.	Score=199	.	.	.	.	.	.	.
ENST0	.	6p25.1	.	.	.	.	.	.	.	.	.	.	.
ENST0	.	6p22.2	.	.	.	.	rs748	.	.	.	.	.	.
ENST0	.	6p22.1	.	.	.	.	.	.	.	.	.	.	.
ENST0	.	6p22.1	.	.	.	.	.	.	.	.	.	.	.
ENST0	.	6p22.1	.	.	.	.	rs940	.	.	.	.	.	.
ENST0	.	6p21.33	.	.	.	.	.	.	.	.	.	.	.
ENST0	.	6p21.33	.	.	.	.	.	.	.	.	.	.	.
ENST0	.	6p21.33	.	.	Scc	.	.	.	.	.	.	.	.
ENST0	.	6p21.33	.	.	.	.	.	.	.	.	.	.	.
ENST0	.	6p21.33	.	.	Scc	.	.	.	.	.	.	.	.
ENST0	.	6p21.33	.	Scc	Scc	.	.	.	.	.	.	.	.
ENST0	.	6p21.33	.	.	Scc	.	rs542	.	.	.	.	.	2E-04
ENST0	.	6p21.33	.	Scc	Scc	.	.	.	.	.	.	.	.
ENST0	.	6p21.33	.	.	Scc	.	.	.	.	.	.	.	.
ENST0	.	6p21.31	.	.	.	.	.	.	.	.	.	.	.
ENST0	.	6p21.2	.	.	.	.	.	.	.	.	.	.	.

ENST0	.	6p21.1	.	.	.	.	rs374	.	.	.	.	.	.
ENST0	.	6p12.3	.	.	Scc	.	rs548	.	.	.	.	.	2E-04
ENST0	.	6p12.3	.	.	.	.	rs144	.	.	.	.	.	0.003
ENST0	.	6q14.1	.	.	.	.	rs900	.	.	.	.	.	.
ENST0	.	6q15	.	.	.	.	.	.	.	.	.	.	.
ENST0	.	6q21	.	.	.	.	rs746	.	.	.	.	.	.
ENST0	.	6q22.31	.	Scc	.	.	.	.	.	.	.	.	.
.	.	6q25.2	.	.	.	Score=143	rs541	.	.	.	.	0.001	8E-04
ENST0	.	6q25.3	.	.	.	.	.	.	.	.	.	.	.
ENST0	.	7p22.3	.	.	.	.	rs369	.	.	.	.	.	.
ENST0	.	7p22.1	.	.	.	.	rs918	.	.	.	.	.	.
.	.	7p22.1	.	.	.	Score=175	.	.	.	.	.	.	.
ENST0	.	7p14.3	.	.	Scc	Score=234	.	.	.	.	.	.	.
ENST0	.	7p13	.	.	.	.	rs148	.	.	.	.	.	0.008
ENST0	.	7p11.2	.	.	Scc	.	rs367	.	.	.	.	.	.
ENST0	.	7p11.2	.	.	Scc	.	.	ID=COSN2	.	.	.	.	.
ENST0	.	7p11.2	.	.	Scc	.	.	.	.	.	.	.	.
ENST0	.	7p11.2	.	.	Scc	.	.	.	.	.	.	.	.
ENST0	.	7q11.21	.	.	Scc	.	rs201	ID=COSN2	.	.	.	.	.
ENST0	.	7q11.21	.	.	Scc	Score=593	rs186	ID=COSN2	.	.	.	0.355	0.359
.	.	7q11.21	.	.	Scc	.	.	ID=COSN1	.	.	.	.	.
.	.	7q11.21	.	.	Scc	.	.	.	.	.	.	.	.
ENST0	.	7q11.21	.	.	Scc	.	rs268	.	.	.	.	.	.
ENST0	.	7q11.22	.	.	.	.	.	.	.	.	.	.	.
ENST0	.	7q11.23	.	Scc	.	.	.	.	.	.	.	.	.
.	.	7q21.3	.	.	Scc	.	.	.	.	.	.	.	.
ENST0	.	7q22.1	.	.	.	.	rs373	.	.	.	.	.	.
ENST0	.	7q32.1	.	.	.	.	rs372	.	.	.	.	.	2E-04
ENST0	.	7q32.1	.	.	.	.	.	.	.	.	.	.	.
ENST0	.	7q32.1	.	.	.	.	rs103	.	.	.	.	.	.
ENST0	.	7q34	.	.	Scc	.	.	.	.	.	.	.	.
ENST0	.	7q36.1	.	.	.	.	.	.	.	.	.	.	.
ENST0	.	7q36.1	.	.	Scc	.	rs867	.	.	.	.	.	.
ENST0	.	8p23.1	.	.	Scc	.	.	.	.	.	.	.	.
.	.	8p23.1	.	.	Scc	.	rs626	.	.	.	.	.	.
ENST0	.	8p23.1	.	.	.	Score=360	.	.	.	.	.	.	.
ENST0	.	8p23.1	.	.	Scc	Score=345	.	.	.	.	.	.	.
ENST0	.	8p23.1	.	.	Scc	.	.	.	.	.	.	.	.
ENST0	.	8p21.3	.	.	.	.	rs760	.	.	.	.	.	.
ENST0	.	8p12	.	.	.	.	.	.	.	.	.	.	.
ENST0	.	8p11.23	.	.	.	.	rs373	ID=COSM1	.	.	.	.	.
ENST0	.	8q11.23	.	.	.	.	.	.	.	.	.	.	.
ENST0	.	8q21.11	.	.	.	.	.	.	.	.	.	.	.
ENST0	.	8q21.13	.	.	.	.	rs987	.	.	.	.	.	.
ENST0	.	8q21.3	.	.	.	.	.	.	.	.	.	.	.
ENST0	.	8q21.3	.	.	.	.	.	.	.	.	.	.	.
ENST0	.	8q21.3	.	.	.	Score=288	.	.	.	.	.	.	.
ENST0	.	8q22.2	.	.	.	.	.	.	.	.	.	.	.
ENST0	.	8q23.3	.	.	.	.	.	ID=COSN5	.	.	.	.	.
ENST0	.	8q24.12	.	.	.	.	.	.	.	.	.	.	.
ENST0	.	8q24.13	.	.	.	.	.	.	.	.	.	.	.
ENST0	.	8q24.13	.	.	.	.	rs143	.	.	.	.	.	.
ENST0	.	8q24.13	.	Scc	.	.	.	.	.	.	.	.	.
ENST0	.	8q24.3	.	.	.	.	.	.	.	.	.	.	.

ENST0	.	9p22.3	.	.	Scc	.	.	.	.	.	.	.	.
ENST0	.	9p22.1	.	.	.	.	rs768	.	.	.	.	.	.
ENST0	.	9p21.3	.	.	.	.	.	.	.	.	.	.	.
ENST0	.	9p13.3	.	.	.	.	.	.	.	.	.	.	.
ENST0	.	9p13.1	.	.	Scc	.	rs376	ID=COSN2	.	.	.	.	.
ENST0	.	9p11.2	.	.	Scc	.	.	.	.	.	.	.	.
ENST0	.	9q13	.	.	Scc	.	.	.	.	.	.	.	.
ENST0	.	9q21.11	.	.	Scc	.	.	.	.	.	.	.	.
ENST0	.	9q21.12	.	.	.	.	.	.	.	.	.	.	.
ENST0	.	9q21.2	.	.	.	.	.	.	.	.	.	.	.
ENST0	.	9q21.31	.	.	.	.	.	.	.	.	.	.	.
ENST0	.	9q22.32	.	Scc	.	.	.	.	.	.	.	.	.
ENST0	.	9q32	.	.	.	.	rs961	.	.	.	.	.	.
ENST0	.	9q33.2	.	.	.	.	.	.	.	.	.	.	.
ENST0	.	9q33.3	.	.	.	.	.	.	.	.	.	.	.
ENST0	.	9q34.11	.	.	.	.	.	.	.	.	.	.	.
ENST0	.	9q34.11	.	.	.	.	rs758	.	.	.	.	.	.
ENST0	.	9q34.11	.	.	.	.	.	.	.	.	.	.	.
ENST0	.	9q34.13	.	.	.	.	.	.	.	.	.	.	.
ENST0	.	9q34.3	.	.	.	.	.	.	.	.	.	.	.
ENST0	.	9q34.3	.	.	.	.	.	.	.	.	.	.	.
ENST0	.	10p14	.	.	.	.	.	.	.	.	.	.	.
ENST0	.	10p12.31	.	.	.	Score=390	rs201	ID=COSM4	.	.	.	.	.
ENST0	.	10q11.21	.	.	.	.	.	.	.	.	.	.	.
ENST0	.	10q11.21	.	.	.	.	.	.	.	.	.	.	.
ENST0	.	10q22.1	.	.	.	.	.	.	.	.	.	.	.
ENST0	.	10q22.1	.	.	.	.	rs376	.	.	.	.	.	.
ENST0	.	10q22.1	.	.	.	.	.	.	.	.	.	.	.
ENST0	.	10q22.1	.	.	.	.	.	.	.	.	.	.	.
ENST0	.	10q22.1	.	Scc	.	.	rs759	.	.	.	.	.	.
ENST0	.	10q22.1	.	.	.	.	rs769	ID=COSM9	.	.	.	.	.
.	.	10q22.3	.	.	Scc	Score=226	.	ID=COSN2	.	.	.	.	.
.	.	10q22.3	.	.	Scc	Score=226	.	ID=COSN2	.	.	.	.	.
ENST0	.	10q23.33	.	.	.	.	.	.	.	.	.	.	.
ENST0	.	10q24.33	.	.	.	.	.	.	.	.	.	.	.
ENST0	.	10q26.13	.	.	.	.	.	.	.	.	.	.	.
ENST0	.	10q26.3	.	.	Scc	.	rs796	.	.	.	.	.	.
ENST0	.	11p15.5	.	.	.	.	rs765	ID=COSM3	.	.	.	.	.
ENST0	.	11p15.4	.	.	Scc	.	rs866	ID=COSN2	.	.	.	.	.
ENST0	.	11p15.4	.	.	.	.	.	.	.	.	.	.	.
ENST0	.	11p14.3	.	.	.	.	.	.	.	.	.	.	.
ENST0	.	11p13	.	Scc	.	.	.	.	.	.	.	.	.
ENST0	.	11p11.2	.	Scc	.	.	.	.	.	.	.	.	.
ENST0	.	11p11.2	.	.	.	.	.	.	.	.	.	.	.
ENST0	.	11q12.1	.	.	.	.	.	.	.	.	.	.	.
ENST0	.	11q12.2	.	.	.	.	rs746	ID=COSM2	.	.	.	.	.
ENST0	.	11q12.2	.	Scc	.	Score=343	.	.	.	.	.	.	.
ENST0	.	11q12.3	.	Scc	.	.	.	.	.	.	.	.	.
ENST0	.	11q13.1	.	Scc	.	.	rs370	ID=COSM6	.	.	.	.	.
ENST0	.	11q13.1	.	.	.	Score=255	.	.	.	.	.	.	.
ENST0	.	11q13.1	.	.	.	.	.	.	.	.	.	.	.
ENST0	.	11q13.1	.	.	.	.	.	.	.	.	.	.	.
ENST0	.	11q13.1	.	Scc	.	.	.	.	.	.	.	.	.
ENST0	.	11q13.1	.	.	.	.	.	.	.	.	.	.	.

ENST0	.	11q13.1	.	.	.	.	.	.	.	.	.	.	.
ENST0	.	11q13.1	.	.	.	.	rs745	.	.	.	.	.	.
ENST0	.	11q13.4	.	.	.	.	.	.	.	.	.	.	.
ENST0	.	11q23.2	.	.	.	.	.	.	.	.	.	.	.
ENST0	.	11q23.3	.	.	Scc	.	rs352	.	.	.	.	.	.
ENST0	.	11q23.3	.	.	Scc	.	rs358	ID=COSN2	.	.	.	.	.
ENST0	.	11q24.2	.	.	.	.	rs920	.	.	.	.	.	.
ENST0	.	11q24.3	.	Scc	.	.	.	.	.	.	.	.	.
ENST0	.	12p13.31	.	.	.	.	.	.	.	.	.	.	.
ENST0	.	12p13.31	.	.	.	.	.	.	.	.	.	.	.
ENST0	.	12p13.31	.	.	.	.	.	.	.	.	.	.	.
ENST0	.	12p11.21	.	Scc	Scc	.	rs712	ID=COSM(	.	.	.	0.131	0.104
ENST0	.	12p11.21	.	.	.	.	.	.	.	.	.	.	.
ENST0	.	12q12	.	.	.	.	.	.	.	.	.	.	.
ENST0	.	12q12	.	.	.	.	.	.	.	.	.	.	.
ENST0	.	12q14.1	.	.	.	.	.	.	.	.	.	.	.
ENST0	.	12q14.1	.	.	.	.	.	.	.	.	.	.	.
ENST0	.	12q14.2	.	.	.	Score=399	rs867	.	.	.	.	.	.
ENST0	.	12q15	.	.	.	.	rs910	.	.	.	.	.	.
ENST0	.	12q21.31	.	.	.	Score=383	rs964	.	.	.	.	.	.
ENST0	.	12q21.32	.	.	.	.	.	.	.	.	.	.	.
ENST0	.	12q22	.	.	.	.	.	.	.	.	.	.	.
ENST0	.	12q23.2	.	.	.	.	.	.	.	.	.	.	.
ENST0	.	12q24.21	.	.	.	Score=672	.	.	.	.	.	.	.
ENST0	.	12q24.21	.	.	.	.	.	.	.	.	.	.	.
ENST0	.	12q24.23	.	.	.	.	.	.	.	.	.	.	.
ENST0	.	12q24.31	.	.	.	.	rs754	.	.	.	.	.	.
ENST0	.	12q24.31	.	.	.	.	.	.	.	.	.	.	.
ENST0	.	12q24.33	.	.	.	.	.	.	.	.	.	.	.
ENST0	.	13q12.11	.	.	.	.	.	.	.	.	.	.	.
ENST0	.	13q13.3	.	.	.	.	.	.	.	.	.	.	.
ENST0	.	13q14.11	.	Scc	.	.	.	.	.	.	.	.	.
ENST0	.	13q32.3	.	.	.	.	.	.	.	.	.	.	.
ENST0	.	13q33.1	.	.	.	.	rs772	.	.	.	.	.	.
ENST0	.	14q11.2	.	.	Scc	.	rs145	.	.	.	.	.	.
.	.	14q11.2	.	.	.	.	.	.	.	.	.	.	.
ENST0	.	14q11.2	.	.	Scc	.	.	.	.	.	.	.	.
ENST0	.	14q11.2	.	.	Scc	.	.	.	.	.	.	.	.
ENST0	.	14q21.1	.	.	.	.	.	.	.	.	.	.	.
.	.	14q21.3	.	.	.	Score=224	.	.	.	.	.	.	.
ENST0	.	14q22.2	.	.	.	.	rs150	.	Lik	.	.	.	2E-04
ENST0	.	14q23.1	.	.	.	.	.	.	.	.	.	.	.
ENST0	.	14q23.3	.	.	.	Score=220	.	.	.	.	.	.	.
ENST0	.	14q23.3	.	.	.	.	.	.	.	.	.	.	.
ENST0	.	14q24.2	.	.	.	.	rs767	.	.	.	.	.	.
ENST0	.	14q24.2	.	.	.	.	.	.	.	.	.	.	.
ENST0	.	14q24.3	.	.	.	.	.	.	.	.	.	.	.
ENST0	.	14q31.3	.	.	.	.	.	.	.	.	.	.	.
ENST0	.	14q32.12	.	.	.	.	.	.	.	.	.	.	.
ENST0	.	14q32.13	.	.	.	.	rs375	.	.	.	.	.	.
ENST0	.	14q32.33	.	.	.	.	.	ID=COSN2	.	.	.	.	.
ENST0	.	14q32.33	.	.	.	.	rs553	.	.	.	.	.	2E-04
.	.	14q32.33	.	.	Scc	.	rs128	ID=COSN1	.	.	.	.	.
ENST0	.	15q11.2	.	.	Scc	.	.	.	.	.	.	.	.

ENST0	.	15q11.2	.	.	Scc	Score=796	.	.	.	.	.	.	.
ENST0	.	15q13.1	.	Scc	.	.	rs540	.	.	.	.	.	2E-04
ENST0	.	15q13.1	.	.	Scc	.	.	.	.	.	.	.	.
ENST0	.	15q13.1	.	.	Scc	.	.	.	.	.	.	.	.
ENST0	.	15q13.2	.	.	Scc	.	.	ID=COSN2	.	.	.	.	.
ENST0	.	15q13.2	.	.	Scc	.	rs201	.	.	.	.	.	.
ENST0	.	15q15.1	.	.	.	.	rs748	.	.	.	.	.	.
ENST0	.	15q15.1	.	.	.	.	.	.	.	.	.	.	.
ENST0	.	15q15.1	.	.	.	.	.	.	.	.	.	.	.
ENST0	.	15q15.1	.	.	.	.	rs775	.	.	.	.	.	.
ENST0	.	15q15.3	.	.	Scc	.	.	.	.	.	.	.	.
ENST0	.	15q15.3	.	.	.	.	rs192	.	.	.	.	.	2E-04
ENST0	.	15q21.1	.	.	.	.	.	.	.	.	.	.	.
ENST0	.	15q21.1	.	.	Scc	.	rs620	.	.	.	.	0.422	0.324
ENST0	.	15q21.1	.	Scc	.	.	.	ID=COSM4	.	.	.	.	.
ENST0	.	15q21.3	.	Scc	.	.	.	.	.	.	.	.	.
ENST0	.	15q22.2	.	.	.	.	.	.	.	.	.	.	.
ENST0	.	15q22.31	.	.	.	.	rs547	.	.	.	.	.	4E-04
ENST0	.	15q22.31	.	.	.	.	.	.	.	.	.	.	.
ENST0	.	15q23	.	.	Scc	.	.	.	.	.	.	.	.
ENST0	.	15q24.1	.	.	.	.	.	.	.	.	.	.	.
.	.	15q24.2	.	.	Scc	.	.	.	.	.	.	.	.
ENST0	.	15q25.1	.	Scc	.	.	.	.	.	.	.	.	.
ENST0	.	15q25.1	.	.	.	.	.	.	.	.	.	.	.
ENST0	.	15q25.2	.	.	Scc	.	rs240	.	.	.	.	.	.
ENST0	.	15q26.1	.	.	.	.	.	.	.	.	.	.	.
ENST0	.	15q26.1	.	.	.	.	.	.	.	.	.	.	.
ENST0	.	15q26.2	.	.	.	.	.	.	.	.	.	.	.
ENST0	.	15q26.3	.	.	.	.	.	.	.	.	.	.	.
ENST0	.	15q26.3	.	.	.	.	rs751	ID=COSM5	.	.	.	.	.
ENST0	.	16p13.3	.	Scc	.	.	rs750	.	.	.	.	.	.
ENST0	.	16p13.3	.	.	.	.	.	.	.	.	.	.	.
ENST0	.	16p13.3	.	.	.	.	.	.	.	.	.	.	.
ENST0	.	16p13.13	.	.	.	.	.	.	.	.	.	.	.
ENST0	.	16p13.13	.	.	.	.	.	.	.	.	.	.	.
ENST0	.	16p13.11	.	.	Scc	.	.	.	.	.	.	.	.
ENST0	.	16p13.11	.	.	Scc	.	rs552	.	.	.	.	0.034	0.021
ENST0	.	16p13.11	.	.	Scc	.	rs620	ID=COSN1	.	.	.	0.04	0.04
ENST0	.	16p12.3	.	.	.	.	rs765	.	.	.	.	.	.
ENST0	.	16p12.2	.	.	.	Score=201	.	.	.	.	.	.	.
ENST0	.	16p12.2	.	.	.	Score=428	.	.	.	.	.	.	.
ENST0	.	16p12.2	.	Scc	Scc	.	.	.	.	.	.	.	.
ENST0	.	16p11.2	.	.	.	.	.	.	.	.	.	.	.
ENST0	.	16p11.2	.	.	.	.	.	.	.	.	.	.	.
ENST0	.	16p11.2	.	.	Scc	.	.	.	.	.	.	.	.
ENST0	.	16p11.2	.	.	.	.	.	.	.	.	.	.	.
ENST0	.	16p11.2	.	.	.	.	.	.	.	.	.	.	.
ENST0	.	16p11.2	.	.	Scc	Score=184	rs789	.	.	.	.	.	.
ENST0	.	16q12.1	.	.	.	.	rs751	.	.	.	.	.	.
ENST0	.	16q21	.	.	.	.	.	.	.	.	.	.	.
ENST0	.	16q22.1	.	.	.	.	.	.	.	.	.	.	.
ENST0	.	16q22.2	.	.	.	.	.	.	.	.	.	.	.
ENST0	.	16q23.1	.	.	Scc	.	rs291	ID=COSN2	.	.	.	.	.
ENST0	.	16q24.3	.	.	.	Score=262	.	.	.	.	.	.	.

ENST0	.	16q24.3	.	.	Scc	.	.	ID=COSN2	.	.	.	.	.
ENST0	.	17p13.2	.	.	.	.	rs780	.	.	.	.	.	.
ENST0	.	17p13.1	.	.	.	.	.	.	.	.	.	.	.
ENST0	.	17p13.1	.	Scc	.	.	.	.	.	.	.	.	.
ENST0	.	17p13.1	.	Scc	.	.	.	.	.	.	.	.	.
ENST0	.	17p13.1	.	.	.	.	.	.	.	.	.	.	.
ENST0	.	17p12	.	Scc	.	.	.	ID=COSM4	.	.	.	.	.
ENST0	.	17p12	.	.	.	.	rs559	.	.	.	.	.	2E-04
ENST0	.	17p11.2	.	.	.	.	.	.	.	.	.	.	.
ENST0	.	17p11.2	.	.	Scc	.	.	.	.	.	.	.	.
ENST0	.	17p11.2	.	.	Scc	.	.	.	.	.	.	.	.
ENST0	.	17p11.2	.	.	Scc	.	.	.	.	.	.	.	.
ENST0	.	17p11.2	.	.	Scc	.	rs784	.	.	.	.	.	.
ENST0	.	17q11.2	.	.	.	.	.	.	.	.	.	.	.
ENST0	.	17q11.2	.	.	.	.	.	.	.	.	.	.	.
ENST0	.	17q11.2	.	.	.	.	.	.	.	.	.	.	.
ENST0	.	17q11.2	.	.	Scc	.	.	.	.	.	.	.	.
ENST0	.	17q11.2	.	.	Scc	.	.	.	.	.	.	.	.
ENST0	.	17q11.2	.	.	.	.	.	.	.	.	.	.	.
ENST0	.	17q12	.	.	Scc	.	.	.	.	.	.	.	.
ENST0	.	17q12	.	.	Scc	.	rs879	.	.	.	.	.	.
ENST0	.	17q12	.	.	.	.	.	.	.	.	.	.	.
ENST0	.	17q12	.	.	.	.	.	.	.	.	.	.	.
ENST0	.	17q12	.	.	.	.	.	.	.	.	.	.	.
ENST0	.	17q12	.	.	Scc	.	.	.	.	.	.	.	.
ENST0	.	17q12	.	.	.	.	.	.	.	.	.	.	.
ENST0	.	17q21.2	.	.	.	.	rs103	.	.	.	.	.	.
ENST0	.	17q21.2	.	.	.	.	.	.	.	.	.	.	.
ENST0	.	17q21.31	.	.	.	.	.	.	.	.	.	.	.
ENST0	.	17q21.33	.	.	.	.	.	.	.	.	.	.	.
ENST0	.	17q21.33	.	.	.	.	.	.	.	.	.	.	.
ENST0	.	17q22	.	.	.	.	.	.	.	.	.	.	.
ENST0	.	17q23.3	.	.	.	.	.	.	.	.	.	.	.
ENST0	.	17q23.3	.	.	.	.	.	.	.	.	.	.	.
ENST0	.	17q24.1	.	.	.	Score=256	.	.	.	.	.	.	.
ENST0	.	17q25.1	.	.	.	.	.	.	.	.	.	.	.
ENST0	.	17q25.3	.	.	.	.	rs140	.	.	.	.	.	1E-03
ENST0	.	17q25.3	.	.	.	.	.	.	.	.	.	.	.
ENST0	.	17q25.3	.	.	Scc	.	rs367	.	.	.	.	.	.
ENST0	.	18p11.31	.	.	.	.	.	.	.	.	.	.	.
ENST0	.	18p11.23	.	Scc	.	.	.	.	.	.	.	.	.
ENST0	.	18p11.22	.	.	.	.	.	.	.	.	.	.	.
ENST0	.	18p11.22	.	.	.	.	.	.	.	.	.	.	.
ENST0	.	18p11.22	.	Scc	.	.	.	.	.	.	.	.	.
ENST0	.	18p11.21	.	Scc	.	.	.	.	.	.	.	.	.
ENST0	.	18p11.21	.	.	Scc	Score=468	.	ID=COSN1	.	.	.	.	.
ENST0	.	18q11.2	.	.	.	Score=917	.	.	.	.	.	.	.
ENST0	.	18q21.1	.	.	.	Score=278	.	.	.	.	.	.	.
ENST0	.	18q21.2	.	.	.	.	.	.	.	.	.	.	.
ENST0	.	18q21.2	.	.	.	Score=355	.	.	.	.	.	.	.
ENST0	.	18q21.33	.	.	.	.	rs746	.	.	.	.	.	.
ENST0	.	18q22.1	.	.	.	.	.	.	.	.	.	.	.
ENST0	.	18q23	.	.	.	.	.	.	.	.	.	.	.
.	.	19p13.3	.	.	.	.	rs954	.	.	.	.	.	.

ENST0	.	19p13.3	.	.	.	.	.	.	.	.	.	.	.
ENST0	.	19p13.3	.	.	.	.	.	.	.	.	.	.	.
ENST0	.	19p13.3	.	.	.	.	.	.	.	.	.	.	.
ENST0	.	19p13.3	.	.	.	.	rs374	.	.	.	.	.	6E-04
ENST0	.	19p13.3	.	.	.	Score=242	rs141	.	.	.	.	0.106	0.023
ENST0	.	19p13.3	.	.	.	.	.	.	.	.	.	.	.
ENST0	.	19p13.3	.	.	.	.	rs762	.	.	.	.	.	.
ENST0	.	19p13.3	.	.	.	Score=227	.	.	.	.	.	.	.
ENST0	.	19p13.3	.	.	.	.	.	.	.	.	.	.	.
ENST0	.	19p13.3	.	.	.	.	.	.	.	.	.	.	.
ENST0	.	19p13.2	.	.	.	.	.	.	.	.	.	.	.
ENST0	.	19p13.2	.	.	.	Score=101	.	.	.	.	.	.	.
ENST0	.	19p13.2	.	.	.	Score=394	.	.	.	.	.	.	.
ENST0	.	19p13.2	.	.	.	.	.	.	.	.	.	.	.
ENST0	.	19p13.2	.	.	.	.	.	.	.	.	.	.	.
ENST0	.	19p13.2	.	.	.	.	.	.	.	.	.	.	.
ENST0	.	19p13.2	.	.	.	.	.	ID=COSM:	.	.	.	.	.
ENST0	.	19p13.2	.	.	.	.	rs774	.	.	.	.	.	.
ENST0	.	19p13.2	.	.	.	Score=216	.	.	.	.	.	.	.
ENST0	.	19p13.2	.	.	.	.	.	.	.	.	.	.	.
ENST0	.	19p13.2	.	.	.	.	.	.	.	.	.	.	.
ENST0	.	19p13.2	.	.	.	.	.	.	.	.	.	.	.
ENST0	.	19p13.11	.	.	.	.	.	.	.	.	.	.	.
ENST0	.	19p13.11	.	.	.	.	rs779	.	.	.	.	.	.
ENST0	.	19p13.11	.	.	.	Score=216	.	.	.	.	.	.	.
ENST0	.	19p13.11	.	.	.	.	.	.	.	.	.	.	.
ENST0	.	19p12	.	.	.	.	.	ID=COSM5	.	.	.	.	.
ENST0	.	19p12	.	.	Scc	.	.	.	.	.	.	.	.
ENST0	.	19q12	.	.	.	.	.	.	.	.	.	.	.
ENST0	.	19q13.11	.	.	.	.	.	.	.	.	.	.	.
ENST0	.	19q13.12	.	.	.	.	.	.	.	.	.	.	.
.	.	19q13.12	.	.	Scc	Score=106	rs868	.	.	.	.	.	.
.	.	19q13.12	.	.	Scc	Score=106	rs879	.	.	.	.	.	.
ENST0	.	19q13.12	.	.	.	.	rs765	ID=COSM:	.	.	.	.	.
ENST0	.	19q13.2	.	.	.	Score=206	rs989	.	.	.	.	.	.
ENST0	.	19q13.2	.	.	Scc	.	.	.	.	.	.	.	.
ENST0	.	19q13.2	.	.	.	.	rs140	.	.	.	.	0.001	4E-04
ENST0	.	19q13.2	.	.	.	.	.	.	.	.	.	.	.
ENST0	.	19q13.2	.	.	.	Score=341	rs144	.	.	.	.	.	1E-03
ENST0	.	19q13.2	.	.	Scc	.	.	.	.	.	.	.	.
ENST0	.	19q13.31	.	.	.	Score=910	.	.	.	.	.	.	.
ENST0	.	19q13.31	.	.	.	.	rs425	.	.	.	.	.	0.002
ENST0	.	19q13.32	.	.	.	.	.	.	.	.	.	.	.
ENST0	.	19q13.32	.	.	.	.	.	.	.	.	.	.	.
ENST0	.	19q13.33	.	.	.	.	.	.	.	.	.	.	.
ENST0	.	19q13.41	.	.	.	.	.	.	.	.	.	.	.
ENST0	.	19q13.41	.	.	Scc	.	.	.	.	.	.	.	.
.	.	19q13.42	.	.	Scc	.	.	.	.	.	.	.	.
ENST0	.	19q13.42	.	.	.	.	.	.	.	.	.	.	.
ENST0	.	19q13.43	.	.	.	.	.	.	.	.	.	.	.
ENST0	.	19q13.43	.	.	.	.	.	.	.	.	.	.	.
ENST0	.	20p13	.	.	.	.	.	.	.	.	.	.	.
ENST0	.	20p13	.	.	.	.	.	.	.	.	.	.	.
ENST0	.	20p13	.	.	.	.	.	.	.	.	.	.	.

ENST0	.	20p13	.	.	.	.	.	.	.	.	.	.	.
ENST0	.	20p12.3	.	.	.	.	rs761	.	.	.	.	.	.
ENST0	.	20p12.1	.	.	.	.	.	.	.	.	.	.	.
ENST0	HBI-43	20p11.23	.	.	.	.	.	.	.	.	.	.	.
ENST0	.	20q11.21	.	.	Scc	.	rs812	ID=COSN1	.	.	.	.	.
ENST0	.	20q11.21	.	.	Scc	.	rs779	ID=COSM4	.	.	.	0.001	6E-04
ENST0	.	20q11.21	.	.	.	.	.	.	.	.	.	.	.
ENST0	.	20q11.22	.	.	.	.	.	.	.	.	.	.	.
ENST0	.	20q11.22	.	Scc	.	.	rs190	.	.	.	.	0.001	2E-04
ENST0	.	20q13.13	.	Scc	.	.	.	.	.	.	.	.	.
ENST0	.	20q13.2	.	Scc	.	.	.	.	.	.	.	.	.
ENST0	.	20q13.32	.	.	.	.	.	.	.	.	.	.	.
ENST0	.	20q13.33	.	.	.	Score=201	.	.	.	.	.	.	.
ENST0	.	20q13.33	.	.	.	.	.	.	.	.	.	.	.
ENST0	.	20q13.33	.	.	.	Score=279	rs187	.	.	.	.	.	.
.	.	21p11.2	.	.	Scc	.	.	.	.	.	.	.	.
ENST0	.	21p11.2	.	.	Scc	.	.	.	.	.	.	.	.
.	.	21p11.2	.	.	Scc	Score=348	.	.	.	.	.	.	.
.	.	21p11.2	.	.	Scc	.	rs370	ID=COSN1	.	.	.	.	.
ENST0	.	21p11.1	.	.	Scc	.	rs746	ID=COSN2	.	.	.	.	.
.	.	21p11.1	.	.	Scc	Score=190	rs463	.	.	.	.	.	.
ENST0	.	21q22.12	.	.	.	.	.	.	.	.	.	.	.
ENST0	.	21q22.13	.	.	.	.	.	.	.	.	.	.	.
ENST0	.	21q22.3	.	.	.	.	.	.	.	.	.	.	.
ENST0	.	21q22.3	.	Scc	.	.	.	.	.	.	.	.	.
ENST0	.	21q22.3	.	.	.	.	rs727	ID=COSN2	.	.	.	.	.
ENST0	.	21q22.3	.	.	.	.	.	.	.	.	.	.	.
ENST0	.	22q11.1	.	.	Scc	.	.	.	.	.	.	.	.
ENST0	.	22q11.1	.	.	Scc	.	.	.	.	.	.	.	.
ENST0	.	22q11.1	.	.	Scc	Score=466	.	.	.	.	.	.	.
ENST0	.	22q11.21	.	.	Scc	.	rs376	.	.	.	.	.	.
ENST0	.	22q11.21	.	.	Scc	.	rs287	ID=COSM5	.	.	.	0.032	0.04
ENST0	.	22q11.21	.	.	.	.	.	.	.	.	.	.	.
.	.	22q11.21	.	.	Scc	Score=493	rs138	.	.	.	.	0.067	0.148
ENST0	.	22q11.23	.	.	Scc	.	rs373	ID=COSN1	.	.	.	.	.
ENST0	.	22q12.2	.	.	Scc	.	rs185	ID=COSN1	.	.	.	0.016	0.03
ENST0	.	22q12.2	.	.	.	.	.	.	.	.	.	.	.
ENST0	.	22q12.2	.	.	.	.	rs566	.	.	.	.	.	.
ENST0	.	22q12.3	.	.	.	.	.	.	.	.	.	.	.
ENST0	.	22q13.1	.	.	.	.	rs760	.	.	.	.	.	.
ENST0	.	22q13.1	.	.	Scc	.	.	.	.	.	.	.	.
ENST0	.	22q13.1	.	.	.	.	.	.	.	.	.	.	.
ENST0	.	22q13.2	.	.	.	.	rs140	.	.	.	.	.	.
ENST0	.	22q13.2	.	.	.	.	.	.	.	.	.	.	.
ENST0	.	22q13.2	.	.	.	.	rs373	.	.	.	.	.	.
ENST0	.	22q13.31	.	.	.	Score=157	.	.	.	.	.	.	.
ENST0	.	22q13.33	.	.	.	Score=699	.	.	.	.	.	.	.
ENST0	.	22q13.33	.	.	.	Score=241	.	.	.	.	.	.	.
ENST0	.	22q13.33	.	.	.	.	rs771	ID=COSM5	.	.	.	.	.
ENST0	.	Xp22.33	.	.	Scc	.	.	.	.	.	.	.	.
.	.	Xp22.33	.	.	Scc	Score=103	rs867	.	.	.	.	.	.
ENST0	.	Xp22.31	.	.	Scc	.	rs764	.	.	.	.	.	.
ENST0	.	Xp22.31	.	.	Scc	.	rs664	.	.	.	.	.	.
ENST0	.	Xp22.2	.	.	.	.	.	.	.	.	.	.	.

ENST0	.	Xp22.2	.	.	.	.	.	.	.	.	.	.	.
ENST0	.	Xp21.1	.	Scc	.	.	.	.	.	.	.	.	.
ENST0	.	Xp11.3	.	.	.	.	.	.	.	.	.	.	.
ENST0	.	Xp11.23	.	.	Scc	.	.	.	.	.	.	.	.
ENST0	.	Xp11.23	.	.	.	Score=228	.	.	.	.	.	.	.
ENST0	.	Xp11.23	.	.	Scc	.	.	.	.	.	.	.	.
ENST0	.	Xp11.22	.	.	.	.	.	.	.	.	.	.	.
ENST0	.	Xp11.22	.	.	.	.	.	.	.	.	.	.	.
ENST0	.	Xp11.22	.	.	.	.	.	.	.	.	.	.	.
ENST0	.	Xp11.21	.	.	Scc	.	.	.	.	.	.	.	.
ENST0	.	Xq13.1	.	.	.	Score=939	.	.	.	.	.	.	.
ENST0	.	Xq13.1	.	.	.	.	.	.	.	.	.	.	.
ENST0	.	Xq13.1	.	Scc	.	.	.	.	.	.	.	.	.
ENST0	.	Xq13.1	.	.	Scc	.	.	.	.	.	.	.	.
ENST0	.	Xq24	.	.	.	.	.	.	.	.	.	.	.
ENST0	.	Xq26.1	.	.	.	.	.	.	.	.	.	.	.
ENST0	.	Xq26.1	.	.	.	Score=227	.	ID=COSM(	.	.	.	.	.
ENST0	.	Xq26.1	.	.	.	.	.	.	.	.	.	.	.
ENST0	.	Xq26.2	.	.	.	.	.	.	.	.	.	.	.
ENST0	.	Xq26.3	.	Scc	.	.	.	.	.	.	.	.	.
ENST0	.	Xq28	.	.	Scc	Score=357	.	.	.	.	.	.	.
ENST0	.	Xq28	.	Scc	.	.	rs782	.	.	.	.	.	.
ENST0	.	Xq28	.	.	.	Score=238	.	.	.	.	.	.	.
ENST0	.	1p36.33	.	.	Scc	.	.	.	.	.	.	.	.
ENST0	.	1p36.33	.	.	Scc	Score=411	rs781	ID=COSN1	.	.	.	.	.
ENST0	.	1p36.33	.	.	Scc	.	rs114	.	.	.	.	.	.
ENST0	.	1p36.32	.	.	.	.	.	.	.	.	.	.	.
ENST0	.	1p36.32	.	.	.	.	.	.	.	.	.	.	.
ENST0	.	1p36.23	.	.	.	.	.	.	.	.	.	.	.
ENST0	.	1p36.13	.	.	Scc	.	rs200	ID=COSN6	.	.	.	.	.
ENST0	.	1p36.13	.	.	Scc	.	rs556	.	.	.	.	0.002	0.001
ENST0	.	1p36.13	.	.	Scc	.	rs617	.	.	.	.	.	.
ENST0	.	1p36.13	.	.	Scc	.	rs100	ID=COSN2	.	.	.	.	.
ENST0	.	1p36.13	.	.	Scc	.	rs538	ID=COSN2	.	.	.	0.025	0.005
ENST0	.	1p36.13	.	Scc	Scc	.	rs666	ID=COSM3	.	.	.	.	.
ENST0	.	1p36.13	.	.	.	.	.	.	.	.	.	.	.
ENST0	.	1p36.11	.	.	.	.	rs766	ID=COSN2	.	.	.	.	.
ENST0	.	1p34.2	.	.	.	.	rs780	ID=COSM3	.	.	.	.	.
.	.	1p34.1	.	.	Scc	Score=724	rs866	ID=COSN2	.	.	.	.	.
.	.	1p32.3	.	.	.	Score=615	.	.	.	.	.	.	.
ENST0	.	1q21.1	.	.	Scc	.	rs758	.	.	.	.	.	.
ENST0	.	1q21.1	.	.	Scc	.	rs769	.	.	.	.	.	.
.	.	1q21.1	.	.	Scc	.	rs201	.	.	.	.	.	.
ENST0	.	1q21.1	.	.	Scc	.	.	ID=COSN2	.	.	.	.	.
ENST0	.	1q21.1	.	.	Scc	.	rs370	.	.	.	.	.	.
ENST0	.	1q21.1	.	.	Scc	.	rs199	ID=COSN2	.	.	.	.	.
.	.	1q21.1	.	.	Scc	.	.	.	.	.	.	.	.
.	.	1q21.1	.	.	Scc	.	.	.	.	.	.	.	.
ENST0	.	1q21.1	.	.	Scc	.	.	ID=COSN2	.	.	.	.	.
ENST0	.	1q21.1	.	.	Scc	Score=250	.	.	.	.	.	.	.
ENST0	.	1q21.1	.	.	Scc	.	.	ID=COSN2	.	.	.	.	.
ENST0	.	1q21.2	.	.	Scc	.	rs879	ID=COSN2	.	.	.	.	.
ENST0	.	1q21.2	.	.	Scc	Score=173	rs200	.	.	.	.	.	.
ENST0	.	1q21.2	.	.	Scc	Score=173	rs370	.	.	.	.	.	.

ENST0	.	1q23.1	.	.	.	.	.	.	.	.	.	.	.
ENST0	.	1q41	.	.	Scc	.	rs183	.	.	.	.	0.404	0.351
ENST0	.	1q42.11	.	.	Scc	Score=411	.	.	.	.	.	.	.
ENST0	.	1q42.11	.	.	Scc	Score=411	.	.	.	.	.	.	.
ENST0	.	1q42.12	.	.	.	.	.	.	.	.	.	.	.
.	.	1q42.13	.	.	Scc	.	rs182	.	.	.	.	.	.
.	.	2p11.2	.	.	Scc	.	rs409	ID=COSN1	.	.	.	0.026	0.052
ENST0	.	2p11.2	.	.	Scc	.	.	.	.	.	.	.	.
.	.	2p11.1	.	.	Scc	.	.	.	.	.	.	.	.
ENST0	.	2p11.1	.	.	Scc	.	.	.	.	.	.	.	.
ENST0	.	2q13	.	.	Scc	.	rs201	ID=COSM2	.	.	.	.	.
.	.	2q13	.	.	Scc	.	rs868	ID=COSN2	.	.	.	.	.
ENST0	.	2q13	.	.	Scc	.	rs274	ID=COSN2	.	.	.	.	.
ENST0	.	2q14.2	.	.	.	Score=216	rs102	.	.	.	.	.	.
ENST0	.	2q21.1	.	.	Scc	Score=488	rs200	.	.	.	.	.	.
ENST0	.	2q21.2	.	.	Scc	.	rs780	ID=COSN1	.	.	.	.	.
ENST0	.	2q31.1	.	.	.	.	.	.	.	.	.	.	.
ENST0	.	2q31.1	.	.	.	.	.	.	.	.	.	.	.
ENST0	.	2q31.3	.	.	.	.	rs187	ID=COSN5	.	.	.	0.006	0.002
ENST0	.	2q34	.	.	.	Score=381	rs867	ID=COSN1	.	.	.	.	.
ENST0	.	3p21.31	.	.	.	.	.	.	.	.	.	.	.
ENST0	.	3q24	.	Scc	.	.	.	.	.	.	.	.	.
ENST0	.	3q25.1	.	.	.	.	.	.	.	.	.	.	.
ENST0	.	3q25.1	.	.	.	.	.	.	.	.	.	.	.
.	.	3q29	.	.	Scc	Score=127	rs376	ID=COSN1	.	.	.	.	.
ENST0	.	3q29	.	.	.	.	rs867	.	.	.	.	.	.
ENST0	.	3q29	.	.	Scc	.	rs112	ID=COSN6	.	.	.	.	.
ENST0	.	4p16.3	.	.	.	.	.	.	.	.	.	.	.
ENST0	.	4p15.31	.	.	.	.	rs144	ID=COSN6	.	.	.	.	4E-04
ENST0	.	5p15.1	.	.	.	.	.	.	.	.	.	.	.
ENST0	.	5p13.2	.	.	Scc	Score=212	.	.	.	.	.	.	.
ENST0	.	5q13.2	.	.	Scc	Score=154	rs375	.	.	.	.	0.772	0.477
ENST0	.	5q14.3	.	.	Scc	Score=215	rs879	ID=COSN1	.	.	.	.	.
ENST0	.	5q21.1	.	.	Scc	.	.	ID=COSN1	.	.	.	.	.
ENST0	.	5q33.1	.	.	.	.	.	.	.	.	.	.	.
ENST0	.	5q34	.	.	.	.	.	.	.	.	.	.	.
ENST0	.	5q35.3	.	.	.	.	.	.	.	.	.	.	.
ENST0	.	5q35.3	.	.	.	.	.	.	.	.	.	.	.
.	.	6p22.2	.	.	.	.	rs371	.	.	.	.	.	.
.	.	6p22.2	.	.	.	.	rs920	.	.	.	.	.	.
.	.	6p11.1	.	.	.	Score=118	rs429	ID=COSN2	.	.	.	.	.
.	.	6p11.1	.	.	.	Score=118	rs443	ID=COSN1	.	.	.	.	.
ENST0	.	6q23.3	.	.	.	.	rs750	.	.	.	.	.	.
ENST0	.	6q25.1	.	.	.	.	.	.	.	.	.	.	.
ENST0	.	6q27	.	.	Scc	.	rs143	.	.	.	.	.	.
ENST0	.	6q27	.	.	.	Score=300	.	.	.	.	.	.	.
ENST0	.	7p22.2	.	.	.	.	.	.	.	.	.	.	.
ENST0	.	7p22.1	.	.	Scc	Score=29;I	rs201	.	.	.	.	.	.
ENST0	.	7p14.2	.	.	.	Score=166	.	.	.	.	.	.	.
ENST0	.	7p11.2	.	.	Scc	.	.	.	.	.	.	.	.
ENST0	.	7p11.2	.	.	Scc	.	.	ID=COSN1	.	.	.	.	.
ENST0	.	7p11.2	.	.	Scc	.	.	.	.	.	.	.	.
ENST0	.	7p11.2	.	.	Scc	.	.	.	.	.	.	.	.
ENST0	.	7p11.2	.	.	Scc	.	.	.	.	.	.	.	.

ENST0	.	7p11.2	.	.	Scc	.	.	.	.	.	.	.	.
ENST0	.	7p11.2	.	.	Scc	Score=498	.	.	.	.	.	.	.
.	.	7q11.21	.	.	.	Score=133	rs459	.	.	.	.	.	.
.	.	7q11.21	.	.	.	Score=133	rs216	ID=COSN1	.	.	.	.	.
ENST0	.	7q11.21	.	.	Scc	.	rs586	ID=COSN1	.	.	.	.	.
.	.	7q11.21	.	.	Scc	.	rs868	ID=COSN2	.	.	.	.	.
ENST0	.	7q11.23	.	.	Scc	.	rs246	ID=COSN2	.	.	.	.	.
ENST0	.	7q22.1	.	.	Scc	.	rs624	.	.	.	.	.	.
ENST0	.	7q32.1	.	.	Scc	.	rs374	.	.	.	.	.	.
ENST0	.	7q36.1	.	.	Scc	.	.	.	.	.	.	.	.
.	.	8p23.1	.	.	Scc	.	rs207	ID=COSN2	.	.	.	.	.
ENST0	.	8p11.23	.	.	.	.	.	.	.	.	.	.	.
.	.	8p11.1	.	.	.	Score=126	rs750	.	.	.	.	.	.
.	.	8p11.1	.	.	.	Score=126	rs796	ID=COSN1	.	.	.	.	.
.	.	8p11.1	.	.	.	Score=126	.	ID=COSN1	.	.	.	.	.
.	.	8q11.1	.	.	.	Score=124	rs436	ID=COSN1	.	.	.	.	.
.	.	8q11.1	.	.	.	Score=124	rs374	.	.	.	.	.	.
ENST0	.	8q23.3	.	.	.	Score=598	.	.	.	.	.	.	.
ENST0	.	8q24.3	.	Scc	.	.	.	.	.	.	.	.	.
ENST0	.	8q24.3	.	.	.	Score=270	rs782	ID=COSN2	.	.	.	.	.
.	.	9p11.2	.	.	Scc	Score=748	rs749	ID=COSN2	.	.	.	.	.
.	.	9q13	.	.	Scc	Score=540	rs492	.	.	.	.	.	.
ENST0	.	9q13	.	Scc	Scc	.	rs456	.	.	.	.	.	.
ENST0	.	9q21.11	.	.	Scc	.	rs143	ID=COSN2	.	.	.	.	.
ENST0	.	9q21.11	.	.	Scc	.	rs377	.	.	.	.	.	.
ENST0	.	9q22.1	.	.	.	Score=22;I	.	.	.	.	.	.	.
ENST0	.	9q33.3	.	.	.	.	.	.	.	.	.	.	.
ENST0	.	9q33.3	.	.	.	Score=983	.	.	.	.	.	.	.
.	.	10p12.2	.	.	.	Score=139	rs139	ID=COSN6	.	.	.	.	.
ENST0	hsa-mir-60	10p12.2	.	.	.	Score=475	.	.	.	.	.	.	.
ENST0	.	10p11.22	.	.	.	Score=101	rs963	.	.	.	.	.	.
ENST0	.	10p11.21	.	.	.	.	.	.	.	.	.	.	.
ENST0	.	10q11.22	.	.	Scc	Score=922	rs694	.	.	.	.	.	.
ENST0	.	10q22.3	.	.	Scc	.	rs527	.	.	.	.	.	1E-03
ENST0	.	10q22.3	.	.	.	.	rs188	.	.	.	.	.	6E-04
.	.	10q22.3	.	.	Scc	.	rs257	ID=COSN2	.	.	.	.	.
ENST0	.	10q23.2	.	.	Scc	.	rs312	.	.	.	.	0.524	0.433
ENST0	.	11p11.12	.	.	Scc	.	.	ID=COSN1	.	.	.	.	.
ENST0	.	11p11.12	.	.	Scc	.	.	ID=COSN2	.	.	.	.	.
.	.	11q13.2	.	.	.	Score=113	rs903	ID=COSN7	.	.	.	.	.
.	.	11q13.4	.	.	Scc	.	rs187	ID=COSN2	.	.	.	.	.
ENST0	.	11q13.5	.	.	Scc	.	rs966	.	.	.	.	.	.
ENST0	.	11q13.5	.	.	.	Score=113	rs568	.	.	.	.	.	4E-04
ENST0	.	11q24.2	.	.	.	.	.	.	.	.	.	.	.
ENST0	.	11q25	.	.	.	.	.	.	.	.	.	.	.
ENST0	.	12q13.13	.	.	Scc	.	rs760	.	.	.	.	.	.
ENST0	.	12q13.2	.	.	.	.	.	ID=COSM1	.	.	.	.	.
ENST0	.	12q23.3	.	.	.	.	rs759	ID=COSN1	.	.	.	.	.
ENST0	.	13q21.31	.	.	Scc	.	rs200	.	.	.	.	.	.
ENST0	.	13q33.3	.	Scc	.	.	rs151	ID=COSM2	.	.	.	.	4E-04
ENST0	.	14q11.2	.	.	Scc	.	rs744	ID=COSN2	.	.	.	.	.
ENST0	.	14q11.2	.	.	Scc	.	.	.	.	.	.	.	.
ENST0	.	14q11.2	.	.	Scc	.	rs200	.	.	.	.	.	.
ENST0	.	14q11.2	.	.	Scc	.	rs202	.	.	.	.	.	.

ENST0	.	14q11.2	.	.	Scc	.	rs770	ID=COSN2	.	.	.	.	.
ENST0	.	14q23.1	.	.	.	.	.	.	.	.	.	.	.
ENST0	.	14q24.3	.	.	.	.	.	.	.	.	.	.	.
ENST0	.	14q32.33	.	.	Scc	.	.	ID=COSN2	.	.	.	.	.
.	.	15q11.1	.	.	Scc	Score=217	rs620	ID=COSN2	.	.	.	0.345	0.258
ENST0	.	15q11.2	.	.	Scc	.	.	.	.	.	.	.	.
ENST0	.	15q11.2	.	.	.	.	.	.	.	.	.	.	.
ENST0	.	15q13.2	.	.	Scc	.	rs775	.	.	.	.	.	.
ENST0	.	15q13.2	.	.	Scc	Score=271	rs244	ID=COSN6	.	.	.	0.026	0.192
ENST0	.	15q13.2	.	.	Scc	Score=271	rs244	ID=COSN1	.	.	.	0.167	0.485
ENST0	.	15q13.3	.	.	Scc	.	.	.	.	.	.	.	.
ENST0	.	15q13.3	.	.	Scc	.	.	.	.	.	.	.	.
ENST0	.	15q21.1	.	.	Scc	.	rs620	.	.	.	.	0.422	0.324
ENST0	.	15q21.2	.	.	.	.	.	.	.	.	.	.	.
ENST0	.	15q22.31	.	.	.	.	.	.	.	.	.	.	.
ENST0	.	15q25.2	.	.	Scc	.	.	.	.	.	.	.	.
.	.	15q25.2	.	.	Scc	.	rs374	.	.	.	.	0.446	0.327
ENST0	.	15q25.2	.	.	Scc	.	rs970	.	.	.	.	.	.
ENST0	.	15q25.2	.	.	Scc	.	rs364	.	.	.	.	.	8E-04
ENST0	.	16p13.11	.	.	Scc	.	rs200	.	.	.	.	.	.
ENST0	.	16p13.11	.	.	Scc	.	rs201	.	.	.	.	.	.
ENST0	.	16p12.3	.	.	Scc	.	.	ID=COSN2	.	.	.	.	.
.	.	16p12.2	.	.	Scc	.	rs566	.	.	.	.	0.001	4E-04
ENST0	.	16p11.2	.	.	Scc	.	rs561	ID=COSM4	.	.	.	.	.
.	.	16p11.2	.	.	Scc	Score=179	rs879	.	.	.	.	.	.
.	.	16q11.2	.	.	.	Score=117	rs431	ID=COSN1	.	.	.	.	.
.	.	16q11.2	.	.	.	Score=114	rs434	.	.	.	.	.	.
.	.	16q11.2	.	.	.	Score=114	rs424	.	.	.	.	.	.
.	.	16q11.2	.	.	.	.	.	.	.	.	.	.	.
.	.	16q11.2	.	.	.	Score=989	rs879	.	.	.	.	.	.
.	.	16q11.2	.	.	.	Score=111	rs997	.	.	.	.	.	.
.	.	16q11.2	.	.	.	Score=122	rs432	ID=COSN2	.	.	.	.	.
.	.	16q11.2	.	.	.	Score=112	rs932	ID=COSN1	.	.	.	.	.
.	.	16q11.2	.	.	.	Score=112	rs438	ID=COSN1	.	.	.	.	.
.	.	16q11.2	.	.	.	Score=112	rs429	.	.	.	.	.	.
.	.	16q11.2	.	.	.	.	rs401	ID=COSN1	.	.	.	.	.
.	.	16q11.2	.	.	.	Score=114	.	.	.	.	.	.	.
.	.	16q11.2	.	.	.	Score=114	rs720	.	.	.	.	.	.
.	.	16q11.2	.	.	.	Score=979	rs409	.	.	.	.	.	.
.	.	16q11.2	.	.	.	Score=979	rs424	.	.	.	.	.	.
.	.	16q11.2	.	.	.	Score=979	.	.	.	.	.	.	.
ENST0	.	16q24.3	.	.	Scc	.	rs620	.	.	.	.	.	.
ENST0	.	17p11.2	.	.	Scc	.	rs254	ID=COSN4	.	.	.	.	.
ENST0	.	17p11.2	.	.	Scc	.	rs712	ID=COSN2	.	.	.	.	.
ENST0	.	17p11.2	.	.	Scc	.	rs712	ID=COSN2	.	.	.	.	.
ENST0	.	17p11.2	.	.	Scc	.	rs784	.	.	.	.	.	.
.	.	17p11.1	.	.	.	Score=113	.	.	.	.	.	.	.
.	.	17p11.1	.	.	.	Score=113	rs143	.	.	.	.	.	.
.	.	17p11.1	.	.	.	Score=113	.	.	.	.	.	.	.
ENST0	.	17q12	.	.	Scc	.	rs620	.	.	.	.	.	.
ENST0	.	17q21.2	.	.	Scc	.	rs748	ID=COSM1	.	.	.	.	.
ENST0	.	17q21.2	.	.	.	.	rs145	ID=COSN1	.	.	.	.	.
ENST0	.	17q21.31	.	.	Scc	.	.	.	.	.	.	.	.
ENST0	.	18p11.21	.	.	Scc	.	rs201	ID=COSN2	.	.	.	.	.

ENST0	.	18p11.21	.	.	Scc	.	rs373	.	.	.	.	.	.
ENST0	.	18q21.33	.	.	.	.	rs752	.	.	.	.	.	.
ENST0	.	19p13.3	.	.	.	.	.	.	.	.	.	.	.
ENST0	.	19q13.12	.	.	.	.	.	.	.	.	.	.	.
.	.	19q13.12	.	.	Scc	Score=123	rs764	.	.	.	.	.	.
.	.	19q13.12	.	.	Scc	Score=259	.	.	.	.	.	.	.
ENST0	.	19q13.31	.	.	Scc	.	.	.	.	.	.	.	.
ENST0	.	19q13.32	.	.	.	.	.	.	.	.	.	.	.
ENST0	.	19q13.42	.	.	.	.	rs349	ID=COSM(	.	.	.	.	.
ENST0	.	19q13.42	.	.	.	.	rs561	ID=COSM!	.	.	.	.	.
ENST0	.	19q13.42	.	.	Scc	.	rs133	ID=COSN2	.	.	.	.	.
ENST0	.	19q13.43	.	.	.	.	rs375	ID=COSN2	.	.	.	.	.
ENST0	.	20q11.22	.	.	.	Score=507	.	ID=COSM2	.	.	.	.	.
ENST0	.	21p11.2	.	.	Scc	.	rs798	ID=COSN1	.	.	.	.	.
.	.	21p11.1	.	.	Scc	Score=970	rs201	ID=COSN2	.	.	.	.	.
ENST0	.	22q11.1	.	.	Scc	.	rs375	ID=COSN1	.	.	.	.	.
ENST0	.	22q11.1	.	.	Scc	.	.	.	.	.	.	.	.
ENST0	.	22q11.1	.	.	Scc	.	rs202	ID=COSN6	.	.	.	.	.
ENST0	.	22q11.21	.	.	Scc	.	.	.	.	.	.	.	.
ENST0	.	22q11.21	.	.	Scc	.	rs622	ID=COSN2	.	.	.	.	.
ENST0	.	22q11.21	.	.	Scc	.	rs201	ID=COSN3	.	.	.	0.114	0.24
ENST0	.	22q11.23	.	.	Scc	.	rs622	ID=COSN2	.	.	.	.	.
ENST0	.	22q12.2	.	.	Scc	.	rs367	ID=COSN2	.	.	.	.	.
ENST0	.	22q13.2	.	.	Scc	.	rs360	.	.	.	.	0.302	0.458
ENST0	.	22q13.2	.	.	Scc	.	.	.	.	.	.	.	.
ENST0	.	22q13.31	.	.	.	.	.	.	.	.	.	.	.
ENST0	.	Xp11.22	.	.	.	.	.	.	.	.	.	.	.
.	.	Xq11.1	.	.	.	Score=120	.	.	.	.	.	.	.
.	.	Xq11.1	.	.	.	Score=120	.	ID=COSN1	.	.	.	.	.
.	.	Xq11.1	.	.	.	Score=120	rs752	.	.	.	.	.	.
.	.	Xq11.1	.	.	.	Score=120	rs775	.	.	.	.	.	.
.	.	Xq11.1	.	.	.	Score=120	.	.	.	.	.	.	.
ENST0	.	Xq13.1	.	Scc	.	.	rs104	ID=COSM1	.	.	.	.	.
ENST0	.	Xq13.1	.	.	.	.	.	.	.	.	.	.	.
ENST0	.	Xq13.1	.	.	.	.	.	.	.	.	.	.	.
ENST0	.	Xq27.1	.	.	Scc	.	rs142	ID=COSN2	.	.	.	.	.
.	.	Xq28	.	.	.	.	.	.	.	.	.	.	.
ENST0	.	1p36.33	.	.	Scc	.	rs374	ID=COSN2	.	.	.	0.092	0.039
ENST0	.	1p36.33	.	.	.	.	.	.	.	.	.	.	.
ENST0	.	1p36.33	.	Scc	.	.	rs115	.	.	.	.	.	0.007
ENST0	.	1p36.33	.	.	.	.	.	.	.	.	.	.	.
ENST0	.	1p36.33	.	.	.	.	.	.	.	.	.	.	.
ENST0	.	1p36.33	.	.	.	.	rs541	.	.	.	.	.	2E-04
ENST0	.	1p36.33	.	.	.	.	rs768	ID=COSN1	.	.	.	.	.
ENST0	.	1p36.33	.	.	.	.	.	.	.	.	.	.	.
ENST0	.	1p36.33	.	Scc	.	.	.	.	.	.	.	.	.
ENST0	.	1p36.33	.	.	.	.	rs780	.	.	.	.	.	.
ENST0	.	1p36.33	.	.	.	.	.	.	.	.	.	.	.
ENST0	.	1p36.33	.	.	Scc	.	rs201	.	.	.	.	0.001	2E-04
ENST0	.	1p36.33	.	Scc	Scc	.	rs746	.	.	.	.	.	.
ENST0	.	1p36.33	.	.	Scc	Score=229	.	.	.	.	.	.	.
ENST0	.	1p36.33	.	.	Scc	Score=161	.	.	.	.	.	.	.
ENST0	.	1p36.33	.	.	.	.	.	.	.	.	.	.	.
ENST0	.	1p36.33	.	.	.	.	.	.	.	.	.	.	.

ENST0	.	1p36.32	.	.	.	.	.	.	.	.	.	.	.
ENST0	.	1p36.32	.	.	.	.	.	.	.	.	.	.	.
ENST0	.	1p36.32	.	.	.	Score=203	.	.	.	.	.	.	.
ENST0	.	1p36.31	.	Scc	.	.	.	.	.	.	.	.	.
ENST0	.	1p36.31	.	.	.	.	.	.	.	.	.	.	.
ENST0	.	1p36.31	.	.	.	.	rs943	.	.	.	.	.	.
ENST0	.	1p36.31	.	Scc	.	.	.	ID=COSM3	.	.	.	.	.
ENST0	.	1p36.31	.	Scc	.	.	.	.	.	.	.	.	.
ENST0	.	1p36.31	.	.	.	.	rs986	.	.	.	.	.	.
ENST0	.	1p36.31	.	.	.	.	rs756	.	.	.	.	.	.
ENST0	.	1p36.23	.	.	.	.	rs143	.	.	.	.	.	4E-04
ENST0	.	1p36.23	.	.	.	Score=296	.	.	.	.	.	.	.
ENST0	.	1p36.23	.	.	.	.	rs774	.	.	.	.	.	.
ENST0	.	1p36.23	.	.	.	.	rs944	.	.	.	.	.	.
ENST0	.	1p36.23	.	Scc	.	.	rs147	.	.	.	.	.	.
ENST0	.	1p36.22	.	.	.	Score=202	.	.	.	.	.	.	.
ENST0	.	1p36.22	.	.	.	.	.	.	.	.	.	.	.
ENST0	.	1p36.22	.	.	.	.	.	.	.	.	.	.	.
ENST0	.	1p36.22	.	.	.	.	rs793	.	Ur	.	.	.	0.007
ENST0	.	1p36.22	.	.	.	.	.	.	.	.	.	.	.
ENST0	.	1p36.22	.	.	.	Score=317	.	.	.	.	.	.	.
ENST0	.	1p36.22	.	.	.	.	.	.	.	.	.	.	.
ENST0	.	1p36.21	.	.	Scc	.	rs202	ID=COSN2	.	.	.	.	.
ENST0	.	1p36.21	.	.	Scc	.	rs587	ID=COSN8	.	.	.	0.307	0.259
ENST0	.	1p36.21	.	.	.	.	rs464	.	.	.	.	.	.
ENST0	.	1p36.13	.	.	.	.	rs374	.	.	.	.	.	.
ENST0	.	1p36.13	.	.	.	.	.	.	.	.	.	.	.
ENST0	.	1p36.13	.	.	Scc	.	rs373	ID=COSN1	.	.	.	.	.
ENST0	.	1p36.13	.	.	Scc	.	rs617	.	.	.	.	.	.
ENST0	.	1p36.13	.	.	Scc	.	rs411	.	.	.	.	.	.
ENST0	.	1p36.13	.	.	Scc	.	rs121	ID=COSN1	.	.	.	.	.
.	.	1p36.13	.	.	Scc	.	rs393	ID=COSN2	.	.	.	.	.
ENST0	.	1p36.13	.	.	.	.	rs578	.	.	.	.	0.019	0.005
ENST0	.	1p36.13	.	.	.	.	rs377	.	.	.	.	.	.
ENST0	.	1p36.13	.	.	.	.	rs376	.	.	.	.	.	.
ENST0	.	1p36.13	.	Scc	.	.	rs347	.	.	.	.	.	0.005
ENST0	.	1p36.13	.	Scc	.	.	.	.	.	.	.	.	.
ENST0	.	1p36.13	.	.	.	.	.	.	.	.	.	.	.
ENST0	.	1p36.13	.	.	.	.	rs371	.	.	.	.	.	.
ENST0	.	1p36.13	.	.	.	Score=203	.	.	.	.	.	.	.
ENST0	.	1p36.12	.	.	.	.	rs100	.	.	.	.	.	.
ENST0	.	1p36.12	.	.	.	.	.	.	.	.	.	.	.
ENST0	.	1p36.12	.	.	.	.	.	.	.	.	.	.	.
.	.	1p36.12	.	.	Scc	.	rs758	ID=COSN1	.	.	.	.	.
ENST0	.	1p36.12	.	.	Scc	.	.	.	.	.	.	.	.
ENST0	.	1p36.12	.	.	.	.	.	.	.	.	.	.	.
ENST0	.	1p36.11	.	.	.	.	.	.	.	.	.	.	.
ENST0	.	1p36.11	.	.	.	.	rs139	.	.	.	.	.	0.002
ENST0	.	1p36.11	.	.	.	.	rs751	ID=COSM3	.	.	.	.	.
ENST0	.	1p36.11	.	.	Scc	.	.	.	.	.	.	.	.
ENST0	.	1p36.11	.	.	.	.	.	.	.	.	.	.	.
ENST0	.	1p36.11	.	.	.	.	.	.	.	.	.	.	.
ENST0	.	1p36.11	.	Scc	.	.	.	.	.	.	.	.	.
ENST0	.	1p36.11	.	.	.	.	rs773	.	.	.	.	.	.

ENST0	.	1p36.11	.	Scc	.	.	rs776	.	.	.	.	.	.
ENST0	.	1p36.11	.	.	.	.	.	.	.	.	.	.	.
ENST0	.	1p36.11	.	.	.	.	rs879	ID=COSM	Pa	.	.	.	.
ENST0	.	1p36.11	.	Scc	.	.	.	.	.	.	.	.	.
ENST0	.	1p36.11	.	.	.	.	rs100	.	.	.	.	.	.
ENST0	.	1p36.11	.	Scc	.	.	rs762	.	.	.	.	.	.
ENST0	.	1p36.11	.	.	.	.	rs946	.	.	.	.	.	.
ENST0	.	1p36.11	.	.	Scc	.	.	.	.	.	.	.	.
ENST0	.	1p35.3	.	.	.	.	.	ID=COSN	.	.	.	.	.
ENST0	.	1p35.3	.	.	.	.	.	.	.	.	.	.	.
ENST0	.	1p35.2	.	.	.	.	rs759	ID=COSM	.	.	.	.	.
ENST0	.	1p35.2	.	.	.	.	rs923	.	.	.	.	.	.
ENST0	.	1p35.1	.	.	.	.	.	.	.	.	.	.	.
ENST0	.	1p35.1	.	.	.	.	.	.	.	.	.	.	.
.	.	1p35.1	.	.	.	Score=625	.	.	.	.	.	.	.
ENST0	.	1p35.1	.	.	.	Score=189	.	.	.	.	.	.	.
ENST0	.	1p34.3	.	.	.	.	rs965	.	.	.	.	.	.
ENST0	.	1p34.3	.	.	.	.	rs772	.	.	.	.	.	.
ENST0	.	1p34.3	.	.	.	Score=196	.	.	.	.	.	.	.
ENST0	.	1p34.3	.	.	.	.	rs181	.	.	.	.	0.009	0.002
ENST0	.	1p34.3	.	.	.	Score=216	.	.	.	.	.	.	.
ENST0	.	1p34.3	.	.	.	.	.	.	.	.	.	.	.
ENST0	.	1p34.3	.	.	.	.	.	.	.	.	.	.	.
ENST0	.	1p34.3	.	.	Scc	.	rs552	.	.	.	.	.	4E-04
ENST0	.	1p34.3	.	.	.	.	.	.	.	.	.	.	.
ENST0	.	1p34.2	.	Scc	.	.	.	.	.	.	.	.	.
ENST0	.	1p34.2	.	.	.	.	.	.	.	.	.	.	.
ENST0	.	1p34.2	.	Scc	.	.	.	.	.	.	.	.	.
ENST0	.	1p34.2	.	.	.	.	rs909	.	.	.	.	.	.
ENST0	.	1p34.2	.	.	.	.	.	.	.	.	.	.	.
ENST0	.	1p34.2	.	.	.	.	rs117	.	.	.	.	.	0.01
ENST0	.	1p34.2	.	.	.	.	rs745	.	.	.	.	.	.
ENST0	.	1p34.2	.	.	.	.	.	.	.	.	.	.	.
ENST0	.	1p34.1	.	.	.	Score=976	.	.	.	.	.	.	.
ENST0	.	1p34.1	.	.	.	.	.	.	.	.	.	.	.
ENST0	.	1p34.1	.	.	.	.	.	.	.	.	.	.	.
ENST0	.	1p34.1	.	.	.	.	rs729	.	.	.	.	0.009	0.013
ENST0	.	1p33	.	.	.	.	.	.	.	.	.	.	.
ENST0	.	1p33	.	Scc	.	.	rs550	.	.	.	.	.	2E-04
ENST0	.	1p33	.	.	Scc	.	.	.	.	.	.	.	.
ENST0	.	1p33	.	.	.	.	.	.	.	.	.	.	.
ENST0	.	1p32.3	.	.	.	Score=180	rs757	.	.	.	.	.	.
ENST0	.	1p32.3	.	.	.	.	rs951	.	.	.	.	.	.
ENST0	.	1p32.3	.	.	.	Score=240	rs775	.	.	.	.	.	.
ENST0	.	1p32.3	.	Scc	.	.	.	.	.	.	.	.	.
ENST0	.	1p32.3	.	.	.	.	rs376	.	.	.	.	.	.
ENST0	.	1p32.2	.	.	.	.	rs781	ID=COSM	.	.	.	.	.
ENST0	.	1p32.2	.	.	.	.	.	.	.	.	.	.	.
ENST0	.	1p31.3	.	.	.	.	.	.	.	.	.	.	.
ENST0	.	1p31.3	.	.	.	.	.	.	.	.	.	.	.
ENST0	.	1p31.1	.	.	.	.	.	.	.	.	.	.	.
ENST0	.	1p31.1	.	.	.	.	.	.	.	.	.	.	.
ENST0	.	1p31.1	.	.	.	.	rs178	.	.	.	.	.	.
ENST0	.	1p31.1	.	.	Scc	.	.	.	.	.	.	.	.

ENST0	.	1p31.1	.	.	.	.	rs139	ID=COSM:	.	.	.	.	.
ENST0	.	1p22.3	.	Scc	.	.	rs763	.	.	.	.	.	.
ENST0	.	1p22.3	.	.	.	.	.	.	.	.	.	.	.
ENST0	.	1p22.1	.	.	.	.	.	.	.	.	.	.	.
ENST0	.	1p22.1	.	.	.	.	.	.	.	.	.	.	.
ENST0	.	1p22.1	.	.	.	.	rs617	ID=COSM4	no	.	.	.	.
ENST0	.	1p21.2	.	.	.	.	.	.	.	.	.	.	.
ENST0	.	1p21.1	.	.	.	.	.	.	.	.	.	.	.
ENST0	.	1p13.3	.	.	.	.	.	.	.	.	.	.	.
ENST0	.	1p13.3	.	.	.	.	rs936	.	.	.	.	.	.
ENST0	.	1p13.3	.	.	.	Score=513	rs777	.	.	.	.	.	.
ENST0	.	1p13.3	.	.	.	.	.	.	.	.	.	.	.
ENST0	.	1p13.2	.	.	.	Score=852	.	.	.	.	.	.	.
ENST0	.	1p13.2	.	.	.	.	.	ID=COSM:	.	.	.	.	.
ENST0	.	1p13.2	.	.	.	Score=472	.	.	.	.	.	.	.
ENST0	.	1p13.2	.	Scc	.	.	rs755	ID=COSM:	.	.	.	.	.
ENST0	.	1p13.2	.	.	.	.	.	.	.	.	.	.	.
ENST0	.	1p13.2	.	.	.	.	.	.	.	.	.	.	.
ENST0	.	1p13.2	.	.	.	.	.	.	.	.	.	.	.
ENST0	.	1p13.2	.	.	.	.	rs372	.	.	.	.	.	.
ENST0	.	1p13.1	.	.	.	.	.	ID=COSN1	.	.	.	.	.
ENST0	.	1p12	.	.	.	.	.	.	.	.	.	.	.
ENST0	.	1p12	.	.	Scc	.	.	.	.	.	.	.	.
ENST0	.	1p12	.	.	.	.	rs104	.	.	.	.	.	.
ENST0	.	1p12	.	.	.	.	.	.	.	.	.	.	.
ENST0	.	1q21.1	.	.	Scc	.	.	.	.	.	.	.	.
ENST0	.	1q21.1	.	.	Scc	.	rs944	ID=COSN1	.	.	.	.	.
ENST0	.	1q21.1	.	.	Scc	.	rs406	ID=COSN2	.	.	.	.	.
ENST0	.	1q21.1	.	.	Scc	.	.	.	.	.	.	.	.
ENST0	.	1q21.1	.	Scc	.	.	rs781	ID=COSN1	.	.	.	.	.
ENST0	.	1q21.1	.	.	Scc	.	rs751	.	.	.	.	.	.
.	.	1q21.1	.	.	Scc	.	.	.	.	.	.	.	.
ENST0	.	1q21.1	.	.	.	.	.	.	.	.	.	.	.
ENST0	.	1q21.1	.	.	Scc	.	.	.	.	.	.	.	.
ENST0	.	1q21.2	.	.	Scc	.	.	.	.	.	.	.	.
ENST0	.	1q21.2	.	.	Scc	.	.	.	.	.	.	.	.
ENST0	.	1q21.3	.	.	.	.	.	.	.	.	.	.	.
ENST0	.	1q21.3	.	.	.	.	.	.	.	.	.	.	.
ENST0	.	1q21.3	.	Scc	.	.	.	.	.	.	.	.	.
ENST0	.	1q21.3	.	.	.	.	.	.	.	.	.	.	.
ENST0	.	1q21.3	.	.	.	.	rs947	.	.	.	.	.	.
ENST0	.	1q21.3	.	.	.	.	.	.	.	.	.	.	.
ENST0	.	1q21.3	.	.	.	.	.	.	.	.	.	.	.
ENST0	.	1q21.3	.	.	.	.	rs770	ID=COSM8	.	.	.	.	.
ENST0	.	1q21.3	.	Scc	.	.	.	.	.	.	.	.	.
ENST0	.	1q22	.	.	.	.	.	.	.	.	.	.	.
ENST0	.	1q22	.	.	.	.	.	.	.	.	.	.	.
ENST0	.	1q22	.	.	.	.	.	.	.	.	.	.	.
ENST0	.	1q22	.	.	.	.	rs932	.	.	.	.	.	.
ENST0	.	1q23.1	.	.	.	.	.	.	.	.	.	.	.
ENST0	.	1q23.1	.	.	.	.	rs371	.	.	.	.	.	.
.	.	1q23.1	.	.	.	Score=362	rs200	ID=COSN6	.	.	.	.	.
ENST0	.	1q23.1	.	.	.	.	rs773	.	.	.	.	.	.
ENST0	.	1q23.2	.	.	.	.	rs762	.	.	.	.	.	.

ENST0	.	1q23.3	.	Scc	.	.	.	.	.	.	.	.	.
ENST0	.	1q23.3	.	.	.	.	rs201	.	.	.	.	.	8E-04
ENST0	.	1q23.3	.	.	.	.	.	.	.	.	.	.	.
ENST0	.	1q23.3	.	Scc	.	.	.	.	.	.	.	.	.
ENST0	.	1q24.2	.	Scc	.	.	.	ID=COSM2	.	.	.	.	.
ENST0	.	1q24.2	.	.	.	.	.	.	.	.	.	.	.
ENST0	.	1q24.2	.	.	.	.	.	.	.	.	.	.	.
ENST0	.	1q24.2	.	.	.	.	.	.	.	.	.	.	.
ENST0	.	1q25.1	.	.	.	.	.	.	.	.	.	.	.
ENST0	.	1q25.2	.	.	.	.	.	.	.	.	.	.	.
ENST0	.	1q25.2	.	Scc	.	.	rs776	ID=COSM1	.	.	.	.	.
ENST0	.	1q25.2	.	.	.	.	.	.	.	.	.	.	.
ENST0	.	1q25.2	.	.	.	.	rs101	.	.	.	.	.	.
ENST0	.	1q25.2	.	.	.	Score=216	.	ID=COSN1	.	.	.	.	.
ENST0	.	1q25.3	.	Scc	.	.	rs773	.	.	.	.	.	.
ENST0	.	1q25.3	.	.	.	.	rs764	.	.	.	.	.	.
ENST0	.	1q25.3	.	.	.	.	.	.	.	.	.	.	.
ENST0	.	1q25.3	.	.	.	.	rs770	.	.	.	.	.	.
ENST0	.	1q31.1	.	.	.	.	.	.	.	.	.	.	.
ENST0	.	1q31.3	.	.	.	.	.	.	.	.	.	.	.
ENST0	.	1q32.1	.	.	.	.	.	.	.	.	.	.	.
ENST0	.	1q32.1	.	Scc	.	.	rs371	.	.	.	.	.	.
ENST0	.	1q32.1	.	.	.	.	rs372	.	.	.	.	.	.
ENST0	.	1q32.1	.	.	.	.	rs369	ID=COSM1	.	.	.	.	.
ENST0	.	1q32.1	.	.	.	.	.	.	.	.	.	.	.
ENST0	.	1q32.1	.	.	.	.	rs104	.	.	.	.	.	.
ENST0	.	1q32.1	.	.	.	.	.	.	.	.	.	.	.
ENST0	.	1q32.2	.	.	.	.	.	.	.	.	.	.	.
ENST0	.	1q32.2	.	.	Scc	.	.	.	.	.	.	.	.
ENST0	.	1q32.2	.	.	.	.	.	.	.	.	.	.	.
ENST0	.	1q32.2	.	.	.	.	.	.	.	.	.	.	.
ENST0	.	1q32.2	.	.	.	.	.	.	.	.	.	.	.
ENST0	.	1q32.3	.	.	.	.	rs918	.	.	.	.	.	.
ENST0	.	1q32.3	.	.	.	.	.	.	.	.	.	.	.
ENST0	.	1q32.3	.	Scc	.	.	.	.	.	.	.	.	.
ENST0	.	1q41	.	.	.	.	.	.	.	.	.	.	.
ENST0	.	1q41	.	.	.	.	.	.	.	.	.	.	.
ENST0	.	1q41	.	.	.	.	rs910	.	.	.	.	.	.
ENST0	.	1q41	.	.	.	.	.	.	.	.	.	.	.
ENST0	.	1q41	.	.	.	.	rs756	ID=COSM1	.	.	.	.	.
ENST0	.	1q41	.	.	.	Score=413	rs190	.	.	.	.	0.002	8E-04
ENST0	.	1q41	.	Scc	.	.	.	.	.	.	.	.	.
ENST0	.	1q41	.	.	.	.	.	.	.	.	.	.	.
ENST0	.	1q41	.	.	.	.	rs779	ID=COSM1	.	.	.	.	.
ENST0	.	1q41	.	Scc	.	.	.	.	.	.	.	.	.
ENST0	.	1q41	.	.	.	.	.	.	.	.	.	.	.
ENST0	.	1q42.11	.	.	.	Score=217	.	.	.	.	.	.	.
ENST0	.	1q42.12	.	.	.	.	.	.	.	.	.	.	.
ENST0	.	1q42.12	.	Scc	Scc	.	.	.	.	.	.	.	.
.	.	1q42.12	.	.	.	Score=837	rs930	.	.	.	.	.	.
ENST0	.	1q42.13	.	.	.	.	.	.	.	.	.	.	.
ENST0	.	1q42.13	.	Scc	.	.	.	.	.	.	.	.	.
ENST0	.	1q42.2	.	.	.	.	.	.	.	.	.	.	.
ENST0	.	1q42.2	.	.	.	.	.	.	.	.	.	.	.

ENST0	.	1q42.2	.	Scc	.	.	rs100	ID=COSM3	.	.	.	.	.
ENST0	.	1q43	.	.	.	.	rs367	ID=COSN1	.	.	.	.	0.001
ENST0	.	1q43	.	.	Scc	.	.	.	.	.	.	.	.
ENST0	.	1q44	.	.	.	.	.	.	.	.	.	.	.
.	.	1q44	.	.	.	Score=144	rs100	.	.	.	.	.	.
ENST0	.	1q44	.	.	.	.	rs182	.	.	.	.	.	0.001
ENST0	.	1q44	.	.	.	.	rs758	.	.	.	.	.	.
ENST0	.	2p25.3	.	.	.	.	rs372	.	.	.	.	.	.
ENST0	.	2p25.3	.	.	.	.	rs777	.	.	.	.	.	.
ENST0	.	2p25.3	.	.	.	.	.	.	.	.	.	.	.
ENST0	.	2p25.3	.	.	.	.	rs569	.	.	.	.	0.001	2E-04
.	.	2p25.1	.	.	.	.	.	.	.	.	.	.	.
ENST0	.	2p25.1	.	.	.	.	rs749	.	.	.	.	.	.
ENST0	.	2p25.1	.	.	.	.	rs369	.	.	.	.	.	.
ENST0	.	2p25.1	.	.	.	Score=247	rs965	.	.	.	.	.	.
ENST0	.	2p24.2	.	Scc	.	Score=644	.	.	.	.	.	.	.
ENST0	.	2p24.2	.	.	.	.	rs376	.	.	.	.	.	2E-04
ENST0	.	2p24.2	.	Scc	.	.	rs368	.	.	.	.	.	.
ENST0	.	2p24.2	.	.	.	.	rs755	.	.	.	.	.	.
ENST0	.	2p24.1	.	Scc	.	.	.	ID=COSM3	.	.	.	.	.
ENST0	.	2p23.3	.	.	.	.	rs565	.	.	.	.	.	2E-04
ENST0	.	2p23.3	.	Scc	.	.	rs773	.	.	.	.	.	.
ENST0	.	2p23.3	.	.	.	.	rs147	.	.	.	.	.	2E-04
ENST0	.	2p23.3	.	.	.	.	.	.	.	.	.	.	.
ENST0	.	2p23.3	.	.	.	.	.	.	.	.	.	.	.
ENST0	.	2p23.3	.	.	.	.	.	.	.	.	.	.	.
ENST0	.	2p23.1	.	.	.	.	.	.	.	.	.	.	.
ENST0	.	2p22.3	.	.	.	.	rs766	ID=COSM1	.	.	.	.	.
ENST0	.	2p22.3	.	.	.	Score=365	rs104	.	.	.	.	.	.
ENST0	.	2p22.3	.	.	.	Score=646	.	.	.	.	.	.	.
ENST0	.	2p22.2	.	.	.	.	.	.	.	.	.	.	.
ENST0	.	2p22.1	.	.	.	Score=270	rs772	ID=COSN1	.	.	.	.	.
ENST0	.	2p21	.	.	.	.	.	.	.	.	.	.	.
ENST0	.	2p16.3	.	.	.	.	.	.	.	.	.	.	.
ENST0	.	2p16.3	.	.	.	.	.	.	.	.	.	.	.
ENST0	.	2p16.2	.	.	.	.	.	.	.	.	.	.	.
ENST0	.	2p16.2	.	Scc	.	.	.	.	.	.	.	.	.
ENST0	.	2p16.1	.	Scc	.	.	.	.	.	.	.	.	.
ENST0	.	2p16.1	.	.	.	Score=324	rs778	ID=COSN1	.	.	.	.	.
ENST0	.	2p16.1	.	.	.	.	rs760	.	.	.	.	.	.
ENST0	.	2p16.1	.	Scc	.	.	.	.	.	.	.	.	.
ENST0	.	2p15	.	.	.	.	.	.	.	.	.	.	.
ENST0	.	2p14	.	.	.	.	.	.	.	.	.	.	.
ENST0	.	2p14	.	.	.	.	.	.	.	.	.	.	.
ENST0	.	2p13.3	.	.	.	.	.	ID=COSM1	.	.	.	.	.
ENST0	.	2p13.3	.	.	.	.	.	.	.	.	.	.	.
ENST0	.	2p13.3	.	.	.	.	.	.	.	.	.	.	.
ENST0	.	2p13.3	.	.	.	.	.	.	.	.	.	.	.
ENST0	.	2p13.2	.	.	.	.	.	.	.	.	.	.	.
ENST0	.	2p12	.	.	.	.	rs767	ID=COSM5	.	.	.	.	.
ENST0	.	2p11.2	.	.	.	.	.	.	.	.	.	.	.
ENST0	.	2p11.2	.	.	.	.	rs188	.	.	.	.	.	2E-04
ENST0	.	2p11.2	.	.	.	.	.	.	.	.	.	.	.
ENST0	.	2p11.2	.	.	.	.	.	.	.	.	.	.	.

ENST0	.	2p11.2	.	.	.	.	.	.	.	.	.	.	.
ENST0	.	2p11.2	.	.	Scc	.	.	.	.	.	.	.	.
ENST0	.	2p11.2	.	.	Scc	.	.	.	.	.	.	.	.
ENST0	.	2p11.2	.	.	Scc	.	rs191	ID=COSN2	.	.	.	.	0.004
.	.	2p11.2	.	.	Scc	.	rs777	.	.	.	.	.	.
.	.	2p11.2	.	.	Scc	Score=221	rs292	.	.	.	.	.	.
ENST0	.	2p11.2	.	.	Scc	.	rs376	.	.	.	.	.	.
ENST0	.	2p11.2	.	.	Scc	.	.	.	.	.	.	.	.
.	.	2p11.2	.	.	Scc	.	.	.	.	.	.	.	.
.	.	2p11.1	.	.	Scc	Score=231	rs868	ID=COSN1	.	.	.	.	.
.	.	2p11.1	.	.	Scc	.	rs375	ID=COSN2	.	.	.	.	.
ENST0	.	2p11.1	.	.	Scc	.	rs121	ID=COSN1	.	.	.	.	.
ENST0	.	2q11.1	.	.	Scc	.	rs293	.	.	.	.	.	2E-04
ENST0	.	2q11.1	.	.	Scc	.	.	.	.	.	.	.	.
ENST0	.	2q11.1	.	Scc	.	.	rs778	.	.	.	.	.	.
ENST0	.	2q11.2	.	.	.	.	.	.	.	.	.	.	.
ENST0	.	2q11.2	.	.	.	Score=255	rs570	.	.	.	.	.	.
ENST0	.	2q11.2	.	.	.	.	.	.	.	.	.	.	.
ENST0	.	2q11.2	.	.	Scc	.	rs557	.	.	.	.	0.001	2E-04
ENST0	.	2q11.2	.	.	Scc	.	.	.	.	.	.	.	.
ENST0	.	2q11.2	.	Scc	.	.	.	.	.	.	.	.	.
ENST0	.	2q11.2	.	.	.	.	rs768	ID=COSM7	.	.	.	.	.
ENST0	.	2q12.1	.	.	.	.	.	.	.	.	.	.	.
ENST0	.	2q12.1	.	.	.	.	rs751	.	.	.	.	.	.
ENST0	.	2q12.2	.	.	Scc	.	rs201	.	.	.	.	.	.
ENST0	.	2q13	.	.	.	.	rs758	.	.	.	.	.	.
ENST0	.	2q13	.	.	.	.	.	.	.	.	.	.	.
.	.	2q13	.	.	.	.	.	.	.	.	.	.	.
ENST0	.	2q13	.	.	.	.	.	.	.	.	.	.	.
ENST0	.	2q14.1	.	.	Scc	.	.	ID=COSN1	.	.	.	.	.
ENST0	.	2q14.1	.	.	.	.	.	.	.	.	.	.	.
ENST0	.	2q14.2	.	.	.	.	rs764	.	.	.	.	.	.
ENST0	.	2q14.3	.	.	.	.	.	.	.	.	.	.	.
ENST0	.	2q14.3	.	.	.	.	.	.	.	.	.	.	.
ENST0	.	2q21.1	.	.	Scc	.	rs781	.	.	.	.	.	.
ENST0	.	2q21.1	.	.	Scc	.	rs134	ID=COSN9	.	.	.	.	.
ENST0	.	2q21.2	.	.	Scc	.	rs182	.	.	.	.	.	8E-04
ENST0	.	2q21.2	.	.	.	.	.	.	.	.	.	.	.
ENST0	.	2q21.2	.	.	.	Score=379	.	.	.	.	.	.	.
ENST0	.	2q22.1	.	.	.	.	.	.	.	.	.	.	.
ENST0	.	2q23.3	.	.	.	.	.	.	.	.	.	.	.
ENST0	.	2q23.3	.	.	.	.	.	.	.	.	.	.	.
ENST0	.	2q23.3	.	.	.	.	.	.	.	.	.	.	.
ENST0	.	2q23.3	.	.	.	.	rs101	.	.	.	.	.	.
ENST0	.	2q23.3	.	.	.	Score=216	rs985	.	.	.	.	.	.
ENST0	.	2q24.2	.	.	.	.	rs200	ID=COSM6	.	.	.	.	.
ENST0	.	2q24.2	.	Scc	.	.	rs372	.	.	.	.	.	.
ENST0	.	2q24.2	.	.	.	.	.	.	.	.	.	.	.
ENST0	.	2q24.2	.	.	.	.	rs102	.	.	.	.	.	.
ENST0	.	2q24.3	.	.	.	.	.	.	.	.	.	.	.
ENST0	.	2q31.1	.	.	.	Score=586	.	.	.	.	.	.	.
ENST0	.	2q31.1	.	.	.	.	.	.	.	.	.	.	.
ENST0	.	2q31.1	.	.	.	.	.	.	.	.	.	.	.
ENST0	.	2q31.1	.	.	.	.	rs921	.	.	.	.	.	.

ENST0	.	2q31.1	.	.	.	Score=395	.	.	.	.	.	.	.
ENST0	.	2q31.1	.	.	.	.	rs963	.	.	.	.	.	.
ENST0	.	2q31.1	.	.	.	.	.	.	.	.	.	.	.
ENST0	.	2q31.1	.	.	.	.	.	.	.	.	.	.	.
ENST0	.	2q31.1	.	.	.	Score=588	.	.	.	.	.	.	.
ENST0	.	2q31.2	.	.	.	.	.	.	.	.	.	.	.
ENST0	.	2q31.2	.	.	.	Score=132	.	ID=COSN2	.	.	.	.	.
ENST0	.	2q31.2	.	.	.	.	rs960	.	.	.	.	.	.
ENST0	.	2q32.1	.	.	.	.	rs377	.	.	.	.	.	.
ENST0	.	2q32.1	.	.	.	.	rs376	.	.	.	.	.	.
ENST0	.	2q32.1	.	.	.	.	.	.	.	.	.	.	.
ENST0	.	2q32.1	.	.	.	.	.	.	.	.	.	.	.
ENST0	.	2q32.2	.	.	.	.	rs376	.	.	.	.	.	.
ENST0	.	2q32.2	.	.	.	.	rs771	.	.	.	.	.	.
ENST0	.	2q32.2	.	Scc	.	.	.	.	.	.	.	.	.
ENST0	.	2q32.3	.	.	.	.	.	.	.	.	.	.	.
ENST0	.	2q32.3	.	.	.	.	.	.	.	.	.	.	.
ENST0	.	2q33.1	.	Scc	.	.	.	.	.	.	.	.	.
ENST0	.	2q33.1	.	Scc	.	.	.	.	.	.	.	.	.
ENST0	.	2q33.1	.	.	.	.	rs548	.	.	.	.	.	.
ENST0	.	2q33.2	.	.	.	.	rs190	.	.	.	.	0.009	0.007
ENST0	.	2q33.2	.	.	.	Score=209	rs777	.	.	.	.	.	.
ENST0	.	2q33.3	.	.	.	.	rs147	ID=COSM:	.	.	.	.	0.002
ENST0	.	2q33.3	.	Scc	.	.	rs537	.	.	.	.	0.001	0.002
ENST0	.	2q33.3	.	Scc	.	.	.	.	.	.	.	.	.
ENST0	.	2q34	.	.	.	.	.	.	.	.	.	.	.
ENST0	.	2q34	.	.	.	.	.	.	.	.	.	.	.
ENST0	.	2q34	.	.	.	.	.	.	.	.	.	.	.
ENST0	.	2q34	.	.	.	.	.	.	.	.	.	.	.
ENST0	.	2q34	.	.	.	.	.	.	.	.	.	.	.
ENST0	.	2q35	.	.	.	.	.	.	.	.	.	.	.
ENST0	.	2q35	.	.	.	.	.	.	.	.	.	.	.
ENST0	.	2q35	.	.	.	.	.	.	.	.	.	.	.
ENST0	.	2q35	.	.	.	.	.	.	.	.	.	.	.
ENST0	.	2q35	.	.	.	.	rs575	.	.	.	.	.	2E-04
ENST0	.	2q35	.	.	.	.	.	.	.	.	.	.	.
ENST0	.	2q35	.	.	.	.	rs373	.	.	.	.	.	.
ENST0	.	2q35	.	.	.	.	.	.	.	.	.	.	.
ENST0	.	2q35	.	.	.	.	rs746	.	.	.	.	.	.
ENST0	.	2q35	.	Scc	.	.	rs529	.	.	.	.	.	0.003
ENST0	.	2q36.1	.	.	.	.	.	.	.	.	.	.	.
ENST0	.	2q36.3	.	.	.	.	.	.	.	.	.	.	.
ENST0	.	2q36.3	.	.	.	.	.	.	.	.	.	.	.
ENST0	.	2q36.3	.	.	.	.	rs499	.	.	.	.	0.426	0.284
ENST0	.	2q37.1	.	.	.	.	rs753	.	.	.	.	.	.
ENST0	.	2q37.1	.	.	Scc	.	.	.	.	.	.	.	.
ENST0	.	2q37.1	.	.	Scc	.	.	.	.	.	.	.	.
ENST0	.	2q37.1	.	.	Scc	.	.	.	.	.	.	.	.
ENST0	.	2q37.1	.	Scc	.	.	rs202	.	.	.	.	.	.
ENST0	.	2q37.1	.	.	.	.	rs105	.	.	.	.	.	.
ENST0	.	2q37.1	.	.	.	.	.	.	.	.	.	.	.
ENST0	.	2q37.3	.	.	.	.	.	.	.	.	.	.	.
ENST0	.	2q37.3	.	.	.	.	rs748	.	.	.	.	.	.
ENST0	.	2q37.3	.	.	.	.	.	.	.	.	.	.	.

ENST0	.	2q37.3	.	.	.	.	rs377	.	.	.	.	.	.
ENST0	.	2q37.3	.	.	.	.	rs150	.	.	.	.	0.001	0.007
ENST0	.	2q37.3	.	.	.	.	.	.	.	.	.	.	.
ENST0	.	2q37.3	.	.	.	.	.	.	.	.	.	.	.
ENST0	.	2q37.3	.	.	.	.	.	.	.	.	.	.	.
ENST0	.	3p26.3	.	.	.	.	rs373	ID=COSM4	.	.	.	.	.
ENST0	.	3p26.3	.	.	.	Score=662	rs569	.	.	.	.	.	0.002
ENST0	.	3p26.1	.	.	.	.	.	.	.	.	.	.	.
ENST0	.	3p25.3	.	.	.	Score=237	.	.	.	.	.	.	.
ENST0	.	3p25.3	.	.	.	Score=215	rs750	.	.	.	.	.	.
ENST0	.	3p25.3	.	.	.	.	.	.	.	.	.	.	.
ENST0	.	3p25.3	.	Scc	.	.	rs756	.	.	.	.	.	.
ENST0	.	3p25.3	.	.	Scc	.	.	.	.	.	.	.	.
ENST0	.	3p25.3	.	Scc	.	.	.	.	.	.	.	.	.
ENST0	.	3p25.2	.	.	.	.	.	.	.	.	.	.	.
.	.	3p25.2	.	.	.	.	.	.	.	.	.	.	.
ENST0	.	3p25.2	.	.	.	Score=802	rs992	.	.	.	.	.	.
ENST0	.	3p25.2	.	.	.	.	.	.	.	.	.	.	.
ENST0	.	3p25.2	.	.	.	.	.	.	.	.	.	.	.
ENST0	.	3p25.1	.	.	.	.	.	.	.	.	.	.	.
ENST0	.	3p24.3	.	.	.	.	.	.	.	.	.	.	.
ENST0	.	3p24.3	.	.	.	.	.	.	.	.	.	.	.
ENST0	.	3p24.2	.	.	.	.	.	.	.	.	.	.	.
ENST0	.	3p24.2	.	.	.	.	.	.	.	.	.	.	.
.	.	3p24.1	.	.	.	Score=995	.	.	.	.	.	.	.
ENST0	.	3p24.1	.	.	.	Score=232	rs897	ID=COSN2	.	.	.	.	.
ENST0	.	3p24.1	.	.	.	.	rs112	.	.	.	.	.	2E-04
ENST0	hsa-mir-46	3p23	.	.	.	Score=244	.	.	.	.	.	.	.
ENST0	.	3p23	.	.	.	.	rs780	.	.	.	.	.	.
ENST0	.	3p22.2	.	.	.	.	.	.	.	.	.	.	.
ENST0	.	3p22.2	.	.	.	.	rs540	.	.	.	.	0.001	4E-04
ENST0	.	3p22.2	.	.	.	.	rs559	.	.	.	.	.	8E-04
ENST0	.	3p22.2	.	.	.	.	.	.	.	.	.	.	.
ENST0	.	3p22.2	.	Scc	.	.	rs373	.	.	.	.	.	.
ENST0	.	3p22.2	.	.	.	Score=273	.	.	.	.	.	.	.
ENST0	.	3p22.2	.	.	.	.	rs748	ID=COSM3	.	.	.	.	.
ENST0	.	3p21.31	.	.	.	.	.	.	.	.	.	.	.
ENST0	.	3p21.31	.	.	.	.	rs774	.	.	.	.	.	.
ENST0	.	3p21.31	.	.	.	Score=209	.	.	.	.	.	.	.
ENST0	.	3p21.31	.	.	.	.	.	.	.	.	.	.	.
ENST0	.	3p21.31	.	.	.	.	rs751	.	.	.	.	.	.
ENST0	.	3p21.31	.	.	.	.	rs530	.	.	.	.	.	.
ENST0	.	3p21.31	.	.	.	.	rs371	.	.	.	.	.	.
ENST0	.	3p21.31	.	.	.	.	.	.	.	.	.	.	.
ENST0	.	3p21.31	.	.	.	.	rs200	.	.	.	.	.	2E-04
ENST0	.	3p21.31	.	.	.	.	.	.	.	.	.	.	.
ENST0	.	3p21.31	.	.	.	.	.	.	.	.	.	.	.
ENST0	.	3p21.31	.	.	.	.	.	.	.	.	.	.	.
ENST0	.	3p21.31	.	Scc	.	.	rs759	.	.	.	.	.	.
ENST0	.	3p21.2	.	.	.	.	rs182	.	.	.	.	.	6E-04
ENST0	.	3p21.2	.	.	.	.	.	.	.	.	.	.	.
ENST0	.	3p21.2	.	.	.	.	rs778	.	.	.	.	.	.
ENST0	.	3p21.2	.	.	.	.	.	.	.	.	.	.	.
ENST0	.	3p21.1	.	.	.	.	.	.	.	.	.	.	.

ENST0	.	3p21.1	.	.	.	Score=160	.	.	.	.	.	.	.
ENST0	.	3p21.1	.	.	.	.	.	.	.	.	.	.	.
ENST0	.	3p21.1	.	.	.	.	rs962	.	.	.	.	.	.
ENST0	.	3p21.1	.	.	.	.	.	.	.	.	.	.	.
ENST0	.	3p21.1	.	.	.	.	.	.	.	.	.	.	.
ENST0	.	3p21.1	.	Scc	.	.	.	.	.	.	.	.	.
ENST0	.	3p21.1	.	.	.	.	rs200	ID=COSN1	.	.	.	.	.
ENST0	.	3p14.3	.	.	.	Score=294	rs985	.	.	.	.	.	.
ENST0	.	3p14.3	.	.	.	Score=294	rs104	.	.	.	.	.	.
ENST0	.	3p14.3	.	.	.	.	.	.	.	.	.	.	.
ENST0	.	3p14.3	.	.	.	.	.	.	.	.	.	.	.
ENST0	.	3p14.3	.	.	.	.	.	.	.	.	.	.	.
ENST0	.	3p14.3	.	.	.	.	.	.	.	.	.	.	.
ENST0	.	3p14.1	.	.	.	.	rs745	.	.	.	.	.	.
ENST0	.	3p14.1	.	.	.	.	.	.	.	.	.	.	.
ENST0	.	3p13	.	.	.	.	.	.	.	.	.	.	.
ENST0	.	3p12.3	.	.	Scc	.	rs779	ID=COSM4	.	.	.	.	.
ENST0	.	3q11.2	.	.	.	.	.	.	.	.	.	.	.
.	.	3q13.11	.	.	.	.	.	.	.	.	.	.	.
ENST0	.	3q13.13	.	.	.	.	.	.	.	.	.	.	.
ENST0	.	3q13.13	.	.	.	.	.	.	.	.	.	.	.
ENST0	.	3q13.2	.	.	.	.	.	.	.	.	.	.	.
ENST0	.	3q13.33	.	Scc	.	.	.	.	.	.	.	.	.
ENST0	.	3q13.33	.	.	.	.	.	.	.	.	.	.	.
ENST0	.	3q13.33	.	.	.	.	rs368	.	.	.	.	.	.
ENST0	.	3q21.1	.	.	.	.	rs100	ID=COSN1	.	.	.	.	.
ENST0	.	3q21.2	.	.	Scc	.	rs146	.	.	.	.	.	.
ENST0	.	3q21.3	.	.	.	.	.	.	.	.	.	.	.
ENST0	.	3q21.3	.	.	.	.	rs552	.	.	.	.	0.001	4E-04
ENST0	.	3q21.3	.	Scc	.	.	rs751	.	.	.	.	.	.
ENST0	.	3q21.3	.	.	.	.	.	.	.	.	.	.	.
ENST0	.	3q21.3	.	.	Scc	.	rs563	.	.	.	.	.	.
ENST0	.	3q21.3	.	.	Scc	.	.	.	.	.	.	.	.
ENST0	.	3q22.1	.	.	.	.	.	.	.	.	.	.	.
ENST0	.	3q22.1	.	.	.	.	.	.	.	.	.	.	.
ENST0	.	3q23	.	.	Scc	.	.	.	.	.	.	.	.
ENST0	.	3q23	.	.	Scc	.	.	.	.	.	.	.	.
ENST0	.	3q23	.	Scc	.	.	rs140	ID=COSM3	.	.	.	.	8E-04
ENST0	.	3q23	.	.	.	.	.	.	.	.	.	.	.
ENST0	.	3q23	.	.	.	.	.	.	.	.	.	.	.
ENST0	.	3q23	.	.	.	.	rs768	ID=COSN1	Lik	.	.	0.002	0.048
ENST0	.	3q23	.	.	.	.	rs770	.	.	.	.	.	.
ENST0	.	3q24	.	.	.	.	.	ID=COSN2	.	.	.	.	.
ENST0	.	3q25.1	.	.	.	.	.	.	.	.	.	.	.
ENST0	.	3q25.1	.	.	.	.	rs760	.	.	.	.	.	.
ENST0	.	3q25.32	.	.	.	.	.	.	.	.	.	.	.
ENST0	hsa-mir-16	3q25.33	.	Scc	.	.	.	.	.	.	.	.	.
ENST0	.	3q26.1	.	.	.	.	.	.	.	.	.	.	.
.	.	3q26.2	.	.	.	Score=109	.	.	.	.	.	.	.
ENST0	.	3q26.2	.	Scc	.	.	rs538	.	.	.	.	.	2E-04
.	.	3q26.32	.	.	.	Score=115	rs936	.	.	.	.	.	.
ENST0	.	3q26.32	.	.	Scc	.	.	ID=COSN8	.	.	.	.	.
ENST0	.	3q26.33	.	Scc	.	.	rs765	.	.	.	.	.	.
ENST0	.	3q26.33	.	.	.	.	rs571	ID=COSN2	.	.	.	0.001	2E-04

ENST0	.	3q27.1	.	.	.	.	.	.	.	.	.	.	.
ENST0	.	3q27.1	.	.	.	.	.	.	.	.	.	.	.
ENST0	.	3q27.1	.	.	.	.	rs901	.	.	.	.	.	.
ENST0	.	3q27.1	.	.	.	Score=265	.	.	.	.	.	.	.
ENST0	.	3q27.1	.	.	.	.	rs374	ID=COSM3	.	.	.	.	.
ENST0	.	3q27.3	.	.	.	.	.	.	.	.	.	.	.
ENST0	.	3q28	.	Scc	.	.	rs753	.	.	.	.	.	.
ENST0	.	3q29	.	.	.	.	rs777	.	.	.	.	.	.
.	.	3q29	.	.	Scc	.	.	.	.	.	.	.	.
.	.	3q29	.	.	Scc	Score=735	.	.	.	.	.	.	.
ENST0	.	3q29	.	.	.	.	.	.	.	.	.	.	.
ENST0	.	3q29	.	.	.	Score=921	.	.	.	.	.	.	.
ENST0	.	3q29	.	.	.	Score=231	rs886	.	.	.	.	.	.
ENST0	.	3q29	.	.	Scc	.	rs763	ID=COSN8	.	.	.	.	.
ENST0	.	4p16.3	.	.	.	Score=653	.	.	.	.	.	.	.
ENST0	.	4p16.3	.	Scc	.	.	rs762	.	.	.	.	.	.
ENST0	.	4p16.3	.	.	.	.	.	.	.	.	.	.	.
ENST0	.	4p16.3	.	.	.	.	rs912	.	.	.	.	.	.
ENST0	.	4p16.3	.	.	.	.	.	.	.	.	.	.	.
ENST0	.	4p16.3	.	.	.	Score=276	.	.	.	.	.	.	.
ENST0	.	4p16.3	.	.	.	.	rs550	.	.	.	.	.	2E-04
ENST0	.	4p16.3	.	.	.	.	.	.	.	.	.	.	.
ENST0	.	4p16.3	.	.	.	Score=255	.	.	.	.	.	.	.
.	.	4p16.3	.	.	.	.	rs933	.	.	.	.	.	.
ENST0	.	4p16.3	.	.	.	Score=186	.	.	.	.	.	.	.
ENST0	.	4p16.1	.	.	.	.	rs560	.	oth	.	.	0.001	4E-04
ENST0	.	4p16.1	.	.	.	.	.	.	.	.	.	.	.
ENST0	.	4p16.1	.	.	.	.	.	ID=COSN4	.	.	.	.	.
ENST0	.	4p16.1	.	.	.	.	rs376	.	.	.	.	.	.
ENST0	.	4p16.1	.	.	.	.	rs200	.	.	.	.	0.008	0.002
ENST0	.	4p16.1	.	.	.	.	rs752	.	.	.	.	.	.
ENST0	.	4p16.1	.	.	.	.	rs780	ID=COSM3	.	.	.	.	.
ENST0	.	4p15.33	.	.	.	Score=200	.	.	.	.	.	.	.
ENST0	.	4p15.32	.	Scc	.	.	.	.	.	.	.	.	.
ENST0	.	4p15.31	.	.	.	.	.	.	.	.	.	.	.
ENST0	.	4p15.2	.	.	.	.	.	.	.	.	.	.	.
.	.	4p15.1	.	.	.	Score=233	rs142	.	.	.	.	0.01	0.003
ENST0	.	4p14	.	.	.	.	rs534	.	.	.	.	.	2E-04
ENST0	.	4p14	.	.	Scc	.	.	.	.	.	.	.	.
ENST0	.	4p14	.	.	Scc	.	.	.	.	.	.	.	.
ENST0	.	4p14	.	.	.	.	rs775	ID=COSM4	.	.	.	.	.
ENST0	.	4p14	.	Scc	.	.	.	.	.	.	.	.	.
ENST0	.	4p12	.	.	.	Score=201	rs285	ID=COSN1	.	.	.	.	.
ENST0	.	4p11	.	.	Scc	.	rs145	ID=COSN2	.	.	.	.	.
.	.	4p11	.	.	Scc	Score=160	rs594	.	.	.	.	.	.
ENST0	.	4q12	.	.	.	.	.	ID=COSM3	.	.	.	.	.
ENST0	.	4q12	.	Scc	.	.	.	.	.	.	.	.	.
ENST0	.	4q12	.	.	.	.	.	.	.	.	.	.	.
ENST0	.	4q12	.	.	.	.	.	.	.	.	.	.	.
ENST0	.	4q13.2	.	.	.	.	.	.	.	.	.	.	.
ENST0	.	4q13.2	.	.	.	.	.	ID=COSM4	.	.	.	.	.
ENST0	.	4q13.3	.	.	.	.	.	.	.	.	.	.	.
ENST0	.	4q13.3	.	.	.	.	rs761	.	.	.	.	.	.
ENST0	.	4q13.3	.	Scc	.	.	.	.	.	.	.	.	.

ENST0	.	4q13.3	.	.	.	Score=24;I	rs119	ID=COSN1	.	.	.	0.126	0.126
ENST0	.	4q13.3	.	.	.	.	.	.	.	.	.	.	.
ENST0	.	4q21.1	.	.	.	.	rs369	.	.	.	.	.	.
ENST0	.	4q21.1	.	.	.	.	.	.	.	.	.	.	.
ENST0	.	4q21.1	.	.	.	.	rs760	.	.	.	.	.	.
ENST0	.	4q21.21	.	Scc	.	.	.	.	.	.	.	.	.
ENST0	.	4q21.22	.	.	.	.	.	.	.	.	.	.	.
ENST0	.	4q21.23	.	.	.	.	rs886	.	.	.	.	.	.
ENST0	.	4q22.1	.	Scc	.	.	.	.	.	.	.	.	.
ENST0	.	4q24	.	Scc	.	.	.	.	.	.	.	.	.
ENST0	.	4q24	.	.	.	.	.	.	.	.	.	.	.
ENST0	.	4q24	.	Scc	.	.	rs371	.	.	.	.	.	.
ENST0	.	4q25	.	.	Scc	.	.	ID=COSM3	.	.	.	.	.
ENST0	.	4q25	.	.	Scc	.	.	.	.	.	.	.	.
ENST0	.	4q26	.	.	Scc	.	.	.	.	.	.	.	.
ENST0	.	4q26	.	.	Scc	.	.	.	.	.	.	.	.
ENST0	.	4q26	.	Scc	.	.	.	.	.	.	.	.	.
ENST0	.	4q27	.	.	.	.	.	.	.	.	.	.	.
ENST0	.	4q28.1	.	.	.	.	rs774	.	.	.	.	.	.
ENST0	.	4q28.2	.	.	.	.	.	.	.	.	.	.	.
ENST0	.	4q28.2	.	.	.	.	rs774	.	.	.	.	.	.
.	.	4q28.3	.	.	Scc	Score=105	.	ID=COSN2	.	.	.	.	.
ENST0	.	4q28.3	.	.	.	.	.	.	.	.	.	.	.
ENST0	.	4q31.21	.	Scc	.	.	.	.	.	.	.	.	.
ENST0	.	4q31.21	.	.	.	.	.	ID=COSM3	.	.	.	.	.
ENST0	.	4q31.21	.	.	.	.	.	.	.	.	.	.	.
ENST0	.	4q31.21	.	.	Scc	.	.	.	.	.	.	.	.
ENST0	.	4q31.3	.	.	.	Score=116	rs866	.	.	.	.	.	.
ENST0	.	4q32.3	.	.	Scc	Score=159	rs409	ID=COSN9	.	.	.	.	.
ENST0	.	4q34.1	.	.	.	Score=403	.	.	.	.	.	.	.
ENST0	.	4q35.1	.	Scc	.	.	.	.	.	.	.	.	.
ENST0	.	4q35.1	.	.	.	.	rs942	.	.	.	.	.	.
ENST0	.	4q35.1	.	.	.	.	.	ID=COSM4	.	.	.	.	.
ENST0	.	4q35.2	.	.	.	.	rs778	.	.	.	.	.	.
ENST0	.	5p15.33	.	Scc	.	.	.	.	.	.	.	.	.
ENST0	.	5p15.33	.	.	.	.	.	.	.	.	.	.	.
ENST0	.	5p15.33	.	.	Scc	.	rs412	ID=COSN1	.	.	.	.	.
ENST0	.	5p15.33	.	.	.	.	rs100	ID=COSN1	.	.	.	.	.
ENST0	.	5p15.33	.	.	.	Score=198	.	.	.	.	.	.	.
.	.	5p15.33	.	.	.	Score=590	rs903	.	.	.	.	.	.
ENST0	.	5p15.32	.	.	.	.	rs749	.	.	.	.	.	.
ENST0	.	5p15.31	.	Scc	.	.	.	.	.	.	.	.	.
ENST0	.	5p15.31	.	.	.	.	.	ID=COSM5	.	.	.	.	.
ENST0	.	5p15.2	.	Scc	.	.	rs149	ID=COSM2	.	.	.	.	2E-04
ENST0	.	5p15.2	.	.	.	Score=245	rs655	.	Be	.	.	0.548	0.261
ENST0	.	5p15.2	.	.	.	.	rs914	.	.	.	.	.	.
ENST0	.	5p15.2	.	.	.	.	rs766	.	.	.	.	.	.
ENST0	.	5p13.3	.	.	Scc	.	rs925	.	.	.	.	.	.
ENST0	.	5p13.3	.	.	.	.	rs569	.	.	.	.	.	2E-04
ENST0	.	5p13.2	.	.	Scc	Score=142	rs879	.	.	.	.	.	.
ENST0	.	5p13.2	.	.	Scc	Score=142	rs976	ID=COSN2	.	.	.	.	.
ENST0	.	5p13.2	.	.	Scc	Score=215	rs976	.	.	.	.	.	.
ENST0	.	5p13.1	.	.	.	.	.	.	.	.	.	.	.
ENST0	.	5p13.1	.	Scc	.	.	.	.	.	.	.	.	.

ENST0	.	5p13.1	.	.	.	.	.	.	.	.	.	.	.
ENST0	.	5p12	.	.	.	.	.	.	.	.	.	.	.
.	.	5q11.1	.	.	.	Score=122	.	.	.	.	.	.	.
.	.	5q11.1	.	.	.	Score=122	rs432	.	.	.	.	.	.
.	.	5q11.1	.	.	.	Score=122	.	.	.	.	.	.	.
ENST0	.	5q11.2	.	.	.	.	rs200	.	.	.	.	.	.
ENST0	.	5q12.1	.	Scc	.	.	.	.	.	.	.	.	.
.	.	5q12.1	.	.	Scc	.	rs558	.	.	.	.	.	2E-04
ENST0	.	5q12.3	.	Scc	.	.	.	.	.	.	.	.	.
ENST0	.	5q12.3	.	.	.	.	.	.	.	.	.	.	.
ENST0	.	5q12.3	.	.	.	.	rs567	.	.	.	.	.	6E-04
ENST0	.	5q12.3	.	Scc	.	.	.	.	.	.	.	.	.
ENST0	.	5q12.3	.	Scc	.	.	rs201	.	.	.	.	.	0.001
ENST0	.	5q13.1	.	.	.	.	.	.	.	.	.	.	.
ENST0	.	5q13.2	.	.	.	Score=175	.	.	.	.	.	.	.
ENST0	.	5q13.2	.	.	Scc	.	.	.	.	.	.	.	.
ENST0	.	5q13.2	.	.	.	.	.	.	.	.	.	.	.
ENST0	.	5q13.3	.	.	.	Score=101	.	.	.	.	.	.	.
ENST0	.	5q14.1	.	.	.	.	.	.	.	.	.	.	.
ENST0	.	5q14.1	.	.	.	.	.	.	.	.	.	.	.
ENST0	.	5q14.2	.	.	Scc	.	.	.	.	.	.	.	.
ENST0	.	5q14.3	.	.	.	.	rs371	ID=COSN2	.	.	.	.	.
.	.	5q14.3	.	.	.	Score=432	rs781	.	.	.	.	.	.
ENST0	.	5q14.3	.	.	.	.	.	.	.	.	.	.	.
ENST0	.	5q15	.	.	.	.	.	.	.	.	.	.	.
ENST0	.	5q15	.	.	.	.	rs763	ID=COSM4	.	.	.	.	.
ENST0	.	5q21.1	.	.	Scc	.	.	.	.	.	.	.	.
ENST0	.	5q21.1	.	Scc	.	.	.	.	.	.	.	.	.
ENST0	.	5q21.1	.	.	.	.	rs111	.	.	.	.	.	8E-04
ENST0	.	5q22.1	.	.	.	.	.	.	.	.	.	.	.
ENST0	.	5q22.2	.	.	.	.	.	.	.	.	.	.	.
ENST0	.	5q23.1	.	.	.	.	.	.	.	.	.	.	.
ENST0	.	5q23.1	.	.	.	.	.	.	.	.	.	.	.
ENST0	.	5q31.1	.	Scc	.	.	rs934	.	.	.	.	.	.
ENST0	.	5q31.1	.	.	.	.	.	.	.	.	.	.	.
ENST0	.	5q31.1	.	Scc	.	.	rs746	.	.	.	.	.	.
.	.	5q31.1	.	.	.	.	.	.	.	.	.	.	.
ENST0	.	5q31.1	.	.	.	.	rs747	.	.	.	.	.	.
ENST0	.	5q31.2	.	.	.	.	rs766	ID=COSM1	.	.	.	.	.
ENST0	.	5q31.2	.	.	.	.	.	.	.	.	.	.	.
ENST0	.	5q31.2	.	.	.	Score=384	.	.	.	.	.	.	.
ENST0	.	5q31.2	.	.	.	Score=384	rs772	.	.	.	.	.	.
ENST0	.	5q31.3	.	Scc	.	.	.	.	.	.	.	.	.
ENST0	.	5q31.3	.	.	Scc	.	.	.	.	.	.	.	.
ENST0	.	5q31.3	.	.	.	.	.	ID=COSM1	.	.	.	.	.
ENST0	.	5q31.3	.	Scc	.	.	rs782	ID=COSM3	.	.	.	.	.
ENST0	.	5q31.3	.	.	Scc	.	.	ID=COSM1	.	.	.	.	.
ENST0	.	5q31.3	.	.	Scc	.	.	.	.	.	.	.	.
ENST0	.	5q31.3	.	.	.	.	.	.	.	.	.	.	.
ENST0	.	5q31.3	.	.	.	.	rs981	.	.	.	.	.	.
ENST0	.	5q31.3	.	Scc	.	.	rs544	ID=COSM4	.	.	.	.	0.002
ENST0	.	5q31.3	.	.	.	.	rs376	ID=COSM3	.	.	.	.	.
ENST0	.	5q31.3	.	Scc	.	.	rs771	.	.	.	.	.	.
ENST0	.	5q31.3	.	Scc	.	.	rs201	ID=COSM7	.	.	.	0.004	8E-04

ENST0	.	5q32	.	.	.	Score=216	rs100	.	.	.	.	.	.
ENST0	.	5q32	.	.	.	.	.	.	.	.	.	.	.
ENST0	.	5q32	.	.	.	.	.	.	.	.	.	.	.
ENST0	.	5q33.1	.	.	.	.	.	.	.	.	.	.	.
ENST0	.	5q33.1	.	Scc	Scc	.	.	.	.	.	.	.	.
ENST0	.	5q33.3	.	.	.	.	rs549	.	.	.	.	0.002	4E-04
ENST0	.	5q33.3	.	.	.	.	rs561	.	.	.	.	.	2E-04
ENST0	.	5q34	.	Scc	.	.	.	.	.	.	.	.	.
ENST0	.	5q34	.	.	.	.	.	.	.	.	.	.	.
ENST0	.	5q34	.	.	.	.	rs149	.	.	.	.	.	.
ENST0	.	5q34	.	.	.	.	rs757	ID=COSM	.	.	.	.	.
ENST0	.	5q34	.	.	.	.	.	.	.	.	.	.	.
ENST0	.	5q35.1	.	.	.	.	.	.	.	.	.	.	.
ENST0	.	5q35.1	.	.	.	.	rs182	.	.	.	.	.	2E-04
ENST0	.	5q35.1	.	.	.	.	.	.	.	.	.	.	.
ENST0	.	5q35.2	.	Scc	.	.	.	.	.	.	.	.	.
ENST0	.	5q35.2	.	.	.	.	.	.	.	.	.	.	.
ENST0	.	5q35.2	.	.	.	.	rs455	.	.	.	.	.	0.003
ENST0	.	5q35.2	.	Scc	.	.	rs558	.	.	.	.	.	4E-04
ENST0	.	5q35.3	.	.	Scc	.	.	.	.	.	.	.	.
ENST0	.	5q35.3	.	.	Scc	.	.	.	.	.	.	.	.
ENST0	.	5q35.3	.	.	.	.	.	.	.	.	.	.	.
ENST0	.	5q35.3	.	.	Scc	.	rs102	.	.	.	.	.	.
ENST0	.	5q35.3	.	Scc	.	Score=22;I	.	.	.	.	.	.	.
ENST0	.	5q35.3	.	.	.	Score=248	.	.	.	.	.	.	.
ENST0	.	6p25.1	.	.	.	.	rs765	.	.	.	.	.	.
ENST0	.	6p25.1	.	.	.	Score=256	rs102	.	.	.	.	.	.
ENST0	.	6p24.3	.	.	.	.	rs186	.	.	.	.	.	0.002
ENST0	.	6p24.3	.	.	Scc	.	.	.	.	.	.	.	.
ENST0	.	6p24.3	.	.	.	.	rs200	.	.	.	.	.	.
ENST0	.	6p24.3	.	.	.	.	.	.	.	.	.	.	.
ENST0	.	6p24.3	.	.	.	.	rs369	ID=COSN1	.	.	.	.	.
ENST0	.	6p24.1	.	.	.	.	rs746	.	.	.	.	.	.
ENST0	.	6p22.3	.	.	.	.	rs767	.	.	.	.	.	.
ENST0	.	6p22.3	.	.	.	.	.	.	.	.	.	.	.
.	.	6p22.3	.	.	Scc	.	.	.	.	.	.	.	.
ENST0	.	6p22.3	.	.	Scc	.	.	.	.	.	.	.	.
ENST0	.	6p22.2	.	.	Scc	.	.	.	.	.	.	.	.
ENST0	.	6p22.1	.	.	.	.	.	.	.	.	.	.	.
.	.	6p22.1	.	.	.	.	.	.	.	.	.	.	.
ENST0	.	6p22.1	.	.	.	Score=205	.	.	.	.	.	.	.
ENST0	.	6p22.1	.	.	.	.	.	.	.	.	.	.	.
ENST0	.	6p22.1	.	.	.	Score=207	rs765	ID=COSN1	.	.	.	.	.
ENST0	.	6p22.1	.	.	Scc	.	.	.	.	.	.	.	.
ENST0	.	6p21.33	.	.	.	.	rs751	.	.	.	.	.	.
ENST0	.	6p21.33	.	.	.	.	.	.	.	.	.	.	.
ENST0	.	6p21.33	.	.	.	.	.	.	.	.	.	.	.
.	.	6p21.33	.	.	.	.	.	.	.	.	.	.	.
ENST0	.	6p21.33	.	.	.	.	rs779	.	.	.	.	.	.
ENST0	.	6p21.33	.	.	.	.	.	.	.	.	.	.	.
ENST0	.	6p21.32	.	.	.	.	.	.	.	.	.	.	.
ENST0	.	6p21.32	.	.	.	.	rs111	.	.	.	.	.	.
ENST0	.	6p21.32	.	Scc	.	.	rs775	.	.	.	.	.	.
ENST0	.	6p21.31	.	Scc	.	.	.	.	.	.	.	.	.

ENST0	.	6p21.31	.	.	.	.	.	.	.	.	.	.	.
ENST0	.	6p21.31	.	Scc	.	.	rs774	.	.	.	.	.	.
ENST0	.	6p21.31	.	.	.	Score=360	.	.	.	.	.	.	.
ENST0	.	6p21.31	.	.	.	.	rs771	.	.	.	.	.	.
ENST0	.	6p21.31	.	.	.	.	.	.	.	.	.	.	.
ENST0	.	6p21.31	.	.	.	.	.	.	.	.	.	.	.
ENST0	.	6p21.2	.	.	.	.	rs100	.	.	.	.	.	.
ENST0	.	6p21.2	.	.	.	.	.	.	.	.	.	.	.
ENST0	.	6p21.1	.	Scc	Scc	.	rs375	.	.	.	.	.	.
ENST0	.	6p21.1	.	.	.	.	rs777	ID=COSM(	.	.	.	.	.
ENST0	.	6p21.1	.	Scc	.	.	.	ID=COSM(	.	.	.	.	.
ENST0	.	6p21.1	.	.	.	.	rs800	.	.	.	.	0.023	0.005
ENST0	.	6p21.1	.	Scc	.	.	rs780	ID=COSM4	.	.	.	.	.
ENST0	.	6p21.1	.	.	.	.	.	.	.	.	.	.	.
ENST0	.	6p21.1	.	.	.	.	rs143	ID=COSM3	.	.	.	.	2E-04
ENST0	.	6p21.1	.	Scc	.	.	.	.	.	.	.	.	.
ENST0	.	6p21.1	.	.	.	.	.	.	.	.	.	.	.
ENST0	.	6p21.1	.	.	.	.	.	.	.	.	.	.	.
ENST0	.	6p21.1	.	.	.	.	rs149	.	.	.	.	.	4E-04
ENST0	.	6p21.1	.	.	.	.	rs143	.	.	.	.	.	.
ENST0	.	6p12.2	.	.	.	.	.	.	.	.	.	.	.
ENST0	.	6p12.1	.	.	.	.	.	.	.	.	.	.	.
ENST0	.	6q12	.	.	Scc	.	.	.	.	.	.	.	.
ENST0	.	6q13	.	.	.	.	.	.	.	.	.	.	.
ENST0	.	6q13	.	.	.	.	rs202	ID=COSN1	.	.	.	0.004	0.001
ENST0	.	6q13	.	.	.	.	.	.	.	.	.	.	.
ENST0	.	6q13	.	Scc	.	.	.	.	.	.	.	.	.
ENST0	.	6q14.1	.	.	.	.	rs776	.	.	.	.	.	.
ENST0	.	6q14.1	.	Scc	.	.	rs149	.	Lik	.	.	.	2E-04
ENST0	.	6q14.2	.	.	.	.	rs100	.	.	.	.	.	.
ENST0	.	6q15	.	.	.	.	rs761	.	.	.	.	.	.
ENST0	.	6q15	.	.	.	.	rs101	.	.	.	.	.	.
ENST0	.	6q15	.	.	.	Score=285	.	.	.	.	.	.	.
ENST0	.	6q16.1	.	.	.	.	.	.	.	.	.	.	.
ENST0	.	6q21	.	.	.	.	rs558	ID=COSN2	.	.	.	.	2E-04
ENST0	.	6q21	.	.	.	.	.	.	.	.	.	.	.
ENST0	.	6q21	.	.	.	.	.	.	.	.	.	.	.
ENST0	.	6q21	.	Scc	.	.	.	.	.	.	.	.	.
ENST0	.	6q21	.	.	.	.	.	.	.	.	.	.	.
ENST0	.	6q21	.	Scc	.	.	.	.	.	.	.	.	.
.	.	6q22.31	.	.	.	.	.	ID=COSN6	.	.	.	.	.
ENST0	.	6q22.31	.	.	.	.	.	.	.	.	.	.	.
ENST0	.	6q22.32	.	.	.	.	.	.	.	.	.	.	.
ENST0	.	6q22.33	.	.	.	.	rs375	.	.	.	.	.	2E-04
ENST0	.	6q22.33	.	.	.	.	.	.	.	.	.	.	.
ENST0	.	6q23.2	.	.	.	.	rs760	.	.	.	.	.	.
ENST0	.	6q23.3	.	.	.	Score=372	rs527	.	.	.	.	.	.
ENST0	.	6q23.3	.	.	.	Score=189	rs100	.	.	.	.	.	.
ENST0	.	6q23.3	.	.	.	.	.	.	.	.	.	.	.
ENST0	.	6q23.3	.	.	.	.	.	.	.	.	.	.	.
ENST0	.	6q24.1	.	.	.	.	rs191	.	.	.	.	0.005	1E-03
ENST0	.	6q24.2	.	Scc	.	.	rs140	.	.	.	.	.	.
ENST0	.	6q24.3	.	.	.	.	.	.	.	.	.	.	.
ENST0	.	6q25.1	.	Scc	.	.	rs773	ID=COSM3	.	.	.	.	.

ENST0	.	6q25.1	.	Scc	.	.	rs777	.	.	.	.	.	.
ENST0	.	6q25.1	.	Scc	.	.	.	.	.	.	.	.	.
ENST0	.	6q25.1	.	.	Scc	.	.	.	.	.	.	.	.
ENST0	.	6q25.1	.	.	.	.	.	.	.	.	.	.	.
ENST0	.	6q25.1	.	.	.	.	.	.	.	.	.	.	.
ENST0	.	6q25.2	.	.	.	.	rs529	.	.	.	.	.	.
ENST0	.	6q25.2	.	.	.	.	.	ID=COSM7	.	.	.	.	.
ENST0	.	6q25.2	.	.	.	.	.	.	.	.	.	.	.
ENST0	.	6q25.2	.	.	.	.	.	.	.	.	.	.	.
ENST0	.	6q25.3	.	.	.	.	.	ID=COSM3	.	.	.	.	.
ENST0	.	6q25.3	.	.	.	.	rs101	.	.	.	.	.	.
ENST0	.	6q25.3	.	Scc	.	.	.	.	.	.	.	.	.
ENST0	.	6q25.3	.	.	Scc	.	.	.	.	.	.	.	.
ENST0	.	6q25.3	.	.	.	Score=137	.	.	.	.	.	.	.
ENST0	.	6q26	.	.	Scc	.	.	.	.	.	.	.	.
ENST0	.	6q26	.	.	.	.	.	.	.	.	.	.	.
ENST0	.	6q26	.	.	.	.	.	.	.	.	.	.	.
ENST0	.	6q26	.	Scc	.	.	rs773	.	.	.	.	.	.
ENST0	.	6q27	.	.	.	.	rs200	ID=COSM3	.	.	.	.	4E-04
ENST0	.	6q27	.	.	Scc	.	rs755	.	.	.	.	.	.
ENST0	.	6q27	.	.	.	.	.	.	.	.	.	.	.
ENST0	.	7p22.3	.	.	.	.	.	.	.	.	.	.	.
.	.	7p22.3	.	.	.	.	.	.	.	.	.	.	.
ENST0	.	7p22.3	.	.	.	.	.	.	.	.	.	.	.
ENST0	.	7p22.3	.	.	.	.	.	.	.	.	.	.	.
ENST0	.	7p22.3	.	.	.	.	.	.	.	.	.	.	.
ENST0	.	7p22.3	.	Scc	.	.	rs778	.	.	.	.	.	.
ENST0	.	7p22.3	.	.	.	.	.	.	.	.	.	.	.
ENST0	.	7p22.3	.	.	.	Score=481	.	.	.	.	.	.	.
ENST0	.	7p22.3	.	.	.	.	rs561	.	.	.	.	.	2E-04
ENST0	.	7p22.2	.	.	.	.	.	.	.	.	.	.	.
ENST0	.	7p22.2	.	.	.	.	rs369	.	.	.	.	.	.
ENST0	.	7p22.2	.	.	.	.	.	.	.	.	.	.	.
ENST0	.	7p22.1	.	.	.	.	rs541	.	.	.	.	.	.
ENST0	.	7p22.1	.	.	Scc	.	.	.	.	.	.	.	.
ENST0	.	7p22.1	.	.	.	.	.	.	.	.	.	.	.
ENST0	.	7p22.1	.	.	.	.	rs774	.	.	.	.	.	.
ENST0	.	7p22.1	.	Scc	.	.	.	.	.	.	.	.	.
ENST0	.	7p22.1	.	.	.	.	.	.	.	.	.	.	.
ENST0	.	7p22.1	.	Scc	.	.	.	.	.	.	.	.	.
ENST0	.	7p22.1	.	.	Scc	.	rs780	ID=COSM5	.	.	.	.	.
ENST0	.	7p22.1	.	.	Scc	.	rs879	.	.	.	.	.	.
ENST0	.	7p22.1	.	.	Scc	.	rs372	ID=COSN1	.	.	.	.	.
ENST0	.	7p21.3	.	.	.	.	.	.	.	.	.	.	.
ENST0	.	7p21.3	.	.	.	.	.	ID=COSM4	.	.	.	.	.
ENST0	.	7p21.3	.	.	.	Score=193	.	.	.	.	.	.	.
ENST0	.	7p21.3	.	.	.	.	.	.	.	.	.	.	.
ENST0	.	7p21.1	.	.	.	.	.	.	.	.	.	.	.
ENST0	.	7p15.3	.	.	.	.	rs371	.	.	.	.	.	.
ENST0	.	7p15.3	.	.	.	Score=840	.	.	.	.	.	.	.
ENST0	.	7p15.3	.	.	.	.	.	.	.	.	.	.	.
ENST0	.	7p15.2	.	.	.	.	rs142	.	.	.	.	.	.
ENST0	.	7p14.3	.	.	.	.	.	.	.	.	.	.	.
.	.	7p14.3	.	.	.	.	.	.	.	.	.	.	.

ENST0	.	7p14.3	.	Scc	Scc	.	.	.	.	.	.	.	.
ENST0	.	7p14.3	.	.	Scc	.	rs750	.	.	.	.	.	.
ENST0	.	7p14.3	.	.	.	.	.	.	.	.	.	.	.
ENST0	.	7p14.2	.	.	Scc	.	.	.	.	.	.	.	.
ENST0	.	7p14.2	.	.	Scc	.	rs769	ID=COSN5	.	.	.	.	.
ENST0	.	7p14.1	.	Scc	Scc	.	.	.	.	.	.	.	.
ENST0	.	7p13	.	.	.	.	rs775	ID=COSM4	.	.	.	.	.
ENST0	.	7p13	.	.	.	.	.	.	.	.	.	.	.
ENST0	.	7p13	.	.	.	.	.	.	.	.	.	.	.
ENST0	.	7p13	.	.	.	Score=218	rs111	.	.	.	.	.	2E-04
ENST0	.	7p13	.	.	.	.	.	.	.	.	.	.	.
ENST0	.	7p13	.	.	.	.	.	.	.	.	.	.	.
ENST0	.	7p12.3	.	.	.	.	rs747	.	.	.	.	.	.
ENST0	.	7p12.3	.	.	.	.	rs102	.	.	.	.	.	.
ENST0	.	7p12.3	.	.	.	.	.	.	.	.	.	.	.
ENST0	.	7p12.3	.	.	.	.	.	.	.	.	.	.	.
ENST0	.	7p11.2	.	.	.	.	.	.	.	.	.	.	.
.	.	7p11.2	.	.	Scc	.	.	.	.	.	.	.	.
ENST0	.	7p11.2	.	.	Scc	.	.	.	.	.	.	.	.
ENST0	.	7p11.2	.	.	Scc	.	.	.	.	.	.	.	.
ENST0	.	7p11.2	.	.	Scc	.	rs374	.	.	.	.	.	.
ENST0	.	7p11.2	.	.	Scc	.	.	.	.	.	.	.	.
ENST0	.	7p11.2	.	.	Scc	.	rs879	ID=COSN1	.	.	.	.	.
ENST0	.	7p11.2	.	.	Scc	.	.	.	.	.	.	.	.
ENST0	.	7q11.21	.	.	Scc	.	.	.	.	.	.	.	.
ENST0	.	7q11.21	.	.	Scc	.	.	.	.	.	.	.	.
.	.	7q11.21	.	.	Scc	.	.	.	.	.	.	.	.
.	.	7q11.21	.	.	Scc	.	.	.	.	.	.	.	.
ENST0	.	7q11.21	.	.	Scc	Score=173	rs615	.	.	.	.	.	.
ENST0	.	7q11.21	.	.	Scc	.	.	ID=COSN1	.	.	.	.	.
ENST0	.	7q11.21	.	.	Scc	.	.	.	.	.	.	.	.
ENST0	.	7q11.21	.	.	Scc	.	rs548	ID=COSN1	.	.	.	0.015	0.019
ENST0	.	7q11.22	.	Scc	.	.	rs770	.	.	.	.	.	.
ENST0	.	7q11.23	.	.	Scc	Score=232	rs557	.	.	.	.	.	0.004
ENST0	.	7q11.23	.	.	Scc	.	.	.	.	.	.	.	.
ENST0	.	7q11.23	.	.	Scc	.	.	.	.	.	.	.	.
ENST0	.	7q11.23	.	.	.	.	.	.	.	.	.	.	.
ENST0	.	7q11.23	.	.	.	.	rs782	.	.	.	.	.	.
ENST0	.	7q11.23	.	.	.	.	.	.	.	.	.	.	.
ENST0	.	7q11.23	.	.	.	.	.	.	.	.	.	.	.
ENST0	.	7q11.23	.	.	.	.	rs782	.	.	.	.	.	.
ENST0	.	7q11.23	.	.	Scc	.	rs370	.	.	.	.	.	.
ENST0	.	7q11.23	.	.	Scc	.	rs879	.	.	.	.	.	.
ENST0	.	7q11.23	.	.	Scc	.	rs149	.	.	.	.	0.569	0.598
ENST0	.	7q11.23	.	.	Scc	.	rs140	.	.	.	.	.	.
ENST0	.	7q21.11	.	.	.	.	.	.	.	.	.	.	.
ENST0	.	7q21.11	.	.	.	.	.	.	.	.	.	.	.
ENST0	.	7q21.11	.	.	.	.	.	.	.	.	.	.	.
ENST0	.	7q21.13	.	.	.	.	rs141	.	.	.	.	.	6E-04
ENST0	.	7q21.3	.	.	.	.	rs987	.	.	.	.	.	.
ENST0	.	7q21.3	.	Scc	.	.	rs764	ID=COSM5	.	.	.	.	.
ENST0	.	7q21.3	.	.	.	.	.	.	.	.	.	.	.
ENST0	.	7q21.3	.	.	.	.	.	.	.	.	.	.	.
ENST0	.	7q21.3	.	.	.	.	.	ID=COSM1	.	.	.	.	.

ENST0	.	7q22.1	.	.	.	.	rs782	.	.	.	.	.	.
ENST0	.	7q22.1	.	Scc	.	.	rs775	ID=COSM3	.	.	.	.	.
ENST0	.	7q22.1	.	.	.	.	.	.	.	.	.	.	.
ENST0	.	7q22.1	.	.	.	.	rs903	ID=COSN1	.	.	.	.	.
ENST0	.	7q22.1	.	Scc	.	.	rs576	.	.	.	.	.	.
ENST0	.	7q22.1	.	Scc	.	.	.	.	.	.	.	.	.
ENST0	.	7q22.1	.	.	Scc	.	rs764	.	.	.	.	.	.
ENST0	.	7q22.1	.	.	.	.	.	.	.	.	.	.	.
ENST0	.	7q22.1	.	.	.	.	.	.	.	.	.	.	.
ENST0	.	7q22.1	.	.	Scc	.	rs759	.	.	.	.	.	.
ENST0	.	7q22.1	.	.	Scc	.	rs472	ID=COSM4	.	.	.	.	.
ENST0	.	7q22.1	.	.	.	.	.	.	.	.	.	.	.
ENST0	.	7q22.2	.	Scc	.	.	.	.	.	.	.	.	.
ENST0	.	7q31.1	.	Scc	.	.	.	.	.	.	.	.	.
ENST0	.	7q31.1	.	.	.	.	.	.	.	.	.	.	.
ENST0	.	7q31.2	.	.	.	.	.	.	.	.	.	.	.
ENST0	.	7q31.32	.	.	.	.	rs145	.	.	.	.	.	0.001
ENST0	.	7q31.33	.	.	.	.	.	.	.	.	.	.	.
ENST0	.	7q31.33	.	.	.	.	rs375	.	.	.	.	.	.
ENST0	.	7q32.1	.	.	Scc	.	.	.	.	.	.	.	.
ENST0	.	7q32.1	.	.	Scc	.	.	.	.	.	.	.	.
ENST0	.	7q32.1	.	.	.	.	rs200	.	.	.	.	.	2E-04
.	.	7q32.1	.	.	.	.	.	.	.	.	.	.	.
ENST0	.	7q32.3	.	.	.	.	rs367	.	.	.	.	.	.
.	.	7q33	.	Scc	.	.	.	.	.	.	.	.	.
ENST0	.	7q33	.	.	.	.	.	.	.	.	.	.	.
ENST0	.	7q33	.	.	.	.	.	.	.	.	.	.	.
ENST0	.	7q33	.	.	.	.	.	.	.	.	.	.	.
ENST0	.	7q34	.	.	.	.	rs182	.	.	.	.	0.007	0.002
ENST0	.	7q34	.	.	.	.	.	.	.	.	.	.	.
.	.	7q34	.	.	.	.	.	.	.	.	.	.	.
ENST0	.	7q34	.	.	.	.	.	.	.	.	.	.	.
ENST0	.	7q34	.	.	.	.	rs782	ID=COSM1	.	.	.	.	.
ENST0	.	7q34	.	.	.	.	.	.	.	.	.	.	.
ENST0	.	7q34	.	.	.	.	.	.	.	.	.	.	.
ENST0	.	7q34	.	.	.	.	.	.	.	.	.	.	.
ENST0	.	7q34	.	.	Scc	.	.	.	.	.	.	.	.
.	.	7q34	.	.	.	.	rs766	.	.	.	.	.	.
ENST0	.	7q34	.	.	.	.	.	.	.	.	.	.	.
ENST0	.	7q34	.	.	.	.	rs373	ID=COSN2	.	.	.	.	.
ENST0	.	7q35	.	.	.	.	rs759	.	.	.	.	.	.
ENST0	.	7q36.1	.	.	.	.	rs801	.	.	.	.	0.013	0.003
ENST0	.	7q36.1	.	.	.	.	rs144	.	.	.	.	.	4E-04
ENST0	.	7q36.1	.	.	.	.	.	.	.	.	.	.	.
ENST0	.	7q36.1	.	.	.	.	.	.	.	.	.	.	.
ENST0	.	7q36.1	.	.	.	.	.	.	.	.	.	.	.
ENST0	.	7q36.1	.	Scc	.	.	.	.	.	.	.	.	.
ENST0	.	7q36.1	.	Scc	.	.	.	.	.	.	.	.	.
ENST0	.	7q36.1	.	.	.	.	.	.	.	.	.	.	.
ENST0	.	7q36.1	.	.	.	.	.	.	.	.	.	.	.
ENST0	.	7q36.1	.	.	.	.	rs376	.	.	.	.	.	.
ENST0	.	7q36.1	.	.	Scc	.	.	.	.	.	.	.	.
ENST0	.	7q36.2	.	.	Scc	.	rs936	.	.	.	.	.	.
ENST0	.	7q36.3	.	.	.	.	.	.	.	.	.	.	.

ENST0	.	7q36.3	.	.	.	Score=183	rs105	.	.	.	.	.	.
ENST0	.	7q36.3	.	.	.	.	rs369	.	.	.	.	.	.
ENST0	.	7q36.3	.	.	.	.	.	.	.	.	.	.	.
ENST0	.	8p23.3	.	.	.	.	.	.	.	.	.	.	.
ENST0	.	8p23.3	.	Scc	.	.	.	.	.	.	.	.	.
ENST0	.	8p23.1	.	.	.	.	rs199	.	.	.	.	.	.
ENST0	.	8p23.1	.	.	.	.	.	.	.	.	.	.	.
ENST0	.	8p23.1	.	.	Scc	.	rs769	.	.	.	.	.	.
.	.	8p23.1	.	.	Scc	Score=212	rs294	ID=COSN2	.	.	.	.	.
ENST0	.	8p23.1	.	.	.	.	rs781	.	.	.	.	.	.
ENST0	.	8p23.1	.	.	.	.	rs760	.	.	.	.	.	.
ENST0	.	8p23.1	.	.	.	.	.	.	.	.	.	.	.
ENST0	.	8p23.1	.	.	.	Score=221	rs751	.	.	.	.	.	.
ENST0	.	8p23.1	.	.	Scc	.	rs762	.	.	.	.	.	.
ENST0	.	8p23.1	.	.	.	.	.	.	.	.	.	.	.
ENST0	.	8p23.1	.	.	Scc	.	.	.	.	.	.	.	.
ENST0	.	8p23.1	.	.	Scc	.	rs398	.	.	.	.	0.425	0.244
ENST0	.	8p23.1	.	.	Scc	Score=305	rs371	.	.	.	.	.	.
ENST0	.	8p22	.	.	.	.	rs103	.	.	.	.	.	.
ENST0	.	8p22	.	Scc	.	.	.	.	.	.	.	.	.
.	.	8p21.3	.	.	.	Score=909	rs147	ID=COSN1	.	.	.	.	.
.	.	8p21.3	.	.	.	Score=909	rs139	.	.	.	.	.	.
ENST0	.	8p21.3	.	.	.	.	.	.	.	.	.	.	.
ENST0	.	8p21.3	.	.	.	.	.	.	.	.	.	.	.
ENST0	.	8p21.3	.	.	.	.	.	.	.	.	.	.	.
ENST0	.	8p21.2	.	.	.	.	.	.	.	.	.	.	.
ENST0	.	8p21.2	.	.	.	.	rs769	.	.	.	.	.	.
ENST0	.	8p21.2	.	.	.	.	rs201	.	.	.	.	0.001	2E-04
ENST0	.	8p21.2	.	.	.	.	.	.	.	.	.	.	.
ENST0	.	8p21.2	.	.	.	.	rs724	.	.	.	.	.	.
ENST0	.	8p12	.	.	.	.	.	.	.	.	.	.	.
ENST0	.	8p12	.	.	.	.	rs141	ID=COSM1	.	.	.	.	.
ENST0	.	8p12	.	.	.	.	rs119	.	.	.	.	.	0.031
ENST0	.	8p11.23	.	.	.	.	rs532	.	.	.	.	.	.
ENST0	.	8p11.23	.	.	.	Score=324	.	.	.	.	.	.	.
ENST0	.	8p11.23	.	.	.	Score=217	rs761	.	.	.	.	.	.
ENST0	.	8p11.23	.	.	.	.	.	.	.	.	.	.	.
ENST0	.	8p11.22	.	.	.	.	rs760	.	.	.	.	.	.
ENST0	.	8p11.22	.	.	.	.	.	.	.	.	.	.	.
ENST0	.	8p11.21	.	.	.	.	.	.	.	.	.	.	.
ENST0	.	8p11.21	.	.	.	.	rs370	.	.	.	.	.	.
ENST0	.	8p11.21	.	.	.	.	rs760	.	.	.	.	.	.
.	.	8p11.1	.	.	.	Score=126	.	.	.	.	.	.	.
.	.	8p11.1	.	.	.	Score=126	rs373	.	.	.	.	.	.
.	.	8p11.1	.	.	.	Score=126	rs200	ID=COSN2	.	.	.	.	.
.	.	8p11.1	.	.	.	Score=126	.	.	.	.	.	.	.
.	.	8q11.1	.	.	.	Score=124	rs449	.	.	.	.	.	.
.	.	8q11.1	.	.	.	Score=124	.	.	.	.	.	.	.
.	.	8q11.1	.	.	.	Score=124	rs440	.	.	.	.	.	.
ENST0	.	8q11.21	.	.	.	.	.	.	.	.	.	.	.
ENST0	.	8q11.21	.	.	.	.	.	.	.	.	.	.	.
ENST0	.	8q11.23	.	.	.	.	.	.	.	.	.	.	.
ENST0	.	8q12.1	.	Scc	.	.	rs187	.	.	.	.	.	.
ENST0	.	8q12.1	.	Scc	.	.	.	.	.	.	.	.	.

ENST0	.	8q12.1	.	.	.	.	.	.	.	.	.	.	.
ENST0	.	8q12.1	.	.	.	.	rs779	.	.	.	.	.	.
ENST0	.	8q12.2	.	.	.	.	rs151	.	Lik	.	.	.	4E-04
ENST0	.	8q13.1	.	.	.	Score=318	rs889	.	.	.	.	.	.
ENST0	.	8q13.2	.	.	.	Score=204	.	.	.	.	.	.	.
ENST0	.	8q13.2	.	.	.	Score=186	.	.	.	.	.	.	.
ENST0	.	8q13.2	.	.	.	.	rs745	.	.	.	.	.	.
ENST0	.	8q21.11	.	.	.	.	.	.	.	.	.	.	.
.	.	8q21.11	.	.	.	Score=972	.	ID=COSN1	.	.	.	.	.
ENST0	.	8q21.13	.	.	.	.	.	.	.	.	.	.	.
ENST0	.	8q21.3	.	.	.	.	.	.	.	.	.	.	.
ENST0	.	8q21.3	.	.	.	.	.	.	.	.	.	.	.
ENST0	.	8q21.3	.	.	.	.	rs103	.	.	.	.	.	.
ENST0	.	8q21.3	.	Scc	.	.	rs755	ID=COSM4	.	.	.	.	.
ENST0	.	8q21.3	.	Scc	.	.	.	.	.	.	.	.	.
ENST0	.	8q21.3	.	Scc	.	.	.	.	.	.	.	.	.
ENST0	.	8q22.1	.	.	.	.	.	.	.	.	.	.	.
ENST0	.	8q22.1	.	.	.	.	rs100	.	.	.	.	.	.
ENST0	.	8q22.1	.	.	.	.	.	.	.	.	.	.	.
ENST0	.	8q22.3	.	.	.	.	.	.	.	.	.	.	.
ENST0	.	8q22.3	.	.	.	.	.	.	.	.	.	.	.
ENST0	.	8q23.1	.	.	.	.	.	.	.	.	.	.	.
ENST0	.	8q23.2	.	.	.	.	.	.	.	.	.	.	.
ENST0	.	8q24.13	.	Scc	.	.	rs149	.	.	.	.	.	6E-04
ENST0	.	8q24.13	.	.	.	.	.	ID=COSM5	.	.	.	.	.
ENST0	.	8q24.13	.	.	.	Score=520	rs762	.	.	.	.	.	.
ENST0	.	8q24.21	.	.	.	.	.	.	.	.	.	.	.
ENST0	.	8q24.22	.	.	.	.	rs359	.	Be	.	.	.	0.017
ENST0	.	8q24.22	.	Scc	.	.	rs168	.	.	.	.	.	0.011
ENST0	.	8q24.3	.	.	.	.	.	.	.	.	.	.	.
ENST0	.	8q24.3	.	.	.	.	.	.	.	.	.	.	.
ENST0	.	8q24.3	.	Scc	.	.	.	.	.	.	.	.	.
ENST0	.	8q24.3	.	.	.	Score=440	.	.	.	.	.	.	.
ENST0	.	8q24.3	.	.	.	.	.	.	.	.	.	.	.
ENST0	.	8q24.3	.	.	.	.	.	.	.	.	.	.	.
ENST0	.	8q24.3	.	.	.	.	rs759	ID=COSM1	.	.	.	.	.
ENST0	.	8q24.3	.	.	.	.	rs564	ID=COSM3	.	.	.	0.001	2E-04
ENST0	.	8q24.3	.	.	.	.	.	.	.	.	.	.	.
ENST0	.	8q24.3	.	.	.	.	rs781	ID=COSM3	.	.	.	.	.
ENST0	.	8q24.3	.	.	.	.	.	ID=COSM1	.	.	.	.	.
ENST0	.	8q24.3	.	.	.	.	.	.	.	.	.	.	.
ENST0	.	8q24.3	.	.	.	.	rs200	.	Lik	.	.	.	1E-03
ENST0	.	8q24.3	.	.	.	.	rs556	.	.	.	.	.	0.002
ENST0	.	8q24.3	.	Scc	.	.	.	.	.	.	.	.	.
ENST0	.	8q24.3	.	.	.	.	.	.	.	.	.	.	.
ENST0	.	8q24.3	.	.	.	.	rs746	.	.	.	.	.	.
ENST0	.	9p24.3	.	.	Scc	.	rs712	ID=COSN2	.	.	.	0.208	0.278
ENST0	.	9p24.2	.	.	.	.	rs898	.	.	.	.	.	.
ENST0	.	9p23	.	Scc	.	.	.	.	.	.	.	.	.
ENST0	.	9p23	.	.	.	.	rs759	.	.	.	.	.	.
ENST0	.	9p22.3	.	.	.	.	.	.	.	.	.	.	.
ENST0	.	9p22.3	.	.	.	.	rs755	ID=COSM4	.	.	.	.	.
ENST0	.	9p22.3	.	.	.	.	.	.	.	.	.	.	.
ENST0	.	9p22.3	.	Scc	.	.	.	.	.	.	.	.	.

ENST0	.	9p22.2	.	Scc	.	.	rs771	.	.	.	.	.	.
ENST0	.	9p22.1	.	.	.	.	.	.	.	.	.	.	.
ENST0	.	9p21.3	.	Scc	.	.	rs755	ID=COSM3	.	.	.	.	.
ENST0	.	9p21.3	.	.	.	Score=301	rs898	.	.	.	.	.	.
ENST0	.	9p21.1	.	.	.	.	rs770	.	.	.	.	.	.
ENST0	.	9p13.3	.	.	.	.	.	.	.	.	.	.	.
ENST0	.	9p13.3	.	.	.	.	.	ID=COSM1	.	.	.	.	.
ENST0	.	9p13.2	.	Scc	.	.	.	.	.	.	.	.	.
ENST0	.	9p13.2	.	.	.	.	.	ID=COSM3	.	.	.	.	.
ENST0	.	9p13.1	.	.	Scc	.	rs161	ID=COSN1	.	.	.	.	.
ENST0	.	9p11.2	.	.	Scc	Score=23;I	rs782	ID=COSM4	.	.	.	.	.
.	.	9p11.2	.	.	Scc	Score=266	rs625	.	.	.	.	.	.
ENST0	.	9q12	.	.	Scc	.	.	.	.	.	.	.	.
ENST0	.	9q13	.	.	Scc	.	rs200	ID=COSN2	.	.	.	.	.
.	.	9q13	.	.	Scc	Score=865	rs201	.	.	.	.	.	.
.	.	9q13	.	.	Scc	.	rs201	.	.	.	.	.	.
ENST0	.	9q13	.	.	Scc	.	rs376	ID=COSN1	.	.	.	.	.
ENST0	.	9q13	.	.	Scc	.	.	.	.	.	.	.	.
ENST0	.	9q21.11	.	.	Scc	.	.	.	.	.	.	.	.
ENST0	.	9q21.11	.	.	Scc	.	rs750	.	.	.	.	.	.
ENST0	.	9q21.12	.	.	.	Score=743	.	.	.	.	.	.	.
ENST0	.	9q21.13	.	Scc	.	.	.	.	.	.	.	.	.
ENST0	.	9q21.13	.	.	.	.	.	.	.	.	.	.	.
ENST0	.	9q21.2	.	.	.	.	rs966	.	.	.	.	.	.
ENST0	.	9q21.31	.	.	.	.	rs529	.	.	.	.	.	.
.	.	9q21.31	.	.	.	.	.	.	.	.	.	.	.
ENST0	.	9q21.32	.	.	Scc	.	rs569	ID=COSM1	.	.	.	.	2E-04
ENST0	.	9q21.32	.	.	Scc	.	.	.	.	.	.	.	.
ENST0	.	9q21.32	.	.	.	.	rs774	.	.	.	.	.	.
ENST0	.	9q21.33	.	.	.	.	.	.	.	.	.	.	.
ENST0	.	9q21.33	.	.	.	.	.	.	.	.	.	.	.
ENST0	.	9q22.1	.	.	Scc	.	rs936	.	.	.	.	.	.
ENST0	.	9q22.1	.	.	.	.	rs144	.	.	.	.	.	8E-04
ENST0	.	9q22.31	.	.	.	.	.	.	.	.	.	.	.
ENST0	.	9q22.31	.	.	.	Score=195	.	.	.	.	.	.	.
ENST0	.	9q22.31	.	.	.	.	rs535	.	.	.	.	.	4E-04
ENST0	.	9q22.31	.	.	.	.	.	ID=COSN2	.	.	.	.	.
ENST0	.	9q22.31	.	Scc	.	.	.	.	.	.	.	.	.
ENST0	.	9q22.32	.	.	.	.	.	.	.	.	.	.	.
ENST0	.	9q22.33	.	.	.	.	rs575	.	.	.	.	.	6E-04
ENST0	.	9q22.33	.	.	.	.	rs566	ID=COSM1	.	.	.	.	2E-04
ENST0	.	9q22.33	.	.	.	.	rs534	.	.	.	.	0.001	6E-04
ENST0	.	9q31.2	.	.	.	Score=139	rs527	.	.	.	.	.	.
ENST0	.	9q31.3	.	.	.	.	.	.	.	.	.	.	.
ENST0	.	9q32	.	Scc	.	.	rs941	.	.	.	.	.	.
ENST0	.	9q32	.	Scc	.	.	rs753	.	.	.	.	.	.
ENST0	.	9q32	.	.	.	Score=939	.	.	.	.	.	.	.
ENST0	.	9q32	.	.	.	.	.	.	.	.	.	.	.
ENST0	.	9q32	.	.	.	.	rs101	.	.	.	.	.	.
ENST0	.	9q32	.	.	.	.	.	.	.	.	.	.	.
ENST0	.	9q32	.	.	.	.	rs529	.	.	.	.	.	4E-04
ENST0	.	9q32	.	.	.	Score=226	.	.	.	.	.	.	.
ENST0	.	9q33.1	.	Scc	.	.	rs539	ID=COSM1	.	.	.	.	.
ENST0	.	9q33.2	.	.	.	.	rs577	.	.	.	.	.	1E-03

ENST0	.	9q33.2	.	.	.	.	rs776	.	.	.	.	.	.
ENST0	.	9q33.2	.	.	.	.	.	.	.	.	.	.	.
ENST0	.	9q33.2	.	Scc	.	.	rs781	.	.	.	.	.	.
ENST0	.	9q33.2	.	Scc	.	.	.	.	.	.	.	.	.
ENST0	.	9q33.3	.	.	.	.	.	.	.	.	.	.	.
ENST0	.	9q33.3	.	.	.	Score=253	rs767	.	.	.	.	.	.
ENST0	.	9q33.3	.	.	.	.	.	.	.	.	.	.	.
ENST0	.	9q33.3	.	.	.	.	.	.	.	.	.	.	.
ENST0	.	9q33.3	.	.	.	.	rs770	.	.	.	.	.	.
ENST0	.	9q34.11	.	.	.	.	rs751	.	.	.	.	.	.
ENST0	.	9q34.11	.	.	.	.	.	.	.	.	.	.	.
ENST0	.	9q34.11	.	.	.	.	rs772	ID=COSM3	.	.	.	.	.
ENST0	.	9q34.11	.	.	.	.	rs375	.	.	.	.	.	.
ENST0	.	9q34.11	.	Scc	.	.	rs770	.	.	.	.	.	.
ENST0	.	9q34.11	.	.	.	.	rs370	.	Lik	.	.	.	.
ENST0	.	9q34.11	.	Scc	.	.	rs102	.	.	.	.	.	.
ENST0	.	9q34.11	.	.	.	.	.	.	.	.	.	.	.
ENST0	.	9q34.11	.	.	.	.	.	.	.	.	.	.	.
ENST0	.	9q34.11	.	.	.	.	rs120	.	.	.	.	.	0.005
ENST0	.	9q34.11	.	.	.	Score=311	rs483	ID=COSN1	.	.	.	.	.
ENST0	.	9q34.11	.	.	.	Score=311	rs483	ID=COSN1	.	.	.	.	.
ENST0	.	9q34.11	.	.	.	.	rs769	.	.	.	.	.	.
ENST0	.	9q34.11	.	.	.	.	.	.	.	.	.	.	.
ENST0	.	9q34.11	.	Scc	.	.	.	ID=COSM4	.	.	.	.	.
ENST0	.	9q34.13	.	.	.	.	rs937	ID=COSM1	.	.	.	.	.
ENST0	.	9q34.13	.	.	.	.	.	.	.	.	.	.	.
ENST0	.	9q34.13	.	.	.	.	.	.	.	.	.	.	.
ENST0	.	9q34.13	.	.	.	.	rs449	.	.	.	.	0.038	0.087
ENST0	.	9q34.2	.	.	.	.	.	.	.	.	.	.	.
ENST0	.	9q34.2	.	.	.	Score=387	.	.	.	.	.	.	.
ENST0	.	9q34.2	.	.	.	.	.	.	.	.	.	.	.
ENST0	.	9q34.2	.	.	.	.	.	.	.	.	.	.	.
ENST0	.	9q34.2	.	.	.	.	rs782	ID=COSM6	.	.	.	.	.
ENST0	.	9q34.2	.	.	.	.	rs780	.	.	.	.	.	.
ENST0	.	9q34.2	.	.	.	.	.	.	.	.	.	.	.
ENST0	.	9q34.3	.	.	.	.	.	.	.	.	.	.	.
ENST0	.	9q34.3	.	.	.	.	rs617	.	Be	.	.	.	2E-04
ENST0	.	9q34.3	.	.	.	.	.	.	.	.	.	.	.
ENST0	.	9q34.3	.	.	.	.	rs747	.	.	.	.	.	.
ENST0	.	9q34.3	.	.	.	.	.	.	.	.	.	.	.
ENST0	.	9q34.3	.	.	.	.	rs994	ID=COSN2	.	.	.	.	.
ENST0	.	9q34.3	.	.	.	.	.	.	.	.	.	.	.
ENST0	.	9q34.3	.	.	.	.	.	.	.	.	.	.	.
ENST0	.	9q34.3	.	.	.	.	.	.	.	.	.	.	.
ENST0	.	9q34.3	.	.	.	Score=445	.	.	.	.	.	.	.
ENST0	.	9q34.3	.	.	.	.	rs139	.	.	.	.	.	.
ENST0	.	9q34.3	.	.	.	.	rs565	.	.	.	.	.	2E-04
ENST0	.	9q34.3	.	.	.	.	rs778	.	.	.	.	.	.
ENST0	.	9q34.3	.	.	.	.	.	.	.	.	.	.	.
ENST0	.	9q34.3	.	.	.	.	rs777	ID=COSM5	.	.	.	.	.
ENST0	.	9q34.3	.	.	.	.	rs112	.	.	.	.	0.001	8E-04
ENST0	.	9q34.3	.	.	.	.	.	.	.	.	.	.	.
ENST0	.	9q34.3	.	.	Scc	.	rs370	.	.	.	.	.	.
ENST0	.	10p15.3	.	.	.	.	rs754	.	.	.	.	.	.

ENST0	.	10p15.3	.	.	.	Score=602	.	.	.	.	.	.	.
ENST0	.	10p15.2	.	.	.	.	rs568	.	.	.	.	0.001	2E-04
ENST0	.	10p15.1	.	.	.	Score=234	.	.	.	.	.	.	.
ENST0	.	10p14	.	.	.	.	rs201	ID=COSM1	.	.	.	.	4E-04
ENST0	.	10p14	.	.	.	Score=128	rs796	.	.	.	.	.	.
ENST0	.	10p14	.	.	.	.	rs370	ID=COSM3	.	.	.	.	.
ENST0	.	10p14	.	.	.	.	.	.	.	.	.	.	.
ENST0	.	10p14	.	.	.	.	.	.	.	.	.	.	.
.	.	10p14	.	.	.	Score=746	.	.	.	.	.	.	.
.	.	10p14	.	.	.	.	.	.	.	.	.	.	.
ENST0	.	10p13	.	.	Scc	Score=181	rs191	.	.	.	.	.	6E-04
ENST0	.	10p13	.	.	.	.	rs765	ID=COSN6	.	.	.	0.03	0.007
ENST0	.	10p13	.	.	.	.	.	.	.	.	.	.	.
ENST0	.	10p12.31	.	.	.	.	.	.	.	.	.	.	.
ENST0	.	10p12.31	.	.	.	.	rs965	.	.	.	.	.	.
ENST0	.	10p12.1	.	.	.	.	.	.	.	.	.	.	.
ENST0	.	10p12.1	.	Scc	Scc	.	rs764	.	.	.	.	.	.
ENST0	.	10p12.1	.	.	.	.	rs199	.	.	.	.	.	0.003
ENST0	.	10p12.1	.	.	.	.	.	.	.	.	.	.	.
ENST0	.	10p12.1	.	.	.	.	.	ID=COSN5	.	.	.	.	.
ENST0	.	10p12.1	.	.	.	.	rs367	ID=COSM2	Ur	.	.	.	.
ENST0	.	10p12.1	.	.	.	.	.	.	.	.	.	.	.
ENST0	.	10p12.1	.	.	Scc	.	.	ID=COSN1	.	.	.	.	.
ENST0	.	10p11.23	.	.	Scc	.	rs776	.	.	.	.	.	.
ENST0	.	10p11.23	.	.	Scc	.	rs573	.	.	.	.	.	2E-04
ENST0	.	10p11.21	.	Scc	.	.	.	ID=COSM1	.	.	.	.	.
.	.	10q11.21	.	.	Scc	.	.	.	.	.	.	.	.
ENST0	.	10q11.22	.	.	Scc	.	.	ID=COSN6	.	.	.	.	.
ENST0	.	10q11.22	.	.	Scc	.	.	.	.	.	.	.	.
ENST0	.	10q11.22	.	.	Scc	.	.	.	.	.	.	.	.
ENST0	.	10q11.22	.	.	Scc	.	.	.	.	.	.	.	.
ENST0	.	10q11.23	.	.	.	.	.	.	.	.	.	.	.
ENST0	.	10q11.23	.	.	.	.	rs904	ID=COSM1	.	.	.	.	.
ENST0	.	10q11.23	.	.	.	.	rs374	.	.	.	.	.	.
ENST0	.	10q11.23	.	.	Scc	.	.	.	.	.	.	.	.
ENST0	.	10q21.1	.	.	.	.	rs751	.	.	.	.	.	.
.	.	10q21.1	.	.	.	Score=474	.	.	.	.	.	.	.
ENST0	.	10q21.1	.	.	.	.	rs138	ID=COSM4	.	.	.	.	6E-04
ENST0	.	10q21.2	.	.	.	.	rs573	.	.	.	.	.	8E-04
ENST0	.	10q21.3	.	.	.	Score=101	.	.	.	.	.	.	.
ENST0	.	10q22.1	.	.	.	.	.	.	.	.	.	.	.
ENST0	.	10q22.1	.	.	.	.	rs754	ID=COSM9	.	.	.	.	.
ENST0	.	10q22.1	.	Scc	.	.	.	.	.	.	.	.	.
ENST0	.	10q22.1	.	.	.	.	rs374	.	.	.	.	.	.
ENST0	.	10q22.1	.	Scc	.	.	rs201	ID=COSM1	Lik	.	.	.	2E-04
ENST0	.	10q22.1	.	Scc	.	.	.	.	.	.	.	.	.
ENST0	.	10q22.2	.	.	.	.	.	.	.	.	.	.	.
ENST0	.	10q22.2	.	.	.	.	rs775	.	.	.	.	.	.
ENST0	.	10q22.2	.	.	Scc	.	rs780	ID=COSN2	.	.	.	.	.
ENST0	.	10q22.2	.	.	Scc	Score=233	rs878	.	.	.	.	.	.
ENST0	.	10q22.2	.	.	Scc	.	.	.	.	.	.	.	.
ENST0	.	10q22.3	.	.	.	.	.	.	.	.	.	.	.
ENST0	.	10q22.3	.	.	.	.	.	.	.	.	.	.	.
ENST0	.	10q23.1	.	Scc	.	.	rs568	.	.	.	.	0.001	2E-04

ENST0	.	10q23.1	.	.	.	.	.	.	.	.	.	.	.
ENST0	.	10q23.1	.	.	.	.	.	.	.	.	.	.	.
ENST0	.	10q23.1	.	.	.	.	.	.	.	.	.	.	.
.	.	10q23.32	.	.	.	Score=895	rs529	.	.	.	.	.	.
ENST0	.	10q23.33	.	.	.	.	.	.	.	.	.	.	.
ENST0	.	10q23.33	.	.	.	.	rs369	.	.	.	.	.	.
ENST0	.	10q23.33	.	.	.	.	rs753	.	.	.	.	.	.
ENST0	.	10q23.33	.	.	.	.	.	.	.	.	.	.	.
ENST0	.	10q24.1	.	.	.	Score=890	rs904	.	.	.	.	.	.
ENST0	.	10q24.1	.	.	.	.	rs782	.	.	.	.	.	.
ENST0	.	10q24.1	.	.	.	.	.	.	.	.	.	.	.
ENST0	.	10q24.2	.	.	.	Score=204	rs376	.	.	.	.	.	.
ENST0	.	10q24.2	.	.	.	.	rs994	.	.	.	.	.	.
ENST0	.	10q24.2	.	.	.	.	rs539	ID=COSM3	.	.	.	.	.
ENST0	.	10q24.2	.	Scc	.	.	rs369	.	.	.	.	.	.
ENST0	.	10q24.31	.	.	.	.	rs944	.	.	.	.	.	.
ENST0	.	10q24.32	.	.	.	.	.	.	.	.	.	.	.
ENST0	.	10q24.32	.	.	.	.	.	.	.	.	.	.	.
ENST0	.	10q24.32	.	.	.	.	.	.	.	.	.	.	.
ENST0	.	10q25.1	.	.	.	.	.	.	.	.	.	.	.
ENST0	.	10q25.1	.	.	.	.	.	.	.	.	.	.	.
ENST0	.	10q25.1	.	.	.	.	.	.	.	.	.	.	.
ENST0	.	10q25.3	.	.	.	Score=297	.	.	.	.	.	.	.
ENST0	.	10q25.3	.	.	.	.	.	.	.	.	.	.	.
ENST0	.	10q26.11	.	.	.	Score=240	.	.	.	.	.	.	.
ENST0	.	10q26.13	.	.	.	.	.	.	.	.	.	.	.
ENST0	.	10q26.13	.	.	.	.	rs750	ID=COSM2	.	.	.	.	.
ENST0	.	10q26.2	.	.	.	.	.	.	.	.	.	.	.
ENST0	.	10q26.2	.	.	.	.	rs376	.	.	.	.	.	.
ENST0	.	10q26.2	.	.	.	.	.	ID=COSM1	.	.	.	.	.
ENST0	.	10q26.3	.	.	.	.	rs545	.	.	.	.	.	2E-04
ENST0	.	10q26.3	.	.	.	.	.	.	.	.	.	.	.
ENST0	.	10q26.3	.	.	.	.	.	.	.	.	.	.	.
ENST0	.	10q26.3	.	.	.	.	rs539	.	.	.	.	.	2E-04
ENST0	.	10q26.3	.	Scc	.	.	.	.	.	.	.	.	.
ENST0	.	10q26.3	.	Scc	.	.	.	.	.	.	.	.	.
ENST0	.	10q26.3	.	.	.	.	rs779	ID=COSN2	.	.	.	.	.
ENST0	.	10q26.3	.	.	.	.	.	.	.	.	.	.	.
ENST0	.	10q26.3	.	.	.	.	.	.	.	.	.	.	.
ENST0	.	11p15.5	.	.	Scc	.	.	.	.	.	.	.	.
ENST0	.	11p15.5	.	.	.	.	rs200	ID=COSN2	.	.	.	.	.
ENST0	.	11p15.5	.	.	.	.	.	.	.	.	.	.	.
ENST0	.	11p15.5	.	.	.	.	rs100	.	.	.	.	.	.
ENST0	.	11p15.5	.	.	.	.	rs779	.	.	.	.	.	.
ENST0	.	11p15.5	.	Scc	.	.	rs374	.	.	.	.	.	.
ENST0	.	11p15.5	.	.	.	.	.	.	.	.	.	.	.
ENST0	.	11p15.5	.	.	.	.	.	.	.	.	.	.	.
ENST0	.	11p15.5	.	.	.	.	rs896	.	.	.	.	.	.
ENST0	.	11p15.5	.	.	.	.	rs758	.	.	.	.	.	.
ENST0	.	11p15.5	.	.	.	.	rs751	.	.	.	.	.	.
ENST0	.	11p15.5	.	.	.	.	.	.	.	.	.	.	.
ENST0	.	11p15.5	.	.	.	.	.	ID=COSM3	.	.	.	.	.
ENST0	.	11p15.5	.	Scc	.	.	.	ID=COSM4	.	.	.	.	.
ENST0	.	11p15.5	.	.	.	.	rs368	ID=COSM1	Ur	.	.	.	.

ENST0	.	11p15.5	.	.	.	.	.	.	.	.	.	.	.
.	.	11p15.5	.	.	.	.	rs101	.	.	.	.	.	.
ENST0	.	11p15.5	.	.	.	Score=138	rs772	.	.	.	.	.	.
ENST0	.	11p15.5	.	.	.	.	.	.	.	.	.	.	.
ENST0	.	11p15.5	.	.	.	.	.	.	.	.	.	.	.
.	.	11p15.4	.	.	Scc	.	rs559	ID=COSN2	.	.	.	0.115	0.18
ENST0	.	11p15.4	.	.	.	.	.	.	.	.	.	.	.
ENST0	.	11p15.4	.	.	.	.	.	ID=COSM4	.	.	.	.	.
ENST0	.	11p15.4	.	.	.	Score=351	rs866	ID=COSN1	.	.	.	.	.
ENST0	.	11p15.4	.	.	.	.	.	.	.	.	.	.	.
ENST0	.	11p15.4	.	.	.	.	rs728	.	.	.	.	0.001	0.01
ENST0	.	11p15.4	.	.	.	.	.	.	.	.	.	.	.
ENST0	.	11p15.4	.	.	.	.	.	.	.	.	.	.	.
ENST0	.	11p15.3	.	.	.	.	rs759	.	.	.	.	.	0.003
ENST0	.	11p15.2	.	Scc	.	.	rs781	.	.	.	.	.	.
ENST0	.	11p15.2	.	.	.	.	.	ID=COSM6	.	.	.	.	.
ENST0	.	11p15.2	.	.	.	.	rs782	ID=COSM3	.	.	.	.	.
ENST0	.	11p15.2	.	Scc	.	.	.	.	.	.	.	.	.
ENST0	.	11p15.2	.	.	.	.	.	.	.	.	.	.	.
ENST0	.	11p15.1	.	.	.	Score=671	.	.	.	.	.	.	.
ENST0	.	11p15.1	.	.	.	.	rs778	.	.	.	.	.	.
ENST0	.	11p15.1	.	Scc	.	.	.	.	.	.	.	.	.
ENST0	.	11p15.1	.	Scc	.	.	.	.	.	.	.	.	.
ENST0	.	11p15.1	.	.	.	.	.	.	.	.	.	.	.
ENST0	.	11p15.1	.	.	Scc	.	.	.	.	.	.	.	.
ENST0	.	11p15.1	.	.	.	.	.	.	.	.	.	.	.
ENST0	.	11p15.1	.	.	.	.	.	.	.	.	.	.	.
ENST0	.	11p15.1	.	Scc	.	.	rs114	.	.	.	.	.	0.005
ENST0	.	11p14.3	.	.	.	.	.	.	.	.	.	.	.
ENST0	.	11p14.3	.	.	Scc	.	.	.	.	.	.	.	.
ENST0	.	11p13	.	.	.	.	.	.	.	.	.	.	.
ENST0	.	11p13	.	.	.	.	.	.	.	.	.	.	.
ENST0	.	11p11.2	.	.	Scc	.	.	.	.	.	.	.	.
ENST0	.	11p11.2	.	Scc	.	.	.	.	.	.	.	.	.
ENST0	.	11p11.2	.	Scc	.	.	.	.	.	.	.	.	.
ENST0	.	11p11.2	.	.	.	Score=457	rs773	.	.	.	.	.	.
ENST0	.	11q11	.	Scc	Scc	.	.	ID=COSM1	.	.	.	.	.
ENST0	.	11q11	.	.	.	.	rs778	.	.	.	.	.	.
ENST0	.	11q12.1	.	.	.	.	.	.	.	.	.	.	.
ENST0	.	11q12.1	.	.	Scc	.	rs755	.	.	.	.	.	.
ENST0	.	11q12.1	.	.	.	.	rs370	.	.	.	.	.	.
ENST0	.	11q12.1	.	.	.	.	rs746	.	.	.	.	.	.
ENST0	.	11q12.2	.	.	.	.	rs761	.	.	.	.	.	.
ENST0	.	11q12.2	.	.	.	.	.	.	.	.	.	.	.
ENST0	.	11q12.2	.	.	.	.	.	.	.	.	.	.	.
ENST0	.	11q12.3	.	.	.	.	rs750	.	.	.	.	.	.
ENST0	.	11q12.3	.	.	.	.	rs368	.	.	.	.	.	.
ENST0	.	11q12.3	.	.	.	.	rs373	.	.	.	.	.	.
ENST0	.	11q12.3	.	.	.	.	.	.	.	.	.	.	.
ENST0	.	11q13.1	.	.	.	.	rs755	.	.	.	.	.	.
ENST0	.	11q13.1	.	.	.	.	rs320	ID=COSN2	.	.	.	.	0.023
ENST0	.	11q13.1	.	.	.	.	.	.	.	.	.	.	.
ENST0	.	11q13.1	.	Scc	.	.	rs769	.	.	.	.	.	.
ENST0	.	11q13.1	.	Scc	.	.	rs759	.	.	.	.	.	.

ENST0	.	11q13.1	.	.	.	.	rs200	ID=COSM3	.	.	.	.	2E-04
ENST0	.	11q13.1	.	.	.	Score=224	.	.	.	.	.	.	.
ENST0	.	11q13.1	.	.	.	.	.	.	.	.	.	.	.
ENST0	.	11q13.1	.	Scc	.	.	.	.	.	.	.	.	.
ENST0	.	11q13.1	.	.	.	.	rs373	.	.	.	.	.	.
ENST0	.	11q13.1	.	.	.	.	.	.	.	.	.	.	.
ENST0	.	11q13.1	.	.	.	.	.	.	.	.	.	.	.
ENST0	.	11q13.1	.	.	.	.	.	.	.	.	.	.	.
ENST0	.	11q13.1	.	Scc	.	.	.	.	.	.	.	.	.
ENST0	.	11q13.2	.	.	.	.	.	.	.	.	.	.	.
ENST0	.	11q13.2	.	.	.	.	.	.	.	.	.	.	.
ENST0	.	11q13.2	.	Scc	.	.	.	.	.	.	.	.	.
ENST0	.	11q13.2	.	.	.	.	.	.	.	.	.	.	.
ENST0	.	11q13.2	.	.	.	.	rs891	.	.	.	.	.	.
ENST0	.	11q13.2	.	.	.	.	rs143	.	.	.	.	.	.
ENST0	.	11q13.3	.	.	.	.	rs762	.	.	.	.	.	.
ENST0	.	11q13.3	.	.	.	.	.	.	.	.	.	.	.
ENST0	.	11q13.3	.	.	.	.	.	.	.	.	.	.	.
ENST0	.	11q13.4	.	Scc	.	.	rs772	ID=COSM5	.	.	.	.	.
ENST0	.	11q13.4	.	.	.	Score=485	rs191	.	Ur	.	.	.	0.002
ENST0	.	11q13.4	.	.	.	.	.	.	.	.	.	.	.
ENST0	.	11q13.4	.	.	.	.	.	.	.	.	.	.	.
ENST0	.	11q13.4	.	.	.	Score=350	.	.	.	.	.	.	.
ENST0	.	11q13.4	.	.	.	.	.	.	.	.	.	.	.
ENST0	.	11q13.4	.	.	.	.	.	.	.	.	.	.	.
ENST0	.	11q13.4	.	Scc	.	.	.	.	.	.	.	.	.
ENST0	.	11q13.4	.	.	.	.	.	.	.	.	.	.	.
ENST0	.	11q13.4	.	.	.	.	rs780	.	.	.	.	.	.
ENST0	.	11q13.5	.	Scc	.	.	rs780	.	.	.	.	.	.
ENST0	.	11q13.5	.	.	.	.	rs745	.	.	.	.	.	.
ENST0	.	11q14.1	.	.	.	.	.	.	.	.	.	.	.
ENST0	.	11q14.1	.	.	.	Score=320	rs372	.	.	.	.	.	.
ENST0	.	11q14.1	.	.	Scc	.	.	.	.	.	.	.	.
ENST0	.	11q14.3	.	Scc	.	.	.	ID=COSM9	.	.	.	.	.
ENST0	.	11q14.3	.	.	Scc	.	.	.	.	.	.	.	.
ENST0	.	11q21	.	.	.	.	rs791	.	.	.	.	.	0.007
ENST0	.	11q22.3	.	.	.	.	rs191	.	Ur	.	.	.	0.001
ENST0	.	11q22.3	.	.	.	.	rs754	.	.	.	.	.	.
ENST0	.	11q22.3	.	Scc	.	.	rs763	.	.	.	.	.	.
ENST0	.	11q22.3	.	.	.	.	.	.	.	.	.	.	.
ENST0	.	11q22.3	.	.	.	.	.	.	.	.	.	.	.
ENST0	.	11q22.3	.	.	.	.	.	.	.	.	.	.	.
ENST0	.	11q23.1	.	.	.	.	rs574	.	.	.	.	.	4E-04
ENST0	.	11q23.1	.	Scc	.	.	rs104	.	.	.	.	.	.
ENST0	.	11q23.1	.	.	.	.	rs973	.	.	.	.	.	.
ENST0	.	11q23.2	.	.	.	.	rs771	.	.	.	.	.	.
ENST0	.	11q23.3	.	.	.	Score=203	rs755	ID=COSN2	.	.	.	.	.
ENST0	.	11q23.3	.	.	.	.	.	.	.	.	.	.	.
ENST0	.	11q23.3	.	.	.	.	rs370	.	.	.	.	.	.
ENST0	.	11q23.3	.	.	.	.	.	.	.	.	.	.	.
ENST0	.	11q23.3	.	.	.	.	.	.	.	.	.	.	.
ENST0	.	11q23.3	.	.	.	.	.	.	.	.	.	.	.
ENST0	.	11q23.3	.	Scc	.	.	.	.	.	.	.	.	.
ENST0	.	11q23.3	.	.	.	.	rs576	.	.	.	.	.	.

ENST0	.	11q23.3	.	.	.	.	.	.	.	.	.	.	.
ENST0	.	11q23.3	.	.	.	.	rs142	ID=COSM4	oth	.	.	0.001	0.002
ENST0	.	11q24.2	.	Scc	.	.	rs146	.	.	.	.	.	0.003
ENST0	.	11q24.2	.	.	.	.	rs186	ID=COSN2	.	.	.	0.008	0.002
ENST0	.	11q24.2	.	.	.	.	.	.	.	.	.	.	.
ENST0	.	11q24.2	.	.	.	.	.	.	.	.	.	.	.
ENST0	.	11q24.2	.	.	.	.	rs103	.	.	.	.	.	.
ENST0	.	11q24.3	.	.	.	Score=784	rs149	.	.	.	.	.	4E-04
ENST0	.	11q24.3	.	.	.	.	rs116	.	.	.	.	.	.
ENST0	.	11q24.3	.	Scc	.	.	rs750	.	.	.	.	.	.
ENST0	.	11q25	.	.	.	.	.	.	.	.	.	.	.
.	.	11q25	.	.	.	Score=920	rs961	.	.	.	.	.	.
ENST0	.	11q25	.	.	.	.	.	.	.	.	.	.	.
ENST0	.	12p13.33	.	.	.	.	rs149	.	.	.	.	.	.
.	.	12p13.33	.	.	.	Score=47;l	.	.	.	.	.	.	.
ENST0	.	12p13.33	.	.	.	.	rs747	.	.	.	.	.	.
ENST0	.	12p13.33	.	.	.	.	.	.	.	.	.	.	.
ENST0	.	12p13.33	.	Scc	.	.	.	ID=COSM1	.	.	.	.	.
ENST0	.	12p13.33	.	.	.	.	rs202	.	Lik	.	.	0.001	4E-04
ENST0	.	12p13.33	.	.	.	.	rs104	.	.	.	.	.	.
ENST0	.	12p13.32	.	.	.	.	.	.	.	.	.	.	.
ENST0	.	12p13.32	.	.	.	.	.	.	.	.	.	.	.
ENST0	.	12p13.32	.	.	.	.	.	.	.	.	.	.	.
ENST0	.	12p13.32	.	.	.	.	.	.	.	.	.	.	.
ENST0	.	12p13.31	.	.	.	Score=22;l	.	.	.	.	.	.	.
ENST0	.	12p13.31	.	.	.	.	rs752	.	.	.	.	.	.
ENST0	.	12p13.31	.	.	.	.	.	.	.	.	.	.	.
ENST0	.	12p13.31	.	.	.	.	.	.	.	.	.	.	.
ENST0	.	12p13.31	.	.	.	.	.	.	.	.	.	.	.
ENST0	.	12p13.31	.	.	.	.	.	.	.	.	.	.	.
ENST0	.	12p13.31	.	.	.	.	.	.	.	.	.	.	.
ENST0	.	12p13.31	.	.	Scc	.	.	.	.	.	.	.	.
ENST0	.	12p13.31	.	.	Scc	.	rs760	ID=COSN1	.	.	.	.	.
ENST0	.	12p13.2	.	.	.	.	.	.	.	.	.	.	.
ENST0	.	12p13.2	.	.	Scc	.	.	.	.	.	.	.	.
ENST0	.	12p13.2	.	.	.	.	.	.	.	.	.	.	.
ENST0	.	12p13.2	.	Scc	.	.	rs752	.	.	.	.	.	.
ENST0	.	12p13.2	.	.	.	.	.	ID=COSM3	.	.	.	.	.
.	.	12p13.1	.	.	.	.	rs755	.	.	.	.	.	.
ENST0	.	12p12.3	.	.	.	.	.	.	.	.	.	.	.
ENST0	.	12p12.3	.	.	.	.	.	.	.	.	.	.	.
.	.	12p12.3	.	.	.	.	rs941	.	.	.	.	.	.
ENST0	.	12p12.1	.	.	.	.	.	.	.	.	.	.	.
ENST0	.	12p11.22	.	.	.	.	.	.	.	.	.	.	.
ENST0	.	12p11.22	.	.	.	.	.	.	.	.	.	.	.
ENST0	.	12p11.21	.	.	.	.	rs371	.	.	.	.	.	.
ENST0	.	12p11.21	.	.	.	.	.	.	.	.	.	.	.
ENST0	.	12p11.21	.	.	Scc	.	.	.	.	.	.	.	.
ENST0	.	12q12	.	.	Scc	.	.	.	.	.	.	.	.
ENST0	.	12q12	.	.	.	.	.	.	.	.	.	.	.
ENST0	.	12q12	.	.	.	.	.	.	.	.	.	.	.
ENST0	.	12q12	.	.	.	.	.	.	.	.	.	.	.
ENST0	.	12q13.11	.	.	.	.	.	.	.	.	.	.	.
ENST0	.	12q13.12	.	.	.	.	.	.	.	.	.	.	.

ENST0	.	12q13.12	.	.	.	.	.	.	.	.	.	.	.
ENST0	.	12q13.12	.	.	.	.	rs555	.	.	.	.	0.001	2E-04
ENST0	.	12q13.12	.	.	.	.	rs777	ID=COSM3	.	.	.	.	.
ENST0	.	12q13.12	.	.	.	.	.	.	.	.	.	.	.
ENST0	.	12q13.12	.	.	.	.	rs790	.	.	.	.	.	0.035
ENST0	.	12q13.12	.	.	.	.	.	.	.	.	.	.	.
ENST0	.	12q13.13	.	.	.	.	.	.	.	.	.	.	.
ENST0	.	12q13.13	.	.	.	.	.	.	.	.	.	.	.
ENST0	.	12q13.13	.	.	.	.	.	.	.	.	.	.	.
ENST0	.	12q13.13	.	.	.	.	rs377	.	.	.	.	.	.
ENST0	.	12q13.13	.	.	.	Score=291	rs530	ID=COSM1	.	.	.	.	2E-04
ENST0	.	12q13.13	.	Scc	.	.	.	.	.	.	.	.	.
ENST0	.	12q13.13	.	.	.	.	.	.	.	.	.	.	.
ENST0	.	12q13.13	.	.	.	Score=210	.	.	.	.	.	.	.
ENST0	.	12q13.13	.	.	.	.	rs903	.	.	.	.	.	.
ENST0	.	12q13.13	.	Scc	.	.	.	.	.	.	.	.	.
ENST0	.	12q13.3	.	.	.	.	rs368	.	.	.	.	.	.
ENST0	.	12q13.3	.	Scc	.	.	rs768	.	.	.	.	.	.
ENST0	.	12q13.3	.	.	.	.	rs192	ID=COSN1	.	.	.	.	0.021
ENST0	.	12q13.3	.	.	.	.	rs945	.	.	.	.	.	.
ENST0	.	12q13.3	.	.	.	.	.	.	.	.	.	.	.
ENST0	.	12q13.3	.	.	.	.	rs370	.	.	.	.	.	.
ENST0	.	12q13.3	.	.	.	.	.	.	.	.	.	.	.
ENST0	.	12q13.3	.	Scc	.	.	rs536	ID=COSM4	.	.	.	.	2E-04
ENST0	.	12q14.2	.	.	Scc	.	.	.	.	.	.	.	.
ENST0	.	12q14.2	.	.	.	.	rs180	.	.	.	.	.	2E-04
ENST0	.	12q14.2	.	.	.	.	.	.	.	.	.	.	.
ENST0	.	12q15	.	.	.	.	.	.	.	.	.	.	.
ENST0	.	12q15	.	Scc	.	.	.	.	.	.	.	.	.
ENST0	.	12q15	.	.	.	Score=692	rs531	.	.	.	.	.	.
ENST0	.	12q21.1	.	.	.	.	.	.	.	.	.	.	.
ENST0	.	12q21.2	.	.	.	.	rs367	.	.	.	.	.	.
ENST0	.	12q21.2	.	.	.	.	.	.	.	.	.	.	.
ENST0	.	12q21.2	.	.	.	.	.	.	.	.	.	.	.
ENST0	.	12q21.2	.	Scc	.	.	.	.	.	.	.	.	.
ENST0	.	12q21.31	.	Scc	.	.	rs767	.	.	.	.	.	.
ENST0	.	12q21.31	.	.	.	.	rs938	.	.	.	.	.	.
ENST0	.	12q21.31	.	Scc	.	.	.	.	.	.	.	.	.
ENST0	.	12q22	.	.	Scc	.	.	.	.	.	.	.	.
ENST0	.	12q22	.	.	.	.	.	.	.	.	.	.	.
ENST0	.	12q23.1	.	.	.	.	rs575	.	.	.	.	.	2E-04
ENST0	.	12q23.1	.	.	.	.	rs149	ID=COSM2	.	.	.	.	2E-04
ENST0	.	12q23.1	.	.	.	.	.	.	.	.	.	.	.
ENST0	.	12q23.1	.	.	.	.	rs759	.	.	.	.	.	.
ENST0	.	12q23.1	.	.	.	.	.	.	.	.	.	.	.
ENST0	.	12q23.2	.	.	.	.	.	.	.	.	.	.	.
ENST0	.	12q23.2	.	.	.	.	.	.	.	.	.	.	.
ENST0	.	12q23.3	.	.	.	.	.	.	.	.	.	.	.
ENST0	.	12q23.3	.	.	.	.	.	.	.	.	.	.	.
ENST0	.	12q23.3	.	.	.	.	.	.	.	.	.	.	.
ENST0	.	12q23.3	.	Scc	.	.	.	.	.	.	.	.	.
ENST0	.	12q24.11	.	.	.	.	.	.	.	.	.	.	.
ENST0	.	12q24.11	.	.	.	.	.	.	.	.	.	.	.
ENST0	.	12q24.12	.	.	.	.	.	.	.	.	.	.	.

ENST0	.	12q24.12	.	.	.	.	.	.	.	.	.	.	.
ENST0	.	12q24.13	.	Scc	.	.	rs369	.	.	.	.	.	.
ENST0	.	12q24.21	.	.	.	.	rs764	.	.	.	.	.	.
ENST0	.	12q24.22	.	.	.	.	.	.	.	.	.	.	.
ENST0	.	12q24.23	.	.	.	.	.	.	.	.	.	.	.
ENST0	.	12q24.23	.	.	.	.	.	.	.	.	.	.	.
ENST0	.	12q24.31	.	Scc	.	.	rs909	.	.	.	.	.	.
ENST0	.	12q24.31	.	.	.	.	rs762	.	.	.	.	.	.
ENST0	.	12q24.31	.	.	.	.	.	.	.	.	.	.	.
ENST0	.	12q24.31	.	.	.	Score=346	rs100	ID=COSN2	.	.	.	.	.
ENST0	.	12q24.31	.	.	.	.	.	.	.	.	.	.	.
ENST0	.	12q24.31	.	.	.	.	.	.	.	.	.	.	.
ENST0	.	12q24.31	.	.	.	Score=184	.	.	.	.	.	.	.
ENST0	.	12q24.31	.	.	.	.	rs548	.	.	.	.	.	2E-04
ENST0	.	12q24.31	.	.	.	.	.	.	.	.	.	.	.
ENST0	.	12q24.31	.	.	.	Score=175	.	.	.	.	.	.	.
ENST0	.	12q24.31	.	Scc	.	.	.	.	.	.	.	.	.
ENST0	.	12q24.31	.	.	.	.	rs368	.	.	.	.	.	.
ENST0	.	12q24.31	.	.	.	.	.	.	.	.	.	.	.
ENST0	.	12q24.31	.	.	.	.	rs186	.	.	.	.	0.005	0.001
ENST0	.	12q24.32	.	.	.	.	.	.	.	.	.	.	.
.	.	12q24.32	.	.	.	Score=161	.	.	.	.	.	.	.
ENST0	.	12q24.32	.	.	.	.	rs775	ID=COSM:	.	.	.	.	.
ENST0	.	12q24.33	.	.	.	.	rs868	.	.	.	.	.	.
ENST0	.	12q24.33	.	.	.	.	.	.	.	.	.	.	.
ENST0	.	12q24.33	.	.	.	.	.	.	.	.	.	.	.
ENST0	.	12q24.33	.	.	.	.	.	.	.	.	.	.	.
ENST0	.	12q24.33	.	Scc	.	.	.	.	.	.	.	.	.
ENST0	.	13q12.11	.	.	.	.	.	.	.	.	.	.	.
ENST0	.	13q12.11	.	.	.	.	.	.	.	.	.	.	.
ENST0	.	13q12.11	.	.	.	.	rs765	ID=COSM(	.	.	.	.	.
ENST0	.	13q12.12	.	.	.	.	.	.	.	.	.	.	.
ENST0	.	13q12.13	.	.	.	.	rs760	.	.	.	.	.	.
.	.	13q12.3	.	.	.	.	.	.	.	.	.	.	.
ENST0	.	13q13.1	.	.	.	.	.	.	.	.	.	.	.
ENST0	.	13q13.2	.	.	.	.	.	.	.	.	.	.	.
ENST0	.	13q13.3	.	Scc	.	.	.	.	.	.	.	.	.
ENST0	.	13q13.3	.	.	.	.	.	.	.	.	.	.	.
ENST0	.	13q13.3	.	Scc	.	.	.	.	.	.	.	.	.
ENST0	.	13q14.11	.	.	.	.	rs113	.	.	.	.	.	.
.	.	13q14.11	.	.	.	Score=843	rs926	.	.	.	.	.	.
ENST0	.	13q14.13	.	.	.	.	.	.	.	.	.	.	.
ENST0	.	13q14.13	.	.	.	.	rs530	.	.	.	.	0.003	6E-04
ENST0	.	13q14.2	.	.	.	.	rs775	.	.	.	.	.	.
.	.	13q21.33	.	.	.	Score=564	rs535	.	.	.	.	.	2E-04
ENST0	.	13q21.33	.	.	.	.	.	.	.	.	.	.	.
ENST0	.	13q22.2	.	.	.	.	.	.	.	.	.	.	.
ENST0	.	13q31.3	.	.	.	.	rs537	.	.	.	.	.	4E-04
ENST0	.	13q32.1	.	.	.	.	.	.	.	.	.	.	.
ENST0	.	13q32.1	.	Scc	.	.	rs149	.	.	.	.	.	.
ENST0	.	13q32.3	.	.	.	.	rs373	.	.	.	.	.	.
ENST0	.	13q32.3	.	.	.	.	rs373	.	.	.	.	.	.
ENST0	.	13q33.3	.	.	.	.	.	.	.	.	.	.	.
ENST0	.	13q33.3	.	Scc	.	.	rs756	.	.	.	.	.	.

ENST0	.	13q34	.	.	.	.	rs148	ID=COSM:	.	.	.	.	.
ENST0	.	13q34	.	.	.	.	rs756	.	.	.	.	.	.
ENST0	.	13q34	.	.	.	.	.	.	.	.	.	.	.
ENST0	.	13q34	.	.	.	.	rs100	.	.	.	.	.	.
ENST0	.	13q34	.	.	.	.	rs752	ID=COSN7	.	.	.	.	.
ENST0	.	13q34	.	.	.	.	rs750	.	.	.	.	.	.
ENST0	.	13q34	.	.	.	.	rs770	.	.	.	.	.	.
ENST0	.	14q11.2	.	.	Scc	.	rs879	.	.	.	.	.	.
ENST0	.	14q11.2	.	.	Scc	.	.	.	.	.	.	.	.
ENST0	.	14q11.2	.	.	Scc	.	rs744	.	.	.	.	.	.
ENST0	.	14q11.2	.	.	.	.	.	.	.	.	.	.	.
ENST0	.	14q11.2	.	.	.	.	rs145	.	.	.	.	.	.
ENST0	.	14q11.2	.	.	.	.	rs549	.	.	.	.	.	8E-04
ENST0	.	14q11.2	.	.	.	.	.	.	.	.	.	.	.
.	.	14q11.2	.	.	.	.	.	.	.	.	.	.	.
ENST0	.	14q11.2	.	.	.	.	rs374	.	.	.	.	.	.
ENST0	.	14q11.2	.	.	.	.	rs757	ID=COSM:	.	.	.	.	.
ENST0	.	14q11.2	.	.	.	.	.	.	.	.	.	.	.
ENST0	.	14q13.2	.	.	.	.	rs777	.	.	.	.	.	.
ENST0	.	14q13.2	.	.	.	Score=205	rs924	.	.	.	.	.	.
ENST0	.	14q13.2	.	.	.	.	.	.	.	.	.	.	.
ENST0	.	14q13.2	.	.	.	.	.	.	.	.	.	.	.
ENST0	.	14q13.3	.	.	.	.	.	.	.	.	.	.	.
ENST0	.	14q13.3	.	.	Scc	.	rs567	.	.	.	.	.	0.006
ENST0	.	14q21.1	.	.	.	.	.	.	.	.	.	.	.
ENST0	.	14q21.1	.	.	.	.	rs865	ID=COSM:	.	.	.	.	.
ENST0	.	14q21.2	.	.	.	.	.	.	.	.	.	.	.
ENST0	.	14q21.3	.	.	.	.	.	.	.	.	.	.	.
.	.	14q22.1	.	.	.	.	.	.	.	.	.	.	.
ENST0	.	14q22.1	.	Scc	.	.	rs746	.	.	.	.	.	.
ENST0	.	14q23.1	.	.	.	.	.	.	.	.	.	.	.
ENST0	.	14q23.1	.	.	.	.	.	.	.	.	.	.	.
ENST0	.	14q23.1	.	.	.	.	.	.	.	.	.	.	.
ENST0	.	14q23.1	.	.	.	.	.	.	.	.	.	.	.
ENST0	.	14q23.1	.	.	.	.	rs140	.	.	.	.	.	.
.	.	14q23.2	.	.	.	.	.	.	.	.	.	.	.
ENST0	.	14q23.3	.	.	.	.	rs540	.	.	.	.	.	2E-04
ENST0	.	14q23.3	.	.	.	Score=461	rs140	.	.	.	.	0.001	2E-04
ENST0	.	14q23.3	.	.	.	.	.	.	.	.	.	.	.
ENST0	.	14q24.1	.	.	.	.	.	.	.	.	.	.	.
ENST0	.	14q24.3	.	.	.	.	.	.	.	.	.	.	.
ENST0	.	14q24.3	.	Scc	.	.	rs754	.	.	.	.	.	.
ENST0	.	14q24.3	.	.	.	.	rs376	.	.	.	.	.	.
ENST0	.	14q24.3	.	Scc	.	.	.	.	.	.	.	.	.
ENST0	.	14q24.3	.	.	.	.	.	.	.	.	.	.	.
ENST0	.	14q24.3	.	.	.	Score=234	.	.	.	.	.	.	.
ENST0	.	14q24.3	.	.	.	.	rs141	.	.	.	.	.	6E-04
ENST0	.	14q32.11	.	.	.	.	.	.	.	.	.	.	.
ENST0	.	14q32.11	.	.	.	.	.	.	.	.	.	.	.
ENST0	.	14q32.11	.	.	.	.	rs751	.	.	.	.	.	.
ENST0	.	14q32.11	.	.	.	.	rs763	.	.	.	.	.	.
ENST0	.	14q32.11	.	.	.	.	.	.	.	.	.	.	.
ENST0	.	14q32.12	.	.	.	.	.	.	.	.	.	.	.
ENST0	.	14q32.12	.	.	.	Score=187	.	.	.	.	.	.	.

ENST0	.	14q32.13	.	.	.	.	.	.	.	.	.	.	.
ENST0	.	14q32.2	.	.	.	.	.	.	.	.	.	.	.
ENST0	.	14q32.2	.	Scc	.	.	.	.	.	.	.	.	.
ENST0	.	14q32.2	.	.	.	.	.	.	.	.	.	.	.
ENST0	hsa-mir-41	14q32.31	.	Scc	.	.	.	.	.	.	.	.	.
ENST0	.	14q32.31	.	.	.	.	.	.	.	.	.	.	.
ENST0	.	14q32.31	.	Scc	.	.	rs757	.	.	.	.	.	.
ENST0	.	14q32.32	.	.	.	.	rs972	.	.	.	.	.	.
ENST0	.	14q32.32	.	.	.	.	rs104	.	.	.	.	.	.
ENST0	.	14q32.33	.	.	.	.	.	.	.	.	.	.	.
ENST0	.	14q32.33	.	Scc	.	.	.	.	.	.	.	.	.
ENST0	.	14q32.33	.	.	.	.	.	.	.	.	.	.	.
ENST0	.	14q32.33	.	.	.	.	rs756	.	.	.	.	.	.
ENST0	.	14q32.33	.	.	.	.	.	.	.	.	.	.	.
ENST0	.	14q32.33	.	.	.	.	rs768	.	.	.	.	.	.
ENST0	.	14q32.33	.	.	.	.	.	.	.	.	.	.	.
ENST0	.	14q32.33	.	.	.	.	rs782	.	.	.	.	.	.
ENST0	.	14q32.33	.	.	.	.	rs782	.	.	.	.	.	.
ENST0	.	14q32.33	.	.	Scc	.	.	.	.	.	.	.	.
.	.	14q32.33	.	.	Scc	.	.	.	.	.	.	.	.
.	.	15q11.1	.	.	Scc	Score=319	rs200	ID=COSN1	.	.	.	.	.
ENST0	.	15q11.2	.	.	Scc	.	.	.	.	.	.	.	.
.	.	15q11.2	.	.	Scc	Score=233	.	.	.	.	.	.	.
ENST0	.	15q11.2	.	.	Scc	.	.	.	.	.	.	.	.
ENST0	.	15q11.2	.	.	.	.	.	.	.	.	.	.	.
ENST0	.	15q11.2	.	.	Scc	.	.	.	.	.	.	.	.
ENST0	.	15q11.2	.	.	Scc	Score=237	.	.	.	.	.	.	.
ENST0	.	15q11.2	.	.	Scc	.	.	.	.	.	.	.	.
ENST0	.	15q11.2	.	.	Scc	Score=486	rs760	.	.	.	.	.	.
ENST0	.	15q11.2	.	.	.	.	rs919	.	.	.	.	.	.
ENST0	.	15q11.2	.	.	.	.	.	.	.	.	.	.	.
ENST0	.	15q11.2	.	.	.	.	rs748	.	.	.	.	.	.
ENST0	.	15q11.2	.	.	.	.	.	.	.	.	.	.	.
ENST0	.	15q12	.	.	.	.	rs920	.	.	.	.	.	.
ENST0	.	15q13.1	.	.	.	.	rs371	ID=COSM(	.	.	.	.	.
ENST0	.	15q13.1	.	.	Scc	.	.	.	.	.	.	.	.
ENST0	.	15q13.1	.	.	Scc	.	rs202	ID=COSM(	.	.	.	.	.
ENST0	.	15q13.1	.	.	.	.	.	.	.	.	.	.	.
ENST0	.	15q13.2	.	.	Scc	.	.	.	.	.	.	.	.
ENST0	.	15q13.2	.	.	Scc	.	.	.	.	.	.	.	.
ENST0	.	15q13.2	.	.	Scc	Score=221	rs199	.	.	.	.	.	.
ENST0	.	15q13.2	.	.	Scc	.	.	ID=COSN2	.	.	.	.	.
ENST0	.	15q13.3	.	.	.	.	.	.	.	.	.	.	.
.	.	15q13.3	.	.	Scc	.	rs112	.	.	.	.	.	.
ENST0	.	15q14	.	.	.	.	.	.	.	.	.	.	.
ENST0	.	15q14	.	.	.	.	.	.	.	.	.	.	.
ENST0	.	15q15.1	.	Scc	.	.	.	.	.	.	.	.	.
ENST0	.	15q15.1	.	.	.	.	.	.	.	.	.	.	.
ENST0	.	15q15.1	.	.	.	.	rs370	.	.	.	.	.	0.001
ENST0	.	15q15.1	.	.	.	.	.	.	.	.	.	.	.
ENST0	.	15q15.2	.	Scc	.	.	rs531	ID=COSM!	.	.	.	.	.
ENST0	.	15q15.3	.	.	.	.	rs755	ID=COSM(	.	.	.	.	.
ENST0	.	15q15.3	.	.	Scc	.	rs536	.	.	.	.	.	2E-04
ENST0	.	15q15.3	.	.	Scc	.	.	.	.	.	.	.	.

ENST0	.	15q15.3	.	.	Scc	.	rs532	.	.	.	.	.	2E-04
ENST0	.	15q21.1	.	.	Scc	.	.	.	.	.	.	.	.
ENST0	.	15q21.1	.	Scc	.	.	.	.	.	.	.	.	.
ENST0	.	15q21.1	.	.	.	.	.	.	.	.	.	.	.
ENST0	.	15q21.1	.	.	.	.	rs773	.	.	.	.	.	.
ENST0	.	15q21.1	.	Scc	.	.	rs140	ID=COSM2	Ur	.	.	0.001	4E-04
ENST0	.	15q21.1	.	.	.	.	.	.	.	.	.	.	.
ENST0	.	15q21.2	.	.	.	.	.	.	.	.	.	.	.
ENST0	.	15q21.2	.	.	.	.	.	.	.	.	.	.	.
ENST0	.	15q21.2	.	.	.	.	.	.	.	.	.	.	.
ENST0	.	15q21.2	.	Scc	.	.	rs368	ID=COSM2	.	.	.	.	.
ENST0	.	15q21.2	.	.	.	.	.	.	.	.	.	.	.
ENST0	.	15q21.3	.	Scc	.	.	.	.	.	.	.	.	.
ENST0	.	15q22.2	.	.	.	.	rs779	.	.	.	.	.	.
ENST0	.	15q22.31	.	.	.	.	rs751	.	.	.	.	.	.
ENST0	.	15q22.31	.	Scc	.	.	.	.	.	.	.	.	.
ENST0	.	15q22.31	.	Scc	.	.	rs536	.	.	.	.	0.001	2E-04
ENST0	.	15q22.31	.	.	.	Score=842	.	.	.	.	.	.	.
ENST0	.	15q22.32	.	.	.	.	.	.	.	.	.	.	.
ENST0	.	15q23	.	.	.	.	.	.	.	.	.	.	.
ENST0	.	15q23	.	.	.	.	rs756	ID=COSN1	.	.	.	.	.
ENST0	.	15q23	.	.	.	.	.	.	.	.	.	.	.
ENST0	.	15q23	.	.	.	Score=233	rs546	.	.	.	.	0.001	2E-04
ENST0	.	15q23	.	.	.	.	.	.	.	.	.	.	.
ENST0	.	15q23	.	.	.	.	.	.	.	.	.	.	.
ENST0	.	15q24.1	.	.	.	.	rs104	.	.	.	.	.	.
ENST0	.	15q24.1	.	.	.	.	.	.	.	.	.	.	.
ENST0	.	15q24.2	.	.	Scc	.	.	.	.	.	.	.	.
ENST0	.	15q24.2	.	.	Scc	.	.	.	.	.	.	.	.
ENST0	.	15q24.3	.	.	.	.	rs974	.	.	.	.	.	.
ENST0	.	15q24.3	.	.	.	.	rs104	.	.	.	.	.	.
ENST0	.	15q24.3	.	.	Scc	.	rs878	ID=COSN2	.	.	.	.	.
ENST0	.	15q24.3	.	.	Scc	.	.	.	.	.	.	.	.
ENST0	.	15q25.1	.	.	Scc	.	.	.	.	.	.	.	.
ENST0	.	15q25.1	.	.	.	.	rs147	ID=COSM3	.	.	.	.	.
ENST0	.	15q25.1	.	.	.	Score=462	.	.	.	.	.	.	.
ENST0	.	15q25.1	.	.	.	.	rs529	.	.	.	.	.	2E-04
ENST0	.	15q25.1	.	.	.	.	.	.	.	.	.	.	.
ENST0	.	15q25.2	.	.	.	.	rs941	.	.	.	.	.	.
ENST0	.	15q25.2	.	.	Scc	.	rs792	ID=COSM5	.	.	.	.	.
ENST0	.	15q25.3	.	.	.	.	.	.	.	.	.	.	.
ENST0	.	15q25.3	.	.	.	.	rs371	.	.	.	.	.	.
ENST0	.	15q26.1	.	.	.	.	.	.	.	.	.	.	.
ENST0	.	15q26.1	.	.	.	Score=444	.	.	.	.	.	.	.
ENST0	.	15q26.1	.	Scc	.	.	rs201	.	.	.	.	.	.
ENST0	.	15q26.1	.	.	.	.	.	ID=COSM4	.	.	.	.	.
ENST0	.	15q26.1	.	.	.	.	rs147	.	.	.	.	.	.
.	.	15q26.3	.	.	.	Score=26;I	rs558	.	.	.	.	0.001	1E-03
ENST0	.	15q26.3	.	.	.	.	.	.	.	.	.	.	.
ENST0	.	15q26.3	.	.	.	.	.	.	.	.	.	.	.
ENST0	.	15q26.3	.	.	.	Score=298	.	.	.	.	.	.	.
ENST0	.	15q26.3	.	Scc	.	.	rs776	ID=COSM2	.	.	.	.	.
ENST0	.	15q26.3	.	.	.	.	.	.	.	.	.	.	.
ENST0	.	15q26.3	.	.	Scc	.	.	.	.	.	.	.	.

ENST0	.	15q26.3	.	.	Scc	.	rs368	.	.	.	.	.	.
ENST0	.	15q26.3	.	.	Scc	.	.	.	.	.	.	.	.
ENST0	.	15q26.3	.	.	Scc	.	.	.	.	.	.	.	.
ENST0	.	16p13.3	.	.	.	.	rs761	.	.	.	.	.	.
ENST0	.	16p13.3	.	.	.	.	.	.	.	.	.	.	.
ENST0	.	16p13.3	.	.	.	.	.	.	.	.	.	.	.
ENST0	.	16p13.3	.	.	.	Score=420	rs892	.	.	.	.	.	.
ENST0	.	16p13.3	.	.	.	.	rs199	.	.	.	.	.	2E-04
ENST0	.	16p13.3	.	.	.	Score=201	rs201	ID=COSN1	.	.	.	.	.
ENST0	.	16p13.3	.	.	.	Score=202	rs776	ID=COSM!	.	.	.	.	.
ENST0	.	16p13.3	.	.	.	.	rs146	.	.	.	.	.	2E-04
ENST0	.	16p13.3	.	.	.	.	rs368	.	.	.	.	.	.
ENST0	.	16p13.3	.	.	.	.	.	.	.	.	.	.	.
ENST0	.	16p13.3	.	.	.	.	.	.	.	.	.	.	.
ENST0	.	16p13.3	.	.	.	.	rs150	.	.	.	.	.	.
ENST0	.	16p13.3	.	.	.	.	rs997	.	.	.	.	.	.
ENST0	.	16p13.3	.	.	.	.	.	.	.	.	.	.	.
ENST0	.	16p13.3	.	.	.	.	rs371	.	.	.	.	.	2E-04
ENST0	.	16p13.3	.	.	.	.	.	.	.	.	.	.	.
ENST0	.	16p13.3	.	.	.	.	rs759	.	.	.	.	.	.
ENST0	.	16p13.3	.	.	.	.	.	.	.	.	.	.	.
.	.	16p13.3	.	.	.	.	rs536	.	.	.	.	0.002	4E-04
ENST0	.	16p13.3	.	Scc	.	.	rs760	.	.	.	.	.	.
ENST0	.	16p13.3	.	.	.	.	.	.	.	.	.	.	.
ENST0	.	16p13.3	.	.	.	.	rs145	ID=COSM(	.	.	.	.	.
ENST0	.	16p13.3	.	.	.	.	.	.	.	.	.	.	.
ENST0	.	16p13.3	.	.	.	.	rs374	.	.	.	.	.	4E-04
ENST0	.	16p13.3	.	.	.	.	.	.	.	.	.	.	.
ENST0	.	16p13.3	.	.	.	.	rs763	.	.	.	.	.	.
ENST0	.	16p13.3	.	.	.	.	.	.	.	.	.	.	.
ENST0	.	16p13.3	.	.	.	.	.	.	.	.	.	.	.
ENST0	.	16p13.3	.	.	.	.	.	.	.	.	.	.	.
ENST0	.	16p13.3	.	.	.	Score=756	rs141	.	.	.	.	0.015	0.003
ENST0	.	16p13.3	.	Scc	.	.	.	.	.	.	.	.	.
ENST0	.	16p13.3	.	.	.	.	rs752	.	.	.	.	.	.
ENST0	.	16p13.3	.	.	.	.	rs534	.	Lik	.	.	0.008	0.002
ENST0	.	16p13.3	.	Scc	.	.	.	.	.	.	.	.	.
ENST0	.	16p13.3	.	.	.	Score=232	rs550	.	.	.	.	0.001	4E-04
ENST0	.	16p13.3	.	.	.	.	rs189	.	.	.	.	.	2E-04
ENST0	.	16p13.3	.	.	.	.	rs371	ID=COSM:	.	.	.	.	.
ENST0	.	16p13.3	.	.	.	.	rs201	.	.	.	.	.	.
ENST0	.	16p13.3	.	.	.	.	.	.	.	.	.	.	.
ENST0	.	16p13.13	.	.	.	.	rs563	ID=COSM:	.	.	.	0.001	2E-04
ENST0	.	16p13.13	.	.	.	.	rs767	.	.	.	.	.	.
ENST0	.	16p13.13	.	.	.	.	rs918	.	.	.	.	.	.
ENST0	.	16p13.12	.	.	.	.	rs780	.	.	.	.	.	.
ENST0	.	16p13.11	.	.	Scc	Score=225	rs566	.	.	.	.	.	2E-04
ENST0	.	16p13.11	.	.	Scc	.	rs750	.	.	.	.	.	.
ENST0	.	16p13.11	.	.	.	.	.	.	.	.	.	.	.
ENST0	.	16p13.11	.	.	.	.	.	.	.	.	.	.	.
ENST0	.	16p13.11	.	.	.	Score=128	.	.	.	.	.	.	.
ENST0	.	16p13.11	.	Scc	Scc	.	.	.	.	.	.	.	.
ENST0	.	16p12.3	.	.	Scc	.	rs178	ID=COSM4	.	.	.	.	.
ENST0	.	16p12.3	.	.	.	.	rs757	.	.	.	.	.	.

ENST0	.	16p12.3	.	.	.	.	rs764	.	.	.	.	.	.
ENST0	.	16p12.2	.	.	.	.	.	.	.	.	.	.	.
ENST0	.	16p12.2	.	.	.	.	rs182	.	.	.	.	.	.
ENST0	.	16p12.2	.	Scc	.	.	.	.	.	.	.	.	.
ENST0	.	16p12.2	.	.	.	.	.	.	.	.	.	.	.
ENST0	.	16p12.1	.	.	.	Score=454	rs972	.	.	.	.	.	.
.	.	16p12.1	.	.	.	.	.	.	.	.	.	.	.
ENST0	.	16p12.1	.	.	.	.	.	.	.	.	.	.	.
ENST0	.	16p12.1	.	.	.	.	.	.	.	.	.	.	.
ENST0	.	16p12.1	.	.	.	.	rs141	.	.	.	.	.	2E-04
ENST0	.	16p11.2	.	.	.	.	rs190	.	.	.	.	0.02	0.006
ENST0	.	16p11.2	.	.	Scc	.	.	.	.	.	.	.	.
ENST0	.	16p11.2	.	Scc	.	.	rs749	.	.	.	.	.	.
ENST0	.	16p11.2	.	.	.	.	.	.	.	.	.	.	.
ENST0	.	16p11.2	.	.	.	.	rs182	.	.	.	.	.	2E-04
ENST0	.	16p11.2	.	.	Scc	.	.	.	.	.	.	.	.
ENST0	.	16p11.2	.	Scc	.	.	.	.	.	.	.	.	.
ENST0	.	16p11.2	.	Scc	.	.	rs781	.	.	.	.	.	.
ENST0	.	16p11.2	.	.	.	Score=100	rs965	.	.	.	.	.	.
ENST0	.	16p11.2	.	.	.	.	.	.	.	.	.	.	.
ENST0	.	16p11.2	.	.	Scc	.	.	.	.	.	.	.	.
ENST0	.	16p11.2	.	.	.	.	.	.	.	.	.	.	.
ENST0	.	16p11.2	.	.	Scc	.	rs747	.	.	.	.	.	2E-04
ENST0	.	16p11.2	.	.	Scc	.	rs460	ID=COSN1	.	.	.	0.19	0.215
ENST0	.	16p11.2	.	.	Scc	.	.	.	.	.	.	.	.
ENST0	.	16p11.2	.	.	Scc	Score=184	.	.	.	.	.	.	.
ENST0	.	16p11.2	.	.	Scc	Score=753	rs374	ID=COSN1	.	.	.	0.1	0.02
.	.	16p11.2	.	.	Scc	.	.	.	.	.	.	.	.
ENST0	.	16p11.2	.	Scc	Scc	.	.	.	.	.	.	.	.
ENST0	.	16p11.2	.	.	.	Score=476	.	.	.	.	.	.	.
.	.	16q11.2	.	.	.	Score=112	rs133	.	.	.	.	.	.
.	.	16q11.2	.	.	.	Score=112	rs288	ID=COSN1	.	.	.	.	.
.	.	16q11.2	.	.	.	Score=114	rs424	ID=COSN1	.	.	.	.	.
.	.	16q11.2	.	.	.	Score=114	rs559	.	.	.	.	.	.
.	.	16q11.2	.	.	.	Score=114	rs424	.	.	.	.	.	.
.	.	16q11.2	.	.	.	Score=114	rs429	ID=COSN2	.	.	.	.	.
.	.	16q11.2	.	.	.	Score=989	rs879	.	.	.	.	.	.
.	.	16q11.2	.	.	.	Score=111	rs879	.	.	.	.	.	.
.	.	16q11.2	.	.	.	Score=112	rs429	.	.	.	.	.	.
.	.	16q11.2	.	.	.	Score=104	rs437	.	.	.	.	.	.
.	.	16q11.2	.	.	.	Score=114	rs720	ID=COSN1	.	.	.	.	.
.	.	16q11.2	.	.	.	Score=114	rs718	.	.	.	.	.	.
.	.	16q11.2	.	.	.	Score=114	rs932	.	.	.	.	.	.
.	.	16q11.2	.	.	.	Score=114	rs720	.	.	.	.	.	.
.	.	16q11.2	.	.	.	Score=114	rs444	.	.	.	.	.	.
.	.	16q11.2	.	.	.	Score=114	rs288	ID=COSN1	.	.	.	.	.
.	.	16q11.2	.	.	.	Score=114	rs288	.	.	.	.	.	.
.	.	16q11.2	.	.	.	Score=112	rs766	.	.	.	.	.	.
.	.	16q11.2	.	.	.	Score=112	rs424	.	.	.	.	.	.
.	.	16q11.2	.	.	.	Score=114	.	.	.	.	.	.	.
ENST0	.	16q11.2	.	.	.	.	rs368	.	.	.	.	.	.
ENST0	.	16q12.1	.	.	.	.	.	.	.	.	.	.	.
ENST0	.	16q12.2	.	.	.	.	.	.	.	.	.	.	.
ENST0	.	16q13	.	.	.	.	.	.	.	.	.	.	.

ENST0	.	16q13	.	.	.	Score=675	.	.	.	.	.	.	.
ENST0	.	16q13	.	.	.	.	.	.	.	.	.	.	.
ENST0	.	16q13	.	.	.	.	rs147	.	.	.	.	.	4E-04
ENST0	.	16q21	.	.	.	Score=575	.	.	.	.	.	.	.
ENST0	.	16q21	.	.	.	.	.	.	.	.	.	.	.
ENST0	.	16q21	.	.	.	.	.	.	.	.	.	.	.
ENST0	.	16q21	.	.	.	Score=507	rs149	.	.	.	.	.	.
ENST0	.	16q22.1	.	.	.	.	.	.	.	.	.	.	.
ENST0	.	16q22.1	.	.	.	Score=223	rs105	.	.	.	.	.	.
ENST0	.	16q22.1	.	.	.	.	rs771	.	.	.	.	.	.
ENST0	.	16q22.1	.	.	.	.	rs561	.	.	.	.	.	2E-04
ENST0	.	16q22.1	.	.	.	.	.	.	.	.	.	.	.
ENST0	.	16q22.1	.	Scc	.	.	rs754	ID=COSM4	.	.	.	.	.
ENST0	.	16q22.1	.	.	.	.	rs369	.	.	.	.	.	.
ENST0	.	16q22.1	.	.	.	.	.	.	.	.	.	.	.
ENST0	.	16q22.1	.	Scc	.	.	rs758	.	.	.	.	.	.
ENST0	.	16q22.1	.	.	.	.	.	.	.	.	.	.	.
ENST0	.	16q22.1	.	.	.	.	.	.	.	.	.	.	.
ENST0	.	16q22.1	.	.	.	.	.	.	.	.	.	.	.
ENST0	.	16q22.1	.	.	.	Score=235	rs867	.	.	.	.	.	.
ENST0	.	16q22.2	.	.	.	.	rs927	.	.	.	.	.	.
ENST0	.	16q22.2	.	.	.	.	rs148	.	.	.	.	.	1E-03
ENST0	.	16q22.2	.	.	.	.	rs377	.	.	.	.	.	.
ENST0	.	16q23.1	.	.	Scc	.	rs750	.	.	.	.	.	.
ENST0	.	16q23.1	.	.	.	.	.	.	.	.	.	.	.
ENST0	.	16q23.1	.	.	.	.	.	.	.	.	.	.	.
ENST0	.	16q23.2	.	.	.	Score=870	rs146	.	.	.	.	0.029	0.01
ENST0	.	16q23.2	.	Scc	.	.	rs773	ID=COSN8	.	.	.	.	.
ENST0	.	16q23.3	.	.	.	Score=228	.	.	.	.	.	.	.
ENST0	.	16q23.3	.	Scc	.	.	rs748	.	Lik	.	.	0.104	0.063
ENST0	.	16q23.3	.	.	.	.	rs140	.	.	.	.	.	6E-04
ENST0	.	16q24.1	.	.	.	.	rs764	ID=COSM1	.	.	.	.	.
ENST0	.	16q24.1	.	.	.	Score=316	rs933	ID=COSM5	.	.	.	.	.
ENST0	.	16q24.1	.	.	.	.	.	.	.	.	.	.	.
ENST0	.	16q24.2	.	.	.	.	rs368	.	.	.	.	0.001	2E-04
ENST0	.	16q24.2	.	.	.	.	rs966	.	.	.	.	.	.
ENST0	.	16q24.2	.	.	.	.	rs560	.	.	.	.	.	2E-04
ENST0	.	16q24.3	.	.	.	.	rs964	.	.	.	.	.	.
ENST0	.	16q24.3	.	Scc	.	.	.	.	.	.	.	.	.
ENST0	.	16q24.3	.	.	.	.	.	.	.	.	.	.	.
ENST0	.	16q24.3	.	.	.	.	rs201	.	.	.	.	0.001	2E-04
ENST0	.	16q24.3	.	.	.	.	rs753	.	.	.	.	.	.
ENST0	.	17p13.3	.	.	.	.	rs762	.	.	.	.	.	.
ENST0	.	17p13.3	.	Scc	.	.	.	.	.	.	.	.	.
ENST0	.	17p13.3	.	.	.	.	.	.	.	.	.	.	.
ENST0	.	17p13.3	.	Scc	.	.	.	.	.	.	.	.	.
ENST0	.	17p13.3	.	.	.	.	.	.	.	.	.	.	.
ENST0	.	17p13.3	.	.	.	.	.	.	.	.	.	.	.
ENST0	.	17p13.3	.	.	.	.	rs780	.	.	.	.	.	.
ENST0	.	17p13.3	.	.	.	.	.	.	.	.	.	.	.
ENST0	.	17p13.2	.	.	.	.	.	.	.	.	.	.	.
ENST0	.	17p13.2	.	.	.	.	.	.	.	.	.	.	.
ENST0	.	17p13.2	.	.	.	.	rs550	.	.	.	.	0.001	4E-04
ENST0	.	17p13.2	.	Scc	.	.	rs376	.	.	.	.	.	.

ENST0	.	17p13.2	.	Scc	Scc	.	rs200	ID=COSM	.	.	.	.	2E-04
ENST0	.	17p13.2	.	.	Scc	.	rs201	ID=COSM	.	.	.	.	.
ENST0	.	17p13.2	.	.	.	Score=186	.	.	.	.	.	.	.
ENST0	.	17p13.2	.	.	.	.	.	.	.	.	.	.	.
ENST0	.	17p13.1	.	.	.	.	.	.	.	.	.	.	.
ENST0	.	17p13.1	.	.	.	Score=573	.	.	.	.	.	.	.
ENST0	.	17p13.1	.	Scc	.	.	rs200	.	.	.	.	.	1E-03
ENST0	.	17p13.1	.	.	.	.	rs745	.	.	.	.	.	.
ENST0	.	17p13.1	.	Scc	.	.	rs147	.	.	.	.	.	.
ENST0	.	17p13.1	.	.	.	.	.	.	.	.	.	.	.
ENST0	.	17p13.1	.	.	.	.	.	.	.	.	.	.	.
ENST0	.	17p12	.	.	.	.	rs753	.	.	.	.	.	.
ENST0	.	17p12	.	.	.	.	.	.	.	.	.	.	.
ENST0	.	17p12	.	.	.	.	.	.	.	.	.	.	.
ENST0	.	17p12	.	.	.	.	rs763	.	.	.	.	.	.
ENST0	.	17p12	.	.	Scc	.	rs620	.	.	.	.	.	.
ENST0	.	17p12	.	Scc	.	.	.	.	.	.	.	.	.
ENST0	.	17p12	.	.	.	.	.	.	.	.	.	.	.
ENST0	.	17p11.2	.	.	.	.	.	.	.	.	.	.	.
ENST0	.	17p11.2	.	.	.	.	.	.	.	.	.	.	.
ENST0	.	17p11.2	.	.	.	.	rs115	.	.	.	.	.	0.002
ENST0	.	17p11.2	.	.	.	.	.	.	.	.	.	.	.
ENST0	.	17p11.2	.	.	Scc	.	.	.	.	.	.	.	.
ENST0	.	17p11.2	.	Scc	Scc	.	.	.	.	.	.	.	.
ENST0	.	17p11.2	.	.	Scc	.	rs286	ID=COSN	.	.	.	0.654	0.875
ENST0	.	17p11.2	.	.	.	.	rs752	.	.	.	.	.	.
ENST0	.	17p11.2	.	.	.	.	.	.	.	.	.	.	.
ENST0	.	17q11.2	.	.	Scc	.	.	.	.	.	.	.	.
ENST0	.	17q11.2	.	.	.	.	.	.	.	.	.	.	.
ENST0	.	17q11.2	.	.	.	.	.	.	.	.	.	.	.
ENST0	.	17q11.2	.	.	.	.	.	.	.	.	.	.	.
ENST0	.	17q11.2	.	.	.	Score=857	.	.	.	.	.	.	.
ENST0	.	17q11.2	.	.	.	.	rs105	.	.	.	.	.	.
ENST0	.	17q11.2	.	.	.	.	.	.	.	.	.	.	.
ENST0	.	17q11.2	.	.	.	.	.	.	.	.	.	.	.
ENST0	.	17q11.2	.	Scc	.	.	.	.	.	.	.	.	.
ENST0	.	17q11.2	.	.	.	Score=959	rs796	ID=COSN1	.	.	.	.	.
ENST0	.	17q11.2	.	Scc	.	.	rs532	.	.	.	.	.	2E-04
ENST0	.	17q11.2	.	.	.	.	.	.	.	.	.	.	.
ENST0	.	17q11.2	.	.	.	.	.	.	.	.	.	.	.
ENST0	.	17q11.2	.	.	.	.	.	.	.	.	.	.	.
ENST0	.	17q12	.	.	.	.	.	.	.	.	.	.	.
ENST0	.	17q12	.	Scc	.	.	.	.	.	.	.	.	.
ENST0	.	17q12	.	.	Scc	.	rs761	ID=COSM	.	.	.	.	.
ENST0	.	17q12	.	.	Scc	.	.	.	.	.	.	.	.
ENST0	.	17q12	.	.	.	.	.	.	.	.	.	.	.
ENST0	.	17q12	.	.	Scc	.	rs370	.	.	.	.	.	.
ENST0	.	17q12	.	.	Scc	Score=173	rs372	.	.	.	.	.	.
ENST0	.	17q12	.	.	Scc	.	.	ID=COSN1	.	.	.	.	.
ENST0	.	17q12	.	.	.	.	rs754	.	.	.	.	.	.
ENST0	.	17q12	.	.	.	Score=308	.	.	.	.	.	.	.
ENST0	.	17q12	.	.	.	.	rs780	.	.	.	.	.	.
ENST0	.	17q12	.	.	.	.	.	.	.	.	.	.	.
ENST0	.	17q12	.	.	.	.	.	.	.	.	.	.	.

ENST0	.	17q12	.	.	.	.	rs105	ID=COSM1	Lik	.	.	.	.
ENST0	.	17q21.2	.	.	.	.	rs199	.	.	.	.	.	8E-04
ENST0	.	17q21.2	.	.	.	.	rs148	ID=COSM6	.	.	.	.	2E-04
ENST0	.	17q21.2	.	Scc	.	.	rs782	.	.	.	.	.	.
ENST0	.	17q21.2	.	.	.	.	rs782	.	.	.	.	.	.
ENST0	.	17q21.2	.	.	.	.	.	.	.	.	.	.	.
ENST0	.	17q21.2	.	.	.	.	.	.	.	.	.	.	.
ENST0	.	17q21.2	.	.	.	.	rs906	.	.	.	.	.	.
ENST0	.	17q21.2	.	.	.	Score=184	rs576	.	.	.	.	0.003	6E-04
ENST0	.	17q21.2	.	.	.	.	.	.	.	.	.	.	.
ENST0	.	17q21.31	.	.	.	.	rs104	.	.	.	.	.	.
ENST0	.	17q21.31	.	.	Scc	.	rs998	.	.	.	.	.	.
ENST0	.	17q21.31	.	.	.	.	.	.	.	.	.	.	.
ENST0	.	17q21.31	.	.	.	.	.	.	.	.	.	.	.
ENST0	.	17q21.31	.	Scc	.	.	.	.	.	.	.	.	.
ENST0	.	17q21.31	.	.	.	.	.	.	.	.	.	.	.
ENST0	.	17q21.31	.	Scc	.	.	rs761	.	.	.	.	.	.
ENST0	.	17q21.31	.	Scc	.	.	.	.	.	.	.	.	.
ENST0	.	17q21.31	.	.	.	.	rs913	.	.	.	.	.	.
ENST0	.	17q21.31	.	.	.	.	.	.	.	.	.	.	.
ENST0	.	17q21.31	.	Scc	.	.	rs780	.	.	.	.	.	.
ENST0	.	17q21.31	.	.	Scc	.	.	.	.	.	.	.	.
ENST0	.	17q21.31	.	.	Scc	.	.	.	.	.	.	.	.
ENST0	.	17q21.31	.	.	Scc	.	.	.	.	.	.	.	.
ENST0	.	17q21.31	.	.	.	.	rs100	.	.	.	.	.	.
ENST0	.	17q21.32	.	.	Scc	.	rs105	.	.	.	.	0.304	0.402
ENST0	.	17q21.32	.	.	Scc	.	rs264	ID=COSM3	.	.	.	.	.
ENST0	.	17q21.32	.	.	.	.	.	.	.	.	.	.	.
ENST0	.	17q21.32	.	.	.	.	rs374	.	.	.	.	.	.
ENST0	.	17q21.32	.	.	.	.	rs937	ID=COSM4	.	.	.	.	.
ENST0	.	17q21.33	.	.	.	.	.	.	.	.	.	.	.
ENST0	.	17q21.33	.	Scc	.	.	rs778	.	.	.	.	.	.
ENST0	.	17q21.33	.	.	.	Score=155	.	.	.	.	.	.	.
ENST0	.	17q21.33	.	.	.	.	.	.	.	.	.	.	.
ENST0	.	17q21.33	.	.	.	.	rs102	.	.	.	.	.	.
ENST0	.	17q21.33	.	.	.	.	.	.	.	.	.	.	.
ENST0	.	17q21.33	.	.	.	Score=198	rs389	ID=COSN2	.	.	.	.	.
ENST0	.	17q22	.	.	.	.	.	.	.	.	.	.	.
ENST0	.	17q22	.	.	.	.	.	.	.	.	.	.	.
ENST0	.	17q23.2	.	.	.	Score=243	rs367	.	.	.	.	.	1E-03
ENST0	.	17q23.3	.	.	Scc	.	.	.	.	.	.	.	.
ENST0	.	17q23.3	.	Scc	.	.	rs763	ID=COSM1	Lik	.	.	.	.
ENST0	.	17q24.1	.	.	Scc	.	.	.	.	.	.	.	.
ENST0	.	17q24.2	.	.	.	.	.	.	.	.	.	.	.
ENST0	.	17q24.3	.	.	.	.	.	.	.	.	.	.	.
ENST0	.	17q25.1	.	.	.	Score=163	.	.	.	.	.	.	.
.	.	17q25.1	.	Scc	.	.	.	.	.	.	.	.	.
ENST0	.	17q25.1	.	Scc	.	.	rs561	.	.	.	.	.	2E-04
ENST0	.	17q25.1	.	.	.	.	rs101	.	.	.	.	.	.
ENST0	.	17q25.1	.	.	.	Score=620	rs377	.	.	.	.	.	.
ENST0	.	17q25.1	.	.	.	.	.	.	.	.	.	.	.
ENST0	.	17q25.1	.	.	.	.	.	.	.	.	.	.	.
ENST0	.	17q25.1	.	.	.	.	.	.	.	.	.	.	.
ENST0	.	17q25.1	.	.	.	.	.	.	.	.	.	.	.

ENST0	.	17q25.1	.	Scc	.	.	rs369	.	.	.	.	.	.
ENST0	.	17q25.1	.	.	.	.	.	.	.	.	.	.	.
ENST0	.	17q25.2	.	.	.	.	.	.	.	.	.	.	.
ENST0	.	17q25.2	.	.	.	.	rs767	.	.	.	.	.	.
ENST0	.	17q25.3	.	.	.	.	.	.	.	.	.	.	.
ENST0	.	17q25.3	.	Scc	.	.	rs752	.	.	.	.	.	.
ENST0	.	17q25.3	.	.	.	.	.	.	.	.	.	.	.
ENST0	.	17q25.3	.	Scc	.	.	.	.	.	.	.	.	.
ENST0	.	17q25.3	.	.	.	.	rs535	ID=COSM9	.	.	.	.	2E-04
ENST0	.	17q25.3	.	.	.	.	rs773	.	.	.	.	.	.
ENST0	.	17q25.3	.	.	.	.	rs367	.	.	.	.	.	.
ENST0	.	17q25.3	.	.	.	.	.	.	.	.	.	.	.
ENST0	.	17q25.3	.	.	.	.	.	.	.	.	.	.	.
ENST0	.	17q25.3	.	.	.	.	.	.	.	.	.	.	.
ENST0	.	17q25.3	.	.	.	.	.	.	.	.	.	.	.
ENST0	.	17q25.3	.	.	.	.	rs367	.	.	.	.	.	.
ENST0	.	17q25.3	.	.	.	.	.	.	.	.	.	.	.
ENST0	.	17q25.3	.	.	.	.	.	ID=COSM2	.	.	.	.	.
ENST0	.	17q25.3	.	.	.	.	.	.	.	.	.	.	.
ENST0	.	17q25.3	.	.	.	.	.	.	.	.	.	.	.
ENST0	.	17q25.3	.	.	.	.	.	.	.	.	.	.	.
ENST0	.	17q25.3	.	.	.	.	rs759	ID=COSM5	.	.	.	.	.
ENST0	.	17q25.3	.	.	.	.	.	.	.	.	.	.	.
ENST0	.	17q25.3	.	Scc	.	.	rs145	ID=COSM7	.	.	.	.	.
ENST0	.	17q25.3	.	.	.	.	.	.	.	.	.	.	.
ENST0	.	18p11.32	.	.	.	.	.	.	.	.	.	.	.
ENST0	.	18p11.32	.	.	.	.	.	.	.	.	.	.	.
ENST0	.	18p11.21	.	.	.	.	.	.	.	.	.	.	.
ENST0	.	18p11.21	.	.	.	Score=238	.	ID=COSN1	.	.	.	.	.
ENST0	.	18p11.21	.	.	.	.	.	.	.	.	.	.	.
ENST0	.	18p11.21	.	.	Scc	.	.	.	.	.	.	.	.
ENST0	.	18p11.21	.	.	Scc	.	.	ID=COSN1	.	.	.	.	.
ENST0	.	18q11.2	.	.	.	.	.	.	.	.	.	.	.
ENST0	.	18q11.2	.	.	.	.	rs139	.	.	.	.	.	2E-04
ENST0	.	18q11.2	.	.	.	Score=173	.	.	.	.	.	.	.
ENST0	.	18q11.2	.	.	.	.	rs545	.	.	.	.	0.001	2E-04
ENST0	.	18q12.1	.	.	.	.	rs779	.	.	.	.	.	.
ENST0	.	18q12.2	.	.	.	.	.	.	.	.	.	.	.
ENST0	.	18q12.3	.	.	.	.	.	.	.	.	.	.	.
ENST0	.	18q21.1	.	.	.	.	rs778	.	.	.	.	.	.
ENST0	.	18q21.1	.	.	.	.	.	.	.	.	.	.	.
ENST0	.	18q21.1	.	.	.	.	rs771	.	.	.	.	.	.
ENST0	.	18q21.1	.	.	.	.	.	.	.	.	.	.	.
ENST0	.	18q21.32	.	.	Scc	.	.	.	.	.	.	.	.
ENST0	.	18q21.33	.	.	.	.	.	.	.	.	.	.	.
ENST0	.	18q21.33	.	.	.	.	rs141	.	.	.	.	.	4E-04
ENST0	.	18q21.33	.	.	Scc	.	.	.	.	.	.	.	.
ENST0	.	18q22.2	.	.	.	.	.	ID=COSM4	.	.	.	.	.
ENST0	.	18q22.3	.	.	.	.	.	.	.	.	.	.	.
ENST0	.	18q23	.	.	.	.	.	.	.	.	.	.	.
ENST0	.	19p13.3	.	.	.	.	rs102	.	.	.	.	.	.
ENST0	.	19p13.3	.	.	.	.	.	ID=COSN2	.	.	.	.	.
ENST0	.	19p13.3	.	.	.	.	rs374	.	.	.	.	.	.
ENST0	.	19p13.3	.	.	.	.	rs778	.	.	.	.	.	.

ENST0	.	19p13.3	.	.	.	.	.	.	.	.	.	.	.
ENST0	.	19p13.3	.	.	.	.	rs377	.	.	.	.	0.001	2E-04
ENST0	.	19p13.3	.	Scc	.	.	.	.	.	.	.	.	.
ENST0	.	19p13.3	.	.	.	.	rs945	.	.	.	.	.	.
ENST0	.	19p13.3	.	.	.	.	.	.	.	.	.	.	.
ENST0	.	19p13.3	.	.	.	.	rs762	ID=COSM4	.	.	.	.	2E-04
ENST0	.	19p13.3	.	Scc	.	.	rs202	.	.	.	.	.	4E-04
ENST0	.	19p13.3	.	.	.	.	.	.	.	.	.	.	.
ENST0	.	19p13.3	.	.	.	Score=196	rs779	.	.	.	.	.	.
ENST0	.	19p13.3	.	.	.	.	.	.	.	.	.	.	.
ENST0	.	19p13.3	.	.	.	.	.	.	.	.	.	.	.
ENST0	.	19p13.3	.	.	.	.	rs375	.	.	.	.	.	.
ENST0	.	19p13.3	.	.	.	Score=32;I	.	.	.	.	.	.	.
ENST0	.	19p13.3	.	.	.	.	.	.	.	.	.	.	.
ENST0	.	19p13.3	.	.	.	.	rs100	.	.	.	.	.	.
ENST0	.	19p13.3	.	Scc	.	.	rs369	.	.	.	.	.	4E-04
ENST0	.	19p13.3	.	.	.	.	rs752	ID=COSN9	.	.	.	.	.
ENST0	.	19p13.3	.	.	.	.	.	ID=COSM6	.	.	.	.	.
ENST0	.	19p13.3	.	.	.	.	.	.	.	.	.	.	.
ENST0	.	19p13.3	.	Scc	.	.	rs376	.	.	.	.	.	.
ENST0	.	19p13.3	.	.	.	.	rs777	.	.	.	.	.	.
ENST0	.	19p13.3	.	.	.	Score=289	.	.	.	.	.	.	.
ENST0	.	19p13.3	.	.	.	Score=557	.	.	.	.	.	.	.
ENST0	.	19p13.3	.	.	.	.	rs759	.	.	.	.	.	.
ENST0	.	19p13.3	.	.	.	.	.	ID=COSM7	.	.	.	.	.
ENST0	.	19p13.3	.	.	.	.	rs150	.	.	.	.	.	6E-04
ENST0	.	19p13.3	.	.	.	Score=216	.	.	.	.	.	.	.
ENST0	.	19p13.3	.	.	.	.	.	.	.	.	.	.	.
ENST0	.	19p13.3	.	.	.	.	.	.	.	.	.	.	.
ENST0	.	19p13.3	.	.	.	.	rs101	.	.	.	.	.	.
ENST0	.	19p13.3	.	.	.	.	rs763	ID=COSM1	.	.	.	.	.
ENST0	.	19p13.3	.	.	.	Score=378	.	.	.	.	.	.	.
ENST0	.	19p13.2	.	.	.	.	rs139	.	.	.	.	.	6E-04
ENST0	.	19p13.2	.	.	.	.	rs377	.	.	.	.	.	.
ENST0	.	19p13.2	.	.	.	.	rs374	.	.	.	.	.	.
ENST0	.	19p13.2	.	.	.	.	rs751	.	.	.	.	.	.
ENST0	.	19p13.2	.	.	.	.	rs141	ID=COSM4	.	.	.	.	2E-04
ENST0	.	19p13.2	.	.	Scc	.	.	.	.	.	.	.	.
ENST0	.	19p13.2	.	.	.	.	.	.	.	.	.	.	.
ENST0	.	19p13.2	.	.	.	.	rs759	.	.	.	.	.	.
ENST0	.	19p13.2	.	.	.	.	rs352	ID=COSM3	.	.	.	0.005	0.015
ENST0	.	19p13.2	.	.	.	.	rs761	.	.	.	.	.	.
ENST0	.	19p13.2	.	.	.	.	rs530	.	.	.	.	0.007	0.002
ENST0	.	19p13.2	.	.	.	.	.	ID=COSM4	.	.	.	.	.
.	.	19p13.2	.	.	Scc	Score=149	rs369	.	.	.	.	.	.
.	.	19p13.2	.	.	Scc	Score=198	rs375	.	.	.	.	.	.
ENST0	.	19p13.2	.	.	Scc	.	rs568	.	.	.	.	0.001	6E-04
ENST0	.	19p13.2	.	.	.	.	rs780	.	.	.	.	.	.
ENST0	.	19p13.2	.	.	.	.	.	.	.	.	.	.	.
ENST0	.	19p13.2	.	.	.	.	.	.	.	.	.	.	.
ENST0	.	19p13.2	.	Scc	.	.	rs756	.	.	.	.	.	.
ENST0	.	19p13.2	.	Scc	.	.	rs781	ID=COSM5	.	.	.	.	.
ENST0	.	19p13.2	.	.	.	.	.	.	.	.	.	.	.
ENST0	.	19p13.2	.	Scc	.	.	.	.	.	.	.	.	.

ENST0	.	19p13.2	.	.	.	.	.	.	.	.	.	.	.
ENST0	.	19p13.2	.	.	.	.	.	.	.	.	.	.	.
ENST0	.	19p13.2	.	.	.	.	.	.	.	.	.	.	.
ENST0	.	19p13.2	.	Scc	.	.	rs138	ID=COSM	.	.	.	.	2E-04
ENST0	.	19p13.2	.	.	.	.	rs200	.	.	.	.	.	.
ENST0	.	19p13.2	.	.	.	Score=216	.	.	.	.	.	.	.
ENST0	.	19p13.2	.	.	.	.	.	.	.	.	.	.	.
ENST0	.	19p13.2	.	Scc	.	.	rs763	ID=COSM	.	.	.	.	.
ENST0	.	19p13.2	.	.	.	.	rs373	ID=COSN	.	.	.	.	.
ENST0	.	19p13.2	.	.	.	.	rs372	.	.	.	.	.	.
ENST0	.	19p13.2	.	.	.	.	.	.	.	.	.	.	.
ENST0	.	19p13.2	.	.	.	Score=224	rs538	ID=COSN	.	.	.	.	8E-04
ENST0	.	19p13.2	.	.	.	Score=232	.	.	.	.	.	.	.
ENST0	.	19p13.2	.	Scc	.	.	rs371	.	.	.	.	.	.
ENST0	.	19p13.12	.	Scc	.	.	.	ID=COSM	.	.	.	.	.
ENST0	.	19p13.12	.	Scc	.	.	.	.	.	.	.	.	.
ENST0	.	19p13.12	.	Scc	.	Score=22;	.	.	.	.	.	.	.
ENST0	.	19p13.12	.	.	Scc	.	.	ID=COSM	.	.	.	.	.
ENST0	.	19p13.11	.	.	.	.	.	.	.	.	.	.	.
ENST0	.	19p13.11	.	.	.	.	rs101	.	.	.	.	.	.
ENST0	.	19p13.11	.	.	.	.	.	.	.	.	.	.	.
ENST0	.	19p13.11	.	.	.	Score=305	rs113	.	.	.	.	.	4E-04
ENST0	.	19p13.11	.	.	.	Score=230	.	.	.	.	.	.	.
ENST0	.	19p13.11	.	.	.	.	rs929	ID=COSM	.	.	.	.	.
ENST0	.	19p13.11	.	.	.	.	rs769	.	.	.	.	.	.
ENST0	.	19p13.11	.	.	.	.	rs104	.	.	.	.	.	.
ENST0	.	19p13.11	.	.	.	Score=231	.	.	.	.	.	.	.
ENST0	.	19p13.11	.	.	.	.	.	.	.	.	.	.	.
ENST0	.	19p13.11	.	.	.	.	.	.	.	.	.	.	.
ENST0	.	19p13.11	.	.	.	.	rs371	.	.	.	.	.	.
ENST0	.	19p13.11	.	.	.	.	rs201	.	.	.	.	.	2E-04
ENST0	.	19p12	.	.	.	.	.	.	.	.	.	.	.
ENST0	.	19p12	.	.	Scc	.	.	.	.	.	.	.	.
ENST0	.	19p12	.	.	Scc	.	.	.	.	.	.	.	.
ENST0	.	19q12	.	.	.	Score=319	.	.	.	.	.	.	.
ENST0	.	19q12	.	Scc	.	.	rs754	ID=COSM	.	.	.	.	.
ENST0	.	19q13.11	.	Scc	.	.	rs375	.	.	.	.	.	.
ENST0	.	19q13.11	.	.	.	.	.	.	.	.	.	.	.
ENST0	.	19q13.12	.	.	.	Score=243	.	.	.	.	.	.	.
ENST0	.	19q13.12	.	Scc	.	.	rs765	.	.	.	.	.	.
ENST0	.	19q13.12	.	.	.	.	.	.	.	.	.	.	.
ENST0	.	19q13.12	.	.	.	.	.	.	.	.	.	.	.
ENST0	.	19q13.12	.	.	.	Score=227	rs558	.	.	.	.	.	2E-04
.	.	19q13.12	.	.	Scc	Score=123	.	.	.	.	.	.	.
.	.	19q13.12	.	.	Scc	.	rs480	.	.	.	.	.	.
.	.	19q13.12	.	.	Scc	Score=123	rs712	.	.	.	.	.	.
.	.	19q13.12	.	.	Scc	Score=123	.	.	.	.	.	.	.
.	.	19q13.13	.	.	.	Score=130	rs772	.	.	.	.	.	.
ENST0	.	19q13.13	.	Scc	.	.	.	.	.	.	.	.	.
ENST0	.	19q13.2	.	.	.	.	rs147	ID=COSM	.	.	.	.	6E-04
ENST0	.	19q13.2	.	.	.	Score=644	.	.	.	.	.	.	.
ENST0	.	19q13.2	.	.	.	.	rs375	.	.	.	.	0.001	2E-04
ENST0	.	19q13.2	.	.	.	Score=141	rs537	.	.	.	.	0.003	0.004
ENST0	.	19q13.2	.	.	.	.	.	.	.	.	.	.	.

ENST0	.	19q13.2	.	.	.	.	.	.	.	.	.	.	.
ENST0	.	19q13.2	.	.	.	Score=242	rs572	.	.	.	.	0.008	0.009
ENST0	.	19q13.2	.	.	Scc	.	rs891	.	.	.	.	.	.
ENST0	.	19q13.2	.	.	Scc	.	.	.	.	.	.	.	.
ENST0	.	19q13.2	.	.	.	.	rs756	ID=COSN2	.	.	.	.	.
ENST0	.	19q13.2	.	Scc	.	.	rs773	ID=COSM1	.	.	.	.	.
ENST0	.	19q13.2	.	.	Scc	.	.	.	.	.	.	.	.
ENST0	.	19q13.2	.	Scc	.	.	rs746	.	.	.	.	.	.
ENST0	.	19q13.2	.	.	.	Score=948	.	.	.	.	.	.	.
ENST0	.	19q13.2	.	.	.	.	.	.	.	.	.	.	.
ENST0	.	19q13.2	.	.	.	.	.	.	.	.	.	.	.
ENST0	.	19q13.2	.	Scc	.	.	.	.	.	.	.	.	.
ENST0	.	19q13.2	.	.	.	.	.	.	.	.	.	.	.
ENST0	.	19q13.2	.	.	.	.	rs104	.	.	.	.	.	.
ENST0	.	19q13.2	.	.	.	.	.	.	.	.	.	.	.
ENST0	.	19q13.2	.	.	.	.	.	.	.	.	.	.	.
ENST0	.	19q13.2	.	.	.	.	.	.	.	.	.	.	.
ENST0	.	19q13.2	.	Scc	.	.	.	.	.	.	.	.	.
ENST0	.	19q13.2	.	.	.	.	.	.	.	.	.	.	.
ENST0	.	19q13.2	.	.	Scc	.	.	.	.	.	.	.	.
ENST0	.	19q13.2	.	.	Scc	.	.	.	.	.	.	.	.
ENST0	.	19q13.31	.	.	.	.	.	.	.	.	.	.	.
ENST0	.	19q13.31	.	.	.	.	.	.	.	.	.	.	.
ENST0	.	19q13.31	.	.	Scc	.	.	.	.	.	.	.	.
ENST0	.	19q13.31	.	.	.	.	rs773	ID=COSM(	.	.	.	.	.
ENST0	.	19q13.31	.	.	.	.	rs907	.	.	.	.	.	.
ENST0	.	19q13.31	.	.	.	.	.	.	.	.	.	.	.
ENST0	.	19q13.32	.	.	.	Score=268	rs563	.	.	.	.	.	2E-04
ENST0	.	19q13.32	.	.	.	.	.	.	.	.	.	.	.
ENST0	.	19q13.32	.	.	.	.	.	.	.	.	.	.	.
ENST0	.	19q13.32	.	.	.	.	.	.	.	.	.	.	.
ENST0	.	19q13.32	.	Scc	.	.	.	.	.	.	.	.	.
ENST0	.	19q13.32	.	.	.	.	rs102	.	.	.	.	.	.
ENST0	.	19q13.32	.	Scc	.	.	rs369	ID=COSM4	.	.	.	.	.
ENST0	.	19q13.32	.	.	.	Score=103	.	.	.	.	.	.	.
ENST0	.	19q13.33	.	.	.	.	.	.	.	.	.	.	.
ENST0	.	19q13.33	.	.	.	.	.	.	.	.	.	.	.
.	.	19q13.33	.	.	Scc	Score=300	rs621	.	.	.	.	.	.
ENST0	.	19q13.33	.	.	.	.	.	.	.	.	.	.	.
ENST0	.	19q13.33	.	.	.	.	rs101	ID=COSN2	.	.	.	.	.
ENST0	.	19q13.33	.	.	.	.	.	.	.	.	.	.	.
ENST0	.	19q13.33	.	.	.	.	.	.	.	.	.	.	.
ENST0	.	19q13.33	.	.	.	.	rs562	.	.	.	.	.	4E-04
ENST0	.	19q13.33	.	.	.	.	.	.	.	.	.	.	.
ENST0	.	19q13.33	.	.	.	.	.	.	.	.	.	.	.
ENST0	.	19q13.33	.	.	.	.	rs750	.	.	.	.	.	.
ENST0	.	19q13.33	.	.	.	.	rs374	ID=COSM4	.	.	.	.	.
ENST0	.	19q13.33	.	.	.	.	rs200	.	.	.	.	0.001	6E-04
ENST0	.	19q13.33	.	.	.	.	.	.	.	.	.	.	.
ENST0	.	19q13.33	.	.	.	.	rs564	.	.	.	.	.	0.015
ENST0	.	19q13.33	.	.	.	.	rs369	.	Lik	.	.	.	.
ENST0	.	19q13.33	.	.	.	.	.	.	.	.	.	.	.
ENST0	.	19q13.33	.	.	.	.	rs773	ID=COSM4	.	.	.	.	.
ENST0	.	19q13.33	.	.	.	.	.	.	.	.	.	.	.

ENST0	.	19q13.33	.	.	Scc	.	.	.	.	.	.	.	.
ENST0	.	19q13.41	.	.	.	.	.	.	.	.	.	.	.
ENST0	.	19q13.41	.	.	.	Score=113	.	.	.	.	.	.	.
ENST0	.	19q13.42	.	.	.	.	rs142	ID=COSM4	.	.	.	.	.
ENST0	hsa-mir-52	19q13.42	.	.	.	.	.	.	.	.	.	.	.
ENST0	.	19q13.42	.	.	.	.	.	.	.	.	.	.	.
ENST0	.	19q13.42	.	.	.	.	.	.	.	.	.	.	.
ENST0	.	19q13.42	.	.	.	.	.	.	.	.	.	.	.
ENST0	.	19q13.42	.	.	Scc	.	rs370	.	.	.	.	.	.
ENST0	.	19q13.42	.	.	.	.	.	.	.	.	.	.	.
ENST0	.	19q13.42	.	.	.	.	rs752	.	.	.	.	.	.
ENST0	.	19q13.42	.	.	.	.	.	.	.	.	.	.	.
ENST0	.	19q13.42	.	.	.	.	rs370	.	.	.	.	.	.
ENST0	.	19q13.43	.	.	.	.	rs200	.	.	.	.	.	.
ENST0	.	19q13.43	.	.	.	.	rs770	ID=COSM4	.	.	.	.	.
ENST0	.	19q13.43	.	.	Scc	.	.	.	.	.	.	.	.
ENST0	.	19q13.43	.	.	.	.	.	.	.	.	.	.	.
ENST0	.	19q13.43	.	.	.	.	rs147	.	.	.	.	.	.
ENST0	.	19q13.43	.	.	.	.	.	.	.	.	.	.	.
ENST0	.	19q13.43	.	.	.	.	rs373	.	.	.	.	.	.
ENST0	.	20p13	.	.	Scc	.	rs102	ID=COSM6	.	.	.	.	.
ENST0	.	20p13	.	.	.	.	rs762	.	.	.	.	.	.
ENST0	.	20p13	.	.	.	.	.	.	.	.	.	.	.
ENST0	.	20p13	.	.	.	Score=236	rs867	.	.	.	.	.	.
ENST0	.	20p13	.	.	.	.	.	.	.	.	.	.	.
ENST0	.	20p13	.	.	.	.	rs763	.	.	.	.	.	.
ENST0	.	20p13	.	.	.	.	.	.	.	.	.	.	.
ENST0	.	20p12.2	.	.	.	Score=230	rs103	.	.	.	.	.	.
ENST0	.	20p12.1	.	.	.	.	.	.	.	.	.	.	.
ENST0	.	20p11.23	.	.	.	.	.	.	.	.	.	.	.
ENST0	.	20p11.23	.	.	.	Score=268	.	.	.	.	.	.	.
ENST0	.	20p11.21	.	.	Scc	.	rs150	ID=COSM1	.	.	.	.	2E-04
ENST0	.	20p11.21	.	.	.	.	.	.	.	.	.	.	.
ENST0	.	20p11.1	.	.	Scc	.	.	ID=COSN1	.	.	.	.	.
ENST0	.	20q11.21	.	.	Scc	.	.	ID=COSN2	.	.	.	.	.
ENST0	.	20q11.21	.	.	Scc	.	.	ID=COSN2	.	.	.	.	.
ENST0	.	20q11.21	.	.	.	.	rs184	ID=COSM5	.	.	.	0.001	2E-04
ENST0	.	20q11.21	.	.	.	.	.	.	.	.	.	.	.
ENST0	.	20q11.22	.	.	.	.	.	.	.	.	.	.	.
ENST0	.	20q11.22	.	.	.	Score=686	.	.	.	.	.	.	.
ENST0	.	20q11.22	.	.	.	.	rs746	ID=COSN2	.	.	.	0.001	0.022
ENST0	.	20q11.22	.	.	.	.	.	.	.	.	.	.	.
ENST0	.	20q11.22	.	Scc	.	.	rs148	.	.	.	.	.	.
ENST0	.	20q11.22	.	.	.	Score=291	rs543	ID=COSN1	.	.	.	.	0.001
ENST0	.	20q11.23	.	.	.	.	.	.	.	.	.	.	.
ENST0	.	20q11.23	.	.	.	.	.	.	.	.	.	.	.
ENST0	.	20q11.23	.	Scc	.	Score=396	rs879	.	.	.	.	.	.
ENST0	.	20q11.23	.	.	.	.	.	.	.	.	.	.	.
ENST0	.	20q11.23	.	.	.	.	rs984	.	.	.	.	.	.
ENST0	.	20q11.23	.	Scc	.	.	rs946	.	.	.	.	.	.
ENST0	.	20q12	.	.	.	.	rs371	.	.	.	.	0.001	2E-04
ENST0	.	20q12	.	.	.	.	.	.	.	.	.	.	.
ENST0	.	20q12	.	Scc	.	.	.	.	.	.	.	.	.
ENST0	.	20q12	.	.	.	.	.	.	.	.	.	.	.

ENST0	.	20q12	.	.	.	.	rs376	ID=COSM4	.	.	.	.	.
ENST0	.	20q13.12	.	.	.	.	.	.	.	.	.	.	.
ENST0	.	20q13.12	.	.	.	.	.	.	.	.	.	.	.
ENST0	.	20q13.12	.	.	.	Score=167	.	.	.	.	.	.	.
ENST0	.	20q13.12	.	.	.	.	.	.	.	.	.	.	.
ENST0	.	20q13.12	.	.	.	.	.	.	.	.	.	.	.
ENST0	.	20q13.12	.	Scc	.	.	.	ID=COSM5	.	.	.	.	.
ENST0	.	20q13.13	.	.	.	.	.	ID=COSM4	.	.	.	.	.
ENST0	.	20q13.2	.	.	.	.	.	.	.	.	.	.	.
ENST0	.	20q13.2	.	.	.	.	.	.	.	.	.	.	.
ENST0	.	20q13.2	.	.	.	.	rs889	.	.	.	.	.	.
.	.	20q13.2	.	.	.	Score=865	rs866	.	.	.	.	.	.
ENST0	.	20q13.32	.	.	.	.	.	.	.	.	.	.	.
ENST0	.	20q13.32	.	.	.	.	.	.	.	.	.	.	.
ENST0	.	20q13.33	.	.	.	.	.	.	.	.	.	.	.
ENST0	.	20q13.33	.	.	.	.	rs770	.	.	.	.	.	.
ENST0	.	20q13.33	.	.	.	.	rs778	.	.	.	.	.	.
ENST0	.	20q13.33	.	.	.	.	rs181	.	.	.	.	0.002	4E-04
ENST0	.	20q13.33	.	Scc	.	.	rs143	.	.	.	.	.	0.002
ENST0	.	20q13.33	.	.	.	.	.	.	.	.	.	.	.
ENST0	.	20q13.33	.	.	.	.	.	.	.	.	.	.	.
ENST0	.	20q13.33	.	.	.	.	rs546	ID=COSM5	.	.	.	.	2E-04
ENST0	.	21p11.2	.	.	Scc	.	rs796	.	.	.	.	.	.
.	.	21p11.2	.	.	Scc	.	.	.	.	.	.	.	.
.	.	21p11.2	.	.	Scc	Score=298	.	.	.	.	.	.	.
.	.	21p11.2	.	.	Scc	Score=348	.	.	.	.	.	.	.
ENST0	.	21p11.2	.	.	Scc	Score=333	rs868	.	.	.	.	.	.
ENST0	.	21p11.2	.	.	Scc	Score=333	rs371	.	.	.	.	.	.
.	.	21p11.2	.	.	Scc	.	rs377	ID=COSN1	.	.	.	.	.
ENST0	.	21p11.1	.	.	Scc	Score=756	.	.	.	.	.	.	.
ENST0	.	21p11.1	.	.	Scc	.	.	.	.	.	.	.	.
ENST0	.	21p11.1	.	.	Scc	.	rs528	.	.	.	.	.	2E-04
.	.	21p11.1	.	.	Scc	Score=144	.	.	.	.	.	.	.
.	.	21p11.1	.	.	Scc	Score=263	rs665	.	.	.	.	.	.
.	.	21p11.1	.	.	Scc	Score=263	rs658	ID=COSN1	.	.	.	.	.
ENST0	.	21p11.1	.	.	Scc	.	rs915	ID=COSN1	.	.	.	.	.
ENST0	.	21q11.2	.	.	Scc	Score=247	.	.	.	.	.	.	.
ENST0	.	21q11.2	.	.	Scc	.	.	ID=COSM6	.	.	.	.	.
ENST0	.	21q21.1	.	.	.	Score=297	.	.	.	.	.	.	.
ENST0	.	21q21.3	.	.	.	.	.	.	.	.	.	.	.
ENST0	.	21q21.3	.	.	.	.	.	.	.	.	.	.	.
ENST0	.	21q21.3	.	.	.	.	rs917	.	.	.	.	.	.
ENST0	.	21q22.11	.	.	.	.	.	.	.	.	.	.	.
ENST0	.	21q22.11	.	.	.	Score=160	.	.	.	.	.	.	.
ENST0	.	21q22.11	.	.	.	.	.	.	.	.	.	.	.
ENST0	.	21q22.11	.	.	.	.	rs103	.	.	.	.	.	.
ENST0	.	21q22.11	.	Scc	.	Score=261	.	.	.	.	.	.	.
ENST0	.	21q22.11	.	.	.	.	rs758	.	.	.	.	.	.
ENST0	.	21q22.11	.	.	.	.	.	.	.	.	.	.	.
ENST0	.	21q22.12	.	.	.	Score=454	.	.	.	.	.	.	.
ENST0	.	21q22.13	.	.	.	.	.	.	.	.	.	.	.
ENST0	.	21q22.13	.	Scc	.	Score=453	rs558	.	.	.	.	.	.
ENST0	.	21q22.2	.	.	.	.	rs759	.	.	.	.	.	.
ENST0	.	21q22.2	.	.	.	.	rs758	ID=COSM1	.	.	.	.	.

ENST0	.	21q22.2	.	.	.	.	.	.	.	.	.	.	.
ENST0	.	21q22.2	.	Scc	.	.	.	.	.	.	.	.	.
ENST0	.	21q22.2	.	Scc	.	.	.	.	.	.	.	.	.
ENST0	.	21q22.2	.	.	.	.	.	ID=COSM4	.	.	.	.	.
ENST0	.	21q22.2	.	.	.	.	.	.	.	.	.	.	.
ENST0	.	21q22.3	.	.	.	.	.	.	.	.	.	.	.
ENST0	.	21q22.3	.	.	.	.	.	.	.	.	.	.	.
ENST0	.	21q22.3	.	.	.	Score=230	rs946	.	.	.	.	.	.
ENST0	.	21q22.3	.	Scc	.	.	.	.	.	.	.	.	.
ENST0	.	21q22.3	.	.	.	.	.	.	.	.	.	.	.
ENST0	.	21q22.3	.	.	.	.	rs101	.	.	.	.	.	.
ENST0	.	21q22.3	.	.	.	.	rs780	.	.	.	.	.	.
ENST0	.	21q22.3	.	Scc	.	.	rs566	.	.	.	.	.	2E-04
ENST0	.	21q22.3	.	.	.	.	rs985	.	.	.	.	.	.
ENST0	.	21q22.3	.	Scc	.	.	rs756	.	.	.	.	.	.
ENST0	.	21q22.3	.	.	.	.	rs758	ID=COSM5	.	.	.	.	.
ENST0	.	21q22.3	.	.	.	.	rs377	.	.	.	.	.	.
ENST0	.	21q22.3	.	.	.	.	.	.	.	.	.	.	.
ENST0	.	21q22.3	.	.	.	.	.	.	.	.	.	.	.
ENST0	.	21q22.3	.	.	.	.	rs751	.	.	.	.	.	.
ENST0	.	21q22.3	.	.	.	.	.	.	.	.	.	.	.
ENST0	.	22q11.1	.	.	Scc	Score=231	rs284	ID=COSN1	.	.	.	.	.
ENST0	.	22q11.1	.	.	Scc	.	rs879	.	.	.	.	.	.
ENST0	.	22q11.1	.	.	Scc	.	.	.	.	.	.	.	.
ENST0	.	22q11.1	.	.	Scc	.	rs124	ID=COSN1	.	.	.	.	.
ENST0	.	22q11.1	.	.	.	.	.	.	.	.	.	.	.
ENST0	.	22q11.21	.	.	.	.	rs555	.	.	.	.	.	4E-04
ENST0	.	22q11.21	.	.	.	Score=21;I	.	.	.	.	.	.	.
ENST0	.	22q11.21	.	.	Scc	.	rs189	.	.	.	.	.	0.002
ENST0	.	22q11.21	.	.	Scc	.	.	.	.	.	.	.	.
ENST0	.	22q11.21	.	.	.	.	.	.	.	.	.	.	.
ENST0	.	22q11.21	.	Scc	Scc	.	rs371	.	.	.	.	.	.
ENST0	.	22q11.21	.	.	.	.	rs772	.	.	.	.	.	.
ENST0	.	22q11.22	.	.	Scc	.	.	.	.	.	.	.	.
ENST0	.	22q11.22	.	.	.	.	rs377	.	.	.	.	.	.
ENST0	.	22q11.23	.	.	.	.	rs368	.	.	.	.	0.001	2E-04
ENST0	.	22q11.23	.	.	.	.	.	.	.	.	.	.	.
ENST0	.	22q11.23	.	.	.	.	.	.	.	.	.	.	.
ENST0	.	22q11.23	.	.	.	.	.	.	.	.	.	.	.
ENST0	.	22q11.23	.	.	Scc	.	rs772	.	.	.	.	.	.
ENST0	.	22q11.23	.	.	Scc	.	.	.	.	.	.	.	.
ENST0	.	22q12.1	.	.	.	.	.	.	.	.	.	.	.
ENST0	.	22q12.2	.	.	.	.	rs192	.	.	.	.	.	4E-04
ENST0	.	22q12.2	.	.	.	.	.	.	.	.	.	.	.
ENST0	.	22q12.2	.	.	.	.	.	.	.	.	.	.	.
ENST0	.	22q12.2	.	.	.	.	rs571	.	.	.	.	.	1E-03
ENST0	.	22q12.2	.	.	.	.	rs101	.	.	.	.	.	.
ENST0	.	22q12.2	.	.	.	.	.	.	.	.	.	.	.
.	.	22q12.3	.	.	Scc	.	.	.	.	.	.	.	.
ENST0	.	22q12.3	.	.	.	.	.	.	.	.	.	.	.
ENST0	.	22q12.3	.	.	.	.	.	.	.	.	.	.	.
ENST0	.	22q12.3	.	.	.	.	rs137	ID=COSM1	.	.	.	.	.
ENST0	.	22q12.3	.	.	.	.	.	.	.	.	.	.	.
ENST0	.	22q12.3	.	.	.	.	rs368	.	.	.	.	0.001	2E-04

ENST0	.	22q12.3	.	.	.	.	rs536	.	.	.	.	.	2E-04
ENST0	.	22q13.1	.	Scc	.	.	.	.	.	.	.	.	.
ENST0	.	22q13.1	.	.	.	.	.	.	.	.	.	.	.
ENST0	.	22q13.1	.	.	.	.	rs752	.	.	.	.	.	.
ENST0	.	22q13.1	.	Scc	.	.	.	ID=COSM4	.	.	.	.	.
ENST0	.	22q13.1	.	Scc	.	.	.	ID=COSM3	.	.	.	.	.
ENST0	.	22q13.1	.	.	.	.	rs571	.	.	.	.	0.001	2E-04
ENST0	.	22q13.1	.	.	Scc	.	.	.	.	.	.	.	.
ENST0	.	22q13.1	.	.	.	Score=701	rs371	.	.	.	.	0.005	1E-03
ENST0	.	22q13.1	.	.	.	.	.	.	.	.	.	.	.
ENST0	.	22q13.2	.	.	.	.	rs201	.	.	.	.	.	.
ENST0	.	22q13.2	.	Scc	.	.	.	.	.	.	.	.	.
ENST0	.	22q13.2	.	.	.	.	rs183	.	.	.	.	.	2E-04
ENST0	.	22q13.2	.	.	.	.	.	.	.	.	.	.	.
ENST0	.	22q13.2	.	.	Scc	.	.	.	.	.	.	.	.
ENST0	.	22q13.2	.	.	.	.	rs191	ID=COSM9	.	.	.	.	2E-04
ENST0	.	22q13.31	.	.	.	.	.	.	.	.	.	.	.
ENST0	.	22q13.31	.	Scc	.	.	rs558	.	.	.	.	0.001	2E-04
ENST0	.	22q13.31	.	.	.	.	rs130	ID=COSM1	.	.	.	.	.
ENST0	.	22q13.31	.	.	.	.	.	.	.	.	.	.	.
ENST0	.	22q13.31	.	.	.	.	.	.	.	.	.	.	.
ENST0	.	22q13.31	.	.	.	.	.	.	.	.	.	.	.
ENST0	.	22q13.31	.	.	.	.	.	.	.	.	.	.	.
ENST0	.	22q13.31	.	.	.	.	.	.	.	.	.	.	.
ENST0	.	22q13.33	.	.	.	.	.	.	.	.	.	.	.
ENST0	.	22q13.33	.	.	.	.	.	.	.	.	.	.	.
ENST0	.	22q13.33	.	Scc	.	.	rs559	.	.	.	.	.	.
ENST0	.	22q13.33	.	.	.	.	.	.	.	.	.	.	.
ENST0	.	22q13.33	.	Scc	.	.	.	.	.	.	.	.	.
ENST0	.	22q13.33	.	.	.	.	rs370	.	.	.	.	0.001	2E-04
ENST0	.	22q13.33	.	.	Scc	.	.	.	.	.	.	.	.
ENST0	.	22q13.33	.	Scc	.	.	.	ID=COSM1	.	.	.	.	.
ENST0	.	22q13.33	.	.	.	.	rs980	.	.	.	.	.	.
ENST0	.	Xp22.33	.	.	Scc	.	.	.	.	.	.	.	.
ENST0	.	Xp22.33	.	.	Scc	.	.	.	.	.	.	.	.
ENST0	.	Xp22.33	.	.	Scc	.	.	.	.	.	.	.	.
ENST0	.	Xp22.32	.	.	.	Score=240	.	.	.	.	.	.	.
ENST0	.	Xp22.2	.	.	.	.	.	.	.	.	.	.	.
ENST0	.	Xp22.2	.	.	.	.	.	.	.	.	.	.	.
ENST0	.	Xp22.2	.	.	.	.	.	.	.	.	.	.	.
ENST0	.	Xp22.13	.	Scc	.	.	.	.	.	.	.	.	.
ENST0	.	Xp22.12	.	Scc	.	.	.	.	.	.	.	.	.
ENST0	.	Xp22.11	.	.	.	.	rs373	.	.	.	.	.	.
ENST0	.	Xp21.3	.	.	.	.	.	.	.	.	.	.	.
ENST0	.	Xp21.3	.	.	.	Score=121	.	.	.	.	.	.	.
ENST0	.	Xp21.3	.	.	.	.	.	.	.	.	.	.	.
ENST0	.	Xp21.2	.	.	.	.	.	.	.	.	.	.	.
ENST0	.	Xp21.2	.	.	.	.	.	.	.	.	.	.	.
ENST0	.	Xp21.2	.	.	.	.	.	.	.	.	.	.	.
ENST0	.	Xp21.2	.	.	.	.	.	.	.	.	.	.	.
ENST0	.	Xp21.1	.	.	.	.	.	.	.	.	.	.	.
ENST0	.	Xp21.1	.	.	.	.	.	.	.	.	.	.	.
ENST0	.	Xp11.4	.	.	.	.	.	.	.	.	.	.	.
ENST0	.	Xp11.4	.	Scc	.	.	.	.	.	.	.	.	.

ENST0	.	Xp11.3	.	.	.	.	.	.	.	.	.	.	.
ENST0	.	Xp11.23	.	.	.	.	.	.	.	.	.	.	.
ENST0	.	Xp11.23	.	.	Scc	.	.	.	.	.	.	.	.
ENST0	.	Xp11.23	.	.	.	.	rs782	.	.	.	.	.	.
ENST0	.	Xp11.23	.	.	.	.	rs367	ID=COSM4	.	.	.	.	.
ENST0	.	Xp11.23	.	Scc	.	.	rs187	ID=COSM5	.	.	.	.	.
ENST0	.	Xp11.23	.	.	.	.	.	.	.	.	.	.	.
ENST0	.	Xp11.23	.	.	.	.	.	.	.	.	.	.	.
ENST0	.	Xp11.23	.	.	.	.	rs782	.	.	.	.	.	.
ENST0	.	Xp11.22	.	.	.	.	.	.	.	.	.	.	.
ENST0	.	Xp11.22	.	Scc	.	.	.	.	.	.	.	.	.
.	.	Xp11.22	.	.	Scc	.	.	.	.	.	.	.	.
ENST0	.	Xp11.22	.	.	.	.	rs782	.	.	.	.	.	.
ENST0	.	Xp11.22	.	.	.	Score=202	.	.	.	.	.	.	.
ENST0	.	Xp11.22	.	.	.	.	.	.	.	.	.	.	.
ENST0	.	Xp11.22	.	.	.	.	.	.	.	.	.	.	.
ENST0	.	Xp11.22	.	.	.	.	.	.	.	.	.	.	.
ENST0	.	Xp11.22	.	.	.	.	.	.	.	.	.	.	.
ENST0	.	Xp11.22	.	.	.	.	.	.	.	.	.	.	.
ENST0	.	Xp11.21	.	.	Scc	.	rs961	.	.	.	.	.	.
.	.	Xq11.1	.	.	.	Score=120	.	.	.	.	.	.	.
.	.	Xq11.1	.	.	.	Score=120	.	.	.	.	.	.	.
.	.	Xq11.1	.	.	.	Score=120	.	.	.	.	.	.	.
.	.	Xq11.1	.	.	.	Score=120	rs712	.	.	.	.	.	.
.	.	Xq11.1	.	.	.	Score=120	.	.	.	.	.	.	.
.	.	Xq11.1	.	.	.	Score=120	rs376	ID=COSN1	.	.	.	.	.
.	.	Xq11.1	.	.	.	Score=120	.	.	.	.	.	.	.
.	.	Xq11.1	.	.	.	Score=120	.	.	.	.	.	.	.
.	.	Xq11.1	.	.	.	Score=120	.	ID=COSN1	.	.	.	.	.
.	.	Xq11.1	.	.	.	Score=120	.	.	.	.	.	.	.
ENST0	.	Xq11.2	.	.	Scc	.	.	.	.	.	.	.	.
ENST0	.	Xq13.1	.	.	.	.	.	.	.	.	.	.	.
ENST0	.	Xq13.1	.	.	.	.	rs768	.	.	.	.	0.001	3E-04
ENST0	.	Xq13.1	.	.	.	.	.	.	.	.	.	.	.
ENST0	.	Xq13.1	.	Scc	.	.	rs750	.	.	.	.	.	.
ENST0	.	Xq13.1	.	.	.	.	rs863	.	Pa	.	.	.	.
ENST0	.	Xq13.1	.	.	.	Score=390	rs768	ID=COSN1	.	.	.	.	.
ENST0	.	Xq13.1	.	.	.	.	.	.	.	.	.	.	.
ENST0	.	Xq13.1	.	.	.	.	.	.	.	.	.	.	.
ENST0	.	Xq13.2	.	Scc	.	.	.	.	.	.	.	.	.
ENST0	.	Xq13.2	.	.	.	.	.	.	.	.	.	.	.
ENST0	.	Xq13.2	.	.	Scc	.	.	.	.	.	.	.	.
ENST0	.	Xq13.2	.	.	Scc	.	.	.	.	.	.	.	.
ENST0	.	Xq13.3	.	.	.	.	.	.	.	.	.	.	.
ENST0	.	Xq21.1	.	.	.	.	.	.	.	.	.	.	.
ENST0	.	Xq21.1	.	.	.	.	.	ID=COSM4	.	.	.	.	.
ENST0	.	Xq21.31	.	Scc	Scc	.	.	.	.	.	.	.	.
ENST0	.	Xq21.31	.	.	Scc	.	.	.	.	.	.	.	.
ENST0	.	Xq21.31	.	.	Scc	.	.	.	.	.	.	.	.
ENST0	.	Xq21.31	.	.	Scc	.	.	.	.	.	.	.	.
.	.	Xq21.33	.	.	.	Score=506	rs967	.	.	.	.	.	.
ENST0	.	Xq22.1	.	.	.	.	.	.	.	.	.	.	.
ENST0	.	Xq22.1	.	.	Scc	.	.	.	.	.	.	.	.
ENST0	.	Xq22.1	.	.	Scc	.	.	.	.	.	.	.	.

ENST0	.	Xq22.3	.	.	.	.	rs150	.	.	.	.	.	0.013
ENST0	.	Xq23	.	.	.	.	.	.	.	.	.	.	.
ENST0	.	Xq24	.	.	.	.	.	.	.	.	.	.	.
ENST0	.	Xq24	.	.	.	Score=114	rs866	.	.	.	.	.	.
ENST0	.	Xq24	.	.	.	.	.	.	.	.	.	.	.
ENST0	.	Xq24	.	.	.	Score=249	.	.	.	.	.	.	.
ENST0	.	Xq25	.	.	.	.	.	.	.	.	.	.	.
ENST0	.	Xq25	.	.	.	.	rs138	ID=COSM4	.	.	.	.	.
ENST0	.	Xq26.1	.	.	.	.	.	.	.	.	.	.	.
ENST0	.	Xq26.2	.	.	.	.	.	.	.	.	.	.	.
ENST0	.	Xq26.3	.	.	.	.	.	.	.	.	.	.	.
ENST0	.	Xq26.3	.	.	.	.	.	.	.	.	.	.	.
ENST0	.	Xq27.1	.	.	.	.	.	.	.	.	.	.	.
ENST0	.	Xq27.2	.	.	Scc	Score=278	.	ID=COSN8	.	.	.	.	.
ENST0	.	Xq28	.	.	.	.	.	.	.	.	.	.	.
ENST0	.	Xq28	.	.	Scc	.	rs782	ID=COSN1	.	.	.	.	.
ENST0	.	Xq28	.	.	.	.	.	.	.	.	.	.	.
ENST0	.	Xq28	.	.	.	.	rs371	.	.	.	.	.	.
ENST0	.	Xq28	.	Scc	.	.	.	.	.	.	.	.	.
ENST0	.	Xq28	.	.	.	.	.	.	.	.	.	.	.
ENST0	.	Xq28	.	.	.	.	rs782	.	.	.	.	.	.
ENST0	.	Xq28	.	.	.	.	.	.	.	.	.	.	.
ENST0	.	Xq28	.	.	.	.	.	.	.	.	.	.	.
ENST0	.	Xq28	.	.	.	.	.	.	.	.	.	.	.
ENST0	.	Xq28	.	.	.	.	.	.	.	.	.	.	.
ENST0	.	Xq28	.	.	.	.	.	.	.	.	.	.	.
ENST0	.	Xq28	.	.	.	.	rs781	ID=COSM1	.	.	.	.	.
ENST0	.	Xq28	.	.	.	.	.	.	.	.	.	.	.
ENST0	.	Xq28	.	.	.	.	.	.	.	.	.	.	.
ENST0	.	Xq28	.	.	.	.	.	.	.	.	.	.	.
ENST0	.	Xq28	.	.	.	.	.	.	.	.	.	.	.
.	.	Yq11.1	.	.	Scc	.	.	.	.	.	.	.	.
.	.	.	.	.	.	.	rs879	.	.	.	.	.	.
.	.	.	.	.	.	.	.	.	.	.	.	.	.
.	.	.	.	.	.	.	.	.	.	.	.	.	.
ENST0	.	1p36.13	.	.	.	.	rs575	.	.	.	0	.	2E-04
ENST0	.	1p36.13	.	.	Scc	.	rs410	.	.	.	.	.	.
ENST0	.	1p36.13	.	.	Scc	.	rs762	ID=COSN1	.	.	.	.	.
ENST0	.	1p36.13	.	.	Scc	Score=214	.	.	.	.	.	.	.
ENST0	.	1p36.12	.	Scc	.	.	.	.	.	.	.	.	.
.	.	1q21.1	.	.	Scc	.	rs377	.	.	.	.	.	.
ENST0	.	1q21.1	.	.	Scc	Score=858	rs372	.	.	.	.	.	.
ENST0	.	1q21.1	.	.	Scc	Score=423	rs618	.	.	.	.	.	.
ENST0	.	1q44	.	.	.	.	.	.	.	.	.	.	.
ENST0	.	2p11.2	.	.	.	.	.	.	.	.	.	.	.
.	.	2p11.1	.	.	Scc	Score=100	rs164	.	.	.	.	.	.
ENST0	.	3q11.2	.	.	.	.	rs793	.	.	.	0.002	0.001	4E-04
ENST0	.	3q22.1	.	.	.	.	rs748	ID=COSM3	.	.	.	.	.
ENST0	.	3q22.1	.	.	.	.	.	.	.	.	.	.	.
ENST0	.	4p16.1	.	.	Scc	.	.	.	.	.	.	.	.
ENST0	.	5p15.33	.	.	Scc	.	rs671	.	.	.	.	.	.
ENST0	.	5p13.2	.	.	Scc	Score=212	rs868	ID=COSN2	.	.	.	.	.
ENST0	.	5q35.3	.	.	.	.	.	.	.	.	.	.	.
ENST0	.	6q24.3	.	.	.	.	.	.	.	.	.	.	.

ENST0	.	7p14.1	.	.	.	.	.	.	.	.	.	.	.
.	.	7p13	.	.	Scc	Score=822	.	.	.	.	.	.	.
ENST0	.	7q31.2	.	.	.	.	.	ID=COSM4	.	.	.	.	.
ENST0	.	7q36.1	.	.	.	.	rs147	.	.	.	0	.	4E-04
ENST0	.	8p21.3	.	.	.	.	rs749	.	.	.	.	.	.
ENST0	.	8p21.3	.	.	.	.	.	ID=COSM3	.	.	.	.	.
.	.	9q21.11	.	.	Scc	.	rs148	ID=COSN1	.	.	.	.	.
ENST0	.	9q34.11	.	.	.	.	.	.	.	.	.	.	.
ENST0	.	10q11.22	.	.	Scc	.	rs202	ID=COSM4	.	.	.	.	.
ENST0	.	10q24.2	.	.	.	.	.	.	.	.	.	.	.
ENST0	.	11p11.2	.	Scc	.	.	rs773	ID=COSM9	.	.	.	.	.
ENST0	.	11q12.1	.	.	.	.	.	.	.	.	.	.	.
ENST0	.	12q13.12	.	Scc	.	.	rs773	.	.	.	.	.	.
ENST0	.	12q13.13	.	.	.	.	rs776	.	.	.	.	.	.
ENST0	.	14q32.33	.	Scc	.	.	.	.	.	.	.	.	.
.	.	14q32.33	.	.	Scc	Score=126	rs715	ID=COSN2	.	.	.	.	.
ENST0	.	15q11.2	.	.	Scc	.	rs201	ID=COSM(	.	.	0.206	0.211	0.075
ENST0	.	15q11.2	.	.	Scc	.	rs373	ID=COSM(	.	.	.	.	.
ENST0	.	15q11.2	.	.	.	.	.	.	.	.	.	.	.
ENST0	.	15q13.2	.	.	Scc	.	.	.	.	.	.	.	.
ENST0	.	15q23	.	.	.	.	.	.	.	.	.	.	.
ENST0	.	16p12.1	.	.	.	.	.	.	.	.	.	.	.
ENST0	.	17p13.2	.	.	.	Score=231	rs778	ID=COSM4	.	.	.	.	.
.	.	17q12	.	.	Scc	.	rs372	ID=COSN1	.	.	.	.	.
ENST0	.	19q13.11	.	.	Scc	.	rs786	ID=COSM1	.	.	.	.	.
.	.	19q13.2	.	.	Scc	.	.	.	.	.	.	.	.
ENST0	.	19q13.2	.	.	.	.	.	.	.	.	.	.	.
ENST0	.	20q11.23	.	.	.	.	.	.	.	.	.	.	.
.	.	21p11.1	.	.	Scc	.	rs926	.	.	.	.	.	.
ENST0	.	Xp21.1	.	.	.	.	rs766	ID=COSM4	.	.	.	.	.
ENST0	.	Xq13.1	.	.	Scc	.	rs339	ID=COSN9	.	.	0.374	0.348	0.144
ENST0	.	1p36.13	.	.	.	.	.	.	.	.	.	.	.
ENST0	.	1p36.13	.	.	Scc	.	rs436	ID=COSN1	.	.	.	.	.
ENST0	.	1p35.2	.	.	.	.	.	.	.	.	.	.	.
ENST0	.	1p35.1	Scc	.	.	.	.	.	.	.	.	.	.
ENST0	.	1p21.2	.	.	.	.	rs372	.	.	.	.	.	.
ENST0	.	1p13.3	.	.	.	.	.	.	.	.	.	.	.
.	.	1q21.1	.	.	Scc	Score=270	rs200	ID=COSN1	.	.	.	.	.
ENST0	.	1q21.1	.	.	Scc	.	rs942	ID=COSN1	.	.	.	.	.
.	.	1q21.3	.	.	Scc	.	.	.	.	.	.	.	.
ENST0	.	1q22	.	.	.	.	.	.	.	.	.	.	.
ENST0	.	1q22	.	Scc	.	.	.	.	.	.	.	.	.
ENST0	.	1q23.1	.	.	.	.	.	.	.	.	.	.	.
ENST0	.	1q23.2	.	.	.	.	.	.	.	.	.	.	.
ENST0	.	1q23.2	.	Scc	.	.	.	.	.	.	.	.	.
ENST0	.	1q23.3	.	.	.	.	.	.	.	.	.	.	.
ENST0	.	1q25.3	.	.	.	.	.	ID=COSM1	.	.	.	.	.
ENST0	.	1q31.3	.	.	.	.	rs966	.	.	.	.	.	.
ENST0	.	1q42.13	.	.	.	.	rs757	.	.	.	.	.	.
ENST0	.	1q42.2	.	Scc	.	.	.	.	.	.	.	.	.
ENST0	.	1q43	.	.	.	.	.	.	.	.	.	.	.
ENST0	.	1q43	.	Scc	.	.	.	.	.	.	.	.	.
ENST0	.	2p22.1	.	.	Scc	.	.	.	.	.	.	.	.
ENST0	.	2p21	.	.	.	.	rs142	.	.	.	.	.	.

ENST0	.	2q23.2	.	.	.	.	.	.	.	.	.	.	.
ENST0	.	2q24.2	.	.	.	.	.	.	.	.	.	.	.
ENST0	.	2q32.3	.	Scc	.	.	.	.	.	.	.	.	.
ENST0	.	2q33.1	.	.	Scc	Score=206	rs185	.	.	.	0	0.003	6E-04
ENST0	.	2q33.3	.	.	.	.	.	.	.	.	.	.	.
ENST0	.	2q36.1	.	.	.	.	.	.	.	.	.	.	.
ENST0	.	2q37.1	.	.	.	.	.	.	.	.	.	.	.
ENST0	.	2q37.3	.	.	.	.	rs778	.	.	.	.	.	.
ENST0	.	3p22.3	.	.	.	.	.	.	.	.	.	.	.
ENST0	.	3q11.2	.	.	.	.	.	.	.	.	.	.	.
ENST0	.	3q22.2	.	.	.	.	.	.	.	.	.	.	.
ENST0	.	3q22.2	.	Scc	.	.	.	.	.	.	.	.	.
ENST0	.	3q22.3	.	.	.	.	.	.	.	.	.	.	.
ENST0	.	3q26.2	.	.	.	.	rs746	ID=COSM(	.	.	.	.	.
ENST0	.	3q26.31	.	.	.	.	.	.	.	.	.	.	.
ENST0	.	3q26.32	.	.	Scc	.	rs104	ID=COSM7	Pa	.	.	.	.
ENST0	.	3q27.1	.	Scc	.	.	.	.	.	.	.	.	.
ENST0	.	4p16.3	.	.	.	.	.	.	.	.	.	.	.
ENST0	.	4q22.3	.	.	.	.	.	.	.	.	.	.	.
ENST0	.	4q25	.	.	.	Score=187	.	.	.	.	.	.	.
ENST0	.	4q31.1	.	Scc	.	.	.	.	.	.	.	.	.
ENST0	.	4q31.21	.	.	.	.	.	.	.	.	.	.	.
.	.	4q34.1	.	.	.	.	.	.	.	.	.	.	.
ENST0	.	5p13.2	.	.	.	Score=288	rs187	ID=COSN2	.	.	.	.	.
ENST0	.	5q12.3	.	Scc	.	.	.	.	.	.	.	.	.
ENST0	.	5q13.3	.	.	.	.	.	.	.	.	.	.	.
ENST0	.	5q31.1	.	.	.	.	rs795	ID=COSM1	.	.	0.033	0.031	0.007
ENST0	.	5q33.3	.	.	.	.	.	.	.	.	.	.	.
ENST0	.	5q34	.	.	.	.	.	.	.	.	.	.	.
ENST0	.	5q35.1	.	.	.	.	.	ID=COSM1	.	.	.	.	.
ENST0	.	6p25.2	.	.	.	.	.	.	.	.	.	.	.
ENST0	.	6p24.2	.	.	.	.	.	.	.	.	.	.	.
ENST0	.	6p22.2	.	.	.	.	.	.	.	.	.	.	.
ENST0	.	6p22.1	.	.	Scc	.	rs178	.	.	.	0.309	0.277	0.213
ENST0	.	6p22.1	.	.	.	.	rs536	.	.	.	0.003	0.003	6E-04
ENST0	.	6q12	.	.	.	.	.	.	.	.	.	.	.
ENST0	.	6q12	.	Scc	.	.	.	.	.	.	.	.	.
ENST0	.	6q25.1	.	.	.	.	.	.	.	.	.	.	.
ENST0	.	6q27	.	.	.	.	.	.	.	.	.	.	.
ENST0	.	7p22.1	.	.	Scc	.	.	.	.	.	.	.	.
.	.	7q11.21	.	.	Scc	.	.	.	.	.	.	.	.
ENST0	.	7q21.13	.	.	.	.	.	.	.	.	.	.	.
ENST0	.	7q32.3	.	.	.	.	.	.	.	.	.	.	.
ENST0	.	7q33	.	.	.	.	rs368	.	.	.	.	.	.
ENST0	.	8p12	.	.	.	.	.	.	.	.	.	.	.
ENST0	.	8q13.2	.	.	.	.	.	.	.	.	.	.	.
ENST0	.	8q24.12	Scc	.	.	.	.	.	.	.	.	.	.
ENST0	.	8q24.3	.	.	.	.	.	.	.	.	.	.	.
ENST0	.	8q24.3	.	Scc	.	.	rs374	ID=COSM4	.	.	.	.	.
.	.	9q13	.	.	Scc	Score=540	rs492	ID=COSN2	.	.	.	.	.
.	.	9q21.33	.	.	.	.	.	.	.	.	.	.	.
ENST0	.	9q22.31	.	.	.	.	.	.	.	.	.	.	.
ENST0	.	9q32	.	.	.	.	.	.	.	.	.	.	.
ENST0	.	9q34.13	.	.	.	.	.	.	.	.	.	.	.

ENST0	.	10p15.3	.	.	.	.	.	.	.	.	.	.	.
ENST0	.	10p15.2	.	.	.	.	.	.	.	.	.	.	.
ENST0	.	10q23.2	.	.	Scc	.	rs312	.	.	.	0.527	0.524	0.433
ENST0	.	10q26.3	.	.	.	.	.	.	.	.	.	.	.
ENST0	.	11p15.4	.	.	Scc	.	.	.	.	.	.	.	.
ENST0	.	11p15.1	.	.	.	.	.	.	.	.	.	.	.
ENST0	.	11p11.2	.	.	.	.	.	.	.	.	.	.	.
ENST0	.	11q12.3	.	.	.	.	.	.	.	.	.	.	.
ENST0	.	11q14.1	.	.	.	.	.	.	.	.	.	.	.
ENST0	.	11q23.3	.	.	.	.	.	.	.	.	.	.	.
ENST0	.	11q24.1	.	.	.	.	.	.	.	.	.	.	.
ENST0	.	12p13.33	.	.	Scc	.	rs538	ID=COSN1	.	.	0.402	0.375	0.205
ENST0	.	12p13.1	.	Scc	.	.	.	.	.	.	.	.	.
ENST0	.	12p11.23	.	.	.	.	.	.	.	.	.	.	.
ENST0	.	12q14.2	.	.	.	.	rs865	ID=COSN2	.	.	.	.	.
ENST0	.	12q21.31	.	.	.	Score=660	.	.	.	.	.	.	.
ENST0	.	12q24.13	.	.	.	.	.	.	.	.	.	.	.
ENST0	.	12q24.22	.	.	.	.	rs770	.	.	.	.	.	.
ENST0	.	13q12.3	.	.	.	.	.	.	.	.	.	.	.
ENST0	.	13q13.1	.	.	.	.	rs370	.	.	.	0	.	4E-04
ENST0	.	13q13.3	.	.	.	.	rs412	ID=COSM5	Ur	.	0	.	4E-04
ENST0	.	13q13.3	.	.	.	.	.	.	.	.	.	.	.
ENST0	.	13q21.2	.	.	.	.	.	ID=COSM5	.	.	.	.	.
ENST0	.	13q22.3	.	.	.	.	.	.	.	.	.	.	.
ENST0	.	13q22.3	.	.	.	.	.	.	.	.	.	.	.
ENST0	.	13q33.1	.	.	.	.	.	.	.	.	.	.	.
ENST0	.	14q11.2	.	.	.	.	rs369	.	.	.	.	.	.
ENST0	.	14q13.2	.	.	.	Score=222	rs766	.	.	.	.	.	.
ENST0	.	14q32.12	.	Scc	.	.	.	.	.	.	.	.	.
ENST0	.	14q32.13	.	.	.	.	.	.	.	.	.	.	.
ENST0	.	14q32.33	.	.	.	.	rs121	ID=COSM3	Pa	.	.	.	.
ENST0	.	14q32.33	.	.	Scc	.	.	.	.	.	.	.	.
ENST0	.	15q11.1	.	.	Scc	.	rs493	ID=COSM3	.	.	.	.	.
.	.	15q11.2	.	.	Scc	Score=254	rs749	.	.	.	.	.	.
ENST0	.	15q11.2	.	.	Scc	.	rs107	ID=COSN1	.	.	.	.	.
ENST0	.	15q11.2	.	.	Scc	Score=559	rs182	.	.	.	0.086	0.088	0.073
ENST0	.	15q11.2	.	.	.	.	.	.	.	.	.	.	.
ENST0	.	15q15.1	.	.	.	Score=227	rs186	.	.	.	0.003	0.002	4E-04
ENST0	.	15q21.1	.	.	.	.	rs995	ID=COSM5	.	.	.	.	.
ENST0	.	15q21.1	.	.	.	Score=512	.	.	.	.	.	.	.
ENST0	.	15q23	.	.	.	.	.	.	.	.	.	.	.
ENST0	.	15q25.1	.	.	.	.	.	.	.	.	.	.	.
ENST0	.	15q25.1	.	.	Scc	Score=444	.	.	.	.	.	.	.
ENST0	.	16p13.3	.	.	.	.	.	ID=COSM4	.	.	.	.	.
.	.	16p11.2	.	.	Scc	.	.	.	.	.	.	.	.
ENST0	.	16q12.2	.	.	.	.	rs775	.	.	.	.	.	.
ENST0	.	16q22.2	.	.	.	Score=212	.	.	.	.	.	.	.
ENST0	.	17p13.2	.	.	.	.	.	.	.	.	.	.	.
ENST0	.	17q12	.	.	.	.	rs781	ID=COSM2	.	.	.	.	.
ENST0	.	17q21.31	.	.	.	.	rs150	.	.	.	0	.	0.001
ENST0	.	17q21.32	.	.	.	.	.	.	.	.	.	.	.
ENST0	.	17q25.3	.	.	.	.	rs374	.	.	.	0.002	0.002	4E-04
ENST0	.	17q25.3	.	.	.	.	rs561	.	.	.	.	.	.
ENST0	.	17q25.3	.	Scc	.	.	rs550	.	.	.	0	.	2E-04

ENST0	.	18p11.21	.	.	Scc	Score=468	rs199	ID=COSN2	.	.	.	.	.
ENST0	.	18q21.31	.	.	.	.	.	.	.	.	.	.	.
ENST0	.	19p13.2	.	.	.	.	.	.	.	.	.	.	.
ENST0	.	19p13.11	.	.	.	.	.	.	.	.	.	.	.
ENST0	.	19p13.11	.	Scc	.	.	.	.	.	.	.	.	.
ENST0	.	19p13.11	.	.	Scc	.	.	.	.	.	.	.	.
ENST0	.	19q13.12	.	.	.	.	.	.	.	.	.	.	.
ENST0	.	19q13.12	.	.	.	.	.	.	.	.	.	.	.
ENST0	.	19q13.2	.	.	.	.	.	ID=COSM1	.	.	.	.	.
ENST0	.	19q13.31	.	.	Scc	.	.	.	.	.	.	.	.
ENST0	.	19q13.42	.	.	.	.	.	.	.	.	.	.	.
ENST0	.	20p13	.	.	.	.	.	.	.	.	.	.	.
ENST0	.	20p13	.	.	.	.	.	.	.	.	.	.	.
ENST0	.	20p12.3	.	Scc	.	.	rs143	.	.	.	.	.	.
ENST0	.	20q11.21	.	.	.	.	.	.	.	.	.	.	.
ENST0	.	20q11.23	.	.	.	Score=579	.	.	.	.	.	.	.
ENST0	.	21p11.1	.	.	Scc	.	rs117	ID=COSN1	.	.	.	.	.
ENST0	.	21q21.1	.	.	.	.	.	.	.	.	.	.	.
ENST0	.	22q11.21	.	.	.	.	.	.	.	.	.	.	.
ENST0	.	22q12.2	.	.	.	.	.	.	.	.	.	.	.
ENST0	.	22q13.1	.	Scc	.	.	.	.	.	.	.	.	.
ENST0	.	Xp22.33	.	.	.	.	rs750	ID=COSM1	.	.	.	.	.
ENST0	.	Xp21.3	.	Scc	.	.	.	.	.	.	.	.	.
ENST0	.	Xp11.3	.	.	Scc	.	.	.	.	.	.	.	.
ENST0	.	Xq13.1	.	.	.	.	.	.	.	.	.	.	.
ENST0	.	Xq24	.	Scc	.	.	.	.	.	.	.	.	.
.	.	Xq27.1	.	.	.	.	.	.	.	.	.	.	.
.	.	Xq27.1	.	.	.	.	.	.	.	.	.	.	.
ENST0	.	Xq28	.	Scc	.	.	.	.	.	.	.	.	.
ENST0	.	Xq28	.	.	.	.	.	.	.	.	.	.	.
.	.	.	.	.	.	.	.	.	.	.	.	.	.
ENST0	.	1p36.33	.	.	Scc	.	rs114	.	.	.	.	.	.
ENST0	.	1p36.11	.	Scc	.	.	.	.	.	.	.	.	.
ENST0	.	1p31.1	.	.	.	.	.	.	.	.	.	.	.
ENST0	.	1p22.3	.	Scc	.	.	.	.	.	.	.	.	.
ENST0	.	1p11.2	.	.	Scc	.	rs199	.	.	.	.	.	.
ENST0	.	1q21.1	.	.	Scc	.	rs866	ID=COSN1	.	.	.	.	.
ENST0	.	2p16.2	.	.	.	.	.	.	.	.	.	.	.
ENST0	.	2p14	.	.	.	.	.	.	.	.	.	.	.
ENST0	.	2p11.2	.	.	.	.	.	.	.	.	.	.	.
ENST0	.	2q14.2	.	.	.	.	rs370	.	.	.	.	.	.
ENST0	.	2q21.3	.	.	.	Score=22;I	rs200	ID=COSN1	.	.	0	.	0.007
ENST0	.	2q24.3	.	.	.	.	rs904	.	.	.	.	.	.
ENST0	.	2q35	.	.	.	.	rs370	.	.	.	.	.	.
ENST0	.	2q37.1	.	.	.	.	rs557	.	.	.	0	.	.
ENST0	.	3p24.3	.	.	.	Score=601	rs144	.	.	.	0.078	0.081	0.102
ENST0	.	3p22.2	.	.	.	.	.	.	.	.	.	.	.
ENST0	.	3q13.31	.	.	.	.	.	.	.	.	.	.	.
ENST0	.	3q13.31	.	.	.	.	rs201	ID=COSM5	.	.	.	.	.
ENST0	.	4p15.33	.	.	.	.	rs116	.	.	.	0	.	0.014
.	.	4p11	.	.	Scc	.	rs469	ID=COSN1	.	.	.	.	.
.	.	4p11	.	.	Scc	Score=430	rs469	ID=COSN1	.	.	.	.	.
ENST0	.	4q13.3	.	.	.	.	rs749	ID=COSM3	.	.	.	.	.
ENST0	.	4q35.1	.	.	.	.	rs775	ID=COSM6	.	.	.	.	.

ENST0	.	5q31.3	.	.	Scc	.	.	ID=COSM3	.	.	.	.	.
ENST0	.	6p21.33	.	.	.	.	rs116	.	.	.	.	.	.
ENST0	.	6q14.1	.	.	.	.	.	.	.	.	.	.	.
ENST0	.	6q14.1	.	.	Scc	.	.	.	.	.	.	.	.
ENST0	.	6q21	.	.	.	.	rs757	.	.	.	.	.	.
ENST0	.	7p22.1	.	.	.	.	.	.	.	.	.	.	.
ENST0	.	7q21.12	.	.	.	.	.	.	.	.	.	.	.
ENST0	.	7q22.1	.	.	Scc	.	.	.	.	.	.	.	.
ENST0	.	8p22	.	Scc	.	.	rs772	.	.	.	.	.	.
ENST0	.	8q24.3	.	.	.	.	.	.	.	.	.	.	.
ENST0	.	9q34.3	.	.	.	.	rs750	ID=COSM3	.	.	.	.	.
ENST0	.	10p14	.	Scc	.	.	.	.	.	.	.	.	.
ENST0	.	10p13	.	.	.	.	rs757	ID=COSM9	.	.	.	.	.
ENST0	.	10p11.1	.	.	Scc	.	rs565	.	.	.	0	0.001	0.002
ENST0	.	10q22.1	.	.	.	Score=246	rs790	ID=COSN1	.	.	0.347	0.355	0.327
ENST0	.	10q23.31	.	.	.	.	.	.	.	.	.	.	.
ENST0	.	10q24.32	.	.	.	.	rs970	.	.	.	.	.	.
ENST0	.	10q24.32	.	.	.	.	rs772	.	.	.	.	.	.
ENST0	.	10q24.33	.	.	.	.	.	ID=COSM4	.	.	.	.	.
ENST0	.	10q26.13	.	.	.	.	rs121	ID=COSM9	Lik	.	.	.	.
ENST0	.	11p15.5	.	.	.	.	.	.	.	.	.	.	.
ENST0	.	11p15.1	.	.	.	.	rs545	.	.	.	0	.	2E-04
ENST0	.	11p15.1	.	Scc	.	.	.	.	.	.	.	.	.
ENST0	.	11q23.3	.	.	.	.	.	.	.	.	.	.	.
ENST0	.	12q24.13	.	.	.	.	rs103	.	.	.	.	.	.
ENST0	.	13q14.2	.	.	.	.	.	.	.	.	.	.	.
.	.	13q22.3	.	.	Scc	.	rs914	.	.	.	.	.	.
.	.	13q31.1	.	.	.	Score=303	rs981	.	.	.	.	.	.
ENST0	.	14q11.2	.	.	.	.	.	.	.	.	.	.	.
ENST0	.	14q13.1	.	Scc	.	.	.	.	.	.	.	.	.
ENST0	.	14q21.2	.	Scc	.	.	rs563	ID=COSM1	.	.	0	.	2E-04
ENST0	.	14q22.1	.	.	.	.	.	.	.	.	.	.	.
ENST0	.	14q31.3	.	.	.	.	.	.	.	.	.	.	.
ENST0	.	14q32.2	.	.	.	.	.	.	.	.	.	.	.
ENST0	.	15q11.1	.	.	Scc	.	.	.	.	.	.	.	.
ENST0	.	15q11.2	.	.	Scc	.	rs778	.	.	.	.	.	.
ENST0	.	15q15.1	.	.	.	.	.	.	.	.	.	.	.
ENST0	.	15q15.2	.	.	.	Score=171	.	.	.	.	.	.	.
ENST0	.	16q22.1	.	.	.	.	.	.	.	.	.	.	.
ENST0	.	17p13.1	.	.	.	.	.	.	.	.	.	.	.
.	.	17p11.2	.	.	Scc	.	rs586	.	.	.	.	.	.
ENST0	.	17q21.32	.	.	.	.	rs779	.	.	.	.	.	.
ENST0	.	18p11.31	.	.	.	.	.	.	.	.	.	.	.
ENST0	.	19p13.2	.	Scc	.	.	rs747	ID=COSM3	.	.	.	.	.
ENST0	.	19p13.12	.	.	.	.	.	.	.	.	.	.	.
ENST0	.	19q13.11	.	.	Scc	Score=758	rs947	.	.	.	.	.	.
ENST0	.	19q13.12	.	.	.	.	.	.	.	.	.	.	.
ENST0	.	19q13.31	.	.	.	.	.	.	.	.	.	.	.
ENST0	.	19q13.41	.	.	.	.	rs371	.	.	.	.	.	.
ENST0	.	19q13.42	.	.	.	.	rs105	.	.	.	.	.	.
ENST0	.	20p13	.	.	.	.	rs558	ID=COSN1	.	.	0	.	0.002
ENST0	.	20p11.1	.	.	Scc	.	rs603	ID=COSN2	.	.	.	.	.
ENST0	.	20q11.22	.	.	.	.	.	.	.	.	.	.	.
ENST0	.	20q13.12	.	Scc	.	.	.	.	.	.	.	.	.

ENST0	.	21p11.1	.	.	Scc	.	rs201	ID=COSN1	.	.	.	.	.
ENST0	.	21p11.1	.	.	Scc	Score=183	rs200	ID=COSN1	.	.	.	.	.
ENST0	.	22q13.31	.	.	.	.	.	ID=COSM3	.	.	.	.	.
ENST0	.	Xq28	.	.	.	.	.	.	.	.	.	.	.
.	.	.	.	.	.	.	.	.	.	.	.	.	.
.	.	.	.	.	.	.	.	.	.	.	.	.	.
ENST0	.	1p36.33	.	.	Scc	Score=237	rs626	ID=COSN2	.	.	.	.	.
ENST0	.	1p32.3	.	.	.	.	.	.	.	.	.	.	.
ENST0	.	1q21.1	.	.	Scc	.	rs745	ID=COSN1	.	.	.	.	.
ENST0	.	1q24.2	.	Scc	.	.	.	.	.	.	.	.	.
ENST0	.	1q42.3	.	.	.	.	.	.	.	.	.	.	.
.	.	2p11.2	.	.	Scc	.	.	ID=COSN2	.	.	.	.	.
.	.	2p11.2	.	.	Scc	.	rs371	ID=COSN1	.	.	.	.	.
ENST0	.	2q31.3	.	Scc	.	.	.	.	.	.	.	.	.
ENST0	.	3q29	.	.	Scc	.	rs775	ID=COSN2	.	.	.	.	.
ENST0	.	4p16.3	.	.	.	.	.	.	.	.	.	.	.
.	.	4p11	.	.	Scc	Score=866	rs469	.	.	.	.	.	.
ENST0	.	9q21.12	.	.	.	Score=239	.	ID=COSN2	.	.	.	.	.
ENST0	.	10p11.1	.	.	Scc	.	rs407	ID=COSN2	.	.	.	.	.
ENST0	.	12q21.2	.	.	.	Score=223	rs921	.	.	.	.	.	.
ENST0	.	14q21.3	.	.	.	.	rs112	ID=COSN1	.	.	.	.	.
ENST0	.	14q24.3	.	.	.	Score=286	.	.	.	.	.	.	.
ENST0	.	15q11.2	.	.	Scc	Score=496	rs285	ID=COSM3	Be	.	.	.	.
ENST0	.	15q11.2	.	.	Scc	.	.	.	.	.	.	.	.
ENST0	.	18q12.1	.	.	.	Score=813	.	ID=COSN1	.	.	.	.	.
ENST0	.	1q21.1	.	.	Scc	.	.	.	.	.	.	.	.
ENST0	.	1q21.2	.	.	Scc	.	rs278	ID=COSN1	.	.	.	.	.
ENST0	.	1q21.2	.	.	Scc	.	rs278	ID=COSN1	.	.	0.319	0.325	0.398
.	.	2p11.2	.	.	Scc	.	rs371	ID=COSN1	.	.	.	.	.
ENST0	.	5p14.3	.	.	.	Score=354	rs199	.	.	.	.	.	.
ENST0	.	7q11.21	.	.	Scc	.	rs201	ID=COSN2	.	.	.	.	.
.	.	7q11.21	.	.	Scc	.	.	.	.	.	.	.	.
ENST0	.	7q22.1	.	.	Scc	.	rs139	ID=COSN1	.	.	.	.	.
.	.	8p23.1	.	.	Scc	.	rs207	ID=COSN2	.	.	.	.	.
ENST0	.	9q13	.	.	Scc	.	rs113	ID=COSN2	.	.	.	.	.
ENST0	.	9q21.11	.	.	Scc	.	rs764	ID=COSN8	.	.	.	.	.
ENST0	.	9q21.13	.	.	.	.	.	.	.	.	.	.	.
ENST0	.	10q11.23	.	Scc	Scc	.	rs199	ID=COSM1	.	.	.	.	.
ENST0	.	11p15.5	.	.	Scc	Score=247	rs371	ID=COSN2	.	.	.	.	.
ENST0	.	11p11.2	.	.	.	.	rs879	.	.	.	.	.	.
ENST0	.	17p11.2	.	.	Scc	.	rs115	ID=COSN1	.	.	.	.	.
ENST0	.	18p11.21	.	.	Scc	.	rs201	ID=COSM1	.	.	.	.	.
.	.	21p11.2	.	.	Scc	Score=348	.	ID=COSN1	.	.	.	.	.
.	.	21p11.2	.	.	Scc	Score=218	rs879	ID=COSN1	.	.	.	.	.
ENST0	.	21p11.1	.	.	.	Score=215	rs104	ID=COSN1	.	.	.	.	.
ENST0	.	Xq27.1	.	.	Scc	.	rs144	.	.	.	0.107	0.101	0.042
ENST0	.	1p36.33	.	.	Scc	.	.	.	.	.	.	.	.
ENST0	.	1p36.31	.	.	.	.	.	.	.	.	.	.	.
ENST0	.	1p36.31	.	.	.	.	.	.	.	.	.	.	.
ENST0	.	1p36.22	.	Scc	.	.	.	.	.	.	.	.	.
ENST0	.	1p36.21	.	.	Scc	.	.	.	.	.	.	.	.
ENST0	.	1p36.21	.	.	Scc	.	.	.	.	.	.	.	.
ENST0	.	1p21.2	.	Scc	.	.	.	.	.	.	.	.	.
ENST0	.	1q21.1	.	.	Scc	.	rs389	ID=COSN1	.	.	.	.	.

ENST0	.	1q21.1	.	.	Scc	.	rs111	ID=COSN1	.	.	.	.	.
ENST0	.	1q21.1	.	.	Scc	.	rs201	ID=COSN1	.	.	.	.	.
ENST0	.	1q21.1	.	.	Scc	.	rs371	ID=COSN2	.	.	.	.	.
ENST0	.	1q21.1	.	.	Scc	.	rs879	.	.	.	.	.	.
ENST0	.	1q21.1	.	.	Scc	.	rs669	ID=COSN1	.	.	.	.	.
ENST0	.	1q21.2	.	.	Scc	.	rs879	.	.	.	.	.	.
ENST0	.	1q21.2	.	.	.	.	.	.	.	.	.	.	.
ENST0	.	1q21.3	.	.	.	.	.	.	.	.	.	.	.
ENST0	.	1q22	.	Scc	.	.	rs483	ID=COSM3	no	.	.	.	.
ENST0	.	1q25.1	.	.	.	.	rs894	.	.	.	.	.	.
ENST0	.	1q25.3	.	.	.	.	rs773	.	.	.	.	.	.
ENST0	.	2p11.2	.	.	Scc	.	.	.	.	.	.	.	.
ENST0	.	2p11.2	.	.	.	.	rs778	.	.	.	.	.	.
ENST0	.	2q11.2	.	Scc	.	.	rs201	ID=COSM1	.	.	0	0.001	4E-04
ENST0	.	2q12.1	.	Scc	.	.	.	.	.	.	.	.	.
ENST0	.	2q31.1	.	.	.	.	rs768	.	.	.	.	.	.
ENST0	.	2q31.3	.	Scc	.	.	rs755	.	.	.	.	.	.
ENST0	.	3p22.1	.	.	.	.	rs622	ID=COSM4	.	.	.	.	.
ENST0	.	3q26.33	.	.	.	.	.	.	.	.	.	.	.
ENST0	.	3q27.1	.	Scc	.	.	.	.	.	.	.	.	.
ENST0	.	3q27.1	.	.	.	.	.	.	.	.	.	.	.
ENST0	.	3q27.2	.	.	.	.	.	.	.	.	.	.	.
ENST0	.	4q25	.	.	.	.	.	.	.	.	.	.	.
ENST0	.	4q26	.	.	.	.	rs574	.	.	.	0	.	2E-04
ENST0	.	4q34.3	.	.	.	.	rs554	ID=COSM3	.	.	0	.	4E-04
ENST0	.	5p13.2	.	.	Scc	Score=189	.	.	.	.	.	.	.
ENST0	.	5q13.2	.	.	Scc	Score=154	rs375	.	.	.	0.777	0.772	0.477
ENST0	.	5q13.2	.	.	.	.	.	.	.	.	.	.	.
ENST0	.	5q31.1	.	.	.	.	.	.	.	.	.	.	.
ENST0	.	5q31.1	.	Scc	.	.	.	.	.	.	.	.	.
ENST0	.	5q35.2	.	.	.	.	.	.	.	.	.	.	.
ENST0	.	5q35.2	.	.	.	Score=316	.	.	.	.	.	.	.
ENST0	.	6p23	.	.	.	Score=479	rs758	.	.	.	.	.	.
ENST0	.	6p22.3	.	.	.	.	rs759	.	.	.	.	.	.
ENST0	.	6p22.3	.	.	.	.	.	.	.	.	.	.	.
ENST0	.	6p22.1	.	.	Scc	.	.	.	.	.	.	.	.
ENST0	.	6p21.33	.	.	.	.	.	.	.	.	.	.	.
ENST0	.	6p21.32	.	.	.	.	.	.	.	.	.	.	.
ENST0	.	6p21.1	.	.	.	.	.	.	.	.	.	.	.
ENST0	.	6q11.1	.	.	.	.	.	.	.	.	.	.	.
ENST0	.	6q12	.	.	Scc	.	.	.	.	.	.	.	.
ENST0	.	6q25.3	.	.	.	Score=239	.	.	.	.	.	.	.
ENST0	.	7p22.3	.	.	.	.	.	.	.	.	.	.	.
ENST0	.	7q11.21	.	.	Scc	.	rs201	ID=COSN2	.	.	.	.	.
ENST0	.	7q21.12	.	.	.	.	rs980	.	.	.	.	.	.
ENST0	.	7q35	.	.	Scc	Score=245	rs120	ID=COSN2	.	.	.	.	.
ENST0	.	7q35	.	.	Scc	Score=245	rs624	ID=COSN2	.	.	.	.	.
ENST0	.	7q36.1	.	.	.	.	.	.	.	.	.	.	.
ENST0	.	7q36.1	.	.	Scc	.	.	.	.	.	.	.	.
ENST0	.	8p21.1	.	.	.	.	.	.	.	.	.	.	.
.	.	8p11.1	.	.	.	Score=126	rs202	ID=COSN2	.	.	.	.	.
ENST0	.	8q24.3	.	.	.	.	.	.	.	.	.	.	.
ENST0	.	9q21.32	.	Scc	.	.	.	.	.	.	.	.	.
ENST0	.	9q22.31	.	.	.	.	.	.	.	.	.	.	.

ENST0	.	9q22.32	.	.	Scc	.	.	.	.	.	.		.	.
ENST0	.	9q34.3	.	.	.	.	rs772	.	.	.	.		.	.
ENST0	.	10q11.21	.	.	.	.	rs535	.	.	.		0	.	2E-04
ENST0	.	10q25.1	.	.	.	.	.	.	.	.	.		.	.
ENST0	.	10q25.1	.	Scc	.	.	.	ID=COSM5	.	.	.		.	.
ENST0	.	10q25.3	.	.	.	.	.	.	.	.	.		.	.
ENST0	.	11p15.5	.	.	.	.	rs147	.	.	.		0	0.001	8E-04
ENST0	.	11p15.4	.	.	.	.	.	.	.	.	.		.	.
ENST0	.	11p15.4	.	Scc	.	.	.	.	.	.	.		.	.
ENST0	.	11p15.4	.	.	.	Score=136	.	.	.	.	.		.	.
ENST0	.	11p15.1	.	.	.	.	rs777	.	.	.	.		.	.
ENST0	.	11p11.2	.	.	.	.	rs780	.	.	.	.		.	.
ENST0	.	11q12.1	.	.	.	.	.	.	.	.	.		.	.
ENST0	.	11q13.1	.	.	.	.	.	.	.	.	.		.	.
ENST0	.	11q23.3	.	.	.	.	rs100	.	.	.	.		.	.
ENST0	.	11q24.2	.	.	.	.	.	.	.	.	.		.	.
ENST0	.	12q13.3	.	.	.	.	.	.	.	.	.		.	.
ENST0	.	12q24.13	.	.	.	Score=441	rs868	ID=COSN1	.	.	.		.	.
ENST0	.	12q24.31	.	.	.	.	.	.	.	.	.		.	.
ENST0	.	12q24.31	.	.	.	.	.	.	.	.	.		.	.
ENST0	.	13q12.11	.	.	Scc	.	.	.	.	.	.		.	.
ENST0	.	13q12.11	.	.	Scc	.	.	ID=COSN5	.	.	.		.	.
ENST0	.	13q12.12	.	.	.	.	rs760	.	.	.	.		.	.
ENST0	.	13q12.13	.	.	.	.	.	.	.	.	.		.	.
ENST0	.	13q13.3	.	.	.	.	.	.	.	.	.		.	.
ENST0	.	13q13.3	.	.	.	.	.	ID=COSN1	.	.	.		.	.
ENST0	.	13q22.2	.	.	.	.	rs184	.	.	.		0	.	2E-04
ENST0	.	14q11.2	.	.	Scc	.	rs867	.	.	.	.		.	.
ENST0	.	14q12	.	Scc	.	.	.	.	.	.	.		.	.
ENST0	.	14q13.1	.	.	.	.	.	.	.	.	.		.	.
ENST0	.	14q21.2	.	.	.	.	.	.	.	.	.		.	.
ENST0	.	14q22.3	.	.	.	.	rs759	.	.	.	.		.	.
ENST0	.	14q23.2	.	.	.	.	.	.	.	.	.		.	.
ENST0	.	14q24.2	.	.	Scc	.	rs112	ID=COSN2	.	.	.		.	.
ENST0	.	14q32.33	.	.	.	.	.	.	.	.	.		.	.
ENST0	.	14q32.33	.	.	.	.	.	.	.	.	.		.	.
ENST0	.	14q32.33	.	.	.	.	.	.	.	.	.		.	.
ENST0	.	15q22.2	.	.	.	.	.	.	.	.	.		.	.
ENST0	.	15q23	.	.	.	.	.	.	.	.	.		.	.
ENST0	.	16p13.3	.	Scc	.	.	.	.	.	.	.		.	.
ENST0	.	16p13.3	.	.	.	Score=210	.	.	.	.	.		.	.
ENST0	.	16p13.3	.	.	.	.	rs748	.	.	.	.		.	.
ENST0	.	16p13.3	.	.	.	Score=346	.	.	.	.	.		.	.
ENST0	.	16p12.3	.	.	Scc	.	.	.	.	.	.		.	.
ENST0	.	16p11.2	.	Scc	.	.	.	.	.	.	.		.	.
ENST0	.	16p11.2	.	.	.	.	.	.	.	.	.		.	.
.	.	16q11.2	.	.	.	Score=108	rs750	.	.	.	.		.	.
.	.	16q11.2	.	.	.	Score=113	rs212	.	.	.	.		.	.
.	.	16q11.2	.	.	.	Score=114	rs443	ID=COSN2	.	.	.		.	.
.	.	16q11.2	.	.	.	Score=114	rs288	ID=COSN1	.	.	.		.	.
ENST0	.	16q22.2	.	.	Scc	.	.	.	.	.	.		.	.
ENST0	.	16q24.3	.	.	.	.	.	.	.	.	.		.	.
ENST0	.	17q11.2	.	.	.	.	.	.	.	.	.		.	.
ENST0	.	17q11.2	.	Scc	.	.	.	.	.	.	.		.	.

ENST0	.	17q12	.	.	.	Score=684	.	.	.	.	.	.	.
ENST0	.	17q21.2	.	.	.	.	rs199	.	.	.	.	.	.
ENST0	.	17q21.2	.	.	.	.	.	.	.	.	.	.	.
ENST0	.	17q21.31	.	.	.	.	rs102	.	.	.	.	.	.
ENST0	.	17q23.3	.	.	.	.	.	.	.	.	.	.	.
ENST0	.	17q24.2	.	.	.	.	rs372	.	.	.	.	.	.
ENST0	.	17q25.3	.	.	.	.	.	.	.	.	.	.	.
ENST0	.	18p11.22	.	.	.	.	.	.	.	.	.	.	.
ENST0	.	18p11.21	.	.	.	.	.	.	.	.	.	.	.
.	.	18p11.21	.	.	Scc	.	.	.	.	.	.	.	.
ENST0	.	18q21.2	.	.	.	.	rs755	.	.	.	.	.	.
ENST0	.	19q13.13	.	.	.	.	rs995	.	.	.	.	.	.
ENST0	.	19q13.33	.	Scc	.	.	.	.	.	.	.	.	.
.	.	20p12.1	.	.	.	.	.	.	.	.	.	.	.
ENST0	.	20q11.23	.	.	.	Score=520	.	.	.	.	.	.	.
ENST0	.	21p11.2	.	.	Scc	.	rs369	.	.	.	.	.	.
ENST0	.	21p11.2	.	.	Scc	.	rs796	ID=COSN1	.	.	.	.	.
ENST0	.	21q22.3	.	.	.	.	.	.	.	.	.	.	.
ENST0	.	21q22.3	.	.	.	.	.	.	.	.	.	.	.
ENST0	.	22q11.1	.	.	Scc	.	rs221	ID=COSN2	.	.	.	.	.
ENST0	.	22q11.21	.	.	.	.	.	.	.	.	.	.	.
ENST0	.	22q11.21	.	.	Scc	.	rs201	.	.	.	.	.	.
ENST0	.	22q13.1	.	.	.	.	rs761	.	.	.	.	.	.
ENST0	.	22q13.1	.	.	.	.	.	.	.	.	.	.	.
ENST0	.	Xq13.1	.	.	Scc	.	rs339	ID=COSN9	.	.	0.374	0.348	0.144
ENST0	.	Xq21.31	.	.	.	.	.	ID=COSM1	.	.	.	.	.
.	.	.	.	.	.	.	rs879	.	.	.	.	.	.
ENST0	.	1p36.13	.	.	.	.	.	.	.	.	.	.	.
ENST0	.	1p36.13	.	.	Scc	.	rs199	.	.	.	.	.	.
ENST0	.	1p34.3	.	.	.	.	.	.	.	.	.	.	.
ENST0	.	1p13.3	.	Scc	.	.	.	.	.	.	.	.	.
ENST0	.	1q21.1	.	.	Scc	.	.	.	.	.	.	.	.
ENST0	.	1q21.1	.	.	Scc	.	rs169	.	.	.	.	.	.
ENST0	.	1q21.1	.	.	Scc	.	rs944	ID=COSN1	.	.	.	.	.
ENST0	.	1q21.1	.	.	Scc	.	rs781	.	.	.	.	.	.
ENST0	.	1q21.2	.	.	Scc	.	rs202	ID=COSN1	.	.	.	.	.
.	.	1q21.2	.	.	.	.	.	.	.	.	.	.	.
ENST0	.	1q21.3	.	.	.	.	.	ID=COSN2	.	.	.	.	.
ENST0	.	1q23.1	.	.	Scc	.	.	.	.	.	.	.	.
ENST0	.	1q25.3	.	.	.	.	rs149	.	.	.	.	.	.
ENST0	.	1q31.1	.	.	.	.	.	.	.	.	.	.	.
ENST0	.	1q41	.	.	.	.	rs763	.	.	.	.	.	.
ENST0	.	1q42.2	.	.	.	.	rs372	.	.	.	.	.	.
ENST0	.	2p21	.	.	.	.	.	.	.	.	.	.	.
ENST0	.	2p11.1	.	.	Scc	.	rs621	ID=COSN1	.	.	.	.	.
ENST0	.	2q13	.	.	.	.	.	.	.	.	.	.	.
ENST0	.	2q13	.	.	Scc	.	rs147	ID=COSM4	.	.	.	.	.
ENST0	.	2q37.1	.	.	Scc	.	.	.	.	.	.	.	.
ENST0	.	3p22.1	.	.	.	.	.	.	.	.	.	.	.
ENST0	.	3p21.31	.	.	.	.	.	.	.	.	.	.	.
ENST0	.	3q13.13	.	.	.	.	.	.	.	.	.	.	.
ENST0	.	3q13.2	.	.	.	.	rs142	.	.	.	.	.	.
ENST0	.	3q25.2	.	.	.	.	.	.	.	.	.	.	.
ENST0	.	4p16.3	.	.	.	.	rs497	ID=COSN1	.	.	.	.	.

ENST0	.	4q24	.	.	.	.	.	.	.	.	.	.	.
ENST0	.	4q24	.	.	.	.	.	ID=COSM5	.	.	.	.	.
ENST0	.	5p13.2	.	.	.	.	.	.	.	.	.	.	.
ENST0	.	5q31.3	.	.	.	Score=317	.	.	.	.	.	.	.
ENST0	.	7p14.1	.	.	.	.	rs964	.	.	.	.	.	.
ENST0	.	7p11.2	.	.	Scc	.	rs624	ID=COSN2	.	.	.	.	.
ENST0	.	7p11.2	.	.	Scc	.	.	.	.	.	.	.	.
ENST0	.	7q22.1	.	Scc	.	.	.	.	.	.	.	.	.
ENST0	.	7q31.32	.	.	.	.	.	ID=COSM4	.	.	.	.	.
ENST0	.	7q36.1	.	.	.	.	rs891	.	.	.	.	.	.
ENST0	.	7q36.2	.	.	.	.	.	.	.	.	.	.	.
ENST0	.	8p21.1	.	.	.	.	.	.	.	.	.	.	.
ENST0	.	8p11.23	.	.	.	.	.	.	.	.	.	.	.
ENST0	.	8q13.1	.	.	.	.	.	.	.	.	.	.	.
ENST0	.	9p24.1	.	.	.	.	.	.	.	.	.	.	.
ENST0	.	9q34.3	.	.	.	.	.	.	.	.	.	.	.
ENST0	.	10q22.2	.	.	.	.	.	.	.	.	.	.	.
ENST0	.	10q23.1	.	.	.	.	.	.	.	.	.	.	.
ENST0	.	10q24.32	.	.	.	.	.	.	.	.	.	.	.
ENST0	.	10q24.33	.	.	.	.	.	.	.	.	.	.	.
ENST0	.	10q26.3	.	.	.	.	.	.	.	.	.	.	.
ENST0	.	11p15.4	.	.	.	.	.	.	.	.	.	.	.
ENST0	.	11p15.4	.	.	.	.	.	ID=COSM5	.	.	.	.	.
ENST0	.	11p14.1	.	.	.	.	.	.	.	.	.	.	.
ENST0	.	11p12	.	Scc	Scc	.	.	.	.	.	.	.	.
ENST0	.	11q21	.	.	.	.	.	.	.	.	.	.	.
ENST0	.	11q23.3	.	.	.	.	rs758	ID=COSM6	.	.	.	.	.
ENST0	.	12p13.31	.	.	.	.	.	.	.	.	.	.	.
.	.	12q13.12	.	.	.	.	.	.	.	.	.	.	.
ENST0	.	12q13.12	.	.	.	.	.	.	.	.	.	.	.
ENST0	.	12q13.13	.	.	.	.	.	.	.	.	.	.	.
ENST0	.	12q13.3	.	.	.	.	.	.	.	.	.	.	.
ENST0	.	12q24.31	.	.	Scc	.	.	.	.	.	.	.	.
ENST0	.	13q31.3	.	.	.	.	.	.	.	.	.	.	.
ENST0	.	14q11.2	.	.	Scc	.	rs714	ID=COSN1	.	.	.	.	.
.	.	14q32.32	.	.	.	Score=111	.	.	.	.	.	.	.
ENST0	.	15q11.2	.	.	Scc	.	.	.	.	.	.	.	.
ENST0	.	15q13.2	.	.	Scc	Score=221	rs199	.	.	.	.	.	.
ENST0	.	15q13.3	.	.	.	.	.	.	.	.	.	.	.
ENST0	.	15q21.3	.	.	.	.	.	.	.	.	.	.	.
ENST0	.	15q23	.	.	.	.	.	.	.	.	.	.	.
ENST0	.	15q26.1	.	.	.	.	.	.	.	.	.	.	.
ENST0	.	16p13.3	.	.	.	.	.	.	.	.	.	.	.
ENST0	.	16q23.1	.	.	.	.	.	.	.	.	.	.	.
ENST0	.	17p13.1	.	.	.	.	.	.	.	.	.	.	.
ENST0	.	17q23.1	.	.	Scc	.	.	.	.	.	.	.	.
ENST0	.	18p11.21	.	.	Scc	.	rs879	ID=COSN1	.	.	.	.	.
ENST0	.	19p13.3	.	.	.	Score=235	rs867	ID=COSN2	.	.	.	.	.
ENST0	.	19p13.2	.	.	.	.	rs766	.	.	.	.	.	.
ENST0	.	19p13.11	.	Scc	.	.	rs768	.	.	.	.	.	.
ENST0	.	19p12	.	.	Scc	.	.	.	.	.	.	.	.
ENST0	.	19q13.12	.	.	.	.	.	.	.	.	.	.	.
ENST0	.	19q13.2	.	.	.	.	.	.	.	.	.	.	.
ENST0	.	19q13.32	.	.	.	.	.	.	.	.	.	.	.

ENST0	.	19q13.32	.	.	.	.	.	.	.	.	.	.	.
ENST0	.	19q13.33	.	.	.	.	.	.	.	.	.	.	.
ENST0	.	19q13.33	.	.	.	.	.	.	.	.	.	.	.
.	.	19q13.33	.	.	Scc	.	.	.	.	.	.	.	.
ENST0	.	19q13.42	.	.	Scc	.	.	.	.	.	.	.	.
ENST0	.	20q13.12	.	.	.	.	.	.	.	.	.	.	.
ENST0	.	20q13.2	.	.	.	.	.	.	.	.	.	.	.
.	.	21p11.1	.	.	Scc	Score=460	rs111	.	.	.	.	.	.
ENST0	.	21q21.3	.	.	.	.	.	.	.	.	.	.	.
ENST0	.	21q22.11	.	Scc	.	.	.	.	.	.	.	.	.
.	.	22q11.22	.	.	Scc	.	rs200	ID=COSN1	.	.	.	.	.
ENST0	.	22q13.31	.	.	.	.	rs939	.	.	.	.	.	.
ENST0	.	Xp11.3	.	.	.	Score=788	rs734	.	.	.	0.207	0.192	0.243
ENST0	.	Xq21.31	.	.	Scc	.	rs182	.	.	.	0	.	5E-04
ENST0	.	Xq25	.	Scc	.	.	.	.	.	.	.	.	.
ENST0	.	Xq26.3	.	.	.	.	.	ID=COSM3	.	.	.	.	.
ENST0	.	Xq27.3	.	.	.	.	.	.	.	.	.	.	.
ENST0	.	Xq28	.	.	.	.	.	.	.	.	.	.	.
.	.	.	.	.	.	.	.	.	.	.	.	.	.
.	.	.	.	.	.	.	.	.	.	.	.	.	.
ENST0	.	1p36.13	.	.	Scc	Score=221	rs181	ID=COSN1	.	.	.	.	.
.	.	1q21.1	.	.	Scc	Score=285	rs369	.	.	.	.	.	.
ENST0	.	1q21.1	.	.	Scc	.	rs377	.	.	.	.	.	.
ENST0	.	1q21.1	.	.	.	.	rs259	ID=COSN1	.	.	.	.	.
ENST0	.	1q21.2	.	.	Scc	.	rs202	.	.	.	.	.	.
.	.	1q21.2	.	.	Scc	.	.	.	.	.	.	.	.
ENST0	.	2p11.2	.	.	.	Score=181	.	.	.	.	.	.	.
ENST0	.	2p11.1	.	.	Scc	.	rs264	ID=COSN5	.	.	.	.	.
ENST0	.	2q12.1	.	.	.	Score=591	.	.	.	.	.	.	.
ENST0	.	2q13	.	.	.	Score=217	.	.	.	.	.	.	.
ENST0	.	2q13	.	.	.	Score=217	.	.	.	.	.	.	.
ENST0	.	2q21.1	.	.	Scc	Score=339	rs621	.	.	.	.	.	.
ENST0	.	3q23	.	.	.	Score=120	.	.	.	.	.	.	.
ENST0	.	4p16.2	.	.	.	.	.	.	.	.	.	.	.
.	.	4q13.2	.	.	.	Score=997	rs142	.	.	.	0.684	0.681	0.468
ENST0	.	5p14.3	.	.	Scc	.	rs287	ID=COSN1	.	.	.	.	.
ENST0	.	7p11.2	.	.	Scc	.	rs624	ID=COSN2	.	.	.	.	.
ENST0	.	7q11.21	.	.	Scc	.	rs368	ID=COSN2	.	.	.	.	.
ENST0	.	8q12.1	.	.	Scc	Score=220	rs149	ID=COSN1	.	.	.	.	.
ENST0	.	10q22.1	.	.	.	.	.	.	.	.	.	.	.
ENST0	.	10q22.3	.	.	.	Score=474	rs771	ID=COSN1	.	.	.	.	.
ENST0	.	10q22.3	.	.	.	.	rs178	ID=COSN8	.	.	.	.	.
ENST0	.	10q26.13	.	.	.	.	.	.	.	.	.	.	.
ENST0	.	11q24.2	.	.	.	Score=203	.	.	.	.	.	.	.
ENST0	.	12p13.31	.	.	Scc	.	rs106	.	.	.	0.269	0.267	0.134
ENST0	.	14q11.2	.	.	Scc	Score=238	rs424	.	.	.	.	.	.
ENST0	.	14q11.2	.	.	Scc	.	rs744	.	.	.	.	.	.
ENST0	.	14q11.2	.	.	Scc	.	rs867	.	.	.	.	.	.
ENST0	.	14q11.2	.	.	.	.	.	.	.	.	.	.	.
ENST0	.	17q12	.	.	Scc	.	rs393	ID=COSN1	.	.	.	.	.
ENST0	.	17q21.33	.	.	.	Score=198	rs389	ID=COSN2	.	.	.	.	.
.	.	21p11.2	.	.	Scc	.	rs196	ID=COSN1	.	.	.	.	.
ENST0	.	22q11.1	.	.	Scc	.	rs371	.	.	.	.	.	.
ENST0	.	22q11.21	.	.	Scc	.	.	.	.	.	.	.	.

ENST0	.	Xq26.3	.	.	Scc	.	rs781	.	.	.	0.254	0.249	0.268
ENST0	.	1p36.33	.	.	Scc	.	.	.	.	.	.	.	.
ENST0	.	1p36.13	.	.	Scc	.	rs453	ID=COSN1	.	.	0.45	0.437	0.444
ENST0	.	1p34.1	.	.	.	.	.	.	.	.	.	.	.
ENST0	.	1q21.1	.	.	Scc	.	rs181	.	.	.	.	.	.
ENST0	.	1q21.1	.	.	Scc	Score=208	.	.	.	.	.	.	.
ENST0	.	1q21.1	.	.	Scc	.	rs879	.	.	.	.	.	.
ENST0	.	1q21.2	.	.	Scc	.	rs145	ID=COSN1	.	.	.	.	.
ENST0	.	1q21.2	.	.	Scc	.	rs200	ID=COSN2	.	.	.	.	.
ENST0	.	1q21.2	.	.	Scc	.	rs797	.	.	.	.	.	.
ENST0	.	1q32.1	.	.	.	.	.	.	.	.	.	.	.
ENST0	.	2q37.3	.	.	.	.	rs199	ID=COSN2	.	.	.	.	.
ENST0	.	3p21.31	.	.	.	Score=841	.	.	.	.	.	.	.
ENST0	.	3q26.2	.	.	.	.	rs187	.	.	.	0.063	0.055	0.011
.	.	3q29	.	.	Scc	Score=578	rs372	.	.	.	.	.	.
ENST0	.	3q29	.	.	.	Score=235	.	.	.	.	.	.	.
ENST0	.	3q29	.	.	Scc	.	rs200	.	.	.	.	.	.
.	.	4p16.1	.	.	Scc	.	.	.	.	.	.	.	.
ENST0	.	5q31.1	.	.	.	.	.	.	.	.	.	.	.
.	.	6q25.3	.	.	.	Score=865	.	.	.	.	.	.	.
ENST0	.	6q26	.	.	Scc	.	rs774	ID=COSN1	.	.	0.359	0.355	0.336
.	.	6q27	.	.	Scc	.	rs375	.	.	.	.	.	.
.	.	7q11.21	.	.	Scc	.	.	ID=COSN1	.	.	.	.	.
.	.	7q11.21	.	.	Scc	.	.	.	.	.	.	.	.
ENST0	.	7q11.22	.	Scc	.	Score=211	.	.	.	.	.	.	.
ENST0	.	9p11.2	.	.	Scc	Score=121	rs280	ID=COSN1	.	.	.	.	.
.	.	10q11.22	.	.	Scc	.	rs199	.	.	.	.	.	.
.	.	10q11.22	.	.	Scc	Score=348	rs145	ID=COSN1	.	.	.	.	.
ENST0	.	11p15.4	.	.	.	Score=364	.	.	.	.	.	.	.
ENST0	.	11q12.1	.	Scc	.	.	.	.	.	.	.	.	.
ENST0	.	12p13.33	.	.	Scc	.	rs371	ID=COSN1	.	.	.	.	.
ENST0	.	12p13.33	.	.	Scc	.	rs376	ID=COSN1	.	.	.	.	.
ENST0	.	12q14.2	.	.	.	.	rs560	ID=COSN2	.	.	.	.	.
ENST0	.	14q11.2	.	.	Scc	Score=143	rs775	ID=COSN1	.	.	.	.	.
ENST0	.	14q11.2	.	.	Scc	.	rs101	ID=COSN1	.	.	.	.	.
ENST0	.	15q11.2	.	.	Scc	.	.	.	.	.	.	.	.
ENST0	.	15q14	.	.	.	.	rs756	.	.	.	.	.	.
ENST0	.	16p12.2	.	.	Scc	Score=247	rs373	.	.	.	.	.	.
ENST0	.	16q12.2	.	.	Scc	.	rs713	.	.	.	0.251	0.262	0.49
ENST0	.	17p11.2	.	.	Scc	.	rs712	.	.	.	.	.	.
ENST0	.	17p11.2	.	.	Scc	.	rs614	ID=COSN2	.	.	.	.	.
ENST0	.	17q23.1	.	.	Scc	.	rs287	ID=COSN2	.	.	.	.	.
ENST0	.	17q23.1	.	.	Scc	.	rs284	ID=COSN2	.	.	.	.	.
ENST0	.	17q25.3	.	.	.	.	.	.	.	.	.	.	.
ENST0	.	18q22.1	.	.	.	.	.	.	.	.	.	.	.
ENST0	.	19p13.2	.	.	.	Score=871	.	.	.	.	.	.	.
ENST0	.	21p11.2	.	.	Scc	.	.	.	.	.	.	.	.
ENST0	.	22q11.21	.	.	Scc	.	.	ID=COSN2	.	.	.	.	.
ENST0	.	22q13.2	.	.	Scc	.	rs360	.	.	.	0.266	0.302	0.458
ENST0	.	Xp11.21	.	.	.	.	.	.	.	.	.	.	.
ENST0	.	1p36.33	.	.	.	.	rs755	.	.	.	.	.	.
ENST0	.	1p36.33	.	.	Scc	.	.	.	.	.	.	.	.
ENST0	.	1p34.3	.	.	.	.	.	.	.	.	.	.	.
ENST0	.	1p34.3	.	.	.	.	.	.	.	.	.	.	.

ENST0	.	1p34.3	.	.	.	.	.	.	.	.	.	.	.
ENST0	.	1p34.2	.	Scc	.	.	.	.	.	.	.	.	.
ENST0	.	1p34.2	.	Scc	.	.	.	.	.	.	.	.	.
ENST0	.	1p22.3	.	.	.	.	.	.	.	.	.	.	.
ENST0	.	1p22.2	.	Scc	Scc	.	.	.	.	.	.	.	.
ENST0	.	1p13.2	.	Scc	.	.	rs143	.	.	.	0.002	0.001	0.015
ENST0	.	1p12	.	.	Scc	Score=239	.	.	.	.	.	.	.
ENST0	.	1q21.1	.	.	Scc	.	rs199	ID=COSN1	.	.	.	.	.
ENST0	.	1q21.1	.	.	Scc	Score=269	.	ID=COSN1	.	.	.	.	.
ENST0	.	1q21.1	.	.	Scc	.	rs375	.	.	.	.	.	.
ENST0	.	1q21.2	.	.	Scc	.	rs284	.	.	.	.	.	.
ENST0	.	1q21.2	.	.	Scc	.	rs782	.	.	.	.	.	.
ENST0	.	1q21.2	.	.	Scc	.	rs376	ID=COSN1	.	.	.	.	.
ENST0	.	1q21.3	.	.	.	.	rs909	ID=COSM(	.	.	.	.	.
ENST0	.	1q21.3	.	.	.	.	.	.	.	.	.	.	.
ENST0	.	1q25.2	.	.	.	.	.	.	.	.	.	.	.
ENST0	.	1q32.1	.	.	.	.	.	.	.	.	.	.	.
ENST0	.	1q32.2	.	.	.	.	.	.	.	.	.	.	.
ENST0	.	2q31.2	.	.	.	.	.	.	.	.	.	.	.
ENST0	.	2q35	.	.	.	.	.	.	.	.	.	.	.
ENST0	.	3p26.1	.	.	.	.	rs779	ID=COSM3	.	.	.	.	.
ENST0	.	3p25.3	.	.	.	.	.	.	.	.	.	.	.
ENST0	.	3p25.1	.	.	.	.	.	.	.	.	.	.	.
ENST0	.	3p21.1	.	Scc	.	.	.	.	.	.	.	.	.
ENST0	.	4p16.3	.	.	.	.	rs778	.	.	.	.	.	.
ENST0	.	4q22.1	.	.	.	.	rs774	.	.	.	.	.	.
ENST0	.	5p15.2	.	.	Scc	.	rs238	ID=COSM(	.	.	.	.	.
ENST0	.	5q14.1	.	.	.	.	rs781	.	.	.	.	.	.
ENST0	.	5q23.1	.	.	.	.	.	.	.	.	.	.	.
ENST0	.	5q23.2	.	.	.	.	rs114	ID=COSM1	.	.	0	.	0.001
ENST0	.	5q33.1	.	.	.	.	.	.	.	.	.	.	.
ENST0	.	5q34	.	Scc	.	.	.	.	.	.	.	.	.
ENST0	.	6p25.2	.	.	.	.	.	.	.	.	.	.	.
ENST0	.	6p21.33	.	.	.	.	.	.	.	.	.	.	.
ENST0	.	6p12.3	.	.	.	.	.	.	.	.	.	.	.
ENST0	.	6q22.33	.	.	Scc	.	rs990	.	.	.	.	.	.
ENST0	.	6q25.3	.	.	.	.	.	ID=COSM!	.	.	.	.	.
ENST0	.	6q27	.	.	.	.	.	.	.	.	.	.	.
ENST0	.	6q27	.	.	.	.	.	.	.	.	.	.	.
ENST0	.	7p22.3	.	Scc	.	.	.	.	.	.	.	.	.
ENST0	.	7p22.1	.	.	Scc	.	.	.	.	.	.	.	.
ENST0	.	7p21.2	.	.	.	.	rs746	.	.	.	.	.	.
ENST0	.	7p13	.	.	.	.	.	.	.	.	.	.	.
.	.	7p12.3	.	.	.	Score=193	.	.	.	.	.	.	.
ENST0	.	7q35	.	.	.	.	rs570	.	.	.	0	.	2E-04
ENST0	.	7q36.1	.	Scc	.	.	rs148	ID=COSM9	.	.	.	.	.
ENST0	.	8p23.3	.	.	.	.	rs758	.	.	.	.	.	.
ENST0	.	8p23.1	.	.	.	.	rs747	.	.	.	.	.	.
.	.	8p11.1	.	.	.	Score=126	.	.	.	.	.	.	.
ENST0	.	8q23.3	.	.	.	Score=113	rs532	.	.	.	.	.	.
ENST0	.	8q24.3	.	.	.	.	rs569	.	.	.	0	.	2E-04
ENST0	.	9q21.2	.	.	.	.	rs368	.	.	.	0	.	2E-04
ENST0	.	9q31.1	.	.	.	.	rs374	.	.	.	0.003	0.003	6E-04
ENST0	.	9q34.11	.	.	.	.	rs752	.	.	.	.	.	.

ENST0	.	9q34.3	.	.	.	.	rs564	ID=COSM	.	.	.	.	.
ENST0	.	9q34.3	.	.	.	.	rs550	.	.	.	0	.	2E-04
ENST0	.	10q25.2	.	.	.	.	.	.	.	.	.	.	.
ENST0	.	11p14.1	.	.	.	Score=228	rs761	ID=COSN	.	.	.	.	.
ENST0	.	11p14.1	.	.	.	.	.	.	.	.	.	.	.
ENST0	.	11p11.2	.	Scc	.	.	rs781	ID=COSM	.	.	.	.	.
.	.	11q12.3	.	.	.	.	.	.	.	.	.	.	.
ENST0	.	11q13.1	.	.	.	.	rs752	.	.	.	.	.	.
ENST0	.	11q13.1	.	.	.	.	.	.	.	.	.	.	.
ENST0	.	11q25	.	.	.	.	.	.	.	.	.	.	.
ENST0	.	12q24.13	.	Scc	.	.	.	.	.	.	.	.	.
ENST0	.	12q24.31	.	.	.	.	rs140	ID=COSM	.	.	0	.	0.001
ENST0	.	13q13.3	.	.	.	.	.	.	.	.	.	.	.
.	.	13q22.2	.	.	.	.	.	.	.	.	.	.	.
ENST0	.	14q11.2	.	.	Scc	Score=520	.	ID=COSN	.	.	.	.	.
ENST0	.	14q22.1	.	.	.	.	.	.	.	.	.	.	.
ENST0	.	14q24.1	.	.	.	.	.	.	.	.	.	.	.
ENST0	.	14q24.3	.	.	.	.	.	.	.	.	.	.	.
ENST0	.	14q24.3	.	.	.	.	.	.	.	.	.	.	.
ENST0	.	16p13.3	.	.	.	.	rs766	.	.	.	.	.	.
ENST0	.	16p13.3	.	.	.	.	rs575	.	.	.	0	.	2E-04
ENST0	.	16p13.3	.	.	.	.	.	.	.	.	.	.	.
ENST0	.	16p12.2	.	.	.	.	.	.	.	.	.	.	.
ENST0	.	16p11.2	.	.	Scc	.	rs756	.	.	.	.	.	.
ENST0	.	16p11.2	.	.	Scc	.	rs752	.	.	.	.	.	.
ENST0	.	17q11.2	.	.	.	Score=216	.	.	.	.	.	.	.
ENST0	.	17q12	.	Scc	Scc	.	rs371	.	.	.	0	.	2E-04
ENST0	.	17q21.2	.	.	.	.	rs774	.	.	.	.	.	.
ENST0	.	17q21.33	.	.	.	.	rs746	ID=COSM	.	.	.	.	.
ENST0	.	17q23.1	.	.	.	Score=216	rs866	.	.	.	.	.	.
ENST0	.	17q23.2	.	.	Scc	.	rs866	ID=COSN	.	.	.	.	.
ENST0	.	17q25.3	.	.	.	.	.	.	.	.	.	.	.
ENST0	.	17q25.3	.	.	.	.	rs199	.	.	.	0	.	1E-03
ENST0	.	17q25.3	.	.	.	.	.	.	.	.	.	.	.
.	.	19q13.12	.	.	Scc	.	rs386	ID=COSN1	.	.	0.174	0.216	0.348
.	.	19q13.12	.	.	Scc	.	.	.	.	.	.	.	.
ENST0	.	19q13.2	.	Scc	.	.	rs757	ID=COSM	.	.	.	.	.
ENST0	.	19q13.32	.	.	.	.	rs756	ID=COSM	.	.	.	.	.
ENST0	.	20p13	.	.	.	.	.	.	.	.	.	.	.
ENST0	.	20q11.21	.	.	.	.	.	.	.	.	.	.	.
ENST0	.	20q11.23	.	.	.	.	rs377	.	.	.	.	.	.
ENST0	.	21q22.3	.	.	.	.	.	.	.	.	.	.	.
.	.	22q11.1	.	.	Scc	.	rs619	.	.	.	0.168	0.168	0.086
ENST0	.	22q11.21	.	.	Scc	.	.	.	.	.	.	.	.
ENST0	.	22q11.23	.	.	Scc	.	rs566	ID=COSN	.	.	.	.	.
ENST0	.	22q13.2	.	.	.	.	.	.	.	.	.	.	.
ENST0	.	22q13.31	.	.	.	.	rs100	.	.	.	.	.	.
ENST0	.	Xp11.23	.	.	.	Score=234	.	.	.	.	.	.	.
ENST0	.	Xp11.23	.	.	.	.	.	.	.	.	.	.	.
ENST0	.	Xp11.22	.	.	.	.	rs797	.	Pa	.	.	.	.
ENST0	.	Xp11.21	.	.	.	.	.	.	.	.	.	.	.
ENST0	.	Xq28	.	.	Scc	.	.	.	.	.	.	.	.
ENST0	.	1p36.13	.	.	Scc	Score=195	rs463	ID=COSN	.	.	.	.	.
ENST0	.	1p35.1	.	.	.	.	.	.	.	.	.	.	.

.	.	1q21.1	.	.	Scc	Score=224	rs745	ID=COSN1	.	.	.	.	.
.	.	1q21.1	.	.	Scc	.	rs201	.	.	.	.	.	.
.	.	1q21.1	.	.	Scc	.	.	.	.	.	.	.	.
ENST0	.	1q21.1	.	.	Scc	.	rs866	ID=COSN1	.	.	.	.	.
ENST0	.	1q21.1	.	.	Scc	.	rs414	ID=COSN1	.	.	.	.	.
ENST0	.	1q21.2	.	.	Scc	.	rs618	ID=COSN2	.	.	.	.	.
ENST0	.	1q23.1	.	.	.	.	.	.	.	.	.	.	.
ENST0	.	1q32.1	.	.	.	.	rs200	.	.	.	0	0.001	2E-04
ENST0	.	2p15	.	.	.	.	.	.	.	.	.	.	.
ENST0	.	2p13.2	.	Scc	.	.	rs183	.	Ur	.	0	.	2E-04
ENST0	.	2p13.2	.	.	.	.	rs200	ID=COSM3	Ur	.	.	.	.
ENST0	.	2p13.1	.	.	.	.	.	.	.	.	.	.	.
ENST0	.	2q11.2	.	.	Scc	.	rs952	ID=COSN6	.	.	.	.	.
ENST0	.	2q23.1	.	Scc	.	.	.	.	.	.	.	.	.
ENST0	.	2q37.3	.	.	.	.	rs373	.	.	.	.	.	.
ENST0	.	3p24.1	.	.	.	.	rs568	.	.	.	.	.	.
ENST0	.	3p21.31	.	.	.	.	rs147	.	.	.	0	.	2E-04
ENST0	.	3p21.2	.	Scc	.	.	rs200	.	.	.	.	.	.
ENST0	.	3q13.33	.	.	.	.	.	.	.	.	.	.	.
ENST0	.	4p16.3	.	.	.	.	.	.	.	.	.	.	.
ENST0	.	4p16.1	.	.	.	.	.	.	.	.	.	.	.
ENST0	.	4p16.1	.	Scc	.	.	.	.	.	.	.	.	.
ENST0	.	4p13	.	Scc	.	.	.	.	.	.	.	.	.
.	.	4p11	.	.	Scc	.	rs293	ID=COSN1	.	.	.	.	.
ENST0	.	4p11	.	.	Scc	.	.	ID=COSN5	.	.	.	.	.
ENST0	.	4q12	.	.	.	.	.	.	.	.	.	.	.
ENST0	.	4q21.21	.	.	.	.	rs766	ID=COSM4	.	.	.	.	.
ENST0	.	5q12.1	.	.	.	.	.	.	.	.	.	.	.
.	.	5q12.1	.	Scc	.	.	.	.	.	.	.	.	.
ENST0	.	5q21.1	.	Scc	.	.	.	.	.	.	.	.	.
ENST0	.	5q31.3	.	Scc	.	.	.	ID=COSN2	.	.	.	.	.
ENST0	.	5q32	.	.	.	.	rs531	.	.	.	0	.	2E-04
ENST0	.	6p24.2	.	.	.	Score=21;I	rs566	.	.	.	0	.	2E-04
ENST0	.	6p22.1	.	.	Scc	.	.	.	.	.	.	.	.
ENST0	.	6p21.33	.	Scc	.	.	.	.	.	.	.	.	.
ENST0	.	7p22.3	.	.	.	.	rs181	.	.	.	0	.	4E-04
.	.	7p11.2	.	.	Scc	.	rs782	.	.	.	.	.	.
ENST0	.	7q21.12	.	.	.	.	rs148	.	.	.	0.003	0.002	4E-04
ENST0	.	7q22.1	.	.	.	.	rs552	.	.	.	0.005	0.004	0.003
ENST0	.	7q33	.	Scc	.	.	.	ID=COSM6	.	.	.	.	.
ENST0	.	7q33	.	.	.	.	rs373	.	.	.	.	.	.
ENST0	.	7q35	.	Scc	.	.	.	.	.	.	.	.	.
ENST0	.	8q11.21	.	.	Scc	.	rs998	.	.	.	.	.	.
ENST0	.	8q21.3	.	.	.	.	.	.	.	.	.	.	.
ENST0	.	9p24.2	.	.	.	.	.	.	.	.	.	.	.
ENST0	.	9p21.1	.	.	.	.	.	.	.	.	.	.	.
ENST0	.	9q13	.	.	Scc	.	rs933	ID=COSN2	.	.	.	.	.
ENST0	.	9q22.31	.	.	.	.	.	.	.	.	.	.	.
.	.	9q32	.	.	Scc	.	.	.	.	.	.	.	.
ENST0	.	9q34.3	.	Scc	.	.	rs945	.	.	.	.	.	.
ENST0	.	9q34.3	.	.	.	.	rs949	.	.	.	.	.	.
ENST0	.	10p12.1	.	Scc	.	.	.	.	.	.	.	.	.
ENST0	.	10p11.1	.	.	Scc	.	.	.	.	.	.	.	.
.	.	10q11.22	.	.	Scc	.	rs299	.	.	.	.	.	.

ENST0	.	10q11.23	.	.	Scc	.	.	.	.	.	.	.	.
ENST0	.	10q24.1	.	.	.	.	.	.	.	.	.	.	.
ENST0	.	10q24.2	.	.	.	.	rs779	.	.	.	.	.	.
ENST0	.	11p15.3	.	.	.	.	.	.	.	.	.	.	.
ENST0	.	11q22.3	.	.	.	.	.	.	.	.	.	.	.
ENST0	.	11q23.3	.	Scc	.	.	.	.	.	.	.	.	.
ENST0	.	11q25	.	.	.	.	.	.	.	.	.	.	.
ENST0	.	12p13.31	.	.	.	.	.	.	.	.	.	.	.
ENST0	.	12p13.2	.	.	.	.	.	ID=COSM6	.	.	.	.	.
ENST0	.	12p12.1	.	Scc	.	.	rs121	ID=COSM5	Pa	.	.	.	.
ENST0	.	12q13.3	.	.	.	.	rs352	.	Be	.	0	.	8E-04
ENST0	.	12q21.1	.	.	.	.	.	.	.	.	.	.	.
ENST0	.	12q23.3	.	.	.	.	rs539	.	.	.	0	.	2E-04
.	.	13q31.1	.	.	Scc	.	.	.	.	.	.	.	.
ENST0	.	14q22.2	.	.	.	Score=27;I	.	.	.	.	.	.	.
ENST0	.	14q32.2	.	.	.	.	rs879	.	.	.	.	.	.
ENST0	.	15q11.2	.	.	Scc	.	.	.	.	.	.	.	.
ENST0	.	15q11.2	.	Scc	Scc	.	.	.	.	.	.	.	.
ENST0	.	15q12	.	.	.	.	.	ID=COSM2	.	.	.	.	.
ENST0	.	15q13.3	.	.	Scc	.	rs783	.	.	.	0.095	0.082	0.146
.	.	15q25.2	.	.	Scc	.	rs201	ID=COSN1	.	.	.	.	.
ENST0	.	16p12.1	.	.	.	.	.	.	.	.	.	.	.
ENST0	.	16p11.2	.	.	Scc	.	.	.	.	.	.	.	.
ENST0	.	16q12.1	.	.	.	.	.	.	.	.	.	.	.
ENST0	.	16q22.1	.	.	Scc	.	rs200	ID=COSN2	.	.	.	.	.
ENST0	.	16q23.3	.	.	.	.	.	.	.	.	.	.	.
ENST0	.	17q12	.	.	.	.	.	.	.	.	.	.	.
ENST0	.	17q12	.	.	Scc	.	.	.	.	.	.	.	.
ENST0	.	17q12	.	.	.	.	.	.	.	.	.	.	.
ENST0	.	17q25.3	.	.	.	.	.	.	.	.	.	.	.
ENST0	.	18q21.33	.	.	Scc	Score=214	.	.	.	.	.	.	.
ENST0	.	19p13.3	.	.	.	Score=235	rs867	ID=COSN2	.	.	.	.	.
ENST0	.	19p13.2	.	.	.	.	.	.	.	.	.	.	.
ENST0	.	19p13.2	.	Scc	.	.	rs768	.	.	.	.	.	.
ENST0	.	19q13.12	.	.	.	.	.	.	.	.	.	.	.
ENST0	.	19q13.2	.	.	Scc	.	.	.	.	.	.	.	.
ENST0	.	19q13.31	.	.	.	.	.	.	.	.	.	.	.
ENST0	.	19q13.42	.	.	.	.	.	.	.	.	.	.	.
ENST0	.	19q13.42	.	.	.	Score=213	.	.	.	.	.	.	.
ENST0	.	19q13.43	.	.	Scc	.	rs770	.	.	.	.	.	.
ENST0	.	20p13	.	Scc	.	.	.	.	.	.	.	.	.
ENST0	.	20p11.21	.	.	.	.	rs200	.	.	.	0	.	2E-04
ENST0	.	20q11.23	.	.	.	.	.	.	.	.	.	.	.
.	.	20q13.2	.	.	.	Score=126	.	.	.	.	.	.	.
ENST0	.	21q21.1	.	Scc	.	.	.	.	.	.	.	.	.
ENST0	.	22q12.3	.	.	.	.	.	.	.	.	.	.	.
ENST0	.	Xp11.23	.	.	Scc	.	.	.	.	.	.	.	.
ENST0	.	Xq22.1	.	.	.	.	.	.	.	.	.	.	.
ENST0	.	Xq22.3	.	.	.	.	.	ID=COSM5	.	.	.	.	.
ENST0	.	Xq22.3	.	.	.	.	rs753	.	.	.	0.002	0.001	3E-04
ENST0	.	Xq25	.	.	.	.	.	.	.	.	.	.	.
ENST0	.	Xq26.3	.	.	.	.	rs776	.	.	.	.	.	.
ENST0	.	Xq27.3	.	.	.	.	.	.	.	.	.	.	.
ENST0	.	Xq28	.	.	.	.	rs398	.	Pa	.	0.002	0.001	5E-04

.	.	.	.	.	.	.	.	.	.	.	.	.	.
ENST0	.	1p36.22	.	.	.	.	.	.	.	.	.	.	.
ENST0	.	1p36.13	.	.	Scc	.	rs101	ID=COSN1	.	.	.	.	.
ENST0	.	1p36.13	.	.	.	.	.	.	.	.	.	.	.
ENST0	.	1p36.12	.	.	.	.	rs145	.	.	.	.	.	.
ENST0	.	1p22.1	.	Scc	.	.	.	.	.	.	.	.	.
ENST0	.	1q21.1	.	.	Scc	.	rs770	.	.	.	.	.	.
ENST0	.	1q21.1	.	.	Scc	.	rs141	ID=COSN7	.	.	.	.	.
ENST0	.	1q21.1	.	.	Scc	.	rs125	ID=COSN1	.	.	.	.	.
ENST0	.	1q21.1	.	.	Scc	.	rs372	.	.	.	.	.	.
ENST0	.	1q21.1	.	.	Scc	.	rs587	ID=COSM3	.	.	0.269	0.314	0.245
ENST0	.	1q21.2	.	.	Scc	.	.	.	.	.	.	.	.
ENST0	.	1q21.2	.	Scc	.	.	rs373	.	.	.	.	.	.
ENST0	.	1q25.3	.	.	.	.	.	.	.	.	.	.	.
ENST0	.	1q32.2	.	.	.	.	.	.	.	.	.	.	.
ENST0	.	2p21	.	Scc	.	.	.	.	.	.	.	.	.
.	.	2p11.2	.	.	Scc	.	rs754	.	.	.	.	.	.
ENST0	.	2q24.1	.	.	.	.	.	.	.	.	.	.	.
ENST0	.	2q31.2	.	.	.	.	.	.	.	.	.	.	.
ENST0	.	2q33.1	.	.	.	.	.	.	.	.	.	.	.
ENST0	.	2q37.2	.	Scc	.	.	rs982	.	.	.	.	.	.
ENST0	.	3p26.1	.	.	.	.	.	.	.	.	.	.	.
ENST0	.	3p26.1	.	.	.	Score=318	rs130	.	.	.	.	.	.
ENST0	.	3p22.3	.	.	.	.	.	.	.	.	.	.	.
ENST0	.	3p22.2	.	.	.	.	rs371	.	.	.	.	.	.
ENST0	.	3p22.1	.	.	.	Score=323	.	.	.	.	.	.	.
ENST0	.	3p21.31	.	.	.	.	.	.	.	.	.	.	.
ENST0	.	3p21.31	.	Scc	.	.	.	.	.	.	.	.	.
ENST0	.	3p21.31	.	Scc	.	.	.	.	.	.	.	.	.
ENST0	.	3p21.31	.	.	.	.	.	.	.	.	.	.	.
ENST0	.	3q13.31	.	.	.	.	.	.	.	.	.	.	.
ENST0	.	3q21.3	.	.	.	.	rs572	.	.	.	0	.	2E-04
ENST0	.	4p16.3	.	.	.	.	rs497	ID=COSN1	.	.	.	.	.
.	.	4q13.3	.	.	.	Score=274	rs145	.	.	.	0.002	0.001	2E-04
ENST0	.	4q22.1	.	.	.	.	.	.	.	.	.	.	.
ENST0	.	4q26	.	.	.	.	.	.	.	.	.	.	.
ENST0	.	4q28.1	.	.	.	.	rs372	ID=COSM1	.	.	.	.	.
ENST0	.	4q34.1	.	Scc	.	.	.	.	.	.	.	.	.
ENST0	.	4q35.2	.	.	.	Score=232	.	.	.	.	.	.	.
ENST0	.	5q32	.	.	.	.	.	.	.	.	.	.	.
ENST0	.	5q33.3	.	.	.	.	.	.	.	.	.	.	.
ENST0	.	6p22.1	.	.	.	.	.	.	.	.	.	.	.
ENST0	.	6p21.2	.	.	.	.	.	.	.	.	.	.	.
ENST0	.	6q16.1	.	.	.	.	rs901	.	.	.	.	.	.
ENST0	.	6q22.31	.	.	.	.	.	.	.	.	.	.	.
ENST0	.	7p22.1	.	.	Scc	.	rs154	ID=COSN1	.	.	.	.	.
ENST0	.	7p22.1	.	.	.	.	.	.	.	.	.	.	.
ENST0	.	7p15.3	.	.	.	.	.	.	.	.	.	.	.
ENST0	.	7p15.2	.	.	.	.	.	.	.	.	.	.	.
ENST0	.	7p13	.	.	.	.	.	.	.	.	.	.	.
.	.	7p12.3	.	.	Scc	.	rs192	.	.	.	0.002	0.001	2E-04
ENST0	.	7q11.21	.	.	Scc	.	.	.	.	.	.	.	.
ENST0	.	7q11.23	.	Scc	.	.	.	.	.	.	.	.	.
ENST0	.	7q21.3	.	.	.	.	.	.	.	.	.	.	.

ENST0	.	7q22.1	.	.	.	.	.	.	.	.	.	.	.
ENST0	.	7q33	.	.	.	.	rs575	.	.	.	0	.	2E-04
ENST0	.	7q33	.	Scc	.	.	.	.	.	.	.	.	.
ENST0	.	8q11.1	.	.	.	.	.	.	.	.	.	.	.
ENST0	.	8q13.3	.	.	.	.	.	.	.	.	.	.	.
ENST0	.	8q13.3	.	.	.	.	rs547	.	.	.	0	.	2E-04
ENST0	.	8q21.3	.	.	.	.	.	.	.	.	.	.	.
ENST0	.	9p21.1	.	.	Scc	.	rs374	ID=COSN2	.	.	.	.	.
ENST0	.	9p13.3	.	Scc	.	.	.	ID=COSM3	.	.	.	.	.
ENST0	.	9q21.11	.	.	.	.	.	.	.	.	.	.	.
ENST0	.	9q21.13	.	Scc	.	.	.	.	.	.	.	.	.
.	.	9q32	.	.	Scc	.	rs286	.	.	.	.	.	.
ENST0	hsa-mir-45	9q32	.	.	.	.	rs371	.	.	.	.	.	.
ENST0	.	9q34.3	.	.	.	.	rs997	.	.	.	.	.	.
ENST0	.	9q34.3	.	.	.	.	rs376	.	.	.	.	.	.
ENST0	.	10p15.2	.	.	.	.	rs926	.	.	.	.	.	.
ENST0	.	10p11.21	.	.	.	.	.	.	.	.	.	.	.
ENST0	.	10q11.22	.	.	.	Score=492	.	.	.	.	.	.	.
ENST0	.	10q22.2	.	Scc	.	.	.	.	.	.	.	.	.
ENST0	.	10q23.33	.	.	.	.	.	.	.	.	.	.	.
ENST0	.	10q26.2	.	.	.	.	.	.	.	.	.	.	.
ENST0	.	11p15.4	.	.	.	.	.	.	.	.	.	.	.
ENST0	.	11p15.1	.	.	.	.	.	.	.	.	.	.	.
ENST0	.	11p15.1	.	.	.	Score=254	.	ID=COSN1	.	.	.	.	.
ENST0	.	11q11	.	.	.	.	.	.	.	.	.	.	.
ENST0	.	11q12.1	.	.	.	.	rs369	.	.	.	.	.	.
ENST0	.	11q13.2	.	.	.	Score=242	.	.	.	.	.	.	.
ENST0	.	11q22.3	.	.	.	.	.	.	.	.	.	.	.
ENST0	.	11q23.3	.	.	.	.	.	.	.	.	.	.	.
ENST0	.	12q13.11	.	.	.	.	.	.	.	.	.	.	.
ENST0	.	12q23.1	.	.	.	.	.	.	.	.	.	.	.
ENST0	.	12q24.13	.	.	.	Score=441	rs868	ID=COSN1	.	.	.	.	.
ENST0	.	12q24.21	.	.	.	.	.	.	.	.	.	.	.
ENST0	.	12q24.31	.	Scc	.	.	rs568	.	.	.	0.002	0.001	2E-04
ENST0	.	13q32.3	.	.	.	.	rs752	.	.	.	.	.	.
ENST0	.	14q11.2	.	.	.	.	.	.	.	.	.	.	.
ENST0	.	14q11.2	.	.	.	Score=303	rs128	ID=COSN2	.	.	.	.	.
.	.	14q32.31	.	.	.	.	rs531	.	.	.	0	.	2E-04
ENST0	.	15q11.2	.	.	Scc	.	.	.	.	.	.	.	.
ENST0	.	15q13.1	.	.	.	.	rs754	.	.	.	.	.	.
ENST0	.	15q13.2	.	.	Scc	.	rs201	.	.	.	.	.	.
ENST0	.	15q13.2	.	.	Scc	.	rs712	.	.	.	.	.	.
ENST0	.	15q14	.	.	.	.	.	.	.	.	.	.	.
ENST0	.	15q21.1	.	.	.	.	.	.	.	.	.	.	.
ENST0	.	15q24.3	.	.	Scc	.	rs186	ID=COSN2	.	.	0.043	0.047	0.04
ENST0	.	15q24.3	.	.	Scc	.	rs878	ID=COSN2	.	.	.	.	.
ENST0	.	15q25.1	.	.	.	.	.	.	.	.	.	.	.
ENST0	.	15q25.2	.	.	.	.	.	.	.	.	.	.	.
ENST0	.	16p13.3	.	.	.	.	rs765	ID=COSM1	.	.	.	.	.
ENST0	.	16p13.3	.	Scc	.	.	rs201	.	.	.	0.003	0.002	4E-04
ENST0	.	16p13.3	.	.	.	.	.	.	.	.	.	.	.
ENST0	.	16q22.2	.	.	.	.	.	ID=COSM4	.	.	.	.	.
ENST0	.	17p13.3	.	.	.	.	.	.	.	.	.	.	.
ENST0	.	17p13.2	.	.	.	.	.	.	.	.	.	.	.

ENST0	.	17p13.2	.	.	.	Score=231	rs778	ID=COSM4	.	.	.	.	.
ENST0	.	17q22	.	.	.	.	.	.	.	.	.	.	.
ENST0	.	17q25.1	.	.	.	.	rs113	.	.	.	0	.	4E-04
ENST0	.	17q25.1	.	.	.	.	.	.	.	.	.	.	.
ENST0	.	17q25.3	.	.	.	.	rs764	.	.	.	.	.	.
ENST0	.	17q25.3	.	.	.	.	.	.	.	.	.	.	.
ENST0	.	17q25.3	.	Scc	.	.	rs372	.	.	.	0	.	2E-04
ENST0	.	17q25.3	.	.	.	.	.	.	.	.	.	.	.
ENST0	.	17q25.3	.	.	.	.	.	.	.	.	.	.	.
ENST0	.	18p11.32	.	.	.	.	.	.	.	.	.	.	.
ENST0	.	18p11.32	.	.	.	.	.	.	.	.	.	.	.
ENST0	.	18q12.2	.	.	.	.	.	.	.	.	.	.	.
ENST0	.	19p13.3	.	.	.	.	rs528	.	.	.	0	.	2E-04
ENST0	.	19p13.3	.	Scc	.	.	.	.	.	.	.	.	.
ENST0	.	19p13.3	.	.	.	Score=337	rs907	.	.	.	.	.	.
ENST0	.	19p13.3	.	.	.	Score=337	.	.	.	.	.	.	.
ENST0	.	19p13.2	.	.	.	Score=175	.	.	.	.	.	.	.
ENST0	.	19p13.13	.	.	.	.	.	.	.	.	.	.	.
ENST0	.	19q13.12	.	.	.	.	.	.	.	.	.	.	.
ENST0	.	19q13.31	.	.	.	.	.	.	.	.	.	.	.
ENST0	.	19q13.33	.	.	.	.	.	.	.	.	.	.	.
ENST0	.	19q13.33	.	Scc	.	.	.	.	.	.	.	.	.
ENST0	.	19q13.33	.	.	.	.	.	.	.	.	.	.	.
ENST0	.	19q13.33	.	.	.	.	.	.	.	.	.	.	.
ENST0	.	19q13.41	.	.	Scc	.	.	.	.	.	.	.	.
ENST0	.	19q13.42	.	.	.	Score=367	.	.	.	.	.	.	.
ENST0	.	20q12	.	.	.	.	rs745	ID=COSM4	.	.	.	.	.
.	.	21p11.2	.	.	Scc	Score=28;I	.	.	.	.	.	.	.
.	.	21p11.2	.	.	Scc	Score=218	rs879	ID=COSN1	.	.	.	.	.
ENST0	.	21p11.1	.	.	Scc	.	.	.	.	.	.	.	.
ENST0	.	22q11.21	.	.	.	.	rs116	.	.	.	0	.	0.004
ENST0	.	22q11.21	.	.	Scc	.	rs376	ID=COSN2	.	.	.	.	.
ENST0	.	22q12.2	.	.	.	.	.	.	.	.	.	.	.
ENST0	.	Xq13.2	.	.	Scc	.	.	.	.	.	.	.	.
ENST0	.	Xq27.1	.	.	.	.	.	.	.	.	.	.	.
ENST0	.	Xq27.2	.	.	.	.	.	.	.	.	.	.	.
ENST0	.	1p36.33	.	.	Scc	.	rs115	ID=COSN1	.	.	0.596	0.581	0.437
ENST0	.	1p36.33	.	.	.	.	.	.	.	.	.	.	.
ENST0	.	1p34.3	.	.	.	.	.	.	.	.	.	.	.
ENST0	.	1p34.1	.	.	.	.	rs531	ID=COSM5	.	.	0	.	0.004
ENST0	.	1p32.3	.	.	.	.	.	.	.	.	.	.	.
ENST0	.	1p32.2	.	Scc	.	.	rs778	.	.	.	.	.	.
ENST0	.	1p21.2	.	.	.	.	.	.	.	.	.	.	.
ENST0	.	1q21.1	.	.	Scc	.	rs773	.	.	.	0.571	0.554	0.317
ENST0	.	1q21.1	.	.	Scc	.	rs171	ID=COSN1	.	.	.	.	.
ENST0	.	1q21.3	.	.	.	.	.	.	.	.	.	.	.
ENST0	.	1q21.3	.	.	.	.	.	.	.	.	.	.	.
ENST0	.	1q23.3	.	.	.	.	.	ID=COSN1	.	.	.	.	.
ENST0	.	1q25.3	.	.	.	.	.	.	.	.	.	.	.
ENST0	.	1q31.3	.	.	.	.	.	.	.	.	.	.	.
.	.	1q31.3	.	.	.	.	.	.	.	.	.	.	.
ENST0	.	1q32.1	.	.	.	.	.	.	.	.	.	.	.
ENST0	.	1q43	.	.	.	.	.	.	.	.	.	.	.
ENST0	.	2p25.3	.	.	.	.	.	.	.	.	.	.	.

ENST0	.	2p25.3	.	.	.	.	.	.	.	.	.	.	.
ENST0	.	2p24.3	.	.	.	.	.	.	.	.	.	.	.
ENST0	.	2p22.3	.	.	.	.	.	.	.	.	.	.	.
ENST0	.	2p13.3	.	.	.	.	.	.	.	.	.	.	.
.	.	2q11.1	.	.	.	Score=788	rs201	ID=COSN2	.	.	.	.	.
ENST0	.	2q11.2	.	Scc	.	.	.	.	.	.	.	.	.
ENST0	.	2q12.2	.	.	Scc	.	rs200	.	.	.	.	.	.
ENST0	.	2q14.2	.	.	.	.	rs724	ID=COSN2	.	.	0.042	0.042	0.057
ENST0	.	2q14.2	.	.	.	.	.	.	.	.	.	.	.
ENST0	.	2q31.2	.	.	.	.	.	ID=COSM3	.	.	.	.	.
ENST0	.	2q33.2	.	.	.	.	.	.	.	.	.	.	.
ENST0	.	2q33.2	.	.	.	.	rs552	.	.	.	.	.	.
ENST0	.	2q33.2	.	.	.	.	.	.	.	.	.	.	.
ENST0	.	2q35	.	.	.	Score=215	rs181	.	.	.	0.002	0.001	2E-04
ENST0	.	2q37.1	.	Scc	.	.	.	.	.	.	.	.	.
ENST0	.	3p21.31	.	.	.	Score=255	.	.	.	.	.	.	.
ENST0	.	3p21.31	.	.	.	.	rs918	.	.	.	.	.	.
ENST0	.	3p21.1	.	.	.	.	rs373	.	.	.	.	.	.
ENST0	.	3q12.1	.	.	.	.	.	.	.	.	.	.	.
ENST0	.	3q12.1	.	.	.	.	.	.	.	.	.	.	.
ENST0	.	3q12.3	.	.	.	.	.	.	.	.	.	.	.
ENST0	.	3q13.33	.	.	.	.	.	.	.	.	.	.	.
ENST0	.	3q22.1	.	.	.	.	rs145	ID=COSM1	.	.	0	.	0.004
ENST0	.	3q26.32	.	.	Scc	.	rs104	ID=COSM7	Pa	.	.	.	.
ENST0	.	4p16.3	.	.	.	.	.	.	.	.	.	.	.
ENST0	.	4p16.3	.	.	Scc	.	rs401	.	.	.	.	.	.
ENST0	.	4p13	.	.	.	.	.	.	.	.	.	.	.
ENST0	.	4p13	.	.	.	.	.	.	.	.	.	.	.
ENST0	.	4q13.3	.	.	.	.	.	.	.	.	.	.	.
ENST0	.	4q31.1	.	.	.	.	.	.	.	.	.	.	.
.	.	4q35.2	.	.	Scc	Score=782	.	.	.	.	.	.	.
ENST0	.	5p14.3	.	.	Scc	.	.	.	.	.	.	.	.
ENST0	.	5q11.2	.	.	.	.	.	.	.	.	.	.	.
ENST0	.	5q23.1	.	.	.	.	.	.	.	.	.	.	.
ENST0	.	5q31.1	.	.	.	.	.	.	.	.	.	.	.
ENST0	.	5q31.3	.	.	.	.	rs773	ID=COSM4	.	.	.	.	.
ENST0	.	5q35.1	.	.	.	.	.	ID=COSM4	.	.	.	.	.
ENST0	.	5q35.3	.	.	.	.	.	.	.	.	.	.	.
ENST0	.	5q35.3	.	.	.	.	rs376	ID=COSM4	.	.	.	.	.
ENST0	.	6p22.2	.	Scc	.	.	.	.	.	.	.	.	.
ENST0	.	6p21.33	.	Scc	.	.	rs141	ID=COSM1	.	.	.	.	.
ENST0	.	6q21	.	.	.	.	rs368	.	.	.	.	.	.
ENST0	.	6q25.1	.	.	Scc	.	.	.	.	.	.	.	.
ENST0	.	6q25.2	.	.	.	.	.	.	.	.	.	.	.
ENST0	.	6q25.3	.	.	.	.	rs201	.	.	.	.	.	.
.	.	6q27	.	.	Scc	.	rs375	.	.	.	.	.	.
ENST0	.	7p13	.	.	.	.	rs767	.	.	.	.	.	.
ENST0	.	7p13	.	.	.	.	.	.	.	.	.	.	.
ENST0	.	7p11.2	.	.	Scc	.	rs624	ID=COSN2	.	.	.	.	.
ENST0	.	7p11.2	.	.	Scc	.	rs201	ID=COSN1	.	.	.	.	.
ENST0	.	7q11.21	.	.	Scc	.	.	.	.	.	.	.	.
.	.	7q11.23	.	.	Scc	.	.	.	.	.	.	.	.
ENST0	.	7q22.1	.	.	Scc	.	rs761	.	.	.	.	.	.
ENST0	.	7q34	.	.	.	.	.	.	.	.	.	.	.

ENST0	.	7q36.1	.	.	Scc	.	rs141	ID=COSM1	.	.	.	.	.
ENST0	.	7q36.3	.	.	.	.	.	.	.	.	.	.	.
ENST0	.	8p23.1	.	.	.	Score=242	.	.	.	.	.	.	.
ENST0	.	8p23.1	.	.	Scc	.	rs748	ID=COSN2	.	.	.	.	.
ENST0	.	8p21.3	.	.	.	.	.	.	.	.	.	.	.
ENST0	.	8p21.3	.	.	.	Score=237	.	ID=COSN2	.	.	.	.	.
ENST0	.	8q21.3	.	Scc	.	.	.	.	.	.	.	.	.
ENST0	.	8q22.3	.	.	.	.	rs535	.	.	.	0	0.001	2E-04
ENST0	.	8q23.3	.	.	.	Score=26;I	rs559	ID=COSN2	.	.	0	0.001	0.002
ENST0	.	8q24.13	.	.	.	.	rs376	.	.	.	.	.	.
ENST0	.	8q24.3	.	.	.	.	.	.	.	.	.	.	.
ENST0	.	8q24.3	.	.	.	.	.	.	.	.	.	.	.
ENST0	.	9p22.3	.	.	.	.	.	.	.	.	.	.	.
ENST0	.	9p22.2	.	Scc	.	.	.	.	.	.	.	.	.
ENST0	.	9p11.2	.	.	Scc	Score=121	rs280	ID=COSN1	.	.	.	.	.
ENST0	.	9q22.1	.	.	Scc	.	rs755	.	.	.	.	.	.
ENST0	.	9q33.3	.	.	.	.	.	.	.	.	.	.	.
ENST0	.	10p15.2	.	.	.	.	rs190	.	.	.	0	.	2E-04
ENST0	.	10p11.22	.	Scc	.	.	.	.	.	.	.	.	.
ENST0	.	10p11.1	.	.	Scc	.	rs407	ID=COSN2	.	.	.	.	.
ENST0	.	10q22.3	.	.	.	Score=266	.	.	.	.	.	.	.
ENST0	.	10q23.31	.	.	.	.	.	.	.	.	.	.	.
ENST0	.	10q24.1	.	.	.	.	.	.	.	.	.	.	.
ENST0	.	11p15.5	.	Scc	.	.	rs748	.	.	.	.	.	.
ENST0	.	11p15.3	.	.	.	.	.	.	.	.	.	.	.
ENST0	.	11p15.1	.	.	.	.	.	.	.	.	.	.	.
ENST0	.	11p15.1	.	.	.	Score=212	.	ID=COSN1	.	.	.	.	.
ENST0	.	11p14.2	.	.	.	.	.	.	.	.	.	.	.
ENST0	.	11p14.1	.	.	.	Score=459	rs147	.	.	.	.	.	.
ENST0	.	11q12.3	.	Scc	.	.	.	.	.	.	.	.	.
ENST0	.	11q13.1	.	Scc	.	.	.	.	.	.	.	.	.
ENST0	.	11q14.3	.	.	Scc	.	rs201	ID=COSN2	.	.	.	.	.
ENST0	.	11q22.3	.	.	.	.	.	.	.	.	.	.	.
ENST0	.	12q12	.	.	.	.	.	.	.	.	.	.	.
ENST0	.	12q13.13	.	.	.	.	rs776	.	.	.	.	.	.
ENST0	.	12q13.3	.	.	.	.	.	.	.	.	.	.	.
ENST0	.	12q23.1	.	.	.	.	.	.	.	.	.	.	.
ENST0	.	12q24.11	.	.	.	.	.	.	.	.	.	.	.
ENST0	.	13q14.11	.	.	.	.	.	.	.	.	.	.	.
ENST0	.	13q21.2	.	.	Scc	.	.	.	.	.	.	.	.
.	.	13q31.3	.	.	.	.	.	.	.	.	.	.	.
.	.	14q11.2	.	.	Scc	.	rs225	ID=COSN1	.	.	.	.	.
ENST0	.	14q11.2	.	.	Scc	.	rs796	ID=COSN1	.	.	.	.	.
ENST0	.	14q11.2	.	Scc	.	.	rs751	ID=COSM1	.	.	.	.	.
ENST0	.	14q11.2	.	.	.	.	rs112	.	.	.	.	.	.
ENST0	.	14q13.2	.	.	.	.	.	.	.	.	.	.	.
ENST0	.	14q32.32	.	Scc	.	.	.	.	.	.	.	.	.
.	.	15q11.1	.	.	Scc	.	rs714	ID=COSN1	.	.	.	.	.
ENST0	.	15q11.2	.	Scc	.	.	.	.	.	.	.	.	.
ENST0	.	15q13.1	.	.	Scc	.	.	.	.	.	.	.	.
ENST0	.	15q15.1	.	.	.	.	.	.	.	.	.	.	.
ENST0	.	15q24.2	.	.	Scc	.	.	.	.	.	.	.	.
ENST0	.	16p13.13	.	.	.	.	.	.	.	.	.	.	.
ENST0	.	16p12.3	.	.	.	.	.	.	.	.	.	.	.

ENST0	.	16p11.2	.	.	.	.	.	.	.	.	.	.	.
ENST0	.	16p11.2	.	.	Scc	.	rs145	ID=COSM	.	.	.	.	.
ENST0	.	16q22.1	.	.	.	.	.	.	.	.	.	.	.
ENST0	.	16q22.1	.	.	.	.	rs113	ID=COSN2	.	.	.	.	.
ENST0	.	17p13.3	.	Scc	.	.	rs767	.	.	.	.	.	.
ENST0	.	17p13.1	.	Scc	.	.	rs760	.	.	.	.	.	.
ENST0	.	17p13.1	.	.	.	.	.	.	.	.	.	.	.
ENST0	.	17q11.2	.	.	.	.	rs554	.	.	.	0	.	4E-04
ENST0	.	17q21.31	.	.	.	.	.	.	.	.	.	.	.
ENST0	.	17q24.2	.	.	.	.	.	.	.	.	.	.	.
ENST0	.	17q25.3	.	.	.	.	rs740	.	.	.	0	.	0.011
ENST0	.	18p11.21	.	.	Scc	.	rs783	ID=COSN2	.	.	.	.	.
ENST0	.	18q12.3	.	.	.	.	.	.	.	.	.	.	.
ENST0	.	18q21.2	.	.	.	.	.	.	.	.	.	.	.
ENST0	.	18q21.32	.	.	Scc	.	rs146	.	.	.	0.013	0.011	0.021
ENST0	.	18q22.1	.	.	.	.	rs563	.	.	.	0	.	2E-04
ENST0	.	19p13.3	.	.	.	.	.	.	.	.	.	.	.
ENST0	.	19p13.2	.	.	.	.	.	.	.	.	.	.	.
ENST0	.	19p13.2	.	.	.	Score=980	.	.	.	.	.	.	.
ENST0	.	19p13.12	.	.	.	.	.	.	.	.	.	.	.
.	.	19p13.11	.	.	.	.	.	.	.	.	.	.	.
ENST0	.	19p12	.	.	.	.	.	.	.	.	.	.	.
ENST0	.	19p12	.	.	Scc	.	.	.	.	.	.	.	.
ENST0	.	19q13.12	.	.	.	Score=321	.	.	.	.	.	.	.
.	.	19q13.12	.	.	Scc	Score=123	rs712	.	.	.	.	.	.
ENST0	.	19q13.41	.	.	.	.	rs182	.	.	.	0	.	0.002
ENST0	.	20p13	.	.	.	.	.	.	.	.	.	.	.
ENST0	.	20p13	.	Scc	.	.	.	.	.	.	.	.	.
ENST0	.	20p12.3	.	.	.	.	.	.	.	.	.	.	.
ENST0	.	20q13.33	.	.	.	.	.	.	.	.	.	.	.
.	.	21p11.2	.	.	Scc	.	rs796	.	.	.	.	.	.
ENST0	.	21p11.1	.	.	Scc	.	rs373	ID=COSN1	.	.	0.061	0.062	0.071
ENST0	.	21p11.1	.	.	Scc	Score=471	rs728	ID=COSN1	.	.	.	.	.
ENST0	.	21q21.1	.	.	.	Score=149	.	.	.	.	.	.	.
ENST0	.	21q22.12	.	.	.	.	rs773	.	.	.	.	.	.
ENST0	.	21q22.3	.	Scc	.	.	rs551	.	.	.	0	.	2E-04
ENST0	.	21q22.3	.	Scc	.	.	.	.	.	.	.	.	.
ENST0	.	21q22.3	.	Scc	.	.	.	.	.	.	.	.	.
ENST0	.	22q11.1	.	.	Scc	.	rs126	.	.	.	.	.	.
ENST0	.	22q11.22	.	.	Scc	.	rs860	ID=COSN1	.	.	.	.	.
ENST0	.	22q12.2	.	.	.	.	.	.	.	.	.	.	.
ENST0	.	Xp22.2	.	.	.	.	.	.	.	.	.	.	.
ENST0	.	Xp22.13	.	.	.	.	rs778	.	.	.	.	.	.
ENST0	.	Xp11.22	.	.	.	.	.	.	.	.	.	.	.
.	.	Xq12	.	.	.	.	.	.	.	.	.	.	.
ENST0	.	Xq13.1	.	.	.	Score=939	.	.	.	.	.	.	.
ENST0	.	Xq13.1	.	Scc	.	.	.	ID=COSM	.	.	.	.	.
ENST0	.	Xq22.1	.	.	Scc	.	.	.	.	.	.	.	.
ENST0	.	Xq22.1	.	.	Scc	.	.	.	.	.	.	.	.
ENST0	.	Xq25	.	.	.	.	rs917	.	.	.	.	.	.
.	.	Xq28	.	.	.	Score=415	rs927	.	.	.	.	.	.
ENST0	.	Xq28	.	.	.	.	.	.	.	.	.	.	.
ENST0	.	1p36.13	.	.	.	.	rs371	ID=COSM	.	.	.	.	.
ENST0	.	1p36.11	.	Scc	.	.	.	.	.	.	.	.	.

ENST0	.	1p34.3	.	.	.	.	rs767	.	.	.	.	.	.
ENST0	.	1p34.3	.	.	.	.	rs760	ID=COSM6	.	.	.	.	.
ENST0	.	1p34.1	.	.	.	.	.	.	.	.	.	.	.
ENST0	.	1p34.1	.	Scc	.	.	.	.	.	.	.	.	.
ENST0	.	1p31.3	.	.	.	.	.	.	.	.	.	.	.
ENST0	.	1p31.3	.	.	.	.	.	.	.	.	.	.	.
ENST0	.	1p13.3	.	.	.	.	.	.	.	.	.	.	.
ENST0	.	1p13.2	.	.	.	Score=180	rs754	ID=COSN8	.	.	.	.	.
ENST0	.	1q21.1	.	.	Scc	Score=239	rs752	.	.	.	.	.	.
ENST0	.	1q21.1	.	.	Scc	Score=208	rs200	ID=COSN6	.	.	.	.	.
ENST0	.	1q21.1	.	.	Scc	.	rs372	ID=COSN2	.	.	.	.	.
ENST0	.	1q21.1	.	.	Scc	Score=423	rs879	.	.	.	.	.	.
ENST0	.	1q32.1	.	.	.	.	.	.	.	.	.	.	.
ENST0	.	1q42.13	.	.	.	.	rs775	.	.	.	.	.	.
ENST0	.	2p25.3	.	.	.	.	rs758	.	.	.	.	.	.
ENST0	.	2q31.1	.	Scc	.	.	.	.	.	.	.	.	.
ENST0	.	2q31.2	.	.	.	.	.	.	.	.	.	.	.
ENST0	.	2q32.3	.	.	.	.	rs141	.	.	.	.	.	.
ENST0	.	3p21.31	.	.	.	.	rs115	ID=COSM1	.	.	0	.	0.01
ENST0	.	3q13.2	.	.	Scc	.	.	.	.	.	.	.	.
ENST0	.	3q23	.	.	.	.	.	.	.	.	.	.	.
ENST0	.	3q27.1	.	.	.	.	.	.	.	.	.	.	.
ENST0	.	4p16.3	.	Scc	.	.	.	.	.	.	.	.	.
ENST0	.	4p16.3	.	.	.	.	.	.	.	.	.	.	.
ENST0	.	4p16.3	.	.	.	.	rs730	.	.	.	0	.	0.016
.	.	4p16.1	.	.	Scc	.	rs879	.	.	.	.	.	.
ENST0	.	4q22.2	.	.	.	Score=194	rs769	.	.	.	.	.	.
ENST0	.	4q32.1	.	.	.	.	.	.	.	.	.	.	.
.	.	4q32.3	.	.	.	Score=543	rs100	.	.	.	.	.	.
ENST0	.	5p15.31	.	.	.	.	.	.	.	.	.	.	.
ENST0	.	5q14.1	.	Scc	.	.	.	.	.	.	.	.	.
ENST0	.	5q22.2	.	.	.	.	.	.	.	.	.	.	.
ENST0	.	5q31.3	.	.	.	.	.	.	.	.	.	.	.
ENST0	.	5q33.3	.	.	.	.	.	.	.	.	.	.	.
ENST0	.	5q34	.	Scc	.	.	.	.	.	.	.	.	.
ENST0	.	6p22.1	.	.	.	.	.	.	.	.	.	.	.
ENST0	.	6p21.33	.	.	.	.	rs758	.	.	.	.	.	.
ENST0	.	6p11.2	.	.	.	.	.	.	.	.	.	.	.
ENST0	.	6q14.3	.	Scc	.	.	.	ID=COSM8	.	.	.	.	.
.	.	6q27	.	.	.	.	rs441	ID=COSN2	.	.	.	.	.
ENST0	.	7q21.11	.	.	.	.	.	.	.	.	.	.	.
ENST0	.	7q21.3	.	.	.	.	.	.	.	.	.	.	.
ENST0	.	7q31.1	.	.	.	.	.	ID=COSM4	.	.	.	.	.
ENST0	.	8p23.1	.	.	Scc	.	.	.	.	.	.	.	.
ENST0	.	8q23.3	.	Scc	.	.	.	.	.	.	.	.	.
ENST0	.	8q24.22	.	.	.	Score=347	.	.	.	.	.	.	.
ENST0	.	9p23	.	Scc	.	.	.	.	.	.	.	.	.
ENST0	.	9q31.2	.	.	.	.	.	.	.	.	.	.	.
ENST0	.	9q33.2	.	.	.	.	.	.	.	.	.	.	.
ENST0	.	10p11.23	.	.	Scc	.	.	.	.	.	.	.	.
.	.	10q11.22	.	.	Scc	.	rs312	ID=COSN1	.	.	.	.	.
ENST0	.	10q22.1	.	.	.	.	.	.	.	.	.	.	.
ENST0	.	10q22.1	.	.	.	Score=246	rs790	ID=COSN1	.	.	0.347	0.355	0.327
ENST0	.	10q23.31	.	.	.	.	.	.	.	.	.	.	.

ENST0	.	10q25.2	.	.	.	.	rs764	ID=COSM4	.	.	.	.	.
ENST0	.	10q25.3	.	.	.	.	.	.	.	.	.	.	.
ENST0	.	11p15.4	.	.	.	.	.	.	.	.	.	.	.
ENST0	.	11p11.12	.	.	Scc	.	.	.	.	.	.	.	.
ENST0	.	11q13.5	.	.	.	.	rs377	.	.	.	.	.	.
ENST0	.	11q14.3	.	.	Scc	.	.	.	.	.	.	.	.
.	.	11q22.3	.	.	.	Score=234	rs769	.	.	.	.	.	.
ENST0	.	11q23.3	.	Scc	.	.	rs758	ID=COSM3	.	.	.	.	.
ENST0	.	12p11.23	.	.	.	.	.	.	.	.	.	.	.
ENST0	.	12q13.12	.	.	.	.	.	.	.	.	.	.	.
ENST0	.	12q13.2	.	.	.	.	.	.	.	.	.	.	.
ENST0	.	13q12.2	.	.	.	.	.	.	.	.	.	.	.
.	.	14q11.2	.	.	Scc	.	rs225	ID=COSN1	.	.	.	.	.
.	.	14q11.2	.	.	Scc	.	rs201	.	.	.	.	.	.
ENST0	.	14q24.1	.	Scc	.	.	.	.	.	.	.	.	.
ENST0	.	15q21.3	.	.	.	.	.	.	.	.	.	.	.
ENST0	.	16p13.3	.	.	.	.	rs776	.	.	.	.	.	.
ENST0	.	16p12.2	.	.	.	.	.	.	.	.	.	.	.
ENST0	.	16q23.1	.	.	Scc	.	rs201	ID=COSN1	.	.	0.324	0.285	0.318
.	.	18p11.21	.	.	Scc	.	rs750	ID=COSN6	.	.	0.623	0.62	0.291
ENST0	.	19p13.2	.	.	.	.	rs758	ID=COSM3	.	.	.	.	.
ENST0	.	19p13.2	.	.	.	.	.	.	.	.	.	.	.
ENST0	.	19q13.2	.	.	.	Score=433	.	.	.	.	.	.	.
ENST0	.	19q13.32	.	.	.	.	.	.	.	.	.	.	.
ENST0	.	19q13.32	.	.	.	.	.	.	.	.	.	.	.
ENST0	.	19q13.42	.	Scc	.	.	rs901	.	.	.	.	.	.
ENST0	.	20p12.2	.	.	.	.	.	.	.	.	.	.	.
ENST0	.	20p12.2	.	.	.	.	rs777	.	.	.	.	.	.
ENST0	.	20q11.23	.	.	.	.	rs779	.	.	.	.	.	.
ENST0	.	21q22.11	.	.	.	.	.	.	.	.	.	.	.
ENST0	.	22q12.2	.	Scc	.	.	.	.	.	.	.	.	.
ENST0	.	Xp22.12	.	.	.	Score=234	.	.	.	.	.	.	.
ENST0	.	Xp21.3	.	.	Scc	.	.	.	.	.	.	.	.
ENST0	.	Xp11.23	.	Scc	.	.	.	.	.	.	.	.	.
ENST0	.	Xp11.22	.	.	.	.	.	.	.	.	.	.	.
ENST0	.	Xq22.1	.	.	.	.	.	.	.	.	.	.	.
ENST0	.	Xq22.3	.	.	.	.	.	.	.	.	.	.	.
ENST0	.	Xq26.3	.	.	.	.	.	.	.	.	.	.	.
ENST0	.	Xq26.3	.	.	.	.	.	ID=COSM4	.	.	.	.	.
ENST0	.	Xq28	.	.	.	.	.	.	.	.	.	.	.
ENST0	.	Xq28	.	.	Scc	.	rs401	.	.	.	.	.	.
.	.	.	.	.	.	.	.	ID=COSM1	.	.	.	.	.

esp6500	ExA	ExA	SIF	Pol	Polyp	Mutat	gerp	CAD	Nov	Nov	INFO	FOR	ACa	ACa	Ori_REF	Ori_ALT	shared_ho
.	.	.	.	.	.	.	.	.	0	0.2	SON	GT:/	0:8,(	0/1::	T	C	0
.	.	.	.	.	.	.	.	.	.	.	SON	GT:/	0:11	0/1:7	A	G	0
.	.	.	.	.	.	.	.	.	.	.	SON	GT:/	0:50	0/1:8	C	T	0
.	.	.	.	.	.	.	.	.	.	.	SON	GT:/	0:13	0/1::	C	T	0
.	##	0	0.9	0.0	0.001	1.000	.	.	.	.	SON	GT:/	0:71	0/1::	G	A	0
.	.	.	.	.	.	.	.	.	.	.	SON	GT:/	0:21	0/1:3	C	T	0
.	.	.	.	.	.	.	.	.	0	.	SON	GT:/	0:8,(	0/1:3	A	G	0
.	.	.	.	.	.	.	.	.	0	.	SON	GT:/	0:14	0/1::	G	T	0
.	.	.	.	.	.	.	.	.	0	0	SON	GT:/	0:30	0/1:6	C	G	0
.	.	.	.	.	.	.	.	.	0	0	SON	GT:/	0:17	0/1:2	T	C	0
.	.	.	.	.	.	.	.	.	0	.	SON	GT:/	0:25	0/1:3	G	A	0
.	.	.	.	.	.	.	.	.	.	.	SON	GT:/	0:11	0/1:2	A	C	0
2E-04	##	0	.	.	.	.	3.3	4.54	.	.	SON	GT:/	0:19	0/1:5	G	A	0
.	.	.	.	.	.	.	.	.	.	.	SON	GT:/	0:49	0/1:8	G	A	0
.	.	.	.	.	.	.	.	.	.	.	SON	GT:/	0:14	0/1:2	A	C	0
.	.	.	.	.	.	.	.	.	.	.	SON	GT:/	0:13	0/1:2	G	A	0
.	.	.	.	.	.	.	.	.	.	.	SON	GT:/	0:10	0/1:2	A	T	0
.	.	.	.	.	.	.	.	.	.	.	DB;S	GT:/	0:23	0/1:2	G	A	0
.	.	.	.	.	.	.	.	.	0	0	SON	GT:/	0:18	0/1:2	A	C	0
.	.	.	.	.	.	.	.	.	0	.	SON	GT:/	0:9,(	0/1::	T	C	0
.	.	.	.	.	.	.	.	.	0	0.1	SON	GT:/	0:17	0/1::	G	A	0
.	.	.	.	.	.	.	.	.	0	0.3	SON	GT:/	0:12	0/1::	C	G	0
.	.	.	.	.	.	.	.	.	0	.	SON	GT:/	0:42	0/1:5	A	T	0
.	.	.	.	.	.	.	.	.	0	0.1	SON	GT:/	0:30	0/1:4	C	G	0
.	.	.	.	.	.	.	.	.	.	.	SON	GT:/	0:43	0/1:7	G	A	0
.	.	.	.	.	.	.	.	.	0	0	SON	GT:/	0:8,(	0/1::	C	T	0
.	.	.	.	.	.	.	4.8	4.02	.	.	SON	GT:/	0:11	0/1:2	T	C	0
.	.	.	.	.	.	.	.	.	0.1	0.5	SON	GT:/	0:8,(	0/1:4	T	C	0
.	.	.	.	.	.	.	.	.	0	.	SON	GT:/	0:15	0/1:3	A	G	0
.	.	.	.	.	.	.	.	.	.	.	SON	GT:/	0:13	0/1::	G	T	0
.	.	.	.	.	.	.	.	.	0	.	SON	GT:/	0:37	0/1:6	G	A	0
.	.	.	.	.	.	.	.	.	.	.	SON	GT:/	0:36	0/1:4	C	T	0
.	.	.	.	.	.	.	.	.	.	.	SON	GT:/	0:57	0/1:6	C	G	0
.	.	.	.	.	.	.	.	.	0	0.4	SON	GT:/	0:14	0/1::	G	A	0
.	.	.	.	.	.	.	.	.	0	.	DB;S	GT:/	0:40	0/1:8	A	C	0
.	##	0	.	.	.	.	.	.	.	.	SON	GT:/	0:12	0/1::	C	T	0
.	.	.	.	.	.	.	.	.	0.2	0.7	SON	GT:/	0:9,(	0/1::	C	T	0
0.004	0	0	0.2:	0.0(	0.001	1,N	.	.	0	0	SON	GT:/	0:21	0/1:3	T	G	0
.	##	.	0.1!	0.0(	0.00:	1,D	3.5	3.54	0	.	SON	GT:/	0:15	0/1::	G	A	0
.	.	.	.	.	.	.	.	.	.	.	SON	GT:/	0:24	0/1:4	G	T	0
.	.	.	.	.	.	.	.	1.82	0	.	SON	GT:/	0:17	0/1:3	A	C	0
.	.	.	.	.	.	.	.	.	.	.	SON	GT:/	0:49	0/1:6	A	C	0
.	0	0	.	.	.	.	.	.	0	0.1	SON	GT:/	0:54	0/1::	A	G	0
.	.	.	.	.	.	.	.	.	0.2	0.1	DB;S	GT:/	0:64	0/1::	A	G	0
.	.	.	.	.	.	.	.	.	0.1	0.1	DB;S	GT:/	0:28	0/1:6	G	T	0
.	.	.	.	.	.	.	.	.	0	0	DB;S	GT:/	0:24	0/1:2	C	A	0
.	.	.	.	.	.	.	.	.	0	.	SON	GT:/	0:14	0/1:9	G	A	0
.	.	.	.	.	.	.	.	.	.	.	SON	GT:/	0:8,(	0/1:6	C	T	0
.	.	.	0.1:	0.1(	0.087	1,D	3.4	2.35	.	.	SON	GT:/	0:10	0/1::	A	G	0
.	.	.	.	.	.	.	.	.	0.1	0.2	SON	GT:/	0:66	0/1:9	T	G	0
.	.	.	.	.	.	.	.	.	0	0	SON	GT:/	0:8,(	0/1::	A	G	0

.	1	1	.	.	.	.	.	.	0	0	SOM	GT:/	0:25	0/1:1	T	C	0
.	.	.	0.0	0.9	0.998	1,N	.	.	.	.	SOM	GT:/	0:12	0/1:1	C	A	0
.	.	.	.	.	.	.	.	.	.	.	SOM	GT:/	0:19	0/1:3	C	T	0
.	.	.	0.0	0.2	0.347	1.000	5.5	2.28	.	.	SOM	GT:/	0:57	0/1:1	A	T	0
.	.	.	.	.	.	.	.	.	.	.	SOM	GT:/	0:20	0/1:3	T	G	0
.	.	.	0.0	0.0	0.006	0.812	4.4	2.83	.	.	SOM	GT:/	0:13	0/1:2	C	T	0
.	.	.	0.8	0.0	0.0,B	0.986	2.4	.	.	.	SOM	GT:/	0:38	0/1:5	T	G	0
.	##	0	.	.	.	.	.	.	.	.	SOM	GT:/	0:54	0/1:9	C	T	0
.	.	.	.	.	.	.	.	.	.	.	SOM	GT:/	0:44	0/1:7	C	T	0
.	.	.	.	.	.	.	.	.	0	.	SOM	GT:/	0:32	0/1:6	A	C	0
.	.	.	.	.	.	.	.	.	0	.	SOM	GT:/	0:29	0/1:5	T	G	0
.	.	.	.	.	.	.	3.4	.	.	.	SOM	GT:/	0:22	0/1:3	T	G	0
.	.	.	0.3	0.1	0.167	1.000	5.7	2.23	.	.	SOM	GT:/	0:22	0/1:3	C	G	0
.	.	.	.	.	.	.	.	.	.	.	SOM	GT:/	0:10	0/1:2	G	A	0
8E-04	0	0	0.0	0.9	1.0,D	1.000	4.9	5.22	.	.	SOM	GT:/	0:57	0/1:1	C	T	0
5E-04	0	0	0.0	0.3	0.932	1,N	.	.	.	.	SOM	GT:/	0:11	0/1:3	C	T	0
.	.	.	.	.	.	.	.	1.45	.	.	SOM	GT:/	0:44	0/1:6	G	A	0
.	0	.	.	.	.	.	.	.	0	0	DB;S	GT:/	0:62	0/1:1	G	T	0
.	.	.	.	.	.	.	.	.	.	.	SOM	GT:/	0:12	0/1:1	C	G	0
.	##	0	.	.	.	.	.	.	.	.	SOM	GT:/	0:39	0/1:5	C	T	0
.	.	.	1.0	0.1	0.478	1.000	5.9	.	.	.	SOM	GT:/	0:27	0/1:4	G	A	0
.	.	.	.	.	.	.	.	.	.	.	SOM	GT:/	0:15	0/1:2	C	T	0
.	.	.	.	.	.	.	.	.	0	0.2	SOM	GT:/	0:16	0/1:1	T	C	0
.	.	.	.	.	.	.	.	.	0	0	SOM	GT:/	0:16	0/1:2	C	T	0
.	.	.	.	.	.	.	.	.	0.2	0.2	DB;S	GT:/	0:24	0/1:3	C	T	0
.	.	.	.	.	.	.	.	.	0	.	SOM	GT:/	0:22	0/1:2	T	G	0
.	.	.	.	.	.	.	.	.	0	0	SOM	GT:/	0:9,(	0/1:2	C	T	0
.	.	.	.	.	.	.	.	.	0	.	SOM	GT:/	0:10	0/1:1	G	A	0
.	.	.	0.1	.	.	0.941	.	.	.	.	SOM	GT:/	0:36	0/1:6	C	T	0
2E-04	##	0	.	.	.	.	.	.	0	.	SOM	GT:/	0:19	0/1:2	C	T	0
.	.	.	.	.	.	.	.	.	.	.	SOM	GT:/	0:44	0/1:5	C	T	0
.	.	.	.	.	.	.	.	.	0	0	DB;S	GT:/	0:51	0/1:1	T	C	0
.	.	.	.	.	.	.	.	.	.	.	SOM	GT:/	0:40	0/1:1	G	A	0
.	.	.	.	.	.	.	.	.	.	.	SOM	GT:/	0:21	0/1:5	C	A	0
.	.	.	.	.	.	.	.	.	0	0	SOM	GT:/	0:36	0/1:1	A	G	0
.	.	.	.	.	.	.	.	2.36	.	.	SOM	GT:/	0:11	0/1:3	T	G	0
.	.	.	.	.	.	.	.	.	0.1	0.2	SOM	GT:/	0:16	0/1:3	T	C	0
.	.	.	.	.	.	.	.	.	.	.	SOM	GT:/	0:67	0/1:8	C	G	0
.	.	.	.	.	.	.	.	.	.	.	SOM	GT:/	0:26	0/1:5	A	G	0
.	##	0	.	.	.	.	.	.	.	.	SOM	GT:/	0:48	0/1:1	C	T	0
.	.	.	.	.	.	.	.	1.72	.	.	SOM	GT:/	0:43	0/1:5	C	T	0
.	##	0	0.0	0.9	1.0,D	1,D	5.7	3.22	.	.	SOM	GT:/	0:71	0/1:1	C	T	0
.	.	.	.	.	.	.	.	1.61	0.1	0	SOM	GT:/	0:9,(	0/1:1	A	C	0
.	.	.	.	.	.	.	.	.	.	0	DB;S	GT:/	0:22	0/1:3	G	C	0
.	.	.	.	.	.	.	.	.	.	.	SOM	GT:/	0:37	0/1:4	G	A	0
.	.	.	0.4	0.0	0.0,B	0.999	.	.	.	.	SOM	GT:/	0:51	0/1:4	T	G	0
.	.	.	.	.	.	.	.	.	.	.	SOM	GT:/	0:15	0/1:1	T	C	0
.	.	.	0.0	0.9	0.998	1,D	5.2	4.56	.	.	SOM	GT:/	0:30	0/1:6	A	G	0
.	.	.	.	.	.	.	.	.	.	.	SOM	GT:/	0:8,(	0/1:1	C	T	0
.	.	.	.	.	.	.	.	.	.	.	SOM	GT:/	0:87	0/1:1	T	A	0
.	.	.	.	.	.	.	.	.	0	0	SOM	GT:/	0:10	0/1:1	T	C	0
.	.	.	0.1	0.0	0.063	1,N	.	.	.	.	SOM	GT:/	0:22	0/1:3	C	T	0
.	##	0	.	.	.	.	.	.	.	.	SOM	GT:/	0:81	0/1:1	G	A	0
.	.	.	.	.	.	.	.	.	.	.	SOM	GT:/	0:65	0/1:7	G	A	0

0.033	0	0	.	.	.	.	.	.	.	.	DB;S	GT:/	0:43	0/1:6	C	T	0
.	.	.	.	.	.	.	.	.	0	0	SOM	GT:/	0:23	0/1:4	C	T	0
.	.	.	.	.	.	.	.	.	.	.	SOM	GT:/	0:11	0/1:2	G	T	0
.	.	.	.	.	.	.	.	.	.	.	DB;S	GT:/	0:19	0/1:4	G	C	0
.	.	.	.	.	.	.	.	.	.	.	SOM	GT:/	0:38	0/1:6	G	A	0
.	.	.	.	.	.	.	.	.	.	.	SOM	GT:/	0:23	0/1:1	C	T	0
.	.	.	.	.	.	.	.	.	0	0	SOM	GT:/	0:8,(	0/1:2	C	T	0
.	.	.	.	.	.	.	.	.	0	0	SOM	GT:/	0:10	0/1:2	G	C	0
.	.	.	.	.	.	.	.	.	.	.	SOM	GT:/	0:12	0/1:5	C	T	0
.	.	.	0.0	0.3	0.883	1,N	.	.	.	.	SOM	GT:/	0:10	0/1:1	A	C	0
.	.	.	.	.	.	.	.	.	0	.	DB;S	GT:/	0:22	0/1:6	G	T	0
.	.	.	.	.	.	.	.	.	.	.	SOM	GT:/	0:52	0/1:6	G	T	0
.	.	.	.	.	.	.	.	.	.	.	SOM	GT:/	0:90	0/1:9	G	A	0
.	.	.	.	.	.	.	.	.	0	.	SOM	GT:/	0:28	0/1:4	G	A	0
.	.	.	.	.	.	.	.	.	0	.	SOM	GT:/	0:30	0/1:4	C	G	0
.	.	.	.	.	.	.	.	.	0	.	SOM	GT:/	0:12	0/1:1	G	A	0
.	.	.	.	.	.	.	.	.	.	.	SOM	GT:/	0:34	0/1:7	G	T	0
.	##	0	.	.	.	.	.	.	.	.	SOM	GT:/	0:41	0/1:7	G	A	0
0.007	0	0	.	.	.	.	.	.	0.1	0.1	SOM	GT:/	0:39	0/1:9	G	C	0
.	##	0	0.0	0.9	1.0,D	1,D	4.6	3.91	.	.	SOM	GT:/	0:49	0/1:8	G	A	0
.	.	.	.	.	.	.	2.4	.	.	.	SOM	GT:/	0:69	0/1:7	G	A	0
.	0	0	.	.	.	.	.	.	0	0	SOM	GT:/	0:88	0/1:1	A	G	0
.	0	0	0.0	1.0	1.0,D	1.000	4.9	4.26	0	0	SOM	GT:/	0:10	0/1:1	C	T	0
.	.	.	.	.	.	.	.	2.11	.	.	SOM	GT:/	0:21	0/1:2	C	T	0
.	.	.	.	.	.	.	.	.	0.3	0.4	SOM	GT:/	0:11	0/1:8	C	T	0
.	.	.	.	.	.	.	.	.	0.3	0.4	SOM	GT:/	0:12	0/1:7	T	C	0
.	.	.	.	.	.	.	.	.	.	.	SOM	GT:/	0:64	0/1:1	C	A	0
.	.	.	.	.	.	.	.	.	.	.	SOM	GT:/	0:10	0/1:1	A	G	0
.	.	.	.	.	.	.	.	.	0	.	SOM	GT:/	0:15	0/1:2	A	C	0
.	.	.	0.0	0.8	0.699	1,D	5.6	4.01	.	.	SOM	GT:/	0:12	0/1:1	T	G	0
.	.	.	.	.	.	.	.	.	.	.	SOM	GT:/	0:10	0/1:1	G	A	0
.	.	.	.	.	.	.	.	.	0	.	SOM	GT:/	0:14	0/1:6	A	G	0
.	.	.	.	.	.	.	.	.	.	.	SOM	GT:/	0:16	0/1:1	T	C	0
2E-04	0	0	.	.	.	.	.	.	0	0	SOM	GT:/	0:35	0/1:5	A	T	0
.	0	0	.	.	.	.	.	.	.	.	DB;S	GT:/	0:38	0/1:1	T	C	0
.	.	.	.	.	.	.	.	.	0	0	DB;S	GT:/	0:55	0/1:8	T	C	0
.	.	.	.	.	.	.	.	.	0.1	0.2	SOM	GT:/	0:12	0/1:2	C	A	0
.	.	.	.	.	.	.	.	.	0	0.1	SOM	GT:/	0:9,(	0/1:1	G	A	0
.	0	0	.	.	.	.	.	.	0	0	DB;S	GT:/	0:50	0/1:1	A	C	0
.	.	.	.	.	.	.	.	.	.	.	SOM	GT:/	0:17	0/1:2	G	A	0
.	.	.	.	.	.	.	.	.	0	0.1	SOM	GT:/	0:9,(	0/1:1	G	A	0
.	.	.	.	.	.	.	3.7	.	.	.	SOM	GT:/	0:85	0/1:9	C	T	0
.	.	.	.	.	.	.	5.6	2.81	.	.	SOM	GT:/	0:39	0/1:6	T	G	0
.	##	0	.	.	.	.	.	.	.	.	SOM	GT:/	0:85	0/1:1	A	G	0
.	##	0	.	.	.	.	.	.	.	.	SOM	GT:/	0:11	0/1:1	C	T	0
.	.	.	0.0	1.0	1.0,D	1,D	5.7	5.41	.	.	SOM	GT:/	0:55	0/1:7	G	A	0
.	.	.	.	.	.	.	.	.	.	.	DB;S	GT:/	0:37	0/1:7	T	G	0
.	.	.	0.1	0.0	0.02,I	1,N	.	.	.	.	SOM	GT:/	0:31	0/1:2	C	T	0
.	.	.	0.0	0.9	1.0,D	1,D	5.3	4.51	.	.	SOM	GT:/	0:18	0/1:2	G	A	0
.	##	0	0.1	0.0	0.002	1,N	.	.	0	.	SOM	GT:/	0:67	0/1:8	G	A	0
.	##	0	.	.	.	.	.	.	.	.	SOM	GT:/	0:12	0/1:1	C	T	0
.	0	0	.	.	.	.	.	.	0	.	DB;S	GT:/	0:45	0/1:7	G	T	0
.	.	.	.	.	.	.	.	.	.	.	SOM	GT:/	0:24	0/1:3	C	T	0
.	.	.	.	.	.	.	.	.	0	0.4	SOM	GT:/	0:8,(	0/1:3	A	C	0

.	.	.	.	.	.	.	.	.	0	.	SON	GT:/	0:54	0/1:8	C	T	0
.	0	0	.	.	.	.	.	.	0	.	SON	GT:/	0:39	0/1:5	T	A	0
.	##	0	.	.	.	.	5.4	2.57	.	.	SON	GT:/	0:94	0/1:1	C	T	0
.	.	.	.	.	.	.	.	.	.	.	SON	GT:/	0:9,(	0/1:3	C	A	0
.	.	.	.	.	.	.	2.7	.	.	.	SON	GT:/	0:90	0/1:1	C	T	0
2E-04	##	0	.	.	.	.	.	.	.	.	SON	GT:/	0:12	0/1:1	C	T	0
#####	##	0	0.1(	0.0!	0.685	1,N	4.4	.	.	.	SON	GT:/	0:44	0/1:5	G	A	0
.	.	.	.	.	.	.	.	.	.	.	SON	GT:/	0:17	0/1:2	G	T	0
.	.	.	.	.	.	.	2.1	.	.	.	SON	GT:/	0:51	0/1:5	A	C	0
.	.	.	.	.	.	.	.	.	0	0	SON	GT:/	0:38	0/1:7	C	T	0
.	.	.	.	.	.	.	.	.	.	.	SON	GT:/	0:20	0/1:9	A	G	0
.	.	.	.	.	.	.	.	.	.	.	SON	GT:/	0:22	0/1:3	G	A	0
.	.	.	.	.	.	.	.	.	.	.	SON	GT:/	0:9,(	0/1:1	G	T	0
.	.	.	.	.	.	.	.	.	0	0	SON	GT:/	0:11	0/1:1	A	T	0
.	.	.	.	.	.	.	.	.	.	.	SON	GT:/	0:8,(	0/1:1	G	A	0
.	.	.	.	.	.	.	.	.	.	.	SON	GT:/	0:8,(	0/1:1	T	G	0
.	.	.	.	.	.	.	.	.	.	.	SON	GT:/	0:84	0/1:1	G	A	0
.	.	.	0.0	0.2!	0.594	0.996	.	2.32	0	.	SON	GT:/	0:43	0/1:7	T	A	0
.	.	.	.	.	.	.	.	.	0	.	SON	GT:/	0:20	0/1:3	T	G	0
.	.	.	.	.	.	.	2.4	.	0	.	SON	GT:/	0:20	0/1:3	T	G	0
.	##	0	.	.	.	.	.	2.22	.	.	SON	GT:/	0:10	0/1:1	C	T	0
0.002	0	0	0.0(	0.0:	0.595	0.989	5.2	2.47	0	.	SON	GT:/	0:43	0/1:8	C	T	0
.	.	.	.	.	.	.	.	.	.	.	SON	GT:/	0:13	0/1:2	G	T	0
.	##	.	0.1(	0.0(	0.002	1,N	.	.	0	.	SON	GT:/	0:89	0/1:1	C	T	0
.	.	.	.	.	.	.	.	.	0.1	0.1	DB;S	GT:/	0:47	0/1:9	T	C	0
.	.	.	.	.	.	.	.	.	0.1	0	SON	GT:/	0:19	0/1:5	T	C	0
.	.	.	.	.	.	.	.	.	0	.	SON	GT:/	0:66	0/1:7	C	G	0
.	.	.	0.0	0.9	0.994	1,D	5.2	4.41	.	.	SON	GT:/	0:46	0/1:3	A	C	0
.	.	.	.	.	.	.	.	.	0	0.1	SON	GT:/	0:9,(	0/1:5	T	C	0
.	.	.	.	.	.	.	.	.	0	.	SON	GT:/	0:8,(	0/1:1	A	G	0
.	.	.	.	.	.	.	.	.	.	.	SON	GT:/	0:25	0/1:2	G	A	0
.	.	.	.	.	.	.	.	1.67	.	.	SON	GT:/	0:22	0/1:4	G	C	0
.	.	.	.	.	.	.	3.6	.	.	.	SON	GT:/	0:47	0/1:7	C	T	0
.	.	.	.	.	.	.	.	1.42	0	.	SON	GT:/	0:55	0/1:1	A	C	0
.	.	.	.	.	.	.	.	.	.	.	SON	GT:/	0:21	0/1:3	G	A	0
.	.	.	.	.	.	.	.	.	.	.	SON	GT:/	0:15	0/1:3	C	T	0
.	.	.	.	.	.	.	.	.	.	.	SON	GT:/	0:15	0/1:5	C	T	0
.	.	.	.	.	.	.	3.2	1.26	.	.	SON	GT:/	0:42	0/1:6	C	T	0
.	.	.	0.0	0.9(	0.993	0.990	3.4	1.91	.	.	SON	GT:/	0:91	0/1:1	C	A	0
.	.	.	.	.	.	.	.	.	0	0	SON	GT:/	0:22	0/1:3	G	A	0
.	.	.	.	.	.	.	.	.	0	0	SON	GT:/	0:16	0/1:2	C	T	0
.	.	.	.	.	.	.	.	.	0	.	SON	GT:/	0:8,(	0/1:5	G	A	0
.	.	.	.	.	.	.	.	.	.	0	SON	GT:/	0:8,(	0/1:7	C	T	0
.	.	.	.	.	.	.	.	.	0	0	SON	GT:/	0:11	0/1:1	G	T	0
.	.	.	0.0(	0.1:	0.164	0.825	3.6	2.01	.	.	SON	GT:/	0:88	0/1:1	C	T	0
.	.	.	.	.	.	.	3.6	.	0	.	SON	GT:/	0:11	0/1:1	A	C	0
.	.	.	.	.	.	.	.	.	0	.	SON	GT:/	0:11	0/1:1	A	C	0
.	.	.	.	.	.	.	.	.	.	.	SON	GT:/	0:33	0/1:4	G	A	0
.	.	.	.	.	.	.	2.8	1.36	.	.	SON	GT:/	0:18	0/1:3	G	A	0
.	.	.	.	.	.	.	.	.	.	.	SON	GT:/	0:13	0/1:1	T	G	0
.	##	0	0.0(	0.3!	0.934	1,D	4.3	1.26	.	.	SON	GT:/	0:89	0/1:1	G	A	0
.	.	.	.	.	.	.	.	1.47	.	.	SON	GT:/	0:24	0/1:4	G	T	0
.	.	.	.	.	.	.	.	.	0.1	0.3	SON	GT:/	0:8,(	0/1:2	C	T	0
.	.	.	.	.	.	.	.	.	0.1	0	SON	GT:/	0:11	0/1:1	T	A	0

.	.	.	.	.	.	.	.	.	0	0.4	DB;S	GT:/	0:22	0/1:2	T	C	0
.	.	.	.	.	.	.	.	1.48	.	.	SON	GT:/	0:47	0/1:1	C	A	0
.	.	.	.	.	.	.	.	.	0	0	SON	GT:/	0:10	0/1:2	T	G	0
.	.	.	.	.	.	.	.	.	0	0.1	SON	GT:/	0:42	0/1:8	G	A	0
.	.	.	.	.	.	.	.	.	0	0	SON	GT:/	0:40	0/1:7	C	T	0
.	.	.	.	.	.	.	.	.	.	.	SON	GT:/	0:36	0/1:5	C	T	0
.	.	.	.	.	.	.	.	.	.	.	SON	GT:/	0:70	0/1:1	C	T	0
.	.	.	.	.	.	.	.	.	.	.	SON	GT:/	0:26	0/1:3	T	C	0
.	.	.	.	.	.	.	.	.	.	.	SON	GT:/	0:18	0/1:5	A	G	0
.	.	.	.	.	.	.	.	.	0	0.2	SON	GT:/	0:8,(	0/1:1	C	T	0
.	.	.	.	.	.	.	.	1.74	.	.	SON	GT:/	0:99	0/1:3	G	A	0
.	.	.	.	.	.	.	.	.	.	.	SON	GT:/	0:20	0/1:6	G	A	0
.	##	0	0.0'	.	.	1,N	.	.	.	.	SON	GT:/	0:11	0/1:3	C	T	0
.	.	.	0.0(	0.9!	1.0,D	1.000	5.1	4.80	.	.	SON	GT:/	0:71	0/1:1	G	A	0
.	.	.	.	.	.	.	.	.	.	.	SON	GT:/	0:51	0/1:1	T	C	0
.	.	.	.	.	.	.	.	.	0	.	SON	GT:/	0:41	0/1:5	C	A	0
.	.	.	.	.	.	.	.	.	.	.	SON	GT:/	0:49	0/1:5	G	A	0
.	.	.	.	.	.	.	.	.	0	.	SON	GT:/	0:93	0/1:1	G	T	0
.	.	.	.	.	.	.	2.5	.	0	.	SON	GT:/	0:8,(	0/1:5	G	A	0
.	.	.	.	.	.	.	.	.	.	.	SON	GT:/	0:16	0/1:5	G	A	0
.	.	.	.	.	.	.	.	.	.	.	SON	GT:/	0:49	0/1:1	C	T	0
.	.	.	.	.	.	.	.	.	0	.	SON	GT:/	0:11	0/1:3	G	A	0
.	.	.	.	.	.	.	.	.	0	.	SON	GT:/	0:10	0/1:1	A	G	0
.	0	0	0.3'	.	.	1,N	.	.	.	.	SON	GT:/	0:24	0/1:4	A	G	0
.	0	0	.	.	.	.	.	.	.	.	SON	GT:/	0:14	0/1:6	A	G	0
.	.	.	.	.	.	.	.	.	.	.	SON	GT:/	0:56	0/1:1	G	A	0
.	.	.	.	.	.	.	.	.	.	.	SON	GT:/	0:23	0/1:7	C	T	0
.	##	0	.	.	.	.	.	1.60	.	0	SON	GT:/	0:89	0/1:2	C	T	0
.	.	.	.	.	.	.	3.8	.	.	.	SON	GT:/	0:10	0/1:2	G	A	0
.	.	.	.	.	.	.	.	.	0	0	SON	GT:/	0:26	0/1:7	C	T	0
.	.	.	.	.	.	.	.	.	.	.	DB;S	GT:/	0:24	0/1:3	T	C	0
.	.	.	.	.	.	.	.	.	0	.	DB;S	GT:/	0:34	0/1:4	T	C	0
.	.	.	.	.	.	.	.	.	.	.	DB;S	GT:/	0:23	0/1:3	T	C	0
.	.	.	.	.	.	.	.	.	.	.	DB;S	GT:/	0:56	0/1:1	C	G	0
.	.	.	.	.	.	.	.	.	0	.	DB;S	GT:/	0:84	0/1:1	T	A	0
.	.	.	.	.	.	.	.	.	.	.	DB;S	GT:/	0:31	0/1:2	T	C	0
.	.	.	.	.	.	.	.	.	0	.	SON	GT:/	0:42	0/1:1	C	T	0
.	.	.	.	.	.	.	.	.	0	.	SON	GT:/	0:25	0/1:5	T	G	0
.	.	.	.	.	.	.	.	.	.	.	SON	GT:/	0:40	0/1:8	A	T	0
.	.	.	.	.	.	.	.	.	0	.	DB;S	GT:/	0:66	0/1:1	A	G	0
.	.	.	.	.	.	.	.	.	.	.	DB;S	GT:/	0:49	0/1:9	A	T	0
.	.	.	.	.	.	.	.	.	0	.	DB;S	GT:/	0:36	0/1:5	C	G	0
.	.	.	.	.	.	.	.	.	0	.	DB;S	GT:/	0:47	0/1:7	T	C	0
.	.	.	.	.	.	.	.	.	0	.	DB;S	GT:/	0:36	0/1:4	T	C	0
.	.	.	.	.	.	.	.	.	0	.	DB;S	GT:/	0:44	0/1:1	A	T	0
.	.	.	.	.	.	.	.	.	0	.	SON	GT:/	0:20	0/1:3	A	G	0
.	.	.	.	.	.	.	.	.	.	.	DB;S	GT:/	0:20	0/1:2	G	A	0
.	.	.	.	.	.	.	.	.	0	.	DB;S	GT:/	0:36	0/1:6	C	G	0
.	.	.	.	.	.	.	.	.	0	.	DB;S	GT:/	0:33	0/1:6	A	C	0
.	.	.	.	.	.	.	.	.	0	.	SON	GT:/	0:20	0/1:4	G	A	0
.	.	.	.	.	.	.	.	.	.	.	SON	GT:/	0:13	0/1:3	T	C	0
.	.	.	.	.	.	.	.	.	.	.	SON	GT:/	0:9,(	0/1:1	A	C	0
.	.	.	.	.	.	1,A	5	5.87	.	.	SON	GT:/	0:76	0/1:7	C	T	0
.	.	.	.	.	.	.	.	.	0	0	SON	GT:/	0:9,(	0/1:5	C	G	0

.	.	.	0.0	0.5	0.998	1,N	2.8	.	.	.	SOM	GT:/	0:49	0/1:4	C	T	0
.	##	0	.	.	.	.	.	1.43	.	0	SOM	GT:/	0:74	0/1:1	G	A	0
.	.	.	0.2	0.0	0.082	1,D	3.4	3.15	.	.	SOM	GT:/	0:25	0/1:4	T	C	0
2E-04	##	0	0.5	0.0	0.093	0.995	.	.	.	.	SOM	GT:/	0:93	0/1:1	G	A	0
.	.	.	.	.	.	.	.	.	.	.	SOM	GT:/	0:21	0/1:3	A	C	0
.	0	0	.	.	.	.	.	.	.	.	SOM	GT:/	0:13	0/1:2	C	T	0
.	.	.	.	.	.	.	.	.	.	.	SOM	GT:/	0:89	0/1:1	G	A	0
.	.	.	.	.	.	.	.	.	.	.	SOM	GT:/	0:63	0/1:1	G	T	0
.	.	.	.	.	.	.	.	.	0	.	SOM	GT:/	0:28	0/1:5	C	G	0
.	.	.	.	.	.	.	.	.	0	0	DB;S	GT:/	0:26	0/1:2	G	T	0
.	.	.	.	.	.	.	.	.	0	.	SOM	GT:/	0:9,(	0/1:1	C	A	0
.	.	.	.	.	.	.	.	.	0	0	SOM	GT:/	0:33	0/1:5	T	G	0
.	.	.	.	.	.	.	.	.	0	0	DB;S	GT:/	0:34	0/1:5	T	G	0
.	.	.	.	.	.	.	.	.	0	.	SOM	GT:/	0:34	0/1:5	G	A	0
.	.	.	.	.	.	.	.	.	.	.	SOM	GT:/	0:10	0/1:5	G	C	0
.	.	.	.	.	.	.	.	.	.	.	SOM	GT:/	0:16	0/1:3	T	A	0
.	.	.	.	.	.	.	.	.	0	0	DB;S	GT:/	0:37	0/1:5	A	T	0
.	.	.	.	.	.	.	2.3	.	0	.	SOM	GT:/	0:44	0/1:8	T	G	0
.	.	.	.	.	.	.	.	.	0	0.2	SOM	GT:/	0:34	0/1:8	G	A	0
.	.	.	.	.	.	.	.	.	0	0.1	SOM	GT:/	0:77	0/1:1	T	C	0
.	.	.	.	.	.	.	2.6	.	.	.	SOM	GT:/	0:54	0/1:6	G	A	0
.	.	.	.	.	.	.	.	.	.	.	SOM	GT:/	0:37	0/1:3	T	G	0
.	.	.	.	.	.	.	.	.	.	.	SOM	GT:/	0:43	0/1:9	A	C	0
.	.	.	.	.	.	.	5.5	2.32	.	.	SOM	GT:/	0:45	0/1:8	C	T	0
.	.	.	.	.	.	.	.	.	0	.	SOM	GT:/	0:8,(	0/1:1	T	C	0
.	.	.	.	.	.	.	.	.	0	.	SOM	GT:/	0:10	0/1:1	C	G	0
.	.	.	.	.	.	.	.	.	.	.	SOM	GT:/	0:13	0/1:3	G	A	0
.	.	.	.	.	.	.	.	.	0	.	SOM	GT:/	0:20	0/1:5	C	G	0
.	.	.	.	.	.	.	.	.	0	.	SOM	GT:/	0:34	0/1:7	C	T	0
.	.	.	.	.	.	.	.	.	0	.	SOM	GT:/	0:10	0/1:1	A	G	0
.	.	.	.	.	.	.	.	.	.	.	SOM	GT:/	0:19	0/1:5	G	T	0
.	.	.	.	.	.	.	3.2	.	.	.	SOM	GT:/	0:43	0/1:7	G	A	0
.	.	.	.	.	.	.	.	.	.	.	SOM	GT:/	0:10	0/1:2	G	A	0
.	.	.	.	.	.	.	.	.	.	.	SOM	GT:/	0:48	0/1:8	G	T	0
.	.	.	0.8	0.0	0.0,B	1,N	.	.	0	.	SOM	GT:/	0:10	0/1:2	G	A	0
.	.	.	.	.	.	.	.	.	.	.	SOM	GT:/	0:10	0/1:1	T	C	0
.	.	.	.	.	.	.	.	.	0	.	DB;S	GT:/	0:20	0/1:3	G	A	0
.	.	.	.	.	.	.	.	.	.	.	SOM	GT:/	0:14	0/1:2	C	T	0
.	.	.	.	.	.	.	.	.	0	.	SOM	GT:/	0:8,(	0/1:7	C	T	0
.	.	.	.	.	.	.	.	.	0.1	0.1	SOM	GT:/	0:28	0/1:2	G	A	0
.	.	.	.	.	.	.	.	.	0	0	SOM	GT:/	0:8,(	0/1:1	G	T	0
.	.	.	.	.	.	.	.	.	0	0	SOM	GT:/	0:10	0/1:1	G	A	0
.	.	.	.	.	.	.	.	.	0	0	SOM	GT:/	0:14	0/1:3	A	T	0
.	.	.	.	.	.	.	.	.	.	.	SOM	GT:/	0:28	0/1:3	G	A	0
.	.	.	.	.	.	.	.	.	0	0	SOM	GT:/	0:11	0/1:3	C	A	0
.	.	.	.	.	.	.	.	.	0	0	SOM	GT:/	0:10	0/1:1	G	A	0
.	##	0	.	.	.	.	.	.	.	.	SOM	GT:/	0:53	0/1:1	C	T	0
.	.	.	.	.	.	.	.	.	.	.	SOM	GT:/	0:17	0/1:2	A	G	0
.	.	.	.	.	.	.	.	.	0	.	DB;S	GT:/	0:36	0/1:8	C	G	0
.	.	.	.	.	.	.	.	.	0	.	SOM	GT:/	0:36	0/1:8	T	G	0
.	.	.	.	.	.	.	.	.		0	SOM	GT:/	0:56	0/1:7	A	C	0
.	.	.	.	.	.	.	3	.	.	.	SOM	GT:/	0:26	0/1:3	C	T	0
.	.	.	.	.	.	.	3.3	3.32	.	.	SOM	GT:/	0:23	0/1:4	A	C	0
.	.	.	.	.	.	.	.	.	.	.	SOM	GT:/	0:32	0/1:7	G	A	0

.	.	.	.	.	.	.	.	.	0	.	SOM	GT:	0:15	0/1:2	C	T	0
.	.	.	.	.	.	.	.	.	.	.	SOM	GT:	0:45	0/1:6	G	A	0
.	.	.	.	.	.	.	.	.	.	.	SOM	GT:	0:37	0/1:4	G	A	0
.	##	0	.	.	.	.	.	.	.	.	SOM	GT:	0:17	0/1:3	C	T	0
.	.	.	.	.	.	.	.	.	.	0	SOM	GT:	0:8,	0/1:5	C	T	0
.	.	.	.	.	.	.	.	.	0	0	SOM	GT:	0:8,	0/1:1	T	C	0
.	.	.	0.0	0.5	0.857	0.996	4.6	4.05	.	.	SOM	GT:	0:12	0/1:1	G	A	0
.	.	.	.	.	.	.	.	1.53	.	.	DB;S	GT:	0:20	0/1:4	G	A	0
.	.	.	.	.	.	.	.	.	.	.	SOM	GT:	0:26	0/1:2	C	T	0
.	0	0	.	.	.	.	.	.	.	.	SOM	GT:	0:45	0/1:9	C	T	0
.	##	0	.	.	.	.	.	.	.	.	SOM	GT:	0:92	0/1:1	C	T	0
.	.	.	.	.	.	.	.	.	0	.	SOM	GT:	0:8,	0/1:2	A	T	0
.	.	.	.	.	.	.	.	.	.	.	SOM	GT:	0:12	0/1:9	T	G	0
.	.	.	.	.	.	.	4.4	.	.	.	SOM	GT:	0:26	0/1:4	C	T	0
.	.	.	.	.	.	.	3.1	1.56	.	.	SOM	GT:	0:22	0/1:4	C	T	0
.	.	.	.	.	.	.	.	.	.	.	SOM	GT:	0:37	0/1:4	C	T	0
.	.	.	.	.	.	.	3.6	1.91	.	.	SOM	GT:	0:15	0/1:2	G	A	0
.	##	0	.	0.9	1.0,D	1,N	.	.	.	.	SOM	GT:	0:79	0/1:1	G	A	0
.	.	.	0.0	0.0	0.48,I	0.992	.	1.45	.	.	SOM	GT:	0:14	0/1:2	C	T	0
.	.	.	0.6	0.0	0.005	0.990	.	.	.	.	SOM	GT:	0:40	0/1:8	C	T	0
.	.	.	.	.	.	.	.	.	.	.	SOM	GT:	0:12	0/1:1	C	T	0
.	.	.	.	.	.	.	.	.	.	.	SOM	GT:	0:32	0/1:3	G	A	0
.	.	.	0.0	0.4	0.884	1,D	5.2	3.32	.	.	SOM	GT:	0:90	0/1:1	C	T	0
.	.	.	0.1	0.0	0.107	1.000	5.3	4.84	.	.	SOM	GT:	0:64	0/1:1	C	T	0
.	.	.	.	.	.	.	.	.	0	0	SOM	GT:	0:21	0/1:9	G	A	0
.	0	0	.	.	.	.	.	.	0	0.2	DB;S	GT:	0:21	0/1:3	G	A	0
.	.	.	.	.	.	.	.	.	.	.	SOM	GT:	0:22	0/1:2	C	T	0
.	.	.	.	.	.	.	.	1.65	0.1	0.2	SOM	GT:	0:9,	0/1:2	C	T	0
.	.	.	.	.	.	.	.	.	.	.	SOM	GT:	0:10	0/1:2	A	C	0
#####	##	0	.	.	.	.	2.3	.	.	.	SOM	GT:	0:36	0/1:4	G	A	0
.	.	.	.	.	.	.	.	.	.	.	SOM	GT:	0:30	0/1:4	T	C	0
.	.	.	.	.	.	.	.	.	.	.	SOM	GT:	0:17	0/1:2	T	C	0
.	.	.	.	.	.	.	2.9	.	.	.	SOM	GT:	0:55	0/1:8	C	T	0
.	.	.	.	.	.	.	.	.	0	0.2	SOM	GT:	0:12	0/1:5	G	C	0
.	.	.	.	.	.	.	.	.	0	0	SOM	GT:	0:15	0/1:3	C	A	0
.	.	.	.	.	.	.	.	.	0	0	SOM	GT:	0:8,	0/1:1	T	G	0
.	.	.	.	.	.	.	.	.	0	.	SOM	GT:	0:33	0/1:6	A	T	0
.	.	.	.	.	.	.	.	.	.	.	SOM	GT:	0:22	0/1:6	T	C	0
.	.	.	.	.	.	.	.	.	0	0	SOM	GT:	0:29	0/1:7	G	C	0
.	.	.	.	.	.	.	.	.	.	.	SOM	GT:	0:9,	0/1:1	G	A	0
.	.	.	.	.	.	.	.	.	.	.	SOM	GT:	0:35	0/1:1	C	A	0
.	.	.	.	.	.	.	.	.	.	.	SOM	GT:	0:35	0/1:1	A	C	0
.	.	.	.	.	.	.	.	.	0	0.3	SOM	GT:	0:8,	0/1:5	A	G	0
.	.	.	.	.	.	.	.	.	0	.	SOM	GT:	0:21	0/1:5	A	C	0
.	.	.	.	.	.	.	.	.	0	0	SOM	GT:	0:23	0/1:3	C	G	0
.	.	.	.	.	.	.	.	.	0	0	SOM	GT:	0:23	0/1:3	C	T	0
.	.	.	.	.	.	.	.	.	.	.	SOM	GT:	0:8,	0/1:1	C	T	0
.	.	.	.	.	.	.	.	.	0.1	0.2	SOM	GT:	0:24	0/1:5	G	A	0
.	.	.	.	.	.	.	.	.	0	0.5	SOM	GT:	0:8,	0/1:6	G	A	0
.	.	.	.	.	.	.	.	.	0	0.5	SOM	GT:	0:10	0/1:7	G	A	0
.	.	.	.	.	.	.	.	.	.	.	SOM	GT:	0:17	0/1:3	A	C	0
.	##	0	0.0	0.9	1.0,D	1,D	5.7	5.14	0	.	SOM	GT:	0:65	0/1:1	C	T	0
.	.	.	.	.	.	.	.	.	.	.	SOM	GT:	0:23	0/1:3	C	T	0
.	.	.	.	.	.	.	.	.	.	.	SOM	GT:	0:19	0/1:3	C	A	0

.	.	.	.	.	.	.	.	.	.	.	SON	GT:/	0:28	0/1:4	G	T	0
.	.	.	.	.	.	.	.	.	.	.	SON	GT:/	0:19	0/1:4	G	A	0
.	.	.	.	.	.	.	.	.	.	.	SON	GT:/	0:24	0/1:4	A	G	0
.	.	.	.	.	.	.	3.7	2.73	.	.	SON	GT:/	0:58	0/1:7	C	A	0
.	.	.	.	.	.	.	.	.	.	.	SON	GT:/	0:39	0/1:6	A	G	0
.	.	.	.	.	.	.	.	.	.	.	SON	GT:/	0:11	0/1:3	C	A	0
.	.	.	.	.	.	.	.	.	.	0	SON	GT:/	0:8,(	0/1:1	C	T	0
.	.	.	.	.	.	.	.	.	0	0.2	SON	GT:/	0:8,(	0/1:1	A	T	0
.	.	.	.	.	.	.	.	.	0.1	0	SON	GT:/	0:13	0/1:4	A	T	0
.	.	.	.	.	.	.	.	.	0.1	0	SON	GT:/	0:36	0/1:1	T	C	0
.	##	0	0.2:	0.0(	0.008	1.000	2.3	.	0	.	SON	GT:/	0:97	0/1:1	G	A	0
.	.	.	.	.	.	.	.	.	0	.	DB;S	GT:/	0:62	0/1:1	A	C	0
.	.	.	.	.	.	.	.	.	0	.	SON	GT:/	0:23	0/1:3	C	T	0
.	.	.	.	.	.	.	.	.	0	0	SON	GT:/	0:8,(	0/1:6	C	T	0
.	.	.	.	.	.	.	3	1.31	.	.	SON	GT:/	0:14	0/1:2	G	C	0
.	.	.	.	.	.	.	.	.	.	0	SON	GT:/	0:11	0/1:1	G	A	0
.	.	.	0.0	0.0!	0.102	1,N	3.6	.	.	.	SON	GT:/	0:52	0/1:6	G	T	0
.	.	.	.	.	.	.	.	.	.	.	SON	GT:/	0:8,(	0/1:1	A	G	0
.	.	.	.	.	.	.	.	.	.	.	SON	GT:/	0:45	0/1:1	T	C	0
0.003	0	0	0.4:	0.0(	0.007	1,N	.	.	0	0	SON	GT:/	0:79	0/1:6	C	T	0
.	.	.	0.0:	0.9(	0.999	1,D	5.1	5.69	.	.	SON	GT:/	0:42	0/1:8	C	T	0
.	.	.	.	.	.	.	.	.	0	0.1	SON	GT:/	0:8,(	0/1:0	T	C	0
.	.	.	.	.	.	1,D	5.2	4.50	.	.	SON	GT:/	0:13	0/1:3	G	A	0
.	.	.	.	.	.	.	5.2	1.86	.	.	SON	GT:/	0:9,(	0/1:1	G	A	0
.	0	0	0.2	0.0(	0.001	1,D	4.8	4.41	0	0	DB;S	GT:/	0:77	0/1:1	G	C	0
.	.	.	.	.	.	.	.	.	0.2	0.3	DB;S	GT:/	0:27	0/1:7	G	A	0
.	##	0	.	.	.	.	.	.	0	.	SON	GT:/	0:34	0/1:3	G	A	0
.	.	.	.	.	.	.	4.1	.	.	.	SON	GT:/	0:54	0/1:8	C	T	0
.	.	.	.	.	.	.	.	.	.	.	SON	GT:/	0:28	0/1:2	C	G	0
.	.	.	0.0:	0.9:	0.995	1,D	5.8	5.36	.	.	DB;S	GT:/	0:19	0/1:6	G	A	0
.	.	.	.	.	.	.	.	.	.	.	SON	GT:/	0:29	0/1:8	C	G	0
.	.	.	.	.	.	.	.	.	.	.	SON	GT:/	0:55	0/1:3	A	G	0
.	.	.	.	.	.	.	.	.	.	.	SON	GT:/	0:8,(	0/1:9	G	T	0
.	.	.	.	.	.	.	.	.	0	0.3	DB;S	GT:/	0:31	0/1:2	A	G	0
.	.	.	.	.	.	.	.	.	.	.	SON	GT:/	0:11	0/1:1	C	T	0
.	.	.	0.0:	.	.	0.738	.	2.06	.	.	SON	GT:/	0:60	0/1:7	G	T	0
.	.	.	.	.	.	.	.	.	.	.	SON	GT:/	0:30	0/1:4	G	T	0
.	.	.	.	.	.	.	.	.	0	.	SON	GT:/	0:57	0/1:9	C	G	0
.	.	.	.	.	.	.	.	.	.	.	SON	GT:/	0:18	0/1:1	C	T	0
.	.	.	.	.	.	.	.	.	0	.	SON	GT:/	0:8,(	0/1:1	A	G	0
.	.	.	.	.	.	.	.	.	.	.	SON	GT:/	0:15	0/1:2	G	A	0
.	##	.	.	.	.	.	2.4	.	0	.	SON	GT:/	0:11	0/1:3	C	T	0
.	.	.	.	0.9(	0.999	.	4	5.22	.	.	SON	GT:/	0:22	0/1:5	C	T	0
.	.	.	.	.	.	.	.	.	.	.	SON	GT:/	0:12	0/1:2	T	C	0
.	.	.	.	.	.	.	.	2.00	.	.	SON	GT:/	0:9,(	0/1:3	G	A	0
.	0	0	0.4:	0.0(	0.004	1,N	.	.	0	0	SON	GT:/	0:60	0/1:1	T	G	0
.	.	.	.	.	.	.	.	.	.	.	SON	GT:/	0:16	0/1:1	G	A	0
.	##	0	.	.	.	.	.	.	.	.	SON	GT:/	0:11	0/1:1	C	T	0
.	.	.	.	.	.	.	.	.	0	.	SON	GT:/	0:11	0/1:2	C	G	0
.	.	.	.	.	.	.	.	1.84	.	.	DB;S	GT:/	0:33	0/1:5	C	T	0
.	.	.	.	.	.	.	.	.	.	.	SON	GT:/	0:16	0/1:2	G	A	0
.	.	.	.	.	.	.	.	.	0	0	SON	GT:/	0:20	0/1:4	G	A	0
.	.	.	.	.	.	.	.	.	0	.	SON	GT:/	0:13	0/1:1	A	G	0
.	.	.	.	.	.	.	.	.	0	.	SON	GT:/	0:12	0/1:1	A	C	0

.	.	.	.	.	.	.	.	.	0	0	SOM	GT:/	0:12	0/1:3	G	C	0
.	.	.	.	.	.	.	3.5	2.17	.	.	SOM	GT:/	0:39	0/1:5	C	A	0
.	.	.	.	.	.	.	.	.	0.2	0.4	SOM	GT:/	0:8,(	0/1:7	T	G	0
.	.	.	.	.	.	.	.	.	.	.	SOM	GT:/	0:20	0/1:2	G	A	0
.	##	0	.	.	.	.	.	.	0	0	DB;S	GT:/	0:44	0/1:9	G	A	0
8E-04	0	0	.	.	.	.	.	.	.	.	SOM	GT:/	0:23	0/1:5	C	A	0
.	0	0	.	.	.	.	.	.	0	0	DB;S	GT:/	0:90	0/1:2	A	G	0
.	0	0	.	.	.	.	.	.	0.1	0.2	SOM	GT:/	0:69	0/1:2	T	C	0
.	.	.	.	.	.	.	.	.	.	.	SOM	GT:/	0:33	0/1:5	C	T	0
.	.	.	.	.	.	.	.	.	0	0	DB;S	GT:/	0:62	0/1:2	T	A	0
.	.	.	.	.	.	1,D	6	5.89	.	.	SOM	GT:/	0:41	0/1:6	G	T	0
.	.	.	.	.	.	.	.	.	.	.	SOM	GT:/	0:15	0/1:3	A	C	0
.	##	0	.	.	.	.	.	.	.	.	SOM	GT:/	0:36	0/1:5	A	G	0
.	0	0	0.0	0.0	0.0,B	1,N	.	.	.	.	SOM	GT:/	0:55	0/1:7	C	T	0
.	.	.	.	.	.	.	.	.	0	.	SOM	GT:/	0:12	0/1:1	G	T	0
.	.	.	.	.	.	.	.	.	0	.	SOM	GT:/	0:8,(	0/1:2	A	G	0
.	.	.	.	.	.	.	.	.	.	.	SOM	GT:/	0:11	0/1:(	C	T	0
.	.	.	.	.	.	.	.	.	.	.	DB;S	GT:/	0:76	0/1:1	G	C	0
.	.	.	.	.	.	.	.	.	0	.	SOM	GT:/	0:38	0/1:9	C	A	0
.	.	.	.	.	.	.	.	.	0	.	SOM	GT:/	0:20	0/1:4	G	A	0
.	.	.	.	.	.	.	.	.	.	.	SOM	GT:/	0:74	0/1:1	G	A	0
.	.	.	.	.	.	.	.	.	.	.	SOM	GT:/	0:75	0/1:7	C	G	0
.	.	.	.	.	.	.	.	.	.	.	DB;S	GT:/	0:10	0/1:2	G	A	0
.	.	.	.	.	.	.	.	.	.	.	SOM	GT:/	0:21	0/1:3	G	T	0
.	.	.	.	.	.	.	.	.	0	0	SOM	GT:/	0:8,(	0/1:1	C	T	0
.	.	.	.	.	.	.	.	.	0	0	SOM	GT:/	0:8,(	0/1:6	T	C	0
.	.	.	.	.	.	.	4.9	.	.	.	SOM	GT:/	0:57	0/1:9	A	G	0
.	.	.	.	.	.	.	.	.	0	0	SOM	GT:/	0:15	0/1:1	T	G	0
.	.	.	.	.	.	.	.	.	0	.	SOM	GT:/	0:14	0/1:2	T	G	0
.	.	.	.	.	.	.	.	.	0	.	SOM	GT:/	0:14	0/1:3	T	G	0
.	.	.	.	.	.	.	.	.	0	0	SOM	GT:/	0:8,(	0/1:2	G	C	0
.	.	.	.	.	.	.	.	.	.	.	SOM	GT:/	0:8,(	0/1:1	C	T	0
.	.	.	.	.	.	.	.	.	0	0	SOM	GT:/	0:10	0/1:2	C	G	0
.	.	.	.	.	.	.	.	.	0	0	SOM	GT:/	0:12	0/1:2	G	A	0
.	##	0	.	.	.	.	.	.	0	.	SOM	GT:/	0:18	0/1:2	G	A	0
.	.	.	.	.	.	.	.	.	0.1	0.1	SOM	GT:/	0:8,(	0/1:2	C	G	0
.	.	.	.	.	.	.	.	.	.	.	SOM	GT:/	0:8,(	0/1:2	G	A	0
.	.	.	.	.	.	.	.	.	.	.	SOM	GT:/	0:20	0/1:3	G	T	0
.	.	.	.	.	.	.	.	.	.	.	SOM	GT:/	0:23	0/1:5	G	C	0
.	.	.	.	.	.	.	.	.	.	.	DB;S	GT:/	0:43	0/1:1	C	T	0
.	.	.	.	.	.	.	.	.	.	.	SOM	GT:/	0:8,(	0/1:7	G	T	0
.	.	.	.	.	.	.	.	.	.	.	SOM	GT:/	0:80	0/1:1	G	A	0
.	.	.	.	.	.	.	2.2	.	.	.	SOM	GT:/	0:17	0/1:3	C	T	0
.	.	.	.	.	.	.	.	.	0	.	SOM	GT:/	0:19	0/1:7	G	A	0
.	.	.	.	.	.	.	.	.	.	.	SOM	GT:/	0:37	0/1:7	C	T	0
0.123	0	0	1.0	0.0	0.004	1.000	.	.	0	0.3	DB;S	GT:/	0:20	0/1:4	T	C	0
.	.	.	.	.	.	.	.	.	0	0	SOM	GT:/	0:12	0/1:2	C	A	0
.	.	.	.	.	.	.	.	.	0	0	SOM	GT:/	0:13	0/1:2	T	A	0
.	.	.	.	.	.	.	.	.	0	0	SOM	GT:/	0:34	0/1:6	G	T	0
.	.	.	.	.	.	.	.	.	0.1	0.2	SOM	GT:/	0:8,(	0/1:2	C	A	0
.	.	.	.	.	.	.	.	.	0.1	0.2	DB;S	GT:/	0:29	0/1:6	A	G	0
.	.	.	.	.	.	.	.	.	0	.	SOM	GT:/	0:22	0/1:5	G	T	0
.	.	.	.	.	.	.	.	.	.	.	SOM	GT:/	0:14	0/1:1	G	A	0
.	.	.	.	.	.	.	.	2.31	.	.	SOM	GT:/	0:82	0/1:1	G	A	0

.	.	.	0.0	1.0	1.0,D	1,D	4.9	4.64	.	.	SOM	GT:/	0:99	0/1:1	G	A	0
.	##	0	.	.	.	.	.	.	.	.	SOM	GT:/	0:50	0/1:6	C	T	0
.	.	.	.	.	.	.	.	.	0	0	SOM	GT:/	0:15	0/1:1	C	T	0
.	.	.	.	.	.	.	.	.	.	.	SOM	GT:/	0:47	0/1:6	T	G	0
.	.	.	.	.	.	.	.	.	0.1	0.3	SOM	GT:/	0:8,(	0/1:1	T	A	0
.	.	.	.	.	.	.	.	.	.	.	SOM	GT:/	0:8,(	0/1:2	G	C	0
.	.	.	.	.	.	.	.	.	.	.	SOM	GT:/	0:43	0/1:1	T	A	0
.	.	.	.	.	.	.	.	.	.	.	SOM	GT:/	0:30	0/1:3	G	A	0
.	.	.	.	.	.	.	.	.	.	.	SOM	GT:/	0:52	0/1:7	C	T	0
.	.	.	.	.	.	.	2.7	.	0.4	0.5	DB;S	GT:/	0:31	0/1:5	C	G	0
.	.	.	.	.	.	.	.	.	.	.	SOM	GT:/	0:16	0/1:3	C	G	0
.	.	.	.	.	.	.	.	.	.	.	SOM	GT:/	0:18	0/1:2	C	T	0
.	.	.	.	.	.	.	.	.	0.1	0	SOM	GT:/	0:9,(	0/1:3	A	C	0
.	.	.	.	.	.	.	.	.	0	0	SOM	GT:/	0:39	0/1:9	T	C	0
.	.	.	.	.	.	.	.	.	.	.	SOM	GT:/	0:11	0/1:4	T	G	0
.	.	.	.	.	.	.	3.5	.	.	.	SOM	GT:/	0:38	0/1:1	G	A	0
.	.	.	.	.	.	.	.	1.64	.	.	SOM	GT:/	0:52	0/1:8	G	A	0
.	.	.	.	.	.	.	.	1.75	.	.	SOM	GT:/	0:22	0/1:3	G	A	0
.	.	.	.	.	.	.	2.9	.	.	.	SOM	GT:/	0:27	0/1:1	G	C	0
.	.	.	.	.	.	.	.	.	.	.	SOM	GT:/	0:12	0/1:2	T	C	0
.	.	.	0.0	0.1	0.912	1,N	2.8	.	.	.	SOM	GT:/	0:90	0/1:1	C	T	0
.	.	.	.	.	.	.	.	.	.	.	SOM	GT:/	0:11	0/1:8	G	A	0
.	##	0	.	.	.	.	.	.	.	.	SOM	GT:/	0:53	0/1:6	G	A	0
.	.	.	.	.	.	.	.	.	.	.	SOM	GT:/	0:79	0/1:1	G	A	0
.	.	.	.	.	.	.	.	.	.	.	SOM	GT:/	0:21	0/1:2	C	A	0
.	.	.	.	.	.	.	.	.	0	0.2	DB;S	GT:/	0:35	0/1:6	A	G	0
.	.	.	.	.	.	.	.	.	.	.	SOM	GT:/	0:13	0/1:1	A	T	0
.	.	.	.	.	.	.	.	.	.	.	SOM	GT:/	0:52	0/1:1	C	T	0
.	.	.	.	.	.	.	.	.	.	.	SOM	GT:/	0:8,(	0/1:1	C	T	0
.	##	0	0.0	0.9	1.0,D	0.969	2.9	3.63	.	.	SOM	GT:/	0:33	0/1:5	G	T	0
.	.	.	.	.	.	.	.	.	.	0	SOM	GT:/	0:13	0/1:1	T	C	0
.	.	.	.	.	.	.	.	.	0.1	0.3	SOM	GT:/	0:61	0/1:1	C	G	0
.	.	.	0.0	0.9	1.0,D	1.000	5.2	3.95	.	.	SOM	GT:/	0:60	0/1:7	T	A	0
.	.	.	.	.	.	.	3.7	2.31	0	.	SOM	GT:/	0:11	0/1:3	T	C	0
.	.	.	.	.	.	.	2.9	.	.	.	SOM	GT:/	0:64	0/1:5	G	A	0
.	.	.	.	.	.	.	5.7	1.53	.	.	SOM	GT:/	0:53	0/1:6	T	C	0
#####	##	0	0.2	0.0	0.009	0.689	4.3	2.22	.	.	SOM	GT:/	0:63	0/1:8	G	A	0
.	.	.	.	.	.	.	4.3	1.40	.	.	SOM	GT:/	0:19	0/1:2	C	T	0
.	.	.	.	.	.	1,A	.	5.99	.	.	SOM	GT:/	0:59	0/1:8	G	A	0
.	.	.	.	.	.	.	.	.	.	.	SOM	GT:/	0:23	0/1:4	C	G	0
.	.	.	0.0	0.9	0.997	1.000	4.1	4.28	.	.	SOM	GT:/	0:51	0/1:9	C	T	0
.	.	.	.	.	.	.	.	.	0	.	SOM	GT:/	0:15	0/1:6	G	A	0
.	.	.	.	.	.	.	.	.	0	.	SOM	GT:/	0:17	0/1:4	A	G	0
.	.	.	.	.	.	.	.	.	.	.	SOM	GT:/	0:37	0/1:5	G	C	0
.	.	.	0.0	0.9	1.0,D	1.000	3.9	3.50	.	.	SOM	GT:/	0:40	0/1:5	C	G	0
.	.	.	.	.	.	.	6	1.44	.	.	SOM	GT:/	0:37	0/1:6	A	G	0
.	.	.	.	.	.	.	.	1.48	.	.	SOM	GT:/	0:24	0/1:3	A	C	0
.	.	.	.	.	.	.	.	.	0	0	SOM	GT:/	0:8,(	0/1:8	T	C	0
.	.	.	.	.	.	.	.	.	.	.	SOM	GT:/	0:91	0/1:1	G	T	0
.	.	.	.	.	.	.	.	.	.	.	SOM	GT:/	0:16	0/1:3	C	G	0
.	.	.	.	.	.	.	.	.	0.1	0.2	SOM	GT:/	0:13	0/1:2	C	A	0
.	##	0	0.0	0.4	0.949	0.981	3.8	4.06	.	.	SOM	GT:/	0:10	0/1:1	G	A	0
.	.	.	.	.	.	.	4.4	1.49	.	.	SOM	GT:/	0:37	0/1:1	T	A	0
.	.	.	.	.	.	.	4.6	1.64	.	.	SOM	GT:/	0:84	0/1:1	G	T	0

.	.	.	.	0.9:	0.998	1,N	2.2	.	.	.	SOM	GT:/	0:22	0/1:2	T	C	0
.	.	.	0.2(	0.0(	0.396	0.996	5.8	3.14	0	.	SOM	GT:/	0:17	0/1:3	G	C	0
.	.	.	.	.	.	.	.	.	.	.	SOM	GT:/	0:75	0/1:1	G	A	0
.	.	.	.	.	.	.	.	.	0	.	SOM	GT:/	0:16	0/1:2	A	C	0
.	.	.	.	.	.	.	.	.	.	.	SOM	GT:/	0:52	0/1:8	C	T	0
.	.	.	.	.	.	.	.	.	.	.	SOM	GT:/	0:16	0/1:1	C	T	0
.	.	.	.	.	.	.	.	.	.	0	SOM	GT:/	0:15	0/1:2	A	G	0
.	.	.	.	.	.	.	.	.	.	.	SOM	GT:/	0:55	0/1:1	G	T	0
.	.	.	.	.	.	.	.	.	0	0	SOM	GT:/	0:66	0/1:1	C	A	0
.	.	.	.	.	.	.	.	.	0	0	SOM	GT:/	0:70	0/1:1	A	G	0
.	.	.	.	.	.	.	.	1.30	.	.	SOM	GT:/	0:46	0/1:8	C	A	0
.	.	.	.	.	.	.	3	.	.	.	SOM	GT:/	0:11	0/1:1	A	G	0
.	.	.	.	.	.	.	.	.	.	.	SOM	GT:/	0:35	0/1:7	G	A	0
.	.	.	.	.	.	.	2.7	.	.	.	SOM	GT:/	0:68	0/1:7	C	T	0
.	.	.	.	.	.	.	.	.	.	.	SOM	GT:/	0:44	0/1:6	C	T	0
.	.	.	.	.	.	.	.	.	.	.	SOM	GT:/	0:12	0/1:1	C	A	0
.	.	.	.	.	.	.	.	.	0	.	SOM	GT:/	0:37	0/1:3	C	T	0
.	.	.	.	.	.	.	.	.	0	0	SOM	GT:/	0:10	0/1:3	T	C	0
.	.	.	.	.	.	.	.	.	.	.	SOM	GT:/	0:9,(	0/1:1	C	A	0
.	.	.	.	.	.	.	.	.	0	0	SOM	GT:/	0:47	0/1:1	G	A	0
.	.	.	.	.	.	.	.	.	.	.	SOM	GT:/	0:44	0/1:1	C	T	0
.	##	0	.	.	.	.	5.4	2.61	.	.	SOM	GT:/	0:48	0/1:1	C	T	0
.	.	.	.	.	.	.	.	.	.	.	SOM	GT:/	0:58	0/1:7	G	A	0
.	0	0	.	.	.	.	.	.	0	0	SOM	GT:/	0:21	0/1:3	C	T	0
.	.	.	.	.	.	.	3.2	.	.	.	SOM	GT:/	0:15	0/1:1	C	G	0
.	.	.	.	.	.	.	3.9	.	.	.	SOM	GT:/	0:12	0/1:1	G	A	0
.	.	.	0.0	0.0(	0.001	0.997	2.8	.	.	.	SOM	GT:/	0:67	0/1:1	C	T	0
.	.	.	.	.	.	.	.	.	0	.	SOM	GT:/	0:11	0/1:3	A	G	0
.	.	.	.	.	.	.	.	.	0	.	SOM	GT:/	0:20	0/1:4	T	C	0
.	.	.	.	.	.	.	.	.	.	.	SOM	GT:/	0:12	0/1:2	C	A	0
.	.	.	.	.	.	.	.	.	.	.	SOM	GT:/	0:17	0/1:2	C	T	0
.	.	.	.	.	.	.	.	.	.	.	SOM	GT:/	0:32	0/1:5	C	T	0
.	.	.	.	.	.	.	.	.	0	.	SOM	GT:/	0:11	0/1:4	G	T	0
.	.	.	.	.	.	.	.	.	0	.	DB;S	GT:/	0:51	0/1:2	G	C	0
.	.	.	.	.	.	.	.	.	0	.	DB;S	GT:/	0:38	0/1:1	A	T	0
.	.	.	.	.	.	.	.	.	0	.	DB;S	GT:/	0:37	0/1:1	T	C	0
.	.	.	.	.	.	.	.	.	.	.	DB;S	GT:/	0:36	0/1:1	A	G	0
.	.	.	.	.	.	.	.	.	0	.	DB;S	GT:/	0:62	0/1:1	T	C	0
.	.	.	.	.	.	.	.	.	.	.	DB;S	GT:/	0:78	0/1:2	G	T	0
.	.	.	.	.	.	.	.	.	0	.	DB;S	GT:/	0:49	0/1:1	C	G	0
.	.	.	.	.	.	.	.	.	0	.	DB;S	GT:/	0:46	0/1:1	C	T	0
.	.	.	.	.	.	.	.	.	0	.	DB;S	GT:/	0:35	0/1:8	T	G	0
.	.	.	.	.	.	.	.	.	0	.	SOM	GT:/	0:50	0/1:1	A	G	0
.	.	.	.	.	.	.	.	.	.	.	DB;S	GT:/	0:52	0/1:1	A	C	0
.	.	.	.	.	.	.	.	.	0	.	DB;S	GT:/	0:36	0/1:1	T	G	0
.	.	.	.	.	.	.	.	.	0	.	SOM	GT:/	0:34	0/1:7	A	G	0
.	.	.	.	.	.	.	.	.	0	.	DB;S	GT:/	0:55	0/1:1	C	G	0
.	.	.	.	.	.	.	.	.	.	.	DB;S	GT:/	0:80	0/1:2	C	T	0
.	.	.	.	.	.	.	.	.	.	.	DB;S	GT:/	0:70	0/1:2	G	T	0
.	.	.	.	.	.	.	.	.	.	.	DB;S	GT:/	0:66	0/1:1	A	T	0
.	.	.	.	.	.	.	.	.	.	.	SOM	GT:/	0:18	0/1:4	G	T	0
.	.	.	.	.	.	.	.	.	.	.	DB;S	GT:/	0:19	0/1:4	C	T	0
.	.	.	.	.	.	.	.	.	.	.	SOM	GT:/	0:17	0/1:4	G	C	0
.	.	.	.	.	.	.	.	.	0	.	DB;S	GT:/	0:44	0/1:1	T	C	0

.	.	.	.	.	.	.	.	.	.	.	SON	GT:/	0:11	0/1:4	G	A	0
.	.	.	.	.	.	.	.	.	0	.	SON	GT:/	0:14	0/1:4	C	G	0
.	.	.	.	.	.	.	.	.	.	.	SON	GT:/	0:14	0/1:3	C	T	0
.	.	.	.	.	.	.	.	.	.	.	SON	GT:/	0:28	0/1:3	C	G	0
.	0	0	0.0	0.0	0.044	1,D	5.2	.	0	0	SON	GT:/	0:12	0/1:3	G	C	0
.	.	.	.	.	.	.	.	.	0	0.1	DB;S	GT:/	0:39	0/1:8	T	C	0
.	.	.	.	.	.	.	.	.	.	.	SON	GT:/	0:15	0/1:1	G	T	0
.	.	.	0.0	0.9	0.988	1,D	5.3	3.11	.	.	SON	GT:/	0:15	0/1:8	C	A	0
.	##	.	0.0	0.8	0.933	1,D	5.3	3.92	.	.	SON	GT:/	0:15	0/1:5	C	T	0
.	.	.	.	.	.	.	.	.	0	0	SON	GT:/	0:18	0/1:5	C	A	0
.	.	.	.	.	.	.	.	.	0	0.1	SON	GT:/	0:9,(	0/1:2	C	A	0
.	.	.	.	.	.	.	.	.	0	0.2	SON	GT:/	0:9,(	0/1:2	A	G	0
.	.	.	0.7	0.0	0.042	1,N	.	.	.	.	SON	GT:/	0:35	0/1:5	G	A	0
.	.	.	.	.	.	.	.	.	0	0	SON	GT:/	0:17	0/1:1	A	C	0
.	.	.	.	.	.	.	.	.	.	.	SON	GT:/	0:94	0/1:1	C	T	0
.	.	.	.	.	.	.	.	2.31	0	0	SON	GT:/	0:36	0/1:5	G	A	0
.	.	.	.	.	.	.	.	.	.	.	SON	GT:/	0:11	0/1:1	C	T	0
.	.	.	.	.	.	.	.	.	.	.	SON	GT:/	0:16	0/1:2	C	T	0
.	.	.	.	.	.	.	.	.	0.1	0.1	SON	GT:/	0:16	0/1:2	T	A	0
.	.	.	.	.	.	.	.	.	0.2	0.2	SON	GT:/	0:10	0/1:3	A	G	0
.	.	.	.	.	.	.	.	.	0	.	SON	GT:/	0:51	0/1:1	C	G	0
.	.	.	.	.	.	.	.	.	0	0	SON	GT:/	0:37	0/1:4	T	G	0
.	.	.	.	.	.	.	.	.	0	.	DB;S	GT:/	0:37	0/1:5	A	G	0
.	.	.	.	.	.	.	.	.	0	.	SON	GT:/	0:10	0/1:4	T	G	0
.	.	.	.	.	.	.	.	.	.	.	SON	GT:/	0:9,(	0/1:4	C	T	0
.	.	.	.	.	.	.	.	.	.	.	SON	GT:/	0:45	0/1:6	G	C	0
.	.	.	.	.	.	.	.	.	.	.	SON	GT:/	0:45	0/1:8	C	T	0
.	.	.	.	.	.	.	.	.	0.1	0	DB;S	GT:/	0:38	0/1:1	T	C	0
.	.	.	.	.	.	.	.	.	0	0	SON	GT:/	0:9,(	0/1:1	T	A	0
.	.	.	.	.	.	.	.	.	0	0	SON	GT:/	0:8,(	0/1:1	T	A	0
.	.	.	.	.	.	.	.	2.77	.	.	SON	GT:/	0:59	0/1:7	C	G	0
.	.	.	.	.	.	.	2.7	2.69	.	.	SON	GT:/	0:28	0/1:6	G	A	0
.	.	.	.	.	.	.	.	.	0	0	SON	GT:/	0:96	0/1:1	G	T	0
.	##	0	.	.	.	.	.	.	0	.	SON	GT:/	0:96	0/1:1	G	A	0
.	.	.	0.6	0.0	0.066	1,D	5.1	2.42	.	.	SON	GT:/	0:53	0/1:8	C	T	0
.	.	.	.	.	.	.	.	.	0	.	SON	GT:/	0:8,(	0/1:8	G	A	0
.	.	.	.	.	.	.	.	.	.	.	DB;S	GT:/	0:26	0/1:5	C	T	0
.	.	.	.	.	.	.	.	.	0	0	SON	GT:/	0:15	0/1:3	A	T	0
.	.	.	.	.	.	.	.	.	0	0	SON	GT:/	0:8,(	0/1:6	G	C	0
.	.	.	.	.	.	.	.	.	0	0	SON	GT:/	0:11	0/1:2	G	A	0
.	.	.	.	.	.	.	.	.	0	0	SON	GT:/	0:10	0/1:2	C	T	0
.	.	.	.	.	.	.	.	.	.	.	SON	GT:/	0:42	0/1:8	C	A	0
#####	##	0	.	.	.	.	.	.	.	.	SON	GT:/	0:10	0/1:1	G	A	0
.	.	.	.	.	.	.	.	.	.	.	SON	GT:/	0:54	0/1:6	G	A	0
.	.	.	.	.	.	.	.	.	.	.	SON	GT:/	0:56	0/1:6	G	T	0
.	.	.	0.1	0.8	0.985	0.645	5.1	2.59	.	.	SON	GT:/	0:15	0/1:2	G	A	0
.	.	.	.	.	.	.	.	.	.	.	SON	GT:/	0:56	0/1:9	T	C	0
.	.	.	.	.	.	.	4.1	1.24	.	.	SON	GT:/	0:56	0/1:7	C	T	0
.	.	.	.	.	.	.	.	.	.	.	SON	GT:/	0:51	0/1:7	G	A	0
.	.	.	.	.	.	.	.	.	.	.	SON	GT:/	0:11	0/1:1	G	C	0
.	.	.	.	.	.	.	.	.	0	.	SON	GT:/	0:18	0/1:3	C	G	0
.	.	.	.	.	.	.	.	.	.	.	SON	GT:/	0:90	0/1:1	G	C	0
.	.	.	.	.	.	.	.	.	0.2	0.2	SON	GT:/	0:9,(	0/1:1	T	A	0
.	##	0	0.0	0.7	0.928	1,N	.	2.55	.	.	SON	GT:/	0:85	0/1:1	G	A	0

.	.	.	.	.	.	.	.	.	0.3	0.5	DB;S	GT:/	0:48	0/1:	T	C	0
.	.	.	.	.	.	.	.	.	.	.	SOM	GT:/	0:8,	0/1:	G	A	0
.	.	.	0.0	0.7	0.976	1.000	5.8	4.40	.	.	SOM	GT:/	0:61	0/1:	T	G	0
.	.	.	.	.	.	.	.	.	.	.	SOM	GT:/	0:46	0/1:	G	A	0
.	##	0	.	.	.	.	.	.	.	.	SOM	GT:/	0:58	0/1:	G	A	0
#####	##	0	.	.	.	.	.	.	.	.	SOM	GT:/	0:45	0/1:	G	A	0
.	.	.	.	.	.	.	.	.	0	.	SOM	GT:/	0:12	0/1:	C	T	0
.	.	.	.	.	.	.	3.1	1.79	.	.	SOM	GT:/	0:25	0/1:	C	A	0
.	.	.	.	.	.	.	.	.	.	.	SOM	GT:/	0:32	0/1:	G	A	0
.	.	.	0.2	0.9	0.998	0.995	3.9	1.92	.	.	SOM	GT:/	0:55	0/1:	G	A	0
.	.	.	.	.	.	.	.	.	0	0.2	SOM	GT:/	0:8,	0/1:	C	G	0
.	.	.	.	.	.	.	.	.	0	0.1	SOM	GT:/	0:8,	0/1:	G	A	0
.	.	.	.	.	.	.	.	.	0.1	0	SOM	GT:/	0:48	0/1:	G	A	0
.	.	.	.	.	.	.	.	.	0	0.1	SOM	GT:/	0:21	0/1:	T	C	0
.	.	.	.	.	.	.	.	.	0	0	SOM	GT:/	0:27	0/1:	G	T	0
.	.	.	.	.	.	.	.	.	0	0	DB;S	GT:/	0:32	0/1:	G	A	0
.	.	.	.	.	.	.	.	.	0	0	SOM	GT:/	0:8,	0/1:	T	A	0
.	.	.	.	.	.	.	.	.	.	.	SOM	GT:/	0:9,	0/1:	A	C	0
.	.	.	.	.	.	.	.	.	.	.	SOM	GT:/	0:11	0/1:	T	G	0
.	.	.	.	.	.	.	4.5	.	.	.	SOM	GT:/	0:70	0/1:	G	A	0
.	.	.	.	.	.	.	2.9	2.16	.	.	SOM	GT:/	0:34	0/1:	G	A	0
.	.	.	.	.	.	.	.	.	.	.	SOM	GT:/	0:14	0/1:	C	T	0
.	.	.	0.0	0.0	0.313	0.997	2	2.54	.	.	SOM	GT:/	0:18	0/1:	G	A	0
.	.	.	.	.	.	.	.	.	0	0.1	SOM	GT:/	0:10	0/1:	C	T	0
.	.	.	.	.	.	.	.	.	.	.	SOM	GT:/	0:22	0/1:	G	T	0
.	0	0	.	.	.	.	.	.	0	0	SOM	GT:/	0:55	0/1:	C	T	0
.	.	.	.	.	.	.	.	.	.	.	SOM	GT:/	0:31	0/1:	G	A	0
0.085	0	0	0.5	0.0	0.002	1,D	.	.	0	.	DB;S	GT:/	0:34	0/1:	G	C	0
.	.	.	.	.	.	.	.	.	.	.	SOM	GT:/	0:18	0/1:	G	A	0
.	.	.	.	0.9	0.986	0.999	3.6	2.28	.	.	SOM	GT:/	0:11	0/1:	G	A	0
.	.	.	.	.	.	.	.	.	.	.	SOM	GT:/	0:8,	0/1:	C	A	0
.	##	0	.	.	.	.	.	.	.	.	SOM	GT:/	0:12	0/1:	G	A	0
.	.	.	.	.	.	.	.	.	.	.	SOM	GT:/	0:21	0/1:	G	C	0
.	.	.	.	.	.	.	.	.	.	.	SOM	GT:/	0:23	0/1:	G	T	0
.	.	.	.	.	.	.	.	.	.	.	SOM	GT:/	0:12	0/1:	G	T	0
.	.	.	.	.	.	.	.	.	.	.	SOM	GT:/	0:9,	0/1:	A	C	0
.	.	.	.	.	.	.	.	.	.	.	SOM	GT:/	0:15	0/1:	C	T	0
.	.	.	.	.	.	.	.	.	.	.	SOM	GT:/	0:21	0/1:	G	C	0
.	.	.	.	.	.	1,A	4.3	12.6	.	.	SOM	GT:/	0:58	0/1:	C	T	0
.	.	.	.	.	.	.	.	1.66	.	.	SOM	GT:/	0:47	0/1:	T	A	0
.	.	.	.	.	.	.	.	.	.	.	SOM	GT:/	0:11	0/1:	G	A	0
.	.	.	.	.	.	.	.	.	.	.	SOM	GT:/	0:52	0/1:	A	G	0
.	.	.	.	.	.	.	.	1.46	.	.	SOM	GT:/	0:39	0/1:	C	A	0
.	.	.	.	.	.	.	.	.	.	.	SOM	GT:/	0:49	0/1:	C	T	0
.	.	.	.	.	.	.	.	.	.	.	SOM	GT:/	0:36	0/1:	T	G	0
.	.	.	.	.	.	.	.	.	0	0	SOM	GT:/	0:13	0/1:	G	C	0
.	.	.	.	.	.	.	.	.	0	.	SOM	GT:/	0:29	0/1:	G	A	0
.	.	.	.	.	.	.	.	.	.	.	SOM	GT:/	0:41	0/1:	G	A	0
.	.	.	.	.	.	.	.	.	.	.	SOM	GT:/	0:11	0/1:	C	A	0
.	.	.	.	.	.	.	.	.	.	.	SOM	GT:/	0:56	0/1:	C	T	0
.	.	.	.	.	.	.	.	.	.	.	SOM	GT:/	0:30	0/1:	C	T	0
.	.	.	.	.	.	.	.	.	0	0	SOM	GT:/	0:8,	0/1:	A	G	0
.	.	.	.	.	.	.	.	.	.	0	SOM	GT:/	0:33	0/1:	G	A	0
.	.	.	0.0	0.9	1.0,D	1,D	4.6	5.20	.	.	SOM	GT:/	0:90	0/1:	C	T	0

.	##	0	.	.	.	.	.	.	.	.	SOM	GT:/	0:41	0/1:9	C	T	0
.	.	.	.	.	.	.	.	.	0	.	SOM	GT:/	0:62	0/1:1	T	C	0
.	.	.	.	.	.	.	.	.	0.1	0.1	SOM	GT:/	0:47	0/1:1	T	C	0
.	.	.	.	.	.	.	.	.	0	0	DB;S	GT:/	0:43	0/1:1	T	C	0
.	.	.	.	.	.	.	.	.	0.1	0.2	DB;S	GT:/	0:19	0/1:3	T	G	0
.	.	.	.	.	.	.	.	.	0.1	0.2	DB;S	GT:/	0:19	0/1:3	G	C	0
.	.	.	.	.	.	.	.	.	0.3	0.3	DB;S	GT:/	0:63	0/1:1	C	T	0
.	##	0	0.2	0.3	0.966	1.000	5.8	3.60	0	.	SOM	GT:/	0:12	0/1:1	C	T	0
.	.	.	.	.	.	.	.	.	.	.	SOM	GT:/	0:49	0/1:9	G	A	0
.	.	.	.	.	.	.	.	1.42	.	.	SOM	GT:/	0:38	0/1:9	G	T	0
.	.	.	.	.	.	.	2.7	.	0	.	SOM	GT:/	0:19	0/1:1	T	G	0
.	.	.	0.0	0.8	0.992	1,N	4.5	1.58	.	.	SOM	GT:/	0:40	0/1:6	G	T	0
.	.	.	.	.	.	.	.	.	.	.	SOM	GT:/	0:66	0/1:1	C	T	0
.	.	.	0.0	0.9	1.0,D	1,D	.	2.95	.	.	SOM	GT:/	0:66	0/1:1	G	A	0
.	.	.	.	.	.	.	.	.	0	.	SOM	GT:/	0:15	0/1:1	T	C	0
.	.	.	.	.	.	.	.	.	0	.	SOM	GT:/	0:8,(	0/1:9	G	A	0
.	.	.	.	.	.	.	.	.	0	0.3	SOM	GT:/	0:14	0/1:1	C	G	0
.	.	.	.	.	.	.	.	.	0	0	SOM	GT:/	0:70	0/1:1	G	A	0
.	.	.	.	.	.	.	.	.	.	.	SOM	GT:/	0:30	0/1:4	C	G	0
.	.	.	.	.	.	.	.	.	0.1	0	DB;S	GT:/	0:20	0/1:1	G	C	0
.	.	.	.	.	.	.	.	.	0	0	SOM	GT:/	0:8,(	0/1:1	A	G	0
.	.	.	.	.	.	.	.	.	.	.	SOM	GT:/	0:27	0/1:4	C	G	0
.	.	.	.	.	.	.	.	.	0	0	DB;S	GT:/	0:34	0/1:6	T	A	0
.	##	0	.	.	.	.	.	.	.	.	SOM	GT:/	0:60	0/1:9	G	A	0
.	.	.	0.0	0.9	0.998	0.921	2.1	2.54	.	.	SOM	GT:/	0:56	0/1:9	G	T	0
.	.	.	.	.	.	.	.	.	.	.	SOM	GT:/	0:20	0/1:4	G	C	0
.	##	0	0.0	1.0	1.0,D	1,D	5	5.82	.	.	SOM	GT:/	0:81	0/1:1	G	A	0
0.002	0	0	1.0	0.0	0.0,B	1.000	3.3	.	0	0.1	SOM	GT:/	0:24	0/1:3	A	G	0
#####	##	0	0.0	0.0	0.047	1,N	.	1.51	.	.	SOM	GT:/	0:27	0/1:7	C	T	0
.	0	0	.	.	.	.	.	.	0	0	SOM	GT:/	0:40	0/1:9	A	G	0
.	.	.	1.0	0.0	0.0,B	1,D	5.4	.	.	.	SOM	GT:/	0:34	0/1:6	C	A	0
.	.	.	.	.	.	.	.	1.35	.	.	SOM	GT:/	0:54	0/1:9	T	A	0
.	.	.	.	.	.	.	.	.	.	.	SOM	GT:/	0:11	0/1:1	G	A	0
.	.	.	.	.	.	.	3.2	1.43	0	0	SOM	GT:/	0:11	0/1:1	G	A	0
.	.	.	.	.	.	.	.	.	.	.	SOM	GT:/	0:51	0/1:3	G	C	0
.	.	.	.	.	.	.	.	.	.	.	SOM	GT:/	0:16	0/1:1	T	C	0
.	.	.	.	.	.	.	.	.	0	0	SOM	GT:/	0:12	0/1:1	G	T	0
.	.	.	0.0	0.7	0.763	1.000	4.2	2.72	.	.	SOM	GT:/	0:13	0/1:1	C	T	0
.	.	.	.	.	.	.	3.3	.	.	.	SOM	GT:/	0:41	0/1:8	C	T	0
.	.	.	.	.	.	.	.	.	.	.	SOM	GT:/	0:9,(	0/1:5	C	A	0
.	.	.	.	.	.	.	.	.	0	.	SOM	GT:/	0:10	0/1:7	G	C	0
.	.	.	.	.	.	.	.	.	0	.	SOM	GT:/	0:10	0/1:7	C	T	0
.	0	0	.	.	.	.	.	.	0	0	DB;S	GT:/	0:55	0/1:9	G	A	0
.	.	.	.	.	.	.	.	1.95	0	0	SOM	GT:/	0:16	0/1:1	G	A	0
.	.	.	.	.	.	.	.	.	.	.	SOM	GT:/	0:14	0/1:2	C	T	0
.	.	.	.	.	.	.	.	.	.	.	SOM	GT:/	0:96	0/1:1	G	A	0
.	.	.	.	.	.	.	.	1.31	.	.	SOM	GT:/	0:22	0/1:4	A	C	0
.	.	.	.	.	.	.	.	.	.	.	SOM	GT:/	0:27	0/1:2	A	C	0
.	.	.	.	.	.	.	.	.	.	.	SOM	GT:/	0:38	0/1:7	T	G	0
.	.	.	0.1	0.6	0.936	1,D	5.6	5.16	.	.	SOM	GT:/	0:42	0/1:6	G	A	0
.	##	0	0.0	0.9	0.991	1,D	5.8	5.70	.	.	DB;S	GT:/	0:26	0/1:3	G	A	0
.	.	.	.	.	.	.	.	1.41	.	.	SOM	GT:/	0:52	0/1:7	C	A	0
.	0	0	0.1	0.5	0.981	0.999	4.2	3.28	.	.	SOM	GT:/	0:20	0/1:3	G	A	0
.	.	.	.	.	.	.	.	.	.	.	SOM	GT:/	0:10	0/1:1	A	G	0

.	.	.	.	.	.	.	.	.	0	.	SOM	GT:/	0:51	0/1:8	A	G	0
.	.	.	.	.	.	.	.	.	0	.	SOM	GT:/	0:8,(	0/1:4	C	T	0
.	.	.	0.2	0.0	0.002	1,N	2.2	1.28	.	.	SOM	GT:/	0:69	0/1:1	C	T	0
.	.	.	.	.	.	.	.	.	.	.	SOM	GT:/	0:26	0/1:4	A	C	0
.	.	.	.	.	.	.	.	.	0.1	0.2	SOM	GT:/	0:13	0/1:2	G	A	0
.	.	.	.	.	.	.	.	.	0	0	SOM	GT:/	0:42	0/1:5	C	T	0
.	##	0	0.2	0.0	0.804	1,D	2.4	1.31	.	.	SOM	GT:/	0:17	0/1:2	G	A	0
.	.	.	.	.	.	.	.	.	0.1	0.2	DB;S	GT:/	0:19	0/1:1	C	T	0
.	.	.	.	.	.	.	.	.	.	.	SOM	GT:/	0:17	0/1:2	G	A	0
.	.	.	.	.	.	.	.	.	.	.	SOM	GT:/	0:15	0/1:2	A	C	0
.	.	.	0.0	0.9	1.0,D	0.917	3.2	.	.	.	SOM	GT:/	0:19	0/1:8	G	C	0
.	.	.	0.0	0.9	0.99,I	0.866	3.5	3.17	.	.	SOM	GT:/	0:75	0/1:1	C	A	0
.	##	0	0.0	0.4	0.99,I	0.995	3.7	2.54	.	.	SOM	GT:/	0:77	0/1:1	C	T	0
.	.	.	.	.	.	.	.	.	.	.	SOM	GT:/	0:30	0/1:5	G	A	0
.	.	.	.	.	.	.	.	.	.	0	SOM	GT:/	0:53	0/1:8	T	A	0
.	.	.	.	.	.	.	.	.	.	.	SOM	GT:/	0:59	0/1:8	T	G	0
.	.	.	.	.	.	.	.	.	.	.	SOM	GT:/	0:27	0/1:4	C	T	0
.	.	.	.	.	.	.	.	.	.	.	SOM	GT:/	0:36	0/1:6	C	T	0
.	.	.	0.0	0.9	1.0,D	1,D	5.6	5.94	.	.	SOM	GT:/	0:79	0/1:1	G	A	0
.	.	.	.	.	.	.	.	.	0	.	SOM	GT:/	0:46	0/1:9	T	G	0
.	.	.	.	.	.	.	.	.	0	.	SOM	GT:/	0:73	0/1:1	C	T	0
.	.	.	.	.	.	.	.	.	0.2	0.2	SOM	GT:/	0:9,(	0/1:8	G	A	0
.	.	.	.	.	.	.	.	.	.	.	SOM	GT:/	0:52	0/1:3	C	T	0
.	.	.	.	.	.	.	.	.	.	.	SOM	GT:/	0:31	0/1:2	G	T	0
.	.	.	.	.	.	.	.	.	.	.	SOM	GT:/	0:12	0/1:4	A	G	0
.	.	.	.	.	.	.	.	.	0	0	SOM	GT:/	0:9,(	0/1:8	C	G	0
.	.	.	.	.	.	.	.	.	.	.	SOM	GT:/	0:21	0/1:1	C	A	0
.	.	.	.	.	.	.	.	.	0	0	SOM	GT:/	0:23	0/1:1	T	C	0
.	.	.	.	.	.	.	.	.	.	0	SOM	GT:/	0:70	0/1:9	C	T	0
.	.	.	.	.	.	.	.	.	.	0	SOM	GT:/	0:85	0/1:1	C	T	0
.	.	.	.	.	.	.	.	.	0	0	SOM	GT:/	0:11	0/1:1	G	A	0
.	.	.	0.0	0.0	0.006	1,D	6.1	4.63	.	.	SOM	GT:/	0:37	0/1:6	G	A	0
.	.	.	0.0	0.6	0.989	0.994	3.7	.	.	.	SOM	GT:/	0:52	0/1:8	C	T	0
.	.	.	0.0	1.0	1.0,D	1,D	5.3	5.07	.	.	SOM	GT:/	0:40	0/1:8	C	T	0
.	.	.	.	.	.	.	.	.	.	.	SOM	GT:/	0:39	0/1:7	G	A	0
.	.	.	.	.	.	.	.	.	0	.	SOM	GT:/	0:32	0/1:6	C	T	0
.	.	.	.	.	.	.	.	1.34	.	.	SOM	GT:/	0:10	0/1:1	A	C	0
.	.	.	.	.	.	.	.	.	.	.	SOM	GT:/	0:28	0/1:5	G	C	0
.	.	.	.	.	.	.	.	.	.	.	SOM	GT:/	0:8,(	0/1:5	C	T	0
.	.	.	.	.	.	.	.	.	.	.	SOM	GT:/	0:53	0/1:6	G	C	0
.	.	.	.	.	.	.	.	.	0	.	SOM	GT:/	0:19	0/1:2	G	A	0
.	.	.	.	.	.	.	.	.	.	.	SOM	GT:/	0:90	0/1:9	T	A	0
.	.	.	.	.	.	.	.	.	.	.	SOM	GT:/	0:10	0/1:1	C	G	0
.	.	.	.	.	.	.	3.2	2.52	.	.	SOM	GT:/	0:19	0/1:4	C	T	0
.	.	.	.	.	.	.	.	.	0	.	SOM	GT:/	0:22	0/1:3	G	A	0
.	.	.	.	.	.	.	.	.	0	0	SOM	GT:/	0:12	0/1:1	T	C	0
.	.	.	.	.	.	.	.	.	.	.	SOM	GT:/	0:46	0/1:7	G	A	0
.	.	.	.	.	.	.	.	.	0	.	SOM	GT:/	0:37	0/1:7	C	T	0
.	.	.	.	.	.	.	.	.	.	.	SOM	GT:/	0:37	0/1:5	G	A	0
.	.	.	.	.	.	.	.	.	.	.	SOM	GT:/	0:94	0/1:1	C	T	0
.	.	.	.	.	.	.	.	.	.	.	SOM	GT:/	0:15	0/1:1	G	A	0
.	0	0	.	.	.	.	.	.	0.1	0	DB;S	GT:/	0:47	0/1:7	C	T	0
.	0	0	.	.	.	.	.	.	0	.	SOM	GT:/	0:47	0/1:7	T	C	0
0.367	0	0	.	.	.	.	.	.	0	0.1	SOM	GT:/	0:23	0/1:2	G	C	0

.	.	.	.	.	.	.	.	.	.	.	SOM	GT:/	0:69	0/1:9	G	A	0
.	.	.	.	.	.	.	.	.	.	.	SOM	GT:/	0:17	0/1:2	G	T	0
.	.	.	.	.	.	.	.	.	0	0	DB;S	GT:/	0:33	0/1:5	T	C	0
.	##	0	.	.	.	.	.	.	.	.	SOM	GT:/	0:12	0/1:2	G	A	0
.	.	.	0.0(	0.9	1.0,D	1,D	5.7	4.61	.	.	SOM	GT:/	0:78	0/1:1	G	A	0
.	.	.	.	.	.	.	.	.	.	.	SOM	GT:/	0:21	0/1:1	G	A	0
.	##	0	.	.	.	.	.	.	.	.	SOM	GT:/	0:62	0/1:1	G	A	0
.	.	.	.	.	.	.	.	.	0	.	DB;S	GT:/	0:22	0/1:3	A	G	0
.	.	.	.	.	.	.	.	.	0.3	0.2	SOM	GT:/	0:10	0/1:2	G	A	0
.	.	.	.	.	.	.	2.9	.	.	.	SOM	GT:/	0:27	0/1:3	G	A	0
2E-04	##	0	0.0(	0.9	0.999	1,D	5.8	5.60	.	.	SOM	GT:/	0:58	0/1:1	G	A	0
.	.	.	.	.	.	.	.	.	.	.	SOM	GT:/	0:22	0/1:3	A	G	0
.	1	1	.	.	.	.	.	.	0.1	0.5	SOM	GT:/	0:48	0/1:9	C	G	0
.	.	.	.	.	.	.	.	.	.	.	SOM	GT:/	0:19	0/1:3	G	A	0
.	.	.	.	.	.	.	.	.	.	.	SOM	GT:/	0:18	0/1:3	C	G	0
.	.	.	.	.	.	.	.	.	.	.	SOM	GT:/	0:12	0/1:2	C	T	0
.	.	.	0.3	0.0	0.049	1,D	5.2	2.12	.	.	SOM	GT:/	0:10	0/1:1	C	A	0
.	.	.	.	.	.	.	.	1.70	.	.	SOM	GT:/	0:39	0/1:8	G	T	0
.	.	.	.	.	.	.	.	.	.	.	SOM	GT:/	0:12	0/1:3	C	T	0
.	.	.	.	.	.	.	.	.	.	.	SOM	GT:/	0:9,(	0/1:7	A	T	0
.	0	0	.	.	.	.	2.1	.	.	.	SOM	GT:/	0:12	0/1:1	T	C	0
.	.	.	.	.	.	.	.	.	.	.	SOM	GT:/	0:11	0/1:1	C	A	0
.	.	.	.	.	.	.	.	.	.	.	SOM	GT:/	0:93	0/1:1	G	C	0
.	.	.	.	.	.	.	2.9	.	.	.	SOM	GT:/	0:31	0/1:3	G	C	0
.	.	.	.	.	.	.	.	.	0	.	SOM	GT:/	0:16	0/1:4	C	A	0
.	##	0	0.0	0.3	0.517	1,D	5.7	4.40	0	.	DB;S	GT:/	0:17	0/1:4	C	T	0
#####	##	0	0.4	0.0	0.375	0.519	4	2.41	.	.	SOM	GT:/	0:49	0/1:7	C	T	0
.	0	0	.	.	.	.	.	.	.	.	DB;S	GT:/	0:11	0/1:2	T	C	0
.	0	0	0.1	0.0	0.075	1.000	.	1.87	.	.	SOM	GT:/	0:45	0/1:5	C	T	0
.	.	.	.	.	.	1,A	.	3.73	.	.	SOM	GT:/	0:51	0/1:7	G	A	0
.	.	.	.	.	.	.	.	.	0	0	SOM	GT:/	0:37	0/1:6	G	A	0
.	.	.	.	.	.	.	2.6	.	.	.	SOM	GT:/	0:15	0/1:3	C	A	0
.	.	.	.	.	.	.	.	.	.	.	SOM	GT:/	0:52	0/1:8	G	A	0
.	.	.	.	.	.	.	.	.	0.1	0.3	SOM	GT:/	0:8,(	0/1:6	T	C	0
.	##	0	0.0	1.0	1.0,D	1,D	5	4.51	.	.	SOM	GT:/	0:84	0/1:1	G	A	0
.	.	.	.	.	.	.	.	.	.	.	SOM	GT:/	0:9,(	0/1:2	C	T	0
.	.	.	.	.	.	.	.	.	.	.	SOM	GT:/	0:31	0/1:7	A	C	0
.	##	0	.	.	.	1,A	4.9	5.89	.	.	SOM	GT:/	0:17	0/1:4	G	A	0
.	.	.	0.0(	0.9	1.0,D	1,D	6.2	4.86	.	.	SOM	GT:/	0:31	0/1:7	C	T	0
.	.	.	.	.	.	.	.	.	.	.	SOM	GT:/	0:83	0/1:1	C	T	0
.	.	.	.	.	.	.	.	.	0	.	SOM	GT:/	0:33	0/1:4	T	G	0
.	.	.	.	.	.	.	.	.	0	0	SOM	GT:/	0:9,(	0/1:1	G	A	0
.	.	.	.	.	.	.	.	.	0	.	SOM	GT:/	0:9,(	0/1:9	C	T	0
.	.	.	.	.	.	.	.	.	0	0	SOM	GT:/	0:8,(	0/1:4	T	G	0
.	.	.	.	.	.	.	.	.	.	.	SOM	GT:/	0:10	0/1:2	T	C	0
.	.	.	.	.	.	.	.	.	0	0	SOM	GT:/	0:10	0/1:1	C	T	0
.	.	.	.	.	.	.	.	3.49	.	.	SOM	GT:/	0:43	0/1:6	G	T	0
.	##	0	.	.	.	.	.	.	.	.	SOM	GT:/	0:10	0/1:2	G	A	0
.	.	.	.	.	.	.	.	.	0	.	SOM	GT:/	0:23	0/1:3	T	A	0
.	.	.	.	.	.	.	.	.	0	0.3	DB;S	GT:/	0:44	0/1:6	G	A	0
.	.	.	.	.	.	.	.	.	0	0	SOM	GT:/	0:8,(	0/1:1	C	T	0
.	.	.	.	.	.	.	.	.	0.2	0.2	DB;S	GT:/	0:22	0/1:5	G	C	0
.	.	.	.	.	.	.	.	.	.	.	SOM	GT:/	0:8,(	0/1:5	G	T	0
.	.	.	.	.	.	.	.	.	0	0.1	SOM	GT:/	0:10	0/1:7	A	C	0

.	.	.	.	.	.	.	.	.	0	0	SOM	GT:/	0:10	0/1:1	C	G	0
.	.	.	.	.	.	.	.	.	0	0.1	SOM	GT:/	0:9,(	0/1:1	A	G	0
.	.	.	.	.	.	.	.	.	0	0.2	SOM	GT:/	0:8,(	0/1:1	G	A	0
.	0	0	.	.	.	.	.	.	0	0	SOM	GT:/	0:74	0/1:1	G	A	0
.	.	.	.	.	.	.	2.8	2.38	.	.	SOM	GT:/	0:22	0/1:3	G	A	0
.	##	0	.	.	.	.	.	.	.	.	SOM	GT:/	0:68	0/1:1	C	T	0
.	.	.	.	.	.	.	.	.	.	.	SOM	GT:/	0:20	0/1:2	C	T	0
.	0	0	1.0	0.0	0.0,B	1.000	.	.	0	0	SOM	GT:/	0:42	0/1:7	A	G	0
.	.	.	.	.	.	.	.	.	.	.	SOM	GT:/	0:9,(	0/1:1	G	A	0
.	.	.	0.5	0.0	0.0,B	1,N	.	.	.	.	SOM	GT:/	0:99	0/1:1	T	C	0
.	.	.	.	.	.	.	.	.	.	.	SOM	GT:/	0:18	0/1:3	A	T	0
.	.	.	.	.	.	.	.	.	.	.	SOM	GT:/	0:38	0/1:7	C	T	0
.	.	.	.	.	.	.	.	.	.	.	SOM	GT:/	0:15	0/1:2	G	A	0
.	.	.	.	.	.	.	.	.	0	.	DB;S	GT:/	0:23	0/1:7	C	G	0
.	.	.	.	.	.	.	.	.	0	.	DB;S	GT:/	0:25	0/1:4	C	T	0
.	.	.	.	.	.	.	.	.	.	.	SOM	GT:/	0:35	0/1:1	T	G	0
.	.	.	.	.	.	.	.	.	0	.	DB;S	GT:/	0:42	0/1:1	G	A	0
.	.	.	.	.	.	.	.	.	0	.	DB;S	GT:/	0:59	0/1:2	C	T	0
.	.	.	.	.	.	.	.	.	0	.	DB;S	GT:/	0:65	0/1:2	T	A	0
.	.	.	.	.	.	.	.	.	0	.	DB;S	GT:/	0:36	0/1:1	C	T	0
.	.	.	.	.	.	.	.	.	0	.	DB;S	GT:/	0:35	0/1:1	G	C	0
.	.	.	.	.	.	.	.	.	.	.	DB;S	GT:/	0:43	0/1:7	C	T	0
.	.	.	.	.	.	.	.	.	0	.	SOM	GT:/	0:8,(	0/1:5	G	A	0
.	.	.	.	.	.	.	.	.	0	.	DB;S	GT:/	0:22	0/1:1	T	C	0
.	.	.	.	.	.	.	.	.	.	.	DB;S	GT:/	0:34	0/1:1	G	T	0
.	.	.	.	.	.	.	.	.	0	.	DB;S	GT:/	0:38	0/1:1	A	G	0
.	.	.	.	.	.	.	.	.	0	.	DB;S	GT:/	0:39	0/1:1	G	C	0
.	.	.	.	.	.	.	.	.	0	0	SOM	GT:/	0:8,(	0/1:2	C	A	0
.	.	.	.	.	.	.	.	.	0	.	SOM	GT:/	0:22	0/1:7	A	G	0
.	.	.	.	.	.	.	.	.	.	.	DB;S	GT:/	0:43	0/1:1	C	T	0
.	.	.	.	.	.	.	.	.	0	.	DB;S	GT:/	0:43	0/1:1	C	T	0
.	.	.	.	.	.	.	.	.	.	.	DB;S	GT:/	0:30	0/1:1	C	T	0
.	.	.	.	.	.	.	.	.	.	.	SOM	GT:/	0:14	0/1:3	A	T	0
.	.	.	.	.	.	.	.	.	.	.	DB;S	GT:/	0:35	0/1:1	A	C	0
.	.	.	.	.	.	.	.	.	0	.	DB;S	GT:/	0:27	0/1:1	A	C	0
.	.	.	.	.	.	.	.	.	0	.	DB;S	GT:/	0:21	0/1:8	T	C	0
.	.	.	.	.	.	.	.	.	.	.	SOM	GT:/	0:33	0/1:6	C	A	0
.	0	0	0.6	0.0	0.0,B	1,N	.	.	0	0	SOM	GT:/	0:79	0/1:1	A	G	0
.	.	.	.	.	.	.	.	.	0	0	SOM	GT:/	0:14	0/1:2	A	C	0
.	.	.	0.1	0.0	0.0,B	1,N	.	1.72	.	.	SOM	GT:/	0:46	0/1:6	C	G	0
.	.	.	.	.	.	.	.	.	.	.	SOM	GT:/	0:35	0/1:8	C	A	0
.	.	.	.	.	.	.	.	.	.	.	SOM	GT:/	0:19	0/1:2	T	G	0
.	.	.	.	.	.	.	3.2	.	.	.	SOM	GT:/	0:58	0/1:1	G	A	0
.	.	.	.	.	.	.	.	.	.	.	SOM	GT:/	0:79	0/1:1	G	T	0
.	.	.	.	.	.	.	.	.	.	.	SOM	GT:/	0:14	0/1:2	G	A	0
.	.	.	.	.	.	.	.	.	0	0	SOM	GT:/	0:16	0/1:3	T	G	0
.	.	.	.	.	.	.	.	.	0	0.2	SOM	GT:/	0:11	0/1:9	A	G	0
.	.	.	.	.	.	.	.	.	0	0.2	SOM	GT:/	0:13	0/1:1	C	A	0
.	.	.	.	0.1	0.458	1.000	4.9	3.16	0	.	SOM	GT:/	0:25	0/1:3	C	T	0
.	.	.	.	.	.	.	.	.	.	.	SOM	GT:/	0:37	0/1:5	A	G	0
.	.	.	.	.	.	.	.	.	0	0	DB;S	GT:/	0:29	0/1:3	A	T	0
.	.	.	.	.	.	.	.	.	.	.	SOM	GT:/	0:18	0/1:0	G	A	0
.	.	.	.	.	.	.	.	.	.	.	SOM	GT:/	0:33	0/1:4	G	A	0
.	##	.	0.0	0.9	1.0,D	1.000	3.4	4.41	.	.	SOM	GT:/	0:13	0/1:2	C	T	0

.	0	0	.	.	.	.	2.5	.	.	.	SON	GT:/	0:88	0/1:1	G	A	0
.	.	.	.	.	.	.	.	.	.	.	SON	GT:/	0:93	0/1:1	G	A	0
.	.	.	.	.	.	.	.	.	.	.	SON	GT:/	0:21	0/1:2	C	T	0
.	.	.	.	.	.	.	4	.	.	.	SON	GT:/	0:55	0/1:1	G	A	0
.	.	.	.	.	.	1,N	.	.	.	.	SON	GT:/	0:10	0/1:1	C	T	0
#####	##	0	.	.	.	.	.	.	0	.	SON	GT:/	0:87	0/1:1	C	T	0
.	.	.	.	.	.	.	.	1.42	.	.	SON	GT:/	0:14	0/1:2	C	T	0
.	0	.	.	.	.	.	.	.	0	.	SON	GT:/	0:17	0/1:1	C	T	0
.	.	.	.	.	.	.	.	.	.	.	SON	GT:/	0:32	0/1:3	G	A	0
.	.	.	.	.	.	.	.	.	.	.	SON	GT:/	0:65	0/1:1	G	T	0
.	.	.	.	.	.	.	.	.	.	.	SON	GT:/	0:11	0/1:2	G	T	0
.	.	.	.	.	.	.	2.4	1.26	.	.	SON	GT:/	0:10	0/1:1	C	A	0
.	.	.	.	.	.	.	.	.	.	.	SON	GT:/	0:11	0/1:1	G	A	0
.	.	.	.	.	.	.	.	.	0	0.1	SON	GT:/	0:8,(	0/1:6	T	C	0
.	.	.	.	.	.	.	.	.	0	0	SON	GT:/	0:26	0/1:3	G	T	0
.	.	.	.	.	.	.	.	.	0	.	SON	GT:/	0:38	0/1:6	C	G	0
.	.	.	.	.	.	.	3.1	.	.	.	SON	GT:/	0:24	0/1:4	C	T	0
.	.	.	.	.	.	.	.	.	0	.	SON	GT:/	0:22	0/1:5	A	C	0
.	.	.	.	.	.	.	.	.	.	.	SON	GT:/	0:19	0/1:2	C	G	0
.	.	.	0.0(	0.8(	0.975	0.810	.	3.51	.	.	DB;S	GT:/	0:37	0/1:5	C	A	0
.	.	.	.	.	.	.	.	.	0	.	DB;S	GT:/	0:34	0/1:7	C	A	0
.	.	.	.	.	.	.	.	.	.	.	SON	GT:/	0:17	0/1:2	A	C	0
.	.	.	.	.	.	.	.	.	0	0	SON	GT:/	0:11	0/1:3	T	C	0
.	.	.	.	.	.	.	.	.	0	.	SON	GT:/	0:85	0/1:1	T	C	0
.	##	0	.	.	.	.	.	.	.	.	SON	GT:/	0:75	0/1:1	G	A	0
.	.	.	.	.	.	.	.	.	.	.	SON	GT:/	0:39	0/1:7	G	T	0
.	##	0	0.0	0.9	0.998	0.766	2.6	3.22	.	.	SON	GT:/	0:17	0/1:2	T	G	0
.	.	.	.	.	.	.	2.9	.	.	.	SON	GT:/	0:42	0/1:7	C	T	0
.	.	.	.	.	.	.	.	.	.	.	SON	GT:/	0:17	0/1:4	C	T	0
.	.	.	.	.	.	.	.	.	.	.	SON	GT:/	0:21	0/1:3	G	T	0
.	.	.	.	.	.	.	.	.	.	.	SON	GT:/	0:87	0/1:1	C	A	0
.	.	.	.	.	.	.	.	.	.	0	SON	GT:/	0:23	0/1:2	A	G	0
.	.	.	.	.	.	.	.	.	.	.	SON	GT:/	0:41	0/1:7	C	A	0
.	.	.	.	.	.	.	.	.	0.1	0.2	SON	GT:/	0:10	0/1:1	C	T	0
.	.	.	.	.	.	.	.	.	0	0	SON	GT:/	0:41	0/1:4	C	T	0
.	.	.	.	.	.	.	.	.	0	.	SON	GT:/	0:54	0/1:1	A	G	0
.	.	.	.	.	.	.	.	.	.	.	SON	GT:/	0:9,(	0/1:1	G	A	0
.	.	.	.	.	.	.	.	.	.	.	SON	GT:/	0:29	0/1:6	C	T	0
.	.	.	.	.	.	.	.	.	.	.	SON	GT:/	0:12	0/1:9	T	G	0
.	.	.	.	.	.	.	.	.	.	.	SON	GT:/	0:56	0/1:1	C	T	0
.	.	.	.	.	.	.	.	.	.	.	SON	GT:/	0:20	0/1:5	C	T	0
.	0	0	.	.	.	.	.	.	0	0	SON	GT:/	0:18	0/1:3	G	A	0
.	.	.	.	.	.	.	.	.	0	0	SON	GT:/	0:22	0/1:4	C	T	0
#####	##	0	.	.	.	.	.	.	.	.	SON	GT:/	0:63	0/1:1	C	T	0
.	.	.	.	.	.	.	3.5	.	.	.	SON	GT:/	0:56	0/1:8	G	A	0
.	.	.	.	.	.	.	.	2.38	.	.	SON	GT:/	0:25	0/1:3	G	A	0
.	.	.	.	.	.	.	.	.	.	.	SON	GT:/	0:26	0/1:6	C	A	0
.	.	.	.	.	.	.	.	.	0	0.1	SON	GT:/	0:31	0/1:1	G	T	0
.	.	.	.	.	.	.	.	.	.	.	SON	GT:/	0:11	0/1:1	T	C	0
.	.	.	0.0(	0.7	0.898	0.976	5	3.18	.	.	SON	GT:/	0:11	0/1:2	C	T	0
.	##	0	0.0	0.8(	0.998	1,D	5.1	3.90	.	.	SON	GT:/	0:48	0/1:9	C	T	0
.	.	.	.	.	.	.	2.6	.	.	.	SON	GT:/	0:93	0/1:1	G	A	0
.	0	0	0.0	0.9	1.0,D	1,D	4.9	3.75	.	.	SON	GT:/	0:43	0/1:8	G	A	0
.	.	.	.	.	.	.	.	.	.	.	SON	GT:/	0:26	0/1:1	G	C	0

.	.	.	.	.	.	.	.	.	0	.	SOM	GT:/	0:13	0/1:4	T	C	0
.	.	.	.	.	.	.	.	.	0	.	SOM	GT:/	0:13	0/1:4	A	G	0
0.003	.	.	.	.	.	.	.	.	0	0	DB;S	GT:/	0:60	0/1:1	A	T	0
.	.	.	0.0	0.4	0.974	0.935	4.8	.	.	.	SOM	GT:/	0:71	0/1:1	G	A	0
.	.	.	0.4	0.0	0.0,B	1.000	.	.	.	.	SOM	GT:/	0:89	0/1:1	T	C	0
.	.	.	.	.	.	.	.	.	.	.	SOM	GT:/	0:65	0/1:1	C	T	0
.	.	.	.	.	.	.	.	.	.	.	SOM	GT:/	0:17	0/1:3	C	A	0
.	.	.	.	.	.	.	.	.	.	.	SOM	GT:/	0:20	0/1:3	G	A	0
.	0	0	.	.	.	.	.	3.39	.	.	SOM	GT:/	0:17	0/1:1	G	A	0
.	.	.	.	.	.	.	.	.	.	.	SOM	GT:/	0:26	0/1:9	T	C	0
.	.	.	.	.	.	.	.	.	.	.	SOM	GT:/	0:29	0/1:1	G	A	0
.	.	.	.	.	.	.	.	.	.	.	SOM	GT:/	0:22	0/1:6	C	A	0
.	##	0	.	.	.	.	.	.	.	.	SOM	GT:/	0:95	0/1:1	C	T	0
.	.	.	.	.	.	.	.	.	.	.	SOM	GT:/	0:40	0/1:8	C	G	0
3E-04	0	0	0.8	0.0	0.877	0.994	4.2	2.67	.	.	SOM	GT:/	0:92	0/1:1	C	T	0
.	.	.	0.0	0.3	0.273	1,D	5.4	4.81	.	.	SOM	GT:/	0:14	0/1:1	C	T	0
.	0	0	.	.	.	.	.	.	0	0	SOM	GT:/	0:17	0/1:2	G	A	0
.	.	.	.	.	.	.	.	.	0	0.4	SOM	GT:/	0:8,(	0/1:3	G	C	0
.	.	.	.	.	.	.	.	.	.	.	SOM	GT:/	0:31	0/1:3	T	A	0
.	.	.	.	.	.	.	.	.	0	0	SOM	GT:/	0:8,(	0/1:1	C	T	0
.	.	.	0.0	0.0	0.103	1,N	3	2.65	.	.	SOM	GT:/	0:40	0/1:5	G	C	0
.	.	.	.	.	.	.	.	.	.	.	SOM	GT:/	0:13	0/1:1	C	T	0
9E-04	0	0	0.2	0.0	0.055	1,D	5	3.03	0	0	SOM	GT:/	0:46	0/1:4	G	A	0
.	.	.	.	.	.	.	.	.	.	.	SOM	GT:/	0:13	0/1:1	G	A	0
.	.	.	.	.	.	.	2.3	1.84	.	.	SOM	GT:/	0:86	0/1:1	G	A	0
.	##	0	0.0	0.0	0.002	1,D	3.2	2.14	.	.	SOM	GT:/	0:93	0/1:9	G	A	0
.	.	.	0.0	0.1	0.236	1.000	6.2	2.89	.	.	SOM	GT:/	0:39	0/1:6	C	T	0
.	.	.	0.0	0.9	1.0,D	1,D	5.9	5.31	.	.	SOM	GT:/	0:30	0/1:5	G	C	0
.	.	.	.	.	.	.	2.3	2.51	.	.	SOM	GT:/	0:37	0/1:7	G	T	0
.	.	.	.	.	.	.	.	.	.	.	SOM	GT:/	0:25	0/1:6	G	T	0
.	.	.	.	.	.	.	.	.	.	.	SOM	GT:/	0:12	0/1:2	G	C	0
.	.	.	.	.	.	.	.	.	0	.	SOM	GT:/	0:18	0/1:3	A	G	0
.	##	0	0.0	0.0	0.029	1,N	.	1.80	.	.	SOM	GT:/	0:23	0/1:3	C	T	0
.	.	.	.	.	.	.	.	.	0	0.1	SOM	GT:/	0:8,(	0/1:6	C	A	0
.	.	.	.	.	.	.	2.2	1.31	.	.	SOM	GT:/	0:41	0/1:5	C	T	0
.	.	.	.	.	.	.	.	.	.	.	SOM	GT:/	0:28	0/1:3	C	G	0
.	.	.	.	.	.	.	.	.	.	.	SOM	GT:/	0:20	0/1:3	G	A	0
.	.	.	.	.	.	.	.	.	.	.	SOM	GT:/	0:10	0/1:1	G	A	0
.	.	.	.	.	.	.	.	.	.	.	SOM	GT:/	0:11	0/1:1	G	A	0
.	.	.	.	.	.	.	.	.	.	.	SOM	GT:/	0:8,(	0/1:6	C	A	0
.	.	.	.	.	.	.	.	.	.	.	SOM	GT:/	0:31	0/1:5	G	A	0
.	.	.	.	.	.	.	.	1.48	.	.	SOM	GT:/	0:56	0/1:6	G	A	0
.	.	.	.	.	.	.	.	.	.	.	SOM	GT:/	0:10	0/1:1	G	A	0
.	0	0	.	.	.	.	.	.	0	0.3	SOM	GT:/	0:11	0/1:2	G	A	0
.	.	.	.	.	.	.	.	.	0	0	SOM	GT:/	0:12	0/1:1	C	T	0
.	.	.	.	.	.	.	.	.	.	.	SOM	GT:/	0:9,(	0/1:6	C	T	0
.	.	.	.	.	.	.	.	.	0	0	SOM	GT:/	0:13	0/1:1	G	A	0
.	.	.	.	.	.	.	.	.	0	0	SOM	GT:/	0:41	0/1:8	A	G	0
.	.	.	.	.	.	.	.	.	0	0.1	SOM	GT:/	0:20	0/1:3	C	G	0
.	.	.	.	.	.	.	.	.	0	.	DB;S	GT:/	0:52	0/1:6	A	T	0
.	.	.	.	.	.	.	.	.	.	.	SOM	GT:/	0:68	0/1:1	T	G	0
.	.	.	.	.	.	.	.	.	.	.	SOM	GT:/	0:9,(	0/1:6	A	G	0
.	.	.	.	.	.	.	.	.	.	.	SOM	GT:/	0:8,(	0/1:1	G	A	0
.	.	.	.	.	.	.	.	.	.	.	SOM	GT:/	0:19	0/1:3	C	A	0

.	.	.	.	.	.	.	.	.	0	.	DB;S	GT:/	0:27	0/1:3	C	T	0
.	.	.	.	.	.	.	.	.	.	.	SOM	GT:/	0:16	0/1:1	T	G	0
.	.	.	.	.	.	.	.	.	.	.	SOM	GT:/	0:18	0/1:1	C	G	0
.	.	.	.	.	.	.	.	.	0	0	SOM	GT:/	0:12	0/1:1	G	A	0
.	.	.	.	.	.	.	.	.	0	0	SOM	GT:/	0:12	0/1:1	G	A	0
.	.	.	.	.	.	.	.	1.97	0	0	SOM	GT:/	0:11	0/1:1	G	C	0
.	.	.	.	.	.	.	.	.	.	.	SOM	GT:/	0:9,(	0/1:8	G	A	0
.	.	.	.	.	.	.	.	.	0	0.2	SOM	GT:/	0:24	0/1:4	C	T	0
.	.	.	.	.	.	.	.	.	0	.	SOM	GT:/	0:14	0/1:1	T	A	0
.	.	.	.	.	.	.	.	1.76	.	.	SOM	GT:/	0:18	0/1:4	C	T	0
.	.	.	.	.	.	.	.	.	.	.	SOM	GT:/	0:62	0/1:9	C	G	0
.	.	.	.	.	.	.	.	.	.	.	SOM	GT:/	0:31	0/1:3	C	A	0
.	.	.	.	.	.	.	.	.	.	.	SOM	GT:/	0:69	0/1:1	A	T	0
.	.	.	.	.	.	.	.	.	.	.	SOM	GT:/	0:19	0/1:3	G	T	0
.	.	.	.	.	.	.	3.3	1.34	.	.	SOM	GT:/	0:51	0/1:7	C	T	0
.	.	.	.	.	.	.	2.8	1.23	.	.	SOM	GT:/	0:85	0/1:1	C	T	0
.	.	.	.	.	.	.	2	.	.	.	SOM	GT:/	0:42	0/1:5	C	T	0
.	.	.	0.3	0.0	0.11,I	1,N	.	.	.	.	SOM	GT:/	0:58	0/1:7	C	G	0
5E-04	0	0	.	.	.	.	.	.	.	.	SOM	GT:/	0:12	0/1:2	G	A	0
#####	0	0	0.0	0.3	0.988	1.000	5.6	3.54	.	.	SOM	GT:/	0:52	0/1:8	G	A	0
.	.	.	0.1	0.5	0.994	0.999	2.6	3.80	.	.	SOM	GT:/	0:25	0/1:6	G	A	0
.	.	.	0.0	1.0	1.0,D	1,D	5.7	5.03	0	.	SOM	GT:/	0:75	0/1:1	C	T	0
.	.	.	.	.	.	.	.	.	.	.	SOM	GT:/	0:13	0/1:1	G	A	0
.	##	0	.	.	.	.	.	.	.	.	SOM	GT:/	0:38	0/1:6	G	A	0
.	.	.	.	.	.	.	.	1.87	.	.	SOM	GT:/	0:28	0/1:4	C	T	0
.	.	.	.	.	.	.	.	.	.	.	SOM	GT:/	0:9,(	0/1:2	C	G	0
.	.	.	.	.	.	.	3.4	.	.	.	DB;S	GT:/	0:40	0/1:7	G	C	0
.	.	.	.	.	.	.	.	.	.	.	SOM	GT:/	0:31	0/1:4	G	C	0
.	.	.	.	.	.	.	.	.	0	.	SOM	GT:/	0:9,(	0/1:5	C	A	0
.	.	.	.	.	.	.	4.1	.	.	.	SOM	GT:/	0:69	0/1:8	C	T	0
.	.	.	.	.	.	.	3.9	.	.	.	SOM	GT:/	0:13	0/1:2	C	T	0
.	.	.	.	.	.	.	.	.	.	.	SOM	GT:/	0:36	0/1:5	A	G	0
.	.	.	.	.	.	.	.	1.61	.	.	SOM	GT:/	0:17	0/1:2	C	G	0
.	.	.	.	.	.	.	.	.	.	.	SOM	GT:/	0:12	0/1:1	C	G	0
.	.	.	.	.	.	.	.	.	0	.	SOM	GT:/	0:27	0/1:3	C	T	0
.	.	.	.	.	.	.	2.6	.	.	0	SOM	GT:/	0:31	0/1:5	C	T	0
#####	##	0	.	.	.	.	.	.	.	.	SOM	GT:/	0:59	0/1:8	C	T	0
.	.	.	.	.	.	.	.	.	.	.	SOM	GT:/	0:77	0/1:1	C	T	0
.	.	.	.	.	.	.	.	1.57	.	.	SOM	GT:/	0:39	0/1:4	G	A	0
.	.	.	.	.	.	.	.	.	0	.	SOM	GT:/	0:17	0/1:2	A	G	0
.	.	.	.	.	.	.	.	.	0	.	SOM	GT:/	0:18	0/1:2	C	A	0
.	.	.	.	.	.	.	.	1.90	.	.	SOM	GT:/	0:27	0/1:3	C	T	0
.	.	.	.	.	.	.	.	.	0.1	0.5	SOM	GT:/	0:8,(	0/1:1	G	A	0
.	.	.	.	.	.	.	.	.	0	0.1	SOM	GT:/	0:8,(	0/1:1	T	C	0
.	.	.	.	.	.	.	3.8	1.39	.	.	SOM	GT:/	0:90	0/1:1	C	T	0
.	.	.	.	.	.	.	.	.	.	.	SOM	GT:/	0:29	0/1:3	G	A	0
.	.	.	.	.	.	.	.	1.92	.	.	SOM	GT:/	0:31	0/1:3	G	C	0
.	.	.	.	.	.	.	.	.	0	0	DB;S	GT:/	0:28	0/1:6	A	T	0
.	.	.	.	.	.	.	5.4	3.63	.	.	SOM	GT:/	0:10	0/1:1	C	T	0
.	.	.	.	.	.	.	.	.	.	.	SOM	GT:/	0:16	0/1:2	C	T	0
.	1	1	.	.	.	.	.	.	0	0	SOM	GT:/	0:17	0/1:4	T	C	0
.	.	.	.	.	.	.	.	.	0	.	SOM	GT:/	0:9,(	0/1:1	A	G	0
.	.	.	.	.	.	.	.	.	.	.	SOM	GT:/	0:18	0/1:1	C	T	0
.	.	.	.	.	.	.	.	.	.	.	SOM	GT:/	0:17	0/1:2	G	T	0

.	.	.	.	.	.	.	.	.	.	.	SOM	GT:/	0:22	0/1:4	G	C	0
.	.	.	.	.	.	.	.	.	.	.	SOM	GT:/	0:14	0/1:2	T	G	0
.	.	.	0.1	0.1	0.291	0.943	2.4	2.90	.	.	SOM	GT:/	0:37	0/1:6	C	T	0
.	.	.	.	.	.	.	.	.	.	.	SOM	GT:/	0:15	0/1:1	T	G	0
.	.	.	.	.	.	.	.	.	.	.	SOM	GT:/	0:9,(	0/1:1	T	C	0
.	.	.	.	.	.	.	.	.	.	.	SOM	GT:/	0:33	0/1:4	C	T	0
.	.	.	0.3	0.0	0.118	1,N	.	.	.	.	SOM	GT:/	0:26	0/1:4	G	A	0
.	.	.	.	.	.	.	.	.	.	.	SOM	GT:/	0:13	0/1:3	G	A	0
.	.	.	.	.	.	.	.	.	.	.	SOM	GT:/	0:25	0/1:2	C	T	0
.	.	.	.	.	.	.	.	.	.	.	SOM	GT:/	0:67	0/1:1	G	A	0
.	.	.	.	.	.	.	.	.	.	.	SOM	GT:/	0:45	0/1:8	G	A	0
.	.	.	.	.	.	.	.	.	.	.	SOM	GT:/	0:16	0/1:2	G	A	0
.	.	.	.	.	.	.	.	.	.	.	SOM	GT:/	0:10	0/1:1	G	T	0
.	.	.	0.0	0.1	0.146	1,D	5.5	4.87	.	.	SOM	GT:/	0:37	0/1:4	C	G	0
.	.	.	.	.	.	.	.	.	.	.	SOM	GT:/	0:55	0/1:9	C	T	0
.	.	.	.	.	.	.	.	.	.	.	SOM	GT:/	0:15	0/1:2	G	A	0
.	.	.	.	.	.	.	4.6	2.01	.	.	SOM	GT:/	0:98	0/1:1	C	T	0
.	.	.	0.1	0.3	0.722	1,D	5.4	4.72	.	.	SOM	GT:/	0:10	0/1:1	C	T	0
.	.	.	.	.	.	.	.	.	.	.	SOM	GT:/	0:16	0/1:2	G	A	0
.	##	.	.	.	.	.	.	1.27	0	.	SOM	GT:/	0:17	0/1:2	C	T	0
#####	.	.	0.1	0.6	0.939	1.000	5.4	4.66	.	.	SOM	GT:/	0:10	0/1:1	G	A	0
.	##	0	.	.	.	.	.	.	.	.	SOM	GT:/	0:52	0/1:1	G	A	0
.	.	.	.	.	.	.	.	.	.	.	SOM	GT:/	0:33	0/1:5	A	T	0
0.001	0	0	.	.	.	.	3.1	.	.	.	SOM	GT:/	0:17	0/1:2	G	A	0
.	.	.	0.1	0.0	0.011	1,N	.	.	.	.	SOM	GT:/	0:79	0/1:2	C	T	0
.	.	.	0.1	0.2	0.437	1,N	3.6	3.56	.	.	SOM	GT:/	0:68	0/1:2	C	G	0
.	.	.	.	.	.	.	.	.	.	.	SOM	GT:/	0:43	0/1:1	C	T	0
.	.	.	0.0	0.9	0.999	1.000	3.5	3.61	.	.	SOM	GT:/	0:11	0/1:3	C	T	0
.	.	.	.	.	.	.	.	.	.	.	SOM	GT:/	0:21	0/1:6	C	T	0
.	.	.	0.1	0.5	0.873	1,N	4.2	1.92	.	.	SOM	GT:/	0:68	0/1:2	C	T	0
.	.	.	.	.	.	.	.	.	.	.	SOM	GT:/	0:41	0/1:1	C	T	0
.	.	.	.	.	.	.	2.9	.	.	.	SOM	GT:/	0:71	0/1:2	G	A	0
.	.	.	.	.	.	.	.	.	.	.	SOM	GT:/	0:32	0/1:1	C	T	0
0.004	0	0	.	.	.	.	.	.	.	.	SOM	GT:/	0:26	0/1:6	G	A	0
.	.	.	.	.	.	1,D	3.4	8.37	.	.	SOM	GT:/	0:59	0/1:1	C	A	0
.	.	.	.	.	.	.	.	.	.	.	SOM	GT:/	0:11	0/1:3	G	A	0
.	.	.	.	.	.	.	.	.	.	.	SOM	GT:/	0:13	0/1:4	T	G	0
.	.	.	.	.	.	.	2.8	.	.	.	SOM	GT:/	0:9,(	0/1:2	G	C	0
.	.	.	.	.	.	.	.	2.10	.	.	SOM	GT:/	0:18	0/1:4	C	T	0
.	.	.	.	.	.	.	.	.	.	.	SOM	GT:/	0:25	0/1:7	G	A	0
.	.	.	.	.	.	.	.	.	.	.	SOM	GT:/	0:40	0/1:1	G	C	0
.	.	.	.	.	.	.	5.6	.	.	.	SOM	GT:/	0:70	0/1:2	C	T	0
.	.	.	0.4	1.0	1.0,D	1,N	.	.	.	.	SOM	GT:/	0:93	0/1:1	C	G	0
.	.	.	.	.	.	.	.	.	.	.	SOM	GT:/	0:22	0/1:6	G	A	0
.	.	.	.	.	.	.	.	.	.	.	SOM	GT:/	0:24	0/1:4	G	A	0
.	.	.	.	.	.	.	.	.	.	.	SOM	GT:/	0:8,(	0/1:1	C	T	0
.	.	.	.	.	.	.	.	.	0	.	SOM	GT:/	0:17	0/1:5	C	T	0
.	.	.	.	.	.	.	.	.	0	0	SOM	GT:/	0:10	0/1:2	C	T	0
.	.	.	.	.	.	.	.	.	0	0	SOM	GT:/	0:11	0/1:3	G	A	0
.	.	.	.	.	.	.	.	.	0	0	SOM	GT:/	0:14	0/1:4	C	T	0
.	.	.	.	.	.	.	.	.	0.4	0.4	DB;	GT:/	0:41	0/1:7	C	T	0
.	0	0	.	.	.	.	2.1	.	.	.	SOM	GT:/	0:84	0/1:1	C	T	0
.	##	0	0.0	0.0	0.0,B	1.000	3	2.06	0	.	SOM	GT:/	0:42	0/1:7	G	A	0
.	.	.	.	.	.	.	.	.	.	.	SOM	GT:/	0:10	0/1:2	G	A	0

.	.	.	.	.	.	.	4	1.61	.	.	SOM	GT:/	0:21	0/1:3	G	A	0
.	.	.	0.0	0.9	0.99,I	1,D	5.6	4.79	.	.	SOM	GT:/	0:57	0/1:1	C	G	0
.	.	.	.	.	.	.	.	.	0	0	DB;S	GT:/	0:20	0/1:1	G	T	0
.	.	.	.	.	.	.	.	.	.	.	SOM	GT:/	0:19	0/1:4	G	A	0
.	.	.	.	.	.	.	.	.	.	.	SOM	GT:/	0:38	0/1:7	C	T	0
.	.	.	.	.	.	.	.	.	.	.	SOM	GT:/	0:14	0/1:1	G	C	0
.	.	.	.	.	.	.	.	.	0	0.1	SOM	GT:/	0:9,	0/1:2	C	A	0
.	.	.	.	.	.	.	.	.	.	.	SOM	GT:/	0:18	0/1:3	C	T	0
.	.	.	.	.	.	.	.	.	.	.	SOM	GT:/	0:28	0/1:5	G	A	0
.	.	.	.	.	.	.	.	1.30	0	0	SOM	GT:/	0:9,	0/1:1	A	T	0
.	.	.	0.0	1.0	1.0,D	1,D	4.7	4.00	.	.	SOM	GT:/	0:17	0/1:2	G	C	0
.	.	.	.	.	.	.	.	.	0	.	SOM	GT:/	0:11	0/1:2	A	G	0
.	.	.	.	.	.	.	3.1	.	.	.	SOM	GT:/	0:26	0/1:3	T	G	0
.	.	.	.	.	.	.	.	.	.	.	SOM	GT:/	0:10	0/1:3	T	C	0
.	.	.	.	.	.	.	.	.	0	0	SOM	GT:/	0:10	0/1:1	G	C	0
.	.	.	0.5	0.0	0.001	1,D	5.8	.	.	.	SOM	GT:/	0:17	0/1:3	G	A	0
.	.	.	.	.	.	.	.	.	.	.	SOM	GT:/	0:10	0/1:2	C	T	0
.	.	.	.	.	.	.	.	.	.	.	SOM	GT:/	0:14	0/1:2	G	A	0
.	.	.	.	.	.	.	.	.	.	.	SOM	GT:/	0:14	0/1:2	A	C	0
.	.	.	.	.	.	.	.	.	.	.	SOM	GT:/	0:14	0/1:3	G	A	0
.	.	.	.	.	.	.	2.5	.	.	.	SOM	GT:/	0:11	0/1:2	T	A	0
.	.	.	.	.	.	.	.	.	.	.	SOM	GT:/	0:11	0/1:1	C	T	0
.	.	.	.	.	.	.	.	.	.	.	SOM	GT:/	0:29	0/1:4	G	C	0
.	.	.	.	.	.	.	.	.	0	0	SOM	GT:/	0:22	0/1:4	C	T	0
.	.	.	.	.	.	.	.	.	.	.	SOM	GT:/	0:26	0/1:4	C	T	0
.	.	.	.	.	.	1,A	.	7.72	.	.	SOM	GT:/	0:37	0/1:5	C	T	0
.	.	.	0.0	0.9	1.0,D	1,D	5.8	5.12	.	.	SOM	GT:/	0:69	0/1:1	C	T	0
.	.	.	.	.	.	.	2.1	1.99	.	.	SOM	GT:/	0:35	0/1:3	C	A	0
.	.	.	.	.	.	.	.	2.08	.	.	SOM	GT:/	0:12	0/1:2	C	A	0
.	.	.	.	.	.	.	.	.	.	.	SOM	GT:/	0:40	0/1:7	A	C	0
.	.	.	.	.	.	.	.	.	.	.	SOM	GT:/	0:24	0/1:4	C	T	0
.	.	.	.	.	.	.	.	.	.	.	SOM	GT:/	0:10	0/1:2	G	C	0
.	.	.	.	.	.	.	.	.	.	.	SOM	GT:/	0:31	0/1:6	G	A	0
.	.	.	.	.	.	.	.	1.83	.	0	SOM	GT:/	0:12	0/1:3	G	A	0
.	.	.	.	.	.	.	.	3.63	.	.	SOM	GT:/	0:83	0/1:8	C	T	0
.	.	.	.	.	.	.	3.2	.	.	.	SOM	GT:/	0:13	0/1:2	G	C	0
.	##	.	0.4	0.4	0.986	1.000	5.9	2.50	.	.	SOM	GT:/	0:31	0/1:6	C	G	0
.	.	.	.	.	.	.	.	.	.	.	SOM	GT:/	0:12	0/1:3	C	G	0
.	.	.	0.0	0.0	0.088	0.994	5.6	2.43	.	.	SOM	GT:/	0:68	0/1:1	C	G	0
.	##	0	.	.	.	.	5.2	1.69	.	.	SOM	GT:/	0:24	0/1:4	G	A	0
.	.	.	0.2	0.0	0.017	1,N	.	.	.	.	SOM	GT:/	0:76	0/1:1	C	T	0
.	.	.	0.0	0.1	0.358	1,N	.	.	.	.	SOM	GT:/	0:92	0/1:1	C	G	0
.	.	.	.	.	.	.	.	.	.	.	SOM	GT:/	0:12	0/1:1	C	T	0
.	.	.	.	.	.	.	4	.	.	.	SOM	GT:/	0:49	0/1:9	G	C	0
.	.	.	.	.	.	.	.	.	.	.	SOM	GT:/	0:15	0/1:3	G	C	0
.	.	.	.	.	.	.	.	.	.	.	SOM	GT:/	0:34	0/1:6	G	A	0
.	.	.	.	.	.	.	.	.	.	.	SOM	GT:/	0:15	0/1:3	G	A	0
.	.	.	.	.	.	.	.	.	.	.	SOM	GT:/	0:12	0/1:2	G	A	0
.	.	.	0.0	0.7	0.988	1.000	3.8	3.27	.	.	SOM	GT:/	0:97	0/1:1	C	T	0
.	0	0	.	.	.	.	.	.	.	.	SOM	GT:/	0:59	0/1:1	C	T	0
.	.	.	.	.	.	.	2.4	1.28	.	.	SOM	GT:/	0:18	0/1:3	C	T	0
.	.	.	.	.	.	.	.	.	.	.	SOM	GT:/	0:13	0/1:2	C	T	0
.	.	.	.	.	.	.	.	.	.	.	SOM	GT:/	0:37	0/1:5	C	A	0
.	.	.	.	.	.	.	.	.	.	.	SOM	GT:/	0:24	0/1:3	T	G	0

#####	.	.	.	.	.	.	.	4.09	0	.	SOM	GT:/	0:13	0/1:2	A	C	0
.	##	0	0.0	0.8	0.997	1.000	5.4	3.50	0	.	SOM	GT:/	0:14	0/1:1	C	T	0
0.002	0	0	0.0	0.0	0.0,B	1,N	.	.	.	.	SOM	GT:/	0:42	0/1:7	G	A	0
.	.	.	.	.	.	.	.	.	.	.	SOM	GT:/	0:48	0/1:1	C	G	0
.	.	.	.	.	.	.	.	.	.	.	SOM	GT:/	0:15	0/1:2	C	T	0
.	##	0	.	.	.	.	.	1.60	.	.	SOM	GT:/	0:21	0/1:3	C	T	0
.	.	.	0.0	0.9	1.0,D	1.000	6.2	5.48	.	.	SOM	GT:/	0:70	0/1:1	C	T	0
.	.	.	.	.	.	.	.	.	.	.	SOM	GT:/	0:8,(	0/1:7	G	A	0
.	.	.	.	.	.	.	.	.	.	.	SOM	GT:/	0:98	0/1:1	G	C	0
2E-04	##	0	.	.	.	.	.	.	.	.	SOM	GT:/	0:57	0/1:8	G	A	0
.	.	.	.	.	.	.	.	.	.	.	SOM	GT:/	0:59	0/1:9	C	T	0
.	.	.	.	.	.	.	.	.	.	.	SOM	GT:/	0:26	0/1:3	G	T	0
.	.	.	.	.	.	.	.	.	.	.	SOM	GT:/	0:14	0/1:1	C	T	0
3E-04	0	0	.	.	.	.	.	.	0	.	SOM	GT:/	0:50	0/1:7	C	T	0
.	.	.	.	.	.	.	.	.	.	.	SOM	GT:/	0:21	0/1:2	G	A	0
.	.	.	.	.	.	.	.	.	.	.	SOM	GT:/	0:69	0/1:4	C	G	0
.	.	.	.	.	.	.	.	.	.	.	SOM	GT:/	0:24	0/1:4	A	T	0
.	.	.	.	.	.	.	.	.	.	.	SOM	GT:/	0:25	0/1:3	T	C	0
.	.	.	.	.	.	.	.	.	0	0	SOM	GT:/	0:10	0/1:2	T	C	0
.	.	.	.	.	.	.	.	.	0.1	0.4	SOM	GT:/	0:8,(	0/1:2	C	T	0
.	.	.	.	.	.	.	.	.	.	.	SOM	GT:/	0:34	0/1:1	C	A	0
.	.	.	.	.	.	.	.	.	0	.	SOM	GT:/	0:20	0/1:3	G	C	0
.	##	0	.	.	.	.	2.1	.	0	.	SOM	GT:/	0:11	0/1:1	T	C	0
.	.	.	.	.	.	.	.	.	0	.	SOM	GT:/	0:12	0/1:3	A	C	0
.	.	.	0.1	.	.	0.519	2.6	.	.	.	SOM	GT:/	0:68	0/1:1	G	T	0
.	.	.	.	.	.	.	.	.	0	0	SOM	GT:/	0:13	0/1:3	A	G	0
#####	0	0	.	.	.	.	.	.	.	.	SOM	GT:/	0:14	0/1:1	G	A	0
.	##	0	0.0	0.7	0.974	0.777	4.5	4.43	.	.	SOM	GT:/	0:75	0/1:1	G	A	0
.	.	.	0.4	0.0	0.014	1,D	5.9	2.69	.	.	SOM	GT:/	0:59	0/1:1	G	C	0
.	.	.	.	.	.	.	3.9	.	.	.	SOM	GT:/	0:48	0/1:8	C	T	0
.	.	.	.	.	.	.	.	.	.	.	SOM	GT:/	0:41	0/1:6	C	T	0
.	.	.	.	.	.	.	.	.	.	.	SOM	GT:/	0:64	0/1:9	G	C	0
.	.	.	.	.	.	.	.	.	0	0	SOM	GT:/	0:9,(	0/1:1	G	T	0
.	.	.	.	.	.	.	.	.	.	.	SOM	GT:/	0:48	0/1:8	G	A	0
.	.	.	.	.	.	.	.	.	.	.	SOM	GT:/	0:16	0/1:1	C	T	0
.	.	.	.	.	.	.	.	.	.	.	SOM	GT:/	0:60	0/1:6	G	A	0
.	.	.	.	.	.	.	.	.	.	.	SOM	GT:/	0:36	0/1:4	A	G	0
.	.	.	.	.	.	.	.	.	.	.	SOM	GT:/	0:19	0/1:2	G	A	0
.	0	0	0.0	0.7	0.982	1,D	5.8	3.58	.	.	SOM	GT:/	0:36	0/1:5	C	T	0
.	.	.	0.3	0.4	0.961	1,N	4.9	3.65	.	.	SOM	GT:/	0:11	0/1:1	G	A	0
2E-04	##	0	0.1	0.0	0.178	0.993	3.2	.	.	.	SOM	GT:/	0:51	0/1:7	G	A	0
.	.	.	.	.	.	.	.	.	.	.	SOM	GT:/	0:29	0/1:3	C	T	0
.	.	.	.	.	.	.	2.8	.	.	.	SOM	GT:/	0:35	0/1:5	G	A	0
.	.	.	.	.	.	.	.	.	.	.	SOM	GT:/	0:39	0/1:5	T	A	0
.	.	.	.	.	.	.	.	.	0	0	SOM	GT:/	0:38	0/1:5	G	A	0
.	.	.	.	.	.	.	.	.	.	.	SOM	GT:/	0:15	0/1:2	G	A	0
.	.	.	.	.	.	.	.	.	.	.	SOM	GT:/	0:11	0/1:1	T	A	0
.	.	.	.	.	.	.	.	.	.	.	SOM	GT:/	0:31	0/1:5	G	C	0
.	.	.	.	.	.	.	.	.	.	.	SOM	GT:/	0:85	0/1:1	G	A	0
.	.	.	0.1	0.7	0.972	1.000	2.2	3.43	.	.	SOM	GT:/	0:32	0/1:5	G	T	0
.	.	.	.	.	.	.	.	.	.	.	SOM	GT:/	0:25	0/1:2	G	A	0
.	.	.	0.1	0.9	0.991	1,D	4.8	2.47	.	.	SOM	GT:/	0:56	0/1:7	G	C	0
.	.	.	.	.	.	.	.	.	.	.	SOM	GT:/	0:12	0/1:2	G	C	0
.	.	.	.	.	.	.	.	.	.	.	SOM	GT:/	0:25	0/1:5	G	A	0

.	.	.	.	.	.	.	.	.	.	.	SOM	GT:/	0:26	0/1:3	C	T	0
.	##	0	0.1	0.0	0.008	0.999	.	2.58	.	.	SOM	GT:/	0:17	0/1:3	C	T	0
.	.	.	0.8	0.0	0.025	1,N	.	.	.	.	SOM	GT:/	0:46	0/1:9	C	T	0
.	.	.	.	.	.	1,A	5.9	8.08	.	.	SOM	GT:/	0:46	0/1:6	G	C	0
.	.	.	.	.	.	.	.	.	0	0.1	SOM	GT:/	0:10	0/1:1	T	C	0
.	.	.	.	.	.	.	.	.	.	.	SOM	GT:/	0:40	0/1:7	C	G	0
.	.	.	.	.	.	.	.	.	.	.	SOM	GT:/	0:26	0/1:3	G	C	0
.	.	.	.	.	.	.	.	.	.	.	SOM	GT:/	0:9,(	0/1:1	G	A	0
.	.	.	.	.	.	.	2.4	.	.	.	SOM	GT:/	0:11	0/1:2	C	G	0
.	.	.	.	.	.	.	.	.	.	.	SOM	GT:/	0:16	0/1:2	C	G	0
.	.	.	.	.	.	.	.	.	.	.	SOM	GT:/	0:54	0/1:9	T	G	0
.	.	.	0.0	0.3	0.673	0.578	6	2.71	.	.	SOM	GT:/	0:22	0/1:4	G	C	0
.	.	.	.	.	.	.	3	1.95	.	.	SOM	GT:/	0:20	0/1:3	C	T	0
.	.	.	.	.	.	.	5.1	1.57	.	.	SOM	GT:/	0:65	0/1:1	C	T	0
.	.	.	.	.	.	.	3	1.29	.	.	SOM	GT:/	0:98	0/1:1	G	A	0
.	.	.	.	.	.	.	.	.	.	.	SOM	GT:/	0:17	0/1:2	G	A	0
.	##	0	.	.	.	.	.	2.08	.	.	SOM	GT:/	0:30	0/1:3	C	T	0
.	.	.	.	.	.	.	.	.	.	.	SOM	GT:/	0:24	0/1:6	C	A	0
.	.	.	.	.	.	.	.	.	.	.	SOM	GT:/	0:20	0/1:3	G	T	0
.	.	.	.	.	.	.	.	.	.	.	SOM	GT:/	0:24	0/1:4	C	T	0
.	.	.	.	.	.	.	.	.	.	.	SOM	GT:/	0:78	0/1:1	C	T	0
.	.	.	.	.	.	1,A	5.2	10.2	.	.	SOM	GT:/	0:32	0/1:5	C	A	0
.	0	0	.	.	.	.	.	.	0	0	DB;S	GT:/	0:45	0/1:8	T	C	0
.	.	.	.	.	.	.	.	.	.	.	SOM	GT:/	0:16	0/1:1	T	G	0
.	.	.	.	.	.	.	.	.	.	.	SOM	GT:/	0:15	0/1:1	T	G	0
.	.	.	.	.	.	.	2	.	.	.	SOM	GT:/	0:29	0/1:2	G	T	0
#####	##	0	.	.	.	.	.	.	.	.	SOM	GT:/	0:72	0/1:1	G	A	0
.	.	.	.	.	.	.	.	2.53	.	.	SOM	GT:/	0:14	0/1:2	G	A	0
.	.	.	.	.	.	.	.	.	.	.	SOM	GT:/	0:48	0/1:7	G	C	0
.	##	0	0.0	0.5	0.996	1.000	4.8	4.62	.	.	SOM	GT:/	0:11	0/1:1	G	A	0
.	##	0	.	.	.	.	4.3	2.58	.	.	SOM	GT:/	0:68	0/1:1	G	A	0
.	.	.	.	.	.	.	.	.	0	.	SOM	GT:/	0:8,(	0/1:1	C	T	0
.	.	.	.	.	.	.	.	.	0	.	SOM	GT:/	0:8,(	0/1:1	A	G	0
.	.	.	.	.	.	.	.	.	.	.	SOM	GT:/	0:12	0/1:2	C	G	0
.	.	.	.	.	.	.	.	1.39	.	.	SOM	GT:/	0:11	0/1:1	G	A	0
.	.	.	.	.	.	.	.	.	.	.	SOM	GT:/	0:24	0/1:1	G	A	0
.	.	.	.	.	.	.	.	.	0.1	0.1	SOM	GT:/	0:21	0/1:3	A	G	0
.	.	.	.	.	.	.	.	.	.	.	SOM	GT:/	0:93	0/1:1	G	A	0
.	.	.	.	.	.	.	.	.	.	.	SOM	GT:/	0:26	0/1:4	C	G	0
.	.	.	0.0	0.9	1.0,D	1,D	5	4.63	.	.	SOM	GT:/	0:28	0/1:5	G	A	0
.	.	.	.	.	.	1,A	4.6	3.58	.	.	SOM	GT:/	0:71	0/1:1	C	T	0
.	.	.	0.0	0.1	0.463	.	2.3	4.08	.	.	SOM	GT:/	0:22	0/1:4	C	T	0
.	.	.	0.0	0.6	0.933	1,D	4.2	3.80	.	.	SOM	GT:/	0:77	0/1:1	A	G	0
.	.	.	.	.	.	.	2.2	.	.	.	SOM	GT:/	0:65	0/1:9	G	T	0
.	.	.	.	.	.	.	.	.	.	.	SOM	GT:/	0:11	0/1:2	C	A	0
.	##	0	0.6	0.9	1.0,D	0.836	4	3.94	.	.	SOM	GT:/	0:77	0/1:1	C	T	0
.	.	.	.	.	.	.	.	2.32	.	.	SOM	GT:/	0:43	0/1:9	T	G	0
.	.	.	.	.	.	.	.	.	.	.	SOM	GT:/	0:29	0/1:3	C	T	0
2E-04	##	0	0.9	0.0	0.016	0.983	3.8	.	.	.	SOM	GT:/	0:53	0/1:8	C	T	0
.	.	.	.	.	.	.	.	1.36	.	.	SOM	GT:/	0:22	0/1:2	C	G	0
.	.	.	0.0	0.9	0.999	1,D	3.1	2.73	.	.	SOM	GT:/	0:14	0/1:2	C	G	0
.	.	.	.	.	.	.	3	1.80	.	.	SOM	GT:/	0:94	0/1:1	G	T	0
.	.	.	0.0	0.4	0.697	1,D	4	4.83	.	.	SOM	GT:/	0:33	0/1:4	G	A	0
.	.	.	.	.	.	.	2.3	.	.	.	SOM	GT:/	0:24	0/1:4	G	A	0

.	.	.	.	.	.	.	.	.	.	.	SOM	GT:/	0:51	0/1:8	C	T	0
.	##	0	.	.	.	.	.	.	.	.	SOM	GT:/	0:93	0/1:1	C	T	0
.	.	.	0.6	0.0	0.017	0.921	5.6	1.76	.	.	SOM	GT:/	0:28	0/1:5	C	G	0
.	.	.	.	.	.	.	3.4	3.20	.	.	SOM	GT:/	0:11	0/1:1	G	A	0
.	.	.	.	.	.	.	.	.	0	0	SOM	GT:/	0:82	0/1:1	T	C	0
.	.	.	.	.	.	.	.	.	0	0	SOM	GT:/	0:89	0/1:1	C	T	0
.	.	.	.	.	.	.	.	.	0	0	SOM	GT:/	0:15	0/1:3	C	T	0
.	.	.	0.3	0.9	1.0,D	1,D	6.2	5.79	.	.	SOM	GT:/	0:35	0/1:4	C	T	0
.	.	.	.	.	.	.	.	2.83	.	.	SOM	GT:/	0:79	0/1:1	C	G	0
.	.	.	.	.	.	.	.	.	.	.	SOM	GT:/	0:47	0/1:6	C	T	0
.	.	.	.	.	.	.	2.7	1.91	.	.	SOM	GT:/	0:65	0/1:8	C	T	0
.	##	0	0.0	0.9	1.0,D	1,D	4	3.20	0	.	SOM	GT:/	0:9,(	0/1:1	G	A	0
.	.	.	0.0	0.1	0.347	1,N	.	2.37	.	.	SOM	GT:/	0:37	0/1:5	G	A	0
.	.	.	.	.	.	.	.	.	.	.	SOM	GT:/	0:17	0/1:2	G	C	0
.	.	.	0.0	0.5	0.96,I	1,N	3.9	.	.	.	SOM	GT:/	0:38	0/1:9	C	G	0
.	.	.	0.3	0.0	0.002	1,N	4.3	.	.	.	SOM	GT:/	0:15	0/1:2	C	T	0
.	.	.	.	.	.	.	3.2	.	.	.	SOM	GT:/	0:15	0/1:4	C	G	0
.	.	.	.	.	.	.	.	.	.	.	SOM	GT:/	0:8,(	0/1:1	G	A	0
.	.	.	.	.	.	.	.	.	.	.	SOM	GT:/	0:12	0/1:2	C	T	0
.	.	.	.	.	.	.	.	2.04	.	.	SOM	GT:/	0:18	0/1:2	C	T	0
.	.	.	.	.	.	.	.	.	.	.	SOM	GT:/	0:24	0/1:4	T	G	0
.	.	.	0.0	.	.	1,N	.	.	.	.	SOM	GT:/	0:36	0/1:8	C	A	0
.	.	.	0.0	0.9	1.0,D	1,D	6	4.89	.	.	SOM	GT:/	0:45	0/1:9	G	C	0
.	.	.	.	.	.	.	.	.	.	.	SOM	GT:/	0:10	0/1:6	C	T	0
.	.	.	.	.	.	.	.	.	.	.	SOM	GT:/	0:43	0/1:7	C	T	0
.	.	.	.	.	.	.	.	.	.	.	SOM	GT:/	0:21	0/1:1	G	A	0
.	##	0	.	.	.	.	.	.	.	.	SOM	GT:/	0:47	0/1:9	G	A	0
.	.	.	.	.	.	.	.	.	.	.	SOM	GT:/	0:37	0/1:6	G	A	0
.	.	.	.	.	.	.	.	.	.	.	SOM	GT:/	0:45	0/1:5	C	G	0
.	.	.	.	.	.	.	2.7	2.30	.	.	SOM	GT:/	0:15	0/1:1	A	C	0
.	.	.	0.0	0.9	0.998	1.000	5.2	4.92	.	.	SOM	GT:/	0:25	0/1:3	G	C	0
.	.	.	.	.	.	1.000	3.6	1.82	.	.	SOM	GT:/	0:65	0/1:1	G	C	0
.	.	.	.	.	.	.	.	.	.	.	SOM	GT:/	0:32	0/1:3	C	A	0
.	0	0	.	.	.	.	5.1	1.83	.	.	SOM	GT:/	0:99	0/1:1	G	T	0
.	.	.	.	.	.	.	.	.	0	0.3	SOM	GT:/	0:8,(	0/1:7	C	T	0
.	.	.	.	.	.	.	.	.	.	.	SOM	GT:/	0:16	0/1:1	A	C	0
.	.	.	.	.	.	.	2.2	.	.	.	SOM	GT:/	0:53	0/1:8	G	A	0
.	.	.	.	.	.	.	.	.	.	.	SOM	GT:/	0:16	0/1:2	G	A	0
.	.	.	.	.	.	.	.	.	.	.	SOM	GT:/	0:16	0/1:2	C	T	0
.	.	.	.	.	.	.	.	.	.	.	SOM	GT:/	0:9,(	0/1:1	G	C	0
8E-04	0	0	.	.	.	.	.	.	.	.	SOM	GT:/	0:14	0/1:4	C	T	0
.	.	.	.	.	.	.	.	.	.	.	SOM	GT:/	0:23	0/1:4	C	A	0
.	.	.	.	.	.	.	.	.	.	.	SOM	GT:/	0:22	0/1:4	C	A	0
.	.	.	.	.	.	.	.	.	.	.	SOM	GT:/	0:41	0/1:7	C	A	0
.	.	.	0.0	0.5	0.994	0.662	4.2	3.13	.	.	SOM	GT:/	0:53	0/1:9	G	A	0
.	.	.	.	.	.	.	.	.	.	.	SOM	GT:/	0:14	0/1:2	C	T	0
.	.	.	.	.	.	.	.	.	.	.	SOM	GT:/	0:39	0/1:4	G	A	0
.	.	.	.	.	.	.	.	.	.	.	SOM	GT:/	0:37	0/1:5	G	C	0
.	.	.	.	.	.	.	.	.	0	.	SOM	GT:/	0:67	0/1:9	G	A	0
#####	.	.	.	.	.	.	.	1.75	.	.	SOM	GT:/	0:53	0/1:6	G	A	0
.	.	.	.	.	.	.	.	.	0	0	SOM	GT:/	0:14	0/1:3	T	G	0
.	.	.	.	.	.	.	.	.	.	.	SOM	GT:/	0:58	0/1:6	G	A	0
.	.	.	.	.	.	.	.	.	0	0.1	DB;	GT:/	0:31	0/1:6	G	A	0
.	.	.	.	.	.	.	.	.	.	.	SOM	GT:/	0:20	0/1:3	C	T	0

.	.	.	.	.	.	.	.	.	.	.	SOM	GT:/	0:9,(	0/1:3	C	A	0
.	##	0	.	.	.	.	4.2	1.36	.	.	SOM	GT:/	0:13	0/1:2	C	T	0
.	.	.	.	.	.	.	.	.	.	.	SOM	GT:/	0:13	0/1:1	T	G	0
.	.	.	.	.	.	.	.	1.97	.	.	SOM	GT:/	0:24	0/1:4	T	G	0
.	.	.	.	.	.	.	.	.	.	.	SOM	GT:/	0:48	0/1:8	T	C	0
.	.	.	.	.	.	.	.	.	0	0	SOM	GT:/	0:60	0/1:1	C	A	0
.	.	.	.	.	.	.	.	1.94	.	.	SOM	GT:/	0:87	0/1:1	C	T	0
.	.	.	.	.	.	.	.	3.36	.	.	SOM	GT:/	0:41	0/1:9	C	T	0
.	.	.	0.0	0.9	1.0,D	1,D	5.8	4.85	.	.	SOM	GT:/	0:76	0/1:1	C	G	0
.	##	0	.	.	.	.	.	.	.	.	SOM	GT:/	0:77	0/1:1	G	A	0
.	.	.	.	.	.	.	.	.	.	.	SOM	GT:/	0:16	0/1:2	G	T	0
.	.	.	.	.	.	.	.	.	.	.	SOM	GT:/	0:29	0/1:7	C	T	0
.	.	.	.	.	.	.	.	.	.	.	SOM	GT:/	0:33	0/1:5	C	T	0
.	.	.	.	.	.	.	.	.	0	0	SOM	GT:/	0:9,(	0/1:1	G	A	0
.	.	.	.	.	.	.	.	.	.	.	SOM	GT:/	0:16	0/1:3	G	A	0
.	.	.	.	.	.	.	3	1.84	.	.	SOM	GT:/	0:23	0/1:3	G	A	0
.	.	.	.	.	.	.	.	.	.	.	SOM	GT:/	0:23	0/1:3	C	T	0
.	.	.	.	.	.	.	.	.	0	.	SOM	GT:/	0:56	0/1:1	C	T	0
.	.	.	.	.	.	.	.	.	.	.	SOM	GT:/	0:52	0/1:8	C	A	0
.	.	.	.	.	.	.	.	.	.	.	SOM	GT:/	0:22	0/1:3	G	C	0
.	.	.	.	.	.	.	.	.	.	.	SOM	GT:/	0:29	0/1:5	G	A	0
.	.	.	.	.	.	.	.	.	.	.	SOM	GT:/	0:11	0/1:2	C	T	0
.	.	.	0.0	0.9	0.999	1,D	4	4.10	.	.	SOM	GT:/	0:97	0/1:2	C	T	0
.	.	.	0.0	0.0	0.214	1,N	.	.	.	.	SOM	GT:/	0:98	0/1:1	C	G	0
.	.	.	.	.	.	.	.	.	0	0	DB;S	GT:/	0:22	0/1:3	T	C	0
.	.	.	.	.	.	.	.	.	.	.	SOM	GT:/	0:27	0/1:8	C	T	0
.	.	.	0.2	0.1	0.587	1,D	5.5	2.88	.	.	SOM	GT:/	0:55	0/1:1	C	G	0
.	.	.	.	.	.	.	.	.	.	.	SOM	GT:/	0:10	0/1:2	C	G	0
.	.	.	.	.	.	.	.	.	0	.	SOM	GT:/	0:14	0/1:3	C	T	0
.	##	0	0.8	0.0	0.102	1,N	.	.	0	.	SOM	GT:/	0:31	0/1:6	C	T	0
.	##	0	.	0.9	0.999	1,D	5.7	3.83	.	.	SOM	GT:/	0:89	0/1:1	G	A	0
.	.	.	.	.	.	.	.	.	.	.	SOM	GT:/	0:28	0/1:5	C	T	0
.	.	.	.	.	.	.	.	.	.	.	SOM	GT:/	0:44	0/1:5	G	A	0
.	.	.	.	.	.	1,A	5.1	5.03	.	.	SOM	GT:/	0:67	0/1:8	G	A	0
.	.	.	0.0	0.2	0.759	0.676	4.4	4.04	.	.	SOM	GT:/	0:57	0/1:1	C	T	0
.	.	.	.	.	.	.	.	.	.	.	SOM	GT:/	0:21	0/1:2	G	A	0
.	.	.	.	.	.	.	.	.	0	.	SOM	GT:/	0:11	0/1:1	C	T	0
.	##	0	.	.	.	.	.	.	0	0	SOM	GT:/	0:10	0/1:6	T	C	0
.	##	0	0.1	0.9	0.997	1.000	5.9	4.60	.	.	SOM	GT:/	0:46	0/1:8	C	T	0
.	.	.	.	.	.	.	.	.	.	.	SOM	GT:/	0:45	0/1:6	G	A	0
.	.	.	.	.	.	.	.	.	.	.	SOM	GT:/	0:8,(	0/1:2	C	T	0
.	.	.	.	.	.	.	.	.	.	.	SOM	GT:/	0:62	0/1:1	A	G	0
.	.	.	0.1	0.9	0.999	1,D	5.8	5.29	.	.	SOM	GT:/	0:72	0/1:1	C	G	0
.	.	.	.	.	.	.	.	.	.	.	SOM	GT:/	0:10	0/1:2	G	A	0
.	.	.	.	.	.	.	.	.	.	.	SOM	GT:/	0:65	0/1:1	G	A	0
.	.	.	.	.	.	.	.	.	.	.	SOM	GT:/	0:89	0/1:1	T	A	0
.	.	.	.	.	.	.	2.1	.	.	.	SOM	GT:/	0:12	0/1:1	C	T	0
.	.	.	.	.	.	.	.	.	0	.	DB;S	GT:/	0:32	0/1:6	A	C	0
.	##	0	.	.	.	.	.	.	.	.	SOM	GT:/	0:11	0/1:2	C	G	0
.	.	.	0.0	0.9	0.993	1,D	5.9	5.60	.	.	SOM	GT:/	0:67	0/1:9	C	T	0
.	.	.	0.0	0.9	0.999	1,D	5	4.69	.	.	SOM	GT:/	0:88	0/1:1	C	G	0
.	.	.	.	.	.	.	2.8	2.61	.	.	SOM	GT:/	0:10	0/1:1	T	A	0
.	.	.	.	.	.	.	.	.	0.1	.	SOM	GT:/	0:22	0/1:3	A	G	0
.	.	.	.	.	.	.	.	.	.	.	SOM	GT:/	0:27	0/1:4	G	A	0

.	.	.	.	.	.	.	.	.	.	.	SON	GT:/	0:13	0/1:2	C	T	0
.	##	0	.	.	.	.	.	.	.	.	SON	GT:/	0:12	0/1:2	C	T	0
.	.	.	.	.	.	.	.	.	.	.	SON	GT:/	0:45	0/1:9	C	T	0
.	.	.	0.0(	0.0!	0.332	0.914	.	2.91	.	.	SON	GT:/	0:42	0/1:5	C	G	0
.	.	.	.	.	.	.	4.3	.	.	.	SON	GT:/	0:30	0/1:5	C	T	0
.	.	.	0.0(	0.9!	0.996	1.00(	3.9	4.25	.	.	SON	GT:/	0:52	0/1:8	A	G	0
.	.	.	0.7:	0.1(	0.652	1,N	.	.	.	.	SON	GT:/	0:23	0/1:4	G	C	0
.	.	.	.	.	.	.	.	.	.	.	SON	GT:/	0:23	0/1:4	G	A	0
.	.	.	.	.	.	.	.	.	.	.	SON	GT:/	0:53	0/1:6	C	G	0
.	.	.	.	.	.	.	.	.	.	.	SON	GT:/	0:70	0/1:1	G	C	0
.	.	.	.	.	.	.	.	.	.	.	SON	GT:/	0:34	0/1:6	C	T	0
.	.	.	.	.	.	.	.	.	.	.	SON	GT:/	0:13	0/1:2	G	A	0
.	.	.	.	.	.	.	.	.	0	.	SON	GT:/	0:35	0/1:5	G	A	0
.	.	.	.	.	.	.	.	.	.	.	SON	GT:/	0:26	0/1:3	C	T	0
.	.	.	.	.	.	.	.	2.06	.	.	SON	GT:/	0:70	0/1:1	G	C	0
.	.	.	.	.	.	.	4.3	2.33	.	.	SON	GT:/	0:10	0/1:1	G	A	0
.	.	.	0.0:	0.6:	0.991	1,D	4.8	2.26	.	.	SON	GT:/	0:24	0/1:3	C	G	0
.	.	.	.	.	.	.	4.7	.	.	.	SON	GT:/	0:17	0/1:2	C	A	0
.	.	.	.	.	.	.	.	.	.	.	SON	GT:/	0:13	0/1:3	G	C	0
.	.	.	.	.	.	.	.	.	.	.	SON	GT:/	0:16	0/1:2	G	T	0
.	.	.	.	.	.	.	.	.	.	.	SON	GT:/	0:49	0/1:5	C	T	0
.	.	.	.	.	.	.	.	1.26	.	.	SON	GT:/	0:10	0/1:1	G	A	0
.	.	.	.	.	.	.	4.3	3.84	.	.	SON	GT:/	0:27	0/1:4	G	C	0
.	.	.	.	.	.	.	3.4	2.30	.	.	SON	GT:/	0:50	0/1:7	G	A	0
.	.	.	.	.	.	.	.	.	0	.	SON	GT:/	0:15	0/1:3	C	A	0
.	.	.	0.0(	0.2	0.412	1,D	5.4	2.54	.	.	SON	GT:/	0:73	0/1:1	G	C	0
.	.	.	.	.	.	.	2	.	.	.	SON	GT:/	0:33	0/1:7	C	T	0
.	.	.	.	.	.	.	2.3	.	.	.	SON	GT:/	0:24	0/1:3	G	A	0
.	.	.	.	.	.	.	.	.	.	.	SON	GT:/	0:36	0/1:5	C	T	0
.	.	.	.	.	.	.	.	1.24	.	.	SON	GT:/	0:45	0/1:8	C	T	0
.	.	.	.	.	.	.	.	.	.	.	SON	GT:/	0:33	0/1:4	C	T	0
.	.	.	.	.	.	.	4.4	1.99	.	.	SON	GT:/	0:17	0/1:3	C	G	0
.	.	.	0.0!	0.0(	0.012	0.992	6	2.11	.	.	SON	GT:/	0:24	0/1:3	G	C	0
.	.	.	0.0!	0.0:	0.029	0.985	3.9	1.98	.	.	SON	GT:/	0:24	0/1:3	G	T	0
.	.	.	.	.	.	.	.	.	.	.	SON	GT:/	0:36	0/1:3	T	G	0
.	.	.	.	.	.	.	.	.	.	.	SON	GT:/	0:11	0/1:1	G	C	0
6E-04	0	0	.	.	.	.	2.4	.	.	.	SON	GT:/	0:49	0/1:9	C	T	0
.	.	.	.	.	.	.	5.3	1.40	.	.	SON	GT:/	0:11	0/1:1	C	T	0
.	.	.	.	.	.	.	.	.	0.1	0	SON	GT:/	0:8,(	0/1:1	A	G	0
.	.	.	.	.	.	.	.	.	.	.	SON	GT:/	0:19	0/1:3	G	C	0
.	.	.	.	.	.	.	.	.	.	.	SON	GT:/	0:11	0/1:2	G	A	0
.	.	.	0.0:	0.0!	0.053	0.868	3.3	2.65	.	.	SON	GT:/	0:42	0/1:7	C	G	0
.	.	.	.	.	.	.	2.3	.	.	.	SON	GT:/	0:14	0/1:3	G	C	0
.	.	.	0.0!	.	.	1,D	5.1	4.06	.	.	SON	GT:/	0:30	0/1:6	C	G	0
.	.	.	1.0:	0.0(	0.001	1,D	.	.	.	.	SON	GT:/	0:37	0/1:6	G	A	0
.	.	.	.	.	.	.	.	.	0	0	SON	GT:/	0:20	0/1:3	C	T	0
.	.	.	.	.	.	.	.	.	.	.	SON	GT:/	0:11	0/1:1	G	T	0
.	.	.	0.0(	.	.	1,D	3.8	3.39	.	.	SON	GT:/	0:85	0/1:1	C	T	0
.	.	.	.	.	.	.	2.3	.	.	.	SON	GT:/	0:11	0/1:1	G	A	0
.	.	.	.	.	.	.	.	.	.	.	SON	GT:/	0:13	0/1:3	C	T	0
.	.	.	.	.	.	.	.	.	.	.	SON	GT:/	0:34	0/1:5	C	T	0
.	.	.	.	.	.	.	3.6	.	.	.	SON	GT:/	0:38	0/1:6	G	A	0
.	.	.	.	.	.	.	.	.	.	.	SON	GT:/	0:29	0/1:4	A	G	0
.	.	.	.	.	.	.	.	.	.	.	SON	GT:/	0:18	0/1:2	C	T	0

.	.	.	.	.	.	.	.	.	.	.	SOM	GT:/	0:59	0/1:8	G	A	0
.	.	.	.	.	.	.	.	.	.	.	SOM	GT:/	0:12	0/1:1	G	C	0
.	.	.	.	.	.	.	.	.	.	.	SOM	GT:/	0:38	0/1:7	G	T	0
9E-04	0	0	.	.	.	.	.	.	.	.	SOM	GT:/	0:50	0/1:7	G	A	0
.	.	.	.	.	.	.	.	.	0	0.1	SOM	GT:/	0:9,(	0/1:1	A	T	0
.	.	.	.	.	.	1,A	.	5.18	.	.	SOM	GT:/	0:58	0/1:1	G	T	0
.	.	.	.	.	.	.	.	.	.	.	SOM	GT:/	0:36	0/1:6	G	A	0
.	.	.	.	.	.	.	.	1.54	.	.	SOM	GT:/	0:13	0/1:2	C	T	0
.	.	.	.	.	.	.	.	.	.	.	SOM	GT:/	0:13	0/1:2	G	C	0
.	.	.	.	.	.	.	.	.	.	.	SOM	GT:/	0:8,(	0/1:1	G	A	0
.	.	.	.	.	.	.	.	.	.	.	SOM	GT:/	0:69	0/1:1	T	A	0
.	.	.	.	.	.	.	.	.	.	.	SOM	GT:/	0:9,(	0/1:5	A	G	0
.	.	.	.	.	.	.	.	.	.	.	SOM	GT:/	0:13	0/1:2	G	C	0
.	.	.	.	.	.	.	.	.	.	.	SOM	GT:/	0:39	0/1:6	C	G	0
.	.	.	.	.	.	1,A	2.1	49.9	.	.	SOM	GT:/	0:95	0/1:1	G	T	0
.	.	.	0.0	0.2	0.376	1,N	.	.	.	.	SOM	GT:/	0:89	0/1:1	G	A	0
.	.	.	.	.	.	.	.	.	.	.	SOM	GT:/	0:62	0/1:8	G	A	0
.	##	0	0.0	0.6	0.7,P	1,N	.	1.70	.	.	SOM	GT:/	0:10	0/1:1	G	A	0
.	.	.	.	.	.	.	.	.	.	.	SOM	GT:/	0:57	0/1:8	C	T	0
.	.	.	0.0	0.9	1.0,D	0.828	.	2.14	.	.	SOM	GT:/	0:89	0/1:1	G	C	0
.	.	.	.	.	.	.	.	2.25	.	.	SOM	GT:/	0:90	0/1:1	G	A	0
.	.	.	.	.	.	.	.	1.68	.	.	SOM	GT:/	0:71	0/1:8	G	T	0
.	.	.	0.1	0.6	0.958	1,N	.	.	.	.	SOM	GT:/	0:31	0/1:7	C	G	0
.	0	0	0.1	0.0	0.013	1,N	.	.	.	.	SOM	GT:/	0:44	0/1:9	G	A	0
.	.	.	.	.	.	.	.	.	.	.	SOM	GT:/	0:9,(	0/1:9	G	A	0
.	.	.	.	.	.	.	.	.	.	.	SOM	GT:/	0:97	0/1:1	A	T	0
.	.	.	.	0.0	0.015	1,N	.	.	.	.	SOM	GT:/	0:18	0/1:3	C	T	0
.	.	.	.	.	.	.	.	.	.	.	SOM	GT:/	0:10	0/1:1	G	A	0
.	.	.	.	.	.	.	.	.	.	.	SOM	GT:/	0:39	0/1:5	C	G	0
.	.	.	0.0	0.9	0.998	0.999	6	4.03	.	.	SOM	GT:/	0:70	0/1:1	G	A	0
.	.	.	.	.	.	.	.	3.43	.	.	SOM	GT:/	0:41	0/1:7	G	C	0
.	.	.	.	.	.	.	.	.	.	.	SOM	GT:/	0:11	0/1:2	T	G	0
.	.	.	.	.	.	.	.	.	.	.	SOM	GT:/	0:11	0/1:2	T	C	0
.	##	0	.	0.7	0.999	1,D	.	.	.	.	SOM	GT:/	0:12	0/1:2	C	T	0
.	.	.	.	.	.	.	.	.	.	.	SOM	GT:/	0:32	0/1:4	C	T	0
.	.	.	.	.	.	.	.	3.20	.	.	SOM	GT:/	0:12	0/1:1	G	A	0
.	.	.	.	.	.	.	.	.	0	0	SOM	GT:/	0:11	0/1:1	C	T	0
.	.	.	.	.	.	.	5.4	1.35	.	.	SOM	GT:/	0:50	0/1:8	G	C	0
.	.	.	.	.	.	.	.	.	.	.	SOM	GT:/	0:68	0/1:7	C	T	0
.	.	.	.	.	.	.	.	.	0	.	SOM	GT:/	0:33	0/1:5	G	A	0
.	.	.	.	.	.	.	.	.	.	.	SOM	GT:/	0:8,(	0/1:1	G	C	0
0.003	0	0	.	.	.	.	.	.	.	0	DB;S	GT:/	0:52	0/1:7	G	A	0
.	.	.	0.5	0.0	0.089	1,N	2.3	.	.	.	SOM	GT:/	0:11	0/1:2	C	G	0
.	.	.	.	.	.	.	.	.	.	.	SOM	GT:/	0:35	0/1:4	G	T	0
.	.	.	.	.	.	.	.	.	.	.	SOM	GT:/	0:13	0/1:3	C	A	0
.	.	.	.	.	.	.	.	.	.	.	SOM	GT:/	0:79	0/1:1	G	A	0
.	.	.	.	.	.	.	.	.	0	.	SOM	GT:/	0:8,(	0/1:1	T	G	0
.	.	.	.	.	.	.	.	.	.	.	SOM	GT:/	0:34	0/1:2	G	A	0
.	.	.	0.4	0.3	0.824	1,N	3.2	.	.	.	SOM	GT:/	0:55	0/1:9	G	C	0
.	.	.	0.0	0.9	1.0,D	0.999	.	1.34	.	.	SOM	GT:/	0:49	0/1:8	G	C	0
.	.	.	0.0	0.0	0.026	1.000	.	.	.	.	SOM	GT:/	0:44	0/1:7	G	C	0
.	.	.	.	.	.	.	.	.	.	.	SOM	GT:/	0:10	0/1:2	C	T	0
.	.	.	.	.	.	.	.	.	.	.	SOM	GT:/	0:20	0/1:2	T	G	0
.	.	.	.	.	.	.	.	.	.	.	SOM	GT:/	0:14	0/1:2	G	A	0

.	.	.	0.0(	0.8(	0.994	1,N	.	1.83	.	.	SON	GT:/	0:80	0/1:1	G	A	0
.	##	0	0.0'	0.8!	0.99,I	0.998	.	3.67	.	.	SON	GT:/	0:23	0/1:5	C	G	0
.	.	.	.	.	.	.	2	.	.	.	SON	GT:/	0:32	0/1:8	C	G	0
.	.	.	.	.	.	.	5.2	3.13	.	.	SON	GT:/	0:32	0/1:7	G	A	0
.	.	.	.	.	.	.	.	.	0.1	0.2	SON	GT:/	0:21	0/1:5	C	G	0
.	0	0	0.0:	0.5	0.526	0.995	.	2.37	0.1	0.3	DB;S	GT:/	0:22	0/1:5	G	A	0
.	.	.	.	.	.	.	.	.	.	.	SON	GT:/	0:30	0/1:3	C	T	0
.	.	.	.	.	.	.	.	1.29	.	.	SON	GT:/	0:91	0/1:1	C	G	0
#####	##	0	0.0(	0.0	0.0,B	0.998	2.7	1.92	.	.	SON	GT:/	0:63	0/1:9	C	T	0
.	.	.	.	.	.	1,A	4	4.42	.	.	SON	GT:/	0:64	0/1:9	C	T	0
.	.	.	.	.	.	1,A	5.5	8.81	.	.	SON	GT:/	0:20	0/1:5	C	T	0
.	.	.	.	.	.	.	4.1	.	.	.	SON	GT:/	0:23	0/1:5	C	T	0
.	.	.	.	.	.	.	.	.	.	.	SON	GT:/	0:15	0/1:1	G	T	0
.	.	.	.	.	.	.	.	.	.	.	SON	GT:/	0:42	0/1:7	G	A	0
.	.	.	.	.	.	.	.	.	0	0	SON	GT:/	0:11	0/1:1	G	A	0
.	.	.	.	.	.	.	.	.	0	0.1	SON	GT:/	0:11	0/1:1	C	T	0
.	.	.	.	.	.	.	.	.	0	0	DB;S	GT:/	0:22	0/1:2	A	C	0
.	.	.	.	.	.	.	.	.	0	0	SON	GT:/	0:23	0/1:5	T	G	0
.	.	.	.	.	.	.	.	.	0.1	0.1	SON	GT:/	0:15	0/1:3	G	A	0
.	.	.	.	.	.	.	.	1.41	.	.	DB;S	GT:/	0:54	0/1:1	C	T	0
.	.	.	.	.	.	.	.	.	0	0	SON	GT:/	0:9,(	0/1:1	C	A	0
.	.	.	.	.	.	.	.	.	.	.	SON	GT:/	0:38	0/1:4	T	C	0
.	.	.	.	.	.	.	.	.	.	.	SON	GT:/	0:32	0/1:2	G	A	0
.	.	.	.	.	.	.	.	.	.	.	SON	GT:/	0:75	0/1:1	G	A	0
.	.	.	0.0(	1.0	1.0,D	1.000	3.7	3.81	.	.	DB;S	GT:/	0:87	0/1:6	G	C	0
.	.	.	.	.	.	.	.	.	0.1	0.4	DB;S	GT:/	0:32	0/1:4	G	A	0
.	.	.	.	.	.	.	.	.	.	.	SON	GT:/	0:42	0/1:4	C	G	0
.	.	.	.	.	.	.	.	.	.	.	SON	GT:/	0:70	0/1:1	C	T	0
.	.	.	.	.	.	.	.	.	.	.	SON	GT:/	0:13	0/1:2	T	A	0
.	.	.	.	.	.	.	.	.	0	0	SON	GT:/	0:11	0/1:2	G	C	0
.	.	.	.	.	.	.	.	.	0	0	SON	GT:/	0:8,(	0/1:1	G	T	0
0.05	0	0	0.5	0.0	0.026	0.999	.	.	0	0	DB;S	GT:/	0:60	0/1:1	C	T	0
.	.	.	.	.	.	.	.	.	.	.	SON	GT:/	0:30	0/1:2	G	A	0
.	.	.	.	.	.	.	.	.	.	0	SON	GT:/	0:8,(	0/1:7	C	G	0
.	.	.	.	.	.	.	.	.	0.2	0.4	SON	GT:/	0:14	0/1:3	G	C	0
.	.	.	.	.	.	.	.	.	0	0	SON	GT:/	0:11	0/1:4	C	T	0
.	.	.	.	.	.	.	3.3	.	.	.	SON	GT:/	0:32	0/1:3	C	G	0
.	.	.	.	.	.	.	.	.	.	.	SON	GT:/	0:12	0/1:1	G	A	0
.	.	.	.	.	.	.	.	1.37	.	.	SON	GT:/	0:17	0/1:2	G	T	0
.	##	0	.	.	.	.	.	2.00	.	.	SON	GT:/	0:12	0/1:1	G	A	0
.	.	.	.	.	.	.	.	.	.	.	SON	GT:/	0:14	0/1:1	G	C	0
.	.	.	0.0:	0.0	0.026	1,N	.	1.58	.	.	SON	GT:/	0:70	0/1:1	G	C	0
#####	##	0	0.0:	0.9:	1.0,D	1.000	4.7	3.81	.	.	SON	GT:/	0:46	0/1:7	C	T	0
.	.	.	0.0(	0.0(	0.016	0.826	4.5	1.39	.	.	SON	GT:/	0:11	0/1:1	G	C	0
#####	##	0	.	.	.	.	.	.	.	.	SON	GT:/	0:88	0/1:1	G	A	0
.	.	.	.	.	.	.	.	.	.	.	SON	GT:/	0:30	0/1:3	C	G	0
.	.	.	.	.	.	.	.	.	.	.	SON	GT:/	0:16	0/1:2	C	T	0
.	.	.	.	.	.	.	.	.	.	.	SON	GT:/	0:9,(	0/1:1	G	A	0
.	##	0	.	.	.	.	.	.	.	.	SON	GT:/	0:12	0/1:1	G	A	0
.	.	.	.	.	.	.	.	.	.	.	SON	GT:/	0:16	0/1:2	C	T	0
.	.	.	.	.	.	.	.	.	.	.	SON	GT:/	0:9,(	0/1:3	G	A	0
.	##	0	.	.	.	.	.	.	.	.	SON	GT:/	0:13	0/1:2	T	C	0
.	.	.	.	.	.	.	.	.	0	0	SON	GT:/	0:19	0/1:1	G	A	0
.	.	.	0.0!	0.0(	0.005	1,N	3.9	.	.	.	SON	GT:/	0:47	0/1:8	C	G	0

.	.	.	.	.	.	1,D	5.6	5.60	.	.	SOM	GT:/	0:67	0/1:1	C	A	0
.	.	.	.	.	.	.	.	.	.	.	SOM	GT:/	0:27	0/1:5	G	T	0
.	.	.	0.0	0.1	0.645	1,D	5.9	4.00	.	.	SOM	GT:/	0:60	0/1:7	G	A	0
.	.	.	.	.	.	.	.	.	.	.	SOM	GT:/	0:53	0/1:7	G	A	0
.	.	.	.	.	.	.	.	.	.	.	SOM	GT:/	0:20	0/1:3	G	A	0
.	.	.	.	.	.	.	.	2.04	.	.	SOM	GT:/	0:32	0/1:4	C	G	0
.	.	.	.	.	.	.	5.1	1.74	.	.	SOM	GT:/	0:64	0/1:8	G	A	0
.	.	.	.	.	.	.	2.6	2.43	.	.	SOM	GT:/	0:34	0/1:6	A	G	0
.	.	.	.	.	.	.	.	.	.	.	SOM	GT:/	0:53	0/1:1	G	A	0
.	.	.	.	.	.	.	.	.	.	.	SOM	GT:/	0:32	0/1:6	C	T	0
.	.	.	.	.	.	.	.	.	0	0	SOM	GT:/	0:14	0/1:2	T	C	0
.	.	.	.	.	.	1,A	4.7	9.66	.	.	SOM	GT:/	0:62	0/1:1	C	G	0
.	.	.	.	.	.	1,A	4.4	6.48	.	.	SOM	GT:/	0:48	0/1:7	G	A	0
.	.	.	.	.	.	.	.	.	.	.	SOM	GT:/	0:23	0/1:4	C	G	0
.	.	.	.	.	.	.	.	.	.	.	SOM	GT:/	0:48	0/1:5	G	A	0
.	.	.	.	.	.	1,A	5.2	5.59	.	.	SOM	GT:/	0:38	0/1:6	C	T	0
.	.	.	0.0	0.7	0.935	1,N	.	1.24	.	.	SOM	GT:/	0:22	0/1:4	G	C	0
.	.	.	0.0	0.5	0.879	1.000	4.9	3.19	.	.	SOM	GT:/	0:74	0/1:9	G	A	0
.	.	.	.	.	.	.	.	.	.	.	SOM	GT:/	0:24	0/1:2	G	A	0
.	.	.	0.4	0.0	0.001	1.000	4.3	1.36	.	.	SOM	GT:/	0:78	0/1:1	C	T	0
.	.	.	0.2	0.0	0.008	0.797	4.1	.	.	.	SOM	GT:/	0:20	0/1:5	G	A	0
.	0	0	0.6	0.8	0.998	0.834	4.1	.	.	.	SOM	GT:/	0:63	0/1:9	G	A	0
.	.	.	.	.	.	.	.	.	0	.	SOM	GT:/	0:15	0/1:2	A	C	0
.	.	.	.	.	.	.	.	.	0	0	SOM	GT:/	0/1:1	0:11	T	C	0
.	.	.	.	.	.	.	.	.	0	0	SOM	GT:/	0/1:1	0:13	G	A	0
.	.	.	.	.	.	.	.	.	0	0	SOM	GT:/	0/1:5	0:17	C	T	0
.	.	.	.	.	.	.	.	.	.	.	SOM	GT:/	0/1:3	0:19	G	T	0
.	.	.	.	.	.	.	.	.	.	.	SOM	GT:/	0/1:4	0:24	G	A	0
.	.	.	.	.	.	.	.	.	0	.	SOM	GT:/	0/1:3	0:20	A	C	0
.	.	.	.	.	.	.	.	.	0.1	0.4	DB;S	GT:/	0/1:4	0:31	C	T	0
.	.	.	.	.	.	.	.	.	0	.	SOM	GT:/	0/1:7	0:40	T	C	0
.	.	.	.	.	.	.	.	.	0	0.1	SOM	GT:/	0/1:1	0:15	C	A	0
.	.	.	.	.	.	.	.	.	0.3	0.4	DB;S	GT:/	0/1:4	0:20	C	G	0
.	.	.	.	.	.	.	.	.	0	0	SOM	GT:/	0/1:1	0:10	G	A	0
.	0	0	0.0	1.0	1.0,D	1.000	4.5	3.37	.	.	DB;S	GT:/	0/1:7	0:46	G	C	0
.	.	.	.	.	.	.	.	.	0	.	SOM	GT:/	0/1:8	0:45	A	C	0
.	##	0	.	.	.	.	.	.	.	.	SOM	GT:/	0/1:1	0:93	C	T	0
.	##	0	0.0	0.9	1.0,D	1.000	4.8	3.42	.	.	SOM	GT:/	0/1:1	0:70	C	T	0
.	.	.	.	.	.	.	.	.	0	0	SOM	GT:/	0/1:2	0:8,(	C	T	0
.	.	.	.	.	.	.	.	.	.	.	SOM	GT:/	0/1:9	0:45	G	C	0
.	.	.	.	.	.	.	.	.	0.3	0.5	DB;S	GT:/	0/1:1	0:19	T	C	0
.	.	.	.	.	.	.	.	.	0.3	0.4	SOM	GT:/	0/1:1	0:17	A	C	0
.	.	.	.	.	.	.	.	.	0	0.1	SOM	GT:/	0/1:9	0:8,(	T	A	0
.	.	.	.	.	.	.	.	.	0	0	SOM	GT:/	0/1:4	0:26	C	A	0
.	.	.	.	.	.	.	.	.	0	0	SOM	GT:/	0/1:1	0:9,(	C	A	0
.	.	.	.	.	.	.	.	.	.	.	SOM	GT:/	0/1:9	0:53	T	A	0
.	.	.	.	.	.	.	.	.	0	.	SOM	GT:/	0/1:4	0:28	A	T	0
.	.	.	.	.	.	.	.	.	0	.	SOM	GT:/	0/1:6	0:45	C	G	0
.	.	.	.	.	.	.	.	.	0	.	SOM	GT:/	0/1:2	0:10	G	A	0
.	.	.	.	.	.	.	.	.	0	.	SOM	GT:/	0/1:1	0:8,(	T	A	0
.	.	.	.	.	.	.	.	.	.	.	SOM	GT:/	0/1:6	0:43	G	T	0
.	.	.	.	.	.	.	.	.	0	.	SOM	GT:/	0/1:1	0:8,(	G	C	0
.	.	.	.	.	.	.	.	.	0	0	SOM	GT:/	0/1:1	0:11	G	A	0
.	.	.	.	.	.	.	.	.	0	0	SOM	GT:/	0/1:1	0:12	G	A	0

.	.	.	.	.	.	.	.	.	0	.	SON	GT:/	0/1:9	0:43	T	G	0
.	.	.	.	.	.	.	.	.	0	0.2	SON	GT:/	0/1:1	0:12	T	A	0
.	.	.	.	.	.	.	.	.	0	0	SON	GT:/	0/1:2	0:10	T	C	0
.	.	.	.	.	.	.	.	.	0	0	SON	GT:/	0/1:2	0:10	A	G	0
.	.	.	.	.	.	.	.	.	.	.	SON	GT:/	0/1:3	0:23	A	C	0
.	.	.	.	.	.	.	.	.	0	0.1	SON	GT:/	0/1:2	0:15	T	C	0
.	.	.	.	.	.	.	.	.	0.2	0.5	SON	GT:/	0/1:1	0:9,(	A	C	0
.	.	.	.	.	.	.	.	.	.	.	SON	GT:/	0/1:3	0:52	G	C	0
.	.	.	.	.	.	.	.	.	.	.	SON	GT:/	0/1:4	0:8,(	G	A	0
.	.	.	.	.	.	.	.	.	.	.	SON	GT:/	0/1:9	0:69	G	T	0
.	0	0	0.0	0.1(	0.431	1,N	4.8	2.17	0.1	.	SON	GT:/	0/1:2	0:9,(	G	A	0
.	.	.	.	.	.	.	.	.	0	.	SON	GT:/	0/1:7	0:33	G	A	0
.	.	.	.	.	.	.	.	.	0	0	SON	GT:/	0/1:7	0:37	A	G	0
.	.	.	.	.	.	.	.	.	.	.	SON	GT:/	0/1:7	0:52	G	T	0
.	.	.	.	.	.	.	.	.	0	.	SON	GT:/	0/1:1	0:14	A	G	0
.	.	.	.	.	.	.	.	.	.	.	SON	GT:/	0/1:1	0:70	C	T	0
.	.	.	.	.	.	.	.	.	.	.	SON	GT:/	0/1:1	0:10	T	C	0
.	.	.	.	.	.	.	.	.	.	.	SON	GT:/	0/1:4	0:25	A	T	0
.	.	.	.	.	.	.	.	.	0	0	SON	GT:/	0/1:4	0:8,(	C	T	0
.	.	.	.	.	.	.	3.9	.	0	.	SON	GT:/	0/1:1	0:12	C	G	0
.	.	.	.	.	.	.	.	1.88	0	.	SON	GT:/	0/1:2	0:20	T	G	0
.	.	.	.	.	.	.	2.5	.	.	.	SON	GT:/	0/1:7	0:25	G	A	0
.	.	.	.	.	.	.	.	.	0	.	SON	GT:/	0/1:2	0:24	T	G	0
.	.	.	.	.	.	.	.	.	0	.	SON	GT:/	0/1:2	0:23	T	G	0
.	.	.	.	.	.	.	.	.	0	0.2	SON	GT:/	0/1:1	0:14	G	A	0
.	.	.	.	.	.	.	.	.	0	0.1	SON	GT:/	0/1:3	0:10	A	G	0
.	.	.	.	.	.	.	.	.	.	.	SON	GT:/	0/1:9	0:34	A	G	0
.	.	.	.	.	.	.	.	.	.	.	SON	GT:/	0/1:1	0:57	C	T	0
4E-04	0	0	.	.	.	.	.	.	0	.	SON	GT:/	0/1:3	0:27	G	A	0
.	.	.	.	.	.	.	.	.	.	.	SON	GT:/	0/1:6	0:32	G	A	0
.	.	.	.	.	.	.	.	.	0	0	SON	GT:/	0/1:4	0:23	C	T	0
.	.	.	.	.	.	.	.	.	0.1	0.4	SON	GT:/	0/1:1	0:9,(	G	T	0
.	.	.	.	.	.	.	.	.	0	.	SON	GT:/	0/1:3	0:20	T	C	0
.	.	.	.	.	.	.	.	.	0	.	SON	GT:/	0/1:1	0:59	G	A	0
.	.	.	.	.	.	.	.	.	0	.	SON	GT:/	0/1:1	0:18	A	C	0
.	.	.	.	.	.	.	.	.	.	.	SON	GT:/	0/1:2	0:15	A	G	0
.	.	.	0.1(	0.1:	0.05,I	0.695	.	1.65	.	.	SON	GT:/	0/1:1	0:74	C	A	0
.	.	.	.	.	.	.	.	.	.	.	SON	GT:/	0/1:9	0:45	C	A	0
#####	##	0	.	.	.	.	2.1	.	.	.	SON	GT:/	0/1:1	0:75	T	C	0
.	.	.	.	.	.	.	.	.	0	.	SON	GT:/	0/1:5	0:35	C	G	0
.	.	.	.	.	.	.	.	.	0	0	DB;S	GT:/	0/1:1	0:59	A	G	0
.	.	.	.	.	.	.	.	.	0	.	DB;S	GT:/	0/1:1	0:43	G	T	0
.	##	0	.	.	.	.	.	.	.	.	SON	GT:/	0/1:1	0:77	C	T	0
.	.	.	.	.	.	.	.	.	.	.	SON	GT:/	0/1:4	0:24	A	C	0
.	.	.	.	.	.	.	.	.	0	.	SON	GT:/	0/1:1	0:9,(	G	A	0
.	.	.	.	.	.	.	.	.	.	.	SON	GT:/	0/1:5	0:8,(	A	G	0
.	.	.	.	.	.	.	.	.	0	.	SON	GT:/	0/1:4	0:20	A	C	0
.	.	.	.	.	.	.	.	.	0	0	SON	GT:/	0/1:9	0:12	T	A	0
.	.	.	.	.	.	.	.	.	.	.	SON	GT:/	0/1:1	0:8,(	T	G	0
.	.	.	.	.	.	.	.	.	.	.	SON	GT:/	0/1:6	0:46	C	G	0
.	.	.	.	.	.	.	.	.	.	.	SON	GT:/	0/1:4	0:27	C	G	0
.	.	.	.	.	.	.	.	.	.	.	SON	GT:/	0/1:2	0:18	C	G	0
.	.	.	.	.	.	.	.	.	.	.	SON	GT:/	0/1:2	0:10	C	A	0
.	.	.	.	.	.	.	.	.	.	.	SON	GT:/	0/1:3	0:20	G	T	0

.	.	.	.	.	.	.	.	.	.	.	SON	GT:/	0/1:3	0:23	C	T	0
.	.	.	.	.	.	.	.	.	.	.	SON	GT:/	0/1:3	0:23	A	C	0
.	.	.	.	.	.	.	.	.	.	.	DB;S	GT:/	0/1:6	0:39	C	A	0
.	.	.	.	.	.	.	.	.	0	.	DB;S	GT:/	0/1:3	0:22	A	C	0
.	.	.	.	.	.	.	.	.	0	0.4	SON	GT:/	0/1:6	0:8,(	G	T	0
.	.	.	.	.	.	.	.	.	0	.	SON	GT:/	0/1:3	0:9,(	G	A	0
.	.	.	.	.	.	.	.	.	0	0	DB;S	GT:/	0/1:4	0:23	C	T	0
.	.	.	.	.	.	.	.	.	0	.	SON	GT:/	0/1:1	0:9,(	G	A	0
.	.	.	.	.	.	.	.	.	0	0.1	SON	GT:/	0/1:2	0:14	C	G	0
.	.	.	.	.	.	.	4.2	3.16	.	.	SON	GT:/	0/1:1	0:87	T	C	0
.	.	.	.	.	.	.	.	.	0.2	0.3	SON	GT:/	0/1:2	0:24	A	C	0
.	.	.	.	.	.	.	.	.	.	.	SON	GT:/	0/1:6	0:25	T	G	0
.	.	.	.	.	.	.	.	.	.	.	SON	GT:/	0/1:9	0:34	T	G	0
.	.	.	.	.	.	.	.	.	.	.	SON	GT:/	0/1:2	0:18	G	A	0
.	.	.	.	.	.	.	.	.	.	.	SON	GT:/	0/1:4	0:20	A	G	0
.	.	.	.	.	.	.	.	.	.	.	SON	GT:/	0/1:5	0:50	C	G	0
.	.	.	.	.	.	.	.	.	0	.	SON	GT:/	0/1:8	0:37	G	C	0
.	.	.	.	.	.	.	.	.	0	.	SON	GT:/	0/1:2	0:9,(	C	T	0
.	.	.	0.0(	0.9!	0.999	1.000	4.8	4.21	.	.	DB;S	GT:/	0/1:2	0:88	G	A	0
.	0	0	.	.	.	.	.	.	0	0	SON	GT:/	0/1:1	0:14	T	G	0
.	.	.	.	.	.	.	.	.	.	.	SON	GT:/	0/1:3	0:18	G	A	0
.	0	0	.	.	.	.	.	.	0	0	DB;S	GT:/	0/1:8	0:48	A	C	0
.	.	.	.	.	.	.	.	.	0.1	0.2	DB;S	GT:/	0/1:7	0:38	G	A	0
.	.	.	.	.	.	.	.	.	0	0.1	SON	GT:/	0/1:1	0:8,(	T	C	0
.	.	.	.	.	.	.	.	.	0	0	SON	GT:/	0/1:9	0:57	T	C	0
.	.	.	.	.	.	.	.	1.65	0	.	SON	GT:/	0/1:3	0:26	C	G	0
.	.	.	.	.	.	.	.	.	.	.	SON	GT:/	0/1:6	0:37	T	G	0
.	.	.	.	.	.	.	.	.	0	0	SON	GT:/	0/1:9	0:8,(	C	T	0
.	.	.	.	.	.	.	.	.	0	0.2	SON	GT:/	0/1:6	0:8,(	T	C	0
.	.	.	.	.	.	.	.	.	0	.	SON	GT:/	0/1:2	0:16	G	T	0
.	.	.	.	.	.	.	.	.	0	.	SON	GT:/	0/1:1	0:13	T	C	0
.	.	.	.	.	.	.	2.5	.	.	.	SON	GT:/	0/1:1	0:50	T	C	0
.	.	.	.	.	.	.	.	.	0	0.1	SON	GT:/	0/1:1	0:12	A	G	0
.	.	.	.	.	.	.	.	.	.	.	SON	GT:/	0/1:7	0:11	G	A	0
8E-04	0	0	.	.	.	.	.	2.28	.	.	SON	GT:/	0/1:9	0:38	G	A	0
.	.	.	.	.	.	.	.	.	0	0.1	SON	GT:/	0/1:2	0:12	T	C	0
.	1	1	.	.	.	.	.	.	0.1	0.5	SON	GT:/	0/1:7	0:41	C	G	0
.	.	.	.	.	.	.	.	.	0	.	SON	GT:/	0/1:4	0:23	T	C	0
.	.	.	.	.	.	.	.	.	0	.	SON	GT:/	0/1:4	0:24	G	A	0
.	.	.	.	.	.	.	.	.	0	.	SON	GT:/	0/1:1	0:8,(	C	T	0
.	.	.	.	.	.	.	.	.	0	0	SON	GT:/	0/1:3	0:20	T	C	0
.	.	.	0.0,	0.0:	0.05:	1,D	5.1	3.04	.	.	DB;S	GT:/	0/1:6	0:39	G	T	0
.	.	.	.	.	.	.	.	.	0	0	SON	GT:/	0/1:6	0:10	G	A	0
.	.	.	.	.	.	.	4.2	2.69	0	.	SON	GT:/	0/1:1	0:67	G	A	0
.	.	.	.	.	.	.	.	.	.	.	SON	GT:/	0/1:6	0:36	T	G	0
.	.	.	.	.	.	.	.	.	0	.	SON	GT:/	0/1:3	0:12	T	C	0
.	.	.	0.0(	0.2(	0.612	1,D	4.6	3.17	.	.	SON	GT:/	0/1:1	0:70	T	C	0
.	.	.	.	.	.	.	3.9	1.70	0	0	SON	GT:/	0/1:5	0:40	C	T	0
.	.	.	.	.	.	.	.	.	.	.	SON	GT:/	0/1:7	0:30	C	T	0
#####	##	0	.	.	.	.	.	.	.	.	SON	GT:/	0/1:3	0:17	G	A	0
.	.	.	.	.	.	.	.	.	0	0	DB;S	GT:/	0/1:7	0:49	T	C	0
.	.	.	.	.	.	.	.	.	.	.	SON	GT:/	0/1:4	0:8,(	C	T	0
.	.	.	.	.	.	.	.	.	0	0	SON	GT:/	0/1:2	0:10	C	T	0
.	.	.	.	.	.	.	.	.	0	0	SON	GT:/	0/1:2	0:10	T	C	0

.	.	.	.	.	.	.	.	.	.	.	SON	GT:/	0/1:1	0:19	G	T	0
.	.	.	0.5·	0.5!	0.99,I	1,D	3.9	2.76	.	.	SON	GT:/	0/1:1	0:92	G	A	0
.	.	.	.	.	.	.	.	.	0	.	SON	GT:/	0/1:1	0:20	A	T	0
.	.	.	.	.	.	.	.	.	.	.	SON	GT:/	0/1:6	0:29	C	T	0
.	.	.	.	.	.	.	.	.	0	0.1	SON	GT:/	0/1:6	0:9,(	C	T	0
.	.	.	.	.	.	.	.	.	.	.	SON	GT:/	0/1:5	0:22	C	T	0
.	.	.	.	.	.	.	.	.	0	.	SON	GT:/	0/1:4	0:27	A	C	0
.	.	.	.	.	.	.	.	.	.	.	SON	GT:/	0/1:1	0:75	G	C	0
.	.	.	.	.	.	.	.	.	0	0	DB;S	GT:/	0/1:7	0:38	T	C	0
.	.	.	.	.	.	.	.	.	0.1	0.1	DB;S	GT:/	0/1:8	0:41	T	C	0
.	.	.	.	.	.	.	.	.	.	.	SON	GT:/	0/1:4	0:38	G	A	0
.	.	.	.	.	.	.	.	.	.	.	SON	GT:/	0/1:7	0:11	G	A	0
.	.	.	.	.	.	.	.	.	0	0	SON	GT:/	0/1:1	0:11	G	A	0
.	.	.	.	.	.	.	2.1	.	.	.	SON	GT:/	0/1:6	0:39	A	C	0
.	.	.	.	.	.	.	.	2.12	.	.	SON	GT:/	0/1:8	0:44	T	A	0
.	.	.	0.0(	.	.	0.993	.	1.82	.	.	SON	GT:/	0/1:2	0:20	C	T	0
.	.	.	.	.	.	.	.	.	0	0.5	SON	GT:/	0/1:1	0:12	T	G	0
.	.	.	.	.	.	.	.	.	.	.	SON	GT:/	0/1:1	0:10	C	G	0
.	.	.	.	.	.	.	.	.	.	.	DB;S	GT:/	0/1:6	0:34	T	C	0
.	.	.	.	.	.	.	.	.	0	0	SON	GT:/	0/1:2	0:13	G	A	0
.	.	.	.	.	.	.	.	.	0	.	SON	GT:/	0/1:1	0:8,(	C	T	0
.	.	.	.	.	.	.	.	.	0	.	SON	GT:/	0/1:1	0:11	C	T	0
.	.	.	.	.	.	.	.	.	.	.	SON	GT:/	0/1:1	0:12	T	C	0
.	0	0	.	.	.	.	.	.	0	.	SON	GT:/	0/1:4	0:18	T	C	0
.	.	.	.	.	.	.	.	.	0	.	SON	GT:/	0/1:1	0:16	A	T	0
.	.	.	.	.	.	.	.	.	0	.	DB;S	GT:/	0/1:1	0:54	C	T	0
.	.	.	.	.	.	.	.	.	0	.	DB;S	GT:/	0/1:2	0:58	G	A	0
.	.	.	.	.	.	.	.	.	.	.	SON	GT:/	0/1:2	0:20	G	A	0
.	.	.	.	.	.	.	.	.	.	.	SON	GT:/	0/1:4	0:18	A	T	0
.	.	.	.	.	.	.	.	.	.	.	SON	GT:/	0/1:9	0:30	A	G	0
.	.	.	.	.	.	.	.	.	0	.	DB;S	GT:/	0/1:1	0:35	A	G	0
.	.	.	.	.	.	.	.	.	0	.	DB;S	GT:/	0/1:1	0:46	G	A	0
.	.	.	.	.	.	.	.	.	0	0.1	DB;S	GT:/	0/1:8	0:35	T	C	0
.	.	.	.	.	.	.	.	.	0	0.2	DB;S	GT:/	0/1:8	0:35	C	T	0
.	.	.	.	.	.	.	.	.	0	0	DB;S	GT:/	0/1:7	0:30	A	G	0
.	.	.	.	.	.	.	.	.	0	.	DB;S	GT:/	0/1:4	0:27	G	C	0
.	.	.	.	.	.	.	.	.	.	.	DB;S	GT:/	0/1:6	0:33	G	C	0
.	.	.	.	.	.	.	.	.	0	.	DB;S	GT:/	0/1:1	0:46	T	A	0
.	.	.	.	.	.	.	.	.	0	.	SON	GT:/	0/1:6	0:39	C	A	0
.	.	.	.	.	.	.	.	.	0	.	DB;S	GT:/	0/1:1	0:50	G	A	0
.	.	.	.	.	.	.	.	.	.	.	SON	GT:/	0/1:5	0:11	C	T	0
.	.	.	.	.	.	.	.	.	0	.	SON	GT:/	0/1:1	0:9,(	G	A	0
.	.	.	.	.	.	.	.	.	.	.	SON	GT:/	0/1:9	0:48	G	A	0
.	.	.	.	.	.	.	.	.	.	.	SON	GT:/	0/1:8	0:38	T	C	0
.	.	.	.	.	.	.	.	.	0	.	DB;S	GT:/	0/1:6	0:38	G	A	0
.	.	.	.	.	.	.	.	.	0	.	SON	GT:/	0/1:8	0:34	G	A	0
.	.	.	.	.	.	.	.	.	.	.	SON	GT:/	0/1:4	0:10	T	A	0
.	.	.	.	.	.	.	.	.	0	.	SON	GT:/	0/1:2	0:13	T	G	0
.	.	.	.	.	.	.	.	.	.	.	SON	GT:/	0/1:1	0:9,(	G	A	0
.	.	.	.	.	.	.	.	.	0	.	SON	GT:/	0/1:4	0:13	C	T	0
.	##	0	.	.	.	.	.	.	.	.	SON	GT:/	0/1:8	0:46	G	T	0
.	.	.	.	.	.	.	.	.	0	0.2	SON	GT:/	0/1:5	0:28	G	A	0
.	.	.	.	.	.	.	.	.	0	.	SON	GT:/	0/1:3	0:23	A	T	0
.	.	.	.	.	.	.	.	.	0	.	SON	GT:/	0/1:8	0:62	C	G	0

.	.	.	.	.	.	.	.	.	.	.	SON	GT:/	0/1:7	0:67	A	T	0
.	0	0	.	.	.	.	.	.	.	.	SON	GT:/	0/1:3	0:27	G	A	0
.	.	.	.	.	.	.	.	.	.	.	SON	GT:/	0/1:4	0:27	A	C	0
.	.	.	.	.	.	.	2.1	.	.	.	DB;S	GT:/	0/1:5	0:44	T	A	0
.	.	.	.	.	.	.	.	.	0	0	SON	GT:/	0/1:3	0:10	G	T	0
.	.	.	.	.	.	.	.	.	0	.	SON	GT:/	0/1:3	0:17	G	A	0
.	.	.	.	.	.	.	.	.	0	.	SON	GT:/	0/1:2	0:16	C	A	0
.	.	.	.	.	.	.	.	.	0	.	SON	GT:/	0/1:1	0:13	T	A	0
.	0	0	.	.	.	.	.	.	0	0	SON	GT:/	0/1:9	0:49	A	G	0
.	0	0	.	.	.	.	.	1.77	0	0	DB;S	GT:/	0/1:7	0:37	C	T	0
.	0	0	.	.	.	.	.	.	0	0	DB;S	GT:/	0/1:7	0:27	G	A	0
.	0	0	.	.	.	.	.	.	0	0	SON	GT:/	0/1:4	0:38	G	A	0
.	0	0	.	.	.	.	.	.	.	.	SON	GT:/	0/1:8	0:58	C	T	0
.	.	.	.	.	.	.	.	.	0.3	0.4	DB;S	GT:/	0/1:3	0:23	A	G	0
.	.	.	.	.	.	.	.	.	0	0	DB;S	GT:/	0/1:3	0:43	T	C	0
.	.	.	.	.	.	.	.	.	0.1	0.2	SON	GT:/	0/1:1	0:11	G	A	0
.	.	.	.	.	.	.	.	.	.	.	SON	GT:/	0/1:1	0:9,(	A	T	0
.	.	.	.	.	.	.	.	.	0	0	SON	GT:/	0/1:8	0:8,(	A	G	0
.	.	.	.	.	.	.	.	.	.	.	SON	GT:/	0/1:5	0:39	T	G	0
.	.	.	.	.	.	.	.	.	0	0	DB;S	GT:/	0/1:6	0:33	C	T	0
.	.	.	.	.	.	.	.	.	0	0.1	SON	GT:/	0/1:1	0:8,(	G	T	0
.	.	.	.	.	.	.	.	.	0	0	DB;S	GT:/	0/1:6	0:33	C	T	0
.	.	.	.	.	.	.	.	.	0	0	DB;S	GT:/	0/1:4	0:22	T	C	0
.	.	.	.	.	.	.	.	.	0.1	0.3	DB;S	GT:/	0/1:6	0:36	C	T	0
.	.	.	.	.	.	.	.	.	0	0	SON	GT:/	0/1:7	0:35	C	A	0
.	.	.	.	.	.	.	.	.	.	.	SON	GT:/	0/1:6	0:24	G	A	0
.	.	.	.	.	.	.	3.1	1.73	.	.	SON	GT:/	0/1:8	0:39	C	T	0
.	.	.	.	.	.	.	.	.	.	.	SON	GT:/	0/1:1	0:51	G	C	0
.	.	.	.	.	.	.	.	.	.	.	SON	GT:/	0/1:6	0:39	C	T	0
.	.	.	.	.	.	.	.	.	.	.	SON	GT:/	0/1:6	0:38	T	C	0
.	.	.	.	.	.	.	.	.	0	.	SON	GT:/	0/1:5	0:35	A	G	0
.	.	.	.	.	.	.	.	.	.	.	SON	GT:/	0/1:2	0:15	A	C	0
.	.	.	0.2	0.0	0.0,B	1.000	5.3	.	.	.	SON	GT:/	0/1:8	0:38	C	T	0
.	.	.	.	.	.	.	.	.	.	.	SON	GT:/	0/1:3	0:23	T	A	0
.	.	.	0.1	0.0	0.001	1,N	.	.	.	.	SON	GT:/	0/1:1	0:71	G	A	0
.	.	.	.	.	.	.	.	.	0	0	SON	GT:/	0/1:5	0:23	G	A	0
.	.	.	.	.	.	.	.	.	.	.	SON	GT:/	0/1:3	0:16	C	T	0
.	.	.	.	.	.	.	.	.	0	0.1	SON	GT:/	0:11	0/1:1	C	T	0
.	.	.	.	.	.	.	.	.	.	.	SON	GT:/	0:43	0/1:7	C	T	0
0.006	0	0	.	.	.	.	.	1.30	.	.	DB;S	GT:/	0:22	0/1:4	C	T	0
.	.	.	0.4	0.0	0.089	1,N	.	1.28	.	.	SON	GT:/	0:15	0/1:3	G	A	0
.	.	.	0.0	0.9	1.0,D	1.000	.	1.51	.	.	SON	GT:/	0:16	0/1:2	C	T	0
.	.	.	.	.	.	.	.	.	.	.	SON	GT:/	0:46	0/1:9	C	T	0
.	##	0	.	.	.	.	.	.	.	.	SON	GT:/	0:69	0/1:1	C	T	0
.	.	.	.	.	.	.	.	.	.	.	SON	GT:/	0:79	0/1:1	G	A	0
.	.	.	0.2	0.0	0.84,I	1,N	2.7	1.91	.	.	SON	GT:/	0:29	0/1:6	C	T	0
.	##	0	.	.	.	.	.	.	.	.	SON	GT:/	0:51	0/1:9	C	T	0
.	.	.	.	.	.	.	.	.	.	.	SON	GT:/	0:95	0/1:1	G	A	0
#####	##	0	.	.	.	.	.	.	0	.	SON	GT:/	0:12	0/1:2	C	T	0
.	##	0	.	.	.	.	.	.	.	.	SON	GT:/	0:31	0/1:5	G	A	0
.	.	.	.	.	.	.	.	.	.	.	SON	GT:/	0:39	0/1:6	C	T	0
.	.	.	.	.	.	.	.	.	.	.	SON	GT:/	0:19	0/1:2	C	T	0
.	.	.	.	.	.	.	.	.	.	.	SON	GT:/	0:65	0/1:1	C	A	0
.	.	.	.	.	.	.	.	.	.	.	SON	GT:/	0:23	0/1:3	G	A	0

.	.	.	.	.	.	.	.	.	.	.	SON	GT:/	0:77	0/1:1	G	A	0
.	.	.	.	.	.	.	.	.	.	.	SON	GT:/	0:13	0/1:2	A	G	0
.	.	.	.	.	.	.	.	.	.	.	SON	GT:/	0:28	0/1:6	C	A	0
.	.	.	0.1	0.9	1.0,D	0.992	5.5	4.01	.	.	SON	GT:/	0:17	0/1:2	G	T	0
.	.	.	.	.	.	.	.	.	.	.	SON	GT:/	0:29	0/1:4	G	A	0
.	.	.	.	.	.	.	.	.	.	.	SON	GT:/	0:37	0/1:5	C	T	0
.	.	.	0.0	0.9	1.0,D	1.000	4.5	6.11	.	.	SON	GT:/	0:98	0/1:1	C	T	0
.	.	.	0.0	0.9	0.997	1,D	4.8	4.48	.	.	SON	GT:/	0:57	0/1:8	C	T	0
.	.	.	.	.	.	.	.	.	.	.	SON	GT:/	0:22	0/1:5	G	A	0
.	##	0	.	.	.	.	.	2.54	.	.	SON	GT:/	0:12	0/1:1	C	T	0
#####	0	0	.	.	.	.	.	.	.	.	SON	GT:/	0:22	0/1:3	C	T	0
.	.	.	.	.	.	.	.	.	.	.	SON	GT:/	0:16	0/1:2	T	A	0
.	##	0	.	.	.	.	2	1.90	.	.	SON	GT:/	0:69	0/1:8	C	T	0
.	.	.	.	.	.	.	.	.	.	.	SON	GT:/	0:29	0/1:3	C	T	0
#####	##	0	0.1	0.5	0.975	1,N	.	1.35	.	.	SON	GT:/	0:64	0/1:1	G	A	0
.	.	.	.	.	.	.	.	.	.	.	SON	GT:/	0:42	0/1:5	C	T	0
.	.	.	.	.	.	.	.	.	.	.	SON	GT:/	0:22	0/1:3	C	T	0
.	.	.	.	.	.	.	.	.	.	.	SON	GT:/	0:17	0/1:3	A	G	0
0.005	0	0	.	.	.	.	.	.	.	.	DB;S	GT:/	0:11	0/1:2	C	T	0
.	.	.	.	.	.	.	3	1.88	.	.	SON	GT:/	0:33	0/1:5	C	T	0
.	.	.	.	.	.	.	.	3.36	.	.	SON	GT:/	0:11	0/1:6	G	T	0
.	.	.	.	0.8	0.994	1.000	6.1	4.05	.	.	SON	GT:/	0:35	0/1:6	C	T	0
.	.	.	.	.	.	.	.	.	0	0	SON	GT:/	0:65	0/1:9	G	C	0
.	0	0	.	.	.	.	.	.	0	.	SON	GT:/	0:9,(	0/1:1	C	T	0
.	##	0	0.3	0.3	0.975	1,N	.	2.01	.	.	DB;S	GT:/	0:77	0/1:1	G	A	0
#####	##	0	.	.	.	.	.	.	.	.	SON	GT:/	0:10	0/1:1	C	T	0
.	.	.	0.0	0.7	0.998	0.969	2.2	4.05	.	.	SON	GT:/	0:45	0/1:1	G	A	0
0.002	0	0	.	.	.	.	.	.	0	0	SON	GT:/	0:22	0/1:3	G	A	0
.	.	.	.	.	.	.	.	.	0	0.1	SON	GT:/	0:22	0/1:3	C	A	0
.	.	.	.	.	.	.	.	.	0.1	0.2	DB;S	GT:/	0:23	0/1:3	T	C	0
.	.	.	.	.	.	.	.	.	0.2	0.3	DB;S	GT:/	0:56	0/1:8	A	T	0
.	.	.	.	.	.	.	.	.	.	.	DB;S	GT:/	0:24	0/1:3	G	A	0
.	.	.	.	.	.	.	.	.	0	0	DB;S	GT:/	0:44	0/1:7	G	A	0
.	##	.	0.0	0.0	0.065	1.000	4	3.56	.	0	SON	GT:/	0:49	0/1:9	C	T	0
#####	##	0	0.0	0.5	0.98,I	0.999	4.7	2.17	.	.	SON	GT:/	0:11	0/1:1	C	T	0
0.006	0	0	.	.	.	.	.	.	.	.	DB;S	GT:/	0:47	0/1:8	G	A	0
.	.	.	.	.	.	.	.	1.26	.	.	SON	GT:/	0:16	0/1:2	G	A	0
.	.	.	.	.	.	.	.	.	.	.	SON	GT:/	0:46	0/1:7	A	G	0
#####	##	0	.	.	.	.	4.6	1.47	.	.	SON	GT:/	0:36	0/1:6	C	T	0
.	.	.	.	.	.	.	2.2	.	.	.	SON	GT:/	0:82	0/1:1	G	A	0
.	.	.	.	.	.	.	.	.	.	.	SON	GT:/	0:26	0/1:5	G	A	0
.	.	.	.	.	.	.	.	.	.	.	SON	GT:/	0:68	0/1:1	C	T	0
.	.	.	.	.	.	.	.	.	.	.	SON	GT:/	0:78	0/1:1	A	G	0
.	.	.	.	.	.	.	.	.	0	.	SON	GT:/	0:32	0/1:5	C	T	0
.	.	.	0.5	0.0	0.055	1,N	.	.	.	.	SON	GT:/	0:23	0/1:4	C	A	0
.	.	.	.	.	.	.	3	.	.	.	SON	GT:/	0:10	0/1:2	G	A	0
.	.	.	.	.	.	.	.	1.26	.	.	SON	GT:/	0:49	0/1:6	G	T	0
.	.	.	.	.	.	.	.	.	.	.	SON	GT:/	0:8,(	0/1:5	C	T	0
.	##	0	.	.	.	.	.	.	.	.	SON	GT:/	0:40	0/1:6	G	A	0
.	.	.	.	.	.	.	.	1.51	.	.	SON	GT:/	0:17	0/1:4	G	T	0
.	.	.	.	.	.	.	.	1.49	.	.	SON	GT:/	0:37	0/1:8	G	A	0
.	.	.	.	.	.	.	2.5	.	.	.	SON	GT:/	0:13	0/1:3	G	A	0
.	.	.	.	.	.	.	2.4	.	.	.	SON	GT:/	0:16	0/1:2	C	T	0
.	##	0	0.0	0.0	0.006	1,N	3.5	1.43	.	.	SON	GT:/	0:14	0/1:2	C	T	0

.	##	0	0.0	1.0	1.0,D	1.000	5.5	4.84	.	.	SOM	GT:	0:75	0/1:	C	T		0
.	.	.	0.5	0.2	0.937	1,D	5.1	4.66	.	0	SOM	GT:	0:74	0/1:	G	A		0
.	.	.	.	.	.	1,D	5	10.3	.	.	SOM	GT:	0:12	0/1:	C	T		0
.	.	.	0.0	0.0	0.008	1.000	2.2	1.43	.	.	SOM	GT:	0:92	0/1:	C	T		0
.	.	.	.	.	.	.	.	.	.	.	SOM	GT:	0:10	0/1:	G	A		0
.	.	.	.	.	.	.	.	3.14	.	.	SOM	GT:	0:67	0/1:	C	T		0
.	.	.	.	.	.	.	.	2.64	.	.	SOM	GT:	0:17	0/1:	G	A		0
.	.	.	.	.	.	.	.	.	0	.	SOM	GT:	0:58	0/1:	C	T		0
.	.	.	.	.	.	.	.	.	.	.	SOM	GT:	0:67	0/1:	G	A		0
.	.	.	.	.	.	.	2.3	.	.	.	SOM	GT:	0:10	0/1:	G	C		0
.	##	0	.	.	.	.	2.3	1.55	0	.	SOM	GT:	0:78	0/1:	G	A		0
.	.	.	0.0	0.2	0.947	1,N	.	1.35	0	.	SOM	GT:	0:79	0/1:	G	A		0
.	.	.	.	.	.	.	3.2	2.43	.	.	SOM	GT:	0:86	0/1:	G	A		0
.	.	.	0.0	0.7	0.772	1.000	.	3.12	.	.	SOM	GT:	0:57	0/1:	C	A		0
.	.	.	.	.	.	.	.	.	.	.	SOM	GT:	0:15	0/1:	G	A		0
.	.	.	.	.	.	.	.	.	.	.	SOM	GT:	0:22	0/1:	C	T		0
.	.	.	.	.	.	.	2.3	.	.	.	SOM	GT:	0:20	0/1:	A	G		0
.	##	0	0.1	0.0	0.161	1.000	3.8	2.32	.	.	SOM	GT:	0:59	0/1:	G	A		0
.	.	.	.	.	.	.	.	.	.	.	SOM	GT:	0:29	0/1:	G	A		0
.	0	0	.	.	.	.	.	.	0	0	SOM	GT:	0:70	0/1:	C	T		0
.	.	.	.	.	.	.	.	.	.	.	SOM	GT:	0:20	0/1:	C	T		0
.	.	.	.	.	.	.	5.8	4.31	.	.	SOM	GT:	0:53	0/1:	G	A		0
.	.	.	.	.	.	.	5.6	1.81	.	.	SOM	GT:	0:30	0/1:	A	C		0
.	.	.	.	.	.	.	.	.	.	.	SOM	GT:	0:46	0/1:	C	T		0
.	.	.	.	.	.	.	.	.	.	.	SOM	GT:	0:73	0/1:	G	T		0
.	.	.	0.9	0.9	1.0,D	1,D	4.4	5.13	.	.	SOM	GT:	0:13	0/1:	G	A		0
.	.	.	.	.	.	.	3.1	.	.	.	SOM	GT:	0:63	0/1:	T	C		0
.	.	.	.	.	.	.	5.4	1.33	.	.	SOM	GT:	0:30	0/1:	G	A		0
.	.	.	.	.	.	.	4.8	2.89	.	.	SOM	GT:	0:54	0/1:	G	T		0
.	.	.	.	.	.	.	.	.	.	.	SOM	GT:	0:43	0/1:	T	A		0
0.014	0	0	.	.	.	.	.	.	.	.	DB;	GT:	0:33	0/1:	C	T		0
.	##	0	0.0	0.0	0.029	1,D	5.4	3.98	.	.	SOM	GT:	0:12	0/1:	G	A		0
.	.	.	.	.	.	.	.	.	.	.	SOM	GT:	0:52	0/1:	G	A		0
.	.	.	.	.	.	.	.	1.80	.	.	SOM	GT:	0:15	0/1:	C	T		0
.	.	.	.	.	.	.	.	.	.	.	SOM	GT:	0:69	0/1:	C	T		0
.	.	.	0.0	0.9	0.998	1,N	.	3.39	.	.	SOM	GT:	0:13	0/1:	C	A		0
.	.	.	.	.	.	.	.	.	0	0	DB;	GT:	0:32	0/1:	C	T		0
.	.	.	.	.	.	.	3.1	.	.	.	SOM	GT:	0:47	0/1:	A	G		0
.	##	0	.	.	.	.	.	.	.	.	SOM	GT:	0:54	0/1:	C	T		0
.	.	.	.	.	.	.	.	.	.	.	SOM	GT:	0:66	0/1:	C	T		0
.	.	.	.	.	.	.	.	.	.	.	SOM	GT:	0:27	0/1:	G	T		0
.	.	.	.	.	.	.	2.3	.	.	.	SOM	GT:	0:48	0/1:	C	T		0
.	.	.	.	.	.	.	.	.	.	.	SOM	GT:	0:8,	0/1:	C	T		0
.	.	.	.	.	.	.	.	.	.	.	SOM	GT:	0:22	0/1:	C	T		0
.	.	.	0.4	0.7	0.98,	1,D	5.2	4.82	.	.	SOM	GT:	0:48	0/1:	G	A		0
2E-04	##	0	0.0	0.1	0.729	1,D	3.9	4.67	0	.	SOM	GT:	0:51	0/1:	G	A		0
.	##	0	0.0	0.9	1.0,D	1,D	2.8	3.49	0	.	SOM	GT:	0:48	0/1:	C	T		0
.	.	.	.	.	.	.	.	.	.	.	SOM	GT:	0:11	0/1:	C	T		0
.	.	.	.	.	.	.	.	.	.	.	SOM	GT:	0:32	0/1:	T	A		0
.	.	.	.	.	.	.	.	.	.	.	SOM	GT:	0:43	0/1:	C	T		0
.	.	.	.	.	.	.	.	.	.	.	SOM	GT:	0:12	0/1:	C	A		0
.	.	.	.	.	.	.	.	.	.	.	SOM	GT:	0:33	0/1:	A	G		0
.	##	0	.	.	.	.	.	.	.	.	DB;	GT:	0:40	0/1:	G	A		0
.	.	.	.	.	.	.	.	.	.	.	SOM	GT:	0:16	0/1:	G	A		0

.	##	.	.	.	.	.	.	.	.	.	SOM	GT:/	0:54	0/1:8	C	T	0
.	##	0	0.1:	0.0(	0.001	0.990	.	1.97	.	.	SOM	GT:/	0:89	0/1:1	C	T	0
.	.	.	.	.	.	.	.	.	.	.	SOM	GT:/	0:24	0/1:4	C	A	0
.	.	.	.	.	.	.	.	.	.	.	SOM	GT:/	0:26	0/1:4	G	T	0
.	.	.	.	.	.	.	3.5	.	.	.	SOM	GT:/	0:71	0/1:1	G	C	0
.	##	0	.	.	.	.	.	1.40	.	.	DB;S	GT:/	0:48	0/1:9	G	A	0
.	.	.	.	.	.	.	.	.	.	.	SOM	GT:/	0:15	0/1:1	T	A	0
.	.	.	.	.	.	.	.	.	.	.	SOM	GT:/	0:18	0/1:2	A	T	0
.	.	.	0.0	0.9	0.995	1,D	5.6	1.99	.	.	SOM	GT:/	0:48	0/1:7	A	C	0
.	.	.	.	.	.	.	.	.	.	.	SOM	GT:/	0:10	0/1:1	C	T	0
.	.	.	.	.	.	.	.	.	0	.	SOM	GT:/	0:31	0/1:3	G	A	0
.	.	.	0.0	0.9	1.0,D	1,D	4.9	4.51	.	.	SOM	GT:/	0:11	0/1:1	C	T	0
.	.	.	.	.	.	.	.	.	.	.	SOM	GT:/	0:56	0/1:8	T	C	0
.	.	.	.	.	.	1,A	.	3.27	.	.	SOM	GT:/	0:19	0/1:4	C	T	0
.	.	.	.	.	.	.	.	.	.	.	SOM	GT:/	0:10	0/1:1	T	C	0
.	##	0	0.0	0.6	0.997	1.000	4.1	3.60	.	.	SOM	GT:/	0:96	0/1:1	C	T	0
.	.	.	.	.	.	.	5.4	2.78	.	.	SOM	GT:/	0:10	0/1:1	G	A	0
.	.	.	0.0	1.0	1.0,D	1,D	5.5	4.45	.	.	SOM	GT:/	0:78	0/1:8	G	T	0
.	.	.	.	.	.	.	.	.	.	.	SOM	GT:/	0:76	0/1:1	T	G	0
#####	##	0	0.0	0.9	1.0,D	1,D	5.5	5.31	.	.	SOM	GT:/	0:27	0/1:3	C	T	0
.	.	.	.	.	.	.	.	.	0	.	SOM	GT:/	0:60	0/1:7	G	A	0
.	.	.	.	.	.	.	.	.	.	.	SOM	GT:/	0:14	0/1:2	G	T	0
.	.	.	.	.	.	.	.	.	.	.	SOM	GT:/	0:17	0/1:2	C	T	0
.	.	.	.	.	.	.	.	.	.	.	SOM	GT:/	0:11	0/1:1	C	T	0
.	.	.	.	.	.	.	2.2	2.04	.	.	SOM	GT:/	0:34	0/1:5	A	G	0
.	.	.	.	.	.	.	.	.	.	.	SOM	GT:/	0:17	0/1:2	T	G	0
.	0	0	.	.	.	.	.	.	0	0.1	DB;S	GT:/	0:72	0/1:1	T	G	0
.	.	.	.	.	.	.	.	.	.	0	DB;S	GT:/	0:46	0/1:6	G	C	0
.	.	.	.	.	.	.	.	.	.	.	SOM	GT:/	0:12	0/1:2	A	G	0
.	##	0	.	.	.	.	6.2	3.22	.	.	SOM	GT:/	0:11	0/1:2	C	T	0
.	.	.	.	.	.	.	.	.	.	0.1	DB;S	GT:/	0:69	0/1:1	T	A	0
.	.	.	.	.	.	.	.	.	.	.	SOM	GT:/	0:48	0/1:3	C	T	0
.	.	.	.	.	.	.	4.9	3.57	.	.	SOM	GT:/	0:51	0/1:8	C	T	0
.	.	.	.	.	.	.	.	.	.	.	SOM	GT:/	0:13	0/1:1	G	A	0
.	.	.	.	.	.	.	.	.	.	.	SOM	GT:/	0:33	0/1:3	G	A	0
.	.	.	.	.	.	.	.	.	.	.	SOM	GT:/	0:88	0/1:1	C	T	0
.	.	.	.	.	.	.	.	.	.	.	DB;S	GT:/	0:90	0/1:1	C	T	0
.	.	.	.	.	.	1,D	5.5	10.0	.	.	SOM	GT:/	0:62	0/1:1	G	A	0
.	.	.	0.0	0.9	0.999	1.000	6	3.34	.	.	SOM	GT:/	0:66	0/1:7	T	C	0
.	.	.	.	.	.	.	.	.	.	.	SOM	GT:/	0:34	0/1:5	G	A	0
.	.	.	.	.	.	.	.	.	.	.	SOM	GT:/	0:32	0/1:3	C	T	0
.	.	.	.	.	.	.	.	.	.	.	SOM	GT:/	0:54	0/1:7	C	T	0
.	.	.	.	.	.	.	.	.	.	.	SOM	GT:/	0:33	0/1:4	A	G	0
.	##	0	0.0	0.2	0.978	0.997	3.8	3.72	.	.	SOM	GT:/	0:53	0/1:1	G	A	0
.	.	.	.	.	.	1,D	4.3	3.54	.	.	SOM	GT:/	0:10	0/1:1	C	A	0
.	.	.	.	.	.	.	.	1.74	.	.	SOM	GT:/	0:60	0/1:1	C	T	0
.	.	.	0.0	0.0	0.0,B	0.999	4.2	2.12	.	.	SOM	GT:/	0:57	0/1:8	C	A	0
.	.	.	.	.	.	.	.	1.62	.	.	SOM	GT:/	0:23	0/1:3	C	A	0
.	.	.	.	.	.	.	.	.	.	.	SOM	GT:/	0:20	0/1:2	C	T	0
.	.	.	.	.	.	.	.	.	.	.	SOM	GT:/	0:37	0/1:4	T	C	0
.	##	0	0.3	0.0	0.007	1,N	.	.	.	0	SOM	GT:/	0:70	0/1:1	C	T	0
.	.	.	.	.	.	.	.	.	0	0	SOM	GT:/	0:12	0/1:2	A	G	0
.	##	0	.	.	.	.	.	.	.	.	SOM	GT:/	0:42	0/1:5	G	A	0
.	##	0	.	.	.	.	.	.	.	.	SOM	GT:/	0:30	0/1:4	C	T	0

.	.	.	0.0	0.9	1.0,D	0.790	3.9	3.05	.	.	SON	GT:/	0:11	0/1:1	G	A	0
.	##	0	.	.	.	.	2.1	.	.	.	SON	GT:/	0:16	0/1:2	C	T	0
.	.	.	0.1	0.6	0.989	1,N	2.9	1.79	.	.	SON	GT:/	0:80	0/1:1	C	A	0
.	.	.	0.1	0.7	0.958	1,D	6.1	3.94	.	.	SON	GT:/	0:72	0/1:1	A	G	0
.	.	.	.	.	.	.	.	.	.	.	SON	GT:/	0:12	0/1:2	C	T	0
.	.	.	.	.	.	.	.	.	.	.	SON	GT:/	0:15	0/1:3	G	A	0
.	.	.	.	.	.	.	.	.	.	.	SON	GT:/	0:19	0/1:2	A	G	0
.	.	.	.	.	.	.	.	.	.	.	SON	GT:/	0:25	0/1:2	C	T	0
.	.	.	.	.	.	.	.	.	.	.	SON	GT:/	0:11	0/1:2	G	A	0
.	.	.	.	.	.	.	.	.	.	.	SON	GT:/	0:34	0/1:3	T	G	0
.	##	0	.	.	.	.	.	.	.	.	SON	GT:/	0:61	0/1:1	C	T	0
.	.	.	.	.	.	1,D	5.4	3.73	.	.	SON	GT:/	0:10	0/1:1	C	A	0
.	.	.	.	.	.	.	.	.	.	.	SON	GT:/	0:22	0/1:3	C	T	0
.	.	.	.	.	.	.	.	.	.	.	DB;S	GT:/	0:36	0/1:4	C	T	0
.	##	.	0.0	0.8	0.996	1,D	4.2	4.18	.	.	SON	GT:/	0:45	0/1:7	C	A	0
.	##	0	0.5	0.0	0.013	1,N	.	.	.	.	SON	GT:/	0:10	0/1:1	G	A	0
.	.	.	0.0	0.9	1.0,D	0.967	4.8	4.07	.	.	SON	GT:/	0:69	0/1:1	G	A	0
.	##	0	0.0	0.9	1.0,D	1,D	5.6	4.70	.	.	DB;S	GT:/	0:70	0/1:1	C	T	0
.	.	.	0.0	0.6	0.976	1.000	5.1	2.99	.	.	SON	GT:/	0:27	0/1:3	C	A	0
.	.	.	.	.	.	.	.	.	.	.	SON	GT:/	0:22	0/1:4	C	T	0
.	.	.	.	.	.	.	.	.	.	.	SON	GT:/	0:39	0/1:4	A	G	0
#####	##	0	0.0	0.9	1.0,D	1.000	4	4.09	0	.	SON	GT:/	0:44	0/1:8	G	A	0
#####	##	.	.	.	.	.	3.4	.	.	.	SON	GT:/	0:60	0/1:1	C	T	0
#####	##	0	1.0	0.0	0.027	0.972	2.8	.	.	.	SON	GT:/	0:19	0/1:2	G	A	0
.	.	.	.	.	.	.	.	2.55	.	.	SON	GT:/	0:20	0/1:3	G	T	0
.	.	.	.	.	.	.	.	.	.	.	SON	GT:/	0:16	0/1:2	C	T	0
.	.	.	.	.	.	.	.	.	.	.	SON	GT:/	0:53	0/1:8	C	T	0
.	.	.	.	.	.	.	.	.	.	.	SON	GT:/	0:30	0/1:5	T	G	0
.	.	.	.	.	.	.	.	.	.	.	SON	GT:/	0:11	0/1:1	G	A	0
.	.	.	.	.	.	.	.	.	.	.	SON	GT:/	0:60	0/1:7	T	C	0
.	.	.	.	.	.	.	.	.	.	.	SON	GT:/	0:20	0/1:4	C	G	0
.	.	.	.	.	.	.	.	.	.	.	SON	GT:/	0:26	0/1:3	T	C	0
.	.	.	.	.	.	.	.	.	.	.	SON	GT:/	0:61	0/1:1	C	T	0
.	.	.	0.2	0.0	0.102	1,N	.	3.19	.	.	SON	GT:/	0:12	0/1:1	C	T	0
.	.	.	0.0	1.0	1.0,D	1,D	6	3.50	.	.	SON	GT:/	0:32	0/1:5	G	T	0
.	.	.	.	.	.	.	2.3	.	.	.	SON	GT:/	0:57	0/1:9	A	G	0
.	.	.	.	.	.	.	.	.	.	.	SON	GT:/	0:38	0/1:6	G	A	0
.	.	.	0.0	0.6	0.976	0.982	3.2	1.82	.	.	SON	GT:/	0:13	0/1:2	T	C	0
.	.	.	.	.	.	.	.	.	.	.	SON	GT:/	0:57	0/1:6	G	T	0
.	##	0	0.2	0.0	0.021	0.998	.	2.30	.	.	SON	GT:/	0:73	0/1:8	C	T	0
.	0	0	.	.	.	.	.	.	.	0	SON	GT:/	0:18	0/1:4	C	T	0
.	.	.	.	.	.	1,D	6.1	4.56	.	.	SON	GT:/	0:54	0/1:7	C	T	0
.	.	.	.	.	.	.	2.6	.	.	.	SON	GT:/	0:88	0/1:1	C	T	0
.	##	0	.	0.1	0.48,I	1.000	4.9	2.72	0	.	SON	GT:/	0:40	0/1:7	C	T	0
.	.	.	0.1	0.5	0.767	1.000	2.9	3.54	.	.	SON	GT:/	0:31	0/1:6	C	A	0
.	.	.	0.0	0.2	0.274	1.000	5.8	3.31	.	.	SON	GT:/	0:52	0/1:9	T	G	0
.	.	.	.	.	.	.	.	.	.	.	SON	GT:/	0:33	0/1:3	C	T	0
.	.	.	.	.	.	.	.	.	.	.	SON	GT:/	0:60	0/1:1	G	A	0
.	.	.	0.0	0.8	0.999	1,N	.	2.96	.	.	SON	GT:/	0:19	0/1:2	G	A	0
.	.	.	.	.	.	.	.	.	.	.	SON	GT:/	0:13	0/1:3	C	T	0
.	.	.	.	.	.	.	.	.	.	.	SON	GT:/	0:68	0/1:1	G	A	0
.	.	.	0.0	0.0	0.223	0.965	3.9	4.16	.	.	SON	GT:/	0:16	0/1:3	A	T	0
.	.	.	0.0	1.0	1.0,D	1,D	5.3	4.11	.	.	SON	GT:/	0:48	0/1:9	G	A	0
.	.	.	.	.	.	.	.	.	0	.	SON	GT:/	0:26	0/1:2	C	T	0

.	.	.	.	.	.	.	.	.	.		0	SOM	GT:/	0:16	0/1:2	C	T	0
3E-04	0	0	.	.	.	.	.	.	.	.		SOM	GT:/	0:46	0/1:9	C	T	0
.	.	.	.	.	.	.	.	.	.	.		SOM	GT:/	0:34	0/1:5	C	T	0
.	.	.	.	.	.	1,D	6.1	4.95	.	.		SOM	GT:/	0:30	0/1:3	C	T	0
.	.	.	.	.	.	.	.	.	.	.		SOM	GT:/	0:53	0/1:6	G	A	0
0.001	0	0	.	.	.	.	.	.	.	.		SOM	GT:/	0:67	0/1:1	C	T	0
.	##	0	0.4	0.9	1.0,D	1.000	3.6	3.57	0	.		SOM	GT:/	0:47	0/1:7	C	T	0
3E-04	0	0	.	.	.	.	.	.	.	.		SOM	GT:/	0:30	0/1:5	G	A	0
.	##	0	.	.	.	.	.	.	.	.		SOM	GT:/	0:18	0/1:3	G	A	0
.	.	.	0.0	0.2	0.617	1.000	3.9	3.19	.	.		SOM	GT:/	0:18	0/1:3	A	G	0
.	##	0	0.0	0.9	0.999	1,D	4.7	4.09	0	.		SOM	GT:/	0:54	0/1:9	G	A	0
.	.	.	.	.	.	.	.	.	.	.		SOM	GT:/	0:73	0/1:8	T	C	0
.	##	0	0.0	0.9	1.0,D	1,D	4.7	4.78	.	.		SOM	GT:/	0:33	0/1:5	G	A	0
#####	##	0	0.0	0.8	0.998	1,D	5.5	3.85	.	.		SOM	GT:/	0:59	0/1:1	C	T	0
.	.	.	.	.	.	.	.	.	.	.		SOM	GT:/	0:46	0/1:5	G	A	0
.	.	.	.	.	.	.	2.1	1.37	.	.		SOM	GT:/	0:17	0/1:2	C	T	0
2E-04	##	0	.	.	.	.	3.1	.	.	.		SOM	GT:/	0:28	0/1:5	C	T	0
#####	##	0	.	.	.	.	.	.	.	.		SOM	GT:/	0:95	0/1:1	C	T	0
.	##	0	0.0	0.9	1.0,D	1,D	5.6	3.16	.	.		SOM	GT:/	0:72	0/1:1	C	T	0
.	.	.	0.0	0.9	1.0,D	1,D	5.2	6.08	.	.		SOM	GT:/	0:92	0/1:1	G	A	0
.	.	.	.	.	.	.	.	.	.	.		SOM	GT:/	0:34	0/1:7	G	A	0
.	##	0	.	.	.	.	.	.	.	.		SOM	GT:/	0:48	0/1:9	G	A	0
2E-04	##	0	0.2	0.0	0.002	0.988	.	.	.	.		SOM	GT:/	0:71	0/1:9	G	A	0
.	.	.	0.0	0.0	0.002	0.777	.	2.96	.	.		SOM	GT:/	0:57	0/1:1	G	A	0
.	.	.	1.0	0.1	0.645	1,D	4.7	.	.	.		SOM	GT:/	0:87	0/1:1	A	T	0
.	.	.	.	.	.	.	3	.	.	.		SOM	GT:/	0:11	0/1:1	G	A	0
.	.	.	.	.	.	.	.	.	.	.		SOM	GT:/	0:23	0/1:3	G	A	0
.	##	0	0.0	0.9	0.999	1,D	5.6	5.41	0	.		SOM	GT:/	0:34	0/1:6	G	A	0
.	.	.	.	.	.	.	.	.	.	.		SOM	GT:/	0:17	0/1:2	G	A	0
.	.	.	.	.	.	.	.	.	.	.		SOM	GT:/	0:19	0/1:3	A	G	0
.	.	.	0.1	0.0	0.005	1,D	5.3	3.98	.	.		SOM	GT:/	0:50	0/1:7	C	T	0
.	0	0	.	.	.	.	.	.	.	.		DB;S	GT:/	0:46	0/1:7	C	T	0
.	.	.	.	.	.	.	2.4	.	.	.		SOM	GT:/	0:11	0/1:1	T	G	0
.	.	.	.	.	.	.	3.2	.	.	.		SOM	GT:/	0:41	0/1:6	T	C	0
.	.	.	.	.	.	.	.	.	.	.		SOM	GT:/	0:34	0/1:3	C	A	0
.	.	.	.	.	.	.	.	.	.	.		SOM	GT:/	0:27	0/1:4	C	T	0
.	.	.	0.6	0.0	0.02,I	0.992	5.6	1.44	.	.		SOM	GT:/	0:81	0/1:1	C	A	0
.	.	.	.	.	.	.	5.2	2.70	.	.		SOM	GT:/	0:39	0/1:5	T	C	0
.	##	.	.	.	.	.	.	.	.	.		SOM	GT:/	0:16	0/1:2	T	C	0
.	##	0	.	.	.	.	.	.	.	.		SOM	GT:/	0:29	0/1:3	C	T	0
.	.	.	.	.	.	.	2.3	1.51	.	.		SOM	GT:/	0:21	0/1:2	T	C	0
.	.	.	.	.	.	.	.	.	.	.		SOM	GT:/	0:24	0/1:4	C	T	0
.	.	.	.	.	.	.	.	.	.	.		SOM	GT:/	0:16	0/1:3	A	T	0
.	.	.	.	.	.	.	.	1.28	.	.		SOM	GT:/	0:18	0/1:4	A	C	0
.	.	.	0.2	0.0	0.094	0.932	4	.	.	.		SOM	GT:/	0:48	0/1:8	C	T	0
.	.	.	.	.	.	.	.	.	.	.		SOM	GT:/	0:22	0/1:3	A	G	0
.	.	.	.	.	.	.	.	.	.	.		SOM	GT:/	0:52	0/1:7	C	A	0
.	.	.	.	.	.	.	.	.	.	.		SOM	GT:/	0:72	0/1:1	C	T	0
.	.	.	.	.	.	.	.	.	.	.		SOM	GT:/	0:58	0/1:9	C	A	0
.	##	0	.	.	.	.	.	.	.	.		SOM	GT:/	0:95	0/1:1	C	T	0
.	.	.	0.8	0.1	0.571	0.954	4.5	.	.	.		SOM	GT:/	0:30	0/1:5	A	G	0
.	.	.	.	.	.	.	.	.	.	.		SOM	GT:/	0:25	0/1:4	G	A	0
.	.	.	.	.	.	.	2.2	.	.	.		SOM	GT:/	0:55	0/1:7	G	A	0
.	.	.	.	.	.	.	5	.	.	.		SOM	GT:/	0:34	0/1:7	G	T	0

.	.	.	.	.	.	.	.	.	.	.	SOM	GT:/	0:49	0/1:6	T	C	0
.	.	.	.	.	.	.	.	.	.	.	SOM	GT:/	0:28	0/1:6	C	T	0
.	.	.	.	.	.	.	.	1.67	.	.	SOM	GT:/	0:25	0/1:2	A	C	0
.	.	.	.	.	.	.	.	.	0	.	SOM	GT:/	0:24	0/1:2	G	A	0
.	.	.	.	.	.	.	.	.	0.2	0.5	SOM	GT:/	0:8,(	0/1:2	G	A	0
.	.	.	.	.	.	.	.	.	0.3	0.5	SOM	GT:/	0:9,(	0/1:3	G	A	0
2E-04	##	0	.	.	.	.	2.1	3.65	.	.	SOM	GT:/	0:16	0/1:2	C	T	0
.	.	.	.	.	.	.	.	1.49	.	.	SOM	GT:/	0:18	0/1:2	T	G	0
.	.	.	.	.	.	.	.	.	.	.	SOM	GT:/	0:18	0/1:2	T	C	0
.	.	.	.	.	.	.	.	.	0	.	SOM	GT:/	0:8,(	0/1:1	G	A	0
.	.	.	.	.	.	.	.	.	0	.	SOM	GT:/	0:10	0/1:1	C	A	0
.	.	.	.	.	.	.	.	.	0.1	0.1	DB;S	GT:/	0:38	0/1:7	G	A	0
.	0	0	.	.	.	.	2.3	1.39	.	.	SOM	GT:/	0:63	0/1:6	G	A	0
.	.	.	.	.	.	.	.	.	.	.	SOM	GT:/	0:61	0/1:1	A	G	0
.	##	0	.	.	.	.	.	.	.	.	SOM	GT:/	0:76	0/1:1	C	T	0
.	.	.	.	.	.	.	3.4	1.32	.	0	SOM	GT:/	0:43	0/1:6	C	T	0
.	.	.	.	.	.	.	.	.	.	.	SOM	GT:/	0:45	0/1:8	G	A	0
.	.	.	.	.	.	.	.	.	.	.	SOM	GT:/	0:70	0/1:1	T	C	0
.	0	0	.	.	.	.	.	.	.	.	SOM	GT:/	0:14	0/1:2	G	A	0
.	.	.	.	.	.	.	.	.	.	.	SOM	GT:/	0:46	0/1:5	T	C	0
.	.	.	.	.	.	.	.	.	.	.	SOM	GT:/	0:30	0/1:3	C	A	0
.	##	0	0.0(	0.9	1.0,D	1,D	5.7	5.60	.	.	SOM	GT:/	0:68	0/1:1	C	T	0
.	.	.	0.7	0.0	0.003	1,N	.	.	.	.	SOM	GT:/	0:29	0/1:4	G	A	0
.	##	0	.	.	.	.	.	.	.	.	SOM	GT:/	0:60	0/1:9	G	A	0
7E-04	0	0	1.0	0.0	0.002	1,N	2.3	.	.	.	SOM	GT:/	0:27	0/1:3	C	T	0
.	##	0	0.0	0.5	0.993	0.854	4	2.75	.	.	SOM	GT:/	0:42	0/1:4	C	T	0
.	.	.	.	.	.	.	.	.	.	.	SOM	GT:/	0:46	0/1:8	G	A	0
.	.	.	.	.	.	.	.	.	.	.	SOM	GT:/	0:18	0/1:2	A	G	0
.	.	.	0.0(	0.9	0.999	1.000	4.5	3.86	.	.	SOM	GT:/	0:58	0/1:8	A	G	0
.	.	.	.	.	.	.	.	.	.	.	SOM	GT:/	0:91	0/1:1	G	A	0
.	.	.	.	.	.	.	.	.	.	.	SOM	GT:/	0:68	0/1:7	T	C	0
.	##	0	.	.	.	.	.	.	.	0	SOM	GT:/	0:37	0/1:7	C	T	0
.	.	.	.	.	.	.	.	.	0	.	SOM	GT:/	0:35	0/1:3	T	A	0
.	.	.	.	.	.	.	.	.	.	.	SOM	GT:/	0:24	0/1:4	G	A	0
.	0	0	.	.	.	.	.	.	.	.	SOM	GT:/	0:62	0/1:8	C	T	0
.	.	.	.	.	.	.	.	.	0.2	0	DB;S	GT:/	0:30	0/1:4	G	C	0
.	.	.	.	.	.	.	.	.	0	.	SOM	GT:/	0:19	0/1:3	C	T	0
.	.	.	.	.	.	.	.	.	.	.	SOM	GT:/	0:9,(	0/1:7	A	G	0
.	.	.	.	.	.	.	.	.	.	.	SOM	GT:/	0:24	0/1:2	C	T	0
.	.	.	.	.	.	.	.	.	.	.	SOM	GT:/	0:14	0/1:2	C	A	0
.	.	.	0.6	0.4	0.976	0.999	5.4	.	.	.	SOM	GT:/	0:43	0/1:8	G	A	0
.	.	.	.	.	.	.	.	1.25	.	.	SOM	GT:/	0:10	0/1:1	G	T	0
.	.	.	.	.	.	.	.	.	.	.	DB;S	GT:/	0:44	0/1:6	G	A	0
.	.	.	.	.	.	.	.	.	0	.	SOM	GT:/	0:27	0/1:4	C	T	0
.	.	.	.	.	.	.	3	.	.	.	SOM	GT:/	0:10	0/1:1	A	C	0
.	##	0	0.0	1.0	1.0,D	1,D	5.1	4.07	.	.	SOM	GT:/	0:56	0/1:8	C	T	0
#####	##	0	0.1(	0.0	0.157	0.999	5.6	3.15	.	.	SOM	GT:/	0:27	0/1:4	G	A	0
.	.	.	.	.	.	.	.	3.88	.	.	SOM	GT:/	0:33	0/1:4	G	T	0
.	.	.	.	.	.	.	.	1.43	.	.	SOM	GT:/	0:29	0/1:2	C	T	0
.	.	.	.	.	.	.	.	.	.	.	SOM	GT:/	0:56	0/1:1	G	A	0
.	.	.	.	.	.	.	.	.	.	.	SOM	GT:/	0:29	0/1:4	T	G	0
.	.	.	.	.	.	.	.	.	.	.	SOM	GT:/	0:86	0/1:1	G	T	0
.	.	.	.	.	.	.	3.5	1.73	.	.	SOM	GT:/	0:35	0/1:5	A	G	0
.	.	.	.	.	.	1,A	3.6	5.01	.	.	SOM	GT:/	0:39	0/1:5	C	T	0

.	.	.	.	.	.	.	.	.	.	.	SON	GT:/	0:21	0/1:3	T	A	0
.	.	.	.	.	.	.	.	.	.	.	SON	GT:/	0:9,(	0/1:1	G	A	0
.	.	.	.	.	.	.	3.5	1.85	.	.	SON	GT:/	0:32	0/1:3	T	C	0
.	.	.	.	.	.	.	.	3.87	.	.	SON	GT:/	0:25	0/1:4	T	C	0
.	.	.	.	.	.	.	.	.	.	.	SON	GT:/	0:13	0/1:1	G	A	0
.	.	.	.	.	.	.	.	.	.	.	SON	GT:/	0:10	0/1:8	A	T	0
.	.	.	.	.	.	.	.	.	.	.	SON	GT:/	0:8,(	0/1:3	G	A	0
.	0	0	.	.	.	.	.	.	.	.	SON	GT:/	0:21	0/1:2	T	A	0
#####	.	.	0.0	0.8	0.998	1,D	6	4.95	.	.	SON	GT:/	0:77	0/1:1	C	T	0
.	.	.	1.0	.	.	1,N	.	.	.	.	SON	GT:/	0:39	0/1:6	T	C	0
.	.	.	0.1	.	.	1,N	2.8	.	.	.	SON	GT:/	0:60	0/1:8	A	G	0
.	.	.	.	.	.	.	.	3.07	.	.	SON	GT:/	0:32	0/1:5	T	C	0
.	##	.	.	.	.	.	.	.	.	.	SON	GT:/	0:23	0/1:4	G	A	0
.	##	0	.	.	.	.	.	.	.	.	SON	GT:/	0:80	0/1:1	C	T	0
.	.	.	.	.	.	.	5.2	4.01	.	.	SON	GT:/	0:35	0/1:5	G	A	0
.	.	.	0.0	0.0	0.075	1,N	.	2.39	.	.	SON	GT:/	0:10	0/1:1	G	A	0
.	.	.	0.0	1.0	1.0,D	1,D	.	4.12	.	.	DB;S	GT:/	0:21	0/1:4	T	A	0
.	.	.	0.0	0.9	1.0,D	0.959	5.5	5.36	.	.	SON	GT:/	0:81	0/1:9	C	T	0
.	.	.	0.0	0.9	0.986	1.000	5.4	4.37	.	.	SON	GT:/	0:66	0/1:7	T	C	0
.	##	0	.	.	.	.	.	.	.	.	SON	GT:/	0:10	0/1:1	G	A	0
.	.	.	.	.	.	.	.	.	0	0	SON	GT:/	0:12	0/1:2	T	A	0
.	##	0	.	.	.	.	.	.	.	.	SON	GT:/	0:74	0/1:1	A	G	0
6E-04	0	0	.	.	.	.	.	.	.	.	SON	GT:/	0:67	0/1:1	G	A	0
.	0	0	0.0	.	.	1,D	5.8	5.44	.	.	SON	GT:/	0:64	0/1:1	G	A	0
.	.	.	0.2	0.0	0.001	1.000	.	.	.	.	SON	GT:/	0:99	0/1:1	G	C	0
.	.	.	.	.	.	.	.	.	.	.	SON	GT:/	0:18	0/1:3	C	A	0
.	.	.	0.0	0.9	1.0,D	1.000	5.4	4.90	.	.	SON	GT:/	0:49	0/1:1	G	A	0
.	.	.	.	.	.	.	.	.	.	.	SON	GT:/	0:87	0/1:1	C	T	0
.	.	.	.	.	.	.	.	.	.	.	SON	GT:/	0:57	0/1:1	A	G	0
.	.	.	.	.	.	.	.	.	.	.	SON	GT:/	0:19	0/1:2	T	C	0
.	.	.	.	.	.	.	.	.	.	.	SON	GT:/	0:35	0/1:4	G	T	0
.	.	.	0.0	.	.	.	4.8	4.78	.	.	SON	GT:/	0:46	0/1:8	A	T	0
.	.	.	.	.	.	.	2.9	.	.	.	SON	GT:/	0:32	0/1:5	C	A	0
.	.	.	.	.	.	.	.	.	.	.	SON	GT:/	0:48	0/1:7	G	T	0
.	0	0	0.0	0.0	0.0,B	1,N	.	.	.	.	SON	GT:/	0:98	0/1:1	G	A	0
.	.	.	.	.	.	.	2.9	.	.	.	SON	GT:/	0:16	0/1:2	G	T	0
#####	##	0	.	.	.	.	.	.	.	.	SON	GT:/	0:66	0/1:9	G	A	0
.	.	.	.	.	.	.	2.7	.	.	.	SON	GT:/	0:61	0/1:1	G	A	0
.	##	0	.	.	.	.	.	.	.	.	SON	GT:/	0:67	0/1:1	C	T	0
.	0	0	.	.	.	.	.	.	.	0	SON	GT:/	0:80	0/1:1	C	T	0
.	.	.	0.0	0.2	0.171	1,D	6.2	2.79	.	.	SON	GT:/	0:66	0/1:1	T	A	0
.	.	.	.	.	.	.	.	.	.	.	SON	GT:/	0:87	0/1:1	C	T	0
.	.	.	.	.	.	.	.	.	.	.	DB;S	GT:/	0:33	0/1:2	A	T	0
0.226	0	0	.	.	.	.	.	2.16	0.4	0.4	DB;S	GT:/	0:53	0/1:7	C	T	0
.	##	0	0.0	0.9	1.0,D	0.979	3.3	2.52	.	.	SON	GT:/	0:13	0/1:2	G	A	0
.	.	.	.	.	.	.	5.3	2.55	.	.	SON	GT:/	0:18	0/1:4	C	A	0
.	.	.	.	.	.	.	.	.	.	.	SON	GT:/	0:15	0/1:2	G	T	0
.	.	.	.	.	.	.	.	.	.	.	SON	GT:/	0:56	0/1:1	G	A	0
#####	##	0	0.0	0.9	0.995	0.986	5.5	3.13	.	.	SON	GT:/	0:51	0/1:9	G	A	0
.	.	.	.	.	.	.	.	.	.	.	SON	GT:/	0:8,(	0/1:1	C	T	0
.	.	.	.	.	.	.	.	.	.	.	SON	GT:/	0:63	0/1:1	C	T	0
.	.	.	.	.	.	.	2.8	.	.	.	SON	GT:/	0:37	0/1:5	C	T	0
.	.	.	0.0	0.9	1.0,D	1,D	4.4	3.71	.	.	SON	GT:/	0:51	0/1:9	C	T	0
.	.	.	.	.	.	.	.	.	.	.	SON	GT:/	0:18	0/1:3	C	T	0

#####	##	0	.	.	.	.	.	.	.	.	SON	GT:/	0:30	0/1:6	G	A	0
0.013	0	0	.	.	.	.	.	.	0	0	SON	GT:/	0:10	0/1:1	C	T	0
.	.	.	.	.	.	.	.	.	.	.	SON	GT:/	0:15	0/1:9	G	T	0
.	.	.	.	.	.	.	.	.	.	.	SON	GT:/	0:20	0/1:2	C	T	0
.	.	.	.	.	.	.	4.6	2.74	.	.	SON	GT:/	0:49	0/1:9	G	A	0
#####	##	0	.	.	.	.	4.8	1.24	.	.	SON	GT:/	0:29	0/1:4	C	T	0
.	.	.	.	.	.	.	.	.	.	.	SON	GT:/	0:8,0	0/1:1	C	T	0
.	.	.	.	.	.	.	.	2.21	.	.	SON	GT:/	0:41	0/1:5	C	T	0
.	.	.	.	.	.	.	2.5	1.28	.	.	SON	GT:/	0:14	0/1:3	G	A	0
.	.	.	.	.	.	.	.	.	.	.	SON	GT:/	0:19	0/1:3	C	T	0
.	.	.	.	.	.	.	4.1	2.41	.	.	SON	GT:/	0:48	0/1:8	T	C	0
.	##	0	0.0	0.9	1.0,D	0.999	5.2	3.90	0	.	SON	GT:/	0:27	0/1:5	C	T	0
.	.	.	.	.	.	.	.	2.23	.	.	SON	GT:/	0:21	0/1:4	C	T	0
.	.	.	0.0	0.9	1.0,D	1,D	4.8	4.39	.	.	SON	GT:/	0:36	0/1:6	C	T	0
.	.	.	.	.	.	.	.	.	.	.	SON	GT:/	0:19	0/1:3	G	A	0
.	.	.	.	.	.	.	.	.	.	.	SON	GT:/	0:13	0/1:8	G	A	0
.	.	.	.	.	.	.	.	.	0	0	SON	GT:/	0:8,0	0/1:1	C	T	0
.	.	.	.	.	.	.	.	.	.	.	SON	GT:/	0:77	0/1:1	G	T	0
.	.	.	.	.	.	.	.	.	.	.	SON	GT:/	0:16	0/1:2	C	A	0
.	.	.	0.0	0.9	1.0,D	1.000	2.3	3.38	.	.	SON	GT:/	0:16	0/1:2	T	A	0
.	.	.	.	.	.	.	.	.	.	.	SON	GT:/	0:31	0/1:3	T	C	0
.	.	.	.	.	.	.	3.8	1.28	.	.	SON	GT:/	0:26	0/1:4	G	T	0
.	.	.	.	.	.	.	2.1	.	.	.	SON	GT:/	0:40	0/1:7	G	A	0
.	.	.	.	.	.	.	.	1.31	.	.	SON	GT:/	0:39	0/1:6	C	T	0
.	.	.	.	.	.	.	.	.	.	.	SON	GT:/	0:20	0/1:3	A	G	0
.	##	0	.	.	.	.	.	.	.	.	SON	GT:/	0:22	0/1:3	A	G	0
2E-04	##	0	0.3	0.0	0.003	1.000	5.4	1.35	.	.	DB;S	GT:/	0:74	0/1:1	G	A	0
.	.	.	.	.	.	.	.	.	.	.	SON	GT:/	0:26	0/1:4	C	T	0
.	##	0	.	.	.	.	.	.	0	.	SON	GT:/	0:62	0/1:7	C	T	0
.	.	.	.	.	.	.	.	.	.	.	SON	GT:/	0:45	0/1:5	G	T	0
.	##	0	0.5	0.0	0.002	1,N	.	.	0	0	SON	GT:/	0:17	0/1:3	G	A	0
.	.	.	.	.	.	.	.	.	.	.	SON	GT:/	0:56	0/1:6	G	A	0
.	.	.	.	.	.	.	.	.	.	.	SON	GT:/	0:9,0	0/1:2	T	C	0
2E-04	##	0	.	.	.	.	.	.	.	.	SON	GT:/	0:36	0/1:7	G	A	0
.	.	.	.	.	.	.	2.3	.	.	.	SON	GT:/	0:30	0/1:5	C	T	0
.	##	0	.	.	.	.	.	.	.	.	SON	GT:/	0:63	0/1:8	G	A	0
.	.	.	.	.	.	.	.	.	.	.	SON	GT:/	0:49	0/1:7	G	A	0
.	##	0	0.1	0.9	0.995	1,D	5	3.50	.	.	SON	GT:/	0:10	0/1:1	G	A	0
.	.	.	.	.	.	.	.	.	0	.	SON	GT:/	0:21	0/1:3	G	A	0
.	.	.	.	.	.	.	2	.	.	.	SON	GT:/	0:47	0/1:5	G	A	0
.	0	0	0.0	0.0	0.022	0.999	2.9	2.38	.	.	SON	GT:/	0:15	0/1:2	C	T	0
.	##	0	.	.	.	.	.	.	0	.	SON	GT:/	0:16	0/1:2	G	A	0
#####	##	0	1.0	0.0	0.608	0.982	3.6	.	.	.	SON	GT:/	0:64	0/1:1	C	T	0
.	.	.	0.0	1.0	1.0,D	1.000	5.3	3.33	.	.	SON	GT:/	0:10	0/1:1	C	T	0
.	##	0	.	.	.	.	.	.		0	SON	GT:/	0:12	0/1:2	C	T	0
.	.	.	0.0	0.8	0.999	0.998	2.3	4.32	.	.	SON	GT:/	0:10	0/1:1	G	A	0
.	.	.	.	.	.	1,A	.	6.60	.	.	SON	GT:/	0:12	0/1:1	C	T	0
.	.	.	.	.	.	.	2.8	.	.	.	SON	GT:/	0:31	0/1:5	G	T	0
.	##	0	0.1	0.6	0.985	1.000	5.1	4.02	.	.	SON	GT:/	0:15	0/1:2	C	T	0
7E-04	0	0	.	.	.	.	.	.	.	.	SON	GT:/	0:72	0/1:8	G	A	0
.	.	.	.	.	.	.	.	.	.	.	SON	GT:/	0:52	0/1:9	A	G	0
.	##	0	0.0	1.0	1.0,D	1,D	4.4	4.28	.	.	SON	GT:/	0:82	0/1:1	G	A	0
.	.	.	.	.	.	1,D	4.5	3.79	.	.	DB;S	GT:/	0:26	0/1:7	A	G	0
.	.	.	.	.	.	.	.	.	.	.	SON	GT:/	0:38	0/1:5	G	A	0

.	.	.	.	.	.	.	.	2.42	.	.	SOM	GT:/	0:20	0/1:2	A	G	0
.	.	.	.	.	.	.	.	.	.	.	SOM	GT:/	0:12	0/1:2	C	T	0
.	.	.	.	.	.	.	.	.	.	.	SOM	GT:/	0:27	0/1:4	C	T	0
.	.	.	.	.	.	.	.	.	.	.	SOM	GT:/	0:24	0/1:2	A	T	0
.	.	.	0.1:	0.9	0.999	1.000	5.6	5.03	.	.	SOM	GT:/	0:66	0/1:1	T	G	0
.	.	.	0.9	0.3	0.58,I	0.999	5.4	1.88	.	.	SOM	GT:/	0:55	0/1:9	C	T	0
.	.	.	.	.	.	.	.	.	.	.	DB;S	GT:/	0:20	0/1:2	C	T	0
.	.	.	.	.	.	.	.	.	.	.	DB;S	GT:/	0:36	0/1:4	T	C	0
.	.	.	.	.	.	.	.	.	.	.	SOM	GT:/	0:36	0/1:4	T	C	0
.	.	.	.	.	.	.	.	.	.	.	SOM	GT:/	0:13	0/1:3	G	A	0
.	.	.	.	.	.	.	.	1.48	.	.	SOM	GT:/	0:67	0/1:7	C	A	0
.	.	.	.	.	.	.	.	.	.	.	SOM	GT:/	0:61	0/1:8	G	T	0
.	.	.	.	.	.	.	4.2	2.40	.	.	SOM	GT:/	0:54	0/1:5	G	T	0
.	##	0	.	.	.	.	.	2.23	.	.	SOM	GT:/	0:98	0/1:1	C	T	0
.	.	.	0.5	0.0	0.0,B	1,N	.	.	.	.	SOM	GT:/	0:10	0/1:1	A	G	0
.	.	.	0.0:	0.9	0.998	1.000	5.9	3.83	.	.	SOM	GT:/	0:76	0/1:8	C	T	0
.	##	0	.	.	.	.	2	.	.	.	SOM	GT:/	0:29	0/1:3	C	T	0
.	.	.	0.3	0.1	0.575	0.907	5	.	.	.	SOM	GT:/	0:25	0/1:4	G	A	0
.	.	.	.	.	.	.	.	.	.	.	SOM	GT:/	0:20	0/1:2	A	G	0
.	.	.	.	.	.	.	.	.	.	.	SOM	GT:/	0:46	0/1:7	G	T	0
.	.	.	.	.	.	.	2.2	4.04	0	.	SOM	GT:/	0:24	0/1:3	C	T	0
.	.	.	.	.	.	.	2.5	.	.	.	SOM	GT:/	0:28	0/1:4	G	T	0
.	.	.	.	.	.	.	.	.	.	.	SOM	GT:/	0:44	0/1:9	A	C	0
.	.	.	.	.	.	.	.	.	.	.	SOM	GT:/	0:31	0/1:5	C	A	0
#####	##	0	0.0	0.5	0.996	1.000	5.5	4.81	.	.	SOM	GT:/	0:28	0/1:5	G	A	0
.	.	.	.	.	.	.	2.7	.	.	.	SOM	GT:/	0:20	0/1:4	G	A	0
.	.	.	.	.	.	.	.	.	.	.	SOM	GT:/	0:25	0/1:6	G	A	0
.	.	.	.	.	.	.	3.2	.	.	.	SOM	GT:/	0:79	0/1:1	C	A	0
.	0	0	.	.	.	.	.	.	0	0	SOM	GT:/	0:55	0/1:8	G	A	0
.	##	0	.	.	.	.	2.6	1.42	.	.	SOM	GT:/	0:12	0/1:2	G	A	0
.	.	.	.	.	.	.	.	.	.	.	SOM	GT:/	0:26	0/1:7	A	G	0
.	.	.	.	.	.	.	.	.	.	.	SOM	GT:/	0:40	0/1:5	C	T	0
.	.	.	.	.	.	.	.	3.01	.	.	SOM	GT:/	0:25	0/1:4	A	G	0
.	.	.	0.0	0.9	0.999	1,D	3.7	4.27	.	.	SOM	GT:/	0:74	0/1:1	G	A	0
.	.	.	.	.	.	.	.	.	.	.	SOM	GT:/	0:58	0/1:9	C	A	0
.	.	.	0.0	0.9	0.998	1,N	.	1.53	.	.	SOM	GT:/	0:35	0/1:5	G	A	0
.	.	.	.	.	.	.	.	.	.	.	SOM	GT:/	0:94	0/1:1	C	T	0
#####	##	0	.	.	.	.	.	.	.	.	SOM	GT:/	0:31	0/1:5	C	T	0
.	.	.	.	.	.	.	4.6	.	.	.	SOM	GT:/	0:56	0/1:5	G	A	0
.	.	.	.	.	.	.	.	.	.	.	SOM	GT:/	0:10	0/1:1	C	T	0
0.016	0	0	.	.	.	.	.	.	0	0	DB;S	GT:/	0:20	0/1:2	A	T	0
.	##	0	0.1	0.0	0.102	1.000	3.9	3.82	.	.	SOM	GT:/	0:31	0/1:6	C	T	0
.	.	.	.	.	.	.	.	.	.	.	SOM	GT:/	0:46	0/1:6	G	A	0
.	.	.	.	.	.	.	.	.	.	.	SOM	GT:/	0:42	0/1:4	C	T	0
.	##	0	.	.	.	.	3.2	.	0	.	SOM	GT:/	0:62	0/1:9	C	T	0
.	.	.	.	.	.	.	.	.	.	.	SOM	GT:/	0:9,(	0/1:1	C	A	0
.	.	.	.	.	.	.	6.1	2.79	.	.	SOM	GT:/	0:22	0/1:5	G	A	0
.	.	.	.	.	.	.	.	.	.	.	SOM	GT:/	0:98	0/1:1	G	A	0
.	.	.	.	.	.	.	.	.	.	.	SOM	GT:/	0:9,(	0/1:1	G	C	0
.	##	0	0.0	0.9	1.0,D	1.000	6.1	4.63	.	.	SOM	GT:/	0:38	0/1:5	C	T	0
.	.	.	.	.	.	.	.	.	.	.	SOM	GT:/	0:9,(	0/1:1	G	A	0
.	.	.	.	.	.	.	.	1.54	.	.	SOM	GT:/	0:34	0/1:4	G	T	0
.	##	0	.	.	.	.	.	.	.	.	SOM	GT:/	0:47	0/1:1	C	T	0
.	.	.	.	.	.	.	.	.	.	0	SOM	GT:/	0:27	0/1:5	C	T	0

.	.	.	.	.	.	.	5.6	.	.	.	SOM	GT:/	0:82	0/1:1	T	G	0
.	.	.	0.2	0.4	0.836	1.000	5.7	4.22	.	.	SOM	GT:/	0:10	0/1:1	C	T	0
.	.	.	.	.	.	.	.	.	.	.	SOM	GT:/	0:59	0/1:8	C	T	0
.	.	.	.	.	.	.	.	.	.	.	SOM	GT:/	0:60	0/1:8	T	C	0
#####	##	0	0.8	0.0	0.083	1,D	5.1	.	.	.	SOM	GT:/	0:63	0/1:6	G	A	0
.	.	.	.	.	.	.	.	.	.	.	SOM	GT:/	0:39	0/1:6	T	A	0
.	##	0	.	.	.	.	.	1.48	.	.	SOM	GT:/	0:54	0/1:6	C	T	0
.	##	0	.	.	.	.	2.5	.	.	.	SOM	GT:/	0:33	0/1:5	G	A	0
.	.	.	.	.	.	.	.	.	.	.	SOM	GT:/	0:24	0/1:5	C	T	0
.	.	.	.	.	.	.	.	.	0	0	SOM	GT:/	0:10	0/1:1	C	T	0
.	.	.	.	.	.	.	3.1	.	.	.	SOM	GT:/	0:43	0/1:5	C	T	0
.	.	.	.	.	.	.	.	.	.	.	SOM	GT:/	0:51	0/1:7	C	T	0
.	.	.	.	.	.	.	.	.	0	.	SOM	GT:/	0:9,(	0/1:1	G	A	0
.	.	.	.	.	.	.	.	.	0	0	DB;S	GT:/	0:75	0/1:1	A	G	0
.	.	.	.	.	.	.	.	.	.	.	SOM	GT:/	0:8,(	0/1:1	G	T	0
.	##	0	0.0	0.9	1.0,D	0.799	3.3	1.95	0	0	SOM	GT:/	0:80	0/1:1	G	A	0
.	.	.	.	.	.	.	.	.	.	.	SOM	GT:/	0:79	0/1:1	C	A	0
.	.	.	.	.	.	.	.	.	.	.	SOM	GT:/	0:45	0/1:6	C	T	0
.	.	.	.	.	.	.	.	.	0	.	SOM	GT:/	0:43	0/1:6	C	T	0
.	.	.	.	.	.	.	.	.	.	.	SOM	GT:/	0:70	0/1:9	G	A	0
.	.	.	.	.	.	.	.	.	.	.	SOM	GT:/	0:19	0/1:2	G	A	0
.	.	.	.	.	.	.	.	.	.	.	SOM	GT:/	0:68	0/1:8	T	C	0
.	.	.	.	.	.	.	.	.	.	.	SOM	GT:/	0:16	0/1:1	G	A	0
.	.	.	.	.	.	.	.	.	.	.	SOM	GT:/	0:10	0/1:1	G	A	0
.	.	.	.	.	.	1,D	.	3.49	.	.	SOM	GT:/	0:9,(	0/1:2	C	T	0
5E-04	0	0	.	.	.	.	.	.	.	.	DB;S	GT:/	0:14	0/1:2	C	T	0
.	.	.	.	.	.	.	.	.	.	.	SOM	GT:/	0:14	0/1:2	G	A	0
.	.	.	.	.	.	.	.	1.69	.	.	SOM	GT:/	0:72	0/1:1	G	A	0
.	##	0	0.2	0.0	0.004	1,D	4.1	1.85	.	.	SOM	GT:/	0:35	0/1:6	G	A	0
.	0	0	.	.	.	.	.	.	0	0	SOM	GT:/	0:36	0/1:7	C	T	0
.	##	0	.	.	.	.	.	.	.	.	SOM	GT:/	0:12	0/1:2	C	T	0
.	.	.	0.0	0.9	1.0,D	0.996	6.2	4.57	.	.	SOM	GT:/	0:62	0/1:1	G	A	0
.	.	.	0.0	0.5	0.959	0.687	3.7	1.42	.	.	SOM	GT:/	0:60	0/1:5	C	A	0
.	.	.	0.0	1.0	1.0,D	1,D	5.4	4.96	.	.	SOM	GT:/	0:59	0/1:8	G	A	0
.	.	.	.	.	.	.	.	.	.	.	SOM	GT:/	0:31	0/1:4	A	T	0
.	.	.	0.1	0.9	0.994	0.879	5.6	3.33	.	.	SOM	GT:/	0:40	0/1:5	T	C	0
.	.	.	.	.	.	.	.	.	0	0	SOM	GT:/	0:8,(	0/1:6	A	G	0
.	##	0	.	.	.	.	2.2	2.78	.	.	SOM	GT:/	0:20	0/1:2	C	T	0
.	.	.	.	.	.	.	.	.	.	.	SOM	GT:/	0:55	0/1:8	C	T	0
.	.	.	.	.	.	.	.	1.70	.	.	SOM	GT:/	0:27	0/1:4	G	T	0
.	##	0	0.0	0.9	0.998	1,D	5.9	5.07	.	.	SOM	GT:/	0:67	0/1:1	G	A	0
.	.	.	0.2	0.1	0.475	0.999	4.1	2.31	.	.	SOM	GT:/	0:56	0/1:6	A	T	0
.	.	.	.	.	.	.	.	.	.	.	DB;S	GT:/	0:37	0/1:5	C	T	0
.	.	.	.	.	.	.	.	.	0.1	0.2	SOM	GT:/	0:20	0/1:2	C	T	0
.	.	.	.	.	.	.	.	.	0	.	SOM	GT:/	0:8,(	0/1:6	C	T	0
.	.	.	0.1	0.0	0.127	1,N	.	1.50	.	.	SOM	GT:/	0:14	0/1:2	C	T	0
.	.	.	.	.	.	.	.	1.36	.	.	SOM	GT:/	0:13	0/1:2	C	T	0
.	.	.	.	.	.	.	.	.	.	.	SOM	GT:/	0:69	0/1:8	G	C	0
.	.	.	.	.	.	.	.	.	.	.	SOM	GT:/	0:9,(	0/1:1	C	A	0
.	.	.	.	.	.	.	.	.	.	.	SOM	GT:/	0:18	0/1:4	G	A	0
.	.	.	.	.	.	1,A	.	5.21	.	.	SOM	GT:/	0:17	0/1:2	C	T	0
.	.	.	.	.	.	.	3.8	.	.	.	SOM	GT:/	0:30	0/1:4	A	T	0
.	##	0	.	.	.	.	.	.	.	.	SOM	GT:/	0:27	0/1:3	C	T	0
.	.	.	.	.	.	1,D	5.6	4.33	.	.	SOM	GT:/	0:44	0/1:7	C	T	0

.	0	0	.	.	.	.	.	.	0	0	DB;S	GT:/	0:20	0/1:4	T	A	0
.	.	.	.	.	.	.	.	.	.	.	SOM	GT:/	0:14	0/1:1	G	T	0
#####	0	0	0.0	1.0	1.0,D	1.000	3.9	3.90	.	.	SOM	GT:/	0:49	0/1:7	C	T	0
.	.	.	.	.	.	.	4.7	1.51	.	.	SOM	GT:/	0:73	0/1:1	G	T	0
.	##	0	0.0	0.9	1.0,D	1.000	5.7	5.10	.	.	SOM	GT:/	0:53	0/1:8	C	T	0
.	.	.	.	.	.	1,A	.	8.91	.	.	SOM	GT:/	0:34	0/1:7	G	T	0
.	.	.	.	.	.	.	.	1.24	.	.	SOM	GT:/	0:8,(	0/1:1	A	G	0
.	.	.	.	.	.	.	.	.	.	.	SOM	GT:/	0:30	0/1:3	T	C	0
.	.	.	0.0	0.9	1.0,D	1,D	5.6	3.37	.	.	SOM	GT:/	0:12	0/1:2	G	T	0
.	.	.	.	.	.	.	3.9	3.01	.	.	SOM	GT:/	0:24	0/1:3	C	A	0
.	.	.	0.0	1.0	1.0,D	1,D	5.5	5.97	.	.	SOM	GT:/	0:86	0/1:1	G	A	0
#####	##	0	0.0	0.9	1.0,D	1,D	5.4	5.22	.	.	SOM	GT:/	0:96	0/1:1	C	T	0
.	.	.	.	.	.	.	.	.	.	.	SOM	GT:/	0:84	0/1:1	C	T	0
.	.	.	.	.	.	.	.	.	.	.	SOM	GT:/	0:15	0/1:1	A	G	0
.	.	.	.	.	.	.	.	.	.	.	SOM	GT:/	0:44	0/1:4	A	G	0
.	.	.	.	.	.	.	.	.	.	.	DB;S	GT:/	0:28	0/1:2	A	G	0
.	.	.	.	.	.	1,A	5.8	7.24	.	.	SOM	GT:/	0:55	0/1:5	G	T	0
.	.	.	0.0	0.3	0.627	1,D	3.9	2.49	.	.	SOM	GT:/	0:34	0/1:6	C	A	0
.	##	0	0.2	0.0	0.006	0.647	4.2	1.72	.	.	SOM	GT:/	0:67	0/1:1	T	C	0
.	.	.	0.0	0.9	1.0,D	1,D	6	4.97	.	.	SOM	GT:/	0:48	0/1:5	G	T	0
.	##	.	0.1	0.5	0.819	1.000	5.1	4.00	.	.	SOM	GT:/	0:33	0/1:8	A	G	0
.	.	.	.	.	.	.	.	.	0	0	SOM	GT:/	0:10	0/1:2	C	A	0
.	.	.	.	.	.	.	.	.	.	0	SOM	GT:/	0:99	0/1:1	G	A	0
.	.	.	.	.	.	.	3.2	.	.	.	SOM	GT:/	0:47	0/1:1	G	T	0
.	.	.	.	.	.	.	.	1.27	.	.	SOM	GT:/	0:95	0/1:1	G	A	0
.	.	.	.	.	.	.	3.1	.	.	.	SOM	GT:/	0:34	0/1:6	A	G	0
.	.	.	.	.	.	.	.	.	.	.	SOM	GT:/	0:36	0/1:5	C	A	0
.	.	.	.	.	.	.	.	.	0	0	SOM	GT:/	0:9,(	0/1:7	T	C	0
.	0	0	.	.	.	.	.	.	0.1	0	SOM	GT:/	0:15	0/1:2	G	A	0
.	.	.	.	.	.	.	.	.	.	.	SOM	GT:/	0:12	0/1:2	G	A	0
.	.	.	0.0	0.6	0.909	1,D	3.7	4.04	.	.	SOM	GT:/	0:53	0/1:7	A	G	0
.	.	.	.	.	.	.	.	.	.	.	SOM	GT:/	0:27	0/1:2	C	T	0
.	.	.	.	.	.	.	.	.	.	.	SOM	GT:/	0:58	0/1:6	G	A	0
.	##	0	.	.	.	.	.	.	.	.	SOM	GT:/	0:58	0/1:1	G	A	0
.	.	.	.	.	.	.	.	.	.	.	SOM	GT:/	0:32	0/1:4	A	G	0
.	.	.	0.0	0.5	0.938	1,N	2.5	.	.	.	SOM	GT:/	0:83	0/1:1	G	A	0
.	.	.	.	.	.	.	.	.	0	0.1	DB;S	GT:/	0:45	0/1:7	G	C	0
.	.	.	.	.	.	.	.	.	.	.	SOM	GT:/	0:45	0/1:5	C	T	0
.	.	.	0.5	0.0	0.0,B	1,N	.	.	.	.	SOM	GT:/	0:38	0/1:7	C	A	0
.	.	.	.	.	.	.	.	.	0	.	SOM	GT:/	0:39	0/1:4	G	A	0
.	##	0	0.1	0.3	0.728	1,N	4.9	3.90	.	.	SOM	GT:/	0:57	0/1:7	G	A	0
.	.	.	0.0	0.9	1.0,D	1,D	5	3.70	.	.	SOM	GT:/	0:11	0/1:2	G	T	0
.	.	.	0.0	0.1	0.569	1.000	5.4	2.84	.	.	SOM	GT:/	0:68	0/1:9	A	G	0
0.002	0	0	.	.	.	.	.	.	.	.	SOM	GT:/	0:70	0/1:1	G	A	0
0.197	0	1	.	.	.	.	.	.	0.5	0.5	DB;S	GT:/	0:52	0/1:7	G	A	0
.	.	.	.	.	.	.	.	.	.	.	SOM	GT:/	0:10	0/1:1	G	C	0
.	0	0	.	.	.	.	.	.	.	.	SOM	GT:/	0:83	0/1:1	G	A	0
.	.	.	.	.	.	.	.	.	.	.	SOM	GT:/	0:40	0/1:7	G	A	0
.	##	0	.	.	.	.	.	.	.	.	SOM	GT:/	0:73	0/1:1	G	A	0
.	.	.	.	.	.	.	.	.	.	.	SOM	GT:/	0:35	0/1:8	G	C	0
.	.	.	.	.	.	.	.	.	.	.	SOM	GT:/	0:36	0/1:8	G	T	0
.	.	.	.	.	.	.	.	.	.	.	DB;S	GT:/	0:35	0/1:8	C	T	0
.	.	.	.	.	.	.	5.5	2.43	.	.	SOM	GT:/	0:49	0/1:7	C	A	0
.	.	.	.	.	.	.	.	.	.	.	SOM	GT:/	0:18	0/1:2	T	A	0

.	.	.	.	.	.	.	.	.	.	.	SON	GT:/	0:37	0/1:4	A	G	0
.	.	.	.	.	.	.	.	.	.	.	SON	GT:/	0:46	0/1:6	G	T	0
.	.	.	.	.	.	.	.	.	.	.	SON	GT:/	0:8,(	0/1:1	T	C	0
.	.	.	.	.	.	.	.	.	0	.	SON	GT:/	0:8,(	0/1:2	T	C	0
.	.	.	.	.	.	.	.	.	.	.	SON	GT:/	0:8,(	0/1:2	A	G	0
#####	##	.	.	0.2(	0.507	1.00(	4.8	1.30	.	.	SON	GT:/	0:10	0/1:2	C	T	0
.	.	.	0.4:	0.1·	0.931	1,D	4.3	1.68	.	.	SON	GT:/	0:45	0/1:8	C	T	0
.	.	.	.	.	.	.	.	.	.	.	SON	GT:/	0:13	0/1:2	C	T	0
.	.	.	.	.	.	1,D	5.3	2.11	.	.	SON	GT:/	0:39	0/1:6	C	A	0
.	.	.	.	.	.	.	.	2.24	.	.	SON	GT:/	0:40	0/1:6	G	A	0
.	.	.	.	.	.	.	2.2	.	0	0	SON	GT:/	0:20	0/1:2	G	A	0
.	.	.	0.4:	0.0(	0.0,B	1,N	.	1.81	.	.	SON	GT:/	0:12	0/1:1	A	G	0
5E-04	0	0	0.0:	0.4:	0.988	1,N	.	2.82	.	.	SON	GT:/	0:11	0/1:2	C	T	0
.	.	.	.	.	.	.	5.2	.	.	.	SON	GT:/	0:53	0/1:7	C	T	0
.	.	.	.	.	.	.	.	.	.	.	SON	GT:/	0:8,(	0/1:1	A	G	0
.	.	.	.	.	.	.	.	.	0	.	SON	GT:/	0:24	0/1:4	C	G	0
.	.	.	.	.	.	.	2.9	1.25	.	.	SON	GT:/	0:34	0/1:5	C	A	0
.	.	.	.	.	.	.	.	.	.	.	SON	GT:/	0:12	0/1:1	A	G	0
.	.	.	.	.	.	.	.	3.64	.	.	SON	GT:/	0:19	0/1:2	C	T	0
.	.	.	.	.	.	.	3.8	.	.	.	SON	GT:/	0:47	0/1:7	G	A	0
.	.	.	.	.	.	.	3.8	.	.	.	SON	GT:/	0:15	0/1:1	G	A	0
#####	##	0	.	.	.	.	.	.	.	.	SON	GT:/	0:20	0/1:2	C	T	0
.	.	.	.	.	.	.	.	.	0	.	SON	GT:/	0:23	0/1:3	C	T	0
.	.	.	0.5!	0.0:	0.04,I	1,N	2.1	.	.	.	SON	GT:/	0:10	0/1:1	G	T	0
.	.	.	0.6!	0.0(	0.0,B	1,N	.	.	.	.	SON	GT:/	0:53	0/1:9	G	A	0
.	##	0	0.0(	0.1:	0.588	0.879	5.8	2.16	0	.	SON	GT:/	0:32	0/1:5	C	T	0
.	.	.	.	.	.	.	.	.	.	.	SON	GT:/	0:25	0/1:4	C	T	0
.	.	.	0.6:	0.3	0.745	1,D	3.6	2.38	.	.	DB;!	GT:/	0:29	0/1:4	A	C	0
.	.	.	.	.	.	.	.	.	.	.	DB;!	GT:/	0:30	0/1:2	C	T	0
.	.	.	.	.	.	.	.	.	.	.	SON	GT:/	0:16	0/1:3	G	T	0
.	.	.	.	.	.	.	.	.	.	.	SON	GT:/	0:96	0/1:1	A	G	0
.	.	.	0.0:	0.6(	0.832	1,D	5.9	5.19	.	.	SON	GT:/	0:43	0/1:6	C	T	0
.	.	.	.	.	.	.	.	.	.	.	SON	GT:/	0:13	0/1:1	G	T	0
.	.	.	0.0(	0.9'	1.0,D	0.617	4.2	4.51	.	.	SON	GT:/	0:13	0/1:1	G	A	0
.	.	.	.	.	.	.	4.9	.	.	.	SON	GT:/	0:23	0/1:3	A	T	0
.	##	0	.	.	.	.	.	1.26	.	.	SON	GT:/	0:57	0/1:8	C	T	0
.	.	.	.	.	.	.	.	2.16	.	.	SON	GT:/	0:16	0/1:2	C	T	0
.	##	0	.	.	.	.	.	.	.	.	SON	GT:/	0:21	0/1:2	T	C	0
.	##	0	0.0·	0.9!	1.0,D	0.977	5.4	3.82	0	.	SON	GT:/	0:51	0/1:6	C	T	0
.	.	.	0.0	0.9!	0.999	1.00(	4.7	3.65	.	.	SON	GT:/	0:36	0/1:6	A	G	0
.	.	.	1.0	0.0	0.0,B	1,N	.	.	.	.	DB;!	GT:/	0:10	0/1:2	A	G	0
.	##	0	0.1·	0.0(	0.003	1.00(	3.6	1.87	.	.	SON	GT:/	0:10	0/1:2	G	A	0
.	.	.	0.4:	0.0:	0.044	0.995	4.2	1.79	.	.	SON	GT:/	0:70	0/1:1	T	C	0
.	.	.	.	.	.	.	.	.	.	.	SON	GT:/	0:11	0/1:1	G	A	0
.	.	.	.	.	.	.	.	.	.	.	SON	GT:/	0:16	0/1:2	C	T	0
.	##	0	.	.	.	.	.	.	0	.	SON	GT:/	0:55	0/1:8	G	A	0
.	.	.	0.0(	1.0	1.0,D	0.813	4.2	3.64	.	.	SON	GT:/	0:14	0/1:2	G	A	0
.	.	.	0.0	0.9·	0.994	1.00(	4.2	2.91	.	.	SON	GT:/	0:12	0/1:1	C	T	0
.	.	.	.	.	.	.	.	.	.	.	SON	GT:/	0:65	0/1:1	C	T	0
.	.	.	0.0·	0.2'	0.479	1,N	3.4	2.43	.	.	SON	GT:/	0:76	0/1:1	C	T	0
.	##	0	.	.	.	.	.	.	0	.	SON	GT:/	0:89	0/1:1	C	T	0
#####	##	0	.	.	.	.	.	.	.	.	SON	GT:/	0:20	0/1:3	C	T	0
.	##	0	0.0(	0.8'	0.998	1,D	4	3.02	.	.	SON	GT:/	0:10	0/1:1	G	A	0
.	##	0	0.0(	0.0(	0.003	1,N	.	.	0	0	SON	GT:/	0:70	0/1:1	G	A	0

.	.	.	.	.	.	.	.	.	0	.	SOM	GT:/	0:19	0/1:4	A	G	0
.	.	.	.	.	.	.	2.7	.	.	.	SOM	GT:/	0:24	0/1:2	T	C	0
.	.	.	.	.	.	.	.	.	.	.	SOM	GT:/	0:23	0/1:5	A	T	0
.	.	.	0.0	0.6	0.978	0.861	5.5	3.81	.	.	SOM	GT:/	0:11	0/1:2	G	A	0
.	.	.	0.0	0.8	0.998	1,D	5.4	3.63	.	.	SOM	GT:/	0:63	0/1:1	C	A	0
.	.	.	.	.	.	.	.	.	0	0	SOM	GT:/	0:16	0/1:2	G	A	0
.	0	0	0.1	0.5	0.96,I	1.000	5.7	3.45	.	.	SOM	GT:/	0:86	0/1:1	C	T	0
.	.	.	0.0	0.5	0.984	0.997	5.2	3.64	.	.	SOM	GT:/	0:29	0/1:7	G	A	0
.	.	.	0.0	0.9	0.997	1.000	5.8	4.50	.	.	SOM	GT:/	0:91	0/1:1	C	A	0
2E-04	##	0	0.1	0.2	0.981	0.995	4.3	.	.	.	SOM	GT:/	0:72	0/1:1	C	T	0
.	##	0	0.0	0.9	1.0,D	1,D	5.6	3.40	0	.	SOM	GT:/	0:48	0/1:6	C	T	0
.	.	.	.	.	.	.	5.1	.	.	.	SOM	GT:/	0:49	0/1:9	G	T	0
.	.	.	0.0	0.0	0.181	1,D	5.8	3.16	.	.	SOM	GT:/	0:14	0/1:2	T	C	0
.	.	.	.	.	.	.	.	.	0	.	SOM	GT:/	0:14	0/1:1	C	T	0
.	.	.	.	.	.	.	.	.	.	.	SOM	GT:/	0:28	0/1:5	C	A	0
.	.	.	.	.	.	.	5.1	3.89	.	.	SOM	GT:/	0:27	0/1:4	T	C	0
.	.	.	.	.	.	.	.	.	.	.	SOM	GT:/	0:12	0/1:2	G	A	0
.	0	0	.	.	.	.	.	.	0	.	DB;S	GT:/	0:88	0/1:1	G	A	0
.	##	0	0.0	0.9	1.0,D	1,D	3.6	3.58	.	.	SOM	GT:/	0:22	0/1:3	C	T	0
.	.	.	.	.	.	.	3.2	3.75	.	.	SOM	GT:/	0:47	0/1:7	C	A	0
.	.	.	.	.	.	.	.	.	.	.	SOM	GT:/	0:33	0/1:3	C	T	0
.	.	.	.	.	.	.	.	.	.	.	SOM	GT:/	0:21	0/1:3	G	A	0
.	.	.	.	.	.	.	.	.	0	0	SOM	GT:/	0:21	0/1:4	G	A	0
.	.	.	.	.	.	.	3.7	3.15	.	.	SOM	GT:/	0:25	0/1:4	G	A	0
.	.	.	.	.	.	.	.	.	.	.	SOM	GT:/	0:8,(	0/1:9	G	T	0
.	##	0	0.1	0.0	0.14,I	0.739	5.2	1.40	0	.	SOM	GT:/	0:83	0/1:1	G	A	0
.	.	.	.	.	.	.	.	.	.	.	SOM	GT:/	0:15	0/1:1	A	T	0
.	.	.	.	.	.	.	.	.	.	0	SOM	GT:/	0:33	0/1:5	G	A	0
.	.	.	.	.	.	.	.	.	.	.	SOM	GT:/	0:37	0/1:4	A	G	0
.	##	0	.	.	.	.	.	.	.	.	SOM	GT:/	0:57	0/1:8	C	T	0
.	.	.	.	.	.	.	.	.	.	.	SOM	GT:/	0:16	0/1:1	T	A	0
#####	##	0	.	.	.	.	.	.	0	.	DB;S	GT:/	0:58	0/1:8	G	A	0
.	##	0	0.0	0.9	1.0,D	1,D	5.9	6.16	.	.	SOM	GT:/	0:90	0/1:1	G	A	0
.	##	0	.	.	.	.	.	.	.	.	SOM	GT:/	0:43	0/1:7	C	T	0
.	.	.	.	.	.	.	.	.	.	.	SOM	GT:/	0:28	0/1:2	T	G	0
.	.	.	.	.	.	.	.	.	.	.	SOM	GT:/	0:17	0/1:5	C	T	0
.	.	.	.	.	.	.	.	.	.	.	SOM	GT:/	0:12	0/1:2	G	A	0
.	.	.	.	.	.	.	.	.	.	.	SOM	GT:/	0:42	0/1:6	T	C	0
.	.	.	.	.	.	.	.	.	.	.	SOM	GT:/	0:19	0/1:3	G	A	0
.	.	.	.	.	.	.	.	.	.	.	SOM	GT:/	0:11	0/1:1	C	T	0
.	.	.	.	.	.	.	.	2.64	.	.	SOM	GT:/	0:18	0/1:2	G	A	0
.	.	.	.	.	.	.	.	.	.	.	SOM	GT:/	0:20	0/1:5	T	C	0
.	0	0	.	.	.	.	.	.	.	.	DB;S	GT:/	0:20	0/1:3	G	A	0
.	.	.	.	.	.	.	.	.	.	.	SOM	GT:/	0:52	0/1:8	A	G	0
.	.	.	.	.	.	.	.	.	.	.	SOM	GT:/	0:57	0/1:9	C	T	0
.	.	.	.	.	.	.	.	.	.	.	SOM	GT:/	0:57	0/1:8	G	T	0
.	.	.	.	.	.	.	.	.	.	.	SOM	GT:/	0:59	0/1:9	G	A	0
.	.	.	.	.	.	.	.	.	.	.	SOM	GT:/	0:29	0/1:6	A	G	0
.	##	0	.	.	.	.	.	.	.	0	SOM	GT:/	0:81	0/1:1	C	T	0
.	.	.	.	.	.	.	.	.	.	.	SOM	GT:/	0:10	0/1:1	G	A	0
.	.	.	.	.	.	.	.	.	.	.	SOM	GT:/	0:15	0/1:1	T	C	0
.	.	.	0.0	1.0	1.0,D	1,D	4.2	4.48	.	.	DB;S	GT:/	0:63	0/1:9	C	T	0
.	0	0	.	.	.	.	.	.	0	.	SOM	GT:/	0:99	0/1:1	C	T	0
.	.	.	0.0	1.0	1.0,D	1,D	5.7	5.41	.	.	SOM	GT:/	0:64	0/1:1	G	A	0

.	.	.	0.0	0.9	0.996	1,D	3.6	2.38	.	.	SOM	GT:/	0:68	0/1:	G	T	0
.	##	0	.	.	.	.	.	1.66	.	.	SOM	GT:/	0:51	0/1:	C	T	0
.	.	.	.	.	.	.	3.3	.	.	.	SOM	GT:/	0:12	0/1:	C	T	0
.	##	0	.	.	.	.	.	.	0	.	SOM	GT:/	0:11	0/1:	C	T	0
.	.	.	.	.	.	.	.	1.32	.	.	SOM	GT:/	0:40	0/1:	G	A	0
.	.	.	.	.	.	.	2.9	2.80	.	.	SOM	GT:/	0:28	0/1:	G	C	0
.	.	.	0.0	0.1	0.865	1,D	4	2.29	.	.	SOM	GT:/	0:60	0/1:	G	A	0
.	.	.	.	.	.	.	.	.	.	.	SOM	GT:/	0:84	0/1:	A	G	0
.	.	.	.	.	.	.	2.1	.	.	.	SOM	GT:/	0:26	0/1:	C	T	0
.	##	0	0.9	0.0	0.014	1,N	.	.	.	.	SOM	GT:/	0:83	0/1:	G	A	0
.	.	.	0.0	0.6	0.991	0.706	4.9	1.80	.	.	SOM	GT:/	0:96	0/1:	C	A	0
.	0	0	.	.	.	.	2.2	.	0	0	DB;S	GT:/	0:71	0/1:	C	A	0
.	##	0	.	.	.	.	.	1.37	0	.	SOM	GT:/	0:60	0/1:	C	T	0
.	.	.	.	.	.	.	.	.	.	.	SOM	GT:/	0:84	0/1:	G	T	0
2E-04	##	0	.	.	.	.	.	.	0	.	SOM	GT:/	0:67	0/1:	C	T	0
.	.	.	0.0	1.0	1.0,D	1,D	5.9	4.56	.	.	SOM	GT:/	0:73	0/1:	C	T	0
.	.	.	.	.	.	.	.	.	.	.	SOM	GT:/	0:23	0/1:	G	T	0
.	.	.	.	.	.	.	.	.	.	.	SOM	GT:/	0:60	0/1:	A	G	0
5E-04	0	0	.	.	.	.	.	.	.	.	SOM	GT:/	0:40	0/1:	G	A	0
3E-04	0	0	0.0	0.4	0.99,I	1,N	2.6	2.98	.	.	SOM	GT:/	0:10	0/1:	G	A	0
.	.	.	.	.	.	.	.	.	.	.	SOM	GT:/	0:39	0/1:	G	A	0
.	.	.	0.0	0.9	1.0,D	.	6	3.85	.	.	SOM	GT:/	0:73	0/1:	C	A	0
.	.	.	.	.	.	.	.	.	.	.	SOM	GT:/	0:10	0/1:	C	G	0
.	.	.	.	.	.	.	3.4	.	.	.	SOM	GT:/	0:34	0/1:	A	G	0
.	0	0	.	.	.	.	.	.	0	0	SOM	GT:/	0:13	0/1:	A	G	0
.	.	.	.	.	.	.	.	.	0	.	SOM	GT:/	0:24	0/1:	A	T	0
.	.	.	.	.	.	.	2.9	.	.	.	SOM	GT:/	0:45	0/1:	C	A	0
.	##	0	0.0	0.0	0.007	0.657	4.8	2.89	.	.	SOM	GT:/	0:66	0/1:	C	T	0
2E-04	##	0	0.0	1.0	1.0,D	1,D	.	3.70	.	.	SOM	GT:/	0:82	0/1:	C	T	0
.	.	.	.	.	.	.	.	.	.	.	SOM	GT:/	0:21	0/1:	T	C	0
.	##	0	.	.	.	.	.	1.51	.	.	SOM	GT:/	0:32	0/1:	G	A	0
.	.	.	.	.	.	.	.	.	.	.	SOM	GT:/	0:12	0/1:	G	A	0
.	.	.	.	.	.	.	2.4	1.98	.	.	SOM	GT:/	0:8,(	0/1:	C	T	0
.	.	.	0.0	0.9	0.999	1,D	4.5	3.86	.	.	SOM	GT:/	0:16	0/1:	T	C	0
.	.	.	.	.	.	.	.	.	0	.	SOM	GT:/	0:65	0/1:	A	C	0
.	.	.	.	.	.	.	5.9	3.72	.	.	SOM	GT:/	0:41	0/1:	T	C	0
.	.	.	.	.	.	.	.	.	.	.	SOM	GT:/	0:28	0/1:	T	C	0
.	.	.	.	.	.	.	2.5	2.14	.	.	SOM	GT:/	0:14	0/1:	T	C	0
.	.	.	.	.	.	.	.	.	.	.	SOM	GT:/	0:35	0/1:	G	T	0
.	.	.	0.0	0.9	1.0,D	0.998	5.8	3.48	.	.	SOM	GT:/	0:70	0/1:	G	T	0
.	.	.	.	.	.	.	.	.	.	.	SOM	GT:/	0:21	0/1:	C	T	0
.	.	.	.	.	.	.	.	.	.	.	SOM	GT:/	0:26	0/1:	G	A	0
.	.	.	.	.	.	.	.	.	.	.	SOM	GT:/	0:23	0/1:	T	C	0
2E-04	##	0	.	.	.	.	.	.	0	.	SOM	GT:/	0:54	0/1:	C	T	0
.	.	.	.	.	.	.	.	.	.	.	SOM	GT:/	0:30	0/1:	A	C	0
.	##	0	0.0	0.9	1.0,D	1,D	5.8	4.37	.	.	SOM	GT:/	0:28	0/1:	G	A	0
.	.	.	.	.	.	.	.	.	0	.	SOM	GT:/	0:16	0/1:	G	A	0
.	.	.	.	.	.	.	.	.	.	.	SOM	GT:/	0:22	0/1:	T	C	0
.	.	.	.	.	.	.	.	.	.	.	SOM	GT:/	0:56	0/1:	T	C	0
.	.	.	.	.	.	.	2.9	.	.	.	SOM	GT:/	0:73	0/1:	C	T	0
2E-04	0	0	.	.	.	.	2.9	2.40	0	0	SOM	GT:/	0:22	0/1:	G	A	0
#####	##	0	0.0	0.3	0.935	1,D	5.2	4.87	.	.	SOM	GT:/	0:22	0/1:	G	A	0
.	.	.	0.3	0.0	0.01,I	1,N	.	.	.	.	SOM	GT:/	0:96	0/1:	A	G	0
.	##	0	0.0	0.9	1.0,D	1,D	5.6	4.88	.	.	SOM	GT:/	0:51	0/1:	C	T	0

.	##	0	0.1	0.0	0.004	0.982	3.1	2.10	.	.	SOM	GT:/	0:34	0/1:4	A	G	0
.	.	.	0.0	0.9	1.0,D	1,D	5.8	4.44	.	.	SOM	GT:/	0:48	0/1:6	T	C	0
.	.	.	.	.	.	.	.	.	.	.	SOM	GT:/	0:36	0/1:5	T	C	0
.	.	.	.	.	.	.	.	.	.	.	SOM	GT:/	0:29	0/1:4	C	T	0
.	.	.	.	.	.	.	2.6	2.16	.	.	SOM	GT:/	0:20	0/1:4	C	T	0
.	##	0	.	.	.	.	.	.	.	.	SOM	GT:/	0:82	0/1:1	G	A	0
.	.	.	.	.	.	.	.	.	.	.	SOM	GT:/	0:71	0/1:1	T	C	0
.	.	.	0.1	0.1	0.525	1,D	4.6	1.33	.	.	SOM	GT:/	0:24	0/1:3	T	C	0
.	.	.	0.0	0.9	1.0,D	1,D	4.6	5.20	.	.	SOM	GT:/	0:53	0/1:6	G	A	0
.	.	.	.	.	.	1,A	4.9	8.66	.	.	SOM	GT:/	0:74	0/1:1	C	T	0
.	.	.	.	.	.	.	.	.	.	.	SOM	GT:/	0:10	0/1:1	G	A	0
.	.	.	0.0	1.0	1.0,D	1,D	5.8	4.61	.	.	SOM	GT:/	0:50	0/1:8	C	T	0
.	.	.	.	.	.	.	.	.	0	.	SOM	GT:/	0:92	0/1:1	G	A	0
.	.	.	.	.	.	.	.	.	.	.	SOM	GT:/	0:70	0/1:1	C	T	0
.	.	.	.	.	.	.	.	.	.	.	SOM	GT:/	0:69	0/1:4	C	T	0
.	.	.	.	.	.	.	2.9	1.44	.	.	SOM	GT:/	0:11	0/1:1	C	T	0
.	.	.	0.3	0.9	0.993	1,D	4.7	.	.	.	SOM	GT:/	0:57	0/1:7	T	C	0
.	##	0	.	.	.	.	5.7	.	.	.	SOM	GT:/	0:30	0/1:6	C	T	0
#####	##	0	0.0	0.0	0.231	1,N	.	3.09	.	.	SOM	GT:/	0:12	0/1:1	G	A	0
.	##	0	.	.	.	.	.	.	0	.	SOM	GT:/	0:14	0/1:2	C	T	0
.	.	.	.	.	.	.	.	.	.	.	SOM	GT:/	0:27	0/1:4	A	G	0
.	.	.	0.0	0.9	0.996	1,D	5.1	3.79	.	.	SOM	GT:/	0:10	0/1:1	T	A	0
.	.	.	.	.	.	.	.	.	.	.	SOM	GT:/	0:11	0/1:1	C	T	0
.	.	.	.	.	.	.	3	.	.	.	SOM	GT:/	0:97	0/1:1	G	T	0
.	.	.	0.3	0.0	0.041	0.967	4.4	1.40	.	.	SOM	GT:/	0:54	0/1:1	C	T	0
.	.	.	0.0	0.0	0.167	1.000	5	3.97	.	.	SOM	GT:/	0:51	0/1:8	C	T	0
.	##	0	.	.	.	.	.	.	.	.	SOM	GT:/	0:90	0/1:1	G	A	0
.	.	.	.	.	.	.	.	.	.	.	SOM	GT:/	0:99	0/1:1	A	G	0
.	.	.	.	.	.	.	.	.	.	.	SOM	GT:/	0:14	0/1:2	G	A	0
.	##	0	.	.	.	.	.	.	.	.	SOM	GT:/	0:18	0/1:2	G	A	0
.	.	.	.	.	.	.	.	.	.	.	SOM	GT:/	0:52	0/1:1	C	T	0
#####	.	.	.	.	.	.	.	.	.	.	SOM	GT:/	0:68	0/1:8	C	T	0
.	.	.	.	.	.	.	2.5	.	.	.	SOM	GT:/	0:9,(	0/1:7	C	A	0
.	.	.	.	.	.	.	.	1.40	.	.	SOM	GT:/	0:42	0/1:6	C	T	0
.	.	.	0.0	1.0	1.0,D	1,D	3.6	3.67	.	.	SOM	GT:/	0:78	0/1:9	C	T	0
.	.	.	0.1	0.0	0.0,B	1.000	4.5	3.16	.	.	SOM	GT:/	0:93	0/1:1	C	A	0
.	##	0	0.0	0.6	0.966	1.000	.	3.84	.	.	SOM	GT:/	0:54	0/1:7	C	A	0
.	.	.	.	.	.	.	.	.	.	.	SOM	GT:/	0:17	0/1:4	G	T	0
.	.	.	0.0	0.9	1.0,D	1.000	3.2	3.89	.	.	SOM	GT:/	0:16	0/1:2	C	T	0
.	.	.	.	.	.	.	.	2.05	.	.	SOM	GT:/	0:29	0/1:5	C	T	0
.	##	0	.	.	.	.	.	.	.	.	SOM	GT:/	0:13	0/1:1	G	A	0
.	.	.	.	.	.	.	.	.	0	0	SOM	GT:/	0:13	0/1:3	A	G	0
.	.	.	.	.	.	.	.	.	0	.	SOM	GT:/	0:12	0/1:3	G	A	0
.	.	.	0.5	0.0	0.011	1,N	.	.	.	.	SOM	GT:/	0:32	0/1:6	T	C	0
.	.	.	0.0	0.9	1.0,D	1,D	5.7	4.60	.	.	SOM	GT:/	0:54	0/1:8	G	A	0
.	.	.	.	.	.	.	.	.	.	.	SOM	GT:/	0:35	0/1:5	G	A	0
.	.	.	0.0	1.0	1.0,D	1,D	5.6	3.42	.	.	SOM	GT:/	0:14	0/1:2	C	A	0
.	.	.	.	.	.	.	.	2.47	.	.	SOM	GT:/	0:40	0/1:4	T	C	0
.	##	0	.	.	.	.	5.1	1.42	.	.	SOM	GT:/	0:57	0/1:9	C	T	0
.	.	.	.	.	.	.	.	.	.	.	SOM	GT:/	0:35	0/1:4	T	C	0
.	.	.	.	.	.	.	.	.	.	.	SOM	GT:/	0:28	0/1:5	A	T	0
#####	##	0	0.1	0.0	0.122	1.000	5.9	3.45	.	.	SOM	GT:/	0:29	0/1:4	C	T	0
.	.	.	0.0	0.0	0.013	1,N	.	.	.	.	SOM	GT:/	0:60	0/1:1	T	C	0
.	.	.	.	.	.	.	.	.	.	.	SOM	GT:/	0:15	0/1:1	C	T	0

.	.	.	.	.	.	.	.	.	.	.	SON	GT:/	0:31	0/1:	G	T	0
.	##	0	.	.	.	.	.	.	0	0	SON	GT:/	0:69	0/1:	C	T	0
.	.	.	.	.	.	.	3.1	.	.	.	SON	GT:/	0:31	0/1:	C	T	0
.	.	.	.	.	.	.	.	.	.	.	SON	GT:/	0:19	0/1:	G	A	0
.	##	0	.	.	.	.	4.1	1.68	.	0	SON	GT:/	0:58	0/1:	C	T	0
.	.	.	.	.	.	.	.	.	.	.	SON	GT:/	0:11	0/1:	G	A	0
.	##	0	.	.	.	.	.	.	.	.	SON	GT:/	0:15	0/1:	G	A	0
.	.	.	.	.	.	.	.	.	.	.	SON	GT:/	0:17	0/1:	G	A	0
.	.	.	0.0	0.9	1.0,D	0.962	.	3.31	.	.	SON	GT:/	0:17	0/1:	G	A	0
.	.	.	.	.	.	.	.	.	.	.	DB;	GT:/	0:44	0/1:	G	A	0
.	.	.	.	.	.	.	.	.	.	.	SON	GT:/	0:46	0/1:	G	A	0
.	.	.	.	.	.	.	.	.	.	.	SON	GT:/	0:11	0/1:	G	T	0
.	##	0	.	.	.	.	.	.	0	.	SON	GT:/	0:21	0/1:	C	T	0
.	.	.	.	.	.	.	.	.	.	.	SON	GT:/	0:28	0/1:	G	A	0
.	.	.	.	.	.	.	.	.	.	.	SON	GT:/	0:42	0/1:	A	C	0
.	.	.	.	.	.	.	.	.	.	.	SON	GT:/	0:33	0/1:	T	C	0
.	.	.	0.4	0.0	0.011	0.982	.	.	.	.	SON	GT:/	0:54	0/1:	G	A	0
.	.	.	.	.	.	.	.	.	0	.	SON	GT:/	0:49	0/1:	C	T	0
.	.	.	.	.	.	.	.	.	.	.	SON	GT:/	0:91	0/1:	G	T	0
.	.	.	.	.	.	.	.	.	.	.	SON	GT:/	0:37	0/1:	C	T	0
.	.	.	.	.	.	.	.	.	.	.	SON	GT:/	0:34	0/1:	T	G	0
.	.	.	.	.	.	.	.	.	.	.	SON	GT:/	0:64	0/1:	C	T	0
.	.	.	.	.	.	.	.	.	.	.	SON	GT:/	0:52	0/1:	C	T	0
.	.	.	.	.	.	.	.	.	.	.	SON	GT:/	0:22	0/1:	G	A	0
.	.	.	.	.	.	.	.	.	.	.	SON	GT:/	0:69	0/1:	C	T	0
.	.	.	.	.	.	.	.	.	.	.	SON	GT:/	0:47	0/1:	C	T	0
.	.	.	.	.	.	.	.	.	0	.	SON	GT:/	0:33	0/1:	G	T	0
.	.	.	.	.	.	.	.	.	.	.	SON	GT:/	0:41	0/1:	G	A	0
.	.	.	.	.	.	.	.	.	0.1	0.5	SON	GT:/	0:9,	0/1:	C	T	0
.	.	.	.	.	.	.	2.1	.	0	0	SON	GT:/	0:18	0/1:	G	T	0
.	.	.	.	.	.	.	2.1	.	.	.	SON	GT:/	0:18	0/1:	G	A	0
.	.	.	.	.	.	.	.	.	0	0	SON	GT:/	0:17	0/1:	T	A	0
.	##	0	0.0	0.9	0.994	0.97	5.3	3.78	.	.	SON	GT:/	0:43	0/1:	C	T	0
.	.	.	.	.	.	.	.	.	.	0	SON	GT:/	0:9,	0/1:	C	T	0
.	.	.	.	.	.	.	.	.	.	.	SON	GT:/	0:75	0/1:	T	C	0
.	.	.	.	.	.	.	.	.	.	.	SON	GT:/	0:12	0/1:	G	A	0
.	.	.	0.0	0.9	1.0,D	1.00	3.5	3.09	.	.	SON	GT:/	0:20	0/1:	C	T	0
.	##	0	0.2	0.7	0.994	0.99	4.8	2.69	.	.	SON	GT:/	0:96	0/1:	C	T	0
.	.	.	.	.	.	.	3	.	.	.	SON	GT:/	0:49	0/1:	G	T	0
.	.	.	.	.	.	.	.	.	0	.	SON	GT:/	0:40	0/1:	G	A	0
.	##	0	0.1	0.9	1.0,D	1.00	4.7	5.21	.	.	SON	GT:/	0:11	0/1:	G	A	0
#####	.	.	.	.	.	.	3.5	.	.	.	SON	GT:/	0:17	0/1:	T	C	0
.	.	.	.	.	.	.	.	.	.	.	SON	GT:/	0:41	0/1:	T	C	0
.	.	.	.	.	.	.	.	.	0.2	0.1	SON	GT:/	0:18	0/1:	T	C	0
.	.	.	.	.	.	.	.	.	0	0	SON	GT:/	0:13	0/1:	T	A	0
.	.	.	.	.	.	.	5.8	3.77	.	.	SON	GT:/	0:41	0/1:	G	A	0
.	.	.	.	.	.	.	.	.	.	.	SON	GT:/	0:26	0/1:	A	G	0
.	.	.	0.0	0.9	1.0,D	1.00	5.1	2.61	.	.	SON	GT:/	0:39	0/1:	C	A	0
4E-04	##	0	0.0	0.9	0.981	0.99	5.9	3.17	.	.	SON	GT:/	0:35	0/1:	G	A	0
.	.	.	.	.	.	.	.	.	.	.	SON	GT:/	0:10	0/1:	G	T	0
.	##	0	0.0	0.9	1.0,D	1,D	5.6	4.80	.	.	SON	GT:/	0:87	0/1:	G	A	0
.	.	.	.	.	.	.	.	.	.	.	SON	GT:/	0:68	0/1:	T	G	0
.	.	.	.	.	.	.	.	.	.	.	SON	GT:/	0:52	0/1:	G	A	0
.	.	.	.	.	.	1,D	5.5	4.26	.	.	SON	GT:/	0:98	0/1:	G	T	0

.	##	0	.	.	.	.	.	.	.	.	SON	GT:/	0:42	0/1:6	C	T	0
.	##	0	0.0(	0.7(	0.998	1,D	6	4.93	0	.	SON	GT:/	0:33	0/1:6	C	T	0
.	.	.	.	.	.	.	.	.	.	.	SON	GT:/	0:30	0/1:4	C	T	0
.	.	.	.	.	.	.	.	.	.	.	SON	GT:/	0:44	0/1:6	G	A	0
.	##	.	0.0(	0.6	0.975	1.000	3.1	4.07	.	.	SON	GT:/	0:90	0/1:1	G	A	0
.	.	.	.	.	.	.	3.1	.	.	.	SON	GT:/	0:11	0/1:1	C	T	0
.	0	0	.	.	.	1,A	.	13.8	.	.	SON	GT:/	0:89	0/1:9	C	T	0
.	.	.	.	.	.	.	.	.	.	.	SON	GT:/	0:10	0/1:2	G	A	0
.	.	.	0.0(	0.9	1.0,D	1,D	4.4	4.77	.	.	SON	GT:/	0:13	0/1:2	G	A	0
.	0	0	.	.	.	.	.	1.45	.	.	SON	GT:/	0:97	0/1:6	C	T	0
.	.	.	.	.	.	.	.	.	.	.	SON	GT:/	0:41	0/1:4	T	C	0
.	.	.	0.0	1.0	1.0,D	1,D	3.7	3.79	.	.	SON	GT:/	0:34	0/1:7	C	T	0
.	.	.	0.0(	0.7	0.999	1.000	5.7	3.34	.	.	SON	GT:/	0:43	0/1:5	G	A	0
.	.	.	.	.	.	.	.	.	.	.	SON	GT:/	0:75	0/1:1	T	C	0
.	.	.	.	.	.	.	.	.	.	.	SON	GT:/	0:23	0/1:3	G	A	0
.	.	.	.	.	.	.	.	.	.	.	SON	GT:/	0:25	0/1:3	G	A	0
.	.	.	.	.	.	.	.	.	.	.	SON	GT:/	0:32	0/1:3	C	T	0
.	.	.	.	.	.	.	.	.	.	.	SON	GT:/	0:48	0/1:7	G	A	0
#####	##	0	0.0(	0.8(	0.989	0.995	5.5	4.90	.	.	SON	GT:/	0:10	0/1:1	C	T	0
.	.	.	.	.	.	.	2.8	2.65	.	.	SON	GT:/	0:16	0/1:1	T	A	0
.	.	.	0.0(	0.9	0.998	1.000	4.9	3.01	.	.	SON	GT:/	0:74	0/1:1	G	T	0
.	##	0	.	.	.	.	.	1.31	.	.	SON	GT:/	0:14	0/1:2	C	T	0
.	.	.	.	.	.	.	.	.	.	.	SON	GT:/	0:64	0/1:9	G	T	0
#####	##	0	0.0	0.8	0.997	0.987	4	3.66	.	.	SON	GT:/	0:91	0/1:1	G	A	0
.	.	.	.	.	.	.	.	.	.	.	SON	GT:/	0:52	0/1:9	C	A	0
.	.	.	.	.	.	.	.	.	.	.	DB;S	GT:/	0:51	0/1:6	G	A	0
.	.	.	1.0	0.0(	0.001	0.993	.	.	.	.	SON	GT:/	0:52	0/1:1	A	G	0
.	.	.	0.6	0.0(	0.001	1,N	.	.	.	.	SON	GT:/	0:16	0/1:4	C	T	0
.	##	0	.	.	.	.	.	.	.	.	SON	GT:/	0:18	0/1:2	G	A	0
.	.	.	.	.	.	.	2.3	3.13	.	.	SON	GT:/	0:29	0/1:4	T	C	0
.	.	.	.	.	.	.	.	.	.	.	SON	GT:/	0:64	0/1:8	C	T	0
.	.	.	.	.	.	.	.	1.64	.	.	SON	GT:/	0:40	0/1:8	C	T	0
.	##	0	0.0	0.2	0.938	1.000	5.6	3.26	.	.	SON	GT:/	0:32	0/1:4	C	T	0
.	.	.	0.1!	.	.	.	5	.	.	.	SON	GT:/	0:34	0/1:6	C	T	0
.	.	.	.	.	.	.	.	.	.	.	SON	GT:/	0:20	0/1:3	C	A	0
.	.	.	.	.	.	.	.	.	.	.	SON	GT:/	0:15	0/1:2	C	T	0
.	.	.	.	.	.	.	.	.	.	.	SON	GT:/	0:12	0/1:1	C	T	0
.	0	0	.	.	.	.	.	.	.	.	SON	GT:/	0:13	0/1:1	C	T	0
.	.	.	.	.	.	.	.	.	.	.	SON	GT:/	0:26	0/1:6	G	A	0
2E-04	0	0	.	.	.	.	.	.	0	.	SON	GT:/	0:51	0/1:7	G	A	0
.	##	0	.	.	.	.	.	.	.	.	SON	GT:/	0:57	0/1:8	C	T	0
.	.	.	.	.	.	.	.	.	0	0	DB;S	GT:/	0:27	0/1:6	C	T	0
5E-04	0	0	.	0.9	1.0,D	.	3.7	.	.	.	SON	GT:/	0:11	0/1:1	G	A	0
.	.	.	.	.	.	.	.	.	.	.	SON	GT:/	0:62	0/1:1	T	G	0
.	.	.	.	.	.	.	.	.	.	.	SON	GT:/	0:68	0/1:1	A	G	0
.	.	.	.	0.2	0.68,I	.	5.3	1.29	.	.	SON	GT:/	0:65	0/1:1	C	T	0
.	.	.	0.0(	0.9	1.0,D	1,D	4.6	5.06	.	.	SON	GT:/	0:99	0/1:1	C	T	0
.	.	.	0.1	0.0	0.297	0.998	4.8	4.97	.	.	SON	GT:/	0:24	0/1:4	C	T	0
.	.	.	.	.	.	.	.	.	.	.	SON	GT:/	0:35	0/1:6	C	T	0
.	.	.	.	.	.	.	.	.	.	.	SON	GT:/	0:18	0/1:4	C	T	0
#####	##	0	0.4	0.0(	0.003	1,N	.	1.38	.	.	SON	GT:/	0:36	0/1:6	C	T	0
.	.	.	.	.	.	.	.	.	.	.	SON	GT:/	0:35	0/1:4	G	C	0
.	.	.	.	.	.	.	.	.	.	.	SON	GT:/	0:21	0/1:3	G	A	0
.	.	.	.	.	.	.	.	.	.	.	SON	GT:/	0:37	0/1:4	G	T	0

.	.	.	.	.	.	.	.	.	.	.	SON	GT:/	0:8,(	0/1:1	G	A	0
.	.	.	.	.	.	.	.	.	.	.	SON	GT:/	0:11	0/1:5	T	A	0
.	.	.	.	.	.	.	.	1.63	.	.	SON	GT:/	0:47	0/1:8	C	T	0
.	.	.	.	.	.	.	.	.	.	.	SON	GT:/	0:10	0/1:1	C	T	0
.	.	.	.	.	.	.	.	1.65	.	.	SON	GT:/	0:84	0/1:1	T	A	0
7E-04	0	0	.	.	.	.	.	.	.	.	SON	GT:/	0:12	0/1:1	G	A	0
.	.	.	.	.	.	.	.	.	.	.	SON	GT:/	0:57	0/1:7	C	A	0
.	0	0	.	.	.	.	.	.	.	.	SON	GT:/	0:16	0/1:2	T	C	0
.	.	.	.	.	.	.	.	.	0	.	SON	GT:/	0:14	0/1:2	C	T	0
.	##	0	.	.	.	.	3.3	.	.	.	SON	GT:/	0:10	0/1:1	C	T	0
.	##	0	0.0(	0.0!	0.487	1,N	.	3.36	.	.	SON	GT:/	0:68	0/1:1	G	A	0
.	.	.	.	.	.	1,D	4.7	.	.	.	SON	GT:/	0:90	0/1:1	G	A	0
.	.	.	.	.	.	.	.	.	.	.	SON	GT:/	0:39	0/1:5	C	T	0
.	##	0	.	.	.	.	.	.	0	.	SON	GT:/	0:92	0/1:1	C	T	0
.	.	.	.	.	.	.	.	.	.	.	SON	GT:/	0:25	0/1:3	C	T	0
.	.	.	.	.	.	.	.	.	.	.	SON	GT:/	0:53	0/1:8	C	T	0
.	.	.	.	.	.	.	.	.	0.3	0.5	SON	GT:/	0:9,(	0/1:5	A	G	0
.	.	.	.	.	.	.	.	.	.	.	SON	GT:/	0:21	0/1:2	A	T	0
.	.	.	.	.	.	.	.	.	.	.	SON	GT:/	0:12	0/1:2	G	A	0
.	.	.	0.9(	0.9'	0.981	1.000	5.6	2.25	.	.	SON	GT:/	0:40	0/1:6	T	C	0
.	.	.	.	.	.	.	.	.	.	.	SON	GT:/	0:11	0/1:8	A	G	0
.	.	.	.	.	.	.	.	.	0	0	SON	GT:/	0:11	0/1:9	G	A	0
.	.	.	.	.	.	.	.	.	.	.	SON	GT:/	0:47	0/1:7	C	T	0
.	.	.	.	.	.	.	.	.	.	.	SON	GT:/	0:43	0/1:8	G	T	0
.	.	.	.	.	.	.	4	2.52	.	.	SON	GT:/	0:17	0/1:4	C	A	0
.	.	.	.	.	.	.	.	.	.	.	SON	GT:/	0:91	0/1:1	G	T	0
.	0	0	.	.	.	.	.	.	.	.	SON	GT:/	0:85	0/1:1	C	T	0
.	0	0	.	.	.	.	.	.	.	.	SON	GT:/	0:43	0/1:5	G	A	0
.	.	.	.	.	.	.	.	.	0	.	SON	GT:/	0:23	0/1:3	G	A	0
#####	0	0	0.2!	0.0(	0.002	1,N	.	.	.	.	DB;!	GT:/	0:85	0/1:1	C	T	0
.	.	.	.	.	.	.	.	.	.	.	SON	GT:/	0:68	0/1:1	G	A	0
2E-04	##	0	.	.	.	1,A	.	5.79	.	.	SON	GT:/	0:64	0/1:1	G	A	0
0.028	0	0	.	.	.	.	.	.	.	.	DB;!	GT:/	0:76	0/1:1	G	A	0
.	##	0	.	.	.	.	.	.	.	0	SON	GT:/	0:18	0/1:4	C	T	0
.	.	.	.	.	.	.	.	.	.	.	SON	GT:/	0:20	0/1:1	C	T	0
.	##	0	.	.	.	.	.	.	.	.	SON	GT:/	0:44	0/1:4	G	A	0
.	.	.	.	.	.	.	5.6	1.92	.	.	SON	GT:/	0:57	0/1:9	C	T	0
.	##	0	0.0:	0.9!	0.999	1,D	3.7	3.34	.	.	SON	GT:/	0:36	0/1:6	G	A	0
.	.	.	.	.	.	.	3.3	.	.	.	SON	GT:/	0:27	0/1:1	T	C	0
.	.	.	.	.	.	.	.	.	.	.	SON	GT:/	0:22	0/1:3	C	T	0
2E-04	##	0	0.0(	.	.	1.000	.	2.48	.	.	SON	GT:/	0:51	0/1:9	G	A	0
.	0	0	.	.	.	.	.	.	.	0	SON	GT:/	0:23	0/1:3	G	A	0
.	.	.	.	.	.	.	.	.	.	.	SON	GT:/	0:12	0/1:5	G	A	0
.	.	.	.	.	.	.	.	.	.	.	SON	GT:/	0:12	0/1:8	T	G	0
.	.	.	.	.	.	.	.	.	0	.	SON	GT:/	0:13	0/1:9	T	C	0
.	.	.	.	.	.	.	.	.	.	.	SON	GT:/	0:12	0/1:9	A	C	0
.	.	.	.	.	.	.	.	.	.	.	SON	GT:/	0:27	0/1:3	A	C	0
.	.	.	.	.	.	.	.	.	.	.	SON	GT:/	0:14	0/1:6	G	A	0
.	.	.	.	.	.	.	.	.	0	.	DB;!	GT:/	0:35	0/1:2	C	G	0
.	.	.	0.7!	0.0(	0.097	0.999	5.2	.	.	.	SON	GT:/	0:35	0/1:6	G	T	0
.	.	.	.	.	.	.	2.5	.	.	.	SON	GT:/	0:30	0/1:3	T	C	0
.	.	.	.	.	.	.	.	.	.	.	SON	GT:/	0:15	0/1:2	A	G	0
.	.	.	.	.	.	.	.	.	.	.	SON	GT:/	0:23	0/1:4	C	T	0
.	.	.	.	.	.	.	4.9	4.20	.	.	SON	GT:/	0:36	0/1:5	G	A	0

.	.	.	.	.	.	.	.	1.75	.	.	SOM	GT:/	0:16	0/1:2	T	A	0
.	##	0	.	.	.	.	.	.	.	.	SOM	GT:/	0:26	0/1:4	G	A	0
6E-04	0	0	.	.	.	.	2.3	.	.	0	SOM	GT:/	0:55	0/1:8	C	T	0
.	.	.	.	.	.	.	.	.	.	.	SOM	GT:/	0:10	0/1:1	G	A	0
.	.	.	.	.	.	.	2.7	.	.	.	SOM	GT:/	0:30	0/1:6	A	G	0
.	.	.	.	.	.	.	.	.	.	.	SOM	GT:/	0:24	0/1:5	G	T	0
.	##	0	.	.	.	.	.	.	.	.	SOM	GT:/	0:25	0/1:5	G	A	0
.	.	.	.	.	.	.	4.1	.	.	.	SOM	GT:/	0:27	0/1:5	G	A	0
.	.	.	.	.	.	.	.	.	.	.	SOM	GT:/	0:8,(	0/1:1	C	T	0
.	.	.	.	.	.	.	.	.	.	.	SOM	GT:/	0:21	0/1:3	T	C	0
.	.	.	.	.	.	.	.	.	.	.	SOM	GT:/	0:81	0/1:1	T	C	0
.	.	.	.	.	.	.	.	.	.	.	SOM	GT:/	0:33	0/1:6	G	A	0
.	.	.	.	.	.	.	.	.	.	.	SOM	GT:/	0:62	0/1:8	C	T	0
.	##	0	0.0	1.0	1.0,D	1,D	5	5.17	.	.	SOM	GT:/	0:78	0/1:1	G	A	0
.	.	.	0.0	0.9	0.997	1,D	5.9	5.77	.	.	SOM	GT:/	0:69	0/1:1	G	A	0
.	.	.	.	.	.	.	.	.	.	.	SOM	GT:/	0:36	0/1:7	T	C	0
.	.	.	.	.	.	.	.	.	0	.	SOM	GT:/	0:17	0/1:2	G	A	0
.	.	.	.	.	.	.	.	.	.	.	SOM	GT:/	0:36	0/1:4	T	C	0
.	.	.	.	.	.	.	.	.	.	.	SOM	GT:/	0:12	0/1:1	T	A	0
.	.	.	.	.	.	.	.	.	.	.	SOM	GT:/	0:31	0/1:3	G	A	0
.	.	.	.	.	.	.	.	.	.	.	SOM	GT:/	0:32	0/1:3	G	A	0
.	.	.	.	.	.	.	2.6	1.45	.	.	SOM	GT:/	0:19	0/1:1	C	T	0
.	.	.	0.0	0.2	0.898	0.687	4.8	3.48	.	.	SOM	GT:/	0:10	0/1:1	C	T	0
0.001	0	0	0.1	0.0	0.108	1.000	3.1	1.59	0	.	SOM	GT:/	0:61	0/1:1	C	T	0
.	.	.	.	.	.	.	.	.	.	.	SOM	GT:/	0:50	0/1:6	A	G	0
.	.	.	.	.	.	.	.	.	.	.	SOM	GT:/	0:8,(	0/1:1	C	T	0
.	.	.	.	.	.	.	.	.	.	.	SOM	GT:/	0:42	0/1:5	C	T	0
0.019	0	0	.	.	.	.	.	.	.	.	DB;S	GT:/	0:65	0/1:7	G	A	0
.	0	.	0.1	0.0	0.014	1,P	.	1.51	0	.	DB;S	GT:/	0:10	0/1:1	G	A	0
.	.	.	.	.	.	.	.	.	.	.	SOM	GT:/	0:22	0/1:4	G	T	0
.	.	.	.	.	.	.	.	.	.	.	SOM	GT:/	0:19	0/1:3	C	T	0
.	.	.	.	.	.	.	.	1.36	.	.	SOM	GT:/	0:71	0/1:8	C	A	0
.	.	.	.	.	.	.	.	.	.	.	SOM	GT:/	0:29	0/1:3	C	A	0
.	.	.	.	.	.	.	.	.	.	0	SOM	GT:/	0:31	0/1:5	C	T	0
.	.	.	.	.	.	.	.	.	.	.	SOM	GT:/	0:60	0/1:1	G	T	0
.	##	0	.	0.0	0.166	1,N	.	.	.	.	SOM	GT:/	0:87	0/1:1	C	T	0
.	##	0	.	.	.	.	.	.	.	.	SOM	GT:/	0:48	0/1:6	G	A	0
.	.	.	0.0	0.0	0.278	0.981	4	1.69	.	.	SOM	GT:/	0:68	0/1:1	G	T	0
.	##	0	1.0	0.0	0.004	1.000	3.3	.	.	.	SOM	GT:/	0:21	0/1:3	C	T	0
.	.	.	0.4	0.8	0.998	1.000	3.8	3.69	.	.	SOM	GT:/	0:67	0/1:1	C	T	0
.	.	.	.	0.1	0.389	1.000	.	.	.	.	SOM	GT:/	0:18	0/1:3	G	A	0
3E-04	0	0	0.3	0.0	0.0,B	0.997	.	1.25	.	.	SOM	GT:/	0:15	0/1:2	G	A	0
.	.	.	.	.	.	.	.	.	0	.	SOM	GT:/	0:17	0/1:2	C	T	0
.	.	.	.	.	.	1,D	3.6	2.41	.	.	SOM	GT:/	0:75	0/1:1	C	T	0
.	.	.	0.0	0.9	1.0,D	1.000	5.2	3.52	.	.	SOM	GT:/	0:42	0/1:6	G	A	0
.	0	0	0.2	0.0	0.002	1,N	.	1.50	.	.	SOM	GT:/	0:70	0/1:1	G	A	0
.	0	0	.	.	.	.	.	.	0	0	SOM	GT:/	0:9,(	0/1:1	A	G	0
.	.	.	.	.	.	.	.	.	.	.	SOM	GT:/	0:19	0/1:3	C	T	0
.	.	.	0.0	0.0	0.101	1,D	6.2	3.93	.	.	SOM	GT:/	0:84	0/1:1	C	A	0
.	##	0	.	.	.	.	.	.	.	.	SOM	GT:/	0:58	0/1:6	G	A	0
.	.	.	0.1	0.2	0.57,I	0.952	3.1	2.96	.	.	SOM	GT:/	0:62	0/1:1	C	T	0
.	##	0	0.0	0.9	1.0,D	1.000	6	5.23	.	.	SOM	GT:/	0:60	0/1:8	C	T	0
.	.	.	.	.	.	.	.	.	.	.	SOM	GT:/	0:18	0/1:3	C	T	0
.	.	.	0.0	1.0	1.0,D	1,D	5.9	3.74	.	.	SOM	GT:/	0:63	0/1:1	G	A	0

.	##	0	0.0(	0.9!	1.0,D	0.999	5.2	4.81	.		.	SOM	GT:/	0:29	0/1:4	G	A	0
.	.	.	.	.	.	.	.	.	.		.	SOM	GT:/	0:26	0/1:4	G	A	0
.	##	0	.	.	.	.	.	1.41	.		.	SOM	GT:/	0:71	0/1:9	C	T	0
.	.	.	.	.	.	.	.	.	.		.	SOM	GT:/	0:26	0/1:3	G	A	0
.	0	0	.	.	.	.	.	.		0	.	SOM	GT:/	0:63	0/1:1	C	T	0
.	.	.	.	.	.	.	.	.	.		.	SOM	GT:/	0:23	0/1:5	G	A	0
.	.	.	0.0	1.0	1.0,D	1,D	5.3	4.47	.		.	SOM	GT:/	0:12	0/1:1	C	T	0
.	.	.	0.1(	0.0	0.024	1,D	5.9	2.74	.		.	SOM	GT:/	0:15	0/1:2	G	A	0
.	.	.	.	.	.	1,D	5.5	13.0	.		.	SOM	GT:/	0:95	0/1:1	G	A	0
.	.	.	.	.	.	.	.	.		0	.	SOM	GT:/	0:12	0/1:2	A	G	0
.	.	.	.	.	.	.	.	.	.		.	SOM	GT:/	0:10	0/1:2	G	C	0
.	.	.	.	.	.	.	.	.	.		.	SOM	GT:/	0:8,(	0/1:2	C	T	0
.	.	.	.	.	.	.	.	.	.		.	SOM	GT:/	0:65	0/1:1	C	T	0
.	0	0	.	.	.	.	.	.		0	0	SOM	GT:/	0:13	0/1:3	C	T	0
.	.	.	.	.	.	.	.	.		0	0.2	SOM	GT:/	0:9,(	0/1:1	G	A	0
.	.	.	.	.	.	.	.	.	.		.	SOM	GT:/	0:9,(	0/1:1	G	C	0
.	.	.	.	.	.	.	.	.		0	.	SOM	GT:/	0:11	0/1:3	A	G	0
.	.	.	.	.	.	.	.	.	.		.	SOM	GT:/	0:36	0/1:4	A	C	0
.	.	.	0.0:	0.0	0.745	1,N	.	.	.		.	SOM	GT:/	0:13	0/1:1	G	A	0
.	##	0	0.0	0.0(	0.003	1.000	.	.	.		.	SOM	GT:/	0:25	0/1:2	G	A	0
.	.	.	.	.	.	.	.	.	.		.	SOM	GT:/	0:10	0/1:1	G	T	0
.	.	.	.	.	.	.	3.5	.	.		.	SOM	GT:/	0:93	0/1:1	G	T	0
.	.	.	.	.	.	.	3.7	.	.		.	SOM	GT:/	0:23	0/1:1	C	A	0
.	.	.	.	.	.	.	.	.	.		.	SOM	GT:/	0:36	0/1:5	T	C	0
.	.	.	.	.	.	.	.	1.82	.		.	SOM	GT:/	0:70	0/1:1	G	A	0
.	.	.	.	.	.	.	.	.	.		.	SOM	GT:/	0:17	0/1:3	A	G	0
.	##	0	1.0	0.0(	0.019	1,N	.	.	.		.	SOM	GT:/	0:10	0/1:1	G	A	0
.	.	.	.	.	.	.	.	.	.		.	SOM	GT:/	0:26	0/1:5	G	A	0
.	0	0	.	.	.	.	4.6	.	.		.	SOM	GT:/	0:26	0/1:5	A	G	0
.	.	.	.	.	.	.	.	.	.		.	SOM	GT:/	0:31	0/1:6	G	A	0
.	.	.	0.0(	1.0	1.0,D	1,D	5.7	4.59	.		.	SOM	GT:/	0:46	0/1:9	C	T	0
.	.	.	.	.	.	.	.	.	.		.	SOM	GT:/	0:13	0/1:2	C	T	0
4E-04	0	0	.	.	.	.	.	.	.		.	SOM	GT:/	0:63	0/1:1	C	T	0
.	.	.	.	.	.	.	.	.	.		.	SOM	GT:/	0:19	0/1:2	C	T	0
.	.	.	.	.	.	.	.	.	.		.	SOM	GT:/	0:36	0/1:7	A	G	0
.	0	0	0.3	0.0	0.006	1,N	.	1.43	.		.	SOM	GT:/	0:14	0/1:2	G	A	0
.	.	.	.	.	.	.	.	.		0	0	SOM	GT:/	0:12	0/1:1	A	G	0
.	.	.	.	.	.	.	6.1	2.42	.		.	SOM	GT:/	0:23	0/1:3	A	G	0
.	.	.	0.4(	0.0(	0.0,B	1,N	4.4	.	.		.	SOM	GT:/	0:52	0/1:8	C	T	0
.	.	.	.	.	.	.	.	.		0	0	SOM	GT:/	0:35	0/1:4	C	T	0
.	##	0	0.0:	0.4!	0.977	0.898	2.2	3.05	.		.	SOM	GT:/	0:94	0/1:1	C	T	0
.	.	.	.	.	.	.	.	.		0	0	SOM	GT:/	0:78	0/1:1	G	A	0
.	.	.	.	.	.	.	.	.	.		.	SOM	GT:/	0:11	0/1:1	G	A	0
.	.	.	.	.	.	.	.	1.27	.		.	SOM	GT:/	0:24	0/1:3	A	G	0
.	.	.	0.0(	0.2(	0.918	0.999	5.1	3.26	.		.	SOM	GT:/	0:86	0/1:1	C	T	0
.	##	0	0.3	0.0:	0.023	0.991	5.8	.	.		.	SOM	GT:/	0:10	0/1:1	C	T	0
.	.	.	.	.	.	.	.	.	.		.	SOM	GT:/	0:12	0/1:2	G	T	0
.	.	.	.	.	.	.	.	.	.		.	SOM	GT:/	0:69	0/1:1	G	T	0
.	.	.	.	.	.	.	2.8	.	.		.	SOM	GT:/	0:82	0/1:1	C	T	0
.	.	.	.	.	.	.	2.8	.	.		.	SOM	GT:/	0:73	0/1:1	C	T	0
.	.	.	.	.	.	.	.	.		0	0	SOM	GT:/	0:90	0/1:1	C	T	0
.	.	.	.	.	.	.	.	.	.		.	SOM	GT:/	0:19	0/1:1	C	A	0
.	.	.	0.1	0.0(	0.02,I	1.000	.	.	.		.	SOM	GT:/	0:48	0/1:1	G	A	0
.	.	.	.	.	.	.	.	.		0	.	SOM	GT:/	0:17	0/1:2	C	T	0

.	##	0	.	.	.	.	.	.	.	.	SOM	GT:/	0:35	0/1:	C	T	0
.	.	.	.	.	.	.	.	.	.	.	SOM	GT:/	0:16	0/1:	A	G	0
.	##	0	.	.	.	.	.	.	.	.	SOM	GT:/	0:56	0/1:	C	T	0
.	.	.	0.0	0.9	0.995	1,D	5.2	4.96	.	.	SOM	GT:/	0:69	0/1:	G	T	0
.	.	.	.	.	.	.	.	.	.	.	SOM	GT:/	0:36	0/1:	C	A	0
.	.	.	.	.	.	.	.	.	.	.	SOM	GT:/	0:8,	0/1:	C	T	0
.	.	.	.	.	.	.	.	.	.	.	SOM	GT:/	0:69	0/1:	T	C	0
.	.	.	.	.	.	.	.	.	.	.	SOM	GT:/	0:64	0/1:	C	T	0
.	##	0	.	.	.	.	.	.	0	0	SOM	GT:/	0:67	0/1:	C	T	0
.	##	0	0.7	0.3	0.86,	1,D	5.5	3.10	0	.	SOM	GT:/	0:59	0/1:	G	A	0
.	.	.	.	.	.	.	.	.	.	.	SOM	GT:/	0:68	0/1:	C	A	0
.	##	0	0.2	0.0	0.006	1.000	2.8	.	0	.	SOM	GT:/	0:11	0/1:	C	T	0
.	.	.	.	.	.	.	.	.	.	.	SOM	GT:/	0:52	0/1:	C	T	0
.	##	0	.	.	.	.	4.8	.	.	.	SOM	GT:/	0:63	0/1:	C	T	0
#####	##	0	0.0	0.9	0.999	1,D	6	4.62	0	.	SOM	GT:/	0:57	0/1:	G	A	0
.	.	.	0.0	0.9	1.0,D	1,D	4.9	4.69	.	.	SOM	GT:/	0:82	0/1:	C	T	0
.	.	.	.	.	.	.	2.4	2.14	.	.	SOM	GT:/	0:94	0/1:	T	C	0
.	.	.	.	.	.	.	.	.	.	.	SOM	GT:/	0:98	0/1:	C	T	0
.	.	.	.	.	.	.	.	.	0	.	DB;	GT:/	0:41	0/1:	C	T	0
.	.	.	.	.	.	.	.	.	0	0	DB;	GT:/	0:23	0/1:	T	C	0
.	.	.	.	.	.	.	.	.	0	0	DB;	GT:/	0:21	0/1:	C	T	0
.	##	0	0.7	0.0	0.005	1.000	.	2.06	.	.	SOM	GT:/	0:56	0/1:	G	A	0
.	.	.	.	.	.	.	.	.	.	.	SOM	GT:/	0:64	0/1:	G	A	0
.	.	.	0.1	0.0	0.032	1.000	.	.	.	.	SOM	GT:/	0:96	0/1:	G	A	0
.	.	.	.	.	.	.	.	.	.	.	SOM	GT:/	0:85	0/1:	C	T	0
.	.	.	.	.	.	.	.	.	.	.	SOM	GT:/	0:80	0/1:	C	T	0
.	.	.	0.1	0.0	0.041	1,N	3	3.01	.	.	SOM	GT:/	0:10	0/1:	C	T	0
0.094	.	.	.	.	.	.	.	.	0.1	0.1	DB;	GT:/	0:35	0/1:	G	A	0
.	.	.	.	.	.	.	.	.	.	.	SOM	GT:/	0:47	0/1:	G	A	0
.	.	.	0.4	0.6	0.94,	1,N	.	3.02	.	.	SOM	GT:/	0:46	0/1:	C	T	0
.	.	.	0.3	0.0	0.047	1,N	2.7	.	.	.	SOM	GT:/	0:18	0/1:	A	G	0
.	.	.	.	.	.	.	.	.	.	.	SOM	GT:/	0:58	0/1:	G	T	0
.	##	0	0.8	0.0	0.016	1,D	.	.	0	.	SOM	GT:/	0:10	0/1:	G	A	0
.	0	0	0.9	0.0	0.007	0.886	3.7	.	.	.	SOM	GT:/	0:71	0/1:	C	T	0
.	.	.	0.1	0.4	0.389	1,N	.	.	.	.	SOM	GT:/	0:68	0/1:	G	T	0
.	.	.	0.0	0.9	1.0,D	0.984	4.5	1.94	.	.	SOM	GT:/	0:15	0/1:	C	T	0
0.002	0	0	.	.	.	.	.	.	.	.	DB;	GT:/	0:67	0/1:	C	T	0
.	.	.	.	.	.	.	.	.	.	.	SOM	GT:/	0:61	0/1:	G	A	0
.	##	0	0.0	0.9	0.999	0.993	4	3.94	.	.	SOM	GT:/	0:43	0/1:	C	T	0
.	.	.	.	.	.	.	.	.	.	.	SOM	GT:/	0:63	0/1:	C	A	0
.	.	.	.	.	.	.	.	1.39	.	.	DB;	GT:/	0:56	0/1:	C	T	0
.	.	.	0.0	0.9	1.0,D	1,D	4.5	4.54	.	.	SOM	GT:/	0:35	0/1:	C	A	0
.	.	.	.	.	.	.	2.9	.	.	.	SOM	GT:/	0:43	0/1:	C	T	0
.	##	0	0.4	0.0	0.001	1.000	3.5	.	.	.	SOM	GT:/	0:70	0/1:	G	A	0
.	.	.	.	.	.	.	.	.	.	.	SOM	GT:/	0:10	0/1:	T	C	0
#####	##	0	.	.	.	.	.	.	.	.	SOM	GT:/	0:12	0/1:	C	T	0
.	.	.	.	.	.	.	.	.	0	.	SOM	GT:/	0:18	0/1:	C	T	0
.	.	.	.	.	.	.	2.1	.	.	.	SOM	GT:/	0:87	0/1:	G	A	0
.	.	.	0.0	0.9	0.999	0.535	.	3.45	.	.	SOM	GT:/	0:60	0/1:	C	A	0
.	.	.	.	.	.	.	.	.	.	.	SOM	GT:/	0:10	0/1:	G	A	0
.	.	.	.	.	.	.	.	.	0	.	DB;	GT:/	0:51	0/1:	C	T	0
.	.	.	.	.	.	.	.	.	.	.	SOM	GT:/	0:11	0/1:	G	A	0
.	.	.	.	.	.	.	.	.	.	.	SOM	GT:/	0:12	0/1:	C	T	0
.	##	0	.	.	.	.	.	.	.	.	SOM	GT:/	0:21	0/1:	C	T	0

.	.	.	.	.	.	.	.	.	.	.	SOM	GT:/	0:21	0/1:4	C	A	0
.	.	.	.	.	.	.	.	.	.	.	SOM	GT:/	0:14	0/1:2	C	T	0
.	.	.	.	.	.	.	.	.	.	.	SOM	GT:/	0:23	0/1:2	A	T	0
.	0	0	0.0	0.9	0.999	0.996	.	2.73	.	0	SOM	GT:/	0:43	0/1:6	G	A	0
.	.	.	.	.	.	.	.	.	.	.	SOM	GT:/	0:10	0/1:1	G	A	0
#####	##	0	0.0	1.0	1.0,D	0.991	4.3	5.25	.	.	SOM	GT:/	0:28	0/1:4	C	T	0
.	.	.	.	.	.	.	3.2	.	.	.	SOM	GT:/	0:57	0/1:7	C	A	0
.	.	.	.	.	.	.	.	.	.	.	SOM	GT:/	0:35	0/1:6	G	A	0
.	.	.	.	.	.	.	.	.	.	.	SOM	GT:/	0:17	0/1:1	C	A	0
.	.	.	.	.	.	.	.	.	.	.	SOM	GT:/	0:15	0/1:2	C	A	0
.	.	.	.	.	.	.	.	.	.	.	SOM	GT:/	0:15	0/1:2	C	T	0
2E-04	0	0	.	.	.	.	.	.	0	0.1	DB;S	GT:/	0:37	0/1:6	G	A	0
.	.	.	0.0	0.9	1.0,D	1.000	4.6	2.46	.	.	SOM	GT:/	0:42	0/1:7	G	T	0
.	.	.	.	.	.	.	.	.	.	.	SOM	GT:/	0:21	0/1:2	C	T	0
.	.	.	.	.	.	.	3	2.07	.	.	SOM	GT:/	0:26	0/1:3	T	C	0
.	.	.	0.7	0.2	0.804	1,N	.	2.47	.	.	SOM	GT:/	0:53	0/1:5	A	T	0
.	##	0	0.0	0.9	1.0,D	1,D	5	5.02	.	.	SOM	GT:/	0:38	0/1:5	T	C	0
0.003	0	0	.	.	.	.	.	.	.	.	SOM	GT:/	0:33	0/1:5	A	G	0
.	.	.	0.0	0.9	0.999	1,D	5.8	4.12	.	.	SOM	GT:/	0:33	0/1:1	A	G	0
.	.	.	.	.	.	.	.	.	.	.	SOM	GT:/	0:59	0/1:9	A	G	0
#####	0	0	0.1	0.7	0.99,I	1,N	.	3.04	.	.	SOM	GT:/	0:62	0/1:9	G	A	0
.	.	.	.	.	.	.	.	.	.	.	SOM	GT:/	0:11	0/1:1	G	T	0
.	.	.	.	.	.	.	.	.	0	.	SOM	GT:/	0:56	0/1:6	G	A	0
.	##	0	.	.	.	.	.	1.99	.	.	SOM	GT:/	0:27	0/1:6	G	A	0
.	##	0	.	.	.	.	.	.	.	.	SOM	GT:/	0:25	0/1:4	C	T	0
.	.	.	0.7	0.9	1.0,D	1.000	5.9	3.84	.	.	SOM	GT:/	0:27	0/1:3	G	A	0
.	.	.	.	.	.	.	.	.	.	.	SOM	GT:/	0:9,(	0/1:9	G	A	0
.	.	.	.	.	.	.	.	.	.	.	SOM	GT:/	0:19	0/1:2	G	A	0
.	.	.	.	.	.	.	.	.	.	.	SOM	GT:/	0:65	0/1:7	A	G	0
.	.	.	.	.	.	.	.	.	.	.	SOM	GT:/	0:13	0/1:1	C	T	0
.	.	.	.	.	.	.	.	.	.	.	SOM	GT:/	0:13	0/1:2	G	A	0
.	.	.	.	.	.	.	.	.	0	.	SOM	GT:/	0:40	0/1:6	G	A	0
.	.	.	0.1	0.0	0.025	1,N	.	.	0	.	SOM	GT:/	0:46	0/1:9	G	A	0
#####	##	0	.	.	.	.	.	.	.	.	SOM	GT:/	0:47	0/1:7	G	A	0
.	.	.	.	.	.	.	.	.	.	.	SOM	GT:/	0:22	0/1:3	C	A	0
.	.	.	.	.	.	.	.	.	.	.	SOM	GT:/	0:53	0/1:1	G	A	0
.	.	.	.	.	.	.	.	.	.	.	SOM	GT:/	0:12	0/1:1	C	A	0
5E-04	0	0	0.3	0.7	0.996	1,D	6	2.56	0	.	SOM	GT:/	0:28	0/1:4	G	A	0
.	.	.	.	.	.	.	3.6	1.58	.	.	SOM	GT:/	0:8,(	0/1:7	A	G	0
.	.	.	.	.	.	.	.	.	0	.	SOM	GT:/	0:11	0/1:2	G	A	0
.	.	.	.	.	.	.	.	.	.	.	SOM	GT:/	0:13	0/1:1	A	G	0
.	##	0	.	.	.	.	.	.	0	0	SOM	GT:/	0:56	0/1:8	C	T	0
.	.	.	0.0	0.9	0.998	1,D	6.2	5.20	.	.	SOM	GT:/	0:48	0/1:9	C	T	0
.	.	.	.	.	.	.	.	.	.	.	SOM	GT:/	0:63	0/1:9	G	A	0
2E-04	##	0	0.0	0.9	1.0,D	1,D	5.2	4.17	.	.	SOM	GT:/	0:10	0/1:1	C	T	0
.	.	.	0.0	0.9	1.0,D	1,D	4.4	3.97	.	.	SOM	GT:/	0:37	0/1:4	C	A	0
.	.	.	.	.	.	.	.	.	.	.	SOM	GT:/	0:31	0/1:7	C	A	0
.	##	0	.	.	.	.	2.1	1.45	.	.	SOM	GT:/	0:28	0/1:4	A	G	0
.	0	0	.	.	.	.	.	.	.	.	SOM	GT:/	0:70	0/1:9	A	G	0
.	.	.	.	.	.	.	.	.	.	.	SOM	GT:/	0:21	0/1:2	A	G	0
.	.	.	.	.	.	.	.	.	.	.	SOM	GT:/	0:14	0/1:1	C	T	0
.	.	.	.	.	.	.	.	2.57	.	.	SOM	GT:/	0:38	0/1:6	A	G	0
.	.	.	.	.	.	.	.	.	.	.	SOM	GT:/	0:39	0/1:5	A	G	0
.	##	0	0.0	0.9	1.0,D	1.000	5.2	4.83	0	.	SOM	GT:/	0:43	0/1:8	C	T	0

.	.	.	.	.	.	.	4.4	1.39	.	.	SOM	GT:/	0:18	0/1:3	C	T	0
.	.	.	.	.	.	.	.	1.28	.	.	SOM	GT:/	0:43	0/1:8	C	T	0
.	.	.	.	.	.	.	.	.	.	.	SOM	GT:/	0:17	0/1:5	C	T	0
.	.	.	.	.	.	.	.	.	.	.	SOM	GT:/	0:8,(	0/1:1	C	T	0
.	.	.	.	.	.	.	.	1.59	.	.	SOM	GT:/	0:10	0/1:1	G	A	0
#####	##	.	.	.	.	1,A	3.9	12.2	.	0	SOM	GT:/	0:48	0/1:5	G	A	0
.	##	0	0.1:	0.0(	0.066	0.723	3.9	3.01	.	.	SOM	GT:/	0:21	0/1:5	G	A	0
.	.	.	.	.	.	.	.	.	.	.	SOM	GT:/	0:40	0/1:3	G	T	0
.	.	.	.	.	.	.	.	2.29	.	.	SOM	GT:/	0:17	0/1:1	T	G	0
.	##	0	0.0	0.3:	0.912	0.999	5.8	3.59	.	.	SOM	GT:/	0:52	0/1:1	C	T	0
.	.	.	.	.	.	.	.	.	.	.	SOM	GT:/	0:11	0/1:4	G	A	0
.	.	.	.	.	.	.	.	.	.	.	SOM	GT:/	0:38	0/1:4	G	A	0
.	.	.	.	.	.	.	.	.	.	.	SOM	GT:/	0:37	0/1:4	C	T	0
.	##	0	0.0	0.8:	0.999	1,D	4.8	4.65	.	.	SOM	GT:/	0:65	0/1:8	G	A	0
#####	##	0	0.0	1.0	1.0,D	1,D	4.9	4.09	.	.	SOM	GT:/	0:47	0/1:7	C	T	0
.	.	.	.	.	.	.	.	.	.	.	SOM	GT:/	0:56	0/1:8	C	T	0
.	.	.	0.0	0.9:	1.0,D	0.998	5.6	4.37	.	.	SOM	GT:/	0:12	0/1:1	G	A	0
.	.	.	.	.	.	.	3	2.85	.	.	SOM	GT:/	0:60	0/1:9	A	G	0
.	.	.	.	.	.	.	.	.	.	0	SOM	GT:/	0:15	0/1:2	C	T	0
.	.	.	.	.	.	.	.	.	.	.	SOM	GT:/	0:36	0/1:6	C	A	0
.	.	.	.	.	.	.	.	.	.	.	SOM	GT:/	0:17	0/1:1	G	A	0
.	.	.	.	.	.	.	.	.	.	.	SOM	GT:/	0:17	0/1:2	T	G	0
.	.	.	.	.	.	.	.	.	.	.	SOM	GT:/	0:18	0/1:3	C	T	0
.	.	.	0.0:	0.4!	0.822	0.954	3.9	1.76	.	.	SOM	GT:/	0:70	0/1:1	C	T	0
.	.	.	.	.	.	.	.	.	.	.	SOM	GT:/	0:9,(	0/1:7	T	A	0
.	.	.	.	.	.	.	.	.	.	.	SOM	GT:/	0:42	0/1:6	A	G	0
.	##	0	.	.	.	.	.	.	0	.	SOM	GT:/	0:55	0/1:7	G	A	0
.	.	.	.	.	.	1,A	5	8.40	.	.	SOM	GT:/	0:67	0/1:1	C	T	0
5E-04	0	0	.	.	.	.	.	1.65	.	.	SOM	GT:/	0:31	0/1:5	C	T	0
.	.	.	0.0(	0.9!	1.0,D	1,D	3.8	4.93	.	.	SOM	GT:/	0:53	0/1:9	C	T	0
.	.	.	.	.	.	.	.	1.50	.	.	SOM	GT:/	0:63	0/1:1	G	A	0
.	.	.	.	.	.	.	.	1.46	.	.	SOM	GT:/	0:57	0/1:1	G	T	0
.	.	.	.	.	.	.	.	.	.	.	SOM	GT:/	0:24	0/1:5	C	T	0
.	0	0	.	.	.	.	.	.	.	.	SOM	GT:/	0:36	0/1:7	C	T	0
.	.	.	0.6'	0.0(	0.001	1,N	.	.	.	.	SOM	GT:/	0:81	0/1:1	G	T	0
.	.	.	.	.	.	.	2.8	.	.	.	SOM	GT:/	0:15	0/1:2	G	A	0
.	##	0	.	.	.	.	.	.	.	.	SOM	GT:/	0:37	0/1:6	G	A	0
.	.	.	.	.	.	.	.	.	.	.	SOM	GT:/	0:19	0/1:2	G	A	0
.	.	.	.	.	.	.	.	.	.	.	SOM	GT:/	0:44	0/1:5	T	C	0
.	.	.	.	.	.	.	.	.	.	.	SOM	GT:/	0:21	0/1:3	G	T	0
.	.	.	.	.	.	.	.	.	.	.	SOM	GT:/	0:12	0/1:4	C	A	0
.	.	.	.	.	.	.	3.2	2.43	.	.	SOM	GT:/	0:29	0/1:4	G	T	0
.	.	.	.	.	.	.	.	.	.	.	SOM	GT:/	0:19	0/1:3	G	A	0
.	##	0	.	.	.	.	.	.	.	.	SOM	GT:/	0:16	0/1:2	C	T	0
#####	##	0	0.1:	0.0(	0.008	1,N	.	.	.	.	SOM	GT:/	0:46	0/1:8	G	A	0
.	.	.	0.1'	0.2!	0.687	1.000	4.7	1.29	.	.	SOM	GT:/	0:56	0/1:8	A	G	0
.	.	.	.	.	.	.	.	.	.	.	SOM	GT:/	0:86	0/1:1	G	T	0
.	.	.	.	.	.	.	.	.	.	.	SOM	GT:/	0:24	0/1:5	G	A	0
.	0	0	.	.	.	.	.	.	.	.	SOM	GT:/	0:51	0/1:9	G	A	0
.	0	0	.	.	.	.	.	.	.	.	SOM	GT:/	0:75	0/1:1	G	A	0
.	.	.	.	.	.	.	.	.	.	.	SOM	GT:/	0:93	0/1:1	T	C	0
.	.	.	.	.	.	.	.	.	.	.	SOM	GT:/	0:28	0/1:4	G	A	0
.	.	.	0.0	0.9!	1.0,D	1.000	3.2	1.94	.	.	SOM	GT:/	0:95	0/1:1	C	T	0
2E-04	##	0	.	.	.	.	.	.	0	.	SOM	GT:/	0:23	0/1:3	C	T	0

.	.	.	.	.	.	.	.	.	.	.	SOM	GT:/	0:10	0/1:2	A	G	0
.	.	.	.	.	.	.	.	.	.	.	SOM	GT:/	0:48	0/1:9	C	T	0
.	.	.	.	.	.	.	.	.	.	.	SOM	GT:/	0:51	0/1:9	G	A	0
.	.	.	.	.	.	.	.	.	.	.	SOM	GT:/	0:72	0/1:9	G	T	0
.	.	.	.	.	.	.	.	.	.	.	SOM	GT:/	0:19	0/1:3	G	A	0
.	.	.	.	.	.	.	.	.	.	.	SOM	GT:/	0:8,(	0/1:6	T	C	0
.	.	.	0.1	0.0	0.027	1,N	4.8	.	.	.	SOM	GT:/	0:83	0/1:1	T	C	0
.	.	.	.	.	.	.	.	.	.	.	SOM	GT:/	0:39	0/1:5	G	A	0
.	.	.	.	.	.	.	.	.	.	.	SOM	GT:/	0:26	0/1:2	C	T	0
.	.	.	.	.	.	.	3.4	1.62	.	.	SOM	GT:/	0:65	0/1:1	G	A	0
0.012	0	0	.	.	.	.	3.9	.	0	0	DB;S	GT:/	0:10	0/1:1	C	T	0
.	.	.	.	.	.	1,D	5.4	16.1	.	.	SOM	GT:/	0:83	0/1:1	G	A	0
.	.	.	0.7	0.0	0.024	0.991	5.2	.	.	.	SOM	GT:/	0:13	0/1:2	C	T	0
0.005	0	0	0.1	0.0	0.008	1,N	.	1.50	0	.	DB;S	GT:/	0:73	0/1:1	C	T	0
.	##	0	.	.	.	.	.	.	0	0	SOM	GT:/	0:37	0/1:4	C	T	0
.	.	.	.	0.9	0.999	.	5.7	5.19	.	.	SOM	GT:/	0:79	0/1:1	G	A	0
.	##	0	0.0	0.9	1.0,D	1,D	4.7	5.02	.	.	SOM	GT:/	0:20	0/1:3	C	T	0
.	.	.	0.0	1.0	1.0,D	1,D	4.9	4.04	.	.	SOM	GT:/	0:47	0/1:8	C	A	0
.	.	.	.	.	.	.	3.1	3.01	.	.	SOM	GT:/	0:21	0/1:3	G	A	0
.	.	.	.	.	.	.	.	.	.	.	SOM	GT:/	0:10	0/1:3	G	A	0
.	##	0	0.0	0.1	0.549	1,D	3.2	2.63	.	.	SOM	GT:/	0:14	0/1:2	G	A	0
.	.	.	0.3	.	.	0.992	2.7	1.67	.	.	SOM	GT:/	0:64	0/1:9	G	A	0
.	.	.	.	.	.	.	.	.	.	.	SOM	GT:/	0:21	0/1:2	G	T	0
.	.	.	.	.	.	.	.	.	.	.	SOM	GT:/	0:55	0/1:1	C	T	0
.	.	.	.	.	.	.	.	.	.	.	SOM	GT:/	0:41	0/1:5	G	T	0
.	.	.	.	.	.	.	.	.	.	.	SOM	GT:/	0:35	0/1:5	C	A	0
.	.	.	.	.	.	.	.	.	.	.	SOM	GT:/	0:59	0/1:7	A	C	0
0.006	0	0	0.0	0.9	1.0,D	1,D	5.8	5.04	.	.	DB;S	GT:/	0:96	0/1:1	G	A	0
.	.	.	0.4	0.0	0.002	1.000	5.6	2.60	.	.	SOM	GT:/	0:31	0/1:4	A	G	0
.	.	.	.	.	.	.	.	.	.	.	SOM	GT:/	0:16	0/1:3	A	T	0
.	.	.	.	.	.	.	.	1.90	.	.	SOM	GT:/	0:19	0/1:4	C	A	0
.	.	.	.	.	.	.	.	.	.	.	SOM	GT:/	0:69	0/1:9	C	T	0
.	.	.	0.0	0.9	1.0,D	1,D	5.4	4.86	.	.	SOM	GT:/	0:52	0/1:6	C	T	0
.	.	.	0.1	0.0	0.703	0.630	2.3	2.96	.	.	SOM	GT:/	0:11	0/1:2	G	A	0
.	.	.	.	.	.	1,D	3.5	11.1	.	.	SOM	GT:/	0:64	0/1:1	G	A	0
.	##	.	.	.	.	.	.	.	.	.	SOM	GT:/	0:75	0/1:1	C	T	0
.	.	.	0.0	0.9	0.999	1.000	3.5	.	.	.	SOM	GT:/	0:22	0/1:5	T	C	0
.	.	.	.	.	.	.	.	.	.	.	SOM	GT:/	0:45	0/1:5	T	C	0
.	.	.	0.0	1.0	1.0,D	1.000	4.4	4.35	.	.	SOM	GT:/	0:53	0/1:1	T	G	0
.	##	0	0.0	1.0	1.0,D	0.909	3.2	.	.	.	SOM	GT:/	0:16	0/1:2	G	A	0
#####	.	.	1.0	0.0	0.006	1,N	.	.	.	.	SOM	GT:/	0:77	0/1:1	C	T	0
.	##	0	.	.	.	.	.	.	0	.	SOM	GT:/	0:35	0/1:4	C	T	0
.	##	0	0.0	1.0	1.0,D	1.000	5	4.11	.	.	SOM	GT:/	0:98	0/1:1	G	A	0
.	.	.	.	.	.	.	.	.	.	.	SOM	GT:/	0:17	0/1:2	C	A	0
.	.	.	.	.	.	.	5.1	.	.	.	SOM	GT:/	0:14	0/1:2	G	T	0
.	##	0	.	.	.	.	.	.	.	0	SOM	GT:/	0:68	0/1:8	G	A	0
#####	##	0	0.0	0.2	0.928	1.000	5	3.02	.	.	SOM	GT:/	0:67	0/1:1	C	T	0
#####	##	0	0.0	0.0	0.006	0.757	3.7	1.63	.	.	SOM	GT:/	0:92	0/1:1	C	T	0
.	.	.	.	.	.	.	4.7	.	.	.	SOM	GT:/	0:15	0/1:3	T	G	0
.	##	0	0.3	0.8	0.997	1,D	5.3	3.80	.	.	SOM	GT:/	0:34	0/1:6	C	T	0
0.023	0	0	.	.	.	.	.	.	.	.	DB;S	GT:/	0:40	0/1:9	G	A	0
.	.	.	.	.	.	.	.	.	.	.	SOM	GT:/	0:32	0/1:7	T	A	0
.	##	0	0.0	0.0	0.218	1.000	5	3.71	.	.	SOM	GT:/	0:45	0/1:9	C	T	0
.	##	0	.	.	.	.	.	1.45	.	.	SOM	GT:/	0:86	0/1:1	G	A	0

7E-04	0	0	.	.	.	.	.	.	.	.	SOM	GT:/	0:25	0/1:4	C	T	0
.	.	.	.	.	.	.	.	.	.	.	SOM	GT:/	0:15	0/1:2	G	A	0
.	.	.	0.2(	0.0	0.0,B	1,N	.	.	.	.	SOM	GT:/	0:86	0/1:1	G	A	0
.	.	.	.	.	.	.	3.1	.	.	.	SOM	GT:/	0:49	0/1:9	G	A	0
#####	##	0	0.1	0.6(	0.998	0.820	2.3	4.16	.	.	SOM	GT:/	0:59	0/1:1	G	A	0
.	.	.	0.0	0.7	0.991	0.964	3.8	3.80	.	.	SOM	GT:/	0:45	0/1:9	G	A	0
.	.	.	.	.	.	.	.	.	.	.	SOM	GT:/	0:12	0/1:2	T	G	0
.	.	.	.	.	.	.	.	.	.	.	SOM	GT:/	0:16	0/1:2	G	A	0
.	.	.	.	.	.	.	2.6	.	.	.	SOM	GT:/	0:13	0/1:1	C	T	0
.	.	.	.	.	.	.	2.1	.	.	.	SOM	GT:/	0:61	0/1:1	C	T	0
.	.	.	0.1	0.9	0.995	0.537	2.3	2.09	.	.	SOM	GT:/	0:59	0/1:1	C	T	0
.	.	.	0.0	0.9	0.999	1.000	5	4.68	.	.	SOM	GT:/	0:67	0/1:1	T	C	0
.	.	.	.	.	.	.	.	.	.	.	SOM	GT:/	0:23	0/1:3	G	A	0
.	.	.	.	.	.	.	.	.	0	.	SOM	GT:/	0:52	0/1:5	C	T	0
#####	##	0	.	.	.	.	.	3.28	.	.	SOM	GT:/	0:24	0/1:4	C	T	0
.	##	0	0.0	0.9	1.0,D	1,D	4.1	3.12	.	.	SOM	GT:/	0:27	0/1:4	C	T	0
.	.	.	.	.	.	.	.	.	.	.	SOM	GT:/	0:25	0/1:2	G	A	0
.	.	.	.	.	.	.	.	.	.	.	SOM	GT:/	0:28	0/1:3	A	G	0
.	##	0	0.0	0.9	1.0,D	1.000	4.8	4.03	.	.	SOM	GT:/	0:74	0/1:1	C	T	0
.	.	.	.	.	.	.	2.5	.	.	.	SOM	GT:/	0:59	0/1:1	G	A	0
.	.	.	.	.	.	.	.	.	.	.	SOM	GT:/	0:28	0/1:3	G	C	0
.	.	.	.	.	.	.	2.4	.	.	.	SOM	GT:/	0:39	0/1:5	C	A	0
.	.	.	.	.	.	.	.	.	.	.	SOM	GT:/	0:38	0/1:5	A	G	0
.	.	.	.	.	.	.	.	.	.	.	SOM	GT:/	0:19	0/1:2	C	A	0
.	.	.	.	.	.	.	.	.	.	.	SOM	GT:/	0:22	0/1:2	T	C	0
.	.	.	.	.	.	.	4.4	.	.	.	SOM	GT:/	0:36	0/1:5	G	A	0
.	.	.	.	.	.	.	.	.	.	.	SOM	GT:/	0:19	0/1:3	C	T	0
.	##	0	0.2	0.0	0.652	1,N	.	.	.	.	SOM	GT:/	0:75	0/1:9	G	A	0
.	##	0	0.1	0.0	0.096	1,N	.	1.64	.	.	SOM	GT:/	0:59	0/1:9	G	A	0
.	##	0	.	.	.	.	.	.	.	.	SOM	GT:/	0:29	0/1:5	C	T	0
.	.	.	.	.	.	.	.	.	.	.	SOM	GT:/	0:11	0/1:2	G	A	0
.	.	.	.	.	.	.	.	.	0	0.1	SOM	GT:/	0:10	0/1:2	G	C	0
.	.	.	.	.	.	.	.	.	.	.	SOM	GT:/	0:44	0/1:6	G	A	0
.	.	.	0.0	0.9	1.0,D	1,D	5.6	4.14	.	.	SOM	GT:/	0:50	0/1:1	G	A	0
.	.	.	.	.	.	.	.	.	.	.	SOM	GT:/	0:20	0/1:2	G	A	0
.	0	0	.	.	.	.	.	.	.	.	DB;S	GT:/	0:30	0/1:4	G	A	0
4E-04	0	0	0.0	0.2	0.647	1,D	5.6	3.66	.	.	SOM	GT:/	0:44	0/1:6	G	A	0
.	.	.	.	.	.	.	.	.	.	.	SOM	GT:/	0:20	0/1:2	G	A	0
.	##	0	0.0	0.2(	0.284	1,D	5.3	3.64	.	.	SOM	GT:/	0:68	0/1:9	C	T	0
.	.	.	.	.	.	.	3.9	1.89	.	.	SOM	GT:/	0:28	0/1:4	A	G	0
.	.	.	.	.	.	.	.	1.33	.	.	SOM	GT:/	0:48	0/1:6	T	C	0
.	.	.	.	.	.	.	.	1.75	.	.	SOM	GT:/	0:99	0/1:1	A	G	0
.	.	.	.	.	.	.	.	.	.	.	SOM	GT:/	0:30	0/1:6	C	T	0
.	.	.	.	.	.	.	5.8	5.38	.	.	SOM	GT:/	0:16	0/1:3	G	A	0
.	.	.	.	.	.	.	.	.	.	.	SOM	GT:/	0:63	0/1:8	C	T	0
.	##	0	.	.	.	.	.	.	.	.	SOM	GT:/	0:35	0/1:5	G	A	0
.	.	.	.	.	.	.	.	.	.	0	SOM	GT:/	0:26	0/1:2	T	G	0
.	.	.	.	.	.	.	4.8	.	.	.	SOM	GT:/	0:83	0/1:1	C	T	0
.	.	.	.	.	.	.	2.7	.	.	.	SOM	GT:/	0:75	0/1:1	C	A	0
.	.	.	0.1	0.2	0.673	0.986	4.9	3.03	.	.	SOM	GT:/	0:70	0/1:1	C	T	0
.	.	.	.	.	.	.	.	.	.	.	SOM	GT:/	0:21	0/1:3	G	T	0
.	.	.	.	.	.	.	.	2.81	.	.	SOM	GT:/	0:78	0/1:1	C	A	0
.	.	.	.	.	.	.	4.7	.	.	.	SOM	GT:/	0:43	0/1:6	G	A	0
.	##	0	.	.	.	.	.	.	.	.	SOM	GT:/	0:38	0/1:6	T	C	0

.	.	.	0.0(	1.0.	1.0,D	1,D	4.7	4.88	.	.	SON	GT:/	0:42	0/1:(	C	A	0
0.001	0	0	0.0:	0.9'	1.0,D	0.839	4.3	3.69	0	.	SON	GT:/	0:10	0/1:2	C	T	0
0.002	0	0	0.0(	0.7'	0.819	1,N	.	1.29	.	.	SON	GT:/	0:25	0/1:4	C	T	0
.	0	0	.	.	.	.	.	.	0	.	SON	GT:/	0:14	0/1:1	C	T	0
.	.	.	.	.	.	.	2.9	.	.	.	SON	GT:/	0:58	0/1:8	C	A	0
.	.	.	0.0	0.9!	0.998	1,D	5.5	3.68	.	.	SON	GT:/	0:48	0/1:6	C	T	0
.	.	.	0.0	1.0	1.0,D	1,D	5.4	5.19	.	.	SON	GT:/	0:78	0/1:1	G	A	0
.	0	0	.	.	.	.	.	.	.	.	SON	GT:/	0:36	0/1:7	C	T	0
.	##	.	.	.	.	.	.	.	.	.	DB;S	GT:/	0:21	0/1:3	G	T	0
.	##	0	0.1'	1.0	1.0,D	1,D	5.9	4.06	.	.	SON	GT:/	0:72	0/1:1	G	A	0
.	.	.	0.0!	0.6	0.566	0.975	.	3.26	.	.	SON	GT:/	0:87	0/1:1	G	T	0
.	.	.	.	.	.	.	.	.	.	.	SON	GT:/	0:14	0/1:1	G	A	0
.	.	.	.	.	.	.	.	.	.	.	SON	GT:/	0:9,(	0/1:1	G	A	0
.	.	.	.	.	.	.	.	.	.	.	SON	GT:/	0:22	0/1:3	C	T	0
.	.	.	.	.	.	.	2	.	.	.	SON	GT:/	0:9,(	0/1:4	G	A	0
.	##	0	0.5(	0.1(	0.642	0.851	4.9	3.51	.	.	SON	GT:/	0:45	0/1:7	G	A	0
.	.	.	.	.	.	.	.	.	.	.	SON	GT:/	0:17	0/1:3	G	T	0
.	.	.	.	.	.	.	3.7	1.29	.	.	SON	GT:/	0:14	0/1:2	C	T	0
#####	##	0	.	.	.	.	.	.	0	.	SON	GT:/	0:13	0/1:1	C	T	0
.	.	.	.	.	.	.	.	.	0	0	SON	GT:/	0:43	0/1:6	G	A	0
.	.	.	.	.	.	.	.	.	.	0	SON	GT:/	0:17	0/1:2	C	T	0
.	.	.	.	.	.	.	.	.	.	.	SON	GT:/	0:91	0/1:1	C	A	0
.	.	.	.	.	.	.	2.3	.	.	.	SON	GT:/	0:28	0/1:4	G	T	0
.	.	.	0.0:	0.9:	0.997	1.00(	4.9	3.64	.	.	SON	GT:/	0:12	0/1:2	A	G	0
.	.	.	0.1:	0.0(	0.04,I	1,N	.	2.00	.	.	SON	GT:/	0:83	0/1:1	G	A	0
.	.	.	.	.	.	.	.	3.44	.	.	SON	GT:/	0:19	0/1:4	C	T	0
.	.	.	.	.	.	.	.	.	.	.	SON	GT:/	0:8,(	0/1:1	C	T	0
.	.	.	.	.	.	.	.	.	.	.	SON	GT:/	0:87	0/1:1	C	A	0
.	.	.	.	.	.	1.00(	2.9	1.47	.	.	SON	GT:/	0:35	0/1:6	T	C	0
.	.	.	0.1:	0.0(	0.0,B	1,N	.	.	.	.	SON	GT:/	0:57	0/1:9	T	A	0
.	.	.	0.1!	0.6(	0.885	1,D	3.1	2.21	.	.	SON	GT:/	0:35	0/1:5	G	A	0
.	.	.	.	.	.	.	2.2	1.34	.	.	SON	GT:/	0:8,(	0/1:1	A	G	0
.	.	.	.	.	.	.	.	.	0	.	DB;S	GT:/	0:21	0/1:2	T	C	0
.	.	.	.	.	.	.	.	.	.	.	SON	GT:/	0:64	0/1:9	C	A	0
.	.	.	.	.	.	.	.	.	.	.	SON	GT:/	0:69	0/1:1	G	A	0
.	.	.	.	.	.	.	.	2.01	.	.	SON	GT:/	0:50	0/1:8	C	A	0
.	##	0	0.0:	0.9!	1.0,D	1,D	.	4.09	.	.	SON	GT:/	0:46	0/1:7	C	T	0
.	.	.	0.0(	0.9:	0.996	1,D	6	3.76	.	.	SON	GT:/	0:51	0/1:7	T	C	0
.	##	0	.	.	.	.	.	.	.	.	SON	GT:/	0:17	0/1:1	C	T	0
.	.	.	0.0(	0.9!	1.0,D	1.00(	4	4.05	.	.	SON	GT:/	0:39	0/1:6	C	T	0
.	.	.	.	.	.	1,A	3.9	7.41	.	.	SON	GT:/	0:25	0/1:4	G	T	0
.	.	.	.	.	.	.	.	.	.	.	SON	GT:/	0:8,(	0/1:2	A	C	0
.	.	.	0.0	0.8(	0.988	0.968	3.7	1.94	.	.	SON	GT:/	0:27	0/1:2	A	G	0
.	.	.	.	.	.	.	.	.	.	.	SON	GT:/	0:44	0/1:6	A	G	0
.	.	.	.	.	.	.	3.1	2.96	.	.	SON	GT:/	0:36	0/1:5	C	T	0
4E-04	##	0	.	.	.	.	.	.	.	.	SON	GT:/	0:62	0/1:1	C	T	0
.	.	.	.	.	.	.	.	.	.	.	SON	GT:/	0:36	0/1:6	C	T	0
.	.	.	.	.	.	.	.	.	.	.	SON	GT:/	0:36	0/1:6	G	A	0
.	.	.	.	.	.	.	.	.	.	.	SON	GT:/	0:10	0/1:1	C	T	0
.	.	.	.	.	.	.	.	.	.	.	SON	GT:/	0:9,(	0/1:1	C	T	0
.	.	.	.	.	.	.	.	.	.	.	SON	GT:/	0:13	0/1:4	C	T	0
.	.	.	.	.	.	.	.	.	.	.	SON	GT:/	0:27	0/1:2	A	T	0
.	.	.	0.0!	0.0(	0.246	1,N	.	.	.	.	SON	GT:/	0:55	0/1:9	A	C	0
.	.	.	.	.	.	.	.	.	.	.	SON	GT:/	0:36	0/1:6	C	T	0

.	.	.	0.0(	0.8(	0.98,I	1,N	.	2.20	.	.	SOM	GT:/	0:15	0/1:2	T	C	0
.	.	.	.	.	.	.	.	1.67	.	.	SOM	GT:/	0:66	0/1:1	G	A	0
.	##	0	0.0(	0.9!	1.0,D	1,D	4.6	5.03	.	.	SOM	GT:/	0:56	0/1:9	G	A	0
.	.	.	.	.	.	.	.	.	.	.	SOM	GT:/	0:40	0/1:6	A	G	0
.	.	.	.	.	.	.	2.5	3.31	.	.	DB;S	GT:/	0:24	0/1:4	G	A	0
.	.	.	.	.	.	.	.	.	.	.	SOM	GT:/	0:77	0/1:9	G	A	0
.	.	.	0.0!	0.9!	0.996	1,D	5.7	5.20	.	.	SOM	GT:/	0:34	0/1:5	T	C	0
.	.	.	.	.	.	.	.	.	.	.	SOM	GT:/	0:58	0/1:1	A	G	0
.	.	.	.	.	.	.	.	.	.	.	SOM	GT:/	0:45	0/1:7	G	T	0
#####	##	.	.	.	.	.	.	.	0	.	SOM	GT:/	0:20	0/1:3	C	T	0
.	##	0	0.1:	0.4	0.995	0.968	.	.	.	.	SOM	GT:/	0:63	0/1:9	G	A	0
.	.	.	.	.	.	.	4.6	.	.	.	SOM	GT:/	0:69	0/1:1	C	T	0
.	.	.	.	.	.	.	.	.	.	.	SOM	GT:/	0:38	0/1:6	G	A	0
.	.	.	.	.	.	.	.	2.01	.	.	SOM	GT:/	0:49	0/1:1	C	T	0
.	.	.	.	.	.	.	.	.	.	.	SOM	GT:/	0:36	0/1:7	G	A	0
.	.	.	.	.	.	.	5.3	2.00	.	.	SOM	GT:/	0:14	0/1:2	G	T	0
#####	##	0	0.0	0.8	0.991	0.999	4.1	4.54	.	.	SOM	GT:/	0:41	0/1:4	C	T	0
.	##	0	0.0(	0.9!	1.0,D	1.000	4.9	4.81	.	.	SOM	GT:/	0:82	0/1:1	C	T	0
.	.	.	.	.	.	.	.	.	0	.	DB;S	GT:/	0:34	0/1:3	T	A	0
.	.	.	.	.	.	.	.	.	.	.	SOM	GT:/	0:14	0/1:2	G	C	0
.	.	.	0.0	0.9!	0.999	1,D	5	4.83	.	.	SOM	GT:/	0:95	0/1:1	G	A	0
#####	##	0	.	.	.	.	.	.	0	.	SOM	GT:/	0:38	0/1:7	G	A	0
.	.	.	.	.	.	.	.	.	.	.	SOM	GT:/	0:26	0/1:3	T	C	0
.	0	0	0.0	0.0	0.068	1.000	.	3.03	.	.	SOM	GT:/	0:10	0/1:1	G	A	0
.	.	.	0.1:	0.2	0.565	0.999	3	2.43	.	.	SOM	GT:/	0:68	0/1:1	T	G	0
.	0	0	.	.	.	.	3.9	.	.	.	SOM	GT:/	0:45	0/1:5	C	T	0
.	.	.	0.0	0.5	0.956	1,D	5.2	4.98	.	.	SOM	GT:/	0:56	0/1:7	A	G	0
.	.	.	0.2	0.0	0.019	1,D	4.9	2.97	.	.	SOM	GT:/	0:58	0/1:7	A	G	0
.	.	.	0.0!	0.0(	0.003	1,N	.	.	.	.	SOM	GT:/	0:55	0/1:8	G	A	0
.	##	0	.	.	.	.	2.9	.	.	.	SOM	GT:/	0:50	0/1:9	C	T	0
.	.	.	0.0(	0.9	0.999	1.000	5.5	4.78	.	.	SOM	GT:/	0:35	0/1:5	A	C	0
#####	##	0	0.4	0.1!	0.736	1.000	5.9	2.50	.	.	SOM	GT:/	0:30	0/1:3	C	T	0
.	.	.	.	.	.	.	.	1.50	.	.	SOM	GT:/	0:58	0/1:7	G	T	0
.	.	.	0.2	0.9	0.999	1,D	5.1	3.90	.	.	SOM	GT:/	0:37	0/1:6	C	A	0
.	.	.	0.0	0.9!	1.0,D	1,D	5.7	4.55	.	.	SOM	GT:/	0:50	0/1:7	G	T	0
.	##	0	.	.	.	.	3.1	1.36	0	.	SOM	GT:/	0:59	0/1:6	C	T	0
.	.	.	.	.	.	.	.	.	0	.	SOM	GT:/	0:38	0/1:3	A	G	0
.	.	.	0.0	0.8(	0.997	0.994	3.3	2.82	.	.	SOM	GT:/	0:32	0/1:6	G	T	0
.	.	.	.	.	.	.	.	.	.	.	SOM	GT:/	0:72	0/1:1	C	T	0
.	.	.	.	.	.	.	.	.	.	.	SOM	GT:/	0:30	0/1:3	G	A	0
.	##	0	0.0	0.2(	0.923	1,D	5.7	3.88	.	.	SOM	GT:/	0:58	0/1:8	G	A	0
.	##	.	.	.	.	.	.	.	.	.	SOM	GT:/	0:26	0/1:5	G	A	0
.	.	.	.	.	.	.	.	.	.	.	SOM	GT:/	0:27	0/1:3	T	C	0
.	##	0	0.5(	0.0(	0.002	1,N	4.1	.	.	.	SOM	GT:/	0:75	0/1:1	C	T	0
.	.	.	.	.	.	.	.	.	.	.	SOM	GT:/	0:53	0/1:8	C	T	0
.	.	.	.	.	.	.	.	.	.	.	SOM	GT:/	0:29	0/1:2	A	G	0
.	.	.	.	.	.	.	4.4	.	.	.	SOM	GT:/	0:23	0/1:4	G	T	0
.	.	.	0.0	0.9!	1.0,D	1,D	5.4	4.00	.	.	SOM	GT:/	0:71	0/1:9	G	T	0
.	.	.	.	.	.	.	.	.	.	.	SOM	GT:/	0:48	0/1:8	G	A	0
.	.	.	.	.	.	.	.	.	.	.	SOM	GT:/	0:75	0/1:1	C	T	0
.	.	.	0.2!	1.0	1.0,D	1,D	6	2.22	.	.	SOM	GT:/	0:23	0/1:5	C	T	0
.	.	.	.	.	.	.	.	.	.	.	SOM	GT:/	0:13	0/1:1	T	C	0
.	.	.	.	.	.	.	.	.	.	.	SOM	GT:/	0:19	0/1:3	C	T	0
.	.	.	.	.	.	.	.	.	.	.	SOM	GT:/	0:9,(	0/1:9	G	T	0

.	.	.	.	.	.	.	3.7	.	.	.	SOM	GT:/	0:22	0/1:2	C	A	0
#####	##	0	.	.	.	.	.	.	.	.	SOM	GT:/	0:59	0/1:8	G	A	0
.	##	0	.	.	.	.	.	.	.	.	SOM	GT:/	0:52	0/1:8	C	T	0
.	.	.	.	.	.	.	.	.	.	.	SOM	GT:/	0:62	0/1:9	T	G	0
.	.	.	.	.	.	.	.	.	.	.	SOM	GT:/	0:16	0/1:2	A	G	0
.	.	.	.	.	.	.	.	3.27	.	.	SOM	GT:/	0:83	0/1:1	G	T	0
.	.	.	.	.	.	.	3.6	2.68	.	.	SOM	GT:/	0:18	0/1:2	C	T	0
.	0	0	.	.	.	.	.	2.56	.	.	SOM	GT:/	0:38	0/1:5	C	T	0
.	.	.	0.7	0.0	0.001	1,N	.	.	.	.	SOM	GT:/	0:68	0/1:8	A	T	0
.	.	.	.	.	.	.	.	.	.	.	SOM	GT:/	0:9,(	0/1:9	G	A	0
.	.	.	.	.	.	.	.	.	.	.	SOM	GT:/	0:40	0/1:5	C	T	0
.	.	.	.	.	.	.	.	.	.	.	SOM	GT:/	0:15	0/1:2	G	T	0
.	.	.	.	.	.	.	.	.	.	.	SOM	GT:/	0:12	0/1:2	G	A	0
.	.	.	.	.	.	.	.	.	.	.	SOM	GT:/	0:57	0/1:9	G	A	0
.	.	.	.	.	.	.	.	.	.	.	SOM	GT:/	0:18	0/1:3	C	A	0
.	.	.	.	.	.	.	.	.	.	.	SOM	GT:/	0:14	0/1:1	G	A	0
.	.	.	.	.	.	.	.	2.11	.	.	SOM	GT:/	0:22	0/1:3	T	C	0
#####	##	0	.	.	.	.	.	.	.	.	SOM	GT:/	0:28	0/1:4	C	T	0
.	.	.	.	.	.	.	.	.	.	.	SOM	GT:/	0:36	0/1:5	T	C	0
.	0	0	.	.	.	.	.	.	0	0	SOM	GT:/	0:11	0/1:1	G	A	0
.	.	.	.	.	.	.	5.5	4.12	.	.	SOM	GT:/	0:18	0/1:2	T	G	0
.	.	.	.	.	.	.	.	.	.	.	SOM	GT:/	0:42	0/1:7	C	T	0
.	##	0	.	.	.	.	.	.	.	.	SOM	GT:/	0:17	0/1:2	C	T	0
.	.	.	0.3	0.8	0.999	1,N	4.4	1.24	0	0	SOM	GT:/	0:40	0/1:7	G	A	0
.	.	.	.	.	.	.	.	.	.	.	SOM	GT:/	0:39	0/1:6	G	T	0
.	.	.	.	.	.	.	.	.	.	.	SOM	GT:/	0:13	0/1:2	T	C	0
.	.	.	.	.	.	.	.	.	.	.	SOM	GT:/	0:46	0/1:6	C	T	0
.	.	.	0.0	0.9	0.999	1,D	5.8	5.60	.	.	SOM	GT:/	0:57	0/1:9	C	T	0
.	.	.	.	.	.	.	.	.	.	.	SOM	GT:/	0:65	0/1:1	C	T	0
.	.	.	.	0.7	0.99,I	1.000	5.2	4.36	.	.	SOM	GT:/	0:34	0/1:4	G	T	0
.	##	0	0.0	0.7	0.958	1,D	4.9	4.00	0	.	SOM	GT:/	0:26	0/1:3	G	A	0
.	.	.	.	.	.	.	.	.	.	.	SOM	GT:/	0:29	0/1:5	G	A	0
.	##	0	.	.	.	.	.	.	.	.	SOM	GT:/	0:57	0/1:8	G	A	0
.	.	.	.	.	.	.	4.7	1.34	.	.	SOM	GT:/	0:18	0/1:2	T	C	0
.	.	.	.	0.3	0.352	1,D	5.9	3.74	.	.	SOM	GT:/	0:27	0/1:6	G	T	0
.	.	.	.	.	.	.	.	.	.	.	SOM	GT:/	0:29	0/1:3	T	G	0
.	.	.	.	.	.	.	.	.	.	.	SOM	GT:/	0:36	0/1:7	C	T	0
.	.	.	0.0	0.0	0.013	1,N	2.1	3.35	.	.	SOM	GT:/	0:57	0/1:7	C	T	0
.	.	.	0.0	0.9	0.998	1,D	5.5	2.69	.	.	SOM	GT:/	0:54	0/1:8	T	C	0
.	##	.	1.0	0.1	0.84,I	0.999	4.1	1.69	.	.	DB;S	GT:/	0:26	0/1:3	C	T	0
.	.	.	.	.	.	.	.	.	.	.	SOM	GT:/	0:9,(	0/1:1	G	A	0
.	.	.	.	.	.	.	.	.	.	.	SOM	GT:/	0:9,(	0/1:1	G	A	0
.	.	.	.	.	.	.	.	.	0	0	SOM	GT:/	0:43	0/1:5	C	T	0
.	##	0	.	.	.	.	.	.	.	.	SOM	GT:/	0:24	0/1:3	C	T	0
.	.	.	.	.	.	.	.	.	.	.	SOM	GT:/	0:8,(	0/1:5	C	T	0
.	.	.	.	.	.	.	4.7	3.36	.	.	SOM	GT:/	0:41	0/1:5	A	C	0
.	.	.	.	.	.	.	.	.	.	.	SOM	GT:/	0:39	0/1:5	G	A	0
.	.	.	.	.	.	.	.	.	.	0	SOM	GT:/	0:9,(	0/1:1	C	T	0
.	.	.	.	.	.	.	.	.	.	.	SOM	GT:/	0:71	0/1:1	C	T	0
#####	##	0	0.0	0.9	1.0,D	1,D	5.9	5.76	.	.	SOM	GT:/	0:11	0/1:1	G	A	0
#####	0	0	.	.	.	.	.	.	.	.	SOM	GT:/	0:15	0/1:3	G	A	0
#####	##	0	.	.	.	.	.	.	.	.	SOM	GT:/	0:21	0/1:3	C	T	0
.	.	.	0.5	0.0	0.004	0.999	5.9	.	.	.	SOM	GT:/	0:56	0/1:1	T	C	0
.	##	0	0.0	0.9	1.0,D	1.000	5.4	5.22	.	.	SOM	GT:/	0:63	0/1:1	G	A	0

#####	0	0	0.0(	0.9!	1.0,D	1.000	3.8	3.80	.	.	SOM	GT:/	0:58	0/1:8	G	A	0
.	##	0	.	.	.	.	.	.	.	.	SOM	GT:/	0:13	0/1:2	G	A	0
.	.	.	.	.	.	.	.	.	.	.	SOM	GT:/	0:38	0/1:6	G	A	0
.	.	.	.	.	.	.	.	.	.	.	SOM	GT:/	0:72	0/1:1	C	T	0
.	.	.	.	.	.	.	.	.	.	.	SOM	GT:/	0:26	0/1:2	G	A	0
.	##	0	0.0	0.9	1.0,D	1.000	4.3	3.99	.	.	SOM	GT:/	0:33	0/1:5	G	A	0
.	##	0	0.7	0.0	0.003	1.000	.	.	.	.	SOM	GT:/	0:82	0/1:1	C	T	0
.	.	.	.	.	.	.	.	.	.	.	SOM	GT:/	0:9,(	0/1:7	C	T	0
.	.	.	.	.	.	.	.	.	.	.	SOM	GT:/	0:33	0/1:7	G	T	0
.	.	.	.	.	.	.	.	.	0	0	SOM	GT:/	0:12	0/1:1	C	T	0
.	.	.	.	.	.	.	2.8	1.66	.	.	SOM	GT:/	0:15	0/1:2	A	G	0
.	##	.	0.1	0.0	0.0,B	1,N	3	.	0	0	SOM	GT:/	0:77	0/1:1	C	A	0
.	0	0	0.0	0.1	0.545	0.999	5.5	3.45	.	.	SOM	GT:/	0:82	0/1:1	C	T	0
.	.	.	.	0.0	0.049	1,N	.	1.36	.	.	SOM	GT:/	0:30	0/1:6	C	T	0
.	.	.	.	.	.	.	3.1	.	.	.	SOM	GT:/	0:28	0/1:3	T	C	0
#####	.	.	.	.	.	.	.	.	.	.	SOM	GT:/	0:30	0/1:6	G	A	0
.	##	0	.	.	.	.	.	.	.	.	SOM	GT:/	0:86	0/1:1	G	A	0
.	.	.	.	.	.	.	.	.	.	.	SOM	GT:/	0:13	0/1:2	A	G	0
.	##	0	.	.	.	.	.	.	.	.	SOM	GT:/	0:17	0/1:4	G	A	0
.	.	.	.	.	.	.	.	.	.	.	SOM	GT:/	0:9,(	0/1:1	C	T	0
.	.	.	.	.	.	.	.	.	.	.	SOM	GT:/	0:9,(	0/1:1	T	A	0
.	.	.	0.0	0.0	0.033	1,N	.	.	.	.	SOM	GT:/	0:18	0/1:5	C	A	0
.	.	.	.	.	.	.	.	.	.	.	SOM	GT:/	0:38	0/1:6	C	A	0
.	0	0	.	.	.	.	.	.	.	.	SOM	GT:/	0:14	0/1:2	G	A	0
.	.	.	.	.	.	.	4.5	6.74	.	.	SOM	GT:/	0:40	0/1:8	C	T	0
.	.	.	0.0	0.1	0.955	1,D	4.8	1.42	.	.	SOM	GT:/	0:36	0/1:5	G	A	0
.	.	.	.	.	.	1,D	5.3	3.23	.	.	SOM	GT:/	0:44	0/1:6	T	C	0
.	.	.	.	.	.	.	.	.	.	.	SOM	GT:/	0:33	0/1:4	G	T	0
.	.	.	.	.	.	.	2.1	.	.	.	SOM	GT:/	0:37	0/1:5	G	T	0
.	##	0	.	.	.	.	.	1.31	.	.	SOM	GT:/	0:15	0/1:2	G	A	0
.	.	.	.	.	.	.	3.5	1.56	.	.	SOM	GT:/	0:67	0/1:1	A	T	0
.	.	.	.	.	.	.	.	.	.	.	SOM	GT:/	0:15	0/1:1	T	C	0
.	.	.	0.0	0.9	1.0,D	1,D	5.4	3.88	.	.	SOM	GT:/	0:32	0/1:6	G	T	0
.	.	.	.	.	.	.	2.1	.	.	.	SOM	GT:/	0:46	0/1:7	T	C	0
#####	##	0	.	.	.	.	.	.	.	.	SOM	GT:/	0:53	0/1:8	C	T	0
.	.	.	.	.	.	.	.	.	.	.	SOM	GT:/	0:26	0/1:2	A	G	0
.	##	0	0.0	0.0	0.417	1,D	3.1	2.69	.	.	SOM	GT:/	0:59	0/1:8	C	T	0
.	.	.	.	.	.	.	.	.	0	.	SOM	GT:/	0:15	0/1:4	C	T	0
.	.	.	.	.	.	.	.	.	.	.	SOM	GT:/	0:16	0/1:2	A	G	0
.	.	.	.	.	.	.	2.6	1.79	.	.	SOM	GT:/	0:10	0/1:1	C	T	0
.	.	.	.	.	.	.	.	.	.	.	SOM	GT:/	0:21	0/1:3	C	T	0
.	##	0	0.1	0.8	0.998	1,D	2.7	2.21	.	.	SOM	GT:/	0:18	0/1:3	C	T	0
#####	##	0	.	.	.	.	.	1.98	.	.	SOM	GT:/	0:27	0/1:4	C	T	0
.	.	.	0.5	0.5	0.979	0.915	4.9	3.01	.	.	SOM	GT:/	0:92	0/1:1	A	G	0
.	.	.	.	.	.	.	2	.	.	.	SOM	GT:/	0:65	0/1:1	C	T	0
.	.	.	.	.	.	.	.	.	.	.	SOM	GT:/	0:21	0/1:3	G	T	0
#####	0	0	.	.	.	.	.	.	.	.	SOM	GT:/	0:95	0/1:1	G	A	0
.	.	.	.	.	.	.	.	.	.	.	SOM	GT:/	0:14	0/1:2	C	A	0
.	.	.	.	.	.	.	.	.	.	.	SOM	GT:/	0:9,(	0/1:1	G	T	0
.	##	0	.	.	.	1,A	5.7	5.57	.	.	SOM	GT:/	0:49	0/1:6	G	A	0
.	0	0	.	.	.	.	.	1.26	.	.	SOM	GT:/	0:34	0/1:5	G	A	0
.	.	.	.	.	.	.	.	.	.	.	SOM	GT:/	0:38	0/1:5	G	A	0
.	.	.	.	.	.	.	.	.	.	.	SOM	GT:/	0:98	0/1:1	G	A	0
.	.	.	.	.	.	.	.	.	.	.	SOM	GT:/	0:15	0/1:2	C	T	0

.	.	.	.	.	.	.	.	.	.	.	SOM	GT:/	0:12	0/1:1	G	A	0
.	.	.	0.0	0.8	0.984	0.999	4.3	3.06	.	.	SOM	GT:/	0:56	0/1:8	A	G	0
.	.	.	0.2	0.9	0.999	1,D	5.4	3.15	.	.	SOM	GT:/	0:38	0/1:6	G	T	0
.	.	.	.	.	.	.	3.5	.	.	.	SOM	GT:/	0:21	0/1:3	C	T	0
.	.	.	.	.	.	.	4.2	2.86	.	.	SOM	GT:/	0:25	0/1:7	T	C	0
.	.	.	.	.	.	.	.	.	.	.	SOM	GT:/	0:20	0/1:2	G	A	0
.	##	0	.	.	.	.	.	.	.	.	SOM	GT:/	0:31	0/1:5	G	A	0
.	.	.	.	.	.	.	.	.	.	.	SOM	GT:/	0:15	0/1:2	C	T	0
.	.	.	.	.	.	.	.	.	.	.	SOM	GT:/	0:81	0/1:1	C	T	0
.	.	.	0.2	0.0	0.02,I	1,D	.	.	.	.	SOM	GT:/	0:13	0/1:3	C	T	0
.	.	.	0.3	.	.	1.000	3.7	1.88	.	.	SOM	GT:/	0:60	0/1:1	G	T	0
.	.	.	.	.	.	.	.	.	.	.	SOM	GT:/	0:12	0/1:1	C	T	0
.	##	0	.	.	.	.	.	.	0	0	SOM	GT:/	0:66	0/1:1	C	T	0
.	.	.	.	.	.	.	.	.	.	.	SOM	GT:/	0:20	0/1:4	G	T	0
.	##	0	.	.	.	.	.	.	.	.	SOM	GT:/	0:13	0/1:2	G	A	0
.	.	.	.	.	.	.	.	.	.	.	SOM	GT:/	0:31	0/1:5	C	A	0
.	##	0	0.3	0.0	0.104	0.999	2.3	1.34	.	.	SOM	GT:/	0:99	0/1:1	G	A	0
.	##	0	.	.	.	.	.	.	.	.	SOM	GT:/	0:84	0/1:1	C	T	0
.	.	.	.	.	.	.	.	.	.	.	SOM	GT:/	0:22	0/1:4	G	A	0
.	.	.	.	.	.	.	.	.	.	.	SOM	GT:/	0:38	0/1:4	C	T	0
.	.	.	.	.	.	.	.	.	0.1	0.2	SOM	GT:/	0:8,(	0/1:9	G	A	0
.	.	.	.	.	.	.	.	.	.	.	SOM	GT:/	0:12	0/1:2	C	T	0
.	.	.	.	.	.	.	.	.	.	.	SOM	GT:/	0:24	0/1:2	A	G	0
.	.	.	.	.	.	.	.	.	.	.	SOM	GT:/	0:96	0/1:1	A	G	0
.	.	.	.	.	.	.	.	.	.	.	SOM	GT:/	0:28	0/1:2	C	T	0
.	.	.	.	.	.	.	.	.	.	.	SOM	GT:/	0:46	0/1:7	G	A	0
.	.	.	.	.	.	.	.	.	.	.	SOM	GT:/	0:40	0/1:5	G	A	0
.	.	.	.	.	.	.	.	.	0	.	SOM	GT:/	0:60	0/1:9	C	T	0
.	0	0	0.1	.	.	1,N	.	.	0	.	SOM	GT:/	0:78	0/1:1	G	A	0
.	.	.	.	.	.	.	.	.	.	.	SOM	GT:/	0:75	0/1:1	G	A	0
.	.	.	.	.	.	.	.	.	.	.	SOM	GT:/	0:11	0/1:7	G	A	0
.	.	.	.	.	.	.	.	.	.	.	SOM	GT:/	0:25	0/1:3	T	C	0
.	.	.	.	.	.	.	.	.	.	.	SOM	GT:/	0:13	0/1:1	A	G	0
.	.	.	.	.	.	.	.	.	.	.	SOM	GT:/	0:24	0/1:4	G	A	0
2E-04	##	0	.	.	.	.	.	.	0	.	SOM	GT:/	0:70	0/1:1	C	T	0
.	.	.	0.0	0.9	0.967	1,D	4	4.18	.	.	SOM	GT:/	0:11	0/1:6	A	G	0
.	##	0	.	.	.	.	.	.	0	.	SOM	GT:/	0:55	0/1:3	C	T	0
.	.	.	.	.	.	.	.	.	.	.	SOM	GT:/	0:24	0/1:2	G	T	0
.	.	.	0.1	.	.	1,N	.	.	0	.	SOM	GT:/	0:14	0/1:2	G	A	0
.	.	.	.	.	.	.	.	.	0	.	SOM	GT:/	0:40	0/1:6	C	T	0
.	.	.	.	.	.	.	.	.	0.2	0.3	SOM	GT:/	0:10	0/1:1	A	G	0
.	.	.	.	.	.	.	.	.	.	.	SOM	GT:/	0:45	0/1:6	T	C	0
.	.	.	.	.	.	.	.	.	.	.	SOM	GT:/	0:16	0/1:3	C	T	0
.	.	.	.	.	.	.	.	.	.	.	SOM	GT:/	0:19	0/1:4	A	T	0
.	.	.	.	.	.	.	.	.	.	.	SOM	GT:/	0:29	0/1:3	C	T	0
.	.	.	.	.	.	.	2.3	.	.	.	SOM	GT:/	0:11	0/1:3	G	A	0
.	.	.	0.1	0.9	1.0,D	1.000	4.5	2.61	.	.	SOM	GT:/	0:76	0/1:1	G	A	0
.	.	.	.	.	.	.	.	2.07	.	.	SOM	GT:/	0:39	0/1:4	G	T	0
6E-04	0	0	.	.	.	.	.	.	.	.	SOM	GT:/	0:86	0/1:1	C	T	0
.	.	.	.	.	.	.	.	.	.	.	SOM	GT:/	0:87	0/1:1	G	A	0
.	##	0	.	.	.	.	.	.	.	.	SOM	GT:/	0:60	0/1:9	G	A	0
.	##	0	.	.	.	1,A	3.8	5.48	.	.	SOM	GT:/	0:55	0/1:1	C	T	0
.	##	0	.	.	.	1,D	4.3	7.65	.	.	SOM	GT:/	0:12	0/1:1	C	T	0
.	.	.	.	.	.	.	.	.	.	.	SOM	GT:/	0:30	0/1:3	G	A	0

.	.	.	.	.	.	.	.	.	.	.	SOM	GT:	0:16	0/1:	G	A	0
.	.	.	.	.	.	.	.	.	.	.	SOM	GT:	0:48	0/1:	G	A	0
.	.	.	0.0	0.9	1.0,D	1,D	4.4	3.92	.	.	SOM	GT:	0:10	0/1:	G	T	0
.	.	.	.	.	.	.	.	.	.	.	SOM	GT:	0:16	0/1:	A	G	0
.	##	0	0.0	0.4	0.969	0.969	5.8	4.26	.	.	DB;	GT:	0:60	0/1:	G	A	0
2E-04	0	0	0.0	0.9	1.0,D	1,D	5.7	5.61	.	.	SOM	GT:	0:10	0/1:	C	T	0
.	.	.	.	.	.	.	.	.	.	.	SOM	GT:	0:39	0/1:	G	T	0
.	.	.	.	.	.	.	.	.	.	.	SOM	GT:	0:15	0/1:	A	G	0
.	.	.	.	.	.	.	.	.	.	.	SOM	GT:	0:42	0/1:	G	T	0
.	.	.	.	.	.	1,D	6	3.25	.	.	SOM	GT:	0:27	0/1:	A	G	0
.	##	0	.	.	.	.	.	.	.	.	SOM	GT:	0:64	0/1:	G	A	0
.	.	.	.	.	.	.	.	.	.	.	SOM	GT:	0:17	0/1:	G	T	0
.	.	.	0.0	0.9	1.0,D	1,D	5.5	3.28	.	.	SOM	GT:	0:67	0/1:	T	C	0
.	##	0	0.0	0.9	1.0,D	1,D	5.6	5.38	.	.	SOM	GT:	0:42	0/1:	G	A	0
.	##	0	0.0	0.9	1.0,D	0.987	2.8	3.48	0	0	SOM	GT:	0:42	0/1:	C	T	0
.	.	.	0.1	0.0	0.001	1,N	2.8	.	.	.	SOM	GT:	0:32	0/1:	C	G	0
.	##	0	.	.	.	.	3.7	.	.	.	SOM	GT:	0:71	0/1:	C	T	0
.	.	.	.	.	.	.	.	.	.	.	SOM	GT:	0:15	0/1:	G	A	0
.	.	.	.	.	.	.	.	.	.	.	SOM	GT:	0:27	0/1:	T	C	0
.	.	.	.	.	.	.	3.3	3.65	.	.	SOM	GT:	0:54	0/1:	T	C	0
.	##	0	.	.	.	.	.	.	.	.	SOM	GT:	0:32	0/1:	C	T	0
.	.	.	.	.	.	.	.	.	.	.	SOM	GT:	0:61	0/1:	C	T	0
.	.	.	.	.	.	.	.	.	0	0	SOM	GT:	0:23	0/1:	C	T	0
.	.	.	.	.	.	.	.	.	.	.	SOM	GT:	0:34	0/1:	C	A	0
.	.	.	0.1	0.9	1.0,D	1,D	6	4.74	.	.	SOM	GT:	0:55	0/1:	G	A	0
.	.	.	.	.	.	.	.	.	.	.	SOM	GT:	0:76	0/1:	C	A	0
.	.	.	.	.	.	.	.	.	.	.	SOM	GT:	0:8,	0/1:	G	A	0
.	.	.	.	.	.	.	.	.	0	.	SOM	GT:	0:69	0/1:	C	T	0
.	.	.	.	.	.	.	.	.	0	.	SOM	GT:	0:64	0/1:	C	T	0
.	.	.	.	.	.	.	.	.	.	.	SOM	GT:	0:14	0/1:	G	A	0
.	.	.	.	.	.	.	.	.	.	.	SOM	GT:	0:12	0/1:	C	T	0
.	.	.	.	.	.	.	.	.	0	0	SOM	GT:	0:46	0/1:	A	G	0
.	.	.	.	.	.	.	.	.	.	.	SOM	GT:	0:41	0/1:	G	T	0
.	.	.	.	.	.	.	2.5	1.34	.	.	SOM	GT:	0:35	0/1:	C	T	0
2E-04	##	0	.	.	.	.	.	.	.	.	SOM	GT:	0:10	0/1:	C	T	0
.	.	.	.	.	.	.	.	.	.	.	SOM	GT:	0:15	0/1:	G	A	0
.	##	0	.	.	.	.	.	.	0	.	SOM	GT:	0:58	0/1:	G	A	0
.	.	.	.	.	.	.	.	.	.	.	SOM	GT:	0:14	0/1:	T	C	0
.	.	.	.	.	.	.	.	.	.	.	SOM	GT:	0:9,	0/1:	C	T	0
.	0	.	1.0	.	.	1,N	.	.	.	.	SOM	GT:	0:29	0/1:	A	G	0
.	.	.	.	.	.	.	.	.	.	.	SOM	GT:	0:23	0/1:	G	A	0
#####	##	0	0.0	0.4	0.901	1.000	5.4	3.98	0	.	SOM	GT:	0:14	0/1:	C	T	0
.	.	.	0.0	0.7	0.999	1,D	5.1	4.69	.	.	SOM	GT:	0:86	0/1:	G	A	0
.	.	.	.	.	.	.	.	.	.	.	SOM	GT:	0:27	0/1:	A	T	0
.	##	.	.	.	.	.	.	.	0	.	SOM	GT:	0:10	0/1:	G	A	0
.	.	.	.	.	.	.	.	1.71	.	.	SOM	GT:	0:46	0/1:	C	T	0
2E-04	##	0	.	.	.	.	.	.	.	.	SOM	GT:	0:10	0/1:	C	T	0
.	.	.	.	.	.	.	.	.	0	.	SOM	GT:	0:18	0/1:	T	C	0
.	.	.	.	.	.	.	.	.	.	.	SOM	GT:	0:80	0/1:	T	C	0
.	.	.	.	.	.	.	.	.	.	.	SOM	GT:	0:35	0/1:	C	T	0
.	.	.	.	.	.	.	.	.	.	.	SOM	GT:	0:45	0/1:	G	T	0
.	##	0	0.0	0.9	1.0,D	1.000	5.7	3.91	.	.	SOM	GT:	0:40	0/1:	G	A	0
.	.	.	.	.	.	.	3.4	.	.	.	SOM	GT:	0:23	0/1:	C	A	0
.	.	.	.	.	.	.	.	.	.	.	SOM	GT:	0:59	0/1:	G	A	0

.	.	.	.	.	.	.	.	.	.	.	SOM	GT:	0:73	0/1:	G	A	0
.	.	.	.	.	.	.	.	.	.	.	SOM	GT:	0:17	0/1:	C	T	0
.	.	.	.	.	.	.	.	.	.	.	SOM	GT:	0:29	0/1:	A	G	0
.	##	0	.	0.0	0.001	0.771	3.5	.	.	.	SOM	GT:	0:68	0/1:	G	A	0
.	.	.	0.1	0.1	0.302	1,N	.	.	.	.	SOM	GT:	0:11	0/1:	G	T	0
.	.	.	.	.	.	.	.	.	.	.	SOM	GT:	0:12	0/1:	G	A	0
.	.	.	.	.	.	.	.	.	.	.	SOM	GT:	0:9,	0/1:	G	C	0
.	##	0	0.0	0.0	0.491	1.00	.	.	.	.	SOM	GT:	0:79	0/1:	C	T	0
.	0	0	.	.	.	.	.	.	0	.	DB;	GT:	0:66	0/1:	C	T	0
.	##	0	0.2	0.0	0.006	1,N	.	1.60	.	.	SOM	GT:	0:46	0/1:	G	A	0
#####	##	0	0.0	0.8	0.999	1,D	4.2	3.34	.	.	SOM	GT:	0:17	0/1:	C	T	0
#####	##	0	.	.	.	.	.	.	0	.	SOM	GT:	0:25	0/1:	C	T	0
.	.	.	.	.	.	.	3	.	.	.	SOM	GT:	0:31	0/1:	G	T	0
.	.	.	.	.	.	.	.	.	.	.	SOM	GT:	0:8,	0/1:	C	A	0
#####	.	.	.	.	.	.	.	.	.	.	SOM	GT:	0:97	0/1:	C	T	0
.	.	.	.	.	.	.	.	.	.	.	SOM	GT:	0:28	0/1:	G	A	0
.	.	.	0.0	0.6	0.958	1,N	2.8	2.07	.	.	SOM	GT:	0:44	0/1:	A	T	0
.	.	.	.	.	.	.	.	.	.	.	SOM	GT:	0:11	0/1:	C	T	0
.	.	.	0.3	0.4	0.833	0.982	2.6	1.55	.	.	SOM	GT:	0:65	0/1:	G	A	0
.	##	0	.	.	.	.	.	.	.	.	SOM	GT:	0:43	0/1:	C	T	0
.	.	.	0.0	0.9	1.0,D	1,D	5.2	5.25	.	.	SOM	GT:	0:12	0/1:	G	A	0
.	.	.	.	.	.	.	.	.	0	.	SOM	GT:	0:83	0/1:	G	A	0
.	0	0	0.0	0.9	1.0,D	1,D	4.8	4.71	.	.	SOM	GT:	0:69	0/1:	C	T	0
.	.	.	.	.	.	.	.	.	0	.	SOM	GT:	0:22	0/1:	C	T	0
#####	##	0	0.0	0.9	1.0,D	1.00	.	3.51	.	.	SOM	GT:	0:20	0/1:	C	T	0
.	.	.	.	.	.	.	.	1.74	.	.	SOM	GT:	0:99	0/1:	T	A	0
#####	##	0	.	.	.	.	.	.	.	0	SOM	GT:	0:45	0/1:	G	A	0
.	.	.	.	.	.	.	.	.	.	.	SOM	GT:	0:30	0/1:	C	A	0
.	##	0	0.4	0.0	0.001	1,N	.	.	0	.	SOM	GT:	0:10	0/1:	G	A	0
.	.	.	.	.	.	.	.	.	.	.	SOM	GT:	0:43	0/1:	G	A	0
.	.	.	.	.	.	.	.	.	.	.	SOM	GT:	0:32	0/1:	T	C	0
.	.	.	.	.	.	.	.	.	.	.	SOM	GT:	0:66	0/1:	G	A	0
.	.	.	.	.	.	.	.	.	0	0	SOM	GT:	0:30	0/1:	C	T	0
.	.	.	.	.	.	.	.	.	.	.	SOM	GT:	0:48	0/1:	A	G	0
.	##	0	.	.	.	.	.	.	.	.	SOM	GT:	0:36	0/1:	C	T	0
.	0	0	0.3	0.0	0.0,B	1,N	.	.	0	0	SOM	GT:	0:52	0/1:	G	A	0
.	.	.	0.0	0.8	0.997	1.00	5.7	3.29	.	.	SOM	GT:	0:61	0/1:	C	A	0
.	.	.	.	.	.	.	.	.	.	.	SOM	GT:	0:14	0/1:	T	A	0
.	.	.	.	.	.	.	.	.	.	.	SOM	GT:	0:28	0/1:	G	A	0
#####	##	0	.	.	.	.	.	.	.	.	SOM	GT:	0:22	0/1:	C	T	0
2E-04	0	0	0.0	0.0	0.003	1,N	.	.	0	.	SOM	GT:	0:90	0/1:	C	T	0
.	.	.	0.0	0.9	0.999	0.964	4.8	2.03	.	.	SOM	GT:	0:10	0/1:	G	T	0
.	##	0	0.2	0.0	0.0,B	1,N	.	.	0	.	SOM	GT:	0:12	0/1:	G	A	0
.	.	.	.	.	.	.	.	.	.	.	SOM	GT:	0:37	0/1:	T	C	0
.	.	.	.	.	.	.	.	.	.	.	SOM	GT:	0:28	0/1:	G	A	0
.	##	0	.	.	.	.	.	2.57	.	.	SOM	GT:	0:27	0/1:	A	G	0
.	.	.	.	.	.	.	.	2.86	.	.	SOM	GT:	0:31	0/1:	G	A	0
.	0	0	.	.	.	.	.	.	.	.	SOM	GT:	0:81	0/1:	G	A	0
.	.	.	.	.	.	.	5.6	2.61	.	.	SOM	GT:	0:14	0/1:	C	T	0
.	.	.	.	.	.	.	.	.	.	.	SOM	GT:	0:16	0/1:	C	T	0
.	.	.	.	.	.	.	.	.	.	.	SOM	GT:	0:22	0/1:	G	A	0
.	.	.	.	.	.	.	2.1	.	.	.	SOM	GT:	0:10	0/1:	T	C	0
.	0	0	0.0	0.0	0.0,B	1.00	4.2	2.45	0	.	DB;	GT:	0:91	0/1:	A	T	0
.	0	0	0.6	0.0	0.004	1,N	2.2	.	0	.	SOM	GT:	0:18	0/1:	G	A	0

.	##	0	.	.	.	.	.	.	.	.	SOM	GT:/	0:72	0/1:1	G	A	0
.	.	.	.	0.9	1.0,D	1.000	3.2	2.86	.	.	SOM	GT:/	0:50	0/1:8	C	A	0
#####	##	.	.	.	.	.	.	.	.	.	SOM	GT:/	0:82	0/1:1	C	T	0
.	.	.	0.0	0.9	1.0,D	1,D	5.7	4.84	.	.	SOM	GT:/	0:60	0/1:8	G	A	0
.	.	.	0.3	0.7	0.998	0.999	4.6	2.75	.	.	SOM	GT:/	0:77	0/1:1	C	T	0
.	.	.	.	.	.	.	.	.	.	.	SOM	GT:/	0:22	0/1:2	C	T	0
.	.	.	.	.	.	.	.	.	.	.	SOM	GT:/	0:19	0/1:3	G	T	0
.	.	.	0.0	0.9	1.0,D	0.731	3.6	4.00	.	.	SOM	GT:/	0:55	0/1:9	G	A	0
.	.	.	.	.	.	.	.	.	.	.	SOM	GT:/	0:55	0/1:7	C	T	0
#####	##	0	0.0	0.9	1.0,D	1.000	5.2	3.22	.	.	SOM	GT:/	0:11	0/1:1	G	A	0
.	.	.	.	.	.	.	.	.	0	0	SOM	GT:/	0:25	0/1:2	A	T	0
.	.	.	0.4	.	.	1,N	.	.	.	.	SOM	GT:/	0:27	0/1:2	G	A	0
.	.	.	0.0	0.9	1.0,D	1,D	5.8	3.66	.	.	SOM	GT:/	0:11	0/1:1	C	T	0
.	.	.	.	.	.	.	.	1.23	.	.	SOM	GT:/	0:52	0/1:9	T	C	0
.	##	0	0.0	0.9	1.0,D	0.999	3.4	4.90	.	.	SOM	GT:/	0:13	0/1:2	G	A	0
.	.	.	.	.	.	.	.	.	.	.	SOM	GT:/	0:68	0/1:9	G	T	0
.	.	.	.	.	.	.	.	.	.	.	SOM	GT:/	0:8,(	0/1:6	G	T	0
.	##	0	0.2	0.4	0.99,I	0.896	4.5	.	0	0	SOM	GT:/	0:13	0/1:1	C	T	0
.	.	.	.	.	.	.	.	.	.	.	SOM	GT:/	0:37	0/1:5	G	A	0
.	.	.	.	.	.	.	3.4	2.32	.	.	SOM	GT:/	0:67	0/1:1	T	C	0
.	.	.	.	.	.	.	.	.	.	.	SOM	GT:/	0:43	0/1:8	G	T	0
.	.	.	.	.	.	.	.	.	.	.	SOM	GT:/	0:10	0/1:2	C	A	0
.	.	.	.	.	.	.	.	.	0	0	DB;S	GT:/	0:48	0/1:6	C	T	0
.	.	.	.	.	.	.	.	.	0	.	SOM	GT:/	0:24	0/1:2	A	G	0
.	.	.	.	.	.	.	2.7	.	.	.	SOM	GT:/	0:23	0/1:3	A	G	0
.	.	.	.	.	.	.	.	.	0	.	SOM	GT:/	0:49	0/1:8	A	G	0
.	.	.	.	.	.	.	.	.	0	0.1	SOM	GT:/	0:8,(	0/1:3	G	C	0
.	.	.	.	.	.	.	.	.	.	.	SOM	GT:/	0:9,(	0/1:5	C	A	0
.	.	.	.	.	.	.	.	.	.	.	SOM	GT:/	0:21	0/1:3	A	C	0
.	.	.	.	.	.	.	.	.	.	.	SOM	GT:/	0:9,(	0/1:1	C	G	0
.	.	.	.	.	.	.	.	.	0	.	DB;S	GT:/	0:19	0/1:1	A	C	0
.	.	.	.	.	.	.	.	.	0	.	DB;S	GT:/	0:19	0/1:1	C	T	0
.	.	.	.	.	.	.	.	.	0	.	DB;S	GT:/	0:22	0/1:2	T	G	0
.	.	.	.	.	.	.	.	.	.	.	DB;S	GT:/	0:19	0/1:1	C	T	0
.	.	.	.	.	.	.	.	.	0	.	DB;S	GT:/	0:28	0/1:4	G	C	0
.	.	.	.	.	.	.	.	.	0	.	DB;S	GT:/	0:39	0/1:6	G	C	0
.	.	.	.	.	.	.	.	.	.	.	SOM	GT:/	0:23	0/1:2	A	G	0
.	.	.	.	.	.	.	.	.	0	.	SOM	GT:/	0:30	0/1:2	A	C	0
.	.	.	.	.	.	.	.	.	0	0	DB;S	GT:/	0:24	0/1:2	A	G	0
.	.	.	.	.	.	.	.	.	.	.	DB;S	GT:/	0:29	0/1:2	A	G	0
.	.	.	.	.	.	.	.	.	0	.	DB;S	GT:/	0:34	0/1:2	T	G	0
.	.	.	.	.	.	.	.	.	.	.	DB;S	GT:/	0:35	0/1:3	A	G	0
.	.	.	.	.	.	.	.	.	0	.	DB;S	GT:/	0:34	0/1:4	G	C	0
.	.	.	.	.	.	.	.	.	0	.	DB;S	GT:/	0:37	0/1:5	T	A	0
.	.	.	.	.	.	.	.	.	0	.	DB;S	GT:/	0:23	0/1:3	A	T	0
.	.	.	.	.	.	.	.	.	0	.	DB;S	GT:/	0:27	0/1:2	T	G	0
.	.	.	.	.	.	.	.	.	0	.	SOM	GT:/	0:10	0/1:6	G	T	0
.	.	.	.	.	.	.	.	.	0	.	SOM	GT:/	0:13	0/1:1	G	A	0
.	.	.	.	.	.	.	.	.	.	.	SOM	GT:/	0:10	0/1:9	T	C	0
.	.	.	.	.	.	.	.	.	.	.	SOM	GT:/	0:10	0/1:2	T	C	0
2E-04	##	0	.	.	.	.	.	.	.	.	SOM	GT:/	0:15	0/1:2	G	A	0
.	.	.	.	.	.	.	.	.	.	.	SOM	GT:/	0:24	0/1:6	C	A	0
.	.	.	.	.	.	.	.	.	.	.	SOM	GT:/	0:49	0/1:9	C	A	0
.	.	.	0.1	0.0	0.002	1.000	.	1.43	.	.	SOM	GT:/	0:50	0/1:9	G	T	0

.	.	.	.	.	.	.	.	.	.	.	SOM	GT:/	0:25	0/1:2	A	C	0
.	.	.	.	.	.	.	.	.	.	.	SOM	GT:/	0:47	0/1:6	G	A	0
8E-04	0	.	.	.	.	.	.	.	.	.	SOM	GT:/	0:20	0/1:3	C	T	0
.	.	.	.	.	.	.	.	.	.	.	SOM	GT:/	0:53	0/1:7	T	C	0
.	.	.	0.0	0.7	0.986	0.671	3.1	3.79	.	.	SOM	GT:/	0:61	0/1:8	G	T	0
.	.	.	.	.	.	.	.	1.73	.	.	SOM	GT:/	0:17	0/1:3	A	G	0
.	.	.	.	.	.	.	.	.	0	.	SOM	GT:/	0:8,	0/1:9	A	G	0
.	.	.	0.1	0.8	0.996	1,N	.	1.95	.	.	SOM	GT:/	0:93	0/1:1	C	T	0
.	.	.	.	.	.	.	.	.	.	.	SOM	GT:/	0:30	0/1:5	A	G	0
.	##	0	.	.	.	.	.	.		0	SOM	GT:/	0:13	0/1:1	C	T	0
.	##	0	0.0	0.9	1.0,D	1.000	5.3	3.77	.	.	SOM	GT:/	0:89	0/1:1	G	A	0
.	.	.	.	.	.	.	2.2	1.30	.	.	SOM	GT:/	0:60	0/1:9	G	T	0
.	##	0	0.0	0.8	0.996	1.000	.	3.55	.	.	SOM	GT:/	0:48	0/1:9	C	T	0
2E-04	##	0	.	.	.	.	.	.	0	.	SOM	GT:/	0:32	0/1:5	G	A	0
.	.	.	.	.	.	.	.	.	.	.	SOM	GT:/	0:19	0/1:3	C	A	0
.	##	0	.	0.0	0.124	0.982	.	1.80	.	.	SOM	GT:/	0:65	0/1:1	C	T	0
.	.	.	.	.	.	.	.	.	.	.	SOM	GT:/	0:41	0/1:5	C	A	0
.	.	.	.	.	.	.	.	.	.	.	SOM	GT:/	0:10	0/1:1	G	A	0
.	.	.	0.5	0.8	0.931	0.991	5.5	3.13	.	.	SOM	GT:/	0:31	0/1:7	C	T	0
.	.	.	.	.	.	.	.	.	0	0	SOM	GT:/	0:38	0/1:5	A	G	0
.	.	.	.	.	.	.	.	.	.	.	SOM	GT:/	0:25	0/1:3	C	T	0
.	.	.	.	.	.	.	.	.	.	.	SOM	GT:/	0:38	0/1:7	C	T	0
2E-04	##	0	.	0.7	0.996	.	4.1	.	.	.	SOM	GT:/	0:25	0/1:4	G	A	0
.	0	0	.	.	.	.	.	.	0	0	SOM	GT:/	0:10	0/1:2	G	T	0
.	.	.	.	.	.	.	.	.	.	.	SOM	GT:/	0:46	0/1:5	C	T	0
.	.	.	.	.	.	.	.	.	.	.	SOM	GT:/	0:38	0/1:5	C	T	0
.	.	.	.	.	.	.	.	.	0	0	SOM	GT:/	0:10	0/1:8	A	G	0
.	0	0	.	0.0	0.205	.	.	.	0	.	SOM	GT:/	0:38	0/1:4	G	A	0
.	.	.	.	.	.	.	.	.	.	.	SOM	GT:/	0:14	0/1:1	T	C	0
.	.	.	.	.	.	.	.	.	0.1	0	DB;S	GT:/	0:34	0/1:5	C	T	0
2E-04	##	0	0.0	0.8	0.984	0.988	5.1	2.66	.	0	SOM	GT:/	0:38	0/1:5	G	A	0
.	##	0	0.0	0.7	0.993	1.000	4	2.25	.	.	SOM	GT:/	0:64	0/1:1	G	A	0
.	.	.	0.0	0.8	0.997	1.000	.	2.53	.	.	SOM	GT:/	0:24	0/1:4	C	T	0
.	.	.	0.0	1.0	1.0,D	1,D	4.7	4.17	.	.	SOM	GT:/	0:14	0/1:2	T	A	0
#####	##	0	.	.	.	.	.	.	0	.	SOM	GT:/	0:11	0/1:2	C	T	0
.	.	.	.	.	.	.	.	.	.	.	SOM	GT:/	0:58	0/1:9	T	C	0
.	##	0	0.1	0.0	0.025	1,N	.	.	.	.	SOM	GT:/	0:11	0/1:1	G	A	0
.	.	.	.	.	.	.	.	.	.	.	SOM	GT:/	0:20	0/1:3	C	T	0
.	.	.	0.0	0.9	1.0,D	1,D	4.7	4.08	.	.	SOM	GT:/	0:13	0/1:2	C	T	0
.	.	.	.	.	.	.	.	.	.	.	SOM	GT:/	0:70	0/1:1	C	A	0
.	##	0	.	.	.	.	.	.	.	.	SOM	GT:/	0:62	0/1:1	G	A	0
.	##	0	.	.	.	.	.	.	.	.	SOM	GT:/	0:11	0/1:1	G	A	0
.	##	0	0.0	0.9	0.994	1.000	4.9	4.09	0	.	SOM	GT:/	0:81	0/1:1	T	C	0
.	.	.	0.6	0.0	0.001	1,D	5.6	3.09	.	.	SOM	GT:/	0:73	0/1:9	C	T	0
.	.	.	.	.	.	.	2.2	.	.	.	SOM	GT:/	0:10	0/1:1	A	G	0
.	.	.	.	.	.	.	.	.	.	.	SOM	GT:/	0:63	0/1:1	T	C	0
.	.	.	.	.	.	.	.	.	.	.	SOM	GT:/	0:13	0/1:2	C	A	0
.	.	.	0.2	0.0	0.142	0.908	5.5	.	.	.	SOM	GT:/	0:82	0/1:1	G	T	0
.	##	0	0.0	0.0	0.492	0.972	3.9	4.11	.	.	SOM	GT:/	0:40	0/1:4	C	T	0
.	.	.	.	.	.	.	.	.	.	.	SOM	GT:/	0:80	0/1:1	A	G	0
.	.	.	.	.	.	.	3.4	2.72	.	.	SOM	GT:/	0:62	0/1:9	T	C	0
.	.	.	.	.	.	.	3.4	.	.	.	SOM	GT:/	0:11	0/1:1	A	C	0
.	##	0	.	.	.	.	3.2	.	.	.	SOM	GT:/	0:32	0/1:5	C	T	0
#####	0	0	.	.	.	.	.	.	.	.	SOM	GT:/	0:58	0/1:7	C	T	0

.	0	0	.	.	.	.	.	.	.	.	SOM	GT:/	0:65	0/1:8	G	A	0
.	0	0	0.5	0.0	0.002	1,N	.	.	.	.	SOM	GT:/	0:15	0/1:2	C	T	0
.	.	.	.	.	.	.	.	.	.	.	SOM	GT:/	0:8,(	0/1:1	C	T	0
.	.	.	0.1	0.0	0.019	0.989	.	.	.	.	SOM	GT:/	0:26	0/1:2	T	C	0
.	.	.	.	.	.	.	.	.	.	0	SOM	GT:/	0:61	0/1:8	C	T	0
.	.	.	.	.	.	.	.	.	.	.	SOM	GT:/	0:15	0/1:2	A	G	0
0.002	0	0	0.3	0.3	0.921	0.997	5.4	3.35	.	.	SOM	GT:/	0:74	0/1:1	G	A	0
.	##	0	.	1.0	1.0,D	1,D	5.6	5.52	.	.	SOM	GT:/	0:68	0/1:9	G	A	0
3E-04	0	0	.	.	.	.	.	.	.	.	SOM	GT:/	0:10	0/1:1	G	A	0
.	.	.	.	.	.	.	.	.	.	.	SOM	GT:/	0:89	0/1:1	C	T	0
.	.	.	.	.	.	.	.	1.68	.	.	SOM	GT:/	0:64	0/1:1	C	T	0
.	##	0	.	.	.	.	.	2.87	.	.	SOM	GT:/	0:14	0/1:8	A	G	0
.	.	.	.	.	.	1,A	4.1	19.1	.	.	SOM	GT:/	0:48	0/1:7	G	T	0
.	.	.	.	.	.	.	3.5	.	.	.	SOM	GT:/	0:90	0/1:1	C	T	0
.	##	0	.	.	.	.	.	.	.	.	SOM	GT:/	0:74	0/1:1	C	T	0
.	0	0	.	.	.	.	.	.	.	.	SOM	GT:/	0:19	0/1:1	G	A	0
.	.	.	.	.	.	.	3.2	.	.	.	SOM	GT:/	0:33	0/1:5	G	A	0
.	.	.	.	.	.	.	.	.	.	.	SOM	GT:/	0:32	0/1:3	T	C	0
.	.	.	.	.	.	.	.	.	.	.	SOM	GT:/	0:68	0/1:1	C	T	0
.	.	.	.	.	.	.	.	.	.	.	SOM	GT:/	0:31	0/1:4	G	A	0
.	.	.	.	.	.	.	.	.	.	.	DB;S	GT:/	0:67	0/1:1	G	A	0
.	.	.	.	.	.	.	.	.	.	.	SOM	GT:/	0:84	0/1:1	A	G	0
.	.	.	.	.	.	.	.	.	.	.	SOM	GT:/	0:19	0/1:3	G	A	0
.	.	.	.	.	.	.	.	.	.	.	SOM	GT:/	0:16	0/1:2	C	A	0
.	1	0	.	.	.	.	2.3	1.54	0.1	0.2	DB;S	GT:/	0:58	0/1:1	T	C	0
.	##	0	.	.	.	.	.	.	.	.	SOM	GT:/	0:50	0/1:9	G	A	0
.	.	.	.	.	.	.	.	.	.	.	SOM	GT:/	0:16	0/1:3	G	A	0
.	.	.	.	.	.	.	.	.	.	.	SOM	GT:/	0:16	0/1:3	C	T	0
.	.	.	.	.	.	.	.	.	0	.	SOM	GT:/	0:17	0/1:1	A	T	0
.	.	.	.	.	.	1,N	.	.	.	.	SOM	GT:/	0:66	0/1:8	G	T	0
.	.	.	.	.	.	.	.	.	.	.	SOM	GT:/	0:86	0/1:1	C	T	0
.	.	.	.	.	.	.	.	.	.	.	SOM	GT:/	0:26	0/1:3	C	T	0
.	.	.	.	.	.	.	.	.	0	.	SOM	GT:/	0:59	0/1:9	C	T	0
.	.	.	.	.	.	.	.	.	.	.	SOM	GT:/	0:69	0/1:1	A	G	0
.	.	.	0.0	0.1	0.22,I	0.940	2.9	2.39	.	.	SOM	GT:/	0:96	0/1:1	C	T	0
.	.	.	0.0	0.9	0.99,I	1.000	4.9	5.48	.	.	SOM	GT:/	0:48	0/1:8	C	T	0
.	.	.	.	.	.	.	.	.	0	0	SOM	GT:/	0:12	0/1:1	A	G	0
.	##	0	1.0	0.0	0.0,B	1,N	.	.	.	.	SOM	GT:/	0:67	0/1:9	C	T	0
.	.	.	.	.	.	.	.	.	.	.	SOM	GT:/	0:53	0/1:8	G	T	0
.	.	.	.	.	.	.	2.5	2.71	.	.	SOM	GT:/	0:14	0/1:1	A	G	0
.	.	.	.	.	.	.	.	.	.	.	SOM	GT:/	0:16	0/1:3	G	T	0
.	.	.	.	.	.	.	.	.	.	.	SOM	GT:/	0:58	0/1:6	G	T	0
.	.	.	0.5	0.5	0.627	1.000	4.1	2.37	.	.	SOM	GT:/	0:63	0/1:9	G	A	0
.	##	0	0.5	0.0	0.248	1,N	.	.	.	.	SOM	GT:/	0:99	0/1:1	C	T	0
.	.	.	.	.	.	.	.	.	.	.	SOM	GT:/	0:16	0/1:2	G	T	0
.	.	.	.	.	.	.	.	.	.	.	SOM	GT:/	0:19	0/1:2	C	A	0
.	.	.	.	.	.	.	.	.	0.1	0.2	SOM	GT:/	0:42	0/1:8	T	G	0
.	.	.	.	.	.	.	.	.	0	0	SOM	GT:/	0:12	0/1:2	T	C	0
.	.	.	.	.	.	.	.	.	.	.	SOM	GT:/	0:36	0/1:5	A	G	0
.	##	0	.	0.8	0.977	1.000	4.1	1.52	.	.	SOM	GT:/	0:44	0/1:7	C	T	0
.	.	.	.	.	.	.	.	.	.	.	SOM	GT:/	0:26	0/1:4	G	T	0
.	##	0	0.0	0.9	1.0,D	1.000	2.6	4.46	.	.	SOM	GT:/	0:62	0/1:1	G	A	0
.	.	.	.	.	.	.	.	.	.	.	SOM	GT:/	0:37	0/1:6	C	T	0
.	.	.	.	.	.	.	.	.	.	.	SOM	GT:/	0:20	0/1:3	C	T	0

.	.	.	0.0(	0.9:	0.999	1,D	5.1	3.24	.	.	SON	GT:/	0:13	0/1:2	G	A	0
#####	##	0	.	.	.	.	.	.	.	0	SON	GT:/	0:59	0/1:9	C	T	0
5E-04	0	0	.	.	.	.	.	.	.	.	SON	GT:/	0:15	0/1:2	C	T	0
.	##	0	.	.	.	.	.	.	.	.	SON	GT:/	0:13	0/1:1	C	T	0
.	##	0	.	.	.	.	.	.	.	.	SON	GT:/	0:57	0/1:7	G	A	0
.	.	.	.	.	.	.	.	.	.	.	SON	GT:/	0:64	0/1:1	A	G	0
.	.	.	.	.	.	.	.	1.44	.	.	SON	GT:/	0:57	0/1:9	G	T	0
.	.	.	.	.	.	.	.	.	.	.	SON	GT:/	0:49	0/1:1	G	A	0
.	.	.	.	.	.	.	.	.	0	0	SON	GT:/	0:14	0/1:3	G	A	0
.	.	.	.	.	.	.	.	.	.	.	SON	GT:/	0:39	0/1:4	G	T	0
.	.	.	.	.	.	.	.	.	.	.	SON	GT:/	0:10	0/1:1	C	T	0
.	.	.	.	.	.	.	.	.	.	.	SON	GT:/	0:22	0/1:2	C	T	0
.	.	.	.	.	.	.	.	.	.	.	SON	GT:/	0:41	0/1:7	A	G	0
.	.	.	.	.	.	.	.	.	.	.	SON	GT:/	0:87	0/1:1	G	A	0
.	.	.	.	.	.	.	2.1	.	.	.	SON	GT:/	0:66	0/1:1	G	T	0
.	.	.	.	.	.	.	.	.	.	.	SON	GT:/	0:65	0/1:9	C	A	0
.	##	0	.	.	.	.	.	.	0	.	SON	GT:/	0:62	0/1:9	G	A	0
.	.	.	.	.	.	.	2.8	.	.	.	SON	GT:/	0:62	0/1:6	T	G	0
.	.	.	.	.	.	.	.	.	.	.	SON	GT:/	0:35	0/1:4	C	T	0
.	.	.	.	.	.	.	.	.	.	.	SON	GT:/	0:19	0/1:3	G	T	0
.	##	0	0.1	0.9:	0.997	1,D	4.5	4.18	.	.	SON	GT:/	0:11	0/1:1	C	T	0
.	.	.	.	.	.	.	.	3.18	.	.	SON	GT:/	0:80	0/1:1	C	T	0
.	.	.	.	.	.	.	.	.	.	.	SON	GT:/	0:59	0/1:1	C	T	0
.	.	.	.	.	.	.	.	.	.	.	SON	GT:/	0:83	0/1:1	G	A	0
.	.	.	.	.	.	.	.	.	.	.	SON	GT:/	0:23	0/1:4	G	A	0
.	.	.	.	.	.	.	.	.	0.2	0.3	SON	GT:/	0:9,(	0/1:7	A	G	0
.	0	0	.	.	.	.	3.4	.	.	.	SON	GT:/	0:46	0/1:8	C	T	0
.	.	.	.	.	.	.	.	.	.	.	SON	GT:/	0:20	0/1:3	A	G	0
#####	##	0	0.0'	0.0(	0.473	1,N	.	2.27	.	.	SON	GT:/	0:55	0/1:9	G	A	0
.	.	.	.	.	.	.	.	.	.	.	SON	GT:/	0:14	0/1:2	G	A	0
.	.	.	.	.	.	.	3.7	.	.	.	SON	GT:/	0:21	0/1:2	G	T	0
.	##	0	0.0	0.9!	1.0,D	1.000	5.3	5.31	.	.	SON	GT:/	0:41	0/1:8	C	T	0
.	.	.	.	.	.	.	.	1.41	.	.	SON	GT:/	0:50	0/1:6	C	A	0
.	.	.	.	0.0!	0.51,I	.	3.5	2.51	.	.	SON	GT:/	0:83	0/1:1	G	A	0
.	.	.	.	.	.	.	.	.	.	.	SON	GT:/	0:63	0/1:1	C	T	0
.	.	.	.	.	.	.	.	.	.	.	SON	GT:/	0:16	0/1:2	G	T	0
.	.	.	.	.	.	.	.	.	0	.	DB;S	GT:/	0:31	0/1:3	A	T	0
.	.	.	.	.	.	.	5.5	3.34	.	.	SON	GT:/	0:10	0/1:1	C	T	0
.	.	.	.	.	.	.	.	.	.	.	SON	GT:/	0:8,(	0/1:9	G	A	0
.	.	.	.	.	.	.	.	.	.	.	SON	GT:/	0:26	0/1:3	A	G	0
.	.	.	.	.	.	.	.	.	.	.	SON	GT:/	0:84	0/1:1	G	T	0
.	##	0	.	.	.	.	.	.	.	.	SON	GT:/	0:10	0/1:2	C	T	0
.	.	.	.	.	.	.	2.8	.	.	.	SON	GT:/	0:21	0/1:2	C	A	0
.	.	.	.	.	.	.	.	.	.	.	SON	GT:/	0:50	0/1:6	C	T	0
.	.	.	0.0(	0.9!	1.0,D	0.978	4.8	2.38	.	.	SON	GT:/	0:38	0/1:4	T	C	0
.	.	.	.	.	.	.	.	.	.	.	SON	GT:/	0:29	0/1:2	C	T	0
.	.	.	.	.	.	.	.	.	.	.	SON	GT:/	0:41	0/1:6	C	T	0
.	##	0	0.0	1.0	1.0,D	1,D	3.9	5.02	.	.	SON	GT:/	0:11	0/1:1	C	T	0
.	.	.	.	.	.	.	3.2	1.45	.	.	SON	GT:/	0:41	0/1:7	G	C	0
.	##	0	.	.	.	.	.	.	.	.	SON	GT:/	0:41	0/1:6	G	A	0
.	.	.	.	.	.	.	4.7	.	.	.	SON	GT:/	0:57	0/1:7	C	T	0
.	.	.	.	.	.	.	.	.	.	.	SON	GT:/	0:63	0/1:8	G	T	0
.	.	.	.	.	.	.	.	.	.	.	SON	GT:/	0:36	0/1:6	G	T	0
.	.	.	.	.	.	.	.	.	.	.	SON	GT:/	0:55	0/1:9	G	C	0

#####	##	0	0.0(	0.9!	1.0,D	1.000	3.4	4.53	.	.	SON	GT:/	0:13	0/1:2	C	T	0
.	.	.	.	.	.	.	.	.	.	.	SON	GT:/	0:34	0/1:(	A	G	0
.	.	.	.	.	.	.	2.6	1.73	.	.	SON	GT:/	0:31	0/1:5	C	A	0
.	0	0	.	.	.	.	.	.	.	.	SON	GT:/	0:43	0/1:5	G	A	0
.	.	.	.	.	.	.	.	.	.	.	SON	GT:/	0:8,(	0/1:1	C	A	0
.	0	0	.	0.3!	0.981	0.987	5.5	2.78	0	0	SON	GT:/	0:70	0/1:1	C	T	0
.	.	.	.	0.9!	1.0,D	1,D	4.6	2.18	.	.	SON	GT:/	0:83	0/1:1	G	A	0
.	.	.	0.0(	0.9!	1.0,D	1.000	4.1	3.94	.	.	SON	GT:/	0:24	0/1:3	G	A	0
.	##	0	0.0(	0.9(	1.0,D	1,N	.	1.46	0	.	SON	GT:/	0:65	0/1:1	G	A	0
.	.	.	.	.	.	.	.	.	.	.	SON	GT:/	0:62	0/1:1	G	A	0
#####	##	0	.	.	.	.	.	.	.	.	SON	GT:/	0:41	0/1:8	C	T	0
.	.	.	.	.	.	.	.	.	.	.	SON	GT:/	0:20	0/1:3	G	A	0
.	.	.	.	.	.	.	.	.	.	.	SON	GT:/	0:32	0/1:3	G	A	0
.	.	.	0.0(	0.9!	1.0,D	1,D	5.3	5.05	.	.	SON	GT:/	0:80	0/1:1	G	A	0
.	.	.	.	.	.	.	.	.	.	.	SON	GT:/	0:22	0/1:2	C	A	0
.	##	.	.	.	.	.	.	.	0	0	SON	GT:/	0:28	0/1:3	C	T	0
.	.	.	.	.	.	.	.	.	.	.	SON	GT:/	0:24	0/1:5	G	A	0
.	.	.	.	.	.	.	.	.	.	.	SON	GT:/	0:66	0/1:1	C	T	0
.	.	.	.	.	.	.	.	.	.	.	SON	GT:/	0:26	0/1:5	C	A	0
.	.	.	.	.	.	1,D	3.8	.	.	.	SON	GT:/	0:18	0/1:3	T	C	0
.	.	.	0.1:	0.8:	0.994	0.964	4.5	2.47	.	.	SON	GT:/	0:10	0/1:1	G	A	0
.	##	0	0.0(	0.9:	1.0,D	1.000	3.3	3.15	.	.	SON	GT:/	0:12	0/1:1	G	A	0
.	.	.	.	.	.	.	.	1.54	.	.	SON	GT:/	0:89	0/1:1	C	T	0
2E-04	0	0	.	.	.	.	2.5	.	0	0	SON	GT:/	0:47	0/1:8	G	A	0
.	.	.	.	.	.	.	2.6	.	.	.	SON	GT:/	0:10	0/1:1	G	A	0
.	.	.	0.3:	0.0(	0.716	0.995	3.1	.	.	.	SON	GT:/	0:15	0/1:3	G	A	0
.	.	.	.	.	.	.	2.2	.	.	.	SON	GT:/	0:45	0/1:7	A	G	0
.	.	.	.	.	.	.	2.3	.	.	.	SON	GT:/	0:41	0/1:5	A	G	0
.	.	.	.	.	.	.	.	.	0	.	SON	GT:/	0:27	0/1:5	G	A	0
.	.	.	0.3(	0.0:	0.131	0.646	4.1	2.86	.	.	SON	GT:/	0:17	0/1:2	A	T	0
.	.	.	.	.	.	.	.	.	.	.	SON	GT:/	0:20	0/1:4	C	A	0
.	.	.	.	.	.	.	.	.	.	.	SON	GT:/	0:11	0/1:1	G	A	0
.	.	.	0.0'	0.8:	0.983	1,D	5.9	3.34	.	.	SON	GT:/	0:22	0/1:3	G	T	0
.	.	.	.	.	.	.	.	.	.	.	SON	GT:/	0:39	0/1:4	C	T	0
.	.	.	.	.	.	.	.	.	.	.	SON	GT:/	0:27	0/1:5	G	T	0
.	##	0	0.0	0.0	0.313	0.999	4.8	1.42	.	.	SON	GT:/	0:81	0/1:1	C	T	0
.	##	0	.	.	.	.	4	.	.	.	SON	GT:/	0:35	0/1:3	C	T	0
.	.	.	.	.	.	.	.	.	.	.	SON	GT:/	0:49	0/1:7	A	G	0
.	.	.	0.0(	0.9!	1.0,D	1,D	5.9	3.90	.	.	SON	GT:/	0:36	0/1:(	A	G	0
.	##	0	.	.	.	.	.	.	0	.	SON	GT:/	0:34	0/1:4	G	A	0
.	.	.	.	.	.	.	3.1	.	.	.	SON	GT:/	0:28	0/1:3	A	G	0
.	##	0	0.0:	0.7'	0.999	1.000	4.8	4.35	.	.	SON	GT:/	0:85	0/1:1	G	A	0
.	.	.	.	.	.	.	.	.	.	.	SON	GT:/	0:18	0/1:5	C	T	0
.	.	.	.	.	.	.	.	.	.	.	SON	GT:/	0:14	0/1:2	G	A	0
.	.	.	0.0(	0.9!	0.999	0.997	.	3.46	.	.	SON	GT:/	0:28	0/1:2	G	T	0
#####	0	0	.	.	.	1,D	.	.	.	.	SON	GT:/	0:41	0/1:5	C	T	0
.	.	.	0.0	0.4	0.529	1,N	3.8	2.23	.	.	SON	GT:/	0:60	0/1:(	T	C	0
.	.	.	.	.	.	.	.	1.56	.	.	SON	GT:/	0:25	0/1:4	A	G	0
.	.	.	0.0'	0.8	0.993	1,N	.	.	.	.	SON	GT:/	0:50	0/1:(	C	A	0
.	.	.	.	.	.	.	.	.	.	.	SON	GT:/	0:42	0/1:4	G	T	0
.	.	.	.	.	.	.	.	1.76	.	.	SON	GT:/	0:56	0/1:7	C	T	0
.	.	.	.	.	.	.	2.7	3.73	.	.	SON	GT:/	0:17	0/1:3	C	T	0
#####	##	0	.	.	.	.	.	.	.	.	SON	GT:/	0:30	0/1:5	C	T	0
.	##	0	.	0.0	0.0,B	1,N	.	.	.	.	SON	GT:/	0:90	0/1:1	G	A	0

.	.	.	0.3	0.2	0.839	0.869	2.8	.	.	.	SOM	GT:/	0:16	0/1:2	G	A	0
#####	##	0	.	.	.	.	.	.	.	0	SOM	GT:/	0:62	0/1:1	C	T	0
.	.	.	0.0	0.9	1.0,D	0.999	.	2.19	.	.	SOM	GT:/	0:78	0/1:1	G	T	0
.	.	.	.	.	.	.	.	.	.	.	SOM	GT:/	0:12	0/1:1	C	T	0
.	.	.	.	.	.	.	.	.	.	.	SOM	GT:/	0:13	0/1:1	C	T	0
6E-04	0	0	0.0	0.2	0.546	1,D	3.5	2.62	.	.	DB;S	GT:/	0:53	0/1:1	G	A	0
2E-04	##	0	.	.	.	.	.	.	.	.	SOM	GT:/	0:46	0/1:6	C	T	0
.	.	.	.	.	.	.	.	.	.	.	SOM	GT:/	0:22	0/1:3	C	T	0
.	0	0	.	.	.	.	.	.	0	.	SOM	GT:/	0:14	0/1:2	G	A	0
.	.	.	0.0	0.1	0.875	0.570	.	.	.	.	SOM	GT:/	0:45	0/1:8	G	A	0
.	.	.	.	.	.	.	.	1.24	.	.	SOM	GT:/	0:56	0/1:9	C	A	0
#####	##	0	0.1	0.9	1.0,D	1,D	2.1	3.16	.	.	SOM	GT:/	0:11	0/1:1	C	T	0
.	.	.	.	.	.	.	.	2.19	.	.	SOM	GT:/	0:11	0/1:1	G	A	0
.	.	.	.	.	.	.	.	.	.	.	SOM	GT:/	0:18	0/1:3	G	A	0
.	.	.	.	.	.	.	.	.	0	.	SOM	GT:/	0:16	0/1:2	C	T	0
#####	##	0	.	.	.	.	.	1.68	.	.	SOM	GT:/	0:19	0/1:2	G	A	0
.	0	0	.	.	.	.	.	.	.	.	SOM	GT:/	0:11	0/1:2	G	A	0
.	.	.	0.5	0.1	0.199	0.996	4.7	1.33	.	.	SOM	GT:/	0:11	0/1:1	G	T	0
.	.	.	.	.	.	.	.	.	.	.	SOM	GT:/	0:33	0/1:7	C	A	0
#####	##	0	.	.	.	.	.	1.31	.	.	SOM	GT:/	0:14	0/1:2	C	T	0
.	.	.	.	.	.	.	.	.	.	.	SOM	GT:/	0:9,	0/1:2	C	T	0
.	.	.	.	.	.	.	.	.	.	.	SOM	GT:/	0:25	0/1:2	G	A	0
.	.	.	.	.	.	.	.	.	.	.	SOM	GT:/	0:27	0/1:4	C	T	0
.	##	0	0.0	0.3	0.815	1,N	.	2.00	.	.	SOM	GT:/	0:82	0/1:1	G	A	0
.	.	.	.	.	.	1,D	3.7	6.30	.	.	SOM	GT:/	0:66	0/1:9	G	A	0
7E-04	0	0	0.0	0.8	0.999	1,N	.	4.56	.	.	SOM	GT:/	0:53	0/1:1	G	A	0
.	.	.	.	.	.	.	.	.	.	.	SOM	GT:/	0:29	0/1:6	G	A	0
.	.	.	0.0	0.3	0.416	1,D	4	4.18	.	.	SOM	GT:/	0:10	0/1:1	T	C	0
.	.	.	.	.	.	.	.	1.51	.	.	SOM	GT:/	0:63	0/1:1	C	T	0
.	.	.	.	.	.	.	.	.	0	.	SOM	GT:/	0:94	0/1:1	G	A	0
.	##	0	.	.	.	.	.	1.52	.	.	SOM	GT:/	0:97	0/1:1	G	A	0
.	.	.	.	.	.	.	.	.	.	.	SOM	GT:/	0:31	0/1:5	T	C	0
#####	##	0	0.0	0.1	0.534	1.000	5.3	3.72	.	.	SOM	GT:/	0:92	0/1:1	C	T	0
#####	0	0	.	.	.	.	.	.	0	.	SOM	GT:/	0:10	0/1:1	C	T	0
#####	##	0	0.0	0.0	0.146	1,D	4.1	2.79	.	.	SOM	GT:/	0:19	0/1:3	G	A	0
.	##	0	.	.	.	.	.	.	.	.	SOM	GT:/	0:76	0/1:1	C	T	0
#####	##	0	0.0	0.9	1.0,D	1.000	5.4	5.41	0	.	SOM	GT:/	0:39	0/1:9	G	A	0
.	.	.	.	.	.	.	.	1.28	.	.	SOM	GT:/	0:10	0/1:2	G	T	0
.	.	.	.	.	.	.	.	.	.	.	SOM	GT:/	0:10	0/1:1	C	A	0
.	##	0	.	.	.	.	.	.	.	.	SOM	GT:/	0:85	0/1:1	C	T	0
0.021	0	0	0.5	0.0	0.084	1,N	.	.	0	0	DB;S	GT:/	0:16	0/1:3	C	T	0
.	##	0	.	.	.	.	.	.	.	.	SOM	GT:/	0:19	0/1:3	C	T	0
.	.	.	.	.	.	.	2.2	.	.	.	SOM	GT:/	0:16	0/1:1	T	G	0
.	.	.	0.1	0.0	0.004	1,N	.	.	0	.	SOM	GT:/	0:78	0/1:1	C	T	0
.	.	.	.	.	.	.	.	.	.	.	SOM	GT:/	0:8,	0/1:6	C	T	0
.	.	.	.	.	.	.	.	.	.	.	SOM	GT:/	0:11	0/1:1	C	G	0
.	0	0	.	.	.	.	.	.	.	0	SOM	GT:/	0:12	0/1:1	G	A	0
.	##	0	0.3	0.0	0.0,B	1,N	.	.	.	.	SOM	GT:/	0:11	0/1:1	C	T	0
.	.	.	.	.	.	.	.	.	.	.	SOM	GT:/	0:11	0/1:2	C	T	0
.	.	.	.	.	.	.	.	.	.	.	SOM	GT:/	0:11	0/1:2	A	G	0
.	.	.	0.1	0.5	0.953	1.000	4.3	2.96	0	.	SOM	GT:/	0:37	0/1:5	G	A	0
.	##	0	0.0	0.9	1.0,D	1.000	4.8	2.99	.	.	SOM	GT:/	0:12	0/1:1	G	A	0
.	.	.	0.0	0.7	0.994	0.671	2.8	3.44	.	.	SOM	GT:/	0:61	0/1:9	C	T	0
.	.	.	1.0	0.1	0.327	0.911	5.5	.	.	.	SOM	GT:/	0:75	0/1:1	G	A	0

.	.	.	.	.	.	.	.	.	.	.	SOM	GT:/	0:31	0/1:	G	A	0
.	.	.	0.2	0.2	0.514	1.000	.	.	0	.	SOM	GT:/	0:47	0/1:	G	T	0
.	.	.	.	.	.	.	.	.	.	.	SOM	GT:/	0:10	0/1:	G	A	0
.	##	0	.	.	.	.	.	1.42	.	.	SOM	GT:/	0:13	0/1:	G	A	0
.	##	0	.	.	.	.	.	.	.	.	SOM	GT:/	0:16	0/1:	G	A	0
.	.	.	.	.	.	.	.	.	.	.	SOM	GT:/	0:9,	0/1:	A	G	0
.	.	.	0.0	0.9	0.993	1,D	4.4	4.77	.	.	SOM	GT:/	0:70	0/1:	C	T	0
.	##	0	.	.	.	.	.	.	.	.	SOM	GT:/	0:25	0/1:	C	T	0
#####	##	0	.	.	.	.	.	.	.	.	SOM	GT:/	0:52	0/1:	G	A	0
#####	##	0	0.0	0.9	1.0,D	1,D	4.8	5.30	.	.	SOM	GT:/	0:49	0/1:	C	T	0
.	.	.	.	.	.	.	.	.	.	.	SOM	GT:/	0:11	0/1:	A	G	0
.	.	.	.	.	.	.	.	.	0	0	SOM	GT:/	0:31	0/1:	A	T	0
.	.	.	.	.	.	.	.	.	.	.	SOM	GT:/	0:39	0/1:	G	T	0
2E-04	##	0	0.2	0.0	0.012	1,N	.	1.25	0	.	SOM	GT:/	0:13	0/1:	G	A	0
.	.	.	0.0	0.9	0.998	1.000	3	3.50	.	.	SOM	GT:/	0:55	0/1:	G	A	0
.	.	.	0.0	0.8	0.99,I	1,D	4.5	4.51	.	.	SOM	GT:/	0:53	0/1:	A	C	0
.	.	.	0.2	0.2	0.641	1,N	2.4	.	.	.	SOM	GT:/	0:11	0/1:	C	G	0
.	.	.	.	.	.	.	.	.	.	.	SOM	GT:/	0:34	0/1:	C	T	0
.	.	.	0.0	0.9	1.0,D	1.000	2.2	3.41	.	.	SOM	GT:/	0:94	0/1:	G	A	0
.	.	.	.	.	.	.	.	.	.	.	SOM	GT:/	0:38	0/1:	G	A	0
.	.	.	.	.	.	.	.	.	.	.	SOM	GT:/	0:71	0/1:	C	T	0
.	.	.	.	.	.	.	.	.	.	.	DB;	GT:/	0:46	0/1:	G	A	0
.	.	.	.	.	.	.	.	.	.	.	SOM	GT:/	0:32	0/1:	T	C	0
.	.	.	0.0	0.9	1.0,D	1,N	.	1.71	.	.	SOM	GT:/	0:80	0/1:	G	A	0
.	##	0	.	.	.	.	.	.	.	.	SOM	GT:/	0:59	0/1:	G	A	0
.	.	.	0.3	0.1	0.804	0.988	4.7	2.67	.	.	SOM	GT:/	0:90	0/1:	C	T	0
.	.	.	.	.	.	.	.	.	.	.	SOM	GT:/	0:28	0/1:	T	C	0
.	.	.	.	.	.	.	.	.	.	.	SOM	GT:/	0:98	0/1:	G	T	0
.	.	.	0.0	0.9	1.0,D	0.999	4.4	4.50	.	.	SOM	GT:/	0:50	0/1:	C	T	0
#####	##	0	.	.	.	.	.	.	0	.	SOM	GT:/	0:24	0/1:	C	T	0
.	.	.	.	.	.	.	.	.	.	.	SOM	GT:/	0:56	0/1:	C	T	0
.	.	.	.	.	.	.	.	.	.	.	SOM	GT:/	0:21	0/1:	C	A	0
.	.	.	.	.	.	.	.	.	0	.	SOM	GT:/	0:17	0/1:	T	C	0
.	.	.	.	.	.	.	.	.	.	.	SOM	GT:/	0:11	0/1:	T	C	0
.	.	.	.	.	.	.	.	.	.	.	SOM	GT:/	0:34	0/1:	C	T	0
.	##	0	0.4	0.9	0.997	1,D	5.8	2.48	.	.	SOM	GT:/	0:72	0/1:	C	T	0
#####	##	0	0.0	0.0	0.022	1.000	2.1	2.96	.	.	SOM	GT:/	0:23	0/1:	C	T	0
.	.	.	.	.	.	.	.	.	.	.	SOM	GT:/	0:84	0/1:	C	G	0
.	.	.	.	.	.	.	.	.	.	.	SOM	GT:/	0:8,	0/1:	A	C	0
.	##	0	0.0	0.0	0.168	1,D	4.9	3.90	.	.	SOM	GT:/	0:58	0/1:	G	A	0
.	.	.	.	.	.	.	.	.	.	.	SOM	GT:/	0:69	0/1:	G	A	0
.	.	.	.	.	.	.	2.4	.	.	.	SOM	GT:/	0:20	0/1:	T	C	0
.	.	.	.	.	.	.	.	.	0	.	SOM	GT:/	0:12	0/1:	C	T	0
.	.	.	.	.	.	.	.	.	.	.	SOM	GT:/	0:9,	0/1:	C	T	0
.	.	.	.	.	.	.	.	.	0	0	DB;	GT:/	0:19	0/1:	C	A	0
.	.	.	.	.	.	.	.	.	0	.	SOM	GT:/	0:42	0/1:	C	G	0
.	.	.	.	.	.	.	.	.	0	.	SOM	GT:/	0:35	0/1:	C	T	0
.	.	.	.	.	.	.	.	.	.	.	SOM	GT:/	0:14	0/1:	G	A	0
.	.	.	0.2	0.0	0.021	0.935	3.2	1.73	.	.	SOM	GT:/	0:32	0/1:	G	T	0
0.002	0	0	0.2	0.0	0.103	1,N	.	2.00	.	.	SOM	GT:/	0:62	0/1:	C	T	0
.	.	.	.	.	.	.	2.2	.	.	.	SOM	GT:/	0:13	0/1:	C	T	0
.	##	0	.	.	.	.	.	.	0	.	SOM	GT:/	0:10	0/1:	G	A	0
.	.	.	.	.	.	.	.	.	0	0	SOM	GT:/	0:45	0/1:	A	G	0
.	.	.	.	.	.	.	.	.	.	.	SOM	GT:/	0:19	0/1:	G	A	0

.	.	.	.	.	.	.	.	.	.	.	SOM	GT:	0:21	0/1:	C	A	0
.	.	.	.	.	.	.	.	.	.	.	SOM	GT:	0:38	0/1:	A	G	0
.	.	.	.	.	.	.	2.5	3.60	.	.	SOM	GT:	0:72	0/1:	G	A	0
.	.	.	.	.	.	.	.	1.35	.	.	SOM	GT:	0:15	0/1:	T	C	0
.	##	0	.	.	.	.	.	.	.	0	SOM	GT:	0:12	0/1:	G	A	0
.	##	0	0.1	0.2	0.968	0.992	4.2	3.28	.	.	SOM	GT:	0:40	0/1:	G	A	0
.	.	.	.	.	.	.	2.4	.	.	.	SOM	GT:	0:62	0/1:	G	A	0
.	##	0	.	0.7	0.986	1.000	4.8	3.90	.	.	SOM	GT:	0:13	0/1:	G	A	0
.	.	.	.	.	.	.	.	.	.	.	SOM	GT:	0:44	0/1:	C	T	0
.	.	.	.	.	.	.	2.4	.	.	.	SOM	GT:	0:26	0/1:	G	A	0
.	.	.	0.0	0.0	0.503	1,N	3	2.64	.	.	SOM	GT:	0:14	0/1:	C	T	0
.	.	.	0.4	0.0	0.009	0.551	3.6	2.75	.	.	SOM	GT:	0:65	0/1:	C	T	0
.	.	.	.	.	.	.	.	2.41	.	.	SOM	GT:	0:9,	0/1:	C	A	0
.	.	.	0.0	0.2	0.748	1.000	3.5	2.25	.	.	SOM	GT:	0:42	0/1:	G	C	0
.	.	.	.	.	.	1,A	4.6	7.89	.	.	SOM	GT:	0:31	0/1:	G	T	0
.	.	.	0.2	0.8	0.991	0.983	4.8	2.51	.	.	SOM	GT:	0:69	0/1:	G	T	0
.	.	.	0.0	0.0	0.149	0.884	2.9	2.50	.	.	SOM	GT:	0:41	0/1:	C	T	0
.	.	.	0.0	0.9	1.0,D	1,D	4.2	3.92	.	.	SOM	GT:	0:65	0/1:	C	A	0
.	.	.	.	.	.	1,A	.	4.13	.	.	SOM	GT:	0:91	0/1:	C	A	0
.	.	.	.	.	.	.	.	.	0	0	SOM	GT:	0:73	0/1:	T	C	0
.	.	.	.	.	.	.	.	.	.	.	SOM	GT:	0:24	0/1:	C	T	0
.	.	.	.	.	.	.	.	.	.	.	SOM	GT:	0:19	0/1:	G	A	0
.	.	.	.	.	.	.	.	.	.	.	SOM	GT:	0:41	0/1:	T	G	0
.	.	.	.	0.0	0.012	1,N	.	2.49	.	.	SOM	GT:	0:59	0/1:	G	T	0
.	##	0	.	0.0	0.316	1,N	.	1.91	0	.	SOM	GT:	0:10	0/1:	C	T	0
.	.	.	.	.	.	.	.	.	.	.	SOM	GT:	0:14	0/1:	C	T	0
.	.	.	.	.	.	.	.	.	.	.	SOM	GT:	0:59	0/1:	C	T	0
.	.	.	.	.	.	.	.	.	.	.	SOM	GT:	0:27	0/1:	C	T	0
.	.	.	.	.	.	.	.	2.03	.	.	SOM	GT:	0:10	0/1:	C	T	0
.	.	.	0.0	0.9	1.0,D	0.989	3.8	2.47	.	.	SOM	GT:	0:10	0/1:	C	A	0
.	.	.	.	.	.	.	.	1.42	.	.	SOM	GT:	0:9,	0/1:	T	C	0
.	.	.	0.0	0.5	0.712	1,D	2.4	3.32	.	.	SOM	GT:	0:12	0/1:	G	T	0
.	.	.	0.0	0.8	0.995	0.997	5.3	2.86	.	.	SOM	GT:	0:10	0/1:	C	T	0
#####	##	0	0.0	0.9	0.997	1,D	4.7	3.93	0	0	SOM	GT:	0:14	0/1:	C	T	0
.	.	.	.	.	.	.	.	.	.	.	SOM	GT:	0:34	0/1:	G	A	0
.	.	.	.	.	.	.	.	.	.	.	SOM	GT:	0:27	0/1:	G	A	0
.	.	.	.	.	.	.	.	.	.	.	SOM	GT:	0:66	0/1:	C	T	0
.	.	.	.	.	.	.	.	.	.	.	SOM	GT:	0:9,	0/1:	C	G	0
.	.	.	0.0	0.2	0.957	1,D	4.1	3.36	.	.	SOM	GT:	0:20	0/1:	G	A	0
.	.	.	.	.	.	.	.	.	.	.	SOM	GT:	0:51	0/1:	G	A	0
.	.	.	.	.	.	.	.	.	.	.	SOM	GT:	0:46	0/1:	G	A	0
.	.	.	.	.	.	.	4.4	2.17	.	.	SOM	GT:	0:47	0/1:	A	G	0
.	.	.	.	.	.	.	.	.	.	.	SOM	GT:	0:17	0/1:	C	T	0
.	.	.	.	.	.	.	.	.	.	.	SOM	GT:	0:23	0/1:	C	T	0
.	.	.	.	.	.	.	2.5	1.43	.	.	SOM	GT:	0:64	0/1:	G	T	0
.	.	.	.	.	.	.	.	.	.	.	SOM	GT:	0:63	0/1:	C	T	0
2E-04	0	0	0.0	0.9	1.0,D	1.000	3.1	4.07	.	.	SOM	GT:	0:17	0/1:	G	A	0
0.002	0	0	.	.	.	.	4.4	1.63	.	.	SOM	GT:	0:10	0/1:	G	A	0
.	.	.	.	.	.	.	.	.	.	.	SOM	GT:	0:17	0/1:	G	A	0
.	.	.	.	.	.	.	.	.	0	0	DB;	GT:	0:20	0/1:	T	G	0
#####	##	0	.	.	.	.	.	.	.	.	SOM	GT:	0:40	0/1:	C	T	0
.	.	.	.	.	.	.	.	.	.	.	SOM	GT:	0:21	0/1:	G	A	0
.	0	0	0.1	0.0	0.001	0.970	.	1.31	.	.	SOM	GT:	0:66	0/1:	G	A	0
.	.	.	.	.	.	1,D	2.8	1.87	.	.	SOM	GT:	0:96	0/1:	G	A	0

.	.	.	.	.	.	.	.	.	.	.	SON	GT:/	0:15	0/1:2	A	G	0
.	.	.	0.0	1.0	1.0,D	1,D	.	2.92	.	.	SON	GT:/	0:13	0/1:1	T	C	0
.	.	.	.	.	.	.	.	.	.	.	SON	GT:/	0:49	0/1:5	A	G	0
0.004	0	0	.	.	.	.	.	.	0	0	SON	GT:/	0:66	0/1:1	T	G	0
.	.	.	.	.	.	.	.	.	.	.	SON	GT:/	0:90	0/1:1	G	A	0
.	.	.	0.3	0.5	0.782	.	4.8	3.58	.	.	SON	GT:/	0:17	0/1:2	T	G	0
.	.	.	.	.	.	.	.	.	.	.	SON	GT:/	0:11	0/1:2	T	C	0
.	.	.	.	.	.	.	.	.	.	.	SON	GT:/	0:22	0/1:4	G	A	0
2E-04	##	0	.	.	.	.	.	.	.	.	SON	GT:/	0:96	0/1:1	G	A	0
.	.	.	.	.	.	.	2.9	1.43	.	.	SON	GT:/	0:88	0/1:1	C	A	0
.	##	.	.	.	.	1,N	.	2.84	.	.	SON	GT:/	0:99	0/1:1	G	A	0
.	.	.	.	.	.	.	.	.	.	.	SON	GT:/	0:10	0/1:1	G	T	0
.	##	0	0.1	0.0	0.864	1,N	.	2.24	0	.	SON	GT:/	0:11	0/1:1	C	T	0
#####	0	0	0.0	0.3	0.529	1,N	.	.	0	.	SON	GT:/	0:47	0/1:8	G	A	0
.	##	0	.	.	.	.	.	.	0	.	SON	GT:/	0:63	0/1:8	C	T	0
.	.	.	.	.	.	.	.	1.27	.	.	SON	GT:/	0:20	0/1:2	G	A	0
.	.	.	.	.	.	.	.	.	.	.	SON	GT:/	0:37	0/1:5	G	A	0
2E-04	0	0	.	.	.	.	.	.	.	.	SON	GT:/	0:32	0/1:6	C	T	0
.	.	.	.	.	.	.	.	.	.	.	SON	GT:/	0:13	0/1:2	C	T	0
#####	##	0	.	.	.	.	.	.	.	.	SON	GT:/	0:89	0/1:1	C	T	0
.	.	.	0.2	0.1	0.555	1,N	.	.	.	.	SON	GT:/	0:20	0/1:3	C	T	0
.	##	0	.	.	.	.	3.8	3.57	0	.	SON	GT:/	0:17	0/1:3	T	C	0
.	.	.	.	.	.	.	.	1.56	.	.	SON	GT:/	0:85	0/1:1	G	A	0
.	.	.	.	.	.	.	.	.	0	.	SON	GT:/	0:24	0/1:3	C	T	0
.	.	.	.	.	.	.	.	.	.	.	SON	GT:/	0:21	0/1:4	C	T	0
.	0	0	.	.	.	1,A	3.4	8.95	.	.	SON	GT:/	0:37	0/1:5	G	A	0
.	.	.	.	.	.	.	.	.	.	.	SON	GT:/	0:12	0/1:1	C	T	0
.	.	.	.	.	.	.	.	.	.	.	SON	GT:/	0:42	0/1:5	G	A	0
.	.	.	.	.	.	.	.	.	.	.	SON	GT:/	0:14	0/1:3	T	C	0
.	.	.	0.0	0.9	0.99,I	1,D	5.6	4.31	.	.	SON	GT:/	0:55	0/1:8	A	G	0
.	.	.	.	.	.	.	.	.	.	.	SON	GT:/	0:30	0/1:4	T	C	0
2E-04	##	0	0.0	0.5	0.928	1,N	.	1.82	0	.	SON	GT:/	0:48	0/1:1	G	A	0
.	.	.	.	.	.	.	.	.	.	.	SON	GT:/	0:10	0/1:1	T	C	0
.	.	.	.	.	.	.	.	.	0	.	SON	GT:/	0:9,(	0/1:2	C	T	0
.	.	.	.	.	.	.	.	.	0	.	SON	GT:/	0:15	0/1:3	C	T	0
.	.	.	.	.	.	.	.	.	0	.	SON	GT:/	0:16	0/1:4	G	A	0
.	##	0	0.3	0.0	0.142	1.000	5	2.85	.	0	SON	GT:/	0:55	0/1:1	C	T	0
.	.	.	.	.	.	.	.	.	.	.	SON	GT:/	0:84	0/1:1	C	A	0
.	.	.	.	.	.	.	2.6	.	.	.	SON	GT:/	0:14	0/1:2	G	T	0
.	.	.	.	.	.	.	.	.	0	0	SON	GT:/	0:10	0/1:6	G	A	0
0.009	0	0	.	.	.	.	.	.	0	0	DB;S	GT:/	0:40	0/1:8	A	T	0
.	.	.	.	.	.	.	.	.	.	.	SON	GT:/	0:14	0/1:2	A	C	0
2E-04	##	0	0.0	0.9	1.0,D	1,D	5	5.37	.	.	SON	GT:/	0:52	0/1:1	C	T	0
.	.	.	.	.	.	.	.	.	0	.	SON	GT:/	0:20	0/1:4	T	C	0
.	.	.	.	.	.	.	.	1.86	.	.	SON	GT:/	0:73	0/1:1	G	A	0
.	.	.	.	.	.	.	.	.	.	.	SON	GT:/	0:44	0/1:6	G	A	0
.	.	.	.	.	.	.	.	2.54	0	0	SON	GT:/	0:9,(	0/1:2	G	A	0
.	.	.	.	.	.	.	.	.	.	.	SON	GT:/	0:15	0/1:2	G	T	0
.	.	.	.	.	.	.	.	.	.	.	SON	GT:/	0:31	0/1:2	C	T	0
.	.	.	0.0	0.9	0.996	1.000	4.4	5.38	.	.	SON	GT:/	0:11	0/1:1	G	A	0
#####	##	0	.	.	.	.	3.8	3.08	0	0	SON	GT:/	0:53	0/1:7	G	A	0
.	.	.	0.6	0.0	0.02,I	0.757	3.1	.	.	.	SON	GT:/	0:29	0/1:3	G	A	0
.	.	.	0.0	0.9	1.0,D	1,D	4.5	2.75	.	.	SON	GT:/	0:79	0/1:1	C	T	0
.	.	.	.	.	.	1,A	4.3	8.44	.	.	SON	GT:/	0:14	0/1:2	G	A	0

2E-04	##	0	.	.	.	.	.	.	.	.	SON	GT:/	0:78	0/1:	G	A	0
.	.	.	.	.	.	.	2.5	.	.	.	SON	GT:/	0:21	0/1:	T	C	0
.	.	.	.	.	.	.	.	.	.	.	SON	GT:/	0:81	0/1:	G	T	0
.	.	.	.	.	.	1,N	.	.	.	.	SON	GT:/	0:27	0/1:	C	A	0
.	.	.	.	.	.	.	.	.	.	.	SON	GT:/	0:29	0/1:	G	T	0
.	.	.	.	.	.	.	.	.	.	.	SON	GT:/	0:63	0/1:	G	T	0
.	.	.	0.0	0.9	1.0,D	1.000	5.6	4.89	.	.	SON	GT:/	0:61	0/1:	C	T	0
.	.	.	0.0	0.9	1.0,D	1,D	5.2	4.06	.	.	SON	GT:/	0:96	0/1:	G	A	0
.	.	.	.	.	.	.	.	.	.	.	SON	GT:/	0:40	0/1:	C	T	0
.	.	.	.	.	.	.	.	.	.	.	SON	GT:/	0:45	0/1:	G	A	0
.	.	.	.	.	.	.	.	.	.	.	SON	GT:/	0:82	0/1:	C	T	0
.	.	.	.	.	.	.	.	.	0	0	SON	GT:/	0:9,(	0/1:	T	C	0
.	.	.	0.0	0.0	0.213	1,N	.	.	.	.	SON	GT:/	0:21	0/1:	G	A	0
.	.	.	0.0	0.0	0.437	1,N	.	2.41	.	.	SON	GT:/	0:64	0/1:	C	T	0
.	.	.	.	.	.	.	.	.	.	.	SON	GT:/	0:16	0/1:	G	A	0
.	##	0	0.0	0.9	1.0,D	1.000	3.6	2.21	.	.	SON	GT:/	0:57	0/1:	C	T	0
.	##	0	.	.	.	.	.	.	.	.	SON	GT:/	0:72	0/1:	C	T	0
.	.	.	.	.	.	.	.	.	0	.	SON	GT:/	0:31	0/1:	G	A	0
0.002	0	0	.	.	.	.	.	3.33	.	.	SON	GT:/	0:17	0/1:	G	A	0
.	.	.	.	.	.	.	.	.	0	.	SON	GT:/	0:13	0/1:	C	T	0
.	.	.	.	.	.	.	.	.	.	.	SON	GT:/	0:12	0/1:	A	G	0
.	##	0	0.0	0.9	1.0,D	1.000	4.6	3.93	.	.	SON	GT:/	0:11	0/1:	G	A	0
.	.	.	.	.	.	.	.	.	.	.	SON	GT:/	0:59	0/1:	A	G	0
.	.	.	.	.	.	.	.	.	.	.	SON	GT:/	0:8,(	0/1:	C	T	0
.	.	.	.	.	.	.	.	.	0.1	0.1	SON	GT:/	0:38	0/1:	G	A	0
.	.	.	.	.	.	.	.	.	0	0	SON	GT:/	0:58	0/1:	C	A	0
.	.	.	.	.	.	.	.	.	0	0.1	SON	GT:/	0:9,(	0/1:	G	A	0
.	.	.	.	.	.	.	.	.	0	0.1	SON	GT:/	0:8,(	0/1:	C	A	0
.	.	.	.	.	.	.	.	.	0.2	0.4	SON	GT:/	0:14	0/1:	C	T	0
.	.	.	.	.	.	.	.	.	.	.	SON	GT:/	0:78	0/1:	G	A	0
.	.	.	.	.	.	.	.	.	.	.	SON	GT:/	0:11	0/1:	C	A	0
.	.	.	.	.	.	.	.	.	.	.	SON	GT:/	0:70	0/1:	G	C	0
.	.	.	.	.	.	.	.	.	.	.	SON	GT:/	0:9,(	0/1:	C	T	0
.	.	.	.	.	.	.	.	.	0.3	0.6	SON	GT:/	0:9,(	0/1:	T	C	0
.	.	.	.	.	.	.	.	.	0.3	0.5	SON	GT:/	0:9,(	0/1:	C	T	0
.	.	.	.	.	.	.	.	.	0.1	0.2	DB;S	GT:/	0:33	0/1:	G	C	0
.	.	.	.	.	.	.	.	.	.	.	SON	GT:/	0:30	0/1:	C	T	0
.	.	.	.	.	.	.	.	.	.	.	SON	GT:/	0:31	0/1:	T	C	0
.	.	.	.	.	.	.	.	.	.	.	SON	GT:/	0:33	0/1:	A	G	0
.	.	.	.	.	.	.	2.5	.	.	.	SON	GT:/	0:24	0/1:	C	G	0
.	.	.	.	.	.	.	.	.	.	.	SON	GT:/	0:75	0/1:	A	G	0
.	.	.	.	.	.	.	.	.	.	.	SON	GT:/	0:18	0/1:	C	T	0
.	.	.	0.5	0.1	0.245	1,N	.	.	.	.	SON	GT:/	0:11	0/1:	C	T	0
.	.	.	.	.	.	.	.	.	.	.	SON	GT:/	0:14	0/1:	A	G	0
.	.	.	.	.	.	.	.	.	.	.	SON	GT:/	0:21	0/1:	G	T	0
.	.	.	.	.	.	.	.	.	.	.	SON	GT:/	0:37	0/1:	C	T	0
.	.	.	.	.	.	.	3.8	.	.	.	SON	GT:/	0:28	0/1:	C	T	0
.	##	0	.	.	.	.	.	1.49	.	.	SON	GT:/	0:22	0/1:	A	G	0
.	.	.	.	.	.	.	.	.	0	.	SON	GT:/	0:47	0/1:	C	T	0
.	.	.	.	.	.	.	.	.	.	.	SON	GT:/	0:12	0/1:	T	G	0
.	.	.	.	.	.	.	.	.	.	.	SON	GT:/	0:10	0/1:	C	T	0
.	.	.	.	.	.	.	.	1.71	.	.	SON	GT:/	0:22	0/1:	C	T	0
.	##	0	.	.	.	.	.	.	.	.	SON	GT:/	0:51	0/1:	G	A	0
.	##	0	0.0	0.9	1.0,D	1.000	4.8	4.76	.	.	SON	GT:/	0:30	0/1:	C	T	0

.	.	.	.	.	.	.	.	.	.	.	SON	GT:/	0:28	0/1:4	C	A	0
.	.	.	.	.	.	.	.	.	.	.	SON	GT:/	0:93	0/1:1	C	A	0
.	.	.	.	.	.	.	.	.	.	.	SON	GT:/	0:42	0/1:7	T	C	0
.	.	.	0.0	0.9	1.0,D	1.000	5	2.65	.	.	SON	GT:/	0:36	0/1:6	G	A	0
.	.	.	0.0	0.9	1.0,D	1.000	5.3	3.79	.	.	SON	GT:/	0:44	0/1:8	T	C	0
.	.	.	.	.	.	.	.	.	.	.	SON	GT:/	0:29	0/1:3	C	T	0
.	.	.	.	.	.	.	.	.	.	.	SON	GT:/	0:16	0/1:3	T	C	0
.	.	.	.	.	.	.	.	.	.	.	SON	GT:/	0:34	0/1:4	G	A	0
.	.	.	0.0	0.9	1.0,D	1.000	5.3	4.28	.	.	SON	GT:/	0:73	0/1:1	G	A	0
.	.	.	.	.	.	.	.	.	.	.	SON	GT:/	0:23	0/1:4	T	C	0
.	.	.	.	.	.	.	.	.	.	.	SON	GT:/	0:12	0/1:1	C	A	0
.	.	.	.	.	.	.	.	.	.	.	SON	GT:/	0:53	0/1:8	G	A	0
.	##	.	0.0	0.9	1.0,D	0.997	.	2.81	.	.	SON	GT:/	0:10	0/1:1	C	T	0
.	.	.	.	.	.	.	.	.	.	.	SON	GT:/	0:18	0/1:4	C	T	0
.	##	0	0.0	0.9	1.0,D	1,D	5	4.64	.	.	SON	GT:/	0:72	0/1:9	G	A	0
.	##	0	.	.	.	.	.	.	.	.	SON	GT:/	0:14	0/1:2	C	T	0
2E-04	.	.	.	.	.	.	.	.	.	.	SON	GT:/	0:35	0/1:9	C	T	0
.	.	.	.	.	.	.	.	.	.	.	SON	GT:/	0:15	0/1:3	G	A	0
.	.	.	.	.	.	.	.	.	.	.	SON	GT:/	0:20	0/1:3	G	A	0
.	##	0	0.0	0.9	1.0,D	0.992	5.5	3.51	.	.	SON	GT:/	0:90	0/1:1	G	A	0
.	.	.	0.4	0.3	0.837	0.999	4.5	1.36	.	.	SON	GT:/	0:36	0/1:7	G	A	0
.	.	.	.	.	.	.	.	.	0.1	0.3	SON	GT:/	0:17	0/1:3	A	G	0
.	.	.	.	.	.	.	.	.	0	0.1	SON	GT:/	0:10	0/1:1	C	G	0
.	.	.	.	.	.	.	.	.	.	.	SON	GT:/	0:23	0/1:2	A	G	0
.	.	.	.	.	.	.	.	.	0.2	0.3	DB;S	GT:/	0:19	0/1:3	C	A	0
.	.	.	.	.	.	.	.	.	.	.	SON	GT:/	0:49	0/1:9	G	A	0
.	##	0	.	.	.	.	.	.	.	.	SON	GT:/	0:16	0/1:2	G	A	0
.	.	.	.	.	.	.	.	2.51	0	.	SON	GT:/	0:8,(	0/1:1	T	A	0
.	0	0	.	.	.	.	.	.	.	.	SON	GT:/	0:59	0/1:6	G	A	0
.	.	.	.	.	.	.	.	.	0	0	SON	GT:/	0:61	0/1:9	C	A	0
.	.	.	.	.	.	.	.	.	.	.	SON	GT:/	0:24	0/1:3	G	A	0
.	##	0	.	.	.	.	.	.	.	.	SON	GT:/	0:14	0/1:2	G	A	0
.	##	0	.	.	.	.	.	.	.	.	SON	GT:/	0:12	0/1:1	G	A	0
.	.	.	.	.	.	.	.	.	.	.	SON	GT:/	0:11	0/1:1	G	A	0
#####	##	0	.	.	.	.	.	.	.	.	SON	GT:/	0:52	0/1:6	G	A	0
#####	##	0	.	.	.	.	.	.	.	.	SON	GT:/	0:59	0/1:1	C	T	0
.	.	.	0.0	0.2	0.745	1,N	.	1.99	.	.	SON	GT:/	0:12	0/1:2	G	T	0
.	.	.	0.0	0.0	0.009	0.766	3.3	2.16	.	.	SON	GT:/	0:11	0/1:1	C	T	0
.	.	.	.	.	.	.	2.3	.	.	.	SON	GT:/	0:44	0/1:7	G	T	0
.	##	0	0.2	0.0	0.127	0.995	3.7	1.77	.	.	SON	GT:/	0:10	0/1:1	G	A	0
.	.	.	.	.	.	.	.	.	.	.	SON	GT:/	0:44	0/1:8	C	T	0
.	.	.	.	.	.	.	.	.	.	.	SON	GT:/	0:22	0/1:3	C	T	0
.	.	.	.	.	.	.	.	.	.	.	SON	GT:/	0:45	0/1:7	C	T	0
.	.	.	.	.	.	.	.	.	.	.	SON	GT:/	0:50	0/1:8	T	C	0
.	.	.	.	.	.	.	.	.	.	.	SON	GT:/	0:20	0/1:4	G	A	0
.	0	0	.	.	.	.	.	.	.	.	SON	GT:/	0:15	0/1:3	G	A	0
.	.	.	.	.	.	.	2.2	.	.	.	SON	GT:/	0:27	0/1:4	C	T	0
.	.	.	.	.	.	.	4	2.97	.	.	SON	GT:/	0:81	0/1:1	G	A	0
.	.	.	.	.	.	.	.	.	.	.	SON	GT:/	0:16	0/1:2	T	C	0
.	.	.	.	.	.	.	.	.	.	.	SON	GT:/	0:18	0/1:4	T	C	0
.	.	.	.	.	.	.	.	.	.	.	SON	GT:/	0:49	0/1:8	G	A	0
#####	##	0	.	.	.	.	.	.	.	.	SON	GT:/	0:72	0/1:1	C	T	0
.	.	.	.	.	.	.	.	.	.	.	SON	GT:/	0:42	0/1:6	C	T	0
#####	0	0	0.2	0.2	0.863	1,N	.	2.01	0	0	SON	GT:/	0:66	0/1:1	G	A	0

.	.	.	.	.	.	.	.	.	.		.	SOM	GT:/	0:47	0/1:	C	T	0
.	.	.	.	.	.	1,A	4.5	7.78	.		.	SOM	GT:/	0:53	0/1:	G	A	0
.	.	.	.	.	.	.	.	.	.		.	SOM	GT:/	0:10	0/1:	C	A	0
.	##	0	.	.	.	.	.	.	.		.	SOM	GT:/	0:75	0/1:	A	G	0
.	.	.	0.0	1.0	1.0,D	1,D	4.9	4.46	.		.	SOM	GT:/	0:17	0/1:	G	A	0
.	.	.	0.0	0.7	0.999	0.997	4.4	3.54	.		.	SOM	GT:/	0:14	0/1:	G	A	0
.	.	.	.	.	.	.	.	.	.		.	SOM	GT:/	0:82	0/1:	C	T	0
.	.	.	.	.	.	.	.	.	.		.	SOM	GT:/	0:26	0/1:	C	T	0
.	.	.	.	.	.	.	.	.		0	.	SOM	GT:/	0:49	0/1:	C	T	0
.	.	.	0.2	0.9	0.999	0.996	5.6	4.50	.		.	SOM	GT:/	0:54	0/1:	C	T	0
.	##	0	0.4	0.0	0.035	1,N	.	.	.		.	SOM	GT:/	0:12	0/1:	G	A	0
.	.	.	0.0	0.7	0.948	1,D	5.4	5.33	.		.	SOM	GT:/	0:86	0/1:	C	T	0
.	##	0	0.0	0.9	1.0,D	1,D	4.4	3.92	.		.	SOM	GT:/	0:69	0/1:	C	T	0
.	.	.	.	.	.	.	.	.	.		.	SOM	GT:/	0:26	0/1:	C	A	0
.	.	.	.	.	.	.	.	.	.		.	SOM	GT:/	0:36	0/1:	G	A	0
.	##	0	.	.	.	.	.	.	.		.	SOM	GT:/	0:82	0/1:	C	T	0
.	.	.	.	.	.	.	.	.	.		.	SOM	GT:/	0:22	0/1:	G	A	0
.	.	.	.	.	.	.	.	.		0	0	SOM	GT:/	0:13	0/1:	C	T	0
.	.	.	.	.	.	.	.	.		0	0	DB;S	GT:/	0:37	0/1:	C	T	0
.	.	.	.	.	.	.	2.2	1.77	.		.	SOM	GT:/	0:26	0/1:	C	T	0
.	.	.	.	.	.	.	5.5	2.28	.		.	SOM	GT:/	0:30	0/1:	A	G	0
.	.	.	.	.	.	.	.	.	.		.	SOM	GT:/	0:16	0/1:	T	C	0
.	.	.	.	.	.	.	.	.	.		.	SOM	GT:/	0:65	0/1:	G	A	0
.	.	.	0.0	0.2	0.937	1,N	2.7	1.74	.		.	SOM	GT:/	0:12	0/1:	G	A	0
.	.	.	.	.	.	.	.	.	.		.	SOM	GT:/	0:75	0/1:	C	A	0
.	.	.	.	.	.	.	.	.	.		.	SOM	GT:/	0:15	0/1:	C	A	0
.	.	.	.	.	.	.	.	.	.		.	SOM	GT:/	0:52	0/1:	G	A	0
.	.	.	.	.	.	.	.	.	.		.	SOM	GT:/	0:8,	0/1:	G	A	0
.	.	.	.	.	.	.	3.7	.	.		.	SOM	GT:/	0:21	0/1:	G	A	0
#####	##	0	.	.	.	.	.	.	.		.	SOM	GT:/	0:54	0/1:	C	T	0
.	.	.	.	.	.	.	.	.	.		.	SOM	GT:/	0:53	0/1:	G	A	0
.	.	.	0.0	0.9	1.0,D	1.000	4.8	4.78	.		.	SOM	GT:/	0:96	0/1:	C	T	0
.	.	.	.	.	.	.	.	.	.		.	SOM	GT:/	0:20	0/1:	C	T	0
.	.	.	.	.	.	.	.	.	.		.	SOM	GT:/	0:19	0/1:	A	G	0
.	.	.	.	.	.	.	.	.	.		.	SOM	GT:/	0:22	0/1:	T	G	0
.	.	.	.	.	.	.	.	.	.		.	SOM	GT:/	0:50	0/1:	A	C	0
.	.	.	.	.	.	.	.	.	.		.	SOM	GT:/	0:70	0/1:	T	C	0
.	.	.	.	.	.	.	.	.	.		.	SOM	GT:/	0:51	0/1:	T	C	0
.	.	.	.	.	.	.	2.1	.	.		.	SOM	GT:/	0:37	0/1:	G	T	0
.	.	.	.	.	.	.	.	.	.		.	SOM	GT:/	0:17	0/1:	C	T	0
.	.	.	0.0	1.0	1.0,D	1,D	5.6	4.37	.		.	SOM	GT:/	0:11	0/1:	C	T	0
.	.	.	.	.	.	.	.	1.86	.		.	SOM	GT:/	0:71	0/1:	G	A	0
.	##	0	0.0	0.4	0.916	1,N	.	2.58	.		.	SOM	GT:/	0:61	0/1:	C	T	0
.	.	.	.	.	.	.	.	.	.		.	SOM	GT:/	0:71	0/1:	C	T	0
.	.	.	.	.	.	.	.	.	.		.	SOM	GT:/	0:94	0/1:	T	C	0
.	.	.	.	.	.	.	.	.	.		.	SOM	GT:/	0:15	0/1:	C	T	0
.	.	.	.	.	.	.	.	.	.		.	SOM	GT:/	0:58	0/1:	T	A	0
.	.	.	.	.	.	.	.	.	.		.	SOM	GT:/	0:64	0/1:	C	A	0
.	.	.	.	.	.	.	3	4.36	.		.	SOM	GT:/	0:12	0/1:	G	A	0
.	.	.	.	.	.	.	.	.	.		.	SOM	GT:/	0:43	0/1:	T	C	0
.	.	.	.	.	.	.	.	.	.		.	SOM	GT:/	0:29	0/1:	A	C	0
.	.	.	.	.	.	.	.	.	.		.	SOM	GT:/	0:45	0/1:	A	C	0
.	.	.	.	.	.	.	5.2	3.80	.		.	SOM	GT:/	0:30	0/1:	A	C	0
.	.	.	.	.	.	.	.	1.99	.		.	SOM	GT:/	0:12	0/1:	G	A	0

.	.	.	.	.	.	.	.	.	.	.	SON	GT:/	0:42	0/1:6	G	A	0
.	.	.	.	.	.	.	.	.	.	.	SON	GT:/	0:99	0/1:1	C	T	0
.	.	.	.	.	.	.	.	.	.	.	SON	GT:/	0:86	0/1:1	G	T	0
.	0	0	.	.	.	.	.	.	.	.	SON	GT:/	0:21	0/1:3	C	T	0
#####	##	0	0.3	0.0	0.507	1,D	3.3	3.27	.	.	SON	GT:/	0:14	0/1:2	G	A	0
.	##	0	0.1	0.8	0.999	1,D	4	4.03	.	.	SON	GT:/	0:12	0/1:1	C	T	0
.	.	.	.	.	.	.	.	.	.	.	SON	GT:/	0:14	0/1:2	G	T	0
.	.	.	.	.	.	.	2.7	1.77	.	.	SON	GT:/	0:38	0/1:5	G	A	0
.	##	0	.	.	.	.	.	.	.	.	SON	GT:/	0:89	0/1:1	C	T	0
.	.	.	.	.	.	.	4.2	1.57	.	.	SON	GT:/	0:65	0/1:1	C	T	0
.	.	.	0.0	0.1	0.703	0.962	2.4	1.63	.	.	SON	GT:/	0:63	0/1:1	G	A	0
.	.	.	.	.	.	.	.	.	.	.	SON	GT:/	0:14	0/1:1	G	A	0
.	##	0	0.3	0.0	0.585	1,D	3.6	1.69	.	.	SON	GT:/	0:11	0/1:1	C	T	0
.	.	.	.	.	.	.	.	.	.	.	SON	GT:/	0:42	0/1:5	G	T	0
.	.	.	0.3	0.0	0.357	1,D	4.6	3.14	.	.	SON	GT:/	0:74	0/1:1	A	C	0
.	.	.	0.0	0.1	0.206	0.984	5.8	1.66	.	.	SON	GT:/	0:10	0/1:1	G	T	0
.	.	.	.	.	.	.	.	.	.	.	SON	GT:/	0:14	0/1:2	T	C	0
.	.	.	.	.	.	1,D	5.1	3.90	.	.	SON	GT:/	0:11	0/1:2	C	T	0
.	.	.	.	.	.	.	.	.	.	.	SON	GT:/	0:94	0/1:1	G	A	0
.	.	.	.	.	.	.	2.4	.	.	.	SON	GT:/	0:22	0/1:4	A	G	0
.	.	.	.	.	.	.	.	.	.	.	SON	GT:/	0:8,(	0/1:3	T	G	0
.	.	.	.	.	.	.	.	.	.	.	SON	GT:/	0:11	0/1:2	G	C	0
.	.	.	.	.	.	.	.	.	.	.	SON	GT:/	0:29	0/1:3	A	G	0
.	.	.	.	.	.	.	.	.	.	.	DB;	GT:/	0:30	0/1:3	C	A	0
.	.	.	.	.	.	.	.	.	.	.	SON	GT:/	0:8,(	0/1:4	A	G	0
.	.	.	.	.	.	.	.	.	.	.	SON	GT:/	0:10	0/1:6	T	G	0
.	.	.	.	.	.	.	.	.	.	.	SON	GT:/	0:10	0/1:3	T	C	0
.	.	.	.	.	.	.	.	.	.	.	SON	GT:/	0:10	0/1:4	T	G	0
.	.	.	.	.	.	.	.	.	.	.	SON	GT:/	0:8,(	0/1:1	A	C	0
.	.	.	.	.	.	.	.	.	0	.	SON	GT:/	0:17	0/1:1	G	A	0
.	.	.	.	.	.	.	.	.	.	.	SON	GT:/	0:9,(	0/1:2	C	A	0
.	.	.	.	.	.	.	.	.	.	.	SON	GT:/	0:14	0/1:1	C	A	0
.	.	.	.	.	.	.	.	.	.	.	SON	GT:/	0:29	0/1:3	G	A	0
.	.	.	.	.	.	.	.	.	.	.	SON	GT:/	0:60	0/1:8	G	C	0
.	##	0	0.0	0.9	0.998	1,D	4.6	3.56	.	.	SON	GT:/	0:96	0/1:1	C	T	0
.	.	.	0.0	0.8	0.999	0.986	2.9	1.95	.	.	SON	GT:/	0:11	0/1:2	C	T	0
.	0	0	.	.	.	.	.	.	.	.	SON	GT:/	0:16	0/1:1	G	A	0
.	.	.	.	.	.	.	.	.	.	.	SON	GT:/	0:99	0/1:1	C	A	0
.	.	.	0.0	0.0	0.288	1.000	3.8	1.91	.	.	SON	GT:/	0:93	0/1:1	G	A	0
.	.	.	0.1	0.9	1.0,D	1,D	4.5	4.69	.	.	SON	GT:/	0:30	0/1:4	C	T	0
.	.	.	.	.	.	.	.	.	.	.	SON	GT:/	0:41	0/1:5	A	G	0
.	.	.	.	.	.	.	.	.	.	.	SON	GT:/	0:77	0/1:1	G	A	0
.	.	.	.	.	.	.	.	.	.	.	SON	GT:/	0:11	0/1:6	G	T	0
.	.	.	.	.	.	1,A	6.1	9.05	.	.	SON	GT:/	0:79	0/1:1	C	A	0
.	.	.	.	.	.	.	2.8	.	.	.	SON	GT:/	0:8,(	0/1:2	A	G	0
.	.	.	0.0	0.5	0.963	0.999	5	4.16	.	.	SON	GT:/	0:40	0/1:6	C	T	0
.	.	.	0.1	0.9	1.0,D	1.000	4.7	2.63	.	.	SON	GT:/	0:66	0/1:1	G	A	0
.	.	.	0.0	0.9	0.996	0.999	5.2	3.37	.	.	SON	GT:/	0:23	0/1:3	C	A	0
.	.	.	.	.	.	.	.	1.55	.	.	SON	GT:/	0:24	0/1:2	C	T	0
.	.	.	.	.	.	.	.	.	.	.	SON	GT:/	0:12	0/1:2	T	C	0
.	.	.	.	.	.	.	.	.	.	.	SON	GT:/	0:8,(	0/1:1	G	A	0
.	.	.	0.0	0.9	1.0,D	1,D	3.9	4.36	.	.	SON	GT:/	0:85	0/1:1	G	A	0
.	.	.	0.0	0.0	0.032	1.000	.	.	.	.	SON	GT:/	0:85	0/1:8	C	A	0
.	.	.	.	.	.	.	.	.	.	.	SON	GT:/	0:21	0/1:5	C	T	0

0.02	0	0	.	.	.	.	.	.	.	.	SOM	GT:/	0:82	0/1:1	C	T	0
.	.	.	.	.	.	.	3.4	.	.	.	SOM	GT:/	0:99	0/1:1	G	A	0
.	.	.	.	.	.	.	.	.	.	.	SOM	GT:/	0:50	0/1:7	C	A	0
.	.	.	.	.	.	.	.	.	.	.	SOM	GT:/	0:15	0/1:5	T	C	0
.	.	.	.	.	.	.	2.4	2.35	.	.	SOM	GT:/	0:12	0/1:2	C	T	0
.	.	.	.	.	.	.	.	.	.	.	SOM	GT:/	0:41	0/1:8	C	T	0
.	.	.	.	.	.	.	.	.	.	.	SOM	GT:/	0:10	0/1:3	G	A	0
#####	0	0	1.0	0.0	0.075	0.926	2.9	.	0	.	SOM	GT:/	0:64	0/1:1	C	T	0
.	.	.	.	.	.	.	.	.	.	.	SOM	GT:/	0:9,(	0/1:1	A	G	0
.	.	.	.	.	.	.	.	.	.	.	SOM	GT:/	0:13	0/1:2	C	A	0
.	.	.	.	.	.	.	.	.	.	.	SOM	GT:/	0:57	0/1:7	C	T	0
.	.	.	.	.	.	.	.	.	.	.	SOM	GT:/	0:32	0/1:4	G	T	0
.	.	.	.	.	.	.	.	1.46	.	.	SOM	GT:/	0:29	0/1:3	A	G	0
.	.	.	.	.	.	.	.	.	.	.	SOM	GT:/	0:16	0/1:2	G	A	0
.	.	.	.	.	.	.	.	.	.	.	SOM	GT:/	0:69	0/1:8	C	T	0
.	0	0	.	.	.	.	.	.	0	0	SOM	GT:/	0:69	0/1:1	C	T	0
.	.	.	0.0	0.6	0.955	1.000	5.3	2.25	.	.	SOM	GT:/	0:61	0/1:1	G	A	0
.	##	0	.	.	.	.	.	.	.	.	SOM	GT:/	0:10	0/1:2	C	T	0
.	.	.	0.0	0.8	0.992	1.000	4.8	4.69	.	.	SOM	GT:/	0:93	0/1:1	G	T	0
.	.	.	.	.	.	.	.	.	.	.	SOM	GT:/	0:36	0/1:7	G	A	0
.	##	0	.	.	.	.	.	.	.	.	SOM	GT:/	0:75	0/1:1	G	A	0
.	.	.	.	.	.	.	.	.	.	0	SOM	GT:/	0:46	0/1:6	G	A	0
.	.	.	.	.	.	.	.	1.53	.	.	SOM	GT:/	0:15	0/1:8	G	A	0
.	.	.	.	.	.	.	4	1.79	.	.	SOM	GT:/	0:86	0/1:1	G	A	0
.	.	.	.	.	.	.	.	.	.	0	SOM	GT:/	0:14	0/1:2	C	T	0
.	.	.	.	.	.	.	5.5	.	.	.	SOM	GT:/	0:18	0/1:2	G	A	0
.	##	0	0.1	0.1	0.6,P	1,D	4.7	3.31	.	.	SOM	GT:/	0:56	0/1:9	C	T	0
.	.	.	.	.	.	.	.	.	.	.	SOM	GT:/	0:36	0/1:9	C	T	0
.	.	.	.	.	.	.	.	1.48	.	.	SOM	GT:/	0:27	0/1:5	G	A	0
.	.	.	.	.	.	.	.	.	.	.	SOM	GT:/	0:37	0/1:5	G	A	0
.	.	.	0.0	0.9	1.0,D	1,D	4.2	2.12	.	.	SOM	GT:/	0:52	0/1:6	A	G	0
.	.	.	.	.	.	.	.	.	.	.	SOM	GT:/	0:9,(	0/1:6	C	T	0
.	.	.	.	.	.	.	.	.	.	.	SOM	GT:/	0:34	0/1:4	C	T	0
.	.	.	.	.	.	.	.	.	.	.	SOM	GT:/	0:27	0/1:3	A	T	0
.	.	.	.	.	.	.	.	.	.	.	SOM	GT:/	0:19	0/1:1	T	C	0
.	.	.	.	.	.	.	.	.	.	.	SOM	GT:/	0/1:6	0:41	C	T	0
.	.	.	.	.	.	.	.	.	0	0.1	SOM	GT:/	0/1:1	0:9,(	C	T	0
.	.	.	.	.	.	.	.	.	0	0	SOM	GT:/	0/1:5	0:17	A	C	0
.	.	.	.	.	.	.	.	.	0	0	SOM	GT:/	0/1:1	0:9,(	T	C	0
.	.	.	.	.	.	1,D	5.6	4.73	.	.	SOM	GT:/	0/1:3	0:18	C	A	0
.	.	.	.	.	.	.	.	.	0	0	SOM	GT:/	0/1:3	0:13	G	C	0
.	.	.	.	.	.	.	.	.	0	0.1	SOM	GT:/	0/1:9	0:8,(	G	T	0
.	.	.	.	.	.	.	.	.	0	.	SOM	GT:/	0/1:2	0:11	T	C	0
.	.	.	.	.	.	.	.	.	.	.	SOM	GT:/	0/1:2	0:82	G	T	0
.	.	.	.	.	.	.	.	.	.	.	SOM	GT:/	0/1:5	0:28	G	T	0
.	.	.	.	.	.	.	.	.	0	0	SOM	GT:/	0/1:1	0:13	G	C	0
#####	0	0	.	.	.	.	.	1.33	0	0	DB;S	GT:/	0/1:1	0:49	G	A	0
.	##	0	.	.	.	.	.	.	.	.	SOM	GT:/	0/1:8	0:25	C	T	0
.	.	.	0.0	1.0	1.0,D	1,D	5.8	4.66	.	.	SOM	GT:/	0/1:4	0:21	C	A	0
.	.	.	.	.	.	.	.	.	.	.	SOM	GT:/	0/1:2	0:18	C	T	0
.	.	.	.	.	.	.	.	.	0	0	DB;S	GT:/	0/1:5	0:35	A	G	0
.	.	.	.	.	.	.	.	.	0	0	SOM	GT:/	0/1:3	0:13	G	T	0
.	.	.	.	.	.	.	3.4	.	.	.	SOM	GT:/	0/1:8	0:58	T	C	0
.	.	.	.	.	.	.	.	.	.	.	SOM	GT:/	0/1:1	0:9,(	A	G	0

.	.	.	.	.	.	.	.	.	.	.	SOM	GT:/	0/1:4	0:24	T	C	0
.	.	.	.	.	.	.	.	.	.	.	SOM	GT:/	0/1:4	0:15	C	T	0
.	.	.	.	.	.	1,D	5.2	4.11	.	.	SOM	GT:/	0/1:4	0:25	C	T	0
2E-04	##	0	0.5	0.0	0.007	1,N	.	.	.	.	SOM	GT:/	0/1:3	0:20	G	A	0
.	##	0	.	.	.	.	2.6	2.20	.	.	SOM	GT:/	0/1:1	0:51	G	A	0
.	.	.	.	.	.	.	.	1.24	.	.	SOM	GT:/	0/1:3	0:20	C	T	0
.	.	.	.	.	.	.	.	.	0	0	SOM	GT:/	0/1:7	0:9,(	G	T	0
.	.	.	.	.	.	.	.	.	.	.	SOM	GT:/	0/1:2	0:12	G	T	0
.	0	0	.	.	.	.	.	.	.	.	SOM	GT:/	0/1:4	0:38	T	C	0
.	.	.	0.5	0.0	0.001	1,N	2.8	.	.	.	SOM	GT:/	0/1:3	0:30	A	C	0
.	##	0	0.0	0.0	0.056	1.000	5.5	3.45	.	.	SOM	GT:/	0/1:3	0:24	G	A	0
.	.	.	.	.	.	.	.	1.63	.	.	SOM	GT:/	0/1:2	0:8,(	A	T	0
.	##	0	.	.	.	.	.	.	0	.	SOM	GT:/	0/1:2	0:12	G	A	0
.	##	0	0.0	0.3	0.978	1,N	.	1.80	0	0	SOM	GT:/	0/1:9	0:44	C	T	0
.	.	.	.	.	.	.	.	.	.	.	SOM	GT:/	0/1:5	0:29	G	A	0
.	.	.	.	.	.	.	.	.	0.1	0.2	DB;S	GT:/	0/1:4	0:35	C	T	0
.	0	0	.	0.0	0.013	1,N	.	.	0.2	0.2	SOM	GT:/	0/1:5	0:40	T	G	0
.	0	0	.	.	.	.	.	.	0.3	0.2	SOM	GT:/	0/1:5	0:42	A	G	0
.	.	.	.	.	.	.	.	.	.	.	SOM	GT:/	0/1:1	0:99	C	A	0
.	.	.	.	.	.	.	.	.	0.1	0.1	SOM	GT:/	0/1:1	0:11	A	G	0
.	.	.	.	.	.	.	.	1.81	.	.	SOM	GT:/	0/1:1	0:9,(	C	A	0
.	.	.	.	.	.	.	.	.	.	.	SOM	GT:/	0/1:7	0:51	C	T	0
.	0	0	.	.	.	.	.	.	.	.	SOM	GT:/	0/1:2	0:9,(	T	C	0
.	.	.	.	.	.	.	.	.	0	0.4	SOM	GT:/	0/1:2	0:13	A	C	0
.	0	0	.	.	.	1,A	4.7	8.33	0.1	0	DB;S	GT:/	0/1:1	0:11	G	A	0
.	.	.	.	.	.	.	4.4	1.68	.	.	SOM	GT:/	0/1:2	0:18	C	T	0
.	.	.	.	.	.	.	3.6	.	.	.	SOM	GT:/	0/1:4	0:22	C	G	0
.	.	.	.	.	.	.	.	.	.	.	SOM	GT:/	0/1:4	0:26	A	T	0
.	.	.	.	.	.	.	.	.	0	.	SOM	GT:/	0/1:1	0:39	A	G	0
.	##	0	.	.	.	.	.	.	0	.	SOM	GT:/	0/1:6	0:26	G	A	0
.	0	0	.	.	.	.	.	.	0.1	0.1	SOM	GT:/	0/1:3	0:12	A	G	0
.	.	.	.	.	.	.	.	.	.	.	SOM	GT:/	0/1:4	0:12	G	A	0
.	.	.	.	.	.	.	.	.	0.3	0.4	DB;S	GT:/	0/1:4	0:33	C	T	0
.	.	.	.	.	.	.	3.1	1.70	.	.	SOM	GT:/	0/1:4	0:26	G	T	0
.	.	.	.	.	.	.	2.4	2.84	.	.	SOM	GT:/	0/1:1	0:84	G	C	0
#####	##	0	.	.	.	.	.	.	.	.	SOM	GT:/	0/1:4	0:14	C	T	0
.	.	.	.	.	.	.	2.6	2.07	.	.	SOM	GT:/	0/1:3	0:16	G	A	0
.	.	.	.	.	.	.	.	.	0	0.1	SOM	GT:/	0/1:1	0:9,(	G	C	0
.	.	.	.	.	.	.	.	.	0	0.2	SOM	GT:/	0/1:3	0:11	G	C	0
.	.	.	.	.	.	.	.	.	.	.	SOM	GT:/	0/1:9	0:56	T	G	0
.	.	.	.	.	.	1,D	.	1.30	.	.	SOM	GT:/	0/1:3	0:21	G	A	0
.	.	.	0.1	0.0	0.008	1.000	4.4	1.76	.	.	SOM	GT:/	0/1:2	0:89	G	C	0
.	.	.	.	.	.	.	.	.	.	.	SOM	GT:/	0/1:5	0:19	C	G	0
.	.	.	.	.	.	.	4	.	.	.	SOM	GT:/	0/1:1	0:85	G	C	0
.	.	.	0.0	0.9	0.998	1,D	5.7	5.56	.	.	SOM	GT:/	0/1:6	0:20	C	T	0
.	.	.	.	.	.	.	.	.	.	.	SOM	GT:/	0/1:7	0:30	G	C	0
.	.	.	0.1	0.3	0.738	0.983	5	2.81	.	.	SOM	GT:/	0/1:2	0:26	C	T	0
.	.	.	.	.	.	.	.	.	.	.	SOM	GT:/	0/1:1	0:9,(	G	A	0
.	##	0	0.0	0.9	1.0,D	1,D	4.8	5.21	.	.	SOM	GT:/	0/1:1	0:10	G	A	0
.	.	.	.	.	.	.	2.5	1.40	.	.	SOM	GT:/	0/1:4	0:35	C	G	0
.	.	.	.	.	.	.	.	.	.	.	SOM	GT:/	0/1:4	0:23	G	A	0
.	.	.	.	.	.	.	.	1.24	.	.	SOM	GT:/	0/1:3	0:12	C	T	0
.	.	.	.	.	.	.	2.5	.	.	.	SOM	GT:/	0/1:5	0:23	C	T	0
.	##	.	0.0	0.9	1.0,D	1,D	5.6	4.51	.	.	SOM	GT:/	0/1:5	0:17	G	A	0

.	.	.	.	.	.	.	.	.	.	.	SOM	GT:/	0/1:9	0:52	T	C	0
.	.	.	0.0	0.7	0.986	1.000	5.9	5.01	.	.	SOM	GT:/	0/1:5	0:31	C	T	0
.	.	.	0.0	0.9	0.999	1,D	4.9	3.98	.	.	SOM	GT:/	0/1:8	0:47	C	G	0
.	.	.	.	.	.	.	.	.	.	.	SOM	GT:/	0/1:1	0:12	C	T	0
.	.	.	.	.	.	.	.	.	.	.	SOM	GT:/	0/1:6	0:37	G	C	0
.	.	.	.	.	.	.	3	1.75	.	.	SOM	GT:/	0/1:3	0:22	G	T	0
.	.	.	0.0	.	.	0.997	2.2	.	.	.	SOM	GT:/	0/1:2	0:17	G	C	0
.	##	0	0.0	0.4	0.962	1,D	5.2	3.79	.	.	SOM	GT:/	0/1:4	0:23	G	A	0
.	.	.	.	.	.	.	.	.	.	.	SOM	GT:/	0/1:4	0:31	G	C	0
.	.	.	.	.	.	.	.	.	.	.	SOM	GT:/	0/1:5	0:36	A	G	0
.	.	.	0.0	0.9	0.994	1,D	3.2	3.19	.	.	SOM	GT:/	0/1:9	0:54	G	T	0
.	.	.	0.4	0.0	0.026	1,D	4.2	.	.	.	SOM	GT:/	0/1:9	0:54	G	A	0
.	.	.	.	.	.	.	.	.	.	.	SOM	GT:/	0/1:3	0:10	G	C	0
.	##	0	.	.	.	0.999	2.3	8.58	.	.	SOM	GT:/	0/1:1	0:61	G	A	0
.	.	.	.	.	.	.	.	.	.	.	SOM	GT:/	0/1:1	0:55	G	C	0
.	##	0	0.0	0.9	0.991	1,D	5.8	5.70	.	.	DB;S	GT:/	0/1:3	0:25	G	A	0
.	.	.	0.0	0.0	0.002	0.812	.	3.23	.	.	SOM	GT:/	0/1:8	0:32	A	C	0
.	.	.	.	.	.	.	.	.	.	.	SOM	GT:/	0/1:6	0:33	C	T	0
.	.	.	0.0	0.3	0.817	1.000	5.6	2.56	.	.	SOM	GT:/	0/1:5	0:39	G	A	0
.	.	.	.	.	.	.	.	.	.	.	SOM	GT:/	0/1:3	0:19	C	T	0
.	.	.	0.0	0.7	0.941	1,D	5.3	5.17	.	.	SOM	GT:/	0/1:3	0:20	C	T	0
.	.	.	0.0	0.9	1.0,D	1,D	6.2	4.90	.	.	SOM	GT:/	0/1:5	0:17	C	G	0
.	.	.	.	.	.	.	.	.	.	.	SOM	GT:/	0/1:3	0:13	C	T	0
.	.	.	.	.	.	.	.	.	.	0	SOM	GT:/	0/1:1	0:14	G	C	0
.	.	.	0.0	0.9	1.0,D	1.000	5.6	4.57	.	.	SOM	GT:/	0/1:4	0:24	G	C	0
.	.	.	0.1	0.0	0.0,B	1,N	.	.	.	.	SOM	GT:/	0/1:8	0:36	C	T	0
.	0	0	0.1	0.1	0.768	0.632	3.9	1.85	0	0	DB;S	GT:/	0/1:6	0:37	C	T	0
.	.	.	0.0	0.3	0.974	1.000	4.5	2.94	.	.	SOM	GT:/	0/1:1	0:77	C	G	0
.	.	.	.	.	.	.	.	.	.	.	SOM	GT:/	0/1:5	0:30	G	A	0
.	.	.	0.6	0.0	0.056	1,D	4.2	2.60	.	.	SOM	GT:/	0/1:1	0:14	G	A	0
.	.	.	.	.	.	.	.	.	.	.	SOM	GT:/	0/1:1	0:8,(	C	A	0
.	.	.	0.3	0.0	0.001	1,N	.	.	.	.	SOM	GT:/	0/1:4	0:21	A	G	0
.	.	.	.	.	.	.	.	.	.	.	SOM	GT:/	0/1:1	0:14	G	A	0
.	.	.	.	.	.	.	.	.	0.2	0.3	DB;S	GT:/	0/1:7	0:39	C	T	0
.	.	.	.	.	.	.	.	.	0	0	SOM	GT:/	0/1:1	0:8,(	A	G	0
.	.	.	0.0	0.9	1.0,D	0.996	5.2	2.21	.	.	SOM	GT:/	0/1:3	0:17	C	G	0
.	.	.	0.0	0.0	0.037	1.000	2.5	.	.	.	SOM	GT:/	0/1:5	0:30	C	T	0
.	.	.	.	.	.	.	.	.	.	.	SOM	GT:/	0/1:2	0:12	C	T	0
.	.	.	.	.	.	.	.	.	.	.	SOM	GT:/	0/1:1	0:72	C	G	0
.	.	.	.	.	.	.	3.7	2.89	.	.	SOM	GT:/	0/1:4	0:27	G	C	0
.	.	.	.	.	.	.	.	.	0	.	SOM	GT:/	0/1:1	0:29	C	A	0
.	.	.	.	.	.	.	.	.	.	.	SOM	GT:/	0/1:6	0:45	C	A	0
.	.	.	.	.	.	.	4.8	1.97	.	.	SOM	GT:/	0/1:8	0:54	C	T	0
.	##	0	.	.	.	.	.	.	.	.	SOM	GT:/	0/1:8	0:44	G	C	0
.	.	.	.	.	.	.	.	.	.	.	SOM	GT:/	0/1:1	0:15	G	A	0
.	.	.	0.0	0.1	0.202	1,D	6.2	3.33	.	.	SOM	GT:/	0/1:4	0:18	A	T	0
.	.	.	.	.	.	.	4	1.65	.	.	SOM	GT:/	0/1:4	0:30	G	A	0
.	.	.	0.2	0.0	0.004	1,N	.	1.51	.	.	SOM	GT:/	0/1:4	0:39	C	T	0
#####	##	0	0.0	0.6	0.992	0.988	3.8	1.87	0	.	SOM	GT:/	0/1:1	0:12	C	T	0
.	0	0	.	.	.	.	.	.	0.1	0	DB;S	GT:/	0/1:5	0:43	C	T	0
.	.	.	.	.	.	.	.	.	.	.	SOM	GT:/	0/1:3	0:22	G	A	0
.	.	.	.	.	.	.	4.9	1.37	.	.	SOM	GT:/	0/1:1	0:12	G	A	0
.	.	.	.	.	.	.	.	.	.	.	SOM	GT:/	0/1:1	0:13	C	T	0
.	.	.	.	.	.	.	.	.	.	.	SOM	GT:/	0/1:1	0:69	T	A	0

.	.	.	0.0	0.9	0.999	1,D	6	2.81	.	.	SON	GT:/	0/1:2	0:12	C	T	0
.	.	.	.	.	.	.	.	.	.	.	SON	GT:/	0/1:1	0:81	G	A	0
.	1	1	.	.	.	.	.	.	0.1	0.5	SON	GT:/	0/1:3	0:44	C	G	0
.	.	.	.	.	.	.	.	.	.	.	SON	GT:/	0/1:6	0:87	G	C	0
.	.	.	.	.	.	.	.	.	.	.	SON	GT:/	0/1:1	0:11	C	T	0
.	.	.	.	.	.	.	.	1.76	.	.	SON	GT:/	0/1:4	0:33	C	G	0
.	.	.	.	.	.	.	.	.	.	.	SON	GT:/	0/1:2	0:13	C	T	0
.	.	.	.	.	.	.	.	.	.	.	SON	GT:/	0/1:2	0:16	G	A	0
.	.	.	.	.	.	.	3.9	1.41	.	.	SON	GT:/	0/1:6	0:43	G	A	0
.	.	.	.	.	.	.	.	.	.	.	SON	GT:/	0/1:6	0:51	G	A	0
.	.	.	.	.	.	.	.	.	.	.	SON	GT:/	0/1:3	0:35	G	A	0
.	.	.	.	.	.	.	.	.	0.1	0	SON	GT:/	0/1:2	0:15	T	C	0
.	.	.	0.0	0.9	1.0,D	1,D	6.1	3.62	.	.	SON	GT:/	0/1:1	0:44	A	G	0
.	.	.	0.0	0.4	0.849	1.000	2.8	.	.	.	SON	GT:/	0/1:3	0:24	C	G	0
.	.	.	.	.	.	.	.	.	0	0	SON	GT:/	0/1:2	0:15	T	A	0
.	.	.	.	.	.	.	.	.	.	.	SON	GT:/	0/1:3	0:16	C	T	0
.	.	.	.	.	.	.	2.5	.	.	.	SON	GT:/	0/1:5	0:29	G	A	0
.	.	.	0.0	1.0	1.0,D	1,D	5	5.03	.	.	DB;S	GT:/	0/1:1	0:52	G	A	0
.	.	.	.	.	.	.	.	.	.	.	SON	GT:/	0/1:3	0:14	T	A	0
#####	##	0	.	0.9	0.997	1,D	6	5.36	.	.	SON	GT:/	0/1:1	0:42	G	A	0
0.003	0	0	0.0	0.6	0.997	1.000	5.1	3.59	.	.	DB;S	GT:/	0/1:2	0:10	C	T	0
.	.	.	0.0	0.3	0.356	0.946	3.2	1.48	.	.	SON	GT:/	0/1:9	0:46	C	G	0
.	.	.	0.1	0.0	0.064	0.996	5.9	1.36	.	.	SON	GT:/	0/1:4	0:31	C	T	0
.	.	.	.	.	.	.	.	.	.	.	SON	GT:/	0/1:6	0:28	C	G	0
.	.	.	0.1	0.4	0.881	1.000	5.7	2.53	.	.	SON	GT:/	0/1:7	0:37	C	G	0
.	.	.	.	0.0	0.27,I	1,N	.	2.37	.	.	SON	GT:/	0/1:6	0:49	G	A	0
2E-04	0	0	.	.	.	.	.	.	.	.	SON	GT:/	0/1:3	0:27	C	T	0
.	.	.	.	.	.	.	.	.	.	.	SON	GT:/	0/1:4	0:14	C	T	0
.	.	.	.	.	.	.	.	.	.	.	SON	GT:/	0/1:3	0:8,(	C	T	0
.	.	.	.	.	.	.	4.1	.	.	.	SON	GT:/	0/1:5	0:43	G	A	0
.	##	0	0.0	1.0	1.0,D	1,D	4.6	4.45	.	.	DB;S	GT:/	0/1:7	0:81	C	T	0
.	.	.	.	.	.	.	.	.	.	.	SON	GT:/	0/1:7	0:35	G	A	0
.	0	0	.	.	.	.	.	.	0	.	DB;S	GT:/	0/1:4	0:58	G	A	0
.	.	.	.	.	.	.	.	.	0	0	SON	GT:/	0/1:8	0:9,(	C	G	0
.	0	0	.	.	.	.	.	.	0.4	0.3	SON	GT:/	0/1:4	0:33	T	C	0
.	.	.	.	.	.	.	.	.	0	0.1	SON	GT:/	0/1:4	0:17	G	C	0
.	.	.	.	.	.	.	.	.	.	.	SON	GT:/	0/1:9	0:58	G	A	0
.	0	0	.	.	.	.	.	.	0	0	SON	GT:/	0/1:8	0:49	C	T	0
.	.	.	0.0	0.8	0.992	1,N	.	1.98	.	.	SON	GT:/	0/1:1	0:81	G	A	0
.	.	.	.	.	.	.	.	.	.	.	SON	GT:/	0/1:1	0:60	G	C	0
.	.	.	.	.	.	.	4.6	.	.	.	SON	GT:/	0/1:4	0:14	G	A	0
.	.	.	.	.	.	.	.	.	.	.	SON	GT:/	0/1:8	0:47	G	A	0
.	.	.	.	.	.	.	.	.	.	.	SON	GT:/	0/1:9	0:76	C	T	0
.	.	.	0.0	0.9	1.0,D	1,D	5.4	3.69	.	.	SON	GT:/	0/1:1	0:10	C	T	0
.	.	.	.	.	.	.	.	.	.	.	SON	GT:/	0/1:1	0:18	G	A	0
.	##	0	0.0	0.5	0.987	1.000	5.8	5.62	.	.	SON	GT:/	0/1:1	0:50	C	T	0
.	.	.	.	.	.	.	.	.	.	.	SON	GT:/	0/1:7	0:44	C	T	0
.	.	.	0.0	0.5	0.899	1.000	2.6	2.10	.	.	SON	GT:/	0/1:1	0:90	G	C	0
.	##	0	.	0.0	0.001	1,N	.	.	.	.	SON	GT:/	0/1:2	0:24	C	T	0
4E-04	0	0	.	.	.	.	.	.	.	.	SON	GT:/	0/1:2	0:17	G	A	0
.	.	.	.	.	.	.	3	.	.	.	SON	GT:/	0/1:2	0:20	G	A	0
.	0	0	.	.	.	.	.	.	.	.	SON	GT:/	0/1:3	0:19	C	T	0
.	.	.	.	.	.	.	.	.	0	.	SON	GT:/	0/1:3	0:25	C	T	0
.	.	.	.	.	.	.	.	2.32	.	.	SON	GT:/	0/1:3	0:32	G	A	0

.	0	0	.	.	.	.	.	.	0	0	SON	GT:/	0/1:2	0:14	C	T	0
.	.	.	.	.	.	.	.	.	.	.	SON	GT:/	0/1:2	0:22	G	C	0
.	.	.	.	.	.	.	2.8	.	.	.	SON	GT:/	0/1:7	0:24	C	G	0
.	.	.	.	.	.	.	.	.	.	.	SON	GT:/	0/1:4	0:13	G	A	0
.	.	.	0.0	0.9	0.999	1.000	3.1	3.49	.	.	SON	GT:/	0/1:7	0:57	G	T	0
.	.	.	0.0	0.1	0.43,I	1,N	.	.	.	.	SON	GT:/	0/1:1	0:65	C	T	0
.	.	.	.	.	.	.	.	.	.	.	SON	GT:/	0/1:3	0:32	C	T	0
.	.	.	.	.	.	1,D	4	6.43	.	.	SON	GT:/	0/1:6	0:37	C	G	0
.	.	.	.	.	.	.	.	1.59	.	0	SON	GT:/	0/1:5	0:62	G	A	0
.	.	.	.	.	.	.	.	.	.	.	SON	GT:/	0/1:1	0:76	G	C	0
.	.	.	.	.	.	.	.	.	.	.	SON	GT:/	0/1:1	0:11	C	G	0
.	.	.	.	.	.	.	.	.	.	.	SON	GT:/	0/1:9	0:48	G	C	0
.	.	.	.	.	.	.	5.9	.	.	.	SON	GT:/	0/1:4	0:11	G	A	0
#####	##	0	0.4(	0.0(	0.005	0.984	.	1.70	0	0	SON	GT:/	0/1:1	0:44	G	A	0
.	.	.	.	.	.	.	.	.	.	.	SON	GT:/	0/1:6	0:17	G	C	0
.	.	.	.	.	.	.	.	.	.	.	SON	GT:/	0/1:1	0:8,(	T	C	0
.	.	.	.	.	.	.	.	.	0	0	DB;S	GT:/	0/1:4	0:28	T	C	0
.	.	.	.	.	.	1,A	5.5	10.7	.	.	SON	GT:/	0/1:5	0:27	C	G	0
.	.	.	.	.	.	.	.	.	.	.	DB;S	GT:/	0/1:1	0:20	G	C	0
.	.	.	0.3	0.1(	0.487	1,N	.	2.74	.	.	SON	GT:/	0/1:2	0:15	C	T	0
.	.	.	.	.	.	.	4.4	.	.	.	SON	GT:/	0/1:4	0:24	C	A	0
.	0	0	0.0	0.9	0.371	1.000	3.5	2.43	.	.	SON	GT:/	0/1:4	0:42	G	A	0
.	.	.	0.0	0.7	0.995	0.973	2.7	2.50	.	.	SON	GT:/	0/1:6	0:16	G	A	0
.	.	.	.	.	.	.	.	.	.	.	SON	GT:/	0/1:8	0:9,(	T	A	0
.	.	.	0.3(	0.9(	0.999	1,D	4.9	4.26	.	.	SON	GT:/	0/1:7	0:34	G	A	0
.	.	.	0.0(	0.0	0.07,I	1,N	.	.	.	.	SON	GT:/	0/1:1	0:15	G	C	0
.	.	.	.	.	.	.	.	.	0	.	SON	GT:/	0/1:6	0:10	T	A	0
.	.	.	.	.	.	.	.	.	.	.	SON	GT:/	0/1:1	0:15	T	A	0
.	.	.	.	.	.	.	.	.	.	.	SON	GT:/	0/1:4	0:16	G	C	0
.	.	.	.	.	.	.	.	.	.	.	SON	GT:/	0/1:6	0:52	C	T	0
.	.	.	.	.	.	.	.	.	.	.	SON	GT:/	0/1:1	0:45	T	C	0
.	0	0	.	.	.	.	.	.	0	0	DB;S	GT:/	0/1:1	0:37	T	C	0
.	.	.	1.0	0.0	0.048	1,D	4.9	.	.	.	SON	GT:/	0/1:7	0:48	C	A	0
.	.	.	.	.	.	.	.	.	.	.	SON	GT:/	0/1:1	0:93	C	T	0
.	.	.	0.4	0.0	0.007	0.988	4.1	1.44	.	.	SON	GT:/	0/1:5	0:31	G	A	0
.	.	.	.	.	.	.	.	1.47	0	0.1	SON	GT:/	0/1:2	0:9,(	T	C	0
.	.	.	.	.	.	.	.	.	0.2	0.2	SON	GT:/	0/1:1	0:13	G	T	0
.	.	.	.	.	.	.	.	1.67	.	.	SON	GT:/	0/1:1	0:49	T	A	0
.	.	.	0.5	0.0(	0.001	1,N	.	.	.	.	SON	GT:/	0/1:1	0:72	G	A	0
.	.	.	.	.	.	.	3.3	.	.	.	SON	GT:/	0/1:3	0:18	C	G	0
#####	##	0	.	.	.	.	.	.	.	.	SON	GT:/	0/1:8	0:85	C	T	0
.	0	0	.	.	.	.	.	.	0	0	SON	GT:/	0/1:1	0:61	T	A	0
.	.	.	.	.	.	.	.	.	.	.	SON	GT:/	0/1:4	0:27	C	T	0
2E-04	##	0	.	.	.	.	.	.	.	.	SON	GT:/	0/1:1	0:12	C	T	0
.	0	0	.	.	.	.	.	.	0	.	SON	GT:/	0/1:1	0:10	G	A	0
.	.	.	.	.	.	.	.	.	0	0.1	SON	GT:/	0/1:1	0:9,(	C	T	0
.	.	.	.	.	.	.	.	.	.	.	SON	GT:/	0/1:4	0:22	G	T	0
.	.	.	.	.	.	.	4.8	.	.	.	SON	GT:/	0/1:6	0:40	C	T	0
#####	##	0	0.4(	0.0(	0.002	1,N	2	1.66	.	.	SON	GT:/	0/1:8	0:64	C	T	0
.	.	.	.	.	.	.	.	.	0	0	DB;S	GT:/	0/1:1	0:72	C	T	0
.	.	.	.	.	.	.	.	.	0.4	0.5	DB;S	GT:/	0/1:4	0:52	C	G	0
.	.	.	.	.	.	.	.	.	0.4	0.4	DB;S	GT:/	0/1:3	0:40	G	T	0
.	##	0	.	.	.	.	.	.	.	.	SON	GT:/	0/1:3	0:12	C	T	0
.	##	0	0.0(	0.3	0.84,I	1,D	2.7	2.81	.	.	SON	GT:/	0/1:1	0:68	G	A	0

.	.	.	.	.	.	.	.	.	.	.	SOM	GT:/	0/1:2	0:33	C	T	0
.	.	.	.	.	.	.	.	.	.	.	DB;S	GT:/	0/1:4	0:33	C	T	0
.	.	.	.	.	.	.	.	.	.	.	SOM	GT:/	0/1:6	0:24	G	T	0
.	.	.	.	.	.	.	.	.	.	.	SOM	GT:/	0/1:1	0:74	T	A	0
.	##	0	.	.	.	.	.	.	.	.	SOM	GT:/	0/1:1	0:46	C	T	0
.	.	.	0.0	0.1	0.557	.	.	1.91	.	.	SOM	GT:/	0/1:4	0:44	G	A	0
.	.	.	1.0	0.0	0.0,B	1.000	4	.	.	.	SOM	GT:/	0/1:4	0:23	C	A	0
.	.	.	.	.	.	.	.	.	.	.	SOM	GT:/	0/1:3	0:23	T	C	0
.	##	0	.	.	.	.	.	.	.	.	SOM	GT:/	0/1:8	0:31	C	T	0
.	.	.	.	.	.	.	.	.	.	.	SOM	GT:/	0/1:5	0:21	G	T	0
.	##	0	0.4	0.2	0.866	0.990	4.5	2.15	.	.	SOM	GT:/	0/1:9	0:81	G	A	0
.	.	.	.	.	.	.	3.9	2.86	.	.	SOM	GT:/	0/1:5	0:24	T	A	0
.	##	0	.	.	.	.	.	.	.	.	SOM	GT:/	0/1:4	0:27	G	A	0
.	.	.	.	.	.	.	.	.	0	.	SOM	GT:/	0/1:3	0:25	C	T	0
.	.	.	.	.	.	.	.	.	0	.	DB;S	GT:/	0/1:4	0:19	T	A	0
.	.	.	.	.	.	.	.	.	.	.	SOM	GT:/	0/1:4	0:32	G	A	0
.	.	.	.	.	.	.	.	.	.	.	SOM	GT:/	0/1:9	0:63	C	T	0
.	##	0	.	.	.	.	.	.	.	.	SOM	GT:/	0/1:1	0:68	A	G	0
.	.	.	.	.	.	.	.	1.46	.	.	SOM	GT:/	0/1:6	0:11	G	A	0
.	.	.	0.0	0.6	0.887	1,D	5.8	4.15	.	.	DB;S	GT:/	0/1:7	0:46	A	G	0
.	.	.	0.0	0.9	1.0,D	1,D	4	4.84	.	.	SOM	GT:/	0/1:1	0:10	G	A	0
.	##	0	.	.	.	.	.	.	0	.	SOM	GT:/	0/1:4	0:38	G	A	0
.	.	.	.	.	.	.	.	1.62	.	.	SOM	GT:/	0/1:1	0:10	A	G	0
.	.	.	.	.	.	.	2.4	.	.	.	SOM	GT:/	0/1:1	0:51	T	C	0
.	.	.	0.2	1.0	1.0,D	1,D	4.8	3.46	.	.	SOM	GT:/	0/1:8	0:55	T	C	0
.	.	.	.	.	.	.	.	.	.	.	SOM	GT:/	0/1:8	0:44	G	A	0
.	.	.	.	.	.	.	.	.	.	.	SOM	GT:/	0/1:1	0:19	C	T	0
.	.	.	.	.	.	.	.	.	0	.	SOM	GT:/	0/1:4	0:21	A	G	0
.	.	.	.	.	.	.	.	1.45	.	.	SOM	GT:/	0/1:2	0:12	G	T	0
.	.	.	.	.	.	.	5.7	4.01	.	.	SOM	GT:/	0/1:4	0:37	C	A	0
.	##	0	.	.	.	.	.	.	.	.	SOM	GT:/	0/1:5	0:22	G	A	0
.	.	.	.	.	.	.	.	.	.	.	SOM	GT:/	0/1:6	0:45	G	A	0
.	.	.	0.0	1.0	1.0,D	1,D	5	4.65	.	.	SOM	GT:/	0/1:4	0:23	G	A	0
.	.	.	0.0	0.9	0.994	1.000	5.1	4.41	.	.	SOM	GT:/	0/1:1	0:66	C	A	0
.	.	.	.	.	.	.	.	.	0	0	SOM	GT:/	0/1:2	0:9,(	G	T	0
.	.	.	.	.	.	.	.	.	0	.	SOM	GT:/	0/1:4	0:17	G	A	0
.	.	.	0.1	0.0	0.104	1.000	3.8	.	.	.	SOM	GT:/	0/1:7	0:48	T	C	0
.	.	.	0.7	0.0	0.0,B	0.999	2.5	.	.	.	SOM	GT:/	0/1:3	0:26	C	A	0
.	.	.	.	.	.	.	.	.	.	.	SOM	GT:/	0/1:2	0:19	C	T	0
.	.	.	.	.	.	.	4.1	1.54	.	.	SOM	GT:/	0/1:1	0:82	A	T	0
.	.	.	.	.	.	.	.	.	0	0	SOM	GT:/	0/1:7	0:15	G	C	0
.	0	0	.	.	.	.	.	.	.	.	SOM	GT:/	0/1:5	0:24	G	A	0
.	.	.	.	.	.	.	.	.	.	.	SOM	GT:/	0/1:8	0:27	C	A	0
.	##	0	.	.	.	.	.	.	.	.	SOM	GT:/	0/1:7	0:25	G	A	0
.	.	.	.	.	.	.	.	.	.	.	SOM	GT:/	0/1:6	0:46	C	A	0
.	.	.	.	.	.	.	.	.	.	.	SOM	GT:/	0/1:9	0:74	T	C	0
.	.	.	0.0	0.6	0.955	0.758	4.4	3.62	.	.	SOM	GT:/	0/1:4	0:13	C	T	0
.	.	.	.	.	.	.	.	.	0	.	SOM	GT:/	0/1:5	0:8,(	C	A	0
#####	##	0	.	.	.	.	.	.	.	.	SOM	GT:/	0/1:3	0:9,(	C	T	0
.	.	.	.	.	.	.	.	.	.	.	SOM	GT:/	0/1:1	0:12	C	T	0
.	0	0	.	.	.	.	.	.	.	.	SOM	GT:/	0/1:6	0:26	T	A	0
.	.	.	.	.	.	.	.	.	0.2	0.2	DB;S	GT:/	0/1:5	0:38	C	A	0
.	.	.	.	.	.	.	.	.	.	.	SOM	GT:/	0/1:1	0:45	G	C	0
.	.	.	.	.	.	.	.	2.11	.	.	SOM	GT:/	0/1:5	0:36	G	A	0

.	.	.	.	.	.	.	.	.	0.1	0.2	SOM	GT:/	0/1:1	0:19	C	T	0
.	.	.	.	.	.	.	.	.	0.1	0.2	SOM	GT:/	0/1:1	0:16	G	A	0
.	.	.	0.2	0.0	0.014	1,N	.	.	.	.	SOM	GT:/	0/1:1	0:57	G	A	0
.	.	.	.	.	.	1,A	.	4.71	.	.	SOM	GT:/	0/1:1	0:98	G	T	0
.	.	.	.	.	.	.	.	.	.	.	SOM	GT:/	0/1:5	0:9,(	G	A	0
.	.	.	.	.	.	.	.	.	.	.	SOM	GT:/	0/1:4	0:8,(	G	A	0
.	.	.	.	.	.	.	.	.	0	0.1	SOM	GT:/	0/1:8	0:9,(	T	A	0
.	.	.	.	.	.	.	3.3	.	.	.	SOM	GT:/	0/1:3	0:24	C	A	0
.	.	.	.	.	.	.	.	.	0	0.1	SOM	GT:/	0/1:1	0:72	C	G	0
.	.	.	.	.	.	.	.	.	.	.	SOM	GT:/	0/1:3	0:13	T	C	0
.	.	.	.	.	.	.	.	.	.	.	SOM	GT:/	0/1:3	0:31	C	A	0
.	.	.	.	.	.	.	.	.	0	0	SOM	GT:/	0/1:1	0:14	G	C	0
.	.	.	.	.	.	.	.	.	0	0	SOM	GT:/	0/1:1	0:8,(	G	A	0
.	.	.	.	.	.	.	5.1	4.13	.	.	SOM	GT:/	0/1:5	0:15	A	T	0
.	.	.	.	.	.	.	.	1.24	0	0.1	SOM	GT:/	0/1:3	0:21	T	C	0
.	.	.	.	.	.	.	.	.	0	.	SOM	GT:/	0/1:1	0:12	T	A	0
.	.	.	.	.	.	.	.	.	0.1	0	SOM	GT:/	0/1:1	0:11	T	G	0
.	.	.	.	.	.	.	.	.	.	.	SOM	GT:/	0/1:1	0:9,(	T	C	0
.	.	.	.	.	.	.	.	.	0.1	0.3	DB;S	GT:/	0/1:2	0:35	G	A	0
.	.	.	.	.	.	.	.	.	0	.	SOM	GT:/	0/1:1	0:17	C	A	0
.	0	0	.	.	.	.	.	.	0	.	DB;S	GT:/	0/1:3	0:45	G	A	0
.	.	.	.	.	.	.	.	.	.	.	SOM	GT:/	0/1:4	0:20	A	C	0
.	0	0	.	0.8	0.997	1,N	.	.	0	0	DB;S	GT:/	0/1:5	0:35	G	A	0
.	.	.	.	.	.	.	.	.	.	.	SOM	GT:/	0/1:1	0:9,(	G	T	0
.	.	.	.	.	.	.	.	.	0	.	SOM	GT:/	0/1:2	0:9,(	G	A	0
.	.	.	.	.	.	.	.	.	.	.	SOM	GT:/	0:12	0/1:2	A	C	0
.	.	.	.	.	.	.	.	.	0	0.1	SOM	GT:/	0:36	0/1:5	G	T	0
.	.	.	.	.	.	.	.	.	0	0.2	SOM	GT:/	0:35	0/1:5	C	T	0
.	.	.	.	.	.	.	.	.	0	0	SOM	GT:/	0:14	0/1:1	G	A	0
.	.	.	.	.	.	.	3.1	.	.	.	SOM	GT:/	0:12	0/1:2	G	T	0
.	.	.	.	.	.	.	.	.	0	0	SOM	GT:/	0:15	0/1:1	T	C	0
.	.	.	.	.	.	.	.	.	0	.	SOM	GT:/	0:17	0/1:2	G	T	0
0.045	0	0	0.0	0.6	0.998	1,D	.	3.26	0	0.1	SOM	GT:/	0:22	0/1:4	G	C	0
.	.	.	.	.	.	.	.	.	0.2	0.3	SOM	GT:/	0:12	0/1:1	A	C	0
.	.	.	.	.	.	.	.	.	0.1	0.2	DB;S	GT:/	0:32	0/1:7	C	A	0
.	.	.	.	.	.	.	.	.	0	.	SOM	GT:/	0:33	0/1:3	A	G	0
.	.	.	.	.	.	.	.	.	0	.	SOM	GT:/	0:8,(	0/1:2	G	C	0
.	0	0	0.1	0.0	0.23	1,N	.	.	0.1	0.1	SOM	GT:/	0:10	0/1:2	C	T	0
.	.	.	.	.	.	.	.	.	0	0.1	SOM	GT:/	0:19	0/1:2	C	G	0
.	.	.	.	.	.	.	.	.	0	.	SOM	GT:/	0:8,(	0/1:1	A	T	0
.	.	.	.	.	.	.	.	.	0	.	DB;S	GT:/	0:23	0/1:3	C	A	0
.	0	0	.	.	.	.	.	1.95	.	.	SOM	GT:/	0:42	0/1:4	G	A	0
.	.	.	.	.	.	.	.	.	0.1	0	SOM	GT:/	0:37	0/1:7	G	A	0
.	.	.	.	.	.	.	.	.	0.2	0	SOM	GT:/	0:36	0/1:5	A	G	0
.	0	0	.	.	.	.	.	.	0.1	0.4	DB;S	GT:/	0:26	0/1:3	A	T	0
.	.	.	.	.	.	.	.	.	0.1	0	SOM	GT:/	0:14	0/1:2	G	C	0
.	.	.	.	.	.	.	.	.	.	.	SOM	GT:/	0/1:6	0:48	G	A	0
.	.	.	.	.	.	.	.	.	.	.	SOM	GT:/	0/1:5	0:58	A	G	0
.	.	.	.	.	.	.	3.5	.	.	.	SOM	GT:/	0/1:3	0:9,(	C	A	0
.	.	.	0.0	0.9	1.0,D	0.99	4.8	3.80	.	.	SOM	GT:/	0/1:5	0:47	C	T	0
.	.	.	.	.	.	.	.	.	0	.	SOM	GT:/	0/1:4	0:25	C	T	0
.	.	.	.	.	.	.	.	.	0	.	SOM	GT:/	0/1:4	0:25	G	T	0
.	.	.	.	.	.	.	5.9	3.35	.	.	SOM	GT:/	0/1:2	0:8,(	T	G	0
.	.	.	.	.	.	.	.	.	0.1	0.2	DB;S	GT:/	0/1:3	0:20	T	C	0

.	.	.	.	.	.	.	.	.	0.1	0.3	SOM	GT:/	0/1:3	0:21	G	A	0
.	.	.	.	.	.	.	.	.	0.1	0.1	SOM	GT:/	0/1:4	0:18	G	A	0
.	.	.	.	.	.	.	.	.	.	0	SOM	GT:/	0/1:7	0:33	T	C	0
.	.	.	.	.	.	.	.	.	0	0	SOM	GT:/	0/1:7	0:38	G	C	0
.	.	.	.	.	.	.	.	1.23	0.1	0.2	SOM	GT:/	0/1:4	0:17	A	G	0
.	.	.	.	.	.	.	.	.	0	0.2	SOM	GT:/	0/1:1	0:12	C	A	0
.	.	.	1.0	0.0	0.004	1.000	3.2	1.46	.	.	SOM	GT:/	0/1:1	0:34	C	G	0
.	.	.	.	.	.	.	.	.	.	.	SOM	GT:/	0/1:5	0:17	C	G	0
.	.	.	0.1	0.9	0.999	1,D	5.8	4.66	.	.	SOM	GT:/	0/1:7	0:22	C	T	0
.	.	.	.	.	.	.	5.4	.	.	.	SOM	GT:/	0/1:5	0:28	G	A	0
.	.	.	.	.	.	.	.	.	0	0	SOM	GT:/	0/1:9	0:22	C	T	0
.	.	.	.	.	.	.	.	.	.	.	SOM	GT:/	0/1:5	0:50	G	C	0
.	##	.	.	.	.	.	4.5	2.39	.	.	SOM	GT:/	0/1:1	0:98	G	T	0
#####	0	0	.	.	.	.	.	.	0	.	SOM	GT:/	0/1:1	0:28	G	A	0
.	.	.	0.2	0.0	0.001	0.994	4.8	1.44	.	.	SOM	GT:/	0/1:2	0:13	A	C	0
.	##	0	0.0	0.0	0.298	0.928	4.9	2.12	.	.	SOM	GT:/	0/1:2	0:21	G	A	0
.	##	0	0.2	0.0	0.001	1,N	.	.	.	.	SOM	GT:/	0/1:1	0:50	C	T	0
.	0	0	.	.	.	.	2.7	.	.	.	DB;S	GT:/	0/1:1	0:29	A	G	0
.	.	.	.	.	.	.	.	.	.	.	SOM	GT:/	0/1:5	0:12	C	G	0
.	.	.	.	.	.	.	5.6	1.28	.	.	SOM	GT:/	0/1:2	0:10	G	A	0
.	.	.	0.4	0.8	0.991	1.000	2.5	2.33	.	.	SOM	GT:/	0/1:2	0:58	G	A	0
.	.	.	.	.	.	.	2.8	.	.	.	SOM	GT:/	0/1:2	0:12	T	G	0
.	.	.	.	.	.	.	.	.	.	.	SOM	GT:/	0/1:2	0:11	C	G	0
.	.	.	.	.	.	.	.	.	.	.	SOM	GT:/	0/1:4	0:20	T	C	0
.	##	0	.	0.9	1.0,D	1,D	5.7	4.68	.	.	SOM	GT:/	0/1:1	0:43	G	A	0
.	.	.	.	.	.	.	.	.	0	0	SOM	GT:/	0/1:3	0:8,(	G	A	0
.	.	.	.	.	.	.	.	.	0.1	0.4	SOM	GT:/	0/1:1	0:15	G	T	0
.	.	.	1.0	0.0	0.001	1,N	.	.	.	.	SOM	GT:/	0/1:3	0:11	C	T	0
.	.	.	.	.	.	.	.	.	.	.	SOM	GT:/	0/1:1	0:11	G	C	0
.	.	.	.	.	.	.	.	.	.	.	SOM	GT:/	0/1:8	0:42	G	A	0
.	.	.	.	.	.	.	.	2.48	.	.	SOM	GT:/	0/1:1	0:13	C	T	0
.	.	.	.	.	.	.	.	.	.	.	SOM	GT:/	0/1:4	0:35	T	G	0
.	.	.	.	.	.	.	.	.	.	.	SOM	GT:/	0/1:7	0:30	C	T	0
.	.	.	.	.	.	.	.	.	.	.	SOM	GT:/	0/1:6	0:46	G	A	0
.	.	.	.	.	.	.	2.4	1.48	.	.	SOM	GT:/	0/1:8	0:68	G	A	0
.	.	.	0.0	0.9	0.996	1.000	.	.	.	.	SOM	GT:/	0/1:4	0:29	C	A	0
.	.	.	.	.	.	.	3.9	.	.	.	SOM	GT:/	0/1:1	0:48	G	C	0
.	.	.	.	.	.	.	.	.	.	.	SOM	GT:/	0/1:1	0:86	C	T	0
.	.	.	.	.	.	.	2.7	1.88	.	.	SOM	GT:/	0/1:1	0:85	C	T	0
.	.	.	.	.	.	.	5.3	4.40	.	.	SOM	GT:/	0/1:4	0:24	A	G	0
.	.	.	.	.	.	.	.	.	.	.	SOM	GT:/	0/1:1	0:88	A	T	0
.	.	.	.	.	.	.	.	.	.	.	SOM	GT:/	0/1:1	0:10	G	T	0
.	.	.	.	.	.	.	.	.	.	.	SOM	GT:/	0/1:5	0:42	G	A	0
.	.	.	.	.	.	.	.	.	0	0	SOM	GT:/	0/1:1	0:18	T	C	0
.	.	.	.	.	.	.	.	1.59	.	.	SOM	GT:/	0/1:3	0:31	C	G	0
.	.	.	.	.	.	.	.	.	0.1	0	SOM	GT:/	0/1:2	0:16	G	A	0
.	.	.	.	.	.	.	.	.	0.1	0	SOM	GT:/	0/1:2	0:13	G	A	0
.	.	.	0.0	0.4	0.988	1.000	.	3.03	.	.	SOM	GT:/	0/1:8	0:59	G	A	0
.	.	.	.	.	.	.	3.7	.	.	.	SOM	GT:/	0/1:2	0:15	G	A	0
.	.	.	.	.	.	.	.	1.91	.	.	SOM	GT:/	0/1:5	0:17	G	A	0
.	.	.	.	.	.	.	.	.	0	.	SOM	GT:/	0/1:2	0:10	A	G	0
.	.	.	.	.	.	.	.	.	.	0	SOM	GT:/	0/1:7	0:50	T	G	0
.	.	.	.	.	.	.	2.9	.	.	.	SOM	GT:/	0/1:9	0:39	G	C	0
.	.	.	.	.	.	.	2.2	.	.	.	SOM	GT:/	0/1:8	0:40	C	G	0

.	.	.	.	.	.	.	.	.	.	.	SOM	GT:/	0/1:3	0:13	T	C	0
.	##	0	.	.	.	.	.	.	.	.	SOM	GT:/	0/1:8	0:38	G	A	0
.	.	.	.	.	.	.	.	.	.	0	SOM	GT:/	0/1:1	0:15	C	T	0
.	.	.	.	.	.	.	.	.	.	.	SOM	GT:/	0/1:7	0:40	A	T	0
.	.	.	.	.	.	1,A	6	7.18	.	.	SOM	GT:/	0/1:5	0:21	G	T	0
.	.	.	0.5	0.0	0.002	1,N	2.9	.	.	.	SOM	GT:/	0/1:2	0:83	C	T	0
0.001	0	0	.	.	.	.	.	.	.	.	SOM	GT:/	0/1:1	0:86	G	A	0
.	.	.	.	.	.	.	.	.	.	.	SOM	GT:/	0/1:3	0:23	G	C	0
.	.	.	0.0	0.9	1.0,D	0.966	5.7	3.84	.	.	SOM	GT:/	0/1:1	0:77	G	A	0
.	.	.	.	.	.	.	.	.	.	.	SOM	GT:/	0/1:2	0:17	G	A	0
.	##	0	.	.	.	.	.	.	.	.	SOM	GT:/	0/1:1	0:12	C	T	0
.	##	0	.	.	.	.	2.1	.	.	.	SOM	GT:/	0/1:2	0:16	G	A	0
.	.	.	.	.	.	.	.	.	.	.	SOM	GT:/	0/1:4	0:18	C	G	0
.	.	.	.	.	.	.	.	.	.	.	SOM	GT:/	0/1:1	0:11	C	T	0
.	.	.	.	.	.	.	.	.	.	.	SOM	GT:/	0/1:3	0:33	G	A	0
.	.	.	0.0	.	.	1,N	.	.	.	.	SOM	GT:/	0/1:1	0:15	G	A	0
.	.	.	0.9	0.0	0.053	1,N	.	1.98	.	.	SOM	GT:/	0/1:6	0:28	G	C	0
.	.	.	.	.	.	.	.	.	.	.	SOM	GT:/	0/1:5	0:15	T	C	0
.	.	.	.	.	.	.	.	.	0	.	DB;S	GT:/	0/1:2	0:19	G	T	0
.	.	.	.	.	.	.	.	.	.	.	SOM	GT:/	0/1:2	0:10	G	T	0
.	.	.	.	.	.	.	.	.	.	.	SOM	GT:/	0/1:6	0:56	G	A	0
.	.	.	.	.	.	.	.	.	.	.	SOM	GT:/	0/1:4	0:30	G	A	0
.	##	0	.	.	.	.	.	.	.	.	SOM	GT:/	0/1:1	0:13	G	A	0
.	.	.	0.0	0.9	0.999	1,D	4.7	4.37	.	.	SOM	GT:/	0/1:8	0:42	G	A	0
.	.	.	.	.	.	.	.	.	.	.	SOM	GT:/	0/1:9	0:57	A	T	0
.	.	.	.	.	.	.	.	.	.	.	SOM	GT:/	0/1:3	0:20	A	T	0
.	##	0	.	.	.	1,A	4.1	26.1	.	.	SOM	GT:/	0/1:4	0:15	G	A	0
.	.	.	.	.	.	.	.	.	0	0	SOM	GT:/	0/1:3	0:34	G	T	0
.	.	.	0.1	0.8	0.975	1,D	5.5	5.27	.	.	SOM	GT:/	0/1:4	0:24	C	T	0
.	.	.	.	.	.	.	.	1.25	.	.	SOM	GT:/	0/1:1	0:65	C	T	0
.	.	.	.	.	.	.	.	.	.	.	SOM	GT:/	0/1:2	0:17	C	G	0
.	##	0	1.0	0.0	0.015	1,D	3.8	.	.	.	SOM	GT:/	0/1:1	0:39	C	T	0
.	.	.	.	.	.	.	.	1.47	.	.	SOM	GT:/	0/1:5	0:22	G	A	0
.	.	.	.	.	.	.	.	.	0.1	0	DB;S	GT:/	0/1:1	0:41	A	G	0
.	.	.	.	.	.	.	.	.	.	.	SOM	GT:/	0/1:3	0:10	C	G	0
.	.	.	.	.	.	.	.	.	.	.	SOM	GT:/	0/1:3	0:11	C	T	0
.	.	.	.	.	.	.	.	.	.	.	SOM	GT:/	0/1:3	0:8,(	C	T	0
.	.	.	.	.	.	.	.	.	.	.	SOM	GT:/	0/1:9	0:91	C	T	0
.	.	.	0.0	0.5	0.948	1,N	4.3	2.60	.	.	SOM	GT:/	0/1:2	0:14	C	A	0
.	.	.	.	.	.	1,D	4.2	3.22	.	.	SOM	GT:/	0/1:1	0:11	G	A	0
.	.	.	.	.	.	.	.	.	.	.	SOM	GT:/	0/1:4	0:31	G	A	0
.	##	0	0.0	0.0	0.507	1.000	.	3.78	.	.	SOM	GT:/	0/1:7	0:47	G	A	0
.	.	.	.	.	.	.	.	.	.	.	SOM	GT:/	0/1:8	0:63	G	C	0
.	.	.	0.1	0.0	0.0,B	1,N	.	.	.	.	SOM	GT:/	0/1:8	0:46	G	A	0
.	.	.	0.7	0.0	0.0,B	0.713	4.8	.	.	.	SOM	GT:/	0/1:3	0:24	G	A	0
.	.	.	.	.	.	.	3	.	.	.	SOM	GT:/	0/1:5	0:43	C	A	0
.	.	.	.	.	.	.	.	.	0	.	DB;S	GT:/	0/1:5	0:19	C	T	0
.	.	.	.	.	.	.	.	.	0	0	DB;S	GT:/	0/1:3	0:22	T	A	0
.	.	.	.	.	.	.	.	.	0.1	0	DB;S	GT:/	0/1:9	0:35	T	G	0
.	.	.	.	.	.	.	.	.	0	.	DB;S	GT:/	0/1:7	0:36	T	G	0
.	.	.	.	.	.	.	.	.	.	.	SOM	GT:/	0/1:4	0:20	C	A	0
.	.	.	0.4	0.0	0.001	1,N	.	.	.	.	SOM	GT:/	0/1:2	0:22	T	C	0
.	.	.	.	.	.	.	.	.	.	.	SOM	GT:/	0/1:2	0:13	G	C	0
.	.	.	0.8	0.0	0.049	1,D	5.8	1.77	.	.	SOM	GT:/	0/1:3	0:25	C	G	0

.	.	.	.	.	.	.	.	.	.	.	SON	GT:/	0/1:9	0:40	G	A	0
#####	##	.	0.0(	0.4	0.801	1,D	.	2.66	.	.	SON	GT:/	0/1:3	0:20	G	T	0
.	.	.	.	.	.	.	2.9	.	.	.	SON	GT:/	0/1:3	0:15	C	G	0
.	.	.	.	.	.	.	.	.	.	.	SON	GT:/	0/1:1	0:9,(	A	T	0
.	.	.	0.0	0.0	0.028	1,D	5.3	4.86	.	.	SON	GT:/	0/1:2	0:17	C	T	0
#####	##	0	.	.	.	.	.	1.52	0	.	SON	GT:/	0/1:2	0:15	C	T	0
.	.	.	.	.	.	.	.	1.30	.	.	SON	GT:/	0/1:1	0:11	G	A	0
.	.	.	1.0	0.0	0.358	1,D	5.9	2.88	.	.	SON	GT:/	0/1:7	0:29	G	A	0
.	.	.	.	.	.	.	.	.	.	.	SON	GT:/	0/1:9	0:66	C	T	0
.	.	.	.	.	.	.	.	.	.	.	SON	GT:/	0/1:3	0:24	G	C	0
.	##	0	0.1	0.0	0.0,B	1,N	.	.	.	.	SON	GT:/	0/1:6	0:30	C	T	0
.	.	.	0.0	0.0	0.661	0.979	4.4	2.60	.	.	SON	GT:/	0/1:2	0:18	G	A	0
.	.	.	0.0	0.7	0.999	1,N	3.3	2.81	.	.	SON	GT:/	0/1:8	0:49	C	T	0
.	.	.	.	.	.	.	.	.	.	.	SON	GT:/	0/1:1	0:8,(	G	T	0
.	.	.	.	.	.	.	.	.	.	.	SON	GT:/	0/1:2	0:9,(	T	G	0
.	.	.	.	.	.	.	.	.	0.1	0.4	SON	GT:/	0/1:4	0:26	C	A	0
.	.	.	.	.	.	.	.	.	0	0.1	SON	GT:/	0/1:4	0:24	C	T	0
.	.	.	.	.	.	.	.	.	.	.	SON	GT:/	0/1:1	0:86	C	T	0
.	.	.	.	.	.	.	.	.	.	.	SON	GT:/	0/1:7	0:22	C	G	0
.	0	0	.	.	.	.	.	.	.	.	SON	GT:/	0/1:9	0:55	T	G	0
.	.	.	.	.	.	.	.	.	.	.	SON	GT:/	0/1:1	0:83	G	T	0
.	.	.	.	.	.	.	.	.	0.1	0.1	SON	GT:/	0/1:3	0:33	G	C	0
.	0	0	0.6	0.0(	0.014	0.644	4.5	.	.	.	SON	GT:/	0/1:7	0:41	G	A	0
.	.	.	.	.	.	.	.	.	.	.	SON	GT:/	0/1:2	0:17	T	A	0
.	0	0	.	.	.	.	.	.	0.1	0.1	SON	GT:/	0/1:2	0:8,(	A	G	0
.	.	.	.	.	.	.	.	.	.	.	SON	GT:/	0/1:6	0:31	C	T	0
.	.	.	.	.	.	.	.	.	0	.	SON	GT:/	0/1:4	0:14	A	G	0
.	.	.	0.0(	0.7	0.985	1,D	5.7	4.01	.	.	SON	GT:/	0/1:1	0:58	G	A	0
.	.	.	.	.	.	.	.	.	0	0	SON	GT:/	0/1:3	0:26	T	C	0
.	.	.	0.0	0.9	0.997	1,D	4.6	1.92	.	.	SON	GT:/	0/1:1	0:93	C	A	0
.	.	.	0.0	0.9	0.995	1,D	5.4	4.16	.	.	SON	GT:/	0/1:4	0:15	A	G	0
.	.	.	.	.	.	.	.	.	0	0	SON	GT:/	0/1:5	0:21	T	C	0
.	.	.	.	.	.	.	.	.	0	0.1	SON	GT:/	0/1:1	0:17	C	G	0
.	0	0	.	.	.	.	.	.	0	0.1	DB;S	GT:/	0/1:9	0:60	T	G	0
.	0	0	.	.	.	.	.	1.48	.	.	SON	GT:/	0/1:6	0:51	C	G	0
.	.	.	.	.	.	.	.	.	0	0	SON	GT:/	0/1:2	0:26	T	A	0
.	.	.	.	.	.	.	.	.	.	.	SON	GT:/	0/1:7	0:47	C	T	0
.	.	.	.	.	.	.	.	.	.	0	SON	GT:/	0/1:7	0:44	C	T	0
.	.	.	.	.	.	.	.	.	.	.	SON	GT:/	0/1:1	0:17	C	G	0
#####	##	0	0.4	0.0(	0.002	1,N	.	.	.	.	SON	GT:/	0/1:1	0:87	T	C	0
.	.	.	0.0	0.9	0.999	0.982	5.5	4.27	.	.	SON	GT:/	0/1:5	0:42	C	A	0
.	##	0	0.0	0.9	1.0,D	0.980	5.6	5.56	.	.	SON	GT:/	0/1:1	0:80	C	T	0
.	.	.	.	.	.	.	.	.	.	.	SON	GT:/	0/1:6	0:39	G	T	0
.	.	.	0.0(	0.0	0.034	1,D	5.3	3.35	.	.	SON	GT:/	0/1:2	0:16	T	C	0
.	.	.	.	.	.	.	.	.	0.1	0.1	SON	GT:/	0/1:6	0:42	A	G	0
.	.	.	.	.	.	.	.	.	.	.	SON	GT:/	0/1:2	0:13	C	G	0
.	0	0	0.1	0.0	0.108	0.889	4.6	3.30	0	0	SON	GT:/	0/1:2	0:10	C	T	0
.	.	.	.	.	.	.	.	.	.	.	SON	GT:/	0/1:3	0:22	C	G	0
.	.	.	.	.	.	.	.	.	.	.	SON	GT:/	0/1:3	0:10	G	A	0
.	.	.	0.0	0.5	0.896	0.994	4.8	2.62	.	.	SON	GT:/	0/1:3	0:19	C	A	0
.	.	.	.	.	.	.	.	.	.	.	SON	GT:/	0/1:3	0:15	G	T	0
2E-04	##	0	.	.	.	.	3.4	.	.	.	SON	GT:/	0/1:7	0:48	G	A	0
.	.	.	.	.	.	.	.	.	.	.	SON	GT:/	0/1:5	0:38	T	C	0
.	.	.	.	.	.	.	.	.	0.2	0.2	DB;S	GT:/	0/1:2	0:19	C	T	0

.	.	.	.	.	.	.	4.3	2.02	.	.	SOM	GT:/	0/1:5	0:47	T	C	0
.	.	.	0.6	0.0(	0.024	1,N	.	.	.	.	SOM	GT:/	0/1:9	0:59	G	A	0
.	.	.	.	.	.	1,A	5.6	8.61	.	.	SOM	GT:/	0/1:8	0:59	C	T	0
.	.	.	.	.	.	.	.	.	.	.	SOM	GT:/	0/1:6	0:28	C	A	0
.	.	.	.	.	.	.	.	.	.	.	SOM	GT:/	0/1:8	0:45	C	T	0
.	.	.	.	.	.	.	.	.	0.1	0	SOM	GT:/	0/1:1	0:11	G	A	0
.	.	.	.	.	.	.	.	.	0	.	SOM	GT:/	0/1:8	0:8,(	C	A	0
.	.	.	.	.	.	.	3.9	4.59	.	.	SOM	GT:/	0/1:1	0:10	C	T	0
.	.	.	0.0(	0.9!	1.0,D	1.000	.	4.03	.	.	SOM	GT:/	0/1:5	0:31	C	A	0
.	##	0	.	.	.	.	.	.	0	.	SOM	GT:/	0/1:8	0:60	C	T	0
.	.	.	.	.	.	.	.	.	.	.	SOM	GT:/	0/1:3	0:24	A	T	0
.	.	.	.	.	.	.	.	.	.	.	SOM	GT:/	0/1:1	0:18	G	A	0
.	.	.	0.0	0.9!	1.0,D	1,D	2.2	2.54	.	.	SOM	GT:/	0/1:9	0:10	C	G	0
.	.	.	.	.	.	.	.	.	.	.	SOM	GT:/	0/1:5	0:29	G	A	0
.	.	.	.	.	.	.	.	.	.	.	SOM	GT:/	0/1:1	0:68	G	A	0
.	.	.	.	.	.	.	.	.	.	.	SOM	GT:/	0/1:8	0:71	G	A	0
.	.	.	.	.	.	.	3.3	.	.	.	SOM	GT:/	0/1:3	0:18	C	G	0
.	.	.	0.6!	0.0	0.014	0.945	5.1	2.33	.	.	SOM	GT:/	0/1:1	0:82	G	C	0
.	.	.	.	.	.	.	5.3	.	.	.	SOM	GT:/	0/1:1	0:92	G	T	0
.	.	.	.	.	.	.	.	.	.	.	SOM	GT:/	0/1:1	0:54	G	C	0
.	.	.	.	.	.	1,D	4	3.48	.	.	SOM	GT:/	0/1:6	0:35	G	A	0
.	.	.	0.2	0.0	0.009	1,N	.	.	.	.	SOM	GT:/	0/1:8	0:40	C	A	0
.	.	.	.	.	.	.	.	.	.	.	SOM	GT:/	0/1:5	0:43	T	C	0
.	.	.	.	.	.	.	3.5	.	.	.	SOM	GT:/	0/1:3	0:21	C	T	0
.	.	.	0.0	0.7	0.983	1,D	5.5	4.92	.	.	SOM	GT:/	0/1:9	0:89	C	T	0
.	.	.	.	.	.	.	.	.	.	.	SOM	GT:/	0/1:5	0:35	T	C	0
.	##	0	.	.	.	.	.	.	.	.	SOM	GT:/	0/1:5	0:40	C	T	0
.	.	.	0.0(	0.0	0.22,I	1,N	5.3	1.44	.	.	SOM	GT:/	0/1:1	0:98	A	G	0
.	.	.	.	.	.	.	.	.	.	.	SOM	GT:/	0/1:1	0:16	G	A	0
.	.	.	0.0	1.0	1.0,D	1,D	4.8	3.94	.	.	SOM	GT:/	0/1:1	0:88	C	G	0
.	.	.	.	.	.	.	3.9	.	.	.	SOM	GT:/	0/1:2	0:16	C	T	0
.	.	.	.	.	.	.	.	.	.	.	SOM	GT:/	0/1:1	0:14	G	C	0
.	.	.	0.2!	0.6	0.846	1,D	2.3	.	.	.	SOM	GT:/	0/1:2	0:12	C	A	0
.	.	.	0.0	0.6	0.934	1,N	4	.	.	.	SOM	GT:/	0/1:7	0:48	C	A	0
.	.	.	.	.	.	.	.	.	0.1	0.1	DB;S	GT:/	0/1:5	0:24	A	G	0
.	.	.	.	.	.	.	.	.	.	.	SOM	GT:/	0/1:4	0:24	G	T	0
.	.	.	0.0(	0.9	0.999	0.994	3.2	3.49	.	.	SOM	GT:/	0/1:1	0:12	T	G	0
.	.	.	.	.	.	.	.	.	0.2	0.3	SOM	GT:/	0/1:7	0:8,(	A	G	0
.	.	.	0.0(	0.2	0.867	.	3	.	.	.	SOM	GT:/	0/1:1	0:79	G	T	0
.	.	.	.	.	.	.	.	.	.	.	SOM	GT:/	0/1:3	0:24	C	G	0
.	.	.	.	.	.	.	.	.	.	.	SOM	GT:/	0/1:5	0:31	C	A	0
.	.	.	0.6	0.0(	0.002	1,N	.	.	.	.	SOM	GT:/	0/1:1	0:80	G	C	0
.	.	.	.	.	.	.	.	.	.	.	SOM	GT:/	0/1:3	0:25	G	A	0
.	.	.	.	.	.	.	.	.	.	.	SOM	GT:/	0/1:5	0:48	G	T	0
.	.	.	.	.	.	.	.	.	.	.	SOM	GT:/	0/1:1	0:11	G	C	0
.	.	.	.	.	.	.	.	.	.	.	SOM	GT:/	0/1:1	0:10	T	C	0
.	.	.	.	.	.	.	.	.	0	.	SOM	GT:/	0/1:1	0:15	G	A	0
.	.	.	.	.	.	.	.	.	.	.	SOM	GT:/	0/1:3	0:16	T	C	0
.	##	0	.	.	.	1.000	.	.	.	.	SOM	GT:/	0/1:1	0:83	G	A	0
.	##	0	0.0	0.9!	1.0,D	1,D	5	4.32	.	.	SOM	GT:/	0/1:2	0:12	G	A	0
.	.	.	.	.	.	.	.	.	.	.	SOM	GT:/	0/1:8	0:65	T	A	0
.	.	.	.	.	.	.	.	.	.	.	SOM	GT:/	0/1:1	0:15	T	G	0
.	.	.	.	.	.	.	2.9	.	.	.	SOM	GT:/	0/1:3	0:21	C	T	0
.	.	.	.	.	.	.	.	.	0	.	SOM	GT:/	0/1:3	0:29	T	G	0

.	.	.	.	.	.	.	.	.	.	.	SOM	GT:/	0/1:1	0:10	G	C	0
.	.	.	.	.	.	.	.	.	.	.	SOM	GT:/	0/1:7	0:52	C	G	0
.	.	.	.	.	.	.	.	.	.	.	SOM	GT:/	0/1:3	0:37	G	A	0
.	.	.	.	.	.	.	.	.	.	.	SOM	GT:/	0/1:5	0:27	A	G	0
.	.	.	.	.	.	.	.	.	.	.	SOM	GT:/	0/1:6	0:29	G	A	0
.	.	.	.	.	.	.	.	.	.	.	SOM	GT:/	0/1:3	0:11	G	C	0
.	.	.	0.0	0.4	0.938	1,D	2.9	1.62	.	.	SOM	GT:/	0/1:1	0:94	A	G	0
.	.	.	.	.	.	.	.	.	0	0	DB;S	GT:/	0/1:4	0:33	G	A	0
.	.	.	0.0	1.0	1.0,D	1,D	5.4	4.70	.	.	SOM	GT:/	0/1:3	0:27	C	A	0
.	.	.	0.0	0.7	0.97,I	0.997	5.9	3.43	.	.	SOM	GT:/	0/1:7	0:52	C	T	0
.	.	.	.	.	.	.	.	.	0.2	0	SOM	GT:/	0/1:3	0:28	T	C	0
.	.	.	.	.	.	.	3.1	4.26	.	.	SOM	GT:/	0/1:8	0:65	G	A	0
.	.	.	.	.	.	.	.	.	0	0	SOM	GT:/	0/1:5	0:23	T	G	0
.	.	.	.	.	.	.	.	.	.	.	SOM	GT:/	0/1:4	0:12	G	C	0
.	.	.	0.1	0.4	0.919	1.000	5.7	2.46	.	.	SOM	GT:/	0/1:1	0:47	T	A	0
.	.	.	0.0	0.9	1.0,D	1.000	2.3	1.66	.	.	SOM	GT:/	0/1:7	0:45	C	T	0
.	.	.	0.0	0.8	0.993	1.000	5.8	2.89	.	.	SOM	GT:/	0/1:6	0:48	C	A	0
.	.	.	0.0	0.9	1.0,D	1,D	4.5	4.02	.	.	SOM	GT:/	0/1:8	0:40	G	A	0
.	.	.	.	.	.	.	.	.	.	.	SOM	GT:/	0/1:1	0:60	G	A	0
.	.	.	.	.	.	.	.	.	.	.	SOM	GT:/	0/1:1	0:56	T	C	0
.	.	.	.	.	.	.	.	.	0	.	SOM	GT:/	0/1:1	0:11	G	A	0
.	.	.	.	.	.	.	.	.	0	0	SOM	GT:/	0/1:3	0:10	G	A	0
.	.	.	.	.	.	.	.	.	0.1	0.4	SOM	GT:/	0/1:2	0:31	G	A	0
.	.	.	.	.	.	.	.	.	0.5	0.5	DB;S	GT:/	0/1:3	0:24	T	A	0
.	.	.	.	.	.	.	.	.	.	0	SOM	GT:/	0/1:1	0:16	T	A	0
.	.	.	.	.	.	.	.	.	0	.	SOM	GT:/	0/1:5	0:37	C	A	0
.	.	.	.	.	.	.	.	1.25	0	0	SOM	GT:/	0/1:1	0:10	G	C	0
.	.	.	.	.	.	.	.	1.90	0.1	0	DB;S	GT:/	0/1:6	0:37	C	T	0
.	.	.	.	.	.	.	.	.	0	.	SOM	GT:/	0/1:1	0:8,(	C	T	0
.	.	.	.	.	.	.	.	.	.	.	SOM	GT:/	0/1:1	0:8,(	C	A	0
.	.	.	.	.	.	.	.	.	.	.	SOM	GT:/	0/1:1	0:8,(	C	G	0
.	.	.	.	.	.	.	.	.	0	0.4	SOM	GT:/	0/1:9	0:9,(	T	A	0
.	.	.	.	.	.	.	.	.	.	.	SOM	GT:/	0/1:4	0:29	G	T	0
.	.	.	.	.	.	.	.	.	.	.	SOM	GT:/	0/1:1	0:8,(	C	A	0
.	.	.	.	.	.	.	.	.	0.2	0.6	SOM	GT:/	0/1:9	0:9,(	C	T	0
.	.	.	.	.	.	.	.	.	0	0	SOM	GT:/	0/1:6	0:33	T	C	0
.	.	.	.	.	.	.	.	.	0	0	DB;S	GT:/	0/1:4	0:25	T	C	0
.	.	.	.	.	.	.	2.1	.	0.1	0	SOM	GT:/	0/1:5	0:44	T	C	0
.	.	.	.	.	.	.	.	.	0	.	SOM	GT:/	0/1:1	0:10	G	C	0
.	.	.	.	.	.	.	.	.	0	.	SOM	GT:/	0/1:3	0:12	T	A	0
.	.	.	.	.	.	.	.	.	0	.	SOM	GT:/	0/1:1	0:10	G	A	0
.	.	.	.	.	.	.	.	.	0	0	DB;S	GT:/	0/1:5	0:25	A	G	0
.	.	.	.	.	.	.	.	.	.	.	SOM	GT:/	0/1:1	0:36	T	G	0
.	.	.	.	.	.	.	.	.	.	.	SOM	GT:/	0/1:3	0:10	T	A	0
.	.	.	.	.	.	.	.	.	0.2	0.2	SOM	GT:/	0/1:8	0:8,(	T	G	0
.	.	.	.	.	.	.	.	.	0.1	0.3	SOM	GT:/	0/1:1	0:10	T	C	0
.	.	.	.	.	.	.	.	.	0	0	SOM	GT:/	0/1:4	0:22	C	T	0
.	.	.	.	.	.	.	.	.	0	0	SOM	GT:/	0/1:4	0:37	G	A	0
.	.	.	.	.	.	.	.	.	0	.	SOM	GT:/	0/1:2	0:9,(	A	T	0
.	.	.	.	.	.	.	.	.	0	0.1	DB;S	GT:/	0/1:4	0:26	G	T	0
.	.	.	.	.	.	.	.	.	0	.	DB;S	GT:/	0/1:6	0:40	A	T	0
.	.	.	.	.	.	.	.	.	0	0	DB;S	GT:/	0/1:2	0:19	G	A	0
.	.	.	.	.	.	.	.	.	0	0	SOM	GT:/	0/1:3	0:12	G	T	0
.	.	.	.	.	.	.	.	.	0	0	SOM	GT:/	0/1:4	0:22	C	A	0

.	.	.	.	.	.	.	.	.	.	0	SOM	GT:/	0/1:1	0:13	C	A	0
.	.	.	.	.	.	.	.	.	.	.	SOM	GT:/	0:87	0/1:1	T	G	0
.	.	.	.	.	.	.	.	.	0.2	0.5	DB;S	GT:/	0:22	0/1:2	G	C	0
.	.	.	.	.	.	.	.	.	.	.	SOM	GT:/	0:16	0/1:2	G	T	0
.	.	.	.	.	.	.	.	.	0.1	0.4	SOM	GT:/	0:12	0/1:1	C	G	0
.	.	.	.	.	.	.	.	.	0	0	SOM	GT:/	0:13	0/1:2	C	G	0
.	.	.	.	.	.	.	.	.	0	.	SOM	GT:/	0:22	0/1:5	G	T	0
.	.	.	.	.	.	.	.	.	0.2	0.5	SOM	GT:/	0:14	0/1:2	C	A	0
.	.	.	.	.	.	.	.	.	0	0	SOM	GT:/	0:11	0/1:2	G	T	0
.	.	.	.	.	.	.	.	.	.	.	SOM	GT:/	0:37	0/1:3	T	G	0
.	.	.	.	.	.	.	.	.	.	.	SOM	GT:/	0:8,(	0/1:3	G	T	0
.	.	.	.	.	.	.	.	.	0	0.1	SOM	GT:/	0:18	0/1:4	C	T	0
.	.	.	.	.	.	.	.	.	.	.	SOM	GT:/	0:13	0/1:2	G	T	0
.	.	.	.	.	.	.	.	.	0.1	0	SOM	GT:/	0:15	0/1:2	C	T	0
.	.	.	.	.	.	.	.	.	0.1	0.4	SOM	GT:/	0:8,(	0/1:1	A	G	0
.	.	.	.	.	.	.	.	.	.	.	SOM	GT:/	0:9,(	0/1:2	T	C	0
.	.	.	.	.	.	.	.	.	0.1	0.3	SOM	GT:/	0:18	0/1:1	G	A	0
.	.	.	.	.	.	.	.	.	.	.	SOM	GT:/	0:10	0/1:3	T	A	0
.	.	.	.	.	.	.	.	.	.	.	SOM	GT:/	0:13	0/1:3	C	A	0
.	.	.	.	.	.	.	.	.	.	.	SOM	GT:/	0:20	0/1:2	C	T	0
.	.	.	.	.	.	.	.	.	0.1	0.3	DB;S	GT:/	0:51	0/1:1	C	G	0
.	.	.	.	.	.	.	.	.	0.1	0.1	SOM	GT:/	0:12	0/1:1	G	C	0
.	.	.	.	.	.	.	.	.	0	.	SOM	GT:/	0:41	0/1:1	C	G	0
.	.	.	.	.	.	.	.	.	0	.	SOM	GT:/	0:46	0/1:1	T	A	0
.	.	.	0.0:	0.1'	0.763	1.000	5.2	2.74	0	.	SOM	GT:/	0:58	0/1:1	T	G	0
.	.	.	.	.	.	.	.	.	0	.	SOM	GT:/	0:8,(	0/1:9	G	T	0
.	.	.	.	.	.	.	.	.	0	0	SOM	GT:/	0:8,(	0/1:5	C	T	0
.	.	.	.	.	.	.	.	.	0	0.1	SOM	GT:/	0:8,(	0/1:9	C	A	0
.	.	.	.	.	.	.	2.7	.	.	.	SOM	GT:/	0:15	0/1:5	A	C	0
.	.	.	.	.	.	.	.	1.80	.	.	SOM	GT:/	0:12	0/1:4	T	C	0
.	.	.	.	.	.	.	.	.	0	0	SOM	GT:/	0:35	0/1:7	T	G	0
.	.	.	.	.	.	.	.	.	0	0.1	SOM	GT:/	0:17	0/1:3	C	T	0
.	.	.	.	.	.	.	.	.	.	.	SOM	GT:/	0:11	0/1:3	A	T	0
.	.	.	.	.	.	.	.	.	0	0.1	SOM	GT:/	0:10	0/1:2	G	A	0
.	.	.	.	.	.	.	.	.	0	0.1	SOM	GT:/	0:14	0/1:4	C	T	0
.	.	.	.	.	.	.	.	.	0	.	SOM	GT:/	0:16	0/1:3	G	A	0
.	##	0	.	.	.	.	.	1.26	0	.	SOM	GT:/	0:12	0/1:1	T	C	0
.	.	.	.	.	.	.	.	.	0	0	SOM	GT:/	0:13	0/1:7	C	T	0
.	.	.	.	.	.	.	.	.	0	0.2	DB;S	GT:/	0:37	0/1:7	G	A	0
.	.	.	.	.	.	.	.	.	0	0	SOM	GT:/	0:18	0/1:1	G	A	0
.	.	.	.	.	.	.	.	.	0	0.1	DB;S	GT:/	0:20	0/1:8	G	T	0
.	.	.	.	.	.	.	.	.	0	0.1	DB;S	GT:/	0:34	0/1:5	T	C	0
.	.	.	.	.	.	.	2.2	.	0	0.1	DB;S	GT:/	0:40	0/1:7	C	T	0
.	.	.	.	.	.	.	.	.	.	.	SOM	GT:/	0:16	0/1:4	G	C	0
.	.	.	.	.	.	.	.	.	.	.	SOM	GT:/	0:11	0/1:3	C	A	0
.	.	.	.	.	.	.	.	.	.	.	SOM	GT:/	0:12	0/1:8	A	G	0
.	.	.	.	.	.	.	.	.	0	0	SOM	GT:/	0:9,(	0/1:1	G	T	0
.	.	.	.	.	.	.	.	.	.	.	SOM	GT:/	0:44	0/1:1	A	T	0
.	.	.	.	.	.	.	.	.	0.1	0.3	DB;S	GT:/	0:40	0/1:1	C	T	0
.	.	.	.	.	.	.	.	.	.	.	SOM	GT:/	0:9,(	0/1:2	C	A	0
.	##	0	0.1:	0.9!	1.0,D	1.000	4.2	2.42	.	.	SOM	GT:/	0/1:9	0:10	C	T	0
.	.	.	.	.	.	.	2.3	.	.	.	SOM	GT:/	0/1:5	0:47	C	T	0
.	.	.	.	.	.	.	2.9	.	.	.	SOM	GT:/	0/1:4	0:33	G	C	0
.	.	.	.	.	.	.	2.6	.	.	.	SOM	GT:/	0/1:2	0:11	G	C	0

.	.	.	.	.	.	.	2.3	.	.	.	SOM	GT:/	0/1:1	0:10	C	T	0
.	.	.	0.0	.	.	0.994	3.2	2.83	.	.	SOM	GT:/	0/1:8	0:85	G	A	0
.	.	.	.	.	.	.	2.1	1.40	.	.	SOM	GT:/	0/1:1	0:13	G	T	0
.	.	.	.	.	.	.	.	.	.	.	SOM	GT:/	0/1:3	0:12	C	T	0
.	.	.	.	.	.	1,A	5.3	7.23	.	.	SOM	GT:/	0/1:3	0:30	C	T	0
.	.	.	.	.	.	.	.	.	0	0	SOM	GT:/	0/1:2	0:8,(	T	C	0
.	.	.	.	.	.	.	.	.	.	0	SOM	GT:/	0/1:2	0:11	G	A	0
.	.	.	.	.	.	.	.	.	0.1	0.4	SOM	GT:/	0/1:1	0:13	C	T	0
.	.	.	.	.	.	.	.	.	0	0	SOM	GT:/	0/1:1	0:9,(	G	C	0
.	.	.	.	.	.	.	.	.	0	.	SOM	GT:/	0/1:8	0:20	A	T	0
.	.	.	.	.	.	.	.	.	.	0	SOM	GT:/	0/1:3	0:57	G	A	0
.	.	.	.	.	.	.	.	.	.	.	SOM	GT:/	0/1:4	0:17	A	C	0
.	.	.	.	.	.	.	.	.	0	0.1	SOM	GT:/	0/1:3	0:16	T	G	0
.	.	.	0.0	0.9	0.996	0.963	4.3	3.06	0	.	SOM	GT:/	0/1:3	0:38	C	T	0
.	.	.	.	.	.	.	5.4	1.82	.	.	SOM	GT:/	0/1:1	0:10	G	C	0
.	.	.	.	.	.	.	.	.	.	.	SOM	GT:/	0/1:1	0:10	G	A	0
.	.	.	.	.	.	.	.	.	.	.	SOM	GT:/	0/1:1	0:10	A	G	0
.	.	.	.	.	.	.	.	.	.	.	SOM	GT:/	0/1:1	0:10	C	T	0
.	.	.	.	.	.	.	.	.	.	.	SOM	GT:/	0/1:3	0:30	G	T	0
.	.	.	.	.	.	.	3	.	.	.	SOM	GT:/	0/1:6	0:47	G	C	0
.	##	0	0.4	0.1	0.796	1,D	5.4	3.42	.	.	SOM	GT:/	0/1:1	0:14	G	A	0
.	.	.	.	.	.	.	2.3	.	.	.	SOM	GT:/	0/1:3	0:17	C	T	0
.	.	.	.	.	.	.	.	.	.	.	SOM	GT:/	0/1:1	0:11	G	C	0
.	.	.	0.1	0.4	0.899	1,N	3.3	1.89	.	.	SOM	GT:/	0/1:1	0:14	C	T	0
.	##	0	.	.	.	.	.	1.59	.	.	SOM	GT:/	0/1:1	0:12	C	T	0
.	##	0	.	.	.	.	.	.	.	.	SOM	GT:/	0/1:2	0:17	G	A	0
.	.	.	.	.	.	.	.	.	0.1	0	SOM	GT:/	0/1:5	0:14	A	G	0
.	##	0	0.0	0.6	0.918	0.976	4.9	3.84	.	.	SOM	GT:/	0/1:2	0:23	C	G	0
.	.	.	.	.	.	.	.	.	.	.	SOM	GT:/	0/1:1	0:15	A	G	0
0.003	0	0	0.2	0.1	0.791	0.946	3.4	.	.	.	DB;S	GT:/	0/1:2	0:10	G	A	0
.	.	.	.	.	.	.	.	.	.	.	SOM	GT:/	0/1:6	0:14	C	T	0
.	.	.	.	.	.	1,D	2.9	6.40	.	.	SOM	GT:/	0/1:1	0:67	G	A	0
.	.	.	.	.	.	1,D	5.1	4.27	.	.	SOM	GT:/	0/1:1	0:55	G	C	0
.	.	.	0.0	0.8	0.919	1.000	4.4	4.65	.	.	SOM	GT:/	0/1:1	0:14	C	T	0
.	.	.	.	.	.	.	.	.	.	.	SOM	GT:/	0/1:1	0:12	T	C	0
.	.	.	.	.	.	.	.	.	.	.	SOM	GT:/	0/1:8	0:23	C	T	0
.	.	.	0.0	0.1	0.693	1.000	5.1	2.94	0	.	SOM	GT:/	0/1:3	0:31	G	A	0
.	.	.	0.0	0.0	0.004	0.859	3.7	2.36	.	.	SOM	GT:/	0/1:2	0:10	G	A	0
.	.	.	.	.	.	.	.	.	.	.	SOM	GT:/	0/1:2	0:21	G	A	0
.	.	.	0.0	0.2	0.891	0.757	4.5	3.26	.	.	SOM	GT:/	0/1:1	0:14	C	G	0
.	.	.	.	.	.	.	2.6	2.09	.	.	SOM	GT:/	0/1:3	0:17	C	G	0
.	##	0	.	.	.	.	.	.	.	.	SOM	GT:/	0/1:4	0:26	T	C	0
.	.	.	0.0	0.6	0.915	1,D	5.1	3.61	.	.	SOM	GT:/	0/1:7	0:25	A	T	0
.	.	.	.	.	.	.	.	.	.	.	SOM	GT:/	0/1:6	0:61	G	A	0
.	0	0	.	.	.	.	4.6	2.69	.	.	SOM	GT:/	0/1:4	0:16	T	G	0
2E-04	##	0	0.0	0.8	0.999	1,N	3.2	1.45	.	.	SOM	GT:/	0/1:3	0:31	C	T	0
.	##	0	.	.	.	.	2.4	.	.	.	SOM	GT:/	0/1:2	0:18	G	A	0
.	##	0	.	.	.	.	.	.	0	.	SOM	GT:/	0/1:8	0:59	G	A	0
.	.	.	.	.	.	.	.	.	.	.	SOM	GT:/	0/1:4	0:8,(	T	G	0
.	.	.	.	.	.	.	.	.	.	.	SOM	GT:/	0/1:2	0:17	C	T	0
.	##	0	.	.	.	.	2.9	2.55	.	.	SOM	GT:/	0/1:1	0:10	G	A	0
#####	##	0	0.1	0.0	0.064	0.758	4.1	2.41	.	.	SOM	GT:/	0/1:1	0:28	G	A	0
.	0	0	.	.	.	.	.	.	0	0	SOM	GT:/	0/1:5	0:29	G	A	0
.	##	0	.	.	.	.	.	.	.	.	SOM	GT:/	0/1:8	0:27	G	A	0

.	##	0	0.1:	0.5!	0.853	0.982	2.8	3.03	0	.	SON	GT:/	0/1:2	0:17	G	A	0
.	.	.	.	.	.	.	.	.	.	.	SON	GT:/	0/1:1	0:10	C	T	0
.	.	.	0.0(	0.9!	1.0,D	1,D	5.2	5.53	.	.	SON	GT:/	0/1:4	0:25	G	A	0
.	.	.	.	.	.	.	.	.	0	0	SON	GT:/	0/1:1	0:10	A	T	0
.	.	.	.	.	.	.	.	.	.	.	SON	GT:/	0/1:2	0:24	C	T	0
.	0	0	.	.	.	.	3.4	1.86	0	0	SON	GT:/	0/1:9	0:10	C	G	0
.	.	.	.	.	.	.	.	2.44	.	.	SON	GT:/	0/1:3	0:41	T	C	0
.	##	0	0.0!	0.0(	0.012	0.998	.	3.22	.	.	SON	GT:/	0/1:2	0:32	G	A	0
.	.	.	.	.	.	.	.	.	.	.	SON	GT:/	0/1:1	0:90	G	C	0
.	.	.	.	.	.	.	.	.	.	.	SON	GT:/	0/1:1	0:97	G	A	0
.	.	.	0.6:	0.9!	1.0,D	1,D	4.7	4.05	.	.	SON	GT:/	0/1:1	0:14	C	G	0
0.002	0	0	0.1(	0.2'	0.881	0.888	4.4	3.43	.	.	SON	GT:/	0/1:1	0:10	G	A	0
.	.	.	.	.	.	.	3.9	1.92	.	.	SON	GT:/	0/1:2	0:21	T	C	0
.	.	.	.	.	.	.	.	2.06	.	.	SON	GT:/	0/1:9	0:29	G	T	0
.	.	.	.	.	.	.	.	.	0.1	0.1	SON	GT:/	0/1:2	0:8,(	C	G	0
.	.	.	.	.	.	.	.	.	.	.	SON	GT:/	0/1:1	0:12	C	A	0
.	.	.	.	.	.	.	.	.	.	.	SON	GT:/	0/1:1	0:88	A	C	0
.	.	.	.	.	.	.	.	2.69	.	.	SON	GT:/	0/1:3	0:21	C	T	0
.	.	.	.	.	.	.	.	4.15	.	.	SON	GT:/	0/1:4	0:30	C	T	0
.	##	0	0.0!	0.5·	0.948	1,N	.	2.41	.	.	SON	GT:/	0/1:6	0:55	C	T	0
.	##	0	.	.	.	.	.	.	.	.	SON	GT:/	0/1:1	0:15	C	T	0
.	.	.	.	.	.	.	.	.	.	.	SON	GT:/	0/1:9	0:41	C	T	0
.	.	.	.	.	.	.	.	.	.	.	SON	GT:/	0/1:2	0:9,(	T	C	0
.	.	.	.	.	.	.	.	.	0	0	SON	GT:/	0/1:5	0:53	A	G	0
.	.	.	.	.	.	.	.	.	0	0	SON	GT:/	0/1:6	0:50	G	A	0
.	.	.	.	.	.	.	.	.	.	.	SON	GT:/	0/1:5	0:23	T	C	0
.	##	0	.	.	.	1.00(	.	.	.	.	SON	GT:/	0/1:1	0:84	C	T	0
.	0	0	.	.	.	.	3.8	.	.	.	SON	GT:/	0/1:1	0:66	C	T	0
.	##	0	.	.	.	.	.	.	.	.	SON	GT:/	0/1:1	0:14	G	A	0
.	.	.	.	.	.	.	.	.	.	.	SON	GT:/	0/1:2	0:13	T	G	0
.	.	.	.	.	.	.	.	.	0	0	SON	GT:/	0/1:1	0:14	T	G	0
.	.	.	0.0	0.0	0.0,B	1,N	.	.	.	.	SON	GT:/	0/1:8	0:56	G	C	0
0.001	0	0	.	.	.	.	.	.	0	0	SON	GT:/	0/1:1	0:16	G	A	0
.	.	.	.	.	.	.	.	.	.	.	SON	GT:/	0/1:2	0:27	G	T	0
.	.	.	.	.	.	.	.	.	0.1	0.2	DB;!	GT:/	0/1:1	0:19	C	G	0
.	.	.	.	.	.	.	.	.	0	.	SON	GT:/	0/1:1	0:9,(	T	C	0
.	##	.	.	.	.	.	.	1.49	0	.	SON	GT:/	0/1:1	0:12	G	A	0
.	##	0	.	.	.	.	.	.	.	.	SON	GT:/	0/1:2	0:16	G	A	0
.	.	.	.	.	.	.	2.4	.	.	.	SON	GT:/	0/1:2	0:16	G	A	0
.	.	.	.	.	.	.	4	3.19	.	.	SON	GT:/	0/1:7	0:38	C	T	0
#####	##	0	0.0:	0.9·	1.0,D	1.00(	4.6	1.33	.	.	SON	GT:/	0/1:1	0:38	G	A	0
.	.	.	.	.	.	.	.	.	.	.	SON	GT:/	0/1:1	0:95	C	A	0
.	.	.	.	.	.	.	.	.	0	0.1	SON	GT:/	0/1:1	0:11	A	G	0
.	.	.	.	.	.	.	.	.	.	.	SON	GT:/	0/1:5	0:83	A	G	0
.	0	0	.	.	.	.	.	.	0	0	SON	GT:/	0/1:4	0:40	G	C	0
.	.	.	.	.	.	.	2.3	.	.	.	SON	GT:/	0/1:8	0:60	C	G	0
.	.	.	.	.	.	.	.	.	.	.	SON	GT:/	0/1:3	0:25	G	A	0
.	.	.	.	.	.	.	.	.	.	.	SON	GT:/	0/1:4	0:30	G	C	0
.	.	.	.	.	.	.	.	.	.	.	SON	GT:/	0/1:2	0:11	G	A	0
.	.	.	.	.	.	1,A	4.6	8.20	.	.	SON	GT:/	0/1:1	0:70	C	T	0
.	.	.	0.1:	0.0(	0.02,I	1,N	.	.	.	.	SON	GT:/	0/1:9	0:37	A	G	0
.	.	.	.	.	.	.	.	.	.	.	SON	GT:/	0/1:1	0:11	C	T	0
.	.	.	.	.	.	.	.	.	0	0	SON	GT:/	0/1:2	0:16	A	G	0
.	.	.	.	.	.	.	.	.	0	.	SON	GT:/	0/1:5	0:36	G	A	0

.	.	.	.	.	.	.	.	.	0.1	0.3	SOM	GT:/	0/1:1	0:8,(	G	T	0
.	.	.	.	.	.	.	.	.	0	0.1	SOM	GT:/	0/1:1	0:9,(	T	A	0
.	.	.	.	.	.	.	.	.	0	.	SOM	GT:/	0/1:4	0:54	A	T	0
.	.	.	.	.	.	.	.	.	0.2	0.2	SOM	GT:/	0/1:1	0:9,(	G	T	0
.	0	0	.	.	.	.	.	.	0.2	0.4	DB;S	GT:/	0/1:6	0:39	G	A	0
.	.	.	.	.	.	.	.	.	.	.	SOM	GT:/	0/1:3	0:40	C	T	0
.	.	.	.	.	.	.	.	.	.	.	SOM	GT:/	0/1:7	0:49	G	T	0
.	##	0	.	.	.	.	.	.	.	.	SOM	GT:/	0/1:6	0:50	G	A	0
.	.	.	.	.	.	.	3.7	1.32	.	.	SOM	GT:/	0/1:2	0:21	G	A	0
.	##	0	.	.	.	.	3.4	2.15	0	0	SOM	GT:/	0/1:1	0:11	C	T	0
.	##	0	0.0	1.0	1.0,D	1.000	3.1	4.05	0	.	SOM	GT:/	0/1:2	0:19	C	T	0
.	.	.	0.0	0.9	0.999	0.996	4.6	3.60	.	.	SOM	GT:/	0/1:4	0:27	G	C	0
.	.	.	.	.	.	.	.	.	0	0	SOM	GT:/	0/1:2	0:15	T	C	0
.	.	.	.	.	.	.	3.3	.	.	.	SOM	GT:/	0/1:4	0:38	A	G	0
#####	##	0	0.0	0.1	0.086	0.583	.	2.62	.	.	SOM	GT:/	0/1:8	0:58	G	A	0
.	0	0	0.4	0.0	0.002	1,N	.	.	0	.	SOM	GT:/	0/1:4	0:34	C	T	0
2E-04	0	0	.	.	.	.	2.2	.	.	.	SOM	GT:/	0/1:1	0:84	C	T	0
2E-04	0	0	0.6	0.0	0.009	1,N	.	2.04	.	.	SOM	GT:/	0/1:1	0:80	G	A	0
.	.	.	.	.	.	1,D	4.1	9.70	.	.	SOM	GT:/	0/1:2	0:14	C	T	0
.	.	.	.	.	.	.	.	.	.	.	SOM	GT:/	0/1:7	0:47	T	G	0
.	.	.	.	.	.	.	.	.	.	.	SOM	GT:/	0/1:9	0:69	T	C	0
.	.	.	.	.	.	.	.	.	.	.	SOM	GT:/	0/1:9	0:55	G	A	0
.	.	.	.	.	.	.	.	.	.	.	SOM	GT:/	0/1:6	0:47	G	A	0
.	.	.	.	.	.	.	.	.	0.2	0.2	DB;S	GT:/	0/1:3	0:26	A	T	0
.	.	.	.	.	.	.	.	.	0	0	SOM	GT:/	0/1:1	0:9,(	T	C	0
.	.	.	.	.	.	.	.	.	.	.	SOM	GT:/	0/1:2	0:25	G	A	0
.	##	0	.	.	.	.	.	.	.	.	SOM	GT:/	0/1:1	0:92	C	T	0
.	.	.	.	.	.	.	.	.	.	.	SOM	GT:/	0/1:5	0:28	C	T	0
.	.	.	.	.	.	.	2.2	1.64	.	.	SOM	GT:/	0/1:3	0:16	C	A	0
.	.	.	0.2	0.0	0.005	0.999	4.3	1.98	.	.	SOM	GT:/	0/1:2	0:13	G	A	0
.	.	.	.	.	.	.	2.7	.	.	.	SOM	GT:/	0/1:6	0:59	G	A	0
.	##	0	.	.	.	.	.	.	0	.	SOM	GT:/	0/1:8	0:55	G	A	0
.	.	.	.	.	.	.	2.3	.	0	.	SOM	GT:/	0/1:3	0:21	A	T	0
.	.	.	.	.	.	.	.	.	.	.	SOM	GT:/	0/1:1	0:14	C	T	0
.	.	.	.	.	.	.	.	.	.	.	SOM	GT:/	0/1:7	0:47	C	A	0
0.001	0	0	.	.	.	.	.	.	.	.	SOM	GT:/	0/1:9	0:58	G	A	0
.	.	.	.	.	.	.	.	.	0	0.1	SOM	GT:/	0/1:7	0:8,(	G	A	0
.	##	0	.	.	.	.	.	3.53	0	.	SOM	GT:/	0/1:6	0:43	G	A	0
.	.	.	.	.	.	.	.	.	.	0	SOM	GT:/	0/1:1	0:58	A	T	0
.	.	.	.	.	.	.	5.4	1.35	.	.	SOM	GT:/	0/1:2	0:15	G	T	0
#####	##	0	.	.	.	.	.	.	0	.	SOM	GT:/	0/1:5	0:25	G	A	0
.	.	.	.	.	.	.	.	.	.	.	SOM	GT:/	0/1:2	0:25	C	T	0
.	.	.	.	.	.	.	.	.	.	.	SOM	GT:/	0/1:9	0:8,(	G	A	0
.	.	.	0.0	0.9	1.0,D	1,D	5.8	4.25	.	.	SOM	GT:/	0/1:8	0:79	C	T	0
.	.	.	0.0	0.9	1.0,D	1,D	5.9	4.98	.	.	SOM	GT:/	0/1:1	0:10	G	A	0
.	.	.	.	.	.	.	.	.	0	.	SOM	GT:/	0/1:6	0:56	G	A	0
.	.	.	.	.	.	.	.	.	0.1	0.3	DB;S	GT:/	0/1:2	0:22	T	C	0
.	.	.	.	.	.	.	.	.	.	.	SOM	GT:/	0/1:1	0:11	G	A	0
.	.	.	.	.	.	.	.	.	.	.	SOM	GT:/	0/1:1	0:9,(	C	A	0
.	.	.	0.0	0.5	0.9,P	1.000	2.7	3.35	.	.	SOM	GT:/	0/1:1	0:85	G	A	0
.	.	.	.	.	.	.	4.9	4.07	.	.	SOM	GT:/	0/1:4	0:25	C	T	0
.	.	.	0.1	0.2	0.598	0.999	2.9	2.50	.	.	SOM	GT:/	0/1:1	0:74	C	G	0
.	.	.	0.1	0.5	0.831	1,N	.	.	.	.	SOM	GT:/	0/1:7	0:65	T	A	0
.	.	.	.	.	.	.	.	.	0	0	SOM	GT:/	0/1:1	0:8,(	G	A	0

.	.	.	.	.	.	.	.	.	.	.	SON	GT:/	0/1:7	0:43	G	C	0
.	.	.	.	.	.	.	.	.	.	.	SON	GT:/	0/1:3	0:28	G	C	0
.	##	0	.	.	.	.	.	.	.	.	SON	GT:/	0/1:5	0:52	G	A	0
.	.	.	.	.	.	.	.	.	.	.	SON	GT:/	0/1:9	0:93	T	A	0
.	.	.	.	.	.	.	.	.	.	.	SON	GT:/	0/1:3	0:20	C	A	0
.	.	.	0.0	1.0	1.0,D	1,D	.	4.09	.	.	SON	GT:/	0/1:8	0:81	T	G	0
.	.	.	.	.	.	.	.	.	.	.	SON	GT:/	0/1:1	0:55	T	C	0
.	.	.	.	.	.	.	.	.	.	.	SON	GT:/	0/1:3	0:14	T	C	0
.	.	.	0.0(	1.0	1.0,D	1,D	5	3.25	.	.	SON	GT:/	0/1:1	0:63	G	A	0
.	##	0	0.0	0.3(	0.517	1,D	5.7	4.40	0	.	DB;S	GT:/	0/1:2	0:19	C	T	0
0.006	0	0	.	.	.	.	.	.	.	.	DB;S	GT:/	0/1:2	0:15	G	A	0
.	.	.	.	.	.	.	.	.	.	.	SON	GT:/	0/1:1	0:86	T	A	0
.	.	.	.	.	.	.	.	.	.	.	SON	GT:/	0/1:1	0:62	G	A	0
.	.	.	.	.	.	.	.	.	.	.	SON	GT:/	0/1:5	0:33	G	A	0
.	.	.	.	.	.	.	.	1.37	.	.	SON	GT:/	0/1:4	0:47	C	T	0
.	.	.	.	.	.	.	.	1.50	.	0	SON	GT:/	0/1:5	0:36	G	A	0
.	.	.	.	.	.	.	.	.	0	.	SON	GT:/	0/1:3	0:31	G	A	0
.	.	.	0.1	0.6	0.623	0.884	.	2.43	.	.	SON	GT:/	0/1:1	0:14	A	G	0
.	.	.	0.0(	0.6	0.978	1,D	4.4	3.66	.	.	SON	GT:/	0/1:1	0:11	C	T	0
.	.	.	.	.	.	.	.	.	0	0	SON	GT:/	0/1:1	0:10	A	C	0
.	.	.	.	.	.	.	.	.	0	.	SON	GT:/	0/1:5	0:41	T	A	0
.	.	.	.	.	.	.	.	.	.	.	SON	GT:/	0/1:1	0:13	G	A	0
.	.	.	.	.	.	.	2.3	2.10	.	.	SON	GT:/	0/1:1	0:70	T	C	0
.	.	.	.	.	.	.	.	.	.	.	SON	GT:/	0/1:1	0:98	C	A	0
.	.	.	.	.	.	.	.	.	0	.	SON	GT:/	0/1:6	0:37	A	C	0
.	.	.	.	.	.	.	.	.	.	.	SON	GT:/	0/1:4	0:30	C	A	0
.	.	.	.	.	.	.	2.2	1.33	.	.	SON	GT:/	0/1:5	0:40	C	T	0
.	.	.	.	.	.	.	.	.	0	.	SON	GT:/	0/1:1	0:53	C	T	0
.	.	.	.	.	.	.	3	1.38	.	.	SON	GT:/	0/1:1	0:66	C	A	0
.	.	.	.	.	.	.	.	.	.	.	SON	GT:/	0/1:1	0:86	C	G	0
.	.	.	.	.	.	.	.	.	.	.	SON	GT:/	0/1:1	0:18	T	A	0
.	.	.	.	.	.	.	.	.	.	.	SON	GT:/	0/1:3	0:13	T	C	0
.	.	.	.	.	.	.	.	.	.	.	SON	GT:/	0/1:2	0:9,(	A	T	0
.	##	0	0.7	0.0(	0.0,B	0.989	.	.	.	.	SON	GT:/	0/1:2	0:10	C	T	0
.	.	.	0.0(	0.9	1.0,D	1.000	4.9	3.96	.	.	SON	GT:/	0/1:9	0:55	A	T	0
.	.	.	.	.	.	.	.	.	.	.	SON	GT:/	0/1:2	0:19	G	A	0
.	.	.	.	.	.	.	.	1.73	.	.	SON	GT:/	0/1:1	0:75	C	T	0
.	.	.	.	.	.	.	.	.	.	.	SON	GT:/	0/1:1	0:72	G	A	0
.	.	.	.	.	.	.	.	.	.	.	SON	GT:/	0/1:4	0:29	G	A	0
.	##	0	.	.	.	.	.	.	.	.	SON	GT:/	0/1:1	0:12	G	A	0
.	.	.	0.0(	0.9	1.0,D	1.000	4	5.08	.	.	SON	GT:/	0/1:1	0:15	G	A	0
2E-04	##	0	.	.	.	.	.	.	.	.	SON	GT:/	0/1:1	0:52	G	A	0
.	.	.	0.0(	0.0	0.014	1.000	.	3.33	.	.	SON	GT:/	0/1:8	0:56	G	T	0
.	.	.	.	.	.	.	.	.	.	.	SON	GT:/	0/1:3	0:26	C	T	0
.	.	.	0.0(	0.4	0.93,I	1.000	.	3.58	.	.	SON	GT:/	0/1:2	0:19	T	G	0
.	.	.	.	.	.	.	.	.	.	.	SON	GT:/	0/1:1	0:12	G	A	0
.	.	.	.	.	.	.	.	.	.	.	SON	GT:/	0/1:1	0:72	C	G	0
.	.	.	.	.	.	.	.	.	.	.	SON	GT:/	0/1:5	0:37	G	A	0
.	.	.	.	0.9	0.999	1,N	2.4	1.61	.	.	SON	GT:/	0/1:1	0:11	C	T	0
.	0	0	.	.	.	.	.	1.38	.	0	SON	GT:/	0/1:9	0:65	C	T	0
.	.	.	.	.	.	.	.	.	.	.	SON	GT:/	0/1:1	0:10	C	T	0
.	##	0	.	.	.	.	.	.	.	.	SON	GT:/	0/1:1	0:52	C	T	0
.	.	.	.	.	.	.	.	.	.	.	SON	GT:/	0/1:4	0:23	C	A	0
.	0	0	0.0	0.9	1.0,D	1,D	4.8	3.91	.	.	SON	GT:/	0/1:2	0:10	G	A	0

.	.	.	.	.	.	.	.	.	.	.	SOM	GT:/	0/1:2	0:15	A	G	0
.	.	.	.	.	.	.	.	.	.	.	SOM	GT:/	0:39	0/1:3	C	T	0
.	.	.	.	.	.	.	.	.	0.3	0.4	DB;S	GT:/	0:34	0/1:8	T	C	0
.	.	.	.	.	.	.	.	.	0	.	SOM	GT:/	0:86	0/1:8	C	T	0
#####	##	0	0.0	0.9	1.0,D	1.000	2.9	3.92	.	.	SOM	GT:/	0:35	0/1:8	G	A	0
.	.	.	.	.	.	1,A	5.9	5.29	.	.	SOM	GT:/	0:30	0/1:6	G	T	0
.	.	.	.	.	.	.	.	.	0.1	0.1	DB;S	GT:/	0:33	0/1:7	T	C	0
.	.	.	.	.	.	.	.	.	0	0.1	SOM	GT:/	0:8,0	0/1:9	A	G	0
.	.	.	.	.	.	.	.	.	0	0	SOM	GT:/	0:8,0	0/1:2	A	G	0
.	0	0	.	.	.	.	.	.	0	0	SOM	GT:/	0:19	0/1:2	C	T	0
.	0	0	.	.	.	.	.	.	0.1	0	SOM	GT:/	0:35	0/1:3	C	T	0
.	.	.	.	.	.	.	.	.	0	.	SOM	GT:/	0:42	0/1:1	A	G	0
#####	##	0	.	.	.	.	.	.	.	.	SOM	GT:/	0:12	0/1:1	C	T	0
.	.	.	.	.	.	.	.	.	.	.	SOM	GT:/	0:95	0/1:9	G	C	0
.	.	.	0.0	0.9	0.992	1,D	3.6	3.92	.	.	SOM	GT:/	0:12	0/1:1	C	G	0
.	.	.	0.0	1.0	1.0,D	1,D	4.4	4.19	.	.	SOM	GT:/	0:78	0/1:2	G	A	0
.	0	0	.	.	.	.	.	.	.	.	SOM	GT:/	0:13	0/1:9	G	A	0
.	.	.	.	.	.	.	.	.	.	.	SOM	GT:/	0:41	0/1:2	G	A	0
.	.	.	.	.	.	.	.	2.12	.	.	SOM	GT:/	0:37	0/1:6	C	A	0
.	.	.	.	.	.	.	.	.	.	.	SOM	GT:/	0:11	0/1:2	G	T	0
.	.	.	.	.	.	.	.	.	.	.	SOM	GT:/	0:34	0/1:5	G	A	0
.	.	.	.	.	.	.	.	.	.	.	SOM	GT:/	0:37	0/1:3	A	G	0
.	.	.	.	.	.	.	.	.	0	.	DB;S	GT:/	0:55	0/1:3	A	G	0
.	.	.	.	.	.	.	.	.	.	.	SOM	GT:/	0:53	0/1:3	A	C	0
#####	##	0	.	.	.	.	.	1.89	.	.	SOM	GT:/	0:98	0/1:4	G	A	0
.	.	.	.	.	.	.	.	2.87	.	.	SOM	GT:/	0:49	0/1:4	G	T	0
.	.	.	0.0	0.4	0.9,P	1,D	6.2	4.76	.	.	SOM	GT:/	0:12	0/1:8	G	A	0
.	.	.	.	.	.	.	5.7	.	.	.	SOM	GT:/	0:20	0/1:2	G	A	0
.	.	.	0.0	0.8	0.998	1.000	5.7	1.95	.	.	SOM	GT:/	0:20	0/1:2	G	T	0
.	.	.	.	.	.	.	.	.	.	.	SOM	GT:/	0:91	0/1:1	C	T	0
.	.	.	.	.	.	.	.	.	.	.	SOM	GT:/	0:10	0/1:1	T	C	0
.	.	.	.	.	.	.	.	.	.	.	SOM	GT:/	0:52	0/1:8	T	C	0
.	.	.	.	.	.	.	.	.	0.2	0.2	DB;S	GT:/	0:36	0/1:2	C	T	0
.	.	.	.	.	.	.	.	.	0	0	SOM	GT:/	0:8,0	0/1:1	C	T	0
.	.	.	0.5	0.0	0.0,B	0.999	2.9	.	.	.	SOM	GT:/	0:55	0/1:8	G	A	0
.	.	.	.	.	.	.	2.2	.	.	.	SOM	GT:/	0:33	0/1:7	A	G	0
#####	##	0	0.2	0.9	0.999	1.000	5.7	4.26	.	.	SOM	GT:/	0:52	0/1:1	C	T	0
.	.	.	0.0	1.0	1.0,D	1,D	5.8	4.93	.	.	SOM	GT:/	0:33	0/1:5	C	T	0
.	.	.	.	.	.	.	.	.	.	.	SOM	GT:/	0:39	0/1:2	A	G	0
.	.	.	.	.	.	.	2.5	.	.	.	SOM	GT:/	0:34	0/1:3	G	C	0
.	.	.	.	.	.	.	.	.	.	.	SOM	GT:/	0:25	0/1:1	C	A	0
.	.	.	.	.	.	.	.	.	.	.	SOM	GT:/	0:31	0/1:1	C	G	0
.	.	.	.	.	.	.	.	.	.	.	SOM	GT:/	0:8,0	0/1:1	C	G	0
.	.	.	.	.	.	.	4.9	4.04	.	.	SOM	GT:/	0:19	0/1:4	C	T	0
.	.	.	.	.	.	.	.	.	.	.	SOM	GT:/	0:34	0/1:2	A	T	0
.	.	.	.	.	.	.	.	.	0.1	0	SOM	GT:/	0:8,0	0/1:1	G	C	0
.	.	.	.	.	.	.	.	.	.	.	SOM	GT:/	0:12	0/1:2	G	C	0
.	.	.	.	.	.	.	.	.	.	.	SOM	GT:/	0:17	0/1:3	C	G	0
.	.	.	.	.	.	.	.	.	.	.	SOM	GT:/	0:11	0/1:6	C	A	0
.	.	.	0.1	0.4	0.876	1.000	5.7	2.17	.	.	SOM	GT:/	0:21	0/1:1	T	C	0
.	.	.	.	.	.	.	.	.	.	.	SOM	GT:/	0:54	0/1:7	G	A	0
.	.	.	.	.	.	.	2.3	.	.	.	SOM	GT:/	0:23	0/1:5	T	G	0
.	.	.	.	.	.	.	4.6	1.39	.	.	SOM	GT:/	0:10	0/1:8	G	A	0
.	.	.	.	.	.	.	.	.	.	.	SOM	GT:/	0:26	0/1:6	T	A	0

.	.	.	0.0	0.9	1.0,D	1,D	4.7	4.84	.	.	SOM	GT:/	0:15	0/1:	G	C	0
.	.	.	.	.	.	.	.	.	0	.	SOM	GT:/	0:16	0/1:	C	T	0
.	.	.	.	.	.	.	5	.	.	.	SOM	GT:/	0:27	0/1:	G	A	0
.	.	.	.	.	.	.	.	.	.	.	SOM	GT:/	0:43	0/1:	G	A	0
.	.	.	0.0	0.9	1.0,D	1,D	3.8	5.83	.	.	SOM	GT:/	0:11	0/1:	G	C	0
.	.	.	.	.	.	.	.	.	.	.	SOM	GT:/	0:21	0/1:	T	C	0
.	.	.	.	.	.	.	.	.	.	.	SOM	GT:/	0:36	0/1:	G	C	0
.	.	.	.	.	.	.	.	.	.	.	SOM	GT:/	0:14	0/1:	C	T	0
.	.	.	.	0.0	0.509	0.937	5.8	2.26	.	.	SOM	GT:/	0:59	0/1:	C	T	0
.	.	.	0.0	0.2	0.957	1,D	5.9	5.10	.	.	SOM	GT:/	0:21	0/1:	C	T	0
.	.	.	0.0	1.0	1.0,D	1.000	5.7	5.01	.	.	SOM	GT:/	0:14	0/1:	C	T	0
.	.	.	.	.	.	.	.	.	0	0.1	SOM	GT:/	0:8,	0/1:	G	A	0
#####	##	0	.	.	.	.	4.5	2.74	.	.	SOM	GT:/	0:82	0/1:	C	T	0
.	.	.	.	.	.	.	.	.	.	.	SOM	GT:/	0:10	0/1:	G	A	0
#####	##	0	0.0	0.9	1.0,D	1.000	5.6	4.24	.	.	SOM	GT:/	0:19	0/1:	C	T	0
.	.	.	.	.	.	.	.	.	.	.	SOM	GT:/	0:29	0/1:	C	T	0
.	.	.	.	.	.	.	4.6	.	.	.	SOM	GT:/	0:15	0/1:	A	T	0
.	.	.	.	.	.	.	.	.	.	.	SOM	GT:/	0:17	0/1:	A	G	0
.	.	.	.	.	.	1,A	5.5	6.00	.	.	SOM	GT:/	0:28	0/1:	T	A	0
.	.	.	.	.	.	.	.	.	.	.	SOM	GT:/	0:30	0/1:	T	G	0
.	.	.	.	.	.	.	.	.	0	.	SOM	GT:/	0:9,	0/1:	A	C	0
.	.	.	.	.	.	.	.	.	0	.	SOM	GT:/	0:25	0/1:	A	C	0
.	.	.	.	.	.	.	.	.	.	.	SOM	GT:/	0:19	0/1:	C	T	0
.	.	.	.	.	.	.	.	.	.	.	SOM	GT:/	0:31	0/1:	T	A	0
.	.	.	.	.	.	.	.	.	.	.	SOM	GT:/	0:9,	0/1:	C	T	0
#####	##	0	.	.	.	.	.	.	.	.	SOM	GT:/	0:58	0/1:	C	T	0
.	.	.	.	.	.	.	.	.	.	.	SOM	GT:/	0:21	0/1:	C	T	0
.	.	.	.	.	.	1,D	5.6	6.86	.	.	SOM	GT:/	0:38	0/1:	G	A	0
.	.	.	.	.	.	.	.	.	.	.	SOM	GT:/	0:59	0/1:	G	A	0
.	.	.	.	.	.	.	.	.	.	.	SOM	GT:/	0:76	0/1:	A	C	0
.	.	.	0.0	0.9	1.0,D	0.957	4.9	3.23	.	.	SOM	GT:/	0:40	0/1:	G	C	0
.	.	.	.	.	.	.	.	.	.	.	SOM	GT:/	0:42	0/1:	T	C	0
.	.	.	.	.	.	.	.	1.39	.	.	SOM	GT:/	0:17	0/1:	G	A	0
.	##	0	0.0	0.7	0.988	1,D	2.5	3.43	0	.	SOM	GT:/	0:59	0/1:	C	T	0
.	##	0	.	.	.	.	.	.	.	.	SOM	GT:/	0:29	0/1:	C	T	0
.	.	.	0.3	0.0	0.059	0.991	4.9	1.32	.	.	SOM	GT:/	0:63	0/1:	T	C	0
.	0	0	.	.	.	.	4	1.94	0.1	0	DB;	GT:/	0:43	0/1:	A	G	0
.	.	.	.	.	.	.	.	.	0	.	SOM	GT:/	0:57	0/1:	G	A	0
.	.	.	0.1	0.4	0.392	0.867	3.2	2.29	.	.	SOM	GT:/	0:15	0/1:	C	G	0
.	##	0	0.0	0.9	0.999	1,D	5.8	4.07	0	.	SOM	GT:/	0:40	0/1:	C	T	0
.	0	0	.	.	.	.	.	1.62	.	.	SOM	GT:/	0:16	0/1:	A	C	0
.	0	0	.	.	.	.	.	.	0	.	SOM	GT:/	0:43	0/1:	C	G	0
.	.	.	.	.	.	.	.	.	.	.	SOM	GT:/	0:18	0/1:	G	A	0
.	.	.	.	.	.	.	.	.	.	.	SOM	GT:/	0:15	0/1:	C	T	0
.	.	.	.	.	.	.	.	.	0	.	SOM	GT:/	0:39	0/1:	T	A	0
.	.	.	.	.	.	.	.	.	0	0	SOM	GT:/	0:38	0/1:	A	G	0
.	.	.	0.1	0.8	0.996	0.999	2.8	2.64	.	.	SOM	GT:/	0:75	0/1:	C	T	0
.	.	.	.	.	.	.	.	.	.	0	SOM	GT:/	0:37	0/1:	A	T	0
.	##	0	.	.	.	.	.	.	.	.	SOM	GT:/	0:78	0/1:	C	T	0
#####	##	0	.	.	.	.	.	.	0	0	SOM	GT:/	0:10	0/1:	G	A	0
.	.	.	.	.	.	.	.	1.69	.	.	SOM	GT:/	0:39	0/1:	T	C	0
.	.	.	.	.	.	.	.	.	0	.	SOM	GT:/	0:90	0/1:	C	T	0
.	.	.	.	.	.	.	2	1.99	.	.	SOM	GT:/	0:19	0/1:	C	G	0
.	.	.	.	.	.	.	.	.	.	.	SOM	GT:/	0:11	0/1:	G	A	0

.	0	0	.	.	.	.	.	.	.	.	SOM	GT:	0:14	0/1:	T	C	0
.	.	.	.	.	.	.	.	.	.	.	SOM	GT:	0:34	0/1:	C	T	0
4E-04	##	0	.	.	.	.	.	.	.	.	DB;	GT:	0:21	0/1:	C	T	0
.	.	.	.	.	.	.	.	.	.	.	SOM	GT:	0:11	0/1:	G	A	0
.	##	0	.	.	.	.	.	.	.	.	SOM	GT:	0:19	0/1:	G	A	0
.	.	.	.	.	.	.	.	.	.	.	SOM	GT:	0:14	0/1:	C	T	0
#####	##	0	0.7	0.0	0.596	1,N	.	.	.	.	SOM	GT:	0:25	0/1:	C	T	0
.	.	.	.	.	.	.	.	.	.	.	SOM	GT:	0:45	0/1:	C	G	0
.	.	.	.	.	.	.	.	.	.	.	SOM	GT:	0:44	0/1:	C	G	0
.	.	.	.	.	.	.	.	.	.	.	SOM	GT:	0:49	0/1:	C	T	0
.	.	.	.	.	.	.	.	.	.	.	SOM	GT:	0:47	0/1:	C	T	0
.	.	.	.	.	.	.	.	.	.	.	SOM	GT:	0:28	0/1:	A	C	0
.	.	.	.	.	.	.	3.5	2.39	0	.	SOM	GT:	0:65	0/1:	C	T	0
.	.	.	.	.	.	.	3.8	2.10	.	.	SOM	GT:	0:44	0/1:	G	A	0
.	.	.	.	.	.	.	.	.	.	.	SOM	GT:	0:66	0/1:	G	A	0
.	.	.	.	.	.	.	.	.	.	.	SOM	GT:	0:62	0/1:	G	T	0
.	.	.	.	.	.	.	.	.	.	.	SOM	GT:	0:8,	0/1:	C	A	0
.	.	.	.	0.9	1.0,D	1,D	4.3	3.43	.	.	SOM	GT:	0:36	0/1:	C	T	0
.	.	.	0.1	0.0	0.018	1,N	.	1.37	.	.	SOM	GT:	0:60	0/1:	A	C	0
.	.	.	.	.	.	.	.	.	.	.	SOM	GT:	0:20	0/1:	C	A	0
.	.	.	.	.	.	.	.	.	.	.	SOM	GT:	0:35	0/1:	G	C	0
.	.	.	.	.	.	1,D	4.7	11.6	.	.	SOM	GT:	0:83	0/1:	G	A	0
.	.	.	0.0	0.7	0.977	1,N	5.1	3.83	.	.	SOM	GT:	0:92	0/1:	G	A	0
.	.	.	.	.	.	1,D	.	.	.	.	SOM	GT:	0:11	0/1:	C	T	0
.	.	.	.	.	.	.	.	.	.	.	SOM	GT:	0:36	0/1:	C	A	0
.	.	.	.	.	.	.	.	.	.	.	SOM	GT:	0:52	0/1:	C	A	0
.	0	0	0.0	0.9	0.999	1.000	3	2.89	.	0	SOM	GT:	0:96	0/1:	C	T	0
.	.	.	.	.	.	.	.	.	0	.	SOM	GT:	0:9,	0/1:	G	A	0
.	.	.	.	.	.	.	.	.	0.2	0	SOM	GT:	0:37	0/1:	A	G	0
.	.	.	.	.	.	.	.	.	0	0	SOM	GT:	0:12	0/1:	A	C	0
.	.	.	.	.	.	.	.	.	.	.	DB;	GT:	0:30	0/1:	C	T	0
.	.	.	.	.	.	.	.	.	0.4	0.4	DB;	GT:	0:52	0/1:	A	G	0
.	.	.	.	.	.	1,A	3.1	4.59	.	.	SOM	GT:	0:17	0/1:	C	T	0
.	.	.	0.1	0.0	0.104	0.946	5.4	2.06	.	.	SOM	GT:	0:83	0/1:	C	T	0
.	.	.	0.0	1.0	1.0,D	0.964	3.8	2.70	.	.	SOM	GT:	0:44	0/1:	G	T	0
.	.	.	.	.	.	.	.	.	.	.	SOM	GT:	0:16	0/1:	T	A	0
.	.	.	.	.	.	.	.	.	0.5	0.5	DB;	GT:	0:53	0/1:	T	C	0
.	.	.	.	.	.	.	.	.	.	.	SOM	GT:	0:39	0/1:	T	G	0
.	.	.	.	.	.	.	3	.	0	.	SOM	GT:	0:11	0/1:	G	A	0
.	0	0	.	.	.	.	.	.	.	.	SOM	GT:	0:15	0/1:	C	T	0
.	.	.	.	.	.	.	4.6	.	.	.	SOM	GT:	0:15	0/1:	T	A	0
.	##	0	.	.	.	.	.	.	.	.	SOM	GT:	0:22	0/1:	C	T	0
.	.	.	.	.	.	.	5.6	.	.	.	SOM	GT:	0:23	0/1:	C	T	0
.	.	.	.	.	.	.	.	.	0	0	SOM	GT:	0:51	0/1:	A	T	0
.	.	.	.	.	.	.	.	.	0.1	0.1	SOM	GT:	0:22	0/1:	A	C	0
.	.	.	.	.	.	.	.	.	.	.	SOM	GT:	0:94	0/1:	C	A	0
.	.	.	.	.	.	.	.	.	.	.	SOM	GT:	0:11	0/1:	C	T	0
.	.	.	.	.	.	.	.	.	.	.	SOM	GT:	0:50	0/1:	C	T	0
.	.	.	.	.	.	.	.	.	.	.	SOM	GT:	0:67	0/1:	T	G	0
.	.	.	.	.	.	.	.	.	.	.	SOM	GT:	0:11	0/1:	G	T	0
.	.	.	.	.	.	.	.	.	.	.	SOM	GT:	0:26	0/1:	A	T	0
.	.	.	0.0	0.8	0.993	1.000	5.4	4.17	.	.	SOM	GT:	0:36	0/1:	G	A	0
.	.	.	.	.	.	.	.	.	.	.	SOM	GT:	0:54	0/1:	G	A	0
.	.	.	0.0	0.9	1.0,D	1.000	5.1	3.49	.	.	SOM	GT:	0:22	0/1:	C	T	0

.	.	.	.	.	.	.	.	.	.	.	SOM	GT:/	0:22	0/1:3	C	T	0
.	.	.	0.0	0.9	1.0,D	1.000	3.1	3.33	.	.	SOM	GT:/	0:48	0/1:4	C	A	0
.	.	.	0.1	0.0	0.002	.	2.1	1.30	.	.	SOM	GT:/	0:56	0/1:5	G	T	0
.	.	.	.	.	.	.	.	.	.	.	SOM	GT:/	0:37	0/1:3	G	A	0
.	.	.	.	.	.	.	.	.	0	0.1	SOM	GT:/	0:9,(	0/1:1	T	C	0
.	.	.	0.0	0.9	1.0,D	1,D	5.5	4.92	.	.	SOM	GT:/	0:95	0/1:1	A	T	0
.	.	.	.	.	.	.	.	.	0	0.1	SOM	GT:/	0:21	0/1:3	G	T	0
.	.	.	.	.	.	.	.	.	0	0	DB;S	GT:/	0:45	0/1:1	G	T	0
.	.	.	.	.	.	.	.	1.59	.	.	SOM	GT:/	0:12	0/1:8	A	T	0
.	.	.	.	.	.	.	.	.	.	.	SOM	GT:/	0:46	0/1:3	G	A	0
.	.	.	.	.	.	.	3.5	2.23	.	.	SOM	GT:/	0:28	0/1:3	C	A	0
.	.	.	.	.	.	.	.	.	.	.	SOM	GT:/	0:68	0/1:5	C	T	0
.	.	.	.	.	.	.	.	.	.	.	SOM	GT:/	0:43	0/1:2	C	G	0
.	.	.	.	.	.	.	.	.	0	0	SOM	GT:/	0:12	0/1:1	G	A	0
.	.	.	.	.	.	.	4.7	3.12	.	.	SOM	GT:/	0:17	0/1:1	G	A	0
.	.	.	.	.	.	.	.	.	.	.	SOM	GT:/	0:8,(	0/1:1	C	G	0
.	.	.	.	.	.	.	.	.	.	.	SOM	GT:/	0:12	0/1:1	C	G	0
2E-04	##	0	.	.	.	.	.	.	.	.	SOM	GT:/	0:96	0/1:1	C	T	0
.	.	.	.	.	.	.	.	2.91	.	.	SOM	GT:/	0:80	0/1:9	T	C	0
.	.	.	0.4	0.0	0.002	1,D	4.7	1.64	.	.	SOM	GT:/	0:60	0/1:6	A	G	0
.	.	.	.	.	.	.	.	1.87	.	.	SOM	GT:/	0:31	0/1:4	T	G	0
.	.	.	.	.	.	.	.	1.92	.	.	SOM	GT:/	0:15	0/1:2	G	A	0
0.01	0	0	.	.	.	.	4.6	.	0	.	SOM	GT:/	0:24	0/1:4	C	T	0
.	##	0	0.0	0.9	0.991	1,D	5.8	5.70	.	.	DB;S	GT:/	0:28	0/1:3	G	A	0
.	.	.	.	.	.	.	.	.	.	.	SOM	GT:/	0:79	0/1:1	T	G	0
.	.	.	.	.	.	.	.	.	.	0	DB;S	GT:/	0:55	0/1:9	C	T	0
.	.	.	.	.	.	.	4.1	.	.	.	SOM	GT:/	0:30	0/1:3	C	T	0
.	.	.	.	.	.	.	.	.	0	.	SOM	GT:/	0:63	0/1:7	G	A	0
.	.	.	.	.	.	.	.	.	.	.	SOM	GT:/	0:19	0/1:1	G	T	0
.	.	.	.	.	.	.	.	.	.	.	SOM	GT:/	0:15	0/1:1	G	C	0
.	.	.	.	.	.	.	.	.	.	.	SOM	GT:/	0:12	0/1:2	C	G	0
.	.	.	.	.	.	.	.	.	.	.	SOM	GT:/	0:76	0/1:5	C	T	0
.	.	.	.	.	.	1,D	5.3	4.66	.	.	SOM	GT:/	0:16	0/1:2	G	A	0
.	.	.	.	.	.	.	.	1.74	.	.	SOM	GT:/	0:91	0/1:7	G	A	0
.	.	.	.	.	.	.	.	.	.	.	SOM	GT:/	0:17	0/1:2	A	T	0
.	##	0	0.0	0.9	1.0,D	1,N	3.2	1.65	.	.	SOM	GT:/	0:25	0/1:2	C	T	0
.	.	.	.	.	.	.	.	.	.	.	SOM	GT:/	0:68	0/1:7	C	T	0
.	.	.	.	.	.	1,A	.	5.99	.	.	SOM	GT:/	0:79	0/1:6	G	A	0
#####	##	0	0.0	0.9	1.0,D	0.877	5	3.10	.	.	SOM	GT:/	0:12	0/1:1	C	T	0
.	.	.	.	.	.	.	.	.	.	.	SOM	GT:/	0:20	0/1:1	C	T	0
.	0	0	0.0	0.8	0.997	0.998	5.5	3.85	.	.	SOM	GT:/	0:27	0/1:3	T	G	0
#####	##	0	.	.	.	.	.	.	.	.	SOM	GT:/	0:33	0/1:4	C	T	0
.	.	.	.	.	.	.	.	.	.	.	SOM	GT:/	0:73	0/1:7	G	A	0
.	.	.	.	.	.	.	6	4.00	.	.	SOM	GT:/	0:62	0/1:6	G	A	0
2E-04	0	0	0.0	1.0	1.0,D	1.000	6	5.21	.	.	SOM	GT:/	0:23	0/1:2	G	A	0
.	.	.	.	.	.	.	.	.	0.1	0.1	SOM	GT:/	0:12	0/1:5	G	C	0
.	##	0	.	.	.	.	.	.	.	.	SOM	GT:/	0:30	0/1:4	A	G	0
.	.	.	0.3	0.0	0.006	0.999	.	1.83	.	.	SOM	GT:/	0:12	0/1:1	T	C	0
.	.	.	.	.	.	.	.	.	0.1	0	SOM	GT:/	0:13	0/1:2	G	A	0
.	.	.	.	.	.	.	.	.	0	0	SOM	GT:/	0:9,(	0/1:2	C	T	0
.	.	.	.	.	.	.	.	.	.	.	SOM	GT:/	0:20	0/1:1	C	G	0
.	.	.	.	.	.	.	.	.	.	.	SOM	GT:/	0:86	0/1:9	C	T	0
.	0	0	0.1	.	.	1,N	.	.	0	.	SOM	GT:/	0:25	0/1:4	G	A	0
.	.	.	.	.	.	.	.	.	.	.	SOM	GT:/	0:10	0/1:2	A	C	0

0.017	0	0	.	.	.	.	2.3	1.34	0	0	SOM	GT:/	0:89	0/1:1	A	T	0
.	.	.	.	.	.	.	.	.	.	.	SOM	GT:/	0:16	0/1:2	G	A	0
.	.	.	.	.	.	.	.	.	.	.	SOM	GT:/	0:23	0/1:3	G	A	0
.	0	0	.	.	.	.	.	.	0	0.2	SOM	GT:/	0:15	0/1:2	C	A	0
.	.	.	.	.	.	.	.	.	0	.	SOM	GT:/	0:13	0/1:2	A	C	0
.	.	.	.	.	.	.	.	.	.	.	SOM	GT:/	0:16	0/1:3	T	C	0
.	.	.	0.0(	0.9!	1.0,D	1,D	5.3	5.19	.	.	SOM	GT:/	0:15	0/1:2	G	A	0
.	.	.	.	.	.	.	.	.	.	.	SOM	GT:/	0:9,(	0/1:7	G	A	0
.	.	.	.	.	.	.	.	.	.	0	SOM	GT:/	0:11	0/1:1	A	T	0
#####	##	0	0.0(	0.9!	1.0,D	1.000	4.8	4.42	.	.	SOM	GT:/	0:61	0/1:6	C	T	0
.	.	.	.	.	.	.	.	.	.	.	SOM	GT:/	0:23	0/1:9	G	A	0
.	.	.	.	1.0.	1.0,D	.	4.4	1.95	.	.	SOM	GT:/	0:58	0/1:2	G	A	0
.	.	.	.	.	.	.	5.6	.	.	.	SOM	GT:/	0:21	0/1:3	G	T	0
.	.	.	.	.	.	.	.	1.54	.	.	SOM	GT:/	0:8,(	0/1:1	T	C	0
.	.	.	.	.	.	.	.	.	0	.	SOM	GT:/	0:10	0/1:5	G	T	0
.	##	0	.	.	.	.	.	.	.	.	SOM	GT:/	0:37	0/1:3	G	A	0
.	.	.	0.4(	0.8:	0.826	1,D	6.1	3.43	.	.	SOM	GT:/	0:17	0/1:2	A	G	0
.	.	.	.	.	.	.	.	.	.	.	SOM	GT:/	0:27	0/1:3	G	A	0
.	.	.	0.0·	0.0.	0.002	1,D	6	4.31	.	.	SOM	GT:/	0:45	0/1:2	G	T	0
.	.	.	.	.	.	.	.	.	0.1	0.3	DB;S	GT:/	0:26	0/1:9	G	A	0
.	.	.	.	.	.	.	.	.	.	.	SOM	GT:/	0:12	0/1:1	C	T	0
.	.	.	.	.	.	.	.	1.27	.	.	SOM	GT:/	0:28	0/1:3	G	C	0
.	.	.	.	.	.	.	.	.	.	.	SOM	GT:/	0:11	0/1:1	G	A	0
.	##	0	.	.	.	.	.	.	.	0	SOM	GT:/	0:90	0/1:1	G	A	0
.	.	.	.	.	.	.	2.4	.	.	.	SOM	GT:/	0:41	0/1:3	C	T	0
.	.	.	.	.	.	.	.	.	.	.	SOM	GT:/	0:68	0/1:4	G	A	0
.	.	.	.	.	.	.	.	.	0	.	SOM	GT:/	0:23	0/1:1	T	A	0
.	.	.	.	.	.	.	.	.	.	.	SOM	GT:/	0:8,(	0/1:1	C	T	0
.	.	.	.	.	.	.	.	.	0	0	SOM	GT:/	0:19	0/1:8	G	T	0
.	.	.	.	.	.	.	.	.	0	.	SOM	GT:/	0:8,(	0/1:2	A	T	0
.	.	.	.	0.9!	1.0,D	.	3.3	2.24	.	.	SOM	GT:/	0:11	0/1:2	G	A	0
.	.	.	.	.	.	.	.	.	0	.	SOM	GT:/	0:46	0/1:5	C	A	0
.	.	.	.	.	.	.	3.8	1.25	.	.	SOM	GT:/	0:30	0/1:5	A	T	0
.	.	.	.	.	.	.	2.3	1.46	.	.	SOM	GT:/	0:68	0/1:3	G	C	0
.	##	.	.	.	.	.	.	1.76	.	0	SOM	GT:/	0:31	0/1:2	A	G	0
.	.	.	0.0(	0.5!	0.594	0.995	4.3	3.78	.	.	SOM	GT:/	0:39	0/1:9	G	A	0
.	.	.	.	.	.	.	.	.	.	.	SOM	GT:/	0:31	0/1:1	C	G	0
.	.	.	.	.	.	.	2.7	.	.	.	SOM	GT:/	0:51	0/1:5	G	A	0
.	.	.	.	.	.	.	.	.	.	.	SOM	GT:/	0:20	0/1:1	C	G	0
.	.	.	.	.	.	.	.	.	.	.	SOM	GT:/	0:9,(	0/1:9	C	A	0
.	.	.	.	.	.	.	.	.	.	.	SOM	GT:/	0:44	0/1:5	G	A	0
.	.	.	.	.	.	.	.	.	0.1	0.2	SOM	GT:/	0:34	0/1:6	C	A	0
.	.	.	.	.	.	.	.	.	0	0	SOM	GT:/	0:48	0/1:3	C	A	0
.	##	0	0.0'	0.0(	0.003	1,N	4.5	1.47	.	.	SOM	GT:/	0:19	0/1:1	C	T	0
.	##	.	.	.	.	.	.	.	.	.	DB;S	GT:/	0:93	0/1:9	C	T	0
.	.	.	.	.	.	1,D	6	3.52	.	.	SOM	GT:/	0:51	0/1:4	C	A	0
.	.	.	0.0.	1.0.	1.0,D	1,D	5.5	3.84	.	.	SOM	GT:/	0:89	0/1:1	T	G	0
.	.	.	.	.	.	.	.	.	0	0.1	DB;S	GT:/	0:20	0/1:2	T	C	0
.	.	.	0.6:	0.0(	0.071	1.000	5.8	1.48	.	.	SOM	GT:/	0:34	0/1:3	T	C	0
.	.	.	.	.	.	.	.	.	.	.	SOM	GT:/	0:70	0/1:9	G	A	0
.	.	.	0.0(	1.0.	1.0,D	1,D	5.6	5.71	.	.	SOM	GT:/	0:44	0/1:3	G	A	0
.	.	.	.	.	.	.	.	.	.	.	SOM	GT:/	0:91	0/1:1	G	T	0
.	.	.	.	.	.	.	.	1.33	.	.	SOM	GT:/	0:8,(	0/1:1	T	C	0
.	.	.	.	.	.	.	.	.	.	.	SOM	GT:/	0:26	0/1:3	A	T	0

.	.	.	0.0	0.0	0.006	1,N	.	1.70	.	.	SOM	GT:/	0:30	0/1:2	C	T	0
2E-04	##	0	.	.	.	.	.	.	.	.	SOM	GT:/	0:74	0/1:8	C	T	0
.	.	.	0.0	0.5	0.73,I	1,D	5.8	5.49	.	.	SOM	GT:/	0:23	0/1:2	C	G	0
.	.	.	.	.	.	.	.	4.49	0	0	DB;S	GT:/	0:63	0/1:5	G	A	0
.	##	0	0.0	0.9	0.999	0.998	5.6	4.76	0	.	SOM	GT:/	0:21	0/1:2	G	A	0
.	##	0	.	.	.	1,A	.	5.58	.	.	SOM	GT:/	0:56	0/1:5	C	T	0
.	.	.	.	.	.	.	.	.	.	.	SOM	GT:/	0:13	0/1:1	G	A	0
.	##	0	.	.	.	.	.	.	0	.	SOM	GT:/	0:51	0/1:5	G	A	0
.	.	.	.	.	.	.	3.9	1.88	.	.	SOM	GT:/	0:46	0/1:5	A	G	0
.	.	.	.	.	.	.	.	.	.	.	SOM	GT:/	0:45	0/1:5	G	T	0
0.011	0	0	.	.	.	.	2.5	.	.	.	DB;S	GT:/	0:12	0/1:2	C	T	0
.	##	0	.	.	.	.	.	.	0.1	0.2	DB;S	GT:/	0:22	0/1:5	T	A	0
.	.	.	.	.	.	.	.	.	.	.	SOM	GT:/	0:29	0/1:9	C	G	0
.	.	.	.	.	.	.	.	.	.	.	SOM	GT:/	0:50	0/1:6	G	A	0
.	.	.	.	.	.	.	.	.	0	0	SOM	GT:/	0:13	0/1:1	G	A	0
.	.	.	.	.	.	.	.	.	.	.	SOM	GT:/	0:10	0/1:5	C	T	0
.	.	.	.	.	.	.	2.9	.	.	.	SOM	GT:/	0:46	0/1:5	C	T	0
.	.	.	.	.	.	.	.	.	.	.	SOM	GT:/	0:54	0/1:6	G	A	0
.	.	.	.	.	.	.	.	.	.	.	SOM	GT:/	0:21	0/1:3	A	G	0
.	.	.	.	.	.	.	.	.	.	.	SOM	GT:/	0:35	0/1:3	G	A	0
.	.	.	.	.	.	.	.	.	.	.	SOM	GT:/	0:66	0/1:5	C	G	0
.	.	.	.	.	.	.	.	.	.	.	SOM	GT:/	0:21	0/1:2	G	A	0
.	.	.	.	.	.	.	.	.	.	.	SOM	GT:/	0:36	0/1:5	A	C	0
.	.	.	.	.	.	.	.	.	.	.	SOM	GT:/	0:38	0/1:5	C	T	0
.	.	.	.	.	.	.	.	.	0	.	SOM	GT:/	0:23	0/1:2	C	G	0
.	.	.	.	.	.	.	.	.	.	.	SOM	GT:/	0:61	0/1:6	G	A	0
.	.	.	.	.	.	.	.	1.50	.	.	SOM	GT:/	0:53	0/1:6	C	T	0
.	.	.	.	.	.	1,A	.	.	.	.	SOM	GT:/	0:26	0/1:5	G	A	0
.	.	.	.	.	.	.	.	1.84	.	.	DB;S	GT:/	0:21	0/1:2	T	A	0
.	.	.	.	.	.	.	.	.	.	.	SOM	GT:/	0:43	0/1:9	G	A	0
.	.	.	.	.	.	.	.	.	0	0.1	SOM	GT:/	0:14	0/1:2	C	G	0
.	.	.	.	.	.	.	.	.	0.1	0.1	SOM	GT:/	0:12	0/1:1	C	A	0
.	.	.	.	.	.	.	.	.	0	0	SOM	GT:/	0:17	0/1:3	T	C	0
.	.	.	.	.	.	.	.	.	.	.	SOM	GT:/	0:8,(	0/1:9	G	T	0
.	##	0	.	.	.	.	.	1.27	.	.	SOM	GT:/	0:34	0/1:2	C	T	0
.	.	.	.	.	.	.	3.3	3.26	.	.	SOM	GT:/	0:90	0/1:1	C	T	0
.	.	.	0.0	0.9	0.999	1,D	4.9	5.51	.	.	SOM	GT:/	0:89	0/1:1	G	A	0
.	.	.	0.0	0.9	1.0,D	1,D	4.2	3.76	.	.	SOM	GT:/	0:10	0/1:1	G	A	0
.	.	.	.	.	.	.	.	.	0.1	0.2	SOM	GT:/	0:8,(	0/1:2	G	A	0
.	.	.	.	.	.	.	.	.	0	0.1	SOM	GT:/	0:34	0/1:4	A	G	0
.	.	.	.	.	.	.	.	.	.	.	SOM	GT:/	0:50	0/1:3	C	A	0
.	.	.	.	.	.	.	.	.	.	.	SOM	GT:/	0:80	0/1:9	T	C	0
.	##	0	0.0	0.1	0.958	1,N	3.7	1.71	.	.	SOM	GT:/	0:63	0/1:7	C	T	0
.	.	.	.	.	.	.	.	.	.	.	SOM	GT:/	0:42	0/1:4	C	T	0
.	.	.	.	.	.	.	.	.	.	.	SOM	GT:/	0:16	0/1:3	C	T	0
.	.	.	.	.	.	.	.	.	0	0	SOM	GT:/	0:20	0/1:3	T	C	0
.	.	.	0.0	1.0	1.0,D	1,D	5.2	4.19	.	.	SOM	GT:/	0:48	0/1:4	C	T	0
.	.	.	.	.	.	.	.	.	.	.	SOM	GT:/	0:45	0/1:2	G	A	0
.	.	.	.	.	.	.	.	.	.	.	SOM	GT:/	0:26	0/1:2	C	T	0
.	.	.	.	.	.	.	.	.	.	.	SOM	GT:/	0:46	0/1:5	C	T	0
.	.	.	.	.	.	.	.	.	0	.	SOM	GT:/	0:10	0/1:1	G	A	0
.	.	.	0.1	0.9	0.999	1,N	4	3.15	.	.	SOM	GT:/	0:15	0/1:2	C	T	0
2E-04	##	0	.	.	.	.	.	.	0	.	SOM	GT:/	0:14	0/1:2	G	A	0
.	.	.	.	.	.	1,A	4.8	8.04	.	.	SOM	GT:/	0:32	0/1:8	T	A	0

.	##	0	.	.	.	.	.	1.66	.	.	SOM	GT:/	0:18	0/1:4	C	T	0
.	##	0	.	.	.	.	.	.	.	.	SOM	GT:/	0:29	0/1:3	G	A	0
.	.	.	.	.	.	.	.	1.72	.	.	SOM	GT:/	0:16	0/1:3	G	A	0
.	.	.	0.6	0.0	0.001	0.987	3.8	1.70	.	.	SOM	GT:/	0:33	0/1:4	G	T	0
.	.	.	.	.	.	.	.	.	.	.	SOM	GT:/	0:24	0/1:6	G	C	0
.	.	.	.	.	.	.	.	.	.	.	SOM	GT:/	0:34	0/1:4	G	T	0
.	.	.	0.0	0.9	0.999	1,D	4.2	5.24	.	.	SOM	GT:/	0:22	0/1:2	G	A	0
.	.	.	.	.	.	.	.	.	0	0	DB;S	GT:/	0:25	0/1:3	T	G	0
.	.	.	.	.	.	.	.	.	0	0	SOM	GT:/	0:27	0/1:4	C	T	0
.	.	.	.	.	.	.	.	.	0.1	0.3	SOM	GT:/	0:9,(	0/1:3	C	T	0
.	.	.	.	.	.	.	.	.	0	0	SOM	GT:/	0:10	0/1:3	A	G	0
.	.	.	.	.	.	.	.	.	.	.	SOM	GT:/	0:35	0/1:4	G	T	0
.	.	.	.	.	.	.	.	1.68	0	.	SOM	GT:/	0:22	0/1:7	T	G	0
.	##	0	.	.	.	.	.	.	.	.	SOM	GT:/	0:31	0/1:5	C	T	0
.	##	0	0.0	1.0	1.0,D	1,D	2.7	3.85	.	.	SOM	GT:/	0:23	0/1:4	C	T	0
.	.	.	.	.	.	.	4.5	.	.	.	SOM	GT:/	0:12	0/1:1	G	A	0
.	.	.	.	.	.	.	.	1.48	.	.	SOM	GT:/	0:78	0/1:9	A	C	0
.	##	0	.	.	.	.	.	.	.	.	SOM	GT:/	0:23	0/1:2	C	T	0
0.011	0	0	0.0	0.1	0.899	0.996	4	3.11	0	.	DB;S	GT:/	0:16	0/1:1	C	T	0
.	.	.	.	.	.	.	.	.	.	.	SOM	GT:/	0:35	0/1:5	A	G	0
.	.	.	.	.	.	.	.	.	.	.	SOM	GT:/	0:41	0/1:7	C	A	0
.	.	.	0.0	0.9	1.0,D	1,D	5.7	5.49	.	.	SOM	GT:/	0:34	0/1:1	C	T	0
.	.	.	0.2	0.0	0.001	1.000	.	.	.	.	SOM	GT:/	0:56	0/1:4	T	A	0
.	.	.	0.0	0.9	1.0,D	1,D	5.8	4.83	.	.	SOM	GT:/	0:17	0/1:1	C	T	0
.	.	.	.	.	.	.	.	.	.	.	DB;S	GT:/	0:25	0/1:2	T	C	0
.	.	.	.	.	.	.	.	.	0	.	SOM	GT:/	0:27	0/1:3	A	G	0
.	.	.	.	.	.	.	3.4	.	.	.	SOM	GT:/	0:26	0/1:3	C	G	0
.	.	.	.	.	.	.	.	.	.	.	SOM	GT:/	0:18	0/1:2	T	G	0
.	.	.	.	.	.	.	.	.	0	0.1	SOM	GT:/	0:9,(	0/1:1	G	A	0
.	.	.	.	.	.	.	.	.	.	.	SOM	GT:/	0:25	0/1:8	G	C	0
.	.	.	.	.	.	.	.	2.50	.	.	SOM	GT:/	0:11	0/1:1	G	A	0
.	.	.	0.0	0.9	0.997	1.000	5.9	4.12	.	.	SOM	GT:/	0:62	0/1:7	G	A	0
.	.	.	.	.	.	.	4.1	.	.	.	SOM	GT:/	0:45	0/1:5	G	C	0
.	.	.	.	.	.	.	2.7	2.12	.	.	SOM	GT:/	0:79	0/1:8	C	A	0
.	.	.	.	.	.	.	2.5	.	.	.	SOM	GT:/	0:12	0/1:2	T	A	0
.	.	.	1.0	0.0	0.0,B	1,N	.	.	.	.	SOM	GT:/	0:31	0/1:5	G	T	0
.	##	0	0.0	0.0	0.313	0.999	2.5	2.21	.	.	SOM	GT:/	0:14	0/1:1	C	T	0
.	.	.	.	.	.	.	2.3	1.83	.	.	SOM	GT:/	0:28	0/1:2	T	A	0
.	.	.	0.0	0.0	0.665	1.000	4.9	1.86	.	.	SOM	GT:/	0:14	0/1:1	G	A	0
.	.	.	.	.	.	.	.	.	0	0	DB;S	GT:/	0:26	0/1:1	T	C	0
.	.	.	.	.	.	.	.	.	.	.	SOM	GT:/	0:40	0/1:3	C	G	0
.	.	.	.	.	.	.	.	.	.	.	SOM	GT:/	0:31	0/1:3	G	C	0
.	.	.	.	.	.	.	5	.	.	.	SOM	GT:/	0:16	0/1:1	C	T	0
.	.	.	0.0	.	.	0.982	.	2.98	.	.	SOM	GT:/	0:14	0/1:1	A	T	0
.	.	.	0.9	0.9	0.999	1.000	5.8	1.57	.	.	SOM	GT:/	0:76	0/1:8	T	C	0
.	.	.	.	.	.	.	.	.	.	.	SOM	GT:/	0:70	0/1:1	T	C	0
.	.	.	.	.	.	1,A	5.9	13.3	.	.	SOM	GT:/	0:43	0/1:9	G	A	0
.	.	.	.	.	.	1,D	.	2.38	.	.	SOM	GT:/	0:48	0/1:6	C	T	0
.	.	.	.	.	.	.	.	.	.	.	SOM	GT:/	0:53	0/1:1	C	T	0
.	.	.	.	.	.	.	.	1.26	0	.	SOM	GT:/	0:10	0/1:2	A	C	0
.	.	.	.	.	.	.	.	.	0.3	0.2	SOM	GT:/	0:8,(	0/1:2	G	A	0
.	.	.	.	.	.	.	2.1	.	.	.	SOM	GT:/	0:20	0/1:7	G	A	0
.	.	.	.	.	.	.	.	.	0	.	DB;S	GT:/	0:19	0/1:2	T	A	0
.	.	.	.	.	.	.	.	.	.	.	SOM	GT:/	0:35	0/1:3	C	A	0

.	##	0	0.9	0.0	0.025	0.979	4.8	.	.	.	SOM	GT:	0:48	0/1:	A	G	0
.	.	.	.	.	.	.	.	.	.	.	SOM	GT:	0:27	0/1:	G	A	0
.	##	0	0.1	0.0	0.019	1,N	.	.	.	.	SOM	GT:	0:52	0/1:	C	T	0
.	.	.	.	.	.	.	.	.	.	.	SOM	GT:	0:53	0/1:	C	A	0
.	.	.	.	.	.	.	.	3.00	.	.	SOM	GT:	0:11	0/1:	C	T	0
.	.	.	.	.	.	.	.	.	.	.	SOM	GT:	0:81	0/1:	C	T	0
.	.	.	.	.	.	.	.	.	.	.	SOM	GT:	0:22	0/1:	G	A	0
.	##	0	0.1	0.1	0.205	1.000	5.1	3.30	.	.	SOM	GT:	0:57	0/1:	G	A	0
.	.	.	.	.	.	.	3.7	.	.	.	SOM	GT:	0:14	0/1:	C	T	0
.	.	.	0.2	0.0	0.358	1,N	.	.	.	.	SOM	GT:	0:17	0/1:	T	G	0
.	.	.	.	.	.	.	.	.	.	.	SOM	GT:	0:19	0/1:	A	G	0
.	.	.	0.0	0.8	0.983	0.886	4.2	3.66	.	.	SOM	GT:	0:11	0/1:	C	T	0
.	.	.	.	.	.	.	.	.	0.1	0.2	SOM	GT:	0:37	0/1:	C	A	0
.	.	.	.	.	.	.	.	.	0	0.1	SOM	GT:	0:15	0/1:	G	T	0
.	.	.	0.0	.	.	1,D	4.6	1.63	.	.	SOM	GT:	0:58	0/1:	G	A	0
.	.	.	0.0	0.5	0.927	1.000	5.1	4.23	.	.	SOM	GT:	0:32	0/1:	C	G	0
.	##	0	0.2	0.0	0.012	1,N	.	.	.	.	SOM	GT:	0:19	0/1:	C	T	0
.	.	.	.	.	.	.	.	.	.	.	SOM	GT:	0:44	0/1:	G	C	0
.	.	.	.	.	.	.	.	.	0.1	0.2	SOM	GT:	0:23	0/1:	C	A	0
.	.	.	.	.	.	.	.	.	0.3	0.6	SOM	GT:	0:9,	0/1:	A	C	0
.	0	0	.	.	.	.	3.2	2.71	.	.	SOM	GT:	0:28	0/1:	C	T	0
.	.	.	0.0	0.9	0.999	0.997	4.6	2.87	.	.	SOM	GT:	0:11	0/1:	C	A	0
.	.	.	.	.	.	.	.	.	.	.	SOM	GT:	0:9,	0/1:	A	T	0
.	.	.	.	.	.	.	.	3.11	.	.	SOM	GT:	0:28	0/1:	G	T	0
.	.	.	.	.	.	.	.	.	.	.	SOM	GT:	0:29	0/1:	G	A	0
.	.	.	.	.	.	.	.	3.37	.	.	SOM	GT:	0:10	0/1:	G	A	0
.	.	.	0.0	0.4	0.883	1.000	4.7	4.04	.	.	SOM	GT:	0:38	0/1:	C	G	0
.	##	.	0.3	0.0	0.002	0.974	.	1.77	.	.	SOM	GT:	0:40	0/1:	G	C	0
.	##	0	0.1	0.0	0.087	0.985	4.1	1.73	.	.	SOM	GT:	0:83	0/1:	C	T	0
.	.	.	0.0	0.9	0.999	1.000	4.1	4.31	.	.	SOM	GT:	0:13	0/1:	G	A	0
.	.	.	0.0	0.9	1.0,D	1,D	4.8	5.03	.	.	SOM	GT:	0:20	0/1:	C	T	0
.	.	.	.	.	.	.	.	.	.	.	SOM	GT:	0:9,	0/1:	G	A	0
.	.	.	.	.	.	.	.	.	.	.	SOM	GT:	0:22	0/1:	G	T	0
.	.	.	.	.	.	.	.	.	0	.	SOM	GT:	0:12	0/1:	C	T	0
.	.	.	.	.	.	.	.	.	.	.	SOM	GT:	0:13	0/1:	C	A	0
.	.	.	.	.	.	.	2	.	.	.	SOM	GT:	0:23	0/1:	C	G	0
.	.	.	.	.	.	.	2.5	2.83	.	.	SOM	GT:	0:11	0/1:	C	T	0
.	.	.	.	.	.	.	.	.	.	.	SOM	GT:	0:28	0/1:	G	C	0
.	.	.	.	.	.	.	2.9	.	.	.	SOM	GT:	0:21	0/1:	C	T	0
.	.	.	.	.	.	.	.	.	.	.	SOM	GT:	0:33	0/1:	A	G	0
.	.	.	.	.	.	.	.	1.29	0.5	0.5	SOM	GT:	0:13	0/1:	G	A	0
.	.	.	.	.	.	.	.	.	.	.	SOM	GT:	0:55	0/1:	G	A	0

shared_he	OMIM	GWAS_Pu	HGM	GO_	GO_	GO_	KEGG	PID	BIO	REACTOME_PATHWAY
0	.	NA	.	.	.	.	.	.	.	.
0	.	NA	.	.	.	.	.	.	.	.
0	.	NA	.	.	.	.	.	.	.	.
0	.	NA	.	.	.	.	.	.	.	.
0	.	NA	.	.	.	.	.	.	.	.
0	Homocysti	NA	.	CAR	.	OXID	KEGG	.	.	REACTOME_METABOLISM_OF_VIT
0	.	NA	.	.	.	.	.	.	.	.
0	.	NA	.	.	.	.	.	.	.	.
0	.	NA	.	.	.	.	.	.	.	.
0	.	NA	.	.	.	.	.	.	.	.
0	Rhabdomy	NA	.	CELI	.	TRAI	.	.	.	.
0	.	NA	.	.	NUC	RNA	KEGG	PID	.	REACTOME_RNA_POL_II_TRANSCF
0	.	NA	.	RNA	ORG	RNA	KEGG	.	.	REACTOME_PROCESSING_OF_CA
0	.	NA	.	.	.	.	.	.	.	.
0	.	NA	.	CAR	.	OXID	KEGG	.	.	REACTOME_BIOLOGICAL_OXIDATI
0	.	NA	.	LIPID	INTF	TRAI	.	.	.	REACTOME_PHOSPHOLIPID_META
0	.	NA	.	.	.	.	.	.	.	.
0	.	NA	.	.	.	.	.	.	.	.
0	.	NA	.	.	.	.	.	.	.	.
0	.	NA	.	.	.	.	.	.	.	.
0	.	NA	.	.	.	.	.	.	.	.
0	.	NA	.	.	.	.	.	.	.	.
0	.	NA	.	.	.	.	.	.	.	.
0	.	NA	.	.	.	.	.	.	.	.
0	.	NA	.	.	.	.	.	.	.	.
0	.	NA	.	.	.	.	.	.	.	.
0	.	NA	.	.	.	.	.	.	.	.
0	.	NA	.	.	.	.	.	.	.	.
0	.	NA	.	.	.	.	.	.	.	.
0	.	NA	.	.	.	.	.	.	.	.
0	.	NA	.	.	.	.	.	.	.	.
0	.	NA	.	.	.	.	.	.	.	.
0	.	NA	.	.	.	.	.	.	.	.
0	.	NA	.	.	.	.	.	.	.	.
0	.	NA	.	.	.	.	.	.	.	.
0	.	NA	.	MUL	.	REC	.	.	.	.
0	.	NA	.	.	.	.	.	.	.	.
0	{Malaria, re	NA	.	SIGN	.	.	KEGG	PID	BIO	REACTOME_IMMUNOREGULATORY
0	.	NA	.	NEG	NUC	TRAI	.	PID	.	REACTOME_RNA_POL_III_TRANSC
0	.	NA	.	.	.	.	.	.	.	.
0	HEM skele	NA	.	.	ORG	DNA	.	.	.	REACTOME_METABOLISM_OF_LIPI
0	.	NA	.	.	.	.	.	.	.	.
0	.	NA	.	.	.	.	.	.	.	.
0	.	NA	.	.	.	.	.	.	.	.
0	.	NA	.	.	.	.	.	.	.	.
0	.	NA	.	.	.	.	.	.	.	.
0	.	NA	.	.	.	.	.	.	.	.
0	.	NA	.	.	.	.	.	.	.	.
0	.	NA	.	.	INTF	INTE	KEGG	PID	BIO	REACTOME_SIGNALING_BY_ILS;RE
0	.	NA	.	.	.	.	.	.	.	.
0	.	NA	.	.	.	.	.	.	.	.

0	.	NA	.	.	.	.	.	.	.	.
0	.	NA	.	.	.	.	.	.	.	.
0	.	NA	.	.	.	.	KEG	.	.	REACTOME_METABOLISM_OF_PRO
0	Mowat-Wil	NA	.	.	.	.	.	.	.	.
0	.	NA	.	CEL	NUC	.	.	PID	.	.
0	.	NA	.	RNA	ORG	LIGA	KEG	.	.	REACTOME_MITOCHONDRIAL_TRN
0	.	NA	.	.	.	.	.	.	.	.
0	.	NA	.	.	INTR	G_PI	KEG	.	.	REACTOME_SIGNALING_BY_GPCR
0	Spinal mus	NA	.	PRO	.	UNF	.	.	.	.
0	.	NA	.	.	.	.	.	.	.	.
0	.	NA	.	RNA	.	TRA	.	PID	.	.
0	.	NA	.	.	.	.	.	.	.	.
0	.	NA	.	N_TE	NUC	HIST	KEG	PID	BIO	REACTOME_PRE_NOTCH_TRANSC
0	Nail disord	NA	.	MEM	CYT	LIPA	KEG	.	.	.
0	.	NA	.	EST	.	.	.	.	.	.
0	.	NA	CM1	EST	.	.	.	.	.	.
0	.	NA	.	.	.	.	.	.	.	.
0	.	NA	.	.	.	.	.	.	.	.
0	.	NA	.	.	.	.	.	.	.	.
0	.	NA	.	.	.	.	.	.	.	.
0	.	NA	.	REG	.	.	KEG	.	.	.
0	.	NA	.	.	.	.	.	.	.	.
0	.	NA	.	.	.	.	.	.	.	.
0	.	NA	.	.	.	.	.	.	.	.
0	.	NA	.	.	.	.	.	.	.	.
0	.	NA	.	.	.	.	.	PID	.	.
0	.	NA	.	.	.	.	.	.	.	.
0	.	NA	.	.	.	.	.	.	.	.
0	.	NA	.	.	.	.	.	.	.	.
0	.	NA	.	.	.	.	.	.	.	.
0	.	NA	.	.	.	.	.	.	.	.
0	.	NA	.	.	.	.	.	.	.	.
0	.	NA	.	.	.	.	.	.	.	.
0	.	NA	.	.	.	.	.	.	.	.
0	.	NA	.	.	.	.	.	.	.	.
0	.	NA	.	.	.	.	.	.	.	REACTOME_METABOLISM_OF_NON
0	.	NA	.	.	.	.	KEG	.	.	.
0	.	NA	.	.	.	.	.	.	.	.
0	.	NA	.	.	.	.	.	.	.	.
0	.	NA	.	BIOF	.	.	KEG	PID	BIO	REACTOME_SIGNALLING_BY_NGF;
0	.	NA	.	CEL	PRO	PRO	KEG	PID	BIO	REACTOME_SIGNALING_BY_WNT;F
0	.	NA	.	.	.	.	.	.	.	.
0	.	NA	.	.	.	.	.	.	.	.
0	.	NA	.	.	.	.	.	.	.	.
0	.	NA	.	.	.	.	.	.	.	.
0	.	NA	.	COF	.	.	KEG	.	.	REACTOME_BIOLOGICAL_OXIDATI
0	.	NA	.	.	.	.	.	.	.	.
0	.	NA	.	.	.	.	.	.	.	.
0	.	NA	.	.	.	.	.	.	.	.
0	.	NA	.	.	.	.	.	.	.	.
0	Coenzyme	NA	.	ISOF	.	TRA	KEG	.	.	.
0	.	NA	.	.	.	.	.	.	.	.
0	.	NA	.	.	.	.	.	.	.	REACTOME_TRANSMEMBRANE_TF
0	.	NA	.	MUS	MEM	.	.	.	BIO	.

0	.	NA	.	.	.	.	.	.	.	.
0	.	NA	.	.	.	.	KEGG	.	.	.
0	.	NA	.	MAC	EUK	TRAI	.	.	.	REACTOME_TRANSLATION;REACT
0	.	NA	.	.	.	.	.	.	.	.
0	.	NA	.	MUS	.	EXO	.	.	.	.
0	.	NA	.	.	.	.	.	.	.	.
0	.	NA	.	.	.	.	.	.	.	.
0	.	NA	.	.	.	.	.	.	.	.
0	.	NA	.	.	.	.	.	.	.	.
0	.	NA	.	.	.	.	.	.	.	.
0	.	NA	.	.	.	.	.	.	.	.
0	.	NA	.	.	.	.	.	.	.	.
0	.	NA	.	.	.	.	.	.	.	.
0	.	NA	.	.	.	.	.	.	.	.
0	.	NA	.	.	.	.	.	.	.	.
0	.	NA	.	.	.	.	.	.	.	.
0	.	NA	.	.	.	.	.	.	.	.
0	.	NA	.	.	.	.	.	.	.	.
0	.	NA	.	.	.	.	KEGG	.	.	.
0	.	NA	.	DNA	.	SING	.	PID	.	REACTOME_MEIOSIS;REACTOME_
0	.	NA	.	.	.	.	.	.	.	.
0	.	NA	.	.	.	.	.	.	.	.
0	.	NA	.	.	.	.	.	.	.	.
0	.	NA	.	.	.	.	.	.	.	.
0	.	NA	.	.	.	.	.	.	.	.
0	.	NA	.	.	.	.	.	.	.	.
0	.	NA	.	.	.	.	.	.	.	.
0	.	NA	.	.	.	.	.	.	.	.
0	.	NA	.	ESTA	ORG	.	.	.	.	.
0	.	NA	.	.	.	.	.	.	.	.
0	.	NA	.	.	.	.	.	.	.	.
0	.	NA	.	.	.	.	.	.	.	.
0	.	NA	.	.	.	.	.	.	.	.
0	.	NA	.	PHO	CYT	PHO	KEGG	.	.	.
0	.	NA	.	.	.	.	.	.	.	.
0	.	NA	.	.	.	.	.	.	.	.
0	.	NA	.	.	.	.	.	.	.	.
0	.	NA	.	.	.	.	.	.	.	.
0	.	NA	.	.	.	.	.	.	.	.
0	.	NA	.	BIOF	.	NUC	.	.	.	.
0	.	NA	.	.	.	.	.	.	.	.
0	Telangiecta	NA	.	REG	INTF	.	.	PID	.	.
0	.	NA	.	.	.	IDEN	.	.	.	.
0	Thrombotic	NA	.	BIOF	PRO	INTE	.	.	.	.
0	Dopamine	NA	.	CELI	CYT	.	KEGG	.	BIO	REACTOME_METABOLISM_OF_AMI
0	.	NA	.	.	.	.	.	.	.	.
0	.	NA	.	BIOF	.	.	.	.	.	REACTOME_MEMBRANE_BINDING_
0	Deafness,	NA	.	PRO	ORG	HYD	.	.	.	.
0	Deafness,	NA	.	PRO	ORG	HYD	.	.	.	.
0	.	NA	.	.	.	.	.	.	.	.
0	.	NA	.	MUS	CYT	ACTI	.	PID	.	.
0	.	NA	.	.	.	.	.	.	.	.
0	.	NA	.	.	.	.	.	.	.	.
0	.	NA	.	.	.	.	.	.	.	.

0	.	NA	.	.	.	.	.	.	.	.
0	.	NA	.	.	.	.	.	.	.	.
0	?Retinitis p	NA	.	LIPID	.	.	.	.	.	.
0	.	NA	.	.	.	.	.	.	.	.
0	.	NA	.	.	.	.	.	.	.	.
0	.	NA	.	.	.	.	.	.	.	.
0	Preeclamp	NA	.	.	.	.	.	.	.	.
0	.	NA	.	.	.	.	.	.	.	.
0	Cholestery	NA	.	GLY	CYT	.	KEG	.	.	.
0	.	NA	.	.	.	.	.	.	.	.
0	Epidermoly	NA	.	SYS	INTR	.	.	PID	.	REACTOME_CELL_CELL_COMMUN
0	.	NA	.	.	.	.	.	.	.	.
0	.	NA	.	.	.	.	.	.	.	.
0	.	NA	.	.	.	.	.	.	.	.
0	.	NA	.	PRO	.	ACID	KEG	PID	BIO	REACTOME_SIGNALLING_BY_NGF;
0	.	NA	.	.	.	.	.	.	.	.
0	.	NA	.	.	.	.	.	.	.	.
0	.	NA	.	SIGN	.	.	.	.	.	.
0	.	NA	.	RNA	NUC	.	.	.	.	.
0	.	NA	.	RNA	NUC	.	.	.	.	.
0	.	NA	.	.	.	.	.	.	.	.
0	.	NA	.	.	.	.	.	.	.	.
0	.	NA	.	ENZ	CYT	NUC	.	PID	.	.
0	.	NA	.	.	.	.	KEG	.	.	REACTOME_SIGNALING_BY_GPCR
0	.	NA	.	.	.	.	.	.	.	.
0	.	NA	.	.	.	.	.	.	.	.
0	.	NA	.	.	.	.	.	.	.	.
0	.	NA	.	.	.	.	.	.	.	.
0	.	NA	.	REG	SYN	.	.	PID	.	.
0	.	NA	.	.	.	.	.	.	.	.
0	.	NA	.	.	.	.	.	.	.	.
0	.	NA	.	.	.	.	.	.	.	REACTOME_TRANSMISSION_ACRC
0	.	NA	.	.	.	.	.	.	.	.
0	.	NA	.	.	.	.	.	.	.	.
0	.	NA	.	.	.	.	.	.	.	.
0	.	NA	.	SULF	.	SULF	KEG	.	.	REACTOME_CHONDROITIN_SULFA
0	.	NA	.	.	.	.	.	.	.	.
0	.	NA	.	SYS	.	PHO	.	PID	.	.
0	.	NA	.	.	.	.	.	.	.	.
0	.	NA	.	.	.	.	.	.	.	.
0	.	NA	.	.	.	.	.	.	.	.
0	.	NA	.	.	.	.	.	.	.	.
0	.	NA	.	.	.	.	.	.	.	.
0	.	NA	.	.	.	.	.	.	.	.
0	.	NA	.	.	.	.	.	.	.	.
0	.	NA	.	.	.	.	.	.	.	.
0	.	NA	.	.	.	.	.	.	.	.
0	.	NA	.	.	.	.	.	.	.	.
0	.	NA	.	.	.	.	.	.	.	.
0	.	NA	.	.	.	.	.	.	.	.
0	Emery-Dre	NA	.	.	.	.	.	.	.	REACTOME_MEIOSIS;REACTOME_
0	.	NA	.	.	.	.	.	.	.	.
0	.	NA	.	.	.	.	.	.	.	.
0	.	NA	.	.	.	.	.	.	.	.

0	.	NA	.	.	.	.	.	.	.	.
0	.	NA	.	.	.	.	.	.	.	.
0	.	NA	.	.	.	.	.	.	.	.
0	.	NA	.	.	.	.	.	.	.	.
0	.	NA	.	.	.	.	.	.	.	.
0	.	NA	.	.	.	.	.	.	.	.
0	.	NA	.	.	.	.	.	.	.	.
0	.	NA	.	.	.	.	.	.	.	.
0	.	NA	.	.	.	.	.	.	.	.
0	.	NA	.	.	.	.	.	.	.	.
0	.	NA	.	.	.	.	.	.	.	.
0	.	NA	.	.	.	.	.	.	.	REACTOME_TRANSMEMBRANE_TF
0	.	NA	.	.	.	.	.	.	.	.
0	.	NA	.	SYS	.	.	.	.	.	.
0	.	NA	.	.	.	.	.	.	.	.
0	.	NA	.	.	.	.	.	.	.	.
0	.	NA	.	.	.	.	.	.	.	.
0	.	NA	.	.	.	.	.	.	.	.
0	.	NA	.	.	.	.	.	.	.	.
0	.	NA	.	.	.	.	.	.	.	.
0	.	NA	.	.	.	.	.	.	.	.
0	.	NA	.	.	.	.	.	.	.	.
0	.	NA	.	.	.	.	.	.	.	.
0	.	NA	.	.	.	.	.	.	.	.
0	.	NA	.	.	.	.	.	.	.	.
0	.	NA	.	SIGN	.	GTP	.	.	.	REACTOME_SIGNALING_BY_GPCR
0	Deafness,	NA	.	.	.	.	.	.	.	.
0	Immunode	NA	.	.	ORG	STR	.	.	.	.
0	.	NA	.	.	.	.	.	.	.	.
0	.	NA	.	.	.	.	.	.	.	.
0	.	NA	.	.	.	.	.	.	.	.
0	.	NA	.	.	.	.	.	.	.	.
0	.	NA	.	.	.	.	.	.	.	.
0	.	NA	.	.	.	.	.	.	.	.
0	.	NA	.	.	.	.	.	.	.	.
0	.	NA	.	.	.	.	.	.	.	.
0	.	NA	.	.	.	.	.	.	.	.
0	.	NA	.	.	.	.	.	.	.	.
0	.	NA	.	.	.	.	.	.	.	.
0	.	NA	.	.	.	.	.	.	.	.
0	.	NA	.	.	.	.	.	.	.	.
0	.	NA	.	.	.	.	.	.	.	.
0	.	NA	.	.	.	.	.	.	.	.
0	.	NA	.	.	.	.	.	.	.	.
0	.	NA	.	.	.	.	.	.	.	.
0	.	NA	.	.	.	.	.	.	.	.
0	.	NA	.	.	.	.	.	.	.	.
0	.	NA	.	.	.	.	.	.	.	.
0	.	NA	.	.	.	.	.	.	.	.
0	.	NA	.	.	.	.	.	.	.	.
0	.	NA	.	.	.	.	.	.	.	.
0	Growth ret	NA	.	PAT	.	.	.	.	.	.
0	Myeloid leu	NA	.	RNA	.	RNA	.	PID	.	.
0	.	NA	.	.	.	.	.	.	.	.

0	Brittle corn	NA	.	.	.	.	.	.	.	.
0	Retinitis pi(	NA	.	RNA	ORG	RNA	KEG(	.	.	REACTOME_PROCESSING_OF_CAI
0	.	NA	.	.	.	.	.	.	.	.
0	.	NA	.	RNA	.	HYD	.	PID	.	.
0	.	NA	.	GAM	ORG	.	.	.	.	.
0	.	NA	.	.	.	.	.	.	.	.
0	Deafness,	NA	.	SEN	.	.	.	.	.	.
0	.	NA	.	.	.	.	.	.	.	.
0	.	NA	.	.	.	.	.	.	.	.
0	.	NA	.	.	.	.	.	.	.	.
0	.	NA	.	.	.	.	.	.	.	.
0	.	NA	.	.	.	.	.	.	.	.
0	.	NA	.	.	.	.	.	.	.	.
0	.	NA	.	.	.	.	.	.	.	.
0	.	NA	.	.	.	.	.	.	.	.
0	.	NA	.	.	.	.	.	.	.	.
0	.	NA	.	.	.	.	.	.	.	.
0	.	NA	.	CELI	.	HYD	.	.	.	.
0	Cortical dy	NA	.	MICF	ORG	STRI	.	PID	.	REACTOME_CELL_CYCLE;REACTO
0	.	NA	.	.	.	.	.	.	.	.
0	.	NA	.	.	.	.	.	.	.	.
0	Amelogene	NA	.	RNA	.	ENZ	KEG(	PID	BIO	REACTOME_SIGNALLING_BY_NGF;
0	.	NA	.	.	.	.	.	.	.	.
0	.	NA	.	.	CELI	.	KEG(	.	.	.
0	.	NA	.	.	.	.	.	.	.	.
0	.	NA	.	.	.	.	.	.	.	.
0	.	NA	.	.	.	.	.	.	.	.
0	.	NA	.	.	.	.	.	.	.	.
0	.	NA	.	.	.	.	.	.	.	.
0	.	NA	.	.	.	OXY	KEG(	.	.	REACTOME_BIOLOGICAL_OXIDATI(
0	.	NA	.	.	.	.	.	.	.	.
0	.	NA	.	RNA	.	TRAI	.	PID	.	.
0	.	NA	.	.	.	.	.	.	.	.
0	.	NA	.	CAR	.	ELE(	KEG(	.	.	.
0	.	NA	.	.	ORG	.	.	.	.	.
0	.	NA	.	.	.	.	.	.	.	.
0	.	NA	.	.	.	.	.	.	.	.
0	.	NA	.	REG	.	TRAI	KEG(	PID	.	.
0	.	NA	.	.	.	.	.	.	.	.
0	.	NA	.	.	.	.	.	.	.	.
0	.	NA	.	.	.	.	.	.	.	.
0	.	NA	.	.	.	.	.	.	.	.
0	.	NA	.	.	.	.	.	.	.	.
0	.	NA	.	.	.	.	.	.	.	.
0	.	NA	.	.	.	.	.	.	.	.
0	.	NA	.	.	.	.	.	.	.	.
0	.	NA	.	DNA	NUC	.	.	.	.	.
0	.	NA	.	CAR	INTF	TRAI	KEG(	.	.	REACTOME_BIOLOGICAL_OXIDATI(
0	.	NA	.	.	.	.	.	.	.	.
0	.	NA	.	.	.	.	.	.	.	.
0	.	NA	.	.	.	.	.	.	.	.
0	.	NA	.	.	ORG	.	.	.	.	.
0	.	NA	.	.	.	.	.	.	.	.
0	.	NA	.	.	.	.	.	.	.	.

0	.	NA	.	.	.	.	.	.	.	.
0	.	NA	.	SYS	INTR	.	.	.	.	.
0	.	NA	.	SYS	INTR	.	.	.	.	.
0	.	NA	.	GAM	ORG	CHR	.	.	.	.
0	.	NA	.	.	.	.	.	.	.	.
0	.	NA	.	.	.	.	.	.	.	.
0	Renal cell (	NA	.	.	.	.	.	PID	.	.
0	Renal cell (	NA	.	.	.	.	.	PID	.	.
0	Aland Islar	NA	.	DET	INTR	CAT	KEG	.	.	.
0	.	NA	.	.	.	.	.	.	.	.
0	.	NA	.	REG	ORG	.	.	.	.	REACTOME_DIABETES_PATHWAYS
0	.	NA	.	.	.	.	.	.	.	.
0	Androgen i	NA	.	EST	CYT	LIGA	KEG	PID	.	REACTOME_GENERIC_TRANSCRIP
0	.	NA	.	.	.	.	.	.	.	.
0	Mental reta	NA	.	.	.	.	.	.	.	.
0	.	NA	.	.	.	.	.	.	.	.
0	.	NA	.	RNA	NUC	RNA	.	.	.	REACTOME_PROCESSING_OF_INT
0	.	NA	.	.	.	.	.	.	.	.
0	.	NA	.	.	.	.	KEG	.	.	REACTOME_HS_GAG_BIOSYNTHES
0	.	NA	.	.	CYT	.	.	PID	.	REACTOME_SIGNALING_BY_RHO_
0	.	NA	.	.	.	.	.	.	.	.
0	.	NA	.	.	.	.	.	.	.	.
0	.	NA	.	REG	MEM	KINA	KEG	PID	BIO	REACTOME_SIGNALLING_BY_NGF;
0	.	NA	.	REG	MEM	KINA	KEG	PID	BIO	REACTOME_SIGNALLING_BY_NGF;
0	.	NA	.	.	.	.	.	.	.	.
0	.	NA	.	.	.	.	.	.	.	.
0	.	NA	.	.	.	.	.	.	.	.
0	.	NA	.	.	.	.	.	.	.	.
0	.	NA	.	.	.	.	.	.	.	.
0	Meier-Gorl	NA	.	DNA	NUC	DNA	KEG	PID	BIO	REACTOME_ACTIVATION_OF_THE_
0	.	NA	.	.	.	.	.	.	.	.
0	Myopia 21,	NA	.	.	.	.	.	.	.	.
0	.	NA	.	EST	INTR	CAT	.	.	.	REACTOME_NEURONAL_SYSTEM;
0	.	NA	.	.	.	.	.	.	.	.
0	.	NA	.	.	.	.	.	.	.	.
0	.	NA	.	.	.	.	.	.	.	.
0	.	NA	.	.	.	.	.	.	.	.
0	.	NA	.	.	.	.	.	.	.	.
0	.	NA	.	.	.	.	.	.	.	.
0	.	NA	.	.	.	.	.	.	.	.
0	.	NA	.	.	.	.	.	.	.	.
0	.	NA	.	.	.	.	.	.	.	.
0	.	NA	.	.	.	.	.	.	.	.
0	.	NA	.	.	.	.	.	.	.	.
0	.	NA	.	.	.	.	.	.	.	.
0	.	NA	.	.	.	.	.	.	.	.
0	.	NA	.	.	.	.	.	.	.	.
0	.	NA	.	.	.	.	.	.	.	.
0	.	NA	.	.	.	.	.	.	.	.
0	.	NA	.	.	.	.	.	.	.	.
0	.	NA	.	.	.	.	.	.	.	.
0	.	NA	.	.	.	.	KEG	.	.	.
0	.	NA	.	STE	CYT	TRA	KEG	.	.	.
0	.	NA	.	.	.	.	.	.	.	.

0	.	NA	.	.	.	.	.	.	.	.
0	.	NA	.	.	.	.	.	.	.	.
0	.	NA	.	.	.	.	.	.	.	.
0	.	NA	.	.	.	.	.	.	.	REACTOME_SIGNALING_BY_GPCR
0	.	NA	.	.	NUC	PRO	.	PID	BIO	REACTOME_DEVELOPMENTAL_BIC
0	.	NA	.	.	.	.	.	.	.	.
0	.	NA	.	.	.	.	.	.	.	.
0	.	NA	.	.	.	.	.	.	.	.
0	.	NA	.	.	.	.	.	.	.	.
0	.	NA	.	.	.	.	.	.	.	.
0	.	NA	.	.	.	.	.	.	.	.
0	.	NA	.	AGIN	.	.	.	PID	.	REACTOME_CELL_CELL_COMMUN
0	.	NA	.	.	.	.	.	.	.	.
0	.	NA	.	.	.	.	.	.	.	.
0	.	NA	.	.	.	.	.	.	.	.
0	Bardet-Bie	NA	.	.	.	.	.	.	.	.
0	Cardiomyo	NA	.	MUS	ORG	NUC	KEGG	.	.	.
0	.	NA	.	.	.	.	.	.	.	.
0	AICA-ribos	NA	.	NUC	.	HYD	KEGG	.	.	REACTOME_METABOLISM_OF_NUC
0	Alport sync	NA	.	REG	PRO	STRI	KEGG	PID	BIO	REACTOME_DEVELOPMENTAL_BIC
0	.	NA	.	.	.	.	KEGG	PID	.	REACTOME_SIGNALING_BY_RHO_
0	.	NA	.	.	.	.	.	.	.	.
0	Spinocereb	NA	.	.	INTR	CATI	KEGG	.	.	.
0	.	NA	.	.	.	.	.	.	.	.
0	T-cell immu	NA	.	.	.	.	.	PID	.	.
0	T-cell immu	NA	.	.	.	.	.	PID	.	.
0	.	NA	.	.	.	.	.	.	.	.
0	Tietz albini	NA	.	.	.	TRAI	KEGG	PID	.	.
0	.	NA	.	.	.	.	.	.	.	.
0	Breast can	NA	.	.	.	PHO	KEGG	PID	BIO	REACTOME_SIGNALLING_BY_NGF;
0	.	NA	.	.	.	.	.	.	.	REACTOME_FACTORS_INVOLVED_
0	.	NA	.	.	.	.	.	.	.	.
0	.	NA	.	.	.	.	.	.	.	.
0	.	NA	.	.	.	.	.	.	.	.
0	.	NA	.	VIRA	.	TRAI	KEGG	PID	BIO	.
0	.	NA	.	.	.	.	.	.	.	.
0	.	NA	.	.	.	.	.	.	.	.
0	.	NA	.	.	.	.	.	.	.	.
0	.	NA	.	.	.	.	.	.	.	.
0	.	NA	.	.	.	.	.	.	.	.
0	.	NA	.	.	.	.	.	.	.	.
0	.	NA	.	.	.	.	.	.	.	.
0	{Alcohol de	NA	.	.	.	.	KEGG	.	.	REACTOME_BIOLOGICAL_OXIDATIO
0	.	NA	.	.	.	.	.	.	.	.
0	Cardiac arr	NA	.	.	.	.	.	.	.	.
0	[Blood grou	NA	.	.	INTR	.	.	.	.	.
0	.	NA	.	.	.	.	.	.	.	.
0	.	NA	.	.	.	.	.	.	.	.
0	.	NA	.	.	.	.	.	.	.	.
0	.	NA	.	.	.	.	.	.	.	.
0	.	NA	.	.	.	.	.	.	.	.
0	.	NA	.	.	.	.	.	.	.	.
0	.	NA	.	.	.	.	.	.	.	.
0	.	NA	.	.	.	.	.	.	.	.

0	.	NA	.	.	.	.	.	.	.	.
0	.	NA	.	.	.	.	.	.	.	.
0	.	NA	.	.	.	.	.	.	.	.
0	Charcot-Ma	NA	.	.	.	.	.	.	.	.
0	.	NA	.	ORG	.	RAS	KEG	PID	.	REACTOME_FACTORS_INVOLVED_
0	.	NA	.	RNA	NUC	TRA	KEG	PID	.	REACTOME_PRE_NOTCH_TRANSC
0	.	NA	.	BIOF	NUC	PHO	.	.	.	.
0	.	NA	.	.	.	.	.	.	.	.
0	.	NA	.	EST	.	CATI	.	.	.	REACTOME_NEURONAL_SYSTEM;F
0	.	NA	.	.	.	.	.	.	.	.
0	.	NA	.	.	CYT	ISON	KEG	.	.	REACTOME_CHONDROITIN_SULFA
0	.	NA	.	RNA	NUC	.	.	.	.	.
0	Ciliary dysk	NA	.	.	.	.	.	.	.	.
0	.	NA	.	.	.	.	.	.	.	.
0	.	NA	.	.	.	.	.	.	.	.
0	.	NA	.	.	.	.	.	.	.	.
0	.	NA	.	.	.	.	.	.	.	.
0	.	NA	.	.	.	.	.	.	.	.
0	.	NA	.	.	.	.	.	.	.	.
0	.	NA	.	.	.	.	.	.	.	.
0	.	NA	.	.	.	.	.	.	.	.
0	.	NA	.	.	.	.	.	.	.	.
0	.	NA	.	.	.	.	.	.	.	.
0	.	NA	.	.	.	.	.	.	.	.
0	.	NA	.	.	.	.	.	.	.	.
0	.	NA	.	.	.	.	.	.	.	.
0	.	NA	.	SIGN	.	.	.	.	.	.
0	.	NA	.	EST	CELL	ANIC	.	.	.	REACTOME_TRANSMEMBRANE_TF
0	.	NA	.	EST	ORG	NUC	.	.	.	.
0	.	NA	.	EST	ORG	NUC	.	.	.	.
0	.	NA	.	BIOF	INTF	PRO	KEG	.	.	.
0	.	NA	.	.	.	.	.	.	.	.
0	.	NA	.	.	.	.	.	.	.	.
0	.	NA	.	.	.	.	.	.	.	.
0	Occult mac	NA	.	.	.	.	.	.	.	.
0	.	NA	.	.	.	.	.	.	.	.
0	.	NA	.	.	.	.	.	.	.	.
0	Spastic pa	NA	.	.	.	.	KEG	.	.	REACTOME_MEMBRANE_TRAFFICK
0	.	NA	.	.	.	.	.	.	.	.
0	.	NA	.	.	.	.	.	.	.	.
0	.	NA	.	.	.	.	.	.	.	.
0	.	NA	.	.	.	.	.	.	.	.
0	.	NA	.	SYS	.	.	.	.	.	.
0	.	NA	.	.	.	.	.	PID	.	REACTOME_TRANSMEMBRANE_TF
0	Lymphopla	NA	.	RNA	.	.	.	PID	.	.
0	.	NA	.	.	.	.	.	.	.	.
0	.	NA	.	.	.	.	.	.	.	.
0	.	NA	.	.	.	.	.	.	.	.
0	.	NA	.	.	.	.	.	.	.	.
0	.	NA	.	.	.	.	.	.	.	.
0	.	NA	.	.	.	.	.	.	.	.
0	.	NA	.	.	.	.	.	.	.	.
0	.	NA	.	.	.	.	.	.	.	.
0	.	NA	.	.	.	GTP	KEG	PID	BIO	REACTOME_SIGNALLING_BY_NGF;

0	.	NA	.	CELL	MEM	CATI	KEGG	.	.	REACTOME_TRANSMISSION_ACRO
0	Megaloblas	NA	.	ESTA	MEM	PRO	.	.	.	REACTOME_METABOLISM_OF_STE
0	.	NA	.	.	.	.	.	.	.	.
0	.	NA	.	.	.	.	.	.	.	.
0	.	NA	.	.	.	.	.	.	.	.
0	.	NA	.	.	.	.	.	.	.	REACTOME_MITOCHONDRIAL_PRO
0	.	NA	.	.	.	.	.	.	.	.
0	.	NA	.	.	.	.	.	.	.	.
0	.	NA	.	ESTA	.	CYT	.	.	.	.
0	.	NA	.	.	.	.	KEGG	.	.	REACTOME_TRANSMEMBRANE_TF
0	.	NA	.	.	.	.	.	.	.	.
0	.	NA	.	.	.	.	.	.	.	.
0	.	NA	.	DIGE	PRO	STRI	KEGG	.	.	REACTOME_O_LINKED_GLYCOSYL
0	{Pulmonar	NA	.	.	.	.	.	.	.	REACTOME_O_LINKED_GLYCOSYL
0	.	NA	.	.	.	.	.	.	.	.
0	.	NA	.	SIGN	ORG	RAS	.	.	.	.
0	Exostoses,	NA	.	SYS	ORG	.	KEGG	.	.	REACTOME_HS_GAG_BIOSYNTHES
0	.	NA	.	.	.	.	.	.	.	.
0	.	NA	.	POS	.	.	KEGG	PID	.	REACTOME_VEGF_LIGAND_RECEP
0	.	NA	.	BIOF	CYT	ACID	KEGG	.	.	REACTOME_DIABETES_PATHWAYS
0	.	NA	.	.	ORG	.	.	.	.	REACTOME_MHC_CLASS_II_ANTIG
0	.	NA	.	.	.	.	.	.	.	.
0	.	NA	.	REG	CYT	RAS	KEGG	PID	.	REACTOME_SIGNALING_BY_RHO_
0	.	NA	.	REG	CYT	RAS	KEGG	PID	.	REACTOME_SIGNALING_BY_RHO_
0	.	NA	.	.	.	.	.	.	.	.
0	Deafness,	NA	.	LYS	ORG	CALI	.	.	.	.
0	.	NA	.	RNA	NUC	.	.	.	.	.
0	.	NA	.	.	.	.	.	.	.	.
0	.	NA	.	GLY	INTF	EXO	KEGG	.	.	.
0	.	NA	.	.	.	.	KEGG	.	.	REACTOME_SIGNALING_BY_GPCR
0	.	NA	.	.	.	.	.	.	.	.
0	.	NA	.	.	.	.	.	.	.	.
0	.	NA	.	.	.	.	.	.	.	.
0	.	NA	.	.	.	.	.	.	.	.
0	.	NA	.	.	.	.	.	.	.	.
0	.	NA	.	.	.	.	.	.	.	.
0	.	NA	.	.	.	.	.	.	.	.
0	Deafness,	NA	.	.	.	.	.	.	.	.
0	.	NA	.	CELL	CYT	.	.	.	.	.
0	.	NA	.	.	.	.	.	.	.	.
0	.	NA	.	.	.	.	.	.	.	.
0	.	NA	.	.	.	.	.	.	.	.
0	.	NA	.	.	.	.	.	.	.	.
0	Spermatog	NA	.	SYS	CELL	OXID	KEGG	.	BIO	REACTOME_LATENT_INFECTION_
0	.	NA	.	.	NUC	TRAI	KEGG	PID	BIO	REACTOME_DEVELOPMENTAL_BIC
0	.	NA	.	DNA	NON	TRAI	KEGG	PID	BIO	REACTOME_TRIF_MEDIATED_TLR3
0	.	NA	.	.	.	.	.	.	.	.
0	.	NA	.	.	.	.	.	.	.	.
0	.	NA	.	.	.	.	.	.	.	.
0	.	NA	.	PRO	CYT	SER	.	.	.	REACTOME_IMMUNE_SYSTEM;REA
0	.	NA	.	.	.	.	.	.	.	.
0	.	NA	.	.	.	.	KEGG	.	.	REACTOME_SIGNALING_BY_GPCR
0	.	NA	.	.	.	.	.	.	.	.
0	.	NA	.	.	.	.	.	.	.	.

0	.	NA	.	.	.	.	.	.	.	.
0	.	NA	.	.	.	.	KEGC	.	.	.
0	Glaucoma	NA	.	EST/	PRO	.	.	.	.	.
0	.	NA	.	.	.	.	.	.	.	.
0	.	NA	.	.	.	.	.	.	.	.
0	.	NA	.	.	.	.	.	.	.	.
0	.	NA	.	.	.	.	.	.	.	.
0	.	NA	.	.	.	.	.	.	.	.
0	.	NA	.	.	.	.	.	.	.	.
0	.	NA	.	.	.	.	.	.	.	.
0	.	NA	.	EST/	.	CATI	KEGC	.	.	.
0	.	NA	.	.	.	.	.	.	.	.
0	Bartter syn	NA	.	.	MEM	CATI	.	.	.	REACTOME_TRANSMEMBRANE_TF
0	.	NA	.	.	.	.	.	.	.	.
0	.	NA	.	.	.	.	.	.	.	.
0	.	NA	.	.	INTF	.	KEGC	.	.	REACTOME_DEVELOPMENTAL_BIC
0	.	NA	.	.	.	.	.	.	.	.
0	.	NA	.	.	.	.	.	.	.	.
0	.	NA	.	.	.	.	.	.	.	.
0	.	NA	.	.	.	.	.	.	.	.
0	.	NA	.	.	.	.	.	.	.	REACTOME_TRANSMEMBRANE_TF
0	Weill-Marc	NA	.	.	.	.	.	.	.	.
0	.	NA	.	.	.	.	.	.	.	.
0	.	NA	.	.	.	.	.	.	.	.
0	Lissencepl	NA	.	CEN	ORG	MICF	.	.	.	.
0	.	NA	.	.	.	.	.	.	.	.
0	Floating-Ha	NA	.	RNA	.	TRAI	.	.	.	.
0	.	NA	.	.	.	.	.	.	.	.
0	.	NA	.	.	.	.	.	.	.	.
0	.	NA	.	.	.	.	.	.	.	.
0	.	NA	.	.	.	.	.	.	.	.
0	.	NA	.	.	.	.	.	.	.	.
0	.	NA	.	.	.	.	.	.	.	.
0	.	NA	.	.	.	.	.	.	.	.
0	.	NA	.	.	.	.	.	.	.	.
0	.	NA	.	.	.	.	.	.	.	.
0	.	NA	.	.	.	.	.	.	.	.
0	.	NA	.	.	.	.	.	.	.	.
0	.	NA	.	.	.	.	.	.	.	.
0	.	NA	.	.	.	.	.	.	.	.
0	.	NA	.	.	.	.	.	.	.	.
0	.	NA	.	.	.	.	.	.	.	.
0	.	NA	.	.	.	.	.	.	.	.
0	.	NA	.	.	.	.	.	.	.	.
0	.	NA	.	.	.	.	.	.	.	.
0	.	NA	.	.	.	.	.	.	.	.
0	.	NA	.	.	.	.	.	.	.	.
0	.	NA	.	.	.	.	.	.	.	.
0	.	NA	.	.	.	.	.	.	.	.
0	.	NA	.	.	.	.	.	.	.	.
0	.	NA	.	.	.	.	.	.	.	.
0	.	NA	.	.	.	.	.	.	.	.
0	.	NA	.	.	.	.	.	.	.	.
0	.	NA	.	.	.	.	.	.	.	.

0	.	NA	.	.	.	.	.	.	.	.
0	.	NA	.	.	.	.	.	.	.	.
0	.	NA	.	.	.	.	.	.	.	.
0	Ciliary dysk	NA	.	.	.	.	.	.	.	.
0	[Anhaptogl	NA	.	DEFI	.	.	.	.	.	.
0	.	NA	.	.	.	.	.	.	.	.
0	.	NA	.	.	.	.	.	.	.	.
0	Combined	NA	.	.	.	.	.	.	.	.
0	Combined	NA	.	.	.	.	.	.	.	.
0	.	NA	.	.	.	.	.	.	.	.
0	.	NA	.	.	.	.	.	.	.	.
0	.	NA	.	.	.	.	.	.	.	.
0	.	NA	.	.	.	.	.	.	.	REACTOME_INTERFERON_ALPHA_
0	.	NA	.	CALC	CELI	IDEN	KEGG	.	.	REACTOME_CELL_CELL_COMMUN
0	.	NA	.	.	.	.	.	.	.	.
0	.	NA	.	.	.	MOT	KEGG	.	.	.
0	.	NA	.	GAM	ORG	.	.	.	.	.
0	.	NA	.	GAM	ORG	.	.	.	.	.
0	.	NA	.	.	.	.	.	.	.	.
0	.	NA	.	.	.	.	.	.	.	.
0	.	NA	.	.	.	.	.	.	.	.
0	.	NA	.	.	.	.	.	.	.	.
0	.	NA	.	.	.	.	.	.	.	.
0	.	NA	.	.	.	.	.	.	.	.
0	.	NA	.	.	.	.	.	.	.	.
0	.	NA	.	.	.	.	.	.	.	.
0	.	NA	.	.	.	.	.	.	.	.
0	.	NA	.	.	.	.	.	.	.	.
0	.	NA	.	.	.	.	.	.	.	.
0	.	NA	.	.	.	.	.	.	.	.
0	Night blind	NA	.	.	.	.	.	.	.	.
0	.	NA	.	.	.	.	.	.	.	.
0	.	NA	.	.	.	.	.	.	.	.
0	Colorectal	NA	.	SIGN	NUC	.	KEGG	PID	.	.
0	.	NA	.	.	CYT	.	.	.	.	.
0	.	NA	.	.	.	.	.	.	.	.
0	.	NA	.	.	.	.	.	.	.	.
0	.	NA	.	PRO	ORG	CHA	.	.	.	REACTOME_PROTEIN_FOLDING;RE
0	.	NA	.	.	.	.	.	.	.	.
0	.	NA	.	.	.	.	.	.	.	.
0	.	NA	.	.	.	.	.	.	.	.
0	Cerebral cr	NA	.	BIOS	.	MET	KEGG	.	.	REACTOME_METABOLISM_OF_AMI
0	.	NA	.	.	.	.	.	.	.	.
0	.	NA	.	.	.	MET	KEGG	.	.	.
0	.	NA	.	.	.	MET	KEGG	.	.	.
0	Spastic pa	NA	.	.	.	.	.	.	.	.
0	.	NA	.	EST	INTR	ACTI	.	.	.	REACTOME_GLUTAMATE_NEUROT
0	.	NA	.	REG	NUC	SEQ	.	.	.	.
0	.	NA	.	.	.	.	.	.	.	.
0	.	NA	.	.	.	.	.	.	.	.
0	.	NA	.	.	.	.	.	.	.	.
0	Central cor	NA	.	EST	INTR	CATI	KEGG	.	.	.
0	.	NA	.	.	.	.	.	.	.	.
0	.	NA	.	REG	INTR	PEPT	KEGG	PID	BIO	REACTOME_SIGNALING_BY_GPCR

0	.	NA	.	.	.	.	.	.	.	.
0	.	NA	.	.	.	.	.	.	.	.
0	.	NA	.	BIOS	RIBC	REC	.	.	.	.
0	.	NA	.	.	.	.	.	.	.	.
0	.	NA	.	.	.	.	KEGG	PID	.	REACTOME_BIOLOGICAL_OXIDATI
0	.	NA	.	.	.	.	KEGG	PID	.	REACTOME_BIOLOGICAL_OXIDATI
0	.	NA	.	.	.	.	.	.	.	.
0	.	NA	.	.	.	.	.	.	.	.
0	.	NA	.	.	.	.	.	.	.	.
0	.	NA	.	.	.	.	.	.	.	.
0	.	NA	.	.	.	.	.	.	.	.
0	.	NA	.	.	.	.	.	.	.	.
0	.	NA	.	.	.	.	.	.	.	.
0	.	NA	.	.	.	.	.	.	.	.
0	.	NA	.	.	.	.	.	.	.	.
0	.	NA	.	.	.	.	.	.	.	.
0	.	NA	.	.	.	.	.	.	.	.
0	.	NA	.	.	.	.	.	.	.	.
0	.	NA	.	.	.	.	.	.	.	.
0	.	NA	.	EST	INTR	CATI	.	.	.	.
0	.	NA	.	.	.	.	.	.	.	.
0	.	NA	.	.	.	.	.	.	.	.
0	Conotrunca	NA	.	SYS	NUC	PRO	.	.	.	.
0	.	NA	.	.	.	.	.	.	.	.
0	.	NA	.	.	.	.	.	.	.	.
0	.	NA	.	.	.	.	.	.	.	.
0	.	NA	.	.	.	.	.	.	.	.
0	.	NA	.	GAM	ORG	CHR	.	.	.	.
0	Opitz GBB	NA	.	PAT	ORG	.	KEGG	.	.	.
0	.	NA	.	.	.	.	.	.	.	.
0	.	NA	.	.	.	.	.	.	.	.
0	.	NA	.	.	.	.	.	.	.	.
0	.	NA	.	BIOF	.	.	.	.	.	.
0	.	NA	.	BIOF	.	.	.	.	.	.
0	.	NA	.	.	.	.	.	.	.	.
0	.	NA	.	.	.	.	.	.	.	.
0	.	NA	.	.	.	.	.	.	.	.
0	.	NA	.	.	.	.	.	.	.	.
0	Lujan-Fryn	NA	.	RNA	NUC	RNA	.	PID	.	REACTOME_DEVELOPMENTAL_BIC
0	.	NA	.	.	.	.	.	.	.	.
0	.	NA	.	.	.	.	.	.	.	.
0	Lissenceph	NA	.	SYS	ORG	MICF	.	PID	.	REACTOME_DEVELOPMENTAL_BIC
0	Dent disea	NA	.	LIPID	ORG	PHO	KEGG	.	.	REACTOME_SIGNALING_BY_RHO_
0	Hypothyroi	NA	.	SIGN	INTR	PRO	.	.	.	.
0	.	NA	.	.	.	.	.	.	.	.
0	.	NA	.	SYS	CYT	ENZ	KEGG	.	.	.
0	.	NA	.	.	.	.	.	.	.	.
0	Cardiac va	NA	.	NUC	CYT	ACTI	KEGG	PID	.	.
0	.	NA	.	.	.	.	.	.	.	.
0	.	NA	.	.	.	.	.	.	.	.
0	.	NA	.	.	.	.	.	.	.	.
0	.	NA	.	.	.	.	.	.	.	.
0	.	NA	.	REG	NUC	NUC	.	PID	BIO	.
0	?Neurobla	NA	.	DNA	.	TRAI	KEGG	PID	BIO	.

0	.	NA	.	.	.	.	.	.	.	.
0	.	NA	.	.	.	.	.	.	.	.
0	.	NA	.	.	.	.	.	.	.	.
0	.	NA	.	.	.	.	.	.	.	.
0	.	NA	.	.	.	.	.	.	.	.
0	.	NA	.	.	.	.	.	.	.	.
0	.	NA	.	.	.	.	.	.	.	.
0	.	NA	.	.	.	.	.	.	.	.
0	.	NA	.	.	.	.	.	.	.	.
0	.	NA	.	.	.	.	.	.	.	REACTOME_IMMUNE_SYSTEM;REA
0	Hypobetali	NA	.	REG	EXTI	INTE	.	PID	.	.
0	.	NA	.	.	.	.	.	.	.	.
0	.	NA	.	SEN	.	.	.	PID	.	.
0	.	NA	.	SYS	.	.	.	.	.	.
0	.	NA	.	.	.	.	.	.	.	.
0	.	NA	.	.	.	.	.	.	.	.
0	.	NA	.	.	.	.	.	.	.	.
0	.	NA	.	.	.	.	.	.	.	.
0	.	NA	.	.	.	.	.	.	.	.
0	.	NA	.	.	.	.	.	.	.	.
0	.	NA	.	.	.	.	.	.	.	.
0	.	NA	.	.	.	.	.	.	.	.
0	.	NA	.	.	.	.	.	.	.	.
0	.	NA	.	.	.	.	.	.	.	.
0	.	NA	.	.	.	.	.	.	.	.
0	.	NA	.	.	.	STR	.	.	.	.
0	.	NA	.	.	.	.	.	.	.	.
0	.	NA	.	ENZ	.	.	KEG(	.	.	REACTOME_DEVELOPMENTAL_BIC
0	.	NA	.	.	.	.	KEG(	.	.	REACTOME_SIGNALING_BY_GPCR
0	.	NA	.	.	.	.	.	.	.	.
0	.	NA	.	.	.	.	.	.	.	.
0	.	NA	.	.	.	.	.	.	.	.
0	.	NA	.	.	.	.	.	.	.	.
0	.	NA	.	.	.	.	.	.	.	.
0	.	NA	.	.	.	.	.	.	.	.
0	.	NA	.	.	.	.	.	.	.	.
0	.	NA	.	BIOF	.	ACIE	.	.	.	.
0	.	NA	.	.	.	IDEN	KEG(	PID	BIO	.
0	AICA-ribos	NA	.	NUC	.	HYD	KEG(	.	.	REACTOME_METABOLISM_OF_NU(
0	.	NA	.	.	.	.	.	.	.	.
0	.	NA	.	.	.	.	.	.	.	REACTOME_FACTORS_INVOLVED_
0	.	NA	.	.	.	.	.	.	.	REACTOME_FACTORS_INVOLVED_
0	.	NA	.	.	.	.	KEG(	.	.	REACTOME_TRANSMISSION_ACRC
0	.	NA	.	.	.	.	.	.	.	.
0	Vesicouret	NA	.	SYS	CELI	IDEN	KEG(	.	.	REACTOME_DEVELOPMENTAL_BIC
0	.	NA	.	DEFI	.	LIPA	KEG(	.	.	REACTOME_PHOSPHOLIPID_META
0	.	NA	.	.	.	.	.	.	.	.
0	.	NA	.	.	.	.	KEG(	.	.	.
0	.	NA	.	.	.	.	.	.	.	.
0	Breast can	NA	.	.	.	PHO	KEG(	PID	BIO	REACTOME_SIGNALLING_BY_NGF;
0	Breast can	NA	CM1	.	.	PHO	KEG(	PID	BIO	REACTOME_SIGNALLING_BY_NGF;
0	.	NA	.	.	.	.	.	.	.	.
0	.	NA	.	.	.	.	.	PID	.	.
0	.	NA	.	.	RIBC	RNA	.	.	.	.

0	.	NA	.	.	.	.	.	.	.	.
0	.	NA	.	.	.	.	.	.	.	.
0	.	NA	.	DETI	INTF	.	KEG(	.	.	REACTOME_GASTRIN_CREB_SIGN
0	.	NA	.	.	.	.	.	.	.	.
0	.	NA	.	.	.	.	.	.	.	.
0	.	NA	.	.	.	.	.	.	.	.
0	.	NA	.	.	.	.	.	.	.	.
0	.	NA	.	.	.	.	.	.	.	.
0	.	NA	.	.	.	.	.	.	.	.
0	.	NA	.	RES	CYT(	.	.	.	.	.
0	{Abacavir I	NA	.	DEFI	CELI	.	KEG(	.	.	REACTOME_ANTIGEN_PROCESSIN
0	.	NA	.	.	.	.	KEG(	PID	.	REACTOME_RNA_POL_I_TRANSCR
0	.	NA	.	REG	.	LIGA	KEG(	PID	BIO	REACTOME_GENERIC_TRANSCRIP
0	.	NA	.	.	.	.	KEG(	.	.	REACTOME_PPARA_ACTIVATES_G
0	.	NA	.	.	.	TRAI	KEG(	.	.	REACTOME_BIOLOGICAL_OXIDATI(
0	.	NA	.	SYS`	.	.	.	PID	.	REACTOME_EXTRACELLULAR_MA`
0	.	NA	.	.	.	.	.	.	.	.
0	.	NA	.	.	.	.	.	.	.	.
0	.	NA	.	.	.	.	.	.	.	.
0	Diabetes m	NA	.	REG	.	CAR	KEG(	PID	BIO	REACTOME_DEVELOPMENTAL_BIC
0	.	NA	.	.	.	.	.	.	.	.
0	.	NA	.	.	.	.	.	.	.	.
0	.	NA	.	.	.	.	.	.	.	.
0	.	NA	.	.	.	.	.	.	.	.
0	.	NA	.	.	.	.	.	.	.	.
0	.	NA	.	.	.	.	.	.	.	.
0	.	NA	.	.	.	.	.	.	.	.
0	.	NA	.	.	.	.	.	.	.	.
0	.	NA	.	.	.	.	.	.	.	.
0	.	NA	.	.	.	.	.	.	.	.
0	.	NA	.	.	.	.	.	.	.	.
0	.	NA	.	.	EXTI	HYD	KEG(	PID	BIO	REACTOME_TRANSMISSION_ACRC
0	Tumoral ca	NA	.	.	.	.	.	.	.	.
0	.	NA	.	GLU	CYT(	PHO	.	.	.	REACTOME_PYRUVATE_METABOL
0	.	NA	.	.	.	.	.	.	.	.
0	.	NA	.	.	.	.	.	.	.	.
0	Premature	NA	.	.	.	.	.	.	.	.
0	.	NA	.	.	.	.	.	.	.	.
0	.	NA	.	.	.	.	.	.	.	.
0	.	NA	.	SIGN	EXTI	REC	.	PID	.	REACTOME_CELL_SURFACE_INTEI
0	.	NA	.	.	.	.	.	.	.	.
0	.	NA	.	.	.	.	.	.	.	.
0	.	NA	.	.	.	.	.	.	.	.
0	.	NA	.	.	.	.	.	.	.	REACTOME_TRANSMEMBRANE_TF
0	Cohen syn	NA	.	.	.	.	.	.	.	.
0	Seizures, t	NA	.	EST/	INTF	CATI	.	.	.	REACTOME_DEVELOPMENTAL_BIC
0	.	NA	.	.	.	.	.	.	.	.
0	Craniosync	NA	.	.	INTF	.	KEG(	.	.	.
0	.	NA	.	.	.	.	.	.	.	.
0	.	NA	.	.	.	.	.	.	.	.
0	.	NA	.	.	.	.	.	.	.	.
0	.	NA	.	.	.	.	.	.	.	.
0	.	NA	.	.	.	.	.	.	.	.
0	.	NA	.	.	ADH	STRI	.	.	.	.

0	.	NA	.	.	.	.	.	.	.	.
0	.	NA	.	.	.	.	.	.	.	REACTOME_TRANSMEMBRANE_TF
0	Maturity-or	NA	.	REG	CYT	.	KEG	.	.	.
0	Aortic valv	NA	.	MUS	NUC	.	KEG	PID	BIO	.
0	.	NA	.	.	MAC	.	.	.	.	.
0	Brugada sy	NA	.	MUS	MEN	CATI	KEG	.	.	REACTOME_DEVELOPMENTAL_BIC
0	.	NA	.	.	NUC	.	.	.	.	.
0	.	NA	.	.	.	.	.	.	.	.
0	.	NA	.	.	.	.	.	.	.	.
0	.	NA	.	.	.	.	.	.	.	.
0	Cardiomyo	NA	.	MAC	ADH	OXID	KEG	PID	BIO	REACTOME_RESPONSE_TO_ELEVA
0	.	NA	.	.	.	.	.	.	.	.
0	.	NA	.	.	.	.	.	.	.	.
0	.	NA	.	.	.	.	.	.	.	.
0	.	NA	.	.	.	.	KEG	.	.	REACTOME_TRANSMEMBRANE_TF
0	.	NA	.	.	.	.	.	.	.	.
0	.	NA	.	.	.	.	.	.	.	.
0	.	NA	.	.	.	.	.	.	.	.
0	.	NA	.	.	.	.	.	.	.	.
0	.	NA	.	.	.	.	.	.	.	.
0	.	NA	.	.	.	.	.	.	.	.
0	.	NA	.	EST/	.	KINA	.	.	.	.
0	.	NA	.	RNA	.	HYD	.	PID	.	.
0	.	NA	.	SYS	.	.	.	.	.	.
0	.	NA	.	.	.	.	.	.	.	.
0	Bladder ca	NA	CM0	.	.	.	KEG	PID	BIO	REACTOME_SIGNALLING_BY_NGF;
0	.	NA	.	SIGN	CYT	CATI	KEG	.	.	REACTOME_SIGNALLING_BY_NGF;
0	Warsaw br	NA	.	POS	ORG	.	.	.	.	REACTOME_DIABETES_PATHWAYS
0	.	NA	.	.	.	.	.	.	.	.
0	.	NA	.	.	.	.	.	.	.	.
0	.	NA	.	.	.	.	.	.	.	.
0	.	NA	.	.	.	.	.	.	.	REACTOME_MEMBRANE_TRAFFICK
0	.	NA	.	.	.	.	.	.	.	.
0	.	NA	.	.	.	.	.	.	.	.
0	.	NA	.	.	.	.	.	.	.	.
0	.	NA	.	.	.	.	.	.	.	.
0	.	NA	.	.	.	.	.	.	.	.
0	Leukemia,	NA	.	ENZ	INTF	TRAI	KEG	.	BIO	.
0	.	NA	.	SYS	INTF	REC	.	.	.	.
0	.	NA	.	.	.	.	.	.	.	.
0	.	NA	.	.	.	.	.	.	.	.
0	.	NA	.	.	.	.	.	.	.	.
0	.	NA	.	.	.	.	.	.	.	.
0	.	NA	.	.	.	.	.	.	.	.
0	.	NA	.	.	.	.	.	.	.	.
0	.	NA	.	.	.	.	.	.	.	.
0	.	NA	.	.	.	.	.	.	.	.
0	.	NA	.	.	.	.	.	.	.	.
0	.	NA	.	.	.	.	.	.	.	.
0	.	NA	.	.	.	.	.	.	.	.
0	.	NA	.	.	.	.	.	.	.	.
0	.	NA	.	.	.	.	.	.	.	.
0	.	NA	.	.	.	.	.	.	.	.

0	.	NA	.	.	.	.	.	.	.	.
0	.	NA	.	.	.	.	.	.	.	.
0	.	NA	.	.	.	.	.	.	.	.
0	.	NA	.	.	.	.	.	.	.	.
0	.	NA	.	.	.	.	.	.	.	REACTOME_TRANSMISSION_ACR
0	.	NA	.	.	EXT	PRO	.	.	.	.
0	.	NA	.	.	.	.	.	.	.	.
0	.	NA	.	.	.	.	.	.	.	.
0	.	NA	.	.	.	.	.	.	.	.
0	Mainzer-Sa	NA	.	.	.	.	.	.	.	.
0	.	NA	.	DEF	EXT	.	.	.	.	.
0	.	NA	.	.	.	.	.	.	.	.
0	.	NA	.	.	.	.	.	.	.	.
0	.	NA	.	.	.	.	.	.	.	.
0	.	NA	.	.	.	.	.	.	.	.
0	.	NA	.	.	.	.	.	.	.	.
0	.	NA	.	.	.	.	.	.	.	.
0	.	NA	.	.	.	.	.	.	.	.
0	.	NA	.	.	.	.	.	.	.	.
0	.	NA	.	.	.	.	.	.	.	.
0	.	NA	.	.	.	.	.	.	.	.
0	.	NA	.	.	.	.	.	.	.	.
0	.	NA	.	.	.	.	.	.	.	.
0	.	NA	.	.	.	.	.	.	.	.
0	.	NA	.	.	.	.	.	.	.	.
0	.	NA	.	.	.	.	.	.	.	.
0	.	NA	.	.	.	.	.	.	.	.
0	.	NA	.	.	.	.	.	.	.	.
0	.	NA	.	.	.	.	.	.	.	.
0	.	NA	.	.	.	.	.	.	.	.
0	.	NA	.	.	.	.	.	.	.	.
0	.	NA	.	.	.	.	.	.	.	.
0	.	NA	.	.	.	.	.	.	.	.
0	.	NA	.	.	.	.	.	.	.	.
0	.	NA	.	.	.	.	.	.	.	.
0	.	NA	.	.	.	.	.	.	.	.
0	.	NA	.	.	.	.	.	.	.	.
0	.	NA	.	.	.	.	.	.	.	REACTOME_PYRUVATE_METABOL
0	.	NA	.	.	.	.	.	.	.	.
0	Fanconi ar	NA	.	DNA	CYT	.	.	PID	.	REACTOME_FANCONI_ANEMIA_PA
0	.	NA	.	.	.	.	KEG	.	.	.
0	.	NA	.	.	.	.	.	.	.	.
0	.	NA	.	NEG	.	TRA	.	.	.	.
0	Mitochond	NA	.	.	.	.	.	.	.	.
0	.	NA	.	.	.	.	.	.	.	.
0	.	NA	.	.	.	.	.	.	.	.
0	.	NA	.	.	.	.	.	.	.	.
0	.	NA	.	.	.	.	.	.	.	.
0	.	NA	.	.	.	.	.	.	.	.
0	.	NA	.	.	.	.	.	.	.	.
0	.	NA	.	.	.	.	.	.	.	.
0	.	NA	.	.	.	.	.	.	.	.
0	Koolen-De	NA	.	.	.	.	.	.	.	.
0	.	NA	.	.	.	CAT	KEG	.	.	.

0	.	NA	.	.	.	.	.	.	.	.
0	.	NA	.	.	.	.	KEG(	.	.	.
0	Amyotroph	NA	.	PRO	ORG	HYD	.	.	.	.
0	.	NA	.	.	.	.	.	.	.	.
0	{Diabetes r	NA	.	REG	INTF	G_PI	KEG(	.	.	REACTOME_GASTRIN_CREB_SIGN
0	.	NA	.	.	.	.	KEG(	PID	BIO	REACTOME_VITAMIN_B5_PANTOTH
0	.	NA	.	DNA	CYT(	PRO	.	.	.	.
0	.	NA	.	.	.	.	KEG(	.	.	.
0	.	NA	.	RNA	.	RNA	.	.	.	REACTOME_DIABETES_PATHWAYS
0	.	NA	.	DNA	.	.	KEG(	.	.	REACTOME_TRANSCRIPTION_COU
0	.	NA	.	EST/	ORG	.	.	.	.	REACTOME_MITOCHONDRIAL_PRC
0	.	NA	.	.	.	.	.	.	.	.
0	.	NA	.	.	.	.	.	.	.	.
0	.	NA	.	.	.	.	.	.	.	.
0	.	NA	.	.	.	.	.	.	.	.
0	.	NA	.	.	.	.	.	.	.	.
0	.	NA	.	EST/	NUC	ACTI	.	.	.	REACTOME_DEVELOPMENTAL_BIC
0	.	NA	.	.	.	.	KEG(	.	.	REACTOME_DIABETES_PATHWAYS
0	.	NA	.	.	.	.	.	.	.	.
0	.	NA	.	LIPIC	CYT(	LIPA	.	.	.	REACTOME_ACYL_CHAIN_REMODE
0	.	NA	.	.	.	.	.	.	.	REACTOME_METABOLISM_OF_AMI
0	.	NA	.	.	.	.	.	.	.	.
0	.	NA	.	.	.	.	.	.	.	.
0	.	NA	.	.	.	.	.	.	.	.
0	Spinocereb	NA	.	DNA	.	PHO	KEG(	PID	BIO	REACTOME_SIGNALLING_BY_NGF;
0	.	NA	.	.	.	.	.	.	.	REACTOME_GENERIC_TRANSCRIP
0	.	NA	.	.	.	.	.	.	.	.
0	.	NA	.	.	.	.	.	.	.	.
0	Spinocereb	NA	.	.	.	.	.	.	.	.
0	.	NA	.	.	.	.	.	.	.	.
0	.	NA	.	.	.	.	.	.	.	.
0	.	NA	.	.	.	.	.	.	.	.
0	.	NA	.	GAM	EXTI	.	.	.	.	.
0	.	NA	.	.	.	.	.	.	.	.
0	.	NA	.	.	.	.	.	.	.	.
0	.	NA	.	.	.	.	.	.	.	.
0	.	NA	.	.	.	.	.	.	.	.
0	.	NA	.	.	.	.	.	.	.	.
0	.	NA	.	.	.	.	.	.	.	.
0	.	NA	.	.	.	.	.	.	.	.
0	.	NA	.	ANA'	.	TRAI	.	.	.	.
0	.	NA	.	.	.	.	.	.	.	.
0	.	NA	.	.	.	.	.	.	.	.
0	.	NA	.	DNA	NUC	.	KEG(	.	BIO	REACTOME_ACTIVATION_OF_THE_
0	.	NA	.	.	.	.	.	.	.	.
0	.	NA	.	.	.	.	.	.	.	.
0	.	NA	.	.	.	.	.	.	.	.
0	.	NA	.	.	.	.	.	.	.	.
0	.	NA	.	ACTI	ORG	CATI	.	.	.	.
0	.	NA	.	.	.	.	.	.	.	.
0	.	NA	.	.	CELI	.	.	.	.	.
0	Neutropeni	NA	.	REG	NON	GTP.	KEG(	PID	.	REACTOME_TCR_SIGNALING;REAC
0	Aland Islar	NA	.	DETI	INTF	CATI	KEG(	.	.	.
0	.	NA	.	.	.	.	.	.	.	.

0	.	NA	.	.	.	.	.	.	.	.
0	.	NA	.	.	.	.	.	.	.	.
0	.	NA	.	.	.	.	.	.	.	.
0	.	NA	.	SYS	ORG	.	.	.	.	REACTOME_SIGNALING_BY_HIPPC
0	Tn polyagg	NA	.	.	.	.	KEG	.	.	REACTOME_O_LINKED_GLYCOSYL
0	.	NA	.	.	.	.	.	.	.	.
0	.	NA	.	.	CYT	.	.	PID	.	REACTOME_SIGNALING_BY_RHO_
0	CHILD syn	NA	.	BIOS	CYT	STEI	KEG	.	.	REACTOME_METABOLISM_OF_LIPI
0	.	NA	.	.	.	.	.	.	.	.
0	.	NA	.	.	.	.	.	.	.	.
0	.	NA	.	.	.	.	.	.	.	.
0	.	NA	.	.	.	.	.	.	.	.
0	.	NA	.	.	.	.	.	.	.	.
0	.	NA	.	REG	CYT	NUC	.	PID	BIO	.
0	.	NA	.	BIOF	.	ACID	KEG	.	.	REACTOME_IMMUNE_SYSTEM;REA
0	.	NA	.	MITC	.	.	KEG	.	.	REACTOME_DNA_REPAIR
0	.	NA	.	.	.	.	.	.	.	.
0	.	NA	.	.	.	.	.	.	.	.
0	.	NA	.	.	.	.	.	.	.	.
0	.	NA	.	.	.	.	.	.	.	.
0	.	NA	.	.	.	.	.	.	.	.
0	.	NA	.	.	.	.	.	.	.	.
0	.	NA	.	.	.	.	.	.	.	.
0	[Rh-negati	NA	.	.	INTR	.	.	.	.	.
0	.	NA	.	MUL	PRO	INTE	.	PID	.	REACTOME_EXTRACELLULAR_MA
0	.	NA	.	.	.	.	.	.	.	.
0	Psychomot	NA	.	.	.	.	.	PID	.	.
0	.	NA	.	.	CYT	.	.	.	.	.
0	.	NA	.	.	CYT	.	.	.	.	.
0	.	NA	.	.	CYT	.	.	.	.	.
0	.	NA	.	.	CYT	.	.	.	.	.
0	.	NA	.	NUC	.	TRAI	.	.	.	.
0	.	NA	.	.	.	.	.	.	.	.
0	.	NA	.	.	.	.	.	.	.	.
0	.	NA	.	.	.	.	.	.	.	.
0	.	NA	.	.	.	.	KEG	.	.	REACTOME_CELL_CELL_COMMUN
0	.	NA	.	.	.	.	.	.	.	.
0	.	NA	.	.	.	.	.	.	.	.
0	.	NA	.	MUL	.	.	.	PID	.	.
0	Cone-rod c	NA	.	EST	CELI	NUC	KEG	.	.	REACTOME_ABC_FAMILY_PROTEI
0	.	NA	.	.	.	.	.	.	.	.
0	.	NA	.	.	MEM	.	.	.	.	.
0	.	NA	.	.	.	.	.	.	.	.
0	.	NA	.	.	.	.	.	.	.	REACTOME_CROSS_PRESENTATI
0	.	NA	.	.	.	.	.	.	.	.
0	.	NA	.	.	.	.	.	.	.	.
0	.	NA	.	.	.	.	.	.	.	.
0	.	NA	.	.	.	.	.	.	.	.
0	.	NA	.	.	.	.	.	.	.	.
0	.	NA	.	.	.	.	.	.	.	.
0	.	NA	.	.	.	.	.	.	.	.
0	.	NA	.	.	.	.	.	.	.	.
0	.	NA	.	.	.	.	.	.	.	.
0	.	NA	.	.	.	.	.	.	.	.

0	.	NA	.	.	.	.	.	.	.	.
0	.	NA	.	.	.	.	.	.	.	.
0	.	NA	.	.	.	.	.	.	.	.
0	.	NA	.	.	.	.	.	.	.	.
0	.	NA	.	.	.	.	.	.	.	.
0	.	NA	.	.	.	.	.	.	.	.
0	.	NA	.	.	.	.	.	.	.	.
0	.	NA	.	.	.	.	.	.	.	.
0	.	NA	.	.	.	.	.	.	.	.
0	.	NA	.	.	.	.	.	.	.	.
0	.	NA	.	RNA	NUC	.	.	.	.	.
0	.	NA	.	.	.	.	.	.	.	.
0	.	NA	.	REG	.	.	.	.	.	.
0	.	NA	.	ACTI	ORG	RAS	KEG(	PID	BIO	REACTOME_SIGNALING_BY_RHO_(
0	.	NA	.	.	.	.	.	.	.	.
0	.	NA	.	.	.	.	.	.	.	.
0	{Myocardia	NA	.	POS	INTF	.	KEG(	.	.	.
0	Microcephi	NA	.	.	.	.	.	.	.	.
0	.	NA	.	.	.	.	.	.	.	.
0	.	NA	.	.	.	.	.	.	.	.
0	.	NA	.	.	.	.	.	.	.	.
0	.	NA	.	.	.	.	KEG(	PID	BIO	.
0	.	NA	.	.	.	.	.	.	.	.
0	.	NA	.	.	.	.	.	PID	.	REACTOME_SIGNALING_BY_RHO_(
0	.	NA	.	.	.	.	KEG(	PID	BIO	.
0	.	NA	.	.	.	.	.	.	.	REACTOME_RNA_POL_I_TRANSCR
0	.	NA	.	.	.	.	.	.	.	.
0	.	NA	.	.	.	LIGA	KEG(	PID	.	REACTOME_METABOLISM_OF_NU(
0	.	NA	.	.	.	.	.	.	.	.
0	Cystinuria	NA	.	EST/	INTF	ACTI	.	.	.	REACTOME_AMINO_ACID_TRANSP
0	Cystinuria	NA	.	EST/	INTF	ACTI	.	.	.	REACTOME_AMINO_ACID_TRANSP
0	.	NA	.	.	.	.	KEG(	PID	.	REACTOME_SIGNALING_BY_RHO_(
0	.	NA	.	.	.	.	.	.	.	.
0	.	NA	.	.	.	.	.	.	.	REACTOME_SIGNALING_BY_RHO_(
0	Miyoshi mu	NA	.	SYS'	MEM	.	.	.	.	.
0	.	NA	.	ISOF	ORG	OXID	KEG(	.	.	.
0	.	NA	.	.	.	.	.	.	.	.
0	.	NA	.	.	.	.	.	.	.	.
0	.	NA	.	.	.	.	.	.	.	.
0	.	NA	.	.	.	.	.	.	.	.
0	.	NA	.	.	.	.	.	.	.	.
0	.	NA	.	.	.	.	.	.	.	.
0	.	NA	.	.	.	.	.	.	.	.
0	.	NA	.	.	.	.	.	.	.	.
0	.	NA	.	.	INTF	CATI	.	.	.	REACTOME_TRANSMEMBRANE_TF
0	.	NA	.	.	.	.	.	.	.	.
0	.	NA	.	.	.	.	KEG(	.	BIO	REACTOME_RNA_POL_I_TRANSCR
0	.	NA	.	.	.	.	.	.	.	.
0	.	NA	.	.	.	.	.	.	.	.
0	.	NA	.	.	.	.	.	.	.	.
0	.	NA	.	.	.	.	.	.	.	.
0	.	NA	.	.	.	.	.	.	.	.
0	.	NA	.	.	.	.	.	.	.	.
0	.	NA	.	NEG	CYT(	.	KEG(	.	.	.

0	.	NA	.	.	.	.	.	.	.	.
0	.	NA	.	.	.	.	.	.	.	.
0	.	NA	.	.	.	.	.	.	.	.
0	.	NA	.	.	NUC	.	.	.	.	REACTOME_RNA_POL_III_TRANSC
0	Carbamoyl	NA	.	.	CYT(	LIGA	KEG(	.	.	REACTOME_METABOLISM_OF_AMI
0	.	NA	.	.	.	.	.	.	.	.
0	.	NA	.	DEFI	NUC	TRAI	.	.	.	.
0	.	NA	.	DEFI	NUC	TRAI	.	.	.	.
0	Brachydac	NA	.	RNA	NUC	TRAI	.	PID	BIO	REACTOME_NOTCH1_INTRACELLU
0	.	NA	.	.	.	.	.	.	.	.
0	.	NA	.	.	.	.	KEG(	.	.	REACTOME_FACTORS_INVOLVED_
0	.	NA	.	.	.	.	.	.	.	.
0	.	NA	.	.	.	.	.	.	.	.
0	.	NA	.	.	.	.	KEG(	.	.	.
0	Gray platel	NA	.	.	.	.	.	.	.	.
0	.	NA	.	.	.	.	.	.	.	.
0	Mitochondı	NA	.	.	.	.	.	.	.	.
0	.	NA	.	.	.	.	.	.	.	.
0	.	NA	.	.	.	.	KEG(	.	.	.
0	.	NA	.	SYS	INTF	LIPC	.	.	.	.
0	.	NA	.	.	.	.	.	.	.	.
0	Robinow s	NA	.	SIGN	EXTI	REC	KEG(	PID	.	REACTOME_SIGNALING_BY_GPCR
0	.	NA	.	.	.	.	KEG(	PID	.	.
0	.	NA	.	.	.	.	.	.	.	.
0	.	NA	.	.	.	.	.	.	.	.
0	.	NA	.	.	.	.	KEG(	.	.	.
0	.	NA	.	.	.	.	KEG(	.	.	.
0	.	NA	.	.	.	.	.	.	.	.
0	.	NA	.	.	.	.	.	.	.	.
0	.	NA	.	.	NUC	END	KEG(	.	.	REACTOME_BASE_EXCISION_REP/
0	.	NA	.	.	NUC	END	KEG(	.	.	REACTOME_BASE_EXCISION_REP/
0	.	NA	.	.	.	.	.	.	.	.
0	Hailey-Hail	NA	.	REG	ORG	CATI	.	.	.	REACTOME_TRANSMEMBRANE_TF
0	.	NA	.	.	.	.	.	.	.	.
0	Blepharopl	NA	.	DNA	NUC	ENZ	.	.	.	.
0	.	NA	.	.	.	.	KEG(	PID	.	.
0	Cutaneous	NA	.	DNA	.	PRO	KEG(	PID	BIO	REACTOME_MEIOSIS;REACTOME_(
0	Bleeding d	NA	.	.	.	.	.	.	.	REACTOME_SIGNALING_BY_GPCR
0	Leukemia,	NA	.	SYS	CYT	PRO	.	.	.	.
0	.	NA	.	.	.	.	.	.	.	.
0	Fanconi-Bi	NA	.	EST/	INTF	SUG	KEG(	PID	.	REACTOME_DEVELOPMENTAL_BIC
0	.	NA	.	.	.	.	.	.	.	.
0	.	NA	.	.	.	.	.	.	.	.
0	.	NA	.	.	ORG	SMA	KEG(	.	.	.
0	.	NA	.	DNA	ORG	.	KEG(	PID	.	REACTOME_CELL_CYCLE;REACTO
0	.	NA	.	.	.	.	.	.	.	.
0	.	NA	.	.	.	.	.	.	.	.
0	.	NA	.	.	.	.	.	.	.	.
0	.	NA	.	.	.	.	.	.	.	.
0	.	NA	.	.	.	.	.	.	.	.
0	.	NA	.	.	.	.	.	.	.	REACTOME_GENERIC_TRANSCRIP
0	.	NA	.	.	INTF	.	.	.	.	REACTOME_TRANSMEMBRANE_TF
0	.	NA	.	REG	NUC	RNA	KEG(	PID	BIO	REACTOME_DEVELOPMENTAL_BIC
0	.	NA	.	.	.	.	.	.	.	.

0	.	NA	.	.	.	NUC	.	.	.	.
0	.	NA	.	.	.	.	.	.	.	.
0	.	NA	.	.	.	.	.	.	.	.
0	.	NA	.	.	.	.	KEGG	.	.	.
0	.	NA	.	.	.	.	KEGG	.	.	.
0	.	NA	.	SIGN	CYT	.	.	.	.	.
0	Wolfram sy	NA	.	.	MAC	.	.	.	.	.
0	.	NA	.	.	EXTI	CAR	.	.	.	REACTOME_EXTRACELLULAR_MAT
0	.	NA	.	.	.	.	.	.	.	.
0	Ceroid lipo	NA	.	.	.	.	KEGG	.	.	.
0	.	NA	.	.	.	.	.	.	.	.
0	[Blood grou	NA	.	.	INTR	.	.	.	.	.
0	.	NA	.	SIGN	CYT	PHO	KEGG	.	.	REACTOME_NITRIC_OXIDE_STIMUL
0	Short rib-p	NA	.	.	CYT	.	.	.	.	.
0	.	NA	.	.	.	.	.	.	.	.
0	.	NA	.	CELL	CYT	CATI	.	.	.	.
0	.	NA	.	.	.	.	.	.	.	.
0	Increased	NA	.	SYS	INTR	REC	KEGG	PID	BIO	REACTOME_GROWTH_HORMONE_
0	.	NA	.	.	.	.	.	.	.	.
0	.	NA	.	.	.	.	.	.	.	.
0	.	NA	.	.	.	.	.	.	.	.
0	.	NA	.	.	.	.	.	.	.	.
0	.	NA	.	.	.	.	.	.	.	REACTOME_RNA_POL_I_TRANSCR
0	.	NA	.	.	.	.	.	.	.	.
0	Sandhoff d	NA	.	.	.	PRO	KEGG	.	.	REACTOME_GLYCOSPHINGOLIPID_
0	Sandhoff d	NA	.	.	.	PRO	KEGG	.	.	REACTOME_GLYCOSPHINGOLIPID_
0	D-bifunctio	NA	.	.	CYT	LIPID	KEGG	.	BIO	REACTOME_BILE_ACID_AND_BILE_
0	Usher sync	NA	.	.	.	.	KEGG	.	.	REACTOME_CYTOSOLIC_TRNA_AM
0	Usher sync	NA	.	.	.	.	KEGG	.	.	REACTOME_CYTOSOLIC_TRNA_AM
0	.	NA	.	SIGN	NUC	HYD	.	.	.	.
0	Hyperekple	NA	.	REG	INTR	EXC	KEGG	.	.	REACTOME_TRANSMEMBRANE_TR
0	.	NA	.	.	.	.	.	.	.	.
0	.	NA	.	.	.	.	.	.	.	REACTOME_DEVELOPMENTAL_BIO
0	.	NA	.	.	.	.	.	.	.	.
0	.	NA	.	.	.	.	KEGG	.	.	.
0	.	NA	.	.	.	.	.	.	.	.
0	.	NA	.	CELL	.	.	.	.	.	.
0	.	NA	.	RES	CYT	.	.	.	.	.
0	.	NA	.	GAM	.	.	.	.	.	.
0	.	NA	.	.	.	.	KEGG	.	.	REACTOME_MEIOSIS;REACTOME_(
0	.	NA	.	.	.	.	.	.	.	.
0	.	NA	.	.	.	.	.	.	.	.
0	.	NA	.	.	.	.	.	.	.	.
0	.	NA	.	RNA	NUC	RNA	KEGG	.	.	.
0	.	NA	.	RNA	NUC	RNA	KEGG	.	.	.
0	.	NA	.	.	.	.	.	.	.	.
0	{Asthma, s	NA	.	RNA	CELL	REC	KEGG	PID	BIO	REACTOME_EXTRINSIC_PATHWAY
0	.	NA	.	.	.	.	.	.	.	.
0	.	NA	.	BIOF	NUC	PHO	.	.	.	.
0	.	NA	.	.	.	.	.	.	.	.
0	Adrenal hy	NA	.	.	.	.	KEGG	.	.	REACTOME_METABOLISM_OF_STE
0	.	NA	.	.	.	.	.	.	.	.
0	.	NA	.	RNA	NUC	SPE	KEGG	.	.	.
0	.	NA	.	.	.	.	.	.	.	.

0	Combined	NA	.	.	.	.	KEGC	.	.	REACTOME_MITOCHONDRIAL_TRN
0	.	NA	.	.	INTF	G_PI	.	.	.	.
0	.	NA	.	.	.	.	.	.	.	REACTOME_DEFENSINS;REACTOM
0	.	NA	.	MITC	ORG	PHO	KEGC	.	.	.
0	.	NA	.	REG	CYT	ENZ	.	.	.	.
0	.	NA	.	.	.	.	.	.	.	.
0	.	NA	.	.	.	.	.	.	.	.
0	.	NA	.	.	.	.	.	.	.	.
0	.	NA	.	.	.	.	.	.	.	.
0	.	NA	.	RNA	NUC	.	.	.	.	.
0	Spastic pa	NA	.	.	.	.	.	.	.	.
0	.	NA	.	.	.	.	.	.	.	.
0	.	NA	.	.	.	.	.	.	.	.
0	.	NA	.	.	.	.	.	.	.	.
0	.	NA	.	.	.	.	.	.	.	.
0	.	NA	.	.	.	.	.	.	.	.
0	.	NA	.	.	.	.	.	.	.	.
0	.	NA	.	.	.	.	.	.	.	.
0	.	NA	.	.	.	.	.	.	.	.
0	.	NA	.	.	.	.	.	.	.	.
0	.	NA	.	.	.	.	.	.	.	.
0	.	NA	.	.	.	.	.	.	.	.
0	.	NA	.	.	.	.	.	.	.	.
0	.	NA	.	.	.	.	KEGC	.	.	REACTOME_O_LINKED_GLYCOSYL
0	{Prostate c	NA	.	REG	CYT	STR	KEGC	PID	.	.
0	.	NA	.	.	.	.	.	.	.	.
0	.	NA	.	.	.	.	.	.	.	.
0	Alopecia, r	NA	.	.	.	.	.	.	.	.
0	Alopecia, r	NA	.	.	.	.	.	.	.	.
0	.	NA	.	.	.	.	.	.	.	.
0	.	NA	.	.	.	.	.	.	.	.
0	Weaver sy	NA	.	RNA	NUC	HIST	.	.	.	.
0	.	NA	.	.	.	.	.	.	.	.
0	.	NA	.	.	.	.	.	.	.	.
0	.	NA	.	.	.	.	.	.	.	.
0	Maturity-or	NA	.	SIGN	.	.	.	PID	.	REACTOME_ANTIGEN_ACTIVATES_
0	.	NA	.	.	.	.	.	.	.	.
0	.	NA	.	.	.	.	.	.	.	.
0	.	NA	.	.	.	.	.	.	.	REACTOME_MEMBRANE_TRAFFICI
0	{?Schizoph	NA	.	NEG	.	TRA	KEGC	PID	BIO	REACTOME_SIGNALING_BY_ERBB
0	.	NA	.	.	.	.	KEGC	.	.	.
0	.	NA	.	EST	.	.	KEGC	.	.	REACTOME_INSULIN_RECEPTOR_I
0	.	NA	.	.	NUC	IDEN	.	PID	BIO	REACTOME_MEIOSIS;REACTOME_
0	.	NA	.	SYS	.	TRA	.	PID	.	REACTOME_NOTCH1_INTRACELLU
0	.	NA	.	.	.	.	.	.	.	.
0	.	NA	.	.	.	.	.	.	.	.
0	.	NA	.	.	.	.	.	.	.	.
0	.	NA	.	.	.	.	.	.	.	.
0	.	NA	.	.	.	.	.	.	.	.
0	.	NA	.	RNA	NUC	RNA	KEGC	.	.	.
0	.	NA	.	.	.	.	.	.	.	.
0	Spastic pa	NA	.	.	.	.	.	.	.	.
0	Spastic pa	NA	.	.	.	.	.	.	.	.
0	Mental reta	NA	.	.	.	.	.	.	.	.

0	.	NA	.	.	.	.	.	.	.	.
0	.	NA	.	.	.	.	.	.	.	.
0	.	NA	.	REG	.	.	KEG(	PID	BIO	REACTOME_TRAF6_MEDIATED_IRF
0	.	NA	.	.	.	.	.	.	.	.
0	.	NA	.	.	.	.	.	.	.	.
0	.	NA	.	.	.	.	.	.	.	.
0	.	NA	.	.	.	.	.	.	.	.
0	.	NA	.	.	.	.	.	.	.	.
0	.	NA	.	.	.	.	.	.	.	.
0	Choreoaca	NA	.	.	.	.	.	.	.	.
0	.	NA	.	.	.	.	.	PID	.	REACTOME_NOTCH1_INTRACELLU
0	.	NA	.	.	.	.	.	.	.	.
0	.	NA	.	.	.	.	.	.	.	.
0	.	NA	.	.	.	.	.	PID	.	.
0	Nail-patella	NA	.	RNA	NUC	TRAI	.	.	.	.
0	.	NA	.	CELI	.	.	.	.	.	.
0	.	NA	.	.	.	.	.	.	.	.
0	.	NA	.	.	.	.	.	.	.	.
0	.	NA	.	ENZ'	.	.	KEG(	PID	BIO	REACTOME_SIGNALLING_BY_NGF;
0	.	NA	.	.	.	.	.	.	.	.
0	.	NA	.	.	.	.	.	.	.	.
0	.	NA	.	BIOF	CYT(	IDEN	.	.	.	REACTOME_METABOLISM_OF_MRI
0	.	NA	.	.	.	.	.	.	.	.
0	.	NA	.	.	.	.	.	.	.	.
0	.	NA	.	.	.	.	.	.	.	.
0	.	NA	.	.	.	.	.	.	.	.
0	.	NA	.	.	.	.	.	.	.	.
0	.	NA	.	.	.	.	.	.	.	.
0	.	NA	.	.	.	.	KEG(	PID	.	REACTOME_DEVELOPMENTAL_BIC
0	.	NA	.	.	.	.	KEG(	PID	.	REACTOME_DEVELOPMENTAL_BIC
0	Deafness,	NA	.	REG	NON	.	.	.	.	.
0	.	NA	.	.	.	.	.	.	.	.
0	.	NA	.	.	.	.	.	.	.	.
0	Microcepha	NA	.	M_P	ORG	MICF	.	.	.	REACTOME_MHC_CLASS_II_ANTIG
0	.	NA	.	.	NUC	TRAI	.	.	.	.
0	{Age-relate	NA	.	.	.	LIPIL	.	PID	.	.
0	.	NA	.	.	.	.	.	.	.	.
0	.	NA	.	.	.	.	.	.	.	REACTOME_O_LINKED_GLYCOSYL
0	.	NA	.	.	.	.	.	.	.	.
0	Charcot-M	NA	.	.	MEM	.	.	.	.	.
0	.	NA	.	.	.	.	.	.	.	.
0	Denys-Dra	NA	.	.	.	.	.	PID	BIO	.
0	.	NA	.	.	NUC	NUC	KEG(	.	.	.
0	.	NA	.	.	.	.	KEG(	.	.	.
0	.	NA	.	.	.	.	.	.	.	.
0	.	NA	.	.	.	.	.	.	.	.
0	.	NA	.	.	.	.	.	.	.	.
0	.	NA	.	.	.	.	.	.	.	.
0	.	NA	.	.	.	.	.	.	.	.
0	.	NA	.	.	.	.	.	.	.	.
0	.	NA	.	.	.	.	.	.	.	.
0	.	NA	.	POS	.	.	KEG(	PID	.	REACTOME_VEGF_LIGAND_RECEF
0	.	NA	.	.	CYT(	DRU	.	.	.	.
0	.	NA	.	.	CYT(	DRU	.	.	.	.

0	.	NA	.	.	.	.	.	.	.	.
0	Glaucoma	NA	.	.	.	.	.	.	.	.
0	.	NA	.	.	.	.	KEG(	.	.	.
0	.	NA	.	.	INTF	.	KEG(	PID	.	REACTOME_DEVELOPMENTAL_BIC
0	.	NA	.	.	ORG	PEP'	.	.	.	.
0	.	NA	.	.	ORG	PEP'	.	.	.	.
0	Megalence	NA	.	.	.	.	.	.	.	.
0	.	NA	.	.	.	.	.	.	.	.
0	.	NA	.	.	.	HYD	KEG(	.	.	REACTOME_GLYCOLYSIS;REACTO
0	.	NA	.	.	.	HYD	KEG(	.	.	REACTOME_GLYCOLYSIS;REACTO
0	.	NA	.	.	.	.	.	.	.	REACTOME_MEMBRANE_TRAFFICI
0	Warsaw br	NA	.	POS	ORG	.	.	.	.	REACTOME_DIABETES_PATHWAYS
0	.	NA	.	.	.	.	.	.	.	.
0	.	NA	.	.	.	.	.	.	.	.
0	.	NA	.	RNA	NUC	RNA	.	.	.	.
0	Vitamin D-(	NA	.	BIOS	.	.	KEG(	.	.	REACTOME_METABOLISM_OF_STE
0	Vitamin D-(	NA	.	BIOS	.	.	KEG(	.	.	REACTOME_METABOLISM_OF_STE
0	.	NA	.	.	.	.	.	.	.	.
0	.	NA	.	.	.	.	.	.	.	.
0	Deafness,	NA	.	.	.	.	.	.	.	.
0	.	NA	.	.	.	.	.	.	.	.
0	Mental reta	NA	.	CELI	.	.	.	PID	BIO	.
0	Arthrogryp	NA	.	.	CYT(	STRI	.	.	.	REACTOME_STRIATED_MUSCLE_C
0	.	NA	.	.	.	.	.	.	.	.
0	Transpositi	NA	.	.	.	.	.	.	.	REACTOME_DEVELOPMENTAL_BIC
0	.	NA	.	.	.	.	.	.	.	.
0	.	NA	.	ACTI	.	LIPA	KEG(	PID	.	REACTOME_ACYL_CHAIN_REMODE
0	.	NA	.	.	.	.	.	.	.	.
0	.	NA	.	.	.	.	.	.	.	.
0	.	NA	.	EST/	.	.	.	PID	.	.
0	.	NA	.	.	ORG	.	.	.	.	.
0	.	NA	.	.	.	.	.	.	.	.
0	.	NA	.	.	.	.	.	PID	.	REACTOME_FACTORS_INVOLVED_
0	Spinocereb	NA	.	SYS'	.	REC	KEG(	.	.	.
0	.	NA	.	.	.	.	.	.	.	.
0	.	NA	.	.	.	.	.	.	.	.
0	.	NA	.	.	.	.	.	.	.	.
0	.	NA	.	.	.	.	.	.	.	.
0	.	NA	.	.	.	.	.	.	.	.
0	.	NA	.	.	.	.	.	.	.	.
0	Dystonia, [	NA	.	REG	.	HYD	KEG(	.	.	REACTOME_TETRAHYDROBIOPTEI
0	.	NA	.	.	.	.	.	.	.	.
0	{Pheochroi	NA	.	RNA	.	TRAI	KEG(	PID	BIO	REACTOME_CELL_CYCLE;REACTO
0	{Pheochroi	NA	.	RNA	.	TRAI	KEG(	PID	BIO	REACTOME_CELL_CYCLE;REACTO
0	Microphtha	NA	.	.	.	.	.	.	.	.
0	.	NA	.	.	.	.	.	.	.	.
0	.	NA	.	.	.	.	.	.	.	.
0	.	NA	.	.	.	.	.	.	.	.
0	.	NA	.	.	.	CYS'	KEG(	.	.	REACTOME_METABOLISM_OF_STE
0	.	NA	.	.	.	.	.	.	.	.
0	.	NA	.	SIGN	.	.	.	PID	BIO	.
0	.	NA	.	.	.	.	.	.	.	.
0	.	NA	.	.	.	.	.	.	.	.
0	.	NA	.	.	.	.	.	.	.	.

0	.	NA	.	.	.	.	.	.	.	.
0	[Skin/hair/e	NA	.	.	.	.	KEG(	.	.	REACTOME_IMMUNE_SYSTEM;REA
0	.	NA	.	.	.	.	.	.	.	.
0	.	NA	.	.	.	.	.	.	.	.
0	.	NA	.	.	.	.	.	.	.	.
0	.	NA	.	.	.	.	.	.	.	.
0	.	NA	.	.	.	.	.	.	.	.
0	.	NA	.	.	.	.	.	.	.	.
0	.	NA	.	.	.	.	.	.	.	.
0	.	NA	.	.	.	.	.	.	.	REACTOME_DEVELOPMENTAL_BIC
0	.	NA	.	.	.	.	.	.	.	.
0	.	NA	.	.	.	.	.	.	.	.
0	.	NA	.	MAC	EUK.	TRAI	.	.	.	REACTOME_TRANSLATION;REACT
0	.	NA	.	.	.	.	.	.	.	.
0	.	NA	.	.	.	.	.	.	.	.
0	.	NA	.	.	.	.	.	.	.	.
0	.	NA	.	SYS	MEM	ENZ	.	.	.	.
0	.	NA	.	.	.	.	.	.	.	.
0	Cardiofacic	NA	.	SIGN	.	PHO	KEG(	PID	BIO	REACTOME_SIGNALLING_BY_NGF;
0	.	NA	.	SIGN	.	.	KEG(	PID	BIO	REACTOME_SIGNALLING_BY_NGF;
0	.	NA	.	.	.	.	.	.	.	.
0	.	NA	.	.	.	.	.	.	.	.
0	.	NA	.	EST/	INTF	CATI	KEG(	.	.	REACTOME_TRANSMISSION_ACRC
0	.	NA	.	.	.	.	.	.	.	.
0	.	NA	.	.	.	.	.	.	.	.
0	Fanconi ar	NA	.	.	.	.	.	PID	.	.
0	Arthrogryp	NA	.	EST/	.	.	.	.	.	.
0	.	NA	.	.	INTF	LIPIC	.	.	.	.
0	{Coronary	NA	.	MUS	NUC	.	.	PID	BIO	REACTOME_SIGNALLING_BY_NGF;
0	.	NA	.	.	.	.	KEG(	.	.	.
0	.	NA	.	.	.	.	.	.	.	.
0	Osteopetrc	NA	.	.	.	.	.	.	.	.
0	.	NA	.	.	ORG	PRO	.	.	.	.
0	.	NA	.	.	.	.	.	.	.	.
0	.	NA	.	SIGN	INTF	REC	KEG(	.	BIO	.
0	.	NA	.	.	.	.	.	.	.	.
0	.	NA	.	.	.	.	.	.	.	.
0	.	NA	.	.	.	.	.	.	.	.
0	.	NA	.	BIOF	CYT	PHO	.	.	.	REACTOME_METABOLISM_OF_MRI
0	.	NA	.	BIOF	CYT	TRAI	.	PID	BIO	REACTOME_INSULIN_RECEPTOR_
0	.	NA	.	.	.	.	.	.	.	REACTOME_RNA_POL_III_TRANSC
0	.	NA	.	.	.	.	.	.	.	.
0	.	NA	.	EST/	.	PRO	.	.	.	.
0	.	NA	.	EST/	.	PRO	.	.	.	.
0	.	NA	.	.	.	.	.	.	.	.
0	.	NA	.	.	.	.	.	.	.	.
0	{Systemic	NA	.	.	INTF	.	KEG(	PID	BIO	REACTOME_CELL_SURFACE_INTEI
0	.	NA	.	.	.	.	.	.	.	.
0	.	NA	.	.	CYT	.	.	.	.	REACTOME_ASSOCIATION_OF_TR
0	.	NA	.	.	.	.	KEG(	.	.	.
0	.	NA	.	RNA	NUC	CATI	.	.	.	.
0	.	NA	.	.	.	.	.	.	.	.
0	.	NA	.	.	.	.	.	.	.	.
0	.	NA	.	.	.	.	.	.	.	.

0	.	NA	.	.	.	.	.	.	.	.
0	Bleeding d	NA	.	EST/	INTF	CATI	KEG(	.	.	REACTOME_ELEVATION_OF_CYTO
0	.	NA	.	.	.	.	.	.	.	.
0	.	NA	.	SYS'	INTF	.	KEG(	.	BIO	.
0	.	NA	.	.	.	.	KEG(	.	.	.
0	.	NA	.	.	.	MOT	KEG(	.	.	.
0	.	NA	.	GAM	ORG	.	.	.	.	.
0	Mitochondi	NA	.	.	.	.	.	.	.	.
0	.	NA	.	RNA	ORG	RNA	KEG(	PID	.	REACTOME_DEVELOPMENTAL_BIC
0	.	NA	.	.	.	.	.	.	.	.
0	.	NA	.	.	.	.	.	.	.	.
0	.	NA	.	.	.	.	.	.	.	.
0	.	NA	.	.	.	.	.	.	.	.
0	.	NA	.	.	.	.	.	.	.	.
0	.	NA	.	.	.	.	.	.	.	.
0	.	NA	.	.	.	.	.	.	.	.
0	.	NA	.	.	NUC	HIST	.	.	.	.
0	.	NA	.	.	NUC	HIST	.	.	.	.
0	.	NA	.	REG	ORG	.	.	.	.	REACTOME_SIGNALING_BY_RHO_(
0	.	NA	.	.	.	.	.	.	.	.
0	.	NA	.	.	.	.	.	.	.	.
0	.	NA	.	.	.	.	.	.	.	.
0	Acetyl-CoA	NA	.	.	.	LIGA	KEG(	.	BIO	REACTOME_TRIGLYCERIDE_BIOSY
0	.	NA	.	EST/	ORG	.	.	.	.	.
0	.	NA	.	.	.	.	.	.	.	.
0	.	NA	.	RNA	NUC	RNA	.	PID	BIO	REACTOME_DEVELOPMENTAL_BIC
0	.	NA	.	.	.	.	.	.	.	.
0	.	NA	.	.	.	.	.	.	.	.
0	Ovalocytos	NA	.	REG	INTF	ANIC	.	.	.	REACTOME_TRANSMEMBRANE_TF
0	.	NA	.	.	.	.	KEG(	.	.	.
0	.	NA	.	.	MEM	CATI	KEG(	PID	.	REACTOME_DEVELOPMENTAL_BIC
0	.	NA	.	.	CYT(	REC	.	.	.	.
0	.	NA	.	.	.	.	KEG(	.	.	.
0	.	NA	.	.	.	.	KEG(	.	.	.
0	.	NA	.	.	.	.	.	.	.	.
0	Hemophag	NA	.	.	.	.	.	.	.	.
0	.	NA	.	.	.	.	.	.	.	.
0	.	NA	.	SYS'	MEM	MOL	KEG(	PID	BIO	.
0	.	NA	.	.	.	.	.	.	.	.
0	.	NA	.	REG	INTF	.	.	.	.	.
0	.	NA	.	.	.	.	.	.	.	.
0	.	NA	.	.	.	.	.	.	.	.
0	.	NA	.	.	CYT(	OXIE	.	.	.	.
0	.	NA	.	.	.	.	.	.	.	.
0	.	NA	.	RNA	NUC	S_AI	.	.	.	REACTOME_RNA_POL_II_TRANSCF
0	.	NA	.	.	.	.	.	.	.	.
0	Scalp-ear-I	NA	.	.	.	.	.	.	.	.
0	.	NA	.	.	.	.	.	.	.	.
0	Pitt-Hopkin	NA	.	RNA	NUC	TRAI	.	PID	.	REACTOME_DEVELOPMENTAL_BIC
0	.	NA	.	.	.	.	.	.	.	.
0	Osteolysis,	NA	.	POS	.	REC	KEG(	.	BIO	.
0	.	NA	.	.	.	.	.	.	.	.
0	.	NA	.	.	.	.	.	.	.	REACTOME_TRANSMEMBRANE_TF
0	.	NA	.	.	.	.	.	.	.	.

0 .	NA	.	.	.	.	.	.	.	REACTOME_SIGNALING_BY_RHO_(
0 .	NA	.	.	.	.	.	.	.	REACTOME_SIGNALING_BY_RHO_(
0 Leukemia,	NA	.	RNA	.	PRO	.	PID	.	REACTOME_DEVELOPMENTAL_BIC
0 Leukemia,	NA	.	RNA	.	PRO	.	PID	.	REACTOME_DEVELOPMENTAL_BIC
0 .	NA	.	.	.	.	.	.	.	.
0 .	NA	.	.	.	MET	KEG(	.	.	.
0 .	NA	.	.	.	MET	KEG(	.	.	.
0 .	NA	.	RNA	.	TRAI	.	PID	.	.
0 .	NA	.	CHR	NUC	CHR	.	.	.	.
0 .	NA	.	.	.	.	.	.	.	.
0 Hemophag	NA	.	.	.	.	.	.	.	.
0 {Reduced 1	NA	.	REG	EXTI	ENZ'	KEG(	.	.	REACTOME_DEVELOPMENTAL_BIC
0 .	NA	.	.	.	.	.	.	.	.
0 .	NA	.	.	.	.	.	.	.	.
0 .	NA	.	.	MEN	.	.	.	.	REACTOME_O_LINKED_GLYCOSYL
0 .	NA	.	.	MEN	.	.	.	.	REACTOME_O_LINKED_GLYCOSYL
0 .	NA	.	.	MEN	.	.	.	.	REACTOME_O_LINKED_GLYCOSYL
0 .	NA	.	.	MEN	.	.	.	.	REACTOME_O_LINKED_GLYCOSYL
0 .	NA	.	.	.	TRAI	.	.	.	.
0 .	NA	.	.	.	.	.	.	.	REACTOME_GENERIC_TRANSCRIP
0 .	NA	.	EST/	.	.	.	.	.	.
0 .	NA	.	RNA	ORG	RNA	.	PID	BIO	REACTOME_SMAD2_SMAD3_SMAD
0 .	NA	.	.	.	.	.	.	.	.
0 .	NA	.	.	.	.	.	.	.	.
0 Thyroid dy:	NA	.	.	INTF	INOF	.	.	.	REACTOME_METABOLISM_OF_AMI
0 .	NA	.	.	.	.	.	.	.	.
0 .	NA	.	.	.	.	.	.	.	.
0 .	NA	.	.	.	.	.	.	.	.
0 .	NA	.	.	.	.	.	.	.	.
0 .	NA	.	.	.	.	.	.	.	.
0 .	NA	.	.	.	.	.	.	.	.
0 .	NA	.	.	.	.	.	.	.	.
0 .	NA	.	.	.	.	.	.	.	.
0 .	NA	.	.	.	.	.	.	.	.
0 .	NA	.	.	.	.	.	.	.	.
0 .	NA	.	.	.	.	.	.	.	.
0 .	NA	.	CELI	MEN	RAS	KEG(	PID	BIO	REACTOME_SIGNALLING_BY_NGF;
0 .	NA	.	.	.	GLU'	KEG(	.	.	REACTOME_TRANSMISSION_ACRC
0 .	NA	.	.	.	GLU'	KEG(	.	.	REACTOME_TRANSMISSION_ACRC
0 .	NA	.	.	.	.	.	.	.	.
0 .	NA	.	BEH	CELI	ENZ'	KEG(	PID	.	REACTOME_METABOLISM_OF_PR(
0 .	NA	.	BEH	CELI	ENZ'	KEG(	PID	.	REACTOME_METABOLISM_OF_PR(
0 .	NA	.	.	.	.	.	.	.	.
0 .	NA	.	SYS'	.	TRAI	.	.	.	.
0 .	NA	.	.	.	.	.	.	.	.
0 .	NA	.	.	.	.	.	.	.	REACTOME_GENERIC_TRANSCRIP
0 .	NA	.	.	.	.	.	.	.	.
0 .	NA	.	.	.	.	.	.	.	.
0 .	NA	.	REG	NUC	REC	KEG(	.	.	.
0 .	NA	.	.	.	SMA	.	.	.	.
0 .	NA	.	.	.	SMA	.	.	.	.
0 .	NA	.	SIGI	INTF	.	.	.	.	REACTOME_CELL_CELL_COMMUN
0 Spinocereb	NA	.	.	.	.	.	.	.	.
0 .	NA	.	.	.	.	.	.	.	.

0	.	NA	.	.	.	.	KEG(	.	.	.
0	Epileptic er	NA	.	.	CYT(	.	KEG(	PID	BIO	REACTOME_GASTRIN_CREB_SIGN
0	.	NA	.	.	.	.	.	.	.	.
0	.	NA	.	.	.	.	.	.	.	.
0	.	NA	.	.	.	.	.	.	.	.
0	.	NA	.	.	.	.	.	.	.	.
0	.	NA	.	SYS	.	.	KEG(	PID	BIO	REACTOME_SIGNALING_BY_ILS;RE
0	.	NA	.	RNA	NUC	TRA	.	PID	.	REACTOME_DEVELOPMENTAL_BIC
0	.	NA	.	RNA	NUC	TRA	.	PID	.	REACTOME_DEVELOPMENTAL_BIC
0	.	NA	.	.	.	.	.	.	.	.
0	.	NA	.	.	.	.	.	PID	.	.
0	.	NA	.	.	.	.	.	.	.	.
0	.	NA	.	.	.	.	.	.	.	.
0	.	NA	.	.	.	.	.	.	.	.
0	.	NA	.	.	.	.	.	.	BIO	.
0	.	NA	.	.	.	.	.	.	.	.
0	.	NA	.	.	.	.	.	.	.	.
0	.	NA	.	.	.	.	.	.	.	.
0	.	NA	.	.	.	.	.	.	.	.
0	.	NA	.	.	.	.	.	.	.	.
0	.	NA	.	.	.	.	.	.	.	.
0	.	NA	.	.	.	.	.	.	.	.
0	.	NA	.	.	.	.	.	.	.	.
0	.	NA	.	.	CYT(	.	.	.	.	.
0	Homocystii	NA	CM9	.	CYT(	HYD	KEG(	.	.	REACTOME_SULFUR_AMINO_ACID
0	.	NA	.	.	.	.	.	.	.	.
0	Knobloch s	NA	.	SYS	PRO	.	.	PID	.	.
0	.	NA	.	.	.	.	.	.	.	.
0	.	NA	.	.	.	.	.	.	.	.
0	.	NA	.	.	.	.	.	.	.	.
0	.	NA	.	.	.	.	.	.	.	.
0	Hyperprolir	NA	CM0	.	.	OXID	KEG(	.	.	REACTOME_METABOLISM_OF_AMI
0	.	NA	.	.	.	.	.	.	.	.
0	.	NA	.	.	.	.	.	.	.	.
0	.	NA	.	.	.	.	.	.	.	.
0	.	NA	.	.	.	.	.	.	.	.
0	.	NA	.	.	.	.	.	.	.	.
0	.	NA	.	ANA	.	.	.	.	.	.
0	.	NA	.	EST/	CYT(	.	.	PID	.	.
0	.	NA	.	PAT	EXTI	.	KEG(	.	.	REACTOME_PRE_NOTCH_EXPRES
0	.	NA	.	.	.	.	.	.	.	.
0	.	NA	.	.	INTF	.	.	.	.	.
0	Nephronop	NA	.	.	.	.	.	.	.	.
0	Colorectal	NA	.	REG	NUC	TRA	KEG(	PID	BIO	REACTOME_DEVELOPMENTAL_BIC
0	.	NA	.	RNA	.	RNA	.	.	.	REACTOME_PPARA_ACTIVATES_G
0	.	NA	.	.	.	.	KEG(	.	.	REACTOME_MEIOSIS;REACTOME_(
0	.	NA	.	.	.	.	.	.	.	.
0	.	NA	.	CELI	.	NUC	.	.	.	.
0	.	NA	.	.	.	.	KEG(	.	.	.
0	.	NA	.	.	.	.	KEG(	.	.	.
0	.	NA	.	.	.	.	.	.	.	.
0	.	NA	.	GAM	ORG	.	.	.	.	.
0	.	NA	.	.	.	.	.	.	.	.
0	.	NA	.	POS	.	RNA	KEG(	.	BIO	REACTOME_TRAFFICKING_AND_PI

0	.	NA	.	POS	.	RNA	KEG(	.	.	REACTOME_TRAFFICKING_AND_PI
0	Becker mu	NA	.	BIOS	PRO	ACTI	KEG(	.	BIO	REACTOME_STRIATED_MUSCLE_C
0	Brunner sy	NA	.	BEH.	.	.	KEG(	.	.	REACTOME_BIOLOGICAL_OXIDATI(
0	?Sarcoma,	NA	.	.	NUC	TRAI	.	.	.	.
0	.	NA	.	.	EXTI	REC	.	.	.	.
0	.	NA	.	.	.	.	.	.	.	.
0	.	NA	.	.	.	.	.	.	.	.
0	.	NA	.	.	.	.	.	.	.	.
0	.	NA	.	.	.	.	.	.	.	.
0	.	NA	.	.	.	.	.	.	.	REACTOME_TCA_CYCLE_AND_RES
0	.	NA	.	.	.	.	.	.	.	.
0	Lujan-Fryn	NA	.	RNA	NUC	RNA	.	PID	.	REACTOME_DEVELOPMENTAL_BIC
0	{Asperger :	NA	.	.	.	.	KEG(	.	.	.
0	.	NA	.	.	.	.	.	.	.	.
0	.	NA	.	.	.	.	.	.	.	.
0	.	NA	.	.	.	.	.	.	.	.
0	.	NA	.	.	.	.	.	.	.	.
0	.	NA	.	.	.	.	.	.	.	.
0	.	NA	.	.	.	.	.	.	.	.
0	Mental reta	NA	.	.	.	.	KEG(	PID	BIO	REACTOME_SIGNALING_BY_RHO_(
0	.	NA	.	MUL	.	DNA	.	.	.	.
0	.	NA	.	DNA	.	HYD	.	.	.	.
0	.	NA	.	.	INTF	.	.	.	.	REACTOME_APOPTOTIC_CLEAVAG
0	.	NA	.	.	.	.	.	.	.	.
0	.	NA	.	.	.	.	.	.	.	.
0	.	NA	.	.	.	.	.	.	.	.
0	.	NA	.	.	.	.	.	.	.	.
0	.	NA	.	.	.	.	.	.	.	.
0	.	NA	.	.	.	.	.	.	.	.
0	.	NA	.	.	.	.	.	.	.	.
0	.	NA	.	.	.	.	.	.	.	.
0	.	NA	.	.	.	.	.	.	.	.
0	.	NA	.	.	.	.	.	.	.	.
0	.	NA	.	.	.	.	.	.	.	.
0	.	NA	.	CEN	ORG	STRI	.	.	.	.
0	Rhabdomy	NA	.	CELI	.	TRAI	.	.	.	.
0	.	NA	.	REG	CYT(	.	.	.	.	REACTOME_INTERFERON_ALPHA_
0	.	NA	.	NUC	.	TRAI	.	.	.	.
0	.	NA	.	.	.	.	.	.	.	.
0	.	NA	.	.	.	.	.	.	.	.
0	.	NA	.	.	.	.	.	.	.	.
0	.	NA	.	.	.	.	.	.	.	.
0	.	NA	.	.	.	.	.	.	.	.
0	.	NA	.	.	.	.	.	.	.	.
0	.	NA	.	.	.	.	.	.	.	.
0	.	NA	.	.	.	.	.	.	.	.
0	.	NA	.	.	.	.	.	.	.	.
0	.	NA	.	.	.	.	.	.	.	.
0	.	NA	.	.	.	.	.	.	.	.
0	.	NA	.	.	.	.	.	.	.	.
0	.	NA	.	.	.	.	.	.	.	.
0	.	NA	.	.	.	.	.	.	.	.
0	.	NA	.	.	.	.	.	.	.	.
0	.	NA	.	.	.	.	.	.	.	.

0	.	NA	.	.	.	.	.	.	.	.
0	.	NA	.	.	.	.	.	.	.	.
0	.	NA	.	.	.	.	.	.	.	.
0	.	NA	.	.	.	.	.	.	.	.
0	.	NA	.	.	.	.	KEG(	PID	.	REACTOME_DEVELOPMENTAL_BIC
0	.	NA	.	.	.	.	.	.	.	.
0	.	NA	.	.	.	.	.	.	.	.
0	.	NA	.	.	.	.	.	.	.	.
0	.	NA	.	.	.	.	.	.	.	.
0	.	NA	.	.	.	.	.	.	.	.
0	.	NA	.	.	.	.	KEG(	.	.	REACTOME_INHIBITION_OF_THE_F
0	.	NA	.	.	.	.	.	.	.	.
0	.	NA	.	.	.	.	.	.	.	.
0	.	NA	.	.	.	.	.	.	.	.
0	.	NA	.	.	.	.	.	.	.	.
0	.	NA	.	.	.	.	.	.	.	.
0	.	NA	.	BIOS	ORG	STEI	KEG(	.	.	.
0	Hypomyeli	NA	.	EST/	CYT(	ACTI	.	.	.	REACTOME_MITOCHONDRIAL_PRC
0	.	NA	.	.	.	.	KEG(	.	.	REACTOME_IMMUNE_SYSTEM;REA
0	.	NA	.	.	.	.	.	.	.	.
0	.	NA	.	.	.	.	.	.	.	.
0	Hypertensi	NA	.	REG	INTF	.	KEG(	PID	BIO	REACTOME_GASTRIN_CREB_SIGN
0	Cerebellar	NA	.	REG	EXTI	OXII	KEG(	PID	.	REACTOME_TRANSMEMBRANE_TF
0	Cerebellar	NA	.	REG	EXTI	OXII	KEG(	PID	.	REACTOME_TRANSMEMBRANE_TF
0	.	NA	.	.	.	.	.	.	.	.
0	.	NA	.	.	.	.	.	.	.	.
0	.	NA	.	.	.	.	.	.	.	.
0	.	NA	.	.	.	.	.	.	.	REACTOME_GENERIC_TRANSCRIP
0	.	NA	.	AXO	EXTI	REC	KEG(	PID	.	REACTOME_DEVELOPMENTAL_BIC
0	Neuropathy	NA	.	.	.	.	.	.	.	.
0	.	NA	.	.	.	.	.	.	.	.
0	.	NA	.	.	.	.	.	.	.	.
0	.	NA	.	.	.	.	.	.	.	.
0	.	NA	.	.	.	.	.	.	.	.
0	Hyperglyci	NA	.	.	.	.	.	.	.	REACTOME_AMINO_ACID_TRANSP
0	.	NA	.	REG	.	.	KEG(	PID	.	.
0	.	NA	.	.	.	.	.	.	.	REACTOME_EXTRACELLULAR_MAT
0	Ehlers-Dar	NA	.	.	.	MET	.	.	.	REACTOME_EXTRACELLULAR_MAT
0	.	NA	.	.	.	.	.	.	.	.
0	.	NA	.	.	.	.	.	.	.	.
0	.	NA	.	.	.	.	.	.	.	.
0	.	NA	.	.	.	.	.	.	.	.
0	.	NA	.	.	.	.	.	.	.	.
0	Emery-Dre	NA	.	ORG	SYN.	ACTI	.	.	.	REACTOME_MEIOSIS;REACTOME_(
0	.	NA	.	.	.	.	.	.	.	.
0	.	NA	.	.	.	.	.	.	.	.
0	.	NA	.	.	.	.	.	.	.	.
0	.	NA	.	.	.	.	.	.	.	.
0	.	NA	.	.	.	.	.	.	.	.
0	.	NA	.	.	.	.	.	.	.	.
0	.	NA	.	.	.	.	.	.	.	.
0	.	NA	.	.	.	.	.	.	.	.
0	.	NA	.	.	.	.	.	.	.	.
0	.	NA	.	.	.	.	.	.	.	.

0	.	NA	.	.	.	.	.	.	.	.
0	.	NA	.	.	.	.	.	.	.	.
0	.	NA	.	.	.	.	.	.	.	.
0	.	NA	.	.	.	.	.	.	.	.
0	.	NA	.	.	.	.	.	.	.	.
0	.	NA	.	.	.	.	.	.	.	.
0	.	NA	.	.	.	.	KEG(	.	.	REACTOME_ACTIVATED_NOTCH1_
0	.	NA	.	.	.	.	KEG(	.	.	.
0	.	NA	.	.	.	.	.	.	.	.
0	.	NA	.	.	.	.	.	.	.	.
0	.	NA	.	.	.	.	.	.	.	.
0	.	NA	.	.	.	.	.	.	.	.
0	.	NA	.	.	.	.	.	.	.	.
0	.	NA	.	.	.	.	.	.	.	.
0	.	NA	.	.	.	.	.	.	.	.
0	.	NA	.	.	.	.	.	.	.	.
0	.	NA	.	.	.	.	.	.	.	.
0	.	NA	.	.	.	.	.	.	.	.
0	Myoclonus	NA	.	.	.	.	.	.	.	.
0	5-oxoprolir	NA	.	.	.	.	KEG(	.	.	REACTOME_BIOLOGICAL_OXIDATI(
0	.	NA	.	.	.	.	.	.	.	.
0	.	NA	.	.	.	.	.	.	.	.
0	.	NA	.	.	.	.	.	.	.	.
0	.	NA	.	.	.	.	.	.	.	.
0	.	NA	.	.	.	.	.	.	.	.
0	.	NA	.	.	.	.	.	.	.	.
0	.	NA	.	.	.	.	.	.	.	.
0	.	NA	.	.	.	.	.	PID	.	REACTOME_SIGNALLING_BY_NGF;
0	.	NA	.	.	.	.	.	.	.	.
0	.	NA	.	.	.	.	.	.	.	.
0	.	NA	.	.	.	.	.	.	.	.
0	.	NA	.	RNA	.	.	.	PID	BIO	.
0	.	NA	.	.	.	.	.	.	.	.
0	.	NA	.	.	.	.	.	.	.	.
0	.	NA	.	NEG	CYT(	.	.	.	.	.
0	.	NA	.	.	.	.	.	.	.	.
0	.	NA	.	.	.	.	.	.	.	.
0	.	NA	.	.	.	.	.	.	.	.
0	.	NA	.	.	.	.	.	.	.	.
0	.	NA	.	.	.	.	.	.	.	.
0	.	NA	.	.	.	.	.	.	.	.
0	.	NA	.	SIGN	.	.	.	.	.	.
0	.	NA	.	.	.	.	.	.	.	.
0	.	NA	.	.	.	.	KEG(	.	.	.
0	.	NA	.	CELI	.	.	.	.	.	.
0	Pachyonyc	NA	.	SYS	ORG	.	.	.	.	.
0	Lethal con(	NA	.	ENZ	INTF	TRA	KEG(	PID	BIO	REACTOME_SIGNALING_BY_ERBB4
0	.	NA	.	DNA	NUC	DAM	KEG(	.	.	REACTOME_BASE_EXCISION_REP/
0	.	NA	.	.	.	.	.	.	.	.
0	.	NA	.	SYS	NUC	ACTI	.	.	.	.
0	.	NA	.	.	.	.	.	.	.	.
0	.	NA	.	.	.	.	.	.	.	.
0	.	NA	.	.	.	.	.	.	.	.
0	.	NA	.	.	.	.	.	.	.	.

0	.	NA	.	.	.	.	.	.	.	.
0	{Cerebral i	NA	.	BIOF	.	PHO	KEG	PID	BIO	REACTOME_GASTRIN_CREB_SIGN
0	Tyrosinemi	NA	.	CAR	CYT	OXID	KEG	.	.	REACTOME_METABOLISM_OF_AMI
0	.	NA	.	.	.	.	.	.	.	.
0	.	NA	.	.	.	.	.	.	.	.
0	.	NA	.	.	.	.	.	.	.	.
0	.	NA	.	.	.	.	.	.	.	.
0	.	NA	.	.	.	.	.	.	.	.
0	.	NA	.	.	.	.	.	.	.	.
0	.	NA	.	.	.	.	.	.	.	.
0	.	NA	.	.	.	.	.	.	.	.
0	.	NA	.	.	.	.	.	.	.	.
0	.	NA	.	.	.	.	.	.	.	.
0	.	NA	.	CAR	ORG	LIGA	KEG	.	.	REACTOME_BILE_ACID_AND_BILE_
0	.	NA	.	EST	CYT	.	.	PID	.	.
0	.	NA	.	.	.	.	.	.	.	.
0	.	NA	.	.	.	.	.	.	.	.
0	.	NA	.	.	.	.	.	.	.	.
0	.	NA	.	.	.	.	.	.	.	.
0	.	NA	.	.	.	.	.	.	.	.
0	.	NA	.	.	.	.	.	.	.	.
0	.	NA	.	.	.	.	.	.	.	.
0	.	NA	.	.	.	.	.	.	.	.
0	.	NA	.	.	.	.	.	.	.	.
0	.	NA	.	.	.	.	.	.	.	.
0	.	NA	.	.	.	.	.	.	.	.
0	.	NA	.	.	.	.	.	.	.	.
0	.	NA	.	.	.	.	.	.	.	.
0	.	NA	.	.	.	.	.	.	.	.
0	.	NA	.	.	.	.	.	.	.	.
0	.	NA	.	.	.	.	.	.	.	.
0	.	NA	.	.	.	.	.	.	.	.
0	.	NA	.	.	.	.	.	.	.	.
0	.	NA	.	.	.	.	.	.	.	.
0	.	NA	.	.	.	.	.	.	.	.
0	.	NA	.	.	.	.	.	.	.	.
0	.	NA	.	.	.	.	.	.	.	.
0	.	NA	.	.	.	.	.	.	.	.
0	.	NA	.	.	.	.	.	.	.	.
0	.	NA	.	.	.	.	.	.	.	.
0	.	NA	.	.	.	.	.	.	.	.
0	.	NA	.	.	.	.	.	.	.	.
0	.	NA	.	.	.	.	.	.	.	.
0	.	NA	.	.	.	.	.	.	.	.
0	.	NA	.	.	.	.	.	.	.	.
0	.	NA	.	.	.	.	.	.	.	.
0	.	NA	.	.	.	.	.	.	.	.
0	.	NA	.	.	.	.	.	.	.	.
0	.	NA	.	.	.	.	.	.	.	.
0	.	NA	.	.	.	.	.	.	.	.
0	.	NA	.	.	.	.	.	.	.	.
0	Cortical dy	NA	.	MICF	ORG	STR	.	PID	.	REACTOME_CELL_CYCLE;REACTO
0	.	NA	.	.	.	.	.	.	.	.
0	.	NA	.	.	.	.	.	.	.	.

0	.	NA	.	.	.	.	.	.	.	.
0	.	NA	.	RES	.	.	.	.	.	.
0	.	NA	.	.	.	.	.	.	.	.
0	.	NA	.	IMMU	INTR	.	KEGG	PID	.	.
0	.	NA	.	.	.	.	.	.	.	.
0	.	NA	.	.	.	.	.	.	.	.
0	.	NA	.	.	.	.	.	.	.	.
0	.	NA	.	SEN	ORG	.	.	.	.	.
0	.	NA	.	.	.	.	.	.	.	.
0	.	NA	.	.	.	.	.	.	.	.
0	.	NA	.	SIGN	INTR	REC	KEGG	.	.	REACTOME_IMMUNOREGULATORY
0	.	NA	.	.	.	.	.	.	.	.
0	.	NA	.	RNA	NUC	TRAI	.	PID	.	REACTOME_DEVELOPMENTAL_BIO
0	.	NA	.	.	.	.	.	.	.	.
0	.	NA	.	.	.	.	.	.	.	.
0	.	NA	.	.	.	.	.	.	.	.
0	.	NA	.	.	.	.	.	.	.	.
0	.	NA	.	.	.	.	.	.	.	.
0	.	NA	.	.	.	.	.	.	.	.
0	.	NA	.	.	.	.	.	.	.	.
0	.	NA	.	.	.	.	.	.	.	.
0	.	NA	.	.	.	.	.	.	.	.
0	.	NA	.	.	.	.	.	.	.	.
0	.	NA	.	.	.	.	.	.	.	.
0	.	NA	.	.	.	.	.	.	.	.
0	.	NA	.	CELL	NON	ACTI	KEGG	PID	.	.
0	Mental reta	NA	.	.	.	.	.	.	.	.
0	.	NA	.	.	.	.	.	.	.	.
0	.	NA	.	.	.	.	.	.	.	.
0	.	NA	.	.	.	.	.	.	.	.
0	.	NA	.	.	.	.	.	.	.	.
0	.	NA	.	.	.	.	.	.	.	.
0	.	NA	.	.	.	.	.	.	.	.
0	.	NA	.	.	.	.	.	.	.	.
0	.	NA	.	.	.	.	.	.	.	.
0	.	NA	.	.	.	.	.	.	.	.
0	.	NA	.	.	.	.	.	.	.	.
0	.	NA	.	.	.	.	.	.	.	.
0	.	NA	.	.	.	.	.	PID	.	.
0	.	NA	.	.	.	.	.	.	.	.
0	.	NA	.	.	.	.	.	.	.	.
0	.	NA	.	BIOF	.	.	KEGG	.	.	REACTOME_ANTIVIRAL_MECHANIS
0	.	NA	.	.	.	.	.	.	.	.
0	.	NA	.	.	.	.	KEGG	.	.	REACTOME_IMMUNE_SYSTEM;REA
0	.	NA	.	.	.	.	.	.	.	.
0	.	NA	.	.	.	.	.	.	.	.
0	.	NA	.	.	.	.	.	.	.	.
0	.	NA	.	POS	.	.	.	.	.	REACTOME_ACTIVATED_NOTCH1_
0	.	NA	.	REG	CYT	NUC	.	PID	BIO	.
0	.	NA	.	REG	CYT	NUC	.	PID	BIO	.
0	.	NA	.	REG	CYT	NUC	.	PID	BIO	.
0	.	NA	.	REG	CYT	NUC	.	PID	BIO	.
0	{Epilepsy, (	NA	.	SIGN	INTR	GAB	KEGG	.	.	.
0	.	NA	.	CELL	MEM	PHO	KEGG	PID	BIO	REACTOME_TGF_BETA_RECEPTOR

0	Peroxisom	NA	.	EST	CYT	.	KEG	.	.	.
0	.	NA	.	.	.	.	.	.	.	.
0	.	NA	.	DNA	CYT	HYD	KEG	PID	BIO	REACTOME_APOPTOSIS_INDUCED
0	Nephronop	NA	.	.	.	.	.	.	.	REACTOME_SIGNALING_BY_HIPP
0	Nephronop	NA	.	.	.	.	.	.	.	REACTOME_SIGNALING_BY_HIPP
0	.	NA	.	.	.	.	.	.	.	.
0	.	NA	.	.	.	.	.	.	.	.
0	.	NA	.	.	.	.	.	.	.	.
0	.	NA	.	.	.	.	.	.	.	.
0	.	NA	.	.	.	TRA	.	.	.	.
0	Cerebellar	NA	.	.	.	.	.	.	.	.
0	Cerebellar	NA	.	.	.	.	.	.	.	.
0	Cerebellar	NA	.	.	.	.	.	.	.	.
0	.	NA	.	.	.	.	.	.	.	REACTOME_TRANSMEMBRANE_T
0	.	NA	.	.	.	.	.	.	.	.
0	.	NA	.	.	.	.	.	.	.	.
0	.	NA	.	.	.	.	.	.	.	.
0	.	NA	.	.	.	.	.	.	.	.
0	Ehlers-Dar	NA	.	SYS	.	PRO	KEG	.	.	REACTOME_EXTRACELLULAR_MA
0	.	NA	.	POS	.	REC	KEG	.	.	.
0	.	NA	.	.	.	.	.	.	.	.
0	.	NA	.	.	.	.	.	.	.	.
0	.	NA	.	.	.	.	.	.	.	.
0	.	NA	.	.	.	.	.	.	.	.
0	.	NA	.	REG	.	ENZ	KEG	PID	BIO	REACTOME_SIGNALLING_BY_NGF;
0	Cataract 6,	NA	.	SIGN	INTR	.	KEG	PID	.	.
0	.	NA	.	.	.	.	.	.	BIO	.
0	.	NA	.	.	.	.	.	.	.	.
0	.	NA	.	.	.	.	.	.	.	.
0	.	NA	.	.	.	.	.	.	.	.
0	.	NA	.	.	.	.	.	.	.	.
0	.	NA	.	.	.	.	.	.	.	.
0	Parkinson	NA	.	.	.	.	.	.	.	.
0	.	NA	.	.	.	.	.	.	.	.
0	.	NA	.	DET	.	TAS	KEG	.	.	REACTOME_SIGNALING_BY_GPCR
0	.	NA	.	.	.	.	.	.	.	REACTOME_IMMUNE_SYSTEM;REA
0	.	NA	.	.	.	.	.	.	.	REACTOME_IMMUNE_SYSTEM;REA
0	.	NA	.	.	.	.	.	.	.	.
0	.	NA	.	.	ORG	ACT	.	.	.	.
0	.	NA	.	.	.	.	.	.	.	.
0	.	NA	.	SIGN	CYT	GTP	.	.	.	REACTOME_RAP1_SIGNALLING;RE
0	Dyssegme	NA	.	.	.	.	KEG	.	.	REACTOME_HS_GAG_DEGRADATI
0	Dyssegme	NA	.	.	.	.	KEG	.	.	REACTOME_HS_GAG_DEGRADATI
0	.	NA	.	.	.	.	.	.	.	.
0	.	NA	.	STE	.	SER	.	.	.	.
0	.	NA	.	.	.	.	.	.	.	.
0	Galactose	NA	.	.	.	.	KEG	.	.	REACTOME_METABOLISM_OF_CA
0	HMG-CoA	NA	.	.	.	.	KEG	.	.	REACTOME_METABOLISM_OF_LIP
0	.	NA	.	RNA	.	TRA	.	PID	.	.
0	Rh-null dis	NA	.	.	INTR	.	.	.	.	.
0	.	NA	.	GLY	ORG	CAT	KEG	.	.	REACTOME_METABOLISM_OF_PR
0	.	NA	.	SIGN	.	TUB	KEG	PID	.	.
0	.	NA	.	.	.	.	.	.	.	.
0	.	NA	.	ENZ	CEL	PRO	.	PID	.	.

0	.	NA	.	.	.	.	.	.	.	.
0	.	NA	.	SIGN	.	PHO	KEGG	PID	BIO	REACTOME_SIGNALLING_BY_NGF;
0	Mental reta	NA	.	RNA	NUC	TRA	.	.	.	.
0	.	NA	.	.	.	.	.	.	.	.
0	.	NA	.	REG	INTR	CATI	KEGG	PID	.	REACTOME_HYALURONAN_UPTAK
0	.	NA	.	REG	INTR	CATI	KEGG	PID	.	REACTOME_HYALURONAN_UPTAK
0	.	NA	.	.	.	.	.	.	.	.
0	.	NA	.	.	.	.	.	.	.	.
0	.	NA	.	ANA	.	.	.	.	.	.
0	.	NA	.	IMMU	.	.	.	.	.	.
0	.	NA	.	NEG	.	.	KEGG	.	.	.
0	.	NA	.	.	.	.	.	.	.	.
0	.	NA	.	.	.	.	.	.	.	.
0	SCID due t	NA	.	REG	ORG	PRO	KEGG	PID	BIO	REACTOME_SIGNALING_BY_SCF_K
0	.	NA	.	.	.	.	.	.	.	.
0	.	NA	.	.	NUC	.	.	.	.	.
0	.	NA	.	MUL	.	DNA	.	.	.	.
0	Palmoplan	NA	.	.	.	.	.	PID	.	.
0	.	NA	.	.	.	.	.	.	.	.
0	.	NA	.	.	.	.	.	.	.	.
0	.	NA	.	.	CYTO	.	.	.	.	.
0	.	NA	.	.	CYTO	.	.	.	.	.
0	.	NA	.	.	CYTO	.	.	.	.	.
0	.	NA	.	.	.	.	.	.	.	.
0	.	NA	.	REG	CYTO	MRN	.	.	.	.
0	Ceroid lipo	NA	.	REG	AXO	THIC	KEGG	.	.	.
0	.	NA	.	.	.	CATI	.	.	.	.
0	.	NA	.	.	.	CATI	.	.	.	.
0	Deafness,	NA	.	ESTA	.	.	.	.	.	REACTOME_NEURONAL_SYSTEM;F
0	Deafness,	NA	.	ESTA	.	.	.	.	.	REACTOME_NEURONAL_SYSTEM;F
0	.	NA	.	.	.	.	.	.	.	.
0	.	NA	.	RNA	.	SING	.	PID	.	REACTOME_PROCESSING_OF_CAF
0	.	NA	.	.	NUC	.	.	.	.	REACTOME_DEVELOPMENTAL_BIC
0	.	NA	.	.	.	.	.	.	.	.
0	.	NA	.	BIOF	.	PHO	.	PID	.	.
0	.	NA	.	.	.	.	.	.	.	.
0	Adenocarc	NA	.	DNA	NUC	.	KEGG	.	.	.
0	Spastic pa	NA	.	CAR	INSC	HYD	.	.	.	.
0	.	NA	.	BIOF	.	NUC	KEGG	PID	BIO	REACTOME_SPRY_REGULATION_C
0	.	NA	.	.	.	.	.	.	.	.
0	.	NA	.	.	.	.	.	.	.	.
0	.	NA	.	.	.	.	.	.	.	.
0	{Myocardia	NA	.	ESTA	.	REC	.	PID	.	REACTOME_PLATELET_HOMEOSTA
0	.	NA	.	RNA	NUC	SPE	.	.	.	.
0	.	NA	.	.	.	.	.	.	.	.
0	Hyperchole	NA	.	REG	EXTI	SER	.	.	.	.
0	.	NA	.	BIOF	.	.	KEGG	PID	BIO	REACTOME_ACTIVATED_AMPK_ST
0	Ciliary dysl	NA	.	.	.	.	.	PID	.	.
0	.	NA	.	.	.	.	KEGG	PID	BIO	REACTOME_IL_7_SIGNALING;REAC
0	.	NA	.	.	.	.	.	.	.	.
0	.	NA	.	CELL	CYTO	.	KEGG	.	.	.
0	.	NA	.	PRO	ORG	LIPID	KEGG	.	.	REACTOME_METABOLISM_OF_PRO
0	.	NA	.	.	INTR	G_PI	.	.	.	.
0	.	NA	.	.	.	.	.	.	.	.

0	.	NA	.	.	INTF	G_PI	.	.	.	.
0	.	NA	.	REG	INTF	LIPIC	KEGC	PID	BIO	REACTOME_GASTRIN_CREB_SIGN
0	.	NA	.	.	.	.	.	.	.	.
0	.	NA	.	ENZ	INTF	CAR	.	PID	BIO	.
0	Cone-rod c	NA	.	EST/	CELI	NUC	KEGC	.	.	REACTOME_ABC_FAMILY_PROTEIN
0	Cone-rod c	NA	.	EST/	CELI	NUC	KEGC	.	.	REACTOME_ABC_FAMILY_PROTEIN
0	.	NA	.	.	.	.	.	.	.	.
0	Fibrochonc	NA	.	SYS	PRO	.	KEGC	PID	.	REACTOME_EXTRACELLULAR_MAT
0	.	NA	.	.	.	.	KEGC	.	.	.
0	.	NA	.	.	.	.	.	.	.	.
0	.	NA	.	.	.	.	.	.	.	.
0	Megakaryc	NA	.	.	.	.	.	.	.	.
0	.	NA	.	AMIN	.	CAR	KEGC	.	.	.
0	.	NA	.	.	.	.	.	.	.	.
0	.	NA	.	.	.	.	.	.	.	.
0	.	NA	.	.	.	.	.	.	.	REACTOME_PRE_NOTCH_TRANSC
0	.	NA	.	POS	.	HYD	.	PID	.	REACTOME_SIGNALING_BY_RHO_(
0	.	NA	.	.	.	.	KEGC	PID	.	.
0	Myoadenyl	NA	.	.	.	HYD	KEGC	.	.	REACTOME_METABOLISM_OF_NUC
0	Myoadenyl	NA	.	.	.	HYD	KEGC	.	.	REACTOME_METABOLISM_OF_NUC
0	Bamforth-L	NA	.	RNA	NUC	HYD	.	.	.	.
0	.	NA	.	.	NUC	.	.	.	.	.
0	.	NA	.	.	.	.	.	.	.	.
0	Alagille syr	NA	.	REG	INTF	SPE(	KEGC	PID	.	REACTOME_PRE_NOTCH_TRANSC
0	Alagille syr	NA	.	REG	INTF	SPE(	KEGC	PID	.	REACTOME_PRE_NOTCH_TRANSC
0	.	NA	.	.	.	.	.	.	.	.
0	.	NA	.	.	.	.	.	.	.	.
0	.	NA	.	.	.	.	.	.	.	.
0	.	NA	.	.	.	.	.	.	.	.
0	.	NA	.	EST/	.	.	KEGC	.	.	.
0	.	NA	.	.	.	.	.	.	.	.
0	.	NA	.	.	.	.	.	.	.	.
0	.	NA	.	RNA	NUC	NUC	KEGC	.	.	REACTOME_RNA_POL_III_TRANSC
0	.	NA	.	.	.	.	.	.	.	.
0	.	NA	.	.	.	.	.	.	.	.
0	.	NA	.	.	.	.	.	.	.	.
0	.	NA	.	.	.	.	KEGC	.	.	REACTOME_MITOCHONDRIAL_TRN
0	.	NA	.	PHO	CYT(	PHO	KEGC	PID	.	REACTOME_SYNTHESIS_OF_PIPS_
0	.	NA	.	.	.	.	.	.	.	.
0	.	NA	.	.	.	.	.	.	.	.
0	.	NA	.	MUL	.	REC	.	.	.	.
0	.	NA	.	.	.	.	.	.	.	.
0	.	NA	.	RNA	NUC	.	.	.	.	.
0	.	NA	.	.	.	.	.	.	.	.
0	.	NA	.	.	.	.	KEGC	.	.	REACTOME_IMMUNE_SYSTEM;REA
0	.	NA	.	.	INTF	.	.	.	.	REACTOME_MITOCHONDRIAL_PRC
0	.	NA	.	RNA	CELI	RNA	KEGC	.	.	.
0	.	NA	.	.	.	.	.	.	.	.
0	.	NA	.	RNA	NUC	TRAI	.	.	.	REACTOME_CELL_CYCLE;REACTO
0	.	NA	.	RNA	.	RAS	KEGC	PID	BIO	REACTOME_SIGNALING_BY_RHO_(
0	.	NA	.	.	.	.	.	.	.	.
0	.	NA	.	.	.	.	.	.	.	.
0	.	NA	.	.	INTF	.	KEGC	.	.	.
0	.	NA	.	DEFI	.	.	.	PID	.	REACTOME_INNATE_IMMUNE_SYS

0	.	NA	.	SYS	.	.	.	.	.	.
0	.	NA	.	.	.	.	.	.	.	.
0	.	NA	.	REG	.	GTP	.	.	.	REACTOME_SIGNALING_BY_GPCR
0	Leukemia,	NA	.	RNA	NUC	.	.	PID	.	.
0	.	NA	.	NEG	NUC	TRA	.	PID	.	REACTOME_RNA_POL_III_TRANSC
0	.	NA	.	.	.	.	.	.	.	.
0	Platelet al	NA	.	.	ORG	.	KEG	PID	BIO	REACTOME_CELL_SURFACE_INTE
0	.	NA	.	.	CYT	.	.	.	.	.
0	.	NA	.	.	.	.	.	.	.	.
0	.	NA	.	PRO	.	MET	.	.	.	REACTOME_DIABETES_PATHWAY
0	.	NA	.	.	.	.	.	.	.	.
0	Leukemia,	NA	.	BIO	.	CAT	KEG	.	.	REACTOME_DEVELOPMENTAL_BIC
0	Nephrotic	NA	.	EST	INT	.	.	PID	.	.
0	.	NA	.	.	.	.	.	.	.	.
0	.	NA	.	CEL	MEM	CAT	KEG	.	.	.
0	.	NA	.	.	.	.	.	.	.	.
0	.	NA	.	.	.	.	.	.	.	.
0	.	NA	.	.	.	.	KEG	.	.	.
0	.	NA	.	NUC	ORG	.	KEG	.	.	REACTOME_METABOLISM_OF_NON
0	Leber cong	NA	.	ANA	.	.	.	.	.	.
0	.	NA	.	.	.	.	.	.	.	.
0	.	NA	.	.	.	.	.	.	.	.
0	Hypokalem	NA	.	SYS	MEM	CAT	KEG	.	.	REACTOME_DEVELOPMENTAL_BIC
0	.	NA	.	.	.	.	.	.	.	.
0	.	NA	.	EST	ORG	.	.	.	.	REACTOME_MITOCHONDRIAL_PRC
0	.	NA	.	.	.	.	KEG	.	.	REACTOME_PHOSPHOLIPID_META
0	?Epilepsy,	NA	.	.	INT	.	KEG	.	.	REACTOME_DEVELOPMENTAL_BIC
0	.	NA	.	.	.	.	KEG	PID	.	REACTOME_REGULATION_OF_CON
0	.	NA	.	.	.	.	.	.	.	.
0	.	NA	.	.	.	.	KEG	.	.	REACTOME_DEVELOPMENTAL_BIC
0	Cortisone	NA	.	.	.	.	KEG	.	.	REACTOME_METABOLISM_OF_STE
0	.	NA	.	.	.	.	.	.	.	.
0	.	NA	.	.	.	.	.	.	.	.
0	Ataxia, pos	NA	.	EST	INT	.	.	.	.	REACTOME_TRANSMEMBRANE_TF
0	.	NA	.	SIG	.	PHO	.	.	.	.
0	Choanal at	NA	.	BIO	.	PRO	.	.	.	.
0	Choanal at	NA	.	BIO	.	PRO	.	.	.	.
0	Retinitis pi	NA	.	REG	PRO	PRO	.	.	.	.
0	Retinitis pi	NA	.	REG	PRO	PRO	.	.	.	.
0	Retinitis pi	NA	.	REG	PRO	PRO	.	.	.	.
0	.	NA	.	RNA	.	PRO	.	.	.	REACTOME_GENERIC_TRANSCRIP
0	Hypermang	NA	.	.	.	.	.	.	.	.
0	.	NA	.	.	.	.	KEG	.	.	REACTOME_MITOCHONDRIAL_TRN
0	.	NA	.	.	.	.	KEG	.	.	REACTOME_MITOCHONDRIAL_TRN
0	.	NA	.	.	.	.	.	.	.	.
0	.	NA	.	.	.	.	.	.	.	.
0	.	NA	.	.	NUC	NUC	.	.	.	.
0	.	NA	.	.	.	.	.	.	.	.
0	Left-right a	NA	.	ENZ	.	.	KEG	.	.	REACTOME_DEVELOPMENTAL_BIC
0	.	NA	.	.	.	.	.	.	.	.
0	Coenzyme	NA	.	.	.	.	.	.	.	.
0	.	NA	.	.	.	.	.	PID	.	REACTOME_SIGNALING_BY_RHO_
0	.	NA	.	.	.	.	.	.	.	.
0	.	NA	.	PRO	.	PRO	.	.	.	.

0	.	NA	.	EST/	INTF	CATI	.	.	.	REACTOME_NEURONAL_SYSTEM;F
0	Arrhythmo(	NA	.	REG	MEM	CATI	KEG(	.	BIO	.
0	.	NA	.	.	.	.	.	.	.	.
0	Megalence	NA	.	BIOF	.	.	KEG(	PID	.	REACTOME_SIGNALLING_BY_NGF;
0	.	NA	.	.	.	.	.	.	.	.
0	.	NA	.	.	.	.	.	.	.	REACTOME_GENERIC_TRANSCRIP
0	.	NA	.	.	.	.	.	.	.	.
0	Thromboc)	NA	.	.	INTF	.	KEG(	.	.	REACTOME_METABOLISM_OF_AMI
0	.	NA	.	.	.	.	.	.	.	.
0	.	NA	.	.	.	.	.	.	.	.
0	.	NA	.	.	.	.	.	.	.	.
0	.	NA	.	.	.	.	.	.	.	.
0	.	NA	.	.	.	.	KEG(	.	.	REACTOME_ACYL_CHAIN_REMODE
0	Inflammatc	NA	.	CELI	INTF	.	KEG(	PID	BIO	REACTOME_SIGNALLING_BY_NGF;
0	.	NA	.	.	.	.	.	.	.	.
0	.	NA	.	.	.	.	.	.	.	.
0	.	NA	.	.	.	.	.	.	.	.
0	.	NA	.	.	.	.	.	.	.	.
0	.	NA	.	EST/	.	CATI	.	.	.	REACTOME_NEURONAL_SYSTEM;F
0	.	NA	.	.	.	.	.	.	.	.
0	.	NA	.	.	.	MOL	.	PID	.	.
0	.	NA	.	.	NUC	PRO	.	PID	BIO	REACTOME_DEVELOPMENTAL_BIC
0	.	NA	.	.	.	.	.	.	.	.
0	.	NA	.	.	.	LIGA	KEG(	PID	.	REACTOME_METABOLISM_OF_NU(
0	.	NA	.	.	.	LIGA	KEG(	PID	.	REACTOME_METABOLISM_OF_NU(
0	.	NA	.	.	.	.	.	.	.	.
0	{Neuroblas	NA	.	SYS	INTF	TRAI	.	.	.	.
0	.	NA	.	CELI	.	.	KEG(	.	.	.
0	.	NA	.	.	.	.	.	.	.	.
0	.	NA	.	.	.	.	.	.	.	.
0	.	NA	.	.	.	.	.	PID	.	.
0	.	NA	.	EST/	INTF	CATI	KEG(	.	.	REACTOME_TRANSMEMBRANE_TF
0	Leigh synd	NA	.	EST/	ORG	MICF	.	.	.	.
0	.	NA	.	.	.	.	.	.	.	.
0	.	NA	.	.	.	.	.	.	.	.
0	.	NA	.	.	ORG	ACTI	.	PID	.	REACTOME_DEVELOPMENTAL_BIC
0	.	NA	.	.	ORG	ACTI	.	PID	.	REACTOME_DEVELOPMENTAL_BIC
0	.	NA	.	SYS	ORG	.	.	PID	.	REACTOME_SIGNALLING_BY_NGF;
0	Doyne hon	NA	.	SEN	PRO	.	.	.	.	.
0	.	NA	.	.	.	.	.	.	.	.
0	.	NA	.	.	.	.	.	.	.	.
0	.	NA	.	.	.	.	.	.	.	.
0	.	NA	.	GLU	.	NUC	KEG(	.	.	REACTOME_BIOLOGICAL_OXIDATI(
0	.	NA	.	.	.	.	.	.	.	REACTOME_SIGNALING_BY_ILS;RE
0	.	NA	.	.	.	.	.	.	.	REACTOME_SIGNALING_BY_RHO_(
0	GAPO syn	NA	.	.	.	.	.	PID	.	.
0	.	NA	.	.	.	ACTI	.	.	.	.
0	.	NA	.	AMIN	.	.	KEG(	.	.	.
0	Miyoshi mu	NA	.	SYS	MEM	.	.	.	.	.
0	.	NA	.	.	.	.	.	.	.	.
0	.	NA	.	.	.	.	.	.	.	.
0	.	NA	.	.	.	.	.	.	.	.
0	.	NA	.	ISOF	ORG	OXID	KEG(	.	.	.
0	Pseudoxar	NA	.	REG	INTF	LYAS	.	.	.	REACTOME_PTM_GAMMA_CARBOX

0	.	NA	.	MUS	MEM	.	KEGG	.	.	.
0	.	NA	.	.	.	.	.	.	.	.
0	.	NA	.	.	.	.	.	.	.	.
0	.	NA	.	.	.	.	.	.	.	.
0	.	NA	.	.	.	.	.	.	.	.
0	.	NA	.	.	.	.	.	.	.	.
0	.	NA	.	.	.	.	.	.	.	.
0	.	NA	.	.	.	.	.	.	.	.
0	.	NA	.	.	.	.	.	.	.	.
0	.	NA	.	.	.	.	.	.	.	.
0	.	NA	.	.	.	.	.	.	.	.
0	.	NA	.	.	.	.	.	.	.	.
0	.	NA	.	.	.	.	.	.	.	.
0	.	NA	.	.	.	.	.	.	.	.
0	.	NA	.	PRO	.	.	.	.	.	.
0	Retinitis pi	NA	.	RNA	ORG	HYD	KEGG	.	.	REACTOME_PROCESSING_OF_CAF
0	.	NA	.	.	.	.	.	.	.	.
0	.	NA	.	.	.	.	.	.	.	.
0	.	NA	.	.	.	.	.	.	.	.
0	.	NA	.	.	.	.	.	.	.	.
0	.	NA	.	.	.	.	.	.	.	.
0	.	NA	.	MUL	NUC	.	.	.	.	.
0	.	NA	.	SIGN	MEM	.	KEGG	PID	BIO	.
0	.	NA	.	ENZY	MEM	SMA	.	PID	.	.
0	.	NA	.	.	.	.	.	.	.	.
0	Colorectal	NA	.	MITO	ORG	.	KEGG	PID	.	REACTOME_CELL_CYCLE;REACTO
0	.	NA	.	.	.	.	.	.	.	.
0	.	NA	.	.	.	.	.	.	.	.
0	.	NA	.	.	.	.	.	.	.	.
0	.	NA	.	.	.	.	.	.	.	.
0	.	NA	.	.	CELL	.	.	.	.	.
0	.	NA	.	SIGN	.	GTP	KEGG	PID	.	REACTOME_SIGNALLING_BY_NGF;
0	Thrombopl	NA	.	REG	.	SER	KEGG	PID	BIO	REACTOME_PTM_GAMMA_CARBOX
0	.	NA	.	.	.	.	.	.	.	.
0	.	NA	.	LIPID	ORG	LIPA	KEGG	.	.	REACTOME_GLYCOSPHINGOLIPID_
0	.	NA	.	.	.	.	.	.	.	.
0	.	NA	.	.	.	.	.	.	.	.
0	.	NA	.	SIGN	INTR	.	.	.	.	REACTOME_SIGNALING_BY_GPCR
0	.	NA	.	.	.	.	.	.	.	.
0	.	NA	.	ESTA	.	.	.	.	.	.
0	Nemaline r	NA	.	.	SAR	STRI	.	.	.	REACTOME_STRIATED_MUSCLE_C
0	.	NA	.	.	.	.	KEGG	PID	.	REACTOME_MEMBRANE_TRAFFICK
0	.	NA	.	.	.	.	.	.	.	.
0	.	NA	.	.	.	.	KEGG	.	.	.
0	.	NA	.	.	.	.	.	.	.	.
0	.	NA	.	.	.	.	.	.	.	.
0	.	NA	.	.	.	.	.	.	.	.
0	.	NA	.	IMMU	.	SER	.	.	.	REACTOME_INTEGRATION_OF_EN
0	.	NA	.	IMMU	.	SER	.	.	.	REACTOME_INTEGRATION_OF_EN
0	.	NA	.	.	.	.	.	.	.	.
0	Cholestasi	NA	.	ESTA	INTR	CATI	KEGG	.	BIO	REACTOME_BILE_ACID_AND_BILE_
0	Bardet-Bie	NA	.	.	.	.	.	.	.	.
0	.	NA	.	.	.	.	.	.	.	.
0	.	NA	.	.	.	.	.	.	.	.

0	.	NA	.	.	.	.	KEGG	.	.	REACTOME_CELL_CYCLE;REACTO
0	.	NA	.	SIGN	CELL	GTP.	KEGG	.	.	REACTOME_INTEGRIN_CELL_SURF
0	Wiskott-Ald	NA	.	MAC	NON	ACTI	.	PID	.	.
0	Multiple pte	NA	.	REG	INTR	CATI	KEGG	PID	BIO	REACTOME_TRANSMISSION_ACRO
0	.	NA	.	.	.	.	.	.	.	.
0	Rhizomelic	NA	.	BIOS	CYT	TRAI	KEGG	.	.	REACTOME_PEROXISOMAL_LIPID_
0	Pigmented	NA	.	SIGN	.	.	KEGG	.	.	REACTOME_SIGNALING_BY_GPCR
0	Cardiomyo	NA	.	MUS	ORG	NUC	KEGG	.	.	.
0	.	NA	.	.	.	.	.	.	.	.
0	.	NA	.	.	.	.	.	.	.	.
0	.	NA	.	.	.	.	.	.	.	.
0	.	NA	.	.	.	.	KEGG	PID	BIO	REACTOME_DEVELOPMENTAL_BIO
0	Ehlers-Dan	NA	.	SYST	PRO	.	KEGG	PID	.	REACTOME_DEVELOPMENTAL_BIO
0	.	NA	.	.	.	.	.	.	.	.
0	Candidiasis	NA	.	RNA	CYT	REC	KEGG	PID	BIO	REACTOME_SIGNALING_BY_SCF_K
0	.	NA	.	.	CYT	LIPID	.	.	.	.
0	.	NA	.	CELL	NUC	NUC	.	.	.	.
0	.	NA	.	.	.	.	.	.	.	.
0	3MC syndr	NA	.	CELL	.	PHO	.	.	.	.
0	.	NA	.	.	.	.	.	.	.	.
0	.	NA	.	.	.	.	.	.	.	.
0	.	NA	.	.	.	.	.	.	.	.
0	.	NA	.	.	.	.	.	.	.	REACTOME_TRANSLATION;REACT
0	.	NA	.	SYST	INTR	INTE	.	.	.	.
0	Cataract 4,	NA	.	SENS	.	.	.	.	.	.
0	.	NA	.	.	.	.	.	.	.	.
0	.	NA	.	.	.	.	.	.	.	.
0	.	NA	.	.	.	.	.	.	.	.
0	.	NA	.	.	.	.	.	.	.	.
0	Carbamoyl	NA	.	.	CYT	LIGA	KEGG	.	.	REACTOME_METABOLISM_OF_AMI
0	.	NA	.	.	.	.	.	PID	BIO	.
0	.	NA	.	.	.	.	.	.	.	.
0	.	NA	.	.	.	.	.	.	.	.
0	{Buruli ulce	NA	.	MUL	INTR	.	KEGG	PID	.	REACTOME_LATENT_INFECTION_O
0	Cholestasis	NA	.	MAC	.	.	.	.	.	.
0	.	NA	.	.	.	.	.	.	.	.
0	.	NA	.	.	.	.	.	.	.	.
0	Spinal mus	NA	.	PRO	.	UNF	.	.	.	.
0	?Muscular	NA	.	ORG	SAR	STRI	KEGG	PID	.	REACTOME_STRIATED_MUSCLE_C
0	.	NA	.	MUS	NUC	.	.	.	.	.
0	.	NA	.	SIGN	INTR	.	KEGG	PID	BIO	.
0	{Coronary	NA	.	ENZ	VESI	PRO	KEGG	PID	BIO	REACTOME_SIGNALLING_BY_NGF;
0	.	NA	.	.	.	.	.	.	.	.
0	.	NA	.	.	.	.	.	.	.	.
0	.	NA	.	REG	INTR	NEU	KEGG	.	.	REACTOME_GASTRIN_CREB_SIGN
0	Perlman sy	NA	.	.	.	.	.	.	.	.
0	Perlman sy	NA	.	.	.	.	.	.	.	.
0	.	NA	.	.	.	.	KEGG	.	.	.
0	.	NA	.	SIGN	.	PHO	KEGG	PID	.	.
0	.	NA	.	.	.	.	.	.	.	.
0	.	NA	.	.	.	.	.	.	.	.
0	.	NA	.	.	.	.	.	.	.	.
0	Advanced	NA	.	RHY	.	.	KEGG	PID	.	REACTOME_BMAL1_CLOCK_NPAS2
0	.	NA	.	.	.	.	.	.	.	.

0	Mental reta	NA	.	EST/	.	.	.	.	.	.	
0	Mental reta	NA	.	EST/	.	.	.	.	.	.	
0	.	NA	.	.	.	.	.	.	.	.	
0	.	NA	.	.	.	.	.	.	.	.	
0	.	NA	.	.	.	.	.	.	.	.	
0	.	NA	.	SIGN	INTR	.	.	.	.	.	
0	.	NA	.	SYS	.	.	.	PID	.	REACTOME_DEVELOPMENTAL_BIO	
0	.	NA	.	.	.	.	.	.	.	.	
0	.	NA	.	.	.	.	.	.	.	.	
0	.	NA	.	.	.	.	.	.	.	.	
0	.	NA	.	.	.	.	.	.	.	.	
0	Atrioventric	NA	.	.	.	.	.	.	.	.	
0	Fanconi ar	NA	.	.	.	.	.	PID	BIO	REACTOME_REGULATION_OF_THE	
0	.	NA	.	.	.	.	.	.	.	REACTOME_TRANSMISSION_ACRO	
0	.	NA	.	EST/	ORG	.	.	.	.	.	
0	.	NA	.	.	.	.	.	.	.	.	
0	Pontocereb	NA	.	.	.	.	.	.	.	.	
0	LEOPARD	NA	.	CELI	ORG	.	KEGG	PID	BIO	REACTOME_SIGNALLING_BY_NGF;	
0	.	NA	.	.	.	.	KEGG	PID	.	.	
0	.	NA	.	.	.	.	.	.	.	.	
0	.	NA	.	.	.	.	.	.	.	.	
0	.	NA	.	.	.	.	.	.	.	REACTOME_NEURONAL_SYSTEM;F	
0	.	NA	.	RNA	NUC	.	.	.	.	REACTOME_GENERIC_TRANSCRIP	
0	.	NA	.	.	NUC	KINA	.	.	BIO	.	
0	.	NA	.	.	.	.	.	.	.	.	
0	.	NA	.	.	.	.	.	.	.	REACTOME_TRANSMEMBRANE_TF	
0	Colorectal	NA	.	POS	INTR	TRAI	KEGG	PID	BIO	REACTOME_TGF_BETA_RECEPTOI	
0	.	NA	.	.	.	.	.	.	.	.	
0	.	NA	.	GLY	ORG	TRAI	KEGG	.	.	.	
0	.	NA	.	.	CELI	.	.	.	.	.	
0	.	NA	.	.	.	.	.	.	.	.	
0	Colorectal	NA	.	DNA	.	SING	KEGG	PID	.	REACTOME_MEIOSIS;REACTOME_I	
0	.	NA	.	EST/	ORG	.	.	.	.	.	
0	Esophagea	NA	.	NEG	CYT	.	.	.	.	.	
0	.	NA	.	.	.	.	KEGG	.	.	.	
0	.	NA	.	EST/	.	.	.	.	.	.	
0	.	NA	.	.	.	.	.	.	.	.	
0	.	NA	.	REG	INTR	.	KEGG	.	.	REACTOME_SIGNALING_BY_GPCR	
0	.	NA	.	.	.	.	.	.	.	.	
0	.	NA	.	.	.	.	KEGG	.	.	.	
0	.	NA	.	.	.	.	.	.	.	.	
0	EBD invers	NA	.	SYS	PRO	.	.	PID	.	REACTOME_EXTRACELLULAR_MA	
0	.	NA	.	NUC	.	.	.	.	.	.	
0	.	NA	.	.	.	.	.	.	.	.	
0	.	NA	.	.	.	.	.	.	.	.	
0	.	NA	.	.	CYT	CYS	.	.	.	.	
0	.	NA	.	CELI	NUC	.	.	.	.	.	
0	.	NA	.	.	.	.	.	.	.	.	
0	.	NA	.	.	.	.	KEGG	.	.	.	
0	.	NA	.	.	.	.	.	.	.	.	
0	.	NA	.	MITC	.	RNA	.	PID	.	.	
0	.	NA	.	MITC	.	RNA	.	PID	.	.	
0	.	NA	.	COF	.	.	KEGG	PID	BIO	REACTOME_PPARA_ACTIVATES_G	
0	.	NA	.	.	.	.	KEGG	.	.	.	

0	.	NA	.	.	.	.	KEG(	.	.	.
0	.	NA	.	.	.	.	KEG(	.	.	.
0	.	NA	.	.	.	.	KEG(	.	.	.
0	.	NA	.	CHR	.	.	.	.	.	.
0	.	NA	.	CHR	.	.	.	.	.	.
0	Sinoatrial r	NA	.	EST/	MEM	CATI	KEG(	.	.	.
0	.	NA	.	.	.	.	.	.	.	.
0	.	NA	.	.	.	.	KEG(	.	.	REACTOME_TRANSMISSION_ACRC
0	.	NA	.	.	.	.	KEG(	.	.	REACTOME_TRANSMISSION_ACRC
0	Hypogonac	NA	.	.	.	.	.	PID	.	REACTOME_SIGNALING_BY_FGFR_
0	.	NA	.	SIGN	NUC	.	KEG(	PID	.	REACTOME_REGULATION_OF_APC
0	.	NA	.	.	.	.	.	.	.	.
0	.	NA	.	BIOF	CYT(	NUC	.	.	.	.
0	.	NA	.	GLY(	PRO	.	.	.	.	.
0	.	NA	.	.	.	.	.	.	.	.
0	Tietz albini	NA	.	.	.	TRAI	KEG(	PID	.	.
0	.	NA	.	.	.	.	.	.	.	.
0	.	NA	.	.	.	.	.	PID	.	.
0	.	NA	.	.	.	.	.	.	.	.
0	.	NA	.	.	.	.	.	.	.	.
0	.	NA	.	GAM	NUC	.	.	.	.	.
0	.	NA	.	.	.	.	.	.	.	.
0	.	NA	.	.	.	.	.	.	.	.
0	.	NA	.	.	.	.	.	.	.	.
0	.	NA	.	.	.	.	.	.	.	.
0	.	NA	.	.	.	.	KEG(	.	.	.
0	.	NA	.	.	.	.	.	.	.	.
0	Urocanase	NA	.	.	.	.	KEG(	.	.	REACTOME_METABOLISM_OF_AMI
0	.	NA	.	MUL	.	REC	KEG(	.	.	REACTOME_DEVELOPMENTAL_BIC
0	.	NA	.	LIPIC	INTF	G_PI	.	.	.	.
0	.	NA	.	.	.	.	.	.	.	.
0	.	NA	.	.	.	.	.	.	.	.
0	.	NA	.	.	.	.	.	.	.	.
0	.	NA	.	.	.	.	.	.	.	.
0	.	NA	.	.	.	.	KEG(	.	.	.
0	.	NA	.	.	.	.	.	.	.	.
0	.	NA	.	.	.	.	.	.	.	.
0	.	NA	.	.	.	.	.	.	.	.
0	.	NA	.	.	.	RAS	KEG(	PID	.	.
0	.	NA	.	.	.	TRAI	KEG(	PID	.	.
0	Cutaneous	NA	.	DNA	.	PRO	KEG(	PID	BIO	REACTOME_MEIOSIS;REACTOME_(
0	.	NA	.	.	.	.	.	.	.	REACTOME_EXTRACELLULAR_MAT
0	.	NA	.	.	.	.	.	.	.	.
0	.	NA	.	.	.	.	.	.	.	.
0	.	NA	.	MAC	CYT(	TRAI	.	PID	.	.
0	Combined	NA	.	.	.	.	.	.	.	.
0	.	NA	.	.	.	.	.	.	.	.
0	.	NA	.	.	.	.	.	.	.	.
0	.	NA	.	.	.	.	.	.	.	.
0	.	NA	.	RNA	.	TRAI	.	PID	BIO	REACTOME_TRANSCRIPTIONAL_A(
0	.	NA	.	.	.	.	.	.	.	.
0	.	NA	.	REG	INTF	CATI	KEG(	.	.	REACTOME_NEURONAL_SYSTEM;F
0	.	NA	.	.	.	CYS	.	.	.	.
0	.	NA	.	.	.	.	.	.	.	.

0	3-Methylcr	NA	.	.	.	.	KEGG	.	.	REACTOME_METABOLISM_OF_AMI
0	.	NA	.	.	.	.	.	.	.	.
0	.	NA	.	.	.	.	.	.	.	.
0	.	NA	.	.	.	.	.	.	.	.
0	.	NA	.	SYS	ORG	CAT	.	.	.	.
0	.	NA	.	CELI	EXTI	REC	.	.	.	REACTOME_SIGNALING_BY_GPCR
0	Leukemia,	NA	.	.	.	.	.	PID	.	.
0	.	NA	.	.	.	.	.	.	.	.
0	.	NA	.	.	.	.	.	.	.	.
0	.	NA	.	.	.	.	.	.	.	.
0	.	NA	.	.	INTR	REC	.	.	.	REACTOME_O_LINKED_GLYCOSYL
0	.	NA	.	.	.	.	.	.	.	.
0	.	NA	.	.	.	.	.	.	.	.
0	.	NA	.	.	.	.	.	.	.	.
0	.	NA	.	.	.	.	.	.	.	.
0	.	NA	.	.	.	.	.	.	.	.
0	.	NA	.	VIRA	.	TRA	KEGG	PID	BIO	.
0	.	NA	.	.	.	.	.	.	.	.
0	{?Bladder (	NA	.	.	.	.	.	PID	.	.
0	.	NA	.	SIGN	INTR	CATI	.	.	.	.
0	.	NA	.	ANA	.	.	KEGG	.	.	.
0	.	NA	.	RNA	.	TRA	.	PID	.	.
0	Fetal akine	NA	.	.	.	.	.	.	.	.
0	.	NA	.	.	.	.	.	.	.	.
0	{Congestiv	NA	.	REG	INTR	AMIN	KEGG	.	.	.
0	Deafness,	NA	.	SYS	INTR	.	.	.	.	REACTOME_DIABETES_PATHWAYS
0	.	NA	.	.	.	.	KEGG	.	BIO	.
0	.	NA	.	.	.	.	.	.	.	.
0	.	NA	.	.	.	.	.	.	.	.
0	.	NA	.	.	NON	.	KEGG	.	.	.
0	.	NA	.	.	.	.	.	.	.	.
0	.	NA	.	.	.	.	.	.	.	.
0	.	NA	.	.	.	.	.	.	.	.
0	COACH sy	NA	.	.	.	.	.	.	.	.
0	.	NA	.	.	.	.	.	.	.	.
0	.	NA	.	.	.	.	.	.	.	.
0	.	NA	.	.	.	.	.	.	.	.
0	.	NA	.	BIOF	.	ACID	KEGG	.	.	REACTOME_NEGATIVE_REGULATO
0	.	NA	.	BIOF	.	ACID	KEGG	.	.	REACTOME_NEGATIVE_REGULATO
0	.	NA	.	.	.	.	.	.	.	.
0	.	NA	.	SIGN	CYT	GTP	KEGG	.	.	REACTOME_SIGNALING_BY_RHO_
0	.	NA	.	.	.	.	.	.	.	.
0	.	NA	.	SIGN	INTR	GAB	KEGG	.	.	REACTOME_TRANSMISSION_ACRO
0	.	NA	.	.	.	.	.	.	.	.
0	.	NA	.	.	.	.	.	.	.	.
0	.	NA	.	.	.	.	.	.	.	.
0	.	NA	.	.	.	.	.	.	.	.
0	.	NA	.	.	.	.	.	PID	.	REACTOME_DIABETES_PATHWAYS
0	.	NA	.	.	.	.	.	.	.	.
0	.	NA	.	RES	INTR	PEP	.	.	.	.
0	.	NA	.	LIPID	.	.	KEGG	.	.	REACTOME_BIOLOGICAL_OXIDATI
0	.	NA	.	.	.	.	.	.	.	.
0	Renal tubu	NA	.	.	INTR	CATI	KEGG	.	.	REACTOME_TRANSMEMBRANE_TR
0	.	NA	.	.	.	.	.	.	.	REACTOME_EXTRACELLULAR_MA

0	.	NA	.	.	EXTI	.	.	.	.	.
0	.	NA	.	.	.	.	.	.	.	.
0	.	NA	.	BIOF	.	PHO	.	.	.	.
0	.	NA	.	.	.	.	.	.	.	.
0	.	NA	.	PAT	ADH	ACTI	.	.	.	.
0	Fraser syn	NA	.	.	.	.	.	.	.	.
0	.	NA	.	.	.	.	.	.	.	REACTOME_G0_AND_EARLY_G1;R
0	.	NA	.	.	.	.	.	.	.	.
0	.	NA	.	.	.	.	.	.	.	REACTOME_SIGNALING_BY_RHO_(
0	.	NA	.	RNA	CYT	TRAI	KEG(	PID	BIO	.
0	Myelodysp	NA	.	.	.	.	.	.	.	.
0	.	NA	.	.	.	.	.	.	.	.
0	.	NA	.	CELI	INTR	MET	KEG(	.	.	.
0	.	NA	.	.	.	.	.	.	.	.
0	.	NA	.	.	.	.	KEG(	.	.	REACTOME_HS_GAG_BIOSYNTHES
0	.	NA	.	.	.	.	.	.	.	.
0	.	NA	.	.	.	.	.	.	.	.
0	.	NA	.	.	.	.	.	.	.	.
0	.	NA	.	.	.	.	.	.	.	.
0	.	NA	.	.	.	.	.	.	.	.
0	.	NA	.	.	.	.	.	.	.	.
0	.	NA	.	.	.	.	.	.	.	.
0	.	NA	.	.	.	.	.	.	.	.
0	.	NA	.	.	.	.	.	.	.	.
0	.	NA	.	.	.	.	.	.	.	.
0	.	NA	.	POS	INTR	.	KEG(	.	BIO	.
0	[Blood grou	NA	.	.	INTR	.	.	.	.	.
0	.	NA	.	.	CYT	.	.	.	.	.
0	.	NA	.	EST	NON	.	.	.	.	.
0	.	NA	.	.	.	.	KEG(	.	.	REACTOME_O_LINKED_GLYCOSYL
0	.	NA	.	.	.	.	.	.	.	.
0	.	NA	.	.	.	.	.	.	.	.
0	.	NA	.	.	NON	MOL	.	.	.	.
0	.	NA	.	.	.	.	.	.	.	.
0	.	NA	.	.	.	CATI	KEG(	PID	.	REACTOME_TRANSMEMBRANE_TF
0	.	NA	.	.	.	.	.	.	.	REACTOME_CELL_CYCLE;REACTO
0	.	NA	.	.	.	.	.	.	.	.
0	.	NA	.	.	INTR	CATI	.	.	.	REACTOME_TRANSMEMBRANE_TF
0	.	NA	.	SYS	.	.	.	.	.	.
0	.	NA	.	.	.	.	.	.	.	.
0	.	NA	.	.	.	.	.	.	.	.
0	.	NA	.	.	.	.	KEG(	.	.	.
0	.	NA	.	ORG	NUC	.	KEG(	.	.	.
0	Mental reta	NA	.	SIGN	CYT	.	.	.	.	.
0	Ciliary dysl	NA	.	.	.	.	.	.	.	.
0	.	NA	.	ENZ	.	PHO	.	PID	BIO	REACTOME_SIGNALING_BY_RHO_(
0	.	NA	.	ENZ	.	PHO	.	PID	BIO	REACTOME_SIGNALING_BY_RHO_(
0	.	NA	.	.	.	.	.	.	.	.
0	.	NA	.	.	.	.	.	.	.	.
0	.	NA	.	.	.	.	.	.	.	.
0	.	NA	.	.	.	.	.	.	.	.
0	.	NA	.	.	.	.	.	.	.	.
0	.	NA	.	.	.	.	.	.	.	.
0	.	NA	.	BIOF	.	.	KEG(	PID	BIO	REACTOME_ACTIVATED_AMPK_ST

0	Succinyl C	NA	.	.	CYT	TRA	KEG	.	.	REACTOME_METABOLISM_OF_LIPI
0	.	NA	.	.	CYT	TRA	KEG	PID	.	REACTOME_PPARA_ACTIVATES_G
0	.	NA	.	.	.	.	.	.	.	.
0	.	NA	.	.	.	.	.	.	.	.
0	.	NA	.	.	.	.	.	.	.	.
0	.	NA	.	.	.	.	.	.	.	.
0	Acrodysos	NA	.	.	INSC	HYD	KEG	.	.	REACTOME_SIGNALING_BY_GPCR
0	.	NA	.	.	.	.	.	.	.	.
0	.	NA	.	.	.	.	.	.	.	REACTOME_CELL_CYCLE;REACTO
0	.	NA	.	ENZ	PRO	INTE	KEG	.	.	.
0	.	NA	.	.	.	.	.	.	.	.
0	.	NA	.	.	.	.	.	.	.	.
0	.	NA	.	.	.	.	.	.	.	.
0	Agammagl	NA	.	ENZ	CYT	HOR	KEG	PID	BIO	REACTOME_SIGNALLING_BY_NGF;
0	.	NA	.	DNA	NUC	.	.	PID	BIO	REACTOME_CELL_CYCLE;REACTO
0	.	NA	.	.	.	.	.	.	.	.
0	.	NA	.	.	ORG	.	.	PID	.	.
0	.	NA	.	.	.	.	.	.	.	.
0	.	NA	.	G_PI	INTR	.	.	.	.	.
0	.	NA	.	COF	CYT	THIC	KEG	.	.	.
0	.	NA	.	.	.	.	.	.	.	.
0	Wagner sy	NA	.	MUL	PRO	CAR	KEG	PID	.	REACTOME_CS_DS_DEGRADATION
0	.	NA	.	.	.	.	.	.	.	.
0	Febrile sei	NA	.	REG	CELI	CATI	.	.	.	.
0	.	NA	.	.	.	.	.	.	.	.
0	.	NA	.	.	.	ARYI	.	.	.	REACTOME_PTM_GAMMA_CARBOX
0	.	NA	.	.	.	.	.	.	.	.
0	.	NA	.	BIOF	ORG	CHR	.	.	.	.
0	.	NA	.	BIOF	ORG	CHR	.	.	.	.
0	.	NA	.	.	.	.	.	.	.	.
0	.	NA	.	.	.	.	.	.	.	.
0	.	NA	.	.	.	.	KEG	.	.	REACTOME_DEVELOPMENTAL_BIO
0	.	NA	.	CELI	CYT	ENZ	.	.	.	.
0	.	NA	.	.	.	.	.	.	.	.
0	.	NA	.	RNA	.	TRA	.	.	.	.
0	.	NA	.	.	.	.	.	.	.	.
0	.	NA	.	.	.	.	.	.	.	.
0	.	NA	.	.	.	.	KEG	.	.	.
0	.	NA	.	REG	.	.	KEG	PID	BIO	REACTOME_CELL_CYCLE;REACTO
0	.	NA	.	REG	.	.	KEG	PID	BIO	REACTOME_CELL_CYCLE;REACTO
0	.	NA	.	NUC	NUC	TRA	KEG	PID	BIO	REACTOME_INTERFERON_ALPHA_
0	.	NA	.	NUC	NUC	TRA	KEG	PID	BIO	REACTOME_INTERFERON_ALPHA_
0	.	NA	.	.	.	.	.	.	.	.
0	.	NA	.	SYS	INTR	.	.	.	.	.
0	.	NA	.	.	.	.	.	.	.	.
0	.	NA	.	SYS	INTR	.	.	.	.	.
0	.	NA	.	SYS	INTR	.	.	.	.	.
0	.	NA	.	.	.	.	.	.	.	.
0	.	NA	.	.	.	.	.	.	.	.
0	.	NA	.	.	.	.	.	.	.	.
0	.	NA	.	.	.	.	.	.	.	.
0	.	NA	.	.	.	.	.	.	.	.
0	.	NA	.	.	.	.	.	.	.	.
0	.	NA	.	.	CYT	.	.	.	.	.

0	.	NA	.	.	.	.	.	.	.	.
0	.	NA	.	BIOF	.	PHO	KEG	PID	BIO	REACTOME_SIGNALING_BY_WNT;F
0	Retinitis pi	NA	.	SEN	.	.	KEG	PID	.	REACTOME_CGMP_EFFECTS;REAC
0	.	NA	.	.	CEL	ACT	.	.	.	.
0	.	NA	.	SIGN	NUC	HYD	.	.	.	.
0	.	NA	.	CEL	CYT	.	KEG	PID	.	.
0	.	NA	.	.	.	.	.	.	.	.
0	.	NA	.	.	.	.	.	.	.	.
0	.	NA	.	.	.	.	.	.	.	.
0	[Memory, e	NA	.	.	.	.	.	.	.	REACTOME_SIGNALING_BY_HIPPO
0	.	NA	.	RNA	CYT	.	KEG	.	.	REACTOME_CYTOSOLIC_TRNA_AM
0	.	NA	.	SYS	EXT	.	KEG	.	.	REACTOME_DEVELOPMENTAL_BIO
0	Frank-ter H	NA	.	.	.	.	.	.	.	.
0	.	NA	.	.	.	.	.	.	.	.
0	.	NA	.	.	.	.	.	.	.	.
0	.	NA	.	.	.	.	.	.	.	.
0	{Cancer pr	NA	.	ENZ	INTR	TRA	KEG	PID	.	REACTOME_NEGATIVE_REGULATI
0	{Cancer pr	NA	.	ENZ	INTR	TRA	KEG	PID	.	REACTOME_NEGATIVE_REGULATI
0	{Cancer pr	NA	.	ENZ	INTR	TRA	KEG	PID	.	REACTOME_NEGATIVE_REGULATI
0	.	NA	.	.	.	.	.	.	.	.
0	.	NA	.	.	.	.	.	.	.	REACTOME_TRANSLATION;REACT
0	Night blind	NA	.	GLU	INTR	GLU	KEG	.	.	REACTOME_SIGNALING_BY_GPCR
0	.	NA	.	.	.	.	.	.	.	.
0	.	NA	.	GLY	.	TRA	KEG	.	.	REACTOME_METABOLISM_OF_PRO
0	.	NA	.	ENE	.	.	KEG	.	.	REACTOME_METABOLISM_OF_PRO
0	.	NA	.	.	ORG	HYD	.	.	.	.
0	.	NA	.	.	ORG	HYD	.	.	.	.
0	.	NA	.	.	.	.	.	.	.	.
0	.	NA	.	.	.	.	.	.	.	.
0	.	NA	.	.	.	.	.	.	.	.
0	.	NA	.	.	.	.	.	.	.	.
0	Adult i phe	NA	.	BIOS	CEL	TRA	KEG	.	.	.
0	.	NA	.	.	NUC	DNA	.	.	.	.
0	Hermansky	NA	.	ORG	AXO	IDEN	.	.	.	REACTOME_MEMBRANE_TRAFFICK
0	.	NA	.	.	.	.	.	.	.	.
0	.	NA	.	.	.	.	.	.	.	.
0	.	NA	.	.	.	.	.	.	.	.
0	.	NA	.	.	.	.	.	.	.	.
0	.	NA	.	.	.	.	.	.	.	.
0	.	NA	.	.	.	.	.	.	.	.
0	.	NA	.	.	.	.	.	.	.	REACTOME_GENERIC_TRANSCRIP
0	.	NA	.	.	.	.	.	.	.	.
0	Diabetes m	NA	.	.	.	.	.	.	.	.
0	Hemochro	NA	.	.	.	.	.	.	.	.
0	.	NA	.	.	.	.	.	.	.	.
0	.	NA	.	.	NUC	.	.	PID	.	REACTOME_HOMOLOGOUS_RECO
0	.	NA	.	.	.	.	.	.	.	.
0	.	NA	.	.	.	.	.	.	.	.
0	.	NA	.	.	.	.	.	.	.	.
0	.	NA	.	.	.	LIGA	KEG	.	.	REACTOME_CYTOSOLIC_TRNA_AM
0	.	NA	.	.	CYT	.	KEG	PID	BIO	.
0	Deafness,	NA	.	.	.	.	KEG	PID	.	REACTOME_EXTRACELLULAR_MAT
0	Deafness,	NA	.	.	.	.	KEG	PID	.	REACTOME_EXTRACELLULAR_MAT
0	{Diabetes,	NA	.	SIGN	BRU	CAT	KEG	.	.	REACTOME_SIGNALLING_BY_NGF;

0	.	NA	.	.	.	.	.	.	.	.	
0	.	NA	.	REG	.	LIGA	KEGG	PID	BIO	REACTOME_GENERIC_TRANSCRIP	
0	.	NA	.	REG	.	LIGA	KEGG	PID	BIO	REACTOME_GENERIC_TRANSCRIP	
0	.	NA	.	REG	.	LIGA	KEGG	PID	BIO	REACTOME_GENERIC_TRANSCRIP	
0	.	NA	.	ANAT	.	.	KEGG	.	.	REACTOME_TRANSLATION;REACT	
0	Spermatog	NA	.	.	.	.	.	.	.	.	
0	.	NA	.	.	.	.	.	.	.	.	
0	.	NA	.	.	.	.	KEGG	.	.	.	
0	.	NA	.	.	.	.	.	.	.	.	
0	.	NA	.	RNA	CYT	TRA	.	.	.	.	
0	.	NA	.	ENZ	.	.	.	PID	.	REACTOME_SIGNALING_BY_FGFR_	
0	Retinitis pi	NA	.	REG	.	ENZ	KEGG	PID	.	.	
0	.	NA	.	RNA	NUC	TRA	.	.	.	.	
0	Choriodal (	NA	.	SEN	INTR	.	KEGG	.	.	.	
0	.	NA	.	SIGN	INTR	TRA	.	.	.	.	
0	.	NA	.	SIGN	INTR	TRA	.	.	.	.	
0	.	NA	.	.	.	.	.	PID	.	.	
0	.	NA	.	.	MEM	.	.	.	.	.	
0	.	NA	.	.	.	.	.	.	.	REACTOME_MICRORNA_MIRNA_BI	
0	.	NA	.	.	.	.	.	.	.	.	
0	.	NA	.	.	.	.	.	.	.	.	
0	Neuropathy	NA	.	ORG	PRO	INTE	.	PID	.	.	
0	.	NA	.	.	.	.	.	.	.	.	
0	.	NA	.	SYS	.	.	.	.	.	REACTOME_EXTRACELLULAR_MAT	
0	.	NA	.	.	.	.	KEGG	.	.	.	
0	Hydatidifor	NA	.	.	.	.	.	.	.	.	
0	.	NA	.	.	.	.	.	.	.	.	
0	.	NA	.	.	.	.	.	.	.	.	
0	Maple syru	NA	CM0	CAR	ORG	LYAS	KEGG	.	.	REACTOME_METABOLISM_OF_AMI	
0	.	NA	.	SEN	CYT	ELE	.	.	.	.	
0	Congenital	NA	.	EST	INTR	SUB	.	.	.	REACTOME_TRANSMEMBRANE_TR	
0	.	NA	.	.	.	.	.	.	.	.	
0	.	NA	.	.	.	.	.	.	.	.	
0	.	NA	.	POS	.	IDEN	.	.	.	.	
0	.	NA	.	.	.	.	.	.	.	.	
0	.	NA	.	SYS	.	LIGA	.	.	.	REACTOME_GENERIC_TRANSCRIP	
0	.	NA	.	RES	NUC	.	KEGG	PID	.	.	
0	.	NA	.	.	.	.	.	.	.	.	
0	.	NA	.	.	CYT	PRO	.	.	.	.	
0	.	NA	.	DNA	.	NUC	.	.	.	REACTOME_DNA_REPAIR	
0	.	NA	.	.	.	.	.	.	.	.	
0	.	NA	.	.	.	.	.	.	.	.	
0	.	NA	.	.	.	.	KEGG	.	.	.	
0	Muscular d	NA	.	MUS	PRO	STR	KEGG	PID	BIO	REACTOME_INTEGRIN_CELL_SURF	
0	Muscular d	NA	.	MUS	PRO	STR	KEGG	PID	BIO	REACTOME_INTEGRIN_CELL_SURF	
0	Cardiomyo	NA	.	ANA	.	.	.	.	.	.	
0	.	NA	.	ISOF	.	OXID	.	.	.	.	
0	.	NA	.	.	.	.	KEGG	.	.	.	
0	.	NA	.	.	.	.	.	.	.	.	
0	.	NA	.	.	.	.	.	.	.	.	
0	.	NA	.	.	INTR	G_PI	.	.	.	.	
0	Hemophag	NA	.	MEM	.	.	KEGG	.	.	REACTOME_BOTULINUM_NEUROT	
0	.	NA	.	.	.	.	.	.	.	.	
0	.	NA	.	BIOF	INTR	SULF	KEGG	.	.	REACTOME_CHONDROITIN_SULFA	

0	.	NA	.	.	.	.	.	.	.	.
0	.	NA	.	INTE	ORG	HYD	.	PID	.	.
0	.	NA	.	.	.	.	.	.	.	.
0	.	NA	.	SIGN	CYT	KINA	.	.	.	.
0	Emery-Dre	NA	.	ORG	SYN	ACTI	.	.	.	REACTOME_MEIOSIS;REACTOME_
0	Emery-Dre	NA	.	ORG	SYN	ACTI	.	.	.	REACTOME_MEIOSIS;REACTOME_
0	Emery-Dre	NA	.	ORG	SYN	ACTI	.	.	.	REACTOME_MEIOSIS;REACTOME_
0	.	NA	.	RES	.	OXID	.	PID	.	.
0	.	NA	.	.	.	.	.	.	.	.
0	Mental reta	NA	.	.	NUC	DNA	.	.	.	.
0	.	NA	.	.	.	.	.	.	.	.
0	{Microvasc	NA	.	RNA	CYT	OXID	KEG	PID	BIO	.
0	.	NA	.	.	.	.	.	.	.	.
0	.	NA	.	.	.	.	.	.	.	.
0	[LPA defici	NA	.	SYS	.	PRO	.	PID	.	REACTOME_METABOLISM_OF_LIPI
0	.	NA	.	REG	.	PHO	KEG	PID	BIO	.
0	Adenocarc	NA	.	BIOF	CYT	ACID	KEG	PID	BIO	REACTOME_IMMUNE_SYSTEM;REA
0	.	NA	.	.	.	.	.	.	.	.
0	.	NA	.	.	.	.	.	.	.	.
0	.	NA	.	.	CYT	.	.	.	.	.
0	.	NA	.	.	.	.	.	.	.	.
0	Raine sync	NA	.	.	.	.	.	.	.	.
0	.	NA	.	.	.	.	.	.	.	.
0	.	NA	.	.	.	.	.	.	.	.
0	.	NA	.	RNA	NUC	.	.	.	.	.
0	.	NA	.	RNA	NUC	.	.	.	.	.
0	.	NA	.	RNA	NUC	.	.	.	.	.
0	.	NA	.	.	.	.	.	.	.	.
0	.	NA	.	RES	.	HYD	.	.	.	.
0	.	NA	.	SULI	.	SULI	KEG	.	.	REACTOME_CHONDROITIN_SULFA
0	Immunode	NA	.	REG	MEM	PRO	KEG	PID	.	REACTOME_DOWNSTREAM_SIGNA
0	.	NA	.	.	.	.	.	.	.	.
0	.	NA	.	.	.	.	.	.	.	.
0	Spastic pa	NA	.	.	.	.	.	.	.	.
0	.	NA	.	.	.	.	.	.	.	.
0	.	NA	.	EST	MEM	LIPID	.	PID	BIO	.
0	.	NA	.	.	.	.	.	PID	.	REACTOME_GASTRIN_CREB_SIGN
0	.	NA	.	.	.	.	.	.	.	.
0	.	NA	.	.	.	.	.	.	.	.
0	.	NA	.	.	.	.	.	.	.	.
0	.	NA	.	.	.	.	.	.	.	.
0	.	NA	.	.	.	.	.	.	.	.
0	.	NA	.	.	.	.	.	.	.	.
0	.	NA	.	.	.	.	.	.	.	REACTOME_EXTRACELLULAR_MA
0	.	NA	.	.	.	.	.	.	.	.
0	.	NA	.	.	.	.	.	.	.	.
0	.	NA	.	.	.	.	.	.	.	.
0	.	NA	.	.	.	.	KEG	.	.	REACTOME_ABC_FAMILY_PROTEIN
0	Ciliary dysk	NA	.	.	.	.	.	.	.	.
0	.	NA	.	.	.	.	.	.	.	.
0	.	NA	.	.	.	.	.	.	.	.
0	.	NA	.	.	.	.	KEG	.	.	REACTOME_METABOLISM_OF_AMI
0	.	NA	.	PRO	CYT	ENZ	KEG	.	.	REACTOME_TRIF_MEDIATED_TLR3
0	.	NA	.	.	.	.	.	.	.	.

0	.	NA	.	.	.	.	.	.	.	.
0	.	NA	.	PRO	CYT	CATI	.	.	.	REACTOME_ASSOCIATION_OF_TR
0	Bardet-Bie	NA	.	.	.	.	.	.	.	.
0	.	NA	.	.	.	.	.	.	.	.
0	.	NA	.	.	.	.	.	.	.	.
0	.	NA	.	.	.	.	.	.	.	.
0	.	NA	.	.	.	.	.	.	.	.
0	.	NA	.	.	.	.	.	.	.	.
0	.	NA	.	.	.	.	.	.	.	.
0	.	NA	.	.	.	.	KEG	.	BIO	.
0	.	NA	.	POS	.	.	.	.	.	.
0	Alpha-keto	NA	.	GEN	ORG	OXID	KEG	.	BIO	REACTOME_PYRUVATE_METABOL
0	.	NA	.	.	.	.	KEG	PID	BIO	REACTOME_SIGNALLING_BY_NGF;
0	.	NA	.	MUS	NUC	ENZ	KEG	PID	BIO	REACTOME_DIABETES_PATHWAYS
0	.	NA	.	.	.	.	.	.	.	.
0	.	NA	.	.	.	.	.	.	.	.
0	.	NA	.	.	.	.	.	.	.	.
0	.	NA	.	.	.	.	.	.	.	.
0	.	NA	.	.	.	.	.	.	.	.
0	.	NA	.	.	.	.	.	.	.	.
0	.	NA	.	.	.	.	.	.	.	.
0	.	NA	.	.	.	.	.	.	.	.
0	.	NA	.	.	.	.	.	.	.	.
0	.	NA	.	.	.	.	.	.	.	.
0	.	NA	.	.	.	.	.	.	.	.
0	.	NA	.	.	.	.	.	.	.	.
0	.	NA	.	.	.	.	.	.	.	.
0	.	NA	.	.	.	.	.	.	.	.
0	.	NA	.	.	.	.	.	.	.	.
0	.	NA	.	.	.	.	.	.	.	.
0	.	NA	.	.	.	.	.	.	.	.
0	.	NA	.	.	.	.	.	.	.	.
0	.	NA	.	.	.	.	KEG	.	.	REACTOME_O_LINKED_GLYCOSYL
0	.	NA	.	.	.	.	.	.	.	.
0	.	NA	.	.	.	.	.	.	.	.
0	.	NA	.	PRO	.	DRU	.	.	.	REACTOME_MEIOSIS;REACTOME_
0	.	NA	.	SYS	INTR	REC	KEG	PID	.	REACTOME_SIGNALING_BY_GPCR
0	.	NA	.	.	.	.	.	.	.	.
0	.	NA	.	.	.	.	.	.	.	.
0	.	NA	.	POS	MEM	PRO	.	PID	.	.
0	.	NA	.	.	.	.	KEG	.	.	.
0	Chronic gra	NA	.	DEFI	CYT	HYD	KEG	PID	.	.
0	.	NA	.	.	.	.	.	.	.	.
0	.	NA	.	.	.	.	.	.	.	.
0	.	NA	.	.	.	.	KEG	.	.	REACTOME_ACTIVATED_NOTCH1_
0	.	NA	.	ESTA	MEM	CATI	KEG	.	.	.
0	.	NA	.	.	.	.	.	.	.	.
0	.	NA	.	ORG	SYN	LIPID	.	.	.	.
0	.	NA	.	.	CYT	.	.	.	.	.
0	.	NA	.	.	.	.	.	.	.	.
0	Ehlers-Dar	NA	.	SYS	PRO	.	KEG	PID	.	REACTOME_DEVELOPMENTAL_BIO
0	Dystonia-1	NA	.	MUS	PRO	.	.	.	.	.
0	.	NA	.	.	.	.	.	.	.	REACTOME_IMMUNE_SYSTEM;REA
0	.	NA	.	PRO	INTR	PHO	.	.	.	.

0	.	NA	.	SIGN	NUC	TRAI	.	PID	BIO	.
0	.	NA	.	SIGN	NUC	TRAI	.	PID	BIO	.
0	.	NA	.	.	.	.	.	.	.	.
0	.	NA	.	.	.	.	.	.	.	.
0	.	NA	.	MUL	.	.	.	.	.	REACTOME_EXTRACELLULAR_MAT
0	.	NA	.	.	NUC	STRI	.	.	.	.
0	.	NA	.	.	.	.	.	.	.	REACTOME_O_LINKED_GLYCOSYL
0	.	NA	.	.	.	.	.	.	.	.
0	.	NA	.	RNA	.	RNA	.	.	.	REACTOME_SIGNALING_BY_FGFR_
0	.	NA	.	.	.	.	.	.	.	.
0	.	NA	.	.	.	.	KEGG	.	.	.
0	.	NA	.	.	CYTO	.	KEGG	.	BIO	REACTOME_SIGNALING_BY_WNT;F
0	.	NA	.	DNA	NUC	NUC	KEGG	.	BIO	REACTOME_ACTIVATION_OF_THE_
0	.	NA	.	.	.	.	.	.	.	.
0	.	NA	.	MUS	.	.	.	.	.	.
0	.	NA	.	.	CYTO	.	.	.	.	.
0	Hyperlysine	NA	.	PRO	CYTO	.	KEGG	.	.	REACTOME_METABOLISM_OF_AMI
0	.	NA	.	.	.	.	.	.	.	.
0	.	NA	.	SIGN	INTR	.	KEGG	.	BIO	.
0	.	NA	.	.	.	.	.	.	.	.
0	Myopathy,	NA	.	.	.	.	KEGG	.	.	REACTOME_CELL_CELL_COMMUN
0	Basal cell c	NA	.	.	INTR	REC	KEGG	PID	BIO	REACTOME_SIGNALING_BY_GPCR
0	.	NA	.	.	.	.	.	.	.	.
0	.	NA	.	.	.	.	.	.	.	.
0	.	NA	.	.	.	.	.	.	.	.
0	.	NA	.	.	CYTO	PHO	KEGG	.	.	REACTOME_GASTRIN_CREB_SIGN
0	.	NA	.	.	CYTO	PHO	KEGG	.	.	REACTOME_GASTRIN_CREB_SIGN
0	.	NA	.	.	.	.	KEGG	.	.	.
0	Renal tubu	NA	.	SYS	CELI	.	KEGG	.	.	REACTOME_INSULIN_RECEPTOR_I
0	.	NA	.	.	.	.	.	.	.	.
0	.	NA	.	.	.	.	.	.	.	.
0	Cataract 3	NA	.	.	.	.	KEGG	.	.	.
0	.	NA	.	BIOF	.	.	KEGG	.	.	REACTOME_METABOLISM_OF_CAF
0	.	NA	.	.	.	.	.	.	.	.
0	.	NA	.	.	.	.	.	.	.	.
0	.	NA	.	.	.	.	.	.	.	.
0	.	NA	.	.	.	.	.	.	.	.
0	.	NA	.	.	.	.	.	.	.	.
0	.	NA	.	.	.	IDEN	KEGG	.	.	.
0	.	NA	.	.	.	.	.	.	.	.
0	Cortical dy	NA	.	.	.	.	KEGG	.	.	.
0	.	NA	.	.	.	.	.	.	.	.
0	.	NA	.	.	.	.	.	PID	.	.
0	.	NA	.	.	.	.	.	.	.	.
0	{Alzheimer	NA	.	.	CYTO	OXID	KEGG	PID	BIO	REACTOME_TETRAHYDROBIOPTEF
0	.	NA	.	ORG	.	.	.	.	.	.
0	.	NA	.	SYS	AXO	PHO	KEGG	PID	BIO	REACTOME_DEVELOPMENTAL_BIC
0	.	NA	.	.	.	.	.	.	.	.
0	Cardiomyo	NA	.	.	.	.	KEGG	.	BIO	REACTOME_ACTIVATED_AMPK_ST
0	Cardiomyo	NA	.	.	.	.	KEGG	.	BIO	REACTOME_ACTIVATED_AMPK_ST
0	.	NA	.	.	.	.	.	.	.	.
0	.	NA	.	.	.	.	.	.	.	.
0	Ventricular	NA	.	.	.	.	.	.	.	.
0	.	NA	.	PRO	.	ACID	KEGG	.	.	REACTOME_IMMUNE_SYSTEM;REA

0	.	NA	.	BIOF	INTF	PRO	KEG	.	.	.
0	.	NA	.	BIOF	INTF	PRO	KEG	.	.	.
0	.	NA	.	.	.	.	.	.	.	.
0	.	NA	.	.	.	.	.	.	.	.
0	Ceroid lipo	NA	.	SYS	CYT	.	.	.	.	.
0	.	NA	.	.	.	.	.	.	.	.
0	.	NA	.	.	.	.	.	.	.	REACTOME_DEFENSINS;REACTOM
0	.	NA	.	.	.	.	.	.	.	.
0	.	NA	.	.	.	.	.	.	.	.
0	.	NA	.	CAL	CELI	IDEN	KEG	.	.	.
0	Occult mac	NA	.	.	.	.	.	.	.	.
0	.	NA	.	.	.	.	.	.	.	.
0	.	NA	.	.	.	.	.	.	.	.
0	.	NA	.	.	.	.	.	.	.	.
0	.	NA	.	.	.	.	.	.	.	.
0	.	NA	.	.	.	.	.	.	.	.
0	.	NA	.	.	.	.	.	.	.	.
0	.	NA	.	.	.	.	.	.	.	.
0	Colorectal	NA	.	REG	.	GTP	.	PID	.	REACTOME_SIGNALING_BY_RHO_
0	Mental reta	NA	.	GLY	ORG	TRA	KEG	.	.	REACTOME_METABOLISM_OF_PR
0	.	NA	.	.	.	.	.	.	.	.
0	.	NA	.	.	.	.	.	.	.	.
0	Alopecia u	NA	.	.	.	.	.	.	.	.
0	.	NA	.	SIGN	.	REC	KEG	PID	BIO	.
0	.	NA	.	.	.	.	.	.	.	.
0	Persistent	NA	.	SYS	.	TRA	.	.	.	.
0	.	NA	.	.	.	.	.	.	.	REACTOME_FACTORS_INVOLVED_
0	.	NA	.	.	.	.	.	.	.	.
0	.	NA	.	.	.	.	.	.	.	.
0	{Hyperchol	NA	.	.	.	.	KEG	.	.	.
0	.	NA	.	EST	CYT	KINA	.	PID	.	.
0	.	NA	.	.	.	.	.	.	.	REACTOME_MEIOSIS;REACTOME_
0	.	NA	.	.	.	.	.	.	.	.
0	.	NA	.	.	.	.	.	.	.	.
0	Spastic pa	NA	.	.	ORG	.	.	.	.	.
0	Hypogona	NA	.	SYS	INTF	TRA	KEG	PID	.	REACTOME_DEVELOPMENTAL_BIC
0	Hypogona	NA	.	SYS	INTF	TRA	KEG	PID	.	REACTOME_DEVELOPMENTAL_BIC
0	.	NA	.	.	.	.	.	PID	.	.
0	.	NA	.	GAM	CELI	.	.	.	.	.
0	.	NA	.	.	.	.	.	.	.	.
0	.	NA	.	NEG	NUC	TRA	.	.	.	.
0	.	NA	.	DNA	ORG	MICF	KEG	.	.	REACTOME_BASE_EXCISION_REP
0	.	NA	.	.	.	.	.	.	.	.
0	.	NA	.	.	.	.	.	.	.	.
0	.	NA	.	.	.	.	.	.	.	.
0	.	NA	.	.	.	.	.	.	.	.
0	.	NA	.	.	.	.	.	.	.	.
0	.	NA	.	.	.	.	.	.	.	.
0	.	NA	.	.	.	.	.	.	.	.
0	.	NA	.	.	.	.	.	.	.	.
0	.	NA	.	.	PRO	.	.	.	.	.
0	.	NA	.	.	.	.	.	.	.	.
0	.	NA	.	.	.	.	.	.	.	.
0	.	NA	.	.	.	.	.	.	.	.

0	.	NA	.	MEM	CYT	REC	.	PID	BIO	.
0	Cerebellar	NA	.	.	.	HYD	KEG	.	.	.
0	CHARGE	NA	.	.	.	.	.	PID	.	.
0	.	NA	.	.	.	HYD	KEG	.	.	REACTOME_SIGNALING_BY_GPCR
0	.	NA	.	.	.	.	.	.	.	.
0	.	NA	.	.	.	.	.	.	.	.
0	.	NA	.	SIG	.	RAS	.	PID	.	.
0	.	NA	.	.	.	.	.	.	.	.
0	.	NA	.	.	.	.	.	.	.	.
0	.	NA	.	.	CYT	LIPI	KEG	PID	.	REACTOME_DEVELOPMENTAL_BIC
0	Achromato	NA	.	SEN	.	.	.	PID	.	.
0	Achromato	NA	.	SEN	.	.	.	PID	.	.
0	.	NA	.	.	.	.	.	.	.	.
0	?DECR de	NA	.	.	CYT	OXI	.	.	.	REACTOME_MITOCHONDRIAL_FAT
0	?DECR de	NA	.	.	CYT	OXI	.	.	.	REACTOME_MITOCHONDRIAL_FAT
0	.	NA	.	GEN	.	TRA	KEG	.	.	.
0	.	NA	.	.	.	.	.	.	.	.
0	.	NA	.	.	.	.	.	.	.	.
0	.	NA	.	.	.	.	.	.	.	.
0	.	NA	.	.	.	.	.	.	.	.
0	.	NA	.	.	.	.	.	.	.	.
0	.	NA	.	.	.	.	.	.	.	.
0	.	NA	.	EST	.	AUX	.	.	.	REACTOME_NEURONAL_SYSTEM;F
0	.	NA	.	.	.	.	.	.	.	.
0	.	NA	.	.	.	.	.	.	.	.
0	.	NA	.	.	.	.	.	.	.	.
0	.	NA	.	.	.	.	.	.	.	.
0	Ciliary dys	NA	.	.	.	.	.	.	.	.
0	Thyroid dy	NA	.	.	.	.	KEG	.	.	.
0	Birk-Barel	NA	.	.	.	.	.	.	.	REACTOME_NEURONAL_SYSTEM;F
0	Mental reta	NA	.	.	.	.	.	.	.	.
0	.	NA	.	.	.	.	.	.	.	.
0	.	NA	.	.	.	.	.	.	.	.
0	.	NA	.	SYS	INTR	G_PI	KEG	.	.	.
0	.	NA	.	SYS	.	.	.	.	.	.
0	.	NA	.	.	.	.	.	.	.	.
0	Amelogene	NA	.	.	.	.	.	.	.	.
0	Amelogene	NA	.	.	.	.	.	.	.	.
0	.	NA	.	.	.	.	.	.	.	.
0	.	NA	.	.	.	.	.	.	.	.
0	.	NA	.	.	.	.	.	.	.	.
0	Epidermoly	NA	.	.	.	STR	.	.	.	REACTOME_APOPTOTIC_CLEAVAG
0	.	NA	.	.	.	TRA	.	PID	.	.
0	.	NA	.	.	.	TRA	.	PID	.	.
0	.	NA	.	.	.	.	.	.	.	.
0	.	NA	.	.	.	.	.	.	.	.
0	.	NA	.	.	.	.	.	.	.	.
0	{?Schizoph	NA	.	EST	INTR	ACT	.	.	.	REACTOME_GLUTAMATE_NEUROT
0	Hydroceph	NA	.	.	.	.	KEG	.	.	.
0	.	NA	.	.	NUC	TRA	.	PID	.	REACTOME_RNA_POL_III_TRANSC
0	Bifid nose	NA	.	.	.	.	.	.	.	.
0	Bifid nose	NA	.	.	.	.	.	.	.	.
0	.	NA	.	.	.	.	.	.	.	.
0	.	NA	.	.	.	.	.	.	.	.

0	.	NA	.	.	.	.	.	.	.	.
0	.	NA	.	.	.	.	.	PID	.	REACTOME_TRANSMEMBRANE_TF
0	.	NA	.	.	.	.	.	.	.	.
0	.	NA	.	.	.	.	.	.	.	.
0	.	NA	.	.	ORG	.	KEG(	.	.	REACTOME_TCA_CYCLE_AND_RES
0	.	NA	.	.	.	.	.	.	.	.
0	.	NA	.	GAM	.	PHO	.	.	.	REACTOME_CELL_CELL_COMMUN
0	.	NA	.	.	.	.	.	.	.	.
0	.	NA	.	.	.	.	.	.	.	.
0	.	NA	.	.	.	.	.	.	.	.
0	.	NA	.	.	.	.	.	.	.	.
0	.	NA	.	.	.	.	.	.	.	.
0	.	NA	.	.	.	.	.	.	.	.
0	.	NA	.	.	.	.	.	.	.	.
0	.	NA	.	.	.	.	.	.	.	.
0	.	NA	.	.	.	.	.	.	.	.
0	.	NA	.	.	.	.	.	.	.	.
0	.	NA	.	.	.	.	.	.	.	.
0	.	NA	.	.	.	.	.	.	.	.
0	.	NA	.	.	.	.	.	.	.	.
0	.	NA	.	.	.	.	.	.	.	.
0	.	NA	.	.	INTF	.	.	.	.	.
0	.	NA	.	.	.	.	.	.	.	.
0	.	NA	.	CELI	.	.	.	.	.	.
0	.	NA	.	.	.	.	.	.	.	.
0	.	NA	.	.	.	.	.	.	.	.
0	.	NA	.	.	.	.	.	.	.	.
0	.	NA	.	.	.	.	.	.	.	.
0	.	NA	.	.	.	.	.	.	.	.
0	Obesity, hy	NA	.	ENZ'	INTF	GRC	KEG(	PID	.	REACTOME_SIGNALLING_BY_NGF;
0	.	NA	.	.	.	.	.	.	.	.
0	.	NA	.	.	.	.	.	.	.	.
0	.	NA	.	SYS'	.	.	KEG(	PID	.	REACTOME_SIGNALLING_BY_NGF;
0	.	NA	.	.	CYT(	.	KEG(	.	.	REACTOME_CYTOSOLIC_TRNA_AM
0	.	NA	.	.	.	.	.	.	.	.
0	.	NA	.	BIOF	.	NUC	.	.	.	.
0	.	NA	.	.	NUC	.	.	.	.	.
0	.	NA	.	.	NUC	.	.	.	.	.
0	.	NA	.	RNA	NUC	TRAI	.	.	.	.
0	.	NA	.	.	NUC	PRO	KEG(	PID	.	.
0	.	NA	.	.	.	.	.	.	.	.
0	.	NA	.	.	.	.	.	.	.	.
0	.	NA	.	.	.	.	.	.	.	.
0	.	NA	.	.	.	.	.	.	.	.
0	.	NA	.	.	.	.	.	.	.	.
0	.	NA	.	.	.	.	.	.	.	.
0	.	NA	.	.	.	.	.	.	.	.
0	Porphyria,	NA	.	COF	.	HYD	KEG(	.	BIO	REACTOME_METABOLISM_OF_POF
0	.	NA	.	.	.	.	.	.	.	REACTOME_EXTRACELLULAR_MAT
0	.	NA	.	.	.	.	.	.	.	REACTOME_EXTRACELLULAR_MAT
0	.	NA	.	.	.	.	.	.	.	REACTOME_EXTRACELLULAR_MAT
0	.	NA	.	.	.	.	.	.	.	.
0	.	NA	.	.	EXTI	MET	.	.	.	REACTOME_DIABETES_PATHWAYS
0	Microcepha	NA	.	.	ORG	KINA	.	.	.	REACTOME_CELL_CYCLE;REACTO

0	.	NA	.	.	.	.	.	.	.	.
0	.	NA	.	.	.	.	.	.	.	.
0	.	NA	.	.	.	.	.	PID	.	.
0	.	NA	.	.	.	.	.	PID	.	.
0	.	NA	.	.	NUC	.	.	.	.	.
0	.	NA	.	POS	CYT	NUC	.	.	.	.
0	.	NA	.	.	.	.	.	PID	.	REACTOME_SIGNALLING_BY_NGF;
0	.	NA	.	.	.	.	.	.	.	.
0	Charcot-M	NA	.	.	.	.	.	.	.	REACTOME_IMMUNE_SYSTEM;REA
0	Epileptic e	NA	.	.	.	.	.	.	.	REACTOME_GLUTAMATE_NEUROT
0	.	NA	.	.	.	.	.	.	.	.
0	.	NA	.	NUC	CYT	ACID	KEGG	.	.	REACTOME_METABOLISM_OF_VIT
0	Telangiecta	NA	.	REG	INTR	.	.	PID	.	.
0	.	NA	.	.	.	STR	.	PID	.	.
0	Epileptic e	NA	.	.	ORG	ACTI	KEGG	PID	BIO	REACTOME_APOPTOTIC_CLEAVAG
0	Epileptic e	NA	.	.	ORG	ACTI	KEGG	PID	BIO	REACTOME_APOPTOTIC_CLEAVAG
0	Epileptic e	NA	.	.	ORG	ACTI	KEGG	PID	BIO	REACTOME_APOPTOTIC_CLEAVAG
0	Epileptic e	NA	.	.	ORG	ACTI	KEGG	PID	BIO	REACTOME_APOPTOTIC_CLEAVAG
0	.	NA	.	REG	UBIQ	ACID	.	.	.	.
0	.	NA	.	AMIN	CYT	.	.	.	.	REACTOME_METABOLISM_OF_AMI
0	.	NA	.	AMIN	CYT	.	.	.	.	REACTOME_METABOLISM_OF_AMI
0	.	NA	.	GLY	ORG	HYD	KEGG	.	.	REACTOME_METABOLISM_OF_PRO
0	?Carnitine	NA	.	GEN	.	.	KEGG	.	.	REACTOME_PEROXISOMAL_LIPID_
0	?Carnitine	NA	.	GEN	.	.	KEGG	.	.	REACTOME_PEROXISOMAL_LIPID_
0	.	NA	.	ENZ	.	.	KEGG	PID	BIO	REACTOME_SIGNALLING_BY_NGF;
0	.	NA	.	SYS	INTR	.	.	.	.	.
0	Amyotroph	NA	.	DNA	NUC	DNA	.	.	.	.
0	.	NA	.	.	.	.	.	.	.	.
0	.	NA	.	.	NUC	.	.	.	.	REACTOME_RNA_POL_III_TRANSC
0	.	NA	.	.	.	GTP	KEGG	PID	BIO	REACTOME_SIGNALLING_BY_NGF;
0	.	NA	.	GLY	.	.	KEGG	.	.	.
0	.	NA	.	.	CYT	.	.	.	.	.
0	.	NA	.	.	.	.	.	.	.	.
0	.	NA	.	.	.	.	.	.	.	.
0	Dopamine	NA	.	CELL	CYT	.	KEGG	.	BIO	REACTOME_METABOLISM_OF_AMI
0	Ehlers-Dar	NA	.	.	PRO	.	KEGG	PID	.	REACTOME_DEVELOPMENTAL_BIO
0	Ehlers-Dar	NA	.	.	PRO	.	KEGG	PID	.	REACTOME_DEVELOPMENTAL_BIO
0	Ehlers-Dar	NA	.	.	PRO	.	KEGG	PID	.	REACTOME_DEVELOPMENTAL_BIO
0	.	NA	.	.	.	.	.	.	.	.
0	.	NA	.	.	.	.	.	.	.	.
0	Aortic valv	NA	.	MUS	NUC	.	KEGG	PID	BIO	.
0	Aortic valv	NA	.	MUS	NUC	.	KEGG	PID	BIO	.
0	Aortic valv	NA	.	MUS	NUC	.	KEGG	PID	BIO	.
0	.	NA	.	.	.	.	.	.	.	.
0	.	NA	.	.	.	.	.	.	.	.
0	.	NA	.	POS	.	.	KEGG	PID	BIO	REACTOME_EXTRINSIC_PATHWAY
0	Mental reta	NA	.	OLIG	INTR	CATI	KEGG	.	.	REACTOME_METABOLISM_OF_PRO
0	Hypophosp	NA	.	REG	MEM	CATI	.	.	.	REACTOME_TRANSMEMBRANE_TR
0	.	NA	.	.	.	.	.	.	.	.
0	.	NA	.	.	.	.	.	.	.	.
0	.	NA	.	.	.	.	.	.	.	.
0	Kleefstra s	NA	.	ONE	.	HIST	KEGG	.	.	.
0	.	NA	.	.	.	.	.	.	.	.
0	.	NA	.	REG	CYT	HYD	.	.	.	.

0	.	NA	.	.	.	HYD	.	.	.	.
0	.	NA	.	PRO	ORG	MET	.	.	.	.
0	.	NA	.	.	.	.	.	.	.	.
0	.	NA	.	.	.	.	.	.	.	.
0	.	NA	.	.	.	.	.	.	.	.
0	.	NA	.	.	.	.	.	.	.	.
0	.	NA	.	MAIN	NUC	.	.	.	.	.
0	.	NA	.	MAIN	NUC	.	.	.	.	.
0	.	NA	.	.	.	.	.	.	.	.
0	.	NA	.	.	.	.	.	.	.	.
0	.	NA	.	.	.	.	.	.	.	.
0	Megalobla	NA	.	EST	MEM	PRO	.	.	.	REACTOME_METABOLISM_OF_STE
0	Megalobla	NA	.	EST	MEM	PRO	.	.	.	REACTOME_METABOLISM_OF_STE
0	.	NA	.	.	.	.	.	.	.	.
0	.	NA	.	NEG	CYT	ENZ	.	.	.	.
0	.	NA	.	SYS	CYT	.	.	.	.	.
0	.	NA	.	.	.	.	.	.	.	.
0	.	NA	.	.	.	.	.	.	.	.
0	Deafness,	NA	.	PRO	ORG	HYD	.	.	.	.
0	.	NA	.	.	.	.	.	.	.	REACTOME_INTEGRIN_CELL_SURF
0	Thromboc	NA	.	.	.	.	.	.	.	.
0	Thromboc	NA	.	.	.	.	.	.	.	.
0	.	NA	.	.	.	.	.	.	.	.
0	.	NA	.	MUS	CYT	ACTI	.	PID	.	.
0	.	NA	.	.	.	.	.	.	.	.
0	.	NA	.	.	NUC	.	.	.	.	.
0	.	NA	.	.	.	.	.	.	.	.
0	.	NA	.	.	.	.	.	.	.	.
0	.	NA	.	.	.	.	.	.	.	.
0	.	NA	.	.	.	.	.	.	.	.
0	.	NA	.	.	.	.	.	.	.	.
0	.	NA	.	.	.	.	.	.	.	.
0	.	NA	.	.	.	.	.	.	.	.
0	Myasthenic	NA	.	.	CYT	.	KEGG	.	.	REACTOME_PHOSPHOLIPID_META
0	.	NA	.	.	.	.	.	.	.	.
0	Deafness,	NA	.	REG	NON	.	.	.	.	.
0	.	NA	.	.	.	.	.	.	.	.
0	.	NA	.	.	.	.	.	.	.	.
0	Thyroid pa	NA	.	.	.	STR	KEGG	.	.	.
0	.	NA	.	.	.	.	.	.	.	.
0	.	NA	.	DNA	ORG	DNA	.	.	.	.
0	.	NA	.	SYS	PRO	CAR	.	PID	.	REACTOME_EXTRACELLULAR_MA
0	.	NA	.	CELL	ORG	NUC	.	PID	.	.
0	Hemophag	NA	.	MUL	.	.	KEGG	PID	BIO	.
0	Deafness,	NA	.	REG	NON	.	.	.	.	.
0	.	NA	.	.	CYT	OXID	KEGG	.	.	.
0	.	NA	.	.	.	.	.	.	.	.
0	.	NA	.	.	PRO	CATI	KEGG	PID	BIO	REACTOME_SIGNALING_BY_GPCR
0	.	NA	.	.	.	.	.	.	.	.
0	.	NA	.	.	.	.	.	.	.	.
0	.	NA	.	.	.	.	.	.	.	.
0	.	NA	.	.	.	.	.	.	.	.
0	.	NA	.	REG	MEM	CAR	.	PID	.	.
0	.	NA	.	.	.	.	.	.	.	.

0	Cone-rod	NA	.	.	.	.	.	.	.	.
0	.	NA	.	.	ORG	.	.	.	.	.
0	.	NA	.	.	.	.	KEG	.	.	.
0	.	NA	.	.	.	.	.	.	.	.
0	.	NA	.	.	.	.	.	.	.	.
0	.	NA	.	SYS	MEM	.	.	PID	.	.
0	.	NA	.	.	.	.	.	.	.	.
0	Rhabdomy	NA	.	.	.	.	KEG	.	.	REACTOME_BIOLOGICAL_OXIDATI
0	.	NA	.	.	.	.	.	.	.	.
0	Agammagl	NA	.	SYS	.	MOL	KEG	PID	BIO	REACTOME_ANTIGEN_ACTIVATES_
0	.	NA	.	.	.	.	.	.	.	.
0	Spastic pa	NA	.	.	.	.	.	.	.	.
0	.	NA	.	.	.	.	.	.	.	.
0	Urofacial s	NA	.	.	.	HYD	KEG	.	.	REACTOME_HS_GAG_DEGRADATI
0	.	NA	.	.	.	.	.	.	.	.
0	.	NA	.	.	.	.	.	.	.	.
0	.	NA	.	.	.	.	.	.	.	.
0	.	NA	.	.	.	.	KEG	.	.	.
0	.	NA	.	.	.	.	.	.	.	.
0	Epidermoly	NA	.	SYS	INTR	.	.	PID	.	REACTOME_CELL_CELL_COMMUN
0	.	NA	.	.	.	.	.	.	.	.
0	.	NA	.	.	.	.	.	.	.	REACTOME_SIGNALLING_BY_NGF;
0	.	NA	.	.	.	REC	.	PID	.	REACTOME_DEVELOPMENTAL_BI
0	.	NA	.	.	.	.	.	.	.	.
0	.	NA	.	RNA	CYT	RNA	.	.	.	.
0	.	NA	.	.	.	.	.	.	.	.
0	.	NA	.	.	.	.	.	.	.	.
0	.	NA	.	CEL	CYT	GTP	KEG	PID	BIO	REACTOME_DEVELOPMENTAL_BI
0	.	NA	.	CEL	CYT	GTP	KEG	PID	BIO	REACTOME_DEVELOPMENTAL_BI
0	.	NA	.	.	.	.	.	.	.	.
0	.	NA	.	.	.	.	KEG	.	BIO	.
0	.	NA	.	.	.	.	.	.	.	.
0	.	NA	.	.	.	.	.	.	.	.
0	.	NA	.	.	.	.	.	.	.	.
0	.	NA	.	.	.	.	.	.	.	.
0	.	NA	.	.	.	.	KEG	PID	.	REACTOME_METABOLISM_OF_AMI
0	.	NA	.	.	.	.	KEG	PID	.	REACTOME_METABOLISM_OF_AMI
0	.	NA	.	.	.	.	KEG	PID	.	REACTOME_METABOLISM_OF_AMI
0	.	NA	.	.	.	.	.	.	.	.
0	.	NA	.	.	.	.	.	.	.	.
0	.	NA	.	.	.	.	.	.	.	.
0	.	NA	.	.	PRO	.	KEG	.	.	REACTOME_SIGNALING_BY_WNT;
0	.	NA	.	SYS	EXT	ENZ	.	.	.	.
0	.	NA	.	.	.	.	.	.	.	.
0	.	NA	.	.	.	.	.	.	.	.
0	.	NA	.	.	.	.	.	.	.	.
0	Epileptic e	NA	.	EST	ORG	ACT	.	.	.	.
0	.	NA	.	.	.	.	KEG	.	.	REACTOME_SIGNALLING_BY_NGF;
0	.	NA	.	.	.	.	.	.	.	.
0	{Pulmonar	NA	.	.	.	.	.	.	.	REACTOME_O_LINKED_GLYCOSYL
0	{Pulmonar	NA	.	.	.	.	.	.	.	REACTOME_O_LINKED_GLYCOSYL
0	{Pulmonar	NA	.	.	.	.	.	.	.	REACTOME_O_LINKED_GLYCOSYL
0	{Pulmonar	NA	.	.	.	.	.	.	.	REACTOME_O_LINKED_GLYCOSYL
0	{Pulmonar	NA	.	.	.	.	.	.	.	REACTOME_O_LINKED_GLYCOSYL

0	Segawa sy	NA	.	ANA	.	OXID	KEGG	PID	BIO	REACTOME_METABOLISM_OF_AMI
0	.	NA	.	.	.	.	.	.	.	.
0	Immunode	NA	.	PHO	INTR	.	KEGG	PID	.	REACTOME_IMMUNOREGULATORY
0	.	NA	.	.	INTR	SUB	KEGG	.	.	.
0	.	NA	.	.	INTR	SUB	KEGG	.	.	.
0	.	NA	.	.	.	.	.	.	.	.
0	.	NA	.	.	.	.	KEGG	.	.	REACTOME_SIGNALING_BY_GPCR
0	.	NA	.	.	.	.	.	.	.	.
0	.	NA	.	.	.	.	.	.	.	.
0	.	NA	.	.	.	.	.	.	.	.
0	.	NA	.	REG	NUC	TRA	KEGG	PID	.	.
0	.	NA	.	.	.	.	.	.	.	.
0	.	NA	.	.	.	.	.	.	.	.
0	.	NA	.	.	.	.	.	.	.	.
0	.	NA	.	RNA	NUC	.	KEGG	PID	.	REACTOME_BMAL1_CLOCK_NPAS2
0	.	NA	.	.	.	.	.	.	.	.
0	Ovarian ca	NA	.	.	.	HYD	KEGG	.	.	.
0	.	NA	.	.	.	HYD	KEGG	.	BIO	REACTOME_INSULIN_RECEPTOR_S
0	Rickets du	NA	.	.	.	.	.	.	.	REACTOME_METABOLISM_OF_STE
0	.	NA	.	.	.	.	.	PID	.	.
0	.	NA	.	.	.	.	.	PID	.	.
0	Deafness,	NA	.	.	.	.	.	.	.	.
0	.	NA	.	.	.	.	KEGG	.	BIO	REACTOME_METABOLISM_OF_AMI
0	.	NA	.	.	.	.	.	.	.	.
0	.	NA	.	REG	.	G_P	.	PID	.	REACTOME_TRIF_MEDIATED_TLR3
0	Glycogen s	NA	.	.	CYT	OXID	KEGG	PID	BIO	REACTOME_PYRUVATE_METABOL
0	Hyperekple	NA	.	CELL	.	CATI	.	.	.	REACTOME_TRANSMEMBRANE_TR
0	.	NA	.	SYS	.	.	.	.	.	.
0	.	NA	.	CELL	ORG	.	.	PID	BIO	REACTOME_APOPTOTIC_CLEAVAG
0	.	NA	.	.	.	.	.	.	.	.
0	Hemolytic	NA	.	REG	CELL	.	KEGG	.	.	.
0	.	NA	.	.	.	.	.	.	.	.
0	.	NA	.	.	.	.	KEGG	.	.	REACTOME_TRIGLYCERIDE_BIOSY
0	.	NA	.	.	.	IDEN	.	.	.	.
0	.	NA	.	EST	ORG	.	.	PID	.	REACTOME_CELL_CYCLE;REACTO
0	.	NA	.	.	.	.	KEGG	.	.	.
0	.	NA	.	.	.	.	KEGG	.	.	REACTOME_SIGNALING_BY_GPCR
0	.	NA	.	.	.	.	.	.	.	.
0	.	NA	.	.	.	.	.	.	.	.
0	.	NA	.	.	.	.	KEGG	.	.	REACTOME_SIGNALING_BY_GPCR
0	.	NA	.	.	.	.	KEGG	.	.	REACTOME_SIGNALING_BY_GPCR
0	.	NA	.	.	.	.	KEGG	.	.	REACTOME_MRNA_DECAY_BY_5_T
0	.	NA	.	.	.	.	.	.	.	.
0	.	NA	.	.	.	.	.	.	.	.
0	.	NA	.	PHO	.	OXID	KEGG	.	.	REACTOME_PPARA_ACTIVATES_G
0	.	NA	.	.	.	.	.	.	.	.
0	.	NA	.	ORG	.	.	.	PID	.	.
0	.	NA	.	RNA	NUC	.	.	.	.	.
0	.	NA	.	.	.	.	.	.	.	REACTOME_PROCESSING_OF_CAP
0	.	NA	.	.	.	.	.	.	.	.
0	.	NA	.	.	.	.	.	.	.	.
0	.	NA	.	CELL	.	.	KEGG	PID	BIO	REACTOME_SIGNALLING_BY_NGF;
0	McArdle di	NA	.	GLU	.	TRA	KEGG	.	BIO	REACTOME_GLYCOGEN_BREAKDO
0	.	NA	.	BIOF	CYT	ACID	KEGG	.	.	REACTOME_DIABETES_PATHWAYS

0	.	NA	.	.	NUC	KINA	KEG(	.	.	.
0	.	NA	.	.	.	.	KEG(	.	.	REACTOME_ACTIVATION_OF_THE_
0	.	NA	.	EST/	.	CATI	.	.	.	REACTOME_NEURONAL_SYSTEM;F
0	.	NA	.	PRO	ORG	PHO	KEG(	PID	BIO	.
0	.	NA	.	.	.	.	.	.	.	.
0	Metastasis	NA	.	CELI	.	GTP	KEG(	PID	.	REACTOME_RAP1_SIGNALLING;RE
0	Metastasis	NA	.	CELI	.	GTP	KEG(	PID	.	REACTOME_RAP1_SIGNALLING;RE
0	Aicardi-Go	NA	.	.	.	.	KEG(	.	.	.
0	.	NA	.	.	.	.	.	.	.	.
0	.	NA	.	.	ORG	.	.	.	.	REACTOME_MHC_CLASS_II_ANTIG
0	.	NA	.	.	PRO	.	.	.	.	.
0	.	NA	.	.	.	.	.	.	.	.
0	.	NA	.	BIOF	.	PHO	KEG(	PID	BIO	REACTOME_DOWNREGULATION_C
0	Mitochondi	NA	.	.	ORG	.	KEG(	.	.	REACTOME_TCA_CYCLE_AND_RES
0	Osteopetrc	NA	.	POS	INTF	.	KEG(	.	.	REACTOME_INSULIN_RECEPTOR_I
0	[Skin/hair/e	NA	.	.	.	.	.	.	.	.
0	[Skin/hair/e	NA	.	.	.	.	.	.	.	.
0	.	NA	.	SIGN	CYT(	.	.	.	.	.
0	Smith-Lem	NA	.	BIOS	ORG	OXID	KEG(	.	.	REACTOME_METABOLISM_OF_LIPI
0	Deafness,	NA	.	.	.	.	.	.	.	.
0	.	NA	.	EST/	CELI	VITA	.	.	.	.
0	.	NA	.	.	.	.	.	.	.	.
0	.	NA	.	.	.	.	.	.	.	.
0	.	NA	.	REG	CYT(	RAS	KEG(	PID	.	REACTOME_SIGNALING_BY_RHO_(
0	.	NA	.	.	.	.	.	.	.	.
0	.	NA	.	BIOF	.	PRO	.	.	.	.
0	.	NA	.	LIPIL	INTF	.	KEG(	.	.	REACTOME_GLYCOSPHINGOLIPID_
0	.	NA	.	.	.	.	.	.	.	REACTOME_TRANSMEMBRANE_TF
0	.	NA	.	.	.	.	.	.	.	.
0	.	NA	.	DNA	CYT(	.	.	.	.	.
0	.	NA	.	.	.	.	.	.	.	.
0	.	NA	.	.	.	.	.	.	.	.
0	.	NA	.	.	MEM	PHO	.	.	.	.
0	.	NA	.	G_PI	INTF	GLU	KEG(	.	.	.
0	.	NA	.	.	.	.	.	.	.	.
0	.	NA	.	.	.	.	.	.	.	.
0	Asphyxiatir	NA	.	ORG	CYT(	.	KEG(	.	.	REACTOME_MHC_CLASS_II_ANTIG
0	.	NA	.	.	.	PHO	KEG(	.	.	REACTOME_NITRIC_OXIDE_STIMUI
0	.	NA	.	.	.	.	.	.	.	.
0	.	NA	.	CELI	.	CATI	KEG(	.	.	REACTOME_SIGNALING_BY_ERBB2
0	Alpha-metl	NA	.	.	CYT(	TRAI	KEG(	.	.	REACTOME_METABOLISM_OF_AMI
0	Ataxia-tela	NA	.	DNA	.	PHO	KEG(	PID	BIO	REACTOME_MEIOSIS;REACTOME_(
0	.	NA	.	.	.	.	.	.	.	.
0	.	NA	.	.	.	.	.	.	.	.
0	.	NA	.	ENZ	CYT(	NUC	.	PID	.	.
0	.	NA	.	MITC	ORG	SEQ	.	.	.	REACTOME_CELL_CYCLE;REACTO
0	.	NA	.	POS	CELI	PRO	KEG(	.	.	REACTOME_CELL_CELL_COMMUN
0	.	NA	.	BIOF	.	NUC	.	PID	.	.
0	.	NA	.	.	.	.	.	.	.	.
0	Atrial fibrilla	NA	.	EST/	INTF	CATI	.	.	.	REACTOME_DEVELOPMENTAL_BIC
0	.	NA	.	.	.	.	.	.	.	.
0	.	NA	.	EST/	ORG	.	.	.	.	REACTOME_COPI_MEDIATED_TRA
0	.	NA	.	.	RIBC	RNA	KEG(	.	.	REACTOME_MRNA_DECAY_BY_5_T
0	Leukemia,	NA	.	.	.	.	KEG(	PID	BIO	REACTOME_SIGNALING_BY_RHO_(

0	Leukemia,	NA	.	.	.	.	KEG(	PID	BIO	REACTOME_SIGNALING_BY_RHO_(
0	Deafness,	NA	.	SEN:	.	.	.	.	.	.
0	.	NA	.	.	.	.	KEG(	.	.	REACTOME_SIGNALING_BY_GPCR
0	.	NA	.	.	.	.	KEG(	.	.	.
0	.	NA	.	SYS	INTR	REC	.	.	.	.
0	.	NA	.	.	.	.	.	.	.	.
0	Leigh synd	NA	.	.	CYT(	.	.	.	.	.
0	.	NA	.	CELI	CYT(	.	KEG(	PID	.	.
0	.	NA	.	.	.	.	.	.	.	.
0	.	NA	.	.	.	.	.	.	.	.
0	{Ovarian ca	NA	.	CELI	INTR	PEP	.	.	.	.
0	.	NA	.	.	.	.	.	.	.	.
0	.	NA	.	.	.	.	.	.	.	.
0	.	NA	.	.	.	.	.	.	.	.
0	.	NA	.	.	.	.	.	.	.	.
0	.	NA	.	.	.	.	.	.	.	.
0	Neuropathy	NA	.	EST/	CYT(	NUC	.	.	.	.
0	Brugada sy	NA	.	REG	ORG	CATI	KEG(	.	.	.
0	Brugada sy	NA	.	REG	ORG	CATI	KEG(	.	.	.
0	Brugada sy	NA	.	REG	ORG	CATI	KEG(	.	.	.
0	.	NA	.	.	.	.	.	.	.	.
0	.	NA	.	POS	MAC	.	KEG(	PID	BIO	REACTOME_CELL_CYCLE;REACTO
0	.	NA	.	.	.	SIN(	.	.	.	.
0	.	NA	.	EST/	INTR	CATI	.	.	.	REACTOME_NEURONAL_SYSTEM;F
0	Periodic fe	NA	.	RNA	INTR	REC	KEG(	PID	BIO	REACTOME_EXTRINSIC_PATHWAY
0	.	NA	.	SYS	.	.	.	.	.	.
0	OKT4 epit(	NA	.	SYS	MEM	KINA	KEG(	PID	BIO	REACTOME_NEF_MEDIATES_DOW
0	.	NA	.	.	.	.	.	.	.	.
0	.	NA	.	.	INTR	.	.	.	.	.
0	.	NA	.	.	.	.	.	.	.	.
0	.	NA	.	.	.	STRI	.	PID	.	.
0	.	NA	.	.	.	.	.	.	.	.
0	.	NA	.	.	.	.	.	.	.	.
0	.	NA	.	.	.	.	.	.	.	.
0	.	NA	.	.	.	.	.	.	.	.
0	Leukemia,	NA	.	.	.	TRAI	KEG(	.	.	.
0	.	NA	.	.	.	.	.	.	.	.
0	.	NA	.	.	.	.	.	.	.	.
0	.	NA	.	.	.	.	.	.	.	.
0	.	NA	.	.	.	.	KEG(	.	BIO	REACTOME_SYNTHESIS_OF_PIPS_
0	.	NA	.	.	.	.	KEG(	.	BIO	REACTOME_SYNTHESIS_OF_PIPS_
0	.	NA	.	.	.	.	.	.	.	.
0	Hyperbiliru	NA	.	EST/	INTR	ACTI	.	.	.	REACTOME_BILE_ACID_AND_BILE_
0	.	NA	.	.	.	.	.	.	.	.
0	.	NA	.	EST/	CYT(	.	.	.	.	.
0	.	NA	.	.	.	.	.	.	.	.
0	.	NA	.	.	.	.	.	.	.	.
0	.	NA	.	.	.	.	.	.	.	.
0	.	NA	.	.	.	.	.	.	.	.
0	.	NA	.	.	.	.	.	.	.	REACTOME_O_LINKED_GLYCOSYL
0	.	NA	.	.	.	.	.	.	.	REACTOME_O_LINKED_GLYCOSYL
0	.	NA	.	.	.	.	.	.	.	.
0	.	NA	.	.	.	.	.	.	.	.
0	.	NA	.	.	.	.	KEG(	PID	.	REACTOME_SIGNALLING_BY_NGF;

0	.	NA	.	.	CYT(	.	.	.	.	.
0	.	NA	.	.	.	.	KEG(	.	.	.
0	.	NA	.	.	.	.	KEG(	.	.	.
0	.	NA	.	.	.	.	.	.	.	.
0	.	NA	.	.	.	.	.	.	.	.
0	.	NA	.	.	.	.	.	.	.	.
0	.	NA	.	RNA	.	TRAI	.	PID	.	.
0	.	NA	.	.	.	.	.	.	.	.
0	.	NA	.	.	.	.	.	.	.	.
0	.	NA	.	.	.	.	.	.	.	.
0	Ichthyosis	NA	.	SYST	ORG	STRI	.	.	.	.
0	White spor	NA	.	ANAT	.	.	.	.	.	.
0	.	NA	.	.	.	.	.	.	.	.
0	.	NA	.	.	INTR	IDEN	KEG(	PID	.	REACTOME_IMMUNOREGULATORY
0	.	NA	.	PRO	CYT(	KINA	KEG(	PID	BIO	.
0	.	NA	.	.	.	.	.	.	.	.
0	.	NA	.	.	.	.	.	.	.	.
0	Deafness,	NA	.	SEN	ORG	.	.	.	.	.
0	.	NA	.	.	.	.	.	.	.	.
0	.	NA	.	LIPIE	INTR	CATI	KEG(	PID	.	.
0	.	NA	.	LIPIE	INTR	CATI	KEG(	PID	.	.
0	.	NA	.	LIPIE	INTR	CATI	KEG(	PID	.	.
0	.	NA	.	LIPIE	INTR	CATI	KEG(	PID	.	.
0	.	NA	.	.	CYT(	.	KEG(	.	.	.
0	Spermatog	NA	.	.	.	.	.	.	.	.
0	.	NA	.	.	.	RNA	.	.	.	.
0	.	NA	.	.	.	RNA	.	.	.	.
0	.	NA	.	.	NUC	TRAI	.	PID	.	.
0	.	NA	.	.	.	.	.	.	.	REACTOME_ASSOCIATION_OF_TR
0	.	NA	.	.	.	.	.	.	.	.
0	.	NA	.	.	.	.	.	.	.	.
0	.	NA	.	.	.	.	.	.	.	.
0	.	NA	.	.	.	.	.	PID	.	.
0	.	NA	.	.	.	.	.	.	.	.
0	.	NA	.	.	.	.	.	.	.	.
0	Deafness,	NA	.	.	.	.	.	.	.	.
0	Deafness,	NA	.	.	.	.	.	.	.	.
0	.	NA	.	.	.	.	.	.	.	.
0	.	NA	.	.	.	.	.	.	.	.
0	.	NA	.	.	NUC	.	.	.	.	.
0	.	NA	.	BIOF	.	.	.	.	.	.
0	.	NA	.	.	.	.	.	.	.	.
0	.	NA	.	.	.	.	.	.	.	.
0	.	NA	.	SIGN	.	PRO	.	PID	BIO	.
0	.	NA	.	.	.	.	.	.	.	.
0	.	NA	.	.	CYT(	HYD	.	.	.	.
0	Arthrogryp	NA	.	.	CYT(	STRI	.	.	.	REACTOME_STRIATED_MUSCLE_C
0	.	NA	.	MUL	INTR	CAR	.	.	.	REACTOME_HYALURONAN_UPTAK
0	.	NA	.	DNA	NUC	DAM	KEG(	.	.	REACTOME_BASE_EXCISION_REP/
0	.	NA	.	.	.	.	.	.	.	.
0	Porokerato	NA	.	.	.	.	.	.	.	.
0	.	NA	.	.	.	DAM	.	.	.	REACTOME_DNA_REPAIR
0	.	NA	.	.	.	.	.	.	.	.
0	.	NA	.	.	.	.	.	.	.	.

0	.	NA	.	SIGN	CYT	ACID	.	.	.	.
0	.	NA	.	.	.	.	.	.	.	.
0	Transpositi	NA	.	.	.	.	.	.	.	REACTOME_DEVELOPMENTAL_BIC
0	Spermatog	NA	.	SYS	CELI	OXID	KEGG	.	BIO	REACTOME_LATENT_INFECTION_C
0	.	NA	.	PRO	.	PHO	KEGG	PID	.	.
0	.	NA	.	.	.	.	.	.	.	.
0	.	NA	.	.	.	.	.	.	.	REACTOME_METABOLISM_OF_PRC
0	.	NA	.	.	.	.	.	.	.	.
0	.	NA	.	.	ORG	RNA	.	.	.	REACTOME_INTERFERON_GAMMA
0	.	NA	.	SIGN	INTR	CATI	KEGG	.	.	.
0	.	NA	.	.	.	.	.	.	.	.
0	.	NA	.	M_P	ORG	PRO	.	PID	.	REACTOME_CELL_CYCLE;REACTO
0	.	NA	.	MITC	ORG	.	.	.	.	REACTOME_CELL_CYCLE;REACTO
0	.	NA	.	.	.	.	.	.	.	REACTOME_MEMBRANE_TRAFFICK
0	.	NA	.	.	.	.	.	.	.	.
0	.	NA	.	.	.	.	.	.	.	.
0	.	NA	.	.	.	.	KEGG	PID	.	.
0	.	NA	.	.	.	.	.	.	.	.
0	.	NA	.	.	.	.	.	.	.	.
0	.	NA	.	.	.	.	.	.	.	.
0	.	NA	.	.	.	.	.	.	.	.
0	.	NA	.	.	.	.	.	.	.	.
0	.	NA	.	.	.	.	.	.	.	.
0	.	NA	.	.	.	.	.	.	.	.
0	.	NA	.	.	.	.	.	.	.	.
0	.	NA	.	.	.	.	.	.	.	.
0	.	NA	.	.	.	.	KEGG	.	.	REACTOME_O_LINKED_GLYCOSYL
0	.	NA	.	MITC	.	.	.	.	.	.
0	.	NA	.	.	.	.	.	.	.	.
0	.	NA	.	.	.	.	.	.	.	REACTOME_SIGNALING_BY_FGFR_
0	.	NA	.	.	.	.	.	.	.	.
0	Muscular d	NA	.	MUS	PRO	.	KEGG	.	.	.
0	.	NA	.	MAC	.	.	KEGG	.	BIO	.
0	Keratosis li	NA	.	.	.	.	KEGG	.	.	.
0	.	NA	.	.	.	.	.	.	.	.
0	.	NA	.	DNA	ORG	HYD	KEGG	PID	.	REACTOME_CELL_CYCLE;REACTO
0	.	NA	.	.	.	.	.	.	.	.
0	.	NA	.	.	.	.	.	.	.	.
0	Pulmonary	NA	.	.	.	.	KEGG	PID	.	.
0	.	NA	.	BIOS	CYT	TRAI	.	.	.	.
0	.	NA	.	.	.	.	.	.	.	.
0	.	NA	.	.	.	.	.	.	.	.
0	.	NA	.	.	.	.	.	.	.	.
0	.	NA	.	.	.	.	.	.	.	.
0	.	NA	.	.	.	.	.	.	.	.
0	.	NA	.	.	.	.	.	.	.	.
0	.	NA	.	.	.	.	.	PID	.	.
0	.	NA	.	.	.	.	.	.	.	.
0	.	NA	.	.	CYT	.	.	.	.	.
0	.	NA	.	.	.	.	.	.	.	.
0	.	NA	.	EST	INTR	.	.	.	.	.
0	Propionica	NA	.	.	.	LIGA	KEGG	.	.	REACTOME_MITOCHONDRIAL_FAT
0	.	NA	.	.	.	.	.	.	.	.
0	.	NA	.	SYS	NUC	ACTI	.	.	.	.

0	Angiopathy	NA	.	.	.	.	KEGG	PID	BIO	REACTOME_DEVELOPMENTAL_BIO
0	.	NA	.	.	.	.	KEGG	.	.	REACTOME_MITOCHONDRIAL_TRN
0	.	NA	.	.	.	.	.	.	.	.
0	.	NA	.	.	.	.	.	.	.	.
0	.	NA	.	.	.	.	.	.	.	.
0	.	NA	.	.	.	.	.	PID	.	.
0	.	NA	.	SIGN	.	REC	.	.	.	.
0	.	NA	.	.	.	.	.	.	.	.
0	.	NA	.	.	.	.	.	.	.	.
0	.	NA	.	.	.	.	.	.	.	.
0	.	NA	.	.	.	.	.	.	.	.
0	.	NA	.	DNA	ORG	NUC	.	.	BIO	.
0	.	NA	.	.	.	.	.	.	.	.
0	.	NA	.	RNA	.	TRAI	.	.	.	REACTOME_RNA_POL_II_TRANSCF
0	.	NA	.	.	.	.	.	.	.	.
0	Lysinuric p	NA	.	EST/	INTR	.	.	.	.	REACTOME_CELL_SURFACE_INTEI
0	.	NA	.	.	.	.	.	.	.	.
0	.	NA	.	.	.	.	.	.	.	.
0	.	NA	.	.	.	.	.	.	.	.
0	.	NA	.	.	.	.	.	.	.	.
0	.	NA	.	.	.	.	.	.	.	.
0	.	NA	.	.	.	.	.	.	.	.
0	.	NA	.	.	.	.	.	.	.	.
0	.	NA	.	.	.	.	.	.	.	.
0	.	NA	.	.	.	.	.	.	.	.
0	.	NA	.	.	.	.	.	.	.	.
0	Fanconi ar	NA	.	.	.	.	.	PID	.	REACTOME_FANCONI_ANEMIA_PA
0	.	NA	.	.	.	.	.	.	.	.
0	.	NA	.	.	.	.	.	.	.	.
0	{Asthma, a	NA	.	SIGN	INTR	RHO	KEGG	.	.	REACTOME_SIGNALING_BY_GPCR
0	.	NA	.	.	.	.	.	.	.	.
0	.	NA	.	.	.	.	KEGG	PID	.	.
0	.	NA	.	.	.	.	KEGG	PID	.	.
0	.	NA	.	.	.	.	.	.	.	.
0	.	NA	.	.	.	.	.	.	.	.
0	.	NA	.	.	.	.	.	.	.	.
0	.	NA	.	.	CYT	LIGA	KEGG	.	.	REACTOME_METABOLISM_OF_VITA
0	.	NA	.	.	.	.	.	.	.	.
0	Molybdenu	NA	.	.	.	.	.	.	BIO	REACTOME_METABOLISM_OF_VITA
0	Bleeding d	NA	.	FOC	ADH	INTE	KEGG	PID	BIO	REACTOME_CELL_CELL_COMMUN
0	.	NA	.	.	.	.	.	.	.	.
0	.	NA	.	.	.	.	.	.	.	.
0	.	NA	.	.	.	.	.	.	.	.
0	Glaucoma	NA	.	EST/	PRO	.	.	.	.	.
0	.	NA	.	.	.	.	.	.	.	.
0	.	NA	.	.	.	.	.	.	.	.
0	.	NA	.	.	.	.	.	.	.	.
0	Spinocereb	NA	.	.	.	.	.	.	.	REACTOME_DOUBLE_STRAND_BR
0	.	NA	.	.	.	.	.	.	.	.
0	.	NA	.	.	.	.	.	.	.	.
0	.	NA	.	SIGN	INTR	.	.	.	.	REACTOME_GASTRIN_CREB_SIGN
0	Hydroceph	NA	.	REG	INSC	PRO	.	.	.	.
0	.	NA	.	.	.	.	.	.	.	.
0	.	NA	.	.	.	.	.	.	.	REACTOME_INTERFERON_ALPHA_

0	Goiter, mul	NA	.	MAC	.	HYD	.	PID	.	REACTOME_MICRORNA_MIRNA_BI
0	.	NA	.	.	.	.	.	.	.	.
0	.	NA	.	.	.	.	.	.	.	.
0	.	NA	.	MUL	EXTI	.	.	PID	.	REACTOME_ACTIVATED_NOTCH1_
0	.	NA	.	.	.	.	.	.	.	.
0	.	NA	.	.	.	.	.	.	.	.
0	.	NA	.	.	.	.	.	.	.	.
0	.	NA	.	BIOF	CELI	NUC	.	.	.	.
0	.	NA	.	.	.	.	.	.	.	.
0	.	NA	.	.	.	.	.	.	.	REACTOME_MHC_CLASS_II_ANTIG
0	.	NA	.	.	.	.	.	.	.	.
0	.	NA	.	CELI	.	REC	.	.	.	.
0	Breast can	NA	.	REG	MEM	PHO	KEG(	PID	BIO	REACTOME_SIGNALLING_BY_NGF;
0	.	NA	.	.	.	.	.	.	.	.
0	.	NA	.	SYS	INTF	REC	KEG(	PID	.	REACTOME_ACTIVATED_NOTCH1_
0	.	NA	.	RNA	.	.	.	.	.	REACTOME_RNA_POL_III_TRANSC
0	.	NA	.	.	.	.	.	.	.	.
0	.	NA	.	.	.	.	.	.	.	.
0	.	NA	.	.	.	.	.	.	.	.
0	.	NA	.	.	.	.	.	.	.	.
0	.	NA	.	.	.	.	.	.	.	.
0	.	NA	.	.	.	.	.	.	.	.
0	.	NA	.	.	.	.	.	.	.	.
0	.	NA	.	.	.	.	.	.	.	.
0	.	NA	.	SYS	CYT	RAS	KEG(	.	.	.
0	.	NA	.	.	.	.	.	.	.	.
0	.	NA	.	.	.	.	.	.	.	.
0	.	NA	.	.	.	.	.	.	.	.
0	.	NA	.	.	.	.	.	.	.	.
0	.	NA	.	.	.	.	.	.	.	.
0	.	NA	.	.	.	.	.	.	.	.
0	.	NA	.	.	.	.	.	.	.	.
0	.	NA	.	.	.	.	.	.	.	.
0	.	NA	.	.	.	.	.	.	.	REACTOME_TRANSMEMBRANE_TF
0	[Skin/hair/e	NA	.	.	.	.	KEG(	.	.	REACTOME_IMMUNE_SYSTEM;REA
0	[Skin/hair/e	NA	.	.	.	.	KEG(	.	.	REACTOME_IMMUNE_SYSTEM;REA
0	.	NA	.	.	.	.	.	.	.	.
0	.	NA	.	.	.	.	.	.	.	.
0	.	NA	.	.	.	.	.	.	.	.
0	.	NA	.	.	.	.	.	.	.	REACTOME_TRANSMISSION_ACRC
0	.	NA	.	.	.	.	.	.	.	.
0	.	NA	.	.	.	.	.	.	.	.
0	.	NA	.	.	.	.	.	.	.	.
0	.	NA	.	.	.	.	.	.	.	.
0	.	NA	.	EST/	.	CATI	KEG(	.	.	.
0	.	NA	.	.	.	.	.	.	.	.
0	.	NA	.	.	.	.	KEG(	.	BIO	REACTOME_DEVELOPMENTAL_BIC
0	Platelet PL	NA	.	REG	.	LIPA	KEG(	PID	.	REACTOME_GASTRIN_CREB_SIGN
0	.	NA	.	.	.	.	.	.	.	REACTOME_DEVELOPMENTAL_BIC
0	.	NA	.	.	.	.	.	.	.	REACTOME_DEVELOPMENTAL_BIC
0	Spinocereb	NA	.	.	.	.	.	.	.	.
0	.	NA	.	.	ORG	.	.	.	.	.
0	.	NA	.	.	.	.	KEG(	.	.	REACTOME_METABOLISM_OF_AMI
0	Deafness,	NA	.	.	.	.	.	.	.	.

0	.	NA	.	.	.	.	.	.	.	.
0	?Cataract,	NA	.	SEN	.	.	KEG(	.	.	.
0	.	NA	.	SIGN	.	.	.	.	.	.
0	.	NA	.	.	.	.	KEG(	.	.	REACTOME_DEVELOPMENTAL_BIC
0	Acromicric	NA	.	SYS	PRO	CATI	.	PID	.	REACTOME_INTEGRIN_CELL_SURF
0	Acromicric	NA	CM0	SYS	PRO	CATI	.	PID	.	REACTOME_INTEGRIN_CELL_SURF
0	.	NA	.	.	.	.	.	.	.	.
0	.	NA	.	.	.	.	.	.	.	.
0	.	NA	.	.	.	.	.	.	.	REACTOME_TRANSMEMBRANE_TF
0	.	NA	.	.	.	.	.	.	.	.
0	.	NA	.	.	.	.	.	.	.	.
0	Griscelli sy	NA	.	.	CYT	.	.	.	.	REACTOME_DIABETES_PATHWAYS
0	.	NA	.	ESTA	INTF	ACTI	.	.	.	REACTOME_TRANSMEMBRANE_TF
0	.	NA	.	MAC	.	.	KEG(	.	.	.
0	Combined	NA	.	.	.	.	KEG(	.	.	.
0	.	NA	.	.	.	.	.	.	.	.
0	.	NA	.	.	.	.	.	.	.	.
0	.	NA	.	.	.	.	.	.	.	.
0	.	NA	.	.	.	.	.	.	.	.
0	.	NA	.	.	.	.	.	.	.	.
0	Ceroid lipo	NA	.	REG	ORG	PRO	.	.	.	.
0	.	NA	.	MUS	INTF	COL	KEG(	PID	.	REACTOME_INTEGRIN_CELL_SURF
0	.	NA	.	.	.	.	.	.	.	.
0	.	NA	.	.	.	.	.	.	.	.
0	.	NA	.	.	.	.	.	.	.	.
0	Bardet-Bie	NA	.	CEN	ORG	MICF	.	.	.	.
0	Adrenal ins	NA	.	BIOS	.	OXY	KEG(	.	.	REACTOME_METABOLISM_OF_STE
0	.	NA	.	.	.	.	.	.	.	.
0	.	NA	.	.	.	.	.	.	.	.
0	.	NA	.	.	.	.	.	.	.	.
0	.	NA	.	.	.	.	.	.	.	.
0	.	NA	.	.	.	.	.	.	.	.
0	.	NA	.	.	.	.	.	.	.	.
0	.	NA	.	.	.	.	.	.	.	.
0	.	NA	.	.	.	.	.	.	.	.
0	Tyrosinemi	NA	.	CAR	.	.	KEG(	.	.	REACTOME_METABOLISM_OF_AMI
0	.	NA	.	.	.	.	.	.	.	.
0	.	NA	.	.	.	.	.	.	.	.
0	.	NA	.	.	.	.	.	.	.	.
0	.	NA	.	.	.	.	.	.	.	.
0	.	NA	.	.	CELI	.	.	PID	BIO	REACTOME_SIGNALING_BY_RHO_(
0	.	NA	.	.	CYT	.	.	.	.	.
0	Mitochondı	NA	.	DNA	CYT	NUC	.	.	.	.
0	.	NA	.	REG	INTF	CATI	.	.	.	REACTOME_TRANSMEMBRANE_TF
0	Acrocallosa	NA	.	.	.	.	.	.	.	.
0	Acrocallosa	NA	.	.	.	.	.	.	.	.
0	.	NA	.	.	.	.	.	.	.	.
0	.	NA	.	.	.	.	.	.	.	.
0	Weill-Marc	NA	.	.	.	.	.	.	.	.
0	.	NA	.	.	.	.	.	.	.	.
0	.	NA	.	.	.	.	.	.	.	.
0	.	NA	.	CELI	.	SER	.	.	.	REACTOME_SIGNALLING_BY_NGF;
0	.	NA	.	.	.	.	KEG(	.	.	.
0	.	NA	.	.	.	.	.	.	.	.

0	.	NA	.	.	.	.	.	.	.	.
0	.	NA	.	.	.	.	.	.	.	.
0	.	NA	.	.	.	.	.	.	.	.
0	.	NA	.	.	.	.	.	.	.	.
0	.	NA	.	.	.	.	.	.	.	.
0	.	NA	.	SIGN	.	GTP.	.	.	.	REACTOME_SIGNALING_BY_GPCR
0	.	NA	.	.	.	.	KEGG	PID	.	.
0	.	NA	.	.	.	.	.	.	.	.
0	.	NA	.	REG	ORG	.	.	.	.	REACTOME_SIGNALING_BY_RHO_(
0	.	NA	.	.	.	.	.	.	.	.
0	.	NA	.	.	.	.	.	.	.	.
0	.	NA	.	REG	.	.	.	.	.	.
0	.	NA	.	.	.	.	.	.	.	.
0	Lipase defi	NA	.	.	.	.	.	.	.	.
0	Somatosta	NA	.	SIGN	INTR	NEU	KEGG	.	.	REACTOME_SIGNALING_BY_GPCR
0	.	NA	.	BIOF	NUC	.	KEGG	PID	.	REACTOME_MEIOSIS;REACTOME_(
0	.	NA	.	REG	.	PRO	.	.	.	.
0	.	NA	.	REG	.	PRO	.	.	.	.
0	.	NA	.	REG	.	PRO	.	.	.	.
0	.	NA	.	REG	.	PRO	.	.	.	.
0	.	NA	.	REG	.	PRO	.	.	.	.
0	.	NA	.	.	.	.	.	.	.	.
0	Osteopetro	NA	.	.	.	.	.	.	.	.
0	.	NA	.	.	.	.	.	.	.	.
0	.	NA	.	.	.	.	.	.	.	.
0	.	NA	.	EST/	CYT	KINA	KEGG	PID	.	.
0	.	NA	.	BIOS	RIBO	STRU	KEGG	.	.	REACTOME_TRANSLATION;REACT(
0	Myopathy,	NA	.	.	.	.	.	.	.	REACTOME_MITOCHONDRIAL_PRO
0	.	NA	.	.	.	.	.	.	.	.
0	.	NA	.	POS	UBIQ	ACID	.	.	.	.
0	.	NA	.	.	.	.	KEGG	.	.	REACTOME_DEVELOPMENTAL_BIO
0	.	NA	.	.	.	.	.	.	.	.
0	.	NA	.	.	.	.	.	.	.	.
0	.	NA	.	.	.	.	.	.	.	REACTOME_GENERIC_TRANSCRIP
0	.	NA	.	.	.	.	.	.	.	.
0	Fanconi ar	NA	.	.	.	.	.	.	.	.
0	Rubinstein	NA	.	RNA	CYT	TRAI	KEGG	PID	BIO	REACTOME_DEVELOPMENTAL_BIO
0	Rubinstein	NA	.	RNA	CYT	TRAI	KEGG	PID	BIO	REACTOME_DEVELOPMENTAL_BIO
0	Rubinstein	NA	.	RNA	CYT	TRAI	KEGG	PID	BIO	REACTOME_DEVELOPMENTAL_BIO
0	.	NA	.	.	INTR	PHO	KEGG	PID	.	REACTOME_SIGNALLING_BY_NGF;
0	.	NA	.	.	.	.	.	.	.	.
0	.	NA	.	.	NON	STRU	.	.	.	.
0	.	NA	.	IMMU	.	RNA	KEGG	.	.	REACTOME_INTERFERON_GAMMA
0	.	NA	.	IMMU	.	RNA	KEGG	.	.	REACTOME_INTERFERON_GAMMA
0	.	NA	.	.	.	.	.	.	.	.
0	.	NA	.	CELL	INTR	STRU	.	.	.	.
0	.	NA	.	.	.	.	.	.	.	.
0	.	NA	.	.	.	.	.	.	.	.
0	.	NA	.	.	.	.	.	.	.	.
0	Lissenceph	NA	.	CEN	ORG	MICF	.	.	.	.
0	.	NA	.	EST/	INTR	HYD	KEGG	PID	.	REACTOME_ABC_FAMILY_PROTEIN
0	.	NA	.	.	.	.	.	.	.	.
0	.	NA	.	BIOF	CYT	PHO	.	.	.	REACTOME_METABOLISM_OF_MRI
0	.	NA	.	.	.	.	.	.	.	.

0	Glomerulo	NA	.	DEFI	MEM	.	.	.	.	.
0	.	NA	.	REPI	.	REC	.	.	.	.
0	.	NA	.	BIOF	CYT	TRAI	.	PID	BIO	REACTOME_INSULIN_RECEPTOR_S
0	.	NA	.	.	ORG	SMA	KEGG	.	.	.
0	.	NA	.	BIOF	.	NUC	.	.	.	.
0	.	NA	.	ONE	.	PRO	KEGG	.	.	.
0	.	NA	.	.	.	.	.	.	.	.
0	.	NA	.	.	NUC	.	.	.	.	.
0	.	NA	.	.	.	.	.	.	.	.
0	.	NA	.	.	.	.	.	.	.	.
0	.	NA	.	EST/	.	PRO	.	.	.	.
0	.	NA	.	.	.	.	.	.	.	.
0	.	NA	.	.	.	.	.	.	.	.
0	Combined	NA	.	BIOF	CYT	TRAI	.	.	.	.
0	Brody myo	NA	.	EST/	.	CATI	KEGG	.	.	REACTOME_PRE_NOTCH_EXPRES
0	.	NA	.	.	.	.	.	.	.	.
0	.	NA	.	.	.	.	.	.	.	.
0	.	NA	.	DNA	.	.	.	.	.	.
0	Branched-	NA	.	CAR	ORG	NUC	.	.	.	.
0	.	NA	.	.	CYT	SER	.	.	.	.
0	.	NA	.	SYS	INTF	REC	KEGG	PID	BIO	REACTOME_CELL_SURFACE_INTEI
0	.	NA	.	MUL	.	.	.	.	.	REACTOME_GENERIC_TRANSCRIP
0	.	NA	.	.	.	.	.	.	.	.
0	.	NA	.	.	.	.	.	.	.	.
0	.	NA	.	.	.	.	.	.	.	.
0	.	NA	.	.	.	.	.	.	.	.
0	.	NA	.	.	.	.	.	.	.	.
0	.	NA	.	.	.	.	.	.	.	.
0	.	NA	.	.	.	.	.	.	.	.
0	.	NA	.	.	.	.	.	.	.	.
0	.	NA	.	.	.	.	.	.	.	.
0	.	NA	.	.	.	.	.	.	.	.
0	.	NA	.	.	.	.	.	.	.	.
0	.	NA	.	.	.	.	.	.	.	.
0	.	NA	.	.	.	.	.	.	.	.
0	.	NA	.	.	.	.	.	.	.	.
0	.	NA	.	.	.	.	.	.	.	.
0	.	NA	.	.	.	.	.	.	.	.
0	.	NA	.	.	.	.	.	.	.	.
0	.	NA	.	.	.	.	.	.	.	.
0	.	NA	.	.	.	.	.	.	.	.
0	.	NA	.	.	.	.	.	.	.	.
0	.	NA	.	.	.	.	.	.	.	.
0	.	NA	.	.	.	.	.	.	.	.
0	.	NA	.	.	.	.	.	.	.	.
0	.	NA	.	.	.	.	.	.	.	.
0	.	NA	.	.	.	.	.	.	.	.
0	.	NA	.	.	.	.	.	.	.	.
0	.	NA	.	.	.	.	.	.	.	.
0	.	NA	.	.	.	.	KEGG	.	.	.
0	Phosphory	NA	.	GEN	.	.	KEGG	.	.	REACTOME_GLYCOGEN_BREAKDC
0	.	NA	.	.	.	.	.	.	.	.
0	.	NA	.	.	.	.	.	.	.	REACTOME_METABOLISM_OF_NON

0	.	NA	.	.	.	.	.	.	.	REACTOME_METABOLISM_OF_NON
0	Hyperalpha	NA	.	REG	EXTI	LIPID	.	.	.	REACTOME_HDL_MEDIATED_LIPID_
0	.	NA	.	.	.	.	.	.	.	.
0	.	NA	.	.	.	.	.	.	.	.
0	.	NA	.	.	.	.	.	.	.	.
0	.	NA	.	.	MAC	.	.	.	.	.
0	.	NA	.	.	.	.	.	.	.	.
0	.	NA	.	.	.	.	.	.	.	.
0	.	NA	.	.	.	.	.	.	.	.
0	.	NA	.	.	.	.	.	.	.	.
0	.	NA	.	.	INTR	CATI	.	.	.	REACTOME_TRANSMEMBRANE_TR
0	.	NA	.	.	.	.	.	.	.	.
0	.	NA	.	.	.	.	.	.	.	.
0	.	NA	.	.	.	.	.	.	.	.
0	.	NA	.	RNA	NUC	CATI	.	.	.	.
0	.	NA	.	PRO	EXTI	.	.	.	.	.
0	.	NA	.	REG	INTR	CATI	.	.	.	REACTOME_TRANSMEMBRANE_TR
0	.	NA	.	.	.	.	.	.	.	.
0	.	NA	.	DNA	ORG	SING	.	PID	.	REACTOME_MEIOSIS;REACTOME_(
0	.	NA	.	.	.	.	.	.	.	.
0	.	NA	.	.	.	.	.	.	.	.
0	.	NA	.	.	.	.	.	.	.	.
0	.	NA	.	.	.	.	.	.	.	.
0	.	NA	.	.	.	.	.	.	.	.
0	.	NA	.	.	.	.	.	.	.	.
0	.	NA	.	REG	ADH	KINA	KEGG	PID	BIO	REACTOME_INTEGRIN_CELL_SURF
0	.	NA	.	.	.	.	.	.	.	.
0	.	NA	.	.	.	.	.	.	.	.
0	Autoinflam	NA	.	.	.	.	KEGG	PID	.	REACTOME_ANTIGEN_ACTIVATES_
0	Malonyl-Co	NA	.	COF	CYTO	LYAS	KEGG	.	.	.
0	.	NA	.	.	.	.	.	.	.	.
0	.	NA	.	RNA	.	RNA	.	.	.	REACTOME_RNA_POL_I_TRANSCR
0	.	NA	.	.	.	.	.	.	.	.
0	Lymphedem	NA	.	SYST	NUC	TRAI	.	.	.	.
0	.	NA	.	.	.	.	.	.	.	.
0	Huntington	NA	.	.	.	.	.	.	.	.
0	Brittle corn	NA	.	.	.	.	.	.	.	.
0	.	NA	.	.	.	LYAS	KEGG	.	.	REACTOME_METABOLISM_OF_LIPI
0	.	NA	.	CELI	.	.	.	PID	.	.
0	.	NA	.	INTE	.	PHO	.	.	.	.
0	.	NA	.	.	.	.	.	.	.	.
0	.	NA	.	.	.	.	.	.	.	.
0	Glomerulos	NA	.	.	ORG	.	.	.	.	.
0	Retinitis pig	NA	.	RNA	ORG	RNA	KEGG	.	.	REACTOME_PROCESSING_OF_CAP
0	.	NA	.	.	.	.	.	.	.	.
0	.	NA	.	RNA	.	TRAI	.	PID	BIO	.
0	.	NA	.	DNA	ORG	TELO	.	PID	.	REACTOME_METABOLISM_OF_MRI
0	.	NA	.	DNA	ORG	TELO	.	PID	.	REACTOME_METABOLISM_OF_MRI
0	.	NA	.	RNA	.	TRAI	.	.	.	.
0	.	NA	.	.	.	.	.	.	.	.
0	.	NA	.	.	.	.	KEGG	.	BIO	REACTOME_TRANSMISSION_ACRO
0	.	NA	.	.	ORG	.	.	.	.	.
0	.	NA	.	BIOF	.	.	KEGG	.	BIO	REACTOME_IMMUNE_SYSTEM;REA
0	.	NA	.	.	.	.	.	PID	BIO	.

0	.	NA	.	EST	CYT	.	.	.	.	.
0	.	NA	.	.	.	.	.	.	.	.
0	.	NA	.	.	.	.	.	.	.	.
0	.	NA	.	.	.	.	.	.	.	.
0	.	NA	.	.	.	.	.	.	.	REACTOME_TRANSMEMBRANE_TF
0	.	NA	.	.	MEM	CAR	.	.	.	.
0	.	NA	.	.	.	.	.	.	.	.
0	.	NA	.	.	NUC	.	KEG	.	BIO	REACTOME_MICRORNA_MIRNA_BI
0	.	NA	.	.	.	.	.	.	.	.
0	.	NA	.	.	.	.	.	PID	.	REACTOME_PHOSPHOLIPID_META
0	.	NA	.	NEG	.	TRA	.	.	.	.
0	.	NA	.	GAM	ORG	.	.	.	.	.
0	.	NA	.	GAM	ORG	.	.	.	.	.
0	.	NA	.	SULI	INTR	SULI	KEG	.	.	REACTOME_HS_GAG_BIOSYNTHES
0	.	NA	.	.	.	.	.	.	.	.
0	.	NA	.	.	.	.	.	.	.	.
0	.	NA	.	RNA	.	TRA	.	PID	BIO	REACTOME_DEVELOPMENTAL_BIC
0	.	NA	.	RNA	.	TRA	.	PID	BIO	REACTOME_DEVELOPMENTAL_BIC
0	.	NA	.	.	.	.	.	.	.	.
0	.	NA	.	.	.	.	.	.	.	.
0	Deafness,	NA	.	SEN	.	.	.	.	.	.
0	Deafness,	NA	.	SEN	.	.	.	.	.	.
0	.	NA	.	.	.	.	.	.	.	.
0	.	NA	.	.	.	.	.	.	.	.
0	.	NA	.	.	.	.	.	.	.	.
0	.	NA	.	.	.	.	.	.	.	.
0	.	NA	.	RNA	.	PHO	KEG	PID	BIO	REACTOME_TRIF_MEDIATED_TLR3
0	{Hypertens	NA	.	MUL	CYT	OXID	KEG	PID	BIO	REACTOME_LATENT_INFECTION_C
0	.	NA	.	.	INTR	CATI	.	.	.	REACTOME_TRANSMEMBRANE_TF
0	.	NA	.	.	INTR	CATI	.	.	.	REACTOME_TRANSMEMBRANE_TF
0	.	NA	.	.	.	.	.	.	.	.
0	.	NA	.	.	.	.	.	.	.	.
0	.	NA	.	.	.	.	.	.	.	.
0	.	NA	.	.	.	.	.	.	.	.
0	.	NA	.	.	.	.	.	.	.	.
0	.	NA	.	CELI	.	.	.	.	.	REACTOME_SIGNALING_BY_FGFR_
0	{Anxiety-re	NA	.	REG	INTR	ACTI	.	.	.	.
0	.	NA	.	.	.	.	.	.	.	.
0	.	NA	.	.	.	.	KEG	.	.	.
0	.	NA	.	.	.	.	.	.	.	.
0	.	NA	.	.	.	.	.	.	.	.
0	.	NA	.	.	.	.	.	.	.	.
0	.	NA	.	.	.	.	.	.	.	.
0	.	NA	.	.	NUC	.	.	.	.	.
0	.	NA	.	.	.	.	.	.	.	.
0	.	NA	.	.	.	.	KEG	.	.	REACTOME_SIGNALLING_BY_NGF;
0	.	NA	.	.	.	.	.	.	.	.
0	.	NA	.	.	.	.	.	.	.	.
0	.	NA	.	.	.	.	.	.	.	.
0	.	NA	.	.	.	TRA	.	.	.	.
0	.	NA	.	.	.	.	.	.	.	.
0	.	NA	.	.	MEM	CATI	KEG	.	.	REACTOME_DEVELOPMENTAL_BIC
0	.	NA	.	BIOS	.	STRI	KEG	.	.	REACTOME_TRANSLATION;REACT
0	.	NA	.	.	.	.	.	.	.	.

0	Adenocarc	NA	.	SYS	EXTI	PHO	KEG(	PID	.	REACTOME_DEVELOPMENTAL_BIC
0	.	NA	.	REG	INTF	.	KEG(	.	BIO	REACTOME_SIGNALING_BY_GPCR
0	.	NA	.	SYS	.	.	.	.	.	.
0	.	NA	.	MEI(	ORG	.	.	.	.	.
0	.	NA	.	.	CYT(	.	.	.	.	.
0	.	NA	.	.	CYT(	.	.	.	.	.
0	Hyper-IgE	NA	.	RNA	CYT(	REC	KEG(	PID	BIO	REACTOME_SIGNALLING_BY_NGF;
0	Hyper-IgE	NA	.	RNA	CYT(	REC	KEG(	PID	BIO	REACTOME_SIGNALLING_BY_NGF;
0	Hyper-IgE	NA	.	RNA	CYT(	REC	KEG(	PID	BIO	REACTOME_SIGNALLING_BY_NGF;
0	.	NA	.	COF	.	PHO	KEG(	.	.	REACTOME_VITAMIN_B5_PANTOTH
0	Pseudohyp	NA	.	EST/	CELI	NUC	.	.	.	.
0	.	NA	.	.	.	.	.	.	.	.
0	.	NA	.	.	.	.	.	.	.	.
0	.	NA	.	.	.	.	.	.	.	.
0	.	NA	.	SIGN	INTF	PHO	.	.	.	.
0	.	NA	.	SIGN	INTF	PHO	.	.	.	.
0	.	NA	.	SIGN	INTF	PHO	.	.	.	.
0	.	NA	.	.	.	.	.	.	.	.
0	.	NA	.	.	.	.	.	.	.	.
0	.	NA	.	.	.	.	.	.	.	.
0	.	NA	.	.	.	.	.	.	.	.
0	Osteopetro	NA	.	.	.	.	.	.	.	.
0	.	NA	.	.	.	.	.	.	.	.
0	.	NA	.	.	.	.	.	.	.	.
0	.	NA	.	.	.	.	KEG(	.	.	REACTOME_TRANSMISSION_ACRC
0	.	NA	.	.	.	.	.	.	.	.
0	.	NA	.	.	CYT(	EXO	.	.	.	REACTOME_IMMUNE_SYSTEM;REA
0	.	NA	.	.	.	.	.	.	.	.
0	.	NA	.	BIOF	LAM	.	.	.	.	.
0	.	NA	.	.	.	.	.	.	.	.
0	.	NA	.	.	.	.	.	.	.	.
0	.	NA	.	.	.	.	.	.	.	.
0	.	NA	.	REG	NUC	.	.	.	.	.
0	.	NA	.	REG	NUC	.	.	.	.	.
0	Caffey dise	NA	.	SYS	.	.	KEG(	PID	.	REACTOME_DEVELOPMENTAL_BIC
0	{Pseudoxa	NA	.	.	.	.	KEG(	.	.	.
0	.	NA	.	GAM	CYT(	.	.	.	.	.
0	.	NA	.	RNA	NUC	DOU	.	.	.	.
0	Fanconi ar	NA	.	DNA	NUC	DNA	KEG(	.	.	REACTOME_MEIOSIS;REACTOME_I
0	Breast can	NA	.	BIOF	NUC	PHO	KEG(	PID	.	.
0	Growth hor	NA	.	EST/	.	HEM	KEG(	.	BIO	REACTOME_GROWTH_HORMONE_
0	Hyperkaler	NA	.	EST/	.	CATI	.	.	.	REACTOME_DEVELOPMENTAL_BIC
0	.	NA	.	.	.	.	.	.	.	.
0	.	NA	.	.	.	.	.	.	.	.
0	.	NA	.	.	.	.	KEG(	.	.	REACTOME_ABCA_TRANSPORTER
0	.	NA	.	.	.	.	.	.	.	.
0	.	NA	.	.	.	.	.	.	.	.
0	.	NA	.	BIOS	CYT(	ELE(	.	PID	.	.
0	.	NA	.	.	.	.	.	.	.	.
0	.	NA	.	.	CYT(	.	.	.	.	.
0	.	NA	.	.	CYT(	.	.	.	.	.
0	.	NA	.	.	.	.	.	.	.	.
0	.	NA	.	DNA	.	.	.	.	.	.
0	.	NA	.	DNA	.	.	.	.	.	.

0	.	NA	.	RES	.	OXID	.	.	.	.
0	.	NA	.	.	.	.	.	.	.	.
0	.	NA	.	.	.	.	KEGG	.	.	.
0	.	NA	.	.	CYTO	.	.	.	.	.
0	.	NA	.	.	.	.	.	.	.	.
0	.	NA	.	.	.	.	.	.	.	.
0	.	NA	.	.	.	.	.	.	.	.
0	.	NA	.	CELL	NUC	TRAI	.	PID	.	.
0	Ciliary dysk	NA	.	.	.	.	.	.	.	.
0	Pityriasis ru	NA	.	POS	CYTO	PRO	.	.	.	.
0	Pityriasis ru	NA	.	POS	CYTO	PRO	.	.	.	.
0	{Moyamoya	NA	.	.	.	.	.	.	.	.
0	.	NA	.	.	.	.	KEGG	PID	.	REACTOME_INSULIN_RECEPTOR_S
0	.	NA	.	SYST	MEM	MOL	KEGG	PID	BIO	.
0	.	NA	.	.	.	.	.	.	.	.
0	.	NA	.	.	.	.	.	.	.	.
0	.	NA	.	.	.	.	.	.	.	.
0	.	NA	.	.	.	.	.	.	.	.
0	.	NA	.	.	.	.	.	.	.	.
0	Alveolar sc	NA	.	.	.	.	.	.	.	.
0	.	NA	.	.	.	.	KEGG	PID	BIO	REACTOME_VITAMIN_B5_PANTOTH
0	.	NA	.	.	.	.	KEGG	PID	BIO	REACTOME_VITAMIN_B5_PANTOTH
0	.	NA	.	.	.	.	.	.	.	.
0	.	NA	.	.	.	.	.	.	.	.
0	Seborrhea	NA	.	.	.	.	.	.	.	.
0	.	NA	.	MUL	CELL	NEU	.	.	.	REACTOME_SIGNALLING_BY_NGF;
0	.	NA	.	M_P	ORG	.	.	PID	.	REACTOME_CELL_CYCLE;REACTO
0	Ataxia, spa	NA	.	.	CYTO	UNF	.	.	.	.
0	.	NA	.	.	.	.	.	.	.	REACTOME_CELL_CYCLE;REACTO
0	Glucocortic	NA	.	SIGN	INTR	NEU	KEGG	.	.	REACTOME_SIGNALING_BY_GPCR
0	.	NA	.	.	.	.	.	.	.	.
0	.	NA	.	.	.	.	.	.	.	.
0	.	NA	.	.	.	.	.	.	.	.
0	.	NA	.	.	.	.	.	.	.	.
0	.	NA	.	.	.	.	.	.	.	.
0	Cardiomyo	NA	.	SYST	PRO	.	KEGG	PID	BIO	REACTOME_CELL_CELL_COMMUN
0	.	NA	.	.	.	.	.	.	.	.
0	.	NA	.	RNA	NUC	DNA	.	.	.	.
0	Vici syndro	NA	.	.	.	.	.	.	.	.
0	.	NA	.	.	.	.	.	.	.	.
0	.	NA	.	.	.	.	.	.	.	.
0	Microvillus	NA	.	.	.	.	.	.	.	REACTOME_TRANSMEMBRANE_TR
0	.	NA	.	.	.	.	.	.	.	.
0	.	NA	.	.	.	.	.	.	.	.
0	.	NA	.	.	CYTO	.	.	.	.	.
0	.	NA	.	.	.	PRO	.	.	.	.
0	.	NA	.	.	.	.	.	.	.	.
0	Polymicrog	NA	.	.	.	.	.	.	.	.
0	.	NA	.	.	.	.	.	.	.	.
0	.	NA	.	.	.	.	.	.	.	REACTOME_TRANSMEMBRANE_TR
0	.	NA	.	.	.	.	.	.	.	.
0	[Blood grou	NA	.	SIGN	.	.	.	PID	.	REACTOME_PYRUVATE_METABOL
0	.	NA	.	RNA	CYTO	NUC	.	.	.	REACTOME_TRANSCRIPTION;REAC
0	.	NA	.	RNA	CYTO	NUC	.	.	.	REACTOME_TRANSCRIPTION;REAC

0	.	NA	.	.	INTF	.	.	.	.	.
0	.	NA	.	RNA	NUC	RNA	.	.	.	REACTOME_PROCESSING_OF_CAI
0	.	NA	.	.	.	.	.	.	.	.
0	.	NA	.	.	.	.	.	.	.	REACTOME_SIGNALING_BY_RHO_(
0	.	NA	.	RNA	NUC	NUC	KEG(	.	.	REACTOME_RNA_POL_III_TRANSC
0	.	NA	.	MEM	CYT	OXID	KEG(	.	.	.
0	.	NA	.	.	.	.	.	.	.	.
0	.	NA	.	.	.	.	.	.	.	.
0	.	NA	.	.	.	.	.	.	.	.
0	.	NA	.	.	.	.	.	.	.	.
0	.	NA	.	.	.	.	.	.	.	.
0	.	NA	.	RESI	.	RNA	.	.	.	.
0	.	NA	.	GAM	.	RNA	.	.	.	.
0	.	NA	.	GAM	.	RNA	.	.	.	.
0	.	NA	.	.	.	.	.	.	.	.
0	.	NA	.	.	.	.	KEG(	.	.	.
0	.	NA	.	.	.	.	.	.	.	.
0	.	NA	.	.	CYT	.	.	.	.	.
0	.	NA	.	.	CYT	.	.	.	.	.
0	.	NA	.	REG	INTF	LIPID	KEG(	PID	.	REACTOME_SIGNALING_BY_GPCR
0	.	NA	.	RNA	.	TRAI	.	PID	.	.
0	.	NA	.	.	.	.	.	.	.	.
0	.	NA	.	.	.	.	.	.	.	.
0	.	NA	.	.	.	.	.	.	.	.
0	Leukemia,	NA	.	SYS	.	.	KEG(	PID	.	.
0	.	NA	.	.	.	.	.	.	.	.
0	.	NA	.	.	.	.	KEG(	.	.	.
0	.	NA	.	.	.	.	.	.	.	.
0	.	NA	.	ORG	NUC	DOU	.	PID	.	.
0	.	NA	.	.	.	.	.	.	.	.
0	.	NA	.	.	.	.	.	.	.	.
0	.	NA	.	.	.	TRAI	KEG(	PID	BIO	REACTOME_SIGNALING_BY_RHO_(
0	.	NA	.	.	.	.	.	PID	BIO	REACTOME_SIGNALING_BY_RHO_(
0	Spastic pai	NA	.	.	.	.	.	.	.	.
0	.	NA	.	DNA	.	.	KEG(	.	.	REACTOME_TRANSCRIPTION_COU
0	.	NA	.	DNA	.	.	KEG(	.	.	REACTOME_TRANSCRIPTION_COU
0	Hemophag	NA	.	.	.	.	.	.	.	.
0	{Dengue fe	NA	.	MUL	.	CAR	.	.	.	.
0	{Dengue fe	NA	.	MUL	.	CAR	.	.	.	.
0	.	NA	.	.	.	.	.	.	.	.
0	.	NA	.	.	.	.	.	.	.	.
0	Methylmal	NA	.	.	CYT	.	.	.	.	.
0	.	NA	.	.	.	.	.	.	.	.
0	.	NA	.	.	.	.	.	.	.	.
0	.	NA	.	.	.	.	.	.	.	.
0	.	NA	.	.	.	.	.	.	.	.
0	.	NA	.	.	MEM	.	.	.	.	REACTOME_O_LINKED_GLYCOSYL
0	.	NA	.	.	MEM	.	.	.	.	REACTOME_O_LINKED_GLYCOSYL
0	.	NA	.	.	MEM	.	.	.	.	REACTOME_O_LINKED_GLYCOSYL
0	.	NA	.	.	.	.	.	.	.	.
0	.	NA	.	M_P	NUC	.	KEG(	PID	BIO	REACTOME_ANTIVIRAL_MECHANIS
0	.	NA	.	.	.	.	.	.	.	.
0	.	NA	.	.	.	.	.	.	.	.
0	.	NA	.	RNA	NUC	.	.	.	.	.

0	.	NA	.	G_PI	INTF	REC	KEG(	.	.	REACTOME_GASTRIN_CREB_SIGN
0	{Malaria, c	NA	.	.	INTF	REC	KEG(	PID	BIO	REACTOME_IMMUNOREGULATORY
0	.	NA	.	.	INTF	.	.	.	.	.
0	Tyrosine ki	NA	.	BIOF	.	PHO	KEG(	PID	BIO	REACTOME_SIGNALING_BY_ILS;RE
0	.	NA	.	SIGN	INSC	HYD	KEG(	.	.	.
0	.	NA	.	EST/	ORG	MICF	KEG(	.	.	.
0	.	NA	.	.	.	.	.	.	.	.
0	Hypercholε	NA	.	GLY(	INTF	LIPC	KEG(	.	.	REACTOME_METABOLISM_OF_LIPI
0	Hypercholε	NA	.	GLY(	INTF	LIPC	KEG(	.	.	REACTOME_METABOLISM_OF_LIPI
0	Adams-Oli	NA	.	.	.	.	.	PID	.	REACTOME_FACTORS_INVOLVED_
0	.	NA	.	.	.	.	.	.	.	.
0	.	NA	.	REG	.	.	.	.	.	.
0	Mannosido	NA	.	GLY(	.	HYD	KEG(	.	.	.
0	.	NA	.	.	.	.	.	.	.	.
0	.	NA	.	IMMU	.	RNA	.	.	.	.
0	.	NA	.	.	.	.	KEG(	PID	BIO	REACTOME_SIGNALLING_BY_NGF;
0	.	NA	.	.	.	.	.	.	.	.
0	.	NA	.	.	.	.	KEG(	.	.	REACTOME_SIGNALING_BY_GPCR
0	.	NA	.	.	.	.	.	.	.	.
0	.	NA	.	.	.	.	.	.	.	REACTOME_SIGNALING_BY_EGFR_
0	.	NA	.	.	.	.	.	.	.	.
0	{Celiac dis	NA	.	EST/	ORG	HYD	.	PID	.	REACTOME_SIGNALING_BY_RHO_(
0	.	NA	.	.	.	.	.	.	.	.
0	.	NA	.	.	.	PRO	.	.	.	.
0	.	NA	.	.	.	.	.	.	.	.
0	.	NA	.	.	.	.	.	.	.	.
0	.	NA	.	.	.	.	.	.	.	.
0	.	NA	.	.	.	.	.	.	.	.
0	.	NA	.	.	.	HYD	KEG(	.	.	REACTOME_SIGNALING_BY_GPCR
0	.	NA	.	DNA	ORG	HYD	.	.	.	.
0	.	NA	.	.	.	.	.	.	.	.
0	.	NA	.	.	.	.	.	.	.	.
0	.	NA	.	.	.	.	.	.	.	.
0	.	NA	.	.	.	.	.	.	.	REACTOME_GENERIC_TRANSCRIP
0	.	NA	.	.	.	.	.	.	.	.
0	.	NA	.	.	.	.	.	.	.	.
0	.	NA	.	.	.	.	.	.	.	.
0	Hemolytic	NA	.	REG	.	.	KEG(	.	BIO	REACTOME_GLYCOLYSIS;REACTO
0	.	NA	.	IMMU	INTF	.	KEG(	PID	.	.
0	.	NA	.	.	.	.	.	.	.	.
0	Microcephε	NA	.	.	.	.	.	.	.	.
0	.	NA	.	RNA	NUC	CAR	.	.	.	.
0	.	NA	.	.	.	.	.	.	.	REACTOME_GENERIC_TRANSCRIP
0	.	NA	.	.	.	.	.	.	.	.
0	.	NA	.	.	.	.	.	.	.	.
0	.	NA	.	.	.	.	.	.	.	.
0	.	NA	.	.	.	.	.	.	.	.
0	.	NA	.	.	.	.	.	.	.	.
0	.	NA	.	.	.	.	.	.	.	.
0	.	NA	.	.	.	.	.	.	.	.
0	.	NA	.	REG	MEM	LIPIL	KEG(	PID	.	.
0	Central cor	NA	.	EST/	INTF	CATI	KEG(	.	.	.
0	Central cor	NA	.	EST/	INTF	CATI	KEG(	.	.	.
0	.	NA	.	SYS	.	NUC	KEG(	PID	BIO	.

0	.	NA	.	.	NUC	RNA	.	.	.	REACTOME_PROCESSING_OF_CAF
0	.	NA	.	MUS	ORG	CATI	.	PID	.	.
0	.	NA	.	.	.	.	.	.	.	.
0	.	NA	.	.	.	.	.	.	.	.
0	Cold-induc	NA	.	MUL	.	.	.	.	.	.
0	.	NA	.	RNA	NUC	TRAI	.	.	BIO	.
0	.	NA	.	.	.	.	.	.	.	.
0	.	NA	.	.	.	.	.	.	.	.
0	.	NA	.	.	.	.	.	.	.	.
0	.	NA	.	.	.	.	.	.	.	.
0	Cutis laxa,	NA	.	REG	PRO	CATI	.	.	.	.
0	Cutis laxa,	NA	.	REG	PRO	CATI	.	.	.	.
0	.	NA	.	SYS	.	.	KEG(	.	.	.
0	.	NA	.	.	.	.	.	.	.	.
0	.	NA	.	RNA	ORG	RNA	.	.	.	REACTOME_PROCESSING_OF_CAF
0	Camurati-E	NA	.	REG	PRO	TRAI	KEG(	PID	BIO	REACTOME_DEVELOPMENTAL_BIC
0	.	NA	.	CELI	MEN	RAS	KEG(	PID	BIO	REACTOME_SIGNALLING_BY_NGF;
0	Carpenter	NA	.	.	.	.	.	.	.	.
0	.	NA	.	IMMI	INTF	.	.	.	.	.
0	.	NA	.	MUL	.	.	.	.	.	.
0	.	NA	.	.	.	.	.	.	.	.
0	.	NA	.	.	.	.	.	.	.	.
0	.	NA	.	.	.	TRAI	.	.	.	REACTOME_GENERIC_TRANSCRIP
0	.	NA	.	.	.	.	.	.	.	REACTOME_GENERIC_TRANSCRIP
0	.	NA	.	.	.	.	.	.	.	.
0	.	NA	.	.	.	.	.	.	.	.
0	.	NA	.	.	.	.	.	.	.	.
0	Deafness,	NA	.	.	.	.	.	.	.	.
0	Leukemia/I	NA	.	POS	CYT	PRO	.	PID	.	.
0	[Blood grou	NA	.	SIGN	INTF	REC	.	.	.	.
0	.	NA	.	.	.	.	KEG(	.	.	REACTOME_MITOCHONDRIAL_PRC
0	.	NA	.	SIGN	INTF	.	.	.	.	REACTOME_GASTRIN_CREB_SIGN
0	.	NA	.	.	.	.	.	.	.	.
0	.	NA	.	SIGN	CYT	STRI	.	.	.	.
0	.	NA	.	.	.	.	KEG(	.	.	REACTOME_TRANSMEMBRANE_TF
0	Cone-rod r	NA	.	SYS	.	.	.	.	.	.
0	.	NA	.	.	.	.	.	.	.	.
0	.	NA	.	.	.	.	.	.	.	.
0	.	NA	.	.	CYT	.	KEG(	PID	.	REACTOME_SPHINGOLIPID_DE_NC
0	.	NA	.	.	.	.	.	.	.	.
0	.	NA	.	RNA	ORG	RNA	KEG(	.	.	REACTOME_PROCESSING_OF_CAF
0	.	NA	.	RNA	ORG	RNA	KEG(	.	.	REACTOME_PROCESSING_OF_CAF
0	.	NA	.	ANA	EXTI	.	.	.	.	.
0	.	NA	.	.	.	.	.	.	.	.
0	.	NA	.	.	.	.	.	.	.	.
0	.	NA	.	.	.	.	KEG(	PID	.	.
0	Deafness,	NA	.	.	.	.	KEG(	.	.	REACTOME_DEVELOPMENTAL_BIC
0	.	NA	.	NEG	CYT	.	.	PID	.	REACTOME_GENERIC_TRANSCRIP
0	Mandibular	NA	.	DNA	NUC	NUC	KEG(	.	.	REACTOME_BASE_EXCISION_REP/
0	Mandibular	NA	.	DNA	NUC	NUC	KEG(	.	.	REACTOME_BASE_EXCISION_REP/
0	Mandibular	NA	.	DNA	NUC	NUC	KEG(	.	.	REACTOME_BASE_EXCISION_REP/
0	.	NA	.	.	.	.	.	.	.	.
0	.	NA	.	.	.	STRI	.	.	.	REACTOME_STRIATED_MUSCLE_C
0	.	NA	.	.	.	STRI	.	.	.	REACTOME_STRIATED_MUSCLE_C

0	.	NA	.	SIGI	INTF	.	.	.	.	.
0	.	NA	.	.	.	.	.	.	.	.
0	.	NA	.	.	.	.	.	.	.	.
0	.	NA	.	.	.	.	.	.	.	.
0	.	NA	.	.	.	.	.	.	.	.
0	.	NA	.	.	.	.	KEGC	.	.	REACTOME_TRANSMISSION_ACRC
0	Retinitis pi	NA	.	RNA	NUC	RIBC	KEGC	.	.	.
0	.	NA	.	.	.	.	.	.	.	.
0	.	NA	.	SIGI	INTF	REC	KEGC	.	.	REACTOME_IMMUNOREGULATORY
0	.	NA	.	.	INTF	.	.	.	.	.
0	.	NA	.	.	.	.	.	.	.	.
0	.	NA	.	CELI	.	.	.	.	.	REACTOME_INTEGRATION_OF_EN
0	.	NA	.	.	.	.	.	.	.	.
0	.	NA	.	.	.	.	.	.	.	.
0	.	NA	.	.	.	.	.	.	.	.
0	.	NA	.	.	.	.	.	.	.	REACTOME_GENERIC_TRANSCRIP
0	.	NA	.	.	.	.	.	.	.	REACTOME_GENERIC_TRANSCRIP
0	.	NA	.	.	.	.	.	.	.	REACTOME_GENERIC_TRANSCRIP
0	.	NA	.	.	.	.	.	.	.	.
0	.	NA	.	.	EXTI	.	.	.	.	.
0	.	NA	.	.	MEM	.	.	.	.	REACTOME_CELL_CELL_COMMUN
0	Spinocereb	NA	.	.	.	.	.	.	.	.
0	Spinocereb	NA	.	RNA	ORG	RNA	.	.	.	REACTOME_ASSOCIATION_OF_TR
0	.	NA	.	.	.	.	.	.	.	.
0	.	NA	.	.	.	.	.	.	.	REACTOME_DEVELOPMENTAL_BIC
0	.	NA	.	.	.	.	.	.	.	.
0	.	NA	.	.	.	.	.	.	.	.
0	.	NA	.	.	.	.	.	.	.	.
0	.	NA	.	.	.	.	.	.	.	.
0	.	NA	.	.	NUC	.	.	.	.	.
0	.	NA	.	.	.	.	.	.	.	.
0	.	NA	.	.	.	.	.	.	.	.
0	Polyneurop	NA	.	.	.	.	.	.	.	.
0	.	NA	.	.	.	.	.	.	.	.
0	.	NA	.	.	.	.	.	.	.	.
0	.	NA	.	.	.	.	.	.	.	.
0	.	NA	.	.	.	.	.	.	.	.
0	.	NA	.	.	CYT(	.	.	.	.	.
0	.	NA	.	.	.	.	.	.	.	.
0	.	NA	.	PHO	ORG	LIPIC	KEGC	.	.	REACTOME_METABOLISM_OF_PRC
0	.	NA	.	EST/	ORG	PRO	.	.	.	.
0	.	NA	.	CEN	ORG	KINA	.	.	.	REACTOME_CELL_CYCLE;REACTO
0	.	NA	.	BIOF	ORG	PRO	.	.	.	REACTOME_METABOLISM_OF_VIT/
0	.	NA	.	.	NUC	.	.	.	.	.
0	Mental reta	NA	.	.	.	.	KEGC	PID	.	REACTOME_TRANSMISSION_ACRC
0	.	NA	.	.	.	.	.	.	.	.
0	.	NA	.	.	CYT(	.	.	.	.	.
0	.	NA	.	.	.	.	.	.	.	.
0	.	NA	.	.	.	.	.	.	.	.
0	.	NA	.	.	.	.	.	.	.	.
0	.	NA	.	.	.	.	.	.	.	.
0	DNA topois	NA	.	RES	NUC	CHR	.	PID	.	.
0	.	NA	.	.	CYT(	.	KEGC	PID	BIO	REACTOME_SIGNALLING_BY_NGF;
0	.	NA	.	ENZ	INTF	PRO	.	.	.	.

0	.	NA	.	ENZ'	INTF	PRO	.	.	.	.
0	.	NA	.	SIGN	CELI	.	.	.	.	.
0	.	NA	.	MUL	EXTI	.	.	.	.	.
0	.	NA	.	.	.	.	.	.	.	REACTOME_TRANSMEMBRANE_TF
0	.	NA	.	.	.	.	.	PID	.	.
0	.	NA	.	.	.	.	.	.	.	REACTOME_TRANSMEMBRANE_TF
0	.	NA	.	.	.	.	.	.	.	.
0	.	NA	.	.	.	.	.	.	.	REACTOME_NEURONAL_SYSTEM;F
0	.	NA	.	.	.	.	.	.	.	REACTOME_TRANSMEMBRANE_TF
0	.	NA	.	.	.	.	.	.	.	.
0	.	NA	.	.	.	TRAI	.	.	.	.
0	.	NA	.	.	.	.	.	.	.	.
0	ACTH-inde	NA	.	REG	EXTI	HYD	KEG(	.	BIO	REACTOME_SIGNALING_BY_GPCR
0	ACTH-inde	NA	.	REG	EXTI	HYD	KEG(	.	BIO	REACTOME_SIGNALING_BY_GPCR
0	.	NA	.	.	.	.	.	.	.	REACTOME_MEIOSIS;REACTOME_(
0	.	NA	.	.	.	.	.	.	.	.
0	Epiphyseal	NA	.	.	PRO	.	.	PID	.	REACTOME_DEVELOPMENTAL_BIC
0	Epiphyseal	NA	.	.	PRO	.	.	PID	.	REACTOME_DEVELOPMENTAL_BIC
0	.	NA	.	.	.	.	KEG(	PID	BIO	REACTOME_COPI_MEDIATED_TRA
0	.	NA	.	.	.	.	.	.	.	.
0	.	NA	.	.	.	.	.	.	.	.
0	.	NA	.	.	.	.	.	.	.	.
0	.	NA	.	.	.	.	.	.	.	.
0	.	NA	.	.	.	.	.	.	.	.
0	.	NA	.	.	.	.	.	.	.	.
0	.	NA	.	.	.	.	.	.	.	.
0	.	NA	.	.	.	.	.	.	.	.
0	.	NA	.	.	.	.	.	.	.	.
0	.	NA	.	.	.	.	.	.	.	.
0	.	NA	.	BIOF	INTF	PRO	.	.	.	.
0	.	NA	.	BIOF	INTF	PRO	.	.	.	.
0	.	NA	.	.	.	.	.	.	.	.
0	.	NA	.	.	.	.	.	.	.	.
0	.	NA	.	.	.	.	.	.	.	.
0	.	NA	.	.	.	.	.	.	.	.
0	.	NA	.	.	.	.	.	.	.	.
0	.	NA	.	.	.	.	.	.	.	.
0	.	NA	.	.	.	.	.	.	.	.
0	.	NA	.	CELI	INTF	.	KEG(	.	.	.
0	.	NA	.	.	.	.	KEG(	PID	.	REACTOME_CELL_SURFACE_INTEI
0	Alzheimer (	NA	.	REG	INTF	PRO	KEG(	PID	BIO	REACTOME_TRIF_MEDIATED_TLR3
0	.	NA	.	.	.	.	.	.	.	.
0	.	NA	.	.	.	.	.	.	.	.
0	.	NA	.	SIGN	.	PHO	.	.	.	.
0	.	NA	.	SIGN	.	PHO	.	.	.	.
0	.	NA	.	EST/	.	PHO	KEG(	PID	BIO	REACTOME_PHOSPHOLIPID_META
0	.	NA	.	.	.	.	.	.	.	.
0	.	NA	.	RES	INTF	REC	KEG(	PID	BIO	REACTOME_INTERFERON_ALPHA_
0	.	NA	.	EST/	.	.	.	PID	.	REACTOME_SIGNALING_BY_RHO_(
0	.	NA	.	.	.	.	.	.	.	.
0	.	NA	.	.	.	.	.	.	.	.
0	.	NA	.	.	.	.	.	.	.	.
0	.	NA	.	.	INTF	.	.	.	.	.
0	.	NA	.	.	NUC	.	.	.	.	.

0	.	NA	.	.	.	.	KEGG	.	.	.	
0	.	NA	.	SYS	INTR	.	.	PID	.	REACTOME_CELL_CELL_COMMUN
0	.	NA	.	SYS	INTR	.	.	PID	.	REACTOME_CELL_CELL_COMMUN
0	.	NA	.	SYS	INTR	.	.	PID	.	REACTOME_CELL_CELL_COMMUN
0	.	NA	.	SYS	INTR	.	.	PID	.	REACTOME_CELL_CELL_COMMUN
0	.	NA	.	EST/	.	.	.	.	.	.
0	.	NA	.	.	.	.	.	PID	.	.
0	.	NA	.	.	CYT	.	.	.	.	.
0	.	NA	.	.	.	.	.	.	.	.
0	.	NA	.	.	.	.	.	.	.	.
0	.	NA	.	RNA	NUC	.	KEGG	.	.	REACTOME_PROCESSING_OF_CAF
0	.	NA	.	.	ORG	.	.	.	.	.
0	.	NA	.	SIGN	.	.	.	.	.	.
0	.	NA	.	EST/	INTR	CATI	.	.	.	.
0	Leukocyte	NA	.	BIOF	.	KINA	KEGG	PID	BIO	REACTOME_IMMUNOREGULATORY
0	.	NA	.	EST/	INTR	COF	.	.	.	REACTOME_METABOLISM_OF_VIT/
0	Bethlem m	NA	.	.	.	.	KEGG	PID	.	REACTOME_DEVELOPMENTAL_BIC
0	Glutamate	NA	.	COF	CYT	.	KEGG	.	.	REACTOME_METABOLISM_OF_AMI
0	.	NA	.	.	.	.	.	.	.	.
0	Microcepha	NA	.	ANA	.	.	.	.	BIO	REACTOME_CELL_CYCLE;REACTO
0	.	NA	.	.	.	.	.	.	.	.
0	.	NA	.	.	.	.	.	.	.	.
0	.	NA	.	.	.	.	.	.	.	.
0	.	NA	.	.	.	.	.	.	.	.
0	.	NA	.	.	.	.	.	.	.	.
0	.	NA	.	.	.	.	.	.	.	.
0	DiGeorge s	NA	.	SYS	.	.	.	.	.	.
0	DiGeorge s	NA	.	SYS	.	.	.	.	.	.
0	.	NA	.	.	.	.	.	.	.	.
0	.	NA	.	.	.	.	.	.	.	.
0	.	NA	.	.	.	.	.	.	.	.
0	.	NA	.	PHO	ORG	PHO	KEGG	PID	.	REACTOME_SYNTHESIS_OF_PIPS_
0	.	NA	.	.	NUC	PRO	.	.	.	.
0	.	NA	.	.	.	.	.	.	.	.
0	.	NA	.	.	.	.	.	.	.	.
0	Mental reta	NA	.	DNA	NUC	.	.	PID	.	.
0	.	NA	.	.	.	.	.	.	.	.
0	.	NA	.	REG	INTR	.	KEGG	PID	.	REACTOME_SIGNALLING_BY_NGF;
0	Beta-ureid	NA	.	.	.	HYD	KEGG	.	.	REACTOME_PYRIMIDINE_CATABOL
0	Glutathioni	NA	.	CAR	.	.	KEGG	.	.	REACTOME_BIOLOGICAL_OXIDATI
0	.	NA	.	.	.	.	.	.	.	.
0	.	NA	.	.	.	.	.	.	.	.
0	.	NA	.	.	.	.	.	.	.	.
0	.	NA	.	.	.	.	.	.	.	.
0	.	NA	.	.	.	.	.	.	.	.
0	.	NA	.	MUS	NON	ACTI	.	.	.	.
0	.	NA	.	.	.	.	.	.	.	.
0	.	NA	.	.	.	.	.	.	.	.
0	.	NA	.	.	.	.	.	.	.	.
0	.	NA	.	.	.	.	.	.	.	.
0	Muscular d	NA	.	REG	ORG	TRA	.	.	.	.
0	.	NA	.	DNA	NUC	.	KEGG	.	BIO	REACTOME_ACTIVATION_OF_THE_
0	Deafness,	NA	.	REG	ORG	HYD	KEGG	.	.	REACTOME_DEVELOPMENTAL_BIC
0	Surfactant	NA	.	RES	INTR	INTE	KEGG	PID	BIO	REACTOME_SIGNALING_BY_ILS;RE

0	.	NA	.	.	.	.	.	.	.	.
0	Infantile ne	NA	CM1	MEM	.	LIPA	KEG(	.	.	REACTOME_ACYL_CHAIN_REMODE
0	.	NA	.	RNA	.	.	.	.	BIO	REACTOME_FACTORS_INVOLVED_
0	.	NA	.	.	.	.	.	.	.	.
0	.	NA	.	EST/	INTF	CATI	.	.	.	REACTOME_TRANSMISSION_ACRO
0	.	NA	.	EST/	INTF	CATI	.	.	.	REACTOME_TRANSMISSION_ACRO
0	.	NA	.	SIGN	.	GTP	.	.	.	.
0	.	NA	.	RNA	ORG	CATI	.	.	.	.
0	.	NA	.	SIGN	.	.	KEG(	.	.	REACTOME_DEVELOPMENTAL_BIC
0	.	NA	.	.	.	.	.	.	.	REACTOME_PRE_NOTCH_TRANSC
0	.	NA	.	REG	INTF	NEU	KEG(	.	.	REACTOME_GASTRIN_CREB_SIGN
0	.	NA	.	RNA	NUC	NUC	KEG(	.	.	REACTOME_RNA_POL_III_TRANSC
0	.	NA	.	RNA	NUC	NUC	KEG(	.	.	REACTOME_RNA_POL_III_TRANSC
0	.	NA	.	.	.	.	.	.	.	REACTOME_CELL_CYCLE;REACTO
0	.	NA	.	.	.	.	.	.	.	.
0	.	NA	.	.	.	.	.	.	.	.
0	.	NA	.	LIPIC	INTF	TRAI	KEG(	.	.	REACTOME_PHOSPHOLIPID_META
0	.	NA	.	.	.	.	.	.	.	.
0	.	NA	.	.	.	.	.	.	.	.
0	.	NA	.	.	.	.	KEG(	.	.	REACTOME_MEIOSIS;REACTOME_(
0	Synpolyda(	NA	.	.	PRO	STRI	.	.	.	.
0	Spinocerel	NA	.	SYS'	CYT(	.	.	.	.	.
0	.	NA	.	EST/	MEM	.	.	.	.	.
0	.	NA	.	SYS'	INTF	.	.	.	.	.
0	.	NA	.	.	.	.	.	.	.	.
0	.	NA	.	CELI	.	NUC	.	.	.	.
0	.	NA	.	CELI	.	NUC	.	.	.	.
0	.	NA	.	.	.	.	.	.	.	REACTOME_NEURONAL_SYSTEM
0	.	NA	.	.	.	.	.	.	.	REACTOME_NEURONAL_SYSTEM
0	.	NA	.	.	.	.	KEG(	.	.	.
0	.	NA	.	.	.	.	.	.	.	.
0	.	NA	.	.	.	.	.	.	.	.
0	.	NA	.	ENZ'	CYT(	KINA	KEG(	.	.	.
0	.	NA	.	BIOS	.	.	KEG(	.	.	.
0	.	NA	.	.	INTF	.	KEG(	.	.	.
0	Chondrody	NA	.	SYS'	.	ARYI	.	.	.	REACTOME_PTM_GAMMA_CARBOX
0	.	NA	.	.	.	.	KEG(	.	.	.
0	.	NA	.	.	.	.	.	.	.	.
0	.	NA	.	.	.	.	.	.	.	.
0	.	NA	.	CELI	EXTI	INTE	.	.	.	.
0	Cataract 4(	NA	.	.	.	.	.	.	.	.
0	.	NA	.	.	.	.	.	.	.	.
0	.	NA	.	.	.	.	.	.	.	.
0	Epileptic er	NA	.	.	.	.	.	.	.	.
0	.	NA	.	.	.	.	.	.	.	.
0	.	NA	.	.	.	.	.	.	.	.
0	Mental reta	NA	.	BEH.	.	.	.	.	.	.
0	.	NA	.	.	.	.	.	.	.	.
0	Glycerol kii	NA	.	.	.	.	KEG(	.	.	REACTOME_TRIGLYCERIDE_BIOSY
0	Glycerol kii	NA	.	.	.	.	KEG(	.	.	REACTOME_TRIGLYCERIDE_BIOSY
0	Becker mu	NA	.	BIOS	PRO	ACTI	KEG(	.	BIO	REACTOME_STRIATED_MUSCLE_C
0	.	NA	.	.	.	.	.	.	.	.
0	.	NA	.	.	CELI	.	.	.	.	.
0	.	NA	.	.	.	.	.	.	.	.

0	.	NA	.	.	.	.	.	.	.	.
0	.	NA	.	.	.	.	.	.	.	.
0	.	NA	.	.	.	.	.	.	.	.
0	Renpennin	NA	.	RNA	NUC	TRA	KEG	.	.	.
0	.	NA	.	.	.	.	KEG	.	.	REACTOME_NEGATIVE_REGULATC
0	Neurodege	NA	.	.	.	.	.	.	.	.
0	.	NA	.	.	.	.	.	.	.	.
0	.	NA	.	.	.	.	.	.	.	.
0	Aland Islar	NA	.	DET	INTF	CAT	KEG	.	.	.
0	.	NA	.	SIGN	NON	KINA	.	.	.	.
0	.	NA	.	CELI	MAC	.	KEG	PID	.	REACTOME_SIGNALLING_BY_NGF;
0	.	NA	.	.	.	.	.	.	.	.
0	Mental reta	NA	.	.	.	.	.	.	.	.
0	Mental reta	NA	.	.	.	.	KEG	.	.	.
0	.	NA	.	DNA	ORG	PRO	KEG	PID	.	REACTOME_MEIOSIS;REACTOME_
0	Mental reta	NA	.	PRO	NUC	ACID	KEG	PID	.	REACTOME_IMMUNE_SYSTEM;REA
0	.	NA	.	.	.	.	.	.	.	.
0	Aarskog-S	NA	.	REG	CYT	GTP	KEG	PID	.	REACTOME_SIGNALING_BY_RHO_
0	.	NA	.	.	.	.	.	.	.	.
0	.	NA	.	.	.	.	.	.	.	.
0	.	NA	.	.	.	.	.	.	.	.
0	.	NA	.	.	.	.	.	.	.	.
0	.	NA	.	.	.	.	.	.	.	.
0	.	NA	.	.	.	.	.	.	.	.
0	.	NA	.	.	.	.	.	.	.	.
0	.	NA	.	.	.	.	.	.	.	.
0	.	NA	.	.	.	.	.	.	.	.
0	.	NA	.	.	.	.	.	.	.	.
0	.	NA	.	.	.	.	.	.	.	.
0	.	NA	.	.	.	.	.	.	.	.
0	.	NA	.	.	.	.	.	.	.	.
0	.	NA	.	.	.	.	.	.	.	REACTOME_SIGNALING_BY_RHO_
0	.	NA	.	.	.	.	.	.	.	.
0	.	NA	.	SIGN	CYT	.	.	PID	.	.
0	Lujan-Fryn	NA	.	RNA	NUC	RNA	.	PID	.	REACTOME_DEVELOPMENTAL_BIC
0	Charcot-M	NA	CM9	SYS	CELI	.	.	.	.	REACTOME_MEMBRANE_TRAFFICK
0	.	NA	.	.	.	.	.	.	.	.
0	.	NA	.	.	.	.	.	.	.	.
0	Cornelia de	NA	.	RNA	NUC	HYD	.	PID	BIO	REACTOME_NOTCH1_INTRACELLU
0	Muscle gly	NA	.	GEN	.	PHO	KEG	.	.	REACTOME_GLYCOGEN_BREAKDC
0	.	NA	.	.	.	.	.	PID	.	.
0	.	NA	.	.	.	.	.	.	.	.
0	.	NA	.	.	.	.	.	.	.	.
0	?Mental re	NA	.	.	.	.	.	.	.	.
0	.	NA	.	.	.	.	.	.	.	.
0	Mental reta	NA	.	.	.	.	.	.	.	.
0	.	NA	.	.	INTF	.	.	.	.	.
0	.	NA	.	.	INTF	.	.	.	.	.
0	.	NA	.	.	INTF	.	.	.	.	.
0	.	NA	.	.	INTF	.	.	.	.	.
0	.	NA	.	.	.	.	.	.	.	.
0	.	NA	.	EST	.	VOL	KEG	PID	BIO	.
0	.	NA	.	EST	CYT	RNA	.	.	.	.
0	.	NA	.	.	.	.	.	.	.	.

0	.	NA	.	SYS	.	.	.	.	.	.
0	Mental reta	NA	.	BEH	.	LIGA	KEG	.	.	REACTOME_TRIGLYCERIDE_BIOSY
0	.	NA	.	.	.	.	.	.	.	.
0	.	NA	.	.	.	.	.	.	.	.
0	.	NA	.	.	.	.	.	.	.	.
0	Mental reta	NA	.	.	.	.	KEG	.	.	.
0	.	NA	.	.	.	.	.	.	.	.
0	.	NA	.	.	.	.	.	.	.	.
0	.	NA	.	.	.	.	.	.	.	.
0	Simpson-G	NA	.	ANA	INTR	.	.	.	.	REACTOME_HS_GAG_DEGRADATIO
0	Mental reta	NA	.	.	ORG	CATI	KEG	.	.	REACTOME_TRANSMEMBRANE_TR
0	.	NA	.	.	INTR	G_PI	.	.	.	.
0	.	NA	.	.	.	.	.	.	.	REACTOME_TRANSMEMBRANE_TR
0	.	NA	.	NEG	NUC	.	.	.	.	.
0	.	NA	.	.	.	.	.	.	.	.
0	.	NA	.	.	.	.	.	.	.	.
0	.	NA	.	.	.	.	.	.	.	.
0	.	NA	.	.	.	.	.	.	.	.
0	.	NA	.	.	MEM	.	.	PID	.	REACTOME_CS_DS_DEGRADATION
0	Spinocereb	NA	.	.	MEM	CATI	KEG	.	.	REACTOME_TRANSMEMBRANE_TR
0	.	NA	.	.	.	.	.	.	.	REACTOME_TRANSLATION;REACT
0	CRASH sy	NA	.	SYS	.	.	KEG	PID	BIO	REACTOME_DEVELOPMENTAL_BIO
0	CRASH sy	NA	.	SYS	.	.	KEG	PID	BIO	REACTOME_DEVELOPMENTAL_BIO
0	.	NA	.	REG	.	PRO	KEG	.	.	.
0	Mental reta	NA	.	RNA	CYT	IDEN	.	.	.	.
0	.	NA	.	.	.	.	.	.	.	.
0	Favism;He	NA	.	GLU	CYT	.	KEG	.	.	REACTOME_METABOLISM_OF_CAR
0	Dyskeratos	NA	.	DNA	NUC	NUC	.	PID	.	REACTOME_CELL_CYCLE;REACTO
0	.	NA	.	SIGN	INTR	PHO	.	.	.	.
0	.	NA	.	.	.	.	.	.	.	.
0	Epsilon-trir	NA	.	.	.	.	KEG	.	.	REACTOME_METABOLISM_OF_AMI
0	.	NA	.	.	.	.	.	.	.	.
0	.	NA	.	.	.	.	.	.	.	.
0	.	NA	.	.	.	.	.	.	.	.
0	.	NA	.	.	.	.	.	.	.	.
0	.	NA	.	ESTA	ORG	.	.	.	.	.
0	.	NA	.	.	.	.	.	.	.	.
0	.	NA	.	.	.	.	.	.	.	.
0	.	NA	.	.	.	.	.	.	.	.
0	.	NA	.	MAC	CYT	RNA	KEG	.	BIO	REACTOME_ANTIVIRAL_MECHANIS
0	.	NA	.	.	.	.	.	.	.	.
0	.	NA	.	.	.	.	.	.	.	.
0	.	NA	.	.	.	.	.	.	.	.
0	.	NA	.	.	.	.	.	.	.	.
0	?Deafness	NA	.	.	.	.	.	.	.	.
0	.	NA	.	.	.	.	.	.	.	.
0	.	NA	.	.	.	.	KEG	PID	.	.
0	.	NA	.	.	.	.	KEG	.	.	.
0	.	NA	.	RES	NUC	PRO	.	PID	.	.
0	.	NA	.	.	.	.	.	.	.	.
0	.	NA	.	.	.	.	.	.	.	.
0	.	NA	.	.	.	.	.	.	.	.
0	.	NA	.	.	.	.	.	.	.	.
0	.	NA	.	.	.	.	.	.	.	.

0	.	NA	.	EST/	COA	.	KEG(	PID	BIO	.
0	.	NA	.	.	.	.	.	.	.	.
0	.	NA	.	.	.	TRAI	.	PID	.	.
0	.	NA	.	.	.	.	.	.	.	.
0	.	NA	.	EST/	ORG	.	.	.	.	.
0	.	NA	.	.	.	.	KEG(	.	.	.
0	.	NA	.	.	.	.	.	.	.	.
0	.	NA	.	.	.	.	.	.	.	.
0	.	NA	.	.	.	.	.	.	.	.
0	Urofacial s	NA	.	.	.	HYD	KEG(	.	.	REACTOME_HS_GAG_DEGRADATI(
0	.	NA	.	EST/	ORG	.	.	PID	.	REACTOME_CELL_CYCLE;REACTO
0	.	NA	.	.	EXTI	CAR	KEG(	.	.	.
0	.	NA	.	.	.	.	.	.	.	.
0	.	NA	.	DNA	CYT(	.	.	.	.	.
0	.	NA	.	SYS	INTF	REC	KEG(	PID	.	REACTOME_ACTIVATED_NOTCH1_
0	.	NA	.	.	.	.	.	.	.	.
0	.	NA	.	.	.	.	.	.	.	.
0	.	NA	.	.	.	.	.	.	.	.
0	.	NA	.	.	.	.	.	.	.	.
0	.	NA	.	.	.	.	.	.	.	.
0	.	NA	.	MICF	ORG	MICF	.	PID	.	REACTOME_CELL_CYCLE;REACTO
0	Immunode	NA	.	.	.	.	KEG(	.	.	.
0	.	NA	.	DNA	NUC	PRO	.	.	.	.
0	.	NA	.	.	.	.	.	.	.	.
0	.	NA	.	.	.	.	.	.	.	.
0	.	NA	.	.	.	.	.	.	.	.
0	.	NA	.	SIGN	INTF	TRAI	.	PID	.	.
0	.	NA	.	.	.	.	.	.	.	.
0	.	NA	.	.	.	.	.	.	.	.
0	Becker mu	NA	.	BIOS	PRO	ACTI	KEG(	.	BIO	REACTOME_STRIATED_MUSCLE_C
0	.	NA	.	.	.	.	.	.	.	.
0	Megakaryc	NA	.	.	.	.	.	PID	BIO	.
0	.	NA	.	.	.	.	.	.	.	.
0	.	NA	.	MUL	PRO	INTE	.	PID	.	REACTOME_EXTRACELLULAR_MA
0	{Bardet-Bie	NA	.	.	.	.	.	.	.	.
0	.	NA	.	CEN	ORG	.	.	.	.	.
0	.	NA	.	.	.	.	.	.	.	.
0	.	NA	.	.	.	.	.	.	.	.
0	.	NA	.	.	.	.	.	.	.	.
0	.	NA	.	.	.	.	.	.	.	.
0	.	NA	.	.	.	.	.	.	.	.
0	.	NA	.	.	.	.	.	.	.	.
0	Elliptocytos	NA	.	ACTI	ORG	ACTI	.	.	.	REACTOME_DEVELOPMENTAL_BIC
0	[Blood grou	NA	.	.	MEM	.	.	.	.	REACTOME_SIGNALING_BY_GPCR
0	.	NA	.	.	.	.	.	.	.	.
0	.	NA	.	.	.	.	.	.	.	.
0	.	NA	.	.	.	.	.	.	.	.
0	Nephropatl	NA	.	.	.	.	.	.	.	.
0	.	NA	.	.	.	.	KEG(	PID	.	REACTOME_SIGNALING_BY_GPCR
0	.	NA	.	PRO	.	PRO	.	.	.	.
0	Homocystil	NA	.	.	.	.	KEG(	.	.	REACTOME_SULFUR_AMINO_ACID
0	.	NA	.	.	.	.	KEG(	.	.	.
0	.	NA	.	.	.	.	KEG(	.	.	.
0	Sitosterole	NA	.	.	.	PRO	KEG(	.	.	REACTOME_ABCA_TRANSPORTER

0	.	NA	.	.	.	.	.	.	.	.
0	.	NA	.	.	.	.	.	.	.	.
0	.	NA	.	.	.	.	.	.	.	REACTOME_TRANSMEMBRANE_TR
0	Leukodystr	NA	.	CELL	ORG	CHA	KEGG	PID	.	REACTOME_MITOCHONDRIAL_PRO
0	.	NA	.	RNA	.	CATI	.	.	.	.
0	.	NA	.	.	CYT	LIPID	.	.	.	.
0	.	NA	.	.	.	.	.	.	.	.
0	.	NA	.	SIGN	.	IDEN	.	.	.	.
0	.	NA	.	RNA	NUC	SPE	.	.	.	.
0	.	NA	.	.	.	.	.	.	.	.
0	Seckel syn	NA	.	.	ORG	.	.	.	.	REACTOME_CELL_CYCLE;REACTO
0	Seckel syn	NA	.	.	ORG	.	.	.	.	REACTOME_CELL_CYCLE;REACTO
0	.	NA	.	.	.	.	.	.	.	.
0	.	NA	.	.	ORG	.	.	.	.	.
0	.	NA	.	POS	INTR	.	KEGG	PID	BIO	REACTOME_EXTRINSIC_PATHWAY
0	Breast can	NA	CM1	.	.	PHO	KEGG	PID	BIO	REACTOME_SIGNALLING_BY_NGF;
0	.	NA	.	.	.	.	.	.	.	.
0	.	NA	.	.	.	.	.	.	.	.
0	Adermatog	NA	.	DNA	.	.	.	.	.	.
0	Spastic pa	NA	.	.	.	.	KEGG	.	.	REACTOME_BIOLOGICAL_OXIDATI
0	.	NA	.	RNA	NUC	TRAI	.	.	.	.
0	.	NA	.	SIGN	.	PHO	KEGG	.	.	REACTOME_SYNTHESIS_OF_PIPS_
0	.	NA	.	.	.	.	.	.	.	.
0	Albinism, c	NA	.	.	.	.	.	.	.	.
0	.	NA	.	.	.	.	.	.	.	.
0	.	NA	.	REG	INTR	PEPT	KEGG	PID	BIO	REACTOME_GASTRIN_CREB_SIGN
0	.	NA	.	.	.	.	.	.	.	.
0	.	NA	.	.	.	.	.	.	.	.
0	{Breast car	NA	.	.	.	.	KEGG	.	.	REACTOME_HYALURONAN_UPTAK
0	.	NA	.	SIGN	.	.	.	.	.	.
0	.	NA	.	.	.	.	.	.	.	.
0	.	NA	.	.	.	.	.	.	.	.
0	.	NA	.	.	INTR	REC	.	.	.	.
0	{Hypersens	NA	.	.	.	.	KEGG	PID	BIO	REACTOME_NEF_MEDIATED_DOW
0	.	NA	.	.	.	.	.	.	.	.
0	Retinitis pi	NA	.	.	.	.	.	.	.	.
0	Retinitis pi	NA	.	.	.	.	.	.	.	.
0	.	NA	.	.	.	.	.	.	.	.
0	.	NA	.	M_P	ORG	.	.	.	.	.
0	.	NA	.	.	.	.	.	.	.	.
0	.	NA	.	.	.	.	.	.	.	.
0	.	NA	.	.	.	.	.	.	.	.
0	.	NA	.	.	.	.	.	.	.	.
0	.	NA	.	.	.	.	KEGG	.	.	.
0	.	NA	.	EST	CYT	KINA	.	PID	.	.
0	.	NA	.	SIGN	.	RAS	.	PID	.	.
0	.	NA	.	SYST	PRO	.	.	.	.	.
0	.	NA	.	.	.	.	.	.	.	.
0	Epidermoly	NA	.	.	.	STRI	.	.	.	REACTOME_APOPTOTIC_CLEAVAG
0	.	NA	.	.	.	.	.	.	.	.
0	.	NA	.	.	.	.	.	.	.	.
0	.	NA	.	.	NUC	.	.	.	.	.
0	.	NA	.	.	.	.	.	.	.	.
0	.	NA	.	.	.	.	.	.	.	.

0	.	NA	.	.	.	.	.	.	.	.	
0	.	NA	.	.	.	PHO	KEG(	.	BIO	REACTOME_GLYCOLYSIS;REACTO	
0	.	NA	.	.	.	.	.	.	.	.	
0	.	NA	.	.	.	.	.	.	.	.	
0	.	NA	.	.	.	.	.	.	.	.	
0	.	NA	.	.	.	.	.	.	.	.	
0	.	NA	.	REG	.	.	.	.	.	.	
0	.	NA	.	.	.	.	.	.	.	.	
0	.	NA	.	.	.	.	.	.	.	.	
0	.	NA	.	BIOF	.	NUC	.	PID	.	.	
0	.	NA	.	.	.	.	.	.	.	.	
0	.	NA	.	.	.	.	.	.	.	.	
0	.	NA	.	DNA	NUC	.	.	.	.	.	
0	.	NA	.	.	.	.	.	.	.	.	
0	.	NA	.	.	.	RNA	.	.	.	.	
0	Deafness,	NA	.	.	.	.	.	.	.	.	
0	.	NA	.	.	.	.	.	.	.	.	
0	Spermatog	NA	.	SYS	CELL	OXID	KEG(	.	BIO	REACTOME_LATENT_INFECTION_(	
0	.	NA	.	.	.	.	.	.	.	.	
0	.	NA	.	.	.	.	.	.	.	.	
0	Fraser syn	NA	.	.	.	.	.	.	.	.	
0	Fraser syn	NA	.	.	.	.	.	.	.	.	
0	.	NA	.	.	.	.	.	.	.	.	
0	.	NA	.	.	.	.	.	.	.	.	
0	.	NA	.	.	.	.	.	.	.	.	
0	.	NA	.	.	.	.	.	.	.	.	
0	.	NA	.	.	.	.	.	.	.	.	
0	.	NA	.	.	.	.	.	.	.	.	
0	.	NA	.	.	.	.	.	.	.	.	
0	.	NA	.	.	.	.	.	.	.	.	
0	Breast can	NA	.	REG	MEM	PHO	KEG(	PID	BIO	REACTOME_SIGNALLING_BY_NGF;	
0	.	NA	.	.	.	.	.	.	.	.	
0	.	NA	.	.	.	.	.	.	.	.	
0	.	NA	.	.	.	.	.	.	.	.	
0	.	NA	.	.	.	.	.	.	.	.	
0	.	NA	.	.	.	.	.	.	.	.	
0	.	NA	.	.	.	.	.	.	.	.	
0	.	NA	.	.	.	.	.	.	.	.	
0	.	NA	.	.	.	.	.	.	.	.	
0	.	NA	.	.	.	.	.	.	.	.	
0	.	NA	.	.	.	.	.	.	.	.	
0	.	NA	.	.	.	.	.	.	.	.	
0	.	NA	.	.	.	.	.	.	.	.	
0	.	NA	.	.	.	.	.	.	.	.	
0	.	NA	.	.	.	.	.	.	.	.	
0	.	NA	.	.	.	.	.	.	.	.	
0	.	NA	.	.	.	.	.	.	.	.	
0	.	NA	.	.	.	.	.	.	.	.	
0	Night blind	NA	.	.	.	.	.	.	.	.	
0	.	NA	.	.	.	.	.	.	.	.	
0	.	NA	.	.	.	.	.	.	.	.	
0	.	NA	.	.	INTR	.	.	.	.	.	
0	.	NA	.	.	.	.	.	.	.	.	
0	.	NA	.	.	.	.	.	.	.	.	

0	.	NA	.	.	.	.	.	.	.	.
0	.	NA	.	.	.	.	.	.	.	.
0	.	NA	.	.	.	.	.	.	.	.
0	.	NA	.	RNA	ORG	.	KEG(	.	.	REACTOME_MRNA_DECAY_BY_5_T
0	.	NA	.	.	.	.	.	.	.	REACTOME_PROCESSING_OF_CAF
0	.	NA	.	.	ORG	.	.	.	.	REACTOME_GENERIC_TRANSCRIP
0	.	NA	.	.	.	.	.	.	.	.
0	.	NA	.	.	.	.	.	.	.	.
0	.	NA	.	.	.	.	.	.	.	.
0	.	NA	.	.	.	.	.	.	.	.
0	.	NA	.	.	.	.	.	.	.	.
0	.	NA	.	.	.	.	.	.	.	.
0	Spinocereb	NA	.	.	.	.	.	.	.	.
0	.	NA	.	.	.	.	KEG(	.	.	REACTOME_PHOSPHOLIPID_META
0	.	NA	.	.	.	.	.	.	.	.
0	.	NA	.	REG	.	TRAI	KEG(	PID	.	.
0	.	NA	.	.	.	.	.	.	.	.
0	.	NA	.	BIOF	.	.	.	.	.	.
0	.	NA	.	MUL	.	.	.	.	.	.
0	.	NA	.	.	.	.	.	.	.	.
0	.	NA	.	.	.	.	.	.	.	.
0	.	NA	.	.	.	ARYI	.	.	.	REACTOME_PTM_GAMMA_CARBOX
0	Epileptic e	NA	.	.	.	.	.	.	.	.
0	.	NA	.	.	.	.	.	.	.	.
0	.	NA	.	EST/	ORG	MICF	.	.	.	REACTOME_DEVELOPMENTAL_BIC
0	.	NA	.	.	.	.	.	.	.	.
0	.	NA	.	.	.	.	.	.	.	.
0	.	NA	.	.	.	.	.	.	.	.
0	.	NA	.	SIGN	INTF	NEU	KEG(	.	BIO	REACTOME_TRANSMISSION_ACRC
0	.	NA	.	PRO	.	KINA	KEG(	PID	BIO	REACTOME_SIGNALLING_BY_NGF;
0	.	NA	.	.	.	.	.	.	.	.
0	.	NA	.	.	.	.	.	.	.	.
0	.	NA	.	.	.	.	.	.	.	.
0	.	NA	.	.	.	.	.	.	.	.
0	.	NA	.	.	.	.	.	PID	.	REACTOME_EXTRACELLULAR_MAT
0	.	NA	.	.	.	.	.	.	.	REACTOME_CROSS_PRESENTATIC
0	.	NA	.	.	.	.	.	.	.	.
0	.	NA	.	.	ORG	ACTI	.	PID	.	REACTOME_DEVELOPMENTAL_BIC
0	.	NA	.	.	.	.	.	.	.	.
0	.	NA	.	.	.	.	.	.	.	.
0	Holoprosen	NA	.	DNA	.	CATI	KEG(	PID	BIO	.
0	.	NA	.	.	.	.	.	.	.	.
0	.	NA	.	.	.	.	.	PID	.	.
0	.	NA	.	.	.	.	.	.	.	REACTOME_DIABETES_PATHWAYS
0	.	NA	.	.	.	.	.	.	.	.
0	.	NA	.	.	.	.	.	.	.	.
0	.	NA	.	.	.	.	.	.	.	.
0	.	NA	.	.	.	.	.	.	.	.
0	{Essential t	NA	.	SIGN	INTF	AMIN	KEG(	.	.	REACTOME_SIGNALING_BY_GPCR
0	Cone-rod c	NA	.	.	.	HYD	.	.	.	.
0	.	NA	.	.	.	.	.	.	.	.
0	.	NA	.	.	.	.	.	.	.	.
0	.	NA	.	.	EXTI	.	.	.	.	.
0	.	NA	.	.	.	.	.	.	.	.

0	.	NA	.	SYS	INTF	.	.	.	.	.
0	.	NA	.	.	.	.	KEG(	PID	.	REACTOME_RNA_POL_I_TRANSCR
0	.	NA	.	.	.	.	.	.	.	.
0	.	NA	.	.	.	.	.	.	.	.
0	.	NA	.	.	.	.	.	.	.	REACTOME_DEVELOPMENTAL_BIC
0	.	NA	.	.	.	.	.	.	.	.
0	{Colchicine	NA	.	EST/	CELI	NUC	KEG(	PID	BIO	REACTOME_ABACAVIR_TRANSPOF
0	.	NA	.	.	.	.	.	.	.	.
0	Thyroid ca	NA	.	.	ORG	.	.	.	.	REACTOME_CELL_CYCLE;REACTO
0	.	NA	.	SIGN	NON	PRO	KEG(	PID	BIO	REACTOME_APOPTOTIC_CLEAVAG
0	.	NA	.	POS	.	.	KEG(	PID	BIO	REACTOME_EXTRINSIC_PATHWAY
0	.	NA	.	.	.	.	.	.	.	.
0	.	NA	.	.	INTF	.	KEG(	PID	.	REACTOME_INTEGRIN_CELL_SURF
0	.	NA	.	.	.	.	.	.	.	.
0	.	NA	.	.	.	.	.	.	.	.
0	.	NA	.	.	.	.	.	.	.	.
0	.	NA	.	.	.	.	.	.	.	.
0	.	NA	.	.	.	.	.	.	.	.
0	.	NA	.	.	ORG	STRI	.	.	.	.
0	Antley-Bixl	NA	.	.	CELI	.	KEG(	PID	.	REACTOME_NEGATIVE_REGULATI(
0	.	NA	.	RNA	NUC	.	.	.	.	.
0	Deafness,	NA	.	REG	CYT(	.	.	.	BIO	.
0	Deafness,	NA	.	REG	CYT(	.	.	.	BIO	.
0	Leukemia,	NA	.	.	.	.	KEG(	PID	BIO	REACTOME_SIGNALING_BY_RHO_(
0	{Diabetes r	NA	.	NUC	CYT(	.	.	.	.	REACTOME_INTERFERON_GAMMA
0	Bladder ca	NA	.	REG	NUC	KINA	KEG(	PID	BIO	REACTOME_CELL_CYCLE;REACTO
0	.	NA	.	.	.	.	.	.	.	.
0	.	NA	.	.	.	.	.	.	.	.
0	.	NA	.	.	.	.	.	.	.	.
0	.	NA	.	.	.	.	.	.	.	.
0	.	NA	.	.	.	.	.	.	.	.
0	.	NA	.	PRO	VESI	.	KEG(	.	.	REACTOME_DIABETES_PATHWAYS
0	.	NA	.	.	.	.	.	.	.	.
0	.	NA	.	.	.	.	.	.	.	.
0	.	NA	.	.	.	.	.	.	.	.
0	.	NA	.	.	.	.	.	.	.	.
0	.	NA	.	.	.	.	.	.	.	.
0	Spinocereb	NA	.	.	.	.	.	.	.	.
0	.	NA	.	.	INTF	CATI	.	.	.	REACTOME_TRANSMEMBRANE_TF
0	.	NA	.	.	.	.	.	.	.	.
0	.	NA	.	.	.	.	.	.	.	.
0	.	NA	.	.	.	.	.	.	.	.
0	.	NA	.	.	CELI	.	KEG(	.	.	.
0	Episodic at	NA	.	REG	INTF	CATI	KEG(	.	.	REACTOME_TRANSMISSION_ACRC
0	.	NA	.	.	.	.	KEG(	.	.	REACTOME_SIGNALING_BY_GPCR
0	.	NA	.	.	.	.	.	.	.	.
0	.	NA	.	SYS	PRO	.	.	.	.	.
0	.	NA	.	EST/	INTF	CATI	.	.	.	REACTOME_NEURONAL_SYSTEM;F
0	.	NA	.	.	.	.	.	.	.	.
0	.	NA	.	.	.	.	.	.	.	.
0	.	NA	.	.	.	.	.	.	.	.
0	.	NA	.	.	.	.	.	.	.	.
0	.	NA	.	CEN	ORG	KINA	.	.	.	REACTOME_CELL_CYCLE;REACTO
0	.	NA	.	.	.	.	.	.	.	REACTOME_TRANSMEMBRANE_TF

0	.	NA	.	BIOF	INTF	PRO	.	.	.	.
0	.	NA	.	BIOF	INTF	PRO	.	.	.	.
0	.	NA	.	EST/	MEM	.	.	.	.	.
0	.	NA	.	PRO	.	KIN/	KEG(	PID	BIO	REACTOME_SIGNALLING_BY_NGF;
0	.	NA	.	.	.	.	.	.	.	.
0	.	NA	.	.	.	.	.	.	.	.
0	.	NA	.	.	.	.	.	.	.	.
0	.	NA	.	.	.	.	.	.	.	.
0	.	NA	.	.	.	.	.	.	.	.
0	.	NA	.	NEG	NUC	TRAI	.	PID	.	REACTOME_RNA_POL_III_TRANSC
0	.	NA	.	.	.	.	.	PID	.	.
0	.	NA	.	.	.	.	.	.	.	.
0	.	NA	.	.	.	.	.	.	.	.
0	.	NA	.	.	.	IDEN	KEG(	PID	BIO	.
0	.	NA	.	.	.	.	.	.	.	.
0	.	NA	.	RNA	.	TRAI	.	PID	.	.
0	.	NA	.	.	.	.	.	.	.	.
0	.	NA	.	EST/	.	.	.	.	.	.
0	.	NA	.	.	.	.	.	.	.	.
0	.	NA	.	.	.	.	.	.	.	.
0	.	NA	.	.	.	.	KEG(	PID	.	REACTOME_SIGNALING_BY_RHO_(
0	Leukoence	NA	.	REG	CYT(	TRAI	.	.	BIO	REACTOME_TRANSLATION;REACT(
0	.	NA	.	.	.	.	.	.	.	.
0	.	NA	.	.	.	.	.	.	.	.
0	.	NA	.	.	ORG	RNA	.	.	.	.
0	.	NA	.	.	.	.	.	.	.	.
0	.	NA	.	.	.	.	.	.	.	.
0	.	NA	.	.	.	.	.	.	.	.
0	.	NA	.	.	.	.	.	.	.	.
0	.	NA	.	.	.	.	.	.	.	REACTOME_CELL_CELL_COMMUN
0	.	NA	.	.	.	.	.	.	.	.
0	.	NA	.	.	.	.	.	.	.	.
0	.	NA	.	.	.	.	KEG(	.	.	.
0	.	NA	.	.	.	.	.	.	.	.
0	.	NA	.	.	.	.	.	.	.	.
0	.	NA	.	.	.	.	.	.	.	.
0	.	NA	.	.	.	.	.	PID	.	.
0	.	NA	.	.	.	.	.	.	.	.
0	.	NA	.	.	.	.	.	.	.	.
0	{Diabetes r	NA	.	EST/	CYT(	KIN/	KEG(	PID	.	.
0	.	NA	.	.	.	.	.	.	.	.
0	.	NA	.	.	.	.	.	.	.	.
0	.	NA	.	.	.	.	.	.	.	.
0	.	NA	.	.	.	.	.	.	.	.
0	.	NA	.	.	.	.	.	.	.	.
0	.	NA	.	.	.	.	.	.	.	.
0	.	NA	.	.	.	.	.	.	.	.
0	Nephronop	NA	.	.	.	.	.	.	.	REACTOME_SIGNALING_BY_HIPPC
0	Nephronop	NA	.	.	.	.	.	.	.	REACTOME_SIGNALING_BY_HIPPC
0	.	NA	.	.	.	.	.	.	.	.
0	.	NA	.	.	.	.	.	.	.	.
0	.	NA	.	.	.	.	.	.	.	.
0	.	NA	.	.	.	.	.	.	.	.
0	.	NA	.	.	.	.	.	.	.	.

0	.	NA	.	.	.	.	.	.	.	.
0	.	NA	.	.	.	.	.	.	.	.
0	.	NA	.	.	.	.	.	.	.	.
0	.	NA	.	.	.	.	.	.	.	.
0	.	NA	.	.	.	.	.	.	.	.
0	.	NA	.	.	.	.	.	.	.	.
0	.	NA	.	SIGN	CYT	.	.	.	.	.
0	.	NA	.	.	.	.	.	.	.	.
0	Noonan sy	NA	CM1	SIGN	MEM	GTP	.	PID	.	REACTOME_SIGNALLING_BY_NGF;
0	.	NA	.	.	.	.	.	.	.	.
0	.	NA	.	CELL	MEM	CATI	KEGG	.	.	.
0	.	NA	.	.	.	.	.	.	.	.
0	.	NA	.	.	.	.	.	.	.	.
0	.	NA	.	.	.	.	.	.	.	.
0	.	NA	.	.	INTR	CATI	.	.	.	REACTOME_TRANSMEMBRANE_TF
0	.	NA	.	.	.	.	.	.	.	.
0	.	NA	.	.	ORG	RNA	.	.	.	.
0	.	NA	.	.	CYT	REC	KEGG	.	.	REACTOME_TRANSLATION;REACT
0	.	NA	.	EST	.	HYD	.	.	.	REACTOME_TRANSMEMBRANE_TF
0	.	NA	.	EST	INTR	NUC	KEGG	.	.	REACTOME_HYALURONAN_METAB
0	.	NA	.	.	.	.	.	.	.	REACTOME_TRANSMEMBRANE_TF
0	.	NA	.	.	.	.	.	.	.	.
0	Sebaceous	NA	.	.	.	SEQ	KEGG	PID	BIO	.
0	.	NA	.	.	.	.	.	.	.	.
0	.	NA	.	SIGN	.	.	KEGG	PID	.	REACTOME_VEGF_LIGAND_RECEF
0	.	NA	.	.	.	.	.	.	.	.
0	.	NA	.	.	.	.	.	.	.	.
0	.	NA	.	.	.	.	.	.	.	.
0	Myelodysp	NA	.	.	.	.	KEGG	.	.	REACTOME_TRIGLYCERIDE_BIOSY
0	.	NA	.	.	.	.	.	.	.	.
0	.	NA	.	.	.	.	KEGG	.	.	.
0	.	NA	.	.	.	.	KEGG	PID	.	REACTOME_DEVELOPMENTAL_BIC
0	.	NA	.	.	.	.	.	.	.	.
0	.	NA	.	SYST	.	.	.	.	.	.
0	.	NA	.	.	.	.	.	.	.	.
0	{Asthma, s	NA	.	DEFI	.	REC	KEGG	.	.	REACTOME_ANTIGEN_PROCESSIN
0	.	NA	.	.	.	.	.	.	.	.
0	Deafness,	NA	.	.	.	.	KEGG	PID	.	REACTOME_EXTRACELLULAR_MAT
0	.	NA	.	.	.	.	.	.	.	.
0	.	NA	.	.	.	.	.	.	.	.
0	.	NA	.	.	.	.	.	.	.	.
0	.	NA	.	.	.	.	KEGG	.	BIO	REACTOME_PHOSPHOLIPID_META
0	.	NA	.	.	.	.	.	.	.	.
0	.	NA	.	.	.	.	.	.	.	.
0	.	NA	.	SYST	.	.	.	.	.	.
0	.	NA	.	.	.	.	.	.	.	.
0	.	NA	.	.	.	.	.	.	.	.
0	.	NA	.	.	.	.	.	.	.	.
0	.	NA	.	.	.	.	.	.	.	.
0	.	NA	.	RNA	NUC	.	.	.	.	.
0	.	NA	.	.	.	.	.	.	.	.
0	.	NA	.	.	.	.	.	.	.	.
0	.	NA	.	.	.	.	.	.	.	.
0	Brachydac	NA	.	SIGN	INTR	TRAI	.	PID	.	.

0	.	NA	.	.	.	.	.	.	.	.
0	Mental reta	NA	.	OLIG	INTR	CATI	KEGG	.	.	REACTOME_METABOLISM_OF_PRO
0	{AIDS, resi	NA	.	REG	.	G_PI	KEGG	PID	BIO	REACTOME_SIGNALING_BY_ERBB4
0	.	NA	.	.	.	.	.	.	.	.
0	.	NA	.	.	.	.	.	.	.	.
0	{Migraine,	NA	.	.	.	.	.	.	.	REACTOME_NEURONAL_SYSTEM;F
0	.	NA	.	SIGN	.	REC	KEGG	PID	.	.
0	.	NA	.	.	.	.	.	.	.	.
0	.	NA	.	REG	EXTI	COF	.	.	.	.
0	.	NA	.	.	.	.	.	.	.	.
0	Diabetes m	NA	.	ESTA	INTR	.	KEGG	PID	.	REACTOME_NEURONAL_SYSTEM;F
0	.	NA	.	NEG	.	.	.	.	.	.
0	.	NA	.	.	.	.	KEGG	.	.	REACTOME_ACTIVATED_NOTCH1_
0	.	NA	.	.	.	.	.	.	.	.
0	.	NA	.	RNA	.	TRAI	.	.	.	.
0	.	NA	.	CELI	RIBO	HYD	.	.	.	.
0	.	NA	.	BIOS	CYT	.	.	PID	.	.
0	.	NA	.	.	.	.	.	.	.	.
0	.	NA	.	.	.	.	.	.	.	.
0	Hawkinsinu	NA	.	CAR	.	.	KEGG	.	.	REACTOME_METABOLISM_OF_AMI
0	.	NA	.	.	.	.	.	.	.	.
0	.	NA	.	.	.	.	.	.	.	.
0	.	NA	.	.	.	.	.	.	.	.
0	.	NA	.	.	CYT	.	KEGG	.	.	REACTOME_PHOSPHOLIPID_META
0	.	NA	.	.	.	.	.	.	.	.
0	Fraser syn	NA	.	.	.	.	.	.	.	.
0	.	NA	.	.	.	.	.	PID	.	.
0	.	NA	.	.	.	.	.	.	.	.
0	.	NA	.	BIOF	INTR	PHO	.	PID	.	REACTOME_SPHINGOLIPID_DE_NO
0	Nemaline r	NA	.	.	.	.	KEGG	PID	.	.
0	.	NA	.	.	.	.	.	.	.	.
0	.	NA	.	.	.	.	.	.	.	.
0	.	NA	.	.	CYT	PRO	KEGG	.	BIO	REACTOME_SIGNALING_BY_WNT;F
0	.	NA	.	.	.	.	.	.	.	.
0	.	NA	.	.	.	.	.	.	.	.
0	.	NA	.	.	.	.	.	.	.	.
0	.	NA	.	.	.	.	.	.	.	.
0	.	NA	.	.	.	.	.	.	.	.
0	.	NA	.	.	.	.	.	.	.	.
0	.	NA	.	REG	.	PRO	.	.	.	.
0	.	NA	.	.	.	.	.	.	.	.
0	.	NA	.	.	.	.	.	.	.	.
0	.	NA	.	ESTA	INTR	HYD	KEGG	.	.	.
0	.	NA	.	.	.	.	KEGG	.	.	.
0	.	NA	.	ESTA	.	PRO	.	.	.	.
0	Glycogen s	NA	.	GLU	.	LYAS	KEGG	PID	BIO	REACTOME_RESPONSE_TO_ELEVA
0	.	NA	.	.	.	.	.	.	.	.
0	.	NA	.	.	.	.	.	.	.	.
0	.	NA	.	.	.	.	.	.	.	.
0	.	NA	.	.	.	.	.	.	.	.
0	Ciliary dysk	NA	.	.	.	.	.	.	.	.
0	.	NA	.	.	.	.	.	.	.	.
0	.	NA	.	.	.	.	.	PID	.	.
0	.	NA	.	.	.	.	.	.	.	.

0	.	NA	.	.	.	.	.	.	.	.
0	.	NA	.	CELI	.	HYD	.	.	.	.
0	Weaver sy	NA	.	ANA	.	CHR	.	.	.	.
0	Mandibulo	NA	.	RNA	ORG	HYD	KEG(	.	.	REACTOME_PROCESSING_OF_CAF
0	.	NA	.	REG	ORG	NUC	KEG(	.	.	REACTOME_DIABETES_PATHWAYS
0	.	NA	.	.	INTF	CATI	KEG(	.	.	REACTOME_TRANSMISSION_ACRO
0	.	NA	.	RNA	.	RNA	.	.	.	.
0	.	NA	.	EST/	CYT	.	.	.	.	.
0	.	NA	.	DNA	CYT	PRO	.	.	.	.
0	.	NA	.	.	.	.	.	.	.	.
0	.	NA	.	.	.	.	.	.	.	.
0	.	NA	.	.	.	.	.	.	.	.
0	.	NA	.	STEI	CYT	SULI	KEG(	.	.	REACTOME_BIOLOGICAL_OXIDATI(
0	.	NA	.	.	.	.	.	.	.	.
0	.	NA	.	.	INTF	.	.	.	.	.
0	.	NA	.	.	.	.	.	.	.	.
0	.	NA	.	.	.	.	.	.	.	.
0	.	NA	.	.	.	.	.	.	.	.
0	.	NA	.	EST/	INTF	CATI	.	.	.	.
0	.	NA	.	.	.	.	.	.	.	.
0	.	NA	.	.	.	.	.	.	.	.
0	.	NA	.	.	.	.	.	.	.	.
0	.	NA	.	.	.	.	.	.	.	.
0	.	NA	.	DNA	SYN	HYD	.	.	.	REACTOME_TRANSMISSION_ACRO
0	.	NA	.	.	.	.	.	.	.	.
0	.	NA	.	.	.	.	.	.	.	.
0	.	NA	.	.	.	.	.	.	.	.
0	.	NA	.	EST/	ORG	.	.	.	.	.
0	.	NA	.	.	.	.	.	.	.	.
0	.	NA	.	.	.	.	.	.	.	.
0	Auriculoco	NA	.	REG	.	HYD	KEG(	PID	.	REACTOME_TRANSMISSION_ACRO
0	.	NA	.	.	.	.	.	.	.	.
0	.	NA	.	.	.	.	.	.	.	.
0	.	NA	.	.	.	.	.	.	.	.
0	.	NA	.	.	.	.	.	.	.	.
0	.	NA	.	.	.	.	.	.	.	.
0	.	NA	.	.	.	.	.	.	.	.
0	.	NA	.	CELI	CELI	LIPI	.	.	.	.
0	.	NA	.	.	.	.	.	.	.	.
0	Prostate ca	NA	.	.	.	RNA	.	.	.	REACTOME_INTERFERON_ALPHA_
0	{Macular d	NA	.	.	.	.	.	.	.	.
0	.	NA	.	MUS	ORG	PRO	.	PID	.	.
0	.	NA	.	PRO	.	PRO	.	.	.	.
0	.	NA	.	.	.	CATI	KEG(	.	.	REACTOME_TCA_CYCLE_AND_RES
0	.	NA	.	.	.	.	.	.	.	.
0	.	NA	.	.	.	.	.	.	.	.
0	.	NA	.	.	.	.	KEG(	.	.	REACTOME_INHIBITION_OF_THE_F
0	.	NA	.	.	.	.	.	.	.	.
0	.	NA	.	.	.	.	.	.	.	.
0	.	NA	.	.	INTF	G_PI	.	.	.	.
0	.	NA	.	.	.	.	.	.	.	.
0	.	NA	.	.	.	.	.	.	.	.
0	Membrano	NA	.	CELI	INTF	MET	KEG(	.	.	.
0	.	NA	.	.	.	.	.	.	.	.

0	.	NA	.	.	.	.	KEG(	PID	BIO	REACTOME_SIGNALING_BY_GPCR
0	Myelodysp	NA	.	.	.	.	.	.	.	.
0	Cornelia de	NA	.	.	.	.	.	.	.	.
0	.	NA	.	.	.	.	.	.	.	REACTOME_TRANSMEMBRANE_TF
0	.	NA	.	.	.	.	.	.	.	.
0	.	NA	.	.	.	.	.	.	.	.
0	.	NA	.	.	.	.	.	.	.	.
0	Cardiomyo	NA	.	SYS	NUC	.	.	.	.	.
0	{H. pylori ir	NA	.	SYS	INTF	PRO	KEG(	.	.	.
0	.	NA	.	.	.	.	.	PID	.	.
0	.	NA	.	.	.	.	.	.	.	.
0	.	NA	.	RNA	NUC	.	.	.	.	.
0	.	NA	.	.	.	.	.	.	.	.
0	.	NA	.	.	.	.	.	.	.	.
0	Erythrocytc	NA	.	SYS	.	PRO	KEG(	PID	BIO	REACTOME_SIGNALING_BY_SCF_K
0	.	NA	.	GEN	CYT(	NUC	.	.	.	.
0	.	NA	.	.	INTF	.	KEG(	.	.	REACTOME_HS_GAG_BIOSYNTHES
0	.	NA	.	.	.	.	KEG(	.	.	.
0	.	NA	.	RNA	ORG	.	.	.	.	.
0	.	NA	.	.	NUC	TRAI	.	.	.	.
0	.	NA	.	.	.	.	.	.	.	.
0	.	NA	.	.	.	.	.	.	.	.
0	.	NA	.	.	.	.	.	.	.	.
0	.	NA	.	.	.	.	.	.	.	.
0	.	NA	.	PRO	.	ACID	KEG(	PID	BIO	REACTOME_SIGNALLING_BY_NGF;
0	.	NA	.	RNA	NUC	RNA	KEG(	.	.	.
0	.	NA	.	SYS	CELI	PRO	.	.	.	REACTOME_CELL_CELL_COMMUN
0	.	NA	.	.	INTF	.	.	.	.	.
0	.	NA	.	.	.	.	.	.	.	.
0	Hypertrigly	NA	.	.	.	OXID	KEG(	.	.	REACTOME_TRIGLYCERIDE_BIOSY
0	.	NA	.	.	.	.	.	.	.	.
0	.	NA	.	.	.	.	.	.	.	.
0	.	NA	.	SIGN	INTF	.	.	.	.	REACTOME_SIGNALING_BY_GPCR
0	.	NA	.	.	INTF	.	.	.	.	REACTOME_HS_GAG_DEGRADATI(
0	.	NA	.	.	.	.	.	.	.	.
0	.	NA	.	.	.	.	.	.	.	.
0	Prader-Wil	NA	.	NEG	.	.	.	PID	.	.
0	.	NA	.	.	.	.	.	.	.	.
0	.	NA	.	.	.	.	.	PID	.	.
0	.	NA	.	.	.	.	.	.	.	.
0	.	NA	.	.	.	.	.	.	.	.
0	.	NA	.	.	INTF	MET	KEG(	PID	BIO	.
0	.	NA	.	.	ORG	PRO	.	.	.	.
0	.	NA	.	.	.	.	.	.	.	.
0	.	NA	.	.	.	MOT	KEG(	.	.	.
0	.	NA	.	.	.	.	.	.	.	.
0	.	NA	.	.	.	.	.	.	.	.
0	.	NA	.	.	.	.	.	.	.	.
0	Mannosidc	NA	.	GLY(	.	HYD	KEG(	.	.	.
0	Cold-induc	NA	.	.	EXTI	REC	.	.	.	.
0	.	NA	.	.	.	.	.	.	.	.
0	.	NA	.	.	.	.	.	.	.	.
0	.	NA	.	SYS	.	NUC	KEG(	PID	BIO	.
0	.	NA	.	.	NUC	.	KEG(	.	.	.

0	.	NA	.	.	.	.	.	.	.	.
0	.	NA	.	SEN	.	.	.	.	.	.
0	.	NA	.	SEN	.	.	.	.	.	.
0	.	NA	.	.	.	.	.	.	.	.
0	.	NA	.	.	.	.	.	.	.	.
0	.	NA	.	.	.	.	.	.	.	.
0	Duane-rad	NA	.	.	.	.	.	PID	.	.
0	.	NA	.	.	.	.	.	.	.	.
0	.	NA	.	PRO	PRO	INTE	.	.	.	.
0	.	NA	.	.	.	.	.	.	.	.
0	.	NA	.	.	.	.	.	.	.	.
0	.	NA	.	.	.	.	KEG	PID	.	REACTOME_CELL_CELL_COMMUN
0	.	NA	.	.	.	.	.	.	.	.
0	.	NA	.	.	.	.	.	.	.	.
0	Dent disea	NA	.	LIPI	ORG	PHO	KEG	.	.	REACTOME_SIGNALING_BY_RHO_
0	.	NA	.	.	INTR	G_PI	.	.	.	.
0	.	NA	.	.	.	.	.	.	.	.
0	Hemophilia	NA	CM0	REG	.	.	KEG	.	BIO	REACTOME_RESPONSE_TO_ELEV
0	.	NA	.	.	.	.	.	.	.	.
0	.	NA	.	.	.	.	.	.	.	.
0	.	NA	.	.	.	.	.	.	.	.
0	.	NA	.	.	.	.	.	.	.	.
0	.	NA	.	.	.	.	.	.	.	.
0	.	NA	.	EST	.	.	KEG	.	.	.
0	.	NA	.	.	.	.	.	.	.	.
0	.	NA	.	.	.	.	.	.	.	.
0	.	NA	.	.	.	.	.	.	.	.
0	.	NA	.	.	.	.	.	.	.	.
0	.	NA	.	.	.	.	.	.	.	.
0	.	NA	.	.	.	.	.	.	.	.
0	.	NA	.	.	.	.	.	.	.	.
0	.	NA	.	.	.	.	.	.	.	.
0	.	NA	.	.	.	TRAI	KEG	PID	.	.
0	.	NA	.	SIGN	EXT	REC	.	.	.	.
0	.	NA	.	.	.	.	.	.	.	.
0	.	NA	.	.	.	.	.	.	.	.
0	.	NA	.	.	.	.	.	.	.	.
0	.	NA	.	.	.	.	.	.	.	.
0	.	NA	.	.	.	.	.	.	.	.
0	Deafness,	NA	.	REG	NON	.	.	.	.	.
0	Albinism, c	NA	.	.	.	.	.	.	.	.
0	Pulmonary	NA	.	.	.	.	.	PID	.	.
0	.	NA	.	MUL	ORG	REC	.	.	.	.
0	.	NA	.	.	.	.	.	.	.	.
0	.	NA	.	.	.	.	.	.	.	.
0	.	NA	.	.	.	.	.	.	.	.
0	.	NA	.	.	.	.	.	.	.	.
0	.	NA	.	.	.	.	.	.	.	.
0	.	NA	.	.	.	.	.	.	.	.
0	.	NA	.	.	.	.	.	.	.	.
0	.	NA	.	GAM	CYT	.	.	.	.	.
0	.	NA	.	.	.	.	.	.	.	.
0	.	NA	.	.	.	.	.	.	.	.
0	.	NA	.	.	.	.	.	.	.	.

0	.	NA	.	.	.	.	.	.	.	.
0	.	NA	.	.	.	.	.	.	.	.
0	.	NA	.	.	.	.	.	.	.	.
0	.	NA	.	.	.	.	.	.	.	.
0	.	NA	.	.	.	.	.	.	.	.
0	.	NA	.	.	.	.	.	.	.	.
0	.	NA	.	.	.	.	.	.	.	.
0	.	NA	.	.	.	.	.	.	.	.
0	.	NA	.	.	.	.	.	.	.	.
0	.	NA	.	.	.	.	.	.	.	.
0	.	NA	.	.	.	.	.	.	.	.
0	.	NA	.	.	ORG	HYD	KEG	.	.	REACTOME_GLYCOSPHINGOLIPID_
0	.	NA	.	.	.	.	.	.	.	.
0	.	NA	.	EST	CYT	NUC	KEG	PID	.	REACTOME_SIGNALLING_BY_NGF;
0	.	NA	.	.	.	.	.	.	.	.
0	.	NA	.	PRO	MEM	.	.	PID	.	.
0	.	NA	.	.	.	.	.	.	.	.
0	.	NA	.	.	.	.	.	.	.	.
0	.	NA	.	DEFI	CYT	.	.	.	.	.
0	.	NA	.	.	.	.	.	.	.	.
0	[LPA defici	NA	.	SYS	.	PRO	.	PID	.	REACTOME_METABOLISM_OF_LIPI
0	.	NA	.	.	.	.	.	.	.	.
0	.	NA	.	.	.	.	.	.	.	.
0	.	NA	.	.	.	.	.	.	.	.
0	.	NA	.	.	.	.	.	.	.	.
0	.	NA	.	.	.	.	.	.	.	.
0	.	NA	.	.	.	.	.	.	.	.
0	.	NA	.	.	.	.	.	.	.	.
0	.	NA	.	REG	EXTI	COF	.	.	.	.
0	.	NA	.	EST	INTR	ACTI	.	.	.	REACTOME_AMINO_ACID_TRANSP
0	.	NA	.	.	.	.	.	.	.	.
0	.	NA	.	.	.	.	.	.	.	.
0	.	NA	.	.	.	RNA	.	.	.	.
0	.	NA	.	.	.	.	.	.	.	.
0	.	NA	.	.	.	.	.	.	.	.
0	.	NA	.	.	.	.	.	.	.	.
0	.	NA	.	.	.	.	KEG	.	.	.
0	.	NA	.	.	.	.	.	.	.	.
0	.	NA	.	ANA	CYT	HYD	.	PID	.	.
0	.	NA	.	.	.	.	.	.	.	.
0	.	NA	.	.	.	.	.	.	.	.
0	.	NA	.	.	.	.	.	.	.	.
0	.	NA	.	.	.	.	.	.	.	.
0	.	NA	.	.	.	.	.	.	.	.
0	.	NA	.	.	.	.	.	.	.	.
0	.	NA	.	.	MEM	.	.	.	.	REACTOME_O_LINKED_GLYCOSYL
0	.	NA	.	.	.	.	.	.	.	.
0	.	NA	.	.	.	.	.	.	.	.
0	.	NA	.	.	.	.	.	.	.	.
0	.	NA	.	.	.	.	.	.	.	.
0	.	NA	.	.	.	.	.	.	.	.
0	.	NA	.	.	.	.	.	.	.	.
0	.	NA	.	.	.	.	.	PID	.	.
0	.	NA	.	.	.	.	.	.	.	.

0	.	NA	.	.	.	.	.	.	.	.
0	.	NA	.	.	.	.	.	.	.	.
0	.	NA	.	.	.	.	.	.	.	.
0	.	NA	.	PRO	ORG	.	.	.	.	.
0	.	NA	.	.	.	.	.	.	.	.
0	.	NA	.	DNA	.	DNA	.	.	.	REACTOME_MEIOSIS;REACTOME_(
0	Alagille syr	NA	.	REG	INTF	SPE(	KEG(	PID	.	REACTOME_PRE_NOTCH_TRANSC
0	.	NA	.	.	.	.	.	.	.	.
0	.	NA	.	.	.	.	.	.	.	.
0	.	NA	.	.	.	.	.	.	.	.
0	.	NA	.	.	.	.	.	.	.	.
0	.	NA	.	.	.	.	.	.	.	.
0	.	NA	.	.	.	.	.	.	.	.
0	.	NA	.	.	.	CATI	.	.	.	.
0	.	NA	.	.	.	.	.	.	.	.
0	.	NA	.	SIGN	.	RAS	.	.	.	.
0	.	NA	.	.	.	.	.	.	.	.
0	.	NA	.	REG	.	.	KEG(	PID	.	REACTOME_REGULATION_OF_COM
0	Cardiomyo	NA	.	MUS	ORG	NUC	KEG(	.	.	.
0	.	NA	.	.	.	.	.	PID	BIO	.
0	Spinocereb	NA	.	.	INTF	CATI	KEG(	.	.	.
0	.	NA	.	.	.	.	.	.	.	.
0	.	NA	.	.	PRO	CATI	.	.	.	.
0	.	NA	.	SYST	INTF	LIPO	.	.	.	.
0	.	NA	.	.	.	.	.	.	.	.
0	.	NA	.	.	.	.	KEG(	PID	.	REACTOME_INTEGRIN_CELL_SURF
0	.	NA	.	.	.	.	.	.	.	.
0	Megalobla:	NA	.	.	.	.	KEG(	PID	BIO	REACTOME_TETRAHYDROBIOPTEF
0	.	NA	.	.	.	.	.	.	.	.
0	.	NA	.	.	.	.	.	.	.	.
0	.	NA	.	.	.	.	.	.	.	.
0	.	NA	.	SIGN	INTF	GAB	KEG(	.	.	REACTOME_TRANSMISSION_ACRO
0	.	NA	.	DNA	NUC	HYD	.	.	.	.
0	.	NA	.	.	.	.	.	.	.	REACTOME_PHOSPHOLIPID_META
0	.	NA	.	.	.	.	KEG(	.	.	REACTOME_PPARA_ACTIVATES_G
0	Muscular d	NA	.	MUS	PRO	STRI	KEG(	PID	BIO	REACTOME_INTEGRIN_CELL_SURF
0	Mental reta	NA	.	.	NUC	DNA	.	.	.	.
0	.	NA	.	SIGN	CELI	PRO	KEG(	PID	.	.
0	Spinocereb	NA	.	RNA	NUC	RNA	KEG(	PID	BIO	REACTOME_RNA_POL_III_TRANSC
0	.	NA	.	.	.	.	.	.	.	.
0	Colorectal	NA	.	DNA	.	SINC	KEG(	PID	.	.
0	Muscular d	NA	.	.	.	.	.	.	.	.
0	.	NA	.	.	.	.	.	.	.	.
0	.	NA	.	.	.	.	.	.	.	.
0	.	NA	.	.	.	.	.	.	.	.
0	.	NA	.	.	.	.	.	.	.	.
0	.	NA	.	.	.	.	.	.	.	.
0	Maturity-or	NA	.	SIGN	.	.	.	PID	.	REACTOME_ANTIGEN_ACTIVATES_
0	.	NA	.	.	.	.	.	.	.	.
0	.	NA	.	.	.	.	.	.	.	.
0	.	NA	.	.	CYT(	ELE(	KEG(	.	.	REACTOME_TCA_CYCLE_AND_RES
0	.	NA	.	SIGN	EXTI	HYD	KEG(	PID	.	REACTOME_GASTRIN_CREB_SIGN
0	.	NA	.	.	.	.	KEG(	.	.	.
0	.	NA	.	REG	UBIQ	ACID	.	.	.	.

0	.	NA	.	.	CYT(	.	KEG(	.	.	.
0	.	NA	.	CELI	MEM	CATI	KEG(	.	.	REACTOME_TRANSMISSION_ACRO
0	Cardiomyo	NA	.	.	.	.	.	.	.	.
0	.	NA	.	.	.	.	.	.	.	.
0	.	NA	.	.	.	.	.	.	.	.
0	.	NA	.	.	NUC	NUC	KEG(	.	.	.
0	.	NA	.	.	.	.	.	.	.	.
0	.	NA	.	.	.	.	.	.	.	.
0	.	NA	.	POS	.	.	KEG(	PID	BIO	.
0	.	NA	.	.	.	.	.	.	.	.
0	.	NA	.	.	.	RAS	.	PID	.	.
0	.	NA	.	.	.	.	.	.	.	.
0	.	NA	.	GLY(	.	TRAI	KEG(	.	.	REACTOME_METABOLISM_OF_PRO
0	.	NA	.	.	.	.	.	.	.	.
0	.	NA	.	.	.	.	.	.	.	.
0	.	NA	.	.	PRO	COL	.	.	.	.
0	Spastic pa	NA	.	.	.	.	.	.	.	.
0	.	NA	.	.	.	.	.	.	.	.
0	.	NA	.	.	.	.	.	.	.	.
0	.	NA	.	.	.	.	.	.	.	.
0	.	NA	.	.	.	.	.	.	.	.
0	.	NA	.	.	.	.	.	.	.	.
0	Congenital	NA	.	REG	ORG	.	.	.	.	.
0	.	NA	.	.	.	.	.	.	.	.
0	.	NA	.	.	.	.	.	.	.	.
0	.	NA	.	.	INTR	.	.	.	.	.
0	.	NA	.	RES	EXTI	G_PI	KEG(	PID	BIO	REACTOME_SIGNALING_BY_GPCR
0	.	NA	.	.	.	.	.	.	.	.
0	.	NA	.	.	.	.	.	.	.	.
0	.	NA	.	.	.	.	KEG(	PID	.	REACTOME_GAP_JUNCTION_DEGR
0	.	NA	.	.	.	.	.	.	.	.
0	Amyotroph	NA	.	PRO	ORG	HYD	.	.	.	.
0	46XY sex r	NA	.	.	.	.	.	.	.	.
0	.	NA	.	PRO	ORG	CHA	.	.	.	REACTOME_PROTEIN_FOLDING;RE
0	.	NA	.	.	.	.	.	.	.	.
0	.	NA	.	.	.	.	.	.	.	.
0	.	NA	.	.	.	GLU	KEG(	.	.	REACTOME_TRANSMISSION_ACRO
0	.	NA	.	LIPID	.	SUB	.	.	.	.
0	.	NA	.	SIGN	INTR	.	.	.	.	REACTOME_CELL_CELL_COMMUN
0	Exocrine p	NA	.	.	.	CATI	KEG(	.	.	.
0	.	NA	.	.	.	.	.	.	.	.
0	.	NA	.	.	.	.	.	.	.	.
0	.	NA	.	.	.	.	.	.	.	.
0	.	NA	.	.	.	.	.	.	.	.
0	.	NA	.	.	.	.	.	.	.	.
0	.	NA	.	.	.	.	.	.	.	.
0	.	NA	.	.	.	.	.	.	.	.
0	Chondrody	NA	.	BIOF	NUC	HYD	.	PID	BIO	REACTOME_NOTCH1_INTRACELLU
0	.	NA	.	.	.	.	.	.	.	.
0	Dent disea	NA	CM0	EST/	INTR	.	.	.	.	.
0	.	NA	.	.	.	.	.	.	.	.
0	.	NA	.	.	.	.	.	.	.	.
0	.	NA	.	.	.	.	.	.	.	.
0	.	NA	.	.	.	.	.	.	.	.

0	.	NA	.	.	.	.	.	.	.	.		
0	.	NA	.	.	.	.	.	.	.	.		
0	.	NA	.	.	.	.	.	.	.	.		
0	.	NA	.	.	.	.	.	.	.	.		
0	.	NA	.	.	.	.	.	.	.	.		
0	.	NA	.	.	.	.	.	.	.	.		
0	.	NA	.	POS	INTF	LIPIE	KEGC	.	.	.		
0	.	NA	.	.	.	.	.	.	.	REACTOME_TRANSMEMBRANE_TF		
0	.	NA	.	.	.	.	.	.	.	.		
0	Miyoshi mu	NA	.	SYS	MEM	.	.	.	.	.		
0	Miyoshi mu	NA	.	SYS	MEM	.	.	.	.	.		
0	.	NA	.	.	.	.	.	.	.	.		
0	.	NA	.	.	.	.	.	.	.	.		
0	.	NA	.	.	.	.	.	.	.	.		
0	.	NA	.	.	INTF	.	.	PID	.	REACTOME_DEVELOPMENTAL_BIC		
0	.	NA	.	.	.	.	.	.	.	.		
0	.	NA	.	.	EXTI	.	KEGC	.	.	.		
0	.	NA	.	.	.	.	.	.	.	.		
0	Adams-Oli	NA	.	.	.	.	.	.	.	REACTOME_SIGNALING_BY_RHO_		
0	Myopia 23,	NA	.	PRO	INTF	CAR	.	PID	.	.		
0	.	NA	.	.	RIBC	RNA	.	.	.	.		
0	.	NA	.	.	.	.	.	.	.	.		
0	.	NA	.	.	.	.	.	.	.	.		
0	.	NA	.	.	.	.	.	.	.	.		
0	.	NA	.	.	.	.	.	.	.	.		
0	.	NA	.	.	.	.	.	.	.	.		
0	.	NA	.	.	.	.	KEGC	.	.	.		
0	Cockayne	NA	.	DNA	ORG	HYD	KEGC	.	.	REACTOME_TRANSCRIPTION_COU		
0	.	NA	.	.	.	.	.	.	.	.		
0	.	NA	.	.	.	.	.	.	.	REACTOME_TRANSMEMBRANE_TF		
0	.	NA	.	.	.	.	.	.	.	.		
0	.	NA	.	.	.	.	KEGC	.	.	REACTOME_DEVELOPMENTAL_BIC		
0	.	NA	.	.	.	.	.	.	.	.		
0	{Asthma, s	NA	.	DEFI	.	REC	KEGC	.	.	REACTOME_ANTIGEN_PROCESSIN		
0	Ehlers-Dar	NA	.	ORG	PRO	INTE	KEGC	.	.	.		
0	.	NA	.	RNA	NUC	.	.	.	.	.		
0	.	NA	.	.	.	.	.	.	.	.		
0	{Colchicine	NA	.	EST	CELI	NUC	KEGC	PID	BIO	REACTOME_ABACAVIR_TRANSPOF		
0	.	NA	.	SYS	INTF	.	KEGC	PID	.	.		
0	.	NA	.	.	CYT	PHO	KEGC	.	.	REACTOME_GASTRIN_CREB_SIGN		
0	.	NA	.	.	CYT	PHO	KEGC	.	.	REACTOME_GASTRIN_CREB_SIGN		
0	.	NA	.	.	.	.	KEGC	.	.	REACTOME_SIGNALING_BY_GPCR		
0	.	NA	.	.	PRO	.	.	.	.	.		
0	.	NA	.	.	.	.	.	.	.	.		
0	Diabetes m	NA	.	RNA	NUC	SPE	.	.	.	.		
0	.	NA	.	.	.	.	.	.	.	.		
0	.	NA	.	.	.	.	.	.	.	.		
0	.	NA	.	.	.	.	.	.	.	.		
0	.	NA	.	.	.	.	.	.	.	.		
0	.	NA	.	.	.	.	.	.	.	.		
0	.	NA	.	CELI	MEM	CATI	KEGC	.	.	REACTOME_TRANSMISSION_ACRC		
0	.	NA	.	.	.	.	.	.	.	.		
0	.	NA	.	.	.	.	.	.	.	REACTOME_GENERIC_TRANSCRIP		
0	.	NA	.	.	.	.	.	.	.	.		

0	.	NA	.	.	.	.	.	.	.	.
0	.	NA	.	.	.	.	.	.	.	.
0	.	NA	.	PHO	INTF	PHO	.	PID	.	REACTOME_SYNTHESIS_OF_PIPS_
0	.	NA	.	.	.	.	KEGG	PID	.	REACTOME_CELL_CELL_COMMUN
0	Ataxia-tela	NA	.	DNA	.	PHO	KEGG	PID	BIO	REACTOME_MEIOSIS;REACTOME_
0	.	NA	.	.	CYT	.	.	.	.	REACTOME_DIABETES_PATHWAYS
0	.	NA	.	.	.	.	.	.	.	.
0	.	NA	.	.	.	.	.	.	.	.
0	Leukemia,	NA	.	.	.	TRA	KEGG	.	.	.
0	Bladder ca	NA	CM0	.	.	.	KEGG	PID	BIO	REACTOME_SIGNALLING_BY_NGF;
0	Spastic pa	NA	.	EST/	ORG	.	.	.	.	REACTOME_MHC_CLASS_II_ANTIG
0	.	NA	.	.	.	.	.	.	.	REACTOME_NEURONAL_SYSTEM;F
0	.	NA	.	.	.	.	.	.	.	.
0	.	NA	.	.	.	.	.	.	.	.
0	.	NA	.	.	.	.	.	.	.	.
0	.	NA	.	.	.	.	.	.	.	.
0	.	NA	.	.	.	.	.	.	.	.
0	Prader-Wil	NA	.	NEG	.	.	.	PID	.	.
0	.	NA	.	SIGN	INTF	.	KEGG	.	BIO	REACTOME_TRANSMISSION_ACRO
0	.	NA	.	.	.	.	.	.	.	.
0	.	NA	.	.	.	.	.	.	.	.
0	.	NA	.	.	.	.	.	.	.	.
0	.	NA	.	.	.	.	.	.	.	.
0	.	NA	.	SYS	.	.	.	.	.	.
0	.	NA	.	.	.	.	.	.	.	.
0	Autoinflam	NA	.	.	.	.	KEGG	PID	.	REACTOME_ANTIGEN_ACTIVATES_
0	.	NA	.	.	.	.	.	PID	.	.
0	.	NA	.	.	.	.	.	.	.	.
0	.	NA	.	.	.	.	.	.	.	.
0	.	NA	.	.	.	.	.	.	.	.
0	.	NA	.	.	.	PRO	.	.	.	.
0	.	NA	.	.	.	.	.	.	.	.
0	.	NA	.	.	.	.	.	.	.	.
0	Adams-Oli	NA	.	.	.	.	.	PID	.	REACTOME_FACTORS_INVOLVED_
0	.	NA	.	.	.	.	.	.	.	.
0	.	NA	.	.	.	.	.	.	.	.
0	.	NA	.	.	.	.	.	.	.	REACTOME_GENERIC_TRANSCRIP
0	.	NA	.	.	.	.	.	.	.	.
0	.	NA	.	REG	CYT	PRO	.	.	.	.
0	.	NA	.	.	.	.	.	.	.	.
0	.	NA	.	.	.	.	.	.	.	.
0	.	NA	.	.	.	.	.	.	.	.
0	.	NA	.	.	.	.	.	.	.	.
0	.	NA	.	.	.	.	.	.	.	.
0	.	NA	.	CELI	INTF	.	KEGG	.	.	.
0	.	NA	.	REG	COA	.	.	.	.	REACTOME_TRANSMISSION_ACRO
0	.	NA	.	.	.	.	.	.	.	.
0	.	NA	.	.	.	.	.	.	.	.
0	.	NA	.	.	.	.	.	.	.	.
0	.	NA	.	.	.	.	.	PID	.	.
0	Lymphopro	NA	.	CELI	CYT	.	KEGG	PID	.	.
0	.	NA	.	.	.	.	.	.	.	.
0	.	NA	.	.	.	.	.	.	.	.
0	Favism;He	NA	CM9	GLU	CYT	.	KEGG	.	.	REACTOME_METABOLISM_OF_CAF

0	.	NA	.	.	.	.	.	.	.	.
0	.	NA	.	NUC	.	.	KEGG	.	.	REACTOME_METABOLISM_OF_CAF
0	.	NA	.	.	.	.	.	.	.	.
0	.	NA	.	DET	.	TAS	KEGG	.	.	REACTOME_SIGNALING_BY_GPCR
0	.	NA	.	SIGN	CYTO	GTP.	.	.	.	REACTOME_RAP1_SIGNALLING;RE
0	.	NA	.	.	.	.	.	.	.	.
0	.	NA	.	.	.	.	.	.	.	.
0	.	NA	.	.	.	.	.	.	.	.
0	.	NA	.	.	.	.	.	.	.	.
0	.	NA	.	.	.	.	.	.	.	.
0	.	NA	.	.	.	.	.	.	.	.
0	.	NA	.	.	.	.	.	.	.	.
0	.	NA	.	.	INTR	HYD	KEGG	.	.	REACTOME_REVERSIBLE_HYDRAT
0	Chronic gra	NA	.	DEFI	.	ELEC	KEGG	PID	BIO	REACTOME_ANTIGEN_PROCESSIN
0	.	NA	.	MUS	INTR	CATI	.	.	.	REACTOME_NEURONAL_SYSTEM;F
0	.	NA	.	.	.	.	.	.	.	.
0	.	NA	.	.	.	.	.	.	.	.
0	.	NA	.	.	.	.	KEGG	.	.	REACTOME_O_LINKED_GLYCOSYL
0	.	NA	.	.	.	.	.	.	.	.
0	Leukodystr	NA	.	CELI	ORG	CHA	KEGG	PID	.	REACTOME_MITOCHONDRIAL_PRC
0	.	NA	.	ESTA	NUC	.	.	.	.	.
0	Multiple su	NA	.	.	.	.	KEGG	.	.	REACTOME_PTM_GAMMA_CARBOX
0	.	NA	.	.	.	.	KEGG	PID	.	REACTOME_BMAL1_CLOCK_NPAS2
0	.	NA	.	.	.	.	.	.	.	.
0	.	NA	.	.	.	.	.	PID	.	REACTOME_CELL_CYCLE;REACTO
0	.	NA	.	.	.	.	.	.	.	.
0	.	NA	.	.	.	.	.	.	.	.
0	.	NA	.	CELI	NUC	.	.	.	.	.
0	.	NA	.	CELI	NUC	.	.	.	.	.
0	.	NA	.	.	.	.	KEGG	.	.	.
0	.	NA	.	.	.	.	.	.	.	.
0	.	NA	.	.	.	.	.	.	.	.
0	.	NA	.	.	.	.	.	.	.	.
0	.	NA	.	.	.	.	.	.	.	.
0	.	NA	.	.	.	TRAI	.	.	.	.
0	.	NA	.	.	.	.	.	.	.	.
0	.	NA	.	.	.	.	.	.	.	.
0	.	NA	.	GLYC	.	TRAI	KEGG	.	.	REACTOME_O_LINKED_GLYCOSYL
0	.	NA	.	.	.	.	.	.	.	.
0	.	NA	.	.	.	.	KEGG	.	.	REACTOME_DEVELOPMENTAL_BIC
0	{Atopy, res	NA	.	.	.	.	.	.	.	.
0	Narcolepsy	NA	.	SYS	.	.	.	.	.	.
0	.	NA	.	.	.	.	.	.	.	.
0	.	NA	.	.	.	.	.	.	.	.
0	.	NA	.	.	.	.	.	.	.	.
0	.	NA	.	.	.	.	.	.	.	.
0	.	NA	.	.	.	.	.	.	.	.
0	.	NA	.	.	.	.	.	.	.	.
0	.	NA	.	.	.	.	.	.	.	.
0	.	NA	.	POS	CYTO	.	.	.	.	.
0	.	NA	.	.	.	.	.	.	.	.
0	.	NA	.	.	.	.	.	.	.	.
0	{Prostate c	NA	.	REG	CYTO	STRI	KEGG	PID	.	.
0	{Coronary	NA	.	.	.	HYD	.	.	.	.

0	.	NA	.	SIGN	NUC	TRAI	.	PID	BIO	.
0	.	NA	.	.	CYT(	.	.	.	.	.
0	.	NA	.	NUC	.	.	.	.	.	REACTOME_METABOLISM_OF_NON
0	.	NA	.	.	.	.	.	.	.	.
0	.	NA	.	.	.	.	.	.	.	.
0	.	NA	.	.	.	.	.	.	.	.
0	.	NA	.	.	.	.	KEG(	.	.	REACTOME_INSULIN_RECEPTOR_I
0	.	NA	.	.	.	.	.	.	.	.
0	.	NA	.	.	.	.	.	.	.	.
0	.	NA	.	SYS	COA	.	.	.	.	.
0	.	NA	.	BIOF	EXTI	.	.	.	.	REACTOME_SIGNALLING_BY_NGF
0	.	NA	.	.	.	.	.	.	.	.
0	.	NA	.	.	.	.	.	.	.	.
0	Candidiasis	NA	.	POS	CYT(	PRO	KEG(	.	.	REACTOME_INNATE_IMMUNE_SYS
0	.	NA	.	.	.	.	.	.	.	.
0	.	NA	.	.	.	.	.	.	.	.
0	.	NA	.	SYS	CELI	.	KEG(	.	.	REACTOME_CELL_CELL_COMMUN
0	.	NA	.	.	.	.	.	.	.	.
0	.	NA	.	.	.	.	.	.	.	.
0	.	NA	.	REP	EXTI	MET	KEG(	.	.	.
0	.	NA	.	CELI	CYT(	GTP.	KEG(	PID	BIO	REACTOME_DEVELOPMENTAL_BIO
0	.	NA	.	.	.	.	.	.	.	.
0	.	NA	.	.	.	.	KEG(	.	BIO	REACTOME_METABOLISM_OF_AMI
0	Hermansky	NA	.	.	.	.	.	.	.	.
0	.	NA	.	.	.	.	.	.	.	.
0	.	NA	.	.	.	.	.	.	.	.
0	.	NA	.	.	.	.	.	.	.	.
0	.	NA	.	.	.	.	.	.	.	.
0	.	NA	.	.	.	.	.	.	.	.
0	Achondrog	NA	.	SYS	.	.	KEG(	PID	.	REACTOME_DEVELOPMENTAL_BIO
0	.	NA	.	.	.	.	.	.	.	.
0	.	NA	.	.	.	.	.	.	.	.
0	Transpositi	NA	.	.	.	.	.	.	.	REACTOME_DEVELOPMENTAL_BIO
0	Diabetes m	NA	CM0	REG	MAC	TRAI	KEG(	PID	BIO	REACTOME_DEVELOPMENTAL_BIO
0	.	NA	.	.	.	.	.	PID	.	REACTOME_FACTORS_INVOLVED_
0	.	NA	.	ONE	.	MET	.	PID	.	REACTOME_METABOLISM_OF_NON
0	.	NA	.	.	.	.	.	.	.	.
0	.	NA	.	.	.	.	.	.	.	.
0	Prader-Wil	NA	.	NEG	.	.	.	PID	.	.
0	.	NA	.	MAC	CELI	.	KEG(	PID	.	REACTOME_APOPTOTIC_CLEAVAG
0	.	NA	.	.	.	.	.	.	.	REACTOME_TRANSMISSION_ACRO
0	.	NA	.	.	.	.	.	.	.	.
0	.	NA	.	.	.	.	.	.	.	.
0	.	NA	.	.	.	.	.	.	.	.
0	.	NA	.	.	.	.	.	.	.	.
0	.	NA	.	.	.	.	.	.	.	.
0	.	NA	.	.	.	.	.	.	.	.
0	.	NA	.	.	.	.	KEG(	PID	.	.
0	.	NA	.	SIGN	.	REC	.	.	.	.
0	.	NA	.	REG	CYT(	.	.	.	.	.
0	.	NA	.	.	.	.	.	.	.	.
0	.	NA	.	AMIN	ORG	SULI	KEG(	.	.	.
0	.	NA	.	.	.	.	.	.	.	.
0	.	NA	.	.	INTF	.	KEG(	PID	.	REACTOME_INTEGRIN_CELL_SURF

0	.	NA	.	DNA	NUC	PRO	.	.	.	.
0	.	NA	.	.	.	.	.	.	.	.
0	.	NA	.	BIOS	CYT	ELE	.	PID	.	.
0	Epidermoly	NA	.	.	INTF	.	KEG	PID	.	REACTOME_CELL_CELL_COMMUN
0	.	NA	.	.	.	.	KEG	PID	.	REACTOME_INSULIN_RECEPTOR_
0	.	NA	.	BIOF	CELI	OXID	.	.	BIO	REACTOME_EXTRACELLULAR_MA
0	.	NA	.	CELI	INTF	REC	KEG	.	BIO	.
0	.	NA	.	.	.	.	.	.	.	REACTOME_O_LINKED_GLYCOSYL
0	.	NA	.	.	.	.	.	.	.	REACTOME_O_LINKED_GLYCOSYL
0	.	NA	.	.	.	.	.	.	.	.
0	.	NA	.	.	.	.	.	.	.	.
0	.	NA	.	NEG	.	TRA	.	.	.	.
0	.	NA	.	RNA	.	TRA	.	PID	.	.
0	.	NA	.	RNA	.	TRA	.	PID	.	.
0	.	NA	.	RNA	.	TRA	.	PID	.	.
0	.	NA	.	RNA	.	TRA	.	PID	.	.
0	.	NA	.	.	.	.	.	.	.	REACTOME_GENERIC_TRANSCRIP
0	.	NA	.	.	.	.	.	.	.	.
0	.	NA	.	.	.	.	.	.	.	.
0	.	NA	.	.	.	.	.	.	.	.
0	.	NA	.	REP	INTF	.	.	.	.	.
0	[Bombay p	NA	.	GLY	INTF	TRA	KEG	.	.	.
0	[Bombay p	NA	.	GLY	INTF	TRA	KEG	.	.	.
0	.	NA	.	POS	EXTI	REC	.	.	.	.
0	.	NA	.	.	.	.	.	.	.	REACTOME_GENERIC_TRANSCRIP
0	.	NA	.	.	.	.	KEG	.	.	.
0	.	NA	.	.	.	.	.	.	.	REACTOME_TRIGLYCERIDE_BIOSY
0	.	NA	.	.	.	.	.	.	.	.
0	.	NA	.	.	.	.	.	.	.	.
0	.	NA	.	BIOF	INTF	PRO	.	.	.	.
0	.	NA	.	.	INTF	.	.	.	.	.
0	.	NA	.	SYS	PRO	.	.	.	.	.
0	.	NA	.	MUS	NON	ACTI	.	.	.	.
0	.	NA	.	.	.	.	.	.	.	.
0	.	NA	.	.	CYT	.	.	PID	.	REACTOME_SIGNALING_BY_RHO_
0	.	NA	.	.	.	.	.	.	.	.
0	.	NA	.	.	.	.	.	.	.	.
0	{Epilepsy,	NA	.	SIGN	INTF	GAB	KEG	.	.	.
0	Palmoplan	NA	.	.	.	.	.	PID	.	.
0	.	NA	.	.	.	.	.	.	.	.
0	.	NA	.	NUC	.	.	KEG	PID	BIO	REACTOME_DOWNREGULATION_C
0	Ciliary dysl	NA	.	.	.	.	.	PID	.	.
0	Glycogen s	NA	.	.	MAC	.	KEG	.	.	REACTOME_GLYCOGEN_BREAKDC
0	.	NA	.	.	.	.	.	.	.	.
0	.	NA	.	.	.	.	.	.	.	.
0	.	NA	.	.	.	.	.	.	.	.
0	.	NA	.	.	.	.	.	.	.	.
0	.	NA	.	.	.	HYD	.	.	.	.
0	Epidermoly	NA	.	SYS	.	.	KEG	PID	.	REACTOME_CELL_CELL_COMMUN
0	.	NA	.	.	.	.	.	.	.	.
0	.	NA	.	.	.	.	.	.	.	.
0	.	NA	.	RNA	NUC	TRA	.	.	.	.
0	Senior-Lok	NA	.	BIOF	.	.	KEG	PID	.	REACTOME_CELL_CYCLE;REACTO
0	.	NA	.	.	.	.	.	.	.	.

0	.	NA	.	.	.	.	.	.	.	.
0	Short statu	NA	.	.	.	.	.	.	.	.
0	.	NA	.	REG	.	NUC	KEG(	PID	.	REACTOME_INNATE_IMMUNE_SYS
0	GAPO syn	NA	.	.	.	.	.	PID	.	.
0	.	NA	.	.	.	.	.	.	.	.
0	Selective T	NA	.	POS	MEM	.	KEG(	PID	BIO	REACTOME_TCR_SIGNALING;REAC
0	.	NA	.	.	.	.	.	.	.	.
0	.	NA	.	.	.	.	.	.	.	.
0	.	NA	.	.	.	.	.	.	.	.
0	Cardiomyo	NA	.	MUS	ORG	NUC	KEG(	.	.	.
0	.	NA	.	.	.	.	.	.	.	.
0	.	NA	.	BIOF	CYT	PRO	KEG(	PID	BIO	.
0	.	NA	.	BIOF	CYT	PRO	KEG(	PID	BIO	.
0	.	NA	.	.	.	.	.	.	.	.
0	Microphtha	NA	.	.	.	.	.	.	.	.
0	.	NA	.	M_P	ORG	DNA	.	.	BIO	REACTOME_MHC_CLASS_II_ANTIG
0	.	NA	.	SYS	INTF	G_PI	KEG(	.	.	REACTOME_SIGNALING_BY_GPCR
0	.	NA	.	SYS	INTF	LIPC	.	.	.	.
0	.	NA	.	REG	INTF	.	.	.	.	.
0	.	NA	.	REG	INTF	.	.	.	.	.
0	.	NA	.	BIOS	.	STR	KEG(	.	.	REACTOME_TRANSLATION;REACT
0	Deafness,	NA	.	.	.	.	.	.	.	.
0	.	NA	.	.	.	.	KEG(	.	.	.
0	Breast can	NA	CM1	.	.	PHO	KEG(	PID	BIO	REACTOME_SIGNALLING_BY_NGF;
0	Myopia 23,	NA	.	PRO	INTF	CAR	.	PID	.	.
0	.	NA	.	.	.	.	.	.	.	.
0	.	NA	.	.	.	.	.	.	.	REACTOME_TRANSMEMBRANE_TR
0	.	NA	.	.	.	.	.	.	.	REACTOME_TRANSMEMBRANE_TR
0	.	NA	.	.	.	.	.	.	.	.
0	.	NA	.	.	.	.	.	.	.	.
0	.	NA	.	.	.	.	.	.	.	.
0	.	NA	.	.	.	.	.	.	.	.
0	.	NA	.	.	.	.	KEG(	.	.	.
0	.	NA	.	.	.	.	.	.	.	.
0	.	NA	.	.	MEM	RAS	.	.	.	.
0	.	NA	.	.	.	.	.	.	.	.
0	.	NA	.	POS	ORG	REC	KEG(	PID	.	REACTOME_NEGATIVE_REGULATI
0	.	NA	.	.	.	.	.	.	.	.
0	.	NA	.	.	.	.	.	.	.	.
0	.	NA	.	.	.	.	.	.	.	.
0	.	NA	.	RNA	NUC	RNA	KEG(	.	.	.
0	Cardiomyo	NA	.	.	PRO	STR	KEG(	PID	BIO	.
0	.	NA	.	.	.	.	KEG(	.	.	.
0	.	NA	.	SIGN	INTF	PEP	KEG(	PID	.	REACTOME_SIGNALING_BY_GPCR
0	.	NA	.	.	.	GTP	KEG(	PID	.	REACTOME_SIGNALING_BY_RHO_
0	.	NA	.	.	.	.	.	.	.	.
0	.	NA	.	.	.	.	.	.	.	.
0	.	NA	.	.	ORG	HYD	.	.	.	.
0	.	NA	.	.	.	.	.	.	.	.
0	.	NA	.	.	.	.	.	.	.	.
0	Shwachma	NA	.	.	.	.	.	.	.	.
0	.	NA	.	.	.	.	.	.	.	.
0	.	NA	.	.	.	.	.	.	.	REACTOME_O_LINKED_GLYCOSYL
0	.	NA	.	.	.	.	.	.	.	.

0	.	NA	.	.	.	.	.	.	.	.	
0	.	NA	.	.	.	.	.	.	.	.	
0	.	NA	.	BIOF	ORG	TRAI	.	PID.	BIO	.	
0	.	NA	.	.	.	.	.	.	.	.	
0	.	NA	.	EST/	ORG	.	.	.	.	.	
0	.	NA	.	ACTI	ADH	STRI	.	.	.	REACTOME_MUSCLE_CONTRACTI(	
0	.	NA	.	.	INTF	CATI	.	.	.	REACTOME_DEGRADATION_OF_TH	
0	.	NA	.	.	.	.	.	.	.	.	
0	.	NA	.	.	.	.	.	.	.	.	
0	.	NA	.	.	.	.	.	.	.	.	
0	.	NA	.	.	.	.	.	.	.	.	
0	5-oxoprolir	NA	.	.	.	.	KEG(	.	.	REACTOME_BIOLOGICAL_OXIDATI(	
0	.	NA	.	.	.	.	.	.	.	REACTOME_HIV_INFECTION;REAC	
0	.	NA	.	.	.	.	.	.	.	.	
0	.	NA	.	.	.	.	.	.	.	.	
0	.	NA	.	.	.	.	.	.	.	.	
0	.	NA	.	.	.	.	.	.	.	.	
0	.	NA	.	.	.	.	.	.	.	.	
0	.	NA	.	HOM	CYT	IDEN	KEG(	PID.	BIO	REACTOME_DEVELOPMENTAL_BIC	
0	.	NA	.	.	.	.	.	.	.	.	
0	Pulmonary	NA	.	.	.	.	.	PID.	.	.	
0	Cowden sy	NA	.	.	.	.	.	.	.	.	
0	.	NA	.	.	.	.	.	.	.	.	
0	.	NA	.	.	.	.	.	.	.	REACTOME_O_LINKED_GLYCOSYL	
0	.	NA	.	.	.	.	.	.	.	.	
0	Diabetes m	NA	.	EST/	.	CATI	KEG(	PID.	.	REACTOME_NEURONAL_SYSTEM;F	
0	Glycogen s	NA	.	.	CYT	OXID	KEG(	PID.	BIO	REACTOME_PYRUVATE_METABOL	
0	Dystonia 2	NA	.	.	.	.	.	.	.	.	
0	.	NA	.	.	.	.	.	.	.	.	
0	.	NA	.	.	.	.	.	.	.	.	
0	.	NA	.	.	.	.	.	.	.	.	
0	.	NA	.	.	.	.	.	.	.	.	
0	Asphyxiatir	NA	.	ORG	CYT	.	KEG(	.	.	REACTOME_MHC_CLASS_II_ANTIG	
0	.	NA	.	.	.	.	.	.	.	.	
0	.	NA	.	.	.	.	.	.	.	REACTOME_TRANSMEMBRANE_TF	
0	.	NA	.	.	.	.	.	.	.	.	
0	.	NA	.	.	.	.	.	.	.	.	
0	Methylmal(	NA	.	.	.	.	KEG(	.	.	.	
0	.	NA	.	.	.	.	.	.	.	.	
0	.	NA	.	.	.	.	.	.	.	.	
0	.	NA	.	.	.	.	.	.	.	.	
0	.	NA	.	.	.	.	.	.	.	.	
0	.	NA	.	.	.	.	.	.	.	.	
0	.	NA	.	PRO	INTF	CATI	KEG(	PID.	.	REACTOME_DEGRADATION_OF_TH	
0	Atrial septa	NA	.	REG	ORG	HYD	KEG(	.	.	REACTOME_STRIATED_MUSCLE_C	
0	.	NA	.	.	.	.	.	.	.	.	
0	.	NA	.	BIOF	CELI	NUC	.	.	.	.	
0	.	NA	.	.	.	.	.	.	.	.	
0	Angelman	NA	.	BIOF	.	.	KEG(	PID.	BIO	REACTOME_IMMUNE_SYSTEM;REA	
0	.	NA	.	SYS	.	.	.	.	.	.	
0	Colorectal	NA	.	MIT(	ORG	.	KEG(	PID.	.	REACTOME_INHIBITION_OF_THE_F	
0	.	NA	.	.	.	.	.	.	.	.	
0	.	NA	.	.	.	.	.	.	.	.	
0	{Pseudoxa	NA	.	BIOS	.	UDP.	KEG(	.	.	.	

0	.	NA	.	POS	INTF	REC	KEG(	.	.	REACTOME_CELL_SURFACE_INTEI
0	.	NA	.	SYS	INTF	REC	KEG(	PID	BIO	REACTOME_CELL_SURFACE_INTEI
0	.	NA	.	REG	INTF	CATI	.	.	.	REACTOME_TRANSMEMBRANE_TF
0	.	NA	.	MUL	UBIQ	ACID	KEG(	.	.	.
0	.	NA	.	.	.	.	.	.	.	.
0	.	NA	.	POS	INTF	G_PI	KEG(	.	.	REACTOME_SIGNALING_BY_GPCR
0	.	NA	.	.	.	MOT	KEG(	.	.	.
0	.	NA	.	.	.	.	.	.	.	.
0	Alexander	NA	.	.	ORG	STRI	.	.	.	REACTOME_SIGNALING_BY_ERBB
0	.	NA	.	.	.	.	KEG(	.	.	REACTOME_ABCA_TRANSPORTER
0	.	NA	.	PRO	ORG	CHA	.	.	.	REACTOME_PROTEIN_FOLDING;RE
0	.	NA	.	.	.	.	.	.	.	.
0	[Blood grou	NA	.	EST/	INTF	SUB	.	.	.	REACTOME_TRANSMEMBRANE_TF
0	.	NA	.	.	.	.	.	.	.	.
0	.	NA	.	.	.	.	.	.	.	.
0	.	NA	.	.	.	.	.	.	.	.
0	.	NA	.	.	INTF	NUC	KEG(	.	.	REACTOME_ABCA_TRANSPORTER
0	.	NA	.	.	.	.	.	.	.	.
0	.	NA	.	.	.	.	.	.	.	.
0	.	NA	.	.	INTF	G_PI	.	.	.	REACTOME_SIGNALING_BY_GPCR
0	.	NA	.	.	.	.	.	.	.	.
0	.	NA	.	.	.	.	.	.	.	.
0	.	NA	.	.	.	.	.	.	.	REACTOME_GENERIC_TRANSCRIP
0	.	NA	.	.	.	.	.	.	.	.
0	.	NA	.	.	.	.	.	.	.	.
0	.	NA	.	.	INTF	CAR	.	.	.	.
0	.	NA	.	SIGN	.	.	.	.	.	REACTOME_GASTRIN_CREB_SIGN
0	.	NA	.	.	.	.	.	.	.	REACTOME_DEVELOPMENTAL_BIC
0	Epileptic er	NA	.	.	CYT(	.	KEG(	PID	BIO	REACTOME_GASTRIN_CREB_SIGN
0	.	NA	.	.	.	.	.	.	.	REACTOME_FACTORS_INVOLVED_
0	.	NA	.	.	.	.	.	.	.	.
0	.	NA	.	.	.	.	.	.	.	.
0	.	NA	.	.	.	.	.	.	.	.
0	.	NA	.	CELI	INTF	.	KEG(	.	.	.
0	.	NA	.	.	.	.	.	.	.	.
0	.	NA	.	EST/	INTF	CATI	.	.	.	.
0	.	NA	.	.	CYT(	.	.	.	.	.
0	Bethlem m	NA	.	.	.	.	KEG(	PID	.	REACTOME_DEVELOPMENTAL_BIC
0	.	NA	.	.	.	.	.	.	.	.
0	.	NA	.	.	.	.	.	.	.	.
0	.	NA	.	.	.	.	.	.	.	.
0	.	NA	.	INOF	.	VOL	.	.	.	.
0	.	NA	.	.	.	.	.	.	.	.
0	.	NA	.	SIGN	NON	KINA	.	.	.	.
0	.	NA	.	.	.	.	.	.	.	.
0	.	NA	.	.	.	.	.	.	.	.
0	.	NA	.	GLY(	CYT(	TRAI	.	.	.	.
0	.	NA	.	.	.	.	.	.	.	.
0	.	NA	.	.	.	.	.	.	.	.
0	.	NA	.	.	.	.	.	.	.	.
0	.	NA	.	.	.	.	.	.	.	.
0	.	NA	.	PRO	.	KINA	KEG(	PID	BIO	REACTOME_SIGNALLING_BY_NGF;
0	.	NA	.	.	.	.	.	.	.	.
0	.	NA	.	.	.	.	.	.	.	.

0	.	NA	.	.	.	.	.	.	.	.
0	.	NA	.	EST/	INTF	.	.	.	.	REACTOME_MEMBRANE_TRAFFICK
0	Epileptic er	NA	.	.	.	TRAI	KEGC	.	.	REACTOME_O_LINKED_GLYCOSYL
0	.	NA	.	BIOF	NUC	PHO	.	.	.	.
0	.	NA	.	ENZ'	INTF	TRAI	.	.	.	.
0	.	NA	.	.	.	.	KEGC	PID	BIO	REACTOME_IL_7_SIGNALING;REAC
0	.	NA	.	.	.	.	KEGC	.	.	REACTOME_METABOLISM_OF_NUC
0	.	NA	.	.	.	.	.	.	.	.
0	.	NA	.	.	.	.	.	.	.	.
0	.	NA	.	.	.	.	.	.	.	.
0	.	NA	.	.	.	.	.	.	.	.
0	.	NA	.	.	.	.	.	.	.	.
0	.	NA	.	BIOF	CYT	PRO	KEGC	PID	BIO	.
0	.	NA	.	.	.	.	.	PID	.	REACTOME_SIGNALING_BY_RHO_(
0	Thrombocy	NA	.	.	INTF	.	KEGC	.	.	REACTOME_METABOLISM_OF_AMI
0	Donnai-Ba	NA	.	GLY(	CYT	.	KEGC	PID	.	REACTOME_METABOLISM_OF_STE
0	.	NA	.	.	.	.	.	.	.	.
0	.	NA	.	CELI	NUC	NUC	.	.	.	.
0	.	NA	.	.	INTF	G_PI	.	.	.	.
0	.	NA	.	.	.	.	.	.	.	.
0	.	NA	.	.	.	.	.	.	.	REACTOME_EXTRACELLULAR_MAT
0	.	NA	.	EST/	INTF	NUC	KEGC	.	.	REACTOME_HYALURONAN_METAB
0	Night blind	NA	.	DETI	.	.	KEGC	PID	.	REACTOME_CGMP_EFFECTS;REAC
0	.	NA	.	.	.	PHO	.	.	.	REACTOME_MEMBRANE_TRAFFICK
0	.	NA	.	.	.	.	.	PID	.	.
0	.	NA	.	.	.	.	.	.	.	.
0	.	NA	.	GLU	INTF	.	KEGC	.	.	.
0	.	NA	.	.	.	.	KEGC	.	.	REACTOME_SIGNALING_BY_GPCR
0	.	NA	.	.	.	.	.	.	.	.
0	.	NA	.	SYS	.	.	KEGC	.	.	REACTOME_DEVELOPMENTAL_BIC
0	.	NA	.	.	.	.	KEGC	PID	.	REACTOME_SIGNALING_BY_RHO_(
0	Adenoma,	NA	.	NEG	ORG	KINA	KEGC	PID	BIO	REACTOME_APOPTOTIC_CLEAVAG
0	.	NA	.	.	.	.	.	.	.	.
0	.	NA	.	.	.	.	.	.	.	REACTOME_DEVELOPMENTAL_BIC
0	[Memory, e	NA	.	.	.	.	.	.	.	REACTOME_SIGNALING_BY_HIPPC
0	.	NA	.	.	.	.	.	.	.	.
0	.	NA	.	RNA	.	TRAI	.	PID	.	.
0	.	NA	.	DNA	.	NUC	KEGC	.	.	REACTOME_ACTIVATION_OF_THE_
0	.	NA	.	.	.	.	.	.	.	.
0	.	NA	.	.	.	.	.	.	.	.
0	{Hypogona	NA	.	.	EXTI	.	KEGC	.	.	REACTOME_DEVELOPMENTAL_BIC
0	{Osteoporc	NA	.	REG	.	HOR	KEGC	.	.	.
0	.	NA	.	.	.	.	.	.	.	.
0	.	NA	.	.	.	.	.	.	.	.
0	.	NA	.	.	.	.	.	.	.	.
0	Charcot-M	NA	.	RES	.	.	.	PID	.	.
0	.	NA	.	.	NUC	TRAI	.	PID	.	REACTOME_RNA_POL_III_TRANSC
0	Cardiomyo	NA	.	MUS	EXTI	.	.	.	.	.
0	.	NA	.	.	.	.	KEGC	.	.	REACTOME_SIGNALING_BY_GPCR
0	.	NA	.	MUS	CYT	ACTI	.	PID	.	.
0	.	NA	.	.	.	.	.	.	.	.
0	.	NA	.	.	.	.	.	.	.	.
0	.	NA	.	.	.	.	.	.	.	.
0	.	NA	.	SIGN	NUC	DNA	.	.	.	REACTOME_PPARA_ACTIVATES_G

0	Neurofibro	NA	.	NUC	NUC	TRA	.	.	.	.
0	.	NA	.	.	.	.	.	.	.	.
0	.	NA	.	.	.	.	.	.	.	.
0	.	NA	.	.	.	.	.	.	.	.
0	.	NA	.	.	.	TRA	KEG	.	.	REACTOME_O_LINKED_GLYCOSYL
0	.	NA	.	.	.	.	.	.	.	.
0	.	NA	.	.	.	.	.	.	.	.
0	.	NA	.	.	.	.	.	.	.	.
0	.	NA	.	SIGN	CYT	CAT	KEG	.	.	REACTOME_SIGNALLING_BY_NGF;
0	.	NA	.	.	.	.	.	.	.	.
0	.	NA	.	.	.	PHO	KEG	.	.	.
0	.	NA	.	RNA	.	TRA	.	.	.	REACTOME_INTEGRATION_OF_EN
0	.	NA	.	.	.	.	.	.	.	.
0	.	NA	.	.	.	.	.	.	.	.
0	.	NA	.	.	.	MRN	.	PID	.	REACTOME_METABOLISM_OF_MRI
0	.	NA	.	.	.	.	.	.	.	.
0	{Epilepsy,	NA	.	MUS	MEM	CAT	KEG	.	.	REACTOME_DEVELOPMENTAL_BIC
0	.	NA	.	BIOF	.	NUC	.	.	.	.
0	.	NA	.	.	.	.	.	.	.	.
0	.	NA	.	.	.	.	.	.	.	.
0	.	NA	.	.	INTR	G_PI	.	.	.	.
0	.	NA	.	MUS	PRO	.	KEG	.	.	REACTOME_EXTRACELLULAR_MA
0	.	NA	.	.	.	.	.	.	.	.
0	.	NA	.	.	.	.	.	.	.	.
0	.	NA	.	.	.	.	.	PID	.	REACTOME_REGULATION_OF_HYP
0	.	NA	.	.	.	.	.	.	.	.
0	.	NA	.	.	.	.	.	.	.	.
0	.	NA	.	.	.	.	.	.	.	.
0	.	NA	.	.	.	.	.	.	.	.
0	.	NA	.	.	.	.	.	.	.	.
0	.	NA	.	.	CELI	PRO	.	.	.	.
0	.	NA	.	MAC	.	.	.	.	BIO	REACTOME_TRANSLATION;REACT
0	.	NA	.	.	.	.	.	.	.	.
0	.	NA	.	BIOF	.	.	KEG	.	BIO	.
0	Mental reta	NA	.	.	.	.	.	.	.	.
0	.	NA	.	.	.	.	.	PID	.	.
0	.	NA	.	RNA	.	TRA	.	.	.	.
0	.	NA	.	.	.	.	.	.	.	.
0	Congenital	NA	CI97	PAT	.	.	.	.	.	.
0	Hemophilia	NA	.	REG	.	.	KEG	.	BIO	REACTOME_RESPONSE_TO_ELEV
0	.	NA	.	.	.	.	.	.	.	.
0	.	NA	.	.	.	.	.	.	.	.

AMINS_AND_COFACTORS

RIPTION;REACTOME_RNA_POL_II_TRANSCRIPTION_PRE_INITIATION_AND_PROMOTER_OPE
PPED_INTRON_CONTAINING_PRE_MRNA;REACTOME_MRNA_PROCESSING;REACTOME_MRN

ONS;REACTOME_CYTOCHROME_P450_ARRANGED_BY_SUBSTRATE_TYPE;REACTOME_PHA
BOLISM;REACTOME_SYNTHESIS_OF_PE;REACTOME_SYNTHESIS_OF_PC;REACTOME_GLYC

/_INTERACTIONS_BETWEEN_A_LYMPHOID_AND_A_NON_LYMPHOID_CELL;REACTOME_IMMU
RIPTION;REACTOME_TRANSCRIPTION;REACTOME_RNA_POL_I_RNA_POL_III_AND_MITOCHO

DS_AND_LIPOPROTEINS;REACTOME_CHOLESTEROL_BIOSYNTHESIS

EACTOME_IL1_SIGNALING;REACTOME_IMMUNE_SYSTEM;REACTOME_CYTOKINE_SIGNALING

OTEINS;REACTOME_POST_TRANSLATIONAL_PROTEIN_MODIFICATION;REACTOME_ASPARA

NA_AMINOACYLATION;REACTOME_TRNA_AMINOACYLATION

;REACTOME_CLASS_B_2_SECRETIN_FAMILY_RECEPTORS;REACTOME_GPCR_DOWNSTREA

RIPTION_AND_TRANSLATION;REACTOME_YAP1_AND_WWTR1_TAZ_STIMULATED_GENE_EX

N_CODING_RNA;REACTOME_ANTIVIRAL_MECHANISM_BY_IFN_STIMULATED_GENES;REACTO

REACTOME_DAG_AND_IP3_SIGNALING;REACTOME_SIGNALING_BY_ERBB2;REACTOME_SIG
REACTOME_SIGNALLING_BY_NGF;REACTOME_CTNNB1_PHOSPHORYLATION_CASCADE;REA

ONS;REACTOME_GLUTATHIONE_CONJUGATION;REACTOME_PHASE_II_CONJUGATION

RANSPORT_OF_SMALL_MOLECULES;REACTOME_SLC_MEDIATED_TRANSMEMBRANE_TRANS

OME_FORMATION_OF_THE_TERNARY_COMPLEX_AND_SUBSEQUENTLY_THE_43S_COMPLE

CELL_CYCLE;REACTOME_CHROMOSOME_MAINTENANCE;REACTOME_MEIOTIC_SYNAPSIS;F

NO_ACIDS_AND_DERIVATIVES;REACTOME_AMINE_DERIVED_HORMONES

_AND_TARGETTING_OF_GAG_PROTEINS;REACTOME_HIV_INFECTION;REACTOME_HIV_LIFE_

ICATION;REACTOME_EXTRACELLULAR_MATRIX_ORGANIZATION;REACTOME_COLLAGEN_F(

REACTOME_TRIF_MEDIATED_TLR3_SIGNALING;REACTOME_TCR_SIGNALING;REACTOME_D

;REACTOME_OLFACTORY_SIGNALING_PATHWAY;REACTOME_GPCR_DOWNSTREAM_SIGNA

)SS_CHEMICAL_SYNAPSES;REACTOME_NEURONAL_SYSTEM;REACTOME_AMINO_ACID_TRA

.TE_BIOSYNTHESIS;REACTOME_CHONDROITIN_SULFATE_DERMATAN_SULFATE_METABOLI

CELL_CYCLE;REACTOME_CHROMOSOME_MAINTENANCE;REACTOME_MEIOTIC_SYNAPSIS

RANSPORT_OF_SMALL_MOLECULES;REACTOME_ION_TRANSPORT_BY_P_TYPE_ATPASES;R

;REACTOME_GPCR_DOWNSTREAM_SIGNALING;REACTOME_G_ALPHA_I_SIGNALLING_EVEN

PPED_INTRON_CONTAINING_PRE_MRNA;REACTOME_MRNA_PROCESSING;REACTOME_MRN

ME_CELL_CYCLE_MITOTIC;REACTOME_RECRUITMENT_OF_MITOTIC_CENTROSOME_PROTE

REACTOME_DAG_AND_IP3_SIGNALING;REACTOME_SIGNALING_BY_ERBB2;REACTOME_SIG

ONS;REACTOME_XENOBIOTICS;REACTOME_CYTOCHROME_P450_ARRANGED_BY_SUBSTRA

ONS;REACTOME_GLUTATHIONE_CONJUGATION;REACTOME_PHASE_II_CONJUGATION

;REACTOME_ACTIVATION_OF_CHAPERONE_GENES_BY_XBP1S;REACTOME_UNFOLDED_PI

'TION_PATHWAY;REACTOME_NUCLEAR_RECEPTOR_TRANSCRIPTION_PATHWAY

RONLESS_PRE_MRNAS;REACTOME_PROCESSING_OF_CAPPED_INTRON_CONTAINING_PRE

SIS;REACTOME_HEPARAN_SULFATE_HEPARIN_HS_GAG_METABOLISM;REACTOME_GLYCOS
GTPASES;REACTOME_SIGNALLING_BY_NGF;REACTOME_NRAGE_SIGNALS_DEATH_THROU(

REACTOME_SIGNALING_BY_SCF_KIT;REACTOME_SIGNALING_BY_ERBB4;REACTOME_SIGN/
REACTOME_SIGNALING_BY_SCF_KIT;REACTOME_SIGNALING_BY_ERBB4;REACTOME_SIGN/

_PRE_REPLICATIVE_COMPLEX;REACTOME_CELL_CYCLE;REACTOME_ORC1_REMOVAL_FRC

REACTOME_VOLTAGE_GATED_POTASSIUM_CHANNELS;REACTOME_POTASSIUM_CHANNEL

;REACTOME_GPCR_DOWNSTREAM_SIGNALING;REACTOME_G_ALPHA_I_SIGNALLING_EVEN
OLOGY;REACTOME_BMAL1_CLOCK_NPAS2_ACTIVATES_CIRCADIAN_EXPRESSION;REACTOM

ICATION;REACTOME_CELL_EXTRACELLULAR_MATRIX_INTERACTIONS;REACTOME_CELL_JU

CLEOTIDES;REACTOME_PURINE_RIBONUCLEOSIDE_MONOPHOSPHATE_BIOSYNTHESIS;REA
OLOGY;REACTOME_EXTRACELLULAR_MATRIX_ORGANIZATION;REACTOME_COLLAGEN_FOR
GTPASES;REACTOME_SIGNALLING_BY_NGF;REACTOME_NRAGE_SIGNALS_DEATH_THROU

REACTOME_SIGNALING_BY_SCF_KIT;REACTOME_CELL_CELL_COMMUNICATION;REACTOME
_IN_MEGAKARYOCYTE_DEVELOPMENT_AND_PLATELET_PRODUCTION;REACTOME_HEMOST

ONS;REACTOME_PHASE1_FUNCTIONALIZATION_OF_COMPOUNDS;REACTOME_ETHANOL_C

_IN_MEGAKARYOCYTE_DEVELOPMENT_AND_PLATELET_PRODUCTION;REACTOME_HEMOST
RIPTION_AND_TRANSLATION;REACTOME_PRE_NOTCH_EXPRESSION_AND_PROCESSING;R

REACTOME_TANDEM_PORE_DOMAIN_POTASSIUM_CHANNELS;REACTOME_POTASSIUM_CH

TE_DERMATAN_SULFATE_METABOLISM;REACTOME_GLYCOSAMINOGLYCAN_METABOLISM

RANSPORT_OF_SMALL_MOLECULES;REACTOME_SLC_MEDIATED_TRANSMEMBRANE_TRANS

KING;REACTOME_MEMBRANE_BINDING_AND_TARGETTING_OF_GAG_PROTEINS;REACTOME

RANSPORT_OF_SMALL_MOLECULES;REACTOME_SLC_MEDIATED_TRANSMEMBRANE_TRANS

REACTOME_SIGNALLING_TO_RAS;REACTOME_NGF_SIGNALLING_VIA_TRKA_FROM_THE_PL

SS_CHEMICAL_SYNAPSES;REACTOME_NEURONAL_SYSTEM
EROID_HORMONES_AND_VITAMINS_A_AND_D;REACTOME_HDL_MEDIATED_LIPID_TRANSPO

TEIN_IMPORT;REACTOME_METABOLISM_OF_PROTEINS

ANSPORT_OF_SMALL_MOLECULES;REACTOME_AQUAPORIN_MEDIATED_TRANSPORT;REA

ATION_OF_MUCINS;REACTOME_TERMINATION_OF_O_GLYCAN_BIOSYNTHESIS;REACTOME
ATION_OF_MUCINS;REACTOME_TERMINATION_OF_O_GLYCAN_BIOSYNTHESIS;REACTOME

IS;REACTOME_HEPARAN_SULFATE_HEPARIN_HS_GAG_METABOLISM;REACTOME_GLYCO

TOR_INTERACTIONS;REACTOME_RESPONSE_TO_ELEVATED_PLATELET_CYTOSOLIC_CA2_
;REACTOME_ACTIVATION_OF_CHAPERONE_GENES_BY_XBP1S;REACTOME_UNFOLDED_P
EN_PRESENTATION;REACTOME_FACTORS_INVOLVED_IN_MEGAKARYOCYTE_DEVELOPME

GTPASES
GTPASES

;REACTOME_OLFACTORY_SIGNALING_PATHWAY;REACTOME_GPCR_DOWNSTREAM_SIGN

F_HOMO_SAPIENS_WITH_MYCOBACTERIUM_TUBERCULOSIS;REACTOME_NITRIC_OXIDE_
LOGY;REACTOME_NOTCH1_INTRACELLULAR_DOMAIN_REGULATES_TRANSCRIPTION;REA
_SIGNALING;REACTOME_RIP_MEDIATED_NFKB_ACTIVATION_VIA_DAI;REACTOME_APOPTO

CTOME_ADAPTIVE_IMMUNE_SYSTEM;REACTOME_CLASS_I_MHC_MEDIATED_ANTIGEN_PR

;REACTOME_OLFACTORY_SIGNALING_PATHWAY;REACTOME_GPCR_DOWNSTREAM_SIGN

RANSPORT_OF_SMALL_MOLECULES;REACTOME_SLC_MEDIATED_TRANSMEMBRANE_TRANS

LOGY;REACTOME_AXON_GUIDANCE;REACTOME_MYOGENESIS;REACTOME_NETRIN1_SIGN

RANSPORT_OF_SMALL_MOLECULES;REACTOME_SLC_MEDIATED_TRANSMEMBRANE_TRANS

_BETA_SIGNALING;REACTOME_INTERFERON_SIGNALING;REACTOME_IMMUNE_SYSTEM;REA
ICATION;REACTOME_CELL_CELL_JUNCTION_ORGANIZATION;REACTOME_TIGHT_JUNCTION

EACTOME_POST_CHAPERONIN_TUBULIN_FOLDING_PATHWAY;REACTOME_METABOLISM_O

NO_ACIDS_AND_DERIVATIVES

TRANSMITTER_RELEASE_CYCLE;REACTOME_TRANSMISSION_ACROSS_CHEMICAL_SYNAPS

;REACTOME_PEPTIDE_LIGAND_BINDING_RECEPTORS;REACTOME_CLASS_A1_RHODOPSIN_

ONS;REACTOME_PHASE1_FUNCTIONALIZATION_OF_COMPOUNDS;REACTOME_ETHANOL_C
ONS;REACTOME_PHASE1_FUNCTIONALIZATION_OF_COMPOUNDS;REACTOME_ETHANOL_C

OLOGY;REACTOME_PPARA_ACTIVATES_GENE_EXPRESSION;REACTOME_GENERIC_TRANSC

OLOGY;REACTOME_AXON_GUIDANCE;REACTOME_L1CAM_INTERACTIONS
GTPASES;REACTOME_MEMBRANE_TRAFFICKING;REACTOME_TRANS_GOLGI_NETWORK_VE

ACTOME_ADAPTIVE_IMMUNE_SYSTEM;REACTOME_CLASS_I_MHC_MEDIATED_ANTIGEN_PRO

OLOGY;REACTOME_SIGNALING_BY_NODAL
;REACTOME_OLFACTORY_SIGNALING_PATHWAY;REACTOME_GPCR_DOWNSTREAM_SIGNA

CLEOTIDES;REACTOME_PURINE_RIBONUCLEOSIDE_MONOPHOSPHATE_BIOSYNTHESIS;REA

_IN_MEGAKARYOCYTE_DEVELOPMENT_AND_PLATELET_PRODUCTION;REACTOME_KINESIN
_IN_MEGAKARYOCYTE_DEVELOPMENT_AND_PLATELET_PRODUCTION;REACTOME_KINESIN
OSS_CHEMICAL_SYNAPSES;REACTOME_NEURONAL_SYSTEM

OLOGY;REACTOME_AXON_GUIDANCE;REACTOME_SIGNALING_BY_ROBO_RECEPTOR
ABOLISM;REACTOME_GASTRIN_CREB_SIGNALLING_PATHWAY_VIA_PKC_AND_MAPK;REACTO

REACTOME_SIGNALING_BY_SCF_KIT;REACTOME_CELL_CELL_COMMUNICATION;REACTOME
REACTOME_SIGNALING_BY_SCF_KIT;REACTOME_CELL_CELL_COMMUNICATION;REACTOME

ALLING_PATHWAY_VIA_PKC_AND_MAPK;REACTOME_SIGNALING_BY_GPCR;REACTOME_PE

IG_CROSS_PRESENTATION;REACTOME_ENDOSOMAL_VACUOLAR_PATHWAY;REACTOME_E
IPTION;REACTOME_TRANSCRIPTION;REACTOME_RNA_POL_I_RNA_POL_III_AND_MITOCHOI
TION_PATHWAY;REACTOME_NUCLEAR_RECEPTOR_TRANSCRIPTION_PATHWAY
ENE_EXPRESSION;REACTOME_METABOLISM_OF_LIPIDS_AND_LIPOPROTEINS;REACTOME_
ONS;REACTOME_GLUTATHIONE_CONJUGATION;REACTOME_PHASE_II_CONJUGATION
TRIX_ORGANIZATION;REACTOME_COLLAGEN_FORMATION

OLOGY;REACTOME_REGULATION_OF_BETA_CELL_DEVELOPMENT;REACTOME_REGULATION

OSS_CHEMICAL_SYNAPSES;REACTOME_NEURONAL_SYSTEM;REACTOME_SIGNALING_BY_G

ISM_AND_CITRIC_ACID_TCA_CYCLE;REACTOME_TCA_CYCLE_AND_RESPIRATORY_ELECTR

RACTIONS_AT_THE_VASCULAR_WALL;REACTOME_TIE2_SIGNALING;REACTOME_HEMOSTA

RANSPORT_OF_SMALL_MOLECULES;REACTOME_SLC_MEDIATED_TRANSMEMBRANE_TRANS

OLOGY;REACTOME_NEURONAL_SYSTEM;REACTOME_AXON_GUIDANCE;REACTOME_L1CAM_

RANSPORT_OF_SMALL_MOLECULES;REACTOME_SLC_MEDIATED_TRANSMEMBRANE_TRANS

OLOGY;REACTOME_TRANSMISSION_ACROSS_CHEMICAL_SYNAPSES;REACTOME_NEURONA

ATED_PLATELET_CYTOSOLIC_CA2_;REACTOME_MUSCLE_CONTRACTION;REACTOME_SMO

RANSPORT_OF_SMALL_MOLECULES;REACTOME_AQUAPORIN_MEDIATED_TRANSPORT;REA

REACTOME_SIGNALING_BY_SCF_KIT;REACTOME_DEVELOPMENTAL_BIOLOGY;REACTOME_
REACTOME_DAG_AND_IP3_SIGNALING;REACTOME_SIGNALING_BY_ERBB2;REACTOME_SIG
S;REACTOME_ACTIVATION_OF_CHAPERONE_GENES_BY_XBP1S;REACTOME_UNFOLDED_PI

KING;REACTOME_TRANS_GOLGI_NETWORK_VESICLE_BUDDING;REACTOME_GOLGI_ASSOC

)SS_CHEMICAL_SYNAPSES;REACTOME_NEURONAL_SYSTEM;REACTOME_NEUROTRANSMIT

ISM_AND_CITRIC_ACID_TCA_CYCLE;REACTOME_TCA_CYCLE_AND_RESPIRATORY_ELECTR

THWAY;REACTOME_DNA_REPAIR

ALLING_PATHWAY_VIA_PKC_AND_MAPK;REACTOME_SIGNALING_BY_GPCR;REACTOME_INT
HENATE_METABOLISM;REACTOME_METABOLISM_OF_VITAMINS_AND_COFACTORS;REACTO

S;REACTOME_PERK_REGULATED_GENE_EXPRESSION;REACTOME_ACTIVATION_OF_GENES
JPLED_NER_TC_NER;REACTOME_NUCLEOTIDE_EXCISION_REPAIR;REACTOME_FORMATION
OTEIN_IMPORT;REACTOME_METABOLISM_OF_PROTEINS

OLOGY;REACTOME_AXON_GUIDANCE;REACTOME_NCAM_SIGNALING_FOR_NEURITE_OUT_C
S;REACTOME_PERK_REGULATED_GENE_EXPRESSION;REACTOME_ACTIVATION_OF_GENES

ELLING_OF_PI;REACTOME_ACYL_CHAIN_REMODELLING_OF_PC;REACTOME_PHOSPHOLIPID
NO_ACIDS_AND_DERIVATIVES;REACTOME_GLYCOPROTEIN_HORMONES;REACTOME_PEPT

REACTOME_DAG_AND_IP3_SIGNALING;REACTOME_SIGNALING_BY_ERBB2;REACTOME_SIG
TION_PATHWAY

_PRE_REPLICATIVE_COMPLEX;REACTOME_CELL_CYCLE;REACTOME_ORC1_REMOVAL_FRO

CTOME_GENERATION_OF_SECOND_MESSENGER_MOLECULES;REACTOME_IMMUNE_SYSTE

)
.ATION_OF_MUCINS;REACTOME_METABOLISM_OF_PROTEINS;REACTOME_POST_TRANSLAT

GTPASES;REACTOME_SIGNALLING_BY_NGF;REACTOME_NRAGE_SIGNALS_DEATH_THROUG
DS_AND_LIPOPROTEINS;REACTOME_CHOLESTEROL_BIOSYNTHESIS

ACTOME_ADAPTIVE_IMMUNE_SYSTEM;REACTOME_CLASS_I_MHC_MEDIATED_ANTIGEN_PRO

TRIX_ORGANIZATION;REACTOME_COLLAGEN_FORMATION

ICATION;REACTOME_CELL_CELL_JUNCTION_ORGANIZATION;REACTOME_TIGHT_JUNCTION

NS_MEDIATED_TRANSPORT;REACTOME_TRANSMEMBRANE_TRANSPORT_OF_SMALL_MOLE

ON_OF_SOLUBLE_EXOGENOUS_ANTIGENS_ENDOSOMES;REACTOME_ANTIGEN_PROCESSIN

GTPASES;REACTOME_SIGNALLING_BY_NGF;REACTOME_NRAGE_SIGNALS_DEATH_THROU(

GTPASES;REACTOME_SIGNALLING_BY_NGF;REACTOME_NRAGE_SIGNALS_DEATH_THROU(

:IPTION_TERMINATION;REACTOME_RNA_POL_I_TRANSCRIPTION;REACTOME_TRANSCRIPTI

CLEOTIDES;REACTOME_PYRIMIDINE_METABOLISM

ORT_ACROSS_THE_PLASMA_MEMBRANE;REACTOME_TRANSMEMBRANE_TRANSPORT_OF_
ORT_ACROSS_THE_PLASMA_MEMBRANE;REACTOME_TRANSMEMBRANE_TRANSPORT_OF_
GTPASES

GTPASES

RANSPORT_OF_SMALL_MOLECULES;REACTOME_SLC_MEDIATED_TRANSMEMBRANE_TRANS

:IPTION_TERMINATION;REACTOME_RNA_POL_I_TRANSCRIPTION;REACTOME_TRANSCRIPTI

RIPTION_INITIATION_FROM_TYPE_2_PROMOTER;REACTOME_RNA_POL_III_TRANSCRIPTION
NO_ACIDS_AND_DERIVATIVES

LAR_DOMAIN_REGULATES_TRANSCRIPTION;REACTOME_SIGNALING_BY_NOTCH1;REACTO

_IN_MEGAKARYOCYTE_DEVELOPMENT_AND_PLATELET_PRODUCTION;REACTOME_HEMOST

;REACTOME_CLASS_B_2_SECRETIN_FAMILY_RECEPTORS;REACTOME_GPCR_LIGAND_BIND

AIR;REACTOME_RESOLUTION_OF_AP_SITES_VIA_THE_MULTIPLE_NUCLEOTIDE_PATCH_RE
AIR;REACTOME_RESOLUTION_OF_AP_SITES_VIA_THE_MULTIPLE_NUCLEOTIDE_PATCH_RE

RANSPORT_OF_SMALL_MOLECULES;REACTOME_ION_TRANSPORT_BY_P_TYPE_ATPASES;R

CELL_CYCLE;REACTOME_CELL_CYCLE_CHECKPOINTS;REACTOME_REGULATION_OF_THE_
;REACTOME_CLASS_A1_RHODOPSIN_LIKE_RECEPTORS;REACTOME_P2Y_RECEPTORS;REA

OLOGY;REACTOME_REGULATION_OF_BETA_CELL_DEVELOPMENT;REACTOME_REGULATION

ME_CELL_CYCLE_MITOTIC;REACTOME_CELL_CYCLE_CHECKPOINTS;REACTOME_TRANSCR

TION_PATHWAY
RANSPORT_OF_SMALL_MOLECULES;REACTOME_SLC_MEDIATED_TRANSMEMBRANE_TRANS
OLOGY;REACTOME_BMAL1_CLOCK_NPAS2_ACTIVATES_CIRCADIAN_EXPRESSION;REACTOM

TRIX_ORGANIZATION;REACTOME_COLLAGEN_FORMATION

LATES_GUANYLATE_CYCLASE;REACTOME_PLATELET_HOMEOSTASIS;REACTOME_HEMOST

_RECEPTOR_SIGNALING;REACTOME_SIGNALING_BY_ERBB4;REACTOME_PROLACTIN_RECE

IPTION_TERMINATION;REACTOME_RNA_POL_I_TRANSCRIPTION;REACTOME_RNA_POL_II_T

_METABOLISM;REACTOME_PHOSPHOLIPID_METABOLISM;REACTOME_CS_DS_DEGRADATIO
_METABOLISM;REACTOME_PHOSPHOLIPID_METABOLISM;REACTOME_CS_DS_DEGRADATIO
_SALT_METABOLISM;REACTOME_SYNTHESIS_OF_BILE_ACIDS_AND_BILE_SALTS_VIA_7ALPH
IINOACYLATION;REACTOME_TRNA_AMINOACYLATION
IINOACYLATION;REACTOME_TRNA_AMINOACYLATION

RANSPORT_OF_SMALL_MOLECULES;REACTOME_ION_CHANNEL_TRANSPORT;REACTOME_L

OLOGY;REACTOME_TRANSCRIPTIONAL_REGULATION_OF_WHITE_ADIPOCYTE_DIFFERENTIA

CELL_CYCLE;REACTOME_RNA_POL_I_TRANSCRIPTION;REACTOME_TRANSCRIPTION;REACT

_FOR_APOPTOSIS;REACTOME_DEVELOPMENTAL_BIOLOGY;REACTOME_TRANSCRIPTIONAL

EROID_HORMONES_AND_VITAMINS_A_AND_D;REACTOME_BIOLOGICAL_OXIDATIONS;REACT

NA_AMINOACYLATION;REACTOME_TRNA_AMINOACYLATION

ME_BETA_DEFENSINS;REACTOME_INNATE_IMMUNE_SYSTEM;REACTOME_IMMUNE_SYSTEM

LATION_OF_MUCINS;REACTOME_METABOLISM_OF_PROTEINS;REACTOME_POST_TRANSLA

_B_CELL_RECEPTOR_LEADING_TO_GENERATION_OF_SECOND_MESSENGERS;REACTOME_

KING;REACTOME_ENDOSOMAL_SORTING_COMPLEX_REQUIRED_FOR_TRANSPORT_ESCRT
4;REACTOME_DOWNREGULATION_OF_ERBB2_ERBB3_SIGNALING;REACTOME_SIGNALING_

RECYCLING;REACTOME_NEF_MEDIATES_DOWN_MODULATION_OF_CELL_SURFACE_RECEP
CELL_CYCLE;REACTOME_CHROMOSOME_MAINTENANCE;REACTOME_MEIOTIC_SYNAPSIS;R
LAR_DOMAIN_REGULATES_TRANSCRIPTION;REACTOME_SIGNALING_BY_NOTCH1;REACTO

F7_ACTIVATION;REACTOME_FACTORS_INVOLVED_IN_MEGAKARYOCYTE_DEVELOPMENT_A

ILAR_DOMAIN_REGULATES_TRANSCRIPTION;REACTOME_SIGNALING_BY_NOTCH1;REACTO

REACTOME_ARMS_MEDIATED_ACTIVATION;REACTOME_PROLONGED_ERK_ACTIVATION_E

NA;REACTOME_METABOLISM_OF_RNA;REACTOME_NONSENSE_MEDIATED_DECAY_ENHAN

OLOGY;REACTOME_REGULATION_OF_APOPTOSIS;REACTOME_AXON_GUIDANCE;REACTOM
OLOGY;REACTOME_REGULATION_OF_APOPTOSIS;REACTOME_AXON_GUIDANCE;REACTOM

EN_PRESENTATION;REACTOME_FACTORS_INVOLVED_IN_MEGAKARYOCYTE_DEVELOPMEN

ATION_OF_MUCINS;REACTOME_TERMINATION_OF_O_GLYCAN_BIOSYNTHESIS;REACTOME_

PTOR_INTERACTIONS;REACTOME_RESPONSE_TO_ELEVATED_PLATELET_CYTOSOLIC_CA2_

)LOGY;REACTOME_AXON_GUIDANCE;REACTOME_NCAM1_INTERACTIONS;REACTOME_NCA

ME_GLUCONEOGENESIS;REACTOME_METABOLISM_OF_CARBOHYDRATES;REACTOME_GLU
ME_GLUCONEOGENESIS;REACTOME_METABOLISM_OF_CARBOHYDRATES;REACTOME_GLU
KING;REACTOME_TRANS_GOLGI_NETWORK_VESICLE_BUDDING;REACTOME_GOLGI_ASSOC
S;REACTOME_ACTIVATION_OF_CHAPERONE_GENES_BY_XBP1S;REACTOME_UNFOLDED_PI

EROID_HORMONES_AND_VITAMINS_A_AND_D;REACTOME_BIOLOGICAL_OXIDATIONS;REACT
EROID_HORMONES_AND_VITAMINS_A_AND_D;REACTOME_BIOLOGICAL_OXIDATIONS;REACT

CONTRACTION;REACTOME_MUSCLE_CONTRACTION

)LOGY;REACTOME_PPARA_ACTIVATES_GENE_EXPRESSION;REACTOME_METABOLISM_OF_

ELLING_OF_PI;REACTOME_ACYL_CHAIN_REMODELLING_OF_PC;REACTOME_PHOSPHOLIPID

_IN_MEGAKARYOCYTE_DEVELOPMENT_AND_PLATELET_PRODUCTION;REACTOME_HEMOST

RIN_BH4_SYNTHESIS_RECYCLING_SALVAGE_AND_REGULATION;REACTOME_ENOS_ACTIVA

ME_CELL_CYCLE_MITOTIC;REACTOME_CYCLIN_E_ASSOCIATED_EVENTS_DURING_G1_S_T
ME_CELL_CYCLE_MITOTIC;REACTOME_CYCLIN_E_ASSOCIATED_EVENTS_DURING_G1_S_T

EROID_HORMONES_AND_VITAMINS_A_AND_D;REACTOME_TRAFFICKING_AND_PROCESSING

ACTOME_ADAPTIVE_IMMUNE_SYSTEM;REACTOME_CLASS_I_MHC_MEDIATED_ANTIGEN_PR

OLOGY;REACTOME_AXON_GUIDANCE;REACTOME_NCAM_SIGNALING_FOR_NEURITE_OUT_

OME_FORMATION_OF_THE_TERNARY_COMPLEX_AND_SUBSEQUENTLY_THE_43S_COMPLE

REACTOME_SIGNALING_BY_SCF_KIT;REACTOME_DEVELOPMENTAL_BIOLOGY;REACTOME_
REACTOME_NGF_SIGNALLING_VIA_TRKA_FROM_THE_PLASMA_MEMBRANE

OSS_CHEMICAL_SYNAPSES;REACTOME_NEURONAL_SYSTEM;REACTOME_NEUROTRANSMIT

REACTOME_DEVELOPMENTAL_BIOLOGY;REACTOME_TRIF_MEDIATED_TLR3_SIGNALING;RE

NA;REACTOME_METABOLISM_OF_RNA;REACTOME_NONSENSE_MEDIATED_DECAY_ENHAN
SIGNALLING_CASCADE;REACTOME_PKB_MEDIATED_EVENTS;REACTOME_SIGNALING_BY_I
RIPTION_INITIATION_FROM_TYPE_2_PROMOTER;REACTOME_RNA_POL_III_TRANSCRIPTIO

RACTIONS_AT_THE_VASCULAR_WALL;REACTOME_INTEGRIN_CELL_SURFACE_INTERACTIO

IC_CCT_WITH_TARGET_PROTEINS_DURING_BIOSYNTHESIS;REACTOME_PROTEIN_FOLDIN

ͻSOLIC_CA2_LEVELS;REACTOME_PLATELET_HOMEOSTASIS;REACTOME_PLATELET_CALCIU

ͻLOGY;REACTOME_PPARA_ACTIVATES_GENE_EXPRESSION;REACTOME_RORA_ACTIVATES

GTPASES

ΥNTHESIS;REACTOME_FATTY_ACYL_COA_BIOSYNTHESIS;REACTOME_INTEGRATION_OF_EN

ͻLOGY;REACTOME_BMAL1_CLOCK_NPAS2_ACTIVATES_CIRCADIAN_EXPRESSION;REACTOM

RANSPORT_OF_SMALL_MOLECULES;REACTOME_SLC_MEDIATED_TRANSMEMBRANE_TRANS

ͻLOGY;REACTOME_AXON_GUIDANCE;REACTOME_NCAM1_INTERACTIONS;REACTOME_NCA

RIPTION;REACTOME_MRNA_CAPPING;REACTOME_MRNA_PROCESSING;REACTOME_TRANS

ͻLOGY;REACTOME_MYOGENESIS

RANSPORT_OF_SMALL_MOLECULES;REACTOME_ION_TRANSPORT_BY_P_TYPE_ATPASES;R

GTPASES
GTPASES
)LOGY;REACTOME_MYOGENESIS
)LOGY;REACTOME_MYOGENESIS

)LOGY;REACTOME_PPARA_ACTIVATES_GENE_EXPRESSION;REACTOME_METABOLISM_OF_

.ATION_OF_MUCINS;REACTOME_TERMINATION_OF_O_GLYCAN_BIOSYNTHESIS;REACTOME_
.ATION_OF_MUCINS;REACTOME_TERMINATION_OF_O_GLYCAN_BIOSYNTHESIS;REACTOME_
.ATION_OF_MUCINS;REACTOME_TERMINATION_OF_O_GLYCAN_BIOSYNTHESIS;REACTOME_
.ATION_OF_MUCINS;REACTOME_TERMINATION_OF_O_GLYCAN_BIOSYNTHESIS;REACTOME_

'TION_PATHWAY

4_HETEROTRIMER_REGULATES_TRANSCRIPTION;REACTOME_TRANSCRIPTIONAL_ACTIVIT

NO_ACIDS_AND_DERIVATIVES;REACTOME_AMINE_DERIVED_HORMONES;REACTOME_TRAN

REACTOME_P75_NTR_RECEPTOR_MEDIATED_SIGNALLING;REACTOME_SIGNALING_BY_GP
)SS_CHEMICAL_SYNAPSES;REACTOME_NEURONAL_SYSTEM;REACTOME_NEUROTRANSMIT
)SS_CHEMICAL_SYNAPSES;REACTOME_NEURONAL_SYSTEM;REACTOME_NEUROTRANSMIT

)TEINS;REACTOME_POST_TRANSLATIONAL_MODIFICATION_SYNTHESIS_OF_GPI_ANCHORE
)TEINS;REACTOME_POST_TRANSLATIONAL_MODIFICATION_SYNTHESIS_OF_GPI_ANCHORE

'TION_PATHWAY

ICATION;REACTOME_SIGNAL_REGULATORY_PROTEIN_SIRP_FAMILY_INTERACTIONS

ALLING_PATHWAY_VIA_PKC_AND_MAPK;REACTOME_TRANSMISSION_ACROSS_CHEMICAL_

EACTOME_REGULATION_OF_SIGNALING_BY_CBL;REACTOME_IL_3_5_AND_GM_CSF_SIGNAL
OLOGY;REACTOME_BMAL1_CLOCK_NPAS2_ACTIVATES_CIRCADIAN_EXPRESSION;REACTOM
OLOGY;REACTOME_BMAL1_CLOCK_NPAS2_ACTIVATES_CIRCADIAN_EXPRESSION;REACTOM

_METABOLISM;REACTOME_METABOLISM_OF_AMINO_ACIDS_AND_DERIVATIVES

NO_ACIDS_AND_DERIVATIVES

SION_AND_PROCESSING;REACTOME_PRE_NOTCH_PROCESSING_IN_GOLGI;REACTOME_SI

OLOGY;REACTOME_BMAL1_CLOCK_NPAS2_ACTIVATES_CIRCADIAN_EXPRESSION;REACTOM
ENE_EXPRESSION;REACTOME_METABOLISM_OF_LIPIDS_AND_LIPOPROTEINS;REACTOME_
CELL_CYCLE;REACTOME_CHROMOSOME_MAINTENANCE;REACTOME_MEIOTIC_SYNAPSIS

ROCESSING_OF_ENDOSOMAL_TLR;REACTOME_TRAF6_MEDIATED_INDUCTION_OF_NFKB_A

ROCESSING_OF_ENDOSOMAL_TLR;REACTOME_TRAF6_MEDIATED_INDUCTION_OF_NFKB_A
CONTRACTION;REACTOME_MUSCLE_CONTRACTION
ONS;REACTOME_TRANSMISSION_ACROSS_CHEMICAL_SYNAPSES;REACTOME_NEURONAL_

SPIRATORY_ELECTRON_TRANSPORT;REACTOME_RESPIRATORY_ELECTRON_TRANSPORT;

OLOGY;REACTOME_PPARA_ACTIVATES_GENE_EXPRESSION;REACTOME_GENERIC_TRANSC

GTPASES;REACTOME_SIGNALLING_BY_NGF;REACTOME_CELL_CELL_COMMUNICATION;REA

GE_OF_CELLULAR_PROTEINS;REACTOME_APOPTOSIS;REACTOME_APOPTOTIC_EXECUTION

_BETA_SIGNALING;REACTOME_INTERFERON_SIGNALING;REACTOME_IMMUNE_SYSTEM;REA

)LOGY;REACTOME_TCR_SIGNALING;REACTOME_GENERATION_OF_SECOND_MESSENGER_

PROTEOLYTIC_ACTIVITY_OF_APC_C_REQUIRED_FOR_THE_ONSET_OF_ANAPHASE_BY_MIT(

)TEIN_IMPORT;REACTOME_GLUCONEOGENESIS;REACTOME_METABOLISM_OF_PROTEINS;I
ACTOME_ADAPTIVE_IMMUNE_SYSTEM;REACTOME_CLASS_I_MHC_MEDIATED_ANTIGEN_PR(

ALLING_PATHWAY_VIA_PKC_AND_MAPK;REACTOME_SIGNALING_BY_GPCR;REACTOME_PE
RANSPORT_OF_SMALL_MOLECULES;REACTOME_SLC_MEDIATED_TRANSMEMBRANE_TRANS
RANSPORT_OF_SMALL_MOLECULES;REACTOME_SLC_MEDIATED_TRANSMEMBRANE_TRANS

'TION_PATHWAY
)LOGY;REACTOME_AXON_GUIDANCE;REACTOME_ACTIVATION_OF_RAC;REACTOME_SIGNA

ORT_ACROSS_THE_PLASMA_MEMBRANE;REACTOME_TRANSMEMBRANE_TRANSPORT_OF_

TRIX_ORGANIZATION;REACTOME_COLLAGEN_FORMATION
TRIX_ORGANIZATION;REACTOME_COLLAGEN_FORMATION

CELL_CYCLE;REACTOME_CHROMOSOME_MAINTENANCE;REACTOME_MEIOTIC_SYNAPSIS

_TRANSMITS_SIGNAL_TO_THE_NUCLEUS;REACTOME_SIGNALING_BY_NOTCH1;REACTOME_

ONS;REACTOME_GLUTATHIONE_CONJUGATION;REACTOME_PHASE_II_CONJUGATION

REACTOME_SIGNALING_BY_SCF_KIT;REACTOME_SIGNALING_BY_ERBB4;REACTOME_SIGN

4;REACTOME_DOWNREGULATION_OF_ERBB2_ERBB3_SIGNALING;REACTOME_SIGNALING_
AIR;REACTOME_RESOLUTION_OF_AP_SITES_VIA_THE_MULTIPLE_NUCLEOTIDE_PATCH_RE

ALLING_PATHWAY_VIA_PKC_AND_MAPK;REACTOME_SIGNALING_BY_GPCR;REACTOME_G_
NO_ACIDS_AND_DERIVATIVES

_SALT_METABOLISM;REACTOME_SYNTHESIS_OF_BILE_ACIDS_AND_BILE_SALTS_VIA_7ALPH

ME_CELL_CYCLE_MITOTIC;REACTOME_RECRUITMENT_OF_MITOTIC_CENTROSOME_PROTE

Y_INTERACTIONS_BETWEEN_A_LYMPHOID_AND_A_NON_LYMPHOID_CELL;REACTOME_IMMU

OLOGY;REACTOME_BMAL1_CLOCK_NPAS2_ACTIVATES_CIRCADIAN_EXPRESSION;REACTOM

SM_BY_IFN_STIMULATED_GENES;REACTOME_INTERFERON_ALPHA_BETA_SIGNALING;REAC

ACTOME_ADAPTIVE_IMMUNE_SYSTEM;REACTOME_CLASS_I_MHC_MEDIATED_ANTIGEN_PRO

_TRANSMITS_SIGNAL_TO_THE_NUCLEUS;REACTOME_SIGNALING_BY_NOTCH1;REACTOME_

R_SIGNALING_IN_EMT_EPITHELIAL_TO_MESENCHYMAL_TRANSITION;REACTOME_GPVI_MEI

_DNA_FRAGMENTATION;REACTOME_APOPTOSIS;REACTOME_APOPTOTIC_EXECUTION_PH
)
)

RANSPORT_OF_SMALL_MOLECULES;REACTOME_SLC_MEDIATED_TRANSMEMBRANE_TRANS

TRIX_ORGANIZATION;REACTOME_COLLAGEN_FORMATION

REACTOME_SIGNALING_BY_SCF_KIT;REACTOME_SIGNALING_BY_ERBB4;REACTOME_SIGN

;REACTOME_CLASS_C_3_METABOTROPIC_GLUTAMATE_PHEROMONE_RECEPTORS;REACT
ACTOME_ADAPTIVE_IMMUNE_SYSTEM;REACTOME_CLASS_I_MHC_MEDIATED_ANTIGEN_PR
ACTOME_ADAPTIVE_IMMUNE_SYSTEM;REACTOME_CLASS_I_MHC_MEDIATED_ANTIGEN_PR

ACTOME_IMMUNE_SYSTEM;REACTOME_ADAPTIVE_IMMUNE_SYSTEM
ON;REACTOME_CHONDROITIN_SULFATE_DERMATAN_SULFATE_METABOLISM;REACTOME_
ON;REACTOME_CHONDROITIN_SULFATE_DERMATAN_SULFATE_METABOLISM;REACTOME_

RBOHYDRATES
DS_AND_LIPOPROTEINS;REACTOME_FATTY_ACID_TRIACYLGLYCEROL_AND_KETONE_BOD

OTEINS;REACTOME_POST_TRANSLATIONAL_PROTEIN_MODIFICATION;REACTOME_ASPARA

REACTOME_DEVELOPMENTAL_BIOLOGY;REACTOME_TRIF_MEDIATED_TLR3_SIGNALING;RE

E_AND_DEGRADATION;REACTOME_HYALURONAN_METABOLISM;REACTOME_GLYCOSAMIN
E_AND_DEGRADATION;REACTOME_HYALURONAN_METABOLISM;REACTOME_GLYCOSAMIN

KIT;REACTOME_REGULATION_OF_KIT_SIGNALING;REACTOME_NEF_MEDIATES_DOWN_MOD

REACTOME_VOLTAGE_GATED_POTASSIUM_CHANNELS;REACTOME_POTASSIUM_CHANNEL
REACTOME_VOLTAGE_GATED_POTASSIUM_CHANNELS;REACTOME_POTASSIUM_CHANNEL

PPED_INTRON_CONTAINING_PRE_MRNA;REACTOME_MRNA_PROCESSING;REACTOME_MRN
OLOGY;REACTOME_PPARA_ACTIVATES_GENE_EXPRESSION;REACTOME_GENERIC_TRANSC

OF_FGF_SIGNALING;REACTOME_NEGATIVE_REGULATION_OF_FGFR_SIGNALING;REACTOME

ASIS;REACTOME_PLATELET_SENSITIZATION_BY_LDL;REACTOME_HEMOSTASIS

IMULATES_FATTY_ACID_OXIDATION_IN_MUSCLE;REACTOME_INSULIN_RECEPTOR_SIGNAL

CTOME_ANTIVIRAL_MECHANISM_BY_IFN_STIMULATED_GENES;REACTOME_SIGNALING_BY_

OTEINS;REACTOME_POST_TRANSLATIONAL_MODIFICATION_SYNTHESIS_OF_GPI_ANCHORE

ALLING_PATHWAY_VIA_PKC_AND_MAPK;REACTOME_SIGNALING_BY_GPCR;REACTOME_CL

NS_MEDIATED_TRANSPORT;REACTOME_TRANSMEMBRANE_TRANSPORT_OF_SMALL_MOLE
NS_MEDIATED_TRANSPORT;REACTOME_TRANSMEMBRANE_TRANSPORT_OF_SMALL_MOLE

TRIX_ORGANIZATION;REACTOME_COLLAGEN_FORMATION

RIPTION_AND_TRANSLATION;REACTOME_PRE_NOTCH_EXPRESSION_AND_PROCESSING;R
GTPASES;REACTOME_DEVELOPMENTAL_BIOLOGY;REACTOME_SIGNALING_BY_GPCR;REAC

CLEOTIDES;REACTOME_PURINE_SALVAGE;REACTOME_PURINE_METABOLISM
CLEOTIDES;REACTOME_PURINE_SALVAGE;REACTOME_PURINE_METABOLISM

RIPTION_AND_TRANSLATION;REACTOME_SIGNALING_BY_NOTCH2;REACTOME_PRE_NOTC
RIPTION_AND_TRANSLATION;REACTOME_SIGNALING_BY_NOTCH2;REACTOME_PRE_NOTC

RIPTION_INITIATION_FROM_TYPE_2_PROMOTER;REACTOME_RNA_POL_III_TRANSCRIPTION

NA_AMINOACYLATION;REACTOME_TRNA_AMINOACYLATION
_AT_THE_GOLGI_MEMBRANE;REACTOME_PHOSPHOLIPID_METABOLISM;REACTOME_PI_MET

ACTOME_ADAPTIVE_IMMUNE_SYSTEM;REACTOME_CLASS_I_MHC_MEDIATED_ANTIGEN_PR
OTEIN_IMPORT;REACTOME_METABOLISM_OF_PROTEINS

ME_CELL_CYCLE_MITOTIC;REACTOME_MITOTIC_M_M_G1_PHASES;REACTOME_DNA_REPL
GTPASES;REACTOME_SIGNALLING_BY_NGF;REACTOME_DEVELOPMENTAL_BIOLOGY;REAC

TEM;REACTOME_IMMUNE_SYSTEM;REACTOME_COMPLEMENT_CASCADE;REACTOME_CRE

;REACTOME_GPCR_DOWNSTREAM_SIGNALING;REACTOME_G_ALPHA_I_SIGNALLING_EVEN

RIPTION;REACTOME_TRANSCRIPTION;REACTOME_RNA_POL_I_RNA_POL_III_AND_MITOCHO

RACTIONS_AT_THE_VASCULAR_WALL;REACTOME_RESPONSE_TO_ELEVATED_PLATELET_C

S;REACTOME_REGULATION_OF_INSULIN_LIKE_GROWTH_FACTOR_IGF_ACTIVITY_BY_INSUL

OLOGY;REACTOME_AXON_GUIDANCE;REACTOME_SIGNALING_BY_ROBO_RECEPTOR

N_CODING_RNA;REACTOME_ANTIVIRAL_MECHANISM_BY_IFN_STIMULATED_GENES;REACTO

OLOGY;REACTOME_AXON_GUIDANCE;REACTOME_NCAM1_INTERACTIONS;REACTOME_NCA

OTEIN_IMPORT;REACTOME_METABOLISM_OF_PROTEINS
BOLISM;REACTOME_SYNTHESIS_OF_PE;REACTOME_GLYCEROPHOSPHOLIPID_BIOSYNTHE
OLOGY;REACTOME_AXON_GUIDANCE;REACTOME_NCAM1_INTERACTIONS;REACTOME_NCA
MPLEMENT_CASCADE;REACTOME_INNATE_IMMUNE_SYSTEM;REACTOME_IMMUNE_SYSTEM

OLOGY;REACTOME_AXON_GUIDANCE;REACTOME_CRMPS_IN_SEMA3A_SIGNALING;REACTO
EROID_HORMONES_AND_VITAMINS_A_AND_D;REACTOME_STEROID_HORMONES;REACTOM

RANSPORT_OF_SMALL_MOLECULES;REACTOME_IRON_UPTAKE_AND_TRANSPORT

TION_PATHWAY;REACTOME_NUCLEAR_RECEPTOR_TRANSCRIPTION_PATHWAY

NA_AMINOACYLATION;REACTOME_TRNA_AMINOACYLATION
NA_AMINOACYLATION;REACTOME_TRNA_AMINOACYLATION

OLOGY;REACTOME_SIGNALING_BY_NODAL;REACTOME_RESPONSE_TO_ELEVATED_PLATEL

GTPASES;REACTOME_SIGNALLING_BY_NGF;REACTOME_NRAGE_SIGNALS_DEATH_THROUG

REACTOME_TANDEM_PORE_DOMAIN_POTASSIUM_CHANNELS;REACTOME_POTASSIUM_CH

REACTOME_SIGNALING_BY_SCF_KIT;REACTOME_DEVELOPMENTAL_BIOLOGY;REACTOME_

TION_PATHWAY

NO_ACIDS_AND_DERIVATIVES;REACTOME_AMINE_DERIVED_HORMONES

ELLING_OF_PC;REACTOME_PHOSPHOLIPID_METABOLISM;REACTOME_ACYL_CHAIN_REMOD
REACTOME_GROWTH_HORMONE_RECEPTOR_SIGNALING;REACTOME_SIGNALING_BY_ERE

REACTOME_INTEGRATION_OF_ENERGY_METABOLISM;REACTOME_REGULATION_OF_INSUL

LOGY;REACTOME_BMAL1_CLOCK_NPAS2_ACTIVATES_CIRCADIAN_EXPRESSION;REACTOM

CLEOTIDES;REACTOME_PYRIMIDINE_METABOLISM
CLEOTIDES;REACTOME_PYRIMIDINE_METABOLISM

RANSPORT_OF_SMALL_MOLECULES;REACTOME_SLC_MEDIATED_TRANSMEMBRANE_TRANS

LOGY;REACTOME_CELL_CELL_COMMUNICATION;REACTOME_AXON_GUIDANCE;REACTOME
LOGY;REACTOME_CELL_CELL_COMMUNICATION;REACTOME_AXON_GUIDANCE;REACTOME
REACTOME_P75_NTR_RECEPTOR_MEDIATED_SIGNALLING

ONS;REACTOME_METABOLISM_OF_CARBOHYDRATES;REACTOME_GLUCURONIDATION;REA
EACTOME_IL1_SIGNALING;REACTOME_IRAK1_RECRUITS_IKK_COMPLEX;REACTOME_TRAF6_
GTPASES

XYLATION_HYPUSINE_FORMATION_AND_ARYLSULFATASE_ACTIVATION;REACTOME_GAMM

PPED_INTRON_CONTAINING_PRE_MRNA;REACTOME_MRNA_PROCESSING;REACTOME_MRN

ME_CELL_CYCLE_MITOTIC;REACTOME_MITOTIC_M_M_G1_PHASES;REACTOME_DNA_REPL

REACTOME_SIGNALLING_TO_RAS;REACTOME_NGF_SIGNALLING_VIA_TRKA_FROM_THE_PL
XYLATION_HYPUSINE_FORMATION_AND_ARYLSULFATASE_ACTIVATION;REACTOME_GAMMA

_METABOLISM;REACTOME_PHOSPHOLIPID_METABOLISM;REACTOME_SPHINGOLIPID_META

;REACTOME_CLASS_A1_RHODOPSIN_LIKE_RECEPTORS;REACTOME_GPCR_LIGAND_BINDIN

CONTRACTION;REACTOME_MUSCLE_CONTRACTION
KING;REACTOME_SIGNALING_BY_EGFR_IN_CANCER;REACTOME_EGFR_DOWNREGULATION

ERGY_METABOLISM;REACTOME_REGULATION_OF_INSULIN_SECRETION;REACTOME_SYNT
ERGY_METABOLISM;REACTOME_REGULATION_OF_INSULIN_SECRETION;REACTOME_SYNT

_SALT_METABOLISM;REACTOME_SYNTHESIS_OF_BILE_ACIDS_AND_BILE_SALTS_VIA_7ALPH

ME_MHC_CLASS_II_ANTIGEN_PRESENTATION;REACTOME_CELL_CYCLE_MITOTIC;REACTOI
FACE_INTERACTIONS;REACTOME_INTEGRATION_OF_ENERGY_METABOLISM;REACTOME_IN

OSS_CHEMICAL_SYNAPSES;REACTOME_NEURONAL_SYSTEM;REACTOME_NEUROTRANSMIT

_METABOLISM;REACTOME_METABOLISM_OF_LIPIDS_AND_LIPOPROTEINS
;REACTOME_GPCR_DOWNSTREAM_SIGNALING;REACTOME_G_ALPHA_S_SIGNALLING_EVEI

OLOGY;REACTOME_ANTIGEN_PROCESSING_CROSS_PRESENTATION;REACTOME_CELL_SUI
OLOGY;REACTOME_EXTRACELLULAR_MATRIX_ORGANIZATION;REACTOME_COLLAGEN_FOF

KIT;REACTOME_GROWTH_HORMONE_RECEPTOR_SIGNALING;REACTOME_ANTIVIRAL_MECI

OME_METABOLISM_OF_PROTEINS

NO_ACIDS_AND_DERIVATIVES

OF_HOMO_SAPIENS_WITH_MYCOBACTERIUM_TUBERCULOSIS;REACTOME_TRANSMEMBRAI

CONTRACTION;REACTOME_MUSCLE_CONTRACTION

REACTOME_GROWTH_HORMONE_RECEPTOR_SIGNALING;REACTOME_INSULIN_RECEPTOI

ALLING_PATHWAY_VIA_PKC_AND_MAPK;REACTOME_SIGNALING_BY_GPCR;REACTOME_PE

2_ACTIVATES_CIRCADIAN_EXPRESSION;REACTOME_CIRCADIAN_CLOCK

)LOGY;REACTOME_AXON_GUIDANCE;REACTOME_L1CAM_INTERACTIONS

E_FANCONI_ANEMIA_PATHWAY;REACTOME_FANCONI_ANEMIA_PATHWAY;REACTOME_DNA_
OSS_CHEMICAL_SYNAPSES;REACTOME_NEURONAL_SYSTEM;REACTOME_NEUROTRANSMIT

REACTOME_SIGNALING_BY_SCF_KIT;REACTOME_DEVELOPMENTAL_BIOLOGY;REACTOME_

REACTOME_VOLTAGE_GATED_POTASSIUM_CHANNELS;REACTOME_POTASSIUM_CHANNEL
TION_PATHWAY;REACTOME_NUCLEAR_RECEPTOR_TRANSCRIPTION_PATHWAY

RANSPORT_OF_SMALL_MOLECULES;REACTOME_SLC_MEDIATED_TRANSMEMBRANE_TRANS
R_SIGNALING_IN_EMT_EPITHELIAL_TO_MESENCHYMAL_TRANSITION;REACTOME_DOWNRE

MEIOTIC_RECOMBINATION

;REACTOME_PEPTIDE_LIGAND_BINDING_RECEPTORS;REACTOME_CLASS_A1_RHODOPSIN_

TRIX_ORGANIZATION;REACTOME_COLLAGEN_FORMATION

ENE_EXPRESSION;REACTOME_METABOLISM_OF_LIPIDS_AND_LIPOPROTEINS;REACTOME_

)SS_CHEMICAL_SYNAPSES;REACTOME_NEURONAL_SYSTEM
)SS_CHEMICAL_SYNAPSES;REACTOME_NEURONAL_SYSTEM
_IN_DISEASE;REACTOME_SIGNALING_BY_FGFR
)PTOSIS;REACTOME_ROLE_OF_DCC_IN_REGULATING_APOPTOSIS;REACTOME_APOPTOSIS

NO_ACIDS_AND_DERIVATIVES
)LOGY;REACTOME_AXON_GUIDANCE;REACTOME_CRMPS_IN_SEMA3A_SIGNALING;REACTO

CELL_CYCLE;REACTOME_CELL_CYCLE_CHECKPOINTS;REACTOME_REGULATION_OF_THE_
TRIX_ORGANIZATION;REACTOME_COLLAGEN_FORMATION

CTIVITY_OF_SMAD2_SMAD3_SMAD4_HETEROTRIMER;REACTOME_DOWNREGULATION_OF_

REACTOME_CGMP_EFFECTS;REACTOME_NITRIC_OXIDE_STIMULATES_GUANYLATE_CYCLA

NO_ACIDS_AND_DERIVATIVES;REACTOME_BRANCHED_CHAIN_AMINO_ACID_CATABOLISM

;REACTOME_PEPTIDE_LIGAND_BINDING_RECEPTORS;REACTOME_CLASS_A1_RHODOPSIN_

ATION_OF_MUCINS;REACTOME_TERMINATION_OF_O_GLYCAN_BIOSYNTHESIS;REACTOME_

S;REACTOME_ACTIVATION_OF_CHAPERONE_GENES_BY_XBP1S;REACTOME_UNFOLDED_PF

ORS_OF_RIG_I_MDA5_SIGNALING;REACTOME_RIG_I_MDA5_MEDIATED_INDUCTION_OF_IFN_
ORS_OF_RIG_I_MDA5_SIGNALING;REACTOME_RIG_I_MDA5_MEDIATED_INDUCTION_OF_IFN_

GTPASES

OSS_CHEMICAL_SYNAPSES;REACTOME_NEURONAL_SYSTEM;REACTOME_NEUROTRANSMIT

S;REACTOME_INSULIN_SYNTHESIS_AND_PROCESSING

ONS;REACTOME_GLUCURONIDATION;REACTOME_PHASE_II_CONJUGATION

RANSPORT_OF_SMALL_MOLECULES;REACTOME_SLC_MEDIATED_TRANSMEMBRANE_TRANS
TRIX_ORGANIZATION;REACTOME_COLLAGEN_FORMATION

EACTOME_CELL_CYCLE;REACTOME_CELL_CYCLE_MITOTIC;REACTOME_MITOTIC_G1_G1_S

GTPASES

SIS;REACTOME_HEPARAN_SULFATE_HEPARIN_HS_GAG_METABOLISM;REACTOME_GLYCOS

ATION_OF_MUCINS;REACTOME_METABOLISM_OF_PROTEINS;REACTOME_POST_TRANSLAT

RANSPORT_OF_SMALL_MOLECULES;REACTOME_SLC_MEDIATED_TRANSMEMBRANE_TRANS
ME_CELL_CYCLE_MITOTIC;REACTOME_RECRUITMENT_OF_MITOTIC_CENTROSOME_PROTE

RANSPORT_OF_SMALL_MOLECULES;REACTOME_SLC_MEDIATED_TRANSMEMBRANE_TRANS

GTPASES;REACTOME_SIGNALLING_BY_NGF;REACTOME_DEVELOPMENTAL_BIOLOGY;REAC
GTPASES;REACTOME_SIGNALLING_BY_NGF;REACTOME_DEVELOPMENTAL_BIOLOGY;REAC

IMULATES_FATTY_ACID_OXIDATION_IN_MUSCLE;REACTOME_INSULIN_RECEPTOR_SIGNAL

DS_AND_LIPOPROTEINS;REACTOME_FATTY_ACID_TRIACYLGLYCEROL_AND_KETONE_BOD
ENE_EXPRESSION;REACTOME_METABOLISM_OF_LIPIDS_AND_LIPOPROTEINS;REACTOME_

;REACTOME_OPIOID_SIGNALLING;REACTOME_DARPP_32_EVENTS;REACTOME_GPCR_DOW

ME_CELL_CYCLE_MITOTIC;REACTOME_MITOTIC_M_M_G1_PHASES;REACTOME_CHROMOS

REACTOME_SIGNALING_BY_SCF_KIT;REACTOME_CELL_CELL_COMMUNICATION;REACTOM
ME_CELL_CYCLE_CHECKPOINTS;REACTOME_ACTIVATION_OF_ATR_IN_RESPONSE_TO_RE

N;REACTOME_CHONDROITIN_SULFATE_BIOSYNTHESIS;REACTOME_CHONDROITIN_SULFAT

XYLATION_HYPUSINE_FORMATION_AND_ARYLSULFATASE_ACTIVATION;REACTOME_GLYCO

OLOGY;REACTOME_AXON_GUIDANCE;REACTOME_OTHER_SEMAPHORIN_INTERACTIONS;RE

ME_CELL_CYCLE_MITOTIC;REACTOME_CELL_CYCLE_CHECKPOINTS;REACTOME_CYCLIN_A
ME_CELL_CYCLE_MITOTIC;REACTOME_CELL_CYCLE_CHECKPOINTS;REACTOME_CYCLIN_A
_BETA_SIGNALING;REACTOME_INTERFERON_SIGNALING;REACTOME_IMMUNE_SYSTEM;REA
_BETA_SIGNALING;REACTOME_INTERFERON_SIGNALING;REACTOME_IMMUNE_SYSTEM;REA

REACTOME_CTNNB1_PHOSPHORYLATION_CASCADE
CTOME_NITRIC_OXIDE_STIMULATES_GUANYLATE_CYCLASE;REACTOME_PLATELET_HOMEO

)
MINOACYLATION;REACTOME_TRNA_AMINOACYLATION
OLOGY;REACTOME_AXON_GUIDANCE;REACTOME_NETRIN1_SIGNALING

ON_OF_FGFR_SIGNALING;REACTOME_INSULIN_RECEPTOR_SIGNALLING_CASCADE;REACTO
ON_OF_FGFR_SIGNALING;REACTOME_INSULIN_RECEPTOR_SIGNALLING_CASCADE;REACTO
ON_OF_FGFR_SIGNALING;REACTOME_INSULIN_RECEPTOR_SIGNALLING_CASCADE;REACTO

OME_SRP_DEPENDENT_COTRANSLATIONAL_PROTEIN_TARGETING_TO_MEMBRANE;REACT
;REACTOME_CLASS_C_3_METABOTROPIC_GLUTAMATE_PHEROMONE_RECEPTORS;REACT

OTEINS;REACTOME_POST_TRANSLATIONAL_PROTEIN_MODIFICATION;REACTOME_ASPARA
OTEINS;REACTOME_POST_TRANSLATIONAL_PROTEIN_MODIFICATION;REACTOME_SYNTHE

KING;REACTOME_TRANS_GOLGI_NETWORK_VESICLE_BUDDING;REACTOME_GOLGI_ASSOC

TION_PATHWAY

MBINATION_REPAIR_OF_REPLICATION_INDEPENDENT_DOUBLE_STRAND_BREAKS;REACTO

MINOACYLATION;REACTOME_TRNA_AMINOACYLATION

TRIX_ORGANIZATION;REACTOME_COLLAGEN_FORMATION
TRIX_ORGANIZATION;REACTOME_COLLAGEN_FORMATION
REACTOME_DAG_AND_IP3_SIGNALING;REACTOME_SIGNALING_BY_ERBB2;REACTOME_SIG

'TION_PATHWAY;REACTOME_NUCLEAR_RECEPTOR_TRANSCRIPTION_PATHWAY
'TION_PATHWAY;REACTOME_NUCLEAR_RECEPTOR_TRANSCRIPTION_PATHWAY
'TION_PATHWAY;REACTOME_NUCLEAR_RECEPTOR_TRANSCRIPTION_PATHWAY
OME_SRP_DEPENDENT_COTRANSLATIONAL_PROTEIN_TARGETING_TO_MEMBRANE;REACT

_IN_DISEASE;REACTOME_FRS2_MEDIATED_CASCADE;REACTOME_DOWNSTREAM_SIGNALI

OGENESIS;REACTOME_REGULATORY_RNA_PATHWAYS

TRIX_ORGANIZATION;REACTOME_COLLAGEN_FORMATION

NO_ACIDS_AND_DERIVATIVES;REACTOME_BRANCHED_CHAIN_AMINO_ACID_CATABOLISM

RANSPORT_OF_SMALL_MOLECULES;REACTOME_SLC_MEDIATED_TRANSMEMBRANE_TRANS

'TION_PATHWAY;REACTOME_NUCLEAR_RECEPTOR_TRANSCRIPTION_PATHWAY

FACE_INTERACTIONS
FACE_INTERACTIONS

OXICITY;REACTOME_PROTEOLYTIC_CLEAVAGE_OF_SNARE_COMPLEX_PROTEINS;REACTO

TE_DERMATAN_SULFATE_METABOLISM;REACTOME_GLYCOSAMINOGLYCAN_METABOLISM

CELL_CYCLE;REACTOME_CHROMOSOME_MAINTENANCE;REACTOME_MEIOTIC_SYNAPSIS
CELL_CYCLE;REACTOME_CHROMOSOME_MAINTENANCE;REACTOME_MEIOTIC_SYNAPSIS
CELL_CYCLE;REACTOME_CHROMOSOME_MAINTENANCE;REACTOME_MEIOTIC_SYNAPSIS

DS_AND_LIPOPROTEINS;REACTOME_LIPID_DIGESTION_MOBILIZATION_AND_TRANSPORT;R

ACTOME_ADAPTIVE_IMMUNE_SYSTEM;REACTOME_CLASS_I_MHC_MEDIATED_ANTIGEN_PRO

TE_BIOSYNTHESIS;REACTOME_CHONDROITIN_SULFATE_DERMATAN_SULFATE_METABOLIS
ALING_EVENTS_OF_B_CELL_RECEPTOR_BCR;REACTOME_ACTIVATION_OF_NF_KAPPAB_IN_

ALLING_PATHWAY_VIA_PKC_AND_MAPK;REACTOME_SIGNALING_BY_GPCR;REACTOME_G_

TRIX_ORGANIZATION;REACTOME_COLLAGEN_FORMATION

NS_MEDIATED_TRANSPORT;REACTOME_TRANSMEMBRANE_TRANSPORT_OF_SMALL_MOLE

NO_ACIDS_AND_DERIVATIVES;REACTOME_BRANCHED_CHAIN_AMINO_ACID_CATABOLISM
B_SIGNALING;REACTOME_TAK1_ACTIVATES_NFKB_BY_PHOSPHORYLATION_AND_ACTIVATIO

IC_CCT_WITH_TARGET_PROTEINS_DURING_BIOSYNTHESIS;REACTOME_PROTEIN_FOLDING

ISM_AND_CITRIC_ACID_TCA_CYCLE;REACTOME_TCA_CYCLE_AND_RESPIRATORY_ELECTR
REACTOME_DAG_AND_IP3_SIGNALING;REACTOME_SIGNALING_BY_ERBB2;REACTOME_SIG
S;REACTOME_REGULATION_OF_INSULIN_LIKE_GROWTH_FACTOR_IGF_ACTIVITY_BY_INSUL

ATION_OF_MUCINS;REACTOME_METABOLISM_OF_PROTEINS;REACTOME_POST_TRANSLA

CELL_CYCLE;REACTOME_CHROMOSOME_MAINTENANCE;REACTOME_MEIOTIC_SYNAPSIS
;REACTOME_CLASS_B_2_SECRETIN_FAMILY_RECEPTORS;REACTOME_GPCR_LIGAND_BIND

TRANSMITS_SIGNAL_TO_THE_NUCLEUS;REACTOME_SIGNALING_BY_NOTCH1;REACTOME_

LOGY;REACTOME_EXTRACELLULAR_MATRIX_ORGANIZATION;REACTOME_CELL_SURFACE

ACTOME_ADAPTIVE_IMMUNE_SYSTEM;REACTOME_CLASS_I_MHC_MEDIATED_ANTIGEN_PR

TRIX_ORGANIZATION;REACTOME_COLLAGEN_FORMATION

ATION_OF_MUCINS;REACTOME_TERMINATION_OF_O_GLYCAN_BIOSYNTHESIS;REACTOME_

_IN_DISEASE;REACTOME_SIGNALING_BY_FGFR1_MUTANTS;REACTOME_SIGNALING_BY_FG

REACTOME_CROSS_PRESENTATION_OF_SOLUBLE_EXOGENOUS_ANTIGENS_ENDOSOMES;
_PRE_REPLICATIVE_COMPLEX;REACTOME_CELL_CYCLE;REACTOME_ORC1_REMOVAL_FRO

NO_ACIDS_AND_DERIVATIVES

ICATION;REACTOME_CELL_EXTRACELLULAR_MATRIX_INTERACTIONS;REACTOME_CELL_JU
;REACTOME_CLASS_B_2_SECRETIN_FAMILY_RECEPTORS;REACTOME_GPCR_LIGAND_BIND

ALLING_PATHWAY_VIA_PKC_AND_MAPK;REACTOME_SIGNALING_BY_GPCR;REACTOME_G_
ALLING_PATHWAY_VIA_PKC_AND_MAPK;REACTOME_SIGNALING_BY_GPCR;REACTOME_G_

RECYCLING;REACTOME_LATENT_INFECTION_OF_HOMO_SAPIENS_WITH_MYCOBACTERIUM

RBOHYDRATES;REACTOME_DIGESTION_OF_DIETARY_CARBOHYDRATE

RIN_BH4_SYNTHESIS_RECYCLING_SALVAGE_AND_REGULATION;REACTOME_LATENT_INFEC

OLOGY;REACTOME_SIGNALING_BY_GPCR;REACTOME_OPIOID_SIGNALLING;REACTOME_DAI

IMULATES_FATTY_ACID_OXIDATION_IN_MUSCLE;REACTOME_INSULIN_RECEPTOR_SIGNAL
IMULATES_FATTY_ACID_OXIDATION_IN_MUSCLE;REACTOME_INSULIN_RECEPTOR_SIGNAL

ACTOME_ADAPTIVE_IMMUNE_SYSTEM;REACTOME_CLASS_I_MHC_MEDIATED_ANTIGEN_PRO

ME_INNATE_IMMUNE_SYSTEM;REACTOME_IMMUNE_SYSTEM

GTPASES
OTEINS;REACTOME_POST_TRANSLATIONAL_PROTEIN_MODIFICATION;REACTOME_ASPARA

_IN_MEGAKARYOCYTE_DEVELOPMENT_AND_PLATELET_PRODUCTION;REACTOME_HEMOST

MEIOTIC_RECOMBINATION

OLOGY;REACTOME_NEGATIVE_REGULATION_OF_FGFR_SIGNALING;REACTOME_INSULIN_RE
OLOGY;REACTOME_NEGATIVE_REGULATION_OF_FGFR_SIGNALING;REACTOME_INSULIN_RE

AIR;REACTOME_RESOLUTION_OF_AP_SITES_VIA_THE_MULTIPLE_NUCLEOTIDE_PATCH_RE

;REACTOME_GPCR_DOWNSTREAM_SIGNALING;REACTOME_G_ALPHA_S_SIGNALLING_EVE

)LOGY;REACTOME_METABOLISM_OF_LIPIDS_AND_LIPOPROTEINS;REACTOME_TRANSCRIPT

TY_ACID_BETA_OXIDATION;REACTOME_METABOLISM_OF_LIPIDS_AND_LIPOPROTEINS;RE/

TY_ACID_BETA_OXIDATION;REACTOME_METABOLISM_OF_LIPIDS_AND_LIPOPROTEINS;RE/

REACTOME_VOLTAGE_GATED_POTASSIUM_CHANNELS;REACTOME_POTASSIUM_CHANNEL

REACTOME_TANDEM_PORE_DOMAIN_POTASSIUM_CHANNELS;REACTOME_POTASSIUM_CH

GE_OF_CELLULAR_PROTEINS;REACTOME_CELL_CELL_COMMUNICATION;REACTOME_CASP/

RANSMITTER_RELEASE_CYCLE;REACTOME_TRANSMISSION_ACROSS_CHEMICAL_SYNAPS

RIPTION;REACTOME_TRANSCRIPTION;REACTOME_RNA_POL_I_RNA_POL_III_AND_MITOCH(

RANSPORT_OF_SMALL_MOLECULES;REACTOME_SLC_MEDIATED_TRANSMEMBRANE_TRANS

SPIRATORY_ELECTRON_TRANSPORT;REACTOME_RESPIRATORY_ELECTRON_TRANSPORT;

ICATION;REACTOME_CELL_EXTRACELLULAR_MATRIX_INTERACTIONS;REACTOME_CELL_JU

REACTOME_NGF_SIGNALLING_VIA_TRKA_FROM_THE_PLASMA_MEMBRANE

REACTOME_INSULIN_RECEPTOR_SIGNALLING_CASCADE;REACTOME_SIGNALLING_TO_RAS
MINOACYLATION;REACTOME_TRNA_AMINOACYLATION

RPHYRINS
TRIX_ORGANIZATION;REACTOME_COLLAGEN_FORMATION
TRIX_ORGANIZATION;REACTOME_COLLAGEN_FORMATION
TRIX_ORGANIZATION;REACTOME_COLLAGEN_FORMATION

S;REACTOME_REGULATION_OF_INSULIN_LIKE_GROWTH_FACTOR_IGF_ACTIVITY_BY_INSUL
ME_CELL_CYCLE_MITOTIC;REACTOME_RECRUITMENT_OF_MITOTIC_CENTROSOME_PROTE

REACTOME_SIGNALING_BY_SCF_KIT;REACTOME_SIGNALING_BY_ERBB4;REACTOME_SIGN

ACTOME_ADAPTIVE_IMMUNE_SYSTEM;REACTOME_CLASS_I_MHC_MEDIATED_ANTIGEN_PR
RANSMITTER_RELEASE_CYCLE;REACTOME_TRANSMISSION_ACROSS_CHEMICAL_SYNAPS

AMINS_AND_COFACTORS

GE_OF_CELLULAR_PROTEINS;REACTOME_DEVELOPMENTAL_BIOLOGY;REACTOME_CELL_C
GE_OF_CELLULAR_PROTEINS;REACTOME_DEVELOPMENTAL_BIOLOGY;REACTOME_CELL_C
GE_OF_CELLULAR_PROTEINS;REACTOME_DEVELOPMENTAL_BIOLOGY;REACTOME_CELL_C
GE_OF_CELLULAR_PROTEINS;REACTOME_DEVELOPMENTAL_BIOLOGY;REACTOME_CELL_C

NO_ACIDS_AND_DERIVATIVES;REACTOME_AMINO_ACID_SYNTHESIS_AND_INTERCONVERS
NO_ACIDS_AND_DERIVATIVES;REACTOME_AMINO_ACID_SYNTHESIS_AND_INTERCONVERS
OTEINS;REACTOME_POST_TRANSLATIONAL_PROTEIN_MODIFICATION;REACTOME_SYNTHE
METABOLISM;REACTOME_METABOLISM_OF_LIPIDS_AND_LIPOPROTEINS
METABOLISM;REACTOME_METABOLISM_OF_LIPIDS_AND_LIPOPROTEINS
REACTOME_ARMS_MEDIATED_ACTIVATION;REACTOME_PROLONGED_ERK_ACTIVATION_E

RIPTION_INITIATION_FROM_TYPE_2_PROMOTER;REACTOME_RNA_POL_III_TRANSCRIPTION
REACTOME_SIGNALLING_TO_RAS;REACTOME_NGF_SIGNALLING_VIA_TRKA_FROM_THE_P

NO_ACIDS_AND_DERIVATIVES;REACTOME_AMINE_DERIVED_HORMONES
OLOGY;REACTOME_EXTRACELLULAR_MATRIX_ORGANIZATION;REACTOME_COLLAGEN_FO
OLOGY;REACTOME_EXTRACELLULAR_MATRIX_ORGANIZATION;REACTOME_COLLAGEN_FO
OLOGY;REACTOME_EXTRACELLULAR_MATRIX_ORGANIZATION;REACTOME_COLLAGEN_FO

_FOR_APOPTOSIS;REACTOME_TRAF6_MEDIATED_IRF7_ACTIVATION;REACTOME_TRAF6_M
OTEINS;REACTOME_POST_TRANSLATIONAL_PROTEIN_MODIFICATION;REACTOME_ASPARA
RANSPORT_OF_SMALL_MOLECULES;REACTOME_SLC_MEDIATED_TRANSMEMBRANE_TRAN

EROID_HORMONES_AND_VITAMINS_A_AND_D;REACTOME_HDL_MEDIATED_LIPID_TRANSPO
EROID_HORMONES_AND_VITAMINS_A_AND_D;REACTOME_HDL_MEDIATED_LIPID_TRANSPO

FACE_INTERACTIONS;REACTOME_P130CAS_LINKAGE_TO_MAPK_SIGNALING_FOR_INTEGRI

BOLISM;REACTOME_SYNTHESIS_OF_PC;REACTOME_GLYCEROPHOSPHOLIPID_BIOSYNTHE

TRIX_ORGANIZATION;REACTOME_COLLAGEN_FORMATION

;REACTOME_OPIOID_SIGNALLING;REACTOME_DARPP_32_EVENTS

ONS;REACTOME_XENOBIOTICS;REACTOME_CYTOCHROME_P450_ARRANGED_BY_SUBSTRA

_B_CELL_RECEPTOR_LEADING_TO_GENERATION_OF_SECOND_MESSENGERS;REACTOME_

ON;REACTOME_HEPARAN_SULFATE_HEPARIN_HS_GAG_METABOLISM;REACTOME_GLYCOS

ICATION;REACTOME_EXTRACELLULAR_MATRIX_ORGANIZATION;REACTOME_COLLAGEN_F

REACTOME_P75_NTR_RECEPTOR_MEDIATED_SIGNALLING
LOGY;REACTOME_AXON_GUIDANCE;REACTOME_NCAM1_INTERACTIONS;REACTOME_NCA

LOGY;REACTOME_AXON_GUIDANCE;REACTOME_NETRIN1_SIGNALING;REACTOME_DCC_M
LOGY;REACTOME_AXON_GUIDANCE;REACTOME_NETRIN1_SIGNALING;REACTOME_DCC_M

NO_ACIDS_AND_DERIVATIVES;REACTOME_BIOLOGICAL_OXIDATIONS;REACTOME_PHASE1_
NO_ACIDS_AND_DERIVATIVES;REACTOME_BIOLOGICAL_OXIDATIONS;REACTOME_PHASE1_
NO_ACIDS_AND_DERIVATIVES;REACTOME_BIOLOGICAL_OXIDATIONS;REACTOME_PHASE1_

REACTOME_CROSS_PRESENTATION_OF_SOLUBLE_EXOGENOUS_ANTIGENS_ENDOSOMES;

REACTOME_DEVELOPMENTAL_BIOLOGY;REACTOME_NEF_MEDIATES_DOWN_MODULATION

ATION_OF_MUCINS;REACTOME_TERMINATION_OF_O_GLYCAN_BIOSYNTHESIS;REACTOME_
ATION_OF_MUCINS;REACTOME_TERMINATION_OF_O_GLYCAN_BIOSYNTHESIS;REACTOME_
ATION_OF_MUCINS;REACTOME_TERMINATION_OF_O_GLYCAN_BIOSYNTHESIS;REACTOME_
ATION_OF_MUCINS;REACTOME_TERMINATION_OF_O_GLYCAN_BIOSYNTHESIS;REACTOME_
ATION_OF_MUCINS;REACTOME_TERMINATION_OF_O_GLYCAN_BIOSYNTHESIS;REACTOME_

NO_ACIDS_AND_DERIVATIVES;REACTOME_AMINE_DERIVED_HORMONES

Y_INTERACTIONS_BETWEEN_A_LYMPHOID_AND_A_NON_LYMPHOID_CELL;REACTOME_IMMU

;REACTOME_OLFACTORY_SIGNALING_PATHWAY;REACTOME_GPCR_DOWNSTREAM_SIGNA

2_ACTIVATES_CIRCADIAN_EXPRESSION;REACTOME_PPARA_ACTIVATES_GENE_EXPRESSIO

SIGNALLING_CASCADE;REACTOME_SIGNALING_BY_GPCR;REACTOME_GPCR_DOWNSTREA

EROID_HORMONES_AND_VITAMINS_A_AND_D;REACTOME_BIOLOGICAL_OXIDATIONS;REACT

NO_ACIDS_AND_DERIVATIVES;REACTOME_AMINE_DERIVED_HORMONES

B_SIGNALING;REACTOME_RIP_MEDIATED_NFKB_ACTIVATION_VIA_DAI;REACTOME_GASTRIN

ISM_AND_CITRIC_ACID_TCA_CYCLE;REACTOME_TCA_CYCLE_AND_RESPIRATORY_ELECTR

RANSPORT_OF_SMALL_MOLECULES;REACTOME_SLC_MEDIATED_TRANSMEMBRANE_TRANS

GE_OF_CELLULAR_PROTEINS;REACTOME_CASPASE_MEDIATED_CLEAVAGE_OF_CYTOSKEL

YNTHESIS;REACTOME_FATTY_ACYL_COA_BIOSYNTHESIS;REACTOME_METABOLISM_OF_LIP

ME_CELL_CYCLE_MITOTIC;REACTOME_RECRUITMENT_OF_MITOTIC_CENTROSOME_PROTE

;REACTOME_OLFACTORY_SIGNALING_PATHWAY;REACTOME_GPCR_DOWNSTREAM_SIGNA

;REACTOME_OLFACTORY_SIGNALING_PATHWAY;REACTOME_GPCR_DOWNSTREAM_SIGNA

;REACTOME_OLFACTORY_SIGNALING_PATHWAY;REACTOME_GPCR_DOWNSTREAM_SIGNA

TO_3_EXORIBONUCLEASE;REACTOME_METABOLISM_OF_MRNA;REACTOME_DEADENYLATIO

ENE_EXPRESSION;REACTOME_ALPHA_LINOLENIC_ACID_ALA_METABOLISM;REACTOME_ME

PPED_INTRON_CONTAINING_PRE_MRNA;REACTOME_TRANSPORT_OF_MATURE_TRANSCRI

REACTOME_SIGNALING_BY_SCF_KIT;REACTOME_SIGNALING_BY_ERBB4;REACTOME_SIGNA

OWN_GLYCOGENOLYSIS;REACTOME_METABOLISM_OF_CARBOHYDRATES;REACTOME_GLU

S;REACTOME_ACTIVATION_OF_CHAPERONE_GENES_BY_XBP1S;REACTOME_UNFOLDED_PR

_PRE_REPLICATIVE_COMPLEX;REACTOME_CELL_CYCLE;REACTOME_PROCESSIVE_SYNTHE
REACTOME_TANDEM_PORE_DOMAIN_POTASSIUM_CHANNELS;REACTOME_POTASSIUM_CH

ACTOME_IMMUNE_SYSTEM;REACTOME_ADAPTIVE_IMMUNE_SYSTEM
ACTOME_IMMUNE_SYSTEM;REACTOME_ADAPTIVE_IMMUNE_SYSTEM

EN_PRESENTATION;REACTOME_FACTORS_INVOLVED_IN_MEGAKARYOCYTE_DEVELOPMEN

OF_TGF_BETA_RECEPTOR_SIGNALING;REACTOME_TGF_BETA_RECEPTOR_SIGNALING_ACT
SPIRATORY_ELECTRON_TRANSPORT;REACTOME_RESPIRATORY_ELECTRON_TRANSPORT;
RECYCLING;REACTOME_LATENT_INFECTION_OF_HOMO_SAPIENS_WITH_MYCOBACTERIUM

DS_AND_LIPOPROTEINS;REACTOME_CHOLESTEROL_BIOSYNTHESIS

GTPASES

_METABOLISM;REACTOME_PHOSPHOLIPID_METABOLISM;REACTOME_SPHINGOLIPID_META
RANSPORT_OF_SMALL_MOLECULES;REACTOME_SLC_MEDIATED_TRANSMEMBRANE_TRANS

EN_PRESENTATION;REACTOME_IMMUNE_SYSTEM;REACTOME_ADAPTIVE_IMMUNE_SYSTE
LATES_GUANYLATE_CYCLASE;REACTOME_PLATELET_HOMEOSTASIS;REACTOME_HEMOST

2;REACTOME_HIV_INFECTION;REACTOME_HOST_INTERACTIONS_OF_HIV_FACTORS;REACT
NO_ACIDS_AND_DERIVATIVES;REACTOME_BRANCHED_CHAIN_AMINO_ACID_CATABOLISM;
CELL_CYCLE;REACTOME_P53_INDEPENDENT_G1_S_DNA_DAMAGE_CHECKPOINT;REACTOM

ME_CELL_CYCLE_MITOTIC;REACTOME_MITOTIC_M_M_G1_PHASES;REACTOME_DNA_REPL
ICATION;REACTOME_ADHERENS_JUNCTIONS_INTERACTIONS;REACTOME_CELL_CELL_JUN

OLOGY;REACTOME_AXON_GUIDANCE;REACTOME_L1CAM_INTERACTIONS;REACTOME_INTE

NSPORT;REACTOME_MEMBRANE_TRAFFICKING
TO_3_EXORIBONUCLEASE;REACTOME_METABOLISM_OF_MRNA;REACTOME_DEADENYLATIO
GTPASES;REACTOME_SIGNALLING_BY_NGF;REACTOME_DEVELOPMENTAL_BIOLOGY;REAC

GTPASES;REACTOME_SIGNALLING_BY_NGF;REACTOME_DEVELOPMENTAL_BIOLOGY;REAC

;REACTOME_OLFACTORY_SIGNALING_PATHWAY;REACTOME_GPCR_DOWNSTREAM_SIGNA

ME_CELL_CYCLE_MITOTIC;REACTOME_G1_PHASE;REACTOME_MITOTIC_G1_G1_S_PHASES

REACTOME_VOLTAGE_GATED_POTASSIUM_CHANNELS;REACTOME_POTASSIUM_CHANNEL
_FOR_APOPTOSIS;REACTOME_APOPTOSIS

N_MODULATION_OF_CELL_SURFACE_RECEPTORS_BY_RECRUITING_THEM_TO_CLATHRIN_

_AT_THE_GOLGI_MEMBRANE;REACTOME_PHOSPHOLIPID_METABOLISM;REACTOME_SYNTH
_AT_THE_GOLGI_MEMBRANE;REACTOME_PHOSPHOLIPID_METABOLISM;REACTOME_SYNTH

_SALT_METABOLISM;REACTOME_RECYCLING_OF_BILE_ACIDS_AND_SALTS;REACTOME_TR

ATION_OF_MUCINS;REACTOME_TERMINATION_OF_O_GLYCAN_BIOSYNTHESIS;REACTOME_
ATION_OF_MUCINS;REACTOME_TERMINATION_OF_O_GLYCAN_BIOSYNTHESIS;REACTOME_

REACTOME_DAG_AND_IP3_SIGNALING;REACTOME_SIGNALING_BY_ERBB2;REACTOME_SIG

Y_INTERACTIONS_BETWEEN_A_LYMPHOID_AND_A_NON_LYMPHOID_CELL;REACTOME_INTE

IC_CCT_WITH_TARGET_PROTEINS_DURING_BIOSYNTHESIS;REACTOME_PREFOLDIN_MEDIA

CONTRACTION;REACTOME_MUSCLE_CONTRACTION
E_AND_DEGRADATION;REACTOME_HYALURONAN_METABOLISM;REACTOME_GLYCOSAMIN
AIR;REACTOME_RESOLUTION_OF_AP_SITES_VIA_THE_MULTIPLE_NUCLEOTIDE_PATCH_RE

OLOGY;REACTOME_PPARA_ACTIVATES_GENE_EXPRESSION;REACTOME_METABOLISM_OF_
OF_HOMO_SAPIENS_WITH_MYCOBACTERIUM_TUBERCULOSIS;REACTOME_NITRIC_OXIDE_S

OTEINS;REACTOME_POST_TRANSLATIONAL_PROTEIN_MODIFICATION;REACTOME_ASPARA

_SIGNALING;REACTOME_INTERFERON_ALPHA_BETA_SIGNALING;REACTOME_INTERFERON

ME_CELL_CYCLE_MITOTIC;REACTOME_MITOTIC_M_M_G1_PHASES;REACTOME_DNA_REPL
ME_CELL_CYCLE_MITOTIC;REACTOME_MITOTIC_M_M_G1_PHASES;REACTOME_DNA_REPL
KING;REACTOME_TRANS_GOLGI_NETWORK_VESICLE_BUDDING;REACTOME_GOLGI_ASSOC

ATION_OF_MUCINS;REACTOME_METABOLISM_OF_PROTEINS;REACTOME_POST_TRANSLAT

_IN_DISEASE;REACTOME_SIGNALING_BY_FGFR1_MUTANTS;REACTOME_SIGNALING_BY_FG

ME_CELL_CYCLE_MITOTIC;REACTOME_CELL_CYCLE_CHECKPOINTS;REACTOME_TRANSCR

TY_ACID_BETA_OXIDATION;REACTOME_METABOLISM_OF_LIPIDS_AND_LIPOPROTEINS;REA

)LOGY;REACTOME_EXTRACELLULAR_MATRIX_ORGANIZATION;REACTOME_COLLAGEN_FOF
IA_AMINOACYLATION;REACTOME_TRNA_AMINOACYLATION

RIPTION;REACTOME_TRANSCRIPTION;REACTOME_FORMATION_OF_RNA_POL_II_ELONGATI

RACTIONS_AT_THE_VASCULAR_WALL;REACTOME_BASIGIN_INTERACTIONS;REACTOME_AN

THWAY;REACTOME_DNA_REPAIR

;REACTOME_CLASS_A1_RHODOPSIN_LIKE_RECEPTORS;REACTOME_EICOSANOID_LIGAND_

AMINS_AND_COFACTORS

AMINS_AND_COFACTORS
ICATION;REACTOME_RESPONSE_TO_ELEVATED_PLATELET_CYTOSOLIC_CA2_;REACTOME_

EAK_REPAIR;REACTOME_DNA_REPAIR

ALLING_PATHWAY_VIA_PKC_AND_MAPK;REACTOME_SIGNALING_BY_GPCR;REACTOME_CL

_BETA_SIGNALING;REACTOME_INTERFERON_SIGNALING;REACTOME_IMMUNE_SYSTEM;REA

OGENESIS;REACTOME_REGULATORY_RNA_PATHWAYS

_TRANSMITS_SIGNAL_TO_THE_NUCLEUS;REACTOME_SIGNALING_BY_NOTCH1;REACTOME_

EN_PRESENTATION;REACTOME_FACTORS_INVOLVED_IN_MEGAKARYOCYTE_DEVELOPMEN

REACTOME_SIGNALING_BY_SCF_KIT;REACTOME_DEVELOPMENTAL_BIOLOGY;REACTOME_

_TRANSMITS_SIGNAL_TO_THE_NUCLEUS;REACTOME_SIGNALING_BY_NOTCH4;REACTOME_
RIPTION_INITIATION_FROM_TYPE_2_PROMOTER;REACTOME_RNA_POL_III_TRANSCRIPTION

RANSPORT_OF_SMALL_MOLECULES;REACTOME_ION_TRANSPORT_BY_P_TYPE_ATPASES;R
ACTOME_ADAPTIVE_IMMUNE_SYSTEM;REACTOME_CLASS_I_MHC_MEDIATED_ANTIGEN_PRO
ACTOME_ADAPTIVE_IMMUNE_SYSTEM;REACTOME_CLASS_I_MHC_MEDIATED_ANTIGEN_PRO

OSS_CHEMICAL_SYNAPSES;REACTOME_NEURONAL_SYSTEM;REACTOME_NEUROTRANSMIT

OLOGY;REACTOME_AXON_GUIDANCE;REACTOME_ACTIVATION_OF_RAC;REACTOME_SIGNA
ALLING_PATHWAY_VIA_PKC_AND_MAPK;REACTOME_TRANSMISSION_ACROSS_CHEMICAL_
OLOGY;REACTOME_AXON_GUIDANCE;REACTOME_NCAM_SIGNALING_FOR_NEURITE_OUT_G
OLOGY;REACTOME_AXON_GUIDANCE;REACTOME_NCAM_SIGNALING_FOR_NEURITE_OUT_G

NO_ACIDS_AND_DERIVATIVES

)LOGY;REACTOME_METABOLISM_OF_PROTEINS;REACTOME_AXON_GUIDANCE;REACTOME_
-ACE_INTERACTIONS
-ACE_INTERACTIONS

RANSPORT_OF_SMALL_MOLECULES;REACTOME_ION_TRANSPORT_BY_P_TYPE_ATPASES;R

;;REACTOME_INSULIN_SYNTHESIS_AND_PROCESSING
RANSPORT_OF_SMALL_MOLECULES;REACTOME_PASSIVE_TRANSPORT_BY_AQUAPORINS;F

-ACE_INTERACTIONS

EROID_HORMONES_AND_VITAMINS_A_AND_D;REACTOME_BIOLOGICAL_OXIDATIONS;REACT

NO_ACIDS_AND_DERIVATIVES

GTPASES;REACTOME_SIGNALLING_BY_NGF;REACTOME_NRAGE_SIGNALS_DEATH_THROU(

RANSPORT_OF_SMALL_MOLECULES;REACTOME_SLC_MEDIATED_TRANSMEMBRANE_TRAN:

REACTOME_DEVELOPMENTAL_BIOLOGY;REACTOME_SIGNALING_BY_NODAL

;REACTOME_GPCR_DOWNSTREAM_SIGNALING;REACTOME_G_ALPHA_I_SIGNALLING_EVEN

GTPASES

;REACTOME_PEPTIDE_LIGAND_BINDING_RECEPTORS;REACTOME_CLASS_A1_RHODOPSIN_
CELL_CYCLE;REACTOME_CHROMOSOME_MAINTENANCE;REACTOME_MEIOTIC_SYNAPSIS

OME_SRP_DEPENDENT_COTRANSLATIONAL_PROTEIN_TARGETING_TO_MEMBRANE;REACT
OTEIN_IMPORT;REACTOME_METABOLISM_OF_PROTEINS

OLOGY;REACTOME_MYOGENESIS

TION_PATHWAY

OLOGY;REACTOME_BMAL1_CLOCK_NPAS2_ACTIVATES_CIRCADIAN_EXPRESSION;REACTOM
OLOGY;REACTOME_BMAL1_CLOCK_NPAS2_ACTIVATES_CIRCADIAN_EXPRESSION;REACTOM
OLOGY;REACTOME_BMAL1_CLOCK_NPAS2_ACTIVATES_CIRCADIAN_EXPRESSION;REACTOM
REACTOME_DAG_AND_IP3_SIGNALING;REACTOME_SIGNALING_BY_ERBB2;REACTOME_SIG

_SIGNALING;REACTOME_INTERFERON_SIGNALING;REACTOME_IMMUNE_SYSTEM;REACTOI
_SIGNALING;REACTOME_INTERFERON_SIGNALING;REACTOME_IMMUNE_SYSTEM;REACTOI

NS_MEDIATED_TRANSPORT;REACTOME_TRANSMEMBRANE_TRANSPORT_OF_SMALL_MOLE

NA;REACTOME_METABOLISM_OF_RNA;REACTOME_NONSENSE_MEDIATED_DECAY_ENHAN

SIGNALLING_CASCADE;REACTOME_PKB_MEDIATED_EVENTS;REACTOME_SIGNALING_BY_I

SION_AND_PROCESSING;REACTOME_PRE_NOTCH_PROCESSING_IN_GOLGI;REACTOME_T

RACTIONS_AT_THE_VASCULAR_WALL;REACTOME_INTEGRIN_CELL_SURFACE_INTERACTIO
TION_PATHWAY

WN_GLYCOGENOLYSIS;REACTOME_METABOLISM_OF_CARBOHYDRATES;REACTOME_GLU

N_CODING_RNA;REACTOME_ANTIVIRAL_MECHANISM_BY_IFN_STIMULATED_GENES;REACT

_CODING_RNA;REACTOME_ANTIVIRAL_MECHANISM_BY_IFN_STIMULATED_GENES;REACT[illegible]
_TRANSPORT;REACTOME_METABOLISM_OF_LIPIDS_AND_LIPOPROTEINS;REACTOME_LIPID

RANSPORT_OF_SMALL_MOLECULES;REACTOME_SLC_MEDIATED_TRANSMEMBRANE_TRAN[illegible]

RANSPORT_OF_SMALL_MOLECULES;REACTOME_SLC_MEDIATED_TRANSMEMBRANE_TRAN[illegible]

CELL_CYCLE;REACTOME_CHROMOSOME_MAINTENANCE;REACTOME_MEIOTIC_SYNAPSIS;[illegible]

FACE_INTERACTIONS;REACTOME_P130CAS_LINKAGE_TO_MAPK_SIGNALING_FOR_INTEGRI[illegible]

_B_CELL_RECEPTOR_LEADING_TO_GENERATION_OF_SECOND_MESSENGERS;REACTOME_

IPTION_TERMINATION;REACTOME_RNA_POL_I_TRANSCRIPTION;REACTOME_TRANSCRIPTI

DS_AND_LIPOPROTEINS;REACTOME_CHOLESTEROL_BIOSYNTHESIS

PPED_INTRON_CONTAINING_PRE_MRNA;REACTOME_MRNA_PROCESSING;REACTOME_MRN[illegible]

NA;REACTOME_METABOLISM_OF_RNA;REACTOME_NONSENSE_MEDIATED_DECAY_ENHAN[illegible]
NA;REACTOME_METABOLISM_OF_RNA;REACTOME_NONSENSE_MEDIATED_DECAY_ENHAN[illegible]

SS_CHEMICAL_SYNAPSES;REACTOME_NEURONAL_SYSTEM;REACTOME_NEUROTRANSMIT

ACTOME_ADAPTIVE_IMMUNE_SYSTEM;REACTOME_CLASS_I_MHC_MEDIATED_ANTIGEN_PR[illegible]

RANSPORT_OF_SMALL_MOLECULES;REACTOME_SLC_MEDIATED_TRANSMEMBRANE_TRANS

OGENESIS;REACTOME_REGULATORY_RNA_PATHWAYS;REACTOME_PROCESSING_OF_CAP

BOLISM;REACTOME_SYNTHESIS_OF_PIPS_AT_THE_PLASMA_MEMBRANE;REACTOME_PI_M

SIS;REACTOME_HEPARAN_SULFATE_HEPARIN_HS_GAG_METABOLISM;REACTOME_GLYCOS

OLOGY;REACTOME_BMAL1_CLOCK_NPAS2_ACTIVATES_CIRCADIAN_EXPRESSION;REACTOM
OLOGY;REACTOME_BMAL1_CLOCK_NPAS2_ACTIVATES_CIRCADIAN_EXPRESSION;REACTOM

3_SIGNALING;REACTOME_MAP_KINASE_ACTIVATION_IN_TLR_CASCADE;REACTOME_ACTIVA
OF_HOMO_SAPIENS_WITH_MYCOBACTERIUM_TUBERCULOSIS;REACTOME_NITRIC_OXIDE_S
RANSPORT_OF_SMALL_MOLECULES;REACTOME_SLC_MEDIATED_TRANSMEMBRANE_TRANS
RANSPORT_OF_SMALL_MOLECULES;REACTOME_SLC_MEDIATED_TRANSMEMBRANE_TRANS

_IN_DISEASE;REACTOME_SIGNALING_BY_FGFR1_MUTANTS;REACTOME_SIGNALING_BY_FG

REACTOME_DEVELOPMENTAL_BIOLOGY;REACTOME_NEF_MEDIATES_DOWN_MODULATION

OLOGY;REACTOME_TRANSMISSION_ACROSS_CHEMICAL_SYNAPSES;REACTOME_NEURONA
OME_SRP_DEPENDENT_COTRANSLATIONAL_PROTEIN_TARGETING_TO_MEMBRANE;REACT

)LOGY;REACTOME_DOWNREGULATION_OF_ERBB2_ERBB3_SIGNALING;REACTOME_SIGNAL
;REACTOME_PEPTIDE_LIGAND_BINDING_RECEPTORS;REACTOME_CLASS_A1_RHODOPSIN_

REACTOME_SIGNALING_BY_SCF_KIT;REACTOME_GROWTH_HORMONE_RECEPTOR_SIGNA
REACTOME_SIGNALING_BY_SCF_KIT;REACTOME_GROWTH_HORMONE_RECEPTOR_SIGNA
REACTOME_SIGNALING_BY_SCF_KIT;REACTOME_GROWTH_HORMONE_RECEPTOR_SIGNA
HENATE_METABOLISM;REACTOME_METABOLISM_OF_VITAMINS_AND_COFACTORS

)SS_CHEMICAL_SYNAPSES;REACTOME_NEURONAL_SYSTEM;REACTOME_NEUROTRANSMIT

ACTOME_ADAPTIVE_IMMUNE_SYSTEM;REACTOME_CLASS_I_MHC_MEDIATED_ANTIGEN_PR

)LOGY;REACTOME_EXTRACELLULAR_MATRIX_ORGANIZATION;REACTOME_CELL_SURFACE_

FACTORS_INVOLVED_IN_MEGAKARYOCYTE_DEVELOPMENT_AND_PLATELET_PRODUCTION

_RECEPTOR_SIGNALING;REACTOME_SIGNALING_BY_ERBB4;REACTOME_PROLACTIN_RECE
)LOGY;REACTOME_AXON_GUIDANCE;REACTOME_L1CAM_INTERACTIONS;REACTOME_INTE

S_IN_LIPID_HOMEOSTASIS;REACTOME_ABC_FAMILY_PROTEINS_MEDIATED_TRANSPORT;R

SIGNALLING_CASCADE;REACTOME_REGULATION_OF_AMPK_ACTIVITY_VIA_LKB1;REACTOM

HENATE_METABOLISM;REACTOME_METABOLISM_OF_VITAMINS_AND_COFACTORS;REACTO
HENATE_METABOLISM;REACTOME_METABOLISM_OF_VITAMINS_AND_COFACTORS;REACTO

REACTOME_NGF_SIGNALLING_VIA_TRKA_FROM_THE_PLASMA_MEMBRANE;REACTOME_SI
ME_CELL_CYCLE_MITOTIC;REACTOME_MITOTIC_M_M_G1_PHASES;REACTOME_DNA_REPL

ME_CELL_CYCLE_MITOTIC;REACTOME_RECRUITMENT_OF_MITOTIC_CENTROSOME_PROTE
;REACTOME_PEPTIDE_LIGAND_BINDING_RECEPTORS;REACTOME_CLASS_A1_RHODOPSIN_

ICATION;REACTOME_CELL_JUNCTION_ORGANIZATION

RANSPORT_OF_SMALL_MOLECULES;REACTOME_AQUAPORIN_MEDIATED_TRANSPORT;REA

RANSPORT_OF_SMALL_MOLECULES;REACTOME_ION_TRANSPORT_BY_P_TYPE_ATPASES;R

ISM_AND_CITRIC_ACID_TCA_CYCLE;REACTOME_TCA_CYCLE_AND_RESPIRATORY_ELECTR
CTOME_RNA_POL_I_RNA_POL_III_AND_MITOCHONDRIAL_TRANSCRIPTION
CTOME_RNA_POL_I_RNA_POL_III_AND_MITOCHONDRIAL_TRANSCRIPTION

PPED_INTRON_CONTAINING_PRE_MRNA;REACTOME_MRNA_PROCESSING;REACTOME_MRN

GTPASES
RIPTION_INITIATION_FROM_TYPE_2_PROMOTER;REACTOME_MICRORNA_MIRNA_BIOGENES

;REACTOME_CLASS_A1_RHODOPSIN_LIKE_RECEPTORS;REACTOME_GPCR_DOWNSTREAM

GTPASES;REACTOME_SIGNALLING_BY_NGF;REACTOME_SIGNALING_BY_SCF_KIT;REACTOM
GTPASES;REACTOME_SIGNALLING_BY_NGF;REACTOME_TGF_BETA_RECEPTOR_SIGNALING

JPLED_NER_TC_NER;REACTOME_NUCLEOTIDE_EXCISION_REPAIR;REACTOME_FORMATION
JPLED_NER_TC_NER;REACTOME_NUCLEOTIDE_EXCISION_REPAIR;REACTOME_FORMATION

.ATION_OF_MUCINS;REACTOME_TERMINATION_OF_O_GLYCAN_BIOSYNTHESIS;REACTOME_
.ATION_OF_MUCINS;REACTOME_TERMINATION_OF_O_GLYCAN_BIOSYNTHESIS;REACTOME_
.ATION_OF_MUCINS;REACTOME_TERMINATION_OF_O_GLYCAN_BIOSYNTHESIS;REACTOME_

SM_BY_IFN_STIMULATED_GENES;REACTOME_INTERFERON_SIGNALING;REACTOME_NEGAT

ALLING_PATHWAY_VIA_PKC_AND_MAPK;REACTOME_SIGNALING_BY_GPCR;REACTOME_CL
Y_INTERACTIONS_BETWEEN_A_LYMPHOID_AND_A_NON_LYMPHOID_CELL;REACTOME_INTE

EACTOME_INTERFERON_ALPHA_BETA_SIGNALING;REACTOME_REGULATION_OF_IFNA_SIGI

DS_AND_LIPOPROTEINS;REACTOME_LIPID_DIGESTION_MOBILIZATION_AND_TRANSPORT;F
DS_AND_LIPOPROTEINS;REACTOME_LIPID_DIGESTION_MOBILIZATION_AND_TRANSPORT;F
_IN_MEGAKARYOCYTE_DEVELOPMENT_AND_PLATELET_PRODUCTION;REACTOME_HEMOST

REACTOME_DAG_AND_IP3_SIGNALING;REACTOME_CELL_CYCLE;REACTOME_SIGNALING_E

;REACTOME_OLFACTORY_SIGNALING_PATHWAY;REACTOME_GPCR_DOWNSTREAM_SIGNA

_IN_CANCER;REACTOME_EGFR_DOWNREGULATION

GTPASES

;REACTOME_OPIOID_SIGNALLING;REACTOME_DARPP_32_EVENTS;REACTOME_GPCR_DOW

'TION_PATHWAY

ME_GLUCONEOGENESIS;REACTOME_METABOLISM_OF_CARBOHYDRATES;REACTOME_GLU

'TION_PATHWAY

PPED_INTRON_CONTAINING_PRE_MRNA;REACTOME_MRNA_PROCESSING;REACTOME_MRN

PPED_INTRON_CONTAINING_PRE_MRNA;REACTOME_MRNA_PROCESSING;REACTOME_MRN
OLOGY;REACTOME_TGF_BETA_RECEPTOR_SIGNALING_IN_EMT_EPITHELIAL_TO_MESENCH
REACTOME_P75_NTR_RECEPTOR_MEDIATED_SIGNALLING;REACTOME_SIGNALING_BY_GP

TION_PATHWAY
TION_PATHWAY

OTEIN_IMPORT;REACTOME_METABOLISM_OF_PROTEINS
ALLING_PATHWAY_VIA_PKC_AND_MAPK;REACTOME_SIGNALING_BY_GPCR;REACTOME_CL

RANSPORT_OF_SMALL_MOLECULES;REACTOME_SLC_MEDIATED_TRANSMEMBRANE_TRAN

OVO_BIOSYNTHESIS;REACTOME_PHOSPHOLIPID_METABOLISM;REACTOME_SPHINGOLIPID_

PPED_INTRON_CONTAINING_PRE_MRNA;REACTOME_MRNA_PROCESSING;REACTOME_MRN
PPED_INTRON_CONTAINING_PRE_MRNA;REACTOME_MRNA_PROCESSING;REACTOME_MRN

OLOGY;REACTOME_AXON_GUIDANCE;REACTOME_SEMA4D_IN_SEMAPHORIN_SIGNALING;RE
TION_PATHWAY;REACTOME_NUCLEAR_RECEPTOR_TRANSCRIPTION_PATHWAY
AIR;REACTOME_RESOLUTION_OF_AP_SITES_VIA_THE_MULTIPLE_NUCLEOTIDE_PATCH_RE
AIR;REACTOME_RESOLUTION_OF_AP_SITES_VIA_THE_MULTIPLE_NUCLEOTIDE_PATCH_RE
AIR;REACTOME_RESOLUTION_OF_AP_SITES_VIA_THE_MULTIPLE_NUCLEOTIDE_PATCH_RE

CONTRACTION;REACTOME_MUSCLE_CONTRACTION
CONTRACTION;REACTOME_MUSCLE_CONTRACTION

)SS_CHEMICAL_SYNAPSES;REACTOME_NEURONAL_SYSTEM;REACTOME_NEUROTRANSMIT

'_INTERACTIONS_BETWEEN_A_LYMPHOID_AND_A_NON_LYMPHOID_CELL;REACTOME_IMMU

ERGY_METABOLISM;REACTOME_REGULATION_OF_INSULIN_SECRETION

'TION_PATHWAY
'TION_PATHWAY
'TION_PATHWAY

ICATION;REACTOME_CELL_SURFACE_INTERACTIONS_AT_THE_VASCULAR_WALL;REACTOM

IC_CCT_WITH_TARGET_PROTEINS_DURING_BIOSYNTHESIS;REACTOME_PROTEIN_FOLDING

)LOGY;REACTOME_AXON_GUIDANCE;REACTOME_NCAM1_INTERACTIONS;REACTOME_NCA

)TEINS;REACTOME_POST_TRANSLATIONAL_MODIFICATION_SYNTHESIS_OF_GPI_ANCHORE

ME_CELL_CYCLE_MITOTIC;REACTOME_RECRUITMENT_OF_MITOTIC_CENTROSOME_PROTE
AMINS_AND_COFACTORS

)SS_CHEMICAL_SYNAPSES;REACTOME_NEURONAL_SYSTEM;REACTOME_NEUROTRANSMIT

REACTOME_DEVELOPMENTAL_BIOLOGY;REACTOME_DAG_AND_IP3_SIGNALING;REACTOMI

RANSPORT_OF_SMALL_MOLECULES;REACTOME_SLC_MEDIATED_TRANSMEMBRANE_TRANS

RANSPORT_OF_SMALL_MOLECULES;REACTOME_SLC_MEDIATED_TRANSMEMBRANE_TRANS

REACTOME_VOLTAGE_GATED_POTASSIUM_CHANNELS;REACTOME_POTASSIUM_CHANNEL
RANSPORT_OF_SMALL_MOLECULES;REACTOME_ION_TRANSPORT_BY_P_TYPE_ATPASES;R

;REACTOME_INTEGRATION_OF_ENERGY_METABOLISM;REACTOME_TRANSMEMBRANE_TRA
;REACTOME_INTEGRATION_OF_ENERGY_METABOLISM;REACTOME_TRANSMEMBRANE_TRA
CELL_CYCLE;REACTOME_CHROMOSOME_MAINTENANCE;REACTOME_MEIOTIC_SYNAPSIS

OLOGY;REACTOME_EXTRACELLULAR_MATRIX_ORGANIZATION;REACTOME_COLLAGEN_FOR
OLOGY;REACTOME_EXTRACELLULAR_MATRIX_ORGANIZATION;REACTOME_COLLAGEN_FOR
NSPORT;REACTOME_MEMBRANE_TRAFFICKING;REACTOME_DIABETES_PATHWAYS;REACT

RACTIONS_AT_THE_VASCULAR_WALL;REACTOME_INTEGRIN_CELL_SURFACE_INTERACTIO
B_SIGNALING;REACTOME_RIP_MEDIATED_NFKB_ACTIVATION_VIA_DAI;REACTOME_GASTRIN

BOLISM;REACTOME_SYNTHESIS_OF_PIPS_AT_THE_PLASMA_MEMBRANE;REACTOME_PI_M

BETA_SIGNALING;REACTOME_REGULATION_OF_IFNA_SIGNALING;REACTOME_INTERFERO
GTPASES;REACTOME_SIGNALLING_BY_NGF;REACTOME_NRAGE_SIGNALS_DEATH_THROU

ICATION;REACTOME_DSCAM_INTERACTIONS
ICATION;REACTOME_DSCAM_INTERACTIONS
ICATION;REACTOME_DSCAM_INTERACTIONS
ICATION;REACTOME_DSCAM_INTERACTIONS

PPED_INTRON_CONTAINING_PRE_MRNA;REACTOME_TRANSPORT_OF_MATURE_TRANSCRI

Y_INTERACTIONS_BETWEEN_A_LYMPHOID_AND_A_NON_LYMPHOID_CELL;REACTOME_CELI
AMINS_AND_COFACTORS
OLOGY;REACTOME_EXTRACELLULAR_MATRIX_ORGANIZATION;REACTOME_COLLAGEN_FOF
NO_ACIDS_AND_DERIVATIVES

ME_CELL_CYCLE_MITOTIC;REACTOME_RECRUITMENT_OF_MITOTIC_CENTROSOME_PROTE

_AT_THE_GOLGI_MEMBRANE;REACTOME_PHOSPHOLIPID_METABOLISM;REACTOME_PI_MET

REACTOME_NGF_SIGNALLING_VIA_TRKA_FROM_THE_PLASMA_MEMBRANE;REACTOME_SI
LISM;REACTOME_METABOLISM_OF_NUCLEOTIDES;REACTOME_PYRIMIDINE_METABOLISM
ONS;REACTOME_GLUTATHIONE_CONJUGATION;REACTOME_PHASE_II_CONJUGATION

_PRE_REPLICATIVE_COMPLEX;REACTOME_CELL_CYCLE;REACTOME_ORC1_REMOVAL_FRC
OLOGY;REACTOME_AXON_GUIDANCE;REACTOME_SEMA4D_IN_SEMAPHORIN_SIGNALING;RE
EACTOME_IL_3_5_AND_GM_CSF_SIGNALING;REACTOME_IL_RECEPTOR_SHC_SIGNALING;RE

ELLING_OF_PC;REACTOME_PHOSPHOLIPID_METABOLISM;REACTOME_ACYL_CHAIN_REMOD
_IN_MEGAKARYOCYTE_DEVELOPMENT_AND_PLATELET_PRODUCTION;REACTOME_HEMOST

OSS_CHEMICAL_SYNAPSES;REACTOME_NEURONAL_SYSTEM;REACTOME_NEUROTRANSMIT
OSS_CHEMICAL_SYNAPSES;REACTOME_NEURONAL_SYSTEM;REACTOME_NEUROTRANSMIT

OLOGY;REACTOME_AXON_GUIDANCE;REACTOME_NCAM1_INTERACTIONS;REACTOME_NCA
RIPTION_AND_TRANSLATION;REACTOME_PRE_NOTCH_EXPRESSION_AND_PROCESSING;R
ALLING_PATHWAY_VIA_PKC_AND_MAPK;REACTOME_SIGNALING_BY_GPCR;REACTOME_PE
RIPTION_INITIATION_FROM_TYPE_2_PROMOTER;REACTOME_RNA_POL_III_TRANSCRIPTION
RIPTION_INITIATION_FROM_TYPE_2_PROMOTER;REACTOME_RNA_POL_III_TRANSCRIPTION
ME_CELL_CYCLE_MITOTIC;REACTOME_MITOTIC_M_M_G1_PHASES;REACTOME_DNA_REPL

BOLISM;REACTOME_GLYCEROPHOSPHOLIPID_BIOSYNTHESIS;REACTOME_METABOLISM_O

CELL_CYCLE;REACTOME_CHROMOSOME_MAINTENANCE;REACTOME_MEIOTIC_SYNAPSIS

XYLATION_HYPUSINE_FORMATION_AND_ARYLSULFATASE_ACTIVATION;REACTOME_GLYCO

NTHESIS;REACTOME_METABOLISM_OF_LIPIDS_AND_LIPOPROTEINS;REACTOME_FATTY_A
NTHESIS;REACTOME_METABOLISM_OF_LIPIDS_AND_LIPOPROTEINS;REACTOME_FATTY_A
CONTRACTION;REACTOME_MUSCLE_CONTRACTION

ORS_OF_RIG_I_MDA5_SIGNALING;REACTOME_RIG_I_MDA5_MEDIATED_INDUCTION_OF_IFN_

REACTOME_NRAGE_SIGNALS_DEATH_THROUGH_JNK;REACTOME_REGULATION_OF_APOP

CELL_CYCLE;REACTOME_PROCESSING_OF_CAPPED_INTRON_CONTAINING_PRE_MRNA;RE
ACTOME_ADAPTIVE_IMMUNE_SYSTEM;REACTOME_CLASS_I_MHC_MEDIATED_ANTIGEN_PRO

GTPASES;REACTOME_SIGNALLING_BY_NGF;REACTOME_NRAGE_SIGNALS_DEATH_THROUG

GTPASES

OLOGY;REACTOME_PPARA_ACTIVATES_GENE_EXPRESSION;REACTOME_GENERIC_TRANSC
KING;REACTOME_GAP_JUNCTION_TRAFFICKING;REACTOME_GAP_JUNCTION_ASSEMBLY

ULAR_DOMAIN_REGULATES_TRANSCRIPTION;REACTOME_SIGNALING_BY_NOTCH1;REACTO
OWN_GLYCOGENOLYSIS;REACTOME_METABOLISM_OF_CARBOHYDRATES;REACTOME_GLU

'NTHESIS;REACTOME_FATTY_ACYL_COA_BIOSYNTHESIS;REACTOME_METABOLISM_OF_LIF

ON;REACTOME_CHONDROITIN_SULFATE_DERMATAN_SULFATE_METABOLISM;REACTOME_I
RANSPORT_OF_SMALL_MOLECULES;REACTOME_SLC_MEDIATED_TRANSMEMBRANE_TRANS

RANSPORT_OF_SMALL_MOLECULES;REACTOME_ION_TRANSPORT_BY_P_TYPE_ATPASES;R

N;REACTOME_CHONDROITIN_SULFATE_BIOSYNTHESIS;REACTOME_CHONDROITIN_SULFAT
RANSPORT_OF_SMALL_MOLECULES;REACTOME_PLATELET_HOMEOSTASIS;REACTOME_PL/
OME_SRP_DEPENDENT_COTRANSLATIONAL_PROTEIN_TARGETING_TO_MEMBRANE;REACT
)LOGY;REACTOME_CELL_SURFACE_INTERACTIONS_AT_THE_VASCULAR_WALL;REACTOME
)LOGY;REACTOME_CELL_SURFACE_INTERACTIONS_AT_THE_VASCULAR_WALL;REACTOME

RBOHYDRATES
ME_CHROMOSOME_MAINTENANCE;REACTOME_TELOMERE_MAINTENANCE;REACTOME_EX

NO_ACIDS_AND_DERIVATIVES

SM_BY_IFN_STIMULATED_GENES;REACTOME_INTERFERON_SIGNALING;REACTOME_IMMUN

ON;REACTOME_HEPARAN_SULFATE_HEPARIN_HS_GAG_METABOLISM;REACTOME_GLYCOS
ME_CELL_CYCLE_MITOTIC;REACTOME_RECRUITMENT_OF_MITOTIC_CENTROSOME_PROTE

_TRANSMITS_SIGNAL_TO_THE_NUCLEUS;REACTOME_SIGNALING_BY_NOTCH4;REACTOME_

ME_MHC_CLASS_II_ANTIGEN_PRESENTATION;REACTOME_CELL_CYCLE_MITOTIC;REACTOM

CONTRACTION;REACTOME_MUSCLE_CONTRACTION

TRIX_ORGANIZATION;REACTOME_COLLAGEN_FORMATION

OLOGY;REACTOME_AXON_GUIDANCE;REACTOME_NCAM_SIGNALING_FOR_NEURITE_OUT_G
;REACTOME_PEPTIDE_LIGAND_BINDING_RECEPTORS;REACTOME_CLASS_A1_RHODOPSIN_

;REACTOME_CLASS_B_2_SECRETIN_FAMILY_RECEPTORS;REACTOME_GPCR_LIGAND_BIND

_METABOLISM;REACTOME_METABOLISM_OF_AMINO_ACIDS_AND_DERIVATIVES;REACTOME

S_IN_LIPID_HOMEOSTASIS;REACTOME_ABC_FAMILY_PROTEINS_MEDIATED_TRANSPORT;R

RANSPORT_OF_SMALL_MOLECULES;REACTOME_SLC_MEDIATED_TRANSMEMBRANE_TRANS
OTEIN_IMPORT;REACTOME_METABOLISM_OF_PROTEINS

ME_CELL_CYCLE_MITOTIC;REACTOME_RECRUITMENT_OF_MITOTIC_CENTROSOME_PROTE
ME_CELL_CYCLE_MITOTIC;REACTOME_RECRUITMENT_OF_MITOTIC_CENTROSOME_PROTE

_FOR_APOPTOSIS;REACTOME_APOPTOSIS
REACTOME_SIGNALING_BY_SCF_KIT;REACTOME_CELL_CELL_COMMUNICATION;REACTOME

ONS;REACTOME_CYTOCHROME_P450_ARRANGED_BY_SUBSTRATE_TYPE;REACTOME_PHA

_AT_THE_EARLY_ENDOSOME_MEMBRANE;REACTOME_PHOSPHOLIPID_METABOLISM;REACT

ALLING_PATHWAY_VIA_PKC_AND_MAPK;REACTOME_SIGNALING_BY_GPCR;REACTOME_PE

E_AND_DEGRADATION;REACTOME_HYALURONAN_METABOLISM;REACTOME_GLYCOSAMIN

NREGULATION_OF_MHC_CLASS_I_COMPLEX_CELL_SURFACE_EXPRESSION;REACTOME_AI

SE_OF_CELLULAR_PROTEINS;REACTOME_CELL_CELL_COMMUNICATION;REACTOME_CASPA

ME_METABOLISM_OF_CARBOHYDRATES;REACTOME_GLUCOSE_METABOLISM

)F_HOMO_SAPIENS_WITH_MYCOBACTERIUM_TUBERCULOSIS;REACTOME_NITRIC_OXIDE_S

REACTOME_SIGNALING_BY_SCF_KIT;REACTOME_DEVELOPMENTAL_BIOLOGY;REACTOME_

TO_3_EXORIBONUCLEASE;REACTOME_METABOLISM_OF_MRNA;REACTOME_DEADENYLATIO
PPED_INTRON_CONTAINING_PRE_MRNA;REACTOME_MRNA_PROCESSING;REACTOME_MRN
TION_PATHWAY

BOLISM;REACTOME_GLYCEROPHOSPHOLIPID_BIOSYNTHESIS;REACTOME_METABOLISM_O

XYLATION_HYPUSINE_FORMATION_AND_ARYLSULFATASE_ACTIVATION;REACTOME_GLYCO

OLOGY;REACTOME_MHC_CLASS_II_ANTIGEN_PRESENTATION;REACTOME_AXON_GUIDANCE

OSS_CHEMICAL_SYNAPSES;REACTOME_NEURONAL_SYSTEM;REACTOME_NEUROTRANSMIT
REACTOME_TRIF_MEDIATED_TLR3_SIGNALING;REACTOME_P75NTR_RECRUITS_SIGNALLIN

TRIX_ORGANIZATION;REACTOME_COLLAGEN_FORMATION
ON_OF_SOLUBLE_EXOGENOUS_ANTIGENS_ENDOSOMES;REACTOME_ANTIGEN_PROCESSIN

OLOGY;REACTOME_CELL_CELL_COMMUNICATION;REACTOME_AXON_GUIDANCE;REACTOME

S;REACTOME_REGULATION_OF_INSULIN_LIKE_GROWTH_FACTOR_IGF_ACTIVITY_BY_INSUL

;REACTOME_CLASS_A1_RHODOPSIN_LIKE_RECEPTORS;REACTOME_AMINE_LIGAND_BINDI

:IPTION;REACTOME_TRANSCRIPTION;REACTOME_RNA_POL_I_RNA_POL_III_AND_MITOCHOI

)LOGY;REACTOME_PPARA_ACTIVATES_GENE_EXPRESSION;REACTOME_METABOLISM_OF_

RT_AND_METABOLISM;REACTOME_ABC_FAMILY_PROTEINS_MEDIATED_TRANSPORT;REACT

ME_CELL_CYCLE_MITOTIC;REACTOME_RECRUITMENT_OF_MITOTIC_CENTROSOME_PROTE
3E_OF_CELLULAR_PROTEINS;REACTOME_DEVELOPMENTAL_BIOLOGY;REACTOME_CELL_C
_FOR_APOPTOSIS;REACTOME_TRAF6_MEDIATED_IRF7_ACTIVATION;REACTOME_TRAF6_M

FACE_INTERACTIONS

ON_OF_FGFR_SIGNALING;REACTOME_INSULIN_RECEPTOR_SIGNALLING_CASCADE;REACT(

GTPASES;REACTOME_SIGNALLING_BY_NGF;REACTOME_DEVELOPMENTAL_BIOLOGY;REAC
_SIGNALING;REACTOME_INTERFERON_ALPHA_BETA_SIGNALING;REACTOME_INTERFERON
ME_ORC1_REMOVAL_FROM_CHROMATIN;REACTOME_CELL_CYCLE_MITOTIC;REACTOME_(

S;REACTOME_INSULIN_SYNTHESIS_AND_PROCESSING

RANSPORT_OF_SMALL_MOLECULES;REACTOME_SLC_MEDIATED_TRANSMEMBRANE_TRANS

)SS_CHEMICAL_SYNAPSES;REACTOME_NEURONAL_SYSTEM;REACTOME_INTEGRATION_OF
;REACTOME_OLFACTORY_SIGNALING_PATHWAY;REACTOME_GPCR_DOWNSTREAM_SIGNA

REACTOME_POTASSIUM_CHANNELS

ME_CELL_CYCLE_MITOTIC;REACTOME_RECRUITMENT_OF_MITOTIC_CENTROSOME_PROTE
RANSPORT_OF_SMALL_MOLECULES;REACTOME_SLC_MEDIATED_TRANSMEMBRANE_TRANS

REACTOME_TRIF_MEDIATED_TLR3_SIGNALING;REACTOME_P75NTR_RECRUITS_SIGNALLIN

RIPTION;REACTOME_TRANSCRIPTION;REACTOME_RNA_POL_I_RNA_POL_III_AND_MITOCHO

GTPASES;REACTOME_SIGNALLING_BY_NGF;REACTOME_DEVELOPMENTAL_BIOLOGY;REAC
OME_METABOLISM_OF_PROTEINS

ICATION;REACTOME_ADHERENS_JUNCTIONS_INTERACTIONS;REACTOME_CELL_CELL_JUN

)
)

REACTOME_NGF_SIGNALLING_VIA_TRKA_FROM_THE_PLASMA_MEMBRANE;REACTOME_SI[illegible]

[illegible]RANSPORT_OF_SMALL_MOLECULES;REACTOME_SLC_MEDIATED_TRANSMEMBRANE_TRANS[illegible]

[illegible]OME_FORMATION_OF_THE_TERNARY_COMPLEX_AND_SUBSEQUENTLY_THE_43S_COMPLE[illegible]
[illegible]RANSPORT_OF_SMALL_MOLECULES;REACTOME_ION_TRANSPORT_BY_P_TYPE_ATPASES;R[illegible]
[illegible]OLISM;REACTOME_GLYCOSAMINOGLYCAN_METABOLISM;REACTOME_ABC_FAMILY_PROTE[illegible]
[illegible]RANSPORT_OF_SMALL_MOLECULES;REACTOME_ION_CHANNEL_TRANSPORT;REACTOME_L[illegible]

[illegible]PTOR_INTERACTIONS;REACTOME_RESPONSE_TO_ELEVATED_PLATELET_CYTOSOLIC_CA2_[illegible]

[illegible]NTHESIS;REACTOME_FATTY_ACYL_COA_BIOSYNTHESIS;REACTOME_METABOLISM_OF_LIP[illegible]

[illegible]OLOGY;REACTOME_REGULATION_OF_APOPTOSIS;REACTOME_AXON_GUIDANCE;REACTOM[illegible]

[illegible]G_CROSS_PRESENTATION;REACTOME_ENDOSOMAL_VACUOLAR_PATHWAY;REACTOME_E[illegible]

[illegible]TRIX_ORGANIZATION;REACTOME_COLLAGEN_FORMATION

[illegible]BOLISM;REACTOME_SYNTHESIS_OF_PIPS_AT_THE_PLASMA_MEMBRANE;REACTOME_PI_M[illegible]

)TEINS;REACTOME_POST_TRANSLATIONAL_PROTEIN_MODIFICATION;REACTOME_ASPARA(
4;REACTOME_NUCLEAR_SIGNALING_BY_ERBB4;REACTOME_SIGNALING_BY_GPCR;REACTO

REACTOME_TANDEM_PORE_DOMAIN_POTASSIUM_CHANNELS;REACTOME_POTASSIUM_CH

REACTOME_INTEGRATION_OF_ENERGY_METABOLISM;REACTOME_REGULATION_OF_INSUL

_TRANSMITS_SIGNAL_TO_THE_NUCLEUS;REACTOME_SIGNALING_BY_NOTCH1;REACTOME_

NO_ACIDS_AND_DERIVATIVES

BOLISM;REACTOME_SYNTHESIS_OF_PIPS_AT_THE_PLASMA_MEMBRANE;REACTOME_PI_M

)VO_BIOSYNTHESIS;REACTOME_PHOSPHOLIPID_METABOLISM;REACTOME_SPHINGOLIPID_

REACTOME_CTNNB1_PHOSPHORYLATION_CASCADE;REACTOME_CELL_CYCLE;REACTOME_

ATED_PLATELET_CYTOSOLIC_CA2_;REACTOME_GLYCOLYSIS;REACTOME_GLUCONEOGENI

PPED_INTRON_CONTAINING_PRE_MRNA;REACTOME_MRNA_PROCESSING;REACTOME_MRN
S;REACTOME_UNFOLDED_PROTEIN_RESPONSE
OSS_CHEMICAL_SYNAPSES;REACTOME_NEURONAL_SYSTEM;REACTOME_NEUROTRANSMIT

ONS;REACTOME_CYTOSOLIC_SULFONATION_OF_SMALL_MOLECULES;REACTOME_PHASE_

OSS_CHEMICAL_SYNAPSES;REACTOME_NEURONAL_SYSTEM;REACTOME_NEUROTRANSMIT

OSS_CHEMICAL_SYNAPSES;REACTOME_NEURONAL_SYSTEM;REACTOME_SIGNALING_BY_G

_BETA_SIGNALING;REACTOME_INTERFERON_SIGNALING;REACTOME_IMMUNE_SYSTEM;REA

SPIRATORY_ELECTRON_TRANSPORT;REACTOME_RESPIRATORY_ELECTRON_TRANSPORT;

PROTEOLYTIC_ACTIVITY_OF_APC_C_REQUIRED_FOR_THE_ONSET_OF_ANAPHASE_BY_MIT

;REACTOME_OPIOID_SIGNALLING;REACTOME_DARPP_32_EVENTS

RANSPORT_OF_SMALL_MOLECULES;REACTOME_SLC_MEDIATED_TRANSMEMBRANE_TRANS

KIT;REACTOME_GROWTH_HORMONE_RECEPTOR_SIGNALING;REACTOME_SIGNALING_BY_E

SIS;REACTOME_HEPARAN_SULFATE_HEPARIN_HS_GAG_METABOLISM;REACTOME_GLYCOS

REACTOME_TRIF_MEDIATED_TLR3_SIGNALING;REACTOME_TCR_SIGNALING;REACTOME_D

ICATION;REACTOME_DSCAM_INTERACTIONS

NTHESIS;REACTOME_PHOSPHOLIPID_METABOLISM;REACTOME_SYNTHESIS_OF_PA;REACT

;REACTOME_CLASS_A1_RHODOPSIN_LIKE_RECEPTORS;REACTOME_GPCR_DOWNSTREAM
ON;REACTOME_CHONDROITIN_SULFATE_DERMATAN_SULFATE_METABOLISM;REACTOME_[illegible]

ICATION;REACTOME_CELL_EXTRACELLULAR_MATRIX_INTERACTIONS;REACTOME_CELL_JU

GTPASES;REACTOME_MEMBRANE_TRAFFICKING;REACTOME_TRANS_GOLGI_NETWORK_VE

ATED_PLATELET_CYTOSOLIC_CA2_;REACTOME_FORMATION_OF_FIBRIN_CLOT_CLOTTING_

_METABOLISM;REACTOME_PHOSPHOLIPID_METABOLISM;REACTOME_SPHINGOLIPID_META

REACTOME_CELL_CELL_COMMUNICATION;REACTOME_P75NTR_RECRUITS_SIGNALLING_C

DS_AND_LIPOPROTEINS;REACTOME_LIPID_DIGESTION_MOBILIZATION_AND_TRANSPORT;F

ORT_ACROSS_THE_PLASMA_MEMBRANE;REACTOME_TRANSMEMBRANE_TRANSPORT_OF_

.ATION_OF_MUCINS;REACTOME_TERMINATION_OF_O_GLYCAN_BIOSYNTHESIS;REACTOME_

CELL_CYCLE;REACTOME_CHROMOSOME_MAINTENANCE;REACTOME_MEIOTIC_SYNAPSIS
RIPTION_AND_TRANSLATION;REACTOME_SIGNALING_BY_NOTCH2;REACTOME_PRE_NOTC

MPLEMENT_CASCADE;REACTOME_INNATE_IMMUNE_SYSTEM;REACTOME_IMMUNE_SYSTEM

FACE_INTERACTIONS;REACTOME_SIGNALING_BY_PDGF

RIN_BH4_SYNTHESIS_RECYCLING_SALVAGE_AND_REGULATION;REACTOME_METABOLISM_

OSS_CHEMICAL_SYNAPSES;REACTOME_NEURONAL_SYSTEM;REACTOME_NEUROTRANSMIT

BOLISM;REACTOME_SYNTHESIS_OF_PC;REACTOME_GLYCEROPHOSPHOLIPID_BIOSYNTHE
ENE_EXPRESSION;REACTOME_METABOLISM_OF_LIPIDS_AND_LIPOPROTEINS;REACTOME_
FACE_INTERACTIONS

RIPTION_INITIATION_FROM_TYPE_2_PROMOTER;REACTOME_RNA_POL_II_TRANSCRIPTION

_B_CELL_RECEPTOR_LEADING_TO_GENERATION_OF_SECOND_MESSENGERS;REACTOME_

SPIRATORY_ELECTRON_TRANSPORT;REACTOME_MITOCHONDRIAL_PROTEIN_IMPORT;REA
ALLING_PATHWAY_VIA_PKC_AND_MAPK;REACTOME_SIGNALING_BY_GPCR;REACTOME_IN

)SS_CHEMICAL_SYNAPSES;REACTOME_NEURONAL_SYSTEM

)TEINS;REACTOME_POST_TRANSLATIONAL_PROTEIN_MODIFICATION;REACTOME_SYNTHE

;REACTOME_PEPTIDE_LIGAND_BINDING_RECEPTORS;REACTOME_CLASS_A1_RHODOPSIN_

RADATION;REACTOME_SIGNALLING_BY_NGF;REACTOME_DEVELOPMENTAL_BIOLOGY;REA(

EACTOME_POST_CHAPERONIN_TUBULIN_FOLDING_PATHWAY;REACTOME_METABOLISM_O

)SS_CHEMICAL_SYNAPSES;REACTOME_NEURONAL_SYSTEM;REACTOME_NEUROTRANSMIT

ICATION;REACTOME_SIGNAL_REGULATORY_PROTEIN_SIRP_FAMILY_INTERACTIONS

ILAR_DOMAIN_REGULATES_TRANSCRIPTION;REACTOME_SIGNALING_BY_NOTCH1;REACTO

RANSPORT_OF_SMALL_MOLECULES;REACTOME_SLC_MEDIATED_TRANSMEMBRANE_TRANS

OLOGY;REACTOME_HS_GAG_DEGRADATION;REACTOME_CHONDROITIN_SULFATE_DERMAT

GTPASES

UPLED_NER_TC_NER;REACTOME_NUCLEOTIDE_EXCISION_REPAIR;REACTOME_FORMATION

RANSPORT_OF_SMALL_MOLECULES;REACTOME_SLC_MEDIATED_TRANSMEMBRANE_TRANS

OLOGY;REACTOME_AXON_GUIDANCE;REACTOME_NETRIN1_SIGNALING;REACTOME_DCC_M

IG_CROSS_PRESENTATION;REACTOME_ENDOSOMAL_VACUOLAR_PATHWAY;REACTOME_E

RT_AND_METABOLISM;REACTOME_ABC_FAMILY_PROTEINS_MEDIATED_TRANSPORT;REACT

ALLING_PATHWAY_VIA_PKC_AND_MAPK;REACTOME_SIGNALING_BY_GPCR;REACTOME_G_
ALLING_PATHWAY_VIA_PKC_AND_MAPK;REACTOME_SIGNALING_BY_GPCR;REACTOME_G_
;REACTOME_OLFACTORY_SIGNALING_PATHWAY;REACTOME_GPCR_DOWNSTREAM_SIGNA

OSS_CHEMICAL_SYNAPSES;REACTOME_NEURONAL_SYSTEM

PTION_PATHWAY

_AT_THE_EARLY_ENDOSOME_MEMBRANE;REACTOME_SYNTHESIS_OF_PIPS_AT_THE_GOLG
ICATION;REACTOME_CELL_EXTRACELLULAR_MATRIX_INTERACTIONS;REACTOME_CELL_JU
CELL_CYCLE;REACTOME_P53_INDEPENDENT_G1_S_DNA_DAMAGE_CHECKPOINT;REACTOM
S;REACTOME_ACTIVATION_OF_CHAPERONE_GENES_BY_XBP1S;REACTOME_UNFOLDED_PI

REACTOME_SIGNALING_BY_SCF_KIT;REACTOME_DEVELOPMENTAL_BIOLOGY;REACTOME_
EN_PRESENTATION;REACTOME_FACTORS_INVOLVED_IN_MEGAKARYOCYTE_DEVELOPMEN
REACTOME_INTEGRATION_OF_ENERGY_METABOLISM;REACTOME_REGULATION_OF_INSUL

OSS_CHEMICAL_SYNAPSES;REACTOME_NEURONAL_SYSTEM;REACTOME_NEUROTRANSMIT

_B_CELL_RECEPTOR_LEADING_TO_GENERATION_OF_SECOND_MESSENGERS;REACTOME_

_IN_MEGAKARYOCYTE_DEVELOPMENT_AND_PLATELET_PRODUCTION;REACTOME_HEMOST

TION_PATHWAY

OSS_CHEMICAL_SYNAPSES;REACTOME_NEURONAL_SYSTEM;REACTOME_NEUROTRANSMIT

RBOHYDRATES

RBOHYDRATES

;REACTOME_CLASS_C_3_METABOTROPIC_GLUTAMATE_PHEROMONE_RECEPTORS;REACT
ACTOME_IMMUNE_SYSTEM;REACTOME_ADAPTIVE_IMMUNE_SYSTEM

TION_OF_CARBON_DIOXIDE
IG_CROSS_PRESENTATION;REACTOME_LATENT_INFECTION_OF_HOMO_SAPIENS_WITH_MY
REACTOME_VOLTAGE_GATED_POTASSIUM_CHANNELS;REACTOME_POTASSIUM_CHANNEL

ATION_OF_MUCINS;REACTOME_METABOLISM_OF_PROTEINS;REACTOME_POST_TRANSLAT

OTEIN_IMPORT;REACTOME_METABOLISM_OF_PROTEINS

XYLATION_HYPUSINE_FORMATION_AND_ARYLSULFATASE_ACTIVATION;REACTOME_GLYCO
2_ACTIVATES_CIRCADIAN_EXPRESSION;REACTOME_CIRCADIAN_CLOCK

ME_CELL_CYCLE_MITOTIC;REACTOME_MITOTIC_M_M_G1_PHASES;REACTOME_DNA_REPL

ATION_OF_MUCINS;REACTOME_METABOLISM_OF_PROTEINS;REACTOME_POST_TRANSLAT

OLOGY;REACTOME_AXON_GUIDANCE;REACTOME_NETRIN1_SIGNALING;REACTOME_DCC_M

N_CODING_RNA;REACTOME_ANTIVIRAL_MECHANISM_BY_IFN_STIMULATED_GENES;REACTO

RECYCLING;REACTOME_LATENT_INFECTION_OF_HOMO_SAPIENS_WITH_MYCOBACTERIUM

TEM;REACTOME_IMMUNE_SYSTEM;REACTOME_NOD1_2_SIGNALING_PATHWAY;REACTOME

ICATION;REACTOME_TGF_BETA_RECEPTOR_SIGNALING_IN_EMT_EPITHELIAL_TO_MESENC

OLOGY;REACTOME_AXON_GUIDANCE;REACTOME_NETRIN1_SIGNALING;REACTOME_DCC_M

NO_ACIDS_AND_DERIVATIVES;REACTOME_AMINE_DERIVED_HORMONES

OLOGY;REACTOME_EXTRACELLULAR_MATRIX_ORGANIZATION;REACTOME_COLLAGEN_FOR

OLOGY;REACTOME_PPARA_ACTIVATES_GENE_EXPRESSION;REACTOME_METABOLISM_OF_
OLOGY;REACTOME_REGULATION_OF_BETA_CELL_DEVELOPMENT;REACTOME_REGULATION
_IN_MEGAKARYOCYTE_DEVELOPMENT_AND_PLATELET_PRODUCTION;REACTOME_HEMOST
N_CODING_RNA;REACTOME_METABOLISM_OF_RNA

GE_OF_CELLULAR_PROTEINS;REACTOME_MEMBRANE_TRAFFICKING;REACTOME_SIGNALIN
OSS_CHEMICAL_SYNAPSES;REACTOME_NEURONAL_SYSTEM;REACTOME_NEUROTRANSMIT

FACE_INTERACTIONS

ICATION;REACTOME_INTEGRIN_CELL_SURFACE_INTERACTIONS;REACTOME_CELL_JUNCTI
SIGNALLING_CASCADE;REACTOME_REGULATION_OF_AMPK_ACTIVITY_VIA_LKB1;REACTOM
TRIX_ORGANIZATION;REACTOME_COLLAGEN_FORMATION;REACTOME_METABOLISM_OF_L

ATION_OF_MUCINS;REACTOME_METABOLISM_OF_PROTEINS;REACTOME_POST_TRANSLAT
ATION_OF_MUCINS;REACTOME_METABOLISM_OF_PROTEINS;REACTOME_POST_TRANSLAT

TION_PATHWAY

TION_PATHWAY

NTHESIS;REACTOME_PHOSPHOLIPID_METABOLISM;REACTOME_SYNTHESIS_OF_PE;REACT

GTPASES;REACTOME_SIGNALLING_BY_NGF;REACTOME_NRAGE_SIGNALS_DEATH_THROU

OF_TGF_BETA_RECEPTOR_SIGNALING;REACTOME_TGF_BETA_RECEPTOR_SIGNALING_ACT

OWN_GLYCOGENOLYSIS;REACTOME_METABOLISM_OF_CARBOHYDRATES;REACTOME_GLU

ICATION;REACTOME_CELL_JUNCTION_ORGANIZATION

ME_CELL_CYCLE_MITOTIC;REACTOME_RECRUITMENT_OF_MITOTIC_CENTROSOME_PROT

TEM;REACTOME_IMMUNE_SYSTEM;REACTOME_INFLAMMASOMES;REACTOME_NUCLEOTID

CTOME_TRANSLOCATION_OF_ZAP_70_TO_IMMUNOLOGICAL_SYNAPSE;REACTOME_GENER

EN_PRESENTATION;REACTOME_FACTORS_INVOLVED_IN_MEGAKARYOCYTE_DEVELOPMEN
;REACTOME_CLASS_B_2_SECRETIN_FAMILY_RECEPTORS;REACTOME_GPCR_DOWNSTREA

OME_SRP_DEPENDENT_COTRANSLATIONAL_PROTEIN_TARGETING_TO_MEMBRANE;REACT

REACTOME_SIGNALING_BY_SCF_KIT;REACTOME_CELL_CELL_COMMUNICATION;REACTOM

RANSPORT_OF_SMALL_MOLECULES;REACTOME_ION_TRANSPORT_BY_P_TYPE_ATPASES;R
RANSPORT_OF_SMALL_MOLECULES;REACTOME_ION_TRANSPORT_BY_P_TYPE_ATPASES;R

ON_OF_FGFR_SIGNALING;REACTOME_INSULIN_RECEPTOR_SIGNALLING_CASCADE;REACT

;REACTOME_PEPTIDE_LIGAND_BINDING_RECEPTORS;REACTOME_CLASS_A1_RHODOPSIN_
GTPASES;REACTOME_SIGNALLING_BY_NGF;REACTOME_PPARA_ACTIVATES_GENE_EXPRE

ATION_OF_MUCINS;REACTOME_TERMINATION_OF_O_GLYCAN_BIOSYNTHESIS;REACTOME_

ON;REACTOME_SMOOTH_MUSCLE_CONTRACTION
HE_EXTRACELLULAR_MATRIX;REACTOME_EXTRACELLULAR_MATRIX_ORGANIZATION

ONS;REACTOME_GLUTATHIONE_CONJUGATION;REACTOME_PHASE_II_CONJUGATION
TOME_HIV_LIFE_CYCLE;REACTOME_EARLY_PHASE_OF_HIV_LIFE_CYCLE;REACTOME_HOST

OLOGY;REACTOME_CELL_CELL_COMMUNICATION;REACTOME_IMMUNOREGULATORY_INTEI

ATION_OF_MUCINS;REACTOME_TERMINATION_OF_O_GLYCAN_BIOSYNTHESIS;REACTOME_

REACTOME_INTEGRATION_OF_ENERGY_METABOLISM;REACTOME_ABC_FAMILY_PROTEINS
ISM_AND_CITRIC_ACID_TCA_CYCLE;REACTOME_TCA_CYCLE_AND_RESPIRATORY_ELECTR

EN_PRESENTATION;REACTOME_IMMUNE_SYSTEM;REACTOME_ADAPTIVE_IMMUNE_SYSTE

RANSPORT_OF_SMALL_MOLECULES;REACTOME_SLC_MEDIATED_TRANSMEMBRANE_TRANS

HE_EXTRACELLULAR_MATRIX;REACTOME_EXTRACELLULAR_MATRIX_ORGANIZATION
CONTRACTION;REACTOME_MUSCLE_CONTRACTION

ACTOME_ADAPTIVE_IMMUNE_SYSTEM;REACTOME_CLASS_I_MHC_MEDIATED_ANTIGEN_PR

PROTEOLYTIC_ACTIVITY_OF_APC_C_REQUIRED_FOR_THE_ONSET_OF_ANAPHASE_BY_MIT

RACTIONS_AT_THE_VASCULAR_WALL;REACTOME_BASIGIN_INTERACTIONS;REACTOME_HE
RACTIONS_AT_THE_VASCULAR_WALL;REACTOME_INTEGRIN_CELL_SURFACE_INTERACTIO
RANSPORT_OF_SMALL_MOLECULES;REACTOME_SLC_MEDIATED_TRANSMEMBRANE_TRANS

;REACTOME_CLASS_B_2_SECRETIN_FAMILY_RECEPTORS;REACTOME_GLUCAGON_TYPE_L

4;REACTOME_NUCLEAR_SIGNALING_BY_ERBB4
S_IN_LIPID_HOMEOSTASIS;REACTOME_ABC_FAMILY_PROTEINS_MEDIATED_TRANSPORT;R
EACTOME_POST_CHAPERONIN_TUBULIN_FOLDING_PATHWAY;REACTOME_METABOLISM_O

RANSPORT_OF_SMALL_MOLECULES;REACTOME_SLC_MEDIATED_TRANSMEMBRANE_TRANS

S_IN_LIPID_HOMEOSTASIS;REACTOME_ABC_FAMILY_PROTEINS_MEDIATED_TRANSPORT;R

;REACTOME_CLASS_B_2_SECRETIN_FAMILY_RECEPTORS;REACTOME_GPCR_LIGAND_BIND

TION_PATHWAY

ALLING_PATHWAY_VIA_PKC_AND_MAPK;REACTOME_SIGNALING_BY_GPCR;REACTOME_PE
OLOGY;REACTOME_AXON_GUIDANCE;REACTOME_NCAM1_INTERACTIONS;REACTOME_NCA
ALLING_PATHWAY_VIA_PKC_AND_MAPK;REACTOME_TRANSMISSION_ACROSS_CHEMICAL_
_IN_MEGAKARYOCYTE_DEVELOPMENT_AND_PLATELET_PRODUCTION;REACTOME_HEMOST

OLOGY;REACTOME_EXTRACELLULAR_MATRIX_ORGANIZATION;REACTOME_COLLAGEN_FOR

REACTOME_TRIF_MEDIATED_TLR3_SIGNALING;REACTOME_P75NTR_RECRUITS_SIGNALLIN

KING;REACTOME_GAP_JUNCTION_TRAFFICKING;REACTOME_GAP_JUNCTION_ASSEMBLY
ATION_OF_MUCINS;REACTOME_TERMINATION_OF_O_GLYCAN_BIOSYNTHESIS;REACTOME_

CTOME_ANTIVIRAL_MECHANISM_BY_IFN_STIMULATED_GENES;REACTOME_SIGNALING_BY_
CLEOTIDES;REACTOME_PURINE_SALVAGE;REACTOME_PURINE_METABOLISM

GTPASES;REACTOME_SIGNALLING_BY_NGF;REACTOME_NRAGE_SIGNALS_DEATH_THROU
NO_ACIDS_AND_DERIVATIVES;REACTOME_AMINE_DERIVED_HORMONES
EROID_HORMONES_AND_VITAMINS_A_AND_D;REACTOME_STEROID_HORMONES;REACTOM

TRIX_ORGANIZATION;REACTOME_COLLAGEN_FORMATION
OLISM;REACTOME_GLYCOSAMINOGLYCAN_METABOLISM;REACTOME_ABC_FAMILY_PROTE
CTOME_NITRIC_OXIDE_STIMULATES_GUANYLATE_CYCLASE;REACTOME_PLATELET_HOME
KING;REACTOME_TRANS_GOLGI_NETWORK_VESICLE_BUDDING;REACTOME_GOLGI_ASSO

;REACTOME_PEPTIDE_LIGAND_BINDING_RECEPTORS;REACTOME_CLASS_A1_RHODOPSIN_

OLOGY;REACTOME_AXON_GUIDANCE;REACTOME_OTHER_SEMAPHORIN_INTERACTIONS;RE
GTPASES;REACTOME_SIGNALLING_BY_NGF;REACTOME_TRANSMISSION_ACROSS_CHEMIC
E_OF_CELLULAR_PROTEINS;REACTOME_SIGNALING_BY_WNT;REACTOME_CTNNB1_PHOS

OLOGY;REACTOME_TRANSCRIPTIONAL_REGULATION_OF_WHITE_ADIPOCYTE_DIFFERENTI
)

_PRE_REPLICATIVE_COMPLEX;REACTOME_CELL_CYCLE;REACTOME_PROCESSIVE_SYNTH

OLOGY;REACTOME_AXON_GUIDANCE;REACTOME_CRMPS_IN_SEMA3A_SIGNALING;REACTO

RIPTION;REACTOME_TRANSCRIPTION;REACTOME_RNA_POL_I_RNA_POL_III_AND_MITOCH

;REACTOME_OLFACTORY_SIGNALING_PATHWAY;REACTOME_GPCR_DOWNSTREAM_SIGN

ENE_EXPRESSION;REACTOME_METABOLISM_OF_LIPIDS_AND_LIPOPROTEINS;REACTOME_

ATION_OF_MUCINS;REACTOME_METABOLISM_OF_PROTEINS;REACTOME_POST_TRANSLA

REACTOME_DAG_AND_IP3_SIGNALING;REACTOME_SIGNALING_BY_ERBB2;REACTOME_SIG

ERGY_METABOLISM;REACTOME_REGULATION_OF_INSULIN_SECRETION;REACTOME_INCR

NA;REACTOME_METABOLISM_OF_RNA;REACTOME_DESTABILIZATION_OF_MRNA_BY_BRF1;

LOGY;REACTOME_AXON_GUIDANCE;REACTOME_NCAM1_INTERACTIONS;REACTOME_NCA

TRIX_ORGANIZATION;REACTOME_COLLAGEN_FORMATION

OXIA_INDUCIBLE_FACTOR_HIF_BY_OXYGEN;REACTOME_OXYGEN_DEPENDENT_PROLINE_

OME_FORMATION_OF_THE_TERNARY_COMPLEX_AND_SUBSEQUENTLY_THE_43S_COMPLE

ATED_PLATELET_CYTOSOLIC_CA2_;REACTOME_FORMATION_OF_FIBRIN_CLOT_CLOTTING_

NING;REACTOME_TRANSCRIPTION;REACTOME_RNA_POL_II_PRE_TRANSCRIPTION_EVENTS
NA_SPLICING

SE1_FUNCTIONALIZATION_OF_COMPOUNDS
EROPHOSPHOLIPID_BIOSYNTHESIS;REACTOME_METABOLISM_OF_LIPIDS_AND_LIPOPROTE

UNE_SYSTEM;REACTOME_ADAPTIVE_IMMUNE_SYSTEM
ONDRIAL_TRANSCRIPTION;REACTOME_RNA_POL_III_TRANSCRIPTION_INITIATION_FROM_TY

G_IN_IMMUNE_SYSTEM

GINE_N_LINKED_GLYCOSYLATION;REACTOME_TRANSPORT_TO_THE_GOLGI_AND_SUBSEQ

AM_SIGNALING;REACTOME_G_ALPHA_S_SIGNALLING_EVENTS;REACTOME_GPCR_LIGAND_I

XPRESSION;REACTOME_PRE_NOTCH_EXPRESSION_AND_PROCESSING;REACTOME_NOTCH

OME_PROCESSING_OF_CAPPED_INTRON_CONTAINING_PRE_MRNA;REACTOME_TRANSPOR

NALING_BY_EGFR_IN_CANCER;REACTOME_NGF_SIGNALLING_VIA_TRKA_FROM_THE_PLAS
ACTOME_SPRY_REGULATION_OF_FGF_SIGNALING;REACTOME_TRIF_MEDIATED_TLR3_SIGN

SPORT;REACTOME_TRANSPORT_OF_GLUCOSE_AND_OTHER_SUGARS_BILE_SALTS_AND_(

:X;REACTOME_ACTIVATION_OF_THE_MRNA_UPON_BINDING_OF_THE_CAP_BINDING_COMP

REACTOME_PACKAGING_OF_TELOMERE_ENDS;REACTOME_TELOMERE_MAINTENANCE

_CYCLE;REACTOME_LATE_PHASE_OF_HIV_LIFE_CYCLE

ORMATION;REACTOME_CELL_JUNCTION_ORGANIZATION

OWNSTREAM_TCR_SIGNALING;REACTOME_P75NTR_RECRUITS_SIGNALLING_COMPLEXES;

LING

NSPORT_ACROSS_THE_PLASMA_MEMBRANE;REACTOME_TRANSMEMBRANE_TRANSPORT

SM;REACTOME_GLYCOSAMINOGLYCAN_METABOLISM;REACTOME_METABOLISM_OF_CARB

:EACTOME_ION_CHANNEL_TRANSPORT

ITS

NA_SPLICING;REACTOME_MRNA_SPLICING_MINOR_PATHWAY

EINS_AND_COMPLEXES;REACTOME_LOSS_OF_NLP_FROM_MITOTIC_CENTROSOMES;REAC

NALING_BY_EGFR_IN_CANCER;REACTOME_NGF_SIGNALLING_VIA_TRKA_FROM_THE_PLAS

ATE_TYPE;REACTOME_PHASE1_FUNCTIONALIZATION_OF_COMPOUNDS

ROTEIN_RESPONSE

E_MRNA;REACTOME_RNA_POL_II_TRANSCRIPTION;REACTOME_MRNA_PROCESSING;REACT

SAMINOGLYCAN_METABOLISM;REACTOME_METABOLISM_OF_CARBOHYDRATES
GH_JNK;REACTOME_CELL_DEATH_SIGNALLING_VIA_NRAGE_NRIF_AND_NADE;REACTOME_

ALING_BY_ERBB2;REACTOME_SIGNALING_BY_EGFR_IN_CANCER;REACTOME_PI3K_EVENTS
ALING_BY_ERBB2;REACTOME_SIGNALING_BY_EGFR_IN_CANCER;REACTOME_PI3K_EVENTS

OM_CHROMATIN;REACTOME_ASSOCIATION_OF_LICENSING_FACTORS_WITH_THE_PRE_REP

S

ITS
ME_PPARA_ACTIVATES_GENE_EXPRESSION;REACTOME_RORA_ACTIVATES_CIRCADIAN_EX

JNCTION_ORGANIZATION

ACTOME_PURINE_METABOLISM
RMATION;REACTOME_INTEGRIN_CELL_SURFACE_INTERACTIONS;REACTOME_SIGNALING_B
GH_JNK;REACTOME_CELL_DEATH_SIGNALLING_VIA_NRAGE_NRIF_AND_NADE;REACTOME_

E_SIGNALING_BY_ERBB4;REACTOME_SIGNALING_BY_ERBB2;REACTOME_SIGNALING_BY_C
TASIS

OXIDATION

TASIS;REACTOME_HIV_INFECTION;REACTOME_HOST_INTERACTIONS_OF_HIV_FACTORS;RE
EACTOME_NOTCH1_INTRACELLULAR_DOMAIN_REGULATES_TRANSCRIPTION;REACTOME_

ANNELS

;REACTOME_METABOLISM_OF_CARBOHYDRATES

SPORT;REACTOME_TRANSPORT_OF_INORGANIC_CATIONS_ANIONS_AND_AMINO_ACIDS_O

E_ENDOSOMAL_SORTING_COMPLEX_REQUIRED_FOR_TRANSPORT_ESCRT;REACTOME_HIV

SPORT;REACTOME_TRANSPORT_OF_INORGANIC_CATIONS_ANIONS_AND_AMINO_ACIDS_O

LASMA_MEMBRANE;REACTOME_SIGNALLING_TO_ERKS;REACTOME_P38MAPK_EVENTS

RT;REACTOME_STEROID_HORMONES;REACTOME_METABOLISM_OF_LIPIDS_AND_LIPOPRC

CTOME_REGULATION_OF_WATER_BALANCE_BY_RENAL_AQUAPORINS

_METABOLISM_OF_PROTEINS;REACTOME_POST_TRANSLATIONAL_PROTEIN_MODIFICATIO
_METABOLISM_OF_PROTEINS;REACTOME_POST_TRANSLATIONAL_PROTEIN_MODIFICATIO

SAMINOGLYCAN_METABOLISM;REACTOME_METABOLISM_OF_CARBOHYDRATES

_;REACTOME_HEMOSTASIS;REACTOME_PLATELET_ACTIVATION_SIGNALING_AND_AGGREG
ROTEIN_RESPONSE
NT_AND_PLATELET_PRODUCTION;REACTOME_KINESINS;REACTOME_HEMOSTASIS;REACTC

ALING

STIMULATES_GUANYLATE_CYCLASE;REACTOME_PLATELET_HOMEOSTASIS;REACTOME_HE
CTOME_SIGNALING_BY_NOTCH1;REACTOME_TRANSCRIPTIONAL_ACTIVITY_OF_SMAD2_SM
SIS_INDUCED_DNA_FRAGMENTATION;REACTOME_TAK1_ACTIVATES_NFKB_BY_PHOSPHOR

OCESSING_PRESENTATION;REACTOME_ANTIGEN_PROCESSING_UBIQUITINATION_PROTEA

ALING

SPORT;REACTOME_TRANSPORT_OF_INORGANIC_CATIONS_ANIONS_AND_AMINO_ACIDS_O

NALING

SPORT;REACTOME_TRANSPORT_OF_VITAMINS_NUCLEOSIDES_AND_RELATED_MOLECULE

ACTOME_CYTOKINE_SIGNALING_IN_IMMUNE_SYSTEM
_INTERACTIONS;REACTOME_CELL_JUNCTION_ORGANIZATION

F_PROTEINS

ES;REACTOME_NEURONAL_SYSTEM;REACTOME_NEUROTRANSMITTER_RELEASE_CYCLE;F

_LIKE_RECEPTORS;REACTOME_GPCR_DOWNSTREAM_SIGNALING;REACTOME_G_ALPHA_I_

ɔXIDATION
ɔXIDATION

ːRIPTION_PATHWAY;REACTOME_METABOLISM_OF_LIPIDS_AND_LIPOPROTEINS;REACTOME

ːSICLE_BUDDING;REACTOME_SYNTHESIS_OF_PIPS_AT_THE_GOLGI_MEMBRANE;REACTOM

OCESSING_PRESENTATION;REACTOME_ANTIGEN_PROCESSING_UBIQUITINATION_PROTEA

ALING

ACTOME_PURINE_METABOLISM

S;REACTOME_HEMOSTASIS
S;REACTOME_HEMOSTASIS

OME_GLYCEROPHOSPHOLIPID_BIOSYNTHESIS;REACTOME_SIGNALING_BY_GPCR;REACTOM

E_SIGNALING_BY_ERBB4;REACTOME_SIGNALING_BY_ERBB2;REACTOME_SIGNALING_BY_C
E_SIGNALING_BY_ERBB4;REACTOME_SIGNALING_BY_ERBB2;REACTOME_SIGNALING_BY_C

:PTIDE_LIGAND_BINDING_RECEPTORS;REACTOME_CLASS_A1_RHODOPSIN_LIKE_RECEPTC

:R_PHAGOSOME_PATHWAY;REACTOME_IMMUNOREGULATORY_INTERACTIONS_BETWEEN_
NDRIAL_TRANSCRIPTION;REACTOME_RNA_POL_I_TRANSCRIPTION_INITIATION

_FATTY_ACID_TRIACYLGLYCEROL_AND_KETONE_BODY_METABOLISM

N_OF_GENE_EXPRESSION_IN_BETA_CELLS;REACTOME_TRANSMEMBRANE_TRANSPORT_O

;PCR;REACTOME_INTEGRATION_OF_ENERGY_METABOLISM;REACTOME_OPIOID_SIGNALLIN

ON_TRANSPORT;REACTOME_REGULATION_OF_PYRUVATE_DEHYDROGENASE_PDH_COMP

SIS

SPORT;REACTOME_TRANSPORT_OF_INORGANIC_CATIONS_ANIONS_AND_AMINO_ACIDS_O

_INTERACTIONS;REACTOME_INTERACTION_BETWEEN_L1_AND_ANKYRINS;REACTOME_VOL

SPORT;REACTOME_TRANSPORT_OF_VITAMINS_NUCLEOSIDES_AND_RELATED_MOLECULE

L_SYSTEM;REACTOME_INTEGRATION_OF_ENERGY_METABOLISM;REACTOME_AXON_GUID

OTH_MUSCLE_CONTRACTION;REACTOME_HEMOSTASIS;REACTOME_PLATELET_ACTIVATIC

CTOME_REGULATION_OF_WATER_BALANCE_BY_RENAL_AQUAPORINS

SIGNALING_BY_ERBB4;REACTOME_SIGNALING_BY_ERBB2;REACTOME_SIGNALING_BY_CO
NALING_BY_EGFR_IN_CANCER;REACTOME_ANTIGEN_ACTIVATES_B_CELL_RECEPTOR_LE
ROTEIN_RESPONSE

CIATED_VESICLE_BIOGENESIS

TTER_RECEPTOR_BINDING_AND_DOWNSTREAM_TRANSMISSION_IN_THE_POSTSYNAPTIC_

ON_TRANSPORT;REACTOME_REGULATION_OF_PYRUVATE_DEHYDROGENASE_PDH_COMP

TEGRATION_OF_ENERGY_METABOLISM;REACTOME_GLUCAGON_SIGNALING_IN_METABOLI
DME_TRIGLYCERIDE_BIOSYNTHESIS;REACTOME_FATTY_ACYL_COA_BIOSYNTHESIS;REACTO

S_BY_ATF4;REACTOME_UNFOLDED_PROTEIN_RESPONSE;REACTOME_METABOLISM_OF_MI
I_OF_TRANSCRIPTION_COUPLED_NER_TC_NER_REPAIR_COMPLEX;REACTOME_DNA_REPA

GROWTH;REACTOME_L1CAM_INTERACTIONS;REACTOME_INTERACTION_BETWEEN_L1_AND
S_BY_ATF4;REACTOME_UNFOLDED_PROTEIN_RESPONSE;REACTOME_METABOLISM_OF_MI

D_METABOLISM;REACTOME_ACYL_CHAIN_REMODELLING_OF_PE;REACTOME_GLYCEROPHO
TIDE_HORMONE_BIOSYNTHESIS

NALING_BY_EGFR_IN_CANCER;REACTOME_NGF_SIGNALLING_VIA_TRKA_FROM_THE_PLAS

OM_CHROMATIN;REACTOME_CELL_CYCLE_MITOTIC;REACTOME_CELL_CYCLE_CHECKPOINT

EM;REACTOME_ADAPTIVE_IMMUNE_SYSTEM

TIONAL_PROTEIN_MODIFICATION

GH_JNK;REACTOME_CELL_DEATH_SIGNALLING_VIA_NRAGE_NRIF_AND_NADE;REACTOME_

OCESSING_PRESENTATION;REACTOME_ANTIGEN_PROCESSING_UBIQUITINATION_PROTEA

_INTERACTIONS;REACTOME_CELL_JUNCTION_ORGANIZATION

ECULES

NG_CROSS_PRESENTATION;REACTOME_INTERFERON_GAMMA_SIGNALING;REACTOME_INT

GH_JNK;REACTOME_CELL_DEATH_SIGNALLING_VIA_NRAGE_NRIF_AND_NADE;REACTOME_

GH_JNK;REACTOME_CELL_DEATH_SIGNALLING_VIA_NRAGE_NRIF_AND_NADE;REACTOME_

ON;REACTOME_RNA_POL_I_RNA_POL_III_AND_MITOCHONDRIAL_TRANSCRIPTION;REACTO

_SMALL_MOLECULES;REACTOME_SLC_MEDIATED_TRANSMEMBRANE_TRANSPORT;REACT(

_SMALL_MOLECULES;REACTOME_SLC_MEDIATED_TRANSMEMBRANE_TRANSPORT;REACT(

SPORT;REACTOME_TRANSPORT_OF_INORGANIC_CATIONS_ANIONS_AND_AMINO_ACIDS_O

ON;REACTOME_RNA_POL_I_RNA_POL_III_AND_MITOCHONDRIAL_TRANSCRIPTION;REACTO

N;REACTOME_TRANSCRIPTION;REACTOME_RNA_POL_I_RNA_POL_III_AND_MITOCHONDRIAL

ME_SIGNALING_BY_NOTCH

TASIS;REACTOME_INNATE_IMMUNE_SYSTEM;REACTOME_IMMUNE_SYSTEM;REACTOME_TC

DING

PLACEMENT_PATHWAY;REACTOME_BASE_FREE_SUGAR_PHOSPHATE_REMOVAL_VIA_THE
PLACEMENT_PATHWAY;REACTOME_BASE_FREE_SUGAR_PHOSPHATE_REMOVAL_VIA_THE

EACTOME_ION_CHANNEL_TRANSPORT

FANCONI_ANEMIA_PATHWAY;REACTOME_FANCONI_ANEMIA_PATHWAY;REACTOME_DNA_F
CTOME_NUCLEOTIDE_LIKE_PURINERGIC_RECEPTORS;REACTOME_GPCR_DOWNSTREAM_

N_OF_GENE_EXPRESSION_IN_BETA_CELLS;REACTOME_INTEGRATION_OF_ENERGY_META

RIPTION_COUPLED_NER_TC_NER;REACTOME_POL_SWITCHING;REACTOME_NUCLEOTIDE_E

SPORT;REACTOME_TRANSPORT_OF_INORGANIC_CATIONS_ANIONS_AND_AMINO_ACIDS_O
IE_PPARA_ACTIVATES_GENE_EXPRESSION;REACTOME_RORA_ACTIVATES_CIRCADIAN_EX

'ASIS

PTOR_SIGNALING;REACTOME_NUCLEAR_SIGNALING_BY_ERBB4;REACTOME_IMMUNE_SYS

TRANSCRIPTION;REACTOME_MRNA_CAPPING;REACTOME_TRANSCRIPTION_COUPLED_NER

ON;REACTOME_HYALURONAN_UPTAKE_AND_DEGRADATION;REACTOME_HYALURONAN_ME
ON;REACTOME_HYALURONAN_UPTAKE_AND_DEGRADATION;REACTOME_HYALURONAN_ME
HA_HYDROXYCHOLESTEROL;REACTOME_SYNTHESIS_OF_BILE_ACIDS_AND_BILE_SALTS;RE

LIGAND_GATED_ION_CHANNEL_TRANSPORT

ATION

TOME_RNA_POL_I_RNA_POL_III_AND_MITOCHONDRIAL_TRANSCRIPTION;REACTOME_CHRO

L_REGULATION_OF_WHITE_ADIPOCYTE_DIFFERENTIATION;REACTOME_APOPTOSIS

TOME_CYTOCHROME_P450_ARRANGED_BY_SUBSTRATE_TYPE;REACTOME_PHASE1_FUNC

TIONAL_PROTEIN_MODIFICATION

_SIGNALING_BY_THE_B_CELL_RECEPTOR_BCR;REACTOME_IMMUNE_SYSTEM;REACTOME_

BY_ERBB2;REACTOME_GRB2_EVENTS_IN_ERBB2_SIGNALING;REACTOME_PI3K_EVENTS_IN_

PTORS_BY_RECRUITING_THEM_TO_CLATHRIN_ADAPTERS;REACTOME_LATENT_INFECTION_
REACTOME_PACKAGING_OF_TELOMERE_ENDS;REACTOME_TELOMERE_MAINTENANCE
ME_SIGNALING_BY_NOTCH

ND_PLATELET_PRODUCTION;REACTOME_TRAF3_DEPENDENT_IRF_ACTIVATION_PATHWAY

ME_SIGNALING_BY_NOTCH

VENTS;REACTOME_NGF_SIGNALLING_VIA_TRKA_FROM_THE_PLASMA_MEMBRANE;REACTO

CED_BY_THE_EXON_JUNCTION_COMPLEX

E_ROLE_OF_DCC_IN_REGULATING_APOPTOSIS;REACTOME_NETRIN1_SIGNALING;REACTOI
E_ROLE_OF_DCC_IN_REGULATING_APOPTOSIS;REACTOME_NETRIN1_SIGNALING;REACTOI

NT_AND_PLATELET_PRODUCTION;REACTOME_KINESINS;REACTOME_HEMOSTASIS;REACTO

_METABOLISM_OF_PROTEINS;REACTOME_POST_TRANSLATIONAL_PROTEIN_MODIFICATIOI

_;REACTOME_HEMOSTASIS;REACTOME_PLATELET_ACTIVATION_SIGNALING_AND_AGGREG

M_SIGNALING_FOR_NEURITE_OUT_GROWTH;REACTOME_L1CAM_INTERACTIONS;REACTOM

JCOSE_METABOLISM
JCOSE_METABOLISM
CIATED_VESICLE_BIOGENESIS
ROTEIN_RESPONSE

TOME_CYTOCHROME_P450_ARRANGED_BY_SUBSTRATE_TYPE;REACTOME_PHASE1_FUNC
TOME_CYTOCHROME_P450_ARRANGED_BY_SUBSTRATE_TYPE;REACTOME_PHASE1_FUNC

LIPIDS_AND_LIPOPROTEINS;REACTOME_FATTY_ACID_TRIACYLGLYCEROL_AND_KETONE_E

D_METABOLISM;REACTOME_SYNTHESIS_OF_PA;REACTOME_ACYL_CHAIN_REMODELLING_C

TASIS

ATION_AND_REGULATION

RANSITION_;REACTOME_G1_S_TRANSITION;REACTOME_MITOTIC_G1_G1_S_PHASES;REAC
RANSITION_;REACTOME_G1_S_TRANSITION;REACTOME_MITOTIC_G1_G1_S_PHASES;REAC

G_OF_ENDOSOMAL_TLR;REACTOME_MHC_CLASS_II_ANTIGEN_PRESENTATION;REACTOME_

OCESSING_PRESENTATION;REACTOME_ANTIGEN_PROCESSING_UBIQUITINATION_PROTEA

GROWTH;REACTOME_L1CAM_INTERACTIONS;REACTOME_INTERACTION_BETWEEN_L1_AND

X;REACTOME_ACTIVATION_OF_THE_MRNA_UPON_BINDING_OF_THE_CAP_BINDING_COMP

_TRIF_MEDIATED_TLR3_SIGNALING;REACTOME_SIGNALING_BY_ERBB4;REACTOME_SIGNAL

TTER_RECEPTOR_BINDING_AND_DOWNSTREAM_TRANSMISSION_IN_THE_POSTSYNAPTIC_

EACTOME_NGF_SIGNALLING_VIA_TRKA_FROM_THE_PLASMA_MEMBRANE;REACTOME_NUC

CED_BY_THE_EXON_JUNCTION_COMPLEX
NSULIN_RECEPTOR;REACTOME_MTORC1_MEDIATED_SIGNALLING;REACTOME_PI3K_CASCA
N;REACTOME_TRANSCRIPTION;REACTOME_RNA_POL_I_RNA_POL_III_AND_MITOCHONDRIAL

NS;REACTOME_HEMOSTASIS

G;REACTOME_METABOLISM_OF_PROTEINS

IM_HOMEOSTASIS;REACTOME_HEMOSTASIS

_CIRCADIAN_EXPRESSION;REACTOME_METABOLISM_OF_LIPIDS_AND_LIPOPROTEINS;REA

NERGY_METABOLISM;REACTOME_METABOLISM_OF_LIPIDS_AND_LIPOPROTEINS;REACTOM

IE_PPARA_ACTIVATES_GENE_EXPRESSION;REACTOME_RORA_ACTIVATES_CIRCADIAN_EX

SPORT;REACTOME_TRANSPORT_OF_INORGANIC_CATIONS_ANIONS_AND_AMINO_ACIDS_O

M_SIGNALING_FOR_NEURITE_OUT_GROWTH

CRIPTION;REACTOME_HIV_INFECTION;REACTOME_HIV_LIFE_CYCLE;REACTOME_LATE_PHA

EACTOME_ION_CHANNEL_TRANSPORT

_LIPIDS_AND_LIPOPROTEINS;REACTOME_FATTY_ACID_TRIACYLGLYCEROL_AND_KETONE_[illegible]

_METABOLISM_OF_PROTEINS;REACTOME_POST_TRANSLATIONAL_PROTEIN_MODIFICATIO[illegible]
_METABOLISM_OF_PROTEINS;REACTOME_POST_TRANSLATIONAL_PROTEIN_MODIFICATIO[illegible]
_METABOLISM_OF_PROTEINS;REACTOME_POST_TRANSLATIONAL_PROTEIN_MODIFICATIO[illegible]
_METABOLISM_OF_PROTEINS;REACTOME_POST_TRANSLATIONAL_PROTEIN_MODIFICATIO[illegible]

Y_OF_SMAD2_SMAD3_SMAD4_HETEROTRIMER;REACTOME_GENERIC_TRANSCRIPTION_PA[illegible]

NSMEMBRANE_TRANSPORT_OF_SMALL_MOLECULES;REACTOME_SLC_MEDIATED_TRANSM[illegible]

CR;REACTOME_G_ALPHA1213_SIGNALLING_EVENTS;REACTOME_GPCR_DOWNSTREAM_SI[illegible]
TTER_RECEPTOR_BINDING_AND_DOWNSTREAM_TRANSMISSION_IN_THE_POSTSYNAPTIC_
TTER_RECEPTOR_BINDING_AND_DOWNSTREAM_TRANSMISSION_IN_THE_POSTSYNAPTIC_

ED_PROTEINS;REACTOME_POST_TRANSLATIONAL_PROTEIN_MODIFICATION;REACTOME_H
ED_PROTEINS;REACTOME_POST_TRANSLATIONAL_PROTEIN_MODIFICATION;REACTOME_H

_SYNAPSES;REACTOME_NEURONAL_SYSTEM;REACTOME_SIGNALING_BY_GPCR;REACTOMI

LING;REACTOME_HIV_INFECTION;REACTOME_HOST_INTERACTIONS_OF_HIV_FACTORS;REA
IE_PPARA_ACTIVATES_GENE_EXPRESSION;REACTOME_RORA_ACTIVATES_CIRCADIAN_EX
IE_PPARA_ACTIVATES_GENE_EXPRESSION;REACTOME_RORA_ACTIVATES_CIRCADIAN_EX

GNALING_BY_NOTCH

IE_PPARA_ACTIVATES_GENE_EXPRESSION;REACTOME_PRE_NOTCH_TRANSCRIPTION_ANI
_FATTY_ACID_TRIACYLGLYCEROL_AND_KETONE_BODY_METABOLISM

ND_MAP_KINASES_UPON_TLR7_8_OR_9_ACTIVATION;REACTOME_TRAF6_MEDIATED_IRF7_

ND_MAP_KINASES_UPON_TLR7_8_OR_9_ACTIVATION;REACTOME_TRAF6_MEDIATED_IRF7_

_SYSTEM;REACTOME_PHASE1_FUNCTIONALIZATION_OF_COMPOUNDS;REACTOME_NEURO

REACTOME_RESPIRATORY_ELECTRON_TRANSPORT_ATP_SYNTHESIS_BY_CHEMIOSMOTIC

RIPTION_PATHWAY;REACTOME_METABOLISM_OF_LIPIDS_AND_LIPOPROTEINS;REACTOME

CTOME_NRAGE_SIGNALS_DEATH_THROUGH_JNK;REACTOME_CELL_DEATH_SIGNALLING_

_PHASE

CTOME_CYTOKINE_SIGNALING_IN_IMMUNE_SYSTEM

_MOLECULES;REACTOME_AXON_GUIDANCE;REACTOME_SIGNALING_BY_ROBO_RECEPTOR

OTIC_SPINDLE_CHECKPOINT_COMPONENTS;REACTOME_CELL_CYCLE;REACTOME_CELL_C

REACTOME_METABOLISM_OF_CARBOHYDRATES;REACTOME_GLUCOSE_METABOLISM
OCESSING_PRESENTATION;REACTOME_ANTIGEN_PROCESSING_UBIQUITINATION_PROTEA

PTIDE_LIGAND_BINDING_RECEPTORS;REACTOME_CLASS_A1_RHODOPSIN_LIKE_RECEPTO
SPORT;REACTOME_TRANSPORT_OF_GLUCOSE_AND_OTHER_SUGARS_BILE_SALTS_AND_
SPORT;REACTOME_TRANSPORT_OF_GLUCOSE_AND_OTHER_SUGARS_BILE_SALTS_AND_

LING_BY_ROBO_RECEPTOR;REACTOME_NETRIN1_SIGNALING

_SMALL_MOLECULES;REACTOME_SLC_MEDIATED_TRANSMEMBRANE_TRANSPORT;REACT

.SIGNALING_BY_NOTCH

ALING_BY_ERBB2;REACTOME_SIGNALING_BY_EGFR_IN_CANCER;REACTOME_PI3K_EVENTS

BY_ERBB2;REACTOME_PI3K_EVENTS_IN_ERBB2_SIGNALING
PLACEMENT_PATHWAY;REACTOME_BASE_FREE_SUGAR_PHOSPHATE_REMOVAL_VIA_THE

ALPHA_Q_SIGNALLING_EVENTS;REACTOME_GPCR_DOWNSTREAM_SIGNALING;REACTOME

HA_HYDROXYCHOLESTEROL;REACTOME_SYNTHESIS_OF_BILE_ACIDS_AND_BILE_SALTS_VI

EINS_AND_COMPLEXES;REACTOME_LOSS_OF_NLP_FROM_MITOTIC_CENTROSOMES;REACT

UNE_SYSTEM;REACTOME_ADAPTIVE_IMMUNE_SYSTEM

ΛE_PPARA_ACTIVATES_GENE_EXPRESSION;REACTOME_RORA_ACTIVATES_CIRCADIAN_EX

CTOME_INTERFERON_SIGNALING;REACTOME_NEGATIVE_REGULATORS_OF_RIG_I_MDA5_S

OCESSING_PRESENTATION;REACTOME_ANTIGEN_PROCESSING_UBIQUITINATION_PROTEA

_SIGNALING_BY_NOTCH

DIATED_ACTIVATION_CASCADE;REACTOME_HEMOSTASIS;REACTOME_SIGNALING_BY_TGF

ASE

SPORT;REACTOME_TRANSPORT_OF_GLUCOSE_AND_OTHER_SUGARS_BILE_SALTS_AND_(

ALING_BY_ERBB2;REACTOME_SIGNALING_BY_EGFR_IN_CANCER;REACTOME_PI3K_EVENTS

OME_GPCR_LIGAND_BINDING
OCESSING_PRESENTATION;REACTOME_ANTIGEN_PROCESSING_UBIQUITINATION_PROTEA
OCESSING_PRESENTATION;REACTOME_ANTIGEN_PROCESSING_UBIQUITINATION_PROTEA

HS_GAG_BIOSYNTHESIS;REACTOME_HEPARAN_SULFATE_HEPARIN_HS_GAG_METABOLISM
HS_GAG_BIOSYNTHESIS;REACTOME_HEPARAN_SULFATE_HEPARIN_HS_GAG_METABOLISM

Y_METABOLISM

GINE_N_LINKED_GLYCOSYLATION;REACTOME_TRANSPORT_TO_THE_GOLGI_AND_SUBSEQ

EACTOME_NGF_SIGNALLING_VIA_TRKA_FROM_THE_PLASMA_MEMBRANE;REACTOME_GAS

OGLYCAN_METABOLISM;REACTOME_TRANSMEMBRANE_TRANSPORT_OF_SMALL_MOLECU
OGLYCAN_METABOLISM;REACTOME_TRANSMEMBRANE_TRANSPORT_OF_SMALL_MOLECU

ULATION_OF_CELL_SURFACE_RECEPTORS_BY_RECRUITING_THEM_TO_CLATHRIN_ADAP

S
S

NA_SPLICING
CRIPTION_PATHWAY;REACTOME_METABOLISM_OF_LIPIDS_AND_LIPOPROTEINS;REACTOME

E_SIGNALING_BY_FGFR_IN_DISEASE;REACTOME_SIGNALING_BY_FGFR

LING_CASCADE;REACTOME_INTEGRATION_OF_ENERGY_METABOLISM;REACTOME_REGUL

ILS;REACTOME_IL_3_5_AND_GM_CSF_SIGNALING;REACTOME_IL_RECEPTOR_SHC_SIGNALI

ED_PROTEINS;REACTOME_POST_TRANSLATIONAL_PROTEIN_MODIFICATION

ASS_A1_RHODOPSIN_LIKE_RECEPTORS;REACTOME_G_ALPHA_Q_SIGNALLING_EVENTS;RE

ECULES
ECULES

EACTOME_SIGNALING_BY_NOTCH
CTOME_AXON_GUIDANCE;REACTOME_G_ALPHA1213_SIGNALLING_EVENTS;REACTOME_GP

H_EXPRESSION_AND_PROCESSING;REACTOME_PRE_NOTCH_PROCESSING_IN_GOLGI;REA
H_EXPRESSION_AND_PROCESSING;REACTOME_PRE_NOTCH_PROCESSING_IN_GOLGI;REA

N;REACTOME_TRANSCRIPTION;REACTOME_RNA_POL_I_RNA_POL_III_AND_MITOCHONDRIAL

TABOLISM;REACTOME_METABOLISM_OF_LIPIDS_AND_LIPOPROTEINS

OCESSING_PRESENTATION;REACTOME_ANTIGEN_PROCESSING_UBIQUITINATION_PROTEA

ICATION;REACTOME_MITOTIC_PROMETAPHASE
TOME_NRAGE_SIGNALS_DEATH_THROUGH_JNK;REACTOME_CELL_DEATH_SIGNALLING_VI

ATION_OF_C4_AND_C2_ACTIVATORS;REACTOME_INITIAL_TRIGGERING_OF_COMPLEMENT

ITS;REACTOME_G_ALPHA_Z_SIGNALLING_EVENTS

ONDRIAL_TRANSCRIPTION;REACTOME_RNA_POL_III_TRANSCRIPTION_INITIATION_FROM_TY

CYTOSOLIC_CA2_;REACTOME_HEMOSTASIS;REACTOME_PLATELET_ACTIVATION_SIGNALIN

.IN_LIKE_GROWTH_FACTOR_BINDING_PROTEINS_IGFBPS

OME_PROCESSING_OF_CAPPED_INTRON_CONTAINING_PRE_MRNA;REACTOME_TRANSPOI

M_SIGNALING_FOR_NEURITE_OUT_GROWTH

SIS;REACTOME_METABOLISM_OF_LIPIDS_AND_LIPOPROTEINS
M_SIGNALING_FOR_NEURITE_OUT_GROWTH;REACTOME_L1CAM_INTERACTIONS
/I;REACTOME_COMPLEMENT_CASCADE

ME_OTHER_SEMAPHORIN_INTERACTIONS;REACTOME_SEMA3A_PAK_DEPENDENT_AXON_I
E_METABOLISM_OF_LIPIDS_AND_LIPOPROTEINS

ET_CYTOSOLIC_CA2_;REACTOME_HEMOSTASIS;REACTOME_PLATELET_ACTIVATION_SIGN/

GH_JNK;REACTOME_CELL_DEATH_SIGNALLING_VIA_NRAGE_NRIF_AND_NADE;REACTOME_

ANNELS

_SIGNALING_BY_ERBB4;REACTOME_DOWNREGULATION_OF_ERBB2_ERBB3_SIGNALING;REA

DELLING_OF_PE;REACTOME_GLYCEROPHOSPHOLIPID_BIOSYNTHESIS;REACTOME_METABO
3B4;REACTOME_SIGNALING_BY_EGFR_IN_CANCER;REACTOME_NUCLEAR_SIGNALING_BY_E

LIN_SECRETION_BY_GLUCAGON_LIKE_PEPTIDE1;REACTOME_REGULATION_OF_INSULIN_SE

ME_PPARA_ACTIVATES_GENE_EXPRESSION;REACTOME_RORA_ACTIVATES_CIRCADIAN_EX

SPORT;REACTOME_TRANSPORT_OF_INORGANIC_CATIONS_ANIONS_AND_AMINO_ACIDS_O

E_NCAM_SIGNALING_FOR_NEURITE_OUT_GROWTH;REACTOME_L1CAM_INTERACTIONS;RE
E_NCAM_SIGNALING_FOR_NEURITE_OUT_GROWTH;REACTOME_L1CAM_INTERACTIONS;RE

ACTOME_PHASE_II_CONJUGATION;REACTOME_GLUCOSE_METABOLISM
_MEDIATED_INDUCTION_OF_NFKB_AND_MAP_KINASES_UPON_TLR7_8_OR_9_ACTIVATION;I

A_CARBOXYLATION_TRANSPORT_AND_AMINO_TERMINAL_CLEAVAGE_OF_PROTEINS;REAC

NA_SPLICING;REACTOME_MRNA_SPLICING_MINOR_PATHWAY

ICATION;REACTOME_MITOTIC_PROMETAPHASE

LASMA_MEMBRANE;REACTOME_SIGNALLING_TO_ERKS;REACTOME_P38MAPK_EVENTS
A_CARBOXYLATION_TRANSPORT_AND_AMINO_TERMINAL_CLEAVAGE_OF_PROTEINS;REAC

BOLISM;REACTOME_METABOLISM_OF_LIPIDS_AND_LIPOPROTEINS

NG

N;REACTOME_ENDOSOMAL_SORTING_COMPLEX_REQUIRED_FOR_TRANSPORT_ESCRT

HESIS_SECRETION_AND_INACTIVATION_OF_GIP;REACTOME_INCRETIN_SYNTHESIS_SECRE
HESIS_SECRETION_AND_INACTIVATION_OF_GIP;REACTOME_INCRETIN_SYNTHESIS_SECRE

HA_HYDROXYCHOLESTEROL;REACTOME_RECYCLING_OF_BILE_ACIDS_AND_SALTS;REACTO

ME_RECRUITMENT_OF_MITOTIC_CENTROSOME_PROTEINS_AND_COMPLEXES;REACTOME_
ITEGRIN_ALPHAIIB_BETA3_SIGNALING;REACTOME_REGULATION_OF_INSULIN_SECRETION_

TTER_RECEPTOR_BINDING_AND_DOWNSTREAM_TRANSMISSION_IN_THE_POSTSYNAPTIC_

NTS;REACTOME_CGMP_EFFECTS;REACTOME_NITRIC_OXIDE_STIMULATES_GUANYLATE_C

RFACE_INTERACTIONS_AT_THE_VASCULAR_WALL;REACTOME_PECAM1_INTERACTIONS;RE
RMATION;REACTOME_SIGNALING_BY_PDGF;REACTOME_AXON_GUIDANCE;REACTOME_NCA

HANISM_BY_IFN_STIMULATED_GENES;REACTOME_SIGNALING_BY_FGFR_IN_DISEASE;REAC

NE_TRANSPORT_OF_SMALL_MOLECULES;REACTOME_SLC_MEDIATED_TRANSMEMBRANE_

R_SIGNALLING_CASCADE;REACTOME_NGF_SIGNALLING_VIA_TRKA_FROM_THE_PLASMA_M

PTIDE_LIGAND_BINDING_RECEPTORS;REACTOME_CLASS_A1_RHODOPSIN_LIKE_RECEPTO

_REPAIR
TTER_RELEASE_CYCLE;REACTOME_TRANSMEMBRANE_TRANSPORT_OF_SMALL_MOLECUL

_SIGNALING_BY_ERBB4;REACTOME_SIGNALING_BY_ERBB2;REACTOME_GRB2_EVENTS_IN_

S

SPORT;REACTOME_TRANSPORT_OF_INORGANIC_CATIONS_ANIONS_AND_AMINO_ACIDS_O
GULATION_OF_TGF_BETA_RECEPTOR_SIGNALING;REACTOME_TGF_BETA_RECEPTOR_SIG

_LIKE_RECEPTORS;REACTOME_CHEMOKINE_RECEPTORS_BIND_CHEMOKINES;REACTOME_

_FATTY_ACID_TRIACYLGLYCEROL_AND_KETONE_BODY_METABOLISM;REACTOME_METABO

;

ME_OTHER_SEMAPHORIN_INTERACTIONS;REACTOME_SEMA3A_PAK_DEPENDENT_AXON_[illegible]

[illegible]FANCONI_ANEMIA_PATHWAY;REACTOME_FANCONI_ANEMIA_PATHWAY;REACTOME_DNA_[illegible]

[illegible]SMAD2_3_SMAD4_TRANSCRIPTIONAL_ACTIVITY;REACTOME_GENERIC_TRANSCRIPTION_PA[illegible]

SE;REACTOME_PLATELET_HOMEOSTASIS;REACTOME_HEMOSTASIS;REACTOME_POTASSIU[illegible]

_LIKE_RECEPTORS;REACTOME_GPCR_DOWNSTREAM_SIGNALING;REACTOME_G_ALPHA_I_

_METABOLISM_OF_PROTEINS;REACTOME_POST_TRANSLATIONAL_PROTEIN_MODIFICATIO

ROTEIN_RESPONSE

_ALPHA_BETA_PATHWAYS;REACTOME_INNATE_IMMUNE_SYSTEM;REACTOME_IMMUNE_SYS

_ALPHA_BETA_PATHWAYS;REACTOME_INNATE_IMMUNE_SYSTEM;REACTOME_IMMUNE_SYS

TTER_RECEPTOR_BINDING_AND_DOWNSTREAM_TRANSMISSION_IN_THE_POSTSYNAPTIC_

SPORT;REACTOME_TRANSPORT_OF_INORGANIC_CATIONS_ANIONS_AND_AMINO_ACIDS_O

;_PHASES

;AMINOGLYCAN_METABOLISM;REACTOME_METABOLISM_OF_CARBOHYDRATES

TIONAL_PROTEIN_MODIFICATION

SPORT;REACTOME_TRANSPORT_OF_INORGANIC_CATIONS_ANIONS_AND_AMINO_ACIDS_O
EINS_AND_COMPLEXES;REACTOME_LOSS_OF_NLP_FROM_MITOTIC_CENTROSOMES;REAC

SPORT;REACTOME_TRANSPORT_OF_INORGANIC_CATIONS_ANIONS_AND_AMINO_ACIDS_O

;TOME_GASTRIN_CREB_SIGNALLING_PATHWAY_VIA_PKC_AND_MAPK;REACTOME_NRAGE_
;TOME_GASTRIN_CREB_SIGNALLING_PATHWAY_VIA_PKC_AND_MAPK;REACTOME_NRAGE_

LING_CASCADE;REACTOME_REGULATION_OF_AMPK_ACTIVITY_VIA_LKB1;REACTOME_ENE

Y_METABOLISM
_FATTY_ACID_TRIACYLGLYCEROL_AND_KETONE_BODY_METABOLISM;REACTOME_CHOLES

NSTREAM_SIGNALING;REACTOME_G_ALPHA_S_SIGNALLING_EVENTS

OME_MAINTENANCE;REACTOME_DEPOSITION_OF_NEW_CENPA_CONTAINING_NUCLEOSON

E_IL_7_SIGNALING;REACTOME_SIGNALING_BY_ERBB4;REACTOME_SIGNALING_BY_ERBB2;F
PLICATION_STRESS;REACTOME_G2_M_CHECKPOINTS

E_DERMATAN_SULFATE_METABOLISM;REACTOME_HEPARAN_SULFATE_HEPARIN_HS_GAG

SPHINGOLIPID_METABOLISM;REACTOME_PHOSPHOLIPID_METABOLISM;REACTOME_THE_

EACTOME_SEMAPHORIN_INTERACTIONS

_B1_ASSOCIATED_EVENTS_DURING_G2_M_TRANSITION;REACTOME_MITOTIC_G2_G2_M_P
_B1_ASSOCIATED_EVENTS_DURING_G2_M_TRANSITION;REACTOME_MITOTIC_G2_G2_M_P
ACTOME_CYTOKINE_SIGNALING_IN_IMMUNE_SYSTEM
ACTOME_CYTOKINE_SIGNALING_IN_IMMUNE_SYSTEM

ꟷSTASIS;REACTOME_HEMOSTASIS

OME_SIGNALING_BY_FGFR_IN_DISEASE;REACTOME_SIGNALING_BY_FGFR_MUTANTS;REAC
OME_SIGNALING_BY_FGFR_IN_DISEASE;REACTOME_SIGNALING_BY_FGFR_MUTANTS;REAC
OME_SIGNALING_BY_FGFR_IN_DISEASE;REACTOME_SIGNALING_BY_FGFR_MUTANTS;REAC

TOME_PEPTIDE_CHAIN_ELONGATION;REACTOME_METABOLISM_OF_PROTEINS;REACTOME_
TOME_GPCR_LIGAND_BINDING

GINE_N_LINKED_GLYCOSYLATION;REACTOME_TRANSPORT_TO_THE_GOLGI_AND_SUBSEQ
SIS_OF_SUBSTRATES_IN_N_GLYCAN_BIOSYTHESIS;REACTOME_ASPARAGINE_N_LINKED_G

CIATED_VESICLE_BIOGENESIS

ME_DOUBLE_STRAND_BREAK_REPAIR;REACTOME_DNA_REPAIR

NALING_BY_EGFR_IN_CANCER;REACTOME_ANTIGEN_ACTIVATES_B_CELL_RECEPTOR_LEA

TOME_PEPTIDE_CHAIN_ELONGATION;REACTOME_METABOLISM_OF_PROTEINS;REACTOME_

NG_OF_ACTIVATED_FGFR;REACTOME_SIGNALING_BY_FGFR

SPORT;REACTOME_TRANSPORT_OF_VITAMINS_NUCLEOSIDES_AND_RELATED_MOLECULE

ME_NEURONAL_SYSTEM

;REACTOME_METABOLISM_OF_CARBOHYDRATES

REACTOME_LIPOPROTEIN_METABOLISM

OCESSING_PRESENTATION;REACTOME_ANTIGEN_PROCESSING_UBIQUITINATION_PROTEA

SM;REACTOME_GLYCOSAMINOGLYCAN_METABOLISM;REACTOME_METABOLISM_OF_CARB
_B_CELLS;REACTOME_SIGNALING_BY_THE_B_CELL_RECEPTOR_BCR;REACTOME_TCR_SIG

_ALPHA_Q_SIGNALLING_EVENTS;REACTOME_GPCR_DOWNSTREAM_SIGNALING;REACTOME

ECULES

ON_OF_IKKS_COMPLEX;REACTOME_MAP_KINASE_ACTIVATION_IN_TLR_CASCADE;REACTO

G;REACTOME_METABOLISM_OF_PROTEINS

ON_TRANSPORT;REACTOME_METABOLISM_OF_AMINO_ACIDS_AND_DERIVATIVES;REACTO
NALING_BY_EGFR_IN_CANCER;REACTOME_NGF_SIGNALLING_VIA_TRKA_FROM_THE_PLAS
IN_LIKE_GROWTH_FACTOR_BINDING_PROTEINS_IGFBPS

TIONAL_PROTEIN_MODIFICATION

DING

SIGNALING_BY_NOTCH

_INTERACTIONS_AT_THE_VASCULAR_WALL;REACTOME_COLLAGEN_FORMATION;REACTOI

OCESSING_PRESENTATION;REACTOME_ANTIGEN_PROCESSING_UBIQUITINATION_PROTEA

_METABOLISM_OF_PROTEINS;REACTOME_POST_TRANSLATIONAL_PROTEIN_MODIFICATIO

FR1_FUSION_MUTANTS;REACTOME_SIGNALING_BY_FGFR_MUTANTS

REACTOME_ANTIGEN_PROCESSING_CROSS_PRESENTATION;REACTOME_ER_PHAGOSOMI
M_CHROMATIN;REACTOME_ASSOCIATION_OF_LICENSING_FACTORS_WITH_THE_PRE_REF

JNCTION_ORGANIZATION
DING

ALPHA_Q_SIGNALLING_EVENTS;REACTOME_GPCR_DOWNSTREAM_SIGNALING;REACTOME
ALPHA_Q_SIGNALLING_EVENTS;REACTOME_GPCR_DOWNSTREAM_SIGNALING;REACTOME

_TUBERCULOSIS;REACTOME_TRANSMEMBRANE_TRANSPORT_OF_SMALL_MOLECULES;RE

CTION_OF_HOMO_SAPIENS_WITH_MYCOBACTERIUM_TUBERCULOSIS;REACTOME_ENOS_A

RPP_32_EVENTS;REACTOME_AXON_GUIDANCE;REACTOME_CRMPS_IN_SEMA3A_SIGNALIN

LING_CASCADE;REACTOME_INTEGRATION_OF_ENERGY_METABOLISM;REACTOME_REGUL
LING_CASCADE;REACTOME_INTEGRATION_OF_ENERGY_METABOLISM;REACTOME_REGUL

OCESSING_PRESENTATION;REACTOME_ANTIGEN_PROCESSING_UBIQUITINATION_PROTEA

GINE_N_LINKED_GLYCOSYLATION

TASIS

ECEPTOR_SIGNALLING_CASCADE;REACTOME_SIGNALING_BY_FGFR_IN_DISEASE;REACTOM

ECEPTOR_SIGNALLING_CASCADE;REACTOME_SIGNALING_BY_FGFR_IN_DISEASE;REACTOM

PLACEMENT_PATHWAY;REACTOME_BASE_FREE_SUGAR_PHOSPHATE_REMOVAL_VIA_THE

\NTS

TIONAL_REGULATION_OF_WHITE_ADIPOCYTE_DIFFERENTIATION;REACTOME_HORMONE_S

\CTOME_FATTY_ACID_TRIACYLGLYCEROL_AND_KETONE_BODY_METABOLISM
\CTOME_FATTY_ACID_TRIACYLGLYCEROL_AND_KETONE_BODY_METABOLISM

S

ANNELS

\SE_MEDIATED_CLEAVAGE_OF_CYTOSKELETAL_PROTEINS;REACTOME_CELL_JUNCTION_(

ES;REACTOME_NEURONAL_SYSTEM;REACTOME_NEUROTRANSMITTER_RELEASE_CYCLE;F

)NDRIAL_TRANSCRIPTION;REACTOME_RNA_POL_III_TRANSCRIPTION_TERMINATION

SPORT;REACTOME_TRANSPORT_OF_INORGANIC_CATIONS_ANIONS_AND_AMINO_ACIDS_O

REACTOME_RESPIRATORY_ELECTRON_TRANSPORT_ATP_SYNTHESIS_BY_CHEMIOSMOTIC

JNCTION_ORGANIZATION

S;REACTOME_NGF_SIGNALLING_VIA_TRKA_FROM_THE_PLASMA_MEMBRANE;REACTOME_S

.IN_LIKE_GROWTH_FACTOR_BINDING_PROTEINS_IGFBPS
EINS_AND_COMPLEXES;REACTOME_LOSS_OF_NLP_FROM_MITOTIC_CENTROSOMES;REACT

ALING_BY_ERBB2;REACTOME_SIGNALING_BY_EGFR_IN_CANCER;REACTOME_PI3K_EVENTS

OCESSING_PRESENTATION;REACTOME_ANTIGEN_PROCESSING_UBIQUITINATION_PROTEA
ES;REACTOME_NEURONAL_SYSTEM;REACTOME_NEUROTRANSMITTER_RELEASE_CYCLE;F

ELL_COMMUNICATION;REACTOME_CASPASE_MEDIATED_CLEAVAGE_OF_CYTOSKELETAL_I
ELL_COMMUNICATION;REACTOME_CASPASE_MEDIATED_CLEAVAGE_OF_CYTOSKELETAL_I
ELL_COMMUNICATION;REACTOME_CASPASE_MEDIATED_CLEAVAGE_OF_CYTOSKELETAL_I
ELL_COMMUNICATION;REACTOME_CASPASE_MEDIATED_CLEAVAGE_OF_CYTOSKELETAL_I

SION_TRANSAMINATION;REACTOME_TRYPTOPHAN_CATABOLISM
SION_TRANSAMINATION;REACTOME_TRYPTOPHAN_CATABOLISM
SIS_OF_SUBSTRATES_IN_N_GLYCAN_BIOSYTHESIS;REACTOME_ASPARAGINE_N_LINKED_G

VENTS;REACTOME_NGF_SIGNALLING_VIA_TRKA_FROM_THE_PLASMA_MEMBRANE;REACTO

N;REACTOME_TRANSCRIPTION;REACTOME_RNA_POL_I_RNA_POL_III_AND_MITOCHONDRIAL
LASMA_MEMBRANE;REACTOME_SIGNALLING_TO_ERKS;REACTOME_P38MAPK_EVENTS

RMATION;REACTOME_SIGNALING_BY_PDGF;REACTOME_AXON_GUIDANCE;REACTOME_NCA
RMATION;REACTOME_SIGNALING_BY_PDGF;REACTOME_AXON_GUIDANCE;REACTOME_NCA
RMATION;REACTOME_SIGNALING_BY_PDGF;REACTOME_AXON_GUIDANCE;REACTOME_NCA

EDIATED_NFKB_ACTIVATION;REACTOME_RIG_I_MDA5_MEDIATED_INDUCTION_OF_IFN_ALP
GINE_N_LINKED_GLYCOSYLATION;REACTOME_CALNEXIN_CALRETICULIN_CYCLE;REACTOM
SPORT;REACTOME_TRANSPORT_OF_INORGANIC_CATIONS_ANIONS_AND_AMINO_ACIDS_O

RT;REACTOME_STEROID_HORMONES;REACTOME_METABOLISM_OF_LIPIDS_AND_LIPOPRC
RT;REACTOME_STEROID_HORMONES;REACTOME_METABOLISM_OF_LIPIDS_AND_LIPOPRC

NS;REACTOME_GRB2_SOS_PROVIDES_LINKAGE_TO_MAPK_SIGNALING_FOR_INTERGRINS_

ESIS;REACTOME_TRANSMISSION_ACROSS_CHEMICAL_SYNAPSES;REACTOME_NEURONAL_

ATE_TYPE;REACTOME_PHASE1_FUNCTIONALIZATION_OF_COMPOUNDS

_SIGNALING_BY_THE_B_CELL_RECEPTOR_BCR;REACTOME_SIGNALING_BY_ILS;REACTOME

SAMINOGLYCAN_METABOLISM;REACTOME_METABOLISM_OF_CARBOHYDRATES

ORMATION;REACTOME_CELL_JUNCTION_ORGANIZATION

M_SIGNALING_FOR_NEURITE_OUT_GROWTH

IEDIATED_ATTRACTIVE_SIGNALING;REACTOME_FACTORS_INVOLVED_IN_MEGAKARYOCYTI
IEDIATED_ATTRACTIVE_SIGNALING;REACTOME_FACTORS_INVOLVED_IN_MEGAKARYOCYTI

_FUNCTIONALIZATION_OF_COMPOUNDS;REACTOME_METABOLISM_OF_POLYAMINES
_FUNCTIONALIZATION_OF_COMPOUNDS;REACTOME_METABOLISM_OF_POLYAMINES
_FUNCTIONALIZATION_OF_COMPOUNDS;REACTOME_METABOLISM_OF_POLYAMINES

REACTOME_ANTIGEN_PROCESSING_CROSS_PRESENTATION;REACTOME_ER_PHAGOSOMI

N_OF_CELL_SURFACE_RECEPTORS_BY_RECRUITING_THEM_TO_CLATHRIN_ADAPTERS;REA

_METABOLISM_OF_PROTEINS;REACTOME_POST_TRANSLATIONAL_PROTEIN_MODIFICATIO
_METABOLISM_OF_PROTEINS;REACTOME_POST_TRANSLATIONAL_PROTEIN_MODIFICATIO
_METABOLISM_OF_PROTEINS;REACTOME_POST_TRANSLATIONAL_PROTEIN_MODIFICATIO
_METABOLISM_OF_PROTEINS;REACTOME_POST_TRANSLATIONAL_PROTEIN_MODIFICATIO
_METABOLISM_OF_PROTEINS;REACTOME_POST_TRANSLATIONAL_PROTEIN_MODIFICATIO

UNE_SYSTEM;REACTOME_ADAPTIVE_IMMUNE_SYSTEM

ALING

ON;REACTOME_RORA_ACTIVATES_CIRCADIAN_EXPRESSION;REACTOME_CIRCADIAN_REPR

M_SIGNALING;REACTOME_G_ALPHA_S_SIGNALLING_EVENTS;REACTOME_CGMP_EFFECTS
TOME_CYTOCHROME_P450_ARRANGED_BY_SUBSTRATE_TYPE;REACTOME_PHASE1_FUNC

_CREB_SIGNALLING_PATHWAY_VIA_PKC_AND_MAPK;REACTOME_SIGNALING_BY_GPCR;R
ON_TRANSPORT;REACTOME_PYRUVATE_METABOLISM
SPORT;REACTOME_TRANSPORT_OF_GLUCOSE_AND_OTHER_SUGARS_BILE_SALTS_AND_

ETAL_PROTEINS;REACTOME_APOPTOSIS;REACTOME_APOPTOTIC_EXECUTION_PHASE

PIDS_AND_LIPOPROTEINS;REACTOME_FATTY_ACID_TRIACYLGLYCEROL_AND_KETONE_BOD

EINS_AND_COMPLEXES;REACTOME_LOSS_OF_NLP_FROM_MITOTIC_CENTROSOMES;REAC

ALING

ALING
ALING
ON_DEPENDENT_MRNA_DECAY;REACTOME_METABOLISM_OF_RNA

ETABOLISM_OF_LIPIDS_AND_LIPOPROTEINS;REACTOME_FATTY_ACID_TRIACYLGLYCEROL_

PT_TO_CYTOPLASM;REACTOME_MRNA_PROCESSING;REACTOME_TRANSPORT_OF_MATU

ALING_BY_ERBB2;REACTOME_SIGNALING_BY_EGFR_IN_CANCER;REACTOME_PI3K_EVENT
COSE_METABOLISM
ROTEIN_RESPONSE

ESIS_ON_THE_LAGGING_STRAND;REACTOME_CELL_CYCLE_MITOTIC;REACTOME_M_G1_TR
ANNELS

NT_AND_PLATELET_PRODUCTION;REACTOME_KINESINS;REACTOME_HEMOSTASIS;REACTO

TIVATES_SMADS;REACTOME_SIGNALING_BY_GPCR;REACTOME_OPIOID_SIGNALLING;REAC
REACTOME_RESPIRATORY_ELECTRON_TRANSPORT_ATP_SYNTHESIS_BY_CHEMIOSMOTIC
I_TUBERCULOSIS;REACTOME_TRANSMEMBRANE_TRANSPORT_OF_SMALL_MOLECULES;RE

BOLISM;REACTOME_METABOLISM_OF_LIPIDS_AND_LIPOPROTEINS
SPORT;REACTOME_TRANSPORT_OF_VITAMINS_NUCLEOSIDES_AND_RELATED_MOLECULE

M
TASIS

TOME_IMMUNE_SYSTEM;REACTOME_ADAPTIVE_IMMUNE_SYSTEM;REACTOME_CLASS_I_MH
REACTOME_METABOLISM_OF_LIPIDS_AND_LIPOPROTEINS;REACTOME_FATTY_ACID_TRIAC
ME_CELL_CYCLE_CHECKPOINTS;REACTOME_HOMOLOGOUS_RECOMBINATION_REPAIR_OF

ICATION;REACTOME_MITOTIC_PROMETAPHASE
CTION_ORGANIZATION;REACTOME_CELL_JUNCTION_ORGANIZATION

RACTION_BETWEEN_L1_AND_ANKYRINS

ON_DEPENDENT_MRNA_DECAY;REACTOME_METABOLISM_OF_RNA
TOME_NRAGE_SIGNALS_DEATH_THROUGH_JNK;REACTOME_CELL_DEATH_SIGNALLING_VI

:TOME_NRAGE_SIGNALS_DEATH_THROUGH_JNK;REACTOME_CELL_DEATH_SIGNALLING_VI

ALING

S

S

_ADAPTERS;REACTOME_DEFENSINS;REACTOME_TCR_SIGNALING;REACTOME_DOWNSTRE

IESIS_OF_PIPS_AT_THE_PLASMA_MEMBRANE;REACTOME_PI_METABOLISM;REACTOME_ME
IESIS_OF_PIPS_AT_THE_PLASMA_MEMBRANE;REACTOME_PI_METABOLISM;REACTOME_ME

ANSMEMBRANE_TRANSPORT_OF_SMALL_MOLECULES;REACTOME_SLC_MEDIATED_TRANS

_METABOLISM_OF_PROTEINS;REACTOME_POST_TRANSLATIONAL_PROTEIN_MODIFICATIO
_METABOLISM_OF_PROTEINS;REACTOME_POST_TRANSLATIONAL_PROTEIN_MODIFICATIO

NALING_BY_EGFR_IN_CANCER;REACTOME_NGF_SIGNALLING_VIA_TRKA_FROM_THE_PLAS

:GRIN_CELL_SURFACE_INTERACTIONS;REACTOME_IMMUNE_SYSTEM;REACTOME_ADAPTIV

ATED_TRANSFER_OF_SUBSTRATE_TO_CCT_TRIC;REACTOME_PROTEIN_FOLDING;REACTO

OGLYCAN_METABOLISM;REACTOME_METABOLISM_OF_CARBOHYDRATES
PLACEMENT_PATHWAY;REACTOME_BASE_FREE_SUGAR_PHOSPHATE_REMOVAL_VIA_THE

_LIPIDS_AND_LIPOPROTEINS;REACTOME_FATTY_ACID_TRIACYLGLYCEROL_AND_KETONE_E
STIMULATES_GUANYLATE_CYCLASE;REACTOME_PLATELET_HOMEOSTASIS;REACTOME_HE

GINE_N_LINKED_GLYCOSYLATION;REACTOME_N_GLYCAN_TRIMMING_IN_THE_ER_AND_CA

I_SIGNALING;REACTOME_IMMUNE_SYSTEM;REACTOME_CYTOKINE_SIGNALING_IN_IMMUNE

ICATION;REACTOME_MITOTIC_PROMETAPHASE
ICATION;REACTOME_MITOTIC_PROMETAPHASE
CIATED_VESICLE_BIOGENESIS

TIONAL_PROTEIN_MODIFICATION

FR1_FUSION_MUTANTS;REACTOME_SIGNALING_BY_FGFR_MUTANTS

RIPTION_COUPLED_NER_TC_NER;REACTOME_POL_SWITCHING;REACTOME_NUCLEOTIDE_E

ACTOME_FATTY_ACID_TRIACYLGLYCEROL_AND_KETONE_BODY_METABOLISM

RMATION;REACTOME_INTEGRIN_CELL_SURFACE_INTERACTIONS;REACTOME_SIGNALING_B

ON_COMPLEX_;REACTOME_ELONGATION_ARREST_AND_RECOVERY;REACTOME_RNA_POL

MINO_ACID_TRANSPORT_ACROSS_THE_PLASMA_MEMBRANE;REACTOME_TRANSMEMBRAN

_BINDING_RECEPTORS;REACTOME_PROSTANOID_LIGAND_RECEPTORS;REACTOME_GPCR_

_CELL_EXTRACELLULAR_MATRIX_INTERACTIONS;REACTOME_CELL_JUNCTION_ORGANIZAT

ASS_A1_RHODOPSIN_LIKE_RECEPTORS;REACTOME_G_ALPHA_Q_SIGNALLING_EVENTS;RE

ACTOME_CYTOKINE_SIGNALING_IN_IMMUNE_SYSTEM

SIGNALING_BY_NOTCH

NT_AND_PLATELET_PRODUCTION;REACTOME_KINESINS;REACTOME_HEMOSTASIS;REACTC

_TETRAHYDROBIOPTERIN_BH4_SYNTHESIS_RECYCLING_SALVAGE_AND_REGULATION;REA(

SIGNALING_BY_NOTCH2;REACTOME_SIGNALING_BY_NOTCH1;REACTOME_SIGNALING_BY_
N;REACTOME_TRANSCRIPTION;REACTOME_RNA_POL_I_RNA_POL_III_AND_MITOCHONDRIAL

EACTOME_ION_CHANNEL_TRANSPORT
OCESSING_PRESENTATION;REACTOME_ANTIGEN_PROCESSING_UBIQUITINATION_PROTEA
OCESSING_PRESENTATION;REACTOME_ANTIGEN_PROCESSING_UBIQUITINATION_PROTEA

TTER_RECEPTOR_BINDING_AND_DOWNSTREAM_TRANSMISSION_IN_THE_POSTSYNAPTIC_

LING_BY_ROBO_RECEPTOR
SYNAPSES;REACTOME_NEURONAL_SYSTEM;REACTOME_SIGNALING_BY_GPCR;REACTOMI
GROWTH;REACTOME_L1CAM_INTERACTIONS;REACTOME_INTERACTION_BETWEEN_L1_ANI
GROWTH;REACTOME_L1CAM_INTERACTIONS;REACTOME_INTERACTION_BETWEEN_L1_ANI

_POST_TRANSLATIONAL_MODIFICATION_SYNTHESIS_OF_GPI_ANCHORED_PROTEINS;REAC

:EACTOME_ION_CHANNEL_TRANSPORT

REACTOME_AQUAPORIN_MEDIATED_TRANSPORT

TOME_CYTOCHROME_P450_ARRANGED_BY_SUBSTRATE_TYPE;REACTOME_PHASE1_FUNC

GH_JNK;REACTOME_CELL_DEATH_SIGNALLING_VIA_NRAGE_NRIF_AND_NADE;REACTOME_

SPORT;REACTOME_TRANSPORT_OF_GLUCOSE_AND_OTHER_SUGARS_BILE_SALTS_AND_(

ITS

_LIKE_RECEPTORS;REACTOME_GPCR_DOWNSTREAM_SIGNALING;REACTOME_G_ALPHA_I_

TOME_PEPTIDE_CHAIN_ELONGATION;REACTOME_METABOLISM_OF_PROTEINS;REACTOME_

IE_PPARA_ACTIVATES_GENE_EXPRESSION;REACTOME_PRE_NOTCH_TRANSCRIPTION_ANI
IE_PPARA_ACTIVATES_GENE_EXPRESSION;REACTOME_PRE_NOTCH_TRANSCRIPTION_ANI
IE_PPARA_ACTIVATES_GENE_EXPRESSION;REACTOME_PRE_NOTCH_TRANSCRIPTION_ANI
NALING_BY_EGFR_IN_CANCER;REACTOME_NGF_SIGNALLING_VIA_TRKA_FROM_THE_PLAS

ME_CYTOKINE_SIGNALING_IN_IMMUNE_SYSTEM
ME_CYTOKINE_SIGNALING_IN_IMMUNE_SYSTEM

ECULES

CED_BY_THE_EXON_JUNCTION_COMPLEX

NSULIN_RECEPTOR;REACTOME_MTORC1_MEDIATED_SIGNALLING;REACTOME_PI3K_CASCA

RANSMEMBRANE_TRANSPORT_OF_SMALL_MOLECULES;REACTOME_PLATELET_HOMEOSTA

ONS;REACTOME_HEMOSTASIS

COSE_METABOLISM

OME_PROCESSING_OF_CAPPED_INTRON_CONTAINING_PRE_MRNA;REACTOME_TRANSPOI

OME_PROCESSING_OF_CAPPED_INTRON_CONTAINING_PRE_MRNA;REACTOME_TRANSPOI
_DIGESTION_MOBILIZATION_AND_TRANSPORT;REACTOME_LIPOPROTEIN_METABOLISM

SPORT;REACTOME_TRANSPORT_OF_INORGANIC_CATIONS_ANIONS_AND_AMINO_ACIDS_O

SPORT;REACTOME_TRANSPORT_OF_INORGANIC_CATIONS_ANIONS_AND_AMINO_ACIDS_O

REACTOME_PACKAGING_OF_TELOMERE_ENDS;REACTOME_TELOMERE_MAINTENANCE

NS;REACTOME_INTEGRIN_ALPHAIIB_BETA3_SIGNALING;REACTOME_SIGNALING_BY_PDGF;I

_SIGNALING_BY_THE_B_CELL_RECEPTOR_BCR;REACTOME_GPVI_MEDIATED_ACTIVATION_

ON;REACTOME_RNA_POL_I_RNA_POL_III_AND_MITOCHONDRIAL_TRANSCRIPTION;REACTO

NA_SPLICING;REACTOME_MRNA_SPLICING_MINOR_PATHWAY

CED_BY_THE_EXON_JUNCTION_COMPLEX
CED_BY_THE_EXON_JUNCTION_COMPLEX

TER_RECEPTOR_BINDING_AND_DOWNSTREAM_TRANSMISSION_IN_THE_POSTSYNAPTIC_

OCESSING_PRESENTATION;REACTOME_ANTIGEN_PROCESSING_UBIQUITINATION_PROTEA

SPORT;REACTOME_TRANSPORT_OF_GLUCOSE_AND_OTHER_SUGARS_BILE_SALTS_AND_(

PED_INTRON_CONTAINING_PRE_MRNA;REACTOME_RNA_POL_II_TRANSCRIPTION;REACTO

ETABOLISM;REACTOME_SIGNALING_BY_GPCR;REACTOME_GPVI_MEDIATED_ACTIVATION_(

SAMINOGLYCAN_METABOLISM;REACTOME_METABOLISM_OF_CARBOHYDRATES

IE_SIGNALING_BY_ERBB4;REACTOME_NUCLEAR_SIGNALING_BY_ERBB4;REACTOME_PPAR/

IE_SIGNALING_BY_ERBB4;REACTOME_NUCLEAR_SIGNALING_BY_ERBB4;REACTOME_PPAR/

ATED_TAK1_MEDIATES_P38_MAPK_ACTIVATION;REACTOME_TRAF6_MEDIATED_INDUCTION_

STIMULATES_GUANYLATE_CYCLASE;REACTOME_PLATELET_HOMEOSTASIS;REACTOME_HE

SPORT;REACTOME_TRANSPORT_OF_GLUCOSE_AND_OTHER_SUGARS_BILE_SALTS_AND_(

SPORT;REACTOME_TRANSPORT_OF_GLUCOSE_AND_OTHER_SUGARS_BILE_SALTS_AND_(

FR1_FUSION_MUTANTS;REACTOME_SIGNALING_BY_FGFR_MUTANTS

N_OF_CELL_SURFACE_RECEPTORS_BY_RECRUITING_THEM_TO_CLATHRIN_ADAPTERS;RE/

L_SYSTEM;REACTOME_AXON_GUIDANCE;REACTOME_NCAM1_INTERACTIONS;REACTOME_

TOME_PEPTIDE_CHAIN_ELONGATION;REACTOME_METABOLISM_OF_PROTEINS;REACTOME_

.ING_BY_ERBB2;REACTOME_GRB2_EVENTS_IN_ERBB2_SIGNALING;REACTOME_PI3K_EVEN
_LIKE_RECEPTORS;REACTOME_CHEMOKINE_RECEPTORS_BIND_CHEMOKINES;REACTOME_

LING;REACTOME_NGF_SIGNALLING_VIA_TRKA_FROM_THE_PLASMA_MEMBRANE;REACTOM
LING;REACTOME_NGF_SIGNALLING_VIA_TRKA_FROM_THE_PLASMA_MEMBRANE;REACTOM
LING;REACTOME_NGF_SIGNALLING_VIA_TRKA_FROM_THE_PLASMA_MEMBRANE;REACTOM

TER_RECEPTOR_BINDING_AND_DOWNSTREAM_TRANSMISSION_IN_THE_POSTSYNAPTIC_

OCESSING_PRESENTATION;REACTOME_ANTIGEN_PROCESSING_UBIQUITINATION_PROTEA

_INTERACTIONS_AT_THE_VASCULAR_WALL;REACTOME_COLLAGEN_FORMATION;REACTOI

I;REACTOME_MEIOTIC_RECOMBINATION;REACTOME_HEMOSTASIS

PTOR_SIGNALING;REACTOME_NUCLEAR_SIGNALING_BY_ERBB4;REACTOME_DIABETES_PA
RACTION_BETWEEN_L1_AND_ANKYRINS

EACTOME_TRANSMEMBRANE_TRANSPORT_OF_SMALL_MOLECULES

IE_ENERGY_DEPENDENT_REGULATION_OF_MTOR_BY_LKB1_AMPK;REACTOME_PKB_MEDI/

)ME_TRIGLYCERIDE_BIOSYNTHESIS;REACTOME_FATTY_ACYL_COA_BIOSYNTHESIS;REACT(
)ME_TRIGLYCERIDE_BIOSYNTHESIS;REACTOME_FATTY_ACYL_COA_BIOSYNTHESIS;REACT(

GNALING_BY_GPCR;REACTOME_CLASS_B_2_SECRETIN_FAMILY_RECEPTORS;REACTOME_(
ICATION;REACTOME_MITOTIC_PROMETAPHASE

EINS_AND_COMPLEXES;REACTOME_LOSS_OF_NLP_FROM_MITOTIC_CENTROSOMES;REAC
_LIKE_RECEPTORS;REACTOME_GPCR_DOWNSTREAM_SIGNALING;REACTOME_G_ALPHA_S

CTOME_REGULATION_OF_WATER_BALANCE_BY_RENAL_AQUAPORINS

:EACTOME_ION_CHANNEL_TRANSPORT

ON_TRANSPORT;REACTOME_CELL_SURFACE_INTERACTIONS_AT_THE_VASCULAR_WALL;F

NA_SPLICING

SIS;REACTOME_REGULATORY_RNA_PATHWAYS;REACTOME_PROCESSING_OF_CAPPED_IN

_SIGNALING;REACTOME_G_ALPHA_I_SIGNALLING_EVENTS;REACTOME_GPCR_LIGAND_BIN

ME_ANTIGEN_ACTIVATES_B_CELL_RECEPTOR_LEADING_TO_GENERATION_OF_SECOND_M
G_IN_EMT_EPITHELIAL_TO_MESENCHYMAL_TRANSITION;REACTOME_NRAGE_SIGNALS_DEA

I_OF_TRANSCRIPTION_COUPLED_NER_TC_NER_REPAIR_COMPLEX;REACTOME_DNA_REPA
I_OF_TRANSCRIPTION_COUPLED_NER_TC_NER_REPAIR_COMPLEX;REACTOME_DNA_REPA

_METABOLISM_OF_PROTEINS;REACTOME_POST_TRANSLATIONAL_PROTEIN_MODIFICATIO
_METABOLISM_OF_PROTEINS;REACTOME_POST_TRANSLATIONAL_PROTEIN_MODIFICATIO
_METABOLISM_OF_PROTEINS;REACTOME_POST_TRANSLATIONAL_PROTEIN_MODIFICATIO

IVE_REGULATORS_OF_RIG_I_MDA5_SIGNALING;REACTOME_RIG_I_MDA5_MEDIATED_INDU

ASS_A1_RHODOPSIN_LIKE_RECEPTORS;REACTOME_G_ALPHA_Q_SIGNALLING_EVENTS;RE
EGRIN_CELL_SURFACE_INTERACTIONS;REACTOME_INTERFERON_GAMMA_SIGNALING;REA

NALING;REACTOME_INTERFERON_SIGNALING;REACTOME_IL_6_SIGNALING;REACTOME_IMN

REACTOME_LIPOPROTEIN_METABOLISM;REACTOME_CHYLOMICRON_MEDIATED_LIPID_TRA
REACTOME_LIPOPROTEIN_METABOLISM;REACTOME_CHYLOMICRON_MEDIATED_LIPID_TRA
TASIS

BY_ERBB2;REACTOME_SIGNALING_BY_EGFR_IN_CANCER;REACTOME_NGF_SIGNALLING_VI

ALING

VNSTREAM_SIGNALING;REACTOME_G_ALPHA_S_SIGNALLING_EVENTS

JCOSE_METABOLISM

NA_SPLICING

NA_SPLICING
YMAL_TRANSITION;REACTOME_DOWNREGULATION_OF_TGF_BETA_RECEPTOR_SIGNALING
CR;REACTOME_G_ALPHA1213_SIGNALLING_EVENTS;REACTOME_GPCR_DOWNSTREAM_SI

ASS_A1_RHODOPSIN_LIKE_RECEPTORS;REACTOME_G_ALPHA_Q_SIGNALLING_EVENTS;RE

SPORT;REACTOME_TRANSPORT_OF_INORGANIC_CATIONS_ANIONS_AND_AMINO_ACIDS_O

METABOLISM;REACTOME_METABOLISM_OF_LIPIDS_AND_LIPOPROTEINS

NA_SPLICING
NA_SPLICING

EACTOME_SEMAPHORIN_INTERACTIONS;REACTOME_SEMA4D_INDUCED_CELL_MIGRATION

PLACEMENT_PATHWAY;REACTOME_CELL_CYCLE;REACTOME_PROCESSIVE_SYNTHESIS_O
PLACEMENT_PATHWAY;REACTOME_CELL_CYCLE;REACTOME_PROCESSIVE_SYNTHESIS_O
PLACEMENT_PATHWAY;REACTOME_CELL_CYCLE;REACTOME_PROCESSIVE_SYNTHESIS_O

TTER_RECEPTOR_BINDING_AND_DOWNSTREAM_TRANSMISSION_IN_THE_POSTSYNAPTIC_

UNE_SYSTEM;REACTOME_ADAPTIVE_IMMUNE_SYSTEM

ME_SIGNAL_REGULATORY_PROTEIN_SIRP_FAMILY_INTERACTIONS;REACTOME_HEMOSTAS

G;REACTOME_METABOLISM_OF_PROTEINS

M_SIGNALING_FOR_NEURITE_OUT_GROWTH

ED_PROTEINS;REACTOME_POST_TRANSLATIONAL_PROTEIN_MODIFICATION

EINS_AND_COMPLEXES;REACTOME_LOSS_OF_NLP_FROM_MITOTIC_CENTROSOMES;REAC

TTER_RECEPTOR_BINDING_AND_DOWNSTREAM_TRANSMISSION_IN_THE_POSTSYNAPTIC_

E_ANTIVIRAL_MECHANISM_BY_IFN_STIMULATED_GENES;REACTOME_SIGNALING_BY_ERBB

SPORT;REACTOME_TRANSPORT_OF_INORGANIC_CATIONS_ANIONS_AND_AMINO_ACIDS_O

SPORT;REACTOME_TRANSPORT_OF_GLUCOSE_AND_OTHER_SUGARS_BILE_SALTS_AND_(

S
:EACTOME_ION_CHANNEL_TRANSPORT

ANSPORT_OF_SMALL_MOLECULES;REACTOME_GLUCAGON_SIGNALING_IN_METABOLIC_RE
ANSPORT_OF_SMALL_MOLECULES;REACTOME_GLUCAGON_SIGNALING_IN_METABOLIC_RE

RMATION;REACTOME_SIGNALING_BY_PDGF;REACTOME_AXON_GUIDANCE;REACTOME_NCA
RMATION;REACTOME_SIGNALING_BY_PDGF;REACTOME_AXON_GUIDANCE;REACTOME_NCA
OME_ACTIVATION_OF_CHAPERONE_GENES_BY_XBP1S;REACTOME_UNFOLDED_PROTEIN_

NS;REACTOME_HEMOSTASIS
_CREB_SIGNALLING_PATHWAY_VIA_PKC_AND_MAPK;REACTOME_RESPONSE_TO_ELEVAT

ETABOLISM;REACTOME_METABOLISM_OF_LIPIDS_AND_LIPOPROTEINS

N_SIGNALING;REACTOME_IMMUNE_SYSTEM;REACTOME_CYTOKINE_SIGNALING_IN_IMMUN
GH_JNK;REACTOME_CELL_DEATH_SIGNALLING_VIA_NRAGE_NRIF_AND_NADE;REACTOME_

PT_TO_CYTOPLASM;REACTOME_RNA_POL_II_TRANSCRIPTION;REACTOME_MRNA_PROCES

L_SURFACE_INTERACTIONS_AT_THE_VASCULAR_WALL;REACTOME_INTEGRIN_CELL_SURF

RMATION;REACTOME_SIGNALING_BY_PDGF;REACTOME_AXON_GUIDANCE;REACTOME_NCA

EINS_AND_COMPLEXES;REACTOME_LOSS_OF_NLP_FROM_MITOTIC_CENTROSOMES;REAC

TABOLISM;REACTOME_METABOLISM_OF_LIPIDS_AND_LIPOPROTEINS

GNALING_BY_GPCR;REACTOME_CLASS_A1_RHODOPSIN_LIKE_RECEPTORS;REACTOME_NU

M_CHROMATIN;REACTOME_CELL_CYCLE_MITOTIC;REACTOME_CELL_CYCLE_CHECKPOINT
EACTOME_SEMAPHORIN_INTERACTIONS;REACTOME_SEMA4D_INDUCED_CELL_MIGRATION
EACTOME_IL_2_SIGNALING;REACTOME_IMMUNE_SYSTEM;REACTOME_CYTOKINE_SIGNALIN

)ELLING_OF_PE;REACTOME_GLYCEROPHOSPHOLIPID_BIOSYNTHESIS;REACTOME_METAB(
TASIS

TTER_RECEPTOR_BINDING_AND_DOWNSTREAM_TRANSMISSION_IN_THE_POSTSYNAPTIC_
TTER_RECEPTOR_BINDING_AND_DOWNSTREAM_TRANSMISSION_IN_THE_POSTSYNAPTIC_

M_SIGNALING_FOR_NEURITE_OUT_GROWTH
EACTOME_REGULATORY_RNA_PATHWAYS;REACTOME_SIGNALING_BY_NOTCH
PTIDE_LIGAND_BINDING_RECEPTORS;REACTOME_CLASS_A1_RHODOPSIN_LIKE_RECEPTO
N;REACTOME_TRANSCRIPTION;REACTOME_RNA_POL_I_RNA_POL_III_AND_MITOCHONDRIAL
N;REACTOME_TRANSCRIPTION;REACTOME_RNA_POL_I_RNA_POL_III_AND_MITOCHONDRIAL
ICATION;REACTOME_MITOTIC_PROMETAPHASE

)F_LIPIDS_AND_LIPOPROTEINS

)SPHINGOLIPID_METABOLISM;REACTOME_PHOSPHOLIPID_METABOLISM;REACTOME_THE_/

CID_TRIACYLGLYCEROL_AND_KETONE_BODY_METABOLISM
CID_TRIACYLGLYCEROL_AND_KETONE_BODY_METABOLISM

_ALPHA_BETA_PATHWAYS;REACTOME_INNATE_IMMUNE_SYSTEM;REACTOME_IMMUNE_SYS

'TOSIS;REACTOME_CELL_DEATH_SIGNALLING_VIA_NRAGE_NRIF_AND_NADE;REACTOME_P

:ACTOME_CELL_CYCLE_MITOTIC;REACTOME_MRNA_PROCESSING;REACTOME_MRNA_SPLI
OCESSING_PRESENTATION;REACTOME_ANTIGEN_PROCESSING_UBIQUITINATION_PROTEA

GH_JNK;REACTOME_CELL_DEATH_SIGNALLING_VIA_NRAGE_NRIF_AND_NADE;REACTOME_

CRIPTION_PATHWAY;REACTOME_METABOLISM_OF_LIPIDS_AND_LIPOPROTEINS;REACTOME

ME_SIGNALING_BY_NOTCH
COSE_METABOLISM

PIDS_AND_LIPOPROTEINS;REACTOME_FATTY_ACID_TRIACYLGLYCEROL_AND_KETONE_BOD

HS_GAG_BIOSYNTHESIS;REACTOME_HEPARAN_SULFATE_HEPARIN_HS_GAG_METABOLISM
SPORT;REACTOME_TRANSPORT_OF_INORGANIC_CATIONS_ANIONS_AND_AMINO_ACIDS_O

EACTOME_ION_CHANNEL_TRANSPORT

E_DERMATAN_SULFATE_METABOLISM;REACTOME_HEPARAN_SULFATE_HEPARIN_HS_GAG
ATELET_CALCIUM_HOMEOSTASIS;REACTOME_ION_TRANSPORT_BY_P_TYPE_ATPASES;REA
TOME_METABOLISM_OF_PROTEINS
_BASIGIN_INTERACTIONS;REACTOME_AXON_GUIDANCE;REACTOME_L1CAM_INTERACTION
_BASIGIN_INTERACTIONS;REACTOME_AXON_GUIDANCE;REACTOME_L1CAM_INTERACTION

TENSION_OF_TELOMERES

E_SYSTEM;REACTOME_CYTOKINE_SIGNALING_IN_IMMUNE_SYSTEM

SAMINOGLYCAN_METABOLISM;REACTOME_METABOLISM_OF_CARBOHYDRATES
EINS_AND_COMPLEXES;REACTOME_LOSS_OF_NLP_FROM_MITOTIC_CENTROSOMES;REAC

_SIGNALING_BY_NOTCH2;REACTOME_SIGNALING_BY_NOTCH1;REACTOME_SIGNALING_BY_

ME_MITOTIC_M_M_G1_PHASES;REACTOME_FACTORS_INVOLVED_IN_MEGAKARYOCYTE_DE

GROWTH;REACTOME_L1CAM_INTERACTIONS;REACTOME_INTERACTION_BETWEEN_L1_AND
_LIKE_RECEPTORS;REACTOME_GPCR_LIGAND_BINDING

DING

E_BIOLOGICAL_OXIDATIONS;REACTOME_PHASE_II_CONJUGATION

REACTOME_TRANSMEMBRANE_TRANSPORT_OF_SMALL_MOLECULES;REACTOME_METABOL

SPORT;REACTOME_TRANSPORT_OF_GLUCOSE_AND_OTHER_SUGARS_BILE_SALTS_AND_(

EINS_AND_COMPLEXES;REACTOME_LOSS_OF_NLP_FROM_MITOTIC_CENTROSOMES;REACT

EINS_AND_COMPLEXES;REACTOME_LOSS_OF_NLP_FROM_MITOTIC_CENTROSOMES;REACT

E_SIGNALING_BY_ERBB4;REACTOME_SIGNALING_BY_ERBB2;REACTOME_SIGNALING_BY_C

.SE1_FUNCTIONALIZATION_OF_COMPOUNDS

TOME_SYNTHESIS_OF_PIPS_AT_THE_PLASMA_MEMBRANE;REACTOME_PI_METABOLISM;RE

.PTIDE_LIGAND_BINDING_RECEPTORS;REACTOME_CLASS_A1_RHODOPSIN_LIKE_RECEPTC

OGLYCAN_METABOLISM;REACTOME_METABOLISM_OF_CARBOHYDRATES

NTIGEN_PROCESSING_CROSS_PRESENTATION;REACTOME_ENDOSOMAL_VACUOLAR_PATI

ASE_MEDIATED_CLEAVAGE_OF_CYTOSKELETAL_PROTEINS;REACTOME_CELL_JUNCTION_(

STIMULATES_GUANYLATE_CYCLASE;REACTOME_PLATELET_HOMEOSTASIS;REACTOME_HE

_TETRAHYDROBIOPTERIN_BH4_SYNTHESIS_RECYCLING_SALVAGE_AND_REGULATION;REA

ON_DEPENDENT_MRNA_DECAY;REACTOME_METABOLISM_OF_RNA
NA_SPLICING

F_LIPIDS_AND_LIPOPROTEINS

SPHINGOLIPID_METABOLISM;REACTOME_PHOSPHOLIPID_METABOLISM;REACTOME_THE_

;REACTOME_L1CAM_INTERACTIONS;REACTOME_RECYCLING_PATHWAY_OF_L1;REACTOM

TTER_RECEPTOR_BINDING_AND_DOWNSTREAM_TRANSMISSION_IN_THE_POSTSYNAPTIC_
G_COMPLEXES;REACTOME_P75NTR_SIGNALS_VIA_NFKB;REACTOME_NFKB_IS_ACTIVATED

NG_CROSS_PRESENTATION;REACTOME_INTERFERON_GAMMA_SIGNALING;REACTOME_INT

E_NCAM_SIGNALING_FOR_NEURITE_OUT_GROWTH;REACTOME_L1CAM_INTERACTIONS;RE

IN_LIKE_GROWTH_FACTOR_BINDING_PROTEINS_IGFBPS

NG_RECEPTORS;REACTOME_GPCR_DOWNSTREAM_SIGNALING;REACTOME_G_ALPHA_I_SI

NDRIAL_TRANSCRIPTION;REACTOME_RNA_POL_I_TRANSCRIPTION_INITIATION

LIPIDS_AND_LIPOPROTEINS;REACTOME_FATTY_ACID_TRIACYLGLYCEROL_AND_KETONE_B

TOME_TRANSMEMBRANE_TRANSPORT_OF_SMALL_MOLECULES

EINS_AND_COMPLEXES;REACTOME_LOSS_OF_NLP_FROM_MITOTIC_CENTROSOMES;REACT
ELL_COMMUNICATION;REACTOME_INTEGRIN_CELL_SURFACE_INTERACTIONS;REACTOME_
EDIATED_NFKB_ACTIVATION;REACTOME_RIG_I_MDA5_MEDIATED_INDUCTION_OF_IFN_ALP

OME_SIGNALING_BY_FGFR_IN_DISEASE;REACTOME_ACTIVATED_POINT_MUTANTS_OF_FGI

TOME_NRAGE_SIGNALS_DEATH_THROUGH_JNK;REACTOME_CELL_DEATH_SIGNALLING_VI
I_SIGNALING;REACTOME_IMMUNE_SYSTEM;REACTOME_CYTOKINE_SIGNALING_IN_IMMUNE
CYCLIN_E_ASSOCIATED_EVENTS_DURING_G1_S_TRANSITION_;REACTOME_G1_PHASE;REA

SPORT;REACTOME_TRANSPORT_OF_INORGANIC_CATIONS_ANIONS_AND_AMINO_ACIDS_O

F_ENERGY_METABOLISM;REACTOME_REGULATION_OF_INSULIN_SECRETION
ALING

EINS_AND_COMPLEXES;REACTOME_LOSS_OF_NLP_FROM_MITOTIC_CENTROSOMES;REACT
SPORT;REACTOME_TRANSPORT_OF_INORGANIC_CATIONS_ANIONS_AND_AMINO_ACIDS_O

IG_COMPLEXES;REACTOME_P75NTR_SIGNALS_VIA_NFKB;REACTOME_NFKB_IS_ACTIVATED

ONDRIAL_TRANSCRIPTION;REACTOME_RNA_POL_III_TRANSCRIPTION_INITIATION_FROM_TY

CTOME_NRAGE_SIGNALS_DEATH_THROUGH_JNK;REACTOME_CELL_DEATH_SIGNALLING_VI

CTION_ORGANIZATION;REACTOME_CELL_JUNCTION_ORGANIZATION

GNALLING_TO_ERKS;REACTOME_SIGNALLING_TO_P38_VIA_RIT_AND_RIN

SPORT;REACTOME_TRANSPORT_OF_INORGANIC_CATIONS_ANIONS_AND_AMINO_ACIDS_O

X;REACTOME_SRP_DEPENDENT_COTRANSLATIONAL_PROTEIN_TARGETING_TO_MEMBRA
EACTOME_ION_CHANNEL_TRANSPORT
INS_MEDIATED_TRANSPORT;REACTOME_TRANSMEMBRANE_TRANSPORT_OF_SMALL_MOI
IGAND_GATED_ION_CHANNEL_TRANSPORT

;REACTOME_HEMOSTASIS;REACTOME_PLATELET_ACTIVATION_SIGNALING_AND_AGGREG

IDS_AND_LIPOPROTEINS;REACTOME_FATTY_ACID_TRIACYLGLYCEROL_AND_KETONE_BOI

E_ROLE_OF_DCC_IN_REGULATING_APOPTOSIS;REACTOME_NETRIN1_SIGNALING;REACTOI

R_PHAGOSOME_PATHWAY;REACTOME_IMMUNOREGULATORY_INTERACTIONS_BETWEEN_

ETABOLISM;REACTOME_METABOLISM_OF_LIPIDS_AND_LIPOPROTEINS

GINE_N_LINKED_GLYCOSYLATION;REACTOME_CALNEXIN_CALRETICULIN_CYCLE;REACTOM
ME_PEPTIDE_LIGAND_BINDING_RECEPTORS;REACTOME_CLASS_A1_RHODOPSIN_LIKE_RE

ANNELS

LIN_SECRETION;REACTOME_POTASSIUM_CHANNELS;REACTOME_INWARDLY_RECTIFYING_

SIGNALING_BY_NOTCH

ETABOLISM;REACTOME_METABOLISM_OF_LIPIDS_AND_LIPOPROTEINS

METABOLISM;REACTOME_METABOLISM_OF_LIPIDS_AND_LIPOPROTEINS

_CELL_CYCLE_MITOTIC;REACTOME_COSTIMULATION_BY_THE_CD28_FAMILY;REACTOME_C

ESIS;REACTOME_METABOLISM_OF_CARBOHYDRATES;REACTOME_HEMOSTASIS;REACTOM

NA_SPLICING;REACTOME_MRNA_SPLICING_MINOR_PATHWAY

TTER_RECEPTOR_BINDING_AND_DOWNSTREAM_TRANSMISSION_IN_THE_POSTSYNAPTIC_

II_CONJUGATION

TTER_RECEPTOR_BINDING_AND_DOWNSTREAM_TRANSMISSION_IN_THE_POSTSYNAPTIC_

GPCR;REACTOME_OPIOID_SIGNALLING;REACTOME_ADENYLATE_CYCLASE_INHIBITORY_PAT

ACTOME_CYTOKINE_SIGNALING_IN_IMMUNE_SYSTEM

REACTOME_RESPIRATORY_ELECTRON_TRANSPORT_ATP_SYNTHESIS_BY_CHEMIOSMOTIC

OTIC_SPINDLE_CHECKPOINT_COMPONENTS;REACTOME_CELL_CYCLE;REACTOME_CELL_C

SPORT;REACTOME_TRANSPORT_OF_INORGANIC_CATIONS_ANIONS_AND_AMINO_ACIDS_O

ERBB4;REACTOME_PROLACTIN_RECEPTOR_SIGNALING;REACTOME_NUCLEAR_SIGNALING_

SAMINOGLYCAN_METABOLISM;REACTOME_METABOLISM_OF_CARBOHYDRATES

OWNSTREAM_TCR_SIGNALING;REACTOME_P75NTR_RECRUITS_SIGNALLING_COMPLEXES;

TOME_GLYCEROPHOSPHOLIPID_BIOSYNTHESIS;REACTOME_METABOLISM_OF_LIPIDS_AND_

_SIGNALING;REACTOME_G_ALPHA_I_SIGNALLING_EVENTS;REACTOME_GPCR_LIGAND_BIN
HS_GAG_BIOSYNTHESIS;REACTOME_HEPARAN_SULFATE_HEPARIN_HS_GAG_METABOLISM

JNCTION_ORGANIZATION

ESICLE_BUDDING;REACTOME_SYNTHESIS_OF_PIPS_AT_THE_GOLGI_MEMBRANE;REACTOM

_CASCADE;REACTOME_INTRINSIC_PATHWAY;REACTOME_HEMOSTASIS;REACTOME_PLATE

BOLISM;REACTOME_METABOLISM_OF_LIPIDS_AND_LIPOPROTEINS

OMPLEXES;REACTOME_P75NTR_SIGNALS_VIA_NFKB;REACTOME_P75_NTR_RECEPTOR_ME

REACTOME_LIPOPROTEIN_METABOLISM

_SMALL_MOLECULES;REACTOME_SLC_MEDIATED_TRANSMEMBRANE_TRANSPORT;REACT

_METABOLISM_OF_PROTEINS;REACTOME_POST_TRANSLATIONAL_PROTEIN_MODIFICATIO

H_EXPRESSION_AND_PROCESSING;REACTOME_PRE_NOTCH_PROCESSING_IN_GOLGI;REA

/I;REACTOME_COMPLEMENT_CASCADE

_OF_VITAMINS_AND_COFACTORS;REACTOME_CELL_CYCLE;REACTOME_ENOS_ACTIVATION

TER_RECEPTOR_BINDING_AND_DOWNSTREAM_TRANSMISSION_IN_THE_POSTSYNAPTIC_

ESIS;REACTOME_TRANSMEMBRANE_TRANSPORT_OF_SMALL_MOLECULES;REACTOME_SLC
_FATTY_ACID_TRIACYLGLYCEROL_AND_KETONE_BODY_METABOLISM

;REACTOME_RNA_POL_III_TRANSCRIPTION;REACTOME_RNA_POL_II_TRANSCRIPTION_PRE

_SIGNALING_BY_THE_B_CELL_RECEPTOR_BCR;REACTOME_IMMUNE_SYSTEM;REACTOME_

CTOME_METABOLISM_OF_PROTEINS;REACTOME_RESPIRATORY_ELECTRON_TRANSPORT
TEGRATION_OF_ENERGY_METABOLISM;REACTOME_G_ALPHA_Q_SIGNALLING_EVENTS;REA

SIS_OF_SUBSTRATES_IN_N_GLYCAN_BIOSYTHESIS;REACTOME_ASPARAGINE_N_LINKED_G

_LIKE_RECEPTORS;REACTOME_CHEMOKINE_RECEPTORS_BIND_CHEMOKINES;REACTOME_

CTOME_MEMBRANE_TRAFFICKING;REACTOME_TRANS_GOLGI_NETWORK_VESICLE_BUDDIN

F_PROTEINS

TTER_RECEPTOR_BINDING_AND_DOWNSTREAM_TRANSMISSION_IN_THE_POSTSYNAPTIC_

ME_SIGNALING_BY_NOTCH

SPORT;REACTOME_TRANSPORT_OF_INORGANIC_CATIONS_ANIONS_AND_AMINO_ACIDS_O

AN_SULFATE_METABOLISM;REACTOME_HS_GAG_BIOSYNTHESIS;REACTOME_HEPARAN_S

_OF_TRANSCRIPTION_COUPLED_NER_TC_NER_REPAIR_COMPLEX;REACTOME_DNA_REPA

SPORT;REACTOME_TRANSPORT_OF_VITAMINS_NUCLEOSIDES_AND_RELATED_MOLECULE

IEDIATED_ATTRACTIVE_SIGNALING

R_PHAGOSOME_PATHWAY;REACTOME_IMMUNOREGULATORY_INTERACTIONS_BETWEEN_

TOME_TRANSMEMBRANE_TRANSPORT_OF_SMALL_MOLECULES

ALPHA_Q_SIGNALLING_EVENTS;REACTOME_GPCR_DOWNSTREAM_SIGNALING;REACTOME
ALPHA_Q_SIGNALLING_EVENTS;REACTOME_GPCR_DOWNSTREAM_SIGNALING;REACTOME
ALING

3I_MEMBRANE;REACTOME_PHOSPHOLIPID_METABOLISM;REACTOME_SYNTHESIS_OF_PIPS
JNCTION_ORGANIZATION
ME_CELL_CYCLE_CHECKPOINTS;REACTOME_HOMOLOGOUS_RECOMBINATION_REPAIR_OF_
ROTEIN_RESPONSE

_SIGNALING_BY_ERBB4;REACTOME_SIGNALING_BY_ERBB2;REACTOME_SIGNALING_BY_CO
NT_AND_PLATELET_PRODUCTION;REACTOME_KINESINS;REACTOME_HEMOSTASIS;REACTC
_IN_SECRETION_BY_GLUCAGON_LIKE_PEPTIDE1;REACTOME_REGULATION_OF_INSULIN_SE

TTER_RECEPTOR_BINDING_AND_DOWNSTREAM_TRANSMISSION_IN_THE_POSTSYNAPTIC_

_SIGNALING_BY_THE_B_CELL_RECEPTOR_BCR;REACTOME_GPVI_MEDIATED_ACTIVATION_

TASIS

TTER_RELEASE_CYCLE;REACTOME_DOPAMINE_NEUROTRANSMITTER_RELEASE_CYCLE

OME_GPCR_LIGAND_BINDING

YCOBACTERIUM_TUBERCULOSIS;REACTOME_IMMUNE_SYSTEM;REACTOME_ADAPTIVE_IMM
S

TIONAL_PROTEIN_MODIFICATION

OSPHINGOLIPID_METABOLISM;REACTOME_PHOSPHOLIPID_METABOLISM;REACTOME_THE_A

ICATION

TIONAL_PROTEIN_MODIFICATION

IEDIATED_ATTRACTIVE_SIGNALING

OME_PROCESSING_OF_CAPPED_INTRON_CONTAINING_PRE_MRNA;REACTOME_TRANSPOF

I_TUBERCULOSIS;REACTOME_TRANSMEMBRANE_TRANSPORT_OF_SMALL_MOLECULES;RE

E_NUCLEOTIDE_BINDING_DOMAIN_LEUCINE_RICH_REPEAT_CONTAINING_RECEPTOR_NLR_

HYMAL_TRANSITION;REACTOME_CELL_CELL_JUNCTION_ORGANIZATION;REACTOME_TIGH

IEDIATED_ATTRACTIVE_SIGNALING;REACTOME_FACTORS_INVOLVED_IN_MEGAKARYOCYTI

RMATION;REACTOME_INTEGRIN_CELL_SURFACE_INTERACTIONS;REACTOME_SIGNALING_B

LIPIDS_AND_LIPOPROTEINS;REACTOME_FATTY_ACID_TRIACYLGLYCEROL_AND_KETONE_B
N_OF_GENE_EXPRESSION_IN_BETA_CELLS
TASIS

G_BY_HIPPO;REACTOME_APOPTOTIC_CLEAVAGE_OF_CELL_ADHESION_PROTEINS;REACTO
TTER_RECEPTOR_BINDING_AND_DOWNSTREAM_TRANSMISSION_IN_THE_POSTSYNAPTIC_

ON_ORGANIZATION
IE_ENERGY_DEPENDENT_REGULATION_OF_MTOR_BY_LKB1_AMPK;REACTOME_PKB_MEDI/
IPIDS_AND_LIPOPROTEINS;REACTOME_LIPID_DIGESTION_MOBILIZATION_AND_TRANSPORT

TIONAL_PROTEIN_MODIFICATION
TIONAL_PROTEIN_MODIFICATION

TOME_SYNTHESIS_OF_PC;REACTOME_GLYCEROPHOSPHOLIPID_BIOSYNTHESIS;REACTOM

GH_JNK;REACTOME_CELL_DEATH_SIGNALLING_VIA_NRAGE_NRIF_AND_NADE;REACTOME_

TIVATES_SMADS;REACTOME_SIGNALING_BY_TGF_BETA_RECEPTOR_COMPLEX

COSE_METABOLISM

EINS_AND_COMPLEXES;REACTOME_LOSS_OF_NLP_FROM_MITOTIC_CENTROSOMES;REACT

E_BINDING_DOMAIN_LEUCINE_RICH_REPEAT_CONTAINING_RECEPTOR_NLR_SIGNALING_F

ATION_OF_SECOND_MESSENGER_MOLECULES;REACTOME_IMMUNE_SYSTEM;REACTOME_

NT_AND_PLATELET_PRODUCTION;REACTOME_KINESINS;REACTOME_HEMOSTASIS;REACTC
AM_SIGNALING;REACTOME_G_ALPHA_S_SIGNALLING_EVENTS;REACTOME_GPCR_LIGAND_I

TOME_PEPTIDE_CHAIN_ELONGATION;REACTOME_METABOLISM_OF_PROTEINS;REACTOME_

E_SIGNALING_BY_ERBB4;REACTOME_SIGNALING_BY_ERBB2;REACTOME_SIGNALING_BY_C

REACTOME_ION_CHANNEL_TRANSPORT
REACTOME_ION_CHANNEL_TRANSPORT

OME_SIGNALING_BY_FGFR_IN_DISEASE;REACTOME_ACTIVATED_POINT_MUTANTS_OF_FGI

_LIKE_RECEPTORS;REACTOME_OPIOID_SIGNALLING;REACTOME_G_PROTEIN_ACTIVATION;
SSION;REACTOME_NRAGE_SIGNALS_DEATH_THROUGH_JNK;REACTOME_CELL_DEATH_SIC

_METABOLISM_OF_PROTEINS;REACTOME_POST_TRANSLATIONAL_PROTEIN_MODIFICATIOI

Γ_INTERACTIONS_OF_HIV_FACTORS;REACTOME_INTERACTIONS_OF_VPR_WITH_HOST_CE

RACTIONS_BETWEEN_A_LYMPHOID_AND_A_NON_LYMPHOID_CELL;REACTOME_CELL_SURF

_METABOLISM_OF_PROTEINS;REACTOME_POST_TRANSLATIONAL_PROTEIN_MODIFICATIO

_MEDIATED_TRANSPORT;REACTOME_TRANSMEMBRANE_TRANSPORT_OF_SMALL_MOLEC
ON_TRANSPORT;REACTOME_PYRUVATE_METABOLISM

M

SPORT;REACTOME_TRANSPORT_OF_INORGANIC_CATIONS_ANIONS_AND_AMINO_ACIDS_O

OCESSING_PRESENTATION;REACTOME_ANTIGEN_PROCESSING_UBIQUITINATION_PROTEA

OTIC_SPINDLE_CHECKPOINT_COMPONENTS;REACTOME_CELL_CYCLE;REACTOME_CELL_C

:MOSTASIS
)NS;REACTOME_HEMOSTASIS
SPORT;REACTOME_TRANSPORT_OF_INORGANIC_CATIONS_ANIONS_AND_AMINO_ACIDS_O

.IGAND_RECEPTORS;REACTOME_GPCR_DOWNSTREAM_SIGNALING;REACTOME_G_ALPHA_

:EACTOME_TRANSMEMBRANE_TRANSPORT_OF_SMALL_MOLECULES
F_PROTEINS

SPORT;REACTOME_TRANSPORT_OF_GLUCOSE_AND_OTHER_SUGARS_BILE_SALTS_AND_(

:EACTOME_TRANSMEMBRANE_TRANSPORT_OF_SMALL_MOLECULES

)ING

.PTIDE_LIGAND_BINDING_RECEPTORS;REACTOME_CLASS_A1_RHODOPSIN_LIKE_RECEPTC
M_SIGNALING_FOR_NEURITE_OUT_GROWTH
_SYNAPSES;REACTOME_NEURONAL_SYSTEM;REACTOME_SIGNALING_BY_GPCR;REACTOMI
TASIS

RMATION;REACTOME_SIGNALING_BY_PDGF;REACTOME_AXON_GUIDANCE;REACTOME_NCA

IG_COMPLEXES;REACTOME_P75NTR_SIGNALS_VIA_NFKB;REACTOME_NFKB_IS_ACTIVATED

_PRE_NOTCH_EXPRESSION_AND_PROCESSING;REACTOME_PRE_NOTCH_PROCESSING_IN

ILS;REACTOME_IL_3_5_AND_GM_CSF_SIGNALING;REACTOME_IL_RECEPTOR_SHC_SIGNALI

GH_JNK;REACTOME_CELL_DEATH_SIGNALLING_VIA_NRAGE_NRIF_AND_NADE;REACTOME_

E_METABOLISM_OF_LIPIDS_AND_LIPOPROTEINS

EINS_MEDIATED_TRANSPORT;REACTOME_TRANSMEMBRANE_TRANSPORT_OF_SMALL_MOL
OSTASIS;REACTOME_HEMOSTASIS
CIATED_VESICLE_BIOGENESIS

_LIKE_RECEPTORS;REACTOME_GPCR_DOWNSTREAM_SIGNALING;REACTOME_G_ALPHA_S

EACTOME_SEMAPHORIN_INTERACTIONS
CAL_SYNAPSES;REACTOME_NRAGE_SIGNALS_DEATH_THROUGH_JNK;REACTOME_NEURON
PHORYLATION_CASCADE;REACTOME_APOPTOSIS;REACTOME_APOPTOTIC_EXECUTION_PI

ATION

ESIS_ON_THE_LAGGING_STRAND;REACTOME_CELL_CYCLE_MITOTIC;REACTOME_M_G1_TR

ME_SEMA3A_PAK_DEPENDENT_AXON_REPULSION;REACTOME_SEMAPHORIN_INTERACTIO

ONDRIAL_TRANSCRIPTION;REACTOME_RNA_POL_III_TRANSCRIPTION_TERMINATION

ALING

FATTY_ACID_TRIACYLGLYCEROL_AND_KETONE_BODY_METABOLISM

TIONAL_PROTEIN_MODIFICATION

NALING_BY_EGFR_IN_CANCER;REACTOME_ANTIGEN_ACTIVATES_B_CELL_RECEPTOR_LEA

ETIN_SYNTHESIS_SECRETION_AND_INACTIVATION;REACTOME_SYNTHESIS_SECRETION_AI

REACTOME_REGULATION_OF_MRNA_STABILITY_BY_PROTEINS_THAT_BIND_AU_RICH_ELEI

M_SIGNALING_FOR_NEURITE_OUT_GROWTH

_HYDROXYLATION_OF_HYPOXIA_INDUCIBLE_FACTOR_ALPHA

X;REACTOME_ACTIVATION_OF_THE_MRNA_UPON_BINDING_OF_THE_CAP_BINDING_COMP

_CASCADE;REACTOME_INTRINSIC_PATHWAY;REACTOME_HEMOSTASIS;REACTOME_PLATE

S;REACTOME_HIV_INFECTION;REACTOME_HIV_LIFE_CYCLE;REACTOME_LATE_PHASE_OF_I

EINS

YPE_3_PROMOTER

QUENT_MODIFICATION;REACTOME_N_GLYCAN_ANTENNAE_ELONGATION;REACTOME_N_GL

BINDING

1_INTRACELLULAR_DOMAIN_REGULATES_TRANSCRIPTION;REACTOME_SIGNALING_BY_NC

RT_OF_MATURE_TRANSCRIPT_TO_CYTOPLASM;REACTOME_TRANSMEMBRANE_TRANSPOF

SMA_MEMBRANE;REACTOME_SIGNALING_BY_FGFR_IN_DISEASE;REACTOME_TRANSMISSIO
NALING;REACTOME_NEGATIVE_REGULATION_OF_FGFR_SIGNALING;REACTOME_CELL_CYC

ORGANIC_ACIDS_METAL_IONS_AND_AMINE_COMPOUNDS;REACTOME_FACILITATIVE_NA_IN

LEX_AND_EIFS_AND_SUBSEQUENT_BINDING_TO_43S;REACTOME_METABOLISM_OF_PROTI

;REACTOME_REGULATED_PROTEOLYSIS_OF_P75NTR;REACTOME_P75NTR_SIGNALS_VIA_N

_OF_SMALL_MOLECULES;REACTOME_SLC_MEDIATED_TRANSMEMBRANE_TRANSPORT;RE

OHYDRATES

TOME_RECRUITMENT_OF_NUMA_TO_MITOTIC_CENTROSOMES;REACTOME_MITOTIC_G2_G

SMA_MEMBRANE;REACTOME_SIGNALING_BY_FGFR_IN_DISEASE;REACTOME_SIGNALING_BY

TOME_MRNA_SPLICING;REACTOME_PROCESSING_OF_CAPPED_INTRONLESS_PRE_MRNA;R

P75_NTR_RECEPTOR_MEDIATED_SIGNALLING;REACTOME_SIGNALING_BY_GPCR;REACTOM

S_IN_ERBB4_SIGNALING;REACTOME_PI3K_EVENTS_IN_ERBB2_SIGNALING;REACTOME_DOW

S_IN_ERBB4_SIGNALING;REACTOME_PI3K_EVENTS_IN_ERBB2_SIGNALING;REACTOME_DOW

PLICATIVE_COMPLEX;REACTOME_E2F_ENABLED_INHIBITION_OF_PRE_REPLICATION_COMP

PRESSION;REACTOME_YAP1_AND_WWTR1_TAZ_STIMULATED_GENE_EXPRESSION;REACT(

;Y_PDGF;REACTOME_AXON_GUIDANCE;REACTOME_NCAM1_INTERACTIONS;REACTOME_N(
P75_NTR_RECEPTOR_MEDIATED_SIGNALLING;REACTOME_SIGNALING_BY_GPCR;REACTOM

:ONSTITUTIVELY_ACTIVE_EGFR;REACTOME_SIGNALING_BY_EGFR_IN_CANCER;REACTOME_

EACTOME_THE_ROLE_OF_NEF_IN_HIV1_REPLICATION_AND_DISEASE_PATHOGENESIS
SIGNALING_BY_NOTCH1;REACTOME_GENERIC_TRANSCRIPTION_PATHWAY;REACTOME_NC

LIGOPEPTIDES

/_INFECTION;REACTOME_HIV_LIFE_CYCLE;REACTOME_LATE_PHASE_OF_HIV_LIFE_CYCLE

LIGOPEPTIDES

)TEINS;REACTOME_LIPID_DIGESTION_MOBILIZATION_AND_TRANSPORT;REACTOME_LIPOPI

N
N

ATION

)ME_IMMUNE_SYSTEM;REACTOME_ADAPTIVE_IMMUNE_SYSTEM

:MOSTASIS
AD3_SMAD4_HETEROTRIMER;REACTOME_DOWNREGULATION_OF_SMAD2_3_SMAD4_TRAN
RYLATION_AND_ACTIVATION_OF_IKKS_COMPLEX;REACTOME_TRAF6_MEDIATED_NFKB_ACT

SOME_DEGRADATION

LIGOPEPTIDES

S;REACTOME_TRANSPORT_OF_ORGANIC_ANIONS

REACTOME_TRANSMEMBRANE_TRANSPORT_OF_SMALL_MOLECULES;REACTOME_SLC_MEI

_SIGNALLING_EVENTS;REACTOME_GPCR_LIGAND_BINDING

E_FATTY_ACID_TRIACYLGLYCEROL_AND_KETONE_BODY_METABOLISM;REACTOME_TRANS

ME_PHOSPHOLIPID_METABOLISM;REACTOME_PI_METABOLISM;REACTOME_GOLGI_ASSOCI

.SOME_DEGRADATION

/IE_G_ALPHA_Q_SIGNALLING_EVENTS;REACTOME_GPCR_DOWNSTREAM_SIGNALING;REAC

:ONSTITUTIVELY_ACTIVE_EGFR;REACTOME_SIGNALING_BY_EGFR_IN_CANCER;REACTOME
:ONSTITUTIVELY_ACTIVE_EGFR;REACTOME_SIGNALING_BY_EGFR_IN_CANCER;REACTOME

)RS;REACTOME_G_ALPHA_Q_SIGNALLING_EVENTS;REACTOME_GPCR_DOWNSTREAM_SIGI

_A_LYMPHOID_AND_A_NON_LYMPHOID_CELL;REACTOME_INTERFERON_GAMMA_SIGNALIN(

)F_SMALL_MOLECULES;REACTOME_SLC_MEDIATED_TRANSMEMBRANE_TRANSPORT;REAC

NG;REACTOME_ADENYLATE_CYCLASE_INHIBITORY_PATHWAY;REACTOME_NEUROTRANSM

PLEX;REACTOME_PYRUVATE_METABOLISM

)LIGOPEPTIDES

_TAGE_GATED_POTASSIUM_CHANNELS;REACTOME_POTASSIUM_CHANNELS

S

ANCE;REACTOME_NCAM1_INTERACTIONS;REACTOME_REGULATION_OF_INSULIN_SECRET

ON_SIGNALING_AND_AGGREGATION

NSTITUTIVELY_ACTIVE_EGFR;REACTOME_GRB2_EVENTS_IN_ERBB2_SIGNALING;REACTOM
ADING_TO_GENERATION_OF_SECOND_MESSENGERS;REACTOME_SIGNALING_BY_THE_B_C

CELL;REACTOME_ACETYLCHOLINE_BINDING_AND_DOWNSTREAM_EVENTS;REACTOME_HI[illegible]

[illegible]LEX;REACTOME_PYRUVATE_METABOLISM

C_REGULATION;REACTOME_G_ALPHA_Q_SIGNALLING_EVENTS;REACTOME_CLASS_B_2_SE
OME_INTEGRATION_OF_ENERGY_METABOLISM;REACTOME_METABOLISM_OF_LIPIDS_AND_

RNA;REACTOME_METABOLISM_OF_RNA;REACTOME_REGULATION_OF_MRNA_STABILITY_BY
IR

_ANKYRINS
RNA;REACTOME_MRNA_DECAY_BY_3_TO_5_EXORIBONUCLEASE;REACTOME_DEADENYLAT

SPHOLIPID_BIOSYNTHESIS;REACTOME_METABOLISM_OF_LIPIDS_AND_LIPOPROTEINS

MA_MEMBRANE;REACTOME_SIGNALING_BY_FGFR_IN_DISEASE;REACTOME_RESPONSE_T

TS;REACTOME_M_G1_TRANSITION;REACTOME_G1_S_TRANSITION;REACTOME_SYNTHESIS_

P75_NTR_RECEPTOR_MEDIATED_SIGNALLING;REACTOME_SIGNALING_BY_GPCR;REACTOM

SOME_DEGRADATION

TERFERON_SIGNALING;REACTOME_IMMUNE_SYSTEM;REACTOME_ADAPTIVE_IMMUNE_SYS

P75_NTR_RECEPTOR_MEDIATED_SIGNALLING;REACTOME_SIGNALING_BY_GPCR;REACTON

P75_NTR_RECEPTOR_MEDIATED_SIGNALLING;REACTOME_SIGNALING_BY_GPCR;REACTON

ME_RNA_POL_I_TRANSCRIPTION_INITIATION

OME_TRANSPORT_OF_INORGANIC_CATIONS_ANIONS_AND_AMINO_ACIDS_OLIGOPEPTIDES

OME_TRANSPORT_OF_INORGANIC_CATIONS_ANIONS_AND_AMINO_ACIDS_OLIGOPEPTIDES

LIGOPEPTIDES

ME_RNA_POL_I_TRANSCRIPTION_INITIATION

_TRANSCRIPTION

)LL_RECEPTOR_CASCADES

_SINGLE_NUCLEOTIDE_REPLACEMENT_PATHWAY;REACTOME_DNA_REPAIR;REACTOME_R
_SINGLE_NUCLEOTIDE_REPLACEMENT_PATHWAY;REACTOME_DNA_REPAIR;REACTOME_R

REPAIR;REACTOME_CHROMOSOME_MAINTENANCE;REACTOME_ACTIVATION_OF_ATR_IN_R
SIGNALING;REACTOME_G_ALPHA_I_SIGNALLING_EVENTS;REACTOME_SIGNAL_AMPLIFICAT

3OLISM;REACTOME_TRANSMEMBRANE_TRANSPORT_OF_SMALL_MOLECULES;REACTOME_

EXCISION_REPAIR;REACTOME_REPAIR_SYNTHESIS_FOR_GAP_FILLING_BY_DNA_POL_IN_TC

)LIGOPEPTIDES
PRESSION;REACTOME_CIRCADIAN_REPRESSION_OF_EXPRESSION_BY_REV_ERBA;REACTC

TEM;REACTOME_CYTOKINE_SIGNALING_IN_IMMUNE_SYSTEM

_TC_NER;REACTOME_RNA_POL_II_TRANSCRIPTION_PRE_INITIATION_AND_PROMOTER_OP

TABOLISM;REACTOME_CHONDROITIN_SULFATE_DERMATAN_SULFATE_METABOLISM;REAC

TABOLISM;REACTOME_CHONDROITIN_SULFATE_DERMATAN_SULFATE_METABOLISM;REAC

EACTOME_ALPHA_LINOLENIC_ACID_ALA_METABOLISM;REACTOME_PEROXISOMAL_LIPID_M

OMOSOME_MAINTENANCE;REACTOME_DEPOSITION_OF_NEW_CENPA_CONTAINING_NUCLE

TIONALIZATION_OF_COMPOUNDS;REACTOME_ENDOGENOUS_STEROLS;REACTOME_STER

ADAPTIVE_IMMUNE_SYSTEM

_ERBB4_SIGNALING;REACTOME_SHC1_EVENTS_IN_ERBB4_SIGNALING;REACTOME_PI3K_EV

_OF_HOMO_SAPIENS_WITH_MYCOBACTERIUM_TUBERCULOSIS;REACTOME_TRANSMEMBR

;REACTOME_INTERFERON_ALPHA_BETA_SIGNALING;REACTOME_REGULATION_OF_IFNA_S

OME_SIGNALLING_TO_ERKS;REACTOME_SIGNALING_BY_PDGF;REACTOME_DOWNSTREAM_

ME_APOPTOSIS
ME_APOPTOSIS

OME_IMMUNE_SYSTEM;REACTOME_ADAPTIVE_IMMUNE_SYSTEM

N

ATION

ME_SIGNAL_TRANSDUCTION_BY_L1;REACTOME_INTERFERON_GAMMA_SIGNALING;REACTC

TIONALIZATION_OF_COMPOUNDS;REACTOME_STEROID_HORMONES;REACTOME_METABOI
TIONALIZATION_OF_COMPOUNDS;REACTOME_STEROID_HORMONES;REACTOME_METABOI

3ODY_METABOLISM;REACTOME_TRANSCRIPTIONAL_REGULATION_OF_WHITE_ADIPOCYTE_

OF_PG;REACTOME_ACYL_CHAIN_REMODELLING_OF_PE;REACTOME_ACYL_CHAIN_REMODE

TOME_S_PHASE
TOME_S_PHASE

_STEROID_HORMONES;REACTOME_METABOLISM_OF_LIPIDS_AND_LIPOPROTEINS;REACTC

ASOME_DEGRADATION

D_ANKYRINS

LEX_AND_EIFS_AND_SUBSEQUENT_BINDING_TO_43S;REACTOME_METABOLISM_OF_PROTI

ING_BY_ERBB2;REACTOME_GRB2_EVENTS_IN_ERBB2_SIGNALING;REACTOME_SIGNALING_

CELL;REACTOME_ACETYLCHOLINE_BINDING_AND_DOWNSTREAM_EVENTS;REACTOME_HI(

LEAR_EVENTS_KINASE_AND_TRANSCRIPTION_FACTOR_ACTIVATION;REACTOME_ERK_MAF

ADE
L_TRANSCRIPTION;REACTOME_RNA_POL_III_TRANSCRIPTION_INITIATION_FROM_TYPE_3_F

CTOME_FATTY_ACID_TRIACYLGLYCEROL_AND_KETONE_BODY_METABOLISM;REACTOME_[illegible]

E_FATTY_ACID_TRIACYLGLYCEROL_AND_KETONE_BODY_METABOLISM

PRESSION;REACTOME_YAP1_AND_WWTR1_TAZ_STIMULATED_GENE_EXPRESSION;REACT[illegible]

[illegible]LIGOPEPTIDES

[illegible]SE_OF_HIV_LIFE_CYCLE

3ODY_METABOLISM;REACTOME_TRANSCRIPTIONAL_REGULATION_OF_WHITE_ADIPOCYTE_

N
N
N
N

THWAY;REACTOME_SIGNALING_BY_TGF_BETA_RECEPTOR_COMPLEX

IEMBRANE_TRANSPORT;REACTOME_TRANSPORT_OF_INORGANIC_CATIONS_ANIONS_AND_

GNALING
CELL;REACTOME_ACTIVATION_OF_KAINATE_RECEPTORS_UPON_GLUTAMATE_BINDING;RE
CELL;REACTOME_ACTIVATION_OF_KAINATE_RECEPTORS_UPON_GLUTAMATE_BINDING;RE

EMOSTASIS
EMOSTASIS

E_INTEGRATION_OF_ENERGY_METABOLISM;REACTOME_OPIOID_SIGNALLING;REACTOME_

ACTOME_THE_ROLE_OF_NEF_IN_HIV1_REPLICATION_AND_DISEASE_PATHOGENESIS;REAC
PRESSION;REACTOME_YAP1_AND_WWTR1_TAZ_STIMULATED_GENE_EXPRESSION;REACT
PRESSION;REACTOME_YAP1_AND_WWTR1_TAZ_STIMULATED_GENE_EXPRESSION;REACT

D_TRANSLATION;REACTOME_RORA_ACTIVATES_CIRCADIAN_EXPRESSION;REACTOME_PRE

_ACTIVATION_IN_TLR7_8_OR_9_SIGNALING;REACTOME_INNATE_IMMUNE_SYSTEM;REACTO

_ACTIVATION_IN_TLR7_8_OR_9_SIGNALING;REACTOME_INNATE_IMMUNE_SYSTEM;REACTO

TRANSMITTER_RELEASE_CYCLE;REACTOME_NOREPINEPHRINE_NEUROTRANSMITTER_RE

C_COUPLING_AND_HEAT_PRODUCTION_BY_UNCOUPLING_PROTEINS_

E_FATTY_ACID_TRIACYLGLYCEROL_AND_KETONE_BODY_METABOLISM;REACTOME_TRANS

_VIA_NRAGE_NRIF_AND_NADE;REACTOME_P75_NTR_RECEPTOR_MEDIATED_SIGNALLING;R

;REACTOME_IMMUNE_SYSTEM;REACTOME_ADAPTIVE_IMMUNE_SYSTEM

:YCLE_MITOTIC;REACTOME_CELL_CYCLE_CHECKPOINTS;REACTOME_REGULATION_OF_MI

.SOME_DEGRADATION

)RS;REACTOME_G_ALPHA_Q_SIGNALLING_EVENTS;REACTOME_GPCR_DOWNSTREAM_SIG
)RGANIC_ACIDS_METAL_IONS_AND_AMINE_COMPOUNDS;REACTOME_METAL_ION_SLC_TR
)RGANIC_ACIDS_METAL_IONS_AND_AMINE_COMPOUNDS;REACTOME_METAL_ION_SLC_TR

)ME_TRANSPORT_OF_INORGANIC_CATIONS_ANIONS_AND_AMINO_ACIDS_OLIGOPEPTIDES

S_IN_ERBB4_SIGNALING;REACTOME_PI3K_EVENTS_IN_ERBB2_SIGNALING;REACTOME_DOW

_SINGLE_NUCLEOTIDE_REPLACEMENT_PATHWAY;REACTOME_DNA_REPAIR;REACTOME_R

E_G_ALPHA_Z_SIGNALLING_EVENTS;REACTOME_EFFECTS_OF_PIP2_HYDROLYSIS;REACTOI

A_24_HYDROXYCHOLESTEROL;REACTOME_SYNTHESIS_OF_BILE_ACIDS_AND_BILE_SALTS

TOME_RECRUITMENT_OF_NUMA_TO_MITOTIC_CENTROSOMES;REACTOME_MITOTIC_G2_G

PRESSION;REACTOME_YAP1_AND_WWTR1_TAZ_STIMULATED_GENE_EXPRESSION;REACT[illegible]

IGNALING;REACTOME_RIG_I_MDA5_MEDIATED_INDUCTION_OF_IFN_ALPHA_BETA_PATHWA[illegible]

[illegible]SOME_DEGRADATION

_BETA_RECEPTOR_COMPLEX;REACTOME_PLATELET_ACTIVATION_SIGNALING_AND_AGGR[illegible]

DRGANIC_ACIDS_METAL_IONS_AND_AMINE_COMPOUNDS;REACTOME_FACILITATIVE_NA_IN

S_IN_ERBB4_SIGNALING;REACTOME_PI3K_EVENTS_IN_ERBB2_SIGNALING;REACTOME_DOW

SOME_DEGRADATION
SOME_DEGRADATION

M;REACTOME_GLYCOSAMINOGLYCAN_METABOLISM;REACTOME_A_TETRASACCHARIDE_LIN
M;REACTOME_GLYCOSAMINOGLYCAN_METABOLISM;REACTOME_A_TETRASACCHARIDE_LIN

QUENT_MODIFICATION

TRIN_CREB_SIGNALLING_PATHWAY_VIA_PKC_AND_MAPK;REACTOME_NUCLEAR_EVENTS_

LES;REACTOME_SLC_MEDIATED_TRANSMEMBRANE_TRANSPORT;REACTOME_TRANSPORT

LES;REACTOME_SLC_MEDIATED_TRANSMEMBRANE_TRANSPORT;REACTOME_TRANSPORT

TERS;REACTOME_CELL_SURFACE_INTERACTIONS_AT_THE_VASCULAR_WALL;REACTOME_

E_FATTY_ACID_TRIACYLGLYCEROL_AND_KETONE_BODY_METABOLISM;REACTOME_TRANS

ATION_OF_AMPK_ACTIVITY_VIA_LKB1;REACTOME_ENERGY_DEPENDENT_REGULATION_OF

NG;REACTOME_REGULATION_OF_IFNG_SIGNALING;REACTOME_INTERFERON_GAMMA_SIG

EACTOME_GPCR_DOWNSTREAM_SIGNALING;REACTOME_G_ALPHA_I_SIGNALLING_EVENTS

CR_DOWNSTREAM_SIGNALING;REACTOME_SEMA4D_IN_SEMAPHORIN_SIGNALING;REACTC

ACTOME_GENERIC_TRANSCRIPTION_PATHWAY;REACTOME_NOTCH_HLH_TRANSCRIPTION_
ACTOME_GENERIC_TRANSCRIPTION_PATHWAY;REACTOME_NOTCH_HLH_TRANSCRIPTION_

_TRANSCRIPTION;REACTOME_RNA_POL_III_TRANSCRIPTION_INITIATION_FROM_TYPE_3_F

SOME_DEGRADATION

A_NRAGE_NRIF_AND_NADE;REACTOME_P75_NTR_RECEPTOR_MEDIATED_SIGNALLING;RE

'YPE_3_PROMOTER

G_AND_AGGREGATION

RT_OF_MATURE_TRANSCRIPT_TO_CYTOPLASM;REACTOME_TRANSMEMBRANE_TRANSPOF

REPULSION;REACTOME_SEMAPHORIN_INTERACTIONS;REACTOME_SEMA3A_PLEXIN_REPUL

ALING_AND_AGGREGATION

P75_NTR_RECEPTOR_MEDIATED_SIGNALLING;REACTOME_SIGNALING_BY_GPCR;REACTOM

ACTOME_SIGNALING_BY_ERBB2;REACTOME_SIGNALING_BY_EGFR_IN_CANCER;REACTOME

OLISM_OF_LIPIDS_AND_LIPOPROTEINS
ERBB4;REACTOME_ACTIVATED_NOTCH1_TRANSMITS_SIGNAL_TO_THE_NUCLEUS;REACTOI

ECRETION;REACTOME_VOLTAGE_GATED_POTASSIUM_CHANNELS;REACTOME_POTASSIUM_

PRESSION;REACTOME_YAP1_AND_WWTR1_TAZ_STIMULATED_GENE_EXPRESSION;REACT(

OLIGOPEPTIDES;REACTOME_PLATELET_HOMEOSTASIS;REACTOME_PLATELET_CALCIUM_H(

EACTOME_INTERACTION_BETWEEN_L1_AND_ANKYRINS;REACTOME_NEPHRIN_INTERACTIO
EACTOME_INTERACTION_BETWEEN_L1_AND_ANKYRINS;REACTOME_NEPHRIN_INTERACTIO

REACTOME_MYD88_MAL_CASCADE_INITIATED_ON_PLASMA_MEMBRANE;REACTOME_INNAT

CTOME_METABOLISM_OF_PROTEINS;REACTOME_POST_TRANSLATIONAL_PROTEIN_MODIFI

:TOME_CELL_SURFACE_INTERACTIONS_AT_THE_VASCULAR_WALL;REACTOME_COMMON_

ETION_AND_INACTIVATION;REACTOME_SYNTHESIS_SECRETION_AND_INACTIVATION_OF_G
ETION_AND_INACTIVATION;REACTOME_SYNTHESIS_SECRETION_AND_INACTIVATION_OF_G

OME_SYNTHESIS_OF_BILE_ACIDS_AND_BILE_SALTS;REACTOME_METABOLISM_OF_LIPIDS_

_LOSS_OF_NLP_FROM_MITOTIC_CENTROSOMES;REACTOME_MITOTIC_G2_G2_M_PHASES;F
_BY_GLUCAGON_LIKE_PEPTIDE1;REACTOME_REGULATION_OF_INSULIN_SECRETION;REAC

CELL;REACTOME_ACETYLCHOLINE_BINDING_AND_DOWNSTREAM_EVENTS;REACTOME_HI(

YCLASE;REACTOME_PLATELET_HOMEOSTASIS;REACTOME_HEMOSTASIS

EACTOME_INTEGRIN_CELL_SURFACE_INTERACTIONS;REACTOME_AXON_GUIDANCE;REACT
AM1_INTERACTIONS;REACTOME_NCAM_SIGNALING_FOR_NEURITE_OUT_GROWTH

CTOME_SIGNALING_BY_FGFR1_MUTANTS;REACTOME_SIGNALING_BY_FGFR1_FUSION_MUT

TRANSPORT;REACTOME_TRANSPORT_OF_GLUCOSE_AND_OTHER_SUGARS_BILE_SALTS_

EMBRANE;REACTOME_PI3K_AKT_ACTIVATION;REACTOME_SIGNALING_BY_INSULIN_RECEF

RS;REACTOME_G_ALPHA_Q_SIGNALLING_EVENTS;REACTOME_GPCR_DOWNSTREAM_SIGI

ES;REACTOME_SLC_MEDIATED_TRANSMEMBRANE_TRANSPORT;REACTOME_TRANSPORT_

ERBB2_SIGNALING;REACTOME_SIGNALING_BY_EGFR_IN_CANCER;REACTOME_SHC1_EVEN

OLIGOPEPTIDES

SNALING_ACTIVATES_SMADS;REACTOME_SIGNALING_BY_TGF_BETA_RECEPTOR_COMPLEX

_GPCR_DOWNSTREAM_SIGNALING;REACTOME_G_ALPHA_I_SIGNALLING_EVENTS;REACTOI

LISM_OF_PORPHYRINS

REPULSION;REACTOME_SEMAPHORIN_INTERACTIONS;REACTOME_SEMA3A_PLEXIN_REPUL

REPAIR;REACTOME_CHROMOSOME_MAINTENANCE;REACTOME_ACTIVATION_OF_ATR_IN_R

ATHWAY;REACTOME_SIGNALING_BY_TGF_BETA_RECEPTOR_COMPLEX

JM_CHANNELS

_SIGNALLING_EVENTS;REACTOME_GPCR_LIGAND_BINDING

N

STEM;REACTOME_ADAPTIVE_IMMUNE_SYSTEM;REACTOME_CLASS_I_MHC_MEDIATED_ANT
STEM;REACTOME_ADAPTIVE_IMMUNE_SYSTEM;REACTOME_CLASS_I_MHC_MEDIATED_ANT

CELL;REACTOME_TRANSMEMBRANE_TRANSPORT_OF_SMALL_MOLECULES;REACTOME_GA

LIGOPEPTIDES

LIGOPEPTIDES
TOME_MITOTIC_G2_G2_M_PHASES

LIGOPEPTIDES

SIGNALS_DEATH_THROUGH_JNK;REACTOME_CELL_DEATH_SIGNALLING_VIA_NRAGE_NRIF
SIGNALS_DEATH_THROUGH_JNK;REACTOME_CELL_DEATH_SIGNALLING_VIA_NRAGE_NRIF

RGY_DEPENDENT_REGULATION_OF_MTOR_BY_LKB1_AMPK;REACTOME_REGULATION_OF_

TEROL_BIOSYNTHESIS

MES_AT_THE_CENTROMERE;REACTOME_DNA_REPLICATION;REACTOME_MITOTIC_PROMET

REACTOME_SIGNALING_BY_CONSTITUTIVELY_ACTIVE_EGFR;REACTOME_SIGNALING_BY_E

G_METABOLISM;REACTOME_GLYCOSAMINOGLYCAN_METABOLISM;REACTOME_A_TETRASA

ACTIVATION_OF_ARYLSULFATASES;REACTOME_METABOLISM_OF_PROTEINS;REACTOME_S

PHASES;REACTOME_ACTIVATION_OF_ATR_IN_RESPONSE_TO_REPLICATION_STRESS;REAC
PHASES;REACTOME_ACTIVATION_OF_ATR_IN_RESPONSE_TO_REPLICATION_STRESS;REAC

:TOME_FRS2_MEDIATED_CASCADE;REACTOME_PI_3K_CASCADE;REACTOME_DOWNSTREA
:TOME_FRS2_MEDIATED_CASCADE;REACTOME_PI_3K_CASCADE;REACTOME_DOWNSTREA
:TOME_FRS2_MEDIATED_CASCADE;REACTOME_PI_3K_CASCADE;REACTOME_DOWNSTREA

_3_UTR_MEDIATED_TRANSLATIONAL_REGULATION;REACTOME_METABOLISM_OF_MRNA;RI

QUENT_MODIFICATION;REACTOME_N_GLYCAN_ANTENNAE_ELONGATION;REACTOME_N_GL
GLYCOSYLATION;REACTOME_BIOSYNTHESIS_OF_THE_N_GLYCAN_PRECURSOR_DOLICHOL_

ADING_TO_GENERATION_OF_SECOND_MESSENGERS;REACTOME_SIGNALING_BY_THE_B_C

_3_UTR_MEDIATED_TRANSLATIONAL_REGULATION;REACTOME_METABOLISM_OF_MRNA;RI

S

.SOME_DEGRADATION

OHYDRATES
NALING;REACTOME_DOWNSTREAM_TCR_SIGNALING;REACTOME_IMMUNE_SYSTEM;REACT

E_EFFECTS_OF_PIP2_HYDROLYSIS;REACTOME_HEMOSTASIS;REACTOME_PLATELET_ACTIV

ME_JNK_C_JUN_KINASES_PHOSPHORYLATION_AND_ACTIVATION_MEDIATED_BY_ACTIVATE

ME_CITRIC_ACID_CYCLE_TCA_CYCLE
SMA_MEMBRANE;REACTOME_SIGNALING_BY_FGFR_IN_DISEASE;REACTOME_TRANSMISSIO

ME_PLATELET_ADHESION_TO_EXPOSED_COLLAGEN;REACTOME_INTEGRIN_CELL_SURFAC

SOME_DEGRADATION

N

E_PATHWAY;REACTOME_CELL_CYCLE;REACTOME_ORC1_REMOVAL_FROM_CHROMATIN;RI
PLICATIVE_COMPLEX;REACTOME_E2F_ENABLED_INHIBITION_OF_PRE_REPLICATION_COMP

E_EFFECTS_OF_PIP2_HYDROLYSIS;REACTOME_HEMOSTASIS;REACTOME_PLATELET_ACTIV
E_EFFECTS_OF_PIP2_HYDROLYSIS;REACTOME_HEMOSTASIS;REACTOME_PLATELET_ACTIV

EACTOME_IRON_UPTAKE_AND_TRANSPORT;REACTOME_TRANSFERRIN_ENDOCYTOSIS_AN

CTIVATION_AND_REGULATION;REACTOME_NITRIC_OXIDE_STIMULATES_GUANYLATE_CYCI

G;REACTOME_SEMAPHORIN_INTERACTIONS;REACTOME_FACTORS_INVOLVED_IN_MEGAKA

ATION_OF_AMPK_ACTIVITY_VIA_LKB1;REACTOME_ENERGY_DEPENDENT_REGULATION_OF
ATION_OF_AMPK_ACTIVITY_VIA_LKB1;REACTOME_ENERGY_DEPENDENT_REGULATION_OF

SOME_DEGRADATION

ME_SIGNALING_BY_FGFR1_MUTANTS;REACTOME_SIGNALING_BY_ACTIVATED_POINT_MUTA
ME_SIGNALING_BY_FGFR1_MUTANTS;REACTOME_SIGNALING_BY_ACTIVATED_POINT_MUTA

_SINGLE_NUCLEOTIDE_REPLACEMENT_PATHWAY;REACTOME_DNA_REPAIR;REACTOME_R

ENSITIVE_LIPASE_HSL_MEDIATED_TRIACYLGLYCEROL_HYDROLYSIS;REACTOME_LIPID_DI

ORGANIZATION;REACTOME_APOPTOSIS;REACTOME_APOPTOTIC_EXECUTION_PHASE

REACTOME_TRANSMEMBRANE_TRANSPORT_OF_SMALL_MOLECULES;REACTOME_SLC_ME

ɔLIGOPEPTIDES

C_COUPLING_AND_HEAT_PRODUCTION_BY_UNCOUPLING_PROTEINS_

SIGNALLING_TO_ERKS;REACTOME_SIGNALING_BY_INSULIN_RECEPTOR;REACTOME_SIGNA

TOME_MITOTIC_G2_G2_M_PHASES

S_IN_ERBB4_SIGNALING;REACTOME_PI3K_EVENTS_IN_ERBB2_SIGNALING;REACTOME_DOV

SOME_DEGRADATION
REACTOME_INTEGRATION_OF_ENERGY_METABOLISM;REACTOME_DOPAMINE_NEUROTRAN

PROTEINS;REACTOME_AXON_GUIDANCE;REACTOME_NCAM_SIGNALING_FOR_NEURITE_OU
PROTEINS;REACTOME_AXON_GUIDANCE;REACTOME_NCAM_SIGNALING_FOR_NEURITE_OU
PROTEINS;REACTOME_AXON_GUIDANCE;REACTOME_NCAM_SIGNALING_FOR_NEURITE_OU
PROTEINS;REACTOME_AXON_GUIDANCE;REACTOME_NCAM_SIGNALING_FOR_NEURITE_OU

GLYCOSYLATION;REACTOME_BIOSYNTHESIS_OF_THE_N_GLYCAN_PRECURSOR_DOLICHOL_

OME_SIGNALLING_TO_ERKS;REACTOME_SIGNALING_BY_PDGF;REACTOME_DOWNSTREAM_

L_TRANSCRIPTION

AM1_INTERACTIONS;REACTOME_NCAM_SIGNALING_FOR_NEURITE_OUT_GROWTH
AM1_INTERACTIONS;REACTOME_NCAM_SIGNALING_FOR_NEURITE_OUT_GROWTH
AM1_INTERACTIONS;REACTOME_NCAM_SIGNALING_FOR_NEURITE_OUT_GROWTH

HA_BETA_PATHWAYS;REACTOME_APOPTOSIS;REACTOME_INNATE_IMMUNE_SYSTEM;REA
ME_N_GLYCAN_TRIMMING_IN_THE_ER_AND_CALNEXIN_CALRETICULIN_CYCLE
LIGOPEPTIDES

TEINS;REACTOME_LIPID_DIGESTION_MOBILIZATION_AND_TRANSPORT;REACTOME_LIPOP
TEINS;REACTOME_LIPID_DIGESTION_MOBILIZATION_AND_TRANSPORT;REACTOME_LIPOP

;REACTOME_INTEGRIN_ALPHAIIB_BETA3_SIGNALING;REACTOME_PLATELET_AGGREGATIO

SYSTEM;REACTOME_NEUROTRANSMITTER_RELEASE_CYCLE;REACTOME_ACETYLCHOLINE

_REGULATION_OF_SIGNALING_BY_CBL;REACTOME_IL_3_5_AND_GM_CSF_SIGNALING;REA[illegible]

E_DEVELOPMENT_AND_PLATELET_PRODUCTION;REACTOME_HEMOSTASIS
E_DEVELOPMENT_AND_PLATELET_PRODUCTION;REACTOME_HEMOSTASIS

E_PATHWAY;REACTOME_CELL_CYCLE;REACTOME_ORC1_REMOVAL_FROM_CHROMATIN;R[illegible]

ACTOME_SIGNALING_BY_EGFR_IN_CANCER;REACTOME_NGF_SIGNALLING_VIA_TRKA_FRO[illegible]

N
N
N
N
N

ESSION_OF_EXPRESSION_BY_REV_ERBA;REACTOME_METABOLISM_OF_LIPIDS_AND_LIPOI

;REACTOME_NITRIC_OXIDE_STIMULATES_GUANYLATE_CYCLASE;REACTOME_PLATELET_H
TIONALIZATION_OF_COMPOUNDS;REACTOME_STEROID_HORMONES;REACTOME_METABOI

EACTOME_PEPTIDE_LIGAND_BINDING_RECEPTORS;REACTOME_CLASS_A1_RHODOPSIN_LI

ORGANIC_ACIDS_METAL_IONS_AND_AMINE_COMPOUNDS;REACTOME_NA_CL_DEPENDENT_

DY_METABOLISM;REACTOME_SYNTHESIS_OF_VERY_LONG_CHAIN_FATTY_ACYL_COAS

TOME_MITOTIC_M_M_G1_PHASES;REACTOME_MITOTIC_G2_G2_M_PHASES;REACTOME_DN

_AND_KETONE_BODY_METABOLISM

RE_MRNA_DERIVED_FROM_AN_INTRONLESS_TRANSCRIPT

S_IN_ERBB4_SIGNALING;REACTOME_PI3K_EVENTS_IN_ERBB2_SIGNALING;REACTOME_DOV

RANSITION;REACTOME_G1_S_TRANSITION;REACTOME_POL_SWITCHING;REACTOME_SYNTH

OME_IMMUNE_SYSTEM;REACTOME_ADAPTIVE_IMMUNE_SYSTEM

TOME_DARPP_32_EVENTS;REACTOME_METABOLISM_OF_LIPIDS_AND_LIPOPROTEINS;REAC
_COUPLING_AND_HEAT_PRODUCTION_BY_UNCOUPLING_PROTEINS_
ACTOME_IRON_UPTAKE_AND_TRANSPORT;REACTOME_TRANSFERRIN_ENDOCYTOSIS_AN

S;REACTOME_TRANSPORT_OF_ORGANIC_ANIONS

IC_MEDIATED_ANTIGEN_PROCESSING_PRESENTATION;REACTOME_ANTIGEN_PROCESSING
YLGLYCEROL_AND_KETONE_BODY_METABOLISM
_REPLICATION_INDEPENDENT_DOUBLE_STRAND_BREAKS;REACTOME_P53_DEPENDENT_G

A_NRAGE_NRIF_AND_NADE;REACTOME_P75_NTR_RECEPTOR_MEDIATED_SIGNALLING;RE

IA_NRAGE_NRIF_AND_NADE;REACTOME_P75_NTR_RECEPTOR_MEDIATED_SIGNALLING;RE/

AM_TCR_SIGNALING;REACTOME_PHOSPHORYLATION_OF_CD3_AND_TCR_ZETA_CHAINS;RI

ETABOLISM_OF_LIPIDS_AND_LIPOPROTEINS
ETABOLISM_OF_LIPIDS_AND_LIPOPROTEINS

MEMBRANE_TRANSPORT;REACTOME_METABOLISM_OF_LIPIDS_AND_LIPOPROTEINS;REAC

N
N

SMA_MEMBRANE;REACTOME_SIGNALING_BY_FGFR_IN_DISEASE;REACTOME_TRANSMISSIO

/E_IMMUNE_SYSTEM

ME_FORMATION_OF_TUBULIN_FOLDING_INTERMEDIATES_BY_CCT_TRIC;REACTOME_META

_SINGLE_NUCLEOTIDE_REPLACEMENT_PATHWAY;REACTOME_DNA_REPAIR;REACTOME_R

3ODY_METABOLISM;REACTOME_TRANSCRIPTIONAL_REGULATION_OF_WHITE_ADIPOCYTE_
EMOSTASIS

LNEXIN_CALRETICULIN_CYCLE

E_SYSTEM

EXCISION_REPAIR;REACTOME_REPAIR_SYNTHESIS_FOR_GAP_FILLING_BY_DNA_POL_IN_TC

3Y_PDGF;REACTOME_AXON_GUIDANCE;REACTOME_NCAM1_INTERACTIONS;REACTOME_NC

._II_PRE_TRANSCRIPTION_EVENTS;REACTOME_HIV_INFECTION;REACTOME_HIV_LIFE_CYC

IE_TRANSPORT_OF_SMALL_MOLECULES;REACTOME_SLC_MEDIATED_TRANSMEMBRANE_T

_DOWNSTREAM_SIGNALING;REACTOME_G_ALPHA_S_SIGNALLING_EVENTS;REACTOME_GF

TION;REACTOME_NEPHRIN_INTERACTIONS;REACTOME_HEMOSTASIS;REACTOME_PLATELE

EACTOME_GPCR_DOWNSTREAM_SIGNALING;REACTOME_GPCR_LIGAND_BINDING

OME_IMMUNE_SYSTEM;REACTOME_ADAPTIVE_IMMUNE_SYSTEM

CTOME_SIGNALING_BY_ERBB4;REACTOME_DOWNREGULATION_OF_ERBB2_ERBB3_SIGNAI

NOTCH3;REACTOME_RECEPTOR_LIGAND_BINDING_INITIATES_THE_SECOND_PROTEOLYTI
_TRANSCRIPTION

SOME_DEGRADATION
SOME_DEGRADATION

CELL;REACTOME_ACETYLCHOLINE_BINDING_AND_DOWNSTREAM_EVENTS;REACTOME_HI

E_INTEGRATION_OF_ENERGY_METABOLISM;REACTOME_OPIOID_SIGNALLING;REACTOME_
_ANKYRINS
_ANKYRINS

CTOME_OTHER_SEMAPHORIN_INTERACTIONS;REACTOME_SEMAPHORIN_INTERACTIONS;R

TIONALIZATION_OF_COMPOUNDS;REACTOME_ENDOGENOUS_STEROLS;REACTOME_STER

P75_NTR_RECEPTOR_MEDIATED_SIGNALLING;REACTOME_SIGNALING_BY_GPCR;REACTOM

ORGANIC_ACIDS_METAL_IONS_AND_AMINE_COMPOUNDS;REACTOME_AMINE_COMPOUND_

_SIGNALLING_EVENTS;REACTOME_GPCR_LIGAND_BINDING

_3_UTR_MEDIATED_TRANSLATIONAL_REGULATION;REACTOME_METABOLISM_OF_MRNA;RI

D_TRANSLATION;REACTOME_RORA_ACTIVATES_CIRCADIAN_EXPRESSION;REACTOME_YAF
D_TRANSLATION;REACTOME_RORA_ACTIVATES_CIRCADIAN_EXPRESSION;REACTOME_YAF
D_TRANSLATION;REACTOME_RORA_ACTIVATES_CIRCADIAN_EXPRESSION;REACTOME_YAF
SMA_MEMBRANE;REACTOME_SIGNALING_BY_FGFR_IN_DISEASE;REACTOME_TRANSMISSIO

ADE

ASIS;REACTOME_PLATELET_CALCIUM_HOMEOSTASIS;REACTOME_ION_TRANSPORT_BY_P_

RT_OF_MATURE_TRANSCRIPT_TO_CYTOPLASM;REACTOME_TRANSMEMBRANE_TRANSPOR

RT_OF_MATURE_TRANSCRIPT_TO_CYTOPLASM;REACTOME_TRANSMEMBRANE_TRANSPOR

LIGOPEPTIDES

LIGOPEPTIDES

REACTOME_DOWNSTREAM_SIGNAL_TRANSDUCTION;REACTOME_PLATELET_AGGREGATIC

CASCADE;REACTOME_HEMOSTASIS;REACTOME_INNATE_IMMUNE_SYSTEM;REACTOME_IM

ME_RNA_POL_I_TRANSCRIPTION_INITIATION

CELL;REACTOME_ACTIVATION_OF_NMDA_RECEPTOR_UPON_GLUTAMATE_BINDING_AND_F

SOME_DEGRADATION

ORGANIC_ACIDS_METAL_IONS_AND_AMINE_COMPOUNDS;REACTOME_BILE_SALT_AND_OR

OME_MRNA_CAPPING;REACTOME_TRANSCRIPTION_COUPLED_NER_TC_NER;REACTOME_R

CASCADE;REACTOME_GPCR_DOWNSTREAM_SIGNALING;REACTOME_G_BETA_GAMMA_SIG

A_ACTIVATES_GENE_EXPRESSION;REACTOME_RORA_ACTIVATES_CIRCADIAN_EXPRESSIO
A_ACTIVATES_GENE_EXPRESSION;REACTOME_RORA_ACTIVATES_CIRCADIAN_EXPRESSIO

_OF_NFKB_AND_MAP_KINASES_UPON_TLR7_8_OR_9_ACTIVATION;REACTOME_NFKB_AND_
EMOSTASIS
ORGANIC_ACIDS_METAL_IONS_AND_AMINE_COMPOUNDS;REACTOME_BILE_SALT_AND_OR
ORGANIC_ACIDS_METAL_IONS_AND_AMINE_COMPOUNDS;REACTOME_BILE_SALT_AND_OR

ACTOME_SIGNALING_BY_EGFR_IN_CANCER;REACTOME_NGF_SIGNALLING_VIA_TRKA_FRO

_NCAM_SIGNALING_FOR_NEURITE_OUT_GROWTH
_3_UTR_MEDIATED_TRANSLATIONAL_REGULATION;REACTOME_METABOLISM_OF_MRNA;R

TS_IN_ERBB2_SIGNALING;REACTOME_AXON_GUIDANCE;REACTOME_SEMA4D_IN_SEMAPHO
_GPCR_DOWNSTREAM_SIGNALING;REACTOME_G_ALPHA_I_SIGNALLING_EVENTS;REACTOI

ME_SIGNALING_BY_FGFR_IN_DISEASE;REACTOME_SIGNALING_BY_FGFR1_MUTANTS;REAC
ME_SIGNALING_BY_FGFR_IN_DISEASE;REACTOME_SIGNALING_BY_FGFR1_MUTANTS;REAC
ME_SIGNALING_BY_FGFR_IN_DISEASE;REACTOME_SIGNALING_BY_FGFR1_MUTANTS;REAC

CELL;REACTOME_TRAFFICKING_OF_AMPA_RECEPTORS;REACTOME_TRAFFICKING_OF_GL

SOME_DEGRADATION

ME_PLATELET_ADHESION_TO_EXPOSED_COLLAGEN;REACTOME_INTEGRIN_CELL_SURFAC

THWAYS;REACTOME_SYNTHESIS_SECRETION_AND_DEACYLATION_OF_GHRELIN;REACTOI

ATED_EVENTS;REACTOME_SIGNALING_BY_INSULIN_RECEPTOR;REACTOME_MTORC1_MED

OME_INTEGRATION_OF_ENERGY_METABOLISM;REACTOME_METABOLISM_OF_LIPIDS_AND_
OME_INTEGRATION_OF_ENERGY_METABOLISM;REACTOME_METABOLISM_OF_LIPIDS_AND_

GLUCAGON_TYPE_LIGAND_RECEPTORS;REACTOME_GPCR_DOWNSTREAM_SIGNALING;RE

TOME_MITOTIC_G2_G2_M_PHASES
_SIGNALLING_EVENTS;REACTOME_GPCR_LIGAND_BINDING

REACTOME_BASIGIN_INTERACTIONS;REACTOME_INTEGRIN_CELL_SURFACE_INTERACTION

ITRON_CONTAINING_PRE_MRNA;REACTOME_RNA_POL_II_TRANSCRIPTION;REACTOME_RN

IDING

ESSENGERS;REACTOME_SIGNALING_BY_THE_B_CELL_RECEPTOR_BCR;REACTOME_NRAG
ATH_THROUGH_JNK;REACTOME_CELL_DEATH_SIGNALLING_VIA_NRAGE_NRIF_AND_NADE;

AIR
AIR

N
N
N

CTION_OF_IFN_ALPHA_BETA_PATHWAYS;REACTOME_INNATE_IMMUNE_SYSTEM;REACTOM

EACTOME_P2Y_RECEPTORS;REACTOME_NUCLEOTIDE_LIKE_PURINERGIC_RECEPTORS;REA
CTOME_INTERFERON_SIGNALING;REACTOME_IMMUNE_SYSTEM;REACTOME_ADAPTIVE_IM

MUNE_SYSTEM;REACTOME_CYTOKINE_SIGNALING_IN_IMMUNE_SYSTEM

NSPORT
NSPORT

A_TRKA_FROM_THE_PLASMA_MEMBRANE;REACTOME_SIGNALING_BY_FGFR_IN_DISEASE;R

;;REACTOME_TGF_BETA_RECEPTOR_SIGNALING_ACTIVATES_SMADS;REACTOME_RESPON
GNALING

EACTOME_GPCR_DOWNSTREAM_SIGNALING;REACTOME_GPCR_LIGAND_BINDING

OLIGOPEPTIDES;REACTOME_PLATELET_HOMEOSTASIS;REACTOME_PLATELET_CALCIUM_HO

_AND_GROWTH_CONE_COLLAPSE

N_THE_LAGGING_STRAND;REACTOME_CELL_CYCLE_MITOTIC;REACTOME_TRANSCRIPTIOI
N_THE_LAGGING_STRAND;REACTOME_CELL_CYCLE_MITOTIC;REACTOME_TRANSCRIPTIOI
N_THE_LAGGING_STRAND;REACTOME_CELL_CYCLE_MITOTIC;REACTOME_TRANSCRIPTIOI

CELL;REACTOME_TRAFFICKING_OF_AMPA_RECEPTORS

IS

TOME_MITOTIC_G2_G2_M_PHASES

CELL;REACTOME_TRAFFICKING_OF_AMPA_RECEPTORS

2;REACTOME_SIGNALING_BY_CONSTITUTIVELY_ACTIVE_EGFR;REACTOME_SIGNALING_BY_

OLIGOPEPTIDES

ORGANIC_ACIDS_METAL_IONS_AND_AMINE_COMPOUNDS;REACTOME_BILE_SALT_AND_OR

EGULATION;REACTOME_REGULATION_OF_INSULIN_SECRETION_BY_GLUCAGON_LIKE_PEPT
EGULATION;REACTOME_REGULATION_OF_INSULIN_SECRETION_BY_GLUCAGON_LIKE_PEPT

AM1_INTERACTIONS;REACTOME_NCAM_SIGNALING_FOR_NEURITE_OUT_GROWTH
AM1_INTERACTIONS;REACTOME_NCAM_SIGNALING_FOR_NEURITE_OUT_GROWTH
_RESPONSE

ED_PLATELET_CYTOSOLIC_CA2_;REACTOME_SIGNALING_BY_GPCR;REACTOME_PEPTIDE_

E_SYSTEM
P75_NTR_RECEPTOR_MEDIATED_SIGNALLING;REACTOME_SIGNALING_BY_GPCR;REACTOM

SSING;REACTOME_MRNA_SPLICING;REACTOME_TRANSCRIPTION;REACTOME_MRNA_3_END

FACE_INTERACTIONS;REACTOME_HEMOSTASIS;REACTOME_IMMUNE_SYSTEM;REACTOME_

AM1_INTERACTIONS;REACTOME_NCAM_SIGNALING_FOR_NEURITE_OUT_GROWTH

TOME_MITOTIC_G2_G2_M_PHASES

JCLEOTIDE_LIKE_PURINERGIC_RECEPTORS;REACTOME_GPCR_DOWNSTREAM_SIGNALING

TS;REACTOME_M_G1_TRANSITION;REACTOME_G1_S_TRANSITION;REACTOME_SYNTHESIS_
_AND_GROWTH_CONE_COLLAPSE
NG_IN_IMMUNE_SYSTEM

)LISM_OF_LIPIDS_AND_LIPOPROTEINS

CELL;REACTOME_INHIBITION_OF_VOLTAGE_GATED_CA2_CHANNELS_VIA_GBETA_GAMMA_
CELL;REACTOME_INHIBITION_OF_VOLTAGE_GATED_CA2_CHANNELS_VIA_GBETA_GAMMA_

)RS;REACTOME_G_ALPHA_Q_SIGNALLING_EVENTS;REACTOME_GPCR_DOWNSTREAM_SIGI
-_TRANSCRIPTION;REACTOME_RNA_POL_III_TRANSCRIPTION_INITIATION_FROM_TYPE_3_F
-_TRANSCRIPTION;REACTOME_RNA_POL_III_TRANSCRIPTION_INITIATION_FROM_TYPE_3_F

ACTIVATION_OF_ARYLSULFATASES;REACTOME_METABOLISM_OF_PROTEINS;REACTOME_S

STEM

75_NTR_RECEPTOR_MEDIATED_SIGNALLING;REACTOME_ROLE_OF_DCC_IN_REGULATING_

CING;REACTOME_MITOTIC_M_M_G1_PHASES;REACTOME_CHROMOSOME_MAINTENANCE;F
SOME_DEGRADATION

P75_NTR_RECEPTOR_MEDIATED_SIGNALLING;REACTOME_SIGNALING_BY_GPCR;REACTOM

E_FATTY_ACID_TRIACYLGLYCEROL_AND_KETONE_BODY_METABOLISM;REACTOME_TRANS

DY_METABOLISM;REACTOME_SYNTHESIS_OF_VERY_LONG_CHAIN_FATTY_ACYL_COAS

M;REACTOME_GLYCOSAMINOGLYCAN_METABOLISM;REACTOME_A_TETRASACCHARIDE_LIN
OLIGOPEPTIDES

G_METABOLISM;REACTOME_GLYCOSAMINOGLYCAN_METABOLISM;REACTOME_A_TETRASA
ACTOME_ION_CHANNEL_TRANSPORT;REACTOME_HEMOSTASIS

IS;REACTOME_INTERACTION_BETWEEN_L1_AND_ANKYRINS;REACTOME_SIGNAL_TRANSDU
IS;REACTOME_INTERACTION_BETWEEN_L1_AND_ANKYRINS;REACTOME_SIGNAL_TRANSDU

TOME_MITOTIC_M_M_G1_PHASES;REACTOME_MITOTIC_G2_G2_M_PHASES;REACTOME_DN

NOTCH3;REACTOME_RECEPTOR_LIGAND_BINDING_INITIATES_THE_SECOND_PROTEOLYTI

EVELOPMENT_AND_PLATELET_PRODUCTION;REACTOME_KINESINS;REACTOME_DNA_REPL

_ANKYRINS

LISM_OF_LIPIDS_AND_LIPOPROTEINS;REACTOME_LIPID_DIGESTION_MOBILIZATION_AND_T

ORGANIC_ACIDS_METAL_IONS_AND_AMINE_COMPOUNDS;REACTOME_ZINC_TRANSPORTER

TOME_MITOTIC_G2_G2_M_PHASES
TOME_MITOTIC_G2_G2_M_PHASES

CONSTITUTIVELY_ACTIVE_EGFR;REACTOME_SIGNALING_BY_EGFR_IN_CANCER;REACTOME_

EACTOME_METABOLISM_OF_LIPIDS_AND_LIPOPROTEINS

ORS;REACTOME_G_ALPHA_Q_SIGNALLING_EVENTS;REACTOME_GPCR_DOWNSTREAM_SIG

HWAY;REACTOME_ER_PHAGOSOME_PATHWAY;REACTOME_NEF_MEDIATES_DOWN_MODU

ORGANIZATION;REACTOME_APOPTOSIS;REACTOME_APOPTOTIC_EXECUTION_PHASE

:MOSTASIS

CTOME_SIGNALING_BY_ERBB4;REACTOME_DOWNREGULATION_OF_ERBB2_ERBB3_SIGNAI

ACTIVATION_OF_ARYLSULFATASES;REACTOME_METABOLISM_OF_PROTEINS;REACTOME_S

E_FACTORS_INVOLVED_IN_MEGAKARYOCYTE_DEVELOPMENT_AND_PLATELET_PRODUCTI

CELL;REACTOME_TRANSMEMBRANE_TRANSPORT_OF_SMALL_MOLECULES;REACTOME_GA
_AND_SIGNALS_SURVIVAL;REACTOME_P75_NTR_RECEPTOR_MEDIATED_SIGNALLING;REA

TERFERON_SIGNALING;REACTOME_IMMUNE_SYSTEM;REACTOME_ADAPTIVE_IMMUNE_SYS

EACTOME_INTERACTION_BETWEEN_L1_AND_ANKYRINS;REACTOME_NEPHRIN_INTERACTIO

GNALLING_EVENTS;REACTOME_GPCR_LIGAND_BINDING

BODY_METABOLISM;REACTOME_TRANSCRIPTIONAL_REGULATION_OF_WHITE_ADIPOCYTE_

TOME_MITOTIC_G2_G2_M_PHASES
_P130CAS_LINKAGE_TO_MAPK_SIGNALING_FOR_INTEGRINS;REACTOME_GRB2_SOS_PROV
HA_BETA_PATHWAYS;REACTOME_APOPTOSIS;REACTOME_INNATE_IMMUNE_SYSTEM;REA

FR2;REACTOME_SIGNALING_BY_FGFR_MUTANTS;REACTOME_FRS2_MEDIATED_CASCADE;

IA_NRAGE_NRIF_AND_NADE;REACTOME_P75_NTR_RECEPTOR_MEDIATED_SIGNALLING;RE
E_SYSTEM
CTOME_G1_S_TRANSITION;REACTOME_SYNTHESIS_OF_DNA;REACTOME_MITOTIC_G1_G1_

LIGOPEPTIDES

TOME_MITOTIC_G2_G2_M_PHASES
LIGOPEPTIDES

)_AND_SIGNALS_SURVIVAL;REACTOME_P75_NTR_RECEPTOR_MEDIATED_SIGNALLING;REA

YPE_3_PROMOTER

IA_NRAGE_NRIF_AND_NADE;REACTOME_P75_NTR_RECEPTOR_MEDIATED_SIGNALLING;RE/

ᴐLIGOPEPTIDES

ᴎE;REACTOME_ACTIVATION_OF_THE_MRNA_UPON_BINDING_OF_THE_CAP_BINDING_COMP

LECULES;REACTOME_METABOLISM_OF_CARBOHYDRATES

ATION

ᴐY_METABOLISM;REACTOME_SYNTHESIS_OF_VERY_LONG_CHAIN_FATTY_ACYL_COAS

ᴍE_APOPTOSIS

_A_LYMPHOID_AND_A_NON_LYMPHOID_CELL;REACTOME_INTERFERON_GAMMA_SIGNALIN

ME_N_GLYCAN_TRIMMING_IN_THE_ER_AND_CALNEXIN_CALRETICULIN_CYCLE
CEPTORS;REACTOME_CHEMOKINE_RECEPTORS_BIND_CHEMOKINES;REACTOME_GPCR_D

K_CHANNELS

CTLA4_INHIBITORY_SIGNALING;REACTOME_MITOTIC_M_M_G1_PHASES;REACTOME_PLATEL

E_GLUCOSE_METABOLISM;REACTOME_PLATELET_ACTIVATION_SIGNALING_AND_AGGREG

CELL;REACTOME_TRAFFICKING_OF_AMPA_RECEPTORS

CELL;REACTOME_TRAFFICKING_OF_AMPA_RECEPTORS;REACTOME_TRAFFICKING_OF_GL

THWAY;REACTOME_NEUROTRANSMITTER_RECEPTOR_BINDING_AND_DOWNSTREAM_TRA

_COUPLING_AND_HEAT_PRODUCTION_BY_UNCOUPLING_PROTEINS_

YCLE_MITOTIC;REACTOME_CELL_CYCLE_CHECKPOINTS;REACTOME_REGULATION_OF_MI

LIGOPEPTIDES

_BY_ERBB4;REACTOME_SIGNALING_BY_ILS;REACTOME_IL_3_5_AND_GM_CSF_SIGNALING;F

;REACTOME_REGULATED_PROTEOLYSIS_OF_P75NTR;REACTOME_P75NTR_SIGNALS_VIA_N

_LIPOPROTEINS;REACTOME_FATTY_ACID_TRIACYLGLYCEROL_AND_KETONE_BODY_META

DING
1;REACTOME_GLYCOSAMINOGLYCAN_METABOLISM;REACTOME_A_TETRASACCHARIDE_LIN

ME_PHOSPHOLIPID_METABOLISM;REACTOME_PI_METABOLISM;REACTOME_GOLGI_ASSOCI

LET_ACTIVATION_SIGNALING_AND_AGGREGATION

EDIATED_SIGNALLING;REACTOME_CELL_CELL_JUNCTION_ORGANIZATION;REACTOME_TIGH

OME_TRANSPORT_OF_INORGANIC_CATIONS_ANIONS_AND_AMINO_ACIDS_OLIGOPEPTIDES

N

ACTOME_GENERIC_TRANSCRIPTION_PATHWAY;REACTOME_NOTCH_HLH_TRANSCRIPTION_

N_AND_REGULATION;REACTOME_CELL_CYCLE_MITOTIC;REACTOME_G1_S_TRANSITION;RE

CELL;REACTOME_TRANSMEMBRANE_TRANSPORT_OF_SMALL_MOLECULES;REACTOME_GA

C_MEDIATED_TRANSMEMBRANE_TRANSPORT;REACTOME_TRANSPORT_OF_GLUCOSE_AND

_INITIATION_AND_PROMOTER_OPENING;REACTOME_TRANSCRIPTION;REACTOME_RNA_PO

ADAPTIVE_IMMUNE_SYSTEM

;REACTOME_RESPIRATORY_ELECTRON_TRANSPORT_ATP_SYNTHESIS_BY_CHEMIOSMOTIC

ACTOME_REGULATION_OF_INSULIN_SECRETION;REACTOME_REGULATION_OF_INSULIN_SE

ɢLYCOSYLATION;REACTOME_BIOSYNTHESIS_OF_THE_N_GLYCAN_PRECURSOR_DOLICHOL_

_GPCR_LIGAND_BINDING

NG;REACTOME_SIGNALING_BY_EGFR_IN_CANCER;REACTOME_NGF_SIGNALLING_VIA_TRKA

CELL;REACTOME_ACTIVATION_OF_KAINATE_RECEPTORS_UPON_GLUTAMATE_BINDING;RE

OLIGOPEPTIDES

ULFATE_HEPARIN_HS_GAG_METABOLISM;REACTOME_GLYCOSAMINOGLYCAN_METABOLIS

AIR

S;REACTOME_TRANSPORT_OF_ORGANIC_ANIONS

_A_LYMPHOID_AND_A_NON_LYMPHOID_CELL;REACTOME_INTERFERON_GAMMA_SIGNALIN

E_EFFECTS_OF_PIP2_HYDROLYSIS;REACTOME_HEMOSTASIS;REACTOME_PLATELET_ACTIV
E_EFFECTS_OF_PIP2_HYDROLYSIS;REACTOME_HEMOSTASIS;REACTOME_PLATELET_ACTIV

;_AT_THE_PLASMA_MEMBRANE;REACTOME_PI_METABOLISM;REACTOME_METABOLISM_OF

_REPLICATION_INDEPENDENT_DOUBLE_STRAND_BREAKS;REACTOME_P53_DEPENDENT_G

NSTITUTIVELY_ACTIVE_EGFR;REACTOME_GRB2_EVENTS_IN_ERBB2_SIGNALING;REACTOM
)ME_IMMUNE_SYSTEM;REACTOME_ADAPTIVE_IMMUNE_SYSTEM
:CRETION;REACTOME_VOLTAGE_GATED_POTASSIUM_CHANNELS;REACTOME_POTASSIUM_

CELL;REACTOME_TRANSMEMBRANE_TRANSPORT_OF_SMALL_MOLECULES;REACTOME_G/

_CASCADE;REACTOME_HEMOSTASIS;REACTOME_INNATE_IMMUNE_SYSTEM;REACTOME_IM

MUNE_SYSTEM;REACTOME_CLASS_I_MHC_MEDIATED_ANTIGEN_PROCESSING_PRESENTAT

ACTIVATION_OF_ARYLSULFATASES;REACTOME_METABOLISM_OF_PROTEINS;REACTOME_S

RT_OF_MATURE_TRANSCRIPT_TO_CYTOPLASM;REACTOME_TRANSMEMBRANE_TRANSPOF

EACTOME_IRON_UPTAKE_AND_TRANSPORT;REACTOME_TRANSFERRIN_ENDOCYTOSIS_AN

_SIGNALING_PATHWAYS

T_JUNCTION_INTERACTIONS;REACTOME_CELL_JUNCTION_ORGANIZATION;REACTOME_SI(

E_DEVELOPMENT_AND_PLATELET_PRODUCTION;REACTOME_HEMOSTASIS

Y_PDGF;REACTOME_AXON_GUIDANCE;REACTOME_NCAM1_INTERACTIONS;REACTOME_N(

BODY_METABOLISM;REACTOME_TRANSCRIPTIONAL_REGULATION_OF_WHITE_ADIPOCYTE_

OME_APOPTOSIS;REACTOME_APOPTOTIC_EXECUTION_PHASE
CELL;REACTOME_ACETYLCHOLINE_BINDING_AND_DOWNSTREAM_EVENTS;REACTOME_HI(

ATED_EVENTS;REACTOME_SIGNALING_BY_INSULIN_RECEPTOR;REACTOME_MTORC1_MED
T;REACTOME_LIPOPROTEIN_METABOLISM;REACTOME_CHYLOMICRON_MEDIATED_LIPID_TI

E_METABOLISM_OF_LIPIDS_AND_LIPOPROTEINS;REACTOME_FATTY_ACID_TRIACYLGLYCEI

P75_NTR_RECEPTOR_MEDIATED_SIGNALLING;REACTOME_SIGNALING_BY_GPCR;REACTOM

TOME_MITOTIC_G2_G2_M_PHASES;REACTOME_SIGNALLING_BY_NGF;REACTOME_SIGNALII

PATHWAYS

_ADAPTIVE_IMMUNE_SYSTEM

OME_IMMUNE_SYSTEM;REACTOME_ADAPTIVE_IMMUNE_SYSTEM
BINDING

_3_UTR_MEDIATED_TRANSLATIONAL_REGULATION;REACTOME_METABOLISM_OF_MRNA;RI

ONSTITUTIVELY_ACTIVE_EGFR;REACTOME_SIGNALING_BY_EGFR_IN_CANCER;REACTOME_

FR2;REACTOME_SIGNALING_BY_FGFR3_MUTANTS;REACTOME_SIGNALING_BY_FGFR_MUTA

REACTOME_GPCR_DOWNSTREAM_SIGNALING;REACTOME_G_ALPHA_I_SIGNALLING_EVEN
GNALLING_VIA_NRAGE_NRIF_AND_NADE;REACTOME_P75_NTR_RECEPTOR_MEDIATED_SIG

N

LLULAR_PROTEINS;REACTOME_INTEGRATION_OF_PROVIRUS;REACTOME_APOBEC3G_MED

FACE_INTERACTIONS_AT_THE_VASCULAR_WALL;REACTOME_PLATELET_ADHESION_TO_EX

N

ULES;REACTOME_REGULATION_OF_INSULIN_SECRETION;REACTOME_POTASSIUM_CHANN

LIGOPEPTIDES

SOME_DEGRADATION

CYCLE_MITOTIC;REACTOME_CELL_CYCLE_CHECKPOINTS;REACTOME_REGULATION_OF_MI

)LIGOPEPTIDES

_S_SIGNALLING_EVENTS;REACTOME_GPCR_LIGAND_BINDING

)RGANIC_ACIDS_METAL_IONS_AND_AMINE_COMPOUNDS;REACTOME_AMINE_COMPOUND_

)RS;REACTOME_G_ALPHA_Q_SIGNALLING_EVENTS;REACTOME_GPCR_DOWNSTREAM_SIGI

E_INTEGRATION_OF_ENERGY_METABOLISM;REACTOME_OPIOID_SIGNALLING;REACTOME_I

\M1_INTERACTIONS;REACTOME_NCAM_SIGNALING_FOR_NEURITE_OUT_GROWTH

)_AND_SIGNALS_SURVIVAL;REACTOME_P75_NTR_RECEPTOR_MEDIATED_SIGNALLING;REA

_GOLGI;REACTOME_KERATAN_SULFATE_BIOSYNTHESIS;REACTOME_KERATAN_SULFATE_

NG;REACTOME_REGULATION_OF_IFNG_SIGNALING;REACTOME_INTERFERON_GAMMA_SIG

P75_NTR_RECEPTOR_MEDIATED_SIGNALLING;REACTOME_SIGNALING_BY_GPCR;REACTOM

LECULES;REACTOME_METABOLISM_OF_CARBOHYDRATES

_SIGNALLING_EVENTS;REACTOME_GPCR_LIGAND_BINDING

IAL_SYSTEM;REACTOME_CELL_DEATH_SIGNALLING_VIA_NRAGE_NRIF_AND_NADE;REACTO
HASE

RANSITION;REACTOME_G1_S_TRANSITION;REACTOME_POL_SWITCHING;REACTOME_SYNTH

NS;REACTOME_SEMA3A_PLEXIN_REPULSION_SIGNALING_BY_INHIBITING_INTEGRIN_ADHE

ADING_TO_GENERATION_OF_SECOND_MESSENGERS;REACTOME_SIGNALING_BY_THE_B_C

ND_INACTIVATION_OF_GLP1

MENTS

LEX_AND_EIFS_AND_SUBSEQUENT_BINDING_TO_43S;REACTOME_METABOLISM_OF_PROTE

LET_ACTIVATION_SIGNALING_AND_AGGREGATION

HIV_LIFE_CYCLE

YCAN_ANTENNAE_ELONGATION_IN_THE_MEDIAL_TRANS_GOLGI

OTCH1;REACTOME_GENERIC_TRANSCRIPTION_PATHWAY;REACTOME_RNA_POL_I_TRANSC

RT_OF_SMALL_MOLECULES;REACTOME_MRNA_PROCESSING;REACTOME_TRANSPORT_OF_

N_ACROSS_CHEMICAL_SYNAPSES;REACTOME_NEURONAL_SYSTEM;REACTOME_SIGNALIN
LE;REACTOME_NGF_SIGNALLING_VIA_TRKA_FROM_THE_PLASMA_MEMBRANE;REACTOME_

IDEPENDENT_GLUCOSE_TRANSPORTERS

EINS;REACTOME_3_UTR_MEDIATED_TRANSLATIONAL_REGULATION

IFKB;REACTOME_NRIF_SIGNALS_CELL_DEATH_FROM_THE_NUCLEUS;REACTOME_NFKB_IS

EACTOME_TRANSPORT_OF_INORGANIC_CATIONS_ANIONS_AND_AMINO_ACIDS_OLIGOPEPT

ı2_M_PHASES

Y_GPCR;REACTOME_INTEGRATION_OF_ENERGY_METABOLISM;REACTOME_OPIOID_SIGNAI

:EACTOME_TRANSCRIPTION;REACTOME_MRNA_3_END_PROCESSING;REACTOME_CLEAVA(

/IE_G_ALPHA1213_SIGNALLING_EVENTS;REACTOME_GPCR_DOWNSTREAM_SIGNALING

VNSTREAM_SIGNALING_EVENTS_OF_B_CELL_RECEPTOR_BCR;REACTOME_SIGNALING_BY_
VNSTREAM_SIGNALING_EVENTS_OF_B_CELL_RECEPTOR_BCR;REACTOME_SIGNALING_BY_

LEX_FORMATION;REACTOME_CELL_CYCLE_MITOTIC;REACTOME_CELL_CYCLE_CHECKPOII

OME_CIRCADIAN_REPRESSION_OF_EXPRESSION_BY_REV_ERBA;REACTOME_GENERIC_TR

CAM_SIGNALING_FOR_NEURITE_OUT_GROWTH
ME_G_ALPHA1213_SIGNALLING_EVENTS;REACTOME_GPCR_DOWNSTREAM_SIGNALING

_PI3K_EVENTS_IN_ERBB4_SIGNALING;REACTOME_PI3K_EVENTS_IN_ERBB2_SIGNALING;RE

OTCH_HLH_TRANSCRIPTION_PATHWAY;REACTOME_SIGNALING_BY_NOTCH

ROTEIN_METABOLISM

ISCRIPTIONAL_ACTIVITY;REACTOME_GENERIC_TRANSCRIPTION_PATHWAY;REACTOME_ME
TIVATION;REACTOME_TRAF6_MEDIATED_INDUCTION_OF_NFKB_AND_MAP_KINASES_UPON_

DIATED_TRANSMEMBRANE_TRANSPORT;REACTOME_TRANSPORT_OF_INORGANIC_CATION

CRIPTIONAL_REGULATION_OF_WHITE_ADIPOCYTE_DIFFERENTIATION

ATED_VESICLE_BIOGENESIS;REACTOME_METABOLISM_OF_LIPIDS_AND_LIPOPROTEINS

CTOME_EFFECTS_OF_PIP2_HYDROLYSIS;REACTOME_METABOLISM_OF_LIPIDS_AND_LIPOP

_PI3K_EVENTS_IN_ERBB4_SIGNALING;REACTOME_PI3K_EVENTS_IN_ERBB2_SIGNALING;RE
_PI3K_EVENTS_IN_ERBB4_SIGNALING;REACTOME_PI3K_EVENTS_IN_ERBB2_SIGNALING;RE

NALING;REACTOME_GPCR_LIGAND_BINDING

G;REACTOME_INTERFERON_ALPHA_BETA_SIGNALING;REACTOME_INTERFERON_SIGNALIN

TOME_GLUCOSE_TRANSPORT;REACTOME_METABOLISM_OF_CARBOHYDRATES;REACTOM

ITTER_RECEPTOR_BINDING_AND_DOWNSTREAM_TRANSMISSION_IN_THE_POSTSYNAPTIC

ION;REACTOME_NCAM_SIGNALING_FOR_NEURITE_OUT_GROWTH;REACTOME_INHIBITION_

E_SIGNALING_BY_EGFR_IN_CANCER;REACTOME_SHC1_EVENTS_IN_ERBB4_SIGNALING;RE
CELL_RECEPTOR_BCR;REACTOME_NGF_SIGNALLING_VIA_TRKA_FROM_THE_PLASMA_MEM

GHLY_CALCIUM_PERMEABLE_POSTSYNAPTIC_NICOTINIC_ACETYLCHOLINE_RECEPTORS

ECRETIN_FAMILY_RECEPTORS;REACTOME_GLUCAGON_TYPE_LIGAND_RECEPTORS;REACT
_LIPOPROTEINS;REACTOME_FATTY_ACID_TRIACYLGLYCEROL_AND_KETONE_BODY_METAB

Y_PROTEINS_THAT_BIND_AU_RICH_ELEMENTS;REACTOME_DESTABILIZATION_OF_MRNA_B

TION_DEPENDENT_MRNA_DECAY;REACTOME_METABOLISM_OF_RNA;REACTOME_DESTABIL

O_ELEVATED_PLATELET_CYTOSOLIC_CA2_;REACTOME_TRANSMISSION_ACROSS_CHEMICA

_OF_DNA;REACTOME_MITOTIC_G1_G1_S_PHASES;REACTOME_MITOTIC_M_M_G1_PHASES;

ME_G_ALPHA1213_SIGNALLING_EVENTS;REACTOME_GPCR_DOWNSTREAM_SIGNALING

TEM;REACTOME_CYTOKINE_SIGNALING_IN_IMMUNE_SYSTEM;REACTOME_CLASS_I_MHC_M

ME_G_ALPHA1213_SIGNALLING_EVENTS;REACTOME_GPCR_DOWNSTREAM_SIGNALING

ME_G_ALPHA1213_SIGNALLING_EVENTS;REACTOME_GPCR_DOWNSTREAM_SIGNALING

S;REACTOME_AMINO_ACID_AND_OLIGOPEPTIDE_SLC_TRANSPORTERS
S;REACTOME_AMINO_ACID_AND_OLIGOPEPTIDE_SLC_TRANSPORTERS

ESOLUTION_OF_AP_SITES_VIA_THE_SINGLE_NUCLEOTIDE_REPLACEMENT_PATHWAY
ESOLUTION_OF_AP_SITES_VIA_THE_SINGLE_NUCLEOTIDE_REPLACEMENT_PATHWAY

RESPONSE_TO_REPLICATION_STRESS;REACTOME_MEIOTIC_SYNAPSIS;REACTOME_G2_M_(
TION;REACTOME_ADP_SIGNALLING_THROUGH_P2RY12;REACTOME_GPCR_LIGAND_BINDIN(

REGULATION_OF_INSULIN_SECRETION;REACTOME_SLC_MEDIATED_TRANSMEMBRANE_TR

C_NER;REACTOME_SYNTHESIS_OF_DNA;REACTOME_DNA_REPAIR;REACTOME_CHROMOSO

OME_METABOLISM_OF_LIPIDS_AND_LIPOPROTEINS;REACTOME_FATTY_ACID_TRIACYLGLY(

PENING;REACTOME_MRNA_PROCESSING;REACTOME_TRANSCRIPTION;REACTOME_NUCLE(

CTOME_KERATAN_SULFATE_KERATIN_METABOLISM;REACTOME_KERATAN_SULFATE_DEGF
CTOME_KERATAN_SULFATE_KERATIN_METABOLISM;REACTOME_KERATAN_SULFATE_DEGF
ETABOLISM;REACTOME_METABOLISM_OF_LIPIDS_AND_LIPOPROTEINS

EOSOMES_AT_THE_CENTROMERE;REACTOME_RNA_POL_I_PROMOTER_OPENING;REACTOM

OID_HORMONES;REACTOME_METABOLISM_OF_LIPIDS_AND_LIPOPROTEINS

VENTS_IN_ERBB2_SIGNALING;REACTOME_NUCLEAR_SIGNALING_BY_ERBB4

:ANE_TRANSPORT_OF_SMALL_MOLECULES;REACTOME_IRON_UPTAKE_AND_TRANSPORT;I

SIGNALING;REACTOME_INTERFERON_SIGNALING;REACTOME_RIG_I_MDA5_MEDIATED_INDU

_SIGNAL_TRANSDUCTION;REACTOME_SIGNALING_BY_ILS;REACTOME_REGULATION_OF_SI

ᴐME_INTERFERON_SIGNALING;REACTOME_IMMUNE_SYSTEM;REACTOME_CYTOKINE_SIGN/

LISM_OF_LIPIDS_AND_LIPOPROTEINS
LISM_OF_LIPIDS_AND_LIPOPROTEINS

_DIFFERENTIATION

ELLING_OF_PS;REACTOME_GLYCEROPHOSPHOLIPID_BIOSYNTHESIS;REACTOME_METABOL

ᴐME_INNATE_IMMUNE_SYSTEM;REACTOME_IMMUNE_SYSTEM;REACTOME_TOLL_RECEPTO

EINS;REACTOME_3_UTR_MEDIATED_TRANSLATIONAL_REGULATION

_BY_EGFR_IN_CANCER;REACTOME_SHC1_EVENTS_IN_ERBB4_SIGNALING;REACTOME_INSU

GHLY_CALCIUM_PERMEABLE_POSTSYNAPTIC_NICOTINIC_ACETYLCHOLINE_RECEPTORS;RE

PK_TARGETS;REACTOME_MYOGENESIS;REACTOME_MAP_KINASE_ACTIVATION_IN_TLR_CA

PROMOTER;REACTOME_RNA_POL_III_TRANSCRIPTION_TERMINATION;REACTOME_RNA_POL

CIRCADIAN_CLOCK;REACTOME_TRANSCRIPTIONAL_REGULATION_OF_WHITE_ADIPOCYTE_

OME_CIRCADIAN_REPRESSION_OF_EXPRESSION_BY_REV_ERBA;REACTOME_GENERIC_TR

_DIFFERENTIATION

_AMINO_ACIDS_OLIGOPEPTIDES;REACTOME_AMINO_ACID_AND_OLIGOPEPTIDE_SLC_TRAN

EACTOME_IONOTROPIC_ACTIVITY_OF_KAINATE_RECEPTORS

EACTOME_IONOTROPIC_ACTIVITY_OF_KAINATE_RECEPTORS

NEUROTRANSMITTER_RECEPTOR_BINDING_AND_DOWNSTREAM_TRANSMISSION_IN_THE_

TOME_IMMUNE_SYSTEM;REACTOME_CYTOKINE_SIGNALING_IN_IMMUNE_SYSTEM
OME_CIRCADIAN_REPRESSION_OF_EXPRESSION_BY_REV_ERBA;REACTOME_GENERIC_TR
OME_CIRCADIAN_REPRESSION_OF_EXPRESSION_BY_REV_ERBA;REACTOME_GENERIC_TR

E_NOTCH_EXPRESSION_AND_PROCESSING;REACTOME_NOTCH1_INTRACELLULAR_DOMAIN

ME_IMMUNE_SYSTEM;REACTOME_TOLL_RECEPTOR_CASCADES

ME_IMMUNE_SYSTEM;REACTOME_TOLL_RECEPTOR_CASCADES

LEASE_CYCLE

CRIPTIONAL_REGULATION_OF_WHITE_ADIPOCYTE_DIFFERENTIATION

EACTOME_SIGNALING_BY_GPCR;REACTOME_G_ALPHA1213_SIGNALLING_EVENTS;REACT

TOTIC_CELL_CYCLE;REACTOME_APC_C_CDH1_MEDIATED_DEGRADATION_OF_CDC20_AND

NALING;REACTOME_GPCR_LIGAND_BINDING
ANSPORTERS;REACTOME_IRON_UPTAKE_AND_TRANSPORT
ANSPORTERS;REACTOME_IRON_UPTAKE_AND_TRANSPORT

S;REACTOME_AMINO_ACID_AND_OLIGOPEPTIDE_SLC_TRANSPORTERS

WNSTREAM_SIGNALING_EVENTS_OF_B_CELL_RECEPTOR_BCR;REACTOME_SIGNALING_BY_

ESOLUTION_OF_AP_SITES_VIA_THE_SINGLE_NUCLEOTIDE_REPLACEMENT_PATHWAY

ME_HEMOSTASIS;REACTOME_PLATELET_ACTIVATION_SIGNALING_AND_AGGREGATION

;REACTOME_PEROXISOMAL_LIPID_METABOLISM;REACTOME_METABOLISM_OF_LIPIDS_AND

2_M_PHASES

OME_CIRCADIAN_REPRESSION_OF_EXPRESSION_BY_REV_ERBA;REACTOME_GENERIC_TR

YS;REACTOME_INNATE_IMMUNE_SYSTEM;REACTOME_IMMUNE_SYSTEM;REACTOME_CYTO

EGATION

IDEPENDENT_GLUCOSE_TRANSPORTERS

VNSTREAM_SIGNALING_EVENTS_OF_B_CELL_RECEPTOR_BCR;REACTOME_SIGNALING_BY_

IKER_SEQUENCE_IS_REQUIRED_FOR_GAG_SYNTHESIS;REACTOME_METABOLISM_OF_LIPII
IKER_SEQUENCE_IS_REQUIRED_FOR_GAG_SYNTHESIS;REACTOME_METABOLISM_OF_LIPII

KINASE_AND_TRANSCRIPTION_FACTOR_ACTIVATION;REACTOME_ERK_MAPK_TARGETS;RE

T_OF_INORGANIC_CATIONS_ANIONS_AND_AMINO_ACIDS_OLIGOPEPTIDES;REACTOME_ME
T_OF_INORGANIC_CATIONS_ANIONS_AND_AMINO_ACIDS_OLIGOPEPTIDES;REACTOME_ME

PECAM1_INTERACTIONS;REACTOME_TCR_SIGNALING;REACTOME_DOWNSTREAM_TCR_SI

CRIPTIONAL_REGULATION_OF_WHITE_ADIPOCYTE_DIFFERENTIATION

_MTOR_BY_LKB1_AMPK;REACTOME_REGULATION_OF_RHEB_GTPASE_ACTIVITY_BY_AMPK

NALING;REACTOME_INTERFERON_ALPHA_BETA_SIGNALING;REACTOME_REGULATION_OF

;REACTOME_GPCR_LIGAND_BINDING

OME_SEMAPHORIN_INTERACTIONS;REACTOME_SEMA4D_INDUCED_CELL_MIGRATION_AND_

_PATHWAY;REACTOME_RECEPTOR_LIGAND_BINDING_INITIATES_THE_SECOND_PROTEOLY

_PATHWAY;REACTOME_RECEPTOR_LIGAND_BINDING_INITIATES_THE_SECOND_PROTEOLY

PROMOTER;REACTOME_RNA_POL_III_TRANSCRIPTION_TERMINATION;REACTOME_RNA_POI

ACTOME_SIGNALING_BY_GPCR;REACTOME_AXON_GUIDANCE;REACTOME_G_ALPHA1213_S

RT_OF_SMALL_MOLECULES;REACTOME_MRNA_PROCESSING;REACTOME_TRANSPORT_OF_

SION_SIGNALING_BY_INHIBITING_INTEGRIN_ADHESION

ME_G_ALPHA1213_SIGNALLING_EVENTS;REACTOME_GPCR_DOWNSTREAM_SIGNALING

E_PI3K_EVENTS_IN_ERBB4_SIGNALING;REACTOME_PI3K_EVENTS_IN_ERBB2_SIGNALING;RE

ME_SIGNALING_BY_NOTCH1;REACTOME_REGULATED_PROTEOLYSIS_OF_P75NTR;REACTO

_CHANNELS

OME_CIRCADIAN_REPRESSION_OF_EXPRESSION_BY_REV_ERBA;REACTOME_GENERIC_TR

OMEOSTASIS;REACTOME_HEMOSTASIS

NS
NS

TE_IMMUNE_SYSTEM;REACTOME_ACTIVATED_TLR4_SIGNALLING;REACTOME_IMMUNE_SYS

CATION

PATHWAY;REACTOME_METABOLISM_OF_PROTEINS;REACTOME_FORMATION_OF_FIBRIN_C

;LP1
;LP1

AND_LIPOPROTEINS

REACTOME_IMMUNE_SYSTEM;REACTOME_ADAPTIVE_IMMUNE_SYSTEM
TOME_RAP1_SIGNALLING;REACTOME_PLATELET_AGGREGATION_PLUG_FORMATION;REAC

GHLY_CALCIUM_PERMEABLE_POSTSYNAPTIC_NICOTINIC_ACETYLCHOLINE_RECEPTORS;RI

TOME_L1CAM_INTERACTIONS;REACTOME_SIGNAL_TRANSDUCTION_BY_L1;REACTOME_HEM

TANTS;REACTOME_SIGNALING_BY_FGFR_MUTANTS;REACTOME_SIGNALING_BY_PDGF;REA

AND_ORGANIC_ACIDS_METAL_IONS_AND_AMINE_COMPOUNDS;REACTOME_METAL_ION_SL

PTOR;REACTOME_SIGNAL_ATTENUATION;REACTOME_SOS_MEDIATED_SIGNALLING;REACTO

NALING;REACTOME_G_ALPHA_I_SIGNALLING_EVENTS;REACTOME_GPCR_LIGAND_BINDING

_OF_GLUCOSE_AND_OTHER_SUGARS_BILE_SALTS_AND_ORGANIC_ACIDS_METAL_IONS_AN

ITS_IN_ERBB4_SIGNALING;REACTOME_INSULIN_RECEPTOR_SIGNALLING_CASCADE;REACT

(

ME_GPCR_LIGAND_BINDING

SION_SIGNALING_BY_INHIBITING_INTEGRIN_ADHESION

RESPONSE_TO_REPLICATION_STRESS;REACTOME_MEIOTIC_SYNAPSIS;REACTOME_G2_M_

TIGEN_PROCESSING_PRESENTATION;REACTOME_ANTIGEN_PROCESSING_UBIQUITINATION
TIGEN_PROCESSING_PRESENTATION;REACTOME_ANTIGEN_PROCESSING_UBIQUITINATION

ABA_A_RECEPTOR_ACTIVATION;REACTOME_GABA_RECEPTOR_ACTIVATION;REACTOME_IC

_AND_NADE;REACTOME_P75_NTR_RECEPTOR_MEDIATED_SIGNALLING;REACTOME_SIGNA
_AND_NADE;REACTOME_P75_NTR_RECEPTOR_MEDIATED_SIGNALLING;REACTOME_SIGNA

_RHEB_GTPASE_ACTIVITY_BY_AMPK;REACTOME_PKB_MEDIATED_EVENTS;REACTOME_SIG

TAPHASE

GFR_IN_CANCER;REACTOME_PI3K_EVENTS_IN_ERBB4_SIGNALING;REACTOME_PI3K_EVEN

CCHARIDE_LINKER_SEQUENCE_IS_REQUIRED_FOR_GAG_SYNTHESIS;REACTOME_METABC

SPHINGOLIPID_METABOLISM;REACTOME_POST_TRANSLATIONAL_PROTEIN_MODIFICATION;

TOME_G2_M_CHECKPOINTS;REACTOME_G2_M_DNA_DAMAGE_CHECKPOINT
TOME_G2_M_CHECKPOINTS;REACTOME_G2_M_DNA_DAMAGE_CHECKPOINT

AM_SIGNALING_OF_ACTIVATED_FGFR;REACTOME_PHOSPHOLIPASE_C_MEDIATED_CASCAD
AM_SIGNALING_OF_ACTIVATED_FGFR;REACTOME_PHOSPHOLIPASE_C_MEDIATED_CASCAD
AM_SIGNALING_OF_ACTIVATED_FGFR;REACTOME_PHOSPHOLIPASE_C_MEDIATED_CASCAD

EACTOME_METABOLISM_OF_RNA;REACTOME_INFLUENZA_LIFE_CYCLE;REACTOME_INFLUE

YCAN_ANTENNAE_ELONGATION_IN_THE_MEDIAL_TRANS_GOLGI
_LIPID_LINKED_OLIGOSACCHARIDE_LLO_AND_TRANSFER_TO_A_NASCENT_PROTEIN

CELL_RECEPTOR_BCR;REACTOME_NGF_SIGNALLING_VIA_TRKA_FROM_THE_PLASMA_MEM

EACTOME_METABOLISM_OF_RNA;REACTOME_INFLUENZA_LIFE_CYCLE;REACTOME_INFLUE

TOME_ADAPTIVE_IMMUNE_SYSTEM

VATION_SIGNALING_AND_AGGREGATION

ED_HUMAN_TAK1;REACTOME_ACTIVATED_TAK1_MEDIATES_P38_MAPK_ACTIVATION;REACT

N_ACROSS_CHEMICAL_SYNAPSES;REACTOME_NEURONAL_SYSTEM;REACTOME_SIGNALIN

E_INTERACTIONS;REACTOME_SIGNALING_BY_PDGF;REACTOME_GPVI_MEDIATED_ACTIVA

EACTOME_DOWNSTREAM_SIGNALING_EVENTS_OF_B_CELL_RECEPTOR_BCR;REACTOME_
LEX_FORMATION;REACTOME_CELL_CYCLE_MITOTIC;REACTOME_CELL_CYCLE_CHECKPOI

/ATION_SIGNALING_AND_AGGREGATION
/ATION_SIGNALING_AND_AGGREGATION

D_RECYCLING;REACTOME_SIGNALING_BY_INSULIN_RECEPTOR

LASE;REACTOME_PLATELET_HOMEOSTASIS;REACTOME_HEMOSTASIS

ARYOCYTE_DEVELOPMENT_AND_PLATELET_PRODUCTION;REACTOME_HEMOSTASIS

_MTOR_BY_LKB1_AMPK;REACTOME_REGULATION_OF_RHEB_GTPASE_ACTIVITY_BY_AMPK
_MTOR_BY_LKB1_AMPK;REACTOME_REGULATION_OF_RHEB_GTPASE_ACTIVITY_BY_AMPK

ANTS_OF_FGFR1;REACTOME_SIGNALING_BY_FGFR_MUTANTS;REACTOME_AXON_GUIDANC
ANTS_OF_FGFR1;REACTOME_SIGNALING_BY_FGFR_MUTANTS;REACTOME_AXON_GUIDANC

ESOLUTION_OF_AP_SITES_VIA_THE_SINGLE_NUCLEOTIDE_REPLACEMENT_PATHWAY

GESTION_MOBILIZATION_AND_TRANSPORT

DIATED_TRANSMEMBRANE_TRANSPORT;REACTOME_TRANSPORT_OF_INORGANIC_CATION

L_ATTENUATION;REACTOME_SHC_MEDIATED_SIGNALLING;REACTOME_SHC_RELATED_EV[illegible]

VNSTREAM_SIGNALING_EVENTS_OF_B_CELL_RECEPTOR_BCR;REACTOME_SIGNALING_BY_

NSMITTER_RELEASE_CYCLE;REACTOME_ACETYLCHOLINE_NEUROTRANSMITTER_RELEASE

JT_GROWTH;REACTOME_L1CAM_INTERACTIONS;REACTOME_INTERACTION_BETWEEN_L1_
JT_GROWTH;REACTOME_L1CAM_INTERACTIONS;REACTOME_INTERACTION_BETWEEN_L1_
JT_GROWTH;REACTOME_L1CAM_INTERACTIONS;REACTOME_INTERACTION_BETWEEN_L1_
JT_GROWTH;REACTOME_L1CAM_INTERACTIONS;REACTOME_INTERACTION_BETWEEN_L1_

_LIPID_LINKED_OLIGOSACCHARIDE_LLO_AND_TRANSFER_TO_A_NASCENT_PROTEIN

_SIGNAL_TRANSDUCTION;REACTOME_SIGNALING_BY_ILS;REACTOME_REGULATION_OF_SI

CTOME_IMMUNE_SYSTEM

ROTEIN_METABOLISM
ROTEIN_METABOLISM

N_PLUG_FORMATION;REACTOME_HEMOSTASIS;REACTOME_PLATELET_ACTIVATION_SIGN

E_NEUROTRANSMITTER_RELEASE_CYCLE;REACTOME_METABOLISM_OF_LIPIDS_AND_LIPO

CTOME_IMMUNE_SYSTEM;REACTOME_ADAPTIVE_IMMUNE_SYSTEM;REACTOME_CYTOKINE

EACTOME_DOWNSTREAM_SIGNALING_EVENTS_OF_B_CELL_RECEPTOR_BCR;REACTOME_/

M_THE_PLASMA_MEMBRANE;REACTOME_MHC_CLASS_II_ANTIGEN_PRESENTATION;REACT

PROTEINS;REACTOME_FATTY_ACID_TRIACYLGLYCEROL_AND_KETONE_BODY_METABOLIS

IOMEOSTASIS;REACTOME_PKB_MEDIATED_EVENTS;REACTOME_SIGNALING_BY_INSULIN_R
LISM_OF_LIPIDS_AND_LIPOPROTEINS

KE_RECEPTORS;REACTOME_G_ALPHA_Q_SIGNALLING_EVENTS;REACTOME_GPCR_DOWN

_NEUROTRANSMITTER_TRANSPORTERS;REACTOME_AMINE_COMPOUND_SLC_TRANSPOR

IA_REPLICATION;REACTOME_MITOTIC_PROMETAPHASE

VNSTREAM_SIGNALING_EVENTS_OF_B_CELL_RECEPTOR_BCR;REACTOME_SIGNALING_BY_

HESIS_OF_DNA;REACTOME_MITOTIC_G1_G1_S_PHASES;REACTOME_MITOTIC_M_M_G1_PH

CTOME_HORMONE_SENSITIVE_LIPASE_HSL_MEDIATED_TRIACYLGLYCEROL_HYDROLYSIS;F

D_RECYCLING;REACTOME_SIGNALING_BY_INSULIN_RECEPTOR

G_UBIQUITINATION_PROTEASOME_DEGRADATION;REACTOME_VIF_MEDIATED_DEGRADATI

G1_DNA_DAMAGE_RESPONSE;REACTOME_REGULATION_OF_THE_FANCONI_ANEMIA_PATHV

ACTOME_SIGNALING_BY_GPCR;REACTOME_AXON_GUIDANCE;REACTOME_G_ALPHA1213_S

ACTOME_SIGNALING_BY_GPCR;REACTOME_AXON_GUIDANCE;REACTOME_G_ALPHA1213_S

EACTOME_TRANSLOCATION_OF_ZAP_70_TO_IMMUNOLOGICAL_SYNAPSE;REACTOME_GEN

TOME_TRANSPORT_OF_VITAMINS_NUCLEOSIDES_AND_RELATED_MOLECULES;REACTOME

N_ACROSS_CHEMICAL_SYNAPSES;REACTOME_NEURONAL_SYSTEM;REACTOME_SIGNALIN

ABOLISM_OF_PROTEINS

ESOLUTION_OF_AP_SITES_VIA_THE_SINGLE_NUCLEOTIDE_REPLACEMENT_PATHWAY

_DIFFERENTIATION

C_NER;REACTOME_SYNTHESIS_OF_DNA;REACTOME_DNA_REPAIR;REACTOME_CHROMOSC

:AM_SIGNALING_FOR_NEURITE_OUT_GROWTH

LE;REACTOME_LATE_PHASE_OF_HIV_LIFE_CYCLE

TRANSPORT;REACTOME_TRANSPORT_OF_INORGANIC_CATIONS_ANIONS_AND_AMINO_ACI

PCR_LIGAND_BINDING

ET_ACTIVATION_SIGNALING_AND_AGGREGATION

LING;REACTOME_SIGNALING_BY_ERBB2;REACTOME_SIGNALING_BY_EGFR_IN_CANCER;RE

C_CLEAVAGE_OF_NOTCH_RECEPTOR;REACTOME_SIGNALING_BY_NOTCH

GHLY_CALCIUM_PERMEABLE_POSTSYNAPTIC_NICOTINIC_ACETYLCHOLINE_RECEPTORS

NEUROTRANSMITTER_RECEPTOR_BINDING_AND_DOWNSTREAM_TRANSMISSION_IN_THE_

EACTOME_POST_TRANSLATIONAL_PROTEIN_MODIFICATION;REACTOME_SYNTHESIS_OF_(

OID_HORMONES;REACTOME_METABOLISM_OF_LIPIDS_AND_LIPOPROTEINS

ME_G_ALPHA1213_SIGNALLING_EVENTS;REACTOME_GPCR_DOWNSTREAM_SIGNALING

_SLC_TRANSPORTERS

EACTOME_METABOLISM_OF_RNA;REACTOME_INFLUENZA_LIFE_CYCLE;REACTOME_INFLUE

P1_AND_WWTR1_TAZ_STIMULATED_GENE_EXPRESSION;REACTOME_PRE_NOTCH_EXPRES
P1_AND_WWTR1_TAZ_STIMULATED_GENE_EXPRESSION;REACTOME_PRE_NOTCH_EXPRES
P1_AND_WWTR1_TAZ_STIMULATED_GENE_EXPRESSION;REACTOME_PRE_NOTCH_EXPRES
N_ACROSS_CHEMICAL_SYNAPSES;REACTOME_NEURONAL_SYSTEM;REACTOME_SIGNALIN

_TYPE_ATPASES;REACTOME_ION_CHANNEL_TRANSPORT;REACTOME_SIGNALING_BY_NOT[illegible]

[illegible]RT_OF_SMALL_MOLECULES;REACTOME_MRNA_PROCESSING;REACTOME_TRANSPORT_OF_[illegible]

RT_OF_SMALL_MOLECULES;REACTOME_MRNA_PROCESSING;REACTOME_TRANSPORT_OF_

N_PLUG_FORMATION;REACTOME_HEMOSTASIS;REACTOME_PLATELET_ACTIVATION_SIGN

MUNE_SYSTEM;REACTOME_TOLL_RECEPTOR_CASCADES;REACTOME_ADAPTIVE_IMMUNE_

POSTSYNAPTIC_EVENTS;REACTOME_POST_NMDA_RECEPTOR_ACTIVATION_EVENTS

GANIC_ANION_SLC_TRANSPORTERS

NA_POL_II_TRANSCRIPTION_PRE_INITIATION_AND_PROMOTER_OPENING;REACTOME_MRN

NALLING_THROUGH_PI3KGAMMA;REACTOME_G_PROTEIN_BETA_GAMMA_SIGNALLING;REA

N;REACTOME_NOTCH1_INTRACELLULAR_DOMAIN_REGULATES_TRANSCRIPTION;REACTOM

N;REACTOME_NOTCH1_INTRACELLULAR_DOMAIN_REGULATES_TRANSCRIPTION;REACTOM

MAP_KINASES_ACTIVATION_MEDIATED_BY_TLR4_SIGNALING_REPERTOIRE;REACTOME_MY

GANIC_ANION_SLC_TRANSPORTERS

GANIC_ANION_SLC_TRANSPORTERS

M_THE_PLASMA_MEMBRANE;REACTOME_MHC_CLASS_II_ANTIGEN_PRESENTATION;REACT

EACTOME_METABOLISM_OF_RNA;REACTOME_INFLUENZA_LIFE_CYCLE;REACTOME_INFLUE

ORIN_SIGNALING;REACTOME_SEMAPHORIN_INTERACTIONS;REACTOME_SEMA4D_INDUCED
ME_GPCR_LIGAND_BINDING

TOME_SIGNALING_BY_FGFR1_FUSION_MUTANTS;REACTOME_SIGNALING_BY_FGFR_MUTAN
TOME_SIGNALING_BY_FGFR1_FUSION_MUTANTS;REACTOME_SIGNALING_BY_FGFR_MUTAN
TOME_SIGNALING_BY_FGFR1_FUSION_MUTANTS;REACTOME_SIGNALING_BY_FGFR_MUTAN

UR2_CONTAINING_AMPA_RECEPTORS

E_INTERACTIONS;REACTOME_SIGNALING_BY_PDGF;REACTOME_GPVI_MEDIATED_ACTIVAT

ME_IMMUNE_SYSTEM;REACTOME_CYTOKINE_SIGNALING_IN_IMMUNE_SYSTEM

IATED_SIGNALLING;REACTOME_PI3K_CASCADE

_LIPOPROTEINS;REACTOME_FATTY_ACID_TRIACYLGLYCEROL_AND_KETONE_BODY_METAB
_LIPOPROTEINS;REACTOME_FATTY_ACID_TRIACYLGLYCEROL_AND_KETONE_BODY_METAB

ACTOME_G_ALPHA_S_SIGNALLING_EVENTS;REACTOME_GPCR_LIGAND_BINDING

S;REACTOME_PYRUVATE_METABOLISM;REACTOME_HEMOSTASIS

A_POL_III_TRANSCRIPTION;REACTOME_MRNA_CAPPING;REACTOME_TRANSCRIPTION_COU

E_SIGNALS_DEATH_THROUGH_JNK;REACTOME_CELL_DEATH_SIGNALLING_VIA_NRAGE_NI
REACTOME_P75_NTR_RECEPTOR_MEDIATED_SIGNALLING;REACTOME_SIGNALING_BY_GP

IE_IMMUNE_SYSTEM;REACTOME_CYTOKINE_SIGNALING_IN_IMMUNE_SYSTEM

ACTOME_GPCR_DOWNSTREAM_SIGNALING;REACTOME_G_ALPHA_S_SIGNALLING_EVENTS
MUNE_SYSTEM;REACTOME_CYTOKINE_SIGNALING_IN_IMMUNE_SYSTEM

REACTOME_SIGNALING_BY_GPCR;REACTOME_INTEGRATION_OF_ENERGY_METABOLISM;R

ISE_TO_ELEVATED_PLATELET_CYTOSOLIC_CA2_;REACTOME_TRANSCRIPTIONAL_REGULA

OMEOSTASIS;REACTOME_HEMOSTASIS

N_COUPLED_NER_TC_NER;REACTOME_POL_SWITCHING;REACTOME_NUCLEOTIDE_EXCISI
N_COUPLED_NER_TC_NER;REACTOME_POL_SWITCHING;REACTOME_NUCLEOTIDE_EXCISI
N_COUPLED_NER_TC_NER;REACTOME_POL_SWITCHING;REACTOME_NUCLEOTIDE_EXCISI

_EGFR_IN_CANCER;REACTOME_ANTIGEN_ACTIVATES_B_CELL_RECEPTOR_LEADING_TO_(

GANIC_ANION_SLC_TRANSPORTERS

TIDE1;REACTOME_REGULATION_OF_INSULIN_SECRETION;REACTOME_CLASS_B_2_SECRET
TIDE1;REACTOME_REGULATION_OF_INSULIN_SECRETION;REACTOME_CLASS_B_2_SECRET

LIGAND_BINDING_RECEPTORS;REACTOME_CLASS_A1_RHODOPSIN_LIKE_RECEPTORS;REA

ME_G_ALPHA1213_SIGNALLING_EVENTS;REACTOME_GPCR_DOWNSTREAM_SIGNALING

D_PROCESSING;REACTOME_CLEAVAGE_OF_GROWING_TRANSCRIPT_IN_THE_TERMINATIO

,ADAPTIVE_IMMUNE_SYSTEM

;;REACTOME_G_ALPHA_S_SIGNALLING_EVENTS;REACTOME_GPCR_LIGAND_BINDING

_OF_DNA;REACTOME_MITOTIC_G1_G1_S_PHASES;REACTOME_MITOTIC_M_M_G1_PHASES;

_SUBUNITS;REACTOME_GABA_B_RECEPTOR_ACTIVATION;REACTOME_GABA_RECEPTOR_A
_SUBUNITS;REACTOME_GABA_B_RECEPTOR_ACTIVATION;REACTOME_GABA_RECEPTOR_A

NALING;REACTOME_G_ALPHA_I_SIGNALLING_EVENTS;REACTOME_GPCR_LIGAND_BINDING
PROMOTER;REACTOME_RNA_POL_III_TRANSCRIPTION_TERMINATION;REACTOME_RNA_POI
PROMOTER;REACTOME_RNA_POL_III_TRANSCRIPTION_TERMINATION;REACTOME_RNA_POI

SPHINGOLIPID_METABOLISM;REACTOME_POST_TRANSLATIONAL_PROTEIN_MODIFICATION;

_APOPTOSIS;REACTOME_APOPTOSIS

REACTOME_DNA_REPLICATION;REACTOME_MITOTIC_PROMETAPHASE;REACTOME_MEIOTIC

ME_G_ALPHA1213_SIGNALLING_EVENTS;REACTOME_GPCR_DOWNSTREAM_SIGNALING

CRIPTIONAL_REGULATION_OF_WHITE_ADIPOCYTE_DIFFERENTIATION

IKER_SEQUENCE_IS_REQUIRED_FOR_GAG_SYNTHESIS;REACTOME_METABOLISM_OF_CAR

CCHARIDE_LINKER_SEQUENCE_IS_REQUIRED_FOR_GAG_SYNTHESIS;REACTOME_METABC

JCTION_BY_L1;REACTOME_RECYCLING_PATHWAY_OF_L1;REACTOME_HEMOSTASIS
JCTION_BY_L1;REACTOME_RECYCLING_PATHWAY_OF_L1;REACTOME_HEMOSTASIS

IA_REPLICATION;REACTOME_MITOTIC_PROMETAPHASE

C_CLEAVAGE_OF_NOTCH_RECEPTOR;REACTOME_SIGNALING_BY_NOTCH

.ICATION;REACTOME_HEMOSTASIS;REACTOME_IMMUNE_SYSTEM;REACTOME_ADAPTIVE_IN

'RANSPORT

RS;REACTOME_METAL_ION_SLC_TRANSPORTERS

_PI3K_EVENTS_IN_ERBB4_SIGNALING;REACTOME_PI3K_EVENTS_IN_ERBB2_SIGNALING;RE

NALING;REACTOME_GPCR_LIGAND_BINDING;REACTOME_THROMBIN_SIGNALLING_THROUG

LATION_OF_CELL_SURFACE_RECEPTORS_BY_RECRUITING_THEM_TO_CLATHRIN_ADAPTE

_ING;REACTOME_SIGNALING_BY_ERBB2;REACTOME_SIGNALING_BY_EGFR_IN_CANCER;RE

;PHINGOLIPID_METABOLISM;REACTOME_POST_TRANSLATIONAL_PROTEIN_MODIFICATION;

ION;REACTOME_KINESINS;REACTOME_HEMOSTASIS;REACTOME_IMMUNE_SYSTEM;REACT

ABA_A_RECEPTOR_ACTIVATION;REACTOME_GABA_RECEPTOR_ACTIVATION;REACTOME_IC
CTOME_TAK1_ACTIVATES_NFKB_BY_PHOSPHORYLATION_AND_ACTIVATION_OF_IKKS_COM

TEM;REACTOME_CYTOKINE_SIGNALING_IN_IMMUNE_SYSTEM;REACTOME_CLASS_I_MHC_M

NS

_DIFFERENTIATION

IDES_LINKAGE_TO_MAPK_SIGNALING_FOR_INTERGRINS_;REACTOME_INTEGRIN_ALPHAIIB_
CTOME_IMMUNE_SYSTEM

REACTOME_PI_3K_CASCADE;REACTOME_DOWNSTREAM_SIGNALING_OF_ACTIVATED_FGF

ACTOME_SIGNALING_BY_GPCR;REACTOME_AXON_GUIDANCE;REACTOME_G_ALPHA1213_S

_S_PHASES;REACTOME_INHIBITION_OF_REPLICATION_INITIATION_OF_DAMAGED_DNA_BY_

CTOME_TAK1_ACTIVATES_NFKB_BY_PHOSPHORYLATION_AND_ACTIVATION_OF_IKKS_COM

ACTOME_SIGNALING_BY_GPCR;REACTOME_AXON_GUIDANCE;REACTOME_G_ALPHA1213_S

PLEX_AND_EIFS_AND_SUBSEQUENT_BINDING_TO_43S;REACTOME_PEPTIDE_CHAIN_ELONG

G;REACTOME_INTERFERON_ALPHA_BETA_SIGNALING;REACTOME_INTERFERON_SIGNALING

DOWNSTREAM_SIGNALING;REACTOME_G_ALPHA_I_SIGNALLING_EVENTS;REACTOME_GPCR

LET_HOMEOSTASIS;REACTOME_PLATELET_SENSITIZATION_BY_LDL;REACTOME_DNA_REPL

ATION

UR2_CONTAINING_AMPA_RECEPTORS

NSMISSION_IN_THE_POSTSYNAPTIC_CELL;REACTOME_PLC_BETA_MEDIATED_EVENTS;REA

TOTIC_CELL_CYCLE;REACTOME_APC_C_CDH1_MEDIATED_DEGRADATION_OF_CDC20_AND

REACTOME_IL_RECEPTOR_SHC_SIGNALING;REACTOME_FACTORS_INVOLVED_IN_MEGAKAR

IFKB;REACTOME_NRIF_SIGNALS_CELL_DEATH_FROM_THE_NUCLEUS;REACTOME_NFKB_IS

BOLISM

IKER_SEQUENCE_IS_REQUIRED_FOR_GAG_SYNTHESIS;REACTOME_METABOLISM_OF_CAR

ATED_VESICLE_BIOGENESIS;REACTOME_METABOLISM_OF_LIPIDS_AND_LIPOPROTEINS

HT_JUNCTION_INTERACTIONS;REACTOME_CELL_JUNCTION_ORGANIZATION

S;REACTOME_AMINO_ACID_AND_OLIGOPEPTIDE_SLC_TRANSPORTERS

_PATHWAY;REACTOME_RECEPTOR_LIGAND_BINDING_INITIATES_THE_SECOND_PROTEOLY

ACTOME_MITOTIC_G1_G1_S_PHASES;REACTOME_E2F_MEDIATED_REGULATION_OF_DNA_

ABA_A_RECEPTOR_ACTIVATION;REACTOME_GABA_RECEPTOR_ACTIVATION;REACTOME_IC

)_OTHER_SUGARS_BILE_SALTS_AND_ORGANIC_ACIDS_METAL_IONS_AND_AMINE_COMPOU

OL_I_RNA_POL_III_AND_MITOCHONDRIAL_TRANSCRIPTION;REACTOME_RNA_POL_II_PRE_T

C_COUPLING_AND_HEAT_PRODUCTION_BY_UNCOUPLING_PROTEINS_
ECRETION_BY_ACETYLCHOLINE;REACTOME_ADP_SIGNALLING_THROUGH_P2RY1;REACTOM

_LIPID_LINKED_OLIGOSACCHARIDE_LLO_AND_TRANSFER_TO_A_NASCENT_PROTEIN

A_FROM_THE_PLASMA_MEMBRANE;REACTOME_MHC_CLASS_II_ANTIGEN_PRESENTATION;F

EACTOME_IONOTROPIC_ACTIVITY_OF_KAINATE_RECEPTORS

M;REACTOME_A_TETRASACCHARIDE_LINKER_SEQUENCE_IS_REQUIRED_FOR_GAG_SYNTH

G;REACTOME_INTERFERON_ALPHA_BETA_SIGNALING;REACTOME_INTERFERON_SIGNALIN

'ATION_SIGNALING_AND_AGGREGATION
'ATION_SIGNALING_AND_AGGREGATION

_LIPIDS_AND_LIPOPROTEINS

31_DNA_DAMAGE_RESPONSE;REACTOME_REGULATION_OF_THE_FANCONI_ANEMIA_PATHW

E_SIGNALING_BY_EGFR_IN_CANCER;REACTOME_SHC1_EVENTS_IN_ERBB4_SIGNALING;RE

_CHANNELS

ABA_A_RECEPTOR_ACTIVATION;REACTOME_GABA_RECEPTOR_ACTIVATION;REACTOME_IC

MUNE_SYSTEM;REACTOME_TOLL_RECEPTOR_CASCADES;REACTOME_ADAPTIVE_IMMUNE_

TION

SPHINGOLIPID_METABOLISM;REACTOME_POST_TRANSLATIONAL_PROTEIN_MODIFICATION;

RT_OF_SMALL_MOLECULES;REACTOME_MRNA_PROCESSING;REACTOME_TRANSPORT_OF_

D_RECYCLING;REACTOME_SIGNALING_BY_INSULIN_RECEPTOR

GNALING_BY_TGF_BETA_RECEPTOR_COMPLEX

CAM_SIGNALING_FOR_NEURITE_OUT_GROWTH

_DIFFERENTIATION

GHLY_CALCIUM_PERMEABLE_POSTSYNAPTIC_NICOTINIC_ACETYLCHOLINE_RECEPTORS

IATED_SIGNALLING;REACTOME_PI3K_CASCADE
RANSPORT

ROL_AND_KETONE_BODY_METABOLISM

ME_G_ALPHA1213_SIGNALLING_EVENTS;REACTOME_GPCR_DOWNSTREAM_SIGNALING

NG_BY_SCF_KIT;REACTOME_DEVELOPMENTAL_BIOLOGY;REACTOME_SIGNALING_BY_ERBE

EACTOME_METABOLISM_OF_RNA;REACTOME_INFLUENZA_LIFE_CYCLE;REACTOME_INFLUE

_PI3K_EVENTS_IN_ERBB4_SIGNALING;REACTOME_PI3K_EVENTS_IN_ERBB2_SIGNALING;RE

ANTS;REACTOME_FRS2_MEDIATED_CASCADE;REACTOME_PI_3K_CASCADE;REACTOME_DC

TS;REACTOME_GPCR_LIGAND_BINDING
NALLING;REACTOME_SIGNALING_BY_GPCR;REACTOME_G_ALPHA1213_SIGNALLING_EVEN

DIATED_RESISTANCE_TO_HIV1_INFECTION

(POSED_COLLAGEN;REACTOME_BASIGIN_INTERACTIONS;REACTOME_INTEGRIN_CELL_SUI

ELS;REACTOME_INWARDLY_RECTIFYING_K_CHANNELS

TOTIC_CELL_CYCLE;REACTOME_MITOTIC_M_M_G1_PHASES;REACTOME_DNA_REPLICATIOI

_SLC_TRANSPORTERS

NALING;REACTOME_GPCR_LIGAND_BINDING

NEUROTRANSMITTER_RECEPTOR_BINDING_AND_DOWNSTREAM_TRANSMISSION_IN_THE_

CTOME_TAK1_ACTIVATES_NFKB_BY_PHOSPHORYLATION_AND_ACTIVATION_OF_IKKS_COM

KERATIN_METABOLISM;REACTOME_GLYCOSAMINOGLYCAN_METABOLISM;REACTOME_MET

NALING;REACTOME_INTERFERON_ALPHA_BETA_SIGNALING;REACTOME_REGULATION_OF

ME_G_ALPHA1213_SIGNALLING_EVENTS;REACTOME_GPCR_DOWNSTREAM_SIGNALING

ME_P75_NTR_RECEPTOR_MEDIATED_SIGNALLING;REACTOME_SIGNALING_BY_GPCR;REAC

HESIS_OF_DNA;REACTOME_MITOTIC_G1_G1_S_PHASES;REACTOME_MITOTIC_M_M_G1_PH

SION

:ELL_RECEPTOR_BCR;REACTOME_NGF_SIGNALLING_VIA_TRKA_FROM_THE_PLASMA_MEM

EINS;REACTOME_3_UTR_MEDIATED_TRANSLATIONAL_REGULATION

:RIPTION;REACTOME_NOTCH_HLH_TRANSCRIPTION_PATHWAY;REACTOME_TRANSCRIPTIC

_MATURE_MRNA_DERIVED_FROM_AN_INTRONLESS_TRANSCRIPT;REACTOME_SLC_MEDIAT

IG_BY_GPCR;REACTOME_OPIOID_SIGNALLING;REACTOME_CA_DEPENDENT_EVENTS;REAC
_SIGNALING_BY_FGFR_IN_DISEASE;REACTOME_NUCLEAR_EVENTS_KINASE_AND_TRANSCI

_ACTIVATED_AND_SIGNALS_SURVIVAL;REACTOME_CELL_DEATH_SIGNALLING_VIA_NRAGE

TIDES;REACTOME_AMINO_ACID_AND_OLIGOPEPTIDE_SLC_TRANSPORTERS

_LING;REACTOME_CA_DEPENDENT_EVENTS;REACTOME_DARPP_32_EVENTS;REACTOME_F

GE_OF_GROWING_TRANSCRIPT_IN_THE_TERMINATION_REGION_

_THE_B_CELL_RECEPTOR_BCR;REACTOME_INSULIN_RECEPTOR_SIGNALLING_CASCADE;R
_THE_B_CELL_RECEPTOR_BCR;REACTOME_INSULIN_RECEPTOR_SIGNALLING_CASCADE;R

NTS;REACTOME_CDC6_ASSOCIATION_WITH_THE_ORC_ORIGIN_COMPLEX;REACTOME_M_G

ANSCRIPTION_PATHWAY;REACTOME_METABOLISM_OF_LIPIDS_AND_LIPOPROTEINS;REAC

ACTOME_INSULIN_RECEPTOR_SIGNALLING_CASCADE;REACTOME_CELL_SURFACE_INTER

ETABOLISM_OF_LIPIDS_AND_LIPOPROTEINS;REACTOME_FATTY_ACID_TRIACYLGLYCEROL_
_TLR7_8_OR_9_ACTIVATION;REACTOME_ADVANCED_GLYCOSYLATION_ENDPRODUCT_REC

\S_ANIONS_AND_AMINO_ACIDS_OLIGOPEPTIDES;REACTOME_AMINO_ACID_AND_OLIGOPEF

ROTEINS;REACTOME_HORMONE_SENSITIVE_LIPASE_HSL_MEDIATED_TRIACYLGLYCEROL_

ACTOME_INSULIN_RECEPTOR_SIGNALLING_CASCADE;REACTOME_CELL_SURFACE_INTER/
ACTOME_INSULIN_RECEPTOR_SIGNALLING_CASCADE;REACTOME_CELL_SURFACE_INTER/

G;REACTOME_IMMUNE_SYSTEM;REACTOME_ADAPTIVE_IMMUNE_SYSTEM;REACTOME_CYT

E_REGULATION_OF_GLUCOKINASE_BY_GLUCOKINASE_REGULATORY_PROTEIN

_CELL;REACTOME_PLC_BETA_MEDIATED_EVENTS;REACTOME_G_PROTEIN_ACTIVATION;R

_OF_INSULIN_SECRETION_BY_ADRENALINE_NORADRENALINE

ACTOME_DOWNSTREAM_SIGNALING_EVENTS_OF_B_CELL_RECEPTOR_BCR;REACTOME_S
IBRANE;REACTOME_SIGNALING_BY_FGFR_IN_DISEASE;REACTOME_GASTRIN_CREB_SIGNA

TOME_GPCR_DOWNSTREAM_SIGNALING;REACTOME_G_ALPHA_S_SIGNALLING_EVENTS;RE
BOLISM

BY_KSRP

LIZATION_OF_MRNA_BY_BRF1;REACTOME_REGULATION_OF_MRNA_STABILITY_BY_PROTEI

AL_SYNAPSES;REACTOME_NEURONAL_SYSTEM;REACTOME_SIGNALING_BY_GPCR;REACT

REACTOME_ASSEMBLY_OF_THE_PRE_REPLICATIVE_COMPLEX;REACTOME_DNA_REPLICAT

[illegible]MEDIATED_ANTIGEN_PROCESSING_PRESENTATION

CHECKPOINTS;REACTOME_G2_M_DNA_DAMAGE_CHECKPOINT
G;REACTOME_HEMOSTASIS;REACTOME_PLATELET_ACTIVATION_SIGNALING_AND_AGGREG

RANSPORT;REACTOME_TRANSPORT_OF_GLUCOSE_AND_OTHER_SUGARS_BILE_SALTS_AN

OME_MAINTENANCE;REACTOME_GLOBAL_GENOMIC_NER_GG_NER;REACTOME_LAGGING_S

CEROL_AND_KETONE_BODY_METABOLISM;REACTOME_CIRCADIAN_CLOCK;REACTOME_TR

OTIDE_EXCISION_REPAIR;REACTOME_FORMATION_OF_RNA_POL_II_ELONGATION_COMPLE

RADATION;REACTOME_GLYCOSAMINOGLYCAN_METABOLISM;REACTOME_SPHINGOLIPID_M
RADATION;REACTOME_GLYCOSAMINOGLYCAN_METABOLISM;REACTOME_SPHINGOLIPID_M

ME_MEIOTIC_RECOMBINATION;REACTOME_MEIOTIC_SYNAPSIS;REACTOME_AMYLOIDS;REA

REACTOME_TRANSFERRIN_ENDOCYTOSIS_AND_RECYCLING;REACTOME_SIGNALING_BY_IN

JCTION_OF_IFN_ALPHA_BETA_PATHWAYS;REACTOME_HEMOSTASIS;REACTOME_INNATE_II

GNALING_BY_CBL;REACTOME_IL_3_5_AND_GM_CSF_SIGNALING;REACTOME_IMMUNE_SYS

ALING_IN_IMMUNE_SYSTEM

.ISM_OF_LIPIDS_AND_LIPOPROTEINS

R_CASCADES;REACTOME_ADAPTIVE_IMMUNE_SYSTEM

JLIN_RECEPTOR_SIGNALLING_CASCADE;REACTOME_ARMS_MEDIATED_ACTIVATION;REAC

EACTOME_PRESYNAPTIC_NICOTINIC_ACETYLCHOLINE_RECEPTORS

SCADE;REACTOME_MAPK_TARGETS_NUCLEAR_EVENTS_MEDIATED_BY_MAP_KINASES;RE

L_III_CHAIN_ELONGATION

DIFFERENTIATION

ANSCRIPTION_PATHWAY;REACTOME_NUCLEAR_RECEPTOR_TRANSCRIPTION_PATHWAY;R

ISPORTERS

_POSTSYNAPTIC_CELL;REACTOME_PLC_BETA_MEDIATED_EVENTS;REACTOME_G_ALPHA_Q

ANSCRIPTION_PATHWAY;REACTOME_METABOLISM_OF_LIPIDS_AND_LIPOPROTEINS;REACT
ANSCRIPTION_PATHWAY;REACTOME_METABOLISM_OF_LIPIDS_AND_LIPOPROTEINS;REACT

N_REGULATES_TRANSCRIPTION;REACTOME_CIRCADIAN_REPRESSION_OF_EXPRESSION_E

OME_GPCR_DOWNSTREAM_SIGNALING;REACTOME_CELL_EXTRACELLULAR_MATRIX_INTEF

_OTHER_APC_C_CDH1_TARGETED_PROTEINS_IN_LATE_MITOSIS_EARLY_G1;REACTOME_A

_THE_B_CELL_RECEPTOR_BCR;REACTOME_NGF_SIGNALLING_VIA_TRKA_FROM_THE_PLAS

)_LIPOPROTEINS

ANSCRIPTION_PATHWAY;REACTOME_METABOLISM_OF_LIPIDS_AND_LIPOPROTEINS;REAC

KINE_SIGNALING_IN_IMMUNE_SYSTEM

_THE_B_CELL_RECEPTOR_BCR;REACTOME_NGF_SIGNALLING_VIA_TRKA_FROM_THE_PLAS

DS_AND_LIPOPROTEINS;REACTOME_METABOLISM_OF_CARBOHYDRATES;REACTOME_LIPII
DS_AND_LIPOPROTEINS;REACTOME_METABOLISM_OF_CARBOHYDRATES;REACTOME_LIPII

EACTOME_TRANSMISSION_ACROSS_CHEMICAL_SYNAPSES;REACTOME_NEURONAL_SYSTE

TABOLISM_OF_CARBOHYDRATES
TABOLISM_OF_CARBOHYDRATES

GNALING;REACTOME_PHOSPHORYLATION_OF_CD3_AND_TCR_ZETA_CHAINS;REACTOME_T

;REACTOME_METABOLISM_OF_LIPIDS_AND_LIPOPROTEINS;REACTOME_FATTY_ACID_TRIA

_IFNA_SIGNALING;REACTOME_INTERFERON_SIGNALING;REACTOME_IL_2_SIGNALING;REA

_GROWTH_CONE_COLLAPSE

'TIC_CLEAVAGE_OF_NOTCH_RECEPTOR;REACTOME_SIGNALING_BY_NOTCH
'TIC_CLEAVAGE_OF_NOTCH_RECEPTOR;REACTOME_SIGNALING_BY_NOTCH

L_III_CHAIN_ELONGATION

SIGNALLING_EVENTS;REACTOME_GPCR_DOWNSTREAM_SIGNALING;REACTOME_SEMA4D_I

_MATURE_MRNA_DERIVED_FROM_AN_INTRONLESS_TRANSCRIPT;REACTOME_SLC_MEDIAT

EACTOME_DOWNSTREAM_SIGNALING_EVENTS_OF_B_CELL_RECEPTOR_BCR;REACTOME_S

ME_P75_NTR_RECEPTOR_MEDIATED_SIGNALLING;REACTOME_RECEPTOR_LIGAND_BINDIN

ANSCRIPTION_PATHWAY;REACTOME_METABOLISM_OF_LIPIDS_AND_LIPOPROTEINS;REACT

STEM;REACTOME_TOLL_RECEPTOR_CASCADES;REACTOME_CYTOKINE_SIGNALING_IN_IMM

CLOT_CLOTTING_CASCADE;REACTOME_POST_TRANSLATIONAL_PROTEIN_MODIFICATION;F

:TOME_HEMOSTASIS;REACTOME_IMMUNE_SYSTEM;REACTOME_ADAPTIVE_IMMUNE_SYSTE

EACTOME_PRESYNAPTIC_NICOTINIC_ACETYLCHOLINE_RECEPTORS

MOSTASIS;REACTOME_IMMUNE_SYSTEM;REACTOME_ADAPTIVE_IMMUNE_SYSTEM;REACTC

CTOME_DOWNSTREAM_SIGNAL_TRANSDUCTION;REACTOME_SIGNALING_BY_ILS;REACTOM

LC_TRANSPORTERS

OME_IMMUNE_SYSTEM;REACTOME_CYTOKINE_SIGNALING_IN_IMMUNE_SYSTEM;REACTOM

ND_AMINE_COMPOUNDS;REACTOME_NA_CL_DEPENDENT_NEUROTRANSMITTER_TRANSPO

TOME_ARMS_MEDIATED_ACTIVATION;REACTOME_PROLONGED_ERK_ACTIVATION_EVENTS;

CHECKPOINTS;REACTOME_G2_M_DNA_DAMAGE_CHECKPOINT

I_PROTEASOME_DEGRADATION
I_PROTEASOME_DEGRADATION

ON_CHANNEL_TRANSPORT;REACTOME_LIGAND_GATED_ION_CHANNEL_TRANSPORT

LLING_BY_GPCR;REACTOME_AXON_GUIDANCE;REACTOME_G_ALPHA_Q_SIGNALLING_EVEN
LLING_BY_GPCR;REACTOME_AXON_GUIDANCE;REACTOME_G_ALPHA_Q_SIGNALLING_EVEN

NALING_BY_INSULIN_RECEPTOR;REACTOME_PI3K_CASCADE

TS_IN_ERBB2_SIGNALING;REACTOME_ANTIGEN_ACTIVATES_B_CELL_RECEPTOR_LEADING

[illegible]LISM_OF_CARBOHYDRATES

[illegible]REACTOME_METABOLISM_OF_LIPIDS_AND_LIPOPROTEINS

DE;REACTOME_SHC_MEDIATED_CASCADE;REACTOME_SIGNALING_BY_INSULIN_RECEPTOR
DE;REACTOME_SHC_MEDIATED_CASCADE;REACTOME_SIGNALING_BY_INSULIN_RECEPTOR
DE;REACTOME_SHC_MEDIATED_CASCADE;REACTOME_SIGNALING_BY_INSULIN_RECEPTOR

ENZA_VIRAL_RNA_TRANSCRIPTION_AND_REPLICATION;REACTOME_NONSENSE_MEDIATED_

BRANE;REACTOME_SIGNALING_BY_FGFR_IN_DISEASE;REACTOME_GASTRIN_CREB_SIGNA

ENZA_VIRAL_RNA_TRANSCRIPTION_AND_REPLICATION;REACTOME_NONSENSE_MEDIATED_

TOME_SIGNALING_BY_ILS;REACTOME_IL1_SIGNALING;REACTOME_TRAF6_MEDIATED_INDU

IG_BY_GPCR;REACTOME_INTEGRATION_OF_ENERGY_METABOLISM;REACTOME_OPIOID_SI

TION_CASCADE;REACTOME_AXON_GUIDANCE;REACTOME_NCAM1_INTERACTIONS;REACTC

ACTIVATION_OF_NF_KAPPAB_IN_B_CELLS;REACTOME_SIGNALING_BY_THE_B_CELL_RECE[illegible]
[illegible]NTS;REACTOME_CDC6_ASSOCIATION_WITH_THE_ORC_ORIGIN_COMPLEX;REACTOME_M_[illegible]

[illegible];REACTOME_METABOLISM_OF_LIPIDS_AND_LIPOPROTEINS;REACTOME_FATTY_ACID_TRIA[illegible]
[illegible];REACTOME_METABOLISM_OF_LIPIDS_AND_LIPOPROTEINS;REACTOME_FATTY_ACID_TRIA[illegible]

:E;REACTOME_NCAM_SIGNALING_FOR_NEURITE_OUT_GROWTH;REACTOME_FRS2_MEDIAT
:E;REACTOME_NCAM_SIGNALING_FOR_NEURITE_OUT_GROWTH;REACTOME_FRS2_MEDIAT

NS_ANIONS_AND_AMINO_ACIDS_OLIGOPEPTIDES;REACTOME_AMINO_ACID_AND_OLIGOPEF

ENTS

_THE_B_CELL_RECEPTOR_BCR;REACTOME_NGF_SIGNALLING_VIA_TRKA_FROM_THE_PLAS

E_CYCLE;REACTOME_NOREPINEPHRINE_NEUROTRANSMITTER_RELEASE_CYCLE;REACTOM

_AND_ANKYRINS;REACTOME_NEPHRIN_INTERACTIONS;REACTOME_APOPTOSIS;REACTOME
_AND_ANKYRINS;REACTOME_NEPHRIN_INTERACTIONS;REACTOME_APOPTOSIS;REACTOME
_AND_ANKYRINS;REACTOME_NEPHRIN_INTERACTIONS;REACTOME_APOPTOSIS;REACTOME
_AND_ANKYRINS;REACTOME_NEPHRIN_INTERACTIONS;REACTOME_APOPTOSIS;REACTOME

GNALING_BY_CBL;REACTOME_IL_3_5_AND_GM_CSF_SIGNALING;REACTOME_IMMUNE_SYST

ALING_AND_AGGREGATION

PROTEINS

E_SIGNALING_IN_IMMUNE_SYSTEM

ACTIVATION_OF_NF_KAPPAB_IN_B_CELLS;REACTOME_SIGNALING_BY_THE_B_CELL_RECE

OME_RETROGRADE_NEUROTROPHIN_SIGNALLING;REACTOME_EGFR_DOWNREGULATION;

M;REACTOME_CIRCADIAN_CLOCK

RECEPTOR;REACTOME_HEMOSTASIS;REACTOME_PI3K_CASCADE

STREAM_SIGNALING;REACTOME_G_ALPHA_I_SIGNALLING_EVENTS;REACTOME_TAK1_ACT

TERS

_THE_B_CELL_RECEPTOR_BCR;REACTOME_NGF_SIGNALLING_VIA_TRKA_FROM_THE_PLAS

ASES;REACTOME_CHROMOSOME_MAINTENANCE;REACTOME_LAGGING_STRAND_SYNTHES

REACTOME_LIPID_DIGESTION_MOBILIZATION_AND_TRANSPORT;REACTOME_SIGNALING_BY

ON_OF_APOBEC3G

WAY;REACTOME_FANCONI_ANEMIA_PATHWAY;REACTOME_DOUBLE_STRAND_BREAK_REP

SIGNALLING_EVENTS;REACTOME_GPCR_DOWNSTREAM_SIGNALING;REACTOME_SEMA4D_I

SIGNALLING_EVENTS;REACTOME_GPCR_DOWNSTREAM_SIGNALING;REACTOME_SEMA4D_I

ERATION_OF_SECOND_MESSENGER_MOLECULES;REACTOME_PD1_SIGNALING;REACTOME

E_TRANSPORT_OF_ORGANIC_ANIONS

G_BY_GPCR;REACTOME_INTEGRATION_OF_ENERGY_METABOLISM;REACTOME_OPIOID_SI

OME_MAINTENANCE;REACTOME_GLOBAL_GENOMIC_NER_GG_NER;REACTOME_LAGGING_S

DS_OLIGOPEPTIDES;REACTOME_AMINO_ACID_AND_OLIGOPEPTIDE_SLC_TRANSPORTERS;

ACTOME_PI3K_EVENTS_IN_ERBB4_SIGNALING;REACTOME_PI3K_EVENTS_IN_ERBB2_SIGN[illegible]

POSTSYNAPTIC_CELL;REACTOME_PLC_BETA_MEDIATED_EVENTS;REACTOME_G_ALPHA_[illegible]

GLYCOSYLPHOSPHATIDYLINOSITOL_GPI

ENZA_VIRAL_RNA_TRANSCRIPTION_AND_REPLICATION;REACTOME_NONSENSE_MEDIATED_

SSION_AND_PROCESSING;REACTOME_NOTCH1_INTRACELLULAR_DOMAIN_REGULATES_TR
SSION_AND_PROCESSING;REACTOME_NOTCH1_INTRACELLULAR_DOMAIN_REGULATES_TR
SSION_AND_PROCESSING;REACTOME_NOTCH1_INTRACELLULAR_DOMAIN_REGULATES_TR
IG_BY_GPCR;REACTOME_INTEGRATION_OF_ENERGY_METABOLISM;REACTOME_OPIOID_SI

CH;REACTOME_HEMOSTASIS

_MATURE_MRNA_DERIVED_FROM_AN_INTRONLESS_TRANSCRIPT;REACTOME_SLC_MEDIAT

_MATURE_MRNA_DERIVED_FROM_AN_INTRONLESS_TRANSCRIPT;REACTOME_SLC_MEDIAT

ALING_AND_AGGREGATION

_SYSTEM;REACTOME_PLATELET_ACTIVATION_SIGNALING_AND_AGGREGATION

IA_PROCESSING;REACTOME_MRNA_SPLICING;REACTOME_MRNA_SPLICING_MINOR_PATHW

ACTOME_METABOLISM_OF_LIPIDS_AND_LIPOPROTEINS;REACTOME_HEMOSTASIS;REACTO

ME_CIRCADIAN_REPRESSION_OF_EXPRESSION_BY_REV_ERBA;REACTOME_SIGNALING_BY_
ME_CIRCADIAN_REPRESSION_OF_EXPRESSION_BY_REV_ERBA;REACTOME_SIGNALING_BY_

YD88_MAL_CASCADE_INITIATED_ON_PLASMA_MEMBRANE;REACTOME_INNATE_IMMUNE_S

OME_RETROGRADE_NEUROTROPHIN_SIGNALLING;REACTOME_EGFR_DOWNREGULATION;

ENZA_VIRAL_RNA_TRANSCRIPTION_AND_REPLICATION;REACTOME_NONSENSE_MEDIATED_

)_CELL_MIGRATION_AND_GROWTH_CONE_COLLAPSE

NTS;REACTOME_SIGNALING_BY_PDGF;REACTOME_DOWNSTREAM_SIGNAL_TRANSDUCTIO
NTS;REACTOME_SIGNALING_BY_PDGF;REACTOME_DOWNSTREAM_SIGNAL_TRANSDUCTIO
NTS;REACTOME_SIGNALING_BY_PDGF;REACTOME_DOWNSTREAM_SIGNAL_TRANSDUCTIO

TION_CASCADE;REACTOME_AXON_GUIDANCE;REACTOME_NCAM1_INTERACTIONS;REACTO

3OLISM
3OLISM

JPLED_NER_TC_NER;REACTOME_RNA_POL_II_TRANSCRIPTION_PRE_INITIATION_AND_PRC

RIF_AND_NADE;REACTOME_P75_NTR_RECEPTOR_MEDIATED_SIGNALLING;REACTOME_SIG
CR;REACTOME_G_ALPHA1213_SIGNALLING_EVENTS;REACTOME_GPCR_DOWNSTREAM_SI(

;REACTOME_GPCR_LIGAND_BINDING

EACTOME_CELL_CYCLE_MITOTIC;REACTOME_GLUCONEOGENESIS;REACTOME_OPIOID_SI

TION_OF_WHITE_ADIPOCYTE_DIFFERENTIATION;REACTOME_HEMOSTASIS;REACTOME_SIG

ON_REPAIR;REACTOME_REPAIR_SYNTHESIS_FOR_GAP_FILLING_BY_DNA_POL_IN_TC_NER
ON_REPAIR;REACTOME_REPAIR_SYNTHESIS_FOR_GAP_FILLING_BY_DNA_POL_IN_TC_NER
ON_REPAIR;REACTOME_REPAIR_SYNTHESIS_FOR_GAP_FILLING_BY_DNA_POL_IN_TC_NER

GENERATION_OF_SECOND_MESSENGERS;REACTOME_SIGNALING_BY_THE_B_CELL_RECEF

TIN_FAMILY_RECEPTORS;REACTOME_GLUCAGON_TYPE_LIGAND_RECEPTORS;REACTOME_
TIN_FAMILY_RECEPTORS;REACTOME_GLUCAGON_TYPE_LIGAND_RECEPTORS;REACTOME_

ACTOME_G_ALPHA_Q_SIGNALLING_EVENTS;REACTOME_GPCR_DOWNSTREAM_SIGNALING

N_REGION_

REACTOME_ASSEMBLY_OF_THE_PRE_REPLICATIVE_COMPLEX;REACTOME_DNA_REPLICAT

.CTIVATION;REACTOME_POTASSIUM_CHANNELS;REACTOME_INWARDLY_RECTIFYING_K_CI
.CTIVATION;REACTOME_POTASSIUM_CHANNELS;REACTOME_INWARDLY_RECTIFYING_K_CI

L_III_CHAIN_ELONGATION
L_III_CHAIN_ELONGATION

;REACTOME_METABOLISM_OF_LIPIDS_AND_LIPOPROTEINS

C_SYNAPSIS

:BOHYDRATES

)LISM_OF_CARBOHYDRATES

MMUNE_SYSTEM

ACTOME_INSULIN_RECEPTOR_SIGNALLING_CASCADE;REACTOME_CELL_SURFACE_INTER/

GH_PROTEINASE_ACTIVATED_RECEPTORS_PARS;REACTOME_HEMOSTASIS;REACTOME_PL

RS;REACTOME_IMMUNOREGULATORY_INTERACTIONS_BETWEEN_A_LYMPHOID_AND_A_N(

ACTOME_PI3K_EVENTS_IN_ERBB4_SIGNALING;REACTOME_PI3K_EVENTS_IN_ERBB2_SIGNA

;REACTOME_METABOLISM_OF_LIPIDS_AND_LIPOPROTEINS

OME_ADAPTIVE_IMMUNE_SYSTEM

ON_CHANNEL_TRANSPORT;REACTOME_LIGAND_GATED_ION_CHANNEL_TRANSPORT
MPLEX;REACTOME_MAP_KINASE_ACTIVATION_IN_TLR_CASCADE;REACTOME_JNK_C_JUN_K

MEDIATED_ANTIGEN_PROCESSING_PRESENTATION

_BETA3_SIGNALING;REACTOME_AXON_GUIDANCE;REACTOME_NCAM_SIGNALING_FOR_NE

R;REACTOME_PHOSPHOLIPASE_C_MEDIATED_CASCADE;REACTOME_SHC_MEDIATED_CAS

SIGNALLING_EVENTS;REACTOME_GPCR_DOWNSTREAM_SIGNALING;REACTOME_SEMA4D_I

_RB1_E2F1;REACTOME_DNA_REPLICATION;REACTOME_E2F_MEDIATED_REGULATION_OF_D

MPLEX;REACTOME_MAP_KINASE_ACTIVATION_IN_TLR_CASCADE;REACTOME_JNK_C_JUN_K

SIGNALLING_EVENTS;REACTOME_GPCR_DOWNSTREAM_SIGNALING;REACTOME_ACTIVATIO

GATION;REACTOME_METABOLISM_OF_PROTEINS;REACTOME_3_UTR_MEDIATED_TRANSLA

G;REACTOME_IMMUNE_SYSTEM;REACTOME_ADAPTIVE_IMMUNE_SYSTEM;REACTOME_CYT

R_LIGAND_BINDING

ICATION;REACTOME_HEMOSTASIS;REACTOME_MITOTIC_PROMETAPHASE;REACTOME_IMM

ACTOME_G_PROTEIN_ACTIVATION;REACTOME_GPCR_DOWNSTREAM_SIGNALING;REACTOM

_OTHER_APC_C_CDH1_TARGETED_PROTEINS_IN_LATE_MITOSIS_EARLY_G1;REACTOME_A

RYOCYTE_DEVELOPMENT_AND_PLATELET_PRODUCTION;REACTOME_REGULATION_OF_IFN

_ACTIVATED_AND_SIGNALS_SURVIVAL;REACTOME_CELL_DEATH_SIGNALLING_VIA_NRAGE

BOHYDRATES

'TIC_CLEAVAGE_OF_NOTCH_RECEPTOR;REACTOME_SIGNALING_BY_NOTCH

REPLICATION;REACTOME_G1_S_SPECIFIC_TRANSCRIPTION

ON_CHANNEL_TRANSPORT;REACTOME_LIGAND_GATED_ION_CHANNEL_TRANSPORT

JNDS;REACTOME_AMINE_COMPOUND_SLC_TRANSPORTERS;REACTOME_METABOLISM_OF

RANSCRIPTION_EVENTS;REACTOME_RNA_POL_III_TRANSCRIPTION_INITIATION_FROM_TYP

ME_GPCR_DOWNSTREAM_SIGNALING;REACTOME_SIGNAL_AMPLIFICATION;REACTOME_THI

REACTOME_RETROGRADE_NEUROTROPHIN_SIGNALLING;REACTOME_EGFR_DOWNREGUL

HESIS;REACTOME_AXON_GUIDANCE;REACTOME_ACTIVATION_OF_RAC;REACTOME_SIGNAI

G;REACTOME_IMMUNE_SYSTEM;REACTOME_ADAPTIVE_IMMUNE_SYSTEM;REACTOME_CYT

WAY;REACTOME_FANCONI_ANEMIA_PATHWAY;REACTOME_DOUBLE_STRAND_BREAK_REP

ACTOME_DOWNSTREAM_SIGNALING_EVENTS_OF_B_CELL_RECEPTOR_BCR;REACTOME_S

N_CHANNEL_TRANSPORT;REACTOME_LIGAND_GATED_ION_CHANNEL_TRANSPORT

_SYSTEM;REACTOME_PLATELET_ACTIVATION_SIGNALING_AND_AGGREGATION

:REACTOME_METABOLISM_OF_LIPIDS_AND_LIPOPROTEINS

_MATURE_MRNA_DERIVED_FROM_AN_INTRONLESS_TRANSCRIPT;REACTOME_SLC_MEDIAT

34;REACTOME_DOWNREGULATION_OF_ERBB2_ERBB3_SIGNALING;REACTOME_SIGNALING_

ENZA_VIRAL_RNA_TRANSCRIPTION_AND_REPLICATION;REACTOME_NONSENSE_MEDIATED_

ACTOME_INSULIN_RECEPTOR_SIGNALLING_CASCADE;REACTOME_CELL_SURFACE_INTERA

OWNSTREAM_SIGNALING_OF_ACTIVATED_FGFR;REACTOME_PHOSPHOLIPASE_C_MEDIATE

TS;REACTOME_GPCR_DOWNSTREAM_SIGNALING;REACTOME_METABOLISM_OF_LIPIDS_AN

RFACE_INTERACTIONS;REACTOME_AXON_GUIDANCE;REACTOME_OTHER_SEMAPHORIN_IN

N;REACTOME_APC_C_CDC20_MEDIATED_DEGRADATION_OF_MITOTIC_PROTEINS;REACTOM

_POSTSYNAPTIC_CELL;REACTOME_PLC_BETA_MEDIATED_EVENTS;REACTOME_G_ALPHA_Q

MPLEX;REACTOME_MAP_KINASE_ACTIVATION_IN_TLR_CASCADE;REACTOME_JNK_C_JUN_K

TABOLISM_OF_PROTEINS;REACTOME_POST_TRANSLATIONAL_PROTEIN_MODIFICATION;RE

_IFNA_SIGNALING;REACTOME_INTERFERON_SIGNALING;REACTOME_IL_2_SIGNALING;REA(

CTOME_NEUROTRANSMITTER_RECEPTOR_BINDING_AND_DOWNSTREAM_TRANSMISSION_

ASES;REACTOME_CHROMOSOME_MAINTENANCE;REACTOME_LAGGING_STRAND_SYNTHES

BRANE;REACTOME_SIGNALING_BY_FGFR_IN_DISEASE;REACTOME_GASTRIN_CREB_SIGNA

ON;REACTOME_RNA_POL_I_RNA_POL_III_AND_MITOCHONDRIAL_TRANSCRIPTION;REACTOM

TED_TRANSMEMBRANE_TRANSPORT;REACTOME_GLUCOSE_TRANSPORT;REACTOME_MET

TOME_NEUROTRANSMITTER_RECEPTOR_BINDING_AND_DOWNSTREAM_TRANSMISSION_I
RIPTION_FACTOR_ACTIVATION;REACTOME_ERKS_ARE_INACTIVATED;REACTOME_ERK_MA

_NRIF_AND_NADE;REACTOME_P75_NTR_RECEPTOR_MEDIATED_SIGNALLING;REACTOME_

PLC_BETA_MEDIATED_EVENTS;REACTOME_PKA_MEDIATED_PHOSPHORYLATION_OF_CREB

EACTOME_NGF_SIGNALLING_VIA_TRKA_FROM_THE_PLASMA_MEMBRANE;REACTOME_SIG
EACTOME_NGF_SIGNALLING_VIA_TRKA_FROM_THE_PLASMA_MEMBRANE;REACTOME_SIG

1_TRANSITION;REACTOME_G1_S_TRANSITION;REACTOME_CDT1_ASSOCIATION_WITH_TH

TOME_FATTY_ACID_TRIACYLGLYCEROL_AND_KETONE_BODY_METABOLISM;REACTOME_CI

ACTIONS_AT_THE_VASCULAR_WALL;REACTOME_NGF_SIGNALLING_VIA_TRKA_FROM_THE_

_AND_KETONE_BODY_METABOLISM;REACTOME_TRANSCRIPTIONAL_REGULATION_OF_WHI
EPTOR_SIGNALING;REACTOME_NFKB_AND_MAP_KINASES_ACTIVATION_MEDIATED_BY_TLI

ꟼTIDE_SLC_TRANSPORTERS

HYDROLYSIS;REACTOME_LIPID_DIGESTION_MOBILIZATION_AND_TRANSPORT;REACTOME_

ACTIONS_AT_THE_VASCULAR_WALL;REACTOME_NGF_SIGNALLING_VIA_TRKA_FROM_THE_
ACTIONS_AT_THE_VASCULAR_WALL;REACTOME_NGF_SIGNALLING_VIA_TRKA_FROM_THE_

TOKINE_SIGNALING_IN_IMMUNE_SYSTEM;REACTOME_ANTIGEN_PRESENTATION_FOLDING_

EACTOME_REGULATION_OF_INSULIN_SECRETION;REACTOME_INHIBITION_OF_INSULIN_SE

SIGNALING_BY_THE_B_CELL_RECEPTOR_BCR;REACTOME_INSULIN_RECEPTOR_SIGNALLING
LLING_PATHWAY_VIA_PKC_AND_MAPK;REACTOME_SIGNALING_BY_GPCR;REACTOME_INTI

:ACTOME_GPCR_LIGAND_BINDING

NS_THAT_BIND_AU_RICH_ELEMENTS;REACTOME_DESTABILIZATION_OF_MRNA_BY_KSRP;F

OME_OPIOID_SIGNALLING;REACTOME_CA_DEPENDENT_EVENTS;REACTOME_NEUROTRANS

TION;REACTOME_ACTIVATION_OF_ATR_IN_RESPONSE_TO_REPLICATION_STRESS;REACTO

GATION

ID_ORGANIC_ACIDS_METAL_IONS_AND_AMINE_COMPOUNDS;REACTOME_FACILITATIVE_NA

STRAND_SYNTHESIS;REACTOME_DNA_REPLICATION;REACTOME_ACTIVATION_OF_ATR_IN_

ANSCRIPTIONAL_REGULATION_OF_WHITE_ADIPOCYTE_DIFFERENTIATION

EX_;REACTOME_FORMATION_OF_TRANSCRIPTION_COUPLED_NER_TC_NER_REPAIR_COMF

ETABOLISM;REACTOME_METABOLISM_OF_LIPIDS_AND_LIPOPROTEINS;REACTOME_METAB
ETABOLISM;REACTOME_METABOLISM_OF_LIPIDS_AND_LIPOPROTEINS;REACTOME_METAB

ACTOME_PACKAGING_OF_TELOMERE_ENDS;REACTOME_TELOMERE_MAINTENANCE

NSULIN_RECEPTOR;REACTOME_HIV_INFECTION;REACTOME_HOST_INTERACTIONS_OF_HIV

MMUNE_SYSTEM;REACTOME_IMMUNE_SYSTEM;REACTOME_CYTOKINE_SIGNALING_IN_IMM

TEM;REACTOME_CYTOKINE_SIGNALING_IN_IMMUNE_SYSTEM

TOME_PROLONGED_ERK_ACTIVATION_EVENTS;REACTOME_SIGNALLING_TO_RAS;REACTO

ACTOME_TRAF6_MEDIATED_INDUCTION_OF_NFKB_AND_MAP_KINASES_UPON_TLR7_8_OR

:EACTOME_METABOLISM_OF_LIPIDS_AND_LIPOPROTEINS;REACTOME_FATTY_ACID_TRIAC'

)_SIGNALLING_EVENTS;REACTOME_REGULATION_OF_INSULIN_SECRETION;REACTOME_RE

TOME_FATTY_ACID_TRIACYLGLYCEROL_AND_KETONE_BODY_METABOLISM;REACTOME_CI
TOME_FATTY_ACID_TRIACYLGLYCEROL_AND_KETONE_BODY_METABOLISM;REACTOME_CI

3Y_REV_ERBA;REACTOME_SIGNALING_BY_NOTCH1;REACTOME_REGULATION_OF_HYPOXIA

RACTIONS;REACTOME_CELL_JUNCTION_ORGANIZATION

APC_C_CDC20_MEDIATED_DEGRADATION_OF_MITOTIC_PROTEINS;REACTOME_AUTODEGR

SMA_MEMBRANE;REACTOME_SIGNALING_BY_FGFR_IN_DISEASE;REACTOME_PI3K_AKT_AC

TOME_FATTY_ACID_TRIACYLGLYCEROL_AND_KETONE_BODY_METABOLISM;REACTOME_CI

SMA_MEMBRANE;REACTOME_SIGNALING_BY_FGFR_IN_DISEASE;REACTOME_PI3K_AKT_AC

D_DIGESTION_MOBILIZATION_AND_TRANSPORT;REACTOME_LIPOPROTEIN_METABOLISM;R
D_DIGESTION_MOBILIZATION_AND_TRANSPORT;REACTOME_LIPOPROTEIN_METABOLISM;R

:M;REACTOME_SIGNALING_BY_GPCR;REACTOME_NEUROTRANSMITTER_RECEPTOR_BINDI

TRANSLOCATION_OF_ZAP_70_TO_IMMUNOLOGICAL_SYNAPSE;REACTOME_GENERATION_O

CYLGLYCEROL_AND_KETONE_BODY_METABOLISM;REACTOME_PKB_MEDIATED_EVENTS;R

CTOME_IL_6_SIGNALING;REACTOME_IMMUNE_SYSTEM;REACTOME_CYTOKINE_SIGNALING_

N_SEMAPHORIN_SIGNALING;REACTOME_SEMAPHORIN_INTERACTIONS;REACTOME_SEMA4

TED_TRANSMEMBRANE_TRANSPORT;REACTOME_GLUCOSE_TRANSPORT;REACTOME_MET

SIGNALING_BY_THE_B_CELL_RECEPTOR_BCR;REACTOME_NGF_SIGNALLING_VIA_TRKA_FR

IG_INITIATES_THE_SECOND_PROTEOLYTIC_CLEAVAGE_OF_NOTCH_RECEPTOR;REACTOME

TOME_FATTY_ACID_TRIACYLGLYCEROL_AND_KETONE_BODY_METABOLISM;REACTOME_CI

IUNE_SYSTEM

REACTOME_HEMOSTASIS

EM;REACTOME_PLATELET_ACTIVATION_SIGNALING_AND_AGGREGATION

OME_CLASS_I_MHC_MEDIATED_ANTIGEN_PROCESSING_PRESENTATION

ME_REGULATION_OF_IFNG_SIGNALING;REACTOME_INTERFERON_GAMMA_SIGNALING;REA

IE_PI3K_CASCADE

ORTERS;REACTOME_AMINE_COMPOUND_SLC_TRANSPORTERS;REACTOME_GABA_SYNTHE

;REACTOME_SIGNALLING_TO_RAS;REACTOME_NGF_SIGNALLING_VIA_TRKA_FROM_THE_PI

ITS;REACTOME_G_ALPHA1213_SIGNALLING_EVENTS;REACTOME_GPCR_DOWNSTREAM_SI
ITS;REACTOME_G_ALPHA1213_SIGNALLING_EVENTS;REACTOME_GPCR_DOWNSTREAM_SI

;_TO_GENERATION_OF_SECOND_MESSENGERS;REACTOME_SIGNALING_BY_THE_B_CELL_

2;REACTOME_FGFR_LIGAND_BINDING_AND_ACTIVATION;REACTOME_FGFR4_LIGAND_BINDI
2;REACTOME_FGFR_LIGAND_BINDING_AND_ACTIVATION;REACTOME_FGFR4_LIGAND_BINDI
2;REACTOME_FGFR_LIGAND_BINDING_AND_ACTIVATION;REACTOME_FGFR4_LIGAND_BINDI

_DECAY_ENHANCED_BY_THE_EXON_JUNCTION_COMPLEX

LLING_PATHWAY_VIA_PKC_AND_MAPK;REACTOME_SIGNALING_BY_GPCR;REACTOME_INTI

_DECAY_ENHANCED_BY_THE_EXON_JUNCTION_COMPLEX

CTION_OF_NFKB_AND_MAP_KINASES_UPON_TLR7_8_OR_9_ACTIVATION;REACTOME_NFKB

GNALLING;REACTOME_CA_DEPENDENT_EVENTS;REACTOME_ADENYLATE_CYCLASE_ACTI\

)ME_NCAM_SIGNALING_FOR_NEURITE_OUT_GROWTH;REACTOME_HEMOSTASIS;REACTOM

PTOR_BCR;REACTOME_P53_INDEPENDENT_G1_S_DNA_DAMAGE_CHECKPOINT;REACTOME
G1_TRANSITION;REACTOME_G1_S_TRANSITION;REACTOME_CDT1_ASSOCIATION_WITH_TH

CYLGLYCEROL_AND_KETONE_BODY_METABOLISM;REACTOME_PKB_MEDIATED_EVENTS;R
CYLGLYCEROL_AND_KETONE_BODY_METABOLISM;REACTOME_PKB_MEDIATED_EVENTS;R

TED_CASCADE;REACTOME_PI_3K_CASCADE;REACTOME_DOWNSTREAM_SIGNALING_OF_A(
TED_CASCADE;REACTOME_PI_3K_CASCADE;REACTOME_DOWNSTREAM_SIGNALING_OF_A(

ꟾTIDE_SLC_TRANSPORTERS

SMA_MEMBRANE;REACTOME_SIGNALING_BY_FGFR_IN_DISEASE;REACTOME_PI3K_AKT_AC

ME_REGULATION_OF_INSULIN_SECRETION;REACTOME_GABA_SYNTHESIS_RELEASE_REUP

E_APOPTOTIC_EXECUTION_PHASE
E_APOPTOTIC_EXECUTION_PHASE
E_APOPTOTIC_EXECUTION_PHASE
E_APOPTOTIC_EXECUTION_PHASE

TEM;REACTOME_CYTOKINE_SIGNALING_IN_IMMUNE_SYSTEM

PTOR_BCR;REACTOME_P53_INDEPENDENT_G1_S_DNA_DAMAGE_CHECKPOINT;REACTOME

;REACTOME_TRANSMISSION_ACROSS_CHEMICAL_SYNAPSES;REACTOME_NEURONAL_SYS

IVATES_NFKB_BY_PHOSPHORYLATION_AND_ACTIVATION_OF_IKKS_COMPLEX;REACTOME_

SMA_MEMBRANE;REACTOME_SIGNALING_BY_FGFR_IN_DISEASE;REACTOME_PI3K_AKT_AC

SIS;REACTOME_INHIBITION_OF_REPLICATION_INITIATION_OF_DAMAGED_DNA_BY_RB1_E2I

Y_TGF_BETA_RECEPTOR_COMPLEX

AIR;REACTOME_AUTODEGRADATION_OF_THE_E3_UBIQUITIN_LIGASE_COP1;REACTOME_D

N_SEMAPHORIN_SIGNALING;REACTOME_SEMAPHORIN_INTERACTIONS;REACTOME_SEMA4

N_SEMAPHORIN_SIGNALING;REACTOME_SEMAPHORIN_INTERACTIONS;REACTOME_SEMA4

E_COSTIMULATION_BY_THE_CD28_FAMILY;REACTOME_HIV_INFECTION;REACTOME_HIV_LI

GNALLING;REACTOME_CA_DEPENDENT_EVENTS;REACTOME_ADENYLATE_CYCLASE_ACTI

STRAND_SYNTHESIS;REACTOME_DNA_REPLICATION;REACTOME_ACTIVATION_OF_ATR_IN_

REACTOME_HEMOSTASIS

ALING;REACTOME_DOWNSTREAM_SIGNALING_EVENTS_OF_B_CELL_RECEPTOR_BCR;REAC

D_SIGNALLING_EVENTS;REACTOME_REGULATION_OF_INSULIN_SECRETION;REACTOME_RE

_DECAY_ENHANCED_BY_THE_EXON_JUNCTION_COMPLEX

ANSCRIPTION;REACTOME_CIRCADIAN_REPRESSION_OF_EXPRESSION_BY_REV_ERBA;REA
ANSCRIPTION;REACTOME_CIRCADIAN_REPRESSION_OF_EXPRESSION_BY_REV_ERBA;REA
ANSCRIPTION;REACTOME_CIRCADIAN_REPRESSION_OF_EXPRESSION_BY_REV_ERBA;REA
GNALLING;REACTOME_CA_DEPENDENT_EVENTS;REACTOME_ADENYLATE_CYCLASE_ACTI\

TED_TRANSMEMBRANE_TRANSPORT;REACTOME_GLUCOSE_TRANSPORT;REACTOME_MET

TED_TRANSMEMBRANE_TRANSPORT;REACTOME_GLUCOSE_TRANSPORT;REACTOME_MET

VAY;REACTOME_TRANSCRIPTION;REACTOME_NUCLEOTIDE_EXCISION_REPAIR;REACTOME

ME_PLATELET_ACTIVATION_SIGNALING_AND_AGGREGATION

_NOTCH1;REACTOME_TRANSCRIPTIONAL_ACTIVITY_OF_SMAD2_SMAD3_SMAD4_HETEROT
_NOTCH1;REACTOME_TRANSCRIPTIONAL_ACTIVITY_OF_SMAD2_SMAD3_SMAD4_HETEROT

YSTEM;REACTOME_ACTIVATED_TLR4_SIGNALLING;REACTOME_IMMUNE_SYSTEM;REACTOM

;REACTOME_TRANSMISSION_ACROSS_CHEMICAL_SYNAPSES;REACTOME_NEURONAL_SYS

_DECAY_ENHANCED_BY_THE_EXON_JUNCTION_COMPLEX

N;REACTOME_SIGNALING_BY_ILS;REACTOME_IL_6_SIGNALING;REACTOME_IMMUNE_SYSTE
N;REACTOME_SIGNALING_BY_ILS;REACTOME_IL_6_SIGNALING;REACTOME_IMMUNE_SYSTE
N;REACTOME_SIGNALING_BY_ILS;REACTOME_IL_6_SIGNALING;REACTOME_IMMUNE_SYSTE

)ME_NCAM_SIGNALING_FOR_NEURITE_OUT_GROWTH;REACTOME_HEMOSTASIS;REACTOM

)MOTER_OPENING;REACTOME_MRNA_PROCESSING;REACTOME_MRNA_SPLICING;REACTOI

;NALING_BY_GPCR;REACTOME_GPVI_MEDIATED_ACTIVATION_CASCADE;REACTOME_G_ALI
GNALING;REACTOME_SIGNALING_BY_TGF_BETA_RECEPTOR_COMPLEX

GNALLING;REACTOME_RECRUITMENT_OF_MITOTIC_CENTROSOME_PROTEINS_AND_COMP

GNALING_BY_TGF_BETA_RECEPTOR_COMPLEX;REACTOME_PLATELET_ACTIVATION_SIGNA

;REACTOME_SYNTHESIS_OF_DNA;REACTOME_DNA_REPAIR;REACTOME_CHROMOSOME_M
;REACTOME_SYNTHESIS_OF_DNA;REACTOME_DNA_REPAIR;REACTOME_CHROMOSOME_M
;REACTOME_SYNTHESIS_OF_DNA;REACTOME_DNA_REPAIR;REACTOME_CHROMOSOME_M

PTOR_BCR;REACTOME_PROLONGED_ERK_ACTIVATION_EVENTS;REACTOME_CELL_SURFA

_GPCR_DOWNSTREAM_SIGNALING;REACTOME_G_ALPHA_I_SIGNALLING_EVENTS;REACTOM
_GPCR_DOWNSTREAM_SIGNALING;REACTOME_G_ALPHA_I_SIGNALLING_EVENTS;REACTOM

;REACTOME_G_ALPHA_I_SIGNALLING_EVENTS;REACTOME_TAK1_ACTIVATES_NFKB_BY_PH

TION;REACTOME_ACTIVATION_OF_ATR_IN_RESPONSE_TO_REPLICATION_STRESS;REACTO

HANNELS
HANNELS

ACTIONS_AT_THE_VASCULAR_WALL;REACTOME_NGF_SIGNALLING_VIA_TRKA_FROM_THE_

LATELET_ACTIVATION_SIGNALING_AND_AGGREGATION

ON_LYMPHOID_CELL;REACTOME_INTERFERON_GAMMA_SIGNALING;REACTOME_INTERFER

ALING;REACTOME_DOWNSTREAM_SIGNALING_EVENTS_OF_B_CELL_RECEPTOR_BCR;REAC

KINASES_PHOSPHORYLATION_AND_ACTIVATION_MEDIATED_BY_ACTIVATED_HUMAN_TAK1;

URITE_OUT_GROWTH;REACTOME_NETRIN1_SIGNALING;REACTOME_DCC_MEDIATED_ATTF

CADE;REACTOME_SIGNALING_BY_INSULIN_RECEPTOR;REACTOME_FGFR_LIGAND_BINDIN(

N_SEMAPHORIN_SIGNALING;REACTOME_SEMAPHORIN_INTERACTIONS;REACTOME_SEMA4

)NA_REPLICATION;REACTOME_S_PHASE

KINASES_PHOSPHORYLATION_AND_ACTIVATION_MEDIATED_BY_ACTIVATED_HUMAN_TAK1;

ON_OF_RAC;REACTOME_SIGNALING_BY_ROBO_RECEPTOR

TIONAL_REGULATION;REACTOME_METABOLISM_OF_MRNA;REACTOME_METABOLISM_OF_F

TOKINE_SIGNALING_IN_IMMUNE_SYSTEM;REACTOME_ANTIGEN_PRESENTATION_FOLDING_

IUNE_SYSTEM;REACTOME_ADAPTIVE_IMMUNE_SYSTEM

ME_G_ALPHA_I_SIGNALLING_EVENTS;REACTOME_G_ALPHA_S_SIGNALLING_EVENTS;REAC

APC_C_CDC20_MEDIATED_DEGRADATION_OF_MITOTIC_PROTEINS;REACTOME_AUTODEGR

NG_SIGNALING;REACTOME_INTERFERON_GAMMA_SIGNALING;REACTOME_INTERFERON_S

_NRIF_AND_NADE;REACTOME_P75_NTR_RECEPTOR_MEDIATED_SIGNALLING;REACTOME_

E_LIPIDS_AND_LIPOPROTEINS

PE_3_PROMOTER;REACTOME_HIV_INFECTION;REACTOME_HIV_LIFE_CYCLE;REACTOME_LA

ROMBOXANE_SIGNALLING_THROUGH_TP_RECEPTOR;REACTOME_THROMBIN_SIGNALLING

ATION;REACTOME_AXON_GUIDANCE;REACTOME_LYSOSOME_VESICLE_BIOGENESIS;REAC

LING_BY_ROBO_RECEPTOR;REACTOME_METABOLISM_OF_CARBOHYDRATES

TOKINE_SIGNALING_IN_IMMUNE_SYSTEM;REACTOME_ANTIGEN_PRESENTATION_FOLDING_

AIR;REACTOME_AUTODEGRADATION_OF_THE_E3_UBIQUITIN_LIGASE_COP1;REACTOME_D

IGNALING_BY_THE_B_CELL_RECEPTOR_BCR;REACTOME_INSULIN_RECEPTOR_SIGNALLIN

TED_TRANSMEMBRANE_TRANSPORT;REACTOME_GLUCOSE_TRANSPORT;REACTOME_MET

_BY_ERBB2;REACTOME_SIGNALING_BY_EGFR_IN_CANCER;REACTOME_PI3K_EVENTS_IN_E

_DECAY_ENHANCED_BY_THE_EXON_JUNCTION_COMPLEX

ACTIONS_AT_THE_VASCULAR_WALL;REACTOME_NGF_SIGNALLING_VIA_TRKA_FROM_THE_

D_CASCADE;REACTOME_SHC_MEDIATED_CASCADE;REACTOME_SIGNALING_BY_INSULIN_F

ID_LIPOPROTEINS;REACTOME_FATTY_ACID_TRIACYLGLYCEROL_AND_KETONE_BODY_MET

NTERACTIONS;REACTOME_SEMAPHORIN_INTERACTIONS;REACTOME_CELL_EXTRACELLUL

ME_MITOTIC_PROMETAPHASE;REACTOME_APC_CDC20_MEDIATED_DEGRADATION_OF_NE

)_SIGNALLING_EVENTS;REACTOME_REGULATION_OF_INSULIN_SECRETION;REACTOME_RE

KINASES_PHOSPHORYLATION_AND_ACTIVATION_MEDIATED_BY_ACTIVATED_HUMAN_TAK1;

ACTOME_SIGNALING_BY_NOTCH;REACTOME_METABOLISM_OF_CARBOHYDRATES

CTOME_IL_6_SIGNALING;REACTOME_IMMUNE_SYSTEM;REACTOME_CYTOKINE_SIGNALING_

IN_THE_POSTSYNAPTIC_CELL;REACTOME_G_ALPHA1213_SIGNALLING_EVENTS;REACTOME

SIS;REACTOME_INHIBITION_OF_REPLICATION_INITIATION_OF_DAMAGED_DNA_BY_RB1_E2F

LLING_PATHWAY_VIA_PKC_AND_MAPK;REACTOME_SIGNALING_BY_GPCR;REACTOME_INTI

ME_SIGNALING_BY_NOTCH;REACTOME_RNA_POL_I_TRANSCRIPTION_INITIATION

ABOLISM_OF_RNA;REACTOME_INTERFERON_SIGNALING;REACTOME_METABOLISM_OF_CA

N_THE_POSTSYNAPTIC_CELL;REACTOME_PLC_BETA_MEDIATED_EVENTS;REACTOME_SIGN
PK_TARGETS;REACTOME_GLYCOLYSIS;REACTOME_SIGNALING_BY_GPCR;REACTOME_INTE

TAK1_ACTIVATES_NFKB_BY_PHOSPHORYLATION_AND_ACTIVATION_OF_IKKS_COMPLEX;RE

;REACTOME_TRANSMEMBRANE_TRANSPORT_OF_SMALL_MOLECULES;REACTOME_GLUCA

NALING_BY_FGFR_IN_DISEASE;REACTOME_PI3K_AKT_ACTIVATION;REACTOME_GAB1_SIGN
NALING_BY_FGFR_IN_DISEASE;REACTOME_PI3K_AKT_ACTIVATION;REACTOME_GAB1_SIGN

E_CDC6_ORC_ORIGIN_COMPLEX;REACTOME_SYNTHESIS_OF_DNA;REACTOME_MITOTIC_G

RCADIAN_CLOCK;REACTOME_TRANSCRIPTIONAL_REGULATION_OF_WHITE_ADIPOCYTE_D

_PLASMA_MEMBRANE;REACTOME_SIGNALING_BY_FGFR_IN_DISEASE;REACTOME_PHOSPH

TE_ADIPOCYTE_DIFFERENTIATION;REACTOME_SIGNALING_BY_NOTCH;REACTOME_SIGNAL
R4_SIGNALING_REPERTOIRE;REACTOME_RIG_I_MDA5_MEDIATED_INDUCTION_OF_IFN_ALF

_HEMOSTASIS;REACTOME_PLATELET_ACTIVATION_SIGNALING_AND_AGGREGATION

_PLASMA_MEMBRANE;REACTOME_SIGNALING_BY_FGFR_IN_DISEASE;REACTOME_PHOSPH

_PLASMA_MEMBRANE;REACTOME_SIGNALING_BY_FGFR_IN_DISEASE;REACTOME_PHOSPH

ASSEMBLY_AND_PEPTIDE_LOADING_OF_CLASS_I_MHC;REACTOME_CLASS_I_MHC_MEDIAT

CRETION_BY_ADRENALINE_NORADRENALINE;REACTOME_GPCR_DOWNSTREAM_SIGNALIN

G_CASCADE;REACTOME_ARMS_MEDIATED_ACTIVATION;REACTOME_PROLONGED_ERK_AC
EGRATION_OF_ENERGY_METABOLISM;REACTOME_OPIOID_SIGNALLING;REACTOME_PLC_E

REACTOME_DESTABILIZATION_OF_MRNA_BY_TRISTETRAPROLIN_TTP

SMITTER_RECEPTOR_BINDING_AND_DOWNSTREAM_TRANSMISSION_IN_THE_POSTSYNAPT

ME_UNWINDING_OF_DNA;REACTOME_G2_M_CHECKPOINTS;REACTOME_S_PHASE;REACTC

A_INDEPENDENT_GLUCOSE_TRANSPORTERS;REACTOME_GLUCOSE_TRANSPORT;REACTO

_RESPONSE_TO_REPLICATION_STRESS;REACTOME_TELOMERE_MAINTENANCE;REACTOME

PLEX;REACTOME_RNA_POL_I_RNA_POL_III_AND_MITOCHONDRIAL_TRANSCRIPTION;REACT[illegible]

OLISM_OF_CARBOHYDRATES
OLISM_OF_CARBOHYDRATES

/_FACTORS;REACTOME_THE_ROLE_OF_NEF_IN_HIV1_REPLICATION_AND_DISEASE_PATHO

[illegible]UNE_SYSTEM

ME_NGF_SIGNALLING_VIA_TRKA_FROM_THE_PLASMA_MEMBRANE;REACTOME_SIGNALLIN[illegible]

_9_ACTIVATION;REACTOME_NFKB_AND_MAP_KINASES_ACTIVATION_MEDIATED_BY_TLR4_[illegible]

YLGLYCEROL_AND_KETONE_BODY_METABOLISM;REACTOME_CIRCADIAN_CLOCK;REACTOI

EGULATION_OF_INSULIN_SECRETION_BY_ACETYLCHOLINE;REACTOME_G_BETA_GAMMA_S

RCADIAN_CLOCK;REACTOME_TRANSCRIPTIONAL_REGULATION_OF_WHITE_ADIPOCYTE_D
RCADIAN_CLOCK;REACTOME_TRANSCRIPTIONAL_REGULATION_OF_WHITE_ADIPOCYTE_D

A_INDUCIBLE_FACTOR_HIF_BY_OXYGEN;REACTOME_METABOLISM_OF_LIPIDS_AND_LIPOPR

ADATION_OF_CDH1_BY_CDH1_APC_C;REACTOME_APC_C_CDC20_MEDIATED_DEGRADATIC

TIVATION;REACTOME_GAB1_SIGNALOSOME;REACTOME_SIGNALING_BY_PDGF;REACTOME_

RCADIAN_CLOCK;REACTOME_TRANSCRIPTIONAL_REGULATION_OF_WHITE_ADIPOCYTE_D

TIVATION;REACTOME_AKT_PHOSPHORYLATES_TARGETS_IN_THE_CYTOSOL;REACTOME_G

EACTOME_CHYLOMICRON_MEDIATED_LIPID_TRANSPORT;REACTOME_AMYLOIDS
EACTOME_CHYLOMICRON_MEDIATED_LIPID_TRANSPORT;REACTOME_AMYLOIDS

NG_AND_DOWNSTREAM_TRANSMISSION_IN_THE_POSTSYNAPTIC_CELL;REACTOME_AXON

F_SECOND_MESSENGER_MOLECULES;REACTOME_GPVI_MEDIATED_ACTIVATION_CASCAD

EACTOME_SIGNALING_BY_INSULIN_RECEPTOR;REACTOME_PI3K_CASCADE

_IN_IMMUNE_SYSTEM

ɪD_INDUCED_CELL_MIGRATION_AND_GROWTH_CONE_COLLAPSE

ABOLISM_OF_RNA;REACTOME_INTERFERON_SIGNALING;REACTOME_METABOLISM_OF_CA

ROM_THE_PLASMA_MEMBRANE;REACTOME_SIGNALING_BY_FGFR_IN_DISEASE;REACTOME_

E_SIGNALING_BY_NOTCH;REACTOME_IMMUNE_SYSTEM;REACTOME_CYTOKINE_SIGNALIN

RCADIAN_CLOCK;REACTOME_TRANSCRIPTIONAL_REGULATION_OF_WHITE_ADIPOCYTE_D

CTOME_INTERFERON_ALPHA_BETA_SIGNALING;REACTOME_REGULATION_OF_IFNA_SIGNA

:SIS_RELEASE_REUPTAKE_AND_DEGRADATION

LASMA_MEMBRANE;REACTOME_SIGNALLING_TO_ERKS;REACTOME_SIGNALING_BY_FGFR_

GNALING;REACTOME_NETRIN1_SIGNALING;REACTOME_DCC_MEDIATED_ATTRACTIVE_SIGN
GNALING;REACTOME_NETRIN1_SIGNALING;REACTOME_DCC_MEDIATED_ATTRACTIVE_SIGN

RECEPTOR_BCR;REACTOME_INSULIN_RECEPTOR_SIGNALLING_CASCADE;REACTOME_CEI

NG_AND_ACTIVATION;REACTOME_SIGNALING_BY_FGFR;REACTOME_PI3K_CASCADE
NG_AND_ACTIVATION;REACTOME_SIGNALING_BY_FGFR;REACTOME_PI3K_CASCADE
NG_AND_ACTIVATION;REACTOME_SIGNALING_BY_FGFR;REACTOME_PI3K_CASCADE

EGRATION_OF_ENERGY_METABOLISM;REACTOME_OPIOID_SIGNALLING;REACTOME_PLC_E

_AND_MAP_KINASES_ACTIVATION_MEDIATED_BY_TLR4_SIGNALING_REPERTOIRE;REACTO

VATING_PATHWAY;REACTOME_ADENYLATE_CYCLASE_INHIBITORY_PATHWAY;REACTOME_

ME_PLATELET_ACTIVATION_SIGNALING_AND_AGGREGATION

E_CDK_MEDIATED_PHOSPHORYLATION_AND_REMOVAL_OF_CDC6;REACTOME_METABOLISM
E_CDC6_ORC_ORIGIN_COMPLEX;REACTOME_SYNTHESIS_OF_DNA;REACTOME_MITOTIC_G

EACTOME_SIGNALING_BY_INSULIN_RECEPTOR;REACTOME_PI3K_CASCADE
EACTOME_SIGNALING_BY_INSULIN_RECEPTOR;REACTOME_PI3K_CASCADE

CTIVATED_FGFR;REACTOME_PHOSPHOLIPASE_C_MEDIATED_CASCADE;REACTOME_SHC_
CTIVATED_FGFR;REACTOME_PHOSPHOLIPASE_C_MEDIATED_CASCADE;REACTOME_SHC_

TIVATION;REACTOME_GAB1_SIGNALOSOME;REACTOME_SIGNALING_BY_PDGF;REACTOME_

TAKE_AND_DEGRADATION

E_CDK_MEDIATED_PHOSPHORYLATION_AND_REMOVAL_OF_CDC6;REACTOME_METABOLISM

STEM;REACTOME_NEUROTRANSMITTER_RECEPTOR_BINDING_AND_DOWNSTREAM_TRANS

_GPCR_LIGAND_BINDING;REACTOME_TRAF6_MEDIATED_NFKB_ACTIVATION;REACTOME_TR

TIVATION;REACTOME_AKT_PHOSPHORYLATES_TARGETS_IN_THE_CYTOSOL;REACTOME_G

F1;REACTOME_DNA_REPLICATION;REACTOME_E2F_MEDIATED_REGULATION_OF_DNA_REP

NA_REPAIR;REACTOME_MEIOTIC_RECOMBINATION;REACTOME_G2_M_CHECKPOINTS;REAC

4D_INDUCED_CELL_MIGRATION_AND_GROWTH_CONE_COLLAPSE

4D_INDUCED_CELL_MIGRATION_AND_GROWTH_CONE_COLLAPSE

FE_CYCLE;REACTOME_EARLY_PHASE_OF_HIV_LIFE_CYCLE;REACTOME_HOST_INTERACTIC

VATING_PATHWAY;REACTOME_ADENYLATE_CYCLASE_INHIBITORY_PATHWAY;REACTOME_

_RESPONSE_TO_REPLICATION_STRESS;REACTOME_TELOMERE_MAINTENANCE;REACTOME

CTOME_SIGNALING_BY_THE_B_CELL_RECEPTOR_BCR;REACTOME_NGF_SIGNALLING_VIA_T

EGULATION_OF_INSULIN_SECRETION_BY_ACETYLCHOLINE;REACTOME_G_BETA_GAMMA_S

ACTOME_SIGNALING_BY_NOTCH1;REACTOME_REGULATION_OF_HYPOXIA_INDUCIBLE_FAC
ACTOME_SIGNALING_BY_NOTCH1;REACTOME_REGULATION_OF_HYPOXIA_INDUCIBLE_FAC
ACTOME_SIGNALING_BY_NOTCH1;REACTOME_REGULATION_OF_HYPOXIA_INDUCIBLE_FAC
VATING_PATHWAY;REACTOME_ADENYLATE_CYCLASE_INHIBITORY_PATHWAY;REACTOME_

ABOLISM_OF_RNA;REACTOME_INTERFERON_SIGNALING;REACTOME_METABOLISM_OF_CA

ABOLISM_OF_RNA;REACTOME_INTERFERON_SIGNALING;REACTOME_METABOLISM_OF_CA

E_FORMATION_OF_RNA_POL_II_ELONGATION_COMPLEX_;REACTOME_ELONGATION_ARRES

RIMER;REACTOME_DOWNREGULATION_OF_SMAD2_3_SMAD4_TRANSCRIPTIONAL_ACTIVITY
RIMER;REACTOME_DOWNREGULATION_OF_SMAD2_3_SMAD4_TRANSCRIPTIONAL_ACTIVITY

ME_TOLL_RECEPTOR_CASCADES

TEM;REACTOME_NEUROTRANSMITTER_RECEPTOR_BINDING_AND_DOWNSTREAM_TRANS

EM;REACTOME_CYTOKINE_SIGNALING_IN_IMMUNE_SYSTEM
EM;REACTOME_CYTOKINE_SIGNALING_IN_IMMUNE_SYSTEM
EM;REACTOME_CYTOKINE_SIGNALING_IN_IMMUNE_SYSTEM

IE_PLATELET_ACTIVATION_SIGNALING_AND_AGGREGATION

ME_MRNA_SPLICING_MINOR_PATHWAY;REACTOME_TRANSCRIPTION;REACTOME_NUCLEO

PHA1213_SIGNALLING_EVENTS;REACTOME_CD28_CO_STIMULATION;REACTOME_GPCR_DC

'LEXES;REACTOME_CA_DEPENDENT_EVENTS;REACTOME_DARPP_32_EVENTS;REACTOME_

LING_AND_AGGREGATION

IAINTENANCE;REACTOME_GLOBAL_GENOMIC_NER_GG_NER;REACTOME_LAGGING_STRAN
IAINTENANCE;REACTOME_GLOBAL_GENOMIC_NER_GG_NER;REACTOME_LAGGING_STRAN
IAINTENANCE;REACTOME_GLOBAL_GENOMIC_NER_GG_NER;REACTOME_LAGGING_STRAN

CE_INTERACTIONS_AT_THE_VASCULAR_WALL;REACTOME_NGF_SIGNALLING_VIA_TRKA_FI

ME_G_ALPHA_S_SIGNALLING_EVENTS;REACTOME_G_ALPHA_Z_SIGNALLING_EVENTS;REAC
ME_G_ALPHA_S_SIGNALLING_EVENTS;REACTOME_G_ALPHA_Z_SIGNALLING_EVENTS;REAC

HOSPHORYLATION_AND_ACTIVATION_OF_IKKS_COMPLEX;REACTOME_GPCR_LIGAND_BIND

ME_UNWINDING_OF_DNA;REACTOME_G2_M_CHECKPOINTS;REACTOME_S_PHASE;REACTC

_PLASMA_MEMBRANE;REACTOME_SIGNALING_BY_FGFR_IN_DISEASE;REACTOME_PHOSPH[illegible]

ON_ALPHA_BETA_SIGNALING;REACTOME_INTERFERON_SIGNALING;REACTOME_HIV_INFE[illegible]

:TOME_SIGNALING_BY_THE_B_CELL_RECEPTOR_BCR;REACTOME_NGF_SIGNALLING_VIA_T

;REACTOME_ACTIVATED_TAK1_MEDIATES_P38_MAPK_ACTIVATION;REACTOME_SIGNALING

RACTIVE_SIGNALING;REACTOME_SIGNAL_REGULATORY_PROTEIN_SIRP_FAMILY_INTERACT

G_AND_ACTIVATION;REACTOME_FGFR2C_LIGAND_BINDING_AND_ACTIVATION;REACTOME_

4D_INDUCED_CELL_MIGRATION_AND_GROWTH_CONE_COLLAPSE

;REACTOME_ACTIVATED_TAK1_MEDIATES_P38_MAPK_ACTIVATION;REACTOME_SIGNALING

RNA;REACTOME_INFLUENZA_LIFE_CYCLE;REACTOME_INFLUENZA_VIRAL_RNA_TRANSCRIP

ASSEMBLY_AND_PEPTIDE_LOADING_OF_CLASS_I_MHC;REACTOME_CLASS_I_MHC_MEDIAT

TOME_G_ALPHA_Z_SIGNALLING_EVENTS;REACTOME_SIGNAL_AMPLIFICATION;REACTOME_

ADATION_OF_CDH1_BY_CDH1_APC_C;REACTOME_APC_C_CDC20_MEDIATED_DEGRADATIC

IGNALING;REACTOME_IL_2_SIGNALING;REACTOME_IL_6_SIGNALING;REACTOME_HEMOSTA

TAK1_ACTIVATES_NFKB_BY_PHOSPHORYLATION_AND_ACTIVATION_OF_IKKS_COMPLEX;RE

TE_PHASE_OF_HIV_LIFE_CYCLE

_THROUGH_PROTEINASE_ACTIVATED_RECEPTORS_PARS;REACTOME_HEMOSTASIS;REAC

TOME_GOLGI_ASSOCIATED_VESICLE_BIOGENESIS;REACTOME_L1CAM_INTERACTIONS;REA

ASSEMBLY_AND_PEPTIDE_LOADING_OF_CLASS_I_MHC;REACTOME_CLASS_I_MHC_MEDIAT

NA_REPAIR;REACTOME_MEIOTIC_RECOMBINATION;REACTOME_G2_M_CHECKPOINTS;REA(

G_CASCADE;REACTOME_ARMS_MEDIATED_ACTIVATION;REACTOME_PROLONGED_ERK_AC

ABOLISM_OF_RNA;REACTOME_INTERFERON_SIGNALING;REACTOME_METABOLISM_OF_CA

RBB4_SIGNALING;REACTOME_PI3K_EVENTS_IN_ERBB2_SIGNALING;REACTOME_DOWNSTR

_PLASMA_MEMBRANE;REACTOME_SIGNALING_BY_FGFR_IN_DISEASE;REACTOME_PHOSPH

RECEPTOR;REACTOME_FGFR_LIGAND_BINDING_AND_ACTIVATION;REACTOME_FGFR2C_LI

TABOLISM

AR_MATRIX_INTERACTIONS;REACTOME_CELL_JUNCTION_ORGANIZATION;REACTOME_L1C/

K2A

EGULATION_OF_INSULIN_SECRETION_BY_ACETYLCHOLINE;REACTOME_G_BETA_GAMMA_S

;REACTOME_ACTIVATED_TAK1_MEDIATES_P38_MAPK_ACTIVATION;REACTOME_SIGNALING

_IN_IMMUNE_SYSTEM

E_GPCR_DOWNSTREAM_SIGNALING;REACTOME_RAS_ACTIVATION_UOPN_CA2_INFUX_THR

F1;REACTOME_DNA_REPLICATION;REACTOME_E2F_MEDIATED_REGULATION_OF_DNA_REF

EGRATION_OF_ENERGY_METABOLISM;REACTOME_OPIOID_SIGNALLING;REACTOME_PLC_E

ARBOHYDRATES;REACTOME_INFLUENZA_LIFE_CYCLE;REACTOME_NEP_NS2_INTERACTS_W

NALING_BY_PDGF;REACTOME_DOWNSTREAM_SIGNAL_TRANSDUCTION;REACTOME_ACTIV
EGRATION_OF_ENERGY_METABOLISM;REACTOME_CELL_CYCLE_MITOTIC;REACTOME_OPIO

EACTOME_MAP_KINASE_ACTIVATION_IN_TLR_CASCADE;REACTOME_JNK_C_JUN_KINASES_

GON_SIGNALING_IN_METABOLIC_REGULATION;REACTOME_SIGNALING_BY_PDGF;REACTO

IALOSOME;REACTOME_SIGNALING_BY_PDGF;REACTOME_DOWNSTREAM_SIGNAL_TRANSD
IALOSOME;REACTOME_SIGNALING_BY_PDGF;REACTOME_DOWNSTREAM_SIGNAL_TRANSD

1_G1_S_PHASES;REACTOME_MITOTIC_M_M_G1_PHASES;REACTOME_ASSEMBLY_OF_THE_

IFFERENTIATION

OLIPID_METABOLISM;REACTOME_GASTRIN_CREB_SIGNALLING_PATHWAY_VIA_PKC_AND_[illegible]

LING_BY_TGF_BETA_RECEPTOR_COMPLEX
PHA_BETA_PATHWAYS;REACTOME_APOPTOSIS;REACTOME_MYD88_MAL_CASCADE_INITIAT

OLIPID_METABOLISM;REACTOME_GASTRIN_CREB_SIGNALLING_PATHWAY_VIA_PKC_AND_
OLIPID_METABOLISM;REACTOME_GASTRIN_CREB_SIGNALLING_PATHWAY_VIA_PKC_AND_

TED_ANTIGEN_PROCESSING_PRESENTATION

IG;REACTOME_G_ALPHA_I_SIGNALLING_EVENTS;REACTOME_G_ALPHA_S_SIGNALLING_EVI

:TIVATION_EVENTS;REACTOME_SIGNALLING_TO_RAS;REACTOME_CELL_SURFACE_INTERA
3ETA_MEDIATED_EVENTS;REACTOME_ELEVATION_OF_CYTOSOLIC_CA2_LEVELS;REACTOM

TIC_CELL;REACTOME_PLC_BETA_MEDIATED_EVENTS;REACTOME_SIGNALING_BY_PDGF;RE

OME_DNA_STRAND_ELONGATION

ME_METABOLISM_OF_CARBOHYDRATES

E_EXTENSION_OF_TELOMERES;REACTOME_G2_M_CHECKPOINTS;REACTOME_S_PHASE;RE

OME_DNA_REPAIR;REACTOME_RNA_POL_II_PRE_TRANSCRIPTION_EVENTS;REACTOME_GL

GENESIS

G_TO_ERKS;REACTOME_SIGNALING_BY_FGFR_IN_DISEASE;REACTOME_SIGNALLING_TO_F

SIGNALING_REPERTOIRE;REACTOME_MYD88_MAL_CASCADE_INITIATED_ON_PLASMA_MEM

ME_TRANSCRIPTIONAL_REGULATION_OF_WHITE_ADIPOCYTE_DIFFERENTIATION

IGNALLING_THROUGH_PLC_BETA;REACTOME_GPCR_DOWNSTREAM_SIGNALING;REACTOM

IFFERENTIATION
IFFERENTIATION

ROTEINS;REACTOME_FATTY_ACID_TRIACYLGLYCEROL_AND_KETONE_BODY_METABOLISM

ON_OF_CYCLIN_B;REACTOME_CONVERSION_FROM_APC_C_CDC20_TO_APC_C_CDH1_IN_LA

_DOWNSTREAM_SIGNAL_TRANSDUCTION;REACTOME_CD28_CO_STIMULATION;REACTOME_

IFFERENTIATION

AB1_SIGNALOSOME;REACTOME_REGULATION_OF_APOPTOSIS;REACTOME_SIGNALING_BY

_GUIDANCE;REACTOME_ACTIVATION_OF_NMDA_RECEPTOR_UPON_GLUTAMATE_BINDING_

)E;REACTOME_CD28_CO_STIMULATION;REACTOME_CD28_DEPENDENT_VAV1_PATHWAY;RE

RBOHYDRATES;REACTOME_INFLUENZA_LIFE_CYCLE;REACTOME_NEP_NS2_INTERACTS_W

_NEGATIVE_REGULATION_OF_THE_PI3K_AKT_NETWORK;REACTOME_PI3K_AKT_ACTIVATIC

G_IN_IMMUNE_SYSTEM

IFFERENTIATION

\LING;REACTOME_INTERFERON_SIGNALING;REACTOME_IL_6_SIGNALING;REACTOME_IMMU

IN_DISEASE;REACTOME_SIGNALLING_TO_P38_VIA_RIT_AND_RIN;REACTOME_GASTRIN_CR

\NALING
\NALING

-L_SURFACE_INTERACTIONS_AT_THE_VASCULAR_WALL;REACTOME_NGF_SIGNALLING_VIA

BETA_MEDIATED_EVENTS;REACTOME_ELEVATION_OF_CYTOSOLIC_CA2_LEVELS;REACTOM

ME_MYD88_MAL_CASCADE_INITIATED_ON_PLASMA_MEMBRANE;REACTOME_INNATE_IMMU

NEUROTRANSMITTER_RECEPTOR_BINDING_AND_DOWNSTREAM_TRANSMISSION_IN_THE_

M_OF_AMINO_ACIDS_AND_DERIVATIVES;REACTOME_REGULATION_OF_ORNITHINE_DECAR
1_G1_S_PHASES;REACTOME_MITOTIC_M_M_G1_PHASES;REACTOME_ASSEMBLY_OF_THE_

MEDIATED_CASCADE;REACTOME_L1CAM_INTERACTIONS;REACTOME_SIGNAL_TRANSDUCT
MEDIATED_CASCADE;REACTOME_L1CAM_INTERACTIONS;REACTOME_SIGNAL_TRANSDUCT

_DOWNSTREAM_SIGNAL_TRANSDUCTION;REACTOME_CD28_CO_STIMULATION;REACTOME_

M_OF_AMINO_ACIDS_AND_DERIVATIVES;REACTOME_REGULATION_OF_ORNITHINE_DECAR

MISSION_IN_THE_POSTSYNAPTIC_CELL;REACTOME_AXON_GUIDANCE;REACTOME_TRAFFI

AF6_MEDIATED_INDUCTION_OF_NFKB_AND_MAP_KINASES_UPON_TLR7_8_OR_9_ACTIVAT

AB1_SIGNALOSOME;REACTOME_NRAGE_SIGNALS_DEATH_THROUGH_JNK;REACTOME_CE

PLICATION;REACTOME_TELOMERE_MAINTENANCE;REACTOME_EXTENSION_OF_TELOMERE

CTOME_G2_M_DNA_DAMAGE_CHECKPOINT

ONS_OF_HIV_FACTORS;REACTOME_INNATE_IMMUNE_SYSTEM;REACTOME_THE_ROLE_OF_

_NEUROTRANSMITTER_RECEPTOR_BINDING_AND_DOWNSTREAM_TRANSMISSION_IN_THE_

E_EXTENSION_OF_TELOMERES;REACTOME_G2_M_CHECKPOINTS;REACTOME_S_PHASE;RE

RKA_FROM_THE_PLASMA_MEMBRANE;REACTOME_SIGNALING_BY_FGFR_IN_DISEASE;REA

IGNALLING_THROUGH_PLC_BETA;REACTOME_GPCR_DOWNSTREAM_SIGNALING;REACTOM

TOR_HIF_BY_OXYGEN;REACTOME_GENERIC_TRANSCRIPTION_PATHWAY;REACTOME_NOT
TOR_HIF_BY_OXYGEN;REACTOME_GENERIC_TRANSCRIPTION_PATHWAY;REACTOME_NOT
TOR_HIF_BY_OXYGEN;REACTOME_GENERIC_TRANSCRIPTION_PATHWAY;REACTOME_NOT
_NEUROTRANSMITTER_RECEPTOR_BINDING_AND_DOWNSTREAM_TRANSMISSION_IN_THE_

ARBOHYDRATES;REACTOME_INFLUENZA_LIFE_CYCLE;REACTOME_NEP_NS2_INTERACTS_W

ARBOHYDRATES;REACTOME_INFLUENZA_LIFE_CYCLE;REACTOME_NEP_NS2_INTERACTS_W

ST_AND_RECOVERY;REACTOME_FORMATION_OF_TRANSCRIPTION_COUPLED_NER_TC_NE

Y;REACTOME_GENERIC_TRANSCRIPTION_PATHWAY;REACTOME_METABOLISM_OF_LIPIDS_

Y;REACTOME_GENERIC_TRANSCRIPTION_PATHWAY;REACTOME_METABOLISM_OF_LIPIDS_

MISSION_IN_THE_POSTSYNAPTIC_CELL;REACTOME_AXON_GUIDANCE;REACTOME_TRAFFI

TIDE_EXCISION_REPAIR;REACTOME_FORMATION_OF_RNA_POL_II_ELONGATION_COMPLEX

[illegible]WNSTREAM_SIGNALING;REACTOME_CD28_DEPENDENT_VAV1_PATHWAY;REACTOME_COS

_LOSS_OF_NLP_FROM_MITOTIC_CENTROSOMES;REACTOME_PLC_BETA_MEDIATED_EVENT

D_SYNTHESIS;REACTOME_DNA_REPLICATION;REACTOME_TELOMERE_MAINTENANCE;REA
D_SYNTHESIS;REACTOME_DNA_REPLICATION;REACTOME_TELOMERE_MAINTENANCE;REA
D_SYNTHESIS;REACTOME_DNA_REPLICATION;REACTOME_TELOMERE_MAINTENANCE;REA

ROM_THE_PLASMA_MEMBRANE;REACTOME_SIGNALLING_TO_ERKS;REACTOME_SIGNALING

CTOME_GPCR_LIGAND_BINDING;REACTOME_PLATELET_HOMEOSTASIS;REACTOME_AQUAP
CTOME_GPCR_LIGAND_BINDING;REACTOME_PLATELET_HOMEOSTASIS;REACTOME_AQUAP

DING;REACTOME_TRAF6_MEDIATED_NFKB_ACTIVATION;REACTOME_TRAF6_MEDIATED_INDU

)ME_DNA_STRAND_ELONGATION

OLIPID_METABOLISM;REACTOME_GASTRIN_CREB_SIGNALLING_PATHWAY_VIA_PKC_AND_[illegible]

CTION;REACTOME_HOST_INTERACTIONS_OF_HIV_FACTORS;REACTOME_THE_ROLE_OF_N[illegible]

TRKA_FROM_THE_PLASMA_MEMBRANE;REACTOME_SIGNALING_BY_FGFR_IN_DISEASE;REA

_BY_ILS;REACTOME_IL1_SIGNALING;REACTOME_IRAK1_RECRUITS_IKK_COMPLEX;REACTO

TIONS;REACTOME_PLATELET_AGGREGATION_PLUG_FORMATION;REACTOME_APOPTOSIS;F

SIGNALING_BY_FGFR;REACTOME_PI3K_CASCADE

_BY_ILS;REACTOME_IL1_SIGNALING;REACTOME_IRAK1_RECRUITS_IKK_COMPLEX;REACTO

TION_AND_REPLICATION;REACTOME_NONSENSE_MEDIATED_DECAY_ENHANCED_BY_THE_

TED_ANTIGEN_PROCESSING_PRESENTATION

_ADP_SIGNALLING_THROUGH_P2RY12;REACTOME_GABA_B_RECEPTOR_ACTIVATION;REAC

ON_OF_CYCLIN_B;REACTOME_CONVERSION_FROM_APC_C_CDC20_TO_APC_C_CDH1_IN_LA

ASIS;REACTOME_IMMUNE_SYSTEM;REACTOME_CYTOKINE_SIGNALING_IN_IMMUNE_SYSTEM

EACTOME_MAP_KINASE_ACTIVATION_IN_TLR_CASCADE;REACTOME_JNK_C_JUN_KINASES_

TOME_PLATELET_ACTIVATION_SIGNALING_AND_AGGREGATION

ACTOME_SIGNAL_TRANSDUCTION_BY_L1;REACTOME_RECYCLING_PATHWAY_OF_L1;REAC

TED_ANTIGEN_PROCESSING_PRESENTATION

:TOME_G2_M_DNA_DAMAGE_CHECKPOINT

:TIVATION_EVENTS;REACTOME_SIGNALLING_TO_RAS;REACTOME_CELL_SURFACE_INTERA

ARBOHYDRATES;REACTOME_INFLUENZA_LIFE_CYCLE;REACTOME_NEP_NS2_INTERACTS_W

EAM_SIGNALING_EVENTS_OF_B_CELL_RECEPTOR_BCR;REACTOME_SIGNALING_BY_THE_I

OLIPID_METABOLISM;REACTOME_GASTRIN_CREB_SIGNALLING_PATHWAY_VIA_PKC_AND_[illegible]

GAND_BINDING_AND_ACTIVATION;REACTOME_FGFR4_LIGAND_BINDING_AND_ACTIVATION;

AM_INTERACTIONS;REACTOME_SIGNAL_TRANSDUCTION_BY_L1;REACTOME_HEMOSTASIS;

IGNALLING_THROUGH_PLC_BETA;REACTOME_GPCR_DOWNSTREAM_SIGNALING;REACTOM

_BY_ILS;REACTOME_IL1_SIGNALING;REACTOME_IRAK1_RECRUITS_IKK_COMPLEX;REACTO

OUGH_NMDA_RECEPTOR;REACTOME_ACTIVATION_OF_NMDA_RECEPTOR_UPON_GLUTAM

PLICATION;REACTOME_TELOMERE_MAINTENANCE;REACTOME_EXTENSION_OF_TELOMERE

BETA_MEDIATED_EVENTS;REACTOME_ELEVATION_OF_CYTOSOLIC_CA2_LEVELS;REACTOM

/ITH_THE_CELLULAR_EXPORT_MACHINERY;REACTOME_HIV_INFECTION;REACTOME_TRANS

ATION_OF_NMDA_RECEPTOR_UPON_GLUTAMATE_BINDING_AND_POSTSYNAPTIC_EVENTS;
OID_SIGNALLING;REACTOME_DARPP_32_EVENTS;REACTOME_G1_PHASE;REACTOME_G1_S

_PHOSPHORYLATION_AND_ACTIVATION_MEDIATED_BY_ACTIVATED_HUMAN_TAK1;REACTO

ME_DOWNSTREAM_SIGNAL_TRANSDUCTION;REACTOME_REGULATION_OF_INSULIN_SECR

UCTION;REACTOME_CD28_CO_STIMULATION;REACTOME_COSTIMULATION_BY_THE_CD28_
UCTION;REACTOME_CD28_CO_STIMULATION;REACTOME_COSTIMULATION_BY_THE_CD28_

_PRE_REPLICATIVE_COMPLEX;REACTOME_DNA_REPLICATION;REACTOME_E2F_MEDIATED_

MAPK;REACTOME_SIGNALING_BY_FGFR1_MUTANTS;REACTOME_SYNTHESIS_OF_PIPS_AT_

ED_ON_PLASMA_MEMBRANE;REACTOME_INNATE_IMMUNE_SYSTEM;REACTOME_ACTIVATE

MAPK;REACTOME_SIGNALING_BY_FGFR1_MUTANTS;REACTOME_SYNTHESIS_OF_PIPS_AT_
MAPK;REACTOME_SIGNALING_BY_FGFR1_MUTANTS;REACTOME_SYNTHESIS_OF_PIPS_AT_

ENTS;REACTOME_G_ALPHA_Z_SIGNALLING_EVENTS;REACTOME_SIGNAL_AMPLIFICATION;[illegible]

ACTIONS_AT_THE_VASCULAR_WALL;REACTOME_NGF_SIGNALLING_VIA_TRKA_FROM_THE_I
E_SIGNALING_BY_PDGF;REACTOME_DOWNSTREAM_SIGNAL_TRANSDUCTION;REACTOME_

ACTOME_DOWNSTREAM_SIGNAL_TRANSDUCTION;REACTOME_TRAFFICKING_OF_AMPA_R

:ACTOME_DNA_STRAND_ELONGATION

.OBAL_GENOMIC_NER_GG_NER;REACTOME_FORMATION_OF_INCISION_COMPLEX_IN_GG_I

P38_VIA_RIT_AND_RIN;REACTOME_GASTRIN_CREB_SIGNALLING_PATHWAY_VIA_PKC_AND_

IBRANE;REACTOME_INNATE_IMMUNE_SYSTEM;REACTOME_ACTIVATED_TLR4_SIGNALLING;

ME_G_PROTEIN_BETA_GAMMA_SIGNALLING;REACTOME_ACTIVATION_OF_KAINATE_RECEP

;REACTOME_TRAF6_MEDIATED_IRF7_ACTIVATION;REACTOME_CIRCADIAN_CLOCK;REACTO

ATE_ANAPHASE;REACTOME_IMMUNE_SYSTEM;REACTOME_PHOSPHORYLATION_OF_THE_A

_COSTIMULATION_BY_THE_CD28_FAMILY;REACTOME_CD28_DEPENDENT_PI3K_AKT_SIGNA

'_PDGF;REACTOME_DOWNSTREAM_SIGNAL_TRANSDUCTION;REACTOME_PI_3K_CASCADE;

_AND_POSTSYNAPTIC_EVENTS;REACTOME_CREB_PHOSPHORYLATION_THROUGH_THE_A[illegible]

[illegible]EACTOME_PD1_SIGNALING;REACTOME_COSTIMULATION_BY_THE_CD28_FAMILY;REACTOM

/ITH_THE_CELLULAR_EXPORT_MACHINERY;REACTOME_HIV_INFECTION;REACTOME_TRAN[illegible]

ON;REACTOME_AKT_PHOSPHORYLATES_TARGETS_IN_THE_CYTOSOL;REACTOME_GAB1_SI

JNE_SYSTEM;REACTOME_CYTOKINE_SIGNALING_IN_IMMUNE_SYSTEM

EB_SIGNALLING_PATHWAY_VIA_PKC_AND_MAPK;REACTOME_SHC1_EVENTS_IN_EGFR_SIG

A_TRKA_FROM_THE_PLASMA_MEMBRANE;REACTOME_SIGNALING_BY_FGFR_IN_DISEASE;R

E_SIGNALING_BY_PDGF;REACTOME_DOWNSTREAM_SIGNAL_TRANSDUCTION;REACTOME_

JNE_SYSTEM;REACTOME_ACTIVATED_TLR4_SIGNALLING;REACTOME_IMMUNE_SYSTEM;RE

_POSTSYNAPTIC_CELL;REACTOME_PLC_BETA_MEDIATED_EVENTS;REACTOME_PKA_MEDIA

BOXYLASE_ODC;REACTOME_REGULATION_OF_APOPTOSIS;REACTOME_CELL_CYCLE_MITC
_PRE_REPLICATIVE_COMPLEX;REACTOME_DNA_REPLICATION;REACTOME_E2F_MEDIATED_

ION_BY_L1;REACTOME_SIGNALING_BY_INSULIN_RECEPTOR;REACTOME_FGFR_LIGAND_BI
ION_BY_L1;REACTOME_SIGNALING_BY_INSULIN_RECEPTOR;REACTOME_FGFR_LIGAND_BI

_COSTIMULATION_BY_THE_CD28_FAMILY;REACTOME_CD28_DEPENDENT_PI3K_AKT_SIGNA

BOXYLASE_ODC;REACTOME_REGULATION_OF_APOPTOSIS;REACTOME_CELL_CYCLE_MITC

CKING_OF_AMPA_RECEPTORS;REACTOME_TRAFFICKING_OF_GLUR2_CONTAINING_AMPA_

ION;REACTOME_ADVANCED_GLYCOSYLATION_ENDPRODUCT_RECEPTOR_SIGNALING;REA[illegible]

LL_DEATH_SIGNALLING_VIA_NRAGE_NRIF_AND_NADE;REACTOME_P75_NTR_RECEPTOR_M[illegible]

S;REACTOME_S_PHASE;REACTOME_DNA_STRAND_ELONGATION

NEF_IN_HIV1_REPLICATION_AND_DISEASE_PATHOGENESIS;REACTOME_IMMUNE_SYSTEM;

_POSTSYNAPTIC_CELL;REACTOME_PLC_BETA_MEDIATED_EVENTS;REACTOME_PKA_MEDIA

:ACTOME_DNA_STRAND_ELONGATION

ACTOME_ENOS_ACTIVATION_AND_REGULATION;REACTOME_NEGATIVE_REGULATION_OF_

ME_G_PROTEIN_BETA_GAMMA_SIGNALLING;REACTOME_ACTIVATION_OF_KAINATE_RECEPT

CH_HLH_TRANSCRIPTION_PATHWAY;REACTOME_METABOLISM_OF_LIPIDS_AND_LIPOPROT
CH_HLH_TRANSCRIPTION_PATHWAY;REACTOME_METABOLISM_OF_LIPIDS_AND_LIPOPROT
CH_HLH_TRANSCRIPTION_PATHWAY;REACTOME_METABOLISM_OF_LIPIDS_AND_LIPOPROT
_POSTSYNAPTIC_CELL;REACTOME_PLC_BETA_MEDIATED_EVENTS;REACTOME_PKA_MEDIA

/ITH_THE_CELLULAR_EXPORT_MACHINERY;REACTOME_HIV_INFECTION;REACTOME_TRAN[illegible]

/ITH_THE_CELLULAR_EXPORT_MACHINERY;REACTOME_HIV_INFECTION;REACTOME_TRANS

R_REPAIR_COMPLEX;REACTOME_DNA_REPAIR;REACTOME_RNA_POL_II_PRE_TRANSCRIPT

_AND_LIPOPROTEINS;REACTOME_FATTY_ACID_TRIACYLGLYCEROL_AND_KETONE_BODY_M
_AND_LIPOPROTEINS;REACTOME_FATTY_ACID_TRIACYLGLYCEROL_AND_KETONE_BODY_M

CKING_OF_AMPA_RECEPTORS;REACTOME_TRAFFICKING_OF_GLUR2_CONTAINING_AMPA_

(_;REACTOME_ELONGATION_ARREST_AND_RECOVERY;REACTOME_FORMATION_OF_TRAN

STIMULATION_BY_THE_CD28_FAMILY;REACTOME_SIGNALING_BY_ILS;REACTOME_REGULAT

S;REACTOME_PKA_MEDIATED_PHOSPHORYLATION_OF_CREB;REACTOME_TRANSMEMBRA

CTOME_REMOVAL_OF_THE_FLAP_INTERMEDIATE_FROM_THE_C_STRAND;REACTOME_EXT
CTOME_REMOVAL_OF_THE_FLAP_INTERMEDIATE_FROM_THE_C_STRAND;REACTOME_EXT
CTOME_REMOVAL_OF_THE_FLAP_INTERMEDIATE_FROM_THE_C_STRAND;REACTOME_EXT

G_BY_FGFR_IN_DISEASE;REACTOME_SIGNALING_BY_FGFR1_MUTANTS;REACTOME_SIGNAL

'ORIN_MEDIATED_TRANSPORT;REACTOME_PROSTACYCLIN_SIGNALLING_THROUGH_PROS
'ORIN_MEDIATED_TRANSPORT;REACTOME_PROSTACYCLIN_SIGNALLING_THROUGH_PROS

UCTION_OF_NFKB_AND_MAP_KINASES_UPON_TLR7_8_OR_9_ACTIVATION;REACTOME_ADV

MAPK;REACTOME_SIGNALING_BY_FGFR1_MUTANTS;REACTOME_SYNTHESIS_OF_PIPS_AT_

EF_IN_HIV1_REPLICATION_AND_DISEASE_PATHOGENESIS;REACTOME_IMMUNE_SYSTEM;RI

\CTOME_ENOS_ACTIVATION_AND_REGULATION;REACTOME_NEGATIVE_REGULATION_OF_[illegible]

ME_TRAF6_MEDIATED_INDUCTION_OF_NFKB_AND_MAP_KINASES_UPON_TLR7_8_OR_9_AC

REACTOME_HEMOSTASIS;REACTOME_PLATELET_ACTIVATION_SIGNALING_AND_AGGREGA

ME_TRAF6_MEDIATED_INDUCTION_OF_NFKB_AND_MAP_KINASES_UPON_TLR7_8_OR_9_AC

_EXON_JUNCTION_COMPLEX

TOME_GABA_RECEPTOR_ACTIVATION;REACTOME_HEMOSTASIS;REACTOME_PLATELET_A(

ATE_ANAPHASE;REACTOME_IMMUNE_SYSTEM;REACTOME_PHOSPHORYLATION_OF_THE_A

M

_PHOSPHORYLATION_AND_ACTIVATION_MEDIATED_BY_ACTIVATED_HUMAN_TAK1;REACTO

TOME_INFLUENZA_LIFE_CYCLE;REACTOME_IMMUNE_SYSTEM;REACTOME_ADAPTIVE_IMMU

ACTIONS_AT_THE_VASCULAR_WALL;REACTOME_NGF_SIGNALLING_VIA_TRKA_FROM_THE_I

/ITH_THE_CELLULAR_EXPORT_MACHINERY;REACTOME_HIV_INFECTION;REACTOME_TRANS

3_CELL_RECEPTOR_BCR;REACTOME_NGF_SIGNALLING_VIA_TRKA_FROM_THE_PLASMA_M

MAPK;REACTOME_SIGNALING_BY_FGFR1_MUTANTS;REACTOME_SYNTHESIS_OF_PIPS_AT_

REACTOME_SIGNALING_BY_FGFR;REACTOME_PI3K_CASCADE

REACTOME_IMMUNE_SYSTEM;REACTOME_ADAPTIVE_IMMUNE_SYSTEM

ME_G_PROTEIN_BETA_GAMMA_SIGNALLING;REACTOME_ACTIVATION_OF_KAINATE_RECEPT

ME_TRAF6_MEDIATED_INDUCTION_OF_NFKB_AND_MAP_KINASES_UPON_TLR7_8_OR_9_AC

ATE_BINDING_AND_POSTSYNAPTIC_EVENTS;REACTOME_CREB_PHOSPHORYLATION_THR[illegible]

[illegible]S;REACTOME_S_PHASE;REACTOME_DNA_STRAND_ELONGATION

E_SIGNALING_BY_PDGF;REACTOME_DOWNSTREAM_SIGNAL_TRANSDUCTION;REACTOME_

SPORT_OF_RIBONUCLEOPROTEINS_INTO_THE_HOST_NUCLEUS;REACTOME_HIV_LIFE_CY(

;REACTOME_POST_NMDA_RECEPTOR_ACTIVATION_EVENTS;REACTOME_DOWNSTREAM_S
;_TRANSITION;REACTOME_COSTIMULATION_BY_THE_CD28_FAMILY;REACTOME_CTLA4_INH

ME_ACTIVATED_TAK1_MEDIATES_P38_MAPK_ACTIVATION;REACTOME_SIGNALING_BY_ILS;

ETION_BY_GLUCAGON_LIKE_PEPTIDE1;REACTOME_REGULATION_OF_INSULIN_SECRETION

_FAMILY;REACTOME_CD28_DEPENDENT_PI3K_AKT_SIGNALING;REACTOME_PI_3K_CASCAD
_FAMILY;REACTOME_CD28_DEPENDENT_PI3K_AKT_SIGNALING;REACTOME_PI_3K_CASCAD

_REGULATION_OF_DNA_REPLICATION;REACTOME_ACTIVATION_OF_ATR_IN_RESPONSE_T[illegible]

THE_PLASMA_MEMBRANE;REACTOME_SIGNALING_BY_FGFR1_FUSION_MUTANTS;REACTOI

ED_TLR4_SIGNALLING;REACTOME_IMMUNE_SYSTEM;REACTOME_TOLL_RECEPTOR_CASCA

THE_PLASMA_MEMBRANE;REACTOME_SIGNALING_BY_FGFR1_FUSION_MUTANTS;REACTO[illegible]
THE_PLASMA_MEMBRANE;REACTOME_SIGNALING_BY_FGFR1_FUSION_MUTANTS;REACTO[illegible]

REACTOME_ADP_SIGNALLING_THROUGH_P2RY12;REACTOME_GABA_B_RECEPTOR_ACTIV

PLASMA_MEMBRANE;REACTOME_SIGNALLING_TO_ERKS;REACTOME_P38MAPK_EVENTS;RE
_REGULATION_OF_INSULIN_SECRETION_BY_GLUCAGON_LIKE_PEPTIDE1;REACTOME_G_AL

ECEPTORS;REACTOME_TRAFFICKING_OF_GLUR2_CONTAINING_AMPA_RECEPTORS;REACT

\NER;REACTOME_HIV_INFECTION;REACTOME_HIV_LIFE_CYCLE;REACTOME_FORMATION_OI

MAPK;REACTOME_SHC1_EVENTS_IN_EGFR_SIGNALING;REACTOME_SIGNALING_BY_GPCR;

REACTOME_IMMUNE_SYSTEM;REACTOME_TOLL_RECEPTOR_CASCADES

TORS_UPON_GLUTAMATE_BINDING

ME_FACTORS_INVOLVED_IN_MEGAKARYOCYTE_DEVELOPMENT_AND_PLATELET_PRODUC

APC_C;REACTOME_ADAPTIVE_IMMUNE_SYSTEM;REACTOME_CLASS_I_MHC_MEDIATED_AN

ALING;REACTOME_PI_3K_CASCADE;REACTOME_DOWNSTREAM_SIGNALING_OF_ACTIVATED

;REACTOME_DOWNSTREAM_SIGNALING_OF_ACTIVATED_FGFR;REACTOME_ROLE_OF_DCC

CTIVATION_OF_RAS;REACTOME_POST_NMDA_RECEPTOR_ACTIVATION_EVENTS;REACTOM

E_CD28_DEPENDENT_PI3K_AKT_SIGNALING;REACTOME_CTLA4_INHIBITORY_SIGNALING;RE

SPORT_OF_RIBONUCLEOPROTEINS_INTO_THE_HOST_NUCLEUS;REACTOME_HIV_LIFE_CYC

GNALOSOME;REACTOME_REGULATION_OF_BETA_CELL_DEVELOPMENT;REACTOME_REGU

;NALING;REACTOME_TRANSMISSION_ACROSS_CHEMICAL_SYNAPSES;REACTOME_NEURON

:EACTOME_PHOSPHOLIPID_METABOLISM;REACTOME_GASTRIN_CREB_SIGNALLING_PATHW

_REGULATION_OF_INSULIN_SECRETION_BY_GLUCAGON_LIKE_PEPTIDE1;REACTOME_G_AL

ACTOME_TOLL_RECEPTOR_CASCADES;REACTOME_NOD1_2_SIGNALING_PATHWAY;REACT

ATED_PHOSPHORYLATION_OF_CREB;REACTOME_TRANSMEMBRANE_TRANSPORT_OF_SMA

)TIC;REACTOME_CELL_CYCLE_CHECKPOINTS;REACTOME_CYCLIN_E_ASSOCIATED_EVENT
_REGULATION_OF_DNA_REPLICATION;REACTOME_ACTIVATION_OF_ATR_IN_RESPONSE_T(

NDING_AND_ACTIVATION;REACTOME_SIGNALING_BY_FGFR;REACTOME_FGFR1_LIGAND_B
NDING_AND_ACTIVATION;REACTOME_SIGNALING_BY_FGFR;REACTOME_FGFR1_LIGAND_B

ALING;REACTOME_PI_3K_CASCADE;REACTOME_DOWNSTREAM_SIGNALING_OF_ACTIVATED

)TIC;REACTOME_CELL_CYCLE_CHECKPOINTS;REACTOME_CYCLIN_E_ASSOCIATED_EVENT

_RECEPTORS;REACTOME_L1CAM_INTERACTIONS;REACTOME_SIGNAL_TRANSDUCTION_BY_

CTOME_NFKB_AND_MAP_KINASES_ACTIVATION_MEDIATED_BY_TLR4_SIGNALING_REPERT[illegible]

IEDIATED_SIGNALLING;REACTOME_SIGNALING_BY_PDGF;REACTOME_DOWNSTREAM_SIGN[illegible]

REACTOME_BINDING_AND_ENTRY_OF_HIV_VIRION;REACTOME_ADAPTIVE_IMMUNE_SYSTE

ATED_PHOSPHORYLATION_OF_CREB;REACTOME_TRANSMEMBRANE_TRANSPORT_OF_SMA

THE_PI3K_AKT_NETWORK;REACTOME_PI3K_AKT_ACTIVATION;REACTOME_AKT_PHOSPHOR

TORS_UPON_GLUTAMATE_BINDING

TEINS;REACTOME_FATTY_ACID_TRIACYLGLYCEROL_AND_KETONE_BODY_METABOLISM;RE
TEINS;REACTOME_FATTY_ACID_TRIACYLGLYCEROL_AND_KETONE_BODY_METABOLISM;RE
TEINS;REACTOME_FATTY_ACID_TRIACYLGLYCEROL_AND_KETONE_BODY_METABOLISM;RE
ATED_PHOSPHORYLATION_OF_CREB;REACTOME_TRANSMEMBRANE_TRANSPORT_OF_SMA

SPORT_OF_RIBONUCLEOPROTEINS_INTO_THE_HOST_NUCLEUS;REACTOME_HIV_LIFE_CY[illegible]

SPORT_OF_RIBONUCLEOPROTEINS_INTO_THE_HOST_NUCLEUS;REACTOME_HIV_LIFE_CY(

TION_EVENTS;REACTOME_INFLUENZA_LIFE_CYCLE;REACTOME_INFLUENZA_VIRAL_RNA_TR

METABOLISM;REACTOME_CIRCADIAN_CLOCK;REACTOME_TRANSCRIPTIONAL_REGULATION
METABOLISM;REACTOME_CIRCADIAN_CLOCK;REACTOME_TRANSCRIPTIONAL_REGULATION

_RECEPTORS;REACTOME_L1CAM_INTERACTIONS;REACTOME_SIGNAL_TRANSDUCTION_BY_

ISCRIPTION_COUPLED_NER_TC_NER_REPAIR_COMPLEX;REACTOME_RNA_POL_I_RNA_POL

TION_OF_SIGNALING_BY_CBL;REACTOME_IL_3_5_AND_GM_CSF_SIGNALING;REACTOME_HE

ANE_TRANSPORT_OF_SMALL_MOLECULES;REACTOME_GLUCAGON_SIGNALING_IN_METAB

TENSION_OF_TELOMERES;REACTOME_S_PHASE;REACTOME_DNA_STRAND_ELONGATION
TENSION_OF_TELOMERES;REACTOME_S_PHASE;REACTOME_DNA_STRAND_ELONGATION
TENSION_OF_TELOMERES;REACTOME_S_PHASE;REACTOME_DNA_STRAND_ELONGATION

LING_BY_FGFR1_FUSION_MUTANTS;REACTOME_SIGNALING_BY_FGFR_MUTANTS;REACTOM

TACYCLIN_RECEPTOR;REACTOME_REGULATION_OF_WATER_BALANCE_BY_RENAL_AQUA[illegible]
TACYCLIN_RECEPTOR;REACTOME_REGULATION_OF_WATER_BALANCE_BY_RENAL_AQUA[illegible]

[illegible]ANCED_GLYCOSYLATION_ENDPRODUCT_RECEPTOR_SIGNALING;REACTOME_NFKB_AND_[illegible]

THE_PLASMA_MEMBRANE;REACTOME_SIGNALING_BY_FGFR1_FUSION_MUTANTS;REACTO

EACTOME_ADAPTIVE_IMMUNE_SYSTEM;REACTOME_CYTOKINE_SIGNALING_IN_IMMUNE_SY

THE_PI3K_AKT_NETWORK;REACTOME_PI3K_AKT_ACTIVATION;REACTOME_AKT_PHOSPHOR

CTIVATION;REACTOME_TRAF6_MEDIATED_IRF7_ACTIVATION_IN_TLR7_8_OR_9_SIGNALING;

TION;REACTOME_APOPTOTIC_EXECUTION_PHASE

CTIVATION;REACTOME_TRAF6_MEDIATED_IRF7_ACTIVATION_IN_TLR7_8_OR_9_SIGNALING;

CTIVATION_SIGNALING_AND_AGGREGATION

APC_C;REACTOME_ADAPTIVE_IMMUNE_SYSTEM;REACTOME_CLASS_I_MHC_MEDIATED_ANT
[truncated: 18,633 more chars]
